# Supplementary figures and images for: Non-invasive Vagal Nerve Stimulation as a Potential Treatment for Repetitive Blast Trauma
Source: bioRxiv. 2026 Jul 19:2026.07.13.737563. Preprint. [Version 1] doi: 10.64898/2026.07.13.737563 (PMC13405043; doi:10.64898/2026.07.13.737563)

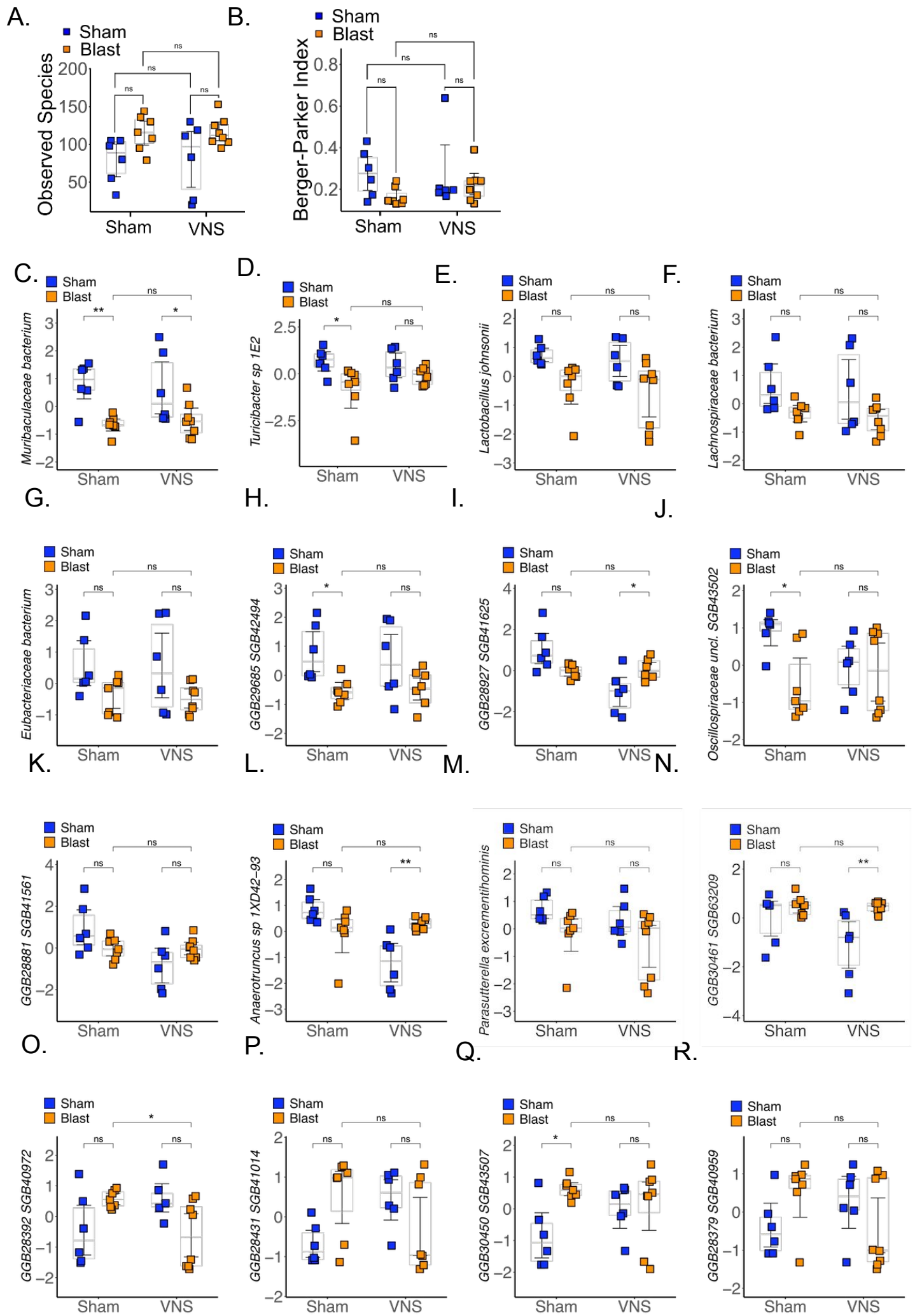

Supplement: Supplement 1 — Supplementary Figure 1: Blast-associated differences in the 4h colonic microbiome are not affected by VNS A-B: Species-level alpha diversity metrics capturing community richness and dominance are not significantly affected by blast or VNS. C-O: Species altered by blast injury. P-R: Species altered by both blast and VNS. Two-way ANOVA post hoc Bonferroni Multiple Comparison Test (a-r) *p ≤ 0.05, **p ≤ 0.01, ***p ≤ 0.001, ****p ≤ 0.0001. [file media-1.pdf]

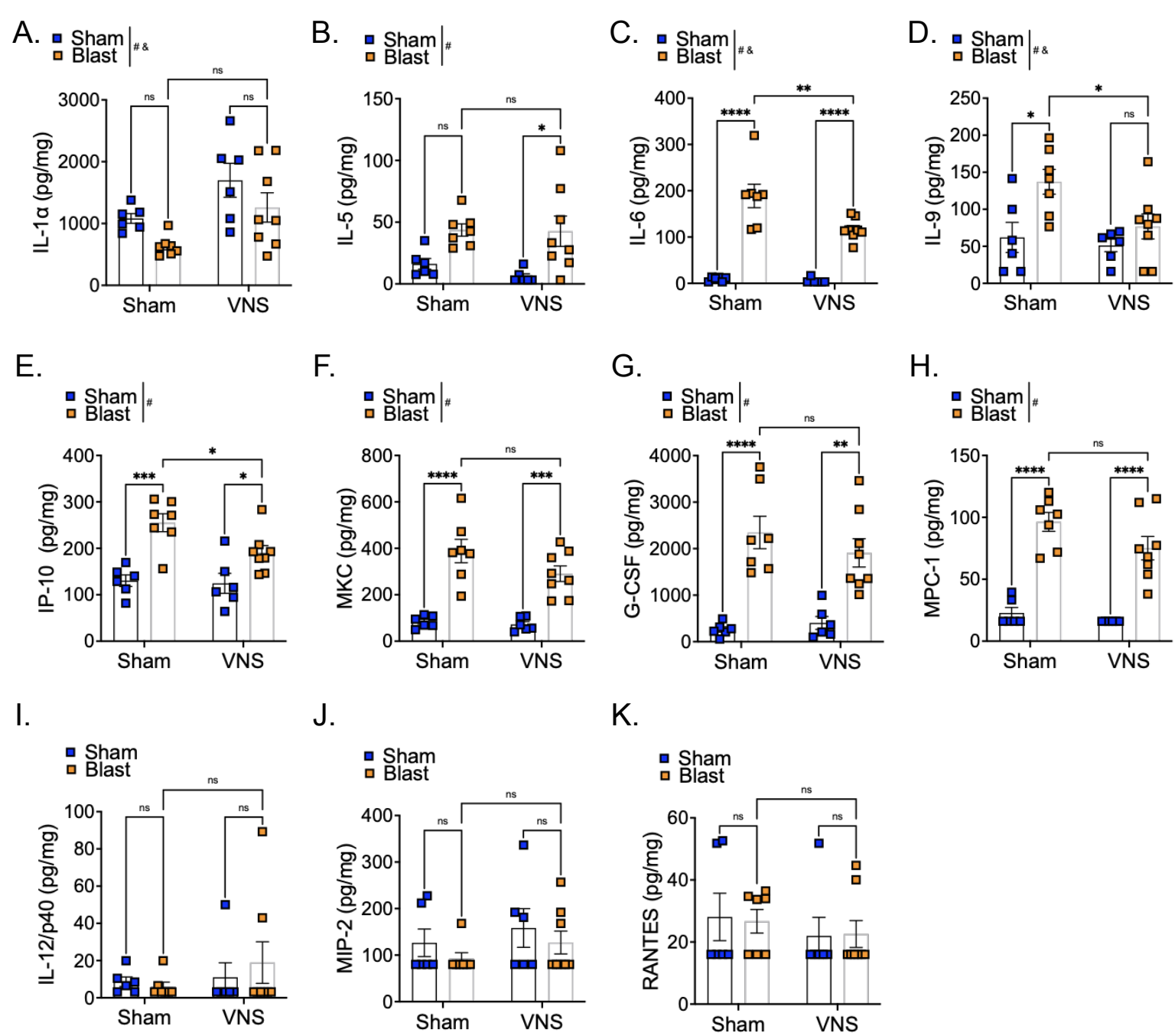

Supplement: Supplement 2 — Supplementary Figure 2: VNS ameliorates some blast-induced changes to serum cytokine levels Two-way ANOVA post hoc Bonferroni Multiple Comparison Test (a-k). *p ≤ 0.05, **p ≤ 0.01, ***p ≤ 0.001, ****p ≤ 0.0001. [file media-2.pdf]

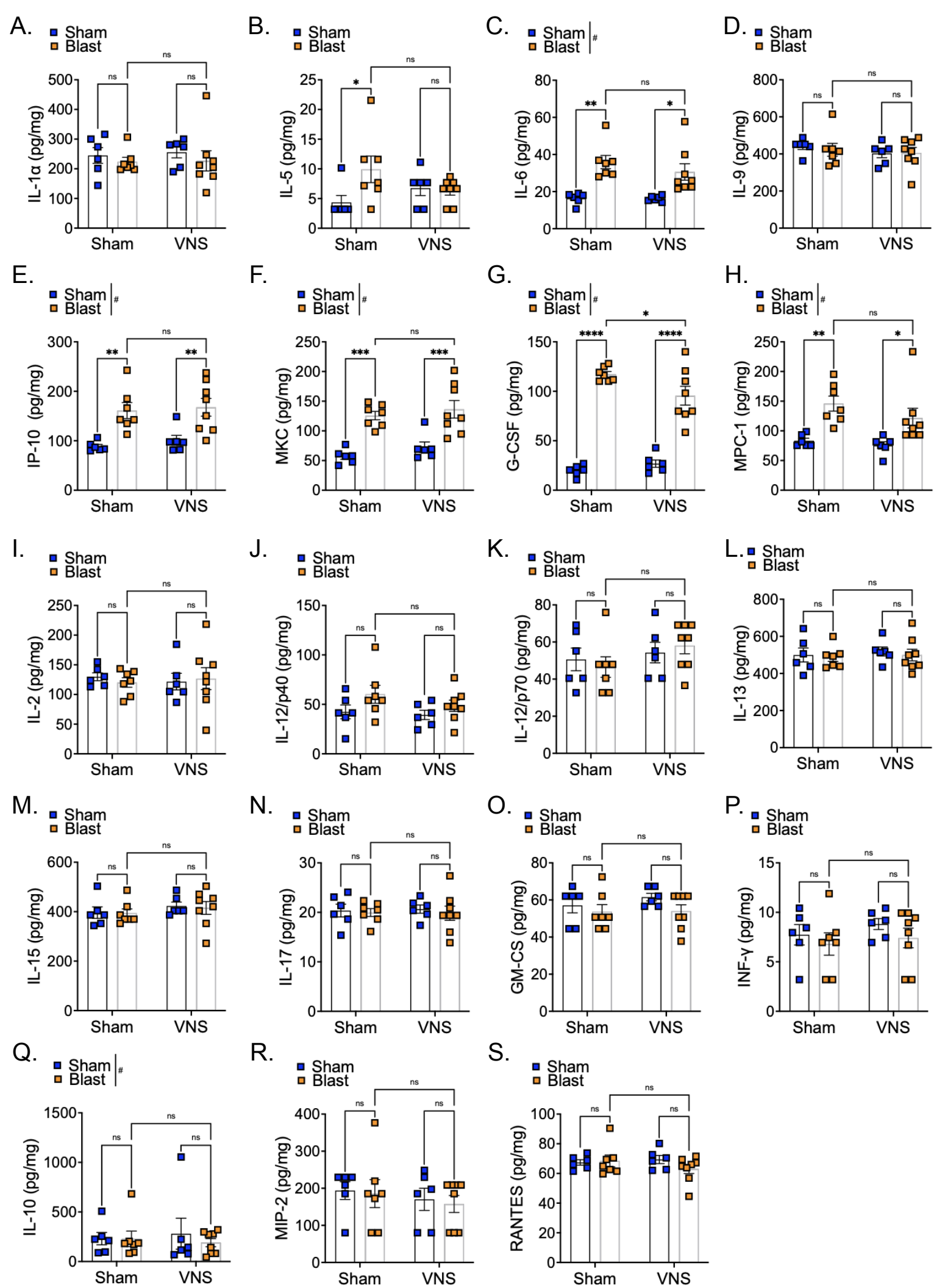

Supplement: Supplement 3 — Supplementary Figure 3: VNS ameliorates some blast-induced changes to brain cytokine levels Two-way ANOVA post hoc Bonferroni Multiple Comparison Test (a-s). *p ≤ 0.05, **p ≤ 0.01, ***p ≤ 0.001, ****p ≤ 0.0001. [file media-3.pdf]

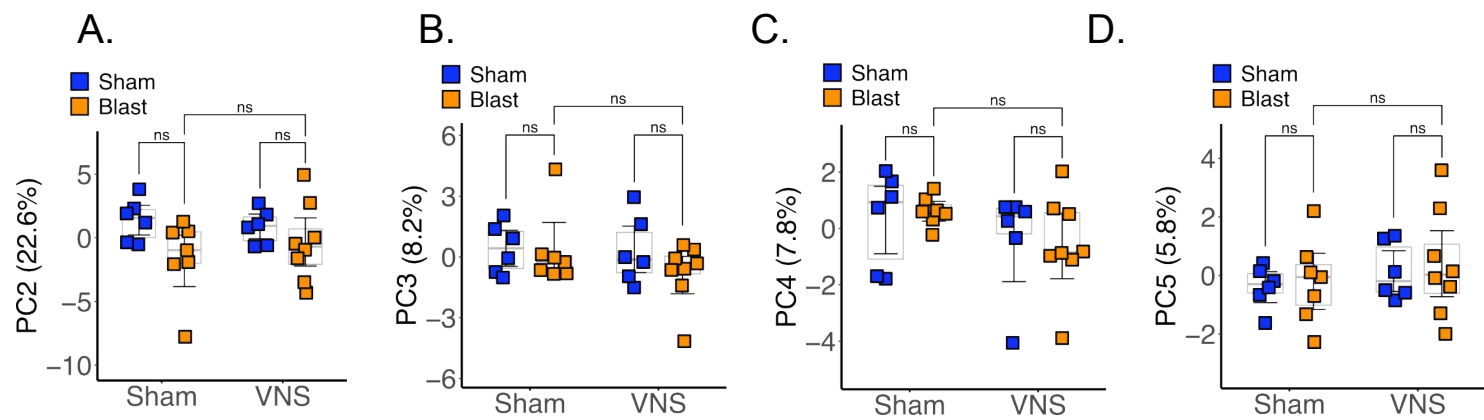

Supplement: Supplement 4 — Supplementary Figure 4: Principal Component Analysis identifies axes of variation in cytokine profiles not significantly explained by blast or VNS Two-way ANOVA post hoc Bonferroni Multiple Comparison Test (a-e); Batch-adjusted linear regression (f-j). *p ≤ 0.05, **p ≤ 0.01, ***p ≤ 0.001, ****p ≤ 0.0001. [file media-4.pdf]

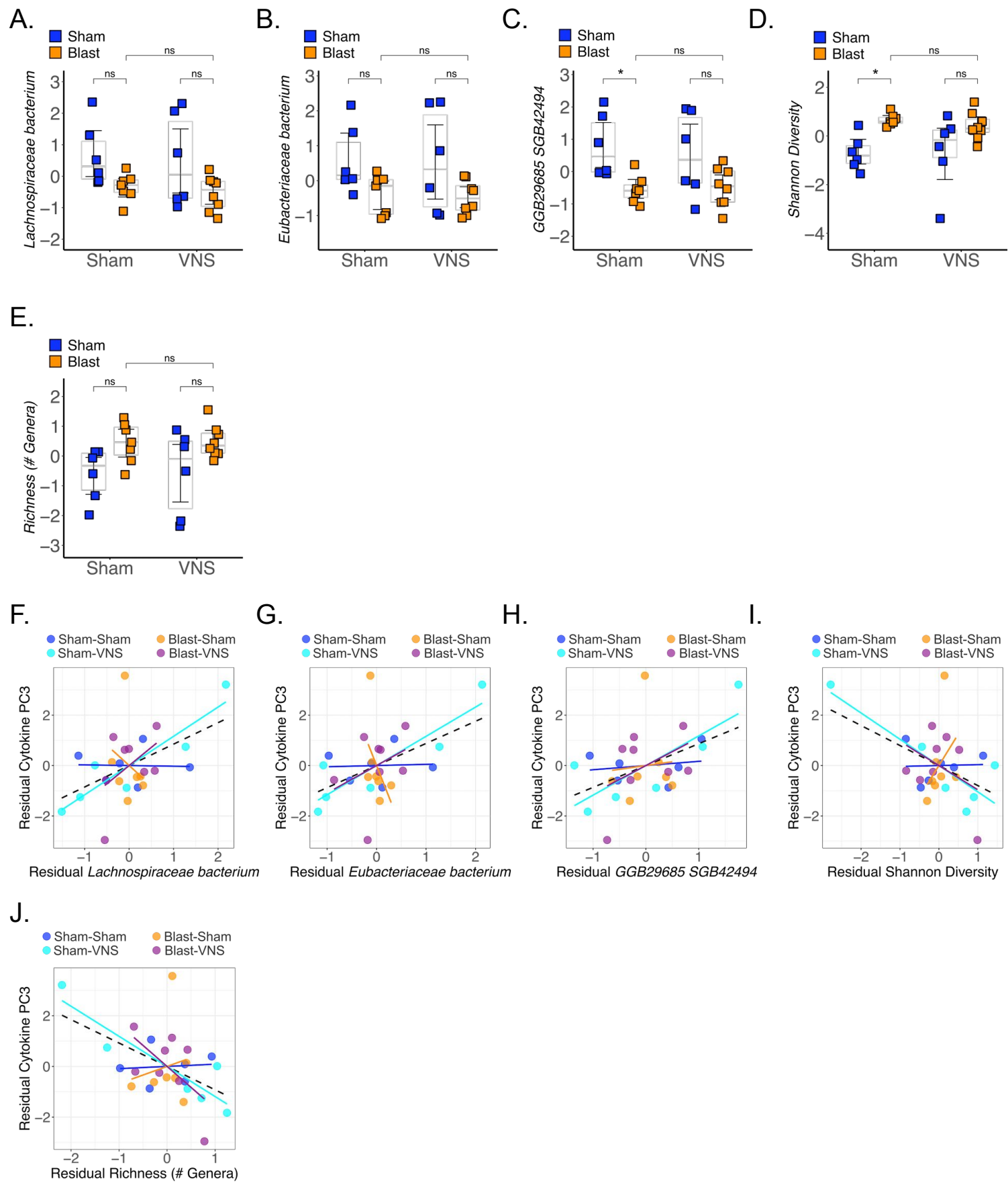

Supplement: Supplement 5 — Supplementary Figure 5: Gut microbial species and alpha diversity metrics mediate Blast effects on Cytokine PC3 A-E: Blast affects relative abundance of PC3-associated species and species-level alpha diversity. F-J: Within-group variation in bacterial features explain within-group differences in cytokine PC3 levels, across all groups. Two-way ANOVA post hoc Bonferroni Multiple Comparison Test (a-e); Batch-adjusted linear regression (f-j). *p ≤ 0.05, **p ≤ 0.01, ***p ≤ 0.001, ****p ≤ 0.0001. [file media-5.pdf]

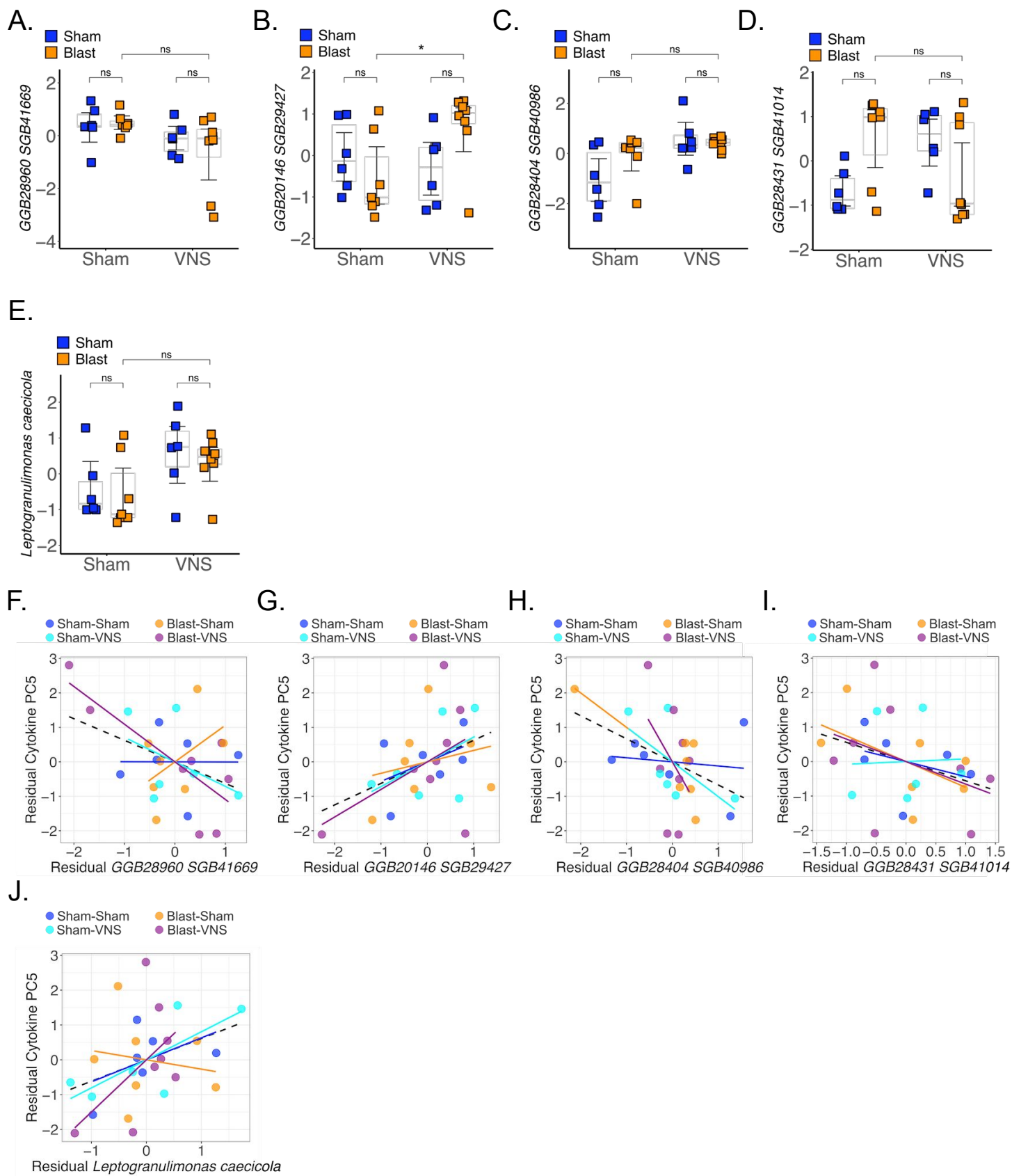

Supplement: Supplement 6 — Supplementary Figure 6: Gut microbial species mediate VNS effects on Cytokine PC5 A-E: VNS affects relative abundance of behavior-associated species. F-J: Variation in bacterial relative abundance explains within-experimental-group differences in behavioral assays, across all groups. Two-way ANOVA post hoc Bonferroni Multiple Comparison Test (a-e); Batch-adjusted linear mixed effects regression (f-j). *p ≤ 0.05, **p ≤ 0.01, ***p ≤ 0.001, ****p ≤ 0.0001. [file media-6.pdf]

A.

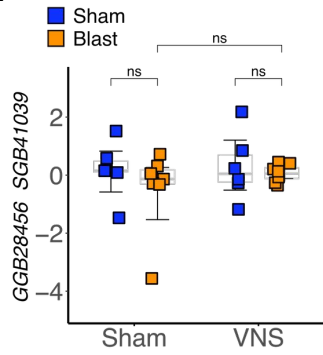

B.

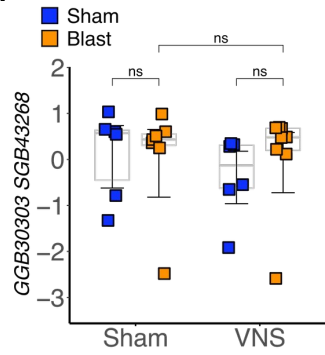

C.

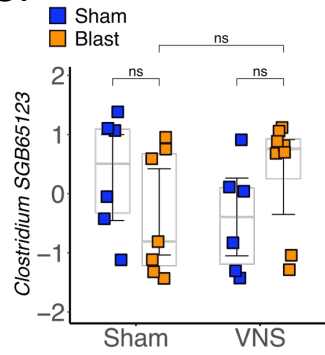

D.

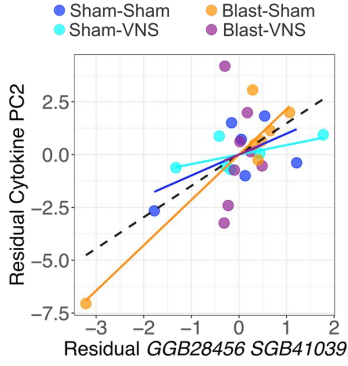

E.

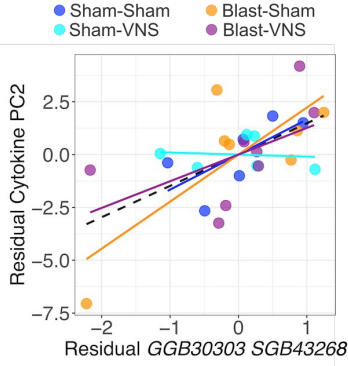

F.

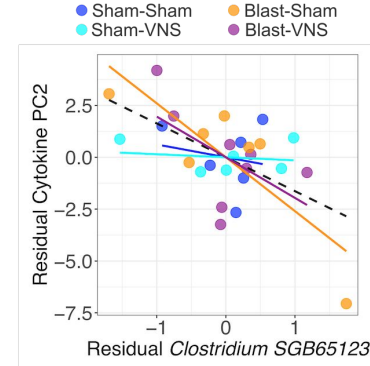

Supplement: Supplement 7 — Supplementary Figure 7: Gut microbial species are associated with Cytokine PC2 independent of blast and VNS A-C: Variation in bacterial relative abundance explains within-treatment-group differences in Cytokine PC2, across all groups. Batch-adjusted linear mixed effects regression (a-c). [file media-7.pdf]

A.

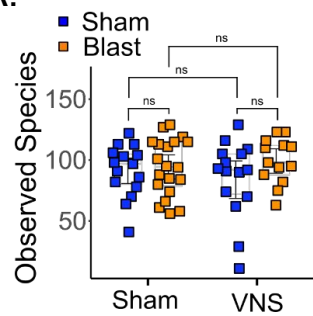

B.

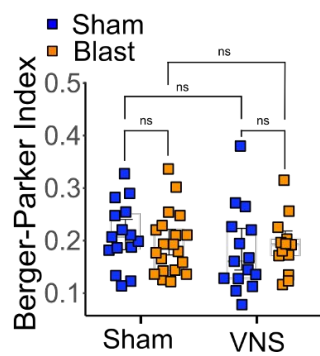

C.

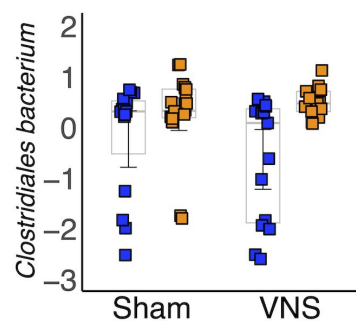

D.

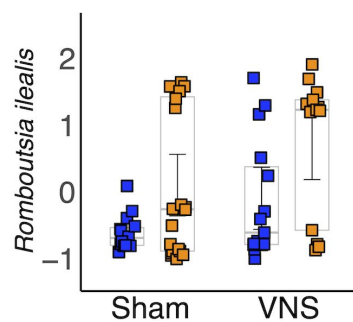

E.

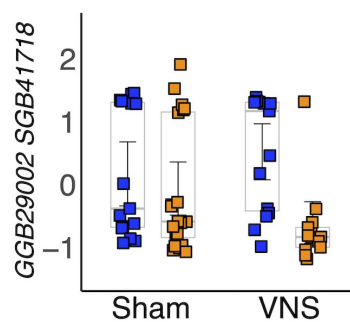

Supplement: Supplement 8 — Supplementary Figure 8: Blast-associated differences in the 24h fecal pellet microbiome are not affected by VNS A-B: Species-level alpha diversity metrics capturing community richness and dominance are not significantly affected by blast or VNS. C-E: Species altered by blast injury. Two-way ANOVA post hoc Bonferroni Multiple Comparison Test (a-e) *p ≤ 0.05, **p ≤ 0.01, ***p ≤ 0.001, ****p ≤ 0.0001. [file media-8.pdf]

A.

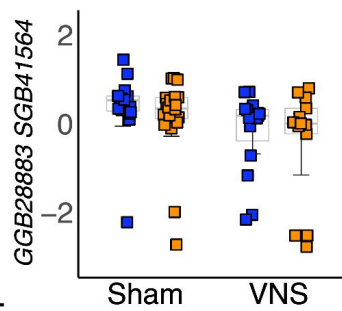

B.

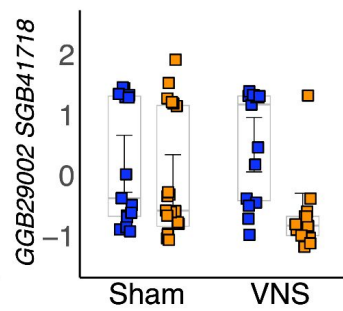

C.

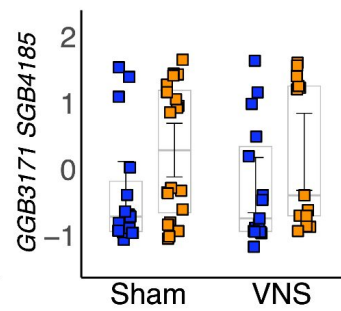

D.

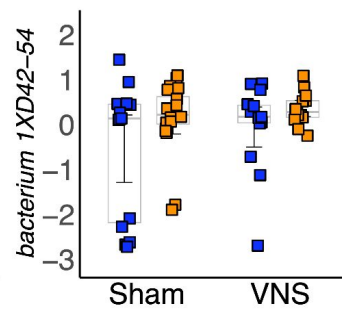

E.

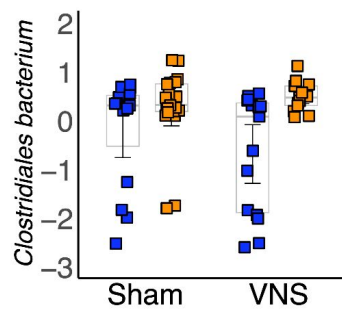

F.

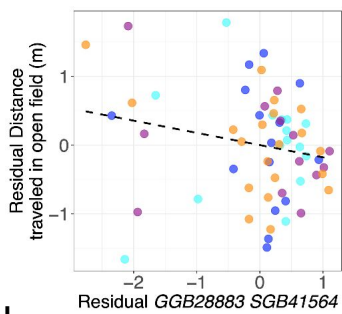

G.

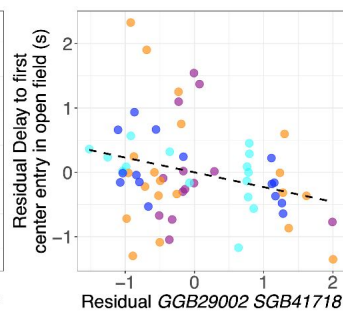

H.

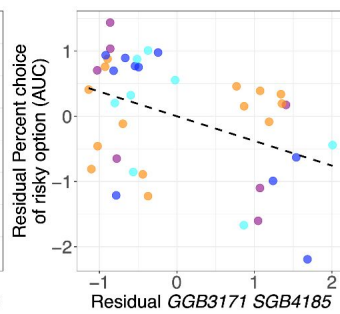

I.

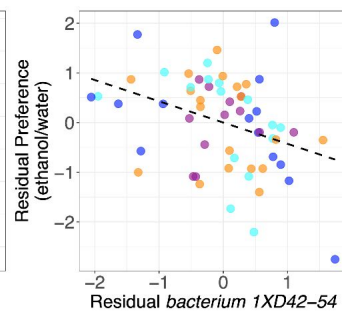

J.

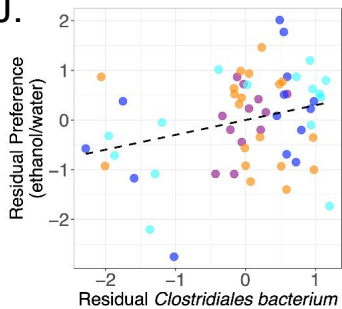

Supplement: Supplement 9 — Supplementary Figure 9: A: VNS affects relative abundance of behavior-associated species. B-E: Blast affects relative abundance of behavior-associated species. F-J: Variation in bacterial relative abundance explains within-experimental-group differences in behavioral assays, across all groups. Two-way ANOVA post hoc Bonferroni Multiple Comparison Test (a-e); Batch-adjusted linear mixed effects regression (f-j). *p ≤ 0.05, **p ≤ 0.01, ***p ≤ 0.001, ****p ≤ 0.0001. [file media-9.pdf]
