# Supplementary material for: Non-invasive Vagal Nerve Stimulation as a Potential Treatment for Repetitive Blast Trauma
Source: bioRxiv. 2026 Jul 19:2026.07.13.737563. Preprint. [Version 1] doi: 10.64898/2026.07.13.737563 (PMC13405043; doi:10.64898/2026.07.13.737563)
Supplement: Supplement 10 [file media-10.pdf]

Species-level feature	n	LRT_F_blast	LRT_F_vns	LRT_F_interact	LRT_p_blast
Acetatifactor_SGB41545	27	-1.737559389	-0.829130954	1.647870903	0.2103509006
Acutalibacter_muris	27	-0.690728962	-1.081850986	0.4367700067	0.7869843388
Akkermansia_muciniphila	27	-0.746179775	0.8790501017	-0.7353449499	0.16660254
Anaerotruncus_sp_1XD42_93	27	-2.167500944	-4.932661639	4.238351262	0.0009175375
Bacteria_unclassified_SGB41677	27	0.3226091387	-0.892429325	0.2981631831	0.7141894931
Bacteria_unclassified_SGB43539	27	2.016693907	0.6767006014	-1.237642783	0.1489024993
Bacteria_unclassified_SGB43546	27	2.178999982	1.536693862	-1.362268113	0.1125519082
Bacteria_unclassified_SGB63211	27	-0.562402231	-0.811577519	1.451468956	0.2950037757
Bacteroides_thetaiotaomicron	27	-2.688563861	-1.611515975	1.444773548	0.0366277544
Clostridia_bacterium	27	-1.687109712	-1.415843682	0.9668606116	0.2483530876
Clostridiaceae_bacterium	27	-1.976156483	-1.648772466	1.265632115	0.1623466052
Clostridiaceae_unclassified_SGB41663	27	-1.327396058	-1.997977181	0.5329468379	0.3624098717
Clostridiales_bacterium	27	-2.380329549	-1.055035497	2.032232708	0.0738247407
Clostridium_SGB65123	27	-1.278513916	-1.546775173	1.919649228	0.1804389829
Clostridium_cocleatum	27	0.1363916586	1.22710568	-0.9159713011	0.5072750859
Eubacteriaceae_bacterium	27	-2.349742056	-0.094316773	0.2257123385	0.0166040620
Eubacteriaceae_unclassified_SGB94922	27	-1.944640614	-1.31025256	1.450688493	0.1742242764
GGB20146_SGB29427	27	-1.009664056	-0.616823623	2.1615213	0.0938444678
GGB20149_SGB29430	27	-1.195873328	-0.016156629	1.304554587	0.4132259254
GGB22635_SGB63107	27	-0.775565824	1.132698371	-0.8669862322	0.1146609038
GGB25041_SGB36960	27	-1.9425547	-1.822629432	1.169068899	0.1675951172
GGB28379_SGB40959	27	2.46669236	1.877464866	-2.814230903	0.0292418367
GGB28382_SGB40962	27	-0.447707979	1.346358344	-1.343939082	0.0725130221
GGB28392_SGB40972	27	2.025461785	2.014129263	-3.120352237	0.0171836690
GGB28404_SGB40986	27	1.99959786	3.16053752	-1.54135182	0.1577679288
GGB28418_SGB41001	27	-0.041585992	0.2096541944	-0.02293525044	0.996265407
GGB28422_SGB41005	27	1.575141745	1.346906441	-0.9369333963	0.2980947557
GGB28431_SGB41014	27	2.612858458	2.312986843	-3.145896704	0.0153775845
GGB28456_SGB41039	27	-1.133730025	0.1756055527	0.5841506241	0.5086215493
GGB28782_SGB41435	27	-0.529075003	-1.325738094	1.401029387	0.3154915999
GGB28792_SGB41445	27	1.646981447	0.1069408407	-0.2260428306	0.1237707265
GGB28798_SGB41451	27	-0.208945716	-0.786117249	-0.06782061124	0.9318373054
GGB28810_SGB41465	27	0.4099870416	-0.002870786	-0.2812842323	0.9195217924
GGB28828_SGB41484	27	2.18871256	1.506310347	-2.764608998	0.0364531763
GGB28851_SGB41518	27	0.0459529763	0.6764984275	0.09092996276	0.9832452673
GGB28865_SGB41537	27	2.467505665	2.617363758	-2.355585473	0.0518382591
GGB28868_SGB41542	27	1.423025439	0.6682990301	-1.550411877	0.2924231839
GGB28869_SGB41543	27	-0.196869533	0.0552246577	-0.481765068	0.6620730776
GGB28875_SGB41555	27	-0.415349022	-0.564469250	-0.1068942592	0.7766440627
GGB28881_SGB41561	27	-2.502061412	-4.068063868	3.298161044	0.0118945489
GGB28892_SGB41573	27	0.6347427618	0.5322266598	-0.08888102133	0.715153028
GGB28893_SGB41574	27	-0.690526180	0.4526941522	0.7523813845	0.7392219927
GGB28898_SGB41580	27	-0.390079089	-0.993294137	0.9355235793	0.6019247005
GGB28901_SGB41592	27	-2.37580037	0.0448243827	1.32598069	0.0720635862
GGB28904_SGB41597	27	-0.621785954	-0.048282861	1.611222842	0.2257525204
GGB28909_SGB41602	27	0.1490523608	-0.022905988	-1.360218857	0.2182830072

GGB28924_SGB41621	27	1.001568899	-0.130095099	-0.7546637238	0.6115781842
GGB28927_SGB41625	27	-2.763553985	-4.998927254	4.089700878	0.0019544839
GGB28934_SGB41635	27	-1.483764113	-1.292771592	1.789347276	0.2164988531
GGB28949_SGB41655	27	-1.761459223	-0.770874578	0.6077940833	0.1595983662
GGB28951_SGB102295	27	-0.817615234	-1.417425515	0.5554203197	0.718725508
GGB28951_SGB41658	27	0.7149686649	2.227075952	-0.3365208725	0.7526278431
GGB28954_SGB41662	27	0.0862181249	-0.016099600	0.4082467004	0.7949390323
GGB28960_SGB41669	27	0.2625165665	-0.982669415	-0.954648455	0.5366388614
GGB28964_SGB94886	27	-1.57805804	-1.223257853	0.4750808505	0.2072130289
GGB28967_SGB41678	27	-0.510056726	-0.614867762	0.5524666292	0.8482050661
GGB28996_SGB41712	27	0.0989955308	0.1422517402	0.8483129055	0.4298847288
GGB29531_SGB42317	27	1.139878452	-0.033994658	0.005846641031	0.2795934449
GGB29685_SGB42494	27	-3.086960459	-0.606392337	0.7640020298	0.0045968812
GGB30141_SGB43066	27	0.9827381462	0.8715180491	-0.8294030133	0.6140281408
GGB30145_SGB43072	27	-0.031208747	-1.442903132	1.140682656	0.2949240353
GGB30286_SGB43248	27	-0.230788931	-0.213833240	0.650091804	0.7667575811
GGB30300_SGB43264	27	-1.141611788	-1.493527393	1.806092231	0.2138469484
GGB30303_SGB43268	27	0.0612920269	-0.863776907	0.427200127	0.7955727513
GGB30450_SGB43507	27	2.903587772	1.664169607	-1.855239558	0.0271094004
GGB30453_SGB43513	27	1.272461974	1.346563515	-1.540777019	0.3157254511
GGB30454_SGB43514	27	-0.885604686	0.2665664068	0.3976172678	0.6428886468
GGB30455_SGB43519	27	0.8320714881	2.025781929	-0.8603659197	0.6637984269
GGB30456_SGB43520	27	2.094670851	1.292716638	-1.983567641	0.1111969489
GGB30457_SGB63218	27	0.0419297937	0.0796109826	-0.3783903328	0.8821640501
GGB30461_SGB43530	27	-0.545894485	-1.240778268	-0.206349993	0.6024683039
GGB30461_SGB43533	27	-0.649134976	-0.787774598	0.433501048	0.8107674231
GGB30461_SGB63209	27	1.186310369	-2.388070782	1.525246869	0.0059141187
GGB30463_SGB43537	27	1.975195137	0.0415191651	-0.1114196225	0.0407681100
GGB30473_SGB43557	27	-0.715707951	-1.892291501	1.45562487	0.3284915799
GGB30475_SGB63182	27	-0.054823336	-0.023471047	0.4892695436	0.811833041
GGB30861_SGB44083	27	0.5087882423	0.0604976815	0.08015704245	0.7194016475
GGB31312_SGB44628	27	0.3705888042	0.4802730455	-0.6041750471	0.8310391405
GGB31438_SGB44768	27	-0.975162992	-0.968230617	1.221833074	0.4812139003
GGB3171_SGB4185	27	-2.393512711	-2.507459011	2.172233609	0.0663422469
GGB31762_SGB45125	27	-1.021684388	-1.100679979	1.293390539	0.4428890562
GGB31823_SGB45199	27	-0.415952333	-1.125005285	0.9094533136	0.6301752034
GGB31838_SGB45216	27	0.5715561169	-0.067387115	0.05382542551	0.6844031648
GGB31841_SGB65084	27	-0.369087597	-1.186547181	1.233063862	0.3717374543
GGB32371_SGB41694	27	-0.143697051	1.376444228	-1.103854608	0.2420180555
GGB3793_SGB5158	27	-0.188176521	-0.096696085	0.4542847466	0.884993491
GGB42601_SGB59797	27	0.9883235619	0.684691121	-0.04068618561	0.4018920133
GGB45514_SGB63186	27	0.260326333	0.8498537703	-0.2941763049	0.9553647659
GGB45564_SGB63259	27	-0.456513487	-1.39485483	1.213998907	0.4152727846
GGB45624_SGB63337	27	0.9587880791	1.387724149	-0.8540069663	0.6203892728
GGB45656_SGB63370	27	0.9827243777	1.978828355	-1.37318629	0.4047054831
GGB47127_SGB65054	27	1.187950687	0.6102248817	-0.5307932959	0.4564936795

GGB74395_SGB43523	27	0.666175050	0.601580936	-0.4573035892	0.802410438
GGB75053_SGB43494	27	0.880361518	0.099461301	-1.831835919	0.177327401
GGB75109_SGB102238	27	0.081862972	-0.029145587	-0.7684505044	0.599792986
Lachnospiraceae_bacterium	27	-2.203964115	-0.387255862	0.1852804211	0.023925903
Lachnospiraceae_bacterium_A2	27	0.265356901	1.313553919	0.3415605907	0.724187807
Lachnospiraceae_unclassified_SGB41414	27	-0.550628961	-0.071448164	0.8166452243	0.719079500
Lachnospiraceae_unclassified_SGB41424	27	1.239724625	-2.049048928	0.02832888319	0.216930413
Lachnospiraceae_unclassified_SGB94868	27	-1.157161566	-0.374208568	-0.1632694205	0.207633504
Lactobacillus_johnsonii	27	-2.904233498	-0.545006746	0.221966001	0.002748365
Leptogranulimonas_caecicola	27	-0.424775229	2.085478283	0.09712389841	0.874577980
Muribaculaceae_bacterium	27	-3.20434493	-0.393571486	0.4976867034	0.001850198
Neglectibacter_sp_X4	27	1.3707582	-0.049643228	-0.8101334702	0.394423067
Oscillibacter_SGB43496	27	0.993141102	0.728529128	0.3318527618	0.220544725
Oscillospiraceae_bacterium	27	1.201927585	-0.248452492	-0.9545140101	0.492783661
Oscillospiraceae_unclassified_SGB43502	27	-3.128709497	-1.94852721	2.128793537	0.017271201
Oscillospiraceae_unclassified_SGB43505	27	-0.010797214	0.697591581	-0.9901098303	0.374299908
Oscillospiraceae_unclassified_SGB94989	27	0.210561514	0.654762590	0.006552710316	0.953368969
Parasutterella_excrementihominis	27	-2.246368465	-0.958588251	0.2381937364	0.023419378
Schaedlerella_arabinosiphila	27	2.167659768	0.713275921	-1.344943283	0.114481983
Turicibacter_sp_1E2	27	-3.247061264	-0.532201248	1.62846818	0.009594137
bacterium_0_1xD8_82	27	0.730564224	-0.014966699	-0.4458468647	0.763436393
bacterium_1XD42_1	27	0.660522350	0.006280943	-1.535207489	0.272519397
bacterium_1XD42_54	27	-2.731523735	-2.13093224	1.693887731	0.038105321
bacterium_1XD42_76	27	-1.502721407	-0.920803995	1.92777027	0.177410768
bacterium_1xD8_48	27	2.076486751	1.089861791	-1.442230665	0.139347611
Berger Parker Index	27	-2.400004014	-0.534745917	1.115422088	0.057475283
Richness (# observed features)	27	2.441438933	0.134471858	-0.2739486566	0.013777874
Shannon Index	27	2.839530142	0.195304147	-0.5263857384	0.006473070

LRT_p_vns	LRT_p_interact	LRT_q_blast	LRT_q_vns	LRT_q_interact	est_intercept	est_blast
0.239098669	0.1135888756	0.499346548	0.717296007	0.5928157147	0.4930595825	-0.96554993
0.491902226	0.6665350338	0.893761146	0.817383170	0.8978474649	0.2279987513	-0.389763961
0.678899621	0.4698968088	0.481170621	0.895252247	0.7941917894	-0.1103214233	-0.399386127
0.000231687	0.000337089034	0.078179359	0.013901240	0.02907734573	0.8338932722	-0.884230098
0.58777865	0.768376414	0.886760580	0.865929823	0.9440157185	0.727861165	0.137934724
0.449260123	0.2288953073	0.481170621	0.816836587	0.6066894195	-0.547433953	1.117385022
0.309908808	0.1868987777	0.416948741	0.772603929	0.5928157147	-1.005997881	1.143442891
0.340871629	0.1607610545	0.576957591	0.772603929	0.5928157147	0.5484779944	-0.270044308
0.275405005	0.1626148411	0.217744694	0.738856224	0.5928157147	0.8184868884	-1.377170165
0.380556079	0.3441231202	0.532185187	0.772603929	0.757210127	0.78033172	-0.93595003
0.277071084	0.2188848206	0.481170621	0.738856224	0.6066894195	0.5655145171	-1.04826086
0.070917324	0.5994132043	0.658927039	0.500592877	0.8978474649	0.6413621793	-0.671498939
0.124851167	0.05437659762	0.316391746	0.601953280	0.5437080554	0.6809451363	-1.250255975
0.179389568	0.06796350693	0.481170621	0.632845883	0.5437080554	0.8492375503	-0.592547765
0.482732384	0.369613781	0.762932324	0.817383170	0.7585775013	-0.5009146985	0.074631012
0.969157578	0.823508827	0.148038868	0.999007775	0.9535244924	0.1354286295	-1.047054478
0.346181024	0.1609762619	0.481170621	0.772603929	0.5928157147	0.7705460849	-1.074513474
0.051218919	0.04180462925	0.388321935	0.409751358	0.4877585812	0.2046435515	-0.505435513
0.180434838	0.2055274741	0.682640193	0.632845883	0.5979782654	0.3051981816	-0.637854067
0.535639339	0.3953075818	0.416948741	0.826524236	0.7721894584	0.1473966249	-0.421797406
0.206256260	0.2548873782	0.481170621	0.668939224	0.6372184455	0.1468927416	-0.845809113
0.032794656	0.01010347507	0.194945578	0.357759888	0.1938341562	-1.083284581	0.867154966
0.370430736	0.1926651073	0.316391746	0.772603929	0.5928157147	0.2289327297	-0.236246045
0.016592400	0.004982143122	0.148038868	0.288021378	0.1195714349	-0.6043400677	0.989085319
0.010090214	0.1374931891	0.481170621	0.288021378	0.5928157147	-1.21957616	0.941802831
0.959497945	0.9819086792	0.996265407	0.999007775	0.9953877755	-0.02550735054	-0.025121584
0.416676405	0.3589651594	0.576957591	0.793669342	0.757210127	-0.4251724064	0.855031643
0.016801247	0.004692592965	0.148038868	0.288021378	0.1195714349	-0.3931090988	1.268171468
0.567597000	0.5650613534	0.762932324	0.851395500	0.8978474649	0.04050973727	-0.651125121
0.359169919	0.1751539938	0.591985220	0.772603929	0.5928157147	0.5002608365	-0.288509786
0.970858107	0.8232549644	0.436837858	0.999007775	0.9535244924	-0.2445948503	0.879403610
0.471552545	0.946541058	0.963969626	0.817383170	0.9953877755	0.3090561082	-0.12167290
0.915105920	0.7811210556	0.959501000	0.983027427	0.9440157185	-0.7135214484	0.172930628
0.029615316	0.01130699244	0.217744694	0.355383798	0.1938341562	-0.3710884395	1.127843444
0.548244701	0.9283710877	0.991507832	0.832776761	0.9948712152	-0.3534930205	0.026967976
0.043832016	0.02782138454	0.270460482	0.404603226	0.417320768	-0.4150402477	1.14205863
0.254323647	0.1353098773	0.576957591	0.726638993	0.5928157147	-0.1682835008	0.804146646
0.806606148	0.6347306827	0.875338584	0.941377455	0.8978474649	-0.3308795206	-0.099385824
0.634070741	0.9158416652	0.893761146	0.874580333	0.9948712152	0.857760516	-0.195555641
0.001988037	0.003276334099	0.148038868	0.079521481	0.1195714349	0.4963207629	-1.030353634
0.784709483	0.9299806264	0.886760580	0.941377455	0.9948712152	-0.6674065486	0.352922207
0.264245168	0.4597968228	0.893761146	0.737428376	0.7882231248	0.2975744393	-0.351514322
0.591718713	0.3596748103	0.855709341	0.865929823	0.757210127	0.6645917313	-0.196403008
0.152665868	0.1984495128	0.316391746	0.631720835	0.5953485384	0.4827366956	-1.21629365
0.085658495	0.1213865217	0.501672267	0.513950974	0.5928157147	0.3631414204	-0.271412487
0.145902665	0.1875366336	0.499346548	0.625297136	0.5928157147	0.4234922075	0.079964967

0.456981189	0.4584536795	0.855709341	0.817383170	0.7882231248	0.5324294399	0.455449925
0.000219425	0.000484622428	0.078179359	0.013901240	0.02907734573	0.7784783317	-1.108579812
0.224102539	0.08733647108	0.499346548	0.689546274	0.5515987647	0.3808768165	-0.837820242
0.744810754	0.5495473988	0.481170621	0.941377455	0.8949850654	0.657170233	-0.975862317
0.298970783	0.5842115419	0.886760580	0.772603929	0.8978474649	0.7041567913	-0.460718028
0.024801176	0.7396669805	0.893761146	0.330682352	0.9289265075	-1.104944222	0.331749997
0.841186745	0.6870387291	0.893761146	0.941377455	0.9059851372	0.226786524	0.047545168
0.043638773	0.3501283184	0.795020535	0.404603226	0.757210127	0.6970955679	0.132356616
0.400370147	0.6394119482	0.499346548	0.774909963	0.8978474649	0.6453720114	-0.853863173
0.820661965	0.5861985137	0.916978449	0.941377455	0.8978474649	0.3497131573	-0.303270173
0.377449394	0.4053994657	0.697110371	0.772603929	0.7721894584	-0.0196178496	0.053462464
0.999007775	0.9953877755	0.576957591	0.999007775	0.9953877755	-0.3405672731	0.651254204
0.748416768	0.4529827042	0.110325149	0.941377455	0.7882231248	0.4695786136	-1.382159056
0.664026053	0.415785445	0.855709341	0.885368071	0.7755275948	0.1741621922	0.490346821
0.367951830	0.2662678912	0.576957591	0.772603929	0.6520846316	0.3928424426	-0.017102403
0.750425732	0.5223641345	0.893761146	0.941377455	0.8706068908	0.1905115758	-0.133483697
0.213511417	0.08460524511	0.499346548	0.674246581	0.5515987647	0.8297433004	-0.531185592
0.660099923	0.6733855986	0.893761146	0.885368071	0.8978474649	0.5331742804	0.032968656
0.186573367	0.07701162963	0.191360473	0.632845883	0.5515987647	-1.038075753	1.461101842
0.310638137	0.1376326802	0.591985220	0.772603929	0.5928157147	-0.3566686451	0.727474260
0.656930027	0.6947440907	0.866816153	0.885368071	0.9061879443	-0.1407191408	-0.503273466
0.104495810	0.3988667773	0.875338584	0.569977148	0.7721894584	-0.7586862338	0.451847563
0.158921661	0.05992185166	0.416948741	0.632845883	0.5437080554	-0.5522432588	1.14649791
0.890757036	0.7087673991	0.931572095	0.971734949	0.9145385795	0.230945138	0.024824711
0.137401205	0.838415279	0.855709341	0.625297136	0.9581888902	0.7020068896	-0.294732404
0.718693859	0.6688718019	0.893761146	0.937426772	0.8978474649	0.8113486528	-0.327268348
0.075178424	0.14144584	0.110966921	0.501189498	0.5928157147	0.2479618484	0.524097573
0.991960140	0.9122938955	0.222371509	0.999007775	0.9948712152	-0.878189359	0.963975216
0.189853765	0.1596190004	0.606445993	0.632845883	0.5928157147	0.3360604264	-0.397676284
0.783129024	0.6294935129	0.893761146	0.941377455	0.8978474649	0.09923559748	-0.031835993
0.981045510	0.936837061	0.886760580	0.999007775	0.9948712152	-0.7711297804	0.233658212
0.833457038	0.551907457	0.906588153	0.941377455	0.8949850654	-0.3838494491	0.213119124
0.483694757	0.2347017435	0.749943740	0.817383170	0.6066894195	0.1064993137	-0.559220344
0.057850794	0.04089105155	0.316391746	0.433880958	0.4877585812	0.2870265978	-1.057635722
0.437364142	0.2092923929	0.708622489	0.816836587	0.5979782654	0.3176836653	-0.595407027
0.537240753	0.3729672715	0.859329822	0.826524236	0.7585775013	0.07883904608	-0.239844412
0.997715556	0.9575600484	0.886760580	0.999007775	0.9953877755	-0.7226635987	0.270246008
0.443951934	0.2305656554	0.660529249	0.816836587	0.6066894195	0.2821383355	-0.211538409
0.400159574	0.2815841683	0.528039393	0.774909963	0.6625509843	-0.2436937208	-0.079653989
0.847239709	0.6540742283	0.931572095	0.941377455	0.8978474649	-0.3069621728	-0.108240899
0.627566595	0.9679130587	0.682640193	0.874580333	0.9953877755	-0.2784167192	0.543202690
0.621527882	0.7713808689	0.971557388	0.874580333	0.9440157185	0.1626706686	0.137427549
0.380878696	0.2376200226	0.682640193	0.772603929	0.6066894195	0.09467760706	-0.258134449
0.385198794	0.4023048237	0.855709341	0.772603929	0.7721894584	-0.4678310475	0.551041127
0.163643553	0.1835292044	0.682640193	0.632845883	0.5928157147	-0.4271534926	0.541175663
0.82583129	0.6008799311	0.720779494	0.941377455	0.8978474649	-0.6989569267	0.664499564

0.835585633	0.6519367304	0.893761146	0.941377455	0.8978474649	-0.6674070068	0.362369771
0.050410443	0.08055040697	0.481170621	0.409751358	0.5515987647	0.312060403	0.457440808
0.510864481	0.4503904215	0.855709341	0.817383170	0.7882231248	0.4154257204	0.046340019
0.917492265	0.8547066334	0.179444278	0.983027427	0.9675924152	0.3233001739	-1.047357365
0.083860739	0.7359225566	0.886760580	0.513950974	0.9289265075	-0.7607993547	0.139218840
0.532128198	0.4228865589	0.886760580	0.826524236	0.7755275948	0.05162971018	-0.322773627
0.022018829	0.9776552592	0.499346548	0.330282438	0.9953877755	0.02875792837	0.588860383
0.754562933	0.8717968425	0.499346548	0.941377455	0.977716085	0.8537068209	-0.597053845
0.832216242	0.8263878934	0.082450979	0.941377455	0.9535244924	0.2366433481	-1.137777585
0.014475087	0.9235073977	0.931572095	0.288021378	0.9948712152	-0.8363794419	-0.19605995
0.883532506	0.6236430334	0.078179359	0.971734949	0.8978474649	0.8753226863	-1.455186082
0.463430059	0.4265401771	0.682640193	0.817383170	0.7755275948	-0.3802660815	0.766483276
0.34795292	0.743141206	0.499346548	0.772603929	0.9289265075	-0.4903039331	0.532443974
0.251973413	0.3501948236	0.758128709	0.726638993	0.757210127	-0.091148803	0.675844532
0.112345497	0.04471120328	0.148038868	0.586150421	0.4877585812	0.6864415035	-1.520440848
0.618204990	0.332885926	0.660529249	0.874580333	0.757210127	-0.3258768721	-0.005746393
0.623615200	0.9948307884	0.971557388	0.874580333	0.9953877755	-0.4107824406	0.123638685
0.504157156	0.8139353891	0.179444278	0.817383170	0.9535244924	0.17216523	-0.920410932
0.386301964	0.1923456044	0.416948741	0.772603929	0.5928157147	-0.2400933694	1.13422369
0.183926772	0.1176630853	0.143912064	0.632845883	0.5928157147	0.3678915024	-1.510200805
0.795139093	0.6600647536	0.893761146	0.941377455	0.8978474649	0.03040147836	0.429864399
0.097091925	0.1389903269	0.573725047	0.554811003	0.5928157147	0.3702698568	0.351211976
0.125406933	0.1043992389	0.217744694	0.601953280	0.5928157147	0.3581191356	-1.178413791
0.141032802	0.06689168433	0.481170621	0.625297136	0.5437080554	0.7698649676	-0.659807983
0.370020407	0.1633234022	0.477763240	0.772603929	0.5928157147	-0.7842887733	1.128531753
0.499505955	0.2767059398	0.287376417	0.817383170	0.6625509843	0.9988602512	-1.094800423
0.958247838	0.7866797654	0.148038868	0.999007775	0.9440157185	-0.2325446079	1.115083509
0.835992445	0.6038872084	0.110966921	0.941377455	0.8978474649	-0.7067915721	1.366784361

est_vns	est_interact	est_batch	se_intercept	se_blast	se_vns	se_interact
-0.477538093	1.280794726	-0.183860328	0.451238152	0.5556931963	0.5759501449	0.7772421515
-0.6327195777	0.3447212754	0.4454459240	0.4582101943	0.5642791645	0.5848491015	0.7892512538
0.4876552937	-0.5505057782	0.5035648044	0.4346305347	0.5352411581	0.5547525588	0.7486361037
-2.085629383	2.418378155	0.0461630185	0.3312658988	0.4079491181	0.4228202814	0.5705940839
-0.3954764188	0.1783085747	-1.3546977755	0.3471903767	0.4275598801	0.4431459239	0.5980234471
0.3886057607	-0.9591343373	0.0194258033	0.4499182315	0.5540677339	0.5742654283	0.774968634
0.8357847083	-0.9998644076	0.4852483670	0.4261161402	0.5247558059	0.5438849780	0.7339703527
-0.4038943833	0.9748033435	-0.992472816	0.3899049288	0.4801622274	0.4976658090	0.6715977903
-0.855562372	1.035114271	0.173489657	0.4159469857	0.5122326402	0.5309053005	0.716454335
-0.8140938177	0.7502300771	-0.1257102422	0.4504847253	0.5547653623	0.5749884881	0.7759444
-0.9064807327	0.9390242135	0.4333505757	0.4307435383	0.5304543787	0.5497912847	0.7419408864
-1.0475750433	0.3770942957	0.3388462683	0.4107859109	0.505876852	0.5243178210	0.7075645610
-0.574352789	1.492988812	-0.2718961927	0.4265133047	0.5252449078	0.5443919105	0.734654455
-0.743010430	1.244403001	-1.0457134895	0.3763471556	0.4634660270	0.4803609753	0.6482449935
0.695926386	-0.7010268573	0.636190548	0.4443265419	0.5471816493	0.5671283224	0.7653371415
-0.0435599960	0.1406778281	0.8872047311	0.3618427048	0.4456040082	0.4618478232	0.6232615774
-0.75037325	1.121160907	-0.2423926090	0.4486867519	0.5525511843	0.5726935957	0.7728474529
-0.3200366155	1.513455455	-0.4285364788	0.4064991019	0.5005977092	0.5188462358	0.7001806809
-0.0089317533	0.9732393759	-0.4871854111	0.4331186472	0.5333792900	0.5528228198	0.7460319295
0.6384829879	-0.6595059353	-0.1011893933	0.4416273629	0.5438576499	0.5636831517	0.7606878989
-0.8225215688	0.7119680032	1.11850697	0.3535654634	0.4354107062	0.4512829399	0.6090043145
0.684074431	-1.383766207	1.364188217	0.2854647312	0.3515456488	0.3643607097	0.4917031525
0.7363430474	-0.9919061008	-0.3854651444	0.4284897569	0.5276788799	0.5469146093	0.7380588258
1.019405222	-2.131250948	0.3276335783	0.3965340033	0.4883258359	0.50612701	0.6830161425
1.542865582	-1.015407516	0.4095889107	0.3824617966	0.4709961187	0.4881655644	0.6587772518
0.1312663238	-0.0193787030	-0.0474751295	0.4905357764	0.604087647	0.6261087415	0.844930954
0.7577916024	-0.7113650619	-0.4818447739	0.4407915685	0.5428283817	0.5626163625	0.7592482717
1.163550049	-2.135638877	-0.5856320001	0.3941239497	0.48535789	0.5030508727	0.6788649082
0.1045304597	0.4692463621	0.265824353	0.4663645556	0.5743211415	0.5952571434	0.8032968599
-0.749291582	1.068591748	-0.5567728944	0.4428065518	0.5453098037	0.5651882410	0.762719011
0.0591824505	-0.1688151972	-0.4665404560	0.4335810272	0.5339487049	0.5534129917	0.7468283632
-0.474457732	-0.0552386880	0.0434535648	0.4728584154	0.582318235	0.6035457588	0.8144823094
-0.0012550255	-0.1659466327	1.385866392	0.3425094221	0.4217953528	0.4371712590	0.5899606649
0.8044968739	-1.992576813	-0.1708158095	0.4184377147	0.5152999367	0.5340844105	0.7207445298
0.4114824449	0.0746385144	0.2139945821	0.4765466713	0.5868602683	0.608253365	0.820835203
1.255579275	-1.524931637	-0.8694961177	0.3758382472	0.4628393144	0.4797114162	0.6473684165
0.3914202	-1.225436873	-0.2457640760	0.4588738655	0.5650964659	0.5856961964	0.7903944040
0.0288954044	-0.3401750873	0.9801076502	0.4099365716	0.5048309031	0.5232337443	0.7061016041
-0.275452842	-0.0703935398	-1.2159008610	0.3823207677	0.4708224437	0.4879855584	0.6585343344
-1.736304606	1.899684441	0.8588794508	0.3343944607	0.4118018963	0.4268135068	0.5759829237
0.3067098100	-0.0691212419	0.6911674061	0.4514939788	0.5560082433	0.5762766764	0.7776828044
0.2388457802	0.5357006625	-0.7993262495	0.4133649112	0.5090528532	0.5276095992	0.7120068007
-0.5183500577	0.6588271236	-1.0008959840	0.4088520683	0.503495353	0.5218495088	0.7042335845
0.0237844237	0.9494824878	-0.2091021430	0.4157183799	0.5119511155	0.5306135137	0.7160605694
-0.0218439777	0.9837070726	-1.0228824560	0.3544538111	0.4365046935	0.4524168069	0.6105344629
-0.0127367833	-1.020683191	-0.3299987400	0.4356439008	0.5364891030	0.5560459963	0.7503815918

-0.061315539	-0.4799926028	-1.269921952	0.3692585304	0.4547364892	0.4713132148	0.6360350812
-2.078383289	2.294625541	0.4884726113	0.3257389898	0.4011428103	0.4157658601	0.5610741737
-0.756584777	1.413194338	0.1207893671	0.4585183478	0.5646586512	0.5852424218	0.7897820378
-0.442638810	0.4709704817	-0.052037078	0.4498696418	0.5540078959	0.5742034095	0.7748849399
-0.827820717	0.4377531323	-0.308769073	0.4575693912	0.5634900249	0.584031195	0.7881474926
1.071047572	-0.2184022922	0.8930612583	0.3767859089	0.4640063458	0.48092099	0.6490007309
-0.009196860	0.3148848474	-0.709748954	0.4477942842	0.5514521234	0.5715544701	0.771310208
-0.513506791	-0.6732148388	-0.633239675	0.4094111561	0.5041838614	0.5225631157	0.7051965938
-0.686014248	0.3595457026	0.1623643965	0.4393757095	0.5410847715	0.5608091922	0.7568095035
-0.378915836	0.4594502972	-0.251075639	0.4828163328	0.5945812649	0.6162558188	0.8316344787
0.0796234122	0.6407826213	-0.501018751	0.4385348553	0.5400492718	0.559735945	0.7553611612
-0.020130404	0.0046721813	-0.025311421	0.4639409724	0.5713365341	0.5921637368	0.7991223215
-0.281404134	0.4784568044	0.6281359025	0.3635780781	0.4477410951	0.4640628143	0.626250698
0.4507043487	-0.5788318566	-1.056675964	0.4051690683	0.4989597923	0.5171486111	0.697889744
-0.819535532	0.8743129343	-0.451631994	0.4449913119	0.5480003039	0.5679768198	0.7664821849
-0.128185317	0.5259073118	-0.42724778	0.4696604446	0.5783799806	0.5994639413	0.808973915
-0.720262571	1.175409056	-1.058067822	0.3778318586	0.4652944177	0.4822560160	0.6508023433
-0.481558074	0.3214029854	-0.824588173	0.4367852861	0.537894703	0.5575028340	0.7523475880
0.8679464434	-1.305770315	0.3389561551	0.4086168409	0.503205674	0.5215492699	0.7038284134
0.7979018539	-1.232065727	-0.199704663	0.4642410622	0.5717060904	0.5925467640	0.7996392159
0.1570071241	0.3160462423	0.5093883945	0.4614608492	0.5682822980	0.5889981637	0.7948503948
1.140181026	-0.6534852193	0.2286278659	0.4409628844	0.5430393542	0.5628350263	0.7595433575
0.7333488732	-1.518538331	-0.031190633	0.4444554241	0.5473403659	0.5672928248	0.7655591367
0.0488522168	-0.3133450066	-0.368081567	0.4807643017	0.5920542185	0.6136366528	0.8280999258
-0.694325589	-0.1558277878	-0.274309183	0.4384195515	0.5399072769	0.5595887738	0.7551625543
-0.411643042	0.3056894229	-1.052300091	0.4093923537	0.5041607067	0.5225391169	0.7051642075
-1.093479964	0.9424867003	-0.522123662	0.3587439246	0.4417879050	0.4578926104	0.6179240353
0.0210016920	-0.0760569331	0.7358397093	0.3963023004	0.488040497	0.5058312690	0.6826170421
-1.089762104	1.131265606	0.2383121055	0.4511953165	0.555640445	0.5758954700	0.7771683688
-0.014126521	0.3973950008	-0.398708456	0.4715455858	0.5807015045	0.6018700929	0.8122210057
0.0287960240	0.0514881081	1.269275514	0.3729191763	0.4592445211	0.4759855802	0.6423404174
0.2862649352	-0.4859752803	0.542095493	0.4669827743	0.5750824688	0.5960462238	0.8043617204
-0.575485486	0.9800289261	0.440722239	0.4656680955	0.5734634611	0.5943681970	0.8020972315
-1.148375788	1.342542885	1.034748896	0.3588154255	0.441875958	0.4579838720	0.6180471933
-0.664826126	1.054261009	0.094394165	0.4732252742	0.5827700163	0.6040140091	0.8151142111
-0.672342267	0.7334790526	0.3856900975	0.4682273439	0.5766151381	0.5976347642	0.8065054480
-0.033023803	0.0355966515	1.202752852	0.38394688	0.4728249772	0.4900610911	0.6613352560
-0.704846597	0.9884771162	-0.191124882	0.4654044337	0.5731387654	0.5940316650	0.8016430831
0.79080263	-0.8558404608	0.2730790128	0.4501221725	0.5543188838	0.5745257339	0.7753199153
-0.057648055	0.3654898957	0.5995958275	0.4670858241	0.5752093732	0.5961777542	0.8045392201
0.3900383147	-0.0312774259	-0.449315840	0.4463067912	0.5496202974	0.5696558678	0.7687480520
0.4649965372	-0.2172124206	-0.863521034	0.4286732991	0.5279049094	0.5471488784	0.7383749708
-0.817468782	0.9601341471	0.3907085488	0.4591589844	0.5654475857	0.5860601157	0.7908855121
0.8266360607	-0.6865055736	-0.131926068	0.4666938962	0.5747267190	0.5956775063	0.8038641378
1.129443386	-1.057687212	-0.302707719	0.4471747384	0.5506891618	0.570763696	0.7702430619
0.3537822271	-0.4152818801	0.55951134	0.454220802	0.5593662862	0.5797571317	0.7823796633

0.339162225	-0.347928075	0.816892004	0.4417070502	0.543955783	0.563784862	0.760825157
0.053564592	-1.331317655	-0.414354312	0.421934063	0.519605638	0.538547070	0.726766869
-0.017099811	-0.6084233491	-0.52344696	0.4596628855	0.566068132	0.586703283	0.791753464
-0.190738421	0.1231519142	0.666644489	0.3858878496	0.475215252	0.492538500	0.664678509
0.714274822	0.2506436155	0.496023349	0.4260282098	0.524647520	0.543772746	0.733818895
-0.04340900	0.669566452	-0.10009255	0.4760030392	0.586190793	0.607559485	0.819898815
-1.00876324	0.0188207891	0.335596102	0.3857072856	0.474992891	0.492308033	0.664367494
-0.20011656	-0.1178272161	-0.79615591	0.4189770478	0.515964117	0.534772803	0.721673512
-0.221298022	0.1216280004	0.984803207	0.3181241034	0.391765188	0.406046391	0.547957794
0.997666039	0.0627013767	0.850323739	0.3748007382	0.461561637	0.478387163	0.645581342
-0.185247652	0.3161233509	-0.13395699	0.3687652638	0.454129038	0.470683620	0.635185446
-0.028770781	-0.633606570	0.326275273	0.4540592877	0.559167383	0.579550978	0.782101460
0.404817887	0.2488454261	-0.18512825	0.4353451324	0.536121174	0.555664655	0.749866973
-0.14479770	-0.750709782	0.027399837	0.4566034982	0.562300541	0.582798351	0.786483775
-0.98143281	1.446968568	0.495154078	0.3946163005	0.485964212	0.503679297	0.679712965
0.384799651	-0.737035260	0.722612185	0.4321697448	0.532210731	0.551611662	0.744397477
0.398482318	0.0053816768	0.278056903	0.4768108363	0.587185583	0.608590539	0.821290218
-0.40708274	0.1365062001	1.058831656	0.3327143305	0.409732840	0.424669026	0.573088957
0.386825390	-0.9843121211	-0.620914837	0.4248917803	0.523248024	0.542322233	0.731861435
-0.25654875	1.059362895	0.602824689	0.3776721384	0.465097724	0.482052153	0.650527230
-0.00912743	-0.366927329	-0.323507932	0.477797431	0.588400560	0.609849806	0.822989593
0.003461437	-1.141747701	-0.47538033	0.4317700681	0.531718535	0.551101524	0.743709048
-0.952823127	1.022112562	1.013047192	0.3503189013	0.431412612	0.447139101	0.603412223
-0.419040597	1.18390168	-1.114913804	0.356541285	0.439075387	0.455081211	0.614130064
0.613911626	-1.096328262	0.340283378	0.4413217698	0.543481316	0.563293099	0.760161525
-0.252825152	0.7116779244	-0.977007052	0.3704193976	0.456166080	0.472794919	0.638034634
0.063656498	-0.175005608	-0.76451494	0.3708790138	0.456732090	0.473381563	0.638826308
0.097434956	-0.354387910	0.004047762	0.3908627202	0.481341733	0.498888312	0.673247553

se_batch	t_intercept	t_blast	t_vns	t_interact	t_batch	t_p_intercept
0.388621075	1.092681504	-1.73755938	-0.829130954	1.647870903	-0.47310951	0.2863550335
0.394625626	0.497585505	-0.690728962	-1.08185098	0.43677006	1.128781038	0.6237132229
0.374318051	-0.25382805	-0.74617977	0.87905010	-0.73534494	1.345285919	0.801985587
0.285297041	2.517292831	-2.167500944	-4.93266163	4.238351262	0.16180686	0.01961137463
0.299011723	2.09643243	0.32260913	-0.89242932	0.29816318	-4.53058414	0.04776309527
0.387484317	-1.216740987	2.016693907	0.67670060	-1.23764278	0.05013313	0.236595477
0.366985176	-2.36085373	2.178999982	1.536693862	-1.36226811	1.322256044	0.02751013538
0.335798895	1.406696745	-0.562402231	-0.81157751	1.451468956	-2.955557122	0.1734871571
0.358227167	1.967767327	-2.688563861	-1.61151597	1.444773548	0.48430066	0.06182717177
0.3879722	1.732204615	-1.687109712	-1.415843682	0.96686061	-0.32401868	0.09723628567
0.370970443	1.312879862	-1.97615648	-1.64877246	1.265632115	1.168153914	0.2027543022
0.353782280	1.561305201	-1.327396058	-1.997977181	0.53294683	0.957781909	0.1327227759
0.367327227	1.596539026	-2.38032954	-1.055035497	2.032232708	-0.74020157	0.1246340893
0.324122496	2.256527086	-1.27851391	-1.54677517	1.919649228	-3.226290984	0.03431061361
0.382668570	-1.12735713	0.13639165	1.22710568	-0.915971301	1.662510581	0.2717375623
0.311630788	0.37427486	-2.34974205	-0.09431677	0.22571233	2.84697393	0.7117830213
0.386423726	1.717336386	-1.944640614	-1.31025256	1.450688493	-0.62727155	0.09996434665
0.350090340	0.50342928	-1.00966405	-0.616823623	2.1615213	-1.22407398	0.6196660522
0.373015964	0.70465260	-1.19587332	-0.01615662	1.304554587	-1.30607120	0.4884197074
0.380343949	0.33375790	-0.775565824	1.132698371	-0.866986232	-0.266047071	0.7417226266
0.304502157	0.415461228	-1.9425547	-1.822629432	1.169068899	3.673231679	0.6818285358
0.245851576	-3.79481057	2.46669236	1.877464866	-2.81423090	5.548828435	0.00099351908
0.369029412	0.53427818	-0.44770797	1.346358344	-1.343939082	-1.04453772	0.5985073233
0.341508071	-1.52405610	2.025461785	2.014129263	-3.120352237	0.95937286	0.1417417613
0.329388625	-3.188752892	1.99959786	3.16053752	-1.54135182	1.243482252	0.00424298328
0.422465477	-0.05199896	-0.041585992	0.20965419	-0.02293525	-0.112376352	0.9589987883
0.379624135	-0.964565651	1.575141745	1.346906441	-0.93693339	-1.26926792	0.3452462483
0.339432454	-0.99742504	2.612858458	2.312986843	-3.145896704	-1.725327071	0.3294031656
0.40164843	0.08686281	-1.13373002	0.17560555	0.58415062	0.66183341	0.9315663221
0.381359505	1.129750304	-0.52907500	-1.325738094	1.401029387	-1.45996857	0.2707492321
0.373414181	-0.56412719	1.646981447	0.10694084	-0.22604283	-1.249391371	0.5783739353
0.407241154	0.65359121	-0.20894571	-0.78611724	-0.067820611	0.10670229	0.5201486819
0.294980332	-2.08321699	0.40998704	-0.00287078	-0.281284232	4.6981654	0.04906233638
0.360372264	-0.88684271	2.18871256	1.506310347	-2.76460899	-0.473998212	0.3847559956
0.410417601	-0.74178048	0.04595297	0.67649842	0.09092996	0.52140693	0.4660661916
0.323684208	-1.10430551	2.467505665	2.617363758	-2.35558547	-2.68624818	0.2813928519
0.395197202	-0.36673149	1.423025439	0.66829903	-1.55041187	-0.62187706	0.7173229524
0.353050802	-0.80714808	-0.19686953	0.05522465	-0.48176506	2.7761094	0.4282217778
0.329267167	2.243562444	-0.415349022	-0.56446925	-0.10689425	-3.69274857	0.03525547755
0.287991461	1.484237394	-2.502061412	-4.06806386	3.298161044	2.98230873	0.1519333233
0.388841402	-1.47821804	0.63474276	0.53222665	-0.088881021	1.777504664	0.1535246957
0.356003400	0.71988316	-0.69052618	0.45269415	0.75238138	-2.24527700	0.4791757771
0.352116792	1.62550659	-0.39007908	-0.993294137	0.93552357	-2.842511367	0.1182956253
0.358030284	1.161210855	-2.37580037	0.04482438	1.32598069	-0.584034792	0.2580010177
0.305267231	1.024509849	-0.621785954	-0.048282861	1.611222842	-3.35077712	0.3167284708
0.375190795	0.97210636	0.14905236	-0.02290598	-1.360218857	-0.87954913	0.3415652828

0.3180175400	1.441887989	1.001568899	-0.130095099	-0.754663723	-3.993244994	0.163419077
0.2805370869	2.389883791	-2.763553985	-4.998927254	4.089700878	1.741205118	0.02585188369
0.3948910189	0.8306686489	-1.483764113	-1.292771592	1.789347276	0.3058802600	0.4150851059
0.3874424699	1.460801468	-1.761459223	-0.770874578	0.6077940833	-0.134309176	0.1582056451
0.3940737463	1.538907114	-0.817615234	-1.417425515	0.5554203197	-0.783531194	0.1380872704
0.3245003655	-2.93255187	0.7149686649	2.227075952	-0.336520872	2.752111718	0.00770654939
0.385655104	0.506452476	0.0862181249	-0.016096960	0.4082467004	-1.840372256	0.617577124
0.3525982969	1.702678487	0.2625165665	-0.982669415	-0.954648455	-1.795923807	0.1027174144
0.3784047517	1.468838621	-1.57805804	-1.223257853	0.4750808505	0.429075998	0.1560313734
0.4158172394	0.724319236	-0.510056726	-0.614867762	0.5524666292	-0.603812481	0.476502694
0.3776805800	-0.044734983	0.0989955308	0.1422517402	0.8483129055	-1.326567415	0.9647221212
0.3995611607	-0.734074577	1.139878452	-0.033994658	0.0058466410	-0.063348053	0.470655165
0.313125349	1.291548204	-3.086960459	-0.606392337	0.7640020298	2.006020606	0.2099187985
0.348944872	0.4298506619	0.9827381462	0.8715180491	-0.829403013	-3.028203161	0.6714852867
0.3832410925	0.8828092417	-0.031208747	-1.442903132	1.140682656	-1.178453989	0.3868843278
0.4044869575	0.405636834	-0.230788931	-0.213833240	0.650091804	-1.056270844	0.6889274643
0.3254011710	2.196064947	-1.141611788	-1.493527393	1.806092231	-3.251579632	0.03892318658
0.3761737943	1.220678208	0.0612920269	-0.863776907	0.427200127	-2.192040451	0.2351302258
0.3519142067	-2.54046248	2.903587772	1.664169607	-1.855239558	0.9631783789	0.01863974064
0.3998196079	-0.768283278	1.272461974	1.346563515	-1.540777019	-0.499486917	0.4504877075
0.3974251974	-0.304942751	-0.885604686	0.2665664068	0.3976172678	1.281721436	0.7632759849
0.3797716788	-1.720521751	0.8320714881	2.025781929	-0.860365919	0.602013996	0.09937445609
0.3827795683	-1.242516637	2.094670851	1.292716638	-1.983567641	-0.081484584	0.2271275071
0.4140499629	0.4803708121	0.0419297937	0.0796109826	-0.378390332	-0.888978627	0.6357058646
0.3775812771	1.601221677	-0.545894485	-1.240778268	-0.206349993	-0.726490428	0.1235906624
0.3525821037	1.981836362	-0.649134976	-0.787774598	0.433501048	-2.984553327	0.06012805345
0.3089620177	0.6911945581	1.186310369	-2.388070782	1.525246869	-1.689928315	0.4966727431
0.341308521	-2.215958268	1.975195137	0.0415191651	-0.111419622	2.155937118	0.03734676942
0.3885841844	0.7448225063	-0.715707951	-1.892291501	1.45562487	0.61328308	0.4642619635
0.4061105029	0.2104475160	-0.054823336	-0.023471047	0.4892695430	-0.981773319	0.8352553444
0.3211702087	-2.067820132	0.5087882423	0.0604976815	0.0801570424	3.95203378	0.05061595134
0.4021808602	-0.821977747	0.3705888042	0.4802730455	-0.604175047	1.347889834	0.4199092252
0.4010486157	0.2287021909	-0.975162992	-0.968230617	1.221833074	1.098924723	0.8212129397
0.3090235960	0.7999282565	-2.393512711	-2.507459011	2.172233609	3.348446226	0.4323055733
0.4075571055	0.6713159300	-1.021684388	-1.100679979	1.293390539	0.2316096658	0.5090073288
0.4032527243	0.1683777063	-0.415952333	-1.125005285	0.9094533130	0.956447593	0.8678245357
0.3306676283	-1.882196826	0.5715561169	-0.067387115	0.0538254255	3.637346838	0.07310406651
0.4008215415	0.6062218473	-0.369087597	-1.186547181	1.233063862	-0.476832860	0.5505720368
0.3876599570	-0.541394616	-0.143697051	1.376444228	-1.103854608	0.704429249	0.5936764321
0.4022696101	-0.657185803	-0.188176521	-0.096696085	0.4542847460	1.490532251	0.5178783828
0.3843740263	-0.623823622	0.9883235619	0.684691121	-0.040686185	-1.168954742	0.5391585193
0.3691874854	0.3794746931	0.260326333	0.8498537703	-0.294176304	-2.338976993	0.7079736401
0.3954427561	0.2061978754	-0.456513487	-1.39485483	1.213998907	0.9880280840	0.8385326433
0.4019320689	-1.00243661	0.9587880791	1.387724149	-0.854006966	-0.328229765	0.3270317882
0.3851215309	-0.955227243	0.9827243772	1.978828355	-1.37318629	-0.786005703	0.3498421095
0.3911898310	-1.538804307	1.187950687	0.6102248817	-0.530793295	1.430280888	0.1381122995

0.380412578	-1.51097204	0.666175050	0.601580936	-0.45730358	2.147384315	0.1450269179
0.363383434	0.739595188	0.880361518	0.099461301	-1.83183591	-1.14026747	0.4673648597
0.395876732	0.903761720	0.081862972	-0.029145587	-0.768450504	-1.322247371	0.3759120142
0.332339254	0.837808638	-2.20396411	-0.387255862	0.185280421	2.00591558	0.411148176
0.366909447	-1.785795722	0.265356901	1.313553919	0.341560590	1.351895821	0.08792543598
0.409949407	0.108465085	-0.550628961	-0.071448164	0.816645224	-0.24415831	0.9146099706
0.332183747	0.074558945	1.239724625	-2.04904892	0.028328883	1.010272493	0.9412394468
0.360836756	2.037598063	-1.15716156	-0.37420856	-0.16326942	-2.20641577	0.05379411254
0.273978897	0.743871167	-2.90423349	-0.54500674	0.221966001	3.594449123	0.464825757
0.322790671	-2.23153093	-0.42477522	2.085478283	0.097123898	2.634288456	0.03615350332
0.317592723	2.373658184	-3.20434493	-0.39357148	0.497686703	-0.42178862	0.02676698259
0.391050730	-0.837481121	1.3707582	-0.04964322	-0.81013347	0.834355361	0.411328246
0.374933486	-1.12624190	0.993141102	0.728529128	0.331852761	-0.493762912	0.272199031
0.393241887	-0.19962370	1.201927585	-0.248452492	-0.95451401	0.069676802	0.8436084935
0.339856482	1.739516342	-3.128709497	-1.94852721	2.128793537	1.456950516	0.09591820973
0.372198738	-0.754048324	-0.01079721	0.697591581	-0.99010983	1.941468655	0.4588156064
0.410645109	-0.861520773	0.210561514	0.654762590	0.006552710	0.677122160	0.3982444261
0.286544478	0.517456611	-2.24636846	-0.958588251	0.238193736	3.69517382	0.6100016148
0.365930717	-0.565069461	2.167659768	0.713275921	-1.344943283	-1.69680982	0.577743949
0.325263615	0.974102839	-3.247061264	-0.53220124	1.62846818	1.853341908	0.340595223
0.411494796	0.063628383	0.730564224	-0.01496669	-0.445846864	-0.78617745	0.9498407282
0.371854524	0.857562587	0.660522350	0.006280943	-1.53520748	-1.278404053	0.4003800966
0.301706111	1.022266096	-2.73152373	-2.13093224	1.693887731	3.35772844	0.3177652991
0.307065032	2.15925897	-1.50272140	-0.92080399	1.92777027	-3.63087192	0.04199991356
0.380080762	-1.77713592	2.076486751	1.089861791	-1.44223066	0.895292295	0.08937588441
0.319017317	2.696565724	-2.40000401	-0.53474591	1.115422088	-3.06255177	0.01317976274
0.319413154	-0.62700934	2.441438933	0.134471858	-0.27394865	-2.39349863	0.5371064229
0.336623776	-1.808285967	2.839530142	0.195304147	-0.52638573	0.012024590	0.08425295881

t_p_blast	t_p_vns	t_p_interact	t_p_batch	t_q_blast	t_q_vns	t_q_interact
0.096269480	0.415936086	0.1135888756	0.640795515	0.312225342	0.805037586	0.5928157147
0.496959682	0.291035077	0.6665350338	0.271149202	0.774677428	0.698484187	0.8978474649
0.463458308	0.388874804	0.4698968088	0.192236684	0.771435749	0.794060801	0.7941917894
0.041292415	0.000062022	0.0003370890	0.872934801	0.190580377	0.003721366	0.0290773457
0.750037207	0.381820795	0.768376414	0.000165072	0.909136008	0.794060801	0.9440157185
0.056095402	0.505649298	0.2288953073	0.960468681	0.221545061	0.839826083	0.6066894195
0.040323350	0.138626929	0.1868987777	0.199666061	0.190580377	0.613233235	0.5928157147
0.579528123	0.425728254	0.1607610545	0.007308493	0.824137168	0.810910961	0.5928157147
0.013418341	0.121322417	0.1626148411	0.632958948	0.161020099	0.613233235	0.5928157147
0.105711902	0.170823696	0.3441231202	0.748984247	0.333827061	0.613233235	0.757210127
0.060808970	0.113402486	0.2188848206	0.255248484	0.221545061	0.613233235	0.6066894195
0.197988745	0.058229805	0.5994132043	0.348580733	0.516492380	0.453314142	0.8978474649
0.026387167	0.302858373	0.0543765976	0.467004283	0.168280905	0.712607937	0.5437080554
0.214392049	0.136182794	0.0679635069	0.003883678	0.541234625	0.613233235	0.5437080554
0.892752081	0.232752993	0.369613781	0.110594004	0.973911362	0.638656179	0.7585775013
0.028170406	0.925711280	0.823508827	0.009377156	0.169022440	0.997735319	0.9535244924
0.064712568	0.203626299	0.1609762619	0.536937710	0.222783661	0.613233235	0.5928157147
0.323632817	0.543682310	0.0418046292	0.233872000	0.627841620	0.840915049	0.4877585812
0.244476819	0.987255065	0.2055274741	0.205020099	0.561857608	0.997735319	0.5979782654
0.446262805	0.269535363	0.3953075818	0.792680408	0.754246995	0.696284431	0.7721894584
0.064978568	0.081980713	0.2548873782	0.001333661	0.222783661	0.491884281	0.6372184455
0.021899879	0.073777180	0.0101034750	0.000014104	0.168280905	0.465961140	0.1938341562
0.658741420	0.191896086	0.1926651073	0.307578822	0.866248951	0.613233235	0.5928157147
0.055119699	0.056383691	0.0049821431	0.347796753	0.221545061	0.453314142	0.1195714349
0.058042175	0.004534043	0.1374931891	0.226778506	0.221545061	0.136021294	0.5928157147
0.967203853	0.835866919	0.9819086792	0.911544080	0.983580975	0.997735319	0.9953877755
0.129495852	0.191722196	0.3589651594	0.217609497	0.379012252	0.613233235	0.757210127
0.015886142	0.030459760	0.0046925929	0.098490188	0.168280905	0.453314142	0.1195714349
0.269111523	0.862210131	0.5650613534	0.514951146	0.566550576	0.997735319	0.8978474649
0.602051461	0.198528572	0.1751539938	0.158432361	0.830415809	0.613233235	0.5928157147
0.113773013	0.915805137	0.8232549644	0.224651686	0.350070812	0.997735319	0.9535244924
0.836413177	0.440184469	0.946541058	0.915992202	0.955900774	0.812648251	0.9953877755
0.68578042	0.997735319	0.7811210556	0.000109686	0.866248951	0.997735319	0.9440157185
0.039520943	0.146212347	0.0113069924	0.640171623	0.190580377	0.613233235	0.1938341562
0.963762313	0.505775153	0.9283710877	0.607292922	0.983580975	0.839826083	0.9948712152
0.021861208	0.015728071	0.0278213845	0.013488141	0.168280905	0.377473711	0.417320768
0.168755498	0.510894201	0.1353098773	0.540414466	0.460242268	0.839826083	0.5928157147
0.845737037	0.956457947	0.6347306827	0.011016512	0.956033509	0.997735319	0.8978474649
0.681909445	0.578145203	0.9158416652	0.001272195	0.866248951	0.857029730	0.9948712152
0.020275649	0.000510897	0.0032763340	0.006870384	0.168280905	0.020435886	0.1195714349
0.532142433	0.599903507	0.9299806264	0.089313706	0.788359160	0.857029730	0.9948712152
0.497084683	0.655201675	0.4597968228	0.035129172	0.774677428	0.914234895	0.7882231248
0.700229152	0.33136677	0.3596748103	0.009473134	0.875286441	0.759672525	0.757210127
0.026644476	0.964651670	0.1984495128	0.565137909	0.168280905	0.997735319	0.5953485384
0.540473291	0.961926464	0.1213865217	0.002891340	0.790936523	0.997735319	0.5928157147
0.882870207	0.981931756	0.1875366336	0.388610181	0.973911362	0.997735319	0.5928157147

0.327441522	0.897673387	0.4584536795	0.000613156	0.627841620	0.997735319	0.7882231248
0.011334001	0.000052821	0.0004846224	0.095615958	0.161020099	0.003721366	0.0290773457
0.152057959	0.209502677	0.0873364710	0.762571532	0.424347793	0.613233235	0.5515987647
0.092054034	0.448981618	0.5495473988	0.894379243	0.306846782	0.816330215	0.8949850654
0.422343986	0.170366426	0.5842115419	0.441669498	0.724018263	0.613233235	0.8978474649
0.482147343	0.036491274	0.7396669805	0.011630882	0.771435749	0.453314142	0.9289265075
0.932072912	0.987306863	0.6870387291	0.079243620	0.983580975	0.997735319	0.9059851372
0.795365828	0.336454286	0.3501283184	0.086254846	0.937686000	0.759672525	0.757210127
0.128824096	0.234173932	0.6394119482	0.672040453	0.379012252	0.638656179	0.8978474649
0.615091020	0.544949898	0.5861985137	0.552144187	0.830515207	0.840915049	0.8978474649
0.922038320	0.888175881	0.4053994657	0.198258405	0.983580975	0.997735319	0.7721894584
0.266595676	0.973187961	0.9953877755	0.950061408	0.566550576	0.997735319	0.9953877755
0.005386755	0.550460878	0.4529827042	0.057303868	0.161020099	0.840915049	0.7882231248
0.336421203	0.392883068	0.415785445	0.006176858	0.627841620	0.794060801	0.7755275948
0.975384467	0.163135781	0.2662678912	0.251205387	0.983580975	0.613233235	0.6520846316
0.819611514	0.832646478	0.5223641345	0.302306288	0.954887201	0.997735319	0.8706068908
0.265889556	0.149503647	0.0846052451	0.003658451	0.566550576	0.613233235	0.5515987647
0.951680060	0.397030400	0.6733855986	0.039249358	0.983580975	0.794060801	0.8978474649
0.008237275	0.110258855	0.0770116296	0.345926374	0.161020099	0.613233235	0.5515987647
0.21649385	0.191830979	0.1376326802	0.622395037	0.541234625	0.613233235	0.5928157147
0.385408447	0.792285605	0.6947440907	0.213284494	0.685022910	0.997735319	0.9061879443
0.414309716	0.055084361	0.3988667773	0.553319263	0.720538637	0.453314142	0.7721894584
0.047934476	0.209521355	0.0599218516	0.935793369	0.213042118	0.613233235	0.5437080554
0.966932885	0.937266398	0.7087673991	0.383632051	0.983580975	0.997735319	0.9145385795
0.590631637	0.227756834	0.838415279	0.475197570	0.824137168	0.638656179	0.9581888902
0.522970796	0.439234358	0.6688718019	0.006834792	0.784456194	0.812648251	0.8978474649
0.248153776	0.025952681	0.14144584	0.105164347	0.561857608	0.444903106	0.5928157147
0.060924891	0.967256524	0.9122938955	0.042288150	0.221545061	0.997735319	0.9948712152
0.481699647	0.071686041	0.1596190004	0.545978077	0.771435749	0.465961140	0.5928157147
0.956774038	0.981486122	0.6294935129	0.336885815	0.983580975	0.997735319	0.8978474649
0.615965445	0.952305483	0.936837061	0.000677915	0.830515207	0.997735319	0.9948712152
0.714488095	0.635774270	0.551907457	0.191410514	0.876233733	0.897563676	0.8949850654
0.340080877	0.343453844	0.2347017435	0.283682054	0.627841620	0.759672525	0.6066894195
0.025651213	0.020037884	0.0408910515	0.002907420	0.168280905	0.400757689	0.4877585812
0.318034491	0.282933820	0.2092923929	0.818981879	0.627841620	0.698484187	0.5979782654
0.681474454	0.272711402	0.3729672715	0.349239171	0.866248951	0.696284431	0.7585775013
0.573416440	0.946882214	0.9575600484	0.001454402	0.824137168	0.997735319	0.9953877755
0.715590882	0.248062225	0.2305656554	0.638183437	0.876233733	0.661499268	0.6066894195
0.887047828	0.182533110	0.2815841683	0.488556028	0.973911362	0.613233235	0.6625509843
0.852463212	0.923843234	0.6540742283	0.150283502	0.956033509	0.997735319	0.8978474649
0.333740213	0.500689253	0.9679130587	0.254932410	0.627841620	0.839826083	0.9953877755
0.797033100	0.404560529	0.7713808689	0.028823970	0.937686000	0.795856779	0.9440157185
0.652495869	0.176984519	0.2376200226	0.333881672	0.866248951	0.613233235	0.6066894195
0.348084781	0.179117425	0.4023048237	0.745841474	0.632881421	0.613233235	0.7721894584
0.336427830	0.060487813	0.1835292044	0.440248461	0.627841620	0.453314142	0.5928157147
0.247520150	0.547965210	0.6008799311	0.166686525	0.561857608	0.840915049	0.8978474649

0.5122249513	0.5536024073	0.6519367304	0.0430385255	0.7834571732	0.8409150491	0.8978474649
0.3881796491	0.9216727727	0.08055040697	0.2664370757	0.6850229102	0.9977353197	0.5515987647
0.9354959088	0.977011262	0.4503904215	0.1996689008	0.9835809754	0.9977353197	0.7882231248
0.0382901315	0.7022877704	0.8547066334	0.0573158737	0.1905803779	0.9576651414	0.9675924152
0.7932051692	0.202531053	0.7359225566	0.1901449524	0.9376860002	0.6132332355	0.9289265075
0.5874364173	0.9436866279	0.4228865589	0.8093708331	0.8241371688	0.9977353197	0.7755275948
0.2281389233	0.0525695459	0.9776552592	0.3233478054	0.5587075672	0.4533141423	0.9953877755
0.2596164629	0.7118316402	0.8717968425	0.0380955613	0.5665505761	0.959772998	0.977716085
0.0082250715	0.5912317089	0.8263878934	0.0016129096	0.1610200991	0.8570297303	0.9535244924
0.6751260948	0.0488377990	0.9235073977	0.0151473681	0.8662489516	0.4533141423	0.9948712152
0.0040899908	0.6976858518	0.6236430334	0.6772723168	0.1610200991	0.9576651414	0.8978474649
0.1842743736	0.9608546525	0.4265401771	0.4130493163	0.4913983296	0.9977353197	0.7755275948
0.3314396693	0.4739740001	0.743141206	0.6263671994	0.6278416206	0.8398260838	0.9289265075
0.242170188	0.8060888763	0.3501948236	0.9450803733	0.561857608	0.9977353197	0.757210127
0.0048855628	0.0642195035	0.04471120328	0.1592560929	0.1610200991	0.4533141423	0.4877585812
0.9914825517	0.4927399347	0.332885926	0.0651174436	0.9914825517	0.8398260838	0.757210127
0.835167472	0.5194082548	0.9948307884	0.5053869291	0.9559007746	0.8409150491	0.9953877755
0.0350489842	0.3481832406	0.8139353891	0.0012647547	0.1905803779	0.7596725251	0.9535244924
0.0412788882	0.4831733423	0.1923456044	0.1038376149	0.1905803779	0.8398260838	0.5928157147
0.0036977407	0.5999208112	0.1176630853	0.0772934368	0.1610200991	0.8570297303	0.5928157147
0.4727544424	0.9881936489	0.6600647536	0.4401499332	0.7714357494	0.9977353197	0.8978474649
0.5157759723	0.9950451727	0.1389903269	0.2144300633	0.7834571732	0.9977353197	0.5928157147
0.0121837068	0.0445158363	0.1043992389	0.0028438958	0.1610200991	0.4533141423	0.5928157147
0.1471303743	0.3671403473	0.06689168433	0.0014773058	0.420372498	0.7867293156	0.5437080554
0.0497361119	0.2875682145	0.1633234022	0.3803223008	0.2131547654	0.698484187	0.5928157147
0.0252958465	0.5981892248	0.2767059398	0.0057023803	0.1682809056	0.8570297303	0.6625509843
0.0231327315	0.894252113	0.7866797654	0.0256519886	0.1682809056	0.9977353197	0.9440157185
0.0095377739	0.8469473757	0.6038872084	0.9905143755	0.1610200991	0.9977353197	0.8978474649

t_q_batch	Genus	Family	Order
0.746557882	Acetatifactor	Lachnospiraceae	Eubacteriales
0.478498591	Acutalibacter	Oscillospiraceae	Eubacteriales
0.427861930	Akkermansia	Akkermansiaceae	Verrucomicrobiales
0.935287286	Anaerotruncus	Oscillospiraceae	Eubacteriales
0.006602889	Bacteria_unclassified	Bacteria_unclassified	Bacteria_unclassified
0.968539846	Bacteria_unclassified	Bacteria_unclassified	Bacteria_unclassified
0.427861930	Bacteria_unclassified	Bacteria_unclassified	Bacteria_unclassified
0.041762821	Bacteria_unclassified	Bacteria_unclassified	Bacteria_unclassified
0.746557882	Bacteroides	Bacteroidaceae	Bacteroidales
0.839982334	Clostridia_unclassified	Clostridia_unclassified	Clostridia_unclassified
0.464088153	Clostridiaceae_unclassified	Clostridiaceae	Eubacteriales
0.537291032	Clostridiaceae_unclassified	Clostridiaceae	Eubacteriales
0.651633884	Eubacteriales_unclassified	Eubacteriales_unclassified	Eubacteriales
0.029127591	Clostridium	Clostridiaceae	Eubacteriales
0.288506097	Erysipelatoclostridium	Erysipelotrichaceae	Erysipelotrichales
0.049425050	Eubacteriaceae_unclassified	Eubacteriaceae	Eubacteriales
0.698929596	Eubacteriaceae_unclassified	Eubacteriaceae	Eubacteriales
0.445470477	GGB20146	FGB77306	OFGB77306
0.431621261	GGB20149	Lachnospiraceae	Eubacteriales
0.872675679	GGB22635	Eggerthellaceae	Eggerthellales
0.017595378	GGB25041	Lachnospiraceae	Eubacteriales
0.001692491	GGB28379	FGB9506	OFGB9506
0.519851531	GGB28382	FGB9508	OFGB9508
0.537291032	GGB28392	FGB9512	OFGB9512
0.438926141	GGB28404	FGB2838	OFGB2838
0.955817950	GGB28418	FGB2838	OFGB2838
0.435218994	GGB28422	FGB2838	OFGB2838
0.274856340	GGB28431	Pumilibacteraceae	Eubacteriales
0.686601527	GGB28456	FGB2833	OFGB2833
0.390014921	GGB28782	Eubacteriaceae	Eubacteriales
0.438926141	GGB28792	Lachnospiraceae	Eubacteriales
0.955817950	GGB28798	Lachnospiraceae	Eubacteriales
0.006581191	GGB28810	FGB9622	OFGB9622
0.746557882	GGB28828	FGB77305	OFGB77305
0.746557882	GGB28851	Clostridiaceae	Eubacteriales
0.062252961	GGB28865	Lachnospiraceae	Eubacteriales
0.698929596	GGB28868	Lachnospiraceae	Eubacteriales
0.055082564	GGB28869	Lachnospiraceae	Eubacteriales
0.017595378	GGB28875	Lachnospiraceae	Eubacteriales
0.041222306	GGB28881	FGB9633	OFGB9633
0.261405970	GGB28892	Bacteria_unclassified	Bacteria_unclassified
0.140516689	GGB28893	Bacteria_unclassified	Bacteria_unclassified
0.049425050	GGB28898	Bacteria_unclassified	Bacteria_unclassified
0.706422386	GGB28901	Bacteria_unclassified	Bacteria_unclassified
0.024920750	GGB28904	Bacteria_unclassified	Bacteria_unclassified
0.575718787	GGB28909	Bacteria_unclassified	Bacteria_unclassified

0.016269970	GGB28924	Lachnospiraceae	Eubacteriales
0.273188452	GGB28927	FGB77359	OFGB77359
0.847301702	GGB28934	FGB9639	OFGB9639
0.949783267	GGB28949	Lachnospiraceae	Eubacteriales
0.623533408	GGB28951	Clostridiaceae	Eubacteriales
0.055828235	GGB28951	Clostridiaceae	Eubacteriales
0.243826523	GGB28954	Clostridiaceae	Eubacteriales
0.258764540	GGB28960	Clostridiaceae	Eubacteriales
0.774025504	GGB28964	Clostridiaceae	Eubacteriales
0.698929596	GGB28967	Clostridiaceae	Eubacteriales
0.427861930	GGB28996	FGB9656	OFGB9656
0.966164143	GGB29531	FGB9827	OFGB9827
0.191052912	GGB29685	Eubacteriaceae	Eubacteriales
0.041179056	GGB30141	FGB77303	OFGB77303
0.464088153	GGB30145	Clostridiaceae	Eubacteriales
0.518239352	GGB30286	Eubacteriales_unclassified	Eubacteriales
0.029127591	GGB30300	FGB72709	OFGB72709
0.147185092	GGB30303	Oscillospiraceae	Eubacteriales
0.537291032	GGB30450	Oscillospiraceae	Eubacteriales
0.746557882	GGB30453	Oscillospiraceae	Eubacteriales
0.435218994	GGB30454	Oscillospiraceae	Eubacteriales
0.698929596	GGB30455	Oscillospiraceae	Eubacteriales
0.966164143	GGB30456	Oscillospiraceae	Eubacteriales
0.575448077	GGB30457	Oscillospiraceae	Eubacteriales
0.655444924	GGB30461	Oscillospiraceae	Eubacteriales
0.041222306	GGB30461	Oscillospiraceae	Eubacteriales
0.280438260	GGB30461	Oscillospiraceae	Eubacteriales
0.151900678	GGB30463	Oscillospiraceae	Eubacteriales
0.698929596	GGB30473	Oscillospiraceae	Eubacteriales
0.537291032	GGB30475	Oscillospiraceae	Eubacteriales
0.016269970	GGB30861	FGB77153	OFGB77153
0.427861930	GGB31312	FGB1791	OFGB1791
0.493360095	GGB31438	FGB10290	OFGB10290
0.024920750	GGB3171	Oscillospiraceae	Eubacteriales
0.885385815	GGB31762	FGB10289	OFGB10289
0.537291032	GGB31823	FGB1765	OFGB1765
0.017595378	GGB31838	FGB1765	OFGB1765
0.746557882	GGB31841	FGB1765	OFGB1765
0.666212766	GGB32371	FGB10667	OFGB10667
0.383702558	GGB3793	Lachnospiraceae	Eubacteriales
0.464088153	GGB42601	Clostridiaceae	Eubacteriales
0.119271600	GGB45514	Oscillospiraceae	Eubacteriales
0.537291032	GGB45564	FGB75721	OFGB75721
0.839982334	GGB45624	Oscillospiraceae	Eubacteriales
0.623533408	GGB45656	Christensenellaceae	Eubacteriales
0.400047661	GGB47127	FGB10299	OFGB10299

0.151900678	GGB74395	Oscillospiraceae	Eubacteriales
0.477200732	GGB75053	Oscillospiraceae	Eubacteriales
0.427861930	GGB75109	Lachnospiraceae	Eubacteriales
0.191052912	Lachnospiraceae_unclassified	Lachnospiraceae	Eubacteriales
0.427861930	Lachnospiraceae_unclassified	Lachnospiraceae	Eubacteriales
0.882949999	Lachnospiraceae_unclassified	Lachnospiraceae	Eubacteriales
0.537291032	Lachnospiraceae_unclassified	Lachnospiraceae	Eubacteriales
0.147185092	Lachnospiraceae_unclassified	Lachnospiraceae	Eubacteriales
0.017595378	Lactobacillus	Lactobacillaceae	Lactobacillales
0.067321635	Leptogranulimonas	Atopobiaceae	Coriobacteriales
0.774025504	Muribaculaceae_unclassified	Muribaculaceae	Bacteroidales
0.604462414	Neglectibacter	Oscillospiraceae	Eubacteriales
0.746557882	Oscillibacter	Oscillospiraceae	Eubacteriales
0.966164143	Oscillospiraceae_unclassified	Oscillospiraceae	Eubacteriales
0.390014921	Oscillospiraceae_unclassified	Oscillospiraceae	Eubacteriales
0.211191709	Oscillospiraceae_unclassified	Oscillospiraceae	Eubacteriales
0.681420578	Oscillospiraceae_unclassified	Oscillospiraceae	Eubacteriales
0.017595378	Parasutterella	Sutterellaceae	Burkholderiales
0.280438260	Schaedlerella	Lachnospiraceae	Eubacteriales
0.243826523	Turicibacter	Turicibacteraceae	Erysipelotrichales
0.623533408	Bacteria_unclassified	Bacteria_unclassified	Bacteria_unclassified
0.435218994	Bacteria_unclassified	Bacteria_unclassified	Bacteria_unclassified
0.024920750	Bacteria_unclassified	Bacteria_unclassified	Bacteria_unclassified
0.017595378	Bacteria_unclassified	Bacteria_unclassified	Bacteria_unclassified
0.575448077	Bacteria_unclassified	Bacteria_unclassified	Bacteria_unclassified
0.040252096			
0.109937094			
0.990514375			

Class	Phylum	Kingdom
Clostridia	Firmicutes	Bacteria
Clostridia	Firmicutes	Bacteria
Verrucomicrobiae	Verrucomicrobia	Bacteria
Clostridia	Firmicutes	Bacteria
Bacteria_unclassified	Bacteria_unclassified	Bacteria
Bacteria_unclassified	Bacteria_unclassified	Bacteria
Bacteria_unclassified	Bacteria_unclassified	Bacteria
Bacteria_unclassified	Bacteria_unclassified	Bacteria
Bacteroidia	Bacteroidota	Bacteria
Clostridia	Firmicutes	Bacteria
Clostridia	Firmicutes	Bacteria
Clostridia	Firmicutes	Bacteria
Clostridia	Firmicutes	Bacteria
Clostridia	Firmicutes	Bacteria
Erysipelotrichia	Firmicutes	Bacteria
Clostridia	Firmicutes	Bacteria
Clostridia	Firmicutes	Bacteria
CFGB77306	Firmicutes	Bacteria
Clostridia	Firmicutes	Bacteria
Coriobacteriia	Actinobacteria	Bacteria
Clostridia	Firmicutes	Bacteria
CFGB9506	Firmicutes	Bacteria
CFGB9508	Firmicutes	Bacteria
CFGB9512	Firmicutes	Bacteria
CFGB2838	Firmicutes	Bacteria
CFGB2838	Firmicutes	Bacteria
CFGB2838	Firmicutes	Bacteria
Clostridia	Firmicutes	Bacteria
CFGB2833	Firmicutes	Bacteria
Clostridia	Firmicutes	Bacteria
Clostridia	Firmicutes	Bacteria
Clostridia	Firmicutes	Bacteria
CFGB9622	Firmicutes	Bacteria
CFGB77305	Firmicutes	Bacteria
Clostridia	Firmicutes	Bacteria
Clostridia	Firmicutes	Bacteria
Clostridia	Firmicutes	Bacteria
Clostridia	Firmicutes	Bacteria
Clostridia	Firmicutes	Bacteria
CFGB9633	Firmicutes	Bacteria
Bacteria_unclassified	Bacteria_unclassified	Bacteria
Bacteria_unclassified	Bacteria_unclassified	Bacteria
Bacteria_unclassified	Bacteria_unclassified	Bacteria
Bacteria_unclassified	Bacteria_unclassified	Bacteria
Bacteria_unclassified	Bacteria_unclassified	Bacteria
Bacteria_unclassified	Bacteria_unclassified	Bacteria

Clostridia	Firmicutes	Bacteria
CFGB77359	Bacteria_unclassified	Bacteria
CFGB9639	Firmicutes	Bacteria
Clostridia	Firmicutes	Bacteria
Clostridia	Firmicutes	Bacteria
Clostridia	Firmicutes	Bacteria
Clostridia	Firmicutes	Bacteria
Clostridia	Firmicutes	Bacteria
Clostridia	Firmicutes	Bacteria
Clostridia	Firmicutes	Bacteria
CFGB9656	Firmicutes	Bacteria
CFGB9827	Firmicutes	Bacteria
Clostridia	Firmicutes	Bacteria
CFGB77303	Bacteria_unclassified	Bacteria
Clostridia	Firmicutes	Bacteria
Clostridia	Firmicutes	Bacteria
CFGB72709	Firmicutes	Bacteria
Clostridia	Firmicutes	Bacteria
Clostridia	Firmicutes	Bacteria
Clostridia	Firmicutes	Bacteria
Clostridia	Firmicutes	Bacteria
Clostridia	Firmicutes	Bacteria
Clostridia	Firmicutes	Bacteria
Clostridia	Firmicutes	Bacteria
Clostridia	Firmicutes	Bacteria
Clostridia	Firmicutes	Bacteria
Clostridia	Firmicutes	Bacteria
Clostridia	Firmicutes	Bacteria
Clostridia	Firmicutes	Bacteria
Clostridia	Firmicutes	Bacteria
CFGB77153	Actinobacteria	Bacteria
CFGB1791	Tenericutes	Bacteria
CFGB10290	Firmicutes	Bacteria
Clostridia	Firmicutes	Bacteria
CFGB10289	Firmicutes	Bacteria
CFGB1765	Firmicutes	Bacteria
CFGB1765	Firmicutes	Bacteria
CFGB1765	Firmicutes	Bacteria
CFGB10667	Firmicutes	Bacteria
Clostridia	Firmicutes	Bacteria
Clostridia	Firmicutes	Bacteria
Clostridia	Firmicutes	Bacteria
CFGB75721	Firmicutes	Bacteria
Clostridia	Firmicutes	Bacteria
Clostridia	Firmicutes	Bacteria
CFGB10299	Firmicutes	Bacteria

Clostridia	Firmicutes	Bacteria
Clostridia	Firmicutes	Bacteria
Clostridia	Firmicutes	Bacteria
Clostridia	Firmicutes	Bacteria
Clostridia	Firmicutes	Bacteria
Clostridia	Firmicutes	Bacteria
Clostridia	Firmicutes	Bacteria
Clostridia	Firmicutes	Bacteria
Bacilli	Firmicutes	Bacteria
Coriobacteriia	Actinobacteria	Bacteria
Bacteroidia	Bacteroidota	Bacteria
Clostridia	Firmicutes	Bacteria
Clostridia	Firmicutes	Bacteria
Clostridia	Firmicutes	Bacteria
Clostridia	Firmicutes	Bacteria
Clostridia	Firmicutes	Bacteria
Clostridia	Firmicutes	Bacteria
Betaproteobacteria	Proteobacteria	Bacteria
Clostridia	Firmicutes	Bacteria
Erysipelotrichia	Firmicutes	Bacteria
Bacteria_unclassified	Bacteria_unclassified	Bacteria
Bacteria_unclassified	Bacteria_unclassified	Bacteria
Bacteria_unclassified	Bacteria_unclassified	Bacteria
Bacteria_unclassified	Bacteria_unclassified	Bacteria
Bacteria_unclassified	Bacteria_unclassified	Bacteria

MetaPhlan Annotation

k__Bacteria|p__Firmicutes|c__Clostridia|o__Eubacteriales|f__Lachnospiraceae|g__Acetatifactor|s__Acetatifactor_SGB4154
k__Bacteria|p__Firmicutes|c__Clostridia|o__Eubacteriales|f__Oscillospiraceae|g__Acutalibacter|s__Acutalibacter_muris
k__Bacteria|p__Verrucomicrobia|c__Verrucomicrobiae|o__Verrucomicrobiales|f__Akkermansiaceae|g__Akkermansia|s__A
k__Bacteria|p__Firmicutes|c__Clostridia|o__Eubacteriales|f__Oscillospiraceae|g__Anaerotruncus|s__Anaerotruncus_sp_1
k__Bacteria|p__Bacteria_unclassified|c__Bacteria_unclassified|o__Bacteria_unclassified|f__Bacteria_unclassified|g__Bacte
k__Bacteria|p__Bacteria_unclassified|c__Bacteria_unclassified|o__Bacteria_unclassified|f__Bacteria_unclassified|g__Bacte
k__Bacteria|p__Bacteria_unclassified|c__Bacteria_unclassified|o__Bacteria_unclassified|f__Bacteria_unclassified|g__Bacte
k__Bacteria|p__Bacteria_unclassified|c__Bacteria_unclassified|o__Bacteria_unclassified|f__Bacteria_unclassified|g__Bacte
k__Bacteria|p__Bacteroidota|c__Bacteroidia|o__Bacteroidales|f__Bacteroidaceae|g__Bacteroides|s__Bacteroides_thetaio
k__Bacteria|p__Firmicutes|c__Clostridia|o__Clostridia_unclassified|f__Clostridia_unclassified|g__Clostridia_unclassified|s_
k__Bacteria|p__Firmicutes|c__Clostridia|o__Eubacteriales|f__Clostridiaceae|g__Clostridiaceae_unclassified|s__Clostridiac
k__Bacteria|p__Firmicutes|c__Clostridia|o__Eubacteriales|f__Clostridiaceae|g__Clostridiaceae_unclassified|s__Clostridiac
k__Bacteria|p__Firmicutes|c__Clostridia|o__Eubacteriales|f__Eubacteriales_unclassified|g__Eubacteriales_unclassified|s_
k__Bacteria|p__Firmicutes|c__Clostridia|o__Eubacteriales|f__Clostridiaceae|g__Clostridium|s__Clostridium_SGB65123
k__Bacteria|p__Firmicutes|c__Erysipelotrichia|o__Erysipelotrichales|f__Erysipelotrichaceae|g__Erysipelatoclostridium|s__
k__Bacteria|p__Firmicutes|c__Clostridia|o__Eubacteriales|f__Eubacteriaceae|g__Eubacteriaceae_unclassified|s__Eubacte
k__Bacteria|p__Firmicutes|c__Clostridia|o__Eubacteriales|f__Eubacteriaceae|g__Eubacteriaceae_unclassified|s__Eubacte
k__Bacteria|p__Firmicutes|c__CFGB77306|o__OFGB77306|f__FGB77306|g__GGB20146|s__GGB20146_SGB29427
k__Bacteria|p__Firmicutes|c__Clostridia|o__Eubacteriales|f__Lachnospiraceae|g__GGB20149|s__GGB20149_SGB29430
k__Bacteria|p__Actinobacteria|c__Coriobacteriia|o__Eggerthellales|f__Eggerthellaceae|g__GGB22635|s__GGB22635_SGB
k__Bacteria|p__Firmicutes|c__Clostridia|o__Eubacteriales|f__Lachnospiraceae|g__GGB25041|s__GGB25041_SGB36960
k__Bacteria|p__Firmicutes|c__CFGB9506|o__OFGB9506|f__FGB9506|g__GGB28379|s__GGB28379_SGB40959
k__Bacteria|p__Firmicutes|c__CFGB9508|o__OFGB9508|f__FGB9508|g__GGB28382|s__GGB28382_SGB40962
k__Bacteria|p__Firmicutes|c__CFGB9512|o__OFGB9512|f__FGB9512|g__GGB28392|s__GGB28392_SGB40972
k__Bacteria|p__Firmicutes|c__CFGB2838|o__OFGB2838|f__FGB2838|g__GGB28404|s__GGB28404_SGB40986
k__Bacteria|p__Firmicutes|c__CFGB2838|o__OFGB2838|f__FGB2838|g__GGB28418|s__GGB28418_SGB41001
k__Bacteria|p__Firmicutes|c__CFGB2838|o__OFGB2838|f__FGB2838|g__GGB28422|s__GGB28422_SGB41005
k__Bacteria|p__Firmicutes|c__Clostridia|o__Eubacteriales|f__Pumilibacteraceae|g__GGB28431|s__GGB28431_SGB41014
k__Bacteria|p__Firmicutes|c__CFGB2833|o__OFGB2833|f__FGB2833|g__GGB28456|s__GGB28456_SGB41039
k__Bacteria|p__Firmicutes|c__Clostridia|o__Eubacteriales|f__Eubacteriaceae|g__GGB28782|s__GGB28782_SGB41435
k__Bacteria|p__Firmicutes|c__Clostridia|o__Eubacteriales|f__Lachnospiraceae|g__GGB28792|s__GGB28792_SGB41445
k__Bacteria|p__Firmicutes|c__Clostridia|o__Eubacteriales|f__Lachnospiraceae|g__GGB28798|s__GGB28798_SGB41451
k__Bacteria|p__Firmicutes|c__CFGB9622|o__OFGB9622|f__FGB9622|g__GGB28810|s__GGB28810_SGB41465
k__Bacteria|p__Firmicutes|c__CFGB77305|o__OFGB77305|f__FGB77305|g__GGB28828|s__GGB28828_SGB41484
k__Bacteria|p__Firmicutes|c__Clostridia|o__Eubacteriales|f__Clostridiaceae|g__GGB28851|s__GGB28851_SGB41518
k__Bacteria|p__Firmicutes|c__Clostridia|o__Eubacteriales|f__Lachnospiraceae|g__GGB28865|s__GGB28865_SGB41537
k__Bacteria|p__Firmicutes|c__Clostridia|o__Eubacteriales|f__Lachnospiraceae|g__GGB28868|s__GGB28868_SGB41542
k__Bacteria|p__Firmicutes|c__Clostridia|o__Eubacteriales|f__Lachnospiraceae|g__GGB28869|s__GGB28869_SGB41543
k__Bacteria|p__Firmicutes|c__Clostridia|o__Eubacteriales|f__Lachnospiraceae|g__GGB28875|s__GGB28875_SGB41555
k__Bacteria|p__Firmicutes|c__CFGB9633|o__OFGB9633|f__FGB9633|g__GGB28881|s__GGB28881_SGB41561
k__Bacteria|p__Bacteria_unclassified|c__Bacteria_unclassified|o__Bacteria_unclassified|f__Bacteria_unclassified|g__GGB2
k__Bacteria|p__Bacteria_unclassified|c__Bacteria_unclassified|o__Bacteria_unclassified|f__Bacteria_unclassified|g__GGB2
k__Bacteria|p__Bacteria_unclassified|c__Bacteria_unclassified|o__Bacteria_unclassified|f__Bacteria_unclassified|g__GGB2
k__Bacteria|p__Bacteria_unclassified|c__Bacteria_unclassified|o__Bacteria_unclassified|f__Bacteria_unclassified|g__GGB2
k__Bacteria|p__Bacteria_unclassified|c__Bacteria_unclassified|o__Bacteria_unclassified|f__Bacteria_unclassified|g__GGB2
k__Bacteria|p__Bacteria_unclassified|c__Bacteria_unclassified|o__Bacteria_unclassified|f__Bacteria_unclassified|g__GGB2

k__Bacteria|p__Firmicutes|c__Clostridia|o__Eubacteriales|f__Lachnospiraceae|g__GGB28924|s__GGB28924_SGB41621
k__Bacteria|p__Bacteria_unclassified|c__CFGB77359|o__OFGB77359|f__FGB77359|g__GGB28927|s__GGB28927_SGB416
k__Bacteria|p__Firmicutes|c__CFGB9639|o__OFGB9639|f__FGB9639|g__GGB28934|s__GGB28934_SGB41635
k__Bacteria|p__Firmicutes|c__Clostridia|o__Eubacteriales|f__Lachnospiraceae|g__GGB28949|s__GGB28949_SGB41655
k__Bacteria|p__Firmicutes|c__Clostridia|o__Eubacteriales|f__Clostridiaceae|g__GGB28951|s__GGB28951_SGB102295
k__Bacteria|p__Firmicutes|c__Clostridia|o__Eubacteriales|f__Clostridiaceae|g__GGB28951|s__GGB28951_SGB41658
k__Bacteria|p__Firmicutes|c__Clostridia|o__Eubacteriales|f__Clostridiaceae|g__GGB28954|s__GGB28954_SGB41662
k__Bacteria|p__Firmicutes|c__Clostridia|o__Eubacteriales|f__Clostridiaceae|g__GGB28960|s__GGB28960_SGB41669
k__Bacteria|p__Firmicutes|c__Clostridia|o__Eubacteriales|f__Clostridiaceae|g__GGB28964|s__GGB28964_SGB94886
k__Bacteria|p__Firmicutes|c__Clostridia|o__Eubacteriales|f__Clostridiaceae|g__GGB28967|s__GGB28967_SGB41678
k__Bacteria|p__Firmicutes|c__CFGB9656|o__OFGB9656|f__FGB9656|g__GGB28996|s__GGB28996_SGB41712
k__Bacteria|p__Firmicutes|c__CFGB9827|o__OFGB9827|f__FGB9827|g__GGB29531|s__GGB29531_SGB42317
k__Bacteria|p__Firmicutes|c__Clostridia|o__Eubacteriales|f__Eubacteriaceae|g__GGB29685|s__GGB29685_SGB42494
k__Bacteria|p__Bacteria_unclassified|c__CFGB77303|o__OFGB77303|f__FGB77303|g__GGB30141|s__GGB30141_SGB430
k__Bacteria|p__Firmicutes|c__Clostridia|o__Eubacteriales|f__Clostridiaceae|g__GGB30145|s__GGB30145_SGB43072
k__Bacteria|p__Firmicutes|c__Clostridia|o__Eubacteriales|f__Eubacteriales_unclassified|g__GGB30286|s__GGB30286_SG
k__Bacteria|p__Firmicutes|c__CFGB72709|o__OFGB72709|f__FGB72709|g__GGB30300|s__GGB30300_SGB43264
k__Bacteria|p__Firmicutes|c__Clostridia|o__Eubacteriales|f__Oscillospiraceae|g__GGB30303|s__GGB30303_SGB43268
k__Bacteria|p__Firmicutes|c__Clostridia|o__Eubacteriales|f__Oscillospiraceae|g__GGB30450|s__GGB30450_SGB43507
k__Bacteria|p__Firmicutes|c__Clostridia|o__Eubacteriales|f__Oscillospiraceae|g__GGB30453|s__GGB30453_SGB43513
k__Bacteria|p__Firmicutes|c__Clostridia|o__Eubacteriales|f__Oscillospiraceae|g__GGB30454|s__GGB30454_SGB43514
k__Bacteria|p__Firmicutes|c__Clostridia|o__Eubacteriales|f__Oscillospiraceae|g__GGB30455|s__GGB30455_SGB43519
k__Bacteria|p__Firmicutes|c__Clostridia|o__Eubacteriales|f__Oscillospiraceae|g__GGB30456|s__GGB30456_SGB43520
k__Bacteria|p__Firmicutes|c__Clostridia|o__Eubacteriales|f__Oscillospiraceae|g__GGB30457|s__GGB30457_SGB63218
k__Bacteria|p__Firmicutes|c__Clostridia|o__Eubacteriales|f__Oscillospiraceae|g__GGB30461|s__GGB30461_SGB43530
k__Bacteria|p__Firmicutes|c__Clostridia|o__Eubacteriales|f__Oscillospiraceae|g__GGB30461|s__GGB30461_SGB43533
k__Bacteria|p__Firmicutes|c__Clostridia|o__Eubacteriales|f__Oscillospiraceae|g__GGB30461|s__GGB30461_SGB63209
k__Bacteria|p__Firmicutes|c__Clostridia|o__Eubacteriales|f__Oscillospiraceae|g__GGB30463|s__GGB30463_SGB43537
k__Bacteria|p__Firmicutes|c__Clostridia|o__Eubacteriales|f__Oscillospiraceae|g__GGB30473|s__GGB30473_SGB43557
k__Bacteria|p__Firmicutes|c__Clostridia|o__Eubacteriales|f__Oscillospiraceae|g__GGB30475|s__GGB30475_SGB63182
k__Bacteria|p__Actinobacteria|c__CFGB77153|o__OFGB77153|f__FGB77153|g__GGB30861|s__GGB30861_SGB44083
k__Bacteria|p__Tenericutes|c__CFGB1791|o__OFGB1791|f__FGB1791|g__GGB31312|s__GGB31312_SGB44628
k__Bacteria|p__Firmicutes|c__CFGB10290|o__OFGB10290|f__FGB10290|g__GGB31438|s__GGB31438_SGB44768
k__Bacteria|p__Firmicutes|c__Clostridia|o__Eubacteriales|f__Oscillospiraceae|g__GGB3171|s__GGB3171_SGB4185
k__Bacteria|p__Firmicutes|c__CFGB10289|o__OFGB10289|f__FGB10289|g__GGB31762|s__GGB31762_SGB45125
k__Bacteria|p__Firmicutes|c__CFGB1765|o__OFGB1765|f__FGB1765|g__GGB31823|s__GGB31823_SGB45199
k__Bacteria|p__Firmicutes|c__CFGB1765|o__OFGB1765|f__FGB1765|g__GGB31838|s__GGB31838_SGB45216
k__Bacteria|p__Firmicutes|c__CFGB1765|o__OFGB1765|f__FGB1765|g__GGB31841|s__GGB31841_SGB65084
k__Bacteria|p__Firmicutes|c__CFGB10667|o__OFGB10667|f__FGB10667|g__GGB32371|s__GGB32371_SGB41694
k__Bacteria|p__Firmicutes|c__Clostridia|o__Eubacteriales|f__Lachnospiraceae|g__GGB3793|s__GGB3793_SGB5158
k__Bacteria|p__Firmicutes|c__Clostridia|o__Eubacteriales|f__Clostridiaceae|g__GGB42601|s__GGB42601_SGB59797
k__Bacteria|p__Firmicutes|c__Clostridia|o__Eubacteriales|f__Oscillospiraceae|g__GGB45514|s__GGB45514_SGB63186
k__Bacteria|p__Firmicutes|c__CFGB75721|o__OFGB75721|f__FGB75721|g__GGB45564|s__GGB45564_SGB63259
k__Bacteria|p__Firmicutes|c__Clostridia|o__Eubacteriales|f__Oscillospiraceae|g__GGB45624|s__GGB45624_SGB63337
k__Bacteria|p__Firmicutes|c__Clostridia|o__Eubacteriales|f__Christensenellaceae|g__GGB45656|s__GGB45656_SGB6337
k__Bacteria|p__Firmicutes|c__CFGB10299|o__OFGB10299|f__FGB10299|g__GGB47127|s__GGB47127_SGB65054

k__Bacteria|p__Firmicutes|c__Clostridia|o__Eubacteriales|f__Oscillospiraceae|g__GGB74395|s__GGB74395_SGB43523
k__Bacteria|p__Firmicutes|c__Clostridia|o__Eubacteriales|f__Oscillospiraceae|g__GGB75053|s__GGB75053_SGB43494
k__Bacteria|p__Firmicutes|c__Clostridia|o__Eubacteriales|f__Lachnospiraceae|g__GGB75109|s__GGB75109_SGB102238
k__Bacteria|p__Firmicutes|c__Clostridia|o__Eubacteriales|f__Lachnospiraceae|g__Lachnospiraceae_unclassified|s__Lachn
k__Bacteria|p__Firmicutes|c__Clostridia|o__Eubacteriales|f__Lachnospiraceae|g__Lachnospiraceae_unclassified|s__Lachn
k__Bacteria|p__Firmicutes|c__Clostridia|o__Eubacteriales|f__Lachnospiraceae|g__Lachnospiraceae_unclassified|s__Lachn
k__Bacteria|p__Firmicutes|c__Clostridia|o__Eubacteriales|f__Lachnospiraceae|g__Lachnospiraceae_unclassified|s__Lachn
k__Bacteria|p__Firmicutes|c__Clostridia|o__Eubacteriales|f__Lachnospiraceae|g__Lachnospiraceae_unclassified|s__Lachn
k__Bacteria|p__Firmicutes|c__Bacilli|o__Lactobacillales|f__Lactobacillaceae|g__Lactobacillus|s__Lactobacillus_johnsonii
k__Bacteria|p__Actinobacteria|c__Coriobacteriia|o__Coriobacteriales|f__Atopobiaceae|g__Leptogranulimonas|s__Leptog
k__Bacteria|p__Bacteroidota|c__Bacteroidia|o__Bacteroidales|f__Muribaculaceae|g__Muribaculaceae_unclassified|s__M
k__Bacteria|p__Firmicutes|c__Clostridia|o__Eubacteriales|f__Oscillospiraceae|g__Neglectibacter|s__Neglectibacter_sp_X4
k__Bacteria|p__Firmicutes|c__Clostridia|o__Eubacteriales|f__Oscillospiraceae|g__Oscillibacter|s__Oscillibacter_SGB43496
k__Bacteria|p__Firmicutes|c__Clostridia|o__Eubacteriales|f__Oscillospiraceae|g__Oscillospiraceae_unclassified|s__Oscillo
k__Bacteria|p__Firmicutes|c__Clostridia|o__Eubacteriales|f__Oscillospiraceae|g__Oscillospiraceae_unclassified|s__Oscillo
k__Bacteria|p__Firmicutes|c__Clostridia|o__Eubacteriales|f__Oscillospiraceae|g__Oscillospiraceae_unclassified|s__Oscillo
k__Bacteria|p__Firmicutes|c__Clostridia|o__Eubacteriales|f__Oscillospiraceae|g__Oscillospiraceae_unclassified|s__Oscillo
k__Bacteria|p__Proteobacteria|c__Betaproteobacteria|o__Burkholderiales|f__Sutterellaceae|g__Parasutterella|s__Parasu
k__Bacteria|p__Firmicutes|c__Clostridia|o__Eubacteriales|f__Lachnospiraceae|g__Schaedlerella|s__Schaedlerella_arabino
k__Bacteria|p__Firmicutes|c__Erysipelotrichia|o__Erysipelotrichales|f__Turicibacteraceae|g__Turicibacter|s__Turicibacter_
k__Bacteria|p__Bacteria_unclassified|c__Bacteria_unclassified|o__Bacteria_unclassified|f__Bacteria_unclassified|g__Bacte
k__Bacteria|p__Bacteria_unclassified|c__Bacteria_unclassified|o__Bacteria_unclassified|f__Bacteria_unclassified|g__Bacte
k__Bacteria|p__Bacteria_unclassified|c__Bacteria_unclassified|o__Bacteria_unclassified|f__Bacteria_unclassified|g__Bacte
k__Bacteria|p__Bacteria_unclassified|c__Bacteria_unclassified|o__Bacteria_unclassified|f__Bacteria_unclassified|g__Bacte
k__Bacteria|p__Bacteria_unclassified|c__Bacteria_unclassified|o__Bacteria_unclassified|f__Bacteria_unclassified|g__Bacte
