# Supplementary material for: Non-invasive Vagal Nerve Stimulation as a Potential Treatment for Repetitive Blast Trauma
Source: bioRxiv. 2026 Jul 19:2026.07.13.737563. Preprint. [Version 1] doi: 10.64898/2026.07.13.737563 (PMC13405043; doi:10.64898/2026.07.13.737563)
Supplement: Supplement 11 [file media-11.pdf]

Cytokine	PC1	PC2	PC3	PC4	PC5
serum_IL-1a	-0.13971	0.036106	-0.087596	-0.357139	0.371858
serum_IL-5	0.240246	0.033876	0.024549	-0.074063	-0.017311
serum_IL-6	0.245772	-0.242198	0.012981	0.057687	0.011426
serum_IL-9	0.174559	-0.121984	0.042901	-0.061347	-0.365543
serum_IL-12(p40)	-0.031778	-0.058041	-0.163105	-0.425547	-0.015543
serum_G-CSF	0.250196	-0.166522	-0.059469	-0.105981	-0.129922
serum_IP-10	0.22272	-0.195961	-0.023552	0.027461	-0.295476
serum_MKC	0.240829	-0.196427	-0.149233	0.006243	-0.205592
serum_MCP-1	0.248431	-0.201177	-0.121352	0.077855	0.051629
serum_MIP-2	-0.094252	-0.036926	-0.047439	-0.490785	-0.180603
serum_RANTES	0.080822	0.113766	0.003994	-0.329662	0.084753
brain_IL-1a	-0.183209	-0.210215	-0.2115	0.204748	0.035073
brain_IL-2	-0.114026	-0.176093	-0.357926	0.056552	-0.112375
brain_IL-5	0.039054	-0.28159	0.273279	-0.044639	0.071742
brain_IL-6	0.2405	-0.036503	-0.019805	0.081688	0.390307
brain_IL-9	-0.123845	-0.268348	0.071079	0.130707	0.069637
brain_IL-10	-0.054829	-0.12279	0.450084	0.002576	0.09619
brain_IL-12(p40)	0.083786	-0.221444	0.293526	0.012208	0.044444
brain_IL-12(p70)	-0.13614	-0.217599	-0.027898	-0.296259	0.128426
brain_IL-13	-0.162908	-0.253525	-0.168449	-0.004867	-0.055385
brain_IL-15	-0.160795	-0.248007	-0.17559	-0.043006	0.087001
brain_IL-17	-0.177583	-0.264139	-0.22875	0.109316	-0.006545
brain_G-CSF	0.306053	-0.093439	-0.050826	-0.010712	0.121084
brain_GM-CSF	-0.191808	-0.194343	0.134021	-0.097393	-0.074245
brain_INF_gamma	-0.20092	-0.245934	0.047837	-0.022614	0.0235
brain_IP-10	0.198248	-0.106163	-0.240737	-0.155292	0.324683
brain_MKC	0.250728	-0.119331	-0.001576	-0.145678	-0.083827
brain_MCP-1	0.241745	-0.065458	-0.002953	0.034903	0.376643
brain_MIP-2	-0.010219	-0.149169	0.386006	-0.244862	-0.10944
brain_RANTES	-0.135069	-0.259359	0.189592	0.148015	0.192105
