# Supplementary material for: Non-invasive Vagal Nerve Stimulation as a Potential Treatment for Repetitive Blast Trauma
Source: bioRxiv. 2026 Jul 19:2026.07.13.737563. Preprint. [Version 1] doi: 10.64898/2026.07.13.737563 (PMC13405043; doi:10.64898/2026.07.13.737563)
Supplement: Supplement 12 [file media-12.pdf]

outcome	Species-level feature	n	LRT_F_microbe	LRT_p_microbe
acute_cyto_PC1	Acetatifactor_SGB41545	27	0.811221818	0.4263375897
acute_cyto_PC1	Acutalibacter_muris	27	-0.5653962247	0.5777957076
acute_cyto_PC1	Akkermansia_muciniphila	27	-0.5296359608	0.6019194146
acute_cyto_PC1	Anaerotruncus_sp_1XD42_93	27	-0.1053420732	0.9171041076
acute_cyto_PC1	Bacteria_unclassified_SGB41677	27	-0.0299959166[illegible]	0.9763535607
acute_cyto_PC1	Bacteria_unclassified_SGB43539	27	1.267139755	0.2189780512
acute_cyto_PC1	Bacteria_unclassified_SGB43546	27	0.5238735197	0.6058519277
acute_cyto_PC1	Bacteria_unclassified_SGB63211	27	2.048051107	0.05326401688
acute_cyto_PC1	Bacteroides_thetaiotaomicron	27	-0.509179467	0.6159351495
acute_cyto_PC1	Clostridia_bacterium	27	-0.5587690197	0.5822293971
acute_cyto_PC1	Clostridiaceae_bacterium	27	-0.4431309337	0.6622024077
acute_cyto_PC1	Clostridiaceae_unclassified_SGB41663	27	-1.444867654	0.1632521935
acute_cyto_PC1	Clostridiales_bacterium	27	0.5961201509	0.5574665105
acute_cyto_PC1	Clostridium_SGB65123	27	-3.01130997	0.0066476783[illegible]
acute_cyto_PC1	Clostridium_cocleatum	27	-0.3568938693	0.7247324894
acute_cyto_PC1	Eubacteriaceae_bacterium	27	-0.1835035817	0.8561631507
acute_cyto_PC1	Eubacteriaceae_unclassified_SGB94922	27	-0.109767717	0.9136358044
acute_cyto_PC1	GGB20146_SGB29427	27	0.3045693121	0.7636919925
acute_cyto_PC1	GGB20149_SGB29430	27	0.4461351552	0.6600657249
acute_cyto_PC1	GGB22635_SGB63107	27	0.3677705653	0.7167250388
acute_cyto_PC1	GGB25041_SGB36960	27	0.2196630048	0.8282547772
acute_cyto_PC1	GGB28379_SGB40959	27	2.176355529	0.04108690661
acute_cyto_PC1	GGB28382_SGB40962	27	-1.573002392	0.1306643628
acute_cyto_PC1	GGB28392_SGB40972	27	-1.591362358	0.1264701801
acute_cyto_PC1	GGB28404_SGB40986	27	0.3398309152	0.7373593019
acute_cyto_PC1	GGB28418_SGB41001	27	0.0349175958	0.9724752282
acute_cyto_PC1	GGB28422_SGB41005	27	0.4177402243	0.6803773997
acute_cyto_PC1	GGB28431_SGB41014	27	-0.1621872213	0.872708849
acute_cyto_PC1	GGB28456_SGB41039	27	1.16296545	0.2578889366
acute_cyto_PC1	GGB28782_SGB41435	27	-0.7729789869	0.4481484807
acute_cyto_PC1	GGB28792_SGB41445	27	0.344766815	0.7336986581
acute_cyto_PC1	GGB28798_SGB41451	27	0.4707769995	0.6426532597
acute_cyto_PC1	GGB28810_SGB41465	27	-0.0498773488[illegible]	0.9606914889
acute_cyto_PC1	GGB28828_SGB41484	27	-1.554575845	0.134989453
acute_cyto_PC1	GGB28851_SGB41518	27	0.9308435064	0.3625128158
acute_cyto_PC1	GGB28865_SGB41537	27	-2.075790327	0.0503856047
acute_cyto_PC1	GGB28868_SGB41542	27	0.3424653491	0.7354047075
acute_cyto_PC1	GGB28869_SGB41543	27	1.035154156	0.3123709457
acute_cyto_PC1	GGB28875_SGB41555	27	-0.0519236560[illegible]	0.9590802748
acute_cyto_PC1	GGB28881_SGB41561	27	-0.6436208003	0.5267884535
acute_cyto_PC1	GGB28892_SGB41573	27	-1.053782381	0.3039568745
acute_cyto_PC1	GGB28893_SGB41574	27	2.21260236	0.03813709284
acute_cyto_PC1	GGB28898_SGB41580	27	-0.4325431875	0.6697561454
acute_cyto_PC1	GGB28901_SGB41592	27	-1.484052936	0.1526502905
acute_cyto_PC1	GGB28904_SGB41597	27	1.300027778	0.2076802308
acute_cyto_PC1	GGB28909_SGB41602	27	-2.385918078	0.02653112285

acute_cyto_PC1	GGB28924_SGB41621	27	-2.054891923	0.05254079686
acute_cyto_PC1	GGB28927_SGB41625	27	-0.9235697201	0.3662013664
acute_cyto_PC1	GGB28934_SGB41635	27	0.6009285837	0.5543189854
acute_cyto_PC1	GGB28949_SGB41655	27	-1.026372543	0.3163940609
acute_cyto_PC1	GGB28951_SGB102295	27	0.5911817779	0.5607087809
acute_cyto_PC1	GGB28951_SGB41658	27	-1.177054193	0.2523431626
acute_cyto_PC1	GGB28954_SGB41662	27	0.2678883809	0.7913988996
acute_cyto_PC1	GGB28960_SGB41669	27	-1.191581955	0.2467184541
acute_cyto_PC1	GGB28964_SGB94886	27	-0.2476769268	0.8067899815
acute_cyto_PC1	GGB28967_SGB41678	27	-0.4790088279	0.636882447
acute_cyto_PC1	GGB28996_SGB41712	27	0.3261232271	0.7475587555
acute_cyto_PC1	GGB29531_SGB42317	27	0.4017377774	0.6919362382
acute_cyto_PC1	GGB29685_SGB42494	27	0.2130394145	0.8333507625
acute_cyto_PC1	GGB30141_SGB43066	27	-0.7716703163	0.4489067099
acute_cyto_PC1	GGB30145_SGB43072	27	1.551965795	0.1356115768
acute_cyto_PC1	GGB30286_SGB43248	27	0.7790528204	0.4446395985
acute_cyto_PC1	GGB30300_SGB43264	27	-1.26996853	0.2179880419
acute_cyto_PC1	GGB30303_SGB43268	27	0.8457922497	0.4072031284
acute_cyto_PC1	GGB30450_SGB43507	27	-0.7977695814	0.4339330774
acute_cyto_PC1	GGB30453_SGB43513	27	0.1317853615	0.8964085344
acute_cyto_PC1	GGB30454_SGB43514	27	0.1369197874	0.892398402
acute_cyto_PC1	GGB30455_SGB43519	27	0.4906613313	0.6287539192
acute_cyto_PC1	GGB30456_SGB43520	27	-0.8573757689	0.400916417
acute_cyto_PC1	GGB30457_SGB63218	27	0.4519449013	0.6559421165
acute_cyto_PC1	GGB30461_SGB43530	27	-0.552541678	0.5864110497
acute_cyto_PC1	GGB30461_SGB43533	27	0.1921807148	0.849446904
acute_cyto_PC1	GGB30461_SGB63209	27	1.136870591	0.268399194
acute_cyto_PC1	GGB30463_SGB43537	27	0.02225685992	0.9824531902
acute_cyto_PC1	GGB30473_SGB43557	27	-0.06014332864	0.9526101765
acute_cyto_PC1	GGB30475_SGB63182	27	0.699893571	0.4916750511
acute_cyto_PC1	GGB30861_SGB44083	27	1.825138121	0.08223849766
acute_cyto_PC1	GGB31312_SGB44628	27	0.5293518882	0.6021129872
acute_cyto_PC1	GGB31438_SGB44768	27	-3.464143267	0.00232035856
acute_cyto_PC1	GGB3171_SGB4185	27	-1.5458549	0.1370774432
acute_cyto_PC1	GGB31762_SGB45125	27	-0.3742329648	0.7119831078
acute_cyto_PC1	GGB31823_SGB45199	27	-0.4338015114	0.668856506
acute_cyto_PC1	GGB31838_SGB45216	27	-0.1235804218	0.9028226785
acute_cyto_PC1	GGB31841_SGB65084	27	-1.997655241	0.05887218089
acute_cyto_PC1	GGB32371_SGB41694	27	-0.2317107619	0.8190056127
acute_cyto_PC1	GGB3793_SGB5158	27	-0.4804734028	0.6358581838
acute_cyto_PC1	GGB42601_SGB59797	27	0.8867956016	0.3852324177
acute_cyto_PC1	GGB45514_SGB63186	27	0.4917523126	0.6279953191
acute_cyto_PC1	GGB45564_SGB63259	27	-0.069750953	0.9450518329
acute_cyto_PC1	GGB45624_SGB63337	27	1.66580504	0.1106008108
acute_cyto_PC1	GGB45656_SGB63370	27	0.9603113865	0.3478258885
acute_cyto_PC1	GGB47127_SGB65054	27	-0.5504710205	0.5878047965

acute_cyto_PC1	GGB74395_SGB43523	27	1.885640797	0.07325377808
acute_cyto_PC1	GGB75053_SGB43494	27	-0.9656016453	0.3452327395
acute_cyto_PC1	GGB75109_SGB102238	27	-1.60283389	0.1239070982
acute_cyto_PC1	Lachnospiraceae_bacterium	27	0.2746479015	0.7862705667
acute_cyto_PC1	Lachnospiraceae_bacterium_A2	27	-0.4828169006	0.6342207918
acute_cyto_PC1	Lachnospiraceae_unclassified_SGB41414	27	0.5779585099	0.5694384296
acute_cyto_PC1	Lachnospiraceae_unclassified_SGB41424	27	2.357147748	0.02819891813
acute_cyto_PC1	Lachnospiraceae_unclassified_SGB94868	27	-0.02781152731	0.9780750794
acute_cyto_PC1	Lactobacillus_johnsonii	27	0.5833192659	0.5658910214
acute_cyto_PC1	Leptogranulimonas_caecicola	27	-1.523024296	0.1426703397
acute_cyto_PC1	Muribaculaceae_bacterium	27	-0.00761838152	0.99399338
acute_cyto_PC1	Neglectibacter_sp_X4	27	0.6379522338	0.5304003737
acute_cyto_PC1	Oscillibacter_SGB43496	27	1.74444352	0.09570201712
acute_cyto_PC1	Oscillospiraceae_bacterium	27	0.8998424088	0.3784074486
acute_cyto_PC1	Oscillospiraceae_unclassified_SGB43502	27	0.4149108539	0.6824153502
acute_cyto_PC1	Oscillospiraceae_unclassified_SGB43505	27	2.885277896	0.00885488484
acute_cyto_PC1	Oscillospiraceae_unclassified_SGB94989	27	1.385488261	0.1804436782
acute_cyto_PC1	Parasutterella_excrementihominis	27	1.801264781	0.08603923696
acute_cyto_PC1	Schaedlerella_arabinosiphila	27	-0.334776754	0.741114287
acute_cyto_PC1	Turicibacter_sp_1E2	27	-2.276558793	0.03339918902
acute_cyto_PC1	bacterium_0_1xD8_82	27	-0.4427230772	0.6624927144
acute_cyto_PC1	bacterium_1XD42_1	27	-0.2006733119	0.8428848194
acute_cyto_PC1	bacterium_1XD42_54	27	0.1996301392	0.8436902412
acute_cyto_PC1	bacterium_1XD42_76	27	3.188114672	0.00442379887
acute_cyto_PC1	bacterium_1xD8_48	27	-0.4870063818	0.6312984042
acute_cyto_PC1	Berger Parker Index	27	0.5546160728	0.5850164374
acute_cyto_PC1	Richness (# observed features)	27	0.1699098669	0.8667072268
acute_cyto_PC1	Shannon Index	27	-0.2057340609	0.8389799902
acute_cyto_PC2	Acetatifactor_SGB41545	27	-0.3501689773	0.729699651
acute_cyto_PC2	Acutalibacter_muris	27	0.6042963891	0.5521200392
acute_cyto_PC2	Akkermansia_muciniphila	27	-0.1032435303	0.9187492969
acute_cyto_PC2	Anaerotruncus_sp_1XD42_93	27	-0.5504887108	0.5877928823
acute_cyto_PC2	Bacteria_unclassified_SGB41677	27	-0.6832335355	0.5019284422
acute_cyto_PC2	Bacteria_unclassified_SGB43539	27	1.06990166	0.2968074174
acute_cyto_PC2	Bacteria_unclassified_SGB43546	27	-0.4402517785	0.6642529128
acute_cyto_PC2	Bacteria_unclassified_SGB63211	27	0.9424632476	0.3566723803
acute_cyto_PC2	Bacteroides_thetaiotaomicron	27	-0.03978911657	0.9686371082
acute_cyto_PC2	Clostridia_bacterium	27	-0.5722310411	0.573241047
acute_cyto_PC2	Clostridiaceae_bacterium	27	-0.5236410097	0.6060108602
acute_cyto_PC2	Clostridiaceae_unclassified_SGB41663	27	-1.758758371	0.09318366282
acute_cyto_PC2	Clostridiales_bacterium	27	-0.2950912952	0.7708218478
acute_cyto_PC2	Clostridium_SGB65123	27	-3.25528208	0.00378456795
acute_cyto_PC2	Clostridium_cocleatum	27	0.2372376377	0.8147715187
acute_cyto_PC2	Eubacteriaceae_bacterium	27	-0.08413454683	0.9337463589
acute_cyto_PC2	Eubacteriaceae_unclassified_SGB94922	27	0.9369028527	0.3594592285
acute_cyto_PC2	GGB20146_SGB29427	27	1.266319437	0.2192657912

acute_cyto_PC2	GGB20149_SGB29430	27	1.232347741	0.2314390826
acute_cyto_PC2	GGB22635_SGB63107	27	1.077431154	0.2935094684
acute_cyto_PC2	GGB25041_SGB36960	27	0.7369272722	0.4693203776
acute_cyto_PC2	GGB28379_SGB40959	27	1.432099219	0.1668328279
acute_cyto_PC2	GGB28382_SGB40962	27	-0.338896761	0.7380528278
acute_cyto_PC2	GGB28392_SGB40972	27	-1.067270996	0.2979659032
acute_cyto_PC2	GGB28404_SGB40986	27	-0.3247224273	0.7486037584
acute_cyto_PC2	GGB28418_SGB41001	27	0.6949720229	0.4946914071
acute_cyto_PC2	GGB28422_SGB41005	27	0.4839059165	0.6334605536
acute_cyto_PC2	GGB28431_SGB41014	27	-0.3898383231	0.7005818042
acute_cyto_PC2	GGB28456_SGB41039	27	3.92989586	0.00076812473[illegible]
acute_cyto_PC2	GGB28782_SGB41435	27	-0.5782861153	0.5692213149
acute_cyto_PC2	GGB28792_SGB41445	27	-0.1055764555	0.9169203831
acute_cyto_PC2	GGB28798_SGB41451	27	-0.5477440335	0.5896428186
acute_cyto_PC2	GGB28810_SGB41465	27	0.125401195	0.9013987003
acute_cyto_PC2	GGB28828_SGB41484	27	-1.386346406	0.1801853188
acute_cyto_PC2	GGB28851_SGB41518	27	0.3025941857	0.7651760368
acute_cyto_PC2	GGB28865_SGB41537	27	-2.21165167	0.03821193107
acute_cyto_PC2	GGB28868_SGB41542	27	1.354382344	0.1900092626
acute_cyto_PC2	GGB28869_SGB41543	27	0.7548553026	0.4587184199
acute_cyto_PC2	GGB28875_SGB41555	27	0.5831572802	0.5659980463
acute_cyto_PC2	GGB28881_SGB41561	27	-0.5503074411	0.5879149708
acute_cyto_PC2	GGB28892_SGB41573	27	-1.128786811	0.2717183239
acute_cyto_PC2	GGB28893_SGB41574	27	2.963520432	0.00741395246[illegible]
acute_cyto_PC2	GGB28898_SGB41580	27	-0.5047567358	0.6189854942
acute_cyto_PC2	GGB28901_SGB41592	27	-0.5855905044	0.5643914995
acute_cyto_PC2	GGB28904_SGB41597	27	1.697022351	0.1044657882
acute_cyto_PC2	GGB28909_SGB41602	27	-1.141357508	0.2665698429
acute_cyto_PC2	GGB28924_SGB41621	27	-2.018021169	0.05654533343
acute_cyto_PC2	GGB28927_SGB41625	27	-0.3588821464	0.7232662676
acute_cyto_PC2	GGB28934_SGB41635	27	0.2199049322	0.828068791
acute_cyto_PC2	GGB28949_SGB41655	27	-1.648304377	0.1141717851
acute_cyto_PC2	GGB28951_SGB102295	27	0.3912223347	0.6995740795
acute_cyto_PC2	GGB28951_SGB41658	27	-1.002985021	0.3272859349
acute_cyto_PC2	GGB28954_SGB41662	27	-1.200312306	0.2433839379
acute_cyto_PC2	GGB28960_SGB41669	27	-0.4872633615	0.6311193475
acute_cyto_PC2	GGB28964_SGB94886	27	0.9154188855	0.3703644085
acute_cyto_PC2	GGB28967_SGB41678	27	-0.5102022612	0.6152307406
acute_cyto_PC2	GGB28996_SGB41712	27	-0.1074183943	0.9154767142
acute_cyto_PC2	GGB29531_SGB42317	27	1.232461785	0.2313973712
acute_cyto_PC2	GGB29685_SGB42494	27	0.04658519237	0.9632840138
acute_cyto_PC2	GGB30141_SGB43066	27	-0.2112526391	0.8347267496
acute_cyto_PC2	GGB30145_SGB43072	27	1.267246398	0.2189406657
acute_cyto_PC2	GGB30286_SGB43248	27	0.266523701	0.7924354436
acute_cyto_PC2	GGB30300_SGB43264	27	0.3887679193	0.7013615732
acute_cyto_PC2	GGB30303_SGB43268	27	3.526249434	0.00200427440[illegible]

acute_cyto_PC2	GGB30450_SGB43507	27	-0.8538722419	0.4028112568
acute_cyto_PC2	GGB30453_SGB43513	27	0.6299998499	0.5354901584
acute_cyto_PC2	GGB30454_SGB43514	27	1.699684715	0.1039562309
acute_cyto_PC2	GGB30455_SGB43519	27	0.8573790641	0.4009146375
acute_cyto_PC2	GGB30456_SGB43520	27	0.3321798111	0.7430462664
acute_cyto_PC2	GGB30457_SGB63218	27	-0.9419613996	0.3569233035
acute_cyto_PC2	GGB30461_SGB43530	27	-0.1408613113	0.8893219555
acute_cyto_PC2	GGB30461_SGB43533	27	0.5928693887	0.559599685
acute_cyto_PC2	GGB30461_SGB63209	27	1.150605812	0.2628283018
acute_cyto_PC2	GGB30463_SGB43537	27	0.4916179085	0.6280887525
acute_cyto_PC2	GGB30473_SGB43557	27	1.128753435	0.2717320899
acute_cyto_PC2	GGB30475_SGB63182	27	0.2843757778	0.7789076763
acute_cyto_PC2	GGB30861_SGB44083	27	0.00493497603(	0.9961090555
acute_cyto_PC2	GGB31312_SGB44628	27	1.38142302	0.1816716013
acute_cyto_PC2	GGB31438_SGB44768	27	-1.90692963	0.07030348686
acute_cyto_PC2	GGB3171_SGB4185	27	-0.7636350447	0.4535793479
acute_cyto_PC2	GGB31762_SGB45125	27	-0.08577777005	0.9324556547
acute_cyto_PC2	GGB31823_SGB45199	27	0.6953887028	0.4944356186
acute_cyto_PC2	GGB31838_SGB45216	27	-0.510882786	0.6147622668
acute_cyto_PC2	GGB31841_SGB65084	27	-0.8430903285	0.4086785656
acute_cyto_PC2	GGB32371_SGB41694	27	-0.5098764025	0.6154551216
acute_cyto_PC2	GGB3793_SGB5158	27	-0.125438139	0.9013698108
acute_cyto_PC2	GGB42601_SGB59797	27	-0.9832324678	0.3366862375
acute_cyto_PC2	GGB45514_SGB63186	27	0.4658515222	0.6461173274
acute_cyto_PC2	GGB45564_SGB63259	27	1.057529367	0.3022840998
acute_cyto_PC2	GGB45624_SGB63337	27	0.4250028761	0.6751577094
acute_cyto_PC2	GGB45656_SGB63370	27	-0.6982020031	0.4927105963
acute_cyto_PC2	GGB47127_SGB65054	27	-1.094504391	0.2861292433
acute_cyto_PC2	GGB74395_SGB43523	27	-0.05380593093	0.9575983741
acute_cyto_PC2	GGB75053_SGB43494	27	-1.188495322	0.2479055584
acute_cyto_PC2	GGB75109_SGB102238	27	-1.452133797	0.1612424576
acute_cyto_PC2	Lachnospiraceae_bacterium	27	0.1064494213	0.9162361358
acute_cyto_PC2	Lachnospiraceae_bacterium_A2	27	0.3156783381	0.7553625359
acute_cyto_PC2	Lachnospiraceae_unclassified_SGB41414	27	0.3037941729	0.7642742957
acute_cyto_PC2	Lachnospiraceae_unclassified_SGB41424	27	1.744387803	0.09571193232
acute_cyto_PC2	Lachnospiraceae_unclassified_SGB94868	27	-1.024969862	0.3170400346
acute_cyto_PC2	Lactobacillus_johnsonii	27	0.1715874077	0.8654046089
acute_cyto_PC2	Leptogranulimonas_caecicola	27	-1.04517865	0.3078227825
acute_cyto_PC2	Muribaculaceae_bacterium	27	0.1735920162	0.8638485361
acute_cyto_PC2	Neglectibacter_sp_X4	27	0.3409411768	0.7365353302
acute_cyto_PC2	Oscillibacter_SGB43496	27	1.250988338	0.2246971229
acute_cyto_PC2	Oscillospiraceae_bacterium	27	1.207185535	0.2407827247
acute_cyto_PC2	Oscillospiraceae_unclassified_SGB43502	27	0.3100020812	0.7596148083
acute_cyto_PC2	Oscillospiraceae_unclassified_SGB43505	27	0.8928920425	0.3820332914
acute_cyto_PC2	Oscillospiraceae_unclassified_SGB94989	27	-0.6778944078	0.5052399228
acute_cyto_PC2	Parasutterella_excrementihominis	27	0.5358307928	0.5977056413

acute_cyto_PC2	Schaedlerella_arabinosiphila	27	-0.2442436395	0.8094126256
acute_cyto_PC2	Turicibacter_sp_1E2	27	-0.82848826	0.4167112574
acute_cyto_PC2	bacterium_0_1xD8_82	27	-1.490223907	0.1510330056
acute_cyto_PC2	bacterium_1XD42_1	27	0.2733842685	0.7872285157
acute_cyto_PC2	bacterium_1XD42_54	27	0.4546979732	0.6539919676
acute_cyto_PC2	bacterium_1XD42_76	27	1.992607704	0.05946189678
acute_cyto_PC2	bacterium_1xD8_48	27	0.1514177317	0.8810913739
acute_cyto_PC2	Berger Parker Index	27	0.3774047316	0.7096601025
acute_cyto_PC2	Richness (# observed features)	27	0.00245786860{	0.9980621059
acute_cyto_PC2	Shannon Index	27	-0.1063925193	0.9162807346
acute_cyto_PC3	Acetatifactor_SGB41545	27	0.1903115271	0.8508927151
acute_cyto_PC3	Acutalibacter_muris	27	-0.4067041552	0.6883405268
acute_cyto_PC3	Akkermansia_muciniphila	27	1.216068945	0.2374519338
acute_cyto_PC3	Anaerotruncus_sp_1XD42_93	27	1.265036412	0.2197164171
acute_cyto_PC3	Bacteria_unclassified_SGB41677	27	1.046019543	0.3074434124
acute_cyto_PC3	Bacteria_unclassified_SGB43539	27	-1.476311818	0.1546990323
acute_cyto_PC3	Bacteria_unclassified_SGB43546	27	0.6745329537	0.5073311112
acute_cyto_PC3	Bacteria_unclassified_SGB63211	27	-0.1139195232	0.9103837051
acute_cyto_PC3	Bacteroides_thetaiotaomicron	27	-0.4255439057	0.6747695356
acute_cyto_PC3	Clostridia_bacterium	27	1.613678119	0.1215242849
acute_cyto_PC3	Clostridiaceae_bacterium	27	2.088713081	0.04909298222
acute_cyto_PC3	Clostridiaceae_unclassified_SGB41663	27	-0.7662039157	0.4520823154
acute_cyto_PC3	Clostridiales_bacterium	27	0.2339847229	0.8172628562
acute_cyto_PC3	Clostridium_SGB65123	27	1.111983076	0.2787140336
acute_cyto_PC3	Clostridium_cocleatum	27	-0.2385669173	0.8137540285
acute_cyto_PC3	Eubacteriaceae_bacterium	27	2.448423639	0.02321785465
acute_cyto_PC3	Eubacteriaceae_unclassified_SGB94922	27	-1.520004105	0.1434240909
acute_cyto_PC3	GGB20146_SGB29427	27	-0.1365575881	0.8926811945
acute_cyto_PC3	GGB20149_SGB29430	27	-1.345693219	0.1927517498
acute_cyto_PC3	GGB22635_SGB63107	27	2.118418649	0.04623409341
acute_cyto_PC3	GGB25041_SGB36960	27	-0.5880709274	0.5627562164
acute_cyto_PC3	GGB28379_SGB40959	27	-0.1777867323	0.8605942371
acute_cyto_PC3	GGB28382_SGB40962	27	-1.824176563	0.08238871481
acute_cyto_PC3	GGB28392_SGB40972	27	-0.6570812837	0.5182659046
acute_cyto_PC3	GGB28404_SGB40986	27	0.2529619094	0.8027574125
acute_cyto_PC3	GGB28418_SGB41001	27	-0.4863103291	0.6317835119
acute_cyto_PC3	GGB28422_SGB41005	27	-0.7236159349	0.4772854309
acute_cyto_PC3	GGB28431_SGB41014	27	-2.087543664	0.04920871562
acute_cyto_PC3	GGB28456_SGB41039	27	-2.201917532	0.03898593793
acute_cyto_PC3	GGB28782_SGB41435	27	1.413258151	0.1722317222
acute_cyto_PC3	GGB28792_SGB41445	27	1.219343553	0.2362329796
acute_cyto_PC3	GGB28798_SGB41451	27	-0.2617273938	0.7960815948
acute_cyto_PC3	GGB28810_SGB41465	27	-0.426566864	0.674035845
acute_cyto_PC3	GGB28828_SGB41484	27	0.07477679813	0.9411000538
acute_cyto_PC3	GGB28851_SGB41518	27	1.283692982	0.2132337182
acute_cyto_PC3	GGB28865_SGB41537	27	0.757076298	0.4574150989

acute_cyto_PC3	GGB28868_SGB41542	27	-1.413940495	0.1720337783
acute_cyto_PC3	GGB28869_SGB41543	27	-1.17740508	0.2522061885
acute_cyto_PC3	GGB28875_SGB41555	27	0.0801714076	0.9368600518
acute_cyto_PC3	GGB28881_SGB41561	27	-0.81998505	0.4214346526
acute_cyto_PC3	GGB28892_SGB41573	27	1.331175908	0.197403228
acute_cyto_PC3	GGB28893_SGB41574	27	-3.268564487	0.00366926147[illegible]
acute_cyto_PC3	GGB28898_SGB41580	27	-0.2791222852	0.7828813756
acute_cyto_PC3	GGB28901_SGB41592	27	-0.9018604315	0.3773589643
acute_cyto_PC3	GGB28904_SGB41597	27	-1.972317699	0.06188576569
acute_cyto_PC3	GGB28909_SGB41602	27	0.8372554805	0.4118764282
acute_cyto_PC3	GGB28924_SGB41621	27	-0.6368323151	0.5311155611
acute_cyto_PC3	GGB28927_SGB41625	27	-0.9668539547	0.3446208297
acute_cyto_PC3	GGB28934_SGB41635	27	0.1732788065	0.8640916272
acute_cyto_PC3	GGB28949_SGB41655	27	-0.2557885878	0.8006028914
acute_cyto_PC3	GGB28951_SGB102295	27	0.6055960653	0.55127267
acute_cyto_PC3	GGB28951_SGB41658	27	0.7060390296	0.4879234862
acute_cyto_PC3	GGB28954_SGB41662	27	0.6178239012	0.5433340329
acute_cyto_PC3	GGB28960_SGB41669	27	0.5826590041	0.5663273256
acute_cyto_PC3	GGB28964_SGB94886	27	-2.517303115	0.02001494513
acute_cyto_PC3	GGB28967_SGB41678	27	1.334395624	0.1963640665
acute_cyto_PC3	GGB28996_SGB41712	27	0.6029555892	0.552994939
acute_cyto_PC3	GGB29531_SGB42317	27	-1.358752407	0.1886416677
acute_cyto_PC3	GGB29685_SGB42494	27	2.421264353	0.02460723615
acute_cyto_PC3	GGB30141_SGB43066	27	-2.090830133	0.04888408709
acute_cyto_PC3	GGB30145_SGB43072	27	0.1704252163	0.8663070142
acute_cyto_PC3	GGB30286_SGB43248	27	-0.5906355332	0.5610680167
acute_cyto_PC3	GGB30300_SGB43264	27	0.02274929599	0.982065034
acute_cyto_PC3	GGB30303_SGB43268	27	-1.885266042	0.07330667194
acute_cyto_PC3	GGB30450_SGB43507	27	-0.1415709371	0.8887682654
acute_cyto_PC3	GGB30453_SGB43513	27	-1.434479821	0.1661604772
acute_cyto_PC3	GGB30454_SGB43514	27	-0.7079392795	0.4867668184
acute_cyto_PC3	GGB30455_SGB43519	27	-1.057668749	0.3022220024
acute_cyto_PC3	GGB30456_SGB43520	27	-1.308199556	0.2049445384
acute_cyto_PC3	GGB30457_SGB63218	27	-0.0991173320[illegible]	0.9219851833
acute_cyto_PC3	GGB30461_SGB43530	27	0.6750569582	0.5070048031
acute_cyto_PC3	GGB30461_SGB43533	27	-1.612397941	0.1218035645
acute_cyto_PC3	GGB30461_SGB63209	27	0.4197563772	0.6789267222
acute_cyto_PC3	GGB30463_SGB43537	27	0.9602554506	0.3478533777
acute_cyto_PC3	GGB30473_SGB43557	27	-0.6786715152	0.5047571718
acute_cyto_PC3	GGB30475_SGB63182	27	-0.6436797334	0.5267509732
acute_cyto_PC3	GGB30861_SGB44083	27	1.25507908	0.2232379076
acute_cyto_PC3	GGB31312_SGB44628	27	-0.5463984523	0.5905508027
acute_cyto_PC3	GGB31438_SGB44768	27	0.25223142	0.8033144609
acute_cyto_PC3	GGB3171_SGB4185	27	-0.9176135124	0.3692404109
acute_cyto_PC3	GGB31762_SGB45125	27	1.426160789	0.1685195711
acute_cyto_PC3	GGB31823_SGB45199	27	-0.5591139602	0.5819982072

acute_cyto_PC3	GGB31838_SGB45216	27	1.563635446	0.1328483636
acute_cyto_PC3	GGB31841_SGB65084	27	0.6843620681	0.5012300742
acute_cyto_PC3	GGB32371_SGB41694	27	1.441998506	0.1640513269
acute_cyto_PC3	GGB3793_SGB5158	27	-0.3768358699	0.7100765252
acute_cyto_PC3	GGB42601_SGB59797	27	2.243849167	0.03575052555
acute_cyto_PC3	GGB45514_SGB63186	27	-0.8883468542	0.3844167313
acute_cyto_PC3	GGB45564_SGB63259	27	-0.736966604	0.4692969601
acute_cyto_PC3	GGB45624_SGB63337	27	-0.5096796176	0.6155906432
acute_cyto_PC3	GGB45656_SGB63370	27	-1.180611779	0.2509569817
acute_cyto_PC3	GGB47127_SGB65054	27	-0.2027366013	0.8412922969
acute_cyto_PC3	GGB74395_SGB43523	27	1.107172814	0.2807406373
acute_cyto_PC3	GGB75053_SGB43494	27	-1.746321996	0.09536824745
acute_cyto_PC3	GGB75109_SGB102238	27	-0.4173732739	0.6806415674
acute_cyto_PC3	Lachnospiraceae_bacterium	27	2.553869563	0.01848733547
acute_cyto_PC3	Lachnospiraceae_bacterium_A2	27	-0.882398847	0.3875504988
acute_cyto_PC3	Lachnospiraceae_unclassified_SGB41414	27	0.8951589635	0.3808481874
acute_cyto_PC3	Lachnospiraceae_unclassified_SGB41424	27	1.305695971	0.2057796704
acute_cyto_PC3	Lachnospiraceae_unclassified_SGB94868	27	1.561076241	0.1334503143
acute_cyto_PC3	Lactobacillus_johnsonii	27	1.718428996	0.1004283297
acute_cyto_PC3	Leptogranulimonas_caecicola	27	1.430635627	0.1672472759
acute_cyto_PC3	Muribaculaceae_bacterium	27	1.69537538	0.1047820704
acute_cyto_PC3	Neglectibacter_sp_X4	27	-0.172652877	0.8645774706
acute_cyto_PC3	Oscillibacter_SGB43496	27	0.6495393602	0.5230316625
acute_cyto_PC3	Oscillospiraceae_bacterium	27	-2.209192031	0.03840617522
acute_cyto_PC3	Oscillospiraceae_unclassified_SGB43502	27	1.82747006	0.08187518489
acute_cyto_PC3	Oscillospiraceae_unclassified_SGB43505	27	0.3485482229	0.7308986096
acute_cyto_PC3	Oscillospiraceae_unclassified_SGB94989	27	0.3126788071	0.7576086032
acute_cyto_PC3	Parasutterella_excrementihominis	27	0.478950048	0.6369235708
acute_cyto_PC3	Schaedlerella_arabinosiphila	27	0.6965191979	0.4937420204
acute_cyto_PC3	Turicibacter_sp_1E2	27	1.205639189	0.2413661101
acute_cyto_PC3	bacterium_0_1xD8_82	27	-0.2616725039	0.79612335
acute_cyto_PC3	bacterium_1XD42_1	27	-1.202341667	0.2426137194
acute_cyto_PC3	bacterium_1XD42_54	27	-0.0191220875	0.9849242331
acute_cyto_PC3	bacterium_1XD42_76	27	0.7868270087	0.4401730268
acute_cyto_PC3	bacterium_1xD8_48	27	-0.6354170628	0.5320201004
acute_cyto_PC3	Berger Parker Index	27	1.916876523	0.06896119119
acute_cyto_PC3	Richness (# observed features)	27	-2.701883533	0.01335399273
acute_cyto_PC3	Shannon Index	27	-2.379238941	0.02690996432
acute_cyto_PC4	Acetatifactor_SGB41545	27	0.6022104862	0.5534814492
acute_cyto_PC4	Acutalibacter_muris	27	2.72339589	0.01273111231
acute_cyto_PC4	Akkermansia_muciniphila	27	0.8478703408	0.406070667
acute_cyto_PC4	Anaerotruncus_sp_1XD42_93	27	-1.443746171	0.1635641806
acute_cyto_PC4	Bacteria_unclassified_SGB41677	27	0.06785558977	0.9465425232
acute_cyto_PC4	Bacteria_unclassified_SGB43539	27	0.196756952	0.8459095008
acute_cyto_PC4	Bacteria_unclassified_SGB43546	27	0.8626800771	0.3980585952
acute_cyto_PC4	Bacteria_unclassified_SGB63211	27	-2.004917184	0.05803288344

acute_cyto_PC4	Bacteroides_thetaiotaomicron	27	0.3379066671	0.7387881338
acute_cyto_PC4	Clostridia_bacterium	27	-0.5136877915	0.6128330794
acute_cyto_PC4	Clostridiaceae_bacterium	27	-0.2631255119	0.7950182484
acute_cyto_PC4	Clostridiaceae_unclassified_SGB41663	27	-0.7656000031	0.4524339813
acute_cyto_PC4	Clostridiales_bacterium	27	0.2851798641	0.7783000156
acute_cyto_PC4	Clostridium_SGB65123	27	-0.2433449652	0.8100994922
acute_cyto_PC4	Clostridium_cocleatum	27	-1.240158691	0.2285954642
acute_cyto_PC4	Eubacteriaceae_bacterium	27	0.5045014686	0.6191617671
acute_cyto_PC4	Eubacteriaceae_unclassified_SGB94922	27	0.2438670853	0.809700411
acute_cyto_PC4	GGB20146_SGB29427	27	-0.2597158674	0.7976121847
acute_cyto_PC4	GGB20149_SGB29430	27	2.050527867	0.0530011472
acute_cyto_PC4	GGB22635_SGB63107	27	1.465279291	0.1576575174
acute_cyto_PC4	GGB25041_SGB36960	27	-0.494745697	0.6259160731
acute_cyto_PC4	GGB28379_SGB40959	27	-1.778356265	0.08982850546
acute_cyto_PC4	GGB28382_SGB40962	27	1.010667784	0.3236794987
acute_cyto_PC4	GGB28392_SGB40972	27	0.3129656763	0.7573936985
acute_cyto_PC4	GGB28404_SGB40986	27	-0.8097050351	0.4271898241
acute_cyto_PC4	GGB28418_SGB41001	27	0.03141968838	0.9752315483
acute_cyto_PC4	GGB28422_SGB41005	27	0.1454222959	0.8857642401
acute_cyto_PC4	GGB28431_SGB41014	27	-1.183174	0.2499621723
acute_cyto_PC4	GGB28456_SGB41039	27	0.8729989646	0.3925368809
acute_cyto_PC4	GGB28782_SGB41435	27	2.913798589	0.0083010268[illegible]
acute_cyto_PC4	GGB28792_SGB41445	27	-1.729578537	0.09837877017
acute_cyto_PC4	GGB28798_SGB41451	27	-1.74546042	0.09552120907
acute_cyto_PC4	GGB28810_SGB41465	27	1.2657596	0.2194623305
acute_cyto_PC4	GGB28828_SGB41484	27	0.1309810204	0.8970370078
acute_cyto_PC4	GGB28851_SGB41518	27	0.6315347447	0.534505723
acute_cyto_PC4	GGB28865_SGB41537	27	-1.02579019	0.3166621381
acute_cyto_PC4	GGB28868_SGB41542	27	1.606382661	0.1231230537
acute_cyto_PC4	GGB28869_SGB41543	27	0.2434268992	0.8100368626
acute_cyto_PC4	GGB28875_SGB41555	27	-0.1607110803	0.873856933
acute_cyto_PC4	GGB28881_SGB41561	27	-0.9021031857	0.3772329684
acute_cyto_PC4	GGB28892_SGB41573	27	0.2372756472	0.8147424199
acute_cyto_PC4	GGB28893_SGB41574	27	0.1532510805	0.8796633326
acute_cyto_PC4	GGB28898_SGB41580	27	-0.746622936	0.4635687616
acute_cyto_PC4	GGB28901_SGB41592	27	0.8331677553	0.4141262171
acute_cyto_PC4	GGB28904_SGB41597	27	0.8143131254	0.4246039737
acute_cyto_PC4	GGB28909_SGB41602	27	-0.0108313065	0.9914602769
acute_cyto_PC4	GGB28924_SGB41621	27	0.4710154836	0.6424857454
acute_cyto_PC4	GGB28927_SGB41625	27	-1.005340608	0.3261772135
acute_cyto_PC4	GGB28934_SGB41635	27	0.4681026567	0.6445330861
acute_cyto_PC4	GGB28949_SGB41655	27	0.07515255214	0.9408046628
acute_cyto_PC4	GGB28951_SGB102295	27	-0.3521298613	0.7282500278
acute_cyto_PC4	GGB28951_SGB41658	27	0.7583156603	0.4566887888
acute_cyto_PC4	GGB28954_SGB41662	27	-1.408452604	0.1736309616
acute_cyto_PC4	GGB28960_SGB41669	27	-0.3082272721	0.7609459855

acute_cyto_PC4	GGB28964_SGB94886	27	1.708382911	0.1023061965
acute_cyto_PC4	GGB28967_SGB41678	27	-0.3727434166	0.7130750465
acute_cyto_PC4	GGB28996_SGB41712	27	-1.285459176	0.2126277585
acute_cyto_PC4	GGB29531_SGB42317	27	0.08047967613	0.9366178187
acute_cyto_PC4	GGB29685_SGB42494	27	0.02217024415	0.9825214644
acute_cyto_PC4	GGB30141_SGB43066	27	-0.0738448248	0.9418327421
acute_cyto_PC4	GGB30145_SGB43072	27	0.8437589321	0.4083131439
acute_cyto_PC4	GGB30286_SGB43248	27	0.03838666721	0.9697419788
acute_cyto_PC4	GGB30300_SGB43264	27	-0.2889320822	0.7754663336
acute_cyto_PC4	GGB30303_SGB43268	27	-0.162425492	0.8725235583
acute_cyto_PC4	GGB30450_SGB43507	27	-0.2060988903	0.8386986541
acute_cyto_PC4	GGB30453_SGB43513	27	-0.1083084215	0.9147792384
acute_cyto_PC4	GGB30454_SGB43514	27	1.814278493	0.08394892999
acute_cyto_PC4	GGB30455_SGB43519	27	-0.1024729246	0.9193535207
acute_cyto_PC4	GGB30456_SGB43520	27	1.812951528	0.08416003484
acute_cyto_PC4	GGB30457_SGB63218	27	0.08957372983	0.9294747727
acute_cyto_PC4	GGB30461_SGB43530	27	-0.920083213	0.3679782556
acute_cyto_PC4	GGB30461_SGB43533	27	-0.3316934091	0.7434083157
acute_cyto_PC4	GGB30461_SGB63209	27	-0.9146294293	0.3707692924
acute_cyto_PC4	GGB30463_SGB43537	27	0.731243059	0.472711939
acute_cyto_PC4	GGB30473_SGB43557	27	0.6342815225	0.5327464705
acute_cyto_PC4	GGB30475_SGB63182	27	0.09688143576	0.9237392219
acute_cyto_PC4	GGB30861_SGB44083	27	-1.034082206	0.3128600922
acute_cyto_PC4	GGB31312_SGB44628	27	0.6692155784	0.5106490299
acute_cyto_PC4	GGB31438_SGB44768	27	-0.7502155098	0.46144833
acute_cyto_PC4	GGB3171_SGB4185	27	-0.7921267067	0.4371440277
acute_cyto_PC4	GGB31762_SGB45125	27	-0.0233330884	0.9816048734
acute_cyto_PC4	GGB31823_SGB45199	27	0.09075304814	0.9285488945
acute_cyto_PC4	GGB31838_SGB45216	27	-1.058671834	0.3017753752
acute_cyto_PC4	GGB31841_SGB65084	27	-0.8713747533	0.3934026916
acute_cyto_PC4	GGB32371_SGB41694	27	-0.4284729349	0.672669652
acute_cyto_PC4	GGB3793_SGB5158	27	-0.6156915393	0.5447140138
acute_cyto_PC4	GGB42601_SGB59797	27	-0.8423939252	0.409059403
acute_cyto_PC4	GGB45514_SGB63186	27	0.6562092896	0.518815686
acute_cyto_PC4	GGB45564_SGB63259	27	0.6053175763	0.5514541828
acute_cyto_PC4	GGB45624_SGB63337	27	0.6479772143	0.5240217956
acute_cyto_PC4	GGB45656_SGB63370	27	-1.358415292	0.1887468887
acute_cyto_PC4	GGB47127_SGB65054	27	2.230691663	0.03673833262
acute_cyto_PC4	GGB74395_SGB43523	27	-1.597338787	0.1251293948
acute_cyto_PC4	GGB75053_SGB43494	27	-0.184662687	0.8552653247
acute_cyto_PC4	GGB75109_SGB102238	27	-0.02388561073	0.9811693666
acute_cyto_PC4	Lachnospiraceae_bacterium	27	0.3213498491	0.751121746
acute_cyto_PC4	Lachnospiraceae_bacterium_A2	27	0.8717941522	0.3931790058
acute_cyto_PC4	Lachnospiraceae_unclassified_SGB41414	27	-0.9059090322	0.375261271
acute_cyto_PC4	Lachnospiraceae_unclassified_SGB41424	27	-0.594908619	0.5582610302
acute_cyto_PC4	Lachnospiraceae_unclassified_SGB94868	27	-0.0026962215[illegible]	0.9978741781

acute_cyto_PC4	Lactobacillus_johnsonii	27	-0.493865462	0.6265271664
acute_cyto_PC4	Leptogranulimonas_caecicola	27	0.03811463546	0.9699562968
acute_cyto_PC4	Muribaculaceae_bacterium	27	0.3329868843	0.7424456627
acute_cyto_PC4	Neglectibacter_sp_X4	27	0.3343993487	0.7413949469
acute_cyto_PC4	Oscillibacter_SGB43496	27	1.161007398	0.258666812
acute_cyto_PC4	Oscillospiraceae_bacterium	27	0.4284370499	0.6726953621
acute_cyto_PC4	Oscillospiraceae_unclassified_SGB43502	27	1.119048648	0.2757566387
acute_cyto_PC4	Oscillospiraceae_unclassified_SGB43505	27	-0.3579761213	0.723934266
acute_cyto_PC4	Oscillospiraceae_unclassified_SGB94989	27	0.1272604688	0.899944963
acute_cyto_PC4	Parasutterella_excrementihominis	27	-1.805812581	0.08530368235
acute_cyto_PC4	Schaedlerella_arabinosiphila	27	-1.502009509	0.1479831614
acute_cyto_PC4	Turicibacter_sp_1E2	27	0.601852887	0.5537150212
acute_cyto_PC4	bacterium_0_1xD8_82	27	-0.9656713329	0.3451986689
acute_cyto_PC4	bacterium_1XD42_1	27	-0.2144353541	0.8322761366
acute_cyto_PC4	bacterium_1XD42_54	27	-0.3086900377	0.7605988196
acute_cyto_PC4	bacterium_1XD42_76	27	-0.4054858278	0.6892219139
acute_cyto_PC4	bacterium_1xD8_48	27	1.142848945	0.2659638145
acute_cyto_PC4	Berger Parker Index	27	0.04234323106	0.966625106
acute_cyto_PC4	Richness (# observed features)	27	0.01267875952	0.990003766
acute_cyto_PC4	Shannon Index	27	-0.5582312714	0.5825899045
acute_cyto_PC5	Acetatifactor_SGB41545	27	-0.3096492217	0.7598794062
acute_cyto_PC5	Acutalibacter_muris	27	0.978737021	0.3388514184
acute_cyto_PC5	Akkermansia_muciniphila	27	1.539382489	0.1386442973
acute_cyto_PC5	Anaerotruncus_sp_1XD42_93	27	1.567419492	0.1319624507
acute_cyto_PC5	Bacteria_unclassified_SGB41677	27	-1.083706231	0.2907811712
acute_cyto_PC5	Bacteria_unclassified_SGB43539	27	-0.9445641512	0.3556232263
acute_cyto_PC5	Bacteria_unclassified_SGB43546	27	-2.652808862	0.01488461554
acute_cyto_PC5	Bacteria_unclassified_SGB63211	27	-0.9325265902	0.361662888
acute_cyto_PC5	Bacteroides_thetaiotaomicron	27	-0.486105574	0.6319262465
acute_cyto_PC5	Clostridia_bacterium	27	1.370435118	0.1850238283
acute_cyto_PC5	Clostridiaceae_bacterium	27	1.201930954	0.2427694518
acute_cyto_PC5	Clostridiaceae_unclassified_SGB41663	27	1.380748843	0.1818758808
acute_cyto_PC5	Clostridiales_bacterium	27	-0.147331557	0.8842756834
acute_cyto_PC5	Clostridium_SGB65123	27	1.341136118	0.1942024975
acute_cyto_PC5	Clostridium_cocleatum	27	-0.00094931903	0.9992515135
acute_cyto_PC5	Eubacteriaceae_bacterium	27	0.1638214416	0.8714381526
acute_cyto_PC5	Eubacteriaceae_unclassified_SGB94922	27	1.749405199	0.09482258756
acute_cyto_PC5	GGB20146_SGB29427	27	2.09766964	0.0482146708
acute_cyto_PC5	GGB20149_SGB29430	27	1.422884055	0.1694561509
acute_cyto_PC5	GGB22635_SGB63107	27	1.101350674	0.2832078842
acute_cyto_PC5	GGB25041_SGB36960	27	-0.8603967561	0.3992871712
acute_cyto_PC5	GGB28379_SGB40959	27	1.47690336	0.1545416901
acute_cyto_PC5	GGB28382_SGB40962	27	0.4135737302	0.6833793254
acute_cyto_PC5	GGB28392_SGB40972	27	-0.1896789541	0.8513821305
acute_cyto_PC5	GGB28404_SGB40986	27	-2.115385234	0.04651898783
acute_cyto_PC5	GGB28418_SGB41001	27	-0.8362145597	0.4124485877

acute_cyto_PC5	GGB28422_SGB41005	27	1.564880941	0.1325562273
acute_cyto_PC5	GGB28431_SGB41014	27	-1.768670637	0.09147343241
acute_cyto_PC5	GGB28456_SGB41039	27	-0.3766995312	0.7101763428
acute_cyto_PC5	GGB28782_SGB41435	27	-0.7432240988	0.4655801956
acute_cyto_PC5	GGB28792_SGB41445	27	-1.465387927	0.1576281629
acute_cyto_PC5	GGB28798_SGB41451	27	-1.92706551	0.06760968239
acute_cyto_PC5	GGB28810_SGB41465	27	0.424256454	0.6756933987
acute_cyto_PC5	GGB28828_SGB41484	27	0.798558248	0.4334854718
acute_cyto_PC5	GGB28851_SGB41518	27	-0.7877056398	0.439669961
acute_cyto_PC5	GGB28865_SGB41537	27	-1.202420784	0.2425837289
acute_cyto_PC5	GGB28868_SGB41542	27	-0.2792224218	0.7828055758
acute_cyto_PC5	GGB28869_SGB41543	27	0.8392004314	0.4108087078
acute_cyto_PC5	GGB28875_SGB41555	27	-0.07408967694	0.9416402419
acute_cyto_PC5	GGB28881_SGB41561	27	0.2202244385	0.8278231804
acute_cyto_PC5	GGB28892_SGB41573	27	-0.2281233779	0.8217569205
acute_cyto_PC5	GGB28893_SGB41574	27	-1.702033883	0.103508377
acute_cyto_PC5	GGB28898_SGB41580	27	0.4805444686	0.6358085023
acute_cyto_PC5	GGB28901_SGB41592	27	0.3336691597	0.7419380615
acute_cyto_PC5	GGB28904_SGB41597	27	0.4884155011	0.6303168527
acute_cyto_PC5	GGB28909_SGB41602	27	0.2822148972	0.7805414054
acute_cyto_PC5	GGB28924_SGB41621	27	2.380298205	0.02684955058
acute_cyto_PC5	GGB28927_SGB41625	27	-0.2681550885	0.7911963677
acute_cyto_PC5	GGB28934_SGB41635	27	-0.8402299452	0.4102442496
acute_cyto_PC5	GGB28949_SGB41655	27	-0.07220443055	0.9431224967
acute_cyto_PC5	GGB28951_SGB102295	27	-0.2889106881	0.7754824814
acute_cyto_PC5	GGB28951_SGB41658	27	0.7917506391	0.4373585409
acute_cyto_PC5	GGB28954_SGB41662	27	-0.8286196058	0.4166385605
acute_cyto_PC5	GGB28960_SGB41669	27	-2.12061281	0.04602900326
acute_cyto_PC5	GGB28964_SGB94886	27	1.198404007	0.2441098883
acute_cyto_PC5	GGB28967_SGB41678	27	-1.204086557	0.24195294
acute_cyto_PC5	GGB28996_SGB41712	27	0.8495098475	0.4051786384
acute_cyto_PC5	GGB29531_SGB42317	27	0.3440851382	0.7342038304
acute_cyto_PC5	GGB29685_SGB42494	27	1.118360637	0.2760436028
acute_cyto_PC5	GGB30141_SGB43066	27	0.4928579722	0.6272269425
acute_cyto_PC5	GGB30145_SGB43072	27	-1.5363527	0.1393828228
acute_cyto_PC5	GGB30286_SGB43248	27	-1.066598718	0.2982624789
acute_cyto_PC5	GGB30300_SGB43264	27	-1.340409195	0.1944347053
acute_cyto_PC5	GGB30303_SGB43268	27	-0.9979576804	0.3296609601
acute_cyto_PC5	GGB30450_SGB43507	27	1.652558436	0.1132949128
acute_cyto_PC5	GGB30453_SGB43513	27	1.242635326	0.2276994246
acute_cyto_PC5	GGB30454_SGB43514	27	-0.8469322003	0.4065816592
acute_cyto_PC5	GGB30455_SGB43519	27	0.7068452854	0.4874325302
acute_cyto_PC5	GGB30456_SGB43520	27	-0.3935107739	0.6979090696
acute_cyto_PC5	GGB30457_SGB63218	27	-0.6616737976	0.5153757219
acute_cyto_PC5	GGB30461_SGB43530	27	-2.129277161	0.04522714171
acute_cyto_PC5	GGB30461_SGB43533	27	0.8501040976	0.4048556272

acute_cyto_PC5	GGB30461_SGB63209	27	1.261052512	0.2211201919
acute_cyto_PC5	GGB30463_SGB43537	27	0.2790963436	0.7829010128
acute_cyto_PC5	GGB30473_SGB43557	27	0.6992152723	0.4920901419
acute_cyto_PC5	GGB30475_SGB63182	27	-0.9755825285	0.340376455
acute_cyto_PC5	GGB30861_SGB44083	27	-1.335965555	0.1958589329
acute_cyto_PC5	GGB31312_SGB44628	27	-0.1581558697	0.8758449468
acute_cyto_PC5	GGB31438_SGB44768	27	0.8521586764	0.4037401113
acute_cyto_PC5	GGB3171_SGB4185	27	0.6304434312	0.535205558
acute_cyto_PC5	GGB31762_SGB45125	27	1.235814588	0.2301736483
acute_cyto_PC5	GGB31823_SGB45199	27	1.235348085	0.2303436188
acute_cyto_PC5	GGB31838_SGB45216	27	-1.225921432	0.233798756
acute_cyto_PC5	GGB31841_SGB65084	27	0.1738115173	0.8636781831
acute_cyto_PC5	GGB32371_SGB41694	27	0.9073681633	0.3745071523
acute_cyto_PC5	GGB3793_SGB5158	27	0.1454115168	0.8857726453
acute_cyto_PC5	GGB42601_SGB59797	27	-1.814659684	0.08388837191
acute_cyto_PC5	GGB45514_SGB63186	27	-0.1321426422	0.8961293948
acute_cyto_PC5	GGB45564_SGB63259	27	0.9779238008	0.3392441183
acute_cyto_PC5	GGB45624_SGB63337	27	-1.331030885	0.1974501358
acute_cyto_PC5	GGB45656_SGB63370	27	-0.1027877003	0.9191067024
acute_cyto_PC5	GGB47127_SGB65054	27	1.287419677	0.2119566975
acute_cyto_PC5	GGB74395_SGB43523	27	-0.06884262351	0.9457662025
acute_cyto_PC5	GGB75053_SGB43494	27	0.420597881	0.6783216138
acute_cyto_PC5	GGB75109_SGB102238	27	0.890149429	0.3834703182
acute_cyto_PC5	Lachnospiraceae_bacterium	27	0.6143630085	0.5455747303
acute_cyto_PC5	Lachnospiraceae_bacterium_A2	27	1.171214928	0.2546307738
acute_cyto_PC5	Lachnospiraceae_unclassified_SGB41414	27	0.7907994898	0.4379013773
acute_cyto_PC5	Lachnospiraceae_unclassified_SGB41424	27	-1.334221959	0.1964200068
acute_cyto_PC5	Lachnospiraceae_unclassified_SGB94868	27	-0.6014257392	0.5539940878
acute_cyto_PC5	Lactobacillus_johnsonii	27	-0.0023232841	0.9981682181
acute_cyto_PC5	Leptogranulimonas_caecicola	27	1.867324302	0.07587850089
acute_cyto_PC5	Muribaculaceae_bacterium	27	0.9501273587	0.3528551549
acute_cyto_PC5	Neglectibacter_sp_X4	27	0.2423160348	0.8108861076
acute_cyto_PC5	Oscillibacter_SGB43496	27	-1.32725454	0.1986746723
acute_cyto_PC5	Oscillospiraceae_bacterium	27	0.1224919858	0.9036740757
acute_cyto_PC5	Oscillospiraceae_unclassified_SGB43502	27	1.026278324	0.3164374223
acute_cyto_PC5	Oscillospiraceae_unclassified_SGB43505	27	-0.6574904754	0.5180080258
acute_cyto_PC5	Oscillospiraceae_unclassified_SGB94989	27	-2.01588025	0.05678601931
acute_cyto_PC5	Parasutterella_excrementihominis	27	-0.0160404996	0.9873535037
acute_cyto_PC5	Schaedlerella_arabinosiphila	27	-0.9128055055	0.3717058445
acute_cyto_PC5	Turicibacter_sp_1E2	27	0.7452989227	0.4643516954
acute_cyto_PC5	bacterium_0_1xD8_82	27	0.5112573272	0.6145045042
acute_cyto_PC5	bacterium_1XD42_1	27	-2.33746808	0.02939495413
acute_cyto_PC5	bacterium_1XD42_54	27	-0.6113728507	0.5475146111
acute_cyto_PC5	bacterium_1XD42_76	27	0.6974615359	0.4931642904
acute_cyto_PC5	bacterium_1xD8_48	27	0.3740792347	0.712095773
acute_cyto_PC5	Berger Parker Index	27	1.431913881	0.1668852645

acute_cyto_PC5	Richness (# observed features)	27	-1.150101141	0.2630314661
acute_cyto_PC5	Shannon Index	27	-1.426489391	0.1684258779

LRT_q_microbe	est_intercept	est_microbe	est_blast	est_vns	est_interact	est_batch
0.9750766376	-3.234967486	0.262133314	5.724219169	-0.478833464	-1.255417694	1.402141897
0.9750766376	-3.064374596	-0.181341115	5.400436133	-0.718750181	-0.857166586	1.43472364
0.9750766376	-3.125495505	-0.179252229	5.399525511	-0.516598809	-1.018358115	1.444211093
0.9907090994	-3.06646382	-0.047075957	5.429490387	-0.702195107	-0.8058312602	1.356119148
0.9907090994	-3.096408584	-0.012793044	5.47288097	-0.609071455	-0.9173976175	1.336615271
0.8476569724	-2.885674582	0.401958192	5.021974301	-0.760215376	-0.5341468226	1.346137619
0.9750766376	-2.923764799	0.180870505	5.264301271	-0.755180910	-0.7388327463	1.266178862
0.5326401688	-3.495201505	0.710113013	5.662878342	-0.31720145	-1.611899267	2.058713842
0.9750766376	-2.958261949	-0.180159507	5.223006067	-0.758149803	-0.7331930502	1.385201791
0.9750766376	-2.963449423	-0.182320821	5.300473186	-0.752438361	-0.782896163	1.331026385
0.9750766376	-3.019971995	-0.151628554	5.312170086	-0.741460471	-0.7772958426	1.419654301
0.7255653047	-2.787135767	-0.496730844	5.13756213	-1.124374943	-0.7323643592	1.522261372
0.9750766376	-3.245472228	0.205232517	5.727709546	-0.486136238	-1.22608858	1.40974792
0.2656465465	-2.264824264	-0.990177459	4.884388924	-1.339724288	0.3125010752	0.318504053
0.9750766376	-3.165118749	-0.118580280	5.479966131	-0.521488961	-1.002806688	1.429385633
0.9878805585	-3.095558218	-0.075035287	5.392550331	-0.607280644	-0.9091229257	1.420517642
0.9907090994	-3.077814362	-0.036215590	5.432202224	-0.631187318	-0.8790752222	1.345167588
0.9854090226	-3.128374738	0.110702700	5.527069441	-0.568583190	-1.087222333	1.401386125
0.9750766376	-3.152052228	0.151809830	5.567948883	-0.602656179	-1.067426032	1.427905514
0.9750766376	-3.123837849	0.122918043	5.522962877	-0.682493187	-0.8386135478	1.366383982
0.9878805585	-3.119218404	0.091891948	5.548839413	-0.528428997	-0.9851028543	1.251164194
0.5326401688	-2.001022572	1.019766727	4.586820583	-1.301608451	0.4914400084	-0.037207773
0.6848176303	-2.988014156	-0.514150981	5.349650229	-0.225420607	-1.429668222	1.155758697
0.6848176303	-3.444981976	-0.561375706	6.026364834	-0.031742781	-2.116111233	1.537871511
0.9750766376	-2.945697437	0.131211737	5.347540779	-0.806454181	-0.7864453424	1.300203107
0.9907090994	-3.105451291	0.010540222	5.471381152	-0.605395684	-0.9194744712	1.354446378
0.9750766376	-3.046300529	0.139754164	5.351622132	-0.70991664	-0.8202624972	1.421285788
0.9879722819	-3.129659575	-0.060897676	5.548345061	-0.533154613	-1.049734173	1.318282351
0.9101962467	-3.120219081	0.357912386	5.704162111	-0.641424854	-1.087627813	1.258804151
0.9750766376	-2.978209381	-0.254888556	5.397578522	-0.794997957	-0.647306919	1.21203094
0.9750766376	-3.077001389	0.117413572	5.367862445	-0.610960930	-0.8998575316	1.408724161
0.9750766376	-3.151044376	0.146653735	5.48896015	-0.534431109	-0.9115777671	1.347573352
0.9907090994	-3.121105264	-0.021562239	5.474845136	-0.604039168	-0.923256908	1.383828362
0.6848176303	-3.299047811	-0.520974642	6.0586942	-0.184889636	-1.95776072	1.264955274
0.9750766376	-3.005521663	0.283452499	5.463472225	-0.720647835	-0.9408352005	1.29328868
0.5326401688	-3.414916548	-0.744979327	6.321926435	0.331368496	-2.055721273	0.706189346
0.9750766376	-3.087174401	0.110205353	5.3824951	-0.647148709	-0.7846290236	1.381030496
0.9750766376	-2.985038758	0.364729087	5.507365266	-0.614551102	-0.7956069777	0.996472210
0.9907090994	-3.088471129	-0.020109359	5.467183866	-0.609551288	-0.921094296	1.329494992
0.9750766376	-2.96563876	-0.282239620	5.180309747	-1.09406606	-0.383512512	1.596355789
0.9750766376	-3.330516343	-0.336820488	5.589987795	-0.500705959	-0.9429601775	1.586745323
0.5326401688	-3.318118286	0.713764740	5.722014894	-0.774491804	-1.302042971	1.924476873
0.9750766376	-3.002067374	-0.155964579	5.440484452	-0.684856356	-0.8169250318	1.197841658
0.7045398025	-2.862951666	-0.502900401	4.8594418	-0.592050911	-0.4421836024	1.248788428
0.8476569724	-3.295455918	0.522484530	5.612925191	-0.592598967	-1.433650455	1.888386239
0.5326401688	-2.801090455	-0.719327730	5.528637383	-0.613174029	-1.653884451	1.116568735

0.5326401688	-2.705384608	-0.751903455	5.813570737	-0.650115473	-1.280586824	0.399087275
0.9750766376	-2.785322348	-0.411569317	5.014858929	-1.459410899	0.0247187396	1.554986318
0.9750766376	-3.17900886	0.192421048	5.632330614	-0.458429271	-1.191607063	1.330703563
0.9750766376	-2.889071268	-0.329669337	5.149404481	-0.749936551	-0.7644142003	1.33679095
0.9750766376	-3.239329856	0.189744263	5.558534968	-0.446937875	-1.002739873	1.41253314
0.9101962467	-3.600779846	-0.448040446	5.619753782	-0.124139475	-1.017531788	1.754073545
0.9878805585	-3.125775968	0.088434816	5.466911717	-0.603198785	-0.9475255106	1.416712498
0.9101962467	-2.814957614	-0.417105693	5.526323063	-0.818198713	-1.200480469	1.089818106
0.9878805585	-3.051928479	-0.083349857	5.399946991	-0.661191297	-0.889710644	1.367479029
0.9750766376	-3.054622301	-0.146113583	5.426804473	-0.659376858	-0.8525467977	1.317260418
0.9750766376	-3.103565275	0.109842238	5.465243928	-0.612758121	-0.9900637244	1.408979001
0.9750766376	-3.062218154	0.127733910	5.387929118	-0.601440772	-0.920275523	1.357179106
0.9878805585	-3.14641978	0.086672678	5.590911792	-0.579622057	-0.9611478598	1.299503758
0.9750766376	-3.057284352	-0.278107386	5.607485438	-0.478667899	-1.080656142	1.060076588
0.6848176303	-3.297878692	0.489149154	5.479481991	-0.203136995	-1.34734816	1.574861388
0.9750766376	-3.151852562	0.242150208	5.50343947	-0.572972006	-1.047027292	1.457404119
0.8476569724	-2.707742048	-0.479640022	5.216338495	-0.949478863	-0.3559055006	0.846454305
0.9750766376	-3.256061936	0.281974953	5.461820029	-0.468224792	-1.010306319	1.586459191
0.9750766376	-3.401382332	-0.284817545	5.887263805	-0.356805732	-1.291585023	1.45048664
0.9907090994	-3.090733728	0.042017756	5.440549529	-0.637538153	-0.8679100897	1.362337121
0.9907090994	-3.099540271	0.043916360	5.493218304	-0.610907289	-0.9335583276	1.331575495
0.9750766376	-2.981424621	0.163829943	5.397090204	-0.790807900	-0.8126182805	1.316489889
0.9750766376	-3.26077703	-0.280776421	5.793025946	-0.398105035	-1.346048486	1.345188385
0.9750766376	-3.137712704	0.138528831	5.467677427	-0.610779548	-0.8762714093	1.404935889
0.9750766376	-2.975651969	-0.185280482	5.416508203	-0.732657087	-0.9485505747	1.303121842
0.9878805585	-3.162068957	0.069450800	5.493845414	-0.575423169	-0.9409091021	1.427029063
0.920225808	-3.218655674	0.455455267	5.232413364	-0.105980897	-1.348939259	1.591749952
0.9907090994	-3.098417013	0.008316123	5.463099828	-0.604187660	-0.9190462282	1.347826646
0.9907090994	-3.099087425	-0.019736685	5.463267553	-0.625520399	-0.8973512934	1.358649471
0.9750766376	-3.127280578	0.217265121	5.478033216	-0.600942907	-1.0060188	1.440571421
0.6452942772	-2.586530815	0.673283981	5.313798033	-0.623400009	-0.9543448454	0.499363108
0.9750766376	-3.041714992	0.166745456	5.435579719	-0.651745485	-0.8386445569	1.263554019
0.2654279322	-3.012135262	-0.878736945	4.979708788	-1.109712466	-0.0584911025	1.741224893
0.6848176303	-2.932217484	-0.604482862	4.831793696	-1.298185591	-0.1081345611	1.979433954
0.9750766376	-3.068642308	-0.116713070	5.401624583	-0.681606006	-0.7966326877	1.364963012
0.9750766376	-3.094952339	-0.136579593	5.438358512	-0.695840341	-0.8195004559	1.406623376
0.9907090994	-3.140150823	-0.047644131	5.483992001	-0.605585498	-0.9179827555	1.411250095
0.5434355159	-2.941335235	-0.582639393	5.347865754	-1.014683502	-0.3437530194	1.242589094
0.9878805585	-3.124272301	-0.076128992	5.465052387	-0.543809100	-0.984832999	1.37473521
0.9750766376	-3.152222202	-0.15149117	5.454718824	-0.612745279	-0.8643102351	1.444779453
0.9750766376	-3.025295168	0.288865468	5.314203865	-0.716680708	-0.9106437587	1.48373781
0.9750766376	-3.133194768	0.168897221	5.447905234	-0.682548731	-0.8829921526	1.499792283
0.9907090994	-3.103590668	-0.022491866	5.465310439	-0.622398506	-0.8980835176	1.362733744
0.6848176303	-2.873328731	0.496742177	5.197390996	-1.014637104	-0.5786624537	1.419479222
0.9750766376	-2.97277419	0.311236958	5.302682497	-0.955536632	-0.5904873757	1.44815981
0.9750766376	-3.230256363	-0.178174383	5.589513165	-0.540977177	-0.9936713198	1.453636567

0.6278895264	-2.715564269	0.584584625	5.259280568	-0.802281130	-0.716285323	0.876403473
0.9750766376	-3.002242264	-0.331595675	5.622801759	-0.586250320	-1.361137904	1.216547881
0.6848176303	-2.903246	-0.487389522	5.493702005	-0.612346376	-1.216217892	1.098823412
0.9878805585	-3.139732074	0.105202324	5.581300795	-0.583945982	-0.932634594	1.283813429
0.9750766376	-3.232691515	-0.166892059	5.494350884	-0.484805311	-0.877848297	1.436728338
0.9750766376	-3.114929908	0.178381092	5.528693077	-0.596268761	-1.039116722	1.371800599
0.5326401688	-3.128862226	0.804720077	4.997248591	0.207759931	-0.934824193	1.083885058
0.9907090994	-3.097328943	-0.009829136	5.465247841	-0.605979080	-0.920836866	1.346120455
0.9750766376	-3.169458752	0.269344599	5.777570612	-0.544406680	-0.952438572	1.088694554
0.6848176303	-3.583301716	-0.571010653	5.359164045	-0.034334169	-0.883875572	1.839489894
0.99399338	-3.1030424	-0.003059150	5.466664731	-0.604578808	-0.918711658	1.353536185
0.9750766376	-3.027362052	0.206061216	5.313173888	-0.598083565	-0.789116986	1.2867133
0.6755436502	-2.833830972	0.554531899	5.175859196	-0.828496539	-1.057671454	1.4566055
0.9750766376	-3.079619456	0.286352255	5.277586759	-0.562548958	-0.704711287	1.346099974
0.9750766376	-3.212158758	0.155058535	5.706873696	-0.451832572	-1.144043554	1.277168113
0.2656465465	-2.833094012	0.836592452	5.475923754	-0.925932592	-0.303080590	0.749414079
0.7733300493	-2.936534428	0.411862092	5.420194277	-0.768131869	-0.921895235	1.239424881
0.6452942772	-3.23417271	0.746100511	6.157835432	-0.300287467	-1.021526073	0.563951139
0.9750766376	-3.133657806	-0.116361656	5.603096512	-0.559000464	-1.034214915	1.281695301
0.5326401688	-2.811633911	-0.799383053	4.263887434	-0.809092837	-0.072841981	1.83583382
0.9750766376	-3.101568171	-0.136571396	5.529823546	-0.605258654	-0.969790504	1.309764049
0.9878805585	-3.080261915	-0.068755877	5.495264252	-0.603774113	-0.998180591	1.321260788
0.9878805585	-3.135910422	0.084302332	5.570459396	-0.523686895	-1.0058452	1.268543738
0.2654279322	-3.942497537	1.086914496	6.188271227	-0.148550808	-2.206478625	2.565761955
0.9750766376	-3.233160013	-0.162491003	5.654492622	-0.504256991	-1.097822206	1.409238967
0.9750766376	-3.325574258	0.220104978	5.712087389	-0.548364032	-1.076322582	1.568990096
0.9879722819	-3.08995554	0.067791738	5.395522915	-0.608327492	-0.907814792	1.405773777
0.9878805585	-3.160753351	-0.077863418	5.577538867	-0.596425489	-0.947272581	1.354261152
0.9703291146	0.5712867148	-0.179274501	-3.201627502	-0.619694890	2.239991968	1.79657415
0.9703291146	0.4138244054	0.302937179	-2.910455024	-0.342410202	1.905949241	1.694593487
0.9743440267	0.4768235015	-0.055022879	-3.050504394	-0.507252288	1.980087719	1.857243204
0.9703291146	0.801666998	-0.382271094	-3.366544627	-1.331360314	2.934854197	1.847182406
0.9703291146	0.8111301872	-0.450960291	-2.966325936	-0.712428647	2.090788219	1.218620715
0.9703291146	0.7766097982	0.536532476	-3.628042372	-0.742584097	2.524984853	1.819113044
0.9703291146	0.2431837289	-0.238280795	-2.756068538	-0.334933042	1.772129646	1.945160986
0.9703291146	0.1820228917	0.548555849	-2.880394634	-0.312525860	1.475644056	2.373962388
0.9850546863	0.501033507	-0.022162606	-3.0590507	-0.553045979	2.033318963	1.833380602
0.9703291146	0.7107818304	-0.292040065	-3.301863927	-0.771832498	2.229475373	1.792823191
0.9703291146	0.641146927	-0.279839364	-3.321873672	-0.787753479	2.273154071	1.950804168
0.9703291146	1.076867116	-0.926112314	-3.650412456	-1.504256634	2.359609803	2.14334532
0.9703291146	0.5918238961	-0.159969117	-3.228531364	-0.625963195	2.249210234	1.786040625
0.151382718	1.870376954	-1.633798753	-3.99663282	-1.748014001	4.043482204	0.121050224
0.9743440267	0.5447771959	0.123540978	-3.037749008	-0.620059913	2.096983676	1.750940016
0.9743440267	0.490188325	-0.053863212	-3.084926737	-0.536430768	2.017955492	1.877323316
0.9703291146	0.1176617372	0.473991074	-2.519219223	-0.178414263	1.478957869	1.944427552
0.9703291146	0.340525233	0.695689993	-2.67690259	-0.311438216	0.9574823167	2.127664158

0.9703291146	0.2886005928	0.636612938	-2.622462867	-0.528398416	1.390801353	2.139684155
0.9703291146	0.4017885687	0.550250966	-2.796434589	-0.885410368	2.37327191	1.88521518
0.9703291146	0.4128576031	0.476783945	-2.625260813	-0.141919408	1.670923219	1.296249453
0.9703291146	1.684714552	1.109423016	-3.990570698	-1.293012406	3.545560211	0.316073812
0.9703291146	0.5247307866	-0.182748367	-3.071702598	-0.399518997	1.829108911	1.759092493
0.9703291146	0.1158510435	-0.607344573	-2.427813418	0.085045742	0.7159744348	2.028522094
0.9703291146	0.243586132	-0.196221916	-2.843726662	-0.231340444	1.811132923	1.90990594
0.9703291146	0.4911718904	0.324541212	-3.02037603	-0.576685818	2.01666732	1.844943255
0.9703291146	0.5904400537	0.252947623	-3.244807242	-0.725766071	2.190316234	1.951417101
0.9703291146	0.3931281134	-0.228347781	-2.738944878	-0.268390414	1.522709733	1.69580785
0.09217496846	0.4228562908	1.48204894	-2.063529723	-0.689003743	1.314932059	1.435570918
0.9703291146	0.6330713897	-0.300198766	-3.115139301	-0.759020895	2.331168056	1.662393083
0.9743440267	0.4690981296	-0.056401736	-2.978929128	-0.530746493	2.000856662	1.803221927
0.9703291146	0.5652533301	-0.266487618	-3.060953341	-0.660521597	1.995657706	1.841115456
0.9743440267	0.5433977663	0.084796417	-3.043192917	-0.533978065	2.024449812	1.712019114
0.9703291146	0.2102389545	-0.734743313	-2.199853591	0.057014211	0.5463456434	1.704029845
0.9703291146	0.5347859108	0.146798391	-3.032487875	-0.594489448	1.999421318	1.798121558
0.9170863458	-0.0267289409	-1.22788729	-1.626209743	1.007625347	0.1379339566	0.761892388
0.9703291146	0.5932547225	0.655804152	-3.555891729	-0.790779479	2.814024721	1.99070872
0.9703291146	0.6221819122	0.420963521	-2.986691213	-0.546248398	2.153579435	1.416946051
0.9703291146	0.1821846791	0.350574571	-2.959972184	-0.437517724	2.035056317	2.255799541
0.9703291146	0.6707866874	-0.378571676	-3.418591722	-1.191400233	2.729544857	2.154683053
0.9703291146	0.1075144158	-0.562444718	-2.830029788	-0.361577173	1.971501255	2.218279076
0.2224185739	0.067834308	1.39480863	-2.538233809	-0.867228642	1.263178225	2.94444277
0.9703291146	0.6718507541	-0.284320495	-3.08437042	-0.681462032	2.197696186	1.544960377
0.9703291146	0.6391720392	-0.323734111	-3.422284764	-0.526384657	2.317758002	1.761842122
0.9703291146	0.1051405237	1.040237106	-2.746195679	-0.511361570	0.9870895338	2.893575905
0.9703291146	0.7323401594	-0.589022539	-2.981427851	-0.541586739	1.409172727	1.635158923
0.9703291146	1.099886012	-1.158824553	-2.500742463	-0.605138439	1.454150919	0.35791888
0.9703291146	0.681006169	-0.254486807	-3.310647956	-1.063005614	2.594330059	1.953845454
0.9743440267	0.4406196185	0.110991490	-2.935538102	-0.450110015	1.853525587	1.816129027
0.9703291146	1.007846343	-0.798807695	-3.808055349	-0.887667775	2.386592977	1.787968
0.9703291146	0.3439080282	0.197378875	-2.937593013	-0.370690164	1.923974911	1.890480111
0.9703291146	-0.1828243679	-0.602490206	-2.828652895	0.111211185	1.87879289	2.36759628
0.9703291146	0.6191431736	-0.600782918	-2.999964694	-0.539609803	2.19955557	1.403130571
0.9703291146	0.6740400561	-0.274203941	-2.992236313	-0.674890073	1.82577997	1.655898804
0.9703291146	0.1773958625	0.473367044	-2.624338333	-0.209347949	1.840181046	1.752677664
0.9703291146	0.5679895151	-0.243330310	-3.102323845	-0.626286195	2.122176316	1.768441305
0.9743440267	0.4817806466	-0.056736971	-3.025495721	-0.529566895	2.046734198	1.801109332
0.9703291146	0.6853167727	0.594370289	-3.415615169	-0.522119572	2.007601126	1.844579976
0.9850546863	0.4689542112	0.029685109	-2.987499476	-0.525730974	1.996175089	1.810889335
0.9743440267	0.5039106526	-0.120674575	-2.969356625	-0.479695930	1.940527843	1.702021695
0.9703291146	0.2330678103	0.635944252	-3.017652844	-0.012905575	1.454363846	2.11674839
0.9703291146	0.4578873237	0.131259111	-3.011008068	-0.517258996	1.941348005	1.885615783
0.9703291146	0.285792513	0.237544781	-2.902348654	-0.362989871	1.731165845	2.080874108
0.1202564645	-0.3074896218	1.482410827	-3.077402113	0.179782416	1.533926867	3.051914055

0.9703291146	-0.01119551405	-0.475966437	-2.333093581	-0.120971110	1.388875287	1.990867372
0.9703291146	0.5939918837	0.311488495	-3.255128882	-0.782621735	2.394152432	1.891741324
0.9703291146	0.5954860567	0.800121092	-2.625849303	-0.659709198	1.757502867	1.42196322
0.9703291146	0.8188422573	0.442803017	-3.228608484	-1.038960085	2.299743359	1.72829851
0.9703291146	0.5782717323	0.172710172	-3.226540871	-0.660741297	2.272645149	1.834922558
0.9703291146	0.5855851459	-0.444657302	-3.01749053	-0.512361992	1.871046987	1.665865462
0.9743440267	0.5351271897	-0.074405944	-3.050458862	-0.585746438	1.998783618	1.809125385
0.9703291146	0.2129126878	0.332755856	-2.91962856	-0.397107853	1.908658186	2.179694637
0.9703291146	0.304178112	0.720738263	-3.406266194	0.254028363	1.331091905	2.20585012
0.9703291146	0.7338599422	0.285776906	-3.304010874	-0.540086285	2.032113447	1.619249623
0.9703291146	0.2937584486	0.562801331	-2.804716277	0.079235075	1.373700343	1.695413249
0.9703291146	0.4690544496	0.139458567	-3.024089217	-0.532114422	1.954957994	1.885138929
0.9980621059	0.4852580359	0.003066062	-3.02924543	-0.534172777	2.010220266	1.825643941
0.9703291146	0.7347835041	0.656220298	-3.168382115	-0.721937348	2.329284976	1.473801552
0.9703291146	0.5761841314	-0.875972099	-3.518390439	-1.038193717	2.868856128	2.215596004
0.9703291146	0.6224984341	-0.486382555	-3.542944585	-1.092634438	2.663367572	2.332819431
0.9743440267	0.4962331112	-0.041989591	-3.053529917	-0.562000264	2.054646121	1.833499191
0.9703291146	0.4560724188	0.340203065	-2.946933215	-0.305351586	1.76084631	1.698322665
0.9703291146	0.2614746487	-0.306392982	-2.945727539	-0.544202748	2.021284696	2.198050652
0.9703291146	0.5993567938	-0.412787186	-3.115849364	-0.825036131	2.41840882	1.750641716
0.9703291146	0.4193287078	-0.260839696	-3.049305942	-0.327811769	1.787140966	1.900765466
0.9743440267	0.4638010619	-0.062196880	-3.03526146	-0.537670119	2.033111121	1.866829688
0.9703291146	0.3439446143	-0.499068770	-2.757433521	-0.339428544	1.994768546	1.605296115
0.9703291146	0.4421454774	0.250495229	-3.062953965	-0.650563901	2.064788807	2.045843519
0.9703291146	0.4336647483	0.519964088	-2.894308376	-0.109030076	1.511142855	1.626381204
0.9703291146	0.5811815734	0.210092660	-3.144298716	-0.707754656	2.154607914	1.857252317
0.9703291146	0.3301316355	-0.357628044	-2.834989425	-0.130163857	1.632119522	1.721278849
0.9703291146	0.1033471001	-0.543018588	-2.667693404	-0.341974161	1.784872352	2.133360677
0.9850546863	0.4640586208	-0.028221284	-3.018302479	-0.524512893	2.000559155	1.852589361
0.9703291146	0.6800300676	-0.631725018	-2.739552216	-0.500246393	1.169351462	1.567777633
0.9703291146	0.7727571374	-0.697750329	-2.996195255	-0.546015886	1.58585054	1.464300324
0.9743440267	0.4622360073	0.063896336	-2.961606721	-0.521897000	2.002509176	1.786939478
0.9703291146	0.6131982143	0.171273161	-3.05237347	-0.656420593	1.967449608	1.744580132
0.9703291146	0.4752759302	0.147546319	-2.980904958	-0.527679647	1.911586066	1.844303907
0.9703291146	0.454731281	0.979292477	-3.605195563	0.453789773	1.991947075	1.50088888
0.9703291146	0.9551092873	-0.553135539	-3.358780721	-0.644776071	1.945203711	1.389153489
0.9743440267	0.4533415048	0.124880751	-2.886442499	-0.506448623	1.995189136	1.706552654
0.9703291146	-0.04396304993	-0.629925518	-3.152032185	0.094370810	2.049875329	2.365176241
0.9743440267	0.3874939075	0.108988145	-2.869930987	-0.513894688	1.975924434	1.844135344
0.9703291146	0.5488667781	0.173491871	-3.161507637	-0.529092990	2.120303722	1.772929511
0.9703291146	0.7978048925	0.642277508	-3.370505809	-0.794089910	1.850550312	1.94843933
0.9703291146	0.5368855193	0.592347543	-3.428863868	-0.448313923	2.455059227	1.813305392
0.9703291146	0.3582378825	0.181597151	-2.752421293	-0.355859083	1.747612762	1.739617049
0.9703291146	0.6360087118	0.469855399	-3.025829045	-0.714884681	2.356678129	1.490012382
0.9703291146	0.3490376135	-0.325856407	-2.988240561	-0.404236470	2.012131786	1.920142242
0.9703291146	0.419087376	0.370610999	-2.687414604	-0.383215145	1.959787433	1.43712096

0.9743440267	0.4509621527	-0.132996389	-2.877681364	-0.482638106	1.879468174	1.746956187
0.9703291146	0.6668938007	-0.500147721	-3.783852512	-0.662396763	2.540216071	2.131037014
0.9703291146	0.5037859282	-0.687210800	-2.733121561	-0.540356961	1.758221708	1.607217474
0.9703291146	0.4286712976	0.1464402391	-3.079960585	-0.534591380	2.177575938	1.899150428
0.9703291146	0.3757243881	0.2992560442	-2.67588157	-0.248946406	1.70450477	1.526375124
0.9703291146	-0.4312902372	1.187460113	-2.245033357	-0.036490492	0.6045421096	3.15345129
0.9743440267	0.5452056525	0.0794502620	-3.118191163	-0.582859926	2.0974817	1.802500015
0.9703291146	0.2478972013	0.2352646452	-2.770961186	-0.474603667	1.842945478	2.059390836
0.9980621059	0.4832507658	0.0015354555	-3.03024118	-0.534182228	2.010646845	1.830709497
0.9743440267	0.4383309434	-0.063049366	-2.942354132	-0.527941274	1.988034199	1.829790828
0.9281860866	-0.1771930248	0.0619588118	-0.175160777	-0.083521029	-0.7895509948	1.145948131
0.8694827707	-0.1170048373	-0.129995456	-0.285652647	-0.195359392	-0.665382276	1.19246231
0.6176478085	-0.1027765652	0.3976296933	-0.076177320	-0.307014947	-0.4912970317	0.9343240451
0.6176478085	-0.5979870776	0.5412484472	0.243603064	1.015734942	-2.019137897	1.109570702
0.6960982921	-0.4609908314	0.4318779564	-0.294556070	0.0576888243	-0.7872020183	1.71962047
0.6078766667	-0.3980358798	-0.459219307	0.2781396720	0.0653465455	-1.150647482	1.143477068
0.7995209302	0.08500816269	0.2302706655	-0.498286459	-0.305565423	-0.4799550328	1.022817899
0.9499656053	-0.1230886732	-0.042946054	-0.246582441	-0.130454392	-0.6683305181	1.091933572
0.8689041286	-0.02404895816	-0.149782094	-0.441260536	-0.241256647	-0.5551528914	1.160542008
0.6078766667	-0.5343039395	0.4967891098	0.2299846789	0.2913242203	-1.082900608	1.197007843
0.421788991	-0.5135316113	0.6487684424	0.4450934615	0.4749873699	-1.319403752	0.8534121862
0.7995209302	0.02684177803	-0.270495240	-0.416622370	-0.396472786	-0.6081922632	1.226212667
0.9252032334	-0.2014990715	0.0805577860	-0.134267250	-0.066840133	-0.8304663487	1.156459719
0.6605662054	-0.5051799003	0.4221860671	0.0151803070	0.2005799280	-1.235564084	1.576042029
0.9252032334	-0.1861349916	-0.078838478	-0.229101308	-0.058242945	-0.7654623661	1.184712659
0.421788991	-0.265484278	0.877514891	0.6838207920	-0.074884177	-0.8336413644	0.3560210009
0.6078766667	0.2176510066	-0.472774636	-0.742987821	-0.467866163	-0.1801380348	1.019959286
0.9396644152	-0.1365399595	-0.049372087	-0.259939510	-0.128909598	-0.6354720196	1.113398623
0.6173390112	-0.01286781294	-0.438324453	-0.514572138	-0.117023728	-0.2835998584	0.9210110848
0.421788991	-0.2410074969	0.6402036547	0.0350511370	-0.521867865	-0.2879763653	1.199338184
0.7995209302	-0.1110123652	-0.242566606	-0.440150149	-0.312624988	-0.5374948133	1.405868803
0.9281860866	-0.2457681013	-0.091503621	-0.155637283	-0.050513435	-0.8368140947	1.259384526
0.5203497778	-0.01350254053	-0.581573017	-0.372379429	0.3151285252	-1.2870603	0.9103802364
0.7995209302	-0.2923970777	-0.241177851	0.0035603680	0.1327492378	-1.224204999	1.213574326
0.9252032334	-0.02822138742	0.0971011531	-0.326435244	-0.26292275	-0.6115972347	1.094784808
0.8492314277	-0.1503410211	-0.144953596	-0.238626567	-0.094081197	-0.7130034881	1.127674673
0.7995209302	-0.2480137427	-0.238421172	-0.031127456	0.0675648397	-0.8797989678	1.019674376
0.421788991	-0.4252476448	-0.708719301	0.6637924932	0.7115216547	-2.223762968	0.7195076607
0.421788991	-0.1212955397	-0.625728552	-0.642412683	-0.047701029	-0.4165736286	1.300890251
0.6078766667	-0.3709766515	0.4484320902	-0.105608057	0.2228976670	-1.189385307	1.384231197
0.6176478085	-0.04890485202	0.3995946227	-0.586390057	-0.136757711	-0.6427368304	1.320983421
0.9252032334	-0.121532512	-0.081251029	-0.244871152	-0.151658902	-0.7146826757	1.138087011
0.8689041286	-0.2767403136	-0.182330433	-0.203454587	-0.113337552	-0.7404515968	1.387241983
0.9570509022	-0.1368943342	0.0262721860	-0.264616017	-0.134244614	-0.6578451256	1.139044069
0.6176478085	-0.01182481164	0.3813903514	-0.245270429	-0.270044157	-0.7386608847	1.052940895
0.7995209302	-0.02536073233	0.2922196270	-0.568717051	-0.480013631	-0.2645795203	1.388640195

0.6078766667	-0.219497128	-0.432921163	0.113146998	0.056345365	-1.240712032	1.028159894
0.6176478085	-0.2819803977	-0.409021261	-0.275636018	-0.101289888	-0.849333318	1.535441231
0.9570509022	-0.1730865464	0.030827844	-0.228956544	-0.104617105	-0.708024394	1.172039966
0.7995209302	0.02950677989	-0.354912451	-0.600670438	-0.729344847	-0.035972812	1.439383376
0.6173390112	0.131200166	0.416303684	-0.381907919	-0.240793146	-0.681419047	0.846820826
0.421788991	0.135019515	-0.946530067	-0.567703979	0.112965989	-0.203137691	0.377970035
0.9252032334	-0.08005546331	-0.100194107	-0.254663527	-0.165044344	-0.644183879	1.034272484
0.7751009975	0.0044397955	-0.312972756	-0.615651880	-0.105664846	-0.413032323	1.06911309
0.4950861255	0.1262590862	-0.751505363	-0.438953043	-0.129524589	0.029066665	0.365854711
0.7995209302	-0.2643661666	0.277980386	-0.257213796	-0.109568146	-0.426464567	1.226289541
0.7995209302	-0.01291605397	-0.251164896	-0.120592070	-0.128509033	-0.830751767	0.815596548
0.758952824	0.1857849698	-0.427023585	-0.708374830	-1.000627408	0.2696647513	1.34314569
0.9281860866	-0.1677922087	0.055526009	-0.188464288	-0.071098589	-0.788663517	1.127849412
0.9252032334	-0.09178881141	-0.083471260	-0.316441561	-0.150056342	-0.670881975	1.130212763
0.7995209302	-0.2824918386	0.192923225	-0.146101895	0.046597120	-0.794647221	1.194125089
0.7995209302	0.1542396426	0.272306308	-0.325322720	-0.404761732	-0.650722153	0.891370149
0.7995209302	-0.1922378661	0.201044693	-0.244543807	-0.111259742	-0.773500403	1.277247625
0.7995209302	-0.2913483641	0.207582334	-0.262459999	-0.006513784	-0.570446967	1.266005734
0.421788991	0.3298567668	-0.738334475	-0.865421722	-0.619616693	-0.444729487	1.254435595
0.6173390112	-0.2830892506	0.390164364	-0.116659889	0.034730733	-0.889455608	1.232517131
0.7995209302	-0.1427115487	0.200434315	-0.245700816	-0.129067986	-0.838629301	1.234977714
0.6173390112	-0.2872487413	-0.412855589	0.033888834	-0.121419672	-0.708265539	1.124106402
0.421788991	-0.5531871918	0.865762496	0.961636371	0.130520423	-1.124424433	0.590739856
0.421788991	-0.02641987838	-0.690297699	0.103500178	0.198011452	-1.109760774	0.405135376
0.9281860866	-0.1687500676	0.056273014	-0.234022699	-0.066990987	-0.759394700	1.159971058
0.7995209302	-0.111705695	-0.183390136	-0.259464697	-0.136616645	-0.613748261	1.056203335
0.9849242331	-0.1539892136	0.008852827	-0.230282609	-0.106732362	-0.720600169	1.143923256
0.5174588607	0.1662799689	-0.586906794	-0.215635575	-0.395738428	-0.521560879	0.650599961
0.9396644152	-0.1995000576	-0.050917689	-0.160589174	-0.068914895	-0.776681282	1.151815228
0.6078766667	-0.3012883823	-0.433580987	0.080433904	0.232846351	-1.24439475	1.047968219
0.7995209302	-0.1780133726	-0.222924425	-0.347177052	-0.078107999	-0.639740048	1.248111479
0.6960982921	-0.4073538474	-0.343633766	-0.079715023	0.278695978	-0.934754063	1.213120619
0.6173390112	-0.3764605722	-0.416151631	0.242131872	0.192075607	-1.342136679	1.121576331
0.9537777759	-0.1396445318	-0.030306362	-0.234232757	-0.111628189	-0.719690822	1.12340115
0.7995209302	-0.30387825	0.223978729	-0.168971314	0.042405440	-0.675292365	1.195995786
0.6078766667	0.2965701378	-0.546267964	-0.413761318	-0.337976129	-0.543206136	0.559718535
0.8689041286	-0.1891252795	0.171323293	-0.324775226	0.074229866	-0.871664401	1.224008309
0.758952824	0.1595773887	0.348695898	-0.571119308	-0.120431926	-0.683673734	0.877972075
0.7995209302	-0.07312132481	-0.218777066	-0.321987554	-0.351523678	-0.462699505	1.186693587
0.7995209302	-0.1269199069	-0.198756621	-0.241312718	-0.115916462	-0.631209587	1.055310418
0.6176478085	0.2213967285	0.477274223	-0.346504145	-0.126852322	-0.734768422	0.528763878
0.8145528314	-0.2122161967	-0.170828844	-0.19857821	-0.064206414	-0.793213070	1.22716191
0.9252032334	-0.1551126097	0.079521364	-0.190515138	-0.067345331	-0.788127712	1.09950953
0.7751009975	-0.04081770085	-0.368697322	-0.624932562	-0.536511801	-0.215202508	1.516065511
0.6078766667	-0.2810559398	0.423101076	0.016932250	0.168179927	-1.156253443	1.094618091
0.8120905217	-0.1329034009	-0.174282144	-0.276785702	-0.230285975	-0.582362173	1.201775261

0.6078766667	0.2629030972	0.566718369	-0.38813848	-0.09439352	-0.730367751	0.452934228
0.7995209302	-0.206975372	0.213837417	-0.18975027	0.037613853	-0.9215678691	1.175426015
0.6078766667	-0.0371485854	0.449314217	-0.19919543	-0.46842758	-0.325653188	1.011858081
0.8875956565	-0.1829343063	-0.11822521	-0.24778190	-0.11992417	-0.666984355	1.205443707
0.421788991	0.03820041955	0.663911488	-0.59562361	-0.37205964	-0.689429033	1.432862313
0.7751009975	-0.0979835636	-0.299132447	-0.193876064	0.025986829	-0.775169758	0.876249203
0.7995209302	-0.1245836479	-0.23300114	-0.29513072	-0.30357988	-0.486482118	1.225591904
0.8394417861	-0.2213061352	-0.159592862	-0.147042872	0.018816492	-0.819755865	1.113501905
0.6176478085	-0.3072183396	-0.375918032	-0.031547412	0.31146941	-1.107798171	1.020763073
0.9281860866	-0.1924693221	-0.065562957	-0.191418547	-0.08991371	-0.737421583	1.171239582
0.6605662054	0.09245134719	0.358244644	-0.36480213	-0.23461177	-0.585551105	0.841909177
0.5715385661	0.03081279178	-0.56866051	0.025143424	-0.08264865	-1.467262264	0.898929425
0.8689041286	-0.0914101212	-0.132956422	-0.22882390	-0.115382252	-0.791088267	1.064960727
0.421788991	-0.4214515403	0.850008516	0.655277576	0.049020560	-0.814874651	0.567902870
0.7751009975	-0.3741581437	-0.29904665	-0.193352174	0.100492776	-0.635240339	1.282890489
0.7751009975	-0.1606548757	0.271379340	-0.14739100	-0.101328414	-0.891900977	1.161719416
0.6173390112	-0.1604097165	0.478688082	-0.516865551	0.369774221	-0.719203762	0.973910509
0.6078766667	-0.5893539503	0.518574176	0.074631602	-0.00933344	-0.649092323	1.547422259
0.5715385661	-0.3226291896	0.743674192	0.611150722	0.051464905	-0.800646080	0.402183634
0.6078766667	0.3014358207	0.535737055	-0.129948523	-0.64759538	-0.743785926	0.679006427
0.5715385661	-0.701584091	0.633983856	0.687579380	0.004335298	-0.910611576	1.219482938
0.9281860866	-0.1678887407	-0.055869042	-0.192162417	-0.11471611	-0.745593467	1.152785051
0.7995209302	-0.0401470287	0.217205294	-0.350634754	-0.201037311	-0.764245019	1.1747672
0.421788991	-0.2050535613	-0.640818867	0.198108824	-0.20589782	-1.191263468	1.152114697
0.5203497778	-0.5808031129	0.632478473	0.726661002	0.507626406	-1.625370946	0.821382068
0.9042044655	-0.1081111153	0.118242584	-0.23430563	-0.15860842	-0.623045521	1.049112831
0.9252032334	-0.1071275842	0.096197039	-0.24687877	-0.151441542	-0.710712176	1.107808113
0.8492314277	-0.1828865444	0.210512340	-0.041227244	-0.027412782	-0.738930715	0.911659234
0.7995209302	-0.0894308025	0.238294112	-0.505263931	-0.20528693	-0.475638691	1.282516714
0.6176478085	-0.3136400507	0.453928429	0.450537975	0.003346051	-1.19106941	0.860917099
0.9252032334	-0.1441995326	-0.080394324	-0.200426445	-0.113842517	-0.739693350	1.108548162
0.6176478085	-0.0000026088	-0.39603826	-0.095891723	-0.111737861	-1.162370249	0.946287564
0.9849242331	-0.1437694622	-0.008025755	-0.244442764	-0.12075584	-0.701991249	1.142686833
0.7995209302	-0.3928463631	0.319799879	-0.02397858	0.020900409	-1.08880609	1.491105664
0.7995209302	-0.3111043527	-0.209694081	0.001661325	0.015624911	-0.940088023	1.205911774
0.5172089339	-0.8477910672	0.701947472	0.533507285	0.064361254	-1.209754995	1.820363995
0.421788991	-0.3612159684	-0.922714705	0.793918847	-0.05437193	-0.871674724	0.429127178
0.421788991	-0.70807465	-0.794337444	0.850702892	-0.03571248	-0.991698062	1.137771653
0.9978741781	0.1856168494	0.205024892	0.454911587	-0.586168482	-0.718519190	0.163014117
0.7638667387	0.106204348	0.791679727	0.565518043	-0.18316441	-0.728833234	-0.227332334
0.9978741781	0.3194963844	0.297222843	0.375656497	-0.829017971	-0.292301496	-0.02435278
0.9978741781	0.8238086101	-0.64408994	-0.31257389	-2.027408595	1.101728666	0.155051309
0.9978741781	0.2646668759	0.030279759	0.252773186	-0.672100747	-0.461323530	0.166338096
0.9978741781	0.3237667597	0.067698436	0.181304598	-0.71038368	-0.390992494	0.124003077
0.9978741781	0.5968327057	0.308277357	-0.095547735	-0.94172917	-0.147688832	-0.02427291
0.9978741781	0.6870635553	-0.729942171	0.059833088	-0.978895221	0.2556256796	-0.59912958

0.9978741781	0.1839579257	0.125534584	0.429832301	-0.576673011	-0.5858670295	0.103539221
0.9978741781	0.4237216032	-0.175585923	0.092610166	-0.827019093	-0.3241945483	0.103245224
0.9978741781	0.3401449641	-0.094495588	0.157893790	-0.769734108	-0.3671907442	0.166267891
0.9978741781	0.4693875701	-0.284833186	0.065684634	-0.982459815	-0.3485154199	0.221832835
0.9978741781	0.2162952341	0.103402020	0.386228811	-0.624686439	-0.6103024497	0.153432789
0.9978741781	0.3716700609	-0.100047064	0.197667152	-0.758411690	-0.3314255226	0.020697608
0.9978741781	0.07759726069	-0.417454463	0.288104866	-0.393558102	-0.7485711799	0.390898757
0.9978741781	0.2576246795	0.214737886	0.481792082	-0.674721696	-0.4861332491	-0.065198295
0.9978741781	0.2219061056	0.084096503	0.347312643	-0.620971911	-0.5502101018	0.145702544
0.9978741781	0.3069329275	-0.098838151	0.206993505	-0.715707505	-0.3063372505	0.082962420
0.9978741781	0.0823481675	0.669591701	0.684051607	-0.678095050	-1.107597399	0.451533482
0.9978741781	0.2145298165	0.489675532	0.463493686	-0.996725175	-0.1329804696	0.174868143
0.9978741781	0.3183712433	-0.215564811	0.074623134	-0.861382385	-0.3024491412	0.366428917
0.9978741781	-0.6881243884	-0.899884243	1.037288907	-0.068487876	-1.701153795	1.352929655
0.9978741781	0.2050020879	0.356891954	0.341264129	-0.946870587	-0.1019210826	0.262887582
0.9978741781	0.3604405911	0.122007885	0.136273608	-0.808451154	-0.1958949676	0.085344293
0.9978741781	-0.1072554522	-0.323031723	0.561182009	-0.185681149	-0.7839332299	0.257628385
0.9978741781	0.2869594817	0.009924372	0.257199132	-0.685378414	-0.4557320681	0.125789334
0.9978741781	0.3084296626	0.051092980	0.213263702	-0.722793509	-0.4195786284	0.149937057
0.9978741781	0.1096556691	-0.450385576	0.828115954	-0.160029518	-1.417785336	-0.138442033
0.9978741781	0.275164135	0.284924142	0.442471084	-0.71385893	-0.5896240069	0.049578397
0.7638667387	-0.143715902	0.860395633	0.505182377	-0.039388473	-1.375336063	0.604363140
0.9978741781	0.1452635255	-0.578273874	0.765485949	-0.649852013	-0.5535458077	-0.144469983
0.9978741781	0.4518952194	-0.534494798	0.191916284	-0.937670868	-0.485449181	0.148543877
0.9978741781	0.6805307351	0.551944722	0.161501669	-0.683382973	-0.3643310214	-0.639603467
0.9978741781	0.3046975205	0.048482198	0.202269487	-0.723079455	-0.3593198855	0.133599699
0.9978741781	0.3586130283	0.203417569	0.251464056	-0.767778437	-0.4711071748	0.081787915
0.9978741781	0.1154237947	-0.412688994	0.728264844	-0.165911929	-1.085246894	-0.233513304
0.9978741781	0.372847904	0.511883615	-0.154679675	-0.884437265	0.1713566674	0.251120777
0.9978741781	0.3171076264	0.091880238	0.266081410	-0.686730595	-0.4246690215	0.035265649
0.9978741781	0.3425403906	-0.065092822	0.244220548	-0.702005681	-0.4605065038	0.046171754
0.9978741781	0.4902632003	-0.410131669	-0.165630839	-1.396189185	0.3231963617	0.477571836
0.9978741781	0.3409804046	0.081320849	0.228249883	-0.709017580	-0.4503033915	0.069111852
0.9978741781	0.2696217328	0.057412875	0.277131265	-0.697788501	-0.4866805053	0.171209792
0.9978741781	0.4723085457	-0.279272521	0.202099853	-0.828836606	-0.2719320776	-0.154204571
0.9978741781	0.139215933	0.305529713	0.628563667	-0.691342526	-0.7460195019	0.189205091
0.9978741781	0.1594325577	0.350479929	0.352074446	-0.676419802	-0.8006939752	0.483817944
0.9978741781	0.2883377937	-0.003852388	0.257257873	-0.684124745	-0.4598564577	0.124046890
0.9978741781	0.182025603	0.196609590	0.167403993	-0.672020455	-0.3615532404	0.374997008
0.9978741781	0.6503405313	-0.467108947	-0.260877732	-1.654907109	0.6159157314	0.353488100
0.9978741781	0.2267690656	0.157366552	0.388794700	-0.565014540	-0.6783139108	0.106309967
0.9978741781	0.2696981268	0.025880981	0.282206091	-0.672619751	-0.4681135677	0.126664944
0.9978741781	0.3704243594	-0.118891166	0.202174513	-0.782496249	-0.4038794091	0.088608258
0.9978741781	0.6266536602	0.307660166	0.154883557	-1.013594352	-0.388730704	-0.149441201
0.9978741781	0.392354592	-0.465848909	0.279098681	-0.688360025	-0.3092356269	-0.205317602
0.9978741781	0.3678408503	-0.116389368	0.27235472	-0.743842409	-0.5342794397	0.051615807

0.9978741781	-0.07761399214	0.564512130	0.738965936	-0.296812313	-0.6588923003	0.033661502
0.9978741781	0.3284019299	-0.119227978	0.220791527	-0.729253047	-0.4011450596	0.095382932
0.9978741781	0.2781272686	-0.437309326	0.280329451	-0.649255617	-0.1757041732	-0.093781999
0.9978741781	0.2958588748	0.026874389	0.239447757	-0.683534686	-0.4560499516	0.125998402
0.9978741781	0.2822696573	0.009448215	0.270008753	-0.681416911	-0.4604449525	0.119383410
0.9978741781	0.2916240523	-0.028236410	0.270795451	-0.671349405	-0.4722685234	0.095481437
0.9978741781	0.1731979959	0.288941135	0.261891404	-0.447278151	-0.7085493612	0.255813234
0.9978741781	0.284293741	0.012663777	0.258640224	-0.682452368	-0.4625843627	0.130728744
0.9978741781	0.3848272344	-0.118254521	0.194134718	-0.769249984	-0.3169269538	0.000196869
0.9978741781	0.3174082078	-0.057583180	0.258848257	-0.711805323	-0.4374169836	0.077835764
0.9978741781	0.2056603083	-0.078073328	0.371022900	-0.616312211	-0.5578702238	0.151781608
0.9978741781	0.2738165893	-0.036139279	0.283240212	-0.655240080	-0.5004503574	0.118100990
0.9978741781	0.3664113943	0.566412335	0.542010116	-0.773006450	-0.6349368796	-0.163205696
0.9978741781	0.2593950086	-0.035998186	0.273215509	-0.643031229	-0.4794486724	0.133548362
0.9978741781	0.6112663763	0.587712088	-0.416860864	-1.115073676	0.4365389444	0.143649286
0.9978741781	0.2800404739	0.028863406	0.256233291	-0.685485719	-0.4468801854	0.135942261
0.9978741781	0.5105160011	-0.318814056	0.162984983	-0.905436435	-0.5056044786	0.037864550
0.9978741781	0.3882965888	-0.125211586	0.215972028	-0.735618156	-0.4176485321	-0.006441989
0.9978741781	0.3827669225	-0.387400666	0.459985566	-1.107690545	-0.0908044137	-0.076952881
0.9978741781	0.5346457024	0.282330186	-0.015209485	-0.690005090	-0.4344512215	-0.082431588
0.9978741781	0.2141966178	0.215763933	0.342754016	-0.448944320	-0.7000107062	0.073899016
0.9978741781	0.2835479206	0.031827456	0.257963075	-0.683626067	-0.4685724616	0.138008049
0.9978741781	-0.03648894958	-0.419119187	0.354880457	-0.672006712	-0.4343447355	0.657295895
0.9978741781	0.3710448533	0.219717694	0.210123774	-0.746973150	-0.3491470212	0.006210201
0.9978741781	0.3129421958	-0.246347677	0.119187184	-0.825845191	-0.21449654	0.233889073
0.9978741781	0.3834526935	-0.337064080	-0.099541195	-1.071151908	-0.0034014064	0.474094858
0.9978741781	0.2891333699	-0.007639778	0.252401039	-0.689154802	-0.4478700693	0.126039324
0.9978741781	0.2843390947	0.030026269	0.26415145	-0.663887748	-0.4779480295	0.113737338
0.9978741781	-0.01412137948	-0.416276282	0.369446820	-0.697822704	-0.4411063478	0.625995660
0.9978741781	0.367115564	-0.284999295	0.196661519	-0.884956462	-0.1742091079	0.070847716
0.9978741781	0.2509190083	-0.146853717	0.245252332	-0.567943372	-0.5816077432	0.165420841
0.9978741781	0.2245695746	-0.202424820	0.235039172	-0.695745075	-0.3819401632	0.246691251
0.9978741781	0.2066229219	-0.287638671	0.413195917	-0.571885575	-0.4649209868	-0.003922438
0.9978741781	0.248511941	0.234795839	0.224682400	-0.793254930	-0.4049238169	0.328069319
0.9978741781	0.2675331891	0.202509851	0.309224586	-0.518530196	-0.650361013	0.046195843
0.9978741781	0.3863615275	0.213015341	0.139569603	-0.860161841	-0.3096881705	0.15342045
0.9978741781	0.09393936151	-0.451282686	0.501173024	-0.174377433	-0.9332403162	-0.011288579
0.9978741781	0.7649273469	0.684192389	-0.197695727	-0.926130785	-0.1717916878	-0.257495227
0.9978741781	-0.06642478324	-0.529109099	0.448682960	-0.504621858	-0.6400163002	0.557543166
0.9978741781	0.3078511083	-0.067758584	0.287945358	-0.680446217	-0.5461325889	0.097242111
0.9978741781	0.2900511089	-0.008051431	0.257322920	-0.684213356	-0.4608230684	0.121103676
0.9978741781	0.245092302	0.128716402	0.391761888	-0.659524515	-0.4717760609	0.039510093
0.9978741781	0.5236841921	0.311485352	0.213585187	-0.906561822	-0.5339962044	-0.029185834
0.9978741781	0.3016422658	-0.289289413	0.163574823	-0.696633445	-0.2622259033	0.096362456
0.9978741781	0.2935219197	-0.236998381	0.396508774	-0.923150935	-0.451463893	0.204853906
0.9978741781	0.2875575852	-0.000997119	0.256354482	-0.684275218	-0.4560418774	0.124524310

0.9978741781	0.3433017138	-0.239158958	-0.015159885	-0.737001083	-0.4268359637	0.360842682
0.9978741781	0.2998847086	0.015756450	0.260039025	-0.699795354	-0.4569123407	0.111920089
0.9978741781	0.164559039	0.13954545	0.460014413	-0.658225211	-0.5000379648	0.144011263
0.9978741781	0.3299845493	0.113810340	0.169716094	-0.680801266	-0.3838134102	0.088184673
0.9978741781	0.4831055171	0.400566193	0.043670761	-0.846232038	-0.5556034546	0.199474291
0.9978741781	0.2999007904	0.144757161	0.159116480	-0.663115173	-0.3472537723	0.121351850
0.9978741781	-0.00630339825	0.426853175	0.905954820	-0.265147964	-1.073567517	-0.08603991
0.9978741781	0.2450084296	-0.127956020	0.256214531	-0.634838246	-0.5502324883	0.217780752
0.9978741781	0.3036877553	0.041339201	0.251838692	-0.700548619	-0.4561468638	0.113823523
0.9978741781	0.4214115373	-0.782418146	-0.463196398	-1.002584601	-0.3491194616	0.953767275
0.9978741781	0.1617384478	-0.520497045	0.847309896	-0.482734205	-0.9682559403	-0.197866164
0.9978741781	0.196639767	0.244818294	0.626674602	-0.621267849	-0.7152758071	-0.02226433
0.9978741781	0.2960222987	-0.306431198	0.388673680	-0.686872610	-0.5683623711	0.026185250
0.9978741781	0.3151685946	-0.076868956	0.283947115	-0.683809601	-0.5436893436	0.088776183
0.9978741781	0.3354912525	-0.13622538	0.096419950	-0.813874371	-0.3166867175	0.263320912
0.9978741781	0.4218416865	-0.175531236	0.141132906	-0.757630392	-0.2481126643	-0.070384024
0.9978741781	0.5920503086	0.389325949	-0.182416879	-0.923087405	-0.0290953479	-0.007162975
0.9978741781	0.2690150861	0.017711437	0.276340306	-0.679597781	-0.4685292288	0.142622373
0.9978741781	0.2879381117	0.005296938	0.251043288	-0.684412863	-0.4549973956	0.129367762
0.9978741781	0.1314444899	-0.219671333	0.557193160	-0.662672011	-0.5337732545	0.126207351
0.9301794211	-0.678170974	-0.091150653	0.047057727	0.475370443	0.2992954962	0.658092324
0.7327832683	-0.7865199948	0.278099408	0.243461361	0.694857292	0.0866834375	0.550973065
0.6974568237	-0.6738015126	0.446986296	0.313588360	0.300923118	0.4286187592	0.449764746
0.6974568237	-1.220139576	0.59603059	0.662096421	1.761997264	-1.258877139	0.647336741
0.7327832683	-0.4287658946	-0.404400999	0.190849175	0.358967293	0.2546583859	0.127010179
0.7327832683	-0.8729714708	-0.273745888	0.440947790	0.625277581	-0.0800088611	0.680169046
0.6974568237	-1.444720562	-0.717304576	0.955265053	1.118410549	-0.5346570957	1.022922188
0.7327832683	-0.5519799202	-0.312015721	0.050810165	0.392876954	0.4867041883	0.365184191
0.867011594	-0.5964770339	-0.154720429	-0.078008125	0.386525374	0.3427035451	0.701693707
0.6974568237	-1.02590416	0.388027905	0.498242965	0.834789471	-0.1085599856	0.723630395
0.6974568237	-0.9263225465	0.359334488	0.511744514	0.844628142	-0.1548735649	0.519133506
0.6974568237	-0.9979144129	0.428464204	0.422781493	0.967746759	0.0209788127	0.529667816
0.957592049	-0.6918142932	-0.045964619	0.077600694	0.492498444	0.2511748823	0.662353708
0.6974568237	-1.109795125	0.455327779	0.404871693	0.857211641	-0.3840610357	1.150993714
0.9992515135	-0.7232561586	-0.000284442	0.135089462	0.519096303	0.1823508182	0.675032272
0.957592049	-0.7312713987	0.060236315	0.198138938	0.521522245	0.1740763061	0.621409369
0.6974568237	-1.096784038	0.484942260	0.656145228	0.882786052	-0.3611480847	0.792397733
0.6974568237	-0.850949126	0.624673721	0.450800518	0.718816815	-0.7628656315	0.942546789
0.6974568237	-0.8505939516	0.417696704	0.401497776	0.522629116	-0.2239686595	0.878347053
0.7327832683	-0.7706962909	0.32282024	0.271232974	0.312783120	0.3954520845	0.707517297
0.7327832683	-0.6763411184	-0.318413000	-0.134248383	0.256996791	0.4092500884	1.030998474
0.6974568237	-0.0129339485	0.655580021	-0.433421236	0.070432822	1.089719699	-0.219483227
0.90115955	-0.7524120642	0.127978149	0.165302566	0.424662531	0.3094925275	0.724182429
0.957592049	-0.7615669403	-0.063628518	0.198002268	0.583761596	0.0469418794	0.695698152
0.6974568237	-1.538472795	-0.668559409	0.764719379	1.550395655	-0.4963100291	0.948685833
0.7327832683	-0.7288085552	-0.223264196	0.129459484	0.548205422	0.1782236496	0.664251816

0.6974568237	-0.5329415986	0.447282268	-0.247372258	0.179951605	0.5007311988	0.890371921
0.6974568237	-0.9422221977	-0.557373312	0.841913166	1.167430097	-1.007797895	0.348435664
0.9090584336	-0.7187720612	-0.107174623	0.065284144	0.530101364	0.2328415223	0.703340938
0.7549949117	-0.6127695897	-0.220573107	0.071430734	0.353624779	0.4182528228	0.552042185
0.6974568237	-0.8279407247	-0.428574221	0.511957952	0.544262424	0.1102003784	0.474904100
0.6974568237	-0.5685422205	-0.500140435	0.074214697	0.281602855	0.1549231186	0.696584197
0.90115955	-0.6059495905	0.164205416	0.106672088	0.519104434	0.2097995561	0.447284545
0.7327832683	-0.63022941	0.250302238	-0.147233504	0.317530983	0.6812966567	0.717606892
0.7327832683	-0.7997793842	-0.216880398	0.14091706	0.608140828	0.1987378509	0.721262543
0.6974568237	-0.8941043703	-0.411985811	0.605580186	1.036179199	-0.445699978	0.316631249
0.9301794211	-0.7367211857	-0.080860622	0.200092033	0.550548833	0.0834606317	0.654978676
0.7327832683	-0.6344101453	0.268084079	0.161711991	0.511151954	0.2737457453	0.412100055
0.9700166179	-0.7009866067	-0.025796326	0.130023617	0.511792680	0.1807343254	0.643485537
0.9460836347	-0.766579987	0.087577053	0.225303569	0.670958793	0.0161814543	0.599633181
0.9460836347	-0.7679526498	-0.067183896	0.158778923	0.539504312	0.1779063857	0.721286632
0.6974568237	-0.5701974008	-0.513875709	-0.045566437	0.641635397	0.4578337783	0.264096969
0.867011594	-0.8265402978	0.155624296	0.165633314	0.599566215	0.0800207124	0.830615046
0.9274225769	-0.774561116	0.106574535	0.264694165	0.516363538	0.0813595652	0.697136276
0.867011594	-0.7893563127	0.182415532	0.184578088	0.522883033	0.0031067704	0.861440961
0.9301794211	-0.7595686288	0.086081753	0.12818471	0.519994756	0.2704124187	0.703258183
0.6974568237	-1.128603428	0.761584014	-0.211795147	0.565595286	0.5481049137	1.642003572
0.9308192561	-0.6379400072	-0.109410456	0.013778011	0.291501487	0.4336062486	0.728295324
0.7327832683	-0.631716945	-0.239964020	-0.065978479	0.337345227	0.5216660148	0.703836415
0.9700166179	-0.7090730679	-0.021365254	0.114218688	0.509441261	0.1926126242	0.673739527
0.9301794211	-0.6640392616	-0.083893837	0.096416831	0.449449295	0.2192750103	0.648947490
0.7327832683	-0.4185123752	0.275671202	0.043614313	0.223641380	0.2427574426	0.428660042
0.7327832683	-0.6681352235	-0.242423811	0.146594315	0.516668814	0.2588858051	0.502791266
0.6974568237	-0.2868551244	-0.625823162	0.217900070	0.197533908	-0.2387632192	0.278555256
0.6974568237	-0.9498376323	0.351307387	0.435036675	0.759900225	0.0562391586	0.617811501
0.6974568237	-0.6108147239	-0.321117323	0.037682928	0.397221912	0.33008767	0.594226575
0.7327832683	-0.718139015	0.253578355	0.121511310	0.498707578	0.0200616170	0.801898823
0.9274153647	-0.689580855	0.098461668	0.070944658	0.520880425	0.1820901893	0.677343517
0.7327832683	-0.9099299963	0.397838218	0.684943931	0.630851672	-0.0077981827	0.424954844
0.867011594	-0.7511568304	0.161017457	0.056113836	0.446327083	0.2757522539	0.844994590
0.6974568237	-0.5519103081	-0.435806701	0.127614892	0.161739275	0.5635816558	0.478027063
0.7327832683	-0.6670131027	-0.294473310	0.095760848	0.481151197	0.337415887	0.549038245
0.6974568237	-0.3469809938	-0.453312106	-0.105724625	0.192394608	0.7153773752	0.19521636
0.7327832683	-0.5646474526	-0.297212807	0.144866941	0.375773125	0.2780753036	0.429773147
0.6974568237	-0.1973307138	0.506497682	-0.604976462	0.079285489	0.8439198585	0.503170806
0.6974568237	-0.6004424608	0.343936081	-0.115136412	0.244471115	0.6063020786	0.743536952
0.7327832683	-0.7569247193	-0.240273227	0.014145094	0.556622960	0.2584876709	0.797243706
0.7685677253	-0.5630976465	0.210911999	0.039768161	0.278420492	0.3203780944	0.626630952
0.9090584336	-0.7879694012	-0.117440499	0.269713521	0.605023210	0.0042123200	0.671188269
0.7868476341	-0.6812319317	-0.181349327	0.139570179	0.527757668	0.125725314	0.608099968
0.6974568237	-0.3114711784	-0.586379570	-0.037756826	0.111760010	0.0911759887	0.514002011
0.7327832683	-0.9436487591	0.271812963	0.224024014	0.630788267	0.0994598720	0.960880119

0.6974568237	-0.8349975228	0.451213952	-0.101411902	1.012291769	-0.2427129292	0.910440794
0.9301794211	-0.6409279193	0.093585463	0.044854167	0.516932899	0.1896680434	0.605987413
0.7685677253	-0.7916547723	0.203954675	0.216176172	0.741160428	-0.0481766894	0.626246444
0.7327832683	-0.6963794527	-0.269401556	0.126491568	0.515092645	0.2896090518	0.567438634
0.6974568237	-1.076199132	-0.457880715	0.242055824	0.532083496	0.2061256319	1.256028093
0.957592049	-0.7404107132	-0.045062031	0.144671815	0.531798031	0.1606511866	0.699279237
0.7327832683	-0.748622688	0.239522773	0.269014242	0.656740231	-0.0521890259	0.569288300
0.8009553077	-0.7896272867	0.231733261	0.380157609	0.785015218	-0.1285616212	0.435065576
0.6974568237	-0.8297538291	0.335680312	0.334934651	0.742067394	-0.171344445	0.643165050
0.6974568237	-0.7498514686	0.339144026	0.216410034	0.746919215	-0.0662048187	0.544046820
0.6974568237	-1.019872094	-0.410645308	0.24604349	0.505337282	0.1971678181	1.16875613
0.957592049	-0.7371315714	0.049684472	0.145578408	0.553918283	0.1334382563	0.684347252
0.7327832683	-0.6589585241	0.263261411	0.156038056	0.310710535	0.4078599882	0.602960146
0.957592049	-0.710397597	0.041425560	0.139552174	0.521286455	0.1674095963	0.650012719
0.6974568237	-0.8632371778	-0.503286947	0.408455058	0.715199545	0.1668086999	0.448716515
0.9596538857	-0.7164405387	-0.041022382	0.140705840	0.537973617	0.1736396492	0.639427623
0.7327832683	-0.749368176	0.277304208	0.206650003	0.745585886	-0.0836990199	0.566506188
0.6974568237	-0.8936992594	-0.364630742	0.335994770	0.820315273	-0.0677708171	0.626747012
0.9674807393	-0.7361821077	-0.030594226	0.151625085	0.553452798	0.1501910982	0.665590204
0.6974568237	-0.4691962656	0.363280485	-0.106331489	0.390376173	0.333414023	0.471591762
0.9700166179	-0.7369604844	-0.020747171	0.142586382	0.525935008	0.1753316968	0.691799511
0.90115955	-0.7643542219	0.132155648	0.074614848	0.511819488	0.3584913677	0.729610575
0.7327832683	-0.8282515348	0.253084613	0.123340288	0.523226051	0.3365328081	0.807327686
0.8009553077	-0.791031926	0.210077984	0.355094959	0.558968295	0.1566787142	0.534803982
0.7105975083	-0.4533317152	0.354603300	0.085700774	0.265614143	0.0936711669	0.498959796
0.7327832683	-0.734366697	0.217956286	0.205418775	0.528359618	0.0366140029	0.696667115
0.6974568237	-0.7103978673	-0.442167099	0.395442922	0.072856433	0.1908721539	0.823240868
0.8009553077	-0.561351513	-0.189482103	0.021937216	0.480979844	0.1602240714	0.523994016
0.9992515135	-0.7228835939	-0.000972278	0.133961998	0.518683188	0.1826684764	0.675808815
0.6974568237	-0.2093655963	0.614252401	0.255498529	-0.093920408	0.1440357489	0.152537914
0.7327832683	-1.017112245	0.335874497	0.623828129	0.581118314	0.0763724483	0.719844052
0.9447216787	-0.6961340663	0.070949296	0.080686785	0.520939618	0.2275041603	0.651702312
0.6974568237	-0.9142657102	-0.389864368	0.342649168	0.676722422	0.279566185	0.602676404
0.9596538857	-0.7198594377	0.035702461	0.110938921	0.524067986	0.2093524069	0.673873071
0.7327832683	-0.9550409664	0.337868978	0.648778030	0.850494055	-0.3063355713	0.507554110
0.7868476341	-0.7884491102	-0.200491163	0.133916133	0.596047282	0.0347811633	0.819728670
0.6974568237	-0.9347556073	-0.515216594	0.198768937	0.724203055	0.1853229494	0.818110844
0.9992515135	-0.7220086473	-0.006418425	0.129160645	0.516285521	0.183426375	0.681647345
0.7327832683	-0.7904602823	-0.280501728	0.453219940	0.627403543	-0.0935510314	0.500683627
0.7549949117	-0.8185142549	0.259317155	0.526689211	0.585425846	-0.0921607521	0.518528529
0.867011594	-0.7274178442	0.141577560	0.074209081	0.520190592	0.2344988963	0.720652776
0.6974568237	-0.4853857407	-0.642039669	0.360560255	0.521120732	-0.5504970961	0.369638282
0.8009553077	-0.6406383726	-0.230301305	-0.136321999	0.299461941	0.4179440776	0.908157404
0.7685677253	-0.9213289387	0.257467568	0.304947392	0.626787716	-0.1222660673	0.961905459
0.9090584336	-0.6349018379	0.112473673	0.008138123	0.449849456	0.3058582868	0.636578391
0.6974568237	-1.213776717	0.491222910	0.672859284	0.643091859	-0.1670422811	1.154779561

0.7173585438	-0.8162310737	-0.400428104	0.58157901[illegible]	0.54438820[illegible]	0.1124730558	0.36871804[illegible]
0.6974568237	-1.051010503	-0.463922942	0.76915085[illegible]	0.564100664	0.0181415381	0.67672916[illegible]

se_intercept	se_microbe	se_blast	se_vns	se_interact	se_batch	t_intercept
0.7022242029	0.323133955	0.898159829	0.886462583	1.248600354	0.591995526	-4.606744503
0.6931857473	0.320732801	0.858040119	0.902929797	1.192462761	0.610612296	-4.420712064
0.6909612271	0.338444219	0.860350063	0.895971002	1.202934214	0.618166823	-4.523402157
0.7880416123	0.446886566	0.941983190	1.286144173	1.6119701	0.598362233	-3.891246061
0.7607474654	0.426492858	0.857323837	0.902385158	1.198718018	0.831626331	-4.070218732
0.6915813096	0.317216937	0.897356898	0.863284296	1.192525557	0.576562834	-4.172574564
0.7725316205	0.345256056	0.937010692	0.926829270	1.237703397	0.617456901	-3.784653885
0.662001679	0.346726217	0.786476469	0.821375188	1.143313631	0.645482985	-5.279747191
0.7485834028	0.353823197	0.979841640	0.931631771	1.244140749	0.597666571	-3.951813437
0.7349514519	0.326290139	0.902286567	0.919200934	1.212501308	0.595181879	-4.03217031
0.7178909612	0.342175513	0.923826454	0.935313928	1.233363302	0.613574439	-4.206727982
0.6981335863	0.343789856	0.847772221	0.918981565	1.148301738	0.582252416	-3.992267127
0.7275474076	0.344280456	0.951145947	0.901058797	1.292900014	0.600507623	-4.46083952
0.6441189253	0.328819506	0.740882927	0.780105909	1.080281601	0.606735286	-3.516158546
0.7121681546	0.332256422	0.853099694	0.913570636	1.215247537	0.632711988	-4.444341871
0.6961942602	0.408903669	0.955882414	0.885970037	1.196754243	0.699169640	-4.446400085
0.7394228877	0.329929345	0.925653869	0.920176138	1.25188306	0.603316806	-4.162454819
0.6969959435	0.363472930	0.872987716	0.892165361	1.314351142	0.616837758	-4.488368644
0.6990337595	0.340277669	0.878530393	0.882334637	1.235896504	0.617999863	-4.509155939
0.6940691014	0.334224799	0.864156831	0.909061462	1.212695519	0.597205824	-4.500759135
0.6964641526	0.418331475	0.924712020	0.949988333	1.23151529	0.758892066	-4.47864889
0.8070076457	0.468566239	0.872944473	0.86255012	1.260242217	0.836985151	-2.479558382
0.661168974	0.326859631	0.812665481	0.872338330	1.177055795	0.579621110	-4.519289733
0.689876385	0.352764223	0.880106542	0.911389232	1.357362001	0.576761903	-4.99362212
0.8375502481	0.386108890	0.927257534	1.066049221	1.255820381	0.617133693	-3.517039656
0.6945670338	0.301859919	0.855330219	0.887360262	1.196308546	0.598318771	-4.471060588
0.7061507624	0.334548018	0.898537527	0.918519597	1.214925992	0.617120320	-4.31395205
0.7096304963	0.375477649	0.978466879	0.987811170	1.439594227	0.636950156	-4.410266457
0.6733195944	0.307758400	0.852915578	0.859864529	1.168529133	0.585528837	-4.634083289
0.7044561972	0.329748363	0.848755833	0.908399489	1.231166409	0.617745312	-4.227671491
0.6975784928	0.340559379	0.903964020	0.884232324	1.194342076	0.617277315	-4.410975138
0.6975837629	0.311514231	0.851687194	0.894159521	1.190189963	0.595186731	-4.517083889
0.7599223241	0.432305241	0.858531873	0.886448532	1.198407298	0.846581310	-4.107137223
0.6693822743	0.335123335	0.893829874	0.881739408	1.31506604	0.569342667	-4.928495925
0.6891037699	0.304511443	0.838244105	0.877748899	1.17260712	0.589804240	-4.361493573
0.6499621346	0.358889487	0.880351085	0.924736849	1.219448471	0.627902340	-5.254023837
0.6947269689	0.321800011	0.891333762	0.892964041	1.256488262	0.601721123	-4.443723275
0.6874330431	0.352342774	0.835035341	0.864773863	1.173066921	0.678001083	-4.342297461
0.7698614802	0.387287054	0.858613305	0.892840069	1.196562799	0.761251692	-4.011723159
0.721408458	0.438518487	0.959985605	1.162073051	1.448269312	0.701949313	-4.110901012
0.7096982458	0.319630024	0.841164437	0.869496875	1.166109585	0.623406418	-4.692862584
0.6327796005	0.322590608	0.778542079	0.802027262	1.091098355	0.597199448	-5.243718798
0.7318179738	0.360575738	0.854476307	0.902151476	1.214494985	0.696339806	-4.102205031
0.6807074093	0.338869584	0.912147103	0.843416450	1.182740159	0.573461937	-4.205847662
0.683931086	0.401902589	0.830049446	0.852891691	1.216924333	0.707214539	-4.81840347
0.6291393612	0.301488863	0.759036561	0.786318534	1.10484007	0.539808052	-4.452257525

0.663014804	0.365909003	0.798043315	0.809209628	1.10564397	0.716815196	-4.08042866
0.7641408474	0.445628855	0.973175410	1.270051979	1.555940406	0.625474467	-3.645037899
0.6993648173	0.320206183	0.889482370	0.911750869	1.269557671	0.594346689	-4.545565893
0.7098626719	0.321198515	0.891558892	0.876674226	1.177166923	0.583942483	-4.069901662
0.7249642977	0.320957565	0.861083345	0.918483919	1.194786259	0.601468732	-4.468261218
0.7933688387	0.380645555	0.837999607	0.950502734	1.161695215	0.671725539	-4.538595003
0.6973909742	0.330118147	0.854007947	0.884995211	1.198805319	0.641466407	-4.482099832
0.7151115945	0.350043646	0.829089911	0.876597486	1.181565442	0.619899012	-3.936389279
0.7267436249	0.336526532	0.901117416	0.914818052	1.200697564	0.599786739	-4.199456829
0.6989690428	0.305033174	0.855701740	0.889240732	1.198071703	0.599832553	-4.370182532
0.692823887	0.336812067	0.853353702	0.884671060	1.21267093	0.620059714	-4.479587574
0.7003131652	0.317953446	0.876853787	0.883137022	1.191759098	0.595933430	-4.372641135
0.7196173343	0.406838700	1.022837286	0.892914356	1.210787757	0.649873010	-4.37235129
0.6877720035	0.360396637	0.861760980	0.889155804	1.198021977	0.702111443	-4.445200351
0.6693933259	0.315180370	0.810141565	0.878487879	1.166138058	0.584163977	-4.926668021
0.6872761957	0.310826432	0.844243313	0.874869342	1.190681578	0.604471831	-4.586005715
0.7390473882	0.377678667	0.848318232	0.896566298	1.235393079	0.701405093	-3.663827369
0.7057596925	0.333385595	0.841186930	0.886436202	1.181328304	0.649297319	-4.613556103
0.7781741637	0.357017304	0.991041035	0.926707935	1.267450335	0.601598065	-4.370978234
0.7035093	0.318834776	0.885871485	0.921929048	1.258702566	0.601298023	-4.393308983
0.6957003646	0.320745167	0.870045677	0.887535298	1.200084343	0.619819256	-4.455280504
0.7355928053	0.333896178	0.863738785	0.960161678	1.209376263	0.599643915	-4.053091058
0.7062465824	0.327483504	0.920761670	0.903870245	1.276754665	0.588051070	-4.617051765
0.6948067197	0.306517080	0.851225946	0.882347910	1.194420955	0.605873963	-4.515950429
0.7286237105	0.335323993	0.854902868	0.910400575	1.188875268	0.600944492	-4.083935131
0.7533364266	0.361382776	0.862715454	0.898127946	1.2003725	0.708369845	-4.197419434
0.6813898652	0.400621910	0.856297113	0.965518964	1.22097989	0.617098039	-4.723662382
0.7681468857	0.373643155	0.928056626	0.886525146	1.196652347	0.658319925	-4.033625691
0.7031863907	0.328160846	0.865147084	0.955844418	1.252506885	0.603204034	-4.407206205
0.6872737098	0.310425942	0.845574667	0.876349915	1.18903218	0.604122279	-4.550269468
0.7051733604	0.368894810	0.799278335	0.823652088	1.111584659	0.726672870	-3.667936086
0.7004711485	0.314999267	0.852319354	0.885249487	1.198245083	0.618262222	-4.342384406
0.5547106668	0.253666455	0.696897743	0.72209006	0.986184454	0.490089627	-5.430101569
0.6676109962	0.391034671	0.909875526	0.952492551	1.249233851	0.696394008	-4.392104835
0.6992943045	0.311872767	0.872473147	0.907561439	1.236862081	0.596906205	-4.388198628
0.6918987133	0.314843517	0.854856433	0.907586612	1.213186261	0.607757078	-4.473129201
0.7481074638	0.385531388	0.861335191	0.886270149	1.195972938	0.756675276	-4.197459557
0.6419757053	0.291661635	0.786485783	0.838243269	1.133921883	0.551155816	-4.581692439
0.6982639074	0.328551819	0.854630929	0.922705764	1.227444458	0.604101065	-4.474343107
0.6975054719	0.315295642	0.851342852	0.881855330	1.195374589	0.624219571	-4.519279532
0.6878986441	0.325740754	0.858182148	0.879578552	1.174583255	0.605232926	-4.397879242
0.692836206	0.343459944	0.851747795	0.895792225	1.191837104	0.664597686	-4.522273433
0.6951348183	0.322459634	0.859263555	0.924763260	1.235607729	0.611220735	-4.464732
0.6674964554	0.298199468	0.820481299	0.868862598	1.142833115	0.563548951	-4.30463519
0.6937327503	0.32410004	0.855314636	0.941710504	1.22004353	0.593611234	-4.285186462
0.7257499952	0.323676227	0.876030399	0.887591107	1.19537157	0.620893486	-4.450921646

0.6747987211	0.310019079	0.798914148	0.826525355	1.111575656	0.608381509	-4.024258174
0.6880180637	0.343408357	0.851558043	0.867647838	1.2567333	0.602359692	-4.363609652
0.6676584324	0.304079870	0.807483869	0.836808177	1.14430217	0.586629988	-4.34840010
0.7042737196	0.383044342	0.943347874	0.887923968	1.195117493	0.649404350	-4.458113353
0.7390904696	0.345663250	0.851973481	0.915540661	1.192894144	0.619088103	-4.37387795
0.6892698979	0.308639961	0.854426408	0.879635367	1.204782275	0.594266661	-4.519172994
0.6177063659	0.341395688	0.786719528	0.860268475	1.063862912	0.544120649	-5.065290564
0.7572365887	0.353419506	0.880950184	0.889301349	1.19703149	0.661029958	-4.090305446
0.6975960323	0.461744733	0.997956003	0.885321759	1.188081291	0.747578121	-4.543401345
0.7298886656	0.374918939	0.814989671	0.920663183	1.135514746	0.651034021	-4.909381231
0.7784164629	0.401548622	1.035862879	0.889615023	1.203043032	0.600577350	-3.986352483
0.6987900927	0.323004146	0.882587549	0.878082668	1.202446724	0.601751642	-4.332291032
0.6675545636	0.317884696	0.817085292	0.838436668	1.120855065	0.562119123	-4.245092652
0.6821465681	0.318224894	0.866411769	0.871108465	1.197972532	0.587020376	-4.514600821
0.7377528623	0.373715302	1.023958593	0.956043271	1.308426952	0.623805847	-4.35397668
0.595295851	0.289952123	0.723806388	0.758441273	1.034687491	0.547838445	-4.759136163
0.6759452141	0.297268554	0.819545118	0.856793878	1.145136837	0.578503438	-4.344337924
0.6503236768	0.414209237	0.882619364	0.842106792	1.11483975	0.708708823	-4.97317386
0.6977072692	0.347579856	0.939739867	0.894309447	1.241231751	0.634411949	-4.491364709
0.6352895718	0.351136573	0.931647856	0.799022933	1.134140401	0.576005149	-4.425751714
0.6913893695	0.308480410	0.861622743	0.882397017	1.196152305	0.603698028	-4.485993433
0.7053816723	0.342625916	0.862933898	0.885652905	1.257575139	0.619390880	-4.366801741
0.7101754765	0.42229261	0.988852912	0.972777970	1.270746664	0.734940729	-4.415683906
0.6276548461	0.340927039	0.737275108	0.741606122	1.061759199	0.620954343	-6.281314582
0.7385670118	0.333652718	0.930153325	0.905021422	1.244597882	0.605553788	-4.37761227
0.7953410138	0.396860079	0.953828479	0.885779904	1.22078352	0.709206601	-4.181318705
0.700241647	0.398986473	0.963591876	0.886256148	1.197544864	0.671083849	-4.412698893
0.7436237672	0.378466345	0.998841198	0.886376231	1.202627695	0.597564544	-4.250473816
1.112587184	0.511965688	1.423022891	1.404490054	1.978252452	0.937943513	0.513475908
1.083449876	0.501305625	1.341117391	1.411279994	1.863820247	0.954387507	0.381950669
1.088045475	0.532942641	1.354779338	1.410871055	1.894241063	0.973417304	0.438238577
1.22454541	0.694421315	1.463756703	1.99855175	2.504855779	0.929800806	0.654664981
1.177329098	0.660038285	1.326790225	1.396526907	1.855130208	1.287020889	0.688957903
1.093299207	0.501478309	1.418603383	1.364739074	1.88522626	0.911470105	0.710336011
1.211052249	0.541237552	1.468896388	1.452935573	1.940274603	0.96795076	0.200803663
1.11129366	0.582044818	1.320247881	1.378831909	1.919265811	1.083563943	0.163793692
1.178448033	0.557001730	1.542503413	1.466609632	1.958572971	0.940869105	0.425163853
1.149544337	0.510353413	1.411274733	1.43773065	1.896484467	0.930929460	0.618316151
1.12120419	0.534410711	1.442834842	1.460776011	1.926270389	0.958282342	0.571837791
1.06930834	0.526571659	1.298504934	1.407573953	1.758816148	0.891816948	1.007068846
1.145588583	0.542100426	1.497664518	1.418797813	2.035786918	0.945553059	0.516611203
0.9831469106	0.501891606	1.130842043	1.190709797	1.648881094	0.926086625	1.902438928
1.116186102	0.520747802	1.337069085	1.431845612	1.904665918	0.9916539	0.488070219
1.090002069	0.640203275	1.496584889	1.387126021	1.873707779	1.094660497	0.449713205
1.133828963	0.505912724	1.419395023	1.410995487	1.91963394	0.925124282	0.103773797
1.053490592	0.549379542	1.319497416	1.348483909	1.986606343	0.932333654	0.323235191

1.061223572	0.516585471	1.333722656	1.33949799	1.876250589	0.938203646	0.271950793
1.060560197	0.510706381	1.32045979	1.389075528	1.853038258	0.912549954	0.378845604
1.077147604	0.646989144	1.430154493	1.469246699	1.904654731	1.173698269	0.383287863
1.334230053	0.774683067	1.443243763	1.426058722	2.083565191	1.383792023	1.262686707
1.090780255	0.539244951	1.340715453	1.439162247	1.941877599	0.956244601	0.481060034
1.112877069	0.569063129	1.419747669	1.470211474	2.189634372	0.930405954	0.104100485
1.310799597	0.604275838	1.451195083	1.668409617	1.965409064	0.965838884	0.185830185
1.074511942	0.466984571	1.323216466	1.372767713	1.850718155	0.925613561	0.457111616
1.103338167	0.522720667	1.403936383	1.435157735	1.898283324	0.964230925	0.535139698
1.107032692	0.585749957	1.526420903	1.540998118	2.24578549	0.993650427	0.355118793
0.8250738823	0.377121682	1.045147614	1.053662735	1.431894863	0.717496646	0.512507182
1.109015158	0.519118059	1.336183979	1.43008012	1.938207393	0.972507471	0.570841061
1.09427288	0.534226465	1.418024383	1.387071794	1.873532737	0.968306553	0.428684780
1.089476459	0.486518522	1.33015302	1.3964857	1.858821858	0.929554223	0.518830237
1.188651463	0.676201028	1.34289405	1.386560589	1.874518676	1.324201279	0.457154837
1.058603728	0.529985369	1.413559447	1.394438814	2.079729126	0.900394727	0.198600240
1.097846765	0.485132889	1.335449927	1.398387053	1.868140895	0.939647561	0.487122545
1.005469948	0.555190180	1.361874043	1.430537354	1.886446496	0.971344175	-0.02658353
1.045348214	0.484209023	1.341180346	1.343633409	1.890624403	0.905403316	0.567518760
1.088042319	0.557674458	1.321661504	1.368730481	1.856684757	1.073113779	0.571836133
1.195017649	0.601166414	1.33278269	1.385911191	1.857364864	1.181653105	0.152453546
1.131712351	0.687927599	1.505981187	1.823006661	2.271978169	1.101185741	0.592718359
1.106353922	0.498273644	1.311297554	1.355465205	1.817856984	0.971832943	0.097179043
0.9232247757	0.47065936	1.13589208	1.170156938	1.591911359	0.871313371	0.073475398
1.14322737	0.563282221	1.33484109	1.409318024	1.897253085	1.087804283	0.587679031
1.11050962	0.552833608	1.488081544	1.375953998	1.929528468	0.935548797	0.575566413
1.043124914	0.612977846	1.265983189	1.300821955	1.856040931	1.078636607	0.100793799
1.076925835	0.516071901	1.299276652	1.345976417	1.891203902	0.924013459	0.680028406
1.040500039	0.574238056	1.252406575	1.269930391	1.735138623	1.124931502	1.057074455
1.215943878	0.709109688	1.548571427	2.020978114	2.475899853	0.995290138	0.560063816
1.102373534	0.504724878	1.402046257	1.43714697	2.001139808	0.936838749	0.399700831
1.07103987	0.4846239	1.34518289	1.322724924	1.776107911	0.881051653	0.940997970
1.139583187	0.504518423	1.353550935	1.443779832	1.878103979	0.945458497	0.301784049
1.252016118	0.600697112	1.322447977	1.499989268	1.833272322	1.060050712	-0.146023973
1.057377928	0.500522168	1.294839175	1.341821787	1.817617851	0.972585602	0.585545770
1.149639178	0.562742785	1.332874829	1.409249719	1.899527199	0.996571998	0.586305746
1.116709141	0.517104302	1.384650682	1.405702983	1.844983429	0.921628084	0.158855924
1.092860421	0.476929110	1.337916999	1.390356284	1.87322337	0.937857353	0.519727409
1.086482428	0.528186743	1.338224361	1.38733606	1.901703566	0.972374071	0.443431604
1.062214889	0.482262652	1.329986634	1.339516864	1.807625961	0.903894706	0.645177147
1.127119919	0.637222008	1.602046288	1.398551021	1.896428745	1.017881005	0.416064167
1.090127765	0.571233458	1.365902606	1.409323766	1.898880754	1.112855968	0.462249168
1.065810999	0.501831572	1.289910965	1.398732268	1.856730147	0.930108455	0.218676491
1.088947604	0.492485701	1.337652518	1.38617761	1.886563014	0.957749091	0.420486093
1.195652303	0.611019504	1.372433844	1.450490966	1.998654759	1.134753506	0.239026439
0.8899499197	0.420393070	1.060721162	1.117779657	1.489633144	0.818751911	-0.34551339

1.214985165	0.557421138	1.547340187	1.446895112	1.978905785	0.939291945	-0.009214527
1.090952204	0.494426301	1.373746515	1.429662021	1.951906447	0.932450223	0.544471042
1.021055762	0.470746771	1.276936447	1.302605369	1.76132297	0.909687639	0.583206205
1.137794215	0.516461196	1.336006805	1.485151017	1.870629111	0.927512303	0.719675180
1.121273642	0.519929768	1.461848903	1.435031201	2.027041812	0.933620327	0.515727571
1.070044039	0.472054695	1.310939034	1.358868725	1.839479939	0.933082257	0.547253313
1.147769228	0.528221297	1.346691291	1.434114414	1.872783482	0.946641712	0.466232389
1.170006294	0.561263345	1.339882789	1.394881905	1.864297718	1.100168728	0.181975677
1.065398271	0.626398941	1.33887736	1.509652973	1.909083082	0.964873735	0.285506481
1.195051658	0.581298812	1.443832725	1.379219737	1.861703013	1.024187344	0.614082192
1.068414379	0.498604313	1.314495839	1.452300463	1.903046452	0.916502186	0.274948048
1.085736211	0.490402413	1.335815735	1.38443363	1.878400519	0.954375855	0.432015111
1.187652207	0.621292238	1.346143704	1.387193952	1.872129674	1.223861658	0.408585975
1.05633989	0.475032114	1.285333358	1.33499338	1.807003875	0.932365379	0.695593824
1.004521145	0.459362572	1.262006595	1.307627161	1.785873603	0.887499417	0.573590843
1.087427609	0.636930637	1.482036356	1.551452423	2.034794795	1.134310362	0.572450459
1.097613481	0.489516004	1.369435275	1.424509916	1.94138074	0.936904953	0.452101873
1.075123509	0.489227196	1.328339294	1.410275355	1.885138741	0.944377999	0.424204675
1.163755617	0.599732445	1.339892616	1.378681425	1.860454937	1.177083702	0.224681750
1.077683883	0.489612053	1.320272785	1.407158019	1.903513369	0.92522464	0.556152693
1.087237669	0.511574364	1.330710252	1.436706743	1.91120268	0.940620626	0.385682652
1.09693582	0.495851427	1.338869024	1.386854641	1.879912428	0.981682345	0.422815130
1.071905597	0.507579628	1.337246783	1.370587341	1.830273074	0.943093241	0.320872113
1.084691961	0.537714739	1.3334811	1.402436271	1.865918833	1.04048224	0.407623079
1.059923687	0.491678155	1.310182963	1.410055228	1.884022877	0.931973652	0.409147142
1.106524695	0.494332326	1.360131298	1.440334124	1.894501542	0.934208454	0.525231452
1.096386277	0.512212860	1.35175286	1.488294265	1.928176207	0.938152639	0.301108872
1.112431881	0.496131941	1.342782159	1.360502448	1.832269311	0.951707493	0.092901958
1.141648627	0.524501374	1.351631548	1.398345177	1.880603478	1.029281611	0.406481127
1.064926367	0.531533449	1.318056402	1.342960467	1.945193736	0.932342844	0.638570035
1.05501852	0.480500027	1.275967464	1.322305063	1.808200007	0.926979234	0.732458362
1.103634028	0.600250668	1.478275826	1.391423644	1.872812084	1.017650835	0.418830876
1.16008263	0.542555951	1.337264757	1.437040338	1.872376703	0.971725904	0.528581498
1.084641202	0.485678570	1.344532947	1.384201987	1.895856034	0.935143269	0.438187235
1.015765417	0.561396081	1.293693143	1.414638111	1.749431791	0.894759983	0.447673521
1.156276081	0.539660296	1.345182791	1.357934751	1.827828846	1.009371631	0.826021832
1.099542806	0.727796714	1.572966722	1.395433929	1.872640004	1.178323998	0.412300005
1.173323887	0.602696504	1.310127001	1.480001205	1.825383293	1.046562037	-0.03746881
1.217091943	0.627840773	1.619621917	1.390956294	1.881016206	0.939031854	0.318376856
1.100875781	0.508861596	1.390430787	1.383333784	1.894337785	0.948001147	0.498572852
1.078168404	0.513416063	1.319675715	1.354160355	1.810294743	0.907879462	0.739963153
1.051831363	0.490684758	1.335957866	1.343199904	1.847205778	0.905152165	0.510429274
1.156416936	0.585793329	1.605040276	1.498583994	2.050940313	0.977806638	0.309782632
1.080368297	0.526217478	1.3135947	1.376451568	1.877794983	0.994240573	0.588696200
1.093016556	0.480689033	1.325220395	1.385452363	1.851708536	0.935451310	0.319334242
1.085926479	0.691656777	1.473819534	1.406170645	1.861586849	1.183419434	0.385926104

1.09303794	0.544523451	1.472209583	1.401037654	1.944530973	0.993878608	0.412576852
1.092213642	0.603687156	1.601723911	1.373710173	1.949856684	0.990289639	0.610589151
1.033554918	0.461146004	1.288036037	1.319091408	1.788122804	0.902465519	0.487430246
1.102785053	0.535657153	1.34910027	1.384618886	1.966077544	0.968348104	0.388717000
1.106807523	0.658142463	1.541125931	1.516073156	1.980456963	1.145404136	0.339466781
1.097126398	0.595932711	1.288740124	1.296310638	1.855930934	1.085414072	-0.393108978
1.161485617	0.524709102	1.462778179	1.423255235	1.957280132	0.952306295	0.469403705
1.24929583	0.623374922	1.498242793	1.391354302	1.9175671	1.113998693	0.198429543
1.096398262	0.624710167	1.508736966	1.387649114	1.875047154	1.050744653	0.440762068
1.164382386	0.592610894	1.564007431	1.387907321	1.883100792	0.935679655	0.376449307
0.7075077211	0.325565207	0.904917562	0.893132306	1.257994793	0.596449686	-0.250446771
0.6908055355	0.319631492	0.855093842	0.899829381	1.188368168	0.608515619	-0.169374492
0.667555207	0.326979563	0.831206067	0.865620362	1.162185326	0.597226683	-0.153959648
0.7544761032	0.427852069	0.901860759	1.231362695	1.543310531	0.572875847	-0.792585842
0.7364614002	0.412877521	0.829954673	0.873577458	1.160450203	0.805077533	-0.625953826
0.6781549292	0.311058478	0.879935584	0.846524469	1.16937383	0.565369426	-0.586939448
0.7638540818	0.341377933	0.926485626	0.916418568	1.22380077	0.610521254	0.111288483
0.7197762618	0.376985902	0.855114286	0.893058705	1.243093542	0.70181594	-0.171009631
0.7446795308	0.351978004	0.974731753	0.9267733	1.237652539	0.594549733	-0.032294372
0.6934415481	0.307861341	0.851325611	0.867284658	1.144019489	0.561566131	-0.770510421
0.6516591352	0.310606779	0.838595248	0.849022899	1.119574568	0.556966739	-0.788037155
0.7169035543	0.353032965	0.870565362	0.943689236	1.179174894	0.597906811	0.037441267
0.7275602218	0.344286520	0.951162699	0.901074667	1.292922786	0.600518200	-0.276951742
0.7437281254	0.379669507	0.855456111	0.900744696	1.247340791	0.700563326	-0.679253457
0.7083325179	0.330466936	0.848505020	0.908650274	1.208702386	0.629304291	-0.262779114
0.6102072475	0.358399942	0.837821295	0.776543802	1.048943024	0.612815138	-0.435072312
0.6970779625	0.311035105	0.872643955	0.867479920	1.180190805	0.568766340	0.312233377
0.693304205	0.361547744	0.868363812	0.88743988	1.307389493	0.613570589	-0.196940907
0.6691359079	0.325723907	0.840955425	0.844596960	1.183036896	0.591567851	-0.019230492
0.627581882	0.302208279	0.781376334	0.821979399	1.096527326	0.539997465	-0.384025581
0.6867197341	0.412478486	0.911774124	0.936696789	1.214284826	0.748274202	-0.161655999
0.8864323352	0.514681946	0.958858584	0.947441230	1.384273689	0.919360189	-0.277255343
0.6448943276	0.318813995	0.792661754	0.850865759	1.148082616	0.565353761	-0.020937601
0.7178026708	0.367044164	0.91573337	0.948282386	1.412308191	0.600109300	-0.407350222
0.8326650318	0.383856816	0.921849078	1.059831228	1.248495502	0.613534110	-0.033892845
0.6858422163	0.298068100	0.844585972	0.876213668	1.181281093	0.590802978	-0.219206426
0.6954656235	0.329485796	0.884941282	0.904620994	1.196542307	0.607782348	-0.356615387
0.6416332666	0.339499150	0.884709582	0.893158497	1.301651424	0.575917201	-0.662758100
0.621722035	0.284174381	0.787555291	0.793971732	1.078982873	0.540658824	-0.195096092
0.6778701798	0.317303735	0.816723980	0.874116698	1.184702467	0.594431744	-0.547267991
0.6712646633	0.327712908	0.869864982	0.850877599	1.149289492	0.593992581	-0.072854798
0.6951814302	0.310441441	0.848754161	0.891080223	1.186091198	0.593137033	-0.174821286
0.7513646342	0.427436935	0.848863714	0.876465997	1.184911711	0.837047730	-0.368316927
0.7017766154	0.351341422	0.937086218	0.924410641	1.378707848	0.596895654	-0.195068247
0.6723409186	0.297104024	0.817853328	0.856397145	1.144082768	0.575456907	-0.017587523
0.699031991	0.385984382	0.946814497	0.994551231	1.311512544	0.675306760	-0.036279787

0.6610065876	0.306180610	0.848070558	0.849621707	1.195501335	0.572515022	-0.332064962
0.6777741662	0.347392132	0.823302557	0.852623234	1.156584575	0.668474731	-0.41603886
0.7643693355	0.384524172	0.852488010	0.886470603	1.188026593	0.755820969	-0.22644360
0.7120469393	0.432827954	0.947528136	1.146993149	1.42947552	0.692840310	0.041439374
0.6943860197	0.312733787	0.823015737	0.850736884	1.140949971	0.609955997	0.188944135
0.5680389384	0.289585862	0.698888232	0.719970609	0.979466390	0.536099047	0.237694118
0.7285414073	0.358961334	0.850650562	0.898112276	1.20905733	0.693222086	-0.10988457
0.6971000121	0.347030145	0.934113170	0.863727366	1.211222572	0.587271885	0.006368950
0.6484055923	0.381026527	0.786934113	0.808589862	1.153713523	0.670479631	0.194722389
0.6928380613	0.332013815	0.835887010	0.865931210	1.216702212	0.594462193	-0.381569924
0.7146344981	0.394397222	0.860175791	0.872211469	1.19172501	0.772623574	-0.01807365
0.7573403509	0.441662966	0.964514603	1.25874911	1.542093264	0.619908037	0.245312387
0.6998824159	0.320443167	0.890140674	0.912425654	1.270497268	0.594786564	-0.239743426
0.721201491	0.326329102	0.905799992	0.890677569	1.195970113	0.593269946	-0.127272076
0.7195657329	0.318567502	0.854671148	0.911644279	1.185889088	0.596989798	-0.392586563
0.8038654592	0.385681664	0.849086712	0.963078305	1.177064956	0.680612765	0.191872459
0.6874400517	0.325407762	0.841822291	0.872367404	1.181699822	0.632313460	-0.279643098
0.7278259713	0.356267272	0.843830773	0.892183012	1.202573168	0.630920550	-0.400299488
0.6334021811	0.293303762	0.785379764	0.797320719	1.046482459	0.522751375	0.520769862
0.6699985422	0.292390320	0.820235065	0.852383951	1.148414658	0.574971009	-0.422522188
0.6837887653	0.332419698	0.842225110	0.873134058	1.196856479	0.611973510	-0.208707068
0.6692472518	0.303849021	0.837956527	0.843961037	1.138892628	0.569497805	-0.429211685
0.6324641573	0.357566283	0.898961005	0.7847731	1.06414871	0.5711666	-0.874653821
0.6300593184	0.330154845	0.789448441	0.814544496	1.097492929	0.643195499	-0.04193236
0.7012753063	0.330191835	0.848727127	0.920328651	1.221679082	0.611986639	-0.240633123
0.6865461909	0.310496281	0.843346583	0.873940081	1.18941687	0.603829778	-0.162706743
0.7614894527	0.389147335	0.874078437	0.923791614	1.272907278	0.722704103	-0.202221072
0.6590320138	0.311312452	0.785492743	0.827746103	1.10311368	0.606307961	0.252309395
0.7839387296	0.359662020	0.998382478	0.933572811	1.276839365	0.606054588	-0.254484247
0.6669295263	0.302256595	0.839809580	0.873992289	1.193254881	0.570032842	-0.451754451
0.6830048005	0.314892013	0.854168553	0.871339013	1.178184472	0.608508416	-0.260632681
0.7157678461	0.324897343	0.840460164	0.934284364	1.176782367	0.583482913	-0.569114482
0.6860322525	0.318110206	0.894407447	0.877999491	1.240211139	0.571219756	-0.548750544
0.6930962392	0.305762494	0.849130392	0.880175739	1.19148052	0.604382418	-0.20147928
0.7209498038	0.331792342	0.845898982	0.900812184	1.176353966	0.594615311	-0.421497097
0.7062443952	0.338792273	0.808785999	0.841984810	1.125335667	0.664088733	0.419925651
0.6941927671	0.408149352	0.872386416	0.98366048	1.243921361	0.628692937	-0.272439138
0.7465300625	0.363128268	0.901939700	0.861576978	1.162976728	0.639793800	0.213758824
0.6907579085	0.322360761	0.849856024	0.938950326	1.230369426	0.592542692	-0.10585665
0.6836337483	0.308781854	0.841096307	0.871708562	1.182734789	0.600922707	-0.18565482
0.7269260649	0.380274224	0.823933925	0.849059543	1.145874061	0.749088776	0.304565676
0.695236318	0.312645183	0.845949717	0.878633752	1.189290239	0.613641763	-0.305243255
0.6894267249	0.315271445	0.866145105	0.897455583	1.225687478	0.609111932	-0.224987811
0.6859909702	0.401800232	0.934925276	0.978715589	1.283626463	0.715566406	-0.059501804
0.6652090909	0.296671371	0.829946798	0.863324806	1.176574577	0.567811623	-0.422507665
0.6850153865	0.311711309	0.846351928	0.898557523	1.201116925	0.601710831	-0.194015205

0.7032925687	0.362436379	0.809737461	0.833178707	1.124328951	0.711347132	0.373817538
0.6877602279	0.312462404	0.842576497	0.898025232	1.214791099	0.590463232	-0.300941176
0.6622181327	0.311591319	0.810513177	0.875073853	1.164081328	0.572916164	-0.056097203
0.6940448094	0.313731307	0.847118928	0.877480019	1.189443756	0.621122515	-0.263577083
0.6248400803	0.295880622	0.779513969	0.798948999	1.066911095	0.549752196	0.061136314
0.6792590544	0.336729337	0.835056535	0.878237849	1.168481291	0.651573910	-0.144250655
0.6815597495	0.316162422	0.842483268	0.906703850	1.211477935	0.599284398	-0.182791968
0.7009036124	0.313123886	0.861545109	0.912347817	1.20003013	0.591753698	-0.315744035
0.6815522568	0.318409522	0.840297103	0.925176041	1.198622122	0.583188664	-0.450762706
0.7251078573	0.323389841	0.875255294	0.886805773	1.194313914	0.620344124	-0.265435438
0.7042877286	0.323567053	0.833827055	0.862644884	1.160152012	0.634968054	0.131269285
0.652405679	0.325633256	0.807480694	0.822737668	1.191683744	0.571181055	0.047229496
0.6994414283	0.318555190	0.845923069	0.876643323	1.19877516	0.614555732	-0.130690172
0.6119514882	0.332831609	0.819685755	0.771527289	1.038451255	0.564274866	-0.688700899
0.7246335114	0.338901914	0.835308478	0.897632254	1.169560572	0.606978447	-0.516341209
0.6770391375	0.303163294	0.839265026	0.864026663	1.183403997	0.583721687	-0.237290382
0.663337571	0.366615270	0.844836074	0.923818229	1.142452594	0.584315930	-0.241822148
0.7117506072	0.332190166	0.828032926	0.835882450	1.125127738	0.621322954	-0.828034348
0.6538124239	0.432763991	0.935320734	0.829755817	1.113513083	0.700657459	-0.493458334
0.7290241082	0.374474845	0.814024311	0.919572652	1.134169723	0.650262867	0.413478535
0.7249135184	0.373948957	0.964664854	0.828469062	1.120354204	0.559297832	-0.967817640
0.700061265	0.323591724	0.884193068	0.879679992	1.204634101	0.602846292	-0.239820068
0.7022343706	0.334398972	0.859533298	0.881993889	1.179084069	0.591321504	-0.057170412
0.6217923438	0.290069337	0.789754328	0.794035475	1.091979617	0.535082624	-0.329778202
0.6832277536	0.346095121	0.948280873	0.885384971	1.211725027	0.577702221	-0.850087119
0.6964943601	0.339243114	0.846851302	0.887374015	1.210581261	0.640969337	-0.155221810
0.6995613189	0.307654492	0.848178302	0.886728455	1.185145511	0.598715132	-0.153135374
0.6900763187	0.439528800	0.936571654	0.893582651	1.182987085	0.752030402	-0.265023649
0.6867503192	0.342121385	0.924981983	0.880265013	1.221739172	0.624449003	-0.130223168
0.6811859586	0.376504374	0.998954596	0.856748209	1.216076181	0.617618542	-0.460432348
0.6885926893	0.307232602	0.858137466	0.878827708	1.191313851	0.601256061	-0.209411942
0.6781303913	0.329389117	0.829595841	0.851437136	1.208990756	0.595461715	-0.000003847
0.7058344094	0.419711275	0.982808382	0.966831701	1.262979011	0.730448280	-0.203687239
0.7482702462	0.406442428	0.878956054	0.884119352	1.265795719	0.740282119	-0.525005992
0.7305038987	0.330010151	0.919998618	0.895141087	1.231010308	0.598942812	-0.425876375
0.7338823437	0.366193369	0.880122951	0.817332717	1.12644948	0.654403826	-1.155213877
0.5993639003	0.341507949	0.824775544	0.758580904	1.025024952	0.574406614	-0.602665539
0.6559835847	0.333861988	0.881122226	0.781911879	1.060891355	0.527138251	-1.079409099
0.7398632845	0.340453873	0.946301023	0.933976806	1.315525091	0.623726372	0.250879930
0.6282680758	0.290695792	0.777683639	0.818369345	1.080787202	0.553427727	0.169043044
0.7156807217	0.350552235	0.891129530	0.928024827	1.245969807	0.640282060	0.446423069
0.7866970581	0.446124089	0.940375982	1.283949758	1.609219761	0.597341309	1.047173879
0.7959678888	0.446238253	0.89701547	0.944163000	1.254215221	0.870128243	0.332509488
0.7501280913	0.344071380	0.973323900	0.936366834	1.293480474	0.625372566	0.431615298
0.7995890136	0.357348414	0.969828853	0.959290833	1.281053114	0.639082908	0.746424344
0.6951274296	0.364075971	0.825830785	0.862475793	1.200523641	0.677782161	0.988399430

0.7859963335	0.371506681	1.028812466	0.978193149	1.306320797	0.627536934	0.234044254
0.7699192089	0.341814477	0.945215848	0.962934972	1.270190086	0.623499634	0.550345540
0.75345641	0.359127427	0.969594272	0.981650853	1.294466062	0.643971883	0.451446108
0.7554994175	0.372039165	0.917433901	0.994494536	1.242658012	0.630096259	0.621294416
0.7662299013	0.362585279	1.001716806	0.948966605	1.361641372	0.632435621	0.282285034
0.8053607686	0.411132665	0.926347636	0.975389280	1.350707744	0.758618909	0.461495115
0.72150777	0.336613746	0.864287534	0.925551512	1.231184707	0.641009588	0.107548752
0.7246956765	0.425643729	0.995015174	0.922240662	1.245748026	0.727792865	0.355493606
0.772852643	0.344845650	0.967503239	0.961777857	1.308481449	0.630593123	0.287126022
0.7297671453	0.380562620	0.914033661	0.934112995	1.376149014	0.645840100	0.420590224
0.6708247653	0.326546013	0.843077944	0.846728670	1.186022808	0.593060932	0.122756600
0.6939881127	0.334185800	0.864055995	0.908955386	1.212554013	0.597136138	0.309126067
0.7253941831	0.435708309	0.963123110	0.989449361	1.282670507	0.790415254	0.438894122
0.8715143312	0.506020228	0.942721699	0.931496492	1.360977382	0.903888034	-0.78957323
0.7142981328	0.353124894	0.877968354	0.942436298	1.271639762	0.626197376	0.286997933
0.7623913091	0.389844302	0.972617115	1.007188019	1.50003829	0.637387034	0.472776364
0.8654050096	0.398949876	0.958095728	1.101503269	1.297585729	0.637657969	-0.123936712
0.7267916038	0.315864768	0.895013428	0.928529510	1.251811509	0.626077885	0.394830485
0.7415991263	0.351342137	0.943643596	0.964628755	1.275914581	0.648099407	0.415898093
0.7194225339	0.380658784	0.991968531	1.001441763	1.459458876	0.645739293	0.152421788
0.7140470974	0.326373975	0.904506416	0.911875692	1.239210685	0.620946086	0.385358523
0.6308266226	0.295283152	0.760044099	0.813453815	1.102485223	0.553178736	-0.227821554
0.6848469424	0.334343807	0.887465714	0.868094142	1.172544061	0.606011347	0.212110935
0.6857281038	0.306219947	0.837212498	0.878962994	1.169962304	0.585071342	0.659000581
0.7665192351	0.436058097	0.865984817	0.894143821	1.208810712	0.853930510	0.887819514
0.7393387543	0.370146744	0.987243151	0.973889122	1.452502293	0.628844107	0.412121667
0.7289071673	0.322100361	0.886662013	0.928448649	1.240338207	0.623871986	0.491987243
0.7286041901	0.402313259	0.986869012	1.036625224	1.366995427	0.703875275	0.158417692
0.6879396231	0.318656088	0.882625606	0.884239958	1.244212619	0.595842426	0.541977655
0.7364081383	0.377444886	0.894526132	0.926383329	1.256640244	0.726304212	0.430613962
0.8051316433	0.405030087	0.897949513	0.933744330	1.251381707	0.796127410	0.425446439
0.7479290663	0.454639420	0.995276849	1.204793487	1.501510971	0.727754559	0.655494247
0.7609829089	0.342727331	0.901949193	0.932329008	1.250375732	0.668455406	0.448078926
0.7348632141	0.374632764	0.904140927	0.931414874	1.267120564	0.693543069	0.366900570
0.7591602298	0.374047605	0.886401335	0.93585775	1.259871068	0.722356523	0.622146059
0.7366291256	0.366708518	0.987082135	0.912705097	1.279905048	0.620573185	0.188990535
0.7324251648	0.430399460	0.888904036	0.913365908	1.303210255	0.757359529	0.217677607
0.7422066014	0.355671634	0.895448578	0.927633594	1.303398967	0.636820909	0.388487239
0.7563444857	0.417416406	0.910380367	0.923118513	1.261280616	0.817718122	0.240664943
0.796718813	0.464627553	1.014665242	1.324198685	1.622275524	0.652140606	0.816273597
0.7342524285	0.336179576	0.933853940	0.957233297	1.332889187	0.623995501	0.308843466
0.7610931468	0.344379270	0.955902303	0.939943417	1.262122538	0.626085353	0.354356267
0.7626332481	0.337634433	0.905825005	0.966208097	1.256867032	0.632720886	0.485717558
0.8456207305	0.405715169	0.893190916	1.013103587	1.23820537	0.715965908	0.741057589
0.6987306089	0.330752279	0.855648431	0.88669522	1.201108132	0.642698615	0.561524838
0.7714252857	0.377608924	0.894379180	0.945627887	1.274611495	0.668714892	0.476832762

0.713592013	0.330436535	0.884810225	0.898262926	1.178968982	0.588932619	-0.10876521
0.7329579572	0.319866087	0.897312128	0.932482028	1.256330587	0.629000736	0.448050159
0.6997866612	0.340196977	0.861929777	0.893561869	1.224858088	0.626291219	0.397445798
0.7354974038	0.333927656	0.920907554	0.927506463	1.251633935	0.625873555	0.402256858
0.7538043994	0.426166502	1.071429508	0.935334292	1.268308995	0.680746712	0.374460082
0.729714849	0.382374938	0.914314308	0.943379768	1.271081727	0.744928760	0.399641110
0.7272993633	0.342445127	0.880223064	0.954481694	1.267015123	0.634697228	0.238138522
0.7294511599	0.329900414	0.896050624	0.928556031	1.263748204	0.641565474	0.389736498
0.800888061	0.409281381	0.919302273	0.971587554	1.33876607	0.760096001	0.480500650
0.7505013425	0.354520582	0.894513993	0.942631843	1.256218636	0.690459536	0.422928234
0.8256853528	0.378814888	1.051548748	0.983287809	1.344834133	0.638328453	0.249078305
0.7362434148	0.333670079	0.927090867	0.964826180	1.317269687	0.629276272	0.371910408
0.6771593198	0.312197017	0.846858171	0.863881679	1.168101008	0.603300511	0.541100718
0.7739225771	0.351294614	0.908745901	1.010193133	1.272393622	0.630889754	0.335169196
0.6991097526	0.324174187	0.911457102	0.894736369	1.263852683	0.582108641	0.874349662
0.7304260438	0.322230704	0.894864115	0.927581548	1.255653044	0.636934142	0.383393330
0.7529203289	0.346505676	0.883410379	0.940758708	1.228519392	0.620983532	0.678047837
0.786917479	0.377491932	0.901172234	0.938163288	1.253880827	0.739946448	0.493440035
0.7204040816	0.423560246	0.905325962	1.02080151	1.290889315	0.652431110	0.531322534
0.7937482867	0.386096227	0.958987625	0.916071950	1.236535314	0.680260927	0.673570842
0.7289210364	0.340170611	0.896809036	0.990825637	1.298345117	0.625279607	0.293854350
0.727332659	0.328519660	0.894860465	0.927429502	1.258336998	0.639334602	0.389846265
0.7747754129	0.405305482	0.878168741	0.904948234	1.221300338	0.798396968	-0.04709616
0.7300955487	0.328321249	0.888365735	0.922688552	1.248921392	0.644409833	0.508214101
0.7180685561	0.328369214	0.902128598	0.934739852	1.27660795	0.634417132	0.435811028
0.7264839662	0.425517884	0.990112482	1.036487671	1.359396967	0.757805194	0.527819898
0.7341605738	0.327422500	0.915973978	0.952811746	1.298531061	0.626667483	0.393828516
0.7270895938	0.330856874	0.898335558	0.953747664	1.274890513	0.638668404	0.391064728
0.7630000293	0.393206156	0.878481779	0.903913117	1.219781156	0.771738401	-0.01850770
0.719909825	0.327068571	0.881963036	0.940003742	1.271576942	0.618064646	0.509946595
0.7284122977	0.342737442	0.891530655	0.962544703	1.280440859	0.630183860	0.344473877
0.7273278666	0.328776355	0.887742685	0.919559748	1.246483769	0.650908569	0.308759756
0.7210815834	0.341453876	0.899579244	0.922007770	1.231242946	0.634428227	0.286545831
0.7217761099	0.357806333	0.887325467	0.933209641	1.241620373	0.692358061	0.344306132
0.7212013895	0.334551414	0.891484721	0.959440572	1.281941269	0.634140647	0.370954899
0.7358566974	0.328738938	0.904509161	0.957845329	1.259873958	0.621263629	0.525049957
0.7110976166	0.332212607	0.876724068	0.965282516	1.250582513	0.608469952	0.132104734
0.6877251918	0.306717598	0.830131834	0.841086832	1.132741505	0.588362514	1.112257274
0.7209979351	0.331244131	0.853610762	0.883112335	1.187678234	0.650033555	-0.09212895
0.7351469362	0.366931648	0.909889318	0.927081255	1.342818865	0.643621011	0.418761329
0.7401221644	0.337082917	0.895123434	0.927630431	1.268498019	0.650299368	0.391896260
0.7364584031	0.400549128	0.986458034	0.928501304	1.249733301	0.679081552	0.332798568
0.7639555131	0.357292316	0.880636221	0.946341976	1.233026396	0.639915935	0.685490428
0.7131569315	0.319336051	0.884037033	0.910119622	1.246534679	0.614861304	0.422967586
0.7208072809	0.398377791	0.918030306	1.003855254	1.241431488	0.634939426	0.407212756
0.7923788023	0.369821175	0.921833760	0.930572489	1.252583925	0.691707367	0.362904187

0.7316107004	0.484259331	1.046616174	0.928489903	1.24601194	0.784029908	0.469240968
0.8047960999	0.413396336	0.898630900	1.015149535	1.252051008	0.717848715	0.372621970
0.8123859883	0.419071911	1.081067177	0.928437173	1.25554295	0.626786107	0.202562625
0.7363002366	0.340342589	0.929963702	0.925217004	1.266992501	0.634052888	0.448165752
0.7245301354	0.345016055	0.886823265	0.909996974	1.216519692	0.610095813	0.666784573
0.7242634795	0.337872650	0.919905534	0.924892211	1.271937432	0.623264032	0.414076919
0.7530079669	0.381442912	1.045131801	0.975812141	1.33548234	0.636704778	-0.008370958
0.7338600197	0.357442892	0.892283339	0.934980021	1.27552675	0.675356180	0.333862620
0.7386370567	0.324839299	0.895555411	0.936258879	1.251344762	0.632157856	0.411146113
0.6802617092	0.433277602	0.923251265	0.880873673	1.166162053	0.741334650	0.619484430
0.6956074664	0.346533788	0.936911649	0.891617955	1.23749617	0.632502638	0.232513961
0.7359514759	0.406774313	1.079267857	0.925628458	1.313845432	0.667273410	0.267191212
0.7112114647	0.317324526	0.886325419	0.907695291	1.230445926	0.621006018	0.416222619
0.7380037292	0.358471468	0.902842333	0.926612034	1.315734699	0.648036088	0.427055558
0.7421430146	0.441301510	1.033364718	1.016566186	1.327947516	0.76802304	0.452057414
0.796963021	0.432891173	0.936153049	0.941652342	1.348165833	0.788455076	0.529311493
0.7540840556	0.340662649	0.949695533	0.924035617	1.270746464	0.618276269	0.785125085
0.8382735928	0.418282622	1.005316226	0.933594383	1.286681524	0.747489637	0.320915615
0.7332261148	0.417780495	1.008981301	0.928002719	1.253954506	0.702694857	0.392700295
0.7731882069	0.393513128	1.038552382	0.921616116	1.250440871	0.621322070	0.170003226
0.6397097801	0.294367455	0.818202541	0.807546623	1.13744564	0.539294041	-1.06012287
0.614101693	0.284141094	0.760148188	0.799916500	1.056417278	0.540948867	-1.28076506
0.5928082578	0.290367273	0.738134937	0.768695822	1.032054055	0.530354502	-1.136626394
0.6705561621	0.380262331	0.801547308	1.094398934	1.371648992	0.509155197	-1.819593414
0.6656246552	0.373164782	0.750125251	0.789552167	1.048831976	0.727640926	-0.644155668
0.6318340484	0.289811854	0.819832222	0.788703228	1.089500599	0.526752277	-1.381646768
0.6050239983	0.270394368	0.733839160	0.725865370	0.969332824	0.483574048	-2.387873152
0.63883347	0.334591768	0.758951990	0.792629351	1.103300849	0.622892885	-0.86404352
0.6733966532	0.318285652	0.881427612	0.838059880	1.11918086	0.537637713	-0.885773683
0.637762799	0.283142120	0.782969821	0.797647462	1.052162325	0.516476102	-1.60859830
0.6272330575	0.298964334	0.807162261	0.817199054	1.07760966	0.536090008	-1.47683948
0.6301520156	0.310312919	0.765219414	0.829494665	1.03648452	0.525554909	-1.583609015
0.6592904957	0.311980814	0.861911507	0.816523425	1.171602953	0.544169307	-1.049331513
0.6650583879	0.339508998	0.764968061	0.805466131	1.115400141	0.626459455	-1.668718335
0.6422316543	0.299628101	0.769323402	0.823855963	1.09590752	0.570578260	-1.12616087
0.6260327973	0.367694941	0.859549950	0.796683242	1.07614706	0.628708322	-1.168103975
0.6212573847	0.277204081	0.777727213	0.773124866	1.051822454	0.506902108	-1.765426157
0.5710502447	0.297794137	0.715240675	0.730952959	1.076850659	0.505376475	-1.490147555
0.603054047	0.293556388	0.757905182	0.761187091	1.066203711	0.533146380	-1.410477147
0.6086942136	0.293113036	0.757860077	0.797241154	1.063526302	0.523745732	-1.266146899
0.6161270172	0.370076942	0.818046495	0.840407185	1.089460009	0.671353871	-1.097730013
0.7645049229	0.443888231	0.826969052	0.817122138	1.19386896	0.792903601	-0.01691807
0.6259419538	0.309444580	0.769366741	0.825860226	1.114342374	0.548738952	-1.202047665
0.6560235225	0.335453761	0.836918913	0.866666532	1.290755011	0.548459671	-1.160883588
0.6855696873	0.316046173	0.758998829	0.872605592	1.027941175	0.505149576	-2.244079374
0.6143418102	0.266993911	0.756536216	0.784866662	1.058130205	0.529210600	-1.186324198

0.6033083703	0.285825111	0.767676309	0.784748230	1.037986588	0.527244144	-0.883365165
0.5955899833	0.315136861	0.821223263	0.829065889	1.208245567	0.534589670	-1.581998059
0.6224553774	0.284509574	0.788484239	0.794908249	1.080255571	0.54129655	-1.154736689
0.6340217018	0.296778734	0.763893653	0.817573885	1.10806921	0.555980536	-0.966480465
0.5990646218	0.292464687	0.776303841	0.759358707	1.025673944	0.530103788	-1.382055783
0.5811844159	0.259534734	0.709574033	0.744959396	0.991593978	0.495873429	-0.978247532
0.6803583561	0.387042825	0.768643472	0.793637253	1.072933895	0.757944135	-0.890632980
0.6260768876	0.313442681	0.836003951	0.824695672	1.229988431	0.532509298	-1.006632608
0.6230707385	0.275331783	0.757919774	0.793639041	1.060242617	0.533286537	-1.283609284
0.6205161701	0.342630314	0.840467551	0.882842457	1.164202428	0.599455776	-1.440904224
0.6251941332	0.289592153	0.802123227	0.803590338	1.130730669	0.541496923	-1.178387874
0.6232615838	0.319451789	0.757085297	0.784047746	1.063561833	0.614710091	-1.017887452
0.6921174117	0.348177069	0.771906679	0.802677071	1.075728516	0.684377079	-1.012814582
0.6542114478	0.397671820	0.870565857	1.05382947	1.313367417	0.636564863	-1.171761805
0.653915392	0.294506847	0.775048234	0.801153720	1.074452432	0.574406171	-1.174391457
0.5922303217	0.301918613	0.728652165	0.75063239	1.021179459	0.558930189	-0.962796702
0.6572799564	0.323849938	0.767445143	0.810264443	1.09079476	0.625415354	-1.257516359
0.6416013475	0.319401815	0.859745027	0.794962892	1.114792743	0.540517036	-1.207231124
0.6355707705	0.373484322	0.771357198	0.792584283	1.130876417	0.657207866	-1.241964466
0.6365122268	0.305022003	0.767931688	0.795533377	1.117787658	0.546134047	-1.193329204
0.5797444244	0.319953194	0.697814226	0.707578122	0.966782224	0.626787834	-1.946725799
0.6996372109	0.408011861	0.891026480	1.162842724	1.424598371	0.572676115	-0.911815434
0.6237664905	0.285593273	0.793333154	0.813194524	1.13232395	0.530100370	-1.012745883
0.6539507293	0.295899491	0.821335747	0.807623463	1.084448018	0.537948574	-1.084291272
0.6558967136	0.290379833	0.779047655	0.830979657	1.080958584	0.544166611	-1.012414375
0.7257003845	0.348179323	0.766524481	0.869431928	1.062611263	0.614432352	-0.576701327
0.6180548142	0.292563451	0.756854824	0.784316934	1.06242757	0.568492303	-1.081029074
0.6028952634	0.295114298	0.698987940	0.739040558	0.996152508	0.522623575	-0.475795949
0.6330615693	0.293146038	0.784957426	0.796891960	1.045919714	0.522470265	-1.500387448
0.611106488	0.266689568	0.748137404	0.777460442	1.047470411	0.524431759	-0.999522564
0.6140149879	0.298499606	0.756284495	0.784039494	1.074729293	0.549527759	-1.169578967
0.6302749279	0.286154959	0.789159743	0.794814592	1.072571411	0.536334197	-1.09409533
0.6292221274	0.355733388	0.894352904	0.78075033	1.058693853	0.568238783	-1.446118877
0.6234691118	0.326701537	0.781191078	0.806024637	1.086013525	0.636467893	-1.204801996
0.6024558029	0.283663185	0.729129599	0.790641466	1.049527405	0.525749158	-0.916100908
0.6104614431	0.276086315	0.749884827	0.777087879	1.057602749	0.536911868	-1.092637562
0.6617740758	0.338189344	0.759619779	0.802823125	1.106222857	0.628067583	-0.524319411
0.6304714341	0.297821052	0.751451713	0.791873933	1.055307859	0.580032292	-0.895595616
0.6680487845	0.306493054	0.850791237	0.795562406	1.088083742	0.516461320	-0.295383688
0.6107144581	0.276779578	0.769022561	0.800324031	1.092676182	0.521985134	-0.983180360
0.6153452429	0.283698302	0.769553238	0.785022765	1.061471618	0.548228591	-1.23008137
0.6573595063	0.298384956	0.771876638	0.858044562	1.080754158	0.535869335	-0.856605314
0.6436180154	0.298442907	0.839110324	0.823716797	1.163534556	0.535903850	-1.224281146
0.6212713715	0.274076634	0.761135861	0.788964010	1.06800859	0.541750874	-1.096512672
0.5983913476	0.275389029	0.702099687	0.747677874	0.976378703	0.493533191	-0.520514174
0.666529804	0.319740799	0.763305135	0.794637061	1.062054109	0.626744702	-1.415763786

0.6085696944	0.357807425	0.764784595	0.862333902	1.090493705	0.551148739	-1.372065567
0.6893527136	0.335315976	0.832859399	0.795588092	1.073903388	0.590791469	-0.92975324
0.6250380459	0.291690819	0.768999301	0.849617022	1.113310022	0.53616719	-1.26657053
0.6113751886	0.276144302	0.752194307	0.779570915	1.057722364	0.537406520	-1.139037805
0.6551646297	0.342733922	0.742595968	0.765241210	1.032754486	0.675139460	-1.642639244
0.6335868632	0.284921653	0.770935887	0.800721695	1.083830998	0.559227632	-1.168601744
0.614652733	0.281077667	0.772204553	0.800119153	1.092751602	0.54304874	-1.21796039
0.6275530798	0.367571854	0.855281282	0.895341205	1.174277469	0.654609056	-1.258263742
0.6090530051	0.27162676	0.759883769	0.790444049	1.077249682	0.519877704	-1.362367187
0.6033128728	0.274533170	0.745406633	0.791385611	1.057858432	0.529944140	-1.24288988
0.6499926922	0.334968700	0.748370530	0.770035253	1.039120324	0.657436830	-1.569051631
0.6291894286	0.285852589	0.770821288	0.821547917	1.111337478	0.540178406	-1.171557464
0.6166225267	0.290137369	0.754707035	0.814822524	1.083931011	0.533469253	-1.068657883
0.6302302443	0.284885004	0.769229827	0.796799341	1.080079296	0.564012855	-1.127203277
0.5856967745	0.277345086	0.730681068	0.748898584	1.000074109	0.515312795	-1.473863636
0.6262278387	0.310440154	0.769861875	0.809671931	1.077255443	0.600704133	-1.144057313
0.6112869573	0.283564229	0.755618321	0.813217385	1.086567482	0.537494675	-1.225886087
0.6132072041	0.273946117	0.753749386	0.798195706	1.049883476	0.517714025	-1.457418069
0.6371056228	0.297644817	0.785498109	0.864841766	1.120455381	0.545156697	-1.155510297
0.6327004808	0.282177204	0.763713205	0.773791696	1.042111156	0.541287785	-0.741577223
0.6559750244	0.301371012	0.776628217	0.803469202	1.080567947	0.591410539	-1.123458146
0.6295172408	0.314209020	0.779151737	0.793873449	1.149875739	0.551142232	-1.214191086
0.624265725	0.284316997	0.755003574	0.782422026	1.069931254	0.548503512	-1.326761188
0.6287064478	0.341944390	0.842128386	0.792651363	1.066883589	0.579724460	-1.258189619
0.6473670138	0.302765352	0.746240888	0.801919180	1.044852223	0.542257319	-0.700270025
0.6155171736	0.275615107	0.763001735	0.785513304	1.075869094	0.530679399	-1.193088883
0.5996286647	0.331404453	0.763695513	0.835091988	1.032728061	0.528196496	-1.184729665
0.6750365063	0.315054862	0.785320656	0.792765279	1.06709048	0.589273366	-0.831586896
0.6322520229	0.418492979	0.904477194	0.802393430	1.076793395	0.677552276	-1.143347222
0.6403926717	0.328947896	0.715058936	0.807775190	0.996282524	0.571206864	-0.326933154
0.6852816109	0.353504711	0.911925448	0.783175646	1.059103071	0.528720336	-1.484225213
0.6334386789	0.292796538	0.800047249	0.795963668	1.089992935	0.545475343	-1.098976254
0.6168456026	0.293737453	0.755017637	0.774747114	1.035712369	0.519419278	-1.48216297
0.6247899393	0.291467730	0.793561651	0.797863436	1.097243936	0.537662201	-1.15216233
0.6499099465	0.329217689	0.902037671	0.842208906	1.152634891	0.549530398	-1.469497384
0.6260547745	0.304933943	0.761205448	0.797629917	1.088149771	0.576145245	-1.259393175
0.5811491933	0.255578968	0.704610336	0.736635249	0.984540367	0.497372863	-1.608460647
0.6282325391	0.400138777	0.852637269	0.813500888	1.076969257	0.684634374	-1.149269741
0.6168448452	0.307296271	0.830826506	0.790661351	1.097376279	0.560885285	-1.281457223
0.6295009868	0.347937112	0.923158935	0.791742454	1.123806424	0.570756747	-1.300258891
0.6206546119	0.276920354	0.773471726	0.792120623	1.073776193	0.541934808	-1.172017142
0.5654837573	0.274673127	0.691788746	0.710001906	1.008161032	0.496547466	-0.858354876
0.6334939051	0.376695342	0.882080997	0.867741756	1.133537123	0.655585116	-1.011277879
0.6796130504	0.369149488	0.798307842	0.802997383	1.149653209	0.672357870	-1.355666931
0.665552802	0.300668047	0.838199028	0.815551648	1.12155782	0.545689171	-0.953946607
0.6875078762	0.343053389	0.824507451	0.765684970	1.055268458	0.613051654	-1.76547318

0.6110521569	0.3481677311	0.8408595761	0.7733740683	1.045014068	0.5856081765	-1.335779711
0.6390035925	0.3252200438	0.85831457	0.7616722608	1.033430414	0.5134933923	-1.644764623

t_microbe	t_blast	t_vns	t_interact	t_batch	t_p_intercept	t_p_microbe
0.811221818	6.373274533	-0.54016207	-1.005459986	2.368500831	0.00015242029	0.426337589
-0.565396224	6.293920311	-0.796020004	-0.718820423	2.349647473	0.00023761175	0.577795707
-0.52963596	6.275963404	-0.576579831	-0.846561767	2.336280497	0.00018593822	0.601919414
-0.105342073	5.763893071	-0.545969202	-0.499904595	2.266384929	0.00084233881	0.917104107
-0.029995916	6.383679922	-0.674957305	-0.765315615	1.607230579	0.00054933890	0.976353560
1.267139755	5.596406857	-0.880608369	-0.447912264	2.334763079	0.00043007288	0.218978051
0.523873519	5.618186977	-0.814800453	-0.596938449	2.050635209	0.00108594605	0.605851927
2.048051107	7.200315026	-0.386183384	-1.409848726	3.189416122	0.00003105461	0.053264016
-0.509179467	5.330459385	-0.813789676	-0.589316804	2.31768323	0.00072896547	0.615935149
-0.558769019	5.874489742	-0.81857876	-0.64568686	2.236335533	0.00060164167	0.582229397
-0.443130933	5.75018182	-0.792739687	-0.630224558	2.313744202	0.00039633826	0.662202407
-1.444867654	6.060073684	-1.223501086	-0.637780415	2.614435476	0.00066182968	0.163252193
0.596120150	6.02190396	-0.539516666	-0.948324361	2.34759371	0.00021589224	0.557466510
-3.01130997	6.592659569	-1.717362055	0.289277420	0.524947305	0.00205258835	0.006647678
-0.356893869	6.42359406	-0.570825003	-0.825187179	2.259141062	0.00022456945	0.724732489
-0.183503581	5.641436905	-0.685441515	-0.759657156	2.031721002	0.00022346805	0.856163150
-0.109767717	5.868502693	-0.685941845	-0.702202346	2.229620614	0.00044060901	0.913635804
0.304569312	6.331211007	-0.637306955	-0.827193204	2.271887713	0.00020215623	0.763691992
0.446135155	6.337798809	-0.683024505	-0.863685614	2.310527234	0.00019236914	0.660065724
0.367770565	6.391158036	-0.750766824	-0.691528528	2.28796158	0.00019626384	0.716725038
0.219663004	6.000613475	-0.556247881	-0.799911184	1.648672123	0.00020690273	0.828254777
2.176355529	5.254424217	-1.509023558	0.389956789	-0.04445452	0.02171505831	0.041086906
-1.573002392	6.582844175	-0.258409609	-1.214613809	1.993989999	0.00018777209	0.130664362
-1.591362358	6.847312844	-0.034829006	-1.558988119	2.666388853	0.00006084721	0.126470180
0.339830915	5.767050233	-0.756488693	-0.626240308	2.106841872	0.00204832450	0.737359301
0.034917595	6.396805614	-0.682243402	-0.76859308	2.263753774	0.00021068610	0.972475228
0.417740224	5.955925013	-0.772892208	-0.675154291	2.303093483	0.00030668844	0.680377399
-0.162187221	5.670447492	-0.53973333	-0.72918754	2.069678982	0.00024361700	0.872708849
1.16296545	6.687839048	-0.74596036	-0.930766535	2.149858504	0.00014280740	0.257888936
-0.772978986	6.359400796	-0.875163369	-0.525767202	1.962023695	0.00037697383	0.448148480
0.344766815	5.938137277	-0.690950685	-0.753433668	2.282157671	0.00024320480	0.733698658
0.470776999	6.444807655	-0.597691011	-0.765909472	2.264118604	0.00018876324	0.642653259
-0.049877348	6.376985301	-0.681414821	-0.770403275	1.63460774	0.00050292782	0.960691488
-1.554575845	6.778352762	-0.209687391	-1.488716658	2.221781971	0.00007097467	0.134989453
0.930843506	6.517758002	-0.821018215	-0.802344778	2.192742256	0.00027373686	0.362512815
-2.075790327	7.181142319	0.358338154	-1.685779531	1.124680226	0.00003298139	0.050385604
0.342465349	6.038697656	-0.724719786	-0.62446188	2.295133813	0.00022490154	0.735404707
1.035154156	6.595367873	-0.710649486	-0.678228124	1.469720676	0.00028659171	0.312370945
-0.051923656	6.367457655	-0.682710497	-0.769783497	1.746459159	0.00063176915	0.959080274
-0.64362080	5.396236901	-0.941477869	-0.264807456	2.274175298	0.00049842181	0.526788453
-1.053782381	6.645535105	-0.575857111	-0.808637704	2.545282302	0.00012415660	0.303956874
2.21260236	7.34965398	-0.965667677	-1.193332357	3.222502761	0.00003378695	0.038137092
-0.432543187	6.367039563	-0.759136768	-0.672645866	1.720197017	0.00050889423	0.669756145
-1.484052936	5.327475999	-0.70196747	-0.373863691	2.177630888	0.00039717365	0.152650290
1.300027778	6.762157621	-0.694811513	-1.178093343	2.670174513	0.00009213042	0.207680230
-2.385918078	7.283756358	-0.779803606	-1.496944667	2.068455128	0.00022036321	0.026531122

-2.054891923	7.284780949	-0.803395623	-1.158227113	0.5567505784	0.000536092390	0.0525407968
-0.923569720	5.153088411	-1.149095409	0.0158866878	2.486090798	0.00151314854	0.3662013664
0.6009285837	6.33214418	-0.502801025	-0.938600183	2.238934929	0.000176360330	0.5543189854
-1.026372543	5.775731165	-0.855433556	-0.649367719	2.289251063	0.000549755455	0.3163940609
0.5911817779	6.455281009	-0.486603919	-0.839262977	2.3484731	0.000212099301	0.5607087809
-1.177054193	6.70615324	-0.130604017	-0.875902538	2.611295004	0.000179318008	0.2523431626
0.2678883809	6.401476393	-0.681584235	-0.790391480	2.208552905	0.000205204827	0.7913988996
-1.191581955	6.665529261	-0.933380173	-1.016008446	1.758057496	0.000756310128	0.2467184541
-0.247676926	5.992500971	-0.722757160	-0.740994793	2.279942083	0.000403291270	0.8067899815
-0.479008827	6.341934596	-0.74150546	-0.711599143	2.196046899	0.000268109891	0.636882447
0.3261232271	6.404429851	-0.692639500	-0.816432306	2.272327919	0.000206439499	0.7475587555
0.4017377774	6.144615206	-0.681027696	-0.772199284	2.277400524	0.000266538861	0.6919362382
0.2130394145	5.466081327	-0.649135108	-0.793820266	1.999627214	0.000266723591	0.8333507625
-0.771670316	6.507007817	-0.538339733	-0.902033654	1.50984092	0.000224109401	0.4489067099
1.551965795	6.763610489	-0.231234829	-1.155393351	2.695923488	0.000071282321	0.1356115768
0.7790528204	6.518783602	-0.654922945	-0.879351215	2.411037279	0.000160145049	0.4446395985
-1.26996853	6.149034994	-1.059016901	-0.288090897	1.20679806	0.001447191645	0.2179880419
0.8457922497	6.492992022	-0.528210367	-0.855229079	2.443347821	0.000149965871	0.4072031284
-0.797769581	5.940484392	-0.385025009	-1.01904192	2.411056025	0.000267600430	0.4339330774
0.1317853615	6.141465911	-0.691526266	-0.689527544	2.265660401	0.000253692121	0.8964085344
0.1369197874	6.313712542	-0.688318864	-0.777910597	2.148328698	0.000218777811	0.892398402
0.4906613313	6.248521303	-0.823619519	-0.671931726	2.195452762	0.000572297497	0.6287539192
-0.857375768	6.291558533	-0.440444895	-1.054273403	2.287536665	0.000148721771	0.400916417
0.4519449013	6.423297418	-0.692220768	-0.733637002	2.318858334	0.000189274589	0.6559421165
-0.552541678	6.33581709	-0.804763428	-0.797855418	2.16845625	0.000531616930	0.5864110497
0.1921807148	6.368085079	-0.640691753	-0.783847598	2.014525423	0.000405261309	0.849446904
1.136870591	6.110511507	-0.109765734	-1.104800554	2.579411777	0.000115384999	0.268399194
0.0222568599	5.886601819	-0.681522417	-0.768014394	2.047373311	0.000599552651	0.9824531902
-0.060143328	6.314842474	-0.654416543	-0.716442000	2.252387903	0.000245405051	0.9526101765
0.699893571	6.478473663	-0.685739700	-0.846082063	2.384569264	0.000174392440	0.4916750511
1.825138121	6.648244793	-0.756872978	-0.858544454	0.6871910717	0.001433151255	0.0822384970
0.5293518882	6.377397964	-0.736228029	-0.699894010	2.043718625	0.000286532147	0.6021129872
-3.464143267	7.145537252	-1.53680618	-0.059310509	3.552870327	0.000021869400	0.0023203585
-1.5458549	5.310389781	-1.362935163	-0.086560703	2.842405206	0.000254423259	0.1370774432
-0.374232964	6.19116428	-0.751030152	-0.644075600	2.286729474	0.000256809618	0.7119831078
-0.433801511	6.361721452	-0.766693042	-0.675494342	2.314450009	0.000209647899	0.668856506
-0.123580421	6.366850047	-0.683296733	-0.767561477	1.865067009	0.000405222420	0.9028226785
-1.997655241	6.799697934	-1.210488099	-0.303154057	2.254515068	0.000161800528	0.0588721808
-0.231710761	6.39463445	-0.589363501	-0.802344246	2.275670891	0.000209041051	0.8190056127
-0.480473402	6.407194011	-0.694836508	-0.723045515	2.314537255	0.000187776671	0.6358581838
0.8867956010	6.192396188	-0.814800118	-0.775290942	2.45151535	0.000250936287	0.3852324177
0.4917523120	6.396148323	-0.761949827	-0.740866473	2.256692001	0.000186439759	0.6279953191
-0.069750953	6.360458796	-0.673035503	-0.726835464	2.229528001	0.000213894525	0.9450518329
1.66580504	6.334563627	-1.167776247	-0.506340292	2.518821511	0.000313597751	0.1106008108
0.9603113865	6.199686376	-1.01468193	-0.483988776	2.439576151	0.000328527424	0.3478258885
-0.550471020	6.38050137	-0.609489181	-0.831265645	2.341201186	0.000221067481	0.5878047965

1.885640797	6.583035963	-0.970667294	-0.64438737	1.440549162	0.00061312608	0.073253778
-0.96560164	6.60295772	-0.67567772	-1.083076181	2.019636933	0.00027235567	0.34523273
-1.60283389	6.803482045	-0.731764331	-1.062846794	1.873111558	0.0002824407	0.12390709
0.27464790	5.91648208	-0.65765313	-0.78037063	1.976909191	0.0002173025	0.78627056
-0.48281690	6.448969367	-0.52952897	-0.735897901	2.320717084	0.0002657520	0.63422079
0.57795850	6.470648637	-0.677859012	-0.862493367	2.308392324	0.0001878244	0.56943842
2.357147748	6.35200781	0.24150592	-0.878707381	1.99199398	0.0000513820	0.02819891
-0.02781152	6.203810312	-0.68141027	-0.76926703	2.036398557	0.0005235813	0.97807507
0.58331926	5.789404135	-0.614925222	-0.80166111	1.456295366	0.0001772734	0.56589102
-1.52302429	6.575744741	-0.037292867	-0.77839198	2.825489657	0.0000742593	0.14267033
-0.00761838	5.277401907	-0.67959599	-0.76365652	2.253724992	0.0006712472	0.99399338
0.63795223	6.019996417	-0.68112443	-0.656259417	2.138279662	0.0002935308	0.53040037
1.74444352	6.334539666	-0.98814444	-0.943629097	2.591275477	0.0003615892	0.09570201
0.89984240	6.09131471	-0.64578520	-0.58825329	2.293106044	0.0001898852	0.37840744
0.41491085	5.573344213	-0.472606822	-0.87436562	2.047380799	0.0002787002	0.68241535
2.885277896	7.565453753	-1.22083624	-0.29291993	1.367947223	0.0001060536	0.00885488
1.385488261	6.61366184	-0.896518857	-0.80505246	2.142467612	0.0002851970	0.18044367
1.801264781	6.976773546	-0.35659071	-0.91629857	0.79574448	0.0000638587	0.08603923
-0.33477675	5.962390984	-0.62506380	-0.83321661	2.020288713	0.0002007153	0.741114287
-2.27655879	4.576715768	-1.012602772	-0.06422659	3.187183003	0.0002347677	0.03339918
-0.44272307	6.417917343	-0.685925544	-0.81075837	2.169568206	0.0002033059	0.66249271
-0.200673311	6.36811726	-0.68172769	-0.79373435	2.133161512	0.0002702853	0.84288481
0.19963013	5.633253772	-0.538341647	-0.79153872	1.726049037	0.0002404836	0.84369024
3.188114672	8.393435709	-0.20030957	-2.078134692	4.131965549	0.0000031407	0.00442379
-0.487006381	6.079097357	-0.55717685	-0.882069801	2.327190407	0.0002633905	0.63129840
0.55461607	5.988589679	-0.619074817	-0.881665392	2.212317387	0.0004211719	0.58501643
0.16990986	5.599386055	-0.686401435	-0.75806328	2.094781117	0.0002422051	0.86670722
-0.20573406	5.584009628	-0.672880734	-0.787669022	2.266301045	0.0003569653	0.83897999
-0.35016897	-2.249877722	-0.441224121	1.13230845	1.915439603	0.6129787052	0.729699651
0.60429638	-2.170171712	-0.24262386	1.022603571	1.775582218	0.7063357006	0.55212003
-0.10324353	-2.25166144	-0.35953128	1.045319816	1.907961976	0.6656882987	0.91874929
-0.55048871	-2.29993455	-0.666162542	1.171665939	1.986643155	0.5197901455	0.58779288
-0.68323353	-2.23571584	-0.51014315	1.127030442	0.94685387	0.4983917522	0.50192844
1.06990166	-2.55747477	-0.54412166	1.339353746	1.995801105	0.4853102145	0.29680741
-0.44025177	-1.87628518	-0.23052160	0.91333960	2.00956605	0.8427841884	0.66425291
0.94246324	-2.181707447	-0.22665986	0.76885861	2.190883522	0.8714597263	0.35667238
-0.03978911	-1.983172727	-0.37709146	1.038163496	1.948603256	0.6750422029	0.96863710
-0.572231041	-2.339632284	-0.536840818	1.17558325	1.925842147	0.5430157332	0.573241047
-0.52364100	-2.30232426	-0.539270547	1.180080473	2.035730058	0.5735026095	0.60601086
-1.758758371	-2.811242652	-1.068687461	1.341589799	2.403346701	0.3253654382	0.09318366
-0.29509129	-2.15571065	-0.44119267	1.104835783	1.888884613	0.6108255146	0.77082184
-3.25528208	-3.53420961	-1.468043687	2.452258211	0.13071155	0.0709169847	0.00378456
0.23723763	-2.27194618	-0.433049421	1.100971911	1.765676529	0.6305573011	0.81477151
-0.08413454	-2.06131089	-0.386721004	1.07698517	1.714982245	0.6575247956	0.93374635
0.93690285	-1.774854204	-0.12644566	0.77043744	2.101801443	0.9183335491	0.35945922
1.266319437	-2.028728937	-0.23095434	0.48196882	2.282084474	0.7497137854	0.21926579

1.232347741	-1.966273014	-0.394474960	0.741266311	2.280618032	0.7883156384	0.2314390820
1.077431154	-2.117773378	-0.637409809	1.28074631	2.065876143	0.70860576	0.2935094684
0.736927272	-1.835648404	-0.096593314	0.877284051	1.104414556	0.7053589626	0.4693203776
1.432099219	-2.765001173	-0.906703479	1.701679519	0.228411355	0.2205435316	0.1668328279
-0.338896761	-2.291092112	-0.277605251	0.941928014	1.839584233	0.6354481273	0.7380528278
-1.067270996	-1.710031628	0.057845924	0.326983556	2.180254849	0.9180774262	0.2979659032
-0.324722427	-1.959575729	-0.138659261	0.921504309	1.977458116	0.8543612009	0.7486037584
0.694972022	-2.282601606	-0.420089876	1.089667443	1.993211133	0.6522843363	0.494691407
0.483905916	-2.311220992	-0.505704741	1.153840529	2.023806797	0.598175016	0.6334605536
-0.389838323	-1.794357554	-0.174166607	0.678029909	1.706644312	0.7260424058	0.7005818042
3.92989586	-1.974390693	-0.653912984	0.918316066	2.000805056	0.6136447125	0.000768124
-0.578286115	-2.331370043	-0.530754105	1.202744383	1.709388494	0.5741658364	0.5692213149
-0.105576455	-2.100760159	-0.382638084	1.067959274	1.86224282	0.6725178811	0.916920383
-0.547744033	-2.301203918	-0.472988443	1.073614288	1.98064342	0.6093037515	0.5896428180
0.125401195	-2.266145208	-0.385109795	1.079983805	1.29286925	0.6522537755	0.901398700
-1.386346406	-1.556251204	0.040886850	0.262700385	1.892536455	0.8444855867	0.1801853188
0.302594185	-2.270761198	-0.425125108	1.070273299	1.913612755	0.6312174614	0.7651760368
-2.21165167	-1.194097025	0.704368427	0.073118403	0.784369132	0.9790429132	0.0382119310
1.354382344	-2.65131512	-0.588538118	1.48841024	2.198698286	0.576379314	0.1900092626
0.754855302	-2.259800414	-0.399091278	1.159905809	1.320406167	0.5735037123	0.4587184199
0.583157280	-2.22089633	-0.315689582	1.095668577	1.909020111	0.8802845003	0.565998046
-0.550307441	-2.270009581	-0.653535863	1.201395724	1.956693564	0.559698895	0.5879149708
-1.128786811	-2.158190396	-0.266755039	1.084519449	2.282572421	0.9235057285	0.2717183239
2.963520432	-2.234573031	-0.741121651	0.793497839	3.379315486	0.9421231884	0.0074139524
-0.504756735	-2.310664875	-0.483540280	1.158356892	1.420255832	0.5630144205	0.6189854942
-0.585590504	-2.299796525	-0.382559779	1.201204357	1.883217773	0.5710250341	0.5643914995
1.697022351	-2.169219704	-0.393106503	0.531825304	2.682623496	0.9206702775	0.1044657882
-1.141357508	-2.29468285	-0.402374612	0.745119405	1.769626737	0.5039148759	0.2665698429
-2.018021169	-1.996749708	-0.476513077	0.838060371	0.318169488	0.302486836	0.0565453334
-0.358882146	-2.137872298	-0.525985712	1.047833197	1.963091342	0.5813618218	0.7232662670
0.219904932	-2.093752676	-0.313196927	0.926234928	1.938571636	0.6934131724	0.828068791
-1.648304377	-2.830882981	-0.671090231	1.343720706	2.029356612	0.3574053511	0.114171785
0.391222334	-2.170286272	-0.256749787	1.024424064	1.999537914	0.7657850145	0.6995740795
-1.002985021	-2.138952113	0.074141320	1.024830227	2.233474545	0.8852950952	0.3272859349
-1.200312306	-2.316862782	-0.402147147	1.210130924	1.442680796	0.5644210138	0.2433839379
-0.487263361	-2.244949225	-0.478900271	0.961176008	1.661594753	0.5639197061	0.6311193475
0.915418885	-1.895307147	-0.148927584	0.997397058	1.901719027	0.8753002039	0.3703644085
-0.510202261	-2.318771528	-0.450450148	1.132900833	1.885618639	0.6086890055	0.6152307406
-0.107418394	-2.260828461	-0.381714936	1.076263532	1.852280294	0.6619884292	0.9154767142
1.232461785	-2.568157515	-0.389782007	1.11062862	2.040702267	0.5257991354	0.2313973712
0.046585192	-1.864802221	-0.375911186	1.052596937	1.77907764	0.6815843354	0.9632840138
-0.211252639	-2.173915338	-0.340373122	1.021932441	1.529417772	0.648656076	0.8347267490
1.267246398	-2.339427236	-0.009226622	0.783293064	2.275808137	0.8290132842	0.218940665
0.266523701	-2.250964302	-0.373154920	1.029039576	1.968799343	0.6784019855	0.7924354430
0.388767919	-2.114745761	-0.250253107	0.866165523	1.833767508	0.8134023678	0.7013615732
3.526249434	-2.901235709	0.160838869	1.029734652	3.727519913	0.7331455264	0.0020042744

-0.853872241	-1.507809078	-0.083607380	0.701840026	2.119540556	0.9927349524	0.4028112568
0.6299998499	-2.369526581	-0.547417308	1.226571302	2.028785319	0.5918526001	0.5354901584
1.699684715	-2.056366478	-0.506453615	0.9978311174	1.563133495	0.5659657198	0.1039562309
0.8573790641	-2.416610807	-0.699565278	1.229395686	1.863369901	0.4796585904	0.4009146375
0.3321798111	-2.20716441	-0.460436885	1.121163429	1.965384113	0.611431992	0.7430462664
-0.941961399	-2.301777926	-0.377050396	1.017160855	1.785336125	0.5899738712	0.3569233035
-0.140861311	-2.265150805	-0.408437731	1.067279607	1.911098319	0.6458491699	0.8893219555
0.5928693887	-2.179017885	-0.284689228	1.023794734	1.981236679	0.857346948	0.559599685
1.150605812	-2.544121138	0.1682693756	0.6972414754	2.286154175	0.778053227	0.2628283018
0.4916179085	-2.288361261	-0.391588280	1.091534704	1.581009209	0.5457567556	0.6280887525
1.128753435	-2.133682126	0.0545583217	0.721842781	1.849873655	0.7860430788	0.2717320899
0.2843757778	-2.26385207	-0.384355313	1.040756737	1.975258403	0.6701338455	0.7789076763
0.0049349760	-2.25031356	-0.385074326	1.073761232	1.491707767	0.6869800342	0.9961090555
1.38142302	-2.465027532	-0.540779721	1.289031534	1.580712439	0.4943097277	0.1816716013
-1.90692963	-2.787933481	-0.793952396	1.606416111	2.496447841	0.5723370694	0.0703034868
-0.763635044	-2.390592222	-0.704265513	1.308912122	2.056597126	0.5730951318	0.4535793479
-0.085777770	-2.229773085	-0.394521833	1.058342693	1.956974595	0.6558308568	0.9324556547
0.6953887028	-2.218509403	-0.216519125	0.9340672237	1.798350518	0.6757305657	0.4944356180
-0.510882786	-2.198480315	-0.394726975	1.086446469	1.867369881	0.8243986549	0.6147622668
-0.843090328	-2.360004235	-0.586313775	1.270497418	1.892126129	0.5839844373	0.4086785656
-0.509876402	-2.291487525	-0.228168880	0.9350870973	2.020756735	0.7036110182	0.6154551216
-0.125438139	-2.267033897	-0.387690320	1.081492463	1.901663707	0.676728301	0.9013698108
-0.983232467	-2.062022924	-0.247651889	1.089874825	1.702160555	0.7514786573	0.3366862375
0.4658515222	-2.296960912	-0.463881258	1.106580185	1.966245497	0.6876760401	0.6461173274
1.057529367	-2.209087171	-0.077323266	0.8020830712	1.745093545	0.6865745397	0.3022840998
0.4250028761	-2.311761167	-0.491382273	1.137295413	1.988049143	0.6049241107	0.6751577094
-0.698202003	-2.097269041	-0.087458414	0.8464576608	1.834753511	0.7662926617	0.4927105963
-1.094504391	-1.98669113	-0.251358725	0.9741321001	2.241613826	0.9268620613	0.2861292433
-0.053805930	-2.233080815	-0.375095435	1.063785736	1.799885804	0.6885018403	0.9575983741
-1.188495322	-2.078478745	-0.372495249	0.6011490994	1.681546271	0.5300060662	0.2479055584
-1.452133797	-2.348175279	-0.412927320	0.8770327029	1.579647386	0.4719855977	0.1612424570
0.1064494213	-2.003419571	-0.375081308	1.069252593	1.755945572	0.6795924874	0.9162361358
0.3156783381	-2.28254985	-0.456786477	1.050776591	1.795341798	0.6026380976	0.7553625359
0.3037941729	-2.21705609	-0.381215785	1.00829706	1.972215347	0.6657249219	0.7642742957
1.744387803	-2.78674706	0.3207815268	1.138625173	1.677420657	0.6589727464	0.0957119323
-1.024969862	-2.496895399	-0.474821099	1.064215457	1.376255729	0.4180778584	0.3170400340
0.1715874077	-1.835030874	-0.362932714	1.065441906	1.448288125	0.6842981099	0.8654046089
-1.04517865	-2.405898193	0.0637640093	1.122983506	2.259948439	0.9704651143	0.3078227825
0.1735920162	-1.771975889	-0.369454231	1.050455827	1.963868781	0.7533437618	0.8638485361
0.3409411768	-2.273761246	-0.382476735	1.119284923	1.870176545	0.6232623216	0.7365353302
1.250988338	-2.554040944	-0.586407590	1.022237024	2.146143195	0.4675149066	0.2246971229
1.207185535	-2.566595815	-0.333765601	1.329066451	2.003315532	0.6150744461	0.2407827247
0.3100020812	-1.714861199	-0.237463555	0.8521031799	1.779101287	0.7597793623	0.7596148083
0.8928920425	-2.30347233	-0.519367842	1.25502419	1.49864371	0.5623443763	0.3820332914
-0.677894407	-2.254900825	-0.291772190	1.08663526	2.052637289	0.7526279714	0.5052399228
0.5358307928	-1.823435326	-0.272523926	1.052751008	1.214380057	0.7034334174	0.5977056413

-0.244243639	-1.954668273	-0.3444861778	0.9665406206	1.757715854	0.6840983672	0.8094126256
-0.82848826	-2.3623625055	-0.4821954277	1.302770656	2.151933059	0.5480236448	0.4167112574
-1.4902239077	-2.12192942	-0.4096433033	0.9832779401	1.780918427	0.631003079	0.1510330056
0.2733842685	-2.2829738111	-0.386092798	1.10757378	1.96122698	0.7013986752	0.7872285157
0.4546979732	-1.7363159733	-0.1642047455	0.8606623631	1.332608357	0.737629611	0.6539919676
1.992607704	-1.7420372922	-0.0281494966	0.3257352401	2.905297962	0.6982012933	0.0594618967
0.1514177311	-2.1316910577	-0.4095259322	1.071630813	1.892773389	0.6436182582	0.8810913739
0.3774047316	-1.8494740633	-0.3411091388	0.9610852614	1.848647443	0.8446174246	0.7096601025
0.0024578686	-2.0084622099	-0.38495483	1.072318017	1.742297229	0.6638892921	0.9980621059
-0.1063925199	-1.8812916558	-0.3803865477	1.055723734	1.955574022	0.7103595524	0.9162807346
0.1903115271	-0.1935654522	-0.0935147333	-0.6276266000	1.921282141	0.804675828	0.8508927151
-0.4067041555	-0.3340599988	-0.2171071500	-0.5599125711	1.959624817	0.8671230291	0.6883405268
1.216068945	-0.0916467322	-0.3546762077	-0.4227355311	1.564437879	0.8791115233	0.2374519338
1.265036412	0.2701116125	0.8248868882	-1.3083160233	1.936843223	0.4368822198	0.2197164171
1.046019543	-0.3549062133	0.0660374461	-0.6783591544	2.135968773	0.5380898461	0.3074434124
-1.4763118188	0.3160909474	0.0771939240	-0.9839860033	2.022530782	0.5635018691	0.1546990323
0.6745329537	-0.5378242733	-0.3334343433	-0.3921839600	1.675319067	0.9124444083	0.5073311112
-0.1139195233	-0.2883619711	-0.1460759433	-0.5376349377	1.555868868	0.8658532107	0.9103837051
-0.4255439055	-0.4526994577	-0.2603189444	-0.4485531066	1.951967924	0.9745422738	0.6747695356
1.613678119	0.2701489010	0.335903809	-0.946575314	2.13155277	0.4495793926	0.1215242849
2.088713081	0.5307607723	0.559451777	-1.1784867133	1.532249818	0.4394802414	0.0490929822
-0.7662039155	-0.4785652966	-0.4201306644	-0.5157778255	2.050842445	0.9704868139	0.4520823154
0.2339847229	-0.1411611811	-0.0741782400	-0.6423170499	1.925769642	0.784524939	0.8172628562
1.111983076	0.0177452786	0.222682331	-0.9905585499	2.249678179	0.5043958315	0.2787140336
-0.2385669177	-0.2700058366	-0.0640983088	-0.6332926733	1.882575211	0.7952816652	0.8137540285
2.448423639	0.8161893187	-0.0964326500	-0.7947441800	0.5809598664	0.6679484637	0.0232178546
-1.5200041055	-0.8514214955	-0.5393394733	-0.1526346700	1.793283485	0.7579423316	0.1434240909
-0.1365575888	-0.2993440155	-0.1452600922	-0.4860617455	1.814621891	0.8457673731	0.8926811945
-1.3456932199	-0.6118899099	-0.1385557066	-0.2397219044	1.556898472	0.9848387783	0.1927517498
2.118418649	0.0448582021	-0.6348916599	-0.2626257999	2.221007062	0.7048203277	0.0462340934
-0.5880709277	-0.4827403388	-0.3337525988	-0.4426431111	1.878815011	0.873121979	0.5627562164
-0.1777867322	-0.1623151599	-0.0533156399	-0.604514917	1.36984888	0.7842949867	0.8605942371
-1.8241765633	-0.4697835199	0.3703622126	-1.1210519888	1.610284213	0.9834930997	0.0823887148
-0.6570812833	0.0038879963	0.1399891421	-0.8668115122	2.022255487	0.6878733197	0.5182659046
0.2529619094	-0.3541092055	-0.2480798299	-0.4898673911	1.784391102	0.9732826873	0.8027574125
-0.4863103299	-0.2825367400	-0.1073724377	-0.6035849499	1.90871528	0.8286058087	0.6317835119
-0.7236159344	-0.0351746000	0.0746885604	-0.7352844622	1.677696595	0.724937938	0.4772854309
-2.0875436644	0.7502942281	0.796635375	-1.7084166522	1.249324831	0.5146946553	0.0492087156
-2.2019175322	-0.8157048666	-0.0600790022	-0.3860799266	2.406120445	0.8471929539	0.0389859379
1.413258151	-0.1293069133	0.2549976085	-1.0039527566	2.32866298	0.5899639681	0.1722317222
1.219343553	-0.6741161788	-0.1607254811	-0.5592471133	2.22390559	0.9426111274	0.2362329796
-0.2617273933	-0.2885065700	-0.1701966877	-0.6025528877	1.918758984	0.8628945975	0.7960815948
-0.4265668644	-0.2396787422	-0.1293119788	-0.6249002266	1.657303321	0.7163236761	0.674035845
0.0747767981	-0.2823817188	-0.1452218400	-0.4771461388	1.90828005	0.8472144756	0.9411005388
1.283692982	-0.2998953733	-0.3153258499	-0.64563588	1.829747598	0.9861339401	0.2132337182
0.757076298	-0.6006636499	-0.4826434433	-0.2017361722	2.056310223	0.9714019264	0.4574150989

-1.413940495	0.133416963	0.066318180	-1.037817354	1.795865353	0.7431317472	0.172033778
-1.17740508	-0.334793104	-0.118797945	-0.734346053	2.296932344	0.6816025597	0.252206188
0.080171407	-0.268574504	-0.118015312	-0.595966789	1.550684637	0.823046018	0.936860051
-0.81998505	-0.633934144	-0.635875504	-0.025165042	2.077511013	0.967337093	0.421434652
1.331175908	-0.464034770	-0.283040680	-0.597238323	1.388331011	0.8519507296	0.197403228
-3.268564487	-0.812295804	0.156903612	-0.207396285	0.705037692	0.8144220696	0.003669261
-0.279122285	-0.299375018	-0.183768053	-0.532798456	1.49197855	0.9135442479	0.782881375
-0.901860431	-0.65907633	-0.122335878	-0.341004480	1.820473816	0.9949784633	0.377358964
-1.972317699	-0.557801518	-0.160185769	0.025194006	0.545661187	0.8474817985	0.061885765
0.837255480	-0.307713594	-0.126532160	-0.350508582	2.062855392	0.7066139065	0.411876428
-0.636832315	-0.140194680	-0.147337014	-0.697100221	1.055619548	0.9857507193	0.531115561
-0.966853954	-0.734436604	-0.794937926	0.174869288	2.166685394	0.8085959748	0.344620829
0.173278806	-0.211724162	-0.077926100	-0.620751840	1.896225436	0.8128537565	0.864091627
-0.255788587	-0.349350368	-0.168474369	-0.560952124	1.905056492	0.8999358882	0.800602891
0.605596065	-0.170945159	0.051113270	-0.670085617	2.000243712	0.698581314	0.55127267
0.706039029	-0.383144284	-0.420279151	-0.552834531	1.30965829	0.8496853015	0.487923486
0.617823901	-0.290493385	-0.127537712	-0.654565896	2.019959567	0.7824871631	0.543334032
0.582659004	-0.311033926	-0.007300950	-0.474355309	2.006600884	0.6929789725	0.566327325
-2.517303115	-1.101914974	-0.777123531	-0.424975577	2.399679188	0.6079750842	0.020014945
1.334395624	-0.142227385	0.040745409	-0.774507362	2.143616131	0.6769387196	0.196364066
0.602955589	-0.291728200	-0.147821500	-0.700693287	2.018024788	0.8366880184	0.552994939
-1.358752407	0.040442234	-0.143868813	-0.621889651	1.973855548	0.6721404569	0.188641667
2.421264353	1.069719783	0.166316128	-1.056642199	1.03426891	0.3916560099	0.024607236
-2.090830133	0.131104418	0.243094702	-1.011178063	0.629879059	0.9669487492	0.048884087
0.170425216	-0.275733733	-0.072790287	-0.621599167	1.895418924	0.8121731312	0.866307014
-0.590635533	-0.307660815	-0.156322668	-0.516007698	1.749173976	0.8723048535	0.561068016
0.022749295	-0.263457601	-0.115537271	-0.566105781	1.582837637	0.8416901362	0.982065034
-1.885266042	-0.274522682	-0.478091563	-0.472807915	1.073051986	0.8032549942	0.073306671
-0.141570937	-0.160849351	-0.073818447	-0.608284255	1.900513995	0.801596871	0.888768265
-1.434479821	0.095776359	0.266416939	-1.042857456	1.838434806	0.6560771157	0.166160477
-0.707939279	-0.406450285	-0.089641343	-0.542988015	2.051099779	0.7969144681	0.486766818
-1.057668749	-0.094846878	0.298298878	-0.794330446	2.079102215	0.5753156276	0.302222002
-1.308199556	0.270717638	0.218765055	-1.082184022	1.963476086	0.5889640871	0.204944538
-0.099117332	-0.275850162	-0.126824888	-0.604030708	1.85875882	0.8422626605	0.921985183
0.675056958	-0.199753537	0.047074674	-0.574055416	2.01137738	0.6776752511	0.507004803
-1.612397941	-0.511583185	-0.401404070	-0.482705873	0.842836969	0.6788049825	0.121803564
0.419756377	-0.372283680	0.075462893	-0.700739153	1.94690959	0.7879452362	0.678926722
0.960255450	-0.633212295	-0.139780808	-0.587865361	1.372273496	0.8327969024	0.347853377
-0.678671515	-0.378873062	-0.374379420	-0.376065509	2.002714069	0.9167007465	0.504757171
-0.643679733	-0.286902600	-0.132976166	-0.533686498	1.756150008	0.8544969855	0.526750973
1.25507908	-0.420548462	-0.149403329	-0.641229649	0.705876118	0.7636947236	0.223237907
-0.546398452	-0.234739968	-0.073075288	-0.666963407	1.999801811	0.763185827	0.590550802
0.25223142	-0.219957531	-0.075040294	-0.643008700	1.805102594	0.8241636408	0.803314460
-0.917613512	-0.668430492	-0.548179478	-0.167651972	2.118692966	0.953115036	0.369240410
1.426160789	0.020401609	0.194804928	-0.982728563	1.927783875	0.6769491527	0.168519571
-0.559113960	-0.327033817	-0.256284065	-0.484852526	1.997263799	0.848028459	0.581998207

1.563635446	-0.479338673	-0.113293253	-0.64960326	0.6367274268	0.7122875792	0.1328483636
0.6843620681	-0.225202432	0.041885074	-0.758622507	1.990684517	0.7664187647	0.5012300742
1.441998506	-0.245764583	-0.535300633	-0.279751234	1.766153836	0.9557946852	0.1640513269
-0.376835869	-0.292499551	-0.136668839	-0.560753168	1.940750297	0.7946748907	0.7100765252
2.243849167	-0.764096133	-0.465686347	-0.646191642	2.606378513	0.9518287676	0.0357505255
-0.888346854	-0.232171183	0.0295897399	-0.663399375	1.34481935	0.8866779252	0.3844167313
-0.736966604	-0.350310489	-0.334817026	-0.401560857	2.045092293	0.8567144552	0.4692969601
-0.509679617	-0.170673445	0.0206242535	-0.683112735	1.881698261	0.7553133659	0.6155906432
-1.180611779	-0.037543165	0.3366596183	-0.924226368	1.750313638	0.6567803028	0.2509569817
-0.202736601	-0.218700244	-0.101390537	-0.617443684	1.888048157	0.7932623168	0.8412922969
1.107172814	-0.437503354	-0.271967965	-0.504719294	1.325907928	0.8968117631	0.2807406373
-1.746321996	0.0311381110	-0.100455658	-1.231251388	1.573808193	0.9627766	0.0953682474
-0.417373273	-0.270502021	-0.131618241	-0.659913796	1.732895278	0.8972642791	0.6806415674
2.553869563	0.7994253550	0.0635370398	-0.784701878	1.006429498	0.4985502318	0.0184873354
-0.882398847	-0.231473976	0.1119531690	-0.543144455	2.113568437	0.6110107935	0.3875504988
0.8951589635	-0.175619148	-0.117274638	-0.753674129	1.990194028	0.8147311394	0.3808481874
1.305695971	-0.611793893	0.4002672920	-0.629526132	1.666753307	0.8112637557	0.2057796704
1.561076241	0.0901312014	-0.011165971	-0.576905450	2.490528071	0.4169625488	0.1334503143
1.718428996	0.6534129948	0.0620241570	-0.719027097	0.574008924	0.6268099033	0.1004283297
1.430635627	-0.159637152	-0.704235155	-0.655797727	1.044202985	0.6834479754	0.1672472759
1.69537538	0.7127650364	0.0052329028	-0.812789002	2.180382022	0.3441504541	0.1047820704
-0.172652877	-0.217330834	-0.130406647	-0.618937706	1.91223711	0.8127951188	0.8645774706
0.6495393602	-0.407936207	-0.227935038	-0.648168387	1.986681003	0.9549499385	0.5230316625
-2.209192031	0.2508486710	-0.259305571	-1.090920974	2.153152885	0.7448344751	0.0384061752
1.82747006	0.766293007	0.5733397593	-1.34136946	1.421808741	0.4048648536	0.0818751848
0.3485482229	-0.276678602	-0.178739094	-0.514666418	1.636759779	0.8781287503	0.7308986096
0.3126788071	-0.291069435	-0.170786830	-0.599683473	1.850309191	0.879753447	0.7576086032
0.478950048	-0.044019316	-0.030677388	-0.624631261	1.212263801	0.7935752634	0.6369235708
0.6965191979	-0.546241916	-0.233210377	-0.389312795	2.053837395	0.8976292203	0.4937420204
1.205639189	0.4510094624	0.0039055254	-0.979436509	1.393930136	0.6499381407	0.2413661101
-0.261672503	-0.233559835	-0.129539062	-0.620905523	1.84372056	0.8361448292	0.79612335
-1.202341667	-0.115588481	-0.131234422	-0.961438492	1.589166088	0.9999969667	0.2426137194
-0.019122087	-0.248718640	-0.124898519	-0.555821786	1.56436378	0.8405587936	0.9849242331
0.7868270087	-0.027280760	0.0236398058	-0.860175203	2.014239742	0.6050781109	0.4401730268
-0.635417062	0.0018057912	0.0174552501	-0.763671934	2.013400527	0.6745310437	0.5320201004
1.916876523	0.6061735860	0.0787454761	-1.073954063	2.781713554	0.2609786169	0.0689611911
-2.701883533	0.9625877653	-0.071675855	-0.850393663	0.7470791032	0.5531842982	0.0133539927
-2.379238941	0.9654766011	-0.045673290	-0.934778153	2.15839327	0.2926475097	0.0269099643
0.6022104862	0.4807260867	-0.627604966	-0.546184329	0.2613551780	0.8043453469	0.5534814492
2.72339589	0.7271826423	-0.223816320	-0.674354057	-0.410771494	0.8673804711	0.0127311123
0.8478703408	0.4215509467	-0.893314431	-0.234597576	-0.038034471	0.6598611084	0.406070667
-1.443746171	-0.332392474	-1.579040444	0.6846353077	0.2595690389	0.3069231726	0.1635641806
0.0678555897	0.2817935644	-0.711848215	-0.367818475	0.1911650359	0.7428009081	0.9465425232
0.196756952	0.186273653	-0.758659592	-0.302279394	0.1982867232	0.6704198663	0.8459095008
0.8626800771	-0.098520203	-0.981693086	-0.115287048	-0.037980848	0.4636861449	0.3980585952
-2.004917184	0.0724519955	-1.134982836	0.2129284840	-0.883955971	0.3342094417	0.0580328834

0.337906667	0.417794608	-0.589528777	-0.448486337	0.164993031	0.8172172445	0.738788133
-0.513687791	0.097977796	-0.858852484	-0.255233096	0.165589871	0.5878893091	0.612833079
-0.263125511	0.162845217	-0.784122079	-0.283661932	0.258191227	0.6562957082	0.795018248
-0.765600003	0.071596040	-0.987898655	-0.280459641	0.352061819	0.5410920501	0.452433981
0.285179864	0.385566867	-0.65828074	-0.448210859	0.242606178	0.7804883618	0.778300015
-0.243344965	0.213383339	-0.777547699	-0.245371749	0.027283275	0.6491880488	0.810099492
-1.240158691	0.333343771	-0.425214693	-0.608008835	0.609817332	0.9153745537	0.228595464
0.504501468	0.484205764	-0.731611307	-0.390234011	-0.089583587	0.7257657409	0.619161767
0.243867085	0.358978274	-0.645650039	-0.420495148	0.231056348	0.7768298754	0.809700411
-0.259715867	0.226461578	-0.766189432	-0.222604708	0.128456595	0.6783271185	0.797612184
2.050527867	0.811374098	-0.800841017	-0.933875294	0.761361029	0.9034670779	0.053001147
1.465279291	0.536416261	-1.096561162	-0.109669728	0.292844683	0.7602717578	0.157657517
-0.494745697	0.077480369	-0.870567427	-0.235796441	0.463590391	0.6652207592	0.625916073
-1.778356265	1.100312964	-0.073524567	-1.249950086	1.496788987	0.4386018349	0.089828505
1.010667784	0.388697529	-1.004705134	-0.080149336	0.419815847	0.7769266087	0.323679498
0.312965676	0.140110230	-0.802681464	-0.130593311	0.133897128	0.6412494845	0.757393698
-0.809705035	0.585726449	-0.168570675	-0.604147543	0.404022842	0.9025440061	0.427189824
0.031419688	0.287369021	-0.738133152	-0.364058058	0.200916431	0.6969495893	0.975231548
0.145422295	0.226000264	-0.74929708	-0.32884539	0.231348857	0.6817039741	0.885764240
-1.183174	0.834820791	-0.159791126	-0.971445896	-0.214393075	0.8803092375	0.249962172
0.872998964	0.489185124	-0.782846758	-0.475806103	0.079843320	0.7038475002	0.392536880
2.913798589	0.664675086	-0.048421277	-1.247487073	1.092527786	0.8219885085	0.008301026
-1.729578537	0.862552702	-0.748596242	-0.472089558	-0.238394848	0.8340657118	0.098378770
-1.74546042	0.229232464	-1.066792202	-0.414927198	0.253890195	0.5170569503	0.095521209
1.2657596	0.186494804	-0.76428753	-0.301396254	-0.749011142	0.3846938922	0.219462330
0.130981020	0.204883150	-0.742465891	-0.247379909	0.212452812	0.6844267913	0.897037007
0.631534744	0.283607567	-0.826947659	-0.379821545	0.131097272	0.6278320176	0.534505723
-1.02579019	0.737954922	-0.16005006	-0.793892117	-0.33175381	0.8756412035	0.316662138
1.606382661	-0.17524947	-1.000223138	0.137722978	0.421455012	0.5935387603	0.123123053
0.243426899	0.297455155	-0.741302842	-0.337940013	0.048554928	0.6711364331	0.810036862
-0.16071108	0.271975812	-0.751817878	-0.36799843	0.057995433	0.6748394584	0.873856933
-0.902103185	-0.166416851	-1.15886183	0.215247419	0.656226512	0.5192667523	0.377232968
0.237275647	0.253062905	-0.760480017	-0.360134461	0.103390371	0.6586848443	0.814742419
0.153251080	0.306513350	-0.749170451	-0.384083819	0.246862523	0.7173643176	0.879663332
-0.74662293	0.228000393	-0.885643791	-0.215841195	-0.213474325	0.54054264	0.463568761
0.833167755	0.636789629	-0.757465394	-0.582870974	0.304887635	0.8519148228	0.414126217
0.814313125	0.396077003	-0.740579209	-0.614401223	0.638822020	0.8297814776	0.424603973
-0.01083130	0.287294970	-0.737494577	-0.352813274	0.194790856	0.701566099	0.991460276
0.471015483	0.183883571	-0.727989359	-0.28665567	0.458589577	0.8121487916	0.642485745
-1.005340608	-0.257107192	-1.249742299	0.379661606	0.542042770	0.4235068276	0.326177213
0.468102656	0.416333522	-0.590257925	-0.508904954	0.170369765	0.7604837287	0.644533086
0.075152552	0.295224826	-0.715596002	-0.370893913	0.202312581	0.7266053753	0.940804662
-0.352129861	0.223193786	-0.809863063	-0.321338215	0.140043200	0.6321967721	0.728250027
0.758315660	0.173404761	-1.000484418	-0.313946873	-0.20872670	0.4668650479	0.456688788
-1.408452604	0.326183828	-0.776320893	-0.257458607	-0.319461715	0.5803836476	0.173630961
-0.308227272	0.304518179	-0.786612175	-0.419170423	0.077186567	0.6384056722	0.760945985

1.708382911	0.8351688475	-0.330429214	-0.558871616	0.0571567967	0.9144212927	0.1023061965
-0.372743416	0.2460587796	-0.782055873	-0.319298967	0.1516420048	0.6587052712	0.7130750465
-1.285459176	0.3252346753	-0.726592796	-0.143445896	-0.149741839	0.6950496942	0.2126277585
0.0804796761	0.2600128064	-0.736959485	-0.364363684	0.2013160668	0.6915600675	0.9366178187
0.0221702441	0.2520079496	-0.728527668	-0.363038466	0.1753712621	0.7118166711	0.9825214644
-0.073844824	0.2961732617	-0.711642784	-0.371548511	0.1281752598	0.6934564938	0.9418327421
0.8437589321	0.2975284508	-0.468608412	-0.559227232	0.4030476628	0.8140819042	0.4083131439
0.0383866672	0.2886446566	-0.734960891	-0.366041559	0.2037652423	0.7006559668	0.9697419788
-0.288932082	0.2111761544	-0.791745407	-0.236730643	0.0002590057	0.6358391348	0.7754663336
-0.162425492	0.2893730664	-0.755125480	-0.348201317	0.1127303776	0.676647066	0.8725235583
-0.206098890	0.3528347129	-0.626787198	-0.414824557	0.2377797946	0.805720154	0.8386986541
-0.108308421	0.3055150499	-0.679127591	-0.379914881	0.187677489	0.7136859725	0.9147792384
1.814278493	0.6400246636	-0.894805931	-0.543563335	-0.270521395	0.5941323519	0.0839489299
-0.102472924	0.3006511604	-0.636542863	-0.376808453	0.2116825661	0.7408224834	0.9193535207
1.812951528	-0.457356537	-1.246259472	0.3454033457	0.2467740142	0.3918178151	0.0841600348
0.0895737298	0.2863376537	-0.739003185	-0.355894637	0.2134322094	0.7052819469	0.9294747721
-0.920083213	0.18449521	-0.962453419	-0.411555960	0.0609751275	0.5051445891	0.3679782556
-0.331693409	0.2396567713	-0.784104607	-0.333084710	-0.008706021	0.6268226125	0.7434083157
-0.914629429	0.5080883408	-1.085118444	-0.070342524	-0.117947902	0.6007707708	0.3707692924
0.731243059	-0.015859939	-0.753221501	-0.351345583	-0.121176427	0.5079305449	0.472711939
0.6342815225	0.3821928666	-0.453101235	-0.539156112	0.1181855532	0.7717538915	0.5327464705
0.0968814357	0.2882718432	-0.737119172	-0.372374381	0.2158620055	0.7005760199	0.9237392219
-1.034082206	0.4041141985	-0.742591329	-0.355641214	0.8232695278	0.9628816034	0.3128600922
0.6692155784	0.2365284542	-0.809561523	-0.279558844	0.0096370369	0.6166003549	0.5106490299
-0.750215509	0.1321177315	-0.883502708	-0.168020683	0.3686676498	0.6674208583	0.46144833
-0.792126706	-0.100535239	-1.033443945	-0.002502143	0.6256157413	0.6031574333	0.4371440277
-0.023333088	0.2755548141	-0.723285376	-0.344905164	0.2011263186	0.697678011	0.9816048734
0.0907530481	0.2940454126	-0.696083223	-0.374893392	0.1780851185	0.6996888068	0.9285488945
-1.058671834	0.4205514893	-0.772001966	-0.361627449	0.8111500726	0.9854085506	0.3017753752
-0.871374753	0.2229815889	-0.941439296	-0.137002411	0.1146283274	0.6154067848	0.3934026916
-0.428472934	0.275091306	-0.590043631	-0.454224604	0.2624961573	0.7339157313	0.672669652
-0.615691539	0.2647604721	-0.756606709	-0.306414068	0.3789952423	0.7605465215	0.5447140138
-0.842393925	0.4593213102	-0.620261123	-0.377602964	-0.006182634	0.777268066	0.409059403
0.6562092896	0.2532130637	-0.850028649	-0.326125300	0.4738434315	0.7340400439	0.518815686
0.6053175763	0.346864706	-0.540450561	-0.507325123	0.0728479455	0.7143869831	0.5514541828
0.6479772143	0.1543042448	-0.898017471	-0.245808851	0.246949029	0.6050480793	0.5240217956
-1.358415292	0.5716428262	-0.180649115	-0.746244495	-0.018552402	0.8961590114	0.1887468887
2.230691663	-0.23814796	-1.101111978	-0.151660098	-0.437647234	0.278598834	0.0367383326
-1.597338787	0.5256294554	-0.571412988	-0.538880213	0.8577144389	0.9274688099	0.1251293948
-0.184662687	0.3164619616	-0.733966104	-0.406706074	0.151085981	0.6796425275	0.8552653247
-0.023885610	0.2874719961	-0.737592615	-0.363282450	0.1862275777	0.6990835866	0.9811693666
0.3213498491	0.397139944	-0.710310811	-0.377501392	0.0581816619	0.7425857879	0.751121746
0.8717941522	0.2425350927	-0.957964293	-0.433077674	-0.045608856	0.5005323652	0.3931790058
-0.905909032	0.1850316415	-0.765430640	-0.210363905	0.1567222651	0.6766188034	0.375261271
-0.594908619	0.4319125111	-0.919605622	-0.363663961	0.3226353542	0.6879727184	0.5582610302
-0.002696221	0.2780918791	-0.735327151	-0.364080895	0.1800245546	0.7203036153	0.9978741781

-0.493865462	-0.014484665	-0.793763163	-0.342561696	0.460240965	0.6437326543	0.626527164
0.038114635	0.289372450	-0.689351992	-0.364931091	0.155910412	0.7131641025	0.969956296
0.332986884	0.425518805	-0.708960423	-0.398264324	0.229761415	0.8414265508	0.742445662
0.334399348	0.182497547	-0.735828743	-0.302932661	0.139080942	0.6586231911	0.741394946
1.161007398	0.049244040	-0.929928408	-0.456715545	0.326955680	0.5121699649	0.258666812
0.428437049	0.172970457	-0.716964815	-0.273011677	0.194703760	0.6830164952	0.672695362
1.119048648	0.866833082	-0.271720296	-0.803879980	-0.135133141	0.993400034	0.275756638
-0.357976121	0.287144811	-0.678985894	-0.431376674	0.322467994	0.7417941517	0.723934266
0.127260468	0.281209503	-0.748242430	-0.364525331	0.180055538	0.6851308872	0.899944963
-1.805812581	-0.501701341	-1.138170695	-0.299374740	1.28655429	0.5422606982	0.085303682
-1.502009509	0.904364779	-0.541413732	-0.782431464	-0.312830575	0.8183899329	0.147983161
0.601852887	0.580647888	-0.671184904	-0.544413969	-0.033366141	0.7919283855	0.553715021
-0.965671332	0.438522546	-0.756721574	-0.461915764	0.042165855	0.6814701963	0.345198668
-0.214435354	0.314503545	-0.737967537	-0.413221103	0.136992654	0.6736854589	0.832276136
-0.308690037	0.093306795	-0.800611295	-0.238478338	0.342855485	0.6558623677	0.760598819
-0.405485827	0.150758368	-0.804575487	-0.184037199	-0.089268274	0.6021405158	0.689221913
1.142848945	-0.192079327	-0.998973835	-0.022896265	-0.011585396	0.4411484808	0.265963814
0.042343231	0.274878987	-0.727936879	-0.364137682	0.190801806	0.7514461549	0.966625106
0.012678759	0.248808662	-0.737511699	-0.362850002	0.184102332	0.6984985754	0.990003766
-0.558231271	0.536509443	-0.719032577	-0.426868048	0.203127101	0.8666347224	0.582589904
-0.309649221	0.057513543	0.588660058	0.263129494	1.220284805	0.3011301266	0.759879406
0.978737021	0.320281447	0.868662280	0.082054164	1.018530767	0.2142411944	0.338851418
1.539382489	0.424838799	0.391472295	0.415306502	0.848045494	0.2684990196	0.138644297
1.567419492	0.826022887	1.610013688	-0.917783737	1.271393762	0.08310798813	0.131962450
-1.083706231	0.254423077	0.454646707	0.242801889	0.174550626	0.5264483409	0.290781171
-0.944564151	0.537851255	0.792791963	-0.073436270	1.29125032	0.1816038449	0.355623226
-2.652808862	1.301736273	1.540796123	-0.551572258	2.115337231	0.02642116863	0.014884615
-0.932526590	0.066947798	0.495662889	0.441134608	0.586271252	0.39732614	0.361662888
-0.486105574	-0.088502021	0.461214506	0.306209261	1.305142272	0.3857703867	0.631926246
1.370435118	0.636350152	1.046564442	-0.103177982	1.401091729	0.1226356512	0.185023828
1.201930954	0.634004509	1.033564757	-0.143719540	0.968370045	0.1545586732	0.242769451
1.380748843	0.552497081	1.166670263	0.020240353	1.007825837	0.1282274631	0.181875880
-0.147331557	0.090033250	0.603165114	0.214385668	1.217183144	0.3059524416	0.884275683
1.341136118	0.529266140	1.064242938	-0.344325791	1.837299611	0.1100156351	0.194202497
-0.000949319	0.175595155	0.630081381	0.166392523	1.183066933	0.2728029745	0.999251513
0.163821441	0.230514745	0.654616813	0.161758845	0.988390556	0.2558558325	0.871438152
1.749405199	0.843670141	1.141841493	-0.343354606	1.563216486	0.09203022303	0.094822587
2.09766964	0.630278077	0.983396819	-0.708422867	1.865038909	0.15105293	0.048214670
1.422884055	0.529746708	0.686597450	-0.210061789	1.647478228	0.1730403653	0.169456150
1.101350674	0.357893208	0.392331879	0.371831033	1.350879356	0.2193263488	0.283207884
-0.860396756	-0.1641085	0.305800326	0.375644892	1.535700496	0.2847501365	0.399287171
1.47690336	-0.524108169	0.086196198	0.912763239	-0.276809471	0.9866616848	0.154541690
0.413573730	0.214855357	0.514206299	0.277735581	1.3197212	0.2427251902	0.683379325
-0.189678954	0.236584769	0.673571177	0.036367768	1.268458171	0.2587160568	0.851382130
-2.115385234	1.007536968	1.776742743	-0.482819485	1.878029551	0.03573346079	0.046518987
-0.836214559	0.171121331	0.698469496	0.168432626	1.255174812	0.2487431277	0.412448587

1.564880941	-0.322235108	0.2293112594	0.482406232	1.688727945	0.3870402502	0.1325562273
-1.768670637	1.025194005	1.408127041	-0.834100221	0.6517815133	0.1285951267	0.0914734324
-0.376699531	0.0827970194	0.6668711329	0.2155429961	1.299363423	0.2611697117	0.7101763428
-0.743224098	0.0935087419	0.4325294458	0.3774609196	0.9929163871	0.344803248	0.4655801956
-1.465387927	0.6594814107	0.7167395585	0.1074419205	0.8958700365	0.1814800369	0.1576281629
-1.92706551	0.1045904916	0.3780110119	0.1562364456	1.404762095	0.3390877527	0.0676096823
0.424256454	0.1387796716	0.6540827459	0.1955381939	0.5901286446	0.3832166978	0.6756933987
0.798558248	-0.176115799	0.385028071	0.5539049306	1.347595046	0.3255702119	0.4334854718
-0.787705639	0.1859260899	0.7662687914	0.1874456353	1.352485939	0.2132624671	0.439669961
-1.202420784	0.7205277408	1.173685282	-0.382837183	0.5281978461	0.1643569447	0.2425837289
-0.279222421	0.2494529844	0.685111315	0.0738112391	1.20957045	0.2518228364	0.7828055758
0.8392004314	0.2135981143	0.6519398299	0.2573858303	0.6703974138	0.3203158469	0.4108087078
-0.074089676	0.1684447368	0.6376072011	0.1680110945	0.9402499832	0.3226767332	0.9416402419
0.2202244385	0.2588012935	0.6366863068	0.0123205846	0.9419828456	0.2544158725	0.8278231804
-0.228123377	0.2048632804	0.6734092327	0.1655786524	1.255708362	0.2533844132	0.8217569205
-1.702033883	-0.062535239	0.8547931125	0.4483382177	0.472504392	0.3466059998	0.103508377
0.4805444686	0.215824305	0.7399636256	0.0733600081	1.328101461	0.2223719648	0.6358085023
0.3336691597	0.3078751915	0.6495442029	0.0729817859	1.289758194	0.2407655416	0.7419380615
0.4884155013	0.2392900313	0.6597191541	0.0027472236	1.310758749	0.2279418744	0.6303168527
0.2822148972	0.1669220217	0.653642916	0.2419175204	1.28770251	0.2460483663	0.7805414054
2.380298205	-0.303512224	0.7993397039	0.5669373099	2.619711937	0.06506776697	0.0268495505
-0.268155088	0.0154630776	0.250680063	0.3043708727	1.271740352	0.3722148868	0.7911963677
-0.840229945	-0.083166168	0.4148395214	0.46070386	1.327741792	0.3227087887	0.4102442496
-0.072204430	0.1390645524	0.6307905659	0.1776135149	1.252423669	0.2905277405	0.9431224967
-0.288910688	0.1237624304	0.5408667844	0.2028523697	1.192552937	0.3228635038	0.7754824814
0.7917506391	0.0568987886	0.2572270159	0.2284536697	0.6976521348	0.5702719989	0.4373585409
-0.828619605	0.1936888169	0.6587500426	0.2436738393	0.8844293288	0.291942908	0.4166385605
-2.12061281	0.3117365239	0.2672842589	-0.239685406	0.5329940513	0.6391320087	0.0460290032
1.198404007	0.5542169049	0.9535799875	0.0537700532	1.182481649	0.1483998961	0.2441098883
-1.204086557	0.0503689934	0.5109223458	0.3151283954	1.133086555	0.3289203945	0.24195294
0.8495098475	0.1606687849	0.636074562	0.0186666699	1.459250804	0.2552744505	0.4051786384
0.3440851382	0.0898989830	0.6553483418	0.1697697585	1.262913162	0.2863044817	0.7342038304
1.118360637	0.7658542030	0.8080069232	-0.007365852	0.7478455484	0.1629046821	0.2760436028
0.4928579722	0.0718311278	0.5537387609	0.253912357	1.327631133	0.2416824003	0.6272269425
-1.5363527	0.1750236073	0.2045671549	0.5369861264	0.9092302965	0.3700148607	0.1393828228
-1.066598718	0.1277007413	0.6191721818	0.3190383981	1.022585414	0.2869296097	0.2982624789
-1.340409195	-0.139180979	0.2396475673	0.6466846811	0.3108206269	0.605547194	0.1944347053
-0.997957680	0.1927827683	0.474536551	0.2635015945	0.7409469317	0.3806201919	0.3296609601
1.652558436	-0.711075098	0.0996596738	0.7756019378	0.9742661959	0.7706015802	0.1132949128
1.242635326	-0.149717859	0.3054651681	0.5548780955	1.424440857	0.3367112797	0.2276994246
-0.846932200	0.0183809170	0.7090532725	0.2435182125	1.454217675	0.2322691914	0.4065816592
0.7068452854	0.0515213954	0.3244825557	0.2964393816	1.169372664	0.4013326147	0.4874325302
-0.393510773	0.3214279623	0.7345039117	0.0036202793	1.252441588	0.2344039723	0.6979090696
-0.661673797	0.1833709149	0.668924896	0.1177193847	1.122471595	0.2852700399	0.5153757219
-2.129277161	-0.053777016	0.1494761509	0.0933817870	1.041474051	0.6081501543	0.0452271417
0.8501040970	0.2934920835	0.7938067561	0.0936485921	1.533128427	0.171505748	0.4048556272

1.261052512	-0.13260191	1.173897682	-0.22257160	1.651896718	0.1845233252	0.22112019
0.279096343	0.05385563	0.64974941	0.17661555	1.025721333	0.3630640965	0.78290101
0.69921527	0.28111361	0.87234649	-0.04327338	1.168005907	0.2191776834	0.49209014
-0.97558252	0.16816342	0.66073866	0.27380441	1.05588342	0.2675144523	0.340376455
-1.33596555	0.32595897	0.69531474	0.19958822	1.860397987	0.1153484049	0.19585893
-0.15815586	0.18765738	0.66414839	0.14822531	1.250437562	0.2556595219	0.87584494
0.85215867	0.34837173	0.82080303	-0.04775927	1.048318978	0.2367472706	0.40374011
0.63044343	0.44448255	0.87677771	-0.10948146	0.66461893	0.2221069439	0.535205558
1.235814588	0.44077089	0.93879812	-0.15905731	1.237146822	0.1875163294	0.23017364
1.235348085	0.29032480	0.943811973	-0.06258381	1.026611635	0.2276074775	0.23034361
-1.22592143	0.32877228	0.65625213	0.18974493	1.777746659	0.1315818558	0.233798756
0.17381151	0.18886142	0.67423734	0.12006996	1.266891167	0.2544961547	0.86367818
0.90736816	0.20675314	0.38132295	0.37627854	1.130262228	0.2973547458	0.37450715
0.14541151	0.181418049	0.65422550	0.15499750	1.152478554	0.2723720261	0.88577264
-1.814659684	0.55900594	0.95500186	0.16679633	0.87076532	0.1553516045	0.08388837
-0.132142642	0.18276764	0.66443407	0.16118707	1.064463498	0.2654735542	0.89612939
0.97792380	0.27348463	0.91683466	-0.07703066	1.053975442	0.2338117846	0.33924411
-1.33103088	0.44576456	1.027711959	-0.06455079	1.210604661	0.1597935083	0.19745013
-0.10278770	0.19303049	0.63994688	0.13404469	1.220915395	0.2608599644	0.91910670
1.287419677	-0.13922960	0.50449775	0.31994094	0.87124035	0.466556686	0.21195669
-0.06884262	0.18359670	0.65458017	0.16225883	1.169744983	0.2739226549	0.94576620
0.420597881	0.09576420	0.64471168	0.31176531	1.323815402	0.2381531031	0.67832161
0.890149429	0.16336384	0.668726127	0.31453685	1.471873322	0.1988350879	0.38347031
0.61436300	0.42166368	0.70518808	0.14685642	0.922514088	0.222133217	0.54557473
1.171214928	0.114843311	0.33122308	0.08965015	0.92015318	0.4914447635	0.25463077
0.79079948	0.26922451	0.67262975	0.03403202	1.312783416	0.2461404506	0.43790137
-1.33422195	0.51780181	0.08724360	0.18482324	1.558588279	0.2493596118	0.19642000
-0.60142573	0.02793408	0.60671154	0.15015040	0.8892206	0.4149983728	0.55399408
-0.00232328	0.14810986	0.64642003	0.16964115	0.99742682	0.2657615721	0.99816821
1.867324302	0.35731114	-0.11627047	0.14457319	0.26704495	0.7469547729	0.07587850
0.95012735	0.68407799	0.742002534	0.07211049	1.361483572	0.1526049493	0.35285515
0.24231603	0.10085252	0.65447662	0.20872076	1.194742018	0.2842186029	0.81088610
-1.32725454	0.45382935	0.87347523	0.26992647	1.160288865	0.1531484299	0.19867472
0.12249198	0.13979874	0.65683920	0.19079841	1.253339122	0.2622024263	0.90367407
1.026278324	0.71923607	1.009837403	-0.26576982	0.92361425	0.1565210068	0.31643742
-0.65749047	0.17592639	0.747272976	0.03196358	1.422781281	0.2217069092	0.51800825
-2.01588025	0.28209767	0.98312299	0.18823296	1.644864254	0.1226658874	0.05678601
-0.01604049	0.15148369	0.63464653	0.17031718	0.99563704	0.2633664144	0.98735350
-0.91280550	0.54550491	0.79351740	-0.08524972	0.89266672	0.2140026959	0.37170584
0.74529892	0.57052928	0.73941449	-0.08200767	0.90849303	0.2076024718	0.46435169
0.51125732	0.09594284	0.65670628	0.21838712	1.329777614	0.2543155818	0.61450450
-2.33746808	0.52119994	0.73397089	-0.54604083	0.74441681	0.4003879066	0.02939495
-0.61137285	-0.15454589	0.34510491	0.36870788	1.385262387	0.3233943031	0.54751461
0.69746153	0.38199222	0.78056009	-0.10635039	1.430645051	0.1896064452	0.49316429
0.37407923	0.00970905	0.55158916	0.272708443	1.166558593	0.3509633057	0.712095773
1.431913881	0.81607423	0.83989092	-0.15829363	1.883657849	0.09202213254	0.16688526

-1.150101141	0.691648198	0.703913184	0.107628269	0.629632672	0.1959186759	0.263031466
-1.426489391	0.896117675	0.740608123	0.017554677	1.317892642	0.1149057812	0.168425877

t_p_blast	t_p_vns	t_p_interact	t_p_batch	t_q_microbe	t_q_blast	t_q_vns
0.000002559	0.594768037	0.3261210949	0.027529537	0.975076637	0.000005033	0.702397450
0.000003053	0.434927067	0.4801742436	0.028649348	0.975076637	0.000005188	0.702397450
0.000003178	0.570352589	0.4067835421	0.029468605	0.975076637	0.000005297	0.702397450
0.000010120	0.590840601	0.6223401172	0.034114898	0.990709099	0.000011906	0.702397450
0.000002501	0.507066849	0.4525996413	0.122936335	0.990709099	0.000005033	0.702397450
0.000014875	0.388497092	0.6588031943	0.029562960	0.847656972	0.000016377	0.702397450
0.000014146	0.424331081	0.5569302067	0.052989780	0.975076637	0.000015865	0.702397450
0.000000426	0.703245748	0.1732235146	0.004410470	0.532640168	0.000005033	0.775145902
0.000027584	0.424898727	0.5619357663	0.030644382	0.975076637	0.000028984	0.702397450
0.000007860	0.422219059	0.5254753497	0.036311601	0.975076637	0.000009724	0.702397450
0.000010443	0.436794515	0.5353459757	0.030898899	0.975076637	0.000012166	0.702397450
0.000005159	0.234692208	0.5305100643	0.016195358	0.725565304	0.000007371	0.702397450
0.000005624	0.595205321	0.3537506627	0.028773841	0.975076637	0.000007791	0.702397450
0.000001577	0.10062636	0.7752056937	0.605118199	0.265646546	0.000005033	0.702397450
0.000002289	0.574176524	0.4185409641	0.034633023	0.975076637	0.000005033	0.702397450
0.000013408	0.500562598	0.4559034085	0.055026614	0.987880558	0.000015323	0.702397450
0.000007968	0.500253391	0.4902636879	0.036819829	0.990709099	0.000009757	0.702397450
0.000002810	0.530812388	0.4174284704	0.033726062	0.985409022	0.000005033	0.702397450
0.000002769	0.502057856	0.3975183258	0.031108202	0.975076637	0.000005033	0.702397450
0.000002460	0.461123444	0.4968081775	0.032613410	0.975076637	0.000005033	0.702397450
0.000005902	0.583920538	0.4327182837	0.114095757	0.987880558	0.000008048	0.702397450
0.000032950	0.146192159	0.7004955247	0.964962110	0.532640168	0.000033508	0.702397450
0.000001612	0.798606576	0.237995127	0.059299879	0.684817630	0.000005033	0.855649903
0.000000906	0.972545031	0.1339431366	0.014445235	0.684817630	0.000005033	0.972545031
0.000010047	0.457759699	0.5379055499	0.047329901	0.975076637	0.000011906	0.702397450
0.000002429	0.502541619	0.4506927005	0.034302267	0.990709099	0.000005033	0.702397450
0.000006531	0.448198735	0.5069442048	0.031596855	0.975076637	0.000008519	0.702397450
0.000012542	0.595058506	0.4739419473	0.051007488	0.987972281	0.000014471	0.702397450
0.000001281	0.463960466	0.362551717	0.043372783	0.910196246	0.000005033	0.702397450
0.000002639	0.391385039	0.604558244	0.063148713	0.975076637	0.000005033	0.702397450
0.000006800	0.497163893	0.459553832	0.033011219	0.975076637	0.000008681	0.702397450
0.000002184	0.556437223	0.4522537525	0.034276231	0.975076637	0.000005033	0.702397450
0.000002538	0.503055075	0.4496415629	0.117034018	0.990709099	0.000005033	0.702397450
0.000001052	0.835932586	0.151426727	0.037421318	0.684817630	0.000005033	0.872240312
0.000001859	0.420858953	0.4313404281	0.039728548	0.975076637	0.000005033	0.702397450
0.000000444	0.723667317	0.1066410966	0.273415956	0.532640168	0.000005033	0.783736364
0.000005415	0.476621918	0.5390501766	0.032127895	0.975076637	0.000007644	0.702397450
0.000001568	0.485119889	0.5050325809	0.156461029	0.975076637	0.000005033	0.702397450
0.000002592	0.502252300	0.4500012858	0.095343915	0.990709099	0.000005033	0.702397450
0.000023662	0.357165180	0.793739578	0.033565616	0.975076637	0.000025128	0.702397450
0.000001405	0.570832102	0.4277901629	0.018835933	0.975076637	0.000005033	0.702397450
0.000000311	0.345200456	0.2460471583	0.004084425	0.532640168	0.000005033	0.702397450
0.000002595	0.456207973	0.5085072178	0.100100908	0.975076637	0.000005033	0.702397450
0.000027777	0.490407161	0.7122537506	0.040979692	0.704539802	0.000028984	0.702397450
0.000001090	0.494789959	0.2519376767	0.014324952	0.847656972	0.000005033	0.702397450
0.000000358	0.444207036	0.14928759	0.051132851	0.532640168	0.000005033	0.702397450

0.000000357	0.430746303	0.2597743306	0.583583141	0.532640168	0.000005033	0.702397450
0.000041791	0.263436687	0.9874747598	0.021411490	0.975076637	0.000042142	0.702397450
0.000002804	0.620336594	0.3586069806	0.036116589	0.975076637	0.000005033	0.715772994
0.000009849	0.401966128	0.5231404027	0.032525627	0.975076637	0.000011819	0.702397450
0.000002134	0.631578875	0.4107744012	0.028720474	0.975076637	0.000005033	0.721804429
0.000001231	0.897331603	0.3909921768	0.016307312	0.910196246	0.000005033	0.920340106
0.000002404	0.502950068	0.4381343621	0.038456795	0.987880558	0.000005033	0.702397450
0.000001345	0.361232356	0.3211889101	0.093305624	0.910196246	0.000005033	0.702397450
0.000006011	0.477802005	0.4669023204	0.033164249	0.987880558	0.000008086	0.702397450
0.000002744	0.466599264	0.4845435713	0.039459613	0.975076637	0.000005033	0.702397450
0.000002388	0.49612468	0.4234180864	0.033695133	0.975076637	0.000005033	0.702397450
0.000004265	0.503295071	0.4486001376	0.033340596	0.975076637	0.000006443	0.702397450
0.000020115	0.523287790	0.4361788076	0.058643208	0.987880558	0.000021552	0.702397450
0.000001904	0.596003139	0.3772690542	0.145984612	0.975076637	0.000005033	0.702397450
0.000001086	0.819370487	0.2609067712	0.013531605	0.684817630	0.000005033	0.862495250
0.000001855	0.519627299	0.3891626298	0.025150147	0.975076637	0.000005033	0.702397450
0.000004223	0.301621841	0.7761013206	0.240928806	0.847656972	0.000006443	0.702397450
0.000001964	0.602891144	0.4020767454	0.023471842	0.975076637	0.000005033	0.702397450
0.000006764	0.704090861	0.3197802594	0.025149141	0.975076637	0.000008681	0.775145902
0.000004295	0.496809569	0.4980405897	0.034166399	0.990709099	0.000006443	0.702397450
0.000002921	0.498785863	0.44529818	0.043508211	0.990709099	0.000005081	0.702397450
0.000003379	0.419411653	0.5089526975	0.039507842	0.975076637	0.000005554	0.702397450
0.000003069	0.664115292	0.3037372915	0.032642384	0.975076637	0.000005188	0.744802197
0.000002291	0.496382231	0.4712817989	0.030568829	0.975076637	0.000005033	0.702397450
0.000002781	0.429973740	0.433884347	0.041756660	0.975076637	0.000005033	0.702397450
0.000002589	0.528653112	0.441881542	0.056938803	0.987880558	0.000005033	0.702397450
0.000004605	0.913637358	0.2817440336	0.017485892	0.920225808	0.000006822	0.929122737
0.000007646	0.502988383	0.4510290446	0.053336157	0.990709099	0.000009558	0.702397450
0.000002914	0.519947004	0.4816094594	0.035122530	0.990709099	0.000005081	0.702397450
0.000002028	0.500381846	0.4070450725	0.026607227	0.975076637	0.000005033	0.702397450
0.000001397	0.457534317	0.4002856266	0.499481836	0.645294277	0.000005033	0.702397450
0.000002536	0.469736810	0.4916747822	0.053726651	0.975076637	0.000005033	0.702397450
0.000000479	0.139272078	0.9532655834	0.001882116	0.265427932	0.000005033	0.702397450
0.000028907	0.187339993	0.9318407499	0.009754459	0.684817630	0.000029904	0.702397450
0.000003842	0.460968316	0.5264992468	0.032697491	0.975076637	0.000005987	0.702397450
0.000002626	0.451797612	0.5067325255	0.030853151	0.975076637	0.000005033	0.702397450
0.000002596	0.501889318	0.4512923956	0.076207596	0.990709099	0.000005033	0.702397450
0.000001005	0.239540344	0.7647552737	0.034967662	0.543435515	0.000005033	0.702397450
0.000002441	0.561905026	0.431340729	0.033461098	0.987880558	0.000005033	0.702397450
0.000002374	0.494774612	0.4776285161	0.030847501	0.975076637	0.000005033	0.702397450
0.000003831	0.42433127	0.446810868	0.023064404	0.975076637	0.000005987	0.702397450
0.000002433	0.454563051	0.4669784906	0.034809818	0.975076637	0.000005033	0.702397450
0.000002633	0.508264255	0.4753517256	0.036826884	0.990709099	0.000005033	0.702397450
0.000002789	0.255985143	0.6178925064	0.019949228	0.684817630	0.000005033	0.702397450
0.000003769	0.321806266	0.633402726	0.023662243	0.975076637	0.000005987	0.702397450
0.000002518	0.548738527	0.415175748	0.029164537	0.975076637	0.000005033	0.702397450

0.000001611	0.342762109	0.5263010378	0.164456207	0.627889526	0.000005033	0.702397450
0.000001542	0.506618390	0.291054277	0.056364289	0.975076637	0.000005033	0.702397450
0.000000996	0.472400312	0.2999215237	0.075040437	0.684817630	0.000005033	0.702397450
0.000007143	0.517905534	0.4438805168	0.061329709	0.987880558	0.000009023	0.702397450
0.000002164	0.601992310	0.469933493	0.030449670	0.975076637	0.000005033	0.702397450
0.000002063	0.505261917	0.3981589651	0.031247823	0.975076637	0.000005033	0.702397450
0.000002683	0.811505581	0.389503763	0.059533956	0.532640168	0.000005033	0.861775839
0.000003734	0.503057891	0.4503011761	0.054516484	0.990709099	0.000005987	0.702397450
0.000009546	0.545210399	0.4317272302	0.160100467	0.975076637	0.000011570	0.702397450
0.000001637	0.970603734	0.4450205518	0.010132874	0.684817630	0.000005033	0.972545031
0.000031225	0.504183210	0.453566819	0.035025110	0.99399338	0.000032026	0.702397450
0.000005648	0.503235090	0.5187840725	0.044407378	0.975076637	0.000007791	0.702397450
0.000002789	0.334331371	0.3560899174	0.017038285	0.675543650	0.000005033	0.702397450
0.000004808	0.525412890	0.5626360835	0.032264485	0.975076637	0.000007037	0.702397450
0.000015689	0.641368468	0.3918093207	0.053335360	0.975076637	0.000016962	0.726077511
0.000000199	0.235678913	0.7724582138	0.185789619	0.265646546	0.000005033	0.702397450
0.000001506	0.380138423	0.4298105966	0.044030625	0.773330049	0.000005033	0.702397450
0.000000686	0.724956137	0.3699135951	0.435083726	0.645294277	0.000005033	0.783736364
0.000006436	0.538662622	0.4140992809	0.056291405	0.975076637	0.000008487	0.702397450
0.000163732	0.322775572	0.949397255	0.004433364	0.532640168	0.000163732	0.702397450
0.000002318	0.500263463	0.4265978687	0.041661786	0.975076637	0.000005033	0.702397450
0.000002589	0.502861158	0.4362277398	0.044871771	0.987880558	0.000005033	0.702397450
0.000013663	0.596001841	0.4374794452	0.099023670	0.987880558	0.000015468	0.702397450
0.000000037	0.843165635	0.0501488580	0.000473937	0.265427932	0.000004538	0.872240312
0.000004943	0.583297110	0.3877243463	0.030038015	0.975076637	0.000007146	0.702397450
0.000006065	0.542525358	0.3879380808	0.038159511	0.975076637	0.000008086	0.702397450
0.000014773	0.499969457	0.4568366339	0.048496371	0.987972281	0.000016377	0.702397450
0.000015307	0.508360755	0.4396909193	0.034120857	0.987880558	0.000016699	0.702397450
0.035306090	0.663560117	0.2702686803	0.069153692	0.970329114	0.065758938	0.977261916
0.041610375	0.810650752	0.3181318793	0.090296997	0.970329114	0.065758938	0.977261916
0.035175563	0.722787804	0.3077590722	0.070163114	0.974344026	0.065758938	0.977261916
0.031806634	0.512559539	0.2544535346	0.060165518	0.970329114	0.065758938	0.977261916
0.036358233	0.615271434	0.2724434445	0.354482159	0.970329114	0.065758938	0.977261916
0.018342793	0.592088725	0.1947722466	0.059088196	0.970329114	0.065758938	0.977261916
0.074584307	0.819917360	0.3714314294	0.057501203	0.970329114	0.085239208	0.977261916
0.040638694	0.822880021	0.4505384171	0.039880546	0.970329114	0.065758938	0.977261916
0.060578312	0.709889408	0.3110006297	0.064829503	0.985054686	0.074177525	0.977261916
0.029261170	0.597019979	0.2529179719	0.067770710	0.970329114	0.065758938	0.977261916
0.031647820	0.595372117	0.2511636376	0.054589130	0.970329114	0.065758938	0.977261916
0.010462418	0.297341721	0.1940576845	0.025565705	0.970329114	0.065758938	0.977261916
0.042858228	0.663582521	0.2817291137	0.072797335	0.970329114	0.065758938	0.977261916
0.001966952	0.156911932	0.0230276756	0.897247569	0.151382718	0.065758938	0.977261916
0.033721952	0.669394152	0.2833689368	0.091987152	0.974344026	0.065758938	0.977261916
0.051870191	0.702853651	0.2937040734	0.101069268	0.974344026	0.068329646	0.977261916
0.090420296	0.900582000	0.4496217357	0.047814281	0.970329114	0.096540743	0.981544683
0.055355158	0.819585542	0.6348131203	0.033016264	0.970329114	0.071426011	0.977261916

0.062624631	0.697208018	0.4667411733	0.033117493	0.970329114	0.075149557	0.977261916
0.046294564	0.530746703	0.2142476599	0.051397930	0.970329114	0.065758938	0.977261916
0.080612022	0.923965279	0.3902586029	0.281907546	0.970329114	0.088851529	0.981544683
0.011603818	0.374850554	0.1035758287	0.821535971	0.970329114	0.065758938	0.977261916
0.032400670	0.784029985	0.3569400004	0.080010196	0.970329114	0.065758938	0.977261916
0.101995969	0.954418255	0.7469171922	0.040759905	0.970329114	0.104611250	0.981544683
0.063452390	0.891040487	0.3672533032	0.061263528	0.970329114	0.075370672	0.981544683
0.032980634	0.678686883	0.2882063292	0.059391121	0.970329114	0.065758938	0.977261916
0.031062954	0.618331061	0.2615288684	0.055899450	0.970329114	0.065758938	0.977261916
0.087166909	0.863402614	0.5051557282	0.102634207	0.970329114	0.094234496	0.981544683
0.061634162	0.520265022	0.3688810729	0.058506823	0.092174968	0.074708075	0.977261916
0.029774952	0.601157781	0.2424610926	0.102116887	0.970329114	0.065758938	0.977261916
0.047914903	0.705833521	0.2976624886	0.076621099	0.974344026	0.066268672	0.977261916
0.031722185	0.641100661	0.2951779623	0.060880747	0.970329114	0.065758938	0.977261916
0.034131930	0.704028990	0.2923974035	0.210099983	0.974344026	0.065758938	0.977261916
0.134591367	0.967772341	0.7953415374	0.072286466	0.970329114	0.136872576	0.984175262
0.033805331	0.675070002	0.2966440168	0.069399115	0.970329114	0.065758938	0.977261916
0.245754332	0.488941682	0.9424038683	0.441582179	0.917086345	0.245754332	0.977261916
0.014933708	0.562448483	0.1515068715	0.039245039	0.970329114	0.065758938	0.977261916
0.034585566	0.693855386	0.2591052064	0.200910540	0.970329114	0.065758938	0.977261916
0.037489837	0.755354120	0.2856309412	0.070019491	0.970329114	0.065758938	0.977261916
0.033858315	0.520503259	0.2429725113	0.063811588	0.970329114	0.065758938	0.977261916
0.042641869	0.792259702	0.2904289409	0.032982644	0.970329114	0.065758938	0.977261916
0.036444375	0.466827025	0.4363624684	0.002832227	0.222418573	0.065758938	0.977261916
0.031099220	0.633715756	0.2597225554	0.170210390	0.970329114	0.065758938	0.977261916
0.031815830	0.705890718	0.2430451445	0.073596361	0.970329114	0.065758938	0.977261916
0.041691500	0.698203093	0.6004285641	0.013936048	0.970329114	0.065758938	0.977261916
0.032158226	0.691474743	0.4644579109	0.091309909	0.970329114	0.065758938	0.977261916
0.058977591	0.638629587	0.411434353	0.753498831	0.970329114	0.073721989	0.977261916
0.044444180	0.604409052	0.3066263099	0.063016672	0.970329114	0.065758938	0.977261916
0.048597026	0.757220474	0.3648469283	0.066111600	0.974344026	0.066268672	0.977261916
0.010010725	0.509477902	0.1933786452	0.055286091	0.970329114	0.065758938	0.977261916
0.041600623	0.799870625	0.3172916404	0.058653560	0.970329114	0.065758938	0.977261916
0.044346687	0.941599640	0.3171043913	0.036527350	0.970329114	0.065758938	0.981544683
0.030697235	0.691639566	0.2396744746	0.163861005	0.970329114	0.065758938	0.977261916
0.035669047	0.636958396	0.3474011672	0.111451161	0.970329114	0.065758938	0.977261916
0.071900967	0.883031675	0.3299265504	0.071015770	0.970329114	0.083768117	0.981544683
0.030574404	0.657001986	0.2700253924	0.073256904	0.970329114	0.065758938	0.977261916
0.034511691	0.706507943	0.2940191571	0.078095457	0.974344026	0.065758938	0.977261916
0.017920696	0.700622820	0.2792835948	0.054050866	0.970329114	0.065758938	0.977261916
0.076246282	0.710753617	0.3044874655	0.089707021	0.985054686	0.086316546	0.977261916
0.041292753	0.736956866	0.3184420302	0.141085472	0.974344026	0.065758938	0.977261916
0.029273821	0.992725416	0.4421999838	0.033451521	0.970329114	0.065758938	0.992725416
0.035226525	0.712773323	0.3151683746	0.062314881	0.970329114	0.065758938	0.977261916
0.046579248	0.804823597	0.3961879144	0.080901018	0.970329114	0.065758938	0.977261916
0.008540774	0.873757532	0.3148494898	0.001244036	0.120256464	0.065758938	0.981544683

0.146500991	0.934160472	0.490485021	0.046129124	0.970329114	0.147732092	0.981544683
0.027469788	0.589863225	0.2335593022	0.055348951	0.970329114	0.065758938	0.977261916
0.052386062	0.617814324	0.3297209053	0.132966249	0.970329114	0.068329646	0.977261916
0.024852909	0.491875927	0.2325207846	0.076455843	0.970329114	0.065758938	0.977261916
0.038566977	0.649934937	0.2748759475	0.062733942	0.970329114	0.065758938	0.977261916
0.031684064	0.709919474	0.3206532561	0.088658989	0.970329114	0.065758938	0.977261916
0.034202665	0.687087170	0.297962106	0.069738164	0.974344026	0.065758938	0.977261916
0.040863381	0.778670779	0.3175819277	0.060809689	0.970329114	0.065758938	0.977261916
0.018883541	0.867981451	0.4932991707	0.032736818	0.970329114	0.065758938	0.981544683
0.032586178	0.699307722	0.2874031996	0.128821243	0.970329114	0.065758938	0.977261916
0.044824333	0.957006066	0.4783523872	0.078455310	0.970329114	0.065758938	0.981544683
0.034295251	0.704579628	0.3098232029	0.061529116	0.970329114	0.065758938	0.977261916
0.035274156	0.704054873	0.2951136014	0.150646211	0.998062105	0.065758938	0.977261916
0.022404774	0.594349704	0.2114062048	0.12888917	0.970329114	0.065758938	0.977261916
0.011023707	0.436103557	0.1231156833	0.020938294	0.970329114	0.065758938	0.977261916
0.026268948	0.489004445	0.2047073287	0.052361896	0.970329114	0.065758938	0.977261916
0.036808217	0.697173944	0.3019218744	0.063776484	0.974344026	0.065758938	0.977261916
0.037675077	0.830672628	0.3608860706	0.086513468	0.970329114	0.065758938	0.977261916
0.039262639	0.697024826	0.2895955185	0.075871868	0.970329114	0.065758938	0.977261916
0.028029097	0.563914410	0.2178033262	0.072343711	0.970329114	0.065758938	0.977261916
0.032373890	0.821722008	0.3603724486	0.056239121	0.970329114	0.065758938	0.977261916
0.034068828	0.702146924	0.2917415842	0.071023366	0.974344026	0.065758938	0.977261916
0.051796278	0.806809098	0.2881170519	0.103484274	0.970329114	0.068329646	0.977261916
0.032005278	0.647505320	0.280991058	0.062628012	0.970329114	0.065758938	0.977261916
0.038414476	0.939098374	0.431488471	0.095586406	0.970329114	0.065758938	0.981544683
0.031027766	0.628252574	0.2682255951	0.059998994	0.970329114	0.065758938	0.977261916
0.048253650	0.931135754	0.406840291	0.080749409	0.970329114	0.066268672	0.981544683
0.060159829	0.803980090	0.3410792443	0.035916614	0.970329114	0.074177525	0.977261916
0.036557133	0.711351148	0.2995057195	0.086263353	0.985054686	0.065758938	0.977261916
0.050114197	0.713257031	0.5541748632	0.107470085	0.970329114	0.067569705	0.977261916
0.028738538	0.683845541	0.3903920003	0.129133189	0.970329114	0.065758938	0.977261916
0.058205092	0.711361498	0.2970929517	0.093673958	0.974344026	0.073522221	0.977261916
0.032984198	0.652514255	0.3053035153	0.087005444	0.970329114	0.065758938	0.977261916
0.037788271	0.706872709	0.3247893879	0.061898211	0.970329114	0.065758938	0.977261916
0.011053030	0.751546339	0.267682739	0.108283254	0.970329114	0.065758938	0.977261916
0.020918067	0.639815276	0.299315558	0.183241990	0.970329114	0.065758938	0.977261916
0.080706805	0.720282618	0.2987733014	0.162303618	0.974344026	0.088851529	0.977261916
0.025427135	0.949761200	0.2741196352	0.034574920	0.970329114	0.065758938	0.981544683
0.090909200	0.715488472	0.3054474726	0.062920677	0.974344026	0.096540743	0.977261916
0.033594605	0.705951379	0.2756581409	0.075464444	0.970329114	0.065758938	0.977261916
0.018480440	0.563852543	0.318301246	0.043702351	0.970329114	0.065758938	0.977261916
0.017981828	0.741866320	0.1980863916	0.058217072	0.970329114	0.065758938	0.977261916
0.101091840	0.814598567	0.4037702167	0.089703040	0.970329114	0.104577766	0.977261916
0.031571782	0.608935346	0.2232574391	0.148848963	0.970329114	0.065758938	0.977261916
0.034939644	0.773323607	0.2895139605	0.052778181	0.970329114	0.065758938	0.977261916
0.082504675	0.787880931	0.3044184678	0.238082472	0.970329114	0.090005100	0.977261916

0.064065071	0.733906615	0.3447738629	0.093365124	0.974344026	0.075370672	0.977261916
0.027889609	0.634654826	0.2067588377	0.043189739	0.970329114	0.065758938	0.977261916
0.045906333	0.686216099	0.3366643848	0.089397662	0.970329114	0.065758938	0.977261916
0.032955011	0.703311822	0.2805712979	0.063247406	0.970329114	0.065758938	0.977261916
0.097157706	0.871140164	0.3991441316	0.196940374	0.970329114	0.101381954	0.981544683
0.096131022	0.977808718	0.7478481454	0.008462534	0.970329114	0.101190550	0.986025598
0.045006001	0.686300884	0.2960476988	0.072253430	0.974344026	0.065758938	0.977261916
0.078515198	0.736410707	0.3474457273	0.078639215	0.970329114	0.088054428	0.977261916
0.057627052	0.704142074	0.295746158	0.096084599	0.998062105	0.073522221	0.977261916
0.073869690	0.707478854	0.3030893707	0.063951599	0.974344026	0.085234258	0.977261916
0.848376163	0.926381114	0.5370142169	0.068373917	0.928186086	0.998576232	0.996920713
0.741647338	0.830220267	0.58146313	0.063446288	0.869482770	0.998576232	0.996920713
0.927847335	0.726369145	0.6767854736	0.132660088	0.617647808	0.998576232	0.996920713
0.789711089	0.418707659	0.2049057526	0.066334749	0.617647808	0.998576232	0.996920713
0.726199336	0.947972669	0.5049511837	0.044616514	0.696098292	0.998576232	0.996920713
0.755053744	0.939200035	0.3363242416	0.056041330	0.607876666	0.998576232	0.996920713
0.596352723	0.742112747	0.6988742357	0.108699486	0.799520930	0.998576232	0.996920713
0.775896677	0.885254574	0.5964811565	0.134682129	0.949965605	0.998576232	0.996920713
0.655407372	0.797153209	0.6583481694	0.064404431	0.868904128	0.998576232	0.996920713
0.78968279	0.740276365	0.3546208452	0.045018644	0.607876666	0.998576232	0.996920713
0.601153241	0.581771836	0.2517843073	0.140388093	0.421788991	0.998576232	0.996920713
0.637192781	0.678657552	0.6113974925	0.052967842	0.799520930	0.998576232	0.996920713
0.889087972	0.941570614	0.5276179887	0.067780265	0.925203233	0.998576232	0.996920713
0.986009579	0.825934357	0.3331782046	0.035320719	0.660566205	0.998576232	0.996920713
0.789791367	0.949498187	0.5333794447	0.073687446	0.925203233	0.998576232	0.996920713
0.423553956	0.924091337	0.4356527917	0.567450919	0.421788991	0.998576232	0.996920713
0.40414013	0.595325403	0.8801434231	0.087343406	0.607876666	0.998576232	0.996920713
0.767620118	0.885890722	0.631956801	0.083894374	0.939664415	0.998576232	0.996920713
0.547178905	0.891121317	0.8128702228	0.134437829	0.617339011	0.998576232	0.996920713
0.964644161	0.532356117	0.7953982609	0.037481264	0.421788991	0.998576232	0.996920713
0.634274255	0.741875992	0.6625496394	0.074222450	0.799520930	0.998576232	0.996920713
0.872609357	0.957984362	0.5519775143	0.185204050	0.928186086	0.998576232	0.996920713
0.643351302	0.714821938	0.2749223051	0.122265866	0.520349777	0.998576232	0.996920713
0.996934533	0.890002550	0.3958418307	0.056071982	0.799520930	0.998576232	0.996920713
0.726787814	0.806482361	0.6293062399	0.088816560	0.925203233	0.998576232	0.996920713
0.780298010	0.915512730	0.5525841778	0.070060840	0.849231427	0.998576232	0.996920713
0.972272723	0.941169421	0.4702990961	0.108228703	0.799520930	0.998576232	0.996920713
0.461401933	0.434577295	0.1022998395	0.225292601	0.421788991	0.998576232	0.996920713
0.423824925	0.952660798	0.7033212118	0.025415098	0.421788991	0.998576232	0.996920713
0.898345296	0.801205620	0.3268301269	0.029945089	0.607876666	0.998576232	0.996920713
0.507590730	0.873845730	0.5819089763	0.037257485	0.617647808	0.998576232	0.996920713
0.775787520	0.866484482	0.5532578527	0.068709715	0.925203233	0.998576232	0.996920713
0.812903246	0.898341337	0.538767928	0.112323587	0.868904128	0.998576232	0.996920713
0.780415243	0.885920550	0.6381862112	0.070119914	0.957050902	0.998576232	0.996920713
0.767205329	0.755626367	0.5255077292	0.081521695	0.617647808	0.998576232	0.996920713
0.554492164	0.634341919	0.8420643784	0.052391958	0.799520930	0.998576232	0.996920713

0.895133894	0.947751833	0.3111580299	0.086919660	0.607876666	0.998576232	0.996920713
0.741102129	0.906564511	0.4708587042	0.032007192	0.617647808	0.998576232	0.996920713
0.790877903	0.907177060	0.5575670521	0.135917817	0.957050902	0.998576232	0.996920713
0.532968785	0.531727001	0.9801609216	0.050211742	0.799520930	0.998576232	0.996920713
0.647397127	0.779916950	0.55673374	0.179588948	0.617339011	0.998576232	0.996920713
0.425734792	0.876819540	0.8376983541	0.488533632	0.421788991	0.998576232	0.996920713
0.767596792	0.855958277	0.5997664642	0.150575714	0.925203233	0.998576232	0.996920713
0.517009269	0.903796195	0.7364883599	0.082969397	0.775100997	0.998576232	0.996920713
0.582878091	0.874265567	0.9801380931	0.591048594	0.495086125	0.998576232	0.996920713
0.761331406	0.900514373	0.7294485169	0.051709983	0.799520930	0.998576232	0.996920713
0.889842151	0.884271429	0.49338576	0.303135881	0.799520930	0.998576232	0.996920713
0.470804688	0.435542535	0.8628573516	0.041908158	0.758952824	0.998576232	0.996920713
0.834363578	0.938627312	0.5414422317	0.071773600	0.928186086	0.998576232	0.996920713
0.730305131	0.867822205	0.5807669886	0.070558816	0.925203233	0.998576232	0.996920713
0.865903271	0.959718332	0.5101053142	0.058571787	0.799520930	0.998576232	0.996920713
0.705463813	0.678550779	0.5862140645	0.204459162	0.799520930	0.998576232	0.996920713
0.774288176	0.899728221	0.519852703	0.056328200	0.799520930	0.998576232	0.996920713
0.758841233	0.994243649	0.6401418611	0.057839819	0.799520930	0.998576232	0.996920713
0.282968063	0.445752332	0.6751772978	0.025766111	0.421788991	0.998576232	0.996920713
0.888256118	0.967883762	0.4472639452	0.043927809	0.617339011	0.998576232	0.996920713
0.773356780	0.883893768	0.4911859164	0.056544927	0.799520930	0.998576232	0.996920713
0.968122593	0.886975733	0.5407080223	0.061699026	0.617339011	0.998576232	0.996920713
0.296887408	0.869499083	0.3026795656	0.312774857	0.421788991	0.998576232	0.996920713
0.896940585	0.810290798	0.3234409509	0.535567671	0.421788991	0.998576232	0.996920713
0.785447683	0.942661849	0.5408954158	0.071885453	0.928186086	0.998576232	0.996920713
0.761371010	0.877271739	0.6112396972	0.094863415	0.799520930	0.998576232	0.996920713
0.794765735	0.90911696	0.5773220163	0.128403402	0.984924233	0.998576232	0.996920713
0.786365478	0.637524323	0.6412273429	0.29542434	0.517458860	0.998576232	0.996920713
0.873749379	0.94185348	0.5495221897	0.071181397	0.939664415	0.998576232	0.996920713
0.924606290	0.792516551	0.3088717169	0.080185549	0.607876666	0.998576232	0.996920713
0.688524149	0.929421686	0.5928552172	0.052940613	0.799520930	0.998576232	0.996920713
0.925335657	0.768406575	0.4358882964	0.050051444	0.696098292	0.998576232	0.996920713
0.789251196	0.828945182	0.2914413151	0.062969149	0.617339011	0.998576232	0.996920713
0.785359465	0.900285504	0.5522933433	0.077133908	0.953777775	0.998576232	0.996920713
0.843594957	0.962898526	0.5720283948	0.057295224	0.799520930	0.998576232	0.996920713
0.614280286	0.692178114	0.6342983225	0.408817092	0.607876666	0.998576232	0.996920713
0.713412191	0.940560701	0.4911578722	0.065044408	0.868904128	0.998576232	0.996920713
0.533430913	0.890165136	0.5628916476	0.184459574	0.758952824	0.998576232	0.996920713
0.708585673	0.711875780	0.7106405986	0.058286374	0.799520930	0.998576232	0.996920713
0.776998606	0.895478225	0.59916258	0.093638248	0.799520930	0.998576232	0.996920713
0.678357143	0.882660920	0.5283104127	0.488022723	0.617647808	0.998576232	0.996920713
0.816684252	0.942437767	0.5120579924	0.058622973	0.814552831	0.998576232	0.996920713
0.828028356	0.940892910	0.527177824	0.085418154	0.925203233	0.998576232	0.996920713
0.511139934	0.589349132	0.8684611047	0.046208407	0.775100997	0.998576232	0.996920713
0.983915605	0.847418000	0.3369284615	0.067515283	0.607876666	0.998576232	0.996920713
0.746879717	0.800225400	0.632801461	0.058917727	0.812090521	0.998576232	0.996920713

0.636651701	0.910874158	0.5229911828	0.531182570	0.607876666	0.998576232	0.996920713
0.823998850	0.966986002	0.4565090731	0.059687966	0.799520930	0.998576232	0.996920713
0.808250510	0.598065697	0.7824053208	0.091905091	0.607876666	0.998576232	0.996920713
0.772775144	0.892594331	0.5809001877	0.065831266	0.887595656	0.998576232	0.996920713
0.453310423	0.646233637	0.5251548038	0.016484043	0.421788991	0.998576232	0.996920713
0.818652669	0.976673660	0.5142920971	0.193029281	0.775100997	0.998576232	0.996920713
0.729595000	0.741084340	0.6920644685	0.053579579	0.799520930	0.998576232	0.996920713
0.866114256	0.983740101	0.5020032293	0.073811916	0.839441786	0.998576232	0.996920713
0.970406532	0.739714635	0.3658673505	0.094662325	0.617647808	0.998576232	0.996920713
0.828995019	0.920202293	0.5435799574	0.072914795	0.928186086	0.998576232	0.996920713
0.666212831	0.788302613	0.6190113473	0.199112769	0.660566205	0.998576232	0.996920713
0.975453442	0.920935474	0.2318403636	0.130477885	0.571538566	0.998576232	0.996920713
0.789414812	0.896539108	0.5164822722	0.097776025	0.868904128	0.998576232	0.996920713
0.432993677	0.949939774	0.4413912469	0.325665583	0.421788991	0.998576232	0.996920713
0.81918714	0.911923747	0.5927494149	0.046690376	0.775100997	0.998576232	0.996920713
0.862275555	0.907756825	0.4594124627	0.059745745	0.775100997	0.998576232	0.996920713
0.547241236	0.693002321	0.5357941844	0.110410049	0.617339011	0.998576232	0.996920713
0.929037091	0.991196428	0.5701366139	0.021207549	0.607876666	0.998576232	0.996920713
0.520580892	0.951130140	0.4800495325	0.572059281	0.571538566	0.998576232	0.996920713
0.874692370	0.489022961	0.5190752831	0.308263371	0.607876666	0.998576232	0.996920713
0.483836571	0.995874159	0.425458154	0.040749280	0.571538566	0.998576232	0.996920713
0.830048205	0.897485837	0.5426139646	0.069584428	0.928186086	0.998576232	0.996920713
0.687449672	0.821901430	0.5239005695	0.060161030	0.799520930	0.998576232	0.996920713
0.804369194	0.797924485	0.2876669926	0.043082438	0.421788991	0.998576232	0.996920713
0.452030450	0.572503932	0.194128005	0.169764415	0.520349777	0.998576232	0.996920713
0.784731837	0.859855737	0.6121606889	0.116580336	0.904204465	0.998576232	0.996920713
0.773853630	0.866026212	0.5551331194	0.078390079	0.925203233	0.998576232	0.996920713
0.965304893	0.975816515	0.5389411041	0.238874356	0.849231427	0.998576232	0.996920713
0.590656476	0.817856203	0.7009645991	0.052651706	0.799520930	0.998576232	0.996920713
0.656605312	0.996920713	0.3385138892	0.177914901	0.617647808	0.998576232	0.996920713
0.817588415	0.898163857	0.5413430319	0.079381936	0.925203233	0.998576232	0.996920713
0.909076864	0.896839004	0.3472722993	0.126965908	0.617647808	0.998576232	0.996920713
0.805994688	0.901791795	0.5842065989	0.132677464	0.984924233	0.998576232	0.996920713
0.978493393	0.981363113	0.3994065109	0.056971066	0.799520930	0.998576232	0.996920713
0.998576232	0.986238213	0.453557829	0.057065936	0.799520930	0.998576232	0.996920713
0.550896355	0.937980609	0.2950291581	0.011178263	0.517208933	0.998576232	0.996920713
0.346708441	0.943538121	0.4046982902	0.463299197	0.421788991	0.998576232	0.996920713
0.345293880	0.964002198	0.3605279848	0.042624212	0.421788991	0.998576232	0.996920713
0.635681542	0.537028120	0.590695354	0.796364753	0.997874178	0.967795776	0.637363585
0.475143480	0.825063280	0.5074425407	0.685401342	0.763866738	0.967795776	0.868487663
0.677636552	0.381812290	0.8167933327	0.970019454	0.997874178	0.967795776	0.636773498
0.742887991	0.129272421	0.5010610689	0.797723940	0.997874178	0.967795776	0.636773498
0.780860074	0.484392482	0.7166898402	0.850232461	0.997874178	0.967795776	0.636773498
0.854017828	0.456487355	0.7654126458	0.844727735	0.997874178	0.967795776	0.636773498
0.922453585	0.337426585	0.9093128759	0.970061700	0.997874178	0.967795776	0.636773498
0.942927839	0.269171605	0.8334361731	0.386728501	0.997874178	0.967795776	0.636773498

0.680338252	0.561796237	0.6583955721	0.870527395	0.997874178	0.967795776	0.642052842
0.922879087	0.400119476	0.8010261646	0.870063501	0.997874178	0.967795776	0.636773498
0.872197177	0.441723973	0.7794472623	0.798772854	0.997874178	0.967795776	0.636773498
0.943600882	0.334448936	0.7818692289	0.728300311	0.997874178	0.967795776	0.636773498
0.703695490	0.517510190	0.6585911623	0.810664272	0.997874178	0.967795776	0.637363585
0.833085969	0.445507544	0.8085506216	0.978491411	0.997874178	0.967795776	0.636773498
0.742180129	0.675005725	0.5497014008	0.548525207	0.997874178	0.967795776	0.729735919
0.633251303	0.472491780	0.7002936386	0.929467033	0.997874178	0.967795776	0.636773498
0.723195407	0.525498736	0.6783954745	0.819507331	0.997874178	0.967795776	0.637363585
0.823032220	0.452090747	0.8259939915	0.899009920	0.997874178	0.967795776	0.636773498
0.426252086	0.432191507	0.3609827837	0.454907050	0.997874178	0.967795776	0.636773498
0.597308145	0.285249317	0.9137125774	0.772514942	0.997874178	0.967795776	0.636773498
0.938974895	0.393833510	0.8158750536	0.647710338	0.997874178	0.967795776	0.636773498
0.283649284	0.942084530	0.2250686404	0.149327833	0.997874178	0.967795776	0.950001207
0.701412863	0.326476058	0.9368773956	0.678883950	0.997874178	0.967795776	0.636773498
0.889908054	0.431150018	0.8973399697	0.894758834	0.997874178	0.967795776	0.636773498
0.564301810	0.867747394	0.5522171283	0.690280894	0.997874178	0.967795776	0.889389515
0.776646372	0.468602733	0.7194544971	0.842697135	0.997874178	0.967795776	0.636773498
0.823386332	0.461989857	0.7455294341	0.819283064	0.997874178	0.967795776	0.636773498
0.413215486	0.874566357	0.3423834452	0.832308678	0.997874178	0.967795776	0.889389515
0.629781050	0.442456377	0.6391248939	0.937117864	0.997874178	0.967795776	0.636773498
0.513491796	0.961838070	0.2259518615	0.286976724	0.763866738	0.967795776	0.961838070
0.398127066	0.462403343	0.6417315435	0.813885718	0.997874178	0.967795776	0.636773498
0.820906064	0.298177102	0.6824035704	0.802049685	0.997874178	0.967795776	0.636773498
0.853846604	0.453198821	0.7660765733	0.462158531	0.997874178	0.967795776	0.636773498
0.839636250	0.466029612	0.8070167777	0.833802441	0.997874178	0.967795776	0.636773498
0.779488360	0.417564543	0.7078919656	0.89694617	0.997874178	0.967795776	0.636773498
0.468708760	0.874371140	0.4361378865	0.743363353	0.997874178	0.967795776	0.889389515
0.862562363	0.328589230	0.8917713512	0.677705496	0.997874178	0.967795776	0.636773498
0.769041655	0.466719494	0.7387633643	0.961732823	0.997874178	0.967795776	0.636773498
0.788296661	0.460504447	0.7165576369	0.954300583	0.997874178	0.967795776	0.636773498
0.869420811	0.259521183	0.831651145	0.518804824	0.997874178	0.967795776	0.636773498
0.802680404	0.455422067	0.722343327	0.918634165	0.997874178	0.967795776	0.636773498
0.762232215	0.462064551	0.7047778127	0.807411884	0.997874178	0.967795776	0.636773498
0.821851283	0.385838801	0.8311942279	0.833015922	0.997874178	0.967795776	0.636773498
0.531142830	0.457186999	0.5661872359	0.763452901	0.997874178	0.967795776	0.636773498
0.696043801	0.467149036	0.5455499617	0.529845284	0.997874178	0.967795776	0.636773498
0.776702291	0.468982681	0.7277450452	0.847428877	0.997874178	0.967795776	0.636773498
0.855868793	0.474659799	0.7771851042	0.651239654	0.997874178	0.967795776	0.636773498
0.799598393	0.225143049	0.7080089248	0.593494696	0.997874178	0.967795776	0.636773498
0.681390313	0.561316418	0.6161242739	0.866350075	0.997874178	0.967795776	0.642052842
0.770721252	0.482122368	0.7144317344	0.841619514	0.997874178	0.967795776	0.636773498
0.825541452	0.427100983	0.7511304368	0.889960363	0.997874178	0.967795776	0.636773498
0.863993868	0.328465781	0.7566587983	0.836672888	0.997874178	0.967795776	0.636773498
0.747513557	0.446215761	0.7993307499	0.752532682	0.997874178	0.967795776	0.636773498
0.763730399	0.440296084	0.6793482012	0.939205817	0.997874178	0.967795776	0.636773498

0.413023889	0.744349593	0.582160629	0.954960655	0.997874178	0.967795776	0.797517421
0.808025775	0.442910948	0.7526543402	0.880916659	0.997874178	0.967795776	0.636773498
0.748221561	0.475497315	0.8873034911	0.882397131	0.997874178	0.967795776	0.636773498
0.797386188	0.469301198	0.7192296539	0.842388643	0.997874178	0.967795776	0.636773498
0.803484893	0.474337209	0.7202047819	0.862467874	0.997874178	0.967795776	0.636773498
0.770006871	0.484517096	0.7139514487	0.899229834	0.997874178	0.967795776	0.636773498
0.768986479	0.644177396	0.5819222985	0.690987139	0.997874178	0.967795776	0.709186125
0.775683285	0.470492009	0.7179957349	0.840498613	0.997874178	0.967795776	0.636773498
0.834785662	0.437361525	0.8151596829	0.999795788	0.997874178	0.967795776	0.636773498
0.775133511	0.458559755	0.7311553262	0.911314997	0.997874178	0.967795776	0.636773498
0.727729206	0.537553825	0.6824775475	0.814356486	0.997874178	0.967795776	0.637363585
0.762981725	0.504473971	0.7078237148	0.852931047	0.997874178	0.967795776	0.636773498
0.529078291	0.381032586	0.5924661667	0.789400109	0.997874178	0.967795776	0.636773498
0.766636864	0.531300492	0.7100965975	0.834395615	0.997874178	0.967795776	0.637363585
0.652111165	0.226393061	0.7332270542	0.807479480	0.997874178	0.967795776	0.636773498
0.777425311	0.468085362	0.7254697656	0.833048346	0.997874178	0.967795776	0.636773498
0.855395037	0.346774322	0.6848350487	0.951955606	0.997874178	0.967795776	0.636773498
0.812920056	0.441734002	0.7423728745	0.993135864	0.997874178	0.967795776	0.636773498
0.616687037	0.290169694	0.9445866076	0.907229824	0.997874178	0.967795776	0.636773498
0.987495846	0.459678588	0.7288296962	0.904703291	0.997874178	0.988580044	0.636773498
0.706158751	0.655122715	0.5954496776	0.907043812	0.997874178	0.967795776	0.714679325
0.775964716	0.46920613	0.7133456715	0.831178215	0.997874178	0.967795776	0.636773498
0.690214747	0.465955242	0.7256567957	0.419606196	0.997874178	0.967795776	0.636773498
0.815314496	0.427270514	0.7825509323	0.992401842	0.997874178	0.967795776	0.636773498
0.896148856	0.386967658	0.8681746513	0.716066076	0.997874178	0.967795776	0.636773498
0.920873059	0.313151596	0.9980271975	0.538307382	0.997874178	0.967795776	0.636773498
0.785583258	0.477484231	0.7335961459	0.842535112	0.997874178	0.967795776	0.636773498
0.771609902	0.49400944	0.7114991724	0.860362842	0.997874178	0.967795776	0.636773498
0.678354967	0.448714481	0.7212435807	0.426377877	0.997874178	0.967795776	0.636773498
0.825704459	0.357184480	0.8923338938	0.909828659	0.997874178	0.967795776	0.636773498
0.785934507	0.561457413	0.6543271002	0.795496857	0.997874178	0.967795776	0.642052842
0.793775288	0.457690475	0.7623067444	0.708496298	0.997874178	0.967795776	0.636773498
0.650722709	0.541759047	0.7095150122	0.995125360	0.997874178	0.967795776	0.637363585
0.802565914	0.404896629	0.7475572093	0.640500847	0.997874178	0.967795776	0.636773498
0.732144744	0.594572625	0.6172132226	0.942616515	0.997874178	0.967795776	0.660636250
0.878843186	0.379357268	0.8082166935	0.807345819	0.997874178	0.967795776	0.636773498
0.573632309	0.858375027	0.4637924659	0.985373315	0.997874178	0.967795776	0.889389515
0.814073275	0.283309371	0.8809025648	0.666110169	0.997874178	0.967795776	0.636773498
0.604652302	0.573785226	0.5956366945	0.400733556	0.997874178	0.967795776	0.649568181
0.754776118	0.471085394	0.688339139	0.881349827	0.997874178	0.967795776	0.636773498
0.776568614	0.468924337	0.7200252159	0.854053502	0.997874178	0.967795776	0.636773498
0.695271776	0.485325516	0.7095893535	0.954154011	0.997874178	0.967795776	0.636773498
0.810718621	0.348980617	0.6693739513	0.964052945	0.997874178	0.967795776	0.636773498
0.854979580	0.452532633	0.8354113617	0.876960693	0.997874178	0.967795776	0.636773498
0.670207239	0.368222106	0.719744466	0.750161642	0.997874178	0.967795776	0.636773498
0.783661483	0.470273648	0.7194376954	0.858859148	0.997874178	0.967795776	0.636773498

0.988580044	0.436211331	0.7353332583	0.650073257	0.997874178	0.988580044	0.636773498
0.775133975	0.498148796	0.7188122912	0.877592659	0.997874178	0.967795776	0.636773498
0.674787542	0.486145912	0.6944554973	0.820500350	0.997874178	0.967795776	0.636773498
0.856942572	0.469974702	0.7649216516	0.890711354	0.997874178	0.967795776	0.636773498
0.961190176	0.362975486	0.6525644192	0.746937977	0.997874178	0.977481534	0.636773498
0.864330959	0.481294798	0.7875110395	0.847496197	0.997874178	0.967795776	0.636773498
0.395830278	0.788490484	0.4304726307	0.893793494	0.997874178	0.967795776	0.837335028
0.776815686	0.504561948	0.6705905985	0.750286615	0.997874178	0.967795776	0.636773498
0.781301886	0.462612173	0.7191107434	0.858835130	0.997874178	0.967795776	0.636773498
0.621096914	0.267868182	0.7675970022	0.212252708	0.997874178	0.967795776	0.636773498
0.376060478	0.593920441	0.4426950372	0.757494904	0.997874178	0.967795776	0.660636250
0.567657346	0.509418798	0.5918911696	0.973697719	0.997874178	0.967795776	0.636773498
0.665485752	0.457623107	0.6488912633	0.966764826	0.997874178	0.967795776	0.636773498
0.756241964	0.468701255	0.6836336381	0.892341512	0.997874178	0.967795776	0.636773498
0.926544314	0.432321614	0.8138218201	0.735115405	0.997874178	0.967795776	0.636773498
0.881605070	0.430079842	0.8557497933	0.929714600	0.997874178	0.967795776	0.636773498
0.849525312	0.329179943	0.9819491885	0.990865756	0.997874178	0.967795776	0.636773498
0.786095419	0.474691256	0.7193959165	0.850513433	0.997874178	0.967795776	0.636773498
0.805925971	0.468972491	0.7203434981	0.855699342	0.997874178	0.967795776	0.636773498
0.597244892	0.480046226	0.6738198914	0.840990973	0.997874178	0.967795776	0.636773498
0.954679863	0.562368177	0.79501522	0.235883483	0.930179421	0.992345060	0.775680244
0.751920016	0.394851380	0.9353807063	0.320017319	0.732783268	0.992345060	0.771430006
0.675275448	0.699392138	0.6821302212	0.405975308	0.697456823	0.992345060	0.818326604
0.418077273	0.122325140	0.3691533239	0.217490551	0.697456823	0.992345060	0.771430006
0.801643494	0.654028258	0.8105146467	0.863104617	0.732783268	0.992345060	0.809107124
0.596334422	0.436764717	0.9421539511	0.210650243	0.732783268	0.992345060	0.771430006
0.207105935	0.138300824	0.5870633551	0.046523508	0.697456823	0.992345060	0.771430006
0.947256566	0.625279615	0.6636238825	0.563942454	0.732783268	0.992345060	0.798229296
0.930316255	0.649386063	0.7624604959	0.205964727	0.867011594	0.992345060	0.809107124
0.531423634	0.307197757	0.9188006905	0.175791886	0.697456823	0.992345060	0.771430006
0.532923748	0.313096405	0.8870921599	0.343881023	0.697456823	0.992345060	0.771430006
0.586441049	0.256421888	0.9840427202	0.325010314	0.697456823	0.992345060	0.771430006
0.929113992	0.552858171	0.83231438	0.237036645	0.957592049	0.992345060	0.771430006
0.602171423	0.299303399	0.734025475	0.080359058	0.697456823	0.992345060	0.771430006
0.862294169	0.535437842	0.8694397163	0.250003682	0.999251513	0.992345060	0.771430006
0.819922621	0.519820555	0.8730419931	0.334213684	0.957592049	0.992345060	0.771430006
0.408361659	0.266373069	0.7347453448	0.132946752	0.697456823	0.992345060	0.771430006
0.535311638	0.336607260	0.486472716	0.076211701	0.697456823	0.992345060	0.771430006
0.601843956	0.499848387	0.8356441191	0.114342738	0.697456823	0.992345060	0.771430006
0.723995407	0.698766608	0.7137441961	0.191111155	0.732783268	0.992345060	0.818326604
0.871214985	0.762767520	0.7109486545	0.139542224	0.732783268	0.992345060	0.840021118
0.605691552	0.932127021	0.3717275661	0.784632704	0.697456823	0.992345060	0.932127021
0.831952874	0.612476781	0.7839312865	0.201135248	0.90115955	0.992345060	0.798229296
0.815271376	0.507930336	0.9713326063	0.218516204	0.957592049	0.992345060	0.771430006
0.325145798	0.090100747	0.6342189869	0.074334635	0.697456823	0.992345060	0.771430006
0.865766480	0.492546758	0.8678546324	0.223203846	0.732783268	0.992345060	0.771430006

0.750460529	0.820845624	0.6345075857	0.106066931	0.697456823	0.992345060	0.864048025
0.316936748	0.173726085	0.4136123237	0.521612323	0.697456823	0.992345060	0.771430006
0.934797086	0.512115766	0.831423688	0.207903882	0.909058433	0.992345060	0.771430006
0.926385816	0.669765972	0.7096189764	0.332054574	0.754994911	0.992345060	0.818326604
0.516754324	0.481430929	0.9154582769	0.380476951	0.697456823	0.992345060	0.771430006
0.917693279	0.709216390	0.8773388576	0.174711706	0.697456823	0.992345060	0.818326604
0.890946501	0.520157799	0.846851271	0.561401477	0.90115955	0.992345060	0.771430006
0.861890261	0.704088627	0.5854943508	0.192148837	0.732783268	0.992345060	0.818326604
0.854286940	0.452044547	0.8531105168	0.190605167	0.732783268	0.992345060	0.771430006
0.479144586	0.253661097	0.7056880986	0.602899683	0.697456823	0.992345060	0.771430006
0.805434185	0.500766724	0.9418591471	0.239885064	0.930179421	0.992345060	0.771430006
0.832920622	0.521512185	0.7993861761	0.509910541	0.732783268	0.992345060	0.771430006
0.867845224	0.530620657	0.8681821006	0.357779906	0.970016617	0.992345060	0.771430006
0.798308369	0.531208841	0.9902861444	0.356912578	0.946083634	0.992345060	0.771430006
0.839651576	0.508031273	0.8700722216	0.223014081	0.946083634	0.992345060	0.771430006
0.950727998	0.402312658	0.6585007344	0.641440358	0.697456823	0.992345060	0.771430006
0.831207224	0.467514626	0.9422139108	0.198399528	0.867011594	0.992345060	0.771430006
0.761210150	0.523028594	0.9425112834	0.211158393	0.927422576	0.992345060	0.771430006
0.813200666	0.516604729	0.9978339659	0.204093591	0.867011594	0.992345060	0.771430006
0.869028261	0.520435626	0.8111908262	0.211860022	0.930179421	0.992345060	0.771430006
0.764486137	0.433042241	0.5767671481	0.016008888	0.697456823	0.992345060	0.771430006
0.987808711	0.804497832	0.7638410517	0.217369702	0.930819256	0.992345060	0.861961963
0.934507079	0.682466762	0.649746474	0.198516339	0.732783268	0.992345060	0.818326604
0.890724146	0.534982894	0.860728571	0.224184293	0.970016617	0.992345060	0.771430006
0.902680319	0.594290749	0.8412029632	0.246345903	0.930179421	0.992345060	0.792387665
0.955163735	0.799507131	0.8215035076	0.493047485	0.732783268	0.992345060	0.861961963
0.848280787	0.517214674	0.8098481119	0.386478845	0.732783268	0.992345060	0.771430006
0.758314645	0.791857712	0.8128981476	0.599633430	0.697456823	0.992345060	0.861961963
0.585284668	0.351144609	0.9576266189	0.250230693	0.697456823	0.992345060	0.771430006
0.960304363	0.614735039	0.755774171	0.269949150	0.697456823	0.992345060	0.798229296
0.873889832	0.531599764	0.985283238	0.159293444	0.732783268	0.992345060	0.771430006
0.929219406	0.519358819	0.8668160393	0.220463713	0.927415364	0.992345060	0.771430006
0.452285936	0.428145204	0.9941924797	0.462846490	0.732783268	0.992345060	0.771430006
0.943416027	0.585606051	0.8020327919	0.198552289	0.867011594	0.992345060	0.791530750
0.862737613	0.839879991	0.5969213676	0.373546212	0.697456823	0.992345060	0.876396512
0.899600773	0.542462443	0.7528491352	0.318140267	0.732783268	0.992345060	0.771430006
0.890633275	0.812927098	0.5248418192	0.759001123	0.697456823	0.992345060	0.863285414
0.848981332	0.640014776	0.7947322864	0.466930730	0.732783268	0.992345060	0.808439717
0.484861549	0.921559782	0.4466311235	0.341014227	0.697456823	0.992345060	0.932127021
0.882415817	0.763019182	0.5848403976	0.169010653	0.697456823	0.992345060	0.840021118
0.985508498	0.486089478	0.8099670652	0.160669791	0.732783268	0.992345060	0.771430006
0.959396991	0.748782753	0.7698064624	0.255355707	0.768567725	0.992345060	0.839756359
0.751063393	0.470764540	0.9971456126	0.224177896	0.909058433	0.992345060	0.771430006
0.856265925	0.510830759	0.9074086929	0.274332199	0.786847634	0.992345060	0.771430006
0.957621137	0.882604172	0.9264854567	0.309498074	0.697456823	0.992345060	0.913038798
0.772026928	0.436186502	0.926276057	0.140172318	0.732783268	0.992345060	0.771430006

0.895770589	0.253577853	0.8260194276	0.113430935	0.697456823	0.992345060	0.771430006
0.957559248	0.522898600	0.8615025939	0.316693846	0.930179421	0.992345060	0.771430006
0.781374429	0.392884542	0.9658924364	0.255894521	0.768567725	0.992345060	0.771430006
0.868063762	0.515963495	0.7869099688	0.303018092	0.732783268	0.992345060	0.771430006
0.747681259	0.494481014	0.8437226086	0.076892267	0.697456823	0.992345060	0.771430006
0.852946606	0.513822122	0.8835790178	0.224894148	0.957592049	0.992345060	0.771430006
0.731029207	0.420978813	0.9623593905	0.306407714	0.732783268	0.992345060	0.771430006
0.661240731	0.390527358	0.9138600803	0.513527005	0.800955307	0.992345060	0.771430006
0.663883001	0.358507679	0.8751435024	0.229688775	0.697456823	0.992345060	0.771430006
0.774415361	0.355998610	0.9506897753	0.316284045	0.697456823	0.992345060	0.771430006
0.745583907	0.518788662	0.851331079	0.089931277	0.697456823	0.992345060	0.771430006
0.852014733	0.507515247	0.9055690528	0.219065216	0.957592049	0.992345060	0.771430006
0.838194181	0.706794384	0.7104845896	0.271110291	0.732783268	0.992345060	0.818326604
0.857779075	0.520067637	0.8783033891	0.262075412	0.957592049	0.992345060	0.771430006
0.5820706	0.350441806	0.8691259223	0.393727874	0.697456823	0.992345060	0.771430006
0.856733302	0.513642940	0.8734866952	0.299205833	0.959653885	0.992345060	0.771430006
0.787152412	0.369639045	0.9393283533	0.303870524	0.732783268	0.992345060	0.771430006
0.660329135	0.315778087	0.9491421959	0.239496584	0.697456823	0.992345060	0.771430006
0.848789784	0.529127873	0.8946435755	0.235649559	0.967480739	0.992345060	0.771430006
0.890595322	0.619164332	0.7521744907	0.393474390	0.697456823	0.992345060	0.798229296
0.856091010	0.519843689	0.8726531575	0.255209075	0.970016617	0.992345060	0.771430006
0.924615826	0.526094908	0.758293072	0.199795037	0.90115955	0.992345060	0.771430006
0.871793921	0.510955046	0.7562170278	0.155883780	0.732783268	0.992345060	0.771430006
0.677555534	0.488441966	0.8846460764	0.366738759	0.800955307	0.992345060	0.771430006
0.909660321	0.743758457	0.929414766	0.367942540	0.710597508	0.992345060	0.839756359
0.790384417	0.508517267	0.9731730189	0.203422332	0.732783268	0.992345060	0.771430006
0.610008796	0.931304446	0.8551409728	0.134037675	0.697456823	0.992345060	0.932127021
0.977978486	0.550545943	0.8820787666	0.383957793	0.800955307	0.992345060	0.771430006
0.883668997	0.525009806	0.8669159182	0.329912445	0.999251513	0.992345060	0.771430006
0.724424683	0.908542917	0.8864263806	0.792039474	0.697456823	0.992345060	0.931838889
0.501405817	0.466304387	0.9431963569	0.187790920	0.732783268	0.992345060	0.771430006
0.920624221	0.519909064	0.8366774622	0.245507539	0.944721678	0.992345060	0.771430006
0.654606983	0.392283230	0.789851603	0.258952700	0.697456823	0.992345060	0.771430006
0.890151139	0.518418498	0.8505160546	0.223857680	0.959653885	0.992345060	0.771430006
0.479923453	0.324067951	0.7930082212	0.366178705	0.732783268	0.992345060	0.771430006
0.862037190	0.463184659	0.974802944	0.169485594	0.786847634	0.992345060	0.771430006
0.780630061	0.336738848	0.8525011106	0.114885067	0.697456823	0.992345060	0.771430006
0.881039985	0.532512923	0.8663909046	0.330761309	0.999251513	0.992345060	0.771430006
0.591154135	0.436351323	0.9328703942	0.382151218	0.732783268	0.992345060	0.771430006
0.574373370	0.467840894	0.9354172319	0.373926474	0.754994911	0.992345060	0.771430006
0.924475652	0.518502296	0.8292358038	0.197855869	0.867011594	0.992345060	0.771430006
0.607680661	0.471082537	0.5907922323	0.464873754	0.697456823	0.992345060	0.771430006
0.878655019	0.733448149	0.7160365255	0.180511730	0.800955307	0.992345060	0.838226456
0.706305335	0.443771447	0.9163137549	0.167244604	0.768567725	0.992345060	0.771430006
0.992345060	0.587051973	0.7877409949	0.256466017	0.909058433	0.992345060	0.791530750
0.423618317	0.410430071	0.8757377399	0.073534036	0.697456823	0.992345060	0.771430006

0.496734528	0.489219355	0.9153122378	0.535725800	0.717358543	0.992345060	0.771430006
0.380347720	0.467131868	0.9861598328	0.201736083	0.697456823	0.992345060	0.771430006

t_q_interact	t_q_batch	Genus	Family
0.6404556554	0.065307203	Acetatifactor	Lachnospiraceae
0.6404556554	0.065307203	Acutalibacter	Oscillospiraceae
0.6404556554	0.065307203	Akkermansia	Akkermansiaceae
0.6979515333	0.065307203	Anaerotruncus	Oscillospiraceae
0.6404556554	0.136595928	Bacteria_unclassified	Bacteria_unclassified
0.7252879204	0.065307203	Bacteria_unclassified	Bacteria_unclassified
0.649195481	0.072440428	Bacteria_unclassified	Bacteria_unclassified
0.6404556554	0.065307203	Bacteria_unclassified	Bacteria_unclassified
0.649195481	0.065307203	Bacteroides	Bacteroidaceae
0.6404556554	0.065307203	Clostridia_unclassified	Clostridia_unclassified
0.6404556554	0.065307203	Clostridiaceae_unclassified	Clostridiaceae
0.6404556554	0.065307203	Clostridiaceae_unclassified	Clostridiaceae
0.6404556554	0.065307203	Eubacteriales_unclassified	Eubacteriales_unclassified
0.8098448563	0.610203226	Clostridium	Clostridiaceae
0.6404556554	0.065307203	Erysipelatoclostridium	Erysipelotrichaceae
0.6404556554	0.072562568	Eubacteriaceae_unclassified	Eubacteriaceae
0.6404556554	0.065307203	Eubacteriaceae_unclassified	Eubacteriaceae
0.6404556554	0.065307203	GGB20146	FGB77306
0.6404556554	0.065307203	GGB20149	Lachnospiraceae
0.6404556554	0.065307203	GGB22635	Eggerthellaceae
0.6404556554	0.129165008	GGB25041	Lachnospiraceae
0.764176936	0.964962110	GGB28379	FGB9506
0.6404556554	0.073650255	GGB28382	FGB9508
0.6404556554	0.065307203	GGB28392	FGB9512
0.6404556554	0.069263270	GGB28404	FGB2838
0.6404556554	0.065307203	GGB28418	FGB2838
0.6404556554	0.065307203	GGB28422	FGB2838
0.6404556554	0.072187554	GGB28431	Pumilibacteraceae
0.6404556554	0.066476697	GGB28456	FGB2833
0.6909237074	0.076543895	GGB28782	Eubacteriaceae
0.6404556554	0.065307203	GGB28792	Lachnospiraceae
0.6404556554	0.065307203	GGB28798	Lachnospiraceae
0.6404556554	0.131253105	GGB28810	FGB9622
0.6404556554	0.065307203	GGB28828	FGB77305
0.6404556554	0.065307203	GGB28851	Clostridiaceae
0.6404556554	0.285303606	GGB28865	Lachnospiraceae
0.6404556554	0.065307203	GGB28868	Lachnospiraceae
0.6404556554	0.170684759	GGB28869	Lachnospiraceae
0.6404556554	0.111080289	GGB28875	Lachnospiraceae
0.8211099083	0.065307203	GGB28881	FGB9633
0.6404556554	0.065307203	GGB28892	Bacteria_unclassified
0.6404556554	0.065307203	GGB28893	Bacteria_unclassified
0.6404556554	0.114401038	GGB28898	Bacteria_unclassified
0.7700040547	0.065931569	GGB28901	Bacteria_unclassified
0.6404556554	0.065307203	GGB28904	Bacteria_unclassified
0.6404556554	0.072187554	GGB28909	Bacteria_unclassified

0.6404556554	0.593474380	GGB28924	Lachnospiraceae
0.9874747598	0.065307203	GGB28927	FGB77359
0.6404556554	0.065307203	GGB28934	FGB9639
0.6404556554	0.065307203	GGB28949	Lachnospiraceae
0.6404556554	0.065307203	GGB28951	Clostridiaceae
0.6404556554	0.065307203	GGB28951	Clostridiaceae
0.6404556554	0.065307203	GGB28954	Clostridiaceae
0.6404556554	0.109771322	GGB28960	Clostridiaceae
0.6404556554	0.065307203	GGB28964	Clostridiaceae
0.6404556554	0.065307203	GGB28967	Clostridiaceae
0.6404556554	0.065307203	GGB28996	FGB9656
0.6404556554	0.065307203	GGB29531	FGB9827
0.6404556554	0.073650255	GGB29685	Eubacteriaceae
0.6404556554	0.160717004	GGB30141	FGB77303
0.6404556554	0.065307203	GGB30145	Clostridiaceae
0.6404556554	0.065307203	GGB30286	Eubacteriales_unclassified
0.8098448563	0.253609269	GGB30300	FGB72709
0.6404556554	0.065307203	GGB30303	Oscillospiraceae
0.6404556554	0.065307203	GGB30450	Oscillospiraceae
0.6404556554	0.065307203	GGB30453	Oscillospiraceae
0.6404556554	0.066476697	GGB30454	Oscillospiraceae
0.6404556554	0.065307203	GGB30455	Oscillospiraceae
0.6404556554	0.065307203	GGB30456	Oscillospiraceae
0.6404556554	0.065307203	GGB30457	Oscillospiraceae
0.6404556554	0.065931569	GGB30461	Oscillospiraceae
0.6404556554	0.072687833	GGB30461	Oscillospiraceae
0.6404556554	0.065307203	GGB30461	Oscillospiraceae
0.6404556554	0.072440428	GGB30463	Oscillospiraceae
0.6404556554	0.065307203	GGB30473	Oscillospiraceae
0.6404556554	0.065307203	GGB30475	Oscillospiraceae
0.6404556554	0.512289062	GGB30861	FGB77153
0.6404556554	0.072440428	GGB31312	FGB1791
0.9612762186	0.065307203	GGB31438	FGB10290
0.9557341024	0.065307203	GGB3171	Oscillospiraceae
0.6404556554	0.065307203	GGB31762	FGB10289
0.6404556554	0.065307203	GGB31823	FGB1765
0.6404556554	0.090543679	GGB31838	FGB1765
0.8098448563	0.065307203	GGB31841	FGB1765
0.6404556554	0.065307203	GGB32371	FGB10667
0.6404556554	0.065307203	GGB3793	Lachnospiraceae
0.6404556554	0.065307203	GGB42601	Clostridiaceae
0.6404556554	0.065307203	GGB45514	Oscillospiraceae
0.6404556554	0.065307203	GGB45564	FGB75721
0.6979515333	0.065307203	GGB45624	Oscillospiraceae
0.7037808067	0.065307203	GGB45656	Christensenellaceae
0.6404556554	0.065307203	GGB47127	FGB10299

0.6404556554	0.176203079	GGB74395	Oscillospiraceae
0.6404556554	0.072687833	GGB75053	Oscillospiraceae
0.6404556554	0.090048525	GGB75109	Lachnospiraceae
0.6404556554	0.075097602	Lachnospiraceae_unclassified	Lachnospiraceae
0.6404556554	0.065307203	Lachnospiraceae_unclassified	Lachnospiraceae
0.6404556554	0.065307203	Lachnospiraceae_unclassified	Lachnospiraceae
0.6404556554	0.073650255	Lachnospiraceae_unclassified	Lachnospiraceae
0.6404556554	0.072562568	Lachnospiraceae_unclassified	Lachnospiraceae
0.6404556554	0.173081586	Lactobacillus	Lactobacillaceae
0.6404556554	0.065307203	Leptogranulimonas	Atopobiaceae
0.6404556554	0.065307203	Muribaculaceae_unclassified	Muribaculaceae
0.6404556554	0.066476697	Neglectibacter	Oscillospiraceae
0.6404556554	0.065307203	Oscillibacter	Oscillospiraceae
0.649195481	0.065307203	Oscillospiraceae_unclassified	Oscillospiraceae
0.6404556554	0.072440428	Oscillospiraceae_unclassified	Oscillospiraceae
0.8098448563	0.197298710	Oscillospiraceae_unclassified	Oscillospiraceae
0.6404556554	0.066476697	Oscillospiraceae_unclassified	Oscillospiraceae
0.6404556554	0.450086613	Parasutterella	Sutterellaceae
0.6404556554	0.072687833	Schaedlerella	Lachnospiraceae
0.9612762186	0.065307203	Turicibacter	Turicibacteraceae
0.6404556554	0.065931569	Bacteria_unclassified	Bacteria_unclassified
0.6404556554	0.066476697	Bacteria_unclassified	Bacteria_unclassified
0.6404556554	0.114258081	Bacteria_unclassified	Bacteria_unclassified
0.6404556554	0.056872449	Bacteria_unclassified	Bacteria_unclassified
0.6404556554	0.065307203	Bacteria_unclassified	Bacteria_unclassified
0.6404556554	0.065307203		
0.6404556554	0.070115235		
0.6404556554	0.065307203		
0.4691765424	0.124463106	Acetatifactor	Lachnospiraceae
0.4691765424	0.124898611	Acutalibacter	Oscillospiraceae
0.4691765424	0.124463106	Akkermansia	Akkermansiaceae
0.4691765424	0.124463106	Anaerotruncus	Oscillospiraceae
0.4691765424	0.366705682	Bacteria_unclassified	Bacteria_unclassified
0.4691765424	0.124463106	Bacteria_unclassified	Bacteria_unclassified
0.4691765424	0.124463106	Bacteria_unclassified	Bacteria_unclassified
0.5052767294	0.124463106	Bacteria_unclassified	Bacteria_unclassified
0.4691765424	0.124463106	Bacteroides	Bacteroidaceae
0.4691765424	0.124463106	Clostridia_unclassified	Clostridia_unclassified
0.4691765424	0.124463106	Clostridiaceae_unclassified	Clostridiaceae
0.4691765424	0.124463106	Clostridiaceae_unclassified	Clostridiaceae
0.4691765424	0.124463106	Eubacteriales_unclassified	Eubacteriales_unclassified
0.4691765424	0.897247569	Clostridium	Clostridiaceae
0.4691765424	0.124898611	Erysipelatoclostridium	Erysipelotrichaceae
0.4691765424	0.129355343	Eubacteriaceae_unclassified	Eubacteriaceae
0.5052767294	0.124463106	Eubacteriaceae_unclassified	Eubacteriaceae
0.6567032279	0.124463106	GGB20146	FGB77306

0.5138434935	0.124463106	GGB20149	Lachnospiraceae
0.4691765424	0.124463106	GGB22635	Eggerthellaceae
0.4829591757	0.294164396	GGB25041	Lachnospiraceae
0.4691765424	0.828439635	GGB28379	FGB9506
0.4691765424	0.124463106	GGB28382	FGB9508
0.7605235377	0.124463106	GGB28392	FGB9512
0.4691765424	0.124463106	GGB28404	FGB2838
0.4691765424	0.124463106	GGB28418	FGB2838
0.4691765424	0.124463106	GGB28422	FGB2838
0.5364485609	0.129355343	GGB28431	Pumilibacteraceae
0.4691765424	0.124463106	GGB28456	FGB2833
0.4691765424	0.129355343	GGB28782	Eubacteriaceae
0.4691765424	0.124463106	GGB28792	Lachnospiraceae
0.4691765424	0.124463106	GGB28798	Lachnospiraceae
0.4691765424	0.223115026	GGB28810	FGB9622
0.8020250797	0.124463106	GGB28828	FGB77305
0.4691765424	0.124463106	GGB28851	Clostridiaceae
0.9424038683	0.452904799	GGB28865	Lachnospiraceae
0.4691765424	0.124463106	GGB28868	Lachnospiraceae
0.4691765424	0.215261293	GGB28869	Lachnospiraceae
0.4691765424	0.124463106	GGB28875	Lachnospiraceae
0.4691765424	0.124463106	GGB28881	FGB9633
0.4691765424	0.124463106	GGB28892	Bacteria_unclassified
0.5034951558	0.124463106	GGB28893	Bacteria_unclassified
0.4691765424	0.187387585	GGB28898	Bacteria_unclassified
0.4691765424	0.124463106	GGB28901	Bacteria_unclassified
0.6265341538	0.124463106	GGB28904	Bacteria_unclassified
0.5138434935	0.124898611	GGB28909	Bacteria_unclassified
0.4840404152	0.766269998	GGB28924	Lachnospiraceae
0.4691765424	0.124463106	GGB28927	FGB77359
0.4691765424	0.124463106	GGB28934	FGB9639
0.4691765424	0.124463106	GGB28949	Lachnospiraceae
0.4691765424	0.124463106	GGB28951	Clostridiaceae
0.4691765424	0.124463106	GGB28951	Clostridiaceae
0.4691765424	0.182067783	GGB28954	Clostridiaceae
0.4691765424	0.135092316	GGB28960	Clostridiaceae
0.4691765424	0.124463106	GGB28964	Clostridiaceae
0.4691765424	0.124463106	GGB28967	Clostridiaceae
0.4691765424	0.124463106	GGB28996	FGB9656
0.4691765424	0.124463106	GGB29531	FGB9827
0.4691765424	0.124898611	GGB29685	Eubacteriaceae
0.4691765424	0.162790929	GGB30141	FGB77303
0.5052767294	0.124463106	GGB30145	Clostridiaceae
0.4691765424	0.124463106	GGB30286	Eubacteriales_unclassified
0.4833746032	0.124463106	GGB30300	FGB72709
0.4691765424	0.124463106	GGB30303	Oscillospiraceae

0.5285348257	0.124463106	GGB30450	Oscillospiraceae
0.4691765424	0.124463106	GGB30453	Oscillospiraceae
0.4691765424	0.154912135	GGB30454	Oscillospiraceae
0.4691765424	0.124463106	GGB30455	Oscillospiraceae
0.4691765424	0.124463106	GGB30456	Oscillospiraceae
0.4691765424	0.124898611	GGB30457	Oscillospiraceae
0.4691765424	0.124463106	GGB30461	Oscillospiraceae
0.4691765424	0.124463106	GGB30461	Oscillospiraceae
0.5285348257	0.124463106	GGB30461	Oscillospiraceae
0.4691765424	0.151921399	GGB30463	Oscillospiraceae
0.5218389679	0.124463106	GGB30473	Oscillospiraceae
0.4691765424	0.124463106	GGB30475	Oscillospiraceae
0.4691765424	0.170542880	GGB30861	FGB77153
0.4691765424	0.151921399	GGB31312	FGB1791
0.4691765424	0.124463106	GGB31438	FGB10290
0.4691765424	0.124463106	GGB3171	Oscillospiraceae
0.4691765424	0.124463106	GGB31762	FGB10289
0.4691765424	0.124898611	GGB31823	FGB1765
0.4691765424	0.124463106	GGB31838	FGB1765
0.4691765424	0.124463106	GGB31841	FGB1765
0.4691765424	0.124463106	GGB32371	FGB10667
0.4691765424	0.124463106	GGB3793	Lachnospiraceae
0.4691765424	0.129355343	GGB42601	Clostridiaceae
0.4691765424	0.124463106	GGB45514	Oscillospiraceae
0.5027050147	0.125327738	GGB45564	FGB75721
0.4691765424	0.124463106	GGB45624	Oscillospiraceae
0.4833746032	0.124463106	GGB45656	Christensenellaceae
0.4691765424	0.124463106	GGB47127	FGB10299
0.4691765424	0.124898611	GGB74395	Oscillospiraceae
0.5833419612	0.13259174	GGB75053	Oscillospiraceae
0.4829591757	0.151921399	GGB75109	Lachnospiraceae
0.4691765424	0.124898611	Lachnospiraceae_unclassified	Lachnospiraceae
0.4691765424	0.124898611	Lachnospiraceae_unclassified	Lachnospiraceae
0.4691765424	0.124463106	Lachnospiraceae_unclassified	Lachnospiraceae
0.4691765424	0.13259174	Lachnospiraceae_unclassified	Lachnospiraceae
0.4691765424	0.199900353	Lachnospiraceae_unclassified	Lachnospiraceae
0.4691765424	0.182022749	Lactobacillus	Lactobacillaceae
0.4691765424	0.124463106	Leptogranulimonas	Atopobiaceae
0.4691765424	0.124463106	Muribaculaceae_unclassified	Muribaculaceae
0.4691765424	0.124463106	Neglectibacter	Oscillospiraceae
0.4691765424	0.124463106	Oscillibacter	Oscillospiraceae
0.4691765424	0.124463106	Oscillospiraceae_unclassified	Oscillospiraceae
0.4833746032	0.124898611	Oscillospiraceae_unclassified	Oscillospiraceae
0.4691765424	0.170113100	Oscillospiraceae_unclassified	Oscillospiraceae
0.4691765424	0.124463106	Oscillospiraceae_unclassified	Oscillospiraceae
0.4691765424	0.250613129	Parasutterella	Sutterellaceae

0.4691765424	0.124898611	Schaedlerella	Lachnospiraceae
0.4691765424	0.124463106	Turicibacter	Turicibacteraceae
0.4691765424	0.124898611	Bacteria_unclassified	Bacteria_unclassified
0.4691765424	0.124463106	Bacteria_unclassified	Bacteria_unclassified
0.4833746032	0.212908512	Bacteria_unclassified	Bacteria_unclassified
0.7605235377	0.124463106	Bacteria_unclassified	Bacteria_unclassified
0.4691765424	0.124463106	Bacteria_unclassified	Bacteria_unclassified
0.4691765424	0.124463106		
0.4691765424	0.125327738		
0.4691765424	0.124463106		
0.7772452641	0.139167094	Acetatifactor	Lachnospiraceae
0.7772452641	0.139167094	Acutalibacter	Oscillospiraceae
0.7884879304	0.173512107	Akkermansia	Akkermansiaceae
0.7772452641	0.139167094	Anaerotruncus	Oscillospiraceae
0.7772452641	0.139167094	Bacteria_unclassified	Bacteria_unclassified
0.7772452641	0.139167094	Bacteria_unclassified	Bacteria_unclassified
0.7887714525	0.159072419	Bacteria_unclassified	Bacteria_unclassified
0.7772452641	0.173512107	Bacteria_unclassified	Bacteria_unclassified
0.7871876904	0.139167094	Bacteroides	Bacteroidaceae
0.7772452641	0.139167094	Clostridia_unclassified	Clostridia_unclassified
0.7772452641	0.177332328	Clostridiaceae_unclassified	Clostridiaceae
0.7772452641	0.139167094	Clostridiaceae_unclassified	Clostridiaceae
0.7772452641	0.139167094	Eubacteriales_unclassified	Eubacteriales_unclassified
0.7772452641	0.139167094	Clostridium	Clostridiaceae
0.7772452641	0.139167094	Erysipelatoclostridium	Erysipelotrichaceae
0.7772452641	0.576866502	Eubacteriaceae_unclassified	Eubacteriaceae
0.8950611082	0.141637955	Eubacteriaceae_unclassified	Eubacteriaceae
0.7772452641	0.141637955	GGB20146	FGB77306
0.8632250154	0.173512107	GGB20149	Lachnospiraceae
0.8522124224	0.139167094	GGB22635	Eggerthellaceae
0.7871876904	0.139167094	GGB25041	Lachnospiraceae
0.7772452641	0.220044416	GGB28379	FGB9506
0.7772452641	0.170603535	GGB28382	FGB9508
0.7772452641	0.139167094	GGB28392	FGB9512
0.7772452641	0.142106496	GGB28404	FGB2838
0.7772452641	0.139167094	GGB28418	FGB2838
0.7772452641	0.159072419	GGB28422	FGB2838
0.7772452641	0.257477259	GGB28431	Pumilibacteraceae
0.7887714525	0.139167094	GGB28456	FGB2833
0.7772452641	0.139167094	GGB28782	Eubacteriaceae
0.7772452641	0.139167094	GGB28792	Lachnospiraceae
0.7772452641	0.139167094	GGB28798	Lachnospiraceae
0.7772452641	0.160462267	GGB28810	FGB9622
0.7772452641	0.139167094	GGB28828	FGB77305
0.7772452641	0.141637955	GGB28851	Clostridiaceae
0.8786758731	0.139167094	GGB28865	Lachnospiraceae

0.7772452641	0.141637955	GGB28868	Lachnospiraceae
0.7772452641	0.139167094	GGB28869	Lachnospiraceae
0.7772452641	0.173512107	GGB28875	Lachnospiraceae
0.9801609216	0.139167094	GGB28881	FGB9633
0.7772452641	0.217683573	GGB28892	Bacteria_unclassified
0.8786758731	0.509774224	GGB28893	Bacteria_unclassified
0.7772452641	0.188219643	GGB28898	Bacteria_unclassified
0.8034418472	0.141637955	GGB28901	Bacteria_unclassified
0.9801609216	0.591048594	GGB28904	Bacteria_unclassified
0.8030625874	0.139167094	GGB28909	Bacteria_unclassified
0.7772452641	0.336817646	GGB28924	Lachnospiraceae
0.8907293381	0.139167094	GGB28927	FGB77359
0.7772452641	0.139167094	GGB28934	FGB9639
0.7772452641	0.139167094	GGB28949	Lachnospiraceae
0.7772452641	0.139167094	GGB28951	Clostridiaceae
0.7772452641	0.235914418	GGB28951	Clostridiaceae
0.7772452641	0.139167094	GGB28954	Clostridiaceae
0.7772452641	0.139167094	GGB28960	Clostridiaceae
0.7884879304	0.139167094	GGB28964	Clostridiaceae
0.7772452641	0.139167094	GGB28967	Clostridiaceae
0.7772452641	0.139167094	GGB28996	FGB9656
0.7772452641	0.139167094	GGB29531	FGB9827
0.7772452641	0.341208935	GGB29685	Eubacteriaceae
0.7772452641	0.549300175	GGB30141	FGB77303
0.7772452641	0.139167094	GGB30145	Clostridiaceae
0.7772452641	0.144096327	GGB30286	Eubacteriales_unclassified
0.7772452641	0.173512107	GGB30300	FGB72709
0.7772452641	0.331317016	GGB30303	Oscillospiraceae
0.7772452641	0.139167094	GGB30450	Oscillospiraceae
0.7772452641	0.141503910	GGB30453	Oscillospiraceae
0.7772452641	0.139167094	GGB30454	Oscillospiraceae
0.7772452641	0.139167094	GGB30455	Oscillospiraceae
0.7772452641	0.139167094	GGB30456	Oscillospiraceae
0.7772452641	0.141503910	GGB30457	Oscillospiraceae
0.7772452641	0.139167094	GGB30461	Oscillospiraceae
0.7772452641	0.438018312	GGB30461	Oscillospiraceae
0.7772452641	0.139167094	GGB30461	Oscillospiraceae
0.7772452641	0.220044416	GGB30463	Oscillospiraceae
0.7896006651	0.139167094	GGB30473	Oscillospiraceae
0.7772452641	0.144096327	GGB30475	Oscillospiraceae
0.7772452641	0.509774224	GGB30861	FGB77153
0.7772452641	0.139167094	GGB31312	FGB1791
0.7772452641	0.141637955	GGB31438	FGB10290
0.8907293381	0.139167094	GGB3171	Oscillospiraceae
0.7772452641	0.139167094	GGB31762	FGB10289
0.7772452641	0.139167094	GGB31823	FGB1765

0.7772452641	0.549300175	GGB31838	FGB1765
0.7772452641	0.139167094	GGB31841	FGB1765
0.8458435901	0.144096327	GGB32371	FGB10667
0.7772452641	0.139167094	GGB3793	Lachnospiraceae
0.7772452641	0.139167094	GGB42601	Clostridiaceae
0.7772452641	0.227093271	GGB45514	Oscillospiraceae
0.7887714525	0.139167094	GGB45564	FGB75721
0.7772452641	0.139167094	GGB45624	Oscillospiraceae
0.7772452641	0.144096327	GGB45656	Christensenellaceae
0.7772452641	0.139167094	GGB47127	FGB10299
0.7772452641	0.231976041	GGB74395	Oscillospiraceae
0.7772452641	0.173512107	GGB75053	Oscillospiraceae
0.7772452641	0.146664038	GGB75109	Lachnospiraceae
0.7772452641	0.352070901	Lachnospiraceae_unclassified	Lachnospiraceae
0.7772452641	0.139167094	Lachnospiraceae_unclassified	Lachnospiraceae
0.7772452641	0.139167094	Lachnospiraceae_unclassified	Lachnospiraceae
0.7772452641	0.159628987	Lachnospiraceae_unclassified	Lachnospiraceae
0.7772452641	0.139167094	Lachnospiraceae_unclassified	Lachnospiraceae
0.7772452641	0.576866502	Lactobacillus	Lactobacillaceae
0.7772452641	0.339372519	Leptogranulimonas	Atopobiaceae
0.7772452641	0.139167094	Muribaculaceae_unclassified	Muribaculaceae
0.7772452641	0.139167094	Neglectibacter	Oscillospiraceae
0.7772452641	0.139167094	Oscillibacter	Oscillospiraceae
0.7772452641	0.139167094	Oscillospiraceae_unclassified	Oscillospiraceae
0.7772452641	0.210017833	Oscillospiraceae_unclassified	Oscillospiraceae
0.7772452641	0.164584004	Oscillospiraceae_unclassified	Oscillospiraceae
0.7772452641	0.141503910	Oscillospiraceae_unclassified	Oscillospiraceae
0.7772452641	0.270423799	Parasutterella	Sutterellaceae
0.7887714525	0.139167094	Schaedlerella	Lachnospiraceae
0.7772452641	0.217683573	Turicibacter	Turicibacteraceae
0.7772452641	0.141503910	Bacteria_unclassified	Bacteria_unclassified
0.7772452641	0.173512107	Bacteria_unclassified	Bacteria_unclassified
0.7772452641	0.173512107	Bacteria_unclassified	Bacteria_unclassified
0.7772452641	0.139167094	Bacteria_unclassified	Bacteria_unclassified
0.7772452641	0.139167094	Bacteria_unclassified	Bacteria_unclassified
0.7772452641	0.139167094		
0.7772452641	0.491999147		
0.7772452641	0.139167094		
0.9369099384	0.999795788	Acetatifactor	Lachnospiraceae
0.9369099384	0.999795788	Acutalibacter	Oscillospiraceae
0.9369099384	0.999795788	Akkermansia	Akkermansiaceae
0.9369099384	0.999795788	Anaerotruncus	Oscillospiraceae
0.9369099384	0.999795788	Bacteria_unclassified	Bacteria_unclassified
0.9369099384	0.999795788	Bacteria_unclassified	Bacteria_unclassified
0.9452199076	0.999795788	Bacteria_unclassified	Bacteria_unclassified
0.9369099384	0.999795788	Bacteria_unclassified	Bacteria_unclassified

0.9369099384	0.999795788	Bacteroides	Bacteroidaceae
0.9369099384	0.999795788	Clostridia_unclassified	Clostridia_unclassified
0.9369099384	0.999795788	Clostridiaceae_unclassified	Clostridiaceae
0.9369099384	0.999795788	Clostridiaceae_unclassified	Clostridiaceae
0.9369099384	0.999795788	Eubacteriales_unclassified	Eubacteriales_unclassified
0.9369099384	0.999795788	Clostridium	Clostridiaceae
0.9369099384	0.999795788	Erysipelatoclostridium	Erysipelotrichaceae
0.9369099384	0.999795788	Eubacteriaceae_unclassified	Eubacteriaceae
0.9369099384	0.999795788	Eubacteriaceae_unclassified	Eubacteriaceae
0.9369099384	0.999795788	GGB20146	FGB77306
0.9369099384	0.999795788	GGB20149	Lachnospiraceae
0.9452199076	0.999795788	GGB22635	Eggerthellaceae
0.9369099384	0.999795788	GGB25041	Lachnospiraceae
0.9369099384	0.999795788	GGB28379	FGB9506
0.9605965501	0.999795788	GGB28382	FGB9508
0.9445683892	0.999795788	GGB28392	FGB9512
0.9369099384	0.999795788	GGB28404	FGB2838
0.9369099384	0.999795788	GGB28418	FGB2838
0.9369099384	0.999795788	GGB28422	FGB2838
0.9369099384	0.999795788	GGB28431	Pumilibacteraceae
0.9369099384	0.999795788	GGB28456	FGB2833
0.9369099384	0.999795788	GGB28782	Eubacteriaceae
0.9369099384	0.999795788	GGB28792	Lachnospiraceae
0.9369099384	0.999795788	GGB28798	Lachnospiraceae
0.9369099384	0.999795788	GGB28810	FGB9622
0.9369099384	0.999795788	GGB28828	FGB77305
0.9369099384	0.999795788	GGB28851	Clostridiaceae
0.9369099384	0.999795788	GGB28865	Lachnospiraceae
0.9445683892	0.999795788	GGB28868	Lachnospiraceae
0.9369099384	0.999795788	GGB28869	Lachnospiraceae
0.9369099384	0.999795788	GGB28875	Lachnospiraceae
0.9369099384	0.999795788	GGB28881	FGB9633
0.9369099384	0.999795788	GGB28892	Bacteria_unclassified
0.9369099384	0.999795788	GGB28893	Bacteria_unclassified
0.9369099384	0.999795788	GGB28898	Bacteria_unclassified
0.9369099384	0.999795788	GGB28901	Bacteria_unclassified
0.9369099384	0.999795788	GGB28904	Bacteria_unclassified
0.9369099384	0.999795788	GGB28909	Bacteria_unclassified
0.9369099384	0.999795788	GGB28924	Lachnospiraceae
0.9369099384	0.999795788	GGB28927	FGB77359
0.9369099384	0.999795788	GGB28934	FGB9639
0.9369099384	0.999795788	GGB28949	Lachnospiraceae
0.9369099384	0.999795788	GGB28951	Clostridiaceae
0.9369099384	0.999795788	GGB28951	Clostridiaceae
0.9369099384	0.999795788	GGB28954	Clostridiaceae
0.9369099384	0.999795788	GGB28960	Clostridiaceae

0.9369099384	0.999795788	GGB28964	Clostridiaceae
0.9369099384	0.999795788	GGB28967	Clostridiaceae
0.9445683892	0.999795788	GGB28996	FGB9656
0.9369099384	0.999795788	GGB29531	FGB9827
0.9369099384	0.999795788	GGB29685	Eubacteriaceae
0.9369099384	0.999795788	GGB30141	FGB77303
0.9369099384	0.999795788	GGB30145	Clostridiaceae
0.9369099384	0.999795788	GGB30286	Eubacteriales_unclassified
0.9369099384	0.999795788	GGB30300	FGB72709
0.9369099384	0.999795788	GGB30303	Oscillospiraceae
0.9369099384	0.999795788	GGB30450	Oscillospiraceae
0.9369099384	0.999795788	GGB30453	Oscillospiraceae
0.9369099384	0.999795788	GGB30454	Oscillospiraceae
0.9369099384	0.999795788	GGB30455	Oscillospiraceae
0.9369099384	0.999795788	GGB30456	Oscillospiraceae
0.9369099384	0.999795788	GGB30457	Oscillospiraceae
0.9369099384	0.999795788	GGB30461	Oscillospiraceae
0.9369099384	0.999795788	GGB30461	Oscillospiraceae
0.9605965501	0.999795788	GGB30461	Oscillospiraceae
0.9369099384	0.999795788	GGB30463	Oscillospiraceae
0.9369099384	0.999795788	GGB30473	Oscillospiraceae
0.9369099384	0.999795788	GGB30475	Oscillospiraceae
0.9369099384	0.999795788	GGB30861	FGB77153
0.9369099384	0.999795788	GGB31312	FGB1791
0.9445683892	0.999795788	GGB31438	FGB10290
0.9980271975	0.999795788	GGB3171	Oscillospiraceae
0.9369099384	0.999795788	GGB31762	FGB10289
0.9369099384	0.999795788	GGB31823	FGB1765
0.9369099384	0.999795788	GGB31838	FGB1765
0.9445683892	0.999795788	GGB31841	FGB1765
0.9369099384	0.999795788	GGB32371	FGB10667
0.9369099384	0.999795788	GGB3793	Lachnospiraceae
0.9369099384	0.999795788	GGB42601	Clostridiaceae
0.9369099384	0.999795788	GGB45514	Oscillospiraceae
0.9369099384	0.999795788	GGB45564	FGB75721
0.9369099384	0.999795788	GGB45624	Oscillospiraceae
0.9369099384	0.999795788	GGB45656	Christensenellaceae
0.9445683892	0.999795788	GGB47127	FGB10299
0.9369099384	0.999795788	GGB74395	Oscillospiraceae
0.9369099384	0.999795788	GGB75053	Oscillospiraceae
0.9369099384	0.999795788	GGB75109	Lachnospiraceae
0.9369099384	0.999795788	Lachnospiraceae_unclassified	Lachnospiraceae
0.9369099384	0.999795788	Lachnospiraceae_unclassified	Lachnospiraceae
0.9369099384	0.999795788	Lachnospiraceae_unclassified	Lachnospiraceae
0.9369099384	0.999795788	Lachnospiraceae_unclassified	Lachnospiraceae
0.9369099384	0.999795788	Lachnospiraceae_unclassified	Lachnospiraceae

0.9369099384	0.999795788	Lactobacillus	Lactobacillaceae
0.9369099384	0.999795788	Leptogranulimonas	Atopobiaceae
0.9369099384	0.999795788	Muribaculaceae_unclassified	Muribaculaceae
0.9369099384	0.999795788	Neglectibacter	Oscillospiraceae
0.9369099384	0.999795788	Oscillibacter	Oscillospiraceae
0.9369099384	0.999795788	Oscillospiraceae_unclassified	Oscillospiraceae
0.9369099384	0.999795788	Oscillospiraceae_unclassified	Oscillospiraceae
0.9369099384	0.999795788	Oscillospiraceae_unclassified	Oscillospiraceae
0.9369099384	0.999795788	Oscillospiraceae_unclassified	Oscillospiraceae
0.9369099384	0.999795788	Parasutterella	Sutterellaceae
0.9369099384	0.999795788	Schaedlerella	Lachnospiraceae
0.9369099384	0.999795788	Turicibacter	Turicibacteraceae
0.9369099384	0.999795788	Bacteria_unclassified	Bacteria_unclassified
0.9369099384	0.999795788	Bacteria_unclassified	Bacteria_unclassified
0.9369099384	0.999795788	Bacteria_unclassified	Bacteria_unclassified
0.9445683892	0.999795788	Bacteria_unclassified	Bacteria_unclassified
0.9902008623	0.999795788	Bacteria_unclassified	Bacteria_unclassified
0.9369099384	0.999795788		
0.9369099384	0.999795788		
0.9369099384	0.999795788		
0.9978339659	0.449272135	Acetatifactor	Lachnospiraceae
0.9978339659	0.460984392	Acutalibacter	Oscillospiraceae
0.9978339659	0.472980942	Akkermansia	Akkermansiaceae
0.9978339659	0.449272135	Anaerotruncus	Oscillospiraceae
0.9978339659	0.863104617	Bacteria_unclassified	Bacteria_unclassified
0.9978339659	0.449272135	Bacteria_unclassified	Bacteria_unclassified
0.9978339659	0.449272135	Bacteria_unclassified	Bacteria_unclassified
0.9978339659	0.598876942	Bacteria_unclassified	Bacteria_unclassified
0.9978339659	0.449272135	Bacteroides	Bacteroidaceae
0.9978339659	0.449272135	Clostridia_unclassified	Clostridia_unclassified
0.9978339659	0.463209264	Clostridiaceae_unclassified	Clostridiaceae
0.9978339659	0.460984392	Clostridiaceae_unclassified	Clostridiaceae
0.9978339659	0.449272135	Eubacteriales_unclassified	Eubacteriales_unclassified
0.9978339659	0.449272135	Clostridium	Clostridiaceae
0.9978339659	0.449272135	Erysipelatoclostridium	Erysipelotrichaceae
0.9978339659	0.460984392	Eubacteriaceae_unclassified	Eubacteriaceae
0.9978339659	0.449272135	Eubacteriaceae_unclassified	Eubacteriaceae
0.9978339659	0.449272135	GGB20146	FGB77306
0.9978339659	0.449272135	GGB20149	Lachnospiraceae
0.9978339659	0.449272135	GGB22635	Eggerthellaceae
0.9978339659	0.449272135	GGB25041	Lachnospiraceae
0.9978339659	0.797931564	GGB28379	FGB9506
0.9978339659	0.449272135	GGB28382	FGB9508
0.9978339659	0.449272135	GGB28392	FGB9512
0.9978339659	0.449272135	GGB28404	FGB2838
0.9978339659	0.449272135	GGB28418	FGB2838

0.9978339659	0.449272135	GGB28422	FGB2838
0.9978339659	0.569031625	GGB28431	Pumilibacteraceae
0.9978339659	0.449272135	GGB28456	FGB2833
0.9978339659	0.460984392	GGB28782	Eubacteriaceae
0.9978339659	0.463209264	GGB28792	Lachnospiraceae
0.9978339659	0.449272135	GGB28798	Lachnospiraceae
0.9978339659	0.598876942	GGB28810	FGB9622
0.9978339659	0.449272135	GGB28828	FGB77305
0.9978339659	0.449272135	GGB28851	Clostridiaceae
0.9978339659	0.629112713	GGB28865	Lachnospiraceae
0.9978339659	0.449272135	GGB28868	Lachnospiraceae
0.9978339659	0.565350831	GGB28869	Lachnospiraceae
0.9978339659	0.463209264	GGB28875	Lachnospiraceae
0.9978339659	0.463209264	GGB28881	FGB9633
0.9978339659	0.449272135	GGB28892	Bacteria_unclassified
0.9978339659	0.663558991	GGB28893	Bacteria_unclassified
0.9978339659	0.449272135	GGB28898	Bacteria_unclassified
0.9978339659	0.449272135	GGB28901	Bacteria_unclassified
0.9978339659	0.449272135	GGB28904	Bacteria_unclassified
0.9978339659	0.449272135	GGB28909	Bacteria_unclassified
0.9978339659	0.449272135	GGB28924	Lachnospiraceae
0.9978339659	0.449272135	GGB28927	FGB77359
0.9978339659	0.449272135	GGB28934	FGB9639
0.9978339659	0.449272135	GGB28949	Lachnospiraceae
0.9978339659	0.449272135	GGB28951	Clostridiaceae
0.9978339659	0.552950450	GGB28951	Clostridiaceae
0.9978339659	0.463209264	GGB28954	Clostridiaceae
0.9978339659	0.629112713	GGB28960	Clostridiaceae
0.9978339659	0.449272135	GGB28964	Clostridiaceae
0.9978339659	0.450957040	GGB28967	Clostridiaceae
0.9978339659	0.449272135	GGB28996	FGB9656
0.9978339659	0.449272135	GGB29531	FGB9827
0.9978339659	0.528600826	GGB29685	Eubacteriaceae
0.9978339659	0.449272135	GGB30141	FGB77303
0.9978339659	0.463209264	GGB30145	Clostridiaceae
0.9978339659	0.460984392	GGB30286	Eubacteriales_unclassified
0.9978339659	0.778462690	GGB30300	FGB72709
0.9978339659	0.528600826	GGB30303	Oscillospiraceae
0.9978339659	0.463209264	GGB30450	Oscillospiraceae
0.9978339659	0.449272135	GGB30453	Oscillospiraceae
0.9978339659	0.449272135	GGB30454	Oscillospiraceae
0.9978339659	0.449272135	GGB30455	Oscillospiraceae
0.9978339659	0.449272135	GGB30456	Oscillospiraceae
0.9978339659	0.450957040	GGB30457	Oscillospiraceae
0.9978339659	0.460984392	GGB30461	Oscillospiraceae
0.9978339659	0.449272135	GGB30461	Oscillospiraceae

0.9978339659	0.449272135	GGB30461	Oscillospiraceae
0.9978339659	0.460984392	GGB30463	Oscillospiraceae
0.9978339659	0.449272135	GGB30473	Oscillospiraceae
0.9978339659	0.460984392	GGB30475	Oscillospiraceae
0.9978339659	0.449272135	GGB30861	FGB77153
0.9978339659	0.449272135	GGB31312	FGB1791
0.9978339659	0.460984392	GGB31438	FGB10290
0.9978339659	0.565350831	GGB3171	Oscillospiraceae
0.9978339659	0.449272135	GGB31762	FGB10289
0.9978339659	0.460984392	GGB31823	FGB1765
0.9978339659	0.449272135	GGB31838	FGB1765
0.9978339659	0.449272135	GGB31841	FGB1765
0.9978339659	0.450957040	GGB32371	FGB10667
0.9978339659	0.449272135	GGB3793	Lachnospiraceae
0.9978339659	0.463209264	GGB42601	Clostridiaceae
0.9978339659	0.460984392	GGB45514	Oscillospiraceae
0.9978339659	0.460984392	GGB45564	FGB75721
0.9978339659	0.449272135	GGB45624	Oscillospiraceae
0.9978339659	0.449272135	GGB45656	Christensenellaceae
0.9978339659	0.463209264	GGB47127	FGB10299
0.9978339659	0.449272135	GGB74395	Oscillospiraceae
0.9978339659	0.449272135	GGB75053	Oscillospiraceae
0.9978339659	0.449272135	GGB75109	Lachnospiraceae
0.9978339659	0.463209264	Lachnospiraceae_unclassified	Lachnospiraceae
0.9978339659	0.463209264	Lachnospiraceae_unclassified	Lachnospiraceae
0.9978339659	0.449272135	Lachnospiraceae_unclassified	Lachnospiraceae
0.9978339659	0.449272135	Lachnospiraceae_unclassified	Lachnospiraceae
0.9978339659	0.463209264	Lachnospiraceae_unclassified	Lachnospiraceae
0.9978339659	0.460984392	Lactobacillus	Lactobacillaceae
0.9978339659	0.798695268	Leptogranulimonas	Atopobiaceae
0.9978339659	0.449272135	Muribaculaceae_unclassified	Muribaculaceae
0.9978339659	0.449272135	Neglectibacter	Oscillospiraceae
0.9978339659	0.449272135	Oscillibacter	Oscillospiraceae
0.9978339659	0.449272135	Oscillospiraceae_unclassified	Oscillospiraceae
0.9978339659	0.463209264	Oscillospiraceae_unclassified	Oscillospiraceae
0.9978339659	0.449272135	Oscillospiraceae_unclassified	Oscillospiraceae
0.9978339659	0.449272135	Oscillospiraceae_unclassified	Oscillospiraceae
0.9978339659	0.460984392	Parasutterella	Sutterellaceae
0.9978339659	0.463209264	Schaedlerella	Lachnospiraceae
0.9978339659	0.463209264	Turicibacter	Turicibacteraceae
0.9978339659	0.449272135	Bacteria_unclassified	Bacteria_unclassified
0.9978339659	0.528600826	Bacteria_unclassified	Bacteria_unclassified
0.9978339659	0.449272135	Bacteria_unclassified	Bacteria_unclassified
0.9978339659	0.449272135	Bacteria_unclassified	Bacteria_unclassified
0.9978339659	0.449272135	Bacteria_unclassified	Bacteria_unclassified
0.9978339659	0.449272135		

0.9978339659	0.579163027[illegible]
0.9978339659	0.449272135[illegible]

Order	Class	Phylum	Kingdom
Eubacteriales	Clostridia	Firmicutes	Bacteria
Eubacteriales	Clostridia	Firmicutes	Bacteria
Verrucomicrobiales	Verrucomicrobiae	Verrucomicrobia	Bacteria
Eubacteriales	Clostridia	Firmicutes	Bacteria
Bacteria_unclassified	Bacteria_unclassified	Bacteria_unclassified	Bacteria
Bacteria_unclassified	Bacteria_unclassified	Bacteria_unclassified	Bacteria
Bacteria_unclassified	Bacteria_unclassified	Bacteria_unclassified	Bacteria
Bacteria_unclassified	Bacteria_unclassified	Bacteria_unclassified	Bacteria
Bacteroidales	Bacteroidia	Bacteroidota	Bacteria
Clostridia_unclassified	Clostridia	Firmicutes	Bacteria
Eubacteriales	Clostridia	Firmicutes	Bacteria
Eubacteriales	Clostridia	Firmicutes	Bacteria
Eubacteriales	Clostridia	Firmicutes	Bacteria
Eubacteriales	Clostridia	Firmicutes	Bacteria
Erysipelotrichales	Erysipelotrichia	Firmicutes	Bacteria
Eubacteriales	Clostridia	Firmicutes	Bacteria
Eubacteriales	Clostridia	Firmicutes	Bacteria
OFGB77306	CFGB77306	Firmicutes	Bacteria
Eubacteriales	Clostridia	Firmicutes	Bacteria
Eggerthellales	Coriobacteriia	Actinobacteria	Bacteria
Eubacteriales	Clostridia	Firmicutes	Bacteria
OFGB9506	CFGB9506	Firmicutes	Bacteria
OFGB9508	CFGB9508	Firmicutes	Bacteria
OFGB9512	CFGB9512	Firmicutes	Bacteria
OFGB2838	CFGB2838	Firmicutes	Bacteria
OFGB2838	CFGB2838	Firmicutes	Bacteria
OFGB2838	CFGB2838	Firmicutes	Bacteria
Eubacteriales	Clostridia	Firmicutes	Bacteria
OFGB2833	CFGB2833	Firmicutes	Bacteria
Eubacteriales	Clostridia	Firmicutes	Bacteria
Eubacteriales	Clostridia	Firmicutes	Bacteria
Eubacteriales	Clostridia	Firmicutes	Bacteria
OFGB9622	CFGB9622	Firmicutes	Bacteria
OFGB77305	CFGB77305	Firmicutes	Bacteria
Eubacteriales	Clostridia	Firmicutes	Bacteria
Eubacteriales	Clostridia	Firmicutes	Bacteria
Eubacteriales	Clostridia	Firmicutes	Bacteria
Eubacteriales	Clostridia	Firmicutes	Bacteria
Eubacteriales	Clostridia	Firmicutes	Bacteria
OFGB9633	CFGB9633	Firmicutes	Bacteria
Bacteria_unclassified	Bacteria_unclassified	Bacteria_unclassified	Bacteria
Bacteria_unclassified	Bacteria_unclassified	Bacteria_unclassified	Bacteria
Bacteria_unclassified	Bacteria_unclassified	Bacteria_unclassified	Bacteria
Bacteria_unclassified	Bacteria_unclassified	Bacteria_unclassified	Bacteria
Bacteria_unclassified	Bacteria_unclassified	Bacteria_unclassified	Bacteria
Bacteria_unclassified	Bacteria_unclassified	Bacteria_unclassified	Bacteria

Eubacteriales	Clostridia	Firmicutes	Bacteria
OFGB77359	CFGB77359	Bacteria_unclassified	Bacteria
OFGB9639	CFGB9639	Firmicutes	Bacteria
Eubacteriales	Clostridia	Firmicutes	Bacteria
Eubacteriales	Clostridia	Firmicutes	Bacteria
Eubacteriales	Clostridia	Firmicutes	Bacteria
Eubacteriales	Clostridia	Firmicutes	Bacteria
Eubacteriales	Clostridia	Firmicutes	Bacteria
Eubacteriales	Clostridia	Firmicutes	Bacteria
Eubacteriales	Clostridia	Firmicutes	Bacteria
OFGB9656	CFGB9656	Firmicutes	Bacteria
OFGB9827	CFGB9827	Firmicutes	Bacteria
Eubacteriales	Clostridia	Firmicutes	Bacteria
OFGB77303	CFGB77303	Bacteria_unclassified	Bacteria
Eubacteriales	Clostridia	Firmicutes	Bacteria
Eubacteriales	Clostridia	Firmicutes	Bacteria
OFGB72709	CFGB72709	Firmicutes	Bacteria
Eubacteriales	Clostridia	Firmicutes	Bacteria
Eubacteriales	Clostridia	Firmicutes	Bacteria
Eubacteriales	Clostridia	Firmicutes	Bacteria
Eubacteriales	Clostridia	Firmicutes	Bacteria
Eubacteriales	Clostridia	Firmicutes	Bacteria
Eubacteriales	Clostridia	Firmicutes	Bacteria
Eubacteriales	Clostridia	Firmicutes	Bacteria
Eubacteriales	Clostridia	Firmicutes	Bacteria
Eubacteriales	Clostridia	Firmicutes	Bacteria
Eubacteriales	Clostridia	Firmicutes	Bacteria
Eubacteriales	Clostridia	Firmicutes	Bacteria
Eubacteriales	Clostridia	Firmicutes	Bacteria
Eubacteriales	Clostridia	Firmicutes	Bacteria
OFGB77153	CFGB77153	Actinobacteria	Bacteria
OFGB1791	CFGB1791	Tenericutes	Bacteria
OFGB10290	CFGB10290	Firmicutes	Bacteria
Eubacteriales	Clostridia	Firmicutes	Bacteria
OFGB10289	CFGB10289	Firmicutes	Bacteria
OFGB1765	CFGB1765	Firmicutes	Bacteria
OFGB1765	CFGB1765	Firmicutes	Bacteria
OFGB1765	CFGB1765	Firmicutes	Bacteria
OFGB10667	CFGB10667	Firmicutes	Bacteria
Eubacteriales	Clostridia	Firmicutes	Bacteria
Eubacteriales	Clostridia	Firmicutes	Bacteria
Eubacteriales	Clostridia	Firmicutes	Bacteria
OFGB75721	CFGB75721	Firmicutes	Bacteria
Eubacteriales	Clostridia	Firmicutes	Bacteria
Eubacteriales	Clostridia	Firmicutes	Bacteria
OFGB10299	CFGB10299	Firmicutes	Bacteria

Eubacteriales	Clostridia	Firmicutes	Bacteria
Eubacteriales	Clostridia	Firmicutes	Bacteria
Eubacteriales	Clostridia	Firmicutes	Bacteria
Eubacteriales	Clostridia	Firmicutes	Bacteria
Eubacteriales	Clostridia	Firmicutes	Bacteria
Eubacteriales	Clostridia	Firmicutes	Bacteria
Eubacteriales	Clostridia	Firmicutes	Bacteria
Eubacteriales	Clostridia	Firmicutes	Bacteria
Lactobacillales	Bacilli	Firmicutes	Bacteria
Coriobacteriales	Coriobacteriia	Actinobacteria	Bacteria
Bacteroidales	Bacteroidia	Bacteroidota	Bacteria
Eubacteriales	Clostridia	Firmicutes	Bacteria
Eubacteriales	Clostridia	Firmicutes	Bacteria
Eubacteriales	Clostridia	Firmicutes	Bacteria
Eubacteriales	Clostridia	Firmicutes	Bacteria
Eubacteriales	Clostridia	Firmicutes	Bacteria
Eubacteriales	Clostridia	Firmicutes	Bacteria
Burkholderiales	Betaproteobacteria	Proteobacteria	Bacteria
Eubacteriales	Clostridia	Firmicutes	Bacteria
Erysipelotrichales	Erysipelotrichia	Firmicutes	Bacteria
Bacteria_unclassified	Bacteria_unclassified	Bacteria_unclassified	Bacteria
Bacteria_unclassified	Bacteria_unclassified	Bacteria_unclassified	Bacteria
Bacteria_unclassified	Bacteria_unclassified	Bacteria_unclassified	Bacteria
Bacteria_unclassified	Bacteria_unclassified	Bacteria_unclassified	Bacteria
Bacteria_unclassified	Bacteria_unclassified	Bacteria_unclassified	Bacteria

Eubacteriales	Clostridia	Firmicutes	Bacteria
Eubacteriales	Clostridia	Firmicutes	Bacteria
Verrucomicrobiales	Verrucomicrobiae	Verrucomicrobia	Bacteria
Eubacteriales	Clostridia	Firmicutes	Bacteria
Bacteria_unclassified	Bacteria_unclassified	Bacteria_unclassified	Bacteria
Bacteria_unclassified	Bacteria_unclassified	Bacteria_unclassified	Bacteria
Bacteria_unclassified	Bacteria_unclassified	Bacteria_unclassified	Bacteria
Bacteria_unclassified	Bacteria_unclassified	Bacteria_unclassified	Bacteria
Bacteroidales	Bacteroidia	Bacteroidota	Bacteria
Clostridia_unclassified	Clostridia	Firmicutes	Bacteria
Eubacteriales	Clostridia	Firmicutes	Bacteria
Eubacteriales	Clostridia	Firmicutes	Bacteria
Eubacteriales	Clostridia	Firmicutes	Bacteria
Eubacteriales	Clostridia	Firmicutes	Bacteria
Erysipelotrichales	Erysipelotrichia	Firmicutes	Bacteria
Eubacteriales	Clostridia	Firmicutes	Bacteria
Eubacteriales	Clostridia	Firmicutes	Bacteria
OFGB77306	CFGB77306	Firmicutes	Bacteria

Eubacteriales	Clostridia	Firmicutes	Bacteria
Eggerthellales	Coriobacteriia	Actinobacteria	Bacteria
Eubacteriales	Clostridia	Firmicutes	Bacteria
OFGB9506	CFGB9506	Firmicutes	Bacteria
OFGB9508	CFGB9508	Firmicutes	Bacteria
OFGB9512	CFGB9512	Firmicutes	Bacteria
OFGB2838	CFGB2838	Firmicutes	Bacteria
OFGB2838	CFGB2838	Firmicutes	Bacteria
OFGB2838	CFGB2838	Firmicutes	Bacteria
Eubacteriales	Clostridia	Firmicutes	Bacteria
OFGB2833	CFGB2833	Firmicutes	Bacteria
Eubacteriales	Clostridia	Firmicutes	Bacteria
Eubacteriales	Clostridia	Firmicutes	Bacteria
Eubacteriales	Clostridia	Firmicutes	Bacteria
OFGB9622	CFGB9622	Firmicutes	Bacteria
OFGB77305	CFGB77305	Firmicutes	Bacteria
Eubacteriales	Clostridia	Firmicutes	Bacteria
Eubacteriales	Clostridia	Firmicutes	Bacteria
Eubacteriales	Clostridia	Firmicutes	Bacteria
Eubacteriales	Clostridia	Firmicutes	Bacteria
Eubacteriales	Clostridia	Firmicutes	Bacteria
OFGB9633	CFGB9633	Firmicutes	Bacteria
Bacteria_unclassified	Bacteria_unclassified	Bacteria_unclassified	Bacteria
Bacteria_unclassified	Bacteria_unclassified	Bacteria_unclassified	Bacteria
Bacteria_unclassified	Bacteria_unclassified	Bacteria_unclassified	Bacteria
Bacteria_unclassified	Bacteria_unclassified	Bacteria_unclassified	Bacteria
Bacteria_unclassified	Bacteria_unclassified	Bacteria_unclassified	Bacteria
Bacteria_unclassified	Bacteria_unclassified	Bacteria_unclassified	Bacteria
Eubacteriales	Clostridia	Firmicutes	Bacteria
OFGB77359	CFGB77359	Bacteria_unclassified	Bacteria
OFGB9639	CFGB9639	Firmicutes	Bacteria
Eubacteriales	Clostridia	Firmicutes	Bacteria
Eubacteriales	Clostridia	Firmicutes	Bacteria
Eubacteriales	Clostridia	Firmicutes	Bacteria
Eubacteriales	Clostridia	Firmicutes	Bacteria
Eubacteriales	Clostridia	Firmicutes	Bacteria
Eubacteriales	Clostridia	Firmicutes	Bacteria
Eubacteriales	Clostridia	Firmicutes	Bacteria
OFGB9656	CFGB9656	Firmicutes	Bacteria
OFGB9827	CFGB9827	Firmicutes	Bacteria
Eubacteriales	Clostridia	Firmicutes	Bacteria
OFGB77303	CFGB77303	Bacteria_unclassified	Bacteria
Eubacteriales	Clostridia	Firmicutes	Bacteria
Eubacteriales	Clostridia	Firmicutes	Bacteria
OFGB72709	CFGB72709	Firmicutes	Bacteria
Eubacteriales	Clostridia	Firmicutes	Bacteria

Eubacteriales	Clostridia	Firmicutes	Bacteria
Eubacteriales	Clostridia	Firmicutes	Bacteria
Eubacteriales	Clostridia	Firmicutes	Bacteria
Eubacteriales	Clostridia	Firmicutes	Bacteria
Eubacteriales	Clostridia	Firmicutes	Bacteria
Eubacteriales	Clostridia	Firmicutes	Bacteria
Eubacteriales	Clostridia	Firmicutes	Bacteria
Eubacteriales	Clostridia	Firmicutes	Bacteria
Eubacteriales	Clostridia	Firmicutes	Bacteria
Eubacteriales	Clostridia	Firmicutes	Bacteria
Eubacteriales	Clostridia	Firmicutes	Bacteria
Eubacteriales	Clostridia	Firmicutes	Bacteria
OFGB77153	CFGB77153	Actinobacteria	Bacteria
OFGB1791	CFGB1791	Tenericutes	Bacteria
OFGB10290	CFGB10290	Firmicutes	Bacteria
Eubacteriales	Clostridia	Firmicutes	Bacteria
OFGB10289	CFGB10289	Firmicutes	Bacteria
OFGB1765	CFGB1765	Firmicutes	Bacteria
OFGB1765	CFGB1765	Firmicutes	Bacteria
OFGB1765	CFGB1765	Firmicutes	Bacteria
OFGB10667	CFGB10667	Firmicutes	Bacteria
Eubacteriales	Clostridia	Firmicutes	Bacteria
Eubacteriales	Clostridia	Firmicutes	Bacteria
Eubacteriales	Clostridia	Firmicutes	Bacteria
OFGB75721	CFGB75721	Firmicutes	Bacteria
Eubacteriales	Clostridia	Firmicutes	Bacteria
Eubacteriales	Clostridia	Firmicutes	Bacteria
OFGB10299	CFGB10299	Firmicutes	Bacteria
Eubacteriales	Clostridia	Firmicutes	Bacteria
Eubacteriales	Clostridia	Firmicutes	Bacteria
Eubacteriales	Clostridia	Firmicutes	Bacteria
Eubacteriales	Clostridia	Firmicutes	Bacteria
Eubacteriales	Clostridia	Firmicutes	Bacteria
Eubacteriales	Clostridia	Firmicutes	Bacteria
Eubacteriales	Clostridia	Firmicutes	Bacteria
Eubacteriales	Clostridia	Firmicutes	Bacteria
Lactobacillales	Bacilli	Firmicutes	Bacteria
Coriobacteriales	Coriobacteriia	Actinobacteria	Bacteria
Bacteroidales	Bacteroidia	Bacteroidota	Bacteria
Eubacteriales	Clostridia	Firmicutes	Bacteria
Eubacteriales	Clostridia	Firmicutes	Bacteria
Eubacteriales	Clostridia	Firmicutes	Bacteria
Eubacteriales	Clostridia	Firmicutes	Bacteria
Eubacteriales	Clostridia	Firmicutes	Bacteria
Eubacteriales	Clostridia	Firmicutes	Bacteria
Burkholderiales	Betaproteobacteria	Proteobacteria	Bacteria

Eubacteriales	Clostridia	Firmicutes	Bacteria
Erysipelotrichales	Erysipelotrichia	Firmicutes	Bacteria
Bacteria_unclassified	Bacteria_unclassified	Bacteria_unclassified	Bacteria
Bacteria_unclassified	Bacteria_unclassified	Bacteria_unclassified	Bacteria
Bacteria_unclassified	Bacteria_unclassified	Bacteria_unclassified	Bacteria
Bacteria_unclassified	Bacteria_unclassified	Bacteria_unclassified	Bacteria
Bacteria_unclassified	Bacteria_unclassified	Bacteria_unclassified	Bacteria

Eubacteriales	Clostridia	Firmicutes	Bacteria
Eubacteriales	Clostridia	Firmicutes	Bacteria
Verrucomicrobiales	Verrucomicrobiae	Verrucomicrobia	Bacteria
Eubacteriales	Clostridia	Firmicutes	Bacteria
Bacteria_unclassified	Bacteria_unclassified	Bacteria_unclassified	Bacteria
Bacteria_unclassified	Bacteria_unclassified	Bacteria_unclassified	Bacteria
Bacteria_unclassified	Bacteria_unclassified	Bacteria_unclassified	Bacteria
Bacteria_unclassified	Bacteria_unclassified	Bacteria_unclassified	Bacteria
Bacteroidales	Bacteroidia	Bacteroidota	Bacteria
Clostridia_unclassified	Clostridia	Firmicutes	Bacteria
Eubacteriales	Clostridia	Firmicutes	Bacteria
Eubacteriales	Clostridia	Firmicutes	Bacteria
Eubacteriales	Clostridia	Firmicutes	Bacteria
Eubacteriales	Clostridia	Firmicutes	Bacteria
Erysipelotrichales	Erysipelotrichia	Firmicutes	Bacteria
Eubacteriales	Clostridia	Firmicutes	Bacteria
Eubacteriales	Clostridia	Firmicutes	Bacteria
OFGB77306	CFGB77306	Firmicutes	Bacteria
Eubacteriales	Clostridia	Firmicutes	Bacteria
Eggerthellales	Coriobacteriia	Actinobacteria	Bacteria
Eubacteriales	Clostridia	Firmicutes	Bacteria
OFGB9506	CFGB9506	Firmicutes	Bacteria
OFGB9508	CFGB9508	Firmicutes	Bacteria
OFGB9512	CFGB9512	Firmicutes	Bacteria
OFGB2838	CFGB2838	Firmicutes	Bacteria
OFGB2838	CFGB2838	Firmicutes	Bacteria
OFGB2838	CFGB2838	Firmicutes	Bacteria
Eubacteriales	Clostridia	Firmicutes	Bacteria
OFGB2833	CFGB2833	Firmicutes	Bacteria
Eubacteriales	Clostridia	Firmicutes	Bacteria
Eubacteriales	Clostridia	Firmicutes	Bacteria
Eubacteriales	Clostridia	Firmicutes	Bacteria
OFGB9622	CFGB9622	Firmicutes	Bacteria
OFGB77305	CFGB77305	Firmicutes	Bacteria
Eubacteriales	Clostridia	Firmicutes	Bacteria
Eubacteriales	Clostridia	Firmicutes	Bacteria

Eubacteriales	Clostridia	Firmicutes	Bacteria
Eubacteriales	Clostridia	Firmicutes	Bacteria
Eubacteriales	Clostridia	Firmicutes	Bacteria
OFGB9633	CFGB9633	Firmicutes	Bacteria
Bacteria_unclassified	Bacteria_unclassified	Bacteria_unclassified	Bacteria
Bacteria_unclassified	Bacteria_unclassified	Bacteria_unclassified	Bacteria
Bacteria_unclassified	Bacteria_unclassified	Bacteria_unclassified	Bacteria
Bacteria_unclassified	Bacteria_unclassified	Bacteria_unclassified	Bacteria
Bacteria_unclassified	Bacteria_unclassified	Bacteria_unclassified	Bacteria
Bacteria_unclassified	Bacteria_unclassified	Bacteria_unclassified	Bacteria
Eubacteriales	Clostridia	Firmicutes	Bacteria
OFGB77359	CFGB77359	Bacteria_unclassified	Bacteria
OFGB9639	CFGB9639	Firmicutes	Bacteria
Eubacteriales	Clostridia	Firmicutes	Bacteria
Eubacteriales	Clostridia	Firmicutes	Bacteria
Eubacteriales	Clostridia	Firmicutes	Bacteria
Eubacteriales	Clostridia	Firmicutes	Bacteria
Eubacteriales	Clostridia	Firmicutes	Bacteria
Eubacteriales	Clostridia	Firmicutes	Bacteria
Eubacteriales	Clostridia	Firmicutes	Bacteria
OFGB9656	CFGB9656	Firmicutes	Bacteria
OFGB9827	CFGB9827	Firmicutes	Bacteria
Eubacteriales	Clostridia	Firmicutes	Bacteria
OFGB77303	CFGB77303	Bacteria_unclassified	Bacteria
Eubacteriales	Clostridia	Firmicutes	Bacteria
Eubacteriales	Clostridia	Firmicutes	Bacteria
OFGB72709	CFGB72709	Firmicutes	Bacteria
Eubacteriales	Clostridia	Firmicutes	Bacteria
Eubacteriales	Clostridia	Firmicutes	Bacteria
Eubacteriales	Clostridia	Firmicutes	Bacteria
Eubacteriales	Clostridia	Firmicutes	Bacteria
Eubacteriales	Clostridia	Firmicutes	Bacteria
Eubacteriales	Clostridia	Firmicutes	Bacteria
Eubacteriales	Clostridia	Firmicutes	Bacteria
Eubacteriales	Clostridia	Firmicutes	Bacteria
Eubacteriales	Clostridia	Firmicutes	Bacteria
Eubacteriales	Clostridia	Firmicutes	Bacteria
Eubacteriales	Clostridia	Firmicutes	Bacteria
Eubacteriales	Clostridia	Firmicutes	Bacteria
Eubacteriales	Clostridia	Firmicutes	Bacteria
OFGB77153	CFGB77153	Actinobacteria	Bacteria
OFGB1791	CFGB1791	Tenericutes	Bacteria
OFGB10290	CFGB10290	Firmicutes	Bacteria
Eubacteriales	Clostridia	Firmicutes	Bacteria
OFGB10289	CFGB10289	Firmicutes	Bacteria
OFGB1765	CFGB1765	Firmicutes	Bacteria

OFGB1765	CFGB1765	Firmicutes	Bacteria
OFGB1765	CFGB1765	Firmicutes	Bacteria
OFGB10667	CFGB10667	Firmicutes	Bacteria
Eubacteriales	Clostridia	Firmicutes	Bacteria
Eubacteriales	Clostridia	Firmicutes	Bacteria
Eubacteriales	Clostridia	Firmicutes	Bacteria
OFGB75721	CFGB75721	Firmicutes	Bacteria
Eubacteriales	Clostridia	Firmicutes	Bacteria
Eubacteriales	Clostridia	Firmicutes	Bacteria
OFGB10299	CFGB10299	Firmicutes	Bacteria
Eubacteriales	Clostridia	Firmicutes	Bacteria
Eubacteriales	Clostridia	Firmicutes	Bacteria
Eubacteriales	Clostridia	Firmicutes	Bacteria
Eubacteriales	Clostridia	Firmicutes	Bacteria
Eubacteriales	Clostridia	Firmicutes	Bacteria
Eubacteriales	Clostridia	Firmicutes	Bacteria
Eubacteriales	Clostridia	Firmicutes	Bacteria
Eubacteriales	Clostridia	Firmicutes	Bacteria
Lactobacillales	Bacilli	Firmicutes	Bacteria
Coriobacteriales	Coriobacteriia	Actinobacteria	Bacteria
Bacteroidales	Bacteroidia	Bacteroidota	Bacteria
Eubacteriales	Clostridia	Firmicutes	Bacteria
Eubacteriales	Clostridia	Firmicutes	Bacteria
Eubacteriales	Clostridia	Firmicutes	Bacteria
Eubacteriales	Clostridia	Firmicutes	Bacteria
Eubacteriales	Clostridia	Firmicutes	Bacteria
Eubacteriales	Clostridia	Firmicutes	Bacteria
Burkholderiales	Betaproteobacteria	Proteobacteria	Bacteria
Eubacteriales	Clostridia	Firmicutes	Bacteria
Erysipelotrichales	Erysipelotrichia	Firmicutes	Bacteria
Bacteria_unclassified	Bacteria_unclassified	Bacteria_unclassified	Bacteria
Bacteria_unclassified	Bacteria_unclassified	Bacteria_unclassified	Bacteria
Bacteria_unclassified	Bacteria_unclassified	Bacteria_unclassified	Bacteria
Bacteria_unclassified	Bacteria_unclassified	Bacteria_unclassified	Bacteria
Bacteria_unclassified	Bacteria_unclassified	Bacteria_unclassified	Bacteria

Eubacteriales	Clostridia	Firmicutes	Bacteria
Eubacteriales	Clostridia	Firmicutes	Bacteria
Verrucomicrobiales	Verrucomicrobiae	Verrucomicrobia	Bacteria
Eubacteriales	Clostridia	Firmicutes	Bacteria
Bacteria_unclassified	Bacteria_unclassified	Bacteria_unclassified	Bacteria
Bacteria_unclassified	Bacteria_unclassified	Bacteria_unclassified	Bacteria
Bacteria_unclassified	Bacteria_unclassified	Bacteria_unclassified	Bacteria
Bacteria_unclassified	Bacteria_unclassified	Bacteria_unclassified	Bacteria

Bacteroidales	Bacteroidia	Bacteroidota	Bacteria
Clostridia_unclassified	Clostridia	Firmicutes	Bacteria
Eubacteriales	Clostridia	Firmicutes	Bacteria
Eubacteriales	Clostridia	Firmicutes	Bacteria
Eubacteriales	Clostridia	Firmicutes	Bacteria
Eubacteriales	Clostridia	Firmicutes	Bacteria
Erysipelotrichales	Erysipelotrichia	Firmicutes	Bacteria
Eubacteriales	Clostridia	Firmicutes	Bacteria
Eubacteriales	Clostridia	Firmicutes	Bacteria
OFGB77306	CFGB77306	Firmicutes	Bacteria
Eubacteriales	Clostridia	Firmicutes	Bacteria
Eggerthellales	Coriobacteriia	Actinobacteria	Bacteria
Eubacteriales	Clostridia	Firmicutes	Bacteria
OFGB9506	CFGB9506	Firmicutes	Bacteria
OFGB9508	CFGB9508	Firmicutes	Bacteria
OFGB9512	CFGB9512	Firmicutes	Bacteria
OFGB2838	CFGB2838	Firmicutes	Bacteria
OFGB2838	CFGB2838	Firmicutes	Bacteria
OFGB2838	CFGB2838	Firmicutes	Bacteria
Eubacteriales	Clostridia	Firmicutes	Bacteria
OFGB2833	CFGB2833	Firmicutes	Bacteria
Eubacteriales	Clostridia	Firmicutes	Bacteria
Eubacteriales	Clostridia	Firmicutes	Bacteria
Eubacteriales	Clostridia	Firmicutes	Bacteria
OFGB9622	CFGB9622	Firmicutes	Bacteria
OFGB77305	CFGB77305	Firmicutes	Bacteria
Eubacteriales	Clostridia	Firmicutes	Bacteria
Eubacteriales	Clostridia	Firmicutes	Bacteria
Eubacteriales	Clostridia	Firmicutes	Bacteria
Eubacteriales	Clostridia	Firmicutes	Bacteria
Eubacteriales	Clostridia	Firmicutes	Bacteria
OFGB9633	CFGB9633	Firmicutes	Bacteria
Bacteria_unclassified	Bacteria_unclassified	Bacteria_unclassified	Bacteria
Bacteria_unclassified	Bacteria_unclassified	Bacteria_unclassified	Bacteria
Bacteria_unclassified	Bacteria_unclassified	Bacteria_unclassified	Bacteria
Bacteria_unclassified	Bacteria_unclassified	Bacteria_unclassified	Bacteria
Bacteria_unclassified	Bacteria_unclassified	Bacteria_unclassified	Bacteria
Bacteria_unclassified	Bacteria_unclassified	Bacteria_unclassified	Bacteria
Eubacteriales	Clostridia	Firmicutes	Bacteria
OFGB77359	CFGB77359	Bacteria_unclassified	Bacteria
OFGB9639	CFGB9639	Firmicutes	Bacteria
Eubacteriales	Clostridia	Firmicutes	Bacteria
Eubacteriales	Clostridia	Firmicutes	Bacteria
Eubacteriales	Clostridia	Firmicutes	Bacteria
Eubacteriales	Clostridia	Firmicutes	Bacteria
Eubacteriales	Clostridia	Firmicutes	Bacteria

Eubacteriales	Clostridia	Firmicutes	Bacteria
Eubacteriales	Clostridia	Firmicutes	Bacteria
OFGB9656	CFGB9656	Firmicutes	Bacteria
OFGB9827	CFGB9827	Firmicutes	Bacteria
Eubacteriales	Clostridia	Firmicutes	Bacteria
OFGB77303	CFGB77303	Bacteria_unclassified	Bacteria
Eubacteriales	Clostridia	Firmicutes	Bacteria
Eubacteriales	Clostridia	Firmicutes	Bacteria
OFGB72709	CFGB72709	Firmicutes	Bacteria
Eubacteriales	Clostridia	Firmicutes	Bacteria
Eubacteriales	Clostridia	Firmicutes	Bacteria
Eubacteriales	Clostridia	Firmicutes	Bacteria
Eubacteriales	Clostridia	Firmicutes	Bacteria
Eubacteriales	Clostridia	Firmicutes	Bacteria
Eubacteriales	Clostridia	Firmicutes	Bacteria
Eubacteriales	Clostridia	Firmicutes	Bacteria
Eubacteriales	Clostridia	Firmicutes	Bacteria
Eubacteriales	Clostridia	Firmicutes	Bacteria
Eubacteriales	Clostridia	Firmicutes	Bacteria
Eubacteriales	Clostridia	Firmicutes	Bacteria
Eubacteriales	Clostridia	Firmicutes	Bacteria
Eubacteriales	Clostridia	Firmicutes	Bacteria
OFGB77153	CFGB77153	Actinobacteria	Bacteria
OFGB1791	CFGB1791	Tenericutes	Bacteria
OFGB10290	CFGB10290	Firmicutes	Bacteria
Eubacteriales	Clostridia	Firmicutes	Bacteria
OFGB10289	CFGB10289	Firmicutes	Bacteria
OFGB1765	CFGB1765	Firmicutes	Bacteria
OFGB1765	CFGB1765	Firmicutes	Bacteria
OFGB1765	CFGB1765	Firmicutes	Bacteria
OFGB10667	CFGB10667	Firmicutes	Bacteria
Eubacteriales	Clostridia	Firmicutes	Bacteria
Eubacteriales	Clostridia	Firmicutes	Bacteria
Eubacteriales	Clostridia	Firmicutes	Bacteria
OFGB75721	CFGB75721	Firmicutes	Bacteria
Eubacteriales	Clostridia	Firmicutes	Bacteria
Eubacteriales	Clostridia	Firmicutes	Bacteria
OFGB10299	CFGB10299	Firmicutes	Bacteria
Eubacteriales	Clostridia	Firmicutes	Bacteria
Eubacteriales	Clostridia	Firmicutes	Bacteria
Eubacteriales	Clostridia	Firmicutes	Bacteria
Eubacteriales	Clostridia	Firmicutes	Bacteria
Eubacteriales	Clostridia	Firmicutes	Bacteria
Eubacteriales	Clostridia	Firmicutes	Bacteria
Eubacteriales	Clostridia	Firmicutes	Bacteria
Eubacteriales	Clostridia	Firmicutes	Bacteria

Lactobacillales	Bacilli	Firmicutes	Bacteria
Coriobacteriales	Coriobacteriia	Actinobacteria	Bacteria
Bacteroidales	Bacteroidia	Bacteroidota	Bacteria
Eubacteriales	Clostridia	Firmicutes	Bacteria
Eubacteriales	Clostridia	Firmicutes	Bacteria
Eubacteriales	Clostridia	Firmicutes	Bacteria
Eubacteriales	Clostridia	Firmicutes	Bacteria
Eubacteriales	Clostridia	Firmicutes	Bacteria
Eubacteriales	Clostridia	Firmicutes	Bacteria
Burkholderiales	Betaproteobacteria	Proteobacteria	Bacteria
Eubacteriales	Clostridia	Firmicutes	Bacteria
Erysipelotrichales	Erysipelotrichia	Firmicutes	Bacteria
Bacteria_unclassified	Bacteria_unclassified	Bacteria_unclassified	Bacteria
Bacteria_unclassified	Bacteria_unclassified	Bacteria_unclassified	Bacteria
Bacteria_unclassified	Bacteria_unclassified	Bacteria_unclassified	Bacteria
Bacteria_unclassified	Bacteria_unclassified	Bacteria_unclassified	Bacteria
Bacteria_unclassified	Bacteria_unclassified	Bacteria_unclassified	Bacteria

Eubacteriales	Clostridia	Firmicutes	Bacteria
Eubacteriales	Clostridia	Firmicutes	Bacteria
Verrucomicrobiales	Verrucomicrobiae	Verrucomicrobia	Bacteria
Eubacteriales	Clostridia	Firmicutes	Bacteria
Bacteria_unclassified	Bacteria_unclassified	Bacteria_unclassified	Bacteria
Bacteria_unclassified	Bacteria_unclassified	Bacteria_unclassified	Bacteria
Bacteria_unclassified	Bacteria_unclassified	Bacteria_unclassified	Bacteria
Bacteria_unclassified	Bacteria_unclassified	Bacteria_unclassified	Bacteria
Bacteroidales	Bacteroidia	Bacteroidota	Bacteria
Clostridia_unclassified	Clostridia	Firmicutes	Bacteria
Eubacteriales	Clostridia	Firmicutes	Bacteria
Eubacteriales	Clostridia	Firmicutes	Bacteria
Eubacteriales	Clostridia	Firmicutes	Bacteria
Eubacteriales	Clostridia	Firmicutes	Bacteria
Erysipelotrichales	Erysipelotrichia	Firmicutes	Bacteria
Eubacteriales	Clostridia	Firmicutes	Bacteria
Eubacteriales	Clostridia	Firmicutes	Bacteria
OFGB77306	CFGB77306	Firmicutes	Bacteria
Eubacteriales	Clostridia	Firmicutes	Bacteria
Eggerthellales	Coriobacteriia	Actinobacteria	Bacteria
Eubacteriales	Clostridia	Firmicutes	Bacteria
OFGB9506	CFGB9506	Firmicutes	Bacteria
OFGB9508	CFGB9508	Firmicutes	Bacteria
OFGB9512	CFGB9512	Firmicutes	Bacteria
OFGB2838	CFGB2838	Firmicutes	Bacteria
OFGB2838	CFGB2838	Firmicutes	Bacteria

OFGB2838	CFGB2838	Firmicutes	Bacteria
Eubacteriales	Clostridia	Firmicutes	Bacteria
OFGB2833	CFGB2833	Firmicutes	Bacteria
Eubacteriales	Clostridia	Firmicutes	Bacteria
Eubacteriales	Clostridia	Firmicutes	Bacteria
Eubacteriales	Clostridia	Firmicutes	Bacteria
OFGB9622	CFGB9622	Firmicutes	Bacteria
OFGB77305	CFGB77305	Firmicutes	Bacteria
Eubacteriales	Clostridia	Firmicutes	Bacteria
Eubacteriales	Clostridia	Firmicutes	Bacteria
Eubacteriales	Clostridia	Firmicutes	Bacteria
Eubacteriales	Clostridia	Firmicutes	Bacteria
Eubacteriales	Clostridia	Firmicutes	Bacteria
OFGB9633	CFGB9633	Firmicutes	Bacteria
Bacteria_unclassified	Bacteria_unclassified	Bacteria_unclassified	Bacteria
Bacteria_unclassified	Bacteria_unclassified	Bacteria_unclassified	Bacteria
Bacteria_unclassified	Bacteria_unclassified	Bacteria_unclassified	Bacteria
Bacteria_unclassified	Bacteria_unclassified	Bacteria_unclassified	Bacteria
Bacteria_unclassified	Bacteria_unclassified	Bacteria_unclassified	Bacteria
Bacteria_unclassified	Bacteria_unclassified	Bacteria_unclassified	Bacteria
Eubacteriales	Clostridia	Firmicutes	Bacteria
OFGB77359	CFGB77359	Bacteria_unclassified	Bacteria
OFGB9639	CFGB9639	Firmicutes	Bacteria
Eubacteriales	Clostridia	Firmicutes	Bacteria
Eubacteriales	Clostridia	Firmicutes	Bacteria
Eubacteriales	Clostridia	Firmicutes	Bacteria
Eubacteriales	Clostridia	Firmicutes	Bacteria
Eubacteriales	Clostridia	Firmicutes	Bacteria
Eubacteriales	Clostridia	Firmicutes	Bacteria
Eubacteriales	Clostridia	Firmicutes	Bacteria
OFGB9656	CFGB9656	Firmicutes	Bacteria
OFGB9827	CFGB9827	Firmicutes	Bacteria
Eubacteriales	Clostridia	Firmicutes	Bacteria
OFGB77303	CFGB77303	Bacteria_unclassified	Bacteria
Eubacteriales	Clostridia	Firmicutes	Bacteria
Eubacteriales	Clostridia	Firmicutes	Bacteria
OFGB72709	CFGB72709	Firmicutes	Bacteria
Eubacteriales	Clostridia	Firmicutes	Bacteria
Eubacteriales	Clostridia	Firmicutes	Bacteria
Eubacteriales	Clostridia	Firmicutes	Bacteria
Eubacteriales	Clostridia	Firmicutes	Bacteria
Eubacteriales	Clostridia	Firmicutes	Bacteria
Eubacteriales	Clostridia	Firmicutes	Bacteria
Eubacteriales	Clostridia	Firmicutes	Bacteria
Eubacteriales	Clostridia	Firmicutes	Bacteria
Eubacteriales	Clostridia	Firmicutes	Bacteria

Eubacteriales	Clostridia	Firmicutes	Bacteria
Eubacteriales	Clostridia	Firmicutes	Bacteria
Eubacteriales	Clostridia	Firmicutes	Bacteria
Eubacteriales	Clostridia	Firmicutes	Bacteria
OFGB77153	CFGB77153	Actinobacteria	Bacteria
OFGB1791	CFGB1791	Tenericutes	Bacteria
OFGB10290	CFGB10290	Firmicutes	Bacteria
Eubacteriales	Clostridia	Firmicutes	Bacteria
OFGB10289	CFGB10289	Firmicutes	Bacteria
OFGB1765	CFGB1765	Firmicutes	Bacteria
OFGB1765	CFGB1765	Firmicutes	Bacteria
OFGB1765	CFGB1765	Firmicutes	Bacteria
OFGB10667	CFGB10667	Firmicutes	Bacteria
Eubacteriales	Clostridia	Firmicutes	Bacteria
Eubacteriales	Clostridia	Firmicutes	Bacteria
Eubacteriales	Clostridia	Firmicutes	Bacteria
OFGB75721	CFGB75721	Firmicutes	Bacteria
Eubacteriales	Clostridia	Firmicutes	Bacteria
Eubacteriales	Clostridia	Firmicutes	Bacteria
OFGB10299	CFGB10299	Firmicutes	Bacteria
Eubacteriales	Clostridia	Firmicutes	Bacteria
Eubacteriales	Clostridia	Firmicutes	Bacteria
Eubacteriales	Clostridia	Firmicutes	Bacteria
Eubacteriales	Clostridia	Firmicutes	Bacteria
Eubacteriales	Clostridia	Firmicutes	Bacteria
Eubacteriales	Clostridia	Firmicutes	Bacteria
Eubacteriales	Clostridia	Firmicutes	Bacteria
Eubacteriales	Clostridia	Firmicutes	Bacteria
Lactobacillales	Bacilli	Firmicutes	Bacteria
Coriobacteriales	Coriobacteriia	Actinobacteria	Bacteria
Bacteroidales	Bacteroidia	Bacteroidota	Bacteria
Eubacteriales	Clostridia	Firmicutes	Bacteria
Eubacteriales	Clostridia	Firmicutes	Bacteria
Eubacteriales	Clostridia	Firmicutes	Bacteria
Eubacteriales	Clostridia	Firmicutes	Bacteria
Eubacteriales	Clostridia	Firmicutes	Bacteria
Eubacteriales	Clostridia	Firmicutes	Bacteria
Burkholderiales	Betaproteobacteria	Proteobacteria	Bacteria
Eubacteriales	Clostridia	Firmicutes	Bacteria
Erysipelotrichales	Erysipelotrichia	Firmicutes	Bacteria
Bacteria_unclassified	Bacteria_unclassified	Bacteria_unclassified	Bacteria
Bacteria_unclassified	Bacteria_unclassified	Bacteria_unclassified	Bacteria
Bacteria_unclassified	Bacteria_unclassified	Bacteria_unclassified	Bacteria
Bacteria_unclassified	Bacteria_unclassified	Bacteria_unclassified	Bacteria
Bacteria_unclassified	Bacteria_unclassified	Bacteria_unclassified	Bacteria

MetaPhlan Annotation

k__Bacteria|p__Firmicutes|c__Clostridia|o__Eubacteriales|f__Lachnospiraceae|g__Acetatifactor|s__Acetatifactor_SGB415[illegible]
k__Bacteria|p__Firmicutes|c__Clostridia|o__Eubacteriales|f__Oscillospiraceae|g__Acutalibacter|s__Acutalibacter_muris
k__Bacteria|p__Verrucomicrobia|c__Verrucomicrobiae|o__Verrucomicrobiales|f__Akkermansiaceae|g__Akkermansia|s__[illegible]
k__Bacteria|p__Firmicutes|c__Clostridia|o__Eubacteriales|f__Oscillospiraceae|g__Anaerotruncus|s__Anaerotruncus_sp_1[illegible]
k__Bacteria|p__Bacteria_unclassified|c__Bacteria_unclassified|o__Bacteria_unclassified|f__Bacteria_unclassified|g__Bact[illegible]
k__Bacteria|p__Bacteria_unclassified|c__Bacteria_unclassified|o__Bacteria_unclassified|f__Bacteria_unclassified|g__Bact[illegible]
k__Bacteria|p__Bacteria_unclassified|c__Bacteria_unclassified|o__Bacteria_unclassified|f__Bacteria_unclassified|g__Bact[illegible]
k__Bacteria|p__Bacteria_unclassified|c__Bacteria_unclassified|o__Bacteria_unclassified|f__Bacteria_unclassified|g__Bact[illegible]
k__Bacteria|p__Bacteroidota|c__Bacteroidia|o__Bacteroidales|f__Bacteroidaceae|g__Bacteroides|s__Bacteroides_thetaio[illegible]
k__Bacteria|p__Firmicutes|c__Clostridia|o__Clostridia_unclassified|f__Clostridia_unclassified|g__Clostridia_unclassified|s_[illegible]
k__Bacteria|p__Firmicutes|c__Clostridia|o__Eubacteriales|f__Clostridiaceae|g__Clostridiaceae_unclassified|s__Clostridiac[illegible]
k__Bacteria|p__Firmicutes|c__Clostridia|o__Eubacteriales|f__Clostridiaceae|g__Clostridiaceae_unclassified|s__Clostridiac[illegible]
k__Bacteria|p__Firmicutes|c__Clostridia|o__Eubacteriales|f__Eubacteriales_unclassified|g__Eubacteriales_unclassified|s_[illegible]
k__Bacteria|p__Firmicutes|c__Clostridia|o__Eubacteriales|f__Clostridiaceae|g__Clostridium|s__Clostridium_SGB65123
k__Bacteria|p__Firmicutes|c__Erysipelotrichia|o__Erysipelotrichales|f__Erysipelotrichaceae|g__Erysipelatoclostridium|s_[illegible]
k__Bacteria|p__Firmicutes|c__Clostridia|o__Eubacteriales|f__Eubacteriaceae|g__Eubacteriaceae_unclassified|s__Eubacte[illegible]
k__Bacteria|p__Firmicutes|c__Clostridia|o__Eubacteriales|f__Eubacteriaceae|g__Eubacteriaceae_unclassified|s__Eubacte[illegible]
k__Bacteria|p__Firmicutes|c__CFGB77306|o__OFGB77306|f__FGB77306|g__GGB20146|s__GGB20146_SGB29427
k__Bacteria|p__Firmicutes|c__Clostridia|o__Eubacteriales|f__Lachnospiraceae|g__GGB20149|s__GGB20149_SGB29430
k__Bacteria|p__Actinobacteria|c__Coriobacteriia|o__Eggerthellales|f__Eggerthellaceae|g__GGB22635|s__GGB22635_SGB[illegible]
k__Bacteria|p__Firmicutes|c__Clostridia|o__Eubacteriales|f__Lachnospiraceae|g__GGB25041|s__GGB25041_SGB36960
k__Bacteria|p__Firmicutes|c__CFGB9506|o__OFGB9506|f__FGB9506|g__GGB28379|s__GGB28379_SGB40959
k__Bacteria|p__Firmicutes|c__CFGB9508|o__OFGB9508|f__FGB9508|g__GGB28382|s__GGB28382_SGB40962
k__Bacteria|p__Firmicutes|c__CFGB9512|o__OFGB9512|f__FGB9512|g__GGB28392|s__GGB28392_SGB40972
k__Bacteria|p__Firmicutes|c__CFGB2838|o__OFGB2838|f__FGB2838|g__GGB28404|s__GGB28404_SGB40986
k__Bacteria|p__Firmicutes|c__CFGB2838|o__OFGB2838|f__FGB2838|g__GGB28418|s__GGB28418_SGB41001
k__Bacteria|p__Firmicutes|c__CFGB2838|o__OFGB2838|f__FGB2838|g__GGB28422|s__GGB28422_SGB41005
k__Bacteria|p__Firmicutes|c__Clostridia|o__Eubacteriales|f__Pumilibacteraceae|g__GGB28431|s__GGB28431_SGB41014
k__Bacteria|p__Firmicutes|c__CFGB2833|o__OFGB2833|f__FGB2833|g__GGB28456|s__GGB28456_SGB41039
k__Bacteria|p__Firmicutes|c__Clostridia|o__Eubacteriales|f__Eubacteriaceae|g__GGB28782|s__GGB28782_SGB41435
k__Bacteria|p__Firmicutes|c__Clostridia|o__Eubacteriales|f__Lachnospiraceae|g__GGB28792|s__GGB28792_SGB41445
k__Bacteria|p__Firmicutes|c__Clostridia|o__Eubacteriales|f__Lachnospiraceae|g__GGB28798|s__GGB28798_SGB41451
k__Bacteria|p__Firmicutes|c__CFGB9622|o__OFGB9622|f__FGB9622|g__GGB28810|s__GGB28810_SGB41465
k__Bacteria|p__Firmicutes|c__CFGB77305|o__OFGB77305|f__FGB77305|g__GGB28828|s__GGB28828_SGB41484
k__Bacteria|p__Firmicutes|c__Clostridia|o__Eubacteriales|f__Clostridiaceae|g__GGB28851|s__GGB28851_SGB41518
k__Bacteria|p__Firmicutes|c__Clostridia|o__Eubacteriales|f__Lachnospiraceae|g__GGB28865|s__GGB28865_SGB41537
k__Bacteria|p__Firmicutes|c__Clostridia|o__Eubacteriales|f__Lachnospiraceae|g__GGB28868|s__GGB28868_SGB41542
k__Bacteria|p__Firmicutes|c__Clostridia|o__Eubacteriales|f__Lachnospiraceae|g__GGB28869|s__GGB28869_SGB41543
k__Bacteria|p__Firmicutes|c__Clostridia|o__Eubacteriales|f__Lachnospiraceae|g__GGB28875|s__GGB28875_SGB41555
k__Bacteria|p__Firmicutes|c__CFGB9633|o__OFGB9633|f__FGB9633|g__GGB28881|s__GGB28881_SGB41561
k__Bacteria|p__Bacteria_unclassified|c__Bacteria_unclassified|o__Bacteria_unclassified|f__Bacteria_unclassified|g__GGB[illegible]
k__Bacteria|p__Bacteria_unclassified|c__Bacteria_unclassified|o__Bacteria_unclassified|f__Bacteria_unclassified|g__GGB[illegible]
k__Bacteria|p__Bacteria_unclassified|c__Bacteria_unclassified|o__Bacteria_unclassified|f__Bacteria_unclassified|g__GGB[illegible]
k__Bacteria|p__Bacteria_unclassified|c__Bacteria_unclassified|o__Bacteria_unclassified|f__Bacteria_unclassified|g__GGB[illegible]
k__Bacteria|p__Bacteria_unclassified|c__Bacteria_unclassified|o__Bacteria_unclassified|f__Bacteria_unclassified|g__GGB[illegible]
k__Bacteria|p__Bacteria_unclassified|c__Bacteria_unclassified|o__Bacteria_unclassified|f__Bacteria_unclassified|g__GGB[illegible]

k__Bacteria|p__Firmicutes|c__Clostridia|o__Eubacteriales|f__Lachnospiraceae|g__GGB28924|s__GGB28924_SGB41621
k__Bacteria|p__Bacteria_unclassified|c__CFGB77359|o__OFGB77359|f__FGB77359|g__GGB28927|s__GGB28927_SGB416[illegible]
k__Bacteria|p__Firmicutes|c__CFGB9639|o__OFGB9639|f__FGB9639|g__GGB28934|s__GGB28934_SGB41635
k__Bacteria|p__Firmicutes|c__Clostridia|o__Eubacteriales|f__Lachnospiraceae|g__GGB28949|s__GGB28949_SGB41655
k__Bacteria|p__Firmicutes|c__Clostridia|o__Eubacteriales|f__Clostridiaceae|g__GGB28951|s__GGB28951_SGB102295
k__Bacteria|p__Firmicutes|c__Clostridia|o__Eubacteriales|f__Clostridiaceae|g__GGB28951|s__GGB28951_SGB41658
k__Bacteria|p__Firmicutes|c__Clostridia|o__Eubacteriales|f__Clostridiaceae|g__GGB28954|s__GGB28954_SGB41662
k__Bacteria|p__Firmicutes|c__Clostridia|o__Eubacteriales|f__Clostridiaceae|g__GGB28960|s__GGB28960_SGB41669
k__Bacteria|p__Firmicutes|c__Clostridia|o__Eubacteriales|f__Clostridiaceae|g__GGB28964|s__GGB28964_SGB94886
k__Bacteria|p__Firmicutes|c__Clostridia|o__Eubacteriales|f__Clostridiaceae|g__GGB28967|s__GGB28967_SGB41678
k__Bacteria|p__Firmicutes|c__CFGB9656|o__OFGB9656|f__FGB9656|g__GGB28996|s__GGB28996_SGB41712
k__Bacteria|p__Firmicutes|c__CFGB9827|o__OFGB9827|f__FGB9827|g__GGB29531|s__GGB29531_SGB42317
k__Bacteria|p__Firmicutes|c__Clostridia|o__Eubacteriales|f__Eubacteriaceae|g__GGB29685|s__GGB29685_SGB42494
k__Bacteria|p__Bacteria_unclassified|c__CFGB77303|o__OFGB77303|f__FGB77303|g__GGB30141|s__GGB30141_SGB430[illegible]
k__Bacteria|p__Firmicutes|c__Clostridia|o__Eubacteriales|f__Clostridiaceae|g__GGB30145|s__GGB30145_SGB43072
k__Bacteria|p__Firmicutes|c__Clostridia|o__Eubacteriales|f__Eubacteriales_unclassified|g__GGB30286|s__GGB30286_SG[illegible]
k__Bacteria|p__Firmicutes|c__CFGB72709|o__OFGB72709|f__FGB72709|g__GGB30300|s__GGB30300_SGB43264
k__Bacteria|p__Firmicutes|c__Clostridia|o__Eubacteriales|f__Oscillospiraceae|g__GGB30303|s__GGB30303_SGB43268
k__Bacteria|p__Firmicutes|c__Clostridia|o__Eubacteriales|f__Oscillospiraceae|g__GGB30450|s__GGB30450_SGB43507
k__Bacteria|p__Firmicutes|c__Clostridia|o__Eubacteriales|f__Oscillospiraceae|g__GGB30453|s__GGB30453_SGB43513
k__Bacteria|p__Firmicutes|c__Clostridia|o__Eubacteriales|f__Oscillospiraceae|g__GGB30454|s__GGB30454_SGB43514
k__Bacteria|p__Firmicutes|c__Clostridia|o__Eubacteriales|f__Oscillospiraceae|g__GGB30455|s__GGB30455_SGB43519
k__Bacteria|p__Firmicutes|c__Clostridia|o__Eubacteriales|f__Oscillospiraceae|g__GGB30456|s__GGB30456_SGB43520
k__Bacteria|p__Firmicutes|c__Clostridia|o__Eubacteriales|f__Oscillospiraceae|g__GGB30457|s__GGB30457_SGB63218
k__Bacteria|p__Firmicutes|c__Clostridia|o__Eubacteriales|f__Oscillospiraceae|g__GGB30461|s__GGB30461_SGB43530
k__Bacteria|p__Firmicutes|c__Clostridia|o__Eubacteriales|f__Oscillospiraceae|g__GGB30461|s__GGB30461_SGB43533
k__Bacteria|p__Firmicutes|c__Clostridia|o__Eubacteriales|f__Oscillospiraceae|g__GGB30461|s__GGB30461_SGB63209
k__Bacteria|p__Firmicutes|c__Clostridia|o__Eubacteriales|f__Oscillospiraceae|g__GGB30463|s__GGB30463_SGB43537
k__Bacteria|p__Firmicutes|c__Clostridia|o__Eubacteriales|f__Oscillospiraceae|g__GGB30473|s__GGB30473_SGB43557
k__Bacteria|p__Firmicutes|c__Clostridia|o__Eubacteriales|f__Oscillospiraceae|g__GGB30475|s__GGB30475_SGB63182
k__Bacteria|p__Actinobacteria|c__CFGB77153|o__OFGB77153|f__FGB77153|g__GGB30861|s__GGB30861_SGB44083
k__Bacteria|p__Tenericutes|c__CFGB1791|o__OFGB1791|f__FGB1791|g__GGB31312|s__GGB31312_SGB44628
k__Bacteria|p__Firmicutes|c__CFGB10290|o__OFGB10290|f__FGB10290|g__GGB31438|s__GGB31438_SGB44768
k__Bacteria|p__Firmicutes|c__Clostridia|o__Eubacteriales|f__Oscillospiraceae|g__GGB3171|s__GGB3171_SGB4185
k__Bacteria|p__Firmicutes|c__CFGB10289|o__OFGB10289|f__FGB10289|g__GGB31762|s__GGB31762_SGB45125
k__Bacteria|p__Firmicutes|c__CFGB1765|o__OFGB1765|f__FGB1765|g__GGB31823|s__GGB31823_SGB45199
k__Bacteria|p__Firmicutes|c__CFGB1765|o__OFGB1765|f__FGB1765|g__GGB31838|s__GGB31838_SGB45216
k__Bacteria|p__Firmicutes|c__CFGB1765|o__OFGB1765|f__FGB1765|g__GGB31841|s__GGB31841_SGB65084
k__Bacteria|p__Firmicutes|c__CFGB10667|o__OFGB10667|f__FGB10667|g__GGB32371|s__GGB32371_SGB41694
k__Bacteria|p__Firmicutes|c__Clostridia|o__Eubacteriales|f__Lachnospiraceae|g__GGB3793|s__GGB3793_SGB5158
k__Bacteria|p__Firmicutes|c__Clostridia|o__Eubacteriales|f__Clostridiaceae|g__GGB42601|s__GGB42601_SGB59797
k__Bacteria|p__Firmicutes|c__Clostridia|o__Eubacteriales|f__Oscillospiraceae|g__GGB45514|s__GGB45514_SGB63186
k__Bacteria|p__Firmicutes|c__CFGB75721|o__OFGB75721|f__FGB75721|g__GGB45564|s__GGB45564_SGB63259
k__Bacteria|p__Firmicutes|c__Clostridia|o__Eubacteriales|f__Oscillospiraceae|g__GGB45624|s__GGB45624_SGB63337
k__Bacteria|p__Firmicutes|c__Clostridia|o__Eubacteriales|f__Christensenellaceae|g__GGB45656|s__GGB45656_SGB6337[illegible]
k__Bacteria|p__Firmicutes|c__CFGB10299|o__OFGB10299|f__FGB10299|g__GGB47127|s__GGB47127_SGB65054

k__Bacteria|p__Firmicutes|c__Clostridia|o__Eubacteriales|f__Oscillospiraceae|g__GGB74395|s__GGB74395_SGB43523
k__Bacteria|p__Firmicutes|c__Clostridia|o__Eubacteriales|f__Oscillospiraceae|g__GGB75053|s__GGB75053_SGB43494
k__Bacteria|p__Firmicutes|c__Clostridia|o__Eubacteriales|f__Lachnospiraceae|g__GGB75109|s__GGB75109_SGB102238
k__Bacteria|p__Firmicutes|c__Clostridia|o__Eubacteriales|f__Lachnospiraceae|g__Lachnospiraceae_unclassified|s__Lachn
k__Bacteria|p__Firmicutes|c__Clostridia|o__Eubacteriales|f__Lachnospiraceae|g__Lachnospiraceae_unclassified|s__Lachn
k__Bacteria|p__Firmicutes|c__Clostridia|o__Eubacteriales|f__Lachnospiraceae|g__Lachnospiraceae_unclassified|s__Lachn
k__Bacteria|p__Firmicutes|c__Clostridia|o__Eubacteriales|f__Lachnospiraceae|g__Lachnospiraceae_unclassified|s__Lachn
k__Bacteria|p__Firmicutes|c__Clostridia|o__Eubacteriales|f__Lachnospiraceae|g__Lachnospiraceae_unclassified|s__Lachn
k__Bacteria|p__Firmicutes|c__Bacilli|o__Lactobacillales|f__Lactobacillaceae|g__Lactobacillus|s__Lactobacillus_johnsonii
k__Bacteria|p__Actinobacteria|c__Coriobacteriia|o__Coriobacteriales|f__Atopobiaceae|g__Leptogranulimonas|s__Leptog
k__Bacteria|p__Bacteroidota|c__Bacteroidia|o__Bacteroidales|f__Muribaculaceae|g__Muribaculaceae_unclassified|s__M
k__Bacteria|p__Firmicutes|c__Clostridia|o__Eubacteriales|f__Oscillospiraceae|g__Neglectibacter|s__Neglectibacter_sp_X
k__Bacteria|p__Firmicutes|c__Clostridia|o__Eubacteriales|f__Oscillospiraceae|g__Oscillibacter|s__Oscillibacter_SGB4349
k__Bacteria|p__Firmicutes|c__Clostridia|o__Eubacteriales|f__Oscillospiraceae|g__Oscillospiraceae_unclassified|s__Oscillo
k__Bacteria|p__Firmicutes|c__Clostridia|o__Eubacteriales|f__Oscillospiraceae|g__Oscillospiraceae_unclassified|s__Oscillo
k__Bacteria|p__Firmicutes|c__Clostridia|o__Eubacteriales|f__Oscillospiraceae|g__Oscillospiraceae_unclassified|s__Oscillo
k__Bacteria|p__Firmicutes|c__Clostridia|o__Eubacteriales|f__Oscillospiraceae|g__Oscillospiraceae_unclassified|s__Oscillo
k__Bacteria|p__Proteobacteria|c__Betaproteobacteria|o__Burkholderiales|f__Sutterellaceae|g__Parasutterella|s__Parasu
k__Bacteria|p__Firmicutes|c__Clostridia|o__Eubacteriales|f__Lachnospiraceae|g__Schaedlerella|s__Schaedlerella_arabinc
k__Bacteria|p__Firmicutes|c__Erysipelotrichia|o__Erysipelotrichales|f__Turicibacteraceae|g__Turicibacter|s__Turicibacter_
k__Bacteria|p__Bacteria_unclassified|c__Bacteria_unclassified|o__Bacteria_unclassified|f__Bacteria_unclassified|g__Bacte
k__Bacteria|p__Bacteria_unclassified|c__Bacteria_unclassified|o__Bacteria_unclassified|f__Bacteria_unclassified|g__Bacte
k__Bacteria|p__Bacteria_unclassified|c__Bacteria_unclassified|o__Bacteria_unclassified|f__Bacteria_unclassified|g__Bacte
k__Bacteria|p__Bacteria_unclassified|c__Bacteria_unclassified|o__Bacteria_unclassified|f__Bacteria_unclassified|g__Bacte
k__Bacteria|p__Bacteria_unclassified|c__Bacteria_unclassified|o__Bacteria_unclassified|f__Bacteria_unclassified|g__Bacte

k__Bacteria|p__Firmicutes|c__Clostridia|o__Eubacteriales|f__Lachnospiraceae|g__Acetatifactor|s__Acetatifactor_SGB415
k__Bacteria|p__Firmicutes|c__Clostridia|o__Eubacteriales|f__Oscillospiraceae|g__Acutalibacter|s__Acutalibacter_muris
k__Bacteria|p__Verrucomicrobia|c__Verrucomicrobiae|o__Verrucomicrobiales|f__Akkermansiaceae|g__Akkermansia|s__A
k__Bacteria|p__Firmicutes|c__Clostridia|o__Eubacteriales|f__Oscillospiraceae|g__Anaerotruncus|s__Anaerotruncus_sp_1
k__Bacteria|p__Bacteria_unclassified|c__Bacteria_unclassified|o__Bacteria_unclassified|f__Bacteria_unclassified|g__Bacte
k__Bacteria|p__Bacteria_unclassified|c__Bacteria_unclassified|o__Bacteria_unclassified|f__Bacteria_unclassified|g__Bacte
k__Bacteria|p__Bacteria_unclassified|c__Bacteria_unclassified|o__Bacteria_unclassified|f__Bacteria_unclassified|g__Bacte
k__Bacteria|p__Bacteria_unclassified|c__Bacteria_unclassified|o__Bacteria_unclassified|f__Bacteria_unclassified|g__Bacte
k__Bacteria|p__Bacteroidota|c__Bacteroidia|o__Bacteroidales|f__Bacteroidaceae|g__Bacteroides|s__Bacteroides_thetaio
k__Bacteria|p__Firmicutes|c__Clostridia|o__Clostridia_unclassified|f__Clostridia_unclassified|g__Clostridia_unclassified|s_
k__Bacteria|p__Firmicutes|c__Clostridia|o__Eubacteriales|f__Clostridiaceae|g__Clostridiaceae_unclassified|s__Clostridiac
k__Bacteria|p__Firmicutes|c__Clostridia|o__Eubacteriales|f__Clostridiaceae|g__Clostridiaceae_unclassified|s__Clostridiac
k__Bacteria|p__Firmicutes|c__Clostridia|o__Eubacteriales|f__Eubacteriales_unclassified|g__Eubacteriales_unclassified|s_
k__Bacteria|p__Firmicutes|c__Clostridia|o__Eubacteriales|f__Clostridiaceae|g__Clostridium|s__Clostridium_SGB65123
k__Bacteria|p__Firmicutes|c__Erysipelotrichia|o__Erysipelotrichales|f__Erysipelotrichaceae|g__Erysipelatoclostridium|s__
k__Bacteria|p__Firmicutes|c__Clostridia|o__Eubacteriales|f__Eubacteriaceae|g__Eubacteriaceae_unclassified|s__Eubacte
k__Bacteria|p__Firmicutes|c__Clostridia|o__Eubacteriales|f__Eubacteriaceae|g__Eubacteriaceae_unclassified|s__Eubacte
k__Bacteria|p__Firmicutes|c__CFGB77306|o__OFGB77306|f__FGB77306|g__GGB20146|s__GGB20146_SGB29427

k__Bacteria|p__Firmicutes|c__Clostridia|o__Eubacteriales|f__Lachnospiraceae|g__GGB20149|s__GGB20149_SGB29430
k__Bacteria|p__Actinobacteria|c__Coriobacteriia|o__Eggerthellales|f__Eggerthellaceae|g__GGB22635|s__GGB22635_SGB
k__Bacteria|p__Firmicutes|c__Clostridia|o__Eubacteriales|f__Lachnospiraceae|g__GGB25041|s__GGB25041_SGB36960
k__Bacteria|p__Firmicutes|c__CFGB9506|o__OFGB9506|f__FGB9506|g__GGB28379|s__GGB28379_SGB40959
k__Bacteria|p__Firmicutes|c__CFGB9508|o__OFGB9508|f__FGB9508|g__GGB28382|s__GGB28382_SGB40962
k__Bacteria|p__Firmicutes|c__CFGB9512|o__OFGB9512|f__FGB9512|g__GGB28392|s__GGB28392_SGB40972
k__Bacteria|p__Firmicutes|c__CFGB2838|o__OFGB2838|f__FGB2838|g__GGB28404|s__GGB28404_SGB40986
k__Bacteria|p__Firmicutes|c__CFGB2838|o__OFGB2838|f__FGB2838|g__GGB28418|s__GGB28418_SGB41001
k__Bacteria|p__Firmicutes|c__CFGB2838|o__OFGB2838|f__FGB2838|g__GGB28422|s__GGB28422_SGB41005
k__Bacteria|p__Firmicutes|c__Clostridia|o__Eubacteriales|f__Pumilibacteraceae|g__GGB28431|s__GGB28431_SGB41014
k__Bacteria|p__Firmicutes|c__CFGB2833|o__OFGB2833|f__FGB2833|g__GGB28456|s__GGB28456_SGB41039
k__Bacteria|p__Firmicutes|c__Clostridia|o__Eubacteriales|f__Eubacteriaceae|g__GGB28782|s__GGB28782_SGB41435
k__Bacteria|p__Firmicutes|c__Clostridia|o__Eubacteriales|f__Lachnospiraceae|g__GGB28792|s__GGB28792_SGB41445
k__Bacteria|p__Firmicutes|c__Clostridia|o__Eubacteriales|f__Lachnospiraceae|g__GGB28798|s__GGB28798_SGB41451
k__Bacteria|p__Firmicutes|c__CFGB9622|o__OFGB9622|f__FGB9622|g__GGB28810|s__GGB28810_SGB41465
k__Bacteria|p__Firmicutes|c__CFGB77305|o__OFGB77305|f__FGB77305|g__GGB28828|s__GGB28828_SGB41484
k__Bacteria|p__Firmicutes|c__Clostridia|o__Eubacteriales|f__Clostridiaceae|g__GGB28851|s__GGB28851_SGB41518
k__Bacteria|p__Firmicutes|c__Clostridia|o__Eubacteriales|f__Lachnospiraceae|g__GGB28865|s__GGB28865_SGB41537
k__Bacteria|p__Firmicutes|c__Clostridia|o__Eubacteriales|f__Lachnospiraceae|g__GGB28868|s__GGB28868_SGB41542
k__Bacteria|p__Firmicutes|c__Clostridia|o__Eubacteriales|f__Lachnospiraceae|g__GGB28869|s__GGB28869_SGB41543
k__Bacteria|p__Firmicutes|c__Clostridia|o__Eubacteriales|f__Lachnospiraceae|g__GGB28875|s__GGB28875_SGB41555
k__Bacteria|p__Firmicutes|c__CFGB9633|o__OFGB9633|f__FGB9633|g__GGB28881|s__GGB28881_SGB41561
k__Bacteria|p__Bacteria_unclassified|c__Bacteria_unclassified|o__Bacteria_unclassified|f__Bacteria_unclassified|g__GGB
k__Bacteria|p__Bacteria_unclassified|c__Bacteria_unclassified|o__Bacteria_unclassified|f__Bacteria_unclassified|g__GGB
k__Bacteria|p__Bacteria_unclassified|c__Bacteria_unclassified|o__Bacteria_unclassified|f__Bacteria_unclassified|g__GGB
k__Bacteria|p__Bacteria_unclassified|c__Bacteria_unclassified|o__Bacteria_unclassified|f__Bacteria_unclassified|g__GGB
k__Bacteria|p__Bacteria_unclassified|c__Bacteria_unclassified|o__Bacteria_unclassified|f__Bacteria_unclassified|g__GGB
k__Bacteria|p__Bacteria_unclassified|c__Bacteria_unclassified|o__Bacteria_unclassified|f__Bacteria_unclassified|g__GGB
k__Bacteria|p__Firmicutes|c__Clostridia|o__Eubacteriales|f__Lachnospiraceae|g__GGB28924|s__GGB28924_SGB41621
k__Bacteria|p__Bacteria_unclassified|c__CFGB77359|o__OFGB77359|f__FGB77359|g__GGB28927|s__GGB28927_SGB416
k__Bacteria|p__Firmicutes|c__CFGB9639|o__OFGB9639|f__FGB9639|g__GGB28934|s__GGB28934_SGB41635
k__Bacteria|p__Firmicutes|c__Clostridia|o__Eubacteriales|f__Lachnospiraceae|g__GGB28949|s__GGB28949_SGB41655
k__Bacteria|p__Firmicutes|c__Clostridia|o__Eubacteriales|f__Clostridiaceae|g__GGB28951|s__GGB28951_SGB102295
k__Bacteria|p__Firmicutes|c__Clostridia|o__Eubacteriales|f__Clostridiaceae|g__GGB28951|s__GGB28951_SGB41658
k__Bacteria|p__Firmicutes|c__Clostridia|o__Eubacteriales|f__Clostridiaceae|g__GGB28954|s__GGB28954_SGB41662
k__Bacteria|p__Firmicutes|c__Clostridia|o__Eubacteriales|f__Clostridiaceae|g__GGB28960|s__GGB28960_SGB41669
k__Bacteria|p__Firmicutes|c__Clostridia|o__Eubacteriales|f__Clostridiaceae|g__GGB28964|s__GGB28964_SGB94886
k__Bacteria|p__Firmicutes|c__Clostridia|o__Eubacteriales|f__Clostridiaceae|g__GGB28967|s__GGB28967_SGB41678
k__Bacteria|p__Firmicutes|c__CFGB9656|o__OFGB9656|f__FGB9656|g__GGB28996|s__GGB28996_SGB41712
k__Bacteria|p__Firmicutes|c__CFGB9827|o__OFGB9827|f__FGB9827|g__GGB29531|s__GGB29531_SGB42317
k__Bacteria|p__Firmicutes|c__Clostridia|o__Eubacteriales|f__Eubacteriaceae|g__GGB29685|s__GGB29685_SGB42494
k__Bacteria|p__Bacteria_unclassified|c__CFGB77303|o__OFGB77303|f__FGB77303|g__GGB30141|s__GGB30141_SGB430
k__Bacteria|p__Firmicutes|c__Clostridia|o__Eubacteriales|f__Clostridiaceae|g__GGB30145|s__GGB30145_SGB43072
k__Bacteria|p__Firmicutes|c__Clostridia|o__Eubacteriales|f__Eubacteriales_unclassified|g__GGB30286|s__GGB30286_SG
k__Bacteria|p__Firmicutes|c__CFGB72709|o__OFGB72709|f__FGB72709|g__GGB30300|s__GGB30300_SGB43264
k__Bacteria|p__Firmicutes|c__Clostridia|o__Eubacteriales|f__Oscillospiraceae|g__GGB30303|s__GGB30303_SGB43268

k__Bacteria|p__Firmicutes|c__Clostridia|o__Eubacteriales|f__Oscillospiraceae|g__GGB30450|s__GGB30450_SGB43507
k__Bacteria|p__Firmicutes|c__Clostridia|o__Eubacteriales|f__Oscillospiraceae|g__GGB30453|s__GGB30453_SGB43513
k__Bacteria|p__Firmicutes|c__Clostridia|o__Eubacteriales|f__Oscillospiraceae|g__GGB30454|s__GGB30454_SGB43514
k__Bacteria|p__Firmicutes|c__Clostridia|o__Eubacteriales|f__Oscillospiraceae|g__GGB30455|s__GGB30455_SGB43519
k__Bacteria|p__Firmicutes|c__Clostridia|o__Eubacteriales|f__Oscillospiraceae|g__GGB30456|s__GGB30456_SGB43520
k__Bacteria|p__Firmicutes|c__Clostridia|o__Eubacteriales|f__Oscillospiraceae|g__GGB30457|s__GGB30457_SGB63218
k__Bacteria|p__Firmicutes|c__Clostridia|o__Eubacteriales|f__Oscillospiraceae|g__GGB30461|s__GGB30461_SGB43530
k__Bacteria|p__Firmicutes|c__Clostridia|o__Eubacteriales|f__Oscillospiraceae|g__GGB30461|s__GGB30461_SGB43533
k__Bacteria|p__Firmicutes|c__Clostridia|o__Eubacteriales|f__Oscillospiraceae|g__GGB30461|s__GGB30461_SGB63209
k__Bacteria|p__Firmicutes|c__Clostridia|o__Eubacteriales|f__Oscillospiraceae|g__GGB30463|s__GGB30463_SGB43537
k__Bacteria|p__Firmicutes|c__Clostridia|o__Eubacteriales|f__Oscillospiraceae|g__GGB30473|s__GGB30473_SGB43557
k__Bacteria|p__Firmicutes|c__Clostridia|o__Eubacteriales|f__Oscillospiraceae|g__GGB30475|s__GGB30475_SGB63182
k__Bacteria|p__Actinobacteria|c__CFGB77153|o__OFGB77153|f__FGB77153|g__GGB30861|s__GGB30861_SGB44083
k__Bacteria|p__Tenericutes|c__CFGB1791|o__OFGB1791|f__FGB1791|g__GGB31312|s__GGB31312_SGB44628
k__Bacteria|p__Firmicutes|c__CFGB10290|o__OFGB10290|f__FGB10290|g__GGB31438|s__GGB31438_SGB44768
k__Bacteria|p__Firmicutes|c__Clostridia|o__Eubacteriales|f__Oscillospiraceae|g__GGB3171|s__GGB3171_SGB4185
k__Bacteria|p__Firmicutes|c__CFGB10289|o__OFGB10289|f__FGB10289|g__GGB31762|s__GGB31762_SGB45125
k__Bacteria|p__Firmicutes|c__CFGB1765|o__OFGB1765|f__FGB1765|g__GGB31823|s__GGB31823_SGB45199
k__Bacteria|p__Firmicutes|c__CFGB1765|o__OFGB1765|f__FGB1765|g__GGB31838|s__GGB31838_SGB45216
k__Bacteria|p__Firmicutes|c__CFGB1765|o__OFGB1765|f__FGB1765|g__GGB31841|s__GGB31841_SGB65084
k__Bacteria|p__Firmicutes|c__CFGB10667|o__OFGB10667|f__FGB10667|g__GGB32371|s__GGB32371_SGB41694
k__Bacteria|p__Firmicutes|c__Clostridia|o__Eubacteriales|f__Lachnospiraceae|g__GGB3793|s__GGB3793_SGB5158
k__Bacteria|p__Firmicutes|c__Clostridia|o__Eubacteriales|f__Clostridiaceae|g__GGB42601|s__GGB42601_SGB59797
k__Bacteria|p__Firmicutes|c__Clostridia|o__Eubacteriales|f__Oscillospiraceae|g__GGB45514|s__GGB45514_SGB63186
k__Bacteria|p__Firmicutes|c__CFGB75721|o__OFGB75721|f__FGB75721|g__GGB45564|s__GGB45564_SGB63259
k__Bacteria|p__Firmicutes|c__Clostridia|o__Eubacteriales|f__Oscillospiraceae|g__GGB45624|s__GGB45624_SGB63337
k__Bacteria|p__Firmicutes|c__Clostridia|o__Eubacteriales|f__Christensenellaceae|g__GGB45656|s__GGB45656_SGB6337
k__Bacteria|p__Firmicutes|c__CFGB10299|o__OFGB10299|f__FGB10299|g__GGB47127|s__GGB47127_SGB65054
k__Bacteria|p__Firmicutes|c__Clostridia|o__Eubacteriales|f__Oscillospiraceae|g__GGB74395|s__GGB74395_SGB43523
k__Bacteria|p__Firmicutes|c__Clostridia|o__Eubacteriales|f__Oscillospiraceae|g__GGB75053|s__GGB75053_SGB43494
k__Bacteria|p__Firmicutes|c__Clostridia|o__Eubacteriales|f__Lachnospiraceae|g__GGB75109|s__GGB75109_SGB102238
k__Bacteria|p__Firmicutes|c__Clostridia|o__Eubacteriales|f__Lachnospiraceae|g__Lachnospiraceae_unclassified|s__Lachn
k__Bacteria|p__Firmicutes|c__Clostridia|o__Eubacteriales|f__Lachnospiraceae|g__Lachnospiraceae_unclassified|s__Lachn
k__Bacteria|p__Firmicutes|c__Clostridia|o__Eubacteriales|f__Lachnospiraceae|g__Lachnospiraceae_unclassified|s__Lachn
k__Bacteria|p__Firmicutes|c__Clostridia|o__Eubacteriales|f__Lachnospiraceae|g__Lachnospiraceae_unclassified|s__Lachn
k__Bacteria|p__Firmicutes|c__Clostridia|o__Eubacteriales|f__Lachnospiraceae|g__Lachnospiraceae_unclassified|s__Lachn
k__Bacteria|p__Firmicutes|c__Bacilli|o__Lactobacillales|f__Lactobacillaceae|g__Lactobacillus|s__Lactobacillus_johnsonii
k__Bacteria|p__Actinobacteria|c__Coriobacteriia|o__Coriobacteriales|f__Atopobiaceae|g__Leptogranulimonas|s__Leptog
k__Bacteria|p__Bacteroidota|c__Bacteroidia|o__Bacteroidales|f__Muribaculaceae|g__Muribaculaceae_unclassified|s__M
k__Bacteria|p__Firmicutes|c__Clostridia|o__Eubacteriales|f__Oscillospiraceae|g__Neglectibacter|s__Neglectibacter_sp_X
k__Bacteria|p__Firmicutes|c__Clostridia|o__Eubacteriales|f__Oscillospiraceae|g__Oscillibacter|s__Oscillibacter_SGB43496
k__Bacteria|p__Firmicutes|c__Clostridia|o__Eubacteriales|f__Oscillospiraceae|g__Oscillospiraceae_unclassified|s__Oscillo
k__Bacteria|p__Firmicutes|c__Clostridia|o__Eubacteriales|f__Oscillospiraceae|g__Oscillospiraceae_unclassified|s__Oscillo
k__Bacteria|p__Firmicutes|c__Clostridia|o__Eubacteriales|f__Oscillospiraceae|g__Oscillospiraceae_unclassified|s__Oscillo
k__Bacteria|p__Firmicutes|c__Clostridia|o__Eubacteriales|f__Oscillospiraceae|g__Oscillospiraceae_unclassified|s__Oscillo
k__Bacteria|p__Proteobacteria|c__Betaproteobacteria|o__Burkholderiales|f__Sutterellaceae|g__Parasutterella|s__Parasu

k__Bacteria|p__Firmicutes|c__Clostridia|o__Eubacteriales|f__Lachnospiraceae|g__Schaedlerella|s__Schaedlerella_arabino
k__Bacteria|p__Firmicutes|c__Erysipelotrichia|o__Erysipelotrichales|f__Turicibacteraceae|g__Turicibacter|s__Turicibacter_
k__Bacteria|p__Bacteria_unclassified|c__Bacteria_unclassified|o__Bacteria_unclassified|f__Bacteria_unclassified|g__Bacte
k__Bacteria|p__Bacteria_unclassified|c__Bacteria_unclassified|o__Bacteria_unclassified|f__Bacteria_unclassified|g__Bacte
k__Bacteria|p__Bacteria_unclassified|c__Bacteria_unclassified|o__Bacteria_unclassified|f__Bacteria_unclassified|g__Bacte
k__Bacteria|p__Bacteria_unclassified|c__Bacteria_unclassified|o__Bacteria_unclassified|f__Bacteria_unclassified|g__Bacte
k__Bacteria|p__Bacteria_unclassified|c__Bacteria_unclassified|o__Bacteria_unclassified|f__Bacteria_unclassified|g__Bacte

k__Bacteria|p__Firmicutes|c__Clostridia|o__Eubacteriales|f__Lachnospiraceae|g__Acetatifactor|s__Acetatifactor_SGB415
k__Bacteria|p__Firmicutes|c__Clostridia|o__Eubacteriales|f__Oscillospiraceae|g__Acutalibacter|s__Acutalibacter_muris
k__Bacteria|p__Verrucomicrobia|c__Verrucomicrobiae|o__Verrucomicrobiales|f__Akkermansiaceae|g__Akkermansia|s__A
k__Bacteria|p__Firmicutes|c__Clostridia|o__Eubacteriales|f__Oscillospiraceae|g__Anaerotruncus|s__Anaerotruncus_sp_1
k__Bacteria|p__Bacteria_unclassified|c__Bacteria_unclassified|o__Bacteria_unclassified|f__Bacteria_unclassified|g__Bacte
k__Bacteria|p__Bacteria_unclassified|c__Bacteria_unclassified|o__Bacteria_unclassified|f__Bacteria_unclassified|g__Bacte
k__Bacteria|p__Bacteria_unclassified|c__Bacteria_unclassified|o__Bacteria_unclassified|f__Bacteria_unclassified|g__Bacte
k__Bacteria|p__Bacteria_unclassified|c__Bacteria_unclassified|o__Bacteria_unclassified|f__Bacteria_unclassified|g__Bacte
k__Bacteria|p__Bacteroidota|c__Bacteroidia|o__Bacteroidales|f__Bacteroidaceae|g__Bacteroides|s__Bacteroides_thetaio
k__Bacteria|p__Firmicutes|c__Clostridia|o__Clostridia_unclassified|f__Clostridia_unclassified|g__Clostridia_unclassified|s_
k__Bacteria|p__Firmicutes|c__Clostridia|o__Eubacteriales|f__Clostridiaceae|g__Clostridiaceae_unclassified|s__Clostridiac
k__Bacteria|p__Firmicutes|c__Clostridia|o__Eubacteriales|f__Clostridiaceae|g__Clostridiaceae_unclassified|s__Clostridiac
k__Bacteria|p__Firmicutes|c__Clostridia|o__Eubacteriales|f__Eubacteriales_unclassified|g__Eubacteriales_unclassified|s_
k__Bacteria|p__Firmicutes|c__Clostridia|o__Eubacteriales|f__Clostridiaceae|g__Clostridium|s__Clostridium_SGB65123
k__Bacteria|p__Firmicutes|c__Erysipelotrichia|o__Erysipelotrichales|f__Erysipelotrichaceae|g__Erysipelatoclostridium|s__
k__Bacteria|p__Firmicutes|c__Clostridia|o__Eubacteriales|f__Eubacteriaceae|g__Eubacteriaceae_unclassified|s__Eubacte
k__Bacteria|p__Firmicutes|c__Clostridia|o__Eubacteriales|f__Eubacteriaceae|g__Eubacteriaceae_unclassified|s__Eubacte
k__Bacteria|p__Firmicutes|c__CFGB77306|o__OFGB77306|f__FGB77306|g__GGB20146|s__GGB20146_SGB29427
k__Bacteria|p__Firmicutes|c__Clostridia|o__Eubacteriales|f__Lachnospiraceae|g__GGB20149|s__GGB20149_SGB29430
k__Bacteria|p__Actinobacteria|c__Coriobacteriia|o__Eggerthellales|f__Eggerthellaceae|g__GGB22635|s__GGB22635_SGB
k__Bacteria|p__Firmicutes|c__Clostridia|o__Eubacteriales|f__Lachnospiraceae|g__GGB25041|s__GGB25041_SGB36960
k__Bacteria|p__Firmicutes|c__CFGB9506|o__OFGB9506|f__FGB9506|g__GGB28379|s__GGB28379_SGB40959
k__Bacteria|p__Firmicutes|c__CFGB9508|o__OFGB9508|f__FGB9508|g__GGB28382|s__GGB28382_SGB40962
k__Bacteria|p__Firmicutes|c__CFGB9512|o__OFGB9512|f__FGB9512|g__GGB28392|s__GGB28392_SGB40972
k__Bacteria|p__Firmicutes|c__CFGB2838|o__OFGB2838|f__FGB2838|g__GGB28404|s__GGB28404_SGB40986
k__Bacteria|p__Firmicutes|c__CFGB2838|o__OFGB2838|f__FGB2838|g__GGB28418|s__GGB28418_SGB41001
k__Bacteria|p__Firmicutes|c__CFGB2838|o__OFGB2838|f__FGB2838|g__GGB28422|s__GGB28422_SGB41005
k__Bacteria|p__Firmicutes|c__Clostridia|o__Eubacteriales|f__Pumilibacteraceae|g__GGB28431|s__GGB28431_SGB41014
k__Bacteria|p__Firmicutes|c__CFGB2833|o__OFGB2833|f__FGB2833|g__GGB28456|s__GGB28456_SGB41039
k__Bacteria|p__Firmicutes|c__Clostridia|o__Eubacteriales|f__Eubacteriaceae|g__GGB28782|s__GGB28782_SGB41435
k__Bacteria|p__Firmicutes|c__Clostridia|o__Eubacteriales|f__Lachnospiraceae|g__GGB28792|s__GGB28792_SGB41445
k__Bacteria|p__Firmicutes|c__Clostridia|o__Eubacteriales|f__Lachnospiraceae|g__GGB28798|s__GGB28798_SGB41451
k__Bacteria|p__Firmicutes|c__CFGB9622|o__OFGB9622|f__FGB9622|g__GGB28810|s__GGB28810_SGB41465
k__Bacteria|p__Firmicutes|c__CFGB77305|o__OFGB77305|f__FGB77305|g__GGB28828|s__GGB28828_SGB41484
k__Bacteria|p__Firmicutes|c__Clostridia|o__Eubacteriales|f__Clostridiaceae|g__GGB28851|s__GGB28851_SGB41518
k__Bacteria|p__Firmicutes|c__Clostridia|o__Eubacteriales|f__Lachnospiraceae|g__GGB28865|s__GGB28865_SGB41537

k__Bacteria|p__Firmicutes|c__Clostridia|o__Eubacteriales|f__Lachnospiraceae|g__GGB28868|s__GGB28868_SGB41542
k__Bacteria|p__Firmicutes|c__Clostridia|o__Eubacteriales|f__Lachnospiraceae|g__GGB28869|s__GGB28869_SGB41543
k__Bacteria|p__Firmicutes|c__Clostridia|o__Eubacteriales|f__Lachnospiraceae|g__GGB28875|s__GGB28875_SGB41555
k__Bacteria|p__Firmicutes|c__CFGB9633|o__OFGB9633|f__FGB9633|g__GGB28881|s__GGB28881_SGB41561
k__Bacteria|p__Bacteria_unclassified|c__Bacteria_unclassified|o__Bacteria_unclassified|f__Bacteria_unclassified|g__GGB
k__Bacteria|p__Bacteria_unclassified|c__Bacteria_unclassified|o__Bacteria_unclassified|f__Bacteria_unclassified|g__GGB
k__Bacteria|p__Bacteria_unclassified|c__Bacteria_unclassified|o__Bacteria_unclassified|f__Bacteria_unclassified|g__GGB
k__Bacteria|p__Bacteria_unclassified|c__Bacteria_unclassified|o__Bacteria_unclassified|f__Bacteria_unclassified|g__GGB
k__Bacteria|p__Bacteria_unclassified|c__Bacteria_unclassified|o__Bacteria_unclassified|f__Bacteria_unclassified|g__GGB
k__Bacteria|p__Bacteria_unclassified|c__Bacteria_unclassified|o__Bacteria_unclassified|f__Bacteria_unclassified|g__GGB
k__Bacteria|p__Firmicutes|c__Clostridia|o__Eubacteriales|f__Lachnospiraceae|g__GGB28924|s__GGB28924_SGB41621
k__Bacteria|p__Bacteria_unclassified|c__CFGB77359|o__OFGB77359|f__FGB77359|g__GGB28927|s__GGB28927_SGB416
k__Bacteria|p__Firmicutes|c__CFGB9639|o__OFGB9639|f__FGB9639|g__GGB28934|s__GGB28934_SGB41635
k__Bacteria|p__Firmicutes|c__Clostridia|o__Eubacteriales|f__Lachnospiraceae|g__GGB28949|s__GGB28949_SGB41655
k__Bacteria|p__Firmicutes|c__Clostridia|o__Eubacteriales|f__Clostridiaceae|g__GGB28951|s__GGB28951_SGB102295
k__Bacteria|p__Firmicutes|c__Clostridia|o__Eubacteriales|f__Clostridiaceae|g__GGB28951|s__GGB28951_SGB41658
k__Bacteria|p__Firmicutes|c__Clostridia|o__Eubacteriales|f__Clostridiaceae|g__GGB28954|s__GGB28954_SGB41662
k__Bacteria|p__Firmicutes|c__Clostridia|o__Eubacteriales|f__Clostridiaceae|g__GGB28960|s__GGB28960_SGB41669
k__Bacteria|p__Firmicutes|c__Clostridia|o__Eubacteriales|f__Clostridiaceae|g__GGB28964|s__GGB28964_SGB94886
k__Bacteria|p__Firmicutes|c__Clostridia|o__Eubacteriales|f__Clostridiaceae|g__GGB28967|s__GGB28967_SGB41678
k__Bacteria|p__Firmicutes|c__CFGB9656|o__OFGB9656|f__FGB9656|g__GGB28996|s__GGB28996_SGB41712
k__Bacteria|p__Firmicutes|c__CFGB9827|o__OFGB9827|f__FGB9827|g__GGB29531|s__GGB29531_SGB42317
k__Bacteria|p__Firmicutes|c__Clostridia|o__Eubacteriales|f__Eubacteriaceae|g__GGB29685|s__GGB29685_SGB42494
k__Bacteria|p__Bacteria_unclassified|c__CFGB77303|o__OFGB77303|f__FGB77303|g__GGB30141|s__GGB30141_SGB430
k__Bacteria|p__Firmicutes|c__Clostridia|o__Eubacteriales|f__Clostridiaceae|g__GGB30145|s__GGB30145_SGB43072
k__Bacteria|p__Firmicutes|c__Clostridia|o__Eubacteriales|f__Eubacteriales_unclassified|g__GGB30286|s__GGB30286_SG
k__Bacteria|p__Firmicutes|c__CFGB72709|o__OFGB72709|f__FGB72709|g__GGB30300|s__GGB30300_SGB43264
k__Bacteria|p__Firmicutes|c__Clostridia|o__Eubacteriales|f__Oscillospiraceae|g__GGB30303|s__GGB30303_SGB43268
k__Bacteria|p__Firmicutes|c__Clostridia|o__Eubacteriales|f__Oscillospiraceae|g__GGB30450|s__GGB30450_SGB43507
k__Bacteria|p__Firmicutes|c__Clostridia|o__Eubacteriales|f__Oscillospiraceae|g__GGB30453|s__GGB30453_SGB43513
k__Bacteria|p__Firmicutes|c__Clostridia|o__Eubacteriales|f__Oscillospiraceae|g__GGB30454|s__GGB30454_SGB43514
k__Bacteria|p__Firmicutes|c__Clostridia|o__Eubacteriales|f__Oscillospiraceae|g__GGB30455|s__GGB30455_SGB43519
k__Bacteria|p__Firmicutes|c__Clostridia|o__Eubacteriales|f__Oscillospiraceae|g__GGB30456|s__GGB30456_SGB43520
k__Bacteria|p__Firmicutes|c__Clostridia|o__Eubacteriales|f__Oscillospiraceae|g__GGB30457|s__GGB30457_SGB63218
k__Bacteria|p__Firmicutes|c__Clostridia|o__Eubacteriales|f__Oscillospiraceae|g__GGB30461|s__GGB30461_SGB43530
k__Bacteria|p__Firmicutes|c__Clostridia|o__Eubacteriales|f__Oscillospiraceae|g__GGB30461|s__GGB30461_SGB43533
k__Bacteria|p__Firmicutes|c__Clostridia|o__Eubacteriales|f__Oscillospiraceae|g__GGB30461|s__GGB30461_SGB63209
k__Bacteria|p__Firmicutes|c__Clostridia|o__Eubacteriales|f__Oscillospiraceae|g__GGB30463|s__GGB30463_SGB43537
k__Bacteria|p__Firmicutes|c__Clostridia|o__Eubacteriales|f__Oscillospiraceae|g__GGB30473|s__GGB30473_SGB43557
k__Bacteria|p__Firmicutes|c__Clostridia|o__Eubacteriales|f__Oscillospiraceae|g__GGB30475|s__GGB30475_SGB63182
k__Bacteria|p__Actinobacteria|c__CFGB77153|o__OFGB77153|f__FGB77153|g__GGB30861|s__GGB30861_SGB44083
k__Bacteria|p__Tenericutes|c__CFGB1791|o__OFGB1791|f__FGB1791|g__GGB31312|s__GGB31312_SGB44628
k__Bacteria|p__Firmicutes|c__CFGB10290|o__OFGB10290|f__FGB10290|g__GGB31438|s__GGB31438_SGB44768
k__Bacteria|p__Firmicutes|c__Clostridia|o__Eubacteriales|f__Oscillospiraceae|g__GGB3171|s__GGB3171_SGB4185
k__Bacteria|p__Firmicutes|c__CFGB10289|o__OFGB10289|f__FGB10289|g__GGB31762|s__GGB31762_SGB45125
k__Bacteria|p__Firmicutes|c__CFGB1765|o__OFGB1765|f__FGB1765|g__GGB31823|s__GGB31823_SGB45199

k__Bacteria|p__Firmicutes|c__CFGB1765|o__OFGB1765|f__FGB1765|g__GGB31838|s__GGB31838_SGB45216
k__Bacteria|p__Firmicutes|c__CFGB1765|o__OFGB1765|f__FGB1765|g__GGB31841|s__GGB31841_SGB65084
k__Bacteria|p__Firmicutes|c__CFGB10667|o__OFGB10667|f__FGB10667|g__GGB32371|s__GGB32371_SGB41694
k__Bacteria|p__Firmicutes|c__Clostridia|o__Eubacteriales|f__Lachnospiraceae|g__GGB3793|s__GGB3793_SGB5158
k__Bacteria|p__Firmicutes|c__Clostridia|o__Eubacteriales|f__Clostridiaceae|g__GGB42601|s__GGB42601_SGB59797
k__Bacteria|p__Firmicutes|c__Clostridia|o__Eubacteriales|f__Oscillospiraceae|g__GGB45514|s__GGB45514_SGB63186
k__Bacteria|p__Firmicutes|c__CFGB75721|o__OFGB75721|f__FGB75721|g__GGB45564|s__GGB45564_SGB63259
k__Bacteria|p__Firmicutes|c__Clostridia|o__Eubacteriales|f__Oscillospiraceae|g__GGB45624|s__GGB45624_SGB63337
k__Bacteria|p__Firmicutes|c__Clostridia|o__Eubacteriales|f__Christensenellaceae|g__GGB45656|s__GGB45656_SGB6337
k__Bacteria|p__Firmicutes|c__CFGB10299|o__OFGB10299|f__FGB10299|g__GGB47127|s__GGB47127_SGB65054
k__Bacteria|p__Firmicutes|c__Clostridia|o__Eubacteriales|f__Oscillospiraceae|g__GGB74395|s__GGB74395_SGB43523
k__Bacteria|p__Firmicutes|c__Clostridia|o__Eubacteriales|f__Oscillospiraceae|g__GGB75053|s__GGB75053_SGB43494
k__Bacteria|p__Firmicutes|c__Clostridia|o__Eubacteriales|f__Lachnospiraceae|g__GGB75109|s__GGB75109_SGB102238
k__Bacteria|p__Firmicutes|c__Clostridia|o__Eubacteriales|f__Lachnospiraceae|g__Lachnospiraceae_unclassified|s__Lachn
k__Bacteria|p__Firmicutes|c__Clostridia|o__Eubacteriales|f__Lachnospiraceae|g__Lachnospiraceae_unclassified|s__Lachn
k__Bacteria|p__Firmicutes|c__Clostridia|o__Eubacteriales|f__Lachnospiraceae|g__Lachnospiraceae_unclassified|s__Lachn
k__Bacteria|p__Firmicutes|c__Clostridia|o__Eubacteriales|f__Lachnospiraceae|g__Lachnospiraceae_unclassified|s__Lachn
k__Bacteria|p__Firmicutes|c__Clostridia|o__Eubacteriales|f__Lachnospiraceae|g__Lachnospiraceae_unclassified|s__Lachn
k__Bacteria|p__Firmicutes|c__Bacilli|o__Lactobacillales|f__Lactobacillaceae|g__Lactobacillus|s__Lactobacillus_johnsonii
k__Bacteria|p__Actinobacteria|c__Coriobacteriia|o__Coriobacteriales|f__Atopobiaceae|g__Leptogranulimonas|s__Leptog
k__Bacteria|p__Bacteroidota|c__Bacteroidia|o__Bacteroidales|f__Muribaculaceae|g__Muribaculaceae_unclassified|s__M
k__Bacteria|p__Firmicutes|c__Clostridia|o__Eubacteriales|f__Oscillospiraceae|g__Neglectibacter|s__Neglectibacter_sp_X
k__Bacteria|p__Firmicutes|c__Clostridia|o__Eubacteriales|f__Oscillospiraceae|g__Oscillibacter|s__Oscillibacter_SGB43496
k__Bacteria|p__Firmicutes|c__Clostridia|o__Eubacteriales|f__Oscillospiraceae|g__Oscillospiraceae_unclassified|s__Oscillo
k__Bacteria|p__Firmicutes|c__Clostridia|o__Eubacteriales|f__Oscillospiraceae|g__Oscillospiraceae_unclassified|s__Oscillo
k__Bacteria|p__Firmicutes|c__Clostridia|o__Eubacteriales|f__Oscillospiraceae|g__Oscillospiraceae_unclassified|s__Oscillo
k__Bacteria|p__Firmicutes|c__Clostridia|o__Eubacteriales|f__Oscillospiraceae|g__Oscillospiraceae_unclassified|s__Oscillo
k__Bacteria|p__Proteobacteria|c__Betaproteobacteria|o__Burkholderiales|f__Sutterellaceae|g__Parasutterella|s__Parasu
k__Bacteria|p__Firmicutes|c__Clostridia|o__Eubacteriales|f__Lachnospiraceae|g__Schaedlerella|s__Schaedlerella_arabino
k__Bacteria|p__Firmicutes|c__Erysipelotrichia|o__Erysipelotrichales|f__Turicibacteraceae|g__Turicibacter|s__Turicibacter_
k__Bacteria|p__Bacteria_unclassified|c__Bacteria_unclassified|o__Bacteria_unclassified|f__Bacteria_unclassified|g__Bacte
k__Bacteria|p__Bacteria_unclassified|c__Bacteria_unclassified|o__Bacteria_unclassified|f__Bacteria_unclassified|g__Bacte
k__Bacteria|p__Bacteria_unclassified|c__Bacteria_unclassified|o__Bacteria_unclassified|f__Bacteria_unclassified|g__Bacte
k__Bacteria|p__Bacteria_unclassified|c__Bacteria_unclassified|o__Bacteria_unclassified|f__Bacteria_unclassified|g__Bacte
k__Bacteria|p__Bacteria_unclassified|c__Bacteria_unclassified|o__Bacteria_unclassified|f__Bacteria_unclassified|g__Bacte

k__Bacteria|p__Firmicutes|c__Clostridia|o__Eubacteriales|f__Lachnospiraceae|g__Acetatifactor|s__Acetatifactor_SGB415
k__Bacteria|p__Firmicutes|c__Clostridia|o__Eubacteriales|f__Oscillospiraceae|g__Acutalibacter|s__Acutalibacter_muris
k__Bacteria|p__Verrucomicrobia|c__Verrucomicrobiae|o__Verrucomicrobiales|f__Akkermansiaceae|g__Akkermansia|s__A
k__Bacteria|p__Firmicutes|c__Clostridia|o__Eubacteriales|f__Oscillospiraceae|g__Anaerotruncus|s__Anaerotruncus_sp_1
k__Bacteria|p__Bacteria_unclassified|c__Bacteria_unclassified|o__Bacteria_unclassified|f__Bacteria_unclassified|g__Bacte
k__Bacteria|p__Bacteria_unclassified|c__Bacteria_unclassified|o__Bacteria_unclassified|f__Bacteria_unclassified|g__Bacte
k__Bacteria|p__Bacteria_unclassified|c__Bacteria_unclassified|o__Bacteria_unclassified|f__Bacteria_unclassified|g__Bacte
k__Bacteria|p__Bacteria_unclassified|c__Bacteria_unclassified|o__Bacteria_unclassified|f__Bacteria_unclassified|g__Bacte

k__Bacteria|p__Bacteroidota|c__Bacteroidia|o__Bacteroidales|f__Bacteroidaceae|g__Bacteroides|s__Bacteroides_thetaio
k__Bacteria|p__Firmicutes|c__Clostridia|o__Clostridia_unclassified|f__Clostridia_unclassified|g__Clostridia_unclassified|s_
k__Bacteria|p__Firmicutes|c__Clostridia|o__Eubacteriales|f__Clostridiaceae|g__Clostridiaceae_unclassified|s__Clostridiac
k__Bacteria|p__Firmicutes|c__Clostridia|o__Eubacteriales|f__Clostridiaceae|g__Clostridiaceae_unclassified|s__Clostridiac
k__Bacteria|p__Firmicutes|c__Clostridia|o__Eubacteriales|f__Eubacteriales_unclassified|g__Eubacteriales_unclassified|s_
k__Bacteria|p__Firmicutes|c__Clostridia|o__Eubacteriales|f__Clostridiaceae|g__Clostridium|s__Clostridium_SGB65123
k__Bacteria|p__Firmicutes|c__Erysipelotrichia|o__Erysipelotrichales|f__Erysipelotrichaceae|g__Erysipelatoclostridium|s__
k__Bacteria|p__Firmicutes|c__Clostridia|o__Eubacteriales|f__Eubacteriaceae|g__Eubacteriaceae_unclassified|s__Eubacte
k__Bacteria|p__Firmicutes|c__Clostridia|o__Eubacteriales|f__Eubacteriaceae|g__Eubacteriaceae_unclassified|s__Eubacte
k__Bacteria|p__Firmicutes|c__CFGB77306|o__OFGB77306|f__FGB77306|g__GGB20146|s__GGB20146_SGB29427
k__Bacteria|p__Firmicutes|c__Clostridia|o__Eubacteriales|f__Lachnospiraceae|g__GGB20149|s__GGB20149_SGB29430
k__Bacteria|p__Actinobacteria|c__Coriobacteriia|o__Eggerthellales|f__Eggerthellaceae|g__GGB22635|s__GGB22635_SGB
k__Bacteria|p__Firmicutes|c__Clostridia|o__Eubacteriales|f__Lachnospiraceae|g__GGB25041|s__GGB25041_SGB36960
k__Bacteria|p__Firmicutes|c__CFGB9506|o__OFGB9506|f__FGB9506|g__GGB28379|s__GGB28379_SGB40959
k__Bacteria|p__Firmicutes|c__CFGB9508|o__OFGB9508|f__FGB9508|g__GGB28382|s__GGB28382_SGB40962
k__Bacteria|p__Firmicutes|c__CFGB9512|o__OFGB9512|f__FGB9512|g__GGB28392|s__GGB28392_SGB40972
k__Bacteria|p__Firmicutes|c__CFGB2838|o__OFGB2838|f__FGB2838|g__GGB28404|s__GGB28404_SGB40986
k__Bacteria|p__Firmicutes|c__CFGB2838|o__OFGB2838|f__FGB2838|g__GGB28418|s__GGB28418_SGB41001
k__Bacteria|p__Firmicutes|c__CFGB2838|o__OFGB2838|f__FGB2838|g__GGB28422|s__GGB28422_SGB41005
k__Bacteria|p__Firmicutes|c__Clostridia|o__Eubacteriales|f__Pumilibacteraceae|g__GGB28431|s__GGB28431_SGB41014
k__Bacteria|p__Firmicutes|c__CFGB2833|o__OFGB2833|f__FGB2833|g__GGB28456|s__GGB28456_SGB41039
k__Bacteria|p__Firmicutes|c__Clostridia|o__Eubacteriales|f__Eubacteriaceae|g__GGB28782|s__GGB28782_SGB41435
k__Bacteria|p__Firmicutes|c__Clostridia|o__Eubacteriales|f__Lachnospiraceae|g__GGB28792|s__GGB28792_SGB41445
k__Bacteria|p__Firmicutes|c__Clostridia|o__Eubacteriales|f__Lachnospiraceae|g__GGB28798|s__GGB28798_SGB41451
k__Bacteria|p__Firmicutes|c__CFGB9622|o__OFGB9622|f__FGB9622|g__GGB28810|s__GGB28810_SGB41465
k__Bacteria|p__Firmicutes|c__CFGB77305|o__OFGB77305|f__FGB77305|g__GGB28828|s__GGB28828_SGB41484
k__Bacteria|p__Firmicutes|c__Clostridia|o__Eubacteriales|f__Clostridiaceae|g__GGB28851|s__GGB28851_SGB41518
k__Bacteria|p__Firmicutes|c__Clostridia|o__Eubacteriales|f__Lachnospiraceae|g__GGB28865|s__GGB28865_SGB41537
k__Bacteria|p__Firmicutes|c__Clostridia|o__Eubacteriales|f__Lachnospiraceae|g__GGB28868|s__GGB28868_SGB41542
k__Bacteria|p__Firmicutes|c__Clostridia|o__Eubacteriales|f__Lachnospiraceae|g__GGB28869|s__GGB28869_SGB41543
k__Bacteria|p__Firmicutes|c__Clostridia|o__Eubacteriales|f__Lachnospiraceae|g__GGB28875|s__GGB28875_SGB41555
k__Bacteria|p__Firmicutes|c__CFGB9633|o__OFGB9633|f__FGB9633|g__GGB28881|s__GGB28881_SGB41561
k__Bacteria|p__Bacteria_unclassified|c__Bacteria_unclassified|o__Bacteria_unclassified|f__Bacteria_unclassified|g__GGB2
k__Bacteria|p__Bacteria_unclassified|c__Bacteria_unclassified|o__Bacteria_unclassified|f__Bacteria_unclassified|g__GGB2
k__Bacteria|p__Bacteria_unclassified|c__Bacteria_unclassified|o__Bacteria_unclassified|f__Bacteria_unclassified|g__GGB2
k__Bacteria|p__Bacteria_unclassified|c__Bacteria_unclassified|o__Bacteria_unclassified|f__Bacteria_unclassified|g__GGB2
k__Bacteria|p__Bacteria_unclassified|c__Bacteria_unclassified|o__Bacteria_unclassified|f__Bacteria_unclassified|g__GGB2
k__Bacteria|p__Bacteria_unclassified|c__Bacteria_unclassified|o__Bacteria_unclassified|f__Bacteria_unclassified|g__GGB2
k__Bacteria|p__Firmicutes|c__Clostridia|o__Eubacteriales|f__Lachnospiraceae|g__GGB28924|s__GGB28924_SGB41621
k__Bacteria|p__Bacteria_unclassified|c__CFGB77359|o__OFGB77359|f__FGB77359|g__GGB28927|s__GGB28927_SGB416
k__Bacteria|p__Firmicutes|c__CFGB9639|o__OFGB9639|f__FGB9639|g__GGB28934|s__GGB28934_SGB41635
k__Bacteria|p__Firmicutes|c__Clostridia|o__Eubacteriales|f__Lachnospiraceae|g__GGB28949|s__GGB28949_SGB41655
k__Bacteria|p__Firmicutes|c__Clostridia|o__Eubacteriales|f__Clostridiaceae|g__GGB28951|s__GGB28951_SGB102295
k__Bacteria|p__Firmicutes|c__Clostridia|o__Eubacteriales|f__Clostridiaceae|g__GGB28951|s__GGB28951_SGB41658
k__Bacteria|p__Firmicutes|c__Clostridia|o__Eubacteriales|f__Clostridiaceae|g__GGB28954|s__GGB28954_SGB41662
k__Bacteria|p__Firmicutes|c__Clostridia|o__Eubacteriales|f__Clostridiaceae|g__GGB28960|s__GGB28960_SGB41669

k__Bacteria|p__Firmicutes|c__Clostridia|o__Eubacteriales|f__Clostridiaceae|g__GGB28964|s__GGB28964_SGB94886
k__Bacteria|p__Firmicutes|c__Clostridia|o__Eubacteriales|f__Clostridiaceae|g__GGB28967|s__GGB28967_SGB41678
k__Bacteria|p__Firmicutes|c__CFGB9656|o__OFGB9656|f__FGB9656|g__GGB28996|s__GGB28996_SGB41712
k__Bacteria|p__Firmicutes|c__CFGB9827|o__OFGB9827|f__FGB9827|g__GGB29531|s__GGB29531_SGB42317
k__Bacteria|p__Firmicutes|c__Clostridia|o__Eubacteriales|f__Eubacteriaceae|g__GGB29685|s__GGB29685_SGB42494
k__Bacteria|p__Bacteria_unclassified|c__CFGB77303|o__OFGB77303|f__FGB77303|g__GGB30141|s__GGB30141_SGB430
k__Bacteria|p__Firmicutes|c__Clostridia|o__Eubacteriales|f__Clostridiaceae|g__GGB30145|s__GGB30145_SGB43072
k__Bacteria|p__Firmicutes|c__Clostridia|o__Eubacteriales|f__Eubacteriales_unclassified|g__GGB30286|s__GGB30286_SG
k__Bacteria|p__Firmicutes|c__CFGB72709|o__OFGB72709|f__FGB72709|g__GGB30300|s__GGB30300_SGB43264
k__Bacteria|p__Firmicutes|c__Clostridia|o__Eubacteriales|f__Oscillospiraceae|g__GGB30303|s__GGB30303_SGB43268
k__Bacteria|p__Firmicutes|c__Clostridia|o__Eubacteriales|f__Oscillospiraceae|g__GGB30450|s__GGB30450_SGB43507
k__Bacteria|p__Firmicutes|c__Clostridia|o__Eubacteriales|f__Oscillospiraceae|g__GGB30453|s__GGB30453_SGB43513
k__Bacteria|p__Firmicutes|c__Clostridia|o__Eubacteriales|f__Oscillospiraceae|g__GGB30454|s__GGB30454_SGB43514
k__Bacteria|p__Firmicutes|c__Clostridia|o__Eubacteriales|f__Oscillospiraceae|g__GGB30455|s__GGB30455_SGB43519
k__Bacteria|p__Firmicutes|c__Clostridia|o__Eubacteriales|f__Oscillospiraceae|g__GGB30456|s__GGB30456_SGB43520
k__Bacteria|p__Firmicutes|c__Clostridia|o__Eubacteriales|f__Oscillospiraceae|g__GGB30457|s__GGB30457_SGB63218
k__Bacteria|p__Firmicutes|c__Clostridia|o__Eubacteriales|f__Oscillospiraceae|g__GGB30461|s__GGB30461_SGB43530
k__Bacteria|p__Firmicutes|c__Clostridia|o__Eubacteriales|f__Oscillospiraceae|g__GGB30461|s__GGB30461_SGB43533
k__Bacteria|p__Firmicutes|c__Clostridia|o__Eubacteriales|f__Oscillospiraceae|g__GGB30461|s__GGB30461_SGB63209
k__Bacteria|p__Firmicutes|c__Clostridia|o__Eubacteriales|f__Oscillospiraceae|g__GGB30463|s__GGB30463_SGB43537
k__Bacteria|p__Firmicutes|c__Clostridia|o__Eubacteriales|f__Oscillospiraceae|g__GGB30473|s__GGB30473_SGB43557
k__Bacteria|p__Firmicutes|c__Clostridia|o__Eubacteriales|f__Oscillospiraceae|g__GGB30475|s__GGB30475_SGB63182
k__Bacteria|p__Actinobacteria|c__CFGB77153|o__OFGB77153|f__FGB77153|g__GGB30861|s__GGB30861_SGB44083
k__Bacteria|p__Tenericutes|c__CFGB1791|o__OFGB1791|f__FGB1791|g__GGB31312|s__GGB31312_SGB44628
k__Bacteria|p__Firmicutes|c__CFGB10290|o__OFGB10290|f__FGB10290|g__GGB31438|s__GGB31438_SGB44768
k__Bacteria|p__Firmicutes|c__Clostridia|o__Eubacteriales|f__Oscillospiraceae|g__GGB3171|s__GGB3171_SGB4185
k__Bacteria|p__Firmicutes|c__CFGB10289|o__OFGB10289|f__FGB10289|g__GGB31762|s__GGB31762_SGB45125
k__Bacteria|p__Firmicutes|c__CFGB1765|o__OFGB1765|f__FGB1765|g__GGB31823|s__GGB31823_SGB45199
k__Bacteria|p__Firmicutes|c__CFGB1765|o__OFGB1765|f__FGB1765|g__GGB31838|s__GGB31838_SGB45216
k__Bacteria|p__Firmicutes|c__CFGB1765|o__OFGB1765|f__FGB1765|g__GGB31841|s__GGB31841_SGB65084
k__Bacteria|p__Firmicutes|c__CFGB10667|o__OFGB10667|f__FGB10667|g__GGB32371|s__GGB32371_SGB41694
k__Bacteria|p__Firmicutes|c__Clostridia|o__Eubacteriales|f__Lachnospiraceae|g__GGB3793|s__GGB3793_SGB5158
k__Bacteria|p__Firmicutes|c__Clostridia|o__Eubacteriales|f__Clostridiaceae|g__GGB42601|s__GGB42601_SGB59797
k__Bacteria|p__Firmicutes|c__Clostridia|o__Eubacteriales|f__Oscillospiraceae|g__GGB45514|s__GGB45514_SGB63186
k__Bacteria|p__Firmicutes|c__CFGB75721|o__OFGB75721|f__FGB75721|g__GGB45564|s__GGB45564_SGB63259
k__Bacteria|p__Firmicutes|c__Clostridia|o__Eubacteriales|f__Oscillospiraceae|g__GGB45624|s__GGB45624_SGB63337
k__Bacteria|p__Firmicutes|c__Clostridia|o__Eubacteriales|f__Christensenellaceae|g__GGB45656|s__GGB45656_SGB6337
k__Bacteria|p__Firmicutes|c__CFGB10299|o__OFGB10299|f__FGB10299|g__GGB47127|s__GGB47127_SGB65054
k__Bacteria|p__Firmicutes|c__Clostridia|o__Eubacteriales|f__Oscillospiraceae|g__GGB74395|s__GGB74395_SGB43523
k__Bacteria|p__Firmicutes|c__Clostridia|o__Eubacteriales|f__Oscillospiraceae|g__GGB75053|s__GGB75053_SGB43494
k__Bacteria|p__Firmicutes|c__Clostridia|o__Eubacteriales|f__Lachnospiraceae|g__GGB75109|s__GGB75109_SGB102238
k__Bacteria|p__Firmicutes|c__Clostridia|o__Eubacteriales|f__Lachnospiraceae|g__Lachnospiraceae_unclassified|s__Lachn
k__Bacteria|p__Firmicutes|c__Clostridia|o__Eubacteriales|f__Lachnospiraceae|g__Lachnospiraceae_unclassified|s__Lachn
k__Bacteria|p__Firmicutes|c__Clostridia|o__Eubacteriales|f__Lachnospiraceae|g__Lachnospiraceae_unclassified|s__Lachn
k__Bacteria|p__Firmicutes|c__Clostridia|o__Eubacteriales|f__Lachnospiraceae|g__Lachnospiraceae_unclassified|s__Lachn
k__Bacteria|p__Firmicutes|c__Clostridia|o__Eubacteriales|f__Lachnospiraceae|g__Lachnospiraceae_unclassified|s__Lachn

k__Bacteria|p__Firmicutes|c__Bacilli|o__Lactobacillales|f__Lactobacillaceae|g__Lactobacillus|s__Lactobacillus_johnsonii
k__Bacteria|p__Actinobacteria|c__Coriobacteriia|o__Coriobacteriales|f__Atopobiaceae|g__Leptogranulimonas|s__Leptog
k__Bacteria|p__Bacteroidota|c__Bacteroidia|o__Bacteroidales|f__Muribaculaceae|g__Muribaculaceae_unclassified|s__M
k__Bacteria|p__Firmicutes|c__Clostridia|o__Eubacteriales|f__Oscillospiraceae|g__Neglectibacter|s__Neglectibacter_sp_X
k__Bacteria|p__Firmicutes|c__Clostridia|o__Eubacteriales|f__Oscillospiraceae|g__Oscillibacter|s__Oscillibacter_SGB4349
k__Bacteria|p__Firmicutes|c__Clostridia|o__Eubacteriales|f__Oscillospiraceae|g__Oscillospiraceae_unclassified|s__Oscillo
k__Bacteria|p__Firmicutes|c__Clostridia|o__Eubacteriales|f__Oscillospiraceae|g__Oscillospiraceae_unclassified|s__Oscillo
k__Bacteria|p__Firmicutes|c__Clostridia|o__Eubacteriales|f__Oscillospiraceae|g__Oscillospiraceae_unclassified|s__Oscillo
k__Bacteria|p__Firmicutes|c__Clostridia|o__Eubacteriales|f__Oscillospiraceae|g__Oscillospiraceae_unclassified|s__Oscillo
k__Bacteria|p__Proteobacteria|c__Betaproteobacteria|o__Burkholderiales|f__Sutterellaceae|g__Parasutterella|s__Parasu
k__Bacteria|p__Firmicutes|c__Clostridia|o__Eubacteriales|f__Lachnospiraceae|g__Schaedlerella|s__Schaedlerella_arabino
k__Bacteria|p__Firmicutes|c__Erysipelotrichia|o__Erysipelotrichales|f__Turicibacteraceae|g__Turicibacter|s__Turicibacter
k__Bacteria|p__Bacteria_unclassified|c__Bacteria_unclassified|o__Bacteria_unclassified|f__Bacteria_unclassified|g__Bacte
k__Bacteria|p__Bacteria_unclassified|c__Bacteria_unclassified|o__Bacteria_unclassified|f__Bacteria_unclassified|g__Bacte
k__Bacteria|p__Bacteria_unclassified|c__Bacteria_unclassified|o__Bacteria_unclassified|f__Bacteria_unclassified|g__Bacte
k__Bacteria|p__Bacteria_unclassified|c__Bacteria_unclassified|o__Bacteria_unclassified|f__Bacteria_unclassified|g__Bacte
k__Bacteria|p__Bacteria_unclassified|c__Bacteria_unclassified|o__Bacteria_unclassified|f__Bacteria_unclassified|g__Bacte

k__Bacteria|p__Firmicutes|c__Clostridia|o__Eubacteriales|f__Lachnospiraceae|g__Acetatifactor|s__Acetatifactor_SGB415
k__Bacteria|p__Firmicutes|c__Clostridia|o__Eubacteriales|f__Oscillospiraceae|g__Acutalibacter|s__Acutalibacter_muris
k__Bacteria|p__Verrucomicrobia|c__Verrucomicrobiae|o__Verrucomicrobiales|f__Akkermansiaceae|g__Akkermansia|s__A
k__Bacteria|p__Firmicutes|c__Clostridia|o__Eubacteriales|f__Oscillospiraceae|g__Anaerotruncus|s__Anaerotruncus_sp_1
k__Bacteria|p__Bacteria_unclassified|c__Bacteria_unclassified|o__Bacteria_unclassified|f__Bacteria_unclassified|g__Bacte
k__Bacteria|p__Bacteria_unclassified|c__Bacteria_unclassified|o__Bacteria_unclassified|f__Bacteria_unclassified|g__Bacte
k__Bacteria|p__Bacteria_unclassified|c__Bacteria_unclassified|o__Bacteria_unclassified|f__Bacteria_unclassified|g__Bacte
k__Bacteria|p__Bacteria_unclassified|c__Bacteria_unclassified|o__Bacteria_unclassified|f__Bacteria_unclassified|g__Bacte
k__Bacteria|p__Bacteroidota|c__Bacteroidia|o__Bacteroidales|f__Bacteroidaceae|g__Bacteroides|s__Bacteroides_thetaio
k__Bacteria|p__Firmicutes|c__Clostridia|o__Clostridia_unclassified|f__Clostridia_unclassified|g__Clostridia_unclassified|s_
k__Bacteria|p__Firmicutes|c__Clostridia|o__Eubacteriales|f__Clostridiaceae|g__Clostridiaceae_unclassified|s__Clostridiac
k__Bacteria|p__Firmicutes|c__Clostridia|o__Eubacteriales|f__Clostridiaceae|g__Clostridiaceae_unclassified|s__Clostridiac
k__Bacteria|p__Firmicutes|c__Clostridia|o__Eubacteriales|f__Eubacteriales_unclassified|g__Eubacteriales_unclassified|s_
k__Bacteria|p__Firmicutes|c__Clostridia|o__Eubacteriales|f__Clostridiaceae|g__Clostridium|s__Clostridium_SGB65123
k__Bacteria|p__Firmicutes|c__Erysipelotrichia|o__Erysipelotrichales|f__Erysipelotrichaceae|g__Erysipelatoclostridium|s__
k__Bacteria|p__Firmicutes|c__Clostridia|o__Eubacteriales|f__Eubacteriaceae|g__Eubacteriaceae_unclassified|s__Eubacte
k__Bacteria|p__Firmicutes|c__Clostridia|o__Eubacteriales|f__Eubacteriaceae|g__Eubacteriaceae_unclassified|s__Eubacte
k__Bacteria|p__Firmicutes|c__CFGB77306|o__OFGB77306|f__FGB77306|g__GGB20146|s__GGB20146_SGB29427
k__Bacteria|p__Firmicutes|c__Clostridia|o__Eubacteriales|f__Lachnospiraceae|g__GGB20149|s__GGB20149_SGB29430
k__Bacteria|p__Actinobacteria|c__Coriobacteriia|o__Eggerthellales|f__Eggerthellaceae|g__GGB22635|s__GGB22635_SGB
k__Bacteria|p__Firmicutes|c__Clostridia|o__Eubacteriales|f__Lachnospiraceae|g__GGB25041|s__GGB25041_SGB36960
k__Bacteria|p__Firmicutes|c__CFGB9506|o__OFGB9506|f__FGB9506|g__GGB28379|s__GGB28379_SGB40959
k__Bacteria|p__Firmicutes|c__CFGB9508|o__OFGB9508|f__FGB9508|g__GGB28382|s__GGB28382_SGB40962
k__Bacteria|p__Firmicutes|c__CFGB9512|o__OFGB9512|f__FGB9512|g__GGB28392|s__GGB28392_SGB40972
k__Bacteria|p__Firmicutes|c__CFGB2838|o__OFGB2838|f__FGB2838|g__GGB28404|s__GGB28404_SGB40986
k__Bacteria|p__Firmicutes|c__CFGB2838|o__OFGB2838|f__FGB2838|g__GGB28418|s__GGB28418_SGB41001

k__Bacteria|p__Firmicutes|c__CFGB2838|o__OFGB2838|f__FGB2838|g__GGB28422|s__GGB28422_SGB41005
k__Bacteria|p__Firmicutes|c__Clostridia|o__Eubacteriales|f__Pumilibacteraceae|g__GGB28431|s__GGB28431_SGB41014
k__Bacteria|p__Firmicutes|c__CFGB2833|o__OFGB2833|f__FGB2833|g__GGB28456|s__GGB28456_SGB41039
k__Bacteria|p__Firmicutes|c__Clostridia|o__Eubacteriales|f__Eubacteriaceae|g__GGB28782|s__GGB28782_SGB41435
k__Bacteria|p__Firmicutes|c__Clostridia|o__Eubacteriales|f__Lachnospiraceae|g__GGB28792|s__GGB28792_SGB41445
k__Bacteria|p__Firmicutes|c__Clostridia|o__Eubacteriales|f__Lachnospiraceae|g__GGB28798|s__GGB28798_SGB41451
k__Bacteria|p__Firmicutes|c__CFGB9622|o__OFGB9622|f__FGB9622|g__GGB28810|s__GGB28810_SGB41465
k__Bacteria|p__Firmicutes|c__CFGB77305|o__OFGB77305|f__FGB77305|g__GGB28828|s__GGB28828_SGB41484
k__Bacteria|p__Firmicutes|c__Clostridia|o__Eubacteriales|f__Clostridiaceae|g__GGB28851|s__GGB28851_SGB41518
k__Bacteria|p__Firmicutes|c__Clostridia|o__Eubacteriales|f__Lachnospiraceae|g__GGB28865|s__GGB28865_SGB41537
k__Bacteria|p__Firmicutes|c__Clostridia|o__Eubacteriales|f__Lachnospiraceae|g__GGB28868|s__GGB28868_SGB41542
k__Bacteria|p__Firmicutes|c__Clostridia|o__Eubacteriales|f__Lachnospiraceae|g__GGB28869|s__GGB28869_SGB41543
k__Bacteria|p__Firmicutes|c__Clostridia|o__Eubacteriales|f__Lachnospiraceae|g__GGB28875|s__GGB28875_SGB41555
k__Bacteria|p__Firmicutes|c__CFGB9633|o__OFGB9633|f__FGB9633|g__GGB28881|s__GGB28881_SGB41561
k__Bacteria|p__Bacteria_unclassified|c__Bacteria_unclassified|o__Bacteria_unclassified|f__Bacteria_unclassified|g__GGB[illegible]
k__Bacteria|p__Bacteria_unclassified|c__Bacteria_unclassified|o__Bacteria_unclassified|f__Bacteria_unclassified|g__GGB[illegible]
k__Bacteria|p__Bacteria_unclassified|c__Bacteria_unclassified|o__Bacteria_unclassified|f__Bacteria_unclassified|g__GGB[illegible]
k__Bacteria|p__Bacteria_unclassified|c__Bacteria_unclassified|o__Bacteria_unclassified|f__Bacteria_unclassified|g__GGB[illegible]
k__Bacteria|p__Bacteria_unclassified|c__Bacteria_unclassified|o__Bacteria_unclassified|f__Bacteria_unclassified|g__GGB[illegible]
k__Bacteria|p__Bacteria_unclassified|c__Bacteria_unclassified|o__Bacteria_unclassified|f__Bacteria_unclassified|g__GGB[illegible]
k__Bacteria|p__Firmicutes|c__Clostridia|o__Eubacteriales|f__Lachnospiraceae|g__GGB28924|s__GGB28924_SGB41621
k__Bacteria|p__Bacteria_unclassified|c__CFGB77359|o__OFGB77359|f__FGB77359|g__GGB28927|s__GGB28927_SGB416[illegible]
k__Bacteria|p__Firmicutes|c__CFGB9639|o__OFGB9639|f__FGB9639|g__GGB28934|s__GGB28934_SGB41635
k__Bacteria|p__Firmicutes|c__Clostridia|o__Eubacteriales|f__Lachnospiraceae|g__GGB28949|s__GGB28949_SGB41655
k__Bacteria|p__Firmicutes|c__Clostridia|o__Eubacteriales|f__Clostridiaceae|g__GGB28951|s__GGB28951_SGB102295
k__Bacteria|p__Firmicutes|c__Clostridia|o__Eubacteriales|f__Clostridiaceae|g__GGB28951|s__GGB28951_SGB41658
k__Bacteria|p__Firmicutes|c__Clostridia|o__Eubacteriales|f__Clostridiaceae|g__GGB28954|s__GGB28954_SGB41662
k__Bacteria|p__Firmicutes|c__Clostridia|o__Eubacteriales|f__Clostridiaceae|g__GGB28960|s__GGB28960_SGB41669
k__Bacteria|p__Firmicutes|c__Clostridia|o__Eubacteriales|f__Clostridiaceae|g__GGB28964|s__GGB28964_SGB94886
k__Bacteria|p__Firmicutes|c__Clostridia|o__Eubacteriales|f__Clostridiaceae|g__GGB28967|s__GGB28967_SGB41678
k__Bacteria|p__Firmicutes|c__CFGB9656|o__OFGB9656|f__FGB9656|g__GGB28996|s__GGB28996_SGB41712
k__Bacteria|p__Firmicutes|c__CFGB9827|o__OFGB9827|f__FGB9827|g__GGB29531|s__GGB29531_SGB42317
k__Bacteria|p__Firmicutes|c__Clostridia|o__Eubacteriales|f__Eubacteriaceae|g__GGB29685|s__GGB29685_SGB42494
k__Bacteria|p__Bacteria_unclassified|c__CFGB77303|o__OFGB77303|f__FGB77303|g__GGB30141|s__GGB30141_SGB430[illegible]
k__Bacteria|p__Firmicutes|c__Clostridia|o__Eubacteriales|f__Clostridiaceae|g__GGB30145|s__GGB30145_SGB43072
k__Bacteria|p__Firmicutes|c__Clostridia|o__Eubacteriales|f__Eubacteriales_unclassified|g__GGB30286|s__GGB30286_SG[illegible]
k__Bacteria|p__Firmicutes|c__CFGB72709|o__OFGB72709|f__FGB72709|g__GGB30300|s__GGB30300_SGB43264
k__Bacteria|p__Firmicutes|c__Clostridia|o__Eubacteriales|f__Oscillospiraceae|g__GGB30303|s__GGB30303_SGB43268
k__Bacteria|p__Firmicutes|c__Clostridia|o__Eubacteriales|f__Oscillospiraceae|g__GGB30450|s__GGB30450_SGB43507
k__Bacteria|p__Firmicutes|c__Clostridia|o__Eubacteriales|f__Oscillospiraceae|g__GGB30453|s__GGB30453_SGB43513
k__Bacteria|p__Firmicutes|c__Clostridia|o__Eubacteriales|f__Oscillospiraceae|g__GGB30454|s__GGB30454_SGB43514
k__Bacteria|p__Firmicutes|c__Clostridia|o__Eubacteriales|f__Oscillospiraceae|g__GGB30455|s__GGB30455_SGB43519
k__Bacteria|p__Firmicutes|c__Clostridia|o__Eubacteriales|f__Oscillospiraceae|g__GGB30456|s__GGB30456_SGB43520
k__Bacteria|p__Firmicutes|c__Clostridia|o__Eubacteriales|f__Oscillospiraceae|g__GGB30457|s__GGB30457_SGB63218
k__Bacteria|p__Firmicutes|c__Clostridia|o__Eubacteriales|f__Oscillospiraceae|g__GGB30461|s__GGB30461_SGB43530
k__Bacteria|p__Firmicutes|c__Clostridia|o__Eubacteriales|f__Oscillospiraceae|g__GGB30461|s__GGB30461_SGB43533

k__Bacteria|p__Firmicutes|c__Clostridia|o__Eubacteriales|f__Oscillospiraceae|g__GGB30461|s__GGB30461_SGB63209
k__Bacteria|p__Firmicutes|c__Clostridia|o__Eubacteriales|f__Oscillospiraceae|g__GGB30463|s__GGB30463_SGB43537
k__Bacteria|p__Firmicutes|c__Clostridia|o__Eubacteriales|f__Oscillospiraceae|g__GGB30473|s__GGB30473_SGB43557
k__Bacteria|p__Firmicutes|c__Clostridia|o__Eubacteriales|f__Oscillospiraceae|g__GGB30475|s__GGB30475_SGB63182
k__Bacteria|p__Actinobacteria|c__CFGB77153|o__OFGB77153|f__FGB77153|g__GGB30861|s__GGB30861_SGB44083
k__Bacteria|p__Tenericutes|c__CFGB1791|o__OFGB1791|f__FGB1791|g__GGB31312|s__GGB31312_SGB44628
k__Bacteria|p__Firmicutes|c__CFGB10290|o__OFGB10290|f__FGB10290|g__GGB31438|s__GGB31438_SGB44768
k__Bacteria|p__Firmicutes|c__Clostridia|o__Eubacteriales|f__Oscillospiraceae|g__GGB3171|s__GGB3171_SGB4185
k__Bacteria|p__Firmicutes|c__CFGB10289|o__OFGB10289|f__FGB10289|g__GGB31762|s__GGB31762_SGB45125
k__Bacteria|p__Firmicutes|c__CFGB1765|o__OFGB1765|f__FGB1765|g__GGB31823|s__GGB31823_SGB45199
k__Bacteria|p__Firmicutes|c__CFGB1765|o__OFGB1765|f__FGB1765|g__GGB31838|s__GGB31838_SGB45216
k__Bacteria|p__Firmicutes|c__CFGB1765|o__OFGB1765|f__FGB1765|g__GGB31841|s__GGB31841_SGB65084
k__Bacteria|p__Firmicutes|c__CFGB10667|o__OFGB10667|f__FGB10667|g__GGB32371|s__GGB32371_SGB41694
k__Bacteria|p__Firmicutes|c__Clostridia|o__Eubacteriales|f__Lachnospiraceae|g__GGB3793|s__GGB3793_SGB5158
k__Bacteria|p__Firmicutes|c__Clostridia|o__Eubacteriales|f__Clostridiaceae|g__GGB42601|s__GGB42601_SGB59797
k__Bacteria|p__Firmicutes|c__Clostridia|o__Eubacteriales|f__Oscillospiraceae|g__GGB45514|s__GGB45514_SGB63186
k__Bacteria|p__Firmicutes|c__CFGB75721|o__OFGB75721|f__FGB75721|g__GGB45564|s__GGB45564_SGB63259
k__Bacteria|p__Firmicutes|c__Clostridia|o__Eubacteriales|f__Oscillospiraceae|g__GGB45624|s__GGB45624_SGB63337
k__Bacteria|p__Firmicutes|c__Clostridia|o__Eubacteriales|f__Christensenellaceae|g__GGB45656|s__GGB45656_SGB6337
k__Bacteria|p__Firmicutes|c__CFGB10299|o__OFGB10299|f__FGB10299|g__GGB47127|s__GGB47127_SGB65054
k__Bacteria|p__Firmicutes|c__Clostridia|o__Eubacteriales|f__Oscillospiraceae|g__GGB74395|s__GGB74395_SGB43523
k__Bacteria|p__Firmicutes|c__Clostridia|o__Eubacteriales|f__Oscillospiraceae|g__GGB75053|s__GGB75053_SGB43494
k__Bacteria|p__Firmicutes|c__Clostridia|o__Eubacteriales|f__Lachnospiraceae|g__GGB75109|s__GGB75109_SGB102238
k__Bacteria|p__Firmicutes|c__Clostridia|o__Eubacteriales|f__Lachnospiraceae|g__Lachnospiraceae_unclassified|s__Lachn
k__Bacteria|p__Firmicutes|c__Clostridia|o__Eubacteriales|f__Lachnospiraceae|g__Lachnospiraceae_unclassified|s__Lachn
k__Bacteria|p__Firmicutes|c__Clostridia|o__Eubacteriales|f__Lachnospiraceae|g__Lachnospiraceae_unclassified|s__Lachn
k__Bacteria|p__Firmicutes|c__Clostridia|o__Eubacteriales|f__Lachnospiraceae|g__Lachnospiraceae_unclassified|s__Lachn
k__Bacteria|p__Firmicutes|c__Clostridia|o__Eubacteriales|f__Lachnospiraceae|g__Lachnospiraceae_unclassified|s__Lachn
k__Bacteria|p__Firmicutes|c__Bacilli|o__Lactobacillales|f__Lactobacillaceae|g__Lactobacillus|s__Lactobacillus_johnsonii
k__Bacteria|p__Actinobacteria|c__Coriobacteriia|o__Coriobacteriales|f__Atopobiaceae|g__Leptogranulimonas|s__Leptog
k__Bacteria|p__Bacteroidota|c__Bacteroidia|o__Bacteroidales|f__Muribaculaceae|g__Muribaculaceae_unclassified|s__M
k__Bacteria|p__Firmicutes|c__Clostridia|o__Eubacteriales|f__Oscillospiraceae|g__Neglectibacter|s__Neglectibacter_sp_X
k__Bacteria|p__Firmicutes|c__Clostridia|o__Eubacteriales|f__Oscillospiraceae|g__Oscillibacter|s__Oscillibacter_SGB43496
k__Bacteria|p__Firmicutes|c__Clostridia|o__Eubacteriales|f__Oscillospiraceae|g__Oscillospiraceae_unclassified|s__Oscillo
k__Bacteria|p__Firmicutes|c__Clostridia|o__Eubacteriales|f__Oscillospiraceae|g__Oscillospiraceae_unclassified|s__Oscillo
k__Bacteria|p__Firmicutes|c__Clostridia|o__Eubacteriales|f__Oscillospiraceae|g__Oscillospiraceae_unclassified|s__Oscillo
k__Bacteria|p__Firmicutes|c__Clostridia|o__Eubacteriales|f__Oscillospiraceae|g__Oscillospiraceae_unclassified|s__Oscillo
k__Bacteria|p__Proteobacteria|c__Betaproteobacteria|o__Burkholderiales|f__Sutterellaceae|g__Parasutterella|s__Parasut
k__Bacteria|p__Firmicutes|c__Clostridia|o__Eubacteriales|f__Lachnospiraceae|g__Schaedlerella|s__Schaedlerella_arabinc
k__Bacteria|p__Firmicutes|c__Erysipelotrichia|o__Erysipelotrichales|f__Turicibacteraceae|g__Turicibacter|s__Turicibacter_
k__Bacteria|p__Bacteria_unclassified|c__Bacteria_unclassified|o__Bacteria_unclassified|f__Bacteria_unclassified|g__Bacte
k__Bacteria|p__Bacteria_unclassified|c__Bacteria_unclassified|o__Bacteria_unclassified|f__Bacteria_unclassified|g__Bacte
k__Bacteria|p__Bacteria_unclassified|c__Bacteria_unclassified|o__Bacteria_unclassified|f__Bacteria_unclassified|g__Bacte
k__Bacteria|p__Bacteria_unclassified|c__Bacteria_unclassified|o__Bacteria_unclassified|f__Bacteria_unclassified|g__Bacte
k__Bacteria|p__Bacteria_unclassified|c__Bacteria_unclassified|o__Bacteria_unclassified|f__Bacteria_unclassified|g__Bacte
