# Supplementary material for: Non-invasive Vagal Nerve Stimulation as a Potential Treatment for Repetitive Blast Trauma
Source: bioRxiv. 2026 Jul 19:2026.07.13.737563. Preprint. [Version 1] doi: 10.64898/2026.07.13.737563 (PMC13405043; doi:10.64898/2026.07.13.737563)
Supplement: Supplement 13 [file media-13.pdf]

Exposure	Mediator (Species-level feature)	Outcome	p_m	p_y
blast	Acetatifactor_SGB41545	acute_cyto_PC1	0.210350900	0.426337589
vns	Acetatifactor_SGB41545	acute_cyto_PC1	0.239098669	0.426337589
blast	Acutalibacter_muris	acute_cyto_PC1	0.786984338	0.577795707
vns	Acutalibacter_muris	acute_cyto_PC1	0.491902226	0.577795707
blast	Akkermansia_muciniphila	acute_cyto_PC1	0.16660254	0.601919414
vns	Akkermansia_muciniphila	acute_cyto_PC1	0.678899621	0.601919414
blast	Anaerotruncus_sp_1XD42_93	acute_cyto_PC1	0.000917537	0.917104107
vns	Anaerotruncus_sp_1XD42_93	acute_cyto_PC1	0.000231687	0.917104107
blast	Bacteria_unclassified_SGB41677	acute_cyto_PC1	0.714189493	0.976353560
vns	Bacteria_unclassified_SGB41677	acute_cyto_PC1	0.58777865	0.976353560
blast	Bacteria_unclassified_SGB43539	acute_cyto_PC1	0.148902499	0.218978051
vns	Bacteria_unclassified_SGB43539	acute_cyto_PC1	0.449260123	0.218978051
blast	Bacteria_unclassified_SGB43546	acute_cyto_PC1	0.112551908	0.605851927
vns	Bacteria_unclassified_SGB43546	acute_cyto_PC1	0.309908808	0.605851927
blast	Bacteria_unclassified_SGB63211	acute_cyto_PC1	0.295003775	0.053264016
vns	Bacteria_unclassified_SGB63211	acute_cyto_PC1	0.340871629	0.053264016
blast	bacterium_0_1xD8_82	acute_cyto_PC1	0.763436393	0.662492714
vns	bacterium_0_1xD8_82	acute_cyto_PC1	0.795139093	0.662492714
blast	bacterium_1XD42_1	acute_cyto_PC1	0.272519397	0.842884819
vns	bacterium_1XD42_1	acute_cyto_PC1	0.097091925	0.842884819
blast	bacterium_1XD42_54	acute_cyto_PC1	0.038105321	0.843690241
vns	bacterium_1XD42_54	acute_cyto_PC1	0.125406933	0.843690241
blast	bacterium_1XD42_76	acute_cyto_PC1	0.177410768	0.004423798
vns	bacterium_1XD42_76	acute_cyto_PC1	0.141032802	0.004423798
blast	bacterium_1xD8_48	acute_cyto_PC1	0.139347611	0.631298404
vns	bacterium_1xD8_48	acute_cyto_PC1	0.370020407	0.631298404
blast	Bacteroides_thetaiotaomicron	acute_cyto_PC1	0.036627754	0.615935149
vns	Bacteroides_thetaiotaomicron	acute_cyto_PC1	0.275405005	0.615935149
blast	Berger Parker Index	acute_cyto_PC1	0.057475283	0.585016437
vns	Berger Parker Index	acute_cyto_PC1	0.499505955	0.585016437
blast	Clostridia_bacterium	acute_cyto_PC1	0.248353087	0.582229397
vns	Clostridia_bacterium	acute_cyto_PC1	0.380556079	0.582229397
blast	Clostridiaceae_bacterium	acute_cyto_PC1	0.162346605	0.662202407
vns	Clostridiaceae_bacterium	acute_cyto_PC1	0.277071084	0.662202407
blast	Clostridiaceae_unclassified_SGB41663	acute_cyto_PC1	0.362409871	0.163252193
vns	Clostridiaceae_unclassified_SGB41663	acute_cyto_PC1	0.070917324	0.163252193
blast	Clostridiales_bacterium	acute_cyto_PC1	0.073824740	0.557466510
vns	Clostridiales_bacterium	acute_cyto_PC1	0.124851167	0.557466510
blast	Clostridium_cocleatum	acute_cyto_PC1	0.507275085	0.724732489
vns	Clostridium_cocleatum	acute_cyto_PC1	0.482732384	0.724732489
blast	Clostridium_SGB65123	acute_cyto_PC1	0.180438982	0.006647678
vns	Clostridium_SGB65123	acute_cyto_PC1	0.179389568	0.006647678
blast	Eubacteriaceae_bacterium	acute_cyto_PC1	0.016604062	0.856163150
vns	Eubacteriaceae_bacterium	acute_cyto_PC1	0.969157578	0.856163150
blast	Eubacteriaceae_unclassified_SGB94922	acute_cyto_PC1	0.174224276	0.913635804
vns	Eubacteriaceae_unclassified_SGB94922	acute_cyto_PC1	0.346181024	0.913635804

blast	GGB20146_SGB29427	acute_cyto_PC1	0.093844467	0.763691992
vns	GGB20146_SGB29427	acute_cyto_PC1	0.051218919	0.763691992
blast	GGB20149_SGB29430	acute_cyto_PC1	0.413225925	0.660065724
vns	GGB20149_SGB29430	acute_cyto_PC1	0.180434838	0.660065724
blast	GGB22635_SGB63107	acute_cyto_PC1	0.114660903	0.716725038
vns	GGB22635_SGB63107	acute_cyto_PC1	0.535639339	0.716725038
blast	GGB25041_SGB36960	acute_cyto_PC1	0.167595117	0.828254777
vns	GGB25041_SGB36960	acute_cyto_PC1	0.206256260	0.828254777
blast	GGB28379_SGB40959	acute_cyto_PC1	0.029241836	0.041086906
vns	GGB28379_SGB40959	acute_cyto_PC1	0.032794656	0.041086906
blast	GGB28382_SGB40962	acute_cyto_PC1	0.072513022	0.130664362
vns	GGB28382_SGB40962	acute_cyto_PC1	0.370430736	0.130664362
blast	GGB28392_SGB40972	acute_cyto_PC1	0.017183669	0.126470180
vns	GGB28392_SGB40972	acute_cyto_PC1	0.016592400	0.126470180
blast	GGB28404_SGB40986	acute_cyto_PC1	0.157767928	0.737359301
vns	GGB28404_SGB40986	acute_cyto_PC1	0.010090214	0.737359301
blast	GGB28418_SGB41001	acute_cyto_PC1	0.996265407	0.972475228
vns	GGB28418_SGB41001	acute_cyto_PC1	0.959497945	0.972475228
blast	GGB28422_SGB41005	acute_cyto_PC1	0.298094755	0.680377399
vns	GGB28422_SGB41005	acute_cyto_PC1	0.416676405	0.680377399
blast	GGB28431_SGB41014	acute_cyto_PC1	0.015377584	0.872708849
vns	GGB28431_SGB41014	acute_cyto_PC1	0.016801247	0.872708849
blast	GGB28456_SGB41039	acute_cyto_PC1	0.508621549	0.257888936
vns	GGB28456_SGB41039	acute_cyto_PC1	0.567597000	0.257888936
blast	GGB28782_SGB41435	acute_cyto_PC1	0.315491599	0.448148480
vns	GGB28782_SGB41435	acute_cyto_PC1	0.359169919	0.448148480
blast	GGB28792_SGB41445	acute_cyto_PC1	0.123770726	0.733698658
vns	GGB28792_SGB41445	acute_cyto_PC1	0.970858107	0.733698658
blast	GGB28798_SGB41451	acute_cyto_PC1	0.931837305	0.642653259
vns	GGB28798_SGB41451	acute_cyto_PC1	0.471552545	0.642653259
blast	GGB28810_SGB41465	acute_cyto_PC1	0.919521792	0.960691488
vns	GGB28810_SGB41465	acute_cyto_PC1	0.915105920	0.960691488
blast	GGB28828_SGB41484	acute_cyto_PC1	0.036453176	0.134989453
vns	GGB28828_SGB41484	acute_cyto_PC1	0.029615316	0.134989453
blast	GGB28851_SGB41518	acute_cyto_PC1	0.983245267	0.362512815
vns	GGB28851_SGB41518	acute_cyto_PC1	0.548244701	0.362512815
blast	GGB28865_SGB41537	acute_cyto_PC1	0.051838259	0.050385604
vns	GGB28865_SGB41537	acute_cyto_PC1	0.043832016	0.050385604
blast	GGB28868_SGB41542	acute_cyto_PC1	0.292423183	0.735404707
vns	GGB28868_SGB41542	acute_cyto_PC1	0.254323647	0.735404707
blast	GGB28869_SGB41543	acute_cyto_PC1	0.662073077	0.312370945
vns	GGB28869_SGB41543	acute_cyto_PC1	0.806606148	0.312370945
blast	GGB28875_SGB41555	acute_cyto_PC1	0.776644062	0.959080274
vns	GGB28875_SGB41555	acute_cyto_PC1	0.634070741	0.959080274
blast	GGB28881_SGB41561	acute_cyto_PC1	0.011894548	0.526788453
vns	GGB28881_SGB41561	acute_cyto_PC1	0.001988037	0.526788453

blast	GGB28892_SGB41573	acute_cyto_PC1	0.715153028	0.303956874
vns	GGB28892_SGB41573	acute_cyto_PC1	0.784709483	0.303956874
blast	GGB28893_SGB41574	acute_cyto_PC1	0.739221992	0.038137092
vns	GGB28893_SGB41574	acute_cyto_PC1	0.264245168	0.038137092
blast	GGB28898_SGB41580	acute_cyto_PC1	0.601924700	0.669756145
vns	GGB28898_SGB41580	acute_cyto_PC1	0.591718713	0.669756145
blast	GGB28901_SGB41592	acute_cyto_PC1	0.072063586	0.152650290
vns	GGB28901_SGB41592	acute_cyto_PC1	0.152665868	0.152650290
blast	GGB28904_SGB41597	acute_cyto_PC1	0.225752520	0.207680230
vns	GGB28904_SGB41597	acute_cyto_PC1	0.085658495	0.207680230
blast	GGB28909_SGB41602	acute_cyto_PC1	0.218283007	0.026531122
vns	GGB28909_SGB41602	acute_cyto_PC1	0.145902665	0.026531122
blast	GGB28924_SGB41621	acute_cyto_PC1	0.611578184	0.052540796
vns	GGB28924_SGB41621	acute_cyto_PC1	0.456981189	0.052540796
blast	GGB28927_SGB41625	acute_cyto_PC1	0.001954483	0.366201366
vns	GGB28927_SGB41625	acute_cyto_PC1	0.000219425	0.366201366
blast	GGB28934_SGB41635	acute_cyto_PC1	0.216498853	0.554318985
vns	GGB28934_SGB41635	acute_cyto_PC1	0.224102539	0.554318985
blast	GGB28949_SGB41655	acute_cyto_PC1	0.159598366	0.316394060
vns	GGB28949_SGB41655	acute_cyto_PC1	0.744810754	0.316394060
blast	GGB28951_SGB102295	acute_cyto_PC1	0.718725508	0.560708780
vns	GGB28951_SGB102295	acute_cyto_PC1	0.298970783	0.560708780
blast	GGB28951_SGB41658	acute_cyto_PC1	0.752627843	0.252343162
vns	GGB28951_SGB41658	acute_cyto_PC1	0.024801176	0.252343162
blast	GGB28954_SGB41662	acute_cyto_PC1	0.794939032	0.791398899
vns	GGB28954_SGB41662	acute_cyto_PC1	0.841186745	0.791398899
blast	GGB28960_SGB41669	acute_cyto_PC1	0.536638861	0.246718454
vns	GGB28960_SGB41669	acute_cyto_PC1	0.043638773	0.246718454
blast	GGB28964_SGB94886	acute_cyto_PC1	0.207213028	0.806789981
vns	GGB28964_SGB94886	acute_cyto_PC1	0.400370147	0.806789981
blast	GGB28967_SGB41678	acute_cyto_PC1	0.848205066	0.636882447
vns	GGB28967_SGB41678	acute_cyto_PC1	0.820661965	0.636882447
blast	GGB28996_SGB41712	acute_cyto_PC1	0.429884728	0.747558755
vns	GGB28996_SGB41712	acute_cyto_PC1	0.377449394	0.747558755
blast	GGB29531_SGB42317	acute_cyto_PC1	0.279593444	0.691936238
vns	GGB29531_SGB42317	acute_cyto_PC1	0.999007775	0.691936238
blast	GGB29685_SGB42494	acute_cyto_PC1	0.004596881	0.833350762
vns	GGB29685_SGB42494	acute_cyto_PC1	0.748416768	0.833350762
blast	GGB30141_SGB43066	acute_cyto_PC1	0.614028140	0.448906709
vns	GGB30141_SGB43066	acute_cyto_PC1	0.664026053	0.448906709
blast	GGB30145_SGB43072	acute_cyto_PC1	0.294924035	0.135611576
vns	GGB30145_SGB43072	acute_cyto_PC1	0.367951830	0.135611576
blast	GGB30286_SGB43248	acute_cyto_PC1	0.766757581	0.444639598
vns	GGB30286_SGB43248	acute_cyto_PC1	0.750425732	0.444639598
blast	GGB30300_SGB43264	acute_cyto_PC1	0.213846948	0.217988041
vns	GGB30300_SGB43264	acute_cyto_PC1	0.213511417	0.217988041

blast	GGB30303_SGB43268	acute_cyto_PC1	0.795572751	0.407203128
vns	GGB30303_SGB43268	acute_cyto_PC1	0.660099923	0.407203128
blast	GGB30450_SGB43507	acute_cyto_PC1	0.027109400	0.433933077
vns	GGB30450_SGB43507	acute_cyto_PC1	0.186573367	0.433933077
blast	GGB30453_SGB43513	acute_cyto_PC1	0.315725451	0.896408534
vns	GGB30453_SGB43513	acute_cyto_PC1	0.310638137	0.896408534
blast	GGB30454_SGB43514	acute_cyto_PC1	0.642888646	0.892398402
vns	GGB30454_SGB43514	acute_cyto_PC1	0.656930027	0.892398402
blast	GGB30455_SGB43519	acute_cyto_PC1	0.663798426	0.628753919
vns	GGB30455_SGB43519	acute_cyto_PC1	0.104495810	0.628753919
blast	GGB30456_SGB43520	acute_cyto_PC1	0.111196948	0.400916417
vns	GGB30456_SGB43520	acute_cyto_PC1	0.158921661	0.400916417
blast	GGB30457_SGB63218	acute_cyto_PC1	0.882164050	0.655942116
vns	GGB30457_SGB63218	acute_cyto_PC1	0.890757036	0.655942116
blast	GGB30461_SGB43530	acute_cyto_PC1	0.602468303	0.586411049
vns	GGB30461_SGB43530	acute_cyto_PC1	0.137401205	0.586411049
blast	GGB30461_SGB43533	acute_cyto_PC1	0.810767423	0.849446904
vns	GGB30461_SGB43533	acute_cyto_PC1	0.718693859	0.849446904
blast	GGB30461_SGB63209	acute_cyto_PC1	0.005914118	0.268399194
vns	GGB30461_SGB63209	acute_cyto_PC1	0.075178424	0.268399194
blast	GGB30463_SGB43537	acute_cyto_PC1	0.040768110	0.982453190
vns	GGB30463_SGB43537	acute_cyto_PC1	0.991960140	0.982453190
blast	GGB30473_SGB43557	acute_cyto_PC1	0.328491579	0.952610176
vns	GGB30473_SGB43557	acute_cyto_PC1	0.189853765	0.952610176
blast	GGB30475_SGB63182	acute_cyto_PC1	0.811833041	0.491675051
vns	GGB30475_SGB63182	acute_cyto_PC1	0.783129024	0.491675051
blast	GGB30861_SGB44083	acute_cyto_PC1	0.719401647	0.082238497
vns	GGB30861_SGB44083	acute_cyto_PC1	0.981045510	0.082238497
blast	GGB31312_SGB44628	acute_cyto_PC1	0.831039140	0.602112987
vns	GGB31312_SGB44628	acute_cyto_PC1	0.833457038	0.602112987
blast	GGB31438_SGB44768	acute_cyto_PC1	0.481213900	0.002320358
vns	GGB31438_SGB44768	acute_cyto_PC1	0.483694757	0.002320358
blast	GGB3171_SGB4185	acute_cyto_PC1	0.066342246	0.137077443
vns	GGB3171_SGB4185	acute_cyto_PC1	0.057850794	0.137077443
blast	GGB31762_SGB45125	acute_cyto_PC1	0.442889056	0.711983107
vns	GGB31762_SGB45125	acute_cyto_PC1	0.437364142	0.711983107
blast	GGB31823_SGB45199	acute_cyto_PC1	0.630175203	0.668856506
vns	GGB31823_SGB45199	acute_cyto_PC1	0.537240753	0.668856506
blast	GGB31838_SGB45216	acute_cyto_PC1	0.684403164	0.902822678
vns	GGB31838_SGB45216	acute_cyto_PC1	0.997715556	0.902822678
blast	GGB31841_SGB65084	acute_cyto_PC1	0.371737454	0.058872180
vns	GGB31841_SGB65084	acute_cyto_PC1	0.443951934	0.058872180
blast	GGB32371_SGB41694	acute_cyto_PC1	0.242018055	0.819005612
vns	GGB32371_SGB41694	acute_cyto_PC1	0.400159574	0.819005612
blast	GGB3793_SGB5158	acute_cyto_PC1	0.884993491	0.635858183
vns	GGB3793_SGB5158	acute_cyto_PC1	0.847239709	0.635858183

blast	GGB42601_SGB59797	acute_cyto_PC1	0.401892013	0.385232417
vns	GGB42601_SGB59797	acute_cyto_PC1	0.627566595	0.385232417
blast	GGB45514_SGB63186	acute_cyto_PC1	0.955364765	0.627995319
vns	GGB45514_SGB63186	acute_cyto_PC1	0.621527882	0.627995319
blast	GGB45564_SGB63259	acute_cyto_PC1	0.415272784	0.945051832
vns	GGB45564_SGB63259	acute_cyto_PC1	0.380878696	0.945051832
blast	GGB45624_SGB63337	acute_cyto_PC1	0.620389272	0.110600810
vns	GGB45624_SGB63337	acute_cyto_PC1	0.385198794	0.110600810
blast	GGB45656_SGB63370	acute_cyto_PC1	0.404705483	0.347825888
vns	GGB45656_SGB63370	acute_cyto_PC1	0.163643553	0.347825888
blast	GGB47127_SGB65054	acute_cyto_PC1	0.456493679	0.587804796
vns	GGB47127_SGB65054	acute_cyto_PC1	0.82583129	0.587804796
blast	GGB74395_SGB43523	acute_cyto_PC1	0.802410438	0.073253778
vns	GGB74395_SGB43523	acute_cyto_PC1	0.835585633	0.073253778
blast	GGB75053_SGB43494	acute_cyto_PC1	0.177327401	0.345232739
vns	GGB75053_SGB43494	acute_cyto_PC1	0.050410443	0.345232739
blast	GGB75109_SGB102238	acute_cyto_PC1	0.599792986	0.123907098
vns	GGB75109_SGB102238	acute_cyto_PC1	0.510864481	0.123907098
blast	Lachnospiraceae_bacterium	acute_cyto_PC1	0.023925903	0.786270566
vns	Lachnospiraceae_bacterium	acute_cyto_PC1	0.917492265	0.786270566
blast	Lachnospiraceae_bacterium_A2	acute_cyto_PC1	0.724187807	0.634220791
vns	Lachnospiraceae_bacterium_A2	acute_cyto_PC1	0.083860739	0.634220791
blast	Lachnospiraceae_unclassified_SGB41414	acute_cyto_PC1	0.719079500	0.569438429
vns	Lachnospiraceae_unclassified_SGB41414	acute_cyto_PC1	0.532128198	0.569438429
blast	Lachnospiraceae_unclassified_SGB41424	acute_cyto_PC1	0.216930413	0.028198918
vns	Lachnospiraceae_unclassified_SGB41424	acute_cyto_PC1	0.022018829	0.028198918
blast	Lachnospiraceae_unclassified_SGB94868	acute_cyto_PC1	0.207633504	0.978075079
vns	Lachnospiraceae_unclassified_SGB94868	acute_cyto_PC1	0.754562933	0.978075079
blast	Lactobacillus_johnsonii	acute_cyto_PC1	0.002748365	0.565891021
vns	Lactobacillus_johnsonii	acute_cyto_PC1	0.832216242	0.565891021
blast	Leptogranulimonas_caecicola	acute_cyto_PC1	0.874577980	0.142670339
vns	Leptogranulimonas_caecicola	acute_cyto_PC1	0.014475087	0.142670339
blast	Muribaculaceae_bacterium	acute_cyto_PC1	0.001850198	0.99399338
vns	Muribaculaceae_bacterium	acute_cyto_PC1	0.883532506	0.99399338
blast	Neglectibacter_sp_X4	acute_cyto_PC1	0.394423067	0.530400373
vns	Neglectibacter_sp_X4	acute_cyto_PC1	0.463430059	0.530400373
blast	Oscillibacter_SGB43496	acute_cyto_PC1	0.220544725	0.095702017
vns	Oscillibacter_SGB43496	acute_cyto_PC1	0.34795292	0.095702017
blast	Oscillospiraceae_bacterium	acute_cyto_PC1	0.492783661	0.378407448
vns	Oscillospiraceae_bacterium	acute_cyto_PC1	0.251973413	0.378407448
blast	Oscillospiraceae_unclassified_SGB43502	acute_cyto_PC1	0.017271201	0.682415350
vns	Oscillospiraceae_unclassified_SGB43502	acute_cyto_PC1	0.112345497	0.682415350
blast	Oscillospiraceae_unclassified_SGB43505	acute_cyto_PC1	0.374299908	0.008854884
vns	Oscillospiraceae_unclassified_SGB43505	acute_cyto_PC1	0.618204990	0.008854884
blast	Oscillospiraceae_unclassified_SGB94989	acute_cyto_PC1	0.953368969	0.180443678
vns	Oscillospiraceae_unclassified_SGB94989	acute_cyto_PC1	0.623615200	0.180443678

blast	Parasutterella_excrementihominis	acute_cyto_PC1	0.023419378	0.086039236
vns	Parasutterella_excrementihominis	acute_cyto_PC1	0.504157156	0.086039236
blast	Richness (# observed features)	acute_cyto_PC1	0.013777874	0.866707226
vns	Richness (# observed features)	acute_cyto_PC1	0.958247838	0.866707226
blast	Schaedlerella_arabinosiphila	acute_cyto_PC1	0.114481983	0.741114287
vns	Schaedlerella_arabinosiphila	acute_cyto_PC1	0.386301964	0.741114287
blast	Shannon Index	acute_cyto_PC1	0.006473070	0.838979990
vns	Shannon Index	acute_cyto_PC1	0.835992445	0.838979990
blast	Turicibacter_sp_1E2	acute_cyto_PC1	0.009594137	0.033399189
vns	Turicibacter_sp_1E2	acute_cyto_PC1	0.183926772	0.033399189
blast	Acetatifactor_SGB41545	acute_cyto_PC2	0.210350900	0.729699651
vns	Acetatifactor_SGB41545	acute_cyto_PC2	0.239098669	0.729699651
blast	Acutalibacter_muris	acute_cyto_PC2	0.786984338	0.552120039
vns	Acutalibacter_muris	acute_cyto_PC2	0.491902226	0.552120039
blast	Akkermansia_muciniphila	acute_cyto_PC2	0.16660254	0.918749296
vns	Akkermansia_muciniphila	acute_cyto_PC2	0.678899621	0.918749296
blast	Anaerotruncus_sp_1XD42_93	acute_cyto_PC2	0.000917537	0.587792882
vns	Anaerotruncus_sp_1XD42_93	acute_cyto_PC2	0.000231687	0.587792882
blast	Bacteria_unclassified_SGB41677	acute_cyto_PC2	0.714189493	0.501928442
vns	Bacteria_unclassified_SGB41677	acute_cyto_PC2	0.58777865	0.501928442
blast	Bacteria_unclassified_SGB43539	acute_cyto_PC2	0.148902499	0.296807417
vns	Bacteria_unclassified_SGB43539	acute_cyto_PC2	0.449260123	0.296807417
blast	Bacteria_unclassified_SGB43546	acute_cyto_PC2	0.112551908	0.664252912
vns	Bacteria_unclassified_SGB43546	acute_cyto_PC2	0.309908808	0.664252912
blast	Bacteria_unclassified_SGB63211	acute_cyto_PC2	0.295003775	0.356672380
vns	Bacteria_unclassified_SGB63211	acute_cyto_PC2	0.340871629	0.356672380
blast	bacterium_0_1xD8_82	acute_cyto_PC2	0.763436393	0.151033005
vns	bacterium_0_1xD8_82	acute_cyto_PC2	0.795139093	0.151033005
blast	bacterium_1XD42_1	acute_cyto_PC2	0.272519397	0.787228515
vns	bacterium_1XD42_1	acute_cyto_PC2	0.097091925	0.787228515
blast	bacterium_1XD42_54	acute_cyto_PC2	0.038105321	0.653991967
vns	bacterium_1XD42_54	acute_cyto_PC2	0.125406933	0.653991967
blast	bacterium_1XD42_76	acute_cyto_PC2	0.177410768	0.059461896
vns	bacterium_1XD42_76	acute_cyto_PC2	0.141032802	0.059461896
blast	bacterium_1xD8_48	acute_cyto_PC2	0.139347611	0.881091373
vns	bacterium_1xD8_48	acute_cyto_PC2	0.370020407	0.881091373
blast	Bacteroides_thetaiotaomicron	acute_cyto_PC2	0.036627754	0.968637108
vns	Bacteroides_thetaiotaomicron	acute_cyto_PC2	0.275405005	0.968637108
blast	Berger Parker Index	acute_cyto_PC2	0.057475283	0.709660102
vns	Berger Parker Index	acute_cyto_PC2	0.499505955	0.709660102
blast	Clostridia_bacterium	acute_cyto_PC2	0.248353087	0.573241047
vns	Clostridia_bacterium	acute_cyto_PC2	0.380556079	0.573241047
blast	Clostridiaceae_bacterium	acute_cyto_PC2	0.162346605	0.606010860
vns	Clostridiaceae_bacterium	acute_cyto_PC2	0.277071084	0.606010860
blast	Clostridiaceae_unclassified_SGB41663	acute_cyto_PC2	0.362409871	0.093183662
vns	Clostridiaceae_unclassified_SGB41663	acute_cyto_PC2	0.070917324	0.093183662

blast	Clostridiales_bacterium	acute_cyto_PC2	0.0738247401	0.7708218478
vns	Clostridiales_bacterium	acute_cyto_PC2	0.1248511673	0.7708218478
blast	Clostridium_cocleatum	acute_cyto_PC2	0.5072750859	0.8147715181
vns	Clostridium_cocleatum	acute_cyto_PC2	0.4827323844	0.8147715181
blast	Clostridium_SGB65123	acute_cyto_PC2	0.1804389829	0.0037845679
vns	Clostridium_SGB65123	acute_cyto_PC2	0.1793895684	0.0037845679
blast	Eubacteriaceae_bacterium	acute_cyto_PC2	0.0166040620	0.9337463589
vns	Eubacteriaceae_bacterium	acute_cyto_PC2	0.9691575780	0.9337463589
blast	Eubacteriaceae_unclassified_SGB94922	acute_cyto_PC2	0.1742242764	0.3594592285
vns	Eubacteriaceae_unclassified_SGB94922	acute_cyto_PC2	0.3461810244	0.3594592285
blast	GGB20146_SGB29427	acute_cyto_PC2	0.0938444678	0.2192657912
vns	GGB20146_SGB29427	acute_cyto_PC2	0.0512189198	0.2192657912
blast	GGB20149_SGB29430	acute_cyto_PC2	0.4132259254	0.2314390820
vns	GGB20149_SGB29430	acute_cyto_PC2	0.1804348381	0.2314390820
blast	GGB22635_SGB63107	acute_cyto_PC2	0.1146609038	0.2935094684
vns	GGB22635_SGB63107	acute_cyto_PC2	0.5356393392	0.2935094684
blast	GGB25041_SGB36960	acute_cyto_PC2	0.1675951172	0.4693203770
vns	GGB25041_SGB36960	acute_cyto_PC2	0.2062562608	0.4693203770
blast	GGB28379_SGB40959	acute_cyto_PC2	0.0292418361	0.1668328279
vns	GGB28379_SGB40959	acute_cyto_PC2	0.0327946564	0.1668328279
blast	GGB28382_SGB40962	acute_cyto_PC2	0.0725130221	0.7380528278
vns	GGB28382_SGB40962	acute_cyto_PC2	0.3704307363	0.7380528278
blast	GGB28392_SGB40972	acute_cyto_PC2	0.0171836690	0.2979659032
vns	GGB28392_SGB40972	acute_cyto_PC2	0.0165924001	0.2979659032
blast	GGB28404_SGB40986	acute_cyto_PC2	0.1577679288	0.7486037584
vns	GGB28404_SGB40986	acute_cyto_PC2	0.0100902140	0.7486037584
blast	GGB28418_SGB41001	acute_cyto_PC2	0.996265407	0.4946914071
vns	GGB28418_SGB41001	acute_cyto_PC2	0.959497945	0.4946914071
blast	GGB28422_SGB41005	acute_cyto_PC2	0.2980947551	0.6334605530
vns	GGB28422_SGB41005	acute_cyto_PC2	0.416676405	0.6334605530
blast	GGB28431_SGB41014	acute_cyto_PC2	0.0153775845	0.7005818042
vns	GGB28431_SGB41014	acute_cyto_PC2	0.0168012470	0.7005818042
blast	GGB28456_SGB41039	acute_cyto_PC2	0.5086215493	0.0007681241
vns	GGB28456_SGB41039	acute_cyto_PC2	0.5675970002	0.0007681241
blast	GGB28782_SGB41435	acute_cyto_PC2	0.3154915999	0.5692213149
vns	GGB28782_SGB41435	acute_cyto_PC2	0.3591699194	0.5692213149
blast	GGB28792_SGB41445	acute_cyto_PC2	0.1237707265	0.9169203831
vns	GGB28792_SGB41445	acute_cyto_PC2	0.9708581071	0.9169203831
blast	GGB28798_SGB41451	acute_cyto_PC2	0.9318373054	0.5896428180
vns	GGB28798_SGB41451	acute_cyto_PC2	0.4715525459	0.5896428180
blast	GGB28810_SGB41465	acute_cyto_PC2	0.9195217924	0.9013987003
vns	GGB28810_SGB41465	acute_cyto_PC2	0.9151059201	0.9013987003
blast	GGB28828_SGB41484	acute_cyto_PC2	0.0364531763	0.1801853188
vns	GGB28828_SGB41484	acute_cyto_PC2	0.0296153165	0.1801853188
blast	GGB28851_SGB41518	acute_cyto_PC2	0.9832452673	0.7651760368
vns	GGB28851_SGB41518	acute_cyto_PC2	0.5482447014	0.7651760368

blast	GGB28865_SGB41537	acute_cyto_PC2	0.051838259	0.038211931
vns	GGB28865_SGB41537	acute_cyto_PC2	0.043832016	0.038211931
blast	GGB28868_SGB41542	acute_cyto_PC2	0.292423183	0.190009262
vns	GGB28868_SGB41542	acute_cyto_PC2	0.254323647	0.190009262
blast	GGB28869_SGB41543	acute_cyto_PC2	0.662073077	0.458718419
vns	GGB28869_SGB41543	acute_cyto_PC2	0.806606148	0.458718419
blast	GGB28875_SGB41555	acute_cyto_PC2	0.776644062	0.565998046
vns	GGB28875_SGB41555	acute_cyto_PC2	0.634070741	0.565998046
blast	GGB28881_SGB41561	acute_cyto_PC2	0.011894548	0.587914970
vns	GGB28881_SGB41561	acute_cyto_PC2	0.001988037	0.587914970
blast	GGB28892_SGB41573	acute_cyto_PC2	0.715153028	0.271718323
vns	GGB28892_SGB41573	acute_cyto_PC2	0.784709483	0.271718323
blast	GGB28893_SGB41574	acute_cyto_PC2	0.739221992	0.007413952
vns	GGB28893_SGB41574	acute_cyto_PC2	0.264245168	0.007413952
blast	GGB28898_SGB41580	acute_cyto_PC2	0.601924700	0.618985494
vns	GGB28898_SGB41580	acute_cyto_PC2	0.591718713	0.618985494
blast	GGB28901_SGB41592	acute_cyto_PC2	0.072063586	0.564391499
vns	GGB28901_SGB41592	acute_cyto_PC2	0.152665868	0.564391499
blast	GGB28904_SGB41597	acute_cyto_PC2	0.225752520	0.104465788
vns	GGB28904_SGB41597	acute_cyto_PC2	0.085658495	0.104465788
blast	GGB28909_SGB41602	acute_cyto_PC2	0.218283007	0.266569842
vns	GGB28909_SGB41602	acute_cyto_PC2	0.145902665	0.266569842
blast	GGB28924_SGB41621	acute_cyto_PC2	0.611578184	0.056545333
vns	GGB28924_SGB41621	acute_cyto_PC2	0.456981189	0.056545333
blast	GGB28927_SGB41625	acute_cyto_PC2	0.001954483	0.723266267
vns	GGB28927_SGB41625	acute_cyto_PC2	0.000219425	0.723266267
blast	GGB28934_SGB41635	acute_cyto_PC2	0.216498853	0.828068791
vns	GGB28934_SGB41635	acute_cyto_PC2	0.224102539	0.828068791
blast	GGB28949_SGB41655	acute_cyto_PC2	0.159598366	0.114171785
vns	GGB28949_SGB41655	acute_cyto_PC2	0.744810754	0.114171785
blast	GGB28951_SGB102295	acute_cyto_PC2	0.718725508	0.699574079
vns	GGB28951_SGB102295	acute_cyto_PC2	0.298970783	0.699574079
blast	GGB28951_SGB41658	acute_cyto_PC2	0.752627843	0.327285934
vns	GGB28951_SGB41658	acute_cyto_PC2	0.024801176	0.327285934
blast	GGB28954_SGB41662	acute_cyto_PC2	0.794939032	0.243383937
vns	GGB28954_SGB41662	acute_cyto_PC2	0.841186745	0.243383937
blast	GGB28960_SGB41669	acute_cyto_PC2	0.536638861	0.631119347
vns	GGB28960_SGB41669	acute_cyto_PC2	0.043638773	0.631119347
blast	GGB28964_SGB94886	acute_cyto_PC2	0.207213028	0.370364408
vns	GGB28964_SGB94886	acute_cyto_PC2	0.400370147	0.370364408
blast	GGB28967_SGB41678	acute_cyto_PC2	0.848205066	0.615230740
vns	GGB28967_SGB41678	acute_cyto_PC2	0.820661965	0.615230740
blast	GGB28996_SGB41712	acute_cyto_PC2	0.429884728	0.915476714
vns	GGB28996_SGB41712	acute_cyto_PC2	0.377449394	0.915476714
blast	GGB29531_SGB42317	acute_cyto_PC2	0.279593444	0.231397371
vns	GGB29531_SGB42317	acute_cyto_PC2	0.999007775	0.231397371

blast	GGB29685_SGB42494	acute_cyto_PC2	0.004596881	0.963284013
vns	GGB29685_SGB42494	acute_cyto_PC2	0.748416768	0.963284013
blast	GGB30141_SGB43066	acute_cyto_PC2	0.614028140	0.834726749
vns	GGB30141_SGB43066	acute_cyto_PC2	0.664026053	0.834726749
blast	GGB30145_SGB43072	acute_cyto_PC2	0.294924035	0.218940665
vns	GGB30145_SGB43072	acute_cyto_PC2	0.367951830	0.218940665
blast	GGB30286_SGB43248	acute_cyto_PC2	0.766757581	0.792435443
vns	GGB30286_SGB43248	acute_cyto_PC2	0.750425732	0.792435443
blast	GGB30300_SGB43264	acute_cyto_PC2	0.213846948	0.701361573
vns	GGB30300_SGB43264	acute_cyto_PC2	0.213511417	0.701361573
blast	GGB30303_SGB43268	acute_cyto_PC2	0.795572751	0.002004274
vns	GGB30303_SGB43268	acute_cyto_PC2	0.660099923	0.002004274
blast	GGB30450_SGB43507	acute_cyto_PC2	0.027109400	0.402811256
vns	GGB30450_SGB43507	acute_cyto_PC2	0.186573367	0.402811256
blast	GGB30453_SGB43513	acute_cyto_PC2	0.315725451	0.535490158
vns	GGB30453_SGB43513	acute_cyto_PC2	0.310638137	0.535490158
blast	GGB30454_SGB43514	acute_cyto_PC2	0.642888646	0.103956230
vns	GGB30454_SGB43514	acute_cyto_PC2	0.656930027	0.103956230
blast	GGB30455_SGB43519	acute_cyto_PC2	0.663798426	0.400914637
vns	GGB30455_SGB43519	acute_cyto_PC2	0.104495810	0.400914637
blast	GGB30456_SGB43520	acute_cyto_PC2	0.111196948	0.743046266
vns	GGB30456_SGB43520	acute_cyto_PC2	0.158921661	0.743046266
blast	GGB30457_SGB63218	acute_cyto_PC2	0.882164050	0.356923303
vns	GGB30457_SGB63218	acute_cyto_PC2	0.890757036	0.356923303
blast	GGB30461_SGB43530	acute_cyto_PC2	0.602468303	0.889321955
vns	GGB30461_SGB43530	acute_cyto_PC2	0.137401205	0.889321955
blast	GGB30461_SGB43533	acute_cyto_PC2	0.810767423	0.559599685
vns	GGB30461_SGB43533	acute_cyto_PC2	0.718693859	0.559599685
blast	GGB30461_SGB63209	acute_cyto_PC2	0.005914118	0.262828301
vns	GGB30461_SGB63209	acute_cyto_PC2	0.075178424	0.262828301
blast	GGB30463_SGB43537	acute_cyto_PC2	0.040768110	0.628088752
vns	GGB30463_SGB43537	acute_cyto_PC2	0.991960140	0.628088752
blast	GGB30473_SGB43557	acute_cyto_PC2	0.328491579	0.271732089
vns	GGB30473_SGB43557	acute_cyto_PC2	0.189853765	0.271732089
blast	GGB30475_SGB63182	acute_cyto_PC2	0.811833041	0.778907676
vns	GGB30475_SGB63182	acute_cyto_PC2	0.783129024	0.778907676
blast	GGB30861_SGB44083	acute_cyto_PC2	0.719401647	0.996109055
vns	GGB30861_SGB44083	acute_cyto_PC2	0.981045510	0.996109055
blast	GGB31312_SGB44628	acute_cyto_PC2	0.831039140	0.181671601
vns	GGB31312_SGB44628	acute_cyto_PC2	0.833457038	0.181671601
blast	GGB31438_SGB44768	acute_cyto_PC2	0.481213900	0.070303486
vns	GGB31438_SGB44768	acute_cyto_PC2	0.483694757	0.070303486
blast	GGB3171_SGB4185	acute_cyto_PC2	0.066342246	0.453579347
vns	GGB3171_SGB4185	acute_cyto_PC2	0.057850794	0.453579347
blast	GGB31762_SGB45125	acute_cyto_PC2	0.442889056	0.932455654
vns	GGB31762_SGB45125	acute_cyto_PC2	0.437364142	0.932455654

blast	GGB31823_SGB45199	acute_cyto_PC2	0.630175203	0.494435618
vns	GGB31823_SGB45199	acute_cyto_PC2	0.537240753	0.494435618
blast	GGB31838_SGB45216	acute_cyto_PC2	0.684403164	0.614762266
vns	GGB31838_SGB45216	acute_cyto_PC2	0.997715556	0.614762266
blast	GGB31841_SGB65084	acute_cyto_PC2	0.371737454	0.408678565
vns	GGB31841_SGB65084	acute_cyto_PC2	0.443951934	0.408678565
blast	GGB32371_SGB41694	acute_cyto_PC2	0.242018055	0.615455121
vns	GGB32371_SGB41694	acute_cyto_PC2	0.400159574	0.615455121
blast	GGB3793_SGB5158	acute_cyto_PC2	0.884993491	0.901369810
vns	GGB3793_SGB5158	acute_cyto_PC2	0.847239709	0.901369810
blast	GGB42601_SGB59797	acute_cyto_PC2	0.401892013	0.336686237
vns	GGB42601_SGB59797	acute_cyto_PC2	0.627566595	0.336686237
blast	GGB45514_SGB63186	acute_cyto_PC2	0.955364765	0.646117327
vns	GGB45514_SGB63186	acute_cyto_PC2	0.621527882	0.646117327
blast	GGB45564_SGB63259	acute_cyto_PC2	0.415272784	0.302284099
vns	GGB45564_SGB63259	acute_cyto_PC2	0.380878696	0.302284099
blast	GGB45624_SGB63337	acute_cyto_PC2	0.620389272	0.675157709
vns	GGB45624_SGB63337	acute_cyto_PC2	0.385198794	0.675157709
blast	GGB45656_SGB63370	acute_cyto_PC2	0.404705483	0.492710596
vns	GGB45656_SGB63370	acute_cyto_PC2	0.163643553	0.492710596
blast	GGB47127_SGB65054	acute_cyto_PC2	0.456493679	0.286129243
vns	GGB47127_SGB65054	acute_cyto_PC2	0.82583129	0.286129243
blast	GGB74395_SGB43523	acute_cyto_PC2	0.802410438	0.957598374
vns	GGB74395_SGB43523	acute_cyto_PC2	0.835585633	0.957598374
blast	GGB75053_SGB43494	acute_cyto_PC2	0.177327401	0.247905558
vns	GGB75053_SGB43494	acute_cyto_PC2	0.050410443	0.247905558
blast	GGB75109_SGB102238	acute_cyto_PC2	0.599792986	0.161242457
vns	GGB75109_SGB102238	acute_cyto_PC2	0.510864481	0.161242457
blast	Lachnospiraceae_bacterium	acute_cyto_PC2	0.023925903	0.916236135
vns	Lachnospiraceae_bacterium	acute_cyto_PC2	0.917492265	0.916236135
blast	Lachnospiraceae_bacterium_A2	acute_cyto_PC2	0.724187807	0.755362535
vns	Lachnospiraceae_bacterium_A2	acute_cyto_PC2	0.083860739	0.755362535
blast	Lachnospiraceae_unclassified_SGB41414	acute_cyto_PC2	0.719079500	0.764274295
vns	Lachnospiraceae_unclassified_SGB41414	acute_cyto_PC2	0.532128198	0.764274295
blast	Lachnospiraceae_unclassified_SGB41424	acute_cyto_PC2	0.216930413	0.095711932
vns	Lachnospiraceae_unclassified_SGB41424	acute_cyto_PC2	0.022018829	0.095711932
blast	Lachnospiraceae_unclassified_SGB94868	acute_cyto_PC2	0.207633504	0.317040034
vns	Lachnospiraceae_unclassified_SGB94868	acute_cyto_PC2	0.754562933	0.317040034
blast	Lactobacillus_johnsonii	acute_cyto_PC2	0.002748365	0.865404608
vns	Lactobacillus_johnsonii	acute_cyto_PC2	0.832216242	0.865404608
blast	Leptogranulimonas_caecicola	acute_cyto_PC2	0.874577980	0.307822782
vns	Leptogranulimonas_caecicola	acute_cyto_PC2	0.014475087	0.307822782
blast	Muribaculaceae_bacterium	acute_cyto_PC2	0.001850198	0.863848536
vns	Muribaculaceae_bacterium	acute_cyto_PC2	0.883532506	0.863848536
blast	Neglectibacter_sp_X4	acute_cyto_PC2	0.394423067	0.736535330
vns	Neglectibacter_sp_X4	acute_cyto_PC2	0.463430059	0.736535330

blast	Oscillibacter_SGB43496	acute_cyto_PC2	0.220544725	0.224697122
vns	Oscillibacter_SGB43496	acute_cyto_PC2	0.34795292	0.224697122
blast	Oscillospiraceae_bacterium	acute_cyto_PC2	0.492783661	0.240782724
vns	Oscillospiraceae_bacterium	acute_cyto_PC2	0.251973413	0.240782724
blast	Oscillospiraceae_unclassified_SGB43502	acute_cyto_PC2	0.017271201	0.759614808
vns	Oscillospiraceae_unclassified_SGB43502	acute_cyto_PC2	0.112345497	0.759614808
blast	Oscillospiraceae_unclassified_SGB43505	acute_cyto_PC2	0.374299908	0.382033291
vns	Oscillospiraceae_unclassified_SGB43505	acute_cyto_PC2	0.618204990	0.382033291
blast	Oscillospiraceae_unclassified_SGB94989	acute_cyto_PC2	0.953368969	0.505239922
vns	Oscillospiraceae_unclassified_SGB94989	acute_cyto_PC2	0.623615200	0.505239922
blast	Parasutterella_excrementihominis	acute_cyto_PC2	0.023419378	0.597705641
vns	Parasutterella_excrementihominis	acute_cyto_PC2	0.504157156	0.597705641
blast	Richness (# observed features)	acute_cyto_PC2	0.013777874	0.998062105
vns	Richness (# observed features)	acute_cyto_PC2	0.958247838	0.998062105
blast	Schaedlerella_arabinosiphila	acute_cyto_PC2	0.114481983	0.809412625
vns	Schaedlerella_arabinosiphila	acute_cyto_PC2	0.386301964	0.809412625
blast	Shannon Index	acute_cyto_PC2	0.006473070	0.916280734
vns	Shannon Index	acute_cyto_PC2	0.835992445	0.916280734
blast	Turicibacter_sp_1E2	acute_cyto_PC2	0.009594137	0.416711257
vns	Turicibacter_sp_1E2	acute_cyto_PC2	0.183926772	0.416711257
blast	Acetatifactor_SGB41545	acute_cyto_PC3	0.210350900	0.850892715
vns	Acetatifactor_SGB41545	acute_cyto_PC3	0.239098669	0.850892715
blast	Acutalibacter_muris	acute_cyto_PC3	0.786984338	0.688340526
vns	Acutalibacter_muris	acute_cyto_PC3	0.491902226	0.688340526
blast	Akkermansia_muciniphila	acute_cyto_PC3	0.16660254	0.237451933
vns	Akkermansia_muciniphila	acute_cyto_PC3	0.678899621	0.237451933
blast	Anaerotruncus_sp_1XD42_93	acute_cyto_PC3	0.000917537	0.219716417
vns	Anaerotruncus_sp_1XD42_93	acute_cyto_PC3	0.000231687	0.219716417
blast	Bacteria_unclassified_SGB41677	acute_cyto_PC3	0.714189493	0.307443412
vns	Bacteria_unclassified_SGB41677	acute_cyto_PC3	0.58777865	0.307443412
blast	Bacteria_unclassified_SGB43539	acute_cyto_PC3	0.148902499	0.154699032
vns	Bacteria_unclassified_SGB43539	acute_cyto_PC3	0.449260123	0.154699032
blast	Bacteria_unclassified_SGB43546	acute_cyto_PC3	0.112551908	0.507331111
vns	Bacteria_unclassified_SGB43546	acute_cyto_PC3	0.309908808	0.507331111
blast	Bacteria_unclassified_SGB63211	acute_cyto_PC3	0.295003775	0.910383705
vns	Bacteria_unclassified_SGB63211	acute_cyto_PC3	0.340871629	0.910383705
blast	bacterium_0_1xD8_82	acute_cyto_PC3	0.763436393	0.79612335
vns	bacterium_0_1xD8_82	acute_cyto_PC3	0.795139093	0.79612335
blast	bacterium_1XD42_1	acute_cyto_PC3	0.272519397	0.242613719
vns	bacterium_1XD42_1	acute_cyto_PC3	0.097091925	0.242613719
blast	bacterium_1XD42_54	acute_cyto_PC3	0.038105321	0.984924233
vns	bacterium_1XD42_54	acute_cyto_PC3	0.125406933	0.984924233
blast	bacterium_1XD42_76	acute_cyto_PC3	0.177410768	0.440173026
vns	bacterium_1XD42_76	acute_cyto_PC3	0.141032802	0.440173026
blast	bacterium_1xD8_48	acute_cyto_PC3	0.139347611	0.532020100
vns	bacterium_1xD8_48	acute_cyto_PC3	0.370020407	0.532020100

blast	Bacteroides_thetaiotaomicron	acute_cyto_PC3	0.036627754	0.674769535
vns	Bacteroides_thetaiotaomicron	acute_cyto_PC3	0.275405005	0.674769535
blast	Berger Parker Index	acute_cyto_PC3	0.057475283	0.068961191
vns	Berger Parker Index	acute_cyto_PC3	0.499505955	0.068961191
blast	Clostridia_bacterium	acute_cyto_PC3	0.248353087	0.121524284
vns	Clostridia_bacterium	acute_cyto_PC3	0.380556079	0.121524284
blast	Clostridiaceae_bacterium	acute_cyto_PC3	0.162346605	0.049092982
vns	Clostridiaceae_bacterium	acute_cyto_PC3	0.277071084	0.049092982
blast	Clostridiaceae_unclassified_SGB41663	acute_cyto_PC3	0.362409871	0.452082315
vns	Clostridiaceae_unclassified_SGB41663	acute_cyto_PC3	0.070917324	0.452082315
blast	Clostridiales_bacterium	acute_cyto_PC3	0.073824740	0.817262856
vns	Clostridiales_bacterium	acute_cyto_PC3	0.124851167	0.817262856
blast	Clostridium_cocleatum	acute_cyto_PC3	0.507275085	0.813754028
vns	Clostridium_cocleatum	acute_cyto_PC3	0.482732384	0.813754028
blast	Clostridium_SGB65123	acute_cyto_PC3	0.180438982	0.278714033
vns	Clostridium_SGB65123	acute_cyto_PC3	0.179389568	0.278714033
blast	Eubacteriaceae_bacterium	acute_cyto_PC3	0.016604062	0.023217854
vns	Eubacteriaceae_bacterium	acute_cyto_PC3	0.969157578	0.023217854
blast	Eubacteriaceae_unclassified_SGB94922	acute_cyto_PC3	0.174224276	0.143424090
vns	Eubacteriaceae_unclassified_SGB94922	acute_cyto_PC3	0.346181024	0.143424090
blast	GGB20146_SGB29427	acute_cyto_PC3	0.093844467	0.892681194
vns	GGB20146_SGB29427	acute_cyto_PC3	0.051218919	0.892681194
blast	GGB20149_SGB29430	acute_cyto_PC3	0.413225925	0.192751749
vns	GGB20149_SGB29430	acute_cyto_PC3	0.180434838	0.192751749
blast	GGB22635_SGB63107	acute_cyto_PC3	0.114660903	0.046234093
vns	GGB22635_SGB63107	acute_cyto_PC3	0.535639339	0.046234093
blast	GGB25041_SGB36960	acute_cyto_PC3	0.167595117	0.562756216
vns	GGB25041_SGB36960	acute_cyto_PC3	0.206256260	0.562756216
blast	GGB28379_SGB40959	acute_cyto_PC3	0.029241836	0.860594237
vns	GGB28379_SGB40959	acute_cyto_PC3	0.032794656	0.860594237
blast	GGB28382_SGB40962	acute_cyto_PC3	0.072513022	0.082388714
vns	GGB28382_SGB40962	acute_cyto_PC3	0.370430736	0.082388714
blast	GGB28392_SGB40972	acute_cyto_PC3	0.017183669	0.518265904
vns	GGB28392_SGB40972	acute_cyto_PC3	0.016592400	0.518265904
blast	GGB28404_SGB40986	acute_cyto_PC3	0.157767928	0.802757412
vns	GGB28404_SGB40986	acute_cyto_PC3	0.010090214	0.802757412
blast	GGB28418_SGB41001	acute_cyto_PC3	0.996265407	0.631783511
vns	GGB28418_SGB41001	acute_cyto_PC3	0.959497945	0.631783511
blast	GGB28422_SGB41005	acute_cyto_PC3	0.298094755	0.477285430
vns	GGB28422_SGB41005	acute_cyto_PC3	0.416676405	0.477285430
blast	GGB28431_SGB41014	acute_cyto_PC3	0.015377584	0.049208715
vns	GGB28431_SGB41014	acute_cyto_PC3	0.016801247	0.049208715
blast	GGB28456_SGB41039	acute_cyto_PC3	0.508621549	0.038985937
vns	GGB28456_SGB41039	acute_cyto_PC3	0.567597000	0.038985937
blast	GGB28782_SGB41435	acute_cyto_PC3	0.315491599	0.172231722
vns	GGB28782_SGB41435	acute_cyto_PC3	0.359169919	0.172231722

blast	GGB28792_SGB41445	acute_cyto_PC3	0.123770726	0.236232979
vns	GGB28792_SGB41445	acute_cyto_PC3	0.970858107	0.236232979
blast	GGB28798_SGB41451	acute_cyto_PC3	0.931837305	0.796081594
vns	GGB28798_SGB41451	acute_cyto_PC3	0.471552545	0.796081594
blast	GGB28810_SGB41465	acute_cyto_PC3	0.919521792	0.674035845
vns	GGB28810_SGB41465	acute_cyto_PC3	0.915105920	0.674035845
blast	GGB28828_SGB41484	acute_cyto_PC3	0.036453176	0.941100053
vns	GGB28828_SGB41484	acute_cyto_PC3	0.029615316	0.941100053
blast	GGB28851_SGB41518	acute_cyto_PC3	0.983245267	0.213233718
vns	GGB28851_SGB41518	acute_cyto_PC3	0.548244701	0.213233718
blast	GGB28865_SGB41537	acute_cyto_PC3	0.051838259	0.457415098
vns	GGB28865_SGB41537	acute_cyto_PC3	0.043832016	0.457415098
blast	GGB28868_SGB41542	acute_cyto_PC3	0.292423183	0.172033778
vns	GGB28868_SGB41542	acute_cyto_PC3	0.254323647	0.172033778
blast	GGB28869_SGB41543	acute_cyto_PC3	0.662073077	0.252206188
vns	GGB28869_SGB41543	acute_cyto_PC3	0.806606148	0.252206188
blast	GGB28875_SGB41555	acute_cyto_PC3	0.776644062	0.936860051
vns	GGB28875_SGB41555	acute_cyto_PC3	0.634070741	0.936860051
blast	GGB28881_SGB41561	acute_cyto_PC3	0.011894548	0.421434652
vns	GGB28881_SGB41561	acute_cyto_PC3	0.001988037	0.421434652
blast	GGB28892_SGB41573	acute_cyto_PC3	0.715153028	0.197403228
vns	GGB28892_SGB41573	acute_cyto_PC3	0.784709483	0.197403228
blast	GGB28893_SGB41574	acute_cyto_PC3	0.739221992	0.003669261
vns	GGB28893_SGB41574	acute_cyto_PC3	0.264245168	0.003669261
blast	GGB28898_SGB41580	acute_cyto_PC3	0.601924700	0.782881375
vns	GGB28898_SGB41580	acute_cyto_PC3	0.591718713	0.782881375
blast	GGB28901_SGB41592	acute_cyto_PC3	0.072063586	0.377358964
vns	GGB28901_SGB41592	acute_cyto_PC3	0.152665868	0.377358964
blast	GGB28904_SGB41597	acute_cyto_PC3	0.225752520	0.061885765
vns	GGB28904_SGB41597	acute_cyto_PC3	0.085658495	0.061885765
blast	GGB28909_SGB41602	acute_cyto_PC3	0.218283007	0.411876428
vns	GGB28909_SGB41602	acute_cyto_PC3	0.145902665	0.411876428
blast	GGB28924_SGB41621	acute_cyto_PC3	0.611578184	0.531115561
vns	GGB28924_SGB41621	acute_cyto_PC3	0.456981189	0.531115561
blast	GGB28927_SGB41625	acute_cyto_PC3	0.001954483	0.344620829
vns	GGB28927_SGB41625	acute_cyto_PC3	0.000219425	0.344620829
blast	GGB28934_SGB41635	acute_cyto_PC3	0.216498853	0.864091627
vns	GGB28934_SGB41635	acute_cyto_PC3	0.224102539	0.864091627
blast	GGB28949_SGB41655	acute_cyto_PC3	0.159598366	0.800602891
vns	GGB28949_SGB41655	acute_cyto_PC3	0.744810754	0.800602891
blast	GGB28951_SGB102295	acute_cyto_PC3	0.718725508	0.55127267
vns	GGB28951_SGB102295	acute_cyto_PC3	0.298970783	0.55127267
blast	GGB28951_SGB41658	acute_cyto_PC3	0.752627843	0.487923486
vns	GGB28951_SGB41658	acute_cyto_PC3	0.024801176	0.487923486
blast	GGB28954_SGB41662	acute_cyto_PC3	0.794939032	0.543334032
vns	GGB28954_SGB41662	acute_cyto_PC3	0.841186745	0.543334032

blast	GGB28960_SGB41669	acute_cyto_PC3	0.536638861	0.566327325
vns	GGB28960_SGB41669	acute_cyto_PC3	0.043638773	0.566327325
blast	GGB28964_SGB94886	acute_cyto_PC3	0.207213028	0.020014945
vns	GGB28964_SGB94886	acute_cyto_PC3	0.400370147	0.020014945
blast	GGB28967_SGB41678	acute_cyto_PC3	0.848205066	0.196364066
vns	GGB28967_SGB41678	acute_cyto_PC3	0.820661965	0.196364066
blast	GGB28996_SGB41712	acute_cyto_PC3	0.429884728	0.552994939
vns	GGB28996_SGB41712	acute_cyto_PC3	0.377449394	0.552994939
blast	GGB29531_SGB42317	acute_cyto_PC3	0.279593444	0.188641667
vns	GGB29531_SGB42317	acute_cyto_PC3	0.999007775	0.188641667
blast	GGB29685_SGB42494	acute_cyto_PC3	0.004596881	0.024607236
vns	GGB29685_SGB42494	acute_cyto_PC3	0.748416768	0.024607236
blast	GGB30141_SGB43066	acute_cyto_PC3	0.614028140	0.048884087
vns	GGB30141_SGB43066	acute_cyto_PC3	0.664026053	0.048884087
blast	GGB30145_SGB43072	acute_cyto_PC3	0.294924035	0.866307014
vns	GGB30145_SGB43072	acute_cyto_PC3	0.367951830	0.866307014
blast	GGB30286_SGB43248	acute_cyto_PC3	0.766757581	0.561068016
vns	GGB30286_SGB43248	acute_cyto_PC3	0.750425732	0.561068016
blast	GGB30300_SGB43264	acute_cyto_PC3	0.213846948	0.982065034
vns	GGB30300_SGB43264	acute_cyto_PC3	0.213511417	0.982065034
blast	GGB30303_SGB43268	acute_cyto_PC3	0.795572751	0.073306671
vns	GGB30303_SGB43268	acute_cyto_PC3	0.660099923	0.073306671
blast	GGB30450_SGB43507	acute_cyto_PC3	0.027109400	0.888768265
vns	GGB30450_SGB43507	acute_cyto_PC3	0.186573367	0.888768265
blast	GGB30453_SGB43513	acute_cyto_PC3	0.315725451	0.166160477
vns	GGB30453_SGB43513	acute_cyto_PC3	0.310638137	0.166160477
blast	GGB30454_SGB43514	acute_cyto_PC3	0.642888646	0.486766818
vns	GGB30454_SGB43514	acute_cyto_PC3	0.656930027	0.486766818
blast	GGB30455_SGB43519	acute_cyto_PC3	0.663798426	0.302222002
vns	GGB30455_SGB43519	acute_cyto_PC3	0.104495810	0.302222002
blast	GGB30456_SGB43520	acute_cyto_PC3	0.111196948	0.204944538
vns	GGB30456_SGB43520	acute_cyto_PC3	0.158921661	0.204944538
blast	GGB30457_SGB63218	acute_cyto_PC3	0.882164050	0.921985183
vns	GGB30457_SGB63218	acute_cyto_PC3	0.890757036	0.921985183
blast	GGB30461_SGB43530	acute_cyto_PC3	0.602468303	0.507004803
vns	GGB30461_SGB43530	acute_cyto_PC3	0.137401205	0.507004803
blast	GGB30461_SGB43533	acute_cyto_PC3	0.810767423	0.121803564
vns	GGB30461_SGB43533	acute_cyto_PC3	0.718693859	0.121803564
blast	GGB30461_SGB63209	acute_cyto_PC3	0.005914118	0.678926722
vns	GGB30461_SGB63209	acute_cyto_PC3	0.075178424	0.678926722
blast	GGB30463_SGB43537	acute_cyto_PC3	0.040768110	0.347853377
vns	GGB30463_SGB43537	acute_cyto_PC3	0.991960140	0.347853377
blast	GGB30473_SGB43557	acute_cyto_PC3	0.328491579	0.504757171
vns	GGB30473_SGB43557	acute_cyto_PC3	0.189853765	0.504757171
blast	GGB30475_SGB63182	acute_cyto_PC3	0.811833041	0.526750973
vns	GGB30475_SGB63182	acute_cyto_PC3	0.783129024	0.526750973

blast	GGB30861_SGB44083	acute_cyto_PC3	0.719401647	0.223237907
vns	GGB30861_SGB44083	acute_cyto_PC3	0.981045510	0.223237907
blast	GGB31312_SGB44628	acute_cyto_PC3	0.831039140	0.590550802
vns	GGB31312_SGB44628	acute_cyto_PC3	0.833457038	0.590550802
blast	GGB31438_SGB44768	acute_cyto_PC3	0.481213900	0.803314460
vns	GGB31438_SGB44768	acute_cyto_PC3	0.483694757	0.803314460
blast	GGB3171_SGB4185	acute_cyto_PC3	0.066342246	0.369240410
vns	GGB3171_SGB4185	acute_cyto_PC3	0.057850794	0.369240410
blast	GGB31762_SGB45125	acute_cyto_PC3	0.442889056	0.168519571
vns	GGB31762_SGB45125	acute_cyto_PC3	0.437364142	0.168519571
blast	GGB31823_SGB45199	acute_cyto_PC3	0.630175203	0.581998207
vns	GGB31823_SGB45199	acute_cyto_PC3	0.537240753	0.581998207
blast	GGB31838_SGB45216	acute_cyto_PC3	0.684403164	0.132848363
vns	GGB31838_SGB45216	acute_cyto_PC3	0.997715556	0.132848363
blast	GGB31841_SGB65084	acute_cyto_PC3	0.371737454	0.501230074
vns	GGB31841_SGB65084	acute_cyto_PC3	0.443951934	0.501230074
blast	GGB32371_SGB41694	acute_cyto_PC3	0.242018055	0.164051326
vns	GGB32371_SGB41694	acute_cyto_PC3	0.400159574	0.164051326
blast	GGB3793_SGB5158	acute_cyto_PC3	0.884993491	0.710076525
vns	GGB3793_SGB5158	acute_cyto_PC3	0.847239709	0.710076525
blast	GGB42601_SGB59797	acute_cyto_PC3	0.401892013	0.035750525
vns	GGB42601_SGB59797	acute_cyto_PC3	0.627566595	0.035750525
blast	GGB45514_SGB63186	acute_cyto_PC3	0.955364765	0.384416731
vns	GGB45514_SGB63186	acute_cyto_PC3	0.621527882	0.384416731
blast	GGB45564_SGB63259	acute_cyto_PC3	0.415272784	0.469296960
vns	GGB45564_SGB63259	acute_cyto_PC3	0.380878696	0.469296960
blast	GGB45624_SGB63337	acute_cyto_PC3	0.620389272	0.615590643
vns	GGB45624_SGB63337	acute_cyto_PC3	0.385198794	0.615590643
blast	GGB45656_SGB63370	acute_cyto_PC3	0.404705483	0.250956981
vns	GGB45656_SGB63370	acute_cyto_PC3	0.163643553	0.250956981
blast	GGB47127_SGB65054	acute_cyto_PC3	0.456493679	0.841292296
vns	GGB47127_SGB65054	acute_cyto_PC3	0.82583129	0.841292296
blast	GGB74395_SGB43523	acute_cyto_PC3	0.802410438	0.280740637
vns	GGB74395_SGB43523	acute_cyto_PC3	0.835585633	0.280740637
blast	GGB75053_SGB43494	acute_cyto_PC3	0.177327401	0.095368247
vns	GGB75053_SGB43494	acute_cyto_PC3	0.050410443	0.095368247
blast	GGB75109_SGB102238	acute_cyto_PC3	0.599792986	0.680641567
vns	GGB75109_SGB102238	acute_cyto_PC3	0.510864481	0.680641567
blast	Lachnospiraceae_bacterium	acute_cyto_PC3	0.023925903	0.018487335
vns	Lachnospiraceae_bacterium	acute_cyto_PC3	0.917492265	0.018487335
blast	Lachnospiraceae_bacterium_A2	acute_cyto_PC3	0.724187807	0.387550498
vns	Lachnospiraceae_bacterium_A2	acute_cyto_PC3	0.083860739	0.387550498
blast	Lachnospiraceae_unclassified_SGB41414	acute_cyto_PC3	0.719079500	0.380848187
vns	Lachnospiraceae_unclassified_SGB41414	acute_cyto_PC3	0.532128198	0.380848187
blast	Lachnospiraceae_unclassified_SGB41424	acute_cyto_PC3	0.216930413	0.205779670
vns	Lachnospiraceae_unclassified_SGB41424	acute_cyto_PC3	0.022018829	0.205779670

blast	Lachnospiraceae_unclassified_SGB94868	acute_cyto_PC3	0.2076335048	0.1334503143
vns	Lachnospiraceae_unclassified_SGB94868	acute_cyto_PC3	0.7545629335	0.1334503143
blast	Lactobacillus_johnsonii	acute_cyto_PC3	0.0027483659	0.1004283291
vns	Lactobacillus_johnsonii	acute_cyto_PC3	0.8322162424	0.1004283291
blast	Leptogranulimonas_caecicola	acute_cyto_PC3	0.8745779805	0.1672472759
vns	Leptogranulimonas_caecicola	acute_cyto_PC3	0.0144750878	0.1672472759
blast	Muribaculaceae_bacterium	acute_cyto_PC3	0.0018501989	0.1047820704
vns	Muribaculaceae_bacterium	acute_cyto_PC3	0.8835325068	0.1047820704
blast	Neglectibacter_sp_X4	acute_cyto_PC3	0.3944230674	0.8645774706
vns	Neglectibacter_sp_X4	acute_cyto_PC3	0.463430059	0.8645774706
blast	Oscillibacter_SGB43496	acute_cyto_PC3	0.2205447256	0.5230316625
vns	Oscillibacter_SGB43496	acute_cyto_PC3	0.34795292	0.5230316625
blast	Oscillospiraceae_bacterium	acute_cyto_PC3	0.4927836617	0.0384061752
vns	Oscillospiraceae_bacterium	acute_cyto_PC3	0.251973413	0.0384061752
blast	Oscillospiraceae_unclassified_SGB43502	acute_cyto_PC3	0.0172712013	0.0818751848
vns	Oscillospiraceae_unclassified_SGB43502	acute_cyto_PC3	0.1123454974	0.0818751848
blast	Oscillospiraceae_unclassified_SGB43505	acute_cyto_PC3	0.3742999083	0.7308986096
vns	Oscillospiraceae_unclassified_SGB43505	acute_cyto_PC3	0.6182049908	0.7308986096
blast	Oscillospiraceae_unclassified_SGB94989	acute_cyto_PC3	0.9533689691	0.7576086032
vns	Oscillospiraceae_unclassified_SGB94989	acute_cyto_PC3	0.6236152007	0.7576086032
blast	Parasutterella_excrementihominis	acute_cyto_PC3	0.0234193782	0.6369235708
vns	Parasutterella_excrementihominis	acute_cyto_PC3	0.5041571561	0.6369235708
blast	Richness (# observed features)	acute_cyto_PC3	0.0137778742	0.0133539927
vns	Richness (# observed features)	acute_cyto_PC3	0.9582478381	0.0133539927
blast	Schaedlerella_arabinosiphila	acute_cyto_PC3	0.114481983	0.4937420204
vns	Schaedlerella_arabinosiphila	acute_cyto_PC3	0.3863019645	0.4937420204
blast	Shannon Index	acute_cyto_PC3	0.0064730704	0.0269099643
vns	Shannon Index	acute_cyto_PC3	0.8359924459	0.0269099643
blast	Turicibacter_sp_1E2	acute_cyto_PC3	0.0095941376	0.2413661101
vns	Turicibacter_sp_1E2	acute_cyto_PC3	0.183926772	0.2413661101
blast	Acetatifactor_SGB41545	acute_cyto_PC4	0.2103509006	0.5534814492
vns	Acetatifactor_SGB41545	acute_cyto_PC4	0.2390986693	0.5534814492
blast	Acutalibacter_muris	acute_cyto_PC4	0.7869843388	0.0127311123
vns	Acutalibacter_muris	acute_cyto_PC4	0.4919022263	0.0127311123
blast	Akkermansia_muciniphila	acute_cyto_PC4	0.16660254	0.406070667
vns	Akkermansia_muciniphila	acute_cyto_PC4	0.6788996217	0.406070667
blast	Anaerotruncus_sp_1XD42_93	acute_cyto_PC4	0.0009175375	0.1635641806
vns	Anaerotruncus_sp_1XD42_93	acute_cyto_PC4	0.0002316873	0.1635641806
blast	Bacteria_unclassified_SGB41677	acute_cyto_PC4	0.7141894931	0.9465425232
vns	Bacteria_unclassified_SGB41677	acute_cyto_PC4	0.58777865	0.9465425232
blast	Bacteria_unclassified_SGB43539	acute_cyto_PC4	0.1489024993	0.8459095008
vns	Bacteria_unclassified_SGB43539	acute_cyto_PC4	0.4492601232	0.8459095008
blast	Bacteria_unclassified_SGB43546	acute_cyto_PC4	0.1125519082	0.3980585952
vns	Bacteria_unclassified_SGB43546	acute_cyto_PC4	0.3099088089	0.3980585952
blast	Bacteria_unclassified_SGB63211	acute_cyto_PC4	0.2950037757	0.0580328834
vns	Bacteria_unclassified_SGB63211	acute_cyto_PC4	0.3408716294	0.0580328834

blast	bacterium_0_1xD8_82	acute_cyto_PC4	0.763436393	0.345198668
vns	bacterium_0_1xD8_82	acute_cyto_PC4	0.795139093	0.345198668
blast	bacterium_1XD42_1	acute_cyto_PC4	0.272519397	0.832276136
vns	bacterium_1XD42_1	acute_cyto_PC4	0.097091925	0.832276136
blast	bacterium_1XD42_54	acute_cyto_PC4	0.038105321	0.760598819
vns	bacterium_1XD42_54	acute_cyto_PC4	0.125406933	0.760598819
blast	bacterium_1XD42_76	acute_cyto_PC4	0.177410768	0.689221913
vns	bacterium_1XD42_76	acute_cyto_PC4	0.141032802	0.689221913
blast	bacterium_1xD8_48	acute_cyto_PC4	0.139347611	0.265963814
vns	bacterium_1xD8_48	acute_cyto_PC4	0.370020407	0.265963814
blast	Bacteroides_thetaiotaomicron	acute_cyto_PC4	0.036627754	0.738788133
vns	Bacteroides_thetaiotaomicron	acute_cyto_PC4	0.275405005	0.738788133
blast	Berger Parker Index	acute_cyto_PC4	0.057475283	0.966625106
vns	Berger Parker Index	acute_cyto_PC4	0.499505955	0.966625106
blast	Clostridia_bacterium	acute_cyto_PC4	0.248353087	0.612833079
vns	Clostridia_bacterium	acute_cyto_PC4	0.380556079	0.612833079
blast	Clostridiaceae_bacterium	acute_cyto_PC4	0.162346605	0.795018248
vns	Clostridiaceae_bacterium	acute_cyto_PC4	0.277071084	0.795018248
blast	Clostridiaceae_unclassified_SGB41663	acute_cyto_PC4	0.362409871	0.452433981
vns	Clostridiaceae_unclassified_SGB41663	acute_cyto_PC4	0.070917324	0.452433981
blast	Clostridiales_bacterium	acute_cyto_PC4	0.073824740	0.778300015
vns	Clostridiales_bacterium	acute_cyto_PC4	0.124851167	0.778300015
blast	Clostridium_cocleatum	acute_cyto_PC4	0.507275085	0.228595464
vns	Clostridium_cocleatum	acute_cyto_PC4	0.482732384	0.228595464
blast	Clostridium_SGB65123	acute_cyto_PC4	0.180438982	0.810099492
vns	Clostridium_SGB65123	acute_cyto_PC4	0.179389568	0.810099492
blast	Eubacteriaceae_bacterium	acute_cyto_PC4	0.016604062	0.619161767
vns	Eubacteriaceae_bacterium	acute_cyto_PC4	0.969157578	0.619161767
blast	Eubacteriaceae_unclassified_SGB94922	acute_cyto_PC4	0.174224276	0.809700411
vns	Eubacteriaceae_unclassified_SGB94922	acute_cyto_PC4	0.346181024	0.809700411
blast	GGB20146_SGB29427	acute_cyto_PC4	0.093844467	0.797612184
vns	GGB20146_SGB29427	acute_cyto_PC4	0.051218919	0.797612184
blast	GGB20149_SGB29430	acute_cyto_PC4	0.413225925	0.053001147
vns	GGB20149_SGB29430	acute_cyto_PC4	0.180434838	0.053001147
blast	GGB22635_SGB63107	acute_cyto_PC4	0.114660903	0.157657517
vns	GGB22635_SGB63107	acute_cyto_PC4	0.535639339	0.157657517
blast	GGB25041_SGB36960	acute_cyto_PC4	0.167595117	0.625916073
vns	GGB25041_SGB36960	acute_cyto_PC4	0.206256260	0.625916073
blast	GGB28379_SGB40959	acute_cyto_PC4	0.029241836	0.089828505
vns	GGB28379_SGB40959	acute_cyto_PC4	0.032794656	0.089828505
blast	GGB28382_SGB40962	acute_cyto_PC4	0.072513022	0.323679498
vns	GGB28382_SGB40962	acute_cyto_PC4	0.370430736	0.323679498
blast	GGB28392_SGB40972	acute_cyto_PC4	0.017183669	0.757393698
vns	GGB28392_SGB40972	acute_cyto_PC4	0.016592400	0.757393698
blast	GGB28404_SGB40986	acute_cyto_PC4	0.157767928	0.427189824
vns	GGB28404_SGB40986	acute_cyto_PC4	0.010090214	0.427189824

blast	GGB28418_SGB41001		acute_cyto_PC4	0.996265407	0.9752315483
vns	GGB28418_SGB41001		acute_cyto_PC4	0.959497945	0.9752315483
blast	GGB28422_SGB41005		acute_cyto_PC4	0.2980947557	0.8857642401
vns	GGB28422_SGB41005		acute_cyto_PC4	0.416676405	0.8857642401
blast	GGB28431_SGB41014		acute_cyto_PC4	0.0153775845	0.2499621723
vns	GGB28431_SGB41014		acute_cyto_PC4	0.0168012470	0.2499621723
blast	GGB28456_SGB41039		acute_cyto_PC4	0.5086215493	0.3925368809
vns	GGB28456_SGB41039		acute_cyto_PC4	0.5675970002	0.3925368809
blast	GGB28782_SGB41435		acute_cyto_PC4	0.3154915999	0.0083010268
vns	GGB28782_SGB41435		acute_cyto_PC4	0.3591699194	0.0083010268
blast	GGB28792_SGB41445		acute_cyto_PC4	0.1237707265	0.0983787701
vns	GGB28792_SGB41445		acute_cyto_PC4	0.9708581077	0.0983787701
blast	GGB28798_SGB41451		acute_cyto_PC4	0.9318373054	0.0955212090
vns	GGB28798_SGB41451		acute_cyto_PC4	0.4715525459	0.0955212090
blast	GGB28810_SGB41465		acute_cyto_PC4	0.9195217924	0.2194623305
vns	GGB28810_SGB41465		acute_cyto_PC4	0.9151059207	0.2194623305
blast	GGB28828_SGB41484		acute_cyto_PC4	0.0364531763	0.8970370078
vns	GGB28828_SGB41484		acute_cyto_PC4	0.0296153165	0.8970370078
blast	GGB28851_SGB41518		acute_cyto_PC4	0.9832452673	0.534505723
vns	GGB28851_SGB41518		acute_cyto_PC4	0.5482447014	0.534505723
blast	GGB28865_SGB41537		acute_cyto_PC4	0.0518382591	0.3166621381
vns	GGB28865_SGB41537		acute_cyto_PC4	0.0438320161	0.3166621381
blast	GGB28868_SGB41542		acute_cyto_PC4	0.2924231839	0.1231230537
vns	GGB28868_SGB41542		acute_cyto_PC4	0.2543236478	0.1231230537
blast	GGB28869_SGB41543		acute_cyto_PC4	0.6620730770	0.8100368620
vns	GGB28869_SGB41543		acute_cyto_PC4	0.8066061480	0.8100368620
blast	GGB28875_SGB41555		acute_cyto_PC4	0.7766440627	0.873856933
vns	GGB28875_SGB41555		acute_cyto_PC4	0.6340707414	0.873856933
blast	GGB28881_SGB41561		acute_cyto_PC4	0.0118945489	0.3772329684
vns	GGB28881_SGB41561		acute_cyto_PC4	0.0019880370	0.3772329684
blast	GGB28892_SGB41573		acute_cyto_PC4	0.715153028	0.8147424199
vns	GGB28892_SGB41573		acute_cyto_PC4	0.7847094835	0.8147424199
blast	GGB28893_SGB41574		acute_cyto_PC4	0.7392219927	0.8796633320
vns	GGB28893_SGB41574		acute_cyto_PC4	0.2642451682	0.8796633320
blast	GGB28898_SGB41580		acute_cyto_PC4	0.6019247005	0.4635687610
vns	GGB28898_SGB41580		acute_cyto_PC4	0.591718713	0.4635687610
blast	GGB28901_SGB41592		acute_cyto_PC4	0.0720635862	0.4141262171
vns	GGB28901_SGB41592		acute_cyto_PC4	0.1526658680	0.4141262171
blast	GGB28904_SGB41597		acute_cyto_PC4	0.2257525204	0.4246039737
vns	GGB28904_SGB41597		acute_cyto_PC4	0.0856584957	0.4246039737
blast	GGB28909_SGB41602		acute_cyto_PC4	0.2182830072	0.9914602769
vns	GGB28909_SGB41602		acute_cyto_PC4	0.1459026652	0.9914602769
blast	GGB28924_SGB41621		acute_cyto_PC4	0.6115781842	0.6424857454
vns	GGB28924_SGB41621		acute_cyto_PC4	0.456981189	0.6424857454
blast	GGB28927_SGB41625		acute_cyto_PC4	0.0019544839	0.3261772135
vns	GGB28927_SGB41625		acute_cyto_PC4	0.0002194259	0.3261772135

blast	GGB28934_SGB41635	acute_cyto_PC4	0.216498853	0.644533086
vns	GGB28934_SGB41635	acute_cyto_PC4	0.224102539	0.644533086
blast	GGB28949_SGB41655	acute_cyto_PC4	0.159598366	0.940804662
vns	GGB28949_SGB41655	acute_cyto_PC4	0.744810754	0.940804662
blast	GGB28951_SGB102295	acute_cyto_PC4	0.718725508	0.728250027
vns	GGB28951_SGB102295	acute_cyto_PC4	0.298970783	0.728250027
blast	GGB28951_SGB41658	acute_cyto_PC4	0.752627843	0.456688788
vns	GGB28951_SGB41658	acute_cyto_PC4	0.024801176	0.456688788
blast	GGB28954_SGB41662	acute_cyto_PC4	0.794939032	0.173630961
vns	GGB28954_SGB41662	acute_cyto_PC4	0.841186745	0.173630961
blast	GGB28960_SGB41669	acute_cyto_PC4	0.536638861	0.760945985
vns	GGB28960_SGB41669	acute_cyto_PC4	0.043638773	0.760945985
blast	GGB28964_SGB94886	acute_cyto_PC4	0.207213028	0.102306196
vns	GGB28964_SGB94886	acute_cyto_PC4	0.400370147	0.102306196
blast	GGB28967_SGB41678	acute_cyto_PC4	0.848205066	0.713075046
vns	GGB28967_SGB41678	acute_cyto_PC4	0.820661965	0.713075046
blast	GGB28996_SGB41712	acute_cyto_PC4	0.429884728	0.212627758
vns	GGB28996_SGB41712	acute_cyto_PC4	0.377449394	0.212627758
blast	GGB29531_SGB42317	acute_cyto_PC4	0.279593444	0.936617818
vns	GGB29531_SGB42317	acute_cyto_PC4	0.999007775	0.936617818
blast	GGB29685_SGB42494	acute_cyto_PC4	0.004596881	0.982521464
vns	GGB29685_SGB42494	acute_cyto_PC4	0.748416768	0.982521464
blast	GGB30141_SGB43066	acute_cyto_PC4	0.614028140	0.941832742
vns	GGB30141_SGB43066	acute_cyto_PC4	0.664026053	0.941832742
blast	GGB30145_SGB43072	acute_cyto_PC4	0.294924035	0.408313143
vns	GGB30145_SGB43072	acute_cyto_PC4	0.367951830	0.408313143
blast	GGB30286_SGB43248	acute_cyto_PC4	0.766757581	0.969741978
vns	GGB30286_SGB43248	acute_cyto_PC4	0.750425732	0.969741978
blast	GGB30300_SGB43264	acute_cyto_PC4	0.213846948	0.775466333
vns	GGB30300_SGB43264	acute_cyto_PC4	0.213511417	0.775466333
blast	GGB30303_SGB43268	acute_cyto_PC4	0.795572751	0.872523558
vns	GGB30303_SGB43268	acute_cyto_PC4	0.660099923	0.872523558
blast	GGB30450_SGB43507	acute_cyto_PC4	0.027109400	0.838698654
vns	GGB30450_SGB43507	acute_cyto_PC4	0.186573367	0.838698654
blast	GGB30453_SGB43513	acute_cyto_PC4	0.315725451	0.914779238
vns	GGB30453_SGB43513	acute_cyto_PC4	0.310638137	0.914779238
blast	GGB30454_SGB43514	acute_cyto_PC4	0.642888646	0.083948929
vns	GGB30454_SGB43514	acute_cyto_PC4	0.656930027	0.083948929
blast	GGB30455_SGB43519	acute_cyto_PC4	0.663798426	0.919353520
vns	GGB30455_SGB43519	acute_cyto_PC4	0.104495810	0.919353520
blast	GGB30456_SGB43520	acute_cyto_PC4	0.111196948	0.084160034
vns	GGB30456_SGB43520	acute_cyto_PC4	0.158921661	0.084160034
blast	GGB30457_SGB63218	acute_cyto_PC4	0.882164050	0.929474772
vns	GGB30457_SGB63218	acute_cyto_PC4	0.890757036	0.929474772
blast	GGB30461_SGB43530	acute_cyto_PC4	0.602468303	0.367978255
vns	GGB30461_SGB43530	acute_cyto_PC4	0.137401205	0.367978255

blast	GGB30461_SGB43533	acute_cyto_PC4	0.810767423	0.743408315
vns	GGB30461_SGB43533	acute_cyto_PC4	0.718693859	0.743408315
blast	GGB30461_SGB63209	acute_cyto_PC4	0.005914118	0.370769292
vns	GGB30461_SGB63209	acute_cyto_PC4	0.075178424	0.370769292
blast	GGB30463_SGB43537	acute_cyto_PC4	0.040768110	0.472711939
vns	GGB30463_SGB43537	acute_cyto_PC4	0.991960140	0.472711939
blast	GGB30473_SGB43557	acute_cyto_PC4	0.328491579	0.532746470
vns	GGB30473_SGB43557	acute_cyto_PC4	0.189853765	0.532746470
blast	GGB30475_SGB63182	acute_cyto_PC4	0.811833041	0.923739221
vns	GGB30475_SGB63182	acute_cyto_PC4	0.783129024	0.923739221
blast	GGB30861_SGB44083	acute_cyto_PC4	0.719401647	0.312860092
vns	GGB30861_SGB44083	acute_cyto_PC4	0.981045510	0.312860092
blast	GGB31312_SGB44628	acute_cyto_PC4	0.831039140	0.510649029
vns	GGB31312_SGB44628	acute_cyto_PC4	0.833457038	0.510649029
blast	GGB31438_SGB44768	acute_cyto_PC4	0.481213900	0.46144833
vns	GGB31438_SGB44768	acute_cyto_PC4	0.483694757	0.46144833
blast	GGB3171_SGB4185	acute_cyto_PC4	0.066342246	0.437144027
vns	GGB3171_SGB4185	acute_cyto_PC4	0.057850794	0.437144027
blast	GGB31762_SGB45125	acute_cyto_PC4	0.442889056	0.981604873
vns	GGB31762_SGB45125	acute_cyto_PC4	0.437364142	0.981604873
blast	GGB31823_SGB45199	acute_cyto_PC4	0.630175203	0.928548894
vns	GGB31823_SGB45199	acute_cyto_PC4	0.537240753	0.928548894
blast	GGB31838_SGB45216	acute_cyto_PC4	0.684403164	0.301775375
vns	GGB31838_SGB45216	acute_cyto_PC4	0.997715556	0.301775375
blast	GGB31841_SGB65084	acute_cyto_PC4	0.371737454	0.393402691
vns	GGB31841_SGB65084	acute_cyto_PC4	0.443951934	0.393402691
blast	GGB32371_SGB41694	acute_cyto_PC4	0.242018055	0.672669652
vns	GGB32371_SGB41694	acute_cyto_PC4	0.400159574	0.672669652
blast	GGB3793_SGB5158	acute_cyto_PC4	0.884993491	0.544714013
vns	GGB3793_SGB5158	acute_cyto_PC4	0.847239709	0.544714013
blast	GGB42601_SGB59797	acute_cyto_PC4	0.401892013	0.409059403
vns	GGB42601_SGB59797	acute_cyto_PC4	0.627566595	0.409059403
blast	GGB45514_SGB63186	acute_cyto_PC4	0.955364765	0.518815686
vns	GGB45514_SGB63186	acute_cyto_PC4	0.621527882	0.518815686
blast	GGB45564_SGB63259	acute_cyto_PC4	0.415272784	0.551454182
vns	GGB45564_SGB63259	acute_cyto_PC4	0.380878696	0.551454182
blast	GGB45624_SGB63337	acute_cyto_PC4	0.620389272	0.524021795
vns	GGB45624_SGB63337	acute_cyto_PC4	0.385198794	0.524021795
blast	GGB45656_SGB63370	acute_cyto_PC4	0.404705483	0.188746888
vns	GGB45656_SGB63370	acute_cyto_PC4	0.163643553	0.188746888
blast	GGB47127_SGB65054	acute_cyto_PC4	0.456493679	0.036738332
vns	GGB47127_SGB65054	acute_cyto_PC4	0.82583129	0.036738332
blast	GGB74395_SGB43523	acute_cyto_PC4	0.802410438	0.125129394
vns	GGB74395_SGB43523	acute_cyto_PC4	0.835585633	0.125129394
blast	GGB75053_SGB43494	acute_cyto_PC4	0.177327401	0.855265324
vns	GGB75053_SGB43494	acute_cyto_PC4	0.050410443	0.855265324

blast	GGB75109_SGB102238	acute_cyto_PC4	0.599792986	0.981169366
vns	GGB75109_SGB102238	acute_cyto_PC4	0.51086448	0.981169366
blast	Lachnospiraceae_bacterium	acute_cyto_PC4	0.023925903	0.751121746
vns	Lachnospiraceae_bacterium	acute_cyto_PC4	0.917492265	0.751121746
blast	Lachnospiraceae_bacterium_A2	acute_cyto_PC4	0.724187807	0.393179005
vns	Lachnospiraceae_bacterium_A2	acute_cyto_PC4	0.083860739	0.393179005
blast	Lachnospiraceae_unclassified_SGB41414	acute_cyto_PC4	0.719079500	0.375261271
vns	Lachnospiraceae_unclassified_SGB41414	acute_cyto_PC4	0.532128198	0.375261271
blast	Lachnospiraceae_unclassified_SGB41424	acute_cyto_PC4	0.216930413	0.558261030
vns	Lachnospiraceae_unclassified_SGB41424	acute_cyto_PC4	0.022018829	0.558261030
blast	Lachnospiraceae_unclassified_SGB94868	acute_cyto_PC4	0.207633504	0.997874178
vns	Lachnospiraceae_unclassified_SGB94868	acute_cyto_PC4	0.754562933	0.997874178
blast	Lactobacillus_johnsonii	acute_cyto_PC4	0.002748365	0.626527166
vns	Lactobacillus_johnsonii	acute_cyto_PC4	0.832216242	0.626527166
blast	Leptogranulimonas_caecicola	acute_cyto_PC4	0.874577980	0.969956296
vns	Leptogranulimonas_caecicola	acute_cyto_PC4	0.014475087	0.969956296
blast	Muribaculaceae_bacterium	acute_cyto_PC4	0.001850198	0.742445662
vns	Muribaculaceae_bacterium	acute_cyto_PC4	0.883532506	0.742445662
blast	Neglectibacter_sp_X4	acute_cyto_PC4	0.394423067	0.741394946
vns	Neglectibacter_sp_X4	acute_cyto_PC4	0.463430059	0.741394946
blast	Oscillibacter_SGB43496	acute_cyto_PC4	0.220544725	0.258666812
vns	Oscillibacter_SGB43496	acute_cyto_PC4	0.34795292	0.258666812
blast	Oscillospiraceae_bacterium	acute_cyto_PC4	0.492783661	0.672695362
vns	Oscillospiraceae_bacterium	acute_cyto_PC4	0.251973413	0.672695362
blast	Oscillospiraceae_unclassified_SGB43502	acute_cyto_PC4	0.017271201	0.275756638
vns	Oscillospiraceae_unclassified_SGB43502	acute_cyto_PC4	0.112345497	0.275756638
blast	Oscillospiraceae_unclassified_SGB43505	acute_cyto_PC4	0.374299908	0.723934266
vns	Oscillospiraceae_unclassified_SGB43505	acute_cyto_PC4	0.618204990	0.723934266
blast	Oscillospiraceae_unclassified_SGB94989	acute_cyto_PC4	0.953368969	0.899944963
vns	Oscillospiraceae_unclassified_SGB94989	acute_cyto_PC4	0.623615200	0.899944963
blast	Parasutterella_excrementihominis	acute_cyto_PC4	0.023419378	0.085303682
vns	Parasutterella_excrementihominis	acute_cyto_PC4	0.504157156	0.085303682
blast	Richness (# observed features)	acute_cyto_PC4	0.013777874	0.990003766
vns	Richness (# observed features)	acute_cyto_PC4	0.958247838	0.990003766
blast	Schaedlerella_arabinosiphila	acute_cyto_PC4	0.114481983	0.147983161
vns	Schaedlerella_arabinosiphila	acute_cyto_PC4	0.386301964	0.147983161
blast	Shannon Index	acute_cyto_PC4	0.006473070	0.582589904
vns	Shannon Index	acute_cyto_PC4	0.835992445	0.582589904
blast	Turicibacter_sp_1E2	acute_cyto_PC4	0.009594137	0.553715021
vns	Turicibacter_sp_1E2	acute_cyto_PC4	0.183926772	0.553715021
blast	Acetatifactor_SGB41545	acute_cyto_PC5	0.210350900	0.759879406
vns	Acetatifactor_SGB41545	acute_cyto_PC5	0.239098669	0.759879406
blast	Acutalibacter_muris	acute_cyto_PC5	0.786984338	0.338851418
vns	Acutalibacter_muris	acute_cyto_PC5	0.491902226	0.338851418
blast	Akkermansia_muciniphila	acute_cyto_PC5	0.16660254	0.138644297
vns	Akkermansia_muciniphila	acute_cyto_PC5	0.678899621	0.138644297

blast	Anaerotruncus_sp_1XD42_93	acute_cyto_PC5	0.000917537	0.131962450
vns	Anaerotruncus_sp_1XD42_93	acute_cyto_PC5	0.000231687	0.131962450
blast	Bacteria_unclassified_SGB41677	acute_cyto_PC5	0.714189493	0.290781171
vns	Bacteria_unclassified_SGB41677	acute_cyto_PC5	0.58777865	0.290781171
blast	Bacteria_unclassified_SGB43539	acute_cyto_PC5	0.148902499	0.355623226
vns	Bacteria_unclassified_SGB43539	acute_cyto_PC5	0.449260123	0.355623226
blast	Bacteria_unclassified_SGB43546	acute_cyto_PC5	0.112551908	0.014884615
vns	Bacteria_unclassified_SGB43546	acute_cyto_PC5	0.309908808	0.014884615
blast	Bacteria_unclassified_SGB63211	acute_cyto_PC5	0.295003775	0.361662888
vns	Bacteria_unclassified_SGB63211	acute_cyto_PC5	0.340871629	0.361662888
blast	bacterium_0_1xD8_82	acute_cyto_PC5	0.763436393	0.614504504
vns	bacterium_0_1xD8_82	acute_cyto_PC5	0.795139093	0.614504504
blast	bacterium_1XD42_1	acute_cyto_PC5	0.272519397	0.029394954
vns	bacterium_1XD42_1	acute_cyto_PC5	0.097091925	0.029394954
blast	bacterium_1XD42_54	acute_cyto_PC5	0.038105321	0.547514611
vns	bacterium_1XD42_54	acute_cyto_PC5	0.125406933	0.547514611
blast	bacterium_1XD42_76	acute_cyto_PC5	0.177410768	0.493164290
vns	bacterium_1XD42_76	acute_cyto_PC5	0.141032802	0.493164290
blast	bacterium_1xD8_48	acute_cyto_PC5	0.139347611	0.712095773
vns	bacterium_1xD8_48	acute_cyto_PC5	0.370020407	0.712095773
blast	Bacteroides_thetaiotaomicron	acute_cyto_PC5	0.036627754	0.631926246
vns	Bacteroides_thetaiotaomicron	acute_cyto_PC5	0.275405005	0.631926246
blast	Berger Parker Index	acute_cyto_PC5	0.057475283	0.166885264
vns	Berger Parker Index	acute_cyto_PC5	0.499505955	0.166885264
blast	Clostridia_bacterium	acute_cyto_PC5	0.248353087	0.185023828
vns	Clostridia_bacterium	acute_cyto_PC5	0.380556079	0.185023828
blast	Clostridiaceae_bacterium	acute_cyto_PC5	0.162346605	0.242769451
vns	Clostridiaceae_bacterium	acute_cyto_PC5	0.277071084	0.242769451
blast	Clostridiaceae_unclassified_SGB41663	acute_cyto_PC5	0.362409871	0.181875880
vns	Clostridiaceae_unclassified_SGB41663	acute_cyto_PC5	0.070917324	0.181875880
blast	Clostridiales_bacterium	acute_cyto_PC5	0.073824740	0.884275683
vns	Clostridiales_bacterium	acute_cyto_PC5	0.124851167	0.884275683
blast	Clostridium_cocleatum	acute_cyto_PC5	0.507275085	0.999251513
vns	Clostridium_cocleatum	acute_cyto_PC5	0.482732384	0.999251513
blast	Clostridium_SGB65123	acute_cyto_PC5	0.180438982	0.194202497
vns	Clostridium_SGB65123	acute_cyto_PC5	0.179389568	0.194202497
blast	Eubacteriaceae_bacterium	acute_cyto_PC5	0.016604062	0.871438152
vns	Eubacteriaceae_bacterium	acute_cyto_PC5	0.969157578	0.871438152
blast	Eubacteriaceae_unclassified_SGB94922	acute_cyto_PC5	0.174224276	0.094822587
vns	Eubacteriaceae_unclassified_SGB94922	acute_cyto_PC5	0.346181024	0.094822587
blast	GGB20146_SGB29427	acute_cyto_PC5	0.093844467	0.048214670
vns	GGB20146_SGB29427	acute_cyto_PC5	0.051218919	0.048214670
blast	GGB20149_SGB29430	acute_cyto_PC5	0.413225925	0.169456150
vns	GGB20149_SGB29430	acute_cyto_PC5	0.180434838	0.169456150
blast	GGB22635_SGB63107	acute_cyto_PC5	0.114660903	0.283207884
vns	GGB22635_SGB63107	acute_cyto_PC5	0.535639339	0.283207884

blast	GGB25041_SGB36960	acute_cyto_PC5	0.167595117	0.399287171
vns	GGB25041_SGB36960	acute_cyto_PC5	0.206256260	0.399287171
blast	GGB28379_SGB40959	acute_cyto_PC5	0.029241836	0.154541690
vns	GGB28379_SGB40959	acute_cyto_PC5	0.032794656	0.154541690
blast	GGB28382_SGB40962	acute_cyto_PC5	0.072513022	0.683379325
vns	GGB28382_SGB40962	acute_cyto_PC5	0.370430736	0.683379325
blast	GGB28392_SGB40972	acute_cyto_PC5	0.017183669	0.851382130
vns	GGB28392_SGB40972	acute_cyto_PC5	0.016592400	0.851382130
blast	GGB28404_SGB40986	acute_cyto_PC5	0.157767928	0.046518987
vns	GGB28404_SGB40986	acute_cyto_PC5	0.010090214	0.046518987
blast	GGB28418_SGB41001	acute_cyto_PC5	0.996265407	0.412448587
vns	GGB28418_SGB41001	acute_cyto_PC5	0.959497945	0.412448587
blast	GGB28422_SGB41005	acute_cyto_PC5	0.298094755	0.132556227
vns	GGB28422_SGB41005	acute_cyto_PC5	0.416676405	0.132556227
blast	GGB28431_SGB41014	acute_cyto_PC5	0.015377584	0.091473432
vns	GGB28431_SGB41014	acute_cyto_PC5	0.016801247	0.091473432
blast	GGB28456_SGB41039	acute_cyto_PC5	0.508621549	0.710176342
vns	GGB28456_SGB41039	acute_cyto_PC5	0.567597000	0.710176342
blast	GGB28782_SGB41435	acute_cyto_PC5	0.315491599	0.465580195
vns	GGB28782_SGB41435	acute_cyto_PC5	0.359169919	0.465580195
blast	GGB28792_SGB41445	acute_cyto_PC5	0.123770726	0.157628162
vns	GGB28792_SGB41445	acute_cyto_PC5	0.970858107	0.157628162
blast	GGB28798_SGB41451	acute_cyto_PC5	0.931837305	0.067609682
vns	GGB28798_SGB41451	acute_cyto_PC5	0.471552545	0.067609682
blast	GGB28810_SGB41465	acute_cyto_PC5	0.919521792	0.675693398
vns	GGB28810_SGB41465	acute_cyto_PC5	0.915105920	0.675693398
blast	GGB28828_SGB41484	acute_cyto_PC5	0.036453176	0.433485471
vns	GGB28828_SGB41484	acute_cyto_PC5	0.029615316	0.433485471
blast	GGB28851_SGB41518	acute_cyto_PC5	0.983245267	0.439669961
vns	GGB28851_SGB41518	acute_cyto_PC5	0.548244701	0.439669961
blast	GGB28865_SGB41537	acute_cyto_PC5	0.051838259	0.242583728
vns	GGB28865_SGB41537	acute_cyto_PC5	0.043832016	0.242583728
blast	GGB28868_SGB41542	acute_cyto_PC5	0.292423183	0.782805575
vns	GGB28868_SGB41542	acute_cyto_PC5	0.254323647	0.782805575
blast	GGB28869_SGB41543	acute_cyto_PC5	0.662073077	0.410808707
vns	GGB28869_SGB41543	acute_cyto_PC5	0.806606148	0.410808707
blast	GGB28875_SGB41555	acute_cyto_PC5	0.776644062	0.941640241
vns	GGB28875_SGB41555	acute_cyto_PC5	0.634070741	0.941640241
blast	GGB28881_SGB41561	acute_cyto_PC5	0.011894548	0.827823180
vns	GGB28881_SGB41561	acute_cyto_PC5	0.001988037	0.827823180
blast	GGB28892_SGB41573	acute_cyto_PC5	0.715153028	0.821756920
vns	GGB28892_SGB41573	acute_cyto_PC5	0.784709483	0.821756920
blast	GGB28893_SGB41574	acute_cyto_PC5	0.739221992	0.103508377
vns	GGB28893_SGB41574	acute_cyto_PC5	0.264245168	0.103508377
blast	GGB28898_SGB41580	acute_cyto_PC5	0.601924700	0.635808502
vns	GGB28898_SGB41580	acute_cyto_PC5	0.591718713	0.635808502

blast	GGB28901_SGB41592	acute_cyto_PC5	0.072063586	0.741938061
vns	GGB28901_SGB41592	acute_cyto_PC5	0.152665868	0.741938061
blast	GGB28904_SGB41597	acute_cyto_PC5	0.225752520	0.630316852
vns	GGB28904_SGB41597	acute_cyto_PC5	0.085658495	0.630316852
blast	GGB28909_SGB41602	acute_cyto_PC5	0.218283007	0.780541405
vns	GGB28909_SGB41602	acute_cyto_PC5	0.145902665	0.780541405
blast	GGB28924_SGB41621	acute_cyto_PC5	0.611578184	0.026849550
vns	GGB28924_SGB41621	acute_cyto_PC5	0.456981189	0.026849550
blast	GGB28927_SGB41625	acute_cyto_PC5	0.001954483	0.791196367
vns	GGB28927_SGB41625	acute_cyto_PC5	0.000219425	0.791196367
blast	GGB28934_SGB41635	acute_cyto_PC5	0.216498853	0.410244249
vns	GGB28934_SGB41635	acute_cyto_PC5	0.224102539	0.410244249
blast	GGB28949_SGB41655	acute_cyto_PC5	0.159598366	0.943122496
vns	GGB28949_SGB41655	acute_cyto_PC5	0.744810754	0.943122496
blast	GGB28951_SGB102295	acute_cyto_PC5	0.718725508	0.775482481
vns	GGB28951_SGB102295	acute_cyto_PC5	0.298970783	0.775482481
blast	GGB28951_SGB41658	acute_cyto_PC5	0.752627843	0.437358540
vns	GGB28951_SGB41658	acute_cyto_PC5	0.024801176	0.437358540
blast	GGB28954_SGB41662	acute_cyto_PC5	0.794939032	0.416638560
vns	GGB28954_SGB41662	acute_cyto_PC5	0.841186745	0.416638560
blast	GGB28960_SGB41669	acute_cyto_PC5	0.536638861	0.046029003
vns	GGB28960_SGB41669	acute_cyto_PC5	0.043638773	0.046029003
blast	GGB28964_SGB94886	acute_cyto_PC5	0.207213028	0.244109888
vns	GGB28964_SGB94886	acute_cyto_PC5	0.400370147	0.244109888
blast	GGB28967_SGB41678	acute_cyto_PC5	0.848205066	0.24195294
vns	GGB28967_SGB41678	acute_cyto_PC5	0.820661965	0.24195294
blast	GGB28996_SGB41712	acute_cyto_PC5	0.429884728	0.405178638
vns	GGB28996_SGB41712	acute_cyto_PC5	0.377449394	0.405178638
blast	GGB29531_SGB42317	acute_cyto_PC5	0.279593444	0.734203830
vns	GGB29531_SGB42317	acute_cyto_PC5	0.999007775	0.734203830
blast	GGB29685_SGB42494	acute_cyto_PC5	0.004596881	0.276043602
vns	GGB29685_SGB42494	acute_cyto_PC5	0.748416768	0.276043602
blast	GGB30141_SGB43066	acute_cyto_PC5	0.614028140	0.627226942
vns	GGB30141_SGB43066	acute_cyto_PC5	0.664026053	0.627226942
blast	GGB30145_SGB43072	acute_cyto_PC5	0.294924035	0.139382822
vns	GGB30145_SGB43072	acute_cyto_PC5	0.367951830	0.139382822
blast	GGB30286_SGB43248	acute_cyto_PC5	0.766757581	0.298262478
vns	GGB30286_SGB43248	acute_cyto_PC5	0.750425732	0.298262478
blast	GGB30300_SGB43264	acute_cyto_PC5	0.213846948	0.194434705
vns	GGB30300_SGB43264	acute_cyto_PC5	0.213511417	0.194434705
blast	GGB30303_SGB43268	acute_cyto_PC5	0.795572751	0.329660960
vns	GGB30303_SGB43268	acute_cyto_PC5	0.660099923	0.329660960
blast	GGB30450_SGB43507	acute_cyto_PC5	0.027109400	0.113294912
vns	GGB30450_SGB43507	acute_cyto_PC5	0.186573367	0.113294912
blast	GGB30453_SGB43513	acute_cyto_PC5	0.315725451	0.227699424
vns	GGB30453_SGB43513	acute_cyto_PC5	0.310638137	0.227699424

blast	GGB30454_SGB43514	acute_cyto_PC5	0.642888646	0.406581659
vns	GGB30454_SGB43514	acute_cyto_PC5	0.656930027	0.406581659
blast	GGB30455_SGB43519	acute_cyto_PC5	0.663798426	0.487432530
vns	GGB30455_SGB43519	acute_cyto_PC5	0.104495810	0.487432530
blast	GGB30456_SGB43520	acute_cyto_PC5	0.111196948	0.697909069
vns	GGB30456_SGB43520	acute_cyto_PC5	0.158921661	0.697909069
blast	GGB30457_SGB63218	acute_cyto_PC5	0.882164050	0.515375721
vns	GGB30457_SGB63218	acute_cyto_PC5	0.890757036	0.515375721
blast	GGB30461_SGB43530	acute_cyto_PC5	0.602468303	0.045227141
vns	GGB30461_SGB43530	acute_cyto_PC5	0.137401205	0.045227141
blast	GGB30461_SGB43533	acute_cyto_PC5	0.810767423	0.404855627
vns	GGB30461_SGB43533	acute_cyto_PC5	0.718693859	0.404855627
blast	GGB30461_SGB63209	acute_cyto_PC5	0.005914118	0.221120191
vns	GGB30461_SGB63209	acute_cyto_PC5	0.075178424	0.221120191
blast	GGB30463_SGB43537	acute_cyto_PC5	0.040768110	0.782901012
vns	GGB30463_SGB43537	acute_cyto_PC5	0.991960140	0.782901012
blast	GGB30473_SGB43557	acute_cyto_PC5	0.328491579	0.492090141
vns	GGB30473_SGB43557	acute_cyto_PC5	0.189853765	0.492090141
blast	GGB30475_SGB63182	acute_cyto_PC5	0.811833041	0.340376455
vns	GGB30475_SGB63182	acute_cyto_PC5	0.783129024	0.340376455
blast	GGB30861_SGB44083	acute_cyto_PC5	0.719401647	0.195858932
vns	GGB30861_SGB44083	acute_cyto_PC5	0.981045510	0.195858932
blast	GGB31312_SGB44628	acute_cyto_PC5	0.831039140	0.875844946
vns	GGB31312_SGB44628	acute_cyto_PC5	0.833457038	0.875844946
blast	GGB31438_SGB44768	acute_cyto_PC5	0.481213900	0.403740111
vns	GGB31438_SGB44768	acute_cyto_PC5	0.483694757	0.403740111
blast	GGB3171_SGB4185	acute_cyto_PC5	0.066342246	0.535205558
vns	GGB3171_SGB4185	acute_cyto_PC5	0.057850794	0.535205558
blast	GGB31762_SGB45125	acute_cyto_PC5	0.442889056	0.230173648
vns	GGB31762_SGB45125	acute_cyto_PC5	0.437364142	0.230173648
blast	GGB31823_SGB45199	acute_cyto_PC5	0.630175203	0.230343618
vns	GGB31823_SGB45199	acute_cyto_PC5	0.537240753	0.230343618
blast	GGB31838_SGB45216	acute_cyto_PC5	0.684403164	0.233798756
vns	GGB31838_SGB45216	acute_cyto_PC5	0.997715556	0.233798756
blast	GGB31841_SGB65084	acute_cyto_PC5	0.371737454	0.863678183
vns	GGB31841_SGB65084	acute_cyto_PC5	0.443951934	0.863678183
blast	GGB32371_SGB41694	acute_cyto_PC5	0.242018055	0.374507152
vns	GGB32371_SGB41694	acute_cyto_PC5	0.400159574	0.374507152
blast	GGB3793_SGB5158	acute_cyto_PC5	0.884993491	0.885772645
vns	GGB3793_SGB5158	acute_cyto_PC5	0.847239709	0.885772645
blast	GGB42601_SGB59797	acute_cyto_PC5	0.401892013	0.083888371
vns	GGB42601_SGB59797	acute_cyto_PC5	0.627566595	0.083888371
blast	GGB45514_SGB63186	acute_cyto_PC5	0.955364765	0.896129394
vns	GGB45514_SGB63186	acute_cyto_PC5	0.621527882	0.896129394
blast	GGB45564_SGB63259	acute_cyto_PC5	0.415272784	0.339244118
vns	GGB45564_SGB63259	acute_cyto_PC5	0.380878696	0.339244118

blast	GGB45624_SGB63337	acute_cyto_PC5	0.620389272	0.197450135
vns	GGB45624_SGB63337	acute_cyto_PC5	0.385198794	0.197450135
blast	GGB45656_SGB63370	acute_cyto_PC5	0.404705483	0.919106702
vns	GGB45656_SGB63370	acute_cyto_PC5	0.163643553	0.919106702
blast	GGB47127_SGB65054	acute_cyto_PC5	0.456493679	0.211956697
vns	GGB47127_SGB65054	acute_cyto_PC5	0.82583129	0.211956697
blast	GGB74395_SGB43523	acute_cyto_PC5	0.802410438	0.945766202
vns	GGB74395_SGB43523	acute_cyto_PC5	0.835585633	0.945766202
blast	GGB75053_SGB43494	acute_cyto_PC5	0.177327401	0.678321613
vns	GGB75053_SGB43494	acute_cyto_PC5	0.050410443	0.678321613
blast	GGB75109_SGB102238	acute_cyto_PC5	0.599792986	0.383470318
vns	GGB75109_SGB102238	acute_cyto_PC5	0.510864481	0.383470318
blast	Lachnospiraceae_bacterium	acute_cyto_PC5	0.023925903	0.545574730
vns	Lachnospiraceae_bacterium	acute_cyto_PC5	0.917492265	0.545574730
blast	Lachnospiraceae_bacterium_A2	acute_cyto_PC5	0.724187807	0.254630773
vns	Lachnospiraceae_bacterium_A2	acute_cyto_PC5	0.083860739	0.254630773
blast	Lachnospiraceae_unclassified_SGB41414	acute_cyto_PC5	0.719079500	0.437901377
vns	Lachnospiraceae_unclassified_SGB41414	acute_cyto_PC5	0.532128198	0.437901377
blast	Lachnospiraceae_unclassified_SGB41424	acute_cyto_PC5	0.216930413	0.196420006
vns	Lachnospiraceae_unclassified_SGB41424	acute_cyto_PC5	0.022018829	0.196420006
blast	Lachnospiraceae_unclassified_SGB94868	acute_cyto_PC5	0.207633504	0.553994087
vns	Lachnospiraceae_unclassified_SGB94868	acute_cyto_PC5	0.754562933	0.553994087
blast	Lactobacillus_johnsonii	acute_cyto_PC5	0.002748365	0.998168218
vns	Lactobacillus_johnsonii	acute_cyto_PC5	0.832216242	0.998168218
blast	Leptogranulimonas_caecicola	acute_cyto_PC5	0.874577980	0.075878500
vns	Leptogranulimonas_caecicola	acute_cyto_PC5	0.014475087	0.075878500
blast	Muribaculaceae_bacterium	acute_cyto_PC5	0.001850198	0.352855154
vns	Muribaculaceae_bacterium	acute_cyto_PC5	0.883532506	0.352855154
blast	Neglectibacter_sp_X4	acute_cyto_PC5	0.394423067	0.810886107
vns	Neglectibacter_sp_X4	acute_cyto_PC5	0.463430059	0.810886107
blast	Oscillibacter_SGB43496	acute_cyto_PC5	0.220544725	0.198674672
vns	Oscillibacter_SGB43496	acute_cyto_PC5	0.34795292	0.198674672
blast	Oscillospiraceae_bacterium	acute_cyto_PC5	0.492783661	0.903674075
vns	Oscillospiraceae_bacterium	acute_cyto_PC5	0.251973413	0.903674075
blast	Oscillospiraceae_unclassified_SGB43502	acute_cyto_PC5	0.017271201	0.316437422
vns	Oscillospiraceae_unclassified_SGB43502	acute_cyto_PC5	0.112345497	0.316437422
blast	Oscillospiraceae_unclassified_SGB43505	acute_cyto_PC5	0.374299908	0.518008025
vns	Oscillospiraceae_unclassified_SGB43505	acute_cyto_PC5	0.618204990	0.518008025
blast	Oscillospiraceae_unclassified_SGB94989	acute_cyto_PC5	0.953368969	0.056786019
vns	Oscillospiraceae_unclassified_SGB94989	acute_cyto_PC5	0.623615200	0.056786019
blast	Parasutterella_excrementihominis	acute_cyto_PC5	0.023419378	0.987353503
vns	Parasutterella_excrementihominis	acute_cyto_PC5	0.504157156	0.987353503
blast	Richness (# observed features)	acute_cyto_PC5	0.013777874	0.263031466
vns	Richness (# observed features)	acute_cyto_PC5	0.958247838	0.263031466
blast	Schaedlerella_arabinosiphila	acute_cyto_PC5	0.114481983	0.371705844
vns	Schaedlerella_arabinosiphila	acute_cyto_PC5	0.386301964	0.371705844

blast	Shannon Index	acute_cyto_PC5	0.006473070	0.16842587
vns	Shannon Index	acute_cyto_PC5	0.83599244	0.16842587
blast	Turicibacter_sp_1E2	acute_cyto_PC5	0.00959413	0.46435169
vns	Turicibacter_sp_1E2	acute_cyto_PC5	0.183926772	0.46435169

p_composite	dact_stat	dact_thr_10	dact_thr_15	dact_thr_20	signif_at
0.284979380	0.290673579	0.001892477	0.002328539	0.002949229	
0.207157594	0.182429176	0.000232278	0.000313216	0.000521262	
0.654198757	0.601665270	0.001892477	0.002328539	0.002949229	
0.338901524	0.334917577	0.000232278	0.000313216	0.000521262	
0.464672568	0.468967998	0.001892477	0.002328539	0.002949229	
0.510656370	0.462185514	0.000232278	0.000313216	0.000521262	
0.911112899	0.874869378	0.001892477	0.002328539	0.002949229	
0.846440848	0.841997280	0.000232278	0.000313216	0.000521262	
0.971180381	0.964241480	0.001892477	0.002328539	0.002949229	
0.937376209	0.954830407	0.000232278	0.000313216	0.000521262	
0.138659486	0.124112140	0.001892477	0.002328539	0.002949229	
0.233901430	0.202502896	0.000232278	0.000313216	0.000521262	
0.476823640	0.473300385	0.001892477	0.002328539	0.002949229	
0.382106246	0.367972319	0.000232278	0.000313216	0.000521262	
0.080019612	0.072316359	0.001892477	0.002328539	0.002949229	
0.128347795	0.116587603	0.000232278	0.000313216	0.000521262	
0.674500717	0.619001935	0.001892477	0.002328539	0.002949229	
0.650898816	0.633703810	0.000232278	0.000313216	0.000521262	
0.816116443	0.769585116	0.001892477	0.002328539	0.002949229	
0.742927949	0.711394795	0.000232278	0.000313216	0.000521262	
0.821437986	0.770463336	0.001892477	0.002328539	0.002949229	
0.75021983	0.712782320	0.000232278	0.000313216	0.000521262	
0.015960153	0.019629421	0.001892477	0.002328539	0.002949229	
0.038851975	0.020035707	0.000232278	0.000313216	0.000521262	
0.514351344	0.502126235	0.001892477	0.002328539	0.002949229	
0.424465542	0.399538993	0.000232278	0.000313216	0.000521262	
0.487885383	0.484550087	0.001892477	0.002328539	0.002949229	
0.386981644	0.380267448	0.000232278	0.000313216	0.000521262	
0.437690012	0.450200465	0.001892477	0.002328539	0.002949229	
0.348666333	0.343328754	0.000232278	0.000313216	0.000521262	
0.432723270	0.447345346	0.001892477	0.002328539	0.002949229	
0.342808590	0.339953856	0.000232278	0.000313216	0.000521262	
0.573531849	0.538092321	0.001892477	0.002328539	0.002949229	
0.478985942	0.439451302	0.000232278	0.000313216	0.000521262	
0.157897499	0.145886115	0.001892477	0.002328539	0.002949229	
0.050081281	0.026885448	0.000232278	0.000313216	0.000521262	
0.392382453	0.420486112	0.001892477	0.002328539	0.002949229	
0.312193875	0.311451228	0.000232278	0.000313216	0.000521262	
0.670647244	0.614409037	0.001892477	0.002328539	0.002949229	
0.552782245	0.526444646	0.000232278	0.000313216	0.000521262	
0.017499687	0.021222866	0.001892477	0.002328539	0.002949229	
0.054507717	0.032366654	0.000232278	0.000313216	0.000521262	
0.840550310	0.787764304	0.001892477	0.002328539	0.002949229	
0.915495678	0.941091732	0.000232278	0.000313216	0.000521262	
0.904127486	0.869973683	0.001892477	0.002328539	0.002949229	
0.833902834	0.835990199	0.000232278	0.000313216	0.000521262	

0.725431196	0.663526651	0.001892477	0.002328539	0.002949229
0.612503649	0.584040370	0.000232278	0.000313216	0.000521262
0.568775514	0.535823971	0.001892477	0.002328539	0.002949229
0.468183113	0.436527261	0.000232278	0.000313216	0.000521262
0.656381120	0.604045112	0.001892477	0.002328539	0.002949229
0.540983943	0.514947145	0.000232278	0.000313216	0.000521262
0.788816833	0.749395260	0.001892477	0.002328539	0.002949229
0.687898307	0.687040487	0.000232278	0.000313216	0.000521262
0.015439219	0.019227941	0.001892477	0.002328539	0.002949229
0.005105552	0.001762015	0.000232278	0.000313216	0.000521262
0.071401441	0.067630660	0.001892477	0.002328539	0.002949229
0.159967935	0.137720025	0.000232278	0.000313216	0.000521262
0.067490670	0.065112100	0.001892477	0.002328539	0.002949229
0.030479706	0.016137769	0.000232278	0.000313216	0.000521262
0.692749566	0.629927868	0.001892477	0.002328539	0.002949229
0.567481531	0.544446189	0.000232278	0.000313216	0.000521262
0.990556109	0.984621234	0.001892477	0.002328539	0.002949229
0.932439417	0.947640042	0.000232278	0.000313216	0.000521262
0.610127185	0.559862164	0.001892477	0.002328539	0.002949229
0.514863730	0.464010459	0.000232278	0.000313216	0.000521262
0.861868600	0.811008607	0.001892477	0.002328539	0.002949229
0.786070888	0.762510245	0.000232278	0.000313216	0.000521262
0.259540036	0.258845860	0.001892477	0.002328539	0.002949229
0.327383425	0.322991840	0.000232278	0.000313216	0.000521262
0.308227723	0.311068739	0.001892477	0.002328539	0.002949229
0.227536636	0.201644379	0.000232278	0.000313216	0.000521262
0.678209353	0.625275241	0.001892477	0.002328539	0.002949229
0.922506647	0.944270022	0.000232278	0.000313216	0.000521262
0.813667156	0.768955932	0.001892477	0.002328539	0.002949229
0.452355938	0.414117418	0.000232278	0.000313216	0.000521262
0.955872388	0.940631370	0.001892477	0.002328539	0.002949229
0.908197694	0.924803934	0.000232278	0.000313216	0.000521262
0.074722396	0.070155183	0.001892477	0.002328539	0.002949229
0.033735907	0.018386757	0.000232278	0.000313216	0.000521262
0.751361835	0.699195194	0.001892477	0.002328539	0.002949229
0.304958154	0.301483010	0.000232278	0.000313216	0.000521262
0.020245148	0.023938332	0.001892477	0.002328539	0.002949229
0.007426844	0.002632926	0.000232278	0.000313216	0.000521262
0.686722258	0.627594561	0.001892477	0.002328539	0.002949229
0.563346684	0.541809812	0.000232278	0.000313216	0.000521262
0.358020885	0.383016249	0.001892477	0.002328539	0.002949229
0.654863854	0.651732456	0.000232278	0.000313216	0.000521262
0.946594559	0.938053973	0.001892477	0.002328539	0.002949229
0.889913552	0.921428124	0.000232278	0.000313216	0.000521262
0.361018871	0.388310137	0.001892477	0.002328539	0.002949229
0.279516153	0.278034851	0.000232278	0.000313216	0.000521262

0.391481730	0.419942571	0.001892477	0.002328539	0.002949229
0.628022645	0.616857639	0.000232278	0.000313216	0.000521262
0.318987754	0.321271904	0.001892477	0.002328539	0.002949229
0.097722027	0.070127891	0.000232278	0.000313216	0.000521262
0.599487188	0.547478708	0.001892477	0.002328539	0.002949229
0.504312788	0.449834769	0.000232278	0.000313216	0.000521262
0.090793707	0.080862254	0.001892477	0.002328539	0.002949229
0.046667440	0.023612183	0.000232278	0.000313216	0.000521262
0.132977220	0.120841522	0.001892477	0.002328539	0.002949229
0.070007841	0.043424416	0.000232278	0.000313216	0.000521262
0.037470659	0.038480710	0.001892477	0.002328539	0.002949229
0.042106386	0.021460021	0.000232278	0.000313216	0.000521262
0.231519125	0.231756307	0.001892477	0.002328539	0.002949229
0.236765527	0.209341329	0.000232278	0.000313216	0.000521262
0.237738517	0.237260028	0.001892477	0.002328539	0.002949229
0.149467714	0.134469861	0.000232278	0.000313216	0.000521262
0.387269449	0.417285791	0.001892477	0.002328539	0.002949229
0.308398697	0.308047959	0.000232278	0.000313216	0.000521262
0.204232876	0.196393182	0.001892477	0.002328539	0.002949229
0.582646414	0.555804265	0.000232278	0.000313216	0.000521262
0.571472326	0.536903936	0.001892477	0.002328539	0.002949229
0.318305662	0.315254016	0.000232278	0.000313216	0.000521262
0.404919517	0.427598850	0.001892477	0.002328539	0.002949229
0.089590317	0.063954216	0.000232278	0.000313216	0.000521262
0.759575969	0.703598930	0.001892477	0.002328539	0.002949229
0.741653265	0.709227725	0.000232278	0.000313216	0.000521262
0.269172670	0.270178878	0.001892477	0.002328539	0.002949229
0.085361360	0.061160352	0.000232278	0.000313216	0.000521262
0.767012807	0.720397246	0.001892477	0.002328539	0.002949229
0.661278165	0.652117234	0.000232278	0.000313216	0.000521262
0.738966668	0.683602533	0.001892477	0.002328539	0.002949229
0.677760161	0.674943606	0.000232278	0.000313216	0.000521262
0.713157846	0.643147161	0.001892477	0.002328539	0.002949229
0.589467317	0.559969101	0.000232278	0.000313216	0.000521262
0.629598925	0.573793342	0.001892477	0.002328539	0.002949229
0.994576308	0.999707478	0.000232278	0.000313216	0.000521262
0.796049155	0.756201320	0.001892477	0.002328539	0.002949229
0.711209412	0.696055260	0.000232278	0.000313216	0.000521262
0.381417723	0.409589542	0.001892477	0.002328539	0.002949229
0.491591161	0.442043533	0.000232278	0.000313216	0.000521262
0.121103445	0.108889061	0.001892477	0.002328539	0.002949229
0.153734526	0.135892112	0.000232278	0.000313216	0.000521262
0.553478036	0.525005017	0.001892477	0.002328539	0.002949229
0.602615565	0.564333845	0.000232278	0.000313216	0.000521262
0.136878256	0.123496746	0.001892477	0.002328539	0.002949229
0.075929012	0.047950285	0.000232278	0.000313216	0.000521262

0.563907406	0.533405853	0.001892477	0.002328539	0.002949229
0.472722662	0.436799212	0.000232278	0.000313216	0.000521262
0.290886039	0.297496208	0.001892477	0.002328539	0.002949229
0.210686438	0.188918422	0.000232278	0.000313216	0.000521262
0.886628709	0.845135218	0.001892477	0.002328539	0.002949229
0.818465689	0.804755307	0.000232278	0.000313216	0.000521262
0.881004150	0.839694905	0.001892477	0.002328539	0.002949229
0.813853797	0.797924236	0.000232278	0.000313216	0.000521262
0.553417194	0.524903513	0.001892477	0.002328539	0.002949229
0.416912333	0.396064740	0.000232278	0.000313216	0.000521262
0.265340556	0.267592923	0.001892477	0.002328539	0.002949229
0.194435428	0.161293811	0.000232278	0.000313216	0.000521262
0.773416489	0.724752777	0.001892477	0.002328539	0.002949229
0.806401368	0.794994797	0.000232278	0.000313216	0.000521262
0.459654237	0.462878522	0.001892477	0.002328539	0.002949229
0.350534795	0.344601731	0.000232278	0.000313216	0.000521262
0.837522512	0.779209415	0.001892477	0.002328539	0.002949229
0.769546063	0.723128183	0.000232278	0.000313216	0.000521262
0.172180381	0.159315626	0.001892477	0.002328539	0.002949229
0.100684831	0.072381704	0.000232278	0.000313216	0.000521262
0.982227077	0.972916780	0.001892477	0.002328539	0.002949229
0.973126203	0.985959334	0.000232278	0.000313216	0.000521262
0.934413459	0.927858652	0.001892477	0.002328539	0.002949229
0.870557428	0.908608612	0.000232278	0.000313216	0.000521262
0.639610606	0.585485681	0.001892477	0.002328539	0.002949229
0.623013437	0.614565873	0.000232278	0.000313216	0.000521262
0.320962505	0.324791362	0.001892477	0.002328539	0.002949229
0.949384063	0.963513578	0.000232278	0.000313216	0.000521262
0.719810682	0.652117951	0.001892477	0.002328539	0.002949229
0.711740718	0.696086204	0.000232278	0.000313216	0.000521262
0.144102321	0.130160716	0.001892477	0.002328539	0.002949229
0.253341680	0.234446633	0.000232278	0.000313216	0.000521262
0.078473927	0.071428664	0.001892477	0.002328539	0.002949229
0.036495119	0.018985153	0.000232278	0.000313216	0.000521262
0.650280269	0.598502018	0.001892477	0.002328539	0.002949229
0.534046057	0.508069293	0.000232278	0.000313216	0.000521262
0.593515976	0.546438080	0.001892477	0.002328539	0.002949229
0.498214074	0.448575122	0.000232278	0.000313216	0.000521262
0.898740000	0.854766031	0.001892477	0.002328539	0.002949229
0.988924383	0.997336868	0.000232278	0.000313216	0.000521262
0.116105036	0.103308670	0.001892477	0.002328539	0.002949229
0.217385910	0.197596144	0.000232278	0.000313216	0.000521262
0.779007018	0.736894620	0.001892477	0.002328539	0.002949229
0.668967030	0.671989358	0.000232278	0.000313216	0.000521262
0.764720495	0.718607230	0.001892477	0.002328539	0.002949229
0.761683655	0.719298223	0.000232278	0.000313216	0.000521262

0.260951683	0.261348072	0.001892477	0.002328539	0.002949229
0.408734417	0.394852630	0.000232278	0.000313216	0.000521262
0.840215773	0.787132081	0.001892477	0.002328539	0.002949229
0.415979852	0.395627644	0.000232278	0.000313216	0.000521262
0.926309491	0.916617735	0.001892477	0.002328539	0.002949229
0.864525231	0.894448897	0.000232278	0.000313216	0.000521262
0.262221349	0.263600110	0.001892477	0.002328539	0.002949229
0.177189504	0.148873911	0.000232278	0.000313216	0.000521262
0.245782773	0.245986504	0.001892477	0.002328539	0.002949229
0.138775690	0.121494318	0.000232278	0.000313216	0.000521262
0.452876495	0.453655558	0.001892477	0.002328539	0.002949229
0.684193146	0.683410955	0.000232278	0.000313216	0.000521262
0.366829693	0.391061040	0.001892477	0.002328539	0.002949229
0.714567100	0.699112189	0.000232278	0.000313216	0.000521262
0.220239212	0.219828347	0.001892477	0.002328539	0.002949229
0.131325834	0.119581287	0.000232278	0.000313216	0.000521262
0.257644954	0.255531629	0.001892477	0.002328539	0.002949229
0.275970317	0.261617290	0.000232278	0.000313216	0.000521262
0.742701027	0.692933846	0.001892477	0.002328539	0.002949229
0.860467105	0.843495820	0.000232278	0.000313216	0.000521262
0.631087016	0.573960084	0.001892477	0.002328539	0.002949229
0.429736793	0.402954094	0.000232278	0.000313216	0.000521262
0.585044281	0.541066897	0.001892477	0.002328539	0.002949229
0.334421081	0.325361691	0.000232278	0.000313216	0.000521262
0.037865227	0.038893563	0.001892477	0.002328539	0.002949229
0.002690811	0.000845396	0.000232278	0.000313216	0.000521262
0.975167082	0.966369258	0.001892477	0.002328539	0.002949229
0.946863753	0.958363499	0.000232278	0.000313216	0.000521262
0.405946274	0.429416895	0.001892477	0.002328539	0.002949229
0.697806034	0.693981981	0.000232278	0.000313216	0.000521262
0.495448499	0.489220642	0.001892477	0.002328539	0.002949229
0.040344583	0.020511971	0.000232278	0.000313216	0.000521262
0.990739122	0.990678263	0.001892477	0.002328539	0.002949229
0.980528694	0.989900365	0.000232278	0.000313216	0.000521262
0.370871838	0.392419342	0.001892477	0.002328539	0.002949229
0.287779566	0.282318386	0.000232278	0.000313216	0.000521262
0.073975006	0.069776983	0.001892477	0.002328539	0.002949229
0.139120623	0.121514889	0.000232278	0.000313216	0.000521262
0.297702627	0.303582614	0.001892477	0.002328539	0.002949229
0.167692584	0.143822578	0.000232278	0.000313216	0.000521262
0.614756459	0.562030043	0.001892477	0.002328539	0.002949229
0.518134628	0.466485471	0.000232278	0.000313216	0.000521262
0.092809015	0.082143371	0.001892477	0.002328539	0.002949229
0.389920018	0.382804470	0.000232278	0.000313216	0.000521262
0.639937021	0.586101887	0.001892477	0.002328539	0.002949229
0.401772259	0.389699977	0.000232278	0.000313216	0.000521262

0.0420921100	0.0423757193	0.0018924774	0.0023285398	0.0029492291
0.2712289903	0.2547646344	0.0002322788	0.0003132165	0.0005212622
0.8521501313	0.8025399992	0.0018924774	0.0023285398	0.0029492291
0.8839361012	0.9200638743	0.0002322788	0.0003132165	0.0005212622
0.7006072198	0.6346377131	0.0018924774	0.0023285398	0.0029492291
0.5769230935	0.5503778027	0.0002322788	0.0003132165	0.0005212622
0.8053197911	0.7639350370	0.0018924774	0.0023285398	0.0029492291
0.7349607630	0.7055623964	0.0002322788	0.0003132165	0.0005212622
0.0113117701	0.0154734030	0.0018924774	0.0023285398	0.0029492291
0.0598737991	0.0340463834	0.0002322788	0.0003132165	0.0005212622
0.6499508043	0.606636208	0.0011481703	0.0015516043	0.0019838328
0.5548292217	0.5458498841	0.0005676314	0.0008404644	0.0011437638
0.6361893414	0.5949222175	0.0011481703	0.0015516043	0.0019838328
0.3310506275	0.3218140067	0.0005676314	0.0008404644	0.0011437638
0.9263731290	0.872260258	0.0011481703	0.0015516043	0.0019838328
0.8750616187	0.8497557719	0.0005676314	0.0008404644	0.0011437638
0.4315194752	0.4363610437	0.0011481703	0.0015516043	0.0019838328
0.3683354040	0.3616535315	0.0005676314	0.0008404644	0.0011437638
0.544102756	0.5077290004	0.0011481703	0.0015516043	0.0019838328
0.366184468	0.3565011668	0.0005676314	0.0008404644	0.0011437638
0.1659068359	0.1665108359	0.0011481703	0.0015516043	0.0019838328
0.2279919551	0.2086154357	0.0005676314	0.0008404644	0.0011437638
0.5612079757	0.5249773518	0.0011481703	0.0015516043	0.0019838328
0.4931773669	0.4564099063	0.0005676314	0.0008404644	0.0011437638
0.2160692912	0.2135566387	0.0011481703	0.0015516043	0.0019838328
0.1589348194	0.1428532047	0.0005676314	0.0008404644	0.0011437638
0.4218240061	0.4216727673	0.0011481703	0.0015516043	0.0019838328
0.6326578457	0.6009604395	0.0005676314	0.0008404644	0.0011437638
0.7579743617	0.6828136727	0.0011481703	0.0015516043	0.0019838328
0.6604477199	0.6309924798	0.0005676314	0.0008404644	0.0011437638
0.5474793098	0.5126010268	0.0011481703	0.0015516043	0.0019838328
0.4805478017	0.4429166650	0.0005676314	0.0008404644	0.0011437638
0.0338917875	0.0421472351	0.0011481703	0.0015516043	0.0019838328
0.0251902409	0.0226693937	0.0005676314	0.0008404644	0.0011437638
0.8550051563	0.8157498680	0.0011481703	0.0015516043	0.0019838328
0.7941231530	0.7836766539	0.0005676314	0.0008404644	0.0011437638
0.9730682959	0.949686698	0.0011481703	0.0015516043	0.0019838328
0.9526289031	0.9405585365	0.0005676314	0.0008404644	0.0011437638
0.6225344834	0.580940927	0.0011481703	0.0015516043	0.0019838328
0.5325916658	0.5178531437	0.0005676314	0.0008404644	0.0011437638
0.4183957730	0.420592057	0.0011481703	0.0015516043	0.0019838328
0.3615081988	0.345294904	0.0005676314	0.0008404644	0.0011437638
0.4664157289	0.4569471459	0.0011481703	0.0015516043	0.0019838328
0.4026915977	0.3834436803	0.0005676314	0.0008404644	0.0011437638
0.1067982471	0.1173943554	0.0011481703	0.0015516043	0.0019838328
0.0151003331	0.0143874768	0.0005676314	0.0008404644	0.0011437638

0.7326593152	0.6604859683	0.0011481703	0.0015516043	0.0019838328
0.6357702906	0.6060682073	0.0005676314	0.0008404644	0.0011437638
0.7958994629	0.7209544869	0.0011481703	0.0015516043	0.0019838328
0.7142360159	0.6743966195	0.0005676314	0.0008404644	0.0011437638
0.0152556441	0.0219485435	0.0011481703	0.0015516043	0.0019838328
0.030997555	0.0304669368	0.0005676314	0.0008404644	0.0011437638
0.9441139961	0.8950979028	0.0011481703	0.0015516043	0.0019838328
0.9498086527	0.9398675662	0.0005676314	0.0008404644	0.0011437638
0.2215168429	0.2157282705	0.0011481703	0.0015516043	0.0019838328
0.1660672373	0.1449070041	0.0005676314	0.0008404644	0.0011437638
0.1005026556	0.1123669456	0.0011481703	0.0015516043	0.0019838328
0.0598346322	0.0595412597	0.0005676314	0.0008404644	0.0011437638
0.1984955012	0.1939251728	0.0011481703	0.0015516043	0.0019838328
0.0694394567	0.0656028193	0.0005676314	0.0008404644	0.0011437638
0.1614525235	0.1640230916	0.0011481703	0.0015516043	0.0019838328
0.3090324149	0.2878851388	0.0005676314	0.0008404644	0.0011437638
0.3055201595	0.3138262472	0.0011481703	0.0015516043	0.0019838328
0.2560767653	0.2370717905	0.0005676314	0.0008404644	0.0011437638
0.0695209577	0.0799872975	0.0011481703	0.0015516043	0.0019838328
0.0393094957	0.0371326294	0.0005676314	0.0008404644	0.0011437638
0.6665568106	0.6172935588	0.0011481703	0.0015516043	0.0019838328
0.5743436902	0.5579811308	0.0005676314	0.0008404644	0.0011437638
0.1677912147	0.1672441971	0.0011481703	0.0015516043	0.0019838328
0.1122021979	0.1027457535	0.0005676314	0.0008404644	0.0011437638
0.6868033433	0.6311389192	0.0011481703	0.0015516043	0.0019838328
0.5907849108	0.5729640887	0.0005676314	0.0008404644	0.0011437638
0.8516827281	0.8068460188	0.0011481703	0.0015516043	0.0019838328
0.9051261848	0.8931994778	0.0005676314	0.0008404644	0.0011437638
0.5235460314	0.488640973	0.0011481703	0.0015516043	0.0019838328
0.4595265903	0.4171681681	0.0005676314	0.0008404644	0.0011437638
0.6034785494	0.5694928444	0.0011481703	0.0015516043	0.0019838328
0.514899665	0.5048161283	0.0005676314	0.0008404644	0.0011437638
0.1594081684	0.1624815936	0.0011481703	0.0015516043	0.0019838328
0.3161894398	0.301307403	0.0005676314	0.0008404644	0.0011437638
0.4113502088	0.4162815505	0.0011481703	0.0015516043	0.0019838328
0.3552549661	0.3407193025	0.0005676314	0.0008404644	0.0011437638
0.9179183101	0.8694332824	0.0011481703	0.0015516043	0.0019838328
0.9593564934	0.9418266513	0.0005676314	0.0008404644	0.0011437638
0.8261689242	0.7647483716	0.0011481703	0.0015516043	0.0019838328
0.3832929592	0.3642811504	0.0005676314	0.0008404644	0.0011437638
0.8959686843	0.8673942385	0.0011481703	0.0015516043	0.0019838328
0.8380607483	0.8425992756	0.0005676314	0.0008404644	0.0011437638
0.0764932601	0.0878976659	0.0011481703	0.0015516043	0.0019838328
0.0447382068	0.0423442696	0.0005676314	0.0008404644	0.0011437638
0.9358495312	0.8921562939	0.0011481703	0.0015516043	0.0019838328
0.6296479098	0.5980213899	0.0005676314	0.0008404644	0.0011437638

0.011383237	0.016060815	0.001148170	0.001551604	0.001983832
0.005332443	0.004384456	0.000567631	0.000840464	0.001143763
0.115896489	0.124990470	0.001148170	0.001551604	0.001983832
0.078777633	0.073290091	0.000567631	0.000840464	0.001143763
0.453215143	0.446644455	0.001148170	0.001551604	0.001983832
0.669449935	0.638627081	0.000567631	0.000840464	0.001143763
0.633454897	0.590010911	0.001148170	0.001551604	0.001983832
0.448799240	0.413609932	0.000567631	0.000840464	0.001143763
0.432936770	0.436507516	0.001148170	0.001551604	0.001983832
0.369626131	0.361797398	0.000567631	0.000840464	0.001143763
0.422217520	0.422261932	0.001148170	0.001551604	0.001983832
0.616936317	0.593616973	0.000567631	0.000840464	0.001143763
0.336729818	0.345050175	0.001148170	0.001551604	0.001983832
0.070316855	0.065928983	0.000567631	0.000840464	0.001143763
0.500157123	0.472185886	0.001148170	0.001551604	0.001983832
0.436160514	0.399457590	0.000567631	0.000840464	0.001143763
0.396360897	0.410804978	0.001148170	0.001551604	0.001983832
0.342013593	0.335080679	0.000567631	0.000840464	0.001143763
0.061500764	0.071253048	0.001148170	0.001551604	0.001983832
0.019705068	0.017235605	0.000567631	0.000840464	0.001143763
0.139636559	0.144594149	0.001148170	0.001551604	0.001983832
0.090320369	0.084239407	0.000567631	0.000840464	0.001143763
0.259111589	0.255583500	0.001148170	0.001551604	0.001983832
0.221545652	0.199136356	0.000567631	0.000840464	0.001143763
0.637885597	0.598173113	0.001148170	0.001551604	0.001983832
0.541975844	0.536457791	0.000567631	0.000840464	0.001143763
0.806497393	0.739303497	0.001148170	0.001551604	0.001983832
0.738490855	0.695413416	0.000567631	0.000840464	0.001143763
0.050616163	0.058893791	0.001148170	0.001551604	0.001983832
0.536840404	0.526116453	0.000567631	0.000840464	0.001143763
0.631023601	0.585912977	0.001148170	0.001551604	0.001983832
0.511259654	0.503714209	0.000567631	0.000840464	0.001143763
0.503620510	0.477515272	0.001148170	0.001551604	0.001983832
0.130862926	0.121818874	0.000567631	0.000840464	0.001143763
0.520027187	0.487018956	0.001148170	0.001551604	0.001983832
0.720161772	0.677488913	0.000567631	0.000840464	0.001143763
0.518701039	0.486151163	0.001148170	0.001551604	0.001983832
0.449315052	0.413875784	0.000567631	0.000840464	0.001143763
0.229891761	0.224824988	0.001148170	0.001551604	0.001983832
0.196789474	0.174701169	0.000567631	0.000840464	0.001143763
0.754935162	0.681217129	0.001148170	0.001551604	0.001983832
0.706213524	0.670423035	0.000567631	0.000840464	0.001143763
0.897242285	0.867544661	0.001148170	0.001551604	0.001983832
0.841440973	0.843633670	0.000567631	0.000840464	0.001143763
0.129746399	0.135911416	0.001148170	0.001551604	0.001983832
0.963010646	0.947907598	0.000567631	0.000840464	0.001143763

0.9642319342	0.9411836591	0.0011481703	0.0015516043	0.0019838328
0.9416040521	0.9310223695	0.0005676314	0.0008404644	0.0011437638
0.819715619	0.7491170259	0.0011481703	0.0015516043	0.0019838328
0.7569715734	0.7066299728	0.0005676314	0.0008404644	0.0011437638
0.1302901254	0.1367602903	0.0011481703	0.0015516043	0.0019838328
0.1529731596	0.1413266424	0.0005676314	0.0008404644	0.0011437638
0.7721858336	0.6904012566	0.0011481703	0.0015516043	0.0019838328
0.6762509193	0.6396697921	0.0005676314	0.0008404644	0.0011437638
0.6116626767	0.5706669721	0.0011481703	0.0015516043	0.0019838328
0.522568351	0.5060851355	0.0005676314	0.0008404644	0.0011437638
0.3783324584	0.3971321773	0.0011481703	0.0015516043	0.0019838328
0.4404268658	0.4074768338	0.0005676314	0.0008404644	0.0011437638
0.2541710714	0.2524918986	0.0011481703	0.0015516043	0.0019838328
0.2030282898	0.1784804386	0.0005676314	0.0008404644	0.0011437638
0.3672215107	0.3803431035	0.0011481703	0.0015516043	0.0019838328
0.3200247211	0.3036430445	0.0005676314	0.0008404644	0.0011437638
0.2889354605	0.2979426079	0.0011481703	0.0015516043	0.0019838328
0.4445300803	0.4103739355	0.0005676314	0.0008404644	0.0011437638
0.4249626777	0.4263995072	0.0011481703	0.0015516043	0.0019838328
0.1986107115	0.1768491817	0.0005676314	0.0008404644	0.0011437638
0.6764307942	0.6238271431	0.0011481703	0.0015516043	0.0019838328
0.58233216	0.5650052431	0.0005676314	0.0008404644	0.0011437638
0.6740789278	0.621111785	0.0011481703	0.0015516043	0.0019838328
0.7861253496	0.7652372028	0.0005676314	0.0008404644	0.0011437638
0.8689510909	0.8284066645	0.0011481703	0.0015516043	0.0019838328
0.8050407866	0.7975928361	0.0005676314	0.0008404644	0.0011437638
0.6742830496	0.6215005333	0.0011481703	0.0015516043	0.0019838328
0.5348849439	0.5201114786	0.0005676314	0.0008404644	0.0011437638
0.1337575446	0.1417407249	0.0011481703	0.0015516043	0.0019838328
0.0865933169	0.0820705336	0.0005676314	0.0008404644	0.0011437638
0.5068094755	0.482133726	0.0011481703	0.0015516043	0.0019838328
0.9698173605	0.9612504698	0.0005676314	0.0008404644	0.0011437638
0.1718223729	0.1696697241	0.0011481703	0.0015516043	0.0019838328
0.0959908209	0.0872210998	0.0005676314	0.0008404644	0.0011437638
0.778693046	0.7048227657	0.0011481703	0.0015516043	0.0019838328
0.657433933	0.6251153052	0.0005676314	0.0008404644	0.0011437638
0.9884258432	0.9944060789	0.0011481703	0.0015516043	0.0019838328
0.9895568136	0.9934726829	0.0005676314	0.0008404644	0.0011437638
0.534198858	0.5005991727	0.0011481703	0.0015516043	0.0019838328
0.6912011837	0.6612854891	0.0005676314	0.0008404644	0.0011437638
0.175663632	0.1715742826	0.0011481703	0.0015516043	0.0019838328
0.2415066133	0.2235338381	0.0005676314	0.0008404644	0.0011437638
0.2898059007	0.2987424882	0.0011481703	0.0015516043	0.0019838328
0.2373736961	0.2223150838	0.0005676314	0.0008404644	0.0011437638
0.9397337359	0.893534727	0.0011481703	0.0015516043	0.0019838328
0.8847771818	0.8741097192	0.0005676314	0.0008404644	0.0011437638

0.430108494	0.434244024	0.001148170	0.001551604	0.001983832
0.317032522	0.302885401	0.000567631	0.000840464	0.001143763
0.559677084	0.523975060	0.001148170	0.001551604	0.001983832
0.974075513	0.971055775	0.000567631	0.000840464	0.001143763
0.263588046	0.258012555	0.001148170	0.001551604	0.001983832
0.230092486	0.211642955	0.000567631	0.000840464	0.001143763
0.488282816	0.467778317	0.001148170	0.001551604	0.001983832
0.426560962	0.394963173	0.000567631	0.000840464	0.001143763
0.889455447	0.846690882	0.001148170	0.001551604	0.001983832
0.830907019	0.819241593	0.000567631	0.000840464	0.001143763
0.232824821	0.227607475	0.001148170	0.001551604	0.001983832
0.410174008	0.390657158	0.000567631	0.000840464	0.001143763
0.853364198	0.813700509	0.001148170	0.001551604	0.001983832
0.474006251	0.433332443	0.000567631	0.000840464	0.001143763
0.226383647	0.221553988	0.001148170	0.001551604	0.001983832
0.181467243	0.155930494	0.000567631	0.000840464	0.001143763
0.579124801	0.538703238	0.001148170	0.001551604	0.001983832
0.501905663	0.470844449	0.000567631	0.000840464	0.001143763
0.323308922	0.336898511	0.001148170	0.001551604	0.001983832
0.274718363	0.259590499	0.000567631	0.000840464	0.001143763
0.242033571	0.237996509	0.001148170	0.001551604	0.001983832
0.686852125	0.656431945	0.000567631	0.000840464	0.001143763
0.962451203	0.933023454	0.001148170	0.001551604	0.001983832
0.928419270	0.920537147	0.000567631	0.000840464	0.001143763
0.126032641	0.131552640	0.001148170	0.001551604	0.001983832
0.079724396	0.073937469	0.000567631	0.000840464	0.001143763
0.282798526	0.285910481	0.001148170	0.001551604	0.001983832
0.270830174	0.254844045	0.000567631	0.000840464	0.001143763
0.901606745	0.868292887	0.001148170	0.001551604	0.001983832
0.869362615	0.847672488	0.000567631	0.000840464	0.001143763
0.702532981	0.640592989	0.001148170	0.001551604	0.001983832
0.601698153	0.582975753	0.000567631	0.000840464	0.001143763
0.721240205	0.652393939	0.001148170	0.001551604	0.001983832
0.622593253	0.596657933	0.000567631	0.000840464	0.001143763
0.055892755	0.065520657	0.001148170	0.001551604	0.001983832
0.015843636	0.014952870	0.000567631	0.000840464	0.001143763
0.187020834	0.181919136	0.001148170	0.001551604	0.001983832
0.562734258	0.553298104	0.000567631	0.000840464	0.001143763
0.840664953	0.792607687	0.001148170	0.001551604	0.001983832
0.783923405	0.757522651	0.000567631	0.000840464	0.001143763
0.635897557	0.594362273	0.001148170	0.001551604	0.001983832
0.119602144	0.108973868	0.000567631	0.000840464	0.001143763
0.832871596	0.790341484	0.001148170	0.001551604	0.001983832
0.799728717	0.787061146	0.000567631	0.000840464	0.001143763
0.662246027	0.615647854	0.001148170	0.001551604	0.001983832
0.567908974	0.555884458	0.000567631	0.000840464	0.001143763

0.105827117	0.116037464	0.001148170	0.001551604	0.001983832
0.138126468	0.12832758	0.000567631	0.000840464	0.001143763
0.244771201	0.242558640	0.001148170	0.001551604	0.001983832
0.082529045	0.075562049	0.000567631	0.000840464	0.001143763
0.705534273	0.645506985	0.001148170	0.001551604	0.001983832
0.60999513	0.589300345	0.000567631	0.000840464	0.001143763
0.240086465	0.234855181	0.001148170	0.001551604	0.001983832
0.399951414	0.382786007	0.000567631	0.000840464	0.001143763
0.824358495	0.758488584	0.001148170	0.001551604	0.001983832
0.429883266	0.397275800	0.000567631	0.000840464	0.001143763
0.453949355	0.447445555	0.001148170	0.001551604	0.001983832
0.393465087	0.373786431	0.000567631	0.000840464	0.001143763
0.990851573	0.996867047	0.001148170	0.001551604	0.001983832
0.998477099	0.997215157	0.000567631	0.000840464	0.001143763
0.782994712	0.713112215	0.001148170	0.001551604	0.001983832
0.698548185	0.665819355	0.000567631	0.000840464	0.001143763
0.902085755	0.868343239	0.001148170	0.001551604	0.001983832
0.860750259	0.845520400	0.000567631	0.000840464	0.001143763
0.267887326	0.264806485	0.001148170	0.001551604	0.001983832
0.214756792	0.190036397	0.000567631	0.000840464	0.001143763
0.846554648	0.669868640	0.004929348	0.016005343	0.019029080
0.710282337	0.600518494	0.005375604	0.006489046	0.007577204
0.860766524	0.683017754	0.004929348	0.016005343	0.019029080
0.624008042	0.521053789	0.005375604	0.006489046	0.007577204
0.178442024	0.154790951	0.004929348	0.016005343	0.019029080
0.584271891	0.484065108	0.005375604	0.006489046	0.007577204
0.111273561	0.109842899	0.004929348	0.016005343	0.019029080
0.053332373	0.066931081	0.005375604	0.006489046	0.007577204
0.603334476	0.468165886	0.004929348	0.016005343	0.019029080
0.505628678	0.412121566	0.005375604	0.006489046	0.007577204
0.103137585	0.104453705	0.004929348	0.016005343	0.019029080
0.304594164	0.268271623	0.005375604	0.006489046	0.007577204
0.399108775	0.331355598	0.004929348	0.016005343	0.019029080
0.385039563	0.322034502	0.005375604	0.006489046	0.007577204
0.929416286	0.751585097	0.004929348	0.016005343	0.019029080
0.831870935	0.695573519	0.005375604	0.006489046	0.007577204
0.907614928	0.728446956	0.004929348	0.016005343	0.019029080
0.854102735	0.714667124	0.005375604	0.006489046	0.007577204
0.219853514	0.185713982	0.004929348	0.016005343	0.019029080
0.103891299	0.106272575	0.005375604	0.006489046	0.007577204
0.953317266	0.782102110	0.004929348	0.016005343	0.019029080
0.854327176	0.714869703	0.005375604	0.006489046	0.007577204
0.348459700	0.293023114	0.004929348	0.016005343	0.019029080
0.242246012	0.225558702	0.005375604	0.006489046	0.007577204
0.437265707	0.356847814	0.004929348	0.016005343	0.019029080
0.436005676	0.358032837	0.005375604	0.006489046	0.007577204

0.591415898	0.459527477	0.004929348	0.016005343	0.019029080	
0.541284534	0.443013679	0.005375604	0.006489046	0.007577204	
0.028146785	0.042491214	0.004929348	0.016005343	0.019029080	
0.338113566	0.290789040	0.005375604	0.006489046	0.007577204	
0.134630943	0.125505066	0.004929348	0.016005343	0.019029080	
0.224433999	0.212322211	0.005375604	0.006489046	0.007577204	
0.050661666	0.064161276	0.004929348	0.016005343	0.019029080	
0.134452508	0.132590659	0.005375604	0.006489046	0.007577204	
0.412012145	0.340511595	0.004929348	0.016005343	0.019029080	
0.223723731	0.211763287	0.005375604	0.006489046	0.007577204	
0.775720716	0.606375810	0.004929348	0.016005343	0.019029080	
0.634395741	0.531020477	0.005375604	0.006489046	0.007577204	
0.871122061	0.693069845	0.004929348	0.016005343	0.019029080	
0.758349660	0.636829321	0.005375604	0.006489046	0.007577204	
0.216418486	0.182852869	0.004929348	0.016005343	0.019029080	
0.151007747	0.147848630	0.005375604	0.006489046	0.007577204	
0.004681174	0.013335436	0.004929348	0.016005343	0.019029080	0.15
0.904757658	0.770279271	0.005375604	0.006489046	0.007577204	
0.107647218	0.107439549	0.004929348	0.016005343	0.019029080	
0.200425638	0.193586203	0.005375604	0.006489046	0.007577204	
0.868284722	0.690331321	0.004929348	0.016005343	0.019029080	
0.695928148	0.587666981	0.005375604	0.006489046	0.007577204	
0.274776276	0.230435338	0.004929348	0.016005343	0.019029080	
0.109871613	0.111188992	0.005375604	0.006489046	0.007577204	
0.033592130	0.048082064	0.004929348	0.016005343	0.019029080	
0.376066932	0.316112289	0.005375604	0.006489046	0.007577204	
0.487373932	0.388157865	0.004929348	0.016005343	0.019029080	
0.398133260	0.330794845	0.005375604	0.006489046	0.007577204	
0.815989467	0.642406396	0.004929348	0.016005343	0.019029080	
0.652245297	0.547669578	0.005375604	0.006489046	0.007577204	
0.037707004	0.051980973	0.004929348	0.016005343	0.019029080	
0.205897981	0.197816662	0.005375604	0.006489046	0.007577204	
0.383580798	0.320248888	0.004929348	0.016005343	0.019029080	
0.262811101	0.2401436	0.005375604	0.006489046	0.007577204	
0.778699539	0.609007872	0.004929348	0.016005343	0.019029080	
0.581342050	0.481384571	0.005375604	0.006489046	0.007577204	
0.983366837	0.843002708	0.004929348	0.016005343	0.019029080	
0.970432192	0.876344874	0.005375604	0.006489046	0.007577204	
0.420800099	0.346397522	0.004929348	0.016005343	0.019029080	
0.402825417	0.334023982	0.005375604	0.006489046	0.007577204	
0.012691633	0.024615356	0.004929348	0.016005343	0.019029080	
0.006421982	0.015912824	0.005375604	0.006489046	0.007577204	
0.260915510	0.219217918	0.004929348	0.016005343	0.019029080	
0.414380086	0.342096193	0.005375604	0.006489046	0.007577204	
0.206227859	0.174816240	0.004929348	0.016005343	0.019029080	
0.218563476	0.207737381	0.005375604	0.006489046	0.007577204	

0.164236772	0.145143233	0.004929348	0.016005343	0.019029080
0.937215175	0.813868011	0.005375604	0.006489046	0.007577204
0.985551363	0.851453722	0.004929348	0.016005343	0.019029080
0.729916616	0.615752096	0.005375604	0.006489046	0.007577204
0.959088379	0.789485431	0.004929348	0.016005343	0.019029080
0.947375055	0.829387646	0.005375604	0.006489046	0.007577204
0.911187994	0.731845425	0.004929348	0.016005343	0.019029080
0.755727866	0.634875457	0.005375604	0.006489046	0.007577204
0.831939662	0.656229367	0.004929348	0.016005343	0.019029080
0.437686732	0.359267452	0.005375604	0.006489046	0.007577204
0.332373208	0.279849976	0.004929348	0.016005343	0.019029080
0.217543673	0.206949227	0.005375604	0.006489046	0.007577204
0.192259246	0.164668982	0.004929348	0.016005343	0.019029080
0.146087669	0.143501274	0.005375604	0.006489046	0.007577204
0.517154178	0.407395588	0.004929348	0.016005343	0.019029080
0.736621598	0.620809821	0.005375604	0.006489046	0.007577204
0.991313536	0.881299235	0.004929348	0.016005343	0.019029080
0.937821649	0.814764448	0.005375604	0.006489046	0.007577204
0.291414308	0.244678490	0.004929348	0.016005343	0.019029080
0.175750028	0.171356665	0.005375604	0.006489046	0.007577204
0.540692451	0.423033004	0.004929348	0.016005343	0.019029080
0.694195962	0.586040789	0.005375604	0.006489046	0.007577204
0.443165860	0.360451873	0.004929348	0.016005343	0.019029080
0.115994310	0.116367801	0.005375604	0.006489046	0.007577204
0.859086792	0.681440270	0.004929348	0.016005343	0.019029080
0.761884659	0.639481498	0.005375604	0.006489046	0.007577204
0.269009447	0.225646069	0.004929348	0.016005343	0.019029080
0.197421034	0.191223152	0.005375604	0.006489046	0.007577204
0.085290499	0.091929085	0.004929348	0.016005343	0.019029080
0.027269241	0.041875461	0.005375604	0.006489046	0.007577204
0.333433318	0.280706627	0.004929348	0.016005343	0.019029080
0.220819921	0.209489549	0.005375604	0.006489046	0.007577204
0.631235295	0.488970725	0.004929348	0.016005343	0.019029080
0.469925374	0.383947480	0.005375604	0.006489046	0.007577204
0.223231333	0.188535515	0.004929348	0.016005343	0.019029080
0.127543118	0.126458410	0.005375604	0.006489046	0.007577204
0.862983730	0.685137483	0.004929348	0.016005343	0.019029080
0.722027372	0.609820762	0.005375604	0.006489046	0.007577204
0.776653850	0.607196068	0.004929348	0.016005343	0.019029080
0.841530842	0.703736343	0.005375604	0.006489046	0.007577204
0.733680613	0.573143810	0.004929348	0.016005343	0.019029080
0.425840274	0.350494870	0.005375604	0.006489046	0.007577204
0.732924947	0.572517171	0.004929348	0.016005343	0.019029080
0.236714200	0.221486812	0.005375604	0.006489046	0.007577204
0.800905408	0.628974503	0.004929348	0.016005343	0.019029080
0.856041245	0.716475137	0.005375604	0.006489046	0.007577204

0.603162288	0.468041971	0.004929348	0.016005343	0.019029080	
0.327539066	0.283816591	0.005375604	0.006489046	0.007577204	
0.054794076	0.067610406	0.004929348	0.016005343	0.019029080	
0.216803459	0.206375245	0.005375604	0.006489046	0.007577204	
0.681234202	0.528322187	0.004929348	0.016005343	0.019029080	
0.743205904	0.625682755	0.005375604	0.006489046	0.007577204	
0.557609510	0.434650152	0.004929348	0.016005343	0.019029080	
0.459715570	0.375760254	0.005375604	0.006489046	0.007577204	
0.194379720	0.166164014	0.004929348	0.016005343	0.019029080	
0.954295327	0.840940967	0.005375604	0.006489046	0.007577204	
0.003574834	0.011437767	0.004929348	0.016005343	0.019029080	0.15
0.615954108	0.513531471	0.005375604	0.006489046	0.007577204	
0.344393357	0.289677358	0.004929348	0.016005343	0.019029080	
0.529682053	0.432867389	0.005375604	0.006489046	0.007577204	
0.882118701	0.703836704	0.004929348	0.016005343	0.019029080	
0.783559849	0.656122233	0.005375604	0.006489046	0.007577204	
0.784189695	0.613988448	0.004929348	0.016005343	0.019029080	
0.735494064	0.619962700	0.005375604	0.006489046	0.007577204	
0.974119675	0.815414610	0.004929348	0.016005343	0.019029080	
0.877666110	0.738422555	0.005375604	0.006489046	0.007577204	
0.556191223	0.433639770	0.004929348	0.016005343	0.019029080	
0.530752016	0.433817228	0.005375604	0.006489046	0.007577204	
0.849061926	0.672183788	0.004929348	0.016005343	0.019029080	
0.741986941	0.624779095	0.005375604	0.006489046	0.007577204	
0.203015168	0.172390628	0.004929348	0.016005343	0.019029080	
0.180142368	0.175474216	0.005375604	0.006489046	0.007577204	
0.634937889	0.491744322	0.004929348	0.016005343	0.019029080	
0.613432250	0.511156253	0.005375604	0.006489046	0.007577204	
0.550187505	0.429452805	0.004929348	0.016005343	0.019029080	
0.138515830	0.136363734	0.005375604	0.006489046	0.007577204	
0.132821720	0.124310437	0.004929348	0.016005343	0.019029080	
0.107683612	0.109410855	0.005375604	0.006489046	0.007577204	
0.992182228	0.886715924	0.004929348	0.016005343	0.019029080	
0.970489599	0.876478997	0.005375604	0.006489046	0.007577204	
0.609767243	0.472879252	0.004929348	0.016005343	0.019029080	
0.305782583	0.269094904	0.005375604	0.006489046	0.007577204	
0.600565020	0.466165295	0.004929348	0.016005343	0.019029080	
0.603432815	0.501545359	0.005375604	0.006489046	0.007577204	
0.587795860	0.456971302	0.004929348	0.016005343	0.019029080	
0.471823318	0.385455461	0.005375604	0.006489046	0.007577204	
0.235984395	0.198808003	0.004929348	0.016005343	0.019029080	
0.964344961	0.862729549	0.005375604	0.006489046	0.007577204	
0.465162231	0.37429366	0.004929348	0.016005343	0.019029080	
0.327137976	0.283548001	0.005375604	0.006489046	0.007577204	
0.808488889	0.635763788	0.004929348	0.016005343	0.019029080	
0.773653700	0.648358759	0.005375604	0.006489046	0.007577204	

0.560885395	0.436968162	0.004929348	0.016005343	0.019029080	
0.944130255	0.824396424	0.005375604	0.006489046	0.007577204	
0.855572543	0.678180758	0.004929348	0.016005343	0.019029080	
0.856407922	0.716822068	0.005375604	0.006489046	0.007577204	
0.854257427	0.676960050	0.004929348	0.016005343	0.019029080	
0.744536859	0.626652716	0.005375604	0.006489046	0.007577204	
0.260381242	0.218798403	0.004929348	0.016005343	0.019029080	
0.161745954	0.157642618	0.005375604	0.006489046	0.007577204	
0.281317859	0.236041559	0.004929348	0.016005343	0.019029080	
0.295553040	0.262050434	0.005375604	0.006489046	0.007577204	
0.674379066	0.522706048	0.004929348	0.016005343	0.019029080	
0.545195143	0.446684171	0.005375604	0.006489046	0.007577204	
0.464035571	0.373590362	0.004929348	0.016005343	0.019029080	
0.946738348	0.828407322	0.005375604	0.006489046	0.007577204	
0.475107570	0.380503012	0.004929348	0.016005343	0.019029080	
0.436956854	0.358729177	0.005375604	0.006489046	0.007577204	
0.157846031	0.140739925	0.004929348	0.016005343	0.019029080	
0.254471895	0.234233692	0.005375604	0.006489046	0.007577204	
0.9467656	0.773943917	0.004929348	0.016005343	0.019029080	
0.892772960	0.755856798	0.005375604	0.006489046	0.007577204	
0.184590582	0.159192501	0.004929348	0.016005343	0.019029080	
0.484890311	0.395627866	0.005375604	0.006489046	0.007577204	
0.879723774	0.701478652	0.004929348	0.016005343	0.019029080	
0.557121200	0.457807449	0.005375604	0.006489046	0.007577204	
0.449881036	0.364645427	0.004929348	0.016005343	0.019029080	
0.378535332	0.317751606	0.005375604	0.006489046	0.007577204	
0.683312853	0.530074935	0.004929348	0.016005343	0.019029080	
0.522827907	0.426873732	0.005375604	0.006489046	0.007577204	
0.297896457	0.250298999	0.004929348	0.016005343	0.019029080	
0.131910306	0.130289091	0.005375604	0.006489046	0.007577204	
0.889631619	0.711056080	0.004929348	0.016005343	0.019029080	
0.904378972	0.769808316	0.005375604	0.006489046	0.007577204	
0.677949567	0.525592548	0.004929348	0.016005343	0.019029080	
0.787048976	0.659007964	0.005375604	0.006489046	0.007577204	
0.080783388	0.088471856	0.004929348	0.016005343	0.019029080	
0.024320777	0.038494568	0.005375604	0.006489046	0.007577204	
0.745413008	0.582284879	0.004929348	0.016005343	0.019029080	
0.622962534	0.520080923	0.005375604	0.006489046	0.007577204	
0.004429370	0.012902288	0.004929348	0.016005343	0.019029080	0.15
0.842816499	0.704835647	0.005375604	0.006489046	0.007577204	
0.657142981	0.509019827	0.004929348	0.016005343	0.019029080	
0.180786159	0.176078850	0.005375604	0.006489046	0.007577204	
0.648721429	0.502398222	0.004929348	0.016005343	0.019029080	
0.462842458	0.378228751	0.005375604	0.006489046	0.007577204	
0.169329289	0.148582417	0.004929348	0.016005343	0.019029080	
0.054510935	0.067913325	0.005375604	0.006489046	0.007577204	

0.119242928	0.115028154	0.004929348	0.016005343	0.019029080	
0.645054865	0.541192178	0.005375604	0.006489046	0.007577204	
0.031697589	0.046199907	0.004929348	0.016005343	0.019029080	
0.735687190	0.620107964	0.005375604	0.006489046	0.007577204	
0.693689124	0.538722602	0.004929348	0.016005343	0.019029080	
0.035678570	0.050929072	0.005375604	0.006489046	0.007577204	
0.033674751	0.048161884	0.004929348	0.016005343	0.019029080	
0.813389884	0.680657402	0.005375604	0.006489046	0.007577204	
0.901576122	0.722722411	0.004929348	0.016005343	0.019029080	
0.816892671	0.683463016	0.005375604	0.006489046	0.007577204	
0.452729505	0.366462472	0.004929348	0.016005343	0.019029080	
0.418154762	0.344800608	0.005375604	0.006489046	0.007577204	
0.249170562	0.209729237	0.004929348	0.016005343	0.019029080	
0.115801597	0.116205044	0.005375604	0.006489046	0.007577204	
0.026112030	0.040226657	0.004929348	0.016005343	0.019029080	
0.041443791	0.056558721	0.005375604	0.006489046	0.007577204	
0.746155206	0.582850026	0.004929348	0.016005343	0.019029080	
0.707561375	0.598121532	0.005375604	0.006489046	0.007577204	
0.986366699	0.855130933	0.004929348	0.016005343	0.019029080	
0.742078883	0.624847270	0.005375604	0.006489046	0.007577204	
0.539725380	0.422390721	0.004929348	0.016005343	0.019029080	
0.580623609	0.480730087	0.005375604	0.006489046	0.007577204	
0.002080287	0.008505740	0.004929348	0.016005343	0.019029080	0.15
0.891611096	0.754514019	0.005375604	0.006489046	0.007577204	
0.384612988	0.320994198	0.004929348	0.016005343	0.019029080	
0.405580966	0.335932695	0.005375604	0.006489046	0.007577204	
0.004389035	0.012832596	0.004929348	0.016005343	0.019029080	0.15
0.722410960	0.610116314	0.005375604	0.006489046	0.007577204	
0.132978712	0.124414590	0.004929348	0.016005343	0.019029080	
0.134096591	0.132260945	0.005375604	0.006489046	0.007577204	
0.344063258	0.339504030	0.001162785	0.001437343	0.001785486	
0.331547431	0.307134294	0.000303578	0.000516412	0.000664109	
0.513438486	0.539249568	0.001162785	0.001437343	0.001785486	
0.261852339	0.242472433	0.000303578	0.000516412	0.000664109	
0.219621909	0.197216959	0.001162785	0.001437343	0.001785486	
0.452432325	0.461989666	0.000303578	0.000516412	0.000664109	
0.056576056	0.044995617	0.001162785	0.001437343	0.001785486	
0.035889323	0.026917037	0.000303578	0.000516412	0.000664109	
0.900831032	0.903403574	0.001162785	0.001437343	0.001785486	
0.850393253	0.897477069	0.000303578	0.000516412	0.000664109	
0.729629055	0.733091334	0.001162785	0.001437343	0.001785486	
0.696610973	0.716858053	0.000303578	0.000516412	0.000664109	
0.212336110	0.190510923	0.001162785	0.001437343	0.001785486	
0.172226635	0.159158612	0.000303578	0.000516412	0.000664109	
0.100916206	0.083456318	0.001162785	0.001437343	0.001785486	
0.108495069	0.116592372	0.000303578	0.000516412	0.000664109	

0.526038643	0.551913701	0.001162785	0.001437343	0.001785486
0.586459729	0.633386516	0.000303578	0.000516412	0.000664109
0.711251956	0.711568429	0.001162785	0.001437343	0.001785486
0.646991954	0.693612935	0.000303578	0.000516412	0.000664109
0.598717037	0.602827103	0.001162785	0.001437343	0.001785486
0.543299834	0.579396570	0.000303578	0.000516412	0.000664109
0.481718328	0.503763599	0.001162785	0.001437343	0.001785486
0.455672281	0.475857101	0.000303578	0.000516412	0.000664109
0.116246322	0.096906373	0.001162785	0.001437343	0.001785486
0.130225403	0.137551085	0.000303578	0.000516412	0.000664109
0.542682517	0.571575231	0.001162785	0.001437343	0.001785486
0.483581608	0.546822099	0.000303578	0.000516412	0.000664109
0.910419227	0.938723038	0.001162785	0.001437343	0.001785486
0.872315377	0.935830226	0.000303578	0.000516412	0.000664109
0.398133525	0.407448562	0.001162785	0.001437343	0.001785486
0.367384527	0.376557772	0.000303578	0.000516412	0.000664109
0.651143853	0.653944926	0.001162785	0.001437343	0.001785486
0.582130405	0.633126104	0.000303578	0.000516412	0.000664109
0.264153792	0.238090580	0.001162785	0.001437343	0.001785486
0.221526848	0.205219858	0.000303578	0.000516412	0.000664109
0.632176694	0.628831285	0.001162785	0.001437343	0.001785486
0.561562567	0.606654065	0.000303578	0.000516412	0.000664109
0.282861808	0.254004281	0.001162785	0.001437343	0.001785486
0.255171258	0.233741882	0.000303578	0.000516412	0.000664109
0.681053863	0.676953400	0.001162785	0.001437343	0.001785486
0.615409649	0.657250676	0.000303578	0.000516412	0.000664109
0.404251990	0.414817960	0.001162785	0.001437343	0.001785486
0.881199842	0.940854731	0.000303578	0.000516412	0.000664109
0.671853671	0.676333733	0.001162785	0.001437343	0.001785486
0.607763463	0.656770637	0.000303578	0.000516412	0.000664109
0.658524280	0.657802639	0.001162785	0.001437343	0.001785486
0.592655489	0.637034028	0.000303578	0.000516412	0.000664109
0.168490002	0.155468288	0.001162785	0.001437343	0.001785486
0.043005274	0.032790167	0.000303578	0.000516412	0.000664109
0.053283667	0.042677436	0.001162785	0.001437343	0.001785486
0.307500287	0.287602798	0.000303578	0.000516412	0.000664109
0.413513104	0.423157878	0.001162785	0.001437343	0.001785486
0.391569108	0.392603102	0.000303578	0.000516412	0.000664109
0.021159926	0.018999648	0.001162785	0.001437343	0.001785486
0.013149047	0.008191783	0.000303578	0.000516412	0.000664109
0.149350765	0.134029075	0.001162785	0.001437343	0.001785486
0.134387432	0.137913040	0.000303578	0.000516412	0.000664109
0.589438386	0.598162196	0.001162785	0.001437343	0.001785486
0.536227339	0.574419200	0.000303578	0.000516412	0.000664109
0.244888825	0.215275404	0.001162785	0.001437343	0.001785486
0.20169474	0.182928425	0.000303578	0.000516412	0.000664109

0.990414667	0.991232598	0.001162785	0.001437343	0.001785486
0.916089261	0.953011302	0.000303578	0.000516412	0.000664109
0.785374181	0.798367844	0.001162785	0.001437343	0.001785486
0.745287008	0.785880729	0.000303578	0.000516412	0.000664109
0.103820670	0.087493943	0.001162785	0.001437343	0.001785486
0.066634097	0.062747851	0.000303578	0.000516412	0.000664109
0.295763037	0.277049968	0.001162785	0.001437343	0.001785486
0.347399147	0.323126488	0.000303578	0.000516412	0.000664109
0.105647730	0.087685918	0.001162785	0.001437343	0.001785486
0.119264362	0.129370501	0.000303578	0.000516412	0.000664109
0.031658559	0.026517573	0.001162785	0.001437343	0.001785486
0.901015190	0.943634702	0.000303578	0.000516412	0.000664109
0.751818797	0.766212660	0.001162785	0.001437343	0.001785486
0.245610647	0.222928877	0.000303578	0.000516412	0.000664109
0.750833889	0.762965448	0.001162785	0.001437343	0.001785486
0.778642694	0.838553414	0.000303578	0.000516412	0.000664109
0.795142611	0.817026728	0.001162785	0.001437343	0.001785486
0.756341252	0.805602045	0.000303578	0.000516412	0.000664109
0.904678627	0.910119096	0.001162785	0.001437343	0.001785486
0.324634263	0.301655003	0.000303578	0.000516412	0.000664109
0.144448424	0.129178378	0.001162785	0.001437343	0.001785486
0.100462934	0.100635403	0.000303578	0.000516412	0.000664109
0.108284930	0.090818639	0.001162785	0.001437343	0.001785486
0.071870560	0.065057964	0.000303578	0.000516412	0.000664109
0.687756817	0.677338744	0.001162785	0.001437343	0.001785486
0.624191313	0.657776361	0.000303578	0.000516412	0.000664109
0.769377475	0.779100049	0.001162785	0.001437343	0.001785486
0.725702390	0.765133867	0.000303578	0.000516412	0.000664109
0.188823065	0.173640374	0.001162785	0.001437343	0.001785486
0.140370199	0.142683933	0.000303578	0.000516412	0.000664109
0.699509691	0.684645325	0.001162785	0.001437343	0.001785486
0.631423187	0.665404662	0.000303578	0.000516412	0.000664109
0.778734655	0.788660901	0.001162785	0.001437343	0.001785486
0.729627744	0.774951487	0.000303578	0.000516412	0.000664109
0.379599048	0.376415991	0.001162785	0.001437343	0.001785486
0.363117169	0.351186322	0.000303578	0.000516412	0.000664109
0.232236597	0.203922679	0.001162785	0.001437343	0.001785486
0.191791714	0.172067315	0.000303578	0.000516412	0.000664109
0.240896613	0.213089678	0.001162785	0.001437343	0.001785486
0.198669906	0.180798796	0.000303578	0.000516412	0.000664109
0.983293924	0.984340669	0.001162785	0.001437343	0.001785486
0.957880463	0.984130843	0.000303578	0.000516412	0.000664109
0.433571225	0.444025886	0.001162785	0.001437343	0.001785486
0.414734524	0.4138874	0.000303578	0.000516412	0.000664109
0.15220159	0.135698280	0.001162785	0.001437343	0.001785486
0.105434494	0.106717971	0.000303578	0.000516412	0.000664109

0.436366515	0.446187422	0.001162785	0.001437343	0.001785486
0.418151958	0.416291534	0.000303578	0.000516412	0.000664109
0.877614304	0.892698511	0.001162785	0.001437343	0.001785486
0.836273539	0.886799029	0.000303578	0.000516412	0.000664109
0.534692805	0.557453751	0.001162785	0.001437343	0.001785486
0.478529210	0.531375323	0.000303578	0.000516412	0.000664109
0.526354775	0.552566647	0.001162785	0.001437343	0.001785486
0.225077369	0.209046139	0.000303578	0.000516412	0.000664109
0.543053289	0.571616723	0.001162785	0.001437343	0.001785486
0.687421808	0.708609958	0.000303578	0.000516412	0.000664109
0.609827498	0.603829724	0.001162785	0.001437343	0.001785486
0.54736547	0.579843377	0.000303578	0.000516412	0.000664109
0.066377602	0.051060313	0.001162785	0.001437343	0.001785486
0.176140738	0.160798931	0.000303578	0.000516412	0.000664109
0.717934895	0.719449800	0.001162785	0.001437343	0.001785486
0.637339834	0.675019799	0.000303578	0.000516412	0.000664109
0.210535824	0.188941015	0.001162785	0.001437343	0.001785486
0.145257851	0.143058122	0.000303578	0.000516412	0.000664109
0.868949558	0.885447849	0.001162785	0.001437343	0.001785486
0.99671111	0.999952160	0.000303578	0.000516412	0.000664109
0.963254980	0.967642763	0.001162785	0.001437343	0.001785486
0.946691099	0.967079366	0.000303578	0.000516412	0.000664109
0.889831749	0.894967452	0.001162785	0.001437343	0.001785486
0.843414613	0.888654772	0.000303578	0.000516412	0.000664109
0.224962838	0.199227016	0.001162785	0.001437343	0.001785486
0.186497518	0.167495888	0.000303578	0.000516412	0.000664109
0.928432639	0.945078592	0.001162785	0.001437343	0.001785486
0.893120354	0.942119673	0.000303578	0.000516412	0.000664109
0.624722411	0.624777654	0.001162785	0.001437343	0.001785486
0.555998678	0.602337012	0.000303578	0.000516412	0.000664109
0.761630337	0.776923092	0.001162785	0.001437343	0.001785486
0.718713003	0.762829983	0.000303578	0.000516412	0.000664109
0.718682838	0.721480304	0.001162785	0.001437343	0.001785486
0.680603352	0.704440704	0.000303578	0.000516412	0.000664109
0.820182158	0.847531205	0.001162785	0.001437343	0.001785486
0.772609779	0.838046472	0.000303578	0.000516412	0.000664109
0.371405016	0.370034449	0.001162785	0.001437343	0.001785486
0.422310268	0.432297939	0.000303578	0.000516412	0.000664109
0.833103641	0.855760377	0.001162785	0.001437343	0.001785486
0.793513095	0.846234745	0.000303578	0.000516412	0.000664109
0.026019155	0.022048661	0.001162785	0.001437343	0.001785486
0.034999895	0.025499176	0.000303578	0.000516412	0.000664109
0.862999045	0.873545706	0.001162785	0.001437343	0.001785486
0.822578581	0.865743584	0.000303578	0.000516412	0.000664109
0.368340205	0.364238552	0.001162785	0.001437343	0.001785486
0.124386383	0.135913376	0.000303578	0.000516412	0.000664109

0.668251282	0.669629848	0.001162785	0.001437343	0.001785486
0.505402574	0.554118026	0.000303578	0.000516412	0.000664109
0.179630760	0.168582372	0.001162785	0.001437343	0.001785486
0.134433623	0.137915815	0.000303578	0.000516412	0.000664109
0.284385876	0.256731393	0.001162785	0.001437343	0.001785486
0.968850383	0.985449593	0.000303578	0.000516412	0.000664109
0.319295278	0.317337649	0.001162785	0.001437343	0.001785486
0.298786912	0.284541402	0.000303578	0.000516412	0.000664109
0.843978256	0.863498659	0.001162785	0.001437343	0.001785486
0.806567187	0.855001018	0.000303578	0.000516412	0.000664109
0.473646578	0.490967647	0.001162785	0.001437343	0.001785486
0.919167536	0.963744199	0.000303578	0.000516412	0.000664109
0.667565897	0.667460155	0.001162785	0.001437343	0.001785486
0.662749201	0.695994740	0.000303578	0.000516412	0.000664109
0.290565760	0.2626989	0.001162785	0.001437343	0.001785486
0.261189139	0.234905761	0.000303578	0.000516412	0.000664109
0.251342850	0.223967793	0.001162785	0.001437343	0.001785486
0.206080009	0.191589895	0.000303578	0.000516412	0.000664109
0.958518248	0.966398582	0.001162785	0.001437343	0.001785486
0.935950113	0.964967096	0.000303578	0.000516412	0.000664109
0.852591724	0.871679337	0.001162785	0.001437343	0.001785486
0.811957837	0.863668839	0.000303578	0.000516412	0.000664109
0.440158776	0.446874452	0.001162785	0.001437343	0.001785486
0.975586233	0.996735821	0.000303578	0.000516412	0.000664109
0.209169686	0.186955683	0.001162785	0.001437343	0.001785486
0.216460929	0.197930674	0.000303578	0.000516412	0.000664109
0.461800172	0.482084504	0.001162785	0.001437343	0.001785486
0.444298552	0.453557289	0.000303578	0.000516412	0.000664109
0.747623797	0.752298544	0.001162785	0.001437343	0.001785486
0.704426066	0.719207079	0.000303578	0.000516412	0.000664109
0.229602371	0.199962128	0.001162785	0.001437343	0.001785486
0.403712529	0.394876457	0.000303578	0.000516412	0.000664109
0.836527143	0.861156380	0.001162785	0.001437343	0.001785486
0.383822150	0.387437252	0.000303578	0.000516412	0.000664109
0.340316302	0.337497317	0.001162785	0.001437343	0.001785486
0.328052598	0.305034048	0.000303578	0.000516412	0.000664109
0.394600259	0.404055098	0.001162785	0.001437343	0.001785486
0.291399422	0.275508062	0.000303578	0.000516412	0.000664109
0.178977409	0.167519281	0.001162785	0.001437343	0.001785486
0.049050298	0.035977778	0.000303578	0.000516412	0.000664109
0.199574505	0.185956553	0.001162785	0.001437343	0.001785486
0.638307621	0.682859889	0.000303578	0.000516412	0.000664109
0.553205148	0.575500506	0.001162785	0.001437343	0.001785486
0.666257792	0.699164065	0.000303578	0.000516412	0.000664109
0.740939902	0.748160976	0.001162785	0.001437343	0.001785486
0.707195789	0.732384451	0.000303578	0.000516412	0.000664109

0.951538330	0.965756591	0.001162785	0.001437343	0.001785486
0.926511090	0.964185359	0.000303578	0.000516412	0.000664109
0.578090881	0.589132852	0.001162785	0.001437343	0.001785486
0.791342219	0.843460671	0.000303578	0.000516412	0.000664109
0.487987196	0.507669638	0.001162785	0.001437343	0.001785486
0.163627177	0.155066770	0.000303578	0.000516412	0.000664109
0.477883454	0.498885867	0.001162785	0.001437343	0.001785486
0.295887482	0.284067808	0.000303578	0.000516412	0.000664109
0.355646825	0.344753061	0.001162785	0.001437343	0.001785486
0.339202958	0.312235657	0.000303578	0.000516412	0.000664109
0.992424866	0.996243349	0.001162785	0.001437343	0.001785486
0.987818020	0.997505312	0.000303578	0.000516412	0.000664109
0.417534238	0.423737822	0.001162785	0.001437343	0.001785486
0.654138091	0.694042617	0.000303578	0.000516412	0.000664109
0.934332301	0.945575272	0.001162785	0.001437343	0.001785486
0.887791995	0.941799649	0.000303578	0.000516412	0.000664109
0.557889684	0.576723425	0.001162785	0.001437343	0.001785486
0.740736992	0.782255668	0.000303578	0.000516412	0.000664109
0.554314989	0.575624687	0.001162785	0.001437343	0.001785486
0.491076242	0.550871292	0.000303578	0.000516412	0.000664109
0.111528061	0.092696836	0.001162785	0.001437343	0.001785486
0.118630405	0.121677854	0.000303578	0.000516412	0.000664109
0.466672285	0.482368675	0.001162785	0.001437343	0.001785486
0.442407584	0.453443719	0.000303578	0.000516412	0.000664109
0.121801335	0.102687650	0.001162785	0.001437343	0.001785486
0.083320091	0.076429825	0.000303578	0.000516412	0.000664109
0.522696012	0.551102247	0.001162785	0.001437343	0.001785486
0.475450883	0.525422960	0.000303578	0.000516412	0.000664109
0.904263389	0.908670103	0.001162785	0.001437343	0.001785486
0.768593399	0.811424496	0.000303578	0.000516412	0.000664109
0.019277169	0.017703732	0.001162785	0.001437343	0.001785486
0.271195983	0.254763898	0.000303578	0.000516412	0.000664109
0.973055900	0.981440742	0.001162785	0.001437343	0.001785486
0.956427356	0.982055708	0.000303578	0.000516412	0.000664109
0.047554320	0.038824717	0.001162785	0.001437343	0.001785486
0.157538614	0.149763492	0.000303578	0.000516412	0.000664109
0.372403511	0.371841324	0.001162785	0.001437343	0.001785486
0.676901736	0.700301951	0.000303578	0.000516412	0.000664109
0.344622597	0.339558545	0.001162785	0.001437343	0.001785486
0.334786202	0.307337966	0.000303578	0.000516412	0.000664109
0.643294874	0.508512860	0.004605957	0.005800995	0.006869736
0.579952597	0.467638327	0.002626182	0.003422277	0.012806334
0.710021700	0.557369896	0.004605957	0.005800995	0.006869736
0.285344294	0.221297552	0.002626182	0.003422277	0.012806334
0.079969752	0.086487989	0.004605957	0.005800995	0.006869736
0.483228053	0.384284412	0.002626182	0.003422277	0.012806334

0.026556281	0.041331279	0.004605957	0.005800995	0.006869736	
0.015335828	0.018210688	0.002626182	0.003422277	0.012806334	0.25
0.600725662	0.477370602	0.004605957	0.005800995	0.006869736	
0.380919898	0.300767275	0.002626182	0.003422277	0.012806334	
0.219522149	0.182668066	0.004605957	0.005800995	0.006869736	
0.244189869	0.190067497	0.002626182	0.003422277	0.012806334	
0.022093592	0.035894967	0.004605957	0.005800995	0.006869736	
0.106500283	0.090021189	0.002626182	0.003422277	0.012806334	
0.278360654	0.220289957	0.004605957	0.005800995	0.006869736	
0.160740504	0.131748796	0.002626182	0.003422277	0.012806334	
0.772324909	0.60375101	0.004605957	0.005800995	0.006869736	
0.667139542	0.536313386	0.002626182	0.003422277	0.012806334	
0.109194757	0.105594448	0.004605957	0.005800995	0.006869736	
0.011378077	0.014166774	0.002626182	0.003422277	0.012806334	0.225
0.373345061	0.285663610	0.004605957	0.005800995	0.006869736	
0.321099068	0.249234974	0.002626182	0.003422277	0.012806334	
0.363790535	0.278185393	0.004605957	0.005800995	0.006869736	
0.265914413	0.206704429	0.002626182	0.003422277	0.012806334	
0.561297731	0.447175901	0.004605957	0.005800995	0.006869736	
0.527819612	0.422543050	0.002626182	0.003422277	0.012806334	
0.451217671	0.352882402	0.004605957	0.005800995	0.006869736	
0.421979297	0.334721767	0.002626182	0.003422277	0.012806334	
0.053981771	0.068129204	0.004605957	0.005800995	0.006869736	
0.283704159	0.220104279	0.002626182	0.003422277	0.012806334	
0.150149090	0.132988663	0.004605957	0.005800995	0.006869736	
0.168791990	0.137094134	0.002626182	0.003422277	0.012806334	
0.140221298	0.126077174	0.004605957	0.005800995	0.006869736	
0.098862971	0.083409284	0.002626182	0.003422277	0.012806334	
0.233023749	0.191323700	0.004605957	0.005800995	0.006869736	
0.036134055	0.036130479	0.002626182	0.003422277	0.012806334	
0.771606982	0.603201653	0.004605957	0.005800995	0.006869736	
0.767105498	0.614815142	0.002626182	0.003422277	0.012806334	
0.980407154	0.834145405	0.004605957	0.005800995	0.006869736	
0.982983206	0.799881786	0.002626182	0.003422277	0.012806334	
0.113107634	0.108253205	0.004605957	0.005800995	0.006869736	
0.051103238	0.046803855	0.002626182	0.003422277	0.012806334	
0.735251408	0.576122564	0.004605957	0.005800995	0.006869736	
0.963736611	0.781764030	0.002626182	0.003422277	0.012806334	
0.068258966	0.078523224	0.004605957	0.005800995	0.006869736	
0.134658908	0.112971537	0.002626182	0.003422277	0.012806334	
0.023933749	0.038060542	0.004605957	0.005800995	0.006869736	
0.004677150	0.007123006	0.002626182	0.003422277	0.012806334	0.175
0.275593159	0.218469397	0.004605957	0.005800995	0.006869736	
0.044760842	0.042077133	0.002626182	0.003422277	0.012806334	
0.152949478	0.134985977	0.004605957	0.005800995	0.006869736	
0.327958534	0.254482632	0.002626182	0.003422277	0.012806334	

0.267696315	0.213328101	0.004605957	0.005800995	0.006869736	
0.182779849	0.145779202	0.002626182	0.003422277	0.012806334	
0.040864352	0.056603980	0.004605957	0.005800995	0.006869736	
0.023776642	0.025903688	0.002626182	0.003422277	0.012806334	
0.510222868	0.405701277	0.004605957	0.005800995	0.006869736	
0.492864596	0.392152217	0.002626182	0.003422277	0.012806334	
0.707070561	0.555119103	0.004605957	0.005800995	0.006869736	
0.702285103	0.565048855	0.002626182	0.003422277	0.012806334	
0.044826078	0.060057932	0.004605957	0.005800995	0.006869736	
0.002032777	0.004146858	0.002626182	0.003422277	0.012806334	0.175
0.968275417	0.798761630	0.004605957	0.005800995	0.006869736	
0.92442045	0.748451286	0.002626182	0.003422277	0.012806334	
0.165566727	0.144162725	0.004605957	0.005800995	0.006869736	
0.201433091	0.158162804	0.002626182	0.003422277	0.012806334	
0.017585451	0.030361242	0.004605957	0.005800995	0.006869736	
0.008150220	0.010962691	0.002626182	0.003422277	0.012806334	0.175
0.675199219	0.531585984	0.004605957	0.005800995	0.006869736	
0.539505767	0.432821021	0.002626182	0.003422277	0.012806334	
0.380199851	0.291166717	0.004605957	0.005800995	0.006869736	
0.256689932	0.199905047	0.002626182	0.003422277	0.012806334	
0.070115494	0.079881974	0.004605957	0.005800995	0.006869736	
0.931476930	0.754533790	0.002626182	0.003422277	0.012806334	
0.818794629	0.639547481	0.004605957	0.005800995	0.006869736	
0.249622156	0.194427919	0.002626182	0.003422277	0.012806334	
0.957820435	0.778054587	0.004605957	0.005800995	0.006869736	
0.865040793	0.695965920	0.002626182	0.003422277	0.012806334	
0.253869208	0.204568281	0.004605957	0.005800995	0.006869736	
0.199531993	0.156930308	0.002626182	0.003422277	0.012806334	
0.964636678	0.790501305	0.004605957	0.005800995	0.006869736	
0.349772191	0.271752143	0.002626182	0.003422277	0.012806334	
0.101143658	0.100203396	0.004605957	0.005800995	0.006869736	
0.063046599	0.055756329	0.002626182	0.003422277	0.012806334	
0.700238700	0.549899602	0.004605957	0.005800995	0.006869736	
0.615072434	0.495334208	0.002626182	0.003422277	0.012806334	
0.580319924	0.461744916	0.004605957	0.005800995	0.006869736	
0.673809369	0.541935575	0.002626182	0.003422277	0.012806334	
0.979229626	0.830413969	0.004605957	0.005800995	0.006869736	
0.898256714	0.725406558	0.002626182	0.003422277	0.012806334	
0.672355669	0.529533758	0.004605957	0.005800995	0.006869736	
0.664711153	0.534308398	0.002626182	0.003422277	0.012806334	
0.877264235	0.687441006	0.004605957	0.005800995	0.006869736	
0.717031805	0.575679115	0.002626182	0.003422277	0.012806334	
0.569380422	0.453371831	0.004605957	0.005800995	0.006869736	
0.084378977	0.071742605	0.002626182	0.003422277	0.012806334	
0.615253259	0.488052366	0.004605957	0.005800995	0.006869736	
0.451926648	0.358241853	0.002626182	0.003422277	0.012806334	

0.577110582	0.459186326	0.004605957	0.005800995	0.006869736	
0.550079674	0.441841599	0.002626182	0.003422277	0.012806334	
0.499316811	0.395661157	0.004605957	0.005800995	0.006869736	
0.403909550	0.321314289	0.002626182	0.003422277	0.012806334	
0.673534379	0.530382332	0.004605957	0.005800995	0.006869736	
0.603351096	0.485882537	0.002626182	0.003422277	0.012806334	
0.417648326	0.323960305	0.004605957	0.005800995	0.006869736	
0.233522953	0.181953248	0.002626182	0.003422277	0.012806334	
0.618522229	0.490384594	0.004605957	0.005800995	0.006869736	
0.607783267	0.489432505	0.002626182	0.003422277	0.012806334	
0.295596488	0.231617622	0.004605957	0.005800995	0.006869736	
0.194812076	0.153818920	0.002626182	0.003422277	0.012806334	
0.878011746	0.688110930	0.004605957	0.005800995	0.006869736	
0.908246934	0.734426641	0.002626182	0.003422277	0.012806334	
0.822123422	0.642214009	0.004605957	0.005800995	0.006869736	
0.607866055	0.489498647	0.002626182	0.003422277	0.012806334	
0.699458121	0.549295511	0.004605957	0.005800995	0.006869736	
0.203081887	0.159250164	0.002626182	0.003422277	0.012806334	
0.746990977	0.584545178	0.004605957	0.005800995	0.006869736	
0.731161840	0.585888901	0.002626182	0.003422277	0.012806334	
0.354274928	0.271114243	0.004605957	0.005800995	0.006869736	
0.003840325	0.006190161	0.002626182	0.003422277	0.012806334	0.175
0.155939772	0.137185676	0.004605957	0.005800995	0.006869736	
0.192234691	0.152077065	0.002626182	0.003422277	0.012806334	
0.759819272	0.594146939	0.004605957	0.005800995	0.006869736	
0.686319893	0.552456332	0.002626182	0.003422277	0.012806334	
0.376044184	0.287841507	0.004605957	0.005800995	0.006869736	
0.204506154	0.160180124	0.002626182	0.003422277	0.012806334	
0.632183892	0.500348551	0.004605957	0.005800995	0.006869736	
0.996801390	0.820750202	0.002626182	0.003422277	0.012806334	
0.109267400	0.105643539	0.004605957	0.005800995	0.006869736	
0.578775538	0.466704283	0.002626182	0.003422277	0.012806334	
0.609139229	0.483672999	0.004605957	0.005800995	0.006869736	
0.489268961	0.389147980	0.002626182	0.003422277	0.012806334	
0.165689188	0.144252067	0.004605957	0.005800995	0.006869736	
0.154730333	0.127525424	0.002626182	0.003422277	0.012806334	
0.670001142	0.527837076	0.004605957	0.005800995	0.006869736	
0.582850338	0.469951238	0.002626182	0.003422277	0.012806334	
0.130886208	0.119847205	0.004605957	0.005800995	0.006869736	
0.061561005	0.054686521	0.002626182	0.003422277	0.012806334	
0.717972232	0.563419016	0.004605957	0.005800995	0.006869736	
0.468819480	0.372713302	0.002626182	0.003422277	0.012806334	
0.026009135	0.040639265	0.004605957	0.005800995	0.006869736	
0.044297800	0.041763472	0.002626182	0.003422277	0.012806334	
0.210990864	0.177112656	0.004605957	0.005800995	0.006869736	
0.117514173	0.099264064	0.002626182	0.003422277	0.012806334	

0.557874868	0.444528811	0.004605957	0.005800995	0.006869736
0.468773722	0.372676052	0.002626182	0.003422277	0.012806334
0.607304604	0.482370996	0.004605957	0.005800995	0.006869736
0.256921015	0.200083729	0.002626182	0.003422277	0.012806334
0.536729506	0.427725854	0.004605957	0.005800995	0.006869736
0.495239993	0.394172511	0.002626182	0.003422277	0.012806334
0.888727946	0.697848467	0.004605957	0.005800995	0.006869736
0.817823568	0.655702208	0.002626182	0.003422277	0.012806334
0.414574336	0.321267816	0.004605957	0.005800995	0.006869736
0.021821149	0.024238043	0.002626182	0.003422277	0.012806334
0.763711940	0.597153418	0.004605957	0.005800995	0.006869736
0.546102656	0.438417146	0.002626182	0.003422277	0.012806334
0.069626338	0.079477273	0.004605957	0.005800995	0.006869736
0.054722869	0.049563969	0.002626182	0.003422277	0.012806334
0.619750992	0.491273676	0.004605957	0.005800995	0.006869736
0.992295198	0.812107389	0.002626182	0.003422277	0.012806334
0.404847681	0.312675167	0.004605957	0.005800995	0.006869736
0.268803717	0.208939298	0.002626182	0.003422277	0.012806334
0.743643679	0.582089170	0.004605957	0.005800995	0.006869736
0.633874277	0.510319320	0.002626182	0.003422277	0.012806334
0.576003519	0.458343191	0.004605957	0.005800995	0.006869736
0.951512737	0.771262806	0.002626182	0.003422277	0.012806334
0.953420565	0.770852409	0.004605957	0.005800995	0.006869736
0.808292762	0.647931541	0.002626182	0.003422277	0.012806334
0.414591545	0.321282786	0.004605957	0.005800995	0.006869736
0.280194365	0.217649711	0.002626182	0.003422277	0.012806334
0.369930093	0.282955575	0.004605957	0.005800995	0.006869736
0.302738420	0.234782044	0.002626182	0.003422277	0.012806334
0.326943569	0.252421192	0.004605957	0.005800995	0.006869736
0.225876522	0.176406218	0.002626182	0.003422277	0.012806334
0.492907591	0.389949819	0.004605957	0.005800995	0.006869736
0.326817736	0.253632843	0.002626182	0.003422277	0.012806334
0.547924864	0.436734017	0.004605957	0.005800995	0.006869736
0.981143291	0.797937828	0.002626182	0.003422277	0.012806334
0.832722857	0.650763620	0.004605957	0.005800995	0.006869736
0.758362771	0.607648173	0.002626182	0.003422277	0.012806334
0.271103623	0.215550284	0.004605957	0.005800995	0.006869736
0.200212622	0.157374208	0.002626182	0.003422277	0.012806334
0.966327137	0.794085126	0.004605957	0.005800995	0.006869736
0.825611262	0.661958868	0.002626182	0.003422277	0.012806334
0.231162966	0.190121584	0.004605957	0.005800995	0.006869736
0.415317082	0.329893138	0.002626182	0.003422277	0.012806334
0.991206720	0.872887650	0.004605957	0.005800995	0.006869736
0.825346437	0.661739972	0.002626182	0.003422277	0.012806334
0.341323186	0.262185372	0.004605957	0.005800995	0.006869736
0.179377177	0.143719264	0.002626182	0.003422277	0.012806334

0.475081628	0.373732027	0.004605957	0.005800995	0.006869736	
0.174113333	0.140494386	0.002626182	0.003422277	0.012806334	
0.910618056	0.718427949	0.004605957	0.005800995	0.006869736	
0.828558510	0.664492575	0.002626182	0.003422277	0.012806334	
0.333303953	0.25675139	0.004605957	0.005800995	0.006869736	
0.692750753	0.557735696	0.002626182	0.003422277	0.012806334	
0.982450290	0.841091717	0.004605957	0.005800995	0.006869736	
0.919161640	0.743851440	0.002626182	0.003422277	0.012806334	
0.534223779	0.425679562	0.004605957	0.005800995	0.006869736	
0.461782328	0.366918374	0.002626182	0.003422277	0.012806334	
0.508057707	0.403703368	0.004605957	0.005800995	0.006869736	
0.306845347	0.238206713	0.002626182	0.003422277	0.012806334	
0.367284880	0.280880560	0.004605957	0.005800995	0.006869736	
0.862526552	0.693882255	0.002626182	0.003422277	0.012806334	
0.600734208	0.477377239	0.004605957	0.005800995	0.006869736	
0.073204793	0.063127767	0.002626182	0.003422277	0.012806334	
0.657063829	0.518562381	0.004605957	0.005800995	0.006869736	
0.332635555	0.257975042	0.002626182	0.003422277	0.012806334	
0.133690835	0.121682873	0.004605957	0.005800995	0.006869736	
0.038390579	0.037692191	0.002626182	0.003422277	0.012806334	
0.424638528	0.330170749	0.004605957	0.005800995	0.006869736	
0.602735755	0.485405410	0.002626182	0.003422277	0.012806334	
0.907122775	0.715141909	0.004605957	0.005800995	0.006869736	
0.996509183	0.820101622	0.002626182	0.003422277	0.012806334	
0.740721255	0.579984921	0.004605957	0.005800995	0.006869736	
0.005643742	0.008264508	0.002626182	0.003422277	0.012806334	0.175
0.168987344	0.146748656	0.004605957	0.005800995	0.006869736	
0.797023235	0.639132229	0.002626182	0.003422277	0.012806334	
0.769513416	0.601603839	0.004605957	0.005800995	0.006869736	
0.674123577	0.542204836	0.002626182	0.003422277	0.012806334	
0.136917082	0.123827759	0.004605957	0.005800995	0.006869736	
0.141984975	0.118306277	0.002626182	0.003422277	0.012806334	
0.914199107	0.721993896	0.004605957	0.005800995	0.006869736	
0.809422211	0.648858492	0.002626182	0.003422277	0.012806334	
0.145746362	0.129867868	0.004605957	0.005800995	0.006869736	
0.110331529	0.093219871	0.002626182	0.003422277	0.012806334	
0.438238875	0.342060398	0.004605957	0.005800995	0.006869736	
0.427848592	0.338892508	0.002626182	0.003422277	0.012806334	
0.845912154	0.660808380	0.004605957	0.005800995	0.006869736	
0.408593535	0.324911974	0.002626182	0.003422277	0.012806334	
0.898804405	0.707240803	0.004605957	0.005800995	0.006869736	
0.965726383	0.783490029	0.002626182	0.003422277	0.012806334	
0.102726332	0.101259294	0.004605957	0.005800995	0.006869736	
0.915166722	0.740399346	0.002626182	0.003422277	0.012806334	
0.221797599	0.184116033	0.004605957	0.005800995	0.006869736	
0.186953416	0.148444580	0.002626182	0.003422277	0.012806334	

0.041148450	0.056854914	0.004605957	0.005800995	0.006869736
0.707481631	0.568869729	0.002626182	0.003422277	0.012806334
0.276327359	0.218950387	0.004605957	0.005800995	0.006869736
0.241677272	0.188068148	0.002626182	0.003422277	0.012806334

Genus	Family	Order
Acetatifactor	Lachnospiraceae	Eubacteriales
Acetatifactor	Lachnospiraceae	Eubacteriales
Acutalibacter	Oscillospiraceae	Eubacteriales
Acutalibacter	Oscillospiraceae	Eubacteriales
Akkermansia	Akkermansiaceae	Verrucomicrobiales
Akkermansia	Akkermansiaceae	Verrucomicrobiales
Anaerotruncus	Oscillospiraceae	Eubacteriales
Anaerotruncus	Oscillospiraceae	Eubacteriales
Bacteria_unclassified	Bacteria_unclassified	Bacteria_unclassified
Bacteria_unclassified	Bacteria_unclassified	Bacteria_unclassified
Bacteria_unclassified	Bacteria_unclassified	Bacteria_unclassified
Bacteria_unclassified	Bacteria_unclassified	Bacteria_unclassified
Bacteria_unclassified	Bacteria_unclassified	Bacteria_unclassified
Bacteria_unclassified	Bacteria_unclassified	Bacteria_unclassified
Bacteria_unclassified	Bacteria_unclassified	Bacteria_unclassified
Bacteria_unclassified	Bacteria_unclassified	Bacteria_unclassified
Bacteria_unclassified	Bacteria_unclassified	Bacteria_unclassified
Bacteria_unclassified	Bacteria_unclassified	Bacteria_unclassified
Bacteria_unclassified	Bacteria_unclassified	Bacteria_unclassified
Bacteria_unclassified	Bacteria_unclassified	Bacteria_unclassified
Bacteria_unclassified	Bacteria_unclassified	Bacteria_unclassified
Bacteria_unclassified	Bacteria_unclassified	Bacteria_unclassified
Bacteria_unclassified	Bacteria_unclassified	Bacteria_unclassified
Bacteria_unclassified	Bacteria_unclassified	Bacteria_unclassified
Bacteria_unclassified	Bacteria_unclassified	Bacteria_unclassified
Bacteria_unclassified	Bacteria_unclassified	Bacteria_unclassified
Bacteroides	Bacteroidaceae	Bacteroidales
Bacteroides	Bacteroidaceae	Bacteroidales
Clostridia_unclassified	Clostridia_unclassified	Clostridia_unclassified
Clostridia_unclassified	Clostridia_unclassified	Clostridia_unclassified
Clostridiaceae_unclassified	Clostridiaceae	Eubacteriales
Clostridiaceae_unclassified	Clostridiaceae	Eubacteriales
Clostridiaceae_unclassified	Clostridiaceae	Eubacteriales
Clostridiaceae_unclassified	Clostridiaceae	Eubacteriales
Eubacteriales_unclassified	Eubacteriales_unclassified	Eubacteriales
Eubacteriales_unclassified	Eubacteriales_unclassified	Eubacteriales
Erysipelatoclostridium	Erysipelotrichaceae	Erysipelotrichales
Erysipelatoclostridium	Erysipelotrichaceae	Erysipelotrichales
Clostridium	Clostridiaceae	Eubacteriales
Clostridium	Clostridiaceae	Eubacteriales
Eubacteriaceae_unclassified	Eubacteriaceae	Eubacteriales
Eubacteriaceae_unclassified	Eubacteriaceae	Eubacteriales
Eubacteriaceae_unclassified	Eubacteriaceae	Eubacteriales
Eubacteriaceae_unclassified	Eubacteriaceae	Eubacteriales

GGB20146	FGB77306	OFGB77306
GGB20146	FGB77306	OFGB77306
GGB20149	Lachnospiraceae	Eubacteriales
GGB20149	Lachnospiraceae	Eubacteriales
GGB22635	Eggerthellaceae	Eggerthellales
GGB22635	Eggerthellaceae	Eggerthellales
GGB25041	Lachnospiraceae	Eubacteriales
GGB25041	Lachnospiraceae	Eubacteriales
GGB28379	FGB9506	OFGB9506
GGB28379	FGB9506	OFGB9506
GGB28382	FGB9508	OFGB9508
GGB28382	FGB9508	OFGB9508
GGB28392	FGB9512	OFGB9512
GGB28392	FGB9512	OFGB9512
GGB28404	FGB2838	OFGB2838
GGB28404	FGB2838	OFGB2838
GGB28418	FGB2838	OFGB2838
GGB28418	FGB2838	OFGB2838
GGB28422	FGB2838	OFGB2838
GGB28422	FGB2838	OFGB2838
GGB28431	Pumilibacteraceae	Eubacteriales
GGB28431	Pumilibacteraceae	Eubacteriales
GGB28456	FGB2833	OFGB2833
GGB28456	FGB2833	OFGB2833
GGB28782	Eubacteriaceae	Eubacteriales
GGB28782	Eubacteriaceae	Eubacteriales
GGB28792	Lachnospiraceae	Eubacteriales
GGB28792	Lachnospiraceae	Eubacteriales
GGB28798	Lachnospiraceae	Eubacteriales
GGB28798	Lachnospiraceae	Eubacteriales
GGB28810	FGB9622	OFGB9622
GGB28810	FGB9622	OFGB9622
GGB28828	FGB77305	OFGB77305
GGB28828	FGB77305	OFGB77305
GGB28851	Clostridiaceae	Eubacteriales
GGB28851	Clostridiaceae	Eubacteriales
GGB28865	Lachnospiraceae	Eubacteriales
GGB28865	Lachnospiraceae	Eubacteriales
GGB28868	Lachnospiraceae	Eubacteriales
GGB28868	Lachnospiraceae	Eubacteriales
GGB28869	Lachnospiraceae	Eubacteriales
GGB28869	Lachnospiraceae	Eubacteriales
GGB28875	Lachnospiraceae	Eubacteriales
GGB28875	Lachnospiraceae	Eubacteriales
GGB28881	FGB9633	OFGB9633
GGB28881	FGB9633	OFGB9633

GGB28892	Bacteria_unclassified	Bacteria_unclassified
GGB28892	Bacteria_unclassified	Bacteria_unclassified
GGB28893	Bacteria_unclassified	Bacteria_unclassified
GGB28893	Bacteria_unclassified	Bacteria_unclassified
GGB28898	Bacteria_unclassified	Bacteria_unclassified
GGB28898	Bacteria_unclassified	Bacteria_unclassified
GGB28901	Bacteria_unclassified	Bacteria_unclassified
GGB28901	Bacteria_unclassified	Bacteria_unclassified
GGB28904	Bacteria_unclassified	Bacteria_unclassified
GGB28904	Bacteria_unclassified	Bacteria_unclassified
GGB28909	Bacteria_unclassified	Bacteria_unclassified
GGB28909	Bacteria_unclassified	Bacteria_unclassified
GGB28924	Lachnospiraceae	Eubacteriales
GGB28924	Lachnospiraceae	Eubacteriales
GGB28927	FGB77359	OFGB77359
GGB28927	FGB77359	OFGB77359
GGB28934	FGB9639	OFGB9639
GGB28934	FGB9639	OFGB9639
GGB28949	Lachnospiraceae	Eubacteriales
GGB28949	Lachnospiraceae	Eubacteriales
GGB28951	Clostridiaceae	Eubacteriales
GGB28951	Clostridiaceae	Eubacteriales
GGB28951	Clostridiaceae	Eubacteriales
GGB28951	Clostridiaceae	Eubacteriales
GGB28954	Clostridiaceae	Eubacteriales
GGB28954	Clostridiaceae	Eubacteriales
GGB28960	Clostridiaceae	Eubacteriales
GGB28960	Clostridiaceae	Eubacteriales
GGB28964	Clostridiaceae	Eubacteriales
GGB28964	Clostridiaceae	Eubacteriales
GGB28967	Clostridiaceae	Eubacteriales
GGB28967	Clostridiaceae	Eubacteriales
GGB28996	FGB9656	OFGB9656
GGB28996	FGB9656	OFGB9656
GGB29531	FGB9827	OFGB9827
GGB29531	FGB9827	OFGB9827
GGB29685	Eubacteriaceae	Eubacteriales
GGB29685	Eubacteriaceae	Eubacteriales
GGB30141	FGB77303	OFGB77303
GGB30141	FGB77303	OFGB77303
GGB30145	Clostridiaceae	Eubacteriales
GGB30145	Clostridiaceae	Eubacteriales
GGB30286	Eubacteriales_unclassified	Eubacteriales
GGB30286	Eubacteriales_unclassified	Eubacteriales
GGB30300	FGB72709	OFGB72709
GGB30300	FGB72709	OFGB72709

GGB30303	Oscillospiraceae	Eubacteriales
GGB30303	Oscillospiraceae	Eubacteriales
GGB30450	Oscillospiraceae	Eubacteriales
GGB30450	Oscillospiraceae	Eubacteriales
GGB30453	Oscillospiraceae	Eubacteriales
GGB30453	Oscillospiraceae	Eubacteriales
GGB30454	Oscillospiraceae	Eubacteriales
GGB30454	Oscillospiraceae	Eubacteriales
GGB30455	Oscillospiraceae	Eubacteriales
GGB30455	Oscillospiraceae	Eubacteriales
GGB30456	Oscillospiraceae	Eubacteriales
GGB30456	Oscillospiraceae	Eubacteriales
GGB30457	Oscillospiraceae	Eubacteriales
GGB30457	Oscillospiraceae	Eubacteriales
GGB30461	Oscillospiraceae	Eubacteriales
GGB30461	Oscillospiraceae	Eubacteriales
GGB30461	Oscillospiraceae	Eubacteriales
GGB30461	Oscillospiraceae	Eubacteriales
GGB30461	Oscillospiraceae	Eubacteriales
GGB30461	Oscillospiraceae	Eubacteriales
GGB30463	Oscillospiraceae	Eubacteriales
GGB30463	Oscillospiraceae	Eubacteriales
GGB30473	Oscillospiraceae	Eubacteriales
GGB30473	Oscillospiraceae	Eubacteriales
GGB30475	Oscillospiraceae	Eubacteriales
GGB30475	Oscillospiraceae	Eubacteriales
GGB30861	FGB77153	OFGB77153
GGB30861	FGB77153	OFGB77153
GGB31312	FGB1791	OFGB1791
GGB31312	FGB1791	OFGB1791
GGB31438	FGB10290	OFGB10290
GGB31438	FGB10290	OFGB10290
GGB3171	Oscillospiraceae	Eubacteriales
GGB3171	Oscillospiraceae	Eubacteriales
GGB31762	FGB10289	OFGB10289
GGB31762	FGB10289	OFGB10289
GGB31823	FGB1765	OFGB1765
GGB31823	FGB1765	OFGB1765
GGB31838	FGB1765	OFGB1765
GGB31838	FGB1765	OFGB1765
GGB31841	FGB1765	OFGB1765
GGB31841	FGB1765	OFGB1765
GGB32371	FGB10667	OFGB10667
GGB32371	FGB10667	OFGB10667
GGB3793	Lachnospiraceae	Eubacteriales
GGB3793	Lachnospiraceae	Eubacteriales

GGB42601	Clostridiaceae	Eubacteriales
GGB42601	Clostridiaceae	Eubacteriales
GGB45514	Oscillospiraceae	Eubacteriales
GGB45514	Oscillospiraceae	Eubacteriales
GGB45564	FGB75721	OFGB75721
GGB45564	FGB75721	OFGB75721
GGB45624	Oscillospiraceae	Eubacteriales
GGB45624	Oscillospiraceae	Eubacteriales
GGB45656	Christensenellaceae	Eubacteriales
GGB45656	Christensenellaceae	Eubacteriales
GGB47127	FGB10299	OFGB10299
GGB47127	FGB10299	OFGB10299
GGB74395	Oscillospiraceae	Eubacteriales
GGB74395	Oscillospiraceae	Eubacteriales
GGB75053	Oscillospiraceae	Eubacteriales
GGB75053	Oscillospiraceae	Eubacteriales
GGB75109	Lachnospiraceae	Eubacteriales
GGB75109	Lachnospiraceae	Eubacteriales
Lachnospiraceae_unclassified	Lachnospiraceae	Eubacteriales
Lachnospiraceae_unclassified	Lachnospiraceae	Eubacteriales
Lachnospiraceae_unclassified	Lachnospiraceae	Eubacteriales
Lachnospiraceae_unclassified	Lachnospiraceae	Eubacteriales
Lachnospiraceae_unclassified	Lachnospiraceae	Eubacteriales
Lachnospiraceae_unclassified	Lachnospiraceae	Eubacteriales
Lachnospiraceae_unclassified	Lachnospiraceae	Eubacteriales
Lachnospiraceae_unclassified	Lachnospiraceae	Eubacteriales
Lachnospiraceae_unclassified	Lachnospiraceae	Eubacteriales
Lachnospiraceae_unclassified	Lachnospiraceae	Eubacteriales
Lactobacillus	Lactobacillaceae	Lactobacillales
Lactobacillus	Lactobacillaceae	Lactobacillales
Leptogranulimonas	Atopobiaceae	Coriobacteriales
Leptogranulimonas	Atopobiaceae	Coriobacteriales
Muribaculaceae_unclassified	Muribaculaceae	Bacteroidales
Muribaculaceae_unclassified	Muribaculaceae	Bacteroidales
Neglectibacter	Oscillospiraceae	Eubacteriales
Neglectibacter	Oscillospiraceae	Eubacteriales
Oscillibacter	Oscillospiraceae	Eubacteriales
Oscillibacter	Oscillospiraceae	Eubacteriales
Oscillospiraceae_unclassified	Oscillospiraceae	Eubacteriales
Oscillospiraceae_unclassified	Oscillospiraceae	Eubacteriales
Oscillospiraceae_unclassified	Oscillospiraceae	Eubacteriales
Oscillospiraceae_unclassified	Oscillospiraceae	Eubacteriales
Oscillospiraceae_unclassified	Oscillospiraceae	Eubacteriales
Oscillospiraceae_unclassified	Oscillospiraceae	Eubacteriales
Oscillospiraceae_unclassified	Oscillospiraceae	Eubacteriales
Oscillospiraceae_unclassified	Oscillospiraceae	Eubacteriales

Parasutterella	Sutterellaceae	Burkholderiales
Parasutterella	Sutterellaceae	Burkholderiales
Schaedlerella	Lachnospiraceae	Eubacteriales
Schaedlerella	Lachnospiraceae	Eubacteriales
Turicibacter	Turicibacteraceae	Erysipelotrichales
Turicibacter	Turicibacteraceae	Erysipelotrichales
Acetatifactor	Lachnospiraceae	Eubacteriales
Acetatifactor	Lachnospiraceae	Eubacteriales
Acutalibacter	Oscillospiraceae	Eubacteriales
Acutalibacter	Oscillospiraceae	Eubacteriales
Akkermansia	Akkermansiaceae	Verrucomicrobiales
Akkermansia	Akkermansiaceae	Verrucomicrobiales
Anaerotruncus	Oscillospiraceae	Eubacteriales
Anaerotruncus	Oscillospiraceae	Eubacteriales
Bacteria_unclassified	Bacteria_unclassified	Bacteria_unclassified
Bacteria_unclassified	Bacteria_unclassified	Bacteria_unclassified
Bacteria_unclassified	Bacteria_unclassified	Bacteria_unclassified
Bacteria_unclassified	Bacteria_unclassified	Bacteria_unclassified
Bacteria_unclassified	Bacteria_unclassified	Bacteria_unclassified
Bacteria_unclassified	Bacteria_unclassified	Bacteria_unclassified
Bacteria_unclassified	Bacteria_unclassified	Bacteria_unclassified
Bacteria_unclassified	Bacteria_unclassified	Bacteria_unclassified
Bacteria_unclassified	Bacteria_unclassified	Bacteria_unclassified
Bacteria_unclassified	Bacteria_unclassified	Bacteria_unclassified
Bacteria_unclassified	Bacteria_unclassified	Bacteria_unclassified
Bacteria_unclassified	Bacteria_unclassified	Bacteria_unclassified
Bacteria_unclassified	Bacteria_unclassified	Bacteria_unclassified
Bacteria_unclassified	Bacteria_unclassified	Bacteria_unclassified
Bacteria_unclassified	Bacteria_unclassified	Bacteria_unclassified
Bacteria_unclassified	Bacteria_unclassified	Bacteria_unclassified
Bacteria_unclassified	Bacteria_unclassified	Bacteria_unclassified
Bacteria_unclassified	Bacteria_unclassified	Bacteria_unclassified
Bacteroides	Bacteroidaceae	Bacteroidales
Bacteroides	Bacteroidaceae	Bacteroidales
Clostridia_unclassified	Clostridia_unclassified	Clostridia_unclassified
Clostridia_unclassified	Clostridia_unclassified	Clostridia_unclassified
Clostridiaceae_unclassified	Clostridiaceae	Eubacteriales
Clostridiaceae_unclassified	Clostridiaceae	Eubacteriales
Clostridiaceae_unclassified	Clostridiaceae	Eubacteriales
Clostridiaceae_unclassified	Clostridiaceae	Eubacteriales

Eubacteriales_unclassified	Eubacteriales_unclassified	Eubacteriales
Eubacteriales_unclassified	Eubacteriales_unclassified	Eubacteriales
Erysipelatoclostridium	Erysipelotrichaceae	Erysipelotrichales
Erysipelatoclostridium	Erysipelotrichaceae	Erysipelotrichales
Clostridium	Clostridiaceae	Eubacteriales
Clostridium	Clostridiaceae	Eubacteriales
Eubacteriaceae_unclassified	Eubacteriaceae	Eubacteriales
Eubacteriaceae_unclassified	Eubacteriaceae	Eubacteriales
Eubacteriaceae_unclassified	Eubacteriaceae	Eubacteriales
Eubacteriaceae_unclassified	Eubacteriaceae	Eubacteriales
GGB20146	FGB77306	OFGB77306
GGB20146	FGB77306	OFGB77306
GGB20149	Lachnospiraceae	Eubacteriales
GGB20149	Lachnospiraceae	Eubacteriales
GGB22635	Eggerthellaceae	Eggerthellales
GGB22635	Eggerthellaceae	Eggerthellales
GGB25041	Lachnospiraceae	Eubacteriales
GGB25041	Lachnospiraceae	Eubacteriales
GGB28379	FGB9506	OFGB9506
GGB28379	FGB9506	OFGB9506
GGB28382	FGB9508	OFGB9508
GGB28382	FGB9508	OFGB9508
GGB28392	FGB9512	OFGB9512
GGB28392	FGB9512	OFGB9512
GGB28404	FGB2838	OFGB2838
GGB28404	FGB2838	OFGB2838
GGB28418	FGB2838	OFGB2838
GGB28418	FGB2838	OFGB2838
GGB28422	FGB2838	OFGB2838
GGB28422	FGB2838	OFGB2838
GGB28431	Pumilibacteraceae	Eubacteriales
GGB28431	Pumilibacteraceae	Eubacteriales
GGB28456	FGB2833	OFGB2833
GGB28456	FGB2833	OFGB2833
GGB28782	Eubacteriaceae	Eubacteriales
GGB28782	Eubacteriaceae	Eubacteriales
GGB28792	Lachnospiraceae	Eubacteriales
GGB28792	Lachnospiraceae	Eubacteriales
GGB28798	Lachnospiraceae	Eubacteriales
GGB28798	Lachnospiraceae	Eubacteriales
GGB28810	FGB9622	OFGB9622
GGB28810	FGB9622	OFGB9622
GGB28828	FGB77305	OFGB77305
GGB28828	FGB77305	OFGB77305
GGB28851	Clostridiaceae	Eubacteriales
GGB28851	Clostridiaceae	Eubacteriales

GGB28865	Lachnospiraceae	Eubacteriales
GGB28865	Lachnospiraceae	Eubacteriales
GGB28868	Lachnospiraceae	Eubacteriales
GGB28868	Lachnospiraceae	Eubacteriales
GGB28869	Lachnospiraceae	Eubacteriales
GGB28869	Lachnospiraceae	Eubacteriales
GGB28875	Lachnospiraceae	Eubacteriales
GGB28875	Lachnospiraceae	Eubacteriales
GGB28881	FGB9633	OFGB9633
GGB28881	FGB9633	OFGB9633
GGB28892	Bacteria_unclassified	Bacteria_unclassified
GGB28892	Bacteria_unclassified	Bacteria_unclassified
GGB28893	Bacteria_unclassified	Bacteria_unclassified
GGB28893	Bacteria_unclassified	Bacteria_unclassified
GGB28898	Bacteria_unclassified	Bacteria_unclassified
GGB28898	Bacteria_unclassified	Bacteria_unclassified
GGB28901	Bacteria_unclassified	Bacteria_unclassified
GGB28901	Bacteria_unclassified	Bacteria_unclassified
GGB28904	Bacteria_unclassified	Bacteria_unclassified
GGB28904	Bacteria_unclassified	Bacteria_unclassified
GGB28909	Bacteria_unclassified	Bacteria_unclassified
GGB28909	Bacteria_unclassified	Bacteria_unclassified
GGB28924	Lachnospiraceae	Eubacteriales
GGB28924	Lachnospiraceae	Eubacteriales
GGB28927	FGB77359	OFGB77359
GGB28927	FGB77359	OFGB77359
GGB28934	FGB9639	OFGB9639
GGB28934	FGB9639	OFGB9639
GGB28949	Lachnospiraceae	Eubacteriales
GGB28949	Lachnospiraceae	Eubacteriales
GGB28951	Clostridiaceae	Eubacteriales
GGB28951	Clostridiaceae	Eubacteriales
GGB28951	Clostridiaceae	Eubacteriales
GGB28951	Clostridiaceae	Eubacteriales
GGB28954	Clostridiaceae	Eubacteriales
GGB28954	Clostridiaceae	Eubacteriales
GGB28960	Clostridiaceae	Eubacteriales
GGB28960	Clostridiaceae	Eubacteriales
GGB28964	Clostridiaceae	Eubacteriales
GGB28964	Clostridiaceae	Eubacteriales
GGB28967	Clostridiaceae	Eubacteriales
GGB28967	Clostridiaceae	Eubacteriales
GGB28996	FGB9656	OFGB9656
GGB28996	FGB9656	OFGB9656
GGB29531	FGB9827	OFGB9827
GGB29531	FGB9827	OFGB9827

GGB29685	Eubacteriaceae	Eubacteriales
GGB29685	Eubacteriaceae	Eubacteriales
GGB30141	FGB77303	OFGB77303
GGB30141	FGB77303	OFGB77303
GGB30145	Clostridiaceae	Eubacteriales
GGB30145	Clostridiaceae	Eubacteriales
GGB30286	Eubacteriales_unclassified	Eubacteriales
GGB30286	Eubacteriales_unclassified	Eubacteriales
GGB30300	FGB72709	OFGB72709
GGB30300	FGB72709	OFGB72709
GGB30303	Oscillospiraceae	Eubacteriales
GGB30303	Oscillospiraceae	Eubacteriales
GGB30450	Oscillospiraceae	Eubacteriales
GGB30450	Oscillospiraceae	Eubacteriales
GGB30453	Oscillospiraceae	Eubacteriales
GGB30453	Oscillospiraceae	Eubacteriales
GGB30454	Oscillospiraceae	Eubacteriales
GGB30454	Oscillospiraceae	Eubacteriales
GGB30455	Oscillospiraceae	Eubacteriales
GGB30455	Oscillospiraceae	Eubacteriales
GGB30456	Oscillospiraceae	Eubacteriales
GGB30456	Oscillospiraceae	Eubacteriales
GGB30457	Oscillospiraceae	Eubacteriales
GGB30457	Oscillospiraceae	Eubacteriales
GGB30461	Oscillospiraceae	Eubacteriales
GGB30461	Oscillospiraceae	Eubacteriales
GGB30461	Oscillospiraceae	Eubacteriales
GGB30461	Oscillospiraceae	Eubacteriales
GGB30461	Oscillospiraceae	Eubacteriales
GGB30461	Oscillospiraceae	Eubacteriales
GGB30463	Oscillospiraceae	Eubacteriales
GGB30463	Oscillospiraceae	Eubacteriales
GGB30473	Oscillospiraceae	Eubacteriales
GGB30473	Oscillospiraceae	Eubacteriales
GGB30475	Oscillospiraceae	Eubacteriales
GGB30475	Oscillospiraceae	Eubacteriales
GGB30861	FGB77153	OFGB77153
GGB30861	FGB77153	OFGB77153
GGB31312	FGB1791	OFGB1791
GGB31312	FGB1791	OFGB1791
GGB31438	FGB10290	OFGB10290
GGB31438	FGB10290	OFGB10290
GGB3171	Oscillospiraceae	Eubacteriales
GGB3171	Oscillospiraceae	Eubacteriales
GGB31762	FGB10289	OFGB10289
GGB31762	FGB10289	OFGB10289

GGB31823	FGB1765	OFGB1765
GGB31823	FGB1765	OFGB1765
GGB31838	FGB1765	OFGB1765
GGB31838	FGB1765	OFGB1765
GGB31841	FGB1765	OFGB1765
GGB31841	FGB1765	OFGB1765
GGB32371	FGB10667	OFGB10667
GGB32371	FGB10667	OFGB10667
GGB3793	Lachnospiraceae	Eubacteriales
GGB3793	Lachnospiraceae	Eubacteriales
GGB42601	Clostridiaceae	Eubacteriales
GGB42601	Clostridiaceae	Eubacteriales
GGB45514	Oscillospiraceae	Eubacteriales
GGB45514	Oscillospiraceae	Eubacteriales
GGB45564	FGB75721	OFGB75721
GGB45564	FGB75721	OFGB75721
GGB45624	Oscillospiraceae	Eubacteriales
GGB45624	Oscillospiraceae	Eubacteriales
GGB45656	Christensenellaceae	Eubacteriales
GGB45656	Christensenellaceae	Eubacteriales
GGB47127	FGB10299	OFGB10299
GGB47127	FGB10299	OFGB10299
GGB74395	Oscillospiraceae	Eubacteriales
GGB74395	Oscillospiraceae	Eubacteriales
GGB75053	Oscillospiraceae	Eubacteriales
GGB75053	Oscillospiraceae	Eubacteriales
GGB75109	Lachnospiraceae	Eubacteriales
GGB75109	Lachnospiraceae	Eubacteriales
Lachnospiraceae_unclassified	Lachnospiraceae	Eubacteriales
Lachnospiraceae_unclassified	Lachnospiraceae	Eubacteriales
Lachnospiraceae_unclassified	Lachnospiraceae	Eubacteriales
Lachnospiraceae_unclassified	Lachnospiraceae	Eubacteriales
Lachnospiraceae_unclassified	Lachnospiraceae	Eubacteriales
Lachnospiraceae_unclassified	Lachnospiraceae	Eubacteriales
Lachnospiraceae_unclassified	Lachnospiraceae	Eubacteriales
Lachnospiraceae_unclassified	Lachnospiraceae	Eubacteriales
Lachnospiraceae_unclassified	Lachnospiraceae	Eubacteriales
Lachnospiraceae_unclassified	Lachnospiraceae	Eubacteriales
Lactobacillus	Lactobacillaceae	Lactobacillales
Lactobacillus	Lactobacillaceae	Lactobacillales
Leptogranulimonas	Atopobiaceae	Coriobacteriales
Leptogranulimonas	Atopobiaceae	Coriobacteriales
Muribaculaceae_unclassified	Muribaculaceae	Bacteroidales
Muribaculaceae_unclassified	Muribaculaceae	Bacteroidales
Neglectibacter	Oscillospiraceae	Eubacteriales
Neglectibacter	Oscillospiraceae	Eubacteriales

Oscillibacter	Oscillospiraceae	Eubacteriales
Oscillibacter	Oscillospiraceae	Eubacteriales
Oscillospiraceae_unclassified	Oscillospiraceae	Eubacteriales
Oscillospiraceae_unclassified	Oscillospiraceae	Eubacteriales
Oscillospiraceae_unclassified	Oscillospiraceae	Eubacteriales
Oscillospiraceae_unclassified	Oscillospiraceae	Eubacteriales
Oscillospiraceae_unclassified	Oscillospiraceae	Eubacteriales
Oscillospiraceae_unclassified	Oscillospiraceae	Eubacteriales
Oscillospiraceae_unclassified	Oscillospiraceae	Eubacteriales
Oscillospiraceae_unclassified	Oscillospiraceae	Eubacteriales
Parasutterella	Sutterellaceae	Burkholderiales
Parasutterella	Sutterellaceae	Burkholderiales
Schaedlerella	Lachnospiraceae	Eubacteriales
Schaedlerella	Lachnospiraceae	Eubacteriales
Turicibacter	Turicibacteraceae	Erysipelotrichales
Turicibacter	Turicibacteraceae	Erysipelotrichales
Acetatifactor	Lachnospiraceae	Eubacteriales
Acetatifactor	Lachnospiraceae	Eubacteriales
Acutalibacter	Oscillospiraceae	Eubacteriales
Acutalibacter	Oscillospiraceae	Eubacteriales
Akkermansia	Akkermansiaceae	Verrucomicrobiales
Akkermansia	Akkermansiaceae	Verrucomicrobiales
Anaerotruncus	Oscillospiraceae	Eubacteriales
Anaerotruncus	Oscillospiraceae	Eubacteriales
Bacteria_unclassified	Bacteria_unclassified	Bacteria_unclassified
Bacteria_unclassified	Bacteria_unclassified	Bacteria_unclassified
Bacteria_unclassified	Bacteria_unclassified	Bacteria_unclassified
Bacteria_unclassified	Bacteria_unclassified	Bacteria_unclassified
Bacteria_unclassified	Bacteria_unclassified	Bacteria_unclassified
Bacteria_unclassified	Bacteria_unclassified	Bacteria_unclassified
Bacteria_unclassified	Bacteria_unclassified	Bacteria_unclassified
Bacteria_unclassified	Bacteria_unclassified	Bacteria_unclassified
Bacteria_unclassified	Bacteria_unclassified	Bacteria_unclassified
Bacteria_unclassified	Bacteria_unclassified	Bacteria_unclassified
Bacteria_unclassified	Bacteria_unclassified	Bacteria_unclassified
Bacteria_unclassified	Bacteria_unclassified	Bacteria_unclassified
Bacteria_unclassified	Bacteria_unclassified	Bacteria_unclassified
Bacteria_unclassified	Bacteria_unclassified	Bacteria_unclassified
Bacteria_unclassified	Bacteria_unclassified	Bacteria_unclassified
Bacteria_unclassified	Bacteria_unclassified	Bacteria_unclassified
Bacteria_unclassified	Bacteria_unclassified	Bacteria_unclassified
Bacteria_unclassified	Bacteria_unclassified	Bacteria_unclassified

Bacteroides	Bacteroidaceae	Bacteroidales
Bacteroides	Bacteroidaceae	Bacteroidales
Clostridia_unclassified	Clostridia_unclassified	Clostridia_unclassified
Clostridia_unclassified	Clostridia_unclassified	Clostridia_unclassified
Clostridiaceae_unclassified	Clostridiaceae	Eubacteriales
Clostridiaceae_unclassified	Clostridiaceae	Eubacteriales
Clostridiaceae_unclassified	Clostridiaceae	Eubacteriales
Clostridiaceae_unclassified	Clostridiaceae	Eubacteriales
Eubacteriales_unclassified	Eubacteriales_unclassified	Eubacteriales
Eubacteriales_unclassified	Eubacteriales_unclassified	Eubacteriales
Erysipelatoclostridium	Erysipelotrichaceae	Erysipelotrichales
Erysipelatoclostridium	Erysipelotrichaceae	Erysipelotrichales
Clostridium	Clostridiaceae	Eubacteriales
Clostridium	Clostridiaceae	Eubacteriales
Eubacteriaceae_unclassified	Eubacteriaceae	Eubacteriales
Eubacteriaceae_unclassified	Eubacteriaceae	Eubacteriales
Eubacteriaceae_unclassified	Eubacteriaceae	Eubacteriales
Eubacteriaceae_unclassified	Eubacteriaceae	Eubacteriales
GGB20146	FGB77306	OFGB77306
GGB20146	FGB77306	OFGB77306
GGB20149	Lachnospiraceae	Eubacteriales
GGB20149	Lachnospiraceae	Eubacteriales
GGB22635	Eggerthellaceae	Eggerthellales
GGB22635	Eggerthellaceae	Eggerthellales
GGB25041	Lachnospiraceae	Eubacteriales
GGB25041	Lachnospiraceae	Eubacteriales
GGB28379	FGB9506	OFGB9506
GGB28379	FGB9506	OFGB9506
GGB28382	FGB9508	OFGB9508
GGB28382	FGB9508	OFGB9508
GGB28392	FGB9512	OFGB9512
GGB28392	FGB9512	OFGB9512
GGB28404	FGB2838	OFGB2838
GGB28404	FGB2838	OFGB2838
GGB28418	FGB2838	OFGB2838
GGB28418	FGB2838	OFGB2838
GGB28422	FGB2838	OFGB2838
GGB28422	FGB2838	OFGB2838
GGB28431	Pumilibacteraceae	Eubacteriales
GGB28431	Pumilibacteraceae	Eubacteriales
GGB28456	FGB2833	OFGB2833
GGB28456	FGB2833	OFGB2833
GGB28782	Eubacteriaceae	Eubacteriales
GGB28782	Eubacteriaceae	Eubacteriales

GGB28792	Lachnospiraceae	Eubacteriales
GGB28792	Lachnospiraceae	Eubacteriales
GGB28798	Lachnospiraceae	Eubacteriales
GGB28798	Lachnospiraceae	Eubacteriales
GGB28810	FGB9622	OFGB9622
GGB28810	FGB9622	OFGB9622
GGB28828	FGB77305	OFGB77305
GGB28828	FGB77305	OFGB77305
GGB28851	Clostridiaceae	Eubacteriales
GGB28851	Clostridiaceae	Eubacteriales
GGB28865	Lachnospiraceae	Eubacteriales
GGB28865	Lachnospiraceae	Eubacteriales
GGB28868	Lachnospiraceae	Eubacteriales
GGB28868	Lachnospiraceae	Eubacteriales
GGB28869	Lachnospiraceae	Eubacteriales
GGB28869	Lachnospiraceae	Eubacteriales
GGB28875	Lachnospiraceae	Eubacteriales
GGB28875	Lachnospiraceae	Eubacteriales
GGB28881	FGB9633	OFGB9633
GGB28881	FGB9633	OFGB9633
GGB28892	Bacteria_unclassified	Bacteria_unclassified
GGB28892	Bacteria_unclassified	Bacteria_unclassified
GGB28893	Bacteria_unclassified	Bacteria_unclassified
GGB28893	Bacteria_unclassified	Bacteria_unclassified
GGB28898	Bacteria_unclassified	Bacteria_unclassified
GGB28898	Bacteria_unclassified	Bacteria_unclassified
GGB28901	Bacteria_unclassified	Bacteria_unclassified
GGB28901	Bacteria_unclassified	Bacteria_unclassified
GGB28904	Bacteria_unclassified	Bacteria_unclassified
GGB28904	Bacteria_unclassified	Bacteria_unclassified
GGB28909	Bacteria_unclassified	Bacteria_unclassified
GGB28909	Bacteria_unclassified	Bacteria_unclassified
GGB28924	Lachnospiraceae	Eubacteriales
GGB28924	Lachnospiraceae	Eubacteriales
GGB28927	FGB77359	OFGB77359
GGB28927	FGB77359	OFGB77359
GGB28934	FGB9639	OFGB9639
GGB28934	FGB9639	OFGB9639
GGB28949	Lachnospiraceae	Eubacteriales
GGB28949	Lachnospiraceae	Eubacteriales
GGB28951	Clostridiaceae	Eubacteriales
GGB28951	Clostridiaceae	Eubacteriales
GGB28951	Clostridiaceae	Eubacteriales
GGB28951	Clostridiaceae	Eubacteriales
GGB28954	Clostridiaceae	Eubacteriales
GGB28954	Clostridiaceae	Eubacteriales

GGB28960	Clostridiaceae	Eubacteriales
GGB28960	Clostridiaceae	Eubacteriales
GGB28964	Clostridiaceae	Eubacteriales
GGB28964	Clostridiaceae	Eubacteriales
GGB28967	Clostridiaceae	Eubacteriales
GGB28967	Clostridiaceae	Eubacteriales
GGB28996	FGB9656	OFGB9656
GGB28996	FGB9656	OFGB9656
GGB29531	FGB9827	OFGB9827
GGB29531	FGB9827	OFGB9827
GGB29685	Eubacteriaceae	Eubacteriales
GGB29685	Eubacteriaceae	Eubacteriales
GGB30141	FGB77303	OFGB77303
GGB30141	FGB77303	OFGB77303
GGB30145	Clostridiaceae	Eubacteriales
GGB30145	Clostridiaceae	Eubacteriales
GGB30286	Eubacteriales_unclassified	Eubacteriales
GGB30286	Eubacteriales_unclassified	Eubacteriales
GGB30300	FGB72709	OFGB72709
GGB30300	FGB72709	OFGB72709
GGB30303	Oscillospiraceae	Eubacteriales
GGB30303	Oscillospiraceae	Eubacteriales
GGB30450	Oscillospiraceae	Eubacteriales
GGB30450	Oscillospiraceae	Eubacteriales
GGB30453	Oscillospiraceae	Eubacteriales
GGB30453	Oscillospiraceae	Eubacteriales
GGB30454	Oscillospiraceae	Eubacteriales
GGB30454	Oscillospiraceae	Eubacteriales
GGB30455	Oscillospiraceae	Eubacteriales
GGB30455	Oscillospiraceae	Eubacteriales
GGB30456	Oscillospiraceae	Eubacteriales
GGB30456	Oscillospiraceae	Eubacteriales
GGB30457	Oscillospiraceae	Eubacteriales
GGB30457	Oscillospiraceae	Eubacteriales
GGB30461	Oscillospiraceae	Eubacteriales
GGB30461	Oscillospiraceae	Eubacteriales
GGB30461	Oscillospiraceae	Eubacteriales
GGB30461	Oscillospiraceae	Eubacteriales
GGB30461	Oscillospiraceae	Eubacteriales
GGB30461	Oscillospiraceae	Eubacteriales
GGB30463	Oscillospiraceae	Eubacteriales
GGB30463	Oscillospiraceae	Eubacteriales
GGB30473	Oscillospiraceae	Eubacteriales
GGB30473	Oscillospiraceae	Eubacteriales
GGB30475	Oscillospiraceae	Eubacteriales
GGB30475	Oscillospiraceae	Eubacteriales

GGB30861	FGB77153	OFGB77153
GGB30861	FGB77153	OFGB77153
GGB31312	FGB1791	OFGB1791
GGB31312	FGB1791	OFGB1791
GGB31438	FGB10290	OFGB10290
GGB31438	FGB10290	OFGB10290
GGB3171	Oscillospiraceae	Eubacteriales
GGB3171	Oscillospiraceae	Eubacteriales
GGB31762	FGB10289	OFGB10289
GGB31762	FGB10289	OFGB10289
GGB31823	FGB1765	OFGB1765
GGB31823	FGB1765	OFGB1765
GGB31838	FGB1765	OFGB1765
GGB31838	FGB1765	OFGB1765
GGB31841	FGB1765	OFGB1765
GGB31841	FGB1765	OFGB1765
GGB32371	FGB10667	OFGB10667
GGB32371	FGB10667	OFGB10667
GGB3793	Lachnospiraceae	Eubacteriales
GGB3793	Lachnospiraceae	Eubacteriales
GGB42601	Clostridiaceae	Eubacteriales
GGB42601	Clostridiaceae	Eubacteriales
GGB45514	Oscillospiraceae	Eubacteriales
GGB45514	Oscillospiraceae	Eubacteriales
GGB45564	FGB75721	OFGB75721
GGB45564	FGB75721	OFGB75721
GGB45624	Oscillospiraceae	Eubacteriales
GGB45624	Oscillospiraceae	Eubacteriales
GGB45656	Christensenellaceae	Eubacteriales
GGB45656	Christensenellaceae	Eubacteriales
GGB47127	FGB10299	OFGB10299
GGB47127	FGB10299	OFGB10299
GGB74395	Oscillospiraceae	Eubacteriales
GGB74395	Oscillospiraceae	Eubacteriales
GGB75053	Oscillospiraceae	Eubacteriales
GGB75053	Oscillospiraceae	Eubacteriales
GGB75109	Lachnospiraceae	Eubacteriales
GGB75109	Lachnospiraceae	Eubacteriales
Lachnospiraceae_unclassified	Lachnospiraceae	Eubacteriales
Lachnospiraceae_unclassified	Lachnospiraceae	Eubacteriales
Lachnospiraceae_unclassified	Lachnospiraceae	Eubacteriales
Lachnospiraceae_unclassified	Lachnospiraceae	Eubacteriales
Lachnospiraceae_unclassified	Lachnospiraceae	Eubacteriales
Lachnospiraceae_unclassified	Lachnospiraceae	Eubacteriales
Lachnospiraceae_unclassified	Lachnospiraceae	Eubacteriales
Lachnospiraceae_unclassified	Lachnospiraceae	Eubacteriales

Lachnospiraceae_unclassified	Lachnospiraceae	Eubacteriales
Lachnospiraceae_unclassified	Lachnospiraceae	Eubacteriales
Lactobacillus	Lactobacillaceae	Lactobacillales
Lactobacillus	Lactobacillaceae	Lactobacillales
Leptogranulimonas	Atopobiaceae	Coriobacteriales
Leptogranulimonas	Atopobiaceae	Coriobacteriales
Muribaculaceae_unclassified	Muribaculaceae	Bacteroidales
Muribaculaceae_unclassified	Muribaculaceae	Bacteroidales
Neglectibacter	Oscillospiraceae	Eubacteriales
Neglectibacter	Oscillospiraceae	Eubacteriales
Oscillibacter	Oscillospiraceae	Eubacteriales
Oscillibacter	Oscillospiraceae	Eubacteriales
Oscillospiraceae_unclassified	Oscillospiraceae	Eubacteriales
Oscillospiraceae_unclassified	Oscillospiraceae	Eubacteriales
Oscillospiraceae_unclassified	Oscillospiraceae	Eubacteriales
Oscillospiraceae_unclassified	Oscillospiraceae	Eubacteriales
Oscillospiraceae_unclassified	Oscillospiraceae	Eubacteriales
Oscillospiraceae_unclassified	Oscillospiraceae	Eubacteriales
Oscillospiraceae_unclassified	Oscillospiraceae	Eubacteriales
Oscillospiraceae_unclassified	Oscillospiraceae	Eubacteriales
Parasutterella	Sutterellaceae	Burkholderiales
Parasutterella	Sutterellaceae	Burkholderiales
Schaedlerella	Lachnospiraceae	Eubacteriales
Schaedlerella	Lachnospiraceae	Eubacteriales
Turicibacter	Turicibacteraceae	Erysipelotrichales
Turicibacter	Turicibacteraceae	Erysipelotrichales
Acetatifactor	Lachnospiraceae	Eubacteriales
Acetatifactor	Lachnospiraceae	Eubacteriales
Acutalibacter	Oscillospiraceae	Eubacteriales
Acutalibacter	Oscillospiraceae	Eubacteriales
Akkermansia	Akkermansiaceae	Verrucomicrobiales
Akkermansia	Akkermansiaceae	Verrucomicrobiales
Anaerotruncus	Oscillospiraceae	Eubacteriales
Anaerotruncus	Oscillospiraceae	Eubacteriales
Bacteria_unclassified	Bacteria_unclassified	Bacteria_unclassified
Bacteria_unclassified	Bacteria_unclassified	Bacteria_unclassified
Bacteria_unclassified	Bacteria_unclassified	Bacteria_unclassified
Bacteria_unclassified	Bacteria_unclassified	Bacteria_unclassified
Bacteria_unclassified	Bacteria_unclassified	Bacteria_unclassified
Bacteria_unclassified	Bacteria_unclassified	Bacteria_unclassified
Bacteria_unclassified	Bacteria_unclassified	Bacteria_unclassified
Bacteria_unclassified	Bacteria_unclassified	Bacteria_unclassified

Bacteria_unclassified	Bacteria_unclassified	Bacteria_unclassified
Bacteria_unclassified	Bacteria_unclassified	Bacteria_unclassified
Bacteria_unclassified	Bacteria_unclassified	Bacteria_unclassified
Bacteria_unclassified	Bacteria_unclassified	Bacteria_unclassified
Bacteria_unclassified	Bacteria_unclassified	Bacteria_unclassified
Bacteria_unclassified	Bacteria_unclassified	Bacteria_unclassified
Bacteria_unclassified	Bacteria_unclassified	Bacteria_unclassified
Bacteria_unclassified	Bacteria_unclassified	Bacteria_unclassified
Bacteria_unclassified	Bacteria_unclassified	Bacteria_unclassified
Bacteria_unclassified	Bacteria_unclassified	Bacteria_unclassified
Bacteroides	Bacteroidaceae	Bacteroidales
Bacteroides	Bacteroidaceae	Bacteroidales

Clostridia_unclassified	Clostridia_unclassified	Clostridia_unclassified
Clostridia_unclassified	Clostridia_unclassified	Clostridia_unclassified
Clostridiaceae_unclassified	Clostridiaceae	Eubacteriales
Clostridiaceae_unclassified	Clostridiaceae	Eubacteriales
Clostridiaceae_unclassified	Clostridiaceae	Eubacteriales
Clostridiaceae_unclassified	Clostridiaceae	Eubacteriales
Eubacteriales_unclassified	Eubacteriales_unclassified	Eubacteriales
Eubacteriales_unclassified	Eubacteriales_unclassified	Eubacteriales
Erysipelatoclostridium	Erysipelotrichaceae	Erysipelotrichales
Erysipelatoclostridium	Erysipelotrichaceae	Erysipelotrichales
Clostridium	Clostridiaceae	Eubacteriales
Clostridium	Clostridiaceae	Eubacteriales
Eubacteriaceae_unclassified	Eubacteriaceae	Eubacteriales
Eubacteriaceae_unclassified	Eubacteriaceae	Eubacteriales
Eubacteriaceae_unclassified	Eubacteriaceae	Eubacteriales
Eubacteriaceae_unclassified	Eubacteriaceae	Eubacteriales
GGB20146	FGB77306	OFGB77306
GGB20146	FGB77306	OFGB77306
GGB20149	Lachnospiraceae	Eubacteriales
GGB20149	Lachnospiraceae	Eubacteriales
GGB22635	Eggerthellaceae	Eggerthellales
GGB22635	Eggerthellaceae	Eggerthellales
GGB25041	Lachnospiraceae	Eubacteriales
GGB25041	Lachnospiraceae	Eubacteriales
GGB28379	FGB9506	OFGB9506
GGB28379	FGB9506	OFGB9506
GGB28382	FGB9508	OFGB9508
GGB28382	FGB9508	OFGB9508
GGB28392	FGB9512	OFGB9512
GGB28392	FGB9512	OFGB9512
GGB28404	FGB2838	OFGB2838
GGB28404	FGB2838	OFGB2838

GGB28418	FGB2838	OFGB2838
GGB28418	FGB2838	OFGB2838
GGB28422	FGB2838	OFGB2838
GGB28422	FGB2838	OFGB2838
GGB28431	Pumilibacteraceae	Eubacteriales
GGB28431	Pumilibacteraceae	Eubacteriales
GGB28456	FGB2833	OFGB2833
GGB28456	FGB2833	OFGB2833
GGB28782	Eubacteriaceae	Eubacteriales
GGB28782	Eubacteriaceae	Eubacteriales
GGB28792	Lachnospiraceae	Eubacteriales
GGB28792	Lachnospiraceae	Eubacteriales
GGB28798	Lachnospiraceae	Eubacteriales
GGB28798	Lachnospiraceae	Eubacteriales
GGB28810	FGB9622	OFGB9622
GGB28810	FGB9622	OFGB9622
GGB28828	FGB77305	OFGB77305
GGB28828	FGB77305	OFGB77305
GGB28851	Clostridiaceae	Eubacteriales
GGB28851	Clostridiaceae	Eubacteriales
GGB28865	Lachnospiraceae	Eubacteriales
GGB28865	Lachnospiraceae	Eubacteriales
GGB28868	Lachnospiraceae	Eubacteriales
GGB28868	Lachnospiraceae	Eubacteriales
GGB28869	Lachnospiraceae	Eubacteriales
GGB28869	Lachnospiraceae	Eubacteriales
GGB28875	Lachnospiraceae	Eubacteriales
GGB28875	Lachnospiraceae	Eubacteriales
GGB28881	FGB9633	OFGB9633
GGB28881	FGB9633	OFGB9633
GGB28892	Bacteria_unclassified	Bacteria_unclassified
GGB28892	Bacteria_unclassified	Bacteria_unclassified
GGB28893	Bacteria_unclassified	Bacteria_unclassified
GGB28893	Bacteria_unclassified	Bacteria_unclassified
GGB28898	Bacteria_unclassified	Bacteria_unclassified
GGB28898	Bacteria_unclassified	Bacteria_unclassified
GGB28901	Bacteria_unclassified	Bacteria_unclassified
GGB28901	Bacteria_unclassified	Bacteria_unclassified
GGB28904	Bacteria_unclassified	Bacteria_unclassified
GGB28904	Bacteria_unclassified	Bacteria_unclassified
GGB28909	Bacteria_unclassified	Bacteria_unclassified
GGB28909	Bacteria_unclassified	Bacteria_unclassified
GGB28924	Lachnospiraceae	Eubacteriales
GGB28924	Lachnospiraceae	Eubacteriales
GGB28927	FGB77359	OFGB77359
GGB28927	FGB77359	OFGB77359

GGB28934	FGB9639	OFGB9639
GGB28934	FGB9639	OFGB9639
GGB28949	Lachnospiraceae	Eubacteriales
GGB28949	Lachnospiraceae	Eubacteriales
GGB28951	Clostridiaceae	Eubacteriales
GGB28951	Clostridiaceae	Eubacteriales
GGB28951	Clostridiaceae	Eubacteriales
GGB28951	Clostridiaceae	Eubacteriales
GGB28954	Clostridiaceae	Eubacteriales
GGB28954	Clostridiaceae	Eubacteriales
GGB28960	Clostridiaceae	Eubacteriales
GGB28960	Clostridiaceae	Eubacteriales
GGB28964	Clostridiaceae	Eubacteriales
GGB28964	Clostridiaceae	Eubacteriales
GGB28967	Clostridiaceae	Eubacteriales
GGB28967	Clostridiaceae	Eubacteriales
GGB28996	FGB9656	OFGB9656
GGB28996	FGB9656	OFGB9656
GGB29531	FGB9827	OFGB9827
GGB29531	FGB9827	OFGB9827
GGB29685	Eubacteriaceae	Eubacteriales
GGB29685	Eubacteriaceae	Eubacteriales
GGB30141	FGB77303	OFGB77303
GGB30141	FGB77303	OFGB77303
GGB30145	Clostridiaceae	Eubacteriales
GGB30145	Clostridiaceae	Eubacteriales
GGB30286	Eubacteriales_unclassified	Eubacteriales
GGB30286	Eubacteriales_unclassified	Eubacteriales
GGB30300	FGB72709	OFGB72709
GGB30300	FGB72709	OFGB72709
GGB30303	Oscillospiraceae	Eubacteriales
GGB30303	Oscillospiraceae	Eubacteriales
GGB30450	Oscillospiraceae	Eubacteriales
GGB30450	Oscillospiraceae	Eubacteriales
GGB30453	Oscillospiraceae	Eubacteriales
GGB30453	Oscillospiraceae	Eubacteriales
GGB30454	Oscillospiraceae	Eubacteriales
GGB30454	Oscillospiraceae	Eubacteriales
GGB30455	Oscillospiraceae	Eubacteriales
GGB30455	Oscillospiraceae	Eubacteriales
GGB30456	Oscillospiraceae	Eubacteriales
GGB30456	Oscillospiraceae	Eubacteriales
GGB30457	Oscillospiraceae	Eubacteriales
GGB30457	Oscillospiraceae	Eubacteriales
GGB30461	Oscillospiraceae	Eubacteriales
GGB30461	Oscillospiraceae	Eubacteriales

GGB30461	Oscillospiraceae	Eubacteriales
GGB30461	Oscillospiraceae	Eubacteriales
GGB30461	Oscillospiraceae	Eubacteriales
GGB30461	Oscillospiraceae	Eubacteriales
GGB30463	Oscillospiraceae	Eubacteriales
GGB30463	Oscillospiraceae	Eubacteriales
GGB30473	Oscillospiraceae	Eubacteriales
GGB30473	Oscillospiraceae	Eubacteriales
GGB30475	Oscillospiraceae	Eubacteriales
GGB30475	Oscillospiraceae	Eubacteriales
GGB30861	FGB77153	OFGB77153
GGB30861	FGB77153	OFGB77153
GGB31312	FGB1791	OFGB1791
GGB31312	FGB1791	OFGB1791
GGB31438	FGB10290	OFGB10290
GGB31438	FGB10290	OFGB10290
GGB3171	Oscillospiraceae	Eubacteriales
GGB3171	Oscillospiraceae	Eubacteriales
GGB31762	FGB10289	OFGB10289
GGB31762	FGB10289	OFGB10289
GGB31823	FGB1765	OFGB1765
GGB31823	FGB1765	OFGB1765
GGB31838	FGB1765	OFGB1765
GGB31838	FGB1765	OFGB1765
GGB31841	FGB1765	OFGB1765
GGB31841	FGB1765	OFGB1765
GGB32371	FGB10667	OFGB10667
GGB32371	FGB10667	OFGB10667
GGB3793	Lachnospiraceae	Eubacteriales
GGB3793	Lachnospiraceae	Eubacteriales
GGB42601	Clostridiaceae	Eubacteriales
GGB42601	Clostridiaceae	Eubacteriales
GGB45514	Oscillospiraceae	Eubacteriales
GGB45514	Oscillospiraceae	Eubacteriales
GGB45564	FGB75721	OFGB75721
GGB45564	FGB75721	OFGB75721
GGB45624	Oscillospiraceae	Eubacteriales
GGB45624	Oscillospiraceae	Eubacteriales
GGB45656	Christensenellaceae	Eubacteriales
GGB45656	Christensenellaceae	Eubacteriales
GGB47127	FGB10299	OFGB10299
GGB47127	FGB10299	OFGB10299
GGB74395	Oscillospiraceae	Eubacteriales
GGB74395	Oscillospiraceae	Eubacteriales
GGB75053	Oscillospiraceae	Eubacteriales
GGB75053	Oscillospiraceae	Eubacteriales

GGB75109	Lachnospiraceae	Eubacteriales
GGB75109	Lachnospiraceae	Eubacteriales
Lachnospiraceae_unclassified	Lachnospiraceae	Eubacteriales
Lachnospiraceae_unclassified	Lachnospiraceae	Eubacteriales
Lachnospiraceae_unclassified	Lachnospiraceae	Eubacteriales
Lachnospiraceae_unclassified	Lachnospiraceae	Eubacteriales
Lachnospiraceae_unclassified	Lachnospiraceae	Eubacteriales
Lachnospiraceae_unclassified	Lachnospiraceae	Eubacteriales
Lachnospiraceae_unclassified	Lachnospiraceae	Eubacteriales
Lachnospiraceae_unclassified	Lachnospiraceae	Eubacteriales
Lachnospiraceae_unclassified	Lachnospiraceae	Eubacteriales
Lachnospiraceae_unclassified	Lachnospiraceae	Eubacteriales
Lactobacillus	Lactobacillaceae	Lactobacillales
Lactobacillus	Lactobacillaceae	Lactobacillales
Leptogranulimonas	Atopobiaceae	Coriobacteriales
Leptogranulimonas	Atopobiaceae	Coriobacteriales
Muribaculaceae_unclassified	Muribaculaceae	Bacteroidales
Muribaculaceae_unclassified	Muribaculaceae	Bacteroidales
Neglectibacter	Oscillospiraceae	Eubacteriales
Neglectibacter	Oscillospiraceae	Eubacteriales
Oscillibacter	Oscillospiraceae	Eubacteriales
Oscillibacter	Oscillospiraceae	Eubacteriales
Oscillospiraceae_unclassified	Oscillospiraceae	Eubacteriales
Oscillospiraceae_unclassified	Oscillospiraceae	Eubacteriales
Oscillospiraceae_unclassified	Oscillospiraceae	Eubacteriales
Oscillospiraceae_unclassified	Oscillospiraceae	Eubacteriales
Oscillospiraceae_unclassified	Oscillospiraceae	Eubacteriales
Oscillospiraceae_unclassified	Oscillospiraceae	Eubacteriales
Oscillospiraceae_unclassified	Oscillospiraceae	Eubacteriales
Oscillospiraceae_unclassified	Oscillospiraceae	Eubacteriales
Parasutterella	Sutterellaceae	Burkholderiales
Parasutterella	Sutterellaceae	Burkholderiales
Schaedlerella	Lachnospiraceae	Eubacteriales
Schaedlerella	Lachnospiraceae	Eubacteriales
Turicibacter	Turicibacteraceae	Erysipelotrichales
Turicibacter	Turicibacteraceae	Erysipelotrichales
Acetatifactor	Lachnospiraceae	Eubacteriales
Acetatifactor	Lachnospiraceae	Eubacteriales
Acutalibacter	Oscillospiraceae	Eubacteriales
Acutalibacter	Oscillospiraceae	Eubacteriales
Akkermansia	Akkermansiaceae	Verrucomicrobiales
Akkermansia	Akkermansiaceae	Verrucomicrobiales

Anaerotruncus	Oscillospiraceae	Eubacteriales
Anaerotruncus	Oscillospiraceae	Eubacteriales
Bacteria_unclassified	Bacteria_unclassified	Bacteria_unclassified
Bacteria_unclassified	Bacteria_unclassified	Bacteria_unclassified
Bacteria_unclassified	Bacteria_unclassified	Bacteria_unclassified
Bacteria_unclassified	Bacteria_unclassified	Bacteria_unclassified
Bacteria_unclassified	Bacteria_unclassified	Bacteria_unclassified
Bacteria_unclassified	Bacteria_unclassified	Bacteria_unclassified
Bacteria_unclassified	Bacteria_unclassified	Bacteria_unclassified
Bacteria_unclassified	Bacteria_unclassified	Bacteria_unclassified
Bacteria_unclassified	Bacteria_unclassified	Bacteria_unclassified
Bacteria_unclassified	Bacteria_unclassified	Bacteria_unclassified
Bacteria_unclassified	Bacteria_unclassified	Bacteria_unclassified
Bacteria_unclassified	Bacteria_unclassified	Bacteria_unclassified
Bacteria_unclassified	Bacteria_unclassified	Bacteria_unclassified
Bacteria_unclassified	Bacteria_unclassified	Bacteria_unclassified
Bacteria_unclassified	Bacteria_unclassified	Bacteria_unclassified
Bacteria_unclassified	Bacteria_unclassified	Bacteria_unclassified
Bacteria_unclassified	Bacteria_unclassified	Bacteria_unclassified
Bacteria_unclassified	Bacteria_unclassified	Bacteria_unclassified
Bacteroides	Bacteroidaceae	Bacteroidales
Bacteroides	Bacteroidaceae	Bacteroidales

Clostridia_unclassified	Clostridia_unclassified	Clostridia_unclassified
Clostridia_unclassified	Clostridia_unclassified	Clostridia_unclassified
Clostridiaceae_unclassified	Clostridiaceae	Eubacteriales
Clostridiaceae_unclassified	Clostridiaceae	Eubacteriales
Clostridiaceae_unclassified	Clostridiaceae	Eubacteriales
Clostridiaceae_unclassified	Clostridiaceae	Eubacteriales
Eubacteriales_unclassified	Eubacteriales_unclassified	Eubacteriales
Eubacteriales_unclassified	Eubacteriales_unclassified	Eubacteriales
Erysipelatoclostridium	Erysipelotrichaceae	Erysipelotrichales
Erysipelatoclostridium	Erysipelotrichaceae	Erysipelotrichales
Clostridium	Clostridiaceae	Eubacteriales
Clostridium	Clostridiaceae	Eubacteriales
Eubacteriaceae_unclassified	Eubacteriaceae	Eubacteriales
Eubacteriaceae_unclassified	Eubacteriaceae	Eubacteriales
Eubacteriaceae_unclassified	Eubacteriaceae	Eubacteriales
Eubacteriaceae_unclassified	Eubacteriaceae	Eubacteriales
GGB20146	FGB77306	OFGB77306
GGB20146	FGB77306	OFGB77306
GGB20149	Lachnospiraceae	Eubacteriales
GGB20149	Lachnospiraceae	Eubacteriales
GGB22635	Eggerthellaceae	Eggerthellales
GGB22635	Eggerthellaceae	Eggerthellales

GGB25041	Lachnospiraceae	Eubacteriales
GGB25041	Lachnospiraceae	Eubacteriales
GGB28379	FGB9506	OFGB9506
GGB28379	FGB9506	OFGB9506
GGB28382	FGB9508	OFGB9508
GGB28382	FGB9508	OFGB9508
GGB28392	FGB9512	OFGB9512
GGB28392	FGB9512	OFGB9512
GGB28404	FGB2838	OFGB2838
GGB28404	FGB2838	OFGB2838
GGB28418	FGB2838	OFGB2838
GGB28418	FGB2838	OFGB2838
GGB28422	FGB2838	OFGB2838
GGB28422	FGB2838	OFGB2838
GGB28431	Pumilibacteraceae	Eubacteriales
GGB28431	Pumilibacteraceae	Eubacteriales
GGB28456	FGB2833	OFGB2833
GGB28456	FGB2833	OFGB2833
GGB28782	Eubacteriaceae	Eubacteriales
GGB28782	Eubacteriaceae	Eubacteriales
GGB28792	Lachnospiraceae	Eubacteriales
GGB28792	Lachnospiraceae	Eubacteriales
GGB28798	Lachnospiraceae	Eubacteriales
GGB28798	Lachnospiraceae	Eubacteriales
GGB28810	FGB9622	OFGB9622
GGB28810	FGB9622	OFGB9622
GGB28828	FGB77305	OFGB77305
GGB28828	FGB77305	OFGB77305
GGB28851	Clostridiaceae	Eubacteriales
GGB28851	Clostridiaceae	Eubacteriales
GGB28865	Lachnospiraceae	Eubacteriales
GGB28865	Lachnospiraceae	Eubacteriales
GGB28868	Lachnospiraceae	Eubacteriales
GGB28868	Lachnospiraceae	Eubacteriales
GGB28869	Lachnospiraceae	Eubacteriales
GGB28869	Lachnospiraceae	Eubacteriales
GGB28875	Lachnospiraceae	Eubacteriales
GGB28875	Lachnospiraceae	Eubacteriales
GGB28881	FGB9633	OFGB9633
GGB28881	FGB9633	OFGB9633
GGB28892	Bacteria_unclassified	Bacteria_unclassified
GGB28892	Bacteria_unclassified	Bacteria_unclassified
GGB28893	Bacteria_unclassified	Bacteria_unclassified
GGB28893	Bacteria_unclassified	Bacteria_unclassified
GGB28898	Bacteria_unclassified	Bacteria_unclassified
GGB28898	Bacteria_unclassified	Bacteria_unclassified

GGB28901	Bacteria_unclassified	Bacteria_unclassified
GGB28901	Bacteria_unclassified	Bacteria_unclassified
GGB28904	Bacteria_unclassified	Bacteria_unclassified
GGB28904	Bacteria_unclassified	Bacteria_unclassified
GGB28909	Bacteria_unclassified	Bacteria_unclassified
GGB28909	Bacteria_unclassified	Bacteria_unclassified
GGB28924	Lachnospiraceae	Eubacteriales
GGB28924	Lachnospiraceae	Eubacteriales
GGB28927	FGB77359	OFGB77359
GGB28927	FGB77359	OFGB77359
GGB28934	FGB9639	OFGB9639
GGB28934	FGB9639	OFGB9639
GGB28949	Lachnospiraceae	Eubacteriales
GGB28949	Lachnospiraceae	Eubacteriales
GGB28951	Clostridiaceae	Eubacteriales
GGB28951	Clostridiaceae	Eubacteriales
GGB28951	Clostridiaceae	Eubacteriales
GGB28951	Clostridiaceae	Eubacteriales
GGB28954	Clostridiaceae	Eubacteriales
GGB28954	Clostridiaceae	Eubacteriales
GGB28960	Clostridiaceae	Eubacteriales
GGB28960	Clostridiaceae	Eubacteriales
GGB28964	Clostridiaceae	Eubacteriales
GGB28964	Clostridiaceae	Eubacteriales
GGB28967	Clostridiaceae	Eubacteriales
GGB28967	Clostridiaceae	Eubacteriales
GGB28996	FGB9656	OFGB9656
GGB28996	FGB9656	OFGB9656
GGB29531	FGB9827	OFGB9827
GGB29531	FGB9827	OFGB9827
GGB29685	Eubacteriaceae	Eubacteriales
GGB29685	Eubacteriaceae	Eubacteriales
GGB30141	FGB77303	OFGB77303
GGB30141	FGB77303	OFGB77303
GGB30145	Clostridiaceae	Eubacteriales
GGB30145	Clostridiaceae	Eubacteriales
GGB30286	Eubacteriales_unclassified	Eubacteriales
GGB30286	Eubacteriales_unclassified	Eubacteriales
GGB30300	FGB72709	OFGB72709
GGB30300	FGB72709	OFGB72709
GGB30303	Oscillospiraceae	Eubacteriales
GGB30303	Oscillospiraceae	Eubacteriales
GGB30450	Oscillospiraceae	Eubacteriales
GGB30450	Oscillospiraceae	Eubacteriales
GGB30453	Oscillospiraceae	Eubacteriales
GGB30453	Oscillospiraceae	Eubacteriales

GGB30454	Oscillospiraceae	Eubacteriales
GGB30454	Oscillospiraceae	Eubacteriales
GGB30455	Oscillospiraceae	Eubacteriales
GGB30455	Oscillospiraceae	Eubacteriales
GGB30456	Oscillospiraceae	Eubacteriales
GGB30456	Oscillospiraceae	Eubacteriales
GGB30457	Oscillospiraceae	Eubacteriales
GGB30457	Oscillospiraceae	Eubacteriales
GGB30461	Oscillospiraceae	Eubacteriales
GGB30461	Oscillospiraceae	Eubacteriales
GGB30461	Oscillospiraceae	Eubacteriales
GGB30461	Oscillospiraceae	Eubacteriales
GGB30461	Oscillospiraceae	Eubacteriales
GGB30461	Oscillospiraceae	Eubacteriales
GGB30463	Oscillospiraceae	Eubacteriales
GGB30463	Oscillospiraceae	Eubacteriales
GGB30473	Oscillospiraceae	Eubacteriales
GGB30473	Oscillospiraceae	Eubacteriales
GGB30475	Oscillospiraceae	Eubacteriales
GGB30475	Oscillospiraceae	Eubacteriales
GGB30861	FGB77153	OFGB77153
GGB30861	FGB77153	OFGB77153
GGB31312	FGB1791	OFGB1791
GGB31312	FGB1791	OFGB1791
GGB31438	FGB10290	OFGB10290
GGB31438	FGB10290	OFGB10290
GGB3171	Oscillospiraceae	Eubacteriales
GGB3171	Oscillospiraceae	Eubacteriales
GGB31762	FGB10289	OFGB10289
GGB31762	FGB10289	OFGB10289
GGB31823	FGB1765	OFGB1765
GGB31823	FGB1765	OFGB1765
GGB31838	FGB1765	OFGB1765
GGB31838	FGB1765	OFGB1765
GGB31841	FGB1765	OFGB1765
GGB31841	FGB1765	OFGB1765
GGB32371	FGB10667	OFGB10667
GGB32371	FGB10667	OFGB10667
GGB3793	Lachnospiraceae	Eubacteriales
GGB3793	Lachnospiraceae	Eubacteriales
GGB42601	Clostridiaceae	Eubacteriales
GGB42601	Clostridiaceae	Eubacteriales
GGB45514	Oscillospiraceae	Eubacteriales
GGB45514	Oscillospiraceae	Eubacteriales
GGB45564	FGB75721	OFGB75721
GGB45564	FGB75721	OFGB75721

GGB45624	Oscillospiraceae	Eubacteriales
GGB45624	Oscillospiraceae	Eubacteriales
GGB45656	Christensenellaceae	Eubacteriales
GGB45656	Christensenellaceae	Eubacteriales
GGB47127	FGB10299	OFGB10299
GGB47127	FGB10299	OFGB10299
GGB74395	Oscillospiraceae	Eubacteriales
GGB74395	Oscillospiraceae	Eubacteriales
GGB75053	Oscillospiraceae	Eubacteriales
GGB75053	Oscillospiraceae	Eubacteriales
GGB75109	Lachnospiraceae	Eubacteriales
GGB75109	Lachnospiraceae	Eubacteriales
Lachnospiraceae_unclassified	Lachnospiraceae	Eubacteriales
Lachnospiraceae_unclassified	Lachnospiraceae	Eubacteriales
Lachnospiraceae_unclassified	Lachnospiraceae	Eubacteriales
Lachnospiraceae_unclassified	Lachnospiraceae	Eubacteriales
Lachnospiraceae_unclassified	Lachnospiraceae	Eubacteriales
Lachnospiraceae_unclassified	Lachnospiraceae	Eubacteriales
Lachnospiraceae_unclassified	Lachnospiraceae	Eubacteriales
Lachnospiraceae_unclassified	Lachnospiraceae	Eubacteriales
Lachnospiraceae_unclassified	Lachnospiraceae	Eubacteriales
Lachnospiraceae_unclassified	Lachnospiraceae	Eubacteriales
Lactobacillus	Lactobacillaceae	Lactobacillales
Lactobacillus	Lactobacillaceae	Lactobacillales
Leptogranulimonas	Atopobiaceae	Coriobacteriales
Leptogranulimonas	Atopobiaceae	Coriobacteriales
Muribaculaceae_unclassified	Muribaculaceae	Bacteroidales
Muribaculaceae_unclassified	Muribaculaceae	Bacteroidales
Neglectibacter	Oscillospiraceae	Eubacteriales
Neglectibacter	Oscillospiraceae	Eubacteriales
Oscillibacter	Oscillospiraceae	Eubacteriales
Oscillibacter	Oscillospiraceae	Eubacteriales
Oscillospiraceae_unclassified	Oscillospiraceae	Eubacteriales
Oscillospiraceae_unclassified	Oscillospiraceae	Eubacteriales
Oscillospiraceae_unclassified	Oscillospiraceae	Eubacteriales
Oscillospiraceae_unclassified	Oscillospiraceae	Eubacteriales
Oscillospiraceae_unclassified	Oscillospiraceae	Eubacteriales
Oscillospiraceae_unclassified	Oscillospiraceae	Eubacteriales
Oscillospiraceae_unclassified	Oscillospiraceae	Eubacteriales
Oscillospiraceae_unclassified	Oscillospiraceae	Eubacteriales
Parasutterella	Sutterellaceae	Burkholderiales
Parasutterella	Sutterellaceae	Burkholderiales
Schaedlerella	Lachnospiraceae	Eubacteriales
Schaedlerella	Lachnospiraceae	Eubacteriales

Turicibacter	Turicibacteraceae	Erysipelotrichales
Turicibacter	Turicibacteraceae	Erysipelotrichales

Class	Phylum	Kingdom
Clostridia	Firmicutes	Bacteria
Clostridia	Firmicutes	Bacteria
Clostridia	Firmicutes	Bacteria
Clostridia	Firmicutes	Bacteria
Verrucomicrobiae	Verrucomicrobia	Bacteria
Verrucomicrobiae	Verrucomicrobia	Bacteria
Clostridia	Firmicutes	Bacteria
Clostridia	Firmicutes	Bacteria
Bacteria_unclassified	Bacteria_unclassified	Bacteria
Bacteria_unclassified	Bacteria_unclassified	Bacteria
Bacteria_unclassified	Bacteria_unclassified	Bacteria
Bacteria_unclassified	Bacteria_unclassified	Bacteria
Bacteria_unclassified	Bacteria_unclassified	Bacteria
Bacteria_unclassified	Bacteria_unclassified	Bacteria
Bacteria_unclassified	Bacteria_unclassified	Bacteria
Bacteria_unclassified	Bacteria_unclassified	Bacteria
Bacteria_unclassified	Bacteria_unclassified	Bacteria
Bacteria_unclassified	Bacteria_unclassified	Bacteria
Bacteria_unclassified	Bacteria_unclassified	Bacteria
Bacteria_unclassified	Bacteria_unclassified	Bacteria
Bacteria_unclassified	Bacteria_unclassified	Bacteria
Bacteria_unclassified	Bacteria_unclassified	Bacteria
Bacteria_unclassified	Bacteria_unclassified	Bacteria
Bacteria_unclassified	Bacteria_unclassified	Bacteria
Bacteria_unclassified	Bacteria_unclassified	Bacteria
Bacteria_unclassified	Bacteria_unclassified	Bacteria
Bacteroidia	Bacteroidota	Bacteria
Bacteroidia	Bacteroidota	Bacteria
Clostridia	Firmicutes	Bacteria
Clostridia	Firmicutes	Bacteria
Clostridia	Firmicutes	Bacteria
Clostridia	Firmicutes	Bacteria
Clostridia	Firmicutes	Bacteria
Clostridia	Firmicutes	Bacteria
Clostridia	Firmicutes	Bacteria
Clostridia	Firmicutes	Bacteria
Erysipelotrichia	Firmicutes	Bacteria
Erysipelotrichia	Firmicutes	Bacteria
Clostridia	Firmicutes	Bacteria
Clostridia	Firmicutes	Bacteria
Clostridia	Firmicutes	Bacteria
Clostridia	Firmicutes	Bacteria
Clostridia	Firmicutes	Bacteria
Clostridia	Firmicutes	Bacteria

CFGB77306	Firmicutes	Bacteria
CFGB77306	Firmicutes	Bacteria
Clostridia	Firmicutes	Bacteria
Clostridia	Firmicutes	Bacteria
Coriobacteriia	Actinobacteria	Bacteria
Coriobacteriia	Actinobacteria	Bacteria
Clostridia	Firmicutes	Bacteria
Clostridia	Firmicutes	Bacteria
CFGB9506	Firmicutes	Bacteria
CFGB9506	Firmicutes	Bacteria
CFGB9508	Firmicutes	Bacteria
CFGB9508	Firmicutes	Bacteria
CFGB9512	Firmicutes	Bacteria
CFGB9512	Firmicutes	Bacteria
CFGB2838	Firmicutes	Bacteria
CFGB2838	Firmicutes	Bacteria
CFGB2838	Firmicutes	Bacteria
CFGB2838	Firmicutes	Bacteria
CFGB2838	Firmicutes	Bacteria
CFGB2838	Firmicutes	Bacteria
Clostridia	Firmicutes	Bacteria
Clostridia	Firmicutes	Bacteria
CFGB2833	Firmicutes	Bacteria
CFGB2833	Firmicutes	Bacteria
Clostridia	Firmicutes	Bacteria
Clostridia	Firmicutes	Bacteria
Clostridia	Firmicutes	Bacteria
Clostridia	Firmicutes	Bacteria
Clostridia	Firmicutes	Bacteria
Clostridia	Firmicutes	Bacteria
CFGB9622	Firmicutes	Bacteria
CFGB9622	Firmicutes	Bacteria
CFGB77305	Firmicutes	Bacteria
CFGB77305	Firmicutes	Bacteria
Clostridia	Firmicutes	Bacteria
Clostridia	Firmicutes	Bacteria
Clostridia	Firmicutes	Bacteria
Clostridia	Firmicutes	Bacteria
Clostridia	Firmicutes	Bacteria
Clostridia	Firmicutes	Bacteria
Clostridia	Firmicutes	Bacteria
Clostridia	Firmicutes	Bacteria
Clostridia	Firmicutes	Bacteria
Clostridia	Firmicutes	Bacteria
CFGB9633	Firmicutes	Bacteria
CFGB9633	Firmicutes	Bacteria

Bacteria_unclassified	Bacteria_unclassified	Bacteria
Bacteria_unclassified	Bacteria_unclassified	Bacteria
Bacteria_unclassified	Bacteria_unclassified	Bacteria
Bacteria_unclassified	Bacteria_unclassified	Bacteria
Bacteria_unclassified	Bacteria_unclassified	Bacteria
Bacteria_unclassified	Bacteria_unclassified	Bacteria
Bacteria_unclassified	Bacteria_unclassified	Bacteria
Bacteria_unclassified	Bacteria_unclassified	Bacteria
Bacteria_unclassified	Bacteria_unclassified	Bacteria
Bacteria_unclassified	Bacteria_unclassified	Bacteria
Bacteria_unclassified	Bacteria_unclassified	Bacteria
Bacteria_unclassified	Bacteria_unclassified	Bacteria
Clostridia	Firmicutes	Bacteria
Clostridia	Firmicutes	Bacteria
CFGB77359	Bacteria_unclassified	Bacteria
CFGB77359	Bacteria_unclassified	Bacteria
CFGB9639	Firmicutes	Bacteria
CFGB9639	Firmicutes	Bacteria
Clostridia	Firmicutes	Bacteria
Clostridia	Firmicutes	Bacteria
Clostridia	Firmicutes	Bacteria
Clostridia	Firmicutes	Bacteria
Clostridia	Firmicutes	Bacteria
Clostridia	Firmicutes	Bacteria
Clostridia	Firmicutes	Bacteria
Clostridia	Firmicutes	Bacteria
Clostridia	Firmicutes	Bacteria
Clostridia	Firmicutes	Bacteria
Clostridia	Firmicutes	Bacteria
Clostridia	Firmicutes	Bacteria
Clostridia	Firmicutes	Bacteria
Clostridia	Firmicutes	Bacteria
CFGB9656	Firmicutes	Bacteria
CFGB9656	Firmicutes	Bacteria
CFGB9827	Firmicutes	Bacteria
CFGB9827	Firmicutes	Bacteria
Clostridia	Firmicutes	Bacteria
Clostridia	Firmicutes	Bacteria
CFGB77303	Bacteria_unclassified	Bacteria
CFGB77303	Bacteria_unclassified	Bacteria
Clostridia	Firmicutes	Bacteria
Clostridia	Firmicutes	Bacteria
Clostridia	Firmicutes	Bacteria
Clostridia	Firmicutes	Bacteria
CFGB72709	Firmicutes	Bacteria
CFGB72709	Firmicutes	Bacteria

Clostridia	Firmicutes	Bacteria
Clostridia	Firmicutes	Bacteria
Clostridia	Firmicutes	Bacteria
Clostridia	Firmicutes	Bacteria
Clostridia	Firmicutes	Bacteria
Clostridia	Firmicutes	Bacteria
Clostridia	Firmicutes	Bacteria
Clostridia	Firmicutes	Bacteria
Clostridia	Firmicutes	Bacteria
Clostridia	Firmicutes	Bacteria
Clostridia	Firmicutes	Bacteria
Clostridia	Firmicutes	Bacteria
Clostridia	Firmicutes	Bacteria
Clostridia	Firmicutes	Bacteria
Clostridia	Firmicutes	Bacteria
Clostridia	Firmicutes	Bacteria
Clostridia	Firmicutes	Bacteria
Clostridia	Firmicutes	Bacteria
Clostridia	Firmicutes	Bacteria
Clostridia	Firmicutes	Bacteria
Clostridia	Firmicutes	Bacteria
Clostridia	Firmicutes	Bacteria
Clostridia	Firmicutes	Bacteria
Clostridia	Firmicutes	Bacteria
Clostridia	Firmicutes	Bacteria
Clostridia	Firmicutes	Bacteria
CFGB77153	Actinobacteria	Bacteria
CFGB77153	Actinobacteria	Bacteria
CFGB1791	Tenericutes	Bacteria
CFGB1791	Tenericutes	Bacteria
CFGB10290	Firmicutes	Bacteria
CFGB10290	Firmicutes	Bacteria
Clostridia	Firmicutes	Bacteria
Clostridia	Firmicutes	Bacteria
CFGB10289	Firmicutes	Bacteria
CFGB10289	Firmicutes	Bacteria
CFGB1765	Firmicutes	Bacteria
CFGB1765	Firmicutes	Bacteria
CFGB1765	Firmicutes	Bacteria
CFGB1765	Firmicutes	Bacteria
CFGB1765	Firmicutes	Bacteria
CFGB1765	Firmicutes	Bacteria
CFGB10667	Firmicutes	Bacteria
CFGB10667	Firmicutes	Bacteria
Clostridia	Firmicutes	Bacteria
Clostridia	Firmicutes	Bacteria

Clostridia	Firmicutes	Bacteria
Clostridia	Firmicutes	Bacteria
Clostridia	Firmicutes	Bacteria
Clostridia	Firmicutes	Bacteria
CFGB75721	Firmicutes	Bacteria
CFGB75721	Firmicutes	Bacteria
Clostridia	Firmicutes	Bacteria
Clostridia	Firmicutes	Bacteria
Clostridia	Firmicutes	Bacteria
Clostridia	Firmicutes	Bacteria
CFGB10299	Firmicutes	Bacteria
CFGB10299	Firmicutes	Bacteria
Clostridia	Firmicutes	Bacteria
Clostridia	Firmicutes	Bacteria
Clostridia	Firmicutes	Bacteria
Clostridia	Firmicutes	Bacteria
Clostridia	Firmicutes	Bacteria
Clostridia	Firmicutes	Bacteria
Clostridia	Firmicutes	Bacteria
Clostridia	Firmicutes	Bacteria
Clostridia	Firmicutes	Bacteria
Clostridia	Firmicutes	Bacteria
Clostridia	Firmicutes	Bacteria
Clostridia	Firmicutes	Bacteria
Clostridia	Firmicutes	Bacteria
Clostridia	Firmicutes	Bacteria
Clostridia	Firmicutes	Bacteria
Clostridia	Firmicutes	Bacteria
Bacilli	Firmicutes	Bacteria
Bacilli	Firmicutes	Bacteria
Coriobacteriia	Actinobacteria	Bacteria
Coriobacteriia	Actinobacteria	Bacteria
Bacteroidia	Bacteroidota	Bacteria
Bacteroidia	Bacteroidota	Bacteria
Clostridia	Firmicutes	Bacteria
Clostridia	Firmicutes	Bacteria
Clostridia	Firmicutes	Bacteria
Clostridia	Firmicutes	Bacteria
Clostridia	Firmicutes	Bacteria
Clostridia	Firmicutes	Bacteria
Clostridia	Firmicutes	Bacteria
Clostridia	Firmicutes	Bacteria
Clostridia	Firmicutes	Bacteria
Clostridia	Firmicutes	Bacteria
Clostridia	Firmicutes	Bacteria
Clostridia	Firmicutes	Bacteria

Betaproteobacteria	Proteobacteria	Bacteria
Betaproteobacteria	Proteobacteria	Bacteria
Clostridia	Firmicutes	Bacteria
Clostridia	Firmicutes	Bacteria
Erysipelotrichia	Firmicutes	Bacteria
Erysipelotrichia	Firmicutes	Bacteria
Clostridia	Firmicutes	Bacteria
Clostridia	Firmicutes	Bacteria
Clostridia	Firmicutes	Bacteria
Clostridia	Firmicutes	Bacteria
Verrucomicrobiae	Verrucomicrobia	Bacteria
Verrucomicrobiae	Verrucomicrobia	Bacteria
Clostridia	Firmicutes	Bacteria
Clostridia	Firmicutes	Bacteria
Bacteria_unclassified	Bacteria_unclassified	Bacteria
Bacteria_unclassified	Bacteria_unclassified	Bacteria
Bacteria_unclassified	Bacteria_unclassified	Bacteria
Bacteria_unclassified	Bacteria_unclassified	Bacteria
Bacteria_unclassified	Bacteria_unclassified	Bacteria
Bacteria_unclassified	Bacteria_unclassified	Bacteria
Bacteria_unclassified	Bacteria_unclassified	Bacteria
Bacteria_unclassified	Bacteria_unclassified	Bacteria
Bacteria_unclassified	Bacteria_unclassified	Bacteria
Bacteria_unclassified	Bacteria_unclassified	Bacteria
Bacteria_unclassified	Bacteria_unclassified	Bacteria
Bacteria_unclassified	Bacteria_unclassified	Bacteria
Bacteria_unclassified	Bacteria_unclassified	Bacteria
Bacteria_unclassified	Bacteria_unclassified	Bacteria
Bacteria_unclassified	Bacteria_unclassified	Bacteria
Bacteria_unclassified	Bacteria_unclassified	Bacteria
Bacteria_unclassified	Bacteria_unclassified	Bacteria
Bacteria_unclassified	Bacteria_unclassified	Bacteria
Bacteroidia	Bacteroidota	Bacteria
Bacteroidia	Bacteroidota	Bacteria
Clostridia	Firmicutes	Bacteria
Clostridia	Firmicutes	Bacteria
Clostridia	Firmicutes	Bacteria
Clostridia	Firmicutes	Bacteria
Clostridia	Firmicutes	Bacteria
Clostridia	Firmicutes	Bacteria

Clostridia	Firmicutes	Bacteria
Clostridia	Firmicutes	Bacteria
Erysipelotrichia	Firmicutes	Bacteria
Erysipelotrichia	Firmicutes	Bacteria
Clostridia	Firmicutes	Bacteria
Clostridia	Firmicutes	Bacteria
Clostridia	Firmicutes	Bacteria
Clostridia	Firmicutes	Bacteria
Clostridia	Firmicutes	Bacteria
Clostridia	Firmicutes	Bacteria
CFGB77306	Firmicutes	Bacteria
CFGB77306	Firmicutes	Bacteria
Clostridia	Firmicutes	Bacteria
Clostridia	Firmicutes	Bacteria
Coriobacteriia	Actinobacteria	Bacteria
Coriobacteriia	Actinobacteria	Bacteria
Clostridia	Firmicutes	Bacteria
Clostridia	Firmicutes	Bacteria
CFGB9506	Firmicutes	Bacteria
CFGB9506	Firmicutes	Bacteria
CFGB9508	Firmicutes	Bacteria
CFGB9508	Firmicutes	Bacteria
CFGB9512	Firmicutes	Bacteria
CFGB9512	Firmicutes	Bacteria
CFGB2838	Firmicutes	Bacteria
CFGB2838	Firmicutes	Bacteria
CFGB2838	Firmicutes	Bacteria
CFGB2838	Firmicutes	Bacteria
CFGB2838	Firmicutes	Bacteria
CFGB2838	Firmicutes	Bacteria
Clostridia	Firmicutes	Bacteria
Clostridia	Firmicutes	Bacteria
CFGB2833	Firmicutes	Bacteria
CFGB2833	Firmicutes	Bacteria
Clostridia	Firmicutes	Bacteria
Clostridia	Firmicutes	Bacteria
Clostridia	Firmicutes	Bacteria
Clostridia	Firmicutes	Bacteria
Clostridia	Firmicutes	Bacteria
Clostridia	Firmicutes	Bacteria
CFGB9622	Firmicutes	Bacteria
CFGB9622	Firmicutes	Bacteria
CFGB77305	Firmicutes	Bacteria
CFGB77305	Firmicutes	Bacteria
Clostridia	Firmicutes	Bacteria
Clostridia	Firmicutes	Bacteria

Clostridia	Firmicutes	Bacteria
Clostridia	Firmicutes	Bacteria
Clostridia	Firmicutes	Bacteria
Clostridia	Firmicutes	Bacteria
Clostridia	Firmicutes	Bacteria
Clostridia	Firmicutes	Bacteria
Clostridia	Firmicutes	Bacteria
Clostridia	Firmicutes	Bacteria
CFGB9633	Firmicutes	Bacteria
CFGB9633	Firmicutes	Bacteria
Bacteria_unclassified	Bacteria_unclassified	Bacteria
Bacteria_unclassified	Bacteria_unclassified	Bacteria
Bacteria_unclassified	Bacteria_unclassified	Bacteria
Bacteria_unclassified	Bacteria_unclassified	Bacteria
Bacteria_unclassified	Bacteria_unclassified	Bacteria
Bacteria_unclassified	Bacteria_unclassified	Bacteria
Bacteria_unclassified	Bacteria_unclassified	Bacteria
Bacteria_unclassified	Bacteria_unclassified	Bacteria
Bacteria_unclassified	Bacteria_unclassified	Bacteria
Bacteria_unclassified	Bacteria_unclassified	Bacteria
Bacteria_unclassified	Bacteria_unclassified	Bacteria
Bacteria_unclassified	Bacteria_unclassified	Bacteria
Clostridia	Firmicutes	Bacteria
Clostridia	Firmicutes	Bacteria
CFGB77359	Bacteria_unclassified	Bacteria
CFGB77359	Bacteria_unclassified	Bacteria
CFGB9639	Firmicutes	Bacteria
CFGB9639	Firmicutes	Bacteria
Clostridia	Firmicutes	Bacteria
Clostridia	Firmicutes	Bacteria
Clostridia	Firmicutes	Bacteria
Clostridia	Firmicutes	Bacteria
Clostridia	Firmicutes	Bacteria
Clostridia	Firmicutes	Bacteria
Clostridia	Firmicutes	Bacteria
Clostridia	Firmicutes	Bacteria
Clostridia	Firmicutes	Bacteria
Clostridia	Firmicutes	Bacteria
Clostridia	Firmicutes	Bacteria
Clostridia	Firmicutes	Bacteria
Clostridia	Firmicutes	Bacteria
Clostridia	Firmicutes	Bacteria
CFGB9656	Firmicutes	Bacteria
CFGB9656	Firmicutes	Bacteria
CFGB9827	Firmicutes	Bacteria
CFGB9827	Firmicutes	Bacteria

Clostridia	Firmicutes	Bacteria
Clostridia	Firmicutes	Bacteria
CFGB77303	Bacteria_unclassified	Bacteria
CFGB77303	Bacteria_unclassified	Bacteria
Clostridia	Firmicutes	Bacteria
Clostridia	Firmicutes	Bacteria
Clostridia	Firmicutes	Bacteria
Clostridia	Firmicutes	Bacteria
CFGB72709	Firmicutes	Bacteria
CFGB72709	Firmicutes	Bacteria
Clostridia	Firmicutes	Bacteria
Clostridia	Firmicutes	Bacteria
Clostridia	Firmicutes	Bacteria
Clostridia	Firmicutes	Bacteria
Clostridia	Firmicutes	Bacteria
Clostridia	Firmicutes	Bacteria
Clostridia	Firmicutes	Bacteria
Clostridia	Firmicutes	Bacteria
Clostridia	Firmicutes	Bacteria
Clostridia	Firmicutes	Bacteria
Clostridia	Firmicutes	Bacteria
Clostridia	Firmicutes	Bacteria
Clostridia	Firmicutes	Bacteria
Clostridia	Firmicutes	Bacteria
Clostridia	Firmicutes	Bacteria
Clostridia	Firmicutes	Bacteria
Clostridia	Firmicutes	Bacteria
Clostridia	Firmicutes	Bacteria
Clostridia	Firmicutes	Bacteria
Clostridia	Firmicutes	Bacteria
Clostridia	Firmicutes	Bacteria
Clostridia	Firmicutes	Bacteria
Clostridia	Firmicutes	Bacteria
Clostridia	Firmicutes	Bacteria
Clostridia	Firmicutes	Bacteria
Clostridia	Firmicutes	Bacteria
Clostridia	Firmicutes	Bacteria
Clostridia	Firmicutes	Bacteria
CFGB77153	Actinobacteria	Bacteria
CFGB77153	Actinobacteria	Bacteria
CFGB1791	Tenericutes	Bacteria
CFGB1791	Tenericutes	Bacteria
CFGB10290	Firmicutes	Bacteria
CFGB10290	Firmicutes	Bacteria
Clostridia	Firmicutes	Bacteria
Clostridia	Firmicutes	Bacteria
CFGB10289	Firmicutes	Bacteria
CFGB10289	Firmicutes	Bacteria

CFGB1765	Firmicutes	Bacteria
CFGB1765	Firmicutes	Bacteria
CFGB1765	Firmicutes	Bacteria
CFGB1765	Firmicutes	Bacteria
CFGB1765	Firmicutes	Bacteria
CFGB1765	Firmicutes	Bacteria
CFGB10667	Firmicutes	Bacteria
CFGB10667	Firmicutes	Bacteria
Clostridia	Firmicutes	Bacteria
Clostridia	Firmicutes	Bacteria
Clostridia	Firmicutes	Bacteria
Clostridia	Firmicutes	Bacteria
Clostridia	Firmicutes	Bacteria
Clostridia	Firmicutes	Bacteria
CFGB75721	Firmicutes	Bacteria
CFGB75721	Firmicutes	Bacteria
Clostridia	Firmicutes	Bacteria
Clostridia	Firmicutes	Bacteria
Clostridia	Firmicutes	Bacteria
Clostridia	Firmicutes	Bacteria
CFGB10299	Firmicutes	Bacteria
CFGB10299	Firmicutes	Bacteria
Clostridia	Firmicutes	Bacteria
Clostridia	Firmicutes	Bacteria
Clostridia	Firmicutes	Bacteria
Clostridia	Firmicutes	Bacteria
Clostridia	Firmicutes	Bacteria
Clostridia	Firmicutes	Bacteria
Clostridia	Firmicutes	Bacteria
Clostridia	Firmicutes	Bacteria
Clostridia	Firmicutes	Bacteria
Clostridia	Firmicutes	Bacteria
Clostridia	Firmicutes	Bacteria
Clostridia	Firmicutes	Bacteria
Clostridia	Firmicutes	Bacteria
Clostridia	Firmicutes	Bacteria
Clostridia	Firmicutes	Bacteria
Bacilli	Firmicutes	Bacteria
Bacilli	Firmicutes	Bacteria
Coriobacteriia	Actinobacteria	Bacteria
Coriobacteriia	Actinobacteria	Bacteria
Bacteroidia	Bacteroidota	Bacteria
Bacteroidia	Bacteroidota	Bacteria
Clostridia	Firmicutes	Bacteria
Clostridia	Firmicutes	Bacteria

Clostridia	Firmicutes	Bacteria
Clostridia	Firmicutes	Bacteria
Clostridia	Firmicutes	Bacteria
Clostridia	Firmicutes	Bacteria
Clostridia	Firmicutes	Bacteria
Clostridia	Firmicutes	Bacteria
Clostridia	Firmicutes	Bacteria
Clostridia	Firmicutes	Bacteria
Clostridia	Firmicutes	Bacteria
Clostridia	Firmicutes	Bacteria
Betaproteobacteria	Proteobacteria	Bacteria
Betaproteobacteria	Proteobacteria	Bacteria
Clostridia	Firmicutes	Bacteria
Clostridia	Firmicutes	Bacteria
Erysipelotrichia	Firmicutes	Bacteria
Erysipelotrichia	Firmicutes	Bacteria
Clostridia	Firmicutes	Bacteria
Clostridia	Firmicutes	Bacteria
Clostridia	Firmicutes	Bacteria
Clostridia	Firmicutes	Bacteria
Verrucomicrobiae	Verrucomicrobia	Bacteria
Verrucomicrobiae	Verrucomicrobia	Bacteria
Clostridia	Firmicutes	Bacteria
Clostridia	Firmicutes	Bacteria
Bacteria_unclassified	Bacteria_unclassified	Bacteria
Bacteria_unclassified	Bacteria_unclassified	Bacteria
Bacteria_unclassified	Bacteria_unclassified	Bacteria
Bacteria_unclassified	Bacteria_unclassified	Bacteria
Bacteria_unclassified	Bacteria_unclassified	Bacteria
Bacteria_unclassified	Bacteria_unclassified	Bacteria
Bacteria_unclassified	Bacteria_unclassified	Bacteria
Bacteria_unclassified	Bacteria_unclassified	Bacteria
Bacteria_unclassified	Bacteria_unclassified	Bacteria
Bacteria_unclassified	Bacteria_unclassified	Bacteria
Bacteria_unclassified	Bacteria_unclassified	Bacteria
Bacteria_unclassified	Bacteria_unclassified	Bacteria
Bacteria_unclassified	Bacteria_unclassified	Bacteria
Bacteria_unclassified	Bacteria_unclassified	Bacteria
Bacteria_unclassified	Bacteria_unclassified	Bacteria
Bacteria_unclassified	Bacteria_unclassified	Bacteria
Bacteria_unclassified	Bacteria_unclassified	Bacteria
Bacteria_unclassified	Bacteria_unclassified	Bacteria

Bacteroidia	Bacteroidota	Bacteria
Bacteroidia	Bacteroidota	Bacteria
Clostridia	Firmicutes	Bacteria
Clostridia	Firmicutes	Bacteria
Clostridia	Firmicutes	Bacteria
Clostridia	Firmicutes	Bacteria
Clostridia	Firmicutes	Bacteria
Clostridia	Firmicutes	Bacteria
Clostridia	Firmicutes	Bacteria
Clostridia	Firmicutes	Bacteria
Erysipelotrichia	Firmicutes	Bacteria
Erysipelotrichia	Firmicutes	Bacteria
Clostridia	Firmicutes	Bacteria
Clostridia	Firmicutes	Bacteria
Clostridia	Firmicutes	Bacteria
Clostridia	Firmicutes	Bacteria
Clostridia	Firmicutes	Bacteria
Clostridia	Firmicutes	Bacteria
CFGB77306	Firmicutes	Bacteria
CFGB77306	Firmicutes	Bacteria
Clostridia	Firmicutes	Bacteria
Clostridia	Firmicutes	Bacteria
Coriobacteriia	Actinobacteria	Bacteria
Coriobacteriia	Actinobacteria	Bacteria
Clostridia	Firmicutes	Bacteria
Clostridia	Firmicutes	Bacteria
CFGB9506	Firmicutes	Bacteria
CFGB9506	Firmicutes	Bacteria
CFGB9508	Firmicutes	Bacteria
CFGB9508	Firmicutes	Bacteria
CFGB9512	Firmicutes	Bacteria
CFGB9512	Firmicutes	Bacteria
CFGB2838	Firmicutes	Bacteria
CFGB2838	Firmicutes	Bacteria
CFGB2838	Firmicutes	Bacteria
CFGB2838	Firmicutes	Bacteria
CFGB2838	Firmicutes	Bacteria
CFGB2838	Firmicutes	Bacteria
Clostridia	Firmicutes	Bacteria
Clostridia	Firmicutes	Bacteria
CFGB2833	Firmicutes	Bacteria
CFGB2833	Firmicutes	Bacteria
Clostridia	Firmicutes	Bacteria
Clostridia	Firmicutes	Bacteria

Clostridia	Firmicutes	Bacteria
Clostridia	Firmicutes	Bacteria
Clostridia	Firmicutes	Bacteria
Clostridia	Firmicutes	Bacteria
CFGB9622	Firmicutes	Bacteria
CFGB9622	Firmicutes	Bacteria
CFGB77305	Firmicutes	Bacteria
CFGB77305	Firmicutes	Bacteria
Clostridia	Firmicutes	Bacteria
Clostridia	Firmicutes	Bacteria
Clostridia	Firmicutes	Bacteria
Clostridia	Firmicutes	Bacteria
Clostridia	Firmicutes	Bacteria
Clostridia	Firmicutes	Bacteria
Clostridia	Firmicutes	Bacteria
Clostridia	Firmicutes	Bacteria
Clostridia	Firmicutes	Bacteria
Clostridia	Firmicutes	Bacteria
CFGB9633	Firmicutes	Bacteria
CFGB9633	Firmicutes	Bacteria
Bacteria_unclassified	Bacteria_unclassified	Bacteria
Bacteria_unclassified	Bacteria_unclassified	Bacteria
Bacteria_unclassified	Bacteria_unclassified	Bacteria
Bacteria_unclassified	Bacteria_unclassified	Bacteria
Bacteria_unclassified	Bacteria_unclassified	Bacteria
Bacteria_unclassified	Bacteria_unclassified	Bacteria
Bacteria_unclassified	Bacteria_unclassified	Bacteria
Bacteria_unclassified	Bacteria_unclassified	Bacteria
Bacteria_unclassified	Bacteria_unclassified	Bacteria
Bacteria_unclassified	Bacteria_unclassified	Bacteria
Bacteria_unclassified	Bacteria_unclassified	Bacteria
Bacteria_unclassified	Bacteria_unclassified	Bacteria
Clostridia	Firmicutes	Bacteria
Clostridia	Firmicutes	Bacteria
CFGB77359	Bacteria_unclassified	Bacteria
CFGB77359	Bacteria_unclassified	Bacteria
CFGB9639	Firmicutes	Bacteria
CFGB9639	Firmicutes	Bacteria
Clostridia	Firmicutes	Bacteria
Clostridia	Firmicutes	Bacteria
Clostridia	Firmicutes	Bacteria
Clostridia	Firmicutes	Bacteria
Clostridia	Firmicutes	Bacteria
Clostridia	Firmicutes	Bacteria
Clostridia	Firmicutes	Bacteria
Clostridia	Firmicutes	Bacteria

Clostridia	Firmicutes	Bacteria
Clostridia	Firmicutes	Bacteria
Clostridia	Firmicutes	Bacteria
Clostridia	Firmicutes	Bacteria
Clostridia	Firmicutes	Bacteria
Clostridia	Firmicutes	Bacteria
CFGB9656	Firmicutes	Bacteria
CFGB9656	Firmicutes	Bacteria
CFGB9827	Firmicutes	Bacteria
CFGB9827	Firmicutes	Bacteria
Clostridia	Firmicutes	Bacteria
Clostridia	Firmicutes	Bacteria
CFGB77303	Bacteria_unclassified	Bacteria
CFGB77303	Bacteria_unclassified	Bacteria
Clostridia	Firmicutes	Bacteria
Clostridia	Firmicutes	Bacteria
Clostridia	Firmicutes	Bacteria
Clostridia	Firmicutes	Bacteria
CFGB72709	Firmicutes	Bacteria
CFGB72709	Firmicutes	Bacteria
Clostridia	Firmicutes	Bacteria
Clostridia	Firmicutes	Bacteria
Clostridia	Firmicutes	Bacteria
Clostridia	Firmicutes	Bacteria
Clostridia	Firmicutes	Bacteria
Clostridia	Firmicutes	Bacteria
Clostridia	Firmicutes	Bacteria
Clostridia	Firmicutes	Bacteria
Clostridia	Firmicutes	Bacteria
Clostridia	Firmicutes	Bacteria
Clostridia	Firmicutes	Bacteria
Clostridia	Firmicutes	Bacteria
Clostridia	Firmicutes	Bacteria
Clostridia	Firmicutes	Bacteria
Clostridia	Firmicutes	Bacteria
Clostridia	Firmicutes	Bacteria
Clostridia	Firmicutes	Bacteria
Clostridia	Firmicutes	Bacteria
Clostridia	Firmicutes	Bacteria
Clostridia	Firmicutes	Bacteria
Clostridia	Firmicutes	Bacteria
Clostridia	Firmicutes	Bacteria
Clostridia	Firmicutes	Bacteria
Clostridia	Firmicutes	Bacteria
Clostridia	Firmicutes	Bacteria
Clostridia	Firmicutes	Bacteria

CFGB77153	Actinobacteria	Bacteria
CFGB77153	Actinobacteria	Bacteria
CFGB1791	Tenericutes	Bacteria
CFGB1791	Tenericutes	Bacteria
CFGB10290	Firmicutes	Bacteria
CFGB10290	Firmicutes	Bacteria
Clostridia	Firmicutes	Bacteria
Clostridia	Firmicutes	Bacteria
CFGB10289	Firmicutes	Bacteria
CFGB10289	Firmicutes	Bacteria
CFGB1765	Firmicutes	Bacteria
CFGB1765	Firmicutes	Bacteria
CFGB1765	Firmicutes	Bacteria
CFGB1765	Firmicutes	Bacteria
CFGB1765	Firmicutes	Bacteria
CFGB1765	Firmicutes	Bacteria
CFGB10667	Firmicutes	Bacteria
CFGB10667	Firmicutes	Bacteria
Clostridia	Firmicutes	Bacteria
Clostridia	Firmicutes	Bacteria
Clostridia	Firmicutes	Bacteria
Clostridia	Firmicutes	Bacteria
Clostridia	Firmicutes	Bacteria
Clostridia	Firmicutes	Bacteria
CFGB75721	Firmicutes	Bacteria
CFGB75721	Firmicutes	Bacteria
Clostridia	Firmicutes	Bacteria
Clostridia	Firmicutes	Bacteria
Clostridia	Firmicutes	Bacteria
Clostridia	Firmicutes	Bacteria
CFGB10299	Firmicutes	Bacteria
CFGB10299	Firmicutes	Bacteria
Clostridia	Firmicutes	Bacteria
Clostridia	Firmicutes	Bacteria
Clostridia	Firmicutes	Bacteria
Clostridia	Firmicutes	Bacteria
Clostridia	Firmicutes	Bacteria
Clostridia	Firmicutes	Bacteria
Clostridia	Firmicutes	Bacteria
Clostridia	Firmicutes	Bacteria
Clostridia	Firmicutes	Bacteria
Clostridia	Firmicutes	Bacteria
Clostridia	Firmicutes	Bacteria
Clostridia	Firmicutes	Bacteria
Clostridia	Firmicutes	Bacteria
Clostridia	Firmicutes	Bacteria

Clostridia	Firmicutes	Bacteria
Clostridia	Firmicutes	Bacteria
Bacilli	Firmicutes	Bacteria
Bacilli	Firmicutes	Bacteria
Coriobacteriia	Actinobacteria	Bacteria
Coriobacteriia	Actinobacteria	Bacteria
Bacteroidia	Bacteroidota	Bacteria
Bacteroidia	Bacteroidota	Bacteria
Clostridia	Firmicutes	Bacteria
Clostridia	Firmicutes	Bacteria
Clostridia	Firmicutes	Bacteria
Clostridia	Firmicutes	Bacteria
Clostridia	Firmicutes	Bacteria
Clostridia	Firmicutes	Bacteria
Clostridia	Firmicutes	Bacteria
Clostridia	Firmicutes	Bacteria
Clostridia	Firmicutes	Bacteria
Clostridia	Firmicutes	Bacteria
Clostridia	Firmicutes	Bacteria
Clostridia	Firmicutes	Bacteria
Betaproteobacteria	Proteobacteria	Bacteria
Betaproteobacteria	Proteobacteria	Bacteria
Clostridia	Firmicutes	Bacteria
Clostridia	Firmicutes	Bacteria
Erysipelotrichia	Firmicutes	Bacteria
Erysipelotrichia	Firmicutes	Bacteria
Clostridia	Firmicutes	Bacteria
Clostridia	Firmicutes	Bacteria
Clostridia	Firmicutes	Bacteria
Clostridia	Firmicutes	Bacteria
Verrucomicrobiae	Verrucomicrobia	Bacteria
Verrucomicrobiae	Verrucomicrobia	Bacteria
Clostridia	Firmicutes	Bacteria
Clostridia	Firmicutes	Bacteria
Bacteria_unclassified	Bacteria_unclassified	Bacteria
Bacteria_unclassified	Bacteria_unclassified	Bacteria
Bacteria_unclassified	Bacteria_unclassified	Bacteria
Bacteria_unclassified	Bacteria_unclassified	Bacteria
Bacteria_unclassified	Bacteria_unclassified	Bacteria
Bacteria_unclassified	Bacteria_unclassified	Bacteria
Bacteria_unclassified	Bacteria_unclassified	Bacteria
Bacteria_unclassified	Bacteria_unclassified	Bacteria

Bacteria_unclassified	Bacteria_unclassified	Bacteria
Bacteria_unclassified	Bacteria_unclassified	Bacteria
Bacteria_unclassified	Bacteria_unclassified	Bacteria
Bacteria_unclassified	Bacteria_unclassified	Bacteria
Bacteria_unclassified	Bacteria_unclassified	Bacteria
Bacteria_unclassified	Bacteria_unclassified	Bacteria
Bacteria_unclassified	Bacteria_unclassified	Bacteria
Bacteria_unclassified	Bacteria_unclassified	Bacteria
Bacteria_unclassified	Bacteria_unclassified	Bacteria
Bacteria_unclassified	Bacteria_unclassified	Bacteria
Bacteroidia	Bacteroidota	Bacteria
Bacteroidia	Bacteroidota	Bacteria
Clostridia	Firmicutes	Bacteria
Clostridia	Firmicutes	Bacteria
Clostridia	Firmicutes	Bacteria
Clostridia	Firmicutes	Bacteria
Clostridia	Firmicutes	Bacteria
Clostridia	Firmicutes	Bacteria
Clostridia	Firmicutes	Bacteria
Clostridia	Firmicutes	Bacteria
Erysipelotrichia	Firmicutes	Bacteria
Erysipelotrichia	Firmicutes	Bacteria
Clostridia	Firmicutes	Bacteria
Clostridia	Firmicutes	Bacteria
Clostridia	Firmicutes	Bacteria
Clostridia	Firmicutes	Bacteria
Clostridia	Firmicutes	Bacteria
Clostridia	Firmicutes	Bacteria
CFGB77306	Firmicutes	Bacteria
CFGB77306	Firmicutes	Bacteria
Clostridia	Firmicutes	Bacteria
Clostridia	Firmicutes	Bacteria
Coriobacteriia	Actinobacteria	Bacteria
Coriobacteriia	Actinobacteria	Bacteria
Clostridia	Firmicutes	Bacteria
Clostridia	Firmicutes	Bacteria
CFGB9506	Firmicutes	Bacteria
CFGB9506	Firmicutes	Bacteria
CFGB9508	Firmicutes	Bacteria
CFGB9508	Firmicutes	Bacteria
CFGB9512	Firmicutes	Bacteria
CFGB9512	Firmicutes	Bacteria
CFGB2838	Firmicutes	Bacteria
CFGB2838	Firmicutes	Bacteria

CFGB2838	Firmicutes	Bacteria
CFGB2838	Firmicutes	Bacteria
CFGB2838	Firmicutes	Bacteria
CFGB2838	Firmicutes	Bacteria
Clostridia	Firmicutes	Bacteria
Clostridia	Firmicutes	Bacteria
CFGB2833	Firmicutes	Bacteria
CFGB2833	Firmicutes	Bacteria
Clostridia	Firmicutes	Bacteria
Clostridia	Firmicutes	Bacteria
Clostridia	Firmicutes	Bacteria
Clostridia	Firmicutes	Bacteria
Clostridia	Firmicutes	Bacteria
Clostridia	Firmicutes	Bacteria
CFGB9622	Firmicutes	Bacteria
CFGB9622	Firmicutes	Bacteria
CFGB77305	Firmicutes	Bacteria
CFGB77305	Firmicutes	Bacteria
Clostridia	Firmicutes	Bacteria
Clostridia	Firmicutes	Bacteria
Clostridia	Firmicutes	Bacteria
Clostridia	Firmicutes	Bacteria
Clostridia	Firmicutes	Bacteria
Clostridia	Firmicutes	Bacteria
Clostridia	Firmicutes	Bacteria
Clostridia	Firmicutes	Bacteria
Clostridia	Firmicutes	Bacteria
Clostridia	Firmicutes	Bacteria
CFGB9633	Firmicutes	Bacteria
CFGB9633	Firmicutes	Bacteria
Bacteria_unclassified	Bacteria_unclassified	Bacteria
Bacteria_unclassified	Bacteria_unclassified	Bacteria
Bacteria_unclassified	Bacteria_unclassified	Bacteria
Bacteria_unclassified	Bacteria_unclassified	Bacteria
Bacteria_unclassified	Bacteria_unclassified	Bacteria
Bacteria_unclassified	Bacteria_unclassified	Bacteria
Bacteria_unclassified	Bacteria_unclassified	Bacteria
Bacteria_unclassified	Bacteria_unclassified	Bacteria
Bacteria_unclassified	Bacteria_unclassified	Bacteria
Bacteria_unclassified	Bacteria_unclassified	Bacteria
Bacteria_unclassified	Bacteria_unclassified	Bacteria
Bacteria_unclassified	Bacteria_unclassified	Bacteria
Clostridia	Firmicutes	Bacteria
Clostridia	Firmicutes	Bacteria
CFGB77359	Bacteria_unclassified	Bacteria
CFGB77359	Bacteria_unclassified	Bacteria

CFGB9639	Firmicutes	Bacteria
CFGB9639	Firmicutes	Bacteria
Clostridia	Firmicutes	Bacteria
Clostridia	Firmicutes	Bacteria
Clostridia	Firmicutes	Bacteria
Clostridia	Firmicutes	Bacteria
Clostridia	Firmicutes	Bacteria
Clostridia	Firmicutes	Bacteria
Clostridia	Firmicutes	Bacteria
Clostridia	Firmicutes	Bacteria
Clostridia	Firmicutes	Bacteria
Clostridia	Firmicutes	Bacteria
Clostridia	Firmicutes	Bacteria
Clostridia	Firmicutes	Bacteria
Clostridia	Firmicutes	Bacteria
Clostridia	Firmicutes	Bacteria
CFGB9656	Firmicutes	Bacteria
CFGB9656	Firmicutes	Bacteria
CFGB9827	Firmicutes	Bacteria
CFGB9827	Firmicutes	Bacteria
Clostridia	Firmicutes	Bacteria
Clostridia	Firmicutes	Bacteria
CFGB77303	Bacteria_unclassified	Bacteria
CFGB77303	Bacteria_unclassified	Bacteria
Clostridia	Firmicutes	Bacteria
Clostridia	Firmicutes	Bacteria
Clostridia	Firmicutes	Bacteria
Clostridia	Firmicutes	Bacteria
CFGB72709	Firmicutes	Bacteria
CFGB72709	Firmicutes	Bacteria
Clostridia	Firmicutes	Bacteria
Clostridia	Firmicutes	Bacteria
Clostridia	Firmicutes	Bacteria
Clostridia	Firmicutes	Bacteria
Clostridia	Firmicutes	Bacteria
Clostridia	Firmicutes	Bacteria
Clostridia	Firmicutes	Bacteria
Clostridia	Firmicutes	Bacteria
Clostridia	Firmicutes	Bacteria
Clostridia	Firmicutes	Bacteria
Clostridia	Firmicutes	Bacteria
Clostridia	Firmicutes	Bacteria
Clostridia	Firmicutes	Bacteria
Clostridia	Firmicutes	Bacteria
Clostridia	Firmicutes	Bacteria
Clostridia	Firmicutes	Bacteria

Clostridia	Firmicutes	Bacteria
Clostridia	Firmicutes	Bacteria
Clostridia	Firmicutes	Bacteria
Clostridia	Firmicutes	Bacteria
Clostridia	Firmicutes	Bacteria
Clostridia	Firmicutes	Bacteria
Clostridia	Firmicutes	Bacteria
Clostridia	Firmicutes	Bacteria
Clostridia	Firmicutes	Bacteria
Clostridia	Firmicutes	Bacteria
CFGB77153	Actinobacteria	Bacteria
CFGB77153	Actinobacteria	Bacteria
CFGB1791	Tenericutes	Bacteria
CFGB1791	Tenericutes	Bacteria
CFGB10290	Firmicutes	Bacteria
CFGB10290	Firmicutes	Bacteria
Clostridia	Firmicutes	Bacteria
Clostridia	Firmicutes	Bacteria
CFGB10289	Firmicutes	Bacteria
CFGB10289	Firmicutes	Bacteria
CFGB1765	Firmicutes	Bacteria
CFGB1765	Firmicutes	Bacteria
CFGB1765	Firmicutes	Bacteria
CFGB1765	Firmicutes	Bacteria
CFGB1765	Firmicutes	Bacteria
CFGB1765	Firmicutes	Bacteria
CFGB10667	Firmicutes	Bacteria
CFGB10667	Firmicutes	Bacteria
Clostridia	Firmicutes	Bacteria
Clostridia	Firmicutes	Bacteria
Clostridia	Firmicutes	Bacteria
Clostridia	Firmicutes	Bacteria
Clostridia	Firmicutes	Bacteria
Clostridia	Firmicutes	Bacteria
CFGB75721	Firmicutes	Bacteria
CFGB75721	Firmicutes	Bacteria
Clostridia	Firmicutes	Bacteria
Clostridia	Firmicutes	Bacteria
Clostridia	Firmicutes	Bacteria
Clostridia	Firmicutes	Bacteria
CFGB10299	Firmicutes	Bacteria
CFGB10299	Firmicutes	Bacteria
Clostridia	Firmicutes	Bacteria
Clostridia	Firmicutes	Bacteria
Clostridia	Firmicutes	Bacteria
Clostridia	Firmicutes	Bacteria

Clostridia	Firmicutes	Bacteria
Clostridia	Firmicutes	Bacteria
Clostridia	Firmicutes	Bacteria
Clostridia	Firmicutes	Bacteria
Clostridia	Firmicutes	Bacteria
Clostridia	Firmicutes	Bacteria
Clostridia	Firmicutes	Bacteria
Clostridia	Firmicutes	Bacteria
Clostridia	Firmicutes	Bacteria
Clostridia	Firmicutes	Bacteria
Clostridia	Firmicutes	Bacteria
Clostridia	Firmicutes	Bacteria
Bacilli	Firmicutes	Bacteria
Bacilli	Firmicutes	Bacteria
Coriobacteriia	Actinobacteria	Bacteria
Coriobacteriia	Actinobacteria	Bacteria
Bacteroidia	Bacteroidota	Bacteria
Bacteroidia	Bacteroidota	Bacteria
Clostridia	Firmicutes	Bacteria
Clostridia	Firmicutes	Bacteria
Clostridia	Firmicutes	Bacteria
Clostridia	Firmicutes	Bacteria
Clostridia	Firmicutes	Bacteria
Clostridia	Firmicutes	Bacteria
Clostridia	Firmicutes	Bacteria
Clostridia	Firmicutes	Bacteria
Clostridia	Firmicutes	Bacteria
Clostridia	Firmicutes	Bacteria
Clostridia	Firmicutes	Bacteria
Clostridia	Firmicutes	Bacteria
Betaproteobacteria	Proteobacteria	Bacteria
Betaproteobacteria	Proteobacteria	Bacteria
Clostridia	Firmicutes	Bacteria
Clostridia	Firmicutes	Bacteria
Erysipelotrichia	Firmicutes	Bacteria
Erysipelotrichia	Firmicutes	Bacteria
Clostridia	Firmicutes	Bacteria
Clostridia	Firmicutes	Bacteria
Clostridia	Firmicutes	Bacteria
Clostridia	Firmicutes	Bacteria
Verrucomicrobiae	Verrucomicrobia	Bacteria
Verrucomicrobiae	Verrucomicrobia	Bacteria

Clostridia	Firmicutes	Bacteria
Clostridia	Firmicutes	Bacteria
Bacteria_unclassified	Bacteria_unclassified	Bacteria
Bacteria_unclassified	Bacteria_unclassified	Bacteria
Bacteria_unclassified	Bacteria_unclassified	Bacteria
Bacteria_unclassified	Bacteria_unclassified	Bacteria
Bacteria_unclassified	Bacteria_unclassified	Bacteria
Bacteria_unclassified	Bacteria_unclassified	Bacteria
Bacteria_unclassified	Bacteria_unclassified	Bacteria
Bacteria_unclassified	Bacteria_unclassified	Bacteria
Bacteria_unclassified	Bacteria_unclassified	Bacteria
Bacteria_unclassified	Bacteria_unclassified	Bacteria
Bacteria_unclassified	Bacteria_unclassified	Bacteria
Bacteria_unclassified	Bacteria_unclassified	Bacteria
Bacteria_unclassified	Bacteria_unclassified	Bacteria
Bacteria_unclassified	Bacteria_unclassified	Bacteria
Bacteria_unclassified	Bacteria_unclassified	Bacteria
Bacteria_unclassified	Bacteria_unclassified	Bacteria
Bacteria_unclassified	Bacteria_unclassified	Bacteria
Bacteria_unclassified	Bacteria_unclassified	Bacteria
Bacteroidia	Bacteroidota	Bacteria
Bacteroidia	Bacteroidota	Bacteria

Clostridia	Firmicutes	Bacteria
Clostridia	Firmicutes	Bacteria
Clostridia	Firmicutes	Bacteria
Clostridia	Firmicutes	Bacteria
Clostridia	Firmicutes	Bacteria
Clostridia	Firmicutes	Bacteria
Clostridia	Firmicutes	Bacteria
Clostridia	Firmicutes	Bacteria
Erysipelotrichia	Firmicutes	Bacteria
Erysipelotrichia	Firmicutes	Bacteria
Clostridia	Firmicutes	Bacteria
Clostridia	Firmicutes	Bacteria
Clostridia	Firmicutes	Bacteria
Clostridia	Firmicutes	Bacteria
Clostridia	Firmicutes	Bacteria
Clostridia	Firmicutes	Bacteria
CFGB77306	Firmicutes	Bacteria
CFGB77306	Firmicutes	Bacteria
Clostridia	Firmicutes	Bacteria
Clostridia	Firmicutes	Bacteria
Coriobacteriia	Actinobacteria	Bacteria
Coriobacteriia	Actinobacteria	Bacteria

Clostridia	Firmicutes	Bacteria
Clostridia	Firmicutes	Bacteria
CFGB9506	Firmicutes	Bacteria
CFGB9506	Firmicutes	Bacteria
CFGB9508	Firmicutes	Bacteria
CFGB9508	Firmicutes	Bacteria
CFGB9512	Firmicutes	Bacteria
CFGB9512	Firmicutes	Bacteria
CFGB2838	Firmicutes	Bacteria
CFGB2838	Firmicutes	Bacteria
CFGB2838	Firmicutes	Bacteria
CFGB2838	Firmicutes	Bacteria
CFGB2838	Firmicutes	Bacteria
CFGB2838	Firmicutes	Bacteria
Clostridia	Firmicutes	Bacteria
Clostridia	Firmicutes	Bacteria
CFGB2833	Firmicutes	Bacteria
CFGB2833	Firmicutes	Bacteria
Clostridia	Firmicutes	Bacteria
Clostridia	Firmicutes	Bacteria
Clostridia	Firmicutes	Bacteria
Clostridia	Firmicutes	Bacteria
Clostridia	Firmicutes	Bacteria
Clostridia	Firmicutes	Bacteria
CFGB9622	Firmicutes	Bacteria
CFGB9622	Firmicutes	Bacteria
CFGB77305	Firmicutes	Bacteria
CFGB77305	Firmicutes	Bacteria
Clostridia	Firmicutes	Bacteria
Clostridia	Firmicutes	Bacteria
Clostridia	Firmicutes	Bacteria
Clostridia	Firmicutes	Bacteria
Clostridia	Firmicutes	Bacteria
Clostridia	Firmicutes	Bacteria
Clostridia	Firmicutes	Bacteria
Clostridia	Firmicutes	Bacteria
Clostridia	Firmicutes	Bacteria
Clostridia	Firmicutes	Bacteria
CFGB9633	Firmicutes	Bacteria
CFGB9633	Firmicutes	Bacteria
Bacteria_unclassified	Bacteria_unclassified	Bacteria
Bacteria_unclassified	Bacteria_unclassified	Bacteria
Bacteria_unclassified	Bacteria_unclassified	Bacteria
Bacteria_unclassified	Bacteria_unclassified	Bacteria
Bacteria_unclassified	Bacteria_unclassified	Bacteria
Bacteria_unclassified	Bacteria_unclassified	Bacteria

Bacteria_unclassified	Bacteria_unclassified	Bacteria
Bacteria_unclassified	Bacteria_unclassified	Bacteria
Bacteria_unclassified	Bacteria_unclassified	Bacteria
Bacteria_unclassified	Bacteria_unclassified	Bacteria
Bacteria_unclassified	Bacteria_unclassified	Bacteria
Bacteria_unclassified	Bacteria_unclassified	Bacteria
Clostridia	Firmicutes	Bacteria
Clostridia	Firmicutes	Bacteria
CFGB77359	Bacteria_unclassified	Bacteria
CFGB77359	Bacteria_unclassified	Bacteria
CFGB9639	Firmicutes	Bacteria
CFGB9639	Firmicutes	Bacteria
Clostridia	Firmicutes	Bacteria
Clostridia	Firmicutes	Bacteria
Clostridia	Firmicutes	Bacteria
Clostridia	Firmicutes	Bacteria
Clostridia	Firmicutes	Bacteria
Clostridia	Firmicutes	Bacteria
Clostridia	Firmicutes	Bacteria
Clostridia	Firmicutes	Bacteria
Clostridia	Firmicutes	Bacteria
Clostridia	Firmicutes	Bacteria
Clostridia	Firmicutes	Bacteria
Clostridia	Firmicutes	Bacteria
Clostridia	Firmicutes	Bacteria
Clostridia	Firmicutes	Bacteria
CFGB9656	Firmicutes	Bacteria
CFGB9656	Firmicutes	Bacteria
CFGB9827	Firmicutes	Bacteria
CFGB9827	Firmicutes	Bacteria
Clostridia	Firmicutes	Bacteria
Clostridia	Firmicutes	Bacteria
CFGB77303	Bacteria_unclassified	Bacteria
CFGB77303	Bacteria_unclassified	Bacteria
Clostridia	Firmicutes	Bacteria
Clostridia	Firmicutes	Bacteria
Clostridia	Firmicutes	Bacteria
Clostridia	Firmicutes	Bacteria
CFGB72709	Firmicutes	Bacteria
CFGB72709	Firmicutes	Bacteria
Clostridia	Firmicutes	Bacteria
Clostridia	Firmicutes	Bacteria
Clostridia	Firmicutes	Bacteria
Clostridia	Firmicutes	Bacteria
Clostridia	Firmicutes	Bacteria
Clostridia	Firmicutes	Bacteria

Clostridia	Firmicutes	Bacteria
Clostridia	Firmicutes	Bacteria
Clostridia	Firmicutes	Bacteria
Clostridia	Firmicutes	Bacteria
Clostridia	Firmicutes	Bacteria
Clostridia	Firmicutes	Bacteria
Clostridia	Firmicutes	Bacteria
Clostridia	Firmicutes	Bacteria
Clostridia	Firmicutes	Bacteria
Clostridia	Firmicutes	Bacteria
Clostridia	Firmicutes	Bacteria
Clostridia	Firmicutes	Bacteria
Clostridia	Firmicutes	Bacteria
Clostridia	Firmicutes	Bacteria
Clostridia	Firmicutes	Bacteria
Clostridia	Firmicutes	Bacteria
Clostridia	Firmicutes	Bacteria
Clostridia	Firmicutes	Bacteria
Clostridia	Firmicutes	Bacteria
Clostridia	Firmicutes	Bacteria
CFGB77153	Actinobacteria	Bacteria
CFGB77153	Actinobacteria	Bacteria
CFGB1791	Tenericutes	Bacteria
CFGB1791	Tenericutes	Bacteria
CFGB10290	Firmicutes	Bacteria
CFGB10290	Firmicutes	Bacteria
Clostridia	Firmicutes	Bacteria
Clostridia	Firmicutes	Bacteria
CFGB10289	Firmicutes	Bacteria
CFGB10289	Firmicutes	Bacteria
CFGB1765	Firmicutes	Bacteria
CFGB1765	Firmicutes	Bacteria
CFGB1765	Firmicutes	Bacteria
CFGB1765	Firmicutes	Bacteria
CFGB1765	Firmicutes	Bacteria
CFGB1765	Firmicutes	Bacteria
CFGB10667	Firmicutes	Bacteria
CFGB10667	Firmicutes	Bacteria
Clostridia	Firmicutes	Bacteria
Clostridia	Firmicutes	Bacteria
Clostridia	Firmicutes	Bacteria
Clostridia	Firmicutes	Bacteria
Clostridia	Firmicutes	Bacteria
Clostridia	Firmicutes	Bacteria
CFGB75721	Firmicutes	Bacteria
CFGB75721	Firmicutes	Bacteria

Clostridia	Firmicutes	Bacteria
Clostridia	Firmicutes	Bacteria
Clostridia	Firmicutes	Bacteria
Clostridia	Firmicutes	Bacteria
CFGB10299	Firmicutes	Bacteria
CFGB10299	Firmicutes	Bacteria
Clostridia	Firmicutes	Bacteria
Clostridia	Firmicutes	Bacteria
Clostridia	Firmicutes	Bacteria
Clostridia	Firmicutes	Bacteria
Clostridia	Firmicutes	Bacteria
Clostridia	Firmicutes	Bacteria
Clostridia	Firmicutes	Bacteria
Clostridia	Firmicutes	Bacteria
Clostridia	Firmicutes	Bacteria
Clostridia	Firmicutes	Bacteria
Clostridia	Firmicutes	Bacteria
Clostridia	Firmicutes	Bacteria
Clostridia	Firmicutes	Bacteria
Clostridia	Firmicutes	Bacteria
Clostridia	Firmicutes	Bacteria
Clostridia	Firmicutes	Bacteria
Bacilli	Firmicutes	Bacteria
Bacilli	Firmicutes	Bacteria
Coriobacteriia	Actinobacteria	Bacteria
Coriobacteriia	Actinobacteria	Bacteria
Bacteroidia	Bacteroidota	Bacteria
Bacteroidia	Bacteroidota	Bacteria
Clostridia	Firmicutes	Bacteria
Clostridia	Firmicutes	Bacteria
Clostridia	Firmicutes	Bacteria
Clostridia	Firmicutes	Bacteria
Clostridia	Firmicutes	Bacteria
Clostridia	Firmicutes	Bacteria
Clostridia	Firmicutes	Bacteria
Clostridia	Firmicutes	Bacteria
Clostridia	Firmicutes	Bacteria
Clostridia	Firmicutes	Bacteria
Clostridia	Firmicutes	Bacteria
Clostridia	Firmicutes	Bacteria
Betaproteobacteria	Proteobacteria	Bacteria
Betaproteobacteria	Proteobacteria	Bacteria
Clostridia	Firmicutes	Bacteria
Clostridia	Firmicutes	Bacteria

Erysipelotrichia	Firmicutes	Bacteria
Erysipelotrichia	Firmicutes	Bacteria

MetaPhlan Annotation
k__Bacteria|p__Firmicutes|c__Clostridia|o__Eubacteriales|f__Lachnospiraceae|g__Acetatifactor|s__Acetatifactor_SGB415
k__Bacteria|p__Firmicutes|c__Clostridia|o__Eubacteriales|f__Lachnospiraceae|g__Acetatifactor|s__Acetatifactor_SGB415
k__Bacteria|p__Firmicutes|c__Clostridia|o__Eubacteriales|f__Oscillospiraceae|g__Acutalibacter|s__Acutalibacter_muris
k__Bacteria|p__Firmicutes|c__Clostridia|o__Eubacteriales|f__Oscillospiraceae|g__Acutalibacter|s__Acutalibacter_muris
k__Bacteria|p__Verrucomicrobia|c__Verrucomicrobiae|o__Verrucomicrobiales|f__Akkermansiaceae|g__Akkermansia|s__A
k__Bacteria|p__Verrucomicrobia|c__Verrucomicrobiae|o__Verrucomicrobiales|f__Akkermansiaceae|g__Akkermansia|s__A
k__Bacteria|p__Firmicutes|c__Clostridia|o__Eubacteriales|f__Oscillospiraceae|g__Anaerotruncus|s__Anaerotruncus_sp_1
k__Bacteria|p__Firmicutes|c__Clostridia|o__Eubacteriales|f__Oscillospiraceae|g__Anaerotruncus|s__Anaerotruncus_sp_1
k__Bacteria|p__Bacteria_unclassified|c__Bacteria_unclassified|o__Bacteria_unclassified|f__Bacteria_unclassified|g__Bacte
k__Bacteria|p__Bacteria_unclassified|c__Bacteria_unclassified|o__Bacteria_unclassified|f__Bacteria_unclassified|g__Bacte
k__Bacteria|p__Bacteria_unclassified|c__Bacteria_unclassified|o__Bacteria_unclassified|f__Bacteria_unclassified|g__Bacte
k__Bacteria|p__Bacteria_unclassified|c__Bacteria_unclassified|o__Bacteria_unclassified|f__Bacteria_unclassified|g__Bacte
k__Bacteria|p__Bacteria_unclassified|c__Bacteria_unclassified|o__Bacteria_unclassified|f__Bacteria_unclassified|g__Bacte
k__Bacteria|p__Bacteria_unclassified|c__Bacteria_unclassified|o__Bacteria_unclassified|f__Bacteria_unclassified|g__Bacte
k__Bacteria|p__Bacteria_unclassified|c__Bacteria_unclassified|o__Bacteria_unclassified|f__Bacteria_unclassified|g__Bacte
k__Bacteria|p__Bacteria_unclassified|c__Bacteria_unclassified|o__Bacteria_unclassified|f__Bacteria_unclassified|g__Bacte
k__Bacteria|p__Bacteria_unclassified|c__Bacteria_unclassified|o__Bacteria_unclassified|f__Bacteria_unclassified|g__Bacte
k__Bacteria|p__Bacteria_unclassified|c__Bacteria_unclassified|o__Bacteria_unclassified|f__Bacteria_unclassified|g__Bacte
k__Bacteria|p__Bacteria_unclassified|c__Bacteria_unclassified|o__Bacteria_unclassified|f__Bacteria_unclassified|g__Bacte
k__Bacteria|p__Bacteria_unclassified|c__Bacteria_unclassified|o__Bacteria_unclassified|f__Bacteria_unclassified|g__Bacte
k__Bacteria|p__Bacteria_unclassified|c__Bacteria_unclassified|o__Bacteria_unclassified|f__Bacteria_unclassified|g__Bacte
k__Bacteria|p__Bacteria_unclassified|c__Bacteria_unclassified|o__Bacteria_unclassified|f__Bacteria_unclassified|g__Bacte
k__Bacteria|p__Bacteria_unclassified|c__Bacteria_unclassified|o__Bacteria_unclassified|f__Bacteria_unclassified|g__Bacte
k__Bacteria|p__Bacteria_unclassified|c__Bacteria_unclassified|o__Bacteria_unclassified|f__Bacteria_unclassified|g__Bacte
k__Bacteria|p__Bacteria_unclassified|c__Bacteria_unclassified|o__Bacteria_unclassified|f__Bacteria_unclassified|g__Bacte
k__Bacteria|p__Bacteroidota|c__Bacteroidia|o__Bacteroidales|f__Bacteroidaceae|g__Bacteroides|s__Bacteroides_thetaio
k__Bacteria|p__Bacteroidota|c__Bacteroidia|o__Bacteroidales|f__Bacteroidaceae|g__Bacteroides|s__Bacteroides_thetaio
k__Bacteria|p__Firmicutes|c__Clostridia|o__Clostridia_unclassified|f__Clostridia_unclassified|g__Clostridia_unclassified|s_
k__Bacteria|p__Firmicutes|c__Clostridia|o__Clostridia_unclassified|f__Clostridia_unclassified|g__Clostridia_unclassified|s_
k__Bacteria|p__Firmicutes|c__Clostridia|o__Eubacteriales|f__Clostridiaceae|g__Clostridiaceae_unclassified|s__Clostridiac
k__Bacteria|p__Firmicutes|c__Clostridia|o__Eubacteriales|f__Clostridiaceae|g__Clostridiaceae_unclassified|s__Clostridiac
k__Bacteria|p__Firmicutes|c__Clostridia|o__Eubacteriales|f__Clostridiaceae|g__Clostridiaceae_unclassified|s__Clostridiac
k__Bacteria|p__Firmicutes|c__Clostridia|o__Eubacteriales|f__Clostridiaceae|g__Clostridiaceae_unclassified|s__Clostridiac
k__Bacteria|p__Firmicutes|c__Clostridia|o__Eubacteriales|f__Eubacteriales_unclassified|g__Eubacteriales_unclassified|s_
k__Bacteria|p__Firmicutes|c__Clostridia|o__Eubacteriales|f__Eubacteriales_unclassified|g__Eubacteriales_unclassified|s_
k__Bacteria|p__Firmicutes|c__Erysipelotrichia|o__Erysipelotrichales|f__Erysipelotrichaceae|g__Erysipelatoclostridium|s__
k__Bacteria|p__Firmicutes|c__Erysipelotrichia|o__Erysipelotrichales|f__Erysipelotrichaceae|g__Erysipelatoclostridium|s__
k__Bacteria|p__Firmicutes|c__Clostridia|o__Eubacteriales|f__Clostridiaceae|g__Clostridium|s__Clostridium_SGB65123
k__Bacteria|p__Firmicutes|c__Clostridia|o__Eubacteriales|f__Clostridiaceae|g__Clostridium|s__Clostridium_SGB65123
k__Bacteria|p__Firmicutes|c__Clostridia|o__Eubacteriales|f__Eubacteriaceae|g__Eubacteriaceae_unclassified|s__Eubacte
k__Bacteria|p__Firmicutes|c__Clostridia|o__Eubacteriales|f__Eubacteriaceae|g__Eubacteriaceae_unclassified|s__Eubacte
k__Bacteria|p__Firmicutes|c__Clostridia|o__Eubacteriales|f__Eubacteriaceae|g__Eubacteriaceae_unclassified|s__Eubacte
k__Bacteria|p__Firmicutes|c__Clostridia|o__Eubacteriales|f__Eubacteriaceae|g__Eubacteriaceae_unclassified|s__Eubacte

k__Bacteria|p__Firmicutes|c__CFGB77306|o__OFGB77306|f__FGB77306|g__GGB20146|s__GGB20146_SGB29427
k__Bacteria|p__Firmicutes|c__CFGB77306|o__OFGB77306|f__FGB77306|g__GGB20146|s__GGB20146_SGB29427
k__Bacteria|p__Firmicutes|c__Clostridia|o__Eubacteriales|f__Lachnospiraceae|g__GGB20149|s__GGB20149_SGB29430
k__Bacteria|p__Firmicutes|c__Clostridia|o__Eubacteriales|f__Lachnospiraceae|g__GGB20149|s__GGB20149_SGB29430
k__Bacteria|p__Actinobacteria|c__Coriobacteriia|o__Eggerthellales|f__Eggerthellaceae|g__GGB22635|s__GGB22635_SGB
k__Bacteria|p__Actinobacteria|c__Coriobacteriia|o__Eggerthellales|f__Eggerthellaceae|g__GGB22635|s__GGB22635_SGB
k__Bacteria|p__Firmicutes|c__Clostridia|o__Eubacteriales|f__Lachnospiraceae|g__GGB25041|s__GGB25041_SGB36960
k__Bacteria|p__Firmicutes|c__Clostridia|o__Eubacteriales|f__Lachnospiraceae|g__GGB25041|s__GGB25041_SGB36960
k__Bacteria|p__Firmicutes|c__CFGB9506|o__OFGB9506|f__FGB9506|g__GGB28379|s__GGB28379_SGB40959
k__Bacteria|p__Firmicutes|c__CFGB9506|o__OFGB9506|f__FGB9506|g__GGB28379|s__GGB28379_SGB40959
k__Bacteria|p__Firmicutes|c__CFGB9508|o__OFGB9508|f__FGB9508|g__GGB28382|s__GGB28382_SGB40962
k__Bacteria|p__Firmicutes|c__CFGB9508|o__OFGB9508|f__FGB9508|g__GGB28382|s__GGB28382_SGB40962
k__Bacteria|p__Firmicutes|c__CFGB9512|o__OFGB9512|f__FGB9512|g__GGB28392|s__GGB28392_SGB40972
k__Bacteria|p__Firmicutes|c__CFGB9512|o__OFGB9512|f__FGB9512|g__GGB28392|s__GGB28392_SGB40972
k__Bacteria|p__Firmicutes|c__CFGB2838|o__OFGB2838|f__FGB2838|g__GGB28404|s__GGB28404_SGB40986
k__Bacteria|p__Firmicutes|c__CFGB2838|o__OFGB2838|f__FGB2838|g__GGB28404|s__GGB28404_SGB40986
k__Bacteria|p__Firmicutes|c__CFGB2838|o__OFGB2838|f__FGB2838|g__GGB28418|s__GGB28418_SGB41001
k__Bacteria|p__Firmicutes|c__CFGB2838|o__OFGB2838|f__FGB2838|g__GGB28418|s__GGB28418_SGB41001
k__Bacteria|p__Firmicutes|c__CFGB2838|o__OFGB2838|f__FGB2838|g__GGB28422|s__GGB28422_SGB41005
k__Bacteria|p__Firmicutes|c__CFGB2838|o__OFGB2838|f__FGB2838|g__GGB28422|s__GGB28422_SGB41005
k__Bacteria|p__Firmicutes|c__Clostridia|o__Eubacteriales|f__Pumilibacteraceae|g__GGB28431|s__GGB28431_SGB41014
k__Bacteria|p__Firmicutes|c__Clostridia|o__Eubacteriales|f__Pumilibacteraceae|g__GGB28431|s__GGB28431_SGB41014
k__Bacteria|p__Firmicutes|c__CFGB2833|o__OFGB2833|f__FGB2833|g__GGB28456|s__GGB28456_SGB41039
k__Bacteria|p__Firmicutes|c__CFGB2833|o__OFGB2833|f__FGB2833|g__GGB28456|s__GGB28456_SGB41039
k__Bacteria|p__Firmicutes|c__Clostridia|o__Eubacteriales|f__Eubacteriaceae|g__GGB28782|s__GGB28782_SGB41435
k__Bacteria|p__Firmicutes|c__Clostridia|o__Eubacteriales|f__Eubacteriaceae|g__GGB28782|s__GGB28782_SGB41435
k__Bacteria|p__Firmicutes|c__Clostridia|o__Eubacteriales|f__Lachnospiraceae|g__GGB28792|s__GGB28792_SGB41445
k__Bacteria|p__Firmicutes|c__Clostridia|o__Eubacteriales|f__Lachnospiraceae|g__GGB28792|s__GGB28792_SGB41445
k__Bacteria|p__Firmicutes|c__Clostridia|o__Eubacteriales|f__Lachnospiraceae|g__GGB28798|s__GGB28798_SGB41451
k__Bacteria|p__Firmicutes|c__Clostridia|o__Eubacteriales|f__Lachnospiraceae|g__GGB28798|s__GGB28798_SGB41451
k__Bacteria|p__Firmicutes|c__CFGB9622|o__OFGB9622|f__FGB9622|g__GGB28810|s__GGB28810_SGB41465
k__Bacteria|p__Firmicutes|c__CFGB9622|o__OFGB9622|f__FGB9622|g__GGB28810|s__GGB28810_SGB41465
k__Bacteria|p__Firmicutes|c__CFGB77305|o__OFGB77305|f__FGB77305|g__GGB28828|s__GGB28828_SGB41484
k__Bacteria|p__Firmicutes|c__CFGB77305|o__OFGB77305|f__FGB77305|g__GGB28828|s__GGB28828_SGB41484
k__Bacteria|p__Firmicutes|c__Clostridia|o__Eubacteriales|f__Clostridiaceae|g__GGB28851|s__GGB28851_SGB41518
k__Bacteria|p__Firmicutes|c__Clostridia|o__Eubacteriales|f__Clostridiaceae|g__GGB28851|s__GGB28851_SGB41518
k__Bacteria|p__Firmicutes|c__Clostridia|o__Eubacteriales|f__Lachnospiraceae|g__GGB28865|s__GGB28865_SGB41537
k__Bacteria|p__Firmicutes|c__Clostridia|o__Eubacteriales|f__Lachnospiraceae|g__GGB28865|s__GGB28865_SGB41537
k__Bacteria|p__Firmicutes|c__Clostridia|o__Eubacteriales|f__Lachnospiraceae|g__GGB28868|s__GGB28868_SGB41542
k__Bacteria|p__Firmicutes|c__Clostridia|o__Eubacteriales|f__Lachnospiraceae|g__GGB28868|s__GGB28868_SGB41542
k__Bacteria|p__Firmicutes|c__Clostridia|o__Eubacteriales|f__Lachnospiraceae|g__GGB28869|s__GGB28869_SGB41543
k__Bacteria|p__Firmicutes|c__Clostridia|o__Eubacteriales|f__Lachnospiraceae|g__GGB28869|s__GGB28869_SGB41543
k__Bacteria|p__Firmicutes|c__Clostridia|o__Eubacteriales|f__Lachnospiraceae|g__GGB28875|s__GGB28875_SGB41555
k__Bacteria|p__Firmicutes|c__Clostridia|o__Eubacteriales|f__Lachnospiraceae|g__GGB28875|s__GGB28875_SGB41555
k__Bacteria|p__Firmicutes|c__CFGB9633|o__OFGB9633|f__FGB9633|g__GGB28881|s__GGB28881_SGB41561
k__Bacteria|p__Firmicutes|c__CFGB9633|o__OFGB9633|f__FGB9633|g__GGB28881|s__GGB28881_SGB41561

k__Bacteria|p__Bacteria_unclassified|c__Bacteria_unclassified|o__Bacteria_unclassified|f__Bacteria_unclassified|g__GGB2
k__Bacteria|p__Bacteria_unclassified|c__Bacteria_unclassified|o__Bacteria_unclassified|f__Bacteria_unclassified|g__GGB2
k__Bacteria|p__Bacteria_unclassified|c__Bacteria_unclassified|o__Bacteria_unclassified|f__Bacteria_unclassified|g__GGB2
k__Bacteria|p__Bacteria_unclassified|c__Bacteria_unclassified|o__Bacteria_unclassified|f__Bacteria_unclassified|g__GGB2
k__Bacteria|p__Bacteria_unclassified|c__Bacteria_unclassified|o__Bacteria_unclassified|f__Bacteria_unclassified|g__GGB2
k__Bacteria|p__Bacteria_unclassified|c__Bacteria_unclassified|o__Bacteria_unclassified|f__Bacteria_unclassified|g__GGB2
k__Bacteria|p__Bacteria_unclassified|c__Bacteria_unclassified|o__Bacteria_unclassified|f__Bacteria_unclassified|g__GGB2
k__Bacteria|p__Bacteria_unclassified|c__Bacteria_unclassified|o__Bacteria_unclassified|f__Bacteria_unclassified|g__GGB2
k__Bacteria|p__Bacteria_unclassified|c__Bacteria_unclassified|o__Bacteria_unclassified|f__Bacteria_unclassified|g__GGB2
k__Bacteria|p__Bacteria_unclassified|c__Bacteria_unclassified|o__Bacteria_unclassified|f__Bacteria_unclassified|g__GGB2
k__Bacteria|p__Bacteria_unclassified|c__Bacteria_unclassified|o__Bacteria_unclassified|f__Bacteria_unclassified|g__GGB2
k__Bacteria|p__Bacteria_unclassified|c__Bacteria_unclassified|o__Bacteria_unclassified|f__Bacteria_unclassified|g__GGB2
k__Bacteria|p__Firmicutes|c__Clostridia|o__Eubacteriales|f__Lachnospiraceae|g__GGB28924|s__GGB28924_SGB41621
k__Bacteria|p__Firmicutes|c__Clostridia|o__Eubacteriales|f__Lachnospiraceae|g__GGB28924|s__GGB28924_SGB41621
k__Bacteria|p__Bacteria_unclassified|c__CFGB77359|o__OFGB77359|f__FGB77359|g__GGB28927|s__GGB28927_SGB416
k__Bacteria|p__Bacteria_unclassified|c__CFGB77359|o__OFGB77359|f__FGB77359|g__GGB28927|s__GGB28927_SGB416
k__Bacteria|p__Firmicutes|c__CFGB9639|o__OFGB9639|f__FGB9639|g__GGB28934|s__GGB28934_SGB41635
k__Bacteria|p__Firmicutes|c__CFGB9639|o__OFGB9639|f__FGB9639|g__GGB28934|s__GGB28934_SGB41635
k__Bacteria|p__Firmicutes|c__Clostridia|o__Eubacteriales|f__Lachnospiraceae|g__GGB28949|s__GGB28949_SGB41655
k__Bacteria|p__Firmicutes|c__Clostridia|o__Eubacteriales|f__Lachnospiraceae|g__GGB28949|s__GGB28949_SGB41655
k__Bacteria|p__Firmicutes|c__Clostridia|o__Eubacteriales|f__Clostridiaceae|g__GGB28951|s__GGB28951_SGB102295
k__Bacteria|p__Firmicutes|c__Clostridia|o__Eubacteriales|f__Clostridiaceae|g__GGB28951|s__GGB28951_SGB102295
k__Bacteria|p__Firmicutes|c__Clostridia|o__Eubacteriales|f__Clostridiaceae|g__GGB28951|s__GGB28951_SGB41658
k__Bacteria|p__Firmicutes|c__Clostridia|o__Eubacteriales|f__Clostridiaceae|g__GGB28951|s__GGB28951_SGB41658
k__Bacteria|p__Firmicutes|c__Clostridia|o__Eubacteriales|f__Clostridiaceae|g__GGB28954|s__GGB28954_SGB41662
k__Bacteria|p__Firmicutes|c__Clostridia|o__Eubacteriales|f__Clostridiaceae|g__GGB28954|s__GGB28954_SGB41662
k__Bacteria|p__Firmicutes|c__Clostridia|o__Eubacteriales|f__Clostridiaceae|g__GGB28960|s__GGB28960_SGB41669
k__Bacteria|p__Firmicutes|c__Clostridia|o__Eubacteriales|f__Clostridiaceae|g__GGB28960|s__GGB28960_SGB41669
k__Bacteria|p__Firmicutes|c__Clostridia|o__Eubacteriales|f__Clostridiaceae|g__GGB28964|s__GGB28964_SGB94886
k__Bacteria|p__Firmicutes|c__Clostridia|o__Eubacteriales|f__Clostridiaceae|g__GGB28964|s__GGB28964_SGB94886
k__Bacteria|p__Firmicutes|c__Clostridia|o__Eubacteriales|f__Clostridiaceae|g__GGB28967|s__GGB28967_SGB41678
k__Bacteria|p__Firmicutes|c__Clostridia|o__Eubacteriales|f__Clostridiaceae|g__GGB28967|s__GGB28967_SGB41678
k__Bacteria|p__Firmicutes|c__CFGB9656|o__OFGB9656|f__FGB9656|g__GGB28996|s__GGB28996_SGB41712
k__Bacteria|p__Firmicutes|c__CFGB9656|o__OFGB9656|f__FGB9656|g__GGB28996|s__GGB28996_SGB41712
k__Bacteria|p__Firmicutes|c__CFGB9827|o__OFGB9827|f__FGB9827|g__GGB29531|s__GGB29531_SGB42317
k__Bacteria|p__Firmicutes|c__CFGB9827|o__OFGB9827|f__FGB9827|g__GGB29531|s__GGB29531_SGB42317
k__Bacteria|p__Firmicutes|c__Clostridia|o__Eubacteriales|f__Eubacteriaceae|g__GGB29685|s__GGB29685_SGB42494
k__Bacteria|p__Firmicutes|c__Clostridia|o__Eubacteriales|f__Eubacteriaceae|g__GGB29685|s__GGB29685_SGB42494
k__Bacteria|p__Bacteria_unclassified|c__CFGB77303|o__OFGB77303|f__FGB77303|g__GGB30141|s__GGB30141_SGB430
k__Bacteria|p__Bacteria_unclassified|c__CFGB77303|o__OFGB77303|f__FGB77303|g__GGB30141|s__GGB30141_SGB430
k__Bacteria|p__Firmicutes|c__Clostridia|o__Eubacteriales|f__Clostridiaceae|g__GGB30145|s__GGB30145_SGB43072
k__Bacteria|p__Firmicutes|c__Clostridia|o__Eubacteriales|f__Clostridiaceae|g__GGB30145|s__GGB30145_SGB43072
k__Bacteria|p__Firmicutes|c__Clostridia|o__Eubacteriales|f__Eubacteriales_unclassified|g__GGB30286|s__GGB30286_SG
k__Bacteria|p__Firmicutes|c__Clostridia|o__Eubacteriales|f__Eubacteriales_unclassified|g__GGB30286|s__GGB30286_SG
k__Bacteria|p__Firmicutes|c__CFGB72709|o__OFGB72709|f__FGB72709|g__GGB30300|s__GGB30300_SGB43264
k__Bacteria|p__Firmicutes|c__CFGB72709|o__OFGB72709|f__FGB72709|g__GGB30300|s__GGB30300_SGB43264

k__Bacteria|p__Firmicutes|c__Clostridia|o__Eubacteriales|f__Oscillospiraceae|g__GGB30303|s__GGB30303_SGB43268
k__Bacteria|p__Firmicutes|c__Clostridia|o__Eubacteriales|f__Oscillospiraceae|g__GGB30303|s__GGB30303_SGB43268
k__Bacteria|p__Firmicutes|c__Clostridia|o__Eubacteriales|f__Oscillospiraceae|g__GGB30450|s__GGB30450_SGB43507
k__Bacteria|p__Firmicutes|c__Clostridia|o__Eubacteriales|f__Oscillospiraceae|g__GGB30450|s__GGB30450_SGB43507
k__Bacteria|p__Firmicutes|c__Clostridia|o__Eubacteriales|f__Oscillospiraceae|g__GGB30453|s__GGB30453_SGB43513
k__Bacteria|p__Firmicutes|c__Clostridia|o__Eubacteriales|f__Oscillospiraceae|g__GGB30453|s__GGB30453_SGB43513
k__Bacteria|p__Firmicutes|c__Clostridia|o__Eubacteriales|f__Oscillospiraceae|g__GGB30454|s__GGB30454_SGB43514
k__Bacteria|p__Firmicutes|c__Clostridia|o__Eubacteriales|f__Oscillospiraceae|g__GGB30454|s__GGB30454_SGB43514
k__Bacteria|p__Firmicutes|c__Clostridia|o__Eubacteriales|f__Oscillospiraceae|g__GGB30455|s__GGB30455_SGB43519
k__Bacteria|p__Firmicutes|c__Clostridia|o__Eubacteriales|f__Oscillospiraceae|g__GGB30455|s__GGB30455_SGB43519
k__Bacteria|p__Firmicutes|c__Clostridia|o__Eubacteriales|f__Oscillospiraceae|g__GGB30456|s__GGB30456_SGB43520
k__Bacteria|p__Firmicutes|c__Clostridia|o__Eubacteriales|f__Oscillospiraceae|g__GGB30456|s__GGB30456_SGB43520
k__Bacteria|p__Firmicutes|c__Clostridia|o__Eubacteriales|f__Oscillospiraceae|g__GGB30457|s__GGB30457_SGB63218
k__Bacteria|p__Firmicutes|c__Clostridia|o__Eubacteriales|f__Oscillospiraceae|g__GGB30457|s__GGB30457_SGB63218
k__Bacteria|p__Firmicutes|c__Clostridia|o__Eubacteriales|f__Oscillospiraceae|g__GGB30461|s__GGB30461_SGB43530
k__Bacteria|p__Firmicutes|c__Clostridia|o__Eubacteriales|f__Oscillospiraceae|g__GGB30461|s__GGB30461_SGB43530
k__Bacteria|p__Firmicutes|c__Clostridia|o__Eubacteriales|f__Oscillospiraceae|g__GGB30461|s__GGB30461_SGB43533
k__Bacteria|p__Firmicutes|c__Clostridia|o__Eubacteriales|f__Oscillospiraceae|g__GGB30461|s__GGB30461_SGB43533
k__Bacteria|p__Firmicutes|c__Clostridia|o__Eubacteriales|f__Oscillospiraceae|g__GGB30461|s__GGB30461_SGB63209
k__Bacteria|p__Firmicutes|c__Clostridia|o__Eubacteriales|f__Oscillospiraceae|g__GGB30461|s__GGB30461_SGB63209
k__Bacteria|p__Firmicutes|c__Clostridia|o__Eubacteriales|f__Oscillospiraceae|g__GGB30463|s__GGB30463_SGB43537
k__Bacteria|p__Firmicutes|c__Clostridia|o__Eubacteriales|f__Oscillospiraceae|g__GGB30463|s__GGB30463_SGB43537
k__Bacteria|p__Firmicutes|c__Clostridia|o__Eubacteriales|f__Oscillospiraceae|g__GGB30473|s__GGB30473_SGB43557
k__Bacteria|p__Firmicutes|c__Clostridia|o__Eubacteriales|f__Oscillospiraceae|g__GGB30473|s__GGB30473_SGB43557
k__Bacteria|p__Firmicutes|c__Clostridia|o__Eubacteriales|f__Oscillospiraceae|g__GGB30475|s__GGB30475_SGB63182
k__Bacteria|p__Firmicutes|c__Clostridia|o__Eubacteriales|f__Oscillospiraceae|g__GGB30475|s__GGB30475_SGB63182
k__Bacteria|p__Actinobacteria|c__CFGB77153|o__OFGB77153|f__FGB77153|g__GGB30861|s__GGB30861_SGB44083
k__Bacteria|p__Actinobacteria|c__CFGB77153|o__OFGB77153|f__FGB77153|g__GGB30861|s__GGB30861_SGB44083
k__Bacteria|p__Tenericutes|c__CFGB1791|o__OFGB1791|f__FGB1791|g__GGB31312|s__GGB31312_SGB44628
k__Bacteria|p__Tenericutes|c__CFGB1791|o__OFGB1791|f__FGB1791|g__GGB31312|s__GGB31312_SGB44628
k__Bacteria|p__Firmicutes|c__CFGB10290|o__OFGB10290|f__FGB10290|g__GGB31438|s__GGB31438_SGB44768
k__Bacteria|p__Firmicutes|c__CFGB10290|o__OFGB10290|f__FGB10290|g__GGB31438|s__GGB31438_SGB44768
k__Bacteria|p__Firmicutes|c__Clostridia|o__Eubacteriales|f__Oscillospiraceae|g__GGB3171|s__GGB3171_SGB4185
k__Bacteria|p__Firmicutes|c__Clostridia|o__Eubacteriales|f__Oscillospiraceae|g__GGB3171|s__GGB3171_SGB4185
k__Bacteria|p__Firmicutes|c__CFGB10289|o__OFGB10289|f__FGB10289|g__GGB31762|s__GGB31762_SGB45125
k__Bacteria|p__Firmicutes|c__CFGB10289|o__OFGB10289|f__FGB10289|g__GGB31762|s__GGB31762_SGB45125
k__Bacteria|p__Firmicutes|c__CFGB1765|o__OFGB1765|f__FGB1765|g__GGB31823|s__GGB31823_SGB45199
k__Bacteria|p__Firmicutes|c__CFGB1765|o__OFGB1765|f__FGB1765|g__GGB31823|s__GGB31823_SGB45199
k__Bacteria|p__Firmicutes|c__CFGB1765|o__OFGB1765|f__FGB1765|g__GGB31838|s__GGB31838_SGB45216
k__Bacteria|p__Firmicutes|c__CFGB1765|o__OFGB1765|f__FGB1765|g__GGB31838|s__GGB31838_SGB45216
k__Bacteria|p__Firmicutes|c__CFGB1765|o__OFGB1765|f__FGB1765|g__GGB31841|s__GGB31841_SGB65084
k__Bacteria|p__Firmicutes|c__CFGB1765|o__OFGB1765|f__FGB1765|g__GGB31841|s__GGB31841_SGB65084
k__Bacteria|p__Firmicutes|c__CFGB10667|o__OFGB10667|f__FGB10667|g__GGB32371|s__GGB32371_SGB41694
k__Bacteria|p__Firmicutes|c__CFGB10667|o__OFGB10667|f__FGB10667|g__GGB32371|s__GGB32371_SGB41694
k__Bacteria|p__Firmicutes|c__Clostridia|o__Eubacteriales|f__Lachnospiraceae|g__GGB3793|s__GGB3793_SGB5158
k__Bacteria|p__Firmicutes|c__Clostridia|o__Eubacteriales|f__Lachnospiraceae|g__GGB3793|s__GGB3793_SGB5158

k__Bacteria|p__Firmicutes|c__Clostridia|o__Eubacteriales|f__Clostridiaceae|g__GGB42601|s__GGB42601_SGB59797
k__Bacteria|p__Firmicutes|c__Clostridia|o__Eubacteriales|f__Clostridiaceae|g__GGB42601|s__GGB42601_SGB59797
k__Bacteria|p__Firmicutes|c__Clostridia|o__Eubacteriales|f__Oscillospiraceae|g__GGB45514|s__GGB45514_SGB63186
k__Bacteria|p__Firmicutes|c__Clostridia|o__Eubacteriales|f__Oscillospiraceae|g__GGB45514|s__GGB45514_SGB63186
k__Bacteria|p__Firmicutes|c__CFGB75721|o__OFGB75721|f__FGB75721|g__GGB45564|s__GGB45564_SGB63259
k__Bacteria|p__Firmicutes|c__CFGB75721|o__OFGB75721|f__FGB75721|g__GGB45564|s__GGB45564_SGB63259
k__Bacteria|p__Firmicutes|c__Clostridia|o__Eubacteriales|f__Oscillospiraceae|g__GGB45624|s__GGB45624_SGB63337
k__Bacteria|p__Firmicutes|c__Clostridia|o__Eubacteriales|f__Oscillospiraceae|g__GGB45624|s__GGB45624_SGB63337
k__Bacteria|p__Firmicutes|c__Clostridia|o__Eubacteriales|f__Christensenellaceae|g__GGB45656|s__GGB45656_SGB6337
k__Bacteria|p__Firmicutes|c__Clostridia|o__Eubacteriales|f__Christensenellaceae|g__GGB45656|s__GGB45656_SGB6337
k__Bacteria|p__Firmicutes|c__CFGB10299|o__OFGB10299|f__FGB10299|g__GGB47127|s__GGB47127_SGB65054
k__Bacteria|p__Firmicutes|c__CFGB10299|o__OFGB10299|f__FGB10299|g__GGB47127|s__GGB47127_SGB65054
k__Bacteria|p__Firmicutes|c__Clostridia|o__Eubacteriales|f__Oscillospiraceae|g__GGB74395|s__GGB74395_SGB43523
k__Bacteria|p__Firmicutes|c__Clostridia|o__Eubacteriales|f__Oscillospiraceae|g__GGB74395|s__GGB74395_SGB43523
k__Bacteria|p__Firmicutes|c__Clostridia|o__Eubacteriales|f__Oscillospiraceae|g__GGB75053|s__GGB75053_SGB43494
k__Bacteria|p__Firmicutes|c__Clostridia|o__Eubacteriales|f__Oscillospiraceae|g__GGB75053|s__GGB75053_SGB43494
k__Bacteria|p__Firmicutes|c__Clostridia|o__Eubacteriales|f__Lachnospiraceae|g__GGB75109|s__GGB75109_SGB102238
k__Bacteria|p__Firmicutes|c__Clostridia|o__Eubacteriales|f__Lachnospiraceae|g__GGB75109|s__GGB75109_SGB102238
k__Bacteria|p__Firmicutes|c__Clostridia|o__Eubacteriales|f__Lachnospiraceae|g__Lachnospiraceae_unclassified|s__Lachn
k__Bacteria|p__Firmicutes|c__Clostridia|o__Eubacteriales|f__Lachnospiraceae|g__Lachnospiraceae_unclassified|s__Lachn
k__Bacteria|p__Firmicutes|c__Clostridia|o__Eubacteriales|f__Lachnospiraceae|g__Lachnospiraceae_unclassified|s__Lachn
k__Bacteria|p__Firmicutes|c__Clostridia|o__Eubacteriales|f__Lachnospiraceae|g__Lachnospiraceae_unclassified|s__Lachn
k__Bacteria|p__Firmicutes|c__Clostridia|o__Eubacteriales|f__Lachnospiraceae|g__Lachnospiraceae_unclassified|s__Lachn
k__Bacteria|p__Firmicutes|c__Clostridia|o__Eubacteriales|f__Lachnospiraceae|g__Lachnospiraceae_unclassified|s__Lachn
k__Bacteria|p__Firmicutes|c__Clostridia|o__Eubacteriales|f__Lachnospiraceae|g__Lachnospiraceae_unclassified|s__Lachn
k__Bacteria|p__Firmicutes|c__Clostridia|o__Eubacteriales|f__Lachnospiraceae|g__Lachnospiraceae_unclassified|s__Lachn
k__Bacteria|p__Firmicutes|c__Clostridia|o__Eubacteriales|f__Lachnospiraceae|g__Lachnospiraceae_unclassified|s__Lachn
k__Bacteria|p__Firmicutes|c__Clostridia|o__Eubacteriales|f__Lachnospiraceae|g__Lachnospiraceae_unclassified|s__Lachn
k__Bacteria|p__Firmicutes|c__Bacilli|o__Lactobacillales|f__Lactobacillaceae|g__Lactobacillus|s__Lactobacillus_johnsonii
k__Bacteria|p__Firmicutes|c__Bacilli|o__Lactobacillales|f__Lactobacillaceae|g__Lactobacillus|s__Lactobacillus_johnsonii
k__Bacteria|p__Actinobacteria|c__Coriobacteriia|o__Coriobacteriales|f__Atopobiaceae|g__Leptogranulimonas|s__Leptog
k__Bacteria|p__Actinobacteria|c__Coriobacteriia|o__Coriobacteriales|f__Atopobiaceae|g__Leptogranulimonas|s__Leptog
k__Bacteria|p__Bacteroidota|c__Bacteroidia|o__Bacteroidales|f__Muribaculaceae|g__Muribaculaceae_unclassified|s__M
k__Bacteria|p__Bacteroidota|c__Bacteroidia|o__Bacteroidales|f__Muribaculaceae|g__Muribaculaceae_unclassified|s__M
k__Bacteria|p__Firmicutes|c__Clostridia|o__Eubacteriales|f__Oscillospiraceae|g__Neglectibacter|s__Neglectibacter_sp_X
k__Bacteria|p__Firmicutes|c__Clostridia|o__Eubacteriales|f__Oscillospiraceae|g__Neglectibacter|s__Neglectibacter_sp_X
k__Bacteria|p__Firmicutes|c__Clostridia|o__Eubacteriales|f__Oscillospiraceae|g__Oscillibacter|s__Oscillibacter_SGB4349
k__Bacteria|p__Firmicutes|c__Clostridia|o__Eubacteriales|f__Oscillospiraceae|g__Oscillibacter|s__Oscillibacter_SGB4349
k__Bacteria|p__Firmicutes|c__Clostridia|o__Eubacteriales|f__Oscillospiraceae|g__Oscillospiraceae_unclassified|s__Oscillo
k__Bacteria|p__Firmicutes|c__Clostridia|o__Eubacteriales|f__Oscillospiraceae|g__Oscillospiraceae_unclassified|s__Oscillo
k__Bacteria|p__Firmicutes|c__Clostridia|o__Eubacteriales|f__Oscillospiraceae|g__Oscillospiraceae_unclassified|s__Oscillo
k__Bacteria|p__Firmicutes|c__Clostridia|o__Eubacteriales|f__Oscillospiraceae|g__Oscillospiraceae_unclassified|s__Oscillo
k__Bacteria|p__Firmicutes|c__Clostridia|o__Eubacteriales|f__Oscillospiraceae|g__Oscillospiraceae_unclassified|s__Oscillo
k__Bacteria|p__Firmicutes|c__Clostridia|o__Eubacteriales|f__Oscillospiraceae|g__Oscillospiraceae_unclassified|s__Oscillo
k__Bacteria|p__Firmicutes|c__Clostridia|o__Eubacteriales|f__Oscillospiraceae|g__Oscillospiraceae_unclassified|s__Oscillo
k__Bacteria|p__Firmicutes|c__Clostridia|o__Eubacteriales|f__Oscillospiraceae|g__Oscillospiraceae_unclassified|s__Oscillo

k__Bacteria|p__Proteobacteria|c__Betaproteobacteria|o__Burkholderiales|f__Sutterellaceae|g__Parasutterella|s__Parasu
k__Bacteria|p__Proteobacteria|c__Betaproteobacteria|o__Burkholderiales|f__Sutterellaceae|g__Parasutterella|s__Parasu

k__Bacteria|p__Firmicutes|c__Clostridia|o__Eubacteriales|f__Lachnospiraceae|g__Schaedlerella|s__Schaedlerella_arabino
k__Bacteria|p__Firmicutes|c__Clostridia|o__Eubacteriales|f__Lachnospiraceae|g__Schaedlerella|s__Schaedlerella_arabino

k__Bacteria|p__Firmicutes|c__Erysipelotrichia|o__Erysipelotrichales|f__Turicibacteraceae|g__Turicibacter|s__Turicibacter_
k__Bacteria|p__Firmicutes|c__Erysipelotrichia|o__Erysipelotrichales|f__Turicibacteraceae|g__Turicibacter|s__Turicibacter_
k__Bacteria|p__Firmicutes|c__Clostridia|o__Eubacteriales|f__Lachnospiraceae|g__Acetatifactor|s__Acetatifactor_SGB415
k__Bacteria|p__Firmicutes|c__Clostridia|o__Eubacteriales|f__Lachnospiraceae|g__Acetatifactor|s__Acetatifactor_SGB415
k__Bacteria|p__Firmicutes|c__Clostridia|o__Eubacteriales|f__Oscillospiraceae|g__Acutalibacter|s__Acutalibacter_muris
k__Bacteria|p__Firmicutes|c__Clostridia|o__Eubacteriales|f__Oscillospiraceae|g__Acutalibacter|s__Acutalibacter_muris
k__Bacteria|p__Verrucomicrobia|c__Verrucomicrobiae|o__Verrucomicrobiales|f__Akkermansiaceae|g__Akkermansia|s__A
k__Bacteria|p__Verrucomicrobia|c__Verrucomicrobiae|o__Verrucomicrobiales|f__Akkermansiaceae|g__Akkermansia|s__A
k__Bacteria|p__Firmicutes|c__Clostridia|o__Eubacteriales|f__Oscillospiraceae|g__Anaerotruncus|s__Anaerotruncus_sp_1
k__Bacteria|p__Firmicutes|c__Clostridia|o__Eubacteriales|f__Oscillospiraceae|g__Anaerotruncus|s__Anaerotruncus_sp_1
k__Bacteria|p__Bacteria_unclassified|c__Bacteria_unclassified|o__Bacteria_unclassified|f__Bacteria_unclassified|g__Bacte
k__Bacteria|p__Bacteria_unclassified|c__Bacteria_unclassified|o__Bacteria_unclassified|f__Bacteria_unclassified|g__Bacte
k__Bacteria|p__Bacteria_unclassified|c__Bacteria_unclassified|o__Bacteria_unclassified|f__Bacteria_unclassified|g__Bacte
k__Bacteria|p__Bacteria_unclassified|c__Bacteria_unclassified|o__Bacteria_unclassified|f__Bacteria_unclassified|g__Bacte
k__Bacteria|p__Bacteria_unclassified|c__Bacteria_unclassified|o__Bacteria_unclassified|f__Bacteria_unclassified|g__Bacte
k__Bacteria|p__Bacteria_unclassified|c__Bacteria_unclassified|o__Bacteria_unclassified|f__Bacteria_unclassified|g__Bacte
k__Bacteria|p__Bacteria_unclassified|c__Bacteria_unclassified|o__Bacteria_unclassified|f__Bacteria_unclassified|g__Bacte
k__Bacteria|p__Bacteria_unclassified|c__Bacteria_unclassified|o__Bacteria_unclassified|f__Bacteria_unclassified|g__Bacte
k__Bacteria|p__Bacteria_unclassified|c__Bacteria_unclassified|o__Bacteria_unclassified|f__Bacteria_unclassified|g__Bacte
k__Bacteria|p__Bacteria_unclassified|c__Bacteria_unclassified|o__Bacteria_unclassified|f__Bacteria_unclassified|g__Bacte
k__Bacteria|p__Bacteria_unclassified|c__Bacteria_unclassified|o__Bacteria_unclassified|f__Bacteria_unclassified|g__Bacte
k__Bacteria|p__Bacteria_unclassified|c__Bacteria_unclassified|o__Bacteria_unclassified|f__Bacteria_unclassified|g__Bacte
k__Bacteria|p__Bacteria_unclassified|c__Bacteria_unclassified|o__Bacteria_unclassified|f__Bacteria_unclassified|g__Bacte
k__Bacteria|p__Bacteria_unclassified|c__Bacteria_unclassified|o__Bacteria_unclassified|f__Bacteria_unclassified|g__Bacte
k__Bacteria|p__Bacteria_unclassified|c__Bacteria_unclassified|o__Bacteria_unclassified|f__Bacteria_unclassified|g__Bacte
k__Bacteria|p__Bacteria_unclassified|c__Bacteria_unclassified|o__Bacteria_unclassified|f__Bacteria_unclassified|g__Bacte
k__Bacteria|p__Bacteria_unclassified|c__Bacteria_unclassified|o__Bacteria_unclassified|f__Bacteria_unclassified|g__Bacte
k__Bacteria|p__Bacteria_unclassified|c__Bacteria_unclassified|o__Bacteria_unclassified|f__Bacteria_unclassified|g__Bacte
k__Bacteria|p__Bacteroidota|c__Bacteroidia|o__Bacteroidales|f__Bacteroidaceae|g__Bacteroides|s__Bacteroides_thetaio
k__Bacteria|p__Bacteroidota|c__Bacteroidia|o__Bacteroidales|f__Bacteroidaceae|g__Bacteroides|s__Bacteroides_thetaio

k__Bacteria|p__Firmicutes|c__Clostridia|o__Clostridia_unclassified|f__Clostridia_unclassified|g__Clostridia_unclassified|s_
k__Bacteria|p__Firmicutes|c__Clostridia|o__Clostridia_unclassified|f__Clostridia_unclassified|g__Clostridia_unclassified|s_
k__Bacteria|p__Firmicutes|c__Clostridia|o__Eubacteriales|f__Clostridiaceae|g__Clostridiaceae_unclassified|s__Clostridiac
k__Bacteria|p__Firmicutes|c__Clostridia|o__Eubacteriales|f__Clostridiaceae|g__Clostridiaceae_unclassified|s__Clostridiac
k__Bacteria|p__Firmicutes|c__Clostridia|o__Eubacteriales|f__Clostridiaceae|g__Clostridiaceae_unclassified|s__Clostridiac
k__Bacteria|p__Firmicutes|c__Clostridia|o__Eubacteriales|f__Clostridiaceae|g__Clostridiaceae_unclassified|s__Clostridiac

k__Bacteria|p__Firmicutes|c__Clostridia|o__Eubacteriales|f__Eubacteriales_unclassified|g__Eubacteriales_unclassified|s__
k__Bacteria|p__Firmicutes|c__Clostridia|o__Eubacteriales|f__Eubacteriales_unclassified|g__Eubacteriales_unclassified|s__
k__Bacteria|p__Firmicutes|c__Erysipelotrichia|o__Erysipelotrichales|f__Erysipelotrichaceae|g__Erysipelatoclostridium|s__
k__Bacteria|p__Firmicutes|c__Erysipelotrichia|o__Erysipelotrichales|f__Erysipelotrichaceae|g__Erysipelatoclostridium|s__
k__Bacteria|p__Firmicutes|c__Clostridia|o__Eubacteriales|f__Clostridiaceae|g__Clostridium|s__Clostridium_SGB65123
k__Bacteria|p__Firmicutes|c__Clostridia|o__Eubacteriales|f__Clostridiaceae|g__Clostridium|s__Clostridium_SGB65123
k__Bacteria|p__Firmicutes|c__Clostridia|o__Eubacteriales|f__Eubacteriaceae|g__Eubacteriaceae_unclassified|s__Eubacte
k__Bacteria|p__Firmicutes|c__Clostridia|o__Eubacteriales|f__Eubacteriaceae|g__Eubacteriaceae_unclassified|s__Eubacte
k__Bacteria|p__Firmicutes|c__Clostridia|o__Eubacteriales|f__Eubacteriaceae|g__Eubacteriaceae_unclassified|s__Eubacte
k__Bacteria|p__Firmicutes|c__Clostridia|o__Eubacteriales|f__Eubacteriaceae|g__Eubacteriaceae_unclassified|s__Eubacte
k__Bacteria|p__Firmicutes|c__CFGB77306|o__OFGB77306|f__FGB77306|g__GGB20146|s__GGB20146_SGB29427
k__Bacteria|p__Firmicutes|c__CFGB77306|o__OFGB77306|f__FGB77306|g__GGB20146|s__GGB20146_SGB29427
k__Bacteria|p__Firmicutes|c__Clostridia|o__Eubacteriales|f__Lachnospiraceae|g__GGB20149|s__GGB20149_SGB29430
k__Bacteria|p__Firmicutes|c__Clostridia|o__Eubacteriales|f__Lachnospiraceae|g__GGB20149|s__GGB20149_SGB29430
k__Bacteria|p__Actinobacteria|c__Coriobacteriia|o__Eggerthellales|f__Eggerthellaceae|g__GGB22635|s__GGB22635_SGB
k__Bacteria|p__Actinobacteria|c__Coriobacteriia|o__Eggerthellales|f__Eggerthellaceae|g__GGB22635|s__GGB22635_SGB
k__Bacteria|p__Firmicutes|c__Clostridia|o__Eubacteriales|f__Lachnospiraceae|g__GGB25041|s__GGB25041_SGB36960
k__Bacteria|p__Firmicutes|c__Clostridia|o__Eubacteriales|f__Lachnospiraceae|g__GGB25041|s__GGB25041_SGB36960
k__Bacteria|p__Firmicutes|c__CFGB9506|o__OFGB9506|f__FGB9506|g__GGB28379|s__GGB28379_SGB40959
k__Bacteria|p__Firmicutes|c__CFGB9506|o__OFGB9506|f__FGB9506|g__GGB28379|s__GGB28379_SGB40959
k__Bacteria|p__Firmicutes|c__CFGB9508|o__OFGB9508|f__FGB9508|g__GGB28382|s__GGB28382_SGB40962
k__Bacteria|p__Firmicutes|c__CFGB9508|o__OFGB9508|f__FGB9508|g__GGB28382|s__GGB28382_SGB40962
k__Bacteria|p__Firmicutes|c__CFGB9512|o__OFGB9512|f__FGB9512|g__GGB28392|s__GGB28392_SGB40972
k__Bacteria|p__Firmicutes|c__CFGB9512|o__OFGB9512|f__FGB9512|g__GGB28392|s__GGB28392_SGB40972
k__Bacteria|p__Firmicutes|c__CFGB2838|o__OFGB2838|f__FGB2838|g__GGB28404|s__GGB28404_SGB40986
k__Bacteria|p__Firmicutes|c__CFGB2838|o__OFGB2838|f__FGB2838|g__GGB28404|s__GGB28404_SGB40986
k__Bacteria|p__Firmicutes|c__CFGB2838|o__OFGB2838|f__FGB2838|g__GGB28418|s__GGB28418_SGB41001
k__Bacteria|p__Firmicutes|c__CFGB2838|o__OFGB2838|f__FGB2838|g__GGB28418|s__GGB28418_SGB41001
k__Bacteria|p__Firmicutes|c__CFGB2838|o__OFGB2838|f__FGB2838|g__GGB28422|s__GGB28422_SGB41005
k__Bacteria|p__Firmicutes|c__CFGB2838|o__OFGB2838|f__FGB2838|g__GGB28422|s__GGB28422_SGB41005
k__Bacteria|p__Firmicutes|c__Clostridia|o__Eubacteriales|f__Pumilibacteraceae|g__GGB28431|s__GGB28431_SGB41014
k__Bacteria|p__Firmicutes|c__Clostridia|o__Eubacteriales|f__Pumilibacteraceae|g__GGB28431|s__GGB28431_SGB41014
k__Bacteria|p__Firmicutes|c__CFGB2833|o__OFGB2833|f__FGB2833|g__GGB28456|s__GGB28456_SGB41039
k__Bacteria|p__Firmicutes|c__CFGB2833|o__OFGB2833|f__FGB2833|g__GGB28456|s__GGB28456_SGB41039
k__Bacteria|p__Firmicutes|c__Clostridia|o__Eubacteriales|f__Eubacteriaceae|g__GGB28782|s__GGB28782_SGB41435
k__Bacteria|p__Firmicutes|c__Clostridia|o__Eubacteriales|f__Eubacteriaceae|g__GGB28782|s__GGB28782_SGB41435
k__Bacteria|p__Firmicutes|c__Clostridia|o__Eubacteriales|f__Lachnospiraceae|g__GGB28792|s__GGB28792_SGB41445
k__Bacteria|p__Firmicutes|c__Clostridia|o__Eubacteriales|f__Lachnospiraceae|g__GGB28792|s__GGB28792_SGB41445
k__Bacteria|p__Firmicutes|c__Clostridia|o__Eubacteriales|f__Lachnospiraceae|g__GGB28798|s__GGB28798_SGB41451
k__Bacteria|p__Firmicutes|c__Clostridia|o__Eubacteriales|f__Lachnospiraceae|g__GGB28798|s__GGB28798_SGB41451
k__Bacteria|p__Firmicutes|c__CFGB9622|o__OFGB9622|f__FGB9622|g__GGB28810|s__GGB28810_SGB41465
k__Bacteria|p__Firmicutes|c__CFGB9622|o__OFGB9622|f__FGB9622|g__GGB28810|s__GGB28810_SGB41465
k__Bacteria|p__Firmicutes|c__CFGB77305|o__OFGB77305|f__FGB77305|g__GGB28828|s__GGB28828_SGB41484
k__Bacteria|p__Firmicutes|c__CFGB77305|o__OFGB77305|f__FGB77305|g__GGB28828|s__GGB28828_SGB41484
k__Bacteria|p__Firmicutes|c__Clostridia|o__Eubacteriales|f__Clostridiaceae|g__GGB28851|s__GGB28851_SGB41518
k__Bacteria|p__Firmicutes|c__Clostridia|o__Eubacteriales|f__Clostridiaceae|g__GGB28851|s__GGB28851_SGB41518

k__Bacteria|p__Firmicutes|c__Clostridia|o__Eubacteriales|f__Lachnospiraceae|g__GGB28865|s__GGB28865_SGB41537
k__Bacteria|p__Firmicutes|c__Clostridia|o__Eubacteriales|f__Lachnospiraceae|g__GGB28865|s__GGB28865_SGB41537
k__Bacteria|p__Firmicutes|c__Clostridia|o__Eubacteriales|f__Lachnospiraceae|g__GGB28868|s__GGB28868_SGB41542
k__Bacteria|p__Firmicutes|c__Clostridia|o__Eubacteriales|f__Lachnospiraceae|g__GGB28868|s__GGB28868_SGB41542
k__Bacteria|p__Firmicutes|c__Clostridia|o__Eubacteriales|f__Lachnospiraceae|g__GGB28869|s__GGB28869_SGB41543
k__Bacteria|p__Firmicutes|c__Clostridia|o__Eubacteriales|f__Lachnospiraceae|g__GGB28869|s__GGB28869_SGB41543
k__Bacteria|p__Firmicutes|c__Clostridia|o__Eubacteriales|f__Lachnospiraceae|g__GGB28875|s__GGB28875_SGB41555
k__Bacteria|p__Firmicutes|c__Clostridia|o__Eubacteriales|f__Lachnospiraceae|g__GGB28875|s__GGB28875_SGB41555
k__Bacteria|p__Firmicutes|c__CFGB9633|o__OFGB9633|f__FGB9633|g__GGB28881|s__GGB28881_SGB41561
k__Bacteria|p__Firmicutes|c__CFGB9633|o__OFGB9633|f__FGB9633|g__GGB28881|s__GGB28881_SGB41561
k__Bacteria|p__Bacteria_unclassified|c__Bacteria_unclassified|o__Bacteria_unclassified|f__Bacteria_unclassified|g__GGB2
k__Bacteria|p__Bacteria_unclassified|c__Bacteria_unclassified|o__Bacteria_unclassified|f__Bacteria_unclassified|g__GGB2
k__Bacteria|p__Bacteria_unclassified|c__Bacteria_unclassified|o__Bacteria_unclassified|f__Bacteria_unclassified|g__GGB2
k__Bacteria|p__Bacteria_unclassified|c__Bacteria_unclassified|o__Bacteria_unclassified|f__Bacteria_unclassified|g__GGB2
k__Bacteria|p__Bacteria_unclassified|c__Bacteria_unclassified|o__Bacteria_unclassified|f__Bacteria_unclassified|g__GGB2
k__Bacteria|p__Bacteria_unclassified|c__Bacteria_unclassified|o__Bacteria_unclassified|f__Bacteria_unclassified|g__GGB2
k__Bacteria|p__Bacteria_unclassified|c__Bacteria_unclassified|o__Bacteria_unclassified|f__Bacteria_unclassified|g__GGB2
k__Bacteria|p__Bacteria_unclassified|c__Bacteria_unclassified|o__Bacteria_unclassified|f__Bacteria_unclassified|g__GGB2
k__Bacteria|p__Bacteria_unclassified|c__Bacteria_unclassified|o__Bacteria_unclassified|f__Bacteria_unclassified|g__GGB2
k__Bacteria|p__Bacteria_unclassified|c__Bacteria_unclassified|o__Bacteria_unclassified|f__Bacteria_unclassified|g__GGB2
k__Bacteria|p__Bacteria_unclassified|c__Bacteria_unclassified|o__Bacteria_unclassified|f__Bacteria_unclassified|g__GGB2
k__Bacteria|p__Bacteria_unclassified|c__Bacteria_unclassified|o__Bacteria_unclassified|f__Bacteria_unclassified|g__GGB2
k__Bacteria|p__Firmicutes|c__Clostridia|o__Eubacteriales|f__Lachnospiraceae|g__GGB28924|s__GGB28924_SGB41621
k__Bacteria|p__Firmicutes|c__Clostridia|o__Eubacteriales|f__Lachnospiraceae|g__GGB28924|s__GGB28924_SGB41621
k__Bacteria|p__Bacteria_unclassified|c__CFGB77359|o__OFGB77359|f__FGB77359|g__GGB28927|s__GGB28927_SGB416
k__Bacteria|p__Bacteria_unclassified|c__CFGB77359|o__OFGB77359|f__FGB77359|g__GGB28927|s__GGB28927_SGB416
k__Bacteria|p__Firmicutes|c__CFGB9639|o__OFGB9639|f__FGB9639|g__GGB28934|s__GGB28934_SGB41635
k__Bacteria|p__Firmicutes|c__CFGB9639|o__OFGB9639|f__FGB9639|g__GGB28934|s__GGB28934_SGB41635
k__Bacteria|p__Firmicutes|c__Clostridia|o__Eubacteriales|f__Lachnospiraceae|g__GGB28949|s__GGB28949_SGB41655
k__Bacteria|p__Firmicutes|c__Clostridia|o__Eubacteriales|f__Lachnospiraceae|g__GGB28949|s__GGB28949_SGB41655
k__Bacteria|p__Firmicutes|c__Clostridia|o__Eubacteriales|f__Clostridiaceae|g__GGB28951|s__GGB28951_SGB102295
k__Bacteria|p__Firmicutes|c__Clostridia|o__Eubacteriales|f__Clostridiaceae|g__GGB28951|s__GGB28951_SGB102295
k__Bacteria|p__Firmicutes|c__Clostridia|o__Eubacteriales|f__Clostridiaceae|g__GGB28951|s__GGB28951_SGB41658
k__Bacteria|p__Firmicutes|c__Clostridia|o__Eubacteriales|f__Clostridiaceae|g__GGB28951|s__GGB28951_SGB41658
k__Bacteria|p__Firmicutes|c__Clostridia|o__Eubacteriales|f__Clostridiaceae|g__GGB28954|s__GGB28954_SGB41662
k__Bacteria|p__Firmicutes|c__Clostridia|o__Eubacteriales|f__Clostridiaceae|g__GGB28954|s__GGB28954_SGB41662
k__Bacteria|p__Firmicutes|c__Clostridia|o__Eubacteriales|f__Clostridiaceae|g__GGB28960|s__GGB28960_SGB41669
k__Bacteria|p__Firmicutes|c__Clostridia|o__Eubacteriales|f__Clostridiaceae|g__GGB28960|s__GGB28960_SGB41669
k__Bacteria|p__Firmicutes|c__Clostridia|o__Eubacteriales|f__Clostridiaceae|g__GGB28964|s__GGB28964_SGB94886
k__Bacteria|p__Firmicutes|c__Clostridia|o__Eubacteriales|f__Clostridiaceae|g__GGB28964|s__GGB28964_SGB94886
k__Bacteria|p__Firmicutes|c__Clostridia|o__Eubacteriales|f__Clostridiaceae|g__GGB28967|s__GGB28967_SGB41678
k__Bacteria|p__Firmicutes|c__Clostridia|o__Eubacteriales|f__Clostridiaceae|g__GGB28967|s__GGB28967_SGB41678
k__Bacteria|p__Firmicutes|c__CFGB9656|o__OFGB9656|f__FGB9656|g__GGB28996|s__GGB28996_SGB41712
k__Bacteria|p__Firmicutes|c__CFGB9656|o__OFGB9656|f__FGB9656|g__GGB28996|s__GGB28996_SGB41712
k__Bacteria|p__Firmicutes|c__CFGB9827|o__OFGB9827|f__FGB9827|g__GGB29531|s__GGB29531_SGB42317
k__Bacteria|p__Firmicutes|c__CFGB9827|o__OFGB9827|f__FGB9827|g__GGB29531|s__GGB29531_SGB42317

k__Bacteria|p__Firmicutes|c__Clostridia|o__Eubacteriales|f__Eubacteriaceae|g__GGB29685|s__GGB29685_SGB42494
k__Bacteria|p__Firmicutes|c__Clostridia|o__Eubacteriales|f__Eubacteriaceae|g__GGB29685|s__GGB29685_SGB42494
k__Bacteria|p__Bacteria_unclassified|c__CFGB77303|o__OFGB77303|f__FGB77303|g__GGB30141|s__GGB30141_SGB430
k__Bacteria|p__Bacteria_unclassified|c__CFGB77303|o__OFGB77303|f__FGB77303|g__GGB30141|s__GGB30141_SGB430
k__Bacteria|p__Firmicutes|c__Clostridia|o__Eubacteriales|f__Clostridiaceae|g__GGB30145|s__GGB30145_SGB43072
k__Bacteria|p__Firmicutes|c__Clostridia|o__Eubacteriales|f__Clostridiaceae|g__GGB30145|s__GGB30145_SGB43072
k__Bacteria|p__Firmicutes|c__Clostridia|o__Eubacteriales|f__Eubacteriales_unclassified|g__GGB30286|s__GGB30286_SG
k__Bacteria|p__Firmicutes|c__Clostridia|o__Eubacteriales|f__Eubacteriales_unclassified|g__GGB30286|s__GGB30286_SG
k__Bacteria|p__Firmicutes|c__CFGB72709|o__OFGB72709|f__FGB72709|g__GGB30300|s__GGB30300_SGB43264
k__Bacteria|p__Firmicutes|c__CFGB72709|o__OFGB72709|f__FGB72709|g__GGB30300|s__GGB30300_SGB43264
k__Bacteria|p__Firmicutes|c__Clostridia|o__Eubacteriales|f__Oscillospiraceae|g__GGB30303|s__GGB30303_SGB43268
k__Bacteria|p__Firmicutes|c__Clostridia|o__Eubacteriales|f__Oscillospiraceae|g__GGB30303|s__GGB30303_SGB43268
k__Bacteria|p__Firmicutes|c__Clostridia|o__Eubacteriales|f__Oscillospiraceae|g__GGB30450|s__GGB30450_SGB43507
k__Bacteria|p__Firmicutes|c__Clostridia|o__Eubacteriales|f__Oscillospiraceae|g__GGB30450|s__GGB30450_SGB43507
k__Bacteria|p__Firmicutes|c__Clostridia|o__Eubacteriales|f__Oscillospiraceae|g__GGB30453|s__GGB30453_SGB43513
k__Bacteria|p__Firmicutes|c__Clostridia|o__Eubacteriales|f__Oscillospiraceae|g__GGB30453|s__GGB30453_SGB43513
k__Bacteria|p__Firmicutes|c__Clostridia|o__Eubacteriales|f__Oscillospiraceae|g__GGB30454|s__GGB30454_SGB43514
k__Bacteria|p__Firmicutes|c__Clostridia|o__Eubacteriales|f__Oscillospiraceae|g__GGB30454|s__GGB30454_SGB43514
k__Bacteria|p__Firmicutes|c__Clostridia|o__Eubacteriales|f__Oscillospiraceae|g__GGB30455|s__GGB30455_SGB43519
k__Bacteria|p__Firmicutes|c__Clostridia|o__Eubacteriales|f__Oscillospiraceae|g__GGB30455|s__GGB30455_SGB43519
k__Bacteria|p__Firmicutes|c__Clostridia|o__Eubacteriales|f__Oscillospiraceae|g__GGB30456|s__GGB30456_SGB43520
k__Bacteria|p__Firmicutes|c__Clostridia|o__Eubacteriales|f__Oscillospiraceae|g__GGB30456|s__GGB30456_SGB43520
k__Bacteria|p__Firmicutes|c__Clostridia|o__Eubacteriales|f__Oscillospiraceae|g__GGB30457|s__GGB30457_SGB63218
k__Bacteria|p__Firmicutes|c__Clostridia|o__Eubacteriales|f__Oscillospiraceae|g__GGB30457|s__GGB30457_SGB63218
k__Bacteria|p__Firmicutes|c__Clostridia|o__Eubacteriales|f__Oscillospiraceae|g__GGB30461|s__GGB30461_SGB43530
k__Bacteria|p__Firmicutes|c__Clostridia|o__Eubacteriales|f__Oscillospiraceae|g__GGB30461|s__GGB30461_SGB43530
k__Bacteria|p__Firmicutes|c__Clostridia|o__Eubacteriales|f__Oscillospiraceae|g__GGB30461|s__GGB30461_SGB43533
k__Bacteria|p__Firmicutes|c__Clostridia|o__Eubacteriales|f__Oscillospiraceae|g__GGB30461|s__GGB30461_SGB43533
k__Bacteria|p__Firmicutes|c__Clostridia|o__Eubacteriales|f__Oscillospiraceae|g__GGB30461|s__GGB30461_SGB63209
k__Bacteria|p__Firmicutes|c__Clostridia|o__Eubacteriales|f__Oscillospiraceae|g__GGB30461|s__GGB30461_SGB63209
k__Bacteria|p__Firmicutes|c__Clostridia|o__Eubacteriales|f__Oscillospiraceae|g__GGB30463|s__GGB30463_SGB43537
k__Bacteria|p__Firmicutes|c__Clostridia|o__Eubacteriales|f__Oscillospiraceae|g__GGB30463|s__GGB30463_SGB43537
k__Bacteria|p__Firmicutes|c__Clostridia|o__Eubacteriales|f__Oscillospiraceae|g__GGB30473|s__GGB30473_SGB43557
k__Bacteria|p__Firmicutes|c__Clostridia|o__Eubacteriales|f__Oscillospiraceae|g__GGB30473|s__GGB30473_SGB43557
k__Bacteria|p__Firmicutes|c__Clostridia|o__Eubacteriales|f__Oscillospiraceae|g__GGB30475|s__GGB30475_SGB63182
k__Bacteria|p__Firmicutes|c__Clostridia|o__Eubacteriales|f__Oscillospiraceae|g__GGB30475|s__GGB30475_SGB63182
k__Bacteria|p__Actinobacteria|c__CFGB77153|o__OFGB77153|f__FGB77153|g__GGB30861|s__GGB30861_SGB44083
k__Bacteria|p__Actinobacteria|c__CFGB77153|o__OFGB77153|f__FGB77153|g__GGB30861|s__GGB30861_SGB44083
k__Bacteria|p__Tenericutes|c__CFGB1791|o__OFGB1791|f__FGB1791|g__GGB31312|s__GGB31312_SGB44628
k__Bacteria|p__Tenericutes|c__CFGB1791|o__OFGB1791|f__FGB1791|g__GGB31312|s__GGB31312_SGB44628
k__Bacteria|p__Firmicutes|c__CFGB10290|o__OFGB10290|f__FGB10290|g__GGB31438|s__GGB31438_SGB44768
k__Bacteria|p__Firmicutes|c__CFGB10290|o__OFGB10290|f__FGB10290|g__GGB31438|s__GGB31438_SGB44768
k__Bacteria|p__Firmicutes|c__Clostridia|o__Eubacteriales|f__Oscillospiraceae|g__GGB3171|s__GGB3171_SGB4185
k__Bacteria|p__Firmicutes|c__Clostridia|o__Eubacteriales|f__Oscillospiraceae|g__GGB3171|s__GGB3171_SGB4185
k__Bacteria|p__Firmicutes|c__CFGB10289|o__OFGB10289|f__FGB10289|g__GGB31762|s__GGB31762_SGB45125
k__Bacteria|p__Firmicutes|c__CFGB10289|o__OFGB10289|f__FGB10289|g__GGB31762|s__GGB31762_SGB45125

k__Bacteria|p__Firmicutes|c__CFGB1765|o__OFGB1765|f__FGB1765|g__GGB31823|s__GGB31823_SGB45199
k__Bacteria|p__Firmicutes|c__CFGB1765|o__OFGB1765|f__FGB1765|g__GGB31823|s__GGB31823_SGB45199
k__Bacteria|p__Firmicutes|c__CFGB1765|o__OFGB1765|f__FGB1765|g__GGB31838|s__GGB31838_SGB45216
k__Bacteria|p__Firmicutes|c__CFGB1765|o__OFGB1765|f__FGB1765|g__GGB31838|s__GGB31838_SGB45216
k__Bacteria|p__Firmicutes|c__CFGB1765|o__OFGB1765|f__FGB1765|g__GGB31841|s__GGB31841_SGB65084
k__Bacteria|p__Firmicutes|c__CFGB1765|o__OFGB1765|f__FGB1765|g__GGB31841|s__GGB31841_SGB65084
k__Bacteria|p__Firmicutes|c__CFGB10667|o__OFGB10667|f__FGB10667|g__GGB32371|s__GGB32371_SGB41694
k__Bacteria|p__Firmicutes|c__CFGB10667|o__OFGB10667|f__FGB10667|g__GGB32371|s__GGB32371_SGB41694
k__Bacteria|p__Firmicutes|c__Clostridia|o__Eubacteriales|f__Lachnospiraceae|g__GGB3793|s__GGB3793_SGB5158
k__Bacteria|p__Firmicutes|c__Clostridia|o__Eubacteriales|f__Lachnospiraceae|g__GGB3793|s__GGB3793_SGB5158
k__Bacteria|p__Firmicutes|c__Clostridia|o__Eubacteriales|f__Clostridiaceae|g__GGB42601|s__GGB42601_SGB59797
k__Bacteria|p__Firmicutes|c__Clostridia|o__Eubacteriales|f__Clostridiaceae|g__GGB42601|s__GGB42601_SGB59797
k__Bacteria|p__Firmicutes|c__Clostridia|o__Eubacteriales|f__Oscillospiraceae|g__GGB45514|s__GGB45514_SGB63186
k__Bacteria|p__Firmicutes|c__Clostridia|o__Eubacteriales|f__Oscillospiraceae|g__GGB45514|s__GGB45514_SGB63186
k__Bacteria|p__Firmicutes|c__CFGB75721|o__OFGB75721|f__FGB75721|g__GGB45564|s__GGB45564_SGB63259
k__Bacteria|p__Firmicutes|c__CFGB75721|o__OFGB75721|f__FGB75721|g__GGB45564|s__GGB45564_SGB63259
k__Bacteria|p__Firmicutes|c__Clostridia|o__Eubacteriales|f__Oscillospiraceae|g__GGB45624|s__GGB45624_SGB63337
k__Bacteria|p__Firmicutes|c__Clostridia|o__Eubacteriales|f__Oscillospiraceae|g__GGB45624|s__GGB45624_SGB63337
k__Bacteria|p__Firmicutes|c__Clostridia|o__Eubacteriales|f__Christensenellaceae|g__GGB45656|s__GGB45656_SGB6337
k__Bacteria|p__Firmicutes|c__Clostridia|o__Eubacteriales|f__Christensenellaceae|g__GGB45656|s__GGB45656_SGB6337
k__Bacteria|p__Firmicutes|c__CFGB10299|o__OFGB10299|f__FGB10299|g__GGB47127|s__GGB47127_SGB65054
k__Bacteria|p__Firmicutes|c__CFGB10299|o__OFGB10299|f__FGB10299|g__GGB47127|s__GGB47127_SGB65054
k__Bacteria|p__Firmicutes|c__Clostridia|o__Eubacteriales|f__Oscillospiraceae|g__GGB74395|s__GGB74395_SGB43523
k__Bacteria|p__Firmicutes|c__Clostridia|o__Eubacteriales|f__Oscillospiraceae|g__GGB74395|s__GGB74395_SGB43523
k__Bacteria|p__Firmicutes|c__Clostridia|o__Eubacteriales|f__Oscillospiraceae|g__GGB75053|s__GGB75053_SGB43494
k__Bacteria|p__Firmicutes|c__Clostridia|o__Eubacteriales|f__Oscillospiraceae|g__GGB75053|s__GGB75053_SGB43494
k__Bacteria|p__Firmicutes|c__Clostridia|o__Eubacteriales|f__Lachnospiraceae|g__GGB75109|s__GGB75109_SGB102238
k__Bacteria|p__Firmicutes|c__Clostridia|o__Eubacteriales|f__Lachnospiraceae|g__GGB75109|s__GGB75109_SGB102238
k__Bacteria|p__Firmicutes|c__Clostridia|o__Eubacteriales|f__Lachnospiraceae|g__Lachnospiraceae_unclassified|s__Lachn
k__Bacteria|p__Firmicutes|c__Clostridia|o__Eubacteriales|f__Lachnospiraceae|g__Lachnospiraceae_unclassified|s__Lachn
k__Bacteria|p__Firmicutes|c__Clostridia|o__Eubacteriales|f__Lachnospiraceae|g__Lachnospiraceae_unclassified|s__Lachn
k__Bacteria|p__Firmicutes|c__Clostridia|o__Eubacteriales|f__Lachnospiraceae|g__Lachnospiraceae_unclassified|s__Lachn
k__Bacteria|p__Firmicutes|c__Clostridia|o__Eubacteriales|f__Lachnospiraceae|g__Lachnospiraceae_unclassified|s__Lachn
k__Bacteria|p__Firmicutes|c__Clostridia|o__Eubacteriales|f__Lachnospiraceae|g__Lachnospiraceae_unclassified|s__Lachn
k__Bacteria|p__Firmicutes|c__Clostridia|o__Eubacteriales|f__Lachnospiraceae|g__Lachnospiraceae_unclassified|s__Lachn
k__Bacteria|p__Firmicutes|c__Clostridia|o__Eubacteriales|f__Lachnospiraceae|g__Lachnospiraceae_unclassified|s__Lachn
k__Bacteria|p__Firmicutes|c__Clostridia|o__Eubacteriales|f__Lachnospiraceae|g__Lachnospiraceae_unclassified|s__Lachn
k__Bacteria|p__Firmicutes|c__Clostridia|o__Eubacteriales|f__Lachnospiraceae|g__Lachnospiraceae_unclassified|s__Lachn
k__Bacteria|p__Firmicutes|c__Bacilli|o__Lactobacillales|f__Lactobacillaceae|g__Lactobacillus|s__Lactobacillus_johnsonii
k__Bacteria|p__Firmicutes|c__Bacilli|o__Lactobacillales|f__Lactobacillaceae|g__Lactobacillus|s__Lactobacillus_johnsonii
k__Bacteria|p__Actinobacteria|c__Coriobacteriia|o__Coriobacteriales|f__Atopobiaceae|g__Leptogranulimonas|s__Leptog
k__Bacteria|p__Actinobacteria|c__Coriobacteriia|o__Coriobacteriales|f__Atopobiaceae|g__Leptogranulimonas|s__Leptog
k__Bacteria|p__Bacteroidota|c__Bacteroidia|o__Bacteroidales|f__Muribaculaceae|g__Muribaculaceae_unclassified|s__M
k__Bacteria|p__Bacteroidota|c__Bacteroidia|o__Bacteroidales|f__Muribaculaceae|g__Muribaculaceae_unclassified|s__M
k__Bacteria|p__Firmicutes|c__Clostridia|o__Eubacteriales|f__Oscillospiraceae|g__Neglectibacter|s__Neglectibacter_sp_X
k__Bacteria|p__Firmicutes|c__Clostridia|o__Eubacteriales|f__Oscillospiraceae|g__Neglectibacter|s__Neglectibacter_sp_X

k__Bacteria|p__Firmicutes|c__Clostridia|o__Eubacteriales|f__Oscillospiraceae|g__Oscillibacter|s__Oscillibacter_SGB43496
k__Bacteria|p__Firmicutes|c__Clostridia|o__Eubacteriales|f__Oscillospiraceae|g__Oscillibacter|s__Oscillibacter_SGB43496
k__Bacteria|p__Firmicutes|c__Clostridia|o__Eubacteriales|f__Oscillospiraceae|g__Oscillospiraceae_unclassified|s__Oscillo
k__Bacteria|p__Firmicutes|c__Clostridia|o__Eubacteriales|f__Oscillospiraceae|g__Oscillospiraceae_unclassified|s__Oscillo
k__Bacteria|p__Firmicutes|c__Clostridia|o__Eubacteriales|f__Oscillospiraceae|g__Oscillospiraceae_unclassified|s__Oscillo
k__Bacteria|p__Firmicutes|c__Clostridia|o__Eubacteriales|f__Oscillospiraceae|g__Oscillospiraceae_unclassified|s__Oscillo
k__Bacteria|p__Firmicutes|c__Clostridia|o__Eubacteriales|f__Oscillospiraceae|g__Oscillospiraceae_unclassified|s__Oscillo
k__Bacteria|p__Firmicutes|c__Clostridia|o__Eubacteriales|f__Oscillospiraceae|g__Oscillospiraceae_unclassified|s__Oscillo
k__Bacteria|p__Firmicutes|c__Clostridia|o__Eubacteriales|f__Oscillospiraceae|g__Oscillospiraceae_unclassified|s__Oscillo
k__Bacteria|p__Firmicutes|c__Clostridia|o__Eubacteriales|f__Oscillospiraceae|g__Oscillospiraceae_unclassified|s__Oscillo
k__Bacteria|p__Proteobacteria|c__Betaproteobacteria|o__Burkholderiales|f__Sutterellaceae|g__Parasutterella|s__Parasu
k__Bacteria|p__Proteobacteria|c__Betaproteobacteria|o__Burkholderiales|f__Sutterellaceae|g__Parasutterella|s__Parasu

k__Bacteria|p__Firmicutes|c__Clostridia|o__Eubacteriales|f__Lachnospiraceae|g__Schaedlerella|s__Schaedlerella_arabino
k__Bacteria|p__Firmicutes|c__Clostridia|o__Eubacteriales|f__Lachnospiraceae|g__Schaedlerella|s__Schaedlerella_arabino

k__Bacteria|p__Firmicutes|c__Erysipelotrichia|o__Erysipelotrichales|f__Turicibacteraceae|g__Turicibacter|s__Turicibacter_
k__Bacteria|p__Firmicutes|c__Erysipelotrichia|o__Erysipelotrichales|f__Turicibacteraceae|g__Turicibacter|s__Turicibacter_
k__Bacteria|p__Firmicutes|c__Clostridia|o__Eubacteriales|f__Lachnospiraceae|g__Acetatifactor|s__Acetatifactor_SGB415
k__Bacteria|p__Firmicutes|c__Clostridia|o__Eubacteriales|f__Lachnospiraceae|g__Acetatifactor|s__Acetatifactor_SGB415
k__Bacteria|p__Firmicutes|c__Clostridia|o__Eubacteriales|f__Oscillospiraceae|g__Acutalibacter|s__Acutalibacter_muris
k__Bacteria|p__Firmicutes|c__Clostridia|o__Eubacteriales|f__Oscillospiraceae|g__Acutalibacter|s__Acutalibacter_muris
k__Bacteria|p__Verrucomicrobia|c__Verrucomicrobiae|o__Verrucomicrobiales|f__Akkermansiaceae|g__Akkermansia|s__A
k__Bacteria|p__Verrucomicrobia|c__Verrucomicrobiae|o__Verrucomicrobiales|f__Akkermansiaceae|g__Akkermansia|s__A
k__Bacteria|p__Firmicutes|c__Clostridia|o__Eubacteriales|f__Oscillospiraceae|g__Anaerotruncus|s__Anaerotruncus_sp_1
k__Bacteria|p__Firmicutes|c__Clostridia|o__Eubacteriales|f__Oscillospiraceae|g__Anaerotruncus|s__Anaerotruncus_sp_1
k__Bacteria|p__Bacteria_unclassified|c__Bacteria_unclassified|o__Bacteria_unclassified|f__Bacteria_unclassified|g__Bacte
k__Bacteria|p__Bacteria_unclassified|c__Bacteria_unclassified|o__Bacteria_unclassified|f__Bacteria_unclassified|g__Bacte
k__Bacteria|p__Bacteria_unclassified|c__Bacteria_unclassified|o__Bacteria_unclassified|f__Bacteria_unclassified|g__Bacte
k__Bacteria|p__Bacteria_unclassified|c__Bacteria_unclassified|o__Bacteria_unclassified|f__Bacteria_unclassified|g__Bacte
k__Bacteria|p__Bacteria_unclassified|c__Bacteria_unclassified|o__Bacteria_unclassified|f__Bacteria_unclassified|g__Bacte
k__Bacteria|p__Bacteria_unclassified|c__Bacteria_unclassified|o__Bacteria_unclassified|f__Bacteria_unclassified|g__Bacte
k__Bacteria|p__Bacteria_unclassified|c__Bacteria_unclassified|o__Bacteria_unclassified|f__Bacteria_unclassified|g__Bacte
k__Bacteria|p__Bacteria_unclassified|c__Bacteria_unclassified|o__Bacteria_unclassified|f__Bacteria_unclassified|g__Bacte
k__Bacteria|p__Bacteria_unclassified|c__Bacteria_unclassified|o__Bacteria_unclassified|f__Bacteria_unclassified|g__Bacte
k__Bacteria|p__Bacteria_unclassified|c__Bacteria_unclassified|o__Bacteria_unclassified|f__Bacteria_unclassified|g__Bacte
k__Bacteria|p__Bacteria_unclassified|c__Bacteria_unclassified|o__Bacteria_unclassified|f__Bacteria_unclassified|g__Bacte
k__Bacteria|p__Bacteria_unclassified|c__Bacteria_unclassified|o__Bacteria_unclassified|f__Bacteria_unclassified|g__Bacte
k__Bacteria|p__Bacteria_unclassified|c__Bacteria_unclassified|o__Bacteria_unclassified|f__Bacteria_unclassified|g__Bacte
k__Bacteria|p__Bacteria_unclassified|c__Bacteria_unclassified|o__Bacteria_unclassified|f__Bacteria_unclassified|g__Bacte
k__Bacteria|p__Bacteria_unclassified|c__Bacteria_unclassified|o__Bacteria_unclassified|f__Bacteria_unclassified|g__Bacte
k__Bacteria|p__Bacteria_unclassified|c__Bacteria_unclassified|o__Bacteria_unclassified|f__Bacteria_unclassified|g__Bacte
k__Bacteria|p__Bacteria_unclassified|c__Bacteria_unclassified|o__Bacteria_unclassified|f__Bacteria_unclassified|g__Bacte
k__Bacteria|p__Bacteria_unclassified|c__Bacteria_unclassified|o__Bacteria_unclassified|f__Bacteria_unclassified|g__Bacte

k__Bacteria|p__Bacteroidota|c__Bacteroidia|o__Bacteroidales|f__Bacteroidaceae|g__Bacteroides|s__Bacteroides_thetaio
k__Bacteria|p__Bacteroidota|c__Bacteroidia|o__Bacteroidales|f__Bacteroidaceae|g__Bacteroides|s__Bacteroides_thetaio

k__Bacteria|p__Firmicutes|c__Clostridia|o__Clostridia_unclassified|f__Clostridia_unclassified|g__Clostridia_unclassified|s_
k__Bacteria|p__Firmicutes|c__Clostridia|o__Clostridia_unclassified|f__Clostridia_unclassified|g__Clostridia_unclassified|s_
k__Bacteria|p__Firmicutes|c__Clostridia|o__Eubacteriales|f__Clostridiaceae|g__Clostridiaceae_unclassified|s__Clostridiac
k__Bacteria|p__Firmicutes|c__Clostridia|o__Eubacteriales|f__Clostridiaceae|g__Clostridiaceae_unclassified|s__Clostridiac
k__Bacteria|p__Firmicutes|c__Clostridia|o__Eubacteriales|f__Clostridiaceae|g__Clostridiaceae_unclassified|s__Clostridiac
k__Bacteria|p__Firmicutes|c__Clostridia|o__Eubacteriales|f__Clostridiaceae|g__Clostridiaceae_unclassified|s__Clostridiac
k__Bacteria|p__Firmicutes|c__Clostridia|o__Eubacteriales|f__Eubacteriales_unclassified|g__Eubacteriales_unclassified|s_
k__Bacteria|p__Firmicutes|c__Clostridia|o__Eubacteriales|f__Eubacteriales_unclassified|g__Eubacteriales_unclassified|s_
k__Bacteria|p__Firmicutes|c__Erysipelotrichia|o__Erysipelotrichales|f__Erysipelotrichaceae|g__Erysipelatoclostridium|s__
k__Bacteria|p__Firmicutes|c__Erysipelotrichia|o__Erysipelotrichales|f__Erysipelotrichaceae|g__Erysipelatoclostridium|s__
k__Bacteria|p__Firmicutes|c__Clostridia|o__Eubacteriales|f__Clostridiaceae|g__Clostridium|s__Clostridium_SGB65123
k__Bacteria|p__Firmicutes|c__Clostridia|o__Eubacteriales|f__Clostridiaceae|g__Clostridium|s__Clostridium_SGB65123
k__Bacteria|p__Firmicutes|c__Clostridia|o__Eubacteriales|f__Eubacteriaceae|g__Eubacteriaceae_unclassified|s__Eubacte
k__Bacteria|p__Firmicutes|c__Clostridia|o__Eubacteriales|f__Eubacteriaceae|g__Eubacteriaceae_unclassified|s__Eubacte
k__Bacteria|p__Firmicutes|c__Clostridia|o__Eubacteriales|f__Eubacteriaceae|g__Eubacteriaceae_unclassified|s__Eubacte
k__Bacteria|p__Firmicutes|c__Clostridia|o__Eubacteriales|f__Eubacteriaceae|g__Eubacteriaceae_unclassified|s__Eubacte
k__Bacteria|p__Firmicutes|c__CFGB77306|o__OFGB77306|f__FGB77306|g__GGB20146|s__GGB20146_SGB29427
k__Bacteria|p__Firmicutes|c__CFGB77306|o__OFGB77306|f__FGB77306|g__GGB20146|s__GGB20146_SGB29427
k__Bacteria|p__Firmicutes|c__Clostridia|o__Eubacteriales|f__Lachnospiraceae|g__GGB20149|s__GGB20149_SGB29430
k__Bacteria|p__Firmicutes|c__Clostridia|o__Eubacteriales|f__Lachnospiraceae|g__GGB20149|s__GGB20149_SGB29430
k__Bacteria|p__Actinobacteria|c__Coriobacteriia|o__Eggerthellales|f__Eggerthellaceae|g__GGB22635|s__GGB22635_SGB
k__Bacteria|p__Actinobacteria|c__Coriobacteriia|o__Eggerthellales|f__Eggerthellaceae|g__GGB22635|s__GGB22635_SGB
k__Bacteria|p__Firmicutes|c__Clostridia|o__Eubacteriales|f__Lachnospiraceae|g__GGB25041|s__GGB25041_SGB36960
k__Bacteria|p__Firmicutes|c__Clostridia|o__Eubacteriales|f__Lachnospiraceae|g__GGB25041|s__GGB25041_SGB36960
k__Bacteria|p__Firmicutes|c__CFGB9506|o__OFGB9506|f__FGB9506|g__GGB28379|s__GGB28379_SGB40959
k__Bacteria|p__Firmicutes|c__CFGB9506|o__OFGB9506|f__FGB9506|g__GGB28379|s__GGB28379_SGB40959
k__Bacteria|p__Firmicutes|c__CFGB9508|o__OFGB9508|f__FGB9508|g__GGB28382|s__GGB28382_SGB40962
k__Bacteria|p__Firmicutes|c__CFGB9508|o__OFGB9508|f__FGB9508|g__GGB28382|s__GGB28382_SGB40962
k__Bacteria|p__Firmicutes|c__CFGB9512|o__OFGB9512|f__FGB9512|g__GGB28392|s__GGB28392_SGB40972
k__Bacteria|p__Firmicutes|c__CFGB9512|o__OFGB9512|f__FGB9512|g__GGB28392|s__GGB28392_SGB40972
k__Bacteria|p__Firmicutes|c__CFGB2838|o__OFGB2838|f__FGB2838|g__GGB28404|s__GGB28404_SGB40986
k__Bacteria|p__Firmicutes|c__CFGB2838|o__OFGB2838|f__FGB2838|g__GGB28404|s__GGB28404_SGB40986
k__Bacteria|p__Firmicutes|c__CFGB2838|o__OFGB2838|f__FGB2838|g__GGB28418|s__GGB28418_SGB41001
k__Bacteria|p__Firmicutes|c__CFGB2838|o__OFGB2838|f__FGB2838|g__GGB28418|s__GGB28418_SGB41001
k__Bacteria|p__Firmicutes|c__CFGB2838|o__OFGB2838|f__FGB2838|g__GGB28422|s__GGB28422_SGB41005
k__Bacteria|p__Firmicutes|c__CFGB2838|o__OFGB2838|f__FGB2838|g__GGB28422|s__GGB28422_SGB41005
k__Bacteria|p__Firmicutes|c__Clostridia|o__Eubacteriales|f__Pumilibacteraceae|g__GGB28431|s__GGB28431_SGB41014
k__Bacteria|p__Firmicutes|c__Clostridia|o__Eubacteriales|f__Pumilibacteraceae|g__GGB28431|s__GGB28431_SGB41014
k__Bacteria|p__Firmicutes|c__CFGB2833|o__OFGB2833|f__FGB2833|g__GGB28456|s__GGB28456_SGB41039
k__Bacteria|p__Firmicutes|c__CFGB2833|o__OFGB2833|f__FGB2833|g__GGB28456|s__GGB28456_SGB41039
k__Bacteria|p__Firmicutes|c__Clostridia|o__Eubacteriales|f__Eubacteriaceae|g__GGB28782|s__GGB28782_SGB41435
k__Bacteria|p__Firmicutes|c__Clostridia|o__Eubacteriales|f__Eubacteriaceae|g__GGB28782|s__GGB28782_SGB41435

k__Bacteria|p__Firmicutes|c__Clostridia|o__Eubacteriales|f__Lachnospiraceae|g__GGB28792|s__GGB28792_SGB41445
k__Bacteria|p__Firmicutes|c__Clostridia|o__Eubacteriales|f__Lachnospiraceae|g__GGB28792|s__GGB28792_SGB41445
k__Bacteria|p__Firmicutes|c__Clostridia|o__Eubacteriales|f__Lachnospiraceae|g__GGB28798|s__GGB28798_SGB41451
k__Bacteria|p__Firmicutes|c__Clostridia|o__Eubacteriales|f__Lachnospiraceae|g__GGB28798|s__GGB28798_SGB41451
k__Bacteria|p__Firmicutes|c__CFGB9622|o__OFGB9622|f__FGB9622|g__GGB28810|s__GGB28810_SGB41465
k__Bacteria|p__Firmicutes|c__CFGB9622|o__OFGB9622|f__FGB9622|g__GGB28810|s__GGB28810_SGB41465
k__Bacteria|p__Firmicutes|c__CFGB77305|o__OFGB77305|f__FGB77305|g__GGB28828|s__GGB28828_SGB41484
k__Bacteria|p__Firmicutes|c__CFGB77305|o__OFGB77305|f__FGB77305|g__GGB28828|s__GGB28828_SGB41484
k__Bacteria|p__Firmicutes|c__Clostridia|o__Eubacteriales|f__Clostridiaceae|g__GGB28851|s__GGB28851_SGB41518
k__Bacteria|p__Firmicutes|c__Clostridia|o__Eubacteriales|f__Clostridiaceae|g__GGB28851|s__GGB28851_SGB41518
k__Bacteria|p__Firmicutes|c__Clostridia|o__Eubacteriales|f__Lachnospiraceae|g__GGB28865|s__GGB28865_SGB41537
k__Bacteria|p__Firmicutes|c__Clostridia|o__Eubacteriales|f__Lachnospiraceae|g__GGB28865|s__GGB28865_SGB41537
k__Bacteria|p__Firmicutes|c__Clostridia|o__Eubacteriales|f__Lachnospiraceae|g__GGB28868|s__GGB28868_SGB41542
k__Bacteria|p__Firmicutes|c__Clostridia|o__Eubacteriales|f__Lachnospiraceae|g__GGB28868|s__GGB28868_SGB41542
k__Bacteria|p__Firmicutes|c__Clostridia|o__Eubacteriales|f__Lachnospiraceae|g__GGB28869|s__GGB28869_SGB41543
k__Bacteria|p__Firmicutes|c__Clostridia|o__Eubacteriales|f__Lachnospiraceae|g__GGB28869|s__GGB28869_SGB41543
k__Bacteria|p__Firmicutes|c__Clostridia|o__Eubacteriales|f__Lachnospiraceae|g__GGB28875|s__GGB28875_SGB41555
k__Bacteria|p__Firmicutes|c__Clostridia|o__Eubacteriales|f__Lachnospiraceae|g__GGB28875|s__GGB28875_SGB41555
k__Bacteria|p__Firmicutes|c__CFGB9633|o__OFGB9633|f__FGB9633|g__GGB28881|s__GGB28881_SGB41561
k__Bacteria|p__Firmicutes|c__CFGB9633|o__OFGB9633|f__FGB9633|g__GGB28881|s__GGB28881_SGB41561
k__Bacteria|p__Bacteria_unclassified|c__Bacteria_unclassified|o__Bacteria_unclassified|f__Bacteria_unclassified|g__GGB[illegible]
k__Bacteria|p__Bacteria_unclassified|c__Bacteria_unclassified|o__Bacteria_unclassified|f__Bacteria_unclassified|g__GGB[illegible]
k__Bacteria|p__Bacteria_unclassified|c__Bacteria_unclassified|o__Bacteria_unclassified|f__Bacteria_unclassified|g__GGB[illegible]
k__Bacteria|p__Bacteria_unclassified|c__Bacteria_unclassified|o__Bacteria_unclassified|f__Bacteria_unclassified|g__GGB[illegible]
k__Bacteria|p__Bacteria_unclassified|c__Bacteria_unclassified|o__Bacteria_unclassified|f__Bacteria_unclassified|g__GGB[illegible]
k__Bacteria|p__Bacteria_unclassified|c__Bacteria_unclassified|o__Bacteria_unclassified|f__Bacteria_unclassified|g__GGB[illegible]
k__Bacteria|p__Bacteria_unclassified|c__Bacteria_unclassified|o__Bacteria_unclassified|f__Bacteria_unclassified|g__GGB[illegible]
k__Bacteria|p__Bacteria_unclassified|c__Bacteria_unclassified|o__Bacteria_unclassified|f__Bacteria_unclassified|g__GGB[illegible]
k__Bacteria|p__Bacteria_unclassified|c__Bacteria_unclassified|o__Bacteria_unclassified|f__Bacteria_unclassified|g__GGB[illegible]
k__Bacteria|p__Bacteria_unclassified|c__Bacteria_unclassified|o__Bacteria_unclassified|f__Bacteria_unclassified|g__GGB[illegible]
k__Bacteria|p__Bacteria_unclassified|c__Bacteria_unclassified|o__Bacteria_unclassified|f__Bacteria_unclassified|g__GGB[illegible]
k__Bacteria|p__Bacteria_unclassified|c__Bacteria_unclassified|o__Bacteria_unclassified|f__Bacteria_unclassified|g__GGB[illegible]
k__Bacteria|p__Firmicutes|c__Clostridia|o__Eubacteriales|f__Lachnospiraceae|g__GGB28924|s__GGB28924_SGB41621
k__Bacteria|p__Firmicutes|c__Clostridia|o__Eubacteriales|f__Lachnospiraceae|g__GGB28924|s__GGB28924_SGB41621
k__Bacteria|p__Bacteria_unclassified|c__CFGB77359|o__OFGB77359|f__FGB77359|g__GGB28927|s__GGB28927_SGB416[illegible]
k__Bacteria|p__Bacteria_unclassified|c__CFGB77359|o__OFGB77359|f__FGB77359|g__GGB28927|s__GGB28927_SGB416[illegible]
k__Bacteria|p__Firmicutes|c__CFGB9639|o__OFGB9639|f__FGB9639|g__GGB28934|s__GGB28934_SGB41635
k__Bacteria|p__Firmicutes|c__CFGB9639|o__OFGB9639|f__FGB9639|g__GGB28934|s__GGB28934_SGB41635
k__Bacteria|p__Firmicutes|c__Clostridia|o__Eubacteriales|f__Lachnospiraceae|g__GGB28949|s__GGB28949_SGB41655
k__Bacteria|p__Firmicutes|c__Clostridia|o__Eubacteriales|f__Lachnospiraceae|g__GGB28949|s__GGB28949_SGB41655
k__Bacteria|p__Firmicutes|c__Clostridia|o__Eubacteriales|f__Clostridiaceae|g__GGB28951|s__GGB28951_SGB102295
k__Bacteria|p__Firmicutes|c__Clostridia|o__Eubacteriales|f__Clostridiaceae|g__GGB28951|s__GGB28951_SGB102295
k__Bacteria|p__Firmicutes|c__Clostridia|o__Eubacteriales|f__Clostridiaceae|g__GGB28951|s__GGB28951_SGB41658
k__Bacteria|p__Firmicutes|c__Clostridia|o__Eubacteriales|f__Clostridiaceae|g__GGB28951|s__GGB28951_SGB41658
k__Bacteria|p__Firmicutes|c__Clostridia|o__Eubacteriales|f__Clostridiaceae|g__GGB28954|s__GGB28954_SGB41662
k__Bacteria|p__Firmicutes|c__Clostridia|o__Eubacteriales|f__Clostridiaceae|g__GGB28954|s__GGB28954_SGB41662

k__Bacteria|p__Firmicutes|c__Clostridia|o__Eubacteriales|f__Clostridiaceae|g__GGB28960|s__GGB28960_SGB41669
k__Bacteria|p__Firmicutes|c__Clostridia|o__Eubacteriales|f__Clostridiaceae|g__GGB28960|s__GGB28960_SGB41669
k__Bacteria|p__Firmicutes|c__Clostridia|o__Eubacteriales|f__Clostridiaceae|g__GGB28964|s__GGB28964_SGB94886
k__Bacteria|p__Firmicutes|c__Clostridia|o__Eubacteriales|f__Clostridiaceae|g__GGB28964|s__GGB28964_SGB94886
k__Bacteria|p__Firmicutes|c__Clostridia|o__Eubacteriales|f__Clostridiaceae|g__GGB28967|s__GGB28967_SGB41678
k__Bacteria|p__Firmicutes|c__Clostridia|o__Eubacteriales|f__Clostridiaceae|g__GGB28967|s__GGB28967_SGB41678
k__Bacteria|p__Firmicutes|c__CFGB9656|o__OFGB9656|f__FGB9656|g__GGB28996|s__GGB28996_SGB41712
k__Bacteria|p__Firmicutes|c__CFGB9656|o__OFGB9656|f__FGB9656|g__GGB28996|s__GGB28996_SGB41712
k__Bacteria|p__Firmicutes|c__CFGB9827|o__OFGB9827|f__FGB9827|g__GGB29531|s__GGB29531_SGB42317
k__Bacteria|p__Firmicutes|c__CFGB9827|o__OFGB9827|f__FGB9827|g__GGB29531|s__GGB29531_SGB42317
k__Bacteria|p__Firmicutes|c__Clostridia|o__Eubacteriales|f__Eubacteriaceae|g__GGB29685|s__GGB29685_SGB42494
k__Bacteria|p__Firmicutes|c__Clostridia|o__Eubacteriales|f__Eubacteriaceae|g__GGB29685|s__GGB29685_SGB42494
k__Bacteria|p__Bacteria_unclassified|c__CFGB77303|o__OFGB77303|f__FGB77303|g__GGB30141|s__GGB30141_SGB430
k__Bacteria|p__Bacteria_unclassified|c__CFGB77303|o__OFGB77303|f__FGB77303|g__GGB30141|s__GGB30141_SGB430
k__Bacteria|p__Firmicutes|c__Clostridia|o__Eubacteriales|f__Clostridiaceae|g__GGB30145|s__GGB30145_SGB43072
k__Bacteria|p__Firmicutes|c__Clostridia|o__Eubacteriales|f__Clostridiaceae|g__GGB30145|s__GGB30145_SGB43072
k__Bacteria|p__Firmicutes|c__Clostridia|o__Eubacteriales|f__Eubacteriales_unclassified|g__GGB30286|s__GGB30286_SG
k__Bacteria|p__Firmicutes|c__Clostridia|o__Eubacteriales|f__Eubacteriales_unclassified|g__GGB30286|s__GGB30286_SG
k__Bacteria|p__Firmicutes|c__CFGB72709|o__OFGB72709|f__FGB72709|g__GGB30300|s__GGB30300_SGB43264
k__Bacteria|p__Firmicutes|c__CFGB72709|o__OFGB72709|f__FGB72709|g__GGB30300|s__GGB30300_SGB43264
k__Bacteria|p__Firmicutes|c__Clostridia|o__Eubacteriales|f__Oscillospiraceae|g__GGB30303|s__GGB30303_SGB43268
k__Bacteria|p__Firmicutes|c__Clostridia|o__Eubacteriales|f__Oscillospiraceae|g__GGB30303|s__GGB30303_SGB43268
k__Bacteria|p__Firmicutes|c__Clostridia|o__Eubacteriales|f__Oscillospiraceae|g__GGB30450|s__GGB30450_SGB43507
k__Bacteria|p__Firmicutes|c__Clostridia|o__Eubacteriales|f__Oscillospiraceae|g__GGB30450|s__GGB30450_SGB43507
k__Bacteria|p__Firmicutes|c__Clostridia|o__Eubacteriales|f__Oscillospiraceae|g__GGB30453|s__GGB30453_SGB43513
k__Bacteria|p__Firmicutes|c__Clostridia|o__Eubacteriales|f__Oscillospiraceae|g__GGB30453|s__GGB30453_SGB43513
k__Bacteria|p__Firmicutes|c__Clostridia|o__Eubacteriales|f__Oscillospiraceae|g__GGB30454|s__GGB30454_SGB43514
k__Bacteria|p__Firmicutes|c__Clostridia|o__Eubacteriales|f__Oscillospiraceae|g__GGB30454|s__GGB30454_SGB43514
k__Bacteria|p__Firmicutes|c__Clostridia|o__Eubacteriales|f__Oscillospiraceae|g__GGB30455|s__GGB30455_SGB43519
k__Bacteria|p__Firmicutes|c__Clostridia|o__Eubacteriales|f__Oscillospiraceae|g__GGB30455|s__GGB30455_SGB43519
k__Bacteria|p__Firmicutes|c__Clostridia|o__Eubacteriales|f__Oscillospiraceae|g__GGB30456|s__GGB30456_SGB43520
k__Bacteria|p__Firmicutes|c__Clostridia|o__Eubacteriales|f__Oscillospiraceae|g__GGB30456|s__GGB30456_SGB43520
k__Bacteria|p__Firmicutes|c__Clostridia|o__Eubacteriales|f__Oscillospiraceae|g__GGB30457|s__GGB30457_SGB63218
k__Bacteria|p__Firmicutes|c__Clostridia|o__Eubacteriales|f__Oscillospiraceae|g__GGB30457|s__GGB30457_SGB63218
k__Bacteria|p__Firmicutes|c__Clostridia|o__Eubacteriales|f__Oscillospiraceae|g__GGB30461|s__GGB30461_SGB43530
k__Bacteria|p__Firmicutes|c__Clostridia|o__Eubacteriales|f__Oscillospiraceae|g__GGB30461|s__GGB30461_SGB43530
k__Bacteria|p__Firmicutes|c__Clostridia|o__Eubacteriales|f__Oscillospiraceae|g__GGB30461|s__GGB30461_SGB43533
k__Bacteria|p__Firmicutes|c__Clostridia|o__Eubacteriales|f__Oscillospiraceae|g__GGB30461|s__GGB30461_SGB43533
k__Bacteria|p__Firmicutes|c__Clostridia|o__Eubacteriales|f__Oscillospiraceae|g__GGB30461|s__GGB30461_SGB63209
k__Bacteria|p__Firmicutes|c__Clostridia|o__Eubacteriales|f__Oscillospiraceae|g__GGB30461|s__GGB30461_SGB63209
k__Bacteria|p__Firmicutes|c__Clostridia|o__Eubacteriales|f__Oscillospiraceae|g__GGB30463|s__GGB30463_SGB43537
k__Bacteria|p__Firmicutes|c__Clostridia|o__Eubacteriales|f__Oscillospiraceae|g__GGB30463|s__GGB30463_SGB43537
k__Bacteria|p__Firmicutes|c__Clostridia|o__Eubacteriales|f__Oscillospiraceae|g__GGB30473|s__GGB30473_SGB43557
k__Bacteria|p__Firmicutes|c__Clostridia|o__Eubacteriales|f__Oscillospiraceae|g__GGB30473|s__GGB30473_SGB43557
k__Bacteria|p__Firmicutes|c__Clostridia|o__Eubacteriales|f__Oscillospiraceae|g__GGB30475|s__GGB30475_SGB63182
k__Bacteria|p__Firmicutes|c__Clostridia|o__Eubacteriales|f__Oscillospiraceae|g__GGB30475|s__GGB30475_SGB63182

k__Bacteria|p__Actinobacteria|c__CFGB77153|o__OFGB77153|f__FGB77153|g__GGB30861|s__GGB30861_SGB44083
k__Bacteria|p__Actinobacteria|c__CFGB77153|o__OFGB77153|f__FGB77153|g__GGB30861|s__GGB30861_SGB44083
k__Bacteria|p__Tenericutes|c__CFGB1791|o__OFGB1791|f__FGB1791|g__GGB31312|s__GGB31312_SGB44628
k__Bacteria|p__Tenericutes|c__CFGB1791|o__OFGB1791|f__FGB1791|g__GGB31312|s__GGB31312_SGB44628
k__Bacteria|p__Firmicutes|c__CFGB10290|o__OFGB10290|f__FGB10290|g__GGB31438|s__GGB31438_SGB44768
k__Bacteria|p__Firmicutes|c__CFGB10290|o__OFGB10290|f__FGB10290|g__GGB31438|s__GGB31438_SGB44768
k__Bacteria|p__Firmicutes|c__Clostridia|o__Eubacteriales|f__Oscillospiraceae|g__GGB3171|s__GGB3171_SGB4185
k__Bacteria|p__Firmicutes|c__Clostridia|o__Eubacteriales|f__Oscillospiraceae|g__GGB3171|s__GGB3171_SGB4185
k__Bacteria|p__Firmicutes|c__CFGB10289|o__OFGB10289|f__FGB10289|g__GGB31762|s__GGB31762_SGB45125
k__Bacteria|p__Firmicutes|c__CFGB10289|o__OFGB10289|f__FGB10289|g__GGB31762|s__GGB31762_SGB45125
k__Bacteria|p__Firmicutes|c__CFGB1765|o__OFGB1765|f__FGB1765|g__GGB31823|s__GGB31823_SGB45199
k__Bacteria|p__Firmicutes|c__CFGB1765|o__OFGB1765|f__FGB1765|g__GGB31823|s__GGB31823_SGB45199
k__Bacteria|p__Firmicutes|c__CFGB1765|o__OFGB1765|f__FGB1765|g__GGB31838|s__GGB31838_SGB45216
k__Bacteria|p__Firmicutes|c__CFGB1765|o__OFGB1765|f__FGB1765|g__GGB31838|s__GGB31838_SGB45216
k__Bacteria|p__Firmicutes|c__CFGB1765|o__OFGB1765|f__FGB1765|g__GGB31841|s__GGB31841_SGB65084
k__Bacteria|p__Firmicutes|c__CFGB1765|o__OFGB1765|f__FGB1765|g__GGB31841|s__GGB31841_SGB65084
k__Bacteria|p__Firmicutes|c__CFGB10667|o__OFGB10667|f__FGB10667|g__GGB32371|s__GGB32371_SGB41694
k__Bacteria|p__Firmicutes|c__CFGB10667|o__OFGB10667|f__FGB10667|g__GGB32371|s__GGB32371_SGB41694
k__Bacteria|p__Firmicutes|c__Clostridia|o__Eubacteriales|f__Lachnospiraceae|g__GGB3793|s__GGB3793_SGB5158
k__Bacteria|p__Firmicutes|c__Clostridia|o__Eubacteriales|f__Lachnospiraceae|g__GGB3793|s__GGB3793_SGB5158
k__Bacteria|p__Firmicutes|c__Clostridia|o__Eubacteriales|f__Clostridiaceae|g__GGB42601|s__GGB42601_SGB59797
k__Bacteria|p__Firmicutes|c__Clostridia|o__Eubacteriales|f__Clostridiaceae|g__GGB42601|s__GGB42601_SGB59797
k__Bacteria|p__Firmicutes|c__Clostridia|o__Eubacteriales|f__Oscillospiraceae|g__GGB45514|s__GGB45514_SGB63186
k__Bacteria|p__Firmicutes|c__Clostridia|o__Eubacteriales|f__Oscillospiraceae|g__GGB45514|s__GGB45514_SGB63186
k__Bacteria|p__Firmicutes|c__CFGB75721|o__OFGB75721|f__FGB75721|g__GGB45564|s__GGB45564_SGB63259
k__Bacteria|p__Firmicutes|c__CFGB75721|o__OFGB75721|f__FGB75721|g__GGB45564|s__GGB45564_SGB63259
k__Bacteria|p__Firmicutes|c__Clostridia|o__Eubacteriales|f__Oscillospiraceae|g__GGB45624|s__GGB45624_SGB63337
k__Bacteria|p__Firmicutes|c__Clostridia|o__Eubacteriales|f__Oscillospiraceae|g__GGB45624|s__GGB45624_SGB63337
k__Bacteria|p__Firmicutes|c__Clostridia|o__Eubacteriales|f__Christensenellaceae|g__GGB45656|s__GGB45656_SGB6337
k__Bacteria|p__Firmicutes|c__Clostridia|o__Eubacteriales|f__Christensenellaceae|g__GGB45656|s__GGB45656_SGB6337
k__Bacteria|p__Firmicutes|c__CFGB10299|o__OFGB10299|f__FGB10299|g__GGB47127|s__GGB47127_SGB65054
k__Bacteria|p__Firmicutes|c__CFGB10299|o__OFGB10299|f__FGB10299|g__GGB47127|s__GGB47127_SGB65054
k__Bacteria|p__Firmicutes|c__Clostridia|o__Eubacteriales|f__Oscillospiraceae|g__GGB74395|s__GGB74395_SGB43523
k__Bacteria|p__Firmicutes|c__Clostridia|o__Eubacteriales|f__Oscillospiraceae|g__GGB74395|s__GGB74395_SGB43523
k__Bacteria|p__Firmicutes|c__Clostridia|o__Eubacteriales|f__Oscillospiraceae|g__GGB75053|s__GGB75053_SGB43494
k__Bacteria|p__Firmicutes|c__Clostridia|o__Eubacteriales|f__Oscillospiraceae|g__GGB75053|s__GGB75053_SGB43494
k__Bacteria|p__Firmicutes|c__Clostridia|o__Eubacteriales|f__Lachnospiraceae|g__GGB75109|s__GGB75109_SGB102238
k__Bacteria|p__Firmicutes|c__Clostridia|o__Eubacteriales|f__Lachnospiraceae|g__GGB75109|s__GGB75109_SGB102238
k__Bacteria|p__Firmicutes|c__Clostridia|o__Eubacteriales|f__Lachnospiraceae|g__Lachnospiraceae_unclassified|s__Lachn
k__Bacteria|p__Firmicutes|c__Clostridia|o__Eubacteriales|f__Lachnospiraceae|g__Lachnospiraceae_unclassified|s__Lachn
k__Bacteria|p__Firmicutes|c__Clostridia|o__Eubacteriales|f__Lachnospiraceae|g__Lachnospiraceae_unclassified|s__Lachn
k__Bacteria|p__Firmicutes|c__Clostridia|o__Eubacteriales|f__Lachnospiraceae|g__Lachnospiraceae_unclassified|s__Lachn
k__Bacteria|p__Firmicutes|c__Clostridia|o__Eubacteriales|f__Lachnospiraceae|g__Lachnospiraceae_unclassified|s__Lachn
k__Bacteria|p__Firmicutes|c__Clostridia|o__Eubacteriales|f__Lachnospiraceae|g__Lachnospiraceae_unclassified|s__Lachn
k__Bacteria|p__Firmicutes|c__Clostridia|o__Eubacteriales|f__Lachnospiraceae|g__Lachnospiraceae_unclassified|s__Lachn
k__Bacteria|p__Firmicutes|c__Clostridia|o__Eubacteriales|f__Lachnospiraceae|g__Lachnospiraceae_unclassified|s__Lachn

k__Bacteria|p__Firmicutes|c__Clostridia|o__Eubacteriales|f__Lachnospiraceae|g__Lachnospiraceae_unclassified|s__Lachn
k__Bacteria|p__Firmicutes|c__Clostridia|o__Eubacteriales|f__Lachnospiraceae|g__Lachnospiraceae_unclassified|s__Lachn
k__Bacteria|p__Firmicutes|c__Bacilli|o__Lactobacillales|f__Lactobacillaceae|g__Lactobacillus|s__Lactobacillus_johnsonii
k__Bacteria|p__Firmicutes|c__Bacilli|o__Lactobacillales|f__Lactobacillaceae|g__Lactobacillus|s__Lactobacillus_johnsonii
k__Bacteria|p__Actinobacteria|c__Coriobacteriia|o__Coriobacteriales|f__Atopobiaceae|g__Leptogranulimonas|s__Leptogr
k__Bacteria|p__Actinobacteria|c__Coriobacteriia|o__Coriobacteriales|f__Atopobiaceae|g__Leptogranulimonas|s__Leptogr
k__Bacteria|p__Bacteroidota|c__Bacteroidia|o__Bacteroidales|f__Muribaculaceae|g__Muribaculaceae_unclassified|s__M
k__Bacteria|p__Bacteroidota|c__Bacteroidia|o__Bacteroidales|f__Muribaculaceae|g__Muribaculaceae_unclassified|s__M
k__Bacteria|p__Firmicutes|c__Clostridia|o__Eubacteriales|f__Oscillospiraceae|g__Neglectibacter|s__Neglectibacter_sp_X4
k__Bacteria|p__Firmicutes|c__Clostridia|o__Eubacteriales|f__Oscillospiraceae|g__Neglectibacter|s__Neglectibacter_sp_X4
k__Bacteria|p__Firmicutes|c__Clostridia|o__Eubacteriales|f__Oscillospiraceae|g__Oscillibacter|s__Oscillibacter_SGB43496
k__Bacteria|p__Firmicutes|c__Clostridia|o__Eubacteriales|f__Oscillospiraceae|g__Oscillibacter|s__Oscillibacter_SGB43496
k__Bacteria|p__Firmicutes|c__Clostridia|o__Eubacteriales|f__Oscillospiraceae|g__Oscillospiraceae_unclassified|s__Oscillo
k__Bacteria|p__Firmicutes|c__Clostridia|o__Eubacteriales|f__Oscillospiraceae|g__Oscillospiraceae_unclassified|s__Oscillo
k__Bacteria|p__Firmicutes|c__Clostridia|o__Eubacteriales|f__Oscillospiraceae|g__Oscillospiraceae_unclassified|s__Oscillo
k__Bacteria|p__Firmicutes|c__Clostridia|o__Eubacteriales|f__Oscillospiraceae|g__Oscillospiraceae_unclassified|s__Oscillo
k__Bacteria|p__Firmicutes|c__Clostridia|o__Eubacteriales|f__Oscillospiraceae|g__Oscillospiraceae_unclassified|s__Oscillo
k__Bacteria|p__Firmicutes|c__Clostridia|o__Eubacteriales|f__Oscillospiraceae|g__Oscillospiraceae_unclassified|s__Oscillo
k__Bacteria|p__Firmicutes|c__Clostridia|o__Eubacteriales|f__Oscillospiraceae|g__Oscillospiraceae_unclassified|s__Oscillo
k__Bacteria|p__Firmicutes|c__Clostridia|o__Eubacteriales|f__Oscillospiraceae|g__Oscillospiraceae_unclassified|s__Oscillo
k__Bacteria|p__Proteobacteria|c__Betaproteobacteria|o__Burkholderiales|f__Sutterellaceae|g__Parasutterella|s__Parasu
k__Bacteria|p__Proteobacteria|c__Betaproteobacteria|o__Burkholderiales|f__Sutterellaceae|g__Parasutterella|s__Parasu

k__Bacteria|p__Firmicutes|c__Clostridia|o__Eubacteriales|f__Lachnospiraceae|g__Schaedlerella|s__Schaedlerella_arabino
k__Bacteria|p__Firmicutes|c__Clostridia|o__Eubacteriales|f__Lachnospiraceae|g__Schaedlerella|s__Schaedlerella_arabino

k__Bacteria|p__Firmicutes|c__Erysipelotrichia|o__Erysipelotrichales|f__Turicibacteraceae|g__Turicibacter|s__Turicibacter_
k__Bacteria|p__Firmicutes|c__Erysipelotrichia|o__Erysipelotrichales|f__Turicibacteraceae|g__Turicibacter|s__Turicibacter_
k__Bacteria|p__Firmicutes|c__Clostridia|o__Eubacteriales|f__Lachnospiraceae|g__Acetatifactor|s__Acetatifactor_SGB415
k__Bacteria|p__Firmicutes|c__Clostridia|o__Eubacteriales|f__Lachnospiraceae|g__Acetatifactor|s__Acetatifactor_SGB415
k__Bacteria|p__Firmicutes|c__Clostridia|o__Eubacteriales|f__Oscillospiraceae|g__Acutalibacter|s__Acutalibacter_muris
k__Bacteria|p__Firmicutes|c__Clostridia|o__Eubacteriales|f__Oscillospiraceae|g__Acutalibacter|s__Acutalibacter_muris
k__Bacteria|p__Verrucomicrobia|c__Verrucomicrobiae|o__Verrucomicrobiales|f__Akkermansiaceae|g__Akkermansia|s__A
k__Bacteria|p__Verrucomicrobia|c__Verrucomicrobiae|o__Verrucomicrobiales|f__Akkermansiaceae|g__Akkermansia|s__A
k__Bacteria|p__Firmicutes|c__Clostridia|o__Eubacteriales|f__Oscillospiraceae|g__Anaerotruncus|s__Anaerotruncus_sp_1
k__Bacteria|p__Firmicutes|c__Clostridia|o__Eubacteriales|f__Oscillospiraceae|g__Anaerotruncus|s__Anaerotruncus_sp_1
k__Bacteria|p__Bacteria_unclassified|c__Bacteria_unclassified|o__Bacteria_unclassified|f__Bacteria_unclassified|g__Bacte
k__Bacteria|p__Bacteria_unclassified|c__Bacteria_unclassified|o__Bacteria_unclassified|f__Bacteria_unclassified|g__Bacte
k__Bacteria|p__Bacteria_unclassified|c__Bacteria_unclassified|o__Bacteria_unclassified|f__Bacteria_unclassified|g__Bacte
k__Bacteria|p__Bacteria_unclassified|c__Bacteria_unclassified|o__Bacteria_unclassified|f__Bacteria_unclassified|g__Bacte
k__Bacteria|p__Bacteria_unclassified|c__Bacteria_unclassified|o__Bacteria_unclassified|f__Bacteria_unclassified|g__Bacte
k__Bacteria|p__Bacteria_unclassified|c__Bacteria_unclassified|o__Bacteria_unclassified|f__Bacteria_unclassified|g__Bacte
k__Bacteria|p__Bacteria_unclassified|c__Bacteria_unclassified|o__Bacteria_unclassified|f__Bacteria_unclassified|g__Bacte
k__Bacteria|p__Bacteria_unclassified|c__Bacteria_unclassified|o__Bacteria_unclassified|f__Bacteria_unclassified|g__Bacte

k__Bacteria|p__Bacteria_unclassified|c__Bacteria_unclassified|o__Bacteria_unclassified|f__Bacteria_unclassified|g__Bacte
k__Bacteria|p__Bacteria_unclassified|c__Bacteria_unclassified|o__Bacteria_unclassified|f__Bacteria_unclassified|g__Bacte
k__Bacteria|p__Bacteria_unclassified|c__Bacteria_unclassified|o__Bacteria_unclassified|f__Bacteria_unclassified|g__Bacte
k__Bacteria|p__Bacteria_unclassified|c__Bacteria_unclassified|o__Bacteria_unclassified|f__Bacteria_unclassified|g__Bacte
k__Bacteria|p__Bacteria_unclassified|c__Bacteria_unclassified|o__Bacteria_unclassified|f__Bacteria_unclassified|g__Bacte
k__Bacteria|p__Bacteria_unclassified|c__Bacteria_unclassified|o__Bacteria_unclassified|f__Bacteria_unclassified|g__Bacte
k__Bacteria|p__Bacteria_unclassified|c__Bacteria_unclassified|o__Bacteria_unclassified|f__Bacteria_unclassified|g__Bacte
k__Bacteria|p__Bacteria_unclassified|c__Bacteria_unclassified|o__Bacteria_unclassified|f__Bacteria_unclassified|g__Bacte
k__Bacteria|p__Bacteria_unclassified|c__Bacteria_unclassified|o__Bacteria_unclassified|f__Bacteria_unclassified|g__Bacte
k__Bacteria|p__Bacteria_unclassified|c__Bacteria_unclassified|o__Bacteria_unclassified|f__Bacteria_unclassified|g__Bacte
k__Bacteria|p__Bacteroidota|c__Bacteroidia|o__Bacteroidales|f__Bacteroidaceae|g__Bacteroides|s__Bacteroides_thetaio
k__Bacteria|p__Bacteroidota|c__Bacteroidia|o__Bacteroidales|f__Bacteroidaceae|g__Bacteroides|s__Bacteroides_thetaio

k__Bacteria|p__Firmicutes|c__Clostridia|o__Clostridia_unclassified|f__Clostridia_unclassified|g__Clostridia_unclassified|s_
k__Bacteria|p__Firmicutes|c__Clostridia|o__Clostridia_unclassified|f__Clostridia_unclassified|g__Clostridia_unclassified|s_
k__Bacteria|p__Firmicutes|c__Clostridia|o__Eubacteriales|f__Clostridiaceae|g__Clostridiaceae_unclassified|s__Clostridiac
k__Bacteria|p__Firmicutes|c__Clostridia|o__Eubacteriales|f__Clostridiaceae|g__Clostridiaceae_unclassified|s__Clostridiac
k__Bacteria|p__Firmicutes|c__Clostridia|o__Eubacteriales|f__Clostridiaceae|g__Clostridiaceae_unclassified|s__Clostridiac
k__Bacteria|p__Firmicutes|c__Clostridia|o__Eubacteriales|f__Clostridiaceae|g__Clostridiaceae_unclassified|s__Clostridiac
k__Bacteria|p__Firmicutes|c__Clostridia|o__Eubacteriales|f__Eubacteriales_unclassified|g__Eubacteriales_unclassified|s_
k__Bacteria|p__Firmicutes|c__Clostridia|o__Eubacteriales|f__Eubacteriales_unclassified|g__Eubacteriales_unclassified|s_
k__Bacteria|p__Firmicutes|c__Erysipelotrichia|o__Erysipelotrichales|f__Erysipelotrichaceae|g__Erysipelatoclostridium|s__
k__Bacteria|p__Firmicutes|c__Erysipelotrichia|o__Erysipelotrichales|f__Erysipelotrichaceae|g__Erysipelatoclostridium|s__
k__Bacteria|p__Firmicutes|c__Clostridia|o__Eubacteriales|f__Clostridiaceae|g__Clostridium|s__Clostridium_SGB65123
k__Bacteria|p__Firmicutes|c__Clostridia|o__Eubacteriales|f__Clostridiaceae|g__Clostridium|s__Clostridium_SGB65123
k__Bacteria|p__Firmicutes|c__Clostridia|o__Eubacteriales|f__Eubacteriaceae|g__Eubacteriaceae_unclassified|s__Eubacte
k__Bacteria|p__Firmicutes|c__Clostridia|o__Eubacteriales|f__Eubacteriaceae|g__Eubacteriaceae_unclassified|s__Eubacte
k__Bacteria|p__Firmicutes|c__Clostridia|o__Eubacteriales|f__Eubacteriaceae|g__Eubacteriaceae_unclassified|s__Eubacte
k__Bacteria|p__Firmicutes|c__Clostridia|o__Eubacteriales|f__Eubacteriaceae|g__Eubacteriaceae_unclassified|s__Eubacte
k__Bacteria|p__Firmicutes|c__CFGB77306|o__OFGB77306|f__FGB77306|g__GGB20146|s__GGB20146_SGB29427
k__Bacteria|p__Firmicutes|c__CFGB77306|o__OFGB77306|f__FGB77306|g__GGB20146|s__GGB20146_SGB29427
k__Bacteria|p__Firmicutes|c__Clostridia|o__Eubacteriales|f__Lachnospiraceae|g__GGB20149|s__GGB20149_SGB29430
k__Bacteria|p__Firmicutes|c__Clostridia|o__Eubacteriales|f__Lachnospiraceae|g__GGB20149|s__GGB20149_SGB29430
k__Bacteria|p__Actinobacteria|c__Coriobacteriia|o__Eggerthellales|f__Eggerthellaceae|g__GGB22635|s__GGB22635_SGB
k__Bacteria|p__Actinobacteria|c__Coriobacteriia|o__Eggerthellales|f__Eggerthellaceae|g__GGB22635|s__GGB22635_SGB
k__Bacteria|p__Firmicutes|c__Clostridia|o__Eubacteriales|f__Lachnospiraceae|g__GGB25041|s__GGB25041_SGB36960
k__Bacteria|p__Firmicutes|c__Clostridia|o__Eubacteriales|f__Lachnospiraceae|g__GGB25041|s__GGB25041_SGB36960
k__Bacteria|p__Firmicutes|c__CFGB9506|o__OFGB9506|f__FGB9506|g__GGB28379|s__GGB28379_SGB40959
k__Bacteria|p__Firmicutes|c__CFGB9506|o__OFGB9506|f__FGB9506|g__GGB28379|s__GGB28379_SGB40959
k__Bacteria|p__Firmicutes|c__CFGB9508|o__OFGB9508|f__FGB9508|g__GGB28382|s__GGB28382_SGB40962
k__Bacteria|p__Firmicutes|c__CFGB9508|o__OFGB9508|f__FGB9508|g__GGB28382|s__GGB28382_SGB40962
k__Bacteria|p__Firmicutes|c__CFGB9512|o__OFGB9512|f__FGB9512|g__GGB28392|s__GGB28392_SGB40972
k__Bacteria|p__Firmicutes|c__CFGB9512|o__OFGB9512|f__FGB9512|g__GGB28392|s__GGB28392_SGB40972
k__Bacteria|p__Firmicutes|c__CFGB2838|o__OFGB2838|f__FGB2838|g__GGB28404|s__GGB28404_SGB40986
k__Bacteria|p__Firmicutes|c__CFGB2838|o__OFGB2838|f__FGB2838|g__GGB28404|s__GGB28404_SGB40986

k__Bacteria|p__Firmicutes|c__CFGB2838|o__OFGB2838|f__FGB2838|g__GGB28418|s__GGB28418_SGB41001
k__Bacteria|p__Firmicutes|c__CFGB2838|o__OFGB2838|f__FGB2838|g__GGB28418|s__GGB28418_SGB41001
k__Bacteria|p__Firmicutes|c__CFGB2838|o__OFGB2838|f__FGB2838|g__GGB28422|s__GGB28422_SGB41005
k__Bacteria|p__Firmicutes|c__CFGB2838|o__OFGB2838|f__FGB2838|g__GGB28422|s__GGB28422_SGB41005
k__Bacteria|p__Firmicutes|c__Clostridia|o__Eubacteriales|f__Pumilibacteraceae|g__GGB28431|s__GGB28431_SGB41014
k__Bacteria|p__Firmicutes|c__Clostridia|o__Eubacteriales|f__Pumilibacteraceae|g__GGB28431|s__GGB28431_SGB41014
k__Bacteria|p__Firmicutes|c__CFGB2833|o__OFGB2833|f__FGB2833|g__GGB28456|s__GGB28456_SGB41039
k__Bacteria|p__Firmicutes|c__CFGB2833|o__OFGB2833|f__FGB2833|g__GGB28456|s__GGB28456_SGB41039
k__Bacteria|p__Firmicutes|c__Clostridia|o__Eubacteriales|f__Eubacteriaceae|g__GGB28782|s__GGB28782_SGB41435
k__Bacteria|p__Firmicutes|c__Clostridia|o__Eubacteriales|f__Eubacteriaceae|g__GGB28782|s__GGB28782_SGB41435
k__Bacteria|p__Firmicutes|c__Clostridia|o__Eubacteriales|f__Lachnospiraceae|g__GGB28792|s__GGB28792_SGB41445
k__Bacteria|p__Firmicutes|c__Clostridia|o__Eubacteriales|f__Lachnospiraceae|g__GGB28792|s__GGB28792_SGB41445
k__Bacteria|p__Firmicutes|c__Clostridia|o__Eubacteriales|f__Lachnospiraceae|g__GGB28798|s__GGB28798_SGB41451
k__Bacteria|p__Firmicutes|c__Clostridia|o__Eubacteriales|f__Lachnospiraceae|g__GGB28798|s__GGB28798_SGB41451
k__Bacteria|p__Firmicutes|c__CFGB9622|o__OFGB9622|f__FGB9622|g__GGB28810|s__GGB28810_SGB41465
k__Bacteria|p__Firmicutes|c__CFGB9622|o__OFGB9622|f__FGB9622|g__GGB28810|s__GGB28810_SGB41465
k__Bacteria|p__Firmicutes|c__CFGB77305|o__OFGB77305|f__FGB77305|g__GGB28828|s__GGB28828_SGB41484
k__Bacteria|p__Firmicutes|c__CFGB77305|o__OFGB77305|f__FGB77305|g__GGB28828|s__GGB28828_SGB41484
k__Bacteria|p__Firmicutes|c__Clostridia|o__Eubacteriales|f__Clostridiaceae|g__GGB28851|s__GGB28851_SGB41518
k__Bacteria|p__Firmicutes|c__Clostridia|o__Eubacteriales|f__Clostridiaceae|g__GGB28851|s__GGB28851_SGB41518
k__Bacteria|p__Firmicutes|c__Clostridia|o__Eubacteriales|f__Lachnospiraceae|g__GGB28865|s__GGB28865_SGB41537
k__Bacteria|p__Firmicutes|c__Clostridia|o__Eubacteriales|f__Lachnospiraceae|g__GGB28865|s__GGB28865_SGB41537
k__Bacteria|p__Firmicutes|c__Clostridia|o__Eubacteriales|f__Lachnospiraceae|g__GGB28868|s__GGB28868_SGB41542
k__Bacteria|p__Firmicutes|c__Clostridia|o__Eubacteriales|f__Lachnospiraceae|g__GGB28868|s__GGB28868_SGB41542
k__Bacteria|p__Firmicutes|c__Clostridia|o__Eubacteriales|f__Lachnospiraceae|g__GGB28869|s__GGB28869_SGB41543
k__Bacteria|p__Firmicutes|c__Clostridia|o__Eubacteriales|f__Lachnospiraceae|g__GGB28869|s__GGB28869_SGB41543
k__Bacteria|p__Firmicutes|c__Clostridia|o__Eubacteriales|f__Lachnospiraceae|g__GGB28875|s__GGB28875_SGB41555
k__Bacteria|p__Firmicutes|c__Clostridia|o__Eubacteriales|f__Lachnospiraceae|g__GGB28875|s__GGB28875_SGB41555
k__Bacteria|p__Firmicutes|c__CFGB9633|o__OFGB9633|f__FGB9633|g__GGB28881|s__GGB28881_SGB41561
k__Bacteria|p__Firmicutes|c__CFGB9633|o__OFGB9633|f__FGB9633|g__GGB28881|s__GGB28881_SGB41561
k__Bacteria|p__Bacteria_unclassified|c__Bacteria_unclassified|o__Bacteria_unclassified|f__Bacteria_unclassified|g__GGB2
k__Bacteria|p__Bacteria_unclassified|c__Bacteria_unclassified|o__Bacteria_unclassified|f__Bacteria_unclassified|g__GGB2
k__Bacteria|p__Bacteria_unclassified|c__Bacteria_unclassified|o__Bacteria_unclassified|f__Bacteria_unclassified|g__GGB2
k__Bacteria|p__Bacteria_unclassified|c__Bacteria_unclassified|o__Bacteria_unclassified|f__Bacteria_unclassified|g__GGB2
k__Bacteria|p__Bacteria_unclassified|c__Bacteria_unclassified|o__Bacteria_unclassified|f__Bacteria_unclassified|g__GGB2
k__Bacteria|p__Bacteria_unclassified|c__Bacteria_unclassified|o__Bacteria_unclassified|f__Bacteria_unclassified|g__GGB2
k__Bacteria|p__Bacteria_unclassified|c__Bacteria_unclassified|o__Bacteria_unclassified|f__Bacteria_unclassified|g__GGB2
k__Bacteria|p__Bacteria_unclassified|c__Bacteria_unclassified|o__Bacteria_unclassified|f__Bacteria_unclassified|g__GGB2
k__Bacteria|p__Bacteria_unclassified|c__Bacteria_unclassified|o__Bacteria_unclassified|f__Bacteria_unclassified|g__GGB2
k__Bacteria|p__Bacteria_unclassified|c__Bacteria_unclassified|o__Bacteria_unclassified|f__Bacteria_unclassified|g__GGB2
k__Bacteria|p__Bacteria_unclassified|c__Bacteria_unclassified|o__Bacteria_unclassified|f__Bacteria_unclassified|g__GGB2
k__Bacteria|p__Bacteria_unclassified|c__Bacteria_unclassified|o__Bacteria_unclassified|f__Bacteria_unclassified|g__GGB2
k__Bacteria|p__Firmicutes|c__Clostridia|o__Eubacteriales|f__Lachnospiraceae|g__GGB28924|s__GGB28924_SGB41621
k__Bacteria|p__Firmicutes|c__Clostridia|o__Eubacteriales|f__Lachnospiraceae|g__GGB28924|s__GGB28924_SGB41621
k__Bacteria|p__Bacteria_unclassified|c__CFGB77359|o__OFGB77359|f__FGB77359|g__GGB28927|s__GGB28927_SGB416
k__Bacteria|p__Bacteria_unclassified|c__CFGB77359|o__OFGB77359|f__FGB77359|g__GGB28927|s__GGB28927_SGB416

k__Bacteria|p__Firmicutes|c__CFGB9639|o__OFGB9639|f__FGB9639|g__GGB28934|s__GGB28934_SGB41635
k__Bacteria|p__Firmicutes|c__CFGB9639|o__OFGB9639|f__FGB9639|g__GGB28934|s__GGB28934_SGB41635
k__Bacteria|p__Firmicutes|c__Clostridia|o__Eubacteriales|f__Lachnospiraceae|g__GGB28949|s__GGB28949_SGB41655
k__Bacteria|p__Firmicutes|c__Clostridia|o__Eubacteriales|f__Lachnospiraceae|g__GGB28949|s__GGB28949_SGB41655
k__Bacteria|p__Firmicutes|c__Clostridia|o__Eubacteriales|f__Clostridiaceae|g__GGB28951|s__GGB28951_SGB102295
k__Bacteria|p__Firmicutes|c__Clostridia|o__Eubacteriales|f__Clostridiaceae|g__GGB28951|s__GGB28951_SGB102295
k__Bacteria|p__Firmicutes|c__Clostridia|o__Eubacteriales|f__Clostridiaceae|g__GGB28951|s__GGB28951_SGB41658
k__Bacteria|p__Firmicutes|c__Clostridia|o__Eubacteriales|f__Clostridiaceae|g__GGB28951|s__GGB28951_SGB41658
k__Bacteria|p__Firmicutes|c__Clostridia|o__Eubacteriales|f__Clostridiaceae|g__GGB28954|s__GGB28954_SGB41662
k__Bacteria|p__Firmicutes|c__Clostridia|o__Eubacteriales|f__Clostridiaceae|g__GGB28954|s__GGB28954_SGB41662
k__Bacteria|p__Firmicutes|c__Clostridia|o__Eubacteriales|f__Clostridiaceae|g__GGB28960|s__GGB28960_SGB41669
k__Bacteria|p__Firmicutes|c__Clostridia|o__Eubacteriales|f__Clostridiaceae|g__GGB28960|s__GGB28960_SGB41669
k__Bacteria|p__Firmicutes|c__Clostridia|o__Eubacteriales|f__Clostridiaceae|g__GGB28964|s__GGB28964_SGB94886
k__Bacteria|p__Firmicutes|c__Clostridia|o__Eubacteriales|f__Clostridiaceae|g__GGB28964|s__GGB28964_SGB94886
k__Bacteria|p__Firmicutes|c__Clostridia|o__Eubacteriales|f__Clostridiaceae|g__GGB28967|s__GGB28967_SGB41678
k__Bacteria|p__Firmicutes|c__Clostridia|o__Eubacteriales|f__Clostridiaceae|g__GGB28967|s__GGB28967_SGB41678
k__Bacteria|p__Firmicutes|c__CFGB9656|o__OFGB9656|f__FGB9656|g__GGB28996|s__GGB28996_SGB41712
k__Bacteria|p__Firmicutes|c__CFGB9656|o__OFGB9656|f__FGB9656|g__GGB28996|s__GGB28996_SGB41712
k__Bacteria|p__Firmicutes|c__CFGB9827|o__OFGB9827|f__FGB9827|g__GGB29531|s__GGB29531_SGB42317
k__Bacteria|p__Firmicutes|c__CFGB9827|o__OFGB9827|f__FGB9827|g__GGB29531|s__GGB29531_SGB42317
k__Bacteria|p__Firmicutes|c__Clostridia|o__Eubacteriales|f__Eubacteriaceae|g__GGB29685|s__GGB29685_SGB42494
k__Bacteria|p__Firmicutes|c__Clostridia|o__Eubacteriales|f__Eubacteriaceae|g__GGB29685|s__GGB29685_SGB42494
k__Bacteria|p__Bacteria_unclassified|c__CFGB77303|o__OFGB77303|f__FGB77303|g__GGB30141|s__GGB30141_SGB430
k__Bacteria|p__Bacteria_unclassified|c__CFGB77303|o__OFGB77303|f__FGB77303|g__GGB30141|s__GGB30141_SGB430
k__Bacteria|p__Firmicutes|c__Clostridia|o__Eubacteriales|f__Clostridiaceae|g__GGB30145|s__GGB30145_SGB43072
k__Bacteria|p__Firmicutes|c__Clostridia|o__Eubacteriales|f__Clostridiaceae|g__GGB30145|s__GGB30145_SGB43072
k__Bacteria|p__Firmicutes|c__Clostridia|o__Eubacteriales|f__Eubacteriales_unclassified|g__GGB30286|s__GGB30286_SG
k__Bacteria|p__Firmicutes|c__Clostridia|o__Eubacteriales|f__Eubacteriales_unclassified|g__GGB30286|s__GGB30286_SG
k__Bacteria|p__Firmicutes|c__CFGB72709|o__OFGB72709|f__FGB72709|g__GGB30300|s__GGB30300_SGB43264
k__Bacteria|p__Firmicutes|c__CFGB72709|o__OFGB72709|f__FGB72709|g__GGB30300|s__GGB30300_SGB43264
k__Bacteria|p__Firmicutes|c__Clostridia|o__Eubacteriales|f__Oscillospiraceae|g__GGB30303|s__GGB30303_SGB43268
k__Bacteria|p__Firmicutes|c__Clostridia|o__Eubacteriales|f__Oscillospiraceae|g__GGB30303|s__GGB30303_SGB43268
k__Bacteria|p__Firmicutes|c__Clostridia|o__Eubacteriales|f__Oscillospiraceae|g__GGB30450|s__GGB30450_SGB43507
k__Bacteria|p__Firmicutes|c__Clostridia|o__Eubacteriales|f__Oscillospiraceae|g__GGB30450|s__GGB30450_SGB43507
k__Bacteria|p__Firmicutes|c__Clostridia|o__Eubacteriales|f__Oscillospiraceae|g__GGB30453|s__GGB30453_SGB43513
k__Bacteria|p__Firmicutes|c__Clostridia|o__Eubacteriales|f__Oscillospiraceae|g__GGB30453|s__GGB30453_SGB43513
k__Bacteria|p__Firmicutes|c__Clostridia|o__Eubacteriales|f__Oscillospiraceae|g__GGB30454|s__GGB30454_SGB43514
k__Bacteria|p__Firmicutes|c__Clostridia|o__Eubacteriales|f__Oscillospiraceae|g__GGB30454|s__GGB30454_SGB43514
k__Bacteria|p__Firmicutes|c__Clostridia|o__Eubacteriales|f__Oscillospiraceae|g__GGB30455|s__GGB30455_SGB43519
k__Bacteria|p__Firmicutes|c__Clostridia|o__Eubacteriales|f__Oscillospiraceae|g__GGB30455|s__GGB30455_SGB43519
k__Bacteria|p__Firmicutes|c__Clostridia|o__Eubacteriales|f__Oscillospiraceae|g__GGB30456|s__GGB30456_SGB43520
k__Bacteria|p__Firmicutes|c__Clostridia|o__Eubacteriales|f__Oscillospiraceae|g__GGB30456|s__GGB30456_SGB43520
k__Bacteria|p__Firmicutes|c__Clostridia|o__Eubacteriales|f__Oscillospiraceae|g__GGB30457|s__GGB30457_SGB63218
k__Bacteria|p__Firmicutes|c__Clostridia|o__Eubacteriales|f__Oscillospiraceae|g__GGB30457|s__GGB30457_SGB63218
k__Bacteria|p__Firmicutes|c__Clostridia|o__Eubacteriales|f__Oscillospiraceae|g__GGB30461|s__GGB30461_SGB43530
k__Bacteria|p__Firmicutes|c__Clostridia|o__Eubacteriales|f__Oscillospiraceae|g__GGB30461|s__GGB30461_SGB43530

k__Bacteria|p__Firmicutes|c__Clostridia|o__Eubacteriales|f__Oscillospiraceae|g__GGB30461|s__GGB30461_SGB43533

k__Bacteria|p__Firmicutes|c__Clostridia|o__Eubacteriales|f__Oscillospiraceae|g__GGB30461|s__GGB30461_SGB43533

k__Bacteria|p__Firmicutes|c__Clostridia|o__Eubacteriales|f__Oscillospiraceae|g__GGB30461|s__GGB30461_SGB63209

k__Bacteria|p__Firmicutes|c__Clostridia|o__Eubacteriales|f__Oscillospiraceae|g__GGB30461|s__GGB30461_SGB63209

k__Bacteria|p__Firmicutes|c__Clostridia|o__Eubacteriales|f__Oscillospiraceae|g__GGB30463|s__GGB30463_SGB43537

k__Bacteria|p__Firmicutes|c__Clostridia|o__Eubacteriales|f__Oscillospiraceae|g__GGB30463|s__GGB30463_SGB43537

k__Bacteria|p__Firmicutes|c__Clostridia|o__Eubacteriales|f__Oscillospiraceae|g__GGB30473|s__GGB30473_SGB43557

k__Bacteria|p__Firmicutes|c__Clostridia|o__Eubacteriales|f__Oscillospiraceae|g__GGB30473|s__GGB30473_SGB43557

k__Bacteria|p__Firmicutes|c__Clostridia|o__Eubacteriales|f__Oscillospiraceae|g__GGB30475|s__GGB30475_SGB63182

k__Bacteria|p__Firmicutes|c__Clostridia|o__Eubacteriales|f__Oscillospiraceae|g__GGB30475|s__GGB30475_SGB63182

k__Bacteria|p__Actinobacteria|c__CFGB77153|o__OFGB77153|f__FGB77153|g__GGB30861|s__GGB30861_SGB44083

k__Bacteria|p__Actinobacteria|c__CFGB77153|o__OFGB77153|f__FGB77153|g__GGB30861|s__GGB30861_SGB44083

k__Bacteria|p__Tenericutes|c__CFGB1791|o__OFGB1791|f__FGB1791|g__GGB31312|s__GGB31312_SGB44628

k__Bacteria|p__Tenericutes|c__CFGB1791|o__OFGB1791|f__FGB1791|g__GGB31312|s__GGB31312_SGB44628

k__Bacteria|p__Firmicutes|c__CFGB10290|o__OFGB10290|f__FGB10290|g__GGB31438|s__GGB31438_SGB44768

k__Bacteria|p__Firmicutes|c__CFGB10290|o__OFGB10290|f__FGB10290|g__GGB31438|s__GGB31438_SGB44768

k__Bacteria|p__Firmicutes|c__Clostridia|o__Eubacteriales|f__Oscillospiraceae|g__GGB3171|s__GGB3171_SGB4185

k__Bacteria|p__Firmicutes|c__Clostridia|o__Eubacteriales|f__Oscillospiraceae|g__GGB3171|s__GGB3171_SGB4185

k__Bacteria|p__Firmicutes|c__CFGB10289|o__OFGB10289|f__FGB10289|g__GGB31762|s__GGB31762_SGB45125

k__Bacteria|p__Firmicutes|c__CFGB10289|o__OFGB10289|f__FGB10289|g__GGB31762|s__GGB31762_SGB45125

k__Bacteria|p__Firmicutes|c__CFGB1765|o__OFGB1765|f__FGB1765|g__GGB31823|s__GGB31823_SGB45199

k__Bacteria|p__Firmicutes|c__CFGB1765|o__OFGB1765|f__FGB1765|g__GGB31823|s__GGB31823_SGB45199

k__Bacteria|p__Firmicutes|c__CFGB1765|o__OFGB1765|f__FGB1765|g__GGB31838|s__GGB31838_SGB45216

k__Bacteria|p__Firmicutes|c__CFGB1765|o__OFGB1765|f__FGB1765|g__GGB31838|s__GGB31838_SGB45216

k__Bacteria|p__Firmicutes|c__CFGB1765|o__OFGB1765|f__FGB1765|g__GGB31841|s__GGB31841_SGB65084

k__Bacteria|p__Firmicutes|c__CFGB1765|o__OFGB1765|f__FGB1765|g__GGB31841|s__GGB31841_SGB65084

k__Bacteria|p__Firmicutes|c__CFGB10667|o__OFGB10667|f__FGB10667|g__GGB32371|s__GGB32371_SGB41694

k__Bacteria|p__Firmicutes|c__CFGB10667|o__OFGB10667|f__FGB10667|g__GGB32371|s__GGB32371_SGB41694

k__Bacteria|p__Firmicutes|c__Clostridia|o__Eubacteriales|f__Lachnospiraceae|g__GGB3793|s__GGB3793_SGB5158

k__Bacteria|p__Firmicutes|c__Clostridia|o__Eubacteriales|f__Lachnospiraceae|g__GGB3793|s__GGB3793_SGB5158

k__Bacteria|p__Firmicutes|c__Clostridia|o__Eubacteriales|f__Clostridiaceae|g__GGB42601|s__GGB42601_SGB59797

k__Bacteria|p__Firmicutes|c__Clostridia|o__Eubacteriales|f__Clostridiaceae|g__GGB42601|s__GGB42601_SGB59797

k__Bacteria|p__Firmicutes|c__Clostridia|o__Eubacteriales|f__Oscillospiraceae|g__GGB45514|s__GGB45514_SGB63186

k__Bacteria|p__Firmicutes|c__Clostridia|o__Eubacteriales|f__Oscillospiraceae|g__GGB45514|s__GGB45514_SGB63186

k__Bacteria|p__Firmicutes|c__CFGB75721|o__OFGB75721|f__FGB75721|g__GGB45564|s__GGB45564_SGB63259

k__Bacteria|p__Firmicutes|c__CFGB75721|o__OFGB75721|f__FGB75721|g__GGB45564|s__GGB45564_SGB63259

k__Bacteria|p__Firmicutes|c__Clostridia|o__Eubacteriales|f__Oscillospiraceae|g__GGB45624|s__GGB45624_SGB63337

k__Bacteria|p__Firmicutes|c__Clostridia|o__Eubacteriales|f__Oscillospiraceae|g__GGB45624|s__GGB45624_SGB63337

k__Bacteria|p__Firmicutes|c__Clostridia|o__Eubacteriales|f__Christensenellaceae|g__GGB45656|s__GGB45656_SGB6337

k__Bacteria|p__Firmicutes|c__Clostridia|o__Eubacteriales|f__Christensenellaceae|g__GGB45656|s__GGB45656_SGB6337

k__Bacteria|p__Firmicutes|c__CFGB10299|o__OFGB10299|f__FGB10299|g__GGB47127|s__GGB47127_SGB65054

k__Bacteria|p__Firmicutes|c__CFGB10299|o__OFGB10299|f__FGB10299|g__GGB47127|s__GGB47127_SGB65054

k__Bacteria|p__Firmicutes|c__Clostridia|o__Eubacteriales|f__Oscillospiraceae|g__GGB74395|s__GGB74395_SGB43523

k__Bacteria|p__Firmicutes|c__Clostridia|o__Eubacteriales|f__Oscillospiraceae|g__GGB74395|s__GGB74395_SGB43523

k__Bacteria|p__Firmicutes|c__Clostridia|o__Eubacteriales|f__Oscillospiraceae|g__GGB75053|s__GGB75053_SGB43494

k__Bacteria|p__Firmicutes|c__Clostridia|o__Eubacteriales|f__Oscillospiraceae|g__GGB75053|s__GGB75053_SGB43494

k__Bacteria|p__Firmicutes|c__Clostridia|o__Eubacteriales|f__Lachnospiraceae|g__GGB75109|s__GGB75109_SGB102238
k__Bacteria|p__Firmicutes|c__Clostridia|o__Eubacteriales|f__Lachnospiraceae|g__GGB75109|s__GGB75109_SGB102238
k__Bacteria|p__Firmicutes|c__Clostridia|o__Eubacteriales|f__Lachnospiraceae|g__Lachnospiraceae_unclassified|s__Lachn
k__Bacteria|p__Firmicutes|c__Clostridia|o__Eubacteriales|f__Lachnospiraceae|g__Lachnospiraceae_unclassified|s__Lachn
k__Bacteria|p__Firmicutes|c__Clostridia|o__Eubacteriales|f__Lachnospiraceae|g__Lachnospiraceae_unclassified|s__Lachn
k__Bacteria|p__Firmicutes|c__Clostridia|o__Eubacteriales|f__Lachnospiraceae|g__Lachnospiraceae_unclassified|s__Lachn
k__Bacteria|p__Firmicutes|c__Clostridia|o__Eubacteriales|f__Lachnospiraceae|g__Lachnospiraceae_unclassified|s__Lachn
k__Bacteria|p__Firmicutes|c__Clostridia|o__Eubacteriales|f__Lachnospiraceae|g__Lachnospiraceae_unclassified|s__Lachn
k__Bacteria|p__Firmicutes|c__Clostridia|o__Eubacteriales|f__Lachnospiraceae|g__Lachnospiraceae_unclassified|s__Lachn
k__Bacteria|p__Firmicutes|c__Clostridia|o__Eubacteriales|f__Lachnospiraceae|g__Lachnospiraceae_unclassified|s__Lachn
k__Bacteria|p__Firmicutes|c__Clostridia|o__Eubacteriales|f__Lachnospiraceae|g__Lachnospiraceae_unclassified|s__Lachn
k__Bacteria|p__Firmicutes|c__Clostridia|o__Eubacteriales|f__Lachnospiraceae|g__Lachnospiraceae_unclassified|s__Lachn
k__Bacteria|p__Firmicutes|c__Bacilli|o__Lactobacillales|f__Lactobacillaceae|g__Lactobacillus|s__Lactobacillus_johnsonii
k__Bacteria|p__Firmicutes|c__Bacilli|o__Lactobacillales|f__Lactobacillaceae|g__Lactobacillus|s__Lactobacillus_johnsonii
k__Bacteria|p__Actinobacteria|c__Coriobacteriia|o__Coriobacteriales|f__Atopobiaceae|g__Leptogranulimonas|s__Leptog
k__Bacteria|p__Actinobacteria|c__Coriobacteriia|o__Coriobacteriales|f__Atopobiaceae|g__Leptogranulimonas|s__Leptog
k__Bacteria|p__Bacteroidota|c__Bacteroidia|o__Bacteroidales|f__Muribaculaceae|g__Muribaculaceae_unclassified|s__M
k__Bacteria|p__Bacteroidota|c__Bacteroidia|o__Bacteroidales|f__Muribaculaceae|g__Muribaculaceae_unclassified|s__M
k__Bacteria|p__Firmicutes|c__Clostridia|o__Eubacteriales|f__Oscillospiraceae|g__Neglectibacter|s__Neglectibacter_sp_X
k__Bacteria|p__Firmicutes|c__Clostridia|o__Eubacteriales|f__Oscillospiraceae|g__Neglectibacter|s__Neglectibacter_sp_X
k__Bacteria|p__Firmicutes|c__Clostridia|o__Eubacteriales|f__Oscillospiraceae|g__Oscillibacter|s__Oscillibacter_SGB43496
k__Bacteria|p__Firmicutes|c__Clostridia|o__Eubacteriales|f__Oscillospiraceae|g__Oscillibacter|s__Oscillibacter_SGB43496
k__Bacteria|p__Firmicutes|c__Clostridia|o__Eubacteriales|f__Oscillospiraceae|g__Oscillospiraceae_unclassified|s__Oscillo
k__Bacteria|p__Firmicutes|c__Clostridia|o__Eubacteriales|f__Oscillospiraceae|g__Oscillospiraceae_unclassified|s__Oscillo
k__Bacteria|p__Firmicutes|c__Clostridia|o__Eubacteriales|f__Oscillospiraceae|g__Oscillospiraceae_unclassified|s__Oscillo
k__Bacteria|p__Firmicutes|c__Clostridia|o__Eubacteriales|f__Oscillospiraceae|g__Oscillospiraceae_unclassified|s__Oscillo
k__Bacteria|p__Firmicutes|c__Clostridia|o__Eubacteriales|f__Oscillospiraceae|g__Oscillospiraceae_unclassified|s__Oscillo
k__Bacteria|p__Firmicutes|c__Clostridia|o__Eubacteriales|f__Oscillospiraceae|g__Oscillospiraceae_unclassified|s__Oscillo
k__Bacteria|p__Firmicutes|c__Clostridia|o__Eubacteriales|f__Oscillospiraceae|g__Oscillospiraceae_unclassified|s__Oscillo
k__Bacteria|p__Firmicutes|c__Clostridia|o__Eubacteriales|f__Oscillospiraceae|g__Oscillospiraceae_unclassified|s__Oscillo
k__Bacteria|p__Proteobacteria|c__Betaproteobacteria|o__Burkholderiales|f__Sutterellaceae|g__Parasutterella|s__Parasu
k__Bacteria|p__Proteobacteria|c__Betaproteobacteria|o__Burkholderiales|f__Sutterellaceae|g__Parasutterella|s__Parasu

k__Bacteria|p__Firmicutes|c__Clostridia|o__Eubacteriales|f__Lachnospiraceae|g__Schaedlerella|s__Schaedlerella_arabino
k__Bacteria|p__Firmicutes|c__Clostridia|o__Eubacteriales|f__Lachnospiraceae|g__Schaedlerella|s__Schaedlerella_arabino

k__Bacteria|p__Firmicutes|c__Erysipelotrichia|o__Erysipelotrichales|f__Turicibacteraceae|g__Turicibacter|s__Turicibacter_
k__Bacteria|p__Firmicutes|c__Erysipelotrichia|o__Erysipelotrichales|f__Turicibacteraceae|g__Turicibacter|s__Turicibacter_
k__Bacteria|p__Firmicutes|c__Clostridia|o__Eubacteriales|f__Lachnospiraceae|g__Acetatifactor|s__Acetatifactor_SGB415
k__Bacteria|p__Firmicutes|c__Clostridia|o__Eubacteriales|f__Lachnospiraceae|g__Acetatifactor|s__Acetatifactor_SGB415
k__Bacteria|p__Firmicutes|c__Clostridia|o__Eubacteriales|f__Oscillospiraceae|g__Acutalibacter|s__Acutalibacter_muris
k__Bacteria|p__Firmicutes|c__Clostridia|o__Eubacteriales|f__Oscillospiraceae|g__Acutalibacter|s__Acutalibacter_muris
k__Bacteria|p__Verrucomicrobia|c__Verrucomicrobiae|o__Verrucomicrobiales|f__Akkermansiaceae|g__Akkermansia|s__A
k__Bacteria|p__Verrucomicrobia|c__Verrucomicrobiae|o__Verrucomicrobiales|f__Akkermansiaceae|g__Akkermansia|s__A

k__Bacteria|p__Firmicutes|c__Clostridia|o__Eubacteriales|f__Oscillospiraceae|g__Anaerotruncus|s__Anaerotruncus_sp_1
k__Bacteria|p__Firmicutes|c__Clostridia|o__Eubacteriales|f__Oscillospiraceae|g__Anaerotruncus|s__Anaerotruncus_sp_1
k__Bacteria|p__Bacteria_unclassified|c__Bacteria_unclassified|o__Bacteria_unclassified|f__Bacteria_unclassified|g__Bacte
k__Bacteria|p__Bacteria_unclassified|c__Bacteria_unclassified|o__Bacteria_unclassified|f__Bacteria_unclassified|g__Bacte
k__Bacteria|p__Bacteria_unclassified|c__Bacteria_unclassified|o__Bacteria_unclassified|f__Bacteria_unclassified|g__Bacte
k__Bacteria|p__Bacteria_unclassified|c__Bacteria_unclassified|o__Bacteria_unclassified|f__Bacteria_unclassified|g__Bacte
k__Bacteria|p__Bacteria_unclassified|c__Bacteria_unclassified|o__Bacteria_unclassified|f__Bacteria_unclassified|g__Bacte
k__Bacteria|p__Bacteria_unclassified|c__Bacteria_unclassified|o__Bacteria_unclassified|f__Bacteria_unclassified|g__Bacte
k__Bacteria|p__Bacteria_unclassified|c__Bacteria_unclassified|o__Bacteria_unclassified|f__Bacteria_unclassified|g__Bacte
k__Bacteria|p__Bacteria_unclassified|c__Bacteria_unclassified|o__Bacteria_unclassified|f__Bacteria_unclassified|g__Bacte
k__Bacteria|p__Bacteria_unclassified|c__Bacteria_unclassified|o__Bacteria_unclassified|f__Bacteria_unclassified|g__Bacte
k__Bacteria|p__Bacteria_unclassified|c__Bacteria_unclassified|o__Bacteria_unclassified|f__Bacteria_unclassified|g__Bacte
k__Bacteria|p__Bacteria_unclassified|c__Bacteria_unclassified|o__Bacteria_unclassified|f__Bacteria_unclassified|g__Bacte
k__Bacteria|p__Bacteria_unclassified|c__Bacteria_unclassified|o__Bacteria_unclassified|f__Bacteria_unclassified|g__Bacte
k__Bacteria|p__Bacteria_unclassified|c__Bacteria_unclassified|o__Bacteria_unclassified|f__Bacteria_unclassified|g__Bacte
k__Bacteria|p__Bacteria_unclassified|c__Bacteria_unclassified|o__Bacteria_unclassified|f__Bacteria_unclassified|g__Bacte
k__Bacteria|p__Bacteria_unclassified|c__Bacteria_unclassified|o__Bacteria_unclassified|f__Bacteria_unclassified|g__Bacte
k__Bacteria|p__Bacteria_unclassified|c__Bacteria_unclassified|o__Bacteria_unclassified|f__Bacteria_unclassified|g__Bacte
k__Bacteria|p__Bacteria_unclassified|c__Bacteria_unclassified|o__Bacteria_unclassified|f__Bacteria_unclassified|g__Bacte
k__Bacteria|p__Bacteria_unclassified|c__Bacteria_unclassified|o__Bacteria_unclassified|f__Bacteria_unclassified|g__Bacte
k__Bacteria|p__Bacteroidota|c__Bacteroidia|o__Bacteroidales|f__Bacteroidaceae|g__Bacteroides|s__Bacteroides_thetaio
k__Bacteria|p__Bacteroidota|c__Bacteroidia|o__Bacteroidales|f__Bacteroidaceae|g__Bacteroides|s__Bacteroides_thetaio

k__Bacteria|p__Firmicutes|c__Clostridia|o__Clostridia_unclassified|f__Clostridia_unclassified|g__Clostridia_unclassified|s_
k__Bacteria|p__Firmicutes|c__Clostridia|o__Clostridia_unclassified|f__Clostridia_unclassified|g__Clostridia_unclassified|s_
k__Bacteria|p__Firmicutes|c__Clostridia|o__Eubacteriales|f__Clostridiaceae|g__Clostridiaceae_unclassified|s__Clostridiac
k__Bacteria|p__Firmicutes|c__Clostridia|o__Eubacteriales|f__Clostridiaceae|g__Clostridiaceae_unclassified|s__Clostridiac
k__Bacteria|p__Firmicutes|c__Clostridia|o__Eubacteriales|f__Clostridiaceae|g__Clostridiaceae_unclassified|s__Clostridiac
k__Bacteria|p__Firmicutes|c__Clostridia|o__Eubacteriales|f__Clostridiaceae|g__Clostridiaceae_unclassified|s__Clostridiac
k__Bacteria|p__Firmicutes|c__Clostridia|o__Eubacteriales|f__Eubacteriales_unclassified|g__Eubacteriales_unclassified|s_
k__Bacteria|p__Firmicutes|c__Clostridia|o__Eubacteriales|f__Eubacteriales_unclassified|g__Eubacteriales_unclassified|s_
k__Bacteria|p__Firmicutes|c__Erysipelotrichia|o__Erysipelotrichales|f__Erysipelotrichaceae|g__Erysipelatoclostridium|s__
k__Bacteria|p__Firmicutes|c__Erysipelotrichia|o__Erysipelotrichales|f__Erysipelotrichaceae|g__Erysipelatoclostridium|s__
k__Bacteria|p__Firmicutes|c__Clostridia|o__Eubacteriales|f__Clostridiaceae|g__Clostridium|s__Clostridium_SGB65123
k__Bacteria|p__Firmicutes|c__Clostridia|o__Eubacteriales|f__Clostridiaceae|g__Clostridium|s__Clostridium_SGB65123
k__Bacteria|p__Firmicutes|c__Clostridia|o__Eubacteriales|f__Eubacteriaceae|g__Eubacteriaceae_unclassified|s__Eubacte
k__Bacteria|p__Firmicutes|c__Clostridia|o__Eubacteriales|f__Eubacteriaceae|g__Eubacteriaceae_unclassified|s__Eubacte
k__Bacteria|p__Firmicutes|c__Clostridia|o__Eubacteriales|f__Eubacteriaceae|g__Eubacteriaceae_unclassified|s__Eubacte
k__Bacteria|p__Firmicutes|c__Clostridia|o__Eubacteriales|f__Eubacteriaceae|g__Eubacteriaceae_unclassified|s__Eubacte
k__Bacteria|p__Firmicutes|c__CFGB77306|o__OFGB77306|f__FGB77306|g__GGB20146|s__GGB20146_SGB29427
k__Bacteria|p__Firmicutes|c__CFGB77306|o__OFGB77306|f__FGB77306|g__GGB20146|s__GGB20146_SGB29427
k__Bacteria|p__Firmicutes|c__Clostridia|o__Eubacteriales|f__Lachnospiraceae|g__GGB20149|s__GGB20149_SGB29430
k__Bacteria|p__Firmicutes|c__Clostridia|o__Eubacteriales|f__Lachnospiraceae|g__GGB20149|s__GGB20149_SGB29430
k__Bacteria|p__Actinobacteria|c__Coriobacteriia|o__Eggerthellales|f__Eggerthellaceae|g__GGB22635|s__GGB22635_SGB
k__Bacteria|p__Actinobacteria|c__Coriobacteriia|o__Eggerthellales|f__Eggerthellaceae|g__GGB22635|s__GGB22635_SGB

k__Bacteria|p__Firmicutes|c__Clostridia|o__Eubacteriales|f__Lachnospiraceae|g__GGB25041|s__GGB25041_SGB36960
k__Bacteria|p__Firmicutes|c__Clostridia|o__Eubacteriales|f__Lachnospiraceae|g__GGB25041|s__GGB25041_SGB36960
k__Bacteria|p__Firmicutes|c__CFGB9506|o__OFGB9506|f__FGB9506|g__GGB28379|s__GGB28379_SGB40959
k__Bacteria|p__Firmicutes|c__CFGB9506|o__OFGB9506|f__FGB9506|g__GGB28379|s__GGB28379_SGB40959
k__Bacteria|p__Firmicutes|c__CFGB9508|o__OFGB9508|f__FGB9508|g__GGB28382|s__GGB28382_SGB40962
k__Bacteria|p__Firmicutes|c__CFGB9508|o__OFGB9508|f__FGB9508|g__GGB28382|s__GGB28382_SGB40962
k__Bacteria|p__Firmicutes|c__CFGB9512|o__OFGB9512|f__FGB9512|g__GGB28392|s__GGB28392_SGB40972
k__Bacteria|p__Firmicutes|c__CFGB9512|o__OFGB9512|f__FGB9512|g__GGB28392|s__GGB28392_SGB40972
k__Bacteria|p__Firmicutes|c__CFGB2838|o__OFGB2838|f__FGB2838|g__GGB28404|s__GGB28404_SGB40986
k__Bacteria|p__Firmicutes|c__CFGB2838|o__OFGB2838|f__FGB2838|g__GGB28404|s__GGB28404_SGB40986
k__Bacteria|p__Firmicutes|c__CFGB2838|o__OFGB2838|f__FGB2838|g__GGB28418|s__GGB28418_SGB41001
k__Bacteria|p__Firmicutes|c__CFGB2838|o__OFGB2838|f__FGB2838|g__GGB28418|s__GGB28418_SGB41001
k__Bacteria|p__Firmicutes|c__CFGB2838|o__OFGB2838|f__FGB2838|g__GGB28422|s__GGB28422_SGB41005
k__Bacteria|p__Firmicutes|c__CFGB2838|o__OFGB2838|f__FGB2838|g__GGB28422|s__GGB28422_SGB41005
k__Bacteria|p__Firmicutes|c__Clostridia|o__Eubacteriales|f__Pumilibacteraceae|g__GGB28431|s__GGB28431_SGB41014
k__Bacteria|p__Firmicutes|c__Clostridia|o__Eubacteriales|f__Pumilibacteraceae|g__GGB28431|s__GGB28431_SGB41014
k__Bacteria|p__Firmicutes|c__CFGB2833|o__OFGB2833|f__FGB2833|g__GGB28456|s__GGB28456_SGB41039
k__Bacteria|p__Firmicutes|c__CFGB2833|o__OFGB2833|f__FGB2833|g__GGB28456|s__GGB28456_SGB41039
k__Bacteria|p__Firmicutes|c__Clostridia|o__Eubacteriales|f__Eubacteriaceae|g__GGB28782|s__GGB28782_SGB41435
k__Bacteria|p__Firmicutes|c__Clostridia|o__Eubacteriales|f__Eubacteriaceae|g__GGB28782|s__GGB28782_SGB41435
k__Bacteria|p__Firmicutes|c__Clostridia|o__Eubacteriales|f__Lachnospiraceae|g__GGB28792|s__GGB28792_SGB41445
k__Bacteria|p__Firmicutes|c__Clostridia|o__Eubacteriales|f__Lachnospiraceae|g__GGB28792|s__GGB28792_SGB41445
k__Bacteria|p__Firmicutes|c__Clostridia|o__Eubacteriales|f__Lachnospiraceae|g__GGB28798|s__GGB28798_SGB41451
k__Bacteria|p__Firmicutes|c__Clostridia|o__Eubacteriales|f__Lachnospiraceae|g__GGB28798|s__GGB28798_SGB41451
k__Bacteria|p__Firmicutes|c__CFGB9622|o__OFGB9622|f__FGB9622|g__GGB28810|s__GGB28810_SGB41465
k__Bacteria|p__Firmicutes|c__CFGB9622|o__OFGB9622|f__FGB9622|g__GGB28810|s__GGB28810_SGB41465
k__Bacteria|p__Firmicutes|c__CFGB77305|o__OFGB77305|f__FGB77305|g__GGB28828|s__GGB28828_SGB41484
k__Bacteria|p__Firmicutes|c__CFGB77305|o__OFGB77305|f__FGB77305|g__GGB28828|s__GGB28828_SGB41484
k__Bacteria|p__Firmicutes|c__Clostridia|o__Eubacteriales|f__Clostridiaceae|g__GGB28851|s__GGB28851_SGB41518
k__Bacteria|p__Firmicutes|c__Clostridia|o__Eubacteriales|f__Clostridiaceae|g__GGB28851|s__GGB28851_SGB41518
k__Bacteria|p__Firmicutes|c__Clostridia|o__Eubacteriales|f__Lachnospiraceae|g__GGB28865|s__GGB28865_SGB41537
k__Bacteria|p__Firmicutes|c__Clostridia|o__Eubacteriales|f__Lachnospiraceae|g__GGB28865|s__GGB28865_SGB41537
k__Bacteria|p__Firmicutes|c__Clostridia|o__Eubacteriales|f__Lachnospiraceae|g__GGB28868|s__GGB28868_SGB41542
k__Bacteria|p__Firmicutes|c__Clostridia|o__Eubacteriales|f__Lachnospiraceae|g__GGB28868|s__GGB28868_SGB41542
k__Bacteria|p__Firmicutes|c__Clostridia|o__Eubacteriales|f__Lachnospiraceae|g__GGB28869|s__GGB28869_SGB41543
k__Bacteria|p__Firmicutes|c__Clostridia|o__Eubacteriales|f__Lachnospiraceae|g__GGB28869|s__GGB28869_SGB41543
k__Bacteria|p__Firmicutes|c__Clostridia|o__Eubacteriales|f__Lachnospiraceae|g__GGB28875|s__GGB28875_SGB41555
k__Bacteria|p__Firmicutes|c__Clostridia|o__Eubacteriales|f__Lachnospiraceae|g__GGB28875|s__GGB28875_SGB41555
k__Bacteria|p__Firmicutes|c__CFGB9633|o__OFGB9633|f__FGB9633|g__GGB28881|s__GGB28881_SGB41561
k__Bacteria|p__Firmicutes|c__CFGB9633|o__OFGB9633|f__FGB9633|g__GGB28881|s__GGB28881_SGB41561
k__Bacteria|p__Bacteria_unclassified|c__Bacteria_unclassified|o__Bacteria_unclassified|f__Bacteria_unclassified|g__GGB[illegible]
k__Bacteria|p__Bacteria_unclassified|c__Bacteria_unclassified|o__Bacteria_unclassified|f__Bacteria_unclassified|g__GGB[illegible]
k__Bacteria|p__Bacteria_unclassified|c__Bacteria_unclassified|o__Bacteria_unclassified|f__Bacteria_unclassified|g__GGB[illegible]
k__Bacteria|p__Bacteria_unclassified|c__Bacteria_unclassified|o__Bacteria_unclassified|f__Bacteria_unclassified|g__GGB[illegible]
k__Bacteria|p__Bacteria_unclassified|c__Bacteria_unclassified|o__Bacteria_unclassified|f__Bacteria_unclassified|g__GGB[illegible]
k__Bacteria|p__Bacteria_unclassified|c__Bacteria_unclassified|o__Bacteria_unclassified|f__Bacteria_unclassified|g__GGB[illegible]

k__Bacteria|p__Bacteria_unclassified|c__Bacteria_unclassified|o__Bacteria_unclassified|f__Bacteria_unclassified|g__GGB2
k__Bacteria|p__Bacteria_unclassified|c__Bacteria_unclassified|o__Bacteria_unclassified|f__Bacteria_unclassified|g__GGB2
k__Bacteria|p__Bacteria_unclassified|c__Bacteria_unclassified|o__Bacteria_unclassified|f__Bacteria_unclassified|g__GGB2
k__Bacteria|p__Bacteria_unclassified|c__Bacteria_unclassified|o__Bacteria_unclassified|f__Bacteria_unclassified|g__GGB2
k__Bacteria|p__Bacteria_unclassified|c__Bacteria_unclassified|o__Bacteria_unclassified|f__Bacteria_unclassified|g__GGB2
k__Bacteria|p__Bacteria_unclassified|c__Bacteria_unclassified|o__Bacteria_unclassified|f__Bacteria_unclassified|g__GGB2
k__Bacteria|p__Firmicutes|c__Clostridia|o__Eubacteriales|f__Lachnospiraceae|g__GGB28924|s__GGB28924_SGB41621
k__Bacteria|p__Firmicutes|c__Clostridia|o__Eubacteriales|f__Lachnospiraceae|g__GGB28924|s__GGB28924_SGB41621
k__Bacteria|p__Bacteria_unclassified|c__CFGB77359|o__OFGB77359|f__FGB77359|g__GGB28927|s__GGB28927_SGB416
k__Bacteria|p__Bacteria_unclassified|c__CFGB77359|o__OFGB77359|f__FGB77359|g__GGB28927|s__GGB28927_SGB416
k__Bacteria|p__Firmicutes|c__CFGB9639|o__OFGB9639|f__FGB9639|g__GGB28934|s__GGB28934_SGB41635
k__Bacteria|p__Firmicutes|c__CFGB9639|o__OFGB9639|f__FGB9639|g__GGB28934|s__GGB28934_SGB41635
k__Bacteria|p__Firmicutes|c__Clostridia|o__Eubacteriales|f__Lachnospiraceae|g__GGB28949|s__GGB28949_SGB41655
k__Bacteria|p__Firmicutes|c__Clostridia|o__Eubacteriales|f__Lachnospiraceae|g__GGB28949|s__GGB28949_SGB41655
k__Bacteria|p__Firmicutes|c__Clostridia|o__Eubacteriales|f__Clostridiaceae|g__GGB28951|s__GGB28951_SGB102295
k__Bacteria|p__Firmicutes|c__Clostridia|o__Eubacteriales|f__Clostridiaceae|g__GGB28951|s__GGB28951_SGB102295
k__Bacteria|p__Firmicutes|c__Clostridia|o__Eubacteriales|f__Clostridiaceae|g__GGB28951|s__GGB28951_SGB41658
k__Bacteria|p__Firmicutes|c__Clostridia|o__Eubacteriales|f__Clostridiaceae|g__GGB28951|s__GGB28951_SGB41658
k__Bacteria|p__Firmicutes|c__Clostridia|o__Eubacteriales|f__Clostridiaceae|g__GGB28954|s__GGB28954_SGB41662
k__Bacteria|p__Firmicutes|c__Clostridia|o__Eubacteriales|f__Clostridiaceae|g__GGB28954|s__GGB28954_SGB41662
k__Bacteria|p__Firmicutes|c__Clostridia|o__Eubacteriales|f__Clostridiaceae|g__GGB28960|s__GGB28960_SGB41669
k__Bacteria|p__Firmicutes|c__Clostridia|o__Eubacteriales|f__Clostridiaceae|g__GGB28960|s__GGB28960_SGB41669
k__Bacteria|p__Firmicutes|c__Clostridia|o__Eubacteriales|f__Clostridiaceae|g__GGB28964|s__GGB28964_SGB94886
k__Bacteria|p__Firmicutes|c__Clostridia|o__Eubacteriales|f__Clostridiaceae|g__GGB28964|s__GGB28964_SGB94886
k__Bacteria|p__Firmicutes|c__Clostridia|o__Eubacteriales|f__Clostridiaceae|g__GGB28967|s__GGB28967_SGB41678
k__Bacteria|p__Firmicutes|c__Clostridia|o__Eubacteriales|f__Clostridiaceae|g__GGB28967|s__GGB28967_SGB41678
k__Bacteria|p__Firmicutes|c__CFGB9656|o__OFGB9656|f__FGB9656|g__GGB28996|s__GGB28996_SGB41712
k__Bacteria|p__Firmicutes|c__CFGB9656|o__OFGB9656|f__FGB9656|g__GGB28996|s__GGB28996_SGB41712
k__Bacteria|p__Firmicutes|c__CFGB9827|o__OFGB9827|f__FGB9827|g__GGB29531|s__GGB29531_SGB42317
k__Bacteria|p__Firmicutes|c__CFGB9827|o__OFGB9827|f__FGB9827|g__GGB29531|s__GGB29531_SGB42317
k__Bacteria|p__Firmicutes|c__Clostridia|o__Eubacteriales|f__Eubacteriaceae|g__GGB29685|s__GGB29685_SGB42494
k__Bacteria|p__Firmicutes|c__Clostridia|o__Eubacteriales|f__Eubacteriaceae|g__GGB29685|s__GGB29685_SGB42494
k__Bacteria|p__Bacteria_unclassified|c__CFGB77303|o__OFGB77303|f__FGB77303|g__GGB30141|s__GGB30141_SGB430
k__Bacteria|p__Bacteria_unclassified|c__CFGB77303|o__OFGB77303|f__FGB77303|g__GGB30141|s__GGB30141_SGB430
k__Bacteria|p__Firmicutes|c__Clostridia|o__Eubacteriales|f__Clostridiaceae|g__GGB30145|s__GGB30145_SGB43072
k__Bacteria|p__Firmicutes|c__Clostridia|o__Eubacteriales|f__Clostridiaceae|g__GGB30145|s__GGB30145_SGB43072
k__Bacteria|p__Firmicutes|c__Clostridia|o__Eubacteriales|f__Eubacteriales_unclassified|g__GGB30286|s__GGB30286_SG
k__Bacteria|p__Firmicutes|c__Clostridia|o__Eubacteriales|f__Eubacteriales_unclassified|g__GGB30286|s__GGB30286_SG
k__Bacteria|p__Firmicutes|c__CFGB72709|o__OFGB72709|f__FGB72709|g__GGB30300|s__GGB30300_SGB43264
k__Bacteria|p__Firmicutes|c__CFGB72709|o__OFGB72709|f__FGB72709|g__GGB30300|s__GGB30300_SGB43264
k__Bacteria|p__Firmicutes|c__Clostridia|o__Eubacteriales|f__Oscillospiraceae|g__GGB30303|s__GGB30303_SGB43268
k__Bacteria|p__Firmicutes|c__Clostridia|o__Eubacteriales|f__Oscillospiraceae|g__GGB30303|s__GGB30303_SGB43268
k__Bacteria|p__Firmicutes|c__Clostridia|o__Eubacteriales|f__Oscillospiraceae|g__GGB30450|s__GGB30450_SGB43507
k__Bacteria|p__Firmicutes|c__Clostridia|o__Eubacteriales|f__Oscillospiraceae|g__GGB30450|s__GGB30450_SGB43507
k__Bacteria|p__Firmicutes|c__Clostridia|o__Eubacteriales|f__Oscillospiraceae|g__GGB30453|s__GGB30453_SGB43513
k__Bacteria|p__Firmicutes|c__Clostridia|o__Eubacteriales|f__Oscillospiraceae|g__GGB30453|s__GGB30453_SGB43513

k__Bacteria|p__Firmicutes|c__Clostridia|o__Eubacteriales|f__Oscillospiraceae|g__GGB30454|s__GGB30454_SGB43514
k__Bacteria|p__Firmicutes|c__Clostridia|o__Eubacteriales|f__Oscillospiraceae|g__GGB30454|s__GGB30454_SGB43514
k__Bacteria|p__Firmicutes|c__Clostridia|o__Eubacteriales|f__Oscillospiraceae|g__GGB30455|s__GGB30455_SGB43519
k__Bacteria|p__Firmicutes|c__Clostridia|o__Eubacteriales|f__Oscillospiraceae|g__GGB30455|s__GGB30455_SGB43519
k__Bacteria|p__Firmicutes|c__Clostridia|o__Eubacteriales|f__Oscillospiraceae|g__GGB30456|s__GGB30456_SGB43520
k__Bacteria|p__Firmicutes|c__Clostridia|o__Eubacteriales|f__Oscillospiraceae|g__GGB30456|s__GGB30456_SGB43520
k__Bacteria|p__Firmicutes|c__Clostridia|o__Eubacteriales|f__Oscillospiraceae|g__GGB30457|s__GGB30457_SGB63218
k__Bacteria|p__Firmicutes|c__Clostridia|o__Eubacteriales|f__Oscillospiraceae|g__GGB30457|s__GGB30457_SGB63218
k__Bacteria|p__Firmicutes|c__Clostridia|o__Eubacteriales|f__Oscillospiraceae|g__GGB30461|s__GGB30461_SGB43530
k__Bacteria|p__Firmicutes|c__Clostridia|o__Eubacteriales|f__Oscillospiraceae|g__GGB30461|s__GGB30461_SGB43530
k__Bacteria|p__Firmicutes|c__Clostridia|o__Eubacteriales|f__Oscillospiraceae|g__GGB30461|s__GGB30461_SGB43533
k__Bacteria|p__Firmicutes|c__Clostridia|o__Eubacteriales|f__Oscillospiraceae|g__GGB30461|s__GGB30461_SGB43533
k__Bacteria|p__Firmicutes|c__Clostridia|o__Eubacteriales|f__Oscillospiraceae|g__GGB30461|s__GGB30461_SGB63209
k__Bacteria|p__Firmicutes|c__Clostridia|o__Eubacteriales|f__Oscillospiraceae|g__GGB30461|s__GGB30461_SGB63209
k__Bacteria|p__Firmicutes|c__Clostridia|o__Eubacteriales|f__Oscillospiraceae|g__GGB30463|s__GGB30463_SGB43537
k__Bacteria|p__Firmicutes|c__Clostridia|o__Eubacteriales|f__Oscillospiraceae|g__GGB30463|s__GGB30463_SGB43537
k__Bacteria|p__Firmicutes|c__Clostridia|o__Eubacteriales|f__Oscillospiraceae|g__GGB30473|s__GGB30473_SGB43557
k__Bacteria|p__Firmicutes|c__Clostridia|o__Eubacteriales|f__Oscillospiraceae|g__GGB30473|s__GGB30473_SGB43557
k__Bacteria|p__Firmicutes|c__Clostridia|o__Eubacteriales|f__Oscillospiraceae|g__GGB30475|s__GGB30475_SGB63182
k__Bacteria|p__Firmicutes|c__Clostridia|o__Eubacteriales|f__Oscillospiraceae|g__GGB30475|s__GGB30475_SGB63182
k__Bacteria|p__Actinobacteria|c__CFGB77153|o__OFGB77153|f__FGB77153|g__GGB30861|s__GGB30861_SGB44083
k__Bacteria|p__Actinobacteria|c__CFGB77153|o__OFGB77153|f__FGB77153|g__GGB30861|s__GGB30861_SGB44083
k__Bacteria|p__Tenericutes|c__CFGB1791|o__OFGB1791|f__FGB1791|g__GGB31312|s__GGB31312_SGB44628
k__Bacteria|p__Tenericutes|c__CFGB1791|o__OFGB1791|f__FGB1791|g__GGB31312|s__GGB31312_SGB44628
k__Bacteria|p__Firmicutes|c__CFGB10290|o__OFGB10290|f__FGB10290|g__GGB31438|s__GGB31438_SGB44768
k__Bacteria|p__Firmicutes|c__CFGB10290|o__OFGB10290|f__FGB10290|g__GGB31438|s__GGB31438_SGB44768
k__Bacteria|p__Firmicutes|c__Clostridia|o__Eubacteriales|f__Oscillospiraceae|g__GGB3171|s__GGB3171_SGB4185
k__Bacteria|p__Firmicutes|c__Clostridia|o__Eubacteriales|f__Oscillospiraceae|g__GGB3171|s__GGB3171_SGB4185
k__Bacteria|p__Firmicutes|c__CFGB10289|o__OFGB10289|f__FGB10289|g__GGB31762|s__GGB31762_SGB45125
k__Bacteria|p__Firmicutes|c__CFGB10289|o__OFGB10289|f__FGB10289|g__GGB31762|s__GGB31762_SGB45125
k__Bacteria|p__Firmicutes|c__CFGB1765|o__OFGB1765|f__FGB1765|g__GGB31823|s__GGB31823_SGB45199
k__Bacteria|p__Firmicutes|c__CFGB1765|o__OFGB1765|f__FGB1765|g__GGB31823|s__GGB31823_SGB45199
k__Bacteria|p__Firmicutes|c__CFGB1765|o__OFGB1765|f__FGB1765|g__GGB31838|s__GGB31838_SGB45216
k__Bacteria|p__Firmicutes|c__CFGB1765|o__OFGB1765|f__FGB1765|g__GGB31838|s__GGB31838_SGB45216
k__Bacteria|p__Firmicutes|c__CFGB1765|o__OFGB1765|f__FGB1765|g__GGB31841|s__GGB31841_SGB65084
k__Bacteria|p__Firmicutes|c__CFGB1765|o__OFGB1765|f__FGB1765|g__GGB31841|s__GGB31841_SGB65084
k__Bacteria|p__Firmicutes|c__CFGB10667|o__OFGB10667|f__FGB10667|g__GGB32371|s__GGB32371_SGB41694
k__Bacteria|p__Firmicutes|c__CFGB10667|o__OFGB10667|f__FGB10667|g__GGB32371|s__GGB32371_SGB41694
k__Bacteria|p__Firmicutes|c__Clostridia|o__Eubacteriales|f__Lachnospiraceae|g__GGB3793|s__GGB3793_SGB5158
k__Bacteria|p__Firmicutes|c__Clostridia|o__Eubacteriales|f__Lachnospiraceae|g__GGB3793|s__GGB3793_SGB5158
k__Bacteria|p__Firmicutes|c__Clostridia|o__Eubacteriales|f__Clostridiaceae|g__GGB42601|s__GGB42601_SGB59797
k__Bacteria|p__Firmicutes|c__Clostridia|o__Eubacteriales|f__Clostridiaceae|g__GGB42601|s__GGB42601_SGB59797
k__Bacteria|p__Firmicutes|c__Clostridia|o__Eubacteriales|f__Oscillospiraceae|g__GGB45514|s__GGB45514_SGB63186
k__Bacteria|p__Firmicutes|c__Clostridia|o__Eubacteriales|f__Oscillospiraceae|g__GGB45514|s__GGB45514_SGB63186
k__Bacteria|p__Firmicutes|c__CFGB75721|o__OFGB75721|f__FGB75721|g__GGB45564|s__GGB45564_SGB63259
k__Bacteria|p__Firmicutes|c__CFGB75721|o__OFGB75721|f__FGB75721|g__GGB45564|s__GGB45564_SGB63259

k__Bacteria|p__Firmicutes|c__Clostridia|o__Eubacteriales|f__Oscillospiraceae|g__GGB45624|s__GGB45624_SGB63337
k__Bacteria|p__Firmicutes|c__Clostridia|o__Eubacteriales|f__Oscillospiraceae|g__GGB45624|s__GGB45624_SGB63337
k__Bacteria|p__Firmicutes|c__Clostridia|o__Eubacteriales|f__Christensenellaceae|g__GGB45656|s__GGB45656_SGB6337
k__Bacteria|p__Firmicutes|c__Clostridia|o__Eubacteriales|f__Christensenellaceae|g__GGB45656|s__GGB45656_SGB6337
k__Bacteria|p__Firmicutes|c__CFGB10299|o__OFGB10299|f__FGB10299|g__GGB47127|s__GGB47127_SGB65054
k__Bacteria|p__Firmicutes|c__CFGB10299|o__OFGB10299|f__FGB10299|g__GGB47127|s__GGB47127_SGB65054
k__Bacteria|p__Firmicutes|c__Clostridia|o__Eubacteriales|f__Oscillospiraceae|g__GGB74395|s__GGB74395_SGB43523
k__Bacteria|p__Firmicutes|c__Clostridia|o__Eubacteriales|f__Oscillospiraceae|g__GGB74395|s__GGB74395_SGB43523
k__Bacteria|p__Firmicutes|c__Clostridia|o__Eubacteriales|f__Oscillospiraceae|g__GGB75053|s__GGB75053_SGB43494
k__Bacteria|p__Firmicutes|c__Clostridia|o__Eubacteriales|f__Oscillospiraceae|g__GGB75053|s__GGB75053_SGB43494
k__Bacteria|p__Firmicutes|c__Clostridia|o__Eubacteriales|f__Lachnospiraceae|g__GGB75109|s__GGB75109_SGB102238
k__Bacteria|p__Firmicutes|c__Clostridia|o__Eubacteriales|f__Lachnospiraceae|g__GGB75109|s__GGB75109_SGB102238
k__Bacteria|p__Firmicutes|c__Clostridia|o__Eubacteriales|f__Lachnospiraceae|g__Lachnospiraceae_unclassified|s__Lachn
k__Bacteria|p__Firmicutes|c__Clostridia|o__Eubacteriales|f__Lachnospiraceae|g__Lachnospiraceae_unclassified|s__Lachn
k__Bacteria|p__Firmicutes|c__Clostridia|o__Eubacteriales|f__Lachnospiraceae|g__Lachnospiraceae_unclassified|s__Lachn
k__Bacteria|p__Firmicutes|c__Clostridia|o__Eubacteriales|f__Lachnospiraceae|g__Lachnospiraceae_unclassified|s__Lachn
k__Bacteria|p__Firmicutes|c__Clostridia|o__Eubacteriales|f__Lachnospiraceae|g__Lachnospiraceae_unclassified|s__Lachn
k__Bacteria|p__Firmicutes|c__Clostridia|o__Eubacteriales|f__Lachnospiraceae|g__Lachnospiraceae_unclassified|s__Lachn
k__Bacteria|p__Firmicutes|c__Clostridia|o__Eubacteriales|f__Lachnospiraceae|g__Lachnospiraceae_unclassified|s__Lachn
k__Bacteria|p__Firmicutes|c__Clostridia|o__Eubacteriales|f__Lachnospiraceae|g__Lachnospiraceae_unclassified|s__Lachn
k__Bacteria|p__Firmicutes|c__Clostridia|o__Eubacteriales|f__Lachnospiraceae|g__Lachnospiraceae_unclassified|s__Lachn
k__Bacteria|p__Firmicutes|c__Clostridia|o__Eubacteriales|f__Lachnospiraceae|g__Lachnospiraceae_unclassified|s__Lachn
k__Bacteria|p__Firmicutes|c__Bacilli|o__Lactobacillales|f__Lactobacillaceae|g__Lactobacillus|s__Lactobacillus_johnsonii
k__Bacteria|p__Firmicutes|c__Bacilli|o__Lactobacillales|f__Lactobacillaceae|g__Lactobacillus|s__Lactobacillus_johnsonii
k__Bacteria|p__Actinobacteria|c__Coriobacteriia|o__Coriobacteriales|f__Atopobiaceae|g__Leptogranulimonas|s__Leptog
k__Bacteria|p__Actinobacteria|c__Coriobacteriia|o__Coriobacteriales|f__Atopobiaceae|g__Leptogranulimonas|s__Leptog
k__Bacteria|p__Bacteroidota|c__Bacteroidia|o__Bacteroidales|f__Muribaculaceae|g__Muribaculaceae_unclassified|s__M
k__Bacteria|p__Bacteroidota|c__Bacteroidia|o__Bacteroidales|f__Muribaculaceae|g__Muribaculaceae_unclassified|s__M
k__Bacteria|p__Firmicutes|c__Clostridia|o__Eubacteriales|f__Oscillospiraceae|g__Neglectibacter|s__Neglectibacter_sp_X
k__Bacteria|p__Firmicutes|c__Clostridia|o__Eubacteriales|f__Oscillospiraceae|g__Neglectibacter|s__Neglectibacter_sp_X
k__Bacteria|p__Firmicutes|c__Clostridia|o__Eubacteriales|f__Oscillospiraceae|g__Oscillibacter|s__Oscillibacter_SGB43496
k__Bacteria|p__Firmicutes|c__Clostridia|o__Eubacteriales|f__Oscillospiraceae|g__Oscillibacter|s__Oscillibacter_SGB43496
k__Bacteria|p__Firmicutes|c__Clostridia|o__Eubacteriales|f__Oscillospiraceae|g__Oscillospiraceae_unclassified|s__Oscillo
k__Bacteria|p__Firmicutes|c__Clostridia|o__Eubacteriales|f__Oscillospiraceae|g__Oscillospiraceae_unclassified|s__Oscillo
k__Bacteria|p__Firmicutes|c__Clostridia|o__Eubacteriales|f__Oscillospiraceae|g__Oscillospiraceae_unclassified|s__Oscillo
k__Bacteria|p__Firmicutes|c__Clostridia|o__Eubacteriales|f__Oscillospiraceae|g__Oscillospiraceae_unclassified|s__Oscillo
k__Bacteria|p__Firmicutes|c__Clostridia|o__Eubacteriales|f__Oscillospiraceae|g__Oscillospiraceae_unclassified|s__Oscillo
k__Bacteria|p__Firmicutes|c__Clostridia|o__Eubacteriales|f__Oscillospiraceae|g__Oscillospiraceae_unclassified|s__Oscillo
k__Bacteria|p__Firmicutes|c__Clostridia|o__Eubacteriales|f__Oscillospiraceae|g__Oscillospiraceae_unclassified|s__Oscillo
k__Bacteria|p__Firmicutes|c__Clostridia|o__Eubacteriales|f__Oscillospiraceae|g__Oscillospiraceae_unclassified|s__Oscillo
k__Bacteria|p__Proteobacteria|c__Betaproteobacteria|o__Burkholderiales|f__Sutterellaceae|g__Parasutterella|s__Parasu
k__Bacteria|p__Proteobacteria|c__Betaproteobacteria|o__Burkholderiales|f__Sutterellaceae|g__Parasutterella|s__Parasu

k__Bacteria|p__Firmicutes|c__Clostridia|o__Eubacteriales|f__Lachnospiraceae|g__Schaedlerella|s__Schaedlerella_arabino
k__Bacteria|p__Firmicutes|c__Clostridia|o__Eubacteriales|f__Lachnospiraceae|g__Schaedlerella|s__Schaedlerella_arabino

k__Bacteria|p__Firmicutes|c__Erysipelotrichia|o__Erysipelotrichales|f__Turicibacteraceae|g__Turicibacter|s__Turicibacter_

k__Bacteria|p__Firmicutes|c__Erysipelotrichia|o__Erysipelotrichales|f__Turicibacteraceae|g__Turicibacter|s__Turicibacter_
