# Supplementary material for: Non-invasive Vagal Nerve Stimulation as a Potential Treatment for Repetitive Blast Trauma
Source: bioRxiv. 2026 Jul 19:2026.07.13.737563. Preprint. [Version 1] doi: 10.64898/2026.07.13.737563 (PMC13405043; doi:10.64898/2026.07.13.737563)
Supplement: Supplement 14 [file media-14.pdf]

			95% Confidence Intervals	
			VNS (–)	
**exposure**	**mediator (Species-level feature)**	**outcome (Cytokine PC)**	**ACME**	**ADE**
Blast	Lachnospiraceae_bacterium	PC3	[-1.741, -0.029]	[-0.756, 2.319]
Blast	Eubacteriaceae_bacterium	PC3	[-1.77, 0.027]	[-0.671, 2.28]
Blast	GGB29685_SGB42494	PC3	[-2.478, -0.062]	[-0.675, 2.835]
Blast	Shannon Index	PC3	[-2.317, 0.697]	[-1.151, 2.439]
Blast	Richness (# observed features)	PC3	[-2.067, -0.046]	[-0.715, 2.463]

			95% Confidence Intervals	
			Blast (–)	
**exposure**	**mediator (Species-level feature)**	**outcome (Cytokine PC)**	**ACME (Blast-)**	**ADE (Blast-)**
VNS	GGB28960_SGB41669	PC5	[-0.393, 0.989]	[-0.915, 1.48]
VNS	GGB20146_SGB29427	PC5	[-0.918, 0.363]	[-0.58, 1.902]
VNS	GGB28404_SGB40986	PC5	[-2.765, 0.025]	[-0.238, 3.563]
VNS	GGB28431_SGB41014	PC5	[-1.641, -0.046]	[-0.141, 2.629]
VNS	Leptogranulimonas_caecicola	PC5	[-0.04, 1.699]	[-1.271, 0.956]

of Blast effect on outcome (at specified VNS condition)					
	VNS (+)			Taxonomic Information	
Total	ACME	ADE	Total	Genus	Family
[-1.347, 1.142]	[-2.284, 0.066]	[-1.203, 0.762]	[-2.575, 0.575]	Lachnospiraceae_unclassifie	Lachnospiraceae
[-1.312, 1.099]	[-2.391, 0.062]	[-1.353, 1.059]	[-2.628, 0.62]	Eubacteriaceae_unclassified	Eubacteriaceae
[-1.351, 1.022]	[-2.403, 0.01]	[-1.424, 1.1]	[-2.666, 0.621]	GGB29685	Eubacteriaceae
[-1.391, 1.04]	[-2.427, 0.109]	[-1.259, 1.065]	[-2.617, 0.654]		
[-1.397, 1.081]	[-2.301, 0.026]	[-1.096, 0.946]	[-2.593, 0.58]		

of VNS effect on outcome (at specified Blast condition)					
	Blast (+)			Taxonomic Information	
Total (Blast-)	ACME (Blast+)	ADE (Blast+)	Total (Blast+)	Genus	Family
[-0.677, 1.777]	[-0.223, 1.896]	[-1.536, 1.323]	[-0.937, 2.053]	GGB28960	Clostridiaceae
[-0.752, 1.859]	[-0.075, 1.95]	[-1.698, 1.327]	[-0.868, 1.968]	GGB20146	FGB77306
[-0.671, 1.777]	[-1.212, 0.031]	[-0.467, 2.377]	[-0.902, 2.047]	GGB28404	FGB2838
[-0.747, 1.661]	[-0.03, 1.305]	[-1.298, 1.558]	[-0.907, 2.141]	GGB28431	Pumilibacteraceae
[-0.709, 1.814]	[-0.062, 1.547]	[-1.595, 1.645]	[-0.87, 2.054]	Leptogranulimonas	Atopobiaceae

Order	Class	Phylum	Kingdom
Eubacteriales	Clostridia	Firmicutes	Bacteria
Eubacteriales	Clostridia	Firmicutes	Bacteria
Eubacteriales	Clostridia	Firmicutes	Bacteria

Order	Class	Phylum	Kingdom
Eubacteriales	Clostridia	Firmicutes	Bacteria
OFGB77306	CFGB77306	Firmicutes	Bacteria
OFGB2838	CFGB2838	Firmicutes	Bacteria
Eubacteriales	Clostridia	Firmicutes	Bacteria
Coriobacteriales	Coriobacteriia	Actinobacteria	Bacteria

MetaPhlan Annotation
k__Bacteria|p__Firmicutes|c__Clostridia|o__Eubacteriales|f__Lachnospiraceae|g__Lachnospiraceae_unclassified|s__Lachn
k__Bacteria|p__Firmicutes|c__Clostridia|o__Eubacteriales|f__Eubacteriaceae|g__Eubacteriaceae_unclassified|s__Eubacte
k__Bacteria|p__Firmicutes|c__Clostridia|o__Eubacteriales|f__Eubacteriaceae|g__GGB29685|s__GGB29685_SGB42494

MetaPhlan Annotation
k__Bacteria|p__Firmicutes|c__Clostridia|o__Eubacteriales|f__Clostridiaceae|g__GGB28960|s__GGB28960_SGB41669
k__Bacteria|p__Firmicutes|c__CFGB77306|o__OFGB77306|f__FGB77306|g__GGB20146|s__GGB20146_SGB29427
k__Bacteria|p__Firmicutes|c__CFGB2838|o__OFGB2838|f__FGB2838|g__GGB28404|s__GGB28404_SGB40986
k__Bacteria|p__Firmicutes|c__Clostridia|o__Eubacteriales|f__Pumilibacteraceae|g__GGB28431|s__GGB28431_SGB41014
k__Bacteria|p__Actinobacteria|c__Coriobacteriia|o__Coriobacteriales|f__Atopobiaceae|g__Leptogranulimonas|s__Leptog
