# Supplementary material for: Non-invasive Vagal Nerve Stimulation as a Potential Treatment for Repetitive Blast Trauma
Source: bioRxiv. 2026 Jul 19:2026.07.13.737563. Preprint. [Version 1] doi: 10.64898/2026.07.13.737563 (PMC13405043; doi:10.64898/2026.07.13.737563)
Supplement: Supplement 15 [file media-15.pdf]

Species-level feature	n	Blast Chi(df=2)	VNS Chi (df=2)	Blast:VNS Chi (df=1	Blast Pr(>Chi)
Acetatifactor_SGB41546	63	-0.4734503914	-0.6735865311	0.05527887593	0.8402457971
Acetatifactor_muris	63	-0.6551189368	-0.5673238041	0.7556277609	0.7395220796
Acutalibacter_muris	63	-1.256302585	-0.1120146742	0.5269336414	0.4221546334
Acutalibacter_sp_1XD8_36	63	0.285010825	0.6121719594	-0.8331188827	0.6605798863
Adlercreutzia_caecimuris	63	0.0032540798	0.6923226625	-0.109097947	0.9896089529
Adlercreutzia_mucosicola	63	-1.293145404	-0.8107578137	1.60909155	0.2718339856
Adlercreutzia_muris	63	0.1453285309	-0.9977873679	0.5602471455	0.6704217743
Akkermansia_muciniphila	63	1.164601827	0.0441076170	0.3446653734	0.1698125404
Alistipes_sp_DSM_112343	63	1.362207172	0.4106786184	-0.6059096635	0.3725159774
Anaerotruncus_sp_1XD42_93	63	0.162751673	0.9054957631	-0.9596841053	0.5202714535
Bacteria_unclassified_SGB102200	63	-1.193792207	-0.7298338535	0.5832040956	0.4742692127
Bacteria_unclassified_SGB41677	63	-0.4715195856	0.1799007488	1.195085048	0.4441438231
Bacteria_unclassified_SGB43546	63	0.1743046341	-0.6360104272	0.6428209411	0.5863583659
Bacteroides_thetaiotaomicron	63	0.0121161501	-0.7991227673	0.2034814256	0.9602014777
Bifidobacterium_pseudolongum	63	-0.0723725935	-0.1327509122	-0.9242887686	0.4280103985
Clostridia_bacterium	63	-1.172211165	-0.6868310968	0.1995598485	0.3745590434
Clostridiaceae_bacterium	63	0.1034305113	-1.201920773	0.02066687185	0.9871697806
Clostridiaceae_unclassified_SGB41663	63	-0.3486267188	-0.1904672991	0.6891000506	0.780382224
Clostridiales_bacterium	63	1.484229444	-1.424419397	1.488073748	0.0023319564
Clostridium_cocleatum	63	2.222775114	1.218321169	0.09951137337	0.0115678325
Coriobacteriaceae_bacterium	63	-1.041914279	0.2056027034	0.01023882737	0.3724483697
Dorea_sp_5_2	63	0.1593344522	-0.0829399946	-0.8581176899	0.5884919176
Dubosiella_newyorkensis	63	-1.915269676	-0.6328732472	0.6214032232	0.1169987394
Erysipelotrichales_bacterium	63	0.2784071645	1.339697821	-0.4645866706	0.8970006656
Eubacteriaceae_bacterium	63	2.751672549	1.510462675	-1.650700676	0.0271233687
Eubacteriaceae_unclassified_SGB9492	63	1.242193062	1.358359623	-0.6975490476	0.4594880799
GGB20149_SGB29430	63	0.9685682607	-0.0204831086	-0.6658386857	0.6280700484
GGB22635_SGB63107	63	1.092976537	0.4376483775	-0.1528866375	0.4117103362
GGB25041_SGB36960	63	-2.252489312	-1.39219352	1.780688688	0.0824966676
GGB27876_SGB40310	63	-1.812623277	-0.5532913055	0.7280341665	0.164666135
GGB27878_SGB40312	63	-1.527971618	-1.626099912	0.3766334312	0.2133541578
GGB27918_SGB40356	63	0.2812774952	-0.1203583756	-0.2447416231	0.9587581408
GGB28382_SGB40962	63	1.114151113	0.4940115299	-0.5441437287	0.5193113079
GGB28399_SGB40980	63	1.161889363	1.030230259	-1.59173109	0.2894655025
GGB28411_SGB40993	63	0.4381956321	0.3093870572	-1.591442774	0.2018022626
GGB28415_SGB40997	63	-2.129244403	0.0888312568	0.1520170106	0.0267267446
GGB28430_SGB41013	63	0.4099890819	-0.5583147446	-0.3724148892	0.9120709472
GGB28439_SGB41022	63	0.5177245932	-0.7763942741	0.6099566625	0.3804444035
GGB28778_SGB41431	63	0.4438702725	-1.272313732	0.05934871732	0.8046989411
GGB28784_SGB41437	63	-0.6744740061	1.534318282	-1.589122652	0.0207266217
GGB28792_SGB41445	63	-1.397060151	-0.0925532547	0.5796802482	0.3385777757
GGB28798_SGB41451	63	0.3906643009	0.9447774686	-1.657947258	0.1640226305
GGB28802_SGB41455	63	0.4123962239	1.468186023	-1.158799927	0.4578986259
GGB28818_SGB41473	63	0.1651347708	-0.0781731024	-0.6862728452	0.7292095104
GGB28828_SGB41484	63	-1.176824424	-0.0692565511	1.56387429	0.2986969933

GGB28851_SGB41518	63	-0.260906186	0.23485215	0.5473952433	0.852552179
GGB28859_SGB41528	63	-0.408183271	0.8331838433	-1.013533439	0.205749329
GGB28864_SGB41535	63	-0.638572657	-0.265440063	0.2215820789	0.785465248
GGB28869_SGB41543	63	-0.349754911	-1.327948185	1.297592468	0.347931625
GGB28883_SGB41564	63	-0.6129437991	-1.554159022	-0.1933407745	0.594877248
GGB28892_SGB41573	63	0.8997775489	0.8742773198	-0.783674421	0.651227207
GGB28893_SGB41574	63	-1.686186836	-1.606896813	1.822989034	0.165594509
GGB28898_SGB41580	63	-1.432725994	0.2309245518	1.422712869	0.304654993
GGB28904_SGB41597	63	-0.752933536	-1.057389145	-0.7915484519	0.172906397
GGB28916_SGB41612	63	-0.645328910	-0.4630564471	0.5573992644	0.801432609
GGB28924_SGB41621	63	0.5930936828	-0.579244489	-0.6488827416	0.796481662
GGB28926_SGB41624	63	-0.091795660	-1.234746851	1.285464272	0.3067808341
GGB28927_SGB41625	63	0.1394694394	-1.227048855	1.266111807	0.193720805
GGB28934_SGB41635	63	-1.167727928	-1.191704148	2.087082539	0.115873311
GGB28946_SGB41652	63	1.180726699	1.280762636	-1.230855982	0.427843225
GGB28949_SGB41655	63	-0.887862344	-0.231163726	-0.09376521063	0.443608116
GGB28949_SGB41656	63	-1.538392369	-0.624118320	0.7023127676	0.282728234
GGB28950_SGB41657	63	-2.62149732	-1.459396162	1.26111009	0.030527036
GGB28951_SGB102295	63	0.3873186134	1.566467804	-0.3192449274	0.925352156
GGB28951_SGB41658	63	-0.213332002	-0.047179104	-0.2519688292	0.847001351
GGB28954_SGB41662	63	-1.146373063	-0.597521626	0.6380008981	0.515758297
GGB28956_SGB41665	63	1.783382845	0.6557111785	-1.761647725	0.163329713
GGB28960_SGB41669	63	1.228625729	1.483860342	-1.964078262	0.155727292
GGB28967_SGB41678	63	1.456567246	1.137338312	-0.0926969036	0.179269529
GGB28991_SGB41705	63	0.5758405906	1.26407252	-1.603233185	0.228893882
GGB29002_SGB41718	63	-0.860710820	1.090754042	-2.052520505	0.002430867
GGB29003_SGB41719	63	-0.041172115	-0.272723564	0.9024894356	0.499398644
GGB29011_SGB41731	63	-0.336193631	-1.277894627	0.4112990586	0.916276033
GGB29531_SGB42317	63	-1.013385715	-1.80711559	1.975506786	0.145082026
GGB29685_SGB42494	63	0.4671803769	1.112099711	-0.1138923044	0.864543852
GGB30141_SGB43066	63	0.1302436989	-0.447176207	-0.09088196888	0.991554803
GGB30286_SGB43248	63	2.39301438	1.182046798	-1.168443427	0.055261633
GGB30303_SGB43268	63	0.813311188	-0.128277481	0.3114034245	0.371936893
GGB30413_SGB43452	63	0.273835828	-0.292185247	-0.07842307499	0.953276950
GGB30454_SGB43514	63	-0.931450420	-1.153409769	-0.154817533	0.379858950
GGB30455_SGB43519	63	-1.39997214	-1.557994347	1.037789879	0.379467796
GGB30461_SGB43527	63	1.34676679	0.559511253	-0.7201983713	0.397503662
GGB30461_SGB43530	63	-1.224896578	-0.789729157	0.3673765275	0.395449
GGB30463_SGB43537	63	-1.426341545	-0.150526143	0.1656987748	0.210054257
GGB30473_SGB43557	63	0.4322874818	-2.470434655	1.804763781	0.020642166
GGB30475_SGB63182	63	1.476781394	-0.127186200	-0.5408442462	0.287965349
GGB30861_SGB44083	63	0.2580569327	0.5739850527	-0.2783889895	0.957552379
GGB31312_SGB44628	63	0.1805007501	-0.222579752	0.4695208346	0.711727128
GGB31438_SGB44768	63	-0.393600043	-1.146891051	0.1185015685	0.906357297
GGB3171_SGB4185	63	1.710840682	0.3357514743	-0.07198001314	0.0853457671
GGB31823_SGB45199	63	1.489891782	0.7051407982	-0.1831198431	0.188977717

GGB31853_SGB45233	63	0.1850728337	1.374462655	-0.9880911527	0.4968427437
GGB32371_SGB41694	63	0.1610064189	2.619816891	-1.191350461	0.3468118508
GGB3793_SGB5158	63	-0.1242037497	-0.0816857986	-0.08652598792	0.9657904747
GGB42598_SGB59794	63	0.5353666761	-0.2871673312	-1.143170137	0.5017400018
GGB45656_SGB63370	63	0.7711268946	0.2415073292	-0.07994670085	0.621044138
GGB47127_SGB65054	63	0.5882221237	0.873303962	0.3569004709	0.4967451202
GGB74395_SGB43521	63	-0.2269015992	-0.7197230491	1.674079714	0.1233397821
GGB75053_SGB43494	63	-1.227698761	-0.982606753	0.4220938725	0.4105959194
GGB75109_SGB102238	63	-0.9660070503	-0.3571691551	0.5237401709	0.6208422921
GGB81440_SGB45230	63	0.9851844807	1.469346382	-2.189691771	0.0796107313
Lachnospiraceae_bacterium	63	-0.5775196456	-0.4403681809	-0.4033409882	0.4848161772
Lachnospiraceae_bacterium_A2	63	-1.30536731	0.4121900628	0.7190113276	0.420834896
Lachnospiraceae_bacterium_MD308	63	-1.253742897	-1.527073537	-0.07314364088	0.2131194519
Lachnospiraceae_bacterium_MD329	63	1.569434144	-0.8172981207	0.7446499022	0.0209761491
Lachnospiraceae_unclassified_SGB414	63	-1.427172411	-1.877633067	2.005384557	0.142639053
Lachnospiraceae_unclassified_SGB414	63	0.0448776106	0.6149767571	-0.04645368359	0.9987579817
Lachnospiraceae_unclassified_SGB414	63	1.659917229	1.593184432	-2.560059828	0.0483863782
Lachnospiraceae_unclassified_SGB415	63	-1.905534628	-1.507502957	1.520310011	0.1654249525
Lactobacillus_johnsonii	63	1.729169167	0.0093960181	-0.6307021833	0.1826784534
Muribaculaceae_bacterium	63	0.5862997367	0.4519000148	-0.4279988911	0.8415252525
Neglectibacter_sp_X4	63	-2.14515562	-1.259408626	1.998620137	0.0847821517
Oscillospiraceae_bacterium	63	0.7143316233	-0.1585713961	-0.2605384149	0.7416725656
Oscillospiraceae_unclassified_SGB4350	63	-0.9535157468	-2.459249644	2.57855137	0.0259412852
Oscillospiraceae_unclassified_SGB4350	63	0.0443117420	0.4774133311	-0.9098576154	0.4959230188
Parasutterella_excrementihominis	63	0.0722400841	0.5907446666	-0.8298439721	0.5671048722
Romboutsia_ilealis	63	2.857654195	1.685300007	0.02578551976	0.0010997742
Schaedlerella_arabinosiphila	63	-0.1376867522	-0.4224508602	0.3336080387	0.9390244043
Turicibacter_sp_1E2	63	0.1707283912	0.1536888746	-1.455449929	0.1991772312
bacterium_1XD42_54	63	2.903031513	1.724849481	-1.278669218	0.0144245959
bacterium_1XD42_76	63	0.2168984617	0.9064029235	-0.8861147937	0.6000316038
bacterium_1xD8_48	63	-0.1464346409	0.2151082771	0.4220521683	0.8997089453
Berger Parker Index	63	-0.80005855	-1.72652844	1.105207518	0.5457417127
Richness (# observed features)	63	0.3862999043	-0.7185444522	0.9243577639	0.2717524423
Shannon Index	63	-0.826127489	-0.1244671288	0.3867142166	0.6947352216

Likelihood Ratio Test Results					
VNS Pr(>Chi)	Blast:VNS Pr(>Ch	Blast Adjusted p-valu	VNS Adjusted p-valu	Blast:VNS Adjusted p-valu	
0.6630009771	0.9567155372	0.9867502072	0.9748269319	0.9867298836	
0.7528971403	0.4518126699	0.9364552596	0.9748269319	0.9634120475	
0.8105784495	0.6011691176	0.7867838207	0.9748269319	0.9634120475	
0.7091849336	0.407256538	0.8878761912	0.9748269319	0.9634120475	
0.6765426913	0.9138311674	0.9987579817	0.9748269319	0.9867298836	
0.2498256574	0.1114340708	0.7669520852	0.8202808144	0.6923511791	
0.5958277454	0.5757855918	0.8915183169	0.9748269319	0.9634120475	
0.8643625099	0.7304679937	0.6504699932	0.9748269319	0.9867298836	
0.8326983074	0.5456838431	0.7867838207	0.9748269319	0.9634120475	
0.6174107217	0.3492927804	0.793096728	0.9748269319	0.9634120475	
0.7641667885	0.56028433	0.793096728	0.9748269319	0.9634120475	
0.1699894243	0.2347115362	0.793096728	0.8202808144	0.870124474	
0.7890099125	0.5236172725	0.8523176191	0.9748269319	0.9634120475	
0.6343063159	0.8395609106	0.9977174325	0.9748269319	0.9867298836	
0.3477990653	0.3569574351	0.7867838207	0.8202808144	0.9634120475	
0.7249401071	0.8418494906	0.7867838207	0.9748269319	0.9867298836	
0.2419370425	0.983839374	0.9987579817	0.8202808144	0.9917735625	
0.719154368	0.4915744456	0.9671862273	0.9748269319	0.9634120475	
0.2964785018	0.1401477562	0.1012861489	0.8202808144	0.7299362302	
0.1960355146	0.9207356632	0.308220099	0.8202808144	0.9867298836	
0.953919137	0.9918495863	0.7867838207	0.9803444972	0.9918495863	
0.4214161244	0.392260953	0.8523176191	0.8953154676	0.9634120475	
0.799186063	0.5419962879	0.6504699932	0.9748269319	0.9634120475	
0.3284081083	0.6425119949	0.9977174325	0.8202808144	0.9867298836	
0.2383186135	0.102464393	0.308220099	0.8202808144	0.6923511791	
0.3726201484	0.4865673277	0.793096728	0.8625466399	0.9634120475	
0.6299827926	0.5066260299	0.862733583	0.9748269319	0.9634120475	
0.8871521383	0.8796922403	0.7867838207	0.9748269319	0.9867298836	
0.2101871969	0.0786399911	0.5926789385	0.8202808144	0.6923511791	
0.7672351949	0.4675262866	0.6504699932	0.9748269319	0.9634120475	
0.1474794298	0.7066228553	0.6504699932	0.8202808144	0.9867298836	
0.8856989297	0.8067114261	0.9977174325	0.9748269319	0.9867298836	
0.8550578348	0.5892195811	0.793096728	0.9748269319	0.9634120475	
0.287117034	0.1161986568	0.7669520852	0.8202808144	0.6923511791	
0.1468484741	0.1150921455	0.6504699932	0.8202808144	0.6923511791	
0.9490516966	0.8792510459	0.308220099	0.9803444972	0.9867298836	
0.4662504826	0.7102045329	0.9977174325	0.9491235182	0.9867298836	
0.7387095529	0.5427880088	0.7867838207	0.9748269319	0.9634120475	
0.2184532434	0.9526874177	0.9671862273	0.8202808144	0.9867298836	
0.2544569976	0.1175944723	0.308220099	0.8202808144	0.6923511791	
0.7612444262	0.5639713269	0.7867838207	0.9748269319	0.9634120475	
0.2489158244	0.1026632614	0.6504699932	0.8202808144	0.6923511791	
0.342689298	0.2505958485	0.793096728	0.8202808144	0.870124474	
0.5694099484	0.4934268922	0.9364552596	0.9748269319	0.9634120475	
0.1067065352	0.1218538075	0.7669520852	0.8202808144	0.6923511791	

0.5783084298	0.5845532243	0.9867502072	0.9748269319	0.9634120475
0.5953667458	0.3132677119	0.6504699932	0.9748269319	0.9634120475
0.9646589853	0.8275909328	0.9671862273	0.9803444972	0.9867298836
0.3814774755	0.2033782479	0.7867838207	0.8669942626	0.870124474
0.0605244586	0.8487218661	0.8523176191	0.8202808144	0.9867298836
0.6676188822	0.4344240852	0.8848195758	0.9748269319	0.9634120475
0.1826993761	0.0721706083	0.6504699932	0.8202808144	0.6923511791
0.0828933309	0.1581963478	0.7669520852	0.8202808144	0.7605593646
0.0560671999	0.429766694	0.6504699932	0.8202808144	0.9634120475
0.8538132969	0.5782729426	0.9671862273	0.9748269319	0.9634120475
0.2786583787	0.5208167584	0.9671862273	0.8202808144	0.9634120475
0.470765265	0.2436965868	0.7669520852	0.9491235182	0.870124474
0.4182433734	0.2127798207	0.6504699932	0.8953154676	0.870124474
0.1113740731	0.04018812587	0.6504699932	0.8202808144	0.6923511791
0.4225889007	0.2250536625	0.7867838207	0.8953154676	0.870124474
0.9089481791	0.9252982663	0.793096728	0.9748269319	0.9867298836
0.7708639493	0.4835409848	0.7669520852	0.9748269319	0.9634120475
0.3364589048	0.2102296777	0.3179899638	0.8202808144	0.870124474
0.1587903977	0.7497999296	0.9977174325	0.8202808144	0.9867298836
0.9202366237	0.8012238941	0.9867502072	0.9748269319	0.9867298836
0.8004849551	0.5245336238	0.793096728	0.9748269319	0.9634120475
0.1534165023	0.08251291477	0.6504699932	0.8202808144	0.6923511791
0.1559574792	0.05400847696	0.6504699932	0.8202808144	0.6923511791
0.3150465193	0.9261468679	0.6504699932	0.8202808144	0.9867298836
0.279982342	0.1124600555	0.6812317928	0.8202808144	0.6923511791
0.1134655877	0.0434459902	0.1012861489	0.8202808144	0.6923511791
0.5839678864	0.3713550509	0.793096728	0.9748269319	0.9634120475
0.3455992366	0.6810658224	0.9977174325	0.8202808144	0.9867298836
0.1355699662	0.05447985183	0.6504699932	0.8202808144	0.6923511791
0.340104263	0.9093514035	0.9914493724	0.8202808144	0.9867298836
0.7601787897	0.927588801	0.9987579817	0.9748269319	0.9867298836
0.4546033673	0.2472501155	0.4934074404	0.9470903485	0.870124474
0.9447221743	0.7558806993	0.7867838207	0.9803444972	0.9867298836
0.8796426046	0.9389931831	0.9977174325	0.9748269319	0.9867298836
0.2001255913	0.8769767752	0.7867838207	0.8202808144	0.9867298836
0.3023489219	0.3029893204	0.7867838207	0.8202808144	0.9634120475
0.7710386558	0.4723848681	0.7867838207	0.9748269319	0.9634120475
0.7038636932	0.7143974322	0.7867838207	0.9748269319	0.9867298836
0.9853614792	0.8685968913	0.6504699932	0.9853614792	0.9867298836
0.0562117431	0.08022178104	0.308220099	0.8202808144	0.6923511791
0.6608559306	0.5933201721	0.7669520852	0.9748269319	0.9634120475
0.8334455291	0.7807794431	0.9977174325	0.9748269319	0.9867298836
0.8841939433	0.6393869554	0.9267280322	0.9748269319	0.9867298836
0.3189600867	0.905742012	0.9977174325	0.8202808144	0.9867298836
0.9185517801	0.942738773	0.5926789385	0.9748269319	0.9867298836
0.7028132582	0.8547230999	0.6504699932	0.9748269319	0.9867298836

0.3962595415	0.3252604076	0.793096728	0.8845079052	0.9634120475
0.0250540925	0.236127701	0.7867838207	0.8202808144	0.870124474
0.9751451519	0.931079469	0.9977174325	0.9830092257	0.9867298836
0.156668604	0.2580707395	0.793096728	0.8202808144	0.8718606065
0.9633746015	0.9363521938	0.8625613028	0.9803444972	0.9867298836
0.2574173127	0.7215181373	0.793096728	0.8202808144	0.9867298836
0.2040214986	0.09887835326	0.6504699932	0.8202808144	0.6923511791
0.5718574569	0.6733052542	0.7867838207	0.9748269319	0.9867298836
0.8718184088	0.6008549705	0.8625613028	0.9748269319	0.9634120475
0.0981405799	0.03158525082	0.5926789385	0.8202808144	0.6923511791
0.5378613321	0.6868886622	0.793096728	0.9748269319	0.9867298836
0.3236483287	0.4731647787	0.7867838207	0.8202808144	0.9634120475
0.0830669656	0.9416934878	0.6504699932	0.8202808144	0.9867298836
0.6991611194	0.4603075655	0.308220099	0.9748269319	0.9634120475
0.119876468	0.04864539177	0.6504699932	0.8202808144	0.6923511791
0.7122365734	0.9630483664	0.9987579817	0.9748269319	0.9867298836
0.0460673576	0.01394245873	0.4652536372	0.8202808144	0.6923511791
0.2735277008	0.1326996547	0.6504699932	0.8202808144	0.7211937753
0.6729515885	0.5408046464	0.6504699932	0.9748269319	0.9634120475
0.8930529806	0.6688780979	0.9867502072	0.9748269319	0.9867298836
0.140656264	0.04955953861	0.5926789385	0.8202808144	0.6923511791
0.856188039	0.7960795889	0.9364552596	0.9748269319	0.9867298836
0.0305351331	0.01200183308	0.308220099	0.8202808144	0.6923511791
0.6425337316	0.3652642266	0.793096728	0.9748269319	0.9634120475
0.7099929171	0.4079042386	0.8439060598	0.9748269319	0.9634120475
0.0569970896	0.9795116329	0.1012861489	0.8202808144	0.9917735625
0.9139116075	0.7388784276	0.9977174325	0.9748269319	0.9867298836
0.1655749312	0.1496824151	0.6504699932	0.8202808144	0.7484120754
0.2345775748	0.2077670838	0.308220099	0.8202808144	0.870124474
0.6429924539	0.3849808138	0.8523176191	0.9748269319	0.9634120475
0.6968168809	0.6732043704	0.9977174325	0.9748269319	0.9867298836
0.2337330546	0.2720400368	0.8219001698	0.8202808144	0.8948685421
0.6607230685	0.3632131264	0.7669520852	0.9748269319	0.9634120475
0.9062773413	0.6991374962	0.9141252915	0.9748269319	0.9867298836

Intercept Estimat	Blast Estimate	VNS Estimate	Blast:VNS Estimat	Intercept Std. Erro	Blast Std. Erro
0.1862036252	-0.1585939777	-0.2414121727	0.02768837327	0.2564678696	0.3349748581
0.1320788204	-0.210843694	-0.1964330009	0.365964301	0.2787717747	0.3218403288
0.1796058645	-0.4146335832	-0.0396196586	0.2605908371	0.2599806818	0.3300427684
-0.0720460121	0.0931080639	0.2148541588	-0.4089721397	0.2718391799	0.3266825531
-0.08417876058	0.0010548158	0.24117751	-0.05315828283	0.2719847962	0.3241518222
0.1090122372	-0.3833676609	-0.259402485	0.7200935783	0.2970369774	0.2964613721
0.07534050249	0.0486005314	-0.3567172486	0.2798318283	0.2527965312	0.3344183769
-0.2427809083	0.3835303967	0.0155285852	0.1695302879	0.248944937	0.3293231967
-0.2528121122	0.449920403	0.1453639231	-0.2998672791	0.2601333911	0.3302877948
-0.1068970553	0.0506544077	0.3038766818	-0.4504865524	0.2956379602	0.3112374014
0.2650911331	-0.3994019262	-0.2610365934	0.2914266764	0.2529078974	0.3345657006
-0.09349046117	-0.1364455461	0.0562094751	0.5222624623	0.2996464452	0.2893740797
0.00901581808	0.0581012266	-0.2270183327	0.3207537912	0.2588255314	0.3333315086
0.09883247034	0.0040541863	-0.286195728	0.10185882	0.2577015618	0.3346101072
0.1279196311	-0.0239936006	-0.0470494745	-0.4576740499	0.2506122327	0.3315288217
0.2922843076	-0.3898508417	-0.2441960002	0.09912726211	0.2514048192	0.3325773149
0.1217954104	0.0328637308	-0.4109114176	0.009883164737	0.276501201	0.3177372944
0.02046925437	-0.1175028811	-0.0686284584	0.3468945298	0.254782047	0.3370449674
-0.1712712765	0.4545159281	-0.4663177181	0.6806123769	0.2314882971	0.3062302328
-0.5658004526	0.6730665607	0.3965381484	0.04530300529	0.2553879661	0.3028046142
-0.05881865091	-0.1933910963	0.0413557631	0.00287954607	0.4227788288	0.1856113311
0.1433831779	0.0450902938	-0.0253644546	-0.3670249921	0.3158803996	0.2829914886
0.3672031281	-0.6141939786	-0.2179055039	0.2992381223	0.262770854	0.3206827667
-0.2126757667	0.0926350809	0.4765384003	-0.2308815058	0.251522044	0.3327323888
-0.5216405645	0.8493352243	0.5011866638	-0.7661107563	0.2611181654	0.3086614447
-0.2688510543	0.3298666093	0.389967474	-0.2800665686	0.3099935256	0.2655518046
-0.1002520089	0.3225158349	-0.0072997648	-0.3316656356	0.2562682992	0.3329820395
-0.250187041	0.3626941195	0.1555119813	-0.07594340189	0.2575501447	0.3318407186
0.416167342	-0.7092584479	-0.4711543561	0.8429083961	0.2649076032	0.3148776085
0.3252687531	-0.5967649727	-0.1947357066	0.3579932643	0.2488724064	0.3292272478
0.1589598841	-0.2237793896	-0.2581122692	0.08358733208	0.3709085894	0.1464552004
-0.06455449758	0.0280698397	-0.0130219460	-0.03702122538	0.4211922243	0.0997941186
-0.1692060535	0.3422611857	0.1636424689	-0.2521240277	0.2927215766	0.3071945821
-0.1963217056	0.326156363	0.3124298741	-0.6751264066	0.3043622289	0.2807120655
0.0778694057	0.1356511277	0.1031175911	-0.7419492816	0.2754196774	0.3095675031
0.1298867135	-0.4699303057	0.0212271521	0.05079583395	0.3380365577	0.2207028489
0.1025410348	0.1246150709	-0.1830395082	-0.1707771485	0.2941111792	0.3039472912
-0.00248189524	0.1659698631	-0.2675588637	0.294015138	0.271187432	0.3205755826
0.09298163417	0.1383495383	-0.4268497528	0.02785119213	0.2746038484	0.3116891283
0.03795657332	-0.1891483353	0.4645308279	-0.6729353303	0.2876608864	0.2804382876
0.1873783265	-0.4181241248	-0.0298784796	0.2617531377	0.2899522637	0.2992885629
0.01993959587	0.1059594045	0.2769962359	-0.6798179453	0.3119108042	0.2712287872
-0.1787713052	0.1354584702	0.5168613722	-0.5703957475	0.2591449273	0.3284668055
0.06161408894	0.0528276641	-0.0269022009	-0.3303494808	0.2763664619	0.3199063641
0.06677732509	-0.3579862672	-0.0227122056	0.7173734013	0.2864071236	0.3041968367

-0.04824489354	-0.0876396606	0.0843348009	0.2746278975	0.25392022	0.3359048775
0.08431351822	-0.113477078	0.250392416	-0.4259851133	0.3212958158	0.278005215
0.1319192454	-0.2069427253	-0.0925118629	0.1080209263	0.2776162037	0.3240707582
0.1550069923	-0.1131513606	-0.461603014	0.6308786115	0.2699727326	0.3235161454
0.365894064	-0.1940106873	-0.5280813505	-0.09187822025	0.2583041955	0.3165227996
-0.07978902738	0.221631913	0.233049718	-0.2921240939	0.3357138957	0.2463185632
0.3347677136	-0.5373333233	-0.550278359	0.8731785322	0.266991347	0.3186677253
0.02982167546	-0.4035053563	0.0702584552	0.6054021629	0.3044176405	0.2816347005
0.3634739045	-0.2399058875	-0.3601762613	-0.376694337	0.2408603096	0.31862824
0.1249573777	-0.2158771894	-0.1659538035	0.2792908071	0.2619631384	0.3345227309
0.08229197167	0.1831792359	-0.1928196932	-0.3021367566	0.2873343203	0.308853797
0.08627837896	-0.0301751771	-0.4350782551	0.6333391021	0.2600368729	0.3287211716
-0.007403762026	0.0417535634	-0.3964447291	0.5721613326	0.299565664	0.2993742833
0.1756310539	-0.3696989799	-0.4053998529	0.9930611173	0.2644733387	0.3165968467
-0.2656703559	0.3879578944	0.4512111358	-0.606354383	0.2612664521	0.3285755244
0.2018382594	-0.2967935866	-0.0826084617	-0.04681423499	0.2526910443	0.3342788308
0.2012822146	-0.4615361389	-0.2020252587	0.3179803862	0.2955793747	0.3000119788
0.3389357668	-0.6320465923	-0.3807371666	0.4600895402	0.3214396109	0.2411013689
-0.3003867302	0.115227992	0.5027718024	-0.1433198595	0.2914606571	0.2975018189
0.05080678249	-0.0701587750	-0.0166605248	-0.1244461837	0.2700334284	0.3288713096
0.2529519639	-0.3573601214	-0.2007380462	0.2998100181	0.2887515532	0.3117310874
-0.236894716	0.5335894813	0.2115369012	-0.794942056	0.28392315	0.2992007482
-0.2373253181	0.4016116375	0.5194696687	-0.9612290826	0.2544297229	0.326878746
-0.4147560344	0.4739015801	0.3955881373	-0.04504537092	0.2459453369	0.3253550988
-0.1350301162	0.190523203	0.4471091555	-0.792263441	0.2501074193	0.3308610163
0.1740001502	-0.2649840273	0.3589923864	-0.9437941061	0.2327251984	0.3078664994
-0.01606083432	-0.0132584405	-0.0944379619	0.4371253956	0.2747778361	0.3220247559
0.2551044445	-0.1005301176	-0.4122720003	0.1856001833	0.2938595898	0.2990244556
0.2417328274	-0.3270412043	-0.6256864191	0.9565335973	0.2598990061	0.3227213482
-0.2296424993	0.1523793025	0.3892167188	-0.0557444768	0.263506357	0.3261680285
0.09867256838	0.0384566528	-0.1425461018	-0.04052016523	0.3028776308	0.2952668972
-0.468304578	0.7716240233	0.4078273497	-0.5634188497	0.2471303504	0.3224485527
-0.1449740372	0.2671629549	-0.0451861662	0.1533899662	0.2619185281	0.3284879869
0.02802320831	0.0853702726	-0.0982233068	-0.03687584641	0.2968704061	0.3117571329
0.3540539676	-0.3043301692	-0.4028699395	-0.07554969753	0.2469825329	0.3267271801
0.3745222909	-0.4540906218	-0.5422286994	0.5051077194	0.2618732528	0.3243568987
-0.2349796394	0.4410470829	0.196621351	-0.3539457954	0.2648781061	0.3274858618
0.2832290556	-0.3582841105	-0.2493747357	0.1622570035	0.2992523001	0.2925015197
0.2465460105	-0.4459389728	-0.0506702241	0.07802123374	0.2784901355	0.3126452949
0.09569724572	0.1299508273	-0.7982636354	0.8156893506	0.2536355586	0.3006120527
-0.224081214	0.4448873185	-0.0413470137	-0.2459256915	0.299830494	0.3012546884
-0.1088221784	0.0871873002	0.2073166847	-0.1404808338	0.2553986954	0.3378607166
-0.005131767126	0.0535660418	-0.0712808830	0.2103146284	0.2956464557	0.2967635415
0.2081340033	-0.0939960435	-0.2964726301	0.04283592046	0.3429560812	0.2388110596
-0.2631342547	0.5170264461	0.1093642054	-0.03279567922	0.2816877152	0.3022060741
-0.3484375405	0.4902040442	0.2480241567	-0.08998806135	0.2487156658	0.3290198994

-0.1806549853	0.0548984985	0.4399747438	-0.4424039564	0.2955213881	0.2966318579
-0.3046639223	0.0509946691	0.8870508155	-0.5635707548	0.2394211783	0.3167244482
-0.02598198852	-0.0358307559	-0.0254597354	-0.03771802252	0.3147146499	0.2884836893
0.05180008554	0.1709260195	-0.0984699878	-0.5482580081	0.2636167733	0.3192690676
-0.1417504503	0.235075737	0.0794164052	-0.03677190635	0.2961674995	0.3048470215
-0.2036200355	0.1594077335	0.2557767649	0.1461931165	0.3053567462	0.2709992145
0.02893440529	-0.0669933525	-0.2292832369	0.745956402	0.2914109425	0.2952528885
0.3045652185	-0.3983775462	-0.3422571016	0.2056184102	0.2643745275	0.3244912831
0.172932547	-0.3248245825	-0.1283921622	0.2630339466	0.2541847953	0.3362548777
-0.1512685845	0.2593575532	0.4181473479	-0.8714941011	0.3039636882	0.263257855
0.2116514492	-0.1921743705	-0.1566536829	-0.2004603044	0.2515415197	0.3327581528
0.04233502942	-0.363129333	0.1238923341	0.3022562315	0.3056164715	0.2781817272
0.3384724004	-0.3206162562	-0.4222551992	-0.02828513019	0.3048817818	0.2557272763
-0.2189550189	0.4886043674	-0.2733594385	0.3483547435	0.2589814162	0.31132518
0.2199680497	-0.3902769739	-0.5550390204	0.8290627242	0.3151185399	0.2734616861
-0.1006650338	0.0149722563	0.2197980371	-0.02321226666	0.2610402954	0.3336241862
-0.2285475353	0.4620926613	0.4792360556	-1.077021707	0.3073738926	0.2783829537
0.2151878806	-0.270110837	-0.2316194046	0.3265915091	0.3910712273	0.1417506841
-0.2348994365	0.5674198304	0.0032961490	-0.3091140064	0.2480550097	0.3281459336
-0.1317291893	0.1980771619	0.1632123563	-0.2159656458	0.255385174	0.3378428294
0.2715843844	-0.6161624254	-0.3905872148	0.8669522589	0.2973279573	0.287234371
-0.06620517248	0.2267946157	-0.0542008088	-0.1245676065	0.2818096345	0.317492056
0.2360393563	-0.2936868619	-0.8145040934	1.194555638	0.2628020617	0.3080042075
0.01227320994	0.0141256003	0.1637859349	-0.4366225838	0.2793681454	0.3187778162
0.0001352241089	0.0171726102	0.1520072575	-0.2985951038	0.3410986492	0.2377158114
-0.6165574123	0.775886241	0.4935272053	0.01056203603	0.2619785709	0.2715115924
0.04896773132	-0.0407209482	-0.1348866452	0.1489861081	0.3036361184	0.2957506631
0.1054452303	0.0545402760	0.0527225602	-0.6983128555	0.2627343205	0.3194563932
-0.5442798437	0.8177437636	0.5245013235	-0.5438414756	0.2874137686	0.2816861477
-0.120128647	0.0710905163	0.3191635069	-0.4364130542	0.2726102811	0.3277594307
-0.05241013265	-0.0493304516	0.0774683596	0.2123560915	0.2546550192	0.3368769255
0.3195058697	-0.2601185666	-0.6025054407	0.5393957355	0.2644431858	0.3251244132
-0.04836798411	0.1280607399	-0.2546876214	0.4577778046	0.251177294	0.3315060099
0.1254652467	-0.2781161792	-0.0447950294	0.1944449381	0.2544838091	0.3366504358

Full model fit coefficient estimates (microbe ~ Blast + VNS + Blast:VNS + (1|I					
VNS Std. Erro	Blast:VNS Std. Err	Intercept t-value	Blast t-value	VNS t-value	Blast:VNS t-valu
0.3583981591	0.5008852441	0.7260310055	-0.4734503914	-0.6735865311	0.05527887593
0.3462449478	0.4843182317	0.4737883544	-0.6551189368	-0.5673238041	0.7556277609
0.35370061	0.4945420383	0.6908431167	-1.256302585	-0.1120146742	0.5269336414
0.3509702716	0.4908928944	-0.2650317446	0.285010825	0.6121719594	-0.8331188827
0.3483599816	0.4872528246	-0.3094980372	0.0032540798	0.6923226625	-0.109097947
0.3199506445	0.4475156049	0.3669988773	-1.293145404	-0.8107578137	1.60909155
0.3575082829	0.4994792575	0.298028229	0.1453285309	-0.9977873679	0.5602471455
0.3520613062	0.4918692188	-0.9752393891	1.164601827	0.0441076170	0.3446653734
0.3539602907	0.4949042689	-0.9718556744	1.362207172	0.4106786184	-0.6059096635
0.3355915004	0.4694112885	-0.3615809526	0.162751673	0.9054957631	-0.9596841053
0.3576657785	0.4996992966	1.048172619	-1.193792207	-0.7298338535	0.5832040956
0.3124471439	0.4370086155	-0.3120025706	-0.4715195856	0.1799007488	1.195085048
0.356941212	0.4989784413	0.03483357314	0.1743046341	-0.6360104272	0.6428209411
0.3581373723	0.5005804324	0.3835152167	0.0121161501	-0.7991227673	0.2034814256
0.3544192184	0.4951634873	0.5104285201	-0.0723725935	-0.1327509122	-0.9242887686
0.3555401049	0.4967294916	1.162604235	-1.172211165	-0.6868310968	0.1995598485
0.3418789548	0.4782129009	0.4404878168	0.1034305113	-1.201920773	0.02066687185
0.3603162263	0.5034022701	0.08034025401	-0.3486267188	-0.1904672991	0.6891000506
0.3273738893	0.4573781224	-0.7398701301	1.484229444	-1.424419397	1.488073748
0.3254791581	0.4552545478	-2.215454632	2.222775114	1.218321169	0.09951137337
0.2011440631	0.2812378767	-0.139123927	-1.041914279	0.2056027034	0.01023882737
0.3058169316	0.4277093882	0.453916033	0.1593344522	-0.0829399946	-0.8581176899
0.3443114477	0.4815522532	1.39742716	-1.915269676	-0.6328732472	0.6214032232
0.3557058859	0.4969611063	-0.8455551781	0.2784071645	1.339697821	-0.4645866706
0.3318100289	0.464112463	-1.997718404	2.751672549	1.510462675	-1.650700676
0.2870870625	0.4015008975	-0.867279579	1.242193062	1.358359623	-0.6975490476
0.3563797356	0.4981171007	-0.3911994158	0.9685682607	-0.0204831086	-0.6658386857
0.3553354456	0.496730147	-0.9714109899	1.092976537	0.4376483775	-0.1528866375
0.3384259079	0.4733608979	1.570990553	-2.252489312	-1.39219352	1.780688688
0.3519587325	0.4917259118	1.306969936	-1.812623277	-0.5532913055	0.7280341665
0.1587308795	0.2219328534	0.4285688945	-1.527971618	-1.626099912	0.3766334312
0.1081931025	0.151266568	-0.153266119	0.2812774952	-0.1203583756	-0.2447416231
0.331252327	0.4633408682	-0.5780443499	1.114151113	0.4940115299	-0.5441437287
0.30326218	0.4241460198	-0.6450265077	1.161889363	1.030230259	-1.59173109
0.3332963959	0.4662117254	0.2827300011	0.4381956321	0.3093870572	-1.591442774
0.2389603939	0.3341457233	0.3842386588	-2.129244403	0.0888312568	0.1520170106
0.3278428699	0.4585669194	0.3486471853	0.4099890819	-0.5583147446	-0.3724148892
0.3446172552	0.482026275	-0.00915195524	0.5177245932	-0.7763942741	0.6099566625
0.3354909579	0.4692804393	0.338602808	0.4438702725	-1.272313732	0.05934871732
0.3027604072	0.4234634308	0.1319490244	-0.6744740061	1.534318282	-1.589122652
0.3228247323	0.4515474497	0.6462385364	-1.397060151	-0.0925532547	0.5796802482
0.2931867504	0.4100359297	0.06392723689	0.3906643009	0.9447774686	-1.657947258
0.3520407934	0.4922297062	-0.6898506835	0.4123962239	1.468186023	-1.158799927
0.3441362829	0.481367554	0.2229434372	0.1651347708	-0.0781731024	-0.6862728452
0.3279430642	0.4587155155	0.233155252	-1.176824424	-0.0692565511	1.56387429

0.3590974189	0.5016994591	-0.1900002038	-0.2609061865	0.23485215	0.5473952433
0.3005248097	0.4202970487	0.2624171062	-0.408183271	0.8331838433	-1.013533439
0.3485226073	0.4874984784	0.4751856831	-0.6385726577	-0.2654400632	0.2215820789
0.3476061938	0.486191641	0.5741579558	-0.349754911	-1.327948185	1.297592468
0.3397859183	0.4752138833	1.41652389	-0.6129437991	-1.554159022	-0.1933407745
0.2665626944	0.3727620631	-0.2376697194	0.8997775489	0.8742773198	-0.783674421
0.3424478502	0.47898178	1.253852297	-1.686186836	-1.606896813	1.822989034
0.3042485294	0.4255265951	0.0979630333	-1.432725994	0.2309245518	1.422712869
0.3406279165	0.4758954882	1.509065172	-0.7529335363	-1.057389145	-0.7915484519
0.3583878479	0.5010605951	0.4770036674	-0.6453289102	-0.4630564471	0.5573992644
0.3328813596	0.4656261251	0.2863979896	0.5930936828	-0.5792444894	-0.6488827416
0.352362312	0.4926928859	0.3317928647	-0.0917956606	-1.234746851	1.285464272
0.3230879745	0.4519042705	-0.02471498879	0.1394694394	-1.227048855	1.266111807
0.340184981	0.4758130542	0.6640784843	-1.167727928	-1.191704148	2.087082539
0.352298797	0.4926282132	-1.016855987	1.180726699	1.280762636	-1.230855982
0.3573591019	0.4992708349	0.7987550963	-0.8878623449	-0.2311637267	-0.09376521063
0.3236970493	0.4527617906	0.6809751689	-1.538392369	-0.6241183205	0.7023127676
0.2608867808	0.3648290058	1.054430615	-2.62149732	-1.459396162	1.26111009
0.3209589123	0.4489338662	-1.03062531	0.3873186134	1.566467804	-0.3192449274
0.3531335556	0.4938951541	0.1881499738	-0.2133320024	-0.0471791042	-0.2519688292
0.3359510975	0.469920997	0.8760194053	-1.146373063	-0.5975216265	0.6380008981
0.3226068247	0.4512491599	-0.8343621012	1.783382845	0.6557111785	-1.761647725
0.3500798922	0.4894046745	-0.9327735589	1.228625729	1.483860342	-1.964078262
0.3478192311	0.485942563	-1.686374865	1.456567246	1.137338312	-0.0926969036
0.3537053044	0.4941660692	-0.539888487	0.5758405906	1.26407252	-1.603233185
0.3291231319	0.4598220109	0.7476635593	-0.8607108206	1.090754042	-2.052520505
0.3462772354	0.4843551386	-0.05845025403	-0.0411721157	-0.2727235644	0.9024894356
0.3226181498	0.4512536058	0.8681167922	-0.3361936314	-1.277894627	0.4112990586
0.3462348631	0.4841965638	0.9301029314	-1.013385715	-1.80711559	1.975506786
0.3499836524	0.4894490203	-0.8714875115	0.4671803769	1.112099711	-0.1138923044
0.3187694234	0.4458548349	0.3257836114	0.1302436989	-0.4471762074	-0.09088196888
0.3450179387	0.4821960881	-1.89496991	2.39301438	1.182046798	-1.168443427
0.3522533005	0.4925763629	-0.5535081394	0.813311188	-0.1282774814	0.3114034245
0.3361679195	0.4702167878	0.09439542553	0.273835828	-0.2921852478	-0.07842307499
0.3492860477	0.4879918707	1.433518247	-0.9314504202	-1.153409769	-0.154817533
0.348029953	0.4867148252	1.430166261	-1.39997214	-1.557994347	1.037789879
0.3514162582	0.4914559787	-0.8871236768	1.34676679	0.559511253	-0.7201983713
0.3157724812	0.4416640459	0.9464557349	-1.224896578	-0.7897291579	0.3673765275
0.3366207551	0.4708618626	0.8852953087	-1.426341545	-0.1505261436	0.1656987748
0.3231267963	0.4519646056	0.3773021664	0.4322874818	-2.470434655	1.804763781
0.3250904071	0.4547070497	-0.747359653	1.476781394	-0.1271862005	-0.5408442462
0.3611882988	0.5046206535	-0.4260874483	0.2580569327	0.5739850527	-0.2783889895
0.3202487302	0.4479346024	-0.01735778335	0.1805007501	-0.2225797524	0.4695208346
0.2585011278	0.3614797761	0.6068823813	-0.3936000439	-1.146891051	0.1185015685
0.3257296358	0.4556220232	-0.934134648	1.710840682	0.3357514743	-0.07198001314
0.3517370677	0.4914162212	-1.4009473	1.489891782	0.7051407982	-0.1831198431

0.3201067284	0.4477359758	-0.611309342	0.1850728337	1.374462655	-0.9880911527
0.3385926775	0.4730520305	-1.272501975	0.1610064189	2.619816891	-1.191350461
0.3116788459	0.4359155374	-0.08255728967	-0.1242037497	-0.0816857986	-0.08652598792
0.3429010794	0.4795944105	0.1964976845	0.5353666761	-0.2871673312	-1.143170137
0.3288364189	0.4599552696	-0.4786158188	0.7711268946	0.2415073292	-0.07994670085
0.2928840083	0.4096187267	-0.6668267134	0.5882221237	0.873303962	0.3569004709
0.3185714799	0.4455919247	0.09929073026	-0.2269015992	-0.7197230491	1.674079714
0.3483154381	0.487139055	1.152021798	-1.227698761	-0.982606753	0.4220938725
0.3594715848	0.502222211	0.680341823	-0.9660070503	-0.3571691551	0.5237401709
0.2845805134	0.3979985277	-0.4976534709	0.9851844807	1.469346382	-2.189691771
0.3557334287	0.4969995867	0.84141755	-0.5775196456	-0.4403681809	-0.4033409882
0.3005708902	0.4203775656	0.1385233892	-1.30536731	0.4121900628	0.7190113276
0.276512682	0.3867066207	1.11017588	-1.253742897	-1.527073537	-0.07314364088
0.33446723	0.4678100977	-0.8454468362	1.569434144	-0.8172981207	0.7446499022
0.2956056911	0.4134183249	0.6980485813	-1.427172411	-1.877633067	2.005384557
0.3574086899	0.4996862436	-0.3856302478	0.0448776106	0.6149767571	-0.04645368359
0.3008038781	0.4207017723	-0.7435489509	1.659917229	1.593184432	-2.560059828
0.1536444114	0.2148190215	0.5502523979	-1.905534628	-1.507502957	1.520310011
0.3508027589	0.4901108868	-0.9469650976	1.729169167	0.0093960181	-0.6307021833
0.3611691767	0.5045939376	-0.5158059383	0.5862997367	0.4519000148	-0.4279988911
0.3101354133	0.4337754048	0.9134169113	-2.14515562	-1.259408626	1.998620137
0.3418069729	0.4781160834	-0.234928705	0.7143316233	-0.1585713961	-0.2605384149
0.3312002486	0.463266178	0.8981640204	-0.9535157468	-2.459249644	2.57855137
0.3430694624	0.4798801224	0.04393203069	0.0443117420	0.4774133311	-0.9098576154
0.2573146507	0.3598207782	0.000396436952	0.0722400841	0.5907446666	-0.8298439721
0.2928423445	0.4096111356	-2.353465057	2.857654195	1.685300007	0.02578551976
0.3192954681	0.446590282	0.1612711017	-0.1376867522	-0.4224508602	0.3336080387
0.3430473444	0.4797917411	0.4013378616	0.1707283912	0.1536888746	-1.455449929
0.3040852719	0.425318345	-1.893715275	2.903031513	1.724849481	-1.278669218
0.3521210034	0.4925017135	-0.4406607355	0.2168984617	0.9064029235	-0.8861147937
0.3601365819	0.5031512867	-0.2058083631	-0.1464346409	0.2151082771	0.4220521683
0.3489693114	0.488049282	1.208221224	-0.80005855	-1.72652844	1.105207518
0.3544493603	0.4952387728	-0.1925651134	0.3862999043	-0.7185444522	0.9243577639
0.3598944542	0.5028130071	0.4930185819	-0.826127489	-0.1244671288	0.3867142166

Batch))

Intercept Pr(>t	Blast Pr(>t)	VNS Pr(>t)	Blast:VNS Pr(>t	Blast Adjusted p-valu	VNS Adjusted p-valu
0.4717721113	0.6375874105	0.5030830517	0.9560935902	0.9715330331	0.9094096952
0.6393388214	0.5149119155	0.5725884718	0.4527962518	0.9446238668	0.9094096952
0.4937732241	0.213837116	0.9111760475	0.6001265862	0.6794305346	0.9762566711
0.7927261947	0.7766207887	0.5427016509	0.4080271324	0.9715330331	0.9094096952
0.7589245883	0.997414402	0.4913573178	0.9134819371	0.997414402	0.9094096952
0.7181529924	0.2010588494	0.4207528074	0.1129215333	0.6794305346	0.9094096952
0.7666622971	0.8849154585	0.3221995489	0.5772977573	0.9715330331	0.856913694
0.3331714454	0.2485713156	0.9649581275	0.7314939168	0.6794305346	0.9806485036
0.3374445177	0.1782112372	0.6827377942	0.5468000845	0.6794305346	0.9482469364
0.7217371056	0.8712768598	0.3688659403	0.3411117346	0.9715330331	0.9040831871
0.2985637064	0.2370351399	0.468196845	0.5618379035	0.6794305346	0.9094096952
0.7589794566	0.6390255376	0.8578449308	0.2368236948	0.9715330331	0.9762566711
0.9723826395	0.8622068001	0.5271209461	0.5227118487	0.9715330331	0.9094096952
0.7032443352	0.990372594	0.4272767101	0.839423832	0.997414402	0.9094096952
0.6115351518	0.9425346728	0.8948134811	0.3588627933	0.9819388528	0.9762566711
0.2493752298	0.2455260069	0.4947096444	0.8424675507	0.6794305346	0.9094096952
0.6638456378	0.9179767377	0.2341448852	0.9835803476	0.9807443779	0.772928993
0.9362213161	0.7285319709	0.849555396	0.4932905013	0.9715330331	0.9762566711
0.4621270535	0.1427343843	0.1592599022	0.1417200842	0.6794305346	0.772928993
0.0344858250б	0.0300302745	0.2278073795	0.92105925	0.5212109185	0.772928993
0.893270234	0.3018461465	0.8378313054	0.9918663807	0.7546153662	0.9762566711
0.657078761	0.8739590166	0.9341816646	0.3943235963	0.9715330331	0.9762566711
0.1717931078	0.0602427903	0.5291811331	0.5366448212	0.6794305346	0.9094096952
0.4010009889	0.781611578	0.1851562804	0.6438291186	0.9715330331	0.772928993
0.0548588263	0.0078373247	0.1361012583	0.1039478603	0.3265551964	0.772928993
0.4014423538	0.2191540565	0.1795479942	0.4882155992	0.6794305346	0.772928993
0.6975480993	0.3365854845	0.9837238137	0.5079763023	0.7817925236	0.9916570702
0.3374482993	0.2787581979	0.6631745033	0.8789859123	0.7111178519	0.9482469364
0.1265270507	0.0279765337	0.1689292277	0.0799482369	0.5212109185	0.772928993
0.1959734672	0.0746550233	0.5820222049	0.4692900302	0.6794305346	0.9094096952
0.6812228107	0.1320333044	0.1094178193	0.7078378771	0.6794305346	0.772928993
0.8829838185	0.7795154295	0.9046219323	0.8075342898	0.9715330331	0.9762566711
0.5697713331	0.2697570467	0.6231132529	0.5883729548	0.702492309	0.9482469364
0.52904787	0.2500304367	0.3071200405	0.1168106813	0.6794305346	0.8345653273
0.7798117931	0.6628440166	0.7580967742	0.1167540956	0.9715330331	0.9762566711
0.7096363272	0.0375271861	0.9295229466	0.8797029514	0.5212109185	0.9762566711
0.7311385894	0.6833064194	0.5787213272	0.7109060082	0.9715330331	0.9094096952
0.9927589906	0.6065637629	0.4405259563	0.5441613632	0.9715330331	0.9094096952
0.7375782808	0.6587465335	0.2081295336	0.9528699051	0.9715330331	0.772928993
0.8967316058	0.5026900618	0.1303110547	0.1173963871	0.9446238668	0.772928993
0.5256324441	0.167645075	0.9265685161	0.5643138282	0.6794305346	0.9762566711
0.9499663114	0.6974718529	0.348639919	0.1026603036	0.9715330331	0.8893875485
0.4943371964	0.6815047686	0.1471384327	0.2509944134	0.9715330331	0.772928993
0.8252205201	0.8693975968	0.9379479524	0.4951577094	0.9715330331	0.9762566711
0.8179144568	0.2440021445	0.9450164577	0.1231259036	0.6794305346	0.9762566711

0.849919849	0.7950158331	0.8150851459	0.5860412737	0.9715330331	0.9762566711
0.7972177278	0.6846453742	0.4081306707	0.3149835856	0.9715330331	0.9094096952
0.6383808149	0.5255545575	0.7915719073	0.825384153	0.9446238668	0.9762566711
0.5702028779	0.7277578393	0.1891652927	0.1993286301	0.9715330331	0.772928993
0.1656247697	0.5422248538	0.1252999104	0.8473308128	0.9446238668	0.772928993
0.8168135946	0.371979503	0.385567217	0.4364168505	0.8146380077	0.9094096952
0.2191770264	0.0969636223	0.1132441231	0.0732034259	0.6794305346	0.772928993
0.9232631499	0.1572770807	0.8181725753	0.1600912349	0.6794305346	0.9762566711
0.1362812815	0.4542942779	0.2943717656	0.431594186	0.8872935116	0.8176993489
0.636031698	0.5211572211	0.6449563288	0.5792680689	0.9446238668	0.9482469364
0.7772372143	0.5553821875	0.5645924176	0.5188907467	0.9446238668	0.9094096952
0.7425437131	0.9271759556	0.2217228225	0.2035256414	0.9819388528	0.772928993
0.9805662211	0.8895590367	0.2246573106	0.2104318127	0.9715330331	0.772928993
0.5116183206	0.2475585865	0.2380034586	0.0410707095	0.6794305346	0.772928993
0.3176173642	0.2424807414	0.2051697968	0.2231230037	0.6794305346	0.772928993
0.4274334461	0.3779920356	0.8179367273	0.9255932377	0.8146380077	0.9762566711
0.5049307657	0.1293681941	0.5349490552	0.4852400673	0.6794305346	0.9094096952
0.3151984334	0.0111729076	0.1498359531	0.2123075888	0.3491533638	0.772928993
0.3159149841	0.6999225808	0.1225421309	0.7506595597	0.9715330331	0.772928993
0.8518969626	0.8317908003	0.9625242264	0.8019087222	0.9715330331	0.9806485036
0.3906028886	0.2562748461	0.552411327	0.5259053849	0.6815820375	0.9094096952
0.4136454563	0.0796777038	0.5145250893	0.0832431009	0.6794305346	0.9094096952
0.3572991307	0.2240367471	0.1429800259	0.0540446994	0.6794305346	0.772928993
0.0966702528	0.1502019837	0.2597041417	0.9264385079	0.6794305346	0.772928993
0.5911772736	0.5667743201	0.2108610377	0.1138854662	0.9446238668	0.772928993
0.4574448822	0.3926612168	0.2795353704	0.0442786897	0.8319093576	0.7941345749
0.953794351	0.9672963029	0.7859913587	0.3703616722	0.9830246981	0.9762566711
0.3962876991	0.7379233157	0.2062447178	0.6823304281	0.9715330331	0.772928993
0.3583486918	0.3149165639	0.0756384753	0.0526953114	0.7718543233	0.772928993
0.3893786197	0.6420580518	0.270437334	0.9096945314	0.9715330331	0.7861550406
0.7486460982	0.8968208456	0.6563789407	0.9278932819	0.9715330331	0.9482469364
0.0648437405	0.0198126012	0.2417096599	0.2470896107	0.4953150309	0.772928993
0.5833229669	0.419246275	0.8983489076	0.7565465861	0.8591112192	0.9762566711
0.9258550357	0.7851835108	0.7711684939	0.9377566636	0.9715330331	0.9762566711
0.1566542989	0.3551764382	0.2530994548	0.877460103	0.7928045496	0.772928993
0.1615064687	0.1666593456	0.1243839941	0.3034362016	0.6794305346	0.772928993
0.3808141221	0.1830870948	0.5778469958	0.4741287596	0.6794305346	0.9094096952
0.3572429263	0.2255224618	0.432833165	0.7146473241	0.6794305346	0.9094096952
0.3843698182	0.159010472	0.8808515258	0.8689478398	0.6794305346	0.9762566711
0.7090090348	0.667113208	0.0163434312	0.0761059050	0.9715330331	0.6999447309
0.4652968507	0.145131803	0.8992252243	0.5906513112	0.6794305346	0.9762566711
0.6714964041	0.7972041132	0.5680216041	0.7816254649	0.9715330331	0.9094096952
0.9863359987	0.8573801603	0.8246237427	0.6404120668	0.9715330331	0.9762566711
0.5579545306	0.6953302106	0.2561407544	0.9060804012	0.9715330331	0.772928993
0.3613020759	0.0924048404	0.7382376279	0.9428589714	0.6794305346	0.9762566711
0.1661365026	0.1412423841	0.4833216116	0.8552920411	0.6794305346	0.9094096952

0.548587347	0.8538104184	0.1744556574	0.3271140384	0.9715330331	0.772928993
0.2078712917	0.872603542	0.0110104044	0.2379850259	0.9715330331	0.6999447309
0.9353513634	0.9015826547	0.9351740161	0.931342549	0.9715330331	0.9762566711
0.8454244841	0.5943741128	0.7749554593	0.2574192036	0.9715330331	0.9762566711
0.6377884532	0.4437328753	0.8099929762	0.9365483203	0.8804223717	0.9762566711
0.5157560599	0.5586574741	0.3860467632	0.7224438426	0.9446238668	0.9094096952
0.921999877	0.821290696	0.474517601	0.09937615863	0.9715330331	0.9094096952
0.2568248324	0.2243190756	0.3296544646	0.6744274021	0.6794305346	0.8584751683
0.4987807408	0.3377343702	0.7221588474	0.6022969057	0.7817925236	0.9762566711
0.6268627924	0.3286155404	0.1470758093	0.03253525753	0.7817925236	0.772928993
0.4032966186	0.5656468272	0.6611777445	0.6880630648	0.9446238668	0.9482469364
0.8917118074	0.1968943778	0.6816947144	0.4749758721	0.6794305346	0.9482469364
0.2875587774	0.2149573465	0.1321194864	0.941940959	0.6794305346	0.772928993
0.4046598148	0.1218555255	0.4169542348	0.4593522963	0.6794305346	0.9094096952
0.4974626098	0.1588925488	0.0654111136	0.04955127073	0.6794305346	0.772928993
0.7018444115	0.9643526587	0.5408343293	0.9630983287	0.9830246981	0.9094096952
0.4692064571	0.1023094199	0.1164747553	0.0130547426	0.6794305346	0.772928993
0.5998803301	0.0617482817	0.1371845111	0.1339422117	0.6794305346	0.772928993
0.3472747416	0.0886764636	0.9925328634	0.5305166572	0.6794305346	0.9925328634
0.6077955002	0.5597689896	0.6528930531	0.6701115396	0.9446238668	0.9482469364
0.3746909509	0.0361211458	0.2128387804	0.0502641679	0.5212109185	0.772928993
0.8161258243	0.4778195828	0.8745354341	0.7953343793	0.9188838131	0.9762566711
0.3769316034	0.3441994847	0.0167986735	0.01236954631	0.7822715562	0.6999447309
0.9652973506	0.9648048601	0.6347919209	0.3665153345	0.9830246981	0.9482469364
0.9996917953	0.9426612987	0.556989603	0.4100363086	0.9819388528	0.9094096952
0.02905014552	0.0058922377	0.0971511679	0.9795144529	0.3265551964	0.772928993
0.8737985282	0.8909606352	0.6742241699	0.7398533071	0.9715330331	0.9482469364
0.6906174469	0.8650087975	0.8783597328	0.1506382462	0.9715330331	0.9762566711
0.07516864414	0.0052018825	0.0897579617	0.2059935391	0.3265551964	0.772928993
0.6630611911	0.8290400029	0.3683366297	0.3790757126	0.9715330331	0.9040831871
0.8376042222	0.884045855	0.8303777408	0.6744237641	0.9715330331	0.9762566711
0.236048894	0.4268609362	0.0893272907	0.2734109383	0.8606067263	0.772928993
0.848277261	0.7006369664	0.475139329	0.3588816154	0.9715330331	0.9094096952
0.6237135778	0.4118495084	0.9013417198	0.7002709377	0.8580198093	0.9762566711

	Taxonomic Information		
Blast:VNS Adjusted p-valu	Genus	Family	Order
0.9867810745	Acetatifactor	Lachnospiraceae	Eubacteriales
0.9652194001	Acetatifactor	Lachnospiraceae	Eubacteriales
0.9652194001	Acutalibacter	Oscillospiraceae	Eubacteriales
0.9652194001	Acutalibacter	Oscillospiraceae	Eubacteriales
0.9867810745	Adlercreutzia	Eggerthellaceae	Eggerthellales
0.6987880186	Adlercreutzia	Eggerthellaceae	Eggerthellales
0.9652194001	Adlercreutzia	Eggerthellaceae	Eggerthellales
0.9867810745	Akkermansia	Akkermansiaceae	Verrucomicrobiales
0.9652194001	Alistipes	Rikenellaceae	Bacteroidales
0.9652194001	Anaerotruncus	Oscillospiraceae	Eubacteriales
0.9652194001	Bacteria_unclassified	Bacteria_unclassified	Bacteria_unclassified
0.8696594716	Bacteria_unclassified	Bacteria_unclassified	Bacteria_unclassified
0.9652194001	Bacteria_unclassified	Bacteria_unclassified	Bacteria_unclassified
0.9867810745	Bacteroides	Bacteroidaceae	Bacteroidales
0.9652194001	Bifidobacterium	Bifidobacteriaceae	Bifidobacteriales
0.9867810745	Clostridia_unclassified	Clostridia_unclassified	Clostridia_unclassifie
0.9915124472	Clostridiaceae_unclassified	Clostridiaceae	Eubacteriales
0.9652194001	Clostridiaceae_unclassified	Clostridiaceae	Eubacteriales
0.7381254386	Eubacteriales_unclassified	Eubacteriales_unclassified	Eubacteriales
0.9867810745	Erysipelatoclostridium	Erysipelotrichaceae	Erysipelotrichales
0.9918663807	Coriobacteriaceae_unclassifie	Coriobacteriaceae	Coriobacteriales
0.9652194001	Dorea	Lachnospiraceae	Eubacteriales
0.9652194001	Dubosiella	Erysipelotrichaceae	Erysipelotrichales
0.9867810745	Erysipelotrichales_unclassifie	Erysipelotrichales_unclassifie	Erysipelotrichales
0.6987880186	Eubacteriaceae_unclassified	Eubacteriaceae	Eubacteriales
0.9652194001	Eubacteriaceae_unclassified	Eubacteriaceae	Eubacteriales
0.9652194001	GGB20149	Lachnospiraceae	Eubacteriales
0.9867810745	GGB22635	Eggerthellaceae	Eggerthellales
0.6987880186	GGB25041	Lachnospiraceae	Eubacteriales
0.9652194001	GGB27876	Muribaculaceae	Bacteroidales
0.9867810745	GGB27878	Muribaculaceae	Bacteroidales
0.9867810745	GGB27918	Muribaculaceae	Bacteroidales
0.9652194001	GGB28382	FGB9508	OFGB9508
0.6987880186	GGB28399	FGB2838	OFGB2838
0.6987880186	GGB28411	FGB2838	OFGB2838
0.9867810745	GGB28415	FGB2838	OFGB2838
0.9867810745	GGB28430	Pumilibacteraceae	Eubacteriales
0.9652194001	GGB28439	FGB28439	OFGB28439
0.9867810745	GGB28778	Clostridia_unclassified	Clostridia_unclassifie
0.6987880186	GGB28784	Eubacteriaceae	Eubacteriales
0.9652194001	GGB28792	Lachnospiraceae	Eubacteriales
0.6987880186	GGB28798	Lachnospiraceae	Eubacteriales
0.8696594716	GGB28802	Lachnospiraceae	Eubacteriales
0.9652194001	GGB28818	Lachnospiraceae	Eubacteriales
0.6995789976	GGB28828	FGB77305	OFGB77305

0.9652194001	GGB28851	Clostridiaceae	Eubacteriales
0.9652194001	GGB28859	Lachnospiraceae	Eubacteriales
0.9867810745	GGB28864	Lachnospiraceae	Eubacteriales
0.8560789869	GGB28869	Lachnospiraceae	Eubacteriales
0.9867810745	GGB28883	FGB9633	OFGB9633
0.9652194001	GGB28892	Bacteria_unclassified	Bacteria_unclassified
0.6987880186	GGB28893	Bacteria_unclassified	Bacteria_unclassified
0.7696693983	GGB28898	Bacteria_unclassified	Bacteria_unclassified
0.9652194001	GGB28904	Bacteria_unclassified	Bacteria_unclassified
0.9652194001	GGB28916	Lachnospiraceae	Eubacteriales
0.9652194001	GGB28924	Lachnospiraceae	Eubacteriales
0.8560789869	GGB28926	Lachnospiraceae	Eubacteriales
0.8560789869	GGB28927	FGB77359	OFGB77359
0.6987880186	GGB28934	FGB9639	OFGB9639
0.8696594716	GGB28946	Lachnospiraceae	Eubacteriales
0.9867810745	GGB28949	Lachnospiraceae	Eubacteriales
0.9652194001	GGB28949	Lachnospiraceae	Eubacteriales
0.8560789869	GGB28950	Clostridiaceae	Eubacteriales
0.9867810745	GGB28951	Clostridiaceae	Eubacteriales
0.9867810745	GGB28951	Clostridiaceae	Eubacteriales
0.9652194001	GGB28954	Clostridiaceae	Eubacteriales
0.6987880186	GGB28956	Clostridiaceae	Eubacteriales
0.6987880186	GGB28960	Clostridiaceae	Eubacteriales
0.9867810745	GGB28967	Clostridiaceae	Eubacteriales
0.6987880186	GGB28991	Eubacteriaceae	Eubacteriales
0.6987880186	GGB29002	FGB9658	OFGB9658
0.9652194001	GGB29003	FGB9659	OFGB9659
0.9867810745	GGB29011	Bacteria_unclassified	Bacteria_unclassified
0.6987880186	GGB29531	FGB9827	OFGB9827
0.9867810745	GGB29685	Eubacteriaceae	Eubacteriales
0.9867810745	GGB30141	FGB77303	OFGB77303
0.8696594716	GGB30286	Eubacteriales_unclassified	Eubacteriales
0.9867810745	GGB30303	Oscillospiraceae	Eubacteriales
0.9867810745	GGB30413	FGB30328	OFGB30328
0.9867810745	GGB30454	Oscillospiraceae	Eubacteriales
0.9652194001	GGB30455	Oscillospiraceae	Eubacteriales
0.9652194001	GGB30461	Oscillospiraceae	Eubacteriales
0.9867810745	GGB30461	Oscillospiraceae	Eubacteriales
0.9867810745	GGB30463	Oscillospiraceae	Eubacteriales
0.6987880186	GGB30473	Oscillospiraceae	Eubacteriales
0.9652194001	GGB30475	Oscillospiraceae	Eubacteriales
0.9867810745	GGB30861	FGB77153	OFGB77153
0.9867810745	GGB31312	FGB1791	OFGB1791
0.9867810745	GGB31438	FGB10290	OFGB10290
0.9867810745	GGB3171	Oscillospiraceae	Eubacteriales
0.9867810745	GGB31823	FGB1765	OFGB1765

0.9652194001	GGB31853	FGB10349	OFGB10349
0.8696594716	GGB32371	FGB10667	OFGB10667
0.9867810745	GGB3793	Lachnospiraceae	Eubacteriales
0.8696594716	GGB42598	Lachnospiraceae	Eubacteriales
0.9867810745	GGB45656	Christensenellaceae	Eubacteriales
0.9867810745	GGB47127	FGB10299	OFGB10299
0.6987880186	GGB74395	Oscillospiraceae	Eubacteriales
0.9867810745	GGB75053	Oscillospiraceae	Eubacteriales
0.9652194001	GGB75109	Lachnospiraceae	Eubacteriales
0.6987880186	GGB81440	Clostridia_unclassified	Clostridia_unclassifie
0.9867810745	Lachnospiraceae_unclassified	Lachnospiraceae	Eubacteriales
0.9652194001	Lachnospiraceae_unclassified	Lachnospiraceae	Eubacteriales
0.9867810745	Lachnospiraceae_unclassified	Lachnospiraceae	Eubacteriales
0.9652194001	Lachnospiraceae_unclassified	Lachnospiraceae	Eubacteriales
0.6987880186	Lachnospiraceae_unclassified	Lachnospiraceae	Eubacteriales
0.9867810745	Lachnospiraceae_unclassified	Lachnospiraceae	Eubacteriales
0.6987880186	Lachnospiraceae_unclassified	Lachnospiraceae	Eubacteriales
0.7279468025	Lachnospiraceae_unclassified	Lachnospiraceae	Eubacteriales
0.9652194001	Lactobacillus	Lactobacillaceae	Lactobacillales
0.9867810745	Muribaculaceae_unclassified	Muribaculaceae	Bacteroidales
0.6987880186	Neglectibacter	Oscillospiraceae	Eubacteriales
0.9867810745	Oscillospiraceae_unclassified	Oscillospiraceae	Eubacteriales
0.6987880186	Oscillospiraceae_unclassified	Oscillospiraceae	Eubacteriales
0.9652194001	Oscillospiraceae_unclassified	Oscillospiraceae	Eubacteriales
0.9652194001	Parasutterella	Sutterellaceae	Burkholderiales
0.9915124472	Romboutsia	Peptostreptococcaceae	Eubacteriales
0.9867810745	Schaedlerella	Lachnospiraceae	Eubacteriales
0.7531912309	Turicibacter	Turicibacteraceae	Erysipelotrichales
0.8560789869	Bacteria_unclassified	Bacteria_unclassified	Bacteria_unclassified
0.9652194001	Bacteria_unclassified	Bacteria_unclassified	Bacteria_unclassified
0.9867810745	Bacteria_unclassified	Bacteria_unclassified	Bacteria_unclassified
0.8993780864			
0.9652194001			
0.9867810745			

Class	Phylum	Kingdom
Clostridia	Firmicutes	Bacteria
Clostridia	Firmicutes	Bacteria
Clostridia	Firmicutes	Bacteria
Clostridia	Firmicutes	Bacteria
Coriobacteriia	Actinobacteria	Bacteria
Coriobacteriia	Actinobacteria	Bacteria
Coriobacteriia	Actinobacteria	Bacteria
Verrucomicrobiae	Verrucomicrobia	Bacteria
Bacteroidia	Bacteroidota	Bacteria
Clostridia	Firmicutes	Bacteria
Bacteria_unclassifie	Bacteria_unclassifie	Bacteria
Bacteria_unclassifie	Bacteria_unclassifie	Bacteria
Bacteria_unclassifie	Bacteria_unclassifie	Bacteria
Bacteroidia	Bacteroidota	Bacteria
Actinomycetia	Actinobacteria	Bacteria
Clostridia	Firmicutes	Bacteria
Clostridia	Firmicutes	Bacteria
Clostridia	Firmicutes	Bacteria
Clostridia	Firmicutes	Bacteria
Erysipelotrichia	Firmicutes	Bacteria
Coriobacteriia	Actinobacteria	Bacteria
Clostridia	Firmicutes	Bacteria
Erysipelotrichia	Firmicutes	Bacteria
Erysipelotrichia	Firmicutes	Bacteria
Clostridia	Firmicutes	Bacteria
Clostridia	Firmicutes	Bacteria
Clostridia	Firmicutes	Bacteria
Coriobacteriia	Actinobacteria	Bacteria
Clostridia	Firmicutes	Bacteria
Bacteroidia	Bacteroidota	Bacteria
Bacteroidia	Bacteroidota	Bacteria
Bacteroidia	Bacteroidota	Bacteria
CFGB9508	Firmicutes	Bacteria
CFGB2838	Firmicutes	Bacteria
CFGB2838	Firmicutes	Bacteria
CFGB2838	Firmicutes	Bacteria
Clostridia	Firmicutes	Bacteria
CFGB28439	Firmicutes	Bacteria
Clostridia	Firmicutes	Bacteria
Clostridia	Firmicutes	Bacteria
Clostridia	Firmicutes	Bacteria
Clostridia	Firmicutes	Bacteria
Clostridia	Firmicutes	Bacteria
Clostridia	Firmicutes	Bacteria
CFGB77305	Firmicutes	Bacteria

Clostridia	Firmicutes	Bacteria
Clostridia	Firmicutes	Bacteria
Clostridia	Firmicutes	Bacteria
Clostridia	Firmicutes	Bacteria
CFGB9633	Firmicutes	Bacteria
Bacteria_unclassifie	Bacteria_unclassifie	Bacteria
Bacteria_unclassifie	Bacteria_unclassifie	Bacteria
Bacteria_unclassifie	Bacteria_unclassifie	Bacteria
Bacteria_unclassifie	Bacteria_unclassifie	Bacteria
Clostridia	Firmicutes	Bacteria
Clostridia	Firmicutes	Bacteria
Clostridia	Firmicutes	Bacteria
CFGB77359	Bacteria_unclassifie	Bacteria
CFGB9639	Firmicutes	Bacteria
Clostridia	Firmicutes	Bacteria
Clostridia	Firmicutes	Bacteria
Clostridia	Firmicutes	Bacteria
Clostridia	Firmicutes	Bacteria
Clostridia	Firmicutes	Bacteria
Clostridia	Firmicutes	Bacteria
Clostridia	Firmicutes	Bacteria
Clostridia	Firmicutes	Bacteria
Clostridia	Firmicutes	Bacteria
Clostridia	Firmicutes	Bacteria
Clostridia	Firmicutes	Bacteria
CFGB9658	Firmicutes	Bacteria
CFGB9659	Firmicutes	Bacteria
Bacteria_unclassifie	Bacteria_unclassifie	Bacteria
CFGB9827	Firmicutes	Bacteria
Clostridia	Firmicutes	Bacteria
CFGB77303	Bacteria_unclassifie	Bacteria
Clostridia	Firmicutes	Bacteria
Clostridia	Firmicutes	Bacteria
CFGB30328	Firmicutes	Bacteria
Clostridia	Firmicutes	Bacteria
Clostridia	Firmicutes	Bacteria
Clostridia	Firmicutes	Bacteria
Clostridia	Firmicutes	Bacteria
Clostridia	Firmicutes	Bacteria
Clostridia	Firmicutes	Bacteria
Clostridia	Firmicutes	Bacteria
CFGB77153	Actinobacteria	Bacteria
CFGB1791	Tenericutes	Bacteria
CFGB10290	Firmicutes	Bacteria
Clostridia	Firmicutes	Bacteria
CFGB1765	Firmicutes	Bacteria

CFGB10349	Firmicutes	Bacteria
CFGB10667	Firmicutes	Bacteria
Clostridia	Firmicutes	Bacteria
Clostridia	Firmicutes	Bacteria
Clostridia	Firmicutes	Bacteria
CFGB10299	Firmicutes	Bacteria
Clostridia	Firmicutes	Bacteria
Clostridia	Firmicutes	Bacteria
Clostridia	Firmicutes	Bacteria
Clostridia	Firmicutes	Bacteria
Clostridia	Firmicutes	Bacteria
Clostridia	Firmicutes	Bacteria
Clostridia	Firmicutes	Bacteria
Clostridia	Firmicutes	Bacteria
Clostridia	Firmicutes	Bacteria
Clostridia	Firmicutes	Bacteria
Clostridia	Firmicutes	Bacteria
Clostridia	Firmicutes	Bacteria
Bacilli	Firmicutes	Bacteria
Bacteroidia	Bacteroidota	Bacteria
Clostridia	Firmicutes	Bacteria
Clostridia	Firmicutes	Bacteria
Clostridia	Firmicutes	Bacteria
Clostridia	Firmicutes	Bacteria
Betaproteobacteria	Proteobacteria	Bacteria
Clostridia	Firmicutes	Bacteria
Clostridia	Firmicutes	Bacteria
Erysipelotrichia	Firmicutes	Bacteria
Bacteria_unclassifie	Bacteria_unclassifie	Bacteria
Bacteria_unclassifie	Bacteria_unclassifie	Bacteria
Bacteria_unclassifie	Bacteria_unclassifie	Bacteria

MetaPhlan Annotation
k__Bacteria|p__Firmicutes|c__Clostridia|o__Eubacteriales|f__Lachnospiraceae|g__Acetatifactor|s__Acetatifactor_SGB415
k__Bacteria|p__Firmicutes|c__Clostridia|o__Eubacteriales|f__Lachnospiraceae|g__Acetatifactor|s__Acetatifactor_muris
k__Bacteria|p__Firmicutes|c__Clostridia|o__Eubacteriales|f__Oscillospiraceae|g__Acutalibacter|s__Acutalibacter_muris
k__Bacteria|p__Firmicutes|c__Clostridia|o__Eubacteriales|f__Oscillospiraceae|g__Acutalibacter|s__Acutalibacter_sp_1XD
k__Bacteria|p__Actinobacteria|c__Coriobacteriia|o__Eggerthellales|f__Eggerthellaceae|g__Adlercreutzia|s__Adlercreutzia
k__Bacteria|p__Actinobacteria|c__Coriobacteriia|o__Eggerthellales|f__Eggerthellaceae|g__Adlercreutzia|s__Adlercreutzia
k__Bacteria|p__Actinobacteria|c__Coriobacteriia|o__Eggerthellales|f__Eggerthellaceae|g__Adlercreutzia|s__Adlercreutzia
k__Bacteria|p__Verrucomicrobia|c__Verrucomicrobiae|o__Verrucomicrobiales|f__Akkermansiaceae|g__Akkermansia|s__
k__Bacteria|p__Bacteroidota|c__Bacteroidia|o__Bacteroidales|f__Rikenellaceae|g__Alistipes|s__Alistipes_sp_DSM_11234
k__Bacteria|p__Firmicutes|c__Clostridia|o__Eubacteriales|f__Oscillospiraceae|g__Anaerotruncus|s__Anaerotruncus_sp_1
k__Bacteria|p__Bacteria_unclassified|c__Bacteria_unclassified|o__Bacteria_unclassified|f__Bacteria_unclassified|g__Bact
k__Bacteria|p__Bacteria_unclassified|c__Bacteria_unclassified|o__Bacteria_unclassified|f__Bacteria_unclassified|g__Bact
k__Bacteria|p__Bacteria_unclassified|c__Bacteria_unclassified|o__Bacteria_unclassified|f__Bacteria_unclassified|g__Bact
k__Bacteria|p__Bacteroidota|c__Bacteroidia|o__Bacteroidales|f__Bacteroidaceae|g__Bacteroides|s__Bacteroides_thetaio
k__Bacteria|p__Actinobacteria|c__Actinomycetia|o__Bifidobacteriales|f__Bifidobacteriaceae|g__Bifidobacterium|s__Bifid
k__Bacteria|p__Firmicutes|c__Clostridia|o__Clostridia_unclassified|f__Clostridia_unclassified|g__Clostridia_unclassified|s_
k__Bacteria|p__Firmicutes|c__Clostridia|o__Eubacteriales|f__Clostridiaceae|g__Clostridiaceae_unclassified|s__Clostridiac
k__Bacteria|p__Firmicutes|c__Clostridia|o__Eubacteriales|f__Clostridiaceae|g__Clostridiaceae_unclassified|s__Clostridiac
k__Bacteria|p__Firmicutes|c__Clostridia|o__Eubacteriales|f__Eubacteriales_unclassified|g__Eubacteriales_unclassified|s__
k__Bacteria|p__Firmicutes|c__Erysipelotrichia|o__Erysipelotrichales|f__Erysipelotrichaceae|g__Erysipelatoclostridium|s__
k__Bacteria|p__Actinobacteria|c__Coriobacteriia|o__Coriobacteriales|f__Coriobacteriaceae|g__Coriobacteriaceae_unclass
k__Bacteria|p__Firmicutes|c__Clostridia|o__Eubacteriales|f__Lachnospiraceae|g__Dorea|s__Dorea_sp_5_2
k__Bacteria|p__Firmicutes|c__Erysipelotrichia|o__Erysipelotrichales|f__Erysipelotrichaceae|g__Dubosiella|s__Dubosiella_
k__Bacteria|p__Firmicutes|c__Erysipelotrichia|o__Erysipelotrichales|f__Erysipelotrichales_unclassified|g__Erysipelotrichal
k__Bacteria|p__Firmicutes|c__Clostridia|o__Eubacteriales|f__Eubacteriaceae|g__Eubacteriaceae_unclassified|s__Eubacte
k__Bacteria|p__Firmicutes|c__Clostridia|o__Eubacteriales|f__Eubacteriaceae|g__Eubacteriaceae_unclassified|s__Eubacte
k__Bacteria|p__Firmicutes|c__Clostridia|o__Eubacteriales|f__Lachnospiraceae|g__GGB20149|s__GGB20149_SGB29430
k__Bacteria|p__Actinobacteria|c__Coriobacteriia|o__Eggerthellales|f__Eggerthellaceae|g__GGB22635|s__GGB22635_SGB
k__Bacteria|p__Firmicutes|c__Clostridia|o__Eubacteriales|f__Lachnospiraceae|g__GGB25041|s__GGB25041_SGB36960
k__Bacteria|p__Bacteroidota|c__Bacteroidia|o__Bacteroidales|f__Muribaculaceae|g__GGB27876|s__GGB27876_SGB403
k__Bacteria|p__Bacteroidota|c__Bacteroidia|o__Bacteroidales|f__Muribaculaceae|g__GGB27878|s__GGB27878_SGB403
k__Bacteria|p__Bacteroidota|c__Bacteroidia|o__Bacteroidales|f__Muribaculaceae|g__GGB27918|s__GGB27918_SGB403
k__Bacteria|p__Firmicutes|c__CFGB9508|o__OFGB9508|f__FGB9508|g__GGB28382|s__GGB28382_SGB40962
k__Bacteria|p__Firmicutes|c__CFGB2838|o__OFGB2838|f__FGB2838|g__GGB28399|s__GGB28399_SGB40980
k__Bacteria|p__Firmicutes|c__CFGB2838|o__OFGB2838|f__FGB2838|g__GGB28411|s__GGB28411_SGB40993
k__Bacteria|p__Firmicutes|c__CFGB2838|o__OFGB2838|f__FGB2838|g__GGB28415|s__GGB28415_SGB40997
k__Bacteria|p__Firmicutes|c__Clostridia|o__Eubacteriales|f__Pumilibacteraceae|g__GGB28430|s__GGB28430_SGB41013
k__Bacteria|p__Firmicutes|c__CFGB28439|o__OFGB28439|f__FGB28439|g__GGB28439|s__GGB28439_SGB41022
k__Bacteria|p__Firmicutes|c__Clostridia|o__Clostridia_unclassified|f__Clostridia_unclassified|g__GGB28778|s__GGB2877
k__Bacteria|p__Firmicutes|c__Clostridia|o__Eubacteriales|f__Eubacteriaceae|g__GGB28784|s__GGB28784_SGB41437
k__Bacteria|p__Firmicutes|c__Clostridia|o__Eubacteriales|f__Lachnospiraceae|g__GGB28792|s__GGB28792_SGB41445
k__Bacteria|p__Firmicutes|c__Clostridia|o__Eubacteriales|f__Lachnospiraceae|g__GGB28798|s__GGB28798_SGB41451
k__Bacteria|p__Firmicutes|c__Clostridia|o__Eubacteriales|f__Lachnospiraceae|g__GGB28802|s__GGB28802_SGB41455
k__Bacteria|p__Firmicutes|c__Clostridia|o__Eubacteriales|f__Lachnospiraceae|g__GGB28818|s__GGB28818_SGB41473
k__Bacteria|p__Firmicutes|c__CFGB77305|o__OFGB77305|f__FGB77305|g__GGB28828|s__GGB28828_SGB41484

k__Bacteria|p__Firmicutes|c__Clostridia|o__Eubacteriales|f__Clostridiaceae|g__GGB28851|s__GGB28851_SGB41518
k__Bacteria|p__Firmicutes|c__Clostridia|o__Eubacteriales|f__Lachnospiraceae|g__GGB28859|s__GGB28859_SGB41528
k__Bacteria|p__Firmicutes|c__Clostridia|o__Eubacteriales|f__Lachnospiraceae|g__GGB28864|s__GGB28864_SGB41535
k__Bacteria|p__Firmicutes|c__Clostridia|o__Eubacteriales|f__Lachnospiraceae|g__GGB28869|s__GGB28869_SGB41543
k__Bacteria|p__Firmicutes|c__CFGB9633|o__OFGB9633|f__FGB9633|g__GGB28883|s__GGB28883_SGB41564
k__Bacteria|p__Bacteria_unclassified|c__Bacteria_unclassified|o__Bacteria_unclassified|f__Bacteria_unclassified|g__GGB
k__Bacteria|p__Bacteria_unclassified|c__Bacteria_unclassified|o__Bacteria_unclassified|f__Bacteria_unclassified|g__GGB
k__Bacteria|p__Bacteria_unclassified|c__Bacteria_unclassified|o__Bacteria_unclassified|f__Bacteria_unclassified|g__GGB
k__Bacteria|p__Bacteria_unclassified|c__Bacteria_unclassified|o__Bacteria_unclassified|f__Bacteria_unclassified|g__GGB
k__Bacteria|p__Firmicutes|c__Clostridia|o__Eubacteriales|f__Lachnospiraceae|g__GGB28916|s__GGB28916_SGB41612
k__Bacteria|p__Firmicutes|c__Clostridia|o__Eubacteriales|f__Lachnospiraceae|g__GGB28924|s__GGB28924_SGB41621
k__Bacteria|p__Firmicutes|c__Clostridia|o__Eubacteriales|f__Lachnospiraceae|g__GGB28926|s__GGB28926_SGB41624
k__Bacteria|p__Bacteria_unclassified|c__CFGB77359|o__OFGB77359|f__FGB77359|g__GGB28927|s__GGB28927_SGB41
k__Bacteria|p__Firmicutes|c__CFGB9639|o__OFGB9639|f__FGB9639|g__GGB28934|s__GGB28934_SGB41635
k__Bacteria|p__Firmicutes|c__Clostridia|o__Eubacteriales|f__Lachnospiraceae|g__GGB28946|s__GGB28946_SGB41652
k__Bacteria|p__Firmicutes|c__Clostridia|o__Eubacteriales|f__Lachnospiraceae|g__GGB28949|s__GGB28949_SGB41655
k__Bacteria|p__Firmicutes|c__Clostridia|o__Eubacteriales|f__Lachnospiraceae|g__GGB28949|s__GGB28949_SGB41656
k__Bacteria|p__Firmicutes|c__Clostridia|o__Eubacteriales|f__Clostridiaceae|g__GGB28950|s__GGB28950_SGB41657
k__Bacteria|p__Firmicutes|c__Clostridia|o__Eubacteriales|f__Clostridiaceae|g__GGB28951|s__GGB28951_SGB102295
k__Bacteria|p__Firmicutes|c__Clostridia|o__Eubacteriales|f__Clostridiaceae|g__GGB28951|s__GGB28951_SGB41658
k__Bacteria|p__Firmicutes|c__Clostridia|o__Eubacteriales|f__Clostridiaceae|g__GGB28954|s__GGB28954_SGB41662
k__Bacteria|p__Firmicutes|c__Clostridia|o__Eubacteriales|f__Clostridiaceae|g__GGB28956|s__GGB28956_SGB41665
k__Bacteria|p__Firmicutes|c__Clostridia|o__Eubacteriales|f__Clostridiaceae|g__GGB28960|s__GGB28960_SGB41669
k__Bacteria|p__Firmicutes|c__Clostridia|o__Eubacteriales|f__Clostridiaceae|g__GGB28967|s__GGB28967_SGB41678
k__Bacteria|p__Firmicutes|c__Clostridia|o__Eubacteriales|f__Eubacteriaceae|g__GGB28991|s__GGB28991_SGB41705
k__Bacteria|p__Firmicutes|c__CFGB9658|o__OFGB9658|f__FGB9658|g__GGB29002|s__GGB29002_SGB41718
k__Bacteria|p__Firmicutes|c__CFGB9659|o__OFGB9659|f__FGB9659|g__GGB29003|s__GGB29003_SGB41719
k__Bacteria|p__Bacteria_unclassified|c__Bacteria_unclassified|o__Bacteria_unclassified|f__Bacteria_unclassified|g__GGB
k__Bacteria|p__Firmicutes|c__CFGB9827|o__OFGB9827|f__FGB9827|g__GGB29531|s__GGB29531_SGB42317
k__Bacteria|p__Firmicutes|c__Clostridia|o__Eubacteriales|f__Eubacteriaceae|g__GGB29685|s__GGB29685_SGB42494
k__Bacteria|p__Bacteria_unclassified|c__CFGB77303|o__OFGB77303|f__FGB77303|g__GGB30141|s__GGB30141_SGB43
k__Bacteria|p__Firmicutes|c__Clostridia|o__Eubacteriales|f__Eubacteriales_unclassified|g__GGB30286|s__GGB30286_SG
k__Bacteria|p__Firmicutes|c__Clostridia|o__Eubacteriales|f__Oscillospiraceae|g__GGB30303|s__GGB30303_SGB43268
k__Bacteria|p__Firmicutes|c__CFGB30328|o__OFGB30328|f__FGB30328|g__GGB30413|s__GGB30413_SGB43452
k__Bacteria|p__Firmicutes|c__Clostridia|o__Eubacteriales|f__Oscillospiraceae|g__GGB30454|s__GGB30454_SGB43514
k__Bacteria|p__Firmicutes|c__Clostridia|o__Eubacteriales|f__Oscillospiraceae|g__GGB30455|s__GGB30455_SGB43519
k__Bacteria|p__Firmicutes|c__Clostridia|o__Eubacteriales|f__Oscillospiraceae|g__GGB30461|s__GGB30461_SGB43527
k__Bacteria|p__Firmicutes|c__Clostridia|o__Eubacteriales|f__Oscillospiraceae|g__GGB30461|s__GGB30461_SGB43530
k__Bacteria|p__Firmicutes|c__Clostridia|o__Eubacteriales|f__Oscillospiraceae|g__GGB30463|s__GGB30463_SGB43537
k__Bacteria|p__Firmicutes|c__Clostridia|o__Eubacteriales|f__Oscillospiraceae|g__GGB30473|s__GGB30473_SGB43557
k__Bacteria|p__Firmicutes|c__Clostridia|o__Eubacteriales|f__Oscillospiraceae|g__GGB30475|s__GGB30475_SGB63182
k__Bacteria|p__Actinobacteria|c__CFGB77153|o__OFGB77153|f__FGB77153|g__GGB30861|s__GGB30861_SGB44083
k__Bacteria|p__Tenericutes|c__CFGB1791|o__OFGB1791|f__FGB1791|g__GGB31312|s__GGB31312_SGB44628
k__Bacteria|p__Firmicutes|c__CFGB10290|o__OFGB10290|f__FGB10290|g__GGB31438|s__GGB31438_SGB44768
k__Bacteria|p__Firmicutes|c__Clostridia|o__Eubacteriales|f__Oscillospiraceae|g__GGB3171|s__GGB3171_SGB4185
k__Bacteria|p__Firmicutes|c__CFGB1765|o__OFGB1765|f__FGB1765|g__GGB31823|s__GGB31823_SGB45199

k__Bacteria|p__Firmicutes|c__CFGB10349|o__OFGB10349|f__FGB10349|g__GGB31853|s__GGB31853_SGB45233
k__Bacteria|p__Firmicutes|c__CFGB10667|o__OFGB10667|f__FGB10667|g__GGB32371|s__GGB32371_SGB41694
k__Bacteria|p__Firmicutes|c__Clostridia|o__Eubacteriales|f__Lachnospiraceae|g__GGB3793|s__GGB3793_SGB5158
k__Bacteria|p__Firmicutes|c__Clostridia|o__Eubacteriales|f__Lachnospiraceae|g__GGB42598|s__GGB42598_SGB59794
k__Bacteria|p__Firmicutes|c__Clostridia|o__Eubacteriales|f__Christensenellaceae|g__GGB45656|s__GGB45656_SGB6337
k__Bacteria|p__Firmicutes|c__CFGB10299|o__OFGB10299|f__FGB10299|g__GGB47127|s__GGB47127_SGB65054
k__Bacteria|p__Firmicutes|c__Clostridia|o__Eubacteriales|f__Oscillospiraceae|g__GGB74395|s__GGB74395_SGB43521
k__Bacteria|p__Firmicutes|c__Clostridia|o__Eubacteriales|f__Oscillospiraceae|g__GGB75053|s__GGB75053_SGB43494
k__Bacteria|p__Firmicutes|c__Clostridia|o__Eubacteriales|f__Lachnospiraceae|g__GGB75109|s__GGB75109_SGB102238
k__Bacteria|p__Firmicutes|c__Clostridia|o__Clostridia_unclassified|f__Clostridia_unclassified|g__GGB81440|s__GGB8144
k__Bacteria|p__Firmicutes|c__Clostridia|o__Eubacteriales|f__Lachnospiraceae|g__Lachnospiraceae_unclassified|s__Lachn
k__Bacteria|p__Firmicutes|c__Clostridia|o__Eubacteriales|f__Lachnospiraceae|g__Lachnospiraceae_unclassified|s__Lachn
k__Bacteria|p__Firmicutes|c__Clostridia|o__Eubacteriales|f__Lachnospiraceae|g__Lachnospiraceae_unclassified|s__Lachn
k__Bacteria|p__Firmicutes|c__Clostridia|o__Eubacteriales|f__Lachnospiraceae|g__Lachnospiraceae_unclassified|s__Lachn
k__Bacteria|p__Firmicutes|c__Clostridia|o__Eubacteriales|f__Lachnospiraceae|g__Lachnospiraceae_unclassified|s__Lachn
k__Bacteria|p__Firmicutes|c__Clostridia|o__Eubacteriales|f__Lachnospiraceae|g__Lachnospiraceae_unclassified|s__Lachn
k__Bacteria|p__Firmicutes|c__Clostridia|o__Eubacteriales|f__Lachnospiraceae|g__Lachnospiraceae_unclassified|s__Lachn
k__Bacteria|p__Firmicutes|c__Clostridia|o__Eubacteriales|f__Lachnospiraceae|g__Lachnospiraceae_unclassified|s__Lachn
k__Bacteria|p__Firmicutes|c__Bacilli|o__Lactobacillales|f__Lactobacillaceae|g__Lactobacillus|s__Lactobacillus_johnsonii
k__Bacteria|p__Bacteroidota|c__Bacteroidia|o__Bacteroidales|f__Muribaculaceae|g__Muribaculaceae_unclassified|s__M
k__Bacteria|p__Firmicutes|c__Clostridia|o__Eubacteriales|f__Oscillospiraceae|g__Neglectibacter|s__Neglectibacter_sp_X
k__Bacteria|p__Firmicutes|c__Clostridia|o__Eubacteriales|f__Oscillospiraceae|g__Oscillospiraceae_unclassified|s__Oscillo
k__Bacteria|p__Firmicutes|c__Clostridia|o__Eubacteriales|f__Oscillospiraceae|g__Oscillospiraceae_unclassified|s__Oscillo
k__Bacteria|p__Firmicutes|c__Clostridia|o__Eubacteriales|f__Oscillospiraceae|g__Oscillospiraceae_unclassified|s__Oscillo
k__Bacteria|p__Proteobacteria|c__Betaproteobacteria|o__Burkholderiales|f__Sutterellaceae|g__Parasutterella|s__Parasu
k__Bacteria|p__Firmicutes|c__Clostridia|o__Eubacteriales|f__Peptostreptococcaceae|g__Romboutsia|s__Romboutsia_ilea
k__Bacteria|p__Firmicutes|c__Clostridia|o__Eubacteriales|f__Lachnospiraceae|g__Schaedlerella|s__Schaedlerella_arabino
k__Bacteria|p__Firmicutes|c__Erysipelotrichia|o__Erysipelotrichales|f__Turicibacteraceae|g__Turicibacter|s__Turicibacter_
k__Bacteria|p__Bacteria_unclassified|c__Bacteria_unclassified|o__Bacteria_unclassified|f__Bacteria_unclassified|g__Bacte
k__Bacteria|p__Bacteria_unclassified|c__Bacteria_unclassified|o__Bacteria_unclassified|f__Bacteria_unclassified|g__Bacte
k__Bacteria|p__Bacteria_unclassified|c__Bacteria_unclassified|o__Bacteria_unclassified|f__Bacteria_unclassified|g__Bacte
