# Supplementary material for: Non-invasive Vagal Nerve Stimulation as a Potential Treatment for Repetitive Blast Trauma
Source: bioRxiv. 2026 Jul 19:2026.07.13.737563. Preprint. [Version 1] doi: 10.64898/2026.07.13.737563 (PMC13405043; doi:10.64898/2026.07.13.737563)
Supplement: Supplement 16 [file media-16.pdf]

			Likelihood Ratio Te:	
**outcome**	**Species-level feature**	**n**	**Microbe Chi (df=1**	**Microbe Pr(>Chi**
EtOH_24h_intakeave_final	Acetatifactor_SGB41546	63	2.643234361	0.01014668495
EtOH_24h_intakeave_final	Acetatifactor_muris	63	0.07887962006	0.93713
EtOH_24h_intakeave_final	Acutalibacter_muris	63	1.653866013	0.1019508547
EtOH_24h_intakeave_final	Acutalibacter_sp_1XD8_36	63	0.8762493561	0.3824095081
EtOH_24h_intakeave_final	Adlercreutzia_caecimuris	63	-2.506293877	0.01451844488
EtOH_24h_intakeave_final	Adlercreutzia_mucosicola	63	0.8430779108	0.4005737629
EtOH_24h_intakeave_final	Adlercreutzia_muris	63	0.4048584518	0.6857845285
EtOH_24h_intakeave_final	Akkermansia_muciniphila	63	-1.481223827	0.14211927
EtOH_24h_intakeave_final	Alistipes_sp_DSM_112343	63	-1.164067037	0.247037984
EtOH_24h_intakeave_final	Anaerotruncus_sp_1XD42_93	63	0.6921483781	0.4897083888
EtOH_24h_intakeave_final	Bacteria_unclassified_SGB102200	63	-0.2215233595	0.8247201987
EtOH_24h_intakeave_final	Bacteria_unclassified_SGB41677	63	-0.4106691382	0.6815264835
EtOH_24h_intakeave_final	Bacteria_unclassified_SGB43546	63	0.320152722	0.748956111
EtOH_24h_intakeave_final	Bacteroides_thetaiotaomicron	63	-0.9335646025	0.3522671042
EtOH_24h_intakeave_final	Bifidobacterium_pseudolongum	63	-1.643744416	0.1040182897
EtOH_24h_intakeave_final	Clostridia_bacterium	63	-1.436975182	0.1542042991
EtOH_24h_intakeave_final	Clostridiaceae_bacterium	63	-2.705183306	0.00859549892:
EtOH_24h_intakeave_final	Clostridiaceae_unclassified_SGB41663	63	1.022121776	0.3088139377
EtOH_24h_intakeave_final	Clostridiales_bacterium	63	0.4240784591	0.6717399822
EtOH_24h_intakeave_final	Clostridium_cocleatum	63	0.2905248716	0.7714928216
EtOH_24h_intakeave_final	Coriobacteriaceae_bacterium	63	-0.5364630185	0.5920819959
EtOH_24h_intakeave_final	Dorea_sp_5_2	63	-1.350288739	0.1801816088
EtOH_24h_intakeave_final	Dubosiella_newyorkensis	63	-1.853825593	0.06753860213
EtOH_24h_intakeave_final	Erysipelotrichales_bacterium	63	0.3876428347	0.6984597667
EtOH_24h_intakeave_final	Eubacteriaceae_bacterium	63	-0.5223830263	0.6018161862
EtOH_24h_intakeave_final	Eubacteriaceae_unclassified_SGB9492:	63	-0.8018527157	0.4238748354
EtOH_24h_intakeave_final	GGB20149_SGB29430	63	1.013870335	0.3127024455
EtOH_24h_intakeave_final	GGB22635_SGB63107	63	-1.393313529	0.1668990797
EtOH_24h_intakeave_final	GGB25041_SGB36960	63	-0.8826170116	0.3789827761
EtOH_24h_intakeave_final	GGB27876_SGB40310	63	-1.102343481	0.2727189781
EtOH_24h_intakeave_final	GGB27878_SGB40312	63	-0.1299359205	0.8966243555
EtOH_24h_intakeave_final	GGB27918_SGB40356	63	0.6806167409	0.4969424086
EtOH_24h_intakeave_final	GGB28382_SGB40962	63	1.136960937	0.2580940175
EtOH_24h_intakeave_final	GGB28399_SGB40980	63	0.2706503609	0.7867234573
EtOH_24h_intakeave_final	GGB28411_SGB40993	63	-0.462376347	0.6441062366
EtOH_24h_intakeave_final	GGB28415_SGB40997	63	-0.3019997873	0.7627396417
EtOH_24h_intakeave_final	GGB28430_SGB41013	63	0.7423300279	0.4589153734
EtOH_24h_intakeave_final	GGB28439_SGB41022	63	1.608962401	0.1113843636
EtOH_24h_intakeave_final	GGB28778_SGB41431	63	0.2801404161	0.7794400064
EtOH_24h_intakeave_final	GGB28784_SGB41437	63	-0.5707917168	0.5686647231
EtOH_24h_intakeave_final	GGB28792_SGB41445	63	0.01198879917	0.9904345571
EtOH_24h_intakeave_final	GGB28798_SGB41451	63	-0.7996461555	0.4251445275
EtOH_24h_intakeave_final	GGB28802_SGB41455	63	-0.06059249991	0.951684485
EtOH_24h_intakeave_final	GGB28818_SGB41473	63	-0.4990469888	0.6181103259
EtOH_24h_intakeave_final	GGB28828_SGB41484	63	-0.4505927261	0.6525574162

EtOH_24h_intakeave_final	GGB28851_SGB41518	63	0.3738803283	0.7086551764
EtOH_24h_intakeave_final	GGB28859_SGB41528	63	-0.5768605856	0.5645725029
EtOH_24h_intakeave_final	GGB28864_SGB41535	63	0.8492169195	0.397172611
EtOH_24h_intakeave_final	GGB28869_SGB41543	63	1.519880099	0.132183137
EtOH_24h_intakeave_final	GGB28883_SGB41564	63	-1.341714031	0.1829224984
EtOH_24h_intakeave_final	GGB28892_SGB41573	63	-1.407206108	0.1627752179
EtOH_24h_intakeave_final	GGB28893_SGB41574	63	1.409591058	0.1620752609
EtOH_24h_intakeave_final	GGB28898_SGB41580	63	-1.944815675	0.05547079326
EtOH_24h_intakeave_final	GGB28904_SGB41597	63	0.9283630238	0.354937354
EtOH_24h_intakeave_final	GGB28916_SGB41612	63	0.3686543291	0.7125408334
EtOH_24h_intakeave_final	GGB28924_SGB41621	63	0.1690292605	0.8657894494
EtOH_24h_intakeave_final	GGB28926_SGB41624	63	-0.7209932954	0.4718705137
EtOH_24h_intakeave_final	GGB28927_SGB41625	63	2.508346013	0.01444196717
EtOH_24h_intakeave_final	GGB28934_SGB41635	63	0.7106768549	0.4782077753
EtOH_24h_intakeave_final	GGB28946_SGB41652	63	0.6858701256	0.4936396379
EtOH_24h_intakeave_final	GGB28949_SGB41655	63	-1.184618857	0.2388848902
EtOH_24h_intakeave_final	GGB28949_SGB41656	63	0.8054138121	0.4218305221
EtOH_24h_intakeave_final	GGB28950_SGB41657	63	-0.4173026666	0.6766781508
EtOH_24h_intakeave_final	GGB28951_SGB102295	63	0.4000679325	0.6893027149
EtOH_24h_intakeave_final	GGB28951_SGB41658	63	2.902957924	0.004983215651
EtOH_24h_intakeave_final	GGB28954_SGB41662	63	-0.2145016379	0.8301879415
EtOH_24h_intakeave_final	GGB28956_SGB41665	63	0.2839554365	0.7765175887
EtOH_24h_intakeave_final	GGB28960_SGB41669	63	-0.02057285725	0.9835864216
EtOH_24h_intakeave_final	GGB28967_SGB41678	63	1.106891304	0.2707651359
EtOH_24h_intakeave_final	GGB28991_SGB41705	63	-0.756447692	0.4504569676
EtOH_24h_intakeave_final	GGB29002_SGB41718	63	1.748620771	0.08417889984
EtOH_24h_intakeave_final	GGB29003_SGB41719	63	-0.526868663	0.5987070189
EtOH_24h_intakeave_final	GGB29011_SGB41731	63	-0.916490799	0.3610809192
EtOH_24h_intakeave_final	GGB29531_SGB42317	63	3.424776826	0.00106378805
EtOH_24h_intakeave_final	GGB29685_SGB42494	63	-2.719328316	0.00827324495
EtOH_24h_intakeave_final	GGB30141_SGB43066	63	0.6620577055	0.5087063797
EtOH_24h_intakeave_final	GGB30286_SGB43248	63	-0.1384540699	0.8898903048
EtOH_24h_intakeave_final	GGB30303_SGB43268	63	1.459516725	0.1479513531
EtOH_24h_intakeave_final	GGB30413_SGB43452	63	-0.6351439783	0.5260284467
EtOH_24h_intakeave_final	GGB30454_SGB43514	63	-1.396380619	0.1659817921
EtOH_24h_intakeave_final	GGB30455_SGB43519	63	1.763591265	0.08162112993
EtOH_24h_intakeave_final	GGB30461_SGB43527	63	-1.679195656	0.09692369261
EtOH_24h_intakeave_final	GGB30461_SGB43530	63	1.180151732	0.2406402822
EtOH_24h_intakeave_final	GGB30463_SGB43537	63	0.9891352589	0.3245562107
EtOH_24h_intakeave_final	GGB30473_SGB43557	63	-0.3354831787	0.7373791736
EtOH_24h_intakeave_final	GGB30475_SGB63182	63	-1.275636235	0.2051152624
EtOH_24h_intakeave_final	GGB30861_SGB44083	63	-2.632621552	0.01043674126
EtOH_24h_intakeave_final	GGB31312_SGB44628	63	-1.922243558	0.05827852977
EtOH_24h_intakeave_final	GGB31438_SGB44768	63	-1.53035277	0.1295884264
EtOH_24h_intakeave_final	GGB3171_SGB4185	63	0.2751050637	0.7833021252
EtOH_24h_intakeave_final	GGB31823_SGB45199	63	-1.003778775	0.317502865

EtOH_24h_intakeave_final	GGB31853_SGB45233	63	2.06763554	0.04213730763
EtOH_24h_intakeave_final	GGB32371_SGB41694	63	-0.688934247	0.4917188186
EtOH_24h_intakeave_final	GGB3793_SGB5158	63	0.4185992032	0.675732129
EtOH_24h_intakeave_final	GGB42598_SGB59794	63	-0.6006072016	0.5487012594
EtOH_24h_intakeave_final	GGB45656_SGB63370	63	1.323001411	0.189013771
EtOH_24h_intakeave_final	GGB47127_SGB65054	63	-1.998784947	0.04922229004
EtOH_24h_intakeave_final	GGB74395_SGB43521	63	-1.637328832	0.105346324
EtOH_24h_intakeave_final	GGB75053_SGB43494	63	1.223982884	0.2238157793
EtOH_24h_intakeave_final	GGB75109_SGB102238	63	-0.8925170102	0.3736937255
EtOH_24h_intakeave_final	GGB81440_SGB45230	63	1.780035132	0.07888645053
EtOH_24h_intakeave_final	Lachnospiraceae_bacterium	63	-1.365260355	0.1754710972
EtOH_24h_intakeave_final	Lachnospiraceae_bacterium_A2	63	-1.156848284	0.2499486122
EtOH_24h_intakeave_final	Lachnospiraceae_bacterium_MD308	63	-1.477078829	0.1432187154
EtOH_24h_intakeave_final	Lachnospiraceae_bacterium_MD329	63	-0.8388782919	0.4029107489
EtOH_24h_intakeave_final	Lachnospiraceae_unclassified_SGB4141	63	0.9097484048	0.3646001881
EtOH_24h_intakeave_final	Lachnospiraceae_unclassified_SGB4141	63	0.2510776692	0.8018050194
EtOH_24h_intakeave_final	Lachnospiraceae_unclassified_SGB4142	63	0.663070971	0.5080602637
EtOH_24h_intakeave_final	Lachnospiraceae_unclassified_SGB4158	63	-0.3894526247	0.6971231456
EtOH_24h_intakeave_final	Lactobacillus_johnsonii	63	-0.05579705059	0.9555040612
EtOH_24h_intakeave_final	Muribaculaceae_bacterium	63	-2.107493665	0.03845406567
EtOH_24h_intakeave_final	Neglectibacter_sp_X4	63	2.044202327	0.04444256131
EtOH_24h_intakeave_final	Oscillospiraceae_bacterium	63	-0.04317403543	0.9655630698
EtOH_24h_intakeave_final	Oscillospiraceae_unclassified_SGB4350	63	0.934605306	0.3517344238
EtOH_24h_intakeave_final	Oscillospiraceae_unclassified_SGB4350	63	0.6632027772	0.5079762489
EtOH_24h_intakeave_final	Parasutterella_excrementihominis	63	-1.275085543	0.2053083017
EtOH_24h_intakeave_final	Romboutsia_ilealis	63	-0.1016791819	0.9190148034
EtOH_24h_intakeave_final	Schaedlerella_arabinosiphila	63	-1.283136531	0.2024995636
EtOH_24h_intakeave_final	Turicibacter_sp_1E2	63	-1.393310511	0.1668999841
EtOH_24h_intakeave_final	bacterium_1XD42_54	63	-2.480615243	0.01550683875
EtOH_24h_intakeave_final	bacterium_1XD42_76	63	-0.3947983951	0.6931806615
EtOH_24h_intakeave_final	bacterium_1xD8_48	63	0.5664291063	0.5716153682
EtOH_24h_intakeave_final	Berger Parker Index	63	-1.824870881	0.07181587317
EtOH_24h_intakeave_final	Richness (# observed features)	63	1.328132768	0.1873283705
EtOH_24h_intakeave_final	Shannon Index	63	0.9345445312	0.3517655168
EtOH_8pm_intakeave_fina	Acetatifactor_SGB41546	63	0.1269479285	0.9033652436
EtOH_8pm_intakeave_fina	Acetatifactor_muris	63	-1.554120016	0.1418367732
EtOH_8pm_intakeave_fina	Acutalibacter_muris	63	-0.07888209967	0.9371657906
EtOH_8pm_intakeave_fina	Acutalibacter_sp_1XD8_36	63	0.5309571262	0.598230628
EtOH_8pm_intakeave_fina	Adlercreutzia_caecimuris	63	-1.539337551	0.1278423439
EtOH_8pm_intakeave_fina	Adlercreutzia_mucosicola	63	0.5737974271	0.6003383763
EtOH_8pm_intakeave_fina	Adlercreutzia_muris	63	-0.07316337356	0.942729343
EtOH_8pm_intakeave_fina	Akkermansia_muciniphila	63	-0.1278160409	0.8984966586
EtOH_8pm_intakeave_fina	Alistipes_sp_DSM_112343	63	-0.09100153497	0.9303283236
EtOH_8pm_intakeave_fina	Anaerotruncus_sp_1XD42_93	63	1.616745063	0.1351392145
EtOH_8pm_intakeave_fina	Bacteria_unclassified_SGB102200	63	-0.5846899654	0.5610027826
EtOH_8pm_intakeave_fina	Bacteria_unclassified_SGB41677	63	1.835199181	0.07990658851

EtOH_8pm_intakeave_fina	Bacteria_unclassified_SGB43546	63	-0.289474308	0.7771851965
EtOH_8pm_intakeave_fina	Bacteroides_thetaiotaomicron	63	-0.9579341796	0.3413404388
EtOH_8pm_intakeave_fina	Bifidobacterium_pseudolongum	63	-1.212773179	0.2294182698
EtOH_8pm_intakeave_fina	Clostridia_bacterium	63	-0.7777415287	0.4390603691
EtOH_8pm_intakeave_fina	Clostridiaceae_bacterium	63	-0.6929243781	0.5055735079
EtOH_8pm_intakeave_fina	Clostridiaceae_unclassified_SGB41663	63	0.8239171355	0.4182340115
EtOH_8pm_intakeave_fina	Clostridiales_bacterium	63	-0.3205314504	0.7567058736
EtOH_8pm_intakeave_fina	Clostridium_cocleatum	63	-0.808616513	0.4213957656
EtOH_8pm_intakeave_fina	Coriobacteriaceae_bacterium	63	-1.115254127	0.2940522676
EtOH_8pm_intakeave_fina	Dorea_sp_5_2	63	-0.5971319561	0.5943998363
EtOH_8pm_intakeave_fina	Dubosiella_newyorkensis	63	-1.893271667	0.06212066834
EtOH_8pm_intakeave_fina	Erysipelotrichales_bacterium	63	-0.9622145803	0.3381752078
EtOH_8pm_intakeave_fina	Eubacteriaceae_bacterium	63	1.378596061	0.1804642666
EtOH_8pm_intakeave_fina	Eubacteriaceae_unclassified_SGB9492	63	-1.086264458	0.2836808213
EtOH_8pm_intakeave_fina	GGB20149_SGB29430	63	-0.05203064248	0.9586771904
EtOH_8pm_intakeave_fina	GGB22635_SGB63107	63	-1.200575448	0.2555200888
EtOH_8pm_intakeave_fina	GGB25041_SGB36960	63	0.9141274723	0.3772106307
EtOH_8pm_intakeave_fina	GGB27876_SGB40310	63	-1.033948322	0.3035189707
EtOH_8pm_intakeave_fina	GGB27878_SGB40312	63	0.120040608	0.9101681622
EtOH_8pm_intakeave_fina	GGB27918_SGB40356	63	-0.1192929489	0.90902181
EtOH_8pm_intakeave_fina	GGB28382_SGB40962	63	1.200277712	0.2578916345
EtOH_8pm_intakeave_fina	GGB28399_SGB40980	63	2.045511993	0.05020343618
EtOH_8pm_intakeave_fina	GGB28411_SGB40993	63	-0.4784546379	0.6336251375
EtOH_8pm_intakeave_fina	GGB28415_SGB40997	63	-0.7385223469	0.552000583
EtOH_8pm_intakeave_fina	GGB28430_SGB41013	63	1.900187735	0.1115038421
EtOH_8pm_intakeave_fina	GGB28439_SGB41022	63	1.63437783	0.1412674171
EtOH_8pm_intakeave_fina	GGB28778_SGB41431	63	1.944177325	0.06304099233
EtOH_8pm_intakeave_fina	GGB28784_SGB41437	63	0.374272144	0.7098434603
EtOH_8pm_intakeave_fina	GGB28792_SGB41445	63	0.7842906854	0.4599351279
EtOH_8pm_intakeave_fina	GGB28798_SGB41451	63	-0.2352053671	0.8146765248
EtOH_8pm_intakeave_fina	GGB28802_SGB41455	63	-0.1410211783	0.8901194529
EtOH_8pm_intakeave_fina	GGB28818_SGB41473	63	-2.130352083	0.04126921894
EtOH_8pm_intakeave_fina	GGB28828_SGB41484	63	0.2458133347	0.8100278231
EtOH_8pm_intakeave_fina	GGB28851_SGB41518	63	-0.7611262568	0.4705578506
EtOH_8pm_intakeave_fina	GGB28859_SGB41528	63	-0.5408020479	0.6011606568
EtOH_8pm_intakeave_fina	GGB28864_SGB41535	63	0.3465868932	0.7307320276
EtOH_8pm_intakeave_fina	GGB28869_SGB41543	63	0.9403416306	0.3767491917
EtOH_8pm_intakeave_fina	GGB28883_SGB41564	63	-1.369408093	0.2008001019
EtOH_8pm_intakeave_fina	GGB28892_SGB41573	63	-1.295178873	0.2558959723
EtOH_8pm_intakeave_fina	GGB28893_SGB41574	63	-0.9739814581	0.3409365904
EtOH_8pm_intakeave_fina	GGB28898_SGB41580	63	-1.23000593	0.221682008
EtOH_8pm_intakeave_fina	GGB28904_SGB41597	63	0.8354969696	0.4060494568
EtOH_8pm_intakeave_fina	GGB28916_SGB41612	63	0.5822121123	0.5612309939
EtOH_8pm_intakeave_fina	GGB28924_SGB41621	63	0.9410525387	0.4221711435
EtOH_8pm_intakeave_fina	GGB28926_SGB41624	63	-0.7530036385	0.4525161498
EtOH_8pm_intakeave_fina	GGB28927_SGB41625	63	1.798080347	0.0793857384

EtOH_8pm_intakeave_fina	GGB28934_SGB41635	63	0.3874243369	0.6994375182
EtOH_8pm_intakeave_fina	GGB28946_SGB41652	63	1.624309639	0.1125450451
EtOH_8pm_intakeave_fina	GGB28949_SGB41655	63	-1.433323615	0.1580025882
EtOH_8pm_intakeave_fina	GGB28949_SGB41656	63	0.4917894739	0.6242686649
EtOH_8pm_intakeave_fina	GGB28950_SGB41657	63	-1.489340947	0.1506172199
EtOH_8pm_intakeave_fina	GGB28951_SGB102295	63	1.179943358	0.2801556966
EtOH_8pm_intakeave_fina	GGB28951_SGB41658	63	2.725497796	0.00858195644З
EtOH_8pm_intakeave_fina	GGB28954_SGB41662	63	0.6012426565	0.6043333808
EtOH_8pm_intakeave_fina	GGB28956_SGB41665	63	1.468584273	0.1670902269
EtOH_8pm_intakeave_fina	GGB28960_SGB41669	63	1.892301327	0.06254288138
EtOH_8pm_intakeave_fina	GGB28967_SGB41678	63	1.370579351	0.1885998892
EtOH_8pm_intakeave_fina	GGB28991_SGB41705	63	-1.200696279	0.236512847
EtOH_8pm_intakeave_fina	GGB29002_SGB41718	63	0.8462623819	0.3996561123
EtOH_8pm_intakeave_fina	GGB29003_SGB41719	63	-0.4535049257	0.6573454362
EtOH_8pm_intakeave_fina	GGB29011_SGB41731	63	-1.286809445	0.2041102158
EtOH_8pm_intakeave_fina	GGB29531_SGB42317	63	3.347517278	0.00192898841
EtOH_8pm_intakeave_fina	GGB29685_SGB42494	63	-2.785511015	0.00984696801
EtOH_8pm_intakeave_fina	GGB30141_SGB43066	63	-0.399380372	0.7409062324
EtOH_8pm_intakeave_fina	GGB30286_SGB43248	63	-1.355290016	0.206012049
EtOH_8pm_intakeave_fina	GGB30303_SGB43268	63	-0.7084879117	0.4894786549
EtOH_8pm_intakeave_fina	GGB30413_SGB43452	63	0.582121701	0.5721068156
EtOH_8pm_intakeave_fina	GGB30454_SGB43514	63	-0.8233265133	0.4128011557
EtOH_8pm_intakeave_fina	GGB30455_SGB43519	63	-0.3009936403	0.7663632045
EtOH_8pm_intakeave_fina	GGB30461_SGB43527	63	-1.423712348	0.1628818294
EtOH_8pm_intakeave_fina	GGB30461_SGB43530	63	-0.4396963662	0.6906625349
EtOH_8pm_intakeave_fina	GGB30463_SGB43537	63	0.1110881217	0.9176070924
EtOH_8pm_intakeave_fina	GGB30473_SGB43557	63	-0.0180628908	0.9857401854
EtOH_8pm_intakeave_fina	GGB30475_SGB63182	63	-0.7060612878	0.5105386436
EtOH_8pm_intakeave_fina	GGB30861_SGB44083	63	-1.379488896	0.1755293023
EtOH_8pm_intakeave_fina	GGB31312_SGB44628	63	-0.5215756988	0.6067032805
EtOH_8pm_intakeave_fina	GGB31438_SGB44768	63	-1.107392309	0.4079368044
EtOH_8pm_intakeave_fina	GGB3171_SGB4185	63	0.113938486	0.9093433478
EtOH_8pm_intakeave_fina	GGB31823_SGB45199	63	-0.2801433176	0.7794655299
EtOH_8pm_intakeave_fina	GGB31853_SGB45233	63	1.571170099	0.1451817329
EtOH_8pm_intakeave_fina	GGB32371_SGB41694	63	-0.7542431616	0.4555430904
EtOH_8pm_intakeave_fina	GGB3793_SGB5158	63	-1.369548524	0.1761896911
EtOH_8pm_intakeave_fina	GGB42598_SGB59794	63	0.7613088249	0.4825346275
EtOH_8pm_intakeave_fina	GGB45656_SGB63370	63	0.8363380493	0.4088445512
EtOH_8pm_intakeave_fina	GGB47127_SGB65054	63	-1.914754799	0.09870239831
EtOH_8pm_intakeave_fina	GGB74395_SGB43521	63	-0.4276743401	0.6878371447
EtOH_8pm_intakeave_fina	GGB75053_SGB43494	63	-0.3808041501	0.7071725039
EtOH_8pm_intakeave_fina	GGB75109_SGB102238	63	-2.144240706	0.03976246471
EtOH_8pm_intakeave_fina	GGB81440_SGB45230	63	0.9337425203	0.3859456233
EtOH_8pm_intakeave_fina	Lachnospiraceae_bacterium	63	-0.9162059812	0.3632274278
EtOH_8pm_intakeave_fina	Lachnospiraceae_bacterium_A2	63	0.1481153175	0.8824398251
EtOH_8pm_intakeave_fina	Lachnospiraceae_bacterium_MD308	63	-1.592137369	0.1316625572

EtOH_8pm_intakeave_fina	Lachnospiraceae_bacterium_MD329	63	-1.295684678	0.2006763306
EtOH_8pm_intakeave_fina	Lachnospiraceae_unclassified_SGB4141	63	1.78788985	0.08834442656
EtOH_8pm_intakeave_fina	Lachnospiraceae_unclassified_SGB4141	63	0.8302654844	0.4102784028
EtOH_8pm_intakeave_fina	Lachnospiraceae_unclassified_SGB4142	63	0.9993086844	0.3225004134
EtOH_8pm_intakeave_fina	Lachnospiraceae_unclassified_SGB4158	63	1.151379959	0.2939987945
EtOH_8pm_intakeave_fina	Lactobacillus_johnsonii	63	-0.3332841997	0.7413871316
EtOH_8pm_intakeave_fina	Muribaculaceae_bacterium	63	-1.857285589	0.06723337548
EtOH_8pm_intakeave_fina	Neglectibacter_sp_X4	63	2.476351109	0.01766820147
EtOH_8pm_intakeave_fina	Oscillospiraceae_bacterium	63	0.6188171623	0.6150398682
EtOH_8pm_intakeave_fina	Oscillospiraceae_unclassified_SGB4350	63	1.069605757	0.2984063192
EtOH_8pm_intakeave_fina	Oscillospiraceae_unclassified_SGB4350	63	0.99480151	0.3547664904
EtOH_8pm_intakeave_fina	Parasutterella_excrementihominis	63	0.2652419208	0.8230381162
EtOH_8pm_intakeave_fina	Romboutsia_ilealis	63	1.093903318	0.2858626563
EtOH_8pm_intakeave_fina	Schaedlerella_arabinosiphila	63	-1.247471065	0.275353565
EtOH_8pm_intakeave_fina	Turicibacter_sp_1E2	63	-0.7201506595	0.4801366629
EtOH_8pm_intakeave_fina	bacterium_1XD42_54	63	-0.8551736252	0.3941583251
EtOH_8pm_intakeave_fina	bacterium_1XD42_76	63	-1.383149714	0.1710571147
EtOH_8pm_intakeave_fina	bacterium_1xD8_48	63	0.3618565104	0.7196012766
EtOH_8pm_intakeave_fina	Berger Parker Index	63	-1.366413049	0.1761792057
EtOH_8pm_intakeave_fina	Richness (# observed features)	63	1.206798294	0.2461953866
EtOH_8pm_intakeave_fina	Shannon Index	63	1.154150513	0.2511436448
EtOH_pref_24h_final	Acetatifactor_SGB41546	63	1.751176327	0.08373762376
EtOH_pref_24h_final	Acetatifactor_muris	63	1.017169183	0.3111439081
EtOH_pref_24h_final	Acutalibacter_muris	63	1.433737522	0.1551190807
EtOH_pref_24h_final	Acutalibacter_sp_1XD8_36	63	-0.09993490134	0.920399309
EtOH_pref_24h_final	Adlercreutzia_caecimuris	63	-2.92050843	0.004742590723
EtOH_pref_24h_final	Adlercreutzia_mucosicola	63	0.6495023356	0.5167488902
EtOH_pref_24h_final	Adlercreutzia_muris	63	0.7655451132	0.4450546575
EtOH_pref_24h_final	Akkermansia_muciniphila	63	-1.392862884	0.1670341845
EtOH_pref_24h_final	Alistipes_sp_DSM_112343	63	-2.788241066	0.006856048457
EtOH_pref_24h_final	Anaerotruncus_sp_1XD42_93	63	0.9318008048	0.3531710923
EtOH_pref_24h_final	Bacteria_unclassified_SGB102200	63	0.2451008255	0.8064258411
EtOH_pref_24h_final	Bacteria_unclassified_SGB41677	63	-0.6806634993	0.4969129587
EtOH_pref_24h_final	Bacteria_unclassified_SGB43546	63	0.3483179185	0.727733522
EtOH_pref_24h_final	Bacteroides_thetaiotaomicron	63	-1.616880923	0.1096713177
EtOH_pref_24h_final	Bifidobacterium_pseudolongum	63	-1.621208574	0.1087441566
EtOH_pref_24h_final	Clostridia_bacterium	63	-2.085255142	0.04047295056
EtOH_pref_24h_final	Clostridiaceae_bacterium	63	-3.998267895	0.000196028189
EtOH_pref_24h_final	Clostridiaceae_unclassified_SGB41663	63	0.2986012269	0.7653289155
EtOH_pref_24h_final	Clostridiales_bacterium	63	2.338330966	0.02216452052
EtOH_pref_24h_final	Clostridium_cocleatum	63	0.5828261859	0.5605641232
EtOH_pref_24h_final	Coriobacteriaceae_bacterium	63	-1.804773612	0.07491658018
EtOH_pref_24h_final	Dorea_sp_5_2	63	0.5962303693	0.5516095073
EtOH_pref_24h_final	Dubosiella_newyorkensis	63	-1.71361528	0.09041991597
EtOH_pref_24h_final	Erysipelotrichales_bacterium	63	-0.1223171371	0.9026538983
EtOH_pref_24h_final	Eubacteriaceae_bacterium	63	-0.9536021644	0.3421029335

EtOH_pref_24h_final	Eubacteriaceae_unclassified_SGB9492;	63	-1.256134705	0.2120340334
EtOH_pref_24h_final	GGB20149_SGB29430	63	0.7716789504	0.4414336778
EtOH_pref_24h_final	GGB22635_SGB63107	63	-0.2443051781	0.8070414992
EtOH_pref_24h_final	GGB25041_SGB36960	63	-0.1953985918	0.845105244
EtOH_pref_24h_final	GGB27876_SGB40310	63	-1.094733476	0.2760104692
EtOH_pref_24h_final	GGB27878_SGB40312	63	-1.543672341	0.1263471145
EtOH_pref_24h_final	GGB27918_SGB40356	63	-0.8529118028	0.395134193
EtOH_pref_24h_final	GGB28382_SGB40962	63	1.805631742	0.07478192925
EtOH_pref_24h_final	GGB28399_SGB40980	63	0.4272480062	0.6694349891
EtOH_pref_24h_final	GGB28411_SGB40993	63	0.3684061778	0.7127255313
EtOH_pref_24h_final	GGB28415_SGB40997	63	-0.8772940625	0.3818459716
EtOH_pref_24h_final	GGB28430_SGB41013	63	2.527416394	0.01374849
EtOH_pref_24h_final	GGB28439_SGB41022	63	1.721569809	0.08896919664
EtOH_pref_24h_final	GGB28778_SGB41431	63	0.5400843377	0.5895904247
EtOH_pref_24h_final	GGB28784_SGB41437	63	0.2713496811	0.7861860792
EtOH_pref_24h_final	GGB28792_SGB41445	63	-0.04046783737	0.9677203682
EtOH_pref_24h_final	GGB28798_SGB41451	63	0.0302135507	0.9758968332
EtOH_pref_24h_final	GGB28802_SGB41455	63	-0.6968714774	0.4867623492
EtOH_pref_24h_final	GGB28818_SGB41473	63	0.8969495152	0.3713409178
EtOH_pref_24h_final	GGB28828_SGB41484	63	-0.7849891372	0.4336360458
EtOH_pref_24h_final	GGB28851_SGB41518	63	-0.3020771661	0.7626807204
EtOH_pref_24h_final	GGB28859_SGB41528	63	-0.07498696339	0.9402264822
EtOH_pref_24h_final	GGB28864_SGB41535	63	1.69283122	0.094302699
EtOH_pref_24h_final	GGB28869_SGB41543	63	1.81819045	0.07283438948
EtOH_pref_24h_final	GGB28883_SGB41564	63	-0.8127464924	0.4176397691
EtOH_pref_24h_final	GGB28892_SGB41573	63	0.03155994124	0.9748230942
EtOH_pref_24h_final	GGB28893_SGB41574	63	1.128033666	0.2618111392
EtOH_pref_24h_final	GGB28898_SGB41580	63	-1.650832592	0.1025669129
EtOH_pref_24h_final	GGB28904_SGB41597	63	1.491448342	0.1394356592
EtOH_pref_24h_final	GGB28916_SGB41612	63	1.272509265	0.2062131865
EtOH_pref_24h_final	GGB28924_SGB41621	63	0.9509860055	0.3434189772
EtOH_pref_24h_final	GGB28926_SGB41624	63	-1.087953313	0.278966344
EtOH_pref_24h_final	GGB28927_SGB41625	63	1.063151096	0.2899667358
EtOH_pref_24h_final	GGB28934_SGB41635	63	0.4712342775	0.6377841813
EtOH_pref_24h_final	GGB28946_SGB41652	63	-0.5736161742	0.5667583928
EtOH_pref_24h_final	GGB28949_SGB41655	63	-1.909184008	0.05995760799
EtOH_pref_24h_final	GGB28949_SGB41656	63	-1.600186346	0.1133081415
EtOH_pref_24h_final	GGB28950_SGB41657	63	-0.9194252273	0.3595561041
EtOH_pref_24h_final	GGB28951_SGB102295	63	-0.616696723	0.5380774745
EtOH_pref_24h_final	GGB28951_SGB41658	63	1.250205144	0.214171622
EtOH_pref_24h_final	GGB28954_SGB41662	63	-0.8980882296	0.3707380036
EtOH_pref_24h_final	GGB28956_SGB41665	63	-0.08155608127	0.9350015243
EtOH_pref_24h_final	GGB28960_SGB41669	63	0.2071230936	0.8359426294
EtOH_pref_24h_final	GGB28967_SGB41678	63	-0.6456072908	0.5192575703
EtOH_pref_24h_final	GGB28991_SGB41705	63	-1.305261613	0.1949287407
EtOH_pref_24h_final	GGB29002_SGB41718	63	1.448232299	0.1510563449

EtOH_pref_24h_final	GGB29003_SGB41719	63	1.17162297	0.244017552
EtOH_pref_24h_final	GGB29011_SGB41731	63	-0.879551068	0.3806302791
EtOH_pref_24h_final	GGB29531_SGB42317	63	2.13702054	0.03590944366
EtOH_pref_24h_final	GGB29685_SGB42494	63	-2.265910606	0.02941465967
EtOH_pref_24h_final	GGB30141_SGB43066	63	1.177231413	0.2417928679
EtOH_pref_24h_final	GGB30286_SGB43248	63	0.3996824091	0.6895861466
EtOH_pref_24h_final	GGB30303_SGB43268	63	1.703385313	0.09231423979
EtOH_pref_24h_final	GGB30413_SGB43452	63	1.088673914	0.2786511469
EtOH_pref_24h_final	GGB30454_SGB43514	63	-1.021874853	0.3089298232
EtOH_pref_24h_final	GGB30455_SGB43519	63	1.21352438	0.2277497993
EtOH_pref_24h_final	GGB30461_SGB43527	63	-0.9262237369	0.3560393559
EtOH_pref_24h_final	GGB30461_SGB43530	63	2.18445958	0.03212892139
EtOH_pref_24h_final	GGB30463_SGB43537	63	0.7234963703	0.4703400877
EtOH_pref_24h_final	GGB30473_SGB43557	63	-0.1067668188	0.9149779944
EtOH_pref_24h_final	GGB30475_SGB63182	63	-0.6750104288	0.5004803345
EtOH_pref_24h_final	GGB30861_SGB44083	63	-3.676387516	0.000480626704
EtOH_pref_24h_final	GGB31312_SGB44628	63	0.01966625909	0.9843096322
EtOH_pref_24h_final	GGB31438_SGB44768	63	-0.3579983632	0.7204874458
EtOH_pref_24h_final	GGB3171_SGB4185	63	0.5896049393	0.5560265887
EtOH_pref_24h_final	GGB31823_SGB45199	63	-0.4499742918	0.6530022331
EtOH_pref_24h_final	GGB31853_SGB45233	63	0.3895341772	0.6970629376
EtOH_pref_24h_final	GGB32371_SGB41694	63	-0.5457471836	0.5857041875
EtOH_pref_24h_final	GGB3793_SGB5158	63	-1.015149119	0.3120976606
EtOH_pref_24h_final	GGB42598_SGB59794	63	-1.218994423	0.2256859588
EtOH_pref_24h_final	GGB45656_SGB63370	63	0.8574914469	0.3926166791
EtOH_pref_24h_final	GGB47127_SGB65054	63	-0.8316530624	0.4069509619
EtOH_pref_24h_final	GGB74395_SGB43521	63	0.1841123181	0.8539457364
EtOH_pref_24h_final	GGB75053_SGB43494	63	0.7492182065	0.4547770632
EtOH_pref_24h_final	GGB75109_SGB102238	63	-1.166353808	0.2461210438
EtOH_pref_24h_final	GGB81440_SGB45230	63	1.105586093	0.2713248738
EtOH_pref_24h_final	Lachnospiraceae_bacterium	63	-2.92523088	0.004679709201
EtOH_pref_24h_final	Lachnospiraceae_bacterium_A2	63	-1.219159133	0.2256240268
EtOH_pref_24h_final	Lachnospiraceae_bacterium_MD308	63	-1.850212251	0.06806036313
EtOH_pref_24h_final	Lachnospiraceae_bacterium_MD329	63	-1.673316022	0.09807214061
EtOH_pref_24h_final	Lachnospiraceae_unclassified_SGB4141	63	-0.3769841354	0.7063510859
EtOH_pref_24h_final	Lachnospiraceae_unclassified_SGB4141	63	0.216592729	0.8285587358
EtOH_pref_24h_final	Lachnospiraceae_unclassified_SGB414[illegible]	63	0.1241997086	0.9011634724
EtOH_pref_24h_final	Lachnospiraceae_unclassified_SGB415[illegible]	63	-0.2127910847	0.8315212206
EtOH_pref_24h_final	Lactobacillus_johnsonii	63	-0.5711781684	0.5684037073
EtOH_pref_24h_final	Muribaculaceae_bacterium	63	-2.067582716	0.04214238537
EtOH_pref_24h_final	Neglectibacter_sp_X4	63	1.227912478	0.2223505874
EtOH_pref_24h_final	Oscillospiraceae_bacterium	63	-0.1211104707	0.9036093976
EtOH_pref_24h_final	Oscillospiraceae_unclassified_SGB4350	63	-1.375765188	0.1722225853
EtOH_pref_24h_final	Oscillospiraceae_unclassified_SGB4350	63	0.944530789	0.3466803967
EtOH_pref_24h_final	Parasutterella_excrementihominis	63	-0.6590260988	0.510642139
EtOH_pref_24h_final	Romboutsia_ilealis	63	0.5636881863	0.5734729949

EtOH_pref_24h_final	Schaedlerella_arabinosiphila	63	-0.246612252	0.8052566644
EtOH_pref_24h_final	Turicibacter_sp_1E2	63	-1.002417467	0.3181541915
EtOH_pref_24h_final	bacterium_1XD42_54	63	-2.870313725	0.00546112408
EtOH_pref_24h_final	bacterium_1XD42_76	63	-0.6066067291	0.5447274353
EtOH_pref_24h_final	bacterium_1xD8_48	63	1.59729399	0.1139480152
EtOH_pref_24h_final	Berger Parker Index	63	-1.114973078	0.2673173421
EtOH_pref_24h_final	Richness (# observed features)	63	1.670838133	0.0985594638
EtOH_pref_24h_final	Shannon Index	63	0.9570894165	0.3403538395
OFB_Cdistance	Acetatifactor_SGB41546	63	-1.097024985	0.2749286589
OFB_Cdistance	Acetatifactor_muris	63	-0.364502659	0.7167870711
OFB_Cdistance	Acutalibacter_muris	63	-0.935597239	0.3538994529
OFB_Cdistance	Acutalibacter_sp_1XD8_36	63	1.788433794	0.07734809396
OFB_Cdistance	Adlercreutzia_caecimuris	63	-1.439504952	0.1538346139
OFB_Cdistance	Adlercreutzia_mucosicola	63	1.2148755	0.2302147754
OFB_Cdistance	Adlercreutzia_muris	63	0.3180812294	0.7505211343
OFB_Cdistance	Akkermansia_muciniphila	63	0.04891594121	0.9611995529
OFB_Cdistance	Alistipes_sp_DSM_112343	63	-0.3400559013	0.7340145132
OFB_Cdistance	Anaerotruncus_sp_1XD42_93	63	0.4097437377	0.6855134038
OFB_Cdistance	Bacteria_unclassified_SGB102200	63	-0.1333212018	0.8957179311
OFB_Cdistance	Bacteria_unclassified_SGB41677	63	-0.87194838	0.4041342254
OFB_Cdistance	Bacteria_unclassified_SGB43546	63	-0.3201214809	0.7587180738
OFB_Cdistance	Bacteroides_thetaiotaomicron	63	-1.569014562	0.1201889326
OFB_Cdistance	Bifidobacterium_pseudolongum	63	-0.7597564102	0.449135987
OFB_Cdistance	Clostridia_bacterium	63	-1.460504214	0.149662105
OFB_Cdistance	Clostridiaceae_bacterium	63	-0.782278713	0.4367078534
OFB_Cdistance	Clostridiaceae_unclassified_SGB41663	63	0.02834144652	0.9777956471
OFB_Cdistance	Clostridiales_bacterium	63	0.8637944696	0.3921130335
OFB_Cdistance	Clostridium_cocleatum	63	0.3728638779	0.7126632451
OFB_Cdistance	Coriobacteriaceae_bacterium	63	0.5826937517	0.5609699392
OFB_Cdistance	Dorea_sp_5_2	63	1.676551846	0.1020232262
OFB_Cdistance	Dubosiella_newyorkensis	63	-0.985712763	0.3408195587
OFB_Cdistance	Erysipelotrichales_bacterium	63	1.14208663	0.2666311715
OFB_Cdistance	Eubacteriaceae_bacterium	63	0.2694487275	0.7908067584
OFB_Cdistance	Eubacteriaceae_unclassified_SGB9492	63	-2.34252918	0.02193379013
OFB_Cdistance	GGB20149_SGB29430	63	-0.6234549376	0.538601681
OFB_Cdistance	GGB22635_SGB63107	63	0.2264119339	0.8219763488
OFB_Cdistance	GGB25041_SGB36960	63	-0.6457937805	0.5212561595
OFB_Cdistance	GGB27876_SGB40310	63	0.2923213017	0.7765272052
OFB_Cdistance	GGB27878_SGB40312	63	-0.1100815996	0.9174226806
OFB_Cdistance	GGB27918_SGB40356	63	-0.9514525948	0.3637891233
OFB_Cdistance	GGB28382_SGB40962	63	1.634818187	0.1187017497
OFB_Cdistance	GGB28399_SGB40980	63	-0.1065674057	0.9158616859
OFB_Cdistance	GGB28411_SGB40993	63	-0.006325946417	0.9950381856
OFB_Cdistance	GGB28415_SGB40997	63	0.3096532356	0.786140247
OFB_Cdistance	GGB28430_SGB41013	63	-0.2912927275	0.7747870695
OFB_Cdistance	GGB28439_SGB41022	63	-0.04583883718	0.9642736767

OFB_Cdistance	GGB28778_SGB41431	63	0.6909227964	0.4909007063
OFB_Cdistance	GGB28784_SGB41437	63	-1.392513287	0.170848058
OFB_Cdistance	GGB28792_SGB41445	63	-0.6698390459	0.5418389817
OFB_Cdistance	GGB28798_SGB41451	63	-1.121923625	0.2661967843
OFB_Cdistance	GGB28802_SGB41455	63	-1.60169579	0.1272459527
OFB_Cdistance	GGB28818_SGB41473	63	0.05919818903	0.9530790465
OFB_Cdistance	GGB28828_SGB41484	63	1.061425841	0.3148378525
OFB_Cdistance	GGB28851_SGB41518	63	-0.03709672199	0.9709621768
OFB_Cdistance	GGB28859_SGB41528	63	0.4475457881	0.6748791925
OFB_Cdistance	GGB28864_SGB41535	63	0.7123777113	0.4975642384
OFB_Cdistance	GGB28869_SGB41543	63	-0.8044450329	0.4223945817
OFB_Cdistance	GGB28883_SGB41564	63	-1.809189771	0.07598617036
OFB_Cdistance	GGB28892_SGB41573	63	-1.288939892	0.2098522383
OFB_Cdistance	GGB28893_SGB41574	63	0.7416334263	0.4741756048
OFB_Cdistance	GGB28898_SGB41580	63	0.895830871	0.3811568824
OFB_Cdistance	GGB28904_SGB41597	63	0.933545938	0.3599627258
OFB_Cdistance	GGB28916_SGB41612	63	0.8387661182	0.4046763302
OFB_Cdistance	GGB28924_SGB41621	63	-0.08336053142	0.9380755815
OFB_Cdistance	GGB28926_SGB41624	63	-0.4103162265	0.6817815824
OFB_Cdistance	GGB28927_SGB41625	63	1.662381646	0.1022900959
OFB_Cdistance	GGB28934_SGB41635	63	-0.208288771	0.8441291442
OFB_Cdistance	GGB28946_SGB41652	63	-0.3405982232	0.7423370841
OFB_Cdistance	GGB28949_SGB41655	63	-0.1914454719	0.8483914157
OFB_Cdistance	GGB28949_SGB41656	63	0.718665579	0.4815249552
OFB_Cdistance	GGB28950_SGB41657	63	-0.3757757144	0.7173287235
OFB_Cdistance	GGB28951_SGB102295	63	-0.2157280117	0.8309215067
OFB_Cdistance	GGB28951_SGB41658	63	0.1298599249	0.9001420322
OFB_Cdistance	GGB28954_SGB41662	63	0.6960902843	0.4902709633
OFB_Cdistance	GGB28956_SGB41665	63	0.7116834037	0.4803977213
OFB_Cdistance	GGB28960_SGB41669	63	0.8544705931	0.3969190854
OFB_Cdistance	GGB28967_SGB41678	63	0.1844274566	0.8542504906
OFB_Cdistance	GGB28991_SGB41705	63	0.1851001282	0.8546185524
OFB_Cdistance	GGB29002_SGB41718	63	1.666375204	0.09949646307
OFB_Cdistance	GGB29003_SGB41719	63	0.5636723936	0.5798437058
OFB_Cdistance	GGB29011_SGB41731	63	-1.429450119	0.1590573472
OFB_Cdistance	GGB29531_SGB42317	63	2.115769879	0.03829558788
OFB_Cdistance	GGB29685_SGB42494	63	-1.201453321	0.2324815169
OFB_Cdistance	GGB30141_SGB43066	63	-0.1349001277	0.8933973747
OFB_Cdistance	GGB30286_SGB43248	63	0.7868163845	0.4332611423
OFB_Cdistance	GGB30303_SGB43268	63	-0.1877435905	0.8530452683
OFB_Cdistance	GGB30413_SGB43452	63	0.6120615268	0.5417115591
OFB_Cdistance	GGB30454_SGB43514	63	-0.2763535252	0.7825333942
OFB_Cdistance	GGB30455_SGB43519	63	-0.3252580793	0.7503977535
OFB_Cdistance	GGB30461_SGB43527	63	0.4218615548	0.6742251546
OFB_Cdistance	GGB30461_SGB43530	63	-0.8836066664	0.3951533267
OFB_Cdistance	GGB30463_SGB43537	63	0.3073879663	0.7613290681

OFB_Cdistance	GGB30473_SGB43557	63	-0.1114972326	0.9117231045
OFB_Cdistance	GGB30475_SGB63182	63	-0.460464426	0.6456022808
OFB_Cdistance	GGB30861_SGB44083	63	-1.710939725	0.09153320834
OFB_Cdistance	GGB31312_SGB44628	63	-1.362975586	0.1783220474
OFB_Cdistance	GGB31438_SGB44768	63	-0.3391168461	0.7388357455
OFB_Cdistance	GGB3171_SGB4185	63	0.1299123722	0.8997336364
OFB_Cdistance	GGB31823_SGB45199	63	1.161624854	0.247931715
OFB_Cdistance	GGB31853_SGB45233	63	0.1161639934	0.9077587857
OFB_Cdistance	GGB32371_SGB41694	63	0.8636173458	0.3998182216
OFB_Cdistance	GGB3793_SGB5158	63	0.5095594259	0.611444888
OFB_Cdistance	GGB42598_SGB59794	63	-0.08895521968	0.9294081678
OFB_Cdistance	GGB45656_SGB63370	63	1.899893571	0.07652807929
OFB_Cdistance	GGB47127_SGB65054	63	-0.8302255858	0.4076980441
OFB_Cdistance	GGB74395_SGB43521	63	-1.24102366	0.2253548287
OFB_Cdistance	GGB75053_SGB43494	63	-0.03923096588	0.9687153911
OFB_Cdistance	GGB75109_SGB102238	63	-0.3749413091	0.7080024591
OFB_Cdistance	GGB81440_SGB45230	63	0.6193845561	0.5554564783
OFB_Cdistance	Lachnospiraceae_bacterium	63	-1.814260349	0.07330615937
OFB_Cdistance	Lachnospiraceae_bacterium_A2	63	0.4854578724	0.6283248353
OFB_Cdistance	Lachnospiraceae_bacterium_MD308	63	0.2566028034	0.7975450374
OFB_Cdistance	Lachnospiraceae_bacterium_MD329	63	-0.8831216906	0.3887480883
OFB_Cdistance	Lachnospiraceae_unclassified_SGB4141	63	-0.7373807906	0.4637614235
OFB_Cdistance	Lachnospiraceae_unclassified_SGB4141	63	0.1288308141	0.8993058601
OFB_Cdistance	Lachnospiraceae_unclassified_SGB4142	63	-0.1056773252	0.9160896425
OFB_Cdistance	Lachnospiraceae_unclassified_SGB4158	63	-1.158351269	0.2598364335
OFB_Cdistance	Lactobacillus_johnsonii	63	0.253416087	0.8005875404
OFB_Cdistance	Muribaculaceae_bacterium	63	0.1811217116	0.8576935066
OFB_Cdistance	Neglectibacter_sp_X4	63	-0.1542307716	0.8793372923
OFB_Cdistance	Oscillospiraceae_bacterium	63	-1.005684608	0.330391997
OFB_Cdistance	Oscillospiraceae_unclassified_SGB4350	63	-0.4527310602	0.6516276948
OFB_Cdistance	Oscillospiraceae_unclassified_SGB4350	63	-0.07755818909	0.9388809505
OFB_Cdistance	Parasutterella_excrementihominis	63	-0.2802371981	0.7927857257
OFB_Cdistance	Romboutsia_ilealis	63	0.8278531487	0.4287836008
OFB_Cdistance	Schaedlerella_arabinosiphila	63	-1.465153727	0.1468669442
OFB_Cdistance	Turicibacter_sp_1E2	63	0.7353280131	0.4645353458
OFB_Cdistance	bacterium_1XD42_54	63	-1.183455045	0.2478069564
OFB_Cdistance	bacterium_1XD42_76	63	-0.06120582285	0.9515012508
OFB_Cdistance	bacterium_1xD8_48	63	0.675516001	0.5001274219
OFB_Cdistance	Berger Parker Index	63	-0.272970109	0.786498134
OFB_Cdistance	Richness (# observed features)	63	-0.0231978421	0.9816188679
OFB_Cdistance	Shannon Index	63	-0.4578632832	0.652558339
OFB_Centries	Acetatifactor_SGB41546	63	-0.9883790317	0.3254504833
OFB_Centries	Acetatifactor_muris	63	-0.850005856	0.3980103038
OFB_Centries	Acutalibacter_muris	63	-1.658185522	0.1009210554
OFB_Centries	Acutalibacter_sp_1XD8_36	63	2.006358204	0.04842804857
OFB_Centries	Adlercreutzia_caecimuris	63	-1.234769352	0.2218181338

OFB_Centries	Adlercreutzia_mucosicola	63	1.322443525	0.1941212822
OFB_Centries	Adlercreutzia_muris	63	0.001948676553	0.9984451821
OFB_Centries	Akkermansia_muciniphila	63	0.2279890475	0.820673657
OFB_Centries	Alistipes_sp_DSM_112343	63	-0.3843455034	0.7013828692
OFB_Centries	Anaerotruncus_sp_1XD42_93	63	0.1934301147	0.8474461831
OFB_Centries	Bacteria_unclassified_SGB102200	63	-0.1697715071	0.8664072849
OFB_Centries	Bacteria_unclassified_SGB41677	63	-0.0627670092	0.9514339953
OFB_Centries	Bacteria_unclassified_SGB43546	63	-0.3091027308	0.7637333326
OFB_Centries	Bacteroides_thetaiotaomicron	63	-1.792404086	0.07690854458
OFB_Centries	Bifidobacterium_pseudolongum	63	-0.7573400789	0.4503396884
OFB_Centries	Clostridia_bacterium	63	-1.29569679	0.1988688031
OFB_Centries	Clostridiaceae_bacterium	63	-0.7696567185	0.4442892504
OFB_Centries	Clostridiaceae_unclassified_SGB41663	63	0.7089827236	0.4849575143
OFB_Centries	Clostridiales_bacterium	63	0.4769450318	0.6353706772
OFB_Centries	Clostridium_cocleatum	63	0.271260193	0.7889158902
OFB_Centries	Coriobacteriaceae_bacterium	63	0.682719051	0.4955796435
OFB_Centries	Dorea_sp_5_2	63	2.438962422	0.02013932312
OFB_Centries	Dubosiella_newyorkensis	63	-0.9692207351	0.3425541493
OFB_Centries	Erysipelotrichales_bacterium	63	0.797716524	0.4318375118
OFB_Centries	Eubacteriaceae_bacterium	63	0.5150091036	0.6102973425
OFB_Centries	Eubacteriaceae_unclassified_SGB9492:	63	-2.008183076	0.05058860908
OFB_Centries	GGB20149_SGB29430	63	-0.2984746279	0.7672991753
OFB_Centries	GGB22635_SGB63107	63	0.02971023131	0.9764327095
OFB_Centries	GGB25041_SGB36960	63	-0.2745972593	0.7844394328
OFB_Centries	GGB27876_SGB40310	63	0.6314079839	0.5359221948
OFB_Centries	GGB27878_SGB40312	63	0.00859048758	0.9933382066
OFB_Centries	GGB27918_SGB40356	63	-0.5969911749	0.5623736048
OFB_Centries	GGB28382_SGB40962	63	1.667978653	0.1092141505
OFB_Centries	GGB28399_SGB40980	63	0.6708169457	0.5073940756
OFB_Centries	GGB28411_SGB40993	63	-0.7264798488	0.4727817822
OFB_Centries	GGB28415_SGB40997	63	0.7548516694	0.506814287
OFB_Centries	GGB28430_SGB41013	63	-0.01024536602	0.9920240824
OFB_Centries	GGB28439_SGB41022	63	0.3351785519	0.7437019052
OFB_Centries	GGB28778_SGB41431	63	0.5796343191	0.5644169504
OFB_Centries	GGB28784_SGB41437	63	-0.7540780355	0.4536086043
OFB_Centries	GGB28792_SGB41445	63	0.07757868879	0.9417744252
OFB_Centries	GGB28798_SGB41451	63	-0.9944653234	0.3296945566
OFB_Centries	GGB28802_SGB41455	63	-2.062695059	0.04800735004
OFB_Centries	GGB28818_SGB41473	63	-0.5657844778	0.5735988937
OFB_Centries	GGB28828_SGB41484	63	0.9211969477	0.3675503245
OFB_Centries	GGB28851_SGB41518	63	-0.08803070152	0.9309415629
OFB_Centries	GGB28859_SGB41528	63	-0.7515163584	0.4722968931
OFB_Centries	GGB28864_SGB41535	63	0.8282250566	0.4216269442
OFB_Centries	GGB28869_SGB41543	63	-0.6606146823	0.5096367801
OFB_Centries	GGB28883_SGB41564	63	-1.377186928	0.1769056149
OFB_Centries	GGB28892_SGB41573	63	-1.339383048	0.187668077

OFB_Centries	GGB28893_SGB41574	63	0.1426920073	0.8883058839
OFB_Centries	GGB28898_SGB41580	63	1.454401013	0.1539663919
OFB_Centries	GGB28904_SGB41597	63	1.266229412	0.2150584201
OFB_Centries	GGB28916_SGB41612	63	0.9037163782	0.3693595747
OFB_Centries	GGB28924_SGB41621	63	0.5884687761	0.5827750578
OFB_Centries	GGB28926_SGB41624	63	-0.3357040357	0.7372073553
OFB_Centries	GGB28927_SGB41625	63	0.8538417734	0.3947577393
OFB_Centries	GGB28934_SGB41635	63	0.1885633556	0.8551878709
OFB_Centries	GGB28946_SGB41652	63	-0.4295024735	0.6745186828
OFB_Centries	GGB28949_SGB41655	63	-0.01067081827	0.9914871894
OFB_Centries	GGB28949_SGB41656	63	0.6356967484	0.528757987
OFB_Centries	GGB28950_SGB41657	63	0.1993954689	0.8463085967
OFB_Centries	GGB28951_SGB102295	63	-0.5537939694	0.5856450536
OFB_Centries	GGB28951_SGB41658	63	-0.150879981	0.8839388217
OFB_Centries	GGB28954_SGB41662	63	0.6476116797	0.5210324892
OFB_Centries	GGB28956_SGB41665	63	0.7496270444	0.4594036035
OFB_Centries	GGB28960_SGB41669	63	1.029542676	0.3062587285
OFB_Centries	GGB28967_SGB41678	63	0.4265515206	0.6713487096
OFB_Centries	GGB28991_SGB41705	63	-0.5372577136	0.5940433579
OFB_Centries	GGB29002_SGB41718	63	1.041976841	0.2995524591
OFB_Centries	GGB29003_SGB41719	63	0.02314461116	0.9817261421
OFB_Centries	GGB29011_SGB41731	63	-1.440214665	0.1535311437
OFB_Centries	GGB29531_SGB42317	63	1.401595052	0.1643484275
OFB_Centries	GGB29685_SGB42494	63	-1.095563086	0.2756902126
OFB_Centries	GGB30141_SGB43066	63	-0.7756900866	0.4400937379
OFB_Centries	GGB30286_SGB43248	63	1.173585658	0.2444696704
OFB_Centries	GGB30303_SGB43268	63	-0.541487251	0.5915626065
OFB_Centries	GGB30413_SGB43452	63	0.5475905362	0.5863474001
OFB_Centries	GGB30454_SGB43514	63	-0.07497532514	0.9402468598
OFB_Centries	GGB30455_SGB43519	63	-0.6833048884	0.5006058143
OFB_Centries	GGB30461_SGB43527	63	0.3754059925	0.7086003264
OFB_Centries	GGB30461_SGB43530	63	-1.285991696	0.2145052302
OFB_Centries	GGB30463_SGB43537	63	0.03536406532	0.9719788907
OFB_Centries	GGB30473_SGB43557	63	-0.06097043946	0.9515138409
OFB_Centries	GGB30475_SGB63182	63	-0.5681751356	0.5704957029
OFB_Centries	GGB30861_SGB44083	63	-1.35364785	0.1796722657
OFB_Centries	GGB31312_SGB44628	63	-1.986923894	0.05142250813
OFB_Centries	GGB31438_SGB44768	63	-0.7209421804	0.4739687758
OFB_Centries	GGB3171_SGB4185	63	0.1664032987	0.8694792801
OFB_Centries	GGB31823_SGB45199	63	0.9638927758	0.3369162068
OFB_Centries	GGB31853_SGB45233	63	0.300435837	0.7645504474
OFB_Centries	GGB32371_SGB41694	63	0.6787498895	0.5027900364
OFB_Centries	GGB3793_SGB5158	63	0.6361050879	0.5255081202
OFB_Centries	GGB42598_SGB59794	63	-0.1226115521	0.903235538
OFB_Centries	GGB45656_SGB63370	63	1.123910576	0.275829535
OFB_Centries	GGB47127_SGB65054	63	-0.7890300239	0.4313710889

OFB_Centries	GGB74395_SGB43521	63	-1.435326145	0.1595204482
OFB_Centries	GGB75053_SGB43494	63	-0.3038188499	0.7617779238
OFB_Centries	GGB75109_SGB102238	63	-0.1320512324	0.8950041349
OFB_Centries	GGB81440_SGB45230	63	-0.1397754322	0.8945518338
OFB_Centries	Lachnospiraceae_bacterium	63	-1.8212301	0.07242942705
OFB_Centries	Lachnospiraceae_bacterium_A2	63	0.2275608306	0.8200845241
OFB_Centries	Lachnospiraceae_bacterium_MD308	63	0.1983349124	0.8428074744
OFB_Centries	Lachnospiraceae_bacterium_MD329	63	-0.525190769	0.6059232348
OFB_Centries	Lachnospiraceae_unclassified_SGB4141	63	-0.3706176629	0.7111933046
OFB_Centries	Lachnospiraceae_unclassified_SGB4141	63	1.487776622	0.146464048
OFB_Centries	Lachnospiraceae_unclassified_SGB4142	63	-0.2722164231	0.7865459707
OFB_Centries	Lachnospiraceae_unclassified_SGB4158	63	-0.6413915636	0.535281593
OFB_Centries	Lactobacillus_johnsonii	63	0.4453642785	0.6565358738
OFB_Centries	Muribaculaceae_bacterium	63	0.198701691	0.8435811933
OFB_Centries	Neglectibacter_sp_X4	63	-0.1933456013	0.8471861896
OFB_Centries	Oscillospiraceae_bacterium	63	-1.139855181	0.266935165
OFB_Centries	Oscillospiraceae_unclassified_SGB4350	63	-0.03936293769	0.9686085963
OFB_Centries	Oscillospiraceae_unclassified_SGB4350	63	0.09744949691	0.9227291823
OFB_Centries	Parasutterella_excrementihominis	63	0.05597449461	0.9573671167
OFB_Centries	Romboutsia_ilealis	63	1.311081375	0.2079905343
OFB_Centries	Schaedlerella_arabinosiphila	63	-1.820760435	0.07229956347
OFB_Centries	Turicibacter_sp_1E2	63	0.9261386811	0.3582024716
OFB_Centries	bacterium_1XD42_54	63	-0.6775256837	0.5129654016
OFB_Centries	bacterium_1XD42_76	63	-0.7843611841	0.4382414261
OFB_Centries	bacterium_1xD8_48	63	0.3577498674	0.7207145956
OFB_Centries	Berger Parker Index	63	-0.04757386599	0.9623959157
OFB_Centries	Richness (# observed features)	63	-0.2733877822	0.7852430771
OFB_Centries	Shannon Index	63	-0.7925520116	0.4354798526
OFB_Clatency	Acetatifactor_SGB41546	63	-0.5871770132	0.5577634466
OFB_Clatency	Acetatifactor_muris	63	0.7820176093	0.4384333551
OFB_Clatency	Acutalibacter_muris	63	-0.0319673238	0.9747726878
OFB_Clatency	Acutalibacter_sp_1XD8_36	63	-0.2332956461	0.8159901459
OFB_Clatency	Adlercreutzia_caecimuris	63	2.383655218	0.01994207195
OFB_Clatency	Adlercreutzia_mucosicola	63	-1.29815407	0.1973879111
OFB_Clatency	Adlercreutzia_muris	63	-0.2320294056	0.8167445486
OFB_Clatency	Akkermansia_muciniphila	63	1.518940182	0.1323482992
OFB_Clatency	Alistipes_sp_DSM_112343	63	0.7023318329	0.4833264089
OFB_Clatency	Anaerotruncus_sp_1XD42_93	63	0.5762525909	0.5651045803
OFB_Clatency	Bacteria_unclassified_SGB102200	63	-1.213833262	0.2295781845
OFB_Clatency	Bacteria_unclassified_SGB41677	63	1.048903697	0.3003578556
OFB_Clatency	Bacteria_unclassified_SGB43546	63	-0.2481120454	0.8062739026
OFB_Clatency	Bacteroides_thetaiotaomicron	63	1.449211734	0.1506322813
OFB_Clatency	Bifidobacterium_pseudolongum	63	2.092765442	0.03990900409
OFB_Clatency	Clostridia_bacterium	63	0.8002325458	0.4257618515
OFB_Clatency	Clostridiaceae_bacterium	63	0.9597075747	0.3406392689
OFB_Clatency	Clostridiaceae_unclassified_SGB41663	63	0.9283632691	0.3561105312

OFB_Clatency	Clostridiales_bacterium	63	-0.3708215471	0.7110596776
OFB_Clatency	Clostridium_cocleatum	63	0.716867379	0.4777248632
OFB_Clatency	Coriobacteriaceae_bacterium	63	1.41668082	0.200816851
OFB_Clatency	Dorea_sp_5_2	63	-0.5809971325	0.5619919552
OFB_Clatency	Dubosiella_newyorkensis	63	1.2813051	0.2087840786
OFB_Clatency	Erysipelotrichales_bacterium	63	-0.1236611966	0.9020454802
OFB_Clatency	Eubacteriaceae_bacterium	63	1.73206169	0.09267304275
OFB_Clatency	Eubacteriaceae_unclassified_SGB9492[illegible]	63	-0.2646553482	0.7940970098
OFB_Clatency	GGB20149_SGB29430	63	-1.531010944	0.1295908698
OFB_Clatency	GGB22635_SGB63107	63	1.160488843	0.2512308597
OFB_Clatency	GGB25041_SGB36960	63	0.1516738413	0.8801421205
OFB_Clatency	GGB27876_SGB40310	63	1.343066132	0.1868124822
OFB_Clatency	GGB27878_SGB40312	63	0.8101573137	0.4463471024
OFB_Clatency	GGB27918_SGB40356	63	2.230561589	0.03929396915
OFB_Clatency	GGB28382_SGB40962	63	-0.7158319111	0.4768925702
OFB_Clatency	GGB28399_SGB40980	63	-0.3317036103	0.7418778416
OFB_Clatency	GGB28411_SGB40993	63	-0.4645364722	0.6449385712
OFB_Clatency	GGB28415_SGB40997	63	1.09102538	0.2970570808
OFB_Clatency	GGB28430_SGB41013	63	-0.4992635676	0.6184612147
OFB_Clatency	GGB28439_SGB41022	63	-0.4079857976	0.6844989969
OFB_Clatency	GGB28778_SGB41431	63	0.5307248102	0.5960282521
OFB_Clatency	GGB28784_SGB41437	63	0.7158341916	0.4871626584
OFB_Clatency	GGB28792_SGB41445	63	1.509991165	0.1395653814
OFB_Clatency	GGB28798_SGB41451	63	0.6076478696	0.544019112
OFB_Clatency	GGB28802_SGB41455	63	1.433187319	0.1620186419
OFB_Clatency	GGB28818_SGB41473	63	-0.311961609	0.7569947566
OFB_Clatency	GGB28828_SGB41484	63	-0.8303257543	0.4234293901
OFB_Clatency	GGB28851_SGB41518	63	-1.332070342	0.1859487227
OFB_Clatency	GGB28859_SGB41528	63	0.3978629004	0.7099541387
OFB_Clatency	GGB28864_SGB41535	63	-1.828087442	0.07284523737
OFB_Clatency	GGB28869_SGB41543	63	0.4142748843	0.6788988597
OFB_Clatency	GGB28883_SGB41564	63	0.2239623791	0.8228432893
OFB_Clatency	GGB28892_SGB41573	63	0.8533818196	0.3962781804
OFB_Clatency	GGB28893_SGB41574	63	-0.8171015995	0.4210962483
OFB_Clatency	GGB28898_SGB41580	63	0.9251849863	0.3699014347
OFB_Clatency	GGB28904_SGB41597	63	-1.3455841	0.1834300824
OFB_Clatency	GGB28916_SGB41612	63	0.4252112359	0.6724506841
OFB_Clatency	GGB28924_SGB41621	63	-0.8370974557	0.4092655574
OFB_Clatency	GGB28926_SGB41624	63	-1.315879568	0.1947947255
OFB_Clatency	GGB28927_SGB41625	63	-0.6296653181	0.5334976101
OFB_Clatency	GGB28934_SGB41635	63	0.6831973744	0.5048072739
OFB_Clatency	GGB28946_SGB41652	63	0.576787747	0.5714516999
OFB_Clatency	GGB28949_SGB41655	63	1.295509354	0.200507977
OFB_Clatency	GGB28949_SGB41656	63	0.2357945096	0.8143513808
OFB_Clatency	GGB28950_SGB41657	63	0.7515355462	0.457939587
OFB_Clatency	GGB28951_SGB102295	63	0.9631626719	0.3381176097

OFB_Clatency	GGB28951_SGB41658	63	1.117198331	0.271926529
OFB_Clatency	GGB28954_SGB41662	63	-0.991118512	0.3238602546
OFB_Clatency	GGB28956_SGB41665	63	0.1265842372	0.899379732
OFB_Clatency	GGB28960_SGB41669	63	0.575215314	0.568835471
OFB_Clatency	GGB28967_SGB41678	63	-2.028226142	0.04764490547
OFB_Clatency	GGB28991_SGB41705	63	0.5168061398	0.6073293258
OFB_Clatency	GGB29002_SGB41718	63	-2.39389046	0.01953826403
OFB_Clatency	GGB29003_SGB41719	63	-0.08821759487	0.9300284713
OFB_Clatency	GGB29011_SGB41731	63	-1.367066828	0.1749113362
OFB_Clatency	GGB29531_SGB42317	63	-1.222084293	0.2254028831
OFB_Clatency	GGB29685_SGB42494	63	2.329232445	0.02265484287
OFB_Clatency	GGB30141_SGB43066	63	-0.2771065112	0.7842829391
OFB_Clatency	GGB30286_SGB43248	63	-0.6159752297	0.538610316
OFB_Clatency	GGB30303_SGB43268	63	-1.789058597	0.07805440486
OFB_Clatency	GGB30413_SGB43452	63	-0.5105372175	0.6119214745
OFB_Clatency	GGB30454_SGB43514	63	-0.9345927976	0.3516943082
OFB_Clatency	GGB30455_SGB43519	63	-1.601555969	0.1147123289
OFB_Clatency	GGB30461_SGB43527	63	-0.2099249898	0.8338967271
OFB_Clatency	GGB30461_SGB43530	63	-0.6148466614	0.5417380418
OFB_Clatency	GGB30463_SGB43537	63	-1.712042572	0.09165626244
OFB_Clatency	GGB30473_SGB43557	63	1.160689901	0.2482865408
OFB_Clatency	GGB30475_SGB63182	63	0.6194173889	0.5362850771
OFB_Clatency	GGB30861_SGB44083	63	1.700156149	0.09304196639
OFB_Clatency	GGB31312_SGB44628	63	-0.02404280957	0.9809167288
OFB_Clatency	GGB31438_SGB44768	63	1.280533723	0.2121177129
OFB_Clatency	GGB3171_SGB4185	63	-0.8057109879	0.4261768125
OFB_Clatency	GGB31823_SGB45199	63	-0.1680652524	0.8666122301
OFB_Clatency	GGB31853_SGB45233	63	-0.24060765	0.8102208765
OFB_Clatency	GGB32371_SGB41694	63	-0.6929897797	0.4938531599
OFB_Clatency	GGB3793_SGB5158	63	-1.014762923	0.3144336536
OFB_Clatency	GGB42598_SGB59794	63	1.184016087	0.238998106
OFB_Clatency	GGB45656_SGB63370	63	-2.973657192	0.00569724008[illegible]
OFB_Clatency	GGB47127_SGB65054	63	0.2779117985	0.7814550249
OFB_Clatency	GGB74395_SGB43521	63	-0.5184839165	0.6045148679
OFB_Clatency	GGB75053_SGB43494	63	-0.76048432	0.448105827
OFB_Clatency	GGB75109_SGB102238	63	-1.419924132	0.1590504823
OFB_Clatency	GGB81440_SGB45230	63	-0.903335435	0.3767188198
OFB_Clatency	Lachnospiraceae_bacterium	63	1.586272633	0.1163223214
OFB_Clatency	Lachnospiraceae_bacterium_A2	63	2.544659452	0.01367016647
OFB_Clatency	Lachnospiraceae_bacterium_MD308	63	2.266545899	0.02916222563
OFB_Clatency	Lachnospiraceae_bacterium_MD329	63	1.409183426	0.1633950825
OFB_Clatency	Lachnospiraceae_unclassified_SGB414[illegible]	63	0.8689367629	0.3947305553
OFB_Clatency	Lachnospiraceae_unclassified_SGB414[illegible]	63	-2.630223252	0.01204365581
OFB_Clatency	Lachnospiraceae_unclassified_SGB414[illegible]	63	0.8597158049	0.3922327518
OFB_Clatency	Lachnospiraceae_unclassified_SGB415[illegible]	63	0.4354516249	0.7266077813
OFB_Clatency	Lactobacillus_johnsonii	63	1.495736424	0.1392004983

OFB_Clatency	Muribaculaceae_bacterium	63	1.878796296	0.06395893975
OFB_Clatency	Neglectibacter_sp_X4	63	0.2133545898	0.8349025754
OFB_Clatency	Oscillospiraceae_bacterium	63	-0.1040487123	0.9174293874
OFB_Clatency	Oscillospiraceae_unclassified_SGB4350	63	0.1200178405	0.9049456263
OFB_Clatency	Oscillospiraceae_unclassified_SGB4350	63	-0.3447151153	0.7331569679
OFB_Clatency	Parasutterella_excrementihominis	63	1.369400326	0.1982449334
OFB_Clatency	Romboutsia_ilealis	63	-0.003276127923	0.9974164499
OFB_Clatency	Schaedlerella_arabinosiphila	63	1.541685179	0.1286996449
OFB_Clatency	Turicibacter_sp_1E2	63	0.3323608815	0.7397542963
OFB_Clatency	bacterium_1XD42_54	63	1.343463026	0.1823548779
OFB_Clatency	bacterium_1XD42_76	63	0.7493200565	0.4585147406
OFB_Clatency	bacterium_1xD8_48	63	-0.4959451592	0.6208522922
OFB_Clatency	Berger Parker Index	63	0.7739073552	0.4405178516
OFB_Clatency	Richness (# observed features)	63	-1.268534043	0.2100343789
OFB_Clatency	Shannon Index	63	-0.8861642271	0.3790876367
OFB_Ctime	Acetatifactor_SGB41546	63	-1.001760818	0.3193417225
OFB_Ctime	Acetatifactor_muris	63	0.4477151478	0.6602640786
OFB_Ctime	Acutalibacter_muris	63	-1.660484903	0.101422053
OFB_Ctime	Acutalibacter_sp_1XD8_36	63	0.3638798653	0.716207136
OFB_Ctime	Adlercreutzia_caecimuris	63	-1.612339213	0.1118266371
OFB_Ctime	Adlercreutzia_mucosicola	63	1.350991436	0.1801681257
OFB_Ctime	Adlercreutzia_muris	63	0.07106372678	0.9434711917
OFB_Ctime	Akkermansia_muciniphila	63	0.3988173307	0.6938934558
OFB_Ctime	Alistipes_sp_DSM_112343	63	0.05037345174	0.9598609979
OFB_Ctime	Anaerotruncus_sp_1XD42_93	63	0.3255636525	0.7451234374
OFB_Ctime	Bacteria_unclassified_SGB102200	63	0.2695344255	0.7921614298
OFB_Ctime	Bacteria_unclassified_SGB41677	63	-1.817904715	0.08029184583
OFB_Ctime	Bacteria_unclassified_SGB43546	63	-0.2068843739	0.8410551671
OFB_Ctime	Bacteroides_thetaiotaomicron	63	-1.119749097	0.2651775294
OFB_Ctime	Bifidobacterium_pseudolongum	63	-0.9052578274	0.3670782778
OFB_Ctime	Clostridia_bacterium	63	-1.346551294	0.1844492297
OFB_Ctime	Clostridiaceae_bacterium	63	-1.012568545	0.314345718
OFB_Ctime	Clostridiaceae_unclassified_SGB41663	63	0.57368602	0.5716007348
OFB_Ctime	Clostridiales_bacterium	63	1.138784294	0.2588960202
OFB_Ctime	Clostridium_cocleatum	63	-0.02650859512	0.9793102559
OFB_Ctime	Coriobacteriaceae_bacterium	63	0.5821497656	0.5619201788
OFB_Ctime	Dorea_sp_5_2	63	1.548544852	0.1257943335
OFB_Ctime	Dubosiella_newyorkensis	63	-1.573974963	0.133746852
OFB_Ctime	Erysipelotrichales_bacterium	63	1.881307025	0.07032699776
OFB_Ctime	Eubacteriaceae_bacterium	63	0.9675778147	0.3541574226
OFB_Ctime	Eubacteriaceae_unclassified_SGB9492[illegible]	63	-2.059581497	0.04688856043
OFB_Ctime	GGB20149_SGB29430	63	-0.479787926	0.6403416468
OFB_Ctime	GGB22635_SGB63107	63	0.3823185651	0.702589549
OFB_Ctime	GGB25041_SGB36960	63	-0.4807616019	0.639257774
OFB_Ctime	GGB27876_SGB40310	63	0.1556192288	0.8810173092
OFB_Ctime	GGB27878_SGB40312	63	-0.1846908066	0.856462496

OFB_Ctime	GGB27918_SGB40356	63	-1.292633915	0.2260075677
OFB_Ctime	GGB28382_SGB40962	63	1.246862136	0.2269447104
OFB_Ctime	GGB28399_SGB40980	63	-0.5769039609	0.5647218801
OFB_Ctime	GGB28411_SGB40993	63	0.05657515714	0.9555048821
OFB_Ctime	GGB28415_SGB40997	63	0.9000596204	0.4019558059
OFB_Ctime	GGB28430_SGB41013	63	-0.0100178978	0.9920445365
OFB_Ctime	GGB28439_SGB41022	63	-0.2099090243	0.8348193272
OFB_Ctime	GGB28778_SGB41431	63	-0.3595872399	0.7193194066
OFB_Ctime	GGB28784_SGB41437	63	-0.4392770858	0.6661751766
OFB_Ctime	GGB28792_SGB41445	63	-0.7037991589	0.5062502222
OFB_Ctime	GGB28798_SGB41451	63	-1.837139921	0.07089736455
OFB_Ctime	GGB28802_SGB41455	63	-2.359648162	0.02606055131
OFB_Ctime	GGB28818_SGB41473	63	0.4401779233	0.6690540598
OFB_Ctime	GGB28828_SGB41484	63	0.6716506475	0.5357387749
OFB_Ctime	GGB28851_SGB41518	63	0.3612968917	0.7210217902
OFB_Ctime	GGB28859_SGB41528	63	1.129578446	0.3079858972
OFB_Ctime	GGB28864_SGB41535	63	1.232148985	0.2523148241
OFB_Ctime	GGB28869_SGB41543	63	0.1361985457	0.8917935461
OFB_Ctime	GGB28883_SGB41564	63	-1.215546098	0.2272496702
OFB_Ctime	GGB28892_SGB41573	63	-2.450152346	0.01870374293
OFB_Ctime	GGB28893_SGB41574	63	1.06043783	0.3002372181
OFB_Ctime	GGB28898_SGB41580	63	2.432495823	0.0216470335
OFB_Ctime	GGB28904_SGB41597	63	0.6621732247	0.5161259991
OFB_Ctime	GGB28916_SGB41612	63	0.4190232414	0.6787733904
OFB_Ctime	GGB28924_SGB41621	63	0.08135429766	0.9382739565
OFB_Ctime	GGB28926_SGB41624	63	0.1341996634	0.8933389516
OFB_Ctime	GGB28927_SGB41625	63	0.5716701404	0.5694284994
OFB_Ctime	GGB28934_SGB41635	63	-0.6879575389	0.5145470996
OFB_Ctime	GGB28946_SGB41652	63	-0.5617529521	0.5908008036
OFB_Ctime	GGB28949_SGB41655	63	0.006392052134	0.9949208784
OFB_Ctime	GGB28949_SGB41656	63	0.04424090139	0.9649965971
OFB_Ctime	GGB28950_SGB41657	63	-0.3333135006	0.7401390197
OFB_Ctime	GGB28951_SGB102295	63	-0.8316014239	0.4073388043
OFB_Ctime	GGB28951_SGB41658	63	-1.153381556	0.2701976199
OFB_Ctime	GGB28954_SGB41662	63	0.2077344078	0.8356300278
OFB_Ctime	GGB28956_SGB41665	63	-0.0668209499	0.9468577916
OFB_Ctime	GGB28960_SGB41669	63	-0.2316307572	0.8194139887
OFB_Ctime	GGB28967_SGB41678	63	0.192476023	0.8476955891
OFB_Ctime	GGB28991_SGB41705	63	-1.648311765	0.1078737586
OFB_Ctime	GGB29002_SGB41718	63	1.213957913	0.227454206
OFB_Ctime	GGB29003_SGB41719	63	0.02559825177	0.9797286821
OFB_Ctime	GGB29011_SGB41731	63	-1.513716934	0.1342716892
OFB_Ctime	GGB29531_SGB42317	63	1.219114188	0.2255453721
OFB_Ctime	GGB29685_SGB42494	63	-1.166067863	0.247080295
OFB_Ctime	GGB30141_SGB43066	63	-0.1393419567	0.8927792095
OFB_Ctime	GGB30286_SGB43248	63	1.8217218	0.07226187933

OFB_Ctime	GGB30303_SGB43268	63	0.1255244816	0.9032898883
OFB_Ctime	GGB30413_SGB43452	63	0.6502198157	0.5165175508
OFB_Ctime	GGB30454_SGB43514	63	-0.1270317208	0.898953702
OFB_Ctime	GGB30455_SGB43519	63	0.2813380192	0.7860094411
OFB_Ctime	GGB30461_SGB43527	63	0.6641591589	0.5117525911
OFB_Ctime	GGB30461_SGB43530	63	-0.2957179066	0.7837552975
OFB_Ctime	GGB30463_SGB43537	63	0.03835966411	0.970873424
OFB_Ctime	GGB30473_SGB43557	63	0.2583025429	0.7986153167
OFB_Ctime	GGB30475_SGB63182	63	-0.01110860444	0.9912118266
OFB_Ctime	GGB30861_SGB44083	63	-0.6643558352	0.513163785
OFB_Ctime	GGB31312_SGB44628	63	-0.5808716281	0.5793112021
OFB_Ctime	GGB31438_SGB44768	63	-1.245364748	0.2492368402
OFB_Ctime	GGB3171_SGB4185	63	0.1325171161	0.8976909592
OFB_Ctime	GGB31823_SGB45199	63	2.341650944	0.02185577779
OFB_Ctime	GGB31853_SGB45233	63	0.2980318512	0.7704272105
OFB_Ctime	GGB32371_SGB41694	63	1.161954569	0.2580898841
OFB_Ctime	GGB3793_SGB5158	63	1.449852332	0.1522085237
OFB_Ctime	GGB42598_SGB59794	63	-0.2608897144	0.7943110635
OFB_Ctime	GGB45656_SGB63370	63	2.092797895	0.05152755498
OFB_Ctime	GGB47127_SGB65054	63	-0.7602106192	0.4581615937
OFB_Ctime	GGB74395_SGB43521	63	-0.7654856399	0.4451799014
OFB_Ctime	GGB75053_SGB43494	63	-0.3329274017	0.7409435884
OFB_Ctime	GGB75109_SGB102238	63	-0.05361550195	0.9572422678
OFB_Ctime	GGB81440_SGB45230	63	0.8049956404	0.4892961624
OFB_Ctime	Lachnospiraceae_bacterium	63	-1.653485755	0.1019292993
OFB_Ctime	Lachnospiraceae_bacterium_A2	63	0.9522526233	0.3428142858
OFB_Ctime	Lachnospiraceae_bacterium_MD308	63	1.182899903	0.2432820103
OFB_Ctime	Lachnospiraceae_bacterium_MD329	63	-1.956854619	0.06035222718
OFB_Ctime	Lachnospiraceae_unclassified_SGB4141	63	-1.193732258	0.2459974185
OFB_Ctime	Lachnospiraceae_unclassified_SGB4141	63	0.6958537923	0.5043903837
OFB_Ctime	Lachnospiraceae_unclassified_SGB4142	63	-0.6008466616	0.5534594918
OFB_Ctime	Lachnospiraceae_unclassified_SGB4158	63	-2.007438672	0.07861071271
OFB_Ctime	Lactobacillus_johnsonii	63	-0.3934027254	0.6954689555
OFB_Ctime	Muribaculaceae_bacterium	63	0.08772806887	0.9307165999
OFB_Ctime	Neglectibacter_sp_X4	63	-1.392996729	0.1775887113
OFB_Ctime	Oscillospiraceae_bacterium	63	-0.4559615879	0.6506742156
OFB_Ctime	Oscillospiraceae_unclassified_SGB4350	63	-1.196565999	0.2350165484
OFB_Ctime	Oscillospiraceae_unclassified_SGB4350	63	-0.1385365455	0.8946303958
OFB_Ctime	Parasutterella_excrementihominis	63	0.3621151045	0.7820241105
OFB_Ctime	Romboutsia_ilealis	63	1.376224343	0.206879804
OFB_Ctime	Schaedlerella_arabinosiphila	63	-1.257618536	0.2252702023
OFB_Ctime	Turicibacter_sp_1E2	63	0.5050256621	0.6185016576
OFB_Ctime	bacterium_1XD42_54	63	-1.250981897	0.2290999082
OFB_Ctime	bacterium_1XD42_76	63	-0.09502429275	0.9253678534
OFB_Ctime	bacterium_1xD8_48	63	2.02010801	0.04675699948
OFB_Ctime	Berger Parker Index	63	0.6866522834	0.4994122149

OFB_Ctime	Richness (# observed features)	63	0.4736440753	0.6421160642
OFB_Ctime	Shannon Index	63	-0.7385991882	0.4722876446
PD_choice_filt	Acetatifactor_SGB41546	63	-0.9299684024	0.3550315504
PD_choice_filt	Acetatifactor_muris	63	-0.4411615223	0.6594934665
PD_choice_filt	Acutalibacter_muris	63	0.4823639393	0.63005687
PD_choice_filt	Acutalibacter_sp_1XD8_36	63	-0.07198233514	0.9426178684
PD_choice_filt	Adlercreutzia_caecimuris	63	2.034380962	0.04733331088
PD_choice_filt	Adlercreutzia_mucosicola	63	-1.099647316	0.2751568637
PD_choice_filt	Adlercreutzia_muris	63	-0.6209325262	0.5356489015
PD_choice_filt	Akkermansia_muciniphila	63	-0.02315555161	0.9815262573
PD_choice_filt	Alistipes_sp_DSM_112343	63	0.05167121379	0.9587913793
PD_choice_filt	Anaerotruncus_sp_1XD42_93	63	0.738505318	0.4617655016
PD_choice_filt	Bacteria_unclassified_SGB102200	63	-1.464755662	0.1484046823
PD_choice_filt	Bacteria_unclassified_SGB41677	63	0.2487427912	0.8036360005
PD_choice_filt	Bacteria_unclassified_SGB43546	63	0.6943609051	0.4887934355
PD_choice_filt	Bacteroides_thetaiotaomicron	63	0.4829969153	0.6296092112
PD_choice_filt	Bifidobacterium_pseudolongum	63	0.4189999744	0.6755599627
PD_choice_filt	Clostridia_bacterium	63	0.8727357119	0.3851102284
PD_choice_filt	Clostridiaceae_bacterium	63	1.78887195	0.07947807304
PD_choice_filt	Clostridiaceae_unclassified_SGB41663	63	0.8309914826	0.4080399042
PD_choice_filt	Clostridiales_bacterium	63	0.4477372343	0.6547570394
PD_choice_filt	Clostridium_cocleatum	63	-1.704789454	0.0940814706
PD_choice_filt	Coriobacteriaceae_bacterium	63	0.6410065216	0.5226097475
PD_choice_filt	Dorea_sp_5_2	63	0.8243960996	0.4117385228
PD_choice_filt	Dubosiella_newyorkensis	63	0.8003786812	0.4253808248
PD_choice_filt	Erysipelotrichales_bacterium	63	-0.4931256114	0.6224652159
PD_choice_filt	Eubacteriaceae_bacterium	63	1.030551016	0.3060068864
PD_choice_filt	Eubacteriaceae_unclassified_SGB9492	63	1.852668169	0.06971773566
PD_choice_filt	GGB20149_SGB29430	63	-0.4545983744	0.6498303588
PD_choice_filt	GGB22635_SGB63107	63	-0.7280039543	0.4681154819
PD_choice_filt	GGB25041_SGB36960	63	0.5243488902	0.6006762588
PD_choice_filt	GGB27876_SGB40310	63	-1.603433621	0.1145859386
PD_choice_filt	GGB27878_SGB40312	63	1.027937131	0.3072189877
PD_choice_filt	GGB27918_SGB40356	63	-0.5847120853	0.5595993683
PD_choice_filt	GGB28382_SGB40962	63	-1.3324099	0.1876343814
PD_choice_filt	GGB28399_SGB40980	63	-1.880030286	0.06585553097
PD_choice_filt	GGB28411_SGB40993	63	1.605358087	0.1141650319
PD_choice_filt	GGB28415_SGB40997	63	-0.2158361988	0.8291656412
PD_choice_filt	GGB28430_SGB41013	63	-0.6584458932	0.5114208509
PD_choice_filt	GGB28439_SGB41022	63	0.6934090082	0.4893858472
PD_choice_filt	GGB28778_SGB41431	63	0.5216670125	0.602533688
PD_choice_filt	GGB28784_SGB41437	63	0.05699135757	0.9545530248
PD_choice_filt	GGB28792_SGB41445	63	0.6552531471	0.513459525
PD_choice_filt	GGB28798_SGB41451	63	-0.1875880123	0.8512327815
PD_choice_filt	GGB28802_SGB41455	63	0.9038180773	0.3685790396
PD_choice_filt	GGB28818_SGB41473	63	-0.9926472027	0.3239066091

PD_choice_filt	GGB28828_SGB41484	63	0.997597252	0.3215295573
PD_choice_filt	GGB28851_SGB41518	63	-1.393966388	0.1684947691
PD_choice_filt	GGB28859_SGB41528	63	0.5811854537	0.5619598544
PD_choice_filt	GGB28864_SGB41535	63	-1.465376005	0.1482374067
PD_choice_filt	GGB28869_SGB41543	63	0.2566585913	0.7975259367
PD_choice_filt	GGB28883_SGB41564	63	-0.624533493	0.5332974063
PD_choice_filt	GGB28892_SGB41573	63	0.2925576931	0.7699828183
PD_choice_filt	GGB28893_SGB41574	63	0.7153493106	0.4758338194
PD_choice_filt	GGB28898_SGB41580	63	-0.429045619	0.6682575557
PD_choice_filt	GGB28904_SGB41597	63	0.6938937079	0.4890841449
PD_choice_filt	GGB28916_SGB41612	63	-0.5750131331	0.5661031726
PD_choice_filt	GGB28924_SGB41621	63	-0.0921512248	0.926581875
PD_choice_filt	GGB28926_SGB41624	63	-0.5498103115	0.5831774684
PD_choice_filt	GGB28927_SGB41625	63	0.1592404318	0.8734998356
PD_choice_filt	GGB28934_SGB41635	63	-0.4354912355	0.663589147
PD_choice_filt	GGB28946_SGB41652	63	-0.1226542089	0.9023902909
PD_choice_filt	GGB28949_SGB41655	63	-0.6250677247	0.5329490057
PD_choice_filt	GGB28949_SGB41656	63	-0.9073319649	0.3667394325
PD_choice_filt	GGB28950_SGB41657	63	-0.2357701883	0.8136761473
PD_choice_filt	GGB28951_SGB102295	63	0.1093504814	0.912931155
PD_choice_filt	GGB28951_SGB41658	63	0.5813540513	0.5618468931
PD_choice_filt	GGB28954_SGB41662	63	0.3826045991	0.7022786813
PD_choice_filt	GGB28956_SGB41665	63	-0.08260283118	0.9341702226
PD_choice_filt	GGB28960_SGB41669	63	1.097035377	0.2762814748
PD_choice_filt	GGB28967_SGB41678	63	-1.007667311	0.3167304264
PD_choice_filt	GGB28991_SGB41705	63	-0.2796854511	0.7798263333
PD_choice_filt	GGB29002_SGB41718	63	0.0127572117	0.9898215044
PD_choice_filt	GGB29003_SGB41719	63	0.4314631963	0.6665049865
PD_choice_filt	GGB29011_SGB41731	63	0.8708460185	0.3861302272
PD_choice_filt	GGB29531_SGB42317	63	0.880433409	0.3809729436
PD_choice_filt	GGB29685_SGB42494	63	-0.6820761225	0.4964696024
PD_choice_filt	GGB30141_SGB43066	63	-0.6512106289	0.5160470968
PD_choice_filt	GGB30286_SGB43248	63	1.123482505	0.2650440549
PD_choice_filt	GGB30303_SGB43268	63	-0.574605138	0.5663775848
PD_choice_filt	GGB30413_SGB43452	63	-0.5239985072	0.6009187763
PD_choice_filt	GGB30454_SGB43514	63	-1.974774425	0.05386573274
PD_choice_filt	GGB30455_SGB43519	63	-1.728793673	0.08970037911
PD_choice_filt	GGB30461_SGB43527	63	-0.1495668955	0.8811232452
PD_choice_filt	GGB30461_SGB43530	63	-0.1628938939	0.8706237862
PD_choice_filt	GGB30463_SGB43537	63	-0.8765312623	0.3830666792
PD_choice_filt	GGB30473_SGB43557	63	-0.9252778571	0.3574372358
PD_choice_filt	GGB30475_SGB63182	63	0.586947926	0.5581054407
PD_choice_filt	GGB30861_SGB44083	63	1.241740935	0.2188006691
PD_choice_filt	GGB31312_SGB44628	63	0.4824116231	0.6300231417
PD_choice_filt	GGB31438_SGB44768	63	1.037711346	0.3027034028
PD_choice_filt	GGB3171_SGB4185	63	-2.852822815	0.00655258972[illegible]

PD_choice_filt	GGB31823_SGB45199	63	-0.4881959599	0.6259376556
PD_choice_filt	GGB31853_SGB45233	63	0.1605656257	0.8724564242
PD_choice_filt	GGB32371_SGB41694	63	0.7226751772	0.4713568109
PD_choice_filt	GGB3793_SGB5158	63	-1.229972424	0.2231142241
PD_choice_filt	GGB42598_SGB59794	63	-0.5159515122	0.6065010953
PD_choice_filt	GGB45656_SGB63370	63	1.026591114	0.3078444439
PD_choice_filt	GGB47127_SGB65054	63	0.7700168018	0.4430137541
PD_choice_filt	GGB74395_SGB43521	63	0.04174055169	0.9667058982
PD_choice_filt	GGB75053_SGB43494	63	-0.228321214	0.8194558021
PD_choice_filt	GGB75109_SGB102238	63	-0.04559219972	0.9636357706
PD_choice_filt	GGB81440_SGB45230	63	-0.7259713709	0.4693503245
PD_choice_filt	Lachnospiraceae_bacterium	63	0.9503846866	0.3446844102
PD_choice_filt	Lachnospiraceae_bacterium_A2	63	-0.1557110429	0.876279863
PD_choice_filt	Lachnospiraceae_bacterium_MD308	63	0.1371259708	0.8909442857
PD_choice_filt	Lachnospiraceae_bacterium_MD329	63	-1.42204684	0.160285117
PD_choice_filt	Lachnospiraceae_unclassified_SGB4141	63	0.9735293507	0.3331985176
PD_choice_filt	Lachnospiraceae_unclassified_SGB4141	63	-0.2768398987	0.7820073724
PD_choice_filt	Lachnospiraceae_unclassified_SGB4142	63	0.8451585042	0.4001649836
PD_choice_filt	Lachnospiraceae_unclassified_SGB4158	63	-0.1489895009	0.8815786412
PD_choice_filt	Lactobacillus_johnsonii	63	-1.084530242	0.2817108294
PD_choice_filt	Muribaculaceae_bacterium	63	-0.4708766077	0.6382054204
PD_choice_filt	Neglectibacter_sp_X4	63	1.379909006	0.1727259155
PD_choice_filt	Oscillospiraceae_bacterium	63	0.4808014533	0.6311625085
PD_choice_filt	Oscillospiraceae_unclassified_SGB4350	63	-0.6154631427	0.539230854
PD_choice_filt	Oscillospiraceae_unclassified_SGB4350	63	-1.030886997	0.3058513257
PD_choice_filt	Parasutterella_excrementihominis	63	1.222116469	0.2260286639
PD_choice_filt	Romboutsia_ilealis	63	0.3747614607	0.7080883536
PD_choice_filt	Schaedlerella_arabinosiphila	63	1.167324661	0.2471429805
PD_choice_filt	Turicibacter_sp_1E2	63	-0.06473011595	0.9483902682
PD_choice_filt	bacterium_1XD42_54	63	2.227966153	0.0306506842
PD_choice_filt	bacterium_1XD42_76	63	0.8479971731	0.3985985537
PD_choice_filt	bacterium_1xD8_48	63	0.7690861123	0.4435610321
PD_choice_filt	Berger Parker Index	63	-0.4749033908	0.6353437792
PD_choice_filt	Richness (# observed features)	63	1.318695898	0.1921173807
PD_choice_filt	Shannon Index	63	1.035721637	0.3036188932
startle_PPI	Acetatifactor_SGB41546	63	1.6440789	0.1037705338
startle_PPI	Acetatifactor_muris	63	1.937298752	0.05621809439
startle_PPI	Acutalibacter_muris	63	-0.5699347208	0.5692189787
startle_PPI	Acutalibacter_sp_1XD8_36	63	-0.01615654923	0.987109513
startle_PPI	Adlercreutzia_caecimuris	63	-0.196016968	0.84462027
startle_PPI	Adlercreutzia_mucosicola	63	-0.8827561233	0.3788351928
startle_PPI	Adlercreutzia_muris	63	1.88661053	0.06277957972
startle_PPI	Akkermansia_muciniphila	63	-1.976350727	0.05157007534
startle_PPI	Alistipes_sp_DSM_112343	63	-3.703468412	0.000427224672
startle_PPI	Anaerotruncus_sp_1XD42_93	63	-0.6549777646	0.5131975944
startle_PPI	Bacteria_unclassified_SGB102200	63	0.9091213447	0.3648508324

startle_PPI	Bacteria_unclassified_SGB41677	63	-1.424884891	0.1574790509
startle_PPI	Bacteria_unclassified_SGB43546	63	0.4389551622	0.6609368392
startle_PPI	Bacteroides_thetaiotaomicron	63	-1.276407436	0.2047019389
startle_PPI	Bifidobacterium_pseudolongum	63	-0.430953788	0.6667325618
startle_PPI	Clostridia_bacterium	63	-1.57834826	0.1180359372
startle_PPI	Clostridiaceae_bacterium	63	-1.005260237	0.3166993457
startle_PPI	Clostridiaceae_unclassified_SGB41663	63	0.7536219159	0.4520921446
startle_PPI	Clostridiales_bacterium	63	0.2192278093	0.8265051483
startle_PPI	Clostridium_cocleatum	63	-0.8895826445	0.3751823172
startle_PPI	Coriobacteriaceae_bacterium	63	-1.657243049	0.1010892466
startle_PPI	Dorea_sp_5_2	63	0.05940311119	0.9526316976
startle_PPI	Dubosiella_newyorkensis	63	-0.5309411171	0.595870244
startle_PPI	Erysipelotrichales_bacterium	63	0.3282983607	0.7427921429
startle_PPI	Eubacteriaceae_bacterium	63	-0.3553081007	0.7224919812
startle_PPI	Eubacteriaceae_unclassified_SGB9492	63	1.137810661	0.2576220309
startle_PPI	GGB20149_SGB29430	63	0.4669418647	0.6408300146
startle_PPI	GGB22635_SGB63107	63	-1.029926979	0.3050656536
startle_PPI	GGB25041_SGB36960	63	-1.762848399	0.08156595798
startle_PPI	GGB27876_SGB40310	63	-2.146066572	0.03500286241
startle_PPI	GGB27878_SGB40312	63	-0.4091281106	0.6826447931
startle_PPI	GGB27918_SGB40356	63	-1.28554402	0.2015206697
startle_PPI	GGB28382_SGB40962	63	-0.5622322861	0.5744370071
startle_PPI	GGB28399_SGB40980	63	-1.697477146	0.0932428364
startle_PPI	GGB28411_SGB40993	63	2.033137472	0.04540073618
startle_PPI	GGB28415_SGB40997	63	-0.2528755989	0.800413907
startle_PPI	GGB28430_SGB41013	63	1.665147555	0.09950657948
startle_PPI	GGB28439_SGB41022	63	0.4933418739	0.622106997
startle_PPI	GGB28778_SGB41431	63	-1.870249331	0.06503136775
startle_PPI	GGB28784_SGB41437	63	0.6434009634	0.5206473959
startle_PPI	GGB28792_SGB41445	63	0.5622813167	0.574403718
startle_PPI	GGB28798_SGB41451	63	0.1016702388	0.9190217358
startle_PPI	GGB28802_SGB41455	63	2.028788991	0.04584955231
startle_PPI	GGB28818_SGB41473	63	-0.839310119	0.4026049268
startle_PPI	GGB28828_SGB41484	63	1.322011668	0.1891902696
startle_PPI	GGB28851_SGB41518	63	1.904845481	0.06034802766
startle_PPI	GGB28859_SGB41528	63	2.055342821	0.04316752283
startle_PPI	GGB28864_SGB41535	63	1.578467362	0.1180087309
startle_PPI	GGB28869_SGB41543	63	1.81652062	0.07291112826
startle_PPI	GGB28883_SGB41564	63	0.6538744385	0.5139051391
startle_PPI	GGB28892_SGB41573	63	-0.7119930441	0.4773526128
startle_PPI	GGB28893_SGB41574	63	1.471592217	0.1445166404
startle_PPI	GGB28898_SGB41580	63	0.04047099663	0.9677178391
startle_PPI	GGB28904_SGB41597	63	-0.4605066333	0.6454302279
startle_PPI	GGB28916_SGB41612	63	-0.5631537466	0.5738115429
startle_PPI	GGB28924_SGB41621	63	-0.6038298925	0.546535992
startle_PPI	GGB28926_SGB41624	63	-0.1233476026	0.9018377434

startle_PPI	GGB28927_SGB41625	63	0.50391594	0.614676557
startle_PPI	GGB28934_SGB41635	63	1.665642092	0.09940823818
startle_PPI	GGB28946_SGB41652	63	-1.45398536	0.1493011204
startle_PPI	GGB28949_SGB41655	63	-0.8349564377	0.4050363445
startle_PPI	GGB28949_SGB41656	63	-1.174386666	0.2427928833
startle_PPI	GGB28950_SGB41657	63	-0.7198817171	0.4725058394
startle_PPI	GGB28951_SGB102295	63	-1.150305092	0.2524856339
startle_PPI	GGB28951_SGB41658	63	0.9758827896	0.3309387896
startle_PPI	GGB28954_SGB41662	63	0.5362549511	0.5922042586
startle_PPI	GGB28956_SGB41665	63	-3.116796939	0.002649290467
startle_PPI	GGB28960_SGB41669	63	0.6987156176	0.4855727492
startle_PPI	GGB28967_SGB41678	63	1.541769537	0.1266326788
startle_PPI	GGB28991_SGB41705	63	0.3407167593	0.7334348773
startle_PPI	GGB29002_SGB41718	63	0.6548653977	0.5132696297
startle_PPI	GGB29003_SGB41719	63	0.8654099446	0.3882175196
startle_PPI	GGB29011_SGB41731	63	1.559926342	0.1223050242
startle_PPI	GGB29531_SGB42317	63	1.071157597	0.2862753734
startle_PPI	GGB29685_SGB42494	63	-0.7888227827	0.4313489761
startle_PPI	GGB30141_SGB43066	63	2.804809512	0.006474774353
startle_PPI	GGB30286_SGB43248	63	0.3297258444	0.7417145215
startle_PPI	GGB30303_SGB43268	63	1.349449692	0.1802947656
startle_PPI	GGB30413_SGB43452	63	-0.7918535576	0.4295897917
startle_PPI	GGB30454_SGB43514	63	-1.387579325	0.1684659819
startle_PPI	GGB30455_SGB43519	63	0.9364128893	0.3507276339
startle_PPI	GGB30461_SGB43527	63	-0.01977082596	0.9842262153
startle_PPI	GGB30461_SGB43530	63	1.21485511	0.2271130332
startle_PPI	GGB30463_SGB43537	63	1.581083924	0.1174123042
startle_PPI	GGB30473_SGB43557	63	-0.6489409337	0.5170752589
startle_PPI	GGB30475_SGB63182	63	-1.513248833	0.1336760022
startle_PPI	GGB30861_SGB44083	63	-1.148945255	0.2530410868
startle_PPI	GGB31312_SGB44628	63	0.6380030004	0.5241404474
startle_PPI	GGB31438_SGB44768	63	-0.9316449454	0.3531691049
startle_PPI	GGB3171_SGB4185	63	-0.5701516877	0.5690723302
startle_PPI	GGB31823_SGB45199	63	-0.3962144238	0.6921286863
startle_PPI	GGB31853_SGB45233	63	1.22301701	0.2240422336
startle_PPI	GGB32371_SGB41694	63	-0.4565392283	0.6482732717
startle_PPI	GGB3793_SGB5158	63	-0.1986566543	0.8425556625
startle_PPI	GGB42598_SGB59794	63	-3.897454639	0.000225155777
startle_PPI	GGB45656_SGB63370	63	0.5173284956	0.6053095031
startle_PPI	GGB47127_SGB65054	63	-0.9505159679	0.3435703186
startle_PPI	GGB74395_SGB43521	63	-1.835755189	0.07000283681
startle_PPI	GGB75053_SGB43494	63	-0.8687585859	0.3863950791
startle_PPI	GGB75109_SGB102238	63	-1.374813351	0.17235808
startle_PPI	GGB81440_SGB45230	63	0.7680021306	0.4435492081
startle_PPI	Lachnospiraceae_bacterium	63	-1.658731762	0.1007896121
startle_PPI	Lachnospiraceae_bacterium_A2	63	-1.125841229	0.2626117913

startle_PPI	Lachnospiraceae_bacterium_MD308	63	0.1076432914	0.9142825809
startle_PPI	Lachnospiraceae_bacterium_MD329	63	-1.303280663	0.1954505754
startle_PPI	Lachnospiraceae_unclassified_SGB4141	63	-2.529553663	0.01356300109
startle_PPI	Lachnospiraceae_unclassified_SGB4141	63	0.1554302228	0.8764939032
startle_PPI	Lachnospiraceae_unclassified_SGB4142	63	-0.1765920164	0.8598460274
startle_PPI	Lachnospiraceae_unclassified_SGB4158	63	-1.52859163	0.1298493933
startle_PPI	Lactobacillus_johnsonii	63	-0.5242744214	0.6004844834
startle_PPI	Muribaculaceae_bacterium	63	-1.604007108	0.1122901132
startle_PPI	Neglectibacter_sp_X4	63	0.8297914037	0.4079325088
startle_PPI	Oscillospiraceae_bacterium	63	-0.4454896748	0.6562189958
startle_PPI	Oscillospiraceae_unclassified_SGB4350	63	-0.1506959121	0.880226309
startle_PPI	Oscillospiraceae_unclassified_SGB4350	63	1.461684378	0.1471939771
startle_PPI	Parasutterella_excrementihominis	63	-0.4930242961	0.6223307765
startle_PPI	Romboutsia_ilealis	63	-0.4808612753	0.6309281171
startle_PPI	Schaedlerella_arabinosiphila	63	-1.539827024	0.1271028145
startle_PPI	Turicibacter_sp_1E2	63	-1.00297174	0.3177935944
startle_PPI	bacterium_1XD42_54	63	-0.2743462059	0.783881503
startle_PPI	bacterium_1XD42_76	63	-1.779478608	0.0787973386
startle_PPI	bacterium_1xD8_48	63	0.351081592	0.7256558296
startle_PPI	Berger Parker Index	63	0.1665168508	0.8677646375
startle_PPI	Richness (# observed features)	63	2.56279297	0.01243599885
startle_PPI	Shannon Index	63	0.936667175	0.3505977329
startle_habituation	Acetatifactor_SGB41546	63	-0.5401624641	0.5902333958
startle_habituation	Acetatifactor_muris	63	1.808727735	0.08225583038
startle_habituation	Acutalibacter_muris	63	-0.3876582122	0.7026534509
startle_habituation	Acutalibacter_sp_1XD8_36	63	-0.2416752419	0.8127144751
startle_habituation	Adlercreutzia_caecimuris	63	0.641458477	0.5238231388
startle_habituation	Adlercreutzia_mucosicola	63	-1.024300524	0.3210901906
startle_habituation	Adlercreutzia_muris	63	0.4653815406	0.6436409553
startle_habituation	Akkermansia_muciniphila	63	-2.242843781	0.0287079963
startle_habituation	Alistipes_sp_DSM_112343	63	-2.366626763	0.02090288402
startle_habituation	Anaerotruncus_sp_1XD42_93	63	-0.1316934952	0.9036972807
startle_habituation	Bacteria_unclassified_SGB102200	63	1.561964048	0.1264589225
startle_habituation	Bacteria_unclassified_SGB41677	63	-0.3133117349	0.7647585713
startle_habituation	Bacteria_unclassified_SGB43546	63	0.2573198942	0.8037535788
startle_habituation	Bacteroides_thetaiotaomicron	63	-2.248879749	0.03104050107
startle_habituation	Bifidobacterium_pseudolongum	63	-0.01004608443	0.9920945847
startle_habituation	Clostridia_bacterium	63	-0.7068517119	0.4938757309
startle_habituation	Clostridiaceae_bacterium	63	-0.4538058262	0.6612819635
startle_habituation	Clostridiaceae_unclassified_SGB41663	63	0.124543435	0.9015736236
startle_habituation	Clostridiales_bacterium	63	-1.354875669	0.1785752658
startle_habituation	Clostridium_cocleatum	63	-2.224893343	0.03101051245
startle_habituation	Coriobacteriaceae_bacterium	63	-1.545400822	0.1270910547
startle_habituation	Dorea_sp_5_2	63	0.1441318787	0.8858584489
startle_habituation	Dubosiella_newyorkensis	63	-0.794503983	0.4820055547
startle_habituation	Erysipelotrichales_bacterium	63	-0.4662338962	0.6459403269

startle_habituation	Eubacteriaceae_bacterium	63	0.2808204486	0.7813572425
startle_habituation	Eubacteriaceae_unclassified_SGB9492.	63	0.6243009244	0.5332259582
startle_habituation	GGB20149_SGB29430	63	-0.4013111543	0.6901424246
startle_habituation	GGB22635_SGB63107	63	-0.5214829839	0.6055076337
startle_habituation	GGB25041_SGB36960	63	-0.8727631186	0.4122800413
startle_habituation	GGB27876_SGB40310	63	-1.949971715	0.05619267284
startle_habituation	GGB27878_SGB40312	63	0.6668896778	0.5780508952
startle_habituation	GGB27918_SGB40356	63	-0.5344178135	0.5992079719
startle_habituation	GGB28382_SGB40962	63	-1.668541197	0.1229408996
startle_habituation	GGB28399_SGB40980	63	-0.07390656215	0.9411447807
startle_habituation	GGB28411_SGB40993	63	1.117143564	0.2742682362
startle_habituation	GGB28415_SGB40997	63	1.828484353	0.07291142053
startle_habituation	GGB28430_SGB41013	63	1.507088518	0.1421543296
startle_habituation	GGB28439_SGB41022	63	0.165414857	0.8688443442
startle_habituation	GGB28778_SGB41431	63	0.9327789226	0.372565146
startle_habituation	GGB28784_SGB41437	63	1.87786062	0.06799480322
startle_habituation	GGB28792_SGB41445	63	1.398132655	0.2328749066
startle_habituation	GGB28798_SGB41451	63	0.6666185269	0.5182868483
startle_habituation	GGB28802_SGB41455	63	3.656195775	0.00067248293
startle_habituation	GGB28818_SGB41473	63	0.3404863832	0.7402622426
startle_habituation	GGB28828_SGB41484	63	2.032449421	0.06355762909
startle_habituation	GGB28851_SGB41518	63	0.2170864461	0.8313974973
startle_habituation	GGB28859_SGB41528	63	0.6632371833	0.5375356189
startle_habituation	GGB28864_SGB41535	63	1.644097719	0.1484954752
startle_habituation	GGB28869_SGB41543	63	0.9604120529	0.3603570436
startle_habituation	GGB28883_SGB41564	63	1.580470949	0.121457029
startle_habituation	GGB28892_SGB41573	63	0.2618297295	0.7981719079
startle_habituation	GGB28893_SGB41574	63	-0.875663561	0.3826751741
startle_habituation	GGB28898_SGB41580	63	1.406539825	0.1636985756
startle_habituation	GGB28904_SGB41597	63	-0.6765055124	0.5163528432
startle_habituation	GGB28916_SGB41612	63	0.3769245685	0.7102913843
startle_habituation	GGB28924_SGB41621	63	-1.235667939	0.2217153737
startle_habituation	GGB28926_SGB41624	63	-0.9131113514	0.388613361
startle_habituation	GGB28927_SGB41625	63	0.3481535104	0.7279684386
startle_habituation	GGB28934_SGB41635	63	1.039331576	0.3356180562
startle_habituation	GGB28946_SGB41652	63	0.2691517524	0.7879935173
startle_habituation	GGB28949_SGB41655	63	0.1387099384	0.890101361
startle_habituation	GGB28949_SGB41656	63	0.1500271537	0.8816912043
startle_habituation	GGB28950_SGB41657	63	-0.4843148302	0.6284995968
startle_habituation	GGB28951_SGB102295	63	-0.9056373387	0.3674906697
startle_habituation	GGB28951_SGB41658	63	0.8224447449	0.4131656052
startle_habituation	GGB28954_SGB41662	63	0.3133645916	0.7548624389
startle_habituation	GGB28956_SGB41665	63	-1.692840493	0.1028361255
startle_habituation	GGB28960_SGB41669	63	2.432826594	0.0190216052
startle_habituation	GGB28967_SGB41678	63	0.597842929	0.5580513302
startle_habituation	GGB28991_SGB41705	63	0.4213548195	0.679717763

startle_habituation	GGB29002_SGB41718	63	0.3452530271	0.7308785659
startle_habituation	GGB29003_SGB41719	63	-0.6768787397	0.5169977742
startle_habituation	GGB29011_SGB41731	63	-0.03890322002	0.9690804104
startle_habituation	GGB29531_SGB42317	63	0.6522237111	0.516081854
startle_habituation	GGB29685_SGB42494	63	-0.9638482915	0.3413918289
startle_habituation	GGB30141_SGB43066	63	2.096088371	0.0454876065
startle_habituation	GGB30286_SGB43248	63	-0.3674270695	0.7232240899
startle_habituation	GGB30303_SGB43268	63	0.637626261	0.5337296769
startle_habituation	GGB30413_SGB43452	63	0.05276728069	0.9581674088
startle_habituation	GGB30454_SGB43514	63	-0.6301698374	0.5293752132
startle_habituation	GGB30455_SGB43519	63	2.160843036	0.04700316645
startle_habituation	GGB30461_SGB43527	63	-0.8567294905	0.4026797198
startle_habituation	GGB30461_SGB43530	63	1.000696775	0.3299897225
startle_habituation	GGB30463_SGB43537	63	1.431319727	0.176235745
startle_habituation	GGB30473_SGB43557	63	-0.712496141	0.5233375857
startle_habituation	GGB30475_SGB63182	63	-1.539758644	0.1711268487
startle_habituation	GGB30861_SGB44083	63	-1.01837543	0.3105070205
startle_habituation	GGB31312_SGB44628	63	0.7371474034	0.4800862301
startle_habituation	GGB31438_SGB44768	63	0.3075609414	0.7683507837
startle_habituation	GGB3171_SGB4185	63	-1.475346438	0.1707888844
startle_habituation	GGB31823_SGB45199	63	-1.796154335	0.07696819616
startle_habituation	GGB31853_SGB45233	63	-0.1247854507	0.9013501256
startle_habituation	GGB32371_SGB41694	63	-1.326569893	0.2035605897
startle_habituation	GGB3793_SGB5158	63	-1.286234305	0.2253065374
startle_habituation	GGB42598_SGB59794	63	-2.255202518	0.02730478375
startle_habituation	GGB45656_SGB63370	63	-0.03540134442	0.9741590441
startle_habituation	GGB47127_SGB65054	63	-0.5729971993	0.591535064
startle_habituation	GGB74395_SGB43521	63	-0.8307619765	0.40771711
startle_habituation	GGB75053_SGB43494	63	1.059888543	0.2945289628
startle_habituation	GGB75109_SGB102238	63	-0.4027008636	0.6916114781
startle_habituation	GGB81440_SGB45230	63	0.7663566438	0.4674294258
startle_habituation	Lachnospiraceae_bacterium	63	-0.5263951162	0.600797878
startle_habituation	Lachnospiraceae_bacterium_A2	63	-1.042444246	0.3277029546
startle_habituation	Lachnospiraceae_bacterium_MD308	63	0.6750159694	0.5036106916
startle_habituation	Lachnospiraceae_bacterium_MD329	63	-1.451095273	0.1514752768
startle_habituation	Lachnospiraceae_unclassified_SGB4141	63	-1.287250905	0.2193774212
startle_habituation	Lachnospiraceae_unclassified_SGB4141	63	-0.5590409134	0.5977285077
startle_habituation	Lachnospiraceae_unclassified_SGB414[illegible]	63	-1.523665245	0.1338604077
startle_habituation	Lachnospiraceae_unclassified_SGB415[illegible]	63	-1.043847695	0.3070195884
startle_habituation	Lactobacillus_johnsonii	63	-0.2304323133	0.8183075298
startle_habituation	Muribaculaceae_bacterium	63	-2.373016447	0.02023268208
startle_habituation	Neglectibacter_sp_X4	63	1.003187477	0.3489764725
startle_habituation	Oscillospiraceae_bacterium	63	-0.2934549106	0.7736363921
startle_habituation	Oscillospiraceae_unclassified_SGB4350	63	-0.9707920929	0.3379221071
startle_habituation	Oscillospiraceae_unclassified_SGB4350	63	1.603743614	0.1124639303
startle_habituation	Parasutterella_excrementihominis	63	-1.472335112	0.1512104697

startle_habituation	Romboutsia_ilealis	63	-2.046965725	0.06145866059
startle_habituation	Schaedlerella_arabinosiphila	63	-1.142780164	0.3538269807
startle_habituation	Turicibacter_sp_1E2	63	-0.8888290442	0.3755851484
startle_habituation	bacterium_1XD42_54	63	-0.2301421334	0.8186205229
startle_habituation	bacterium_1XD42_76	63	-1.288693985	0.2006233
startle_habituation	bacterium_1xD8_48	63	0.3768332785	0.7066193127
startle_habituation	Berger Parker Index	63	-1.815426166	0.0747301899
startle_habituation	Richness (# observed features)	63	1.699161377	0.09294646481
startle_habituation	Shannon Index	63	1.944141934	0.05777189944

st Results					
Microbe Adjusted p-valu	Microbe Estimate	Blast Estimate	VNS Estimate	Blast:VNS Estimate	Microbe Std. Erro
0.2153727604	0.3148338033	0.960831046	0.738839469	-1.080249443	0.1191093034
0.9761770834	0.009552748091	0.8632601954	0.6412276672	-1.056740335	0.1211054019
0.5486787709	0.1971153136	0.9450869445	0.6388254149	-1.113242142	0.1191845724
0.7354029002	0.1069322161	0.8571653925	0.62389001	-1.004091917	0.1220340025
0.2153727604	-0.2889847642	0.8436943497	0.7123352018	-1.02128563	0.115303623
0.7406447589	0.1053084105	0.8913269603	0.6754258438	-1.12830884	0.1249094646
0.8559555964	0.04926325013	0.8581097481	0.6546882057	-1.065014855	0.1216801821
0.5762614969	-0.1816894201	0.9489421913	0.6559377954	-1.027357119	0.1226616914
0.6376240108	-0.146913028	0.9294962612	0.6656230587	-1.071785185	0.1262066731
0.7754670422	0.08272856977	0.8649098453	0.624648569	-1.037345685	0.119524328
0.9023781973	-0.02750883994	0.8540821184	0.6336459213	-1.050857953	0.1241803122
0.8559555964	-0.05046694283	0.8552383502	0.640548536	-1.025580025	0.1228895433
0.8780395729	0.03936799383	0.8643837284	0.6508737499	-1.069937465	0.1229662943
0.7350810244	-0.1118724238	0.8603211853	0.6032485863	-1.03572227	0.1198336178
0.5486787709	-0.1973658474	0.8681949129	0.6409037025	-1.145331575	0.1200708854
0.5762614969	-0.1733875235	0.791061364	0.5971830415	-1.018255325	0.12066146
0.2153727604	-0.3121794085	0.8472068822	0.4837655036	-0.9945557746	0.1154004639
0.7215974205	0.125116097	0.8922532146	0.6543055951	-1.104504796	0.12240821
0.8559555964	0.05636183019	0.8333196035	0.6626145002	-1.091830339	0.1329042515
0.8780395729	0.03772743626	0.8346079949	0.6265013063	-1.052014239	0.1298595747
0.8358558141	-0.06583152002	0.8210502944	0.6109155811	-1.003364398	0.1227139947
0.5762614969	-0.1656362709	0.860006263	0.6136187177	-1.10511407	0.1226672979
0.526118124	-0.2234383286	0.7412088396	0.6031679639	-0.9990083334	0.120528236
0.8559555964	0.04752179282	0.8572611271	0.6191777731	-1.040846115	0.1225916967
0.8358558141	-0.06965992239	0.9094374885	0.6597582528	-1.092405389	0.1333502792
0.7484938865	-0.09981296499	0.8849785618	0.6977619976	-1.078627063	0.1244779285
0.7215974205	0.1269640464	0.8199896351	0.6531570093	-1.00388475	0.1252271045
0.5762614969	-0.1688613034	0.9323965159	0.6775691499	-1.064473677	0.1211940456
0.7354029002	-0.1088152566	0.7799514571	0.5880363616	-0.9511147857	0.1232870601
0.6555744666	-0.1370380564	0.7800700722	0.619928237	-0.9923810497	0.1243152056
0.9498139359	-0.0160706726	0.857926176	0.637978923	-1.054503602	0.1236815234
0.7754670422	0.08889284655	0.8569837846	0.5879082249	-1.031284821	0.1306063181
0.6452350437	0.1380984383	0.8167838387	0.6369607383	-1.034282585	0.1214627818
0.8780395729	0.03390381431	0.8455859341	0.623406832	-1.021893746	0.1252679442
0.8559555964	-0.05993392106	0.8824553144	0.6500993561	-1.117410627	0.1296215117
0.8780395729	-0.03800434462	0.8495809016	0.6448410998	-1.063860388	0.1258422894
0.7754670422	0.09153628343	0.8518137996	0.6677457193	-1.043476745	0.1233094176
0.5569218181	0.1956233859	0.856811949	0.7130614677	-1.141240596	0.1215835657
0.8780395729	0.03736663209	0.8546339038	0.6537171052	-1.061879176	0.1333853666
0.8212864485	-0.07398707388	0.85532467	0.6881570918	-1.118862535	0.1296218423
0.9904345571	0.001509548649	0.8625944091	0.6400092582	-1.055049258	0.1259132485
0.7484938865	-0.09646500085	0.8652136927	0.6654531915	-1.100898738	0.1206346084
0.9790000627	-0.007521474665	0.8617476165	0.6429361354	-1.057984599	0.1241321067
0.8490526455	-0.06146551168	0.8656589344	0.630948579	-1.069132524	0.1231657801
0.8559555964	-0.05565387852	0.8495905186	0.6378603027	-1.02424777	0.1235125986

0.8564192709	0.04575326194	0.8715595177	0.6394112918	-1.071573164	0.122374082
0.8212864485	-0.07168086638	0.8566595234	0.6391527334	-1.089444248	0.1242602947
0.7406447589	0.1046833777	0.8595415952	0.6421518308	-1.028387172	0.1232704805
0.5762614969	0.1855107635	0.9023412088	0.7350347694	-1.17604909	0.122056183
0.5762614969	-0.1650650198	0.8223980688	0.5495378623	-1.060047233	0.1230254852
0.5762614969	-0.1689312638	0.9140060392	0.6865545867	-1.109564967	0.1200472787
0.5762614969	0.1759215617	0.9878131893	0.7489446297	-1.226871267	0.1248032617
0.4856544148	-0.238349872	0.7451342327	0.6102041593	-0.8996297758	0.1225565359
0.7350810244	0.1176884173	0.8900469265	0.6802353973	-1.00193287	0.1267698242
0.8564192709	0.04561396206	0.8706919362	0.6452374747	-1.062593925	0.1237309817
0.9329627687	0.02038475489	0.8593201943	0.6436547574	-1.051482074	0.120598971
0.7754670422	-0.08947607683	0.8615245505	0.6017052498	-0.9947984157	0.1241011219
0.2153727604	0.2893182618	0.8263340282	0.6928203638	-1.15225712	0.1153422456
0.7754670422	0.08997955231	0.9088995281	0.680509543	-1.15743858	0.1266110634
0.7754670422	0.0853007414	0.835928716	0.5984602125	-1.015725428	0.1243686497
0.6376240108	-0.1420788651	0.814593004	0.6290022321	-1.054946142	0.1199363527
0.7484938865	0.1010913231	0.9075153048	0.65629966	-1.081269865	0.1255147622
0.8559555964	-0.05286562498	0.8209506298	0.6192360586	-1.022175582	0.1266841293
0.8559555964	0.050213126	0.8638336157	0.6163281732	-1.050453264	0.1255114992
0.2153727604	0.3311156562	0.8681677116	0.6338912592	-0.9740978422	0.1140614728
0.9023781973	-0.02661414541	0.8525949487	0.6315567973	-1.047236398	0.1240743225
0.8780395729	0.03509057993	0.8407760896	0.633041025	-1.025970925	0.1235777711
0.9904345571	-0.00258276364	0.8624424785	0.6409628262	-1.05664012	0.1255422915
0.6555744666	0.1376303263	0.7927373429	0.5877641392	-1.035393658	0.1243395135
0.7754670422	-0.09183386283	0.8803126506	0.6823097781	-1.128559963	0.1214014714
0.526118124	0.2267853812	0.9270903238	0.5719772736	-0.8540687005	0.1296938621
0.8358558141	-0.06470454942	0.8725556872	0.6401692489	-1.042559897	0.1228096373
0.7350810244	-0.1131017006	0.8658493872	0.5983020905	-1.052257103	0.1234073498
0.132973507	0.3870917018	0.9785021771	0.8555324073	-1.398368442	0.1130268399
0.2153727604	-0.3143962736	0.9215908136	0.7806834698	-1.064357016	0.1156154157
0.7754670422	0.08140428983	0.8562103157	0.6621309578	-1.044118084	0.1229564872
0.9498139359	-0.01764704365	0.8766030672	0.6488364616	-1.066176548	0.1274577458
0.5762614969	0.1766713335	0.8304609941	0.6625911009	-1.090053773	0.1210478307
0.7922115161	-0.07886231977	0.8948730875	0.6551855071	-1.089539999	0.1241644768
0.5762614969	-0.1702827074	0.8136233285	0.5696542519	-1.07317079	0.1219457683
0.526118124	0.2151448042	0.9648961091	0.7752844129	-1.182139697	0.1219924415
0.5486787709	-0.2020796255	0.9522679441	0.6770502282	-1.143144797	0.1203431088
0.6376240108	0.1455861415	0.875431205	0.672565755	-1.025392225	0.1233622233
0.7244558275	0.123512134	0.9160278738	0.6616183375	-1.050593841	0.1248688012
0.8778323496	-0.04374457325	0.8644204635	0.6030329682	-1.010953299	0.130392747
0.5832622209	-0.154070515	0.916091029	0.6235919688	-1.051541467	0.1207793498
0.2153727604	-0.3000911327	0.8918360227	0.7119204347	-1.081081458	0.113989469
0.4856544148	-0.2259052137	0.8626682614	0.6361555011	-0.9736263988	0.1175216391
0.5762614969	-0.1853764931	0.8511550506	0.6436877615	-1.053335081	0.1211331771
0.8780395729	0.03514017344	0.8390662922	0.6380134605	-1.045305676	0.1277336482
0.7215974205	-0.1240137515	0.9085221355	0.660061831	-1.05383816	0.1235468956

0.4629433469	0.2465644919	0.8519521383	0.5524752574	-0.9150754448	0.119249494
0.7754670422	-0.08939978913	0.8654147595	0.7131042784	-1.106755314	0.1297653434
0.8559555964	0.05131439435	0.8658240093	0.6391789638	-1.051716671	0.1225859819
0.8165197312	-0.08022140017	0.8817681008	0.6378927679	-1.089215708	0.1335671633
0.5762614969	0.162784033	0.7977432398	0.6336954419	-1.018301227	0.1230414659
0.4732912503	-0.245893686	0.8969864249	0.7181350554	-0.9925581097	0.1230215819
0.5486787709	-0.2064994227	0.8928431105	0.6184416687	-0.9146442629	0.126119701
0.621710498	0.1514010048	0.9034313247	0.6849542018	-1.081838717	0.1236953611
0.7354029002	-0.1068558537	0.8269190264	0.6264254995	-1.019723281	0.1197241649
0.526118124	0.2261662831	0.7722621276	0.5771882387	-0.7948219643	0.1270572019
0.5762614969	-0.1653387341	0.8218040317	0.6120718586	-1.065264242	0.1211041787
0.6376240108	-0.1403263637	0.8082300845	0.6315678153	-1.00158222	0.1213005765
0.5762614969	-0.1879824063	0.7749325174	0.5306699018	-1.019017149	0.1272663331
0.7406447589	-0.1062476102	0.906789223	0.6175348814	-1.004655293	0.1266543803
0.7350810244	0.1183497308	0.9129721279	0.686536243	-1.174342079	0.1300906164
0.8869524551	0.03081577877	0.8571503937	0.6303446268	-1.048645674	0.1227340483
0.7754670422	0.08136361726	0.8367133141	0.6076753483	-0.9905210197	0.1227072528
0.8559555964	-0.04828329297	0.856134228	0.6516887302	-1.048700594	0.1239773207
0.9790000627	-0.006973926818	0.865799015	0.6403033384	-1.056308466	0.1249873738
0.4629433469	-0.2436695149	0.9003438889	0.6745166552	-1.088165946	0.1156205207
0.4629433469	0.2462954241	1.023957447	0.6945041243	-1.266883032	0.1204848565
0.9812632823	-0.0052071602910	.8626933188	0.6395141262	-1.054104782	0.1206086075
0.7350810244	0.1173022534	0.9032573836	0.7346658369	-1.201005887	0.1255099373
0.7754670422	0.08180741236	0.869552359	0.6396916965	-1.024015486	0.1233520353
0.5832622209	-0.1575016988	0.8660904844	0.7158144808	-1.096600454	0.1235224567
0.9653516843	-0.01406900233	0.8704989005	0.6465110356	-1.051176061	0.1383665965
0.5832622209	-0.1526381984	0.8500960818	0.6252964579	-1.006686154	0.1189570982
0.5762614969	-0.1700128169	0.8791209152	0.6617390744	-1.177005471	0.1220207667
0.2153727604	-0.300126169	1.068798832	0.811961813	-1.144625707	0.1209886014
0.8559555964	-0.05094062145	0.8637848434	0.6539211174	-1.061698275	0.1290294542
0.8212864485	0.07208972602	0.8802005926	0.6474399519	-1.0850347	0.1272705184
0.526118124	-0.218830784	0.7974578533	0.5091642783	-0.9440667475	0.1199157629
0.5762614969	0.1610379964	0.847243299	0.6874876144	-1.121317256	0.1212514292
0.7350810244	0.1124264197	0.8970005124	0.6510641739	-1.073349245	0.1203007625
0.9558407212	0.01596086659	0.7957626027	0.4993691844	-1.023258924	0.1257276647
0.6959866521	-0.1853323559	0.7848514702	0.4835542986	-1.009099735	0.1192522804
0.9580582754	-0.0096543148690	.7884592527	0.4956641813	-1.021618307	0.1223891721
0.8243251094	0.06593492154	0.7919305192	0.4886619035	-0.9971942161	0.1241812536
0.6959866521	-0.1853463399	0.7836903635	0.5422556264	-1.008953828	0.1204065605
0.8243251094	0.07302564364	0.8247432854	0.5273318966	-1.091741709	0.1272672901
0.9580582754	-0.0088841132830	.7942057236	0.4939280719	-1.024120869	0.1214284259
0.9558407212	-0.01596294395	0.8008682113	0.4973848679	-1.023142074	0.1248899891
0.9580582754	-0.01163848207	0.7970553087	0.4972507539	-1.024758452	0.1278932501
0.6959866521	0.1975959001	0.7558505421	0.4190374883	-0.9128392785	0.1222183414
0.8243251094	-0.07202594767	0.7755050476	0.4825960132	-1.019668636	0.1231865637
0.6959866521	0.2206982788	0.844551739	0.5082627847	-1.183623888	0.120258488

0.8938824884	-0.03561235078	0.7938358848	0.4877520755	-1.015836706	0.1230242194
0.732936013	-0.1150298957	0.7905677671	0.4583373845	-1.005809367	0.1200812103
0.7158466291	-0.1458273198	0.7906082134	0.4905587726	-1.080918617	0.1202428634
0.7518157004	-0.09442433273	0.7538796587	0.4715161819	-1.005284701	0.1214083719
0.7782601275	-0.08534462623	0.7838793193	0.4509096658	-1.002921476	0.123165859
0.732936013	0.1010195991	0.8089481991	0.5044932844	-1.054383486	0.1226089309
0.892341832	-0.04253622099	0.8185252789	0.4818630279	-1.002883741	0.1327052959
0.732936013	-0.1057430494	0.8655284197	0.5329547728	-1.026358282	0.1307703315
0.7158466291	-0.1505095783	0.7030755494	0.4412744563	-0.9187735762	0.1349554103
0.8243251094	-0.07524324672	0.8052455663	0.4937865664	-1.066943152	0.1260077374
0.6959866521	-0.2301105221	0.6586861351	0.4551674152	-0.9501044073	0.1215412063
0.732936013	-0.1178408308	0.806779015	0.545019288	-1.062146237	0.1224683487
0.6959866521	0.1845548056	0.6585414467	0.4266057486	-0.9136109231	0.1338715602
0.7158466291	-0.143004054	0.8262208341	0.56855557	-1.058241682	0.1316475494
0.9664084581	-0.006567543474	0.795191529	0.4954517559	-1.027818179	0.1262245316
0.7158466291	-0.1461108568	0.8400627662	0.5235830187	-1.016726495	0.1217006869
0.732936013	0.1135249953	0.8890757673	0.5577547767	-1.147189699	0.1241894579
0.7158466291	-0.1282943041	0.7147818324	0.4734238813	-0.9647673751	0.1240819307
0.9558407212	0.01624221776	0.7984623878	0.4995330806	-1.02770908	0.1353060271
0.9558407212	-0.0170819964	0.7920805956	0.5029353288	-1.026285521	0.1431936803
0.7158466291	0.1511115162	0.7184666384	0.472519274	-0.9600777245	0.1258971275
0.6959866521	0.2489541012	0.6988841196	0.3913832547	-0.8213046264	0.1217074757
0.8337172861	-0.0626813045	0.8156070719	0.5082220491	-1.092717848	0.1310078313
0.8243251094	-0.1038566144	0.7332963584	0.4926836966	-1.008702513	0.1406275854
0.6959866521	0.2426353928	0.7089838659	0.5269469513	-0.905344463	0.1276902215
0.6959866521	0.2007723077	0.7536598642	0.5475436957	-1.063509459	0.1228432643
0.6959866521	0.2527623523	0.7693659587	0.6058872654	-1.110469731	0.1300099271
0.878519134	0.04977310965	0.7963545043	0.4648444641	-0.9813138218	0.1329864123
0.7564722499	0.1009645504	0.8407425197	0.500713935	-1.055231014	0.1287335834
0.9174285189	-0.02982487093	0.7958341558	0.5048411317	-1.042601049	0.1268035305
0.9558407212	-0.01769246271	0.7911500416	0.5022517547	-1.030860578	0.1254596148
0.6959866521	-0.2545997496	0.8333575434	0.475033088	-1.120363614	0.1195106441
0.9174285189	0.03104335502	0.7975867397	0.4945911665	-1.039067959	0.1262883279
0.7635041575	-0.09308536677	0.7552999462	0.4861263382	-0.9633419546	0.122299508
0.8243251094	-0.06865103692	0.7940456758	0.502036045	-1.067502341	0.1269430047
0.8826037281	0.04323503967	0.7916859493	0.4960368755	-1.014653689	0.1247451664
0.732936013	0.1175599155	0.8091535994	0.5504699802	-1.091761456	0.1250183037
0.6959866521	-0.169088803	0.7764555852	0.419175716	-1.064483833	0.1234758315
0.7158466291	-0.1758048438	0.814625155	0.5266498534	-1.043743484	0.1357378872
0.732936013	-0.1235022636	0.7142379374	0.4241885958	-0.9169023211	0.1268014525
0.7158466291	-0.1604593047	0.7112565324	0.482977603	-0.9161734302	0.1304540904
0.732936013	0.1059585154	0.812085716	0.5286752329	-0.9672081252	0.1268209452
0.8243251094	0.07234436808	0.8068372485	0.5015218705	-1.036967076	0.1242577517
0.732936013	0.1175275166	0.746186985	0.4990741763	-0.9589686718	0.1248894315
0.7564722499	-0.09357188283	0.7899344205	0.4521995207	-0.9571331929	0.1242648482
0.6959866521	0.2151085209	0.7827174007	0.5487888877	-1.122733622	0.1196323185

0.878519134	0.04952449939	0.8170724119	0.51829722	-1.079017685	0.1278301198
0.6959866521	0.198744285	0.7252541984	0.4038315347	-0.9268881441	0.1223561569
0.6959866521	-0.1706118131	0.7448846652	0.4904975392	-1.037932021	0.1190323046
0.8301445012	0.0637684134	0.8170167682	0.5058657664	-1.035951114	0.1296660803
0.6959866521	-0.1889119665	0.6662436395	0.433645951	-0.936168778	0.1268426594
0.7158466291	0.1472287017	0.8231204573	0.4428530211	-1.04751857	0.1247760756
0.410290334	0.3151893622	0.8177575211	0.499441974	-0.9750535302	0.1156446953
0.8243251094	0.07609057022	0.8047216602	0.5084835149	-1.024783923	0.1265555087
0.6959866521	0.1791896359	0.7101866117	0.4772006431	-0.9143644927	0.1220152219
0.6959866521	0.2311362675	0.7202124998	0.3800754663	-0.8036642025	0.1221455929
0.6959866521	0.16947743	0.6953311169	0.4299962052	-0.9872258524	0.123653862
0.7158466291	-0.1444758023	0.8105415138	0.5571133814	-1.125037535	0.1203266844
0.732936013	0.1118372314	0.8273723855	0.463905144	-0.9296243802	0.1321543221
0.8559185367	-0.05627319915	0.7962042431	0.4923406702	-1.00602477	0.1240850892
0.6959866521	-0.1635338194	0.7934657924	0.4308765302	-1.016424931	0.1270847212
0.2411235518	0.3861009211	0.8905867875	0.7004923754	-1.354618966	0.1153394857
0.410290334	-0.324337361	0.8144999062	0.6170159587	-0.9877415345	0.1164372926
0.8826037281	-0.0499844832	0.807389725	0.4902243538	-1.046932781	0.125155082
0.6959866521	-0.1709111707	0.9617276384	0.5993196465	-1.17453303	0.1261067143
0.7648103982	-0.08738133567	0.8142517789	0.4907488592	-1.015494873	0.1233349705
0.8243251094	0.07308705886	0.7716311857	0.488475649	-1.006304715	0.1255528848
0.732936013	-0.1005365797	0.7630045433	0.4540780296	-1.034815188	0.1221102176
0.8938824884	-0.03785948463	0.7765352999	0.4740237084	-1.004748149	0.1257816763
0.6959866521	-0.1750115516	0.8634885775	0.5137974816	-1.088713792	0.1229262019
0.878519134	-0.05591798046	0.7959197115	0.4900757365	-1.04766601	0.1271740791
0.9558407212	0.014158954	0.7973026765	0.4969048228	-1.022177999	0.1274569574
0.9857401854	-0.00237562483	0.7929298689	0.4938851689	-1.022553939	0.1315196364
0.7782601275	-0.08860141853	0.8173868829	0.4823249752	-1.016119372	0.1254868665
0.6959866521	-0.1625518138	0.8034972789	0.5244065361	-1.030117184	0.1178348113
0.8243251094	-0.06521726604	0.7912707978	0.4920291943	-1.000131447	0.1250389276
0.732936013	-0.1554597848	0.7480628797	0.4560674384	-0.9728303452	0.1403836596
0.9558407212	0.01473699931	0.7836088165	0.4949558086	-1.021487968	0.1293417161
0.8938824884	-0.03459029948	0.8054310273	0.5016342071	-1.024104018	0.1234735841
0.6959866521	0.2017857413	0.7573067641	0.4033190626	-0.8764073052	0.1284302326
0.7564722499	-0.09774827143	0.7922044714	0.5743291024	-1.075300549	0.1295978226
0.6959866521	-0.1718432146	0.7740241263	0.4888426886	-1.021041899	0.1254743527
0.7635041575	0.1021123142	0.7829115678	0.5084162515	-1.002590082	0.1341273224
0.732936013	0.1062148378	0.7461464128	0.4834408213	-0.993938374	0.1269998871
0.6959866521	-0.2608016095	0.8000721051	0.5549014354	-0.93040176	0.1362062702
0.878519134	-0.05719817616	0.7929145359	0.4852642768	-0.9752717172	0.1337423614
0.878519134	-0.0481381433	0.7826458313	0.4843646654	-1.020622487	0.1264118138
0.6959866521	-0.2476748338	0.7026488783	0.4618222123	-0.9347595704	0.1155070105
0.732936013	0.1287654371	0.7334719785	0.4460601116	-0.8674037468	0.1379025099
0.732936013	-0.1114132788	0.7638736776	0.4755193915	-1.029234967	0.1216028721
0.9558407212	0.01895382956	0.7995357583	0.4961175871	-1.031014805	0.1279667078
0.6959866521	-0.2029153362	0.7230597669	0.3938400446	-1.020516655	0.1274483849

0.6959866521	-0.163727847	0.8638121777	0.4551778166	-0.9501993209	0.1263639602
0.6959866521	0.2291611876	0.9160652655	0.6021510197	-1.291130669	0.1281741085
0.732936013	0.102109749	0.7802876782	0.4686661651	-1.008916544	0.1229844561
0.732936013	0.125762546	0.7558029764	0.4477103604	-0.9264932164	0.1258495477
0.7158466291	0.141975735	0.8327720005	0.4766047914	-1.074643676	0.1233091943
0.8826037281	-0.04162294409	0.814388741	0.4956001874	-1.032053009	0.1248872408
0.6959866521	-0.2153193791	0.8302496104	0.5272994683	-1.059719338	0.115932294
0.5521312959	0.2951780256	1.011190084	0.5772027875	-1.31338953	0.1191987778
0.8266664895	0.07700155132	0.7550810788	0.4839900674	-0.9937012812	0.124433445
0.7158466291	0.134948218	0.8515859464	0.613955255	-1.209592358	0.1261663161
0.732936013	0.1245986899	0.790898347	0.4768462106	-0.9586451244	0.1252497997
0.9185693261	0.03448117875	0.7987074315	0.4853446443	-1.025283348	0.1299989785
0.7158466291	0.151966993	0.7060297333	0.4305050377	-1.068916963	0.1389217771
0.7158466291	-0.1575350141	0.7572497296	0.4616781346	-0.9487447832	0.1262835015
0.7635041575	-0.08974973939	0.7964646587	0.5023990015	-1.082573961	0.1246263378
0.732936013	-0.1112598196	0.8764624786	0.561044191	-1.068695255	0.1301020242
0.6959866521	-0.1784687627	0.7993506178	0.553749094	-1.051995757	0.1290306906
0.8818643096	0.04598480911	0.8026515628	0.4982876031	-1.041385692	0.1270802315
0.6959866521	-0.1665870408	0.7477236935	0.3976611743	-0.9466709835	0.121915581
0.7158466291	0.1462071626	0.7627377037	0.5205959566	-1.060183133	0.1211529411
0.7158466291	0.1381391312	0.8371665855	0.5067289098	-1.048795261	0.119689009
0.4747833966	0.2153146553	0.9178246629	0.5773444613	-1.05331381	0.1229542976
0.6844246943	0.1223404944	0.8709567447	0.5281242959	-1.068097215	0.120275463
0.570290738	0.1720911899	0.9228445976	0.5087641386	-1.087046266	0.1200297734
0.9668059968	-0.01229211541	0.8504878867	0.5114252295	-1.041254376	0.1230012263
0.1365281022	-0.3317132292	0.8293973102	0.5928802392	-0.997720102	0.1135806443
0.7727047178	0.08145457832	0.8729520315	0.5371692085	-1.092821239	0.1254107551
0.7319977919	0.09297707155	0.843346898	0.5377334338	-1.055916493	0.1214521129
0.5965506588	-0.1714783741	0.9323884234	0.5248471771	-1.010165708	0.1231121714
0.1428343429	-0.3353581527	1.00491226	0.5685878808	-1.075648853	0.1202758817
0.6914540463	0.1111962326	0.854394931	0.4892587175	-1.012848109	0.1193347677
0.9255063064	0.03048325153	0.8583306442	0.5163970723	-1.039192418	0.1243702524
0.7723461953	-0.08357764668	0.8394038539	0.5108694285	-0.9881066529	0.122788495
0.88317175	0.04289408797	0.8529847767	0.5216846863	-1.052647816	0.123146372
0.4747833966	-0.1913509546	0.8477507736	0.4471118515	-1.003899082	0.1183457309
0.4747833966	-0.1950900492	0.8564728004	0.5107081542	-1.125580993	0.1203361815
0.405215244	-0.2475060652	0.7492522887	0.4487908984	-0.9841925307	0.1186934204
0.02450352368	-0.4373640483	0.8281897171	0.293778809	-0.9540433789	0.1093883801
0.9111058518	0.03690045378	0.8590096775	0.5138822103	-1.050332906	0.1235777031
0.3463206331	0.298442096	0.7001117247	0.6305038092	-1.234780107	0.1276303912
0.7791752649	0.07564773658	0.795809655	0.4829710091	-1.031122994	0.1297946771
0.4747833966	-0.216560905	0.7165102366	0.4146417822	-0.8682825882	0.1199933906
0.7791752649	0.07414196104	0.8506987966	0.5213092972	-1.012702438	0.1243511986
0.4747833966	-0.2076943328	0.7380471111	0.4755652096	-0.984197197	0.1212024281
0.9653946556	-0.01503629806	0.8513553896	0.5161175804	-1.039718534	0.1229287933
0.6914540463	-0.1267020626	0.9369457117	0.5459342492	-1.104995175	0.1328667943

0.6470164754	-0.1554211051	0.8863447745	0.5998819025	-1.073539242	0.1237296482
0.7319977919	0.09713656181	0.8181239286	0.519832025	-0.9970042868	0.12587691
0.9255063064	-0.03011731255	0.8625986246	0.5163382066	-1.037328268	0.1232774221
0.9348509336	-0.02427758786	0.8317542398	0.4980538251	-1.012496975	0.1242464833
0.6579394906	-0.1363312818	0.7688443561	0.4898480246	-0.9740001588	0.1245337653
0.5094641713	-0.1875717164	0.8068400822	0.488563929	-1.039101031	0.1215100585
0.6956587904	-0.1113332819	0.8557845997	0.5745663786	-1.064188208	0.1305331706
0.4747833966	0.2162293363	0.7797698207	0.5051824727	-1.004317398	0.1197527332
0.862673955	0.05355855534	0.8246432226	0.4837317196	-0.9844685552	0.1253570633
0.8820860536	0.04786182506	0.8333385364	0.5013586017	-0.9850121433	0.1299159133
0.6917499485	-0.1099606891	0.8151364627	0.5242389312	-1.06346154	0.1253407424
0.24550875	0.2981214319	0.818031867	0.600675492	-1.000588191	0.1179550123
0.4747833966	0.2090286243	0.844836022	0.5879004549	-1.12850579	0.1214174547
0.7840298201	0.07202844671	0.8365054026	0.5364617244	-1.050307762	0.1333651833
0.9255063064	0.03530373631	0.8529803988	0.486515385	-1.00463796	0.1301042115
0.983766969	-0.00510363453	0.8466877038	0.5088271722	-1.032605575	0.1261158209
0.983766969	0.003670082907	0.8498389465	0.5086217956	-1.033718055	0.12147142
0.7701935905	-0.08629848529	0.851417809	0.5457980151	-1.078979444	0.1238370174
0.6917499485	0.1101460121	0.8427412779	0.525426383	-1.008726308	0.1228006819
0.7319977919	-0.09678170339	0.8290530785	0.5062582337	-0.9834175466	0.1232905002
0.9111058518	-0.03704129946	0.8419302355	0.5098979118	-1.021425501	0.1226219775
0.9713083494	-0.009358416003	0.8493277244	0.5095164699	-1.040097019	0.1248005731
0.4747833966	0.2054185034	0.8458934134	0.5142505307	-0.9848533647	0.1213461218
0.4747833966	0.2205260496	0.8983806831	0.6228313598	-1.180349508	0.1212887515
0.7151365909	-0.101087038	0.8259546228	0.4543328755	-1.039079117	0.1243770831
0.983766969	0.003856909185	0.8487796042	0.5085306129	-1.034230775	0.1222090103
0.6579394906	0.1418302965	0.9517126746	0.5976075843	-1.174709133	0.1257323259
0.4747833966	-0.2043487481	0.7501051325	0.4842407718	-0.9029790929	0.1237852639
0.5446705437	0.1872922614	0.8952118599	0.573978459	-0.952324269	0.1255774378
0.6470164754	0.1557943061	0.8809542079	0.5282346915	-1.064182665	0.1224307833
0.6914540463	0.114044944	0.8370983277	0.531270076	-1.020319159	0.1199228415
0.6579394906	-0.1344995112	0.8498292358	0.4523632664	-0.9462111553	0.1236261792
0.6712192959	0.127905323	0.8343751001	0.5330467438	-1.078847596	0.1203077563
0.8391897122	0.05990045517	0.8814488637	0.5367119081	-1.104225661	0.1271139602
0.7791752649	-0.07153921171	0.8715237282	0.5442531274	-1.067757941	0.124716169
0.4747833966	-0.2252765666	0.7754077702	0.4925077951	-1.036686506	0.1179962569
0.4747833966	-0.1980747856	0.7600537102	0.4772326483	-0.9824447066	0.1237823245
0.6914540463	-0.116020159	0.76071742	0.464509844	-0.9652251553	0.1261877046
0.7791752649	-0.07738648668	0.8465670179	0.5457432533	-1.041261011	0.1254854839
0.6470164754	0.1505837628	0.852952474	0.5069199394	-0.9990687702	0.120447243
0.6917499485	-0.1109101571	0.8123571582	0.4753250009	-1.006497589	0.1234958364
0.9713083494	-0.0101010798	0.8559762224	0.5115386925	-1.043619351	0.1238544035
0.9329716846	0.02603565161	0.8417039109	0.4976862008	-1.010843207	0.1257013458
0.7727047178	-0.08094077973	0.8904868098	0.5401898367	-1.046551859	0.1253715391
0.6470164754	-0.1572556126	0.8819814667	0.582424274	-1.16283756	0.120478233
0.570290738	0.1895804499	0.9047036838	0.4529181257	-0.868200363	0.1309047244

0.654577244	0.1428282783	0.8258404891	0.5087417406	-1.061177385	0.1219063487
0.6917499485	-0.1087783736	0.8540414714	0.4697050276	-1.033631471	0.1236748809
0.405215244	0.2549979493	0.9269708583	0.6517253936	-1.262220585	0.1193240517
0.4016115174	-0.2676726775	0.8962423607	0.628014	-1.039667027	0.1181302902
0.654577244	0.1438595875	0.8404120858	0.549095684	-1.017687415	0.1222016215
0.8795741666	0.05096556119	0.8067092776	0.4834462003	-1.000890592	0.1275151471
0.4747833966	0.2052552246	0.8137731063	0.5360989556	-1.077155775	0.1204984116
0.6579394906	0.1345259969	0.793254636	0.4833211754	-0.9752016493	0.1235686785
0.6844246943	-0.1257378187	0.8145331385	0.4578163953	-1.049507054	0.1230462012
0.6470164754	0.1502429163	0.9220954695	0.6042250609	-1.12484332	0.1238070852
0.6914540463	-0.1134305812	0.9008566871	0.5305182698	-1.085424116	0.1224656384
0.4016115174	0.2627817049	0.8749012389	0.5687744998	-0.9835068374	0.1202959795
0.7537501405	0.09082805039	0.8899845163	0.5256571138	-1.032846999	0.1255404369
0.9668059968	-0.01395593643	0.8508684248	0.49787466	-1.021699558	0.130714173
0.7723461953	-0.08244696957	0.8791233758	0.5009352949	-1.034075167	0.1221417715
0.03003916901	-0.4005085717	0.8903000411	0.6058773177	-1.071378714	0.1089407931
0.9843096322	0.002385167378	0.8499645507	0.5096367892	-1.036345608	0.1212822107
0.8829503013	-0.04422817558	0.8474783535	0.5105305794	-1.035289868	0.1235429548
0.7791752649	0.07526433217	0.8016652463	0.5058124955	-1.016456278	0.1276521399
0.8502633244	-0.05605415091	0.8711744719	0.518765383	-1.035333817	0.1245718965
0.8801299717	0.04809534153	0.8480893924	0.4925684618	-1.008358091	0.1234688619
0.7840298201	-0.07103780053	0.8529894207	0.5678616936	-1.077260085	0.1301661331
0.6844246943	-0.1237683215	0.839840319	0.511050869	-1.041471718	0.1219213208
0.6470164754	-0.1615824816	0.8905541736	0.505794992	-1.106034612	0.1325539137
0.6956587904	0.106557478	0.808160988	0.5056148643	-1.011999327	0.12426652
0.7065120866	-0.1052291561	0.8651096883	0.5431297452	-1.009117856	0.1265301131
0.9363440092	0.0237650363	0.8463824998	0.5120543641	-1.051555137	0.129079013
0.7382744533	0.09354025705	0.8758067519	0.5375078506	-1.052574318	0.1248504858
0.654577244	-0.1392230843	0.8047610077	0.4921974064	-0.9905820199	0.119366082
0.6579394906	0.1429192991	0.7935072401	0.4700447366	-0.871591819	0.1292701672
0.1365281022	-0.3370666424	0.7688011446	0.4531092336	-1.058061908	0.1152273637
0.6470164754	-0.1479460307	0.7936846084	0.5009392326	-0.9800256914	0.1213508776
0.4747833966	-0.2335316655	0.7422808378	0.3740490002	-0.991793738	0.1262188515
0.4747833966	-0.208703436	0.9386982682	0.4658999738	-0.9381842179	0.1247244593
0.8820860536	-0.04940077136	0.8285413294	0.4900822881	-0.9853437894	0.1310420432
0.9329716846	0.02662997727	0.8461114544	0.5014438098	-1.030699712	0.122949544
0.9653946556	0.01531877127	0.8452859197	0.5035537819	-1.023506768	0.1233398326
0.9329716846	-0.02644744017	0.8469696855	0.5161209721	-1.032485552	0.1242882906
0.7791752649	-0.07131403973	0.8926897866	0.5149386875	-1.057110505	0.1248542813
0.405215244	-0.2397527427	0.8880751472	0.5437757083	-1.068920193	0.1159579933
0.6470164754	0.1513680132	0.9497436169	0.5432301266	-1.166208781	0.1232726403
0.9653946556	-0.01462908665	0.8529856089	0.5088484322	-1.035242492	0.1207912624
0.5979950877	-0.1715221647	0.789095224	0.3708557927	-0.8208240287	0.1246740113
0.6914540463	0.1162644711	0.8612463187	0.5094714307	-0.9926044126	0.1230923041
0.7727047178	-0.08233637021	0.852311608	0.5493460901	-1.05766322	0.1249364333
0.7791752649	0.07792274322	0.8008094405	0.4723240788	-1.052213698	0.1382373183

0.9255063064	-0.02976918454	0.8477277025	0.5067735519	-1.026229318	0.1207125125
0.6856771369	-0.1234508212	0.8626820845	0.525542768	-1.124671151	0.1231531025
0.1365281022	-0.3424823965	1.086390991	0.7060787394	-1.138688373	0.1193188025
0.7791752649	-0.07825918694	0.853298462	0.531321551	-1.047022648	0.1290114059
0.4747833966	0.1999546832	0.9015071147	0.5308417683	-1.121035762	0.1251833941
0.6579394906	-0.1361822995	0.8100451562	0.4283133235	-0.9669603944	0.1221395405
0.4747833966	0.2012524414	0.8320063466	0.569218289	-1.119379435	0.120449993
0.6914540463	0.1152848841	0.8862537	0.5211684442	-1.05513656	0.1204536192
0.9895351491	-0.1119390071	-1.025329847	0.0921884002	-0.2429689496	0.1020387034
0.9895351491	-0.03845475724	-1.021211375	0.1073008809	-0.220809011	0.1054992503
0.9895351491	-0.09705512073	-1.052050811	0.1035474353	-0.2099743342	0.1037360059
0.9895351491	0.1827187371	-1.031471552	0.0743040397	-0.1591722607	0.1021669003
0.9895351491	-0.1493483789	-1.009738512	0.1480142301	-0.2472714191	0.1037498195
0.9895351491	0.1328366404	-0.955296624	0.1510076975	-0.3420810776	0.1093417724
0.9895351491	0.03240258738	-1.014410472	0.1231753262	-0.2425110025	0.1018689076
0.9895351491	0.005105109347	-1.014320654	0.1141681588	-0.2366801754	0.1043649416
0.9895351491	-0.03543354703	-0.995893317	0.1194941546	-0.2468710823	0.1041991828
0.9895351491	0.04387252328	-1.01471687	0.1040472839	-0.2170267834	0.1070730782
0.9895351491	-0.01357219405	-1.018630336	0.1115909198	-0.230923245	0.1018007178
0.9895351491	-0.09813768688	-1.031424266	0.1238274704	-0.1760052305	0.1125498815
0.9895351491	-0.033116974	-1.007824485	0.1032817269	-0.229249567	0.1034512708
0.9895351491	-0.1586404206	-1.007519622	0.0726445678	-0.2281150366	0.1011083163
0.9895351491	-0.07741951329	-1.017552807	0.1073022016	-0.2639342092	0.1019004411
0.9895351491	-0.1480091113	-1.060631889	0.0733464046	-0.2344580807	0.1013411052
0.9895351491	-0.08321429084	-1.011145743	0.0814781759	-0.2330530095	0.1063742237
0.9895351491	0.002895276329	-1.012227566	0.1142705367	-0.2365942983	0.102156971
0.9895351491	0.09505309756	-1.05840072	0.15965313	-0.2941118309	0.1100413361
0.9895351491	0.04200405796	-1.039280732	0.0953189367	-0.2394162835	0.1126525267
0.9895351491	0.07234386587	-0.982066727	0.1299065823	-0.2653429389	0.1241541816
0.9895351491	0.1864771645	-1.031323447	0.1291911718	-0.1597765195	0.1112266018
0.9895351491	-0.1044516626	-1.084101555	0.0965606820	-0.1930726018	0.1059656185
0.9895351491	0.1172278338	-1.037668866	0.0651251232	-0.19205213	0.1026435568
0.9895351491	0.02977779405	-1.03649989	0.0973993745	-0.2144215863	0.1105137676
0.9895351491	-0.263000158	-0.935072769	0.2254619489	-0.3035983926	0.1122718813
0.9895351491	-0.06424395261	-0.994676650	0.1184257759	-0.2534580049	0.1030450619
0.9895351491	0.02353917472	-1.021007958	0.1083307582	-0.2334179537	0.1039661396
0.9895351491	-0.06983801804	-1.060304821	0.0788902618	-0.1791052601	0.108142909
0.9895351491	0.03049327862	-0.995349338	0.1242912182	-0.2456937553	0.1043142544
0.9895351491	-0.01538708559	-1.017505774	0.1114095275	-0.2337637381	0.1397789063
0.9895351491	-0.1246992627	-1.001241717	0.1436121667	-0.2795933543	0.1310619818
0.9895351491	0.1745472807	-1.080629148	0.0987946946	-0.1834636881	0.1067686194
0.9895351491	-0.01220485067	-1.008140802	0.1190113105	-0.2450848958	0.1145270507
0.9950381856	-0.000691019988	-1.01226428	0.1139516995	-0.2363194952	0.109235827
0.9895351491	0.03854549382	-0.991175076	0.1077313916	-0.2380414941	0.1244795448
0.9895351491	-0.03174031063	-1.007111828	0.1059675329	-0.2419665403	0.1089636219
0.9895351491	-0.004879890518	-1.011298035	0.1122474934	-0.2347023057	0.1064575547

0.9895351491	0.0748104055	-1.022038596	0.1447367351	-0.2386784	0.1082760706
0.9895351491	-0.1593332623	-1.030376686	0.1978562073	-0.367121261	0.1144213587
0.9895351491	-0.07356278495	-1.038000682	0.1021129211	-0.2202542549	0.1098215839
0.9895351491	-0.1280057056	-1.000589873	0.1428854361	-0.3141146202	0.1140948482
0.9895351491	-0.1637668209	-1.002981032	0.2114153792	-0.3105285462	0.1022458958
0.9895351491	0.006292384658	-1.012518492	0.113827037	-0.2339486023	0.106293533
0.9895351491	0.1149919909	-0.964151205	0.1094217796	-0.3274421283	0.1083372822
0.9895351491	-0.003784199066	-1.013079488	0.1142418062	-0.2341008378	0.1020089879
0.9895351491	0.05031023915	-1.004005913	0.1011302113	-0.2205030722	0.1124136133
0.9895351491	0.07457212735	-0.9922531509	0.1139360262	-0.2493661	0.1046806016
0.9895351491	-0.0846073917	-1.022989076	0.07431706296	-0.1805597513	0.1051748575
0.9895351491	-0.1914618018	-1.042770165	0.00777224363	-0.26358144	0.1058273736
0.9895351491	-0.1549777095	-0.9794753918	0.1568006965	-0.2735418238	0.1202365684
0.9895351491	0.0791439055	-0.9742868171	0.1638128958	-0.2999988983	0.1067156667
0.9895351491	0.1008710184	-0.9690081562	0.1079337874	-0.3027215388	0.1126005161
0.9895351491	0.1000883364	-0.9977011887	0.1498723944	-0.179623957	0.1072130812
0.9895351491	0.08599654417	-0.9917074386	0.1219111534	-0.2598490522	0.1025274416
0.9895351491	-0.009033893316	-1.010141963	0.1111239337	-0.2390803797	0.1083713499
0.9895351491	-0.04298730705	-1.01536255	0.09209817133	-0.2044402449	0.1047662858
0.9895351491	0.1794486558	-1.026140032	0.1832821823	-0.3239603756	0.1079467258
0.9895351491	-0.02249534847	-1.019030553	0.1024958313	-0.2155595687	0.1080007739
0.9895351491	-0.03570290951	-0.9968912239	0.1253493312	-0.2588033464	0.1048241214
0.9895351491	-0.01941506847	-1.017980308	0.1133326245	-0.237238255	0.1014130461
0.9895351491	0.07902319565	-0.9768745147	0.1336113269	-0.2586008858	0.1099582309
0.9895351491	-0.04590857035	-1.040922259	0.09463211023	-0.217087325	0.1221701366
0.9895351491	-0.02398111122	-1.010663266	0.1266933639	-0.2380836996	0.1111636409
0.9895351491	0.01353624641	-1.010531881	0.1128993947	-0.2352117395	0.1042372881
0.9895351491	0.07463175688	-0.9882908583	0.131260151	-0.2553612121	0.1072156279
0.9895351491	0.07886764944	-1.052507353	0.09637334884	-0.1766386131	0.1108184468
0.9895351491	0.08978906063	-1.044688307	0.06029336039	-0.1504174816	0.1050815105
0.9895351491	0.01958731688	-1.021060936	0.108119436	-0.2364132214	0.1062060782
0.9895351491	0.01925063505	-1.01463125	0.1045443376	-0.222382083	0.1040011978
0.9895351491	0.1820691789	-0.9665469971	0.04930374861	-0.06406125007	0.1092606146
0.9895351491	0.05950851468	-1.009157221	0.1163164803	-0.2648442818	0.1055728742
0.9895351491	-0.1558853657	-1.030631505	0.05449235913	-0.2020192026	0.1090526795
0.9895351491	0.2172702105	-0.9423385149	0.2596347598	-0.4477485557	0.1026908515
0.9895351491	-0.1250551013	-0.9949289773	0.1634355204	-0.2421753776	0.1040865251
0.9895351491	-0.01492012161	-1.011934344	0.1118747172	-0.2360165551	0.1106012416
0.9895351491	0.08370084532	-1.076744217	0.08379629547	-0.1917290834	0.1063791336
0.9895351491	-0.01965070383	-1.00812815	0.1142668942	-0.231690138	0.1046677747
0.9895351491	0.0652323743	-1.02018713	0.1158678194	-0.2286125654	0.1065781322
0.9895351491	-0.02855119569	-1.020655808	0.1038508788	-0.2392579391	0.1033140275
0.9895351491	-0.03439868336	-1.029685007	0.09748114248	-0.2159761229	0.1057581212
0.9895351491	0.04415800757	-1.032642767	0.1069915356	-0.2196964347	0.1046741687
0.9895351491	-0.09863076706	-1.049728714	0.09569575414	-0.2175262161	0.1116229322
0.9895351491	0.03320488489	-0.9966255352	0.1142587448	-0.2395239014	0.1080227222

0.9895351491	-0.01266293623	-1.010315482	0.103475537	-0.2260454584	0.1135717536
0.9895351491	-0.05029541315	-0.9918549136	0.1093678105	-0.2447090154	0.1092275761
0.9895351491	-0.170529379	-0.9882104123	0.1379361603	-0.2657352335	0.09967000973
0.9895351491	-0.1483779647	-1.004147626	0.0991549762	-0.2018287127	0.1088632594
0.9895351491	-0.04062674639	-1.013829463	0.1047585698	-0.2388029733	0.1198016166
0.9895351491	0.01438840015	-1.01935535	0.111397902	-0.2360025444	0.1107546565
0.9895351491	0.1180010473	-1.069411101	0.08172321743	-0.2256316079	0.1015827501
0.9895351491	0.01288271041	-1.012737157	0.1081597355	-0.2304351771	0.1109010635
0.9895351491	0.09375919685	-1.013465482	0.02088673263	-0.1879336199	0.1085656712
0.9895351491	0.05660371155	-1.010241828	0.114378986	-0.2308030463	0.1110836316
0.9895351491	-0.009542147516	-1.010973238	0.1130436394	-0.2406339295	0.1072691131
0.9895351491	0.1987201356	-1.041137307	0.09514973089	-0.2576084816	0.104595404
0.9895351491	-0.09641015383	-0.9986419605	0.1366840479	-0.2160806545	0.1161252501
0.9895351491	-0.1376432185	-1.024137558	0.08992360778	-0.1293246224	0.1109110349
0.9895351491	-0.004147402689	-1.014020917	0.1124761202	-0.234938788	0.1057175779
0.9895351491	-0.03829999592	-1.022805435	0.1114708695	-0.2290157539	0.1021493097
0.9895351491	0.07293796884	-1.030525071	0.07952814784	-0.1725356713	0.1177587787
0.9895351491	-0.1824029165	-1.039738265	0.0881916504	-0.279793235	0.1005384462
0.9895351491	0.0556698383	-0.9927393759	0.1103152604	-0.253881775	0.114674911
0.9895351491	0.03108071063	-1.002424328	0.1277713623	-0.2348127562	0.1211238156
0.9895351491	-0.09654479726	-0.9602160298	0.08175740737	-0.2083611437	0.10932219
0.9895351491	-0.08428585711	-1.045527459	0.06914687824	-0.1686672087	0.1143043841
0.9895351491	0.01329933622	-1.013258049	0.1118887891	-0.2345034644	0.103231019
0.9895351491	-0.01210437429	-1.007266025	0.118705841	-0.2476131396	0.1145408843
0.9895351491	-0.1499988316	-1.040807395	0.1072518267	-0.2164204522	0.1294933891
0.9895351491	0.0267054011	-1.026576656	0.117453784	-0.2308885283	0.1053816331
0.9895351491	0.01817978582	-1.016282292	0.1123204946	-0.2314164308	0.1003733106
0.9895351491	-0.0174838995	-1.022719931	0.1070057518	-0.2217558306	0.1133619402
0.9895351491	-0.1061884555	-0.983834848	0.09938827218	-0.2531196505	0.1055882278
0.9895351491	-0.05001251278	-1.026324265	0.07222589138	-0.1769191094	0.1104684816
0.9895351491	-0.008251285325	-1.012485264	0.115499394	-0.2390807056	0.1063883185
0.9895351491	-0.03370203865	-1.009482607	0.1202929912	-0.2503767899	0.120262545
0.9895351491	0.1010244262	-1.094828617	0.07119820924	-0.233128292	0.1220318198
0.9895351491	-0.1586087817	-1.018085669	0.09060293405	-0.2097921369	0.1082540206
0.9895351491	0.07850756492	-1.018220796	0.1116861985	-0.1787078931	0.1067653666
0.9895351491	-0.1349467908	-0.9047965275	0.1763579682	-0.3038964769	0.1140278132
0.9895351491	-0.006390482825	-1.01196695	0.1162142625	-0.2385595632	0.10440972
0.9895351491	0.06851722693	-1.009409584	0.1045851004	-0.2464613033	0.1014294655
0.9895351491	-0.02881269398	-1.01936424	0.09511943758	-0.2210213092	0.1055525606
0.9895351491	-0.002420323823	-1.012024686	0.1135461614	-0.2348543623	0.1043340071
0.9895351491	-0.04666794142	-1.026515095	0.1184459544	-0.2270590197	0.1019254942
0.9984451821	-0.09983792187	-0.9456276181	0.1207231185	-0.2873828813	0.1010117765
0.9984451821	-0.08876308784	-0.9569311561	0.1229296983	-0.2414484902	0.1044264427
0.9984451821	-0.1675387226	-1.010643101	0.1218597245	-0.2232929715	0.1010373811
0.9984451821	0.2019911604	-0.9622380211	0.09485795133	-0.1870091514	0.1006755224
0.9984451821	-0.1278311358	-0.9307629804	0.1685545645	-0.2922777476	0.103526327

0.9984451821	0.1453095432	-0.8746063842	0.1762118782	-0.3910819583	0.1098795831
0.9984451821	0.0001954718104	-0.9361665487	0.1390033851	-0.2770020805	0.1003100335
0.9984451821	0.02344326723	-0.9450277048	0.1405845249	-0.2811357767	0.102826287
0.9984451821	-0.03960572049	-0.9175589835	0.1456178098	-0.2894218291	0.1030471806
0.9984451821	0.02074387607	-0.9375586074	0.133122141	-0.2671737873	0.107242226
0.9984451821	-0.01699943122	-0.9438989013	0.1358536987	-0.2709758441	0.1001312382
0.9984451821	-0.007126622963	-0.9374596569	0.139540797	-0.272717115	0.113540904
0.9984451821	-0.03160050327	-0.9322043227	0.1298930832	-0.2707465504	0.1022330123
0.9984451821	-0.177730402	-0.9301608609	0.0967980477	-0.2704821351	0.09915755232
0.9984451821	-0.07586935311	-0.9417690037	0.130721707	-0.3028940006	0.1001787113
0.9984451821	-0.1295279319	-0.9779325127	0.1062200519	-0.2770720689	0.09996778023
0.9984451821	-0.08150663564	-0.9327343025	0.109061826	-0.277881393	0.1058999859
0.9984451821	0.07098882936	-0.9336617225	0.1480132998	-0.2953275895	0.1001277281
0.9984451821	0.05184095264	-0.9611070043	0.1631155593	-0.3084831344	0.108693768
0.9984451821	0.0303774059	-0.9557946689	0.1254646443	-0.2795049041	0.1119862283
0.9984451821	0.09083551471	-0.9048297516	0.1526754429	-0.3023841666	0.1330496265
0.9984451821	0.2689043507	-0.9599647355	0.1562686376	-0.1644295485	0.110253585
0.9984451821	-0.1017881103	-1.005993049	0.1178134611	-0.2336505187	0.1050205661
0.9984451821	0.08142240546	-0.953884871	0.1025416393	-0.2460652197	0.102069348
0.9984451821	0.05648741862	-0.9824190164	0.1080760661	-0.2358410116	0.1096823692
0.9984451821	-0.2319453107	-0.8599385609	0.2307731457	-0.3462842728	0.1155000824
0.9984451821	-0.03045362725	-0.9276014804	0.1408425082	-0.2854769341	0.1020308743
0.9984451821	0.003055456125	-0.9373340142	0.1381993565	-0.2765613	0.1028418827
0.9984451821	-0.0295700069	-0.9566155302	0.12441433	-0.2531104834	0.1076850038
0.9984451821	0.06475251805	-0.8987421851	0.1606786305	-0.2996550054	0.1025525804
0.9984451821	0.001319058968	-0.9357616307	0.139227474	-0.2771220475	0.1535487894
0.9984451821	-0.09148782549	-0.9298914424	0.1544315266	-0.2996851815	0.1532482042
0.9984451821	0.1774386655	-1.004756804	0.1159087598	-0.2195376292	0.1063794583
0.9984451821	0.0773435736	-0.9612755303	0.1095058177	-0.2225236961	0.1152975847
0.9984451821	-0.0787493056	-0.9283641846	0.1492916696	-0.3318918269	0.1083984721
0.9984451821	0.09709764759	-0.8832565283	0.1282588045	-0.2856089072	0.1286314272
0.9984451821	-0.001121823828	-0.9359656517	0.1386985911	-0.2772178903	0.1094957297
0.9984451821	0.03538958376	-0.9435196559	0.1498500307	-0.2847625186	0.1055842731
0.9984451821	0.0626217386	-0.9435317209	0.1646904327	-0.2805528131	0.1080366302
0.9984451821	-0.088086687	-0.9497642568	0.1819124809	-0.3421609181	0.1168137551
0.9984451821	0.008625365622	-0.9330649265	0.1397362283	-0.2785413329	0.1111821527
0.9984451821	-0.1160054975	-0.9221829601	0.1656957891	-0.3550334253	0.1166511237
0.9984451821	-0.2054064271	-0.9231276484	0.2544243862	-0.3698127534	0.09958157712
0.9984451821	-0.05965609161	-0.9343144674	0.1393475668	-0.2953403973	0.1054396046
0.9984451821	0.1005631859	-0.8964754835	0.1381873078	-0.3543278578	0.1091657828
0.9984451821	-0.00884330546	-0.9379859761	0.1395663395	-0.272626279	0.1004570599
0.9984451821	-0.08668278282	-0.9495645716	0.161712121	-0.3070431353	0.1153438403
0.9984451821	0.08622958596	-0.9125105107	0.143591263	-0.2952981933	0.1041137131
0.9984451821	-0.06911164487	-0.9454989684	0.1048250796	-0.2302414642	0.1046171796
0.9984451821	-0.1461713589	-0.9562675368	0.0605723084	-0.305038021	0.1061376317
0.9984451821	-0.1649787327	-0.9002496942	0.1805125033	-0.3199375742	0.1231751686

0.9984451821	0.01518568075	-0.9286271335	0.1481158545	-0.2895690699	0.1064227846
0.9984451821	0.1646544224	-0.8661094007	0.1289618674	-0.3842403427	0.1132111577
0.9984451821	0.1329791728	-0.9179295764	0.1825845348	-0.1979142805	0.1050198104
0.9984451821	0.09158394349	-0.9144034896	0.1460412714	-0.3009867858	0.101341467
0.9984451821	0.06364040571	-0.9523708239	0.155033412	-0.251093022	0.1081457646
0.9984451821	-0.03489339617	-0.9395399175	0.1199319079	-0.2493741583	0.1039409494
0.9984451821	0.09378681774	-0.9400723999	0.1738412285	-0.3290399054	0.1098409807
0.9984451821	0.02020184653	-0.9296272008	0.1485976745	-0.295637246	0.1071355909
0.9984451821	-0.04463921248	-0.9181423785	0.153149878	-0.3036271485	0.1039323758
0.9984451821	-0.001063656766	-0.9364474727	0.1389235562	-0.2770669375	0.09967902538
0.9984451821	0.07010159819	-0.904900056	0.154572296	-0.2973940211	0.1102752191
0.9984451821	0.02544745831	-0.9204214818	0.1491684969	-0.2875568444	0.1276230521
0.9984451821	-0.06169455553	-0.9311616713	0.1717086406	-0.2829632523	0.1114034441
0.9984451821	-0.01558063513	-0.9382025138	0.1398936916	-0.2776424521	0.1032650921
0.9984451821	0.06940117236	-0.9127724988	0.1544188163	-0.2956639927	0.1071647942
0.9984451821	0.08327606084	-0.9781982071	0.1195283499	-0.2143661123	0.11108999
0.9984451821	0.1067873888	-0.974938527	0.0747610538	-0.1729698034	0.10372313
0.9984451821	0.04469915076	-0.955306958	0.126757868	-0.2797015014	0.1047919152
0.9984451821	-0.05488574477	-0.9299484577	0.1642440549	-0.3146119314	0.1021590633
0.9984451821	0.1138580745	-0.9066530097	0.0968092427	-0.1709989264	0.1092712141
0.9984451821	0.002434767639	-0.9360037822	0.1391349807	-0.2781977048	0.105198036
0.9984451821	-0.1573345456	-0.952938207	0.0753412134	-0.2459107077	0.1092438158
0.9984451821	0.1453801347	-0.8864130997	0.234757621	-0.4237367404	0.1037247773
0.9984451821	-0.1131477879	-0.9207171445	0.1830052871	-0.28305938	0.103278204
0.9984451821	-0.08610928785	-0.9335384283	0.1270655661	-0.2789886243	0.1110099115
0.9984451821	0.1224125794	-1.029972269	0.0962850920	-0.2140968854	0.1043064718
0.9984451821	-0.05594628536	-0.9241544551	0.1395623403	-0.2652948317	0.1033196724
0.9984451821	0.05847514682	-0.9404915066	0.1430107871	-0.2754608157	0.1067862627
0.9984451821	-0.007608496115	-0.9382771451	0.1363152311	-0.2780519017	0.1014800016
0.9984451821	-0.07129962487	-0.971385501	0.1033928275	-0.2359471007	0.104345258
0.9984451821	0.03891115898	-0.9540995631	0.1333369417	-0.2629523966	0.1036508733
0.9984451821	-0.143343817	-0.9925108433	0.1087989214	-0.2467171496	0.1114655853
0.9984451821	0.003812594269	-0.9343461636	0.1390485081	-0.2774130143	0.1078098413
0.9984451821	-0.006883482172	-0.9349312094	0.1335927281	-0.2719353228	0.1128986806
0.9984451821	-0.06230260793	-0.9095609735	0.1358078383	-0.2903940443	0.1096538796
0.9984451821	-0.1340850026	-0.9162064858	0.1571874334	-0.3003924183	0.09905456774
0.9984451821	-0.2146791752	-0.9223759013	0.1200133147	-0.2334183373	0.1080459981
0.9984451821	-0.0897284591	-0.9416513267	0.1180005267	-0.2796998465	0.1244599935
0.9984451821	0.0184356643	-0.9451300744	0.1364185431	-0.277135493	0.1107890555
0.9984451821	0.09638066895	-0.9827706551	0.1121562889	-0.2684750688	0.09999106889
0.9984451821	0.03349900122	-0.9374068235	0.1239940832	-0.2628804651	0.1115013494
0.9984451821	0.07280521742	-0.9386661601	0.0676849783	-0.2379036098	0.1072636895
0.9984451821	0.07142508008	-0.9333955671	0.1399230762	-0.2726214734	0.1122850319
0.9984451821	-0.01304034166	-0.9345091071	0.1376401412	-0.283025673	0.1063549187
0.9984451821	0.1212826713	-0.9587905832	0.1269655149	-0.281731502	0.1079113178
0.9984451821	-0.09328662333	-0.9217703461	0.1615569275	-0.2609539852	0.1182294976

0.9984451821	-0.1588410966	-0.9507996575	0.1068744665	-0.1527707147	0.1106655077
0.9984451821	-0.03177829723	-0.94933244	0.1287589917	-0.2696771848	0.1045962001
0.9984451821	-0.01332695751	-0.9395356802	0.138340089	-0.2750084252	0.1009226288
0.9984451821	-0.01701566743	-0.9322813284	0.1467250363	-0.2910488258	0.1217357525
0.9984451821	-0.1803420231	-0.9612632695	0.1150860325	-0.3223666746	0.09902209672
0.9984451821	0.02650087059	-0.9263673151	0.1361808103	-0.2856980892	0.1164562043
0.9984451821	0.02460129562	-0.9282413121	0.1496746678	-0.2762781514	0.1240391585
0.9984451821	-0.05724248983	-0.9041504912	0.1209463176	-0.2620567722	0.108993709
0.9984451821	-0.04338576221	-0.9533741347	0.1157606977	-0.2414889548	0.1170633959
0.9984451821	0.1486669431	-0.9462079307	0.1154696908	-0.2611073686	0.09992558082
0.9984451821	-0.03162960222	-0.9219427904	0.1525965164	-0.3097749306	0.1161928508
0.9984451821	-0.09443219443	-0.9551848537	0.1292448343	-0.2611091175	0.1472301786
0.9984451821	0.04635456317	-0.9597098343	0.1461472795	-0.2708664699	0.1040823555
0.9984451821	0.01959925562	-0.9401500988	0.137177135	-0.2724690297	0.09863658188
0.9984451821	-0.02210823291	-0.9495828663	0.1304445918	-0.2583994378	0.114345673
0.9984451821	-0.1199966104	-0.9033470624	0.1281322267	-0.3001690347	0.1052735579
0.9984451821	-0.004330226247	-0.937355768	0.1353648168	-0.2718276738	0.1100077002
0.9984451821	0.01032527941	-0.9360882689	0.1368771632	-0.2726441934	0.1059551844
0.9984451821	0.007068843606	-0.9366307274	0.1376581622	-0.2741495818	0.1262868679
0.9984451821	0.1588030021	-1.066154592	0.06664698851	-0.2688834679	0.1211236809
0.9984451821	-0.1975268101	-0.9453046268	0.1131499894	-0.2446867082	0.1084858866
0.9984451821	0.09767494472	-0.9438588877	0.136117971	-0.2047930647	0.1054647071
0.9984451821	-0.07881692912	-0.8699690248	0.1764674863	-0.3220959338	0.1163305407
0.9984451821	-0.08088792296	-0.9297922945	0.1713694457	-0.3142943971	0.1031258617
0.9984451821	0.03583656757	-0.9348750043	0.1336938852	-0.2816894638	0.1001721338
0.9984451821	-0.004981976777	-0.9374341698	0.1356881932	-0.2743584426	0.104720873
0.9984451821	-0.02816590084	-0.9319604453	0.1358059297	-0.2666589477	0.1030254557
0.9984451821	-0.07935677231	-0.9591611677	0.1474499673	-0.264061031	0.1001281571
0.8210512929	-0.05861490428	0.5799123017	-0.4886058046	0.4620084624	0.09982493008
0.8072442617	0.08101468549	0.6039003884	-0.4676503397	0.4382282093	0.1035970092
0.9888273476	-0.003251149668	0.5836476368	-0.4809821234	0.4707784297	0.1017022785
0.9075027994	-0.02399696094	0.5884146779	-0.4742698959	0.4586695784	0.1028607321
0.4719758931	0.2355316878	0.5748598798	-0.5465701024	0.5022320594	0.09881113929
0.6628678528	-0.1438797548	0.5311662463	-0.5136107606	0.5700746217	0.1108341129
0.9075027994	-0.02280742449	0.5868821122	-0.485599823	0.4730393107	0.0982954054
0.6628678528	0.1509897178	0.5327591855	-0.4687203569	0.4352588096	0.09940465042
0.811937764	0.07122290069	0.5498038548	-0.4921149031	0.49439165	0.1014091877
0.8210512929	0.06174020775	0.5800981525	-0.5024188487	0.5017313285	0.1071408766
0.6832684063	-0.1181598093	0.5336634382	-0.4997970287	0.5061234264	0.09734434948
0.7821819156	0.1190886032	0.6014272401	-0.4927618312	0.4095341113	0.1135362604
0.9075027994	-0.02492521402	0.5872479654	-0.4878020552	0.4760570141	0.100459508
0.6628678528	0.142750752	0.5773179213	-0.4480501821	0.4698031853	0.09850234349
0.5542917235	0.1989268353	0.6008995797	-0.4561194048	0.5346511972	0.09505452992
0.8072442617	0.07903332379	0.6102243655	-0.45996754	0.4698159099	0.0987629461
0.8072442617	0.1008199269	0.575927947	-0.4482176334	0.479688903	0.1050527573
0.8072442617	0.09142411927	0.5901902478	-0.4689807395	0.4421936563	0.09847882

0.8978026233	-0.03949606293	0.6034667346	-0.4983453582	0.4943801568	0.1065096223
0.811937764	0.07908457833	0.5312967256	-0.5178078539	0.4676950771	0.1103196779
0.6628678528	0.2174753154	0.6381553016	-0.4932225142	0.4547238322	0.1535104537
0.8210512929	-0.0673678931	0.5880249668	-0.4814507673	0.4447547404	0.1159521955
0.6628678528	0.1320821262	0.6741369938	-0.4529691918	0.4159250704	0.1030840556
0.9426516941	-0.0125219992	0.5877814901	-0.4747904957	0.4651369121	0.1012605372
0.6628678528	0.1830073923	0.4314470191	-0.5906605278	0.6125525777	0.1056587033
0.9075027994	-0.03258334311	0.595974438	-0.469061075	0.4607826067	0.1231161332
0.6628678528	-0.1512869412	0.6311851118	-0.4704546889	0.4211967444	0.09881506193
0.6978634991	0.1161286406	0.5356179477	-0.5141793099	0.4940236008	0.1000687264
0.9403227783	0.01618126375	0.5965669839	-0.4721109767	0.4562045972	0.1066846043
0.6628678528	0.1349231198	0.6685322507	-0.4305779243	0.4119171378	0.1004590293
0.8072442617	0.1487186887	0.6213698934	-0.4511218408	0.4594228401	0.1835676679
0.5542917235	0.3754698486	0.5697571329	-0.5057219398	0.5227832146	0.1683297384
0.811937764	-0.0774855406	0.6122194854	-0.4680721957	0.4475510595	0.1082454406
0.9003371864	-0.03896973178	0.5977090846	-0.4698559002	0.4441560017	0.1174835925
0.8576310788	-0.05032141553	0.5904713733	-0.473052226	0.4329445271	0.108326081
0.7821819156	0.1538074617	0.6579404906	-0.4930378349	0.465890106	0.1409751455
0.8344788873	-0.05469324974	0.5919714549	-0.4908384038	0.4595509172	0.1095478487
0.8820863362	-0.0426619564	0.5922016211	-0.4929615802	0.4814097242	0.1045672586
0.8344788873	0.05698482827	0.5775598197	-0.4557360918	0.4674161699	0.107371706
0.811937764	0.08454561101	0.6014922622	-0.5125568323	0.5233831348	0.1181078132
0.6628678528	0.1658820951	0.6523542275	-0.4683545333	0.4286958702	0.1098563348
0.8210512929	0.07321434614	0.5773801101	-0.5005581462	0.5193080168	0.1204881146
0.6628678528	0.14314897	0.5716464482	-0.5629688355	0.5423998288	0.09988154943
0.9075027994	-0.03273925249	0.5864985891	-0.478902763	0.4583156422	0.104946415
0.8072442617	-0.09105479999	0.5514340805	-0.4739636027	0.5339560213	0.1096615389
0.6628678528	-0.1296108582	0.5602973881	-0.4770763641	0.5317211468	0.09730031069
0.8978026233	0.04682050716	0.5908091321	-0.496616415	0.4905465506	0.1176800026
0.6628678528	-0.1854002525	0.5373155177	-0.4942952535	0.5056722884	0.1014176063
0.8820863362	0.04301546165	0.5920216849	-0.4583305058	0.4385454561	0.103833139
0.9075027994	0.02375090364	0.588679988	-0.4687421663	0.4741611565	0.1060486307
0.8072442617	0.1100210766	0.5602907384	-0.508124607	0.5022435679	0.1289236237
0.8072442617	-0.08542849973	0.5406415494	-0.5347321619	0.5459008086	0.1045506456
0.8072442617	0.107650087	0.6292551144	-0.4958123655	0.4061690998	0.1163552031
0.6628678528	-0.1388272664	0.5638721431	-0.5235364907	0.3894017562	0.1031724932
0.8820863362	0.04259616103	0.5945244004	-0.4792390205	0.4608009267	0.100176471
0.8072442617	-0.09001117839	0.6040389427	-0.5008119101	0.4385891263	0.1075277171
0.6628678528	-0.1333371977	0.5679895482	-0.5658564874	0.5867780162	0.1013293321
0.8210512929	-0.06972839014	0.5877383538	-0.5125559288	0.5123557292	0.1107388134
0.8194923277	0.07247245023	0.6118807573	-0.4431502453	0.3965323607	0.10607835
0.8210512929	0.05934420663	0.5644975287	-0.4951492772	0.4982213076	0.102887426
0.6628678528	0.1242203891	0.6184850502	-0.4830939633	0.4850259471	0.09588536645
0.9075027994	0.02619434667	0.5967002205	-0.4744313897	0.4614919339	0.1110897226
0.8072442617	0.09925834877	0.6483947478	-0.4396254612	0.4228528691	0.1320740573
0.8072442617	0.1070854212	0.5717187281	-0.5365801736	0.4864685908	0.1111810334

0.7389307852	0.112593333	0.5950143946	-0.4894858927	0.4835850967	0.1007818665
0.8072442617	-0.1055538062	0.5455219195	-0.5030152554	0.5030648972	0.1064996818
0.9426516941	0.0141353918	0.5775867544	-0.4838613546	0.4810751723	0.1116678673
0.8210512929	0.05905926737	0.5636275986	-0.5191972539	0.5295200722	0.1026733224
0.5955613183	-0.2022989023	0.6637548872	-0.4366866896	0.4987852608	0.09974178824
0.8344788873	0.05186914792	0.5789518924	-0.5044826395	0.5061169162	0.1003648059
0.4719758931	-0.2468409757	0.5239176597	-0.3939701521	0.2389428046	0.103112895
0.9528980239	-0.009202587627	0.5846213515	-0.4811838278	0.47411719	0.1043169182
0.6628678528	-0.1501023199	0.5691836949	-0.5436081778	0.497823885	0.1097988166
0.6832684063	-0.1253175591	0.5408674859	-0.5670307232	0.6008665096	0.1025441206
0.4719758931	0.2296440822	0.5545675473	-0.5708636763	0.48405877	0.09859217041
0.9075027994	-0.03115493961	0.5861057307	-0.4833134458	0.4680667453	0.1124294751
0.8210512929	-0.06378047466	0.6326927577	-0.4585784964	0.4395459373	0.1035438952
0.6628678528	-0.1790529053	0.6259763055	-0.4762757832	0.500915103	0.1000821916
0.8344788873	-0.05462028217	0.5875726524	-0.4901115153	0.4724249785	0.106985897
0.8072442617	-0.09207251245	0.560542735	-0.5108519577	0.4539658071	0.09851618018
0.6628678528	-0.1631608291	0.5086508839	-0.560269045	0.5567169409	0.1018764453
0.9075027994	-0.02149290849	0.5946664815	-0.4776648793	0.4627332198	0.1023837539
0.8210512929	-0.06964621223	0.5591982111	-0.4955560091	0.4814948596	0.1132741163
0.6628678528	-0.1799460853	0.501645245	-0.4827192491	0.4866892461	0.105106081
0.6978634991	0.1286821468	0.5599247765	-0.386366824	0.3812069231	0.1108669479
0.8210512929	0.0682812245	0.5532648231	-0.4787699901	0.488960777	0.1102345942
0.6628678528	0.1641561867	0.5605053171	-0.4995204357	0.4954510971	0.09655359406
0.9888273476	-0.00269679101	0.5853060173	-0.4806902457	0.4701378597	0.1121662176
0.6628678528	0.1662755294	0.598825984	-0.4283296416	0.4644527372	0.1298486142
0.8072442617	-0.0889436267	0.628522343	-0.466601953	0.4680872734	0.1103914779
0.9338493859	-0.01652659387	0.593175995	-0.4755589599	0.4679777958	0.09833438877
0.9075027994	-0.026995723	0.5867244593	-0.4678440507	0.4574364744	0.1121981076
0.8122584867	-0.07311521644	0.590025303	-0.4059784655	0.425851929	0.1055069188
0.8021266674	-0.1150649318	0.5815062614	-0.4868959374	0.4664179903	0.11339095
0.694761936	0.1231497293	0.5679832459	-0.4631003121	0.5274633387	0.1040101825
0.4719758931	-0.3080227448	0.6549042464	-0.4246221975	0.4518504794	0.1035838111
0.9075027994	0.03367967646	0.579714747	-0.488570937	0.464620791	0.121188365
0.8344788873	-0.05838964801	0.580414319	-0.4943092351	0.5140252474	0.112616122
0.8072442617	-0.07818182571	0.5536618424	-0.5063544914	0.4863620642	0.1028053093
0.6628678528	-0.1387113291	0.5544013811	-0.4851336703	0.4831039649	0.09768925393
0.8072442617	-0.111670727	0.613965362	-0.4282709701	0.3706260482	0.1236204434
0.6628678528	0.1553782336	0.6028289584	-0.4608793982	0.5140154424	0.09795178356
0.4719758931	0.2868399863	0.6896805252	-0.5271791124	0.3871621608	0.1127223472
0.5207540292	0.278288939	0.6751931409	-0.3757836693	0.4803753411	0.1227810736
0.6628678528	0.1502957701	0.5018721762	-0.4336585188	0.4268980147	0.1066545116
0.8072442617	0.1034916724	0.6252369067	-0.4179635991	0.3827165216	0.1191015006
0.4719758931	-0.2483873231	0.5955884351	-0.4503226811	0.4562322694	0.09443583275
0.8072442617	0.1011282054	0.5379903317	-0.531112471	0.5800275969	0.1176298084
0.9003371864	0.0754818601	0.6034003632	-0.460795889	0.4476392721	0.1733415511
0.6628678528	0.1515307298	0.5117335359	-0.4482895431	0.4797411292	0.101308444

0.6628678528	0.1769052032	0.5545980479	-0.4961122736	0.5017320976	0.09415879921
0.9075027994	0.02464364947	0.6001214198	-0.4690590777	0.4478237021	0.1155055979
0.9477576316	-0.01098713159	0.5879466122	-0.4810071354	0.4676048384	0.1055960363
0.9426516941	0.01310091824	0.5886293257	-0.469120106	0.4539462456	0.1091580901
0.9003371864	-0.0363013333	0.5854412137	-0.4710230103	0.4525159149	0.1053082145
0.6628678528	0.1775710026	0.5804954392	-0.4996092142	0.5227958104	0.1296706297
0.9974164499	-0.000402724322	0.5854543977	-0.4802341152	0.4695949982	0.1229269222
0.6628678528	0.1701259452	0.5913910045	-0.4521457957	0.4441642543	0.1103506394
0.9003371864	0.03486647894	0.5829608765	-0.4817364961	0.4946124228	0.1049054834
0.6628678528	0.1562628613	0.4549479671	-0.5617376448	0.5577845653	0.1163134812
0.8072442617	0.07664616439	0.5753285063	-0.5152873264	0.512097207	0.1022876189
0.8344788873	-0.04875591793	0.5832398876	-0.4712280047	0.4751944744	0.09830909128
0.8072442617	0.07986021053	0.6090631496	-0.4275226655	0.4230578117	0.1031909181
0.6628678528	-0.1277041516	0.6072570539	-0.4877904566	0.5084344299	0.1006706539
0.8072442617	-0.08755466166	0.5632059963	-0.4686344399	0.4771212026	0.09880184618
0.8677764199	-0.10749473	-0.5718154454	0.4728064328	-0.7780620408	0.1073057841
0.9949208784	0.04957521283	-0.5457177013	0.4969355139	-0.7924472426	0.1107293624
0.804159583	-0.1784587885	-0.6321783173	0.4726333415	-0.7230567695	0.1074739
0.9949208784	0.03999834017	-0.561650036	0.4814636024	-0.7562867732	0.1099218286
0.804159583	-0.175052581	-0.5543936899	0.527105468	-0.7854928619	0.108570566
0.804159583	0.1548537885	-0.4941934197	0.5363537663	-0.8925377551	0.1146223316
0.9949208784	0.007617889605	-0.5582364224	0.4926352703	-0.7743543096	0.1071980031
0.9949208784	0.04365721251	-0.5751165291	0.494608217	-0.7798390039	0.1094666885
0.9949208784	0.005521280813	-0.5604803117	0.4898626134	-0.7708752006	0.1096069581
0.9949208784	0.03659411844	-0.5588233293	0.4817813048	-0.7584812941	0.1124023464
0.9949208784	0.02888916835	-0.5444651963	0.4947315047	-0.7828590722	0.1071817386
0.7720369792	-0.21143013	-0.5949918854	0.5131746817	-0.6512483174	0.116304297
0.9949208784	-0.0224928846	-0.5551388854	0.4832083635	-0.7674881982	0.1087220082
0.804159583	-0.1200955099	-0.554444645	0.4595095855	-0.7664529962	0.1072521606
0.9364241781	-0.09684978107	-0.5636013273	0.4820978628	-0.8090521179	0.1069858532
0.804159583	-0.1438714885	-0.6048961637	0.4488786068	-0.7703211535	0.1068444174
0.8677764199	-0.1127710306	-0.5559187013	0.4466896228	-0.7695331813	0.111371256
0.9949208784	0.06136013823	-0.5548604731	0.4998635638	-0.7900758134	0.1069577018
0.804159583	0.1312090175	-0.6203569651	0.5537405442	-0.8550140939	0.1152184994
0.9949208784	-0.003139993328	-0.5558497821	0.4920940621	-0.7724134439	0.1184518951
0.9949208784	0.07533842203	-0.5254856659	0.5071519312	-0.8045554996	0.1294141585
0.804159583	0.1800922557	-0.5735223719	0.5024481297	-0.7043867621	0.1162977329
0.804159583	-0.1730621459	-0.6768842254	0.4662840134	-0.7031603103	0.1099522864
0.7720369792	0.1991478434	-0.6007093552	0.4116927169	-0.7006553823	0.10585611
0.9222849546	0.1115990691	-0.6466283659	0.424023693	-0.6935357243	0.1153385985
0.7720369792	-0.2402769767	-0.4837915093	0.5815622441	-0.8386261772	0.11666301
0.9949208784	-0.05202400088	-0.5439750758	0.4962664378	-0.7869438162	0.1084312424
0.9949208784	0.04173616047	-0.5739049158	0.4816370478	-0.7674859062	0.1091659267
0.9949208784	-0.05470037797	-0.5943266086	0.4604618215	-0.7289904379	0.1137785916
0.9949208784	0.01707940432	-0.5483539444	0.4969744415	-0.7783669542	0.1097512464
0.9949208784	-0.02688436401	-0.566218087	0.4859542785	-0.7701372193	0.1455641702

0.804159583	-0.1694750468	-0.5417236412	0.5302169961	-0.8361277727	0.1311083091
0.804159583	0.1407290966	-0.6109122797	0.480495921	-0.7355998335	0.1128666054
0.9949208784	-0.06912924233	-0.5325274213	0.5184583838	-0.8272697248	0.1198279905
0.9949208784	0.006489545572	-0.5585823745	0.4895803411	-0.7680224129	0.1147066292
0.9949208784	0.1143660899	-0.4986855426	0.471920175	-0.7712511799	0.1270650158
0.9949208784	-0.001147466391	-0.557684455	0.490347423	-0.7728581023	0.1145416347
0.9949208784	-0.0234671172	-0.553440907	0.483358258	-0.7663347041	0.1117966094
0.9949208784	-0.04100547425	-0.5520053172	0.4729496489	-0.7717500193	0.1140348425
0.9949208784	-0.05358133412	-0.5638005749	0.5171556261	-0.8163825347	0.1219761646
0.9949208784	-0.08113007217	-0.5885259814	0.4775147519	-0.751258958	0.1152744659
0.7720369792	-0.21536702	-0.5392940614	0.5373678987	-0.900958968	0.1172295139
0.7720369792	-0.2475179926	-0.5415702714	0.6425704178	-0.8908580204	0.1048961436
0.9949208784	0.04906265753	-0.5582309509	0.4879030464	-0.758844172	0.1114609682
0.9949208784	0.07683549082	-0.5252292361	0.4835701377	-0.8330626212	0.1143980001
0.9949208784	0.0387524993	-0.5509144342	0.486464596	-0.7892232199	0.1072594318
0.8677764199	0.1300166542	-0.5353833489	0.4532182234	-0.7343312688	0.1151019256
0.804159583	0.1343802021	-0.5223830378	0.4831395069	-0.7927003181	0.1090616505
0.9949208784	0.01512481282	-0.5558935416	0.4974505371	-0.7825616774	0.111049738
0.804159583	-0.1368724063	-0.5817067684	0.417660692	-0.7900090594	0.1126015759
0.7720369792	-0.3029229265	-0.4919090292	0.5773916812	-0.8522696518	0.123634323
0.8677764199	0.1183594953	-0.4987740187	0.565086779	-0.872276783	0.1116137994
0.7720369792	0.2708966256	-0.4401736205	0.4697325778	-0.9540419166	0.1113657105
0.9949208784	0.07492780522	-0.5465030733	0.5190650522	-0.7318885383	0.1131543868
0.9949208784	0.04533141921	-0.5465357256	0.4935518759	-0.7856186213	0.1081835439
0.9949208784	0.009261737616	-0.5599013579	0.4934814365	-0.7697179866	0.1138444788
0.9949208784	0.01478942595	-0.556926476	0.4982444262	-0.7833233453	0.1102046426
0.9949208784	0.06581231482	-0.5626392194	0.5152518742	-0.8048253287	0.1151228832
0.9949208784	-0.07782582809	-0.5816836982	0.4499518602	-0.700523995	0.1131259179
0.9949208784	-0.06180528489	-0.5309969489	0.5090485804	-0.8119853773	0.1100221808
0.9949208784	0.0006820376366	-0.5576446585	0.4906179211	-0.7726275375	0.1067008877
0.9949208784	0.005115377357	-0.5554334335	0.4918503484	-0.774298788	0.1156255229
0.9949208784	-0.04284765039	-0.5860526116	0.4742139821	-0.7530653227	0.1285505997
0.9949208784	-0.09653685401	-0.549110319	0.5400453887	-0.7844436519	0.1160854843
0.804159583	-0.1247720985	-0.5737688742	0.5034911599	-0.7811589159	0.1081793773
0.9949208784	0.02344332417	-0.5495877121	0.4949283403	-0.7796894731	0.1128523889
0.9949208784	-0.007813335882	-0.5537789932	0.492302073	-0.7786806326	0.1169294345
0.9949208784	-0.02568054794	-0.548826086	0.5068068467	-0.7970985455	0.1108684712
0.9949208784	0.02148559405	-0.5673016945	0.4842451	-0.773486718	0.1116273794
0.804159583	-0.1756207858	-0.5377642089	0.5787737277	-0.8956735147	0.1065458547
0.804159583	0.1409767951	-0.521284015	0.4394294399	-0.6409625259	0.1161298869
0.9949208784	0.002845556923	-0.5577414608	0.4907546272	-0.7739876516	0.1111621586
0.804159583	-0.1726795036	-0.57568123	0.4230763048	-0.7382714523	0.1140764827
0.804159583	0.1348073614	-0.5120773516	0.5763448243	-0.9060650839	0.1105781253
0.804159583	-0.1277340485	-0.5373129812	0.5370494913	-0.7818874235	0.1095425511
0.9949208784	-0.01617719512	-0.5577650555	0.4893978223	-0.7725993448	0.1160970859
0.7720369792	0.1994680345	-0.7101688284	0.4180110028	-0.6691741362	0.1094942348

0.9949208784	0.0138163659	-0.5606513559	0.4897001531	-0.7756413679	0.1100690935
0.9949208784	0.07277192984	-0.5679202446	0.4950093682	-0.7631229181	0.1119189666
0.9949208784	-0.01381194307	-0.5618187717	0.4857180183	-0.7743994415	0.1087283001
0.9949208784	0.03130654265	-0.5418952456	0.5043606913	-0.7905193743	0.1112773266
0.9949208784	0.07286334247	-0.5921212717	0.4816112176	-0.7455973018	0.1097076529
0.9949208784	-0.03472378075	-0.5712484393	0.4862595986	-0.7664858571	0.1174219754
0.9949208784	0.004355342554	-0.5556798994	0.4903698247	-0.7732308888	0.1135396426
0.9949208784	0.03080073933	-0.5629415671	0.5165865225	-0.7964627172	0.1192428808
0.9949208784	-0.001275839056	-0.5573078314	0.4904478668	-0.7729152914	0.1148514256
0.9949208784	-0.07094766438	-0.5466197877	0.4976299239	-0.7861067596	0.1067916629
0.9949208784	-0.06706191	-0.5519709745	0.4787318839	-0.7590339356	0.115450483
0.804159583	-0.1487984158	-0.5570372712	0.4497117845	-0.7911691922	0.1194817952
0.9949208784	0.01540847409	-0.5655005166	0.4877064437	-0.7725297838	0.1162753503
0.7720369792	0.2425511748	-0.6753894375	0.4247420294	-0.7513543143	0.1035812683
0.9949208784	0.03461638984	-0.5581057673	0.473588858	-0.7588473245	0.1161499675
0.804159583	0.1321025013	-0.5600594924	0.357863971	-0.7032009368	0.1136899022
0.804159583	0.1670927882	-0.5523253715	0.4950007232	-0.7581965704	0.1152481425
0.9949208784	-0.02938901493	-0.5532034032	0.4877307079	-0.7880800369	0.1126491897
0.7720369792	0.2278892371	-0.5925027521	0.4642827931	-0.7927333595	0.1088921379
0.9949208784	-0.09193122563	-0.5417307507	0.505618734	-0.7562086926	0.120928626
0.9949208784	-0.08942135295	-0.5619823619	0.471807004	-0.7076924352	0.1168164996
0.9949208784	-0.03694063159	-0.5734645553	0.4796925282	-0.7641069413	0.1109570177
0.9949208784	-0.005764284697	-0.5593978586	0.4901958718	-0.7716747844	0.1075115309
0.9949208784	0.09784417161	-0.579819518	0.4352016518	-0.6885921318	0.121546213
0.804159583	-0.1754844793	-0.5844488446	0.4663732337	-0.8148741632	0.1061300219
0.9117401219	0.1138487192	-0.5168200248	0.4826733096	-0.8110855649	0.119557265
0.804159583	0.1471767873	-0.5073959962	0.5504898408	-0.7713678421	0.1244203224
0.7720369792	-0.2197559038	-0.4421396471	0.4121309014	-0.7050075204	0.1123005775
0.804159583	-0.1407750242	-0.6108002692	0.4101949201	-0.6634869864	0.1179284746
0.9949208784	0.0750734143	-0.5638453007	0.4832239595	-0.7650971242	0.1078867646
0.9949208784	-0.07169383828	-0.5274280451	0.5164324851	-0.8419826412	0.1193213558
0.7720369792	-0.2344239342	-0.5850026432	0.472919568	-0.768930499	0.1167776319
0.9949208784	-0.04356953516	-0.534428546	0.4844908029	-0.7810036002	0.110750466
0.9949208784	0.009264233832	-0.5598141927	0.4899106253	-0.7705345916	0.1056017071
0.804159583	-0.1619164031	-0.6536286834	0.4246793713	-0.6434503417	0.1162360253
0.9949208784	-0.05092258819	-0.545689218	0.4853498794	-0.7790123809	0.1116817503
0.804159583	-0.1375198984	-0.5967461108	0.3756502919	-0.6100454293	0.1149288033
0.9949208784	-0.01547929888	-0.5584762379	0.4946929889	-0.7785228637	0.1117344079
0.9949208784	0.0468217943	-0.5632986649	0.4877998464	-0.7522444291	0.1293008596
0.804159583	0.1754371135	-0.7026251143	0.424010041	-0.76761113	0.1274771184
0.804159583	-0.1424356881	-0.5579945724	0.459133942	-0.7531302602	0.1132582608
0.9949208784	0.05677458931	-0.5628096688	0.4910467239	-0.7308977319	0.1124192166
0.804159583	-0.1489362697	-0.4392545895	0.5535544765	-0.8457881173	0.1190554956
0.9949208784	-0.01042221318	-0.5573403936	0.4947644137	-0.7770683735	0.1096794607
0.7720369792	0.2094970418	-0.5490908919	0.4628673551	-0.8051621571	0.1037058616
0.9949208784	0.07584617485	-0.5395118444	0.5421493575	-0.8120147314	0.1104579081

0.9949208784	0.05190748147	-0.5637786353	0.4955538058	-0.7944667373	0.1095917466
0.9949208784	-0.07882627338	-0.5819447429	0.5009587142	-0.75856604	0.1067240184
0.8886403667	-0.135294183	1.015254398	0.1769695848	-0.9678863909	0.145482559
0.8886403667	-0.06271159722	1.005197719	0.2317137512	-0.9640794046	0.1421511035
0.8886403667	0.08201883058	1.051331066	0.2998129067	-1.05523865	0.170035162
0.9824246933	-0.01085485698	1.01717441	0.257173985	-0.9945967516	0.1507989003
0.8886403667	0.2780610459	0.963988131	0.1768897216	-0.9608681144	0.1366809124
0.8886403667	-0.1878437925	0.8751522265	0.1572015989	-0.7687504509	0.1708218534
0.8886403667	-0.1013430959	1.020398652	0.1688772906	-0.9393297999	0.1632111246
0.9894417916	-0.003329172291	1.016423004	0.2489478045	-0.9860203428	0.1437742597
0.9824246933	0.007151425249	1.012957634	0.2492771876	-0.9873369103	0.1384025016
0.8886403667	0.1172432812	1.031720716	0.2377358272	-1.001097256	0.1587575314
0.8886403667	-0.2056808883	0.9295266667	0.2573355986	-1.034865383	0.1404199306
0.9824246933	0.03691866958	1.026444272	0.2589177198	-1.017936691	0.1484210634
0.8886403667	0.1003551393	1.022762969	0.2898705062	-1.008817958	0.144528787
0.8886403667	0.07274704688	1.023986727	0.3135175931	-1.06466766	0.1506159658
0.8888946878	0.05734486266	1.023088633	0.2625161917	-0.9504578241	0.1368612558
0.8886403667	0.1326483792	1.074380043	0.3366844994	-1.064239649	0.1519914648
0.8886403667	0.284608699	1.04529923	0.3569552362	-1.050715289	0.159099537
0.8886403667	0.1184344209	1.034603306	0.3049968452	-1.092084339	0.1425218229
0.8886403667	0.08468421382	0.9706955512	0.3071449271	-1.043081299	0.1891381983
0.8886403667	-0.2670277655	1.187891955	0.2957959337	-0.7909363046	0.1566338675
0.8886403667	0.1160638261	1.072570697	0.2685644954	-1.054844457	0.1810649693
0.8886403667	0.1235729294	1.00566264	0.2351039928	-0.8554629578	0.1498950923
0.8886403667	0.1171386908	1.07065967	0.240484282	-0.971345879	0.1463540866
0.8886403667	-0.07234043976	1.015058534	0.2734476799	-0.9695265784	0.1466977948
0.8886403667	0.1586884533	0.8139198341	0.06901174838	-0.7915668076	0.1539840831
0.8886403667	0.2674195702	0.9782598503	0.1366553722	-0.8718530201	0.14434294
0.8886403667	-0.06905156511	1.044020518	0.248666384	-0.9963540236	0.151895759
0.8886403667	-0.121232799	1.057588182	0.2658435719	-1.01013718	0.1665276656
0.8886403667	0.07892343358	1.088759506	0.2713698459	-1.056243153	0.1505170223
0.8886403667	-0.2315181608	0.8848025517	0.208652101	-0.9117233355	0.1443889899
0.8886403667	0.1479982937	1.056952744	0.1989022376	-0.9121742702	0.1439760169
0.8886403667	-0.08778160854	1.031313139	0.2967733171	-1.036589449	0.1501279189
0.8886403667	-0.1898841856	1.012186148	0.20902465	-0.9507027043	0.1425118394
0.8886403667	-0.2683032194	1.115415202	0.413521646	-1.207372334	0.1427121794
0.8886403667	0.2268009211	0.9775773871	0.1663716893	-0.7889278635	0.1412774651
0.9824246933	-0.03323196	0.9978455829	0.2640932299	-1.008658886	0.1539684269
0.8886403667	-0.1046901718	1.060794165	0.1977479658	-1.022635828	0.1589958611
0.8886403667	0.1010078015	0.9951180066	0.2915516538	-1.020171823	0.1456684299
0.8886403667	0.07812491255	1.005687822	0.2916507859	-0.9946784382	0.1497601165
0.9824246933	0.008827927856	1.014425015	0.2461612477	-0.9792920459	0.1548994134
0.8886403667	0.09199427991	1.036888257	0.2376963886	-0.9995376225	0.1403950219
0.9824246933	-0.02685470679	1.01496851	0.2610353789	-1.018925612	0.1431579047
0.8886403667	0.1451067718	0.9346689356	0.1529659532	-0.9118107035	0.1605486496
0.8886403667	-0.1459014274	1.015778665	0.2158275781	-1.007499116	0.1469821574

0.8886403667	0.1462559089	1.044654986	0.2030638294	-1.002371879	0.1466081714
0.8886403667	-0.203726362	1.02536181	0.2349371866	-0.880707308	0.1461486904
0.8886403667	0.08251082429	1.033478297	0.2457985948	-0.9434487696	0.1419698717
0.8886403667	-0.2081541162	1.015670563	0.3157475339	-1.065866179	0.1420482631
0.9824246933	0.03779698553	1.008585258	0.2647656369	-0.9880450609	0.1472656159
0.8886403667	-0.08696822981	0.9917772551	0.2218489669	-1.048145394	0.1392531078
0.9775092155	0.04440505842	1.014045688	0.2315509012	-0.9745036846	0.1517822278
0.8886403667	0.1085398618	1.037303979	0.3471345098	-1.13640705	0.1517298755
0.8886403667	-0.06330458806	1.01268153	0.2637903917	-0.9723797946	0.1475474524
0.8886403667	0.1075217381	1.05861809	0.3199975061	-0.9305316337	0.1549541909
0.8886403667	-0.08550622032	0.9999645962	0.256040862	-0.953024033	0.1487030737
0.9824246933	-0.01389101106	1.016065858	0.2503657801	-0.9992766475	0.1507414697
0.8886403667	-0.08205512788	1.021651752	0.2268663574	-0.9808940184	0.1492426136
0.9824246933	0.03387918195	1.024624803	0.2795701899	-1.022528504	0.2127548988
0.8886403667	-0.06619351325	1.007642683	0.2253073779	-0.9227836998	0.1519973489
0.9824246933	-0.01782552639	1.01659367	0.2491673479	-0.9904810676	0.1453315508
0.8886403667	-0.08514411875	1.001351461	0.253271229	-0.9917967414	0.1362158297
0.8886403667	-0.1531015507	0.9089501911	0.2279456583	-0.9273871844	0.1687381869
0.9824246933	-0.03718038548	0.9923608656	0.2357584087	-0.9703787873	0.1576975687
0.9824246933	0.01682659945	1.013118531	0.2448007624	-0.9971034806	0.1538776897
0.8886403667	0.08911134249	1.045564192	0.2962775078	-1.085213378	0.153282397
0.9124849918	0.05387590073	1.034076984	0.2800650641	-1.01581967	0.140813521
0.9824246933	-0.01435774013	1.018178101	0.2448874469	-0.9835200489	0.173816562
0.8886403667	0.1619534872	0.9835864458	0.1574616475	-0.8382110979	0.1476283178
0.8886403667	-0.1565875358	1.12822807	0.279448573	-1.025802012	0.1553960659
0.9775092155	-0.04002079668	1.022075128	0.2746234454	-1.006667984	0.1430921648
0.9898215044	0.001972977232	1.015939305	0.2486513065	-0.9851816311	0.1546558352
0.8886403667	0.06482682975	1.051358846	0.2723767476	-1.054276636	0.1502488052
0.8886403667	0.1313298986	1.036605139	0.2751382634	-0.969823808	0.1508072561
0.8886403667	0.1389376243	1.06792653	0.3723040147	-1.13469035	0.157805943
0.8886403667	-0.1575247939	1.023749529	0.2795017726	-0.9839611462	0.2309489934
0.8886403667	-0.09539040779	1.015540012	0.2303996588	-1.004476999	0.1464816506
0.8886403667	0.1604995379	0.9120740878	0.1745526769	-0.8772396172	0.1428589561
0.8886403667	-0.08788916091	1.023170116	0.1869251818	-0.9565607987	0.1529557519
0.8886403667	-0.07485423485	1.033074567	0.251477966	-1.016011248	0.1428520002
0.8886403667	-0.2697305303	0.8854132595	0.1156965403	-1.075637714	0.1365880208
0.8886403667	-0.2470403693	0.9159736692	0.1369929426	-1.014029095	0.1428975437
0.9824246933	-0.0231276118	1.028467661	0.2663542099	-0.9954126229	0.1546305533
0.9824246933	-0.02264712892	1.0120841	0.2413189139	-0.9947926425	0.1390299439
0.8886403667	-0.1308334394	0.9747460861	0.2340008736	-1.04252777	0.1492627189
0.8886403667	-0.1806466259	1.006555601	0.0356497596[illegible]	-0.769159915	0.1952350038
0.8886403667	0.1051922801	1.034627524	0.2828389892	-1.017665267	0.1792191017
0.8886403667	0.1823290689	0.9375599168	0.2183098815	-0.9428492531	0.1468334205
0.8886403667	0.07887204872	1.003696139	0.242601427	-0.9943479629	0.1634953325
0.8886403667	0.151669941	1.054919572	0.2561809753	-0.9894811312	0.1461581214
0.8190737159	-0.3784135434	1.103819493	0.1636669737	-0.8751712821	0.1326453019

0.8886403667	-0.07253233389	1.061849372	0.3009062859	-1.024988692	0.1485721715
0.9824246933	0.02543682465	1.005957736	0.2366534598	-0.9814066364	0.1584201135
0.8886403667	0.1214946683	1.003119808	0.1230588211	-0.9317740359	0.1681179487
0.8886403667	-0.1950569718	1.084297552	0.4476280926	-1.116749274	0.1585864594
0.8886403667	-0.06999238955	1.030782099	0.2418306043	-1.013679784	0.1356569133
0.8886403667	0.1444945139	0.9813658858	0.2458067766	-1.025079081	0.1407517676
0.8886403667	0.1183720649	1.024385248	0.1860685679	-1.025362327	0.1537266
0.9824246933	0.006157394664	1.014688559	0.2486658945	-0.9886687873	0.1475158908
0.9824246933	-0.03243938496	0.9999617006	0.2302693026	-0.9747066418	0.1420778402
0.9824246933	-0.005995387161	1.012391426	0.2472019117	-0.9829098373	0.1315002829
0.8886403667	-0.1035812012	1.07111957	0.2938191607	-1.115575811	0.1426794573
0.8886403667	0.133160399	1.050372978	0.3041453972	-1.026221348	0.140112105
0.9824246933	-0.02175297174	1.016484234	0.2541106682	-0.9839620093	0.139700893
0.9824246933	0.0201940531	1.0152958	0.2546883711	-0.9734799235	0.1472664367
0.8886403667	-0.227176032	1.127725454	0.2588383297	-0.9424201523	0.1597528475
0.8886403667	0.1310426114	1.088386873	0.2991101132	-1.089970474	0.134605712
0.9775092155	-0.03993718014	1.025239797	0.2652011874	-0.9980712984	0.144260926
0.8886403667	0.1179421626	0.9994325998	0.229864893	-0.9251902785	0.1395503471
0.9824246933	-0.02259663182	1.008535472	0.2500679711	-0.9804716549	0.1516659341
0.8886403667	-0.1509377769	1.10298093	0.2221010193	-0.9776237291	0.1391734145
0.8886403667	-0.06987508145	1.028945888	0.2523171782	-0.995410395	0.1483936138
0.8886403667	0.2246764266	1.175355291	0.3800335867	-1.214823289	0.1628197408
0.8886403667	0.06748286573	0.9952314859	0.2737562695	-1.015502869	0.1403549537
0.8886403667	-0.0843696175	0.9713644186	0.1692079135	-0.8606394734	0.1370831357
0.8886403667	-0.1502619635	1.016881519	0.3010450048	-1.092553323	0.1457598786
0.8886403667	0.1871705495	1.036268626	0.1790497484	-0.9636088096	0.1531527921
0.9124849918	0.06183703294	0.9707403248	0.2259395281	-1.009291565	0.1650037142
0.8886403667	0.1913281297	1.106167238	0.3019940842	-1.081685895	0.1639030992
0.9824246933	-0.009010888178	1.015077822	0.2517004542	-0.9972856963	0.1392070452
0.8886403667	0.348208305	0.7045306052	-0.0984768841	-0.7036862607	0.1562897644
0.8886403667	0.1263191439	1.044432525	0.2423963833	-0.976539593	0.1489617512
0.8886403667	0.1052610443	1.026494685	0.2508741098	-1.050340648	0.1368650956
0.8886403667	-0.07510081097	0.9847795951	0.2178451818	-0.9190701634	0.158139134
0.8886403667	0.1908763379	0.9685742921	0.3239855185	-1.055368314	0.1447462893
0.8886403667	0.147752013	1.050239967	0.2641762017	-1.01587481	0.1426561035
0.4914558906	0.1908960625	0.2764841154	-0.3019499935	0.7835683561	0.1161112538
0.4914558906	0.2220572234	0.2846028767	-0.3041100705	0.7222536461	0.1146220856
0.8159616578	-0.06786190196	0.2177097855	-0.3504747533	0.8077048259	0.1190696048
0.987109513	-0.001911645511	0.2448007227	-0.3493785087	0.7913910178	0.1183201613
0.9261187171	-0.02323018698	0.2433817019	-0.3446320223	0.7933142287	0.1185111025
0.7352604538	-0.105436092	0.2001193568	-0.3908621446	0.8785639058	0.1194396608
0.4914558906	0.2186674759	0.2340823521	-0.2717616522	0.7309184493	0.1159049377
0.4914558906	-0.2320094786	0.333692395	-0.3461613336	0.8314412026	0.1173928673
0.026701542	-0.4005007638	0.4198325957	-0.2925144251	0.6755845335	0.1081420763
0.7989945845	-0.07709693981	0.2397446547	-0.3386627418	0.7772040854	0.1177092475
0.7352604538	0.1075564258	0.2876679512	-0.3216879496	0.7607637572	0.1183081075

0.5180231938	-0.1682504504	0.2216151809	-0.3569114016	0.8898869374	0.1180800298
0.8170742179	0.05202974114	0.2421181173	-0.3381382855	0.7741755883	0.1185308788
0.5686164971	-0.1493613286	0.244442716	-0.3935278197	0.8092271804	0.1170169683
0.8170742179	-0.05171260101	0.2434689361	-0.3521971633	0.7684410534	0.1199956989
0.4914558906	-0.1854450468	0.1724138	-0.3950490512	0.8104912287	0.1174931106
0.7093607019	-0.1200898202	0.2380516774	-0.4100946513	0.8109946551	0.1194614248
0.7741303846	0.0886835412	0.2551302792	-0.3436778978	0.7613447336	0.1176764361
0.9224387816	0.02851071809	0.2317511321	-0.3364690596	0.7727038213	0.1300506454
0.7352604538	-0.1122126235	0.3205928913	-0.3096147032	0.7955785008	0.1261407517
0.4914558906	-0.195999485	0.1265793736	-0.4399154375	0.9361963864	0.1182684007
0.9760570672	0.00707144377	0.2438636128	-0.3491348175	0.7945217372	0.1190416399
0.8170742179	-0.06426303269	0.2079651287	-0.3623169827	0.805399354	0.1210360822
0.851825852	0.03927612454	0.2410713606	-0.3684806941	0.8011766997	0.1196354574
0.851825852	-0.04411975362	0.2830546772	-0.3298596029	0.7573753148	0.1241732275
0.6312783444	0.1354886973	0.2170269265	-0.4167175805	0.8152149643	0.1190784213
0.8170742179	0.05552490204	0.2265106612	-0.3489227416	0.8109193808	0.1189118094
0.7061704944	-0.1218279633	0.2874707059	-0.3330700223	0.7855245469	0.118287962
0.4914558906	-0.2102954507	0.0941988613	-0.4470803736	0.9783023362	0.1192929867
0.4914558906	-0.2506989529	0.0951013538	-0.3985841503	0.8818571054	0.1168178826
0.8284524188	-0.04884903034	0.2279414111	-0.3589331458	0.7931993799	0.1193978831
0.5686164971	-0.152761122	0.2486258486	-0.2685073746	0.7238923664	0.1188299425
0.8159616578	-0.06663167945	0.2656684431	-0.3452777397	0.7877125836	0.1185127235
0.4914558906	-0.2015845858	0.3358805542	-0.2456165193	0.5895108484	0.1187554049
0.4914558906	0.2397084574	0.1993603126	-0.3675192283	0.9775359768	0.1179007621
0.9013670124	-0.03082885137	0.2318428071	-0.3476056658	0.7850998524	0.1219131127
0.4914558906	0.1936144007	0.2227052546	-0.304363927	0.8019561146	0.1162746209
0.8170742179	0.05897267434	0.2353373698	-0.3337036712	0.7717455233	0.1195371353
0.4914558906	-0.219979004	0.2791172372	-0.4412287739	0.7914351402	0.1176201485
0.7989945845	0.08113461449	0.2508421074	-0.4064378794	0.8690294282	0.1261027246
0.8159616578	0.06739273289	0.2773186759	-0.345545513	0.7616089507	0.1198558993
0.9494026196	0.01207254506	0.2445853901	-0.3523198184	0.7972782194	0.1187421727
0.4914558906	0.2356266571	0.2095396687	-0.4708070828	0.9335237087	0.1161415298
0.7390081682	-0.09929390539	0.2518174174	-0.3580711144	0.759390929	0.1183041919
0.5630662785	0.1568595615	0.2960399686	-0.33459295	0.6859571325	0.1186521764
0.4914558906	0.2196898915	0.2639632551	-0.3682916158	0.7317755959	0.1153321326
0.4914558906	0.2369494364	0.2586110371	-0.3599962418	0.8803633536	0.1152846299
0.4914558906	0.1827158492	0.2675863228	-0.3368174026	0.8020432945	0.1157552279
0.4914558906	0.2116102592	0.2597275987	-0.2621925274	0.6763594216	0.1164920766
0.7989945845	0.08013328889	0.2633782496	-0.3050384025	0.7924673531	0.1225514933
0.7955876879	-0.08388078089	0.2680482306	-0.3326171006	0.7720945995	0.1178112365
0.5043956771	0.1748801405	0.3412172457	-0.2608469545	0.6410726661	0.1188373644
0.9834530885	0.004847935501	0.2463911044	-0.3494007937	0.7892789465	0.1197878952
0.8170742179	-0.05748414486	0.2309189228	-0.3704685369	0.7704546171	0.1248280496
0.8159616578	-0.06628785993	0.2307948831	-0.3627523852	0.8114390631	0.1177082818
0.8159616578	-0.07153464418	0.2510063891	-0.3659058275	0.7853103965	0.1184682061
0.947308554	-0.0147857509	0.2447904084	-0.355332925	0.8001984976	0.1198705981

0.8170742179	0.05986001572	0.2388186219	-0.3360833335	0.7720298899	0.1187896849
0.4914558906	0.198793442	0.3185790935	-0.2779161065	0.5912362758	0.1193494347
0.5043956771	-0.1712626084	0.3131833441	-0.2622640771	0.6824330672	0.1177883995
0.7390081682	-0.09896722156	0.215336871	-0.3579396425	0.7874754941	0.1185298024
0.6312783444	-0.1400661237	0.1656865294	-0.3741477535	0.844621577	0.1192674677
0.7955876879	-0.08777258367	0.1818559247	-0.3769051929	0.833525873	0.1219263965
0.6312783444	-0.1371816683	0.2534366986	-0.2821254594	0.776459995	0.1192567688
0.7237071821	0.1143727649	0.2498714906	-0.3439141215	0.8101794574	0.1171992847
0.8170742179	0.06333354942	0.2638911771	-0.3366308952	0.7748640957	0.1181034306
0.1103871028	-0.3526992659	0.4367769968	-0.2996812979	0.5233020624	0.1131608099
0.7986393901	0.08423200734	0.2100511126	-0.3914346683	0.8721608739	0.1205526329
0.4914558906	0.1853299529	0.1568815501	-0.4230784434	0.8004568254	0.1202060025
0.851825852	0.04098965886	0.2369002265	-0.3680909643	0.8245831771	0.120304205
0.7989945845	0.08445835894	0.2670898237	-0.3800840204	0.8718198703	0.1289705629
0.7352604538	0.1021849798	0.2408963566	-0.3416801445	0.754965943	0.1180769651
0.4914558906	0.1824683592	0.2600654973	-0.2727396479	0.7514148225	0.11697242
0.6751777676	0.1285804521	0.2840973767	-0.2727549557	0.6768314925	0.1200387809
0.7594172115	-0.09402804918	0.2596763923	-0.3130447941	0.7882189967	0.1192004735
0.2023366985	0.3132945563	0.2219423176	-0.2833442266	0.8037122278	0.1116990494
0.851825852	0.04057060605	0.2133021345	-0.3665527874	0.8153684126	0.1230434518
0.5496791634	0.158992701	0.1988264639	-0.338856919	0.7705085013	0.1178203989
0.7594172115	-0.09329986511	0.2689990027	-0.3422756267	0.7581456057	0.117824646
0.5386189999	-0.16667166	0.1939864931	-0.4169111141	0.7795165754	0.1201168517
0.7237071821	0.1120654883	0.2943859289	-0.2876341889	0.7391267088	0.1196752945
0.987109513	-0.002355873817	0.2457267001	-0.3492254002	0.7912560708	0.1191590995
0.6040240245	0.1432828737	0.2769157858	-0.3114966515	0.7939948187	0.1179423558
0.4914558906	0.1860407757	0.319266067	-0.3346893432	0.7901965912	0.1176666039
0.7989945845	-0.08304961981	0.2493285575	-0.421490814	0.8719248301	0.1279771633
0.4914558906	-0.1777207871	0.300296096	-0.3769041171	0.7894712048	0.1174432012
0.6312783444	-0.1340864239	0.2564003409	-0.3219657597	0.7732719963	0.1167039277
0.7989945845	0.07539917502	0.2461770245	-0.3443517372	0.7641808066	0.1181799693
0.7237071821	-0.1090389113	0.2370338088	-0.3561246214	0.7848200432	0.1170391272
0.8159616578	-0.06970626091	0.2872395637	-0.3444867999	0.7843577432	0.1222591504
0.8318854403	-0.04791746802	0.2681990442	-0.337879423	0.7877965688	0.1209382222
0.6040240245	0.1441172634	0.2387930792	-0.4062987138	0.858165795	0.1178374972
0.8170742179	-0.05733310471	0.2476333903	-0.2989067353	0.7597973078	0.1255819898
0.9261187171	-0.02342676656	0.243134915	-0.3479890722	0.7883289681	0.1179259091
0.026701542	-0.4245415619	0.3318918079	-0.3750435162	0.5245780528	0.1089279033
0.8170742179	0.06119125502	0.2307854694	-0.3452554224	0.7908099513	0.1182831712
0.7237071821	-0.1140002756	0.2562702056	-0.3258372386	0.8291194724	0.1199351504
0.4914558906	-0.2166040424	0.2463941415	-0.3944657423	0.9409787943	0.1179917909
0.7352604538	-0.1035109009	0.2046678797	-0.3861263585	0.8114100681	0.119148061
0.5386189999	-0.1605026954	0.1925744866	-0.3703714007	0.8343262263	0.1167450806
0.7700507085	0.09370373288	0.2081668851	-0.3834961231	0.8918703835	0.1220097304
0.4914558906	-0.1943978361	0.2073514258	-0.3802172495	0.7531395195	0.1171966683
0.6312783444	-0.1330604842	0.1981123817	-0.3485474574	0.8432592194	0.1181876101

0.9494026196	0.01321703256	0.2487895124	-0.3434918037	0.7928655949	0.1227854741
0.5681702775	-0.1599659187	0.3113935459	-0.3903327694	0.860338738	0.1227409592
0.2825625227	-0.2894610513	0.1168425215	-0.4746446169	1.024253502	0.1144316705
0.932443124	0.0183913571	0.2445986813	-0.3539963437	0.7921946733	0.1183254889
0.932443124	-0.02112375464	0.2510237257	-0.3426645317	0.7765341489	0.1196189673
0.4914558906	-0.1786430892	0.2043269168	-0.3193725848	0.8134710802	0.1168677662
0.8170742179	-0.06351444052	0.2807490607	-0.3495547595	0.7724753657	0.1211473189
0.4914558906	-0.18540696	0.281434592	-0.3195034058	0.7520670351	0.1155898618
0.7390081682	0.1007516792	0.3109623021	-0.3241829587	0.7096398675	0.1214180801
0.8170742179	-0.05258656117	0.2534711951	-0.3552898635	0.7930544254	0.1180421548
0.932443124	-0.01875812288	0.2386613349	-0.3643995474	0.8144066777	0.1244766538
0.5043956771	0.1707244776	0.2396959469	-0.361853252	0.8616467231	0.1167998237
0.8170742179	-0.05873229452	0.2442683212	-0.3258609316	0.7712871249	0.1191265724
0.8170742179	-0.06370351356	0.2898303686	-0.3195616402	0.8033583356	0.1324779449
0.4914558906	-0.1781160448	0.2331625743	-0.3720679765	0.8358584303	0.1156727619
0.7093607019	-0.1207113359	0.2495365919	-0.3427111505	0.7117004752	0.120353676
0.8907744352	-0.03388592884	0.266943068	-0.3326571793	0.7817755521	0.1235152086
0.4914558906	-0.2055797872	0.2503058974	-0.3002168391	0.7182472424	0.1155281026
0.851825852	0.04147997951	0.2467559337	-0.3529774985	0.7833000426	0.1181491154
0.932443124	0.01998930311	0.2495322196	-0.338186949	0.7814456756	0.1200437254
0.2825625227	0.2929463692	0.2073939344	-0.2743300765	0.6573211566	0.1143074656
0.7237071821	0.1100852501	0.2753261967	-0.3448328406	0.7707030493	0.1175286729
0.8699822324	-0.06410056781	0.4344804538	-0.1505733855	0.444343664	0.1186690525
0.5411567788	0.2114545954	0.504108316	-0.0820671561	0.3296651613	0.1169079189
0.9135982073	-0.04657499484	0.4281132219	-0.136520321	0.4485453796	0.1201444813
0.9136389765	-0.02902954186	0.4449479419	-0.1311689938	0.4355595564	0.12011798
0.8505310426	0.07728174369	0.4428625371	-0.1559157465	0.4507898119	0.1204781704
0.8069640727	-0.125966299	0.40308298	-0.1745003983	0.5193067837	0.1229778723
0.8971393429	0.05495420092	0.4393458327	-0.1200661269	0.4343883886	0.1180841871
0.4850078292	-0.2600882446	0.5310258679	-0.1475551519	0.5130000288	0.1159636025
0.4850078292	-0.2727205319	0.5757579679	-0.0916156444	0.3427500753	0.1152359705
0.9413513341	-0.01599376912	0.4466972951	-0.1313444019	0.4326514705	0.121446918
0.6107874066	0.1822049679	0.5304997201	-0.0851215234	0.3631126938	0.1166511919
0.9136389765	-0.03932614418	0.4432806806	-0.1339782877	0.455665095	0.1255176229
0.9136389765	0.03068034333	0.439660419	-0.1306971721	0.4399369101	0.1192303589
0.4850078292	-0.2584110471	0.4679874798	-0.1972883905	0.4196325585	0.114906565
0.9920945847	-0.001196997586	0.4445199591	-0.1361888659	0.4422459085	0.1191506596
0.8505310426	-0.0841860034	0.4214493343	-0.152000595	0.4309411248	0.1190999498
0.9083543454	-0.05533932926	0.4492154347	-0.1583903933	0.4357718959	0.121944951
0.9413513341	0.0147210117	0.4453805574	-0.135220099	0.4391895584	0.1181998208
0.6200530061	-0.1723406074	0.5239339397	-0.2148066848	0.55539469	0.1272003117
0.4850078292	-0.2774606269	0.6169999413	-0.0354424859	0.4863999035	0.1247073833
0.6107874066	-0.1983547202	0.3500856492	-0.2015641713	0.5423108773	0.1283516337
0.9413513341	0.01809339808	0.4434885057	-0.1346174462	0.4478500441	0.1255336311
0.8505310426	-0.09719451451	0.3686961458	-0.167130789	0.5070687213	0.1223335774
0.8971393429	-0.05591295108	0.4554574182	-0.1075067908	0.4196728453	0.1199246806

0.9136389765	0.03552382421	0.4161790299	-0.1520545968	0.4657773967	0.1265001334
0.8505310426	0.08095229453	0.4227737579	-0.1734491782	0.4625144205	0.1296687084
0.9135982073	-0.04791339347	0.4577554919	-0.1367579332	0.4314342683	0.1193921299
0.8699822324	-0.06241578186	0.4710263879	-0.1234871378	0.4298249755	0.1196890096
0.8069640727	-0.1075737969	0.3816658283	-0.1783353328	0.5055810846	0.1232565797
0.5345013622	-0.2295838925	0.3167102741	-0.1825623707	0.5050760503	0.1177370373
0.8699822324	0.08688851795	0.4899613034	-0.1085742147	0.4011295689	0.130289193
0.8699822324	-0.07665403567	0.4430633924	-0.1116377344	0.427593713	0.1434346568
0.6107874066	-0.1969698019	0.5358353013	-0.1048230056	0.3620355632	0.1180491092
0.9722570048	-0.009463969036	0.4482945864	-0.1319764	0.4346413419	0.1280531628
0.7972913844	0.1373541198	0.4307127653	-0.1426965947	0.5270635099	0.1229511803
0.5345013622	0.2356250137	0.5576499949	-0.1431480857	0.4540647839	0.1288635658
0.6107874066	0.1843097187	0.4099577405	-0.1060175742	0.4925231785	0.1222952179
0.9413513341	0.02016645204	0.4408933002	-0.1308912244	0.4372992614	0.1219143939
0.8069640727	0.1153410486	0.4169263121	-0.0939692894	0.4648577618	0.1236531463
0.5345013622	0.2430958254	0.4644888365	-0.2834303705	0.6662576609	0.1294536042
0.6930800793	0.1761191692	0.4921751123	-0.1472904732	0.4516543215	0.1259674242
0.8505310426	0.08604950934	0.4324188674	-0.1610963867	0.5056717107	0.1290835851
0.08406036695	0.4020307682	0.4217209815	-0.322219176	0.6014159289	0.1099587639
0.9136389765	0.04129225694	0.4456344641	-0.131818521	0.4484802785	0.1212743269
0.5345013622	0.2391833662	0.5599984439	-0.0925599481	0.198268323	0.1176823215
0.919687497	0.02568137742	0.4497800795	-0.1365669042	0.4293915897	0.1183002342
0.8505310426	0.08211176439	0.4619200568	-0.1375240954	0.4494894929	0.1238045249
0.6107874066	0.1918900641	0.50575404	-0.1020965468	0.370564228	0.1167145127
0.8069640727	0.1154134285	0.4681207713	-0.0783061325	0.3462176033	0.1201707414
0.6107874066	0.1931462194	0.4716835484	-0.0405013074	0.4831700242	0.1222080163
0.9136389765	0.03419334097	0.4331181488	-0.1450759598	0.4574015556	0.1305938063
0.8069640727	-0.1070075294	0.3870443157	-0.1933751116	0.5348974088	0.122201647
0.6200530061	0.1771290643	0.5078577562	-0.1357035183	0.3438378326	0.1259324913
0.8505310426	-0.08454720724	0.4350983131	-0.1597750319	0.3865688745	0.1249763759
0.9135982073	0.04470347228	0.4516266825	-0.1312522663	0.4366710031	0.1186005796
0.6869101751	-0.1495957332	0.4698929231	-0.1644396711	0.402051293	0.1210646715
0.8069640727	-0.1094423789	0.4258881181	-0.196118271	0.5462055216	0.1198565528
0.9135982073	0.04308438683	0.4419922093	-0.1229205048	0.4225896205	0.12375112
0.8069640727	0.1279172374	0.4758980323	-0.0937957582	0.3505644655	0.1230764468
0.9136389765	0.03244201678	0.4317409125	-0.1505499881	0.4628974597	0.1205342952
0.9413513341	0.01637949645	0.4498765641	-0.1347722804	0.4425365696	0.1180845197
0.9413513341	0.01870550418	0.4535540668	-0.1329819976	0.4377491786	0.1246807909
0.892755109	-0.06483643975	0.400088529	-0.1578279868	0.4729311056	0.1338725055
0.8069640727	-0.1126842905	0.4562838357	-0.0790063230	0.4238635678	0.1244254026
0.8069640727	0.09794725062	0.4487851818	-0.1354336842	0.4605065334	0.1190928038
0.9136389765	0.03821432523	0.4582757311	-0.127670791	0.4293512878	0.1219484468
0.6107874066	-0.2061845136	0.5735707949	-0.0935072778	0.2454871071	0.121797957
0.4850078292	0.2826484984	0.3187929758	-0.3048579713	0.7559748318	0.1161811117
0.8699822324	0.07322057248	0.4168774538	-0.1591129528	0.4308369228	0.1224745982
0.9135982073	0.05070128366	0.4398203997	-0.1564307593	0.4730487624	0.1203291889

0.9135982073	0.04461671324	0.455131715	-0.1529141952	0.4870118305	0.1292290284
0.8505310426	-0.08183574241	0.4361824886	-0.1484407462	0.4951628674	0.1209016292
0.9820151654	-0.004845954977	0.4440772628	-0.1381483915	0.4438554416	0.124564367
0.8505310426	0.07962134763	0.4727216875	-0.0851181396	0.3620249112	0.1220767449
0.8069640727	-0.1158758008	0.4684551126	-0.0874086526	0.4232659757	0.1202220327
0.5345013622	0.2457400054	0.4516317522	-0.0732234048	0.3994602695	0.117237426
0.9135982073	-0.04532170456	0.4749472492	-0.1210004964	0.4278305717	0.1233488448
0.8505310426	0.07664708375	0.4301991393	-0.1289010916	0.4174309949	0.1202069118
0.9817289025	0.006410484091	0.4437384111	-0.135927156	0.4434069947	0.1214859664
0.8505310426	-0.07572925362	0.4233490354	-0.1643461159	0.4327024816	0.1201727679
0.5345013622	0.2537395992	0.5942060917	0.02498176878	0.2401687141	0.1174262059
0.8069640727	-0.1027389993	0.4825343579	-0.1208146434	0.4227931777	0.1199199986
0.8069640727	0.1232196857	0.4874303952	-0.0983643542	0.4156936872	0.1231338891
0.6200530061	0.1719986242	0.5361933636	-0.1116894651	0.3921856035	0.120167857
0.8505310426	-0.09221000803	0.4671899836	-0.2049505901	0.4934595873	0.1294182561
0.6200530061	-0.1829651691	0.5359632185	-0.1449299335	0.3646556646	0.1188271745
0.8069640727	-0.119001316	0.4584970319	-0.1145401164	0.423600616	0.1168540722
0.8505310426	0.09228416536	0.4333963146	-0.1349647284	0.4367878443	0.1251909251
0.9136389765	0.04045384042	0.4435405232	-0.132700041	0.4529286498	0.1315311373
0.6200530061	-0.1846707563	0.5232622079	-0.1301018505	0.4788186161	0.1251711134
0.5345013622	-0.2088511524	0.5403654104	-0.0848239888	0.4370861062	0.1162768412
0.9413513341	-0.0155921101	0.4446523624	-0.1300479635	0.4371113729	0.1249513466
0.6696072029	-0.1642106435	0.4395942458	0.0076738840[illegible]	0.3810301117	0.1237858965
0.6869101751	-0.161113682	0.4191193092	-0.1439527224	0.4665097413	0.125259979
0.4850078292	-0.267318257	0.4849335803	-0.1651243968	0.3076481154	0.1185340363
0.9820151654	-0.004355132517	0.4450031637	-0.1364595673	0.4439769527	0.1230216702
0.8699822324	-0.07293028889	0.4626630822	-0.114441638	0.441826448	0.1272786132
0.8069640727	-0.1043502895	0.4408927648	-0.1590859985	0.5193292307	0.1256079268
0.8069640727	0.1277062042	0.4901907443	-0.0955311657	0.4279793233	0.1204902205
0.9135982073	-0.04763324551	0.4337316769	-0.1389524021	0.4460135247	0.1182844384
0.8505310426	0.09871557771	0.421118469	-0.1666764992	0.5173853244	0.1288115377
0.8699822324	-0.06280800307	0.4364714124	-0.1442324964	0.4233930233	0.1193172222
0.8069640727	-0.128689899	0.4148806686	-0.1226547587	0.4546730082	0.1234501505
0.8505310426	0.08900874909	0.4765898118	-0.0929654024	0.4379057176	0.1318616938
0.6107874066	-0.1798789471	0.5332984178	-0.1808781072	0.4984393263	0.1239608111
0.6869101751	-0.1585543818	0.3929023152	-0.1990448185	0.5339019547	0.1231728649
0.8699822324	-0.06647323653	0.454170498	-0.1167239465	0.4222943564	0.1189058527
0.6107874066	-0.1885610736	0.5189740394	-0.0604172746	0.2653899815	0.123754922
0.8069640727	-0.138860343	0.422259894	-0.1193029433	0.4434340075	0.1330273982
0.9136389765	-0.02803272807	0.4588988981	-0.1383915124	0.4381059852	0.1216527651
0.4850078292	-0.2654352742	0.4933640971	-0.0998608247	0.3909783731	0.1118556404
0.8069640727	0.128798104	0.511491281	-0.1022104159	0.365768124	0.1283888674
0.9136389765	-0.03557439063	0.4542785952	-0.1373679214	0.4348648581	0.1212260874
0.8069640727	-0.1219032602	0.4130636545	-0.230033287	0.5750066306	0.1255709241
0.6107874066	0.1908362152	0.4427861211	-0.1579290571	0.518763162	0.1189942167
0.6107874066	-0.1845998187	0.4580625454	-0.0635720585	0.349525842	0.125378942

0.5345013622	-0.2656756725	0.6699046199	0.01026617630	0.407047859	0.1297899956
0.8069640727	-0.1353037337	0.4683475818	-0.1351674767	0.4033792386	0.1183987419
0.8069640727	-0.1088095423	0.4502790155	-0.1302084295	0.3672840872	0.122418977
0.9136389765	-0.02991914998	0.4672272573	-0.1202598869	0.4284831716	0.1300029227
0.6696072029	-0.1523553835	0.4549457434	-0.0901771284	0.3763414457	0.1182246408
0.9135982073	0.04441163757	0.4471916571	-0.1406547325	0.4334844622	0.1178548714
0.5345013622	-0.2148041473	0.3799834582	-0.2710149263	0.5769670266	0.1183216103
0.5809154051	0.2001086754	0.4151348371	-0.0971831352	0.3627552328	0.1177690819
0.5345013622	0.2226926525	0.4931719281	-0.1482829887	0.4330072808	0.1145454704

Full model fit coefficient estimates (outcome ~ micr					
Blast Std. Erro	VNS Std. Erro	Blast:VNS Std. Erro	Microbe t-valu	Blast t-value	VNS t-value
0.3147126229	0.331791147	0.4645238408	2.643234361	3.053042605	2.226820926
0.3307937059	0.3487978707	0.4917491689	0.07887962006	2.609663303	1.838393296
0.3268006651	0.3406556494	0.4812238318	1.653866013	2.891937029	1.875282022
0.3281051482	0.3466006504	0.4909455065	0.8762493561	2.612471634	1.800025503
0.3142056869	0.3326788288	0.4670678256	-2.506293877	2.685165752	2.141209901
0.3301055375	0.3488598602	0.4956908862	0.8430779108	2.70012726	1.936095037
0.3298222229	0.3498019545	0.4907737734	0.4048584518	2.601734172	1.871596763
0.3295972194	0.342308069	0.4823216686	-1.481223827	2.879096471	1.916220664
0.3316576853	0.3451800853	0.485500593	-1.164067037	2.802577183	1.928335634
0.3288822411	0.3476393094	0.4893728631	0.6921483781	2.629846605	1.796829507
0.3317737073	0.3492908636	0.4907450992	-0.2215233595	2.574291149	1.814092458
0.3300613671	0.3478499959	0.4949552876	-0.4106691382	2.591149512	1.841450463
0.3299892972	0.3497567467	0.4927554878	0.320152722	2.619429587	1.860932651
0.3277890448	0.3480377881	0.4875904634	-0.9335646025	2.624618482	1.733284738
0.3229911455	0.3407464993	0.483216723	-1.643744416	2.687983634	1.880881253
0.3283123449	0.3437704973	0.4831301546	-1.436975182	2.409477975	1.737156173
0.3117421058	0.3338744472	0.4637992004	-2.705183306	2.717653042	1.448944379
0.3286898075	0.3456321438	0.4889808574	1.022121776	2.714575245	1.893069284
0.3363520116	0.3519554272	0.4979528874	0.4240784591	2.477522283	1.882665954
0.3427785835	0.3510771335	0.4904270742	0.2905248716	2.434831215	1.784511854
0.3379403187	0.3516421706	0.4986269708	-0.5364630185	2.429571877	1.737321722
0.3252554518	0.3437051189	0.484892834	-1.350288739	2.644094844	1.785305728
0.3275944456	0.3393418979	0.4781647368	-1.853825593	2.262580607	1.777463873
0.3299373437	0.3519347393	0.491309342	0.3876428347	2.598254316	1.759353948
0.3418907549	0.3496417512	0.4950362834	-0.5223830263	2.660023634	1.886955006
0.3296925133	0.3539467438	0.489056219	-0.8018527157	2.684254346	1.971375666
0.3299310975	0.3456393117	0.4890879293	1.013870335	2.485336003	1.889706949
0.3288894856	0.3439025104	0.483019746	-1.393313529	2.834984263	1.970236126
0.3408364569	0.3510243727	0.5013520996	-0.8826170116	2.288345162	1.675200947
0.3351323745	0.3453287873	0.4890449679	-1.102343481	2.327647615	1.79518262
0.3313347894	0.3485605615	0.4906511723	-0.1299359205	2.589303036	1.830324464
0.3289615704	0.3552678756	0.4899885567	0.6806167409	2.605118232	1.654830806
0.3290299968	0.3446496243	0.4858279769	1.136960937	2.482399315	1.848139947
0.3352343021	0.3533390614	0.5047280214	0.2706503609	2.522372945	1.764330356
0.3326372525	0.3484299147	0.5085646254	-0.462376347	2.652905854	1.865796617
0.332308085	0.348472086	0.4913853406	-0.3019997873	2.556606173	1.850481361
0.3289178858	0.348785208	0.4886897559	0.7423300279	2.589746062	1.914489789
0.32327198	0.3440813034	0.4834952787	1.608962401	2.65043679	2.072363307
0.3308840607	0.3516424857	0.4911604303	0.2801404161	2.582880245	1.859039029
0.3294501873	0.3575806127	0.5023305036	-0.5707917168	2.596218496	1.924480991
0.3399765506	0.3488525064	0.4958644647	0.01198879917	2.537217368	1.834612756
0.3284435312	0.3479762046	0.4916021828	-0.7996461555	2.634284468	1.912352577
0.3301545807	0.3521931811	0.4946698015	-0.0605924999	2.610133758	1.825521248
0.3295743733	0.3480630877	0.4906131355	-0.4990469888	2.626596618	1.812742004
0.3306807459	0.3477717544	0.4943739832	-0.4505927261	2.569216772	1.834134873

0.3308436555	0.3479298423	0.4923428501	0.3738803283	2.634354636	1.837759267
0.3293587651	0.347373253	0.4931596566	-0.5768605856	2.600992031	1.839959547
0.3282008495	0.34627004	0.4887374223	0.8492169195	2.61894994	1.854482793
0.325086188	0.3475135321	0.4881610612	1.519880099	2.77569839	2.115125604
0.3266249005	0.3497508403	0.4835342087	-1.341714031	2.517867032	1.571226711
0.3269671823	0.3443318323	0.4844125017	-1.407206108	2.795406049	1.993874868
0.3369343855	0.3513464954	0.4980848481	1.409591058	2.931767228	2.131641099
0.3257729503	0.3381896529	0.4825323231	-1.944815675	2.287280856	1.804325336
0.3292389736	0.3485926836	0.4904689422	0.9283630238	2.703346195	1.951376002
0.3307013382	0.3482543187	0.4906888499	0.3686543291	2.632864871	1.852776664
0.3303603538	0.3490038172	0.4908600454	0.1690292605	2.601160171	1.844262801
0.3287371974	0.3508317863	0.4954987016	-0.7209932954	2.620709057	1.715081909
0.314414765	0.3320651109	0.4684810378	2.508346013	2.628165468	2.086399146
0.3354400857	0.3515789276	0.5097995335	0.7106768549	2.709573384	1.935581144
0.3309980994	0.3521674166	0.4920094327	0.6858701256	2.5254789	1.699362815
0.328769323	0.3444502571	0.4850750069	-1.184618857	2.477703809	1.826104696
0.3332947097	0.3470721289	0.4892393628	0.8054138121	2.722861415	1.890960424
0.3437846188	0.3512961288	0.4959748718	-0.4173026666	2.387979523	1.76271814
0.3297650253	0.3527747138	0.4901504581	0.4000679325	2.619542854	1.747087161
0.3091682904	0.3261857582	0.4603325761	2.902957924	2.808074885	1.943344378
0.3327039293	0.3503046124	0.4915978424	-0.2145016379	2.562623623	1.802878909
0.3380051693	0.3489080188	0.5003567096	0.2839554365	2.487465181	1.814349315
0.3325575176	0.3530365001	0.5049047795	-0.02057285725	2.593363352	1.815570985
0.3327100107	0.3480179856	0.486076516	1.106891304	2.382667541	1.688890125
0.3295226611	0.3512133979	0.4981871611	-0.756447692	2.671478337	1.942721383
0.3242229651	0.341988545	0.492161073	1.748620771	2.859422138	1.672504187
0.330050127	0.3475312798	0.4900809865	-0.526868663	2.643706564	1.842047856
0.3279036093	0.3488687347	0.487319852	-0.916490799	2.640560709	1.714977672
0.303882857	0.3247522876	0.4599178	3.424776826	3.219997951	2.634415337
0.3122986733	0.3327276246	0.4630262193	-2.719328316	2.950991766	2.346313958
0.3290603092	0.3487059861	0.4891686172	0.6620577055	2.601985994	1.898823032
0.347386661	0.3543657148	0.4982098296	-0.1384540699	2.523421782	1.830979789
0.3251506254	0.3426665338	0.4828542611	1.459516725	2.554080876	1.933632367
0.3331923933	0.348014513	0.4922289183	-0.6351439783	2.685754853	1.882638461
0.3267338836	0.3464668079	0.4831212148	-1.396380619	2.490171266	1.644181315
0.3272025723	0.3482238525	0.4839385575	1.763591265	2.948925805	2.226396634
0.3271478627	0.3411489136	0.4824916619	-1.679195656	2.910818173	1.984617864
0.3266013712	0.3454783899	0.4857298475	1.180151732	2.680427219	1.946766497
0.3320856002	0.3462322964	0.4867754127	0.9891352589	2.758408895	1.910908787
0.3299544062	0.3648391608	0.5069153213	-0.3354831787	2.619817912	1.652873466
0.3285565999	0.3439381396	0.4841969847	-1.275636235	2.788228966	1.813093394
0.3128077472	0.3309421292	0.4647256967	-2.632621552	2.851067567	2.15119313
0.3204391364	0.3380842075	0.4781090813	-1.922243558	2.692143884	1.881648083
0.3239695374	0.3417375402	0.4814092843	-1.53035277	2.627268778	1.883573461
0.3399842774	0.3481733087	0.4914685258	0.2751050637	2.467956161	1.832459423
0.3307374321	0.3460354193	0.4866454936	-1.003778775	2.746958909	1.907497887

0.3190236646	0.3391883907	0.478864804	2.06763554	2.670498251	1.62881535
0.3289060807	0.362920432	0.4947032782	-0.688934247	2.631191122	1.964905295
0.329829914	0.3478289435	0.4900352125	0.4185992032	2.625062108	1.837624429
0.3308747701	0.3473053108	0.4927084034	-0.6006072016	2.664960222	1.83669166
0.3290074643	0.3433920079	0.4844699583	1.323001411	2.424696478	1.845399506
0.3201756371	0.3395556653	0.4761498481	-1.998784947	2.801544905	2.114925854
0.3235829442	0.3410521425	0.487612458	-1.637328832	2.759240332	1.813334654
0.3278960173	0.3460382883	0.4852264394	1.223982884	2.755237262	1.97941738
0.3302847228	0.3463673485	0.4890166447	-0.8925170102	2.503655087	1.808558174
0.325663815	0.3413012044	0.499954146	1.780035132	2.371347666	1.691140351
0.3264517634	0.3436456591	0.4833333951	-1.365260355	2.517382731	1.781113314
0.3297820584	0.344585665	0.4874578384	-1.156848284	2.450800654	1.832832528
0.329581978	0.3500509064	0.4826167269	-1.477078829	2.351258774	1.515979225
0.332625583	0.3473224384	0.4914229837	-0.8388782919	2.726155983	1.777987291
0.3327269636	0.3497521576	0.5049414266	0.9097484048	2.743907851	1.962922109
0.3304650942	0.350173425	0.4909545394	0.2510776692	2.593769838	1.800092702
0.3310930961	0.3504255313	0.4982674205	0.663070971	2.527124014	1.734106947
0.3300425838	0.349235052	0.4902983648	-0.3894526247	2.59401141	1.866046167
0.3385308317	0.3484501802	0.4921619381	-0.0557970505	2.557518943	1.837574996
0.319106632	0.3365189056	0.4737759905	-2.107493665	2.821451511	2.004394535
0.3289579956	0.3378646045	0.4857438726	2.044202327	3.112730077	2.055569347
0.3310843599	0.3483829757	0.4907112287	-0.0431740354	2.605660138	1.835664113
0.3307943062	0.3604195286	0.5118795178	0.934605306	2.730571133	2.038363015
0.3291719094	0.3470637487	0.4910386994	0.6632027772	2.641635978	1.843153308
0.325792706	0.3488421027	0.4853388312	-1.275085543	2.658409683	2.051972727
0.3414785391	0.3545365264	0.4915694351	-0.1016791819	2.549205297	1.823538585
0.325843266	0.3438357669	0.4855309255	-1.283136531	2.608911003	1.818590496
0.3251858265	0.3431934854	0.4909408335	-1.393310511	2.703441674	1.928180756
0.3253311748	0.3389212383	0.4687545974	-2.480615243	3.285264111	2.395724202
0.3297756723	0.3497207578	0.4904467082	-0.3947983951	2.619310386	1.869837872
0.3309085131	0.347668912	0.4924269235	0.5664291063	2.659951491	1.862231363
0.3232782256	0.3465241607	0.4814356792	-1.824870881	2.466784925	1.469347122
0.3255895065	0.3451973195	0.486289782	1.328132768	2.602182447	1.991578658
0.3299599988	0.3460363188	0.4876124421	0.9345445312	2.718512897	1.881490868
0.3311726215	0.3492117685	0.4901402932	0.1269479285	2.402863495	1.429989563
0.3257319918	0.3434606618	0.4842245586	-1.554120016	2.409500725	1.407888449
0.3336035529	0.3474677615	0.4918757147	-0.0788820996	2.363461797	1.426504085
0.3288694617	0.3473691641	0.493115304	0.5309571262	2.40803909	1.406750955
0.3226995087	0.3419918632	0.4806552123	-1.539337551	2.428545264	1.585580491
0.3313799848	0.3500799636	0.4979735422	0.5737974271	2.488814422	1.506318417
0.3294968367	0.3490970686	0.4909433674	-0.0731633735	2.410359175	1.414873158
0.3347184491	0.3476132039	0.4906323861	-0.1278160409	2.392662291	1.430857235
0.3345431722	0.3481667479	0.4904743334	-0.0910015349	2.382518536	1.428197141
0.3204115319	0.3402970978	0.4802969115	1.616745063	2.358999183	1.231387194
0.3303636031	0.3476948523	0.4894292943	-0.5846899654	2.347428834	1.387987225
0.3229947798	0.3404025555	0.4843583348	1.835199181	2.614753525	1.493122705

0.3294020411	0.3492587875	0.492390786	-0.289474308	2.409930073	1.39653487
0.3265079215	0.3465261026	0.4865271969	-0.9579341796	2.421282043	1.322663375
0.3249565905	0.3429374159	0.4867299261	-1.212773179	2.432965622	1.430461507
0.3307285689	0.3467432938	0.4880548079	-0.7777415287	2.279451277	1.359842253
0.327229423	0.3499724509	0.4877850026	-0.6929243781	2.395503779	1.288414744
0.3280276133	0.3453360096	0.4892362811	0.8239171355	2.466097872	1.460876568
0.3363027052	0.3514264538	0.4978204454	-0.3205314504	2.433894424	1.37116322
0.3403679422	0.3487112381	0.4874506976	-0.808616513	2.542919918	1.528355598
0.3312200335	0.3448604034	0.4896197349	-1.115254127	2.122684253	1.279574146
0.3295830444	0.3482221904	0.4917417028	-0.5971319561	2.443225099	1.418021539
0.3272537227	0.3380324338	0.4776500521	-1.893271667	2.01276896	1.346519948
0.3270383288	0.348628586	0.4881734815	-0.9622145803	2.466924956	1.563323576
0.3349670542	0.3439074263	0.4863549953	1.378596061	1.96598871	1.240466812
0.3275986183	0.3507998424	0.4865542035	-1.086264458	2.522052255	1.620740665
0.33189476	0.3476947016	0.4928449652	-0.05203064248	2.395914684	1.424962053
0.3282898548	0.3435646081	0.4832656957	-1.200575448	2.558905656	1.523972512
0.3410210341	0.3512219826	0.5017741656	0.9141274723	2.6070995	1.588040625
0.3345709858	0.3449338317	0.4891124134	-1.033948322	2.136413086	1.37250637
0.3308638505	0.3481657326	0.4904611172	0.120040608	2.413265718	1.434756594
0.3291987366	0.353917129	0.4910823838	-0.1192929489	2.406086377	1.421053935
0.3275058533	0.3421574677	0.4842551943	1.200277712	2.193752054	1.380999448
0.3257059972	0.3432961682	0.4903822267	2.045511993	2.145751462	1.140074638
0.3317644462	0.3477716977	0.5082553907	-0.4784546379	2.458392035	1.461366904
0.3307491714	0.3446071889	0.4866553766	-0.7385223469	2.217076933	1.429696514
0.317626205	0.3360594982	0.4743673818	1.900187735	2.232132786	1.568016837
0.3199580874	0.3406018202	0.4798473359	1.63437783	2.355495591	1.607577127
0.3225107351	0.342743849	0.4787311636	1.944177325	2.385551472	1.767755328
0.3290644383	0.3559652391	0.5016975082	0.374272144	2.420056414	1.305870386
0.3353135451	0.3451126822	0.4904319293	0.7842906854	2.507332412	1.450870863
0.3293542361	0.3492188644	0.4951295915	-0.2352053671	2.416347107	1.445629613
0.3291336472	0.3510993779	0.4932689368	-0.1410211783	2.403734922	1.430511662
0.3197937413	0.3377337745	0.476053428	-2.130352083	2.605921992	1.406531191
0.3302115123	0.3471058904	0.4950447633	0.2458133347	2.415381385	1.424899952
0.3286619278	0.3449243038	0.4907662274	-0.7611262568	2.298105994	1.409371079
0.3292647355	0.3472741384	0.4936149013	-0.5408020479	2.41157218	1.445647659
0.3288100277	0.3470020417	0.4901524541	0.3465868932	2.407730552	1.429492671
0.3272709085	0.350456792	0.4941794019	0.9403416306	2.472427516	1.570721392
0.3278205415	0.3510311359	0.4853042308	-1.369408093	2.368538535	1.194126883
0.3247029654	0.3427388358	0.4833329713	-1.295178873	2.508831892	1.536592293
0.3397230713	0.3547298714	0.5029741955	-0.9739814581	2.102412222	1.195807374
0.3322369203	0.3434884083	0.4926509496	-1.23000593	2.140811238	1.406095785
0.3282158395	0.3477245533	0.4914429741	0.8354969696	2.474242917	1.520385109
0.3292963687	0.3465947463	0.4894019739	0.5822121123	2.450185684	1.446997901
0.3262381808	0.3437483565	0.4864083074	0.9410525387	2.287246034	1.451859091
0.3277945095	0.3507328793	0.4963276558	-0.7530036385	2.409846405	1.289298915
0.3222563799	0.3406051384	0.4809497452	1.798080347	2.428865492	1.611217289

0.3353591479	0.3521205749	0.5105270521	0.3874243369	2.43641009	1.471931086
0.3231502736	0.3430871522	0.481492106	1.624309639	2.244324878	1.177052338
0.3268755823	0.3425065582	0.4829289396	-1.433323615	2.2788018	1.432082182
0.3327053688	0.3473779102	0.4899533508	0.4917894739	2.455676538	1.456240456
0.3389451333	0.346373288	0.4891086662	-1.489340947	1.965638607	1.251961297
0.3278327962	0.3507076614	0.4872784646	1.179943358	2.510793511	1.262741223
0.3120315792	0.3292031345	0.4647771228	2.725497796	2.620752435	1.517123993
0.3298591821	0.3471939449	0.488676304	0.6012426565	2.43959151	1.464551794
0.3337313447	0.3444963358	0.4940300703	1.468584273	2.128018908	1.385212537
0.3215173364	0.3424048119	0.4892566345	1.892301327	2.240042505	1.110017888
0.3284240485	0.3432861663	0.4815828098	1.370579351	2.117174793	1.252588212
0.3254462111	0.3471571589	0.4925140845	-1.200696279	2.490554464	1.604787247
0.3297658488	0.3479316347	0.501429087	0.8462623819	2.508969284	1.333322693
0.328789911	0.3465204123	0.4898877193	-0.4535049257	2.421620057	1.420812895
0.3243104774	0.3454385573	0.4830838502	-1.286809445	2.446623984	1.247331895
0.3022821996	0.3238402113	0.4615231213	3.347517278	2.946209829	2.163080282
0.3079298099	0.3286875295	0.4588617746	-2.785511015	2.645083003	1.877211343
0.3300088741	0.3496034559	0.4910414185	-0.399380372	2.446569739	1.402229714
0.3437044184	0.3506094954	0.4929288857	-1.355290016	2.798124164	1.709365132
0.3292074282	0.347007408	0.489804782	-0.7084879117	2.473370006	1.41423165
0.3324571274	0.3477301734	0.4919825835	0.582121701	2.320994565	1.404754854
0.3288617687	0.348705274	0.4876119505	-0.8233265133	2.320137565	1.302182856
0.3349811935	0.3560239503	0.4963050823	-0.3009936403	2.318145958	1.331437697
0.3279281007	0.3413694791	0.4844482274	-1.423712348	2.63316433	1.505106675
0.3299109869	0.3488450987	0.4909525145	-0.4396963662	2.412528661	1.404852006
0.3340965283	0.3478966192	0.4900963559	0.1110881217	2.386444063	1.428311732
0.3295521206	0.3635326843	0.5050087724	-0.0180628908	2.406083346	1.358571568
0.3308852051	0.3451837893	0.4873734105	-0.7060612878	2.470303508	1.397299034
0.3237271537	0.342262408	0.4820886388	-1.379488896	2.482020028	1.532176844
0.3282468125	0.3463854025	0.4902840543	-0.5215756988	2.410597049	1.420467464
0.3237551431	0.3421175454	0.482945882	-1.107392309	2.310582228	1.333072345
0.3386399565	0.3475128107	0.4910454539	0.113938486	2.313988062	1.424280755
0.3322375509	0.3478798946	0.4899918305	-0.2801433176	2.424262474	1.441975276
0.3208599017	0.3422097323	0.4841207019	1.571170099	2.360241215	1.178572742
0.3273870938	0.3619522353	0.4933820762	-0.7542431616	2.419779174	1.586753849
0.3238623565	0.3416627271	0.4822855291	-1.369548524	2.389978677	1.430775586
0.3306428856	0.3472199949	0.4926800147	0.7613088249	2.367846404	1.464248197
0.330630002	0.3451785756	0.4877924565	0.8363380493	2.256741398	1.400552802
0.3175727889	0.3375331947	0.4736309464	-1.914754799	2.519334569	1.643990707
0.3285874373	0.346670121	0.4978366294	-0.4276743401	2.413100581	1.399786851
0.33096704	0.3491435569	0.4905273485	-0.3808041501	2.364724389	1.387293724
0.3174378999	0.3334808031	0.4717674308	-2.144240706	2.213500274	1.384853965
0.3290621988	0.3463517232	0.5068338998	0.9337425203	2.228976714	1.287881889
0.3278149977	0.3452897046	0.4866872046	-0.9162059812	2.330197468	1.377160643
0.3326843325	0.3474092699	0.4922549784	0.1481153175	2.40328648	1.428049364
0.330053438	0.3505516467	0.4833070998	-1.592137369	2.19073545	1.123486506

0.3296732495	0.3433469039	0.4856894227	-1.295684678	2.620207066	1.325708231
0.3278251968	0.344599574	0.4975025792	1.78788985	2.794371129	1.747393396
0.3284216208	0.3477759119	0.4886552918	0.8302654844	2.375871833	1.347609622
0.3297933628	0.3489666443	0.4982009379	0.9993086844	2.291747081	1.282960328
0.3282639506	0.3473529886	0.4876560968	1.151379959	2.536897515	1.372105055
0.3364154774	0.346997337	0.4906249367	-0.3332841997	2.420782621	1.428253576
0.3210445613	0.3385719337	0.477402821	-1.857285589	2.586088383	1.557422266
0.3254466341	0.3342581721	0.4805589482	2.476351109	3.107084167	1.72681728
0.3287592371	0.3450612486	0.4875280311	0.6188171623	2.29676004	1.40262075
0.3302402262	0.3604077704	0.5117329706	1.069605757	2.578686298	1.703501715
0.3255483907	0.3434562183	0.4869191684	0.99480151	2.429434055	1.388375534
0.3297753716	0.3526200338	0.4920401193	0.2652419208	2.421974169	1.376395547
0.3389486348	0.3513617496	0.4872821483	1.093903318	2.082999195	1.225247308
0.3231156865	0.3413721919	0.4830388375	-1.247471065	2.343587022	1.352418696
0.3276197498	0.3457773425	0.496306168	-0.7201506595	2.431064242	1.452955239
0.3412768304	0.3535854824	0.4899477739	-0.8551736252	2.568186295	1.586728582
0.3238683016	0.3445118565	0.482879646	-1.383149714	2.468134775	1.607344083
0.3303975674	0.3470867311	0.4925289847	0.3618565104	2.429350703	1.4356285
0.3267423368	0.3505562051	0.4872864772	-1.366413049	2.288419985	1.134372088
0.3244608771	0.3433554281	0.4853830279	1.206798294	2.350784817	1.516201329
0.3279520406	0.3439100829	0.4854674457	1.154150513	2.552710402	1.473434293
0.3248719319	0.3425017716	0.4795192395	1.751176327	2.825189168	1.68566854
0.3285267667	0.3464075484	0.488379197	1.017169183	2.651098276	1.524575023
0.3291181818	0.3430714191	0.484636445	1.433737522	2.803991541	1.48296859
0.330705662	0.349347757	0.4948366695	-0.0999349013	2.571736696	1.463943075
0.3095105207	0.3277076189	0.460088445	-2.92050843	2.679706358	1.809174414
0.3314303269	0.3502599151	0.4976802078	0.6495023356	2.63389304	1.533630271
0.3292040264	0.3491463093	0.4898539001	0.7655451132	2.56177577	1.540137814
0.3308076783	0.343565209	0.4840930141	-1.392862884	2.818521106	1.527649376
0.3160721977	0.3289591437	0.462685613	-2.788241066	3.179375686	1.728445285
0.3283606483	0.3470879688	0.4885967392	0.9318008048	2.602001596	1.409610132
0.3322811723	0.349825122	0.4914957191	0.2451008255	2.583145588	1.476157771
0.3297899679	0.3475639697	0.4945483012	-0.6806634993	2.545268006	1.469857273
0.3304725494	0.3502689473	0.4934771027	0.3483179185	2.581106292	1.489383202
0.3237191267	0.3437164561	0.4815364073	-1.616880923	2.618784939	1.300815959
0.3237047931	0.3414993773	0.4842843883	-1.621208574	2.645845285	1.495487805
0.3229574312	0.3381634545	0.4752500967	-2.085255142	2.319972283	1.327141926
0.2940842645	0.3148229869	0.4377799482	-3.998267895	2.816164675	0.9331555229
0.3318301236	0.3489343277	0.4936526009	0.2986012269	2.588703123	1.472718989
0.3230050081	0.3379892543	0.4781932941	2.338330966	2.167494953	1.865455191
0.3426072791	0.350901682	0.4901819822	0.5828261859	2.322804282	1.376371314
0.3304480859	0.3438461638	0.4875722693	-1.804773612	2.168298947	1.205893291
0.3297203571	0.3484232898	0.4915491424	0.5962303693	2.580061493	1.496195325
0.3294268925	0.3412400558	0.4808394204	-1.71361528	2.240397272	1.393638295
0.3308445892	0.3529024722	0.4926603203	-0.1223171371	2.573278867	1.462493524
0.34065117	0.3483740636	0.4932414425	-0.9536021644	2.750454994	1.567092118

0.3277106165	0.3518190463	0.4861163313	-1.256134705	2.704656883	1.705086489
0.3316431154	0.3474328398	0.4916258146	0.7716789504	2.466880483	1.496208664
0.3345432338	0.3498143388	0.4913230581	-0.2443051781	2.578436917	1.476034998
0.3434888551	0.3537560535	0.5052536348	-0.1953985918	2.421488288	1.407901915
0.3357215738	0.3459359131	0.4899047625	-1.094733476	2.290124961	1.416008012
0.3255175755	0.3424409163	0.4820368555	-1.543672341	2.478637539	1.426710144
0.3287773321	0.3550689041	0.4897141335	-0.8529118028	2.602930665	1.618182758
0.3243976536	0.3397973758	0.4789881084	1.805631742	2.403746796	1.486716816
0.3354727971	0.3535904366	0.5050870989	0.4272480062	2.458152285	1.368056569
0.3333927518	0.3492212829	0.509719698	0.3684061778	2.499570047	1.435647328
0.3309836645	0.3470832435	0.4894269145	-0.8772940625	2.462769466	1.510412678
0.3146354433	0.3336400763	0.4674696166	2.527416394	2.599935527	1.800369724
0.3228303163	0.3436112094	0.4828347132	1.721569809	2.616966188	1.710946671
0.3308339929	0.3515892768	0.4910861102	0.5400843377	2.528474765	1.525819357
0.3306761893	0.3589112981	0.5041998551	0.2713496811	2.579503533	1.355530984
0.3405235135	0.3494137491	0.496662224	-0.04046783737	2.486429484	1.456231112
0.3307218603	0.3503900269	0.4950123019	0.0302135507	2.569648543	1.451587535
0.3293697307	0.3513559405	0.4934938627	-0.6968714774	2.584991059	1.55340483
0.3285974217	0.3470313303	0.4891588195	0.8969495152	2.56466187	1.514060366
0.330086121	0.3471463967	0.4934850077	-0.7849891372	2.51162659	1.458342182
0.3315138518	0.3486346504	0.4933401983	-0.3020771661	2.539653263	1.462556608
0.3307908025	0.3488836167	0.4953038932	-0.07498696339	2.567567532	1.460419594
0.3230773507	0.3408644655	0.4811078089	1.69283122	2.618238053	1.508665709
0.3230421998	0.3453285314	0.4850917354	1.81819045	2.781001007	1.803590793
0.3302133075	0.3535933163	0.4888464721	-0.8127464924	2.501276006	1.284902329
0.33285499	0.3505323311	0.4931354801	0.03155994124	2.549998136	1.450738114
0.3394426027	0.3539619996	0.5017927064	1.128033666	2.803751406	1.688338254
0.3290390865	0.3415802766	0.4873700983	-1.650832592	2.279683974	1.417648515
0.3261421793	0.3453138499	0.4858556322	1.491448342	2.74485153	1.662193564
0.3272262395	0.3445947686	0.4855325593	1.272509265	2.692186938	1.532915586
0.328508212	0.3470471521	0.4881080735	0.9509860055	2.548180828	1.530829666
0.3274790998	0.3494891312	0.4936023973	-1.087953313	2.59506404	1.294355749
0.3279503943	0.3463605916	0.4886492562	1.063151096	2.544211304	1.538993629
0.3367724475	0.3529753926	0.5118244478	0.4712342775	2.617342571	1.52053633
0.3319229966	0.3531514665	0.4933842386	-0.5736161742	2.625680465	1.541132287
0.3234511356	0.3388784142	0.4772284117	-1.909184008	2.397294938	1.453346612
0.3286943558	0.34228161	0.4824865578	-1.600186346	2.312341836	1.39426903
0.3424374636	0.349919539	0.4940313436	-0.9194252273	2.221478374	1.327476154
0.3296966733	0.3527015925	0.4900488622	-0.616696723	2.56771477	1.547322907
0.326477182	0.3444473784	0.4861044514	1.250205144	2.612594451	1.471690514
0.3311527251	0.3486713466	0.4893058088	-0.8980882296	2.453119351	1.363246523
0.3387618038	0.3496890596	0.5014767728	-0.08155608127	2.526779031	1.462838709
0.3329788474	0.3534837756	0.5055444627	0.2071230936	2.527799941	1.407946376
0.3354715244	0.3509065564	0.4901109812	-0.6456072908	2.654433372	1.539412208
0.3270166949	0.3485424772	0.4943985288	-1.305261613	2.697053332	1.671028102
0.3272500115	0.3451814564	0.4967560418	1.448232299	2.764564254	1.31211604

0.3276225446	0.3449751202	0.4864763463	1.17162297	2.520707145	1.474720091
0.3286144617	0.3496250368	0.4883762981	-0.879551068	2.598916271	1.343453638
0.3208134792	0.3428456355	0.4855418006	2.13702054	2.889438626	1.900929532
0.3183071907	0.3391531859	0.4721756448	-2.265910606	2.815652259	1.851711929
0.3270401119	0.3465651783	0.4861654682	1.177231413	2.569752319	1.584393697
0.3475431085	0.3545253054	0.4984342012	0.3996824091	2.321177598	1.363643703
0.3236748124	0.3411112185	0.4806626534	1.703385313	2.514168774	1.571625108
0.3315935828	0.3463445791	0.4898669773	1.088673914	2.392249661	1.395492248
0.3296823148	0.3495933081	0.487480878	-1.021874853	2.4706607	1.309568532
0.3320697272	0.3534037	0.4911371682	1.21352438	2.776812801	1.709730433
0.3329178734	0.3471658652	0.4910015204	-0.9262237369	2.705942694	1.528140647
0.3184834936	0.3368913124	0.473656734	2.18445958	2.747085034	1.688302662
0.3338717994	0.3480945868	0.4893936468	0.7234963703	2.665647467	1.510098501
0.3307677637	0.3657385113	0.5081648981	-0.1067668188	2.572404322	1.361285849
0.3322628018	0.3478178491	0.4896588496	-0.6750104288	2.645867582	1.440223083
0.2989532663	0.3162844634	0.4441426602	-3.676387516	2.978057581	1.915608852
0.330692859	0.3489025542	0.4934080799	0.01966625909	2.570253719	1.460685177
0.3304144651	0.3485359378	0.4909862588	-0.3579983632	2.564894831	1.464786049
0.3397673293	0.3479511351	0.4911549138	0.5896049393	2.359453594	1.453688304
0.3334813795	0.3489062857	0.4906829248	-0.4499742918	2.612363164	1.48683301
0.3303115801	0.3511897884	0.4958083289	0.3895341772	2.567543627	1.40257057
0.329921931	0.3640413382	0.4962312052	-0.5457471836	2.585428068	1.559882447
0.3280415765	0.3459430153	0.487378242	-1.015149119	2.560164257	1.477268933
0.3283647317	0.3446706291	0.4889706841	-1.218994423	2.712088381	1.467473435
0.3322832049	0.3468109673	0.4892935508	0.8574914469	2.432145158	1.457897564
0.329306931	0.3492396707	0.4897294703	-0.8316530624	2.627061889	1.555177693
0.3311755955	0.3490546965	0.4990539489	0.1841123181	2.555691033	1.466974573
0.3309580626	0.3492697546	0.4897577093	0.7492182065	2.64627713	1.5389476
0.3292968747	0.3453313989	0.4875540455	-1.166353808	2.443876846	1.425290049
0.3313359274	0.3472456744	0.5086618868	1.105586093	2.394872317	1.353637414
0.3106100587	0.3269695873	0.4598787052	-2.92523088	2.475132801	1.385784034
0.3299188129	0.3447285582	0.4876599781	-1.219159133	2.405696727	1.453141089
0.326869312	0.3471697683	0.4786444892	-1.850212251	2.270879555	1.077423884
0.3275571351	0.3420300443	0.4839348291	-1.673316022	2.865754299	1.362160961
0.3351603855	0.3523100944	0.508634351	-0.3769841354	2.472074163	1.391053778
0.331045323	0.3507882576	0.4918165547	0.216592729	2.555877989	1.429477181
0.3327999455	0.3522320432	0.5008360861	0.1241997086	2.53992205	1.4296081
0.3308704231	0.3501110313	0.4915281705	-0.2127910847	2.559822899	1.474163696
0.3381703481	0.348079134	0.4916378609	-0.5711781684	2.639763632	1.479372468
0.3200380387	0.3375011351	0.4751588453	-2.067582716	2.77490498	1.611181865
0.3365694398	0.3456821302	0.4969830351	1.227912478	2.82183557	1.571472978
0.3315857686	0.3489105822	0.4914543834	-0.1211104707	2.572443361	1.458392087
0.3285911375	0.358019049	0.5084702786	-1.375765188	2.401450112	1.035854918
0.3284788022	0.3463329682	0.4900047639	0.944530789	2.621923585	1.471045143
0.3295220947	0.3528353406	0.4908945637	-0.6590260988	2.586508224	1.556947468
0.3411594899	0.354205277	0.4911101535	0.5636881863	2.347316913	1.333475556

0.3306516374	0.3489096483	0.4926957599	-0.246612252	2.563809177	1.452449235
0.3282035059	0.3463782734	0.4954966964	-1.002417467	2.628497468	1.51725096
0.3208411844	0.3342436876	0.4622851787	-2.870313725	3.386070877	2.112466939
0.329729544	0.3496718396	0.4903781056	-0.6066067291	2.587873843	1.519486246
0.3254819052	0.3419674483	0.4843515561	1.59729399	2.769761084	1.552316663
0.3292732579	0.3529502772	0.490363662	-1.114973078	2.460100044	1.213523125
0.3234374558	0.3429156669	0.4830755499	1.670838133	2.572387124	1.659936667
0.3303792528	0.3464759997	0.4882320125	0.9570894165	2.682534368	1.504197822
0.2678625381	0.2884700695	0.4026322417	-1.097024985	-3.827820995	0.3195770029
0.270496856	0.2907491427	0.4074863318	-0.364502659	-3.775316985	0.3690496898
0.2725534394	0.2891332408	0.4054522531	-0.935597239	-3.859979947	0.358130511
0.2635917735	0.2841957133	0.3985815116	1.788433794	-3.913140151	0.2614537667
0.2661566018	0.2873141313	0.4003799736	-1.439504952	-3.793775937	0.5151651587
0.2714269889	0.2899137147	0.4113541242	1.2148755	-3.5195344	0.5208711758
0.2699361386	0.2917498896	0.4065476862	0.3180812294	-3.757964671	0.4221949367
0.2727128184	0.2904789707	0.4067894214	0.04891594121	-3.719372856	0.3930341621
0.2741712812	0.290611616	0.4072479743	-0.3400559013	-3.632376496	0.4111816186
0.2693600455	0.2911720183	0.4074869083	0.4097437377	-3.767139512	0.3573395702
0.2732862716	0.2911861657	0.4073206508	-0.1333212018	-3.727338115	0.3832287826
0.2675358966	0.2875593829	0.4068195963	-0.87194838	-3.855274298	0.4306153017
0.2704962437	0.2917652513	0.4077023435	-0.3201214809	-3.725835415	0.353989128
0.26500476	0.2862123644	0.3987075455	-1.569014562	-3.801892548	0.2538135205
0.268647414	0.2891328771	0.4060120975	-0.7597564102	-3.787688824	0.3711172618
0.2688694695	0.287549591	0.400750366	-1.460504214	-3.944783655	0.2550739314
0.2684098267	0.292065185	0.4039109713	-0.782278713	-3.767171102	0.2789725723
0.2701575379	0.2906085068	0.4075614058	0.02834144652	-3.746804822	0.3932112586
0.2726871594	0.292540012	0.4093902936	0.8637944696	-3.881373521	0.5457480121
0.2805689224	0.2939483949	0.4063366854	0.3728638779	-3.704190481	0.3242709889
0.2742654221	0.2912709108	0.4084180645	0.5826937517	-3.58071652	0.4459991626
0.2628252896	0.2829142773	0.3978066481	1.676551846	-3.923988625	0.4566442281
0.2750758328	0.2881434206	0.40327129	-0.985712763	-3.94110069	0.3351132636
0.2665505424	0.2904675025	0.4018728778	1.14208663	-3.892953496	0.22420795
0.2860072833	0.2958761772	0.4151590612	0.2694487275	-3.6240332	0.3291896478
0.2615793459	0.2834079513	0.3914237265	-2.34252918	-3.574719426	0.795538544
0.2705522072	0.2891132285	0.4057032631	-0.6234549376	-3.676468437	0.4096172856
0.2731808167	0.2912667488	0.4065849686	0.2264119339	-3.737480439	0.3719297127
0.2802656982	0.2943451014	0.4155314772	-0.6457937805	-3.783212957	0.2680196188
0.276864018	0.2913434544	0.4075674579	0.2923213017	-3.595083775	0.4266140747
0.2726564628	0.2920528043	0.4061243781	-0.1100815996	-3.731823424	0.3814704939
0.2699001013	0.2927317425	0.4072233553	-0.9514525948	-3.709675218	0.4905930784
0.264730327	0.2830020789	0.3959925211	1.634818187	-4.081999824	0.3490952963
0.2732681355	0.2937848201	0.4161652529	-0.1065674057	-3.689199986	0.405096868
0.2705075078	0.2906927424	0.4144301039	-0.006325946[illegible]	-3.742093107	0.3920004969
0.2766505726	0.2911281249	0.40722185	0.3096532356	-3.582768932	0.3700480385
0.2705521188	0.2913727974	0.4067550422	-0.2912927275	-3.722431863	0.3636836857
0.2706983566	0.2919304934	0.4075724274	-0.045838837[illegible]	-3.735885386	0.3845007492

0.2696664004	0.2932154277	0.4050437873	0.6909227964	-3.790010897	0.4936190985
0.2676337825	0.2942761808	0.4116104604	-1.392513287	-3.849950019	0.6723486991
0.2751604268	0.291040161	0.4086334684	-0.6698390459	-3.772347259	0.3508550872
0.2681460947	0.2895882003	0.4086199922	-1.121923625	-3.731510148	0.4934090408
0.2626582369	0.2876907955	0.3990640619	-1.60169579	-3.818578257	0.734870154
0.2701571211	0.2905329063	0.4078790109	0.05919818903	-3.747887482	0.3917870733
0.2723666831	0.2898286485	0.4124251382	1.061425841	-3.539901408	0.3775395573
0.2704696123	0.2904906181	0.408356949	-0.03709672199	-3.745631459	0.3932719304
0.2707894669	0.2914199943	0.4093291701	0.4475457881	-3.707699286	0.3470256444
0.2711160957	0.2906609473	0.4064506811	0.7123777113	-3.659882857	0.3919894546
0.2689159695	0.2931741605	0.4098760198	-0.8044450329	-3.804121704	0.2534911768
0.2652939776	0.2896298467	0.3978841931	-1.809189771	-3.930621324	0.02683509218
0.2668004221	0.286779589	0.4007987503	-1.288939892	-3.671191312	0.5467637953
0.2738002942	0.2944464735	0.413937265	0.7416334263	-3.558384844	0.5563418501
0.2729484677	0.2896158962	0.410867258	0.895830871	-3.550150563	0.3726790857
0.2679857431	0.2895922704	0.4061649012	0.933545938	-3.722963681	0.5175289872
0.269952492	0.2895296529	0.4054550532	0.8387661182	-3.67363691	0.4210662094
0.2708200603	0.2914441237	0.4075104929	-0.08336053142	-3.729937739	0.3812872679
0.2698282785	0.2948846221	0.4129952835	-0.4103162265	-3.762995324	0.3123193427
0.2633394003	0.2859911135	0.400357308	1.662381646	-3.896644525	0.640866704
0.2733276868	0.2941391694	0.4205842975	-0.208288771	-3.728237577	0.3484603274
0.2731785526	0.2936779246	0.4109694076	-0.3405982232	-3.649229467	0.4268258549
0.2714102435	0.2903863101	0.4062351703	-0.1914454719	-3.75070703	0.3902822568
0.2734816852	0.2894301397	0.4053896613	0.718665579	-3.571992449	0.4616358442
0.2826624174	0.2938693361	0.4107255834	-0.3757757144	-3.682563352	0.3220210434
0.2700104202	0.295445407	0.4062153525	-0.2157280117	-3.743052824	0.4288215721
0.2703460346	0.2906311167	0.4067109574	0.1298599249	-3.737920116	0.3884628597
0.271120851	0.2897540832	0.4054025327	0.6960902843	-3.645204176	0.4530053539
0.2758912932	0.2905307324	0.4144490981	0.7116834037	-3.81493501	0.3317148173
0.2720847475	0.295350486	0.4176161977	0.8544705931	-3.839569534	0.2041417341
0.2736703468	0.2922758741	0.4061411653	0.1844274566	-3.73098857	0.3699225477
0.2706065311	0.294168758	0.4136097443	0.1851001282	-3.749470664	0.3553889894
0.2654650511	0.286760778	0.4101206125	1.666375204	-3.640957607	0.1719333758
0.2700156356	0.2904840592	0.4086721775	0.5636723936	-3.737402904	0.4004229376
0.2650399056	0.2885808006	0.3992594756	-1.429450119	-3.888589918	0.1888287752
0.2622901659	0.2875726862	0.4045868064	2.115769879	-3.592732925	0.9028491655
0.2672265239	0.2898256188	0.4015142528	-1.201453321	-3.723167008	0.5639098469
0.2699942744	0.2909294488	0.4061843225	-0.1349001277	-3.747984459	0.3845424302
0.2803098233	0.2915839193	0.4076743064	0.7868163845	-3.841264657	0.2873831165
0.2710748984	0.2902622803	0.4063670063	-0.1877435905	-3.719002222	0.3936677343
0.2698395847	0.2898531998	0.4055972892	0.6120615268	-3.780717092	0.3997465597
0.2713994211	0.2926601029	0.4062252796	-0.2763535252	-3.760714756	0.3548515078
0.2737952187	0.2953498609	0.4092403127	-0.3252580793	-3.760785205	0.330053118
0.2734603132	0.2905057376	0.4071263741	0.4218615548	-3.776207066	0.3682940534
0.2696025352	0.2889346474	0.4024712084	-0.8836066664	-3.893615885	0.3312020728
0.274297151	0.2905829494	0.4064395	0.3073879663	-3.633379099	0.3932052623

0.2705637357	0.304093881	0.4164217774	-0.1114972326	-3.7341127	0.3402749724
0.2732269007	0.2902100309	0.4061640081	-0.460464426	-3.630151025	0.3768574442
0.2650090324	0.2850630865	0.398379642	-1.710939725	-3.728968795	0.4838794178
0.2670392957	0.2871511481	0.4024841914	-1.362975586	-3.760299111	0.3453058671
0.2703308873	0.2913351333	0.4064284081	-0.3391168461	-3.750327878	0.3595809698
0.2767480547	0.2908475656	0.4065515035	0.1299123722	-3.683333389	0.3830112924
0.2718353872	0.2887961697	0.4022546622	1.161624854	-3.934039318	0.2829788827
0.2701355049	0.2942528302	0.4092390732	0.1161639934	-3.748996849	0.3675741554
0.269675966	0.3069305383	0.4100208558	0.8636173458	-3.758086034	0.0680503567
0.2693779061	0.2897267972	0.4053397297	0.5095594259	-3.75027723	0.3947822123
0.270562687	0.2906685035	0.4101612642	-0.0889552196	-3.736558244	0.3889091458
0.2677691763	0.2861378043	0.4001540507	1.899893571	-3.888189526	0.3325311422
0.2691296177	0.2902675122	0.4048740151	-0.8302255858	-3.710635675	0.4708899278
0.2656003261	0.2869361031	0.4079787088	-1.24102366	-3.855934865	0.313392448
0.2733658438	0.2926649727	0.4069236845	-0.0392309658	-3.709391424	0.384316986
0.2710604621	0.2901964701	0.406156021	-0.3749413091	-3.773347936	0.3841220726
0.2728934215	0.2942684969	0.4215442275	0.6193845561	-3.776291365	0.2702570907
0.2634992149	0.2834026626	0.3966559477	-1.814260349	-3.945887524	0.3111885033
0.272607372	0.2899366207	0.4072204435	0.4854578724	-3.641645376	0.3804806034
0.2726911939	0.295305763	0.4061175258	0.2566028034	-3.67604217	0.4326748012
0.2748879074	0.2912487279	0.406924105	-0.8831216906	-3.493118482	0.2807133544
0.2734228057	0.295324094	0.4158155827	-0.7373807906	-3.823848769	0.2341389668
0.2699019404	0.2910729714	0.4061293381	0.1288308141	-3.754171044	0.3844011643
0.2740111392	0.2944957815	0.4207678023	-0.1056773252	-3.67600393	0.4030816347
0.2703809869	0.2887830806	0.4045905727	-1.158351269	-3.849410447	0.3713923493
0.275240873	0.2904835984	0.4063462272	0.253416087	-3.729739135	0.4043387809
0.2704819351	0.2905574812	0.4065483562	0.1811217116	-3.757301914	0.38656893
0.279469046	0.2931902966	0.4177449574	-0.1542307716	-3.659510582	0.3649703045
0.2703424697	0.2895864577	0.4050425023	-1.005684608	-3.639216765	0.3432075967
0.2717282454	0.303825115	0.4267859303	-0.4527310602	-3.777024591	0.2377219256
0.2699886915	0.2909002824	0.4088408915	-0.0775581890	-3.750102491	0.3970411892
0.2705002611	0.2927753567	0.4089121333	-0.2802371981	-3.731909918	0.4108712994
0.2831310652	0.2939005824	0.4025273464	0.8278531487	-3.866861505	0.2422526987
0.2661114727	0.2864421421	0.4006385529	-1.465153727	-3.82578646	0.3163044843
0.268639713	0.2889964457	0.4111467199	0.7353280131	-3.790283965	0.3864621872
0.2820014604	0.2944505158	0.4068741778	-1.183455045	-3.208481708	0.5989392401
0.2701553958	0.2925845077	0.4090608172	-0.0612058228	-3.745869844	0.3971989613
0.2691469108	0.2897802083	0.4051899922	0.675516001	-3.750403753	0.3609118131
0.2716181413	0.2976621775	0.410433605	-0.272970109	-3.752931358	0.3195550015
0.2705301956	0.2909439771	0.4081724467	-0.0231978421	-3.740893633	0.3902681285
0.2704842421	0.2896271897	0.4053163112	-0.4578632832	-3.795101286	0.4089600653
0.2641218531	0.2849642598	0.3979563391	-0.9883790317	-3.580270269	0.4236430161
0.2647166177	0.2850579675	0.3996837463	-0.850005856	-3.61492665	0.4312445618
0.2640197587	0.2805673541	0.3935567897	-1.658185522	-3.827907071	0.4343332278
0.257313115	0.2780636986	0.3900845226	2.006358204	-3.739560734	0.3411374869
0.2632328329	0.2848110296	0.3968529541	-1.234769352	-3.535892427	0.5918119278

0.2668198555	0.2853453439	0.4050624235	1.322443525	-3.27789093	0.6175389994
0.265722301	0.2875875758	0.4009269967	0.00194867655	-3.523101167	0.4833428033
0.2678988967	0.2861171004	0.4007169386	0.2279890475	-3.527553552	0.4913531025
0.2697769064	0.2863207657	0.4013086023	-0.3843455034	-3.401176905	0.508582776
0.2654747514	0.287928788	0.403051209	0.1934301147	-3.531630042	0.462343977
0.2688584446	0.28689118	0.4014595541	-0.1697715071	-3.510765312	0.4735373834
0.2659923524	0.2862698569	0.4049379161	-0.0627670092	-3.524385752	0.4874449532
0.2659985955	0.2876035645	0.4017192664	-0.3091027308	-3.504546033	0.4516393369
0.2587345455	0.279971498	0.3901513086	-1.792404086	-3.595039305	0.3457425076
0.2642942335	0.2850783826	0.4000020416	-0.7573400789	-3.56333542	0.4585465437
0.2647883085	0.2840749074	0.3959348915	-1.29569679	-3.693261678	0.373915644
0.2640517067	0.2874781745	0.3981170726	-0.7696567185	-3.532392629	0.3793742818
0.2639668194	0.2847073834	0.3991703338	0.7089827236	-3.537041983	0.519878684
0.2695855153	0.2896698555	0.405310261	0.4769450318	-3.565128501	0.5631085049
0.2763450336	0.2901800899	0.4006634788	0.271260193	-3.458700366	0.432368204
0.2685987195	0.2859831936	0.4007690915	0.682719051	-3.368704636	0.5338615916
0.251116377	0.2710481816	0.3812481516	2.438962422	-3.82278825	0.576534536
0.271354171	0.2843476852	0.3981212902	-0.9692207351	-3.70730638	0.4143288909
0.264232555	0.2883198439	0.3989912863	0.797716524	-3.610020238	0.355652382
0.2812533514	0.2918169701	0.4089641993	0.5150091036	-3.493003769	0.3703556583
0.2617278995	0.2836024183	0.39193311	-2.008183076	-3.285620533	0.8137206556
0.2670457938	0.2859302446	0.4012105324	-0.2984746279	-3.473567089	0.4925764618
0.268945972	0.2872855129	0.4008839031	0.02971023131	-3.485213061	0.4810522993
0.2763579441	0.2908511486	0.41031273	-0.2745972593	-3.46150907	0.427759459
0.2715004025	0.2864017767	0.4004248386	0.6314079839	-3.310279384	0.5610252574
0.2687234107	0.2886976523	0.4006830804	0.00859048758	-3.482248265	0.4822604994
0.2658422619	0.2880130027	0.4017652796	-0.5969911749	-3.497906751	0.5361963701
0.2602953831	0.2787101176	0.3900207431	1.667978653	-3.860063872	0.4158756803
0.2683614479	0.2888161958	0.4089023984	0.6708169457	-3.582017975	0.3791540061
0.2642550633	0.284795684	0.4059051956	-0.7264798488	-3.513136789	0.5242062224
0.2733347545	0.287330974	0.4018872028	0.7548516694	-3.231409522	0.4463800149
0.2659932456	0.2870638162	0.4009372557	-0.0102453660[illegible]	-3.518757214	0.483162918
0.2655153916	0.2870478508	0.4006485529	0.3351785519	-3.553540343	0.5220385045
0.2656775228	0.2896137627	0.3999154811	0.5796343191	-3.551417188	0.5686554089
0.2656116941	0.2914239079	0.4079736389	-0.7540780355	-3.575762205	0.6242194824
0.2695546197	0.2861848801	0.401407547	0.07757868879	-3.461505975	0.488272575
0.2649049634	0.2868060906	0.405538518	-0.9944653234	-3.481184152	0.5777275817
0.2547891222	0.2796453287	0.387663028	-2.062695059	-3.62310463	0.9098109644
0.2646894128	0.2853107287	0.4006190829	-0.5657844778	-3.529852054	0.488406333
0.2678342248	0.2855077406	0.4069060938	0.9211969477	-3.347128188	0.4840054689
0.2660683577	0.2862435884	0.4027870063	-0.0880307015[illegible]	-3.525357108	0.4875789193
0.2634488208	0.2849481565	0.3999241778	-0.7515163584	-3.604360683	0.5675141857
0.2664716647	0.2861348794	0.4002649595	0.8282250566	-3.424418547	0.5018306865
0.2651687287	0.2899864993	0.4055031789	-0.6606146823	-3.565650343	0.3614826201
0.2637116319	0.2884637446	0.3967334158	-1.377186928	-3.626186414	0.2099823968
0.2625083215	0.2827573243	0.3951318796	-1.339383048	-3.429413929	0.6384008043

0.2713596152	0.2925200889	0.4110049112	0.1426920073	-3.422127249	0.506344214
0.2664921921	0.2829984194	0.4017994219	1.454401013	-3.250036686	0.4556981897
0.2616412943	0.2832886013	0.3980388682	1.266229412	-3.508351305	0.6445177602
0.2653373265	0.2850969522	0.399293442	0.9037163782	-3.446192444	0.5122512545
0.2644425704	0.2851189942	0.3992270955	0.5884687761	-3.601427798	0.543749856
0.2655796396	0.2916324541	0.408496078	-0.3357040357	-3.537695581	0.4112433517
0.2643130245	0.2878332248	0.4029298262	0.8538417734	-3.556663172	0.6039651212
0.2682323122	0.2896912189	0.413889946	0.1885633556	-3.465753969	0.512951946
0.2684391465	0.2888444584	0.4045475697	-0.4295024735	-3.420299873	0.530215739
0.26702403	0.2863433227	0.4006302337	-0.0106708182[illegible]	-3.506978278	0.4851642945
0.2693082539	0.2858306324	0.400191949	0.6356967484	-3.360090316	0.5407828219
0.2781652576	0.2897521324	0.4044608211	0.1993954689	-3.308901658	0.5148141469
0.2647040573	0.2905219206	0.3990893422	-0.5537939694	-3.517746123	0.5910350592
0.2654736384	0.2860587592	0.4002994286	-0.150879981	-3.534070348	0.4890383081
0.267069924	0.285740907	0.3998579781	0.6476116797	-3.41772853	0.5404155042
0.2716973518	0.2866444344	0.4092250836	0.7496270444	-3.600322935	0.4169916997
0.2667151567	0.2908281416	0.4108392713	1.029542676	-3.655354795	0.2570626538
0.2684456173	0.2872982472	0.39970503	0.4265515206	-3.558661034	0.4412065485
0.2650030286	0.2888112761	0.4057852397	-0.5372577136	-3.509199358	0.5686898972
0.2649543651	0.2868133282	0.4101701649	1.041976841	-3.421921392	0.337533975
0.2656703397	0.2865508918	0.403519512	0.02314461116	-3.523177572	0.4855506811
0.2611964701	0.2849474907	0.3940906902	-1.440214665	-3.648357907	0.2644038496
0.2638691315	0.2898979135	0.4080064708	1.401595052	-3.359290625	0.8097941034
0.2633695257	0.2862506961	0.3965744533	-1.095563086	-3.495913744	0.6393182254
0.2640850862	0.2851522338	0.3982062667	-0.7756900866	-3.534991096	0.4456060694
0.2736977969	0.2852624115	0.3988189637	1.173585658	-3.763173401	0.3375316487
0.2657480566	0.285275478	0.3994312217	-0.541487251	-3.477558658	0.4892195475
0.2655408336	0.2861350686	0.4000636024	0.5475905362	-3.541796167	0.499801677
0.2670717459	0.2885055962	0.4007625823	-0.0749753251[illegible]	-3.513202574	0.4724873033
0.2683817798	0.2899380501	0.4020248719	-0.6833048884	-3.61941672	0.3566031691
0.2690813841	0.2863032302	0.4013002913	0.3754059925	-3.545765777	0.465719306
0.263384034	0.2823731992	0.3934104583	-1.285991696	-3.768302992	0.3853018688
0.2700698463	0.2863954906	0.4006461242	0.03536406532	-3.459646371	0.4855122119
0.2662918071	0.2991110911	0.4093865353	-0.0609704394[illegible]	-3.510927428	0.4466324791
0.2690561309	0.2855905874	0.4001243178	-0.5681751356	-3.380562155	0.4755333136
0.2626792528	0.2830846366	0.3957535477	-1.35364785	-3.487928628	0.5552665639
0.2589032992	0.2789715945	0.3908637664	-1.986923894	-3.562627067	0.4301990491
0.2651627837	0.2868592849	0.399455407	-0.7209421804	-3.551219796	0.4113533462
0.271837395	0.2866781266	0.4006557478	0.1664032987	-3.476821408	0.4758596155
0.2682235802	0.2856488123	0.3978045009	0.9638927758	-3.663997977	0.3926369864
0.2656401407	0.2903072601	0.4034019027	0.300435837	-3.528859836	0.4271132703
0.2653906363	0.3035576705	0.4046710514	0.6787498895	-3.536922678	0.2229723869
0.2647226698	0.2853212934	0.3991421946	0.6361050879	-3.525937419	0.4904053061
0.265986256	0.2865082622	0.4038504717	-0.1226115521	-3.513373665	0.4804054868
0.2658443332	0.2851150665	0.3987347044	1.123910576	-3.606586499	0.445313243
0.2650406426	0.2865264873	0.3992614363	-0.7890300239	-3.477845274	0.5638463968

0.2601879903	0.2816788439	0.4009427808	-1.435326145	-3.654279571	0.3794195722
0.2686394967	0.2881139983	0.4006814229	-0.3038188499	-3.533852809	0.4469029357
0.2667346487	0.2863137846	0.4006606346	-0.1320512324	-3.522360836	0.4831764883
0.2675192828	0.290265964	0.414917721	-0.1397754322	-3.484912634	0.5054848123
0.2587806163	0.2790073695	0.3906531166	-1.8212301	-3.714587604	0.4124838447
0.2689285226	0.2864848112	0.402156454	0.2275608306	-3.444659964	0.4753508911
0.2685218424	0.2913105393	0.4004032113	0.1983349124	-3.456855888	0.5137976408
0.2717109329	0.2879141659	0.4018665106	-0.525190769	-3.327619104	0.4200776894
0.2695535192	0.292722101	0.4116582604	-0.3706176629	-3.536863987	0.3954627865
0.2595913136	0.2806623057	0.3917099092	1.487776622	-3.644990726	0.4114185927
0.2703267622	0.2910198041	0.4174063452	-0.2722164231	-3.410475466	0.5243509695
0.2681164033	0.2865520934	0.4018829534	-0.6413915636	-3.562575217	0.4510343398
0.2701795883	0.2861983124	0.3999420379	0.4453642785	-3.552118205	0.5106503888
0.2659886151	0.2863241293	0.400655633	0.198701691	-3.534550148	0.479097362
0.2750053061	0.2894629574	0.4124200559	-0.1933456013	-3.452961981	0.4506434707
0.2655534641	0.2848926038	0.3986805229	-1.139855181	-3.401752131	0.4497562414
0.2674354721	0.3003474811	0.4214492476	-0.0393629376	-3.504979203	0.4506940306
0.2656709312	0.2870194755	0.4033258454	0.09744949691	-3.52348774	0.4768915523
0.2655282746	0.2876818568	0.4025249087	0.05597449461	-3.527423695	0.4785083207
0.2766969666	0.2870182512	0.3926282186	1.311081375	-3.853148827	0.2322047056
0.2587676726	0.279289654	0.3905168569	-1.820760435	-3.653101708	0.4051349121
0.2633755844	0.2839371382	0.4041585938	0.9261386811	-3.583699263	0.4793947417
0.2812961287	0.2926548308	0.4048215795	-0.6775256837	-3.092715953	0.6029884618
0.2639142453	0.286954074	0.4010269295	-0.7843611841	-3.523084908	0.5972016475
0.2653463422	0.2863963393	0.4002842016	0.3577498674	-3.523225519	0.4668142249
0.2673242246	0.2938597469	0.404840596	-0.0475738659	-3.506731092	0.4617447426
0.2658801081	0.2863327083	0.401666703	-0.2733877822	-3.505190561	0.474294154
0.2647732528	0.2842739687	0.3976584433	-0.7925520116	-3.62257576	0.518689657
0.2597951194	0.2810397262	0.3927112941	-0.5871770132	2.232190901	-1.738564904
0.2594643698	0.2800452764	0.3927938846	0.7820176093	2.327488698	-1.669909758
0.2644094031	0.2815717618	0.3950087711	-0.0319673238	2.207363392	-1.708204403
0.2606319804	0.2823348668	0.3961331972	-0.2332956461	2.257645731	-1.679813412
0.2483124067	0.269672506	0.3756480953	2.383655218	2.315067086	-2.026792091
0.2604245325	0.2791460452	0.3963096322	-1.29815407	2.039616779	-1.839935652
0.2603208028	0.2822666427	0.3936513213	-0.2320294056	2.254457216	-1.720358517
0.2581497897	0.2766200838	0.3873975846	1.518940182	2.063759905	-1.694455263
0.2640733999	0.2807137733	0.3934859174	0.7023318329	2.082011497	-1.75308428
0.2596027653	0.2829288236	0.3961522163	0.5762525909	2.23456076	-1.775778241
0.2614733323	0.2795250758	0.3912409336	-1.213833262	2.040986106	-1.788022156
0.2575548752	0.2780871198	0.3930821546	1.048903697	2.335142131	-1.771969272
0.2599901569	0.2820240181	0.3936623676	-0.2481120454	2.258731532	-1.729647207
0.2559930519	0.2775746815	0.3869146984	1.449211734	2.255209339	-1.614160844
0.2510263856	0.2716015065	0.3806308358	2.092765442	2.393770592	-1.679369937
0.2612092538	0.2812369695	0.3919598399	0.8002325458	2.336151405	-1.635515917
0.2580111724	0.2810650412	0.3899412057	0.9597075747	2.232182202	-1.594711429
0.2590101627	0.2800501537	0.392492949	0.9283632691	2.278637416	-1.674631252

0.2645258889	0.2848798883	0.3984860886	-0.3708215471	2.281314457	-1.749317444
0.2693623206	0.2837105155	0.3909978129	0.716867379	1.972424073	-1.825127465
0.2553208378	0.2734001527	0.3825431455	1.41668082	2.499425065	-1.804031597
0.2597374318	0.2807091903	0.3948637111	-0.5809971325	2.263920771	-1.715122924
0.2647694374	0.2776193457	0.3888958046	1.2813051	2.546128437	-1.631619694
0.2609902988	0.2853541599	0.3949711853	-0.1236611966	2.252120071	-1.663863936
0.2675134436	0.2789097841	0.3900527531	1.73206169	1.612804999	-2.117747607
0.2629485909	0.2849378066	0.394289087	-0.2646553482	2.26650554	-1.646187568
0.2577469089	0.2767053041	0.3881888479	-1.531010944	2.448856184	-1.700201196
0.2602264597	0.2786826668	0.388613969	1.160488843	2.058276273	-1.845035128
0.2710763382	0.2860187092	0.4031364936	0.1516738413	2.200734258	-1.650629702
0.2654016987	0.2806020486	0.3920666073	1.343066132	2.518944882	-1.534478905
0.2608737192	0.2818920437	0.3891751383	0.8101573137	2.381879997	-1.600335486
0.256283508	0.2775241567	0.3876687294	2.230561589	2.223151764	-1.822262774
0.2614624552	0.2804413297	0.3924551727	-0.7158319111	2.341519684	-1.669055685
0.2624677393	0.2830294558	0.4002820434	-0.3317036103	2.27726686	-1.660095409
0.2604565501	0.2814744911	0.4009761748	-0.4645364722	2.267062868	-1.680622014
0.2642732906	0.2767384349	0.3870247904	1.09102538	2.489621592	-1.78160231
0.2598997147	0.2812175826	0.3929176503	-0.4992635676	2.277691823	-1.745404392
0.2602353506	0.282111539	0.3936019861	-0.4079857976	2.275638647	-1.747399564
0.2600820399	0.2844970016	0.3926027909	0.5307248102	2.220683212	-1.601901213
0.261434152	0.2862221336	0.4009245569	0.7158341916	2.300740962	-1.790765885
0.2612629186	0.278082144	0.3897747878	1.509991165	2.496926204	-1.684230877
0.2598275559	0.2824914646	0.4007630014	0.6076478696	2.222166575	-1.77194078
0.2546336057	0.2800639874	0.3879895951	1.433187319	2.24497645	-2.010143613
0.2603570543	0.2813636664	0.3950830052	-0.311961609	2.252670244	-1.70207749
0.2633950708	0.2813165117	0.4013554407	-0.8303257543	2.093562643	-1.684805488
0.25747591	0.2775821589	0.3910085877	-1.332070342	2.17611577	-1.718685257
0.2593223012	0.2815828021	0.3947990119	0.3978629004	2.278281233	-1.76366032
0.2562941114	0.2757449665	0.3858837327	-1.828087442	2.096480152	-1.792581238
0.2603884917	0.2859753186	0.3999438681	0.4142748843	2.273609256	-1.602692526
0.2606500195	0.2859204361	0.3938342598	0.2239623791	2.258507362	-1.639414701
0.2599563492	0.280844002	0.3923400972	0.8533818196	2.155326231	-1.809277048
0.2637679494	0.2856590407	0.4008875858	-0.8171015995	2.049686289	-1.871924517
0.2613701969	0.2781550765	0.3950482964	0.9251849863	2.407524354	-1.782503386
0.2562587845	0.2780519202	0.3913782776	-1.3455841	2.200401224	-1.882873135
0.260499099	0.280674208	0.3930293582	0.4252112359	2.282251274	-1.70745657
0.2589189147	0.2795966512	0.3919064424	-0.8370974557	2.332927061	-1.791194236
0.2559993454	0.2830889207	0.3965141603	-1.315879568	2.218714846	-1.99886483
0.2588708315	0.2837105378	0.3970446184	-0.6296653181	2.270392344	-1.80661576
0.2633073066	0.285311383	0.4073060657	0.6831973744	2.323827489	-1.553216141
0.262701534	0.2830998329	0.3967439165	0.576787747	2.148817025	-1.749027091
0.2572290065	0.2767443217	0.3871925787	1.295509354	2.4044141	-1.745632793
0.2648978459	0.2822330098	0.3949014874	0.2357945096	2.252567281	-1.680991851
0.273246457	0.2851710092	0.3974778692	0.7515355462	2.37293012	-1.541620456
0.2583404548	0.2845117152	0.3903742351	0.9631626719	2.213043747	-1.885968644

0.2567196629	0.2774483313	0.3881559861	1.117198331	2.317759333	-1.764241617
0.2610735558	0.2797130795	0.3914602048	-0.991118512	2.089533418	-1.798325828
0.2669462953	0.2824079685	0.4035603981	0.1265842372	2.163681477	-1.71334172
0.2617952821	0.2871608205	0.4050789841	0.575215314	2.152932604	-1.808036532
0.2534892719	0.2721224503	0.379158685	-2.028226142	2.618473288	-1.60474334
0.2596446069	0.2837350266	0.3983123545	0.5168061398	2.229785935	-1.77800621
0.2493466598	0.2709066331	0.3873460563	-2.39389046	2.101161732	-1.454265433
0.2603484008	0.2815932599	0.3968875006	-0.08821759487	2.245534636	-1.708790289
0.2564891995	0.2806668275	0.3879014512	-1.367066828	2.219133187	-1.93684513
0.2592408563	0.2858943059	0.4025468883	-1.222084293	2.086351255	-1.983357876
0.2494301053	0.2718857234	0.376648631	2.329232445	2.223338464	-2.099645649
0.2604130674	0.2818540644	0.3936291821	-0.2771065112	2.250677113	-1.714764862
0.2706085054	0.2826916691	0.3951590782	-0.6159752297	2.33803722	-1.622186101
0.2558223917	0.2753371507	0.3855017092	-1.789058597	2.446917571	-1.729791211
0.2593915896	0.2808108646	0.3921354971	-0.5105372175	2.265195465	-1.745343849
0.2598712743	0.2813923309	0.3911889722	-0.9345927976	2.157001525	-1.815443783
0.2606000289	0.2819491486	0.3911551538	-1.601555969	1.951845078	-1.98712799
0.2640089302	0.2815187097	0.3945813193	-0.2099249898	2.252448358	-1.696742926
0.2632977063	0.282352644	0.393433316	-0.6148466614	2.123824848	-1.755096046
0.2599515793	0.2760029254	0.3861154016	-1.712042572	1.929764175	-1.748964249
0.2583623112	0.2899110838	0.3965869555	1.160689901	2.167207647	-1.332708012
0.2645501853	0.2805113126	0.393545992	0.6194173889	2.091341658	-1.70677605
0.2553760097	0.2758573878	0.3857133147	1.700156149	2.194823694	-1.810792307
0.2603168335	0.2814052995	0.3939973166	-0.02404280957	2.248437065	-1.708177659
0.2589562214	0.2816014413	0.3909439242	1.280533723	2.31246031	-1.52104918
0.2653059501	0.280950415	0.3925350377	-0.8057109879	2.369047293	-1.660798234
0.2645988555	0.282662914	0.3934995382	-0.1680652524	2.241793503	-1.682424317
0.2603113337	0.2857560112	0.396563548	-0.24060765	2.253933592	-1.63721508
0.2600804559	0.2991323764	0.3978233484	-0.6929897797	2.268626072	-1.357186642
0.2575178805	0.2783971937	0.3893367078	-1.014762923	2.258119942	-1.74892545
0.2577681054	0.2785829309	0.3921174645	1.184016087	2.20346596	-1.662342738
0.2509311951	0.2696504881	0.3769810018	-2.973657192	2.609895697	-1.574713254
0.2609103757	0.2829486778	0.3937200012	0.2779117985	2.22189227	-1.72671221
0.2598269847	0.2819375909	0.4017824155	-0.5184839165	2.233849265	-1.753257639
0.2622435808	0.2819030957	0.3920844896	-0.76048432	2.111250314	-1.796200535
0.2568480019	0.276670838	0.387033043	-1.419924132	2.158480413	-1.753468757
0.2618139717	0.2854156023	0.4072783548	-0.903335435	2.3450443	-1.500517024
0.2552114009	0.2759275647	0.3864737986	1.586272633	2.362076914	-1.670291254
0.2493922972	0.2664904269	0.3735711318	2.544659452	2.765444374	-1.978229082
0.250758235	0.2728158521	0.3746445044	2.266545899	2.692606051	-1.377426079
0.2636798297	0.2794449312	0.3896133381	1.409183426	1.903339276	-1.551856807
0.2640070446	0.2883938518	0.4049305456	0.8689367629	2.368258421	-1.449280547
0.2440503211	0.2645399978	0.3692361166	-2.630223252	2.44043291	-1.702285797
0.2641589849	0.2850712795	0.4110691067	0.8597158049	2.036615684	-1.863086566
0.2653513512	0.2839035244	0.3982061116	0.4354516249	2.273967555	-1.623072098
0.2611120538	0.2778577259	0.3877501859	1.495736424	1.959823487	-1.613378004

0.2541303594	0.2742695337		0.3837540229	1.878796296	2.18233685	-1.808849372
0.2699151809	0.2853251752		0.4063763361	0.2133545898	2.223370386	-1.643945639
0.2615393301	0.2812377279		0.3937981481	-0.1040487123	2.248023699	-1.710322221
0.2619489126	0.2957000689		0.4143287876	0.1200178405	2.247114981	-1.586472765
0.2603520151	0.2823005285		0.396530167	-0.3447151153	2.248652516	-1.668516218
0.2597409802	0.28163		0.3944747327	1.369400326	2.234901242	-1.773991458
0.2776463638	0.2878745556		0.3934235679	-0.0032761279	2.108633406	-1.668206189
0.2566164611	0.2776674538		0.3880971256	1.541685179	2.304571585	-1.628371599
0.2601334274	0.2811304772		0.4002853508	0.3323608815	2.241007172	-1.713569091
0.274483997	0.2843007855		0.3937819611	1.343463026	1.657466271	-1.975856816
0.2587831003	0.2826108446		0.3947165338	0.7493200565	2.22320741	-1.823310521
0.2599632121	0.281454881		0.3931112678	-0.4959451592	2.243547781	-1.674257711
0.2611051978	0.288102027		0.3964434672	0.7739073552	2.332635101	-1.483928003
0.2585292497	0.2788791109		0.3910783749	-1.268534043	2.348891101	-1.749110771
0.2603706683	0.2803397657		0.3919163008	-0.8861642271	2.163093101	-1.671665947
0.2816205976	0.3033182881		0.4233713368	-1.001760818	-2.030446105	1.55877984
0.2849850903	0.3061540926		0.4290057284	0.4477151478	-1.91489913	1.623154895
0.2825874729	0.2997158843		0.4202699779	-1.660484903	-2.237106659	1.576937915
0.2839519884	0.3060619906		0.429230225	0.3638798653	-1.977975358	1.573091783
0.2791737073	0.3012023342		0.4197384189	-1.612339213	-1.985837761	1.750004592
0.2840756555	0.3034489237		0.4305785093	1.350991436	-1.739654244	1.767525683
0.2840675434	0.3069853339		0.4277560335	0.07106372678	-1.965153835	1.60475181
0.2860234591	0.3046753585		0.4266717605	0.3988173307	-2.010732025	1.623394223
0.2885537405	0.3058184202		0.428547464	0.05037345174	-1.942377564	1.601808724
0.2836384459	0.3064216033		0.4288040219	0.3255636525	-1.970195992	1.572282436
0.2877230735	0.3064627807		0.4286390531	0.2695344255	-1.892323718	1.614328185
0.2749859673	0.2957038883		0.418325397	-1.817904715	-2.163717266	1.735434338
0.2843020371	0.3066450402		0.4284973477	-0.2068843739	-1.952637734	1.575790572
0.2811970975	0.3036633949		0.4230061258	-1.119749097	-1.971729616	1.513220208
0.2820315134	0.3034585504		0.4261659129	-0.9052578274	-1.998362951	1.588677802
0.283526939	0.3031231884		0.4224483125	-1.346551294	-2.133469806	1.480845491
0.2812748939	0.3060505964		0.4232076703	-1.012568545	-1.97642489	1.459528679
0.2827885183	0.3042495925		0.4266860855	0.57368602	-1.962103965	1.64293914
0.2854938434	0.3062341281		0.4285591786	1.138784294	-2.172925894	1.808226103
0.2949277617	0.3090123277		0.4271471839	-0.0265085951	-1.884698066	1.592473885
0.2885786785	0.3063892785		0.4296398758	0.5821497656	-1.820944183	1.655253518
0.2782430008	0.2992443565		0.4206951508	1.548544852	-2.061228387	1.679056326
0.2849827307	0.2985536483		0.4178982497	-1.573974963	-2.375176292	1.561809799
0.2746182478	0.2993819151		0.4142390083	1.881307025	-2.18743423	1.375142239
0.3001545526	0.3100187863		0.4352780517	0.9675778147	-2.154318035	1.367735479
0.2792709543	0.302524282		0.4175742508	-2.059581497	-1.732337366	1.922365505
0.2847634479	0.3042573478		0.4269550979	-0.479787926	-1.910270015	1.63107462
0.2869393496	0.305899215		0.4270183524	0.3823185651	-2.000091366	1.574495861
0.2953635307	0.3101008486		0.4378192634	-0.4807616019	-2.012186837	1.484877657
0.2913957664	0.3065385439		0.4288511991	0.1556192288	-1.881818501	1.621246174
0.2866108988	0.3069215477		0.4268614056	-0.1846908066	-1.975563697	1.583317568

0.2832532035	0.3073487308	0.4271203636	-1.292633915	-1.912506671	1.725131562
0.281497413	0.3007256523	0.4207685805	1.246862136	-2.170223425	1.597788274
0.2865886269	0.3080758839	0.4364305378	-0.5769039609	-1.858159646	1.682891816
0.2844987619	0.3056444443	0.4357519066	0.05657515714	-1.963391232	1.601796958
0.2900505545	0.3054077983	0.42721128	0.9000596204	-1.7193056	1.545213245
0.2843283796	0.3062163197	0.4274793766	-0.0100178978	-1.961409747	1.601310549
0.2846242564	0.3068726543	0.4284423874	-0.2099090243	-1.944461494	1.575110233
0.2841545697	0.308938928	0.4267695012	-0.3595872399	-1.942623403	1.530883958
0.28460019	0.3128742597	0.4376614726	-0.4392770858	-1.981026699	1.652918417
0.2889024764	0.3055664703	0.429032355	-0.7037991589	-2.037109508	1.56271973
0.2777998842	0.2998423855	0.4228821719	-1.837139921	-1.941304126	1.792167901
0.2691313732	0.2949603152	0.4090733405	-2.359648162	-2.01228963	2.178497868
0.2842690051	0.3055317324	0.4289027807	0.4401779233	-1.963741882	1.596898111
0.2874339984	0.3058734249	0.4352747762	0.6716506475	-1.827303795	1.580948518
0.2844659192	0.3054198549	0.4292486272	0.3612968917	-1.936662345	1.592773319
0.2845888461	0.3056211718	0.4292975044	1.129578446	-1.881252046	1.482941187
0.2843243507	0.3045798805	0.4258297505	1.232148985	-1.837278575	1.586248922
0.284278003	0.3097976598	0.4331011651	0.1361985457	-1.955457459	1.605727226
0.2817596461	0.3077220563	0.4228347035	-1.215546098	-2.064549614	1.357266025
0.2705387409	0.2910056651	0.4066933533	-2.450152346	-1.818257258	1.984125226
0.2864934778	0.3080470698	0.4330722581	1.06043783	-1.740961164	1.834416991
0.2789337107	0.2958081286	0.4190911777	2.432495823	-1.578058168	1.587963725
0.2829957486	0.3057194653	0.4286567002	0.6621732247	-1.931135277	1.697847573
0.2849637212	0.3055927719	0.4279423691	0.4190232414	-1.917913351	1.615063971
0.2844112833	0.306077768	0.4279800388	0.08135429766	-1.968632719	1.612274683
0.2839958044	0.3102639527	0.4345275815	0.1341996634	-1.961037689	1.605872748
0.2830060671	0.3069048706	0.4296235536	0.5716701404	-1.988081829	1.678865093
0.2874000688	0.3089282634	0.4418365453	-0.6879575389	-2.023951145	1.456493023
0.2873085773	0.308816667	0.4320482758	-0.5617529521	-1.848176459	1.648384413
0.2855334672	0.3054224546	0.4272603099	0.00639205213	-1.952992285	1.606358386
0.289644909	0.3061988765	0.4289298161	0.04424090139	-1.917635754	1.606310102
0.2968495117	0.3086365755	0.431350671	-0.3333135006	-1.974241454	1.536480184
0.2824287831	0.3089673524	0.4248310114	-0.8316014239	-1.944243476	1.747904381
0.2792734245	0.3005790989	0.4206343023	-1.153381556	-2.054505814	1.675070428
0.2865664873	0.3061706205	0.4283427872	0.2077344078	-1.91783665	1.616511537
0.2907011298	0.3061681736	0.436783612	-0.0668209499	-1.904977093	1.607946598
0.2867648856	0.3114943071	0.4403866839	-0.2316307572	-1.913853869	1.627018007
0.2877962467	0.3073049179	0.4269755541	0.192476023	-1.971192123	1.575780509
0.2768550801	0.3012822932	0.423489457	-1.648311765	-1.942403255	1.92103466
0.282298433	0.304802055	0.4359259948	1.213957913	-1.8465707	1.441687917
0.2839883507	0.3055822466	0.4299499252	0.02559825177	-1.963958942	1.605965767
0.2785803078	0.3031612956	0.419468305	-1.513716934	-2.066482138	1.395548545
0.2832357424	0.3101332953	0.4362017973	1.219114188	-1.807954559	1.858377778
0.2818924601	0.3055250534	0.4232514372	-1.166067863	-1.906092064	1.757791989
0.2837611892	0.3057351377	0.4268492956	-0.1393419567	-1.965614315	1.600724817
0.2885928751	0.3001677633	0.419675158	1.8217218	-2.460798203	1.392591257

0.2854834172	0.3055394489	0.4277362687	0.1255244816	-1.963866628	1.602739531
0.2832555819	0.3042837431	0.4257831414	0.6502198157	-2.004974591	1.626801889
0.2855663095	0.3078801537	0.4273132714	-0.1270317208	-1.967384643	1.577620423
0.2888009837	0.3113831926	0.4313323338	0.2813380192	-1.876362187	1.619742822
0.286539676	0.3044167813	0.4266257454	0.6641591589	-2.06645474	1.582078411
0.2855764526	0.3060176441	0.426236716	-0.2957179066	-2.000334531	1.588992034
0.2883947657	0.3055114316	0.4273178327	0.03835966411	-1.926802999	1.605078482
0.2840413159	0.3192398087	0.4371596186	0.2583025429	-1.981900292	1.61817702
0.287649613	0.3055399964	0.4275912101	-0.0111086044	-1.937453785	1.605183847
0.284002692	0.3054547256	0.4268627169	-0.6643558352	-1.924699319	1.629144623
0.2843549175	0.3056697906	0.4284665416	-0.5808716281	-1.941133916	1.566173363
0.2837846015	0.3049445378	0.4258510905	-1.245364748	-1.962887586	1.474733038
0.2910244656	0.3057577462	0.4273974614	0.1325171161	-1.943137376	1.595074695
0.2771149988	0.2943324315	0.4099698046	2.341650944	-2.437217186	1.443069074
0.2842013772	0.3093823343	0.4303467853	0.2980318512	-1.963768694	1.530755979
0.2826300867	0.3213830971	0.429487148	1.161954569	-1.981598983	1.113512111
0.2785894029	0.299703053	0.4192957027	1.449852332	-1.982578539	1.651637239
0.2843618767	0.305422708	0.4310207584	-0.2608897144	-1.945420426	1.596903882
0.2804028226	0.2995020894	0.4188037929	2.092797895	-2.113041326	1.550182151
0.2841890646	0.3062299352	0.4272953836	-0.7602106192	-1.906233625	1.651108125
0.2825755636	0.3049438451	0.4333284719	-0.7654856399	-1.988786131	1.547193071
0.2869473155	0.3071970805	0.4271273505	-0.3329274017	-1.998501203	1.561513955
0.2854182163	0.3054794279	0.4275500549	-0.0536155019	-1.959923462	1.604677196
0.2880682293	0.3098807608	0.4442583019	0.8049956404	-2.012785372	1.404416494
0.2782176081	0.2991773638	0.4187206952	-1.653485755	-2.100689631	1.558852006
0.2851572038	0.3032350336	0.4259161893	0.9522526233	-1.812403888	1.59174652
0.2846373594	0.3080279436	0.4237004511	1.182899903	-1.782605057	1.787142538
0.283787827	0.3006667126	0.4202920029	-1.956854619	-1.557993702	1.37072341
0.2867346566	0.3090352091	0.4352897345	-1.193732258	-2.130193387	1.327340406
0.2817306732	0.3039627316	0.4241416777	0.6958537923	-2.001362842	1.589747391
0.287661497	0.3090350555	0.4410925136	-0.6008466616	-1.8335024	1.671112956
0.2832259587	0.302325578	0.4226490972	-2.007438672	-2.065497972	1.564272435
0.2896210718	0.3054423119	0.4273500292	-0.3934027254	-1.845268173	1.586194132
0.2845484413	0.3055970173	0.4275835939	0.08772806887	-1.967377471	1.603126332
0.291271005	0.3049657077	0.4344671198	-1.392996729	-2.244056814	1.392547951
0.2848571836	0.3052458747	0.4270021944	-0.4559615879	-1.915658967	1.590029284
0.2832988961	0.3165184406	0.4446910188	-1.196565999	-2.106418765	1.186819609
0.2836709487	0.305616476	0.4295243633	-0.1385365455	-1.968746678	1.618672512
0.2822667176	0.305643336	0.4272466508	0.3621151045	-1.995625519	1.595977366
0.2937860241	0.304863414	0.4173304531	1.376224343	-2.391621986	1.390819696
0.2825557558	0.3037747206	0.424915967	-1.257618536	-1.974812266	1.511429065
0.2829608619	0.3043767692	0.4330162049	0.5050256621	-1.989001818	1.613285814
0.2962656177	0.3096298027	0.4277329538	-1.250981897	-1.482637752	1.787794559
0.2839838856	0.3074889642	0.429906514	-0.0950242927	-1.962577533	1.60904771
0.2752307293	0.2962715772	0.4142789167	2.02010801	-1.995020299	1.562307662
0.283799544	0.3111895303	0.4290204835	0.6866522834	-1.9010314	1.742183797

0.2845275727	0.3058963739	0.4291427723	0.4736440753	-1.981455189	1.620005492
0.2831473624	0.3032412617	0.4243591538	-0.7385991882	-2.055271637	1.652013685
0.3649984522	0.4252489645	0.5846228207	-0.9299684024	2.781530693	0.4161552399
0.3688014528	0.4235110302	0.5914312834	-0.4411615223	2.725579607	0.5471256583
0.375480604	0.4345344822	0.6059987829	0.4823639393	2.799961048	0.6899634414
0.3700318146	0.4381382192	0.6014781977	-0.0719823351	2.748883664	0.5869699874
0.3517763846	0.4035327213	0.5618146667	2.034380962	2.740343506	0.4383528578
0.3850964403	0.4246297227	0.6145313144	-1.099647316	2.272553405	0.3702086558
0.3673085329	0.4399856622	0.5927012704	-0.6209325262	2.77804233	0.3838245313
0.3728707836	0.4227745928	0.5909887721	-0.0231555516	2.725938982	0.5888428697
0.3715209047	0.4228223385	0.5909647809	0.05167121379	2.726515846	0.5895553874
0.3671530716	0.4201152358	0.5869738014	0.738505318	2.810056066	0.5658824221
0.3640015494	0.4116395655	0.5760674833	-1.464755662	2.553633819	0.6251478724
0.3714985632	0.424354809	0.6036932168	0.2487427912	2.762983153	0.6101444223
0.3669254805	0.4243053619	0.5879920418	0.6943609051	2.787386058	0.6831648436
0.3683754543	0.4422406711	0.6108346914	0.4829969153	2.779736584	0.7089298058
0.3686782692	0.4230732561	0.5956208273	0.4189999744	2.775017458	0.6204981948
0.3717122323	0.4306164779	0.5917942031	0.8727357119	2.890354284	0.7818662701
0.3551549627	0.410889124	0.5690221672	1.78887195	2.943220116	0.8687385852
0.366544561	0.4244801484	0.5992077352	0.8309914826	2.822585344	0.7185185134
0.3812550053	0.4413051959	0.6026443564	0.4477372343	2.546053266	0.6959920933
0.3701309215	0.4087729647	0.5812965997	-1.704789454	3.209383183	0.7236191219
0.3778529827	0.4216785326	0.59725118	0.6410065216	2.838592643	0.6368939242
0.3660316168	0.4194663335	0.6068098831	0.8243960996	2.74747479	0.5604835811
0.372533679	0.4194706666	0.5862359137	0.8003786812	2.873994301	0.5733041691
0.3678782254	0.4243901788	0.5898893657	-0.4931256114	2.759224286	0.6443308389
0.4131704544	0.4521733096	0.6127480438	1.030551016	1.969937166	0.1526223395
0.3543442915	0.4098117467	0.569696489	1.852668169	2.760760858	0.3334588951
0.3734751541	0.4216538349	0.5895706448	-0.4545983744	2.795421614	0.5897405962
0.3711399746	0.4205710805	0.5876548766	-0.7280039543	2.849566887	0.6321014075
0.3935901004	0.4234633066	0.6035226842	0.5243488902	2.766226857	0.6408343808
0.3665626637	0.4102554196	0.5740608603	-1.603433621	2.413782524	0.5085907244
0.366383522	0.4199803491	0.5873414301	1.027937131	2.884826093	0.4735989148
0.3684501685	0.4288214884	0.5943757274	-0.5847120853	2.799057313	0.6920672708
0.3609074213	0.4145443068	0.5783445248	-1.3324099	2.804558976	0.5042275254
0.3573574485	0.4141860288	0.5777282687	-1.880030286	3.121287122	0.9983959314
0.3581702354	0.4126704374	0.5852027463	1.605358087	2.72936523	0.4031587296
0.3774518609	0.4283508003	0.5992872374	-0.2158361988	2.643636676	0.6165349283
0.3734687365	0.4275519992	0.590036576	-0.6584458932	2.840382771	0.4625120831
0.3679093061	0.4246680974	0.5891301824	0.6934090082	2.704791616	0.6865400428
0.3681928604	0.4292031634	0.5888873643	0.5216670125	2.731415871	0.6795168599
0.3692490497	0.4254477594	0.6038399753	0.05699135757	2.747265065	0.5785933578
0.3685000618	0.4208077984	0.587840701	0.6552531471	2.813807553	0.5648573755
0.3688586933	0.4275125104	0.6153425794	-0.1875880123	2.751645898	0.6105912051
0.3759260982	0.4316597383	0.5904352909	0.9038180773	2.486310315	0.3543669693
0.3644485062	0.4188537868	0.5837873869	-0.9926472027	2.787166494	0.5152814296

0.3655985234	0.4199955442	0.5835484185	0.997597252	2.857382946	0.4834904374
0.3602339544	0.4127364179	0.5815046043	-1.393966388	2.846377464	0.5692184562
0.3687811466	0.4209837945	0.5928152733	0.5811854537	2.802416302	0.583867118
0.3592654092	0.4141113513	0.5776540694	-1.465376005	2.827075852	0.7624701252
0.3696076843	0.4269193419	0.5902606349	0.2566585913	2.728799483	0.6201771877
0.369099648	0.4228927171	0.5960416115	-0.624533493	2.687017613	0.5245986937
0.3686395707	0.4264430634	0.5914852258	0.2925576931	2.750778182	0.5429819853
0.3679273385	0.4419072113	0.6232106236	0.7153493106	2.819317486	0.7855371012
0.3682016798	0.4232030632	0.5902456251	-0.429045619	2.750344676	0.6233187201
0.3720688949	0.4325002472	0.5926135977	0.6938937079	2.845220616	0.7398782039
0.3684210075	0.4211722637	0.5910994074	-0.5750131331	2.71418995	0.6079243198
0.3691067455	0.4230278599	0.6068849288	-0.0921512248	2.752769682	0.5918422963
0.3677894403	0.4230403871	0.5885354772	-0.5498103115	2.777816979	0.5362758837
0.3736354787	0.4644846334	0.63254517	0.1592404318	2.742311321	0.6018933024
0.3685364745	0.4252063665	0.6071477974	-0.4354912355	2.734173557	0.5298777149
0.3691306887	0.4226939236	0.5915334259	-0.1226542089	2.754021003	0.5894746387
0.3678549405	0.4207258116	0.5878511346	-0.6250677247	2.722136775	0.6019864291
0.3834996424	0.4190126603	0.5881984879	-0.9073319649	2.370146124	0.5440066133
0.3812561551	0.4261220007	0.5942187068	-0.2357701883	2.602871724	0.5532650469
0.3694505398	0.4243526657	0.5986429611	0.1093504814	2.74223048	0.5768804633
0.3711317577	0.4287696834	0.612233578	0.5813540513	2.817231807	0.6909945347
0.3716274012	0.4297744526	0.5946018339	0.3826045991	2.782563881	0.651655915
0.3707652842	0.4254956441	0.591724761	-0.0826028311[illegible]	2.746152739	0.5755345567
0.3645975221	0.4246512897	0.5972983964	1.097035377	2.697732118	0.3708022353
0.3811922549	0.4184714123	0.5844897258	-1.007667311	2.959735029	0.6677841421
0.3694758217	0.4322543734	0.5945527258	-0.2796854511	2.766284199	0.6353283213
0.3737236789	0.4231705985	0.5987995992	0.0127572117	2.718423698	0.5875911685
0.3775714191	0.4252631662	0.6099378626	0.4314631963	2.784529742	0.6404898643
0.366313535	0.4198014858	0.5853751453	0.8708460185	2.829830294	0.6554008803
0.3702872772	0.4414752847	0.6086968777	0.880433409	2.884048673	0.8433179106
0.3670565362	0.4226600882	0.587244457	-0.6820761225	2.789078597	0.6612920888
0.3670335407	0.4214464984	0.5881927704	-0.6512106289	2.766886127	0.5466877995
0.3746094839	0.4213172432	0.5894598315	1.123482505	2.43473304	0.4143022385
0.3677336799	0.4345818279	0.5905333105	-0.574605138	2.782367164	0.4301265488
0.3693123748	0.4213178552	0.5913572829	-0.5239985072	2.797292042	0.5968841882
0.3579351182	0.4086994454	0.5650449055	-1.974774425	2.473669708	0.2830846521
0.3602477807	0.4125540297	0.5695374279	-1.728793673	2.542621268	0.3320606097
0.3794552854	0.4384868845	0.5936007279	-0.1495668955	2.710379064	0.6074393997
0.3693887661	0.4251713216	0.592750898	-0.1628938939	2.739888683	0.567580412
0.3683419015	0.41900976	0.5884815367	-0.8765312623	2.646307906	0.55846163
0.3651574199	0.4774986749	0.629762066	-0.9252778571	2.756497736	0.0746593897[illegible]
0.3688942354	0.424870476	0.5905358891	0.586947926	2.804672517	0.6657063862
0.367296699	0.4153822839	0.5804452019	1.241740935	2.552595543	0.5255637757
0.3686971645	0.421714793	0.5891993319	0.4824116231	2.722277889	0.5752736946
0.366039963	0.4171100042	0.5827425495	1.037711346	2.881979233	0.6141808462
0.3370933578	0.3857036755	0.5387293591	-2.852822815	3.274521635	0.4243334562

0.380114958	0.4347437601	0.5942007052	-0.4881959599	2.793495361	0.6921463021
0.3733510586	0.4294419244	0.5913590672	0.1605656257	2.694401723	0.5510720923
0.3669569583	0.4546291317	0.5916634422	0.7226751772	2.733617077	0.270679577
0.3664018262	0.4451609871	0.5891956233	-1.229972424	2.95931263	1.005542052
0.3690104634	0.4215556084	0.5910835465	-0.5159515122	2.793368214	0.5736624052
0.365623232	0.4171810498	0.5841165043	1.026591114	2.684090616	0.5892088739
0.3664444833	0.4274488417	0.5884613916	0.7700168018	2.795471879	0.4353002039
0.3692069821	0.4227928755	0.5931380162	0.04174055169	2.748291901	0.5881506262
0.3747561759	0.4302836572	0.5925605415	-0.228321214	2.668299457	0.5351569801
0.374067554	0.4243698287	0.595739812	-0.0455921997	2.706440095	0.5825152849
0.3745646887	0.4244778251	0.6131467693	-0.7259713709	2.859638409	0.6921896582
0.3666967506	0.4219819588	0.5855002374	0.9503846866	2.864418559	0.7207545035
0.3690028752	0.4239674036	0.5907548906	-0.1557110429	2.754678358	0.5993636916
0.3689351991	0.4247797079	0.5980884915	0.1371259708	2.751962409	0.5995775372
0.3684131081	0.4122766136	0.5768122484	-1.42204684	3.061035097	0.6278268551
0.372291742	0.4208976269	0.5932863335	0.9735293507	2.92347842	0.7106481342
0.3704457974	0.4264460036	0.5916416083	-0.2768398987	2.767583824	0.6218869099
0.3661643045	0.4195541884	0.5898567477	0.8451585042	2.729464853	0.5478789138
0.3716085096	0.4227233495	0.5919108192	-0.1489895009	2.713973028	0.5915641314
0.3724844896	0.417266313	0.5820707696	-1.084530242	2.961145929	0.53227642
0.3691372542	0.4216355174	0.5893618686	-0.4708766077	2.78743442	0.5984248665
0.378565379	0.4236108663	0.6000885515	1.379909006	3.104761704	0.8971289855
0.3702676086	0.4246835981	0.592077808	0.4808014533	2.68787078	0.6446122965
0.3740824536	0.4401985909	0.6223935706	-0.6154631427	2.596658596	0.3843899482
0.364099605	0.4201815685	0.5918588654	-1.030886997	2.792866306	0.7164640893
0.3625644221	0.4188160206	0.5800296082	1.222116469	2.858164131	0.4275140863
0.3869812428	0.4264290713	0.5928111934	0.3747614607	2.508494514	0.5298408184
0.371019515	0.4180544928	0.5863738744	1.167324661	2.981426025	0.7223797121
0.3690071758	0.4249756257	0.6140313847	-0.0647301159	2.750834912	0.5922703303
0.3744924347	0.4276232097	0.5706683383	2.227966153	1.881294627	-0.2302889131
0.3672903976	0.4189946345	0.5854698054	0.8479971731	2.843615111	0.5785190629
0.3665513597	0.4196072501	0.5921555971	0.7690861123	2.800411615	0.5978783963
0.3734895324	0.426589488	0.6058627201	-0.4749033908	2.636699317	0.5106670182
0.3627874153	0.4175496833	0.5803395381	1.318695898	2.669812268	0.775920882
0.3656204043	0.4173334101	0.5834580713	1.035721637	2.872487296	0.6330099514
0.3102547099	0.33230716	0.4625182218	1.6440789	0.8911520327	-0.9086472693
0.3078962377	0.3292623542	0.4602514249	1.937298752	0.9243467177	-0.9236102051
0.3189509169	0.3371930848	0.471887544	-0.5699347208	0.6825808423	-1.039388911
0.3162746939	0.3388996743	0.4743888764	-0.0161565492	0.7740129937	-1.030920167
0.3162013786	0.3389685459	0.4722024307	-0.196016968	0.7697047464	-1.01670797
0.3183207572	0.3391976172	0.4795201692	-0.8827561233	0.6286720305	-1.152313945
0.3077054103	0.3314843539	0.4606476383	1.88661053	0.7607352495	-0.8198325171
0.3101412677	0.3280476919	0.4587443225	-1.976350727	1.075936774	-1.055216489
0.290441134	0.3067416986	0.4291629931	-3.703468412	1.445499781	-0.9536180651
0.3152451373	0.3373398451	0.4712561479	-0.6549777646	0.7605023086	-1.003921555
0.3177046765	0.3372798583	0.4705033396	0.9091213447	0.905457088	-0.9537715984

0.3116713552	0.332777661	0.4699124571	-1.424884891	0.7110540549	-1.072522117
0.3157978988	0.3385806332	0.47335201	0.4389551622	0.7666869166	-0.9986935232
0.3122139988	0.3355271563	0.46650805	-1.276407436	0.7829332346	-1.172864289
0.3157732211	0.3376089502	0.4747985673	-0.430953788	0.7710246462	-1.043210386
0.3135165988	0.3328060325	0.46338273	-1.57834826	0.5499351571	-1.187024911
0.3137889948	0.3407072068	0.4689397883	-1.005260237	0.7586361578	-1.203657108
0.3151128755	0.3366422397	0.471960387	0.7536219159	0.8096472694	-1.020899511
0.3215837948	0.3433286055	0.4803519178	0.2192278093	0.7206555052	-0.980020465
0.3256290443	0.3389735692	0.4693838205	-0.8895826445	0.9845340792	-0.9133889225
0.3176509133	0.3353639781	0.4704402662	-1.657243049	0.398485785	-1.311755186
0.3165369364	0.3382154145	0.4740369734	0.05940311119	0.7704112374	-1.03228535
0.323020812	0.3381327024	0.4719175593	-0.5309411171	0.6438134046	-1.071523044
0.3161493293	0.3425476638	0.4727105275	0.3282983607	0.7625237137	-1.075706341
0.3338340574	0.3423354757	0.4818539758	-0.3553081007	0.8478903543	-0.9635565881
0.3139693831	0.339772416	0.4679678961	1.137810661	0.6912359555	-1.226460892
0.3180762848	0.3374802688	0.4732090404	0.4669418647	0.7121268453	-1.033905605
0.3163326153	0.3356399942	0.4684232896	-1.029926979	0.9087608803	-0.9923430703
0.3202923683	0.3346027298	0.4730144537	-1.762848399	0.2941027345	-1.336152798
0.3131228116	0.3271327031	0.4578486812	-2.146066572	0.303719021	-1.218417317
0.3184543689	0.3383537557	0.471687673	-0.4091281106	0.7157741683	-1.060822112
0.3121722688	0.339643673	0.4692408326	-1.28554402	0.7964379718	-0.7905560913
0.3176298933	0.337308397	0.4711907377	-0.5622322861	0.8364088165	-1.02362628
0.3138622279	0.33622881	0.477034345	-1.697477146	1.070152839	-0.7305040853
0.307145959	0.3276022875	0.4665359567	2.033137472	0.6490735324	-1.121845733
0.3201343105	0.3379952906	0.4728797477	-0.2528755989	0.7242048086	-1.028433459
0.3097700068	0.3319781134	0.4622816899	1.665147555	0.7189374366	-0.9168192561
0.3161873472	0.3389746594	0.473200252	0.4933418739	0.7442972398	-0.9844501999
0.3083453236	0.332662197	0.4597168569	-1.870249331	0.9052098923	-1.32635682
0.3153354078	0.3482767732	0.485705775	0.6434009634	0.795477137	-1.166996799
0.3207215984	0.3372973048	0.4742379649	0.5622813167	0.8646710334	-1.024453822
0.3162016009	0.3389645604	0.4749969634	0.1016702388	0.7735109164	-1.03940016
0.30686534	0.332918691	0.462873261	2.028788991	0.6828391525	-1.414180386
0.3145859173	0.3363302758	0.4713025533	-0.839310119	0.8004726327	-1.064641337
0.3143353362	0.3336625669	0.472757095	1.322011668	0.9417966566	-1.002788395
0.3076602652	0.3288689765	0.4603568193	1.904845481	0.8579699264	-1.119873391
0.3062027671	0.3273025253	0.459237359	2.055342821	0.8445744614	-1.099888372
0.3104900511	0.3316674611	0.4632777661	1.578467362	0.8618193139	-1.015527425
0.3083663084	0.3330465012	0.4647915159	1.81652062	0.8422697021	-0.7872550123
0.3164481535	0.3437909032	0.470712099	0.6538744385	0.8322951064	-0.8872788653
0.3166616699	0.3375671578	0.4712565986	-0.7119930441	0.8464814537	-0.9853360817
0.3177672068	0.3378414249	0.4755984688	1.471592217	1.073796283	-0.7720987876
0.3189385668	0.3381737164	0.4774472716	0.04047099663	0.7725346824	-1.033199142
0.317111503	0.3404729782	0.47385226	-0.4605066333	0.728194722	-1.088099675
0.3163985045	0.3379990264	0.4723708051	-0.5631537466	0.7294436599	-1.073235
0.3154864434	0.3381432325	0.4710800573	-0.6038298925	0.7956170362	-1.082103063
0.3161876754	0.3410198363	0.4767819092	-0.1233476026	0.7741933903	-1.041971426

0.3158062564	0.3384702604	0.4730384706	0.50391594	0.7562187798	-0.9929478975
0.3126455727	0.33365252	0.4777109958	1.665642092	1.018978426	-0.8329507191
0.3145942062	0.3379274015	0.4706596724	-1.45398536	0.9955152953	-0.7760959188
0.3164513461	0.3363464045	0.4697473449	-0.8349564377	0.6804738663	-1.064199402
0.3199748577	0.3350621679	0.4693547315	-1.174386666	0.5178110886	-1.116651742
0.3268115003	0.338781463	0.4738811779	-0.7198817171	0.556455096	-1.112531924
0.3130476625	0.3396916336	0.4676210662	-1.150305092	0.8095786327	-0.8305340242
0.313906375	0.335585932	0.4691420548	0.9758827896	0.7960064225	-1.024816861
0.3175270504	0.3381779767	0.4723280051	0.5362549511	0.8310825071	-0.9954252445
0.3007263187	0.3150778874	0.4480061113	-3.116796939	1.452406955	-0.9511340206
0.318888529	0.3419968165	0.4842358344	0.6987156176	0.6586976123	-1.144556468
0.315606736	0.3352458494	0.4636721218	1.541769537	0.4970792198	-1.261994576
0.3167645779	0.3420042562	0.4814015703	0.3407167593	0.7478747404	-1.076275975
0.317001898	0.3400805744	0.4861905085	0.6548653977	0.8425496041	-1.117629318
0.3143930327	0.3361970738	0.4714812964	0.8654099446	0.7662267655	-1.016309097
0.310445605	0.3353682786	0.464174573	1.559926342	0.8377167952	-0.8132541606
0.3155342243	0.3426490232	0.4802760968	1.071157597	0.9003694521	-0.7960184833
0.3152464824	0.3396070895	0.4700168829	-0.7888227827	0.8237249479	-0.9217852155
0.2982673497	0.3196215637	0.4453391648	2.804809512	0.7441053062	-0.8864990939
0.3299990001	0.3415838267	0.4771428368	0.3297258444	0.6463720631	-1.073097608
0.3136003986	0.3333745646	0.4658998713	1.349449692	0.6340121531	-1.016445029
0.3161546935	0.3365219611	0.4719263892	-0.7918535576	0.8508461467	-1.017097445
0.3136385974	0.3365062614	0.4653389451	-1.387579325	0.6185032541	-1.238940139
0.3184962846	0.3422238779	0.4724534721	0.9364128893	0.9242994131	-0.8404854467
0.3203805821	0.3391542102	0.4742690509	-0.0197708259	0.7669837494	-1.029695017
0.3137071246	0.3356487371	0.4668719983	1.21485511	0.8827207421	-0.928043568
0.3136965099	0.3316821682	0.4632073545	1.581083924	1.017754603	-1.009066435
0.3152538965	0.3546006371	0.4865385056	-0.6489409337	0.7908817631	-1.188635242
0.3127945495	0.3325615907	0.4639530455	-1.513248833	0.9600426111	-1.133336283
0.3131286875	0.3354455488	0.4677219286	-1.148945255	0.818833761	-0.9598152693
0.3152169125	0.3370788518	0.4728185343	0.6380030004	0.7809765743	-1.021576214
0.3141771346	0.335823378	0.4691512749	-0.9316449454	0.7544591338	-1.060452145
0.3241130745	0.3373171368	0.4712886062	-0.5701516877	0.8862325722	-1.021254963
0.3213478525	0.3389683012	0.4718444952	-0.3962144238	0.8346066176	-0.9967876694
0.312574195	0.3372983806	0.4699117687	1.22301701	0.7639564719	-1.204567639
0.3157683193	0.3554102153	0.4768088693	-0.4565392283	0.7842249369	-0.8410189758
0.3162255538	0.3380711989	0.4725416382	-0.1986566543	0.7688654889	-1.029336641
0.2847312024	0.3035189754	0.4294742914	-3.897454639	1.165632024	-1.23565097
0.3167014542	0.3374557253	0.471313003	0.5173284956	0.7287161657	-1.023113246
0.3142172608	0.336603222	0.470569397	-0.9505159679	0.8155828387	-0.9680158041
0.308093648	0.3302634173	0.4672503497	-1.835755189	0.7997378172	-1.194397325
0.3177088736	0.3386484938	0.4700277573	-0.8687585859	0.6441994441	-1.140198068
0.3138848107	0.3334361563	0.4663888857	-1.374813351	0.6135196098	-1.110771564
0.3183313409	0.339341899	0.4877269741	0.7680021306	0.6539314805	-1.130117219
0.3103564776	0.3314191554	0.4629153492	-1.658731762	0.668107292	-1.147239812
0.3158151064	0.3347103866	0.4698275009	-1.125841229	0.627304957	-1.041340429

0.3184595278	0.3430133359	0.4723152632	0.1076432914	0.7812280389	-1.001394896
0.3162136361	0.3350410278	0.4689964721	-1.303280663	0.9847568552	-1.165029764
0.3055055158	0.3258588448	0.4592689109	-2.529553663	0.3824563403	-1.456595776
0.3161653692	0.3390891634	0.4722161232	0.1554302228	0.7736415977	-1.04396242
0.3181623734	0.3403580543	0.4803552317	-0.1765920164	0.7889799258	-1.00677662
0.3116409931	0.3325538682	0.4639945535	-1.52859163	0.6556483945	-0.9603634639
0.322938619	0.3373241126	0.4727648725	-0.5242744214	0.8693573457	-1.036257849
0.3108038046	0.3318970864	0.4636203853	-1.604007108	0.9055056207	-0.9626580615
0.3244874006	0.3376369065	0.4801446815	0.8297914037	0.9583185709	-0.960152615
0.3163402179	0.3377555509	0.4715689385	-0.4454896748	0.8012613661	-1.05191421
0.3187055848	0.3516742244	0.494860881	-0.1506959121	0.7488457882	-1.036184975
0.311014654	0.3325712864	0.4669256919	1.461684378	0.7706902033	-1.088047185
0.3156181595	0.3408741323	0.4732857935	-0.4930242961	0.773936207	-0.9559567615
0.3292982176	0.3432359361	0.472022086	-0.4808612753	0.8801455736	-0.9310261736
0.3105278834	0.332187402	0.464531435	-1.539827024	0.7508587369	-1.120054446
0.3137672615	0.335465565	0.4753888232	-1.00297174	0.7952919966	-1.021598597
0.326261738	0.3435631691	0.4735247307	-0.2743462059	0.8181868632	-0.9682562312
0.3085816142	0.3310435195	0.4627315275	-1.779478608	0.8111497442	-0.9068802783
0.3159700442	0.3378527177	0.4725115607	0.351081592	0.7809472393	-1.04476738
0.3174793146	0.3450613819	0.4765245043	0.1665168508	0.7859794578	-0.9800776524
0.3012798119	0.3230490373	0.452525182	2.56279297	0.6883764735	-0.8491901995
0.315742483	0.3357705892	0.46960797	0.936667175	0.8719960461	-1.026989414
0.3132965366	0.3366683445	0.4695085985	-0.5401624641	1.386802607	-0.4472454507
0.3095261827	0.3314197708	0.463707161	1.808727735	1.628645149	-0.2476229949
0.317880021	0.3366622649	0.4718251962	-0.3876582122	1.346776122	-0.4055112059
0.3135369255	0.3372283283	0.4727334985	-0.2416752419	1.419124529	-0.3889619668
0.3124221261	0.3364682146	0.4688833397	0.641458477	1.417513358	-0.4633892289
0.3169693027	0.3380315092	0.4788925085	-1.024300524	1.271678287	-0.5162252438
0.3130649302	0.3378862651	0.4704888837	0.4653815406	1.403369686	-0.3553447988
0.3037159302	0.3229077134	0.4521312306	-2.242843781	1.748429421	-0.4569576564
0.3062790385	0.3239775151	0.4537216271	-2.366626763	1.879847771	-0.2827839591
0.3141125948	0.3378374392	0.472503735	-0.1316934952	1.422092914	-0.3887798885
0.3131513204	0.3328546477	0.4650083708	1.561964048	1.694068284	-0.2557318158
0.314666904	0.3368957681	0.476448247	-0.3133117349	1.408729914	-0.3976846858
0.3133405802	0.3372894301	0.4714484751	0.2573198942	1.403139098	-0.3874926412
0.3050322016	0.3281668567	0.4565582576	-2.248879749	1.534223198	-0.6011831678
0.3138461159	0.3367877507	0.4733497844	-0.0100460843	1.416362786	-0.4043759479
0.3167694339	0.3375204752	0.4702576511	-0.7068517119	1.330460863	-0.4503448122
0.3140619094	0.3413781947	0.4709945865	-0.4538058262	1.430340392	-0.4639733755
0.3138860881	0.3366936937	0.472244881	0.124543435	1.418924171	-0.4016116177
0.3148299151	0.3369476555	0.4715775361	-1.354875669	1.664180926	-0.6375075811
0.3117880082	0.3263776045	0.4513337255	-2.224893343	1.978908505	-0.1085934985
0.3156069417	0.3341240993	0.4687256004	-1.545400822	1.10924572	-0.6032613981
0.3140767901	0.3367640406	0.4730061501	0.1441318787	1.412038456	-0.3997381845
0.3193493139	0.3344136144	0.4677899389	-0.794503983	1.154523056	-0.4997726821
0.3141618606	0.3412567986	0.471676216	-0.4662338962	1.449754013	-0.3150319385

0.3319917296	0.3421537702	0.4807961431	0.2808204486	1.253582523	-0.444404271
0.3145592851	0.3407158607	0.4701504233	0.6243009244	1.344019325	-0.509072803
0.3153859666	0.3360181854	0.4714831283	-0.4013111543	1.451413634	-0.4069956303
0.3167449982	0.3369305143	0.4704156606	-0.5214829839	1.487083902	-0.3665062455
0.3259175501	0.3411367411	0.4820589583	-0.8727631186	1.171050249	-0.5227678854
0.3141765353	0.3291722907	0.4607534334	-1.949971715	1.008064698	-0.5546103845
0.317994787	0.3385948377	0.471930514	0.6668896778	1.540784074	-0.3206611638
0.3125709725	0.3391897073	0.4712760493	-0.5344178135	1.417480929	-0.3291306665
0.3134753121	0.3330780864	0.4654242269	-1.668541197	1.709338122	-0.314710003
0.3179644891	0.3413151781	0.4838517688	-0.0739065621	1.409888845	-0.3866701759
0.312756169	0.3346407	0.4769968897	1.117143564	1.377151941	-0.4264173327
0.3122959443	0.3292269175	0.4605672776	1.828484353	1.785645972	-0.4348006741
0.3070750372	0.3303878852	0.4610786502	1.507088518	1.335040921	-0.3208882011
0.3143347005	0.3381320352	0.4721393909	0.165414857	1.402623699	-0.3871009272
0.3106268826	0.3372243981	0.4659257673	0.9327789226	1.342209369	-0.2786550735
0.3041047689	0.3344838617	0.467777752	1.87786062	1.52739741	-0.8473663544
0.3101234901	0.328652586	0.46120396	1.398132655	1.587029451	-0.4481646562
0.3118513206	0.3361622287	0.4735735338	0.6666185269	1.386618683	-0.479222152
0.2905294388	0.315195846	0.4382323163	3.656195775	1.451560239	-1.022282432
0.3141065998	0.3368238507	0.4726029084	0.3404863832	1.418736392	-0.3913574431
0.3117659805	0.3309352318	0.4688928106	2.032449421	1.79621408	-0.2796920341
0.3143208555	0.3368457556	0.4727572532	0.2170864461	1.430958435	-0.4054286032
0.3144556394	0.3370354512	0.4733313321	0.6632371833	1.468951416	-0.4080404447
0.3130631391	0.3344160502	0.4671170339	1.644097719	1.615501721	-0.3052979865
0.3135978567	0.3398254349	0.4746880883	0.9604120529	1.492742254	-0.2304304636
0.3073365773	0.335317017	0.4604688719	1.580470949	1.534745888	-0.1207851238
0.3151970648	0.3374349927	0.4715427595	0.2618297295	1.374118598	-0.4299375079
0.3189227816	0.3410697311	0.4799632114	-0.875663561	1.213598833	-0.5669664998
0.3121348307	0.331061511	0.4692594583	1.406539825	1.627046091	-0.4099042438
0.3150448617	0.3391275023	0.4736393804	-0.6765055124	1.381067797	-0.4711355782
0.3141468478	0.3364085691	0.4709383552	0.3769245685	1.437629203	-0.3901573216
0.3112788144	0.3343006514	0.4666339301	-1.235667939	1.509556389	-0.4918915665
0.3101342569	0.3380118965	0.4733207749	-0.9131113514	1.373237908	-0.5802111494
0.3137099966	0.3384181483	0.4735748671	0.3481535104	1.408919748	-0.3632207831
0.3123272454	0.3358336365	0.4802847717	1.039331576	1.523716036	-0.2792923283
0.3169954737	0.3405704983	0.4759809514	0.2691517524	1.361978162	-0.4420523471
0.3156630809	0.3367496338	0.4708980533	0.1387099384	1.425179539	-0.4002150763
0.3203087372	0.3373508158	0.4726983741	0.1500271537	1.415990306	-0.3941949784
0.32662423	0.3390987598	0.4742688912	-0.4843148302	1.22491993	-0.4654336893
0.3122937176	0.3403624568	0.4683799954	-0.9056373387	1.461072733	-0.2321240827
0.311905103	0.3346025099	0.4681756354	0.8224447449	1.438851681	-0.4047599172
0.3162583348	0.3374348218	0.4718693214	0.3133645916	1.449055031	-0.3783568937
0.3163173061	0.3319173428	0.4725263423	-1.692840493	1.813276681	-0.2817185659
0.3012218437	0.3267097358	0.4620274081	2.432826594	1.058332862	-0.9331156616
0.318500153	0.3391586025	0.4702983844	0.597842929	1.308876777	-0.4691402536
0.3145643604	0.3408694981	0.4796146143	0.4213548195	1.398188909	-0.4589168587

0.3151849013	0.3394352065	0.4854472305	0.3452530271	1.444014968	-0.4504959776
0.3115359545	0.3346980887	0.4706098135	-0.6768787397	1.400103206	-0.4435064053
0.3139773384	0.340585421	0.4714249897	-0.03890322002	1.414360874	-0.4056203905
0.3155791244	0.3443117083	0.4837498652	0.6522237111	1.497949804	-0.2472124462
0.3129153625	0.3381652715	0.4683447697	-0.9638482915	1.497066519	-0.2584790931
0.3068906868	0.3291921797	0.45896296	2.096088371	1.471637204	-0.2224336099
0.3267445276	0.3392251401	0.474232749	-0.3674270695	1.453573692	-0.3566967248
0.3153297278	0.3363471581	0.4706087992	0.637626261	1.364283483	-0.3832382361
0.3146200274	0.3366903881	0.4715863535	0.05276728069	1.410394675	-0.4037155821
0.3148866204	0.3387805338	0.4696338203	-0.6301698374	1.344449106	-0.4851108593
0.3125107018	0.3357923764	0.4635745322	2.160843036	1.901394379	0.0743964739
0.3154546151	0.3346811893	0.4688777997	-0.8567294905	1.529647483	-0.3609842659
0.3144695395	0.3366916763	0.4686649247	1.000696775	1.550008296	-0.2921496466
0.3156966039	0.3339127337	0.4666083367	1.431319727	1.698445143	-0.3344869898
0.3148577397	0.3541202188	0.4855234579	-0.712496141	1.483812924	-0.5787599216
0.3151755028	0.3350842083	0.4675742569	-1.539758644	1.700523085	-0.432517946
0.3116121964	0.3346370882	0.4673928334	-1.01837543	1.471370624	-0.3422815952
0.3112598203	0.3343452264	0.4687221289	0.7371474034	1.392394027	-0.4036687763
0.3131719167	0.3364797803	0.4699064013	0.3075609414	1.416284473	-0.3943774597
0.3131173443	0.329002458	0.4598881531	-1.475346438	1.67113773	-0.3954433998
0.3104766102	0.3291256685	0.458439297	-1.796154335	1.740438386	-0.2577252307
0.3137322783	0.3404169366	0.4738635279	-0.1247854507	1.417298739	-0.3820255386
0.3076340274	0.3499359884	0.4675785093	-1.326569893	1.428951958	0.0219293935
0.3075660554	0.3305085203	0.4623923531	-1.286234305	1.362696896	-0.4355492025
0.301752651	0.3233789283	0.4567583237	-2.255202518	1.60705657	-0.5106220053
0.3149885768	0.3365923644	0.4707120354	-0.03540134442	1.412759689	-0.4054149223
0.3146844826	0.3380691449	0.4722413011	-0.5729971993	1.470244349	-0.3385154774
0.3119330141	0.3357475515	0.4763830681	-0.8307619765	1.413421295	-0.4738262358
0.3140137759	0.3356517575	0.4665306865	1.059888543	1.561048534	-0.2846139299
0.315763095	0.3367940465	0.4713628196	-0.4027008636	1.373598384	-0.4125738076
0.3171538412	0.3398588594	0.487804945	0.7663566438	1.32780504	-0.490428584
0.3140944044	0.3366902	0.4708989818	-0.5263951162	1.389618555	-0.4283834112
0.3165660084	0.3357665679	0.4716221585	-1.042444246	1.310566067	-0.365297711
0.3159911848	0.3413092382	0.4697206051	0.6750159694	1.508237681	-0.2723788049
0.3147259127	0.3334390236	0.46629797	-1.451095273	1.694485253	-0.5424623227
0.3166744746	0.3390519134	0.4779105818	-1.287250905	1.240713562	-0.5870629559
0.314239984	0.3378109175	0.4709713594	-0.5590409134	1.445298247	-0.3455304149
0.3123707589	0.334831123	0.4752327728	-1.523665245	1.661404036	-0.1804410359
0.3138157323	0.3350114855	0.4685708571	-1.043847695	1.345566365	-0.3561159794
0.3202921765	0.3365354845	0.4712604018	-0.2304323133	1.432750881	-0.4112241317
0.3011661854	0.3227326237	0.4514166101	-2.373016447	1.638178922	-0.3094227773
0.3198633508	0.3351326034	0.4774727085	1.003187477	1.599093112	-0.3049849967
0.3148818515	0.3368796722	0.4709436653	-0.2934549106	1.442695389	-0.4077655398
0.3147250638	0.3496019959	0.4916806329	-0.9707920929	1.31245872	-0.6579861947
0.3078605682	0.3305146882	0.4644613567	1.603743614	1.438268381	-0.477827651
0.3103054559	0.3354222373	0.4669801867	-1.472335112	1.476166586	-0.1895284554

0.3226168267	0.3362717519	0.4624448582	-2.046965725	2.076471419	0.0305294045[illegible]
0.3154378253	0.3375884792	0.4721239666	-1.142780164	1.484754028	-0.4003912606
0.3119249557	0.3346344041	0.4755155114	-0.8888290442	1.443549184	-0.3891065231
0.3277088133	0.3431181511	0.4737385242	-0.2301421334	1.425739066	-0.3504911837
0.3100665668	0.3343266249	0.4675105256	-1.288693985	1.467251849	-0.2697276309
0.3135891773	0.3366114864	0.4708312362	0.3768332785	1.426043019	-0.4178548213
0.3063731533	0.3350218722	0.4621957828	-1.815426166	1.240263561	-0.8089469637
0.3072122435	0.3299285392	0.4627600795	1.699161377	1.351296525	-0.2945581351
0.3048468888	0.3257821369	0.4559858115	1.944141934	1.617769268	-0.4551599733

obe + Blast + VNS + Blast:VNS + (1|Batch))

Blast:VNS t-value	Microbe Pr(>t	Blast Pr(>t)	VNS Pr(>t)	Blast:VNS Pr(>t	Microbe Adjusted p-valu
-2.325498388	0.0104612000	0.0033741515	0.0297240381	0.0234441675	0.2213566899
-2.14894178	0.9373908607	0.0114265040	0.0709555961	0.03568337829	0.9764488133
-2.313356214	0.1033778047	0.0053261470	0.0656224952	0.02414729275	0.5562319781
-2.045220709	0.3843892261	0.0113427499	0.0768843534	0.04522589771	0.7392100501
-2.186589557	0.0149290403	0.0093601367	0.0363285327	0.03268317212	0.2213566899
-2.276234789	0.4025333416	0.0089936506	0.0575710314	0.02641450713	0.7442416032
-2.170072879	0.6870213735	0.0116660243	0.0661395955	0.03397102758	0.8574168621
-2.130024807	0.1437796996	0.0055199821	0.0601044443	0.03727985259	0.5818748723
-2.207587798	0.2490048423	0.0068168906	0.0585490947	0.031107891	0.6426517028
-2.119745011	0.4915141665	0.0108368721	0.0773963697	0.03817327138	0.7781542416
-2.141351905	0.8254371636	0.0125306718	0.0746642780	0.03631659247	0.903133968
-2.072066005	0.6827780987	0.0119928767	0.0705001785	0.04256522111	0.8574168621
-2.171335462	0.7499648254	0.0111376410	0.0676553643	0.03387103314	0.8790053953
-2.12416433	0.3542703914	0.0109868854	0.0881818389	0.03778692558	0.7391035993
-2.370223382	0.1054594933	0.0092900849	0.0648433756	0.02100881605	0.5562319781
-2.107621136	0.1559195133	0.0190602692	0.0874909328	0.03925089299	0.5818748723
-2.144367161	0.0088728017	0.0085810809	0.1525595899	0.03606386734	0.2213566899
-2.258789437	0.3108268522	0.0086522554	0.0631744598	0.02754367837	0.7261735405
-2.192637832	0.6730252228	0.0160632277	0.0645966887	0.03222240345	0.8574168621
-2.145098209	0.7724165504	0.0178894494	0.0793964345	0.03600282614	0.8790053953
-2.012254564	0.5936221525	0.0181268795	0.0874614887	0.04868879185	0.8379543986
-2.2790893	0.1819949263	0.0104374651	0.0792662436	0.02623370047	0.5818748723
-2.089255557	0.0686817272	0.0272947230	0.0805600954	0.04093372306	0.5341946883
-2.118514806	0.6996521595	0.0117725769	0.0836153947	0.0382814291	0.8574168621
-2.206717822	0.603327167	0.0100068758	0.0640070878	0.0311718057	0.8379543986
-2.205527751	0.4258025937	0.0093828972	0.0532977359	0.03125942404	0.7518844513
-2.052564967	0.3147157819	0.0157477662	0.0636311920	0.0444840808	0.7261735405
-2.203789153	0.1686653941	0.0062366794	0.0534313949	0.03138781673	0.5818748723
-1.897099436	0.3809658094	0.0256549468	0.0990995403	0.06263067566	0.7392100501
-2.029222494	0.2747145198	0.0233216257	0.0776613201	0.04687890396	0.6603714418
-2.149192057	0.8970517631	0.0120507407	0.0721694762	0.03566266342	0.9502666982
-2.104712052	0.4987321994	0.0115632468	0.1031811378	0.0395133703	0.7781542416
-2.128907009	0.2600750207	0.0158656674	0.0695122334	0.03737610838	0.6501875518
-2.024642387	0.7875888342	0.0143271819	0.0827663959	0.04736163303	0.8790053953
-2.197185119	0.6454838731	0.0101972402	0.0669604169	0.03187975002	0.8574168621
-2.16502264	0.7636965784	0.0131182774	0.0691692032	0.03437358899	0.8790053953
-2.135253978	0.4607837117	0.0120368342	0.0603295004	0.03683242564	0.7781542416
-2.360396566	0.1128741809	0.0102640433	0.0425365312	0.02152365157	0.5643709044
-2.161980303	0.7803333756	0.0122539962	0.0679275590	0.03461810939	0.8790053953
-2.227343406	0.5702725284	0.0118353228	0.0590402045	0.02968721411	0.8235845603
-2.127696847	0.9904743265	0.0137911058	0.0715221897	0.03748056224	0.9904743265
-2.239409783	0.4270703684	0.0107110011	0.0606083780	0.02884790047	0.7518844513
-2.138769329	0.9518851263	0.0114124343	0.0729003186	0.03653428121	0.9791894213
-2.179176313	0.6195711599	0.0109299047	0.0748750540	0.0332558081	0.8510592856
-2.071807588	0.65390721	0.0126967822	0.0715940803	0.04259017209	0.8574168621

-2.176477559	0.7098113057	0.0107090216	0.0710503546	0.0334664481	0.8577920996
-2.209110646	0.5661917457	0.0116886746	0.0707219680	0.03099628902	0.8235845603
-2.104171126	0.3991362818	0.0111516711	0.0685862708	0.0395623444	0.7442416032
-2.409141539	0.1337936252	0.0073354134	0.0385807850	0.01907625727	0.5818748723
-2.192290047	0.1847447441	0.0144935919	0.1213890237	0.03224874293	0.5818748723
-2.290537431	0.1645256214	0.0069518245	0.0507168065	0.02551953724	0.5818748723
-2.463177251	0.1638228992	0.0047642565	0.0371410529	0.0166572335	0.5818748723
-1.864392773	0.0564884583	0.0257209150	0.0761999643	0.0671603724	0.4943906211
-2.042805943	0.3569390786	0.0089165391	0.0556855561	0.04547212859	0.7391035993
-2.165514716	0.7136830269	0.0107511194	0.0688343132	0.03433418206	0.8577920996
-2.142121942	0.8663421886	0.0116835395	0.0700834053	0.03625190479	0.9335583929
-2.007671085	0.4737136465	0.0111002940	0.0914910596	0.04918788881	0.7781542416
-2.459559783	0.0148509757	0.0108849051	0.0412010094	0.01681011574	0.2213566899
-2.270379833	0.4800379809	0.0087690787	0.0576353736	0.02678882082	0.7781542416
-2.064442998	0.4954367862	0.0142134823	0.0944304611	0.04330659619	0.7781542416
-2.174810343	0.24083948	0.0160558348	0.072811213	0.03359715955	0.6426517028
-2.210103985	0.4237613288	0.0084618577	0.0634605969	0.03092368211	0.7518844513
-2.060942278	0.677946481	0.0201063849	0.0830406600	0.0436507893	0.8574168621
-2.14312411	0.6905272937	0.0111343302	0.0857390759	0.03616786745	0.8574168621
-2.116074101	0.0051648257	0.0067150825	0.0566698767	0.03849680293	0.2213566899
-2.130270534	0.8308832506	0.0129155935	0.0764296163	0.03725872154	0.903133968
-2.050478999	0.7774221447	0.0156627794	0.0746242446	0.0446936993	0.8790053953
-2.092751274	0.9836546565	0.0119238332	0.0744340829	0.0406086647	0.9904743265
-2.130104261	0.2727590001	0.0203726546	0.0964315503	0.0372730189	0.6603714418
-2.265333294	0.4523408486	0.0097073229	0.0567468448	0.02711521112	0.7781542416
-1.735343869	0.0854711501	0.0058296684	0.0996321893	0.08781380184	0.5341946883
-2.127321659	0.6002273837	0.0104481690	0.0704114741	0.03751299786	0.8379543986
-2.159274035	0.3630786502	0.0105352623	0.0915103003	0.0348368976	0.7391035993
-3.040474714	0.0011153631	0.0020708341	0.0107073095	0.0034983056	0.1394203961
-2.298697075	0.0085425659	0.0045131986	0.0222810813	0.02502109524	0.2213566899
-2.134474795	0.5104691825	0.0116583477	0.0623993269	0.03689879674	0.7781542416
-2.14001508	0.890345208	0.0142886955	0.0720702425	0.03642913335	0.9502666982
-2.257521287	0.1496390042	0.0132041919	0.0578799285	0.02762740115	0.5818748723
-2.213482302	0.5277493682	0.0093454520	0.0646004829	0.03067786705	0.7948032653
-2.221328225	0.1677446231	0.0155553620	0.1053689383	0.03011360288	0.5818748723
-2.442747491	0.0828920337	0.0045395772	0.0297539715	0.017537337	0.5341946883
-2.369252958	0.0983148113	0.0050525471	0.0517654092	0.02105916338	0.5562319781
-2.111034004	0.2425976491	0.0094790253	0.0562486763	0.03894489656	0.6426517028
-2.158272201	0.3265692181	0.0076878642	0.0607973924	0.03491819729	0.7289491475
-1.994323818	0.7384308639	0.0111262939	0.1035804478	0.0506664182	0.8790053953
-2.171722459	0.2070008349	0.0070893286	0.0748201618	0.03384043535	0.5886203368
-2.326278632	0.0107580094	0.0059659683	0.0354974168	0.02339961449	0.2213566899
-2.036410595	0.0593268745	0.0091875385	0.0647372844	0.04612985738	0.4943906211
-2.188024028	0.1311850956	0.0109106043	0.0644715551	0.03257337133	0.5818748723
-2.12690258	0.7841806708	0.0164571851	0.0718466075	0.03754925688	0.8790053953
-2.16551509	0.3195163578	0.0079297678	0.0612459213	0.03433415217	0.7261735405

-1.910926502	0.0429947552	0.0097326291	0.1085920275	0.06079507026	0.4721833344
-2.237210389	0.4935202008	0.0107985960	0.0540604771	0.0289993073	0.7781542416
-2.146206321	0.6770037078	0.0109740832	0.0710705204	0.03591047301	0.8574168621
-2.210670043	0.5503637252	0.0098767586	0.0712101535	0.03088237377	0.8189936387
-2.101887247	0.1908549581	0.0183494993	0.0699155409	0.03976970361	0.5818748723
-2.084549882	0.0501680495	0.0068361644	0.0385984917	0.0413748599	0.482385092
-1.875760654	0.1067965398	0.0076705652	0.0747824932	0.06555558693	0.5562319781
-2.229554347	0.2257433436	0.0077541834	0.0523626207	0.02953183322	0.6270648433
-2.085252705	0.3756815645	0.0150299663	0.0755312214	0.04130871215	0.7392100501
-1.589789725	0.0801339314	0.0209506205	0.0959986753	0.1171380983	0.5341946883
-2.203994701	0.1772684659	0.0145115814	0.0799557916	0.03137261323	0.5818748723
-2.054705334	0.2519194675	0.0171855383	0.0717903078	0.04426988024	0.6426517028
-2.111441839	0.1448843916	0.0220124606	0.1347757046	0.03890846958	0.5818748723
-2.044379946	0.4048674322	0.0083872287	0.0804731906	0.04531149829	0.7442416032
-2.32569961	0.3665953852	0.0079953962	0.0542961211	0.02343267024	0.7391035993
-2.135932331	0.802611948	0.0119111954	0.0768736181	0.03677472823	0.8878450753
-1.987930535	0.5098245799	0.0141535908	0.0880347294	0.05138804356	0.7781542416
-2.1389029	0.6983202595	0.0119036906	0.0669249250	0.03652299443	0.8574168621
-2.146262001	0.9556888751	0.0130873485	0.0710779147	0.03590583796	0.9791894213
-2.296794198	0.0392623624	0.0064731938	0.0495473656	0.02513655597	0.4721833344
-2.608129724	0.0453296001	0.0028386966	0.0441836659	0.01147247793	0.4721833344
-2.148116285	0.9657061631	0.0115468653	0.0713642414	0.03575177729	0.9814087024
-2.346266739	0.3537380101	0.0082881624	0.0459281955	0.02228366046	0.7391035993
-2.085406888	0.5097407616	0.0105054192	0.0702475779	0.04129421323	0.7781542416
-2.259453361	0.2071943585	0.0100497541	0.0445435082	0.02749993587	0.5886203368
-2.138408098	0.9193503678	0.0133715105	0.0732037997	0.03656482047	0.9657041679
-2.073371853	0.2043784651	0.0114490356	0.0739658147	0.04243933087	0.5886203368
-2.397448716	0.1686663019	0.0089142611	0.0585687600	0.01963944182	0.5818748723
-2.441844225	0.0159376816	0.0017041626	0.0197237508	0.01757719671	0.2213566899
-2.164757674	0.6943916551	0.0111411262	0.0663876066	0.03439482467	0.8574168621
-2.203443086	0.573214854	0.0100087889	0.0674692160	0.03141342855	0.8235845603
-1.960940554	0.0729997520	0.0165060127	0.1469627859	0.05453244844	0.5341946883
-2.305862259	0.1891644391	0.0116523621	0.0509751980	0.02459054595	0.5818748723
-2.201234326	0.3537690857	0.0085612928	0.0647590232	0.0315773281	0.7391035993
-2.087685787	0.8994116466	0.0194707101	0.1580433647	0.04113504581	0.9499253008
-2.083949931	0.1254151359	0.0190591885	0.1643243331	0.04143139834	0.6443053671
-2.076984645	0.9373888988	0.0214111153	0.1589987686	0.04212909397	0.957242324
-2.022233356	0.5975365874	0.0191877482	0.1647538694	0.04764608834	0.8118703633
-2.099121786	0.1290725025	0.0182436778	0.1181769677	0.04007515906	0.6443053671
-2.192368904	0.5691777254	0.0156638248	0.1373560911	0.0322812046	0.7905246186
-2.086026489	0.9419264468	0.0190829494	0.1624093429	0.04128830377	0.957242324
-2.085353726	0.8987252864	0.0199408997	0.1577565431	0.04134398555	0.9499253008
-2.089321259	0.9277951185	0.0204425890	0.1585150385	0.04097557642	0.957242324
-1.900572868	0.1115146049	0.0217244689	0.2230703685	0.06223264998	0.6443053671
-2.083382928	0.5610737606	0.0222861433	0.1703782954	0.04152601979	0.7904682604
-2.443694685	0.0714340533	0.0112751078	0.1406452731	0.01749562612	0.6159663638

-2.063070096	0.7732243122	0.0191061993	0.1677872719	0.04350060331	0.8949355466
-2.067324034	0.3419486154	0.0185759685	0.1910829869	0.04308288574	0.7118714722
-2.220776982	0.2302424509	0.0180451628	0.1578737212	0.03021858779	0.6536180385
-2.059778297	0.4398967569	0.0263196848	0.1790704945	0.04382457874	0.7281989247
-2.0560728	0.4910635341	0.0198213773	0.2027149164	0.04421135408	0.7395535153
-2.155162091	0.4132902064	0.0166174964	0.1493700299	0.03523453981	0.7281989247
-2.014549122	0.7497093284	0.0179780114	0.1755559792	0.04850993184	0.8840911892
-2.105563265	0.4219837995	0.0136326037	0.1317497367	0.03948656011	0.7281989247
-1.876504378	0.2794887157	0.0382196636	0.2059261997	0.06574122442	0.6822233369
-2.169722733	0.5539961134	0.0175734547	0.161465159	0.03402748798	0.7904682604
-1.989122378	0.0632069882	0.0486601050	0.1832926065	0.05128115988	0.6159663638
-2.175755704	0.3398098115	0.0165504466	0.1233398025	0.0335483374	0.7118714722
-1.87848574	0.1732175009	0.0540865581	0.219702029	0.06527736672	0.6443053671
-2.174971821	0.2854815212	0.0143735151	0.1104490806	0.03361896071	0.6822233369
-2.085479718	0.958679205	0.0197808018	0.1594492747	0.04133344958	0.9664104889
-2.103866474	0.2346781765	0.0131104072	0.1328797658	0.039669362	0.6536180385
-2.286266965	0.3644781938	0.0115331591	0.1175914496	0.02581580995	0.7118714722
-1.972485974	0.3053994996	0.0368171174	0.1750953733	0.05321767433	0.706943286
-2.095393588	0.9071861887	0.0189345176	0.1566161825	0.04041058251	0.9499253008
-2.089843894	0.9072187268	0.0192967881	0.1608030157	0.04094033468	0.9499253008
-1.982586322	0.2353024939	0.0322134912	0.1725365841	0.05206294823	0.6536180385
-1.674825436	0.0451962738	0.0359483572	0.2587859034	0.09917356944	0.6159663638
-2.149938532	0.6341166691	0.0169297081	0.1492173333	0.03564539612	0.8343640383
-2.07272448	0.4696719247	0.0304755973	0.1582333987	0.04267376628	0.7365881039
-1.908530176	0.0623454447	0.0295607008	0.1224362135	0.0613055069	0.6159663638
-2.216349618	0.1074338676	0.0219292666	0.1133801196	0.03059957199	0.6443053671
-2.319610285	0.0565671091	0.0202277053	0.0821862499	0.02378282689	0.6159663638
-1.955987036	0.7097563078	0.0186232314	0.1967988345	0.05522327811	0.8697993968
-2.15163604	0.4362059266	0.0150231458	0.1521671808	0.03558420185	0.7281989247
-2.105713467	0.8155982288	0.0187924960	0.1535469497	0.03942551684	0.9102658803
-2.089855048	0.8883342771	0.0194039420	0.1578650073	0.04096387516	0.9499253008
-2.353440912	0.0372517111	0.0115389571	0.1647249066	0.02189483994	0.6159663638
-2.098937381	0.8067616018	0.0188401996	0.159470736	0.04006535813	0.9085153174
-1.96293449	0.4495697534	0.0251155863	0.1640116017	0.05432212635	0.7281989247
-2.162621788	0.5914406828	0.0190166932	0.153562513	0.03460031372	0.8118703633
-2.070077749	0.7301179339	0.0192107215	0.1581455836	0.0428230311	0.8775455936
-2.209241121	0.3508272053	0.0163410892	0.1215850561	0.03101038683	0.7118714722
-2.193436129	0.1759757688	0.0210962977	0.2371279887	0.03216201596	0.6443053671
-2.159470895	0.2056994087	0.0149764152	0.1298375541	0.03495218796	0.6536180385
-1.822960958	0.3340839867	0.0398066817	0.2365064016	0.07332272916	0.7118714722
-1.859680634	0.2282348604	0.0363703603	0.164961605	0.06783705132	0.6536180385
-1.968098388	0.4067902709	0.0162820335	0.1337908516	0.05370418489	0.7281989247
-2.118845309	0.5626074009	0.0172713993	0.1532101309	0.03829792901	0.7904682604
-1.971530209	0.3508468321	0.0258286941	0.1519570582	0.0533591798	0.7118714722
-1.928430104	0.454396129	0.0191062595	0.2022686709	0.05854216819	0.7281989247
-2.334409433	0.0787691698	0.0182130841	0.1124176207	0.0229442151	0.6159663638

-2.113536746	0.6998164247	0.017888489£	0.1463237617	0.03877639522	0.8697993968
-1.925032898	0.1095695879	0.0286292981	0.2440040008	0.05906754582	0.6443053671
-2.149243783	0.1571796819	0.0263224172	0.1573981822	0.03569053628	0.6443053671
-2.114387241	0.6252196916	0.017086041€	0.1506255075	0.03870911228	0.8314091643
-1.91403024	0.1513286624	0.054015653C	0.2154779211	0.06041453644	0.6443053671
-2.149732948	0.2426798861	0.0147583534	0.2115677711	0.03561793101	0.6594562121
-2.097894845	0.0085039130	0.0111320625	0.1345570338	0.04016659679	0.3543297105
-2.097060804	0.5500394772	0.0177816565	0.148437872	0.0403047418	0.7904682604
-1.850827607	0.1471691112	0.0374527391	0.1711160879	0.06911859743	0.6443053671
-1.642623004	0.0632896669	0.0289154382	0.2714682952	0.1057789383	0.6159663638
-2.049960739	0.1756573319	0.0385490382	0.2153856502	0.04483368249	0.6443053671
-2.284274847	0.2346536899	0.0156228603	0.1139105255	0.0259938048	0.6536180385
-1.853949849	0.4008041678	0.014884611£	0.1875503858	0.06870830413	0.7281989247
-2.053582342	0.6518322766	0.0185771351	0.1606527125	0.04442557401	0.8487399435
-2.104034177	0.204260505	0.017439810€	0.2172032487	0.03963163221	0.6536180385
-2.935105315	0.0014150994	0.004629275C	0.034642366€	0.00474845235	0.1768874274
-2.152590582	0.0071585520	0.010515645C	0.0655309243	0.03551169449	0.3543297105
-2.132066138	0.6917076187	0.0174398787	0.1661408852	0.03714442827	0.8697993968
-2.382763649	0.1804055028	0.006900392£	0.0925512294	0.02036780922	0.6443053671
-2.073264513	0.4814028008	0.0162974934	0.162558999	0.042476583	0.7365881039
-2.045407192	0.5628134014	0.0238492921	0.1653712888	0.04529949925	0.7904682604
-2.122210473	0.4138057896	0.0238400978	0.1979493518	0.03800194563	0.7281989247
-2.024456701	0.764463568	0.0239158615	0.1881934872	0.04740460371	0.8930649159
-2.247327434	0.159767487	0.0107934115	0.1376796202	0.02836311005	0.6443053671
-2.133945705	0.6626500001	0.0189703093	0.1653305898	0.03698935023	0.8539304125
-2.085667413	0.9119282888	0.0202385661	0.1585083201	0.04132547642	0.9499253008
-2.024824112	0.9856491496	0.0192772734	0.179545883	0.04750996987	0.9856491496
-2.084888814	0.4832017962	0.0163975107	0.1676104904	0.04141009589	0.7365881039
-2.136779632	0.1730174759	0.0159512798	0.1308676111	0.0367617425	0.6443053671
-2.039902049	0.6044479639	0.0190773175	0.1607497127	0.04584208404	0.812430059
-2.014367202	0.2789299007	0.0245475792	0.1877768934	0.04868748848	0.6822233369
-2.080230985	0.9096804071	0.024234886C	0.1596384103	0.04184086751	0.9499253008
-2.090043047	0.780402435	0.0184440813	0.1545998134	0.04090548107	0.8949569209
-1.810307433	0.1222854218	0.0216592267	0.243334194€	0.07535858536	0.6443053671
-2.179447939	0.453682115	0.018649626€	0.117887463	0.03328786629	0.7281989247
-2.117090057	0.1776606498	0.0200911788	0.1577939794	0.03846686381	0.6443053671
-2.034972096	0.4495531969	0.021219520€	0.1484306157	0.04633822216	0.7281989247
-2.037625553	0.4066266869	0.027760297£	0.166589912£	0.04606114485	0.7281989247
-1.964402384	0.0611785149	0.014587444€	0.1056301924	0.05433042731	0.6159663638
-1.959019605	0.6709409825	0.018985447	0.1668433162	0.05478763371	0.8557920696
-2.080663779	0.7047315842	0.0213632304	0.170595407	0.04178546139	0.8697993968
-1.981399116	0.0361703349	0.030827103€	0.171373315	0.05224880477	0.6159663638
-1.7114162	0.3559479761	0.0297295891	0.202823632	0.09230285367	0.7118714722
-2.114777125	0.3633239186	0.0232667161	0.173684170€	0.03866072792	0.7118714722
-2.09447309	0.8830970021	0.0194131217	0.1585610071	0.04050062844	0.9499253008
-2.111528375	0.1166091267	0.0323667114	0.2657061294	0.03890074419	0.6443053671

-1.956392865	0.2000478928	0.0111366019	0.1900545871	0.05517535158	0.6536180385
-2.595224071	0.0788436945	0.0069714996	0.0856855203	0.01186608235	0.6159663638
-2.06467946	0.4097185115	0.0207701637	0.1829527964	0.04331261977	0.7281989247
-1.859677784	0.324282011	0.0254662397	0.2044717814	0.06784577392	0.7118714722
-2.203691666	0.2541435213	0.0138024626	0.1751390992	0.03139502972	0.6618320868
-2.103547806	0.7400884532	0.0186472436	0.1585090188	0.0396873466	0.8810576824
-2.219759271	0.0684452291	0.0122021805	0.1247249956	0.03026181143	0.6159663638
-2.733045624	0.0161109988	0.0028857180	0.0893460930	0.00823311060	0.5034687151
-2.038244404	0.5386566508	0.0252287440	0.1660666855	0.04604207669	0.7904682604
-2.363717852	0.2892626948	0.0124342617	0.0936608231	0.02136279973	0.6822233369
-1.96879726	0.323900551	0.0182322244	0.1702863831	0.05369891727	0.7118714722
-2.08373933	0.7936181259	0.0185507189	0.1740372986	0.04148803657	0.9018387794
-2.193630459	0.2793944084	0.0415276211	0.2253275953	0.03218750443	0.6822233369
-1.964116981	0.2178254332	0.0225831357	0.1815008531	0.05430624171	0.6536180385
-2.181262355	0.4742359431	0.0181327853	0.1515586832	0.03313165408	0.7365881039
-2.181243211	0.3971396584	0.0127361788	0.1178739824	0.03310324091	0.7281989247
-2.178587907	0.1719380601	0.0165149256	0.1132609285	0.03334175354	0.6443053671
-2.114364281	0.7187584883	0.0182039329	0.1564085609	0.03869986955	0.8722797188
-1.942740108	0.1769181676	0.0257086915	0.2611915749	0.05677825997	0.6443053671
-2.184219621	0.2323285295	0.0221331843	0.1348950255	0.03295186509	0.6536180385
-2.160382268	0.2530826144	0.0133075511	0.1459635289	0.03479056905	0.6618320868
-2.196603856	0.0850262291	0.0064070565	0.0970540706	0.03192337091	0.4810582845
-2.187024388	0.3131571074	0.0102461071	0.1326191717	0.03264985396	0.6888398003
-2.243013867	0.1568381911	0.0067905641	0.1433166841	0.02860130241	0.5766109966
-2.10423851	0.9207291752	0.0126140441	0.1484292979	0.0395562407	0.967152495
-2.168539795	0.0049172416	0.0094972320	0.0754342729	0.03409279283	0.1414074649
-2.195830219	0.5184925581	0.0107220493	0.1303770933	0.03198150987	0.7752904146
-2.15557433	0.4469480759	0.0129439786	0.1287845249	0.03513795807	0.735111967
-2.08671821	0.1688010093	0.0065254864	0.1318545547	0.04117107829	0.6028607473
-2.324794251	0.0070890947	0.0023352397	0.0890518353	0.0234844394	0.1476894731
-2.072973533	0.3551738624	0.0116578722	0.1638172884	0.04247769661	0.6952978415
-2.114346835	0.8072147369	0.0122455379	0.1451307674	0.03864985833	0.9264082432
-1.997998275	0.4987028156	0.0135080286	0.1468249337	0.05025562434	0.7750958893
-2.133123929	0.7288206422	0.0123106808	0.1416244841	0.03701411141	0.8844910707
-2.084783345	0.111150124	0.0111565012	0.1982952704	0.04135287682	0.4810582845
-2.324214904	0.1102169301	0.0103893339	0.140028718	0.02351762036	0.4810582845
-2.07089391	0.0413084829	0.0237618814	0.1894899863	0.04267849246	0.4134605685
-2.179276102	0.0001824908	0.0066002339	0.3545762691	0.03328484707	0.02281135096
-2.127676233	0.7662760547	0.0120695950	0.1460535229	0.03748234377	0.9122333985
-2.582177797	0.0227209164	0.0341759984	0.0670090012	0.01227641371	0.3550143195
-2.103551398	0.5621944543	0.0235985853	0.1738218103	0.03961851785	0.7813413209
-1.78082849	0.0761289079	0.0341119569	0.2325925239	0.0800028188	0.4810582845
-2.060226234	0.5532641908	0.0123441774	0.1398446905	0.04372148225	0.7813413209
-2.046831344	0.0917620856	0.0287801487	0.1685677124	0.04506230439	0.4810582845
-2.110416633	0.9030566344	0.0125636507	0.1488246091	0.03900009544	0.9658207475
-2.24027237	0.3441111855	0.0078551750	0.122352491	0.0287887105	0.6952978415

-2.208399867	0.2139362871	0.0088853145	0.0933512981	0.03104833455	0.6525141531
-2.027973832	0.4433333154	0.0165020241	0.1398412228	0.04701008658	0.735111967
-2.111295717	0.8078279881	0.0123964263	0.1451636332	0.03892151752	0.9264082432
-2.003938033	0.8457411307	0.0184973263	0.1643203628	0.0495976282	0.9355543481
-1.988141846	0.278008657	0.0255449657	0.1619436648	0.05136405217	0.6626573547
-2.155646438	0.1279260483	0.0160178561	0.1588466623	0.03513206867	0.5158308398
-2.173080447	0.3971002465	0.0116295909	0.1108687324	0.0337332581	0.6991201523
-2.096748083	0.0759930400	0.0193342717	0.1423259664	0.0402397615	0.4810582845
-1.949106515	0.6707280829	0.0168699401	0.1763961953	0.05596220357	0.8643403131
-1.932458461	0.7138670608	0.0151874224	0.156295781	0.0580276701	0.8834988376
-2.17287098	0.3838262455	0.0166744046	0.1361870128	0.03374977051	0.6953374013
-2.140434705	0.0141429704	0.0117209885	0.0768293764	0.03639377439	0.2525530445
-2.337250738	0.0903000283	0.0112098686	0.0922569279	0.02278102352	0.4810582845
-2.138744592	0.591137952	0.0141045866	0.1323092724	0.03653637181	0.7860877021
-1.992539168	0.7870535268	0.0123620997	0.18032919	0.05086697516	0.9264082432
-2.079090225	0.9678545076	0.0157040678	0.1505419271	0.04189184615	0.9838679628
-2.088267405	0.9759970191	0.0126825708	0.1518252872	0.04102601768	0.9838679628
-2.186409042	0.4885745013	0.0121868565	0.1255857532	0.03269701251	0.7730609198
-2.062165227	0.3733307713	0.0128475864	0.1352608858	0.04353027986	0.6953374013
-1.992801263	0.4355485939	0.0147269459	0.1499612939	0.05083747858	0.735111967
-2.070428287	0.7636378798	0.0137048932	0.1488073882	0.04272356277	0.9122333985
-2.099916906	0.9404745444	0.0127511990	0.1493916291	0.03994935495	0.971564612
-2.047053377	0.0956744536	0.0111725241	0.1366323317	0.04503979254	0.4810582845
-2.433250088	0.074027712	0.0072303332	0.0763165168	0.01796053339	0.4810582845
-2.125573521	0.4195766738	0.0151214867	0.2037646979	0.03766445036	0.7184532086
-2.097254844	0.9749277402	0.013344172	0.1520609659	0.0401931965	0.9838679628
-2.341024725	0.2637962775	0.0067950273	0.0965379577	0.0225716426	0.6626573547
-1.852758501	0.1039981391	0.0261961716	0.1614659234	0.06883695776	0.4810582845
-1.96009721	0.1410830531	0.0079750446	0.1016903106	0.05463329521	0.5511056763
-2.191784351	0.2081014989	0.0091864827	0.1305529464	0.03228707546	0.6525141531
-2.090355015	0.3454266754	0.0134069112	0.1310672796	0.04083124429	0.6952978415
-1.916950081	0.2809667184	0.0118710391	0.2005021922	0.06000981626	0.6626573547
-2.207815897	0.2919737104	0.0135448820	0.1290634093	0.03109115231	0.6758650705
-2.157430474	0.6391823968	0.0111988054	0.1336289721	0.03498663247	0.8410294695
-2.164150893	0.5683715408	0.0109562612	0.1285425216	0.03444349846	0.7813413209
-2.172306762	0.0610238487	0.0196469466	0.1513381243	0.03379428356	0.4810582845
-2.03621156	0.1148101179	0.0242068728	0.1683781272	0.04615045822	0.4810582845
-1.953773111	0.3615548776	0.0301028941	0.1893801263	0.05539462092	0.6952978415
-2.124810588	0.5397678274	0.0127463322	0.1270441829	0.03773071514	0.7813413209
-2.055255341	0.2160787526	0.0113390998	0.1463303941	0.04421498165	0.6525141531
-2.056990885	0.3727283609	0.0170854304	0.1778987124	0.04404213574	0.6953374013
-2.081092101	0.935271178	0.0141661312	0.1487303981	0.04170163724	0.971564612
-1.999513953	0.8366150725	0.0141290495	0.1643072538	0.05008701133	0.9337221791
-2.135336483	0.5209951586	0.0101561105	0.1289613289	0.03682540397	0.7752904146
-2.352024718	0.1967871682	0.0090678577	0.0999247276	0.021971111	0.6525141531
-1.747739917	0.152757888	0.0075606398	0.1944788322	0.08562495284	0.5766109966

-2.181354537	0.2459800514	0.0143885000	0.1455159931	0.03308664497	0.6598047917
-2.116465264	0.3826117382	0.0117522400	0.1841843367	0.03846221486	0.6953374013
-2.599612606	0.0366823352	0.0053633609	0.0621175672	0.01173088147	0.4134605685
-2.201865002	0.0271610365	0.0066182375	0.0690853801	0.03155988953	0.3772366194
-2.093294323	0.2437520173	0.0126791572	0.1183612538	0.04055836967	0.6598047917
-2.008069651	0.6908097347	0.0236922592	0.1777742762	0.04914431473	0.8811348657
-2.240980794	0.0936709797	0.0146314772	0.121296509	0.02874017921	0.4810582845
-1.990747886	0.2806512966	0.0198946007	0.1680109218	0.05106896251	0.6626573547
-2.15291943	0.3109427542	0.0163449323	0.1953343562	0.03535539909	0.6888398003
-2.290283434	0.2296849819	0.0073132136	0.0924831838	0.02553519331	0.6525141531
-2.210632903	0.3580404047	0.0088547799	0.1317326967	0.03088508261	0.6952978415
-2.076412657	0.0328468094	0.0079270655	0.0965448234	0.04214743268	0.4105851175
-2.110462622	0.4721862843	0.0098587674	0.1362670146	0.03899598122	0.756708789
-2.010566966	0.9153301611	0.0125922057	0.1785139638	0.04887204581	0.967152495
-2.111827791	0.5022621362	0.0103887221	0.1550021408	0.0388740244	0.7750958893
-2.412240053	0.0005069474	0.0041805141	0.0601839132	0.01892946357	0.0316842142
-2.100382321	0.9843748613	0.0126626761	0.1493189236	0.03990685585	0.9843748613
-2.108592348	0.7216009473	0.0128398342	0.1481997861	0.03916360216	0.8843148864
-2.069522771	0.5576693329	0.021573642	0.1512436369	0.04281133086	0.7813413209
-2.10998542	0.6543505512	0.0113459744	0.1422953408	0.03903869039	0.8520189469
-2.033765937	0.6982602639	0.0127519893	0.1658981308	0.04640423795	0.8816417474
-2.170883398	0.5872631305	0.0121729981	0.1240471769	0.0339068062	0.7860877021
-2.136885951	0.3141109489	0.0129980859	0.1448335811	0.03669375136	0.6888398003
-2.261965078	0.2276171955	0.0087101591	0.1474699622	0.02733500721	0.6525141531
-2.068286667	0.3945856025	0.0180103589	0.1500834369	0.04293139396	0.6991201523
-2.060561834	0.4089024807	0.0109165416	0.125163152	0.04368833705	0.7099001401
-2.107097116	0.8545461868	0.0131493532	0.1476052269	0.0392980612	0.9370023978
-2.149173557	0.4566530978	0.0103774908	0.1290746384	0.03566419429	0.741319964
-2.031737874	0.2480866017	0.0174876146	0.1592549485	0.04661560038	0.6598047917
-1.713499363	0.2733192267	0.0197655177	0.1809295322	0.09178353469	0.6626573547
-2.300741252	0.0048525263	0.0161608284	0.1709423085	0.02489758553	0.1414074649
-2.009649624	0.2275551433	0.0192406538	0.1513949792	0.04897190756	0.6525141531
-2.072088492	0.0692085566	0.0267566907	0.2856038107	0.04256305044	0.4810582845
-1.938658186	0.0994715946	0.0057282862	0.1782391571	0.05725102414	0.4810582845
-1.937234061	0.707515449	0.0162865408	0.1693462777	0.05742863735	0.8834988376
-2.095699509	0.8292605048	0.0131429992	0.1580534555	0.04033626132	0.9337221791
-2.043596291	0.9015723114	0.0136954101	0.1580160024	0.04539141057	0.9658207475
-2.100562316	0.8322112386	0.0130095735	0.145665294	0.0398904303	0.9337221791
-2.150181239	0.5700122456	0.0105574343	0.1442722882	0.03558089388	0.7813413209
-2.249606007	0.0429998991	0.0073512564	0.1123887837	0.02815506748	0.4134605685
-2.34657664	0.2242752097	0.0064663690	0.1213318295	0.02226673829	0.6525141531
-2.106487453	0.9040082196	0.0125909297	0.1499475891	0.03935300058	0.9658207475
-1.614300901	0.1740084994	0.0194450608	0.3044267312	0.1117095028	0.6041961785
-2.025703597	0.3486866076	0.0110649481	0.1465043418	0.04724940511	0.6952978415
-2.15456291	0.5124003862	0.0121388058	0.1247424209	0.03522065681	0.7752904146
-2.142520758	0.5750672122	0.0222263620	0.187416315	0.03621844148	0.7813413209

-2.082886442	0.8060501283	0.0128759973	0.1515864932	0.04153178469	0.9264082432
-2.269785368	0.3201676749	0.0108754039	0.1344549084	0.02682708695	0.6900165407
-2.463173006	0.0056562985	0.0012557926	0.0388170406	0.01665741216	0.1414074649
-2.135133351	0.5464004326	0.0120957021	0.1338925227	0.03684269386	0.7813413209
-2.314508436	0.1154539883	0.0074547365	0.1258457062	0.02407977451	0.4810582845
-1.971925062	0.26930805	0.0167872031	0.2296854576	0.0532333984	0.6626573547
-2.317193315	0.0999624275	0.0125927678	0.1021454122	0.02392309571	0.4810582845
-2.1611376	0.3423627857	0.0094259880	0.1377764794	0.03468610833	0.6952978415
-0.6034512998	0.2769133459	0.0003145141	0.7503952408	0.5484629448	0.989483196
-0.5418807792	0.7167033487	0.0003713051	0.713381288	0.5898861645	0.989483196
-0.5178768463	0.3530993229	0.0002802697	0.7214883786	0.6064217147	0.989483196
-0.3993468239	0.0785274036	0.0002369731	0.7946299287	0.6910393252	0.989483196
-0.6175918763	0.1549622896	0.0003501098	0.6083090651	0.5391561309	0.989483196
-0.8315975396	0.2292741643	0.0008364868	0.6043703151	0.4089084654	0.989483196
-0.5965130555	0.7515355661	0.0003931123	0.6743894809	0.5530630019	0.989483196
-0.581824804	0.9611468914	0.0004466459	0.6956764044	0.5628446879	0.989483196
-0.6061935182	0.7349613248	0.0005840115	0.6823919048	0.5466491535	0.989483196
-0.5325981743	0.6834035337	0.0003821156	0.7220695374	0.5962400637	0.989483196
-0.5669323286	0.8943907575	0.0004337167	0.7029005269	0.5728603611	0.989483196
-0.4326370513	0.3867393086	0.0002889777	0.6682917541	0.6668323238	0.989483196
-0.5622964171	0.7499566817	0.0004371333	0.7245787483	0.5759998662	0.989483196
-0.5721362415	0.1217754493	0.0003412657	0.8005035379	0.5693509957	0.989483196
-0.6500648892	0.4504289078	0.0003572388	0.7118458548	0.5181252199	0.989483196
-0.5850477021	0.1493716015	0.0002148341	0.7995299872	0.5606863879	0.989483196
-0.5769910352	0.4369812637	0.0003819768	0.7812312387	0.566091129	0.989483196
-0.5805120282	0.9774827193	0.0004087564	0.695547755	0.5637321004	0.989483196
-0.7184142748	0.3912161602	0.0002636484	0.5872563441	0.4752849144	0.989483196
-0.5892066656	0.7105018753	0.0004643552	0.7468419616	0.5579154738	0.989483196
-0.6496846293	0.5660231264	0.0007036711	0.657226842	0.5184239281	0.989483196
-0.4016436634	0.0990987815	0.0002303733	0.6495741212	0.6893676316	0.989483196
-0.4787660479	0.3280855759	0.0002142904	0.738703932	0.6338312255	0.989483196
-0.4778927383	0.2578609584	0.0002538700	0.8233553185	0.6344538257	0.989483196
-0.5164805645	0.7884652689	0.0006009158	0.7431362435	0.6073974703	0.989483196
-0.775625932	0.0235080752	0.0006958242	0.4294030081	0.4409646012	0.989483196
-0.6247374079	0.5353063443	0.0005120463	0.683537979	0.5345004224	0.989483196
-0.5740939083	0.8216257667	0.0004174843	0.7112391304	0.5680279352	0.989483196
-0.4310269376	0.5207595769	0.0003605996	0.7895919305	0.6679816642	0.989483196
-0.602829668	0.7710364933	0.0006601039	0.671170273	0.5488772848	0.989483196
-0.5755964199	0.9143761289	0.0004272959	0.7041784091	0.5670338134	0.989483196
-0.6865847714	0.3646432215	0.0004568206	0.6255279717	0.494966086	0.989483196
-0.4633008916	0.1070892023	0.0001360109	0.7282391605	0.6448219373	0.989483196
-0.588912443	0.91551651	0.0004935388	0.6868589838	0.5581627988	0.989483196
-0.5702276282	0.9949726927	0.0004155065	0.6964367397	0.5706395601	0.9949726927
-0.5845499059	0.7596078641	0.0006767336	0.7126502664	0.5610448429	0.989483196
-0.5948704139	0.7718244944	0.0004411720	0.7173654503	0.5541443061	0.989483196
-0.5758542283	0.9635837904	0.0004229445	0.7019576847	0.5668563971	0.989483196

-0.589265673	0.4921570332	0.0003547901	0.6233564332	0.5578759241	0.989483196
-0.8919143131	0.1690667899	0.000289408	0.503988829	0.3760347135	0.989483196
-0.5390019955	0.5056447941	0.000378776	0.726920129	0.5918839789	0.989483196
-0.768720636	0.2676656958	0.000427181	0.623496315	0.4449818318	0.989483196
-0.7781420977	0.1143905112	0.0003262312	0.465288221	0.4395684427	0.989483196
-0.5735735255	0.9529816402	0.000406562	0.696594009	0.5683809237	0.989483196
-0.7939431863	0.2927808185	0.0007814002	0.707091595	0.4302906602	0.989483196
-0.5732750193	0.9705307846	0.0004087547	0.695504892	0.5685708475	0.989483196
-0.5386937661	0.6564726489	0.000462606	0.729767314	0.5920636475	0.989483196
-0.6135211764	0.4788639542	0.000536168	0.696442649	0.5418232741	0.989483196
-0.4405228472	0.4241712725	0.000338046	0.800739532	0.6611097907	0.989483196
-0.6624576812	0.0752046349	0.000224857	0.978680018	0.5101810293	0.989483196
-0.6824917085	0.2047672778	0.000522392	0.5865616971	0.4975472757	0.989483196
-0.7247448433	0.4610808199	0.000744723	0.580022031	0.471408727	0.989483196
-0.7367867185	0.374500196	0.000753331	0.710685776	0.4640678466	0.989483196
-0.4422439174	0.3542363164	0.000442569	0.606691980	0.6598810723	0.989483196
-0.64088251	0.4048174516	0.0005146397	0.6752019421	0.5240195583	0.989483196
-0.5866852114	0.9338347881	0.000430934	0.7043353581	0.5595918046	0.989483196
-0.4950183527	0.6829743424	0.0003861274	0.7558507874	0.6223429929	0.989483196
-0.8091781245	0.1015748943	0.000252222	0.524007967	0.421554613	0.989483196
-0.5125240528	0.8356762185	0.000433731	0.728697091	0.6101495955	0.989483196
-0.6297387144	0.734545116	0.000559754	0.671050878	0.5312589865	0.989483196
-0.5839924074	0.8488385311	0.000403416	0.697700499	0.5613959788	0.989483196
-0.6379069584	0.4750709199	0.0007174297	0.6460040491	0.5259581498	0.989483196
-0.5285459046	0.709340501	0.0004935327	0.7485319394	0.5990401795	0.989483196
-0.58610217	0.829924691	0.000412449	0.669567801	0.5599880608	0.989483196
-0.5783265368	0.8970928835	0.0004203937	0.699040947	0.5651952552	0.989483196
-0.6298954533	0.4889547616	0.000561054	0.652168120	0.5311342512	0.989483196
-0.4262009831	0.4793741782	0.0003249561	0.7412449981	0.6714563812	0.989483196
-0.3601811482	0.3961190696	0.000302346	0.838917847	0.7199546783	0.989483196
-0.5820961813	0.8542835808	0.0004320574	0.712745262	0.5626640235	0.989483196
-0.5376616147	0.853767301	0.000405309	0.723536755	0.5927971767	0.989483196
-0.1562010007	0.1008053843	0.000572633	0.8640640792	0.8763951327	0.989483196
-0.6480604659	0.5749781847	0.000420298	0.690254318	0.5193785447	0.989483196
-0.5059847417	0.1579640977	0.000257877	0.8508598534	0.6147145765	0.989483196
-1.10668106	0.0383892346	0.000665362	0.370168654	0.2727729905	0.989483196
-0.6031551207	0.2341157683	0.0004403387	0.574902689	0.548661561	0.989483196
-0.5810577662	0.8931561033	0.0004066964	0.701929408	0.5633619156	0.989483196
-0.4702996493	0.4344209838	0.000301114	0.774807249	0.6398423828	0.989483196
-0.5701499739	0.8516885786	0.000447524	0.6952139642	0.5706893679	0.989483196
-0.5636442143	0.5427371169	0.000367834	0.690748707	0.5750908294	0.989483196
-0.5889784589	0.7832601769	0.000390832	0.723942663	0.5580666347	0.989483196
-0.5277488953	0.7460751906	0.0003894414	0.742506398	0.5996022906	0.989483196
-0.5396271249	0.6745770698	0.0003704421	0.713945288	0.5914394153	0.989483196
-0.5404764654	0.3804407507	0.0002513694	0.741645547	0.5908649081	0.989483196
-0.5893223996	0.759566193	0.000581419	0.6955511632	0.557836974	0.989483196

-0.5428281388	0.9115767114	0.0004229212	0.7348503454	0.5892913179	0.989483196
-0.6024881835	0.6468330096	0.0005834740	0.7076015624	0.5490885023	0.989483196
-0.6670401934	0.0921767758	0.0004303648	0.6302184404	0.5072738793	0.989483196
-0.5014574908	0.1782096163	0.0003894774	0.731059157	0.6178703605	0.989483196
-0.5875646696	0.7367377	0.0004026679	0.720397827	0.5590097381	0.989483196
-0.5804985158	0.8970555469	0.0005049785	0.7030554973	0.5637393527	0.989483196
-0.560917322	0.250164556	0.0002216635	0.778157674	0.5769243235	0.989483196
-0.5630820521	0.9079151621	0.0004047604	0.7144621169	0.5754543575	0.989483196
-0.4583513674	0.3911631669	0.0003930429	0.9459660638	0.6483350984	0.989483196
-0.5694064249	0.6123846461	0.0004037614	0.694394718	0.5711929786	0.989483196
-0.5866812652	0.9294008769	0.0004222961	0.6987073908	0.5596179526	0.989483196
-0.6437732698	0.0626533801	0.0002554639	0.7406272862	0.5221265981	0.989483196
-0.5336985	0.4103242029	0.0004566656	0.6394049383	0.595511783	0.989483196
-0.3169886554	0.2193093974	0.0002872749	0.7550645702	0.7523374899	0.989483196
-0.5773534375	0.9688313107	0.0004588095	0.702093352	0.5658419424	0.989483196
-0.5638615262	0.7090032934	0.0003763150	0.7022377654	0.574939009	0.989483196
-0.4092943516	0.538838553	0.0003730435	0.7878698659	0.6837665463	0.989483196
-0.7053801579	0.0745846056	0.0002137063	0.756729052	0.4832768018	0.989483196
-0.6234504652	0.6293527009	0.0005676728	0.7049193217	0.5353365865	0.989483196
-0.5781891725	0.7986461161	0.0005083366	0.6667827589	0.5652821476	0.989483196
-0.5120393243	0.3805292478	0.0008969099	0.7798838131	0.61049271	0.989483196
-0.405629841	0.4643406162	0.0003164451	0.8156503039	0.6864220354	0.989483196
-0.5774107959	0.8979099159	0.0003988404	0.7020346355	0.5658081636	0.989483196
-0.5884792948	0.9162399433	0.0005025633	0.6882836449	0.5583239431	0.989483196
-0.5349122466	0.2700010589	0.0002874583	0.7116349937	0.5946430755	0.989483196
-0.5682064035	0.8007880076	0.0004341444	0.6873851659	0.5720079904	0.989483196
-0.5692223995	0.8568958413	0.0003950190	0.7004362321	0.5713168528	0.989483196
-0.5308402332	0.8779639747	0.0005365851	0.7163899618	0.5974465294	0.989483196
-0.6249212097	0.3184602952	0.0005726355	0.7326321851	0.5343621236	0.989483196
-0.4145382893	0.6522977827	0.0003706348	0.8128890386	0.6799359341	0.989483196
-0.5847768914	0.9384254456	0.0004039170	0.6927298696	0.5608665854	0.989483196
-0.6122997326	0.7811689693	0.0004290494	0.6826411879	0.5426329077	0.989483196
-0.5791613765	0.4109303178	0.0002707781	0.8094038839	0.5646518691	0.989483196
-0.5236444055	0.148378217	0.0003153887	0.752857581	0.602437256	0.989483196
-0.4346572268	0.4648878016	0.0003546372	0.7005131521	0.6653530081	0.989483196
-0.7469052929	0.2417273883	0.0021067620	0.5514190627	0.4579561597	0.989483196
-0.5831884971	0.9513893479	0.0004084814	0.6926039384	0.5619155849	0.989483196
-0.6082610825	0.5019270821	0.0004028910	0.71941983	0.5452929343	0.989483196
-0.5385068534	0.7857730117	0.0003982329	0.7503954175	0.5921944222	0.989483196
-0.5753802448	0.9815673304	0.0004151183	0.6977179349	0.5671814781	0.989483196
-0.5602020284	0.6486817875	0.0003503482	0.6840162833	0.5774199196	0.989483196
-0.722146761	0.3269068325	0.0006966553	0.673349925	0.473015272	0.9984517566
-0.6040988467	0.3985733636	0.0006234790	0.6678435908	0.5480609368	0.9984517566
-0.5673716661	0.1024134451	0.0003136906	0.6656050708	0.572576955	0.9984517566
-0.4794067453	0.0491988056	0.0004194284	0.734195605	0.6333955325	0.9984517566
-0.7364887789	0.2215663033	0.0007989324	0.5561992733	0.4643051886	0.9984517566

-0.9654856528	0.1908749258	0.0017582651	0.5392352939	0.3381992661	0.9984517566
-0.6909040369	0.9984517566	0.0008316960	0.6306258654	0.4923047739	0.9984517566
-0.7015819637	0.8204320567	0.0008225800	0.6249754936	0.4856612186	0.9984517566
-0.7211951785	0.7020569444	0.0012066160	0.612914973	0.4735957649	0.9984517566
-0.6628780197	0.8472445139	0.0008097072	0.6454938625	0.509923675	0.9984517566
-0.6749766977	0.8657728038	0.0008639785	0.6375599033	0.5022955761	0.9984517566
-0.6734788325	0.9501541927	0.0008306444	0.6277293122	0.5032497621	0.9984517566
-0.673969543	0.7583034453	0.0008825932	0.6531629889	0.5029367166	0.9984517566
-0.6932749657	0.0780801630	0.0006645213	0.7307542213	0.4908261671	0.9984517566
-0.7572311367	0.451884704	0.0007336053	0.6482199375	0.4518991606	0.9984517566
-0.6997919982	0.2001083533	0.0004881362	0.7097884622	0.486768248	0.9984517566
-0.6979891396	0.4443957433	0.0008079608	0.7057694339	0.487892696	0.9984517566
-0.7398535525	0.4810957718	0.0007984674	0.6050727805	0.4622980621	0.9984517566
-0.7611036878	0.6351839922	0.0007300155	0.5754730657	0.4496001767	0.9984517566
-0.6976051447	0.7870890522	0.0010109749	0.6670171841	0.4881300344	0.9984517566
-0.7545096988	0.5009070493	0.0013528156	0.5954479864	0.4535679831	0.9984517566
-0.4312927102	0.0175835431	0.0003236968	0.5664456281	0.6678247589	0.9984517566
-0.5868827529	0.3362380903	0.0004619169	0.6801186924	0.5594893012	0.9984517566
-0.6167182797	0.4281511389	0.0006332131	0.7233548585	0.5397619507	0.9984517566
-0.5766788681	0.608370111	0.0009103211	0.7124112266	0.5663148412	0.9984517566
-0.8835290102	0.0496899656	0.0017019786	0.4190183704	0.3804520954	0.9984517566
-0.7115389829	0.7663694291	0.0009710019	0.6241173713	0.4795218626	0.9984517566
-0.6898787851	0.9763951351	0.0009318313	0.6322273339	0.4929353101	0.9984517566
-0.6168721194	0.7845368253	0.0010037521	0.6703574027	0.5396634071	0.9984517566
-0.7483427015	0.5301750956	0.0015940528	0.57687138	0.4571916034	0.9984517566
-0.6916240316	0.9932593245	0.0009403715	0.6313551712	0.4918551284	0.9984517566
-0.7459210559	0.5635361803	0.0008979429	0.5938493687	0.4586471641	0.9984517566
-0.5628870594	0.1002952369	0.0002851744	0.678999009	0.5756284148	0.9984517566
-0.544197581	0.5049018451	0.0006953900	0.7059335281	0.5883664522	0.9984517566
-0.8176584842	0.470249058	0.0008607329	0.6020774099	0.4168142138	0.9984517566
-0.7106693251	0.456224044	0.0019972424	0.656943481	0.4800682391	0.9984517566
-0.6914246217	0.9918579565	0.0008446801	0.6307506217	0.4919766011	0.9984517566
-0.7107538928	0.7386217056	0.0007584064	0.6035775306	0.4800134637	0.9984517566
-0.701530264	0.564237243	0.0007618183	0.5717125191	0.4856928048	0.9984517566
-0.8386838889	0.453649202	0.0007021762	0.5349099768	0.4050439391	0.9984517566
-0.6939115493	0.9384094662	0.0010131549	0.6271550435	0.4904577694	0.9984517566
-0.8754616628	0.3243106478	0.0009458470	0.5655995467	0.3847477051	0.9984517566
-0.9539541475	0.0434825500	0.0006118771	0.3666100844	0.3440025826	0.9984517566
-0.737210008	0.5735700049	0.0008154808	0.6270512104	0.4638803797	0.9984517566
-0.8707853316	0.3604677728	0.0014226194	0.6301469397	0.3873241991	0.9984517566
-0.6768497362	0.9301487231	0.0008255218	0.6276345993	0.5010998467	0.9984517566
-0.7677533702	0.4552780785	0.0006486099	0.5724883798	0.4456690079	0.9984517566
-0.7377567941	0.4106875364	0.0011244880	0.6176263526	0.4635362101	0.9984517566
-0.5677920081	0.511301948	0.0007270782	0.7189997712	0.5722781091	0.9984517566
-0.7688740318	0.1734162113	0.0006034351	0.8343978319	0.4449813319	0.9984517566
-0.8096982063	0.1864537826	0.0011142328	0.5256542459	0.4213336509	0.9984517566

-0.7045391965	0.8869967647	0.0011376521	0.6144638112	0.4838281317	0.9984517566
-0.9562988936	0.1511269885	0.0018979412	0.6502482579	0.3427346469	0.9984517566
-0.4972235032	0.2103476088	0.0008743920	0.5217256786	0.6208496431	0.9984517566
-0.7537984705	0.3696971226	0.0010547427	0.6103633421	0.4539302231	0.9984517566
-0.6289478465	0.558326264	0.0006537957	0.5886587307	0.5317889937	0.9984517566
-0.6104689169	0.7382288072	0.0007934585	0.6823355892	0.543818647	0.9984517566
-0.8166183886	0.3964518035	0.0007498744	0.5481180417	0.4173424379	0.9984517566
-0.7142895081	0.8510520958	0.0009950099	0.6098669438	0.4778295524	0.9984517566
-0.7505350946	0.6690477102	0.0011463866	0.5979528025	0.4559004248	0.9984517566
-0.6915777048	0.9915225386	0.0008755858	0.6293328063	0.4918781977	0.9984517566
-0.7431284458	0.5272809591	0.0013800147	0.5906683962	0.4603272819	0.9984517566
-0.7109634096	0.8427719053	0.0015910281	0.6085628344	0.4798668263	0.9984517566
-0.7090223225	0.5816829751	0.0008462786	0.5567161595	0.4810725908	0.9984517566
-0.6935869309	0.8805640239	0.0008061469	0.6266102077	0.4906388401	0.9984517566
-0.7394225172	0.5195908025	0.0011474818	0.5909199417	0.4625438958	0.9984517566
-0.5238342439	0.4562738074	0.0006503307	0.6781693547	0.602309535	0.9984517566
-0.4210157488	0.3072688239	0.0005495004	0.7980012496	0.6752421707	0.9984517566
-0.6997697811	0.6712140238	0.0007484998	0.6606676003	0.4867847444	0.9984517566
-0.775316351	0.5930861266	0.0008704956	0.5717026156	0.4412318951	0.9984517566
-0.4168975245	0.3016204052	0.0011376523	0.7368946259	0.6782449936	0.9984517566
-0.6894281356	0.9816088858	0.0008320360	0.62906013	0.4932069881	0.9984517566
-0.6239952219	0.1547697076	0.0005615617	0.7923763778	0.5350082426	0.9984517566
-1.038553971	0.1660923701	0.0013737607	0.4212446842	0.303149733	0.9984517566
-0.7137610043	0.2775524279	0.0009057877	0.5250592811	0.4781537718	0.9984517566
-0.7006133444	0.4408664885	0.0008027394	0.6574959125	0.486267322	0.9984517566
-0.5368272448	0.2451837939	0.0003901946	0.7369047568	0.5933912355	0.9984517566
-0.6641815092	0.5901413427	0.0009604890	0.6264798673	0.509132105	0.9984517566
-0.6885425569	0.5859092538	0.0007872729	0.6190413932	0.4937794163	0.9984517566
-0.6938070419	0.9404932577	0.0008591145	0.6383061707	0.4904876053	0.9984517566
-0.5868967747	0.4969949575	0.0006151383	0.722650387	0.5594840476	0.9984517566
-0.6552509487	0.7086562945	0.0007750727	0.6431136634	0.5148258427	0.9984517566
-0.6271240238	0.203163227	0.0003811613	0.7013923348	0.5329821565	0.9984517566
-0.6924140721	0.9719016415	0.0010080550	0.6290885458	0.4913579926	0.9984517566
-0.6642507735	0.9515776377	0.0008610135	0.6567787405	0.5091323191	0.9984517566
-0.7257595487	0.5719443596	0.0012769698	0.6361420557	0.4708011772	0.9984517566
-0.7590391042	0.1809265214	0.0009263417	0.5807836859	0.4508061507	0.9984517566
-0.5971859185	0.0513578194	0.0007339269	0.6685915982	0.5526366663	0.9984517566
-0.7002029304	0.4748142934	0.0007606472	0.6822609149	0.4865116717	0.9984517566
-0.6917047729	0.868372489	0.0009661603	0.6359079091	0.4918031287	0.9984517566
-0.6748919838	0.339126387	0.0005343434	0.6959813656	0.5023452528	0.9984517566
-0.6516589618	0.7648411691	0.0008168911	0.6708168381	0.5171108974	0.9984517566
-0.5878938191	0.4998954532	0.0007967485	0.8243056738	0.5588109039	0.9984517566
-0.6830184258	0.5270965501	0.0008251984	0.6256425528	0.4972369535	0.9984517566
-0.700817983	0.9028132521	0.0008589711	0.6326903372	0.4861540907	0.9984517566
-0.7065637851	0.2653549744	0.0006390245	0.6576926916	0.4825724179	0.9984517566
-0.6535917608	0.4332696252	0.0009553462	0.5749566789	0.5158860755	0.9984517566

-0.3810287205	0.1561426986	0.0005521651	0.7057240198	0.7045255625	0.9984517566
-0.6730463888	0.7622950221	0.0008039618	0.6565620984	0.5035120041	0.9984517566
-0.6863874348	0.895383396	0.0008364988	0.6307363612	0.4951280314	0.9984517566
-0.7014615454	0.8893238648	0.0009435638	0.6150659605	0.4857518151	0.9984517566
-0.8251992904	0.0735740459	0.0004556595	0.6814626368	0.412537087	0.9984517566
-0.7104152784	0.8207623763	0.0010569119	0.6362632141	0.4802132385	0.9984517566
-0.6899998393	0.8435041593	0.0010173644	0.6092798512	0.4928639941	0.9984517566
-0.6520990559	0.6013177965	0.0015012519	0.6759308304	0.516845976	0.9984517566
-0.5866248245	0.7122632057	0.0007955355	0.6938800966	0.559634622	0.9984517566
-0.6665835161	0.1420227889	0.0005694276	0.6822475526	0.5076191346	0.9984517566
-0.7421423612	0.7863945659	0.0011627206	0.6019370548	0.4607988382	0.9984517566
-0.6497143392	0.5315609252	0.0007251219	0.6535769735	0.5183269226	0.9984517566
-0.6772643139	0.6576345291	0.0007639489	0.6114677597	0.5008589776	0.9984517566
-0.6800579031	0.8431866251	0.0008039276	0.6336205152	0.4990978182	0.9984517566
-0.6265443063	0.8473203139	0.0010299777	0.6538549494	0.5333137217	0.9984517566
-0.7529061929	0.2586651414	0.0012058298	0.6545107964	0.4544479014	0.9984517566
-0.6449831748	0.968726724	0.0008815449	0.65382224	0.5213953959	0.9984517566
-0.675989889	0.9226804776	0.0008311328	0.6351693592	0.5016451516	0.9984517566
-0.6810748251	0.955615686	0.0008240864	0.6340603863	0.4984655684	0.9984517566
-0.6848297071	0.1945923947	0.0002868562	0.8171741247	0.496125121	0.9984517566
-0.626571437	0.0734545266	0.0005530304	0.6868230073	0.5333283012	0.9984517566
-0.5067146111	0.3580026607	0.0006890517	0.633409946	0.6142172635	0.9984517566
-0.7956491208	0.5006196769	0.0029799131	0.548773638	0.4293326901	0.9984517566
-0.7837239198	0.4358084359	0.0008320724	0.5526083746	0.4362777422	0.9984517566
-0.7037236609	0.721795411	0.0008315952	0.6423254029	0.4843393368	0.9984517566
-0.6776949873	0.9622092246	0.0008732351	0.6459243268	0.5005699899	0.9984517566
-0.6638811376	0.7854822254	0.0008788857	0.6370248219	0.5093324077	0.9984517566
-0.6640397946	0.4311632975	0.0006113492	0.6058950313	0.5092310344	0.9984517566
1.176458302	0.5593357919	0.0294925099	0.0873822162	0.244177017	0.8151231524
1.115669634	0.4372743286	0.0234595695	0.1002928154	0.2691318144	0.8038399796
1.191817661	0.9746060135	0.0312589227	0.0929023039	0.2381470678	0.9888060224
1.157867055	0.8163278565	0.0277525825	0.0983233866	0.2516187706	0.907007274
1.33697486	0.0203528032	0.0241815945	0.0472557734	0.1864191002	0.4797907818
1.438457649	0.1990168261	0.0459703064	0.0708737335	0.1556587345	0.654914271
1.201670832	0.8173333639	0.0279690420	0.0906716982	0.2343449489	0.907007274
1.123545491	0.1341657621	0.0435458605	0.0954926736	0.2657941887	0.654914271
1.256440518	0.4852231269	0.0417528510	0.0848259699	0.2139495688	0.8070010522
1.266511477	0.5665386284	0.0293195654	0.0809245393	0.2103018749	0.8151231524
1.293636179	0.2297506659	0.0458203650	0.0789602796	0.2008809335	0.6837817438
1.041853736	0.2982750352	0.0230367760	0.0816198091	0.3017901845	0.7767579042
1.209302827	0.8049136926	0.0276933636	0.0889754074	0.2314310354	0.907007274
1.214229356	0.1525996021	0.0279210548	0.1118904089	0.2295494125	0.654914271
1.404644992	0.0407824642	0.0199393759	0.0984144566	0.165438272	0.5884208072
1.198632773	0.4268449464	0.0229826939	0.1073163516	0.2355106844	0.8038399796
1.230156998	0.3410023091	0.0294881479	0.1162128942	0.2235706024	0.8038399796
1.126628281	0.3570405032	0.0264016079	0.0993460204	0.2645098478	0.8038399796

1.240645962	0.7121334035	0.026229717€	0.085491359€	0.2197059983	0.8901667544
1.196157783	0.47624425	0.053327609€	0.073073151C	0.2364781557	0.8054082189
1.188686394	0.1637549057	0.015428791€	0.0765496894	0.2395557615	0.654914271
1.126349999	0.5633160331	0.027347363€	0.091617982€	0.2646169841	0.8151231524
1.06950259	0.2050682882	0.0135592012	0.108142165	0.289237514	0.654914271
1.17764771	0.9020035843	0.0281167671	0.101487982	0.2437028236	0.9425708261
1.570435211	0.0884510219	0.112235317	0.038456126€	0.1217358252	0.654914271
1.168641542	0.7921389862	0.027151440	0.105114238€	0.2472958472	0.907007274
1.085030512	0.1311456623	0.0173825311	0.094401731	0.2823619483	0.654914271
1.271245092	0.250521666	0.044063635	0.070093476€	0.2086829843	0.6958935166
1.131638054	0.8799538267	0.031750617€	0.104167946€	0.2624152874	0.9401216098
1.050630505	0.1844185553	0.014547278C	0.130274558€	0.2977428153	0.654914271
1.180504084	0.4244260348	0.020544187€	0.114876642€	0.2426550472	0.8038399796
1.348530781	0.0470017797	0.030080338	0.073542570	0.1826455056	0.5884208072
1.14038772	0.4768016656	0.0226642982	0.100443577€	0.258766432	0.8054082189
1.10960761	0.7412173087	0.026506702€	0.102298890€	0.2717709137	0.8995355688
1.079726314	0.6439194774	0.027150013€	0.0981728444	0.2847105203	0.8562759008
1.203773292	0.279645281	0.015653204	0.080061333€	0.2335788594	0.7437374496
1.169585833	0.6193668797	0.026459110	0.086173230€	0.2469136184	0.8357540956
1.22308764	0.6847377084	0.026591907€	0.0858196501	0.226213229	0.8733899342
1.190557431	0.5975521447	0.030303955	0.1145581574	0.2386422916	0.8357540956
1.305440452	0.4767504934	0.025019488€	0.078601945€	0.1969376745	0.8054082189
1.09985531	0.1361086775	0.015408875€	0.0974711091	0.2759219737	0.654914271
1.295798302	0.5456052708	0.030191250	0.0815927024	0.2000671118	0.8151231524
1.397975192	0.1571187127	0.0286234474	0.049055607	0.1674400451	0.654914271
1.160048992	0.756145199	0.028094012	0.0940559047	0.2507479174	0.907007274
1.330381919	0.4095509597	0.040698049€	0.0973741221	0.1885562177	0.8038399796
1.359870764	0.1880383131	0.0336373034	0.0909655471	0.1790745883	0.654914271
1.242522235	0.6920767545	0.026462820C	0.083028432	0.2190448208	0.8738342859
1.310426549	0.0724659096	0.040407025	0.078209383C	0.1951645186	0.654914271
1.096517514	0.6801489267	0.026709466C	0.114351259€	0.2773136231	0.8733899342
1.203961171	0.8235544612	0.0277070281	0.106515531€	0.2334513411	0.907007274
1.280122963	0.3967645869	0.0353152231	0.075541753€	0.2055580557	0.8038399796
1.36173039	0.4171265698	0.0449510313	0.066209167€	0.178516097	0.8038399796
1.028150491	0.3584272	0.0192748117	0.079884713C	0.3081379412	0.8038399796
0.9949498438	0.1836370688	0.0317951834	0.064714633	0.3238472142	0.654914271
1.172433858	0.6722292896	0.0261756044	0.093054019	0.2457933919	0.8733899342
1.119116909	0.4058050715	0.023148997€	0.078455247C	0.2676623959	0.8038399796
1.479841264	0.1932344686	0.0304467367	0.0502265047	0.144226577	0.654914271
1.290423558	0.5312245427	0.0269355447	0.0759239247	0.2019463716	0.8151231524
0.9735488717	0.4971126481	0.0236751847	0.125744807€	0.3342897695	0.8070010522
1.255775544	0.5662373977	0.035870890	0.0855827691	0.2142305272	0.8151231524
1.252673666	0.2003313494	0.0194302657	0.086139695€	0.2153246689	0.654914271
1.168625464	0.8143672225	0.028119506€	0.0980980244	0.247309939	0.907007274
1.063840032	0.4552243088	0.020949649C	0.1285052232	0.2917609236	0.8038399796
1.246159575	0.3392153345	0.030850439€	0.0642499447	0.2176804571	0.8038399796

1.245852477	0.268416587	0.0240308948	0.0829313466	0.2178089628	0.7293928996
1.285098437	0.3255279336	0.0410440906	0.0772874942	0.2038238854	0.8038399796
1.192077257	0.8996820288	0.0346131704	0.0919312950	0.2380186197	0.9425708261
1.307202035	0.5673257141	0.0355193299	0.0757151089	0.1962566895	0.8151231524
1.315505303	0.0470736645	0.0112759398	0.1139829036	0.1934949469	0.5884208072
1.270653322	0.6072466917	0.0296658593	0.0806138296	0.2089125828	0.8357540956
0.6168716597	0.0199164341	0.0400035833	0.1512264507	0.5397187363	0.4797907818
1.194588364	0.9299967395	0.0285706649	0.0927932478	0.2370561059	0.9528655118
1.283377217	0.1765473344	0.0304129929	0.0575925805	0.2044292607	0.654914271
1.492662165	0.2265306289	0.0413605806	0.0520113139	0.1408751994	0.6837817438
1.285173316	0.0232760688	0.0301187656	0.0400929341	0.2038096721	0.4797907818
1.189105805	0.7826128	0.0282307540	0.0916959097	0.2392151379	0.907007274
1.112326558	0.540295151	0.0228733192	0.1101595436	0.2705676237	0.8151231524
1.299384908	0.0787047259	0.0174725756	0.0889393524	0.1989104317	0.654914271
1.204749333	0.6114942538	0.0272732801	0.0861639079	0.2331609405	0.8357540956
1.160477006	0.3539257833	0.0351791433	0.0745980954	0.2505705702	0.8038399796
1.423263724	0.1145507699	0.0557970364	0.0516046719	0.1599662513	0.654914271
1.172719531	0.8344466921	0.0281042948	0.0950722645	0.2456745885	0.907007274
1.223828385	0.5408877343	0.0379297230	0.0844780169	0.2259192785	0.8151231524
1.260476127	0.0919742440	0.0585175576	0.0855414491	0.2125023238	0.654914271
0.961219016	0.2503331802	0.0343332159	0.187856815	0.340452552	0.6958935166
1.24244888	0.5379014848	0.0408348087	0.0931739108	0.219007831	0.8151231524
1.284506078	0.0944179546	0.0321997875	0.0753134757	0.2040319234	0.654914271
1.193251426	0.9808955742	0.0283751487	0.0929112672	0.2376045417	0.9888060224
1.188029046	0.2055251867	0.0243218510	0.1335420402	0.2396162366	0.654914271
1.192472591	0.4235046966	0.0212204061	0.1021079077	0.2378998352	0.8038399796
1.189271525	0.8671258194	0.0288265214	0.0978155642	0.2391439658	0.9344028227
1.153501063	0.81065099	0.0280036549	0.1069215948	0.253390635	0.907007274
1.070454841	0.4910555905	0.0270439399	0.179889463	0.2888118427	0.8070010522
1.19798103	0.3141136576	0.0277340692	0.0855607722	0.2357729789	0.8013103511
1.345166656	0.2411068466	0.0315609778	0.1017886271	0.18378695	0.6958935166
1.198602787	0.0041672126	0.0115129095	0.1206840185	0.2355044342	0.4797907818
1.180079218	0.7819902624	0.0302093881	0.0894828027	0.2427583819	0.907007274
1.279362231	0.605964118	0.0293693708	0.0847884056	0.205785223	0.8357540956
1.240452191	0.4499829649	0.0390806291	0.0776347664	0.2197674192	0.8038399796
1.248224082	0.1609112565	0.0350649625	0.0847643414	0.2169335637	0.654914271
0.9100067405	0.3697950515	0.0224942166	0.1388147688	0.3665548898	0.8038399796
1.330013688	0.1180762383	0.0215663944	0.1002142623	0.1886793858	0.654914271
1.036381368	0.0134201954	0.0076199585	0.0526348208	0.3043240807	0.4797907818
1.282216436	0.0268682837	0.0092648902	0.1736643353	0.2048710596	0.4797907818
1.095696612	0.1639299511	0.0619177271	0.1260843787	0.2777165805	0.654914271
0.9451411501	0.388184424	0.0212347179	0.1525143167	0.3484407998	0.8038399796
1.235611168	0.0108941715	0.0177700482	0.0940567368	0.22158489	0.4797907818
1.411022107	0.3932114497	0.0462010528	0.0674082857	0.1633848651	0.8038399796
1.124139633	0.6689182782	0.0266680255	0.1099331911	0.2655458255	0.8733899342
1.237242809	0.1400178659	0.0548624247	0.1120165038	0.2209459501	0.654914271

1.307431499	0.0653379778	0.0331628114	0.0756187776	0.1961898055	0.654914271
1.101992568	0.8317437891	0.0301090633	0.1055140739	0.2749554996	0.907007274
1.187422645	0.9174742615	0.0283907297	0.0925124956	0.2398591567	0.9478039892
1.095618405	0.904867993	0.0284737609	0.1179928241	0.2777358927	0.9425708261
1.141189126	0.7315015047	0.0283649801	0.1005440483	0.258439099	0.8995355688
1.325296064	0.1764691243	0.0293126632	0.0813105697	0.1902297965	0.654914271
1.193611762	0.9973965969	0.0392469904	0.1006050696	0.2374617992	0.9973965969
1.144466746	0.1281850916	0.0248017428	0.1088160796	0.2570971663	0.654914271
1.235649573	0.7407869613	0.0288792248	0.0919058649	0.2215270668	0.8995355688
1.416480744	0.1839854945	0.1026769576	0.0528641302	0.1618840384	0.654914271
1.297379672	0.4565811084	0.0301234422	0.0733365065	0.199576521	0.8038399796
1.208804004	0.6218010472	0.0287064219	0.0994130410	0.2316102266	0.8357540956
1.067132761	0.4420294698	0.0231543338	0.1431573724	0.2902845154	0.8038399796
1.300083212	0.2095725667	0.0222616355	0.0855246107	0.1986896947	0.654914271
1.217405863	0.379142648	0.0346867250	0.0999223636	0.2283460746	0.8038399796
-1.837776848	0.3203968605	0.0468282121	0.1243004567	0.0710133703	0.8706436426
-1.847171704	0.6558967896	0.0603227881	0.1097603641	0.06959392964	0.9949216742
-1.720457819	0.1018415948	0.0290084484	0.1200040613	0.09042430495	0.7766647204
-1.761960666	0.7171681283	0.0525750244	0.1209068421	0.08310777236	0.9949216742
-1.871386622	0.1118871444	0.0516824335	0.0851713477	0.06611478629	0.7766647204
-2.072880406	0.1818576058	0.0871066981	0.0822237276	0.04246050775	0.7766647204
-1.810270923	0.9435853741	0.0540887937	0.1137976889	0.07523525968	0.9949216742
-1.827725845	0.6914303912	0.0489411007	0.1097185786	0.07252952144	0.9949216742
-1.798809386	0.9599862299	0.0568046080	0.1144081158	0.07702773568	0.9949216742
-1.768829711	0.7458544442	0.0534915193	0.1210458485	0.08189770898	0.9949216742
-1.826382982	0.7884542828	0.063327748	0.1116860552	0.07272863679	0.9949216742
-1.556798421	0.0740316806	0.0345762524	0.0878176311	0.1248080055	0.7118430836
-1.7911154	0.836780664	0.0556043493	0.1202950992	0.07829885216	0.9949216742
-1.811919378	0.2671726638	0.0533152490	0.1354539279	0.07496787016	0.7766647204
-1.898443994	0.369019049	0.0502692774	0.1173428346	0.06243820171	0.941375125
-1.823468412	0.1831969472	0.0370645909	0.1438335038	0.07316391551	0.7766647204
-1.818334674	0.315139745	0.0527741356	0.1496775538	0.07398031172	0.8706436426
-1.851655913	0.568302405	0.0544734054	0.1056005339	0.06898127835	0.9949216742
-1.995089912	0.2594237402	0.0337993833	0.0755697446	0.05057316974	0.7766647204
-1.808307471	0.9789355206	0.0643436973	0.1164597551	0.07554284459	0.9949216742
-1.872627623	0.5665374318	0.0738115913	0.1031766441	0.06609436009	0.9949216742
-1.674340103	0.1272855521	0.0436903917	0.0983151882	0.09922187691	0.7766647204
-1.682611283	0.1205811334	0.0207673266	0.1235960526	0.09763095661	0.7766647204
-1.691427819	0.0647046404	0.0326848025	0.1742083913	0.09593829458	0.7118430836
-1.593316552	0.336960152	0.0352641647	0.1763805415	0.1162765304	0.8961706171
-2.008328281	0.0462753175	0.0882869331	0.0592387924	0.04901389877	0.7118430836
-1.843153578	0.6330923249	0.0609693384	0.1080949755	0.07023109614	0.9949216742
-1.797313633	0.70353157	0.0500358756	0.1205657602	0.0772658055	0.9949216742
-1.665048797	0.6323514514	0.0487269587	0.142751368	0.101085025	0.9949216742
-1.815004729	0.8768488224	0.0647689570	0.1101525554	0.07448788001	0.9949216742
-1.804185643	0.8568634003	0.0528395146	0.1185052114	0.07619154713	0.9949216742

-1.957592857	0.2266453748	0.0605894475	0.0897122520	0.05487310033	0.7766647204
-1.748228997	0.2171091903	0.0340111873	0.1153207303	0.08550941494	0.7766647204
-1.895535837	0.5663597394	0.0681551754	0.0976333287	0.06290879579	0.9949216742
-1.762522209	0.9550633178	0.0543228586	0.1144145669	0.08304166105	0.9949216742
-1.80531558	0.3781060918	0.0906099364	0.1275087944	0.07600465458	0.9452652295
-1.807942429	0.9920399718	0.0545352551	0.1145328053	0.07557866342	0.9949216742
-1.788652866	0.8344161797	0.0565923149	0.1204486247	0.07868733347	0.9949216742
-1.808353261	0.7203613022	0.0568206573	0.1309954314	0.07552181503	0.9949216742
-1.865328766	0.6620842926	0.0521995376	0.1036936171	0.06709824426	0.9949216742
-1.751054318	0.4843948778	0.0461485180	0.1233430112	0.08504677955	0.9949216742
-2.130520102	0.0730616250	0.0569682463	0.0780678330	0.03710434542	0.7118430836
-2.177746463	0.0215283678	0.0488064186	0.0333352162	0.03341677025	0.708748979
-1.769268482	0.6613168655	0.0542675955	0.115508095	0.08188386945	0.9949216742
-1.913877548	0.504413425	0.0726700789	0.1191205091	0.06032378958	0.9949216742
-1.838615594	0.7191420241	0.0575447508	0.116432092	0.07084453735	0.9949216742
-1.710541667	0.2650782068	0.0648357259	0.1432195133	0.09222482643	0.7766647204
-1.861542828	0.2225175186	0.0711547404	0.1178879431	0.06750080982	0.7766647204
-1.80687964	0.8920991375	0.0552356965	0.1134885281	0.07569299393	0.9949216742
-1.868363814	0.2287138194	0.0433731721	0.179774942	0.0665433925	0.7766647204
-2.095607526	0.0184818006	0.0741613454	0.0518400650	0.04036637729	0.708748979
-2.014159916	0.2930145806	0.0869123585	0.0714922846	0.04845478791	0.8324277859
-2.276454307	0.0192895080	0.1197263117	0.1174496699	0.02628628918	0.708748979
-1.707400206	0.5103627356	0.0582913864	0.0947239569	0.09284247078	0.9949216742
-1.835804721	0.6766445809	0.0599431473	0.1115206458	0.07131004307	0.9949216742
-1.798490389	0.9354233137	0.0536785039	0.1121423806	0.07707308002	0.9949216742
-1.802701091	0.8936749059	0.054569585	0.113409101	0.076313227	0.9949216742
-1.873326827	0.5696939431	0.0514462873	0.0982705500	0.06579013109	0.9949216742
-1.585482239	0.494004831	0.0474922253	0.1503791328	0.1180499219	0.9949216742
-1.87938576	0.5762847303	0.0696083099	0.104566277	0.06505395976	0.9949216742
-1.808329769	0.9949216742	0.0555631612	0.1134123706	0.07552566721	0.9949216742
-1.805187606	0.9648580204	0.0600392383	0.113412029	0.07603583091	0.9949216742
-1.745830883	0.7408341924	0.0529120092	0.1295634173	0.08587855167	0.9949216742
-1.846483969	0.4089188211	0.0566108037	0.0855247552	0.06971560462	0.9949216742
-1.857097511	0.2531691596	0.0443859610	0.0991377470	0.06822074256	0.7766647204
-1.820246532	0.8361175404	0.0599056176	0.1111877722	0.07365813795	0.9949216742
-1.782760642	0.9469421875	0.0616025024	0.1130360515	0.07959268046	0.9949216742
-1.809996929	0.817586333	0.0605019858	0.1088734937	0.07523919707	0.9949216742
-1.811548016	0.848001799	0.0534273004	0.1203491122	0.07501950217	0.9949216742
-2.11498421	0.104497805	0.0568851995	0.0594844563	0.03861787631	0.7766647204
-1.470347108	0.2294706438	0.0698143955	0.1545468676	0.1466507865	0.7766647204
-1.800180919	0.979658694	0.0542346489	0.1134925708	0.07678649825	0.9949216742
-1.760017249	0.1353257733	0.0431701742	0.1679519953	0.0834762293	0.7766647204
-2.077169534	0.227388129	0.0756668607	0.0679366231	0.0419775732	0.7766647204
-1.847335543	0.2480085415	0.0614956411	0.0838409827	0.06958457545	0.7766647204
-1.810004966	0.8896664883	0.0540618033	0.1146823321	0.07528195653	0.9949216742
-1.594504996	0.0733788068	0.0168103150	0.1688811144	0.1160746743	0.7118430836

-1.813363572	0.9005121593	0.0542778066	0.114218873	0.07474322978	0.9949216742
-1.792280727	0.5179547673	0.0495885478	0.1089773381	0.07812525529	0.9949216742
-1.812252259	0.8993562777	0.0538425872	0.1199036145	0.07490625663	0.9949216742
-1.8327385	0.7793790325	0.0655222472	0.1105130744	0.07174818128	0.9949216742
-1.747661293	0.5090397503	0.0431667210	0.1188820752	0.08562049655	0.9949216742
-1.798263332	0.7685030226	0.0500167644	0.1173212315	0.07715723675	0.9949216742
-1.809498293	0.9695228398	0.0587717931	0.1137127721	0.07535849662	0.9949216742
-1.821903679	0.7970158917	0.0521126214	0.1109380601	0.07354909891	0.9949216742
-1.807603321	0.9911735326	0.0573592059	0.1136823781	0.07561791508	0.9949216742
-1.84159152	0.5089713994	0.0590618696	0.1084915944	0.07043082109	0.9949216742
-1.771512736	0.5636771557	0.0570138779	0.1225302164	0.08153598694	0.9949216742
-1.857854095	0.2250301489	0.0543285613	0.1453881611	0.06801627639	0.7766647204
-1.807520759	0.8950049986	0.0568337126	0.1159152337	0.07566612442	0.9949216742
-1.832706472	0.0226799673	0.0178247067	0.1541542902	0.07176730528	0.708748979
-1.763339127	0.766744124	0.0542600555	0.1309970038	0.08288216361	0.9949216742
-1.637303794	0.249745554	0.0521631596	0.2698102776	0.1067299699	0.7766647204
-1.808262201	0.1527169993	0.0520873499	0.1038233522	0.07556982895	0.7766647204
-1.828403903	0.7950313618	0.0564864824	0.1154925087	0.0724603296	0.9949216742
-1.892851433	0.0410875652	0.0387625020	0.1262582772	0.06309507241	0.7118430836
-1.769756289	0.4510436441	0.0614548607	0.1038685702	0.08181964424	0.9949216742
-1.633154711	0.4469893072	0.0513466246	0.1270254689	0.107572625	0.9949216742
-1.788944071	0.7403035908	0.0502494353	0.1236364872	0.07863515043	0.9949216742
-1.804875886	0.9574164881	0.0547491174	0.113785984	0.07608734558	0.9949216742
-1.549981461	0.4263172719	0.0487898988	0.1652445519	0.1263721716	0.9949216742
-1.946104342	0.1034012464	0.0399498464	0.1242606487	0.05628612962	0.7766647204
-1.904331381	0.3453877136	0.0749481958	0.1166456526	0.06163336324	0.8994471709
-1.820549966	0.2437320952	0.0797116427	0.0788861531	0.07360619647	0.7766647204
-1.677423114	0.0548049791	0.1244095086	0.1754830072	0.09861479492	0.7118430836
-1.524242209	0.2387719631	0.0372828421	0.18925848	0.1325523358	0.7766647204
-1.803871594	0.4891437992	0.0499740522	0.117155658	0.07626782887	0.9949216742
-1.908857247	0.5506726702	0.0716150169	0.0997675378	0.06085227572	0.9949216742
-1.819311822	0.0739883434	0.0431339845	0.1229138034	0.0736931561	0.7118430836
-1.827550127	0.69537366	0.0700493729	0.1178873881	0.07256866484	0.9949216742
-1.802067718	0.9303917819	0.0538403607	0.1141342248	0.07652736348	0.9949216742
-1.481010443	0.1693078947	0.0285251255	0.1687583278	0.1436859221	0.7766647204
-1.824375591	0.6499938854	0.0602081391	0.1170495874	0.07302197505	0.9949216742
-1.371841129	0.2359597391	0.0394203954	0.2398680679	0.1751358198	0.7766647204
-1.812523177	0.890258633	0.0536963506	0.1107214414	0.07487209543	0.9949216742
-1.760679522	0.719949355	0.0508118346	0.1160108311	0.08352952519	0.9949216742
-1.839336488	0.1736444608	0.0199128938	0.1694244897	0.0708703566	0.7766647204
-1.772421652	0.2141077646	0.0529484251	0.1358648855	0.0813448946	0.7766647204
-1.687922354	0.615314975	0.0513357548	0.1119060241	0.09656586005	0.9949216742
-1.977374223	0.216557478	0.1431898649	0.0787458090	0.05246367004	0.7766647204
-1.807528726	0.9245975092	0.0543942527	0.1127618468	0.07561335556	0.9949216742
-1.943526751	0.0478137925	0.0506400071	0.1234192381	0.0566108524	0.7118430836
-1.89271786	0.4948386924	0.0621537923	0.0865246053	0.06317990472	0.9949216742

-1.851287703	0.6374214491	0.0521769873	0.1104665224	0.06903838904	0.9949216742
-1.78755668	0.4629981817	0.0443064657	0.1037397975	0.07889587939	0.9949216742
-1.655574084	0.3581083128	0.0082915420	0.6795777155	0.1058342316	0.8912910305
-1.630078475	0.6615322152	0.0095626019	0.58740976	0.1111363072	0.8912910305
-1.741321401	0.6322437985	0.0079084745	0.4943027056	0.08951137551	0.8912910305
-1.653587371	0.9429838554	0.0090127033	0.5606066728	0.106239765	0.9826407449
-1.710293752	0.0487534429	0.0092107273	0.6635491531	0.09515697716	0.8912910305
-1.250954073	0.2782276095	0.0286438242	0.7132300484	0.2184039647	0.8912910305
-1.58482839	0.5382563276	0.0083659593	0.7031931488	0.1210816467	0.8912910305
-1.668424832	0.981644266	0.0095538909	0.5593620778	0.1032418083	0.989560752
-1.670720392	0.95905443	0.0095399235	0.5588889512	0.1027842829	0.9826407449
-1.705522892	0.4646287096	0.0077056954	0.5747154742	0.0960508018	0.8912910305
-1.796430824	0.1510030151	0.0146830796	0.5355149835	0.08017500852	0.8912910305
-1.686182091	0.8048650177	0.0086943919	0.5453052631	0.09974609489	0.9826407449
-1.71570002	0.4915717897	0.0081680107	0.4985413152	0.09415248389	0.8912910305
-1.742971831	0.6317983373	0.0083297386	0.4825846067	0.08921908876	0.8912910305
-1.595743098	0.6775149515	0.0084309907	0.5385391992	0.118618827	0.8914670415
-1.798327262	0.3881489601	0.0062569563	0.43901438	0.07986891915	0.8912910305
-1.846527868	0.0814048813	0.0054466845	0.3903050448	0.07241432248	0.8912910305
-1.822547131	0.4110372141	0.0074607492	0.4767205427	0.07604578538	0.8912910305
-1.730840566	0.6568201039	0.0149582497	0.4905609315	0.09138625071	0.8912910305
-1.360641547	0.0961888290	0.0026615023	0.4736178072	0.1814390533	0.8912910305
-1.766165547	0.5252675705	0.0071583552	0.527914973	0.08519534086	0.8912910305
-1.409770971	0.4147282122	0.0090450971	0.5783554845	0.16653259	0.8912910305
-1.656919776	0.4283402572	0.0065297049	0.5697300297	0.1055602703	0.8912910305
-1.643573583	0.6246891767	0.0087782168	0.5231327765	0.108303331	0.8912910305
-1.291830819	0.3091004166	0.0559764651	0.8794829987	0.204019025	0.8912910305
-1.530381593	0.0715085500	0.0087438601	0.7405741526	0.1339940931	0.8912910305
-1.689965456	0.651918528	0.0080012666	0.5587660018	0.09901410932	0.8912910305
-1.718929291	0.4709597289	0.0069576845	0.5310089506	0.09355670369	0.8912910305
-1.750129996	0.6030036159	0.0086226531	0.5253782411	0.08796062358	0.8912910305
-1.588199786	0.1169081399	0.0205827122	0.6139043009	0.1203165193	0.8912910305
-1.553056235	0.3103128519	0.0063479066	0.6384264399	0.128488003	0.8912910305
-1.743996939	0.562108977	0.0079268679	0.4929951332	0.08903794703	0.8912910305
-1.643834537	0.1904588894	0.0078155078	0.6169384214	0.1082491403	0.8912910305
-2.089861963	0.0675886695	0.0033841446	0.3242419884	0.04320001858	0.8912910305
-1.348127411	0.1164832223	0.0094712094	0.6890332267	0.1853957921	0.8912910305
-1.683097558	0.8302403295	0.0117522052	0.541123971	0.1003461865	0.9826407449
-1.73317362	0.5141202242	0.0071252575	0.6462843517	0.09096608655	0.8912910305
-1.731657711	0.4921622229	0.0100791838	0.4964345294	0.09123890549	0.8912910305
-1.689080966	0.6048524036	0.0094220414	0.5008239145	0.09918483573	0.8912910305
-1.621774122	0.9548430749	0.0090499285	0.566190226	0.1129096123	0.9826407449
-1.70035457	0.5161514472	0.0076315801	0.5754057153	0.0970269562	0.8912910305
-1.655867229	0.8521722061	0.0089495040	0.5450123934	0.1057745022	0.9826407449
-1.544302513	0.3716410771	0.0172974842	0.7249728311	0.1305916384	0.8912910305
-1.725798018	0.32700105	0.0081726118	0.6092649832	0.09229991031	0.8912910305

-1.71771844	0.3246243643	0.0068179758	0.6314511142	0.09377972887	0.8912910305
-1.51453196	0.1712199348	0.0070154575	0.5724718926	0.1379542671	0.8912910305
-1.591471765	0.5644594707	0.0078587038	0.5626716984	0.1195777184	0.8912910305
-1.845163455	0.1508345772	0.0073747396	0.4503666231	0.07261691037	0.8912910305
-1.673913187	0.798791508	0.0094848161	0.5387483147	0.1021507169	0.9826407449
-1.758510436	0.5359140743	0.0105412087	0.6028315451	0.08650620125	0.8912910305
-1.647553721	0.771411131	0.0089693127	0.5902322175	0.1074792215	0.9792063282
-1.823471885	0.4786542016	0.0075239269	0.4368852722	0.07590293841	0.8912910305
-1.647415505	0.6702508549	0.0089792242	0.5367036285	0.1075077531	0.8912910305
-1.570216474	0.4918615288	0.0070365229	0.4638046798	0.1244435917	0.8912910305
-1.612290625	0.5685851022	0.0098425180	0.5467617602	0.1149629706	0.8912910305
-1.646566919	0.9270496256	0.0089239099	0.5573717614	0.1076830618	0.9826407449
-1.666669311	0.5855845827	0.0083707880	0.5948137672	0.103592833	0.8912910305
-1.616530411	0.8743012671	0.0091647445	0.5507284826	0.1140412344	0.9826407449
-1.519866668	0.6656067289	0.0093562933	0.5992006083	0.1366110055	0.8912910305
-1.674429583	0.9030108141	0.0088954910	0.5589425599	0.1020485493	0.9826407449
-1.687156293	0.5355670374	0.0096464270	0.5506671196	0.09955718402	0.8912910305
-1.576656866	0.3698037061	0.0228219814	0.5895336898	0.1229525601	0.8912910305
-1.63303305	0.8148447686	0.0130057197	0.5832398931	0.1105109229	0.9826407449
-1.665606289	0.9134852362	0.0091666293	0.5673353841	0.103805868	0.9826407449
-1.772547958	0.564346989	0.0075645096	0.4936616197	0.08411520812	0.8912910305
-1.708403191	0.7040902616	0.0082696194	0.5184451736	0.09551034426	0.9147784963
-1.662124207	0.9345898602	0.0090755928	0.5682359998	0.1045062279	0.9826407449
-1.403337265	0.2793535563	0.0102604031	0.7127914006	0.1684284739	0.8912910305
-1.755038569	0.3198255247	0.0052144418	0.5082041125	0.087106277	0.8912910305
-1.693151742	0.7811968836	0.0086213899	0.5289246523	0.09840110317	0.9792063282
-1.645261006	0.9898865319	0.0097375942	0.5601937221	0.1079533086	0.9898865319
-1.728498427	0.668507421	0.0082280555	0.5255997704	0.09180967962	0.8912910305
-1.656756041	0.3891673362	0.0073224399	0.5160573641	0.105593573	0.8912910305
-1.864130393	0.3840180408	0.0063607954	0.4041934693	0.06984351797	0.8912910305
-1.675556294	0.4992219425	0.0081326229	0.5123131239	0.1018259254	0.8912910305
-1.707734351	0.5187294845	0.0086081399	0.5877077001	0.09563561902	0.8912910305
-1.488209324	0.2681009649	0.0195799596	0.6809227002	0.1447376829	0.8912910305
-1.619825303	0.5688583363	0.0082737892	0.6694711145	0.1133291004	0.8912910305
-1.718100508	0.603245007	0.0079629106	0.5540342265	0.0937093085	0.8912910305
-1.903632266	0.0554038139	0.0178332883	0.7786091658	0.06435640708	0.8912910305
-1.780443296	0.0917562130	0.0150843743	0.7416215853	0.08279498693	0.8912910305
-1.676906001	0.8818771279	0.0099378559	0.5470801579	0.1015597692	0.9826407449
-1.678264252	0.8714431179	0.0092213858	0.5735729858	0.1012925095	0.9826407449
-1.771555614	0.3861085987	0.0116740816	0.5797216186	0.08428238858	0.8912910305
-1.221350025	0.3605116813	0.0088394891	0.940867365	0.2292854943	0.8912910305
-1.723291142	0.5606213417	0.0078132250	0.5095172285	0.09275695991	0.8912910305
-1.624355322	0.2217483436	0.0147204926	0.6021669888	0.1123559602	0.8912910305
-1.687625747	0.6322102358	0.0096429779	0.5684106383	0.09946625652	0.8912910305
-1.697973028	0.3057958157	0.0063952239	0.5426623036	0.09747952982	0.8912910305
-1.624510095	0.0068991647	0.0022241076	0.6736543281	0.1123228333	0.8623955959

-1.724987337	0.6281447404	0.0080409469	0.4929460513	0.09244750655	0.8912910305
-1.659578234	0.8732643523	0.0103469367	0.5847276995	0.1050207688	0.9826407449
-1.574837939	0.474191144	0.0093695264	0.788064912	0.1233721885	0.8912910305
-1.895379445	0.2260755655	0.0052202642	0.3208342737	0.06547123033	0.8912910305
-1.714951787	0.6088012432	0.0080435726	0.5694899333	0.09429097814	0.8912910305
-1.754922304	0.3109384647	0.0106191448	0.5591190182	0.08712643248	0.8912910305
-1.742446219	0.4459292722	0.0080002336	0.6657441786	0.08931208644	0.8912910305
-1.666844411	0.966918493	0.0090262964	0.5598219352	0.1035577768	0.9826407449
-1.644906425	0.820589478	0.0110488673	0.5955798321	0.1080267821	0.9826407449
-1.649897854	0.9638679433	0.0100372954	0.5635725636	0.1069962744	0.9826407449
-1.819427039	0.4721908132	0.0067781497	0.4929191263	0.07652942897	0.8912910305
-1.752725759	0.3477696251	0.0066944602	0.4753589526	0.08750795554	0.8912910305
-1.665601123	0.8770639717	0.0088805954	0.5523966279	0.1038069041	0.9826407449
-1.62765199	0.8916367071	0.0089422886	0.5522555084	0.1116520801	0.9826407449
-1.633842129	0.1629614235	0.0039812961	0.5337765803	0.1103401725	0.8912910305
-1.837174417	0.3362901483	0.0057370232	0.4815307607	0.07381281672	0.8912910305
-1.686952514	0.7833650626	0.0085928054	0.5376350282	0.09959667506	0.9792063282
-1.568499949	0.4031776655	0.0094688153	0.5868973789	0.1248434521	0.8912910305
-1.656451653	0.882329679	0.0098479221	0.5575561913	0.1056555064	0.9826407449
-1.679561628	0.284788802	0.0051950377	0.5975541754	0.1010377715	0.8912910305
-1.688963009	0.6403520464	0.0081669975	0.5530163886	0.0992076225	0.8912910305
-2.024406709	0.1754747492	0.0035380909	0.3751549499	0.04981531774	0.8912910305
-1.715151041	0.6333440018	0.0105185914	0.522952244	0.09425408059	0.8912910305
-1.382789788	0.5418240748	0.013207219	0.7027774984	0.1745961974	0.8912910305
-1.845969347	0.3089448101	0.0080539449	0.477973523	0.07249719347	0.8912910305
-1.661309692	0.2289988066	0.0068041573	0.6713562747	0.1046706146	0.8912910305
-1.702551464	0.7098681132	0.0163924801	0.5992259501	0.09661102083	0.9147784963
-1.844703425	0.2501669663	0.0049235075	0.4743706861	0.07268532409	0.8912910305
-1.624160786	0.9487195469	0.0089680164	0.5570880251	0.1123976095	0.9826407449
-1.233091471	0.0317211330	0.0674120665	0.8190709865	0.2249226552	0.8912910305
-1.667958935	0.4016141527	0.0070658558	0.566239872	0.1033348708	0.8912910305
-1.773757865	0.4464751037	0.0078993191	0.5533773011	0.08391175199	0.8912910305
-1.516961075	0.6375046231	0.0119573520	0.6124628727	0.1373413182	0.8912910305
-1.818535951	0.1949624084	0.0110070149	0.4424758519	0.07666803329	0.8912910305
-1.741127357	0.3067116463	0.0065553801	0.5304216748	0.08954579276	0.8912910305
1.694135105	0.1051415257	0.3762384812	0.3669993155	0.09517836115	0.4971223625
1.569258903	0.0571951581	0.3588328663	0.3592133468	0.1215970309	0.4971223625
1.711646845	0.5707488806	0.4973740174	0.3025966002	0.0918810885	0.8181148269
1.668232661	0.9871605578	0.441818093	0.3065198616	0.1002328558	0.9871605578
1.680029956	0.8452277854	0.4443515429	0.3131811022	0.09790423364	0.9267848524
1.832172998	0.380724078	0.5318365927	0.253545951	0.0716530428	0.7388208805
1.586719194	0.0638229964	0.4496531734	0.4154025498	0.1175831567	0.4971223625
1.812428322	0.0524971028	0.286058715	0.2953562945	0.0746854818	0.4971223625
1.574191029	0.0004498818	0.1532738308	0.3439217072	0.1204522394	0.02811761644
1.649217923	0.5148664917	0.4497913461	0.3192568832	0.1040810961	0.8015000474
1.616914681	0.3667509895	0.3686731581	0.3438445785	0.1108935209	0.7388208805

1.893729192	0.1591257904	0.4796751242	0.2875768431	0.06285465464	0.5234401001
1.635517695	0.66219584	0.4461312122	0.3217637147	0.1069276005	0.818594287
1.734647838	0.206496827	0.4365995748	0.245265876	0.08769361077	0.5736022972
1.618456976	0.6679729382	0.4435744797	0.3008374958	0.1105602652	0.818594287
1.749075173	0.1194940315	0.5843083175	0.2396744983	0.08514837903	0.4971223625
1.729421719	0.3186170976	0.4508991731	0.2332254411	0.08863095756	0.7136413882
1.613153889	0.4538836637	0.4211904796	0.3112064612	0.1117095495	0.7771980543
1.608620248	0.8271813153	0.4737871338	0.3308245667	0.1126997144	0.9231934322
1.694942318	0.3770743948	0.3286190499	0.3645204153	0.09502426595	0.7388208805
1.990043059	0.1024425394	0.6916194805	0.1943628845	0.05093000098	0.4971223625
1.676075458	0.9528190666	0.4439355122	0.305885099	0.09867981619	0.9762490436
1.706652652	0.5973264518	0.522033395	0.2880220734	0.09281172198	0.818594287
1.694856901	0.743774771	0.4485931379	0.2861609886	0.09504056218	0.8529527191
1.571794263	0.7235456327	0.399708946	0.3389523414	0.1210074671	0.8529527191
1.742031817	0.2595082993	0.4919566179	0.2245895363	0.08638320492	0.6358253148
1.713659951	0.6421523594	0.4790152245	0.3051328955	0.09150813736	0.818594287
1.676954508	0.3069822203	0.366939795	0.3248263976	0.09850697833	0.7106069915
2.068229266	0.0827759108	0.7696462239	0.186305674	0.04272944555	0.4971223625
1.926088556	0.0357266229	0.762342792	0.2276085777	0.05860820785	0.4971223625
1.681619905	0.6838332374	0.4767754673	0.2928207287	0.09759381088	0.8298947056
1.542688351	0.2033077661	0.4287686166	0.4321690003	0.1279149166	0.5736022972
1.671748871	0.5759528381	0.4060863131	0.3099264	0.09953412172	0.8181148269
1.23578282	0.0945417028	0.2886334701	0.467790095	0.2211274643	0.4971223625
2.095306831	0.0462569946	0.5186501513	0.2661857261	0.04016901192	0.4971223625
1.660252646	0.8011876486	0.4716209025	0.307678389	0.1018334887	0.902238343
1.73477802	0.100849215	0.4748377239	0.3627337582	0.08767036655	0.4971223625
1.630906831	0.623486455	0.4594639613	0.3286599472	0.1078996911	0.818594287
1.72157085	0.0660964908	0.3688030609	0.1895098643	0.09005460057	0.4971223625
1.789209585	0.5222991621	0.4293229953	0.247609928	0.07838806565	0.8015000474
1.605963687	0.5759196394	0.3904998751	0.3095386221	0.1132832015	0.8181148269
1.678491192	0.9193413267	0.4421129013	0.3025914119	0.09820543149	0.9497327755
2.016801979	0.0467111394	0.4972118665	0.1622320186	0.04798297497	0.4971223625
1.611259951	0.404468951	0.4264453716	0.2911017909	0.112122335	0.7423732369
1.450971629	0.1909443739	0.3498942385	0.3197991173	0.1517490225	0.5682868272
1.589583482	0.0613674088	0.3941614375	0.2670190005	0.1169349777	0.4971223625
1.91701162	0.0439968188	0.4015443802	0.2755659302	0.05977400707	0.4971223625
1.731236319	0.1194666698	0.3920554939	0.3137387802	0.08830456169	0.4971223625
1.455188829	0.0740483052	0.4028231714	0.4340843795	0.1505819315	0.4971223625
1.683549997	0.5155724283	0.4083863116	0.3783035944	0.09721806123	0.8015000474
1.638374087	0.4790975012	0.4004881776	0.3282281858	0.106328971	0.7984958353
1.347928364	0.1461088911	0.2870097096	0.4429426886	0.1825084353	0.5098455047
1.65312275	0.9678456141	0.4426864533	0.3054607164	0.1032811712	0.9835829412
1.625938467	0.6467382588	0.4691924452	0.2806962872	0.1089551576	0.818594287
1.717801046	0.57532907	0.468433738	0.2872594423	0.0907448683	0.8181148269
1.66704233	0.5481249181	0.4292422503	0.2833313011	0.1004703013	0.8181148269
1.67833234	0.9022245245	0.4417121964	0.3014070493	0.09823656831	0.9477148366

1.632065758	0.6160781522	0.452336581	0.3245338685	0.1076546875	0.818594287
1.237644268	0.1007502063	0.3121104411	0.4080192237	0.2204408574	0.4971223625
1.44995016	0.1509142694	0.3232940831	0.4405962786	0.1520327681	0.5098455047
1.676380937	0.4068974404	0.498697721	0.2913003385	0.09861972515	0.7423732369
1.799537791	0.2446603018	0.6064037237	0.2683840291	0.07672259677	0.6358253148
1.758934332	0.4742601393	0.5798710469	0.2701367575	0.08344447214	0.7984958353
1.660446997	0.2543659215	0.4212296472	0.4093733663	0.1017942597	0.6358253148
1.72693846	0.3328549812	0.4290175575	0.3093686089	0.08907923957	0.7276160897
1.640521179	0.5936708673	0.4090657958	0.3233375147	0.1058807868	0.818594287
1.168069027	0.0027535773	0.1513510133	0.3451711591	0.24718038	0.1147323911
1.801107667	0.4873011906	0.5124902314	0.2567228179	0.07647205191	0.8014822213
1.726342361	0.1281379808	0.6208635182	0.2116029094	0.08918712618	0.4971223625
1.712880115	0.7344504268	0.4573183885	0.2859082121	0.09165246289	0.8529527191
1.793165138	0.5149383634	0.4026677352	0.2679693077	0.07774668892	0.8015000474
1.601263823	0.3900974249	0.4464029364	0.3133694511	0.1143214472	0.7388208805
1.618819441	0.1237870297	0.4053566774	0.4191352853	0.1104820623	0.4971223625
1.409255004	0.2881850515	0.3713526137	0.429010599	0.1636769106	0.6796817253
1.677001455	0.4331740892	0.4132033802	0.36015725	0.09849775447	0.7626304388
1.804719394	0.0066870624	0.4595792346	0.3787202193	0.07589822984	0.2089707006
1.708856028	0.7427009565	0.52038625	0.2873205954	0.09240018728	0.8529527191
1.653807071	0.1820221728	0.5283682863	0.3133052551	0.1031415011	0.5549456488
1.60649123	0.431417548	0.3980772	0.3129972651	0.1131671384	0.7626304388
1.675158685	0.1701538589	0.5384735257	0.2199637926	0.09886033518	0.5439359075
1.564443384	0.3526364838	0.358857296	0.4038148765	0.1227231459	0.7276160897
1.668369608	0.9842886749	0.4459559804	0.3070902739	0.1002055672	0.9871605578
1.700669181	0.2289550228	0.3807430517	0.3569270039	0.09393688793	0.6089229331
1.705924104	0.118866826	0.3126872423	0.316802732	0.09294812721	0.4971223625
1.792098303	0.5187352942	0.4319803083	0.239044527	0.07791923947	0.8015000474
1.701618757	0.1352172826	0.3407039495	0.2613676432	0.09375757999	0.4971223625
1.653272915	0.2549220487	0.4159679727	0.3408174764	0.1032505092	0.6358253148
1.616224304	0.5257840311	0.4377411369	0.3108884554	0.1110429589	0.8015000474
1.67285071	0.3550766518	0.4533845573	0.2929876118	0.0993159869	0.7276160897
1.664283271	0.5706026249	0.3788626857	0.3110393949	0.1010224398	0.8181148269
1.669610596	0.6932856435	0.4070929564	0.3226808321	0.09995855972	0.8332760139
1.826227501	0.2258785426	0.4477449808	0.2328760597	0.07255510735	0.6089229331
1.59350498	0.6495724024	0.4358469284	0.4035181795	0.1160522182	0.818594287
1.668274083	0.8431710366	0.4448460515	0.3072572646	0.1002246014	0.9267848524
1.221442269	0.0002381904	0.2481574476	0.2211761579	0.2264697486	0.02811761644
1.67788698	0.6067385586	0.4688755929	0.3101669679	0.09832390711	0.818594287
1.761949412	0.3454824939	0.4178116268	0.3367380947	0.0829290722	0.7276160897
2.013864291	0.0711141003	0.4268679336	0.2368001621	0.04829917503	0.4971223625
1.726302448	0.3882768154	0.5217847051	0.2585200499	0.08919435375	0.7388208805
1.788906751	0.1740594904	0.5417417042	0.270888128	0.07843735014	0.5439359075
1.828626323	0.445355096	0.5155359187	0.2627111571	0.07219001333	0.7731859305
1.626948687	0.1021408994	0.5065055065	0.2556207399	0.1087398745	0.4971223625
1.794827288	0.264503331	0.5327263758	0.3016974028	0.07747848684	0.6358253148

1.678678748	0.9146207347	0.4375943281	0.3204667731	0.09816867869		0.9497327755
1.834424754	0.1972219953	0.3285104467	0.2483993379	0.07131386639		0.5733197537
2.23018253	0.0139349255	0.7034101493	0.1501941275	0.02930614178		0.2903109496
1.677610387	0.8769790973	0.4420361571	0.300492142	0.09837818126		0.9329418089
1.616583099	0.8603953355	0.4330829101	0.3178934238	0.1109652744		0.9329418089
1.753191011	0.131371418	0.5144376567	0.3405437685	0.08443358895		0.4971223625
1.633952543	0.6019274845	0.3879518374	0.3040431047	0.1072567758		0.818594287
1.622161274	0.1137145011	0.3686476573	0.3393996649	0.1097631566		0.4971223625
1.477970901	0.4097900268	0.341565497	0.3406490261	0.1443978631		0.7423732369
1.681735926	0.6574930388	0.4259920818	0.2968570269	0.09757119052		0.818594287
1.645728545	0.8806970676	0.4567370071	0.3040768276	0.1048001712		0.9329418089
1.84536156	0.1487980386	0.4437713007	0.2807192781	0.06968559141		0.5098455047
1.629643517	0.623709563	0.4418631729	0.3427480698	0.1081672774		0.818594287
1.701950734	0.6322809	0.382125617	0.3553941288	0.09369495945		0.818594287
1.799358165	0.1286105918	0.4555331793	0.2669424319	0.07675130803		0.4971223625
1.497091308	0.3197113419	0.4294298658	0.3108779406	0.139363097		0.7136413882
1.650970903	0.7847161759	0.4163344471	0.3366189806	0.1037213675		0.8917229272
1.552189984	0.0799846887	0.4203336398	0.3679258304	0.1256262568		0.4971223625
1.657737308	0.7266985101	0.437758265	0.3001227833	0.1023423133		0.8529527191
1.639885606	0.8682835073	0.4348258356	0.3307965616	0.1060132961		0.9329418089
1.452562603	0.0127847938	0.4937428283	0.3989908592	0.1513079017		0.2903109496
1.641162626	0.3525066497	0.386521701	0.3083525128	0.105747189		0.7276160897
0.946401547	0.5910063055	0.1706525217	0.656284424	0.3476569006		0.8721887272
0.710933945	0.0756608910	0.1085748952	0.8052499018	0.4797878427		0.5394055412
0.9506600817	0.6995767795	0.1830732504	0.6865143005	0.3454923922		0.9092262502
0.9213638504	0.8098190913	0.1610386562	0.6986543111	0.3604629346		0.9140125553
0.9614114508	0.5235662389	0.1615206918	0.6447278879	0.3401243099		0.8382203167
1.08439112	0.3106230187	0.2083651442	0.6075665337	0.2824135342		0.7740437187
0.9232702486	0.6433357581	0.1656681796	0.7235643593	0.3594994102		0.8935218862
1.134626396	0.0285355540	0.0855607812	0.6493286691	0.2609710535		0.4637289028
0.7554193029	0.0210377875	0.0649228796	0.7782927167	0.452870819		0.4637289028
0.9156572497	0.8956876649	0.1602231742	0.6987796883	0.3634028065		0.9388421843
0.7808734565	0.1235114339	0.0954025216	0.7990163646	0.4378644738		0.5716165492
0.9563789938	0.7554642993	0.1640828845	0.6922476189	0.3426278491		0.9140125553
0.9331601083	0.7977756378	0.1657504325	0.6997382484	0.3544191369		0.9140125553
0.919121605	0.0280255118	0.1301984883	0.5499304634	0.3616030129		0.4637289028
0.9342898699	0.99201813	0.1618461548	0.6873451875	0.3538423112		0.99201813
0.9163936489	0.4823558287	0.1884485966	0.654051541	0.3630435359		0.8382203167
0.925216358	0.6515511713	0.1578135081	0.6443348013	0.358481781		0.8949878727
0.930003852	0.901288497	0.1611114174	0.689368982	0.3560227051		0.9388421843
1.1777378	0.18057431	0.1012869571	0.5261802575	0.2434750241		0.6100483445
1.077694566	0.0296786497	0.0524059154	0.9138794911	0.2854215764		0.4637289028
1.156990096	0.1369358019	0.2718805264	0.5486186015	0.2518904814		0.5716165492
0.946816535	0.8860413532	0.1631159322	0.6907421378	0.3474321257		0.9388421843
1.08396671	0.4298893321	0.252842562	0.6190488026	0.282656187		0.826710254
0.8897477359	0.6426655509	0.1523085954	0.7538075726	0.3770599343		0.8935218862

0.9687627561	0.7797804658	0.2148291015	0.6582991857	0.3364624692	0.9140125553
0.9837583836	0.5364610027	0.183926231	0.6125345262	0.3290838791	0.8382203167
0.9150577028	0.6895616223	0.1518912517	0.685432523	0.3637510736	0.9092262502
0.9137131508	0.6038630563	0.1421770898	0.7152448731	0.3644345116	0.8721887272
1.048795123	0.3862079837	0.2461754839	0.6030116652	0.2983883225	0.7914098027
1.096195956	0.0556967097	0.3174518121	0.5811667874	0.2772583996	0.5041041973
0.8499759117	0.5278771591	0.1286202436	0.7495492622	0.3986346227	0.8382203167
0.9073105108	0.6070433541	0.1615537501	0.7432275058	0.3678433686	0.8721887272
0.7778614483	0.1017374275	0.0924897027	0.7540432925	0.4396041013	0.5714001441
0.8982944157	0.9414018242	0.1637902421	0.700364694	0.3726307909	0.9725225456
1.104962153	0.2684183983	0.1735894913	0.6712939004	0.2734696813	0.762552268
0.9858815552	0.0838504850	0.0790558607	0.6652339289	0.3280693358	0.5516479282
1.068197754	0.1371879718	0.186939456	0.7493975812	0.2896391392	0.5716165492
0.9262079584	0.8691520828	0.1658806261	0.7000240295	0.3579760825	0.9388421843
0.9977077775	0.3545477698	0.1846355116	0.7814522261	0.322373141	0.7914012718
1.424303866	0.0654852201	0.1319109599	0.4002144012	0.159577371	0.5394055412
0.97929411	0.1671217257	0.1179441893	0.655660451	0.3314227023	0.5802837697
1.067778654	0.5090192606	0.1707162257	0.6334867062	0.2897425213	0.8382203167
1.372367821	0.0005239178	0.1515857053	0.3105568136	0.1748154429	0.06548973439
0.9489579316	0.7346548102	0.1611607363	0.6968939825	0.346360624	0.9092262502
0.4228435978	0.0463285985	0.0772553071	0.7806300357	0.6738492284	0.4825895682
0.90827076	0.8288621232	0.157615708	0.6865779704	0.3672638292	0.9168828796
0.9496297043	0.5112753475	0.1470714158	0.684659392	0.3459993674	0.8382203167
0.7933006102	0.1051376265	0.1111995425	0.7611456388	0.4305803755	0.5714001441
0.7293581024	0.3405792362	0.1407221209	0.8185156362	0.4685162783	0.7740437187
1.04930008	0.119008837	0.1301622133	0.9042616316	0.298173045	0.5716165492
0.9700107706	0.7954885504	0.1745236682	0.6687474714	0.3358423572	0.9140125553
1.114455017	0.3845636174	0.229654878	0.572787653	0.2694077056	0.7914098027
0.7327243521	0.166387421	0.1089011259	0.6833114019	0.4665003435	0.5802837697
0.8161670895	0.501219776	0.1724202103	0.6392276605	0.417523241	0.8382203167
0.9272360135	0.707500704	0.1557431636	0.6977832281	0.3574521987	0.9092262502
0.861598926	0.2217569893	0.1363610768	0.6245530145	0.3922344917	0.6599910397
1.153985945	0.3646741791	0.1748429333	0.5638786714	0.2529333001	0.7914098027
0.8923396276	0.7290919104	0.164027379	0.7176747779	0.3756502326	0.9092262502
0.7299095997	0.3026233288	0.1328890459	0.7809638511	0.4682495189	0.7740437187
0.9725125729	0.7886926274	0.1783193692	0.66004022	0.3346434638	0.9140125553
0.9397714994	0.8901501179	0.1593045434	0.690392987	0.3510183195	0.9388421843
0.9260644896	0.8812774652	0.1620151824	0.6948054034	0.3580486289	0.9388421843
0.9971792675	0.6320372725	0.225279552	0.6432522226	0.322566747	0.8935218862
0.9049565992	0.3691929666	0.1491950252	0.8172097529	0.3690203315	0.7914098027
0.9836191774	0.4139283514	0.1553934471	0.6870675846	0.3291740985	0.8084538113
0.9098944736	0.7551252526	0.1524746361	0.7064736147	0.3664304624	0.9140125553
0.5195204693	0.0966913166	0.0747275025	0.7791009736	0.6052385284	0.5714001441
1.636212092	0.0178604135	0.2941999948	0.3544090854	0.1069312285	0.4637289028
0.9160927128	0.5521020249	0.195617897	0.6406523886	0.3631980011	0.8520092977
0.9863101504	0.6749545757	0.1672266647	0.6479189879	0.3278800682	0.9092262502

1.003223007	0.7310773145	0.1539470131	0.6539442154	0.3196995493	0.9092262502
1.052172847	0.5009737692	0.1666767431	0.6589726639	0.296847269	0.8382203167
0.9415186959	0.9691143342	0.1624245765	0.6864318575	0.3501346231	0.9797266688
0.7483721181	0.5166299171	0.1393512046	0.8055614262	0.4570500143	0.8382203167
0.9037486978	0.3388117732	0.1396198456	0.7969026014	0.3696554758	0.7740437187
0.8703540466	0.0423161691	0.1463090671	0.8247134291	0.3874659934	0.4825895682
0.9021531571	0.7145460771	0.1513164207	0.7225574569	0.3705397671	0.9092262502
0.8870021036	0.5260278499	0.1775991064	0.702870612	0.3785320797	0.8382203167
0.9402456018	0.9580944141	0.1636543082	0.687829582	0.3508151317	0.9797266688
0.9213614159	0.5310444533	0.1838825092	0.6293399057	0.3604651042	0.8382203167
0.5180800441	0.0345167946	0.0618259047	0.9409306387	0.6062171525	0.4793999259
0.9017129368	0.3948436499	0.1313662161	0.7193641692	0.3707570116	0.7960557457
0.8869741799	0.3222768253	0.1262941721	0.7711557269	0.3785366028	0.7740437187
0.8405027785	0.1579641677	0.0945055159	0.7391503963	0.4038640337	0.5802837697
1.016345512	0.4788363505	0.1430882191	0.5649208494	0.3135914354	0.8382203167
0.7798882407	0.1303867506	0.0940814237	0.6668792933	0.4384187212	0.5716165492
0.9063053296	0.3124706744	0.146404712	0.7333146658	0.3683172681	0.7740437187
0.9318694753	0.4642780636	0.168985843	0.6878731107	0.3551032872	0.8382203167
0.9638699292	0.7611158981	0.1618946851	0.6946673376	0.3389148247	0.9140125553
1.041163189	0.1451862082	0.1000766841	0.6939123138	0.3019638414	0.5802837697
0.9534219887	0.0776743979	0.0869302533	0.7974844184	0.344128742	0.5394055412
0.9224414778	0.9011784834	0.1615730202	0.7037547447	0.3599084128	0.9388421843
0.8149008223	0.1895564783	0.1582460066	0.9825750489	0.4183037289	0.623541047
1.008904533	0.2040907339	0.1781493319	0.6647162056	0.3170428234	0.6303394336
0.6735468178	0.0276052432	0.1133345153	0.6114561517	0.5031627877	0.4637289028
0.9432028911	0.9718888555	0.1629288741	0.6865950504	0.349296527	0.9797266688
0.9355946778	0.5705573029	0.1467045893	0.7361263445	0.3531612582	0.8697519862
1.090150481	0.4097628455	0.1627066034	0.6373078638	0.2798708461	0.8084538113
0.9173658577	0.2932419645	0.123764093	0.776904061	0.362550782	0.7740437187
0.9462212677	0.6885546229	0.1747233506	0.68136263	0.347756998	0.9092262502
1.060639769	0.4492622567	0.189314104	0.6255593114	0.2930010065	0.8382203167
0.8991164553	0.6005011718	0.1697981485	0.6698755358	0.3720959591	0.8721887272
0.9640620145	0.305116306	0.194924597	0.7161426572	0.3387770246	0.7740437187
0.9322684865	0.5046939752	0.1366804569	0.7862406873	0.3548460212	0.8382203167
1.068928793	0.1517446407	0.0952276544	0.5894586205	0.2893011202	0.5802837697
1.11715868	0.2067513342	0.2194972998	0.559272587	0.2681983428	0.6303394336
0.8966455135	0.5781166891	0.1535930403	0.7308834554	0.3734088556	0.8706576643
0.5584420871	0.1356381449	0.1016968879	0.8573911724	0.5785220864	0.5716165492
0.9463542188	0.3239604976	0.1834501369	0.7229847848	0.3476562172	0.7740437187
0.9296473533	0.8185078305	0.1571896846	0.6823416534	0.3562210951	0.9140125553
0.8661142818	0.0209104811	0.1066457841	0.7580554753	0.3898120782	0.4637289028
0.7660503261	0.3201710687	0.115083017	0.7614092307	0.446576965	0.7740437187
0.923390397	0.7701912765	0.1542772714	0.6848705309	0.3594150256	0.9140125553
1.169471791	0.3354294137	0.1943738562	0.5129722201	0.2466877904	0.7740437187
1.116913505	0.1139284743	0.1555560851	0.6344679569	0.268369725	0.5716165492
0.748481096	0.1557507146	0.1451125493	0.8503111381	0.4570055973	0.5802837697

0.8802084223	0.0448380181	0.0419355115	0.9757413545	0.3820918371	0.4825895682
0.8543926323	0.26214896	0.1428887837	0.6902604559	0.3962009179	0.7620609302
0.7723913909	0.3774842019	0.154065811	0.6985480219	0.4428295683	0.7914098027
0.9044718756	0.8189552495	0.1588934167	0.7271607468	0.3692332158	0.9140125553
0.8049903159	0.2022279643	0.147511372	0.788267102	0.4239070295	0.6303394336
0.9206790649	0.7075969828	0.1590413155	0.6775125048	0.3608295876	0.9092262502
1.248317376	0.0742192347	0.2197132068	0.4216637827	0.2166706089	0.5394055412
0.7838948278	0.0942610257	0.1816785951	0.7693377371	0.4361430924	0.5714001441
0.9496069175	0.0564596701	0.1110119618	0.6506148552	0.3460734485	0.5041041973

			Taxonomic Information
Blast Adjusted p-valu	VNS Adjusted p-valu	Blast:VNS adjusted p-valu	Genus
0.01711752944	0.0987256072	0.05120735202	Acetatifactor
0.01711752944	0.0987256072	0.05120735202	Acetatifactor
0.01711752944	0.0987256072	0.05120735202	Acutalibacter
0.01711752944	0.0987256072	0.05120735202	Acutalibacter
0.01711752944	0.0987256072	0.05120735202	Adlercreutzia
0.01711752944	0.0987256072	0.05120735202	Adlercreutzia
0.01711752944	0.0987256072	0.05120735202	Adlercreutzia
0.01711752944	0.0987256072	0.05120735202	Akkermansia
0.01711752944	0.0987256072	0.05120735202	Alistipes
0.01711752944	0.0987256072	0.05120735202	Anaerotruncus
0.01737393672	0.0987256072	0.05120735202	Bacteria_unclassified
0.01711752944	0.0987256072	0.05120735202	Bacteria_unclassified
0.01711752944	0.0987256072	0.05120735202	Bacteria_unclassified
0.01711752944	0.1002066352	0.05120735202	Bacteroides
0.01711752944	0.0987256072	0.05120735202	Bifidobacterium
0.02036353548	0.1002066352	0.05120735202	Clostridia_unclassified
0.01711752944	0.1525595899	0.05120735202	Clostridiaceae_unclassified
0.01711752944	0.0987256072	0.05120735202	Clostridiaceae_unclassified
0.01825366792	0.0987256072	0.05120735202	Eubacteriales_unclassified
0.01961562443	0.0987256072	0.05120735202	Erysipelatoclostridium
0.01970312993	0.1002066352	0.05292259984	Coriobacteriaceae_unclassifie
0.01711752944	0.0987256072	0.05120735202	Dorea
0.02729472302	0.0987256072	0.05120735202	Dubosiella
0.01711752944	0.09954213661	0.05120735202	Erysipelotrichales_unclassifie
0.01711752944	0.0987256072	0.05120735202	Eubacteriaceae_unclassified
0.01711752944	0.0987256072	0.05120735202	Eubacteriaceae_unclassified
0.01825366792	0.0987256072	0.05120735202	GGB20149
0.01711752944	0.0987256072	0.05120735202	GGB22635
0.02592834182	0.1064446468	0.06470111122	GGB25041
0.02389510834	0.0987256072	0.05185719465	GGB27876
0.01711752944	0.0987256072	0.05120735202	GGB27878
0.01711752944	0.1088029914	0.05120735202	GGB27918
0.01825366792	0.0987256072	0.05120735202	GGB28382
0.01761114253	0.09954213661	0.05193161517	GGB28399
0.01711752944	0.0987256072	0.05120735202	GGB28411
0.01737393672	0.0987256072	0.05120735202	GGB28415
0.01711752944	0.0987256072	0.05120735202	GGB28430
0.01711752944	0.0987256072	0.05120735202	GGB28439
0.01721066887	0.0987256072	0.05120735202	GGB28778
0.01711752944	0.0987256072	0.05120735202	GGB28784
0.01761114253	0.0987256072	0.05120735202	GGB28792
0.01711752944	0.0987256072	0.05120735202	GGB28798
0.01711752944	0.0987256072	0.05120735202	GGB28802
0.01711752944	0.0987256072	0.05120735202	GGB28818
0.01737393672	0.0987256072	0.05120735202	GGB28828

0.01711752944	0.0987256072	0.05120735202	GGB28851
0.01711752944	0.0987256072	0.05120735202	GGB28859
0.01711752944	0.0987256072	0.05120735202	GGB28864
0.01711752944	0.0987256072	0.05120735202	GGB28869
0.01761114253	0.1243739997	0.05120735202	GGB28883
0.01711752944	0.0987256072	0.05120735202	GGB28892
0.01711752944	0.0987256072	0.05120735202	GGB28893
0.02592834182	0.0987256072	0.06825241097	GGB28898
0.01711752944	0.0987256072	0.05120735202	GGB28904
0.01711752944	0.0987256072	0.05120735202	GGB28916
0.01711752944	0.0987256072	0.05120735202	GGB28924
0.01711752944	0.1021320316	0.05300419053	GGB28926
0.01711752944	0.0987256072	0.05120735202	GGB28927
0.01711752944	0.0987256072	0.05120735202	GGB28934
0.01761114253	0.1044584747	0.05120735202	GGB28946
0.01825366792	0.0987256072	0.05120735202	GGB28949
0.01711752944	0.0987256072	0.05120735202	GGB28949
0.02129913663	0.09954213661	0.05120735202	GGB28950
0.01711752944	0.1002066352	0.05120735202	GGB28951
0.01711752944	0.0987256072	0.05120735202	GGB28951
0.01737393672	0.0987256072	0.05120735202	GGB28954
0.01825366792	0.0987256072	0.05120735202	GGB28956
0.01711752944	0.0987256072	0.05120735202	GGB28960
0.02139984729	0.1048169025	0.05120735202	GGB28967
0.01711752944	0.0987256072	0.05120735202	GGB28991
0.01711752944	0.1064446468	0.08852197766	GGB29002
0.01711752944	0.0987256072	0.05120735202	GGB29003
0.01711752944	0.1021320316	0.05120735202	GGB29011
0.01711752944	0.0987256072	0.05120735202	GGB29531
0.01711752944	0.0987256072	0.05120735202	GGB29685
0.01711752944	0.0987256072	0.05120735202	GGB30141
0.01761114253	0.0987256072	0.05120735202	GGB30286
0.01737393672	0.0987256072	0.05120735202	GGB30303
0.01711752944	0.0987256072	0.05120735202	GGB30413
0.01825366792	0.1097593108	0.05120735202	GGB30454
0.01711752944	0.0987256072	0.05120735202	GGB30455
0.01711752944	0.0987256072	0.05120735202	GGB30461
0.01711752944	0.0987256072	0.05120735202	GGB30461
0.01711752944	0.0987256072	0.05120735202	GGB30463
0.01711752944	0.1088029914	0.05413078867	GGB30473
0.01711752944	0.0987256072	0.05120735202	GGB30475
0.01711752944	0.0987256072	0.05120735202	GGB30861
0.01711752944	0.0987256072	0.05148421583	GGB31312
0.01711752944	0.0987256072	0.05120735202	GGB31438
0.01842188925	0.0987256072	0.05120735202	GGB3171
0.01711752944	0.0987256072	0.05120735202	GGB31823

0.01711752944	0.1121818466	0.06332819819	GGB31853
0.01711752944	0.0987256072	0.05120735202	GGB32371
0.01711752944	0.0987256072	0.05120735202	GGB3793
0.01711752944	0.0987256072	0.05120735202	GGB42598
0.01977316736	0.0987256072	0.05120735202	GGB45656
0.01711752944	0.0987256072	0.05120735202	GGB47127
0.01711752944	0.0987256072	0.06716760956	GGB74395
0.01711752944	0.0987256072	0.05120735202	GGB75053
0.01806486337	0.0987256072	0.05120735202	GGB75109
0.02182356312	0.1048169025	0.1171380983	GGB81440
0.01761114253	0.0987256072	0.05120735202	Lachnospiraceae_unclassified
0.01901055128	0.0987256072	0.05120735202	Lachnospiraceae_unclassified
0.02274014528	0.1369671795	0.05120735202	Lachnospiraceae_unclassified
0.01711752944	0.0987256072	0.05120735202	Lachnospiraceae_unclassified
0.01711752944	0.0987256072	0.05120735202	Lachnospiraceae_unclassified
0.01711752944	0.0987256072	0.05120735202	Lachnospiraceae_unclassified
0.01761114253	0.1002066352	0.05443648682	Lachnospiraceae_unclassified
0.01711752944	0.0987256072	0.05120735202	Lachnospiraceae_unclassified
0.01737393672	0.0987256072	0.05120735202	Lactobacillus
0.01711752944	0.0987256072	0.05120735202	Muribaculaceae_unclassified
0.01711752944	0.0987256072	0.05120735202	Neglectibacter
0.01711752944	0.0987256072	0.05120735202	Oscillospiraceae_unclassified
0.01711752944	0.0987256072	0.05120735202	Oscillospiraceae_unclassified
0.01711752944	0.0987256072	0.05120735202	Oscillospiraceae_unclassified
0.01711752944	0.0987256072	0.05120735202	Parasutterella
0.01741082102	0.0987256072	0.05120735202	Romboutsia
0.01711752944	0.0987256072	0.05120735202	Schaedlerella
0.01711752944	0.0987256072	0.05120735202	Turicibacter
0.01711752944	0.0987256072	0.05120735202	Bacteria_unclassified
0.01711752944	0.0987256072	0.05120735202	Bacteria_unclassified
0.01711752944	0.0987256072	0.05120735202	Bacteria_unclassified
0.01842188925	0.1481479697	0.05728198365	
0.01711752944	0.0987256072	0.05120735202	
0.01711752944	0.0987256072	0.05120735202	
0.03061283424	0.2318968287	0.06391583115	Acetatifactor
0.03061283424	0.2318968287	0.06391583115	Acetatifactor
0.03061283424	0.2318968287	0.06391583115	Acutalibacter
0.03061283424	0.2318968287	0.06391583115	Acutalibacter
0.03061283424	0.2318968287	0.06391583115	Adlercreutzia
0.03061283424	0.2318968287	0.06391583115	Adlercreutzia
0.03061283424	0.2318968287	0.06391583115	Adlercreutzia
0.03061283424	0.2318968287	0.06391583115	Akkermansia
0.03061283424	0.2318968287	0.06391583115	Alistipes
0.03061283424	0.2403775523	0.0682375548	Anaerotruncus
0.03061283424	0.2318968287	0.06391583115	Bacteria_unclassified
0.03061283424	0.2318968287	0.06391583115	Bacteria_unclassified

0.03061283424	0.2318968287	0.06391583115	Bacteria_unclassified
0.03061283424	0.2318968287	0.06391583115	Bacteroides
0.03061283424	0.2318968287	0.06391583115	Bifidobacterium
0.03133621099	0.2318968287	0.06391583115	Clostridia_unclassified
0.03061283424	0.2340070451	0.06391583115	Clostridiaceae_unclassified
0.03061283424	0.2318968287	0.06391583115	Clostridiaceae_unclassified
0.03061283424	0.2318968287	0.06391583115	Eubacteriales_unclassified
0.03061283424	0.2318968287	0.06391583115	Erysipelatoclostridium
0.04014670546	0.2340070451	0.07084183666	Coriobacteriaceae_unclassifie
0.03061283424	0.2318968287	0.06391583115	Dorea
0.04945132624	0.2318968287	0.06391583115	Dubosiella
0.03061283424	0.2318968287	0.06391583115	Erysipelotrichales_unclassifie
0.0540865581	0.2388065533	0.07084183666	Eubacteriaceae_unclassified
0.03061283424	0.2318968287	0.06391583115	Eubacteriaceae_unclassified
0.03061283424	0.2318968287	0.06391583115	GGB20149
0.03061283424	0.2318968287	0.06391583115	GGB22635
0.03061283424	0.2318968287	0.06391583115	GGB25041
0.03933452723	0.2318968287	0.06391583115	GGB27876
0.03061283424	0.2318968287	0.06391583115	GGB27878
0.03061283424	0.2318968287	0.06391583115	GGB27918
0.03548981522	0.2318968287	0.06391583115	GGB28382
0.0390743013	0.2651494912	0.09997335629	GGB28399
0.03061283424	0.2318968287	0.06391583115	GGB28411
0.03431936643	0.2318968287	0.06391583115	GGB28415
0.03378362403	0.2318968287	0.06781582621	GGB28430
0.03061283424	0.2318968287	0.06391583115	GGB28439
0.03061283424	0.2318968287	0.06391583115	GGB28778
0.03061283424	0.2340070451	0.06391583115	GGB28784
0.03061283424	0.2318968287	0.06391583115	GGB28792
0.03061283424	0.2318968287	0.06391583115	GGB28798
0.03061283424	0.2318968287	0.06391583115	GGB28802
0.03061283424	0.2318968287	0.06391583115	GGB28818
0.03061283424	0.2318968287	0.06391583115	GGB28828
0.03133621099	0.2318968287	0.06391583115	GGB28851
0.03061283424	0.2318968287	0.06391583115	GGB28859
0.03061283424	0.2318968287	0.06391583115	GGB28864
0.03061283424	0.2318968287	0.06391583115	GGB28869
0.03061283424	0.2490840217	0.06391583115	GGB28883
0.03061283424	0.2318968287	0.06391583115	GGB28892
0.04112260514	0.2490840217	0.07574662104	GGB28893
0.03919219869	0.2318968287	0.07187052322	GGB28898
0.03061283424	0.2318968287	0.06391583115	GGB28904
0.03061283424	0.2318968287	0.06391583115	GGB28916
0.03133621099	0.2318968287	0.06391583115	GGB28924
0.03061283424	0.2340070451	0.06651750655	GGB28926
0.03061283424	0.2318968287	0.06391583115	GGB28927

0.03061283424	0.2318968287	0.06391583115	GGB28934
0.03344544171	0.2520702488	0.06651750655	GGB28946
0.03133621099	0.2318968287	0.06391583115	GGB28949
0.03061283424	0.2318968287	0.06391583115	GGB28949
0.0540865581	0.238161457	0.06742693799	GGB28950
0.03061283424	0.238161457	0.06391583115	GGB28951
0.03061283424	0.2318968287	0.06391583115	GGB28951
0.03061283424	0.2318968287	0.06391583115	GGB28954
0.03967451176	0.2318968287	0.07199853899	GGB28956
0.03346694243	0.2714682952	0.1057789383	GGB28960
0.04015524817	0.238161457	0.06391583115	GGB28967
0.03061283424	0.2318968287	0.06391583115	GGB28991
0.03061283424	0.2318968287	0.07199853899	GGB29002
0.03061283424	0.2318968287	0.06391583115	GGB29003
0.03061283424	0.238161457	0.06391583115	GGB29011
0.03061283424	0.2318968287	0.06391583115	GGB29531
0.03061283424	0.2318968287	0.06391583115	GGB29685
0.03061283424	0.2318968287	0.06391583115	GGB30141
0.03061283424	0.2318968287	0.06391583115	GGB30286
0.03061283424	0.2318968287	0.06391583115	GGB30303
0.0311404447	0.2318968287	0.06391583115	GGB30413
0.0311404447	0.2340070451	0.06391583115	GGB30454
0.0311404447	0.2318968287	0.06391583115	GGB30455
0.03061283424	0.2318968287	0.06391583115	GGB30461
0.03061283424	0.2318968287	0.06391583115	GGB30461
0.03061283424	0.2318968287	0.06391583115	GGB30463
0.03061283424	0.2318968287	0.06391583115	GGB30473
0.03061283424	0.2318968287	0.06391583115	GGB30475
0.03061283424	0.2318968287	0.06391583115	GGB30861
0.03061283424	0.2318968287	0.06391583115	GGB31312
0.03131068776	0.2318968287	0.06391583115	GGB31438
0.03123052325	0.2318968287	0.06391583115	GGB3171
0.03061283424	0.2318968287	0.06391583115	GGB31823
0.03061283424	0.2520702488	0.07721166532	GGB31853
0.03061283424	0.2318968287	0.06391583115	GGB32371
0.03061283424	0.2318968287	0.06391583115	GGB3793
0.03061283424	0.2318968287	0.06391583115	GGB42598
0.03273620042	0.2318968287	0.06391583115	GGB45656
0.03061283424	0.2318968287	0.06391583115	GGB47127
0.03061283424	0.2318968287	0.06391583115	GGB74395
0.03061283424	0.2318968287	0.06391583115	GGB75053
0.03440524964	0.2318968287	0.06391583115	GGB75109
0.03378362403	0.2340070451	0.09380371308	GGB81440
0.0311404447	0.2318968287	0.06391583115	Lachnospiraceae_unclassified
0.03061283424	0.2318968287	0.06391583115	Lachnospiraceae_unclassified
0.03548981522	0.2678489208	0.06391583115	Lachnospiraceae_unclassified

0.03061283424	0.2318968287	0.06391583115	Lachnospiraceae_unclassified
0.03061283424	0.2318968287	0.06391583115	Lachnospiraceae_unclassified
0.03061283424	0.2318968287	0.06391583115	Lachnospiraceae_unclassified
0.03133621099	0.2340070451	0.07187052322	Lachnospiraceae_unclassified
0.03061283424	0.2318968287	0.06391583115	Lachnospiraceae_unclassified
0.03061283424	0.2318968287	0.06391583115	Lactobacillus
0.03061283424	0.2318968287	0.06391583115	Muribaculaceae_unclassified
0.03061283424	0.2318968287	0.06391583115	Neglectibacter
0.03133621099	0.2318968287	0.06391583115	Oscillospiraceae_unclassified
0.03061283424	0.2318968287	0.06391583115	Oscillospiraceae_unclassified
0.03061283424	0.2318968287	0.06391583115	Oscillospiraceae_unclassified
0.03061283424	0.2318968287	0.06391583115	Parasutterella
0.04254879213	0.2407346103	0.06391583115	Romboutsia
0.03068360841	0.2318968287	0.06391583115	Schaedlerella
0.03061283424	0.2318968287	0.06391583115	Turicibacter
0.03061283424	0.2318968287	0.06391583115	Bacteria_unclassified
0.03061283424	0.2318968287	0.06391583115	Bacteria_unclassified
0.03061283424	0.2318968287	0.06391583115	Bacteria_unclassified
0.03133621099	0.2654385924	0.06511268346	
0.03061283424	0.2318968287	0.06391583115	
0.03061283424	0.2318968287	0.06391583115	
0.02012234551	0.2037494731	0.05732595108	Acetatifactor
0.02012234551	0.2037494731	0.05732595108	Acetatifactor
0.02012234551	0.2037494731	0.05732595108	Acutalibacter
0.02012234551	0.2037494731	0.05732595108	Acutalibacter
0.02012234551	0.2037494731	0.05732595108	Adlercreutzia
0.02012234551	0.2037494731	0.05732595108	Adlercreutzia
0.02012234551	0.2037494731	0.05732595108	Adlercreutzia
0.02012234551	0.2037494731	0.05732595108	Akkermansia
0.02012234551	0.2037494731	0.05732595108	Alistipes
0.02012234551	0.2037494731	0.05732595108	Anaerotruncus
0.02012234551	0.2037494731	0.05732595108	Bacteria_unclassified
0.02012234551	0.2037494731	0.05732595108	Bacteria_unclassified
0.02012234551	0.2037494731	0.05732595108	Bacteria_unclassified
0.02012234551	0.2100585492	0.05732595108	Bacteroides
0.02012234551	0.2037494731	0.05732595108	Bifidobacterium
0.02538662549	0.2059673764	0.05732595108	Clostridia_unclassified
0.02012234551	0.3545762691	0.05732595108	Clostridiaceae_unclassified
0.02012234551	0.2037494731	0.05732595108	Clostridiaceae_unclassified
0.03417599845	0.2037494731	0.05732595108	Eubacteriales_unclassified
0.02538662549	0.2037494731	0.05732595108	Erysipelatoclostridium
0.03417599845	0.2383120122	0.08197010123	Coriobacteriaceae_unclassifie
0.02012234551	0.2037494731	0.05732595108	Dorea
0.0294878573	0.2037494731	0.05732595108	Dubosiella
0.02012234551	0.2037494731	0.05732595108	Erysipelotrichales_unclassifie
0.02012234551	0.2037494731	0.05732595108	Eubacteriaceae_unclassified

0.02012234551	0.2037494731	0.05732595108	Eubacteriaceae_unclassified
0.02067394623	0.2037494731	0.05732595108	GGB20149
0.02012234551	0.2037494731	0.05732595108	GGB22635
0.02181288487	0.2037494731	0.05732595108	GGB25041
0.0268329472	0.2037494731	0.05732595108	GGB27876
0.02067394623	0.2037494731	0.05732595108	GGB27878
0.02012234551	0.2037494731	0.05732595108	GGB27918
0.02220379551	0.2037494731	0.05732595108	GGB28382
0.02067394623	0.2037494731	0.06030409867	GGB28399
0.02041320215	0.2037494731	0.06095343498	GGB28411
0.02067394623	0.2037494731	0.05732595108	GGB28415
0.02012234551	0.2037494731	0.05732595108	GGB28430
0.02012234551	0.2037494731	0.05732595108	GGB28439
0.02012234551	0.2037494731	0.05732595108	GGB28778
0.02012234551	0.2037494731	0.05732595108	GGB28784
0.02067394623	0.2037494731	0.05732595108	GGB28792
0.02012234551	0.2037494731	0.05732595108	GGB28798
0.02012234551	0.2037494731	0.05732595108	GGB28802
0.02012234551	0.2037494731	0.05732595108	GGB28818
0.02022932138	0.2037494731	0.05732595108	GGB28828
0.02012234551	0.2037494731	0.05732595108	GGB28851
0.02012234551	0.2037494731	0.05732595108	GGB28859
0.02012234551	0.2037494731	0.05732595108	GGB28864
0.02012234551	0.2037494731	0.05732595108	GGB28869
0.02041320215	0.2122548936	0.05732595108	GGB28883
0.02012234551	0.2037494731	0.05732595108	GGB28892
0.02012234551	0.2037494731	0.05732595108	GGB28893
0.02728767883	0.2037494731	0.07111255967	GGB28898
0.02012234551	0.2037494731	0.05990492896	GGB28904
0.02012234551	0.2037494731	0.05732595108	GGB28916
0.02012234551	0.2037494731	0.05732595108	GGB28924
0.02012234551	0.2106115464	0.06251022527	GGB28926
0.02012234551	0.2037494731	0.05732595108	GGB28927
0.02012234551	0.2037494731	0.05732595108	GGB28934
0.02012234551	0.2037494731	0.05732595108	GGB28946
0.02220379551	0.2037494731	0.05732595108	GGB28949
0.02564287376	0.2037494731	0.05732595108	GGB28949
0.03059237209	0.2059673764	0.06021154448	GGB28950
0.02012234551	0.2037494731	0.05732595108	GGB28951
0.02012234551	0.2037494731	0.05732595108	GGB28951
0.02073474572	0.2037494731	0.05732595108	GGB28954
0.02012234551	0.2037494731	0.05732595108	GGB28956
0.02012234551	0.2037494731	0.05732595108	GGB28960
0.02012234551	0.2037494731	0.05732595108	GGB28967
0.02012234551	0.2037494731	0.05732595108	GGB28991
0.02012234551	0.2086905514	0.0870172285	GGB29002

0.02020856749	0.2037494731	0.05732595108	GGB29003
0.02012234551	0.2055628758	0.05732595108	GGB29011
0.02012234551	0.2037494731	0.05732595108	GGB29531
0.02012234551	0.2037494731	0.05732595108	GGB29685
0.02012234551	0.2037494731	0.05732595108	GGB30141
0.02538662549	0.2037494731	0.05732595108	GGB30286
0.02022932138	0.2037494731	0.05732595108	GGB30303
0.02220379551	0.2037494731	0.05732595108	GGB30413
0.02067394623	0.2086905514	0.05732595108	GGB30454
0.02012234551	0.2037494731	0.05732595108	GGB30455
0.02012234551	0.2037494731	0.05732595108	GGB30461
0.02012234551	0.2037494731	0.05732595108	GGB30461
0.02012234551	0.2037494731	0.05732595108	GGB30463
0.02012234551	0.2037494731	0.05732595108	GGB30473
0.02012234551	0.2037494731	0.05732595108	GGB30475
0.02012234551	0.2037494731	0.05732595108	GGB30861
0.02012234551	0.2037494731	0.05732595108	GGB31312
0.02012234551	0.2037494731	0.05732595108	GGB31438
0.02386464823	0.2037494731	0.05732595108	GGB3171
0.02012234551	0.2037494731	0.05732595108	GGB31823
0.02012234551	0.2037494731	0.05732595108	GGB31853
0.02012234551	0.2037494731	0.05732595108	GGB32371
0.02012234551	0.2037494731	0.05732595108	GGB3793
0.02012234551	0.2037494731	0.05732595108	GGB42598
0.02144090351	0.2037494731	0.05732595108	GGB45656
0.02012234551	0.2037494731	0.05732595108	GGB47127
0.02012234551	0.2037494731	0.05732595108	GGB74395
0.02012234551	0.2037494731	0.05732595108	GGB75053
0.02101876757	0.2037494731	0.05732595108	GGB75109
0.02220379551	0.2037494731	0.09252372449	GGB81440
0.02067394623	0.2037494731	0.05732595108	Lachnospiraceae_unclassified
0.02220379551	0.2037494731	0.05732595108	Lachnospiraceae_unclassified
0.02764120945	0.2902477751	0.05732595108	Lachnospiraceae_unclassified
0.02012234551	0.2037494731	0.06083542092	Lachnospiraceae_unclassified
0.02067394623	0.2037494731	0.06083542092	Lachnospiraceae_unclassified
0.02012234551	0.2037494731	0.05732595108	Lachnospiraceae_unclassified
0.02012234551	0.2037494731	0.05732595108	Lachnospiraceae_unclassified
0.02012234551	0.2037494731	0.05732595108	Lachnospiraceae_unclassified
0.02012234551	0.2037494731	0.05732595108	Lactobacillus
0.02012234551	0.2037494731	0.05732595108	Muribaculaceae_unclassified
0.02012234551	0.2037494731	0.05732595108	Neglectibacter
0.02012234551	0.2037494731	0.05732595108	Oscillospiraceae_unclassified
0.02220379551	0.3068817855	0.1117095028	Oscillospiraceae_unclassified
0.02012234551	0.2037494731	0.05732595108	Oscillospiraceae_unclassified
0.02012234551	0.2037494731	0.05732595108	Parasutterella
0.02437101104	0.2059673764	0.05732595108	Romboutsia

0.02012234551	0.2037494731	0.05732595108	Schaedlerella
0.02012234551	0.2037494731	0.05732595108	Turicibacter
0.02012234551	0.2037494731	0.05732595108	Bacteria_unclassified
0.02012234551	0.2037494731	0.05732595108	Bacteria_unclassified
0.02012234551	0.2037494731	0.05732595108	Bacteria_unclassified
0.02067394623	0.2372783653	0.05888650266	
0.02012234551	0.2037494731	0.05732595108	
0.02012234551	0.2037494731	0.05732595108	
0.0006174936392	0.8567755293	0.7080320955	Acetatifactor
0.0006174936392	0.8567755293	0.7080320955	Acetatifactor
0.0006174936392	0.8567755293	0.7080320955	Acutalibacter
0.0006174936392	0.8567755293	0.7080320955	Acutalibacter
0.0006174936392	0.8567755293	0.7080320955	Adlercreutzia
0.000850088246	0.8567755293	0.7080320955	Adlercreutzia
0.0006174936392	0.8567755293	0.7080320955	Adlercreutzia
0.0006174936392	0.8567755293	0.7080320955	Akkermansia
0.0006517985519	0.8567755293	0.7080320955	Alistipes
0.0006174936392	0.8567755293	0.7080320955	Anaerotruncus
0.0006174936392	0.8567755293	0.7080320955	Bacteria_unclassified
0.0006174936392	0.8567755293	0.7080320955	Bacteria_unclassified
0.0006174936392	0.8567755293	0.7080320955	Bacteria_unclassified
0.0006174936392	0.8567755293	0.7080320955	Bacteroides
0.0006174936392	0.8567755293	0.7080320955	Bifidobacterium
0.0006174936392	0.8567755293	0.7080320955	Clostridia_unclassified
0.0006174936392	0.8567755293	0.7080320955	Clostridiaceae_unclassified
0.0006174936392	0.8567755293	0.7080320955	Clostridiaceae_unclassified
0.0006174936392	0.8567755293	0.7080320955	Eubacteriales_unclassified
0.0006174936392	0.8567755293	0.7080320955	Erysipelatoclostridium
0.0007454143061	0.8567755293	0.7080320955	Coriobacteriaceae_unclassifie
0.0006174936392	0.8567755293	0.7080320955	Dorea
0.0006174936392	0.8567755293	0.7080320955	Dubosiella
0.0006174936392	0.8576617901	0.7080320955	Erysipelotrichales_unclassifie
0.0006647299644	0.8567755293	0.7080320955	Eubacteriaceae_unclassified
0.0007434019507	0.8567755293	0.7080320955	Eubacteriaceae_unclassified
0.0006369303364	0.8567755293	0.7080320955	GGB20149
0.0006174936392	0.8567755293	0.7080320955	GGB22635
0.0006174936392	0.8567755293	0.7080320955	GGB25041
0.0007232202525	0.8567755293	0.7080320955	GGB27876
0.0006174936392	0.8567755293	0.7080320955	GGB27878
0.0006174936392	0.8567755293	0.7080320955	GGB27918
0.0006174936392	0.8567755293	0.7080320955	GGB28382
0.0006369303364	0.8567755293	0.7080320955	GGB28399
0.0006174936392	0.8567755293	0.7080320955	GGB28411
0.0007292388671	0.8567755293	0.7080320955	GGB28415
0.0006174936392	0.8567755293	0.7080320955	GGB28430
0.0006174936392	0.8567755293	0.7080320955	GGB28439

0.0006174936392	0.8567755293	0.7080320955	GGB28778
0.0006174936392	0.8567755293	0.7080320955	GGB28784
0.0006174936392	0.8567755293	0.7080320955	GGB28792
0.0006174936392	0.8567755293	0.7080320955	GGB28798
0.0006174936392	0.8567755293	0.7080320955	GGB28802
0.0006174936392	0.8567755293	0.7080320955	GGB28818
0.0008006150434	0.8567755293	0.7080320955	GGB28828
0.0006174936392	0.8567755293	0.7080320955	GGB28851
0.0006174936392	0.8567755293	0.7080320955	GGB28859
0.0006449340524	0.8567755293	0.7080320955	GGB28864
0.0006174936392	0.8567755293	0.7080320955	GGB28869
0.0006174936392	0.9786800182	0.7080320955	GGB28883
0.0006401874772	0.8567755293	0.7080320955	GGB28892
0.0007757537257	0.8567755293	0.7080320955	GGB28893
0.0007782354297	0.8567755293	0.7080320955	GGB28898
0.0006174936392	0.8567755293	0.7080320955	GGB28904
0.0006369303364	0.8567755293	0.7080320955	GGB28916
0.0006174936392	0.8567755293	0.7080320955	GGB28924
0.0006174936392	0.8567755293	0.7080320955	GGB28926
0.0006174936392	0.8567755293	0.7080320955	GGB28927
0.0006174936392	0.8567755293	0.7080320955	GGB28934
0.0006517985519	0.8567755293	0.7080320955	GGB28946
0.0006174936392	0.8567755293	0.7080320955	GGB28949
0.0007536026659	0.8567755293	0.7080320955	GGB28949
0.0006369303364	0.8567755293	0.7080320955	GGB28950
0.0006174936392	0.8567755293	0.7080320955	GGB28951
0.0006174936392	0.8567755293	0.7080320955	GGB28951
0.0006517985519	0.8567755293	0.7080320955	GGB28954
0.0006174936392	0.8567755293	0.7080320955	GGB28956
0.0006174936392	0.8666506689	0.7316612585	GGB28960
0.0006174936392	0.8567755293	0.7080320955	GGB28967
0.0006174936392	0.8567755293	0.7080320955	GGB28991
0.0006517985519	0.8781139016	0.8763951327	GGB29002
0.0006174936392	0.8567755293	0.7080320955	GGB29003
0.0006174936392	0.8717826367	0.7080320955	GGB29011
0.0007232202525	0.8567755293	0.7080320955	GGB29531
0.0006174936392	0.8567755293	0.7080320955	GGB29685
0.0006174936392	0.8567755293	0.7080320955	GGB30141
0.0006174936392	0.8567755293	0.7080320955	GGB30286
0.0006174936392	0.8567755293	0.7080320955	GGB30303
0.0006174936392	0.8567755293	0.7080320955	GGB30413
0.0006174936392	0.8567755293	0.7080320955	GGB30454
0.0006174936392	0.8567755293	0.7080320955	GGB30455
0.0006174936392	0.8567755293	0.7080320955	GGB30461
0.0006174936392	0.8567755293	0.7080320955	GGB30461
0.0006517985519	0.8567755293	0.7080320955	GGB30463

0.0006174936392	0.8567755293	0.7080320955	GGB30473
0.0006517985519	0.8567755293	0.7080320955	GGB30475
0.0006174936392	0.8567755293	0.7080320955	GGB30861
0.0006174936392	0.8567755293	0.7080320955	GGB31312
0.0006174936392	0.8567755293	0.7080320955	GGB31438
0.0006369303364	0.8567755293	0.7080320955	GGB3171
0.0006174936392	0.8567755293	0.7080320955	GGB31823
0.0006174936392	0.8567755293	0.7080320955	GGB31853
0.0006174936392	0.9535948224	0.7080320955	GGB32371
0.0006174936392	0.8567755293	0.7080320955	GGB3793
0.0006174936392	0.8567755293	0.7080320955	GGB42598
0.0006174936392	0.8567755293	0.7080320955	GGB45656
0.0006174936392	0.8567755293	0.7080320955	GGB47127
0.0006174936392	0.8567755293	0.7584047277	GGB74395
0.0006174936392	0.8567755293	0.7080320955	GGB75053
0.0006174936392	0.8567755293	0.7080320955	GGB75109
0.0006174936392	0.8567755293	0.7080320955	GGB81440
0.0006174936392	0.8567755293	0.7080320955	Lachnospiraceae_unclassified
0.0006517985519	0.8567755293	0.7080320955	Lachnospiraceae_unclassified
0.0006369303364	0.8567755293	0.7080320955	Lachnospiraceae_unclassified
0.0009041431421	0.8567755293	0.7080320955	Lachnospiraceae_unclassified
0.0006174936392	0.8567755293	0.7080320955	Lachnospiraceae_unclassified
0.0006174936392	0.8567755293	0.7080320955	Lachnospiraceae_unclassified
0.0006369303364	0.8567755293	0.7080320955	Lachnospiraceae_unclassified
0.0006174936392	0.8567755293	0.7080320955	Lachnospiraceae_unclassified
0.0006174936392	0.8567755293	0.7080320955	Lactobacillus
0.0006174936392	0.8567755293	0.7080320955	Muribaculaceae_unclassified
0.0006449340524	0.8567755293	0.7080320955	Neglectibacter
0.0006517985519	0.8567755293	0.7080320955	Oscillospiraceae_unclassified
0.0006174936392	0.8567755293	0.7080320955	Oscillospiraceae_unclassified
0.0006174936392	0.8567755293	0.7080320955	Oscillospiraceae_unclassified
0.0006174936392	0.8567755293	0.7080320955	Parasutterella
0.0006174936392	0.8567755293	0.7080320955	Romboutsia
0.0006174936392	0.8567755293	0.7080320955	Schaedlerella
0.0006174936392	0.8567755293	0.7080320955	Turicibacter
0.002106762016	0.8567755293	0.7080320955	Bacteria_unclassified
0.0006174936392	0.8567755293	0.7080320955	Bacteria_unclassified
0.0006174936392	0.8567755293	0.7080320955	Bacteria_unclassified
0.0006174936392	0.8567755293	0.7080320955	
0.0006174936392	0.8567755293	0.7080320955	
0.0006174936392	0.8567755293	0.7080320955	
0.001271705588	0.7676091217	0.6256830596	Acetatifactor
0.001271705588	0.7676091217	0.6256830596	Acetatifactor
0.001271705588	0.7676091217	0.6256830596	Acutalibacter
0.001271705588	0.7676091217	0.6543342278	Acutalibacter
0.001271705588	0.7676091217	0.6256830596	Adlercreutzia

0.001801501178	0.7676091217	0.6256830596	Adlercreutzia
0.001271705588	0.7676091217	0.6256830596	Adlercreutzia
0.001271705588	0.7676091217	0.6256830596	Akkermansia
0.00134666968	0.7676091217	0.6256830596	Alistipes
0.001271705588	0.7676091217	0.6256830596	Anaerotruncus
0.001271705588	0.7676091217	0.6256830596	Bacteria_unclassified
0.001271705588	0.7676091217	0.6256830596	Bacteria_unclassified
0.001271705588	0.7676091217	0.6256830596	Bacteria_unclassified
0.001271705588	0.7676091217	0.6256830596	Bacteroides
0.001271705588	0.7676091217	0.6256830596	Bifidobacterium
0.001271705588	0.7676091217	0.6256830596	Clostridia_unclassified
0.001271705588	0.7676091217	0.6256830596	Clostridiaceae_unclassified
0.001271705588	0.7676091217	0.6256830596	Clostridiaceae_unclassified
0.001271705588	0.7676091217	0.6256830596	Eubacteriales_unclassified
0.001271705588	0.7676091217	0.6256830596	Erysipelatoclostridium
0.00148335049	0.7676091217	0.6256830596	Coriobacteriaceae_unclassifie
0.001271705588	0.7676091217	0.6837147113	Dorea
0.001271705588	0.7676091217	0.6256830596	Dubosiella
0.001271705588	0.7676091217	0.6256830596	Erysipelotrichales_unclassifie
0.001271705588	0.7676091217	0.6256830596	Eubacteriaceae_unclassified
0.001758242386	0.7676091217	0.6256830596	Eubacteriaceae_unclassified
0.001271705588	0.7676091217	0.6256830596	GGB20149
0.001271705588	0.7676091217	0.6256830596	GGB22635
0.001271705588	0.7676091217	0.6256830596	GGB25041
0.001660471766	0.7676091217	0.6256830596	GGB27876
0.001271705588	0.7676091217	0.6256830596	GGB27878
0.001271705588	0.7676091217	0.6256830596	GGB27918
0.001271705588	0.7676091217	0.6256830596	GGB28382
0.001271705588	0.7676091217	0.6339649952	GGB28399
0.001271705588	0.7676091217	0.6256830596	GGB28411
0.002013349252	0.7676091217	0.6256830596	GGB28415
0.001271705588	0.7676091217	0.6256830596	GGB28430
0.001271705588	0.7676091217	0.6256830596	GGB28439
0.001271705588	0.7676091217	0.6256830596	GGB28778
0.001271705588	0.7676091217	0.6256830596	GGB28784
0.001271705588	0.7676091217	0.6256830596	GGB28792
0.001271705588	0.7676091217	0.6256830596	GGB28798
0.001271705588	0.7676091217	0.6256830596	GGB28802
0.001271705588	0.7676091217	0.6256830596	GGB28818
0.001519892564	0.7676091217	0.6256830596	GGB28828
0.001271705588	0.7676091217	0.6256830596	GGB28851
0.001271705588	0.7676091217	0.6256830596	GGB28859
0.001315919575	0.7676091217	0.6256830596	GGB28864
0.001271705588	0.7676091217	0.6256830596	GGB28869
0.001271705588	0.8343978319	0.6256830596	GGB28883
0.001315919575	0.7676091217	0.6256830596	GGB28892

0.001315919575	0.7676091217	0.6256830596	GGB28893
0.001928802043	0.7676091217	0.6256830596	GGB28898
0.001271705588	0.7676091217	0.6467183782	GGB28904
0.001282660148	0.7676091217	0.6256830596	GGB28916
0.001271705588	0.7676091217	0.6256830596	GGB28924
0.001271705588	0.7676091217	0.6256830596	GGB28926
0.001271705588	0.7676091217	0.6256830596	GGB28927
0.001271705588	0.7676091217	0.6256830596	GGB28934
0.001315919575	0.7676091217	0.6256830596	GGB28946
0.001271705588	0.7676091217	0.6256830596	GGB28949
0.001487084829	0.7676091217	0.6256830596	GGB28949
0.001660471766	0.7676091217	0.6256830596	GGB28950
0.001271705588	0.7676091217	0.6256830596	GGB28951
0.001271705588	0.7676091217	0.6256830596	GGB28951
0.001315919575	0.7676091217	0.6256830596	GGB28954
0.001271705588	0.7676091217	0.6380397617	GGB28956
0.001271705588	0.8176242312	0.6837147113	GGB28960
0.001271705588	0.7676091217	0.6256830596	GGB28967
0.001271705588	0.7676091217	0.6256830596	GGB28991
0.001315919575	0.7676091217	0.6837147113	GGB29002
0.001271705588	0.7676091217	0.6256830596	GGB29003
0.001271705588	0.8176242312	0.6256830596	GGB29011
0.001487084829	0.7676091217	0.6256830596	GGB29531
0.001271705588	0.7676091217	0.6256830596	GGB29685
0.001271705588	0.7676091217	0.6256830596	GGB30141
0.001271705588	0.7676091217	0.6339649952	GGB30286
0.001271705588	0.7676091217	0.6256830596	GGB30303
0.001271705588	0.7676091217	0.6256830596	GGB30413
0.001271705588	0.7676091217	0.6256830596	GGB30454
0.001271705588	0.7676091217	0.6256830596	GGB30455
0.001271705588	0.7676091217	0.6256830596	GGB30461
0.001271705588	0.7676091217	0.6256830596	GGB30461
0.001271705588	0.7676091217	0.6256830596	GGB30463
0.001271705588	0.7676091217	0.6256830596	GGB30473
0.001412577259	0.7676091217	0.6256830596	GGB30475
0.001271705588	0.7676091217	0.6256830596	GGB30861
0.001271705588	0.7676091217	0.6256830596	GGB31312
0.001271705588	0.7676091217	0.6256830596	GGB31438
0.001271705588	0.7676091217	0.6256830596	GGB3171
0.001271705588	0.7676091217	0.6256830596	GGB31823
0.001271705588	0.7676091217	0.6256830596	GGB31853
0.001271705588	0.8309533002	0.6256830596	GGB32371
0.001271705588	0.7676091217	0.6256830596	GGB3793
0.001271705588	0.7676091217	0.6256830596	GGB42598
0.001271705588	0.7676091217	0.6256830596	GGB45656
0.001271705588	0.7676091217	0.6256830596	GGB47127

0.001271705588	0.7676091217	0.7045255625	GGB74395
0.001271705588	0.7676091217	0.6256830596	GGB75053
0.001271705588	0.7676091217	0.6256830596	GGB75109
0.001271705588	0.7676091217	0.6256830596	GGB81440
0.001271705588	0.7676091217	0.6256830596	Lachnospiraceae_unclassified
0.001282660148	0.7676091217	0.6256830596	Lachnospiraceae_unclassified
0.001271705588	0.7676091217	0.6256830596	Lachnospiraceae_unclassified
0.001590309304	0.7676091217	0.6256830596	Lachnospiraceae_unclassified
0.001271705588	0.7676091217	0.6256830596	Lachnospiraceae_unclassified
0.001271705588	0.7676091217	0.6256830596	Lachnospiraceae_unclassified
0.001321273517	0.7676091217	0.6256830596	Lachnospiraceae_unclassified
0.001271705588	0.7676091217	0.6256830596	Lachnospiraceae_unclassified
0.001271705588	0.7676091217	0.6256830596	Lactobacillus
0.001271705588	0.7676091217	0.6256830596	Muribaculaceae_unclassified
0.001274724956	0.7676091217	0.6256830596	Neglectibacter
0.00134666968	0.7676091217	0.6256830596	Oscillospiraceae_unclassified
0.001271705588	0.7676091217	0.6256830596	Oscillospiraceae_unclassified
0.001271705588	0.7676091217	0.6256830596	Oscillospiraceae_unclassified
0.001271705588	0.7676091217	0.6256830596	Parasutterella
0.001271705588	0.8304615089	0.6256830596	Romboutsia
0.001271705588	0.7676091217	0.6256830596	Schaedlerella
0.001271705588	0.7676091217	0.6451862011	Turicibacter
0.002979913103	0.7676091217	0.6256830596	Bacteria_unclassified
0.001271705588	0.7676091217	0.6256830596	Bacteria_unclassified
0.001271705588	0.7676091217	0.6256830596	Bacteria_unclassified
0.001271705588	0.7676091217	0.6256830596	
0.001271705588	0.7676091217	0.6256830596	
0.001271705588	0.7676091217	0.6256830596	
0.04367470257	0.1305416393	0.3171314387	Acetatifactor
0.04367470257	0.1305416393	0.3171314387	Acetatifactor
0.04367470257	0.1305416393	0.3171314387	Acutalibacter
0.04367470257	0.1305416393	0.3171314387	Acutalibacter
0.04367470257	0.1305416393	0.3171314387	Adlercreutzia
0.04894179322	0.1305416393	0.3171314387	Adlercreutzia
0.04367470257	0.1305416393	0.3171314387	Adlercreutzia
0.0481701997	0.1305416393	0.3171314387	Akkermansia
0.04659916416	0.1305416393	0.3171314387	Alistipes
0.04367470257	0.1305416393	0.3171314387	Anaerotruncus
0.04894179322	0.1305416393	0.3171314387	Bacteria_unclassified
0.04367470257	0.1305416393	0.3223772041	Bacteria_unclassified
0.04367470257	0.1305416393	0.3171314387	Bacteria_unclassified
0.04367470257	0.1305416393	0.3171314387	Bacteroides
0.04367470257	0.1305416393	0.3171314387	Bifidobacterium
0.04367470257	0.1305416393	0.3171314387	Clostridia_unclassified
0.04367470257	0.1308703764	0.3171314387	Clostridiaceae_unclassified
0.04367470257	0.1305416393	0.3171314387	Clostridiaceae_unclassified

0.04367470257	0.1305416393	0.3171314387	Eubacteriales_unclassified
0.05601639705	0.1305416393	0.3171314387	Erysipelatoclostridium
0.04367470257	0.1305416393	0.3171314387	Coriobacteriaceae_unclassifie
0.04367470257	0.1305416393	0.3171314387	Dorea
0.04367470257	0.1305416393	0.3171314387	Dubosiella
0.04367470257	0.1305416393	0.3171314387	Erysipelotrichales_unclassifie
0.112235317	0.1305416393	0.3171314387	Eubacteriaceae_unclassified
0.04367470257	0.1305416393	0.3171314387	Eubacteriaceae_unclassified
0.04367470257	0.1305416393	0.3171314387	GGB20149
0.04831538925	0.1305416393	0.3171314387	GGB22635
0.04367470257	0.1305416393	0.3171314387	GGB25041
0.04367470257	0.139182221	0.320843551	GGB27876
0.04367470257	0.1305416393	0.3171314387	GGB27878
0.04367470257	0.1305416393	0.3171314387	GGB27918
0.04367470257	0.1305416393	0.3171314387	GGB28382
0.04367470257	0.1305416393	0.3171314387	GGB28399
0.04367470257	0.1305416393	0.3171314387	GGB28411
0.04367470257	0.1305416393	0.3171314387	GGB28415
0.04367470257	0.1305416393	0.3171314387	GGB28430
0.04367470257	0.1305416393	0.3171314387	GGB28439
0.04367470257	0.1305416393	0.3171314387	GGB28778
0.04367470257	0.1305416393	0.3171314387	GGB28784
0.04367470257	0.1305416393	0.3171314387	GGB28792
0.04367470257	0.1305416393	0.3171314387	GGB28798
0.04367470257	0.1305416393	0.3171314387	GGB28802
0.04367470257	0.1305416393	0.3171314387	GGB28818
0.04657723048	0.1305416393	0.3171314387	GGB28828
0.04395942468	0.1305416393	0.3171314387	GGB28851
0.04367470257	0.1305416393	0.3171314387	GGB28859
0.04657723048	0.1305416393	0.3171314387	GGB28864
0.04367470257	0.1305416393	0.3171314387	GGB28869
0.04367470257	0.1305416393	0.3171314387	GGB28883
0.04395942468	0.1305416393	0.3171314387	GGB28892
0.04885981673	0.1305416393	0.3171314387	GGB28893
0.04367470257	0.1305416393	0.3236743079	GGB28898
0.04367470257	0.1305416393	0.3373408481	GGB28904
0.04367470257	0.1305416393	0.3171314387	GGB28916
0.04367470257	0.1305416393	0.3171314387	GGB28924
0.04367470257	0.1305416393	0.3171314387	GGB28926
0.04367470257	0.1305416393	0.3171314387	GGB28927
0.04367470257	0.1370482377	0.345340671	GGB28934
0.04395942468	0.1305416393	0.3171314387	GGB28946
0.04367470257	0.1305416393	0.3171314387	GGB28949
0.04367470257	0.1305416393	0.3171314387	GGB28949
0.04367470257	0.138475456	0.3171314387	GGB28950
0.04367470257	0.1305416393	0.3171314387	GGB28951

0.04367470257	0.1305416393	0.3171314387	GGB28951
0.04657723048	0.1305416393	0.3171314387	GGB28954
0.04395942468	0.1305416393	0.3171314387	GGB28956
0.04395942468	0.1305416393	0.3171314387	GGB28960
0.04367470257	0.1305416393	0.3171314387	GGB28967
0.04367470257	0.1305416393	0.3171314387	GGB28991
0.04657723048	0.1562256722	0.5397187363	GGB29002
0.04367470257	0.1305416393	0.3171314387	GGB29003
0.04367470257	0.1305416393	0.3171314387	GGB29011
0.04657723048	0.1305416393	0.3171314387	GGB29531
0.04367470257	0.1305416393	0.3171314387	GGB29685
0.04367470257	0.1305416393	0.3171314387	GGB30141
0.04367470257	0.1305416393	0.3171314387	GGB30286
0.04367470257	0.1305416393	0.3171314387	GGB30303
0.04367470257	0.1305416393	0.3171314387	GGB30413
0.04395942468	0.1305416393	0.3171314387	GGB30454
0.05764156657	0.1305416393	0.3171314387	GGB30455
0.04367470257	0.1305416393	0.3171314387	GGB30461
0.04603121726	0.1305416393	0.3171314387	GGB30461
0.05995651403	0.1305416393	0.3171314387	GGB30463
0.04395942468	0.187856815	0.3488243361	GGB30473
0.04657723048	0.1305416393	0.3171314387	GGB30475
0.04374971132	0.1305416393	0.3171314387	GGB30861
0.04367470257	0.1305416393	0.3171314387	GGB31312
0.04367470257	0.1414640256	0.3171314387	GGB31438
0.04367470257	0.1305416393	0.3171314387	GGB3171
0.04367470257	0.1305416393	0.3171314387	GGB31823
0.04367470257	0.1305416393	0.3171314387	GGB31853
0.04367470257	0.1813401845	0.3171314387	GGB32371
0.04367470257	0.1305416393	0.3171314387	GGB3793
0.04367470257	0.1305416393	0.3171314387	GGB42598
0.04367470257	0.1335000204	0.3171314387	GGB45656
0.04367470257	0.1305416393	0.3171314387	GGB47127
0.04367470257	0.1305416393	0.3171314387	GGB74395
0.04657723048	0.1305416393	0.3171314387	GGB75053
0.04395942468	0.1305416393	0.3171314387	GGB75109
0.04367470257	0.1458138328	0.3695109776	GGB81440
0.04367470257	0.1305416393	0.3171314387	Lachnospiraceae_unclassified
0.04367470257	0.1305416393	0.3223772041	Lachnospiraceae_unclassified
0.04367470257	0.1764881456	0.3171314387	Lachnospiraceae_unclassified
0.06292451942	0.1370482377	0.3171314387	Lachnospiraceae_unclassified
0.04367470257	0.1562646687	0.3541065039	Lachnospiraceae_unclassified
0.04367470257	0.1305416393	0.3171314387	Lachnospiraceae_unclassified
0.04894179322	0.1305416393	0.3171314387	Lachnospiraceae_unclassified
0.04367470257	0.1305416393	0.3171314387	Lachnospiraceae_unclassified
0.05714835907	0.1305416393	0.3171314387	Lactobacillus

0.04395942468	0.1305416393	0.3171314387	Muribaculaceae_unclassified
0.04367470257	0.1305416393	0.3171314387	Neglectibacter
0.04367470257	0.1305416393	0.3171314387	Oscillospiraceae_unclassified
0.04367470257	0.1316884198	0.3171314387	Oscillospiraceae_unclassified
0.04367470257	0.1305416393	0.3171314387	Oscillospiraceae_unclassified
0.04367470257	0.1305416393	0.3171314387	Parasutterella
0.04657723048	0.1305416393	0.3171314387	Romboutsia
0.04367470257	0.1305416393	0.3171314387	Schaedlerella
0.04367470257	0.1305416393	0.3171314387	Turicibacter
0.1035049976	0.1305416393	0.3171314387	Bacteria_unclassified
0.04367470257	0.1305416393	0.3171314387	Bacteria_unclassified
0.04367470257	0.1305416393	0.3171314387	Bacteria_unclassified
0.04367470257	0.1491222629	0.3171314387	
0.04367470257	0.1305416393	0.3171314387	
0.04395942468	0.1305416393	0.3171314387	
0.07692301036	0.1589769463	0.102236371	Acetatifactor
0.07692301036	0.1589769463	0.102236371	Acetatifactor
0.07692301036	0.1589769463	0.1066324351	Acutalibacter
0.07692301036	0.1589769463	0.102236371	Acutalibacter
0.07692301036	0.1589769463	0.102236371	Adlercreutzia
0.09073614391	0.1589769463	0.102236371	Adlercreutzia
0.07692301036	0.1589769463	0.102236371	Adlercreutzia
0.07692301036	0.1589769463	0.102236371	Akkermansia
0.07692301036	0.1589769463	0.102236371	Alistipes
0.07692301036	0.1589769463	0.102236371	Anaerotruncus
0.07718538808	0.1589769463	0.102236371	Bacteria_unclassified
0.07692301036	0.1589769463	0.130008339	Bacteria_unclassified
0.07692301036	0.1589769463	0.102236371	Bacteria_unclassified
0.07692301036	0.1617439113	0.102236371	Bacteroides
0.07692301036	0.1589769463	0.102236371	Bifidobacterium
0.07692301036	0.1664739627	0.102236371	Clostridia_unclassified
0.07692301036	0.1693458703	0.102236371	Clostridiaceae_unclassified
0.07692301036	0.1589769463	0.102236371	Clostridiaceae_unclassified
0.07692301036	0.1589769463	0.102236371	Eubacteriales_unclassified
0.07718538808	0.1589769463	0.102236371	Erysipelatoclostridium
0.08061015811	0.1589769463	0.102236371	Coriobacteriaceae_unclassifie
0.07692301036	0.1589769463	0.1097587134	Dorea
0.07692301036	0.1589769463	0.1097587134	Dubosiella
0.07692301036	0.1822113032	0.1097339319	Erysipelotrichales_unclassifie
0.07692301036	0.1822113032	0.1231742907	Eubacteriaceae_unclassified
0.09120550943	0.1589769463	0.102236371	Eubacteriaceae_unclassified
0.07692301036	0.1589769463	0.102236371	GGB20149
0.07692301036	0.1589769463	0.102236371	GGB22635
0.07692301036	0.1664739627	0.1108388432	GGB25041
0.07718538808	0.1589769463	0.102236371	GGB27876
0.07692301036	0.1589769463	0.102236371	GGB27878

0.07692301036	0.1589769463	0.102236371	GGB27918
0.07692301036	0.1589769463	0.102236371	GGB28382
0.07960156012	0.1589769463	0.102236371	GGB28399
0.07692301036	0.1589769463	0.102236371	GGB28411
0.09283804963	0.1589769463	0.102236371	GGB28415
0.07692301036	0.1589769463	0.102236371	GGB28430
0.07692301036	0.1589769463	0.102236371	GGB28439
0.07692301036	0.1589769463	0.102236371	GGB28778
0.07692301036	0.1589769463	0.102236371	GGB28784
0.07692301036	0.1589769463	0.102236371	GGB28792
0.07692301036	0.1589769463	0.102236371	GGB28798
0.07692301036	0.1589769463	0.102236371	GGB28802
0.07692301036	0.1589769463	0.102236371	GGB28818
0.08038725545	0.1589769463	0.102236371	GGB28828
0.07692301036	0.1589769463	0.102236371	GGB28851
0.07718538808	0.1664739627	0.1074565634	GGB28859
0.07992747427	0.1589769463	0.102236371	GGB28864
0.07692301036	0.1589769463	0.102236371	GGB28869
0.07692301036	0.1841956373	0.102236371	GGB28883
0.08061015811	0.1589769463	0.102236371	GGB28892
0.09073614391	0.1589769463	0.102236371	GGB28893
0.121673081	0.1589769463	0.102236371	GGB28898
0.07692301036	0.1589769463	0.1074565634	GGB28904
0.07692301036	0.1589769463	0.102236371	GGB28916
0.07692301036	0.1589769463	0.102236371	GGB28924
0.07692301036	0.1589769463	0.102236371	GGB28926
0.07692301036	0.1589769463	0.102236371	GGB28927
0.07692301036	0.1693458703	0.1240020188	GGB28934
0.07960156012	0.1589769463	0.102236371	GGB28946
0.07692301036	0.1589769463	0.102236371	GGB28949
0.07692301036	0.1589769463	0.102236371	GGB28949
0.07692301036	0.1589769463	0.102236371	GGB28950
0.07692301036	0.1589769463	0.102236371	GGB28951
0.07692301036	0.1589769463	0.102236371	GGB28951
0.07692301036	0.1589769463	0.102236371	GGB28954
0.07692301036	0.1589769463	0.102236371	GGB28956
0.07692301036	0.1589769463	0.102236371	GGB28960
0.07692301036	0.1589769463	0.102236371	GGB28967
0.07692301036	0.1589769463	0.102236371	GGB28991
0.07960156012	0.1709589243	0.1478334542	GGB29002
0.07692301036	0.1589769463	0.102236371	GGB29003
0.07692301036	0.179475095	0.102236371	GGB29011
0.08084066323	0.1589769463	0.102236371	GGB29531
0.07692301036	0.1589769463	0.102236371	GGB29685
0.07692301036	0.1589769463	0.102236371	GGB30141
0.07692301036	0.179475095	0.1231742907	GGB30286

0.07692301036	0.1589769463	0.102236371	GGB30303
0.07692301036	0.1589769463	0.102236371	GGB30413
0.07692301036	0.1589769463	0.102236371	GGB30454
0.077266801	0.1589769463	0.102236371	GGB30455
0.07692301036	0.1589769463	0.102236371	GGB30461
0.07692301036	0.1589769463	0.102236371	GGB30461
0.07692301036	0.1589769463	0.102236371	GGB30463
0.07692301036	0.1589769463	0.102236371	GGB30473
0.07692301036	0.1589769463	0.102236371	GGB30475
0.07692301036	0.1589769463	0.102236371	GGB30861
0.07692301036	0.1589769463	0.102236371	GGB31312
0.07692301036	0.1667295426	0.102236371	GGB31438
0.07692301036	0.1589769463	0.102236371	GGB3171
0.07692301036	0.1709589243	0.102236371	GGB31823
0.07692301036	0.1589769463	0.102236371	GGB31853
0.07692301036	0.2698102776	0.115918777	GGB32371
0.07692301036	0.1589769463	0.102236371	GGB3793
0.07692301036	0.1589769463	0.102236371	GGB42598
0.07692301036	0.1589769463	0.102236371	GGB45656
0.07692301036	0.1589769463	0.102236371	GGB47127
0.07692301036	0.1589769463	0.115918777	GGB74395
0.07692301036	0.1589769463	0.102236371	GGB75053
0.07692301036	0.1589769463	0.102236371	GGB75109
0.07692301036	0.179475095	0.1305497641	GGB81440
0.07692301036	0.1589769463	0.102236371	Lachnospiraceae_unclassified
0.08076314209	0.1589769463	0.102236371	Lachnospiraceae_unclassified
0.08444029956	0.1589769463	0.102236371	Lachnospiraceae_unclassified
0.1254128111	0.1822113032	0.1097587134	Lachnospiraceae_unclassified
0.07692301036	0.1923358537	0.1358118195	Lachnospiraceae_unclassified
0.07692301036	0.1589769463	0.102236371	Lachnospiraceae_unclassified
0.07992747427	0.1589769463	0.102236371	Lachnospiraceae_unclassified
0.07692301036	0.1589769463	0.102236371	Lachnospiraceae_unclassified
0.07960156012	0.1589769463	0.102236371	Lactobacillus
0.07692301036	0.1589769463	0.102236371	Muribaculaceae_unclassified
0.07692301036	0.179475095	0.1460222786	Neglectibacter
0.07692301036	0.1589769463	0.102236371	Oscillospiraceae_unclassified
0.07692301036	0.2418024878	0.1751358198	Oscillospiraceae_unclassified
0.07692301036	0.1589769463	0.102236371	Oscillospiraceae_unclassified
0.07692301036	0.1589769463	0.102236371	Parasutterella
0.07692301036	0.179475095	0.102236371	Romboutsia
0.07692301036	0.1617439113	0.102236371	Schaedlerella
0.07692301036	0.1589769463	0.1097339319	Turicibacter
0.1431898649	0.1589769463	0.102236371	Bacteria_unclassified
0.07692301036	0.1589769463	0.102236371	Bacteria_unclassified
0.07692301036	0.1589769463	0.102236371	Bacteria_unclassified
0.07692301036	0.1589769463	0.102236371	

0.07692301036	0.1589769463	0.102236371	
0.07692301036	0.1589769463	0.102236371	
0.01259897985	0.7599583708	0.1422809042	Acetatifactor
0.01259897985	0.756051987	0.1422809042	Acetatifactor
0.01259897985	0.756051987	0.1422809042	Acutalibacter
0.01259897985	0.756051987	0.1422809042	Acutalibacter
0.01259897985	0.7599583708	0.1422809042	Adlercreutzia
0.02910957752	0.7619979149	0.2219552486	Adlercreutzia
0.01259897985	0.7619979149	0.1431690965	Adlercreutzia
0.01259897985	0.756051987	0.1422809042	Akkermansia
0.01259897985	0.756051987	0.1422809042	Alistipes
0.01259897985	0.756051987	0.1422809042	Anaerotruncus
0.0161408911	0.756051987	0.1422809042	Bacteria_unclassified
0.01259897985	0.756051987	0.1422809042	Bacteria_unclassified
0.01259897985	0.756051987	0.1422809042	Bacteria_unclassified
0.01259897985	0.756051987	0.1422809042	Bacteroides
0.01259897985	0.756051987	0.1431690965	Bifidobacterium
0.01259897985	0.756051987	0.1422809042	Clostridia_unclassified
0.01259897985	0.756051987	0.1422809042	Clostridiaceae_unclassified
0.01259897985	0.756051987	0.1422809042	Clostridiaceae_unclassified
0.01625471369	0.756051987	0.1422809042	Eubacteriales_unclassified
0.01259897985	0.756051987	0.1889990139	Erysipelatoclostridium
0.01259897985	0.756051987	0.1422809042	Coriobacteriaceae_unclassifie
0.01259897985	0.756051987	0.1779194338	Dorea
0.01259897985	0.756051987	0.1422809042	Dubosiella
0.01259897985	0.756051987	0.1422809042	Erysipelotrichales_unclassifie
0.05642788826	0.8865756036	0.2090358863	Eubacteriaceae_unclassified
0.01259897985	0.7725224847	0.1495469789	Eubacteriaceae_unclassified
0.01259897985	0.756051987	0.1422809042	GGB20149
0.01259897985	0.756051987	0.1422809042	GGB22635
0.01259897985	0.756051987	0.1422809042	GGB25041
0.02126313253	0.756051987	0.1431690965	GGB27876
0.01259897985	0.7599583708	0.1460090943	GGB27878
0.01259897985	0.756051987	0.1422809042	GGB27918
0.01259897985	0.756051987	0.1422809042	GGB28382
0.01259897985	0.756051987	0.1422809042	GGB28399
0.01259897985	0.7619979149	0.1915245786	GGB28411
0.01347729962	0.756051987	0.1422809042	GGB28415
0.01259897985	0.7599583708	0.1422809042	GGB28430
0.01259897985	0.756051987	0.1422809042	GGB28439
0.01259897985	0.756051987	0.1422809042	GGB28778
0.01259897985	0.756051987	0.1422809042	GGB28784
0.01259897985	0.756051987	0.1422809042	GGB28792
0.01259897985	0.756051987	0.1422809042	GGB28798
0.01832360615	0.7679796939	0.1470626559	GGB28802
0.01259897985	0.756051987	0.1422809042	GGB28818

0.01259897985	0.7599583708	0.1422809042	GGB28828
0.01259897985	0.756051987	0.1499502903	GGB28851
0.01259897985	0.756051987	0.1431690965	GGB28859
0.01259897985	0.756051987	0.1422809042	GGB28864
0.01259897985	0.756051987	0.1422809042	GGB28869
0.01264183914	0.756051987	0.1422809042	GGB28883
0.01259897985	0.756051987	0.1422809042	GGB28892
0.01259897985	0.756051987	0.1422809042	GGB28893
0.01259897985	0.756051987	0.1422809042	GGB28898
0.01259897985	0.756051987	0.1431690965	GGB28904
0.01259897985	0.756051987	0.1422809042	GGB28916
0.01259897985	0.756051987	0.1422809042	GGB28924
0.01259897985	0.756051987	0.1422809042	GGB28926
0.01259897985	0.756051987	0.1422809042	GGB28927
0.01259897985	0.756051987	0.1499502903	GGB28934
0.01259897985	0.756051987	0.1422809042	GGB28946
0.01259897985	0.756051987	0.1422809042	GGB28949
0.02338317771	0.756051987	0.1431690965	GGB28949
0.01464608076	0.756051987	0.1422809042	GGB28950
0.01259897985	0.756051987	0.1422809042	GGB28951
0.01259897985	0.756051987	0.1422809042	GGB28951
0.01259897985	0.756051987	0.1422809042	GGB28954
0.01259897985	0.756051987	0.1422809042	GGB28956
0.01264183914	0.7619979149	0.1784199936	GGB28960
0.01259897985	0.756051987	0.1422809042	GGB28967
0.01259897985	0.756051987	0.1422809042	GGB28991
0.01259897985	0.756051987	0.1422809042	GGB29002
0.01259897985	0.756051987	0.1422809042	GGB29003
0.01259897985	0.756051987	0.1422809042	GGB29011
0.01259897985	0.756051987	0.1422809042	GGB29531
0.01259897985	0.756051987	0.1422809042	GGB29685
0.01259897985	0.756051987	0.1422809042	GGB30141
0.02039579126	0.7599583708	0.1559673307	GGB30286
0.01259897985	0.7599583708	0.1422809042	GGB30303
0.01259897985	0.756051987	0.1422809042	GGB30413
0.01873244574	0.8043483118	0.1422809042	GGB30454
0.01625471369	0.7725224847	0.1422809042	GGB30455
0.01259897985	0.756051987	0.1422809042	GGB30461
0.01259897985	0.756051987	0.1422809042	GGB30461
0.01347729962	0.756051987	0.1422809042	GGB30463
0.01259897985	0.940867365	0.2292854943	GGB30473
0.01259897985	0.756051987	0.1422809042	GGB30475
0.0161408911	0.756051987	0.1422809042	GGB30861
0.01259897985	0.756051987	0.1422809042	GGB31312
0.01259897985	0.756051987	0.1422809042	GGB31438
0.01259897985	0.7599583708	0.1422809042	GGB3171

0.01259897985	0.756051987	0.1422809042	GGB31823
0.01264183914	0.756051987	0.1422809042	GGB31853
0.01259897985	0.8074435573	0.1431690965	GGB32371
0.01259897985	0.756051987	0.1422809042	GGB3793
0.01259897985	0.756051987	0.1422809042	GGB42598
0.01264183914	0.756051987	0.1422809042	GGB45656
0.01259897985	0.7599583708	0.1422809042	GGB47127
0.01259897985	0.756051987	0.1422809042	GGB74395
0.01290755531	0.756051987	0.1422809042	GGB75053
0.01259897985	0.756051987	0.1422809042	GGB75109
0.01259897985	0.756051987	0.1422809042	GGB81440
0.01259897985	0.756051987	0.1422809042	Lachnospiraceae_unclassified
0.01259897985	0.756051987	0.1422809042	Lachnospiraceae_unclassified
0.01259897985	0.756051987	0.1422809042	Lachnospiraceae_unclassified
0.01259897985	0.756051987	0.1422809042	Lachnospiraceae_unclassified
0.01259897985	0.756051987	0.1422809042	Lachnospiraceae_unclassified
0.01259897985	0.756051987	0.1422809042	Lachnospiraceae_unclassified
0.01259897985	0.756051987	0.1431690965	Lachnospiraceae_unclassified
0.01259897985	0.756051987	0.1422809042	Lachnospiraceae_unclassified
0.01259897985	0.756051987	0.1422809042	Lactobacillus
0.01259897985	0.756051987	0.1422809042	Muribaculaceae_unclassified
0.01259897985	0.756051987	0.1422809042	Neglectibacter
0.01264183914	0.756051987	0.1422809042	Oscillospiraceae_unclassified
0.01474019978	0.7619979149	0.183399367	Oscillospiraceae_unclassified
0.01259897985	0.756051987	0.1422809042	Oscillospiraceae_unclassified
0.01259897985	0.7599583708	0.1422809042	Parasutterella
0.01751333351	0.756051987	0.1422809042	Romboutsia
0.01259897985	0.756051987	0.1422809042	Schaedlerella
0.01259897985	0.756051987	0.1422809042	Turicibacter
0.06741206655	0.8323892139	0.2267365476	Bacteria_unclassified
0.01259897985	0.756051987	0.1422809042	Bacteria_unclassified
0.01259897985	0.756051987	0.1422809042	Bacteria_unclassified
0.01358790005	0.756051987	0.1499502903	
0.01290755531	0.756051987	0.1422809042	
0.01259897985	0.756051987	0.1422809042	
0.578783872	0.4180975346	0.1373635008	Acetatifactor
0.578783872	0.4180975346	0.1381784442	Acetatifactor
0.578783872	0.4180975346	0.1373635008	Acutalibacter
0.578783872	0.4180975346	0.1373635008	Acutalibacter
0.578783872	0.4180975346	0.1373635008	Adlercreutzia
0.578783872	0.4180975346	0.1373635008	Adlercreutzia
0.578783872	0.440045074	0.1373635008	Adlercreutzia
0.578783872	0.4180975346	0.1373635008	Akkermansia
0.578783872	0.4180975346	0.1381784442	Alistipes
0.578783872	0.4180975346	0.1373635008	Anaerotruncus
0.578783872	0.4180975346	0.1373635008	Bacteria_unclassified

0.578783872	0.4180975346	0.1373635008	Bacteria_unclassified
0.578783872	0.4180975346	0.1373635008	Bacteria_unclassified
0.578783872	0.4180975346	0.1373635008	Bacteroides
0.578783872	0.4180975346	0.1373635008	Bifidobacterium
0.613769241	0.4180975346	0.1373635008	Clostridia_unclassified
0.578783872	0.4180975346	0.1373635008	Clostridiaceae_unclassified
0.578783872	0.4180975346	0.1373635008	Clostridiaceae_unclassified
0.578783872	0.4180975346	0.1373635008	Eubacteriales_unclassified
0.578783872	0.4180975346	0.1373635008	Erysipelatoclostridium
0.7086265169	0.4180975346	0.1373635008	Coriobacteriaceae_unclassifie
0.578783872	0.4180975346	0.1373635008	Dorea
0.578783872	0.4180975346	0.1373635008	Dubosiella
0.578783872	0.4180975346	0.1373635008	Erysipelotrichales_unclassifie
0.578783872	0.4180975346	0.1381784442	Eubacteriaceae_unclassified
0.578783872	0.4180975346	0.1373635008	Eubacteriaceae_unclassified
0.578783872	0.4180975346	0.1373635008	GGB20149
0.578783872	0.4180975346	0.1373635008	GGB22635
0.7696462239	0.4180975346	0.1373635008	GGB25041
0.7684907177	0.4180975346	0.1373635008	GGB27876
0.578783872	0.4180975346	0.1373635008	GGB27878
0.578783872	0.4447585855	0.1414988016	GGB27918
0.578783872	0.4180975346	0.1373635008	GGB28382
0.578783872	0.467790095	0.2247230329	GGB28399
0.578783872	0.4180975346	0.1373635008	GGB28411
0.578783872	0.4180975346	0.1373635008	GGB28415
0.578783872	0.4180975346	0.1373635008	GGB28430
0.578783872	0.4180975346	0.1373635008	GGB28439
0.578783872	0.4180975346	0.1373635008	GGB28778
0.578783872	0.4180975346	0.1373635008	GGB28784
0.578783872	0.4180975346	0.1373635008	GGB28792
0.578783872	0.4180975346	0.1373635008	GGB28798
0.578783872	0.4180975346	0.1373635008	GGB28802
0.578783872	0.4180975346	0.1373635008	GGB28818
0.578783872	0.4180975346	0.1596982858	GGB28828
0.578783872	0.4180975346	0.1373635008	GGB28851
0.578783872	0.4180975346	0.1373635008	GGB28859
0.578783872	0.4180975346	0.1373635008	GGB28864
0.578783872	0.4447585855	0.1596982858	GGB28869
0.578783872	0.4226788162	0.1373635008	GGB28883
0.578783872	0.4180975346	0.1373635008	GGB28892
0.578783872	0.446514807	0.188541772	GGB28893
0.578783872	0.4180975346	0.1373635008	GGB28898
0.578783872	0.4180975346	0.1373635008	GGB28904
0.578783872	0.4180975346	0.1373635008	GGB28916
0.578783872	0.4180975346	0.1373635008	GGB28924
0.578783872	0.4180975346	0.1373635008	GGB28926

0.578783872	0.4180975346	0.1373635008	GGB28927
0.578783872	0.4373647075	0.2247230329	GGB28934
0.578783872	0.446514807	0.1596982858	GGB28946
0.578783872	0.4180975346	0.1373635008	GGB28949
0.6316705456	0.4180975346	0.1373635008	GGB28949
0.613769241	0.4180975346	0.1373635008	GGB28950
0.578783872	0.4373647075	0.1373635008	GGB28951
0.578783872	0.4180975346	0.1373635008	GGB28951
0.578783872	0.4180975346	0.1373635008	GGB28954
0.578783872	0.4180975346	0.24718038	GGB28956
0.578783872	0.4180975346	0.1373635008	GGB28960
0.641387932	0.4180975346	0.1373635008	GGB28967
0.578783872	0.4180975346	0.1373635008	GGB28991
0.578783872	0.4180975346	0.1373635008	GGB29002
0.578783872	0.4180975346	0.1373635008	GGB29003
0.578783872	0.4402681568	0.1373635008	GGB29011
0.578783872	0.4447585855	0.1704967819	GGB29531
0.578783872	0.4180975346	0.1373635008	GGB29685
0.578783872	0.4226788162	0.1373635008	GGB30141
0.578783872	0.4180975346	0.1373635008	GGB30286
0.578783872	0.4180975346	0.1373635008	GGB30303
0.578783872	0.4180975346	0.1373635008	GGB30413
0.578783872	0.4180975346	0.1373635008	GGB30454
0.578783872	0.4373647075	0.1382017409	GGB30455
0.578783872	0.4180975346	0.1373635008	GGB30461
0.578783872	0.4180975346	0.1373635008	GGB30461
0.578783872	0.4180975346	0.1373635008	GGB30463
0.578783872	0.4180975346	0.1373635008	GGB30473
0.578783872	0.4180975346	0.1373635008	GGB30475
0.578783872	0.4180975346	0.1373635008	GGB30861
0.578783872	0.4180975346	0.1373635008	GGB31312
0.578783872	0.4180975346	0.1373635008	GGB31438
0.578783872	0.4180975346	0.1373635008	GGB3171
0.578783872	0.4180975346	0.1373635008	GGB31823
0.578783872	0.4180975346	0.1373635008	GGB31853
0.578783872	0.4373647075	0.1373635008	GGB32371
0.578783872	0.4180975346	0.1373635008	GGB3793
0.578783872	0.4180975346	0.2282961175	GGB42598
0.578783872	0.4180975346	0.1373635008	GGB45656
0.578783872	0.4180975346	0.1373635008	GGB47127
0.578783872	0.4180975346	0.1373635008	GGB74395
0.578783872	0.4180975346	0.1373635008	GGB75053
0.578783872	0.4180975346	0.1373635008	GGB75109
0.578783872	0.4180975346	0.1373635008	GGB81440
0.578783872	0.4180975346	0.1373635008	Lachnospiraceae_unclassified
0.578783872	0.4180975346	0.1373635008	Lachnospiraceae_unclassified

0.578783872	0.4180975346	0.1373635008	Lachnospiraceae_unclassified
0.578783872	0.4180975346	0.1373635008	Lachnospiraceae_unclassified
0.7148477127	0.4180975346	0.1373635008	Lachnospiraceae_unclassified
0.578783872	0.4180975346	0.1373635008	Lachnospiraceae_unclassified
0.578783872	0.4180975346	0.1373635008	Lachnospiraceae_unclassified
0.578783872	0.4180975346	0.1373635008	Lachnospiraceae_unclassified
0.578783872	0.4180975346	0.1373635008	Lactobacillus
0.578783872	0.4180975346	0.1373635008	Muribaculaceae_unclassified
0.578783872	0.4180975346	0.156954199	Neglectibacter
0.578783872	0.4180975346	0.1373635008	Oscillospiraceae_unclassified
0.578783872	0.4180975346	0.1373635008	Oscillospiraceae_unclassified
0.578783872	0.4180975346	0.1373635008	Oscillospiraceae_unclassified
0.578783872	0.4180975346	0.1373635008	Parasutterella
0.578783872	0.4180975346	0.1373635008	Romboutsia
0.578783872	0.4180975346	0.1373635008	Schaedlerella
0.578783872	0.4180975346	0.1528104133	Turicibacter
0.578783872	0.4180975346	0.1373635008	Bacteria_unclassified
0.578783872	0.4180975346	0.1402078759	Bacteria_unclassified
0.578783872	0.4180975346	0.1373635008	Bacteria_unclassified
0.578783872	0.4180975346	0.1373635008	
0.578783872	0.4373647075	0.1596982858	
0.578783872	0.4180975346	0.1373635008	
0.2131915586	0.8736371071	0.488522822	Acetatifactor
0.2131915586	0.8736371071	0.4997790029	Acetatifactor
0.2131915586	0.8736371071	0.488522822	Acutalibacter
0.2131915586	0.8736371071	0.488522822	Acutalibacter
0.2131915586	0.8736371071	0.488522822	Adlercreutzia
0.2264838524	0.8736371071	0.488522822	Adlercreutzia
0.2131915586	0.8736371071	0.488522822	Adlercreutzia
0.2131915586	0.8736371071	0.488522822	Akkermansia
0.2131915586	0.8736371071	0.4921389478	Alistipes
0.2131915586	0.8736371071	0.488522822	Anaerotruncus
0.2131915586	0.8736371071	0.4921389478	Bacteria_unclassified
0.2131915586	0.8736371071	0.488522822	Bacteria_unclassified
0.2131915586	0.8736371071	0.488522822	Bacteria_unclassified
0.2131915586	0.8736371071	0.488522822	Bacteroides
0.2131915586	0.8736371071	0.488522822	Bifidobacterium
0.2131915586	0.8736371071	0.488522822	Clostridia_unclassified
0.2131915586	0.8736371071	0.488522822	Clostridiaceae_unclassified
0.2131915586	0.8736371071	0.488522822	Clostridiaceae_unclassified
0.2131915586	0.8736371071	0.488522822	Eubacteriales_unclassified
0.2131915586	0.9363519376	0.488522822	Erysipelatoclostridium
0.276301348	0.8736371071	0.488522822	Coriobacteriaceae_unclassifie
0.2131915586	0.8736371071	0.488522822	Dorea
0.2590600021	0.8736371071	0.488522822	Dubosiella
0.2131915586	0.8736371071	0.488522822	Erysipelotrichales_unclassifie

0.2314968767	0.8736371071	0.488522822	Eubacteriaceae_unclassified
0.2131915586	0.8736371071	0.488522822	Eubacteriaceae_unclassified
0.2131915586	0.8736371071	0.488522822	GGB20149
0.2131915586	0.8736371071	0.488522822	GGB22635
0.2543135164	0.8736371071	0.488522822	GGB25041
0.3174518121	0.8736371071	0.488522822	GGB27876
0.2131915586	0.8736371071	0.488522822	GGB27878
0.2131915586	0.8736371071	0.488522822	GGB27918
0.2131915586	0.8736371071	0.4921389478	GGB28382
0.2131915586	0.8736371071	0.488522822	GGB28399
0.2131915586	0.8736371071	0.488522822	GGB28411
0.2131915586	0.8736371071	0.488522822	GGB28415
0.2131915586	0.8736371071	0.488522822	GGB28430
0.2131915586	0.8736371071	0.488522822	GGB28439
0.2131915586	0.8736371071	0.488522822	GGB28778
0.2131915586	0.8736371071	0.488522822	GGB28784
0.2131915586	0.8736371071	0.488522822	GGB28792
0.2131915586	0.8736371071	0.488522822	GGB28798
0.2131915586	0.8736371071	0.488522822	GGB28802
0.2131915586	0.8736371071	0.488522822	GGB28818
0.2131915586	0.8736371071	0.6738492284	GGB28828
0.2131915586	0.8736371071	0.488522822	GGB28851
0.2131915586	0.8736371071	0.488522822	GGB28859
0.2131915586	0.8736371071	0.4921389478	GGB28864
0.2131915586	0.8736371071	0.4921389478	GGB28869
0.2131915586	0.9341545781	0.488522822	GGB28883
0.2131915586	0.8736371071	0.488522822	GGB28892
0.2392238313	0.8736371071	0.488522822	GGB28893
0.2131915586	0.8736371071	0.4921389478	GGB28898
0.2131915586	0.8736371071	0.4921389478	GGB28904
0.2131915586	0.8736371071	0.488522822	GGB28916
0.2131915586	0.8736371071	0.488522822	GGB28924
0.2131915586	0.8736371071	0.488522822	GGB28926
0.2131915586	0.8736371071	0.488522822	GGB28927
0.2131915586	0.8736371071	0.4921389478	GGB28934
0.2131915586	0.8736371071	0.488522822	GGB28946
0.2131915586	0.8736371071	0.488522822	GGB28949
0.2131915586	0.8736371071	0.488522822	GGB28949
0.2366381849	0.8736371071	0.488522822	GGB28950
0.2131915586	0.8736371071	0.488522822	GGB28951
0.2131915586	0.8736371071	0.488522822	GGB28951
0.2131915586	0.8736371071	0.488522822	GGB28954
0.2131915586	0.8736371071	0.6111060006	GGB28956
0.2965725754	0.8736371071	0.488522822	GGB28960
0.2144933081	0.8736371071	0.488522822	GGB28967
0.2131915586	0.8736371071	0.488522822	GGB28991

0.2131915586	0.8736371071	0.488522822	GGB29002
0.2131915586	0.8736371071	0.488522822	GGB29003
0.2131915586	0.8736371071	0.488522822	GGB29011
0.2131915586	0.8736371071	0.4921389478	GGB29531
0.2131915586	0.8736371071	0.488522822	GGB29685
0.2131915586	0.8736371071	0.488522822	GGB30141
0.2131915586	0.8736371071	0.488522822	GGB30286
0.2131915586	0.8736371071	0.488522822	GGB30303
0.2131915586	0.8736371071	0.488522822	GGB30413
0.2131915586	0.8736371071	0.488522822	GGB30454
0.2131915586	0.9562303239	0.6111060006	GGB30455
0.2131915586	0.8736371071	0.488522822	GGB30461
0.2131915586	0.8736371071	0.488522822	GGB30461
0.2131915586	0.8736371071	0.4901262545	GGB30463
0.2131915586	0.8736371071	0.488522822	GGB30473
0.2131915586	0.8736371071	0.4921389478	GGB30475
0.2131915586	0.8736371071	0.488522822	GGB30861
0.2131915586	0.8736371071	0.488522822	GGB31312
0.2131915586	0.8736371071	0.488522822	GGB31438
0.2131915586	0.8736371071	0.488522822	GGB3171
0.2131915586	0.8736371071	0.488522822	GGB31823
0.2131915586	0.8736371071	0.488522822	GGB31853
0.2131915586	0.9825750489	0.4921389478	GGB32371
0.2131915586	0.8736371071	0.488522822	GGB3793
0.2131915586	0.8736371071	0.5197962683	GGB42598
0.2131915586	0.8736371071	0.488522822	GGB45656
0.2131915586	0.8736371071	0.488522822	GGB47127
0.2131915586	0.8736371071	0.488522822	GGB74395
0.2131915586	0.8736371071	0.488522822	GGB75053
0.2131915586	0.8736371071	0.488522822	GGB75109
0.2131915586	0.8736371071	0.488522822	GGB81440
0.2131915586	0.8736371071	0.488522822	Lachnospiraceae_unclassified
0.2144933081	0.8736371071	0.488522822	Lachnospiraceae_unclassified
0.2131915586	0.8736371071	0.488522822	Lachnospiraceae_unclassified
0.2131915586	0.8736371071	0.488522822	Lachnospiraceae_unclassified
0.2327470411	0.8736371071	0.488522822	Lachnospiraceae_unclassified
0.2131915586	0.8736371071	0.488522822	Lachnospiraceae_unclassified
0.2131915586	0.8931158046	0.5927480394	Lachnospiraceae_unclassified
0.2131915586	0.8736371071	0.488522822	Lachnospiraceae_unclassified
0.2131915586	0.8736371071	0.488522822	Lactobacillus
0.2131915586	0.8736371071	0.488522822	Muribaculaceae_unclassified
0.2131915586	0.8736371071	0.4921389478	Neglectibacter
0.2131915586	0.8736371071	0.488522822	Oscillospiraceae_unclassified
0.2144933081	0.8736371071	0.488522822	Oscillospiraceae_unclassified
0.2131915586	0.8736371071	0.488522822	Oscillospiraceae_unclassified
0.2131915586	0.8931158046	0.4921389478	Parasutterella

0.2131915586	0.9825750489	0.488522822	Romboutsia
0.2131915586	0.8736371071	0.488522822	Schaedlerella
0.2131915586	0.8736371071	0.4921389478	Turicibacter
0.2131915586	0.8736371071	0.488522822	Bacteria_unclassified
0.2131915586	0.8736371071	0.4921389478	Bacteria_unclassified
0.2131915586	0.8736371071	0.488522822	Bacteria_unclassified
0.2327470411	0.8736371071	0.488522822	
0.2131915586	0.8736371071	0.4921389478	
0.2131915586	0.8736371071	0.488522822	

Family	Order	Class	Phylum	Kingdom
Lachnospiraceae	Eubacteriales	Clostridia	Firmicutes	Bacteria
Lachnospiraceae	Eubacteriales	Clostridia	Firmicutes	Bacteria
Oscillospiraceae	Eubacteriales	Clostridia	Firmicutes	Bacteria
Oscillospiraceae	Eubacteriales	Clostridia	Firmicutes	Bacteria
Eggerthellaceae	Eggerthellales	Coriobacteriia	Actinobacteria	Bacteria
Eggerthellaceae	Eggerthellales	Coriobacteriia	Actinobacteria	Bacteria
Eggerthellaceae	Eggerthellales	Coriobacteriia	Actinobacteria	Bacteria
Akkermansiaceae	Verrucomicrobiales	Verrucomicrobiae	Verrucomicrobia	Bacteria
Rikenellaceae	Bacteroidales	Bacteroidia	Bacteroidota	Bacteria
Oscillospiraceae	Eubacteriales	Clostridia	Firmicutes	Bacteria
Bacteria_unclassified	Bacteria_unclassified	Bacteria_unclassifie	Bacteria_unclassifie	Bacteria
Bacteria_unclassified	Bacteria_unclassified	Bacteria_unclassifie	Bacteria_unclassifie	Bacteria
Bacteria_unclassified	Bacteria_unclassified	Bacteria_unclassifie	Bacteria_unclassifie	Bacteria
Bacteroidaceae	Bacteroidales	Bacteroidia	Bacteroidota	Bacteria
Bifidobacteriaceae	Bifidobacteriales	Actinomycetia	Actinobacteria	Bacteria
Clostridia_unclassified	Clostridia_unclassifie	Clostridia	Firmicutes	Bacteria
Clostridiaceae	Eubacteriales	Clostridia	Firmicutes	Bacteria
Clostridiaceae	Eubacteriales	Clostridia	Firmicutes	Bacteria
Eubacteriales_unclassified	Eubacteriales	Clostridia	Firmicutes	Bacteria
Erysipelotrichaceae	Erysipelotrichales	Erysipelotrichia	Firmicutes	Bacteria
Coriobacteriaceae	Coriobacteriales	Coriobacteriia	Actinobacteria	Bacteria
Lachnospiraceae	Eubacteriales	Clostridia	Firmicutes	Bacteria
Erysipelotrichaceae	Erysipelotrichales	Erysipelotrichia	Firmicutes	Bacteria
Erysipelotrichales_unclassifie	Erysipelotrichales	Erysipelotrichia	Firmicutes	Bacteria
Eubacteriaceae	Eubacteriales	Clostridia	Firmicutes	Bacteria
Eubacteriaceae	Eubacteriales	Clostridia	Firmicutes	Bacteria
Lachnospiraceae	Eubacteriales	Clostridia	Firmicutes	Bacteria
Eggerthellaceae	Eggerthellales	Coriobacteriia	Actinobacteria	Bacteria
Lachnospiraceae	Eubacteriales	Clostridia	Firmicutes	Bacteria
Muribaculaceae	Bacteroidales	Bacteroidia	Bacteroidota	Bacteria
Muribaculaceae	Bacteroidales	Bacteroidia	Bacteroidota	Bacteria
Muribaculaceae	Bacteroidales	Bacteroidia	Bacteroidota	Bacteria
FGB9508	OFGB9508	CFGB9508	Firmicutes	Bacteria
FGB2838	OFGB2838	CFGB2838	Firmicutes	Bacteria
FGB2838	OFGB2838	CFGB2838	Firmicutes	Bacteria
FGB2838	OFGB2838	CFGB2838	Firmicutes	Bacteria
Pumilibacteraceae	Eubacteriales	Clostridia	Firmicutes	Bacteria
FGB28439	OFGB28439	CFGB28439	Firmicutes	Bacteria
Clostridia_unclassified	Clostridia_unclassifie	Clostridia	Firmicutes	Bacteria
Eubacteriaceae	Eubacteriales	Clostridia	Firmicutes	Bacteria
Lachnospiraceae	Eubacteriales	Clostridia	Firmicutes	Bacteria
Lachnospiraceae	Eubacteriales	Clostridia	Firmicutes	Bacteria
Lachnospiraceae	Eubacteriales	Clostridia	Firmicutes	Bacteria
Lachnospiraceae	Eubacteriales	Clostridia	Firmicutes	Bacteria
FGB77305	OFGB77305	CFGB77305	Firmicutes	Bacteria

Clostridiaceae	Eubacteriales	Clostridia	Firmicutes	Bacteria
Lachnospiraceae	Eubacteriales	Clostridia	Firmicutes	Bacteria
Lachnospiraceae	Eubacteriales	Clostridia	Firmicutes	Bacteria
Lachnospiraceae	Eubacteriales	Clostridia	Firmicutes	Bacteria
FGB9633	OFGB9633	CFGB9633	Firmicutes	Bacteria
Bacteria_unclassified	Bacteria_unclassified	Bacteria_unclassifie	Bacteria_unclassifie	Bacteria
Bacteria_unclassified	Bacteria_unclassified	Bacteria_unclassifie	Bacteria_unclassifie	Bacteria
Bacteria_unclassified	Bacteria_unclassified	Bacteria_unclassifie	Bacteria_unclassifie	Bacteria
Bacteria_unclassified	Bacteria_unclassified	Bacteria_unclassifie	Bacteria_unclassifie	Bacteria
Lachnospiraceae	Eubacteriales	Clostridia	Firmicutes	Bacteria
Lachnospiraceae	Eubacteriales	Clostridia	Firmicutes	Bacteria
Lachnospiraceae	Eubacteriales	Clostridia	Firmicutes	Bacteria
FGB77359	OFGB77359	CFGB77359	Bacteria_unclassifie	Bacteria
FGB9639	OFGB9639	CFGB9639	Firmicutes	Bacteria
Lachnospiraceae	Eubacteriales	Clostridia	Firmicutes	Bacteria
Lachnospiraceae	Eubacteriales	Clostridia	Firmicutes	Bacteria
Lachnospiraceae	Eubacteriales	Clostridia	Firmicutes	Bacteria
Clostridiaceae	Eubacteriales	Clostridia	Firmicutes	Bacteria
Clostridiaceae	Eubacteriales	Clostridia	Firmicutes	Bacteria
Clostridiaceae	Eubacteriales	Clostridia	Firmicutes	Bacteria
Clostridiaceae	Eubacteriales	Clostridia	Firmicutes	Bacteria
Clostridiaceae	Eubacteriales	Clostridia	Firmicutes	Bacteria
Clostridiaceae	Eubacteriales	Clostridia	Firmicutes	Bacteria
Clostridiaceae	Eubacteriales	Clostridia	Firmicutes	Bacteria
Eubacteriaceae	Eubacteriales	Clostridia	Firmicutes	Bacteria
FGB9658	OFGB9658	CFGB9658	Firmicutes	Bacteria
FGB9659	OFGB9659	CFGB9659	Firmicutes	Bacteria
Bacteria_unclassified	Bacteria_unclassified	Bacteria_unclassifie	Bacteria_unclassifie	Bacteria
FGB9827	OFGB9827	CFGB9827	Firmicutes	Bacteria
Eubacteriaceae	Eubacteriales	Clostridia	Firmicutes	Bacteria
FGB77303	OFGB77303	CFGB77303	Bacteria_unclassifie	Bacteria
Eubacteriales_unclassified	Eubacteriales	Clostridia	Firmicutes	Bacteria
Oscillospiraceae	Eubacteriales	Clostridia	Firmicutes	Bacteria
FGB30328	OFGB30328	CFGB30328	Firmicutes	Bacteria
Oscillospiraceae	Eubacteriales	Clostridia	Firmicutes	Bacteria
Oscillospiraceae	Eubacteriales	Clostridia	Firmicutes	Bacteria
Oscillospiraceae	Eubacteriales	Clostridia	Firmicutes	Bacteria
Oscillospiraceae	Eubacteriales	Clostridia	Firmicutes	Bacteria
Oscillospiraceae	Eubacteriales	Clostridia	Firmicutes	Bacteria
Oscillospiraceae	Eubacteriales	Clostridia	Firmicutes	Bacteria
Oscillospiraceae	Eubacteriales	Clostridia	Firmicutes	Bacteria
FGB77153	OFGB77153	CFGB77153	Actinobacteria	Bacteria
FGB1791	OFGB1791	CFGB1791	Tenericutes	Bacteria
FGB10290	OFGB10290	CFGB10290	Firmicutes	Bacteria
Oscillospiraceae	Eubacteriales	Clostridia	Firmicutes	Bacteria
FGB1765	OFGB1765	CFGB1765	Firmicutes	Bacteria

FGB10349	OFGB10349	CFGB10349	Firmicutes	Bacteria
FGB10667	OFGB10667	CFGB10667	Firmicutes	Bacteria
Lachnospiraceae	Eubacteriales	Clostridia	Firmicutes	Bacteria
Lachnospiraceae	Eubacteriales	Clostridia	Firmicutes	Bacteria
Christensenellaceae	Eubacteriales	Clostridia	Firmicutes	Bacteria
FGB10299	OFGB10299	CFGB10299	Firmicutes	Bacteria
Oscillospiraceae	Eubacteriales	Clostridia	Firmicutes	Bacteria
Oscillospiraceae	Eubacteriales	Clostridia	Firmicutes	Bacteria
Lachnospiraceae	Eubacteriales	Clostridia	Firmicutes	Bacteria
Clostridia_unclassified	Clostridia_unclassifie	Clostridia	Firmicutes	Bacteria
Lachnospiraceae	Eubacteriales	Clostridia	Firmicutes	Bacteria
Lachnospiraceae	Eubacteriales	Clostridia	Firmicutes	Bacteria
Lachnospiraceae	Eubacteriales	Clostridia	Firmicutes	Bacteria
Lachnospiraceae	Eubacteriales	Clostridia	Firmicutes	Bacteria
Lachnospiraceae	Eubacteriales	Clostridia	Firmicutes	Bacteria
Lachnospiraceae	Eubacteriales	Clostridia	Firmicutes	Bacteria
Lachnospiraceae	Eubacteriales	Clostridia	Firmicutes	Bacteria
Lachnospiraceae	Eubacteriales	Clostridia	Firmicutes	Bacteria
Lactobacillaceae	Lactobacillales	Bacilli	Firmicutes	Bacteria
Muribaculaceae	Bacteroidales	Bacteroidia	Bacteroidota	Bacteria
Oscillospiraceae	Eubacteriales	Clostridia	Firmicutes	Bacteria
Oscillospiraceae	Eubacteriales	Clostridia	Firmicutes	Bacteria
Oscillospiraceae	Eubacteriales	Clostridia	Firmicutes	Bacteria
Oscillospiraceae	Eubacteriales	Clostridia	Firmicutes	Bacteria
Sutterellaceae	Burkholderiales	Betaproteobacteria	Proteobacteria	Bacteria
Peptostreptococcaceae	Eubacteriales	Clostridia	Firmicutes	Bacteria
Lachnospiraceae	Eubacteriales	Clostridia	Firmicutes	Bacteria
Turicibacteraceae	Erysipelotrichales	Erysipelotrichia	Firmicutes	Bacteria
Bacteria_unclassified	Bacteria_unclassified	Bacteria_unclassifie	Bacteria_unclassifie	Bacteria
Bacteria_unclassified	Bacteria_unclassified	Bacteria_unclassifie	Bacteria_unclassifie	Bacteria
Bacteria_unclassified	Bacteria_unclassified	Bacteria_unclassifie	Bacteria_unclassifie	Bacteria

Lachnospiraceae	Eubacteriales	Clostridia	Firmicutes	Bacteria
Lachnospiraceae	Eubacteriales	Clostridia	Firmicutes	Bacteria
Oscillospiraceae	Eubacteriales	Clostridia	Firmicutes	Bacteria
Oscillospiraceae	Eubacteriales	Clostridia	Firmicutes	Bacteria
Eggerthellaceae	Eggerthellales	Coriobacteriia	Actinobacteria	Bacteria
Eggerthellaceae	Eggerthellales	Coriobacteriia	Actinobacteria	Bacteria
Eggerthellaceae	Eggerthellales	Coriobacteriia	Actinobacteria	Bacteria
Akkermansiaceae	Verrucomicrobiales	Verrucomicrobiae	Verrucomicrobia	Bacteria
Rikenellaceae	Bacteroidales	Bacteroidia	Bacteroidota	Bacteria
Oscillospiraceae	Eubacteriales	Clostridia	Firmicutes	Bacteria
Bacteria_unclassified	Bacteria_unclassified	Bacteria_unclassifie	Bacteria_unclassifie	Bacteria
Bacteria_unclassified	Bacteria_unclassified	Bacteria_unclassifie	Bacteria_unclassifie	Bacteria

Bacteria_unclassified	Bacteria_unclassified	Bacteria_unclassifie	Bacteria_unclassifie	Bacteria
Bacteroidaceae	Bacteroidales	Bacteroidia	Bacteroidota	Bacteria
Bifidobacteriaceae	Bifidobacteriales	Actinomycetia	Actinobacteria	Bacteria
Clostridia_unclassified	Clostridia_unclassifie	Clostridia	Firmicutes	Bacteria
Clostridiaceae	Eubacteriales	Clostridia	Firmicutes	Bacteria
Clostridiaceae	Eubacteriales	Clostridia	Firmicutes	Bacteria
Eubacteriales_unclassified	Eubacteriales	Clostridia	Firmicutes	Bacteria
Erysipelotrichaceae	Erysipelotrichales	Erysipelotrichia	Firmicutes	Bacteria
Coriobacteriaceae	Coriobacteriales	Coriobacteriia	Actinobacteria	Bacteria
Lachnospiraceae	Eubacteriales	Clostridia	Firmicutes	Bacteria
Erysipelotrichaceae	Erysipelotrichales	Erysipelotrichia	Firmicutes	Bacteria
Erysipelotrichales_unclassifie	Erysipelotrichales	Erysipelotrichia	Firmicutes	Bacteria
Eubacteriaceae	Eubacteriales	Clostridia	Firmicutes	Bacteria
Eubacteriaceae	Eubacteriales	Clostridia	Firmicutes	Bacteria
Lachnospiraceae	Eubacteriales	Clostridia	Firmicutes	Bacteria
Eggerthellaceae	Eggerthellales	Coriobacteriia	Actinobacteria	Bacteria
Lachnospiraceae	Eubacteriales	Clostridia	Firmicutes	Bacteria
Muribaculaceae	Bacteroidales	Bacteroidia	Bacteroidota	Bacteria
Muribaculaceae	Bacteroidales	Bacteroidia	Bacteroidota	Bacteria
Muribaculaceae	Bacteroidales	Bacteroidia	Bacteroidota	Bacteria
FGB9508	OFGB9508	CFGB9508	Firmicutes	Bacteria
FGB2838	OFGB2838	CFGB2838	Firmicutes	Bacteria
FGB2838	OFGB2838	CFGB2838	Firmicutes	Bacteria
FGB2838	OFGB2838	CFGB2838	Firmicutes	Bacteria
Pumilibacteraceae	Eubacteriales	Clostridia	Firmicutes	Bacteria
FGB28439	OFGB28439	CFGB28439	Firmicutes	Bacteria
Clostridia_unclassified	Clostridia_unclassifie	Clostridia	Firmicutes	Bacteria
Eubacteriaceae	Eubacteriales	Clostridia	Firmicutes	Bacteria
Lachnospiraceae	Eubacteriales	Clostridia	Firmicutes	Bacteria
Lachnospiraceae	Eubacteriales	Clostridia	Firmicutes	Bacteria
Lachnospiraceae	Eubacteriales	Clostridia	Firmicutes	Bacteria
Lachnospiraceae	Eubacteriales	Clostridia	Firmicutes	Bacteria
FGB77305	OFGB77305	CFGB77305	Firmicutes	Bacteria
Clostridiaceae	Eubacteriales	Clostridia	Firmicutes	Bacteria
Lachnospiraceae	Eubacteriales	Clostridia	Firmicutes	Bacteria
Lachnospiraceae	Eubacteriales	Clostridia	Firmicutes	Bacteria
Lachnospiraceae	Eubacteriales	Clostridia	Firmicutes	Bacteria
FGB9633	OFGB9633	CFGB9633	Firmicutes	Bacteria
Bacteria_unclassified	Bacteria_unclassified	Bacteria_unclassifie	Bacteria_unclassifie	Bacteria
Bacteria_unclassified	Bacteria_unclassified	Bacteria_unclassifie	Bacteria_unclassifie	Bacteria
Bacteria_unclassified	Bacteria_unclassified	Bacteria_unclassifie	Bacteria_unclassifie	Bacteria
Bacteria_unclassified	Bacteria_unclassified	Bacteria_unclassifie	Bacteria_unclassifie	Bacteria
Lachnospiraceae	Eubacteriales	Clostridia	Firmicutes	Bacteria
Lachnospiraceae	Eubacteriales	Clostridia	Firmicutes	Bacteria
Lachnospiraceae	Eubacteriales	Clostridia	Firmicutes	Bacteria
FGB77359	OFGB77359	CFGB77359	Bacteria_unclassifie	Bacteria

FGB9639	OFGB9639	CFGB9639	Firmicutes	Bacteria
Lachnospiraceae	Eubacteriales	Clostridia	Firmicutes	Bacteria
Lachnospiraceae	Eubacteriales	Clostridia	Firmicutes	Bacteria
Lachnospiraceae	Eubacteriales	Clostridia	Firmicutes	Bacteria
Clostridiaceae	Eubacteriales	Clostridia	Firmicutes	Bacteria
Clostridiaceae	Eubacteriales	Clostridia	Firmicutes	Bacteria
Clostridiaceae	Eubacteriales	Clostridia	Firmicutes	Bacteria
Clostridiaceae	Eubacteriales	Clostridia	Firmicutes	Bacteria
Clostridiaceae	Eubacteriales	Clostridia	Firmicutes	Bacteria
Clostridiaceae	Eubacteriales	Clostridia	Firmicutes	Bacteria
Clostridiaceae	Eubacteriales	Clostridia	Firmicutes	Bacteria
Eubacteriaceae	Eubacteriales	Clostridia	Firmicutes	Bacteria
FGB9658	OFGB9658	CFGB9658	Firmicutes	Bacteria
FGB9659	OFGB9659	CFGB9659	Firmicutes	Bacteria
Bacteria_unclassified	Bacteria_unclassified	Bacteria_unclassified	Bacteria_unclassified	Bacteria
FGB9827	OFGB9827	CFGB9827	Firmicutes	Bacteria
Eubacteriaceae	Eubacteriales	Clostridia	Firmicutes	Bacteria
FGB77303	OFGB77303	CFGB77303	Bacteria_unclassified	Bacteria
Eubacteriales_unclassified	Eubacteriales	Clostridia	Firmicutes	Bacteria
Oscillospiraceae	Eubacteriales	Clostridia	Firmicutes	Bacteria
FGB30328	OFGB30328	CFGB30328	Firmicutes	Bacteria
Oscillospiraceae	Eubacteriales	Clostridia	Firmicutes	Bacteria
Oscillospiraceae	Eubacteriales	Clostridia	Firmicutes	Bacteria
Oscillospiraceae	Eubacteriales	Clostridia	Firmicutes	Bacteria
Oscillospiraceae	Eubacteriales	Clostridia	Firmicutes	Bacteria
Oscillospiraceae	Eubacteriales	Clostridia	Firmicutes	Bacteria
Oscillospiraceae	Eubacteriales	Clostridia	Firmicutes	Bacteria
Oscillospiraceae	Eubacteriales	Clostridia	Firmicutes	Bacteria
FGB77153	OFGB77153	CFGB77153	Actinobacteria	Bacteria
FGB1791	OFGB1791	CFGB1791	Tenericutes	Bacteria
FGB10290	OFGB10290	CFGB10290	Firmicutes	Bacteria
Oscillospiraceae	Eubacteriales	Clostridia	Firmicutes	Bacteria
FGB1765	OFGB1765	CFGB1765	Firmicutes	Bacteria
FGB10349	OFGB10349	CFGB10349	Firmicutes	Bacteria
FGB10667	OFGB10667	CFGB10667	Firmicutes	Bacteria
Lachnospiraceae	Eubacteriales	Clostridia	Firmicutes	Bacteria
Lachnospiraceae	Eubacteriales	Clostridia	Firmicutes	Bacteria
Christensenellaceae	Eubacteriales	Clostridia	Firmicutes	Bacteria
FGB10299	OFGB10299	CFGB10299	Firmicutes	Bacteria
Oscillospiraceae	Eubacteriales	Clostridia	Firmicutes	Bacteria
Oscillospiraceae	Eubacteriales	Clostridia	Firmicutes	Bacteria
Lachnospiraceae	Eubacteriales	Clostridia	Firmicutes	Bacteria
Clostridia_unclassified	Clostridia_unclassified	Clostridia	Firmicutes	Bacteria
Lachnospiraceae	Eubacteriales	Clostridia	Firmicutes	Bacteria
Lachnospiraceae	Eubacteriales	Clostridia	Firmicutes	Bacteria
Lachnospiraceae	Eubacteriales	Clostridia	Firmicutes	Bacteria

Lachnospiraceae	Eubacteriales	Clostridia	Firmicutes	Bacteria
Lachnospiraceae	Eubacteriales	Clostridia	Firmicutes	Bacteria
Lachnospiraceae	Eubacteriales	Clostridia	Firmicutes	Bacteria
Lachnospiraceae	Eubacteriales	Clostridia	Firmicutes	Bacteria
Lachnospiraceae	Eubacteriales	Clostridia	Firmicutes	Bacteria
Lactobacillaceae	Lactobacillales	Bacilli	Firmicutes	Bacteria
Muribaculaceae	Bacteroidales	Bacteroidia	Bacteroidota	Bacteria
Oscillospiraceae	Eubacteriales	Clostridia	Firmicutes	Bacteria
Oscillospiraceae	Eubacteriales	Clostridia	Firmicutes	Bacteria
Oscillospiraceae	Eubacteriales	Clostridia	Firmicutes	Bacteria
Oscillospiraceae	Eubacteriales	Clostridia	Firmicutes	Bacteria
Sutterellaceae	Burkholderiales	Betaproteobacteria	Proteobacteria	Bacteria
Peptostreptococcaceae	Eubacteriales	Clostridia	Firmicutes	Bacteria
Lachnospiraceae	Eubacteriales	Clostridia	Firmicutes	Bacteria
Turicibacteraceae	Erysipelotrichales	Erysipelotrichia	Firmicutes	Bacteria
Bacteria_unclassified	Bacteria_unclassified	Bacteria_unclassifie	Bacteria_unclassifie	Bacteria
Bacteria_unclassified	Bacteria_unclassified	Bacteria_unclassifie	Bacteria_unclassifie	Bacteria
Bacteria_unclassified	Bacteria_unclassified	Bacteria_unclassifie	Bacteria_unclassifie	Bacteria

Lachnospiraceae	Eubacteriales	Clostridia	Firmicutes	Bacteria
Lachnospiraceae	Eubacteriales	Clostridia	Firmicutes	Bacteria
Oscillospiraceae	Eubacteriales	Clostridia	Firmicutes	Bacteria
Oscillospiraceae	Eubacteriales	Clostridia	Firmicutes	Bacteria
Eggerthellaceae	Eggerthellales	Coriobacteriia	Actinobacteria	Bacteria
Eggerthellaceae	Eggerthellales	Coriobacteriia	Actinobacteria	Bacteria
Eggerthellaceae	Eggerthellales	Coriobacteriia	Actinobacteria	Bacteria
Akkermansiaceae	Verrucomicrobiales	Verrucomicrobiae	Verrucomicrobia	Bacteria
Rikenellaceae	Bacteroidales	Bacteroidia	Bacteroidota	Bacteria
Oscillospiraceae	Eubacteriales	Clostridia	Firmicutes	Bacteria
Bacteria_unclassified	Bacteria_unclassified	Bacteria_unclassifie	Bacteria_unclassifie	Bacteria
Bacteria_unclassified	Bacteria_unclassified	Bacteria_unclassifie	Bacteria_unclassifie	Bacteria
Bacteria_unclassified	Bacteria_unclassified	Bacteria_unclassifie	Bacteria_unclassifie	Bacteria
Bacteroidaceae	Bacteroidales	Bacteroidia	Bacteroidota	Bacteria
Bifidobacteriaceae	Bifidobacteriales	Actinomycetia	Actinobacteria	Bacteria
Clostridia_unclassified	Clostridia_unclassifie	Clostridia	Firmicutes	Bacteria
Clostridiaceae	Eubacteriales	Clostridia	Firmicutes	Bacteria
Clostridiaceae	Eubacteriales	Clostridia	Firmicutes	Bacteria
Eubacteriales_unclassified	Eubacteriales	Clostridia	Firmicutes	Bacteria
Erysipelotrichaceae	Erysipelotrichales	Erysipelotrichia	Firmicutes	Bacteria
Coriobacteriaceae	Coriobacteriales	Coriobacteriia	Actinobacteria	Bacteria
Lachnospiraceae	Eubacteriales	Clostridia	Firmicutes	Bacteria
Erysipelotrichaceae	Erysipelotrichales	Erysipelotrichia	Firmicutes	Bacteria
Erysipelotrichales_unclassifie	Erysipelotrichales	Erysipelotrichia	Firmicutes	Bacteria
Eubacteriaceae	Eubacteriales	Clostridia	Firmicutes	Bacteria

Eubacteriaceae	Eubacteriales	Clostridia	Firmicutes	Bacteria
Lachnospiraceae	Eubacteriales	Clostridia	Firmicutes	Bacteria
Eggerthellaceae	Eggerthellales	Coriobacteriia	Actinobacteria	Bacteria
Lachnospiraceae	Eubacteriales	Clostridia	Firmicutes	Bacteria
Muribaculaceae	Bacteroidales	Bacteroidia	Bacteroidota	Bacteria
Muribaculaceae	Bacteroidales	Bacteroidia	Bacteroidota	Bacteria
Muribaculaceae	Bacteroidales	Bacteroidia	Bacteroidota	Bacteria
FGB9508	OFGB9508	CFGB9508	Firmicutes	Bacteria
FGB2838	OFGB2838	CFGB2838	Firmicutes	Bacteria
FGB2838	OFGB2838	CFGB2838	Firmicutes	Bacteria
FGB2838	OFGB2838	CFGB2838	Firmicutes	Bacteria
Pumilibacteraceae	Eubacteriales	Clostridia	Firmicutes	Bacteria
FGB28439	OFGB28439	CFGB28439	Firmicutes	Bacteria
Clostridia_unclassified	Clostridia_unclassifie	Clostridia	Firmicutes	Bacteria
Eubacteriaceae	Eubacteriales	Clostridia	Firmicutes	Bacteria
Lachnospiraceae	Eubacteriales	Clostridia	Firmicutes	Bacteria
Lachnospiraceae	Eubacteriales	Clostridia	Firmicutes	Bacteria
Lachnospiraceae	Eubacteriales	Clostridia	Firmicutes	Bacteria
Lachnospiraceae	Eubacteriales	Clostridia	Firmicutes	Bacteria
FGB77305	OFGB77305	CFGB77305	Firmicutes	Bacteria
Clostridiaceae	Eubacteriales	Clostridia	Firmicutes	Bacteria
Lachnospiraceae	Eubacteriales	Clostridia	Firmicutes	Bacteria
Lachnospiraceae	Eubacteriales	Clostridia	Firmicutes	Bacteria
Lachnospiraceae	Eubacteriales	Clostridia	Firmicutes	Bacteria
FGB9633	OFGB9633	CFGB9633	Firmicutes	Bacteria
Bacteria_unclassified	Bacteria_unclassified	Bacteria_unclassifie	Bacteria_unclassifie	Bacteria
Bacteria_unclassified	Bacteria_unclassified	Bacteria_unclassifie	Bacteria_unclassifie	Bacteria
Bacteria_unclassified	Bacteria_unclassified	Bacteria_unclassifie	Bacteria_unclassifie	Bacteria
Bacteria_unclassified	Bacteria_unclassified	Bacteria_unclassifie	Bacteria_unclassifie	Bacteria
Lachnospiraceae	Eubacteriales	Clostridia	Firmicutes	Bacteria
Lachnospiraceae	Eubacteriales	Clostridia	Firmicutes	Bacteria
Lachnospiraceae	Eubacteriales	Clostridia	Firmicutes	Bacteria
FGB77359	OFGB77359	CFGB77359	Bacteria_unclassifie	Bacteria
FGB9639	OFGB9639	CFGB9639	Firmicutes	Bacteria
Lachnospiraceae	Eubacteriales	Clostridia	Firmicutes	Bacteria
Lachnospiraceae	Eubacteriales	Clostridia	Firmicutes	Bacteria
Lachnospiraceae	Eubacteriales	Clostridia	Firmicutes	Bacteria
Clostridiaceae	Eubacteriales	Clostridia	Firmicutes	Bacteria
Clostridiaceae	Eubacteriales	Clostridia	Firmicutes	Bacteria
Clostridiaceae	Eubacteriales	Clostridia	Firmicutes	Bacteria
Clostridiaceae	Eubacteriales	Clostridia	Firmicutes	Bacteria
Clostridiaceae	Eubacteriales	Clostridia	Firmicutes	Bacteria
Clostridiaceae	Eubacteriales	Clostridia	Firmicutes	Bacteria
Clostridiaceae	Eubacteriales	Clostridia	Firmicutes	Bacteria
Eubacteriaceae	Eubacteriales	Clostridia	Firmicutes	Bacteria
FGB9658	OFGB9658	CFGB9658	Firmicutes	Bacteria

FGB9659	OFGB9659	CFGB9659	Firmicutes	Bacteria
Bacteria_unclassified	Bacteria_unclassified	Bacteria_unclassifie	Bacteria_unclassifie	Bacteria
FGB9827	OFGB9827	CFGB9827	Firmicutes	Bacteria
Eubacteriaceae	Eubacteriales	Clostridia	Firmicutes	Bacteria
FGB77303	OFGB77303	CFGB77303	Bacteria_unclassifie	Bacteria
Eubacteriales_unclassified	Eubacteriales	Clostridia	Firmicutes	Bacteria
Oscillospiraceae	Eubacteriales	Clostridia	Firmicutes	Bacteria
FGB30328	OFGB30328	CFGB30328	Firmicutes	Bacteria
Oscillospiraceae	Eubacteriales	Clostridia	Firmicutes	Bacteria
Oscillospiraceae	Eubacteriales	Clostridia	Firmicutes	Bacteria
Oscillospiraceae	Eubacteriales	Clostridia	Firmicutes	Bacteria
Oscillospiraceae	Eubacteriales	Clostridia	Firmicutes	Bacteria
Oscillospiraceae	Eubacteriales	Clostridia	Firmicutes	Bacteria
Oscillospiraceae	Eubacteriales	Clostridia	Firmicutes	Bacteria
Oscillospiraceae	Eubacteriales	Clostridia	Firmicutes	Bacteria
FGB77153	OFGB77153	CFGB77153	Actinobacteria	Bacteria
FGB1791	OFGB1791	CFGB1791	Tenericutes	Bacteria
FGB10290	OFGB10290	CFGB10290	Firmicutes	Bacteria
Oscillospiraceae	Eubacteriales	Clostridia	Firmicutes	Bacteria
FGB1765	OFGB1765	CFGB1765	Firmicutes	Bacteria
FGB10349	OFGB10349	CFGB10349	Firmicutes	Bacteria
FGB10667	OFGB10667	CFGB10667	Firmicutes	Bacteria
Lachnospiraceae	Eubacteriales	Clostridia	Firmicutes	Bacteria
Lachnospiraceae	Eubacteriales	Clostridia	Firmicutes	Bacteria
Christensenellaceae	Eubacteriales	Clostridia	Firmicutes	Bacteria
FGB10299	OFGB10299	CFGB10299	Firmicutes	Bacteria
Oscillospiraceae	Eubacteriales	Clostridia	Firmicutes	Bacteria
Oscillospiraceae	Eubacteriales	Clostridia	Firmicutes	Bacteria
Lachnospiraceae	Eubacteriales	Clostridia	Firmicutes	Bacteria
Clostridia_unclassified	Clostridia_unclassifie	Clostridia	Firmicutes	Bacteria
Lachnospiraceae	Eubacteriales	Clostridia	Firmicutes	Bacteria
Lachnospiraceae	Eubacteriales	Clostridia	Firmicutes	Bacteria
Lachnospiraceae	Eubacteriales	Clostridia	Firmicutes	Bacteria
Lachnospiraceae	Eubacteriales	Clostridia	Firmicutes	Bacteria
Lachnospiraceae	Eubacteriales	Clostridia	Firmicutes	Bacteria
Lachnospiraceae	Eubacteriales	Clostridia	Firmicutes	Bacteria
Lachnospiraceae	Eubacteriales	Clostridia	Firmicutes	Bacteria
Lachnospiraceae	Eubacteriales	Clostridia	Firmicutes	Bacteria
Lactobacillaceae	Lactobacillales	Bacilli	Firmicutes	Bacteria
Muribaculaceae	Bacteroidales	Bacteroidia	Bacteroidota	Bacteria
Oscillospiraceae	Eubacteriales	Clostridia	Firmicutes	Bacteria
Oscillospiraceae	Eubacteriales	Clostridia	Firmicutes	Bacteria
Oscillospiraceae	Eubacteriales	Clostridia	Firmicutes	Bacteria
Oscillospiraceae	Eubacteriales	Clostridia	Firmicutes	Bacteria
Sutterellaceae	Burkholderiales	Betaproteobacteria	Proteobacteria	Bacteria
Peptostreptococcaceae	Eubacteriales	Clostridia	Firmicutes	Bacteria

Lachnospiraceae	Eubacteriales	Clostridia	Firmicutes	Bacteria
Turicibacteraceae	Erysipelotrichales	Erysipelotrichia	Firmicutes	Bacteria
Bacteria_unclassified	Bacteria_unclassified	Bacteria_unclassifie	Bacteria_unclassifie	Bacteria
Bacteria_unclassified	Bacteria_unclassified	Bacteria_unclassifie	Bacteria_unclassifie	Bacteria
Bacteria_unclassified	Bacteria_unclassified	Bacteria_unclassifie	Bacteria_unclassifie	Bacteria

Lachnospiraceae	Eubacteriales	Clostridia	Firmicutes	Bacteria
Lachnospiraceae	Eubacteriales	Clostridia	Firmicutes	Bacteria
Oscillospiraceae	Eubacteriales	Clostridia	Firmicutes	Bacteria
Oscillospiraceae	Eubacteriales	Clostridia	Firmicutes	Bacteria
Eggerthellaceae	Eggerthellales	Coriobacteriia	Actinobacteria	Bacteria
Eggerthellaceae	Eggerthellales	Coriobacteriia	Actinobacteria	Bacteria
Eggerthellaceae	Eggerthellales	Coriobacteriia	Actinobacteria	Bacteria
Akkermansiaceae	Verrucomicrobiales	Verrucomicrobiae	Verrucomicrobia	Bacteria
Rikenellaceae	Bacteroidales	Bacteroidia	Bacteroidota	Bacteria
Oscillospiraceae	Eubacteriales	Clostridia	Firmicutes	Bacteria
Bacteria_unclassified	Bacteria_unclassified	Bacteria_unclassifie	Bacteria_unclassifie	Bacteria
Bacteria_unclassified	Bacteria_unclassified	Bacteria_unclassifie	Bacteria_unclassifie	Bacteria
Bacteria_unclassified	Bacteria_unclassified	Bacteria_unclassifie	Bacteria_unclassifie	Bacteria
Bacteroidaceae	Bacteroidales	Bacteroidia	Bacteroidota	Bacteria
Bifidobacteriaceae	Bifidobacteriales	Actinomycetia	Actinobacteria	Bacteria
Clostridia_unclassified	Clostridia_unclassifie	Clostridia	Firmicutes	Bacteria
Clostridiaceae	Eubacteriales	Clostridia	Firmicutes	Bacteria
Clostridiaceae	Eubacteriales	Clostridia	Firmicutes	Bacteria
Eubacteriales_unclassified	Eubacteriales	Clostridia	Firmicutes	Bacteria
Erysipelotrichaceae	Erysipelotrichales	Erysipelotrichia	Firmicutes	Bacteria
Coriobacteriaceae	Coriobacteriales	Coriobacteriia	Actinobacteria	Bacteria
Lachnospiraceae	Eubacteriales	Clostridia	Firmicutes	Bacteria
Erysipelotrichaceae	Erysipelotrichales	Erysipelotrichia	Firmicutes	Bacteria
Erysipelotrichales_unclassifie	Erysipelotrichales	Erysipelotrichia	Firmicutes	Bacteria
Eubacteriaceae	Eubacteriales	Clostridia	Firmicutes	Bacteria
Eubacteriaceae	Eubacteriales	Clostridia	Firmicutes	Bacteria
Lachnospiraceae	Eubacteriales	Clostridia	Firmicutes	Bacteria
Eggerthellaceae	Eggerthellales	Coriobacteriia	Actinobacteria	Bacteria
Lachnospiraceae	Eubacteriales	Clostridia	Firmicutes	Bacteria
Muribaculaceae	Bacteroidales	Bacteroidia	Bacteroidota	Bacteria
Muribaculaceae	Bacteroidales	Bacteroidia	Bacteroidota	Bacteria
Muribaculaceae	Bacteroidales	Bacteroidia	Bacteroidota	Bacteria
FGB9508	OFGB9508	CFGB9508	Firmicutes	Bacteria
FGB2838	OFGB2838	CFGB2838	Firmicutes	Bacteria
FGB2838	OFGB2838	CFGB2838	Firmicutes	Bacteria
FGB2838	OFGB2838	CFGB2838	Firmicutes	Bacteria
Pumilibacteraceae	Eubacteriales	Clostridia	Firmicutes	Bacteria
FGB28439	OFGB28439	CFGB28439	Firmicutes	Bacteria

Clostridia_unclassified	Clostridia_unclassifie	Clostridia	Firmicutes	Bacteria
Eubacteriaceae	Eubacteriales	Clostridia	Firmicutes	Bacteria
Lachnospiraceae	Eubacteriales	Clostridia	Firmicutes	Bacteria
Lachnospiraceae	Eubacteriales	Clostridia	Firmicutes	Bacteria
Lachnospiraceae	Eubacteriales	Clostridia	Firmicutes	Bacteria
Lachnospiraceae	Eubacteriales	Clostridia	Firmicutes	Bacteria
FGB77305	OFGB77305	CFGB77305	Firmicutes	Bacteria
Clostridiaceae	Eubacteriales	Clostridia	Firmicutes	Bacteria
Lachnospiraceae	Eubacteriales	Clostridia	Firmicutes	Bacteria
Lachnospiraceae	Eubacteriales	Clostridia	Firmicutes	Bacteria
Lachnospiraceae	Eubacteriales	Clostridia	Firmicutes	Bacteria
FGB9633	OFGB9633	CFGB9633	Firmicutes	Bacteria
Bacteria_unclassified	Bacteria_unclassified	Bacteria_unclassifie	Bacteria_unclassifie	Bacteria
Bacteria_unclassified	Bacteria_unclassified	Bacteria_unclassifie	Bacteria_unclassifie	Bacteria
Bacteria_unclassified	Bacteria_unclassified	Bacteria_unclassifie	Bacteria_unclassifie	Bacteria
Bacteria_unclassified	Bacteria_unclassified	Bacteria_unclassifie	Bacteria_unclassifie	Bacteria
Lachnospiraceae	Eubacteriales	Clostridia	Firmicutes	Bacteria
Lachnospiraceae	Eubacteriales	Clostridia	Firmicutes	Bacteria
Lachnospiraceae	Eubacteriales	Clostridia	Firmicutes	Bacteria
FGB77359	OFGB77359	CFGB77359	Bacteria_unclassifie	Bacteria
FGB9639	OFGB9639	CFGB9639	Firmicutes	Bacteria
Lachnospiraceae	Eubacteriales	Clostridia	Firmicutes	Bacteria
Lachnospiraceae	Eubacteriales	Clostridia	Firmicutes	Bacteria
Lachnospiraceae	Eubacteriales	Clostridia	Firmicutes	Bacteria
Clostridiaceae	Eubacteriales	Clostridia	Firmicutes	Bacteria
Clostridiaceae	Eubacteriales	Clostridia	Firmicutes	Bacteria
Clostridiaceae	Eubacteriales	Clostridia	Firmicutes	Bacteria
Clostridiaceae	Eubacteriales	Clostridia	Firmicutes	Bacteria
Clostridiaceae	Eubacteriales	Clostridia	Firmicutes	Bacteria
Clostridiaceae	Eubacteriales	Clostridia	Firmicutes	Bacteria
Clostridiaceae	Eubacteriales	Clostridia	Firmicutes	Bacteria
Eubacteriaceae	Eubacteriales	Clostridia	Firmicutes	Bacteria
FGB9658	OFGB9658	CFGB9658	Firmicutes	Bacteria
FGB9659	OFGB9659	CFGB9659	Firmicutes	Bacteria
Bacteria_unclassified	Bacteria_unclassified	Bacteria_unclassifie	Bacteria_unclassifie	Bacteria
FGB9827	OFGB9827	CFGB9827	Firmicutes	Bacteria
Eubacteriaceae	Eubacteriales	Clostridia	Firmicutes	Bacteria
FGB77303	OFGB77303	CFGB77303	Bacteria_unclassifie	Bacteria
Eubacteriales_unclassified	Eubacteriales	Clostridia	Firmicutes	Bacteria
Oscillospiraceae	Eubacteriales	Clostridia	Firmicutes	Bacteria
FGB30328	OFGB30328	CFGB30328	Firmicutes	Bacteria
Oscillospiraceae	Eubacteriales	Clostridia	Firmicutes	Bacteria
Oscillospiraceae	Eubacteriales	Clostridia	Firmicutes	Bacteria
Oscillospiraceae	Eubacteriales	Clostridia	Firmicutes	Bacteria
Oscillospiraceae	Eubacteriales	Clostridia	Firmicutes	Bacteria
Oscillospiraceae	Eubacteriales	Clostridia	Firmicutes	Bacteria

Oscillospiraceae	Eubacteriales	Clostridia	Firmicutes	Bacteria
Oscillospiraceae	Eubacteriales	Clostridia	Firmicutes	Bacteria
FGB77153	OFGB77153	CFGB77153	Actinobacteria	Bacteria
FGB1791	OFGB1791	CFGB1791	Tenericutes	Bacteria
FGB10290	OFGB10290	CFGB10290	Firmicutes	Bacteria
Oscillospiraceae	Eubacteriales	Clostridia	Firmicutes	Bacteria
FGB1765	OFGB1765	CFGB1765	Firmicutes	Bacteria
FGB10349	OFGB10349	CFGB10349	Firmicutes	Bacteria
FGB10667	OFGB10667	CFGB10667	Firmicutes	Bacteria
Lachnospiraceae	Eubacteriales	Clostridia	Firmicutes	Bacteria
Lachnospiraceae	Eubacteriales	Clostridia	Firmicutes	Bacteria
Christensenellaceae	Eubacteriales	Clostridia	Firmicutes	Bacteria
FGB10299	OFGB10299	CFGB10299	Firmicutes	Bacteria
Oscillospiraceae	Eubacteriales	Clostridia	Firmicutes	Bacteria
Oscillospiraceae	Eubacteriales	Clostridia	Firmicutes	Bacteria
Lachnospiraceae	Eubacteriales	Clostridia	Firmicutes	Bacteria
Clostridia_unclassified	Clostridia_unclassifie	Clostridia	Firmicutes	Bacteria
Lachnospiraceae	Eubacteriales	Clostridia	Firmicutes	Bacteria
Lachnospiraceae	Eubacteriales	Clostridia	Firmicutes	Bacteria
Lachnospiraceae	Eubacteriales	Clostridia	Firmicutes	Bacteria
Lachnospiraceae	Eubacteriales	Clostridia	Firmicutes	Bacteria
Lachnospiraceae	Eubacteriales	Clostridia	Firmicutes	Bacteria
Lachnospiraceae	Eubacteriales	Clostridia	Firmicutes	Bacteria
Lachnospiraceae	Eubacteriales	Clostridia	Firmicutes	Bacteria
Lachnospiraceae	Eubacteriales	Clostridia	Firmicutes	Bacteria
Lactobacillaceae	Lactobacillales	Bacilli	Firmicutes	Bacteria
Muribaculaceae	Bacteroidales	Bacteroidia	Bacteroidota	Bacteria
Oscillospiraceae	Eubacteriales	Clostridia	Firmicutes	Bacteria
Oscillospiraceae	Eubacteriales	Clostridia	Firmicutes	Bacteria
Oscillospiraceae	Eubacteriales	Clostridia	Firmicutes	Bacteria
Oscillospiraceae	Eubacteriales	Clostridia	Firmicutes	Bacteria
Sutterellaceae	Burkholderiales	Betaproteobacteria	Proteobacteria	Bacteria
Peptostreptococcaceae	Eubacteriales	Clostridia	Firmicutes	Bacteria
Lachnospiraceae	Eubacteriales	Clostridia	Firmicutes	Bacteria
Turicibacteraceae	Erysipelotrichales	Erysipelotrichia	Firmicutes	Bacteria
Bacteria_unclassified	Bacteria_unclassified	Bacteria_unclassifie	Bacteria_unclassifie	Bacteria
Bacteria_unclassified	Bacteria_unclassified	Bacteria_unclassifie	Bacteria_unclassifie	Bacteria
Bacteria_unclassified	Bacteria_unclassified	Bacteria_unclassifie	Bacteria_unclassifie	Bacteria
Lachnospiraceae	Eubacteriales	Clostridia	Firmicutes	Bacteria
Lachnospiraceae	Eubacteriales	Clostridia	Firmicutes	Bacteria
Oscillospiraceae	Eubacteriales	Clostridia	Firmicutes	Bacteria
Oscillospiraceae	Eubacteriales	Clostridia	Firmicutes	Bacteria
Eggerthellaceae	Eggerthellales	Coriobacteriia	Actinobacteria	Bacteria

Eggerthellaceae	Eggerthellales	Coriobacteriia	Actinobacteria	Bacteria
Eggerthellaceae	Eggerthellales	Coriobacteriia	Actinobacteria	Bacteria
Akkermansiaceae	Verrucomicrobiales	Verrucomicrobiae	Verrucomicrobia	Bacteria
Rikenellaceae	Bacteroidales	Bacteroidia	Bacteroidota	Bacteria
Oscillospiraceae	Eubacteriales	Clostridia	Firmicutes	Bacteria
Bacteria_unclassified	Bacteria_unclassified	Bacteria_unclassifie	Bacteria_unclassifie	Bacteria
Bacteria_unclassified	Bacteria_unclassified	Bacteria_unclassifie	Bacteria_unclassifie	Bacteria
Bacteria_unclassified	Bacteria_unclassified	Bacteria_unclassifie	Bacteria_unclassifie	Bacteria
Bacteroidaceae	Bacteroidales	Bacteroidia	Bacteroidota	Bacteria
Bifidobacteriaceae	Bifidobacteriales	Actinomycetia	Actinobacteria	Bacteria
Clostridia_unclassified	Clostridia_unclassifie	Clostridia	Firmicutes	Bacteria
Clostridiaceae	Eubacteriales	Clostridia	Firmicutes	Bacteria
Clostridiaceae	Eubacteriales	Clostridia	Firmicutes	Bacteria
Eubacteriales_unclassified	Eubacteriales	Clostridia	Firmicutes	Bacteria
Erysipelotrichaceae	Erysipelotrichales	Erysipelotrichia	Firmicutes	Bacteria
Coriobacteriaceae	Coriobacteriales	Coriobacteriia	Actinobacteria	Bacteria
Lachnospiraceae	Eubacteriales	Clostridia	Firmicutes	Bacteria
Erysipelotrichaceae	Erysipelotrichales	Erysipelotrichia	Firmicutes	Bacteria
Erysipelotrichales_unclassifie	Erysipelotrichales	Erysipelotrichia	Firmicutes	Bacteria
Eubacteriaceae	Eubacteriales	Clostridia	Firmicutes	Bacteria
Eubacteriaceae	Eubacteriales	Clostridia	Firmicutes	Bacteria
Lachnospiraceae	Eubacteriales	Clostridia	Firmicutes	Bacteria
Eggerthellaceae	Eggerthellales	Coriobacteriia	Actinobacteria	Bacteria
Lachnospiraceae	Eubacteriales	Clostridia	Firmicutes	Bacteria
Muribaculaceae	Bacteroidales	Bacteroidia	Bacteroidota	Bacteria
Muribaculaceae	Bacteroidales	Bacteroidia	Bacteroidota	Bacteria
Muribaculaceae	Bacteroidales	Bacteroidia	Bacteroidota	Bacteria
FGB9508	OFGB9508	CFGB9508	Firmicutes	Bacteria
FGB2838	OFGB2838	CFGB2838	Firmicutes	Bacteria
FGB2838	OFGB2838	CFGB2838	Firmicutes	Bacteria
FGB2838	OFGB2838	CFGB2838	Firmicutes	Bacteria
Pumilibacteraceae	Eubacteriales	Clostridia	Firmicutes	Bacteria
FGB28439	OFGB28439	CFGB28439	Firmicutes	Bacteria
Clostridia_unclassified	Clostridia_unclassifie	Clostridia	Firmicutes	Bacteria
Eubacteriaceae	Eubacteriales	Clostridia	Firmicutes	Bacteria
Lachnospiraceae	Eubacteriales	Clostridia	Firmicutes	Bacteria
Lachnospiraceae	Eubacteriales	Clostridia	Firmicutes	Bacteria
Lachnospiraceae	Eubacteriales	Clostridia	Firmicutes	Bacteria
Lachnospiraceae	Eubacteriales	Clostridia	Firmicutes	Bacteria
FGB77305	OFGB77305	CFGB77305	Firmicutes	Bacteria
Clostridiaceae	Eubacteriales	Clostridia	Firmicutes	Bacteria
Lachnospiraceae	Eubacteriales	Clostridia	Firmicutes	Bacteria
Lachnospiraceae	Eubacteriales	Clostridia	Firmicutes	Bacteria
Lachnospiraceae	Eubacteriales	Clostridia	Firmicutes	Bacteria
FGB9633	OFGB9633	CFGB9633	Firmicutes	Bacteria
Bacteria_unclassified	Bacteria_unclassified	Bacteria_unclassifie	Bacteria_unclassifie	Bacteria

Bacteria_unclassified	Bacteria_unclassified	Bacteria_unclassified	Bacteria_unclassified	Bacteria
Bacteria_unclassified	Bacteria_unclassified	Bacteria_unclassified	Bacteria_unclassified	Bacteria
Bacteria_unclassified	Bacteria_unclassified	Bacteria_unclassified	Bacteria_unclassified	Bacteria
Lachnospiraceae	Eubacteriales	Clostridia	Firmicutes	Bacteria
Lachnospiraceae	Eubacteriales	Clostridia	Firmicutes	Bacteria
Lachnospiraceae	Eubacteriales	Clostridia	Firmicutes	Bacteria
FGB77359	OFGB77359	CFGB77359	Bacteria_unclassified	Bacteria
FGB9639	OFGB9639	CFGB9639	Firmicutes	Bacteria
Lachnospiraceae	Eubacteriales	Clostridia	Firmicutes	Bacteria
Lachnospiraceae	Eubacteriales	Clostridia	Firmicutes	Bacteria
Lachnospiraceae	Eubacteriales	Clostridia	Firmicutes	Bacteria
Clostridiaceae	Eubacteriales	Clostridia	Firmicutes	Bacteria
Clostridiaceae	Eubacteriales	Clostridia	Firmicutes	Bacteria
Clostridiaceae	Eubacteriales	Clostridia	Firmicutes	Bacteria
Clostridiaceae	Eubacteriales	Clostridia	Firmicutes	Bacteria
Clostridiaceae	Eubacteriales	Clostridia	Firmicutes	Bacteria
Clostridiaceae	Eubacteriales	Clostridia	Firmicutes	Bacteria
Clostridiaceae	Eubacteriales	Clostridia	Firmicutes	Bacteria
Eubacteriaceae	Eubacteriales	Clostridia	Firmicutes	Bacteria
FGB9658	OFGB9658	CFGB9658	Firmicutes	Bacteria
FGB9659	OFGB9659	CFGB9659	Firmicutes	Bacteria
Bacteria_unclassified	Bacteria_unclassified	Bacteria_unclassified	Bacteria_unclassified	Bacteria
FGB9827	OFGB9827	CFGB9827	Firmicutes	Bacteria
Eubacteriaceae	Eubacteriales	Clostridia	Firmicutes	Bacteria
FGB77303	OFGB77303	CFGB77303	Bacteria_unclassified	Bacteria
Eubacteriales_unclassified	Eubacteriales	Clostridia	Firmicutes	Bacteria
Oscillospiraceae	Eubacteriales	Clostridia	Firmicutes	Bacteria
FGB30328	OFGB30328	CFGB30328	Firmicutes	Bacteria
Oscillospiraceae	Eubacteriales	Clostridia	Firmicutes	Bacteria
Oscillospiraceae	Eubacteriales	Clostridia	Firmicutes	Bacteria
Oscillospiraceae	Eubacteriales	Clostridia	Firmicutes	Bacteria
Oscillospiraceae	Eubacteriales	Clostridia	Firmicutes	Bacteria
Oscillospiraceae	Eubacteriales	Clostridia	Firmicutes	Bacteria
Oscillospiraceae	Eubacteriales	Clostridia	Firmicutes	Bacteria
Oscillospiraceae	Eubacteriales	Clostridia	Firmicutes	Bacteria
FGB77153	OFGB77153	CFGB77153	Actinobacteria	Bacteria
FGB1791	OFGB1791	CFGB1791	Tenericutes	Bacteria
FGB10290	OFGB10290	CFGB10290	Firmicutes	Bacteria
Oscillospiraceae	Eubacteriales	Clostridia	Firmicutes	Bacteria
FGB1765	OFGB1765	CFGB1765	Firmicutes	Bacteria
FGB10349	OFGB10349	CFGB10349	Firmicutes	Bacteria
FGB10667	OFGB10667	CFGB10667	Firmicutes	Bacteria
Lachnospiraceae	Eubacteriales	Clostridia	Firmicutes	Bacteria
Lachnospiraceae	Eubacteriales	Clostridia	Firmicutes	Bacteria
Christensenellaceae	Eubacteriales	Clostridia	Firmicutes	Bacteria
FGB10299	OFGB10299	CFGB10299	Firmicutes	Bacteria

Oscillospiraceae	Eubacteriales	Clostridia	Firmicutes	Bacteria
Oscillospiraceae	Eubacteriales	Clostridia	Firmicutes	Bacteria
Lachnospiraceae	Eubacteriales	Clostridia	Firmicutes	Bacteria
Clostridia_unclassified	Clostridia_unclassifie	Clostridia	Firmicutes	Bacteria
Lachnospiraceae	Eubacteriales	Clostridia	Firmicutes	Bacteria
Lachnospiraceae	Eubacteriales	Clostridia	Firmicutes	Bacteria
Lachnospiraceae	Eubacteriales	Clostridia	Firmicutes	Bacteria
Lachnospiraceae	Eubacteriales	Clostridia	Firmicutes	Bacteria
Lachnospiraceae	Eubacteriales	Clostridia	Firmicutes	Bacteria
Lachnospiraceae	Eubacteriales	Clostridia	Firmicutes	Bacteria
Lachnospiraceae	Eubacteriales	Clostridia	Firmicutes	Bacteria
Lachnospiraceae	Eubacteriales	Clostridia	Firmicutes	Bacteria
Lactobacillaceae	Lactobacillales	Bacilli	Firmicutes	Bacteria
Muribaculaceae	Bacteroidales	Bacteroidia	Bacteroidota	Bacteria
Oscillospiraceae	Eubacteriales	Clostridia	Firmicutes	Bacteria
Oscillospiraceae	Eubacteriales	Clostridia	Firmicutes	Bacteria
Oscillospiraceae	Eubacteriales	Clostridia	Firmicutes	Bacteria
Oscillospiraceae	Eubacteriales	Clostridia	Firmicutes	Bacteria
Sutterellaceae	Burkholderiales	Betaproteobacteria	Proteobacteria	Bacteria
Peptostreptococcaceae	Eubacteriales	Clostridia	Firmicutes	Bacteria
Lachnospiraceae	Eubacteriales	Clostridia	Firmicutes	Bacteria
Turicibacteraceae	Erysipelotrichales	Erysipelotrichia	Firmicutes	Bacteria
Bacteria_unclassified	Bacteria_unclassified	Bacteria_unclassifie	Bacteria_unclassifie	Bacteria
Bacteria_unclassified	Bacteria_unclassified	Bacteria_unclassifie	Bacteria_unclassifie	Bacteria
Bacteria_unclassified	Bacteria_unclassified	Bacteria_unclassifie	Bacteria_unclassifie	Bacteria

Lachnospiraceae	Eubacteriales	Clostridia	Firmicutes	Bacteria
Lachnospiraceae	Eubacteriales	Clostridia	Firmicutes	Bacteria
Oscillospiraceae	Eubacteriales	Clostridia	Firmicutes	Bacteria
Oscillospiraceae	Eubacteriales	Clostridia	Firmicutes	Bacteria
Eggerthellaceae	Eggerthellales	Coriobacteriia	Actinobacteria	Bacteria
Eggerthellaceae	Eggerthellales	Coriobacteriia	Actinobacteria	Bacteria
Eggerthellaceae	Eggerthellales	Coriobacteriia	Actinobacteria	Bacteria
Akkermansiaceae	Verrucomicrobiales	Verrucomicrobiae	Verrucomicrobia	Bacteria
Rikenellaceae	Bacteroidales	Bacteroidia	Bacteroidota	Bacteria
Oscillospiraceae	Eubacteriales	Clostridia	Firmicutes	Bacteria
Bacteria_unclassified	Bacteria_unclassified	Bacteria_unclassifie	Bacteria_unclassifie	Bacteria
Bacteria_unclassified	Bacteria_unclassified	Bacteria_unclassifie	Bacteria_unclassifie	Bacteria
Bacteria_unclassified	Bacteria_unclassified	Bacteria_unclassifie	Bacteria_unclassifie	Bacteria
Bacteroidaceae	Bacteroidales	Bacteroidia	Bacteroidota	Bacteria
Bifidobacteriaceae	Bifidobacteriales	Actinomycetia	Actinobacteria	Bacteria
Clostridia_unclassified	Clostridia_unclassifie	Clostridia	Firmicutes	Bacteria
Clostridiaceae	Eubacteriales	Clostridia	Firmicutes	Bacteria
Clostridiaceae	Eubacteriales	Clostridia	Firmicutes	Bacteria

Eubacteriales_unclassified	Eubacteriales	Clostridia	Firmicutes	Bacteria
Erysipelotrichaceae	Erysipelotrichales	Erysipelotrichia	Firmicutes	Bacteria
Coriobacteriaceae	Coriobacteriales	Coriobacteriia	Actinobacteria	Bacteria
Lachnospiraceae	Eubacteriales	Clostridia	Firmicutes	Bacteria
Erysipelotrichaceae	Erysipelotrichales	Erysipelotrichia	Firmicutes	Bacteria
Erysipelotrichales_unclassifie	Erysipelotrichales	Erysipelotrichia	Firmicutes	Bacteria
Eubacteriaceae	Eubacteriales	Clostridia	Firmicutes	Bacteria
Eubacteriaceae	Eubacteriales	Clostridia	Firmicutes	Bacteria
Lachnospiraceae	Eubacteriales	Clostridia	Firmicutes	Bacteria
Eggerthellaceae	Eggerthellales	Coriobacteriia	Actinobacteria	Bacteria
Lachnospiraceae	Eubacteriales	Clostridia	Firmicutes	Bacteria
Muribaculaceae	Bacteroidales	Bacteroidia	Bacteroidota	Bacteria
Muribaculaceae	Bacteroidales	Bacteroidia	Bacteroidota	Bacteria
Muribaculaceae	Bacteroidales	Bacteroidia	Bacteroidota	Bacteria
FGB9508	OFGB9508	CFGB9508	Firmicutes	Bacteria
FGB2838	OFGB2838	CFGB2838	Firmicutes	Bacteria
FGB2838	OFGB2838	CFGB2838	Firmicutes	Bacteria
FGB2838	OFGB2838	CFGB2838	Firmicutes	Bacteria
Pumilibacteraceae	Eubacteriales	Clostridia	Firmicutes	Bacteria
FGB28439	OFGB28439	CFGB28439	Firmicutes	Bacteria
Clostridia_unclassified	Clostridia_unclassifie	Clostridia	Firmicutes	Bacteria
Eubacteriaceae	Eubacteriales	Clostridia	Firmicutes	Bacteria
Lachnospiraceae	Eubacteriales	Clostridia	Firmicutes	Bacteria
Lachnospiraceae	Eubacteriales	Clostridia	Firmicutes	Bacteria
Lachnospiraceae	Eubacteriales	Clostridia	Firmicutes	Bacteria
Lachnospiraceae	Eubacteriales	Clostridia	Firmicutes	Bacteria
FGB77305	OFGB77305	CFGB77305	Firmicutes	Bacteria
Clostridiaceae	Eubacteriales	Clostridia	Firmicutes	Bacteria
Lachnospiraceae	Eubacteriales	Clostridia	Firmicutes	Bacteria
Lachnospiraceae	Eubacteriales	Clostridia	Firmicutes	Bacteria
Lachnospiraceae	Eubacteriales	Clostridia	Firmicutes	Bacteria
FGB9633	OFGB9633	CFGB9633	Firmicutes	Bacteria
Bacteria_unclassified	Bacteria_unclassified	Bacteria_unclassifie	Bacteria_unclassifie	Bacteria
Bacteria_unclassified	Bacteria_unclassified	Bacteria_unclassifie	Bacteria_unclassifie	Bacteria
Bacteria_unclassified	Bacteria_unclassified	Bacteria_unclassifie	Bacteria_unclassifie	Bacteria
Bacteria_unclassified	Bacteria_unclassified	Bacteria_unclassifie	Bacteria_unclassifie	Bacteria
Lachnospiraceae	Eubacteriales	Clostridia	Firmicutes	Bacteria
Lachnospiraceae	Eubacteriales	Clostridia	Firmicutes	Bacteria
Lachnospiraceae	Eubacteriales	Clostridia	Firmicutes	Bacteria
FGB77359	OFGB77359	CFGB77359	Bacteria_unclassifie	Bacteria
FGB9639	OFGB9639	CFGB9639	Firmicutes	Bacteria
Lachnospiraceae	Eubacteriales	Clostridia	Firmicutes	Bacteria
Lachnospiraceae	Eubacteriales	Clostridia	Firmicutes	Bacteria
Lachnospiraceae	Eubacteriales	Clostridia	Firmicutes	Bacteria
Clostridiaceae	Eubacteriales	Clostridia	Firmicutes	Bacteria
Clostridiaceae	Eubacteriales	Clostridia	Firmicutes	Bacteria

Clostridiaceae	Eubacteriales	Clostridia	Firmicutes	Bacteria
Clostridiaceae	Eubacteriales	Clostridia	Firmicutes	Bacteria
Clostridiaceae	Eubacteriales	Clostridia	Firmicutes	Bacteria
Clostridiaceae	Eubacteriales	Clostridia	Firmicutes	Bacteria
Clostridiaceae	Eubacteriales	Clostridia	Firmicutes	Bacteria
Eubacteriaceae	Eubacteriales	Clostridia	Firmicutes	Bacteria
FGB9658	OFGB9658	CFGB9658	Firmicutes	Bacteria
FGB9659	OFGB9659	CFGB9659	Firmicutes	Bacteria
Bacteria_unclassified	Bacteria_unclassified	Bacteria_unclassifie	Bacteria_unclassifie	Bacteria
FGB9827	OFGB9827	CFGB9827	Firmicutes	Bacteria
Eubacteriaceae	Eubacteriales	Clostridia	Firmicutes	Bacteria
FGB77303	OFGB77303	CFGB77303	Bacteria_unclassifie	Bacteria
Eubacteriales_unclassified	Eubacteriales	Clostridia	Firmicutes	Bacteria
Oscillospiraceae	Eubacteriales	Clostridia	Firmicutes	Bacteria
FGB30328	OFGB30328	CFGB30328	Firmicutes	Bacteria
Oscillospiraceae	Eubacteriales	Clostridia	Firmicutes	Bacteria
Oscillospiraceae	Eubacteriales	Clostridia	Firmicutes	Bacteria
Oscillospiraceae	Eubacteriales	Clostridia	Firmicutes	Bacteria
Oscillospiraceae	Eubacteriales	Clostridia	Firmicutes	Bacteria
Oscillospiraceae	Eubacteriales	Clostridia	Firmicutes	Bacteria
Oscillospiraceae	Eubacteriales	Clostridia	Firmicutes	Bacteria
Oscillospiraceae	Eubacteriales	Clostridia	Firmicutes	Bacteria
FGB77153	OFGB77153	CFGB77153	Actinobacteria	Bacteria
FGB1791	OFGB1791	CFGB1791	Tenericutes	Bacteria
FGB10290	OFGB10290	CFGB10290	Firmicutes	Bacteria
Oscillospiraceae	Eubacteriales	Clostridia	Firmicutes	Bacteria
FGB1765	OFGB1765	CFGB1765	Firmicutes	Bacteria
FGB10349	OFGB10349	CFGB10349	Firmicutes	Bacteria
FGB10667	OFGB10667	CFGB10667	Firmicutes	Bacteria
Lachnospiraceae	Eubacteriales	Clostridia	Firmicutes	Bacteria
Lachnospiraceae	Eubacteriales	Clostridia	Firmicutes	Bacteria
Christensenellaceae	Eubacteriales	Clostridia	Firmicutes	Bacteria
FGB10299	OFGB10299	CFGB10299	Firmicutes	Bacteria
Oscillospiraceae	Eubacteriales	Clostridia	Firmicutes	Bacteria
Oscillospiraceae	Eubacteriales	Clostridia	Firmicutes	Bacteria
Lachnospiraceae	Eubacteriales	Clostridia	Firmicutes	Bacteria
Clostridia_unclassified	Clostridia_unclassifie	Clostridia	Firmicutes	Bacteria
Lachnospiraceae	Eubacteriales	Clostridia	Firmicutes	Bacteria
Lachnospiraceae	Eubacteriales	Clostridia	Firmicutes	Bacteria
Lachnospiraceae	Eubacteriales	Clostridia	Firmicutes	Bacteria
Lachnospiraceae	Eubacteriales	Clostridia	Firmicutes	Bacteria
Lachnospiraceae	Eubacteriales	Clostridia	Firmicutes	Bacteria
Lachnospiraceae	Eubacteriales	Clostridia	Firmicutes	Bacteria
Lachnospiraceae	Eubacteriales	Clostridia	Firmicutes	Bacteria
Lachnospiraceae	Eubacteriales	Clostridia	Firmicutes	Bacteria
Lactobacillaceae	Lactobacillales	Bacilli	Firmicutes	Bacteria

Muribaculaceae	Bacteroidales	Bacteroidia	Bacteroidota	Bacteria
Oscillospiraceae	Eubacteriales	Clostridia	Firmicutes	Bacteria
Oscillospiraceae	Eubacteriales	Clostridia	Firmicutes	Bacteria
Oscillospiraceae	Eubacteriales	Clostridia	Firmicutes	Bacteria
Oscillospiraceae	Eubacteriales	Clostridia	Firmicutes	Bacteria
Sutterellaceae	Burkholderiales	Betaproteobacteria	Proteobacteria	Bacteria
Peptostreptococcaceae	Eubacteriales	Clostridia	Firmicutes	Bacteria
Lachnospiraceae	Eubacteriales	Clostridia	Firmicutes	Bacteria
Turicibacteraceae	Erysipelotrichales	Erysipelotrichia	Firmicutes	Bacteria
Bacteria_unclassified	Bacteria_unclassified	Bacteria_unclassifie	Bacteria_unclassifie	Bacteria
Bacteria_unclassified	Bacteria_unclassified	Bacteria_unclassifie	Bacteria_unclassifie	Bacteria
Bacteria_unclassified	Bacteria_unclassified	Bacteria_unclassifie	Bacteria_unclassifie	Bacteria

Lachnospiraceae	Eubacteriales	Clostridia	Firmicutes	Bacteria
Lachnospiraceae	Eubacteriales	Clostridia	Firmicutes	Bacteria
Oscillospiraceae	Eubacteriales	Clostridia	Firmicutes	Bacteria
Oscillospiraceae	Eubacteriales	Clostridia	Firmicutes	Bacteria
Eggerthellaceae	Eggerthellales	Coriobacteriia	Actinobacteria	Bacteria
Eggerthellaceae	Eggerthellales	Coriobacteriia	Actinobacteria	Bacteria
Eggerthellaceae	Eggerthellales	Coriobacteriia	Actinobacteria	Bacteria
Akkermansiaceae	Verrucomicrobiales	Verrucomicrobiae	Verrucomicrobia	Bacteria
Rikenellaceae	Bacteroidales	Bacteroidia	Bacteroidota	Bacteria
Oscillospiraceae	Eubacteriales	Clostridia	Firmicutes	Bacteria
Bacteria_unclassified	Bacteria_unclassified	Bacteria_unclassifie	Bacteria_unclassifie	Bacteria
Bacteria_unclassified	Bacteria_unclassified	Bacteria_unclassifie	Bacteria_unclassifie	Bacteria
Bacteria_unclassified	Bacteria_unclassified	Bacteria_unclassifie	Bacteria_unclassifie	Bacteria
Bacteroidaceae	Bacteroidales	Bacteroidia	Bacteroidota	Bacteria
Bifidobacteriaceae	Bifidobacteriales	Actinomycetia	Actinobacteria	Bacteria
Clostridia_unclassified	Clostridia_unclassifie	Clostridia	Firmicutes	Bacteria
Clostridiaceae	Eubacteriales	Clostridia	Firmicutes	Bacteria
Clostridiaceae	Eubacteriales	Clostridia	Firmicutes	Bacteria
Eubacteriales_unclassified	Eubacteriales	Clostridia	Firmicutes	Bacteria
Erysipelotrichaceae	Erysipelotrichales	Erysipelotrichia	Firmicutes	Bacteria
Coriobacteriaceae	Coriobacteriales	Coriobacteriia	Actinobacteria	Bacteria
Lachnospiraceae	Eubacteriales	Clostridia	Firmicutes	Bacteria
Erysipelotrichaceae	Erysipelotrichales	Erysipelotrichia	Firmicutes	Bacteria
Erysipelotrichales_unclassifie	Erysipelotrichales	Erysipelotrichia	Firmicutes	Bacteria
Eubacteriaceae	Eubacteriales	Clostridia	Firmicutes	Bacteria
Eubacteriaceae	Eubacteriales	Clostridia	Firmicutes	Bacteria
Lachnospiraceae	Eubacteriales	Clostridia	Firmicutes	Bacteria
Eggerthellaceae	Eggerthellales	Coriobacteriia	Actinobacteria	Bacteria
Lachnospiraceae	Eubacteriales	Clostridia	Firmicutes	Bacteria
Muribaculaceae	Bacteroidales	Bacteroidia	Bacteroidota	Bacteria
Muribaculaceae	Bacteroidales	Bacteroidia	Bacteroidota	Bacteria

Muribaculaceae	Bacteroidales	Bacteroidia	Bacteroidota	Bacteria
FGB9508	OFGB9508	CFGB9508	Firmicutes	Bacteria
FGB2838	OFGB2838	CFGB2838	Firmicutes	Bacteria
FGB2838	OFGB2838	CFGB2838	Firmicutes	Bacteria
FGB2838	OFGB2838	CFGB2838	Firmicutes	Bacteria
Pumilibacteraceae	Eubacteriales	Clostridia	Firmicutes	Bacteria
FGB28439	OFGB28439	CFGB28439	Firmicutes	Bacteria
Clostridia_unclassified	Clostridia_unclassifie	Clostridia	Firmicutes	Bacteria
Eubacteriaceae	Eubacteriales	Clostridia	Firmicutes	Bacteria
Lachnospiraceae	Eubacteriales	Clostridia	Firmicutes	Bacteria
Lachnospiraceae	Eubacteriales	Clostridia	Firmicutes	Bacteria
Lachnospiraceae	Eubacteriales	Clostridia	Firmicutes	Bacteria
Lachnospiraceae	Eubacteriales	Clostridia	Firmicutes	Bacteria
FGB77305	OFGB77305	CFGB77305	Firmicutes	Bacteria
Clostridiaceae	Eubacteriales	Clostridia	Firmicutes	Bacteria
Lachnospiraceae	Eubacteriales	Clostridia	Firmicutes	Bacteria
Lachnospiraceae	Eubacteriales	Clostridia	Firmicutes	Bacteria
Lachnospiraceae	Eubacteriales	Clostridia	Firmicutes	Bacteria
FGB9633	OFGB9633	CFGB9633	Firmicutes	Bacteria
Bacteria_unclassified	Bacteria_unclassified	Bacteria_unclassifie	Bacteria_unclassifie	Bacteria
Bacteria_unclassified	Bacteria_unclassified	Bacteria_unclassifie	Bacteria_unclassifie	Bacteria
Bacteria_unclassified	Bacteria_unclassified	Bacteria_unclassifie	Bacteria_unclassifie	Bacteria
Bacteria_unclassified	Bacteria_unclassified	Bacteria_unclassifie	Bacteria_unclassifie	Bacteria
Lachnospiraceae	Eubacteriales	Clostridia	Firmicutes	Bacteria
Lachnospiraceae	Eubacteriales	Clostridia	Firmicutes	Bacteria
Lachnospiraceae	Eubacteriales	Clostridia	Firmicutes	Bacteria
FGB77359	OFGB77359	CFGB77359	Bacteria_unclassifie	Bacteria
FGB9639	OFGB9639	CFGB9639	Firmicutes	Bacteria
Lachnospiraceae	Eubacteriales	Clostridia	Firmicutes	Bacteria
Lachnospiraceae	Eubacteriales	Clostridia	Firmicutes	Bacteria
Lachnospiraceae	Eubacteriales	Clostridia	Firmicutes	Bacteria
Clostridiaceae	Eubacteriales	Clostridia	Firmicutes	Bacteria
Clostridiaceae	Eubacteriales	Clostridia	Firmicutes	Bacteria
Clostridiaceae	Eubacteriales	Clostridia	Firmicutes	Bacteria
Clostridiaceae	Eubacteriales	Clostridia	Firmicutes	Bacteria
Clostridiaceae	Eubacteriales	Clostridia	Firmicutes	Bacteria
Clostridiaceae	Eubacteriales	Clostridia	Firmicutes	Bacteria
Clostridiaceae	Eubacteriales	Clostridia	Firmicutes	Bacteria
Eubacteriaceae	Eubacteriales	Clostridia	Firmicutes	Bacteria
FGB9658	OFGB9658	CFGB9658	Firmicutes	Bacteria
FGB9659	OFGB9659	CFGB9659	Firmicutes	Bacteria
Bacteria_unclassified	Bacteria_unclassified	Bacteria_unclassifie	Bacteria_unclassifie	Bacteria
FGB9827	OFGB9827	CFGB9827	Firmicutes	Bacteria
Eubacteriaceae	Eubacteriales	Clostridia	Firmicutes	Bacteria
FGB77303	OFGB77303	CFGB77303	Bacteria_unclassifie	Bacteria
Eubacteriales_unclassified	Eubacteriales	Clostridia	Firmicutes	Bacteria

Oscillospiraceae	Eubacteriales	Clostridia	Firmicutes	Bacteria
FGB30328	OFGB30328	CFGB30328	Firmicutes	Bacteria
Oscillospiraceae	Eubacteriales	Clostridia	Firmicutes	Bacteria
Oscillospiraceae	Eubacteriales	Clostridia	Firmicutes	Bacteria
Oscillospiraceae	Eubacteriales	Clostridia	Firmicutes	Bacteria
Oscillospiraceae	Eubacteriales	Clostridia	Firmicutes	Bacteria
Oscillospiraceae	Eubacteriales	Clostridia	Firmicutes	Bacteria
Oscillospiraceae	Eubacteriales	Clostridia	Firmicutes	Bacteria
Oscillospiraceae	Eubacteriales	Clostridia	Firmicutes	Bacteria
FGB77153	OFGB77153	CFGB77153	Actinobacteria	Bacteria
FGB1791	OFGB1791	CFGB1791	Tenericutes	Bacteria
FGB10290	OFGB10290	CFGB10290	Firmicutes	Bacteria
Oscillospiraceae	Eubacteriales	Clostridia	Firmicutes	Bacteria
FGB1765	OFGB1765	CFGB1765	Firmicutes	Bacteria
FGB10349	OFGB10349	CFGB10349	Firmicutes	Bacteria
FGB10667	OFGB10667	CFGB10667	Firmicutes	Bacteria
Lachnospiraceae	Eubacteriales	Clostridia	Firmicutes	Bacteria
Lachnospiraceae	Eubacteriales	Clostridia	Firmicutes	Bacteria
Christensenellaceae	Eubacteriales	Clostridia	Firmicutes	Bacteria
FGB10299	OFGB10299	CFGB10299	Firmicutes	Bacteria
Oscillospiraceae	Eubacteriales	Clostridia	Firmicutes	Bacteria
Oscillospiraceae	Eubacteriales	Clostridia	Firmicutes	Bacteria
Lachnospiraceae	Eubacteriales	Clostridia	Firmicutes	Bacteria
Clostridia_unclassified	Clostridia_unclassifie	Clostridia	Firmicutes	Bacteria
Lachnospiraceae	Eubacteriales	Clostridia	Firmicutes	Bacteria
Lachnospiraceae	Eubacteriales	Clostridia	Firmicutes	Bacteria
Lachnospiraceae	Eubacteriales	Clostridia	Firmicutes	Bacteria
Lachnospiraceae	Eubacteriales	Clostridia	Firmicutes	Bacteria
Lachnospiraceae	Eubacteriales	Clostridia	Firmicutes	Bacteria
Lachnospiraceae	Eubacteriales	Clostridia	Firmicutes	Bacteria
Lachnospiraceae	Eubacteriales	Clostridia	Firmicutes	Bacteria
Lachnospiraceae	Eubacteriales	Clostridia	Firmicutes	Bacteria
Lactobacillaceae	Lactobacillales	Bacilli	Firmicutes	Bacteria
Muribaculaceae	Bacteroidales	Bacteroidia	Bacteroidota	Bacteria
Oscillospiraceae	Eubacteriales	Clostridia	Firmicutes	Bacteria
Oscillospiraceae	Eubacteriales	Clostridia	Firmicutes	Bacteria
Oscillospiraceae	Eubacteriales	Clostridia	Firmicutes	Bacteria
Oscillospiraceae	Eubacteriales	Clostridia	Firmicutes	Bacteria
Sutterellaceae	Burkholderiales	Betaproteobacteria	Proteobacteria	Bacteria
Peptostreptococcaceae	Eubacteriales	Clostridia	Firmicutes	Bacteria
Lachnospiraceae	Eubacteriales	Clostridia	Firmicutes	Bacteria
Turicibacteraceae	Erysipelotrichales	Erysipelotrichia	Firmicutes	Bacteria
Bacteria_unclassified	Bacteria_unclassified	Bacteria_unclassifie	Bacteria_unclassifie	Bacteria
Bacteria_unclassified	Bacteria_unclassified	Bacteria_unclassifie	Bacteria_unclassifie	Bacteria
Bacteria_unclassified	Bacteria_unclassified	Bacteria_unclassifie	Bacteria_unclassifie	Bacteria

Lachnospiraceae	Eubacteriales	Clostridia	Firmicutes	Bacteria
Lachnospiraceae	Eubacteriales	Clostridia	Firmicutes	Bacteria
Oscillospiraceae	Eubacteriales	Clostridia	Firmicutes	Bacteria
Oscillospiraceae	Eubacteriales	Clostridia	Firmicutes	Bacteria
Eggerthellaceae	Eggerthellales	Coriobacteriia	Actinobacteria	Bacteria
Eggerthellaceae	Eggerthellales	Coriobacteriia	Actinobacteria	Bacteria
Eggerthellaceae	Eggerthellales	Coriobacteriia	Actinobacteria	Bacteria
Akkermansiaceae	Verrucomicrobiales	Verrucomicrobiae	Verrucomicrobia	Bacteria
Rikenellaceae	Bacteroidales	Bacteroidia	Bacteroidota	Bacteria
Oscillospiraceae	Eubacteriales	Clostridia	Firmicutes	Bacteria
Bacteria_unclassified	Bacteria_unclassified	Bacteria_unclassifie	Bacteria_unclassifie	Bacteria
Bacteria_unclassified	Bacteria_unclassified	Bacteria_unclassifie	Bacteria_unclassifie	Bacteria
Bacteria_unclassified	Bacteria_unclassified	Bacteria_unclassifie	Bacteria_unclassifie	Bacteria
Bacteroidaceae	Bacteroidales	Bacteroidia	Bacteroidota	Bacteria
Bifidobacteriaceae	Bifidobacteriales	Actinomycetia	Actinobacteria	Bacteria
Clostridia_unclassified	Clostridia_unclassifie	Clostridia	Firmicutes	Bacteria
Clostridiaceae	Eubacteriales	Clostridia	Firmicutes	Bacteria
Clostridiaceae	Eubacteriales	Clostridia	Firmicutes	Bacteria
Eubacteriales_unclassified	Eubacteriales	Clostridia	Firmicutes	Bacteria
Erysipelotrichaceae	Erysipelotrichales	Erysipelotrichia	Firmicutes	Bacteria
Coriobacteriaceae	Coriobacteriales	Coriobacteriia	Actinobacteria	Bacteria
Lachnospiraceae	Eubacteriales	Clostridia	Firmicutes	Bacteria
Erysipelotrichaceae	Erysipelotrichales	Erysipelotrichia	Firmicutes	Bacteria
Erysipelotrichales_unclassifie	Erysipelotrichales	Erysipelotrichia	Firmicutes	Bacteria
Eubacteriaceae	Eubacteriales	Clostridia	Firmicutes	Bacteria
Eubacteriaceae	Eubacteriales	Clostridia	Firmicutes	Bacteria
Lachnospiraceae	Eubacteriales	Clostridia	Firmicutes	Bacteria
Eggerthellaceae	Eggerthellales	Coriobacteriia	Actinobacteria	Bacteria
Lachnospiraceae	Eubacteriales	Clostridia	Firmicutes	Bacteria
Muribaculaceae	Bacteroidales	Bacteroidia	Bacteroidota	Bacteria
Muribaculaceae	Bacteroidales	Bacteroidia	Bacteroidota	Bacteria
Muribaculaceae	Bacteroidales	Bacteroidia	Bacteroidota	Bacteria
FGB9508	OFGB9508	CFGB9508	Firmicutes	Bacteria
FGB2838	OFGB2838	CFGB2838	Firmicutes	Bacteria
FGB2838	OFGB2838	CFGB2838	Firmicutes	Bacteria
FGB2838	OFGB2838	CFGB2838	Firmicutes	Bacteria
Pumilibacteraceae	Eubacteriales	Clostridia	Firmicutes	Bacteria
FGB28439	OFGB28439	CFGB28439	Firmicutes	Bacteria
Clostridia_unclassified	Clostridia_unclassifie	Clostridia	Firmicutes	Bacteria
Eubacteriaceae	Eubacteriales	Clostridia	Firmicutes	Bacteria
Lachnospiraceae	Eubacteriales	Clostridia	Firmicutes	Bacteria
Lachnospiraceae	Eubacteriales	Clostridia	Firmicutes	Bacteria
Lachnospiraceae	Eubacteriales	Clostridia	Firmicutes	Bacteria
Lachnospiraceae	Eubacteriales	Clostridia	Firmicutes	Bacteria

FGB77305	OFGB77305	CFGB77305	Firmicutes	Bacteria
Clostridiaceae	Eubacteriales	Clostridia	Firmicutes	Bacteria
Lachnospiraceae	Eubacteriales	Clostridia	Firmicutes	Bacteria
Lachnospiraceae	Eubacteriales	Clostridia	Firmicutes	Bacteria
Lachnospiraceae	Eubacteriales	Clostridia	Firmicutes	Bacteria
FGB9633	OFGB9633	CFGB9633	Firmicutes	Bacteria
Bacteria_unclassified	Bacteria_unclassified	Bacteria_unclassifie	Bacteria_unclassifie	Bacteria
Bacteria_unclassified	Bacteria_unclassified	Bacteria_unclassifie	Bacteria_unclassifie	Bacteria
Bacteria_unclassified	Bacteria_unclassified	Bacteria_unclassifie	Bacteria_unclassifie	Bacteria
Bacteria_unclassified	Bacteria_unclassified	Bacteria_unclassifie	Bacteria_unclassifie	Bacteria
Lachnospiraceae	Eubacteriales	Clostridia	Firmicutes	Bacteria
Lachnospiraceae	Eubacteriales	Clostridia	Firmicutes	Bacteria
Lachnospiraceae	Eubacteriales	Clostridia	Firmicutes	Bacteria
FGB77359	OFGB77359	CFGB77359	Bacteria_unclassifie	Bacteria
FGB9639	OFGB9639	CFGB9639	Firmicutes	Bacteria
Lachnospiraceae	Eubacteriales	Clostridia	Firmicutes	Bacteria
Lachnospiraceae	Eubacteriales	Clostridia	Firmicutes	Bacteria
Lachnospiraceae	Eubacteriales	Clostridia	Firmicutes	Bacteria
Clostridiaceae	Eubacteriales	Clostridia	Firmicutes	Bacteria
Clostridiaceae	Eubacteriales	Clostridia	Firmicutes	Bacteria
Clostridiaceae	Eubacteriales	Clostridia	Firmicutes	Bacteria
Clostridiaceae	Eubacteriales	Clostridia	Firmicutes	Bacteria
Clostridiaceae	Eubacteriales	Clostridia	Firmicutes	Bacteria
Clostridiaceae	Eubacteriales	Clostridia	Firmicutes	Bacteria
Clostridiaceae	Eubacteriales	Clostridia	Firmicutes	Bacteria
Eubacteriaceae	Eubacteriales	Clostridia	Firmicutes	Bacteria
FGB9658	OFGB9658	CFGB9658	Firmicutes	Bacteria
FGB9659	OFGB9659	CFGB9659	Firmicutes	Bacteria
Bacteria_unclassified	Bacteria_unclassified	Bacteria_unclassifie	Bacteria_unclassifie	Bacteria
FGB9827	OFGB9827	CFGB9827	Firmicutes	Bacteria
Eubacteriaceae	Eubacteriales	Clostridia	Firmicutes	Bacteria
FGB77303	OFGB77303	CFGB77303	Bacteria_unclassifie	Bacteria
Eubacteriales_unclassified	Eubacteriales	Clostridia	Firmicutes	Bacteria
Oscillospiraceae	Eubacteriales	Clostridia	Firmicutes	Bacteria
FGB30328	OFGB30328	CFGB30328	Firmicutes	Bacteria
Oscillospiraceae	Eubacteriales	Clostridia	Firmicutes	Bacteria
Oscillospiraceae	Eubacteriales	Clostridia	Firmicutes	Bacteria
Oscillospiraceae	Eubacteriales	Clostridia	Firmicutes	Bacteria
Oscillospiraceae	Eubacteriales	Clostridia	Firmicutes	Bacteria
Oscillospiraceae	Eubacteriales	Clostridia	Firmicutes	Bacteria
Oscillospiraceae	Eubacteriales	Clostridia	Firmicutes	Bacteria
Oscillospiraceae	Eubacteriales	Clostridia	Firmicutes	Bacteria
FGB77153	OFGB77153	CFGB77153	Actinobacteria	Bacteria
FGB1791	OFGB1791	CFGB1791	Tenericutes	Bacteria
FGB10290	OFGB10290	CFGB10290	Firmicutes	Bacteria
Oscillospiraceae	Eubacteriales	Clostridia	Firmicutes	Bacteria

FGB1765	OFGB1765	CFGB1765	Firmicutes	Bacteria
FGB10349	OFGB10349	CFGB10349	Firmicutes	Bacteria
FGB10667	OFGB10667	CFGB10667	Firmicutes	Bacteria
Lachnospiraceae	Eubacteriales	Clostridia	Firmicutes	Bacteria
Lachnospiraceae	Eubacteriales	Clostridia	Firmicutes	Bacteria
Christensenellaceae	Eubacteriales	Clostridia	Firmicutes	Bacteria
FGB10299	OFGB10299	CFGB10299	Firmicutes	Bacteria
Oscillospiraceae	Eubacteriales	Clostridia	Firmicutes	Bacteria
Oscillospiraceae	Eubacteriales	Clostridia	Firmicutes	Bacteria
Lachnospiraceae	Eubacteriales	Clostridia	Firmicutes	Bacteria
Clostridia_unclassified	Clostridia_unclassifie	Clostridia	Firmicutes	Bacteria
Lachnospiraceae	Eubacteriales	Clostridia	Firmicutes	Bacteria
Lachnospiraceae	Eubacteriales	Clostridia	Firmicutes	Bacteria
Lachnospiraceae	Eubacteriales	Clostridia	Firmicutes	Bacteria
Lachnospiraceae	Eubacteriales	Clostridia	Firmicutes	Bacteria
Lachnospiraceae	Eubacteriales	Clostridia	Firmicutes	Bacteria
Lachnospiraceae	Eubacteriales	Clostridia	Firmicutes	Bacteria
Lachnospiraceae	Eubacteriales	Clostridia	Firmicutes	Bacteria
Lachnospiraceae	Eubacteriales	Clostridia	Firmicutes	Bacteria
Lactobacillaceae	Lactobacillales	Bacilli	Firmicutes	Bacteria
Muribaculaceae	Bacteroidales	Bacteroidia	Bacteroidota	Bacteria
Oscillospiraceae	Eubacteriales	Clostridia	Firmicutes	Bacteria
Oscillospiraceae	Eubacteriales	Clostridia	Firmicutes	Bacteria
Oscillospiraceae	Eubacteriales	Clostridia	Firmicutes	Bacteria
Oscillospiraceae	Eubacteriales	Clostridia	Firmicutes	Bacteria
Sutterellaceae	Burkholderiales	Betaproteobacteria	Proteobacteria	Bacteria
Peptostreptococcaceae	Eubacteriales	Clostridia	Firmicutes	Bacteria
Lachnospiraceae	Eubacteriales	Clostridia	Firmicutes	Bacteria
Turicibacteraceae	Erysipelotrichales	Erysipelotrichia	Firmicutes	Bacteria
Bacteria_unclassified	Bacteria_unclassified	Bacteria_unclassifie	Bacteria_unclassifie	Bacteria
Bacteria_unclassified	Bacteria_unclassified	Bacteria_unclassifie	Bacteria_unclassifie	Bacteria
Bacteria_unclassified	Bacteria_unclassified	Bacteria_unclassifie	Bacteria_unclassifie	Bacteria

Lachnospiraceae	Eubacteriales	Clostridia	Firmicutes	Bacteria
Lachnospiraceae	Eubacteriales	Clostridia	Firmicutes	Bacteria
Oscillospiraceae	Eubacteriales	Clostridia	Firmicutes	Bacteria
Oscillospiraceae	Eubacteriales	Clostridia	Firmicutes	Bacteria
Eggerthellaceae	Eggerthellales	Coriobacteriia	Actinobacteria	Bacteria
Eggerthellaceae	Eggerthellales	Coriobacteriia	Actinobacteria	Bacteria
Eggerthellaceae	Eggerthellales	Coriobacteriia	Actinobacteria	Bacteria
Akkermansiaceae	Verrucomicrobiales	Verrucomicrobiae	Verrucomicrobia	Bacteria
Rikenellaceae	Bacteroidales	Bacteroidia	Bacteroidota	Bacteria
Oscillospiraceae	Eubacteriales	Clostridia	Firmicutes	Bacteria
Bacteria_unclassified	Bacteria_unclassified	Bacteria_unclassifie	Bacteria_unclassifie	Bacteria

Bacteria_unclassified	Bacteria_unclassified	Bacteria_unclassifie	Bacteria_unclassifie	Bacteria
Bacteria_unclassified	Bacteria_unclassified	Bacteria_unclassifie	Bacteria_unclassifie	Bacteria
Bacteroidaceae	Bacteroidales	Bacteroidia	Bacteroidota	Bacteria
Bifidobacteriaceae	Bifidobacteriales	Actinomycetia	Actinobacteria	Bacteria
Clostridia_unclassified	Clostridia_unclassifie	Clostridia	Firmicutes	Bacteria
Clostridiaceae	Eubacteriales	Clostridia	Firmicutes	Bacteria
Clostridiaceae	Eubacteriales	Clostridia	Firmicutes	Bacteria
Eubacteriales_unclassified	Eubacteriales	Clostridia	Firmicutes	Bacteria
Erysipelotrichaceae	Erysipelotrichales	Erysipelotrichia	Firmicutes	Bacteria
Coriobacteriaceae	Coriobacteriales	Coriobacteriia	Actinobacteria	Bacteria
Lachnospiraceae	Eubacteriales	Clostridia	Firmicutes	Bacteria
Erysipelotrichaceae	Erysipelotrichales	Erysipelotrichia	Firmicutes	Bacteria
Erysipelotrichales_unclassifie	Erysipelotrichales	Erysipelotrichia	Firmicutes	Bacteria
Eubacteriaceae	Eubacteriales	Clostridia	Firmicutes	Bacteria
Eubacteriaceae	Eubacteriales	Clostridia	Firmicutes	Bacteria
Lachnospiraceae	Eubacteriales	Clostridia	Firmicutes	Bacteria
Eggerthellaceae	Eggerthellales	Coriobacteriia	Actinobacteria	Bacteria
Lachnospiraceae	Eubacteriales	Clostridia	Firmicutes	Bacteria
Muribaculaceae	Bacteroidales	Bacteroidia	Bacteroidota	Bacteria
Muribaculaceae	Bacteroidales	Bacteroidia	Bacteroidota	Bacteria
Muribaculaceae	Bacteroidales	Bacteroidia	Bacteroidota	Bacteria
FGB9508	OFGB9508	CFGB9508	Firmicutes	Bacteria
FGB2838	OFGB2838	CFGB2838	Firmicutes	Bacteria
FGB2838	OFGB2838	CFGB2838	Firmicutes	Bacteria
FGB2838	OFGB2838	CFGB2838	Firmicutes	Bacteria
Pumilibacteraceae	Eubacteriales	Clostridia	Firmicutes	Bacteria
FGB28439	OFGB28439	CFGB28439	Firmicutes	Bacteria
Clostridia_unclassified	Clostridia_unclassifie	Clostridia	Firmicutes	Bacteria
Eubacteriaceae	Eubacteriales	Clostridia	Firmicutes	Bacteria
Lachnospiraceae	Eubacteriales	Clostridia	Firmicutes	Bacteria
Lachnospiraceae	Eubacteriales	Clostridia	Firmicutes	Bacteria
Lachnospiraceae	Eubacteriales	Clostridia	Firmicutes	Bacteria
Lachnospiraceae	Eubacteriales	Clostridia	Firmicutes	Bacteria
FGB77305	OFGB77305	CFGB77305	Firmicutes	Bacteria
Clostridiaceae	Eubacteriales	Clostridia	Firmicutes	Bacteria
Lachnospiraceae	Eubacteriales	Clostridia	Firmicutes	Bacteria
Lachnospiraceae	Eubacteriales	Clostridia	Firmicutes	Bacteria
Lachnospiraceae	Eubacteriales	Clostridia	Firmicutes	Bacteria
FGB9633	OFGB9633	CFGB9633	Firmicutes	Bacteria
Bacteria_unclassified	Bacteria_unclassified	Bacteria_unclassifie	Bacteria_unclassifie	Bacteria
Bacteria_unclassified	Bacteria_unclassified	Bacteria_unclassifie	Bacteria_unclassifie	Bacteria
Bacteria_unclassified	Bacteria_unclassified	Bacteria_unclassifie	Bacteria_unclassifie	Bacteria
Bacteria_unclassified	Bacteria_unclassified	Bacteria_unclassifie	Bacteria_unclassifie	Bacteria
Lachnospiraceae	Eubacteriales	Clostridia	Firmicutes	Bacteria
Lachnospiraceae	Eubacteriales	Clostridia	Firmicutes	Bacteria
Lachnospiraceae	Eubacteriales	Clostridia	Firmicutes	Bacteria

FGB77359	OFGB77359	CFGB77359	Bacteria_unclassifie	Bacteria
FGB9639	OFGB9639	CFGB9639	Firmicutes	Bacteria
Lachnospiraceae	Eubacteriales	Clostridia	Firmicutes	Bacteria
Lachnospiraceae	Eubacteriales	Clostridia	Firmicutes	Bacteria
Lachnospiraceae	Eubacteriales	Clostridia	Firmicutes	Bacteria
Clostridiaceae	Eubacteriales	Clostridia	Firmicutes	Bacteria
Clostridiaceae	Eubacteriales	Clostridia	Firmicutes	Bacteria
Clostridiaceae	Eubacteriales	Clostridia	Firmicutes	Bacteria
Clostridiaceae	Eubacteriales	Clostridia	Firmicutes	Bacteria
Clostridiaceae	Eubacteriales	Clostridia	Firmicutes	Bacteria
Clostridiaceae	Eubacteriales	Clostridia	Firmicutes	Bacteria
Clostridiaceae	Eubacteriales	Clostridia	Firmicutes	Bacteria
Eubacteriaceae	Eubacteriales	Clostridia	Firmicutes	Bacteria
FGB9658	OFGB9658	CFGB9658	Firmicutes	Bacteria
FGB9659	OFGB9659	CFGB9659	Firmicutes	Bacteria
Bacteria_unclassified	Bacteria_unclassified	Bacteria_unclassifie	Bacteria_unclassifie	Bacteria
FGB9827	OFGB9827	CFGB9827	Firmicutes	Bacteria
Eubacteriaceae	Eubacteriales	Clostridia	Firmicutes	Bacteria
FGB77303	OFGB77303	CFGB77303	Bacteria_unclassifie	Bacteria
Eubacteriales_unclassified	Eubacteriales	Clostridia	Firmicutes	Bacteria
Oscillospiraceae	Eubacteriales	Clostridia	Firmicutes	Bacteria
FGB30328	OFGB30328	CFGB30328	Firmicutes	Bacteria
Oscillospiraceae	Eubacteriales	Clostridia	Firmicutes	Bacteria
Oscillospiraceae	Eubacteriales	Clostridia	Firmicutes	Bacteria
Oscillospiraceae	Eubacteriales	Clostridia	Firmicutes	Bacteria
Oscillospiraceae	Eubacteriales	Clostridia	Firmicutes	Bacteria
Oscillospiraceae	Eubacteriales	Clostridia	Firmicutes	Bacteria
Oscillospiraceae	Eubacteriales	Clostridia	Firmicutes	Bacteria
Oscillospiraceae	Eubacteriales	Clostridia	Firmicutes	Bacteria
FGB77153	OFGB77153	CFGB77153	Actinobacteria	Bacteria
FGB1791	OFGB1791	CFGB1791	Tenericutes	Bacteria
FGB10290	OFGB10290	CFGB10290	Firmicutes	Bacteria
Oscillospiraceae	Eubacteriales	Clostridia	Firmicutes	Bacteria
FGB1765	OFGB1765	CFGB1765	Firmicutes	Bacteria
FGB10349	OFGB10349	CFGB10349	Firmicutes	Bacteria
FGB10667	OFGB10667	CFGB10667	Firmicutes	Bacteria
Lachnospiraceae	Eubacteriales	Clostridia	Firmicutes	Bacteria
Lachnospiraceae	Eubacteriales	Clostridia	Firmicutes	Bacteria
Christensenellaceae	Eubacteriales	Clostridia	Firmicutes	Bacteria
FGB10299	OFGB10299	CFGB10299	Firmicutes	Bacteria
Oscillospiraceae	Eubacteriales	Clostridia	Firmicutes	Bacteria
Oscillospiraceae	Eubacteriales	Clostridia	Firmicutes	Bacteria
Lachnospiraceae	Eubacteriales	Clostridia	Firmicutes	Bacteria
Clostridia_unclassified	Clostridia_unclassifie	Clostridia	Firmicutes	Bacteria
Lachnospiraceae	Eubacteriales	Clostridia	Firmicutes	Bacteria
Lachnospiraceae	Eubacteriales	Clostridia	Firmicutes	Bacteria

Lachnospiraceae	Eubacteriales	Clostridia	Firmicutes	Bacteria
Lachnospiraceae	Eubacteriales	Clostridia	Firmicutes	Bacteria
Lachnospiraceae	Eubacteriales	Clostridia	Firmicutes	Bacteria
Lachnospiraceae	Eubacteriales	Clostridia	Firmicutes	Bacteria
Lachnospiraceae	Eubacteriales	Clostridia	Firmicutes	Bacteria
Lachnospiraceae	Eubacteriales	Clostridia	Firmicutes	Bacteria
Lactobacillaceae	Lactobacillales	Bacilli	Firmicutes	Bacteria
Muribaculaceae	Bacteroidales	Bacteroidia	Bacteroidota	Bacteria
Oscillospiraceae	Eubacteriales	Clostridia	Firmicutes	Bacteria
Oscillospiraceae	Eubacteriales	Clostridia	Firmicutes	Bacteria
Oscillospiraceae	Eubacteriales	Clostridia	Firmicutes	Bacteria
Oscillospiraceae	Eubacteriales	Clostridia	Firmicutes	Bacteria
Sutterellaceae	Burkholderiales	Betaproteobacteria	Proteobacteria	Bacteria
Peptostreptococcaceae	Eubacteriales	Clostridia	Firmicutes	Bacteria
Lachnospiraceae	Eubacteriales	Clostridia	Firmicutes	Bacteria
Turicibacteraceae	Erysipelotrichales	Erysipelotrichia	Firmicutes	Bacteria
Bacteria_unclassified	Bacteria_unclassified	Bacteria_unclassifie	Bacteria_unclassifie	Bacteria
Bacteria_unclassified	Bacteria_unclassified	Bacteria_unclassifie	Bacteria_unclassifie	Bacteria
Bacteria_unclassified	Bacteria_unclassified	Bacteria_unclassifie	Bacteria_unclassifie	Bacteria

Lachnospiraceae	Eubacteriales	Clostridia	Firmicutes	Bacteria
Lachnospiraceae	Eubacteriales	Clostridia	Firmicutes	Bacteria
Oscillospiraceae	Eubacteriales	Clostridia	Firmicutes	Bacteria
Oscillospiraceae	Eubacteriales	Clostridia	Firmicutes	Bacteria
Eggerthellaceae	Eggerthellales	Coriobacteriia	Actinobacteria	Bacteria
Eggerthellaceae	Eggerthellales	Coriobacteriia	Actinobacteria	Bacteria
Eggerthellaceae	Eggerthellales	Coriobacteriia	Actinobacteria	Bacteria
Akkermansiaceae	Verrucomicrobiales	Verrucomicrobiae	Verrucomicrobia	Bacteria
Rikenellaceae	Bacteroidales	Bacteroidia	Bacteroidota	Bacteria
Oscillospiraceae	Eubacteriales	Clostridia	Firmicutes	Bacteria
Bacteria_unclassified	Bacteria_unclassified	Bacteria_unclassifie	Bacteria_unclassifie	Bacteria
Bacteria_unclassified	Bacteria_unclassified	Bacteria_unclassifie	Bacteria_unclassifie	Bacteria
Bacteria_unclassified	Bacteria_unclassified	Bacteria_unclassifie	Bacteria_unclassifie	Bacteria
Bacteroidaceae	Bacteroidales	Bacteroidia	Bacteroidota	Bacteria
Bifidobacteriaceae	Bifidobacteriales	Actinomycetia	Actinobacteria	Bacteria
Clostridia_unclassified	Clostridia_unclassifie	Clostridia	Firmicutes	Bacteria
Clostridiaceae	Eubacteriales	Clostridia	Firmicutes	Bacteria
Clostridiaceae	Eubacteriales	Clostridia	Firmicutes	Bacteria
Eubacteriales_unclassified	Eubacteriales	Clostridia	Firmicutes	Bacteria
Erysipelotrichaceae	Erysipelotrichales	Erysipelotrichia	Firmicutes	Bacteria
Coriobacteriaceae	Coriobacteriales	Coriobacteriia	Actinobacteria	Bacteria
Lachnospiraceae	Eubacteriales	Clostridia	Firmicutes	Bacteria
Erysipelotrichaceae	Erysipelotrichales	Erysipelotrichia	Firmicutes	Bacteria
Erysipelotrichales_unclassifie	Erysipelotrichales	Erysipelotrichia	Firmicutes	Bacteria

Eubacteriaceae	Eubacteriales	Clostridia	Firmicutes	Bacteria
Eubacteriaceae	Eubacteriales	Clostridia	Firmicutes	Bacteria
Lachnospiraceae	Eubacteriales	Clostridia	Firmicutes	Bacteria
Eggerthellaceae	Eggerthellales	Coriobacteriia	Actinobacteria	Bacteria
Lachnospiraceae	Eubacteriales	Clostridia	Firmicutes	Bacteria
Muribaculaceae	Bacteroidales	Bacteroidia	Bacteroidota	Bacteria
Muribaculaceae	Bacteroidales	Bacteroidia	Bacteroidota	Bacteria
Muribaculaceae	Bacteroidales	Bacteroidia	Bacteroidota	Bacteria
FGB9508	OFGB9508	CFGB9508	Firmicutes	Bacteria
FGB2838	OFGB2838	CFGB2838	Firmicutes	Bacteria
FGB2838	OFGB2838	CFGB2838	Firmicutes	Bacteria
FGB2838	OFGB2838	CFGB2838	Firmicutes	Bacteria
Pumilibacteraceae	Eubacteriales	Clostridia	Firmicutes	Bacteria
FGB28439	OFGB28439	CFGB28439	Firmicutes	Bacteria
Clostridia_unclassified	Clostridia_unclassifie	Clostridia	Firmicutes	Bacteria
Eubacteriaceae	Eubacteriales	Clostridia	Firmicutes	Bacteria
Lachnospiraceae	Eubacteriales	Clostridia	Firmicutes	Bacteria
Lachnospiraceae	Eubacteriales	Clostridia	Firmicutes	Bacteria
Lachnospiraceae	Eubacteriales	Clostridia	Firmicutes	Bacteria
Lachnospiraceae	Eubacteriales	Clostridia	Firmicutes	Bacteria
FGB77305	OFGB77305	CFGB77305	Firmicutes	Bacteria
Clostridiaceae	Eubacteriales	Clostridia	Firmicutes	Bacteria
Lachnospiraceae	Eubacteriales	Clostridia	Firmicutes	Bacteria
Lachnospiraceae	Eubacteriales	Clostridia	Firmicutes	Bacteria
Lachnospiraceae	Eubacteriales	Clostridia	Firmicutes	Bacteria
FGB9633	OFGB9633	CFGB9633	Firmicutes	Bacteria
Bacteria_unclassified	Bacteria_unclassified	Bacteria_unclassifie	Bacteria_unclassifie	Bacteria
Bacteria_unclassified	Bacteria_unclassified	Bacteria_unclassifie	Bacteria_unclassifie	Bacteria
Bacteria_unclassified	Bacteria_unclassified	Bacteria_unclassifie	Bacteria_unclassifie	Bacteria
Bacteria_unclassified	Bacteria_unclassified	Bacteria_unclassifie	Bacteria_unclassifie	Bacteria
Lachnospiraceae	Eubacteriales	Clostridia	Firmicutes	Bacteria
Lachnospiraceae	Eubacteriales	Clostridia	Firmicutes	Bacteria
Lachnospiraceae	Eubacteriales	Clostridia	Firmicutes	Bacteria
FGB77359	OFGB77359	CFGB77359	Bacteria_unclassifie	Bacteria
FGB9639	OFGB9639	CFGB9639	Firmicutes	Bacteria
Lachnospiraceae	Eubacteriales	Clostridia	Firmicutes	Bacteria
Lachnospiraceae	Eubacteriales	Clostridia	Firmicutes	Bacteria
Lachnospiraceae	Eubacteriales	Clostridia	Firmicutes	Bacteria
Clostridiaceae	Eubacteriales	Clostridia	Firmicutes	Bacteria
Clostridiaceae	Eubacteriales	Clostridia	Firmicutes	Bacteria
Clostridiaceae	Eubacteriales	Clostridia	Firmicutes	Bacteria
Clostridiaceae	Eubacteriales	Clostridia	Firmicutes	Bacteria
Clostridiaceae	Eubacteriales	Clostridia	Firmicutes	Bacteria
Clostridiaceae	Eubacteriales	Clostridia	Firmicutes	Bacteria
Clostridiaceae	Eubacteriales	Clostridia	Firmicutes	Bacteria
Eubacteriaceae	Eubacteriales	Clostridia	Firmicutes	Bacteria

FGB9658	OFGB9658	CFGB9658	Firmicutes	Bacteria
FGB9659	OFGB9659	CFGB9659	Firmicutes	Bacteria
Bacteria_unclassified	Bacteria_unclassified	Bacteria_unclassifie	Bacteria_unclassifie	Bacteria
FGB9827	OFGB9827	CFGB9827	Firmicutes	Bacteria
Eubacteriaceae	Eubacteriales	Clostridia	Firmicutes	Bacteria
FGB77303	OFGB77303	CFGB77303	Bacteria_unclassifie	Bacteria
Eubacteriales_unclassified	Eubacteriales	Clostridia	Firmicutes	Bacteria
Oscillospiraceae	Eubacteriales	Clostridia	Firmicutes	Bacteria
FGB30328	OFGB30328	CFGB30328	Firmicutes	Bacteria
Oscillospiraceae	Eubacteriales	Clostridia	Firmicutes	Bacteria
Oscillospiraceae	Eubacteriales	Clostridia	Firmicutes	Bacteria
Oscillospiraceae	Eubacteriales	Clostridia	Firmicutes	Bacteria
Oscillospiraceae	Eubacteriales	Clostridia	Firmicutes	Bacteria
Oscillospiraceae	Eubacteriales	Clostridia	Firmicutes	Bacteria
Oscillospiraceae	Eubacteriales	Clostridia	Firmicutes	Bacteria
Oscillospiraceae	Eubacteriales	Clostridia	Firmicutes	Bacteria
FGB77153	OFGB77153	CFGB77153	Actinobacteria	Bacteria
FGB1791	OFGB1791	CFGB1791	Tenericutes	Bacteria
FGB10290	OFGB10290	CFGB10290	Firmicutes	Bacteria
Oscillospiraceae	Eubacteriales	Clostridia	Firmicutes	Bacteria
FGB1765	OFGB1765	CFGB1765	Firmicutes	Bacteria
FGB10349	OFGB10349	CFGB10349	Firmicutes	Bacteria
FGB10667	OFGB10667	CFGB10667	Firmicutes	Bacteria
Lachnospiraceae	Eubacteriales	Clostridia	Firmicutes	Bacteria
Lachnospiraceae	Eubacteriales	Clostridia	Firmicutes	Bacteria
Christensenellaceae	Eubacteriales	Clostridia	Firmicutes	Bacteria
FGB10299	OFGB10299	CFGB10299	Firmicutes	Bacteria
Oscillospiraceae	Eubacteriales	Clostridia	Firmicutes	Bacteria
Oscillospiraceae	Eubacteriales	Clostridia	Firmicutes	Bacteria
Lachnospiraceae	Eubacteriales	Clostridia	Firmicutes	Bacteria
Clostridia_unclassified	Clostridia_unclassifie	Clostridia	Firmicutes	Bacteria
Lachnospiraceae	Eubacteriales	Clostridia	Firmicutes	Bacteria
Lachnospiraceae	Eubacteriales	Clostridia	Firmicutes	Bacteria
Lachnospiraceae	Eubacteriales	Clostridia	Firmicutes	Bacteria
Lachnospiraceae	Eubacteriales	Clostridia	Firmicutes	Bacteria
Lachnospiraceae	Eubacteriales	Clostridia	Firmicutes	Bacteria
Lachnospiraceae	Eubacteriales	Clostridia	Firmicutes	Bacteria
Lachnospiraceae	Eubacteriales	Clostridia	Firmicutes	Bacteria
Lachnospiraceae	Eubacteriales	Clostridia	Firmicutes	Bacteria
Lactobacillaceae	Lactobacillales	Bacilli	Firmicutes	Bacteria
Muribaculaceae	Bacteroidales	Bacteroidia	Bacteroidota	Bacteria
Oscillospiraceae	Eubacteriales	Clostridia	Firmicutes	Bacteria
Oscillospiraceae	Eubacteriales	Clostridia	Firmicutes	Bacteria
Oscillospiraceae	Eubacteriales	Clostridia	Firmicutes	Bacteria
Oscillospiraceae	Eubacteriales	Clostridia	Firmicutes	Bacteria
Sutterellaceae	Burkholderiales	Betaproteobacteria	Proteobacteria	Bacteria

Peptostreptococcaceae	Eubacteriales	Clostridia	Firmicutes	Bacteria
Lachnospiraceae	Eubacteriales	Clostridia	Firmicutes	Bacteria
Turicibacteraceae	Erysipelotrichales	Erysipelotrichia	Firmicutes	Bacteria
Bacteria_unclassified	Bacteria_unclassified	Bacteria_unclassifie	Bacteria_unclassifie	Bacteria
Bacteria_unclassified	Bacteria_unclassified	Bacteria_unclassifie	Bacteria_unclassifie	Bacteria
Bacteria_unclassified	Bacteria_unclassified	Bacteria_unclassifie	Bacteria_unclassifie	Bacteria

MetaPhlan Annotation
k__Bacteria|p__Firmicutes|c__Clostridia|o__Eubacteriales|f__Lachnospiraceae|g__Acetatifactor|s__Acetatifactor_SGB415[illegible]
k__Bacteria|p__Firmicutes|c__Clostridia|o__Eubacteriales|f__Lachnospiraceae|g__Acetatifactor|s__Acetatifactor_muris
k__Bacteria|p__Firmicutes|c__Clostridia|o__Eubacteriales|f__Oscillospiraceae|g__Acutalibacter|s__Acutalibacter_muris
k__Bacteria|p__Firmicutes|c__Clostridia|o__Eubacteriales|f__Oscillospiraceae|g__Acutalibacter|s__Acutalibacter_sp_1XD[illegible]
k__Bacteria|p__Actinobacteria|c__Coriobacteriia|o__Eggerthellales|f__Eggerthellaceae|g__Adlercreutzia|s__Adlercreutzia[illegible]
k__Bacteria|p__Actinobacteria|c__Coriobacteriia|o__Eggerthellales|f__Eggerthellaceae|g__Adlercreutzia|s__Adlercreutzia[illegible]
k__Bacteria|p__Actinobacteria|c__Coriobacteriia|o__Eggerthellales|f__Eggerthellaceae|g__Adlercreutzia|s__Adlercreutzia[illegible]
k__Bacteria|p__Verrucomicrobia|c__Verrucomicrobiae|o__Verrucomicrobiales|f__Akkermansiaceae|g__Akkermansia|s__[illegible]
k__Bacteria|p__Bacteroidota|c__Bacteroidia|o__Bacteroidales|f__Rikenellaceae|g__Alistipes|s__Alistipes_sp_DSM_11234[illegible]
k__Bacteria|p__Firmicutes|c__Clostridia|o__Eubacteriales|f__Oscillospiraceae|g__Anaerotruncus|s__Anaerotruncus_sp_1[illegible]
k__Bacteria|p__Bacteria_unclassified|c__Bacteria_unclassified|o__Bacteria_unclassified|f__Bacteria_unclassified|g__Bact[illegible]
k__Bacteria|p__Bacteria_unclassified|c__Bacteria_unclassified|o__Bacteria_unclassified|f__Bacteria_unclassified|g__Bact[illegible]
k__Bacteria|p__Bacteria_unclassified|c__Bacteria_unclassified|o__Bacteria_unclassified|f__Bacteria_unclassified|g__Bact[illegible]
k__Bacteria|p__Bacteroidota|c__Bacteroidia|o__Bacteroidales|f__Bacteroidaceae|g__Bacteroides|s__Bacteroides_thetaio[illegible]
k__Bacteria|p__Actinobacteria|c__Actinomycetia|o__Bifidobacteriales|f__Bifidobacteriaceae|g__Bifidobacterium|s__Bifid[illegible]
k__Bacteria|p__Firmicutes|c__Clostridia|o__Clostridia_unclassified|f__Clostridia_unclassified|g__Clostridia_unclassified|s_[illegible]
k__Bacteria|p__Firmicutes|c__Clostridia|o__Eubacteriales|f__Clostridiaceae|g__Clostridiaceae_unclassified|s__Clostridia[illegible]
k__Bacteria|p__Firmicutes|c__Clostridia|o__Eubacteriales|f__Clostridiaceae|g__Clostridiaceae_unclassified|s__Clostridia[illegible]
k__Bacteria|p__Firmicutes|c__Clostridia|o__Eubacteriales|f__Eubacteriales_unclassified|g__Eubacteriales_unclassified|s_[illegible]
k__Bacteria|p__Firmicutes|c__Erysipelotrichia|o__Erysipelotrichales|f__Erysipelotrichaceae|g__Erysipelatoclostridium|s_[illegible]
k__Bacteria|p__Actinobacteria|c__Coriobacteriia|o__Coriobacteriales|f__Coriobacteriaceae|g__Coriobacteriaceae_unclass[illegible]
k__Bacteria|p__Firmicutes|c__Clostridia|o__Eubacteriales|f__Lachnospiraceae|g__Dorea|s__Dorea_sp_5_2
k__Bacteria|p__Firmicutes|c__Erysipelotrichia|o__Erysipelotrichales|f__Erysipelotrichaceae|g__Dubosiella|s__Dubosiella_[illegible]
k__Bacteria|p__Firmicutes|c__Erysipelotrichia|o__Erysipelotrichales|f__Erysipelotrichales_unclassified|g__Erysipelotrichal[illegible]
k__Bacteria|p__Firmicutes|c__Clostridia|o__Eubacteriales|f__Eubacteriaceae|g__Eubacteriaceae_unclassified|s__Eubacte[illegible]
k__Bacteria|p__Firmicutes|c__Clostridia|o__Eubacteriales|f__Eubacteriaceae|g__Eubacteriaceae_unclassified|s__Eubacte[illegible]
k__Bacteria|p__Firmicutes|c__Clostridia|o__Eubacteriales|f__Lachnospiraceae|g__GGB20149|s__GGB20149_SGB29430
k__Bacteria|p__Actinobacteria|c__Coriobacteriia|o__Eggerthellales|f__Eggerthellaceae|g__GGB22635|s__GGB22635_SGB[illegible]
k__Bacteria|p__Firmicutes|c__Clostridia|o__Eubacteriales|f__Lachnospiraceae|g__GGB25041|s__GGB25041_SGB36960
k__Bacteria|p__Bacteroidota|c__Bacteroidia|o__Bacteroidales|f__Muribaculaceae|g__GGB27876|s__GGB27876_SGB403[illegible]
k__Bacteria|p__Bacteroidota|c__Bacteroidia|o__Bacteroidales|f__Muribaculaceae|g__GGB27878|s__GGB27878_SGB403[illegible]
k__Bacteria|p__Bacteroidota|c__Bacteroidia|o__Bacteroidales|f__Muribaculaceae|g__GGB27918|s__GGB27918_SGB403[illegible]
k__Bacteria|p__Firmicutes|c__CFGB9508|o__OFGB9508|f__FGB9508|g__GGB28382|s__GGB28382_SGB40962
k__Bacteria|p__Firmicutes|c__CFGB2838|o__OFGB2838|f__FGB2838|g__GGB28399|s__GGB28399_SGB40980
k__Bacteria|p__Firmicutes|c__CFGB2838|o__OFGB2838|f__FGB2838|g__GGB28411|s__GGB28411_SGB40993
k__Bacteria|p__Firmicutes|c__CFGB2838|o__OFGB2838|f__FGB2838|g__GGB28415|s__GGB28415_SGB40997
k__Bacteria|p__Firmicutes|c__Clostridia|o__Eubacteriales|f__Pumilibacteraceae|g__GGB28430|s__GGB28430_SGB41013
k__Bacteria|p__Firmicutes|c__CFGB28439|o__OFGB28439|f__FGB28439|g__GGB28439|s__GGB28439_SGB41022
k__Bacteria|p__Firmicutes|c__Clostridia|o__Clostridia_unclassified|f__Clostridia_unclassified|g__GGB28778|s__GGB2877[illegible]
k__Bacteria|p__Firmicutes|c__Clostridia|o__Eubacteriales|f__Eubacteriaceae|g__GGB28784|s__GGB28784_SGB41437
k__Bacteria|p__Firmicutes|c__Clostridia|o__Eubacteriales|f__Lachnospiraceae|g__GGB28792|s__GGB28792_SGB41445
k__Bacteria|p__Firmicutes|c__Clostridia|o__Eubacteriales|f__Lachnospiraceae|g__GGB28798|s__GGB28798_SGB41451
k__Bacteria|p__Firmicutes|c__Clostridia|o__Eubacteriales|f__Lachnospiraceae|g__GGB28802|s__GGB28802_SGB41455
k__Bacteria|p__Firmicutes|c__Clostridia|o__Eubacteriales|f__Lachnospiraceae|g__GGB28818|s__GGB28818_SGB41473
k__Bacteria|p__Firmicutes|c__CFGB77305|o__OFGB77305|f__FGB77305|g__GGB28828|s__GGB28828_SGB41484

k__Bacteria|p__Firmicutes|c__Clostridia|o__Eubacteriales|f__Clostridiaceae|g__GGB28851|s__GGB28851_SGB41518
k__Bacteria|p__Firmicutes|c__Clostridia|o__Eubacteriales|f__Lachnospiraceae|g__GGB28859|s__GGB28859_SGB41528
k__Bacteria|p__Firmicutes|c__Clostridia|o__Eubacteriales|f__Lachnospiraceae|g__GGB28864|s__GGB28864_SGB41535
k__Bacteria|p__Firmicutes|c__Clostridia|o__Eubacteriales|f__Lachnospiraceae|g__GGB28869|s__GGB28869_SGB41543
k__Bacteria|p__Firmicutes|c__CFGB9633|o__OFGB9633|f__FGB9633|g__GGB28883|s__GGB28883_SGB41564
k__Bacteria|p__Bacteria_unclassified|c__Bacteria_unclassified|o__Bacteria_unclassified|f__Bacteria_unclassified|g__GGB2
k__Bacteria|p__Bacteria_unclassified|c__Bacteria_unclassified|o__Bacteria_unclassified|f__Bacteria_unclassified|g__GGB2
k__Bacteria|p__Bacteria_unclassified|c__Bacteria_unclassified|o__Bacteria_unclassified|f__Bacteria_unclassified|g__GGB2
k__Bacteria|p__Bacteria_unclassified|c__Bacteria_unclassified|o__Bacteria_unclassified|f__Bacteria_unclassified|g__GGB2
k__Bacteria|p__Firmicutes|c__Clostridia|o__Eubacteriales|f__Lachnospiraceae|g__GGB28916|s__GGB28916_SGB41612
k__Bacteria|p__Firmicutes|c__Clostridia|o__Eubacteriales|f__Lachnospiraceae|g__GGB28924|s__GGB28924_SGB41621
k__Bacteria|p__Firmicutes|c__Clostridia|o__Eubacteriales|f__Lachnospiraceae|g__GGB28926|s__GGB28926_SGB41624
k__Bacteria|p__Bacteria_unclassified|c__CFGB77359|o__OFGB77359|f__FGB77359|g__GGB28927|s__GGB28927_SGB416
k__Bacteria|p__Firmicutes|c__CFGB9639|o__OFGB9639|f__FGB9639|g__GGB28934|s__GGB28934_SGB41635
k__Bacteria|p__Firmicutes|c__Clostridia|o__Eubacteriales|f__Lachnospiraceae|g__GGB28946|s__GGB28946_SGB41652
k__Bacteria|p__Firmicutes|c__Clostridia|o__Eubacteriales|f__Lachnospiraceae|g__GGB28949|s__GGB28949_SGB41655
k__Bacteria|p__Firmicutes|c__Clostridia|o__Eubacteriales|f__Lachnospiraceae|g__GGB28949|s__GGB28949_SGB41656
k__Bacteria|p__Firmicutes|c__Clostridia|o__Eubacteriales|f__Clostridiaceae|g__GGB28950|s__GGB28950_SGB41657
k__Bacteria|p__Firmicutes|c__Clostridia|o__Eubacteriales|f__Clostridiaceae|g__GGB28951|s__GGB28951_SGB102295
k__Bacteria|p__Firmicutes|c__Clostridia|o__Eubacteriales|f__Clostridiaceae|g__GGB28951|s__GGB28951_SGB41658
k__Bacteria|p__Firmicutes|c__Clostridia|o__Eubacteriales|f__Clostridiaceae|g__GGB28954|s__GGB28954_SGB41662
k__Bacteria|p__Firmicutes|c__Clostridia|o__Eubacteriales|f__Clostridiaceae|g__GGB28956|s__GGB28956_SGB41665
k__Bacteria|p__Firmicutes|c__Clostridia|o__Eubacteriales|f__Clostridiaceae|g__GGB28960|s__GGB28960_SGB41669
k__Bacteria|p__Firmicutes|c__Clostridia|o__Eubacteriales|f__Clostridiaceae|g__GGB28967|s__GGB28967_SGB41678
k__Bacteria|p__Firmicutes|c__Clostridia|o__Eubacteriales|f__Eubacteriaceae|g__GGB28991|s__GGB28991_SGB41705
k__Bacteria|p__Firmicutes|c__CFGB9658|o__OFGB9658|f__FGB9658|g__GGB29002|s__GGB29002_SGB41718
k__Bacteria|p__Firmicutes|c__CFGB9659|o__OFGB9659|f__FGB9659|g__GGB29003|s__GGB29003_SGB41719
k__Bacteria|p__Bacteria_unclassified|c__Bacteria_unclassified|o__Bacteria_unclassified|f__Bacteria_unclassified|g__GGB2
k__Bacteria|p__Firmicutes|c__CFGB9827|o__OFGB9827|f__FGB9827|g__GGB29531|s__GGB29531_SGB42317
k__Bacteria|p__Firmicutes|c__Clostridia|o__Eubacteriales|f__Eubacteriaceae|g__GGB29685|s__GGB29685_SGB42494
k__Bacteria|p__Bacteria_unclassified|c__CFGB77303|o__OFGB77303|f__FGB77303|g__GGB30141|s__GGB30141_SGB430
k__Bacteria|p__Firmicutes|c__Clostridia|o__Eubacteriales|f__Eubacteriales_unclassified|g__GGB30286|s__GGB30286_SG
k__Bacteria|p__Firmicutes|c__Clostridia|o__Eubacteriales|f__Oscillospiraceae|g__GGB30303|s__GGB30303_SGB43268
k__Bacteria|p__Firmicutes|c__CFGB30328|o__OFGB30328|f__FGB30328|g__GGB30413|s__GGB30413_SGB43452
k__Bacteria|p__Firmicutes|c__Clostridia|o__Eubacteriales|f__Oscillospiraceae|g__GGB30454|s__GGB30454_SGB43514
k__Bacteria|p__Firmicutes|c__Clostridia|o__Eubacteriales|f__Oscillospiraceae|g__GGB30455|s__GGB30455_SGB43519
k__Bacteria|p__Firmicutes|c__Clostridia|o__Eubacteriales|f__Oscillospiraceae|g__GGB30461|s__GGB30461_SGB43527
k__Bacteria|p__Firmicutes|c__Clostridia|o__Eubacteriales|f__Oscillospiraceae|g__GGB30461|s__GGB30461_SGB43530
k__Bacteria|p__Firmicutes|c__Clostridia|o__Eubacteriales|f__Oscillospiraceae|g__GGB30463|s__GGB30463_SGB43537
k__Bacteria|p__Firmicutes|c__Clostridia|o__Eubacteriales|f__Oscillospiraceae|g__GGB30473|s__GGB30473_SGB43557
k__Bacteria|p__Firmicutes|c__Clostridia|o__Eubacteriales|f__Oscillospiraceae|g__GGB30475|s__GGB30475_SGB63182
k__Bacteria|p__Actinobacteria|c__CFGB77153|o__OFGB77153|f__FGB77153|g__GGB30861|s__GGB30861_SGB44083
k__Bacteria|p__Tenericutes|c__CFGB1791|o__OFGB1791|f__FGB1791|g__GGB31312|s__GGB31312_SGB44628
k__Bacteria|p__Firmicutes|c__CFGB10290|o__OFGB10290|f__FGB10290|g__GGB31438|s__GGB31438_SGB44768
k__Bacteria|p__Firmicutes|c__Clostridia|o__Eubacteriales|f__Oscillospiraceae|g__GGB3171|s__GGB3171_SGB4185
k__Bacteria|p__Firmicutes|c__CFGB1765|o__OFGB1765|f__FGB1765|g__GGB31823|s__GGB31823_SGB45199

k__Bacteria|p__Firmicutes|c__CFGB10349|o__OFGB10349|f__FGB10349|g__GGB31853|s__GGB31853_SGB45233
k__Bacteria|p__Firmicutes|c__CFGB10667|o__OFGB10667|f__FGB10667|g__GGB32371|s__GGB32371_SGB41694
k__Bacteria|p__Firmicutes|c__Clostridia|o__Eubacteriales|f__Lachnospiraceae|g__GGB3793|s__GGB3793_SGB5158
k__Bacteria|p__Firmicutes|c__Clostridia|o__Eubacteriales|f__Lachnospiraceae|g__GGB42598|s__GGB42598_SGB59794
k__Bacteria|p__Firmicutes|c__Clostridia|o__Eubacteriales|f__Christensenellaceae|g__GGB45656|s__GGB45656_SGB6337
k__Bacteria|p__Firmicutes|c__CFGB10299|o__OFGB10299|f__FGB10299|g__GGB47127|s__GGB47127_SGB65054
k__Bacteria|p__Firmicutes|c__Clostridia|o__Eubacteriales|f__Oscillospiraceae|g__GGB74395|s__GGB74395_SGB43521
k__Bacteria|p__Firmicutes|c__Clostridia|o__Eubacteriales|f__Oscillospiraceae|g__GGB75053|s__GGB75053_SGB43494
k__Bacteria|p__Firmicutes|c__Clostridia|o__Eubacteriales|f__Lachnospiraceae|g__GGB75109|s__GGB75109_SGB102238
k__Bacteria|p__Firmicutes|c__Clostridia|o__Clostridia_unclassified|f__Clostridia_unclassified|g__GGB81440|s__GGB8144
k__Bacteria|p__Firmicutes|c__Clostridia|o__Eubacteriales|f__Lachnospiraceae|g__Lachnospiraceae_unclassified|s__Lachn
k__Bacteria|p__Firmicutes|c__Clostridia|o__Eubacteriales|f__Lachnospiraceae|g__Lachnospiraceae_unclassified|s__Lachn
k__Bacteria|p__Firmicutes|c__Clostridia|o__Eubacteriales|f__Lachnospiraceae|g__Lachnospiraceae_unclassified|s__Lachn
k__Bacteria|p__Firmicutes|c__Clostridia|o__Eubacteriales|f__Lachnospiraceae|g__Lachnospiraceae_unclassified|s__Lachn
k__Bacteria|p__Firmicutes|c__Clostridia|o__Eubacteriales|f__Lachnospiraceae|g__Lachnospiraceae_unclassified|s__Lachn
k__Bacteria|p__Firmicutes|c__Clostridia|o__Eubacteriales|f__Lachnospiraceae|g__Lachnospiraceae_unclassified|s__Lachn
k__Bacteria|p__Firmicutes|c__Clostridia|o__Eubacteriales|f__Lachnospiraceae|g__Lachnospiraceae_unclassified|s__Lachn
k__Bacteria|p__Firmicutes|c__Clostridia|o__Eubacteriales|f__Lachnospiraceae|g__Lachnospiraceae_unclassified|s__Lachn
k__Bacteria|p__Firmicutes|c__Bacilli|o__Lactobacillales|f__Lactobacillaceae|g__Lactobacillus|s__Lactobacillus_johnsonii
k__Bacteria|p__Bacteroidota|c__Bacteroidia|o__Bacteroidales|f__Muribaculaceae|g__Muribaculaceae_unclassified|s__M
k__Bacteria|p__Firmicutes|c__Clostridia|o__Eubacteriales|f__Oscillospiraceae|g__Neglectibacter|s__Neglectibacter_sp_X
k__Bacteria|p__Firmicutes|c__Clostridia|o__Eubacteriales|f__Oscillospiraceae|g__Oscillospiraceae_unclassified|s__Oscillo
k__Bacteria|p__Firmicutes|c__Clostridia|o__Eubacteriales|f__Oscillospiraceae|g__Oscillospiraceae_unclassified|s__Oscillo
k__Bacteria|p__Firmicutes|c__Clostridia|o__Eubacteriales|f__Oscillospiraceae|g__Oscillospiraceae_unclassified|s__Oscillo
k__Bacteria|p__Proteobacteria|c__Betaproteobacteria|o__Burkholderiales|f__Sutterellaceae|g__Parasutterella|s__Parasu
k__Bacteria|p__Firmicutes|c__Clostridia|o__Eubacteriales|f__Peptostreptococcaceae|g__Romboutsia|s__Romboutsia_ilea
k__Bacteria|p__Firmicutes|c__Clostridia|o__Eubacteriales|f__Lachnospiraceae|g__Schaedlerella|s__Schaedlerella_arabinc
k__Bacteria|p__Firmicutes|c__Erysipelotrichia|o__Erysipelotrichales|f__Turicibacteraceae|g__Turicibacter|s__Turicibacter_
k__Bacteria|p__Bacteria_unclassified|c__Bacteria_unclassified|o__Bacteria_unclassified|f__Bacteria_unclassified|g__Bacte
k__Bacteria|p__Bacteria_unclassified|c__Bacteria_unclassified|o__Bacteria_unclassified|f__Bacteria_unclassified|g__Bacte
k__Bacteria|p__Bacteria_unclassified|c__Bacteria_unclassified|o__Bacteria_unclassified|f__Bacteria_unclassified|g__Bacte

k__Bacteria|p__Firmicutes|c__Clostridia|o__Eubacteriales|f__Lachnospiraceae|g__Acetatifactor|s__Acetatifactor_SGB415
k__Bacteria|p__Firmicutes|c__Clostridia|o__Eubacteriales|f__Lachnospiraceae|g__Acetatifactor|s__Acetatifactor_muris
k__Bacteria|p__Firmicutes|c__Clostridia|o__Eubacteriales|f__Oscillospiraceae|g__Acutalibacter|s__Acutalibacter_muris
k__Bacteria|p__Firmicutes|c__Clostridia|o__Eubacteriales|f__Oscillospiraceae|g__Acutalibacter|s__Acutalibacter_sp_1XD
k__Bacteria|p__Actinobacteria|c__Coriobacteriia|o__Eggerthellales|f__Eggerthellaceae|g__Adlercreutzia|s__Adlercreutzia
k__Bacteria|p__Actinobacteria|c__Coriobacteriia|o__Eggerthellales|f__Eggerthellaceae|g__Adlercreutzia|s__Adlercreutzia
k__Bacteria|p__Actinobacteria|c__Coriobacteriia|o__Eggerthellales|f__Eggerthellaceae|g__Adlercreutzia|s__Adlercreutzia
k__Bacteria|p__Verrucomicrobia|c__Verrucomicrobiae|o__Verrucomicrobiales|f__Akkermansiaceae|g__Akkermansia|s__A
k__Bacteria|p__Bacteroidota|c__Bacteroidia|o__Bacteroidales|f__Rikenellaceae|g__Alistipes|s__Alistipes_sp_DSM_11234
k__Bacteria|p__Firmicutes|c__Clostridia|o__Eubacteriales|f__Oscillospiraceae|g__Anaerotruncus|s__Anaerotruncus_sp_1
k__Bacteria|p__Bacteria_unclassified|c__Bacteria_unclassified|o__Bacteria_unclassified|f__Bacteria_unclassified|g__Bacte
k__Bacteria|p__Bacteria_unclassified|c__Bacteria_unclassified|o__Bacteria_unclassified|f__Bacteria_unclassified|g__Bacte

k__Bacteria|p__Bacteria_unclassified|c__Bacteria_unclassified|o__Bacteria_unclassified|f__Bacteria_unclassified|g__Bacte
k__Bacteria|p__Bacteroidota|c__Bacteroidia|o__Bacteroidales|f__Bacteroidaceae|g__Bacteroides|s__Bacteroides_thetaio
k__Bacteria|p__Actinobacteria|c__Actinomycetia|o__Bifidobacteriales|f__Bifidobacteriaceae|g__Bifidobacterium|s__Bifid
k__Bacteria|p__Firmicutes|c__Clostridia|o__Clostridia_unclassified|f__Clostridia_unclassified|g__Clostridia_unclassified|s_
k__Bacteria|p__Firmicutes|c__Clostridia|o__Eubacteriales|f__Clostridiaceae|g__Clostridiaceae_unclassified|s__Clostridiac
k__Bacteria|p__Firmicutes|c__Clostridia|o__Eubacteriales|f__Clostridiaceae|g__Clostridiaceae_unclassified|s__Clostridiac
k__Bacteria|p__Firmicutes|c__Clostridia|o__Eubacteriales|f__Eubacteriales_unclassified|g__Eubacteriales_unclassified|s_
k__Bacteria|p__Firmicutes|c__Erysipelotrichia|o__Erysipelotrichales|f__Erysipelotrichaceae|g__Erysipelatoclostridium|s_
k__Bacteria|p__Actinobacteria|c__Coriobacteriia|o__Coriobacteriales|f__Coriobacteriaceae|g__Coriobacteriaceae_unclass
k__Bacteria|p__Firmicutes|c__Clostridia|o__Eubacteriales|f__Lachnospiraceae|g__Dorea|s__Dorea_sp_5_2
k__Bacteria|p__Firmicutes|c__Erysipelotrichia|o__Erysipelotrichales|f__Erysipelotrichaceae|g__Dubosiella|s__Dubosiella_
k__Bacteria|p__Firmicutes|c__Erysipelotrichia|o__Erysipelotrichales|f__Erysipelotrichales_unclassified|g__Erysipelotrichal
k__Bacteria|p__Firmicutes|c__Clostridia|o__Eubacteriales|f__Eubacteriaceae|g__Eubacteriaceae_unclassified|s__Eubacte
k__Bacteria|p__Firmicutes|c__Clostridia|o__Eubacteriales|f__Eubacteriaceae|g__Eubacteriaceae_unclassified|s__Eubacte
k__Bacteria|p__Firmicutes|c__Clostridia|o__Eubacteriales|f__Lachnospiraceae|g__GGB20149|s__GGB20149_SGB29430
k__Bacteria|p__Actinobacteria|c__Coriobacteriia|o__Eggerthellales|f__Eggerthellaceae|g__GGB22635|s__GGB22635_SGB
k__Bacteria|p__Firmicutes|c__Clostridia|o__Eubacteriales|f__Lachnospiraceae|g__GGB25041|s__GGB25041_SGB36960
k__Bacteria|p__Bacteroidota|c__Bacteroidia|o__Bacteroidales|f__Muribaculaceae|g__GGB27876|s__GGB27876_SGB4031
k__Bacteria|p__Bacteroidota|c__Bacteroidia|o__Bacteroidales|f__Muribaculaceae|g__GGB27878|s__GGB27878_SGB4031
k__Bacteria|p__Bacteroidota|c__Bacteroidia|o__Bacteroidales|f__Muribaculaceae|g__GGB27918|s__GGB27918_SGB4035
k__Bacteria|p__Firmicutes|c__CFGB9508|o__OFGB9508|f__FGB9508|g__GGB28382|s__GGB28382_SGB40962
k__Bacteria|p__Firmicutes|c__CFGB2838|o__OFGB2838|f__FGB2838|g__GGB28399|s__GGB28399_SGB40980
k__Bacteria|p__Firmicutes|c__CFGB2838|o__OFGB2838|f__FGB2838|g__GGB28411|s__GGB28411_SGB40993
k__Bacteria|p__Firmicutes|c__CFGB2838|o__OFGB2838|f__FGB2838|g__GGB28415|s__GGB28415_SGB40997
k__Bacteria|p__Firmicutes|c__Clostridia|o__Eubacteriales|f__Pumilibacteraceae|g__GGB28430|s__GGB28430_SGB41013
k__Bacteria|p__Firmicutes|c__CFGB28439|o__OFGB28439|f__FGB28439|g__GGB28439|s__GGB28439_SGB41022
k__Bacteria|p__Firmicutes|c__Clostridia|o__Clostridia_unclassified|f__Clostridia_unclassified|g__GGB28778|s__GGB2877
k__Bacteria|p__Firmicutes|c__Clostridia|o__Eubacteriales|f__Eubacteriaceae|g__GGB28784|s__GGB28784_SGB41437
k__Bacteria|p__Firmicutes|c__Clostridia|o__Eubacteriales|f__Lachnospiraceae|g__GGB28792|s__GGB28792_SGB41445
k__Bacteria|p__Firmicutes|c__Clostridia|o__Eubacteriales|f__Lachnospiraceae|g__GGB28798|s__GGB28798_SGB41451
k__Bacteria|p__Firmicutes|c__Clostridia|o__Eubacteriales|f__Lachnospiraceae|g__GGB28802|s__GGB28802_SGB41455
k__Bacteria|p__Firmicutes|c__Clostridia|o__Eubacteriales|f__Lachnospiraceae|g__GGB28818|s__GGB28818_SGB41473
k__Bacteria|p__Firmicutes|c__CFGB77305|o__OFGB77305|f__FGB77305|g__GGB28828|s__GGB28828_SGB41484
k__Bacteria|p__Firmicutes|c__Clostridia|o__Eubacteriales|f__Clostridiaceae|g__GGB28851|s__GGB28851_SGB41518
k__Bacteria|p__Firmicutes|c__Clostridia|o__Eubacteriales|f__Lachnospiraceae|g__GGB28859|s__GGB28859_SGB41528
k__Bacteria|p__Firmicutes|c__Clostridia|o__Eubacteriales|f__Lachnospiraceae|g__GGB28864|s__GGB28864_SGB41535
k__Bacteria|p__Firmicutes|c__Clostridia|o__Eubacteriales|f__Lachnospiraceae|g__GGB28869|s__GGB28869_SGB41543
k__Bacteria|p__Firmicutes|c__CFGB9633|o__OFGB9633|f__FGB9633|g__GGB28883|s__GGB28883_SGB41564
k__Bacteria|p__Bacteria_unclassified|c__Bacteria_unclassified|o__Bacteria_unclassified|f__Bacteria_unclassified|g__GGB2
k__Bacteria|p__Bacteria_unclassified|c__Bacteria_unclassified|o__Bacteria_unclassified|f__Bacteria_unclassified|g__GGB2
k__Bacteria|p__Bacteria_unclassified|c__Bacteria_unclassified|o__Bacteria_unclassified|f__Bacteria_unclassified|g__GGB2
k__Bacteria|p__Bacteria_unclassified|c__Bacteria_unclassified|o__Bacteria_unclassified|f__Bacteria_unclassified|g__GGB2
k__Bacteria|p__Firmicutes|c__Clostridia|o__Eubacteriales|f__Lachnospiraceae|g__GGB28916|s__GGB28916_SGB41612
k__Bacteria|p__Firmicutes|c__Clostridia|o__Eubacteriales|f__Lachnospiraceae|g__GGB28924|s__GGB28924_SGB41621
k__Bacteria|p__Firmicutes|c__Clostridia|o__Eubacteriales|f__Lachnospiraceae|g__GGB28926|s__GGB28926_SGB41624
k__Bacteria|p__Bacteria_unclassified|c__CFGB77359|o__OFGB77359|f__FGB77359|g__GGB28927|s__GGB28927_SGB416

k__Bacteria|p__Firmicutes|c__CFGB9639|o__OFGB9639|f__FGB9639|g__GGB28934|s__GGB28934_SGB41635
k__Bacteria|p__Firmicutes|c__Clostridia|o__Eubacteriales|f__Lachnospiraceae|g__GGB28946|s__GGB28946_SGB41652
k__Bacteria|p__Firmicutes|c__Clostridia|o__Eubacteriales|f__Lachnospiraceae|g__GGB28949|s__GGB28949_SGB41655
k__Bacteria|p__Firmicutes|c__Clostridia|o__Eubacteriales|f__Lachnospiraceae|g__GGB28949|s__GGB28949_SGB41656
k__Bacteria|p__Firmicutes|c__Clostridia|o__Eubacteriales|f__Clostridiaceae|g__GGB28950|s__GGB28950_SGB41657
k__Bacteria|p__Firmicutes|c__Clostridia|o__Eubacteriales|f__Clostridiaceae|g__GGB28951|s__GGB28951_SGB102295
k__Bacteria|p__Firmicutes|c__Clostridia|o__Eubacteriales|f__Clostridiaceae|g__GGB28951|s__GGB28951_SGB41658
k__Bacteria|p__Firmicutes|c__Clostridia|o__Eubacteriales|f__Clostridiaceae|g__GGB28954|s__GGB28954_SGB41662
k__Bacteria|p__Firmicutes|c__Clostridia|o__Eubacteriales|f__Clostridiaceae|g__GGB28956|s__GGB28956_SGB41665
k__Bacteria|p__Firmicutes|c__Clostridia|o__Eubacteriales|f__Clostridiaceae|g__GGB28960|s__GGB28960_SGB41669
k__Bacteria|p__Firmicutes|c__Clostridia|o__Eubacteriales|f__Clostridiaceae|g__GGB28967|s__GGB28967_SGB41678
k__Bacteria|p__Firmicutes|c__Clostridia|o__Eubacteriales|f__Eubacteriaceae|g__GGB28991|s__GGB28991_SGB41705
k__Bacteria|p__Firmicutes|c__CFGB9658|o__OFGB9658|f__FGB9658|g__GGB29002|s__GGB29002_SGB41718
k__Bacteria|p__Firmicutes|c__CFGB9659|o__OFGB9659|f__FGB9659|g__GGB29003|s__GGB29003_SGB41719
k__Bacteria|p__Bacteria_unclassified|c__Bacteria_unclassified|o__Bacteria_unclassified|f__Bacteria_unclassified|g__GGB2
k__Bacteria|p__Firmicutes|c__CFGB9827|o__OFGB9827|f__FGB9827|g__GGB29531|s__GGB29531_SGB42317
k__Bacteria|p__Firmicutes|c__Clostridia|o__Eubacteriales|f__Eubacteriaceae|g__GGB29685|s__GGB29685_SGB42494
k__Bacteria|p__Bacteria_unclassified|c__CFGB77303|o__OFGB77303|f__FGB77303|g__GGB30141|s__GGB30141_SGB430
k__Bacteria|p__Firmicutes|c__Clostridia|o__Eubacteriales|f__Eubacteriales_unclassified|g__GGB30286|s__GGB30286_SG
k__Bacteria|p__Firmicutes|c__Clostridia|o__Eubacteriales|f__Oscillospiraceae|g__GGB30303|s__GGB30303_SGB43268
k__Bacteria|p__Firmicutes|c__CFGB30328|o__OFGB30328|f__FGB30328|g__GGB30413|s__GGB30413_SGB43452
k__Bacteria|p__Firmicutes|c__Clostridia|o__Eubacteriales|f__Oscillospiraceae|g__GGB30454|s__GGB30454_SGB43514
k__Bacteria|p__Firmicutes|c__Clostridia|o__Eubacteriales|f__Oscillospiraceae|g__GGB30455|s__GGB30455_SGB43519
k__Bacteria|p__Firmicutes|c__Clostridia|o__Eubacteriales|f__Oscillospiraceae|g__GGB30461|s__GGB30461_SGB43527
k__Bacteria|p__Firmicutes|c__Clostridia|o__Eubacteriales|f__Oscillospiraceae|g__GGB30461|s__GGB30461_SGB43530
k__Bacteria|p__Firmicutes|c__Clostridia|o__Eubacteriales|f__Oscillospiraceae|g__GGB30463|s__GGB30463_SGB43537
k__Bacteria|p__Firmicutes|c__Clostridia|o__Eubacteriales|f__Oscillospiraceae|g__GGB30473|s__GGB30473_SGB43557
k__Bacteria|p__Firmicutes|c__Clostridia|o__Eubacteriales|f__Oscillospiraceae|g__GGB30475|s__GGB30475_SGB63182
k__Bacteria|p__Actinobacteria|c__CFGB77153|o__OFGB77153|f__FGB77153|g__GGB30861|s__GGB30861_SGB44083
k__Bacteria|p__Tenericutes|c__CFGB1791|o__OFGB1791|f__FGB1791|g__GGB31312|s__GGB31312_SGB44628
k__Bacteria|p__Firmicutes|c__CFGB10290|o__OFGB10290|f__FGB10290|g__GGB31438|s__GGB31438_SGB44768
k__Bacteria|p__Firmicutes|c__Clostridia|o__Eubacteriales|f__Oscillospiraceae|g__GGB3171|s__GGB3171_SGB4185
k__Bacteria|p__Firmicutes|c__CFGB1765|o__OFGB1765|f__FGB1765|g__GGB31823|s__GGB31823_SGB45199
k__Bacteria|p__Firmicutes|c__CFGB10349|o__OFGB10349|f__FGB10349|g__GGB31853|s__GGB31853_SGB45233
k__Bacteria|p__Firmicutes|c__CFGB10667|o__OFGB10667|f__FGB10667|g__GGB32371|s__GGB32371_SGB41694
k__Bacteria|p__Firmicutes|c__Clostridia|o__Eubacteriales|f__Lachnospiraceae|g__GGB3793|s__GGB3793_SGB5158
k__Bacteria|p__Firmicutes|c__Clostridia|o__Eubacteriales|f__Lachnospiraceae|g__GGB42598|s__GGB42598_SGB59794
k__Bacteria|p__Firmicutes|c__Clostridia|o__Eubacteriales|f__Christensenellaceae|g__GGB45656|s__GGB45656_SGB6337
k__Bacteria|p__Firmicutes|c__CFGB10299|o__OFGB10299|f__FGB10299|g__GGB47127|s__GGB47127_SGB65054
k__Bacteria|p__Firmicutes|c__Clostridia|o__Eubacteriales|f__Oscillospiraceae|g__GGB74395|s__GGB74395_SGB43521
k__Bacteria|p__Firmicutes|c__Clostridia|o__Eubacteriales|f__Oscillospiraceae|g__GGB75053|s__GGB75053_SGB43494
k__Bacteria|p__Firmicutes|c__Clostridia|o__Eubacteriales|f__Lachnospiraceae|g__GGB75109|s__GGB75109_SGB102238
k__Bacteria|p__Firmicutes|c__Clostridia|o__Clostridia_unclassified|f__Clostridia_unclassified|g__GGB81440|s__GGB8144
k__Bacteria|p__Firmicutes|c__Clostridia|o__Eubacteriales|f__Lachnospiraceae|g__Lachnospiraceae_unclassified|s__Lachn
k__Bacteria|p__Firmicutes|c__Clostridia|o__Eubacteriales|f__Lachnospiraceae|g__Lachnospiraceae_unclassified|s__Lachn
k__Bacteria|p__Firmicutes|c__Clostridia|o__Eubacteriales|f__Lachnospiraceae|g__Lachnospiraceae_unclassified|s__Lachn

k__Bacteria|p__Firmicutes|c__Clostridia|o__Eubacteriales|f__Lachnospiraceae|g__Lachnospiraceae_unclassified|s__Lachn
k__Bacteria|p__Firmicutes|c__Clostridia|o__Eubacteriales|f__Lachnospiraceae|g__Lachnospiraceae_unclassified|s__Lachn
k__Bacteria|p__Firmicutes|c__Clostridia|o__Eubacteriales|f__Lachnospiraceae|g__Lachnospiraceae_unclassified|s__Lachn
k__Bacteria|p__Firmicutes|c__Clostridia|o__Eubacteriales|f__Lachnospiraceae|g__Lachnospiraceae_unclassified|s__Lachn
k__Bacteria|p__Firmicutes|c__Clostridia|o__Eubacteriales|f__Lachnospiraceae|g__Lachnospiraceae_unclassified|s__Lachn
k__Bacteria|p__Firmicutes|c__Bacilli|o__Lactobacillales|f__Lactobacillaceae|g__Lactobacillus|s__Lactobacillus_johnsonii
k__Bacteria|p__Bacteroidota|c__Bacteroidia|o__Bacteroidales|f__Muribaculaceae|g__Muribaculaceae_unclassified|s__M
k__Bacteria|p__Firmicutes|c__Clostridia|o__Eubacteriales|f__Oscillospiraceae|g__Neglectibacter|s__Neglectibacter_sp_X
k__Bacteria|p__Firmicutes|c__Clostridia|o__Eubacteriales|f__Oscillospiraceae|g__Oscillospiraceae_unclassified|s__Oscillo
k__Bacteria|p__Firmicutes|c__Clostridia|o__Eubacteriales|f__Oscillospiraceae|g__Oscillospiraceae_unclassified|s__Oscillo
k__Bacteria|p__Firmicutes|c__Clostridia|o__Eubacteriales|f__Oscillospiraceae|g__Oscillospiraceae_unclassified|s__Oscillo
k__Bacteria|p__Proteobacteria|c__Betaproteobacteria|o__Burkholderiales|f__Sutterellaceae|g__Parasutterella|s__Parasu
k__Bacteria|p__Firmicutes|c__Clostridia|o__Eubacteriales|f__Peptostreptococcaceae|g__Romboutsia|s__Romboutsia_ilea
k__Bacteria|p__Firmicutes|c__Clostridia|o__Eubacteriales|f__Lachnospiraceae|g__Schaedlerella|s__Schaedlerella_arabinc
k__Bacteria|p__Firmicutes|c__Erysipelotrichia|o__Erysipelotrichales|f__Turicibacteraceae|g__Turicibacter|s__Turicibacter_
k__Bacteria|p__Bacteria_unclassified|c__Bacteria_unclassified|o__Bacteria_unclassified|f__Bacteria_unclassified|g__Bacte
k__Bacteria|p__Bacteria_unclassified|c__Bacteria_unclassified|o__Bacteria_unclassified|f__Bacteria_unclassified|g__Bacte
k__Bacteria|p__Bacteria_unclassified|c__Bacteria_unclassified|o__Bacteria_unclassified|f__Bacteria_unclassified|g__Bacte

k__Bacteria|p__Firmicutes|c__Clostridia|o__Eubacteriales|f__Lachnospiraceae|g__Acetatifactor|s__Acetatifactor_SGB415
k__Bacteria|p__Firmicutes|c__Clostridia|o__Eubacteriales|f__Lachnospiraceae|g__Acetatifactor|s__Acetatifactor_muris
k__Bacteria|p__Firmicutes|c__Clostridia|o__Eubacteriales|f__Oscillospiraceae|g__Acutalibacter|s__Acutalibacter_muris
k__Bacteria|p__Firmicutes|c__Clostridia|o__Eubacteriales|f__Oscillospiraceae|g__Acutalibacter|s__Acutalibacter_sp_1XD
k__Bacteria|p__Actinobacteria|c__Coriobacteriia|o__Eggerthellales|f__Eggerthellaceae|g__Adlercreutzia|s__Adlercreutzia
k__Bacteria|p__Actinobacteria|c__Coriobacteriia|o__Eggerthellales|f__Eggerthellaceae|g__Adlercreutzia|s__Adlercreutzia
k__Bacteria|p__Actinobacteria|c__Coriobacteriia|o__Eggerthellales|f__Eggerthellaceae|g__Adlercreutzia|s__Adlercreutzia
k__Bacteria|p__Verrucomicrobia|c__Verrucomicrobiae|o__Verrucomicrobiales|f__Akkermansiaceae|g__Akkermansia|s__A
k__Bacteria|p__Bacteroidota|c__Bacteroidia|o__Bacteroidales|f__Rikenellaceae|g__Alistipes|s__Alistipes_sp_DSM_11234
k__Bacteria|p__Firmicutes|c__Clostridia|o__Eubacteriales|f__Oscillospiraceae|g__Anaerotruncus|s__Anaerotruncus_sp_1
k__Bacteria|p__Bacteria_unclassified|c__Bacteria_unclassified|o__Bacteria_unclassified|f__Bacteria_unclassified|g__Bacte
k__Bacteria|p__Bacteria_unclassified|c__Bacteria_unclassified|o__Bacteria_unclassified|f__Bacteria_unclassified|g__Bacte
k__Bacteria|p__Bacteria_unclassified|c__Bacteria_unclassified|o__Bacteria_unclassified|f__Bacteria_unclassified|g__Bacte
k__Bacteria|p__Bacteroidota|c__Bacteroidia|o__Bacteroidales|f__Bacteroidaceae|g__Bacteroides|s__Bacteroides_thetaio
k__Bacteria|p__Actinobacteria|c__Actinomycetia|o__Bifidobacteriales|f__Bifidobacteriaceae|g__Bifidobacterium|s__Bifid
k__Bacteria|p__Firmicutes|c__Clostridia|o__Clostridia_unclassified|f__Clostridia_unclassified|g__Clostridia_unclassified|s_
k__Bacteria|p__Firmicutes|c__Clostridia|o__Eubacteriales|f__Clostridiaceae|g__Clostridiaceae_unclassified|s__Clostridiac
k__Bacteria|p__Firmicutes|c__Clostridia|o__Eubacteriales|f__Clostridiaceae|g__Clostridiaceae_unclassified|s__Clostridiac
k__Bacteria|p__Firmicutes|c__Clostridia|o__Eubacteriales|f__Eubacteriales_unclassified|g__Eubacteriales_unclassified|s_
k__Bacteria|p__Firmicutes|c__Erysipelotrichia|o__Erysipelotrichales|f__Erysipelotrichaceae|g__Erysipelatoclostridium|s_
k__Bacteria|p__Actinobacteria|c__Coriobacteriia|o__Coriobacteriales|f__Coriobacteriaceae|g__Coriobacteriaceae_unclass
k__Bacteria|p__Firmicutes|c__Clostridia|o__Eubacteriales|f__Lachnospiraceae|g__Dorea|s__Dorea_sp_5_2
k__Bacteria|p__Firmicutes|c__Erysipelotrichia|o__Erysipelotrichales|f__Erysipelotrichaceae|g__Dubosiella|s__Dubosiella_
k__Bacteria|p__Firmicutes|c__Erysipelotrichia|o__Erysipelotrichales|f__Erysipelotrichales_unclassified|g__Erysipelotrichal
k__Bacteria|p__Firmicutes|c__Clostridia|o__Eubacteriales|f__Eubacteriaceae|g__Eubacteriaceae_unclassified|s__Eubacte

k__Bacteria|p__Firmicutes|c__Clostridia|o__Eubacteriales|f__Eubacteriaceae|g__Eubacteriaceae_unclassified|s__Eubacte
k__Bacteria|p__Firmicutes|c__Clostridia|o__Eubacteriales|f__Lachnospiraceae|g__GGB20149|s__GGB20149_SGB29430
k__Bacteria|p__Actinobacteria|c__Coriobacteriia|o__Eggerthellales|f__Eggerthellaceae|g__GGB22635|s__GGB22635_SGB
k__Bacteria|p__Firmicutes|c__Clostridia|o__Eubacteriales|f__Lachnospiraceae|g__GGB25041|s__GGB25041_SGB36960
k__Bacteria|p__Bacteroidota|c__Bacteroidia|o__Bacteroidales|f__Muribaculaceae|g__GGB27876|s__GGB27876_SGB4031
k__Bacteria|p__Bacteroidota|c__Bacteroidia|o__Bacteroidales|f__Muribaculaceae|g__GGB27878|s__GGB27878_SGB4031
k__Bacteria|p__Bacteroidota|c__Bacteroidia|o__Bacteroidales|f__Muribaculaceae|g__GGB27918|s__GGB27918_SGB4035
k__Bacteria|p__Firmicutes|c__CFGB9508|o__OFGB9508|f__FGB9508|g__GGB28382|s__GGB28382_SGB40962
k__Bacteria|p__Firmicutes|c__CFGB2838|o__OFGB2838|f__FGB2838|g__GGB28399|s__GGB28399_SGB40980
k__Bacteria|p__Firmicutes|c__CFGB2838|o__OFGB2838|f__FGB2838|g__GGB28411|s__GGB28411_SGB40993
k__Bacteria|p__Firmicutes|c__CFGB2838|o__OFGB2838|f__FGB2838|g__GGB28415|s__GGB28415_SGB40997
k__Bacteria|p__Firmicutes|c__Clostridia|o__Eubacteriales|f__Pumilibacteraceae|g__GGB28430|s__GGB28430_SGB41013
k__Bacteria|p__Firmicutes|c__CFGB28439|o__OFGB28439|f__FGB28439|g__GGB28439|s__GGB28439_SGB41022
k__Bacteria|p__Firmicutes|c__Clostridia|o__Clostridia_unclassified|f__Clostridia_unclassified|g__GGB28778|s__GGB2877
k__Bacteria|p__Firmicutes|c__Clostridia|o__Eubacteriales|f__Eubacteriaceae|g__GGB28784|s__GGB28784_SGB41437
k__Bacteria|p__Firmicutes|c__Clostridia|o__Eubacteriales|f__Lachnospiraceae|g__GGB28792|s__GGB28792_SGB41445
k__Bacteria|p__Firmicutes|c__Clostridia|o__Eubacteriales|f__Lachnospiraceae|g__GGB28798|s__GGB28798_SGB41451
k__Bacteria|p__Firmicutes|c__Clostridia|o__Eubacteriales|f__Lachnospiraceae|g__GGB28802|s__GGB28802_SGB41455
k__Bacteria|p__Firmicutes|c__Clostridia|o__Eubacteriales|f__Lachnospiraceae|g__GGB28818|s__GGB28818_SGB41473
k__Bacteria|p__Firmicutes|c__CFGB77305|o__OFGB77305|f__FGB77305|g__GGB28828|s__GGB28828_SGB41484
k__Bacteria|p__Firmicutes|c__Clostridia|o__Eubacteriales|f__Clostridiaceae|g__GGB28851|s__GGB28851_SGB41518
k__Bacteria|p__Firmicutes|c__Clostridia|o__Eubacteriales|f__Lachnospiraceae|g__GGB28859|s__GGB28859_SGB41528
k__Bacteria|p__Firmicutes|c__Clostridia|o__Eubacteriales|f__Lachnospiraceae|g__GGB28864|s__GGB28864_SGB41535
k__Bacteria|p__Firmicutes|c__Clostridia|o__Eubacteriales|f__Lachnospiraceae|g__GGB28869|s__GGB28869_SGB41543
k__Bacteria|p__Firmicutes|c__CFGB9633|o__OFGB9633|f__FGB9633|g__GGB28883|s__GGB28883_SGB41564
k__Bacteria|p__Bacteria_unclassified|c__Bacteria_unclassified|o__Bacteria_unclassified|f__Bacteria_unclassified|g__GGB
k__Bacteria|p__Bacteria_unclassified|c__Bacteria_unclassified|o__Bacteria_unclassified|f__Bacteria_unclassified|g__GGB
k__Bacteria|p__Bacteria_unclassified|c__Bacteria_unclassified|o__Bacteria_unclassified|f__Bacteria_unclassified|g__GGB
k__Bacteria|p__Bacteria_unclassified|c__Bacteria_unclassified|o__Bacteria_unclassified|f__Bacteria_unclassified|g__GGB
k__Bacteria|p__Firmicutes|c__Clostridia|o__Eubacteriales|f__Lachnospiraceae|g__GGB28916|s__GGB28916_SGB41612
k__Bacteria|p__Firmicutes|c__Clostridia|o__Eubacteriales|f__Lachnospiraceae|g__GGB28924|s__GGB28924_SGB41621
k__Bacteria|p__Firmicutes|c__Clostridia|o__Eubacteriales|f__Lachnospiraceae|g__GGB28926|s__GGB28926_SGB41624
k__Bacteria|p__Bacteria_unclassified|c__CFGB77359|o__OFGB77359|f__FGB77359|g__GGB28927|s__GGB28927_SGB416
k__Bacteria|p__Firmicutes|c__CFGB9639|o__OFGB9639|f__FGB9639|g__GGB28934|s__GGB28934_SGB41635
k__Bacteria|p__Firmicutes|c__Clostridia|o__Eubacteriales|f__Lachnospiraceae|g__GGB28946|s__GGB28946_SGB41652
k__Bacteria|p__Firmicutes|c__Clostridia|o__Eubacteriales|f__Lachnospiraceae|g__GGB28949|s__GGB28949_SGB41655
k__Bacteria|p__Firmicutes|c__Clostridia|o__Eubacteriales|f__Lachnospiraceae|g__GGB28949|s__GGB28949_SGB41656
k__Bacteria|p__Firmicutes|c__Clostridia|o__Eubacteriales|f__Clostridiaceae|g__GGB28950|s__GGB28950_SGB41657
k__Bacteria|p__Firmicutes|c__Clostridia|o__Eubacteriales|f__Clostridiaceae|g__GGB28951|s__GGB28951_SGB102295
k__Bacteria|p__Firmicutes|c__Clostridia|o__Eubacteriales|f__Clostridiaceae|g__GGB28951|s__GGB28951_SGB41658
k__Bacteria|p__Firmicutes|c__Clostridia|o__Eubacteriales|f__Clostridiaceae|g__GGB28954|s__GGB28954_SGB41662
k__Bacteria|p__Firmicutes|c__Clostridia|o__Eubacteriales|f__Clostridiaceae|g__GGB28956|s__GGB28956_SGB41665
k__Bacteria|p__Firmicutes|c__Clostridia|o__Eubacteriales|f__Clostridiaceae|g__GGB28960|s__GGB28960_SGB41669
k__Bacteria|p__Firmicutes|c__Clostridia|o__Eubacteriales|f__Clostridiaceae|g__GGB28967|s__GGB28967_SGB41678
k__Bacteria|p__Firmicutes|c__Clostridia|o__Eubacteriales|f__Eubacteriaceae|g__GGB28991|s__GGB28991_SGB41705
k__Bacteria|p__Firmicutes|c__CFGB9658|o__OFGB9658|f__FGB9658|g__GGB29002|s__GGB29002_SGB41718

k__Bacteria|p__Firmicutes|c__CFGB9659|o__OFGB9659|f__FGB9659|g__GGB29003|s__GGB29003_SGB41719
k__Bacteria|p__Bacteria_unclassified|c__Bacteria_unclassified|o__Bacteria_unclassified|f__Bacteria_unclassified|g__GGB2
k__Bacteria|p__Firmicutes|c__CFGB9827|o__OFGB9827|f__FGB9827|g__GGB29531|s__GGB29531_SGB42317
k__Bacteria|p__Firmicutes|c__Clostridia|o__Eubacteriales|f__Eubacteriaceae|g__GGB29685|s__GGB29685_SGB42494
k__Bacteria|p__Bacteria_unclassified|c__CFGB77303|o__OFGB77303|f__FGB77303|g__GGB30141|s__GGB30141_SGB430
k__Bacteria|p__Firmicutes|c__Clostridia|o__Eubacteriales|f__Eubacteriales_unclassified|g__GGB30286|s__GGB30286_SG
k__Bacteria|p__Firmicutes|c__Clostridia|o__Eubacteriales|f__Oscillospiraceae|g__GGB30303|s__GGB30303_SGB43268
k__Bacteria|p__Firmicutes|c__CFGB30328|o__OFGB30328|f__FGB30328|g__GGB30413|s__GGB30413_SGB43452
k__Bacteria|p__Firmicutes|c__Clostridia|o__Eubacteriales|f__Oscillospiraceae|g__GGB30454|s__GGB30454_SGB43514
k__Bacteria|p__Firmicutes|c__Clostridia|o__Eubacteriales|f__Oscillospiraceae|g__GGB30455|s__GGB30455_SGB43519
k__Bacteria|p__Firmicutes|c__Clostridia|o__Eubacteriales|f__Oscillospiraceae|g__GGB30461|s__GGB30461_SGB43527
k__Bacteria|p__Firmicutes|c__Clostridia|o__Eubacteriales|f__Oscillospiraceae|g__GGB30461|s__GGB30461_SGB43530
k__Bacteria|p__Firmicutes|c__Clostridia|o__Eubacteriales|f__Oscillospiraceae|g__GGB30463|s__GGB30463_SGB43537
k__Bacteria|p__Firmicutes|c__Clostridia|o__Eubacteriales|f__Oscillospiraceae|g__GGB30473|s__GGB30473_SGB43557
k__Bacteria|p__Firmicutes|c__Clostridia|o__Eubacteriales|f__Oscillospiraceae|g__GGB30475|s__GGB30475_SGB63182
k__Bacteria|p__Actinobacteria|c__CFGB77153|o__OFGB77153|f__FGB77153|g__GGB30861|s__GGB30861_SGB44083
k__Bacteria|p__Tenericutes|c__CFGB1791|o__OFGB1791|f__FGB1791|g__GGB31312|s__GGB31312_SGB44628
k__Bacteria|p__Firmicutes|c__CFGB10290|o__OFGB10290|f__FGB10290|g__GGB31438|s__GGB31438_SGB44768
k__Bacteria|p__Firmicutes|c__Clostridia|o__Eubacteriales|f__Oscillospiraceae|g__GGB3171|s__GGB3171_SGB4185
k__Bacteria|p__Firmicutes|c__CFGB1765|o__OFGB1765|f__FGB1765|g__GGB31823|s__GGB31823_SGB45199
k__Bacteria|p__Firmicutes|c__CFGB10349|o__OFGB10349|f__FGB10349|g__GGB31853|s__GGB31853_SGB45233
k__Bacteria|p__Firmicutes|c__CFGB10667|o__OFGB10667|f__FGB10667|g__GGB32371|s__GGB32371_SGB41694
k__Bacteria|p__Firmicutes|c__Clostridia|o__Eubacteriales|f__Lachnospiraceae|g__GGB3793|s__GGB3793_SGB5158
k__Bacteria|p__Firmicutes|c__Clostridia|o__Eubacteriales|f__Lachnospiraceae|g__GGB42598|s__GGB42598_SGB59794
k__Bacteria|p__Firmicutes|c__Clostridia|o__Eubacteriales|f__Christensenellaceae|g__GGB45656|s__GGB45656_SGB6337
k__Bacteria|p__Firmicutes|c__CFGB10299|o__OFGB10299|f__FGB10299|g__GGB47127|s__GGB47127_SGB65054
k__Bacteria|p__Firmicutes|c__Clostridia|o__Eubacteriales|f__Oscillospiraceae|g__GGB74395|s__GGB74395_SGB43521
k__Bacteria|p__Firmicutes|c__Clostridia|o__Eubacteriales|f__Oscillospiraceae|g__GGB75053|s__GGB75053_SGB43494
k__Bacteria|p__Firmicutes|c__Clostridia|o__Eubacteriales|f__Lachnospiraceae|g__GGB75109|s__GGB75109_SGB102238
k__Bacteria|p__Firmicutes|c__Clostridia|o__Clostridia_unclassified|f__Clostridia_unclassified|g__GGB81440|s__GGB8144
k__Bacteria|p__Firmicutes|c__Clostridia|o__Eubacteriales|f__Lachnospiraceae|g__Lachnospiraceae_unclassified|s__Lachn
k__Bacteria|p__Firmicutes|c__Clostridia|o__Eubacteriales|f__Lachnospiraceae|g__Lachnospiraceae_unclassified|s__Lachn
k__Bacteria|p__Firmicutes|c__Clostridia|o__Eubacteriales|f__Lachnospiraceae|g__Lachnospiraceae_unclassified|s__Lachn
k__Bacteria|p__Firmicutes|c__Clostridia|o__Eubacteriales|f__Lachnospiraceae|g__Lachnospiraceae_unclassified|s__Lachn
k__Bacteria|p__Firmicutes|c__Clostridia|o__Eubacteriales|f__Lachnospiraceae|g__Lachnospiraceae_unclassified|s__Lachn
k__Bacteria|p__Firmicutes|c__Clostridia|o__Eubacteriales|f__Lachnospiraceae|g__Lachnospiraceae_unclassified|s__Lachn
k__Bacteria|p__Firmicutes|c__Clostridia|o__Eubacteriales|f__Lachnospiraceae|g__Lachnospiraceae_unclassified|s__Lachn
k__Bacteria|p__Firmicutes|c__Clostridia|o__Eubacteriales|f__Lachnospiraceae|g__Lachnospiraceae_unclassified|s__Lachn
k__Bacteria|p__Firmicutes|c__Bacilli|o__Lactobacillales|f__Lactobacillaceae|g__Lactobacillus|s__Lactobacillus_johnsonii
k__Bacteria|p__Bacteroidota|c__Bacteroidia|o__Bacteroidales|f__Muribaculaceae|g__Muribaculaceae_unclassified|s__M
k__Bacteria|p__Firmicutes|c__Clostridia|o__Eubacteriales|f__Oscillospiraceae|g__Neglectibacter|s__Neglectibacter_sp_X
k__Bacteria|p__Firmicutes|c__Clostridia|o__Eubacteriales|f__Oscillospiraceae|g__Oscillospiraceae_unclassified|s__Oscillo
k__Bacteria|p__Firmicutes|c__Clostridia|o__Eubacteriales|f__Oscillospiraceae|g__Oscillospiraceae_unclassified|s__Oscillo
k__Bacteria|p__Firmicutes|c__Clostridia|o__Eubacteriales|f__Oscillospiraceae|g__Oscillospiraceae_unclassified|s__Oscillo
k__Bacteria|p__Proteobacteria|c__Betaproteobacteria|o__Burkholderiales|f__Sutterellaceae|g__Parasutterella|s__Parasu
k__Bacteria|p__Firmicutes|c__Clostridia|o__Eubacteriales|f__Peptostreptococcaceae|g__Romboutsia|s__Romboutsia_ilea

k__Bacteria|p__Firmicutes|c__Clostridia|o__Eubacteriales|f__Lachnospiraceae|g__Schaedlerella|s__Schaedlerella_arabino
k__Bacteria|p__Firmicutes|c__Erysipelotrichia|o__Erysipelotrichales|f__Turicibacteraceae|g__Turicibacter|s__Turicibacter_
k__Bacteria|p__Bacteria_unclassified|c__Bacteria_unclassified|o__Bacteria_unclassified|f__Bacteria_unclassified|g__Bacte
k__Bacteria|p__Bacteria_unclassified|c__Bacteria_unclassified|o__Bacteria_unclassified|f__Bacteria_unclassified|g__Bacte
k__Bacteria|p__Bacteria_unclassified|c__Bacteria_unclassified|o__Bacteria_unclassified|f__Bacteria_unclassified|g__Bacte

k__Bacteria|p__Firmicutes|c__Clostridia|o__Eubacteriales|f__Lachnospiraceae|g__Acetatifactor|s__Acetatifactor_SGB415
k__Bacteria|p__Firmicutes|c__Clostridia|o__Eubacteriales|f__Lachnospiraceae|g__Acetatifactor|s__Acetatifactor_muris
k__Bacteria|p__Firmicutes|c__Clostridia|o__Eubacteriales|f__Oscillospiraceae|g__Acutalibacter|s__Acutalibacter_muris
k__Bacteria|p__Firmicutes|c__Clostridia|o__Eubacteriales|f__Oscillospiraceae|g__Acutalibacter|s__Acutalibacter_sp_1XD
k__Bacteria|p__Actinobacteria|c__Coriobacteriia|o__Eggerthellales|f__Eggerthellaceae|g__Adlercreutzia|s__Adlercreutzia
k__Bacteria|p__Actinobacteria|c__Coriobacteriia|o__Eggerthellales|f__Eggerthellaceae|g__Adlercreutzia|s__Adlercreutzia
k__Bacteria|p__Actinobacteria|c__Coriobacteriia|o__Eggerthellales|f__Eggerthellaceae|g__Adlercreutzia|s__Adlercreutzia
k__Bacteria|p__Verrucomicrobia|c__Verrucomicrobiae|o__Verrucomicrobiales|f__Akkermansiaceae|g__Akkermansia|s__A
k__Bacteria|p__Bacteroidota|c__Bacteroidia|o__Bacteroidales|f__Rikenellaceae|g__Alistipes|s__Alistipes_sp_DSM_11234
k__Bacteria|p__Firmicutes|c__Clostridia|o__Eubacteriales|f__Oscillospiraceae|g__Anaerotruncus|s__Anaerotruncus_sp_1
k__Bacteria|p__Bacteria_unclassified|c__Bacteria_unclassified|o__Bacteria_unclassified|f__Bacteria_unclassified|g__Bacte
k__Bacteria|p__Bacteria_unclassified|c__Bacteria_unclassified|o__Bacteria_unclassified|f__Bacteria_unclassified|g__Bacte
k__Bacteria|p__Bacteria_unclassified|c__Bacteria_unclassified|o__Bacteria_unclassified|f__Bacteria_unclassified|g__Bacte
k__Bacteria|p__Bacteroidota|c__Bacteroidia|o__Bacteroidales|f__Bacteroidaceae|g__Bacteroides|s__Bacteroides_thetaio
k__Bacteria|p__Actinobacteria|c__Actinomycetia|o__Bifidobacteriales|f__Bifidobacteriaceae|g__Bifidobacterium|s__Bifid
k__Bacteria|p__Firmicutes|c__Clostridia|o__Clostridia_unclassified|f__Clostridia_unclassified|g__Clostridia_unclassified|s_
k__Bacteria|p__Firmicutes|c__Clostridia|o__Eubacteriales|f__Clostridiaceae|g__Clostridiaceae_unclassified|s__Clostridiac
k__Bacteria|p__Firmicutes|c__Clostridia|o__Eubacteriales|f__Clostridiaceae|g__Clostridiaceae_unclassified|s__Clostridiac
k__Bacteria|p__Firmicutes|c__Clostridia|o__Eubacteriales|f__Eubacteriales_unclassified|g__Eubacteriales_unclassified|s_
k__Bacteria|p__Firmicutes|c__Erysipelotrichia|o__Erysipelotrichales|f__Erysipelotrichaceae|g__Erysipelatoclostridium|s_
k__Bacteria|p__Actinobacteria|c__Coriobacteriia|o__Coriobacteriales|f__Coriobacteriaceae|g__Coriobacteriaceae_unclass
k__Bacteria|p__Firmicutes|c__Clostridia|o__Eubacteriales|f__Lachnospiraceae|g__Dorea|s__Dorea_sp_5_2
k__Bacteria|p__Firmicutes|c__Erysipelotrichia|o__Erysipelotrichales|f__Erysipelotrichaceae|g__Dubosiella|s__Dubosiella_
k__Bacteria|p__Firmicutes|c__Erysipelotrichia|o__Erysipelotrichales|f__Erysipelotrichales_unclassified|g__Erysipelotrichal
k__Bacteria|p__Firmicutes|c__Clostridia|o__Eubacteriales|f__Eubacteriaceae|g__Eubacteriaceae_unclassified|s__Eubacte
k__Bacteria|p__Firmicutes|c__Clostridia|o__Eubacteriales|f__Eubacteriaceae|g__Eubacteriaceae_unclassified|s__Eubacte
k__Bacteria|p__Firmicutes|c__Clostridia|o__Eubacteriales|f__Lachnospiraceae|g__GGB20149|s__GGB20149_SGB29430
k__Bacteria|p__Actinobacteria|c__Coriobacteriia|o__Eggerthellales|f__Eggerthellaceae|g__GGB22635|s__GGB22635_SGB
k__Bacteria|p__Firmicutes|c__Clostridia|o__Eubacteriales|f__Lachnospiraceae|g__GGB25041|s__GGB25041_SGB36960
k__Bacteria|p__Bacteroidota|c__Bacteroidia|o__Bacteroidales|f__Muribaculaceae|g__GGB27876|s__GGB27876_SGB4031
k__Bacteria|p__Bacteroidota|c__Bacteroidia|o__Bacteroidales|f__Muribaculaceae|g__GGB27878|s__GGB27878_SGB4031
k__Bacteria|p__Bacteroidota|c__Bacteroidia|o__Bacteroidales|f__Muribaculaceae|g__GGB27918|s__GGB27918_SGB4035
k__Bacteria|p__Firmicutes|c__CFGB9508|o__OFGB9508|f__FGB9508|g__GGB28382|s__GGB28382_SGB40962
k__Bacteria|p__Firmicutes|c__CFGB2838|o__OFGB2838|f__FGB2838|g__GGB28399|s__GGB28399_SGB40980
k__Bacteria|p__Firmicutes|c__CFGB2838|o__OFGB2838|f__FGB2838|g__GGB28411|s__GGB28411_SGB40993
k__Bacteria|p__Firmicutes|c__CFGB2838|o__OFGB2838|f__FGB2838|g__GGB28415|s__GGB28415_SGB40997
k__Bacteria|p__Firmicutes|c__Clostridia|o__Eubacteriales|f__Pumilibacteraceae|g__GGB28430|s__GGB28430_SGB41013
k__Bacteria|p__Firmicutes|c__CFGB28439|o__OFGB28439|f__FGB28439|g__GGB28439|s__GGB28439_SGB41022

k__Bacteria|p__Firmicutes|c__Clostridia|o__Clostridia_unclassified|f__Clostridia_unclassified|g__GGB28778|s__GGB2877[illegible]
k__Bacteria|p__Firmicutes|c__Clostridia|o__Eubacteriales|f__Eubacteriaceae|g__GGB28784|s__GGB28784_SGB41437
k__Bacteria|p__Firmicutes|c__Clostridia|o__Eubacteriales|f__Lachnospiraceae|g__GGB28792|s__GGB28792_SGB41445
k__Bacteria|p__Firmicutes|c__Clostridia|o__Eubacteriales|f__Lachnospiraceae|g__GGB28798|s__GGB28798_SGB41451
k__Bacteria|p__Firmicutes|c__Clostridia|o__Eubacteriales|f__Lachnospiraceae|g__GGB28802|s__GGB28802_SGB41455
k__Bacteria|p__Firmicutes|c__Clostridia|o__Eubacteriales|f__Lachnospiraceae|g__GGB28818|s__GGB28818_SGB41473
k__Bacteria|p__Firmicutes|c__CFGB77305|o__OFGB77305|f__FGB77305|g__GGB28828|s__GGB28828_SGB41484
k__Bacteria|p__Firmicutes|c__Clostridia|o__Eubacteriales|f__Clostridiaceae|g__GGB28851|s__GGB28851_SGB41518
k__Bacteria|p__Firmicutes|c__Clostridia|o__Eubacteriales|f__Lachnospiraceae|g__GGB28859|s__GGB28859_SGB41528
k__Bacteria|p__Firmicutes|c__Clostridia|o__Eubacteriales|f__Lachnospiraceae|g__GGB28864|s__GGB28864_SGB41535
k__Bacteria|p__Firmicutes|c__Clostridia|o__Eubacteriales|f__Lachnospiraceae|g__GGB28869|s__GGB28869_SGB41543
k__Bacteria|p__Firmicutes|c__CFGB9633|o__OFGB9633|f__FGB9633|g__GGB28883|s__GGB28883_SGB41564
k__Bacteria|p__Bacteria_unclassified|c__Bacteria_unclassified|o__Bacteria_unclassified|f__Bacteria_unclassified|g__GGB[illegible]
k__Bacteria|p__Bacteria_unclassified|c__Bacteria_unclassified|o__Bacteria_unclassified|f__Bacteria_unclassified|g__GGB[illegible]
k__Bacteria|p__Bacteria_unclassified|c__Bacteria_unclassified|o__Bacteria_unclassified|f__Bacteria_unclassified|g__GGB[illegible]
k__Bacteria|p__Bacteria_unclassified|c__Bacteria_unclassified|o__Bacteria_unclassified|f__Bacteria_unclassified|g__GGB[illegible]
k__Bacteria|p__Firmicutes|c__Clostridia|o__Eubacteriales|f__Lachnospiraceae|g__GGB28916|s__GGB28916_SGB41612
k__Bacteria|p__Firmicutes|c__Clostridia|o__Eubacteriales|f__Lachnospiraceae|g__GGB28924|s__GGB28924_SGB41621
k__Bacteria|p__Firmicutes|c__Clostridia|o__Eubacteriales|f__Lachnospiraceae|g__GGB28926|s__GGB28926_SGB41624
k__Bacteria|p__Bacteria_unclassified|c__CFGB77359|o__OFGB77359|f__FGB77359|g__GGB28927|s__GGB28927_SGB416[illegible]
k__Bacteria|p__Firmicutes|c__CFGB9639|o__OFGB9639|f__FGB9639|g__GGB28934|s__GGB28934_SGB41635
k__Bacteria|p__Firmicutes|c__Clostridia|o__Eubacteriales|f__Lachnospiraceae|g__GGB28946|s__GGB28946_SGB41652
k__Bacteria|p__Firmicutes|c__Clostridia|o__Eubacteriales|f__Lachnospiraceae|g__GGB28949|s__GGB28949_SGB41655
k__Bacteria|p__Firmicutes|c__Clostridia|o__Eubacteriales|f__Lachnospiraceae|g__GGB28949|s__GGB28949_SGB41656
k__Bacteria|p__Firmicutes|c__Clostridia|o__Eubacteriales|f__Clostridiaceae|g__GGB28950|s__GGB28950_SGB41657
k__Bacteria|p__Firmicutes|c__Clostridia|o__Eubacteriales|f__Clostridiaceae|g__GGB28951|s__GGB28951_SGB102295
k__Bacteria|p__Firmicutes|c__Clostridia|o__Eubacteriales|f__Clostridiaceae|g__GGB28951|s__GGB28951_SGB41658
k__Bacteria|p__Firmicutes|c__Clostridia|o__Eubacteriales|f__Clostridiaceae|g__GGB28954|s__GGB28954_SGB41662
k__Bacteria|p__Firmicutes|c__Clostridia|o__Eubacteriales|f__Clostridiaceae|g__GGB28956|s__GGB28956_SGB41665
k__Bacteria|p__Firmicutes|c__Clostridia|o__Eubacteriales|f__Clostridiaceae|g__GGB28960|s__GGB28960_SGB41669
k__Bacteria|p__Firmicutes|c__Clostridia|o__Eubacteriales|f__Clostridiaceae|g__GGB28967|s__GGB28967_SGB41678
k__Bacteria|p__Firmicutes|c__Clostridia|o__Eubacteriales|f__Eubacteriaceae|g__GGB28991|s__GGB28991_SGB41705
k__Bacteria|p__Firmicutes|c__CFGB9658|o__OFGB9658|f__FGB9658|g__GGB29002|s__GGB29002_SGB41718
k__Bacteria|p__Firmicutes|c__CFGB9659|o__OFGB9659|f__FGB9659|g__GGB29003|s__GGB29003_SGB41719
k__Bacteria|p__Bacteria_unclassified|c__Bacteria_unclassified|o__Bacteria_unclassified|f__Bacteria_unclassified|g__GGB[illegible]
k__Bacteria|p__Firmicutes|c__CFGB9827|o__OFGB9827|f__FGB9827|g__GGB29531|s__GGB29531_SGB42317
k__Bacteria|p__Firmicutes|c__Clostridia|o__Eubacteriales|f__Eubacteriaceae|g__GGB29685|s__GGB29685_SGB42494
k__Bacteria|p__Bacteria_unclassified|c__CFGB77303|o__OFGB77303|f__FGB77303|g__GGB30141|s__GGB30141_SGB430[illegible]
k__Bacteria|p__Firmicutes|c__Clostridia|o__Eubacteriales|f__Eubacteriales_unclassified|g__GGB30286|s__GGB30286_SG[illegible]
k__Bacteria|p__Firmicutes|c__Clostridia|o__Eubacteriales|f__Oscillospiraceae|g__GGB30303|s__GGB30303_SGB43268
k__Bacteria|p__Firmicutes|c__CFGB30328|o__OFGB30328|f__FGB30328|g__GGB30413|s__GGB30413_SGB43452
k__Bacteria|p__Firmicutes|c__Clostridia|o__Eubacteriales|f__Oscillospiraceae|g__GGB30454|s__GGB30454_SGB43514
k__Bacteria|p__Firmicutes|c__Clostridia|o__Eubacteriales|f__Oscillospiraceae|g__GGB30455|s__GGB30455_SGB43519
k__Bacteria|p__Firmicutes|c__Clostridia|o__Eubacteriales|f__Oscillospiraceae|g__GGB30461|s__GGB30461_SGB43527
k__Bacteria|p__Firmicutes|c__Clostridia|o__Eubacteriales|f__Oscillospiraceae|g__GGB30461|s__GGB30461_SGB43530
k__Bacteria|p__Firmicutes|c__Clostridia|o__Eubacteriales|f__Oscillospiraceae|g__GGB30463|s__GGB30463_SGB43537

k__Bacteria|p__Firmicutes|c__Clostridia|o__Eubacteriales|f__Oscillospiraceae|g__GGB30473|s__GGB30473_SGB43557
k__Bacteria|p__Firmicutes|c__Clostridia|o__Eubacteriales|f__Oscillospiraceae|g__GGB30475|s__GGB30475_SGB63182
k__Bacteria|p__Actinobacteria|c__CFGB77153|o__OFGB77153|f__FGB77153|g__GGB30861|s__GGB30861_SGB44083
k__Bacteria|p__Tenericutes|c__CFGB1791|o__OFGB1791|f__FGB1791|g__GGB31312|s__GGB31312_SGB44628
k__Bacteria|p__Firmicutes|c__CFGB10290|o__OFGB10290|f__FGB10290|g__GGB31438|s__GGB31438_SGB44768
k__Bacteria|p__Firmicutes|c__Clostridia|o__Eubacteriales|f__Oscillospiraceae|g__GGB3171|s__GGB3171_SGB4185
k__Bacteria|p__Firmicutes|c__CFGB1765|o__OFGB1765|f__FGB1765|g__GGB31823|s__GGB31823_SGB45199
k__Bacteria|p__Firmicutes|c__CFGB10349|o__OFGB10349|f__FGB10349|g__GGB31853|s__GGB31853_SGB45233
k__Bacteria|p__Firmicutes|c__CFGB10667|o__OFGB10667|f__FGB10667|g__GGB32371|s__GGB32371_SGB41694
k__Bacteria|p__Firmicutes|c__Clostridia|o__Eubacteriales|f__Lachnospiraceae|g__GGB3793|s__GGB3793_SGB5158
k__Bacteria|p__Firmicutes|c__Clostridia|o__Eubacteriales|f__Lachnospiraceae|g__GGB42598|s__GGB42598_SGB59794
k__Bacteria|p__Firmicutes|c__Clostridia|o__Eubacteriales|f__Christensenellaceae|g__GGB45656|s__GGB45656_SGB6337
k__Bacteria|p__Firmicutes|c__CFGB10299|o__OFGB10299|f__FGB10299|g__GGB47127|s__GGB47127_SGB65054
k__Bacteria|p__Firmicutes|c__Clostridia|o__Eubacteriales|f__Oscillospiraceae|g__GGB74395|s__GGB74395_SGB43521
k__Bacteria|p__Firmicutes|c__Clostridia|o__Eubacteriales|f__Oscillospiraceae|g__GGB75053|s__GGB75053_SGB43494
k__Bacteria|p__Firmicutes|c__Clostridia|o__Eubacteriales|f__Lachnospiraceae|g__GGB75109|s__GGB75109_SGB102238
k__Bacteria|p__Firmicutes|c__Clostridia|o__Clostridia_unclassified|f__Clostridia_unclassified|g__GGB81440|s__GGB8144
k__Bacteria|p__Firmicutes|c__Clostridia|o__Eubacteriales|f__Lachnospiraceae|g__Lachnospiraceae_unclassified|s__Lachn
k__Bacteria|p__Firmicutes|c__Clostridia|o__Eubacteriales|f__Lachnospiraceae|g__Lachnospiraceae_unclassified|s__Lachn
k__Bacteria|p__Firmicutes|c__Clostridia|o__Eubacteriales|f__Lachnospiraceae|g__Lachnospiraceae_unclassified|s__Lachn
k__Bacteria|p__Firmicutes|c__Clostridia|o__Eubacteriales|f__Lachnospiraceae|g__Lachnospiraceae_unclassified|s__Lachn
k__Bacteria|p__Firmicutes|c__Clostridia|o__Eubacteriales|f__Lachnospiraceae|g__Lachnospiraceae_unclassified|s__Lachn
k__Bacteria|p__Firmicutes|c__Clostridia|o__Eubacteriales|f__Lachnospiraceae|g__Lachnospiraceae_unclassified|s__Lachn
k__Bacteria|p__Firmicutes|c__Clostridia|o__Eubacteriales|f__Lachnospiraceae|g__Lachnospiraceae_unclassified|s__Lachn
k__Bacteria|p__Firmicutes|c__Clostridia|o__Eubacteriales|f__Lachnospiraceae|g__Lachnospiraceae_unclassified|s__Lachn
k__Bacteria|p__Firmicutes|c__Bacilli|o__Lactobacillales|f__Lactobacillaceae|g__Lactobacillus|s__Lactobacillus_johnsonii
k__Bacteria|p__Bacteroidota|c__Bacteroidia|o__Bacteroidales|f__Muribaculaceae|g__Muribaculaceae_unclassified|s__M
k__Bacteria|p__Firmicutes|c__Clostridia|o__Eubacteriales|f__Oscillospiraceae|g__Neglectibacter|s__Neglectibacter_sp_X
k__Bacteria|p__Firmicutes|c__Clostridia|o__Eubacteriales|f__Oscillospiraceae|g__Oscillospiraceae_unclassified|s__Oscillo
k__Bacteria|p__Firmicutes|c__Clostridia|o__Eubacteriales|f__Oscillospiraceae|g__Oscillospiraceae_unclassified|s__Oscillo
k__Bacteria|p__Firmicutes|c__Clostridia|o__Eubacteriales|f__Oscillospiraceae|g__Oscillospiraceae_unclassified|s__Oscillo
k__Bacteria|p__Proteobacteria|c__Betaproteobacteria|o__Burkholderiales|f__Sutterellaceae|g__Parasutterella|s__Parasu
k__Bacteria|p__Firmicutes|c__Clostridia|o__Eubacteriales|f__Peptostreptococcaceae|g__Romboutsia|s__Romboutsia_ilea
k__Bacteria|p__Firmicutes|c__Clostridia|o__Eubacteriales|f__Lachnospiraceae|g__Schaedlerella|s__Schaedlerella_arabino
k__Bacteria|p__Firmicutes|c__Erysipelotrichia|o__Erysipelotrichales|f__Turicibacteraceae|g__Turicibacter|s__Turicibacter
k__Bacteria|p__Bacteria_unclassified|c__Bacteria_unclassified|o__Bacteria_unclassified|f__Bacteria_unclassified|g__Bact
k__Bacteria|p__Bacteria_unclassified|c__Bacteria_unclassified|o__Bacteria_unclassified|f__Bacteria_unclassified|g__Bact
k__Bacteria|p__Bacteria_unclassified|c__Bacteria_unclassified|o__Bacteria_unclassified|f__Bacteria_unclassified|g__Bact

k__Bacteria|p__Firmicutes|c__Clostridia|o__Eubacteriales|f__Lachnospiraceae|g__Acetatifactor|s__Acetatifactor_SGB415
k__Bacteria|p__Firmicutes|c__Clostridia|o__Eubacteriales|f__Lachnospiraceae|g__Acetatifactor|s__Acetatifactor_muris
k__Bacteria|p__Firmicutes|c__Clostridia|o__Eubacteriales|f__Oscillospiraceae|g__Acutalibacter|s__Acutalibacter_muris
k__Bacteria|p__Firmicutes|c__Clostridia|o__Eubacteriales|f__Oscillospiraceae|g__Acutalibacter|s__Acutalibacter_sp_1XD
k__Bacteria|p__Actinobacteria|c__Coriobacteriia|o__Eggerthellales|f__Eggerthellaceae|g__Adlercreutzia|s__Adlercreutzia

k__Bacteria|p__Actinobacteria|c__Coriobacteriia|o__Eggerthellales|f__Eggerthellaceae|g__Adlercreutzia|s__Adlercreutzia
k__Bacteria|p__Actinobacteria|c__Coriobacteriia|o__Eggerthellales|f__Eggerthellaceae|g__Adlercreutzia|s__Adlercreutzia
k__Bacteria|p__Verrucomicrobia|c__Verrucomicrobiae|o__Verrucomicrobiales|f__Akkermansiaceae|g__Akkermansia|s__A
k__Bacteria|p__Bacteroidota|c__Bacteroidia|o__Bacteroidales|f__Rikenellaceae|g__Alistipes|s__Alistipes_sp_DSM_11234
k__Bacteria|p__Firmicutes|c__Clostridia|o__Eubacteriales|f__Oscillospiraceae|g__Anaerotruncus|s__Anaerotruncus_sp_1
k__Bacteria|p__Bacteria_unclassified|c__Bacteria_unclassified|o__Bacteria_unclassified|f__Bacteria_unclassified|g__Bacte
k__Bacteria|p__Bacteria_unclassified|c__Bacteria_unclassified|o__Bacteria_unclassified|f__Bacteria_unclassified|g__Bacte
k__Bacteria|p__Bacteria_unclassified|c__Bacteria_unclassified|o__Bacteria_unclassified|f__Bacteria_unclassified|g__Bacte
k__Bacteria|p__Bacteroidota|c__Bacteroidia|o__Bacteroidales|f__Bacteroidaceae|g__Bacteroides|s__Bacteroides_thetaio
k__Bacteria|p__Actinobacteria|c__Actinomycetia|o__Bifidobacteriales|f__Bifidobacteriaceae|g__Bifidobacterium|s__Bifid
k__Bacteria|p__Firmicutes|c__Clostridia|o__Clostridia_unclassified|f__Clostridia_unclassified|g__Clostridia_unclassified|s_
k__Bacteria|p__Firmicutes|c__Clostridia|o__Eubacteriales|f__Clostridiaceae|g__Clostridiaceae_unclassified|s__Clostridiac
k__Bacteria|p__Firmicutes|c__Clostridia|o__Eubacteriales|f__Clostridiaceae|g__Clostridiaceae_unclassified|s__Clostridiac
k__Bacteria|p__Firmicutes|c__Clostridia|o__Eubacteriales|f__Eubacteriales_unclassified|g__Eubacteriales_unclassified|s_
k__Bacteria|p__Firmicutes|c__Erysipelotrichia|o__Erysipelotrichales|f__Erysipelotrichaceae|g__Erysipelatoclostridium|s__
k__Bacteria|p__Actinobacteria|c__Coriobacteriia|o__Coriobacteriales|f__Coriobacteriaceae|g__Coriobacteriaceae_unclass
k__Bacteria|p__Firmicutes|c__Clostridia|o__Eubacteriales|f__Lachnospiraceae|g__Dorea|s__Dorea_sp_5_2
k__Bacteria|p__Firmicutes|c__Erysipelotrichia|o__Erysipelotrichales|f__Erysipelotrichaceae|g__Dubosiella|s__Dubosiella_
k__Bacteria|p__Firmicutes|c__Erysipelotrichia|o__Erysipelotrichales|f__Erysipelotrichales_unclassified|g__Erysipelotrichal
k__Bacteria|p__Firmicutes|c__Clostridia|o__Eubacteriales|f__Eubacteriaceae|g__Eubacteriaceae_unclassified|s__Eubacte
k__Bacteria|p__Firmicutes|c__Clostridia|o__Eubacteriales|f__Eubacteriaceae|g__Eubacteriaceae_unclassified|s__Eubacte
k__Bacteria|p__Firmicutes|c__Clostridia|o__Eubacteriales|f__Lachnospiraceae|g__GGB20149|s__GGB20149_SGB29430
k__Bacteria|p__Actinobacteria|c__Coriobacteriia|o__Eggerthellales|f__Eggerthellaceae|g__GGB22635|s__GGB22635_SGB
k__Bacteria|p__Firmicutes|c__Clostridia|o__Eubacteriales|f__Lachnospiraceae|g__GGB25041|s__GGB25041_SGB36960
k__Bacteria|p__Bacteroidota|c__Bacteroidia|o__Bacteroidales|f__Muribaculaceae|g__GGB27876|s__GGB27876_SGB4031
k__Bacteria|p__Bacteroidota|c__Bacteroidia|o__Bacteroidales|f__Muribaculaceae|g__GGB27878|s__GGB27878_SGB4031
k__Bacteria|p__Bacteroidota|c__Bacteroidia|o__Bacteroidales|f__Muribaculaceae|g__GGB27918|s__GGB27918_SGB4035
k__Bacteria|p__Firmicutes|c__CFGB9508|o__OFGB9508|f__FGB9508|g__GGB28382|s__GGB28382_SGB40962
k__Bacteria|p__Firmicutes|c__CFGB2838|o__OFGB2838|f__FGB2838|g__GGB28399|s__GGB28399_SGB40980
k__Bacteria|p__Firmicutes|c__CFGB2838|o__OFGB2838|f__FGB2838|g__GGB28411|s__GGB28411_SGB40993
k__Bacteria|p__Firmicutes|c__CFGB2838|o__OFGB2838|f__FGB2838|g__GGB28415|s__GGB28415_SGB40997
k__Bacteria|p__Firmicutes|c__Clostridia|o__Eubacteriales|f__Pumilibacteraceae|g__GGB28430|s__GGB28430_SGB41013
k__Bacteria|p__Firmicutes|c__CFGB28439|o__OFGB28439|f__FGB28439|g__GGB28439|s__GGB28439_SGB41022
k__Bacteria|p__Firmicutes|c__Clostridia|o__Clostridia_unclassified|f__Clostridia_unclassified|g__GGB28778|s__GGB2877
k__Bacteria|p__Firmicutes|c__Clostridia|o__Eubacteriales|f__Eubacteriaceae|g__GGB28784|s__GGB28784_SGB41437
k__Bacteria|p__Firmicutes|c__Clostridia|o__Eubacteriales|f__Lachnospiraceae|g__GGB28792|s__GGB28792_SGB41445
k__Bacteria|p__Firmicutes|c__Clostridia|o__Eubacteriales|f__Lachnospiraceae|g__GGB28798|s__GGB28798_SGB41451
k__Bacteria|p__Firmicutes|c__Clostridia|o__Eubacteriales|f__Lachnospiraceae|g__GGB28802|s__GGB28802_SGB41455
k__Bacteria|p__Firmicutes|c__Clostridia|o__Eubacteriales|f__Lachnospiraceae|g__GGB28818|s__GGB28818_SGB41473
k__Bacteria|p__Firmicutes|c__CFGB77305|o__OFGB77305|f__FGB77305|g__GGB28828|s__GGB28828_SGB41484
k__Bacteria|p__Firmicutes|c__Clostridia|o__Eubacteriales|f__Clostridiaceae|g__GGB28851|s__GGB28851_SGB41518
k__Bacteria|p__Firmicutes|c__Clostridia|o__Eubacteriales|f__Lachnospiraceae|g__GGB28859|s__GGB28859_SGB41528
k__Bacteria|p__Firmicutes|c__Clostridia|o__Eubacteriales|f__Lachnospiraceae|g__GGB28864|s__GGB28864_SGB41535
k__Bacteria|p__Firmicutes|c__Clostridia|o__Eubacteriales|f__Lachnospiraceae|g__GGB28869|s__GGB28869_SGB41543
k__Bacteria|p__Firmicutes|c__CFGB9633|o__OFGB9633|f__FGB9633|g__GGB28883|s__GGB28883_SGB41564
k__Bacteria|p__Bacteria_unclassified|c__Bacteria_unclassified|o__Bacteria_unclassified|f__Bacteria_unclassified|g__GGB2

k__Bacteria|p__Bacteria_unclassified|c__Bacteria_unclassified|o__Bacteria_unclassified|f__Bacteria_unclassified|g__GGB2
k__Bacteria|p__Bacteria_unclassified|c__Bacteria_unclassified|o__Bacteria_unclassified|f__Bacteria_unclassified|g__GGB2
k__Bacteria|p__Bacteria_unclassified|c__Bacteria_unclassified|o__Bacteria_unclassified|f__Bacteria_unclassified|g__GGB2
k__Bacteria|p__Firmicutes|c__Clostridia|o__Eubacteriales|f__Lachnospiraceae|g__GGB28916|s__GGB28916_SGB41612
k__Bacteria|p__Firmicutes|c__Clostridia|o__Eubacteriales|f__Lachnospiraceae|g__GGB28924|s__GGB28924_SGB41621
k__Bacteria|p__Firmicutes|c__Clostridia|o__Eubacteriales|f__Lachnospiraceae|g__GGB28926|s__GGB28926_SGB41624
k__Bacteria|p__Bacteria_unclassified|c__CFGB77359|o__OFGB77359|f__FGB77359|g__GGB28927|s__GGB28927_SGB416
k__Bacteria|p__Firmicutes|c__CFGB9639|o__OFGB9639|f__FGB9639|g__GGB28934|s__GGB28934_SGB41635
k__Bacteria|p__Firmicutes|c__Clostridia|o__Eubacteriales|f__Lachnospiraceae|g__GGB28946|s__GGB28946_SGB41652
k__Bacteria|p__Firmicutes|c__Clostridia|o__Eubacteriales|f__Lachnospiraceae|g__GGB28949|s__GGB28949_SGB41655
k__Bacteria|p__Firmicutes|c__Clostridia|o__Eubacteriales|f__Lachnospiraceae|g__GGB28949|s__GGB28949_SGB41656
k__Bacteria|p__Firmicutes|c__Clostridia|o__Eubacteriales|f__Clostridiaceae|g__GGB28950|s__GGB28950_SGB41657
k__Bacteria|p__Firmicutes|c__Clostridia|o__Eubacteriales|f__Clostridiaceae|g__GGB28951|s__GGB28951_SGB102295
k__Bacteria|p__Firmicutes|c__Clostridia|o__Eubacteriales|f__Clostridiaceae|g__GGB28951|s__GGB28951_SGB41658
k__Bacteria|p__Firmicutes|c__Clostridia|o__Eubacteriales|f__Clostridiaceae|g__GGB28954|s__GGB28954_SGB41662
k__Bacteria|p__Firmicutes|c__Clostridia|o__Eubacteriales|f__Clostridiaceae|g__GGB28956|s__GGB28956_SGB41665
k__Bacteria|p__Firmicutes|c__Clostridia|o__Eubacteriales|f__Clostridiaceae|g__GGB28960|s__GGB28960_SGB41669
k__Bacteria|p__Firmicutes|c__Clostridia|o__Eubacteriales|f__Clostridiaceae|g__GGB28967|s__GGB28967_SGB41678
k__Bacteria|p__Firmicutes|c__Clostridia|o__Eubacteriales|f__Eubacteriaceae|g__GGB28991|s__GGB28991_SGB41705
k__Bacteria|p__Firmicutes|c__CFGB9658|o__OFGB9658|f__FGB9658|g__GGB29002|s__GGB29002_SGB41718
k__Bacteria|p__Firmicutes|c__CFGB9659|o__OFGB9659|f__FGB9659|g__GGB29003|s__GGB29003_SGB41719
k__Bacteria|p__Bacteria_unclassified|c__Bacteria_unclassified|o__Bacteria_unclassified|f__Bacteria_unclassified|g__GGB2
k__Bacteria|p__Firmicutes|c__CFGB9827|o__OFGB9827|f__FGB9827|g__GGB29531|s__GGB29531_SGB42317
k__Bacteria|p__Firmicutes|c__Clostridia|o__Eubacteriales|f__Eubacteriaceae|g__GGB29685|s__GGB29685_SGB42494
k__Bacteria|p__Bacteria_unclassified|c__CFGB77303|o__OFGB77303|f__FGB77303|g__GGB30141|s__GGB30141_SGB430
k__Bacteria|p__Firmicutes|c__Clostridia|o__Eubacteriales|f__Eubacteriales_unclassified|g__GGB30286|s__GGB30286_SG
k__Bacteria|p__Firmicutes|c__Clostridia|o__Eubacteriales|f__Oscillospiraceae|g__GGB30303|s__GGB30303_SGB43268
k__Bacteria|p__Firmicutes|c__CFGB30328|o__OFGB30328|f__FGB30328|g__GGB30413|s__GGB30413_SGB43452
k__Bacteria|p__Firmicutes|c__Clostridia|o__Eubacteriales|f__Oscillospiraceae|g__GGB30454|s__GGB30454_SGB43514
k__Bacteria|p__Firmicutes|c__Clostridia|o__Eubacteriales|f__Oscillospiraceae|g__GGB30455|s__GGB30455_SGB43519
k__Bacteria|p__Firmicutes|c__Clostridia|o__Eubacteriales|f__Oscillospiraceae|g__GGB30461|s__GGB30461_SGB43527
k__Bacteria|p__Firmicutes|c__Clostridia|o__Eubacteriales|f__Oscillospiraceae|g__GGB30461|s__GGB30461_SGB43530
k__Bacteria|p__Firmicutes|c__Clostridia|o__Eubacteriales|f__Oscillospiraceae|g__GGB30463|s__GGB30463_SGB43537
k__Bacteria|p__Firmicutes|c__Clostridia|o__Eubacteriales|f__Oscillospiraceae|g__GGB30473|s__GGB30473_SGB43557
k__Bacteria|p__Firmicutes|c__Clostridia|o__Eubacteriales|f__Oscillospiraceae|g__GGB30475|s__GGB30475_SGB63182
k__Bacteria|p__Actinobacteria|c__CFGB77153|o__OFGB77153|f__FGB77153|g__GGB30861|s__GGB30861_SGB44083
k__Bacteria|p__Tenericutes|c__CFGB1791|o__OFGB1791|f__FGB1791|g__GGB31312|s__GGB31312_SGB44628
k__Bacteria|p__Firmicutes|c__CFGB10290|o__OFGB10290|f__FGB10290|g__GGB31438|s__GGB31438_SGB44768
k__Bacteria|p__Firmicutes|c__Clostridia|o__Eubacteriales|f__Oscillospiraceae|g__GGB3171|s__GGB3171_SGB4185
k__Bacteria|p__Firmicutes|c__CFGB1765|o__OFGB1765|f__FGB1765|g__GGB31823|s__GGB31823_SGB45199
k__Bacteria|p__Firmicutes|c__CFGB10349|o__OFGB10349|f__FGB10349|g__GGB31853|s__GGB31853_SGB45233
k__Bacteria|p__Firmicutes|c__CFGB10667|o__OFGB10667|f__FGB10667|g__GGB32371|s__GGB32371_SGB41694
k__Bacteria|p__Firmicutes|c__Clostridia|o__Eubacteriales|f__Lachnospiraceae|g__GGB3793|s__GGB3793_SGB5158
k__Bacteria|p__Firmicutes|c__Clostridia|o__Eubacteriales|f__Lachnospiraceae|g__GGB42598|s__GGB42598_SGB59794
k__Bacteria|p__Firmicutes|c__Clostridia|o__Eubacteriales|f__Christensenellaceae|g__GGB45656|s__GGB45656_SGB6337
k__Bacteria|p__Firmicutes|c__CFGB10299|o__OFGB10299|f__FGB10299|g__GGB47127|s__GGB47127_SGB65054

k__Bacteria|p__Firmicutes|c__Clostridia|o__Eubacteriales|f__Oscillospiraceae|g__GGB74395|s__GGB74395_SGB43521
k__Bacteria|p__Firmicutes|c__Clostridia|o__Eubacteriales|f__Oscillospiraceae|g__GGB75053|s__GGB75053_SGB43494
k__Bacteria|p__Firmicutes|c__Clostridia|o__Eubacteriales|f__Lachnospiraceae|g__GGB75109|s__GGB75109_SGB102238
k__Bacteria|p__Firmicutes|c__Clostridia|o__Clostridia_unclassified|f__Clostridia_unclassified|g__GGB81440|s__GGB8144
k__Bacteria|p__Firmicutes|c__Clostridia|o__Eubacteriales|f__Lachnospiraceae|g__Lachnospiraceae_unclassified|s__Lachn
k__Bacteria|p__Firmicutes|c__Clostridia|o__Eubacteriales|f__Lachnospiraceae|g__Lachnospiraceae_unclassified|s__Lachn
k__Bacteria|p__Firmicutes|c__Clostridia|o__Eubacteriales|f__Lachnospiraceae|g__Lachnospiraceae_unclassified|s__Lachn
k__Bacteria|p__Firmicutes|c__Clostridia|o__Eubacteriales|f__Lachnospiraceae|g__Lachnospiraceae_unclassified|s__Lachn
k__Bacteria|p__Firmicutes|c__Clostridia|o__Eubacteriales|f__Lachnospiraceae|g__Lachnospiraceae_unclassified|s__Lachn
k__Bacteria|p__Firmicutes|c__Clostridia|o__Eubacteriales|f__Lachnospiraceae|g__Lachnospiraceae_unclassified|s__Lachn
k__Bacteria|p__Firmicutes|c__Clostridia|o__Eubacteriales|f__Lachnospiraceae|g__Lachnospiraceae_unclassified|s__Lachn
k__Bacteria|p__Firmicutes|c__Clostridia|o__Eubacteriales|f__Lachnospiraceae|g__Lachnospiraceae_unclassified|s__Lachn
k__Bacteria|p__Firmicutes|c__Bacilli|o__Lactobacillales|f__Lactobacillaceae|g__Lactobacillus|s__Lactobacillus_johnsonii
k__Bacteria|p__Bacteroidota|c__Bacteroidia|o__Bacteroidales|f__Muribaculaceae|g__Muribaculaceae_unclassified|s__M
k__Bacteria|p__Firmicutes|c__Clostridia|o__Eubacteriales|f__Oscillospiraceae|g__Neglectibacter|s__Neglectibacter_sp_X
k__Bacteria|p__Firmicutes|c__Clostridia|o__Eubacteriales|f__Oscillospiraceae|g__Oscillospiraceae_unclassified|s__Oscillo
k__Bacteria|p__Firmicutes|c__Clostridia|o__Eubacteriales|f__Oscillospiraceae|g__Oscillospiraceae_unclassified|s__Oscillo
k__Bacteria|p__Firmicutes|c__Clostridia|o__Eubacteriales|f__Oscillospiraceae|g__Oscillospiraceae_unclassified|s__Oscillo
k__Bacteria|p__Proteobacteria|c__Betaproteobacteria|o__Burkholderiales|f__Sutterellaceae|g__Parasutterella|s__Parasu
k__Bacteria|p__Firmicutes|c__Clostridia|o__Eubacteriales|f__Peptostreptococcaceae|g__Romboutsia|s__Romboutsia_ilea
k__Bacteria|p__Firmicutes|c__Clostridia|o__Eubacteriales|f__Lachnospiraceae|g__Schaedlerella|s__Schaedlerella_arabino
k__Bacteria|p__Firmicutes|c__Erysipelotrichia|o__Erysipelotrichales|f__Turicibacteraceae|g__Turicibacter|s__Turicibacter_
k__Bacteria|p__Bacteria_unclassified|c__Bacteria_unclassified|o__Bacteria_unclassified|f__Bacteria_unclassified|g__Bact
k__Bacteria|p__Bacteria_unclassified|c__Bacteria_unclassified|o__Bacteria_unclassified|f__Bacteria_unclassified|g__Bact
k__Bacteria|p__Bacteria_unclassified|c__Bacteria_unclassified|o__Bacteria_unclassified|f__Bacteria_unclassified|g__Bact

k__Bacteria|p__Firmicutes|c__Clostridia|o__Eubacteriales|f__Lachnospiraceae|g__Acetatifactor|s__Acetatifactor_SGB415
k__Bacteria|p__Firmicutes|c__Clostridia|o__Eubacteriales|f__Lachnospiraceae|g__Acetatifactor|s__Acetatifactor_muris
k__Bacteria|p__Firmicutes|c__Clostridia|o__Eubacteriales|f__Oscillospiraceae|g__Acutalibacter|s__Acutalibacter_muris
k__Bacteria|p__Firmicutes|c__Clostridia|o__Eubacteriales|f__Oscillospiraceae|g__Acutalibacter|s__Acutalibacter_sp_1XD
k__Bacteria|p__Actinobacteria|c__Coriobacteriia|o__Eggerthellales|f__Eggerthellaceae|g__Adlercreutzia|s__Adlercreutzia
k__Bacteria|p__Actinobacteria|c__Coriobacteriia|o__Eggerthellales|f__Eggerthellaceae|g__Adlercreutzia|s__Adlercreutzia
k__Bacteria|p__Actinobacteria|c__Coriobacteriia|o__Eggerthellales|f__Eggerthellaceae|g__Adlercreutzia|s__Adlercreutzia
k__Bacteria|p__Verrucomicrobia|c__Verrucomicrobiae|o__Verrucomicrobiales|f__Akkermansiaceae|g__Akkermansia|s__
k__Bacteria|p__Bacteroidota|c__Bacteroidia|o__Bacteroidales|f__Rikenellaceae|g__Alistipes|s__Alistipes_sp_DSM_11234
k__Bacteria|p__Firmicutes|c__Clostridia|o__Eubacteriales|f__Oscillospiraceae|g__Anaerotruncus|s__Anaerotruncus_sp_1
k__Bacteria|p__Bacteria_unclassified|c__Bacteria_unclassified|o__Bacteria_unclassified|f__Bacteria_unclassified|g__Bact
k__Bacteria|p__Bacteria_unclassified|c__Bacteria_unclassified|o__Bacteria_unclassified|f__Bacteria_unclassified|g__Bact
k__Bacteria|p__Bacteria_unclassified|c__Bacteria_unclassified|o__Bacteria_unclassified|f__Bacteria_unclassified|g__Bact
k__Bacteria|p__Bacteroidota|c__Bacteroidia|o__Bacteroidales|f__Bacteroidaceae|g__Bacteroides|s__Bacteroides_thetaio
k__Bacteria|p__Actinobacteria|c__Actinomycetia|o__Bifidobacteriales|f__Bifidobacteriaceae|g__Bifidobacterium|s__Bifid
k__Bacteria|p__Firmicutes|c__Clostridia|o__Clostridia_unclassified|f__Clostridia_unclassified|g__Clostridia_unclassified|s
k__Bacteria|p__Firmicutes|c__Clostridia|o__Eubacteriales|f__Clostridiaceae|g__Clostridiaceae_unclassified|s__Clostridiac
k__Bacteria|p__Firmicutes|c__Clostridia|o__Eubacteriales|f__Clostridiaceae|g__Clostridiaceae_unclassified|s__Clostridiac

k__Bacteria|p__Firmicutes|c__Clostridia|o__Eubacteriales|f__Eubacteriales_unclassified|g__Eubacteriales_unclassified|s_
k__Bacteria|p__Firmicutes|c__Erysipelotrichia|o__Erysipelotrichales|f__Erysipelotrichaceae|g__Erysipelatoclostridium|s__
k__Bacteria|p__Actinobacteria|c__Coriobacteriia|o__Coriobacteriales|f__Coriobacteriaceae|g__Coriobacteriaceae_unclass
k__Bacteria|p__Firmicutes|c__Clostridia|o__Eubacteriales|f__Lachnospiraceae|g__Dorea|s__Dorea_sp_5_2
k__Bacteria|p__Firmicutes|c__Erysipelotrichia|o__Erysipelotrichales|f__Erysipelotrichaceae|g__Dubosiella|s__Dubosiella_
k__Bacteria|p__Firmicutes|c__Erysipelotrichia|o__Erysipelotrichales|f__Erysipelotrichales_unclassified|g__Erysipelotrichal
k__Bacteria|p__Firmicutes|c__Clostridia|o__Eubacteriales|f__Eubacteriaceae|g__Eubacteriaceae_unclassified|s__Eubacte
k__Bacteria|p__Firmicutes|c__Clostridia|o__Eubacteriales|f__Eubacteriaceae|g__Eubacteriaceae_unclassified|s__Eubacte
k__Bacteria|p__Firmicutes|c__Clostridia|o__Eubacteriales|f__Lachnospiraceae|g__GGB20149|s__GGB20149_SGB29430
k__Bacteria|p__Actinobacteria|c__Coriobacteriia|o__Eggerthellales|f__Eggerthellaceae|g__GGB22635|s__GGB22635_SGB
k__Bacteria|p__Firmicutes|c__Clostridia|o__Eubacteriales|f__Lachnospiraceae|g__GGB25041|s__GGB25041_SGB36960
k__Bacteria|p__Bacteroidota|c__Bacteroidia|o__Bacteroidales|f__Muribaculaceae|g__GGB27876|s__GGB27876_SGB4031
k__Bacteria|p__Bacteroidota|c__Bacteroidia|o__Bacteroidales|f__Muribaculaceae|g__GGB27878|s__GGB27878_SGB4031
k__Bacteria|p__Bacteroidota|c__Bacteroidia|o__Bacteroidales|f__Muribaculaceae|g__GGB27918|s__GGB27918_SGB4035
k__Bacteria|p__Firmicutes|c__CFGB9508|o__OFGB9508|f__FGB9508|g__GGB28382|s__GGB28382_SGB40962
k__Bacteria|p__Firmicutes|c__CFGB2838|o__OFGB2838|f__FGB2838|g__GGB28399|s__GGB28399_SGB40980
k__Bacteria|p__Firmicutes|c__CFGB2838|o__OFGB2838|f__FGB2838|g__GGB28411|s__GGB28411_SGB40993
k__Bacteria|p__Firmicutes|c__CFGB2838|o__OFGB2838|f__FGB2838|g__GGB28415|s__GGB28415_SGB40997
k__Bacteria|p__Firmicutes|c__Clostridia|o__Eubacteriales|f__Pumilibacteraceae|g__GGB28430|s__GGB28430_SGB41013
k__Bacteria|p__Firmicutes|c__CFGB28439|o__OFGB28439|f__FGB28439|g__GGB28439|s__GGB28439_SGB41022
k__Bacteria|p__Firmicutes|c__Clostridia|o__Clostridia_unclassified|f__Clostridia_unclassified|g__GGB28778|s__GGB2877
k__Bacteria|p__Firmicutes|c__Clostridia|o__Eubacteriales|f__Eubacteriaceae|g__GGB28784|s__GGB28784_SGB41437
k__Bacteria|p__Firmicutes|c__Clostridia|o__Eubacteriales|f__Lachnospiraceae|g__GGB28792|s__GGB28792_SGB41445
k__Bacteria|p__Firmicutes|c__Clostridia|o__Eubacteriales|f__Lachnospiraceae|g__GGB28798|s__GGB28798_SGB41451
k__Bacteria|p__Firmicutes|c__Clostridia|o__Eubacteriales|f__Lachnospiraceae|g__GGB28802|s__GGB28802_SGB41455
k__Bacteria|p__Firmicutes|c__Clostridia|o__Eubacteriales|f__Lachnospiraceae|g__GGB28818|s__GGB28818_SGB41473
k__Bacteria|p__Firmicutes|c__CFGB77305|o__OFGB77305|f__FGB77305|g__GGB28828|s__GGB28828_SGB41484
k__Bacteria|p__Firmicutes|c__Clostridia|o__Eubacteriales|f__Clostridiaceae|g__GGB28851|s__GGB28851_SGB41518
k__Bacteria|p__Firmicutes|c__Clostridia|o__Eubacteriales|f__Lachnospiraceae|g__GGB28859|s__GGB28859_SGB41528
k__Bacteria|p__Firmicutes|c__Clostridia|o__Eubacteriales|f__Lachnospiraceae|g__GGB28864|s__GGB28864_SGB41535
k__Bacteria|p__Firmicutes|c__Clostridia|o__Eubacteriales|f__Lachnospiraceae|g__GGB28869|s__GGB28869_SGB41543
k__Bacteria|p__Firmicutes|c__CFGB9633|o__OFGB9633|f__FGB9633|g__GGB28883|s__GGB28883_SGB41564
k__Bacteria|p__Bacteria_unclassified|c__Bacteria_unclassified|o__Bacteria_unclassified|f__Bacteria_unclassified|g__GGB
k__Bacteria|p__Bacteria_unclassified|c__Bacteria_unclassified|o__Bacteria_unclassified|f__Bacteria_unclassified|g__GGB
k__Bacteria|p__Bacteria_unclassified|c__Bacteria_unclassified|o__Bacteria_unclassified|f__Bacteria_unclassified|g__GGB
k__Bacteria|p__Bacteria_unclassified|c__Bacteria_unclassified|o__Bacteria_unclassified|f__Bacteria_unclassified|g__GGB
k__Bacteria|p__Firmicutes|c__Clostridia|o__Eubacteriales|f__Lachnospiraceae|g__GGB28916|s__GGB28916_SGB41612
k__Bacteria|p__Firmicutes|c__Clostridia|o__Eubacteriales|f__Lachnospiraceae|g__GGB28924|s__GGB28924_SGB41621
k__Bacteria|p__Firmicutes|c__Clostridia|o__Eubacteriales|f__Lachnospiraceae|g__GGB28926|s__GGB28926_SGB41624
k__Bacteria|p__Bacteria_unclassified|c__CFGB77359|o__OFGB77359|f__FGB77359|g__GGB28927|s__GGB28927_SGB416
k__Bacteria|p__Firmicutes|c__CFGB9639|o__OFGB9639|f__FGB9639|g__GGB28934|s__GGB28934_SGB41635
k__Bacteria|p__Firmicutes|c__Clostridia|o__Eubacteriales|f__Lachnospiraceae|g__GGB28946|s__GGB28946_SGB41652
k__Bacteria|p__Firmicutes|c__Clostridia|o__Eubacteriales|f__Lachnospiraceae|g__GGB28949|s__GGB28949_SGB41655
k__Bacteria|p__Firmicutes|c__Clostridia|o__Eubacteriales|f__Lachnospiraceae|g__GGB28949|s__GGB28949_SGB41656
k__Bacteria|p__Firmicutes|c__Clostridia|o__Eubacteriales|f__Clostridiaceae|g__GGB28950|s__GGB28950_SGB41657
k__Bacteria|p__Firmicutes|c__Clostridia|o__Eubacteriales|f__Clostridiaceae|g__GGB28951|s__GGB28951_SGB102295

k__Bacteria|p__Firmicutes|c__Clostridia|o__Eubacteriales|f__Clostridiaceae|g__GGB28951|s__GGB28951_SGB41658
k__Bacteria|p__Firmicutes|c__Clostridia|o__Eubacteriales|f__Clostridiaceae|g__GGB28954|s__GGB28954_SGB41662
k__Bacteria|p__Firmicutes|c__Clostridia|o__Eubacteriales|f__Clostridiaceae|g__GGB28956|s__GGB28956_SGB41665
k__Bacteria|p__Firmicutes|c__Clostridia|o__Eubacteriales|f__Clostridiaceae|g__GGB28960|s__GGB28960_SGB41669
k__Bacteria|p__Firmicutes|c__Clostridia|o__Eubacteriales|f__Clostridiaceae|g__GGB28967|s__GGB28967_SGB41678
k__Bacteria|p__Firmicutes|c__Clostridia|o__Eubacteriales|f__Eubacteriaceae|g__GGB28991|s__GGB28991_SGB41705
k__Bacteria|p__Firmicutes|c__CFGB9658|o__OFGB9658|f__FGB9658|g__GGB29002|s__GGB29002_SGB41718
k__Bacteria|p__Firmicutes|c__CFGB9659|o__OFGB9659|f__FGB9659|g__GGB29003|s__GGB29003_SGB41719
k__Bacteria|p__Bacteria_unclassified|c__Bacteria_unclassified|o__Bacteria_unclassified|f__Bacteria_unclassified|g__GGB2
k__Bacteria|p__Firmicutes|c__CFGB9827|o__OFGB9827|f__FGB9827|g__GGB29531|s__GGB29531_SGB42317
k__Bacteria|p__Firmicutes|c__Clostridia|o__Eubacteriales|f__Eubacteriaceae|g__GGB29685|s__GGB29685_SGB42494
k__Bacteria|p__Bacteria_unclassified|c__CFGB77303|o__OFGB77303|f__FGB77303|g__GGB30141|s__GGB30141_SGB430
k__Bacteria|p__Firmicutes|c__Clostridia|o__Eubacteriales|f__Eubacteriales_unclassified|g__GGB30286|s__GGB30286_SG
k__Bacteria|p__Firmicutes|c__Clostridia|o__Eubacteriales|f__Oscillospiraceae|g__GGB30303|s__GGB30303_SGB43268
k__Bacteria|p__Firmicutes|c__CFGB30328|o__OFGB30328|f__FGB30328|g__GGB30413|s__GGB30413_SGB43452
k__Bacteria|p__Firmicutes|c__Clostridia|o__Eubacteriales|f__Oscillospiraceae|g__GGB30454|s__GGB30454_SGB43514
k__Bacteria|p__Firmicutes|c__Clostridia|o__Eubacteriales|f__Oscillospiraceae|g__GGB30455|s__GGB30455_SGB43519
k__Bacteria|p__Firmicutes|c__Clostridia|o__Eubacteriales|f__Oscillospiraceae|g__GGB30461|s__GGB30461_SGB43527
k__Bacteria|p__Firmicutes|c__Clostridia|o__Eubacteriales|f__Oscillospiraceae|g__GGB30461|s__GGB30461_SGB43530
k__Bacteria|p__Firmicutes|c__Clostridia|o__Eubacteriales|f__Oscillospiraceae|g__GGB30463|s__GGB30463_SGB43537
k__Bacteria|p__Firmicutes|c__Clostridia|o__Eubacteriales|f__Oscillospiraceae|g__GGB30473|s__GGB30473_SGB43557
k__Bacteria|p__Firmicutes|c__Clostridia|o__Eubacteriales|f__Oscillospiraceae|g__GGB30475|s__GGB30475_SGB63182
k__Bacteria|p__Actinobacteria|c__CFGB77153|o__OFGB77153|f__FGB77153|g__GGB30861|s__GGB30861_SGB44083
k__Bacteria|p__Tenericutes|c__CFGB1791|o__OFGB1791|f__FGB1791|g__GGB31312|s__GGB31312_SGB44628
k__Bacteria|p__Firmicutes|c__CFGB10290|o__OFGB10290|f__FGB10290|g__GGB31438|s__GGB31438_SGB44768
k__Bacteria|p__Firmicutes|c__Clostridia|o__Eubacteriales|f__Oscillospiraceae|g__GGB3171|s__GGB3171_SGB4185
k__Bacteria|p__Firmicutes|c__CFGB1765|o__OFGB1765|f__FGB1765|g__GGB31823|s__GGB31823_SGB45199
k__Bacteria|p__Firmicutes|c__CFGB10349|o__OFGB10349|f__FGB10349|g__GGB31853|s__GGB31853_SGB45233
k__Bacteria|p__Firmicutes|c__CFGB10667|o__OFGB10667|f__FGB10667|g__GGB32371|s__GGB32371_SGB41694
k__Bacteria|p__Firmicutes|c__Clostridia|o__Eubacteriales|f__Lachnospiraceae|g__GGB3793|s__GGB3793_SGB5158
k__Bacteria|p__Firmicutes|c__Clostridia|o__Eubacteriales|f__Lachnospiraceae|g__GGB42598|s__GGB42598_SGB59794
k__Bacteria|p__Firmicutes|c__Clostridia|o__Eubacteriales|f__Christensenellaceae|g__GGB45656|s__GGB45656_SGB6337
k__Bacteria|p__Firmicutes|c__CFGB10299|o__OFGB10299|f__FGB10299|g__GGB47127|s__GGB47127_SGB65054
k__Bacteria|p__Firmicutes|c__Clostridia|o__Eubacteriales|f__Oscillospiraceae|g__GGB74395|s__GGB74395_SGB43521
k__Bacteria|p__Firmicutes|c__Clostridia|o__Eubacteriales|f__Oscillospiraceae|g__GGB75053|s__GGB75053_SGB43494
k__Bacteria|p__Firmicutes|c__Clostridia|o__Eubacteriales|f__Lachnospiraceae|g__GGB75109|s__GGB75109_SGB102238
k__Bacteria|p__Firmicutes|c__Clostridia|o__Clostridia_unclassified|f__Clostridia_unclassified|g__GGB81440|s__GGB8144
k__Bacteria|p__Firmicutes|c__Clostridia|o__Eubacteriales|f__Lachnospiraceae|g__Lachnospiraceae_unclassified|s__Lachn
k__Bacteria|p__Firmicutes|c__Clostridia|o__Eubacteriales|f__Lachnospiraceae|g__Lachnospiraceae_unclassified|s__Lachn
k__Bacteria|p__Firmicutes|c__Clostridia|o__Eubacteriales|f__Lachnospiraceae|g__Lachnospiraceae_unclassified|s__Lachn
k__Bacteria|p__Firmicutes|c__Clostridia|o__Eubacteriales|f__Lachnospiraceae|g__Lachnospiraceae_unclassified|s__Lachn
k__Bacteria|p__Firmicutes|c__Clostridia|o__Eubacteriales|f__Lachnospiraceae|g__Lachnospiraceae_unclassified|s__Lachn
k__Bacteria|p__Firmicutes|c__Clostridia|o__Eubacteriales|f__Lachnospiraceae|g__Lachnospiraceae_unclassified|s__Lachn
k__Bacteria|p__Firmicutes|c__Clostridia|o__Eubacteriales|f__Lachnospiraceae|g__Lachnospiraceae_unclassified|s__Lachn
k__Bacteria|p__Firmicutes|c__Clostridia|o__Eubacteriales|f__Lachnospiraceae|g__Lachnospiraceae_unclassified|s__Lachn
k__Bacteria|p__Firmicutes|c__Bacilli|o__Lactobacillales|f__Lactobacillaceae|g__Lactobacillus|s__Lactobacillus_johnsonii

k__Bacteria|p__Bacteroidota|c__Bacteroidia|o__Bacteroidales|f__Muribaculaceae|g__Muribaculaceae_unclassified|s__M
k__Bacteria|p__Firmicutes|c__Clostridia|o__Eubacteriales|f__Oscillospiraceae|g__Neglectibacter|s__Neglectibacter_sp_X
k__Bacteria|p__Firmicutes|c__Clostridia|o__Eubacteriales|f__Oscillospiraceae|g__Oscillospiraceae_unclassified|s__Oscillo
k__Bacteria|p__Firmicutes|c__Clostridia|o__Eubacteriales|f__Oscillospiraceae|g__Oscillospiraceae_unclassified|s__Oscillo
k__Bacteria|p__Firmicutes|c__Clostridia|o__Eubacteriales|f__Oscillospiraceae|g__Oscillospiraceae_unclassified|s__Oscillo
k__Bacteria|p__Proteobacteria|c__Betaproteobacteria|o__Burkholderiales|f__Sutterellaceae|g__Parasutterella|s__Parasu
k__Bacteria|p__Firmicutes|c__Clostridia|o__Eubacteriales|f__Peptostreptococcaceae|g__Romboutsia|s__Romboutsia_ilea
k__Bacteria|p__Firmicutes|c__Clostridia|o__Eubacteriales|f__Lachnospiraceae|g__Schaedlerella|s__Schaedlerella_arabino
k__Bacteria|p__Firmicutes|c__Erysipelotrichia|o__Erysipelotrichales|f__Turicibacteraceae|g__Turicibacter|s__Turicibacter_
k__Bacteria|p__Bacteria_unclassified|c__Bacteria_unclassified|o__Bacteria_unclassified|f__Bacteria_unclassified|g__Bacte
k__Bacteria|p__Bacteria_unclassified|c__Bacteria_unclassified|o__Bacteria_unclassified|f__Bacteria_unclassified|g__Bacte
k__Bacteria|p__Bacteria_unclassified|c__Bacteria_unclassified|o__Bacteria_unclassified|f__Bacteria_unclassified|g__Bacte

k__Bacteria|p__Firmicutes|c__Clostridia|o__Eubacteriales|f__Lachnospiraceae|g__Acetatifactor|s__Acetatifactor_SGB415
k__Bacteria|p__Firmicutes|c__Clostridia|o__Eubacteriales|f__Lachnospiraceae|g__Acetatifactor|s__Acetatifactor_muris
k__Bacteria|p__Firmicutes|c__Clostridia|o__Eubacteriales|f__Oscillospiraceae|g__Acutalibacter|s__Acutalibacter_muris
k__Bacteria|p__Firmicutes|c__Clostridia|o__Eubacteriales|f__Oscillospiraceae|g__Acutalibacter|s__Acutalibacter_sp_1XD
k__Bacteria|p__Actinobacteria|c__Coriobacteriia|o__Eggerthellales|f__Eggerthellaceae|g__Adlercreutzia|s__Adlercreutzia
k__Bacteria|p__Actinobacteria|c__Coriobacteriia|o__Eggerthellales|f__Eggerthellaceae|g__Adlercreutzia|s__Adlercreutzia
k__Bacteria|p__Actinobacteria|c__Coriobacteriia|o__Eggerthellales|f__Eggerthellaceae|g__Adlercreutzia|s__Adlercreutzia
k__Bacteria|p__Verrucomicrobia|c__Verrucomicrobiae|o__Verrucomicrobiales|f__Akkermansiaceae|g__Akkermansia|s__A
k__Bacteria|p__Bacteroidota|c__Bacteroidia|o__Bacteroidales|f__Rikenellaceae|g__Alistipes|s__Alistipes_sp_DSM_11234
k__Bacteria|p__Firmicutes|c__Clostridia|o__Eubacteriales|f__Oscillospiraceae|g__Anaerotruncus|s__Anaerotruncus_sp_1
k__Bacteria|p__Bacteria_unclassified|c__Bacteria_unclassified|o__Bacteria_unclassified|f__Bacteria_unclassified|g__Bacte
k__Bacteria|p__Bacteria_unclassified|c__Bacteria_unclassified|o__Bacteria_unclassified|f__Bacteria_unclassified|g__Bacte
k__Bacteria|p__Bacteria_unclassified|c__Bacteria_unclassified|o__Bacteria_unclassified|f__Bacteria_unclassified|g__Bacte
k__Bacteria|p__Bacteroidota|c__Bacteroidia|o__Bacteroidales|f__Bacteroidaceae|g__Bacteroides|s__Bacteroides_thetaio
k__Bacteria|p__Actinobacteria|c__Actinomycetia|o__Bifidobacteriales|f__Bifidobacteriaceae|g__Bifidobacterium|s__Bifid
k__Bacteria|p__Firmicutes|c__Clostridia|o__Clostridia_unclassified|f__Clostridia_unclassified|g__Clostridia_unclassified|s_
k__Bacteria|p__Firmicutes|c__Clostridia|o__Eubacteriales|f__Clostridiaceae|g__Clostridiaceae_unclassified|s__Clostridiac
k__Bacteria|p__Firmicutes|c__Clostridia|o__Eubacteriales|f__Clostridiaceae|g__Clostridiaceae_unclassified|s__Clostridiac
k__Bacteria|p__Firmicutes|c__Clostridia|o__Eubacteriales|f__Eubacteriales_unclassified|g__Eubacteriales_unclassified|s_
k__Bacteria|p__Firmicutes|c__Erysipelotrichia|o__Erysipelotrichales|f__Erysipelotrichaceae|g__Erysipelatoclostridium|s_
k__Bacteria|p__Actinobacteria|c__Coriobacteriia|o__Coriobacteriales|f__Coriobacteriaceae|g__Coriobacteriaceae_unclass
k__Bacteria|p__Firmicutes|c__Clostridia|o__Eubacteriales|f__Lachnospiraceae|g__Dorea|s__Dorea_sp_5_2
k__Bacteria|p__Firmicutes|c__Erysipelotrichia|o__Erysipelotrichales|f__Erysipelotrichaceae|g__Dubosiella|s__Dubosiella_
k__Bacteria|p__Firmicutes|c__Erysipelotrichia|o__Erysipelotrichales|f__Erysipelotrichales_unclassified|g__Erysipelotrichal
k__Bacteria|p__Firmicutes|c__Clostridia|o__Eubacteriales|f__Eubacteriaceae|g__Eubacteriaceae_unclassified|s__Eubacte
k__Bacteria|p__Firmicutes|c__Clostridia|o__Eubacteriales|f__Eubacteriaceae|g__Eubacteriaceae_unclassified|s__Eubacte
k__Bacteria|p__Firmicutes|c__Clostridia|o__Eubacteriales|f__Lachnospiraceae|g__GGB20149|s__GGB20149_SGB29430
k__Bacteria|p__Actinobacteria|c__Coriobacteriia|o__Eggerthellales|f__Eggerthellaceae|g__GGB22635|s__GGB22635_SGB
k__Bacteria|p__Firmicutes|c__Clostridia|o__Eubacteriales|f__Lachnospiraceae|g__GGB25041|s__GGB25041_SGB36960
k__Bacteria|p__Bacteroidota|c__Bacteroidia|o__Bacteroidales|f__Muribaculaceae|g__GGB27876|s__GGB27876_SGB4031
k__Bacteria|p__Bacteroidota|c__Bacteroidia|o__Bacteroidales|f__Muribaculaceae|g__GGB27878|s__GGB27878_SGB4031

k__Bacteria|p__Bacteroidota|c__Bacteroidia|o__Bacteroidales|f__Muribaculaceae|g__GGB27918|s__GGB27918_SGB4035
k__Bacteria|p__Firmicutes|c__CFGB9508|o__OFGB9508|f__FGB9508|g__GGB28382|s__GGB28382_SGB40962
k__Bacteria|p__Firmicutes|c__CFGB2838|o__OFGB2838|f__FGB2838|g__GGB28399|s__GGB28399_SGB40980
k__Bacteria|p__Firmicutes|c__CFGB2838|o__OFGB2838|f__FGB2838|g__GGB28411|s__GGB28411_SGB40993
k__Bacteria|p__Firmicutes|c__CFGB2838|o__OFGB2838|f__FGB2838|g__GGB28415|s__GGB28415_SGB40997
k__Bacteria|p__Firmicutes|c__Clostridia|o__Eubacteriales|f__Pumilibacteraceae|g__GGB28430|s__GGB28430_SGB41013
k__Bacteria|p__Firmicutes|c__CFGB28439|o__OFGB28439|f__FGB28439|g__GGB28439|s__GGB28439_SGB41022
k__Bacteria|p__Firmicutes|c__Clostridia|o__Clostridia_unclassified|f__Clostridia_unclassified|g__GGB28778|s__GGB2877
k__Bacteria|p__Firmicutes|c__Clostridia|o__Eubacteriales|f__Eubacteriaceae|g__GGB28784|s__GGB28784_SGB41437
k__Bacteria|p__Firmicutes|c__Clostridia|o__Eubacteriales|f__Lachnospiraceae|g__GGB28792|s__GGB28792_SGB41445
k__Bacteria|p__Firmicutes|c__Clostridia|o__Eubacteriales|f__Lachnospiraceae|g__GGB28798|s__GGB28798_SGB41451
k__Bacteria|p__Firmicutes|c__Clostridia|o__Eubacteriales|f__Lachnospiraceae|g__GGB28802|s__GGB28802_SGB41455
k__Bacteria|p__Firmicutes|c__Clostridia|o__Eubacteriales|f__Lachnospiraceae|g__GGB28818|s__GGB28818_SGB41473
k__Bacteria|p__Firmicutes|c__CFGB77305|o__OFGB77305|f__FGB77305|g__GGB28828|s__GGB28828_SGB41484
k__Bacteria|p__Firmicutes|c__Clostridia|o__Eubacteriales|f__Clostridiaceae|g__GGB28851|s__GGB28851_SGB41518
k__Bacteria|p__Firmicutes|c__Clostridia|o__Eubacteriales|f__Lachnospiraceae|g__GGB28859|s__GGB28859_SGB41528
k__Bacteria|p__Firmicutes|c__Clostridia|o__Eubacteriales|f__Lachnospiraceae|g__GGB28864|s__GGB28864_SGB41535
k__Bacteria|p__Firmicutes|c__Clostridia|o__Eubacteriales|f__Lachnospiraceae|g__GGB28869|s__GGB28869_SGB41543
k__Bacteria|p__Firmicutes|c__CFGB9633|o__OFGB9633|f__FGB9633|g__GGB28883|s__GGB28883_SGB41564
k__Bacteria|p__Bacteria_unclassified|c__Bacteria_unclassified|o__Bacteria_unclassified|f__Bacteria_unclassified|g__GGB
k__Bacteria|p__Bacteria_unclassified|c__Bacteria_unclassified|o__Bacteria_unclassified|f__Bacteria_unclassified|g__GGB
k__Bacteria|p__Bacteria_unclassified|c__Bacteria_unclassified|o__Bacteria_unclassified|f__Bacteria_unclassified|g__GGB
k__Bacteria|p__Bacteria_unclassified|c__Bacteria_unclassified|o__Bacteria_unclassified|f__Bacteria_unclassified|g__GGB
k__Bacteria|p__Firmicutes|c__Clostridia|o__Eubacteriales|f__Lachnospiraceae|g__GGB28916|s__GGB28916_SGB41612
k__Bacteria|p__Firmicutes|c__Clostridia|o__Eubacteriales|f__Lachnospiraceae|g__GGB28924|s__GGB28924_SGB41621
k__Bacteria|p__Firmicutes|c__Clostridia|o__Eubacteriales|f__Lachnospiraceae|g__GGB28926|s__GGB28926_SGB41624
k__Bacteria|p__Bacteria_unclassified|c__CFGB77359|o__OFGB77359|f__FGB77359|g__GGB28927|s__GGB28927_SGB416
k__Bacteria|p__Firmicutes|c__CFGB9639|o__OFGB9639|f__FGB9639|g__GGB28934|s__GGB28934_SGB41635
k__Bacteria|p__Firmicutes|c__Clostridia|o__Eubacteriales|f__Lachnospiraceae|g__GGB28946|s__GGB28946_SGB41652
k__Bacteria|p__Firmicutes|c__Clostridia|o__Eubacteriales|f__Lachnospiraceae|g__GGB28949|s__GGB28949_SGB41655
k__Bacteria|p__Firmicutes|c__Clostridia|o__Eubacteriales|f__Lachnospiraceae|g__GGB28949|s__GGB28949_SGB41656
k__Bacteria|p__Firmicutes|c__Clostridia|o__Eubacteriales|f__Clostridiaceae|g__GGB28950|s__GGB28950_SGB41657
k__Bacteria|p__Firmicutes|c__Clostridia|o__Eubacteriales|f__Clostridiaceae|g__GGB28951|s__GGB28951_SGB102295
k__Bacteria|p__Firmicutes|c__Clostridia|o__Eubacteriales|f__Clostridiaceae|g__GGB28951|s__GGB28951_SGB41658
k__Bacteria|p__Firmicutes|c__Clostridia|o__Eubacteriales|f__Clostridiaceae|g__GGB28954|s__GGB28954_SGB41662
k__Bacteria|p__Firmicutes|c__Clostridia|o__Eubacteriales|f__Clostridiaceae|g__GGB28956|s__GGB28956_SGB41665
k__Bacteria|p__Firmicutes|c__Clostridia|o__Eubacteriales|f__Clostridiaceae|g__GGB28960|s__GGB28960_SGB41669
k__Bacteria|p__Firmicutes|c__Clostridia|o__Eubacteriales|f__Clostridiaceae|g__GGB28967|s__GGB28967_SGB41678
k__Bacteria|p__Firmicutes|c__Clostridia|o__Eubacteriales|f__Eubacteriaceae|g__GGB28991|s__GGB28991_SGB41705
k__Bacteria|p__Firmicutes|c__CFGB9658|o__OFGB9658|f__FGB9658|g__GGB29002|s__GGB29002_SGB41718
k__Bacteria|p__Firmicutes|c__CFGB9659|o__OFGB9659|f__FGB9659|g__GGB29003|s__GGB29003_SGB41719
k__Bacteria|p__Bacteria_unclassified|c__Bacteria_unclassified|o__Bacteria_unclassified|f__Bacteria_unclassified|g__GGB
k__Bacteria|p__Firmicutes|c__CFGB9827|o__OFGB9827|f__FGB9827|g__GGB29531|s__GGB29531_SGB42317
k__Bacteria|p__Firmicutes|c__Clostridia|o__Eubacteriales|f__Eubacteriaceae|g__GGB29685|s__GGB29685_SGB42494
k__Bacteria|p__Bacteria_unclassified|c__CFGB77303|o__OFGB77303|f__FGB77303|g__GGB30141|s__GGB30141_SGB430
k__Bacteria|p__Firmicutes|c__Clostridia|o__Eubacteriales|f__Eubacteriales_unclassified|g__GGB30286|s__GGB30286_SG

k__Bacteria|p__Firmicutes|c__Clostridia|o__Eubacteriales|f__Oscillospiraceae|g__GGB30303|s__GGB30303_SGB43268
k__Bacteria|p__Firmicutes|c__CFGB30328|o__OFGB30328|f__FGB30328|g__GGB30413|s__GGB30413_SGB43452
k__Bacteria|p__Firmicutes|c__Clostridia|o__Eubacteriales|f__Oscillospiraceae|g__GGB30454|s__GGB30454_SGB43514
k__Bacteria|p__Firmicutes|c__Clostridia|o__Eubacteriales|f__Oscillospiraceae|g__GGB30455|s__GGB30455_SGB43519
k__Bacteria|p__Firmicutes|c__Clostridia|o__Eubacteriales|f__Oscillospiraceae|g__GGB30461|s__GGB30461_SGB43527
k__Bacteria|p__Firmicutes|c__Clostridia|o__Eubacteriales|f__Oscillospiraceae|g__GGB30461|s__GGB30461_SGB43530
k__Bacteria|p__Firmicutes|c__Clostridia|o__Eubacteriales|f__Oscillospiraceae|g__GGB30463|s__GGB30463_SGB43537
k__Bacteria|p__Firmicutes|c__Clostridia|o__Eubacteriales|f__Oscillospiraceae|g__GGB30473|s__GGB30473_SGB43557
k__Bacteria|p__Firmicutes|c__Clostridia|o__Eubacteriales|f__Oscillospiraceae|g__GGB30475|s__GGB30475_SGB63182
k__Bacteria|p__Actinobacteria|c__CFGB77153|o__OFGB77153|f__FGB77153|g__GGB30861|s__GGB30861_SGB44083
k__Bacteria|p__Tenericutes|c__CFGB1791|o__OFGB1791|f__FGB1791|g__GGB31312|s__GGB31312_SGB44628
k__Bacteria|p__Firmicutes|c__CFGB10290|o__OFGB10290|f__FGB10290|g__GGB31438|s__GGB31438_SGB44768
k__Bacteria|p__Firmicutes|c__Clostridia|o__Eubacteriales|f__Oscillospiraceae|g__GGB3171|s__GGB3171_SGB4185
k__Bacteria|p__Firmicutes|c__CFGB1765|o__OFGB1765|f__FGB1765|g__GGB31823|s__GGB31823_SGB45199
k__Bacteria|p__Firmicutes|c__CFGB10349|o__OFGB10349|f__FGB10349|g__GGB31853|s__GGB31853_SGB45233
k__Bacteria|p__Firmicutes|c__CFGB10667|o__OFGB10667|f__FGB10667|g__GGB32371|s__GGB32371_SGB41694
k__Bacteria|p__Firmicutes|c__Clostridia|o__Eubacteriales|f__Lachnospiraceae|g__GGB3793|s__GGB3793_SGB5158
k__Bacteria|p__Firmicutes|c__Clostridia|o__Eubacteriales|f__Lachnospiraceae|g__GGB42598|s__GGB42598_SGB59794
k__Bacteria|p__Firmicutes|c__Clostridia|o__Eubacteriales|f__Christensenellaceae|g__GGB45656|s__GGB45656_SGB6337
k__Bacteria|p__Firmicutes|c__CFGB10299|o__OFGB10299|f__FGB10299|g__GGB47127|s__GGB47127_SGB65054
k__Bacteria|p__Firmicutes|c__Clostridia|o__Eubacteriales|f__Oscillospiraceae|g__GGB74395|s__GGB74395_SGB43521
k__Bacteria|p__Firmicutes|c__Clostridia|o__Eubacteriales|f__Oscillospiraceae|g__GGB75053|s__GGB75053_SGB43494
k__Bacteria|p__Firmicutes|c__Clostridia|o__Eubacteriales|f__Lachnospiraceae|g__GGB75109|s__GGB75109_SGB102238
k__Bacteria|p__Firmicutes|c__Clostridia|o__Clostridia_unclassified|f__Clostridia_unclassified|g__GGB81440|s__GGB8144
k__Bacteria|p__Firmicutes|c__Clostridia|o__Eubacteriales|f__Lachnospiraceae|g__Lachnospiraceae_unclassified|s__Lachn
k__Bacteria|p__Firmicutes|c__Clostridia|o__Eubacteriales|f__Lachnospiraceae|g__Lachnospiraceae_unclassified|s__Lachn
k__Bacteria|p__Firmicutes|c__Clostridia|o__Eubacteriales|f__Lachnospiraceae|g__Lachnospiraceae_unclassified|s__Lachn
k__Bacteria|p__Firmicutes|c__Clostridia|o__Eubacteriales|f__Lachnospiraceae|g__Lachnospiraceae_unclassified|s__Lachn
k__Bacteria|p__Firmicutes|c__Clostridia|o__Eubacteriales|f__Lachnospiraceae|g__Lachnospiraceae_unclassified|s__Lachn
k__Bacteria|p__Firmicutes|c__Clostridia|o__Eubacteriales|f__Lachnospiraceae|g__Lachnospiraceae_unclassified|s__Lachn
k__Bacteria|p__Firmicutes|c__Clostridia|o__Eubacteriales|f__Lachnospiraceae|g__Lachnospiraceae_unclassified|s__Lachn
k__Bacteria|p__Firmicutes|c__Clostridia|o__Eubacteriales|f__Lachnospiraceae|g__Lachnospiraceae_unclassified|s__Lachn
k__Bacteria|p__Firmicutes|c__Bacilli|o__Lactobacillales|f__Lactobacillaceae|g__Lactobacillus|s__Lactobacillus_johnsonii
k__Bacteria|p__Bacteroidota|c__Bacteroidia|o__Bacteroidales|f__Muribaculaceae|g__Muribaculaceae_unclassified|s__M
k__Bacteria|p__Firmicutes|c__Clostridia|o__Eubacteriales|f__Oscillospiraceae|g__Neglectibacter|s__Neglectibacter_sp_X
k__Bacteria|p__Firmicutes|c__Clostridia|o__Eubacteriales|f__Oscillospiraceae|g__Oscillospiraceae_unclassified|s__Oscillo
k__Bacteria|p__Firmicutes|c__Clostridia|o__Eubacteriales|f__Oscillospiraceae|g__Oscillospiraceae_unclassified|s__Oscillo
k__Bacteria|p__Firmicutes|c__Clostridia|o__Eubacteriales|f__Oscillospiraceae|g__Oscillospiraceae_unclassified|s__Oscillo
k__Bacteria|p__Proteobacteria|c__Betaproteobacteria|o__Burkholderiales|f__Sutterellaceae|g__Parasutterella|s__Parasu
k__Bacteria|p__Firmicutes|c__Clostridia|o__Eubacteriales|f__Peptostreptococcaceae|g__Romboutsia|s__Romboutsia_ilea
k__Bacteria|p__Firmicutes|c__Clostridia|o__Eubacteriales|f__Lachnospiraceae|g__Schaedlerella|s__Schaedlerella_arabino
k__Bacteria|p__Firmicutes|c__Erysipelotrichia|o__Erysipelotrichales|f__Turicibacteraceae|g__Turicibacter|s__Turicibacter_
k__Bacteria|p__Bacteria_unclassified|c__Bacteria_unclassified|o__Bacteria_unclassified|f__Bacteria_unclassified|g__Bacte
k__Bacteria|p__Bacteria_unclassified|c__Bacteria_unclassified|o__Bacteria_unclassified|f__Bacteria_unclassified|g__Bacte
k__Bacteria|p__Bacteria_unclassified|c__Bacteria_unclassified|o__Bacteria_unclassified|f__Bacteria_unclassified|g__Bacte

k__Bacteria|p__Firmicutes|c__Clostridia|o__Eubacteriales|f__Lachnospiraceae|g__Acetatifactor|s__Acetatifactor_SGB415
k__Bacteria|p__Firmicutes|c__Clostridia|o__Eubacteriales|f__Lachnospiraceae|g__Acetatifactor|s__Acetatifactor_muris
k__Bacteria|p__Firmicutes|c__Clostridia|o__Eubacteriales|f__Oscillospiraceae|g__Acutalibacter|s__Acutalibacter_muris
k__Bacteria|p__Firmicutes|c__Clostridia|o__Eubacteriales|f__Oscillospiraceae|g__Acutalibacter|s__Acutalibacter_sp_1XD
k__Bacteria|p__Actinobacteria|c__Coriobacteriia|o__Eggerthellales|f__Eggerthellaceae|g__Adlercreutzia|s__Adlercreutzia
k__Bacteria|p__Actinobacteria|c__Coriobacteriia|o__Eggerthellales|f__Eggerthellaceae|g__Adlercreutzia|s__Adlercreutzia
k__Bacteria|p__Actinobacteria|c__Coriobacteriia|o__Eggerthellales|f__Eggerthellaceae|g__Adlercreutzia|s__Adlercreutzia
k__Bacteria|p__Verrucomicrobia|c__Verrucomicrobiae|o__Verrucomicrobiales|f__Akkermansiaceae|g__Akkermansia|s__A
k__Bacteria|p__Bacteroidota|c__Bacteroidia|o__Bacteroidales|f__Rikenellaceae|g__Alistipes|s__Alistipes_sp_DSM_1123
k__Bacteria|p__Firmicutes|c__Clostridia|o__Eubacteriales|f__Oscillospiraceae|g__Anaerotruncus|s__Anaerotruncus_sp_1
k__Bacteria|p__Bacteria_unclassified|c__Bacteria_unclassified|o__Bacteria_unclassified|f__Bacteria_unclassified|g__Bact
k__Bacteria|p__Bacteria_unclassified|c__Bacteria_unclassified|o__Bacteria_unclassified|f__Bacteria_unclassified|g__Bact
k__Bacteria|p__Bacteria_unclassified|c__Bacteria_unclassified|o__Bacteria_unclassified|f__Bacteria_unclassified|g__Bact
k__Bacteria|p__Bacteroidota|c__Bacteroidia|o__Bacteroidales|f__Bacteroidaceae|g__Bacteroides|s__Bacteroides_thetaio
k__Bacteria|p__Actinobacteria|c__Actinomycetia|o__Bifidobacteriales|f__Bifidobacteriaceae|g__Bifidobacterium|s__Bifid
k__Bacteria|p__Firmicutes|c__Clostridia|o__Clostridia_unclassified|f__Clostridia_unclassified|g__Clostridia_unclassified|s_
k__Bacteria|p__Firmicutes|c__Clostridia|o__Eubacteriales|f__Clostridiaceae|g__Clostridiaceae_unclassified|s__Clostridiac
k__Bacteria|p__Firmicutes|c__Clostridia|o__Eubacteriales|f__Clostridiaceae|g__Clostridiaceae_unclassified|s__Clostridiac
k__Bacteria|p__Firmicutes|c__Clostridia|o__Eubacteriales|f__Eubacteriales_unclassified|g__Eubacteriales_unclassified|s_
k__Bacteria|p__Firmicutes|c__Erysipelotrichia|o__Erysipelotrichales|f__Erysipelotrichaceae|g__Erysipelatoclostridium|s_
k__Bacteria|p__Actinobacteria|c__Coriobacteriia|o__Coriobacteriales|f__Coriobacteriaceae|g__Coriobacteriaceae_unclass
k__Bacteria|p__Firmicutes|c__Clostridia|o__Eubacteriales|f__Lachnospiraceae|g__Dorea|s__Dorea_sp_5_2
k__Bacteria|p__Firmicutes|c__Erysipelotrichia|o__Erysipelotrichales|f__Erysipelotrichaceae|g__Dubosiella|s__Dubosiella_
k__Bacteria|p__Firmicutes|c__Erysipelotrichia|o__Erysipelotrichales|f__Erysipelotrichales_unclassified|g__Erysipelotrichal
k__Bacteria|p__Firmicutes|c__Clostridia|o__Eubacteriales|f__Eubacteriaceae|g__Eubacteriaceae_unclassified|s__Eubacte
k__Bacteria|p__Firmicutes|c__Clostridia|o__Eubacteriales|f__Eubacteriaceae|g__Eubacteriaceae_unclassified|s__Eubacte
k__Bacteria|p__Firmicutes|c__Clostridia|o__Eubacteriales|f__Lachnospiraceae|g__GGB20149|s__GGB20149_SGB29430
k__Bacteria|p__Actinobacteria|c__Coriobacteriia|o__Eggerthellales|f__Eggerthellaceae|g__GGB22635|s__GGB22635_SGB
k__Bacteria|p__Firmicutes|c__Clostridia|o__Eubacteriales|f__Lachnospiraceae|g__GGB25041|s__GGB25041_SGB36960
k__Bacteria|p__Bacteroidota|c__Bacteroidia|o__Bacteroidales|f__Muribaculaceae|g__GGB27876|s__GGB27876_SGB403
k__Bacteria|p__Bacteroidota|c__Bacteroidia|o__Bacteroidales|f__Muribaculaceae|g__GGB27878|s__GGB27878_SGB403
k__Bacteria|p__Bacteroidota|c__Bacteroidia|o__Bacteroidales|f__Muribaculaceae|g__GGB27918|s__GGB27918_SGB403
k__Bacteria|p__Firmicutes|c__CFGB9508|o__OFGB9508|f__FGB9508|g__GGB28382|s__GGB28382_SGB40962
k__Bacteria|p__Firmicutes|c__CFGB2838|o__OFGB2838|f__FGB2838|g__GGB28399|s__GGB28399_SGB40980
k__Bacteria|p__Firmicutes|c__CFGB2838|o__OFGB2838|f__FGB2838|g__GGB28411|s__GGB28411_SGB40993
k__Bacteria|p__Firmicutes|c__CFGB2838|o__OFGB2838|f__FGB2838|g__GGB28415|s__GGB28415_SGB40997
k__Bacteria|p__Firmicutes|c__Clostridia|o__Eubacteriales|f__Pumilibacteraceae|g__GGB28430|s__GGB28430_SGB41013
k__Bacteria|p__Firmicutes|c__CFGB28439|o__OFGB28439|f__FGB28439|g__GGB28439|s__GGB28439_SGB41022
k__Bacteria|p__Firmicutes|c__Clostridia|o__Clostridia_unclassified|f__Clostridia_unclassified|g__GGB28778|s__GGB2877
k__Bacteria|p__Firmicutes|c__Clostridia|o__Eubacteriales|f__Eubacteriaceae|g__GGB28784|s__GGB28784_SGB41437
k__Bacteria|p__Firmicutes|c__Clostridia|o__Eubacteriales|f__Lachnospiraceae|g__GGB28792|s__GGB28792_SGB41445
k__Bacteria|p__Firmicutes|c__Clostridia|o__Eubacteriales|f__Lachnospiraceae|g__GGB28798|s__GGB28798_SGB41451
k__Bacteria|p__Firmicutes|c__Clostridia|o__Eubacteriales|f__Lachnospiraceae|g__GGB28802|s__GGB28802_SGB41455
k__Bacteria|p__Firmicutes|c__Clostridia|o__Eubacteriales|f__Lachnospiraceae|g__GGB28818|s__GGB28818_SGB41473

k__Bacteria|p__Firmicutes|c__CFGB77305|o__OFGB77305|f__FGB77305|g__GGB28828|s__GGB28828_SGB41484
k__Bacteria|p__Firmicutes|c__Clostridia|o__Eubacteriales|f__Clostridiaceae|g__GGB28851|s__GGB28851_SGB41518
k__Bacteria|p__Firmicutes|c__Clostridia|o__Eubacteriales|f__Lachnospiraceae|g__GGB28859|s__GGB28859_SGB41528
k__Bacteria|p__Firmicutes|c__Clostridia|o__Eubacteriales|f__Lachnospiraceae|g__GGB28864|s__GGB28864_SGB41535
k__Bacteria|p__Firmicutes|c__Clostridia|o__Eubacteriales|f__Lachnospiraceae|g__GGB28869|s__GGB28869_SGB41543
k__Bacteria|p__Firmicutes|c__CFGB9633|o__OFGB9633|f__FGB9633|g__GGB28883|s__GGB28883_SGB41564
k__Bacteria|p__Bacteria_unclassified|c__Bacteria_unclassified|o__Bacteria_unclassified|f__Bacteria_unclassified|g__GGB2
k__Bacteria|p__Bacteria_unclassified|c__Bacteria_unclassified|o__Bacteria_unclassified|f__Bacteria_unclassified|g__GGB2
k__Bacteria|p__Bacteria_unclassified|c__Bacteria_unclassified|o__Bacteria_unclassified|f__Bacteria_unclassified|g__GGB2
k__Bacteria|p__Bacteria_unclassified|c__Bacteria_unclassified|o__Bacteria_unclassified|f__Bacteria_unclassified|g__GGB2
k__Bacteria|p__Firmicutes|c__Clostridia|o__Eubacteriales|f__Lachnospiraceae|g__GGB28916|s__GGB28916_SGB41612
k__Bacteria|p__Firmicutes|c__Clostridia|o__Eubacteriales|f__Lachnospiraceae|g__GGB28924|s__GGB28924_SGB41621
k__Bacteria|p__Firmicutes|c__Clostridia|o__Eubacteriales|f__Lachnospiraceae|g__GGB28926|s__GGB28926_SGB41624
k__Bacteria|p__Bacteria_unclassified|c__CFGB77359|o__OFGB77359|f__FGB77359|g__GGB28927|s__GGB28927_SGB416
k__Bacteria|p__Firmicutes|c__CFGB9639|o__OFGB9639|f__FGB9639|g__GGB28934|s__GGB28934_SGB41635
k__Bacteria|p__Firmicutes|c__Clostridia|o__Eubacteriales|f__Lachnospiraceae|g__GGB28946|s__GGB28946_SGB41652
k__Bacteria|p__Firmicutes|c__Clostridia|o__Eubacteriales|f__Lachnospiraceae|g__GGB28949|s__GGB28949_SGB41655
k__Bacteria|p__Firmicutes|c__Clostridia|o__Eubacteriales|f__Lachnospiraceae|g__GGB28949|s__GGB28949_SGB41656
k__Bacteria|p__Firmicutes|c__Clostridia|o__Eubacteriales|f__Clostridiaceae|g__GGB28950|s__GGB28950_SGB41657
k__Bacteria|p__Firmicutes|c__Clostridia|o__Eubacteriales|f__Clostridiaceae|g__GGB28951|s__GGB28951_SGB102295
k__Bacteria|p__Firmicutes|c__Clostridia|o__Eubacteriales|f__Clostridiaceae|g__GGB28951|s__GGB28951_SGB41658
k__Bacteria|p__Firmicutes|c__Clostridia|o__Eubacteriales|f__Clostridiaceae|g__GGB28954|s__GGB28954_SGB41662
k__Bacteria|p__Firmicutes|c__Clostridia|o__Eubacteriales|f__Clostridiaceae|g__GGB28956|s__GGB28956_SGB41665
k__Bacteria|p__Firmicutes|c__Clostridia|o__Eubacteriales|f__Clostridiaceae|g__GGB28960|s__GGB28960_SGB41669
k__Bacteria|p__Firmicutes|c__Clostridia|o__Eubacteriales|f__Clostridiaceae|g__GGB28967|s__GGB28967_SGB41678
k__Bacteria|p__Firmicutes|c__Clostridia|o__Eubacteriales|f__Eubacteriaceae|g__GGB28991|s__GGB28991_SGB41705
k__Bacteria|p__Firmicutes|c__CFGB9658|o__OFGB9658|f__FGB9658|g__GGB29002|s__GGB29002_SGB41718
k__Bacteria|p__Firmicutes|c__CFGB9659|o__OFGB9659|f__FGB9659|g__GGB29003|s__GGB29003_SGB41719
k__Bacteria|p__Bacteria_unclassified|c__Bacteria_unclassified|o__Bacteria_unclassified|f__Bacteria_unclassified|g__GGB2
k__Bacteria|p__Firmicutes|c__CFGB9827|o__OFGB9827|f__FGB9827|g__GGB29531|s__GGB29531_SGB42317
k__Bacteria|p__Firmicutes|c__Clostridia|o__Eubacteriales|f__Eubacteriaceae|g__GGB29685|s__GGB29685_SGB42494
k__Bacteria|p__Bacteria_unclassified|c__CFGB77303|o__OFGB77303|f__FGB77303|g__GGB30141|s__GGB30141_SGB430
k__Bacteria|p__Firmicutes|c__Clostridia|o__Eubacteriales|f__Eubacteriales_unclassified|g__GGB30286|s__GGB30286_SG
k__Bacteria|p__Firmicutes|c__Clostridia|o__Eubacteriales|f__Oscillospiraceae|g__GGB30303|s__GGB30303_SGB43268
k__Bacteria|p__Firmicutes|c__CFGB30328|o__OFGB30328|f__FGB30328|g__GGB30413|s__GGB30413_SGB43452
k__Bacteria|p__Firmicutes|c__Clostridia|o__Eubacteriales|f__Oscillospiraceae|g__GGB30454|s__GGB30454_SGB43514
k__Bacteria|p__Firmicutes|c__Clostridia|o__Eubacteriales|f__Oscillospiraceae|g__GGB30455|s__GGB30455_SGB43519
k__Bacteria|p__Firmicutes|c__Clostridia|o__Eubacteriales|f__Oscillospiraceae|g__GGB30461|s__GGB30461_SGB43527
k__Bacteria|p__Firmicutes|c__Clostridia|o__Eubacteriales|f__Oscillospiraceae|g__GGB30461|s__GGB30461_SGB43530
k__Bacteria|p__Firmicutes|c__Clostridia|o__Eubacteriales|f__Oscillospiraceae|g__GGB30463|s__GGB30463_SGB43537
k__Bacteria|p__Firmicutes|c__Clostridia|o__Eubacteriales|f__Oscillospiraceae|g__GGB30473|s__GGB30473_SGB43557
k__Bacteria|p__Firmicutes|c__Clostridia|o__Eubacteriales|f__Oscillospiraceae|g__GGB30475|s__GGB30475_SGB63182
k__Bacteria|p__Actinobacteria|c__CFGB77153|o__OFGB77153|f__FGB77153|g__GGB30861|s__GGB30861_SGB44083
k__Bacteria|p__Tenericutes|c__CFGB1791|o__OFGB1791|f__FGB1791|g__GGB31312|s__GGB31312_SGB44628
k__Bacteria|p__Firmicutes|c__CFGB10290|o__OFGB10290|f__FGB10290|g__GGB31438|s__GGB31438_SGB44768
k__Bacteria|p__Firmicutes|c__Clostridia|o__Eubacteriales|f__Oscillospiraceae|g__GGB3171|s__GGB3171_SGB4185

k__Bacteria|p__Firmicutes|c__CFGB1765|o__OFGB1765|f__FGB1765|g__GGB31823|s__GGB31823_SGB45199
k__Bacteria|p__Firmicutes|c__CFGB10349|o__OFGB10349|f__FGB10349|g__GGB31853|s__GGB31853_SGB45233
k__Bacteria|p__Firmicutes|c__CFGB10667|o__OFGB10667|f__FGB10667|g__GGB32371|s__GGB32371_SGB41694
k__Bacteria|p__Firmicutes|c__Clostridia|o__Eubacteriales|f__Lachnospiraceae|g__GGB3793|s__GGB3793_SGB5158
k__Bacteria|p__Firmicutes|c__Clostridia|o__Eubacteriales|f__Lachnospiraceae|g__GGB42598|s__GGB42598_SGB59794
k__Bacteria|p__Firmicutes|c__Clostridia|o__Eubacteriales|f__Christensenellaceae|g__GGB45656|s__GGB45656_SGB6337
k__Bacteria|p__Firmicutes|c__CFGB10299|o__OFGB10299|f__FGB10299|g__GGB47127|s__GGB47127_SGB65054
k__Bacteria|p__Firmicutes|c__Clostridia|o__Eubacteriales|f__Oscillospiraceae|g__GGB74395|s__GGB74395_SGB43521
k__Bacteria|p__Firmicutes|c__Clostridia|o__Eubacteriales|f__Oscillospiraceae|g__GGB75053|s__GGB75053_SGB43494
k__Bacteria|p__Firmicutes|c__Clostridia|o__Eubacteriales|f__Lachnospiraceae|g__GGB75109|s__GGB75109_SGB102238
k__Bacteria|p__Firmicutes|c__Clostridia|o__Clostridia_unclassified|f__Clostridia_unclassified|g__GGB81440|s__GGB8144
k__Bacteria|p__Firmicutes|c__Clostridia|o__Eubacteriales|f__Lachnospiraceae|g__Lachnospiraceae_unclassified|s__Lachn
k__Bacteria|p__Firmicutes|c__Clostridia|o__Eubacteriales|f__Lachnospiraceae|g__Lachnospiraceae_unclassified|s__Lachn
k__Bacteria|p__Firmicutes|c__Clostridia|o__Eubacteriales|f__Lachnospiraceae|g__Lachnospiraceae_unclassified|s__Lachn
k__Bacteria|p__Firmicutes|c__Clostridia|o__Eubacteriales|f__Lachnospiraceae|g__Lachnospiraceae_unclassified|s__Lachn
k__Bacteria|p__Firmicutes|c__Clostridia|o__Eubacteriales|f__Lachnospiraceae|g__Lachnospiraceae_unclassified|s__Lachn
k__Bacteria|p__Firmicutes|c__Clostridia|o__Eubacteriales|f__Lachnospiraceae|g__Lachnospiraceae_unclassified|s__Lachn
k__Bacteria|p__Firmicutes|c__Clostridia|o__Eubacteriales|f__Lachnospiraceae|g__Lachnospiraceae_unclassified|s__Lachn
k__Bacteria|p__Firmicutes|c__Clostridia|o__Eubacteriales|f__Lachnospiraceae|g__Lachnospiraceae_unclassified|s__Lachn
k__Bacteria|p__Firmicutes|c__Bacilli|o__Lactobacillales|f__Lactobacillaceae|g__Lactobacillus|s__Lactobacillus_johnsonii
k__Bacteria|p__Bacteroidota|c__Bacteroidia|o__Bacteroidales|f__Muribaculaceae|g__Muribaculaceae_unclassified|s__M
k__Bacteria|p__Firmicutes|c__Clostridia|o__Eubacteriales|f__Oscillospiraceae|g__Neglectibacter|s__Neglectibacter_sp_X
k__Bacteria|p__Firmicutes|c__Clostridia|o__Eubacteriales|f__Oscillospiraceae|g__Oscillospiraceae_unclassified|s__Oscillo
k__Bacteria|p__Firmicutes|c__Clostridia|o__Eubacteriales|f__Oscillospiraceae|g__Oscillospiraceae_unclassified|s__Oscillo
k__Bacteria|p__Firmicutes|c__Clostridia|o__Eubacteriales|f__Oscillospiraceae|g__Oscillospiraceae_unclassified|s__Oscillo
k__Bacteria|p__Proteobacteria|c__Betaproteobacteria|o__Burkholderiales|f__Sutterellaceae|g__Parasutterella|s__Parasu
k__Bacteria|p__Firmicutes|c__Clostridia|o__Eubacteriales|f__Peptostreptococcaceae|g__Romboutsia|s__Romboutsia_ilea
k__Bacteria|p__Firmicutes|c__Clostridia|o__Eubacteriales|f__Lachnospiraceae|g__Schaedlerella|s__Schaedlerella_arabino
k__Bacteria|p__Firmicutes|c__Erysipelotrichia|o__Erysipelotrichales|f__Turicibacteraceae|g__Turicibacter|s__Turicibacter_
k__Bacteria|p__Bacteria_unclassified|c__Bacteria_unclassified|o__Bacteria_unclassified|f__Bacteria_unclassified|g__Bacte
k__Bacteria|p__Bacteria_unclassified|c__Bacteria_unclassified|o__Bacteria_unclassified|f__Bacteria_unclassified|g__Bacte
k__Bacteria|p__Bacteria_unclassified|c__Bacteria_unclassified|o__Bacteria_unclassified|f__Bacteria_unclassified|g__Bacte

k__Bacteria|p__Firmicutes|c__Clostridia|o__Eubacteriales|f__Lachnospiraceae|g__Acetatifactor|s__Acetatifactor_SGB415
k__Bacteria|p__Firmicutes|c__Clostridia|o__Eubacteriales|f__Lachnospiraceae|g__Acetatifactor|s__Acetatifactor_muris
k__Bacteria|p__Firmicutes|c__Clostridia|o__Eubacteriales|f__Oscillospiraceae|g__Acutalibacter|s__Acutalibacter_muris
k__Bacteria|p__Firmicutes|c__Clostridia|o__Eubacteriales|f__Oscillospiraceae|g__Acutalibacter|s__Acutalibacter_sp_1XD
k__Bacteria|p__Actinobacteria|c__Coriobacteriia|o__Eggerthellales|f__Eggerthellaceae|g__Adlercreutzia|s__Adlercreutzia
k__Bacteria|p__Actinobacteria|c__Coriobacteriia|o__Eggerthellales|f__Eggerthellaceae|g__Adlercreutzia|s__Adlercreutzia
k__Bacteria|p__Actinobacteria|c__Coriobacteriia|o__Eggerthellales|f__Eggerthellaceae|g__Adlercreutzia|s__Adlercreutzia
k__Bacteria|p__Verrucomicrobia|c__Verrucomicrobiae|o__Verrucomicrobiales|f__Akkermansiaceae|g__Akkermansia|s__A
k__Bacteria|p__Bacteroidota|c__Bacteroidia|o__Bacteroidales|f__Rikenellaceae|g__Alistipes|s__Alistipes_sp_DSM_11234
k__Bacteria|p__Firmicutes|c__Clostridia|o__Eubacteriales|f__Oscillospiraceae|g__Anaerotruncus|s__Anaerotruncus_sp_1
k__Bacteria|p__Bacteria_unclassified|c__Bacteria_unclassified|o__Bacteria_unclassified|f__Bacteria_unclassified|g__Bacte

k__Bacteria|p__Bacteria_unclassified|c__Bacteria_unclassified|o__Bacteria_unclassified|f__Bacteria_unclassified|g__Bacte
k__Bacteria|p__Bacteria_unclassified|c__Bacteria_unclassified|o__Bacteria_unclassified|f__Bacteria_unclassified|g__Bacte
k__Bacteria|p__Bacteroidota|c__Bacteroidia|o__Bacteroidales|f__Bacteroidaceae|g__Bacteroides|s__Bacteroides_thetaio
k__Bacteria|p__Actinobacteria|c__Actinomycetia|o__Bifidobacteriales|f__Bifidobacteriaceae|g__Bifidobacterium|s__Bifid
k__Bacteria|p__Firmicutes|c__Clostridia|o__Clostridia_unclassified|f__Clostridia_unclassified|g__Clostridia_unclassified|s_
k__Bacteria|p__Firmicutes|c__Clostridia|o__Eubacteriales|f__Clostridiaceae|g__Clostridiaceae_unclassified|s__Clostridiac
k__Bacteria|p__Firmicutes|c__Clostridia|o__Eubacteriales|f__Clostridiaceae|g__Clostridiaceae_unclassified|s__Clostridiac
k__Bacteria|p__Firmicutes|c__Clostridia|o__Eubacteriales|f__Eubacteriales_unclassified|g__Eubacteriales_unclassified|s_
k__Bacteria|p__Firmicutes|c__Erysipelotrichia|o__Erysipelotrichales|f__Erysipelotrichaceae|g__Erysipelatoclostridium|s_
k__Bacteria|p__Actinobacteria|c__Coriobacteriia|o__Coriobacteriales|f__Coriobacteriaceae|g__Coriobacteriaceae_unclass
k__Bacteria|p__Firmicutes|c__Clostridia|o__Eubacteriales|f__Lachnospiraceae|g__Dorea|s__Dorea_sp_5_2
k__Bacteria|p__Firmicutes|c__Erysipelotrichia|o__Erysipelotrichales|f__Erysipelotrichaceae|g__Dubosiella|s__Dubosiella_
k__Bacteria|p__Firmicutes|c__Erysipelotrichia|o__Erysipelotrichales|f__Erysipelotrichales_unclassified|g__Erysipelotrichal
k__Bacteria|p__Firmicutes|c__Clostridia|o__Eubacteriales|f__Eubacteriaceae|g__Eubacteriaceae_unclassified|s__Eubacte
k__Bacteria|p__Firmicutes|c__Clostridia|o__Eubacteriales|f__Eubacteriaceae|g__Eubacteriaceae_unclassified|s__Eubacte
k__Bacteria|p__Firmicutes|c__Clostridia|o__Eubacteriales|f__Lachnospiraceae|g__GGB20149|s__GGB20149_SGB29430
k__Bacteria|p__Actinobacteria|c__Coriobacteriia|o__Eggerthellales|f__Eggerthellaceae|g__GGB22635|s__GGB22635_SGB
k__Bacteria|p__Firmicutes|c__Clostridia|o__Eubacteriales|f__Lachnospiraceae|g__GGB25041|s__GGB25041_SGB36960
k__Bacteria|p__Bacteroidota|c__Bacteroidia|o__Bacteroidales|f__Muribaculaceae|g__GGB27876|s__GGB27876_SGB4031
k__Bacteria|p__Bacteroidota|c__Bacteroidia|o__Bacteroidales|f__Muribaculaceae|g__GGB27878|s__GGB27878_SGB4031
k__Bacteria|p__Bacteroidota|c__Bacteroidia|o__Bacteroidales|f__Muribaculaceae|g__GGB27918|s__GGB27918_SGB4035
k__Bacteria|p__Firmicutes|c__CFGB9508|o__OFGB9508|f__FGB9508|g__GGB28382|s__GGB28382_SGB40962
k__Bacteria|p__Firmicutes|c__CFGB2838|o__OFGB2838|f__FGB2838|g__GGB28399|s__GGB28399_SGB40980
k__Bacteria|p__Firmicutes|c__CFGB2838|o__OFGB2838|f__FGB2838|g__GGB28411|s__GGB28411_SGB40993
k__Bacteria|p__Firmicutes|c__CFGB2838|o__OFGB2838|f__FGB2838|g__GGB28415|s__GGB28415_SGB40997
k__Bacteria|p__Firmicutes|c__Clostridia|o__Eubacteriales|f__Pumilibacteraceae|g__GGB28430|s__GGB28430_SGB41013
k__Bacteria|p__Firmicutes|c__CFGB28439|o__OFGB28439|f__FGB28439|g__GGB28439|s__GGB28439_SGB41022
k__Bacteria|p__Firmicutes|c__Clostridia|o__Clostridia_unclassified|f__Clostridia_unclassified|g__GGB28778|s__GGB2877
k__Bacteria|p__Firmicutes|c__Clostridia|o__Eubacteriales|f__Eubacteriaceae|g__GGB28784|s__GGB28784_SGB41437
k__Bacteria|p__Firmicutes|c__Clostridia|o__Eubacteriales|f__Lachnospiraceae|g__GGB28792|s__GGB28792_SGB41445
k__Bacteria|p__Firmicutes|c__Clostridia|o__Eubacteriales|f__Lachnospiraceae|g__GGB28798|s__GGB28798_SGB41451
k__Bacteria|p__Firmicutes|c__Clostridia|o__Eubacteriales|f__Lachnospiraceae|g__GGB28802|s__GGB28802_SGB41455
k__Bacteria|p__Firmicutes|c__Clostridia|o__Eubacteriales|f__Lachnospiraceae|g__GGB28818|s__GGB28818_SGB41473
k__Bacteria|p__Firmicutes|c__CFGB77305|o__OFGB77305|f__FGB77305|g__GGB28828|s__GGB28828_SGB41484
k__Bacteria|p__Firmicutes|c__Clostridia|o__Eubacteriales|f__Clostridiaceae|g__GGB28851|s__GGB28851_SGB41518
k__Bacteria|p__Firmicutes|c__Clostridia|o__Eubacteriales|f__Lachnospiraceae|g__GGB28859|s__GGB28859_SGB41528
k__Bacteria|p__Firmicutes|c__Clostridia|o__Eubacteriales|f__Lachnospiraceae|g__GGB28864|s__GGB28864_SGB41535
k__Bacteria|p__Firmicutes|c__Clostridia|o__Eubacteriales|f__Lachnospiraceae|g__GGB28869|s__GGB28869_SGB41543
k__Bacteria|p__Firmicutes|c__CFGB9633|o__OFGB9633|f__FGB9633|g__GGB28883|s__GGB28883_SGB41564
k__Bacteria|p__Bacteria_unclassified|c__Bacteria_unclassified|o__Bacteria_unclassified|f__Bacteria_unclassified|g__GGB2
k__Bacteria|p__Bacteria_unclassified|c__Bacteria_unclassified|o__Bacteria_unclassified|f__Bacteria_unclassified|g__GGB2
k__Bacteria|p__Bacteria_unclassified|c__Bacteria_unclassified|o__Bacteria_unclassified|f__Bacteria_unclassified|g__GGB2
k__Bacteria|p__Bacteria_unclassified|c__Bacteria_unclassified|o__Bacteria_unclassified|f__Bacteria_unclassified|g__GGB2
k__Bacteria|p__Firmicutes|c__Clostridia|o__Eubacteriales|f__Lachnospiraceae|g__GGB28916|s__GGB28916_SGB41612
k__Bacteria|p__Firmicutes|c__Clostridia|o__Eubacteriales|f__Lachnospiraceae|g__GGB28924|s__GGB28924_SGB41621
k__Bacteria|p__Firmicutes|c__Clostridia|o__Eubacteriales|f__Lachnospiraceae|g__GGB28926|s__GGB28926_SGB41624

k__Bacteria|p__Bacteria_unclassified|c__CFGB77359|o__OFGB77359|f__FGB77359|g__GGB28927|s__GGB28927_SGB416
k__Bacteria|p__Firmicutes|c__CFGB9639|o__OFGB9639|f__FGB9639|g__GGB28934|s__GGB28934_SGB41635
k__Bacteria|p__Firmicutes|c__Clostridia|o__Eubacteriales|f__Lachnospiraceae|g__GGB28946|s__GGB28946_SGB41652
k__Bacteria|p__Firmicutes|c__Clostridia|o__Eubacteriales|f__Lachnospiraceae|g__GGB28949|s__GGB28949_SGB41655
k__Bacteria|p__Firmicutes|c__Clostridia|o__Eubacteriales|f__Lachnospiraceae|g__GGB28949|s__GGB28949_SGB41656
k__Bacteria|p__Firmicutes|c__Clostridia|o__Eubacteriales|f__Clostridiaceae|g__GGB28950|s__GGB28950_SGB41657
k__Bacteria|p__Firmicutes|c__Clostridia|o__Eubacteriales|f__Clostridiaceae|g__GGB28951|s__GGB28951_SGB102295
k__Bacteria|p__Firmicutes|c__Clostridia|o__Eubacteriales|f__Clostridiaceae|g__GGB28951|s__GGB28951_SGB41658
k__Bacteria|p__Firmicutes|c__Clostridia|o__Eubacteriales|f__Clostridiaceae|g__GGB28954|s__GGB28954_SGB41662
k__Bacteria|p__Firmicutes|c__Clostridia|o__Eubacteriales|f__Clostridiaceae|g__GGB28956|s__GGB28956_SGB41665
k__Bacteria|p__Firmicutes|c__Clostridia|o__Eubacteriales|f__Clostridiaceae|g__GGB28960|s__GGB28960_SGB41669
k__Bacteria|p__Firmicutes|c__Clostridia|o__Eubacteriales|f__Clostridiaceae|g__GGB28967|s__GGB28967_SGB41678
k__Bacteria|p__Firmicutes|c__Clostridia|o__Eubacteriales|f__Eubacteriaceae|g__GGB28991|s__GGB28991_SGB41705
k__Bacteria|p__Firmicutes|c__CFGB9658|o__OFGB9658|f__FGB9658|g__GGB29002|s__GGB29002_SGB41718
k__Bacteria|p__Firmicutes|c__CFGB9659|o__OFGB9659|f__FGB9659|g__GGB29003|s__GGB29003_SGB41719
k__Bacteria|p__Bacteria_unclassified|c__Bacteria_unclassified|o__Bacteria_unclassified|f__Bacteria_unclassified|g__GGB2
k__Bacteria|p__Firmicutes|c__CFGB9827|o__OFGB9827|f__FGB9827|g__GGB29531|s__GGB29531_SGB42317
k__Bacteria|p__Firmicutes|c__Clostridia|o__Eubacteriales|f__Eubacteriaceae|g__GGB29685|s__GGB29685_SGB42494
k__Bacteria|p__Bacteria_unclassified|c__CFGB77303|o__OFGB77303|f__FGB77303|g__GGB30141|s__GGB30141_SGB430
k__Bacteria|p__Firmicutes|c__Clostridia|o__Eubacteriales|f__Eubacteriales_unclassified|g__GGB30286|s__GGB30286_SG
k__Bacteria|p__Firmicutes|c__Clostridia|o__Eubacteriales|f__Oscillospiraceae|g__GGB30303|s__GGB30303_SGB43268
k__Bacteria|p__Firmicutes|c__CFGB30328|o__OFGB30328|f__FGB30328|g__GGB30413|s__GGB30413_SGB43452
k__Bacteria|p__Firmicutes|c__Clostridia|o__Eubacteriales|f__Oscillospiraceae|g__GGB30454|s__GGB30454_SGB43514
k__Bacteria|p__Firmicutes|c__Clostridia|o__Eubacteriales|f__Oscillospiraceae|g__GGB30455|s__GGB30455_SGB43519
k__Bacteria|p__Firmicutes|c__Clostridia|o__Eubacteriales|f__Oscillospiraceae|g__GGB30461|s__GGB30461_SGB43527
k__Bacteria|p__Firmicutes|c__Clostridia|o__Eubacteriales|f__Oscillospiraceae|g__GGB30461|s__GGB30461_SGB43530
k__Bacteria|p__Firmicutes|c__Clostridia|o__Eubacteriales|f__Oscillospiraceae|g__GGB30463|s__GGB30463_SGB43537
k__Bacteria|p__Firmicutes|c__Clostridia|o__Eubacteriales|f__Oscillospiraceae|g__GGB30473|s__GGB30473_SGB43557
k__Bacteria|p__Firmicutes|c__Clostridia|o__Eubacteriales|f__Oscillospiraceae|g__GGB30475|s__GGB30475_SGB63182
k__Bacteria|p__Actinobacteria|c__CFGB77153|o__OFGB77153|f__FGB77153|g__GGB30861|s__GGB30861_SGB44083
k__Bacteria|p__Tenericutes|c__CFGB1791|o__OFGB1791|f__FGB1791|g__GGB31312|s__GGB31312_SGB44628
k__Bacteria|p__Firmicutes|c__CFGB10290|o__OFGB10290|f__FGB10290|g__GGB31438|s__GGB31438_SGB44768
k__Bacteria|p__Firmicutes|c__Clostridia|o__Eubacteriales|f__Oscillospiraceae|g__GGB3171|s__GGB3171_SGB4185
k__Bacteria|p__Firmicutes|c__CFGB1765|o__OFGB1765|f__FGB1765|g__GGB31823|s__GGB31823_SGB45199
k__Bacteria|p__Firmicutes|c__CFGB10349|o__OFGB10349|f__FGB10349|g__GGB31853|s__GGB31853_SGB45233
k__Bacteria|p__Firmicutes|c__CFGB10667|o__OFGB10667|f__FGB10667|g__GGB32371|s__GGB32371_SGB41694
k__Bacteria|p__Firmicutes|c__Clostridia|o__Eubacteriales|f__Lachnospiraceae|g__GGB3793|s__GGB3793_SGB5158
k__Bacteria|p__Firmicutes|c__Clostridia|o__Eubacteriales|f__Lachnospiraceae|g__GGB42598|s__GGB42598_SGB59794
k__Bacteria|p__Firmicutes|c__Clostridia|o__Eubacteriales|f__Christensenellaceae|g__GGB45656|s__GGB45656_SGB6337
k__Bacteria|p__Firmicutes|c__CFGB10299|o__OFGB10299|f__FGB10299|g__GGB47127|s__GGB47127_SGB65054
k__Bacteria|p__Firmicutes|c__Clostridia|o__Eubacteriales|f__Oscillospiraceae|g__GGB74395|s__GGB74395_SGB43521
k__Bacteria|p__Firmicutes|c__Clostridia|o__Eubacteriales|f__Oscillospiraceae|g__GGB75053|s__GGB75053_SGB43494
k__Bacteria|p__Firmicutes|c__Clostridia|o__Eubacteriales|f__Lachnospiraceae|g__GGB75109|s__GGB75109_SGB102238
k__Bacteria|p__Firmicutes|c__Clostridia|o__Clostridia_unclassified|f__Clostridia_unclassified|g__GGB81440|s__GGB8144
k__Bacteria|p__Firmicutes|c__Clostridia|o__Eubacteriales|f__Lachnospiraceae|g__Lachnospiraceae_unclassified|s__Lachn
k__Bacteria|p__Firmicutes|c__Clostridia|o__Eubacteriales|f__Lachnospiraceae|g__Lachnospiraceae_unclassified|s__Lachr

k__Bacteria|p__Firmicutes|c__Clostridia|o__Eubacteriales|f__Lachnospiraceae|g__Lachnospiraceae_unclassified|s__Lachn
k__Bacteria|p__Firmicutes|c__Clostridia|o__Eubacteriales|f__Lachnospiraceae|g__Lachnospiraceae_unclassified|s__Lachn
k__Bacteria|p__Firmicutes|c__Clostridia|o__Eubacteriales|f__Lachnospiraceae|g__Lachnospiraceae_unclassified|s__Lachn
k__Bacteria|p__Firmicutes|c__Clostridia|o__Eubacteriales|f__Lachnospiraceae|g__Lachnospiraceae_unclassified|s__Lachn
k__Bacteria|p__Firmicutes|c__Clostridia|o__Eubacteriales|f__Lachnospiraceae|g__Lachnospiraceae_unclassified|s__Lachn
k__Bacteria|p__Firmicutes|c__Clostridia|o__Eubacteriales|f__Lachnospiraceae|g__Lachnospiraceae_unclassified|s__Lachn
k__Bacteria|p__Firmicutes|c__Bacilli|o__Lactobacillales|f__Lactobacillaceae|g__Lactobacillus|s__Lactobacillus_johnsonii
k__Bacteria|p__Bacteroidota|c__Bacteroidia|o__Bacteroidales|f__Muribaculaceae|g__Muribaculaceae_unclassified|s__M
k__Bacteria|p__Firmicutes|c__Clostridia|o__Eubacteriales|f__Oscillospiraceae|g__Neglectibacter|s__Neglectibacter_sp_X
k__Bacteria|p__Firmicutes|c__Clostridia|o__Eubacteriales|f__Oscillospiraceae|g__Oscillospiraceae_unclassified|s__Oscillo
k__Bacteria|p__Firmicutes|c__Clostridia|o__Eubacteriales|f__Oscillospiraceae|g__Oscillospiraceae_unclassified|s__Oscillo
k__Bacteria|p__Firmicutes|c__Clostridia|o__Eubacteriales|f__Oscillospiraceae|g__Oscillospiraceae_unclassified|s__Oscillo
k__Bacteria|p__Proteobacteria|c__Betaproteobacteria|o__Burkholderiales|f__Sutterellaceae|g__Parasutterella|s__Parasu
k__Bacteria|p__Firmicutes|c__Clostridia|o__Eubacteriales|f__Peptostreptococcaceae|g__Romboutsia|s__Romboutsia_ilea
k__Bacteria|p__Firmicutes|c__Clostridia|o__Eubacteriales|f__Lachnospiraceae|g__Schaedlerella|s__Schaedlerella_arabino
k__Bacteria|p__Firmicutes|c__Erysipelotrichia|o__Erysipelotrichales|f__Turicibacteraceae|g__Turicibacter|s__Turicibacter_
k__Bacteria|p__Bacteria_unclassified|c__Bacteria_unclassified|o__Bacteria_unclassified|f__Bacteria_unclassified|g__Bacte
k__Bacteria|p__Bacteria_unclassified|c__Bacteria_unclassified|o__Bacteria_unclassified|f__Bacteria_unclassified|g__Bacte
k__Bacteria|p__Bacteria_unclassified|c__Bacteria_unclassified|o__Bacteria_unclassified|f__Bacteria_unclassified|g__Bacte

k__Bacteria|p__Firmicutes|c__Clostridia|o__Eubacteriales|f__Lachnospiraceae|g__Acetatifactor|s__Acetatifactor_SGB415
k__Bacteria|p__Firmicutes|c__Clostridia|o__Eubacteriales|f__Lachnospiraceae|g__Acetatifactor|s__Acetatifactor_muris
k__Bacteria|p__Firmicutes|c__Clostridia|o__Eubacteriales|f__Oscillospiraceae|g__Acutalibacter|s__Acutalibacter_muris
k__Bacteria|p__Firmicutes|c__Clostridia|o__Eubacteriales|f__Oscillospiraceae|g__Acutalibacter|s__Acutalibacter_sp_1XD
k__Bacteria|p__Actinobacteria|c__Coriobacteriia|o__Eggerthellales|f__Eggerthellaceae|g__Adlercreutzia|s__Adlercreutzia
k__Bacteria|p__Actinobacteria|c__Coriobacteriia|o__Eggerthellales|f__Eggerthellaceae|g__Adlercreutzia|s__Adlercreutzia
k__Bacteria|p__Actinobacteria|c__Coriobacteriia|o__Eggerthellales|f__Eggerthellaceae|g__Adlercreutzia|s__Adlercreutzia
k__Bacteria|p__Verrucomicrobia|c__Verrucomicrobiae|o__Verrucomicrobiales|f__Akkermansiaceae|g__Akkermansia|s__A
k__Bacteria|p__Bacteroidota|c__Bacteroidia|o__Bacteroidales|f__Rikenellaceae|g__Alistipes|s__Alistipes_sp_DSM_11234
k__Bacteria|p__Firmicutes|c__Clostridia|o__Eubacteriales|f__Oscillospiraceae|g__Anaerotruncus|s__Anaerotruncus_sp_1
k__Bacteria|p__Bacteria_unclassified|c__Bacteria_unclassified|o__Bacteria_unclassified|f__Bacteria_unclassified|g__Bacte
k__Bacteria|p__Bacteria_unclassified|c__Bacteria_unclassified|o__Bacteria_unclassified|f__Bacteria_unclassified|g__Bacte
k__Bacteria|p__Bacteria_unclassified|c__Bacteria_unclassified|o__Bacteria_unclassified|f__Bacteria_unclassified|g__Bacte
k__Bacteria|p__Bacteroidota|c__Bacteroidia|o__Bacteroidales|f__Bacteroidaceae|g__Bacteroides|s__Bacteroides_thetaio
k__Bacteria|p__Actinobacteria|c__Actinomycetia|o__Bifidobacteriales|f__Bifidobacteriaceae|g__Bifidobacterium|s__Bifid
k__Bacteria|p__Firmicutes|c__Clostridia|o__Clostridia_unclassified|f__Clostridia_unclassified|g__Clostridia_unclassified|s_
k__Bacteria|p__Firmicutes|c__Clostridia|o__Eubacteriales|f__Clostridiaceae|g__Clostridiaceae_unclassified|s__Clostridiac
k__Bacteria|p__Firmicutes|c__Clostridia|o__Eubacteriales|f__Clostridiaceae|g__Clostridiaceae_unclassified|s__Clostridiac
k__Bacteria|p__Firmicutes|c__Clostridia|o__Eubacteriales|f__Eubacteriales_unclassified|g__Eubacteriales_unclassified|s_
k__Bacteria|p__Firmicutes|c__Erysipelotrichia|o__Erysipelotrichales|f__Erysipelotrichaceae|g__Erysipelatoclostridium|s_
k__Bacteria|p__Actinobacteria|c__Coriobacteriia|o__Coriobacteriales|f__Coriobacteriaceae|g__Coriobacteriaceae_unclass
k__Bacteria|p__Firmicutes|c__Clostridia|o__Eubacteriales|f__Lachnospiraceae|g__Dorea|s__Dorea_sp_5_2
k__Bacteria|p__Firmicutes|c__Erysipelotrichia|o__Erysipelotrichales|f__Erysipelotrichaceae|g__Dubosiella|s__Dubosiella_
k__Bacteria|p__Firmicutes|c__Erysipelotrichia|o__Erysipelotrichales|f__Erysipelotrichales_unclassified|g__Erysipelotrichal

k__Bacteria|p__Firmicutes|c__Clostridia|o__Eubacteriales|f__Eubacteriaceae|g__Eubacteriaceae_unclassified|s__Eubacte
k__Bacteria|p__Firmicutes|c__Clostridia|o__Eubacteriales|f__Eubacteriaceae|g__Eubacteriaceae_unclassified|s__Eubacte
k__Bacteria|p__Firmicutes|c__Clostridia|o__Eubacteriales|f__Lachnospiraceae|g__GGB20149|s__GGB20149_SGB29430
k__Bacteria|p__Actinobacteria|c__Coriobacteriia|o__Eggerthellales|f__Eggerthellaceae|g__GGB22635|s__GGB22635_SGB
k__Bacteria|p__Firmicutes|c__Clostridia|o__Eubacteriales|f__Lachnospiraceae|g__GGB25041|s__GGB25041_SGB36960
k__Bacteria|p__Bacteroidota|c__Bacteroidia|o__Bacteroidales|f__Muribaculaceae|g__GGB27876|s__GGB27876_SGB4031
k__Bacteria|p__Bacteroidota|c__Bacteroidia|o__Bacteroidales|f__Muribaculaceae|g__GGB27878|s__GGB27878_SGB4031
k__Bacteria|p__Bacteroidota|c__Bacteroidia|o__Bacteroidales|f__Muribaculaceae|g__GGB27918|s__GGB27918_SGB4035
k__Bacteria|p__Firmicutes|c__CFGB9508|o__OFGB9508|f__FGB9508|g__GGB28382|s__GGB28382_SGB40962
k__Bacteria|p__Firmicutes|c__CFGB2838|o__OFGB2838|f__FGB2838|g__GGB28399|s__GGB28399_SGB40980
k__Bacteria|p__Firmicutes|c__CFGB2838|o__OFGB2838|f__FGB2838|g__GGB28411|s__GGB28411_SGB40993
k__Bacteria|p__Firmicutes|c__CFGB2838|o__OFGB2838|f__FGB2838|g__GGB28415|s__GGB28415_SGB40997
k__Bacteria|p__Firmicutes|c__Clostridia|o__Eubacteriales|f__Pumilibacteraceae|g__GGB28430|s__GGB28430_SGB41013
k__Bacteria|p__Firmicutes|c__CFGB28439|o__OFGB28439|f__FGB28439|g__GGB28439|s__GGB28439_SGB41022
k__Bacteria|p__Firmicutes|c__Clostridia|o__Clostridia_unclassified|f__Clostridia_unclassified|g__GGB28778|s__GGB2877
k__Bacteria|p__Firmicutes|c__Clostridia|o__Eubacteriales|f__Eubacteriaceae|g__GGB28784|s__GGB28784_SGB41437
k__Bacteria|p__Firmicutes|c__Clostridia|o__Eubacteriales|f__Lachnospiraceae|g__GGB28792|s__GGB28792_SGB41445
k__Bacteria|p__Firmicutes|c__Clostridia|o__Eubacteriales|f__Lachnospiraceae|g__GGB28798|s__GGB28798_SGB41451
k__Bacteria|p__Firmicutes|c__Clostridia|o__Eubacteriales|f__Lachnospiraceae|g__GGB28802|s__GGB28802_SGB41455
k__Bacteria|p__Firmicutes|c__Clostridia|o__Eubacteriales|f__Lachnospiraceae|g__GGB28818|s__GGB28818_SGB41473
k__Bacteria|p__Firmicutes|c__CFGB77305|o__OFGB77305|f__FGB77305|g__GGB28828|s__GGB28828_SGB41484
k__Bacteria|p__Firmicutes|c__Clostridia|o__Eubacteriales|f__Clostridiaceae|g__GGB28851|s__GGB28851_SGB41518
k__Bacteria|p__Firmicutes|c__Clostridia|o__Eubacteriales|f__Lachnospiraceae|g__GGB28859|s__GGB28859_SGB41528
k__Bacteria|p__Firmicutes|c__Clostridia|o__Eubacteriales|f__Lachnospiraceae|g__GGB28864|s__GGB28864_SGB41535
k__Bacteria|p__Firmicutes|c__Clostridia|o__Eubacteriales|f__Lachnospiraceae|g__GGB28869|s__GGB28869_SGB41543
k__Bacteria|p__Firmicutes|c__CFGB9633|o__OFGB9633|f__FGB9633|g__GGB28883|s__GGB28883_SGB41564
k__Bacteria|p__Bacteria_unclassified|c__Bacteria_unclassified|o__Bacteria_unclassified|f__Bacteria_unclassified|g__GGB
k__Bacteria|p__Bacteria_unclassified|c__Bacteria_unclassified|o__Bacteria_unclassified|f__Bacteria_unclassified|g__GGB
k__Bacteria|p__Bacteria_unclassified|c__Bacteria_unclassified|o__Bacteria_unclassified|f__Bacteria_unclassified|g__GGB
k__Bacteria|p__Bacteria_unclassified|c__Bacteria_unclassified|o__Bacteria_unclassified|f__Bacteria_unclassified|g__GGB
k__Bacteria|p__Firmicutes|c__Clostridia|o__Eubacteriales|f__Lachnospiraceae|g__GGB28916|s__GGB28916_SGB41612
k__Bacteria|p__Firmicutes|c__Clostridia|o__Eubacteriales|f__Lachnospiraceae|g__GGB28924|s__GGB28924_SGB41621
k__Bacteria|p__Firmicutes|c__Clostridia|o__Eubacteriales|f__Lachnospiraceae|g__GGB28926|s__GGB28926_SGB41624
k__Bacteria|p__Bacteria_unclassified|c__CFGB77359|o__OFGB77359|f__FGB77359|g__GGB28927|s__GGB28927_SGB416
k__Bacteria|p__Firmicutes|c__CFGB9639|o__OFGB9639|f__FGB9639|g__GGB28934|s__GGB28934_SGB41635
k__Bacteria|p__Firmicutes|c__Clostridia|o__Eubacteriales|f__Lachnospiraceae|g__GGB28946|s__GGB28946_SGB41652
k__Bacteria|p__Firmicutes|c__Clostridia|o__Eubacteriales|f__Lachnospiraceae|g__GGB28949|s__GGB28949_SGB41655
k__Bacteria|p__Firmicutes|c__Clostridia|o__Eubacteriales|f__Lachnospiraceae|g__GGB28949|s__GGB28949_SGB41656
k__Bacteria|p__Firmicutes|c__Clostridia|o__Eubacteriales|f__Clostridiaceae|g__GGB28950|s__GGB28950_SGB41657
k__Bacteria|p__Firmicutes|c__Clostridia|o__Eubacteriales|f__Clostridiaceae|g__GGB28951|s__GGB28951_SGB102295
k__Bacteria|p__Firmicutes|c__Clostridia|o__Eubacteriales|f__Clostridiaceae|g__GGB28951|s__GGB28951_SGB41658
k__Bacteria|p__Firmicutes|c__Clostridia|o__Eubacteriales|f__Clostridiaceae|g__GGB28954|s__GGB28954_SGB41662
k__Bacteria|p__Firmicutes|c__Clostridia|o__Eubacteriales|f__Clostridiaceae|g__GGB28956|s__GGB28956_SGB41665
k__Bacteria|p__Firmicutes|c__Clostridia|o__Eubacteriales|f__Clostridiaceae|g__GGB28960|s__GGB28960_SGB41669
k__Bacteria|p__Firmicutes|c__Clostridia|o__Eubacteriales|f__Clostridiaceae|g__GGB28967|s__GGB28967_SGB41678
k__Bacteria|p__Firmicutes|c__Clostridia|o__Eubacteriales|f__Eubacteriaceae|g__GGB28991|s__GGB28991_SGB41705

k__Bacteria|p__Firmicutes|c__CFGB9658|o__OFGB9658|f__FGB9658|g__GGB29002|s__GGB29002_SGB41718
k__Bacteria|p__Firmicutes|c__CFGB9659|o__OFGB9659|f__FGB9659|g__GGB29003|s__GGB29003_SGB41719
k__Bacteria|p__Bacteria_unclassified|c__Bacteria_unclassified|o__Bacteria_unclassified|f__Bacteria_unclassified|g__GGB2
k__Bacteria|p__Firmicutes|c__CFGB9827|o__OFGB9827|f__FGB9827|g__GGB29531|s__GGB29531_SGB42317
k__Bacteria|p__Firmicutes|c__Clostridia|o__Eubacteriales|f__Eubacteriaceae|g__GGB29685|s__GGB29685_SGB42494
k__Bacteria|p__Bacteria_unclassified|c__CFGB77303|o__OFGB77303|f__FGB77303|g__GGB30141|s__GGB30141_SGB430
k__Bacteria|p__Firmicutes|c__Clostridia|o__Eubacteriales|f__Eubacteriales_unclassified|g__GGB30286|s__GGB30286_SG
k__Bacteria|p__Firmicutes|c__Clostridia|o__Eubacteriales|f__Oscillospiraceae|g__GGB30303|s__GGB30303_SGB43268
k__Bacteria|p__Firmicutes|c__CFGB30328|o__OFGB30328|f__FGB30328|g__GGB30413|s__GGB30413_SGB43452
k__Bacteria|p__Firmicutes|c__Clostridia|o__Eubacteriales|f__Oscillospiraceae|g__GGB30454|s__GGB30454_SGB43514
k__Bacteria|p__Firmicutes|c__Clostridia|o__Eubacteriales|f__Oscillospiraceae|g__GGB30455|s__GGB30455_SGB43519
k__Bacteria|p__Firmicutes|c__Clostridia|o__Eubacteriales|f__Oscillospiraceae|g__GGB30461|s__GGB30461_SGB43527
k__Bacteria|p__Firmicutes|c__Clostridia|o__Eubacteriales|f__Oscillospiraceae|g__GGB30461|s__GGB30461_SGB43530
k__Bacteria|p__Firmicutes|c__Clostridia|o__Eubacteriales|f__Oscillospiraceae|g__GGB30463|s__GGB30463_SGB43537
k__Bacteria|p__Firmicutes|c__Clostridia|o__Eubacteriales|f__Oscillospiraceae|g__GGB30473|s__GGB30473_SGB43557
k__Bacteria|p__Firmicutes|c__Clostridia|o__Eubacteriales|f__Oscillospiraceae|g__GGB30475|s__GGB30475_SGB63182
k__Bacteria|p__Actinobacteria|c__CFGB77153|o__OFGB77153|f__FGB77153|g__GGB30861|s__GGB30861_SGB44083
k__Bacteria|p__Tenericutes|c__CFGB1791|o__OFGB1791|f__FGB1791|g__GGB31312|s__GGB31312_SGB44628
k__Bacteria|p__Firmicutes|c__CFGB10290|o__OFGB10290|f__FGB10290|g__GGB31438|s__GGB31438_SGB44768
k__Bacteria|p__Firmicutes|c__Clostridia|o__Eubacteriales|f__Oscillospiraceae|g__GGB3171|s__GGB3171_SGB4185
k__Bacteria|p__Firmicutes|c__CFGB1765|o__OFGB1765|f__FGB1765|g__GGB31823|s__GGB31823_SGB45199
k__Bacteria|p__Firmicutes|c__CFGB10349|o__OFGB10349|f__FGB10349|g__GGB31853|s__GGB31853_SGB45233
k__Bacteria|p__Firmicutes|c__CFGB10667|o__OFGB10667|f__FGB10667|g__GGB32371|s__GGB32371_SGB41694
k__Bacteria|p__Firmicutes|c__Clostridia|o__Eubacteriales|f__Lachnospiraceae|g__GGB3793|s__GGB3793_SGB5158
k__Bacteria|p__Firmicutes|c__Clostridia|o__Eubacteriales|f__Lachnospiraceae|g__GGB42598|s__GGB42598_SGB59794
k__Bacteria|p__Firmicutes|c__Clostridia|o__Eubacteriales|f__Christensenellaceae|g__GGB45656|s__GGB45656_SGB6337
k__Bacteria|p__Firmicutes|c__CFGB10299|o__OFGB10299|f__FGB10299|g__GGB47127|s__GGB47127_SGB65054
k__Bacteria|p__Firmicutes|c__Clostridia|o__Eubacteriales|f__Oscillospiraceae|g__GGB74395|s__GGB74395_SGB43521
k__Bacteria|p__Firmicutes|c__Clostridia|o__Eubacteriales|f__Oscillospiraceae|g__GGB75053|s__GGB75053_SGB43494
k__Bacteria|p__Firmicutes|c__Clostridia|o__Eubacteriales|f__Lachnospiraceae|g__GGB75109|s__GGB75109_SGB102238
k__Bacteria|p__Firmicutes|c__Clostridia|o__Clostridia_unclassified|f__Clostridia_unclassified|g__GGB81440|s__GGB8144
k__Bacteria|p__Firmicutes|c__Clostridia|o__Eubacteriales|f__Lachnospiraceae|g__Lachnospiraceae_unclassified|s__Lachn
k__Bacteria|p__Firmicutes|c__Clostridia|o__Eubacteriales|f__Lachnospiraceae|g__Lachnospiraceae_unclassified|s__Lachn
k__Bacteria|p__Firmicutes|c__Clostridia|o__Eubacteriales|f__Lachnospiraceae|g__Lachnospiraceae_unclassified|s__Lachn
k__Bacteria|p__Firmicutes|c__Clostridia|o__Eubacteriales|f__Lachnospiraceae|g__Lachnospiraceae_unclassified|s__Lachn
k__Bacteria|p__Firmicutes|c__Clostridia|o__Eubacteriales|f__Lachnospiraceae|g__Lachnospiraceae_unclassified|s__Lachn
k__Bacteria|p__Firmicutes|c__Clostridia|o__Eubacteriales|f__Lachnospiraceae|g__Lachnospiraceae_unclassified|s__Lachn
k__Bacteria|p__Firmicutes|c__Clostridia|o__Eubacteriales|f__Lachnospiraceae|g__Lachnospiraceae_unclassified|s__Lachn
k__Bacteria|p__Firmicutes|c__Clostridia|o__Eubacteriales|f__Lachnospiraceae|g__Lachnospiraceae_unclassified|s__Lachn
k__Bacteria|p__Firmicutes|c__Bacilli|o__Lactobacillales|f__Lactobacillaceae|g__Lactobacillus|s__Lactobacillus_johnsonii
k__Bacteria|p__Bacteroidota|c__Bacteroidia|o__Bacteroidales|f__Muribaculaceae|g__Muribaculaceae_unclassified|s__M
k__Bacteria|p__Firmicutes|c__Clostridia|o__Eubacteriales|f__Oscillospiraceae|g__Neglectibacter|s__Neglectibacter_sp_X
k__Bacteria|p__Firmicutes|c__Clostridia|o__Eubacteriales|f__Oscillospiraceae|g__Oscillospiraceae_unclassified|s__Oscillo
k__Bacteria|p__Firmicutes|c__Clostridia|o__Eubacteriales|f__Oscillospiraceae|g__Oscillospiraceae_unclassified|s__Oscillo
k__Bacteria|p__Firmicutes|c__Clostridia|o__Eubacteriales|f__Oscillospiraceae|g__Oscillospiraceae_unclassified|s__Oscillo
k__Bacteria|p__Proteobacteria|c__Betaproteobacteria|o__Burkholderiales|f__Sutterellaceae|g__Parasutterella|s__Parasu

k__Bacteria|p__Firmicutes|c__Clostridia|o__Eubacteriales|f__Peptostreptococcaceae|g__Romboutsia|s__Romboutsia_ilea
k__Bacteria|p__Firmicutes|c__Clostridia|o__Eubacteriales|f__Lachnospiraceae|g__Schaedlerella|s__Schaedlerella_arabino
k__Bacteria|p__Firmicutes|c__Erysipelotrichia|o__Erysipelotrichales|f__Turicibacteraceae|g__Turicibacter|s__Turicibacter_
k__Bacteria|p__Bacteria_unclassified|c__Bacteria_unclassified|o__Bacteria_unclassified|f__Bacteria_unclassified|g__Bacte
k__Bacteria|p__Bacteria_unclassified|c__Bacteria_unclassified|o__Bacteria_unclassified|f__Bacteria_unclassified|g__Bacte
k__Bacteria|p__Bacteria_unclassified|c__Bacteria_unclassified|o__Bacteria_unclassified|f__Bacteria_unclassified|g__Bacte

GB102200
GB41677
GB43546

rium

GB102200
GB41677

GB43546

rium

GB102200
GB41677
GB43546

rium

GB102200
GB41677
GB43546

rium

GB102200
GB41677
GB43546

rium

GB102200
GB41677
GB43546

rium

GB102200
GB41677
GB43546

rium

GB102200
GB41677
GB43546

rium

GB41677
GB43546

rium

GB102200
GB41677
GB43546

rium
