# Supplementary material for: Non-invasive Vagal Nerve Stimulation as a Potential Treatment for Repetitive Blast Trauma
Source: bioRxiv. 2026 Jul 19:2026.07.13.737563. Preprint. [Version 1] doi: 10.64898/2026.07.13.737563 (PMC13405043; doi:10.64898/2026.07.13.737563)
Supplement: Supplement 17 [file media-17.pdf]

Exposure	Mediator (Species-level feature)	Outcome	p_m
blast	Acetatifactor_muris	EtOH_24h_intakeave_final	0.7395220796
vns	Acetatifactor_muris	EtOH_24h_intakeave_final	0.7528971403
blast	Acetatifactor_SGB41546	EtOH_24h_intakeave_final	0.8402457971
vns	Acetatifactor_SGB41546	EtOH_24h_intakeave_final	0.6630009771
blast	Acutalibacter_muris	EtOH_24h_intakeave_final	0.4221546334
vns	Acutalibacter_muris	EtOH_24h_intakeave_final	0.8105784495
blast	Acutalibacter_sp_1XD8_36	EtOH_24h_intakeave_final	0.6605798863
vns	Acutalibacter_sp_1XD8_36	EtOH_24h_intakeave_final	0.7091849336
blast	Adlercreutzia_caecimuris	EtOH_24h_intakeave_final	0.9896089529
vns	Adlercreutzia_caecimuris	EtOH_24h_intakeave_final	0.6765426913
blast	Adlercreutzia_mucosicola	EtOH_24h_intakeave_final	0.2718339856
vns	Adlercreutzia_mucosicola	EtOH_24h_intakeave_final	0.2498256574
blast	Adlercreutzia_muris	EtOH_24h_intakeave_final	0.6704217743
vns	Adlercreutzia_muris	EtOH_24h_intakeave_final	0.5958277454
blast	Akkermansia_muciniphila	EtOH_24h_intakeave_final	0.1698125404
vns	Akkermansia_muciniphila	EtOH_24h_intakeave_final	0.8643625099
blast	Alistipes_sp_DSM_112343	EtOH_24h_intakeave_final	0.3725159774
vns	Alistipes_sp_DSM_112343	EtOH_24h_intakeave_final	0.8326983074
blast	Anaerotruncus_sp_1XD42_93	EtOH_24h_intakeave_final	0.5202714535
vns	Anaerotruncus_sp_1XD42_93	EtOH_24h_intakeave_final	0.6174107217
blast	Bacteria_unclassified_SGB102200	EtOH_24h_intakeave_final	0.4742692127
vns	Bacteria_unclassified_SGB102200	EtOH_24h_intakeave_final	0.7641667885
blast	Bacteria_unclassified_SGB41677	EtOH_24h_intakeave_final	0.4441438231
vns	Bacteria_unclassified_SGB41677	EtOH_24h_intakeave_final	0.1699894243
blast	Bacteria_unclassified_SGB43546	EtOH_24h_intakeave_final	0.5863583659
vns	Bacteria_unclassified_SGB43546	EtOH_24h_intakeave_final	0.7890099125
blast	bacterium_1XD42_54	EtOH_24h_intakeave_final	0.0144245959
vns	bacterium_1XD42_54	EtOH_24h_intakeave_final	0.2345775748
blast	bacterium_1XD42_76	EtOH_24h_intakeave_final	0.6000316038
vns	bacterium_1XD42_76	EtOH_24h_intakeave_final	0.6429924539
blast	bacterium_1xD8_48	EtOH_24h_intakeave_final	0.8997089453
vns	bacterium_1xD8_48	EtOH_24h_intakeave_final	0.6968168809
blast	Bacteroides_thetaiotaomicron	EtOH_24h_intakeave_final	0.9602014777
vns	Bacteroides_thetaiotaomicron	EtOH_24h_intakeave_final	0.6343063159
blast	Berger Parker Index	EtOH_24h_intakeave_final	0.5457417127
vns	Berger Parker Index	EtOH_24h_intakeave_final	0.2337330546
blast	Bifidobacterium_pseudolongum	EtOH_24h_intakeave_final	0.4280103985
vns	Bifidobacterium_pseudolongum	EtOH_24h_intakeave_final	0.3477990653
blast	Clostridia_bacterium	EtOH_24h_intakeave_final	0.3745590434
vns	Clostridia_bacterium	EtOH_24h_intakeave_final	0.7249401071
blast	Clostridiaceae_bacterium	EtOH_24h_intakeave_final	0.9871697806
vns	Clostridiaceae_bacterium	EtOH_24h_intakeave_final	0.2419370425
blast	Clostridiaceae_unclassified_SGB41663	EtOH_24h_intakeave_final	0.780382224
vns	Clostridiaceae_unclassified_SGB41663	EtOH_24h_intakeave_final	0.719154368
blast	Clostridiales_bacterium	EtOH_24h_intakeave_final	0.0023319564
vns	Clostridiales_bacterium	EtOH_24h_intakeave_final	0.2964785018

blast	Clostridium_cocleatum	EtOH_24h_intakeave_final	0.011567832
vns	Clostridium_cocleatum	EtOH_24h_intakeave_final	0.196035514
blast	Coriobacteriaceae_bacterium	EtOH_24h_intakeave_final	0.372448369
vns	Coriobacteriaceae_bacterium	EtOH_24h_intakeave_final	0.953919137
blast	Dorea_sp_5_2	EtOH_24h_intakeave_final	0.588491917
vns	Dorea_sp_5_2	EtOH_24h_intakeave_final	0.421416124
blast	Dubosiella_newyorkensis	EtOH_24h_intakeave_final	0.116998739
vns	Dubosiella_newyorkensis	EtOH_24h_intakeave_final	0.799186063
blast	Erysipelotrichales_bacterium	EtOH_24h_intakeave_final	0.897000665
vns	Erysipelotrichales_bacterium	EtOH_24h_intakeave_final	0.328408108
blast	Eubacteriaceae_bacterium	EtOH_24h_intakeave_final	0.027123368
vns	Eubacteriaceae_bacterium	EtOH_24h_intakeave_final	0.238318613
blast	Eubacteriaceae_unclassified_SGB94922	EtOH_24h_intakeave_final	0.459488079
vns	Eubacteriaceae_unclassified_SGB94922	EtOH_24h_intakeave_final	0.372620148
blast	GGB20149_SGB29430	EtOH_24h_intakeave_final	0.628070048
vns	GGB20149_SGB29430	EtOH_24h_intakeave_final	0.629982792
blast	GGB22635_SGB63107	EtOH_24h_intakeave_final	0.411710336
vns	GGB22635_SGB63107	EtOH_24h_intakeave_final	0.887152138
blast	GGB25041_SGB36960	EtOH_24h_intakeave_final	0.082496667
vns	GGB25041_SGB36960	EtOH_24h_intakeave_final	0.210187196
blast	GGB27876_SGB40310	EtOH_24h_intakeave_final	0.164666135
vns	GGB27876_SGB40310	EtOH_24h_intakeave_final	0.767235194
blast	GGB27878_SGB40312	EtOH_24h_intakeave_final	0.213354157
vns	GGB27878_SGB40312	EtOH_24h_intakeave_final	0.147479429
blast	GGB27918_SGB40356	EtOH_24h_intakeave_final	0.958758140
vns	GGB27918_SGB40356	EtOH_24h_intakeave_final	0.885698929
blast	GGB28382_SGB40962	EtOH_24h_intakeave_final	0.519311307
vns	GGB28382_SGB40962	EtOH_24h_intakeave_final	0.855057834
blast	GGB28399_SGB40980	EtOH_24h_intakeave_final	0.289465502
vns	GGB28399_SGB40980	EtOH_24h_intakeave_final	0.287117034
blast	GGB28411_SGB40993	EtOH_24h_intakeave_final	0.201802262
vns	GGB28411_SGB40993	EtOH_24h_intakeave_final	0.146848474
blast	GGB28415_SGB40997	EtOH_24h_intakeave_final	0.026726744
vns	GGB28415_SGB40997	EtOH_24h_intakeave_final	0.949051696
blast	GGB28430_SGB41013	EtOH_24h_intakeave_final	0.912070947
vns	GGB28430_SGB41013	EtOH_24h_intakeave_final	0.466250482
blast	GGB28439_SGB41022	EtOH_24h_intakeave_final	0.380444403
vns	GGB28439_SGB41022	EtOH_24h_intakeave_final	0.738709552
blast	GGB28778_SGB41431	EtOH_24h_intakeave_final	0.804698941
vns	GGB28778_SGB41431	EtOH_24h_intakeave_final	0.218453243
blast	GGB28784_SGB41437	EtOH_24h_intakeave_final	0.020726621
vns	GGB28784_SGB41437	EtOH_24h_intakeave_final	0.254456997
blast	GGB28792_SGB41445	EtOH_24h_intakeave_final	0.338577775
vns	GGB28792_SGB41445	EtOH_24h_intakeave_final	0.761244426
blast	GGB28798_SGB41451	EtOH_24h_intakeave_final	0.164022630
vns	GGB28798_SGB41451	EtOH_24h_intakeave_final	0.248915824

blast	GGB28802_SGB41455	EtOH_24h_intakeave_final	0.4578986259
vns	GGB28802_SGB41455	EtOH_24h_intakeave_final	0.342689298
blast	GGB28818_SGB41473	EtOH_24h_intakeave_final	0.7292095104
vns	GGB28818_SGB41473	EtOH_24h_intakeave_final	0.5694099484
blast	GGB28828_SGB41484	EtOH_24h_intakeave_final	0.2986969933
vns	GGB28828_SGB41484	EtOH_24h_intakeave_final	0.1067065352
blast	GGB28851_SGB41518	EtOH_24h_intakeave_final	0.852552179
vns	GGB28851_SGB41518	EtOH_24h_intakeave_final	0.5783084298
blast	GGB28859_SGB41528	EtOH_24h_intakeave_final	0.2057493298
vns	GGB28859_SGB41528	EtOH_24h_intakeave_final	0.5953667458
blast	GGB28864_SGB41535	EtOH_24h_intakeave_final	0.7854652488
vns	GGB28864_SGB41535	EtOH_24h_intakeave_final	0.9646589853
blast	GGB28869_SGB41543	EtOH_24h_intakeave_final	0.3479316259
vns	GGB28869_SGB41543	EtOH_24h_intakeave_final	0.3814774755
blast	GGB28883_SGB41564	EtOH_24h_intakeave_final	0.5948772487
vns	GGB28883_SGB41564	EtOH_24h_intakeave_final	0.0605244586
blast	GGB28892_SGB41573	EtOH_24h_intakeave_final	0.6512272078
vns	GGB28892_SGB41573	EtOH_24h_intakeave_final	0.6676188822
blast	GGB28893_SGB41574	EtOH_24h_intakeave_final	0.165594509
vns	GGB28893_SGB41574	EtOH_24h_intakeave_final	0.1826993761
blast	GGB28898_SGB41580	EtOH_24h_intakeave_final	0.3046549939
vns	GGB28898_SGB41580	EtOH_24h_intakeave_final	0.0828933309
blast	GGB28904_SGB41597	EtOH_24h_intakeave_final	0.1729063974
vns	GGB28904_SGB41597	EtOH_24h_intakeave_final	0.0560671999
blast	GGB28916_SGB41612	EtOH_24h_intakeave_final	0.8014326092
vns	GGB28916_SGB41612	EtOH_24h_intakeave_final	0.8538132969
blast	GGB28924_SGB41621	EtOH_24h_intakeave_final	0.7964816629
vns	GGB28924_SGB41621	EtOH_24h_intakeave_final	0.2786583787
blast	GGB28926_SGB41624	EtOH_24h_intakeave_final	0.3067808341
vns	GGB28926_SGB41624	EtOH_24h_intakeave_final	0.470765265
blast	GGB28927_SGB41625	EtOH_24h_intakeave_final	0.1937208057
vns	GGB28927_SGB41625	EtOH_24h_intakeave_final	0.4182433734
blast	GGB28934_SGB41635	EtOH_24h_intakeave_final	0.1158733118
vns	GGB28934_SGB41635	EtOH_24h_intakeave_final	0.1113740731
blast	GGB28946_SGB41652	EtOH_24h_intakeave_final	0.4278432256
vns	GGB28946_SGB41652	EtOH_24h_intakeave_final	0.4225889007
blast	GGB28949_SGB41655	EtOH_24h_intakeave_final	0.4436081164
vns	GGB28949_SGB41655	EtOH_24h_intakeave_final	0.9089481791
blast	GGB28949_SGB41656	EtOH_24h_intakeave_final	0.2827282346
vns	GGB28949_SGB41656	EtOH_24h_intakeave_final	0.7708639493
blast	GGB28950_SGB41657	EtOH_24h_intakeave_final	0.0305270365
vns	GGB28950_SGB41657	EtOH_24h_intakeave_final	0.3364589048
blast	GGB28951_SGB102295	EtOH_24h_intakeave_final	0.9253521562
vns	GGB28951_SGB102295	EtOH_24h_intakeave_final	0.1587903977
blast	GGB28951_SGB41658	EtOH_24h_intakeave_final	0.8470013515
vns	GGB28951_SGB41658	EtOH_24h_intakeave_final	0.9202366237

blast	GGB28954_SGB41662	EtOH_24h_intakeave_final	0.515758297
vns	GGB28954_SGB41662	EtOH_24h_intakeave_final	0.800484955
blast	GGB28956_SGB41665	EtOH_24h_intakeave_final	0.163329713
vns	GGB28956_SGB41665	EtOH_24h_intakeave_final	0.153416502
blast	GGB28960_SGB41669	EtOH_24h_intakeave_final	0.155727292
vns	GGB28960_SGB41669	EtOH_24h_intakeave_final	0.155957479
blast	GGB28967_SGB41678	EtOH_24h_intakeave_final	0.179269529
vns	GGB28967_SGB41678	EtOH_24h_intakeave_final	0.315046519
blast	GGB28991_SGB41705	EtOH_24h_intakeave_final	0.228893882
vns	GGB28991_SGB41705	EtOH_24h_intakeave_final	0.279982342
blast	GGB29002_SGB41718	EtOH_24h_intakeave_final	0.002430867
vns	GGB29002_SGB41718	EtOH_24h_intakeave_final	0.113465587
blast	GGB29003_SGB41719	EtOH_24h_intakeave_final	0.499398644
vns	GGB29003_SGB41719	EtOH_24h_intakeave_final	0.583967886
blast	GGB29011_SGB41731	EtOH_24h_intakeave_final	0.916276033
vns	GGB29011_SGB41731	EtOH_24h_intakeave_final	0.345599236
blast	GGB29531_SGB42317	EtOH_24h_intakeave_final	0.145082026
vns	GGB29531_SGB42317	EtOH_24h_intakeave_final	0.135569966
blast	GGB29685_SGB42494	EtOH_24h_intakeave_final	0.864543852
vns	GGB29685_SGB42494	EtOH_24h_intakeave_final	0.340104263
blast	GGB30141_SGB43066	EtOH_24h_intakeave_final	0.991554803
vns	GGB30141_SGB43066	EtOH_24h_intakeave_final	0.760178789
blast	GGB30286_SGB43248	EtOH_24h_intakeave_final	0.055261633
vns	GGB30286_SGB43248	EtOH_24h_intakeave_final	0.454603367
blast	GGB30303_SGB43268	EtOH_24h_intakeave_final	0.371936893
vns	GGB30303_SGB43268	EtOH_24h_intakeave_final	0.944722174
blast	GGB30413_SGB43452	EtOH_24h_intakeave_final	0.953276950
vns	GGB30413_SGB43452	EtOH_24h_intakeave_final	0.879642604
blast	GGB30454_SGB43514	EtOH_24h_intakeave_final	0.379858950
vns	GGB30454_SGB43514	EtOH_24h_intakeave_final	0.200125591
blast	GGB30455_SGB43519	EtOH_24h_intakeave_final	0.379467796
vns	GGB30455_SGB43519	EtOH_24h_intakeave_final	0.302348921
blast	GGB30461_SGB43527	EtOH_24h_intakeave_final	0.397503662
vns	GGB30461_SGB43527	EtOH_24h_intakeave_final	0.771038655
blast	GGB30461_SGB43530	EtOH_24h_intakeave_final	0.395449
vns	GGB30461_SGB43530	EtOH_24h_intakeave_final	0.703863693
blast	GGB30463_SGB43537	EtOH_24h_intakeave_final	0.210054257
vns	GGB30463_SGB43537	EtOH_24h_intakeave_final	0.985361479
blast	GGB30473_SGB43557	EtOH_24h_intakeave_final	0.020642166
vns	GGB30473_SGB43557	EtOH_24h_intakeave_final	0.056211743
blast	GGB30475_SGB63182	EtOH_24h_intakeave_final	0.287965349
vns	GGB30475_SGB63182	EtOH_24h_intakeave_final	0.660855930
blast	GGB30861_SGB44083	EtOH_24h_intakeave_final	0.957552379
vns	GGB30861_SGB44083	EtOH_24h_intakeave_final	0.833445529
blast	GGB31312_SGB44628	EtOH_24h_intakeave_final	0.711727128
vns	GGB31312_SGB44628	EtOH_24h_intakeave_final	0.884193943

blast	GGB31438_SGB44768	EtOH_24h_intakeave_final	0.906357297
vns	GGB31438_SGB44768	EtOH_24h_intakeave_final	0.318960086
blast	GGB3171_SGB4185	EtOH_24h_intakeave_final	0.085345767
vns	GGB3171_SGB4185	EtOH_24h_intakeave_final	0.918551780
blast	GGB31823_SGB45199	EtOH_24h_intakeave_final	0.188977717
vns	GGB31823_SGB45199	EtOH_24h_intakeave_final	0.702813258
blast	GGB31853_SGB45233	EtOH_24h_intakeave_final	0.496842743
vns	GGB31853_SGB45233	EtOH_24h_intakeave_final	0.396259541
blast	GGB32371_SGB41694	EtOH_24h_intakeave_final	0.346811850
vns	GGB32371_SGB41694	EtOH_24h_intakeave_final	0.025054092
blast	GGB3793_SGB5158	EtOH_24h_intakeave_final	0.965790474
vns	GGB3793_SGB5158	EtOH_24h_intakeave_final	0.975145151
blast	GGB42598_SGB59794	EtOH_24h_intakeave_final	0.501740001
vns	GGB42598_SGB59794	EtOH_24h_intakeave_final	0.156668604
blast	GGB45656_SGB63370	EtOH_24h_intakeave_final	0.621044138
vns	GGB45656_SGB63370	EtOH_24h_intakeave_final	0.963374601
blast	GGB47127_SGB65054	EtOH_24h_intakeave_final	0.496745120
vns	GGB47127_SGB65054	EtOH_24h_intakeave_final	0.257417312
blast	GGB74395_SGB43521	EtOH_24h_intakeave_final	0.123339782
vns	GGB74395_SGB43521	EtOH_24h_intakeave_final	0.204021498
blast	GGB75053_SGB43494	EtOH_24h_intakeave_final	0.410595919
vns	GGB75053_SGB43494	EtOH_24h_intakeave_final	0.571857456
blast	GGB75109_SGB102238	EtOH_24h_intakeave_final	0.620842292
vns	GGB75109_SGB102238	EtOH_24h_intakeave_final	0.871818408
blast	GGB81440_SGB45230	EtOH_24h_intakeave_final	0.079610731
vns	GGB81440_SGB45230	EtOH_24h_intakeave_final	0.098140579
blast	Lachnospiraceae_bacterium	EtOH_24h_intakeave_final	0.484816177
vns	Lachnospiraceae_bacterium	EtOH_24h_intakeave_final	0.537861332
blast	Lachnospiraceae_bacterium_A2	EtOH_24h_intakeave_final	0.420834896
vns	Lachnospiraceae_bacterium_A2	EtOH_24h_intakeave_final	0.323648328
blast	Lachnospiraceae_bacterium_MD308	EtOH_24h_intakeave_final	0.213119451
vns	Lachnospiraceae_bacterium_MD308	EtOH_24h_intakeave_final	0.083066965
blast	Lachnospiraceae_bacterium_MD329	EtOH_24h_intakeave_final	0.020976149
vns	Lachnospiraceae_bacterium_MD329	EtOH_24h_intakeave_final	0.699161119
blast	Lachnospiraceae_unclassified_SGB41414	EtOH_24h_intakeave_final	0.142639053
vns	Lachnospiraceae_unclassified_SGB41414	EtOH_24h_intakeave_final	0.119876468
blast	Lachnospiraceae_unclassified_SGB41418	EtOH_24h_intakeave_final	0.998757981
vns	Lachnospiraceae_unclassified_SGB41418	EtOH_24h_intakeave_final	0.712236573
blast	Lachnospiraceae_unclassified_SGB41424	EtOH_24h_intakeave_final	0.048386378
vns	Lachnospiraceae_unclassified_SGB41424	EtOH_24h_intakeave_final	0.046067357
blast	Lachnospiraceae_unclassified_SGB41589	EtOH_24h_intakeave_final	0.165424952
vns	Lachnospiraceae_unclassified_SGB41589	EtOH_24h_intakeave_final	0.273527700
blast	Lactobacillus_johnsonii	EtOH_24h_intakeave_final	0.182678453
vns	Lactobacillus_johnsonii	EtOH_24h_intakeave_final	0.672951588
blast	Muribaculaceae_bacterium	EtOH_24h_intakeave_final	0.841525252
vns	Muribaculaceae_bacterium	EtOH_24h_intakeave_final	0.893052980

blast	Neglectibacter_sp_X4	EtOH_24h_intakeave_final	0.0847821517
vns	Neglectibacter_sp_X4	EtOH_24h_intakeave_final	0.140656264
blast	Oscillospiraceae_bacterium	EtOH_24h_intakeave_final	0.7416725656
vns	Oscillospiraceae_bacterium	EtOH_24h_intakeave_final	0.856188039
blast	Oscillospiraceae_unclassified_SGB43502	EtOH_24h_intakeave_final	0.0259412852
vns	Oscillospiraceae_unclassified_SGB43502	EtOH_24h_intakeave_final	0.0305351331
blast	Oscillospiraceae_unclassified_SGB43505	EtOH_24h_intakeave_final	0.4959230188
vns	Oscillospiraceae_unclassified_SGB43505	EtOH_24h_intakeave_final	0.6425337316
blast	Parasutterella_excrementihominis	EtOH_24h_intakeave_final	0.5671048722
vns	Parasutterella_excrementihominis	EtOH_24h_intakeave_final	0.7099929171
blast	Richness (# observed features)	EtOH_24h_intakeave_final	0.2717524423
vns	Richness (# observed features)	EtOH_24h_intakeave_final	0.6607230685
blast	Romboutsia_ilealis	EtOH_24h_intakeave_final	0.0010997742
vns	Romboutsia_ilealis	EtOH_24h_intakeave_final	0.0569970896
blast	Schaedlerella_arabinosiphila	EtOH_24h_intakeave_final	0.9390244043
vns	Schaedlerella_arabinosiphila	EtOH_24h_intakeave_final	0.9139116075
blast	Shannon Index	EtOH_24h_intakeave_final	0.6947352216
vns	Shannon Index	EtOH_24h_intakeave_final	0.9062773413
blast	Turicibacter_sp_1E2	EtOH_24h_intakeave_final	0.1991772312
vns	Turicibacter_sp_1E2	EtOH_24h_intakeave_final	0.1655749312
blast	Acetatifactor_muris	EtOH_8pm_intakeave_final	0.7395220796
vns	Acetatifactor_muris	EtOH_8pm_intakeave_final	0.7528971403
blast	Acetatifactor_SGB41546	EtOH_8pm_intakeave_final	0.8402457971
vns	Acetatifactor_SGB41546	EtOH_8pm_intakeave_final	0.6630009771
blast	Acutalibacter_muris	EtOH_8pm_intakeave_final	0.4221546334
vns	Acutalibacter_muris	EtOH_8pm_intakeave_final	0.8105784495
blast	Acutalibacter_sp_1XD8_36	EtOH_8pm_intakeave_final	0.6605798863
vns	Acutalibacter_sp_1XD8_36	EtOH_8pm_intakeave_final	0.7091849336
blast	Adlercreutzia_caecimuris	EtOH_8pm_intakeave_final	0.9896089529
vns	Adlercreutzia_caecimuris	EtOH_8pm_intakeave_final	0.6765426913
blast	Adlercreutzia_mucosicola	EtOH_8pm_intakeave_final	0.2718339856
vns	Adlercreutzia_mucosicola	EtOH_8pm_intakeave_final	0.2498256574
blast	Adlercreutzia_muris	EtOH_8pm_intakeave_final	0.6704217743
vns	Adlercreutzia_muris	EtOH_8pm_intakeave_final	0.5958277454
blast	Akkermansia_muciniphila	EtOH_8pm_intakeave_final	0.1698125404
vns	Akkermansia_muciniphila	EtOH_8pm_intakeave_final	0.8643625099
blast	Alistipes_sp_DSM_112343	EtOH_8pm_intakeave_final	0.3725159774
vns	Alistipes_sp_DSM_112343	EtOH_8pm_intakeave_final	0.8326983074
blast	Anaerotruncus_sp_1XD42_93	EtOH_8pm_intakeave_final	0.5202714535
vns	Anaerotruncus_sp_1XD42_93	EtOH_8pm_intakeave_final	0.6174107217
blast	Bacteria_unclassified_SGB102200	EtOH_8pm_intakeave_final	0.4742692127
vns	Bacteria_unclassified_SGB102200	EtOH_8pm_intakeave_final	0.7641667885
blast	Bacteria_unclassified_SGB41677	EtOH_8pm_intakeave_final	0.4441438231
vns	Bacteria_unclassified_SGB41677	EtOH_8pm_intakeave_final	0.1699894243
blast	Bacteria_unclassified_SGB43546	EtOH_8pm_intakeave_final	0.5863583659
vns	Bacteria_unclassified_SGB43546	EtOH_8pm_intakeave_final	0.7890099125

blast	bacterium_1XD42_54	EtOH_8pm_intakeave_final	0.0144245959
vns	bacterium_1XD42_54	EtOH_8pm_intakeave_final	0.2345775748
blast	bacterium_1XD42_76	EtOH_8pm_intakeave_final	0.6000316038
vns	bacterium_1XD42_76	EtOH_8pm_intakeave_final	0.6429924539
blast	bacterium_1xD8_48	EtOH_8pm_intakeave_final	0.8997089453
vns	bacterium_1xD8_48	EtOH_8pm_intakeave_final	0.6968168809
blast	Bacteroides_thetaiotaomicron	EtOH_8pm_intakeave_final	0.9602014777
vns	Bacteroides_thetaiotaomicron	EtOH_8pm_intakeave_final	0.6343063159
blast	Berger Parker Index	EtOH_8pm_intakeave_final	0.5457417127
vns	Berger Parker Index	EtOH_8pm_intakeave_final	0.2337330546
blast	Bifidobacterium_pseudolongum	EtOH_8pm_intakeave_final	0.4280103985
vns	Bifidobacterium_pseudolongum	EtOH_8pm_intakeave_final	0.3477990653
blast	Clostridia_bacterium	EtOH_8pm_intakeave_final	0.3745590434
vns	Clostridia_bacterium	EtOH_8pm_intakeave_final	0.7249401071
blast	Clostridiaceae_bacterium	EtOH_8pm_intakeave_final	0.9871697806
vns	Clostridiaceae_bacterium	EtOH_8pm_intakeave_final	0.2419370425
blast	Clostridiaceae_unclassified_SGB41663	EtOH_8pm_intakeave_final	0.780382224
vns	Clostridiaceae_unclassified_SGB41663	EtOH_8pm_intakeave_final	0.719154368
blast	Clostridiales_bacterium	EtOH_8pm_intakeave_final	0.0023319564
vns	Clostridiales_bacterium	EtOH_8pm_intakeave_final	0.2964785018
blast	Clostridium_cocleatum	EtOH_8pm_intakeave_final	0.0115678325
vns	Clostridium_cocleatum	EtOH_8pm_intakeave_final	0.1960355146
blast	Coriobacteriaceae_bacterium	EtOH_8pm_intakeave_final	0.3724483697
vns	Coriobacteriaceae_bacterium	EtOH_8pm_intakeave_final	0.953919137
blast	Dorea_sp_5_2	EtOH_8pm_intakeave_final	0.5884919176
vns	Dorea_sp_5_2	EtOH_8pm_intakeave_final	0.4214161244
blast	Dubosiella_newyorkensis	EtOH_8pm_intakeave_final	0.1169987394
vns	Dubosiella_newyorkensis	EtOH_8pm_intakeave_final	0.799186063
blast	Erysipelotrichales_bacterium	EtOH_8pm_intakeave_final	0.8970006656
vns	Erysipelotrichales_bacterium	EtOH_8pm_intakeave_final	0.3284081083
blast	Eubacteriaceae_bacterium	EtOH_8pm_intakeave_final	0.0271233687
vns	Eubacteriaceae_bacterium	EtOH_8pm_intakeave_final	0.2383186135
blast	Eubacteriaceae_unclassified_SGB94922	EtOH_8pm_intakeave_final	0.4594880799
vns	Eubacteriaceae_unclassified_SGB94922	EtOH_8pm_intakeave_final	0.3726201484
blast	GGB20149_SGB29430	EtOH_8pm_intakeave_final	0.6280700484
vns	GGB20149_SGB29430	EtOH_8pm_intakeave_final	0.6299827926
blast	GGB22635_SGB63107	EtOH_8pm_intakeave_final	0.4117103362
vns	GGB22635_SGB63107	EtOH_8pm_intakeave_final	0.8871521383
blast	GGB25041_SGB36960	EtOH_8pm_intakeave_final	0.0824966676
vns	GGB25041_SGB36960	EtOH_8pm_intakeave_final	0.2101871969
blast	GGB27876_SGB40310	EtOH_8pm_intakeave_final	0.164666135
vns	GGB27876_SGB40310	EtOH_8pm_intakeave_final	0.7672351949
blast	GGB27878_SGB40312	EtOH_8pm_intakeave_final	0.2133541578
vns	GGB27878_SGB40312	EtOH_8pm_intakeave_final	0.1474794298
blast	GGB27918_SGB40356	EtOH_8pm_intakeave_final	0.9587581408
vns	GGB27918_SGB40356	EtOH_8pm_intakeave_final	0.8856989297

blast	GGB28382_SGB40962	EtOH_8pm_intakeave_final	0.519311307
vns	GGB28382_SGB40962	EtOH_8pm_intakeave_final	0.855057834
blast	GGB28399_SGB40980	EtOH_8pm_intakeave_final	0.289465502
vns	GGB28399_SGB40980	EtOH_8pm_intakeave_final	0.287117034
blast	GGB28411_SGB40993	EtOH_8pm_intakeave_final	0.201802262
vns	GGB28411_SGB40993	EtOH_8pm_intakeave_final	0.146848474
blast	GGB28415_SGB40997	EtOH_8pm_intakeave_final	0.026726744
vns	GGB28415_SGB40997	EtOH_8pm_intakeave_final	0.949051696
blast	GGB28430_SGB41013	EtOH_8pm_intakeave_final	0.912070947
vns	GGB28430_SGB41013	EtOH_8pm_intakeave_final	0.466250482
blast	GGB28439_SGB41022	EtOH_8pm_intakeave_final	0.380444403
vns	GGB28439_SGB41022	EtOH_8pm_intakeave_final	0.738709552
blast	GGB28778_SGB41431	EtOH_8pm_intakeave_final	0.804698941
vns	GGB28778_SGB41431	EtOH_8pm_intakeave_final	0.218453243
blast	GGB28784_SGB41437	EtOH_8pm_intakeave_final	0.020726621
vns	GGB28784_SGB41437	EtOH_8pm_intakeave_final	0.254456997
blast	GGB28792_SGB41445	EtOH_8pm_intakeave_final	0.338577775
vns	GGB28792_SGB41445	EtOH_8pm_intakeave_final	0.761244426
blast	GGB28798_SGB41451	EtOH_8pm_intakeave_final	0.164022630
vns	GGB28798_SGB41451	EtOH_8pm_intakeave_final	0.248915824
blast	GGB28802_SGB41455	EtOH_8pm_intakeave_final	0.457898625
vns	GGB28802_SGB41455	EtOH_8pm_intakeave_final	0.342689298
blast	GGB28818_SGB41473	EtOH_8pm_intakeave_final	0.729209510
vns	GGB28818_SGB41473	EtOH_8pm_intakeave_final	0.569409948
blast	GGB28828_SGB41484	EtOH_8pm_intakeave_final	0.298696993
vns	GGB28828_SGB41484	EtOH_8pm_intakeave_final	0.106706535
blast	GGB28851_SGB41518	EtOH_8pm_intakeave_final	0.852552179
vns	GGB28851_SGB41518	EtOH_8pm_intakeave_final	0.578308429
blast	GGB28859_SGB41528	EtOH_8pm_intakeave_final	0.205749329
vns	GGB28859_SGB41528	EtOH_8pm_intakeave_final	0.595366745
blast	GGB28864_SGB41535	EtOH_8pm_intakeave_final	0.785465248
vns	GGB28864_SGB41535	EtOH_8pm_intakeave_final	0.964658985
blast	GGB28869_SGB41543	EtOH_8pm_intakeave_final	0.347931625
vns	GGB28869_SGB41543	EtOH_8pm_intakeave_final	0.381477475
blast	GGB28883_SGB41564	EtOH_8pm_intakeave_final	0.594877248
vns	GGB28883_SGB41564	EtOH_8pm_intakeave_final	0.060524458
blast	GGB28892_SGB41573	EtOH_8pm_intakeave_final	0.651227207
vns	GGB28892_SGB41573	EtOH_8pm_intakeave_final	0.667618882
blast	GGB28893_SGB41574	EtOH_8pm_intakeave_final	0.165594509
vns	GGB28893_SGB41574	EtOH_8pm_intakeave_final	0.182699376
blast	GGB28898_SGB41580	EtOH_8pm_intakeave_final	0.304654993
vns	GGB28898_SGB41580	EtOH_8pm_intakeave_final	0.082893330
blast	GGB28904_SGB41597	EtOH_8pm_intakeave_final	0.172906397
vns	GGB28904_SGB41597	EtOH_8pm_intakeave_final	0.056067199
blast	GGB28916_SGB41612	EtOH_8pm_intakeave_final	0.801432609
vns	GGB28916_SGB41612	EtOH_8pm_intakeave_final	0.853813296

blast	GGB28924_SGB41621	EtOH_8pm_intakeave_final	0.7964816629
vns	GGB28924_SGB41621	EtOH_8pm_intakeave_final	0.2786583787
blast	GGB28926_SGB41624	EtOH_8pm_intakeave_final	0.3067808341
vns	GGB28926_SGB41624	EtOH_8pm_intakeave_final	0.470765265
blast	GGB28927_SGB41625	EtOH_8pm_intakeave_final	0.1937208057
vns	GGB28927_SGB41625	EtOH_8pm_intakeave_final	0.4182433734
blast	GGB28934_SGB41635	EtOH_8pm_intakeave_final	0.1158733118
vns	GGB28934_SGB41635	EtOH_8pm_intakeave_final	0.1113740731
blast	GGB28946_SGB41652	EtOH_8pm_intakeave_final	0.4278432256
vns	GGB28946_SGB41652	EtOH_8pm_intakeave_final	0.4225889007
blast	GGB28949_SGB41655	EtOH_8pm_intakeave_final	0.4436081164
vns	GGB28949_SGB41655	EtOH_8pm_intakeave_final	0.9089481791
blast	GGB28949_SGB41656	EtOH_8pm_intakeave_final	0.2827282346
vns	GGB28949_SGB41656	EtOH_8pm_intakeave_final	0.7708639493
blast	GGB28950_SGB41657	EtOH_8pm_intakeave_final	0.0305270365
vns	GGB28950_SGB41657	EtOH_8pm_intakeave_final	0.3364589048
blast	GGB28951_SGB102295	EtOH_8pm_intakeave_final	0.9253521562
vns	GGB28951_SGB102295	EtOH_8pm_intakeave_final	0.1587903977
blast	GGB28951_SGB41658	EtOH_8pm_intakeave_final	0.8470013515
vns	GGB28951_SGB41658	EtOH_8pm_intakeave_final	0.9202366237
blast	GGB28954_SGB41662	EtOH_8pm_intakeave_final	0.5157582975
vns	GGB28954_SGB41662	EtOH_8pm_intakeave_final	0.8004849551
blast	GGB28956_SGB41665	EtOH_8pm_intakeave_final	0.163329713
vns	GGB28956_SGB41665	EtOH_8pm_intakeave_final	0.1534165023
blast	GGB28960_SGB41669	EtOH_8pm_intakeave_final	0.155727292
vns	GGB28960_SGB41669	EtOH_8pm_intakeave_final	0.1559574792
blast	GGB28967_SGB41678	EtOH_8pm_intakeave_final	0.1792695292
vns	GGB28967_SGB41678	EtOH_8pm_intakeave_final	0.3150465193
blast	GGB28991_SGB41705	EtOH_8pm_intakeave_final	0.2288938824
vns	GGB28991_SGB41705	EtOH_8pm_intakeave_final	0.279982342
blast	GGB29002_SGB41718	EtOH_8pm_intakeave_final	0.0024308675
vns	GGB29002_SGB41718	EtOH_8pm_intakeave_final	0.1134655877
blast	GGB29003_SGB41719	EtOH_8pm_intakeave_final	0.4993986443
vns	GGB29003_SGB41719	EtOH_8pm_intakeave_final	0.5839678864
blast	GGB29011_SGB41731	EtOH_8pm_intakeave_final	0.916276033
vns	GGB29011_SGB41731	EtOH_8pm_intakeave_final	0.3455992366
blast	GGB29531_SGB42317	EtOH_8pm_intakeave_final	0.1450820268
vns	GGB29531_SGB42317	EtOH_8pm_intakeave_final	0.1355699662
blast	GGB29685_SGB42494	EtOH_8pm_intakeave_final	0.8645438528
vns	GGB29685_SGB42494	EtOH_8pm_intakeave_final	0.340104263
blast	GGB30141_SGB43066	EtOH_8pm_intakeave_final	0.9915548039
vns	GGB30141_SGB43066	EtOH_8pm_intakeave_final	0.7601787897
blast	GGB30286_SGB43248	EtOH_8pm_intakeave_final	0.0552616333
vns	GGB30286_SGB43248	EtOH_8pm_intakeave_final	0.4546033673
blast	GGB30303_SGB43268	EtOH_8pm_intakeave_final	0.3719368939
vns	GGB30303_SGB43268	EtOH_8pm_intakeave_final	0.9447221743

blast	GGB30413_SGB43452	EtOH_8pm_intakeave_final	0.953276950
vns	GGB30413_SGB43452	EtOH_8pm_intakeave_final	0.879642604
blast	GGB30454_SGB43514	EtOH_8pm_intakeave_final	0.379858950
vns	GGB30454_SGB43514	EtOH_8pm_intakeave_final	0.200125591
blast	GGB30455_SGB43519	EtOH_8pm_intakeave_final	0.379467796
vns	GGB30455_SGB43519	EtOH_8pm_intakeave_final	0.302348921
blast	GGB30461_SGB43527	EtOH_8pm_intakeave_final	0.397503662
vns	GGB30461_SGB43527	EtOH_8pm_intakeave_final	0.771038655
blast	GGB30461_SGB43530	EtOH_8pm_intakeave_final	0.395449
vns	GGB30461_SGB43530	EtOH_8pm_intakeave_final	0.703863693
blast	GGB30463_SGB43537	EtOH_8pm_intakeave_final	0.210054257
vns	GGB30463_SGB43537	EtOH_8pm_intakeave_final	0.985361479
blast	GGB30473_SGB43557	EtOH_8pm_intakeave_final	0.020642166
vns	GGB30473_SGB43557	EtOH_8pm_intakeave_final	0.056211743
blast	GGB30475_SGB63182	EtOH_8pm_intakeave_final	0.287965349
vns	GGB30475_SGB63182	EtOH_8pm_intakeave_final	0.660855930
blast	GGB30861_SGB44083	EtOH_8pm_intakeave_final	0.957552379
vns	GGB30861_SGB44083	EtOH_8pm_intakeave_final	0.833445529
blast	GGB31312_SGB44628	EtOH_8pm_intakeave_final	0.711727128
vns	GGB31312_SGB44628	EtOH_8pm_intakeave_final	0.884193943
blast	GGB31438_SGB44768	EtOH_8pm_intakeave_final	0.906357297
vns	GGB31438_SGB44768	EtOH_8pm_intakeave_final	0.318960086
blast	GGB3171_SGB4185	EtOH_8pm_intakeave_final	0.085345767
vns	GGB3171_SGB4185	EtOH_8pm_intakeave_final	0.918551780
blast	GGB31823_SGB45199	EtOH_8pm_intakeave_final	0.188977717
vns	GGB31823_SGB45199	EtOH_8pm_intakeave_final	0.702813258
blast	GGB31853_SGB45233	EtOH_8pm_intakeave_final	0.496842743
vns	GGB31853_SGB45233	EtOH_8pm_intakeave_final	0.396259541
blast	GGB32371_SGB41694	EtOH_8pm_intakeave_final	0.346811850
vns	GGB32371_SGB41694	EtOH_8pm_intakeave_final	0.025054092
blast	GGB3793_SGB5158	EtOH_8pm_intakeave_final	0.965790474
vns	GGB3793_SGB5158	EtOH_8pm_intakeave_final	0.975145151
blast	GGB42598_SGB59794	EtOH_8pm_intakeave_final	0.501740001
vns	GGB42598_SGB59794	EtOH_8pm_intakeave_final	0.156668604
blast	GGB45656_SGB63370	EtOH_8pm_intakeave_final	0.621044138
vns	GGB45656_SGB63370	EtOH_8pm_intakeave_final	0.963374601
blast	GGB47127_SGB65054	EtOH_8pm_intakeave_final	0.496745120
vns	GGB47127_SGB65054	EtOH_8pm_intakeave_final	0.257417312
blast	GGB74395_SGB43521	EtOH_8pm_intakeave_final	0.123339782
vns	GGB74395_SGB43521	EtOH_8pm_intakeave_final	0.204021498
blast	GGB75053_SGB43494	EtOH_8pm_intakeave_final	0.410595919
vns	GGB75053_SGB43494	EtOH_8pm_intakeave_final	0.571857456
blast	GGB75109_SGB102238	EtOH_8pm_intakeave_final	0.620842292
vns	GGB75109_SGB102238	EtOH_8pm_intakeave_final	0.871818408
blast	GGB81440_SGB45230	EtOH_8pm_intakeave_final	0.079610731
vns	GGB81440_SGB45230	EtOH_8pm_intakeave_final	0.098140579

blast	Lachnospiraceae_bacterium	EtOH_8pm_intakeave_final	0.484816177
vns	Lachnospiraceae_bacterium	EtOH_8pm_intakeave_final	0.537861332
blast	Lachnospiraceae_bacterium_A2	EtOH_8pm_intakeave_final	0.420834896
vns	Lachnospiraceae_bacterium_A2	EtOH_8pm_intakeave_final	0.323648328
blast	Lachnospiraceae_bacterium_MD308	EtOH_8pm_intakeave_final	0.213119451
vns	Lachnospiraceae_bacterium_MD308	EtOH_8pm_intakeave_final	0.083066965
blast	Lachnospiraceae_bacterium_MD329	EtOH_8pm_intakeave_final	0.020976149
vns	Lachnospiraceae_bacterium_MD329	EtOH_8pm_intakeave_final	0.699161119
blast	Lachnospiraceae_unclassified_SGB41414	EtOH_8pm_intakeave_final	0.142639053
vns	Lachnospiraceae_unclassified_SGB41414	EtOH_8pm_intakeave_final	0.119876468
blast	Lachnospiraceae_unclassified_SGB41418	EtOH_8pm_intakeave_final	0.998757981
vns	Lachnospiraceae_unclassified_SGB41418	EtOH_8pm_intakeave_final	0.712236573
blast	Lachnospiraceae_unclassified_SGB41424	EtOH_8pm_intakeave_final	0.048386378
vns	Lachnospiraceae_unclassified_SGB41424	EtOH_8pm_intakeave_final	0.046067357
blast	Lachnospiraceae_unclassified_SGB41589	EtOH_8pm_intakeave_final	0.165424952
vns	Lachnospiraceae_unclassified_SGB41589	EtOH_8pm_intakeave_final	0.273527700
blast	Lactobacillus_johnsonii	EtOH_8pm_intakeave_final	0.182678453
vns	Lactobacillus_johnsonii	EtOH_8pm_intakeave_final	0.672951588
blast	Muribaculaceae_bacterium	EtOH_8pm_intakeave_final	0.841525252
vns	Muribaculaceae_bacterium	EtOH_8pm_intakeave_final	0.893052980
blast	Neglectibacter_sp_X4	EtOH_8pm_intakeave_final	0.084782151
vns	Neglectibacter_sp_X4	EtOH_8pm_intakeave_final	0.140656264
blast	Oscillospiraceae_bacterium	EtOH_8pm_intakeave_final	0.741672565
vns	Oscillospiraceae_bacterium	EtOH_8pm_intakeave_final	0.856188039
blast	Oscillospiraceae_unclassified_SGB43502	EtOH_8pm_intakeave_final	0.025941285
vns	Oscillospiraceae_unclassified_SGB43502	EtOH_8pm_intakeave_final	0.030535133
blast	Oscillospiraceae_unclassified_SGB43505	EtOH_8pm_intakeave_final	0.495923018
vns	Oscillospiraceae_unclassified_SGB43505	EtOH_8pm_intakeave_final	0.642533731
blast	Parasutterella_excrementihominis	EtOH_8pm_intakeave_final	0.567104872
vns	Parasutterella_excrementihominis	EtOH_8pm_intakeave_final	0.709992917
blast	Richness (# observed features)	EtOH_8pm_intakeave_final	0.271752442
vns	Richness (# observed features)	EtOH_8pm_intakeave_final	0.660723068
blast	Romboutsia_ilealis	EtOH_8pm_intakeave_final	0.001099774
vns	Romboutsia_ilealis	EtOH_8pm_intakeave_final	0.056997089
blast	Schaedlerella_arabinosiphila	EtOH_8pm_intakeave_final	0.939024404
vns	Schaedlerella_arabinosiphila	EtOH_8pm_intakeave_final	0.913911607
blast	Shannon Index	EtOH_8pm_intakeave_final	0.694735221
vns	Shannon Index	EtOH_8pm_intakeave_final	0.906277341
blast	Turicibacter_sp_1E2	EtOH_8pm_intakeave_final	0.199177231
vns	Turicibacter_sp_1E2	EtOH_8pm_intakeave_final	0.165574931
blast	Acetatifactor_muris	EtOH_pref_24h_final	0.739522079
vns	Acetatifactor_muris	EtOH_pref_24h_final	0.752897140
blast	Acetatifactor_SGB41546	EtOH_pref_24h_final	0.840245797
vns	Acetatifactor_SGB41546	EtOH_pref_24h_final	0.663000977
blast	Acutalibacter_muris	EtOH_pref_24h_final	0.422154633
vns	Acutalibacter_muris	EtOH_pref_24h_final	0.810578449

blast	Acutalibacter_sp_1XD8_36	EtOH_pref_24h_final	0.6605798863
vns	Acutalibacter_sp_1XD8_36	EtOH_pref_24h_final	0.7091849336
blast	Adlercreutzia_caecimuris	EtOH_pref_24h_final	0.9896089529
vns	Adlercreutzia_caecimuris	EtOH_pref_24h_final	0.6765426913
blast	Adlercreutzia_mucosicola	EtOH_pref_24h_final	0.2718339856
vns	Adlercreutzia_mucosicola	EtOH_pref_24h_final	0.2498256574
blast	Adlercreutzia_muris	EtOH_pref_24h_final	0.6704217743
vns	Adlercreutzia_muris	EtOH_pref_24h_final	0.5958277454
blast	Akkermansia_muciniphila	EtOH_pref_24h_final	0.1698125404
vns	Akkermansia_muciniphila	EtOH_pref_24h_final	0.8643625099
blast	Alistipes_sp_DSM_112343	EtOH_pref_24h_final	0.3725159774
vns	Alistipes_sp_DSM_112343	EtOH_pref_24h_final	0.8326983074
blast	Anaerotruncus_sp_1XD42_93	EtOH_pref_24h_final	0.5202714535
vns	Anaerotruncus_sp_1XD42_93	EtOH_pref_24h_final	0.6174107217
blast	Bacteria_unclassified_SGB102200	EtOH_pref_24h_final	0.4742692127
vns	Bacteria_unclassified_SGB102200	EtOH_pref_24h_final	0.7641667885
blast	Bacteria_unclassified_SGB41677	EtOH_pref_24h_final	0.4441438231
vns	Bacteria_unclassified_SGB41677	EtOH_pref_24h_final	0.1699894243
blast	Bacteria_unclassified_SGB43546	EtOH_pref_24h_final	0.5863583659
vns	Bacteria_unclassified_SGB43546	EtOH_pref_24h_final	0.7890099125
blast	bacterium_1XD42_54	EtOH_pref_24h_final	0.0144245959
vns	bacterium_1XD42_54	EtOH_pref_24h_final	0.2345775748
blast	bacterium_1XD42_76	EtOH_pref_24h_final	0.6000316038
vns	bacterium_1XD42_76	EtOH_pref_24h_final	0.6429924539
blast	bacterium_1xD8_48	EtOH_pref_24h_final	0.8997089453
vns	bacterium_1xD8_48	EtOH_pref_24h_final	0.6968168809
blast	Bacteroides_thetaiotaomicron	EtOH_pref_24h_final	0.9602014777
vns	Bacteroides_thetaiotaomicron	EtOH_pref_24h_final	0.6343063159
blast	Berger Parker Index	EtOH_pref_24h_final	0.5457417127
vns	Berger Parker Index	EtOH_pref_24h_final	0.2337330546
blast	Bifidobacterium_pseudolongum	EtOH_pref_24h_final	0.4280103985
vns	Bifidobacterium_pseudolongum	EtOH_pref_24h_final	0.3477990653
blast	Clostridia_bacterium	EtOH_pref_24h_final	0.3745590434
vns	Clostridia_bacterium	EtOH_pref_24h_final	0.7249401071
blast	Clostridiaceae_bacterium	EtOH_pref_24h_final	0.9871697806
vns	Clostridiaceae_bacterium	EtOH_pref_24h_final	0.2419370425
blast	Clostridiaceae_unclassified_SGB41663	EtOH_pref_24h_final	0.780382224
vns	Clostridiaceae_unclassified_SGB41663	EtOH_pref_24h_final	0.719154368
blast	Clostridiales_bacterium	EtOH_pref_24h_final	0.0023319564
vns	Clostridiales_bacterium	EtOH_pref_24h_final	0.2964785018
blast	Clostridium_cocleatum	EtOH_pref_24h_final	0.0115678325
vns	Clostridium_cocleatum	EtOH_pref_24h_final	0.1960355146
blast	Coriobacteriaceae_bacterium	EtOH_pref_24h_final	0.3724483697
vns	Coriobacteriaceae_bacterium	EtOH_pref_24h_final	0.953919137
blast	Dorea_sp_5_2	EtOH_pref_24h_final	0.5884919176
vns	Dorea_sp_5_2	EtOH_pref_24h_final	0.4214161244

blast	Dubosiella_newyorkensis	EtOH_pref_24h_final	0.1169987394
vns	Dubosiella_newyorkensis	EtOH_pref_24h_final	0.799186063
blast	Erysipelotrichales_bacterium	EtOH_pref_24h_final	0.8970006656
vns	Erysipelotrichales_bacterium	EtOH_pref_24h_final	0.3284081083
blast	Eubacteriaceae_bacterium	EtOH_pref_24h_final	0.0271233687
vns	Eubacteriaceae_bacterium	EtOH_pref_24h_final	0.2383186135
blast	Eubacteriaceae_unclassified_SGB94922	EtOH_pref_24h_final	0.4594880799
vns	Eubacteriaceae_unclassified_SGB94922	EtOH_pref_24h_final	0.3726201484
blast	GGB20149_SGB29430	EtOH_pref_24h_final	0.6280700484
vns	GGB20149_SGB29430	EtOH_pref_24h_final	0.6299827926
blast	GGB22635_SGB63107	EtOH_pref_24h_final	0.4117103362
vns	GGB22635_SGB63107	EtOH_pref_24h_final	0.8871521383
blast	GGB25041_SGB36960	EtOH_pref_24h_final	0.0824966676
vns	GGB25041_SGB36960	EtOH_pref_24h_final	0.2101871969
blast	GGB27876_SGB40310	EtOH_pref_24h_final	0.164666135
vns	GGB27876_SGB40310	EtOH_pref_24h_final	0.7672351949
blast	GGB27878_SGB40312	EtOH_pref_24h_final	0.2133541578
vns	GGB27878_SGB40312	EtOH_pref_24h_final	0.1474794298
blast	GGB27918_SGB40356	EtOH_pref_24h_final	0.9587581408
vns	GGB27918_SGB40356	EtOH_pref_24h_final	0.8856989297
blast	GGB28382_SGB40962	EtOH_pref_24h_final	0.5193113079
vns	GGB28382_SGB40962	EtOH_pref_24h_final	0.8550578348
blast	GGB28399_SGB40980	EtOH_pref_24h_final	0.2894655025
vns	GGB28399_SGB40980	EtOH_pref_24h_final	0.287117034
blast	GGB28411_SGB40993	EtOH_pref_24h_final	0.2018022626
vns	GGB28411_SGB40993	EtOH_pref_24h_final	0.1468484741
blast	GGB28415_SGB40997	EtOH_pref_24h_final	0.0267267446
vns	GGB28415_SGB40997	EtOH_pref_24h_final	0.9490516966
blast	GGB28430_SGB41013	EtOH_pref_24h_final	0.9120709472
vns	GGB28430_SGB41013	EtOH_pref_24h_final	0.4662504826
blast	GGB28439_SGB41022	EtOH_pref_24h_final	0.3804444035
vns	GGB28439_SGB41022	EtOH_pref_24h_final	0.7387095529
blast	GGB28778_SGB41431	EtOH_pref_24h_final	0.8046989411
vns	GGB28778_SGB41431	EtOH_pref_24h_final	0.2184532434
blast	GGB28784_SGB41437	EtOH_pref_24h_final	0.0207266217
vns	GGB28784_SGB41437	EtOH_pref_24h_final	0.2544569976
blast	GGB28792_SGB41445	EtOH_pref_24h_final	0.3385777757
vns	GGB28792_SGB41445	EtOH_pref_24h_final	0.7612444262
blast	GGB28798_SGB41451	EtOH_pref_24h_final	0.1640226305
vns	GGB28798_SGB41451	EtOH_pref_24h_final	0.2489158244
blast	GGB28802_SGB41455	EtOH_pref_24h_final	0.4578986259
vns	GGB28802_SGB41455	EtOH_pref_24h_final	0.342689298
blast	GGB28818_SGB41473	EtOH_pref_24h_final	0.7292095104
vns	GGB28818_SGB41473	EtOH_pref_24h_final	0.5694099484
blast	GGB28828_SGB41484	EtOH_pref_24h_final	0.2986969933
vns	GGB28828_SGB41484	EtOH_pref_24h_final	0.1067065352

blast	GGB28851_SGB41518	EtOH_pref_24h_final	0.852552179
vns	GGB28851_SGB41518	EtOH_pref_24h_final	0.5783084298
blast	GGB28859_SGB41528	EtOH_pref_24h_final	0.2057493298
vns	GGB28859_SGB41528	EtOH_pref_24h_final	0.5953667458
blast	GGB28864_SGB41535	EtOH_pref_24h_final	0.7854652488
vns	GGB28864_SGB41535	EtOH_pref_24h_final	0.9646589853
blast	GGB28869_SGB41543	EtOH_pref_24h_final	0.3479316259
vns	GGB28869_SGB41543	EtOH_pref_24h_final	0.3814774755
blast	GGB28883_SGB41564	EtOH_pref_24h_final	0.5948772487
vns	GGB28883_SGB41564	EtOH_pref_24h_final	0.0605244586
blast	GGB28892_SGB41573	EtOH_pref_24h_final	0.6512272078
vns	GGB28892_SGB41573	EtOH_pref_24h_final	0.6676188822
blast	GGB28893_SGB41574	EtOH_pref_24h_final	0.165594509
vns	GGB28893_SGB41574	EtOH_pref_24h_final	0.1826993761
blast	GGB28898_SGB41580	EtOH_pref_24h_final	0.3046549939
vns	GGB28898_SGB41580	EtOH_pref_24h_final	0.0828933309
blast	GGB28904_SGB41597	EtOH_pref_24h_final	0.1729063974
vns	GGB28904_SGB41597	EtOH_pref_24h_final	0.0560671999
blast	GGB28916_SGB41612	EtOH_pref_24h_final	0.8014326092
vns	GGB28916_SGB41612	EtOH_pref_24h_final	0.8538132969
blast	GGB28924_SGB41621	EtOH_pref_24h_final	0.7964816629
vns	GGB28924_SGB41621	EtOH_pref_24h_final	0.2786583787
blast	GGB28926_SGB41624	EtOH_pref_24h_final	0.3067808341
vns	GGB28926_SGB41624	EtOH_pref_24h_final	0.470765265
blast	GGB28927_SGB41625	EtOH_pref_24h_final	0.1937208057
vns	GGB28927_SGB41625	EtOH_pref_24h_final	0.4182433734
blast	GGB28934_SGB41635	EtOH_pref_24h_final	0.1158733118
vns	GGB28934_SGB41635	EtOH_pref_24h_final	0.1113740731
blast	GGB28946_SGB41652	EtOH_pref_24h_final	0.4278432256
vns	GGB28946_SGB41652	EtOH_pref_24h_final	0.4225889007
blast	GGB28949_SGB41655	EtOH_pref_24h_final	0.4436081164
vns	GGB28949_SGB41655	EtOH_pref_24h_final	0.9089481791
blast	GGB28949_SGB41656	EtOH_pref_24h_final	0.2827282346
vns	GGB28949_SGB41656	EtOH_pref_24h_final	0.7708639493
blast	GGB28950_SGB41657	EtOH_pref_24h_final	0.0305270365
vns	GGB28950_SGB41657	EtOH_pref_24h_final	0.3364589048
blast	GGB28951_SGB102295	EtOH_pref_24h_final	0.9253521562
vns	GGB28951_SGB102295	EtOH_pref_24h_final	0.1587903977
blast	GGB28951_SGB41658	EtOH_pref_24h_final	0.8470013515
vns	GGB28951_SGB41658	EtOH_pref_24h_final	0.9202366237
blast	GGB28954_SGB41662	EtOH_pref_24h_final	0.5157582975
vns	GGB28954_SGB41662	EtOH_pref_24h_final	0.8004849551
blast	GGB28956_SGB41665	EtOH_pref_24h_final	0.163329713
vns	GGB28956_SGB41665	EtOH_pref_24h_final	0.1534165023
blast	GGB28960_SGB41669	EtOH_pref_24h_final	0.155727292
vns	GGB28960_SGB41669	EtOH_pref_24h_final	0.1559574792

blast	GGB28967_SGB41678	EtOH_pref_24h_final	0.179269529
vns	GGB28967_SGB41678	EtOH_pref_24h_final	0.315046519
blast	GGB28991_SGB41705	EtOH_pref_24h_final	0.228893882
vns	GGB28991_SGB41705	EtOH_pref_24h_final	0.279982342
blast	GGB29002_SGB41718	EtOH_pref_24h_final	0.002430867
vns	GGB29002_SGB41718	EtOH_pref_24h_final	0.113465587
blast	GGB29003_SGB41719	EtOH_pref_24h_final	0.499398644
vns	GGB29003_SGB41719	EtOH_pref_24h_final	0.583967886
blast	GGB29011_SGB41731	EtOH_pref_24h_final	0.916276033
vns	GGB29011_SGB41731	EtOH_pref_24h_final	0.345599236
blast	GGB29531_SGB42317	EtOH_pref_24h_final	0.145082026
vns	GGB29531_SGB42317	EtOH_pref_24h_final	0.135569966
blast	GGB29685_SGB42494	EtOH_pref_24h_final	0.864543852
vns	GGB29685_SGB42494	EtOH_pref_24h_final	0.340104263
blast	GGB30141_SGB43066	EtOH_pref_24h_final	0.991554803
vns	GGB30141_SGB43066	EtOH_pref_24h_final	0.760178789
blast	GGB30286_SGB43248	EtOH_pref_24h_final	0.055261633
vns	GGB30286_SGB43248	EtOH_pref_24h_final	0.454603367
blast	GGB30303_SGB43268	EtOH_pref_24h_final	0.371936893
vns	GGB30303_SGB43268	EtOH_pref_24h_final	0.944722174
blast	GGB30413_SGB43452	EtOH_pref_24h_final	0.953276950
vns	GGB30413_SGB43452	EtOH_pref_24h_final	0.879642604
blast	GGB30454_SGB43514	EtOH_pref_24h_final	0.379858950
vns	GGB30454_SGB43514	EtOH_pref_24h_final	0.200125591
blast	GGB30455_SGB43519	EtOH_pref_24h_final	0.379467796
vns	GGB30455_SGB43519	EtOH_pref_24h_final	0.302348921
blast	GGB30461_SGB43527	EtOH_pref_24h_final	0.397503662
vns	GGB30461_SGB43527	EtOH_pref_24h_final	0.771038655
blast	GGB30461_SGB43530	EtOH_pref_24h_final	0.395449
vns	GGB30461_SGB43530	EtOH_pref_24h_final	0.703863693
blast	GGB30463_SGB43537	EtOH_pref_24h_final	0.210054257
vns	GGB30463_SGB43537	EtOH_pref_24h_final	0.985361479
blast	GGB30473_SGB43557	EtOH_pref_24h_final	0.020642166
vns	GGB30473_SGB43557	EtOH_pref_24h_final	0.056211743
blast	GGB30475_SGB63182	EtOH_pref_24h_final	0.287965349
vns	GGB30475_SGB63182	EtOH_pref_24h_final	0.660855930
blast	GGB30861_SGB44083	EtOH_pref_24h_final	0.957552379
vns	GGB30861_SGB44083	EtOH_pref_24h_final	0.833445529
blast	GGB31312_SGB44628	EtOH_pref_24h_final	0.711727128
vns	GGB31312_SGB44628	EtOH_pref_24h_final	0.884193943
blast	GGB31438_SGB44768	EtOH_pref_24h_final	0.906357297
vns	GGB31438_SGB44768	EtOH_pref_24h_final	0.318960086
blast	GGB3171_SGB4185	EtOH_pref_24h_final	0.085345767
vns	GGB3171_SGB4185	EtOH_pref_24h_final	0.918551780
blast	GGB31823_SGB45199	EtOH_pref_24h_final	0.188977717
vns	GGB31823_SGB45199	EtOH_pref_24h_final	0.702813258

blast	GGB31853_SGB45233	EtOH_pref_24h_final	0.496842743
vns	GGB31853_SGB45233	EtOH_pref_24h_final	0.396259541
blast	GGB32371_SGB41694	EtOH_pref_24h_final	0.346811850
vns	GGB32371_SGB41694	EtOH_pref_24h_final	0.025054092
blast	GGB3793_SGB5158	EtOH_pref_24h_final	0.965790474
vns	GGB3793_SGB5158	EtOH_pref_24h_final	0.975145151
blast	GGB42598_SGB59794	EtOH_pref_24h_final	0.501740001
vns	GGB42598_SGB59794	EtOH_pref_24h_final	0.156668604
blast	GGB45656_SGB63370	EtOH_pref_24h_final	0.621044138
vns	GGB45656_SGB63370	EtOH_pref_24h_final	0.963374601
blast	GGB47127_SGB65054	EtOH_pref_24h_final	0.496745120
vns	GGB47127_SGB65054	EtOH_pref_24h_final	0.257417312
blast	GGB74395_SGB43521	EtOH_pref_24h_final	0.123339782
vns	GGB74395_SGB43521	EtOH_pref_24h_final	0.204021498
blast	GGB75053_SGB43494	EtOH_pref_24h_final	0.410595919
vns	GGB75053_SGB43494	EtOH_pref_24h_final	0.571857456
blast	GGB75109_SGB102238	EtOH_pref_24h_final	0.620842292
vns	GGB75109_SGB102238	EtOH_pref_24h_final	0.871818408
blast	GGB81440_SGB45230	EtOH_pref_24h_final	0.079610731
vns	GGB81440_SGB45230	EtOH_pref_24h_final	0.098140579
blast	Lachnospiraceae_bacterium	EtOH_pref_24h_final	0.484816177
vns	Lachnospiraceae_bacterium	EtOH_pref_24h_final	0.537861332
blast	Lachnospiraceae_bacterium_A2	EtOH_pref_24h_final	0.420834896
vns	Lachnospiraceae_bacterium_A2	EtOH_pref_24h_final	0.323648328
blast	Lachnospiraceae_bacterium_MD308	EtOH_pref_24h_final	0.213119451
vns	Lachnospiraceae_bacterium_MD308	EtOH_pref_24h_final	0.083066965
blast	Lachnospiraceae_bacterium_MD329	EtOH_pref_24h_final	0.020976149
vns	Lachnospiraceae_bacterium_MD329	EtOH_pref_24h_final	0.699161119
blast	Lachnospiraceae_unclassified_SGB41414	EtOH_pref_24h_final	0.142639053
vns	Lachnospiraceae_unclassified_SGB41414	EtOH_pref_24h_final	0.119876468
blast	Lachnospiraceae_unclassified_SGB41418	EtOH_pref_24h_final	0.998757981
vns	Lachnospiraceae_unclassified_SGB41418	EtOH_pref_24h_final	0.712236573
blast	Lachnospiraceae_unclassified_SGB41424	EtOH_pref_24h_final	0.048386378
vns	Lachnospiraceae_unclassified_SGB41424	EtOH_pref_24h_final	0.046067357
blast	Lachnospiraceae_unclassified_SGB41589	EtOH_pref_24h_final	0.165424952
vns	Lachnospiraceae_unclassified_SGB41589	EtOH_pref_24h_final	0.273527700
blast	Lactobacillus_johnsonii	EtOH_pref_24h_final	0.182678453
vns	Lactobacillus_johnsonii	EtOH_pref_24h_final	0.672951588
blast	Muribaculaceae_bacterium	EtOH_pref_24h_final	0.841525252
vns	Muribaculaceae_bacterium	EtOH_pref_24h_final	0.893052980
blast	Neglectibacter_sp_X4	EtOH_pref_24h_final	0.084782151
vns	Neglectibacter_sp_X4	EtOH_pref_24h_final	0.140656264
blast	Oscillospiraceae_bacterium	EtOH_pref_24h_final	0.741672565
vns	Oscillospiraceae_bacterium	EtOH_pref_24h_final	0.856188039
blast	Oscillospiraceae_unclassified_SGB43502	EtOH_pref_24h_final	0.025941285
vns	Oscillospiraceae_unclassified_SGB43502	EtOH_pref_24h_final	0.030535133

blast	Oscillospiraceae_unclassified_SGB43505	EtOH_pref_24h_final	0.495923018
vns	Oscillospiraceae_unclassified_SGB43505	EtOH_pref_24h_final	0.642533731
blast	Parasutterella_excrementihominis	EtOH_pref_24h_final	0.567104872
vns	Parasutterella_excrementihominis	EtOH_pref_24h_final	0.709992917
blast	Richness (# observed features)	EtOH_pref_24h_final	0.271752442
vns	Richness (# observed features)	EtOH_pref_24h_final	0.660723068
blast	Romboutsia_ilealis	EtOH_pref_24h_final	0.001099774
vns	Romboutsia_ilealis	EtOH_pref_24h_final	0.056997089
blast	Schaedlerella_arabinosiphila	EtOH_pref_24h_final	0.939024404
vns	Schaedlerella_arabinosiphila	EtOH_pref_24h_final	0.913911607
blast	Shannon Index	EtOH_pref_24h_final	0.694735221
vns	Shannon Index	EtOH_pref_24h_final	0.906277341
blast	Turicibacter_sp_1E2	EtOH_pref_24h_final	0.199177231
vns	Turicibacter_sp_1E2	EtOH_pref_24h_final	0.165574931
blast	Acetatifactor_muris	OFB_Cdistance	0.739522079
vns	Acetatifactor_muris	OFB_Cdistance	0.752897140
blast	Acetatifactor_SGB41546	OFB_Cdistance	0.840245797
vns	Acetatifactor_SGB41546	OFB_Cdistance	0.663000977
blast	Acutalibacter_muris	OFB_Cdistance	0.422154633
vns	Acutalibacter_muris	OFB_Cdistance	0.810578449
blast	Acutalibacter_sp_1XD8_36	OFB_Cdistance	0.660579886
vns	Acutalibacter_sp_1XD8_36	OFB_Cdistance	0.709184933
blast	Adlercreutzia_caecimuris	OFB_Cdistance	0.989608952
vns	Adlercreutzia_caecimuris	OFB_Cdistance	0.676542691
blast	Adlercreutzia_mucosicola	OFB_Cdistance	0.271833985
vns	Adlercreutzia_mucosicola	OFB_Cdistance	0.249825657
blast	Adlercreutzia_muris	OFB_Cdistance	0.670421774
vns	Adlercreutzia_muris	OFB_Cdistance	0.595827745
blast	Akkermansia_muciniphila	OFB_Cdistance	0.169812540
vns	Akkermansia_muciniphila	OFB_Cdistance	0.864362509
blast	Alistipes_sp_DSM_112343	OFB_Cdistance	0.372515977
vns	Alistipes_sp_DSM_112343	OFB_Cdistance	0.832698307
blast	Anaerotruncus_sp_1XD42_93	OFB_Cdistance	0.520271453
vns	Anaerotruncus_sp_1XD42_93	OFB_Cdistance	0.617410721
blast	Bacteria_unclassified_SGB102200	OFB_Cdistance	0.474269212
vns	Bacteria_unclassified_SGB102200	OFB_Cdistance	0.764166788
blast	Bacteria_unclassified_SGB41677	OFB_Cdistance	0.444143823
vns	Bacteria_unclassified_SGB41677	OFB_Cdistance	0.169989424
blast	Bacteria_unclassified_SGB43546	OFB_Cdistance	0.586358365
vns	Bacteria_unclassified_SGB43546	OFB_Cdistance	0.789009912
blast	bacterium_1XD42_54	OFB_Cdistance	0.014424595
vns	bacterium_1XD42_54	OFB_Cdistance	0.234577574
blast	bacterium_1XD42_76	OFB_Cdistance	0.600031603
vns	bacterium_1XD42_76	OFB_Cdistance	0.642992453
blast	bacterium_1xD8_48	OFB_Cdistance	0.899708945
vns	bacterium_1xD8_48	OFB_Cdistance	0.696816880

blast	Bacteroides_thetaiotaomicron	OFB_Cdistance	0.960201477
vns	Bacteroides_thetaiotaomicron	OFB_Cdistance	0.634306315
blast	Berger Parker Index	OFB_Cdistance	0.545741712
vns	Berger Parker Index	OFB_Cdistance	0.233733054
blast	Bifidobacterium_pseudolongum	OFB_Cdistance	0.428010398
vns	Bifidobacterium_pseudolongum	OFB_Cdistance	0.347799065
blast	Clostridia_bacterium	OFB_Cdistance	0.374559043
vns	Clostridia_bacterium	OFB_Cdistance	0.724940107
blast	Clostridiaceae_bacterium	OFB_Cdistance	0.987169780
vns	Clostridiaceae_bacterium	OFB_Cdistance	0.241937042
blast	Clostridiaceae_unclassified_SGB41663	OFB_Cdistance	0.780382224
vns	Clostridiaceae_unclassified_SGB41663	OFB_Cdistance	0.719154368
blast	Clostridiales_bacterium	OFB_Cdistance	0.002331956
vns	Clostridiales_bacterium	OFB_Cdistance	0.296478501
blast	Clostridium_cocleatum	OFB_Cdistance	0.011567832
vns	Clostridium_cocleatum	OFB_Cdistance	0.196035514
blast	Coriobacteriaceae_bacterium	OFB_Cdistance	0.372448369
vns	Coriobacteriaceae_bacterium	OFB_Cdistance	0.953919137
blast	Dorea_sp_5_2	OFB_Cdistance	0.588491917
vns	Dorea_sp_5_2	OFB_Cdistance	0.421416124
blast	Dubosiella_newyorkensis	OFB_Cdistance	0.116998739
vns	Dubosiella_newyorkensis	OFB_Cdistance	0.799186063
blast	Erysipelotrichales_bacterium	OFB_Cdistance	0.897000665
vns	Erysipelotrichales_bacterium	OFB_Cdistance	0.328408108
blast	Eubacteriaceae_bacterium	OFB_Cdistance	0.027123368
vns	Eubacteriaceae_bacterium	OFB_Cdistance	0.238318613
blast	Eubacteriaceae_unclassified_SGB94922	OFB_Cdistance	0.459488079
vns	Eubacteriaceae_unclassified_SGB94922	OFB_Cdistance	0.372620148
blast	GGB20149_SGB29430	OFB_Cdistance	0.628070048
vns	GGB20149_SGB29430	OFB_Cdistance	0.629982792
blast	GGB22635_SGB63107	OFB_Cdistance	0.411710336
vns	GGB22635_SGB63107	OFB_Cdistance	0.887152138
blast	GGB25041_SGB36960	OFB_Cdistance	0.082496667
vns	GGB25041_SGB36960	OFB_Cdistance	0.210187196
blast	GGB27876_SGB40310	OFB_Cdistance	0.164666135
vns	GGB27876_SGB40310	OFB_Cdistance	0.767235194
blast	GGB27878_SGB40312	OFB_Cdistance	0.213354157
vns	GGB27878_SGB40312	OFB_Cdistance	0.147479429
blast	GGB27918_SGB40356	OFB_Cdistance	0.958758140
vns	GGB27918_SGB40356	OFB_Cdistance	0.885698929
blast	GGB28382_SGB40962	OFB_Cdistance	0.519311307
vns	GGB28382_SGB40962	OFB_Cdistance	0.855057834
blast	GGB28399_SGB40980	OFB_Cdistance	0.289465502
vns	GGB28399_SGB40980	OFB_Cdistance	0.287117034
blast	GGB28411_SGB40993	OFB_Cdistance	0.201802262
vns	GGB28411_SGB40993	OFB_Cdistance	0.146848474

blast	GGB28415_SGB40997	OFB_Cdistance	0.026726744
vns	GGB28415_SGB40997	OFB_Cdistance	0.949051696
blast	GGB28430_SGB41013	OFB_Cdistance	0.912070947
vns	GGB28430_SGB41013	OFB_Cdistance	0.466250482
blast	GGB28439_SGB41022	OFB_Cdistance	0.380444403
vns	GGB28439_SGB41022	OFB_Cdistance	0.738709552
blast	GGB28778_SGB41431	OFB_Cdistance	0.804698941
vns	GGB28778_SGB41431	OFB_Cdistance	0.218453243
blast	GGB28784_SGB41437	OFB_Cdistance	0.020726621
vns	GGB28784_SGB41437	OFB_Cdistance	0.254456997
blast	GGB28792_SGB41445	OFB_Cdistance	0.338577775
vns	GGB28792_SGB41445	OFB_Cdistance	0.761244426
blast	GGB28798_SGB41451	OFB_Cdistance	0.164022630
vns	GGB28798_SGB41451	OFB_Cdistance	0.248915824
blast	GGB28802_SGB41455	OFB_Cdistance	0.457898625
vns	GGB28802_SGB41455	OFB_Cdistance	0.342689298
blast	GGB28818_SGB41473	OFB_Cdistance	0.729209510
vns	GGB28818_SGB41473	OFB_Cdistance	0.569409948
blast	GGB28828_SGB41484	OFB_Cdistance	0.298696993
vns	GGB28828_SGB41484	OFB_Cdistance	0.106706535
blast	GGB28851_SGB41518	OFB_Cdistance	0.852552179
vns	GGB28851_SGB41518	OFB_Cdistance	0.578308429
blast	GGB28859_SGB41528	OFB_Cdistance	0.205749329
vns	GGB28859_SGB41528	OFB_Cdistance	0.595366745
blast	GGB28864_SGB41535	OFB_Cdistance	0.785465248
vns	GGB28864_SGB41535	OFB_Cdistance	0.964658985
blast	GGB28869_SGB41543	OFB_Cdistance	0.347931625
vns	GGB28869_SGB41543	OFB_Cdistance	0.381477475
blast	GGB28883_SGB41564	OFB_Cdistance	0.594877248
vns	GGB28883_SGB41564	OFB_Cdistance	0.060524458
blast	GGB28892_SGB41573	OFB_Cdistance	0.651227207
vns	GGB28892_SGB41573	OFB_Cdistance	0.667618882
blast	GGB28893_SGB41574	OFB_Cdistance	0.165594509
vns	GGB28893_SGB41574	OFB_Cdistance	0.182699376
blast	GGB28898_SGB41580	OFB_Cdistance	0.304654993
vns	GGB28898_SGB41580	OFB_Cdistance	0.082893330
blast	GGB28904_SGB41597	OFB_Cdistance	0.172906397
vns	GGB28904_SGB41597	OFB_Cdistance	0.056067199
blast	GGB28916_SGB41612	OFB_Cdistance	0.801432609
vns	GGB28916_SGB41612	OFB_Cdistance	0.853813296
blast	GGB28924_SGB41621	OFB_Cdistance	0.796481662
vns	GGB28924_SGB41621	OFB_Cdistance	0.278658378
blast	GGB28926_SGB41624	OFB_Cdistance	0.306780834
vns	GGB28926_SGB41624	OFB_Cdistance	0.470765265
blast	GGB28927_SGB41625	OFB_Cdistance	0.193720805
vns	GGB28927_SGB41625	OFB_Cdistance	0.418243373

blast	GGB28934_SGB41635	OFB_Cdistance	0.115873311
vns	GGB28934_SGB41635	OFB_Cdistance	0.111374073
blast	GGB28946_SGB41652	OFB_Cdistance	0.427843225
vns	GGB28946_SGB41652	OFB_Cdistance	0.422588900
blast	GGB28949_SGB41655	OFB_Cdistance	0.443608116
vns	GGB28949_SGB41655	OFB_Cdistance	0.908948179
blast	GGB28949_SGB41656	OFB_Cdistance	0.282728234
vns	GGB28949_SGB41656	OFB_Cdistance	0.770863949
blast	GGB28950_SGB41657	OFB_Cdistance	0.030527036
vns	GGB28950_SGB41657	OFB_Cdistance	0.336458904
blast	GGB28951_SGB102295	OFB_Cdistance	0.925352156
vns	GGB28951_SGB102295	OFB_Cdistance	0.158790397
blast	GGB28951_SGB41658	OFB_Cdistance	0.847001351
vns	GGB28951_SGB41658	OFB_Cdistance	0.920236623
blast	GGB28954_SGB41662	OFB_Cdistance	0.515758297
vns	GGB28954_SGB41662	OFB_Cdistance	0.800484955
blast	GGB28956_SGB41665	OFB_Cdistance	0.163329713
vns	GGB28956_SGB41665	OFB_Cdistance	0.153416502
blast	GGB28960_SGB41669	OFB_Cdistance	0.155727292
vns	GGB28960_SGB41669	OFB_Cdistance	0.155957479
blast	GGB28967_SGB41678	OFB_Cdistance	0.179269529
vns	GGB28967_SGB41678	OFB_Cdistance	0.315046519
blast	GGB28991_SGB41705	OFB_Cdistance	0.228893882
vns	GGB28991_SGB41705	OFB_Cdistance	0.279982342
blast	GGB29002_SGB41718	OFB_Cdistance	0.002430867
vns	GGB29002_SGB41718	OFB_Cdistance	0.113465587
blast	GGB29003_SGB41719	OFB_Cdistance	0.499398644
vns	GGB29003_SGB41719	OFB_Cdistance	0.583967886
blast	GGB29011_SGB41731	OFB_Cdistance	0.916276033
vns	GGB29011_SGB41731	OFB_Cdistance	0.345599236
blast	GGB29531_SGB42317	OFB_Cdistance	0.145082026
vns	GGB29531_SGB42317	OFB_Cdistance	0.135569966
blast	GGB29685_SGB42494	OFB_Cdistance	0.864543852
vns	GGB29685_SGB42494	OFB_Cdistance	0.340104263
blast	GGB30141_SGB43066	OFB_Cdistance	0.991554803
vns	GGB30141_SGB43066	OFB_Cdistance	0.760178789
blast	GGB30286_SGB43248	OFB_Cdistance	0.055261633
vns	GGB30286_SGB43248	OFB_Cdistance	0.454603367
blast	GGB30303_SGB43268	OFB_Cdistance	0.371936893
vns	GGB30303_SGB43268	OFB_Cdistance	0.944722174
blast	GGB30413_SGB43452	OFB_Cdistance	0.953276950
vns	GGB30413_SGB43452	OFB_Cdistance	0.879642604
blast	GGB30454_SGB43514	OFB_Cdistance	0.379858950
vns	GGB30454_SGB43514	OFB_Cdistance	0.200125591
blast	GGB30455_SGB43519	OFB_Cdistance	0.379467796
vns	GGB30455_SGB43519	OFB_Cdistance	0.302348921

blast	GGB30461_SGB43527	OFB_Cdistance	0.397503662
vns	GGB30461_SGB43527	OFB_Cdistance	0.771038655
blast	GGB30461_SGB43530	OFB_Cdistance	0.395449
vns	GGB30461_SGB43530	OFB_Cdistance	0.703863693
blast	GGB30463_SGB43537	OFB_Cdistance	0.210054257
vns	GGB30463_SGB43537	OFB_Cdistance	0.985361479
blast	GGB30473_SGB43557	OFB_Cdistance	0.020642166
vns	GGB30473_SGB43557	OFB_Cdistance	0.056211743
blast	GGB30475_SGB63182	OFB_Cdistance	0.287965349
vns	GGB30475_SGB63182	OFB_Cdistance	0.660855930
blast	GGB30861_SGB44083	OFB_Cdistance	0.957552379
vns	GGB30861_SGB44083	OFB_Cdistance	0.833445529
blast	GGB31312_SGB44628	OFB_Cdistance	0.711727128
vns	GGB31312_SGB44628	OFB_Cdistance	0.884193943
blast	GGB31438_SGB44768	OFB_Cdistance	0.906357297
vns	GGB31438_SGB44768	OFB_Cdistance	0.318960086
blast	GGB3171_SGB4185	OFB_Cdistance	0.085345767
vns	GGB3171_SGB4185	OFB_Cdistance	0.918551780
blast	GGB31823_SGB45199	OFB_Cdistance	0.188977717
vns	GGB31823_SGB45199	OFB_Cdistance	0.702813258
blast	GGB31853_SGB45233	OFB_Cdistance	0.496842743
vns	GGB31853_SGB45233	OFB_Cdistance	0.396259541
blast	GGB32371_SGB41694	OFB_Cdistance	0.346811850
vns	GGB32371_SGB41694	OFB_Cdistance	0.025054092
blast	GGB3793_SGB5158	OFB_Cdistance	0.965790474
vns	GGB3793_SGB5158	OFB_Cdistance	0.975145151
blast	GGB42598_SGB59794	OFB_Cdistance	0.501740001
vns	GGB42598_SGB59794	OFB_Cdistance	0.156668604
blast	GGB45656_SGB63370	OFB_Cdistance	0.621044138
vns	GGB45656_SGB63370	OFB_Cdistance	0.963374601
blast	GGB47127_SGB65054	OFB_Cdistance	0.496745120
vns	GGB47127_SGB65054	OFB_Cdistance	0.257417312
blast	GGB74395_SGB43521	OFB_Cdistance	0.123339782
vns	GGB74395_SGB43521	OFB_Cdistance	0.204021498
blast	GGB75053_SGB43494	OFB_Cdistance	0.410595919
vns	GGB75053_SGB43494	OFB_Cdistance	0.571857456
blast	GGB75109_SGB102238	OFB_Cdistance	0.620842292
vns	GGB75109_SGB102238	OFB_Cdistance	0.871818408
blast	GGB81440_SGB45230	OFB_Cdistance	0.079610731
vns	GGB81440_SGB45230	OFB_Cdistance	0.098140579
blast	Lachnospiraceae_bacterium	OFB_Cdistance	0.484816177
vns	Lachnospiraceae_bacterium	OFB_Cdistance	0.537861332
blast	Lachnospiraceae_bacterium_A2	OFB_Cdistance	0.420834896
vns	Lachnospiraceae_bacterium_A2	OFB_Cdistance	0.323648328
blast	Lachnospiraceae_bacterium_MD308	OFB_Cdistance	0.213119451
vns	Lachnospiraceae_bacterium_MD308	OFB_Cdistance	0.083066965

blast	Lachnospiraceae_bacterium_MD329	OFB_Cdistance	0.020976149
vns	Lachnospiraceae_bacterium_MD329	OFB_Cdistance	0.699161119
blast	Lachnospiraceae_unclassified_SGB41414	OFB_Cdistance	0.142639053
vns	Lachnospiraceae_unclassified_SGB41414	OFB_Cdistance	0.119876468
blast	Lachnospiraceae_unclassified_SGB41418	OFB_Cdistance	0.998757981
vns	Lachnospiraceae_unclassified_SGB41418	OFB_Cdistance	0.712236573
blast	Lachnospiraceae_unclassified_SGB41424	OFB_Cdistance	0.048386378
vns	Lachnospiraceae_unclassified_SGB41424	OFB_Cdistance	0.046067357
blast	Lachnospiraceae_unclassified_SGB41589	OFB_Cdistance	0.165424952
vns	Lachnospiraceae_unclassified_SGB41589	OFB_Cdistance	0.273527700
blast	Lactobacillus_johnsonii	OFB_Cdistance	0.182678453
vns	Lactobacillus_johnsonii	OFB_Cdistance	0.672951588
blast	Muribaculaceae_bacterium	OFB_Cdistance	0.841525252
vns	Muribaculaceae_bacterium	OFB_Cdistance	0.893052980
blast	Neglectibacter_sp_X4	OFB_Cdistance	0.084782151
vns	Neglectibacter_sp_X4	OFB_Cdistance	0.140656264
blast	Oscillospiraceae_bacterium	OFB_Cdistance	0.741672565
vns	Oscillospiraceae_bacterium	OFB_Cdistance	0.856188039
blast	Oscillospiraceae_unclassified_SGB43502	OFB_Cdistance	0.025941285
vns	Oscillospiraceae_unclassified_SGB43502	OFB_Cdistance	0.030535133
blast	Oscillospiraceae_unclassified_SGB43505	OFB_Cdistance	0.495923018
vns	Oscillospiraceae_unclassified_SGB43505	OFB_Cdistance	0.642533731
blast	Parasutterella_excrementihominis	OFB_Cdistance	0.567104872
vns	Parasutterella_excrementihominis	OFB_Cdistance	0.709992917
blast	Richness (# observed features)	OFB_Cdistance	0.271752442
vns	Richness (# observed features)	OFB_Cdistance	0.660723068
blast	Romboutsia_ilealis	OFB_Cdistance	0.001099774
vns	Romboutsia_ilealis	OFB_Cdistance	0.056997089
blast	Schaedlerella_arabinosiphila	OFB_Cdistance	0.939024404
vns	Schaedlerella_arabinosiphila	OFB_Cdistance	0.913911607
blast	Shannon Index	OFB_Cdistance	0.694735221
vns	Shannon Index	OFB_Cdistance	0.906277341
blast	Turicibacter_sp_1E2	OFB_Cdistance	0.199177231
vns	Turicibacter_sp_1E2	OFB_Cdistance	0.165574931
blast	Acetatifactor_muris	OFB_Centries	0.739522079
vns	Acetatifactor_muris	OFB_Centries	0.752897140
blast	Acetatifactor_SGB41546	OFB_Centries	0.840245797
vns	Acetatifactor_SGB41546	OFB_Centries	0.663000977
blast	Acutalibacter_muris	OFB_Centries	0.422154633
vns	Acutalibacter_muris	OFB_Centries	0.810578449
blast	Acutalibacter_sp_1XD8_36	OFB_Centries	0.660579886
vns	Acutalibacter_sp_1XD8_36	OFB_Centries	0.709184933
blast	Adlercreutzia_caecimuris	OFB_Centries	0.989608952
vns	Adlercreutzia_caecimuris	OFB_Centries	0.676542691
blast	Adlercreutzia_mucosicola	OFB_Centries	0.271833985
vns	Adlercreutzia_mucosicola	OFB_Centries	0.249825657

blast	Adlercreutzia_muris	OFB_Centries	0.6704217743
vns	Adlercreutzia_muris	OFB_Centries	0.5958277454
blast	Akkermansia_muciniphila	OFB_Centries	0.1698125404
vns	Akkermansia_muciniphila	OFB_Centries	0.8643625099
blast	Alistipes_sp_DSM_112343	OFB_Centries	0.3725159774
vns	Alistipes_sp_DSM_112343	OFB_Centries	0.8326983074
blast	Anaerotruncus_sp_1XD42_93	OFB_Centries	0.5202714535
vns	Anaerotruncus_sp_1XD42_93	OFB_Centries	0.6174107217
blast	Bacteria_unclassified_SGB102200	OFB_Centries	0.4742692127
vns	Bacteria_unclassified_SGB102200	OFB_Centries	0.7641667885
blast	Bacteria_unclassified_SGB41677	OFB_Centries	0.4441438231
vns	Bacteria_unclassified_SGB41677	OFB_Centries	0.1699894243
blast	Bacteria_unclassified_SGB43546	OFB_Centries	0.5863583659
vns	Bacteria_unclassified_SGB43546	OFB_Centries	0.7890099125
blast	bacterium_1XD42_54	OFB_Centries	0.0144245959
vns	bacterium_1XD42_54	OFB_Centries	0.2345775748
blast	bacterium_1XD42_76	OFB_Centries	0.6000316038
vns	bacterium_1XD42_76	OFB_Centries	0.6429924539
blast	bacterium_1xD8_48	OFB_Centries	0.8997089453
vns	bacterium_1xD8_48	OFB_Centries	0.6968168809
blast	Bacteroides_thetaiotaomicron	OFB_Centries	0.9602014777
vns	Bacteroides_thetaiotaomicron	OFB_Centries	0.6343063159
blast	Berger Parker Index	OFB_Centries	0.5457417127
vns	Berger Parker Index	OFB_Centries	0.2337330546
blast	Bifidobacterium_pseudolongum	OFB_Centries	0.4280103985
vns	Bifidobacterium_pseudolongum	OFB_Centries	0.3477990653
blast	Clostridia_bacterium	OFB_Centries	0.3745590434
vns	Clostridia_bacterium	OFB_Centries	0.7249401071
blast	Clostridiaceae_bacterium	OFB_Centries	0.9871697806
vns	Clostridiaceae_bacterium	OFB_Centries	0.2419370425
blast	Clostridiaceae_unclassified_SGB41663	OFB_Centries	0.780382224
vns	Clostridiaceae_unclassified_SGB41663	OFB_Centries	0.719154368
blast	Clostridiales_bacterium	OFB_Centries	0.0023319564
vns	Clostridiales_bacterium	OFB_Centries	0.2964785018
blast	Clostridium_cocleatum	OFB_Centries	0.0115678325
vns	Clostridium_cocleatum	OFB_Centries	0.1960355146
blast	Coriobacteriaceae_bacterium	OFB_Centries	0.3724483697
vns	Coriobacteriaceae_bacterium	OFB_Centries	0.953919137
blast	Dorea_sp_5_2	OFB_Centries	0.5884919176
vns	Dorea_sp_5_2	OFB_Centries	0.4214161244
blast	Dubosiella_newyorkensis	OFB_Centries	0.1169987394
vns	Dubosiella_newyorkensis	OFB_Centries	0.799186063
blast	Erysipelotrichales_bacterium	OFB_Centries	0.8970006656
vns	Erysipelotrichales_bacterium	OFB_Centries	0.3284081083
blast	Eubacteriaceae_bacterium	OFB_Centries	0.0271233687
vns	Eubacteriaceae_bacterium	OFB_Centries	0.2383186135

blast	Eubacteriaceae_unclassified_SGB94922	OFB_Centries	0.459488079
vns	Eubacteriaceae_unclassified_SGB94922	OFB_Centries	0.372620148
blast	GGB20149_SGB29430	OFB_Centries	0.628070048
vns	GGB20149_SGB29430	OFB_Centries	0.629982792
blast	GGB22635_SGB63107	OFB_Centries	0.411710336
vns	GGB22635_SGB63107	OFB_Centries	0.887152138
blast	GGB25041_SGB36960	OFB_Centries	0.082496667
vns	GGB25041_SGB36960	OFB_Centries	0.210187196
blast	GGB27876_SGB40310	OFB_Centries	0.164666135
vns	GGB27876_SGB40310	OFB_Centries	0.767235194
blast	GGB27878_SGB40312	OFB_Centries	0.213354157
vns	GGB27878_SGB40312	OFB_Centries	0.147479429
blast	GGB27918_SGB40356	OFB_Centries	0.958758140
vns	GGB27918_SGB40356	OFB_Centries	0.885698929
blast	GGB28382_SGB40962	OFB_Centries	0.519311307
vns	GGB28382_SGB40962	OFB_Centries	0.855057834
blast	GGB28399_SGB40980	OFB_Centries	0.289465502
vns	GGB28399_SGB40980	OFB_Centries	0.287117034
blast	GGB28411_SGB40993	OFB_Centries	0.201802262
vns	GGB28411_SGB40993	OFB_Centries	0.146848474
blast	GGB28415_SGB40997	OFB_Centries	0.026726744
vns	GGB28415_SGB40997	OFB_Centries	0.949051696
blast	GGB28430_SGB41013	OFB_Centries	0.912070947
vns	GGB28430_SGB41013	OFB_Centries	0.466250482
blast	GGB28439_SGB41022	OFB_Centries	0.380444403
vns	GGB28439_SGB41022	OFB_Centries	0.738709552
blast	GGB28778_SGB41431	OFB_Centries	0.804698941
vns	GGB28778_SGB41431	OFB_Centries	0.218453243
blast	GGB28784_SGB41437	OFB_Centries	0.020726621
vns	GGB28784_SGB41437	OFB_Centries	0.254456997
blast	GGB28792_SGB41445	OFB_Centries	0.338577775
vns	GGB28792_SGB41445	OFB_Centries	0.761244426
blast	GGB28798_SGB41451	OFB_Centries	0.164022630
vns	GGB28798_SGB41451	OFB_Centries	0.248915824
blast	GGB28802_SGB41455	OFB_Centries	0.457898625
vns	GGB28802_SGB41455	OFB_Centries	0.342689298
blast	GGB28818_SGB41473	OFB_Centries	0.729209510
vns	GGB28818_SGB41473	OFB_Centries	0.569409948
blast	GGB28828_SGB41484	OFB_Centries	0.298696993
vns	GGB28828_SGB41484	OFB_Centries	0.106706535
blast	GGB28851_SGB41518	OFB_Centries	0.852552179
vns	GGB28851_SGB41518	OFB_Centries	0.578308429
blast	GGB28859_SGB41528	OFB_Centries	0.205749329
vns	GGB28859_SGB41528	OFB_Centries	0.595366745
blast	GGB28864_SGB41535	OFB_Centries	0.785465248
vns	GGB28864_SGB41535	OFB_Centries	0.964658985

blast	GGB28869_SGB41543	OFB_Centries	0.3479316259
vns	GGB28869_SGB41543	OFB_Centries	0.3814774755
blast	GGB28883_SGB41564	OFB_Centries	0.5948772487
vns	GGB28883_SGB41564	OFB_Centries	0.0605244586
blast	GGB28892_SGB41573	OFB_Centries	0.6512272078
vns	GGB28892_SGB41573	OFB_Centries	0.6676188822
blast	GGB28893_SGB41574	OFB_Centries	0.165594509
vns	GGB28893_SGB41574	OFB_Centries	0.1826993761
blast	GGB28898_SGB41580	OFB_Centries	0.3046549939
vns	GGB28898_SGB41580	OFB_Centries	0.0828933309
blast	GGB28904_SGB41597	OFB_Centries	0.1729063974
vns	GGB28904_SGB41597	OFB_Centries	0.0560671999
blast	GGB28916_SGB41612	OFB_Centries	0.8014326092
vns	GGB28916_SGB41612	OFB_Centries	0.8538132969
blast	GGB28924_SGB41621	OFB_Centries	0.7964816629
vns	GGB28924_SGB41621	OFB_Centries	0.2786583787
blast	GGB28926_SGB41624	OFB_Centries	0.3067808341
vns	GGB28926_SGB41624	OFB_Centries	0.470765265
blast	GGB28927_SGB41625	OFB_Centries	0.1937208057
vns	GGB28927_SGB41625	OFB_Centries	0.4182433734
blast	GGB28934_SGB41635	OFB_Centries	0.1158733118
vns	GGB28934_SGB41635	OFB_Centries	0.1113740731
blast	GGB28946_SGB41652	OFB_Centries	0.4278432256
vns	GGB28946_SGB41652	OFB_Centries	0.4225889007
blast	GGB28949_SGB41655	OFB_Centries	0.4436081164
vns	GGB28949_SGB41655	OFB_Centries	0.9089481791
blast	GGB28949_SGB41656	OFB_Centries	0.2827282346
vns	GGB28949_SGB41656	OFB_Centries	0.7708639493
blast	GGB28950_SGB41657	OFB_Centries	0.0305270365
vns	GGB28950_SGB41657	OFB_Centries	0.3364589048
blast	GGB28951_SGB102295	OFB_Centries	0.9253521562
vns	GGB28951_SGB102295	OFB_Centries	0.1587903977
blast	GGB28951_SGB41658	OFB_Centries	0.8470013515
vns	GGB28951_SGB41658	OFB_Centries	0.9202366237
blast	GGB28954_SGB41662	OFB_Centries	0.5157582975
vns	GGB28954_SGB41662	OFB_Centries	0.8004849551
blast	GGB28956_SGB41665	OFB_Centries	0.163329713
vns	GGB28956_SGB41665	OFB_Centries	0.1534165023
blast	GGB28960_SGB41669	OFB_Centries	0.155727292
vns	GGB28960_SGB41669	OFB_Centries	0.1559574792
blast	GGB28967_SGB41678	OFB_Centries	0.1792695292
vns	GGB28967_SGB41678	OFB_Centries	0.3150465193
blast	GGB28991_SGB41705	OFB_Centries	0.2288938824
vns	GGB28991_SGB41705	OFB_Centries	0.279982342
blast	GGB29002_SGB41718	OFB_Centries	0.0024308675
vns	GGB29002_SGB41718	OFB_Centries	0.1134655877

blast	GGB29003_SGB41719	OFB_Centries	0.499398644
vns	GGB29003_SGB41719	OFB_Centries	0.583967886
blast	GGB29011_SGB41731	OFB_Centries	0.916276033
vns	GGB29011_SGB41731	OFB_Centries	0.345599236
blast	GGB29531_SGB42317	OFB_Centries	0.145082026
vns	GGB29531_SGB42317	OFB_Centries	0.135569966
blast	GGB29685_SGB42494	OFB_Centries	0.864543852
vns	GGB29685_SGB42494	OFB_Centries	0.340104263
blast	GGB30141_SGB43066	OFB_Centries	0.991554803
vns	GGB30141_SGB43066	OFB_Centries	0.760178789
blast	GGB30286_SGB43248	OFB_Centries	0.055261633
vns	GGB30286_SGB43248	OFB_Centries	0.454603367
blast	GGB30303_SGB43268	OFB_Centries	0.371936893
vns	GGB30303_SGB43268	OFB_Centries	0.944722174
blast	GGB30413_SGB43452	OFB_Centries	0.953276950
vns	GGB30413_SGB43452	OFB_Centries	0.879642604
blast	GGB30454_SGB43514	OFB_Centries	0.379858950
vns	GGB30454_SGB43514	OFB_Centries	0.200125591
blast	GGB30455_SGB43519	OFB_Centries	0.379467796
vns	GGB30455_SGB43519	OFB_Centries	0.302348921
blast	GGB30461_SGB43527	OFB_Centries	0.397503662
vns	GGB30461_SGB43527	OFB_Centries	0.771038655
blast	GGB30461_SGB43530	OFB_Centries	0.395449
vns	GGB30461_SGB43530	OFB_Centries	0.703863693
blast	GGB30463_SGB43537	OFB_Centries	0.210054257
vns	GGB30463_SGB43537	OFB_Centries	0.985361479
blast	GGB30473_SGB43557	OFB_Centries	0.020642166
vns	GGB30473_SGB43557	OFB_Centries	0.056211743
blast	GGB30475_SGB63182	OFB_Centries	0.287965349
vns	GGB30475_SGB63182	OFB_Centries	0.660855930
blast	GGB30861_SGB44083	OFB_Centries	0.957552379
vns	GGB30861_SGB44083	OFB_Centries	0.833445529
blast	GGB31312_SGB44628	OFB_Centries	0.711727128
vns	GGB31312_SGB44628	OFB_Centries	0.884193943
blast	GGB31438_SGB44768	OFB_Centries	0.906357297
vns	GGB31438_SGB44768	OFB_Centries	0.318960086
blast	GGB3171_SGB4185	OFB_Centries	0.085345767
vns	GGB3171_SGB4185	OFB_Centries	0.918551780
blast	GGB31823_SGB45199	OFB_Centries	0.188977717
vns	GGB31823_SGB45199	OFB_Centries	0.702813258
blast	GGB31853_SGB45233	OFB_Centries	0.496842743
vns	GGB31853_SGB45233	OFB_Centries	0.396259541
blast	GGB32371_SGB41694	OFB_Centries	0.346811850
vns	GGB32371_SGB41694	OFB_Centries	0.025054092
blast	GGB3793_SGB5158	OFB_Centries	0.965790474
vns	GGB3793_SGB5158	OFB_Centries	0.975145151

blast	GGB42598_SGB59794	OFB_Centries	0.5017400018
vns	GGB42598_SGB59794	OFB_Centries	0.156668604
blast	GGB45656_SGB63370	OFB_Centries	0.621044138
vns	GGB45656_SGB63370	OFB_Centries	0.9633746015
blast	GGB47127_SGB65054	OFB_Centries	0.4967451202
vns	GGB47127_SGB65054	OFB_Centries	0.2574173127
blast	GGB74395_SGB43521	OFB_Centries	0.1233397821
vns	GGB74395_SGB43521	OFB_Centries	0.2040214986
blast	GGB75053_SGB43494	OFB_Centries	0.4105959194
vns	GGB75053_SGB43494	OFB_Centries	0.5718574569
blast	GGB75109_SGB102238	OFB_Centries	0.6208422921
vns	GGB75109_SGB102238	OFB_Centries	0.8718184088
blast	GGB81440_SGB45230	OFB_Centries	0.0796107313
vns	GGB81440_SGB45230	OFB_Centries	0.0981405799
blast	Lachnospiraceae_bacterium	OFB_Centries	0.4848161772
vns	Lachnospiraceae_bacterium	OFB_Centries	0.5378613321
blast	Lachnospiraceae_bacterium_A2	OFB_Centries	0.420834896
vns	Lachnospiraceae_bacterium_A2	OFB_Centries	0.3236483287
blast	Lachnospiraceae_bacterium_MD308	OFB_Centries	0.2131194519
vns	Lachnospiraceae_bacterium_MD308	OFB_Centries	0.0830669656
blast	Lachnospiraceae_bacterium_MD329	OFB_Centries	0.0209761491
vns	Lachnospiraceae_bacterium_MD329	OFB_Centries	0.6991611194
blast	Lachnospiraceae_unclassified_SGB41414	OFB_Centries	0.142639053
vns	Lachnospiraceae_unclassified_SGB41414	OFB_Centries	0.119876468
blast	Lachnospiraceae_unclassified_SGB41418	OFB_Centries	0.9987579817
vns	Lachnospiraceae_unclassified_SGB41418	OFB_Centries	0.7122365734
blast	Lachnospiraceae_unclassified_SGB41424	OFB_Centries	0.0483863782
vns	Lachnospiraceae_unclassified_SGB41424	OFB_Centries	0.0460673576
blast	Lachnospiraceae_unclassified_SGB41589	OFB_Centries	0.1654249525
vns	Lachnospiraceae_unclassified_SGB41589	OFB_Centries	0.2735277008
blast	Lactobacillus_johnsonii	OFB_Centries	0.1826784534
vns	Lactobacillus_johnsonii	OFB_Centries	0.6729515885
blast	Muribaculaceae_bacterium	OFB_Centries	0.8415252525
vns	Muribaculaceae_bacterium	OFB_Centries	0.8930529806
blast	Neglectibacter_sp_X4	OFB_Centries	0.0847821517
vns	Neglectibacter_sp_X4	OFB_Centries	0.140656264
blast	Oscillospiraceae_bacterium	OFB_Centries	0.7416725656
vns	Oscillospiraceae_bacterium	OFB_Centries	0.856188039
blast	Oscillospiraceae_unclassified_SGB43502	OFB_Centries	0.0259412852
vns	Oscillospiraceae_unclassified_SGB43502	OFB_Centries	0.0305351331
blast	Oscillospiraceae_unclassified_SGB43505	OFB_Centries	0.4959230188
vns	Oscillospiraceae_unclassified_SGB43505	OFB_Centries	0.6425337316
blast	Parasutterella_excrementihominis	OFB_Centries	0.5671048722
vns	Parasutterella_excrementihominis	OFB_Centries	0.7099929171
blast	Richness (# observed features)	OFB_Centries	0.2717524423
vns	Richness (# observed features)	OFB_Centries	0.6607230685

blast	Romboutsia_ilealis	OFB_Centries	0.001099774
vns	Romboutsia_ilealis	OFB_Centries	0.056997089
blast	Schaedlerella_arabinosiphila	OFB_Centries	0.939024404
vns	Schaedlerella_arabinosiphila	OFB_Centries	0.913911607
blast	Shannon Index	OFB_Centries	0.694735221
vns	Shannon Index	OFB_Centries	0.906277341
blast	Turicibacter_sp_1E2	OFB_Centries	0.199177231
vns	Turicibacter_sp_1E2	OFB_Centries	0.165574931
blast	Acetatifactor_muris	OFB_Clatency	0.739522079
vns	Acetatifactor_muris	OFB_Clatency	0.752897140
blast	Acetatifactor_SGB41546	OFB_Clatency	0.840245797
vns	Acetatifactor_SGB41546	OFB_Clatency	0.663000977
blast	Acutalibacter_muris	OFB_Clatency	0.422154633
vns	Acutalibacter_muris	OFB_Clatency	0.810578449
blast	Acutalibacter_sp_1XD8_36	OFB_Clatency	0.660579886
vns	Acutalibacter_sp_1XD8_36	OFB_Clatency	0.709184933
blast	Adlercreutzia_caecimuris	OFB_Clatency	0.989608952
vns	Adlercreutzia_caecimuris	OFB_Clatency	0.676542691
blast	Adlercreutzia_mucosicola	OFB_Clatency	0.271833985
vns	Adlercreutzia_mucosicola	OFB_Clatency	0.249825657
blast	Adlercreutzia_muris	OFB_Clatency	0.670421774
vns	Adlercreutzia_muris	OFB_Clatency	0.595827745
blast	Akkermansia_muciniphila	OFB_Clatency	0.169812540
vns	Akkermansia_muciniphila	OFB_Clatency	0.864362509
blast	Alistipes_sp_DSM_112343	OFB_Clatency	0.372515977
vns	Alistipes_sp_DSM_112343	OFB_Clatency	0.832698307
blast	Anaerotruncus_sp_1XD42_93	OFB_Clatency	0.520271453
vns	Anaerotruncus_sp_1XD42_93	OFB_Clatency	0.617410721
blast	Bacteria_unclassified_SGB102200	OFB_Clatency	0.474269212
vns	Bacteria_unclassified_SGB102200	OFB_Clatency	0.764166788
blast	Bacteria_unclassified_SGB41677	OFB_Clatency	0.444143823
vns	Bacteria_unclassified_SGB41677	OFB_Clatency	0.169989424
blast	Bacteria_unclassified_SGB43546	OFB_Clatency	0.586358365
vns	Bacteria_unclassified_SGB43546	OFB_Clatency	0.789009912
blast	bacterium_1XD42_54	OFB_Clatency	0.014424595
vns	bacterium_1XD42_54	OFB_Clatency	0.234577574
blast	bacterium_1XD42_76	OFB_Clatency	0.600031603
vns	bacterium_1XD42_76	OFB_Clatency	0.642992453
blast	bacterium_1xD8_48	OFB_Clatency	0.899708945
vns	bacterium_1xD8_48	OFB_Clatency	0.696816880
blast	Bacteroides_thetaiotaomicron	OFB_Clatency	0.960201477
vns	Bacteroides_thetaiotaomicron	OFB_Clatency	0.634306315
blast	Berger Parker Index	OFB_Clatency	0.545741712
vns	Berger Parker Index	OFB_Clatency	0.233733054
blast	Bifidobacterium_pseudolongum	OFB_Clatency	0.428010398
vns	Bifidobacterium_pseudolongum	OFB_Clatency	0.347799065

blast	Clostridia_bacterium	OFB_Clatency	0.374559043
vns	Clostridia_bacterium	OFB_Clatency	0.724940107
blast	Clostridiaceae_bacterium	OFB_Clatency	0.987169780
vns	Clostridiaceae_bacterium	OFB_Clatency	0.241937042
blast	Clostridiaceae_unclassified_SGB41663	OFB_Clatency	0.780382224
vns	Clostridiaceae_unclassified_SGB41663	OFB_Clatency	0.719154368
blast	Clostridiales_bacterium	OFB_Clatency	0.002331956
vns	Clostridiales_bacterium	OFB_Clatency	0.296478501
blast	Clostridium_cocleatum	OFB_Clatency	0.011567832
vns	Clostridium_cocleatum	OFB_Clatency	0.196035514
blast	Coriobacteriaceae_bacterium	OFB_Clatency	0.372448369
vns	Coriobacteriaceae_bacterium	OFB_Clatency	0.953919137
blast	Dorea_sp_5_2	OFB_Clatency	0.588491917
vns	Dorea_sp_5_2	OFB_Clatency	0.421416124
blast	Dubosiella_newyorkensis	OFB_Clatency	0.116998739
vns	Dubosiella_newyorkensis	OFB_Clatency	0.799186063
blast	Erysipelotrichales_bacterium	OFB_Clatency	0.897000665
vns	Erysipelotrichales_bacterium	OFB_Clatency	0.328408108
blast	Eubacteriaceae_bacterium	OFB_Clatency	0.027123368
vns	Eubacteriaceae_bacterium	OFB_Clatency	0.238318613
blast	Eubacteriaceae_unclassified_SGB94922	OFB_Clatency	0.459488079
vns	Eubacteriaceae_unclassified_SGB94922	OFB_Clatency	0.372620148
blast	GGB20149_SGB29430	OFB_Clatency	0.628070048
vns	GGB20149_SGB29430	OFB_Clatency	0.629982792
blast	GGB22635_SGB63107	OFB_Clatency	0.411710336
vns	GGB22635_SGB63107	OFB_Clatency	0.887152138
blast	GGB25041_SGB36960	OFB_Clatency	0.082496667
vns	GGB25041_SGB36960	OFB_Clatency	0.210187196
blast	GGB27876_SGB40310	OFB_Clatency	0.164666135
vns	GGB27876_SGB40310	OFB_Clatency	0.767235194
blast	GGB27878_SGB40312	OFB_Clatency	0.213354157
vns	GGB27878_SGB40312	OFB_Clatency	0.147479429
blast	GGB27918_SGB40356	OFB_Clatency	0.958758140
vns	GGB27918_SGB40356	OFB_Clatency	0.885698929
blast	GGB28382_SGB40962	OFB_Clatency	0.519311307
vns	GGB28382_SGB40962	OFB_Clatency	0.855057834
blast	GGB28399_SGB40980	OFB_Clatency	0.289465502
vns	GGB28399_SGB40980	OFB_Clatency	0.287117034
blast	GGB28411_SGB40993	OFB_Clatency	0.201802262
vns	GGB28411_SGB40993	OFB_Clatency	0.146848474
blast	GGB28415_SGB40997	OFB_Clatency	0.026726744
vns	GGB28415_SGB40997	OFB_Clatency	0.949051696
blast	GGB28430_SGB41013	OFB_Clatency	0.912070947
vns	GGB28430_SGB41013	OFB_Clatency	0.466250482
blast	GGB28439_SGB41022	OFB_Clatency	0.380444403
vns	GGB28439_SGB41022	OFB_Clatency	0.738709552

blast	GGB28778_SGB41431	OFB_Clatency	0.804698941
vns	GGB28778_SGB41431	OFB_Clatency	0.218453243
blast	GGB28784_SGB41437	OFB_Clatency	0.020726621
vns	GGB28784_SGB41437	OFB_Clatency	0.254456997
blast	GGB28792_SGB41445	OFB_Clatency	0.338577775
vns	GGB28792_SGB41445	OFB_Clatency	0.761244426
blast	GGB28798_SGB41451	OFB_Clatency	0.164022630
vns	GGB28798_SGB41451	OFB_Clatency	0.248915824
blast	GGB28802_SGB41455	OFB_Clatency	0.457898625
vns	GGB28802_SGB41455	OFB_Clatency	0.342689298
blast	GGB28818_SGB41473	OFB_Clatency	0.729209510
vns	GGB28818_SGB41473	OFB_Clatency	0.569409948
blast	GGB28828_SGB41484	OFB_Clatency	0.298696993
vns	GGB28828_SGB41484	OFB_Clatency	0.106706535
blast	GGB28851_SGB41518	OFB_Clatency	0.852552179
vns	GGB28851_SGB41518	OFB_Clatency	0.578308429
blast	GGB28859_SGB41528	OFB_Clatency	0.205749329
vns	GGB28859_SGB41528	OFB_Clatency	0.595366745
blast	GGB28864_SGB41535	OFB_Clatency	0.785465248
vns	GGB28864_SGB41535	OFB_Clatency	0.964658985
blast	GGB28869_SGB41543	OFB_Clatency	0.347931625
vns	GGB28869_SGB41543	OFB_Clatency	0.381477475
blast	GGB28883_SGB41564	OFB_Clatency	0.594877248
vns	GGB28883_SGB41564	OFB_Clatency	0.060524458
blast	GGB28892_SGB41573	OFB_Clatency	0.651227207
vns	GGB28892_SGB41573	OFB_Clatency	0.667618882
blast	GGB28893_SGB41574	OFB_Clatency	0.165594509
vns	GGB28893_SGB41574	OFB_Clatency	0.182699376
blast	GGB28898_SGB41580	OFB_Clatency	0.304654993
vns	GGB28898_SGB41580	OFB_Clatency	0.082893330
blast	GGB28904_SGB41597	OFB_Clatency	0.172906397
vns	GGB28904_SGB41597	OFB_Clatency	0.056067199
blast	GGB28916_SGB41612	OFB_Clatency	0.801432609
vns	GGB28916_SGB41612	OFB_Clatency	0.853813296
blast	GGB28924_SGB41621	OFB_Clatency	0.796481662
vns	GGB28924_SGB41621	OFB_Clatency	0.278658378
blast	GGB28926_SGB41624	OFB_Clatency	0.306780834
vns	GGB28926_SGB41624	OFB_Clatency	0.470765265
blast	GGB28927_SGB41625	OFB_Clatency	0.193720805
vns	GGB28927_SGB41625	OFB_Clatency	0.418243373
blast	GGB28934_SGB41635	OFB_Clatency	0.115873311
vns	GGB28934_SGB41635	OFB_Clatency	0.111374073
blast	GGB28946_SGB41652	OFB_Clatency	0.427843225
vns	GGB28946_SGB41652	OFB_Clatency	0.422588900
blast	GGB28949_SGB41655	OFB_Clatency	0.443608116
vns	GGB28949_SGB41655	OFB_Clatency	0.908948179

blast	GGB28949_SGB41656	OFB_Clatency	0.282728234
vns	GGB28949_SGB41656	OFB_Clatency	0.770863949
blast	GGB28950_SGB41657	OFB_Clatency	0.030527036
vns	GGB28950_SGB41657	OFB_Clatency	0.336458904
blast	GGB28951_SGB102295	OFB_Clatency	0.925352156
vns	GGB28951_SGB102295	OFB_Clatency	0.158790397
blast	GGB28951_SGB41658	OFB_Clatency	0.847001351
vns	GGB28951_SGB41658	OFB_Clatency	0.920236623
blast	GGB28954_SGB41662	OFB_Clatency	0.515758297
vns	GGB28954_SGB41662	OFB_Clatency	0.800484955
blast	GGB28956_SGB41665	OFB_Clatency	0.163329713
vns	GGB28956_SGB41665	OFB_Clatency	0.153416502
blast	GGB28960_SGB41669	OFB_Clatency	0.155727292
vns	GGB28960_SGB41669	OFB_Clatency	0.155957479
blast	GGB28967_SGB41678	OFB_Clatency	0.179269529
vns	GGB28967_SGB41678	OFB_Clatency	0.315046519
blast	GGB28991_SGB41705	OFB_Clatency	0.228893882
vns	GGB28991_SGB41705	OFB_Clatency	0.279982342
blast	GGB29002_SGB41718	OFB_Clatency	0.002430867
vns	GGB29002_SGB41718	OFB_Clatency	0.113465587
blast	GGB29003_SGB41719	OFB_Clatency	0.499398644
vns	GGB29003_SGB41719	OFB_Clatency	0.583967886
blast	GGB29011_SGB41731	OFB_Clatency	0.916276033
vns	GGB29011_SGB41731	OFB_Clatency	0.345599236
blast	GGB29531_SGB42317	OFB_Clatency	0.145082026
vns	GGB29531_SGB42317	OFB_Clatency	0.135569966
blast	GGB29685_SGB42494	OFB_Clatency	0.864543852
vns	GGB29685_SGB42494	OFB_Clatency	0.340104263
blast	GGB30141_SGB43066	OFB_Clatency	0.991554803
vns	GGB30141_SGB43066	OFB_Clatency	0.760178789
blast	GGB30286_SGB43248	OFB_Clatency	0.055261633
vns	GGB30286_SGB43248	OFB_Clatency	0.454603367
blast	GGB30303_SGB43268	OFB_Clatency	0.371936893
vns	GGB30303_SGB43268	OFB_Clatency	0.944722174
blast	GGB30413_SGB43452	OFB_Clatency	0.953276950
vns	GGB30413_SGB43452	OFB_Clatency	0.879642604
blast	GGB30454_SGB43514	OFB_Clatency	0.379858950
vns	GGB30454_SGB43514	OFB_Clatency	0.200125591
blast	GGB30455_SGB43519	OFB_Clatency	0.379467796
vns	GGB30455_SGB43519	OFB_Clatency	0.302348921
blast	GGB30461_SGB43527	OFB_Clatency	0.397503662
vns	GGB30461_SGB43527	OFB_Clatency	0.771038655
blast	GGB30461_SGB43530	OFB_Clatency	0.395449
vns	GGB30461_SGB43530	OFB_Clatency	0.703863693
blast	GGB30463_SGB43537	OFB_Clatency	0.210054257
vns	GGB30463_SGB43537	OFB_Clatency	0.985361479

blast	GGB30473_SGB43557	OFB_Clatency	0.020642166
vns	GGB30473_SGB43557	OFB_Clatency	0.056211743
blast	GGB30475_SGB63182	OFB_Clatency	0.287965349
vns	GGB30475_SGB63182	OFB_Clatency	0.660855930
blast	GGB30861_SGB44083	OFB_Clatency	0.957552379
vns	GGB30861_SGB44083	OFB_Clatency	0.833445529
blast	GGB31312_SGB44628	OFB_Clatency	0.711727128
vns	GGB31312_SGB44628	OFB_Clatency	0.884193943
blast	GGB31438_SGB44768	OFB_Clatency	0.906357297
vns	GGB31438_SGB44768	OFB_Clatency	0.318960086
blast	GGB3171_SGB4185	OFB_Clatency	0.085345767
vns	GGB3171_SGB4185	OFB_Clatency	0.918551780
blast	GGB31823_SGB45199	OFB_Clatency	0.188977717
vns	GGB31823_SGB45199	OFB_Clatency	0.702813258
blast	GGB31853_SGB45233	OFB_Clatency	0.496842743
vns	GGB31853_SGB45233	OFB_Clatency	0.396259541
blast	GGB32371_SGB41694	OFB_Clatency	0.346811850
vns	GGB32371_SGB41694	OFB_Clatency	0.025054092
blast	GGB3793_SGB5158	OFB_Clatency	0.965790474
vns	GGB3793_SGB5158	OFB_Clatency	0.975145151
blast	GGB42598_SGB59794	OFB_Clatency	0.501740001
vns	GGB42598_SGB59794	OFB_Clatency	0.156668604
blast	GGB45656_SGB63370	OFB_Clatency	0.621044138
vns	GGB45656_SGB63370	OFB_Clatency	0.963374601
blast	GGB47127_SGB65054	OFB_Clatency	0.496745120
vns	GGB47127_SGB65054	OFB_Clatency	0.257417312
blast	GGB74395_SGB43521	OFB_Clatency	0.123339782
vns	GGB74395_SGB43521	OFB_Clatency	0.204021498
blast	GGB75053_SGB43494	OFB_Clatency	0.410595919
vns	GGB75053_SGB43494	OFB_Clatency	0.571857456
blast	GGB75109_SGB102238	OFB_Clatency	0.620842292
vns	GGB75109_SGB102238	OFB_Clatency	0.871818408
blast	GGB81440_SGB45230	OFB_Clatency	0.079610731
vns	GGB81440_SGB45230	OFB_Clatency	0.098140579
blast	Lachnospiraceae_bacterium	OFB_Clatency	0.484816177
vns	Lachnospiraceae_bacterium	OFB_Clatency	0.537861332
blast	Lachnospiraceae_bacterium_A2	OFB_Clatency	0.420834896
vns	Lachnospiraceae_bacterium_A2	OFB_Clatency	0.323648328
blast	Lachnospiraceae_bacterium_MD308	OFB_Clatency	0.213119451
vns	Lachnospiraceae_bacterium_MD308	OFB_Clatency	0.083066965
blast	Lachnospiraceae_bacterium_MD329	OFB_Clatency	0.020976149
vns	Lachnospiraceae_bacterium_MD329	OFB_Clatency	0.699161119
blast	Lachnospiraceae_unclassified_SGB41414	OFB_Clatency	0.142639053
vns	Lachnospiraceae_unclassified_SGB41414	OFB_Clatency	0.119876468
blast	Lachnospiraceae_unclassified_SGB41418	OFB_Clatency	0.998757981
vns	Lachnospiraceae_unclassified_SGB41418	OFB_Clatency	0.712236573

blast	Lachnospiraceae_unclassified_SGB41424	OFB_Clatency	0.048386378
vns	Lachnospiraceae_unclassified_SGB41424	OFB_Clatency	0.046067357
blast	Lachnospiraceae_unclassified_SGB41589	OFB_Clatency	0.165424952
vns	Lachnospiraceae_unclassified_SGB41589	OFB_Clatency	0.273527700
blast	Lactobacillus_johnsonii	OFB_Clatency	0.182678453
vns	Lactobacillus_johnsonii	OFB_Clatency	0.672951588
blast	Muribaculaceae_bacterium	OFB_Clatency	0.841525252
vns	Muribaculaceae_bacterium	OFB_Clatency	0.893052980
blast	Neglectibacter_sp_X4	OFB_Clatency	0.084782151
vns	Neglectibacter_sp_X4	OFB_Clatency	0.140656264
blast	Oscillospiraceae_bacterium	OFB_Clatency	0.741672565
vns	Oscillospiraceae_bacterium	OFB_Clatency	0.856188039
blast	Oscillospiraceae_unclassified_SGB43502	OFB_Clatency	0.025941285
vns	Oscillospiraceae_unclassified_SGB43502	OFB_Clatency	0.030535133
blast	Oscillospiraceae_unclassified_SGB43505	OFB_Clatency	0.495923018
vns	Oscillospiraceae_unclassified_SGB43505	OFB_Clatency	0.642533731
blast	Parasutterella_excrementihominis	OFB_Clatency	0.567104872
vns	Parasutterella_excrementihominis	OFB_Clatency	0.709992917
blast	Richness (# observed features)	OFB_Clatency	0.271752442
vns	Richness (# observed features)	OFB_Clatency	0.660723068
blast	Romboutsia_ilealis	OFB_Clatency	0.001099774
vns	Romboutsia_ilealis	OFB_Clatency	0.056997089
blast	Schaedlerella_arabinosiphila	OFB_Clatency	0.939024404
vns	Schaedlerella_arabinosiphila	OFB_Clatency	0.913911607
blast	Shannon Index	OFB_Clatency	0.694735221
vns	Shannon Index	OFB_Clatency	0.906277341
blast	Turicibacter_sp_1E2	OFB_Clatency	0.199177231
vns	Turicibacter_sp_1E2	OFB_Clatency	0.165574931
blast	Acetatifactor_muris	OFB_Ctime	0.739522079
vns	Acetatifactor_muris	OFB_Ctime	0.752897140
blast	Acetatifactor_SGB41546	OFB_Ctime	0.840245797
vns	Acetatifactor_SGB41546	OFB_Ctime	0.663000977
blast	Acutalibacter_muris	OFB_Ctime	0.422154633
vns	Acutalibacter_muris	OFB_Ctime	0.810578449
blast	Acutalibacter_sp_1XD8_36	OFB_Ctime	0.660579886
vns	Acutalibacter_sp_1XD8_36	OFB_Ctime	0.709184933
blast	Adlercreutzia_caecimuris	OFB_Ctime	0.989608952
vns	Adlercreutzia_caecimuris	OFB_Ctime	0.676542691
blast	Adlercreutzia_mucosicola	OFB_Ctime	0.271833985
vns	Adlercreutzia_mucosicola	OFB_Ctime	0.249825657
blast	Adlercreutzia_muris	OFB_Ctime	0.670421774
vns	Adlercreutzia_muris	OFB_Ctime	0.595827745
blast	Akkermansia_muciniphila	OFB_Ctime	0.169812540
vns	Akkermansia_muciniphila	OFB_Ctime	0.864362509
blast	Alistipes_sp_DSM_112343	OFB_Ctime	0.372515977
vns	Alistipes_sp_DSM_112343	OFB_Ctime	0.832698307

blast	Anaerotruncus_sp_1XD42_93	OFB_Ctime	0.5202714535
vns	Anaerotruncus_sp_1XD42_93	OFB_Ctime	0.6174107217
blast	Bacteria_unclassified_SGB102200	OFB_Ctime	0.4742692127
vns	Bacteria_unclassified_SGB102200	OFB_Ctime	0.7641667885
blast	Bacteria_unclassified_SGB41677	OFB_Ctime	0.4441438231
vns	Bacteria_unclassified_SGB41677	OFB_Ctime	0.1699894243
blast	Bacteria_unclassified_SGB43546	OFB_Ctime	0.5863583659
vns	Bacteria_unclassified_SGB43546	OFB_Ctime	0.7890099125
blast	bacterium_1XD42_54	OFB_Ctime	0.0144245959
vns	bacterium_1XD42_54	OFB_Ctime	0.2345775748
blast	bacterium_1XD42_76	OFB_Ctime	0.6000316038
vns	bacterium_1XD42_76	OFB_Ctime	0.6429924539
blast	bacterium_1xD8_48	OFB_Ctime	0.8997089453
vns	bacterium_1xD8_48	OFB_Ctime	0.6968168809
blast	Bacteroides_thetaiotaomicron	OFB_Ctime	0.9602014777
vns	Bacteroides_thetaiotaomicron	OFB_Ctime	0.6343063159
blast	Berger Parker Index	OFB_Ctime	0.5457417127
vns	Berger Parker Index	OFB_Ctime	0.2337330546
blast	Bifidobacterium_pseudolongum	OFB_Ctime	0.4280103985
vns	Bifidobacterium_pseudolongum	OFB_Ctime	0.3477990653
blast	Clostridia_bacterium	OFB_Ctime	0.3745590434
vns	Clostridia_bacterium	OFB_Ctime	0.7249401071
blast	Clostridiaceae_bacterium	OFB_Ctime	0.9871697806
vns	Clostridiaceae_bacterium	OFB_Ctime	0.2419370425
blast	Clostridiaceae_unclassified_SGB41663	OFB_Ctime	0.780382224
vns	Clostridiaceae_unclassified_SGB41663	OFB_Ctime	0.719154368
blast	Clostridiales_bacterium	OFB_Ctime	0.0023319564
vns	Clostridiales_bacterium	OFB_Ctime	0.2964785018
blast	Clostridium_cocleatum	OFB_Ctime	0.0115678325
vns	Clostridium_cocleatum	OFB_Ctime	0.1960355146
blast	Coriobacteriaceae_bacterium	OFB_Ctime	0.3724483697
vns	Coriobacteriaceae_bacterium	OFB_Ctime	0.953919137
blast	Dorea_sp_5_2	OFB_Ctime	0.5884919176
vns	Dorea_sp_5_2	OFB_Ctime	0.4214161244
blast	Dubosiella_newyorkensis	OFB_Ctime	0.1169987394
vns	Dubosiella_newyorkensis	OFB_Ctime	0.799186063
blast	Erysipelotrichales_bacterium	OFB_Ctime	0.8970006656
vns	Erysipelotrichales_bacterium	OFB_Ctime	0.3284081083
blast	Eubacteriaceae_bacterium	OFB_Ctime	0.0271233687
vns	Eubacteriaceae_bacterium	OFB_Ctime	0.2383186135
blast	Eubacteriaceae_unclassified_SGB94922	OFB_Ctime	0.4594880799
vns	Eubacteriaceae_unclassified_SGB94922	OFB_Ctime	0.3726201484
blast	GGB20149_SGB29430	OFB_Ctime	0.6280700484
vns	GGB20149_SGB29430	OFB_Ctime	0.6299827926
blast	GGB22635_SGB63107	OFB_Ctime	0.4117103362
vns	GGB22635_SGB63107	OFB_Ctime	0.8871521383

blast	GGB25041_SGB36960	OFB_Ctime	0.082496667
vns	GGB25041_SGB36960	OFB_Ctime	0.210187196
blast	GGB27876_SGB40310	OFB_Ctime	0.164666135
vns	GGB27876_SGB40310	OFB_Ctime	0.767235194
blast	GGB27878_SGB40312	OFB_Ctime	0.213354157
vns	GGB27878_SGB40312	OFB_Ctime	0.147479429
blast	GGB27918_SGB40356	OFB_Ctime	0.958758140
vns	GGB27918_SGB40356	OFB_Ctime	0.885698929
blast	GGB28382_SGB40962	OFB_Ctime	0.519311307
vns	GGB28382_SGB40962	OFB_Ctime	0.855057834
blast	GGB28399_SGB40980	OFB_Ctime	0.289465502
vns	GGB28399_SGB40980	OFB_Ctime	0.287117034
blast	GGB28411_SGB40993	OFB_Ctime	0.201802262
vns	GGB28411_SGB40993	OFB_Ctime	0.146848474
blast	GGB28415_SGB40997	OFB_Ctime	0.026726744
vns	GGB28415_SGB40997	OFB_Ctime	0.949051696
blast	GGB28430_SGB41013	OFB_Ctime	0.912070947
vns	GGB28430_SGB41013	OFB_Ctime	0.466250482
blast	GGB28439_SGB41022	OFB_Ctime	0.380444403
vns	GGB28439_SGB41022	OFB_Ctime	0.738709552
blast	GGB28778_SGB41431	OFB_Ctime	0.804698941
vns	GGB28778_SGB41431	OFB_Ctime	0.218453243
blast	GGB28784_SGB41437	OFB_Ctime	0.020726621
vns	GGB28784_SGB41437	OFB_Ctime	0.254456997
blast	GGB28792_SGB41445	OFB_Ctime	0.338577775
vns	GGB28792_SGB41445	OFB_Ctime	0.761244426
blast	GGB28798_SGB41451	OFB_Ctime	0.164022630
vns	GGB28798_SGB41451	OFB_Ctime	0.248915824
blast	GGB28802_SGB41455	OFB_Ctime	0.457898625
vns	GGB28802_SGB41455	OFB_Ctime	0.342689298
blast	GGB28818_SGB41473	OFB_Ctime	0.729209510
vns	GGB28818_SGB41473	OFB_Ctime	0.569409948
blast	GGB28828_SGB41484	OFB_Ctime	0.298696993
vns	GGB28828_SGB41484	OFB_Ctime	0.106706535
blast	GGB28851_SGB41518	OFB_Ctime	0.852552179
vns	GGB28851_SGB41518	OFB_Ctime	0.578308429
blast	GGB28859_SGB41528	OFB_Ctime	0.205749329
vns	GGB28859_SGB41528	OFB_Ctime	0.595366745
blast	GGB28864_SGB41535	OFB_Ctime	0.785465248
vns	GGB28864_SGB41535	OFB_Ctime	0.964658985
blast	GGB28869_SGB41543	OFB_Ctime	0.347931625
vns	GGB28869_SGB41543	OFB_Ctime	0.381477475
blast	GGB28883_SGB41564	OFB_Ctime	0.594877248
vns	GGB28883_SGB41564	OFB_Ctime	0.060524458
blast	GGB28892_SGB41573	OFB_Ctime	0.651227207
vns	GGB28892_SGB41573	OFB_Ctime	0.667618882

blast	GGB28893_SGB41574	OFB_Ctime	0.165594509
vns	GGB28893_SGB41574	OFB_Ctime	0.182699376
blast	GGB28898_SGB41580	OFB_Ctime	0.304654993
vns	GGB28898_SGB41580	OFB_Ctime	0.082893330
blast	GGB28904_SGB41597	OFB_Ctime	0.172906397
vns	GGB28904_SGB41597	OFB_Ctime	0.056067199
blast	GGB28916_SGB41612	OFB_Ctime	0.801432609
vns	GGB28916_SGB41612	OFB_Ctime	0.853813296
blast	GGB28924_SGB41621	OFB_Ctime	0.796481662
vns	GGB28924_SGB41621	OFB_Ctime	0.278658378
blast	GGB28926_SGB41624	OFB_Ctime	0.306780834
vns	GGB28926_SGB41624	OFB_Ctime	0.470765265
blast	GGB28927_SGB41625	OFB_Ctime	0.193720805
vns	GGB28927_SGB41625	OFB_Ctime	0.418243373
blast	GGB28934_SGB41635	OFB_Ctime	0.115873311
vns	GGB28934_SGB41635	OFB_Ctime	0.111374073
blast	GGB28946_SGB41652	OFB_Ctime	0.427843225
vns	GGB28946_SGB41652	OFB_Ctime	0.422588900
blast	GGB28949_SGB41655	OFB_Ctime	0.443608116
vns	GGB28949_SGB41655	OFB_Ctime	0.908948179
blast	GGB28949_SGB41656	OFB_Ctime	0.282728234
vns	GGB28949_SGB41656	OFB_Ctime	0.770863949
blast	GGB28950_SGB41657	OFB_Ctime	0.030527036
vns	GGB28950_SGB41657	OFB_Ctime	0.336458904
blast	GGB28951_SGB102295	OFB_Ctime	0.925352156
vns	GGB28951_SGB102295	OFB_Ctime	0.158790397
blast	GGB28951_SGB41658	OFB_Ctime	0.847001351
vns	GGB28951_SGB41658	OFB_Ctime	0.920236623
blast	GGB28954_SGB41662	OFB_Ctime	0.515758297
vns	GGB28954_SGB41662	OFB_Ctime	0.800484955
blast	GGB28956_SGB41665	OFB_Ctime	0.163329713
vns	GGB28956_SGB41665	OFB_Ctime	0.153416502
blast	GGB28960_SGB41669	OFB_Ctime	0.155727292
vns	GGB28960_SGB41669	OFB_Ctime	0.155957479
blast	GGB28967_SGB41678	OFB_Ctime	0.179269529
vns	GGB28967_SGB41678	OFB_Ctime	0.315046519
blast	GGB28991_SGB41705	OFB_Ctime	0.228893882
vns	GGB28991_SGB41705	OFB_Ctime	0.279982342
blast	GGB29002_SGB41718	OFB_Ctime	0.002430867
vns	GGB29002_SGB41718	OFB_Ctime	0.113465587
blast	GGB29003_SGB41719	OFB_Ctime	0.499398644
vns	GGB29003_SGB41719	OFB_Ctime	0.583967886
blast	GGB29011_SGB41731	OFB_Ctime	0.916276033
vns	GGB29011_SGB41731	OFB_Ctime	0.345599236
blast	GGB29531_SGB42317	OFB_Ctime	0.145082026
vns	GGB29531_SGB42317	OFB_Ctime	0.135569966

blast	GGB29685_SGB42494	OFB_Ctime	0.8645438528
vns	GGB29685_SGB42494	OFB_Ctime	0.340104263
blast	GGB30141_SGB43066	OFB_Ctime	0.9915548039
vns	GGB30141_SGB43066	OFB_Ctime	0.7601787897
blast	GGB30286_SGB43248	OFB_Ctime	0.0552616333
vns	GGB30286_SGB43248	OFB_Ctime	0.4546033673
blast	GGB30303_SGB43268	OFB_Ctime	0.3719368939
vns	GGB30303_SGB43268	OFB_Ctime	0.9447221743
blast	GGB30413_SGB43452	OFB_Ctime	0.9532769507
vns	GGB30413_SGB43452	OFB_Ctime	0.8796426046
blast	GGB30454_SGB43514	OFB_Ctime	0.3798589507
vns	GGB30454_SGB43514	OFB_Ctime	0.2001255913
blast	GGB30455_SGB43519	OFB_Ctime	0.3794677965
vns	GGB30455_SGB43519	OFB_Ctime	0.3023489219
blast	GGB30461_SGB43527	OFB_Ctime	0.3975036627
vns	GGB30461_SGB43527	OFB_Ctime	0.7710386558
blast	GGB30461_SGB43530	OFB_Ctime	0.395449
vns	GGB30461_SGB43530	OFB_Ctime	0.7038636932
blast	GGB30463_SGB43537	OFB_Ctime	0.2100542578
vns	GGB30463_SGB43537	OFB_Ctime	0.9853614792
blast	GGB30473_SGB43557	OFB_Ctime	0.0206421669
vns	GGB30473_SGB43557	OFB_Ctime	0.0562117431
blast	GGB30475_SGB63182	OFB_Ctime	0.2879653492
vns	GGB30475_SGB63182	OFB_Ctime	0.6608559306
blast	GGB30861_SGB44083	OFB_Ctime	0.9575523797
vns	GGB30861_SGB44083	OFB_Ctime	0.8334455291
blast	GGB31312_SGB44628	OFB_Ctime	0.7117271287
vns	GGB31312_SGB44628	OFB_Ctime	0.8841939433
blast	GGB31438_SGB44768	OFB_Ctime	0.9063572977
vns	GGB31438_SGB44768	OFB_Ctime	0.3189600867
blast	GGB3171_SGB4185	OFB_Ctime	0.0853457671
vns	GGB3171_SGB4185	OFB_Ctime	0.9185517801
blast	GGB31823_SGB45199	OFB_Ctime	0.1889777175
vns	GGB31823_SGB45199	OFB_Ctime	0.7028132582
blast	GGB31853_SGB45233	OFB_Ctime	0.4968427437
vns	GGB31853_SGB45233	OFB_Ctime	0.3962595415
blast	GGB32371_SGB41694	OFB_Ctime	0.3468118508
vns	GGB32371_SGB41694	OFB_Ctime	0.0250540925
blast	GGB3793_SGB5158	OFB_Ctime	0.9657904747
vns	GGB3793_SGB5158	OFB_Ctime	0.9751451519
blast	GGB42598_SGB59794	OFB_Ctime	0.5017400018
vns	GGB42598_SGB59794	OFB_Ctime	0.156668604
blast	GGB45656_SGB63370	OFB_Ctime	0.621044138
vns	GGB45656_SGB63370	OFB_Ctime	0.9633746015
blast	GGB47127_SGB65054	OFB_Ctime	0.4967451202
vns	GGB47127_SGB65054	OFB_Ctime	0.2574173127

blast	GGB74395_SGB43521	OFB_Ctime	0.123339782
vns	GGB74395_SGB43521	OFB_Ctime	0.204021498
blast	GGB75053_SGB43494	OFB_Ctime	0.410595919
vns	GGB75053_SGB43494	OFB_Ctime	0.571857456
blast	GGB75109_SGB102238	OFB_Ctime	0.620842292
vns	GGB75109_SGB102238	OFB_Ctime	0.871818408
blast	GGB81440_SGB45230	OFB_Ctime	0.079610731
vns	GGB81440_SGB45230	OFB_Ctime	0.098140579
blast	Lachnospiraceae_bacterium	OFB_Ctime	0.484816177
vns	Lachnospiraceae_bacterium	OFB_Ctime	0.537861332
blast	Lachnospiraceae_bacterium_A2	OFB_Ctime	0.420834896
vns	Lachnospiraceae_bacterium_A2	OFB_Ctime	0.323648328
blast	Lachnospiraceae_bacterium_MD308	OFB_Ctime	0.213119451
vns	Lachnospiraceae_bacterium_MD308	OFB_Ctime	0.083066965
blast	Lachnospiraceae_bacterium_MD329	OFB_Ctime	0.020976149
vns	Lachnospiraceae_bacterium_MD329	OFB_Ctime	0.699161119
blast	Lachnospiraceae_unclassified_SGB41414	OFB_Ctime	0.142639053
vns	Lachnospiraceae_unclassified_SGB41414	OFB_Ctime	0.119876468
blast	Lachnospiraceae_unclassified_SGB41418	OFB_Ctime	0.998757981
vns	Lachnospiraceae_unclassified_SGB41418	OFB_Ctime	0.712236573
blast	Lachnospiraceae_unclassified_SGB41424	OFB_Ctime	0.048386378
vns	Lachnospiraceae_unclassified_SGB41424	OFB_Ctime	0.046067357
blast	Lachnospiraceae_unclassified_SGB41589	OFB_Ctime	0.165424952
vns	Lachnospiraceae_unclassified_SGB41589	OFB_Ctime	0.273527700
blast	Lactobacillus_johnsonii	OFB_Ctime	0.182678453
vns	Lactobacillus_johnsonii	OFB_Ctime	0.672951588
blast	Muribaculaceae_bacterium	OFB_Ctime	0.841525252
vns	Muribaculaceae_bacterium	OFB_Ctime	0.893052980
blast	Neglectibacter_sp_X4	OFB_Ctime	0.084782151
vns	Neglectibacter_sp_X4	OFB_Ctime	0.140656264
blast	Oscillospiraceae_bacterium	OFB_Ctime	0.741672565
vns	Oscillospiraceae_bacterium	OFB_Ctime	0.856188039
blast	Oscillospiraceae_unclassified_SGB43502	OFB_Ctime	0.025941285
vns	Oscillospiraceae_unclassified_SGB43502	OFB_Ctime	0.030535133
blast	Oscillospiraceae_unclassified_SGB43505	OFB_Ctime	0.495923018
vns	Oscillospiraceae_unclassified_SGB43505	OFB_Ctime	0.642533731
blast	Parasutterella_excrementihominis	OFB_Ctime	0.567104872
vns	Parasutterella_excrementihominis	OFB_Ctime	0.709992917
blast	Richness (# observed features)	OFB_Ctime	0.271752442
vns	Richness (# observed features)	OFB_Ctime	0.660723068
blast	Romboutsia_ilealis	OFB_Ctime	0.001099774
vns	Romboutsia_ilealis	OFB_Ctime	0.056997089
blast	Schaedlerella_arabinosiphila	OFB_Ctime	0.939024404
vns	Schaedlerella_arabinosiphila	OFB_Ctime	0.913911607
blast	Shannon Index	OFB_Ctime	0.694735221
vns	Shannon Index	OFB_Ctime	0.906277341

blast	Turicibacter_sp_1E2	OFB_Ctime	0.1991772311
vns	Turicibacter_sp_1E2	OFB_Ctime	0.1655749311
blast	Acetatifactor_muris	PD_choice_filt	0.7395220790
vns	Acetatifactor_muris	PD_choice_filt	0.7528971403
blast	Acetatifactor_SGB41546	PD_choice_filt	0.8402457971
vns	Acetatifactor_SGB41546	PD_choice_filt	0.6630009771
blast	Acutalibacter_muris	PD_choice_filt	0.4221546334
vns	Acutalibacter_muris	PD_choice_filt	0.8105784495
blast	Acutalibacter_sp_1XD8_36	PD_choice_filt	0.6605798863
vns	Acutalibacter_sp_1XD8_36	PD_choice_filt	0.7091849330
blast	Adlercreutzia_caecimuris	PD_choice_filt	0.9896089529
vns	Adlercreutzia_caecimuris	PD_choice_filt	0.6765426913
blast	Adlercreutzia_mucosicola	PD_choice_filt	0.2718339850
vns	Adlercreutzia_mucosicola	PD_choice_filt	0.2498256574
blast	Adlercreutzia_muris	PD_choice_filt	0.6704217743
vns	Adlercreutzia_muris	PD_choice_filt	0.5958277454
blast	Akkermansia_muciniphila	PD_choice_filt	0.1698125404
vns	Akkermansia_muciniphila	PD_choice_filt	0.8643625099
blast	Alistipes_sp_DSM_112343	PD_choice_filt	0.3725159774
vns	Alistipes_sp_DSM_112343	PD_choice_filt	0.8326983074
blast	Anaerotruncus_sp_1XD42_93	PD_choice_filt	0.5202714535
vns	Anaerotruncus_sp_1XD42_93	PD_choice_filt	0.6174107217
blast	Bacteria_unclassified_SGB102200	PD_choice_filt	0.4742692127
vns	Bacteria_unclassified_SGB102200	PD_choice_filt	0.7641667885
blast	Bacteria_unclassified_SGB41677	PD_choice_filt	0.4441438231
vns	Bacteria_unclassified_SGB41677	PD_choice_filt	0.1699894243
blast	Bacteria_unclassified_SGB43546	PD_choice_filt	0.5863583659
vns	Bacteria_unclassified_SGB43546	PD_choice_filt	0.7890099125
blast	bacterium_1XD42_54	PD_choice_filt	0.0144245959
vns	bacterium_1XD42_54	PD_choice_filt	0.2345775748
blast	bacterium_1XD42_76	PD_choice_filt	0.6000316038
vns	bacterium_1XD42_76	PD_choice_filt	0.6429924539
blast	bacterium_1xD8_48	PD_choice_filt	0.8997089453
vns	bacterium_1xD8_48	PD_choice_filt	0.6968168809
blast	Bacteroides_thetaiotaomicron	PD_choice_filt	0.9602014777
vns	Bacteroides_thetaiotaomicron	PD_choice_filt	0.6343063159
blast	Berger Parker Index	PD_choice_filt	0.5457417127
vns	Berger Parker Index	PD_choice_filt	0.2337330540
blast	Bifidobacterium_pseudolongum	PD_choice_filt	0.4280103985
vns	Bifidobacterium_pseudolongum	PD_choice_filt	0.3477990653
blast	Clostridia_bacterium	PD_choice_filt	0.3745590434
vns	Clostridia_bacterium	PD_choice_filt	0.7249401071
blast	Clostridiaceae_bacterium	PD_choice_filt	0.9871697800
vns	Clostridiaceae_bacterium	PD_choice_filt	0.2419370425
blast	Clostridiaceae_unclassified_SGB41663	PD_choice_filt	0.780382224
vns	Clostridiaceae_unclassified_SGB41663	PD_choice_filt	0.719154368

blast	Clostridiales_bacterium	PD_choice_filt	0.0023319564
vns	Clostridiales_bacterium	PD_choice_filt	0.2964785018
blast	Clostridium_cocleatum	PD_choice_filt	0.0115678325
vns	Clostridium_cocleatum	PD_choice_filt	0.1960355146
blast	Coriobacteriaceae_bacterium	PD_choice_filt	0.3724483697
vns	Coriobacteriaceae_bacterium	PD_choice_filt	0.953919137
blast	Dorea_sp_5_2	PD_choice_filt	0.5884919176
vns	Dorea_sp_5_2	PD_choice_filt	0.4214161244
blast	Dubosiella_newyorkensis	PD_choice_filt	0.1169987394
vns	Dubosiella_newyorkensis	PD_choice_filt	0.799186063
blast	Erysipelotrichales_bacterium	PD_choice_filt	0.8970006656
vns	Erysipelotrichales_bacterium	PD_choice_filt	0.3284081083
blast	Eubacteriaceae_bacterium	PD_choice_filt	0.0271233687
vns	Eubacteriaceae_bacterium	PD_choice_filt	0.2383186135
blast	Eubacteriaceae_unclassified_SGB94922	PD_choice_filt	0.4594880799
vns	Eubacteriaceae_unclassified_SGB94922	PD_choice_filt	0.3726201484
blast	GGB20149_SGB29430	PD_choice_filt	0.6280700484
vns	GGB20149_SGB29430	PD_choice_filt	0.6299827926
blast	GGB22635_SGB63107	PD_choice_filt	0.4117103362
vns	GGB22635_SGB63107	PD_choice_filt	0.8871521383
blast	GGB25041_SGB36960	PD_choice_filt	0.0824966676
vns	GGB25041_SGB36960	PD_choice_filt	0.2101871969
blast	GGB27876_SGB40310	PD_choice_filt	0.164666135
vns	GGB27876_SGB40310	PD_choice_filt	0.7672351949
blast	GGB27878_SGB40312	PD_choice_filt	0.2133541578
vns	GGB27878_SGB40312	PD_choice_filt	0.1474794298
blast	GGB27918_SGB40356	PD_choice_filt	0.9587581408
vns	GGB27918_SGB40356	PD_choice_filt	0.8856989297
blast	GGB28382_SGB40962	PD_choice_filt	0.5193113079
vns	GGB28382_SGB40962	PD_choice_filt	0.8550578348
blast	GGB28399_SGB40980	PD_choice_filt	0.2894655025
vns	GGB28399_SGB40980	PD_choice_filt	0.287117034
blast	GGB28411_SGB40993	PD_choice_filt	0.2018022626
vns	GGB28411_SGB40993	PD_choice_filt	0.1468484741
blast	GGB28415_SGB40997	PD_choice_filt	0.0267267446
vns	GGB28415_SGB40997	PD_choice_filt	0.9490516966
blast	GGB28430_SGB41013	PD_choice_filt	0.9120709472
vns	GGB28430_SGB41013	PD_choice_filt	0.4662504826
blast	GGB28439_SGB41022	PD_choice_filt	0.3804444035
vns	GGB28439_SGB41022	PD_choice_filt	0.7387095529
blast	GGB28778_SGB41431	PD_choice_filt	0.8046989411
vns	GGB28778_SGB41431	PD_choice_filt	0.2184532434
blast	GGB28784_SGB41437	PD_choice_filt	0.0207266217
vns	GGB28784_SGB41437	PD_choice_filt	0.2544569976
blast	GGB28792_SGB41445	PD_choice_filt	0.3385777757
vns	GGB28792_SGB41445	PD_choice_filt	0.7612444262

blast	GGB28798_SGB41451	PD_choice_filt	0.1640226305
vns	GGB28798_SGB41451	PD_choice_filt	0.2489158244
blast	GGB28802_SGB41455	PD_choice_filt	0.4578986259
vns	GGB28802_SGB41455	PD_choice_filt	0.342689298
blast	GGB28818_SGB41473	PD_choice_filt	0.7292095104
vns	GGB28818_SGB41473	PD_choice_filt	0.5694099484
blast	GGB28828_SGB41484	PD_choice_filt	0.2986969933
vns	GGB28828_SGB41484	PD_choice_filt	0.1067065352
blast	GGB28851_SGB41518	PD_choice_filt	0.852552179
vns	GGB28851_SGB41518	PD_choice_filt	0.5783084298
blast	GGB28859_SGB41528	PD_choice_filt	0.2057493298
vns	GGB28859_SGB41528	PD_choice_filt	0.5953667458
blast	GGB28864_SGB41535	PD_choice_filt	0.7854652488
vns	GGB28864_SGB41535	PD_choice_filt	0.9646589853
blast	GGB28869_SGB41543	PD_choice_filt	0.3479316259
vns	GGB28869_SGB41543	PD_choice_filt	0.3814774755
blast	GGB28883_SGB41564	PD_choice_filt	0.5948772487
vns	GGB28883_SGB41564	PD_choice_filt	0.0605244586
blast	GGB28892_SGB41573	PD_choice_filt	0.6512272078
vns	GGB28892_SGB41573	PD_choice_filt	0.6676188822
blast	GGB28893_SGB41574	PD_choice_filt	0.165594509
vns	GGB28893_SGB41574	PD_choice_filt	0.1826993761
blast	GGB28898_SGB41580	PD_choice_filt	0.3046549939
vns	GGB28898_SGB41580	PD_choice_filt	0.0828933309
blast	GGB28904_SGB41597	PD_choice_filt	0.1729063974
vns	GGB28904_SGB41597	PD_choice_filt	0.0560671999
blast	GGB28916_SGB41612	PD_choice_filt	0.8014326092
vns	GGB28916_SGB41612	PD_choice_filt	0.8538132969
blast	GGB28924_SGB41621	PD_choice_filt	0.7964816629
vns	GGB28924_SGB41621	PD_choice_filt	0.2786583787
blast	GGB28926_SGB41624	PD_choice_filt	0.3067808341
vns	GGB28926_SGB41624	PD_choice_filt	0.470765265
blast	GGB28927_SGB41625	PD_choice_filt	0.1937208057
vns	GGB28927_SGB41625	PD_choice_filt	0.4182433734
blast	GGB28934_SGB41635	PD_choice_filt	0.1158733118
vns	GGB28934_SGB41635	PD_choice_filt	0.1113740731
blast	GGB28946_SGB41652	PD_choice_filt	0.4278432256
vns	GGB28946_SGB41652	PD_choice_filt	0.4225889007
blast	GGB28949_SGB41655	PD_choice_filt	0.4436081164
vns	GGB28949_SGB41655	PD_choice_filt	0.9089481791
blast	GGB28949_SGB41656	PD_choice_filt	0.2827282346
vns	GGB28949_SGB41656	PD_choice_filt	0.7708639493
blast	GGB28950_SGB41657	PD_choice_filt	0.0305270365
vns	GGB28950_SGB41657	PD_choice_filt	0.3364589048
blast	GGB28951_SGB102295	PD_choice_filt	0.9253521562
vns	GGB28951_SGB102295	PD_choice_filt	0.1587903977

blast	GGB28951_SGB41658	PD_choice_filt	0.8470013515
vns	GGB28951_SGB41658	PD_choice_filt	0.9202366237
blast	GGB28954_SGB41662	PD_choice_filt	0.5157582975
vns	GGB28954_SGB41662	PD_choice_filt	0.8004849551
blast	GGB28956_SGB41665	PD_choice_filt	0.163329713
vns	GGB28956_SGB41665	PD_choice_filt	0.1534165023
blast	GGB28960_SGB41669	PD_choice_filt	0.155727292
vns	GGB28960_SGB41669	PD_choice_filt	0.1559574792
blast	GGB28967_SGB41678	PD_choice_filt	0.1792695292
vns	GGB28967_SGB41678	PD_choice_filt	0.3150465193
blast	GGB28991_SGB41705	PD_choice_filt	0.2288938824
vns	GGB28991_SGB41705	PD_choice_filt	0.279982342
blast	GGB29002_SGB41718	PD_choice_filt	0.0024308675
vns	GGB29002_SGB41718	PD_choice_filt	0.1134655877
blast	GGB29003_SGB41719	PD_choice_filt	0.4993986443
vns	GGB29003_SGB41719	PD_choice_filt	0.5839678864
blast	GGB29011_SGB41731	PD_choice_filt	0.916276033
vns	GGB29011_SGB41731	PD_choice_filt	0.3455992366
blast	GGB29531_SGB42317	PD_choice_filt	0.1450820268
vns	GGB29531_SGB42317	PD_choice_filt	0.1355699662
blast	GGB29685_SGB42494	PD_choice_filt	0.8645438528
vns	GGB29685_SGB42494	PD_choice_filt	0.340104263
blast	GGB30141_SGB43066	PD_choice_filt	0.9915548039
vns	GGB30141_SGB43066	PD_choice_filt	0.7601787897
blast	GGB30286_SGB43248	PD_choice_filt	0.0552616333
vns	GGB30286_SGB43248	PD_choice_filt	0.4546033673
blast	GGB30303_SGB43268	PD_choice_filt	0.3719368939
vns	GGB30303_SGB43268	PD_choice_filt	0.9447221743
blast	GGB30413_SGB43452	PD_choice_filt	0.9532769507
vns	GGB30413_SGB43452	PD_choice_filt	0.8796426046
blast	GGB30454_SGB43514	PD_choice_filt	0.3798589507
vns	GGB30454_SGB43514	PD_choice_filt	0.2001255913
blast	GGB30455_SGB43519	PD_choice_filt	0.3794677965
vns	GGB30455_SGB43519	PD_choice_filt	0.3023489219
blast	GGB30461_SGB43527	PD_choice_filt	0.3975036627
vns	GGB30461_SGB43527	PD_choice_filt	0.7710386558
blast	GGB30461_SGB43530	PD_choice_filt	0.395449
vns	GGB30461_SGB43530	PD_choice_filt	0.7038636932
blast	GGB30463_SGB43537	PD_choice_filt	0.2100542578
vns	GGB30463_SGB43537	PD_choice_filt	0.9853614792
blast	GGB30473_SGB43557	PD_choice_filt	0.0206421669
vns	GGB30473_SGB43557	PD_choice_filt	0.0562117431
blast	GGB30475_SGB63182	PD_choice_filt	0.2879653492
vns	GGB30475_SGB63182	PD_choice_filt	0.6608559306
blast	GGB30861_SGB44083	PD_choice_filt	0.9575523797
vns	GGB30861_SGB44083	PD_choice_filt	0.8334455291

blast	GGB31312_SGB44628	PD_choice_filt	0.711727128
vns	GGB31312_SGB44628	PD_choice_filt	0.884193943
blast	GGB31438_SGB44768	PD_choice_filt	0.906357297
vns	GGB31438_SGB44768	PD_choice_filt	0.318960086
blast	GGB3171_SGB4185	PD_choice_filt	0.085345767
vns	GGB3171_SGB4185	PD_choice_filt	0.918551780
blast	GGB31823_SGB45199	PD_choice_filt	0.188977717
vns	GGB31823_SGB45199	PD_choice_filt	0.702813258
blast	GGB31853_SGB45233	PD_choice_filt	0.496842743
vns	GGB31853_SGB45233	PD_choice_filt	0.396259541
blast	GGB32371_SGB41694	PD_choice_filt	0.346811850
vns	GGB32371_SGB41694	PD_choice_filt	0.025054092
blast	GGB3793_SGB5158	PD_choice_filt	0.965790474
vns	GGB3793_SGB5158	PD_choice_filt	0.975145151
blast	GGB42598_SGB59794	PD_choice_filt	0.501740001
vns	GGB42598_SGB59794	PD_choice_filt	0.156668604
blast	GGB45656_SGB63370	PD_choice_filt	0.621044138
vns	GGB45656_SGB63370	PD_choice_filt	0.963374601
blast	GGB47127_SGB65054	PD_choice_filt	0.496745120
vns	GGB47127_SGB65054	PD_choice_filt	0.257417312
blast	GGB74395_SGB43521	PD_choice_filt	0.123339782
vns	GGB74395_SGB43521	PD_choice_filt	0.204021498
blast	GGB75053_SGB43494	PD_choice_filt	0.410595919
vns	GGB75053_SGB43494	PD_choice_filt	0.571857456
blast	GGB75109_SGB102238	PD_choice_filt	0.620842292
vns	GGB75109_SGB102238	PD_choice_filt	0.871818408
blast	GGB81440_SGB45230	PD_choice_filt	0.079610731
vns	GGB81440_SGB45230	PD_choice_filt	0.098140579
blast	Lachnospiraceae_bacterium	PD_choice_filt	0.484816177
vns	Lachnospiraceae_bacterium	PD_choice_filt	0.537861332
blast	Lachnospiraceae_bacterium_A2	PD_choice_filt	0.420834896
vns	Lachnospiraceae_bacterium_A2	PD_choice_filt	0.323648328
blast	Lachnospiraceae_bacterium_MD308	PD_choice_filt	0.213119451
vns	Lachnospiraceae_bacterium_MD308	PD_choice_filt	0.083066965
blast	Lachnospiraceae_bacterium_MD329	PD_choice_filt	0.020976149
vns	Lachnospiraceae_bacterium_MD329	PD_choice_filt	0.699161119
blast	Lachnospiraceae_unclassified_SGB41414	PD_choice_filt	0.142639053
vns	Lachnospiraceae_unclassified_SGB41414	PD_choice_filt	0.119876468
blast	Lachnospiraceae_unclassified_SGB41418	PD_choice_filt	0.998757981
vns	Lachnospiraceae_unclassified_SGB41418	PD_choice_filt	0.712236573
blast	Lachnospiraceae_unclassified_SGB41424	PD_choice_filt	0.048386378
vns	Lachnospiraceae_unclassified_SGB41424	PD_choice_filt	0.046067357
blast	Lachnospiraceae_unclassified_SGB41589	PD_choice_filt	0.165424952
vns	Lachnospiraceae_unclassified_SGB41589	PD_choice_filt	0.273527700
blast	Lactobacillus_johnsonii	PD_choice_filt	0.182678453
vns	Lactobacillus_johnsonii	PD_choice_filt	0.672951588

blast	Muribaculaceae_bacterium	PD_choice_filt	0.84152525
vns	Muribaculaceae_bacterium	PD_choice_filt	0.89305298
blast	Neglectibacter_sp_X4	PD_choice_filt	0.08478215
vns	Neglectibacter_sp_X4	PD_choice_filt	0.140656264
blast	Oscillospiraceae_bacterium	PD_choice_filt	0.74167256
vns	Oscillospiraceae_bacterium	PD_choice_filt	0.856188039
blast	Oscillospiraceae_unclassified_SGB43502	PD_choice_filt	0.02594128
vns	Oscillospiraceae_unclassified_SGB43502	PD_choice_filt	0.03053513
blast	Oscillospiraceae_unclassified_SGB43505	PD_choice_filt	0.49592301
vns	Oscillospiraceae_unclassified_SGB43505	PD_choice_filt	0.64253373
blast	Parasutterella_excrementihominis	PD_choice_filt	0.56710487
vns	Parasutterella_excrementihominis	PD_choice_filt	0.70999291
blast	Richness (# observed features)	PD_choice_filt	0.27175244
vns	Richness (# observed features)	PD_choice_filt	0.66072306
blast	Romboutsia_ilealis	PD_choice_filt	0.00109977
vns	Romboutsia_ilealis	PD_choice_filt	0.05699708
blast	Schaedlerella_arabinosiphila	PD_choice_filt	0.93902440
vns	Schaedlerella_arabinosiphila	PD_choice_filt	0.91391160
blast	Shannon Index	PD_choice_filt	0.69473522
vns	Shannon Index	PD_choice_filt	0.90627734
blast	Turicibacter_sp_1E2	PD_choice_filt	0.19917723
vns	Turicibacter_sp_1E2	PD_choice_filt	0.16557493
blast	Acetatifactor_muris	startle_habituation	0.73952207
vns	Acetatifactor_muris	startle_habituation	0.75289714
blast	Acetatifactor_SGB41546	startle_habituation	0.84024579
vns	Acetatifactor_SGB41546	startle_habituation	0.66300097
blast	Acutalibacter_muris	startle_habituation	0.42215463
vns	Acutalibacter_muris	startle_habituation	0.81057844
blast	Acutalibacter_sp_1XD8_36	startle_habituation	0.66057988
vns	Acutalibacter_sp_1XD8_36	startle_habituation	0.70918493
blast	Adlercreutzia_caecimuris	startle_habituation	0.98960895
vns	Adlercreutzia_caecimuris	startle_habituation	0.67654269
blast	Adlercreutzia_mucosicola	startle_habituation	0.27183398
vns	Adlercreutzia_mucosicola	startle_habituation	0.24982565
blast	Adlercreutzia_muris	startle_habituation	0.67042177
vns	Adlercreutzia_muris	startle_habituation	0.59582774
blast	Akkermansia_muciniphila	startle_habituation	0.16981254
vns	Akkermansia_muciniphila	startle_habituation	0.86436250
blast	Alistipes_sp_DSM_112343	startle_habituation	0.37251597
vns	Alistipes_sp_DSM_112343	startle_habituation	0.83269830
blast	Anaerotruncus_sp_1XD42_93	startle_habituation	0.52027145
vns	Anaerotruncus_sp_1XD42_93	startle_habituation	0.61741072
blast	Bacteria_unclassified_SGB102200	startle_habituation	0.47426921
vns	Bacteria_unclassified_SGB102200	startle_habituation	0.76416678
blast	Bacteria_unclassified_SGB41677	startle_habituation	0.44414382
vns	Bacteria_unclassified_SGB41677	startle_habituation	0.16998942

blast	Bacteria_unclassified_SGB43546	startle_habituation	0.586358365
vns	Bacteria_unclassified_SGB43546	startle_habituation	0.789009912
blast	bacterium_1XD42_54	startle_habituation	0.014424595
vns	bacterium_1XD42_54	startle_habituation	0.234577574
blast	bacterium_1XD42_76	startle_habituation	0.600031603
vns	bacterium_1XD42_76	startle_habituation	0.642992453
blast	bacterium_1xD8_48	startle_habituation	0.899708945
vns	bacterium_1xD8_48	startle_habituation	0.696816880
blast	Bacteroides_thetaiotaomicron	startle_habituation	0.960201477
vns	Bacteroides_thetaiotaomicron	startle_habituation	0.634306315
blast	Berger Parker Index	startle_habituation	0.545741712
vns	Berger Parker Index	startle_habituation	0.233733054
blast	Bifidobacterium_pseudolongum	startle_habituation	0.428010398
vns	Bifidobacterium_pseudolongum	startle_habituation	0.347799065
blast	Clostridia_bacterium	startle_habituation	0.374559043
vns	Clostridia_bacterium	startle_habituation	0.724940107
blast	Clostridiaceae_bacterium	startle_habituation	0.987169780
vns	Clostridiaceae_bacterium	startle_habituation	0.241937042
blast	Clostridiaceae_unclassified_SGB41663	startle_habituation	0.780382224
vns	Clostridiaceae_unclassified_SGB41663	startle_habituation	0.719154368
blast	Clostridiales_bacterium	startle_habituation	0.002331956
vns	Clostridiales_bacterium	startle_habituation	0.296478501
blast	Clostridium_cocleatum	startle_habituation	0.011567832
vns	Clostridium_cocleatum	startle_habituation	0.196035514
blast	Coriobacteriaceae_bacterium	startle_habituation	0.372448369
vns	Coriobacteriaceae_bacterium	startle_habituation	0.953919137
blast	Dorea_sp_5_2	startle_habituation	0.588491917
vns	Dorea_sp_5_2	startle_habituation	0.421416124
blast	Dubosiella_newyorkensis	startle_habituation	0.116998739
vns	Dubosiella_newyorkensis	startle_habituation	0.799186063
blast	Erysipelotrichales_bacterium	startle_habituation	0.897000665
vns	Erysipelotrichales_bacterium	startle_habituation	0.328408108
blast	Eubacteriaceae_bacterium	startle_habituation	0.027123368
vns	Eubacteriaceae_bacterium	startle_habituation	0.238318613
blast	Eubacteriaceae_unclassified_SGB94922	startle_habituation	0.459488079
vns	Eubacteriaceae_unclassified_SGB94922	startle_habituation	0.372620148
blast	GGB20149_SGB29430	startle_habituation	0.628070048
vns	GGB20149_SGB29430	startle_habituation	0.629982792
blast	GGB22635_SGB63107	startle_habituation	0.411710336
vns	GGB22635_SGB63107	startle_habituation	0.887152138
blast	GGB25041_SGB36960	startle_habituation	0.082496667
vns	GGB25041_SGB36960	startle_habituation	0.210187196
blast	GGB27876_SGB40310	startle_habituation	0.164666135
vns	GGB27876_SGB40310	startle_habituation	0.767235194
blast	GGB27878_SGB40312	startle_habituation	0.213354157
vns	GGB27878_SGB40312	startle_habituation	0.147479429

blast	GGB27918_SGB40356	startle_habituation	0.9587581408
vns	GGB27918_SGB40356	startle_habituation	0.8856989297
blast	GGB28382_SGB40962	startle_habituation	0.5193113079
vns	GGB28382_SGB40962	startle_habituation	0.8550578348
blast	GGB28399_SGB40980	startle_habituation	0.2894655025
vns	GGB28399_SGB40980	startle_habituation	0.287117034
blast	GGB28411_SGB40993	startle_habituation	0.2018022626
vns	GGB28411_SGB40993	startle_habituation	0.1468484741
blast	GGB28415_SGB40997	startle_habituation	0.0267267446
vns	GGB28415_SGB40997	startle_habituation	0.9490516966
blast	GGB28430_SGB41013	startle_habituation	0.9120709472
vns	GGB28430_SGB41013	startle_habituation	0.4662504826
blast	GGB28439_SGB41022	startle_habituation	0.3804444035
vns	GGB28439_SGB41022	startle_habituation	0.7387095529
blast	GGB28778_SGB41431	startle_habituation	0.8046989411
vns	GGB28778_SGB41431	startle_habituation	0.2184532434
blast	GGB28784_SGB41437	startle_habituation	0.0207266217
vns	GGB28784_SGB41437	startle_habituation	0.2544569976
blast	GGB28792_SGB41445	startle_habituation	0.3385777757
vns	GGB28792_SGB41445	startle_habituation	0.7612444262
blast	GGB28798_SGB41451	startle_habituation	0.1640226305
vns	GGB28798_SGB41451	startle_habituation	0.2489158244
blast	GGB28802_SGB41455	startle_habituation	0.4578986259
vns	GGB28802_SGB41455	startle_habituation	0.342689298
blast	GGB28818_SGB41473	startle_habituation	0.7292095104
vns	GGB28818_SGB41473	startle_habituation	0.5694099484
blast	GGB28828_SGB41484	startle_habituation	0.2986969933
vns	GGB28828_SGB41484	startle_habituation	0.1067065352
blast	GGB28851_SGB41518	startle_habituation	0.852552179
vns	GGB28851_SGB41518	startle_habituation	0.5783084298
blast	GGB28859_SGB41528	startle_habituation	0.2057493298
vns	GGB28859_SGB41528	startle_habituation	0.5953667458
blast	GGB28864_SGB41535	startle_habituation	0.7854652488
vns	GGB28864_SGB41535	startle_habituation	0.9646589853
blast	GGB28869_SGB41543	startle_habituation	0.3479316259
vns	GGB28869_SGB41543	startle_habituation	0.3814774755
blast	GGB28883_SGB41564	startle_habituation	0.5948772487
vns	GGB28883_SGB41564	startle_habituation	0.0605244586
blast	GGB28892_SGB41573	startle_habituation	0.6512272078
vns	GGB28892_SGB41573	startle_habituation	0.6676188822
blast	GGB28893_SGB41574	startle_habituation	0.165594509
vns	GGB28893_SGB41574	startle_habituation	0.1826993761
blast	GGB28898_SGB41580	startle_habituation	0.3046549939
vns	GGB28898_SGB41580	startle_habituation	0.0828933309
blast	GGB28904_SGB41597	startle_habituation	0.1729063974
vns	GGB28904_SGB41597	startle_habituation	0.0560671999

blast	GGB28916_SGB41612	startle_habituation	0.801432609
vns	GGB28916_SGB41612	startle_habituation	0.853813296
blast	GGB28924_SGB41621	startle_habituation	0.796481662
vns	GGB28924_SGB41621	startle_habituation	0.278658378
blast	GGB28926_SGB41624	startle_habituation	0.306780834
vns	GGB28926_SGB41624	startle_habituation	0.470765265
blast	GGB28927_SGB41625	startle_habituation	0.193720805
vns	GGB28927_SGB41625	startle_habituation	0.418243373
blast	GGB28934_SGB41635	startle_habituation	0.115873311
vns	GGB28934_SGB41635	startle_habituation	0.111374073
blast	GGB28946_SGB41652	startle_habituation	0.427843225
vns	GGB28946_SGB41652	startle_habituation	0.422588900
blast	GGB28949_SGB41655	startle_habituation	0.443608116
vns	GGB28949_SGB41655	startle_habituation	0.908948179
blast	GGB28949_SGB41656	startle_habituation	0.282728234
vns	GGB28949_SGB41656	startle_habituation	0.770863949
blast	GGB28950_SGB41657	startle_habituation	0.030527036
vns	GGB28950_SGB41657	startle_habituation	0.336458904
blast	GGB28951_SGB102295	startle_habituation	0.925352156
vns	GGB28951_SGB102295	startle_habituation	0.158790397
blast	GGB28951_SGB41658	startle_habituation	0.847001351
vns	GGB28951_SGB41658	startle_habituation	0.920236623
blast	GGB28954_SGB41662	startle_habituation	0.515758297
vns	GGB28954_SGB41662	startle_habituation	0.800484955
blast	GGB28956_SGB41665	startle_habituation	0.163329713
vns	GGB28956_SGB41665	startle_habituation	0.153416502
blast	GGB28960_SGB41669	startle_habituation	0.155727292
vns	GGB28960_SGB41669	startle_habituation	0.155957479
blast	GGB28967_SGB41678	startle_habituation	0.179269529
vns	GGB28967_SGB41678	startle_habituation	0.315046519
blast	GGB28991_SGB41705	startle_habituation	0.228893882
vns	GGB28991_SGB41705	startle_habituation	0.279982342
blast	GGB29002_SGB41718	startle_habituation	0.002430867
vns	GGB29002_SGB41718	startle_habituation	0.113465587
blast	GGB29003_SGB41719	startle_habituation	0.499398644
vns	GGB29003_SGB41719	startle_habituation	0.583967886
blast	GGB29011_SGB41731	startle_habituation	0.916276033
vns	GGB29011_SGB41731	startle_habituation	0.345599236
blast	GGB29531_SGB42317	startle_habituation	0.145082026
vns	GGB29531_SGB42317	startle_habituation	0.135569966
blast	GGB29685_SGB42494	startle_habituation	0.864543852
vns	GGB29685_SGB42494	startle_habituation	0.340104263
blast	GGB30141_SGB43066	startle_habituation	0.991554803
vns	GGB30141_SGB43066	startle_habituation	0.760178789
blast	GGB30286_SGB43248	startle_habituation	0.055261633
vns	GGB30286_SGB43248	startle_habituation	0.454603367

blast	GGB30303_SGB43268	startle_habituation	0.371936893
vns	GGB30303_SGB43268	startle_habituation	0.944722174
blast	GGB30413_SGB43452	startle_habituation	0.953276950
vns	GGB30413_SGB43452	startle_habituation	0.879642604
blast	GGB30454_SGB43514	startle_habituation	0.379858950
vns	GGB30454_SGB43514	startle_habituation	0.200125591
blast	GGB30455_SGB43519	startle_habituation	0.379467796
vns	GGB30455_SGB43519	startle_habituation	0.302348921
blast	GGB30461_SGB43527	startle_habituation	0.397503662
vns	GGB30461_SGB43527	startle_habituation	0.771038655
blast	GGB30461_SGB43530	startle_habituation	0.395449
vns	GGB30461_SGB43530	startle_habituation	0.703863693
blast	GGB30463_SGB43537	startle_habituation	0.210054257
vns	GGB30463_SGB43537	startle_habituation	0.985361479
blast	GGB30473_SGB43557	startle_habituation	0.020642166
vns	GGB30473_SGB43557	startle_habituation	0.056211743
blast	GGB30475_SGB63182	startle_habituation	0.287965349
vns	GGB30475_SGB63182	startle_habituation	0.660855930
blast	GGB30861_SGB44083	startle_habituation	0.957552379
vns	GGB30861_SGB44083	startle_habituation	0.833445529
blast	GGB31312_SGB44628	startle_habituation	0.711727128
vns	GGB31312_SGB44628	startle_habituation	0.884193943
blast	GGB31438_SGB44768	startle_habituation	0.906357297
vns	GGB31438_SGB44768	startle_habituation	0.318960086
blast	GGB3171_SGB4185	startle_habituation	0.085345767
vns	GGB3171_SGB4185	startle_habituation	0.918551780
blast	GGB31823_SGB45199	startle_habituation	0.188977717
vns	GGB31823_SGB45199	startle_habituation	0.702813258
blast	GGB31853_SGB45233	startle_habituation	0.496842743
vns	GGB31853_SGB45233	startle_habituation	0.396259541
blast	GGB32371_SGB41694	startle_habituation	0.346811850
vns	GGB32371_SGB41694	startle_habituation	0.025054092
blast	GGB3793_SGB5158	startle_habituation	0.965790474
vns	GGB3793_SGB5158	startle_habituation	0.975145151
blast	GGB42598_SGB59794	startle_habituation	0.501740001
vns	GGB42598_SGB59794	startle_habituation	0.156668604
blast	GGB45656_SGB63370	startle_habituation	0.621044138
vns	GGB45656_SGB63370	startle_habituation	0.963374601
blast	GGB47127_SGB65054	startle_habituation	0.496745120
vns	GGB47127_SGB65054	startle_habituation	0.257417312
blast	GGB74395_SGB43521	startle_habituation	0.123339782
vns	GGB74395_SGB43521	startle_habituation	0.204021498
blast	GGB75053_SGB43494	startle_habituation	0.410595919
vns	GGB75053_SGB43494	startle_habituation	0.571857456
blast	GGB75109_SGB102238	startle_habituation	0.620842292
vns	GGB75109_SGB102238	startle_habituation	0.871818408

blast	GGB81440_SGB45230	startle_habituation	0.079610731
vns	GGB81440_SGB45230	startle_habituation	0.098140579
blast	Lachnospiraceae_bacterium	startle_habituation	0.484816177
vns	Lachnospiraceae_bacterium	startle_habituation	0.537861332
blast	Lachnospiraceae_bacterium_A2	startle_habituation	0.420834896
vns	Lachnospiraceae_bacterium_A2	startle_habituation	0.323648328
blast	Lachnospiraceae_bacterium_MD308	startle_habituation	0.213119451
vns	Lachnospiraceae_bacterium_MD308	startle_habituation	0.083066965
blast	Lachnospiraceae_bacterium_MD329	startle_habituation	0.020976149
vns	Lachnospiraceae_bacterium_MD329	startle_habituation	0.699161119
blast	Lachnospiraceae_unclassified_SGB41414	startle_habituation	0.142639053
vns	Lachnospiraceae_unclassified_SGB41414	startle_habituation	0.119876468
blast	Lachnospiraceae_unclassified_SGB41418	startle_habituation	0.998757981
vns	Lachnospiraceae_unclassified_SGB41418	startle_habituation	0.712236573
blast	Lachnospiraceae_unclassified_SGB41424	startle_habituation	0.048386378
vns	Lachnospiraceae_unclassified_SGB41424	startle_habituation	0.046067357
blast	Lachnospiraceae_unclassified_SGB41589	startle_habituation	0.165424952
vns	Lachnospiraceae_unclassified_SGB41589	startle_habituation	0.273527700
blast	Lactobacillus_johnsonii	startle_habituation	0.182678453
vns	Lactobacillus_johnsonii	startle_habituation	0.672951588
blast	Muribaculaceae_bacterium	startle_habituation	0.841525252
vns	Muribaculaceae_bacterium	startle_habituation	0.893052980
blast	Neglectibacter_sp_X4	startle_habituation	0.084782151
vns	Neglectibacter_sp_X4	startle_habituation	0.140656264
blast	Oscillospiraceae_bacterium	startle_habituation	0.741672565
vns	Oscillospiraceae_bacterium	startle_habituation	0.856188039
blast	Oscillospiraceae_unclassified_SGB43502	startle_habituation	0.025941285
vns	Oscillospiraceae_unclassified_SGB43502	startle_habituation	0.030535133
blast	Oscillospiraceae_unclassified_SGB43505	startle_habituation	0.495923018
vns	Oscillospiraceae_unclassified_SGB43505	startle_habituation	0.642533731
blast	Parasutterella_excrementihominis	startle_habituation	0.567104872
vns	Parasutterella_excrementihominis	startle_habituation	0.709992917
blast	Richness (# observed features)	startle_habituation	0.271752442
vns	Richness (# observed features)	startle_habituation	0.660723068
blast	Romboutsia_ilealis	startle_habituation	0.001099774
vns	Romboutsia_ilealis	startle_habituation	0.056997089
blast	Schaedlerella_arabinosiphila	startle_habituation	0.939024404
vns	Schaedlerella_arabinosiphila	startle_habituation	0.913911607
blast	Shannon Index	startle_habituation	0.694735221
vns	Shannon Index	startle_habituation	0.906277341
blast	Turicibacter_sp_1E2	startle_habituation	0.199177231
vns	Turicibacter_sp_1E2	startle_habituation	0.165574931
blast	Acetatifactor_muris	startle_PPI	0.739522079
vns	Acetatifactor_muris	startle_PPI	0.752897140
blast	Acetatifactor_SGB41546	startle_PPI	0.840245797
vns	Acetatifactor_SGB41546	startle_PPI	0.663000977

blast	Acutalibacter_muris	startle_PPI	0.4221546334
vns	Acutalibacter_muris	startle_PPI	0.8105784495
blast	Acutalibacter_sp_1XD8_36	startle_PPI	0.6605798863
vns	Acutalibacter_sp_1XD8_36	startle_PPI	0.7091849336
blast	Adlercreutzia_caecimuris	startle_PPI	0.9896089529
vns	Adlercreutzia_caecimuris	startle_PPI	0.6765426913
blast	Adlercreutzia_mucosicola	startle_PPI	0.2718339856
vns	Adlercreutzia_mucosicola	startle_PPI	0.2498256574
blast	Adlercreutzia_muris	startle_PPI	0.6704217743
vns	Adlercreutzia_muris	startle_PPI	0.5958277454
blast	Akkermansia_muciniphila	startle_PPI	0.1698125404
vns	Akkermansia_muciniphila	startle_PPI	0.8643625099
blast	Alistipes_sp_DSM_112343	startle_PPI	0.3725159774
vns	Alistipes_sp_DSM_112343	startle_PPI	0.8326983074
blast	Anaerotruncus_sp_1XD42_93	startle_PPI	0.5202714535
vns	Anaerotruncus_sp_1XD42_93	startle_PPI	0.6174107217
blast	Bacteria_unclassified_SGB102200	startle_PPI	0.4742692127
vns	Bacteria_unclassified_SGB102200	startle_PPI	0.7641667885
blast	Bacteria_unclassified_SGB41677	startle_PPI	0.4441438231
vns	Bacteria_unclassified_SGB41677	startle_PPI	0.1699894243
blast	Bacteria_unclassified_SGB43546	startle_PPI	0.5863583659
vns	Bacteria_unclassified_SGB43546	startle_PPI	0.7890099125
blast	bacterium_1XD42_54	startle_PPI	0.0144245959
vns	bacterium_1XD42_54	startle_PPI	0.2345775748
blast	bacterium_1XD42_76	startle_PPI	0.6000316038
vns	bacterium_1XD42_76	startle_PPI	0.6429924539
blast	bacterium_1xD8_48	startle_PPI	0.8997089453
vns	bacterium_1xD8_48	startle_PPI	0.6968168809
blast	Bacteroides_thetaiotaomicron	startle_PPI	0.9602014777
vns	Bacteroides_thetaiotaomicron	startle_PPI	0.6343063159
blast	Berger Parker Index	startle_PPI	0.5457417127
vns	Berger Parker Index	startle_PPI	0.2337330546
blast	Bifidobacterium_pseudolongum	startle_PPI	0.4280103985
vns	Bifidobacterium_pseudolongum	startle_PPI	0.3477990653
blast	Clostridia_bacterium	startle_PPI	0.3745590434
vns	Clostridia_bacterium	startle_PPI	0.7249401071
blast	Clostridiaceae_bacterium	startle_PPI	0.9871697806
vns	Clostridiaceae_bacterium	startle_PPI	0.2419370425
blast	Clostridiaceae_unclassified_SGB41663	startle_PPI	0.780382224
vns	Clostridiaceae_unclassified_SGB41663	startle_PPI	0.719154368
blast	Clostridiales_bacterium	startle_PPI	0.0023319564
vns	Clostridiales_bacterium	startle_PPI	0.2964785018
blast	Clostridium_cocleatum	startle_PPI	0.0115678325
vns	Clostridium_cocleatum	startle_PPI	0.1960355146
blast	Coriobacteriaceae_bacterium	startle_PPI	0.3724483697
vns	Coriobacteriaceae_bacterium	startle_PPI	0.953919137

blast	Dorea_sp_5_2	startle_PPI	0.5884919176
vns	Dorea_sp_5_2	startle_PPI	0.4214161244
blast	Dubosiella_newyorkensis	startle_PPI	0.1169987394
vns	Dubosiella_newyorkensis	startle_PPI	0.799186063
blast	Erysipelotrichales_bacterium	startle_PPI	0.8970006656
vns	Erysipelotrichales_bacterium	startle_PPI	0.3284081083
blast	Eubacteriaceae_bacterium	startle_PPI	0.0271233687
vns	Eubacteriaceae_bacterium	startle_PPI	0.2383186135
blast	Eubacteriaceae_unclassified_SGB94922	startle_PPI	0.4594880799
vns	Eubacteriaceae_unclassified_SGB94922	startle_PPI	0.3726201484
blast	GGB20149_SGB29430	startle_PPI	0.6280700484
vns	GGB20149_SGB29430	startle_PPI	0.6299827926
blast	GGB22635_SGB63107	startle_PPI	0.4117103362
vns	GGB22635_SGB63107	startle_PPI	0.8871521383
blast	GGB25041_SGB36960	startle_PPI	0.0824966676
vns	GGB25041_SGB36960	startle_PPI	0.2101871969
blast	GGB27876_SGB40310	startle_PPI	0.164666135
vns	GGB27876_SGB40310	startle_PPI	0.7672351949
blast	GGB27878_SGB40312	startle_PPI	0.2133541578
vns	GGB27878_SGB40312	startle_PPI	0.1474794298
blast	GGB27918_SGB40356	startle_PPI	0.9587581408
vns	GGB27918_SGB40356	startle_PPI	0.8856989297
blast	GGB28382_SGB40962	startle_PPI	0.5193113079
vns	GGB28382_SGB40962	startle_PPI	0.8550578348
blast	GGB28399_SGB40980	startle_PPI	0.2894655025
vns	GGB28399_SGB40980	startle_PPI	0.287117034
blast	GGB28411_SGB40993	startle_PPI	0.2018022626
vns	GGB28411_SGB40993	startle_PPI	0.1468484741
blast	GGB28415_SGB40997	startle_PPI	0.0267267446
vns	GGB28415_SGB40997	startle_PPI	0.9490516966
blast	GGB28430_SGB41013	startle_PPI	0.9120709472
vns	GGB28430_SGB41013	startle_PPI	0.4662504826
blast	GGB28439_SGB41022	startle_PPI	0.3804444035
vns	GGB28439_SGB41022	startle_PPI	0.7387095529
blast	GGB28778_SGB41431	startle_PPI	0.8046989411
vns	GGB28778_SGB41431	startle_PPI	0.2184532434
blast	GGB28784_SGB41437	startle_PPI	0.0207266217
vns	GGB28784_SGB41437	startle_PPI	0.2544569976
blast	GGB28792_SGB41445	startle_PPI	0.3385777757
vns	GGB28792_SGB41445	startle_PPI	0.7612444262
blast	GGB28798_SGB41451	startle_PPI	0.1640226305
vns	GGB28798_SGB41451	startle_PPI	0.2489158244
blast	GGB28802_SGB41455	startle_PPI	0.4578986259
vns	GGB28802_SGB41455	startle_PPI	0.342689298
blast	GGB28818_SGB41473	startle_PPI	0.7292095104
vns	GGB28818_SGB41473	startle_PPI	0.5694099484

blast	GGB28828_SGB41484	startle_PPI	0.298696993
vns	GGB28828_SGB41484	startle_PPI	0.106706535
blast	GGB28851_SGB41518	startle_PPI	0.852552179
vns	GGB28851_SGB41518	startle_PPI	0.578308429
blast	GGB28859_SGB41528	startle_PPI	0.205749329
vns	GGB28859_SGB41528	startle_PPI	0.595366745
blast	GGB28864_SGB41535	startle_PPI	0.785465248
vns	GGB28864_SGB41535	startle_PPI	0.964658985
blast	GGB28869_SGB41543	startle_PPI	0.347931625
vns	GGB28869_SGB41543	startle_PPI	0.381477475
blast	GGB28883_SGB41564	startle_PPI	0.594877248
vns	GGB28883_SGB41564	startle_PPI	0.060524458
blast	GGB28892_SGB41573	startle_PPI	0.651227207
vns	GGB28892_SGB41573	startle_PPI	0.667618882
blast	GGB28893_SGB41574	startle_PPI	0.165594509
vns	GGB28893_SGB41574	startle_PPI	0.182699376
blast	GGB28898_SGB41580	startle_PPI	0.304654993
vns	GGB28898_SGB41580	startle_PPI	0.082893330
blast	GGB28904_SGB41597	startle_PPI	0.172906397
vns	GGB28904_SGB41597	startle_PPI	0.056067199
blast	GGB28916_SGB41612	startle_PPI	0.801432609
vns	GGB28916_SGB41612	startle_PPI	0.853813296
blast	GGB28924_SGB41621	startle_PPI	0.796481662
vns	GGB28924_SGB41621	startle_PPI	0.278658378
blast	GGB28926_SGB41624	startle_PPI	0.306780834
vns	GGB28926_SGB41624	startle_PPI	0.470765265
blast	GGB28927_SGB41625	startle_PPI	0.193720805
vns	GGB28927_SGB41625	startle_PPI	0.418243373
blast	GGB28934_SGB41635	startle_PPI	0.115873311
vns	GGB28934_SGB41635	startle_PPI	0.111374073
blast	GGB28946_SGB41652	startle_PPI	0.427843225
vns	GGB28946_SGB41652	startle_PPI	0.422588900
blast	GGB28949_SGB41655	startle_PPI	0.443608116
vns	GGB28949_SGB41655	startle_PPI	0.908948179
blast	GGB28949_SGB41656	startle_PPI	0.282728234
vns	GGB28949_SGB41656	startle_PPI	0.770863949
blast	GGB28950_SGB41657	startle_PPI	0.030527036
vns	GGB28950_SGB41657	startle_PPI	0.336458904
blast	GGB28951_SGB102295	startle_PPI	0.925352156
vns	GGB28951_SGB102295	startle_PPI	0.158790397
blast	GGB28951_SGB41658	startle_PPI	0.847001351
vns	GGB28951_SGB41658	startle_PPI	0.920236623
blast	GGB28954_SGB41662	startle_PPI	0.515758297
vns	GGB28954_SGB41662	startle_PPI	0.800484955
blast	GGB28956_SGB41665	startle_PPI	0.163329713
vns	GGB28956_SGB41665	startle_PPI	0.153416502

blast	GGB28960_SGB41669	startle_PPI	0.155727292
vns	GGB28960_SGB41669	startle_PPI	0.155957479
blast	GGB28967_SGB41678	startle_PPI	0.179269529
vns	GGB28967_SGB41678	startle_PPI	0.315046519
blast	GGB28991_SGB41705	startle_PPI	0.228893882
vns	GGB28991_SGB41705	startle_PPI	0.279982342
blast	GGB29002_SGB41718	startle_PPI	0.002430867
vns	GGB29002_SGB41718	startle_PPI	0.113465587
blast	GGB29003_SGB41719	startle_PPI	0.499398644
vns	GGB29003_SGB41719	startle_PPI	0.583967886
blast	GGB29011_SGB41731	startle_PPI	0.916276033
vns	GGB29011_SGB41731	startle_PPI	0.345599236
blast	GGB29531_SGB42317	startle_PPI	0.145082026
vns	GGB29531_SGB42317	startle_PPI	0.135569966
blast	GGB29685_SGB42494	startle_PPI	0.864543852
vns	GGB29685_SGB42494	startle_PPI	0.340104263
blast	GGB30141_SGB43066	startle_PPI	0.991554803
vns	GGB30141_SGB43066	startle_PPI	0.760178789
blast	GGB30286_SGB43248	startle_PPI	0.055261633
vns	GGB30286_SGB43248	startle_PPI	0.454603367
blast	GGB30303_SGB43268	startle_PPI	0.371936893
vns	GGB30303_SGB43268	startle_PPI	0.944722174
blast	GGB30413_SGB43452	startle_PPI	0.953276950
vns	GGB30413_SGB43452	startle_PPI	0.879642604
blast	GGB30454_SGB43514	startle_PPI	0.379858950
vns	GGB30454_SGB43514	startle_PPI	0.200125591
blast	GGB30455_SGB43519	startle_PPI	0.379467796
vns	GGB30455_SGB43519	startle_PPI	0.302348921
blast	GGB30461_SGB43527	startle_PPI	0.397503662
vns	GGB30461_SGB43527	startle_PPI	0.771038655
blast	GGB30461_SGB43530	startle_PPI	0.395449
vns	GGB30461_SGB43530	startle_PPI	0.703863693
blast	GGB30463_SGB43537	startle_PPI	0.210054257
vns	GGB30463_SGB43537	startle_PPI	0.985361479
blast	GGB30473_SGB43557	startle_PPI	0.020642166
vns	GGB30473_SGB43557	startle_PPI	0.056211743
blast	GGB30475_SGB63182	startle_PPI	0.287965349
vns	GGB30475_SGB63182	startle_PPI	0.660855930
blast	GGB30861_SGB44083	startle_PPI	0.957552379
vns	GGB30861_SGB44083	startle_PPI	0.833445529
blast	GGB31312_SGB44628	startle_PPI	0.711727128
vns	GGB31312_SGB44628	startle_PPI	0.884193943
blast	GGB31438_SGB44768	startle_PPI	0.906357297
vns	GGB31438_SGB44768	startle_PPI	0.318960086
blast	GGB3171_SGB4185	startle_PPI	0.085345767
vns	GGB3171_SGB4185	startle_PPI	0.918551780

blast	GGB31823_SGB45199	startle_PPI	0.188977717
vns	GGB31823_SGB45199	startle_PPI	0.702813258
blast	GGB31853_SGB45233	startle_PPI	0.496842743
vns	GGB31853_SGB45233	startle_PPI	0.396259541
blast	GGB32371_SGB41694	startle_PPI	0.346811850
vns	GGB32371_SGB41694	startle_PPI	0.025054092
blast	GGB3793_SGB5158	startle_PPI	0.965790474
vns	GGB3793_SGB5158	startle_PPI	0.975145151
blast	GGB42598_SGB59794	startle_PPI	0.501740001
vns	GGB42598_SGB59794	startle_PPI	0.156668604
blast	GGB45656_SGB63370	startle_PPI	0.621044138
vns	GGB45656_SGB63370	startle_PPI	0.963374601
blast	GGB47127_SGB65054	startle_PPI	0.496745120
vns	GGB47127_SGB65054	startle_PPI	0.257417312
blast	GGB74395_SGB43521	startle_PPI	0.123339782
vns	GGB74395_SGB43521	startle_PPI	0.204021498
blast	GGB75053_SGB43494	startle_PPI	0.410595919
vns	GGB75053_SGB43494	startle_PPI	0.571857456
blast	GGB75109_SGB102238	startle_PPI	0.620842292
vns	GGB75109_SGB102238	startle_PPI	0.871818408
blast	GGB81440_SGB45230	startle_PPI	0.079610731
vns	GGB81440_SGB45230	startle_PPI	0.098140579
blast	Lachnospiraceae_bacterium	startle_PPI	0.484816177
vns	Lachnospiraceae_bacterium	startle_PPI	0.537861332
blast	Lachnospiraceae_bacterium_A2	startle_PPI	0.420834896
vns	Lachnospiraceae_bacterium_A2	startle_PPI	0.323648328
blast	Lachnospiraceae_bacterium_MD308	startle_PPI	0.213119451
vns	Lachnospiraceae_bacterium_MD308	startle_PPI	0.083066965
blast	Lachnospiraceae_bacterium_MD329	startle_PPI	0.020976149
vns	Lachnospiraceae_bacterium_MD329	startle_PPI	0.699161119
blast	Lachnospiraceae_unclassified_SGB41414	startle_PPI	0.142639053
vns	Lachnospiraceae_unclassified_SGB41414	startle_PPI	0.119876468
blast	Lachnospiraceae_unclassified_SGB41418	startle_PPI	0.998757981
vns	Lachnospiraceae_unclassified_SGB41418	startle_PPI	0.712236573
blast	Lachnospiraceae_unclassified_SGB41424	startle_PPI	0.048386378
vns	Lachnospiraceae_unclassified_SGB41424	startle_PPI	0.046067357
blast	Lachnospiraceae_unclassified_SGB41589	startle_PPI	0.165424952
vns	Lachnospiraceae_unclassified_SGB41589	startle_PPI	0.273527700
blast	Lactobacillus_johnsonii	startle_PPI	0.182678453
vns	Lactobacillus_johnsonii	startle_PPI	0.672951588
blast	Muribaculaceae_bacterium	startle_PPI	0.841525252
vns	Muribaculaceae_bacterium	startle_PPI	0.893052980
blast	Neglectibacter_sp_X4	startle_PPI	0.084782151
vns	Neglectibacter_sp_X4	startle_PPI	0.140656264
blast	Oscillospiraceae_bacterium	startle_PPI	0.741672565
vns	Oscillospiraceae_bacterium	startle_PPI	0.856188039

blast	Oscillospiraceae_unclassified_SGB43502	startle_PPI	0.0259412852
vns	Oscillospiraceae_unclassified_SGB43502	startle_PPI	0.0305351331
blast	Oscillospiraceae_unclassified_SGB43505	startle_PPI	0.4959230188
vns	Oscillospiraceae_unclassified_SGB43505	startle_PPI	0.6425337316
blast	Parasutterella_excrementihominis	startle_PPI	0.5671048722
vns	Parasutterella_excrementihominis	startle_PPI	0.7099929171
blast	Richness (# observed features)	startle_PPI	0.2717524423
vns	Richness (# observed features)	startle_PPI	0.6607230685
blast	Romboutsia_ilealis	startle_PPI	0.0010997742
vns	Romboutsia_ilealis	startle_PPI	0.0569970896
blast	Schaedlerella_arabinosiphila	startle_PPI	0.9390244043
vns	Schaedlerella_arabinosiphila	startle_PPI	0.9139116075
blast	Shannon Index	startle_PPI	0.6947352216
vns	Shannon Index	startle_PPI	0.9062773413
blast	Turicibacter_sp_1E2	startle_PPI	0.1991772312
vns	Turicibacter_sp_1E2	startle_PPI	0.1655749312

p_y	p_composite	dact_stat	dact_thr_10	dact_thr_15	dact_thr_20	signif_at
0.93713	0.882395964	0.816354558	0.003097121	0.004400596	0.005669218	
0.93713	0.817043014	0.803960468	0.007197031	0.010056868	0.012376928	
0.010146684	0.819624877	0.714775437	0.003097121	0.004400596	0.005669218	
0.010146684	0.588941984	0.573638851	0.007197031	0.010056868	0.012376928	
0.101950854	0.341697162	0.289204687	0.003097121	0.004400596	0.005669218	
0.101950854	0.783135489	0.749263989	0.007197031	0.010056868	0.012376928	
0.382409508	0.678849487	0.539672052	0.003097121	0.004400596	0.005669218	
0.382409508	0.652340244	0.627070677	0.007197031	0.010056868	0.012376928	
0.014518444	0.951941334	0.907077160	0.003097121	0.004400596	0.005669218	
0.014518444	0.610492800	0.589024138	0.007197031	0.010056868	0.012376928	
0.400573762	0.256067769	0.233128323	0.003097121	0.004400596	0.005669218	
0.400573762	0.213013119	0.214479704	0.007197031	0.010056868	0.012376928	
0.685784528	0.719393457	0.583597398	0.003097121	0.004400596	0.005669218	
0.685784528	0.558188394	0.546302599	0.007197031	0.010056868	0.012376928	
0.14211927	0.095651013	0.105567532	0.003097121	0.004400596	0.005669218	
0.14211927	0.841104495	0.817608644	0.007197031	0.010056868	0.012376928	
0.247037984	0.293937423	0.259628695	0.003097121	0.004400596	0.005669218	
0.247037984	0.798601700	0.777220610	0.007197031	0.010056868	0.012376928	
0.489708388	0.522880156	0.408007218	0.003097121	0.004400596	0.005669218	
0.489708388	0.537905925	0.523414541	0.007197031	0.010056868	0.012376928	
0.824720198	0.727508646	0.592898736	0.003097121	0.004400596	0.005669218	
0.824720198	0.763944001	0.731390155	0.007197031	0.010056868	0.012376928	
0.681526483	0.598592159	0.472081626	0.003097121	0.004400596	0.005669218	
0.681526483	0.318374440	0.288466520	0.007197031	0.010056868	0.012376928	
0.748956111	0.723422227	0.588180017	0.003097121	0.004400596	0.005669218	
0.748956111	0.761108388	0.723169560	0.007197031	0.010056868	0.012376928	
0.015506838	0.002738188	0.008270156	0.003097121	0.004400596	0.005669218	
0.015506838	0.139730194	0.162772707	0.007197031	0.010056868	0.012376928	
0.693180661	0.694023573	0.554889371	0.003097121	0.004400596	0.005669218	
0.693180661	0.595965903	0.578688424	0.007197031	0.010056868	0.012376928	
0.571615368	0.893965040	0.833759045	0.003097121	0.004400596	0.005669218	
0.571615368	0.627612815	0.612883250	0.007197031	0.010056868	0.012376928	
0.352267104	0.942921080	0.894752303	0.003097121	0.004400596	0.005669218	
0.352267104	0.554203191	0.541873857	0.007197031	0.010056868	0.012376928	
0.071815873	0.511206758	0.398748259	0.003097121	0.004400596	0.005669218	
0.071815873	0.137809687	0.162164104	0.007197031	0.010056868	0.012376928	
0.104018289	0.350052885	0.294371331	0.003097121	0.004400596	0.005669218	
0.104018289	0.279339173	0.257169133	0.007197031	0.010056868	0.012376928	
0.154204299	0.284958845	0.253854254	0.003097121	0.004400596	0.005669218	
0.154204299	0.678725241	0.645333532	0.007197031	0.010056868	0.012376928	
0.008595498	0.948899643	0.903310972	0.003097121	0.004400596	0.005669218	
0.008595498	0.148481313	0.168584234	0.007197031	0.010056868	0.012376928	
0.308813937	0.783554351	0.667247005	0.003097121	0.004400596	0.005669218	
0.308813937	0.672223456	0.638674636	0.007197031	0.010056868	0.012376928	
0.671739982	0.284260480	0.253401763	0.003097121	0.004400596	0.005669218	
0.671739982	0.395124644	0.359052682	0.007197031	0.010056868	0.012376928	

0.771492821	0.402935990	0.329160501	0.003097121	0.004400596	0.005669218
0.771492821	0.393093369	0.356473271	0.007197031	0.010056868	0.012376928
0.592081995	0.484590200	0.380388656	0.003097121	0.004400596	0.005669218
0.592081995	0.963186636	0.936928252	0.007197031	0.010056868	0.012376928
0.180181608	0.569413180	0.449272653	0.003097121	0.004400596	0.005669218
0.180181608	0.357599763	0.324066476	0.007197031	0.010056868	0.012376928
0.067538602	0.052362868	0.067585513	0.003097121	0.004400596	0.005669218
0.067538602	0.765608396	0.735058521	0.007197031	0.010056868	0.012376928
0.698459766	0.898731392	0.840465586	0.003097121	0.004400596	0.005669218
0.698459766	0.428527369	0.392881743	0.007197031	0.010056868	0.012376928
0.601816186	0.241249151	0.220524909	0.003097121	0.004400596	0.005669218
0.601816186	0.318374017	0.288466073	0.007197031	0.010056868	0.012376928
0.423874835	0.428585883	0.347361155	0.003097121	0.004400596	0.005669218
0.423874835	0.325217906	0.295863914	0.007197031	0.010056868	0.012376928
0.312702445	0.634388466	0.500057492	0.003097121	0.004400596	0.005669218
0.312702445	0.546894472	0.537053705	0.007197031	0.010056868	0.012376928
0.166899079	0.335431258	0.285555264	0.003097121	0.004400596	0.005669218
0.166899079	0.881779500	0.847273748	0.007197031	0.010056868	0.012376928
0.378982776	0.135774870	0.133113318	0.003097121	0.004400596	0.005669218
0.378982776	0.172367162	0.183942478	0.007197031	0.010056868	0.012376928
0.272718978	0.136426244	0.133582545	0.003097121	0.004400596	0.005669218
0.272718978	0.730231870	0.696073773	0.007197031	0.010056868	0.012376928
0.896624355	0.667057288	0.527871447	0.003097121	0.004400596	0.005669218
0.896624355	0.441245291	0.410958376	0.007197031	0.010056868	0.012376928
0.496942408	0.949764064	0.904414856	0.003097121	0.004400596	0.005669218
0.496942408	0.877049479	0.845701337	0.007197031	0.010056868	0.012376928
0.258094017	0.497880977	0.388578012	0.003097121	0.004400596	0.005669218
0.258094017	0.825717791	0.805742355	0.007197031	0.010056868	0.012376928
0.786723457	0.601507533	0.474212189	0.003097121	0.004400596	0.005669218
0.786723457	0.447653104	0.420630463	0.007197031	0.010056868	0.012376928
0.644106236	0.405517542	0.330937636	0.003097121	0.004400596	0.005669218
0.644106236	0.273155687	0.254702328	0.007197031	0.010056868	0.012376928
0.762739641	0.403913124	0.329827583	0.003097121	0.004400596	0.005669218
0.762739641	0.956404292	0.930473406	0.007197031	0.010056868	0.012376928
0.458915373	0.898783119	0.840531586	0.003097121	0.004400596	0.005669218
0.458915373	0.405005681	0.367165009	0.007197031	0.010056868	0.012376928
0.111384363	0.287024317	0.255208758	0.003097121	0.004400596	0.005669218
0.111384363	0.689003650	0.661613837	0.007197031	0.010056868	0.012376928
0.779440006	0.833108249	0.733528462	0.003097121	0.004400596	0.005669218
0.779440006	0.414877779	0.374862075	0.007197031	0.010056868	0.012376928
0.568664723	0.215368590	0.197728965	0.003097121	0.004400596	0.005669218
0.568664723	0.309771115	0.282594690	0.007197031	0.010056868	0.012376928
0.990434557	0.788267037	0.673374766	0.003097121	0.004400596	0.005669218
0.990434557	0.888094087	0.850121335	0.007197031	0.010056868	0.012376928
0.425144527	0.20765665	0.192271487	0.003097121	0.004400596	0.005669218
0.425144527	0.222873896	0.222073786	0.007197031	0.010056868	0.012376928

0.951684485	0.805602033	0.694435577	0.003097121	0.004400596	0.005669218
0.951684485	0.576683008	0.568846606	0.007197031	0.010056868	0.012376928
0.618110325	0.760573832	0.633437855	0.003097121	0.004400596	0.005669218
0.618110325	0.511860326	0.495088229	0.007197031	0.010056868	0.012376928
0.652557416	0.488947625	0.382944869	0.003097121	0.004400596	0.005669218
0.652557416	0.240217972	0.235008951	0.007197031	0.010056868	0.012376928
0.708655176	0.864784955	0.785729355	0.003097121	0.004400596	0.005669218
0.708655176	0.559048686	0.548570576	0.007197031	0.010056868	0.012376928
0.564572502	0.333284615	0.284172007	0.003097121	0.004400596	0.005669218
0.564572502	0.518962465	0.499569244	0.007197031	0.010056868	0.012376928
0.397172611	0.794023708	0.680257617	0.003097121	0.004400596	0.005669218
0.397172611	0.977741218	0.951419346	0.007197031	0.010056868	0.012376928
0.132183137	0.253457436	0.230846654	0.003097121	0.004400596	0.005669218
0.132183137	0.315207184	0.287228694	0.007197031	0.010056868	0.012376928
0.182922498	0.578128093	0.455881613	0.003097121	0.004400596	0.005669218
0.182922498	0.021873817	0.049881853	0.007197031	0.010056868	0.012376928
0.162775217	0.648905089	0.512213702	0.003097121	0.004400596	0.005669218
0.162775217	0.597385009	0.579020093	0.007197031	0.010056868	0.012376928
0.162075260	0.094210156	0.104516863	0.003097121	0.004400596	0.005669218
0.162075260	0.095542857	0.123133325	0.007197031	0.010056868	0.012376928
0.055470793	0.206526095	0.191510513	0.003097121	0.004400596	0.005669218
0.055470793	0.023633771	0.052539991	0.007197031	0.010056868	0.012376928
0.354937354	0.173897117	0.166821490	0.003097121	0.004400596	0.005669218
0.354937354	0.050009366	0.084387467	0.007197031	0.010056868	0.012376928
0.712540833	0.826458629	0.724300379	0.003097121	0.004400596	0.005669218
0.712540833	0.822285791	0.804599377	0.007197031	0.010056868	0.012376928
0.865789449	0.862778494	0.781394557	0.003097121	0.004400596	0.005669218
0.865789449	0.479641728	0.467897365	0.007197031	0.010056868	0.012376928
0.471870513	0.331420140	0.282975625	0.003097121	0.004400596	0.005669218
0.471870513	0.412616142	0.371995742	0.007197031	0.010056868	0.012376928
0.014441967	0.102577518	0.110653554	0.003097121	0.004400596	0.005669218
0.014441967	0.350276121	0.320931473	0.007197031	0.010056868	0.012376928
0.478207775	0.210880591	0.194496189	0.003097121	0.004400596	0.005669218
0.478207775	0.134505102	0.158775722	0.007197031	0.010056868	0.012376928
0.493639637	0.436256612	0.352075159	0.003097121	0.004400596	0.005669218
0.493639637	0.385607329	0.351519016	0.007197031	0.010056868	0.012376928
0.238884890	0.388247456	0.318629477	0.003097121	0.004400596	0.005669218
0.238884890	0.908420264	0.876082509	0.007197031	0.010056868	0.012376928
0.421830522	0.275497474	0.247750029	0.003097121	0.004400596	0.005669218
0.421830522	0.740651380	0.700632691	0.007197031	0.010056868	0.012376928
0.676678150	0.312194967	0.270260290	0.003097121	0.004400596	0.005669218
0.676678150	0.423211864	0.385709348	0.007197031	0.010056868	0.012376928
0.689302714	0.927526913	0.876074961	0.003097121	0.004400596	0.005669218
0.689302714	0.313021158	0.286018834	0.007197031	0.010056868	0.012376928
0.004983215	0.825133659	0.722620273	0.003097121	0.004400596	0.005669218
0.004983215	0.930536792	0.890881134	0.007197031	0.010056868	0.012376928

0.830187941	0.749027859	0.617232306	0.003097121	0.004400596	0.005669218
0.830187941	0.791054592	0.756805968	0.007197031	0.010056868	0.012376928
0.776517588	0.520301017	0.405830678	0.003097121	0.004400596	0.005669218
0.776517588	0.371449991	0.334018245	0.007197031	0.010056868	0.012376928
0.983586421	0.715824630	0.579110603	0.003097121	0.004400596	0.005669218
0.983586421	0.500319438	0.481534973	0.007197031	0.010056868	0.012376928
0.270765135	0.144883328	0.139968638	0.003097121	0.004400596	0.005669218
0.270765135	0.231598091	0.229000400	0.007197031	0.010056868	0.012376928
0.450456967	0.258418768	0.235186672	0.003097121	0.004400596	0.005669218
0.450456967	0.262448593	0.249604454	0.007197031	0.010056868	0.012376928
0.084178899	0.004214518	0.011019006	0.003097121	0.004400596	0.005669218
0.084178899	0.040760709	0.073313307	0.007197031	0.010056868	0.012376928
0.598707018	0.564263354	0.445325952	0.003097121	0.004400596	0.005669218
0.598707018	0.511509835	0.494359476	0.007197031	0.010056868	0.012376928
0.361080919	0.896945939	0.838106147	0.003097121	0.004400596	0.005669218
0.361080919	0.284197302	0.259872395	0.007197031	0.010056868	0.012376928
0.001063788	0.062394214	0.078985945	0.003097121	0.004400596	0.005669218
0.001063788	0.052835634	0.088694729	0.007197031	0.010056868	0.012376928
0.008273244	0.839721727	0.744514781	0.003097121	0.004400596	0.005669218
0.008273244	0.263257250	0.250339195	0.007197031	0.010056868	0.012376928
0.508706379	0.986024317	0.949242425	0.003097121	0.004400596	0.005669218
0.508706379	0.711423790	0.687764697	0.007197031	0.010056868	0.012376928
0.889890304	0.565281289	0.446154900	0.003097121	0.004400596	0.005669218
0.889890304	0.616282655	0.590413812	0.007197031	0.010056868	0.012376928
0.147951353	0.280942179	0.251234120	0.003097121	0.004400596	0.005669218
0.147951353	0.943309917	0.923981250	0.007197031	0.010056868	0.012376928
0.526028446	0.946212098	0.899499467	0.003097121	0.004400596	0.005669218
0.526028446	0.861832001	0.837820035	0.007197031	0.010056868	0.012376928
0.165981792	0.293069393	0.259099681	0.003097121	0.004400596	0.005669218
0.165981792	0.109678962	0.136261437	0.007197031	0.010056868	0.012376928
0.081621129	0.282165017	0.252032388	0.003097121	0.004400596	0.005669218
0.081621129	0.217171563	0.218056922	0.007197031	0.010056868	0.012376928
0.096923692	0.308575491	0.268079684	0.003097121	0.004400596	0.005669218
0.096923692	0.738819014	0.700520360	0.007197031	0.010056868	0.012376928
0.240640282	0.32396174	0.277873903	0.003097121	0.004400596	0.005669218
0.240640282	0.643789351	0.620728495	0.007197031	0.010056868	0.012376928
0.324556210	0.181268593	0.173138703	0.003097121	0.004400596	0.005669218
0.324556210	0.993709402	0.979916341	0.007197031	0.010056868	0.012376928
0.737379173	0.371953431	0.308138914	0.003097121	0.004400596	0.005669218
0.737379173	0.267591818	0.251955643	0.007197031	0.010056868	0.012376928
0.205115262	0.205939585	0.191119167	0.003097121	0.004400596	0.005669218
0.205115262	0.581996160	0.571410898	0.007197031	0.010056868	0.012376928
0.010436741	0.916438712	0.863898967	0.003097121	0.004400596	0.005669218
0.010436741	0.803552344	0.777930334	0.007197031	0.010056868	0.012376928
0.058278529	0.707173780	0.569175726	0.003097121	0.004400596	0.005669218
0.058278529	0.866051226	0.843294216	0.007197031	0.010056868	0.012376928

0.129588426	0.876273999	0.806871339	0.003097121	0.004400596	0.005669218
0.129588426	0.236012104	0.232199855	0.007197031	0.010056868	0.012376928
0.783302125	0.472706963	0.373597514	0.003097121	0.004400596	0.005669218
0.783302125	0.929211607	0.889409319	0.007197031	0.010056868	0.012376928
0.317502865	0.166916282	0.160465084	0.003097121	0.004400596	0.005669218
0.317502865	0.638465592	0.619584048	0.007197031	0.010056868	0.012376928
0.042137307	0.433676178	0.350470694	0.003097121	0.004400596	0.005669218
0.042137307	0.329780020	0.300606511	0.007197031	0.010056868	0.012376928
0.491718818	0.378153633	0.312193648	0.003097121	0.004400596	0.005669218
0.491718818	0.083044936	0.112239133	0.007197031	0.010056868	0.012376928
0.675732129	0.971419935	0.928048544	0.003097121	0.004400596	0.005669218
0.675732129	0.988257395	0.966126050	0.007197031	0.010056868	0.012376928
0.548701259	0.532702970	0.417203453	0.003097121	0.004400596	0.005669218
0.548701259	0.213557531	0.214979092	0.007197031	0.010056868	0.012376928
0.189013771	0.613922890	0.482928449	0.003097121	0.004400596	0.005669218
0.189013771	0.968281273	0.949450023	0.007197031	0.010056868	0.012376928
0.049222290	0.434433900	0.350947955	0.003097121	0.004400596	0.005669218
0.049222290	0.167935377	0.181005079	0.007197031	0.010056868	0.012376928
0.105346324	0.058596574	0.074324390	0.003097121	0.004400596	0.005669218
0.105346324	0.113293535	0.139168154	0.007197031	0.010056868	0.012376928
0.223815779	0.341637526	0.289170467	0.003097121	0.004400596	0.005669218
0.223815779	0.489126614	0.474146670	0.007197031	0.010056868	0.012376928
0.373693725	0.631555711	0.497495665	0.003097121	0.004400596	0.005669218
0.373693725	0.851898542	0.827491674	0.007197031	0.010056868	0.012376928
0.078886450	0.034513930	0.047312729	0.003097121	0.004400596	0.005669218
0.078886450	0.031500720	0.062815863	0.007197031	0.010056868	0.012376928
0.175471097	0.433107214	0.350170012	0.003097121	0.004400596	0.005669218
0.175471097	0.460212039	0.438610195	0.007197031	0.010056868	0.012376928
0.249948612	0.358404192	0.299921523	0.003097121	0.004400596	0.005669218
0.249948612	0.243484228	0.236338242	0.007197031	0.010056868	0.012376928
0.143218715	0.136432599	0.133739590	0.003097121	0.004400596	0.005669218
0.143218715	0.028182006	0.058188038	0.007197031	0.010056868	0.012376928
0.402910748	0.106755810	0.113729723	0.003097121	0.004400596	0.005669218
0.402910748	0.632479102	0.615430090	0.007197031	0.010056868	0.012376928
0.364600188	0.162493274	0.156125411	0.003097121	0.004400596	0.005669218
0.364600188	0.097755633	0.125463799	0.007197031	0.010056868	0.012376928
0.801805019	0.998087864	0.982455935	0.003097121	0.004400596	0.005669218
0.801805019	0.706699585	0.685300264	0.007197031	0.010056868	0.012376928
0.508060263	0.186849926	0.177445384	0.003097121	0.004400596	0.005669218
0.508060263	0.102717300	0.131398567	0.007197031	0.010056868	0.012376928
0.697123145	0.431294271	0.349005328	0.003097121	0.004400596	0.005669218
0.697123145	0.395244684	0.359206015	0.007197031	0.010056868	0.012376928
0.955504061	0.704062595	0.565840707	0.003097121	0.004400596	0.005669218
0.955504061	0.794717115	0.769921661	0.007197031	0.010056868	0.012376928
0.038454065	0.822273544	0.718600936	0.003097121	0.004400596	0.005669218
0.038454065	0.889295835	0.854887692	0.007197031	0.010056868	0.012376928

0.044442561	0.034621596	0.047413563	0.003097121	0.004400596	0.005669218
0.044442561	0.056760223	0.092351874	0.007197031	0.010056868	0.012376928
0.965563069	0.901068114	0.843465175	0.003097121	0.004400596	0.005669218
0.965563069	0.922705952	0.887603203	0.007197031	0.010056868	0.012376928
0.351734423	0.079550964	0.095026096	0.003097121	0.004400596	0.005669218
0.351734423	0.034973767	0.068159656	0.007197031	0.010056868	0.012376928
0.507976248	0.502707861	0.392218691	0.003097121	0.004400596	0.005669218
0.507976248	0.564084518	0.551168053	0.007197031	0.010056868	0.012376928
0.205308301	0.546549762	0.430142494	0.003097121	0.004400596	0.005669218
0.205308301	0.656810828	0.627837035	0.007197031	0.010056868	0.012376928
0.187328370	0.187511640	0.177921173	0.003097121	0.004400596	0.005669218
0.187328370	0.579257990	0.571243158	0.007197031	0.010056868	0.012376928
0.919014803	0.564686553	0.445667887	0.003097121	0.004400596	0.005669218
0.919014803	0.413371979	0.372952552	0.007197031	0.010056868	0.012376928
0.202499563	0.909916079	0.854909085	0.003097121	0.004400596	0.005669218
0.202499563	0.916326199	0.882643234	0.007197031	0.010056868	0.012376928
0.351765516	0.711604395	0.573983240	0.003097121	0.004400596	0.005669218
0.351765516	0.901603233	0.872653618	0.007197031	0.010056868	0.012376928
0.166899984	0.125904183	0.126412560	0.003097121	0.004400596	0.005669218
0.166899984	0.081540955	0.110654100	0.007197031	0.010056868	0.012376928
0.141836773	0.638409505	0.524954626	0.005492180	0.007205648	0.008582503
0.141836773	0.614956504	0.574437662	0.003110558	0.003920326	0.004492969
0.903365243	0.948081795	0.840942349	0.005492180	0.007205648	0.008582503
0.903365243	0.845610299	0.810849417	0.003110558	0.003920326	0.004492969
0.937165790	0.864530919	0.750178573	0.005492180	0.007205648	0.008582503
0.937165790	0.923513855	0.876508834	0.003110558	0.003920326	0.004492969
0.598230628	0.650385581	0.537497745	0.005492180	0.007205648	0.008582503
0.598230628	0.553523499	0.511791167	0.003110558	0.003920326	0.004492969
0.127842343	0.923484062	0.812182648	0.005492180	0.007205648	0.008582503
0.127842343	0.493976888	0.466591162	0.003110558	0.003920326	0.004492969
0.600338376	0.469650024	0.381230472	0.005492180	0.007205648	0.008582503
0.600338376	0.385116898	0.356583284	0.003110558	0.003920326	0.004492969
0.942729343	0.941381140	0.832586262	0.005492180	0.007205648	0.008582503
0.942729343	0.924128711	0.877964908	0.003110558	0.003920326	0.004492969
0.898496658	0.743884089	0.630041325	0.005492180	0.007205648	0.008582503
0.898496658	0.845157884	0.810477392	0.003110558	0.003920326	0.004492969
0.930328323	0.843498814	0.727289220	0.005492180	0.007205648	0.008582503
0.930328323	0.917900508	0.865128618	0.003110558	0.003920326	0.004492969
0.135139214	0.389333863	0.320114398	0.005492180	0.007205648	0.008582503
0.135139214	0.423953030	0.390779727	0.003110558	0.003920326	0.004492969
0.561002782	0.508147828	0.412907015	0.005492180	0.007205648	0.008582503
0.561002782	0.645651219	0.591720519	0.003110558	0.003920326	0.004492969
0.079906588	0.283553599	0.249502199	0.005492180	0.007205648	0.008582503
0.079906588	0.044216656	0.034620031	0.003110558	0.003920326	0.004492969
0.777185196	0.747445236	0.633235240	0.005492180	0.007205648	0.008582503
0.777185196	0.683313632	0.629972758	0.003110558	0.003920326	0.004492969

0.394158325	0.152285312	0.159899861	0.005492180	0.007205648	0.008582503
0.394158325	0.215465398	0.158923615	0.003110558	0.003920326	0.004492969
0.171057114	0.487814448	0.395839824	0.005492180	0.007205648	0.008582503
0.171057114	0.453245829	0.422792479	0.003110558	0.003920326	0.004492969
0.719601276	0.929759273	0.819172583	0.005492180	0.007205648	0.008582503
0.719601276	0.575486321	0.525705233	0.003110558	0.003920326	0.004492969
0.341340438	0.928332783	0.817576733	0.005492180	0.007205648	0.008582503
0.341340438	0.442456037	0.411964315	0.003110558	0.003920326	0.004492969
0.176179205	0.429259070	0.349528081	0.005492180	0.007205648	0.008582503
0.176179205	0.078188742	0.061971396	0.003110558	0.003920326	0.004492969
0.229418269	0.310789657	0.267515340	0.005492180	0.007205648	0.008582503
0.229418269	0.186687966	0.130267003	0.003110558	0.003920326	0.004492969
0.439060369	0.356343225	0.297778690	0.005492180	0.007205648	0.008582503
0.439060369	0.585800305	0.533953297	0.003110558	0.003920326	0.004492969
0.505573507	0.974382090	0.884602161	0.005492180	0.007205648	0.008582503
0.505573507	0.320360924	0.255563444	0.003110558	0.003920326	0.004492969
0.418234011	0.736429165	0.623402249	0.005492180	0.007205648	0.008582503
0.418234011	0.575274048	0.525680093	0.003110558	0.003920326	0.004492969
0.756705873	0.535294334	0.434540839	0.005492180	0.007205648	0.008582503
0.756705873	0.608926028	0.562315473	0.003110558	0.003920326	0.004492969
0.421395765	0.172519069	0.175429988	0.005492180	0.007205648	0.008582503
0.421395765	0.246967654	0.178734232	0.003110558	0.003920326	0.004492969
0.294052267	0.270750086	0.241462062	0.005492180	0.007205648	0.008582503
0.294052267	0.951598919	0.912014068	0.003110558	0.003920326	0.004492969
0.594399836	0.583411930	0.473650996	0.005492180	0.007205648	0.008582503
0.594399836	0.385174285	0.356629763	0.003110558	0.003920326	0.004492969
0.062120668	0.027990386	0.055056841	0.005492180	0.007205648	0.008582503
0.062120668	0.688889800	0.645179992	0.003110558	0.003920326	0.004492969
0.338175207	0.854840839	0.739656274	0.005492180	0.007205648	0.008582503
0.338175207	0.171809028	0.123262471	0.003110558	0.003920326	0.004492969
0.180464266	0.032418387	0.060477246	0.005492180	0.007205648	0.008582503
0.180464266	0.084101961	0.064237139	0.003110558	0.003920326	0.004492969
0.283680821	0.362532309	0.301802961	0.005492180	0.007205648	0.008582503
0.283680821	0.203107076	0.148480430	0.003110558	0.003920326	0.004492969
0.958677190	0.945542257	0.837747484	0.005492180	0.007205648	0.008582503
0.958677190	0.948465440	0.908457466	0.003110558	0.003920326	0.004492969
0.255520088	0.301381549	0.260984518	0.005492180	0.007205648	0.008582503
0.255520088	0.814371012	0.791298965	0.003110558	0.003920326	0.004492969
0.377210630	0.166356107	0.170936448	0.005492180	0.007205648	0.008582503
0.377210630	0.198344802	0.145381044	0.003110558	0.003920326	0.004492969
0.303518970	0.148431527	0.156649133	0.005492180	0.007205648	0.008582503
0.303518970	0.652360957	0.596096777	0.003110558	0.003920326	0.004492969
0.910168162	0.771822327	0.656142575	0.005492180	0.007205648	0.008582503
0.910168162	0.834977027	0.802079185	0.003110558	0.003920326	0.004492969
0.90902181	0.989740516	0.929303997	0.005492180	0.007205648	0.008582503
0.90902181	0.871066780	0.829604803	0.003110558	0.003920326	0.004492969

0.257891634	0.421937627	0.343878017	0.005492180	0.007205648	0.008582503
0.257891634	0.747690794	0.736339149	0.003110558	0.003920326	0.004492969
0.050203436	0.128561553	0.140146644	0.005492180	0.007205648	0.008582503
0.050203436	0.128912590	0.090673628	0.003110558	0.003920326	0.004492969
0.633625137	0.476342235	0.386674923	0.005492180	0.007205648	0.008582503
0.633625137	0.428333981	0.391929146	0.003110558	0.003920326	0.004492969
0.552000583	0.312814225	0.268917275	0.005492180	0.007205648	0.008582503
0.552000583	0.944118670	0.903185226	0.003110558	0.003920326	0.004492969
0.111503842	0.829004108	0.712393409	0.005492180	0.007205648	0.008582503
0.111503842	0.295655561	0.227455455	0.003110558	0.003920326	0.004492969
0.141267417	0.230450391	0.216327152	0.005492180	0.007205648	0.008582503
0.141267417	0.600336747	0.553553781	0.003110558	0.003920326	0.004492969
0.063040992	0.690044384	0.578835797	0.005492180	0.007205648	0.008582503
0.063040992	0.070254372	0.054614117	0.003110558	0.003920326	0.004492969
0.709843460	0.489094365	0.396904540	0.005492180	0.007205648	0.008582503
0.709843460	0.520746529	0.494610752	0.003110558	0.003920326	0.004492969
0.459935127	0.360047650	0.300177435	0.005492180	0.007205648	0.008582503
0.459935127	0.635859715	0.587223065	0.003110558	0.003920326	0.004492969
0.814676524	0.653350977	0.540659846	0.005492180	0.007205648	0.008582503
0.814676524	0.702042923	0.647921235	0.003110558	0.003920326	0.004492969
0.890119452	0.825930002	0.709320371	0.005492180	0.007205648	0.008582503
0.890119452	0.781667964	0.775217826	0.003110558	0.003920326	0.004492969
0.041269218	0.605998283	0.494206371	0.005492180	0.007205648	0.008582503
0.041269218	0.354064547	0.334076249	0.003110558	0.003920326	0.004492969
0.810027823	0.688490386	0.577304212	0.005492180	0.007205648	0.008582503
0.810027823	0.684496159	0.634977560	0.003110558	0.003920326	0.004492969
0.470557850	0.830749920	0.714137879	0.005492180	0.007205648	0.008582503
0.470557850	0.368904116	0.344665909	0.003110558	0.003920326	0.004492969
0.601160656	0.445164201	0.361616365	0.005492180	0.007205648	0.008582503
0.601160656	0.412083991	0.371354200	0.003110558	0.003920326	0.004492969
0.730732027	0.807635533	0.691387206	0.005492180	0.007205648	0.008582503
0.730732027	0.972756022	0.932661371	0.003110558	0.003920326	0.004492969
0.376749191	0.287633156	0.252072580	0.005492180	0.007205648	0.008582503
0.376749191	0.212490727	0.15533991	0.003110558	0.003920326	0.004492969
0.200800101	0.489424717	0.397169320	0.005492180	0.007205648	0.008582503
0.200800101	0.052749185	0.041329632	0.003110558	0.003920326	0.004492969
0.255895972	0.566869202	0.460109997	0.005492180	0.007205648	0.008582503
0.255895972	0.479913851	0.454847024	0.003110558	0.003920326	0.004492969
0.340936590	0.173699436	0.176313595	0.005492180	0.007205648	0.008582503
0.340936590	0.165399724	0.119237159	0.003110558	0.003920326	0.004492969
0.221682008	0.183207064	0.183552501	0.005492180	0.007205648	0.008582503
0.221682008	0.065498119	0.050714611	0.003110558	0.003920326	0.004492969
0.406049456	0.229319385	0.215573426	0.005492180	0.007205648	0.008582503
0.406049456	0.217236268	0.160929852	0.003110558	0.003920326	0.004492969
0.561230993	0.790278124	0.674884017	0.005492180	0.007205648	0.008582503
0.561230993	0.743279507	0.734551022	0.003110558	0.003920326	0.004492969

0.422171143	0.756763558	0.641650904	0.005492180	0.007205648	0.008582503
0.422171143	0.250487525	0.182667841	0.003110558	0.003920326	0.004492969
0.452516149	0.338111826	0.285602979	0.005492180	0.007205648	0.008582503
0.452516149	0.305874876	0.232038264	0.003110558	0.003920326	0.004492969
0.079385738	0.072004555	0.093798551	0.005492180	0.007205648	0.008582503
0.079385738	0.252778538	0.184739539	0.003110558	0.003920326	0.004492969
0.699437518	0.512847254	0.416766987	0.005492180	0.007205648	0.008582503
0.699437518	0.502833077	0.474798728	0.003110558	0.003920326	0.004492969
0.112545045	0.274733695	0.244018845	0.005492180	0.007205648	0.008582503
0.112545045	0.262525338	0.188454224	0.003110558	0.003920326	0.004492969
0.158002588	0.306805623	0.264722499	0.005492180	0.007205648	0.008582503
0.158002588	0.871509028	0.829655250	0.003110558	0.003920326	0.004492969
0.624268664	0.497290066	0.403814665	0.005492180	0.007205648	0.008582503
0.624268664	0.666550945	0.601920805	0.003110558	0.003920326	0.004492969
0.150617219	0.024643224	0.050676605	0.005492180	0.007205648	0.008582503
0.150617219	0.169324229	0.122285384	0.003110558	0.003920326	0.004492969
0.280155696	0.876206814	0.762235596	0.005492180	0.007205648	0.008582503
0.280155696	0.111099856	0.081979497	0.003110558	0.003920326	0.004492969
0.008581956	0.727658705	0.615386375	0.005492180	0.007205648	0.008582503
0.008581956	0.901813878	0.849780072	0.003110558	0.003920326	0.004492969
0.604333380	0.565748036	0.459207179	0.005492180	0.007205648	0.008582503
0.604333380	0.700762675	0.647768848	0.003110558	0.003920326	0.004492969
0.167090226	0.076803779	0.097279268	0.005492180	0.007205648	0.008582503
0.167090226	0.041615152	0.033106128	0.003110558	0.003920326	0.004492969
0.062542881	0.044460222	0.072228767	0.005492180	0.007205648	0.008582503
0.062542881	0.032193091	0.029650667	0.003110558	0.003920326	0.004492969
0.188599889	0.093709827	0.110243793	0.005492180	0.007205648	0.008582503
0.188599889	0.148312461	0.108074597	0.003110558	0.003920326	0.004492969
0.236512847	0.134550912	0.145092934	0.005492180	0.007205648	0.008582503
0.236512847	0.123240549	0.086690313	0.003110558	0.003920326	0.004492969
0.399656112	0.151774677	0.159474359	0.005492180	0.007205648	0.008582503
0.399656112	0.214903804	0.158274287	0.003110558	0.003920326	0.004492969
0.657345436	0.609885727	0.497788829	0.005492180	0.007205648	0.008582503
0.657345436	0.460875903	0.438834962	0.003110558	0.003920326	0.004492969
0.204110215	0.851538053	0.735997165	0.005492180	0.007205648	0.008582503
0.204110215	0.182700657	0.128689358	0.003110558	0.003920326	0.004492969
0.001928988	0.028156081	0.055261691	0.005492180	0.007205648	0.008582503
0.001928988	0.020396850	0.023068774	0.003110558	0.003920326	0.004492969
0.009846968	0.750319675	0.635831304	0.005492180	0.007205648	0.008582503
0.009846968	0.173116096	0.124658090	0.003110558	0.003920326	0.004492969
0.740906232	0.992096386	0.937293978	0.005492180	0.007205648	0.008582503
0.740906232	0.631182033	0.585904978	0.003110558	0.003920326	0.004492969
0.206012049	0.052456844	0.079086853	0.005492180	0.007205648	0.008582503
0.206012049	0.285664436	0.216787799	0.003110558	0.003920326	0.004492969
0.489478654	0.403532747	0.330153792	0.005492180	0.007205648	0.008582503
0.489478654	0.934261131	0.895078352	0.003110558	0.003920326	0.004492969

0.572106815	0.958489870	0.855069854	0.005492180	0.007205648	0.008582503
0.572106815	0.788122395	0.778578078	0.003110558	0.003920326	0.004492969
0.412801155	0.334315224	0.283116674	0.005492180	0.007205648	0.008582503
0.412801155	0.236495725	0.172006427	0.003110558	0.003920326	0.004492969
0.766363204	0.670807183	0.559383628	0.005492180	0.007205648	0.008582503
0.766363204	0.620827714	0.576680378	0.003110558	0.003920326	0.004492969
0.162881829	0.256270563	0.232428675	0.005492180	0.007205648	0.008582503
0.162881829	0.663329391	0.601725012	0.003110558	0.003920326	0.004492969
0.690662534	0.606561736	0.494730804	0.005492180	0.007205648	0.008582503
0.690662534	0.544497806	0.504452344	0.003110558	0.003920326	0.004492969
0.917607092	0.778985394	0.663326093	0.005492180	0.007205648	0.008582503
0.917607092	0.998089927	0.972431821	0.003110558	0.003920326	0.004492969
0.985740185	0.798518659	0.682842266	0.005492180	0.007205648	0.008582503
0.985740185	0.975154075	0.936050574	0.003110558	0.003920326	0.004492969
0.510538643	0.387709842	0.319004291	0.005492180	0.007205648	0.008582503
0.510538643	0.471031690	0.446206114	0.003110558	0.003920326	0.004492969
0.175529302	0.894185188	0.781095826	0.005492180	0.007205648	0.008582503
0.175529302	0.732873075	0.700359542	0.003110558	0.003920326	0.004492969
0.606703280	0.700846887	0.589504358	0.005492180	0.007205648	0.008582503
0.606703280	0.800097764	0.786501433	0.003110558	0.003920326	0.004492969
0.407936804	0.878694533	0.764802150	0.005492180	0.007205648	0.008582503
0.407936804	0.238135857	0.172922279	0.003110558	0.003920326	0.004492969
0.909343347	0.728306073	0.615981331	0.005492180	0.007205648	0.008582503
0.909343347	0.900744309	0.847639292	0.003110558	0.003920326	0.004492969
0.779465529	0.627144965	0.513579085	0.005492180	0.007205648	0.008582503
0.779465529	0.675570600	0.612155847	0.003110558	0.003920326	0.004492969
0.145181732	0.364587240	0.303182125	0.005492180	0.007205648	0.008582503
0.145181732	0.226539286	0.166736322	0.003110558	0.003920326	0.004492969
0.455543090	0.359525438	0.299840542	0.005492180	0.007205648	0.008582503
0.455543090	0.271059516	0.200676433	0.003110558	0.003920326	0.004492969
0.176189691	0.903832877	0.791570962	0.005492180	0.007205648	0.008582503
0.176189691	0.980042162	0.952053740	0.003110558	0.003920326	0.004492969
0.482534627	0.461759274	0.374567087	0.005492180	0.007205648	0.008582503
0.482534627	0.300626131	0.230275358	0.003110558	0.003920326	0.004492969
0.408844551	0.569846688	0.462499051	0.005492180	0.007205648	0.008582503
0.408844551	0.962418775	0.929910826	0.003110558	0.003920326	0.004492969
0.098702398	0.350346041	0.293808469	0.005492180	0.007205648	0.008582503
0.098702398	0.103349214	0.074008520	0.003110558	0.003920326	0.004492969
0.687837144	0.503133536	0.408797464	0.005492180	0.007205648	0.008582503
0.687837144	0.491410405	0.463043837	0.003110558	0.003920326	0.004492969
0.707172503	0.627637269	0.514063104	0.005492180	0.007205648	0.008582503
0.707172503	0.538477116	0.503670703	0.003110558	0.003920326	0.004492969
0.039762464	0.478592261	0.388497070	0.005492180	0.007205648	0.008582503
0.039762464	0.770701706	0.764577143	0.003110558	0.003920326	0.004492969
0.385945623	0.172060147	0.175087067	0.005492180	0.007205648	0.008582503
0.385945623	0.199909161	0.147307432	0.003110558	0.003920326	0.004492969

0.363227427	0.413357234	0.337278831	0.005492180	0.007205648	0.008582503
0.363227427	0.334599025	0.299600700	0.003110558	0.003920326	0.004492969
0.882439825	0.805796441	0.689702688	0.005492180	0.007205648	0.008582503
0.882439825	0.769111604	0.761380416	0.003110558	0.003920326	0.004492969
0.131662557	0.098358761	0.114096294	0.005492180	0.007205648	0.008582503
0.131662557	0.017318092	0.020095969	0.003110558	0.003920326	0.004492969
0.200676330	0.038142740	0.066434973	0.005492180	0.007205648	0.008582503
0.200676330	0.524839977	0.497440341	0.003110558	0.003920326	0.004492969
0.088344426	0.043589124	0.071448838	0.005492180	0.007205648	0.008582503
0.088344426	0.015907781	0.018678956	0.003110558	0.003920326	0.004492969
0.410278402	0.972661769	0.880450097	0.005492180	0.007205648	0.008582503
0.410278402	0.565102845	0.515889440	0.003110558	0.003920326	0.004492969
0.322500413	0.117453438	0.130648453	0.005492180	0.007205648	0.008582503
0.322500413	0.144086200	0.102011450	0.003110558	0.003920326	0.004492969
0.293998794	0.143097641	0.152171488	0.005492180	0.007205648	0.008582503
0.293998794	0.134533488	0.094212986	0.003110558	0.003920326	0.004492969
0.741387131	0.585575875	0.475461892	0.005492180	0.007205648	0.008582503
0.741387131	0.606284626	0.555328134	0.003110558	0.003920326	0.004492969
0.067233375	0.733601727	0.620875696	0.005492180	0.007205648	0.008582503
0.067233375	0.829138266	0.801431233	0.003110558	0.003920326	0.004492969
0.017668201	0.013444558	0.033079586	0.005492180	0.007205648	0.008582503
0.017668201	0.022983759	0.024636735	0.003110558	0.003920326	0.004492969
0.615039868	0.734669075	0.621826136	0.005492180	0.007205648	0.008582503
0.615039868	0.757417338	0.738598201	0.003110558	0.003920326	0.004492969
0.298406319	0.095326846	0.111566114	0.005492180	0.007205648	0.008582503
0.298406319	0.124010362	0.087004289	0.003110558	0.003920326	0.004492969
0.354766490	0.422577214	0.344366579	0.005492180	0.007205648	0.008582503
0.354766490	0.449477928	0.422391728	0.003110558	0.003920326	0.004492969
0.823038116	0.788106758	0.672699709	0.005492180	0.007205648	0.008582503
0.823038116	0.723304232	0.679518825	0.003110558	0.003920326	0.004492969
0.246195386	0.163751845	0.169008858	0.005492180	0.007205648	0.008582503
0.246195386	0.464198607	0.445767892	0.003110558	0.003920326	0.004492969
0.285862656	0.077543263	0.097823741	0.005492180	0.007205648	0.008582503
0.285862656	0.110113410	0.081014506	0.003110558	0.003920326	0.004492969
0.275353565	0.891411941	0.778043167	0.005492180	0.007205648	0.008582503
0.275353565	0.884167697	0.838656867	0.003110558	0.003920326	0.004492969
0.251143644	0.613972783	0.501391664	0.005492180	0.007205648	0.008582503
0.251143644	0.861212304	0.824987311	0.003110558	0.003920326	0.004492969
0.480136662	0.315578272	0.270837082	0.005492180	0.007205648	0.008582503
0.480136662	0.299192887	0.228413100	0.003110558	0.003920326	0.004492969
0.311143908	0.639554055	0.5222124	0.003882898	0.005253553	0.009464192
0.311143908	0.675398337	0.641582462	0.005675962	0.007312185	0.008680667
0.083737623	0.702794202	0.580555072	0.003882898	0.005253553	0.009464192
0.083737623	0.537788242	0.529026305	0.005675962	0.007312185	0.008680667
0.155119080	0.269213909	0.224827722	0.003882898	0.005253553	0.009464192
0.155119080	0.768329624	0.718608952	0.005675962	0.007312185	0.008680667

0.920399309	0.925678019	0.783715291	0.003882898	0.005253553	0.009464192	
0.920399309	0.826916717	0.792875305	0.005675962	0.007312185	0.008680667	
0.004742590	0.881864675	0.739907458	0.003882898	0.005253553	0.009464192	
0.004742590	0.561536058	0.545247827	0.005675962	0.007312185	0.008680667	
0.516748890	0.395508908	0.309442986	0.003882898	0.005253553	0.009464192	
0.516748890	0.299671720	0.260664661	0.005675962	0.007312185	0.008680667	
0.445054657	0.600966247	0.483598856	0.003882898	0.005253553	0.009464192	
0.445054657	0.464261032	0.451782574	0.005675962	0.007312185	0.008680667	
0.167034184	0.082166135	0.090262305	0.003882898	0.005253553	0.009464192	
0.167034184	0.828543614	0.794185567	0.005675962	0.007312185	0.008680667	
0.006856048	0.174207826	0.161879187	0.003882898	0.005253553	0.009464192	
0.006856048	0.782703891	0.749118061	0.005675962	0.007312185	0.008680667	
0.353171092	0.433712301	0.336914117	0.003882898	0.005253553	0.009464192	
0.353171092	0.479233483	0.476035059	0.005675962	0.007312185	0.008680667	
0.806425841	0.738192188	0.614526376	0.003882898	0.005253553	0.009464192	
0.806425841	0.746277096	0.696666723	0.005675962	0.007312185	0.008680667	
0.496912958	0.432976507	0.336376727	0.003882898	0.005253553	0.009464192	
0.496912958	0.254223460	0.216646175	0.005675962	0.007312185	0.008680667	
0.727733522	0.683970298	0.563247772	0.003882898	0.005253553	0.009464192	
0.727733522	0.743050255	0.689853683	0.005675962	0.007312185	0.008680667	
0.005461124	0.001077198	0.004704348	0.003882898	0.005253553	0.009464192	0.15
0.005461124	0.120794110	0.126852474	0.005675962	0.007312185	0.008680667	
0.544727435	0.558347187	0.441650309	0.003882898	0.005253553	0.009464192	
0.544727435	0.514420400	0.505805286	0.005675962	0.007312185	0.008680667	
0.113948015	0.781506137	0.654083141	0.003882898	0.005253553	0.009464192	
0.113948015	0.580263028	0.570172959	0.005675962	0.007312185	0.008680667	
0.109671317	0.865336221	0.725317270	0.003882898	0.005253553	0.009464192	
0.109671317	0.495733429	0.495238899	0.005675962	0.007312185	0.008680667	
0.267317342	0.436645199	0.339071034	0.003882898	0.005253553	0.009464192	
0.267317342	0.135241196	0.136635676	0.005675962	0.007312185	0.008680667	
0.108744156	0.260559326	0.219136045	0.003882898	0.005253553	0.009464192	
0.108744156	0.247627131	0.211806884	0.005675962	0.007312185	0.008680667	
0.040472950	0.186426732	0.170143316	0.003882898	0.005253553	0.009464192	
0.040472950	0.643428311	0.605339411	0.005675962	0.007312185	0.008680667	
0.000196028	0.877313543	0.735907659	0.003882898	0.005253553	0.009464192	
0.000196028	0.129055301	0.131895132	0.005675962	0.007312185	0.008680667	
0.765328915	0.786513577	0.658286312	0.003882898	0.005253553	0.009464192	
0.765328915	0.672087250	0.639864085	0.005675962	0.007312185	0.008680667	
0.022164520	0.001318174	0.005421417	0.003882898	0.005253553	0.009464192	0.175
0.022164520	0.183805824	0.171353266	0.005675962	0.007312185	0.008680667	
0.560564123	0.352373307	0.280260471	0.003882898	0.005253553	0.009464192	
0.560564123	0.304659306	0.267514051	0.005675962	0.007312185	0.008680667	
0.074916580	0.194927563	0.175993764	0.003882898	0.005253553	0.009464192	
0.074916580	0.957170663	0.927619603	0.005675962	0.007312185	0.008680667	
0.551609507	0.549288120	0.433290079	0.003882898	0.005253553	0.009464192	
0.551609507	0.382344383	0.351681888	0.005675962	0.007312185	0.008680667	

0.090419915	0.038443613	0.053895656	0.003882898	0.005253553	0.009464192
0.090419915	0.749422922	0.702983863	0.005675962	0.007312185	0.008680667
0.902653898	0.955942527	0.820201609	0.003882898	0.005253553	0.009464192
0.902653898	0.657012993	0.621136333	0.005675962	0.007312185	0.008680667
0.342102933	0.141592036	0.137588640	0.003882898	0.005253553	0.009464192
0.342102933	0.178740907	0.165890198	0.005675962	0.007312185	0.008680667
0.212034033	0.325331277	0.262478417	0.003882898	0.005253553	0.009464192
0.212034033	0.270049489	0.232567558	0.005675962	0.007312185	0.008680667
0.441433677	0.561228809	0.444524673	0.003882898	0.005253553	0.009464192
0.441433677	0.491118000	0.490561542	0.005675962	0.007312185	0.008680667
0.807041499	0.723283545	0.600148895	0.003882898	0.005253553	0.009464192
0.807041499	0.877525848	0.827891246	0.005675962	0.007312185	0.008680667
0.845105244	0.681913383	0.561252962	0.003882898	0.005253553	0.009464192
0.845105244	0.519007622	0.513441708	0.005675962	0.007312185	0.008680667
0.276010469	0.139511395	0.135935120	0.003882898	0.005253553	0.009464192
0.276010469	0.708291019	0.660359994	0.005675962	0.007312185	0.008680667
0.126347114	0.095168599	0.100791436	0.003882898	0.005253553	0.009464192
0.126347114	0.052095125	0.072168228	0.005675962	0.007312185	0.008680667
0.395134193	0.924983175	0.782929058	0.003882898	0.005253553	0.009464192
0.395134193	0.866410416	0.825352262	0.005675962	0.007312185	0.008680667
0.074781929	0.349371624	0.278267683	0.003882898	0.005253553	0.009464192
0.074781929	0.811026355	0.780772256	0.005675962	0.007312185	0.008680667
0.669434989	0.554682275	0.438163519	0.003882898	0.005253553	0.009464192
0.669434989	0.400295486	0.384402171	0.005675962	0.007312185	0.008680667
0.712725531	0.573258746	0.456764027	0.003882898	0.005253553	0.009464192
0.712725531	0.389664153	0.364238724	0.005675962	0.007312185	0.008680667
0.381845971	0.172239927	0.16049123	0.003882898	0.005253553	0.009464192
0.381845971	0.951294461	0.920421998	0.005675962	0.007312185	0.008680667
0.01374849	0.773914700	0.647675900	0.003882898	0.005253553	0.009464192
0.01374849	0.352671743	0.316947649	0.005675962	0.007312185	0.008680667
0.088969196	0.206611187	0.183918077	0.003882898	0.005253553	0.009464192
0.088969196	0.658657721	0.622987872	0.005675962	0.007312185	0.008680667
0.589590424	0.773490347	0.647303231	0.003882898	0.005253553	0.009464192
0.589590424	0.335289221	0.296541009	0.005675962	0.007312185	0.008680667
0.786186079	0.602296505	0.484962431	0.003882898	0.005253553	0.009464192
0.786186079	0.475284537	0.473422115	0.005675962	0.007312185	0.008680667
0.967720368	0.905848172	0.762023650	0.003882898	0.005253553	0.009464192
0.967720368	0.915436673	0.867355117	0.005675962	0.007312185	0.008680667
0.975896833	0.870661630	0.729967782	0.003882898	0.005253553	0.009464192
0.975896833	0.729673015	0.671967004	0.005675962	0.007312185	0.008680667
0.486762349	0.427536728	0.332454282	0.003882898	0.005253553	0.009464192
0.486762349	0.323512458	0.279725032	0.005675962	0.007312185	0.008680667
0.371340917	0.641933040	0.524503406	0.003882898	0.005253553	0.009464192
0.371340917	0.426633432	0.422671933	0.005675962	0.007312185	0.008680667
0.433636045	0.319030216	0.258160168	0.003882898	0.005253553	0.009464192
0.433636045	0.163009507	0.155940382	0.005675962	0.007312185	0.008680667

0.762680720	0.876743693	0.735394864	0.003882898	0.005253553	0.009464192
0.762680720	0.607418722	0.581095181	0.005675962	0.007312185	0.008680667
0.940226482	0.831678265	0.697568176	0.003882898	0.005253553	0.009464192
0.940226482	0.801073197	0.769502427	0.005675962	0.007312185	0.008680667
0.094302699	0.641409810	0.524007917	0.003882898	0.005253553	0.009464192
0.094302699	0.974470161	0.944298071	0.005675962	0.007312185	0.008680667
0.072834389	0.172440938	0.160634028	0.003882898	0.005253553	0.009464192
0.072834389	0.278021849	0.239978863	0.005675962	0.007312185	0.008680667
0.417639769	0.524065879	0.410825629	0.003882898	0.005253553	0.009464192
0.417639769	0.124798338	0.129281209	0.005675962	0.007312185	0.008680667
0.974823094	0.970330160	0.845601126	0.003882898	0.005253553	0.009464192
0.974823094	0.888281483	0.838190414	0.005675962	0.007312185	0.008680667
0.261811139	0.131284846	0.129294476	0.003882898	0.005253553	0.009464192
0.261811139	0.106147608	0.114468605	0.005675962	0.007312185	0.008680667
0.102566912	0.147220075	0.141972225	0.003882898	0.005253553	0.009464192
0.102566912	0.021372616	0.039571882	0.005675962	0.007312185	0.008680667
0.139435659	0.076442989	0.085807223	0.003882898	0.005253553	0.009464192
0.139435659	0.016804937	0.034231697	0.005675962	0.007312185	0.008680667
0.206213186	0.684864024	0.564090813	0.003882898	0.005253553	0.009464192
0.206213186	0.804876357	0.779129819	0.005675962	0.007312185	0.008680667
0.343418977	0.710017719	0.587390862	0.003882898	0.005253553	0.009464192
0.343418977	0.198432833	0.182568726	0.005675962	0.007312185	0.008680667
0.278966344	0.200655294	0.179839013	0.003882898	0.005253553	0.009464192
0.278966344	0.361669950	0.321557033	0.005675962	0.007312185	0.008680667
0.289966735	0.157721043	0.149855396	0.003882898	0.005253553	0.009464192
0.289966735	0.310566231	0.272543827	0.005675962	0.007312185	0.008680667
0.637784181	0.475011660	0.368734259	0.003882898	0.005253553	0.009464192
0.637784181	0.330254093	0.289248610	0.005675962	0.007312185	0.008680667
0.566758392	0.493928126	0.385030238	0.003882898	0.005253553	0.009464192
0.566758392	0.388604837	0.362331364	0.005675962	0.007312185	0.008680667
0.059957607	0.261410459	0.219692668	0.003882898	0.005253553	0.009464192
0.059957607	0.901576000	0.859351304	0.005675962	0.007312185	0.008680667
0.113308141	0.134992348	0.132349720	0.003882898	0.005253553	0.009464192
0.113308141	0.715185502	0.664997624	0.005675962	0.007312185	0.008680667
0.359556104	0.155577581	0.148305821	0.003882898	0.005253553	0.009464192
0.359556104	0.250225883	0.212511473	0.005675962	0.007312185	0.008680667
0.538077474	0.915791265	0.772418258	0.003882898	0.005253553	0.009464192
0.538077474	0.275649559	0.237770657	0.005675962	0.007312185	0.008680667
0.214171622	0.738812496	0.615142602	0.003882898	0.005253553	0.009464192
0.214171622	0.928512057	0.876410087	0.005675962	0.007312185	0.008680667
0.370738003	0.433959435	0.337090886	0.003882898	0.005253553	0.009464192
0.370738003	0.758809158	0.705030418	0.005675962	0.007312185	0.008680667
0.935001524	0.8131711	0.681284107	0.003882898	0.005253553	0.009464192
0.935001524	0.619247837	0.586838312	0.005675962	0.007312185	0.008680667
0.835942629	0.690226771	0.569036257	0.003882898	0.005253553	0.009464192
0.835942629	0.484142743	0.482498982	0.005675962	0.007312185	0.008680667

0.519257570	0.365623155	0.289080014	0.003882898	0.005253553	0.009464192
0.519257570	0.329595578	0.288314919	0.005675962	0.007312185	0.008680667
0.194928740	0.122442922	0.122304623	0.003882898	0.005253553	0.009464192
0.194928740	0.169691636	0.159221932	0.005675962	0.007312185	0.008680667
0.151056344	0.029246919	0.043685953	0.003882898	0.005253553	0.009464192
0.151056344	0.038712858	0.059228103	0.005675962	0.007312185	0.008680667
0.244017552	0.379324048	0.298303616	0.003882898	0.005253553	0.009464192
0.244017552	0.446884535	0.438442267	0.005675962	0.007312185	0.008680667
0.380630279	0.869487761	0.728932971	0.003882898	0.005253553	0.009464192
0.380630279	0.262326494	0.225547970	0.005675962	0.007312185	0.008680667
0.035909443	0.037628307	0.053065833	0.003882898	0.005253553	0.009464192
0.035909443	0.043214215	0.065291425	0.005675962	0.007312185	0.008680667
0.029414659	0.719351753	0.596296043	0.003882898	0.005253553	0.009464192
0.029414659	0.232677582	0.205473665	0.005675962	0.007312185	0.008680667
0.241792867	0.932596591	0.791654705	0.003882898	0.005253553	0.009464192
0.241792867	0.685356615	0.651036384	0.005675962	0.007312185	0.008680667
0.689586146	0.511795906	0.400167585	0.003882898	0.005253553	0.009464192
0.689586146	0.472844747	0.467848365	0.005675962	0.007312185	0.008680667
0.092314239	0.199863833	0.179294787	0.003882898	0.005253553	0.009464192
0.092314239	0.940688072	0.913481175	0.005675962	0.007312185	0.008680667
0.278651146	0.895395041	0.752019222	0.003882898	0.005253553	0.009464192
0.278651146	0.848928097	0.816398360	0.005675962	0.007312185	0.008680667
0.308929823	0.275861393	0.229301476	0.003882898	0.005253553	0.009464192
0.308929823	0.140058376	0.137621747	0.005675962	0.007312185	0.008680667
0.227749799	0.249813031	0.212170083	0.003882898	0.005253553	0.009464192
0.227749799	0.191954617	0.176016240	0.005675962	0.007312185	0.008680667
0.356039355	0.307243034	0.250357755	0.003882898	0.005253553	0.009464192
0.356039355	0.722040773	0.665471866	0.005675962	0.007312185	0.008680667
0.032128921	0.203323342	0.181657090	0.003882898	0.005253553	0.009464192
0.032128921	0.600845359	0.578832065	0.005675962	0.007312185	0.008680667
0.470340087	0.323899911	0.261508296	0.003882898	0.005253553	0.009464192
0.470340087	0.994884592	0.977177278	0.005675962	0.007312185	0.008680667
0.914977994	0.747738573	0.623908124	0.003882898	0.005253553	0.009464192
0.914977994	0.524903888	0.525710513	0.005675962	0.007312185	0.008680667
0.500480334	0.383913863	0.301457642	0.003882898	0.005253553	0.009464192
0.500480334	0.534746543	0.526881189	0.005675962	0.007312185	0.008680667
0.000480626	0.833661004	0.699368700	0.003882898	0.005253553	0.009464192
0.000480626	0.789578076	0.750157562	0.005675962	0.007312185	0.008680667
0.984309632	0.981278321	0.871610025	0.003882898	0.005253553	0.009464192
0.984309632	0.965120837	0.935981158	0.005675962	0.007312185	0.008680667
0.720487445	0.929417417	0.787986718	0.003882898	0.005253553	0.009464192
0.720487445	0.450265101	0.439765817	0.005675962	0.007312185	0.008680667
0.556026588	0.373627099	0.294429285	0.003882898	0.005253553	0.009464192
0.556026588	0.923438272	0.874219162	0.005675962	0.007312185	0.008680667
0.653002233	0.511027175	0.399502017	0.003882898	0.005253553	0.009464192
0.653002233	0.599828939	0.578146191	0.005675962	0.007312185	0.008680667

0.697062937	0.630180296	0.513010479	0.003882898	0.005253553	0.009464192
0.697062937	0.455461298	0.450738922	0.005675962	0.007312185	0.008680667
0.585704187	0.488885211	0.380702605	0.003882898	0.005253553	0.009464192
0.585704187	0.254023915	0.216436978	0.005675962	0.007312185	0.008680667
0.312097660	0.917429593	0.774274662	0.003882898	0.005253553	0.009464192
0.312097660	0.985344081	0.960914998	0.005675962	0.007312185	0.008680667
0.225685958	0.376297565	0.296252710	0.003882898	0.005253553	0.009464192
0.225685958	0.080443972	0.093453618	0.005675962	0.007312185	0.008680667
0.392616679	0.543644771	0.428191122	0.003882898	0.005253553	0.009464192
0.392616679	0.968976009	0.942596830	0.005675962	0.007312185	0.008680667
0.406950961	0.424417116	0.330203554	0.003882898	0.005253553	0.009464192
0.406950961	0.229025259	0.202739327	0.005675962	0.007312185	0.008680667
0.853945736	0.702825897	0.580584601	0.003882898	0.005253553	0.009464192
0.853945736	0.522212321	0.519996537	0.005675962	0.007312185	0.008680667
0.454777063	0.380390107	0.299029604	0.003882898	0.005253553	0.009464192
0.454777063	0.433151371	0.425410330	0.005675962	0.007312185	0.008680667
0.246121043	0.508758795	0.397543244	0.003882898	0.005253553	0.009464192
0.246121043	0.838728258	0.805013887	0.005675962	0.007312185	0.008680667
0.271324873	0.110746655	0.113234149	0.003882898	0.005253553	0.009464192
0.271324873	0.068601278	0.083697869	0.005675962	0.007312185	0.008680667
0.004679709	0.288650041	0.237673185	0.003882898	0.005253553	0.009464192
0.004679709	0.402361157	0.388726100	0.005675962	0.007312185	0.008680667
0.225624026	0.290017979	0.238606401	0.003882898	0.005253553	0.009464192
0.225624026	0.214149524	0.192533899	0.005675962	0.007312185	0.008680667
0.068060363	0.080046688	0.088560213	0.003882898	0.005253553	0.009464192
0.068060363	0.019795889	0.037434919	0.005675962	0.007312185	0.008680667
0.098072140	0.017236500	0.030357771	0.003882898	0.005253553	0.009464192
0.098072140	0.587147347	0.573058282	0.005675962	0.007312185	0.008680667
0.706351085	0.552968726	0.436607509	0.003882898	0.005253553	0.009464192
0.706351085	0.380189134	0.348016052	0.005675962	0.007312185	0.008680667
0.828558735	0.996123422	0.922771095	0.003882898	0.005253553	0.009464192
0.828558735	0.746658115	0.697628935	0.005675962	0.007312185	0.008680667
0.901163472	0.738513821	0.614847682	0.003882898	0.005253553	0.009464192
0.901163472	0.515589541	0.506585468	0.005675962	0.007312185	0.008680667
0.831521220	0.687659865	0.566669303	0.003882898	0.005253553	0.009464192
0.831521220	0.524614003	0.525099125	0.005675962	0.007312185	0.008680667
0.568403707	0.420616661	0.327489180	0.003882898	0.005253553	0.009464192
0.568403707	0.558724969	0.541467343	0.005675962	0.007312185	0.008680667
0.042142385	0.694910426	0.573312029	0.003882898	0.005253553	0.009464192
0.042142385	0.880784070	0.835789510	0.005675962	0.007312185	0.008680667
0.222350587	0.084198833	0.091909807	0.003882898	0.005253553	0.009464192
0.222350587	0.072251020	0.086148726	0.005675962	0.007312185	0.008680667
0.903609397	0.925931918	0.784005261	0.003882898	0.005253553	0.009464192
0.903609397	0.879740176	0.833284791	0.005675962	0.007312185	0.008680667
0.172222585	0.041919151	0.057234443	0.003882898	0.005253553	0.009464192
0.172222585	0.014381055	0.030182647	0.005675962	0.007312185	0.008680667

0.346680396	0.406023330	0.317052143	0.003882898	0.005253553	0.009464192
0.346680396	0.50734297	0.505069930	0.005675962	0.007312185	0.008680667
0.510642139	0.519668456	0.406981997	0.003882898	0.005253553	0.009464192
0.510642139	0.620302332	0.586961774	0.005675962	0.007312185	0.008680667
0.098559463	0.124041853	0.123531842	0.003882898	0.005253553	0.009464192
0.098559463	0.525988408	0.526320770	0.005675962	0.007312185	0.008680667
0.573472994	0.36387695	0.287928422	0.003882898	0.005253553	0.009464192
0.573472994	0.257989876	0.220695074	0.005675962	0.007312185	0.008680667
0.805256664	0.969733719	0.844323861	0.003882898	0.005253553	0.009464192
0.805256664	0.916714867	0.867510555	0.005675962	0.007312185	0.008680667
0.340353839	0.601992832	0.484650450	0.003882898	0.005253553	0.009464192
0.340353839	0.895713334	0.855654462	0.005675962	0.007312185	0.008680667
0.318154191	0.179952328	0.165804317	0.003882898	0.005253553	0.009464192
0.318154191	0.123009562	0.127281380	0.005675962	0.007312185	0.008680667
0.716787071	0.514913366	0.581611261	0.003303901	0.004786220	0.005872570
0.716787071	0.479725178	0.568323788	0.005915167	0.005975504	0.006080996
0.274928658	0.564573957	0.620636377	0.003303901	0.004786220	0.005872570
0.274928658	0.336156677	0.440508225	0.005915167	0.005975504	0.006080996
0.353899452	0.200887337	0.213773672	0.003303901	0.004786220	0.005872570
0.353899452	0.602483297	0.658201900	0.005915167	0.005975504	0.006080996
0.077348093	0.364633667	0.365222827	0.003303901	0.004786220	0.005872570
0.077348093	0.398787061	0.503729803	0.005915167	0.005975504	0.006080996
0.153834613	0.770077557	0.815217235	0.003303901	0.004786220	0.005872570
0.153834613	0.359896338	0.458540390	0.005915167	0.005975504	0.006080996
0.230214775	0.081152671	0.105429761	0.003303901	0.004786220	0.005872570
0.230214775	0.059209518	0.062892899	0.005915167	0.005975504	0.006080996
0.750521134	0.544903967	0.601400227	0.003303901	0.004786220	0.005872570
0.750521134	0.473058294	0.564628321	0.005915167	0.005975504	0.006080996
0.961199552	0.934288110	0.931533387	0.003303901	0.004786220	0.005872570
0.961199552	0.918940978	0.925730142	0.005915167	0.005975504	0.006080996
0.734014513	0.510834004	0.578197263	0.003303901	0.004786220	0.005872570
0.734014513	0.623402177	0.694953184	0.005915167	0.005975504	0.006080996
0.685513403	0.463489385	0.513565853	0.003303901	0.004786220	0.005872570
0.685513403	0.373115692	0.471231551	0.005915167	0.005975504	0.006080996
0.895717931	0.784830708	0.821466345	0.003303901	0.004786220	0.005872570
0.895717931	0.757189213	0.803970496	0.005915167	0.005975504	0.006080996
0.404134225	0.232189753	0.239081977	0.003303901	0.004786220	0.005872570
0.404134225	0.151308676	0.163898595	0.005915167	0.005975504	0.006080996
0.758718073	0.554323994	0.612852465	0.003303901	0.004786220	0.005872570
0.758718073	0.562609908	0.62408437	0.005915167	0.005975504	0.006080996
0.247806956	0.074125507	0.098702446	0.003303901	0.004786220	0.005872570
0.247806956	0.054706918	0.061890672	0.005915167	0.005975504	0.006080996
0.951501250	0.916934666	0.915183986	0.003303901	0.004786220	0.005872570
0.951501250	0.894382171	0.906949124	0.005915167	0.005975504	0.006080996
0.500127421	0.700441850	0.748506142	0.003303901	0.004786220	0.005872570
0.500127421	0.375946833	0.486750709	0.005915167	0.005975504	0.006080996

0.120188932	0.734843251	0.762587490	0.003303901	0.004786220	0.005872570
0.120188932	0.298127482	0.403098997	0.005915167	0.005975504	0.006080996
0.786498134	0.605215350	0.652708820	0.003303901	0.004786220	0.005872570
0.786498134	0.552358752	0.619599545	0.005915167	0.005975504	0.006080996
0.449135987	0.246886348	0.251633715	0.003303901	0.004786220	0.005872570
0.449135987	0.185103680	0.202520069	0.005915167	0.005975504	0.006080996
0.149662105	0.113209162	0.142542561	0.003303901	0.004786220	0.005872570
0.149662105	0.437342863	0.526412761	0.005915167	0.005975504	0.006080996
0.436707853	0.870452901	0.867932081	0.003303901	0.004786220	0.005872570
0.436707853	0.181157215	0.191392394	0.005915167	0.005975504	0.006080996
0.977795647	0.976838905	0.961206973	0.003303901	0.004786220	0.005872570
0.977795647	0.972025076	0.957781277	0.005915167	0.005975504	0.006080996
0.392113033	0.172989999	0.201427043	0.003303901	0.004786220	0.005872570
0.392113033	0.136890092	0.154441222	0.005915167	0.005975504	0.006080996
0.712663245	0.481963160	0.548855337	0.003303901	0.004786220	0.005872570
0.712663245	0.416291054	0.508797599	0.005915167	0.005975504	0.006080996
0.560969939	0.362691781	0.364316254	0.003303901	0.004786220	0.005872570
0.560969939	0.908937482	0.911476609	0.005915167	0.005975504	0.006080996
0.102023226	0.303697170	0.298051326	0.003303901	0.004786220	0.005872570
0.102023226	0.163370638	0.178114989	0.005915167	0.005975504	0.006080996
0.340819558	0.129615307	0.161207287	0.003303901	0.004786220	0.005872570
0.340819558	0.583893643	0.639838369	0.005915167	0.005975504	0.006080996
0.266631171	0.650903563	0.697911390	0.003303901	0.004786220	0.005872570
0.266631171	0.096354472	0.108446924	0.005915167	0.005975504	0.006080996
0.790806758	0.609482617	0.658488738	0.003303901	0.004786220	0.005872570
0.790806758	0.564642184	0.626404454	0.005915167	0.005975504	0.006080996
0.021933790	0.142908334	0.173749682	0.003303901	0.004786220	0.005872570
0.021933790	0.123392110	0.139240329	0.005915167	0.005975504	0.006080996
0.538601681	0.397361669	0.423925994	0.003303901	0.004786220	0.005872570
0.538601681	0.297083702	0.398046903	0.005915167	0.005975504	0.006080996
0.821976348	0.657178257	0.705323074	0.003303901	0.004786220	0.005872570
0.821976348	0.736734944	0.788748045	0.005915167	0.005975504	0.006080996
0.521256159	0.319966250	0.321700115	0.003303901	0.004786220	0.005872570
0.521256159	0.229842643	0.272439427	0.005915167	0.005975504	0.006080996
0.776527205	0.578099472	0.637865707	0.003303901	0.004786220	0.005872570
0.776527205	0.538739228	0.604538262	0.005915167	0.005975504	0.006080996
0.917422680	0.859678694	0.857029390	0.003303901	0.004786220	0.005872570
0.917422680	0.831182534	0.842729277	0.005915167	0.005975504	0.006080996
0.363789123	0.766330661	0.809090320	0.003303901	0.004786220	0.005872570
0.363789123	0.726097265	0.785712082	0.005915167	0.005975504	0.006080996
0.118701749	0.233549887	0.240007048	0.003303901	0.004786220	0.005872570
0.118701749	0.670652105	0.732097660	0.005915167	0.005975504	0.006080996
0.915861685	0.844443233	0.854503904	0.003303901	0.004786220	0.005872570
0.915861685	0.817668920	0.840005606	0.005915167	0.005975504	0.006080996
0.995038185	0.992385725	0.991290231	0.003303901	0.004786220	0.005872570
0.995038185	0.991178441	0.991242877	0.005915167	0.005975504	0.006080996

0.786140247	0.594402559	0.651667966	0.003303901	0.004786220	0.005872570	
0.786140247	0.887974559	0.902434314	0.005915167	0.005975504	0.006080996	
0.774787069	0.783987649	0.821368215	0.003303901	0.004786220	0.005872570	
0.774787069	0.530309197	0.601536040	0.005915167	0.005975504	0.006080996	
0.964273676	0.945090103	0.937094158	0.003303901	0.004786220	0.005872570	
0.964273676	0.933313335	0.931526706	0.005915167	0.005975504	0.006080996	
0.490900706	0.563274795	0.617017148	0.003303901	0.004786220	0.005872570	
0.490900706	0.219760399	0.241692857	0.005915167	0.005975504	0.006080996	
0.170848058	0.044368722	0.057541585	0.003303901	0.004786220	0.005872570	
0.170848058	0.060647155	0.065173668	0.005915167	0.005975504	0.006080996	
0.541838981	0.343777352	0.343577959	0.003303901	0.004786220	0.005872570	
0.541838981	0.498800698	0.580796159	0.005915167	0.005975504	0.006080996	
0.266196784	0.086654569	0.110091961	0.003303901	0.004786220	0.005872570	
0.266196784	0.067398306	0.071375840	0.005915167	0.005975504	0.006080996	
0.127245952	0.165183667	0.193644010	0.003303901	0.004786220	0.005872570	
0.127245952	0.108142211	0.117905890	0.005915167	0.005975504	0.006080996	
0.953079046	0.926646070	0.918032754	0.003303901	0.004786220	0.005872570	
0.953079046	0.901386062	0.909882157	0.005915167	0.005975504	0.006080996	
0.314837852	0.113401538	0.142564566	0.003303901	0.004786220	0.005872570	
0.314837852	0.085396058	0.099544417	0.005915167	0.005975504	0.006080996	
0.970962176	0.967148151	0.949259026	0.003303901	0.004786220	0.005872570	
0.970962176	0.955256969	0.944316819	0.005915167	0.005975504	0.006080996	
0.674879192	0.446409543	0.499551127	0.003303901	0.004786220	0.005872570	
0.674879192	0.358224214	0.456732170	0.005915167	0.005975504	0.006080996	
0.497564238	0.531049772	0.593862838	0.003303901	0.004786220	0.005872570	
0.497564238	0.93910472	0.932029181	0.005915167	0.005975504	0.006080996	
0.422394581	0.217314031	0.227560594	0.003303901	0.004786220	0.005872570	
0.422394581	0.170669671	0.179221054	0.005915167	0.005975504	0.006080996	
0.075986170	0.304265195	0.298895264	0.003303901	0.004786220	0.005872570	
0.075986170	0.000762570	0.005910408	0.005915167	0.005975504	0.006080996	0.1
0.209852238	0.372157610	0.381899175	0.003303901	0.004786220	0.005872570	
0.209852238	0.340793176	0.446592443	0.005915167	0.005975504	0.006080996	
0.474175604	0.268438163	0.274874718	0.003303901	0.004786220	0.005872570	
0.474175604	0.204820767	0.225499379	0.005915167	0.005975504	0.006080996	
0.381156882	0.164089353	0.192760486	0.003303901	0.004786220	0.005872570	
0.381156882	0.126478069	0.145744619	0.005915167	0.005975504	0.006080996	
0.359962725	0.145743759	0.175823982	0.003303901	0.004786220	0.005872570	
0.359962725	0.118264129	0.129989193	0.005915167	0.005975504	0.006080996	
0.404676330	0.531591805	0.595572080	0.003303901	0.004786220	0.005872570	
0.404676330	0.652534538	0.730255635	0.005915167	0.005975504	0.006080996	
0.938075581	0.894891422	0.892400235	0.003303901	0.004786220	0.005872570	
0.938075581	0.861523513	0.881202530	0.005915167	0.005975504	0.006080996	
0.681781582	0.454872492	0.508523998	0.003303901	0.004786220	0.005872570	
0.681781582	0.367048999	0.465978673	0.005915167	0.005975504	0.006080996	
0.102290095	0.038487894	0.050673940	0.003303901	0.004786220	0.005872570	
0.102290095	0.160131986	0.175448052	0.005915167	0.005975504	0.006080996	

0.844129144	0.683028904	0.738984911	0.003303901	0.004786220	0.005872570
0.844129144	0.630770908	0.713509515	0.005915167	0.005975504	0.006080996
0.742337084	0.527215577	0.589746737	0.003303901	0.004786220	0.005872570
0.742337084	0.460772320	0.552229272	0.005915167	0.005975504	0.006080996
0.848391415	0.695915013	0.745936286	0.003303901	0.004786220	0.005872570
0.848391415	0.800477366	0.827944131	0.005915167	0.005975504	0.006080996
0.481524955	0.280941760	0.282080745	0.003303901	0.004786220	0.005872570
0.481524955	0.520200196	0.595483617	0.005915167	0.005975504	0.006080996
0.717328723	0.495267392	0.555144669	0.003303901	0.004786220	0.005872570
0.717328723	0.428237703	0.515614285	0.005915167	0.005975504	0.006080996
0.830921506	0.840971163	0.852130943	0.003303901	0.004786220	0.005872570
0.830921506	0.612347030	0.691420262	0.005915167	0.005975504	0.006080996
0.900142032	0.813796307	0.829079950	0.003303901	0.004786220	0.005872570
0.900142032	0.851873274	0.848655822	0.005915167	0.005975504	0.006080996
0.490270963	0.314148539	0.311375248	0.003303901	0.004786220	0.005872570
0.490270963	0.593996503	0.642066919	0.005915167	0.005975504	0.006080996
0.480397721	0.275462544	0.280868450	0.003303901	0.004786220	0.005872570
0.480397721	0.208105121	0.231415784	0.005915167	0.005975504	0.006080996
0.396919085	0.183543575	0.205575352	0.003303901	0.004786220	0.005872570
0.396919085	0.145042305	0.158097636	0.005915167	0.005975504	0.006080996
0.854250490	0.711909098	0.754824488	0.003303901	0.004786220	0.005872570
0.854250490	0.658612551	0.730913197	0.005915167	0.005975504	0.006080996
0.854618552	0.719055634	0.755450900	0.003303901	0.004786220	0.005872570
0.854618552	0.665138025	0.731507471	0.005915167	0.005975504	0.006080996
0.099496463	0.012063550	0.027821360	0.003303901	0.004786220	0.005872570
0.099496463	0.007844734	0.013087401	0.005915167	0.005975504	0.006080996
0.579843705	0.377642465	0.385443118	0.003303901	0.004786220	0.005872570
0.579843705	0.274913852	0.342182304	0.005915167	0.005975504	0.006080996
0.159057347	0.653507389	0.704377160	0.003303901	0.004786220	0.005872570
0.159057347	0.111062583	0.119943488	0.005915167	0.005975504	0.006080996
0.038295587	0.009585328	0.024643235	0.003303901	0.004786220	0.005872570
0.038295587	0.011169368	0.018553081	0.005915167	0.005975504	0.006080996
0.232481516	0.585570753	0.645309705	0.003303901	0.004786220	0.005872570
0.232481516	0.103612264	0.116243495	0.005915167	0.005975504	0.006080996
0.893397374	0.979390582	0.966215773	0.003303901	0.004786220	0.005872570
0.893397374	0.750086787	0.799812445	0.005915167	0.005975504	0.006080996
0.433261142	0.227804305	0.236879664	0.003303901	0.004786220	0.005872570
0.433261142	0.189149868	0.207552086	0.005915167	0.005975504	0.006080996
0.853045268	0.705803975	0.753129974	0.003303901	0.004786220	0.005872570
0.853045268	0.881026783	0.894297754	0.005915167	0.005975504	0.006080996
0.541711559	0.818171513	0.836285144	0.003303901	0.004786220	0.005872570
0.541711559	0.713830044	0.775192466	0.005915167	0.005975504	0.006080996
0.782533394	0.589120679	0.646773348	0.003303901	0.004786220	0.005872570
0.782533394	0.539918867	0.613341171	0.005915167	0.005975504	0.006080996
0.750397753	0.538040230	0.600936449	0.003303901	0.004786220	0.005872570
0.750397753	0.465630574	0.564149535	0.005915167	0.005975504	0.006080996

0.674225154	0.442394743	0.498906181	0.003303901	0.004786220	0.005872570
0.674225154	0.526140948	0.595945872	0.005915167	0.005975504	0.006080996
0.395153326	0.181545925	0.204530043	0.003303901	0.004786220	0.005872570
0.395153326	0.390802484	0.496523115	0.005915167	0.005975504	0.006080996
0.761329068	0.558560214	0.616173427	0.003303901	0.004786220	0.005872570
0.761329068	0.9881362	0.972683935	0.005915167	0.005975504	0.006080996
0.911723104	0.831454740	0.847356478	0.003303901	0.004786220	0.005872570
0.911723104	0.801338342	0.832206954	0.005915167	0.005975504	0.006080996
0.645602280	0.416764678	0.462850265	0.003303901	0.004786220	0.005872570
0.645602280	0.334125997	0.438037019	0.005915167	0.005975504	0.006080996
0.091533208	0.702912884	0.752789442	0.003303901	0.004786220	0.005872570
0.091533208	0.624823797	0.695556428	0.005915167	0.005975504	0.006080996
0.178322047	0.403956278	0.441620541	0.003303901	0.004786220	0.005872570
0.178322047	0.717652239	0.782861445	0.005915167	0.005975504	0.006080996
0.738835745	0.764374938	0.805860347	0.003303901	0.004786220	0.005872570
0.738835745	0.452626200	0.546936054	0.005915167	0.005975504	0.006080996
0.899733636	0.798813285	0.827648566	0.003303901	0.004786220	0.005872570
0.899733636	0.844525044	0.845555658	0.005915167	0.005975504	0.006080996
0.247931715	0.077617158	0.098951428	0.003303901	0.004786220	0.005872570
0.247931715	0.384180433	0.494897220	0.005915167	0.005975504	0.006080996
0.907758785	0.824805205	0.841269410	0.003303901	0.004786220	0.005872570
0.907758785	0.789653145	0.825330031	0.005915167	0.005975504	0.006080996
0.399818221	0.190075626	0.208194144	0.003303901	0.004786220	0.005872570
0.399818221	0.147083554	0.160279482	0.005915167	0.005975504	0.006080996
0.611444888	0.871187368	0.869455760	0.003303901	0.004786220	0.005872570
0.611444888	0.963332203	0.952494657	0.005915167	0.005975504	0.006080996
0.929408167	0.878762686	0.877423007	0.003303901	0.004786220	0.005872570
0.929408167	0.853243218	0.864885619	0.005915167	0.005975504	0.006080996
0.076528079	0.324672823	0.324483317	0.003303901	0.004786220	0.005872570
0.076528079	0.920315835	0.929130525	0.005915167	0.005975504	0.006080996
0.407698044	0.273362522	0.279440925	0.003303901	0.004786220	0.005872570
0.407698044	0.158391582	0.166882810	0.005915167	0.005975504	0.006080996
0.225354828	0.064236718	0.085822144	0.003303901	0.004786220	0.005872570
0.225354828	0.040869826	0.051214175	0.005915167	0.005975504	0.006080996
0.968715391	0.954885541	0.944881281	0.003303901	0.004786220	0.005872570
0.968715391	0.947440829	0.939950081	0.005915167	0.005975504	0.006080996
0.708002459	0.479366271	0.543235319	0.003303901	0.004786220	0.005872570
0.708002459	0.701730650	0.761647158	0.005915167	0.005975504	0.006080996
0.555456478	0.352926703	0.357996425	0.003303901	0.004786220	0.005872570
0.555456478	0.247118398	0.309185496	0.005915167	0.005975504	0.006080996
0.073306159	0.177388860	0.203183428	0.003303901	0.004786220	0.005872570
0.073306159	0.231390866	0.289905980	0.005915167	0.005975504	0.006080996
0.628324835	0.406448875	0.441919480	0.003303901	0.004786220	0.005872570
0.628324835	0.290640570	0.395744071	0.005915167	0.005975504	0.006080996
0.797545037	0.627801698	0.668584596	0.003303901	0.004786220	0.005872570
0.797545037	0.575583150	0.636958698	0.005915167	0.005975504	0.006080996

0.388748088	0.168166522	0.198670654	0.003303901	0.004786220	0.005872570
0.388748088	0.380269823	0.48991418	0.005915167	0.005975504	0.006080996
0.463761423	0.255627382	0.264954650	0.003303901	0.004786220	0.005872570
0.463761423	0.189857369	0.215658295	0.005915167	0.005975504	0.006080996
0.899305860	0.989635892	0.978873934	0.003303901	0.004786220	0.005872570
0.899305860	0.763688139	0.810362572	0.005915167	0.005975504	0.006080996
0.916089642	0.846012346	0.854642501	0.003303901	0.004786220	0.005872570
0.916089642	0.819449290	0.84018239	0.005915167	0.005975504	0.006080996
0.259836433	0.082899465	0.106144689	0.003303901	0.004786220	0.005872570
0.259836433	0.071499514	0.075350767	0.005915167	0.005975504	0.006080996
0.800587540	0.635806421	0.673052514	0.003303901	0.004786220	0.005872570
0.800587540	0.599238460	0.642413949	0.005915167	0.005975504	0.006080996
0.857693506	0.733871651	0.760890747	0.003303901	0.004786220	0.005872570
0.857693506	0.745677692	0.799294372	0.005915167	0.005975504	0.006080996
0.879337292	0.751411868	0.794539499	0.003303901	0.004786220	0.005872570
0.879337292	0.703628028	0.774254067	0.005915167	0.005975504	0.006080996
0.330391997	0.452005957	0.506882627	0.003303901	0.004786220	0.005872570
0.330391997	0.679099166	0.734244538	0.005915167	0.005975504	0.006080996
0.651627694	0.421929616	0.470046402	0.003303901	0.004786220	0.005872570
0.651627694	0.315078296	0.425300815	0.005915167	0.005975504	0.006080996
0.938880950	0.900440488	0.893470064	0.003303901	0.004786220	0.005872570
0.938880950	0.871949338	0.883078853	0.005915167	0.005975504	0.006080996
0.792785725	0.621523524	0.661931615	0.003303901	0.004786220	0.005872570
0.792785725	0.574573682	0.630011985	0.005915167	0.005975504	0.006080996
0.981618867	0.981802728	0.967456007	0.003303901	0.004786220	0.005872570
0.981618867	0.979426331	0.965217943	0.005915167	0.005975504	0.006080996
0.428783600	0.221473603	0.232842120	0.003303901	0.004786220	0.005872570
0.428783600	0.172975870	0.184341157	0.005915167	0.005975504	0.006080996
0.146866944	0.680573278	0.735725878	0.003303901	0.004786220	0.005872570
0.146866944	0.809289115	0.836295204	0.005915167	0.005975504	0.006080996
0.652558339	0.466014691	0.517332025	0.003303901	0.004786220	0.005872570
0.652558339	0.784512274	0.822897455	0.005915167	0.005975504	0.006080996
0.464535345	0.260201940	0.265740716	0.003303901	0.004786220	0.005872570
0.464535345	0.193890933	0.216423197	0.005915167	0.005975504	0.006080996
0.398010303	0.428452872	0.458302866	0.011796907	0.012671120	0.013958825
0.398010303	0.495273626	0.568005011	0.003230790	0.003257201	0.003343834
0.325450483	0.460377344	0.478515735	0.011796907	0.012671120	0.013958825
0.325450483	0.378973063	0.440558747	0.003230790	0.003257201	0.003343834
0.100921055	0.077124323	0.132260601	0.011796907	0.012671120	0.013958825
0.100921055	0.600477763	0.657948922	0.003230790	0.003257201	0.003343834
0.048428048	0.141929266	0.204263723	0.011796907	0.012671120	0.013958825
0.048428048	0.438252875	0.503700883	0.003230790	0.003257201	0.003343834
0.221818133	0.525986985	0.525810841	0.011796907	0.012671120	0.013958825
0.221818133	0.403721963	0.458608373	0.003230790	0.003257201	0.003343834
0.194121282	0.089848510	0.146302089	0.011796907	0.012671120	0.013958825
0.194121282	0.058904357	0.062856806	0.003230790	0.003257201	0.003343834

0.998445182	0.996403889	0.998494643	0.011796907	0.012671120	0.013958825
0.998445182	0.993251227	0.998487054	0.003230790	0.003257201	0.003343834
0.820673657	0.742597297	0.761976107	0.011796907	0.012671120	0.013958825
0.820673657	0.696253044	0.748807584	0.003230790	0.003257201	0.003343834
0.701382869	0.600217390	0.617977409	0.011796907	0.012671120	0.013958825
0.701382869	0.623150335	0.694920552	0.003230790	0.003257201	0.003343834
0.847446183	0.787704766	0.796253994	0.011796907	0.012671120	0.013958825
0.847446183	0.662799951	0.719629890	0.003230790	0.003257201	0.003343834
0.866407284	0.805494778	0.820583273	0.011796907	0.012671120	0.013958825
0.866407284	0.702558899	0.752292157	0.003230790	0.003257201	0.003343834
0.951433995	0.907446206	0.9333952	0.011796907	0.012671120	0.013958825
0.951433995	0.867929638	0.906348070	0.003230790	0.003257201	0.003343834
0.763733332	0.674792446	0.692141799	0.011796907	0.012671120	0.013958825
0.763733332	0.587905884	0.624089385	0.003230790	0.003257201	0.003343834
0.512965401	0.370713331	0.413047066	0.011796907	0.012671120	0.013958825
0.512965401	0.234762125	0.263881046	0.003230790	0.003257201	0.003343834
0.438241426	0.359610755	0.407560057	0.011796907	0.012671120	0.013958825
0.438241426	0.360820377	0.414520529	0.003230790	0.003257201	0.003343834
0.720714595	0.733756601	0.757118940	0.011796907	0.012671120	0.013958825
0.720714595	0.471013380	0.520847059	0.003230790	0.003257201	0.003343834
0.076908544	0.376816573	0.415900079	0.011796907	0.012671120	0.013958825
0.076908544	0.343428235	0.403055717	0.003230790	0.003257201	0.003343834
0.962395915	0.929836595	0.948465650	0.011796907	0.012671120	0.013958825
0.962395915	0.898498486	0.927402027	0.003230790	0.003257201	0.003343834
0.450339688	0.283447793	0.351754157	0.011796907	0.012671120	0.013958825
0.450339688	0.177844460	0.203603973	0.003230790	0.003257201	0.003343834
0.198868803	0.117560420	0.175813631	0.011796907	0.012671120	0.013958825
0.198868803	0.472516475	0.526461967	0.003230790	0.003257201	0.003343834
0.444289250	0.638501739	0.657362390	0.011796907	0.012671120	0.013958825
0.444289250	0.173791552	0.198079164	0.003230790	0.003257201	0.003343834
0.484957514	0.531789336	0.535353457	0.011796907	0.012671120	0.013958825
0.484957514	0.463698516	0.518387117	0.003230790	0.003257201	0.003343834
0.635370677	0.536049467	0.542703097	0.011796907	0.012671120	0.013958825
0.635370677	0.350139221	0.404627746	0.003230790	0.003257201	0.003343834
0.788915890	0.717721578	0.722316414	0.011796907	0.012671120	0.013958825
0.788915890	0.577463711	0.623373233	0.003230790	0.003257201	0.003343834
0.495579643	0.337527703	0.395959907	0.011796907	0.012671120	0.013958825
0.495579643	0.885530874	0.911411218	0.003230790	0.003257201	0.003343834
0.020139323	0.096801934	0.151201180	0.011796907	0.012671120	0.013958825
0.020139323	0.145880759	0.178033105	0.003230790	0.003257201	0.003343834
0.342554149	0.183562272	0.252586826	0.011796907	0.012671120	0.013958825
0.342554149	0.590305705	0.639840103	0.003230790	0.003257201	0.003343834
0.431837511	0.568181157	0.581843585	0.011796907	0.012671120	0.013958825
0.431837511	0.161232476	0.187243882	0.003230790	0.003257201	0.003343834
0.610297342	0.514341792	0.515190667	0.011796907	0.012671120	0.013958825
0.610297342	0.331763024	0.373311462	0.003230790	0.003257201	0.003343834

0.050588609	0.060264671	0.115264371	0.011796907	0.012671120	0.013958825
0.050588609	0.129571368	0.139268983	0.003230790	0.003257201	0.003343834
0.767299175	0.688979707	0.696506785	0.011796907	0.012671120	0.013958825
0.767299175	0.544246935	0.590145306	0.003230790	0.003257201	0.003343834
0.976432709	0.953488230	0.967639670	0.011796907	0.012671120	0.013958825
0.976432709	0.949930684	0.955284421	0.003230790	0.003257201	0.003343834
0.784439432	0.697758743	0.716884245	0.011796907	0.012671120	0.013958825
0.784439432	0.558627674	0.616339850	0.003230790	0.003257201	0.003343834
0.535922194	0.410101779	0.436603022	0.011796907	0.012671120	0.013958825
0.535922194	0.541178468	0.589953001	0.003230790	0.003257201	0.003343834
0.993338206	0.984623441	0.990904595	0.011796907	0.012671120	0.013958825
0.993338206	0.983244334	0.987861610	0.003230790	0.003257201	0.003343834
0.562373604	0.693652254	0.706069790	0.011796907	0.012671120	0.013958825
0.562373604	0.741853364	0.785910666	0.003230790	0.003257201	0.003343834
0.109214150	0.116269958	0.173921495	0.011796907	0.012671120	0.013958825
0.109214150	0.670583769	0.732088172	0.003230790	0.003257201	0.003343834
0.507394075	0.360876106	0.40770541	0.011796907	0.012671120	0.013958825
0.507394075	0.227366278	0.258243259	0.003230790	0.003257201	0.003343834
0.472781782	0.309619817	0.373279917	0.011796907	0.012671120	0.013958825
0.472781782	0.200816626	0.224142243	0.003230790	0.003257201	0.003343834
0.506814287	0.354134013	0.406859587	0.011796907	0.012671120	0.013958825
0.506814287	0.862976692	0.902154988	0.003230790	0.003257201	0.003343834
0.992024082	0.982252002	0.989771232	0.011796907	0.012671120	0.013958825
0.992024082	0.977866096	0.985570054	0.003230790	0.003257201	0.003343834
0.743701905	0.651964991	0.667838597	0.011796907	0.012671120	0.013958825
0.743701905	0.492867540	0.554574935	0.003230790	0.003257201	0.003343834
0.564416950	0.587705117	0.598471023	0.011796907	0.012671120	0.013958825
0.564416950	0.265028489	0.319349364	0.003230790	0.003257201	0.003343834
0.453608604	0.285452537	0.354490195	0.011796907	0.012671120	0.013958825
0.453608604	0.180517239	0.206468831	0.003230790	0.003257201	0.003343834
0.941774425	0.894983902	0.920178860	0.011796907	0.012671120	0.013958825
0.941774425	0.850218097	0.888642086	0.003230790	0.003257201	0.003343834
0.329694556	0.172196160	0.241460156	0.011796907	0.012671120	0.013958825
0.329694556	0.101425730	0.109277111	0.003230790	0.003257201	0.003343834
0.048007350	0.058904758	0.113130769	0.011796907	0.012671120	0.013958825
0.048007350	0.111376805	0.117826651	0.003230790	0.003257201	0.003343834
0.573598893	0.549718521	0.557587149	0.011796907	0.012671120	0.013958825
0.573598893	0.285169030	0.330158699	0.003230790	0.003257201	0.003343834
0.367550324	0.203647565	0.274866188	0.011796907	0.012671120	0.013958825
0.367550324	0.124238221	0.135567497	0.003230790	0.003257201	0.003343834
0.930941562	0.882397485	0.906078367	0.011796907	0.012671120	0.013958825
0.930941562	0.834258609	0.868161443	0.003230790	0.003257201	0.003343834
0.472296893	0.305575339	0.372809627	0.011796907	0.012671120	0.013958825
0.472296893	0.320166470	0.355529225	0.003230790	0.003257201	0.003343834
0.421626944	0.499388795	0.500543894	0.011796907	0.012671120	0.013958825
0.421626944	0.915351941	0.931953243	0.003230790	0.003257201	0.003343834

0.509636780	0.367070437	0.410021858	0.011796907	0.012671120	0.013958825
0.509636780	0.232041560	0.260620761	0.003230790	0.003257201	0.003343834
0.176905614	0.180110828	0.248289822	0.011796907	0.012671120	0.013958825
0.176905614	0.024310792	0.031533026	0.003230790	0.003257201	0.003343834
0.187668077	0.211847541	0.282890823	0.011796907	0.012671120	0.013958825
0.187668077	0.383316283	0.446570258	0.003230790	0.003257201	0.003343834
0.888305883	0.830324563	0.848784062	0.011796907	0.012671120	0.013958825
0.888305883	0.752885094	0.790158348	0.003230790	0.003257201	0.003343834
0.153966391	0.073973172	0.129810356	0.011796907	0.012671120	0.013958825
0.153966391	0.015080814	0.023942509	0.003230790	0.003257201	0.003343834
0.215058420	0.093904554	0.147708008	0.011796907	0.012671120	0.013958825
0.215058420	0.041835579	0.046521249	0.003230790	0.003257201	0.003343834
0.369359574	0.460950264	0.479334868	0.011796907	0.012671120	0.013958825
0.369359574	0.66591647	0.730220318	0.003230790	0.003257201	0.003343834
0.582775057	0.590606430	0.604214732	0.011796907	0.012671120	0.013958825
0.582775057	0.291321411	0.340488201	0.003230790	0.003257201	0.003343834
0.737207355	0.642160655	0.660021067	0.011796907	0.012671120	0.013958825
0.737207355	0.479491207	0.544682657	0.003230790	0.003257201	0.003343834
0.394757739	0.222505023	0.299381833	0.011796907	0.012671120	0.013958825
0.394757739	0.145731935	0.175740520	0.003230790	0.003257201	0.003343834
0.855187870	0.792338969	0.805767113	0.011796907	0.012671120	0.013958825
0.855187870	0.673265419	0.732312856	0.003230790	0.003257201	0.003343834
0.674518682	0.57972623	0.587129234	0.011796907	0.012671120	0.013958825
0.674518682	0.400078602	0.456072561	0.003230790	0.003257201	0.003343834
0.991487189	0.970550380	0.988554660	0.011796907	0.012671120	0.013958825
0.991487189	0.973480369	0.984947282	0.003230790	0.003257201	0.003343834
0.528757987	0.397319512	0.429371524	0.011796907	0.012671120	0.013958825
0.528757987	0.551785258	0.595530850	0.003230790	0.003257201	0.003343834
0.846308596	0.768599453	0.794310981	0.011796907	0.012671120	0.013958825
0.846308596	0.645881959	0.717421008	0.003230790	0.003257201	0.003343834
0.585645053	0.683324808	0.694823029	0.011796907	0.012671120	0.013958825
0.585645053	0.299480558	0.343724564	0.003230790	0.003257201	0.003343834
0.883938821	0.827594807	0.843749430	0.011796907	0.012671120	0.013958825
0.883938821	0.822292381	0.848639619	0.003230790	0.003257201	0.003343834
0.521032489	0.385183495	0.421725193	0.011796907	0.012671120	0.013958825
0.521032489	0.598043122	0.642097680	0.003230790	0.003257201	0.003343834
0.459403603	0.293775617	0.360226160	0.011796907	0.012671120	0.013958825
0.459403603	0.186682011	0.211664491	0.003230790	0.003257201	0.003343834
0.306258728	0.155652154	0.221428727	0.011796907	0.012671120	0.013958825
0.306258728	0.086106540	0.094256624	0.003230790	0.003257201	0.003343834
0.671348709	0.570357826	0.583272131	0.011796907	0.012671120	0.013958825
0.671348709	0.388754520	0.451695485	0.003230790	0.003257201	0.003343834
0.594043357	0.494542806	0.497809913	0.011796907	0.012671120	0.013958825
0.594043357	0.313970735	0.353761536	0.003230790	0.003257201	0.003343834
0.299552459	0.148639393	0.215626576	0.011796907	0.012671120	0.013958825
0.299552459	0.081120430	0.090144693	0.003230790	0.003257201	0.003343834

0.981726142	0.962526109	0.975049571	0.011796907	0.012671120	0.013958825
0.981726142	0.955263773	0.965351912	0.003230790	0.003257201	0.003343834
0.153531143	0.394679718	0.428859669	0.011796907	0.012671120	0.013958825
0.153531143	0.116543481	0.119937962	0.003230790	0.003257201	0.003343834
0.164348427	0.056335625	0.109558300	0.011796907	0.012671120	0.013958825
0.164348427	0.021332247	0.027310324	0.003230790	0.003257201	0.003343834
0.275690212	0.437626754	0.465253100	0.011796907	0.012671120	0.013958825
0.275690212	0.109395954	0.116286704	0.003230790	0.003257201	0.003343834
0.440093737	0.638929407	0.658320169	0.011796907	0.012671120	0.013958825
0.440093737	0.500903579	0.579072064	0.003230790	0.003257201	0.003343834
0.244469670	0.112606042	0.170643231	0.011796907	0.012671120	0.013958825
0.244469670	0.184012285	0.207363294	0.003230790	0.003257201	0.003343834
0.591562606	0.489189029	0.495288027	0.011796907	0.012671120	0.013958825
0.591562606	0.856275727	0.894036271	0.003230790	0.003257201	0.003343834
0.586347400	0.696912172	0.716256494	0.011796907	0.012671120	0.013958825
0.586347400	0.721228211	0.775237101	0.003230790	0.003257201	0.003343834
0.940246859	0.887529139	0.918153637	0.011796907	0.012671120	0.013958825
0.940246859	0.838300329	0.885204529	0.003230790	0.003257201	0.003343834
0.500605814	0.345270068	0.400985428	0.011796907	0.012671120	0.013958825
0.500605814	0.216198241	0.251409136	0.003230790	0.003257201	0.003343834
0.708600326	0.610019610	0.626403468	0.011796907	0.012671120	0.013958825
0.708600326	0.557260730	0.595980247	0.003230790	0.003257201	0.003343834
0.214505230	0.129609552	0.191650551	0.011796907	0.012671120	0.013958825
0.214505230	0.428778558	0.496342467	0.003230790	0.003257201	0.003343834
0.971978890	0.943608432	0.961294574	0.011796907	0.012671120	0.013958825
0.971978890	0.966175811	0.972894585	0.003230790	0.003257201	0.003343834
0.951513840	0.902376495	0.933080382	0.011796907	0.012671120	0.013958825
0.951513840	0.868539457	0.906386315	0.003230790	0.003257201	0.003343834
0.570495702	0.449515387	0.472771525	0.011796907	0.012671120	0.013958825
0.570495702	0.375831064	0.437961912	0.003230790	0.003257201	0.003343834
0.179672265	0.453730646	0.475523535	0.011796907	0.012671120	0.013958825
0.179672265	0.625575361	0.695644567	0.003230790	0.003257201	0.003343834
0.051422508	0.164295849	0.234187434	0.011796907	0.012671120	0.013958825
0.051422508	0.727740924	0.782734545	0.003230790	0.003257201	0.003343834
0.473968775	0.595302320	0.613881043	0.011796907	0.012671120	0.013958825
0.473968775	0.205912085	0.225439329	0.003230790	0.003257201	0.003343834
0.869479280	0.810154330	0.824170601	0.011796907	0.012671120	0.013958825
0.869479280	0.814895787	0.845525403	0.003230790	0.003257201	0.003343834
0.336916206	0.178601182	0.247743714	0.011796907	0.012671120	0.013958825
0.336916206	0.424230665	0.494986205	0.003230790	0.003257201	0.003343834
0.764550447	0.680512022	0.693042065	0.011796907	0.012671120	0.013958825
0.764550447	0.530642053	0.585698196	0.003230790	0.003257201	0.003343834
0.502790036	0.350104328	0.403139962	0.011796907	0.012671120	0.013958825
0.502790036	0.217522238	0.253325664	0.003230790	0.003257201	0.003343834
0.525508120	0.664474426	0.689371159	0.011796907	0.012671120	0.013958825
0.525508120	0.939252458	0.952408720	0.003230790	0.003257201	0.003343834

0.903235538	0.858809958	0.868776837	0.011796907	0.012671120	0.013958825
0.903235538	0.780696918	0.816894341	0.003230790	0.003257201	0.003343834
0.275829535	0.238568917	0.320397093	0.011796907	0.012671120	0.013958825
0.275829535	0.906507398	0.929329826	0.003230790	0.003257201	0.003343834
0.431371088	0.291118199	0.358021684	0.011796907	0.012671120	0.013958825
0.431371088	0.157250663	0.186769804	0.003230790	0.003257201	0.003343834
0.159520448	0.052593161	0.106014318	0.011796907	0.012671120	0.013958825
0.159520448	0.036260875	0.041988313	0.003230790	0.003257201	0.003343834
0.761777923	0.666381592	0.689599592	0.011796907	0.012671120	0.013958825
0.761777923	0.513960696	0.581639240	0.003230790	0.003257201	0.003343834
0.895004134	0.850331981	0.858036283	0.011796907	0.012671120	0.013958825
0.895004134	0.779520045	0.802799224	0.003230790	0.003257201	0.003343834
0.894551833	0.838580690	0.856899904	0.011796907	0.012671120	0.013958825
0.894551833	0.766208576	0.801215675	0.003230790	0.003257201	0.003343834
0.072429427	0.082559861	0.137961162	0.011796907	0.012671120	0.013958825
0.072429427	0.257179261	0.289905103	0.003230790	0.003257201	0.003343834
0.820084524	0.737964861	0.761487	0.011796907	0.012671120	0.013958825
0.820084524	0.608035312	0.673682359	0.003230790	0.003257201	0.003343834
0.842807474	0.755635348	0.790027379	0.011796907	0.012671120	0.013958825
0.842807474	0.630681016	0.711250313	0.003230790	0.003257201	0.003343834
0.605923234	0.506278833	0.510432103	0.011796907	0.012671120	0.013958825
0.605923234	0.415543919	0.490131355	0.003230790	0.003257201	0.003343834
0.711193304	0.614924729	0.629176988	0.011796907	0.012671120	0.013958825
0.711193304	0.450001408	0.506626986	0.003230790	0.003257201	0.003343834
0.146464048	0.471588168	0.487884189	0.011796907	0.012671120	0.013958825
0.146464048	0.455828339	0.508139637	0.003230790	0.003257201	0.003343834
0.786545970	0.710820555	0.719437794	0.011796907	0.012671120	0.013958825
0.786545970	0.569736509	0.619487177	0.003230790	0.003257201	0.003343834
0.535281593	0.405355510	0.435944934	0.011796907	0.012671120	0.013958825
0.535281593	0.25036683	0.287335193	0.003230790	0.003257201	0.003343834
0.656535873	0.556173281	0.566519944	0.011796907	0.012671120	0.013958825
0.656535873	0.396681645	0.454193327	0.003230790	0.003257201	0.003343834
0.843581193	0.767737097	0.791641933	0.011796907	0.012671120	0.013958825
0.843581193	0.765067910	0.799280260	0.003230790	0.003257201	0.003343834
0.847186189	0.776066983	0.795486271	0.011796907	0.012671120	0.013958825
0.847186189	0.651023916	0.718712282	0.003230790	0.003257201	0.003343834
0.266935165	0.320763360	0.380934049	0.011796907	0.012671120	0.013958825
0.266935165	0.685451613	0.734181081	0.003230790	0.003257201	0.003343834
0.968608596	0.934014249	0.956472144	0.011796907	0.012671120	0.013958825
0.968608596	0.919875869	0.939201756	0.003230790	0.003257201	0.003343834
0.922729182	0.870437528	0.894705089	0.011796907	0.012671120	0.013958825
0.922729182	0.827661096	0.852994406	0.003230790	0.003257201	0.003343834
0.957367116	0.921799366	0.941608093	0.011796907	0.012671120	0.013958825
0.957367116	0.894781149	0.918219156	0.003230790	0.003257201	0.003343834
0.785243077	0.705908786	0.718060274	0.011796907	0.012671120	0.013958825
0.785243077	0.568427147	0.618052656	0.003230790	0.003257201	0.003343834

0.207990534	0.085197897	0.142099445	0.011796907	0.012671120	0.013958825
0.207990534	0.036914856	0.043525049	0.003230790	0.003257201	0.003343834
0.072299563	0.339100332	0.397025495	0.011796907	0.012671120	0.013958825
0.072299563	0.801394427	0.836220637	0.003230790	0.003257201	0.003343834
0.435479852	0.426232279	0.455045458	0.011796907	0.012671120	0.013958825
0.435479852	0.790042968	0.822680376	0.003230790	0.003257201	0.003343834
0.358202471	0.195126180	0.266444264	0.011796907	0.012671120	0.013958825
0.358202471	0.122055738	0.128832788	0.003230790	0.003257201	0.003343834
0.438433355	0.676152909	0.574000386	0.005238192	0.006352035	0.007313854
0.438433355	0.683264405	0.641709751	0.010564931	0.012312513	0.012954696
0.557763446	0.823971936	0.710368731	0.005238192	0.006352035	0.007313854
0.557763446	0.545800452	0.529500331	0.010564931	0.012312513	0.012954696
0.974772687	0.930695334	0.821333091	0.005238192	0.006352035	0.007313854
0.974772687	0.938150381	0.895315228	0.010564931	0.012312513	0.012954696
0.815990145	0.806902891	0.694537422	0.005238192	0.006352035	0.007313854
0.815990145	0.741832062	0.683993914	0.010564931	0.012312513	0.012954696
0.019942071	0.902585776	0.790054163	0.005238192	0.006352035	0.007313854
0.019942071	0.566471401	0.545263026	0.010564931	0.012312513	0.012954696
0.197387911	0.139935062	0.148737423	0.005238192	0.006352035	0.007313854
0.197387911	0.122838937	0.137575366	0.010564931	0.012312513	0.012954696
0.816744548	0.810483380	0.697854932	0.005238192	0.006352035	0.007313854
0.816744548	0.678645388	0.639390837	0.010564931	0.012312513	0.012954696
0.132348299	0.069189037	0.085252680	0.005238192	0.006352035	0.007313854
0.132348299	0.832639690	0.794150881	0.010564931	0.012312513	0.012954696
0.483326408	0.383277103	0.318739744	0.005238192	0.006352035	0.007313854
0.483326408	0.792340013	0.749594532	0.010564931	0.012312513	0.012954696
0.565104580	0.512541912	0.419397293	0.005238192	0.006352035	0.007313854
0.565104580	0.481097400	0.476246992	0.010564931	0.012312513	0.012954696
0.229578184	0.339266155	0.289026273	0.005238192	0.006352035	0.007313854
0.229578184	0.706601034	0.656266822	0.010564931	0.012312513	0.012954696
0.300357855	0.325223997	0.280425515	0.005238192	0.006352035	0.007313854
0.300357855	0.099975240	0.122425032	0.010564931	0.012312513	0.012954696
0.806273902	0.774372221	0.665174657	0.005238192	0.006352035	0.007313854
0.806273902	0.769596174	0.706456802	0.010564931	0.012312513	0.012954696
0.182354877	0.033602943	0.058303678	0.005238192	0.006352035	0.007313854
0.182354877	0.106685904	0.127029368	0.010564931	0.012312513	0.012954696
0.458514740	0.536299345	0.440531687	0.005238192	0.006352035	0.007313854
0.458514740	0.516039888	0.505719073	0.010564931	0.012312513	0.012954696
0.620852292	0.906867881	0.794610373	0.005238192	0.006352035	0.007313854
0.620852292	0.590027628	0.570679863	0.010564931	0.012312513	0.012954696
0.150632281	0.891006031	0.777396990	0.005238192	0.006352035	0.007313854
0.150632281	0.49763987	0.49527986	0.010564931	0.012312513	0.012954696
0.440517851	0.476818933	0.389174066	0.005238192	0.006352035	0.007313854
0.440517851	0.225595099	0.210367326	0.010564931	0.012312513	0.012954696
0.039909004	0.226080080	0.216556554	0.005238192	0.006352035	0.007313854
0.039909004	0.227189281	0.211738049	0.010564931	0.012312513	0.012954696

0.425761851	0.323166210	0.279136652	0.005238192	0.006352035	0.007313854
0.425761851	0.652801414	0.6057247	0.010564931	0.012312513	0.012954696
0.340639268	0.954144288	0.850939814	0.005238192	0.006352035	0.007313854
0.340639268	0.161012474	0.166736523	0.010564931	0.012312513	0.012954696
0.356110531	0.705767861	0.601836976	0.005238192	0.006352035	0.007313854
0.356110531	0.642363430	0.598327660	0.010564931	0.012312513	0.012954696
0.711059677	0.512301930	0.419200570	0.005238192	0.006352035	0.007313854
0.711059677	0.420896868	0.422665979	0.010564931	0.012312513	0.012954696
0.477724863	0.235776219	0.223236328	0.005238192	0.006352035	0.007313854
0.477724863	0.233499232	0.215824557	0.010564931	0.012312513	0.012954696
0.200816851	0.216397274	0.210349348	0.005238192	0.006352035	0.007313854
0.200816851	0.958097001	0.927745503	0.010564931	0.012312513	0.012954696
0.561991955	0.547750302	0.450806106	0.005238192	0.006352035	0.007313854
0.561991955	0.375064044	0.358629416	0.010564931	0.012312513	0.012954696
0.208784078	0.082998667	0.095226129	0.005238192	0.006352035	0.007313854
0.208784078	0.757263901	0.703102227	0.010564931	0.012312513	0.012954696
0.902045480	0.955127882	0.852464304	0.005238192	0.006352035	0.007313854
0.902045480	0.663306304	0.620476917	0.010564931	0.012312513	0.012954696
0.092673042	0.013614092	0.030100795	0.005238192	0.006352035	0.007313854
0.092673042	0.109798536	0.129497575	0.010564931	0.012312513	0.012954696
0.794097009	0.725775591	0.619945615	0.005238192	0.006352035	0.007313854
0.794097009	0.540599651	0.528196192	0.010564931	0.012312513	0.012954696
0.129590869	0.490853813	0.400673938	0.005238192	0.006352035	0.007313854
0.129590869	0.489664132	0.490249699	0.010564931	0.012312513	0.012954696
0.251230859	0.274773423	0.247209076	0.005238192	0.006352035	0.007313854
0.251230859	0.876256832	0.827335436	0.010564931	0.012312513	0.012954696
0.880142120	0.726266322	0.620402996	0.005238192	0.006352035	0.007313854
0.880142120	0.574221334	0.549745112	0.010564931	0.012312513	0.012954696
0.186812482	0.086720651	0.098155984	0.005238192	0.006352035	0.007313854
0.186812482	0.714248403	0.660270797	0.010564931	0.012312513	0.012954696
0.446347102	0.282241228	0.252229209	0.005238192	0.006352035	0.007313854
0.446347102	0.176136031	0.178973560	0.010564931	0.012312513	0.012954696
0.039293969	0.867160881	0.753249577	0.005238192	0.006352035	0.007313854
0.039293969	0.866903759	0.824996422	0.010564931	0.012312513	0.012954696
0.476892570	0.458606168	0.374364760	0.005238192	0.006352035	0.007313854
0.476892570	0.818901516	0.781174367	0.010564931	0.012312513	0.012954696
0.741877841	0.620320891	0.522582722	0.005238192	0.006352035	0.007313854
0.741877841	0.449943454	0.445818330	0.010564931	0.012312513	0.012954696
0.644938571	0.498933062	0.407494636	0.005238192	0.006352035	0.007313854
0.644938571	0.333902793	0.308951784	0.010564931	0.012312513	0.012954696
0.297057080	0.104947587	0.114421648	0.005238192	0.006352035	0.007313854
0.297057080	0.950597754	0.920337209	0.010564931	0.012312513	0.012954696
0.618461214	0.920337459	0.809507681	0.005238192	0.006352035	0.007313854
0.618461214	0.414363352	0.416615218	0.010564931	0.012312513	0.012954696
0.684498996	0.588283965	0.489413634	0.005238192	0.006352035	0.007313854
0.684498996	0.670382841	0.623583402	0.010564931	0.012312513	0.012954696

0.596028252	0.787264890	0.677051459	0.005238192	0.006352035	0.007313854
0.596028252	0.324693327	0.301127132	0.010564931	0.012312513	0.012954696
0.487162658	0.250832229	0.232422285	0.005238192	0.006352035	0.007313854
0.487162658	0.266036718	0.244666435	0.010564931	0.012312513	0.012954696
0.139565381	0.170292681	0.176353197	0.005238192	0.006352035	0.007313854
0.139565381	0.697951894	0.652333181	0.010564931	0.012312513	0.012954696
0.544019112	0.374187397	0.312145936	0.005238192	0.006352035	0.007313854
0.544019112	0.306747228	0.277684425	0.010564931	0.012312513	0.012954696
0.162018641	0.297750097	0.263024943	0.005238192	0.006352035	0.007313854
0.162018641	0.218929088	0.207699310	0.010564931	0.012312513	0.012954696
0.756994756	0.757921563	0.649394474	0.005238192	0.006352035	0.007313854
0.756994756	0.591499326	0.572345611	0.010564931	0.012312513	0.012954696
0.423429390	0.290943798	0.258368988	0.005238192	0.006352035	0.007313854
0.423429390	0.138576243	0.150681512	0.010564931	0.012312513	0.012954696
0.185948722	0.759097547	0.650511682	0.005238192	0.006352035	0.007313854
0.185948722	0.434186786	0.432173704	0.010564931	0.012312513	0.012954696
0.709954138	0.568412396	0.469653058	0.005238192	0.006352035	0.007313854
0.709954138	0.561730439	0.541277579	0.010564931	0.012312513	0.012954696
0.072845237	0.650116586	0.550822669	0.005238192	0.006352035	0.007313854
0.072845237	0.973932365	0.944276614	0.010564931	0.012312513	0.012954696
0.678898859	0.574770441	0.475883118	0.005238192	0.006352035	0.007313854
0.678898859	0.431323548	0.429812086	0.010564931	0.012312513	0.012954696
0.822843289	0.796679979	0.685403083	0.005238192	0.006352035	0.007313854
0.822843289	0.432142342	0.431275273	0.010564931	0.012312513	0.012954696
0.396278180	0.574967011	0.476072821	0.005238192	0.006352035	0.007313854
0.396278180	0.552879623	0.534872814	0.010564931	0.012312513	0.012954696
0.421096248	0.235570221	0.223105912	0.005238192	0.006352035	0.007313854
0.421096248	0.176949995	0.179894076	0.010564931	0.012312513	0.012954696
0.369901434	0.240644567	0.225958351	0.005238192	0.006352035	0.007313854
0.369901434	0.093099539	0.115623476	0.010564931	0.012312513	0.012954696
0.183430082	0.087629010	0.098882442	0.005238192	0.006352035	0.007313854
0.183430082	0.019633000	0.042798267	0.010564931	0.012312513	0.012954696
0.672450684	0.800362759	0.688640575	0.005238192	0.006352035	0.007313854
0.672450684	0.813309694	0.779596057	0.010564931	0.012312513	0.012954696
0.409265557	0.737441737	0.630424527	0.005238192	0.006352035	0.007313854
0.409265557	0.230239514	0.212371594	0.010564931	0.012312513	0.012954696
0.194794725	0.160618948	0.168126005	0.005238192	0.006352035	0.007313854
0.194794725	0.347852160	0.321472861	0.010564931	0.012312513	0.012954696
0.533497610	0.373109522	0.311367562	0.005238192	0.006352035	0.007313854
0.533497610	0.362098828	0.338602667	0.010564931	0.012312513	0.012954696
0.504807273	0.308031043	0.269623303	0.005238192	0.006352035	0.007313854
0.504807273	0.203202542	0.197952666	0.010564931	0.012312513	0.012954696
0.571451699	0.491478273	0.401195141	0.005238192	0.006352035	0.007313854
0.571451699	0.379242142	0.365541239	0.010564931	0.012312513	0.012954696
0.200507977	0.293314430	0.260059846	0.005238192	0.006352035	0.007313854
0.200507977	0.90464502	0.859491855	0.010564931	0.012312513	0.012954696

0.8143513808	0.7007125348	0.597026696	0.0052381923	0.0063520359	0.0073138541	
0.8143513808	0.7698177260	0.7070608339	0.0105649310	0.0123125137	0.0129546961	
0.457939587	0.2220609527	0.2139621704	0.0052381923	0.0063520359	0.0073138541	
0.457939587	0.2831443058	0.2608667007	0.0105649310	0.0123125137	0.0129546961	
0.3381176097	0.8842614058	0.7701439499	0.0052381923	0.0063520359	0.0073138541	
0.3381176097	0.1152297748	0.1324483875	0.0105649310	0.0123125137	0.0129546961	
0.271926529	0.7701730659	0.6611438198	0.0052381923	0.0063520359	0.0073138541	
0.271926529	0.9302052763	0.8764678422	0.0105649310	0.0123125137	0.0129546961	
0.3238602540	0.4141015279	0.3408477727	0.0052381923	0.0063520359	0.0073138541	
0.3238602540	0.7652490508	0.7049835403	0.0105649310	0.0123125137	0.0129546961	
0.899379732	0.7725701123	0.6634426737	0.0052381923	0.0063520359	0.0073138541	
0.899379732	0.5730313560	0.5475963221	0.0105649310	0.0123125137	0.0129546961	
0.568835471	0.3989556201	0.330105015	0.0052381923	0.0063520359	0.0073138541	
0.568835471	0.2799877503	0.257096103	0.0105649310	0.0123125137	0.0129546961	
0.0476449054	0.0509439131	0.0725124635	0.0052381923	0.0063520359	0.0073138541	
0.0476449054	0.1822799590	0.1856188382	0.0105649310	0.0123125137	0.0129546961	
0.6073293258	0.4670475923	0.3810897194	0.0052381923	0.0063520359	0.0073138541	
0.6073293258	0.3588688005	0.3339096121	0.0105649310	0.0123125137	0.0129546961	
0.0195382640	0.0006581661	0.0047321591	0.0052381923	0.0063520359	0.0073138541	0.1
0.0195382640	0.0281527590	0.0531304370	0.0105649310	0.0123125137	0.0129546961	
0.9300284713	0.8981989588	0.7853609670	0.0052381923	0.0063520359	0.0073138541	
0.9300284713	0.8026744145	0.7534889575	0.0105649310	0.0123125137	0.0129546961	
0.1749113362	0.8401592238	0.7260659624	0.0052381923	0.0063520359	0.0073138541	
0.1749113362	0.2239631890	0.2100779054	0.0105649310	0.0123125137	0.0129546961	
0.2254028831	0.0997149660	0.109608888	0.0052381923	0.0063520359	0.0073138541	
0.2254028831	0.0614585154	0.0849372651	0.0105649310	0.0123125137	0.0129546961	
0.0226548428	0.7394487644	0.6321471314	0.0052381923	0.0063520359	0.0073138541	
0.0226548428	0.2130498563	0.2054669059	0.0105649310	0.0123125137	0.0129546961	
0.7842829391	0.9923679240	0.9455227128	0.0052381923	0.0063520359	0.0073138541	
0.7842829391	0.7374274389	0.6739156359	0.0105649310	0.0123125137	0.0129546961	
0.538610316	0.3250388297	0.2803102633	0.0052381923	0.0063520359	0.0073138541	
0.538610316	0.3737137689	0.3564406007	0.0105649310	0.0123125137	0.0129546961	
0.0780544048	0.1817967260	0.1854591497	0.0052381923	0.0063520359	0.0073138541	
0.0780544048	0.9406649924	0.9134669161	0.0105649310	0.0123125137	0.0129546961	
0.6119214749	0.9601475798	0.8606573188	0.0052381923	0.0063520359	0.0073138541	
0.6119214749	0.8554671849	0.8167316304	0.0105649310	0.0123125137	0.0129546961	
0.3516943082	0.2720456208	0.2454498721	0.0052381923	0.0063520359	0.0073138541	
0.3516943082	0.1440090494	0.1546152627	0.0105649310	0.0123125137	0.0129546961	
0.1147123289	0.1980373219	0.1977920163	0.0052381923	0.0063520359	0.0073138541	
0.1147123289	0.171945651	0.1759032034	0.0105649310	0.0123125137	0.0129546961	
0.8338967271	0.7560470943	0.6476233908	0.0052381923	0.0063520359	0.0073138541	
0.8338967271	0.7828716324	0.7264796099	0.0105649310	0.0123125137	0.0129546961	
0.5417380418	0.4518861810	0.368963813	0.0052381923	0.0063520359	0.0073138541	
0.5417380418	0.6107671509	0.5793416745	0.0105649310	0.0123125137	0.0129546961	
0.0916562624	0.0835611859	0.0956654570	0.0052381923	0.0063520359	0.0073138541	
0.0916562624	0.9915310590	0.9767985948	0.0105649310	0.0123125137	0.0129546961	

0.248286540	0.074120446	0.088586583	0.005238192	0.006352035	0.007313854
0.248286540	0.034060394	0.059720707	0.010564931	0.012312513	0.012954696
0.536285077	0.409157902	0.337430491	0.005238192	0.006352035	0.007313854
0.536285077	0.538876225	0.526916993	0.010564931	0.012312513	0.012954696
0.093041966	0.876653219	0.762430582	0.005238192	0.006352035	0.007313854
0.093041966	0.796027189	0.750250123	0.010564931	0.012312513	0.012954696
0.980916728	0.979093021	0.902488922	0.005238192	0.006352035	0.007313854
0.980916728	0.964499300	0.931977071	0.010564931	0.012312513	0.012954696
0.212117712	0.835044193	0.721111732	0.005238192	0.006352035	0.007313854
0.212117712	0.188880383	0.188837474	0.010564931	0.012312513	0.012954696
0.426176812	0.209931951	0.206145539	0.005238192	0.006352035	0.007313854
0.426176812	0.923695549	0.874089312	0.010564931	0.012312513	0.012954696
0.866612230	0.738989894	0.631752628	0.005238192	0.006352035	0.007313854
0.866612230	0.784540522	0.732601969	0.010564931	0.012312513	0.012954696
0.810220876	0.755149166	0.646774638	0.005238192	0.006352035	0.007313854
0.810220876	0.576088921	0.553188758	0.010564931	0.012312513	0.012954696
0.493853159	0.384960556	0.319956683	0.005238192	0.006352035	0.007313854
0.493853159	0.145886532	0.156850056	0.010564931	0.012312513	0.012954696
0.314433653	0.927325816	0.817457662	0.005238192	0.006352035	0.007313854
0.314433653	0.984914827	0.960917334	0.010564931	0.012312513	0.012954696
0.238998106	0.374713435	0.312526611	0.005238192	0.006352035	0.007313854
0.238998106	0.073421231	0.097178496	0.010564931	0.012312513	0.012954696
0.005697240	0.452332398	0.369317678	0.005238192	0.006352035	0.007313854
0.005697240	0.965504748	0.942209911	0.010564931	0.012312513	0.012954696
0.781455024	0.721304913	0.615967104	0.005238192	0.006352035	0.007313854
0.781455024	0.473295833	0.470151553	0.010564931	0.012312513	0.012954696
0.604514867	0.429698196	0.351921948	0.005238192	0.006352035	0.007313854
0.604514867	0.324934552	0.301476049	0.010564931	0.012312513	0.012954696
0.448105827	0.361467873	0.303408826	0.005238192	0.006352035	0.007313854
0.448105827	0.427741820	0.425403659	0.010564931	0.012312513	0.012954696
0.159050482	0.489787004	0.39980016	0.005238192	0.006352035	0.007313854
0.159050482	0.842138254	0.804926816	0.010564931	0.012312513	0.012954696
0.376718819	0.166255070	0.173093147	0.005238192	0.006352035	0.007313854
0.376718819	0.101710043	0.124783192	0.010564931	0.012312513	0.012954696
0.116322321	0.315908539	0.274576772	0.005238192	0.006352035	0.007313854
0.116322321	0.394211460	0.388837742	0.010564931	0.012312513	0.012954696
0.013670166	0.209954877	0.206161305	0.005238192	0.006352035	0.007313854
0.013670166	0.192885598	0.192321946	0.010564931	0.012312513	0.012954696
0.029162225	0.068299593	0.084652252	0.005238192	0.006352035	0.007313854
0.029162225	0.015833645	0.037396020	0.010564931	0.012312513	0.012954696
0.163395082	0.028619714	0.052587855	0.005238192	0.006352035	0.007313854
0.163395082	0.593209950	0.573123605	0.010564931	0.012312513	0.012954696
0.394730555	0.201496377	0.20025868	0.005238192	0.006352035	0.007313854
0.394730555	0.128841765	0.141832644	0.010564931	0.012312513	0.012954696
0.012043655	0.912603547	0.800736989	0.005238192	0.006352035	0.007313854
0.012043655	0.629021918	0.589275234	0.010564931	0.012312513	0.012954696

0.392232751	0.168312748	0.174807203	0.005238192	0.006352035	0.007313854
0.392232751	0.089821795	0.111127094	0.010564931	0.012312513	0.012954696
0.726607781	0.574732726	0.475846725	0.005238192	0.006352035	0.007313854
0.726607781	0.429623548	0.426913008	0.010564931	0.012312513	0.012954696
0.139200498	0.079271356	0.092361682	0.005238192	0.006352035	0.007313854
0.139200498	0.559684292	0.541038140	0.010564931	0.012312513	0.012954696
0.063958939	0.718063689	0.613108248	0.005238192	0.006352035	0.007313854
0.063958939	0.883934004	0.835811326	0.010564931	0.012312513	0.012954696
0.834902575	0.671232552	0.569519389	0.005238192	0.006352035	0.007313854
0.834902575	0.476094645	0.475334794	0.010564931	0.012312513	0.012954696
0.917429387	0.939179896	0.831492846	0.005238192	0.006352035	0.007313854
0.917429387	0.893554983	0.848398653	0.010564931	0.012312513	0.012954696
0.904945626	0.742988688	0.635240784	0.005238192	0.006352035	0.007313854
0.904945626	0.506555698	0.504474950	0.010564931	0.012312513	0.012954696
0.733156967	0.667455537	0.566109021	0.005238192	0.006352035	0.007313854
0.733156967	0.612575579	0.580258133	0.010564931	0.012312513	0.012954696
0.198244933	0.439609277	0.359127892	0.005238192	0.006352035	0.007313854
0.198244933	0.623386239	0.586649377	0.010564931	0.012312513	0.012954696
0.210034378	0.142440187	0.151221827	0.005238192	0.006352035	0.007313854
0.210034378	0.531125768	0.526432245	0.010564931	0.012312513	0.012954696
0.997416449	0.857145926	0.743607533	0.005238192	0.006352035	0.007313854
0.997416449	0.663424567	0.620699997	0.010564931	0.012312513	0.012954696
0.128699644	0.859198000	0.745658646	0.005238192	0.006352035	0.007313854
0.128699644	0.912741315	0.866833998	0.010564931	0.012312513	0.012954696
0.379087636	0.613697533	0.515669625	0.005238192	0.006352035	0.007313854
0.379087636	0.898108661	0.855693195	0.010564931	0.012312513	0.012954696
0.739754296	0.596427828	0.497565000	0.005238192	0.006352035	0.007313854
0.739754296	0.401484685	0.395311578	0.010564931	0.012312513	0.012954696
0.660264078	0.598332212	0.600538975	0.008643055	0.009628871	0.010456710
0.660264078	0.515441172	0.568267265	0.002262659	0.002725363	0.003224299
0.319341722	0.515450445	0.526406649	0.008643055	0.009628871	0.010456710
0.319341722	0.373645199	0.440552638	0.002262659	0.002725363	0.003224299
0.101422053	0.123682792	0.142800197	0.008643055	0.009628871	0.010456710
0.101422053	0.613463165	0.657949423	0.002262659	0.002725363	0.003224299
0.716207136	0.607380393	0.608465329	0.008643055	0.009628871	0.010456710
0.716207136	0.465725062	0.514378053	0.002262659	0.002725363	0.003224299
0.111826637	0.569120347	0.575482508	0.008643055	0.009628871	0.010456710
0.111826637	0.397456941	0.458498382	0.002262659	0.002725363	0.003224299
0.180168125	0.094736284	0.123760274	0.008643055	0.009628871	0.010456710
0.180168125	0.082776602	0.062842852	0.002262659	0.002725363	0.003224299
0.943471191	0.903311304	0.915697185	0.008643055	0.009628871	0.010456710
0.943471191	0.856381139	0.891677188	0.002262659	0.002725363	0.003224299
0.693893455	0.573170302	0.580780426	0.008643055	0.009628871	0.010456710
0.693893455	0.695228245	0.748680804	0.002262659	0.002725363	0.003224299
0.959860997	0.934901039	0.939685320	0.008643055	0.009628871	0.010456710
0.959860997	0.911051378	0.923125694	0.002262659	0.002725363	0.003224299

0.745123437	0.652279856	0.644355975	0.008643055	0.009628871	0.010456710
0.745123437	0.509677573	0.556571471	0.002262659	0.002725363	0.003224299
0.792161429	0.705200650	0.704826793	0.008643055	0.009628871	0.010456710
0.792161429	0.587754876	0.629076059	0.002262659	0.002725363	0.003224299
0.080291845	0.123965234	0.143120997	0.008643055	0.009628871	0.010456710
0.080291845	0.031517223	0.029146685	0.002262659	0.002725363	0.003224299
0.841055167	0.768356170	0.770344793	0.008643055	0.009628871	0.010456710
0.841055167	0.656662369	0.709003859	0.002262659	0.002725363	0.003224299
0.229099908	0.113214093	0.134920658	0.008643055	0.009628871	0.010456710
0.229099908	0.065104244	0.055490316	0.002262659	0.002725363	0.003224299
0.925367853	0.875943742	0.889134717	0.008643055	0.009628871	0.010456710
0.925367853	0.834990345	0.857874024	0.002262659	0.002725363	0.003224299
0.046756999	0.452219463	0.454440274	0.008643055	0.009628871	0.010456710
0.046756999	0.416967138	0.486297339	0.002262659	0.002725363	0.003224299
0.265177529	0.623235918	0.61643605	0.008643055	0.009628871	0.010456710
0.265177529	0.324496124	0.403243986	0.002262659	0.002725363	0.003224299
0.499412214	0.388485013	0.392449584	0.008643055	0.009628871	0.010456710
0.499412214	0.219135125	0.250145705	0.002262659	0.002725363	0.003224299
0.367078277	0.266771166	0.269434087	0.008643055	0.009628871	0.010456710
0.367078277	0.164376472	0.135461339	0.002262659	0.002725363	0.003224299
0.184449229	0.155273475	0.161274587	0.008643055	0.009628871	0.010456710
0.184449229	0.484016647	0.526447548	0.002262659	0.002725363	0.003224299
0.314345718	0.662953959	0.667417398	0.008643055	0.009628871	0.010456710
0.314345718	0.124225861	0.099369513	0.002262659	0.002725363	0.003224299
0.571600734	0.594313281	0.59232548	0.008643055	0.009628871	0.010456710
0.571600734	0.472833073	0.518473760	0.002262659	0.002725363	0.003224299
0.258896020	0.148458407	0.156568287	0.008643055	0.009628871	0.010456710
0.258896020	0.113230802	0.088454876	0.002262659	0.002725363	0.003224299
0.979310255	0.958043642	0.968515595	0.008643055	0.009628871	0.010456710
0.979310255	0.952208616	0.960223923	0.002262659	0.002725363	0.003224299
0.561920178	0.419831542	0.431004151	0.008643055	0.009628871	0.010456710
0.561920178	0.886077721	0.911477559	0.002262659	0.002725363	0.003224299
0.125794333	0.248551383	0.243997974	0.008643055	0.009628871	0.010456710
0.125794333	0.187171938	0.178138760	0.002262659	0.002725363	0.003224299
0.133746852	0.049876172	0.072072580	0.008643055	0.009628871	0.010456710
0.133746852	0.601438467	0.639631296	0.002262659	0.002725363	0.003224299
0.070326997	0.461583469	0.462841703	0.008643055	0.009628871	0.010456710
0.070326997	0.136805470	0.108250620	0.002262659	0.002725363	0.003224299
0.354157422	0.233594897	0.232195243	0.008643055	0.009628871	0.010456710
0.354157422	0.159552352	0.126019956	0.002262659	0.002725363	0.003224299
0.046888560	0.113412870	0.134943107	0.008643055	0.009628871	0.010456710
0.046888560	0.165303981	0.139265283	0.002262659	0.002725363	0.003224299
0.640341646	0.505718437	0.518140798	0.008643055	0.009628871	0.010456710
0.640341646	0.336989735	0.411307749	0.002262659	0.002725363	0.003224299
0.702589549	0.591585591	0.591557272	0.008643055	0.009628871	0.010456710
0.702589549	0.735808564	0.788628658	0.002262659	0.002725363	0.003224299

0.639257774	0.495318315	0.516349725	0.008643055	0.009628871	0.010456710
0.639257774	0.328681136	0.409499946	0.002262659	0.002725363	0.003224299
0.881017309	0.800474592	0.825274876	0.008643055	0.009628871	0.010456710
0.881017309	0.715545600	0.777839751	0.002262659	0.002725363	0.003224299
0.856462496	0.785150278	0.791110789	0.008643055	0.009628871	0.010456710
0.856462496	0.682595092	0.734531949	0.002262659	0.002725363	0.003224299
0.226007567	0.596464520	0.596678115	0.008643055	0.009628871	0.010456710
0.226007567	0.725019273	0.785574300	0.002262659	0.002725363	0.003224299
0.226944710	0.253446829	0.250258434	0.008643055	0.009628871	0.010456710
0.226944710	0.671542227	0.732205903	0.002262659	0.002725363	0.003224299
0.564721880	0.425137028	0.433912103	0.008643055	0.009628871	0.010456710
0.564721880	0.265571772	0.319762640	0.002262659	0.002725363	0.003224299
0.955504882	0.917332547	0.933031856	0.008643055	0.009628871	0.010456710
0.955504882	0.890352811	0.914091933	0.002262659	0.002725363	0.003224299
0.401955805	0.269801895	0.273775953	0.008643055	0.009628871	0.010456710
0.401955805	0.877338268	0.902050130	0.002262659	0.002725363	0.003224299
0.992044536	0.990191460	0.988747447	0.008643055	0.009628871	0.010456710
0.992044536	0.985817588	0.985610657	0.002262659	0.002725363	0.003224299
0.834819327	0.750222212	0.761655228	0.008643055	0.009628871	0.010456710
0.834819327	0.643298583	0.698496838	0.002262659	0.002725363	0.003224299
0.719319406	0.674396846	0.681841961	0.008643055	0.009628871	0.010456710
0.719319406	0.470989418	0.518358181	0.002262659	0.002725363	0.003224299
0.666175176	0.537639667	0.547590137	0.008643055	0.009628871	0.010456710
0.666175176	0.377748732	0.444709998	0.002262659	0.002725363	0.003224299
0.506250222	0.356636924	0.373276301	0.008643055	0.009628871	0.010456710
0.506250222	0.525685989	0.580760571	0.002262659	0.002725363	0.003224299
0.070897364	0.027998168	0.047597951	0.008643055	0.009628871	0.010456710
0.070897364	0.077854936	0.062278900	0.002262659	0.002725363	0.003224299
0.026060551	0.095181342	0.124444103	0.008643055	0.009628871	0.010456710
0.026060551	0.145698045	0.117804704	0.002262659	0.002725363	0.003224299
0.669054059	0.596401418	0.596552576	0.008643055	0.009628871	0.010456710
0.669054059	0.391011224	0.448871799	0.002262659	0.002725363	0.003224299
0.535738774	0.396644895	0.403385343	0.008643055	0.009628871	0.010456710
0.535738774	0.249040601	0.287658480	0.002262659	0.002725363	0.003224299
0.721021790	0.727808857	0.724980170	0.008643055	0.009628871	0.010456710
0.721021790	0.481605435	0.521171752	0.002262659	0.002725363	0.003224299
0.307985897	0.193313234	0.194522001	0.008643055	0.009628871	0.010456710
0.307985897	0.304602462	0.355364914	0.002262659	0.002725363	0.003224299
0.252314824	0.443733875	0.447575400	0.008643055	0.009628871	0.010456710
0.252314824	0.921729639	0.931783931	0.002262659	0.002725363	0.003224299
0.891793546	0.812927918	0.840675975	0.008643055	0.009628871	0.010456710
0.891793546	0.740061805	0.796568999	0.002262659	0.002725363	0.003224299
0.227249670	0.289822130	0.295380158	0.008643055	0.009628871	0.010456710
0.227249670	0.055771524	0.051930186	0.002262659	0.002725363	0.003224299
0.018703742	0.238936537	0.235564641	0.008643055	0.009628871	0.010456710
0.018703742	0.381914524	0.446401294	0.002262659	0.002725363	0.003224299

0.300237218	0.185305932	0.188352236	0.008643055	0.009628871	0.010456710
0.300237218	0.115290185	0.090625323	0.002262659	0.002725363	0.003224299
0.021647033	0.039721355	0.059907758	0.008643055	0.009628871	0.010456710
0.021647033	0.006543555	0.006975844	0.002262659	0.002725363	0.003224299
0.516125999	0.376510060	0.383104264	0.008643055	0.009628871	0.010456710
0.516125999	0.241430114	0.266958240	0.002262659	0.002725363	0.003224299
0.678773390	0.660301742	0.660119269	0.008643055	0.009628871	0.010456710
0.678773390	0.668362530	0.730529732	0.002262659	0.002725363	0.003224299
0.938273956	0.895675367	0.908181937	0.008643055	0.009628871	0.010456710
0.938273956	0.846541361	0.881574949	0.002262659	0.002725363	0.003224299
0.893338951	0.827397170	0.842827348	0.008643055	0.009628871	0.010456710
0.893338951	0.762099231	0.799418586	0.002262659	0.002725363	0.003224299
0.569428499	0.431447880	0.438859722	0.008643055	0.009628871	0.010456710
0.569428499	0.274188393	0.325236487	0.002262659	0.002725363	0.003224299
0.514547099	0.371065459	0.381442502	0.008643055	0.009628871	0.010456710
0.514547099	0.237833353	0.265384638	0.002262659	0.002725363	0.003224299
0.590800803	0.459979699	0.462292532	0.008643055	0.009628871	0.010456710
0.590800803	0.301169829	0.350058979	0.002262659	0.002725363	0.003224299
0.994920878	0.994482126	0.992669380	0.008643055	0.009628871	0.010456710
0.994920878	0.997294881	0.991771423	0.002262659	0.002725363	0.003224299
0.964996597	0.942795978	0.947264304	0.008643055	0.009628871	0.010456710
0.964996597	0.933589071	0.932954293	0.002262659	0.002725363	0.003224299
0.740139019	0.633569363	0.637591812	0.008643055	0.009628871	0.010456710
0.740139019	0.495921790	0.548882366	0.002262659	0.002725363	0.003224299
0.407338804	0.655497048	0.647697654	0.008643055	0.009628871	0.010456710
0.407338804	0.180441633	0.166491030	0.002262659	0.002725363	0.003224299
0.270197619	0.491191260	0.509558578	0.008643055	0.009628871	0.010456710
0.270197619	0.824781029	0.848025877	0.002262659	0.002725363	0.003224299
0.835630027	0.758437466	0.762891127	0.008643055	0.009628871	0.010456710
0.835630027	0.650415008	0.699913658	0.002262659	0.002725363	0.003224299
0.946857791	0.907498714	0.920184793	0.008643055	0.009628871	0.010456710
0.946857791	0.867840480	0.897639951	0.002262659	0.002725363	0.003224299
0.819413988	0.734479119	0.740649873	0.008643055	0.009628871	0.010456710
0.819413988	0.619882041	0.672414656	0.002262659	0.002725363	0.003224299
0.847695589	0.775865053	0.779017377	0.008643055	0.009628871	0.010456710
0.847695589	0.659166572	0.719750553	0.002262659	0.002725363	0.003224299
0.107873758	0.055796811	0.078512599	0.008643055	0.009628871	0.010456710
0.107873758	0.105961119	0.078777967	0.002262659	0.002725363	0.003224299
0.227454206	0.109655104	0.133739948	0.008643055	0.009628871	0.010456710
0.227454206	0.058074067	0.052076335	0.002262659	0.002725363	0.003224299
0.979728682	0.969862321	0.969635871	0.008643055	0.009628871	0.010456710
0.979728682	0.963142891	0.961431987	0.002262659	0.002725363	0.003224299
0.134271689	0.492111706	0.511342674	0.008643055	0.009628871	0.010456710
0.134271689	0.152330140	0.119918703	0.002262659	0.002725363	0.003224299
0.225545372	0.103284903	0.132530636	0.008643055	0.009628871	0.010456710
0.225545372	0.051155065	0.051231830	0.002262659	0.002725363	0.003224299

0.247080295	0.493876250	0.514801254	0.008643055	0.009628871	0.010456710
0.247080295	0.144552753	0.116258094	0.002262659	0.002725363	0.003224299
0.892779209	0.939886224	0.941985014	0.008643055	0.009628871	0.010456710
0.892779209	0.752316813	0.798707675	0.002262659	0.002725363	0.003224299
0.072261879	0.018794385	0.036562420	0.008643055	0.009628871	0.010456710
0.072261879	0.197211244	0.207191086	0.002262659	0.002725363	0.003224299
0.903289888	0.860205329	0.857071283	0.008643055	0.009628871	0.010456710
0.903289888	0.866336953	0.894347998	0.002262659	0.002725363	0.003224299
0.516517550	0.728381635	0.726654504	0.008643055	0.009628871	0.010456710
0.516517550	0.706620135	0.775167272	0.002262659	0.002725363	0.003224299
0.898953702	0.851532617	0.850887724	0.008643055	0.009628871	0.010456710
0.898953702	0.781195741	0.809216837	0.002262659	0.002725363	0.003224299
0.786009441	0.695236665	0.696682989	0.008643055	0.009628871	0.010456710
0.786009441	0.571830270	0.618899199	0.002262659	0.002725363	0.003224299
0.511752591	0.363824049	0.378890427	0.008643055	0.009628871	0.010456710
0.511752591	0.555713335	0.5957834	0.002262659	0.002725363	0.003224299
0.783755297	0.688045852	0.693759849	0.008643055	0.009628871	0.010456710
0.783755297	0.568894038	0.615759985	0.002262659	0.002725363	0.003224299
0.970873424	0.950823593	0.956001761	0.008643055	0.009628871	0.010456710
0.970873424	0.974065977	0.972893479	0.002262659	0.002725363	0.003224299
0.798615316	0.717191410	0.712860549	0.008643055	0.009628871	0.010456710
0.798615316	0.594467717	0.638641251	0.002262659	0.002725363	0.003224299
0.991211826	0.977216655	0.986853956	0.008643055	0.009628871	0.010456710
0.991211826	0.979446427	0.984152953	0.002262659	0.002725363	0.003224299
0.513163785	0.729342640	0.729450817	0.008643055	0.009628871	0.010456710
0.513163785	0.635077891	0.695978059	0.002262659	0.002725363	0.003224299
0.579311202	0.533835297	0.541219891	0.008643055	0.009628871	0.010456710
0.579311202	0.720868334	0.783262434	0.002262659	0.002725363	0.003224299
0.249236840	0.552998881	0.555341443	0.008643055	0.009628871	0.010456710
0.249236840	0.129748718	0.102303733	0.002262659	0.002725363	0.003224299
0.897690959	0.841470983	0.848793957	0.008643055	0.009628871	0.010456710
0.897690959	0.822487318	0.845553615	0.002262659	0.002725363	0.003224299
0.021855777	0.012590035	0.029435048	0.008643055	0.009628871	0.010456710
0.021855777	0.430065715	0.494671145	0.002262659	0.002725363	0.003224299
0.770427210	0.670282513	0.676593853	0.008643055	0.009628871	0.010456710
0.770427210	0.542503902	0.594724773	0.002262659	0.002725363	0.003224299
0.258089884	0.181088821	0.184937269	0.008643055	0.009628871	0.010456710
0.258089884	0.090335731	0.066893532	0.002262659	0.002725363	0.003224299
0.152208523	0.566041816	0.569463763	0.008643055	0.009628871	0.010456710
0.152208523	0.944083090	0.952035420	0.002262659	0.002725363	0.003224299
0.794311063	0.712782841	0.707676271	0.008643055	0.009628871	0.010456710
0.794311063	0.589137104	0.631881045	0.002262659	0.002725363	0.003224299
0.051527554	0.231973361	0.230371674	0.008643055	0.009628871	0.010456710
0.051527554	0.912435328	0.929105524	0.002262659	0.002725363	0.003224299
0.458161593	0.328302515	0.345908536	0.008643055	0.009628871	0.010456710
0.458161593	0.202185607	0.210627624	0.002262659	0.002725363	0.003224299

0.445179901	0.301336032	0.313572704	0.008643055	0.009628871	0.010456710
0.445179901	0.194827904	0.198834346	0.002262659	0.002725363	0.003224299
0.740943588	0.643175700	0.638982884	0.008643055	0.009628871	0.010456710
0.740943588	0.503660154	0.550310202	0.002262659	0.002725363	0.003224299
0.957242267	0.928738442	0.936034038	0.008643055	0.009628871	0.010456710
0.957242267	0.903750590	0.91814182	0.002262659	0.002725363	0.003224299
0.489296162	0.334296562	0.356103544	0.008643055	0.009628871	0.010456710
0.489296162	0.214995575	0.239998171	0.002262659	0.002725363	0.003224299
0.101929299	0.170988164	0.173410076	0.008643055	0.009628871	0.010456710
0.101929299	0.253141794	0.289934603	0.002262659	0.002725363	0.003224299
0.342814285	0.256799504	0.254855240	0.008643055	0.009628871	0.010456710
0.342814285	0.150868263	0.118188097	0.002262659	0.002725363	0.003224299
0.243282010	0.127569510	0.145310663	0.008643055	0.009628871	0.010456710
0.243282010	0.071741662	0.059512485	0.002262659	0.002725363	0.003224299
0.060352227	0.013141896	0.030127957	0.008643055	0.009628871	0.010456710
0.060352227	0.422426927	0.489585784	0.002262659	0.002725363	0.003224299
0.245997418	0.130675404	0.147215957	0.008643055	0.009628871	0.010456710
0.245997418	0.074132808	0.060880603	0.002262659	0.002725363	0.003224299
0.504390383	0.762987352	0.768390273	0.008643055	0.009628871	0.010456710
0.504390383	0.458689297	0.508497563	0.002262659	0.002725363	0.003224299
0.553459491	0.408285725	0.421698767	0.008643055	0.009628871	0.010456710
0.553459491	0.255802690	0.306916935	0.002262659	0.002725363	0.003224299
0.078610712	0.030465085	0.051445312	0.008643055	0.009628871	0.010456710
0.078610712	0.100689328	0.075169541	0.002262659	0.002725363	0.003224299
0.695468955	0.579792528	0.582695960	0.008643055	0.009628871	0.010456710
0.695468955	0.416500670	0.485045488	0.002262659	0.002725363	0.003224299
0.930716599	0.886731727	0.897167079	0.008643055	0.009628871	0.010456710
0.930716599	0.844241915	0.868057158	0.002262659	0.002725363	0.003224299
0.177588711	0.072591236	0.099779647	0.008643055	0.009628871	0.010456710
0.177588711	0.033452064	0.031855995	0.002262659	0.002725363	0.003224299
0.650674215	0.596991665	0.597764677	0.008643055	0.009628871	0.010456710
0.650674215	0.683120736	0.734564820	0.002262659	0.002725363	0.003224299
0.235016548	0.118840297	0.139157812	0.008643055	0.009628871	0.010456710
0.235016548	0.065209130	0.055498329	0.002262659	0.002725363	0.003224299
0.894630395	0.836578465	0.844850665	0.008643055	0.009628871	0.010456710
0.894630395	0.770562981	0.801900709	0.002262659	0.002725363	0.003224299
0.782024110	0.682417897	0.691677934	0.008643055	0.009628871	0.010456710
0.782024110	0.562798958	0.613053726	0.002262659	0.002725363	0.003224299
0.642116064	0.508864235	0.519826203	0.008643055	0.009628871	0.010456710
0.642116064	0.369591916	0.437857812	0.002262659	0.002725363	0.003224299
0.206879804	0.090117302	0.119371276	0.008643055	0.009628871	0.010456710
0.206879804	0.043630410	0.043063130	0.002262659	0.002725363	0.003224299
0.225270202	0.569553317	0.576340762	0.008643055	0.009628871	0.010456710
0.225270202	0.809939539	0.836373608	0.002262659	0.002725363	0.003224299
0.472287644	0.469895823	0.478512717	0.008643055	0.009628871	0.010456710
0.472287644	0.797599572	0.822717184	0.002262659	0.002725363	0.003224299

0.618501657	0.478841452	0.492856911	0.008643055	0.009628871	0.010456710
0.618501657	0.315727350	0.383328377	0.002262659	0.002725363	0.003224299
0.659493466	0.727650768	0.637712876	0.006047564	0.007230944	0.008662615
0.659493466	0.563611879	0.568266494	0.003263504	0.003726758	0.004421434
0.355031550	0.402167313	0.426068086	0.006047564	0.007230944	0.008662615
0.355031550	0.448145715	0.440588328	0.003263504	0.003726758	0.004421434
0.63005687	0.637434373	0.583861982	0.006047564	0.007230944	0.008662615
0.63005687	0.656574612	0.658478058	0.003263504	0.003726758	0.004421434
0.942617868	0.939585863	0.932460563	0.006047564	0.007230944	0.008662615
0.942617868	0.887480722	0.890180248	0.003263504	0.003726758	0.004421434
0.047333310	0.150674116	0.234721433	0.006047564	0.007230944	0.008662615
0.047333310	0.483469411	0.458433889	0.003263504	0.003726758	0.004421434
0.275156863	0.153337877	0.235539584	0.006047564	0.007230944	0.008662615
0.275156863	0.062593864	0.076236282	0.003263504	0.003726758	0.004421434
0.535648901	0.549509573	0.519082614	0.006047564	0.007230944	0.008662615
0.535648901	0.334952978	0.356142178	0.003263504	0.003726758	0.004421434
0.981526257	0.984343490	0.978069577	0.006047564	0.007230944	0.008662615
0.981526257	0.981080316	0.965239682	0.003263504	0.003726758	0.004421434
0.958791379	0.961413815	0.951261801	0.006047564	0.007230944	0.008662615
0.958791379	0.934082544	0.921072398	0.003263504	0.003726758	0.004421434
0.461765501	0.399627123	0.424069149	0.006047564	0.007230944	0.008662615
0.461765501	0.357679980	0.382275175	0.003263504	0.003726758	0.004421434
0.148404682	0.096727003	0.164184272	0.006047564	0.007230944	0.008662615
0.148404682	0.577595024	0.584863452	0.003263504	0.003726758	0.004421434
0.803636000	0.805648172	0.772519108	0.006047564	0.007230944	0.008662615
0.803636000	0.646474606	0.646804446	0.003263504	0.003726758	0.004421434
0.488793435	0.447418145	0.460384333	0.006047564	0.007230944	0.008662615
0.488793435	0.623595866	0.623814445	0.003263504	0.003726758	0.004421434
0.030650684	0.004800626	0.024722864	0.006047564	0.007230944	0.008662615
0.030650684	0.042691759	0.055291866	0.003263504	0.003726758	0.004421434
0.398598553	0.352247444	0.391486459	0.006047564	0.007230944	0.008662615
0.398598553	0.417082373	0.414480886	0.003263504	0.003726758	0.004421434
0.443561032	0.546961116	0.517643771	0.006047564	0.007230944	0.008662615
0.443561032	0.492553845	0.486694143	0.003263504	0.003726758	0.004421434
0.629609211	0.763429187	0.689044946	0.006047564	0.007230944	0.008662615
0.629609211	0.398564862	0.403608417	0.003263504	0.003726758	0.004421434
0.635343779	0.653783194	0.589553108	0.006047564	0.007230944	0.008662615
0.635343779	0.400462812	0.404530794	0.003263504	0.003726758	0.004421434
0.675559962	0.722301197	0.632152233	0.006047564	0.007230944	0.008662615
0.675559962	0.480442775	0.457404622	0.003263504	0.003726758	0.004421434
0.385110228	0.287197601	0.338124719	0.006047564	0.007230944	0.008662615
0.385110228	0.549384406	0.526648209	0.003263504	0.003726758	0.004421434
0.079478073	0.173854134	0.259470463	0.006047564	0.007230944	0.008662615
0.079478073	0.046496964	0.058854947	0.003263504	0.003726758	0.004421434
0.408039904	0.439215732	0.449011588	0.006047564	0.007230944	0.008662615
0.408039904	0.543624518	0.518310199	0.003263504	0.003726758	0.004421434

0.654757039	0.678051482	0.609549319	0.006047564	0.007230944	0.008662615
0.654757039	0.426598138	0.429658016	0.003263504	0.003726758	0.004421434
0.094081470	0.033205677	0.077047008	0.006047564	0.007230944	0.008662615
0.094081470	0.029671030	0.038720039	0.003263504	0.003726758	0.004421434
0.522609747	0.471672888	0.473084436	0.006047564	0.007230944	0.008662615
0.522609747	0.916282100	0.911438248	0.003263504	0.003726758	0.004421434
0.411738522	0.365520241	0.399243857	0.006047564	0.007230944	0.008662615
0.411738522	0.182692870	0.178424704	0.003263504	0.003726758	0.004421434
0.425380824	0.337858866	0.376611427	0.006047564	0.007230944	0.008662615
0.425380824	0.635722779	0.639922930	0.003263504	0.003726758	0.004421434
0.622465215	0.739352498	0.659791212	0.006047564	0.007230944	0.008662615
0.622465215	0.361343906	0.388413818	0.003263504	0.003726758	0.004421434
0.306006886	0.182916489	0.263560675	0.006047564	0.007230944	0.008662615
0.306006886	0.086768047	0.09418454	0.003263504	0.003726758	0.004421434
0.069717735	0.049020520	0.098459535	0.006047564	0.007230944	0.008662615
0.069717735	0.141622327	0.139288112	0.003263504	0.003726758	0.004421434
0.649830358	0.674860460	0.604948256	0.006047564	0.007230944	0.008662615
0.649830358	0.424075170	0.423559308	0.003263504	0.003726758	0.004421434
0.468115481	0.387368022	0.418730516	0.006047564	0.007230944	0.008662615
0.468115481	0.797801796	0.788394184	0.003263504	0.003726758	0.004421434
0.600676258	0.577070297	0.552785897	0.006047564	0.007230944	0.008662615
0.600676258	0.336839740	0.361622831	0.003263504	0.003726758	0.004421434
0.114585938	0.048195778	0.097256404	0.006047564	0.007230944	0.008662615
0.114585938	0.583418349	0.589531665	0.003263504	0.003726758	0.004421434
0.307218987	0.188957053	0.264865245	0.006047564	0.007230944	0.008662615
0.307218987	0.088464890	0.094838204	0.003263504	0.003726758	0.004421434
0.559599368	0.724485169	0.632481687	0.006047564	0.007230944	0.008662615
0.559599368	0.791437712	0.785907892	0.003263504	0.003726758	0.004421434
0.187634381	0.127671903	0.204563663	0.006047564	0.007230944	0.008662615
0.187634381	0.705414501	0.732166593	0.003263504	0.003726758	0.004421434
0.065855530	0.028076405	0.069731945	0.006047564	0.007230944	0.008662615
0.065855530	0.071813271	0.082789163	0.003263504	0.003726758	0.004421434
0.114165031	0.049820107	0.099678658	0.006047564	0.007230944	0.008662615
0.114165031	0.011011145	0.021825487	0.003263504	0.003726758	0.004421434
0.829165641	0.825452861	0.800862371	0.006047564	0.007230944	0.008662615
0.829165641	0.911117475	0.902477340	0.003263504	0.003726758	0.004421434
0.511420850	0.615960002	0.576423434	0.006047564	0.007230944	0.008662615
0.511420850	0.244881838	0.262528958	0.003263504	0.003726758	0.004421434
0.489385847	0.426764328	0.439788823	0.006047564	0.007230944	0.008662615
0.489385847	0.556318310	0.546919899	0.003263504	0.003726758	0.004421434
0.602533688	0.685076249	0.612339726	0.006047564	0.007230944	0.008662615
0.602533688	0.346549504	0.363867832	0.003263504	0.003726758	0.004421434
0.954553024	0.950562771	0.945897441	0.006047564	0.007230944	0.008662615
0.954553024	0.921761194	0.912380487	0.003263504	0.003726758	0.004421434
0.513459525	0.456530228	0.463834334	0.006047564	0.007230944	0.008662615
0.513459525	0.574448360	0.580767780	0.003263504	0.003726758	0.004421434

0.851232781	0.837272127	0.826069697	0.006047564	0.007230944	0.008662615
0.851232781	0.692395371	0.725697397	0.003263504	0.003726758	0.004421434
0.368579039	0.283416135	0.337255360	0.006047564	0.007230944	0.008662615
0.368579039	0.140823536	0.136561776	0.003263504	0.003726758	0.004421434
0.323906609	0.327893755	0.366203798	0.006047564	0.007230944	0.008662615
0.323906609	0.306337175	0.325121005	0.003263504	0.003726758	0.004421434
0.321529557	0.208418147	0.278198594	0.006047564	0.007230944	0.008662615
0.321529557	0.100455979	0.103809492	0.003263504	0.003726758	0.004421434
0.168494769	0.213646347	0.281017411	0.006047564	0.007230944	0.008662615
0.168494769	0.313939359	0.335187443	0.003263504	0.003726758	0.004421434
0.561959854	0.531231939	0.512933408	0.006047564	0.007230944	0.008662615
0.561959854	0.330511364	0.355618888	0.003263504	0.003726758	0.004421434
0.148237406	0.162929009	0.242766522	0.006047564	0.007230944	0.008662615
0.148237406	0.951755851	0.931679854	0.003263504	0.003726758	0.004421434
0.797525936	0.797871395	0.765578204	0.006047564	0.007230944	0.008662615
0.797525936	0.629006025	0.637226623	0.003263504	0.003726758	0.004421434
0.533297406	0.508132918	0.498008590	0.006047564	0.007230944	0.008662615
0.533297406	0.260806914	0.284999945	0.003263504	0.003726758	0.004421434
0.769982818	0.778886752	0.73521219	0.006047564	0.007230944	0.008662615
0.769982818	0.593964900	0.594311142	0.003263504	0.003726758	0.004421434
0.475833819	0.402591155	0.426116214	0.006047564	0.007230944	0.008662615
0.475833819	0.223766521	0.227076356	0.003263504	0.003726758	0.004421434
0.668257555	0.711929541	0.624224331	0.006047564	0.007230944	0.008662615
0.668257555	0.468290284	0.447319311	0.003263504	0.003726758	0.004421434
0.489084144	0.420558647	0.439280882	0.006047564	0.007230944	0.008662615
0.489084144	0.229098252	0.239748452	0.003263504	0.003726758	0.004421434
0.566103172	0.624356619	0.582142816	0.006047564	0.007230944	0.008662615
0.566103172	0.701646285	0.730417062	0.003263504	0.003726758	0.004421434
0.926581875	0.924571092	0.913772775	0.006047564	0.007230944	0.008662615
0.926581875	0.869094800	0.859759211	0.003263504	0.003726758	0.004421434
0.583177468	0.562782825	0.534867947	0.006047564	0.007230944	0.008662615
0.583177468	0.320802092	0.341149902	0.003263504	0.003726758	0.004421434
0.873499835	0.8610699	0.851593981	0.006047564	0.007230944	0.008662615
0.873499835	0.748222701	0.764293706	0.003263504	0.003726758	0.004421434
0.663589147	0.695470246	0.619057302	0.006047564	0.007230944	0.008662615
0.663589147	0.451503147	0.441125519	0.003263504	0.003726758	0.004421434
0.902390290	0.903453908	0.885201723	0.006047564	0.007230944	0.008662615
0.902390290	0.818956011	0.815633216	0.003263504	0.003726758	0.004421434
0.532949005	0.484597376	0.483609741	0.006047564	0.007230944	0.008662615
0.532949005	0.834311023	0.827628689	0.003263504	0.003726758	0.004421434
0.366739432	0.259950661	0.320573836	0.006047564	0.007230944	0.008662615
0.366739432	0.598519427	0.595368831	0.003263504	0.003726758	0.004421434
0.813676147	0.809928213	0.783385219	0.006047564	0.007230944	0.008662615
0.813676147	0.660681752	0.663219007	0.003263504	0.003726758	0.004421434
0.912931155	0.917325139	0.902525598	0.006047564	0.007230944	0.008662615
0.912931155	0.838638	0.834515015	0.003263504	0.003726758	0.004421434

0.561846893	0.662567712	0.593806773	0.006047564	0.007230944	0.008662615
0.561846893	0.863920418	0.848317527	0.003263504	0.003726758	0.004421434
0.702278681	0.744002574	0.660977772	0.006047564	0.007230944	0.008662615
0.702278681	0.644327881	0.642278927	0.003263504	0.003726758	0.004421434
0.934170222	0.927456076	0.922034308	0.006047564	0.007230944	0.008662615
0.934170222	0.875565027	0.873761591	0.003263504	0.003726758	0.004421434
0.276281474	0.155214380	0.236447197	0.006047564	0.007230944	0.008662615
0.276281474	0.063998021	0.076763692	0.003263504	0.003726758	0.004421434
0.316730426	0.201111456	0.273627243	0.006047564	0.007230944	0.008662615
0.316730426	0.097086253	0.100949939	0.003263504	0.003726758	0.004421434
0.779826333	0.782432182	0.745715782	0.006047564	0.007230944	0.008662615
0.779826333	0.609412720	0.609188918	0.003263504	0.003726758	0.004421434
0.989821504	0.991057564	0.987808956	0.006047564	0.007230944	0.008662615
0.989821504	0.990913421	0.980849897	0.003263504	0.003726758	0.004421434
0.666504986	0.706034176	0.622549167	0.006047564	0.007230944	0.008662615
0.666504986	0.460671542	0.445479369	0.003263504	0.003726758	0.004421434
0.386130227	0.476253219	0.477732811	0.006047564	0.007230944	0.008662615
0.386130227	0.15916675	0.149828281	0.003263504	0.003726758	0.004421434
0.380972943	0.275796108	0.333951513	0.006047564	0.007230944	0.008662615
0.380972943	0.145170504	0.145656926	0.003263504	0.003726758	0.004421434
0.496469602	0.573211875	0.547527440	0.006047564	0.007230944	0.008662615
0.496469602	0.240288584	0.24731864	0.003263504	0.003726758	0.004421434
0.516047096	0.683862369	0.610465418	0.006047564	0.007230944	0.008662615
0.516047096	0.568400986	0.579148018	0.003263504	0.003726758	0.004421434
0.265044054	0.143651007	0.226140175	0.006047564	0.007230944	0.008662615
0.265044054	0.198719815	0.207383869	0.003263504	0.003726758	0.004421434
0.566377584	0.546843350	0.517630718	0.006047564	0.007230944	0.008662615
0.566377584	0.893871055	0.894011086	0.003263504	0.003726758	0.004421434
0.600918776	0.746633361	0.663435686	0.006047564	0.007230944	0.008662615
0.600918776	0.763577789	0.775251673	0.003263504	0.003726758	0.004421434
0.053865732	0.029480123	0.072331009	0.006047564	0.007230944	0.008662615
0.053865732	0.030742528	0.040304243	0.003263504	0.003726758	0.004421434
0.089700379	0.050647678	0.100938932	0.006047564	0.007230944	0.008662615
0.089700379	0.076013006	0.091806919	0.003263504	0.003726758	0.004421434
0.881123245	0.877894621	0.860571734	0.006047564	0.007230944	0.008662615
0.881123245	0.771955691	0.778030335	0.003263504	0.003726758	0.004421434
0.870623786	0.848416406	0.848491633	0.006047564	0.007230944	0.008662615
0.870623786	0.729665343	0.759560264	0.003263504	0.003726758	0.004421434
0.383066679	0.281043279	0.336011413	0.006047564	0.007230944	0.008662615
0.383066679	0.985130154	0.972305672	0.003263504	0.003726758	0.004421434
0.357437235	0.247396512	0.311522706	0.006047564	0.007230944	0.008662615
0.357437235	0.132622471	0.128175026	0.003263504	0.003726758	0.004421434
0.558105440	0.517909740	0.509068654	0.006047564	0.007230944	0.008662615
0.558105440	0.444464737	0.437949522	0.003263504	0.003726758	0.004421434
0.218800669	0.315299541	0.359379399	0.006047564	0.007230944	0.008662615
0.218800669	0.685904908	0.695683696	0.003263504	0.003726758	0.004421434

0.630023141	0.675746104	0.606041341	0.006047564	0.007230944	0.008662615	
0.630023141	0.784922718	0.783313146	0.003263504	0.003726758	0.004421434	
0.302703402	0.371827624	0.407365789	0.006047564	0.007230944	0.008662615	
0.302703402	0.099491656	0.102357200	0.003263504	0.003726758	0.004421434	
0.006552589	0.001060125	0.006784197	0.006047564	0.007230944	0.008662615	0.15
0.006552589	0.852097342	0.844662477	0.003263504	0.003726758	0.004421434	
0.625937655	0.619155282	0.579298691	0.006047564	0.007230944	0.008662615	
0.625937655	0.510866084	0.495275226	0.003263504	0.003726758	0.004421434	
0.872456424	0.856409878	0.850698024	0.006047564	0.007230944	0.008662615	
0.872456424	0.741089487	0.762448928	0.003263504	0.003726758	0.004421434	
0.471356810	0.396845732	0.421867709	0.006047564	0.007230944	0.008662615	
0.471356810	0.218758667	0.222673654	0.003263504	0.003726758	0.004421434	
0.223114224	0.327718523	0.366007418	0.006047564	0.007230944	0.008662615	
0.223114224	0.968046171	0.952106326	0.003263504	0.003726758	0.004421434	
0.606501095	0.599068289	0.559271332	0.006047564	0.007230944	0.008662615	
0.606501095	0.350768233	0.368606748	0.003263504	0.003726758	0.004421434	
0.307844443	0.266494189	0.324035763	0.006047564	0.007230944	0.008662615	
0.307844443	0.937597989	0.929361841	0.003263504	0.003726758	0.004421434	
0.443013754	0.369439648	0.404258891	0.006047564	0.007230944	0.008662615	
0.443013754	0.191700581	0.196961617	0.003263504	0.003726758	0.004421434	
0.966705898	0.975475915	0.960392117	0.006047564	0.007230944	0.008662615	
0.966705898	0.959969294	0.935691020	0.003263504	0.003726758	0.004421434	
0.819455802	0.820356641	0.790276799	0.006047564	0.007230944	0.008662615	
0.819455802	0.669066952	0.672899124	0.003263504	0.003726758	0.004421434	
0.963635770	0.971510711	0.957248238	0.006047564	0.007230944	0.008662615	
0.963635770	0.949823400	0.930429352	0.003263504	0.003726758	0.004421434	
0.469350324	0.389148276	0.419617815	0.006047564	0.007230944	0.008662615	
0.469350324	0.211849678	0.220857218	0.003263504	0.003726758	0.004421434	
0.344684410	0.265838477	0.323241689	0.006047564	0.007230944	0.008662615	
0.344684410	0.272250437	0.290177358	0.003263504	0.003726758	0.004421434	
0.876279863	0.870313344	0.855018004	0.006047564	0.007230944	0.008662615	
0.876279863	0.754403624	0.769066326	0.003263504	0.003726758	0.004421434	
0.890944285	0.892312281	0.871724892	0.006047564	0.007230944	0.008662615	
0.890944285	0.801853229	0.794755731	0.003263504	0.003726758	0.004421434	
0.160285117	0.070450688	0.133387333	0.006047564	0.007230944	0.008662615	
0.160285117	0.495881997	0.489685717	0.003263504	0.003726758	0.004421434	
0.333198517	0.221575829	0.288905703	0.006047564	0.007230944	0.008662615	
0.333198517	0.110871327	0.111474327	0.003263504	0.003726758	0.004421434	
0.782007372	0.837585530	0.826108157	0.006047564	0.007230944	0.008662615	
0.782007372	0.619114682	0.613029774	0.003263504	0.003726758	0.004421434	
0.400164983	0.306240841	0.352206776	0.006047564	0.007230944	0.008662615	
0.400164983	0.166427422	0.160578246	0.003263504	0.003726758	0.004421434	
0.881578641	0.881599071	0.860864518	0.006047564	0.007230944	0.008662615	
0.881578641	0.775023546	0.778336006	0.003263504	0.003726758	0.004421434	
0.281710829	0.161143367	0.241423540	0.006047564	0.007230944	0.008662615	
0.281710829	0.474442084	0.453818502	0.003263504	0.003726758	0.004421434	

0.638205420	0.736310710	0.653038811	0.006047564	0.007230944	0.008662615
0.638205420	0.813454254	0.799074884	0.003263504	0.003726758	0.004421434
0.172725915	0.080140154	0.144232362	0.006047564	0.007230944	0.008662615
0.172725915	0.025102236	0.030147624	0.003263504	0.003726758	0.004421434
0.631162508	0.692425859	0.615687318	0.006047564	0.007230944	0.008662615
0.631162508	0.715764284	0.734545308	0.003263504	0.003726758	0.004421434
0.539230854	0.497766223	0.489564607	0.006047564	0.007230944	0.008662615
0.539230854	0.274004546	0.291339679	0.003263504	0.003726758	0.004421434
0.305851325	0.227848101	0.294364911	0.006047564	0.007230944	0.008662615
0.305851325	0.411202186	0.413797981	0.003263504	0.003726758	0.004421434
0.226028663	0.165033891	0.245711623	0.006047564	0.007230944	0.008662615
0.226028663	0.530437508	0.505025963	0.003263504	0.003726758	0.004421434
0.192117380	0.099712125	0.168735535	0.006047564	0.007230944	0.008662615
0.192117380	0.436379645	0.437407813	0.003263504	0.003726758	0.004421434
0.708088353	0.748172736	0.666749606	0.006047564	0.007230944	0.008662615
0.708088353	0.517510420	0.502154202	0.003263504	0.003726758	0.004421434
0.247142980	0.335589004	0.375006775	0.006047564	0.007230944	0.008662615
0.247142980	0.846659246	0.836395480	0.003263504	0.003726758	0.004421434
0.303618893	0.292163841	0.340121255	0.006047564	0.007230944	0.008662615
0.303618893	0.825227961	0.822548515	0.003263504	0.003726758	0.004421434
0.948390268	0.943858399	0.938800212	0.006047564	0.007230944	0.008662615
0.948390268	0.898221603	0.900558066	0.003263504	0.003726758	0.004421434
0.082255830	0.410392841	0.361777598	0.002545798	0.004602761	0.006730984
0.082255830	0.583119300	0.567689256	0.000966233	0.001005035	0.002118303
0.590233395	0.756273187	0.660541403	0.002545798	0.004602761	0.006730984
0.590233395	0.450074825	0.440823530	0.000966233	0.001005035	0.002118303
0.702653450	0.657887816	0.577716658	0.002545798	0.004602761	0.006730984
0.702653450	0.702582890	0.658550654	0.000966233	0.001005035	0.002118303
0.812714475	0.828276052	0.722049260	0.002545798	0.004602761	0.006730984
0.812714475	0.709228275	0.662026717	0.000966233	0.001005035	0.002118303
0.523823138	0.878332064	0.798114392	0.002545798	0.004602761	0.006730984
0.523823138	0.465695838	0.458910378	0.000966233	0.001005035	0.002118303
0.321090190	0.210832057	0.190567256	0.002545798	0.004602761	0.006730984
0.321090190	0.135039949	0.103669826	0.000966233	0.001005035	0.002118303
0.643640955	0.608637892	0.527806017	0.002545798	0.004602761	0.006730984
0.643640955	0.423852922	0.415513148	0.000966233	0.001005035	0.002118303
0.028707996	0.020517960	0.028954790	0.002545798	0.004602761	0.006730984
0.028707996	0.765996090	0.748015619	0.000966233	0.001005035	0.002118303
0.020902884	0.100063006	0.091994561	0.002545798	0.004602761	0.006730984
0.020902884	0.730982316	0.694240072	0.000966233	0.001005035	0.002118303
0.903697280	0.940096621	0.852000448	0.002545798	0.004602761	0.006730984
0.903697280	0.863922016	0.818189883	0.000966233	0.001005035	0.002118303
0.126458922	0.204935705	0.186016609	0.002545798	0.004602761	0.006730984
0.126458922	0.608011491	0.584841506	0.000966233	0.001005035	0.002118303
0.764758571	0.752003039	0.657260975	0.002545798	0.004602761	0.006730984
0.764758571	0.614494055	0.585790420	0.000966233	0.001005035	0.002118303

0.8037535788	0.8158006064	0.7096996797	0.0025457983	0.0046027613	0.0067309843
0.8037535788	0.6964766557	0.6476125789	0.0009662331	0.0010050358	0.0021183039
0.8186205229	0.8365096438	0.7295463701	0.0025457983	0.0046027613	0.0067309843
0.8186205229	0.7182197514	0.6711927586	0.0009662331	0.0010050358	0.0021183039
0.2006233	0.3497778353	0.296872107	0.0025457983	0.0046027613	0.0067309843
0.2006233	0.4155511274	0.4142829115	0.0009662331	0.0010050358	0.0021183039
0.7066193127	0.8686733027	0.7692331457	0.0025457983	0.0046027613	0.0067309843
0.7066193127	0.5171758728	0.5007142897	0.0009662331	0.0010050358	0.0021183039
0.0310405010	0.6476823737	0.5665685285	0.0025457983	0.0046027613	0.0067309843
0.0310405010	0.4039711177	0.4030098497	0.0009662331	0.0010050358	0.0021183039
0.0747301899	0.2399667260	0.2091382279	0.0025457983	0.0046027613	0.0067309843
0.0747301899	0.0804679620	0.0549396040	0.0009662331	0.0010050358	0.0021183039
0.9920945847	0.99437673	0.9878168433	0.0025457983	0.0046027613	0.0067309843
0.9920945847	0.9928419074	0.9855915580	0.0009662331	0.0010050358	0.0021183039
0.4938757309	0.3952703107	0.344272794	0.0025457983	0.0046027613	0.0067309843
0.4938757309	0.5560980497	0.5267569747	0.0009662331	0.0010050358	0.0021183039
0.6612819635	0.9354861550	0.8502024607	0.0025457983	0.0046027613	0.0067309843
0.6612819635	0.441980397	0.4381970547	0.0009662331	0.0010050358	0.0021183039
0.9015736230	0.9342457097	0.8491108309	0.0025457983	0.0046027613	0.0067309843
0.9015736230	0.8574249267	0.8144557267	0.0009662331	0.0010050358	0.0021183039
0.1785752658	0.0972102870	0.0905659135	0.0025457983	0.0046027613	0.0067309843
0.1785752658	0.1173449494	0.0883745557	0.0009662331	0.0010050358	0.0021183039
0.0310105124	0.0055514683	0.0129927639	0.0025457983	0.0046027613	0.0067309843
0.0310105124	0.0573888187	0.038656969	0.0009662331	0.0010050358	0.0021183039
0.1270910547	0.1502729235	0.1344395431	0.0025457983	0.0046027613	0.0067309843
0.1270910547	0.9284125473	0.9110427301	0.0009662331	0.0010050358	0.0021183039
0.8858584489	0.905019366	0.8257789864	0.0025457983	0.0046027613	0.0067309843
0.8858584489	0.81725553	0.7860524661	0.0009662331	0.0010050358	0.0021183039
0.4820055547	0.3815927759	0.3323168335	0.0025457983	0.0046027613	0.0067309843
0.4820055547	0.6809589498	0.639979555	0.0009662331	0.0010050358	0.0021183039
0.6459403269	0.8513460428	0.7420392479	0.0025457983	0.0046027613	0.0067309843
0.6459403269	0.4269258145	0.4182132544	0.0009662331	0.0010050358	0.0021183039
0.7813572425	0.7780354467	0.6788815047	0.0025457983	0.0046027613	0.0067309843
0.7813572425	0.6528594585	0.6115388163	0.0009662331	0.0010050358	0.0021183039
0.5332259587	0.4561957355	0.3843478248	0.0025457983	0.0046027613	0.0067309843
0.5332259587	0.3068085887	0.2852357680	0.0009662331	0.0010050358	0.0021183039
0.6901424240	0.6390535888	0.5624629797	0.0025457983	0.0046027613	0.0067309843
0.6901424240	0.4774609837	0.4776166915	0.0009662331	0.0010050358	0.0021183039
0.6055076337	0.5490404440	0.4625984605	0.0025457983	0.0046027613	0.0067309843
0.6055076337	0.8273217085	0.7885315763	0.0009662331	0.0010050358	0.0021183039
0.4122800413	0.3206839277	0.2669794127	0.0025457983	0.0046027613	0.0067309843
0.4122800413	0.2205685084	0.1705972997	0.0009662331	0.0010050358	0.0021183039
0.0561926728	0.0331710359	0.0389106968	0.0025457983	0.0046027613	0.0067309843
0.0561926728	0.6197972715	0.5894732727	0.0009662331	0.0010050358	0.0021183039
0.5780508957	0.5005497443	0.4319194147	0.0025457983	0.0046027613	0.0067309843
0.5780508957	0.3369444369	0.3348683678	0.0009662331	0.0010050358	0.0021183039

0.599207971	0.876420784	0.792172250	0.002545798	0.004602761	0.006730984
0.599207971	0.815578949	0.785947501	0.000966233	0.001005035	0.002118303
0.122940899	0.242285066	0.211506211	0.002545798	0.004602761	0.006730984
0.122940899	0.752794924	0.732101899	0.000966233	0.001005035	0.002118303
0.941144780	0.956470525	0.908199476	0.002545798	0.004602761	0.006730984
0.941144780	0.906735188	0.88698176	0.000966233	0.001005035	0.002118303
0.274268236	0.170274593	0.155042936	0.002545798	0.004602761	0.006730984
0.274268236	0.103536185	0.075644182	0.000966233	0.001005035	0.002118303
0.072911420	0.024468975	0.032380940	0.002545798	0.004602761	0.006730984
0.072911420	0.920284543	0.901721086	0.000966233	0.001005035	0.002118303
0.142154329	0.630621371	0.556897850	0.002545798	0.004602761	0.006730984
0.142154329	0.245856178	0.217997917	0.000966233	0.001005035	0.002118303
0.868844344	0.882224635	0.800852478	0.002545798	0.004602761	0.006730984
0.868844344	0.778702162	0.756498048	0.000966233	0.001005035	0.002118303
0.372565146	0.614675021	0.538354988	0.002545798	0.004602761	0.006730984
0.372565146	0.188973160	0.139395806	0.000966233	0.001005035	0.002118303
0.067994803	0.021639117	0.029992623	0.002545798	0.004602761	0.006730984
0.067994803	0.094436156	0.065070815	0.000966233	0.001005035	0.002118303
0.232874906	0.177317614	0.162269486	0.002545798	0.004602761	0.006730984
0.232874906	0.602683443	0.580487195	0.000966233	0.001005035	0.002118303
0.518286848	0.427883067	0.368651516	0.002545798	0.004602761	0.006730984
0.518286848	0.288468010	0.269388459	0.000966233	0.001005035	0.002118303
0.000672482	0.142830455	0.126529582	0.002545798	0.004602761	0.006730984
0.000672482	0.161940729	0.117779316	0.000966233	0.001005035	0.002118303
0.740262242	0.725123773	0.625627019	0.002545798	0.004602761	0.006730984
0.740262242	0.580695962	0.54929786	0.000966233	0.001005035	0.002118303
0.063557629	0.081233121	0.079253684	0.002545798	0.004602761	0.006730984
0.063557629	0.015563370	0.011556548	0.000966233	0.001005035	0.002118303
0.831397497	0.868780618	0.769518681	0.002545798	0.004602761	0.006730984
0.831397497	0.728748578	0.692631504	0.000966233	0.001005035	0.002118303
0.537535618	0.465363910	0.388586721	0.002545798	0.004602761	0.006730984
0.537535618	0.363606393	0.355594464	0.000966233	0.001005035	0.002118303
0.148495475	0.497912961	0.430357049	0.002545798	0.004602761	0.006730984
0.148495475	0.951795549	0.931680112	0.000966233	0.001005035	0.002118303
0.360357043	0.260283098	0.222405068	0.002545798	0.004602761	0.006730984
0.360357043	0.198906047	0.146266898	0.000966233	0.001005035	0.002118303
0.121457029	0.312231744	0.261505053	0.002545798	0.004602761	0.006730984
0.121457029	0.020680629	0.014933791	0.000966233	0.001005035	0.002118303
0.798171907	0.806503549	0.702167027	0.002545798	0.004602761	0.006730984
0.798171907	0.676018031	0.638544185	0.000966233	0.001005035	0.002118303
0.382675174	0.287095935	0.241099837	0.002545798	0.004602761	0.006730984
0.382675174	0.200670512	0.147005663	0.000966233	0.001005035	0.002118303
0.163698575	0.136213138	0.121472884	0.002545798	0.004602761	0.006730984
0.163698575	0.043213785	0.027043815	0.000966233	0.001005035	0.002118303
0.516352843	0.418032952	0.366686198	0.002545798	0.004602761	0.006730984
0.516352843	0.277155513	0.267192678	0.000966233	0.001005035	0.002118303

0.710291384	0.773568805	0.670294522	0.002545798	0.004602761	0.006730984
0.710291384	0.750609690	0.730561250	0.000966233	0.001005035	0.002118303
0.221715373	0.555051026	0.470112454	0.002545798	0.004602761	0.006730984
0.221715373	0.106790276	0.078150865	0.000966233	0.001005035	0.002118303
0.388613361	0.295792734	0.246364331	0.002545798	0.004602761	0.006730984
0.388613361	0.255612825	0.222479313	0.000966233	0.001005035	0.002118303
0.727968438	0.698999691	0.609343924	0.002545798	0.004602761	0.006730984
0.727968438	0.561412668	0.531084259	0.000966233	0.001005035	0.002118303
0.335618056	0.226874397	0.201946783	0.002545798	0.004602761	0.006730984
0.335618056	0.148468403	0.113086471	0.000966233	0.001005035	0.002118303
0.787993517	0.791966355	0.688185520	0.002545798	0.004602761	0.006730984
0.787993517	0.660928357	0.622144365	0.000966233	0.001005035	0.002118303
0.890101361	0.912641297	0.831852412	0.002545798	0.004602761	0.006730984
0.890101361	0.880861297	0.827985841	0.000966233	0.001005035	0.002118303
0.881691204	0.893782208	0.819386837	0.002545798	0.004602761	0.006730984
0.881691204	0.800197764	0.779031934	0.000966233	0.001005035	0.002118303
0.628499596	0.565594257	0.488437411	0.002545798	0.004602761	0.006730984
0.628499596	0.396323044	0.395976701	0.000966233	0.001005035	0.002118303
0.367490669	0.757894544	0.661687587	0.002545798	0.004602761	0.006730984
0.367490669	0.183463218	0.135575673	0.000966233	0.001005035	0.002118303
0.413165605	0.683286249	0.596560017	0.002545798	0.004602761	0.006730984
0.413165605	0.899560969	0.848168845	0.000966233	0.001005035	0.002118303
0.754862438	0.739697606	0.644351114	0.002545798	0.004602761	0.006730984
0.754862438	0.689613879	0.642331510	0.000966233	0.001005035	0.002118303
0.102836125	0.051340148	0.057303737	0.002545798	0.004602761	0.006730984
0.102836125	0.037378366	0.023792875	0.000966233	0.001005035	0.002118303
0.019021605	0.013169144	0.022314963	0.002545798	0.004602761	0.006730984
0.019021605	0.038282182	0.024497714	0.000966233	0.001005035	0.002118303
0.558051330	0.483068575	0.410252573	0.002545798	0.004602761	0.006730984
0.558051330	0.324454514	0.312294385	0.000966233	0.001005035	0.002118303
0.679717763	0.622540686	0.549325741	0.002545798	0.004602761	0.006730984
0.679717763	0.469182482	0.462975937	0.000966233	0.001005035	0.002118303
0.730878565	0.705565080	0.612863944	0.002545798	0.004602761	0.006730984
0.730878565	0.564910637	0.535027822	0.000966233	0.001005035	0.002118303
0.516997774	0.425856249	0.367670527	0.002545798	0.004602761	0.006730984
0.516997774	0.349219215	0.342119458	0.000966233	0.001005035	0.002118303
0.969080410	0.981013007	0.952018545	0.002545798	0.004602761	0.006730984
0.969080410	0.961186635	0.940431521	0.000966233	0.001005035	0.002118303
0.516081854	0.414051123	0.366382111	0.002545798	0.004602761	0.006730984
0.516081854	0.275100155	0.266992131	0.000966233	0.001005035	0.002118303
0.341391828	0.670746471	0.585882919	0.002545798	0.004602761	0.006730984
0.341391828	0.161820050	0.117229876	0.000966233	0.001005035	0.002118303
0.045487606	0.697290576	0.609095154	0.002545798	0.004602761	0.006730984
0.045487606	0.595072786	0.578677458	0.000966233	0.001005035	0.002118303
0.723224089	0.688027298	0.603176748	0.002545798	0.004602761	0.006730984
0.723224089	0.549935076	0.524230911	0.000966233	0.001005035	0.002118303

0.533729676	0.460026964	0.384784228	0.002545798	0.004602761	0.006730984
0.533729676	0.916317919	0.893978438	0.000966233	0.001005035	0.002118303
0.958167408	0.972548591	0.935071110	0.002545798	0.004602761	0.006730984
0.958167408	0.942205682	0.919922593	0.000966233	0.001005035	0.002118303
0.529375213	0.448882669	0.380272814	0.002545798	0.004602761	0.006730984
0.529375213	0.301039178	0.280967617	0.000966233	0.001005035	0.002118303
0.047003166	0.114497757	0.105578219	0.002545798	0.004602761	0.006730984
0.047003166	0.119208807	0.091764222	0.000966233	0.001005035	0.002118303
0.402679719	0.309939986	0.258759965	0.002545798	0.004602761	0.006730984
0.402679719	0.640442289	0.595674327	0.000966233	0.001005035	0.002118303
0.329989722	0.263763367	0.226219285	0.002545798	0.004602761	0.006730984
0.329989722	0.508544976	0.496457952	0.000966233	0.001005035	0.002118303
0.176235745	0.105136671	0.097178026	0.002545798	0.004602761	0.006730984
0.176235745	0.983511779	0.972098841	0.000966233	0.001005035	0.002118303
0.523337585	0.433784487	0.373685013	0.002545798	0.004602761	0.006730984
0.523337585	0.291250129	0.274461777	0.000966233	0.001005035	0.002118303
0.171126848	0.131569925	0.118493130	0.002545798	0.004602761	0.006730984
0.171126848	0.433621990	0.437562543	0.000966233	0.001005035	0.002118303
0.310507020	0.776057238	0.675304296	0.002545798	0.004602761	0.006730984
0.310507020	0.740044626	0.695775402	0.000966233	0.001005035	0.002118303
0.480086230	0.574965416	0.496679522	0.002545798	0.004602761	0.006730984
0.480086230	0.804978230	0.783163209	0.000966233	0.001005035	0.002118303
0.768350783	0.884592421	0.801136801	0.002545798	0.004602761	0.006730984
0.768350783	0.628094553	0.591450237	0.000966233	0.001005035	0.002118303
0.170788884	0.089145536	0.085902205	0.002545798	0.004602761	0.006730984
0.170788884	0.890176773	0.844826713	0.000966233	0.001005035	0.002118303
0.076968196	0.046297243	0.052403802	0.002545798	0.004602761	0.006730984
0.076968196	0.497688628	0.494726257	0.000966233	0.001005035	0.002118303
0.901350125	0.925946848	0.848496122	0.002545798	0.004602761	0.006730984
0.901350125	0.847618900	0.813729658	0.000966233	0.001005035	0.002118303
0.203560589	0.167677014	0.153938123	0.002545798	0.004602761	0.006730984
0.203560589	0.062884208	0.041665528	0.000966233	0.001005035	0.002118303
0.225306537	0.745084194	0.650739150	0.002545798	0.004602761	0.006730984
0.225306537	0.975899063	0.952108518	0.000966233	0.001005035	0.002118303
0.027304783	0.177496030	0.162469471	0.002545798	0.004602761	0.006730984
0.027304783	0.039520083	0.024729024	0.000966233	0.001005035	0.002118303
0.974159044	0.986825039	0.959676167	0.002545798	0.004602761	0.006730984
0.974159044	0.973996521	0.950923376	0.000966233	0.001005035	0.002118303
0.591535064	0.521352164	0.447059009	0.002545798	0.004602761	0.006730984
0.591535064	0.356589305	0.350762684	0.000966233	0.001005035	0.002118303
0.40771711	0.314398521	0.262950128	0.002545798	0.004602761	0.006730984
0.40771711	0.216700985	0.166844980	0.000966233	0.001005035	0.002118303
0.294528962	0.254823003	0.219375586	0.002545798	0.004602761	0.006730984
0.294528962	0.333497650	0.327887337	0.000966233	0.001005035	0.002118303
0.691611478	0.645648965	0.564261295	0.002545798	0.004602761	0.006730984
0.691611478	0.785350125	0.761630767	0.000966233	0.001005035	0.002118303

0.467429425	0.364632296	0.318145542	0.002545798	0.004602761	0.006730984
0.467429425	0.249303779	0.219055838	0.000966233	0.001005035	0.002118303
0.600797878	0.541705597	0.457378821	0.002545798	0.004602761	0.006730984
0.600797878	0.382840429	0.362096749	0.000966233	0.001005035	0.002118303
0.327702954	0.282869718	0.237763222	0.002545798	0.004602761	0.006730984
0.327702954	0.141452444	0.108040577	0.000966233	0.001005035	0.002118303
0.503610691	0.403634252	0.353831633	0.002545798	0.004602761	0.006730984
0.503610691	0.270318079	0.254210406	0.000966233	0.001005035	0.002118303
0.151475276	0.075557809	0.074377942	0.002545798	0.004602761	0.006730984
0.151475276	0.490266350	0.489676907	0.000966233	0.001005035	0.002118303
0.219377421	0.127630102	0.116769479	0.002545798	0.004602761	0.006730984
0.219377421	0.072079324	0.048465706	0.000966233	0.001005035	0.002118303
0.597728507	0.918149295	0.838600664	0.002545798	0.004602761	0.006730984
0.597728507	0.539229821	0.508590901	0.000966233	0.001005035	0.002118303
0.133860407	0.062169615	0.064343714	0.002545798	0.004602761	0.006730984
0.133860407	0.025243475	0.018098536	0.000966233	0.001005035	0.002118303
0.307019588	0.195898813	0.179529876	0.002545798	0.004602761	0.006730984
0.307019588	0.123909842	0.094841574	0.000966233	0.001005035	0.002118303
0.818307529	0.834162466	0.729282018	0.002545798	0.004602761	0.006730984
0.818307529	0.717027433	0.671118472	0.000966233	0.001005035	0.002118303
0.020232682	0.5046381	0.433833448	0.002545798	0.004602761	0.006730984
0.020232682	0.837103274	0.798456911	0.000966233	0.001005035	0.002118303
0.348976472	0.244022015	0.212746118	0.002545798	0.004602761	0.006730984
0.348976472	0.172231518	0.122274211	0.000966233	0.001005035	0.002118303
0.773636392	0.772816278	0.669304189	0.002545798	0.004602761	0.006730984
0.773636392	0.764885555	0.734687782	0.000966233	0.001005035	0.002118303
0.337922107	0.230327158	0.203709594	0.002545798	0.004602761	0.006730984
0.337922107	0.153441102	0.114559807	0.000966233	0.001005035	0.002118303
0.112463930	0.213256600	0.193045279	0.002545798	0.004602761	0.006730984
0.112463930	0.409705204	0.413604593	0.000966233	0.001005035	0.002118303
0.151210469	0.302899703	0.254016054	0.002545798	0.004602761	0.006730984
0.151210469	0.524205534	0.504951145	0.000966233	0.001005035	0.002118303
0.092946464	0.084768322	0.081759972	0.002545798	0.004602761	0.006730984
0.092946464	0.430222668	0.437308642	0.000966233	0.001005035	0.002118303
0.061458660	0.017545607	0.026850864	0.002545798	0.004602761	0.006730984
0.061458660	0.006010513	0.003895622	0.000966233	0.001005035	0.002118303
0.353826980	0.774191210	0.671529915	0.002545798	0.004602761	0.006730984
0.353826980	0.884187340	0.836502164	0.000966233	0.001005035	0.002118303
0.057771899	0.360982993	0.313397711	0.002545798	0.004602761	0.006730984
0.057771899	0.866789987	0.822302668	0.000966233	0.001005035	0.002118303
0.375585148	0.279082892	0.235071758	0.002545798	0.004602761	0.006730984
0.375585148	0.193502502	0.141605363	0.000966233	0.001005035	0.002118303
0.056218094	0.615549501	0.486919703	0.004666501	0.006313121	0.007603344
0.056218094	0.699404478	0.678536143	0.006886504	0.008698764	0.010396563
0.103770533	0.735031024	0.601744647	0.004666501	0.006313121	0.007603344
0.103770533	0.592277619	0.573732475	0.006886504	0.008698764	0.010396563

0.569218978	0.519424606	0.412303658	0.004666501	0.006313121	0.007603344
0.569218978	0.787165015	0.749731257	0.006886504	0.008698764	0.010396563
0.987109513	0.977220493	0.883297436	0.004666501	0.006313121	0.007603344
0.987109513	0.840686716	0.816252145	0.006886504	0.008698764	0.010396563
0.84462027	0.994780391	0.950081455	0.004666501	0.006313121	0.007603344
0.84462027	0.720980274	0.692123595	0.006886504	0.008698764	0.010396563
0.378835192	0.264789918	0.238488049	0.004666501	0.006313121	0.007603344
0.378835192	0.207422844	0.207680671	0.006886504	0.008698764	0.010396563
0.062779579	0.533390824	0.422947694	0.004666501	0.006313121	0.007603344
0.062779579	0.523310793	0.499563707	0.006886504	0.008698764	0.010396563
0.051570075	0.059652013	0.076585277	0.004666501	0.006313121	0.007603344
0.051570075	0.841383308	0.817518095	0.006886504	0.008698764	0.010396563
0.000427224	0.177772252	0.175690677	0.004666501	0.006313121	0.007603344
0.000427224	0.797131623	0.776974000	0.006886504	0.008698764	0.010396563
0.513197594	0.508289948	0.403762756	0.004666501	0.006313121	0.007603344
0.513197594	0.542278056	0.523438030	0.006886504	0.008698764	0.010396563
0.364850832	0.404345635	0.333313355	0.004666501	0.006313121	0.007603344
0.364850832	0.723641072	0.692445276	0.006886504	0.008698764	0.010396563
0.157479050	0.299035321	0.261779437	0.004666501	0.006313121	0.007603344
0.157479050	0.085848672	0.113709695	0.006886504	0.008698764	0.010396563
0.660936839	0.667348131	0.535477603	0.004666501	0.006313121	0.007603344
0.660936839	0.760177	0.723081541	0.006886504	0.008698764	0.010396563
0.783881503	0.601135297	0.475115236	0.004666501	0.006313121	0.007603344
0.783881503	0.428135658	0.387318510	0.006886504	0.008698764	0.010396563
0.078797338	0.450586740	0.364538288	0.004666501	0.006313121	0.007603344
0.078797338	0.568866988	0.551249988	0.006886504	0.008698764	0.010396563
0.725655829	0.943427237	0.816429128	0.004666501	0.006313121	0.007603344
0.725655829	0.662080193	0.629446337	0.006886504	0.008698764	0.010396563
0.204701938	0.898350861	0.761302872	0.004666501	0.006313121	0.007603344
0.204701938	0.556724247	0.541726292	0.006886504	0.008698764	0.010396563
0.867764637	0.854520481	0.718373141	0.004666501	0.006313121	0.007603344
0.867764637	0.469634228	0.442313783	0.006886504	0.008698764	0.010396563
0.666732561	0.622481041	0.492903796	0.004666501	0.006313121	0.007603344
0.666732561	0.428018430	0.387159095	0.006886504	0.008698764	0.010396563
0.118035937	0.217408821	0.205231797	0.004666501	0.006313121	0.007603344
0.118035937	0.679154842	0.645297363	0.006886504	0.008698764	0.010396563
0.316699345	0.945905777	0.820430698	0.004666501	0.006313121	0.007603344
0.316699345	0.176530270	0.185598315	0.006886504	0.008698764	0.010396563
0.452092144	0.757214760	0.622755133	0.004666501	0.006313121	0.007603344
0.452092144	0.674156144	0.638817915	0.006886504	0.008698764	0.010396563
0.826505148	0.644668699	0.513291772	0.004666501	0.006313121	0.007603344
0.826505148	0.475300316	0.451957910	0.006886504	0.008698764	0.010396563
0.375182317	0.157126093	0.158264520	0.004666501	0.006313121	0.007603344
0.375182317	0.158183779	0.174301199	0.006886504	0.008698764	0.010396563
0.101089246	0.209961091	0.199806112	0.004666501	0.006313121	0.007603344
0.101089246	0.959211786	0.936437259	0.006886504	0.008698764	0.010396563

0.952631697	0.947247687	0.822632472	0.004666501	0.006313121	0.007603344
0.952631697	0.636105448	0.616805166	0.006886504	0.008698764	0.010396563
0.595870244	0.416070537	0.341436700	0.004666501	0.006313121	0.007603344
0.595870244	0.769963385	0.735586853	0.006886504	0.008698764	0.010396563
0.742792142	0.944087284	0.817491003	0.004666501	0.006313121	0.007603344
0.742792142	0.45046194	0.418483724	0.006886504	0.008698764	0.010396563
0.722491981	0.531681755	0.421652631	0.004666501	0.006313121	0.007603344
0.722491981	0.389994693	0.352511525	0.006886504	0.008698764	0.010396563
0.257622030	0.351627490	0.296795187	0.004666501	0.006313121	0.007603344
0.257622030	0.308931024	0.279368021	0.006886504	0.008698764	0.010396563
0.640830014	0.662735142	0.531125247	0.004666501	0.006313121	0.007603344
0.640830014	0.560587158	0.542895748	0.006886504	0.008698764	0.010396563
0.305065653	0.318702028	0.274701342	0.004666501	0.006313121	0.007603344
0.305065653	0.883794780	0.847411915	0.006886504	0.008698764	0.010396563
0.081565957	0.028455517	0.047455452	0.004666501	0.006313121	0.007603344
0.081565957	0.118704149	0.143865347	0.006886504	0.008698764	0.010396563
0.035002862	0.052063362	0.070273398	0.004666501	0.006313121	0.007603344
0.035002862	0.728077647	0.695836057	0.006886504	0.008698764	0.010396563
0.682644793	0.559106720	0.442202797	0.004666501	0.006313121	0.007603344
0.682644793	0.303341849	0.275571868	0.006886504	0.008698764	0.010396563
0.201520669	0.895819084	0.758832302	0.004666501	0.006313121	0.007603344
0.201520669	0.875548347	0.845405916	0.006886504	0.008698764	0.010396563
0.574437007	0.563404292	0.445448096	0.004666501	0.006313121	0.007603344
0.574437007	0.829291882	0.806058698	0.006886504	0.008698764	0.010396563
0.093242836	0.145347703	0.147761459	0.004666501	0.006313121	0.007603344
0.093242836	0.203530914	0.205337939	0.006886504	0.008698764	0.010396563
0.045400736	0.077176003	0.090169965	0.004666501	0.006313121	0.007603344
0.045400736	0.061989491	0.096780274	0.006886504	0.008698764	0.010396563
0.800413907	0.624670182	0.494822075	0.004666501	0.006313121	0.007603344
0.800413907	0.956777773	0.930511081	0.006886504	0.008698764	0.010396563
0.099506579	0.814350166	0.680164633	0.004666501	0.006313121	0.007603344
0.099506579	0.405607194	0.366805601	0.006886504	0.008698764	0.010396563
0.622106997	0.558130265	0.441466873	0.004666501	0.006313121	0.007603344
0.622106997	0.693717092	0.662124560	0.006886504	0.008698764	0.010396563
0.065031367	0.687768006	0.554885788	0.004666501	0.006313121	0.007603344
0.065031367	0.125957437	0.150225705	0.006886504	0.008698764	0.010396563
0.520647395	0.290194464	0.255867268	0.004666501	0.006313121	0.007603344
0.520647395	0.289830022	0.261624330	0.006886504	0.008698764	0.010396563
0.574403718	0.490537034	0.391202455	0.004666501	0.006313121	0.007603344
0.574403718	0.718328132	0.68911829	0.006886504	0.008698764	0.010396563
0.919021735	0.792382876	0.658288443	0.004666501	0.006313121	0.007603344
0.919021735	0.512881185	0.488108896	0.006886504	0.008698764	0.010396563
0.045849552	0.273824360	0.24482221	0.004666501	0.006313121	0.007603344
0.045849552	0.272708688	0.252633810	0.006886504	0.008698764	0.010396563
0.402604926	0.692948718	0.559991430	0.004666501	0.006313121	0.007603344
0.402604926	0.489306130	0.471739649	0.006886504	0.008698764	0.010396563

0.189190269	0.178241139	0.176055913	0.004666501	0.006313121	0.007603344
0.189190269	0.046931303	0.078530294	0.006886504	0.008698764	0.010396563
0.060348027	0.737970696	0.604597980	0.004666501	0.006313121	0.007603344
0.060348027	0.500356622	0.480821661	0.006886504	0.008698764	0.010396563
0.043167522	0.079024079	0.091558086	0.004666501	0.006313121	0.007603344
0.043167522	0.518452186	0.499047839	0.006886504	0.008698764	0.010396563
0.118008730	0.680411397	0.547761272	0.004666501	0.006313121	0.007603344
0.118008730	0.975505614	0.951140183	0.006886504	0.008698764	0.010396563
0.072911128	0.180188649	0.17756411	0.004666501	0.006313121	0.007603344
0.072911128	0.317622285	0.287169422	0.006886504	0.008698764	0.010396563
0.513905139	0.588169690	0.464584720	0.004666501	0.006313121	0.007603344
0.513905139	0.116829053	0.142467977	0.006886504	0.008698764	0.010396563
0.477352612	0.635892912	0.505017352	0.004666501	0.006313121	0.007603344
0.477352612	0.602280057	0.579334670	0.006886504	0.008698764	0.010396563
0.144516640	0.086373265	0.096976255	0.004666501	0.006313121	0.007603344
0.144516640	0.095380684	0.123115767	0.006886504	0.008698764	0.010396563
0.967717839	0.891334967	0.754428575	0.004666501	0.006313121	0.007603344
0.967717839	0.454902001	0.425294842	0.006886504	0.008698764	0.010396563
0.645430227	0.500878137	0.398402056	0.004666501	0.006313121	0.007603344
0.645430227	0.198369725	0.200917821	0.006886504	0.008698764	0.010396563
0.573811542	0.807648664	0.673599897	0.004666501	0.006313121	0.007603344
0.573811542	0.822113759	0.804460648	0.006886504	0.008698764	0.010396563
0.546535992	0.795893988	0.661929335	0.004666501	0.006313121	0.007603344
0.546535992	0.318092895	0.287222199	0.006886504	0.008698764	0.010396563
0.901837743	0.816838879	0.682598513	0.004666501	0.006313121	0.007603344
0.901837743	0.623081063	0.608685522	0.006886504	0.008698764	0.010396563
0.614676557	0.473451095	0.379439159	0.004666501	0.006313121	0.007603344
0.614676557	0.439403693	0.402691608	0.006886504	0.008698764	0.010396563
0.099408238	0.045517147	0.064796217	0.004666501	0.006313121	0.007603344
0.099408238	0.040153699	0.071885525	0.006886504	0.008698764	0.010396563
0.149301120	0.279049655	0.248388156	0.004666501	0.006313121	0.007603344
0.149301120	0.364663324	0.325135193	0.006886504	0.008698764	0.010396563
0.405036344	0.386542426	0.320813711	0.004666501	0.006313121	0.007603344
0.405036344	0.910466696	0.876248660	0.006886504	0.008698764	0.010396563
0.242792883	0.183177458	0.179858979	0.004666501	0.006313121	0.007603344
0.242792883	0.737486929	0.700453653	0.006886504	0.008698764	0.010396563
0.472505839	0.245465911	0.225259925	0.004666501	0.006313121	0.007603344
0.472505839	0.324258415	0.291652556	0.006886504	0.008698764	0.010396563
0.252485633	0.868980546	0.732089440	0.004666501	0.006313121	0.007603344
0.252485633	0.092475469	0.121026322	0.006886504	0.008698764	0.010396563
0.330938789	0.797491267	0.663534908	0.004666501	0.006313121	0.007603344
0.330938789	0.933724095	0.891207090	0.006886504	0.008698764	0.010396563
0.592204258	0.580236663	0.458181217	0.004666501	0.006313121	0.007603344
0.592204258	0.776858712	0.737193642	0.006886504	0.008698764	0.010396563
0.002649290	0.042269453	0.061905977	0.004666501	0.006313121	0.007603344
0.002649290	0.067552497	0.1014672	0.006886504	0.008698764	0.010396563

0.485572749	0.314345012	0.271714859	0.004666501	0.006313121	0.007603344
0.485572749	0.179323181	0.188372418	0.006886504	0.008698764	0.010396563
0.126632678	0.089015521	0.098955981	0.004666501	0.006313121	0.007603344
0.126632678	0.234266768	0.228856268	0.006886504	0.008698764	0.010396563
0.733434877	0.621631281	0.492138826	0.004666501	0.006313121	0.007603344
0.733434877	0.425580380	0.383893527	0.006886504	0.008698764	0.010396563
0.513269629	0.274229914	0.245098999	0.004666501	0.006313121	0.007603344
0.513269629	0.156523670	0.173970907	0.006886504	0.008698764	0.010396563
0.388217519	0.440386786	0.357715340	0.004666501	0.006313121	0.007603344
0.388217519	0.510837875	0.487176346	0.006886504	0.008698764	0.010396563
0.122305024	0.825161672	0.690434429	0.004666501	0.006313121	0.007603344
0.122305024	0.278699882	0.255257379	0.006886504	0.008698764	0.010396563
0.286275373	0.147761494	0.149929348	0.004666501	0.006313121	0.007603344
0.286275373	0.086611984	0.114409690	0.006886504	0.008698764	0.010396563
0.431348976	0.842430004	0.706707503	0.004666501	0.006313121	0.007603344
0.431348976	0.306457988	0.278918682	0.006886504	0.008698764	0.010396563
0.006474774	0.888101376	0.751283614	0.004666501	0.006313121	0.007603344
0.006474774	0.707231576	0.687262465	0.006886504	0.008698764	0.010396563
0.741714521	0.566348315	0.447654573	0.004666501	0.006313121	0.007603344
0.741714521	0.515593364	0.493559907	0.006886504	0.008698764	0.010396563
0.180294765	0.235848643	0.218486856	0.004666501	0.006313121	0.007603344
0.180294765	0.943688772	0.924013594	0.006886504	0.008698764	0.010396563
0.429589791	0.937247560	0.807103629	0.004666501	0.006313121	0.007603344
0.429589791	0.862060788	0.837723597	0.006886504	0.008698764	0.010396563
0.168465981	0.239056341	0.220764219	0.004666501	0.006313121	0.007603344
0.168465981	0.109518517	0.136263921	0.006886504	0.008698764	0.010396563
0.350727633	0.302866985	0.264253043	0.004666501	0.006313121	0.007603344
0.350727633	0.237861046	0.230964030	0.006886504	0.008698764	0.010396563
0.984226215	0.932977069	0.801067962	0.004666501	0.006313121	0.007603344
0.984226215	0.889113729	0.851087916	0.006886504	0.008698764	0.010396563
0.227113033	0.274197269	0.245076587	0.004666501	0.006313121	0.007603344
0.227113033	0.644530102	0.620714968	0.006886504	0.008698764	0.010396563
0.117412304	0.104213950	0.111491714	0.004666501	0.006313121	0.007603344
0.117412304	0.992091531	0.979709197	0.006886504	0.008698764	0.010396563
0.517075258	0.286276107	0.253279451	0.004666501	0.006313121	0.007603344
0.517075258	0.115681088	0.141190850	0.006886504	0.008698764	0.010396563
0.133676002	0.155230164	0.156616904	0.004666501	0.006313121	0.007603344
0.133676002	0.583500232	0.571339458	0.006886504	0.008698764	0.010396563
0.253041086	0.906637297	0.769772592	0.004666501	0.006313121	0.007603344
0.253041086	0.806149561	0.778172938	0.006886504	0.008698764	0.010396563
0.524140447	0.705075824	0.572327378	0.004666501	0.006313121	0.007603344
0.524140447	0.870794161	0.843760078	0.006886504	0.008698764	0.010396563
0.353169104	0.871426610	0.734550208	0.004666501	0.006313121	0.007603344
0.353169104	0.256118715	0.241620587	0.006886504	0.008698764	0.010396563
0.569072330	0.372669796	0.311149015	0.004666501	0.006313121	0.007603344
0.569072330	0.928030028	0.889195089	0.006886504	0.008698764	0.010396563

0.692128686	0.560378921	0.443163574	0.004666501	0.006313121	0.007603344
0.692128686	0.642422605	0.619958674	0.006886504	0.008698764	0.010396563
0.224042233	0.380257538	0.316375206	0.004666501	0.006313121	0.007603344
0.224042233	0.334214592	0.300788416	0.006886504	0.008698764	0.010396563
0.648273271	0.573444960	0.452947928	0.004666501	0.006313121	0.007603344
0.648273271	0.174671739	0.183784022	0.006886504	0.008698764	0.010396563
0.842555662	0.988351410	0.921016072	0.004666501	0.006313121	0.007603344
0.842555662	0.989632541	0.966292873	0.006886504	0.008698764	0.010396563
0.000225155	0.306155495	0.266377831	0.004666501	0.006313121	0.007603344
0.000225155	0.071843451	0.103819408	0.006886504	0.008698764	0.010396563
0.605309503	0.640156812	0.5090076	0.004666501	0.006313121	0.007603344
0.605309503	0.971638892	0.949866319	0.006886504	0.008698764	0.010396563
0.343570318	0.421305877	0.344988041	0.004666501	0.006313121	0.007603344
0.343570318	0.199478637	0.202010183	0.006886504	0.008698764	0.010396563
0.070002836	0.041185011	0.060800458	0.004666501	0.006313121	0.007603344
0.070002836	0.112818907	0.139132810	0.006886504	0.008698764	0.010396563
0.386395079	0.346611613	0.293464538	0.004666501	0.006313121	0.007603344
0.386395079	0.495047807	0.474309249	0.006886504	0.008698764	0.010396563
0.17235808	0.509854908	0.404923396	0.004666501	0.006313121	0.007603344
0.17235808	0.851294836	0.827290338	0.006886504	0.008698764	0.010396563
0.443549208	0.239154250	0.220833543	0.004666501	0.006313121	0.007603344
0.443549208	0.111308026	0.138022257	0.006886504	0.008698764	0.010396563
0.100789612	0.323129010	0.277755853	0.004666501	0.006313121	0.007603344
0.100789612	0.463890177	0.438535513	0.006886504	0.008698764	0.010396563
0.262611791	0.312883040	0.270744223	0.004666501	0.006313121	0.007603344
0.262611791	0.247791590	0.236350905	0.006886504	0.008698764	0.010396563
0.914282580	0.801871654	0.667883468	0.004666501	0.006313121	0.007603344
0.914282580	0.426500879	0.385119517	0.006886504	0.008698764	0.010396563
0.195450575	0.052667574	0.070773409	0.004666501	0.006313121	0.007603344
0.195450575	0.631691768	0.615222630	0.006886504	0.008698764	0.010396563
0.013563001	0.035923325	0.055405949	0.004666501	0.006313121	0.007603344
0.013563001	0.045448590	0.077687590	0.006886504	0.008698764	0.010396563
0.876493903	0.997485857	0.968843984	0.004666501	0.006313121	0.007603344
0.876493903	0.769366195	0.735515062	0.006886504	0.008698764	0.010396563
0.859846027	0.693907901	0.560973147	0.004666501	0.006313121	0.007603344
0.859846027	0.362103789	0.324234337	0.006886504	0.008698764	0.010396563
0.129849393	0.081481184	0.093379431	0.004666501	0.006313121	0.007603344
0.129849393	0.186051672	0.194173431	0.006886504	0.008698764	0.010396563
0.600484483	0.450974653	0.364787354	0.004666501	0.006313121	0.007603344
0.600484483	0.610462480	0.585516973	0.006886504	0.008698764	0.010396563
0.112290113	0.738582448	0.605162988	0.004666501	0.006313121	0.007603344
0.112290113	0.890699454	0.854961528	0.006886504	0.008698764	0.010396563
0.407932508	0.210068454	0.199886556	0.004666501	0.006313121	0.007603344
0.407932508	0.128216974	0.151365263	0.006886504	0.008698764	0.010396563
0.656218995	0.767664997	0.633030298	0.004666501	0.006313121	0.007603344
0.656218995	0.837124930	0.807592225	0.006886504	0.008698764	0.010396563

0.880226309	0.708098281	0.575443943	0.004666501	0.006313121	0.007603344
0.880226309	0.370376322	0.329120648	0.006886504	0.008698764	0.010396563
0.147193977	0.352281981	0.297235694	0.004666501	0.006313121	0.007603344
0.147193977	0.564551833	0.550807271	0.006886504	0.008698764	0.010396563
0.622330776	0.627812364	0.497646821	0.004666501	0.006313121	0.007603344
0.622330776	0.661030439	0.628254058	0.006886504	0.008698764	0.010396563
0.012435998	0.112020533	0.118481091	0.004666501	0.006313121	0.007603344
0.012435998	0.580185253	0.571068266	0.006886504	0.008698764	0.010396563
0.630928117	0.406525936	0.334865013	0.004666501	0.006313121	0.007603344
0.630928117	0.185010431	0.194057297	0.006886504	0.008698764	0.010396563
0.127102814	0.853939393	0.717824739	0.004666501	0.006313121	0.007603344
0.127102814	0.916361846	0.882567837	0.006886504	0.008698764	0.010396563
0.350597732	0.645995098	0.514586255	0.004666501	0.006313121	0.007603344
0.350597732	0.902314751	0.872652450	0.006886504	0.008698764	0.010396563
0.317793594	0.186703492	0.182480305	0.004666501	0.006313121	0.007603344
0.317793594	0.114711525	0.140059859	0.006886504	0.008698764	0.010396563

Species	Genus
Acetatifactor_muris	Acetatifactor
Acetatifactor_muris	Acetatifactor
Acetatifactor_SGB41546	Acetatifactor
Acetatifactor_SGB41546	Acetatifactor
Acutalibacter_muris	Acutalibacter
Acutalibacter_muris	Acutalibacter
Acutalibacter_sp_1XD8_36	Acutalibacter
Acutalibacter_sp_1XD8_36	Acutalibacter
Adlercreutzia_caecimuris	Adlercreutzia
Adlercreutzia_caecimuris	Adlercreutzia
Adlercreutzia_mucosicola	Adlercreutzia
Adlercreutzia_mucosicola	Adlercreutzia
Adlercreutzia_muris	Adlercreutzia
Adlercreutzia_muris	Adlercreutzia
Akkermansia_muciniphila	Akkermansia
Akkermansia_muciniphila	Akkermansia
Alistipes_sp_DSM_112343	Alistipes
Alistipes_sp_DSM_112343	Alistipes
Anaerotruncus_sp_1XD42_93	Anaerotruncus
Anaerotruncus_sp_1XD42_93	Anaerotruncus
Bacteria_unclassified_SGB102200	Bacteria_unclassified
Bacteria_unclassified_SGB102200	Bacteria_unclassified
Bacteria_unclassified_SGB41677	Bacteria_unclassified
Bacteria_unclassified_SGB41677	Bacteria_unclassified
Bacteria_unclassified_SGB43546	Bacteria_unclassified
Bacteria_unclassified_SGB43546	Bacteria_unclassified
bacterium_1XD42_54	Bacteria_unclassified
bacterium_1XD42_54	Bacteria_unclassified
bacterium_1XD42_76	Bacteria_unclassified
bacterium_1XD42_76	Bacteria_unclassified
bacterium_1xD8_48	Bacteria_unclassified
bacterium_1xD8_48	Bacteria_unclassified
Bacteroides_thetaiotaomicron	Bacteroides
Bacteroides_thetaiotaomicron	Bacteroides
berger_parker	
berger_parker	
Bifidobacterium_pseudolongum	Bifidobacterium
Bifidobacterium_pseudolongum	Bifidobacterium
Clostridia_bacterium	Clostridia_unclassified
Clostridia_bacterium	Clostridia_unclassified
Clostridiaceae_bacterium	Clostridiaceae_unclassified
Clostridiaceae_bacterium	Clostridiaceae_unclassified
Clostridiaceae_unclassified_SGB41663	Clostridiaceae_unclassified
Clostridiaceae_unclassified_SGB41663	Clostridiaceae_unclassified
Clostridiales_bacterium	Eubacteriales_unclassified
Clostridiales_bacterium	Eubacteriales_unclassified

Clostridium_cocleatum	Erysipelatoclostridium
Clostridium_cocleatum	Erysipelatoclostridium
Coriobacteriaceae_bacterium	Coriobacteriaceae_unclassified
Coriobacteriaceae_bacterium	Coriobacteriaceae_unclassified
Dorea_sp_5_2	Dorea
Dorea_sp_5_2	Dorea
Dubosiella_newyorkensis	Dubosiella
Dubosiella_newyorkensis	Dubosiella
Erysipelotrichales_bacterium	Erysipelotrichales_unclassified
Erysipelotrichales_bacterium	Erysipelotrichales_unclassified
Eubacteriaceae_bacterium	Eubacteriaceae_unclassified
Eubacteriaceae_bacterium	Eubacteriaceae_unclassified
Eubacteriaceae_unclassified_SGB94922	Eubacteriaceae_unclassified
Eubacteriaceae_unclassified_SGB94922	Eubacteriaceae_unclassified
GGB20149_SGB29430	GGB20149
GGB20149_SGB29430	GGB20149
GGB22635_SGB63107	GGB22635
GGB22635_SGB63107	GGB22635
GGB25041_SGB36960	GGB25041
GGB25041_SGB36960	GGB25041
GGB27876_SGB40310	GGB27876
GGB27876_SGB40310	GGB27876
GGB27878_SGB40312	GGB27878
GGB27878_SGB40312	GGB27878
GGB27918_SGB40356	GGB27918
GGB27918_SGB40356	GGB27918
GGB28382_SGB40962	GGB28382
GGB28382_SGB40962	GGB28382
GGB28399_SGB40980	GGB28399
GGB28399_SGB40980	GGB28399
GGB28411_SGB40993	GGB28411
GGB28411_SGB40993	GGB28411
GGB28415_SGB40997	GGB28415
GGB28415_SGB40997	GGB28415
GGB28430_SGB41013	GGB28430
GGB28430_SGB41013	GGB28430
GGB28439_SGB41022	GGB28439
GGB28439_SGB41022	GGB28439
GGB28778_SGB41431	GGB28778
GGB28778_SGB41431	GGB28778
GGB28784_SGB41437	GGB28784
GGB28784_SGB41437	GGB28784
GGB28792_SGB41445	GGB28792
GGB28792_SGB41445	GGB28792
GGB28798_SGB41451	GGB28798
GGB28798_SGB41451	GGB28798

GGB28802_SGB41455	GGB28802
GGB28802_SGB41455	GGB28802
GGB28818_SGB41473	GGB28818
GGB28818_SGB41473	GGB28818
GGB28828_SGB41484	GGB28828
GGB28828_SGB41484	GGB28828
GGB28851_SGB41518	GGB28851
GGB28851_SGB41518	GGB28851
GGB28859_SGB41528	GGB28859
GGB28859_SGB41528	GGB28859
GGB28864_SGB41535	GGB28864
GGB28864_SGB41535	GGB28864
GGB28869_SGB41543	GGB28869
GGB28869_SGB41543	GGB28869
GGB28883_SGB41564	GGB28883
GGB28883_SGB41564	GGB28883
GGB28892_SGB41573	GGB28892
GGB28892_SGB41573	GGB28892
GGB28893_SGB41574	GGB28893
GGB28893_SGB41574	GGB28893
GGB28898_SGB41580	GGB28898
GGB28898_SGB41580	GGB28898
GGB28904_SGB41597	GGB28904
GGB28904_SGB41597	GGB28904
GGB28916_SGB41612	GGB28916
GGB28916_SGB41612	GGB28916
GGB28924_SGB41621	GGB28924
GGB28924_SGB41621	GGB28924
GGB28926_SGB41624	GGB28926
GGB28926_SGB41624	GGB28926
GGB28927_SGB41625	GGB28927
GGB28927_SGB41625	GGB28927
GGB28934_SGB41635	GGB28934
GGB28934_SGB41635	GGB28934
GGB28946_SGB41652	GGB28946
GGB28946_SGB41652	GGB28946
GGB28949_SGB41655	GGB28949
GGB28949_SGB41655	GGB28949
GGB28949_SGB41656	GGB28949
GGB28949_SGB41656	GGB28949
GGB28950_SGB41657	GGB28950
GGB28950_SGB41657	GGB28950
GGB28951_SGB102295	GGB28951
GGB28951_SGB102295	GGB28951
GGB28951_SGB41658	GGB28951
GGB28951_SGB41658	GGB28951

GGB28954_SGB41662	GGB28954
GGB28954_SGB41662	GGB28954
GGB28956_SGB41665	GGB28956
GGB28956_SGB41665	GGB28956
GGB28960_SGB41669	GGB28960
GGB28960_SGB41669	GGB28960
GGB28967_SGB41678	GGB28967
GGB28967_SGB41678	GGB28967
GGB28991_SGB41705	GGB28991
GGB28991_SGB41705	GGB28991
GGB29002_SGB41718	GGB29002
GGB29002_SGB41718	GGB29002
GGB29003_SGB41719	GGB29003
GGB29003_SGB41719	GGB29003
GGB29011_SGB41731	GGB29011
GGB29011_SGB41731	GGB29011
GGB29531_SGB42317	GGB29531
GGB29531_SGB42317	GGB29531
GGB29685_SGB42494	GGB29685
GGB29685_SGB42494	GGB29685
GGB30141_SGB43066	GGB30141
GGB30141_SGB43066	GGB30141
GGB30286_SGB43248	GGB30286
GGB30286_SGB43248	GGB30286
GGB30303_SGB43268	GGB30303
GGB30303_SGB43268	GGB30303
GGB30413_SGB43452	GGB30413
GGB30413_SGB43452	GGB30413
GGB30454_SGB43514	GGB30454
GGB30454_SGB43514	GGB30454
GGB30455_SGB43519	GGB30455
GGB30455_SGB43519	GGB30455
GGB30461_SGB43527	GGB30461
GGB30461_SGB43527	GGB30461
GGB30461_SGB43530	GGB30461
GGB30461_SGB43530	GGB30461
GGB30463_SGB43537	GGB30463
GGB30463_SGB43537	GGB30463
GGB30473_SGB43557	GGB30473
GGB30473_SGB43557	GGB30473
GGB30475_SGB63182	GGB30475
GGB30475_SGB63182	GGB30475
GGB30861_SGB44083	GGB30861
GGB30861_SGB44083	GGB30861
GGB31312_SGB44628	GGB31312
GGB31312_SGB44628	GGB31312

GGB31438_SGB44768	GGB31438
GGB31438_SGB44768	GGB31438
GGB3171_SGB4185	GGB3171
GGB3171_SGB4185	GGB3171
GGB31823_SGB45199	GGB31823
GGB31823_SGB45199	GGB31823
GGB31853_SGB45233	GGB31853
GGB31853_SGB45233	GGB31853
GGB32371_SGB41694	GGB32371
GGB32371_SGB41694	GGB32371
GGB3793_SGB5158	GGB3793
GGB3793_SGB5158	GGB3793
GGB42598_SGB59794	GGB42598
GGB42598_SGB59794	GGB42598
GGB45656_SGB63370	GGB45656
GGB45656_SGB63370	GGB45656
GGB47127_SGB65054	GGB47127
GGB47127_SGB65054	GGB47127
GGB74395_SGB43521	GGB74395
GGB74395_SGB43521	GGB74395
GGB75053_SGB43494	GGB75053
GGB75053_SGB43494	GGB75053
GGB75109_SGB102238	GGB75109
GGB75109_SGB102238	GGB75109
GGB81440_SGB45230	GGB81440
GGB81440_SGB45230	GGB81440
Lachnospiraceae_bacterium	Lachnospiraceae_unclassified
Lachnospiraceae_bacterium	Lachnospiraceae_unclassified
Lachnospiraceae_bacterium_A2	Lachnospiraceae_unclassified
Lachnospiraceae_bacterium_A2	Lachnospiraceae_unclassified
Lachnospiraceae_bacterium_MD308	Lachnospiraceae_unclassified
Lachnospiraceae_bacterium_MD308	Lachnospiraceae_unclassified
Lachnospiraceae_bacterium_MD329	Lachnospiraceae_unclassified
Lachnospiraceae_bacterium_MD329	Lachnospiraceae_unclassified
Lachnospiraceae_unclassified_SGB41414	Lachnospiraceae_unclassified
Lachnospiraceae_unclassified_SGB41414	Lachnospiraceae_unclassified
Lachnospiraceae_unclassified_SGB41418	Lachnospiraceae_unclassified
Lachnospiraceae_unclassified_SGB41418	Lachnospiraceae_unclassified
Lachnospiraceae_unclassified_SGB41424	Lachnospiraceae_unclassified
Lachnospiraceae_unclassified_SGB41424	Lachnospiraceae_unclassified
Lachnospiraceae_unclassified_SGB41589	Lachnospiraceae_unclassified
Lachnospiraceae_unclassified_SGB41589	Lachnospiraceae_unclassified
Lactobacillus_johnsonii	Lactobacillus
Lactobacillus_johnsonii	Lactobacillus
Muribaculaceae_bacterium	Muribaculaceae_unclassified
Muribaculaceae_bacterium	Muribaculaceae_unclassified

Neglectibacter_sp_X4	Neglectibacter
Neglectibacter_sp_X4	Neglectibacter
Oscillospiraceae_bacterium	Oscillospiraceae_unclassified
Oscillospiraceae_bacterium	Oscillospiraceae_unclassified
Oscillospiraceae_unclassified_SGB43502	Oscillospiraceae_unclassified
Oscillospiraceae_unclassified_SGB43502	Oscillospiraceae_unclassified
Oscillospiraceae_unclassified_SGB43505	Oscillospiraceae_unclassified
Oscillospiraceae_unclassified_SGB43505	Oscillospiraceae_unclassified
Parasutterella_excrementihominis	Parasutterella
Parasutterella_excrementihominis	Parasutterella
richness	
richness	
Romboutsia_ilealis	Romboutsia
Romboutsia_ilealis	Romboutsia
Schaedlerella_arabinosiphila	Schaedlerella
Schaedlerella_arabinosiphila	Schaedlerella
shannon	
shannon	
Turicibacter_sp_1E2	Turicibacter
Turicibacter_sp_1E2	Turicibacter
Acetatifactor_muris	Acetatifactor
Acetatifactor_muris	Acetatifactor
Acetatifactor_SGB41546	Acetatifactor
Acetatifactor_SGB41546	Acetatifactor
Acutalibacter_muris	Acutalibacter
Acutalibacter_muris	Acutalibacter
Acutalibacter_sp_1XD8_36	Acutalibacter
Acutalibacter_sp_1XD8_36	Acutalibacter
Adlercreutzia_caecimuris	Adlercreutzia
Adlercreutzia_caecimuris	Adlercreutzia
Adlercreutzia_mucosicola	Adlercreutzia
Adlercreutzia_mucosicola	Adlercreutzia
Adlercreutzia_muris	Adlercreutzia
Adlercreutzia_muris	Adlercreutzia
Akkermansia_muciniphila	Akkermansia
Akkermansia_muciniphila	Akkermansia
Alistipes_sp_DSM_112343	Alistipes
Alistipes_sp_DSM_112343	Alistipes
Anaerotruncus_sp_1XD42_93	Anaerotruncus
Anaerotruncus_sp_1XD42_93	Anaerotruncus
Bacteria_unclassified_SGB102200	Bacteria_unclassified
Bacteria_unclassified_SGB102200	Bacteria_unclassified
Bacteria_unclassified_SGB41677	Bacteria_unclassified
Bacteria_unclassified_SGB41677	Bacteria_unclassified
Bacteria_unclassified_SGB43546	Bacteria_unclassified
Bacteria_unclassified_SGB43546	Bacteria_unclassified

bacterium_1XD42_54	Bacteria_unclassified
bacterium_1XD42_54	Bacteria_unclassified
bacterium_1XD42_76	Bacteria_unclassified
bacterium_1XD42_76	Bacteria_unclassified
bacterium_1xD8_48	Bacteria_unclassified
bacterium_1xD8_48	Bacteria_unclassified
Bacteroides_thetaiotaomicron	Bacteroides
Bacteroides_thetaiotaomicron	Bacteroides
berger_parker	
berger_parker	
Bifidobacterium_pseudolongum	Bifidobacterium
Bifidobacterium_pseudolongum	Bifidobacterium
Clostridia_bacterium	Clostridia_unclassified
Clostridia_bacterium	Clostridia_unclassified
Clostridiaceae_bacterium	Clostridiaceae_unclassified
Clostridiaceae_bacterium	Clostridiaceae_unclassified
Clostridiaceae_unclassified_SGB41663	Clostridiaceae_unclassified
Clostridiaceae_unclassified_SGB41663	Clostridiaceae_unclassified
Clostridiales_bacterium	Eubacteriales_unclassified
Clostridiales_bacterium	Eubacteriales_unclassified
Clostridium_cocleatum	Erysipelatoclostridium
Clostridium_cocleatum	Erysipelatoclostridium
Coriobacteriaceae_bacterium	Coriobacteriaceae_unclassified
Coriobacteriaceae_bacterium	Coriobacteriaceae_unclassified
Dorea_sp_5_2	Dorea
Dorea_sp_5_2	Dorea
Dubosiella_newyorkensis	Dubosiella
Dubosiella_newyorkensis	Dubosiella
Erysipelotrichales_bacterium	Erysipelotrichales_unclassified
Erysipelotrichales_bacterium	Erysipelotrichales_unclassified
Eubacteriaceae_bacterium	Eubacteriaceae_unclassified
Eubacteriaceae_bacterium	Eubacteriaceae_unclassified
Eubacteriaceae_unclassified_SGB94922	Eubacteriaceae_unclassified
Eubacteriaceae_unclassified_SGB94922	Eubacteriaceae_unclassified
GGB20149_SGB29430	GGB20149
GGB20149_SGB29430	GGB20149
GGB22635_SGB63107	GGB22635
GGB22635_SGB63107	GGB22635
GGB25041_SGB36960	GGB25041
GGB25041_SGB36960	GGB25041
GGB27876_SGB40310	GGB27876
GGB27876_SGB40310	GGB27876
GGB27878_SGB40312	GGB27878
GGB27878_SGB40312	GGB27878
GGB27918_SGB40356	GGB27918
GGB27918_SGB40356	GGB27918

GGB28382_SGB40962	GGB28382
GGB28382_SGB40962	GGB28382
GGB28399_SGB40980	GGB28399
GGB28399_SGB40980	GGB28399
GGB28411_SGB40993	GGB28411
GGB28411_SGB40993	GGB28411
GGB28415_SGB40997	GGB28415
GGB28415_SGB40997	GGB28415
GGB28430_SGB41013	GGB28430
GGB28430_SGB41013	GGB28430
GGB28439_SGB41022	GGB28439
GGB28439_SGB41022	GGB28439
GGB28778_SGB41431	GGB28778
GGB28778_SGB41431	GGB28778
GGB28784_SGB41437	GGB28784
GGB28784_SGB41437	GGB28784
GGB28792_SGB41445	GGB28792
GGB28792_SGB41445	GGB28792
GGB28798_SGB41451	GGB28798
GGB28798_SGB41451	GGB28798
GGB28802_SGB41455	GGB28802
GGB28802_SGB41455	GGB28802
GGB28818_SGB41473	GGB28818
GGB28818_SGB41473	GGB28818
GGB28828_SGB41484	GGB28828
GGB28828_SGB41484	GGB28828
GGB28851_SGB41518	GGB28851
GGB28851_SGB41518	GGB28851
GGB28859_SGB41528	GGB28859
GGB28859_SGB41528	GGB28859
GGB28864_SGB41535	GGB28864
GGB28864_SGB41535	GGB28864
GGB28869_SGB41543	GGB28869
GGB28869_SGB41543	GGB28869
GGB28883_SGB41564	GGB28883
GGB28883_SGB41564	GGB28883
GGB28892_SGB41573	GGB28892
GGB28892_SGB41573	GGB28892
GGB28893_SGB41574	GGB28893
GGB28893_SGB41574	GGB28893
GGB28898_SGB41580	GGB28898
GGB28898_SGB41580	GGB28898
GGB28904_SGB41597	GGB28904
GGB28904_SGB41597	GGB28904
GGB28916_SGB41612	GGB28916
GGB28916_SGB41612	GGB28916

GGB28924_SGB41621	GGB28924
GGB28924_SGB41621	GGB28924
GGB28926_SGB41624	GGB28926
GGB28926_SGB41624	GGB28926
GGB28927_SGB41625	GGB28927
GGB28927_SGB41625	GGB28927
GGB28934_SGB41635	GGB28934
GGB28934_SGB41635	GGB28934
GGB28946_SGB41652	GGB28946
GGB28946_SGB41652	GGB28946
GGB28949_SGB41655	GGB28949
GGB28949_SGB41655	GGB28949
GGB28949_SGB41656	GGB28949
GGB28949_SGB41656	GGB28949
GGB28950_SGB41657	GGB28950
GGB28950_SGB41657	GGB28950
GGB28951_SGB102295	GGB28951
GGB28951_SGB102295	GGB28951
GGB28951_SGB41658	GGB28951
GGB28951_SGB41658	GGB28951
GGB28954_SGB41662	GGB28954
GGB28954_SGB41662	GGB28954
GGB28956_SGB41665	GGB28956
GGB28956_SGB41665	GGB28956
GGB28960_SGB41669	GGB28960
GGB28960_SGB41669	GGB28960
GGB28967_SGB41678	GGB28967
GGB28967_SGB41678	GGB28967
GGB28991_SGB41705	GGB28991
GGB28991_SGB41705	GGB28991
GGB29002_SGB41718	GGB29002
GGB29002_SGB41718	GGB29002
GGB29003_SGB41719	GGB29003
GGB29003_SGB41719	GGB29003
GGB29011_SGB41731	GGB29011
GGB29011_SGB41731	GGB29011
GGB29531_SGB42317	GGB29531
GGB29531_SGB42317	GGB29531
GGB29685_SGB42494	GGB29685
GGB29685_SGB42494	GGB29685
GGB30141_SGB43066	GGB30141
GGB30141_SGB43066	GGB30141
GGB30286_SGB43248	GGB30286
GGB30286_SGB43248	GGB30286
GGB30303_SGB43268	GGB30303
GGB30303_SGB43268	GGB30303

GGB30413_SGB43452	GGB30413
GGB30413_SGB43452	GGB30413
GGB30454_SGB43514	GGB30454
GGB30454_SGB43514	GGB30454
GGB30455_SGB43519	GGB30455
GGB30455_SGB43519	GGB30455
GGB30461_SGB43527	GGB30461
GGB30461_SGB43527	GGB30461
GGB30461_SGB43530	GGB30461
GGB30461_SGB43530	GGB30461
GGB30463_SGB43537	GGB30463
GGB30463_SGB43537	GGB30463
GGB30473_SGB43557	GGB30473
GGB30473_SGB43557	GGB30473
GGB30475_SGB63182	GGB30475
GGB30475_SGB63182	GGB30475
GGB30861_SGB44083	GGB30861
GGB30861_SGB44083	GGB30861
GGB31312_SGB44628	GGB31312
GGB31312_SGB44628	GGB31312
GGB31438_SGB44768	GGB31438
GGB31438_SGB44768	GGB31438
GGB3171_SGB4185	GGB3171
GGB3171_SGB4185	GGB3171
GGB31823_SGB45199	GGB31823
GGB31823_SGB45199	GGB31823
GGB31853_SGB45233	GGB31853
GGB31853_SGB45233	GGB31853
GGB32371_SGB41694	GGB32371
GGB32371_SGB41694	GGB32371
GGB3793_SGB5158	GGB3793
GGB3793_SGB5158	GGB3793
GGB42598_SGB59794	GGB42598
GGB42598_SGB59794	GGB42598
GGB45656_SGB63370	GGB45656
GGB45656_SGB63370	GGB45656
GGB47127_SGB65054	GGB47127
GGB47127_SGB65054	GGB47127
GGB74395_SGB43521	GGB74395
GGB74395_SGB43521	GGB74395
GGB75053_SGB43494	GGB75053
GGB75053_SGB43494	GGB75053
GGB75109_SGB102238	GGB75109
GGB75109_SGB102238	GGB75109
GGB81440_SGB45230	GGB81440
GGB81440_SGB45230	GGB81440

Lachnospiraceae_bacterium	Lachnospiraceae_unclassified
Lachnospiraceae_bacterium	Lachnospiraceae_unclassified
Lachnospiraceae_bacterium_A2	Lachnospiraceae_unclassified
Lachnospiraceae_bacterium_A2	Lachnospiraceae_unclassified
Lachnospiraceae_bacterium_MD308	Lachnospiraceae_unclassified
Lachnospiraceae_bacterium_MD308	Lachnospiraceae_unclassified
Lachnospiraceae_bacterium_MD329	Lachnospiraceae_unclassified
Lachnospiraceae_bacterium_MD329	Lachnospiraceae_unclassified
Lachnospiraceae_unclassified_SGB41414	Lachnospiraceae_unclassified
Lachnospiraceae_unclassified_SGB41414	Lachnospiraceae_unclassified
Lachnospiraceae_unclassified_SGB41418	Lachnospiraceae_unclassified
Lachnospiraceae_unclassified_SGB41418	Lachnospiraceae_unclassified
Lachnospiraceae_unclassified_SGB41424	Lachnospiraceae_unclassified
Lachnospiraceae_unclassified_SGB41424	Lachnospiraceae_unclassified
Lachnospiraceae_unclassified_SGB41589	Lachnospiraceae_unclassified
Lachnospiraceae_unclassified_SGB41589	Lachnospiraceae_unclassified
Lactobacillus_johnsonii	Lactobacillus
Lactobacillus_johnsonii	Lactobacillus
Muribaculaceae_bacterium	Muribaculaceae_unclassified
Muribaculaceae_bacterium	Muribaculaceae_unclassified
Neglectibacter_sp_X4	Neglectibacter
Neglectibacter_sp_X4	Neglectibacter
Oscillospiraceae_bacterium	Oscillospiraceae_unclassified
Oscillospiraceae_bacterium	Oscillospiraceae_unclassified
Oscillospiraceae_unclassified_SGB43502	Oscillospiraceae_unclassified
Oscillospiraceae_unclassified_SGB43502	Oscillospiraceae_unclassified
Oscillospiraceae_unclassified_SGB43505	Oscillospiraceae_unclassified
Oscillospiraceae_unclassified_SGB43505	Oscillospiraceae_unclassified
Parasutterella_excrementihominis	Parasutterella
Parasutterella_excrementihominis	Parasutterella
richness	
richness	
Romboutsia_ilealis	Romboutsia
Romboutsia_ilealis	Romboutsia
Schaedlerella_arabinosiphila	Schaedlerella
Schaedlerella_arabinosiphila	Schaedlerella
shannon	
shannon	
Turicibacter_sp_1E2	Turicibacter
Turicibacter_sp_1E2	Turicibacter
Acetatifactor_muris	Acetatifactor
Acetatifactor_muris	Acetatifactor
Acetatifactor_SGB41546	Acetatifactor
Acetatifactor_SGB41546	Acetatifactor
Acutalibacter_muris	Acutalibacter
Acutalibacter_muris	Acutalibacter

Acutalibacter_sp_1XD8_36	Acutalibacter
Acutalibacter_sp_1XD8_36	Acutalibacter
Adlercreutzia_caecimuris	Adlercreutzia
Adlercreutzia_caecimuris	Adlercreutzia
Adlercreutzia_mucosicola	Adlercreutzia
Adlercreutzia_mucosicola	Adlercreutzia
Adlercreutzia_muris	Adlercreutzia
Adlercreutzia_muris	Adlercreutzia
Akkermansia_muciniphila	Akkermansia
Akkermansia_muciniphila	Akkermansia
Alistipes_sp_DSM_112343	Alistipes
Alistipes_sp_DSM_112343	Alistipes
Anaerotruncus_sp_1XD42_93	Anaerotruncus
Anaerotruncus_sp_1XD42_93	Anaerotruncus
Bacteria_unclassified_SGB102200	Bacteria_unclassified
Bacteria_unclassified_SGB102200	Bacteria_unclassified
Bacteria_unclassified_SGB41677	Bacteria_unclassified
Bacteria_unclassified_SGB41677	Bacteria_unclassified
Bacteria_unclassified_SGB43546	Bacteria_unclassified
Bacteria_unclassified_SGB43546	Bacteria_unclassified
bacterium_1XD42_54	Bacteria_unclassified
bacterium_1XD42_54	Bacteria_unclassified
bacterium_1XD42_76	Bacteria_unclassified
bacterium_1XD42_76	Bacteria_unclassified
bacterium_1xD8_48	Bacteria_unclassified
bacterium_1xD8_48	Bacteria_unclassified
Bacteroides_thetaiotaomicron	Bacteroides
Bacteroides_thetaiotaomicron	Bacteroides
berger_parker	
berger_parker	
Bifidobacterium_pseudolongum	Bifidobacterium
Bifidobacterium_pseudolongum	Bifidobacterium
Clostridia_bacterium	Clostridia_unclassified
Clostridia_bacterium	Clostridia_unclassified
Clostridiaceae_bacterium	Clostridiaceae_unclassified
Clostridiaceae_bacterium	Clostridiaceae_unclassified
Clostridiaceae_unclassified_SGB41663	Clostridiaceae_unclassified
Clostridiaceae_unclassified_SGB41663	Clostridiaceae_unclassified
Clostridiales_bacterium	Eubacteriales_unclassified
Clostridiales_bacterium	Eubacteriales_unclassified
Clostridium_cocleatum	Erysipelatoclostridium
Clostridium_cocleatum	Erysipelatoclostridium
Coriobacteriaceae_bacterium	Coriobacteriaceae_unclassified
Coriobacteriaceae_bacterium	Coriobacteriaceae_unclassified
Dorea_sp_5_2	Dorea
Dorea_sp_5_2	Dorea

Dubosiella_newyorkensis	Dubosiella
Dubosiella_newyorkensis	Dubosiella
Erysipelotrichales_bacterium	Erysipelotrichales_unclassified
Erysipelotrichales_bacterium	Erysipelotrichales_unclassified
Eubacteriaceae_bacterium	Eubacteriaceae_unclassified
Eubacteriaceae_bacterium	Eubacteriaceae_unclassified
Eubacteriaceae_unclassified_SGB94922	Eubacteriaceae_unclassified
Eubacteriaceae_unclassified_SGB94922	Eubacteriaceae_unclassified
GGB20149_SGB29430	GGB20149
GGB20149_SGB29430	GGB20149
GGB22635_SGB63107	GGB22635
GGB22635_SGB63107	GGB22635
GGB25041_SGB36960	GGB25041
GGB25041_SGB36960	GGB25041
GGB27876_SGB40310	GGB27876
GGB27876_SGB40310	GGB27876
GGB27878_SGB40312	GGB27878
GGB27878_SGB40312	GGB27878
GGB27918_SGB40356	GGB27918
GGB27918_SGB40356	GGB27918
GGB28382_SGB40962	GGB28382
GGB28382_SGB40962	GGB28382
GGB28399_SGB40980	GGB28399
GGB28399_SGB40980	GGB28399
GGB28411_SGB40993	GGB28411
GGB28411_SGB40993	GGB28411
GGB28415_SGB40997	GGB28415
GGB28415_SGB40997	GGB28415
GGB28430_SGB41013	GGB28430
GGB28430_SGB41013	GGB28430
GGB28439_SGB41022	GGB28439
GGB28439_SGB41022	GGB28439
GGB28778_SGB41431	GGB28778
GGB28778_SGB41431	GGB28778
GGB28784_SGB41437	GGB28784
GGB28784_SGB41437	GGB28784
GGB28792_SGB41445	GGB28792
GGB28792_SGB41445	GGB28792
GGB28798_SGB41451	GGB28798
GGB28798_SGB41451	GGB28798
GGB28802_SGB41455	GGB28802
GGB28802_SGB41455	GGB28802
GGB28818_SGB41473	GGB28818
GGB28818_SGB41473	GGB28818
GGB28828_SGB41484	GGB28828
GGB28828_SGB41484	GGB28828

GGB28851_SGB41518	GGB28851
GGB28851_SGB41518	GGB28851
GGB28859_SGB41528	GGB28859
GGB28859_SGB41528	GGB28859
GGB28864_SGB41535	GGB28864
GGB28864_SGB41535	GGB28864
GGB28869_SGB41543	GGB28869
GGB28869_SGB41543	GGB28869
GGB28883_SGB41564	GGB28883
GGB28883_SGB41564	GGB28883
GGB28892_SGB41573	GGB28892
GGB28892_SGB41573	GGB28892
GGB28893_SGB41574	GGB28893
GGB28893_SGB41574	GGB28893
GGB28898_SGB41580	GGB28898
GGB28898_SGB41580	GGB28898
GGB28904_SGB41597	GGB28904
GGB28904_SGB41597	GGB28904
GGB28916_SGB41612	GGB28916
GGB28916_SGB41612	GGB28916
GGB28924_SGB41621	GGB28924
GGB28924_SGB41621	GGB28924
GGB28926_SGB41624	GGB28926
GGB28926_SGB41624	GGB28926
GGB28927_SGB41625	GGB28927
GGB28927_SGB41625	GGB28927
GGB28934_SGB41635	GGB28934
GGB28934_SGB41635	GGB28934
GGB28946_SGB41652	GGB28946
GGB28946_SGB41652	GGB28946
GGB28949_SGB41655	GGB28949
GGB28949_SGB41655	GGB28949
GGB28949_SGB41656	GGB28949
GGB28949_SGB41656	GGB28949
GGB28950_SGB41657	GGB28950
GGB28950_SGB41657	GGB28950
GGB28951_SGB102295	GGB28951
GGB28951_SGB102295	GGB28951
GGB28951_SGB41658	GGB28951
GGB28951_SGB41658	GGB28951
GGB28954_SGB41662	GGB28954
GGB28954_SGB41662	GGB28954
GGB28956_SGB41665	GGB28956
GGB28956_SGB41665	GGB28956
GGB28960_SGB41669	GGB28960
GGB28960_SGB41669	GGB28960

GGB28967_SGB41678	GGB28967
GGB28967_SGB41678	GGB28967
GGB28991_SGB41705	GGB28991
GGB28991_SGB41705	GGB28991
GGB29002_SGB41718	GGB29002
GGB29002_SGB41718	GGB29002
GGB29003_SGB41719	GGB29003
GGB29003_SGB41719	GGB29003
GGB29011_SGB41731	GGB29011
GGB29011_SGB41731	GGB29011
GGB29531_SGB42317	GGB29531
GGB29531_SGB42317	GGB29531
GGB29685_SGB42494	GGB29685
GGB29685_SGB42494	GGB29685
GGB30141_SGB43066	GGB30141
GGB30141_SGB43066	GGB30141
GGB30286_SGB43248	GGB30286
GGB30286_SGB43248	GGB30286
GGB30303_SGB43268	GGB30303
GGB30303_SGB43268	GGB30303
GGB30413_SGB43452	GGB30413
GGB30413_SGB43452	GGB30413
GGB30454_SGB43514	GGB30454
GGB30454_SGB43514	GGB30454
GGB30455_SGB43519	GGB30455
GGB30455_SGB43519	GGB30455
GGB30461_SGB43527	GGB30461
GGB30461_SGB43527	GGB30461
GGB30461_SGB43530	GGB30461
GGB30461_SGB43530	GGB30461
GGB30463_SGB43537	GGB30463
GGB30463_SGB43537	GGB30463
GGB30473_SGB43557	GGB30473
GGB30473_SGB43557	GGB30473
GGB30475_SGB63182	GGB30475
GGB30475_SGB63182	GGB30475
GGB30861_SGB44083	GGB30861
GGB30861_SGB44083	GGB30861
GGB31312_SGB44628	GGB31312
GGB31312_SGB44628	GGB31312
GGB31438_SGB44768	GGB31438
GGB31438_SGB44768	GGB31438
GGB3171_SGB4185	GGB3171
GGB3171_SGB4185	GGB3171
GGB31823_SGB45199	GGB31823
GGB31823_SGB45199	GGB31823

GGB31853_SGB45233	GGB31853
GGB31853_SGB45233	GGB31853
GGB32371_SGB41694	GGB32371
GGB32371_SGB41694	GGB32371
GGB3793_SGB5158	GGB3793
GGB3793_SGB5158	GGB3793
GGB42598_SGB59794	GGB42598
GGB42598_SGB59794	GGB42598
GGB45656_SGB63370	GGB45656
GGB45656_SGB63370	GGB45656
GGB47127_SGB65054	GGB47127
GGB47127_SGB65054	GGB47127
GGB74395_SGB43521	GGB74395
GGB74395_SGB43521	GGB74395
GGB75053_SGB43494	GGB75053
GGB75053_SGB43494	GGB75053
GGB75109_SGB102238	GGB75109
GGB75109_SGB102238	GGB75109
GGB81440_SGB45230	GGB81440
GGB81440_SGB45230	GGB81440
Lachnospiraceae_bacterium	Lachnospiraceae_unclassified
Lachnospiraceae_bacterium	Lachnospiraceae_unclassified
Lachnospiraceae_bacterium_A2	Lachnospiraceae_unclassified
Lachnospiraceae_bacterium_A2	Lachnospiraceae_unclassified
Lachnospiraceae_bacterium_MD308	Lachnospiraceae_unclassified
Lachnospiraceae_bacterium_MD308	Lachnospiraceae_unclassified
Lachnospiraceae_bacterium_MD329	Lachnospiraceae_unclassified
Lachnospiraceae_bacterium_MD329	Lachnospiraceae_unclassified
Lachnospiraceae_unclassified_SGB41414	Lachnospiraceae_unclassified
Lachnospiraceae_unclassified_SGB41414	Lachnospiraceae_unclassified
Lachnospiraceae_unclassified_SGB41418	Lachnospiraceae_unclassified
Lachnospiraceae_unclassified_SGB41418	Lachnospiraceae_unclassified
Lachnospiraceae_unclassified_SGB41424	Lachnospiraceae_unclassified
Lachnospiraceae_unclassified_SGB41424	Lachnospiraceae_unclassified
Lachnospiraceae_unclassified_SGB41589	Lachnospiraceae_unclassified
Lachnospiraceae_unclassified_SGB41589	Lachnospiraceae_unclassified
Lactobacillus_johnsonii	Lactobacillus
Lactobacillus_johnsonii	Lactobacillus
Muribaculaceae_bacterium	Muribaculaceae_unclassified
Muribaculaceae_bacterium	Muribaculaceae_unclassified
Neglectibacter_sp_X4	Neglectibacter
Neglectibacter_sp_X4	Neglectibacter
Oscillospiraceae_bacterium	Oscillospiraceae_unclassified
Oscillospiraceae_bacterium	Oscillospiraceae_unclassified
Oscillospiraceae_unclassified_SGB43502	Oscillospiraceae_unclassified
Oscillospiraceae_unclassified_SGB43502	Oscillospiraceae_unclassified

Oscillospiraceae_unclassified_SGB43505	Oscillospiraceae_unclassified
Oscillospiraceae_unclassified_SGB43505	Oscillospiraceae_unclassified
Parasutterella_excrementihominis	Parasutterella
Parasutterella_excrementihominis	Parasutterella
richness	
richness	
Romboutsia_ilealis	Romboutsia
Romboutsia_ilealis	Romboutsia
Schaedlerella_arabinosiphila	Schaedlerella
Schaedlerella_arabinosiphila	Schaedlerella
shannon	
shannon	
Turicibacter_sp_1E2	Turicibacter
Turicibacter_sp_1E2	Turicibacter
Acetatifactor_muris	Acetatifactor
Acetatifactor_muris	Acetatifactor
Acetatifactor_SGB41546	Acetatifactor
Acetatifactor_SGB41546	Acetatifactor
Acutalibacter_muris	Acutalibacter
Acutalibacter_muris	Acutalibacter
Acutalibacter_sp_1XD8_36	Acutalibacter
Acutalibacter_sp_1XD8_36	Acutalibacter
Adlercreutzia_caecimuris	Adlercreutzia
Adlercreutzia_caecimuris	Adlercreutzia
Adlercreutzia_mucosicola	Adlercreutzia
Adlercreutzia_mucosicola	Adlercreutzia
Adlercreutzia_muris	Adlercreutzia
Adlercreutzia_muris	Adlercreutzia
Akkermansia_muciniphila	Akkermansia
Akkermansia_muciniphila	Akkermansia
Alistipes_sp_DSM_112343	Alistipes
Alistipes_sp_DSM_112343	Alistipes
Anaerotruncus_sp_1XD42_93	Anaerotruncus
Anaerotruncus_sp_1XD42_93	Anaerotruncus
Bacteria_unclassified_SGB102200	Bacteria_unclassified
Bacteria_unclassified_SGB102200	Bacteria_unclassified
Bacteria_unclassified_SGB41677	Bacteria_unclassified
Bacteria_unclassified_SGB41677	Bacteria_unclassified
Bacteria_unclassified_SGB43546	Bacteria_unclassified
Bacteria_unclassified_SGB43546	Bacteria_unclassified
bacterium_1XD42_54	Bacteria_unclassified
bacterium_1XD42_54	Bacteria_unclassified
bacterium_1XD42_76	Bacteria_unclassified
bacterium_1XD42_76	Bacteria_unclassified
bacterium_1xD8_48	Bacteria_unclassified
bacterium_1xD8_48	Bacteria_unclassified

Bacteroides_thetaiotaomicron	Bacteroides
Bacteroides_thetaiotaomicron	Bacteroides
berger_parker	
berger_parker	
Bifidobacterium_pseudolongum	Bifidobacterium
Bifidobacterium_pseudolongum	Bifidobacterium
Clostridia_bacterium	Clostridia_unclassified
Clostridia_bacterium	Clostridia_unclassified
Clostridiaceae_bacterium	Clostridiaceae_unclassified
Clostridiaceae_bacterium	Clostridiaceae_unclassified
Clostridiaceae_unclassified_SGB41663	Clostridiaceae_unclassified
Clostridiaceae_unclassified_SGB41663	Clostridiaceae_unclassified
Clostridiales_bacterium	Eubacteriales_unclassified
Clostridiales_bacterium	Eubacteriales_unclassified
Clostridium_cocleatum	Erysipelatoclostridium
Clostridium_cocleatum	Erysipelatoclostridium
Coriobacteriaceae_bacterium	Coriobacteriaceae_unclassified
Coriobacteriaceae_bacterium	Coriobacteriaceae_unclassified
Dorea_sp_5_2	Dorea
Dorea_sp_5_2	Dorea
Dubosiella_newyorkensis	Dubosiella
Dubosiella_newyorkensis	Dubosiella
Erysipelotrichales_bacterium	Erysipelotrichales_unclassified
Erysipelotrichales_bacterium	Erysipelotrichales_unclassified
Eubacteriaceae_bacterium	Eubacteriaceae_unclassified
Eubacteriaceae_bacterium	Eubacteriaceae_unclassified
Eubacteriaceae_unclassified_SGB94922	Eubacteriaceae_unclassified
Eubacteriaceae_unclassified_SGB94922	Eubacteriaceae_unclassified
GGB20149_SGB29430	GGB20149
GGB20149_SGB29430	GGB20149
GGB22635_SGB63107	GGB22635
GGB22635_SGB63107	GGB22635
GGB25041_SGB36960	GGB25041
GGB25041_SGB36960	GGB25041
GGB27876_SGB40310	GGB27876
GGB27876_SGB40310	GGB27876
GGB27878_SGB40312	GGB27878
GGB27878_SGB40312	GGB27878
GGB27918_SGB40356	GGB27918
GGB27918_SGB40356	GGB27918
GGB28382_SGB40962	GGB28382
GGB28382_SGB40962	GGB28382
GGB28399_SGB40980	GGB28399
GGB28399_SGB40980	GGB28399
GGB28411_SGB40993	GGB28411
GGB28411_SGB40993	GGB28411

GGB28415_SGB40997	GGB28415
GGB28415_SGB40997	GGB28415
GGB28430_SGB41013	GGB28430
GGB28430_SGB41013	GGB28430
GGB28439_SGB41022	GGB28439
GGB28439_SGB41022	GGB28439
GGB28778_SGB41431	GGB28778
GGB28778_SGB41431	GGB28778
GGB28784_SGB41437	GGB28784
GGB28784_SGB41437	GGB28784
GGB28792_SGB41445	GGB28792
GGB28792_SGB41445	GGB28792
GGB28798_SGB41451	GGB28798
GGB28798_SGB41451	GGB28798
GGB28802_SGB41455	GGB28802
GGB28802_SGB41455	GGB28802
GGB28818_SGB41473	GGB28818
GGB28818_SGB41473	GGB28818
GGB28828_SGB41484	GGB28828
GGB28828_SGB41484	GGB28828
GGB28851_SGB41518	GGB28851
GGB28851_SGB41518	GGB28851
GGB28859_SGB41528	GGB28859
GGB28859_SGB41528	GGB28859
GGB28864_SGB41535	GGB28864
GGB28864_SGB41535	GGB28864
GGB28869_SGB41543	GGB28869
GGB28869_SGB41543	GGB28869
GGB28883_SGB41564	GGB28883
GGB28883_SGB41564	GGB28883
GGB28892_SGB41573	GGB28892
GGB28892_SGB41573	GGB28892
GGB28893_SGB41574	GGB28893
GGB28893_SGB41574	GGB28893
GGB28898_SGB41580	GGB28898
GGB28898_SGB41580	GGB28898
GGB28904_SGB41597	GGB28904
GGB28904_SGB41597	GGB28904
GGB28916_SGB41612	GGB28916
GGB28916_SGB41612	GGB28916
GGB28924_SGB41621	GGB28924
GGB28924_SGB41621	GGB28924
GGB28926_SGB41624	GGB28926
GGB28926_SGB41624	GGB28926
GGB28927_SGB41625	GGB28927
GGB28927_SGB41625	GGB28927

GGB28934_SGB41635	GGB28934
GGB28934_SGB41635	GGB28934
GGB28946_SGB41652	GGB28946
GGB28946_SGB41652	GGB28946
GGB28949_SGB41655	GGB28949
GGB28949_SGB41655	GGB28949
GGB28949_SGB41656	GGB28949
GGB28949_SGB41656	GGB28949
GGB28950_SGB41657	GGB28950
GGB28950_SGB41657	GGB28950
GGB28951_SGB102295	GGB28951
GGB28951_SGB102295	GGB28951
GGB28951_SGB41658	GGB28951
GGB28951_SGB41658	GGB28951
GGB28954_SGB41662	GGB28954
GGB28954_SGB41662	GGB28954
GGB28956_SGB41665	GGB28956
GGB28956_SGB41665	GGB28956
GGB28960_SGB41669	GGB28960
GGB28960_SGB41669	GGB28960
GGB28967_SGB41678	GGB28967
GGB28967_SGB41678	GGB28967
GGB28991_SGB41705	GGB28991
GGB28991_SGB41705	GGB28991
GGB29002_SGB41718	GGB29002
GGB29002_SGB41718	GGB29002
GGB29003_SGB41719	GGB29003
GGB29003_SGB41719	GGB29003
GGB29011_SGB41731	GGB29011
GGB29011_SGB41731	GGB29011
GGB29531_SGB42317	GGB29531
GGB29531_SGB42317	GGB29531
GGB29685_SGB42494	GGB29685
GGB29685_SGB42494	GGB29685
GGB30141_SGB43066	GGB30141
GGB30141_SGB43066	GGB30141
GGB30286_SGB43248	GGB30286
GGB30286_SGB43248	GGB30286
GGB30303_SGB43268	GGB30303
GGB30303_SGB43268	GGB30303
GGB30413_SGB43452	GGB30413
GGB30413_SGB43452	GGB30413
GGB30454_SGB43514	GGB30454
GGB30454_SGB43514	GGB30454
GGB30455_SGB43519	GGB30455
GGB30455_SGB43519	GGB30455

GGB30461_SGB43527	GGB30461
GGB30461_SGB43527	GGB30461
GGB30461_SGB43530	GGB30461
GGB30461_SGB43530	GGB30461
GGB30463_SGB43537	GGB30463
GGB30463_SGB43537	GGB30463
GGB30473_SGB43557	GGB30473
GGB30473_SGB43557	GGB30473
GGB30475_SGB63182	GGB30475
GGB30475_SGB63182	GGB30475
GGB30861_SGB44083	GGB30861
GGB30861_SGB44083	GGB30861
GGB31312_SGB44628	GGB31312
GGB31312_SGB44628	GGB31312
GGB31438_SGB44768	GGB31438
GGB31438_SGB44768	GGB31438
GGB3171_SGB4185	GGB3171
GGB3171_SGB4185	GGB3171
GGB31823_SGB45199	GGB31823
GGB31823_SGB45199	GGB31823
GGB31853_SGB45233	GGB31853
GGB31853_SGB45233	GGB31853
GGB32371_SGB41694	GGB32371
GGB32371_SGB41694	GGB32371
GGB3793_SGB5158	GGB3793
GGB3793_SGB5158	GGB3793
GGB42598_SGB59794	GGB42598
GGB42598_SGB59794	GGB42598
GGB45656_SGB63370	GGB45656
GGB45656_SGB63370	GGB45656
GGB47127_SGB65054	GGB47127
GGB47127_SGB65054	GGB47127
GGB74395_SGB43521	GGB74395
GGB74395_SGB43521	GGB74395
GGB75053_SGB43494	GGB75053
GGB75053_SGB43494	GGB75053
GGB75109_SGB102238	GGB75109
GGB75109_SGB102238	GGB75109
GGB81440_SGB45230	GGB81440
GGB81440_SGB45230	GGB81440
Lachnospiraceae_bacterium	Lachnospiraceae_unclassified
Lachnospiraceae_bacterium	Lachnospiraceae_unclassified
Lachnospiraceae_bacterium_A2	Lachnospiraceae_unclassified
Lachnospiraceae_bacterium_A2	Lachnospiraceae_unclassified
Lachnospiraceae_bacterium_MD308	Lachnospiraceae_unclassified
Lachnospiraceae_bacterium_MD308	Lachnospiraceae_unclassified

Lachnospiraceae_bacterium_MD329	Lachnospiraceae_unclassified
Lachnospiraceae_bacterium_MD329	Lachnospiraceae_unclassified
Lachnospiraceae_unclassified_SGB41414	Lachnospiraceae_unclassified
Lachnospiraceae_unclassified_SGB41414	Lachnospiraceae_unclassified
Lachnospiraceae_unclassified_SGB41418	Lachnospiraceae_unclassified
Lachnospiraceae_unclassified_SGB41418	Lachnospiraceae_unclassified
Lachnospiraceae_unclassified_SGB41424	Lachnospiraceae_unclassified
Lachnospiraceae_unclassified_SGB41424	Lachnospiraceae_unclassified
Lachnospiraceae_unclassified_SGB41589	Lachnospiraceae_unclassified
Lachnospiraceae_unclassified_SGB41589	Lachnospiraceae_unclassified
Lactobacillus_johnsonii	Lactobacillus
Lactobacillus_johnsonii	Lactobacillus
Muribaculaceae_bacterium	Muribaculaceae_unclassified
Muribaculaceae_bacterium	Muribaculaceae_unclassified
Neglectibacter_sp_X4	Neglectibacter
Neglectibacter_sp_X4	Neglectibacter
Oscillospiraceae_bacterium	Oscillospiraceae_unclassified
Oscillospiraceae_bacterium	Oscillospiraceae_unclassified
Oscillospiraceae_unclassified_SGB43502	Oscillospiraceae_unclassified
Oscillospiraceae_unclassified_SGB43502	Oscillospiraceae_unclassified
Oscillospiraceae_unclassified_SGB43505	Oscillospiraceae_unclassified
Oscillospiraceae_unclassified_SGB43505	Oscillospiraceae_unclassified
Parasutterella_excrementihominis	Parasutterella
Parasutterella_excrementihominis	Parasutterella
richness	
richness	
Romboutsia_ilealis	Romboutsia
Romboutsia_ilealis	Romboutsia
Schaedlerella_arabinosiphila	Schaedlerella
Schaedlerella_arabinosiphila	Schaedlerella
shannon	
shannon	
Turicibacter_sp_1E2	Turicibacter
Turicibacter_sp_1E2	Turicibacter
Acetatifactor_muris	Acetatifactor
Acetatifactor_muris	Acetatifactor
Acetatifactor_SGB41546	Acetatifactor
Acetatifactor_SGB41546	Acetatifactor
Acutalibacter_muris	Acutalibacter
Acutalibacter_muris	Acutalibacter
Acutalibacter_sp_1XD8_36	Acutalibacter
Acutalibacter_sp_1XD8_36	Acutalibacter
Adlercreutzia_caecimuris	Adlercreutzia
Adlercreutzia_caecimuris	Adlercreutzia
Adlercreutzia_mucosicola	Adlercreutzia
Adlercreutzia_mucosicola	Adlercreutzia

Adlercreutzia_muris	Adlercreutzia
Adlercreutzia_muris	Adlercreutzia
Akkermansia_muciniphila	Akkermansia
Akkermansia_muciniphila	Akkermansia
Alistipes_sp_DSM_112343	Alistipes
Alistipes_sp_DSM_112343	Alistipes
Anaerotruncus_sp_1XD42_93	Anaerotruncus
Anaerotruncus_sp_1XD42_93	Anaerotruncus
Bacteria_unclassified_SGB102200	Bacteria_unclassified
Bacteria_unclassified_SGB102200	Bacteria_unclassified
Bacteria_unclassified_SGB41677	Bacteria_unclassified
Bacteria_unclassified_SGB41677	Bacteria_unclassified
Bacteria_unclassified_SGB43546	Bacteria_unclassified
Bacteria_unclassified_SGB43546	Bacteria_unclassified
bacterium_1XD42_54	Bacteria_unclassified
bacterium_1XD42_54	Bacteria_unclassified
bacterium_1XD42_76	Bacteria_unclassified
bacterium_1XD42_76	Bacteria_unclassified
bacterium_1xD8_48	Bacteria_unclassified
bacterium_1xD8_48	Bacteria_unclassified
Bacteroides_thetaiotaomicron	Bacteroides
Bacteroides_thetaiotaomicron	Bacteroides
berger_parker	
berger_parker	
Bifidobacterium_pseudolongum	Bifidobacterium
Bifidobacterium_pseudolongum	Bifidobacterium
Clostridia_bacterium	Clostridia_unclassified
Clostridia_bacterium	Clostridia_unclassified
Clostridiaceae_bacterium	Clostridiaceae_unclassified
Clostridiaceae_bacterium	Clostridiaceae_unclassified
Clostridiaceae_unclassified_SGB41663	Clostridiaceae_unclassified
Clostridiaceae_unclassified_SGB41663	Clostridiaceae_unclassified
Clostridiales_bacterium	Eubacteriales_unclassified
Clostridiales_bacterium	Eubacteriales_unclassified
Clostridium_cocleatum	Erysipelatoclostridium
Clostridium_cocleatum	Erysipelatoclostridium
Coriobacteriaceae_bacterium	Coriobacteriaceae_unclassified
Coriobacteriaceae_bacterium	Coriobacteriaceae_unclassified
Dorea_sp_5_2	Dorea
Dorea_sp_5_2	Dorea
Dubosiella_newyorkensis	Dubosiella
Dubosiella_newyorkensis	Dubosiella
Erysipelotrichales_bacterium	Erysipelotrichales_unclassified
Erysipelotrichales_bacterium	Erysipelotrichales_unclassified
Eubacteriaceae_bacterium	Eubacteriaceae_unclassified
Eubacteriaceae_bacterium	Eubacteriaceae_unclassified

Eubacteriaceae_unclassified_SGB94922	Eubacteriaceae_unclassified
Eubacteriaceae_unclassified_SGB94922	Eubacteriaceae_unclassified
GGB20149_SGB29430	GGB20149
GGB20149_SGB29430	GGB20149
GGB22635_SGB63107	GGB22635
GGB22635_SGB63107	GGB22635
GGB25041_SGB36960	GGB25041
GGB25041_SGB36960	GGB25041
GGB27876_SGB40310	GGB27876
GGB27876_SGB40310	GGB27876
GGB27878_SGB40312	GGB27878
GGB27878_SGB40312	GGB27878
GGB27918_SGB40356	GGB27918
GGB27918_SGB40356	GGB27918
GGB28382_SGB40962	GGB28382
GGB28382_SGB40962	GGB28382
GGB28399_SGB40980	GGB28399
GGB28399_SGB40980	GGB28399
GGB28411_SGB40993	GGB28411
GGB28411_SGB40993	GGB28411
GGB28415_SGB40997	GGB28415
GGB28415_SGB40997	GGB28415
GGB28430_SGB41013	GGB28430
GGB28430_SGB41013	GGB28430
GGB28439_SGB41022	GGB28439
GGB28439_SGB41022	GGB28439
GGB28778_SGB41431	GGB28778
GGB28778_SGB41431	GGB28778
GGB28784_SGB41437	GGB28784
GGB28784_SGB41437	GGB28784
GGB28792_SGB41445	GGB28792
GGB28792_SGB41445	GGB28792
GGB28798_SGB41451	GGB28798
GGB28798_SGB41451	GGB28798
GGB28802_SGB41455	GGB28802
GGB28802_SGB41455	GGB28802
GGB28818_SGB41473	GGB28818
GGB28818_SGB41473	GGB28818
GGB28828_SGB41484	GGB28828
GGB28828_SGB41484	GGB28828
GGB28851_SGB41518	GGB28851
GGB28851_SGB41518	GGB28851
GGB28859_SGB41528	GGB28859
GGB28859_SGB41528	GGB28859
GGB28864_SGB41535	GGB28864
GGB28864_SGB41535	GGB28864

GGB28869_SGB41543	GGB28869
GGB28869_SGB41543	GGB28869
GGB28883_SGB41564	GGB28883
GGB28883_SGB41564	GGB28883
GGB28892_SGB41573	GGB28892
GGB28892_SGB41573	GGB28892
GGB28893_SGB41574	GGB28893
GGB28893_SGB41574	GGB28893
GGB28898_SGB41580	GGB28898
GGB28898_SGB41580	GGB28898
GGB28904_SGB41597	GGB28904
GGB28904_SGB41597	GGB28904
GGB28916_SGB41612	GGB28916
GGB28916_SGB41612	GGB28916
GGB28924_SGB41621	GGB28924
GGB28924_SGB41621	GGB28924
GGB28926_SGB41624	GGB28926
GGB28926_SGB41624	GGB28926
GGB28927_SGB41625	GGB28927
GGB28927_SGB41625	GGB28927
GGB28934_SGB41635	GGB28934
GGB28934_SGB41635	GGB28934
GGB28946_SGB41652	GGB28946
GGB28946_SGB41652	GGB28946
GGB28949_SGB41655	GGB28949
GGB28949_SGB41655	GGB28949
GGB28949_SGB41656	GGB28949
GGB28949_SGB41656	GGB28949
GGB28950_SGB41657	GGB28950
GGB28950_SGB41657	GGB28950
GGB28951_SGB102295	GGB28951
GGB28951_SGB102295	GGB28951
GGB28951_SGB41658	GGB28951
GGB28951_SGB41658	GGB28951
GGB28954_SGB41662	GGB28954
GGB28954_SGB41662	GGB28954
GGB28956_SGB41665	GGB28956
GGB28956_SGB41665	GGB28956
GGB28960_SGB41669	GGB28960
GGB28960_SGB41669	GGB28960
GGB28967_SGB41678	GGB28967
GGB28967_SGB41678	GGB28967
GGB28991_SGB41705	GGB28991
GGB28991_SGB41705	GGB28991
GGB29002_SGB41718	GGB29002
GGB29002_SGB41718	GGB29002

GGB29003_SGB41719	GGB29003
GGB29003_SGB41719	GGB29003
GGB29011_SGB41731	GGB29011
GGB29011_SGB41731	GGB29011
GGB29531_SGB42317	GGB29531
GGB29531_SGB42317	GGB29531
GGB29685_SGB42494	GGB29685
GGB29685_SGB42494	GGB29685
GGB30141_SGB43066	GGB30141
GGB30141_SGB43066	GGB30141
GGB30286_SGB43248	GGB30286
GGB30286_SGB43248	GGB30286
GGB30303_SGB43268	GGB30303
GGB30303_SGB43268	GGB30303
GGB30413_SGB43452	GGB30413
GGB30413_SGB43452	GGB30413
GGB30454_SGB43514	GGB30454
GGB30454_SGB43514	GGB30454
GGB30455_SGB43519	GGB30455
GGB30455_SGB43519	GGB30455
GGB30461_SGB43527	GGB30461
GGB30461_SGB43527	GGB30461
GGB30461_SGB43530	GGB30461
GGB30461_SGB43530	GGB30461
GGB30463_SGB43537	GGB30463
GGB30463_SGB43537	GGB30463
GGB30473_SGB43557	GGB30473
GGB30473_SGB43557	GGB30473
GGB30475_SGB63182	GGB30475
GGB30475_SGB63182	GGB30475
GGB30861_SGB44083	GGB30861
GGB30861_SGB44083	GGB30861
GGB31312_SGB44628	GGB31312
GGB31312_SGB44628	GGB31312
GGB31438_SGB44768	GGB31438
GGB31438_SGB44768	GGB31438
GGB3171_SGB4185	GGB3171
GGB3171_SGB4185	GGB3171
GGB31823_SGB45199	GGB31823
GGB31823_SGB45199	GGB31823
GGB31853_SGB45233	GGB31853
GGB31853_SGB45233	GGB31853
GGB32371_SGB41694	GGB32371
GGB32371_SGB41694	GGB32371
GGB3793_SGB5158	GGB3793
GGB3793_SGB5158	GGB3793

GGB42598_SGB59794	GGB42598
GGB42598_SGB59794	GGB42598
GGB45656_SGB63370	GGB45656
GGB45656_SGB63370	GGB45656
GGB47127_SGB65054	GGB47127
GGB47127_SGB65054	GGB47127
GGB74395_SGB43521	GGB74395
GGB74395_SGB43521	GGB74395
GGB75053_SGB43494	GGB75053
GGB75053_SGB43494	GGB75053
GGB75109_SGB102238	GGB75109
GGB75109_SGB102238	GGB75109
GGB81440_SGB45230	GGB81440
GGB81440_SGB45230	GGB81440
Lachnospiraceae_bacterium	Lachnospiraceae_unclassified
Lachnospiraceae_bacterium	Lachnospiraceae_unclassified
Lachnospiraceae_bacterium_A2	Lachnospiraceae_unclassified
Lachnospiraceae_bacterium_A2	Lachnospiraceae_unclassified
Lachnospiraceae_bacterium_MD308	Lachnospiraceae_unclassified
Lachnospiraceae_bacterium_MD308	Lachnospiraceae_unclassified
Lachnospiraceae_bacterium_MD329	Lachnospiraceae_unclassified
Lachnospiraceae_bacterium_MD329	Lachnospiraceae_unclassified
Lachnospiraceae_unclassified_SGB41414	Lachnospiraceae_unclassified
Lachnospiraceae_unclassified_SGB41414	Lachnospiraceae_unclassified
Lachnospiraceae_unclassified_SGB41418	Lachnospiraceae_unclassified
Lachnospiraceae_unclassified_SGB41418	Lachnospiraceae_unclassified
Lachnospiraceae_unclassified_SGB41424	Lachnospiraceae_unclassified
Lachnospiraceae_unclassified_SGB41424	Lachnospiraceae_unclassified
Lachnospiraceae_unclassified_SGB41589	Lachnospiraceae_unclassified
Lachnospiraceae_unclassified_SGB41589	Lachnospiraceae_unclassified
Lactobacillus_johnsonii	Lactobacillus
Lactobacillus_johnsonii	Lactobacillus
Muribaculaceae_bacterium	Muribaculaceae_unclassified
Muribaculaceae_bacterium	Muribaculaceae_unclassified
Neglectibacter_sp_X4	Neglectibacter
Neglectibacter_sp_X4	Neglectibacter
Oscillospiraceae_bacterium	Oscillospiraceae_unclassified
Oscillospiraceae_bacterium	Oscillospiraceae_unclassified
Oscillospiraceae_unclassified_SGB43502	Oscillospiraceae_unclassified
Oscillospiraceae_unclassified_SGB43502	Oscillospiraceae_unclassified
Oscillospiraceae_unclassified_SGB43505	Oscillospiraceae_unclassified
Oscillospiraceae_unclassified_SGB43505	Oscillospiraceae_unclassified
Parasutterella_excrementihominis	Parasutterella
Parasutterella_excrementihominis	Parasutterella
richness	
richness	

Romboutsia_ilealis	Romboutsia
Romboutsia_ilealis	Romboutsia
Schaedlerella_arabinosiphila	Schaedlerella
Schaedlerella_arabinosiphila	Schaedlerella
shannon	
shannon	
Turicibacter_sp_1E2	Turicibacter
Turicibacter_sp_1E2	Turicibacter
Acetatifactor_muris	Acetatifactor
Acetatifactor_muris	Acetatifactor
Acetatifactor_SGB41546	Acetatifactor
Acetatifactor_SGB41546	Acetatifactor
Acutalibacter_muris	Acutalibacter
Acutalibacter_muris	Acutalibacter
Acutalibacter_sp_1XD8_36	Acutalibacter
Acutalibacter_sp_1XD8_36	Acutalibacter
Adlercreutzia_caecimuris	Adlercreutzia
Adlercreutzia_caecimuris	Adlercreutzia
Adlercreutzia_mucosicola	Adlercreutzia
Adlercreutzia_mucosicola	Adlercreutzia
Adlercreutzia_muris	Adlercreutzia
Adlercreutzia_muris	Adlercreutzia
Akkermansia_muciniphila	Akkermansia
Akkermansia_muciniphila	Akkermansia
Alistipes_sp_DSM_112343	Alistipes
Alistipes_sp_DSM_112343	Alistipes
Anaerotruncus_sp_1XD42_93	Anaerotruncus
Anaerotruncus_sp_1XD42_93	Anaerotruncus
Bacteria_unclassified_SGB102200	Bacteria_unclassified
Bacteria_unclassified_SGB102200	Bacteria_unclassified
Bacteria_unclassified_SGB41677	Bacteria_unclassified
Bacteria_unclassified_SGB41677	Bacteria_unclassified
Bacteria_unclassified_SGB43546	Bacteria_unclassified
Bacteria_unclassified_SGB43546	Bacteria_unclassified
bacterium_1XD42_54	Bacteria_unclassified
bacterium_1XD42_54	Bacteria_unclassified
bacterium_1XD42_76	Bacteria_unclassified
bacterium_1XD42_76	Bacteria_unclassified
bacterium_1xD8_48	Bacteria_unclassified
bacterium_1xD8_48	Bacteria_unclassified
Bacteroides_thetaiotaomicron	Bacteroides
Bacteroides_thetaiotaomicron	Bacteroides
berger_parker	
berger_parker	
Bifidobacterium_pseudolongum	Bifidobacterium
Bifidobacterium_pseudolongum	Bifidobacterium

Clostridia_bacterium	Clostridia_unclassified
Clostridia_bacterium	Clostridia_unclassified
Clostridiaceae_bacterium	Clostridiaceae_unclassified
Clostridiaceae_bacterium	Clostridiaceae_unclassified
Clostridiaceae_unclassified_SGB41663	Clostridiaceae_unclassified
Clostridiaceae_unclassified_SGB41663	Clostridiaceae_unclassified
Clostridiales_bacterium	Eubacteriales_unclassified
Clostridiales_bacterium	Eubacteriales_unclassified
Clostridium_cocleatum	Erysipelatoclostridium
Clostridium_cocleatum	Erysipelatoclostridium
Coriobacteriaceae_bacterium	Coriobacteriaceae_unclassified
Coriobacteriaceae_bacterium	Coriobacteriaceae_unclassified
Dorea_sp_5_2	Dorea
Dorea_sp_5_2	Dorea
Dubosiella_newyorkensis	Dubosiella
Dubosiella_newyorkensis	Dubosiella
Erysipelotrichales_bacterium	Erysipelotrichales_unclassified
Erysipelotrichales_bacterium	Erysipelotrichales_unclassified
Eubacteriaceae_bacterium	Eubacteriaceae_unclassified
Eubacteriaceae_bacterium	Eubacteriaceae_unclassified
Eubacteriaceae_unclassified_SGB94922	Eubacteriaceae_unclassified
Eubacteriaceae_unclassified_SGB94922	Eubacteriaceae_unclassified
GGB20149_SGB29430	GGB20149
GGB20149_SGB29430	GGB20149
GGB22635_SGB63107	GGB22635
GGB22635_SGB63107	GGB22635
GGB25041_SGB36960	GGB25041
GGB25041_SGB36960	GGB25041
GGB27876_SGB40310	GGB27876
GGB27876_SGB40310	GGB27876
GGB27878_SGB40312	GGB27878
GGB27878_SGB40312	GGB27878
GGB27918_SGB40356	GGB27918
GGB27918_SGB40356	GGB27918
GGB28382_SGB40962	GGB28382
GGB28382_SGB40962	GGB28382
GGB28399_SGB40980	GGB28399
GGB28399_SGB40980	GGB28399
GGB28411_SGB40993	GGB28411
GGB28411_SGB40993	GGB28411
GGB28415_SGB40997	GGB28415
GGB28415_SGB40997	GGB28415
GGB28430_SGB41013	GGB28430
GGB28430_SGB41013	GGB28430
GGB28439_SGB41022	GGB28439
GGB28439_SGB41022	GGB28439

GGB28778_SGB41431	GGB28778
GGB28778_SGB41431	GGB28778
GGB28784_SGB41437	GGB28784
GGB28784_SGB41437	GGB28784
GGB28792_SGB41445	GGB28792
GGB28792_SGB41445	GGB28792
GGB28798_SGB41451	GGB28798
GGB28798_SGB41451	GGB28798
GGB28802_SGB41455	GGB28802
GGB28802_SGB41455	GGB28802
GGB28818_SGB41473	GGB28818
GGB28818_SGB41473	GGB28818
GGB28828_SGB41484	GGB28828
GGB28828_SGB41484	GGB28828
GGB28851_SGB41518	GGB28851
GGB28851_SGB41518	GGB28851
GGB28859_SGB41528	GGB28859
GGB28859_SGB41528	GGB28859
GGB28864_SGB41535	GGB28864
GGB28864_SGB41535	GGB28864
GGB28869_SGB41543	GGB28869
GGB28869_SGB41543	GGB28869
GGB28883_SGB41564	GGB28883
GGB28883_SGB41564	GGB28883
GGB28892_SGB41573	GGB28892
GGB28892_SGB41573	GGB28892
GGB28893_SGB41574	GGB28893
GGB28893_SGB41574	GGB28893
GGB28898_SGB41580	GGB28898
GGB28898_SGB41580	GGB28898
GGB28904_SGB41597	GGB28904
GGB28904_SGB41597	GGB28904
GGB28916_SGB41612	GGB28916
GGB28916_SGB41612	GGB28916
GGB28924_SGB41621	GGB28924
GGB28924_SGB41621	GGB28924
GGB28926_SGB41624	GGB28926
GGB28926_SGB41624	GGB28926
GGB28927_SGB41625	GGB28927
GGB28927_SGB41625	GGB28927
GGB28934_SGB41635	GGB28934
GGB28934_SGB41635	GGB28934
GGB28946_SGB41652	GGB28946
GGB28946_SGB41652	GGB28946
GGB28949_SGB41655	GGB28949
GGB28949_SGB41655	GGB28949

GGB28949_SGB41656	GGB28949
GGB28949_SGB41656	GGB28949
GGB28950_SGB41657	GGB28950
GGB28950_SGB41657	GGB28950
GGB28951_SGB102295	GGB28951
GGB28951_SGB102295	GGB28951
GGB28951_SGB41658	GGB28951
GGB28951_SGB41658	GGB28951
GGB28954_SGB41662	GGB28954
GGB28954_SGB41662	GGB28954
GGB28956_SGB41665	GGB28956
GGB28956_SGB41665	GGB28956
GGB28960_SGB41669	GGB28960
GGB28960_SGB41669	GGB28960
GGB28967_SGB41678	GGB28967
GGB28967_SGB41678	GGB28967
GGB28991_SGB41705	GGB28991
GGB28991_SGB41705	GGB28991
GGB29002_SGB41718	GGB29002
GGB29002_SGB41718	GGB29002
GGB29003_SGB41719	GGB29003
GGB29003_SGB41719	GGB29003
GGB29011_SGB41731	GGB29011
GGB29011_SGB41731	GGB29011
GGB29531_SGB42317	GGB29531
GGB29531_SGB42317	GGB29531
GGB29685_SGB42494	GGB29685
GGB29685_SGB42494	GGB29685
GGB30141_SGB43066	GGB30141
GGB30141_SGB43066	GGB30141
GGB30286_SGB43248	GGB30286
GGB30286_SGB43248	GGB30286
GGB30303_SGB43268	GGB30303
GGB30303_SGB43268	GGB30303
GGB30413_SGB43452	GGB30413
GGB30413_SGB43452	GGB30413
GGB30454_SGB43514	GGB30454
GGB30454_SGB43514	GGB30454
GGB30455_SGB43519	GGB30455
GGB30455_SGB43519	GGB30455
GGB30461_SGB43527	GGB30461
GGB30461_SGB43527	GGB30461
GGB30461_SGB43530	GGB30461
GGB30461_SGB43530	GGB30461
GGB30463_SGB43537	GGB30463
GGB30463_SGB43537	GGB30463

GGB30473_SGB43557	GGB30473
GGB30473_SGB43557	GGB30473
GGB30475_SGB63182	GGB30475
GGB30475_SGB63182	GGB30475
GGB30861_SGB44083	GGB30861
GGB30861_SGB44083	GGB30861
GGB31312_SGB44628	GGB31312
GGB31312_SGB44628	GGB31312
GGB31438_SGB44768	GGB31438
GGB31438_SGB44768	GGB31438
GGB3171_SGB4185	GGB3171
GGB3171_SGB4185	GGB3171
GGB31823_SGB45199	GGB31823
GGB31823_SGB45199	GGB31823
GGB31853_SGB45233	GGB31853
GGB31853_SGB45233	GGB31853
GGB32371_SGB41694	GGB32371
GGB32371_SGB41694	GGB32371
GGB3793_SGB5158	GGB3793
GGB3793_SGB5158	GGB3793
GGB42598_SGB59794	GGB42598
GGB42598_SGB59794	GGB42598
GGB45656_SGB63370	GGB45656
GGB45656_SGB63370	GGB45656
GGB47127_SGB65054	GGB47127
GGB47127_SGB65054	GGB47127
GGB74395_SGB43521	GGB74395
GGB74395_SGB43521	GGB74395
GGB75053_SGB43494	GGB75053
GGB75053_SGB43494	GGB75053
GGB75109_SGB102238	GGB75109
GGB75109_SGB102238	GGB75109
GGB81440_SGB45230	GGB81440
GGB81440_SGB45230	GGB81440
Lachnospiraceae_bacterium	Lachnospiraceae_unclassified
Lachnospiraceae_bacterium	Lachnospiraceae_unclassified
Lachnospiraceae_bacterium_A2	Lachnospiraceae_unclassified
Lachnospiraceae_bacterium_A2	Lachnospiraceae_unclassified
Lachnospiraceae_bacterium_MD308	Lachnospiraceae_unclassified
Lachnospiraceae_bacterium_MD308	Lachnospiraceae_unclassified
Lachnospiraceae_bacterium_MD329	Lachnospiraceae_unclassified
Lachnospiraceae_bacterium_MD329	Lachnospiraceae_unclassified
Lachnospiraceae_unclassified_SGB41414	Lachnospiraceae_unclassified
Lachnospiraceae_unclassified_SGB41414	Lachnospiraceae_unclassified
Lachnospiraceae_unclassified_SGB41418	Lachnospiraceae_unclassified
Lachnospiraceae_unclassified_SGB41418	Lachnospiraceae_unclassified

Lachnospiraceae_unclassified_SGB41424	Lachnospiraceae_unclassified
Lachnospiraceae_unclassified_SGB41424	Lachnospiraceae_unclassified
Lachnospiraceae_unclassified_SGB41589	Lachnospiraceae_unclassified
Lachnospiraceae_unclassified_SGB41589	Lachnospiraceae_unclassified
Lactobacillus_johnsonii	Lactobacillus
Lactobacillus_johnsonii	Lactobacillus
Muribaculaceae_bacterium	Muribaculaceae_unclassified
Muribaculaceae_bacterium	Muribaculaceae_unclassified
Neglectibacter_sp_X4	Neglectibacter
Neglectibacter_sp_X4	Neglectibacter
Oscillospiraceae_bacterium	Oscillospiraceae_unclassified
Oscillospiraceae_bacterium	Oscillospiraceae_unclassified
Oscillospiraceae_unclassified_SGB43502	Oscillospiraceae_unclassified
Oscillospiraceae_unclassified_SGB43502	Oscillospiraceae_unclassified
Oscillospiraceae_unclassified_SGB43505	Oscillospiraceae_unclassified
Oscillospiraceae_unclassified_SGB43505	Oscillospiraceae_unclassified
Parasutterella_excrementihominis	Parasutterella
Parasutterella_excrementihominis	Parasutterella
richness	
richness	
Romboutsia_ilealis	Romboutsia
Romboutsia_ilealis	Romboutsia
Schaedlerella_arabinosiphila	Schaedlerella
Schaedlerella_arabinosiphila	Schaedlerella
shannon	
shannon	
Turicibacter_sp_1E2	Turicibacter
Turicibacter_sp_1E2	Turicibacter
Acetatifactor_muris	Acetatifactor
Acetatifactor_muris	Acetatifactor
Acetatifactor_SGB41546	Acetatifactor
Acetatifactor_SGB41546	Acetatifactor
Acutalibacter_muris	Acutalibacter
Acutalibacter_muris	Acutalibacter
Acutalibacter_sp_1XD8_36	Acutalibacter
Acutalibacter_sp_1XD8_36	Acutalibacter
Adlercreutzia_caecimuris	Adlercreutzia
Adlercreutzia_caecimuris	Adlercreutzia
Adlercreutzia_mucosicola	Adlercreutzia
Adlercreutzia_mucosicola	Adlercreutzia
Adlercreutzia_muris	Adlercreutzia
Adlercreutzia_muris	Adlercreutzia
Akkermansia_muciniphila	Akkermansia
Akkermansia_muciniphila	Akkermansia
Alistipes_sp_DSM_112343	Alistipes
Alistipes_sp_DSM_112343	Alistipes

Anaerotruncus_sp_1XD42_93	Anaerotruncus
Anaerotruncus_sp_1XD42_93	Anaerotruncus
Bacteria_unclassified_SGB102200	Bacteria_unclassified
Bacteria_unclassified_SGB102200	Bacteria_unclassified
Bacteria_unclassified_SGB41677	Bacteria_unclassified
Bacteria_unclassified_SGB41677	Bacteria_unclassified
Bacteria_unclassified_SGB43546	Bacteria_unclassified
Bacteria_unclassified_SGB43546	Bacteria_unclassified
bacterium_1XD42_54	Bacteria_unclassified
bacterium_1XD42_54	Bacteria_unclassified
bacterium_1XD42_76	Bacteria_unclassified
bacterium_1XD42_76	Bacteria_unclassified
bacterium_1xD8_48	Bacteria_unclassified
bacterium_1xD8_48	Bacteria_unclassified
Bacteroides_thetaiotaomicron	Bacteroides
Bacteroides_thetaiotaomicron	Bacteroides
berger_parker	
berger_parker	
Bifidobacterium_pseudolongum	Bifidobacterium
Bifidobacterium_pseudolongum	Bifidobacterium
Clostridia_bacterium	Clostridia_unclassified
Clostridia_bacterium	Clostridia_unclassified
Clostridiaceae_bacterium	Clostridiaceae_unclassified
Clostridiaceae_bacterium	Clostridiaceae_unclassified
Clostridiaceae_unclassified_SGB41663	Clostridiaceae_unclassified
Clostridiaceae_unclassified_SGB41663	Clostridiaceae_unclassified
Clostridiales_bacterium	Eubacteriales_unclassified
Clostridiales_bacterium	Eubacteriales_unclassified
Clostridium_cocleatum	Erysipelatoclostridium
Clostridium_cocleatum	Erysipelatoclostridium
Coriobacteriaceae_bacterium	Coriobacteriaceae_unclassified
Coriobacteriaceae_bacterium	Coriobacteriaceae_unclassified
Dorea_sp_5_2	Dorea
Dorea_sp_5_2	Dorea
Dubosiella_newyorkensis	Dubosiella
Dubosiella_newyorkensis	Dubosiella
Erysipelotrichales_bacterium	Erysipelotrichales_unclassified
Erysipelotrichales_bacterium	Erysipelotrichales_unclassified
Eubacteriaceae_bacterium	Eubacteriaceae_unclassified
Eubacteriaceae_bacterium	Eubacteriaceae_unclassified
Eubacteriaceae_unclassified_SGB94922	Eubacteriaceae_unclassified
Eubacteriaceae_unclassified_SGB94922	Eubacteriaceae_unclassified
GGB20149_SGB29430	GGB20149
GGB20149_SGB29430	GGB20149
GGB22635_SGB63107	GGB22635
GGB22635_SGB63107	GGB22635

GGB25041_SGB36960	GGB25041
GGB25041_SGB36960	GGB25041
GGB27876_SGB40310	GGB27876
GGB27876_SGB40310	GGB27876
GGB27878_SGB40312	GGB27878
GGB27878_SGB40312	GGB27878
GGB27918_SGB40356	GGB27918
GGB27918_SGB40356	GGB27918
GGB28382_SGB40962	GGB28382
GGB28382_SGB40962	GGB28382
GGB28399_SGB40980	GGB28399
GGB28399_SGB40980	GGB28399
GGB28411_SGB40993	GGB28411
GGB28411_SGB40993	GGB28411
GGB28415_SGB40997	GGB28415
GGB28415_SGB40997	GGB28415
GGB28430_SGB41013	GGB28430
GGB28430_SGB41013	GGB28430
GGB28439_SGB41022	GGB28439
GGB28439_SGB41022	GGB28439
GGB28778_SGB41431	GGB28778
GGB28778_SGB41431	GGB28778
GGB28784_SGB41437	GGB28784
GGB28784_SGB41437	GGB28784
GGB28792_SGB41445	GGB28792
GGB28792_SGB41445	GGB28792
GGB28798_SGB41451	GGB28798
GGB28798_SGB41451	GGB28798
GGB28802_SGB41455	GGB28802
GGB28802_SGB41455	GGB28802
GGB28818_SGB41473	GGB28818
GGB28818_SGB41473	GGB28818
GGB28828_SGB41484	GGB28828
GGB28828_SGB41484	GGB28828
GGB28851_SGB41518	GGB28851
GGB28851_SGB41518	GGB28851
GGB28859_SGB41528	GGB28859
GGB28859_SGB41528	GGB28859
GGB28864_SGB41535	GGB28864
GGB28864_SGB41535	GGB28864
GGB28869_SGB41543	GGB28869
GGB28869_SGB41543	GGB28869
GGB28883_SGB41564	GGB28883
GGB28883_SGB41564	GGB28883
GGB28892_SGB41573	GGB28892
GGB28892_SGB41573	GGB28892

GGB28893_SGB41574	GGB28893
GGB28893_SGB41574	GGB28893
GGB28898_SGB41580	GGB28898
GGB28898_SGB41580	GGB28898
GGB28904_SGB41597	GGB28904
GGB28904_SGB41597	GGB28904
GGB28916_SGB41612	GGB28916
GGB28916_SGB41612	GGB28916
GGB28924_SGB41621	GGB28924
GGB28924_SGB41621	GGB28924
GGB28926_SGB41624	GGB28926
GGB28926_SGB41624	GGB28926
GGB28927_SGB41625	GGB28927
GGB28927_SGB41625	GGB28927
GGB28934_SGB41635	GGB28934
GGB28934_SGB41635	GGB28934
GGB28946_SGB41652	GGB28946
GGB28946_SGB41652	GGB28946
GGB28949_SGB41655	GGB28949
GGB28949_SGB41655	GGB28949
GGB28949_SGB41656	GGB28949
GGB28949_SGB41656	GGB28949
GGB28950_SGB41657	GGB28950
GGB28950_SGB41657	GGB28950
GGB28951_SGB102295	GGB28951
GGB28951_SGB102295	GGB28951
GGB28951_SGB41658	GGB28951
GGB28951_SGB41658	GGB28951
GGB28954_SGB41662	GGB28954
GGB28954_SGB41662	GGB28954
GGB28956_SGB41665	GGB28956
GGB28956_SGB41665	GGB28956
GGB28960_SGB41669	GGB28960
GGB28960_SGB41669	GGB28960
GGB28967_SGB41678	GGB28967
GGB28967_SGB41678	GGB28967
GGB28991_SGB41705	GGB28991
GGB28991_SGB41705	GGB28991
GGB29002_SGB41718	GGB29002
GGB29002_SGB41718	GGB29002
GGB29003_SGB41719	GGB29003
GGB29003_SGB41719	GGB29003
GGB29011_SGB41731	GGB29011
GGB29011_SGB41731	GGB29011
GGB29531_SGB42317	GGB29531
GGB29531_SGB42317	GGB29531

GGB29685_SGB42494	GGB29685
GGB29685_SGB42494	GGB29685
GGB30141_SGB43066	GGB30141
GGB30141_SGB43066	GGB30141
GGB30286_SGB43248	GGB30286
GGB30286_SGB43248	GGB30286
GGB30303_SGB43268	GGB30303
GGB30303_SGB43268	GGB30303
GGB30413_SGB43452	GGB30413
GGB30413_SGB43452	GGB30413
GGB30454_SGB43514	GGB30454
GGB30454_SGB43514	GGB30454
GGB30455_SGB43519	GGB30455
GGB30455_SGB43519	GGB30455
GGB30461_SGB43527	GGB30461
GGB30461_SGB43527	GGB30461
GGB30461_SGB43530	GGB30461
GGB30461_SGB43530	GGB30461
GGB30463_SGB43537	GGB30463
GGB30463_SGB43537	GGB30463
GGB30473_SGB43557	GGB30473
GGB30473_SGB43557	GGB30473
GGB30475_SGB63182	GGB30475
GGB30475_SGB63182	GGB30475
GGB30861_SGB44083	GGB30861
GGB30861_SGB44083	GGB30861
GGB31312_SGB44628	GGB31312
GGB31312_SGB44628	GGB31312
GGB31438_SGB44768	GGB31438
GGB31438_SGB44768	GGB31438
GGB3171_SGB4185	GGB3171
GGB3171_SGB4185	GGB3171
GGB31823_SGB45199	GGB31823
GGB31823_SGB45199	GGB31823
GGB31853_SGB45233	GGB31853
GGB31853_SGB45233	GGB31853
GGB32371_SGB41694	GGB32371
GGB32371_SGB41694	GGB32371
GGB3793_SGB5158	GGB3793
GGB3793_SGB5158	GGB3793
GGB42598_SGB59794	GGB42598
GGB42598_SGB59794	GGB42598
GGB45656_SGB63370	GGB45656
GGB45656_SGB63370	GGB45656
GGB47127_SGB65054	GGB47127
GGB47127_SGB65054	GGB47127

GGB74395_SGB43521	GGB74395
GGB74395_SGB43521	GGB74395
GGB75053_SGB43494	GGB75053
GGB75053_SGB43494	GGB75053
GGB75109_SGB102238	GGB75109
GGB75109_SGB102238	GGB75109
GGB81440_SGB45230	GGB81440
GGB81440_SGB45230	GGB81440
Lachnospiraceae_bacterium	Lachnospiraceae_unclassified
Lachnospiraceae_bacterium	Lachnospiraceae_unclassified
Lachnospiraceae_bacterium_A2	Lachnospiraceae_unclassified
Lachnospiraceae_bacterium_A2	Lachnospiraceae_unclassified
Lachnospiraceae_bacterium_MD308	Lachnospiraceae_unclassified
Lachnospiraceae_bacterium_MD308	Lachnospiraceae_unclassified
Lachnospiraceae_bacterium_MD329	Lachnospiraceae_unclassified
Lachnospiraceae_bacterium_MD329	Lachnospiraceae_unclassified
Lachnospiraceae_unclassified_SGB41414	Lachnospiraceae_unclassified
Lachnospiraceae_unclassified_SGB41414	Lachnospiraceae_unclassified
Lachnospiraceae_unclassified_SGB41418	Lachnospiraceae_unclassified
Lachnospiraceae_unclassified_SGB41418	Lachnospiraceae_unclassified
Lachnospiraceae_unclassified_SGB41424	Lachnospiraceae_unclassified
Lachnospiraceae_unclassified_SGB41424	Lachnospiraceae_unclassified
Lachnospiraceae_unclassified_SGB41589	Lachnospiraceae_unclassified
Lachnospiraceae_unclassified_SGB41589	Lachnospiraceae_unclassified
Lactobacillus_johnsonii	Lactobacillus
Lactobacillus_johnsonii	Lactobacillus
Muribaculaceae_bacterium	Muribaculaceae_unclassified
Muribaculaceae_bacterium	Muribaculaceae_unclassified
Neglectibacter_sp_X4	Neglectibacter
Neglectibacter_sp_X4	Neglectibacter
Oscillospiraceae_bacterium	Oscillospiraceae_unclassified
Oscillospiraceae_bacterium	Oscillospiraceae_unclassified
Oscillospiraceae_unclassified_SGB43502	Oscillospiraceae_unclassified
Oscillospiraceae_unclassified_SGB43502	Oscillospiraceae_unclassified
Oscillospiraceae_unclassified_SGB43505	Oscillospiraceae_unclassified
Oscillospiraceae_unclassified_SGB43505	Oscillospiraceae_unclassified
Parasutterella_excrementihominis	Parasutterella
Parasutterella_excrementihominis	Parasutterella
richness	
richness	
Romboutsia_ilealis	Romboutsia
Romboutsia_ilealis	Romboutsia
Schaedlerella_arabinosiphila	Schaedlerella
Schaedlerella_arabinosiphila	Schaedlerella
shannon	
shannon	

Turicibacter_sp_1E2	Turicibacter
Turicibacter_sp_1E2	Turicibacter
Acetatifactor_muris	Acetatifactor
Acetatifactor_muris	Acetatifactor
Acetatifactor_SGB41546	Acetatifactor
Acetatifactor_SGB41546	Acetatifactor
Acutalibacter_muris	Acutalibacter
Acutalibacter_muris	Acutalibacter
Acutalibacter_sp_1XD8_36	Acutalibacter
Acutalibacter_sp_1XD8_36	Acutalibacter
Adlercreutzia_caecimuris	Adlercreutzia
Adlercreutzia_caecimuris	Adlercreutzia
Adlercreutzia_mucosicola	Adlercreutzia
Adlercreutzia_mucosicola	Adlercreutzia
Adlercreutzia_muris	Adlercreutzia
Adlercreutzia_muris	Adlercreutzia
Akkermansia_muciniphila	Akkermansia
Akkermansia_muciniphila	Akkermansia
Alistipes_sp_DSM_112343	Alistipes
Alistipes_sp_DSM_112343	Alistipes
Anaerotruncus_sp_1XD42_93	Anaerotruncus
Anaerotruncus_sp_1XD42_93	Anaerotruncus
Bacteria_unclassified_SGB102200	Bacteria_unclassified
Bacteria_unclassified_SGB102200	Bacteria_unclassified
Bacteria_unclassified_SGB41677	Bacteria_unclassified
Bacteria_unclassified_SGB41677	Bacteria_unclassified
Bacteria_unclassified_SGB43546	Bacteria_unclassified
Bacteria_unclassified_SGB43546	Bacteria_unclassified
bacterium_1XD42_54	Bacteria_unclassified
bacterium_1XD42_54	Bacteria_unclassified
bacterium_1XD42_76	Bacteria_unclassified
bacterium_1XD42_76	Bacteria_unclassified
bacterium_1xD8_48	Bacteria_unclassified
bacterium_1xD8_48	Bacteria_unclassified
Bacteroides_thetaiotaomicron	Bacteroides
Bacteroides_thetaiotaomicron	Bacteroides
berger_parker	
berger_parker	
Bifidobacterium_pseudolongum	Bifidobacterium
Bifidobacterium_pseudolongum	Bifidobacterium
Clostridia_bacterium	Clostridia_unclassified
Clostridia_bacterium	Clostridia_unclassified
Clostridiaceae_bacterium	Clostridiaceae_unclassified
Clostridiaceae_bacterium	Clostridiaceae_unclassified
Clostridiaceae_unclassified_SGB41663	Clostridiaceae_unclassified
Clostridiaceae_unclassified_SGB41663	Clostridiaceae_unclassified

Clostridiales_bacterium	Eubacteriales_unclassified
Clostridiales_bacterium	Eubacteriales_unclassified
Clostridium_cocleatum	Erysipelatoclostridium
Clostridium_cocleatum	Erysipelatoclostridium
Coriobacteriaceae_bacterium	Coriobacteriaceae_unclassified
Coriobacteriaceae_bacterium	Coriobacteriaceae_unclassified
Dorea_sp_5_2	Dorea
Dorea_sp_5_2	Dorea
Dubosiella_newyorkensis	Dubosiella
Dubosiella_newyorkensis	Dubosiella
Erysipelotrichales_bacterium	Erysipelotrichales_unclassified
Erysipelotrichales_bacterium	Erysipelotrichales_unclassified
Eubacteriaceae_bacterium	Eubacteriaceae_unclassified
Eubacteriaceae_bacterium	Eubacteriaceae_unclassified
Eubacteriaceae_unclassified_SGB94922	Eubacteriaceae_unclassified
Eubacteriaceae_unclassified_SGB94922	Eubacteriaceae_unclassified
GGB20149_SGB29430	GGB20149
GGB20149_SGB29430	GGB20149
GGB22635_SGB63107	GGB22635
GGB22635_SGB63107	GGB22635
GGB25041_SGB36960	GGB25041
GGB25041_SGB36960	GGB25041
GGB27876_SGB40310	GGB27876
GGB27876_SGB40310	GGB27876
GGB27878_SGB40312	GGB27878
GGB27878_SGB40312	GGB27878
GGB27918_SGB40356	GGB27918
GGB27918_SGB40356	GGB27918
GGB28382_SGB40962	GGB28382
GGB28382_SGB40962	GGB28382
GGB28399_SGB40980	GGB28399
GGB28399_SGB40980	GGB28399
GGB28411_SGB40993	GGB28411
GGB28411_SGB40993	GGB28411
GGB28415_SGB40997	GGB28415
GGB28415_SGB40997	GGB28415
GGB28430_SGB41013	GGB28430
GGB28430_SGB41013	GGB28430
GGB28439_SGB41022	GGB28439
GGB28439_SGB41022	GGB28439
GGB28778_SGB41431	GGB28778
GGB28778_SGB41431	GGB28778
GGB28784_SGB41437	GGB28784
GGB28784_SGB41437	GGB28784
GGB28792_SGB41445	GGB28792
GGB28792_SGB41445	GGB28792

GGB28798_SGB41451	GGB28798
GGB28798_SGB41451	GGB28798
GGB28802_SGB41455	GGB28802
GGB28802_SGB41455	GGB28802
GGB28818_SGB41473	GGB28818
GGB28818_SGB41473	GGB28818
GGB28828_SGB41484	GGB28828
GGB28828_SGB41484	GGB28828
GGB28851_SGB41518	GGB28851
GGB28851_SGB41518	GGB28851
GGB28859_SGB41528	GGB28859
GGB28859_SGB41528	GGB28859
GGB28864_SGB41535	GGB28864
GGB28864_SGB41535	GGB28864
GGB28869_SGB41543	GGB28869
GGB28869_SGB41543	GGB28869
GGB28883_SGB41564	GGB28883
GGB28883_SGB41564	GGB28883
GGB28892_SGB41573	GGB28892
GGB28892_SGB41573	GGB28892
GGB28893_SGB41574	GGB28893
GGB28893_SGB41574	GGB28893
GGB28898_SGB41580	GGB28898
GGB28898_SGB41580	GGB28898
GGB28904_SGB41597	GGB28904
GGB28904_SGB41597	GGB28904
GGB28916_SGB41612	GGB28916
GGB28916_SGB41612	GGB28916
GGB28924_SGB41621	GGB28924
GGB28924_SGB41621	GGB28924
GGB28926_SGB41624	GGB28926
GGB28926_SGB41624	GGB28926
GGB28927_SGB41625	GGB28927
GGB28927_SGB41625	GGB28927
GGB28934_SGB41635	GGB28934
GGB28934_SGB41635	GGB28934
GGB28946_SGB41652	GGB28946
GGB28946_SGB41652	GGB28946
GGB28949_SGB41655	GGB28949
GGB28949_SGB41655	GGB28949
GGB28949_SGB41656	GGB28949
GGB28949_SGB41656	GGB28949
GGB28950_SGB41657	GGB28950
GGB28950_SGB41657	GGB28950
GGB28951_SGB102295	GGB28951
GGB28951_SGB102295	GGB28951

GGB28951_SGB41658	GGB28951
GGB28951_SGB41658	GGB28951
GGB28954_SGB41662	GGB28954
GGB28954_SGB41662	GGB28954
GGB28956_SGB41665	GGB28956
GGB28956_SGB41665	GGB28956
GGB28960_SGB41669	GGB28960
GGB28960_SGB41669	GGB28960
GGB28967_SGB41678	GGB28967
GGB28967_SGB41678	GGB28967
GGB28991_SGB41705	GGB28991
GGB28991_SGB41705	GGB28991
GGB29002_SGB41718	GGB29002
GGB29002_SGB41718	GGB29002
GGB29003_SGB41719	GGB29003
GGB29003_SGB41719	GGB29003
GGB29011_SGB41731	GGB29011
GGB29011_SGB41731	GGB29011
GGB29531_SGB42317	GGB29531
GGB29531_SGB42317	GGB29531
GGB29685_SGB42494	GGB29685
GGB29685_SGB42494	GGB29685
GGB30141_SGB43066	GGB30141
GGB30141_SGB43066	GGB30141
GGB30286_SGB43248	GGB30286
GGB30286_SGB43248	GGB30286
GGB30303_SGB43268	GGB30303
GGB30303_SGB43268	GGB30303
GGB30413_SGB43452	GGB30413
GGB30413_SGB43452	GGB30413
GGB30454_SGB43514	GGB30454
GGB30454_SGB43514	GGB30454
GGB30455_SGB43519	GGB30455
GGB30455_SGB43519	GGB30455
GGB30461_SGB43527	GGB30461
GGB30461_SGB43527	GGB30461
GGB30461_SGB43530	GGB30461
GGB30461_SGB43530	GGB30461
GGB30463_SGB43537	GGB30463
GGB30463_SGB43537	GGB30463
GGB30473_SGB43557	GGB30473
GGB30473_SGB43557	GGB30473
GGB30475_SGB63182	GGB30475
GGB30475_SGB63182	GGB30475
GGB30861_SGB44083	GGB30861
GGB30861_SGB44083	GGB30861

GGB31312_SGB44628	GGB31312
GGB31312_SGB44628	GGB31312
GGB31438_SGB44768	GGB31438
GGB31438_SGB44768	GGB31438
GGB3171_SGB4185	GGB3171
GGB3171_SGB4185	GGB3171
GGB31823_SGB45199	GGB31823
GGB31823_SGB45199	GGB31823
GGB31853_SGB45233	GGB31853
GGB31853_SGB45233	GGB31853
GGB32371_SGB41694	GGB32371
GGB32371_SGB41694	GGB32371
GGB3793_SGB5158	GGB3793
GGB3793_SGB5158	GGB3793
GGB42598_SGB59794	GGB42598
GGB42598_SGB59794	GGB42598
GGB45656_SGB63370	GGB45656
GGB45656_SGB63370	GGB45656
GGB47127_SGB65054	GGB47127
GGB47127_SGB65054	GGB47127
GGB74395_SGB43521	GGB74395
GGB74395_SGB43521	GGB74395
GGB75053_SGB43494	GGB75053
GGB75053_SGB43494	GGB75053
GGB75109_SGB102238	GGB75109
GGB75109_SGB102238	GGB75109
GGB81440_SGB45230	GGB81440
GGB81440_SGB45230	GGB81440
Lachnospiraceae_bacterium	Lachnospiraceae_unclassified
Lachnospiraceae_bacterium	Lachnospiraceae_unclassified
Lachnospiraceae_bacterium_A2	Lachnospiraceae_unclassified
Lachnospiraceae_bacterium_A2	Lachnospiraceae_unclassified
Lachnospiraceae_bacterium_MD308	Lachnospiraceae_unclassified
Lachnospiraceae_bacterium_MD308	Lachnospiraceae_unclassified
Lachnospiraceae_bacterium_MD329	Lachnospiraceae_unclassified
Lachnospiraceae_bacterium_MD329	Lachnospiraceae_unclassified
Lachnospiraceae_unclassified_SGB41414	Lachnospiraceae_unclassified
Lachnospiraceae_unclassified_SGB41414	Lachnospiraceae_unclassified
Lachnospiraceae_unclassified_SGB41418	Lachnospiraceae_unclassified
Lachnospiraceae_unclassified_SGB41418	Lachnospiraceae_unclassified
Lachnospiraceae_unclassified_SGB41424	Lachnospiraceae_unclassified
Lachnospiraceae_unclassified_SGB41424	Lachnospiraceae_unclassified
Lachnospiraceae_unclassified_SGB41589	Lachnospiraceae_unclassified
Lachnospiraceae_unclassified_SGB41589	Lachnospiraceae_unclassified
Lactobacillus_johnsonii	Lactobacillus
Lactobacillus_johnsonii	Lactobacillus

Muribaculaceae_bacterium	Muribaculaceae_unclassified
Muribaculaceae_bacterium	Muribaculaceae_unclassified
Neglectibacter_sp_X4	Neglectibacter
Neglectibacter_sp_X4	Neglectibacter
Oscillospiraceae_bacterium	Oscillospiraceae_unclassified
Oscillospiraceae_bacterium	Oscillospiraceae_unclassified
Oscillospiraceae_unclassified_SGB43502	Oscillospiraceae_unclassified
Oscillospiraceae_unclassified_SGB43502	Oscillospiraceae_unclassified
Oscillospiraceae_unclassified_SGB43505	Oscillospiraceae_unclassified
Oscillospiraceae_unclassified_SGB43505	Oscillospiraceae_unclassified
Parasutterella_excrementihominis	Parasutterella
Parasutterella_excrementihominis	Parasutterella
richness	
richness	
Romboutsia_ilealis	Romboutsia
Romboutsia_ilealis	Romboutsia
Schaedlerella_arabinosiphila	Schaedlerella
Schaedlerella_arabinosiphila	Schaedlerella
shannon	
shannon	
Turicibacter_sp_1E2	Turicibacter
Turicibacter_sp_1E2	Turicibacter
Acetatifactor_muris	Acetatifactor
Acetatifactor_muris	Acetatifactor
Acetatifactor_SGB41546	Acetatifactor
Acetatifactor_SGB41546	Acetatifactor
Acutalibacter_muris	Acutalibacter
Acutalibacter_muris	Acutalibacter
Acutalibacter_sp_1XD8_36	Acutalibacter
Acutalibacter_sp_1XD8_36	Acutalibacter
Adlercreutzia_caecimuris	Adlercreutzia
Adlercreutzia_caecimuris	Adlercreutzia
Adlercreutzia_mucosicola	Adlercreutzia
Adlercreutzia_mucosicola	Adlercreutzia
Adlercreutzia_muris	Adlercreutzia
Adlercreutzia_muris	Adlercreutzia
Akkermansia_muciniphila	Akkermansia
Akkermansia_muciniphila	Akkermansia
Alistipes_sp_DSM_112343	Alistipes
Alistipes_sp_DSM_112343	Alistipes
Anaerotruncus_sp_1XD42_93	Anaerotruncus
Anaerotruncus_sp_1XD42_93	Anaerotruncus
Bacteria_unclassified_SGB102200	Bacteria_unclassified
Bacteria_unclassified_SGB102200	Bacteria_unclassified
Bacteria_unclassified_SGB41677	Bacteria_unclassified
Bacteria_unclassified_SGB41677	Bacteria_unclassified

Bacteria_unclassified_SGB43546	Bacteria_unclassified
Bacteria_unclassified_SGB43546	Bacteria_unclassified
bacterium_1XD42_54	Bacteria_unclassified
bacterium_1XD42_54	Bacteria_unclassified
bacterium_1XD42_76	Bacteria_unclassified
bacterium_1XD42_76	Bacteria_unclassified
bacterium_1xD8_48	Bacteria_unclassified
bacterium_1xD8_48	Bacteria_unclassified
Bacteroides_thetaiotaomicron	Bacteroides
Bacteroides_thetaiotaomicron	Bacteroides
berger_parker	
berger_parker	
Bifidobacterium_pseudolongum	Bifidobacterium
Bifidobacterium_pseudolongum	Bifidobacterium
Clostridia_bacterium	Clostridia_unclassified
Clostridia_bacterium	Clostridia_unclassified
Clostridiaceae_bacterium	Clostridiaceae_unclassified
Clostridiaceae_bacterium	Clostridiaceae_unclassified
Clostridiaceae_unclassified_SGB41663	Clostridiaceae_unclassified
Clostridiaceae_unclassified_SGB41663	Clostridiaceae_unclassified
Clostridiales_bacterium	Eubacteriales_unclassified
Clostridiales_bacterium	Eubacteriales_unclassified
Clostridium_cocleatum	Erysipelatoclostridium
Clostridium_cocleatum	Erysipelatoclostridium
Coriobacteriaceae_bacterium	Coriobacteriaceae_unclassified
Coriobacteriaceae_bacterium	Coriobacteriaceae_unclassified
Dorea_sp_5_2	Dorea
Dorea_sp_5_2	Dorea
Dubosiella_newyorkensis	Dubosiella
Dubosiella_newyorkensis	Dubosiella
Erysipelotrichales_bacterium	Erysipelotrichales_unclassified
Erysipelotrichales_bacterium	Erysipelotrichales_unclassified
Eubacteriaceae_bacterium	Eubacteriaceae_unclassified
Eubacteriaceae_bacterium	Eubacteriaceae_unclassified
Eubacteriaceae_unclassified_SGB94922	Eubacteriaceae_unclassified
Eubacteriaceae_unclassified_SGB94922	Eubacteriaceae_unclassified
GGB20149_SGB29430	GGB20149
GGB20149_SGB29430	GGB20149
GGB22635_SGB63107	GGB22635
GGB22635_SGB63107	GGB22635
GGB25041_SGB36960	GGB25041
GGB25041_SGB36960	GGB25041
GGB27876_SGB40310	GGB27876
GGB27876_SGB40310	GGB27876
GGB27878_SGB40312	GGB27878
GGB27878_SGB40312	GGB27878

GGB27918_SGB40356	GGB27918
GGB27918_SGB40356	GGB27918
GGB28382_SGB40962	GGB28382
GGB28382_SGB40962	GGB28382
GGB28399_SGB40980	GGB28399
GGB28399_SGB40980	GGB28399
GGB28411_SGB40993	GGB28411
GGB28411_SGB40993	GGB28411
GGB28415_SGB40997	GGB28415
GGB28415_SGB40997	GGB28415
GGB28430_SGB41013	GGB28430
GGB28430_SGB41013	GGB28430
GGB28439_SGB41022	GGB28439
GGB28439_SGB41022	GGB28439
GGB28778_SGB41431	GGB28778
GGB28778_SGB41431	GGB28778
GGB28784_SGB41437	GGB28784
GGB28784_SGB41437	GGB28784
GGB28792_SGB41445	GGB28792
GGB28792_SGB41445	GGB28792
GGB28798_SGB41451	GGB28798
GGB28798_SGB41451	GGB28798
GGB28802_SGB41455	GGB28802
GGB28802_SGB41455	GGB28802
GGB28818_SGB41473	GGB28818
GGB28818_SGB41473	GGB28818
GGB28828_SGB41484	GGB28828
GGB28828_SGB41484	GGB28828
GGB28851_SGB41518	GGB28851
GGB28851_SGB41518	GGB28851
GGB28859_SGB41528	GGB28859
GGB28859_SGB41528	GGB28859
GGB28864_SGB41535	GGB28864
GGB28864_SGB41535	GGB28864
GGB28869_SGB41543	GGB28869
GGB28869_SGB41543	GGB28869
GGB28883_SGB41564	GGB28883
GGB28883_SGB41564	GGB28883
GGB28892_SGB41573	GGB28892
GGB28892_SGB41573	GGB28892
GGB28893_SGB41574	GGB28893
GGB28893_SGB41574	GGB28893
GGB28898_SGB41580	GGB28898
GGB28898_SGB41580	GGB28898
GGB28904_SGB41597	GGB28904
GGB28904_SGB41597	GGB28904

GGB28916_SGB41612	GGB28916
GGB28916_SGB41612	GGB28916
GGB28924_SGB41621	GGB28924
GGB28924_SGB41621	GGB28924
GGB28926_SGB41624	GGB28926
GGB28926_SGB41624	GGB28926
GGB28927_SGB41625	GGB28927
GGB28927_SGB41625	GGB28927
GGB28934_SGB41635	GGB28934
GGB28934_SGB41635	GGB28934
GGB28946_SGB41652	GGB28946
GGB28946_SGB41652	GGB28946
GGB28949_SGB41655	GGB28949
GGB28949_SGB41655	GGB28949
GGB28949_SGB41656	GGB28949
GGB28949_SGB41656	GGB28949
GGB28950_SGB41657	GGB28950
GGB28950_SGB41657	GGB28950
GGB28951_SGB102295	GGB28951
GGB28951_SGB102295	GGB28951
GGB28951_SGB41658	GGB28951
GGB28951_SGB41658	GGB28951
GGB28954_SGB41662	GGB28954
GGB28954_SGB41662	GGB28954
GGB28956_SGB41665	GGB28956
GGB28956_SGB41665	GGB28956
GGB28960_SGB41669	GGB28960
GGB28960_SGB41669	GGB28960
GGB28967_SGB41678	GGB28967
GGB28967_SGB41678	GGB28967
GGB28991_SGB41705	GGB28991
GGB28991_SGB41705	GGB28991
GGB29002_SGB41718	GGB29002
GGB29002_SGB41718	GGB29002
GGB29003_SGB41719	GGB29003
GGB29003_SGB41719	GGB29003
GGB29011_SGB41731	GGB29011
GGB29011_SGB41731	GGB29011
GGB29531_SGB42317	GGB29531
GGB29531_SGB42317	GGB29531
GGB29685_SGB42494	GGB29685
GGB29685_SGB42494	GGB29685
GGB30141_SGB43066	GGB30141
GGB30141_SGB43066	GGB30141
GGB30286_SGB43248	GGB30286
GGB30286_SGB43248	GGB30286

GGB30303_SGB43268	GGB30303
GGB30303_SGB43268	GGB30303
GGB30413_SGB43452	GGB30413
GGB30413_SGB43452	GGB30413
GGB30454_SGB43514	GGB30454
GGB30454_SGB43514	GGB30454
GGB30455_SGB43519	GGB30455
GGB30455_SGB43519	GGB30455
GGB30461_SGB43527	GGB30461
GGB30461_SGB43527	GGB30461
GGB30461_SGB43530	GGB30461
GGB30461_SGB43530	GGB30461
GGB30463_SGB43537	GGB30463
GGB30463_SGB43537	GGB30463
GGB30473_SGB43557	GGB30473
GGB30473_SGB43557	GGB30473
GGB30475_SGB63182	GGB30475
GGB30475_SGB63182	GGB30475
GGB30861_SGB44083	GGB30861
GGB30861_SGB44083	GGB30861
GGB31312_SGB44628	GGB31312
GGB31312_SGB44628	GGB31312
GGB31438_SGB44768	GGB31438
GGB31438_SGB44768	GGB31438
GGB3171_SGB4185	GGB3171
GGB3171_SGB4185	GGB3171
GGB31823_SGB45199	GGB31823
GGB31823_SGB45199	GGB31823
GGB31853_SGB45233	GGB31853
GGB31853_SGB45233	GGB31853
GGB32371_SGB41694	GGB32371
GGB32371_SGB41694	GGB32371
GGB3793_SGB5158	GGB3793
GGB3793_SGB5158	GGB3793
GGB42598_SGB59794	GGB42598
GGB42598_SGB59794	GGB42598
GGB45656_SGB63370	GGB45656
GGB45656_SGB63370	GGB45656
GGB47127_SGB65054	GGB47127
GGB47127_SGB65054	GGB47127
GGB74395_SGB43521	GGB74395
GGB74395_SGB43521	GGB74395
GGB75053_SGB43494	GGB75053
GGB75053_SGB43494	GGB75053
GGB75109_SGB102238	GGB75109
GGB75109_SGB102238	GGB75109

GGB81440_SGB45230	GGB81440
GGB81440_SGB45230	GGB81440
Lachnospiraceae_bacterium	Lachnospiraceae_unclassified
Lachnospiraceae_bacterium	Lachnospiraceae_unclassified
Lachnospiraceae_bacterium_A2	Lachnospiraceae_unclassified
Lachnospiraceae_bacterium_A2	Lachnospiraceae_unclassified
Lachnospiraceae_bacterium_MD308	Lachnospiraceae_unclassified
Lachnospiraceae_bacterium_MD308	Lachnospiraceae_unclassified
Lachnospiraceae_bacterium_MD329	Lachnospiraceae_unclassified
Lachnospiraceae_bacterium_MD329	Lachnospiraceae_unclassified
Lachnospiraceae_unclassified_SGB41414	Lachnospiraceae_unclassified
Lachnospiraceae_unclassified_SGB41414	Lachnospiraceae_unclassified
Lachnospiraceae_unclassified_SGB41418	Lachnospiraceae_unclassified
Lachnospiraceae_unclassified_SGB41418	Lachnospiraceae_unclassified
Lachnospiraceae_unclassified_SGB41424	Lachnospiraceae_unclassified
Lachnospiraceae_unclassified_SGB41424	Lachnospiraceae_unclassified
Lachnospiraceae_unclassified_SGB41589	Lachnospiraceae_unclassified
Lachnospiraceae_unclassified_SGB41589	Lachnospiraceae_unclassified
Lactobacillus_johnsonii	Lactobacillus
Lactobacillus_johnsonii	Lactobacillus
Muribaculaceae_bacterium	Muribaculaceae_unclassified
Muribaculaceae_bacterium	Muribaculaceae_unclassified
Neglectibacter_sp_X4	Neglectibacter
Neglectibacter_sp_X4	Neglectibacter
Oscillospiraceae_bacterium	Oscillospiraceae_unclassified
Oscillospiraceae_bacterium	Oscillospiraceae_unclassified
Oscillospiraceae_unclassified_SGB43502	Oscillospiraceae_unclassified
Oscillospiraceae_unclassified_SGB43502	Oscillospiraceae_unclassified
Oscillospiraceae_unclassified_SGB43505	Oscillospiraceae_unclassified
Oscillospiraceae_unclassified_SGB43505	Oscillospiraceae_unclassified
Parasutterella_excrementihominis	Parasutterella
Parasutterella_excrementihominis	Parasutterella
richness	
richness	
Romboutsia_ilealis	Romboutsia
Romboutsia_ilealis	Romboutsia
Schaedlerella_arabinosiphila	Schaedlerella
Schaedlerella_arabinosiphila	Schaedlerella
shannon	
shannon	
Turicibacter_sp_1E2	Turicibacter
Turicibacter_sp_1E2	Turicibacter
Acetatifactor_muris	Acetatifactor
Acetatifactor_muris	Acetatifactor
Acetatifactor_SGB41546	Acetatifactor
Acetatifactor_SGB41546	Acetatifactor

Acutalibacter_muris	Acutalibacter
Acutalibacter_muris	Acutalibacter
Acutalibacter_sp_1XD8_36	Acutalibacter
Acutalibacter_sp_1XD8_36	Acutalibacter
Adlercreutzia_caecimuris	Adlercreutzia
Adlercreutzia_caecimuris	Adlercreutzia
Adlercreutzia_mucosicola	Adlercreutzia
Adlercreutzia_mucosicola	Adlercreutzia
Adlercreutzia_muris	Adlercreutzia
Adlercreutzia_muris	Adlercreutzia
Akkermansia_muciniphila	Akkermansia
Akkermansia_muciniphila	Akkermansia
Alistipes_sp_DSM_112343	Alistipes
Alistipes_sp_DSM_112343	Alistipes
Anaerotruncus_sp_1XD42_93	Anaerotruncus
Anaerotruncus_sp_1XD42_93	Anaerotruncus
Bacteria_unclassified_SGB102200	Bacteria_unclassified
Bacteria_unclassified_SGB102200	Bacteria_unclassified
Bacteria_unclassified_SGB41677	Bacteria_unclassified
Bacteria_unclassified_SGB41677	Bacteria_unclassified
Bacteria_unclassified_SGB43546	Bacteria_unclassified
Bacteria_unclassified_SGB43546	Bacteria_unclassified
bacterium_1XD42_54	Bacteria_unclassified
bacterium_1XD42_54	Bacteria_unclassified
bacterium_1XD42_76	Bacteria_unclassified
bacterium_1XD42_76	Bacteria_unclassified
bacterium_1xD8_48	Bacteria_unclassified
bacterium_1xD8_48	Bacteria_unclassified
Bacteroides_thetaiotaomicron	Bacteroides
Bacteroides_thetaiotaomicron	Bacteroides
berger_parker	
berger_parker	
Bifidobacterium_pseudolongum	Bifidobacterium
Bifidobacterium_pseudolongum	Bifidobacterium
Clostridia_bacterium	Clostridia_unclassified
Clostridia_bacterium	Clostridia_unclassified
Clostridiaceae_bacterium	Clostridiaceae_unclassified
Clostridiaceae_bacterium	Clostridiaceae_unclassified
Clostridiaceae_unclassified_SGB41663	Clostridiaceae_unclassified
Clostridiaceae_unclassified_SGB41663	Clostridiaceae_unclassified
Clostridiales_bacterium	Eubacteriales_unclassified
Clostridiales_bacterium	Eubacteriales_unclassified
Clostridium_cocleatum	Erysipelatoclostridium
Clostridium_cocleatum	Erysipelatoclostridium
Coriobacteriaceae_bacterium	Coriobacteriaceae_unclassified
Coriobacteriaceae_bacterium	Coriobacteriaceae_unclassified

Dorea_sp_5_2	Dorea
Dorea_sp_5_2	Dorea
Dubosiella_newyorkensis	Dubosiella
Dubosiella_newyorkensis	Dubosiella
Erysipelotrichales_bacterium	Erysipelotrichales_unclassified
Erysipelotrichales_bacterium	Erysipelotrichales_unclassified
Eubacteriaceae_bacterium	Eubacteriaceae_unclassified
Eubacteriaceae_bacterium	Eubacteriaceae_unclassified
Eubacteriaceae_unclassified_SGB94922	Eubacteriaceae_unclassified
Eubacteriaceae_unclassified_SGB94922	Eubacteriaceae_unclassified
GGB20149_SGB29430	GGB20149
GGB20149_SGB29430	GGB20149
GGB22635_SGB63107	GGB22635
GGB22635_SGB63107	GGB22635
GGB25041_SGB36960	GGB25041
GGB25041_SGB36960	GGB25041
GGB27876_SGB40310	GGB27876
GGB27876_SGB40310	GGB27876
GGB27878_SGB40312	GGB27878
GGB27878_SGB40312	GGB27878
GGB27918_SGB40356	GGB27918
GGB27918_SGB40356	GGB27918
GGB28382_SGB40962	GGB28382
GGB28382_SGB40962	GGB28382
GGB28399_SGB40980	GGB28399
GGB28399_SGB40980	GGB28399
GGB28411_SGB40993	GGB28411
GGB28411_SGB40993	GGB28411
GGB28415_SGB40997	GGB28415
GGB28415_SGB40997	GGB28415
GGB28430_SGB41013	GGB28430
GGB28430_SGB41013	GGB28430
GGB28439_SGB41022	GGB28439
GGB28439_SGB41022	GGB28439
GGB28778_SGB41431	GGB28778
GGB28778_SGB41431	GGB28778
GGB28784_SGB41437	GGB28784
GGB28784_SGB41437	GGB28784
GGB28792_SGB41445	GGB28792
GGB28792_SGB41445	GGB28792
GGB28798_SGB41451	GGB28798
GGB28798_SGB41451	GGB28798
GGB28802_SGB41455	GGB28802
GGB28802_SGB41455	GGB28802
GGB28818_SGB41473	GGB28818
GGB28818_SGB41473	GGB28818

GGB28828_SGB41484	GGB28828
GGB28828_SGB41484	GGB28828
GGB28851_SGB41518	GGB28851
GGB28851_SGB41518	GGB28851
GGB28859_SGB41528	GGB28859
GGB28859_SGB41528	GGB28859
GGB28864_SGB41535	GGB28864
GGB28864_SGB41535	GGB28864
GGB28869_SGB41543	GGB28869
GGB28869_SGB41543	GGB28869
GGB28883_SGB41564	GGB28883
GGB28883_SGB41564	GGB28883
GGB28892_SGB41573	GGB28892
GGB28892_SGB41573	GGB28892
GGB28893_SGB41574	GGB28893
GGB28893_SGB41574	GGB28893
GGB28898_SGB41580	GGB28898
GGB28898_SGB41580	GGB28898
GGB28904_SGB41597	GGB28904
GGB28904_SGB41597	GGB28904
GGB28916_SGB41612	GGB28916
GGB28916_SGB41612	GGB28916
GGB28924_SGB41621	GGB28924
GGB28924_SGB41621	GGB28924
GGB28926_SGB41624	GGB28926
GGB28926_SGB41624	GGB28926
GGB28927_SGB41625	GGB28927
GGB28927_SGB41625	GGB28927
GGB28934_SGB41635	GGB28934
GGB28934_SGB41635	GGB28934
GGB28946_SGB41652	GGB28946
GGB28946_SGB41652	GGB28946
GGB28949_SGB41655	GGB28949
GGB28949_SGB41655	GGB28949
GGB28949_SGB41656	GGB28949
GGB28949_SGB41656	GGB28949
GGB28950_SGB41657	GGB28950
GGB28950_SGB41657	GGB28950
GGB28951_SGB102295	GGB28951
GGB28951_SGB102295	GGB28951
GGB28951_SGB41658	GGB28951
GGB28951_SGB41658	GGB28951
GGB28954_SGB41662	GGB28954
GGB28954_SGB41662	GGB28954
GGB28956_SGB41665	GGB28956
GGB28956_SGB41665	GGB28956

GGB28960_SGB41669	GGB28960
GGB28960_SGB41669	GGB28960
GGB28967_SGB41678	GGB28967
GGB28967_SGB41678	GGB28967
GGB28991_SGB41705	GGB28991
GGB28991_SGB41705	GGB28991
GGB29002_SGB41718	GGB29002
GGB29002_SGB41718	GGB29002
GGB29003_SGB41719	GGB29003
GGB29003_SGB41719	GGB29003
GGB29011_SGB41731	GGB29011
GGB29011_SGB41731	GGB29011
GGB29531_SGB42317	GGB29531
GGB29531_SGB42317	GGB29531
GGB29685_SGB42494	GGB29685
GGB29685_SGB42494	GGB29685
GGB30141_SGB43066	GGB30141
GGB30141_SGB43066	GGB30141
GGB30286_SGB43248	GGB30286
GGB30286_SGB43248	GGB30286
GGB30303_SGB43268	GGB30303
GGB30303_SGB43268	GGB30303
GGB30413_SGB43452	GGB30413
GGB30413_SGB43452	GGB30413
GGB30454_SGB43514	GGB30454
GGB30454_SGB43514	GGB30454
GGB30455_SGB43519	GGB30455
GGB30455_SGB43519	GGB30455
GGB30461_SGB43527	GGB30461
GGB30461_SGB43527	GGB30461
GGB30461_SGB43530	GGB30461
GGB30461_SGB43530	GGB30461
GGB30463_SGB43537	GGB30463
GGB30463_SGB43537	GGB30463
GGB30473_SGB43557	GGB30473
GGB30473_SGB43557	GGB30473
GGB30475_SGB63182	GGB30475
GGB30475_SGB63182	GGB30475
GGB30861_SGB44083	GGB30861
GGB30861_SGB44083	GGB30861
GGB31312_SGB44628	GGB31312
GGB31312_SGB44628	GGB31312
GGB31438_SGB44768	GGB31438
GGB31438_SGB44768	GGB31438
GGB3171_SGB4185	GGB3171
GGB3171_SGB4185	GGB3171

GGB31823_SGB45199	GGB31823
GGB31823_SGB45199	GGB31823
GGB31853_SGB45233	GGB31853
GGB31853_SGB45233	GGB31853
GGB32371_SGB41694	GGB32371
GGB32371_SGB41694	GGB32371
GGB3793_SGB5158	GGB3793
GGB3793_SGB5158	GGB3793
GGB42598_SGB59794	GGB42598
GGB42598_SGB59794	GGB42598
GGB45656_SGB63370	GGB45656
GGB45656_SGB63370	GGB45656
GGB47127_SGB65054	GGB47127
GGB47127_SGB65054	GGB47127
GGB74395_SGB43521	GGB74395
GGB74395_SGB43521	GGB74395
GGB75053_SGB43494	GGB75053
GGB75053_SGB43494	GGB75053
GGB75109_SGB102238	GGB75109
GGB75109_SGB102238	GGB75109
GGB81440_SGB45230	GGB81440
GGB81440_SGB45230	GGB81440
Lachnospiraceae_bacterium	Lachnospiraceae_unclassified
Lachnospiraceae_bacterium	Lachnospiraceae_unclassified
Lachnospiraceae_bacterium_A2	Lachnospiraceae_unclassified
Lachnospiraceae_bacterium_A2	Lachnospiraceae_unclassified
Lachnospiraceae_bacterium_MD308	Lachnospiraceae_unclassified
Lachnospiraceae_bacterium_MD308	Lachnospiraceae_unclassified
Lachnospiraceae_bacterium_MD329	Lachnospiraceae_unclassified
Lachnospiraceae_bacterium_MD329	Lachnospiraceae_unclassified
Lachnospiraceae_unclassified_SGB41414	Lachnospiraceae_unclassified
Lachnospiraceae_unclassified_SGB41414	Lachnospiraceae_unclassified
Lachnospiraceae_unclassified_SGB41418	Lachnospiraceae_unclassified
Lachnospiraceae_unclassified_SGB41418	Lachnospiraceae_unclassified
Lachnospiraceae_unclassified_SGB41424	Lachnospiraceae_unclassified
Lachnospiraceae_unclassified_SGB41424	Lachnospiraceae_unclassified
Lachnospiraceae_unclassified_SGB41589	Lachnospiraceae_unclassified
Lachnospiraceae_unclassified_SGB41589	Lachnospiraceae_unclassified
Lactobacillus_johnsonii	Lactobacillus
Lactobacillus_johnsonii	Lactobacillus
Muribaculaceae_bacterium	Muribaculaceae_unclassified
Muribaculaceae_bacterium	Muribaculaceae_unclassified
Neglectibacter_sp_X4	Neglectibacter
Neglectibacter_sp_X4	Neglectibacter
Oscillospiraceae_bacterium	Oscillospiraceae_unclassified
Oscillospiraceae_bacterium	Oscillospiraceae_unclassified

Oscillospiraceae_unclassified_SGB43502	Oscillospiraceae_unclassified
Oscillospiraceae_unclassified_SGB43502	Oscillospiraceae_unclassified
Oscillospiraceae_unclassified_SGB43505	Oscillospiraceae_unclassified
Oscillospiraceae_unclassified_SGB43505	Oscillospiraceae_unclassified
Parasutterella_excrementihominis	Parasutterella
Parasutterella_excrementihominis	Parasutterella
richness	
richness	
Romboutsia_ilealis	Romboutsia
Romboutsia_ilealis	Romboutsia
Schaedlerella_arabinosiphila	Schaedlerella
Schaedlerella_arabinosiphila	Schaedlerella
shannon	
shannon	
Turicibacter_sp_1E2	Turicibacter
Turicibacter_sp_1E2	Turicibacter

Family	Order	Class
Lachnospiraceae	Eubacteriales	Clostridia
Lachnospiraceae	Eubacteriales	Clostridia
Lachnospiraceae	Eubacteriales	Clostridia
Lachnospiraceae	Eubacteriales	Clostridia
Oscillospiraceae	Eubacteriales	Clostridia
Oscillospiraceae	Eubacteriales	Clostridia
Oscillospiraceae	Eubacteriales	Clostridia
Oscillospiraceae	Eubacteriales	Clostridia
Eggerthellaceae	Eggerthellales	Coriobacteriia
Eggerthellaceae	Eggerthellales	Coriobacteriia
Eggerthellaceae	Eggerthellales	Coriobacteriia
Eggerthellaceae	Eggerthellales	Coriobacteriia
Eggerthellaceae	Eggerthellales	Coriobacteriia
Eggerthellaceae	Eggerthellales	Coriobacteriia
Akkermansiaceae	Verrucomicrobiales	Verrucomicrobiae
Akkermansiaceae	Verrucomicrobiales	Verrucomicrobiae
Rikenellaceae	Bacteroidales	Bacteroidia
Rikenellaceae	Bacteroidales	Bacteroidia
Oscillospiraceae	Eubacteriales	Clostridia
Oscillospiraceae	Eubacteriales	Clostridia
Bacteria_unclassified	Bacteria_unclassified	Bacteria_unclassified
Bacteria_unclassified	Bacteria_unclassified	Bacteria_unclassified
Bacteria_unclassified	Bacteria_unclassified	Bacteria_unclassified
Bacteria_unclassified	Bacteria_unclassified	Bacteria_unclassified
Bacteria_unclassified	Bacteria_unclassified	Bacteria_unclassified
Bacteria_unclassified	Bacteria_unclassified	Bacteria_unclassified
Bacteria_unclassified	Bacteria_unclassified	Bacteria_unclassified
Bacteria_unclassified	Bacteria_unclassified	Bacteria_unclassified
Bacteria_unclassified	Bacteria_unclassified	Bacteria_unclassified
Bacteria_unclassified	Bacteria_unclassified	Bacteria_unclassified
Bacteria_unclassified	Bacteria_unclassified	Bacteria_unclassified
Bacteria_unclassified	Bacteria_unclassified	Bacteria_unclassified
Bacteroidaceae	Bacteroidales	Bacteroidia
Bacteroidaceae	Bacteroidales	Bacteroidia
Bifidobacteriaceae	Bifidobacteriales	Actinomycetia
Bifidobacteriaceae	Bifidobacteriales	Actinomycetia
Clostridia_unclassified	Clostridia_unclassified	Clostridia
Clostridia_unclassified	Clostridia_unclassified	Clostridia
Clostridiaceae	Eubacteriales	Clostridia
Clostridiaceae	Eubacteriales	Clostridia
Clostridiaceae	Eubacteriales	Clostridia
Clostridiaceae	Eubacteriales	Clostridia
Eubacteriales_unclassified	Eubacteriales	Clostridia
Eubacteriales_unclassified	Eubacteriales	Clostridia

Erysipelotrichaceae	Erysipelotrichales	Erysipelotrichia
Erysipelotrichaceae	Erysipelotrichales	Erysipelotrichia
Coriobacteriaceae	Coriobacteriales	Coriobacteriia
Coriobacteriaceae	Coriobacteriales	Coriobacteriia
Lachnospiraceae	Eubacteriales	Clostridia
Lachnospiraceae	Eubacteriales	Clostridia
Erysipelotrichaceae	Erysipelotrichales	Erysipelotrichia
Erysipelotrichaceae	Erysipelotrichales	Erysipelotrichia
Erysipelotrichales_unclassified	Erysipelotrichales	Erysipelotrichia
Erysipelotrichales_unclassified	Erysipelotrichales	Erysipelotrichia
Eubacteriaceae	Eubacteriales	Clostridia
Eubacteriaceae	Eubacteriales	Clostridia
Eubacteriaceae	Eubacteriales	Clostridia
Eubacteriaceae	Eubacteriales	Clostridia
Lachnospiraceae	Eubacteriales	Clostridia
Lachnospiraceae	Eubacteriales	Clostridia
Eggerthellaceae	Eggerthellales	Coriobacteriia
Eggerthellaceae	Eggerthellales	Coriobacteriia
Lachnospiraceae	Eubacteriales	Clostridia
Lachnospiraceae	Eubacteriales	Clostridia
Muribaculaceae	Bacteroidales	Bacteroidia
Muribaculaceae	Bacteroidales	Bacteroidia
Muribaculaceae	Bacteroidales	Bacteroidia
Muribaculaceae	Bacteroidales	Bacteroidia
Muribaculaceae	Bacteroidales	Bacteroidia
Muribaculaceae	Bacteroidales	Bacteroidia
FGB9508	OFGB9508	CFGB9508
FGB9508	OFGB9508	CFGB9508
FGB2838	OFGB2838	CFGB2838
FGB2838	OFGB2838	CFGB2838
FGB2838	OFGB2838	CFGB2838
FGB2838	OFGB2838	CFGB2838
FGB2838	OFGB2838	CFGB2838
FGB2838	OFGB2838	CFGB2838
Pumilibacteraceae	Eubacteriales	Clostridia
Pumilibacteraceae	Eubacteriales	Clostridia
FGB28439	OFGB28439	CFGB28439
FGB28439	OFGB28439	CFGB28439
Clostridia_unclassified	Clostridia_unclassified	Clostridia
Clostridia_unclassified	Clostridia_unclassified	Clostridia
Eubacteriaceae	Eubacteriales	Clostridia
Eubacteriaceae	Eubacteriales	Clostridia
Lachnospiraceae	Eubacteriales	Clostridia
Lachnospiraceae	Eubacteriales	Clostridia
Lachnospiraceae	Eubacteriales	Clostridia
Lachnospiraceae	Eubacteriales	Clostridia

Lachnospiraceae	Eubacteriales	Clostridia
Lachnospiraceae	Eubacteriales	Clostridia
Lachnospiraceae	Eubacteriales	Clostridia
Lachnospiraceae	Eubacteriales	Clostridia
FGB77305	OFGB77305	CFGB77305
FGB77305	OFGB77305	CFGB77305
Clostridiaceae	Eubacteriales	Clostridia
Clostridiaceae	Eubacteriales	Clostridia
Lachnospiraceae	Eubacteriales	Clostridia
Lachnospiraceae	Eubacteriales	Clostridia
Lachnospiraceae	Eubacteriales	Clostridia
Lachnospiraceae	Eubacteriales	Clostridia
Lachnospiraceae	Eubacteriales	Clostridia
Lachnospiraceae	Eubacteriales	Clostridia
FGB9633	OFGB9633	CFGB9633
FGB9633	OFGB9633	CFGB9633
Bacteria_unclassified	Bacteria_unclassified	Bacteria_unclassified
Bacteria_unclassified	Bacteria_unclassified	Bacteria_unclassified
Bacteria_unclassified	Bacteria_unclassified	Bacteria_unclassified
Bacteria_unclassified	Bacteria_unclassified	Bacteria_unclassified
Bacteria_unclassified	Bacteria_unclassified	Bacteria_unclassified
Bacteria_unclassified	Bacteria_unclassified	Bacteria_unclassified
Bacteria_unclassified	Bacteria_unclassified	Bacteria_unclassified
Bacteria_unclassified	Bacteria_unclassified	Bacteria_unclassified
Lachnospiraceae	Eubacteriales	Clostridia
Lachnospiraceae	Eubacteriales	Clostridia
Lachnospiraceae	Eubacteriales	Clostridia
Lachnospiraceae	Eubacteriales	Clostridia
Lachnospiraceae	Eubacteriales	Clostridia
Lachnospiraceae	Eubacteriales	Clostridia
FGB77359	OFGB77359	CFGB77359
FGB77359	OFGB77359	CFGB77359
FGB9639	OFGB9639	CFGB9639
FGB9639	OFGB9639	CFGB9639
Lachnospiraceae	Eubacteriales	Clostridia
Lachnospiraceae	Eubacteriales	Clostridia
Lachnospiraceae	Eubacteriales	Clostridia
Lachnospiraceae	Eubacteriales	Clostridia
Lachnospiraceae	Eubacteriales	Clostridia
Lachnospiraceae	Eubacteriales	Clostridia
Clostridiaceae	Eubacteriales	Clostridia
Clostridiaceae	Eubacteriales	Clostridia
Clostridiaceae	Eubacteriales	Clostridia
Clostridiaceae	Eubacteriales	Clostridia
Clostridiaceae	Eubacteriales	Clostridia
Clostridiaceae	Eubacteriales	Clostridia

Clostridiaceae	Eubacteriales	Clostridia
Clostridiaceae	Eubacteriales	Clostridia
Clostridiaceae	Eubacteriales	Clostridia
Clostridiaceae	Eubacteriales	Clostridia
Clostridiaceae	Eubacteriales	Clostridia
Clostridiaceae	Eubacteriales	Clostridia
Clostridiaceae	Eubacteriales	Clostridia
Clostridiaceae	Eubacteriales	Clostridia
Eubacteriaceae	Eubacteriales	Clostridia
Eubacteriaceae	Eubacteriales	Clostridia
FGB9658	OFGB9658	CFGB9658
FGB9658	OFGB9658	CFGB9658
FGB9659	OFGB9659	CFGB9659
FGB9659	OFGB9659	CFGB9659
Bacteria_unclassified	Bacteria_unclassified	Bacteria_unclassified
Bacteria_unclassified	Bacteria_unclassified	Bacteria_unclassified
FGB9827	OFGB9827	CFGB9827
FGB9827	OFGB9827	CFGB9827
Eubacteriaceae	Eubacteriales	Clostridia
Eubacteriaceae	Eubacteriales	Clostridia
FGB77303	OFGB77303	CFGB77303
FGB77303	OFGB77303	CFGB77303
Eubacteriales_unclassified	Eubacteriales	Clostridia
Eubacteriales_unclassified	Eubacteriales	Clostridia
Oscillospiraceae	Eubacteriales	Clostridia
Oscillospiraceae	Eubacteriales	Clostridia
FGB30328	OFGB30328	CFGB30328
FGB30328	OFGB30328	CFGB30328
Oscillospiraceae	Eubacteriales	Clostridia
Oscillospiraceae	Eubacteriales	Clostridia
Oscillospiraceae	Eubacteriales	Clostridia
Oscillospiraceae	Eubacteriales	Clostridia
Oscillospiraceae	Eubacteriales	Clostridia
Oscillospiraceae	Eubacteriales	Clostridia
Oscillospiraceae	Eubacteriales	Clostridia
Oscillospiraceae	Eubacteriales	Clostridia
Oscillospiraceae	Eubacteriales	Clostridia
Oscillospiraceae	Eubacteriales	Clostridia
Oscillospiraceae	Eubacteriales	Clostridia
Oscillospiraceae	Eubacteriales	Clostridia
Oscillospiraceae	Eubacteriales	Clostridia
Oscillospiraceae	Eubacteriales	Clostridia
FGB77153	OFGB77153	CFGB77153
FGB77153	OFGB77153	CFGB77153
FGB1791	OFGB1791	CFGB1791
FGB1791	OFGB1791	CFGB1791

FGB10290	OFGB10290	CFGB10290
FGB10290	OFGB10290	CFGB10290
Oscillospiraceae	Eubacteriales	Clostridia
Oscillospiraceae	Eubacteriales	Clostridia
FGB1765	OFGB1765	CFGB1765
FGB1765	OFGB1765	CFGB1765
FGB10349	OFGB10349	CFGB10349
FGB10349	OFGB10349	CFGB10349
FGB10667	OFGB10667	CFGB10667
FGB10667	OFGB10667	CFGB10667
Lachnospiraceae	Eubacteriales	Clostridia
Lachnospiraceae	Eubacteriales	Clostridia
Lachnospiraceae	Eubacteriales	Clostridia
Lachnospiraceae	Eubacteriales	Clostridia
Christensenellaceae	Eubacteriales	Clostridia
Christensenellaceae	Eubacteriales	Clostridia
FGB10299	OFGB10299	CFGB10299
FGB10299	OFGB10299	CFGB10299
Oscillospiraceae	Eubacteriales	Clostridia
Oscillospiraceae	Eubacteriales	Clostridia
Oscillospiraceae	Eubacteriales	Clostridia
Oscillospiraceae	Eubacteriales	Clostridia
Lachnospiraceae	Eubacteriales	Clostridia
Lachnospiraceae	Eubacteriales	Clostridia
Clostridia_unclassified	Clostridia_unclassified	Clostridia
Clostridia_unclassified	Clostridia_unclassified	Clostridia
Lachnospiraceae	Eubacteriales	Clostridia
Lachnospiraceae	Eubacteriales	Clostridia
Lachnospiraceae	Eubacteriales	Clostridia
Lachnospiraceae	Eubacteriales	Clostridia
Lachnospiraceae	Eubacteriales	Clostridia
Lachnospiraceae	Eubacteriales	Clostridia
Lachnospiraceae	Eubacteriales	Clostridia
Lachnospiraceae	Eubacteriales	Clostridia
Lachnospiraceae	Eubacteriales	Clostridia
Lachnospiraceae	Eubacteriales	Clostridia
Lachnospiraceae	Eubacteriales	Clostridia
Lachnospiraceae	Eubacteriales	Clostridia
Lachnospiraceae	Eubacteriales	Clostridia
Lachnospiraceae	Eubacteriales	Clostridia
Lachnospiraceae	Eubacteriales	Clostridia
Lachnospiraceae	Eubacteriales	Clostridia
Lactobacillaceae	Lactobacillales	Bacilli
Lactobacillaceae	Lactobacillales	Bacilli
Muribaculaceae	Bacteroidales	Bacteroidia
Muribaculaceae	Bacteroidales	Bacteroidia

Oscillospiraceae	Eubacteriales	Clostridia
Oscillospiraceae	Eubacteriales	Clostridia
Oscillospiraceae	Eubacteriales	Clostridia
Oscillospiraceae	Eubacteriales	Clostridia
Oscillospiraceae	Eubacteriales	Clostridia
Oscillospiraceae	Eubacteriales	Clostridia
Oscillospiraceae	Eubacteriales	Clostridia
Oscillospiraceae	Eubacteriales	Clostridia
Sutterellaceae	Burkholderiales	Betaproteobacteria
Sutterellaceae	Burkholderiales	Betaproteobacteria
Peptostreptococcaceae	Eubacteriales	Clostridia
Peptostreptococcaceae	Eubacteriales	Clostridia
Lachnospiraceae	Eubacteriales	Clostridia
Lachnospiraceae	Eubacteriales	Clostridia
Turicibacteraceae	Erysipelotrichales	Erysipelotrichia
Turicibacteraceae	Erysipelotrichales	Erysipelotrichia
Lachnospiraceae	Eubacteriales	Clostridia
Lachnospiraceae	Eubacteriales	Clostridia
Lachnospiraceae	Eubacteriales	Clostridia
Lachnospiraceae	Eubacteriales	Clostridia
Oscillospiraceae	Eubacteriales	Clostridia
Oscillospiraceae	Eubacteriales	Clostridia
Oscillospiraceae	Eubacteriales	Clostridia
Oscillospiraceae	Eubacteriales	Clostridia
Eggerthellaceae	Eggerthellales	Coriobacteriia
Eggerthellaceae	Eggerthellales	Coriobacteriia
Eggerthellaceae	Eggerthellales	Coriobacteriia
Eggerthellaceae	Eggerthellales	Coriobacteriia
Eggerthellaceae	Eggerthellales	Coriobacteriia
Eggerthellaceae	Eggerthellales	Coriobacteriia
Akkermansiaceae	Verrucomicrobiales	Verrucomicrobiae
Akkermansiaceae	Verrucomicrobiales	Verrucomicrobiae
Rikenellaceae	Bacteroidales	Bacteroidia
Rikenellaceae	Bacteroidales	Bacteroidia
Oscillospiraceae	Eubacteriales	Clostridia
Oscillospiraceae	Eubacteriales	Clostridia
Bacteria_unclassified	Bacteria_unclassified	Bacteria_unclassified
Bacteria_unclassified	Bacteria_unclassified	Bacteria_unclassified
Bacteria_unclassified	Bacteria_unclassified	Bacteria_unclassified
Bacteria_unclassified	Bacteria_unclassified	Bacteria_unclassified
Bacteria_unclassified	Bacteria_unclassified	Bacteria_unclassified
Bacteria_unclassified	Bacteria_unclassified	Bacteria_unclassified

Bacteria_unclassified	Bacteria_unclassified	Bacteria_unclassified
Bacteria_unclassified	Bacteria_unclassified	Bacteria_unclassified
Bacteria_unclassified	Bacteria_unclassified	Bacteria_unclassified
Bacteria_unclassified	Bacteria_unclassified	Bacteria_unclassified
Bacteria_unclassified	Bacteria_unclassified	Bacteria_unclassified
Bacteria_unclassified	Bacteria_unclassified	Bacteria_unclassified
Bacteroidaceae	Bacteroidales	Bacteroidia
Bacteroidaceae	Bacteroidales	Bacteroidia

Bifidobacteriaceae	Bifidobacteriales	Actinomycetia
Bifidobacteriaceae	Bifidobacteriales	Actinomycetia
Clostridia_unclassified	Clostridia_unclassified	Clostridia
Clostridia_unclassified	Clostridia_unclassified	Clostridia
Clostridiaceae	Eubacteriales	Clostridia
Clostridiaceae	Eubacteriales	Clostridia
Clostridiaceae	Eubacteriales	Clostridia
Clostridiaceae	Eubacteriales	Clostridia
Eubacteriales_unclassified	Eubacteriales	Clostridia
Eubacteriales_unclassified	Eubacteriales	Clostridia
Erysipelotrichaceae	Erysipelotrichales	Erysipelotrichia
Erysipelotrichaceae	Erysipelotrichales	Erysipelotrichia
Coriobacteriaceae	Coriobacteriales	Coriobacteriia
Coriobacteriaceae	Coriobacteriales	Coriobacteriia
Lachnospiraceae	Eubacteriales	Clostridia
Lachnospiraceae	Eubacteriales	Clostridia
Erysipelotrichaceae	Erysipelotrichales	Erysipelotrichia
Erysipelotrichaceae	Erysipelotrichales	Erysipelotrichia
Erysipelotrichales_unclassified	Erysipelotrichales	Erysipelotrichia
Erysipelotrichales_unclassified	Erysipelotrichales	Erysipelotrichia
Eubacteriaceae	Eubacteriales	Clostridia
Eubacteriaceae	Eubacteriales	Clostridia
Eubacteriaceae	Eubacteriales	Clostridia
Eubacteriaceae	Eubacteriales	Clostridia
Lachnospiraceae	Eubacteriales	Clostridia
Lachnospiraceae	Eubacteriales	Clostridia
Eggerthellaceae	Eggerthellales	Coriobacteriia
Eggerthellaceae	Eggerthellales	Coriobacteriia
Lachnospiraceae	Eubacteriales	Clostridia
Lachnospiraceae	Eubacteriales	Clostridia
Muribaculaceae	Bacteroidales	Bacteroidia
Muribaculaceae	Bacteroidales	Bacteroidia
Muribaculaceae	Bacteroidales	Bacteroidia
Muribaculaceae	Bacteroidales	Bacteroidia
Muribaculaceae	Bacteroidales	Bacteroidia
Muribaculaceae	Bacteroidales	Bacteroidia

FGB9508	OFGB9508	CFGB9508
FGB9508	OFGB9508	CFGB9508
FGB2838	OFGB2838	CFGB2838
FGB2838	OFGB2838	CFGB2838
FGB2838	OFGB2838	CFGB2838
FGB2838	OFGB2838	CFGB2838
FGB2838	OFGB2838	CFGB2838
FGB2838	OFGB2838	CFGB2838
Pumilibacteraceae	Eubacteriales	Clostridia
Pumilibacteraceae	Eubacteriales	Clostridia
FGB28439	OFGB28439	CFGB28439
FGB28439	OFGB28439	CFGB28439
Clostridia_unclassified	Clostridia_unclassified	Clostridia
Clostridia_unclassified	Clostridia_unclassified	Clostridia
Eubacteriaceae	Eubacteriales	Clostridia
Eubacteriaceae	Eubacteriales	Clostridia
Lachnospiraceae	Eubacteriales	Clostridia
Lachnospiraceae	Eubacteriales	Clostridia
Lachnospiraceae	Eubacteriales	Clostridia
Lachnospiraceae	Eubacteriales	Clostridia
Lachnospiraceae	Eubacteriales	Clostridia
Lachnospiraceae	Eubacteriales	Clostridia
Lachnospiraceae	Eubacteriales	Clostridia
Lachnospiraceae	Eubacteriales	Clostridia
FGB77305	OFGB77305	CFGB77305
FGB77305	OFGB77305	CFGB77305
Clostridiaceae	Eubacteriales	Clostridia
Clostridiaceae	Eubacteriales	Clostridia
Lachnospiraceae	Eubacteriales	Clostridia
Lachnospiraceae	Eubacteriales	Clostridia
Lachnospiraceae	Eubacteriales	Clostridia
Lachnospiraceae	Eubacteriales	Clostridia
Lachnospiraceae	Eubacteriales	Clostridia
Lachnospiraceae	Eubacteriales	Clostridia
FGB9633	OFGB9633	CFGB9633
FGB9633	OFGB9633	CFGB9633
Bacteria_unclassified	Bacteria_unclassified	Bacteria_unclassified
Bacteria_unclassified	Bacteria_unclassified	Bacteria_unclassified
Bacteria_unclassified	Bacteria_unclassified	Bacteria_unclassified
Bacteria_unclassified	Bacteria_unclassified	Bacteria_unclassified
Bacteria_unclassified	Bacteria_unclassified	Bacteria_unclassified
Bacteria_unclassified	Bacteria_unclassified	Bacteria_unclassified
Bacteria_unclassified	Bacteria_unclassified	Bacteria_unclassified
Bacteria_unclassified	Bacteria_unclassified	Bacteria_unclassified
Lachnospiraceae	Eubacteriales	Clostridia
Lachnospiraceae	Eubacteriales	Clostridia

Lachnospiraceae	Eubacteriales	Clostridia
Lachnospiraceae	Eubacteriales	Clostridia
Lachnospiraceae	Eubacteriales	Clostridia
Lachnospiraceae	Eubacteriales	Clostridia
FGB77359	OFGB77359	CFGB77359
FGB77359	OFGB77359	CFGB77359
FGB9639	OFGB9639	CFGB9639
FGB9639	OFGB9639	CFGB9639
Lachnospiraceae	Eubacteriales	Clostridia
Lachnospiraceae	Eubacteriales	Clostridia
Lachnospiraceae	Eubacteriales	Clostridia
Lachnospiraceae	Eubacteriales	Clostridia
Lachnospiraceae	Eubacteriales	Clostridia
Lachnospiraceae	Eubacteriales	Clostridia
Clostridiaceae	Eubacteriales	Clostridia
Clostridiaceae	Eubacteriales	Clostridia
Clostridiaceae	Eubacteriales	Clostridia
Clostridiaceae	Eubacteriales	Clostridia
Clostridiaceae	Eubacteriales	Clostridia
Clostridiaceae	Eubacteriales	Clostridia
Clostridiaceae	Eubacteriales	Clostridia
Clostridiaceae	Eubacteriales	Clostridia
Clostridiaceae	Eubacteriales	Clostridia
Clostridiaceae	Eubacteriales	Clostridia
Clostridiaceae	Eubacteriales	Clostridia
Clostridiaceae	Eubacteriales	Clostridia
Clostridiaceae	Eubacteriales	Clostridia
Clostridiaceae	Eubacteriales	Clostridia
Eubacteriaceae	Eubacteriales	Clostridia
Eubacteriaceae	Eubacteriales	Clostridia
FGB9658	OFGB9658	CFGB9658
FGB9658	OFGB9658	CFGB9658
FGB9659	OFGB9659	CFGB9659
FGB9659	OFGB9659	CFGB9659
Bacteria_unclassified	Bacteria_unclassified	Bacteria_unclassified
Bacteria_unclassified	Bacteria_unclassified	Bacteria_unclassified
FGB9827	OFGB9827	CFGB9827
FGB9827	OFGB9827	CFGB9827
Eubacteriaceae	Eubacteriales	Clostridia
Eubacteriaceae	Eubacteriales	Clostridia
FGB77303	OFGB77303	CFGB77303
FGB77303	OFGB77303	CFGB77303
Eubacteriales_unclassified	Eubacteriales	Clostridia
Eubacteriales_unclassified	Eubacteriales	Clostridia
Oscillospiraceae	Eubacteriales	Clostridia
Oscillospiraceae	Eubacteriales	Clostridia

FGB30328	OFGB30328	CFGB30328
FGB30328	OFGB30328	CFGB30328
Oscillospiraceae	Eubacteriales	Clostridia
Oscillospiraceae	Eubacteriales	Clostridia
Oscillospiraceae	Eubacteriales	Clostridia
Oscillospiraceae	Eubacteriales	Clostridia
Oscillospiraceae	Eubacteriales	Clostridia
Oscillospiraceae	Eubacteriales	Clostridia
Oscillospiraceae	Eubacteriales	Clostridia
Oscillospiraceae	Eubacteriales	Clostridia
Oscillospiraceae	Eubacteriales	Clostridia
Oscillospiraceae	Eubacteriales	Clostridia
Oscillospiraceae	Eubacteriales	Clostridia
Oscillospiraceae	Eubacteriales	Clostridia
Oscillospiraceae	Eubacteriales	Clostridia
Oscillospiraceae	Eubacteriales	Clostridia
FGB77153	OFGB77153	CFGB77153
FGB77153	OFGB77153	CFGB77153
FGB1791	OFGB1791	CFGB1791
FGB1791	OFGB1791	CFGB1791
FGB10290	OFGB10290	CFGB10290
FGB10290	OFGB10290	CFGB10290
Oscillospiraceae	Eubacteriales	Clostridia
Oscillospiraceae	Eubacteriales	Clostridia
FGB1765	OFGB1765	CFGB1765
FGB1765	OFGB1765	CFGB1765
FGB10349	OFGB10349	CFGB10349
FGB10349	OFGB10349	CFGB10349
FGB10667	OFGB10667	CFGB10667
FGB10667	OFGB10667	CFGB10667
Lachnospiraceae	Eubacteriales	Clostridia
Lachnospiraceae	Eubacteriales	Clostridia
Lachnospiraceae	Eubacteriales	Clostridia
Lachnospiraceae	Eubacteriales	Clostridia
Christensenellaceae	Eubacteriales	Clostridia
Christensenellaceae	Eubacteriales	Clostridia
FGB10299	OFGB10299	CFGB10299
FGB10299	OFGB10299	CFGB10299
Oscillospiraceae	Eubacteriales	Clostridia
Oscillospiraceae	Eubacteriales	Clostridia
Oscillospiraceae	Eubacteriales	Clostridia
Oscillospiraceae	Eubacteriales	Clostridia
Lachnospiraceae	Eubacteriales	Clostridia
Lachnospiraceae	Eubacteriales	Clostridia
Clostridia_unclassified	Clostridia_unclassified	Clostridia
Clostridia_unclassified	Clostridia_unclassified	Clostridia

Lachnospiraceae	Eubacteriales	Clostridia
Lachnospiraceae	Eubacteriales	Clostridia
Lachnospiraceae	Eubacteriales	Clostridia
Lachnospiraceae	Eubacteriales	Clostridia
Lachnospiraceae	Eubacteriales	Clostridia
Lachnospiraceae	Eubacteriales	Clostridia
Lachnospiraceae	Eubacteriales	Clostridia
Lachnospiraceae	Eubacteriales	Clostridia
Lachnospiraceae	Eubacteriales	Clostridia
Lachnospiraceae	Eubacteriales	Clostridia
Lachnospiraceae	Eubacteriales	Clostridia
Lachnospiraceae	Eubacteriales	Clostridia
Lachnospiraceae	Eubacteriales	Clostridia
Lachnospiraceae	Eubacteriales	Clostridia
Lachnospiraceae	Eubacteriales	Clostridia
Lachnospiraceae	Eubacteriales	Clostridia
Lactobacillaceae	Lactobacillales	Bacilli
Lactobacillaceae	Lactobacillales	Bacilli
Muribaculaceae	Bacteroidales	Bacteroidia
Muribaculaceae	Bacteroidales	Bacteroidia
Oscillospiraceae	Eubacteriales	Clostridia
Oscillospiraceae	Eubacteriales	Clostridia
Oscillospiraceae	Eubacteriales	Clostridia
Oscillospiraceae	Eubacteriales	Clostridia
Oscillospiraceae	Eubacteriales	Clostridia
Oscillospiraceae	Eubacteriales	Clostridia
Oscillospiraceae	Eubacteriales	Clostridia
Oscillospiraceae	Eubacteriales	Clostridia
Sutterellaceae	Burkholderiales	Betaproteobacteria
Sutterellaceae	Burkholderiales	Betaproteobacteria

Peptostreptococcaceae	Eubacteriales	Clostridia
Peptostreptococcaceae	Eubacteriales	Clostridia
Lachnospiraceae	Eubacteriales	Clostridia
Lachnospiraceae	Eubacteriales	Clostridia

Turicibacteraceae	Erysipelotrichales	Erysipelotrichia
Turicibacteraceae	Erysipelotrichales	Erysipelotrichia
Lachnospiraceae	Eubacteriales	Clostridia
Lachnospiraceae	Eubacteriales	Clostridia
Lachnospiraceae	Eubacteriales	Clostridia
Lachnospiraceae	Eubacteriales	Clostridia
Oscillospiraceae	Eubacteriales	Clostridia
Oscillospiraceae	Eubacteriales	Clostridia

Oscillospiraceae	Eubacteriales	Clostridia
Oscillospiraceae	Eubacteriales	Clostridia
Eggerthellaceae	Eggerthellales	Coriobacteriia
Eggerthellaceae	Eggerthellales	Coriobacteriia
Eggerthellaceae	Eggerthellales	Coriobacteriia
Eggerthellaceae	Eggerthellales	Coriobacteriia
Eggerthellaceae	Eggerthellales	Coriobacteriia
Eggerthellaceae	Eggerthellales	Coriobacteriia
Akkermansiaceae	Verrucomicrobiales	Verrucomicrobiae
Akkermansiaceae	Verrucomicrobiales	Verrucomicrobiae
Rikenellaceae	Bacteroidales	Bacteroidia
Rikenellaceae	Bacteroidales	Bacteroidia
Oscillospiraceae	Eubacteriales	Clostridia
Oscillospiraceae	Eubacteriales	Clostridia
Bacteria_unclassified	Bacteria_unclassified	Bacteria_unclassified
Bacteria_unclassified	Bacteria_unclassified	Bacteria_unclassified
Bacteria_unclassified	Bacteria_unclassified	Bacteria_unclassified
Bacteria_unclassified	Bacteria_unclassified	Bacteria_unclassified
Bacteria_unclassified	Bacteria_unclassified	Bacteria_unclassified
Bacteria_unclassified	Bacteria_unclassified	Bacteria_unclassified
Bacteria_unclassified	Bacteria_unclassified	Bacteria_unclassified
Bacteria_unclassified	Bacteria_unclassified	Bacteria_unclassified
Bacteria_unclassified	Bacteria_unclassified	Bacteria_unclassified
Bacteria_unclassified	Bacteria_unclassified	Bacteria_unclassified
Bacteria_unclassified	Bacteria_unclassified	Bacteria_unclassified
Bacteria_unclassified	Bacteria_unclassified	Bacteria_unclassified
Bacteroidaceae	Bacteroidales	Bacteroidia
Bacteroidaceae	Bacteroidales	Bacteroidia

Bifidobacteriaceae	Bifidobacteriales	Actinomycetia
Bifidobacteriaceae	Bifidobacteriales	Actinomycetia
Clostridia_unclassified	Clostridia_unclassified	Clostridia
Clostridia_unclassified	Clostridia_unclassified	Clostridia
Clostridiaceae	Eubacteriales	Clostridia
Clostridiaceae	Eubacteriales	Clostridia
Clostridiaceae	Eubacteriales	Clostridia
Clostridiaceae	Eubacteriales	Clostridia
Eubacteriales_unclassified	Eubacteriales	Clostridia
Eubacteriales_unclassified	Eubacteriales	Clostridia
Erysipelotrichaceae	Erysipelotrichales	Erysipelotrichia
Erysipelotrichaceae	Erysipelotrichales	Erysipelotrichia
Coriobacteriaceae	Coriobacteriales	Coriobacteriia
Coriobacteriaceae	Coriobacteriales	Coriobacteriia
Lachnospiraceae	Eubacteriales	Clostridia
Lachnospiraceae	Eubacteriales	Clostridia

Erysipelotrichaceae	Erysipelotrichales	Erysipelotrichia
Erysipelotrichaceae	Erysipelotrichales	Erysipelotrichia
Erysipelotrichales_unclassified	Erysipelotrichales	Erysipelotrichia
Erysipelotrichales_unclassified	Erysipelotrichales	Erysipelotrichia
Eubacteriaceae	Eubacteriales	Clostridia
Eubacteriaceae	Eubacteriales	Clostridia
Eubacteriaceae	Eubacteriales	Clostridia
Eubacteriaceae	Eubacteriales	Clostridia
Lachnospiraceae	Eubacteriales	Clostridia
Lachnospiraceae	Eubacteriales	Clostridia
Eggerthellaceae	Eggerthellales	Coriobacteriia
Eggerthellaceae	Eggerthellales	Coriobacteriia
Lachnospiraceae	Eubacteriales	Clostridia
Lachnospiraceae	Eubacteriales	Clostridia
Muribaculaceae	Bacteroidales	Bacteroidia
Muribaculaceae	Bacteroidales	Bacteroidia
Muribaculaceae	Bacteroidales	Bacteroidia
Muribaculaceae	Bacteroidales	Bacteroidia
Muribaculaceae	Bacteroidales	Bacteroidia
Muribaculaceae	Bacteroidales	Bacteroidia
FGB9508	OFGB9508	CFGB9508
FGB9508	OFGB9508	CFGB9508
FGB2838	OFGB2838	CFGB2838
FGB2838	OFGB2838	CFGB2838
FGB2838	OFGB2838	CFGB2838
FGB2838	OFGB2838	CFGB2838
FGB2838	OFGB2838	CFGB2838
FGB2838	OFGB2838	CFGB2838
Pumilibacteraceae	Eubacteriales	Clostridia
Pumilibacteraceae	Eubacteriales	Clostridia
FGB28439	OFGB28439	CFGB28439
FGB28439	OFGB28439	CFGB28439
Clostridia_unclassified	Clostridia_unclassified	Clostridia
Clostridia_unclassified	Clostridia_unclassified	Clostridia
Eubacteriaceae	Eubacteriales	Clostridia
Eubacteriaceae	Eubacteriales	Clostridia
Lachnospiraceae	Eubacteriales	Clostridia
Lachnospiraceae	Eubacteriales	Clostridia
Lachnospiraceae	Eubacteriales	Clostridia
Lachnospiraceae	Eubacteriales	Clostridia
Lachnospiraceae	Eubacteriales	Clostridia
Lachnospiraceae	Eubacteriales	Clostridia
Lachnospiraceae	Eubacteriales	Clostridia
Lachnospiraceae	Eubacteriales	Clostridia
FGB77305	OFGB77305	CFGB77305
FGB77305	OFGB77305	CFGB77305

Clostridiaceae	Eubacteriales	Clostridia
Clostridiaceae	Eubacteriales	Clostridia
Lachnospiraceae	Eubacteriales	Clostridia
Lachnospiraceae	Eubacteriales	Clostridia
Lachnospiraceae	Eubacteriales	Clostridia
Lachnospiraceae	Eubacteriales	Clostridia
Lachnospiraceae	Eubacteriales	Clostridia
Lachnospiraceae	Eubacteriales	Clostridia
FGB9633	OFGB9633	CFGB9633
FGB9633	OFGB9633	CFGB9633
Bacteria_unclassified	Bacteria_unclassified	Bacteria_unclassified
Bacteria_unclassified	Bacteria_unclassified	Bacteria_unclassified
Bacteria_unclassified	Bacteria_unclassified	Bacteria_unclassified
Bacteria_unclassified	Bacteria_unclassified	Bacteria_unclassified
Bacteria_unclassified	Bacteria_unclassified	Bacteria_unclassified
Bacteria_unclassified	Bacteria_unclassified	Bacteria_unclassified
Bacteria_unclassified	Bacteria_unclassified	Bacteria_unclassified
Bacteria_unclassified	Bacteria_unclassified	Bacteria_unclassified
Lachnospiraceae	Eubacteriales	Clostridia
Lachnospiraceae	Eubacteriales	Clostridia
Lachnospiraceae	Eubacteriales	Clostridia
Lachnospiraceae	Eubacteriales	Clostridia
Lachnospiraceae	Eubacteriales	Clostridia
Lachnospiraceae	Eubacteriales	Clostridia
FGB77359	OFGB77359	CFGB77359
FGB77359	OFGB77359	CFGB77359
FGB9639	OFGB9639	CFGB9639
FGB9639	OFGB9639	CFGB9639
Lachnospiraceae	Eubacteriales	Clostridia
Lachnospiraceae	Eubacteriales	Clostridia
Lachnospiraceae	Eubacteriales	Clostridia
Lachnospiraceae	Eubacteriales	Clostridia
Lachnospiraceae	Eubacteriales	Clostridia
Lachnospiraceae	Eubacteriales	Clostridia
Clostridiaceae	Eubacteriales	Clostridia
Clostridiaceae	Eubacteriales	Clostridia
Clostridiaceae	Eubacteriales	Clostridia
Clostridiaceae	Eubacteriales	Clostridia
Clostridiaceae	Eubacteriales	Clostridia
Clostridiaceae	Eubacteriales	Clostridia
Clostridiaceae	Eubacteriales	Clostridia
Clostridiaceae	Eubacteriales	Clostridia
Clostridiaceae	Eubacteriales	Clostridia
Clostridiaceae	Eubacteriales	Clostridia
Clostridiaceae	Eubacteriales	Clostridia
Clostridiaceae	Eubacteriales	Clostridia

Clostridiaceae	Eubacteriales	Clostridia
Clostridiaceae	Eubacteriales	Clostridia
Eubacteriaceae	Eubacteriales	Clostridia
Eubacteriaceae	Eubacteriales	Clostridia
FGB9658	OFGB9658	CFGB9658
FGB9658	OFGB9658	CFGB9658
FGB9659	OFGB9659	CFGB9659
FGB9659	OFGB9659	CFGB9659
Bacteria_unclassified	Bacteria_unclassified	Bacteria_unclassified
Bacteria_unclassified	Bacteria_unclassified	Bacteria_unclassified
FGB9827	OFGB9827	CFGB9827
FGB9827	OFGB9827	CFGB9827
Eubacteriaceae	Eubacteriales	Clostridia
Eubacteriaceae	Eubacteriales	Clostridia
FGB77303	OFGB77303	CFGB77303
FGB77303	OFGB77303	CFGB77303
Eubacteriales_unclassified	Eubacteriales	Clostridia
Eubacteriales_unclassified	Eubacteriales	Clostridia
Oscillospiraceae	Eubacteriales	Clostridia
Oscillospiraceae	Eubacteriales	Clostridia
FGB30328	OFGB30328	CFGB30328
FGB30328	OFGB30328	CFGB30328
Oscillospiraceae	Eubacteriales	Clostridia
Oscillospiraceae	Eubacteriales	Clostridia
Oscillospiraceae	Eubacteriales	Clostridia
Oscillospiraceae	Eubacteriales	Clostridia
Oscillospiraceae	Eubacteriales	Clostridia
Oscillospiraceae	Eubacteriales	Clostridia
Oscillospiraceae	Eubacteriales	Clostridia
Oscillospiraceae	Eubacteriales	Clostridia
Oscillospiraceae	Eubacteriales	Clostridia
Oscillospiraceae	Eubacteriales	Clostridia
Oscillospiraceae	Eubacteriales	Clostridia
Oscillospiraceae	Eubacteriales	Clostridia
Oscillospiraceae	Eubacteriales	Clostridia
Oscillospiraceae	Eubacteriales	Clostridia
FGB77153	OFGB77153	CFGB77153
FGB77153	OFGB77153	CFGB77153
FGB1791	OFGB1791	CFGB1791
FGB1791	OFGB1791	CFGB1791
FGB10290	OFGB10290	CFGB10290
FGB10290	OFGB10290	CFGB10290
Oscillospiraceae	Eubacteriales	Clostridia
Oscillospiraceae	Eubacteriales	Clostridia
FGB1765	OFGB1765	CFGB1765
FGB1765	OFGB1765	CFGB1765

FGB10349	OFGB10349	CFGB10349
FGB10349	OFGB10349	CFGB10349
FGB10667	OFGB10667	CFGB10667
FGB10667	OFGB10667	CFGB10667
Lachnospiraceae	Eubacteriales	Clostridia
Lachnospiraceae	Eubacteriales	Clostridia
Lachnospiraceae	Eubacteriales	Clostridia
Lachnospiraceae	Eubacteriales	Clostridia
Christensenellaceae	Eubacteriales	Clostridia
Christensenellaceae	Eubacteriales	Clostridia
FGB10299	OFGB10299	CFGB10299
FGB10299	OFGB10299	CFGB10299
Oscillospiraceae	Eubacteriales	Clostridia
Oscillospiraceae	Eubacteriales	Clostridia
Oscillospiraceae	Eubacteriales	Clostridia
Oscillospiraceae	Eubacteriales	Clostridia
Lachnospiraceae	Eubacteriales	Clostridia
Lachnospiraceae	Eubacteriales	Clostridia
Clostridia_unclassified	Clostridia_unclassified	Clostridia
Clostridia_unclassified	Clostridia_unclassified	Clostridia
Lachnospiraceae	Eubacteriales	Clostridia
Lachnospiraceae	Eubacteriales	Clostridia
Lachnospiraceae	Eubacteriales	Clostridia
Lachnospiraceae	Eubacteriales	Clostridia
Lachnospiraceae	Eubacteriales	Clostridia
Lachnospiraceae	Eubacteriales	Clostridia
Lachnospiraceae	Eubacteriales	Clostridia
Lachnospiraceae	Eubacteriales	Clostridia
Lachnospiraceae	Eubacteriales	Clostridia
Lachnospiraceae	Eubacteriales	Clostridia
Lachnospiraceae	Eubacteriales	Clostridia
Lachnospiraceae	Eubacteriales	Clostridia
Lachnospiraceae	Eubacteriales	Clostridia
Lachnospiraceae	Eubacteriales	Clostridia
Lachnospiraceae	Eubacteriales	Clostridia
Lachnospiraceae	Eubacteriales	Clostridia
Lactobacillaceae	Lactobacillales	Bacilli
Lactobacillaceae	Lactobacillales	Bacilli
Muribaculaceae	Bacteroidales	Bacteroidia
Muribaculaceae	Bacteroidales	Bacteroidia
Oscillospiraceae	Eubacteriales	Clostridia
Oscillospiraceae	Eubacteriales	Clostridia
Oscillospiraceae	Eubacteriales	Clostridia
Oscillospiraceae	Eubacteriales	Clostridia
Oscillospiraceae	Eubacteriales	Clostridia
Oscillospiraceae	Eubacteriales	Clostridia

Oscillospiraceae	Eubacteriales	Clostridia
Oscillospiraceae	Eubacteriales	Clostridia
Sutterellaceae	Burkholderiales	Betaproteobacteria
Sutterellaceae	Burkholderiales	Betaproteobacteria
Peptostreptococcaceae	Eubacteriales	Clostridia
Peptostreptococcaceae	Eubacteriales	Clostridia
Lachnospiraceae	Eubacteriales	Clostridia
Lachnospiraceae	Eubacteriales	Clostridia
Turicibacteraceae	Erysipelotrichales	Erysipelotrichia
Turicibacteraceae	Erysipelotrichales	Erysipelotrichia
Lachnospiraceae	Eubacteriales	Clostridia
Lachnospiraceae	Eubacteriales	Clostridia
Lachnospiraceae	Eubacteriales	Clostridia
Lachnospiraceae	Eubacteriales	Clostridia
Oscillospiraceae	Eubacteriales	Clostridia
Oscillospiraceae	Eubacteriales	Clostridia
Oscillospiraceae	Eubacteriales	Clostridia
Oscillospiraceae	Eubacteriales	Clostridia
Eggerthellaceae	Eggerthellales	Coriobacteriia
Eggerthellaceae	Eggerthellales	Coriobacteriia
Eggerthellaceae	Eggerthellales	Coriobacteriia
Eggerthellaceae	Eggerthellales	Coriobacteriia
Eggerthellaceae	Eggerthellales	Coriobacteriia
Eggerthellaceae	Eggerthellales	Coriobacteriia
Akkermansiaceae	Verrucomicrobiales	Verrucomicrobiae
Akkermansiaceae	Verrucomicrobiales	Verrucomicrobiae
Rikenellaceae	Bacteroidales	Bacteroidia
Rikenellaceae	Bacteroidales	Bacteroidia
Oscillospiraceae	Eubacteriales	Clostridia
Oscillospiraceae	Eubacteriales	Clostridia
Bacteria_unclassified	Bacteria_unclassified	Bacteria_unclassified
Bacteria_unclassified	Bacteria_unclassified	Bacteria_unclassified
Bacteria_unclassified	Bacteria_unclassified	Bacteria_unclassified
Bacteria_unclassified	Bacteria_unclassified	Bacteria_unclassified
Bacteria_unclassified	Bacteria_unclassified	Bacteria_unclassified
Bacteria_unclassified	Bacteria_unclassified	Bacteria_unclassified
Bacteria_unclassified	Bacteria_unclassified	Bacteria_unclassified
Bacteria_unclassified	Bacteria_unclassified	Bacteria_unclassified
Bacteria_unclassified	Bacteria_unclassified	Bacteria_unclassified
Bacteria_unclassified	Bacteria_unclassified	Bacteria_unclassified
Bacteria_unclassified	Bacteria_unclassified	Bacteria_unclassified
Bacteria_unclassified	Bacteria_unclassified	Bacteria_unclassified

Bacteroidaceae	Bacteroidales	Bacteroidia
Bacteroidaceae	Bacteroidales	Bacteroidia
Bifidobacteriaceae	Bifidobacteriales	Actinomycetia
Bifidobacteriaceae	Bifidobacteriales	Actinomycetia
Clostridia_unclassified	Clostridia_unclassified	Clostridia
Clostridia_unclassified	Clostridia_unclassified	Clostridia
Clostridiaceae	Eubacteriales	Clostridia
Clostridiaceae	Eubacteriales	Clostridia
Clostridiaceae	Eubacteriales	Clostridia
Clostridiaceae	Eubacteriales	Clostridia
Eubacteriales_unclassified	Eubacteriales	Clostridia
Eubacteriales_unclassified	Eubacteriales	Clostridia
Erysipelotrichaceae	Erysipelotrichales	Erysipelotrichia
Erysipelotrichaceae	Erysipelotrichales	Erysipelotrichia
Coriobacteriaceae	Coriobacteriales	Coriobacteriia
Coriobacteriaceae	Coriobacteriales	Coriobacteriia
Lachnospiraceae	Eubacteriales	Clostridia
Lachnospiraceae	Eubacteriales	Clostridia
Erysipelotrichaceae	Erysipelotrichales	Erysipelotrichia
Erysipelotrichaceae	Erysipelotrichales	Erysipelotrichia
Erysipelotrichales_unclassified	Erysipelotrichales	Erysipelotrichia
Erysipelotrichales_unclassified	Erysipelotrichales	Erysipelotrichia
Eubacteriaceae	Eubacteriales	Clostridia
Eubacteriaceae	Eubacteriales	Clostridia
Eubacteriaceae	Eubacteriales	Clostridia
Eubacteriaceae	Eubacteriales	Clostridia
Lachnospiraceae	Eubacteriales	Clostridia
Lachnospiraceae	Eubacteriales	Clostridia
Eggerthellaceae	Eggerthellales	Coriobacteriia
Eggerthellaceae	Eggerthellales	Coriobacteriia
Lachnospiraceae	Eubacteriales	Clostridia
Lachnospiraceae	Eubacteriales	Clostridia
Muribaculaceae	Bacteroidales	Bacteroidia
Muribaculaceae	Bacteroidales	Bacteroidia
Muribaculaceae	Bacteroidales	Bacteroidia
Muribaculaceae	Bacteroidales	Bacteroidia
Muribaculaceae	Bacteroidales	Bacteroidia
Muribaculaceae	Bacteroidales	Bacteroidia
FGB9508	OFGB9508	CFGB9508
FGB9508	OFGB9508	CFGB9508
FGB2838	OFGB2838	CFGB2838
FGB2838	OFGB2838	CFGB2838
FGB2838	OFGB2838	CFGB2838
FGB2838	OFGB2838	CFGB2838

FGB2838	OFGB2838	CFGB2838
FGB2838	OFGB2838	CFGB2838
Pumilibacteraceae	Eubacteriales	Clostridia
Pumilibacteraceae	Eubacteriales	Clostridia
FGB28439	OFGB28439	CFGB28439
FGB28439	OFGB28439	CFGB28439
Clostridia_unclassified	Clostridia_unclassified	Clostridia
Clostridia_unclassified	Clostridia_unclassified	Clostridia
Eubacteriaceae	Eubacteriales	Clostridia
Eubacteriaceae	Eubacteriales	Clostridia
Lachnospiraceae	Eubacteriales	Clostridia
Lachnospiraceae	Eubacteriales	Clostridia
Lachnospiraceae	Eubacteriales	Clostridia
Lachnospiraceae	Eubacteriales	Clostridia
Lachnospiraceae	Eubacteriales	Clostridia
Lachnospiraceae	Eubacteriales	Clostridia
Lachnospiraceae	Eubacteriales	Clostridia
Lachnospiraceae	Eubacteriales	Clostridia
FGB77305	OFGB77305	CFGB77305
FGB77305	OFGB77305	CFGB77305
Clostridiaceae	Eubacteriales	Clostridia
Clostridiaceae	Eubacteriales	Clostridia
Lachnospiraceae	Eubacteriales	Clostridia
Lachnospiraceae	Eubacteriales	Clostridia
Lachnospiraceae	Eubacteriales	Clostridia
Lachnospiraceae	Eubacteriales	Clostridia
Lachnospiraceae	Eubacteriales	Clostridia
Lachnospiraceae	Eubacteriales	Clostridia
FGB9633	OFGB9633	CFGB9633
FGB9633	OFGB9633	CFGB9633
Bacteria_unclassified	Bacteria_unclassified	Bacteria_unclassified
Bacteria_unclassified	Bacteria_unclassified	Bacteria_unclassified
Bacteria_unclassified	Bacteria_unclassified	Bacteria_unclassified
Bacteria_unclassified	Bacteria_unclassified	Bacteria_unclassified
Bacteria_unclassified	Bacteria_unclassified	Bacteria_unclassified
Bacteria_unclassified	Bacteria_unclassified	Bacteria_unclassified
Bacteria_unclassified	Bacteria_unclassified	Bacteria_unclassified
Bacteria_unclassified	Bacteria_unclassified	Bacteria_unclassified
Lachnospiraceae	Eubacteriales	Clostridia
Lachnospiraceae	Eubacteriales	Clostridia
Lachnospiraceae	Eubacteriales	Clostridia
Lachnospiraceae	Eubacteriales	Clostridia
Lachnospiraceae	Eubacteriales	Clostridia
Lachnospiraceae	Eubacteriales	Clostridia
FGB77359	OFGB77359	CFGB77359
FGB77359	OFGB77359	CFGB77359

FGB9639	OFGB9639	CFGB9639
FGB9639	OFGB9639	CFGB9639
Lachnospiraceae	Eubacteriales	Clostridia
Lachnospiraceae	Eubacteriales	Clostridia
Lachnospiraceae	Eubacteriales	Clostridia
Lachnospiraceae	Eubacteriales	Clostridia
Lachnospiraceae	Eubacteriales	Clostridia
Lachnospiraceae	Eubacteriales	Clostridia
Clostridiaceae	Eubacteriales	Clostridia
Clostridiaceae	Eubacteriales	Clostridia
Clostridiaceae	Eubacteriales	Clostridia
Clostridiaceae	Eubacteriales	Clostridia
Clostridiaceae	Eubacteriales	Clostridia
Clostridiaceae	Eubacteriales	Clostridia
Clostridiaceae	Eubacteriales	Clostridia
Clostridiaceae	Eubacteriales	Clostridia
Clostridiaceae	Eubacteriales	Clostridia
Clostridiaceae	Eubacteriales	Clostridia
Clostridiaceae	Eubacteriales	Clostridia
Clostridiaceae	Eubacteriales	Clostridia
Clostridiaceae	Eubacteriales	Clostridia
Clostridiaceae	Eubacteriales	Clostridia
Eubacteriaceae	Eubacteriales	Clostridia
Eubacteriaceae	Eubacteriales	Clostridia
FGB9658	OFGB9658	CFGB9658
FGB9658	OFGB9658	CFGB9658
FGB9659	OFGB9659	CFGB9659
FGB9659	OFGB9659	CFGB9659
Bacteria_unclassified	Bacteria_unclassified	Bacteria_unclassified
Bacteria_unclassified	Bacteria_unclassified	Bacteria_unclassified
FGB9827	OFGB9827	CFGB9827
FGB9827	OFGB9827	CFGB9827
Eubacteriaceae	Eubacteriales	Clostridia
Eubacteriaceae	Eubacteriales	Clostridia
FGB77303	OFGB77303	CFGB77303
FGB77303	OFGB77303	CFGB77303
Eubacteriales_unclassified	Eubacteriales	Clostridia
Eubacteriales_unclassified	Eubacteriales	Clostridia
Oscillospiraceae	Eubacteriales	Clostridia
Oscillospiraceae	Eubacteriales	Clostridia
FGB30328	OFGB30328	CFGB30328
FGB30328	OFGB30328	CFGB30328
Oscillospiraceae	Eubacteriales	Clostridia
Oscillospiraceae	Eubacteriales	Clostridia
Oscillospiraceae	Eubacteriales	Clostridia
Oscillospiraceae	Eubacteriales	Clostridia

Oscillospiraceae	Eubacteriales	Clostridia
Oscillospiraceae	Eubacteriales	Clostridia
Oscillospiraceae	Eubacteriales	Clostridia
Oscillospiraceae	Eubacteriales	Clostridia
Oscillospiraceae	Eubacteriales	Clostridia
Oscillospiraceae	Eubacteriales	Clostridia
Oscillospiraceae	Eubacteriales	Clostridia
Oscillospiraceae	Eubacteriales	Clostridia
Oscillospiraceae	Eubacteriales	Clostridia
Oscillospiraceae	Eubacteriales	Clostridia
FGB77153	OFGB77153	CFGB77153
FGB77153	OFGB77153	CFGB77153
FGB1791	OFGB1791	CFGB1791
FGB1791	OFGB1791	CFGB1791
FGB10290	OFGB10290	CFGB10290
FGB10290	OFGB10290	CFGB10290
Oscillospiraceae	Eubacteriales	Clostridia
Oscillospiraceae	Eubacteriales	Clostridia
FGB1765	OFGB1765	CFGB1765
FGB1765	OFGB1765	CFGB1765
FGB10349	OFGB10349	CFGB10349
FGB10349	OFGB10349	CFGB10349
FGB10667	OFGB10667	CFGB10667
FGB10667	OFGB10667	CFGB10667
Lachnospiraceae	Eubacteriales	Clostridia
Lachnospiraceae	Eubacteriales	Clostridia
Lachnospiraceae	Eubacteriales	Clostridia
Lachnospiraceae	Eubacteriales	Clostridia
Christensenellaceae	Eubacteriales	Clostridia
Christensenellaceae	Eubacteriales	Clostridia
FGB10299	OFGB10299	CFGB10299
FGB10299	OFGB10299	CFGB10299
Oscillospiraceae	Eubacteriales	Clostridia
Oscillospiraceae	Eubacteriales	Clostridia
Oscillospiraceae	Eubacteriales	Clostridia
Oscillospiraceae	Eubacteriales	Clostridia
Lachnospiraceae	Eubacteriales	Clostridia
Lachnospiraceae	Eubacteriales	Clostridia
Clostridia_unclassified	Clostridia_unclassified	Clostridia
Clostridia_unclassified	Clostridia_unclassified	Clostridia
Lachnospiraceae	Eubacteriales	Clostridia
Lachnospiraceae	Eubacteriales	Clostridia
Lachnospiraceae	Eubacteriales	Clostridia
Lachnospiraceae	Eubacteriales	Clostridia
Lachnospiraceae	Eubacteriales	Clostridia
Lachnospiraceae	Eubacteriales	Clostridia

Lachnospiraceae	Eubacteriales	Clostridia
Lachnospiraceae	Eubacteriales	Clostridia
Lachnospiraceae	Eubacteriales	Clostridia
Lachnospiraceae	Eubacteriales	Clostridia
Lachnospiraceae	Eubacteriales	Clostridia
Lachnospiraceae	Eubacteriales	Clostridia
Lachnospiraceae	Eubacteriales	Clostridia
Lachnospiraceae	Eubacteriales	Clostridia
Lachnospiraceae	Eubacteriales	Clostridia
Lachnospiraceae	Eubacteriales	Clostridia
Lactobacillaceae	Lactobacillales	Bacilli
Lactobacillaceae	Lactobacillales	Bacilli
Muribaculaceae	Bacteroidales	Bacteroidia
Muribaculaceae	Bacteroidales	Bacteroidia
Oscillospiraceae	Eubacteriales	Clostridia
Oscillospiraceae	Eubacteriales	Clostridia
Oscillospiraceae	Eubacteriales	Clostridia
Oscillospiraceae	Eubacteriales	Clostridia
Oscillospiraceae	Eubacteriales	Clostridia
Oscillospiraceae	Eubacteriales	Clostridia
Oscillospiraceae	Eubacteriales	Clostridia
Oscillospiraceae	Eubacteriales	Clostridia
Sutterellaceae	Burkholderiales	Betaproteobacteria
Sutterellaceae	Burkholderiales	Betaproteobacteria
Peptostreptococcaceae	Eubacteriales	Clostridia
Peptostreptococcaceae	Eubacteriales	Clostridia
Lachnospiraceae	Eubacteriales	Clostridia
Lachnospiraceae	Eubacteriales	Clostridia
Turicibacteraceae	Erysipelotrichales	Erysipelotrichia
Turicibacteraceae	Erysipelotrichales	Erysipelotrichia
Lachnospiraceae	Eubacteriales	Clostridia
Lachnospiraceae	Eubacteriales	Clostridia
Lachnospiraceae	Eubacteriales	Clostridia
Lachnospiraceae	Eubacteriales	Clostridia
Oscillospiraceae	Eubacteriales	Clostridia
Oscillospiraceae	Eubacteriales	Clostridia
Oscillospiraceae	Eubacteriales	Clostridia
Oscillospiraceae	Eubacteriales	Clostridia
Eggerthellaceae	Eggerthellales	Coriobacteriia
Eggerthellaceae	Eggerthellales	Coriobacteriia
Eggerthellaceae	Eggerthellales	Coriobacteriia
Eggerthellaceae	Eggerthellales	Coriobacteriia

Eggerthellaceae	Eggerthellales	Coriobacteriia
Eggerthellaceae	Eggerthellales	Coriobacteriia
Akkermansiaceae	Verrucomicrobiales	Verrucomicrobiae
Akkermansiaceae	Verrucomicrobiales	Verrucomicrobiae
Rikenellaceae	Bacteroidales	Bacteroidia
Rikenellaceae	Bacteroidales	Bacteroidia
Oscillospiraceae	Eubacteriales	Clostridia
Oscillospiraceae	Eubacteriales	Clostridia
Bacteria_unclassified	Bacteria_unclassified	Bacteria_unclassified
Bacteria_unclassified	Bacteria_unclassified	Bacteria_unclassified
Bacteria_unclassified	Bacteria_unclassified	Bacteria_unclassified
Bacteria_unclassified	Bacteria_unclassified	Bacteria_unclassified
Bacteria_unclassified	Bacteria_unclassified	Bacteria_unclassified
Bacteria_unclassified	Bacteria_unclassified	Bacteria_unclassified
Bacteria_unclassified	Bacteria_unclassified	Bacteria_unclassified
Bacteria_unclassified	Bacteria_unclassified	Bacteria_unclassified
Bacteria_unclassified	Bacteria_unclassified	Bacteria_unclassified
Bacteria_unclassified	Bacteria_unclassified	Bacteria_unclassified
Bacteria_unclassified	Bacteria_unclassified	Bacteria_unclassified
Bacteria_unclassified	Bacteria_unclassified	Bacteria_unclassified
Bacteroidaceae	Bacteroidales	Bacteroidia
Bacteroidaceae	Bacteroidales	Bacteroidia

Bifidobacteriaceae	Bifidobacteriales	Actinomycetia
Bifidobacteriaceae	Bifidobacteriales	Actinomycetia
Clostridia_unclassified	Clostridia_unclassified	Clostridia
Clostridia_unclassified	Clostridia_unclassified	Clostridia
Clostridiaceae	Eubacteriales	Clostridia
Clostridiaceae	Eubacteriales	Clostridia
Clostridiaceae	Eubacteriales	Clostridia
Clostridiaceae	Eubacteriales	Clostridia
Eubacteriales_unclassified	Eubacteriales	Clostridia
Eubacteriales_unclassified	Eubacteriales	Clostridia
Erysipelotrichaceae	Erysipelotrichales	Erysipelotrichia
Erysipelotrichaceae	Erysipelotrichales	Erysipelotrichia
Coriobacteriaceae	Coriobacteriales	Coriobacteriia
Coriobacteriaceae	Coriobacteriales	Coriobacteriia
Lachnospiraceae	Eubacteriales	Clostridia
Lachnospiraceae	Eubacteriales	Clostridia
Erysipelotrichaceae	Erysipelotrichales	Erysipelotrichia
Erysipelotrichaceae	Erysipelotrichales	Erysipelotrichia
Erysipelotrichales_unclassified	Erysipelotrichales	Erysipelotrichia
Erysipelotrichales_unclassified	Erysipelotrichales	Erysipelotrichia
Eubacteriaceae	Eubacteriales	Clostridia
Eubacteriaceae	Eubacteriales	Clostridia

Eubacteriaceae	Eubacteriales	Clostridia
Eubacteriaceae	Eubacteriales	Clostridia
Lachnospiraceae	Eubacteriales	Clostridia
Lachnospiraceae	Eubacteriales	Clostridia
Eggerthellaceae	Eggerthellales	Coriobacteriia
Eggerthellaceae	Eggerthellales	Coriobacteriia
Lachnospiraceae	Eubacteriales	Clostridia
Lachnospiraceae	Eubacteriales	Clostridia
Muribaculaceae	Bacteroidales	Bacteroidia
Muribaculaceae	Bacteroidales	Bacteroidia
Muribaculaceae	Bacteroidales	Bacteroidia
Muribaculaceae	Bacteroidales	Bacteroidia
Muribaculaceae	Bacteroidales	Bacteroidia
Muribaculaceae	Bacteroidales	Bacteroidia
FGB9508	OFGB9508	CFGB9508
FGB9508	OFGB9508	CFGB9508
FGB2838	OFGB2838	CFGB2838
FGB2838	OFGB2838	CFGB2838
FGB2838	OFGB2838	CFGB2838
FGB2838	OFGB2838	CFGB2838
FGB2838	OFGB2838	CFGB2838
FGB2838	OFGB2838	CFGB2838
Pumilibacteraceae	Eubacteriales	Clostridia
Pumilibacteraceae	Eubacteriales	Clostridia
FGB28439	OFGB28439	CFGB28439
FGB28439	OFGB28439	CFGB28439
Clostridia_unclassified	Clostridia_unclassified	Clostridia
Clostridia_unclassified	Clostridia_unclassified	Clostridia
Eubacteriaceae	Eubacteriales	Clostridia
Eubacteriaceae	Eubacteriales	Clostridia
Lachnospiraceae	Eubacteriales	Clostridia
Lachnospiraceae	Eubacteriales	Clostridia
Lachnospiraceae	Eubacteriales	Clostridia
Lachnospiraceae	Eubacteriales	Clostridia
Lachnospiraceae	Eubacteriales	Clostridia
Lachnospiraceae	Eubacteriales	Clostridia
Lachnospiraceae	Eubacteriales	Clostridia
Lachnospiraceae	Eubacteriales	Clostridia
FGB77305	OFGB77305	CFGB77305
FGB77305	OFGB77305	CFGB77305
Clostridiaceae	Eubacteriales	Clostridia
Clostridiaceae	Eubacteriales	Clostridia
Lachnospiraceae	Eubacteriales	Clostridia
Lachnospiraceae	Eubacteriales	Clostridia
Lachnospiraceae	Eubacteriales	Clostridia
Lachnospiraceae	Eubacteriales	Clostridia

Lachnospiraceae	Eubacteriales	Clostridia
Lachnospiraceae	Eubacteriales	Clostridia
FGB9633	OFGB9633	CFGB9633
FGB9633	OFGB9633	CFGB9633
Bacteria_unclassified	Bacteria_unclassified	Bacteria_unclassified
Bacteria_unclassified	Bacteria_unclassified	Bacteria_unclassified
Bacteria_unclassified	Bacteria_unclassified	Bacteria_unclassified
Bacteria_unclassified	Bacteria_unclassified	Bacteria_unclassified
Bacteria_unclassified	Bacteria_unclassified	Bacteria_unclassified
Bacteria_unclassified	Bacteria_unclassified	Bacteria_unclassified
Bacteria_unclassified	Bacteria_unclassified	Bacteria_unclassified
Bacteria_unclassified	Bacteria_unclassified	Bacteria_unclassified
Lachnospiraceae	Eubacteriales	Clostridia
Lachnospiraceae	Eubacteriales	Clostridia
Lachnospiraceae	Eubacteriales	Clostridia
Lachnospiraceae	Eubacteriales	Clostridia
Lachnospiraceae	Eubacteriales	Clostridia
Lachnospiraceae	Eubacteriales	Clostridia
FGB77359	OFGB77359	CFGB77359
FGB77359	OFGB77359	CFGB77359
FGB9639	OFGB9639	CFGB9639
FGB9639	OFGB9639	CFGB9639
Lachnospiraceae	Eubacteriales	Clostridia
Lachnospiraceae	Eubacteriales	Clostridia
Lachnospiraceae	Eubacteriales	Clostridia
Lachnospiraceae	Eubacteriales	Clostridia
Lachnospiraceae	Eubacteriales	Clostridia
Lachnospiraceae	Eubacteriales	Clostridia
Clostridiaceae	Eubacteriales	Clostridia
Clostridiaceae	Eubacteriales	Clostridia
Clostridiaceae	Eubacteriales	Clostridia
Clostridiaceae	Eubacteriales	Clostridia
Clostridiaceae	Eubacteriales	Clostridia
Clostridiaceae	Eubacteriales	Clostridia
Clostridiaceae	Eubacteriales	Clostridia
Clostridiaceae	Eubacteriales	Clostridia
Clostridiaceae	Eubacteriales	Clostridia
Clostridiaceae	Eubacteriales	Clostridia
Clostridiaceae	Eubacteriales	Clostridia
Clostridiaceae	Eubacteriales	Clostridia
Clostridiaceae	Eubacteriales	Clostridia
Clostridiaceae	Eubacteriales	Clostridia
Eubacteriaceae	Eubacteriales	Clostridia
Eubacteriaceae	Eubacteriales	Clostridia
FGB9658	OFGB9658	CFGB9658
FGB9658	OFGB9658	CFGB9658

FGB9659	OFGB9659	CFGB9659
FGB9659	OFGB9659	CFGB9659
Bacteria_unclassified	Bacteria_unclassified	Bacteria_unclassified
Bacteria_unclassified	Bacteria_unclassified	Bacteria_unclassified
FGB9827	OFGB9827	CFGB9827
FGB9827	OFGB9827	CFGB9827
Eubacteriaceae	Eubacteriales	Clostridia
Eubacteriaceae	Eubacteriales	Clostridia
FGB77303	OFGB77303	CFGB77303
FGB77303	OFGB77303	CFGB77303
Eubacteriales_unclassified	Eubacteriales	Clostridia
Eubacteriales_unclassified	Eubacteriales	Clostridia
Oscillospiraceae	Eubacteriales	Clostridia
Oscillospiraceae	Eubacteriales	Clostridia
FGB30328	OFGB30328	CFGB30328
FGB30328	OFGB30328	CFGB30328
Oscillospiraceae	Eubacteriales	Clostridia
Oscillospiraceae	Eubacteriales	Clostridia
Oscillospiraceae	Eubacteriales	Clostridia
Oscillospiraceae	Eubacteriales	Clostridia
Oscillospiraceae	Eubacteriales	Clostridia
Oscillospiraceae	Eubacteriales	Clostridia
Oscillospiraceae	Eubacteriales	Clostridia
Oscillospiraceae	Eubacteriales	Clostridia
Oscillospiraceae	Eubacteriales	Clostridia
Oscillospiraceae	Eubacteriales	Clostridia
Oscillospiraceae	Eubacteriales	Clostridia
Oscillospiraceae	Eubacteriales	Clostridia
Oscillospiraceae	Eubacteriales	Clostridia
Oscillospiraceae	Eubacteriales	Clostridia
FGB77153	OFGB77153	CFGB77153
FGB77153	OFGB77153	CFGB77153
FGB1791	OFGB1791	CFGB1791
FGB1791	OFGB1791	CFGB1791
FGB10290	OFGB10290	CFGB10290
FGB10290	OFGB10290	CFGB10290
Oscillospiraceae	Eubacteriales	Clostridia
Oscillospiraceae	Eubacteriales	Clostridia
FGB1765	OFGB1765	CFGB1765
FGB1765	OFGB1765	CFGB1765
FGB10349	OFGB10349	CFGB10349
FGB10349	OFGB10349	CFGB10349
FGB10667	OFGB10667	CFGB10667
FGB10667	OFGB10667	CFGB10667
Lachnospiraceae	Eubacteriales	Clostridia
Lachnospiraceae	Eubacteriales	Clostridia

Lachnospiraceae	Eubacteriales	Clostridia
Lachnospiraceae	Eubacteriales	Clostridia
Christensenellaceae	Eubacteriales	Clostridia
Christensenellaceae	Eubacteriales	Clostridia
FGB10299	OFGB10299	CFGB10299
FGB10299	OFGB10299	CFGB10299
Oscillospiraceae	Eubacteriales	Clostridia
Oscillospiraceae	Eubacteriales	Clostridia
Oscillospiraceae	Eubacteriales	Clostridia
Oscillospiraceae	Eubacteriales	Clostridia
Lachnospiraceae	Eubacteriales	Clostridia
Lachnospiraceae	Eubacteriales	Clostridia
Clostridia_unclassified	Clostridia_unclassified	Clostridia
Clostridia_unclassified	Clostridia_unclassified	Clostridia
Lachnospiraceae	Eubacteriales	Clostridia
Lachnospiraceae	Eubacteriales	Clostridia
Lachnospiraceae	Eubacteriales	Clostridia
Lachnospiraceae	Eubacteriales	Clostridia
Lachnospiraceae	Eubacteriales	Clostridia
Lachnospiraceae	Eubacteriales	Clostridia
Lachnospiraceae	Eubacteriales	Clostridia
Lachnospiraceae	Eubacteriales	Clostridia
Lachnospiraceae	Eubacteriales	Clostridia
Lachnospiraceae	Eubacteriales	Clostridia
Lachnospiraceae	Eubacteriales	Clostridia
Lachnospiraceae	Eubacteriales	Clostridia
Lachnospiraceae	Eubacteriales	Clostridia
Lachnospiraceae	Eubacteriales	Clostridia
Lachnospiraceae	Eubacteriales	Clostridia
Lachnospiraceae	Eubacteriales	Clostridia
Lactobacillaceae	Lactobacillales	Bacilli
Lactobacillaceae	Lactobacillales	Bacilli
Muribaculaceae	Bacteroidales	Bacteroidia
Muribaculaceae	Bacteroidales	Bacteroidia
Oscillospiraceae	Eubacteriales	Clostridia
Oscillospiraceae	Eubacteriales	Clostridia
Oscillospiraceae	Eubacteriales	Clostridia
Oscillospiraceae	Eubacteriales	Clostridia
Oscillospiraceae	Eubacteriales	Clostridia
Oscillospiraceae	Eubacteriales	Clostridia
Oscillospiraceae	Eubacteriales	Clostridia
Oscillospiraceae	Eubacteriales	Clostridia
Sutterellaceae	Burkholderiales	Betaproteobacteria
Sutterellaceae	Burkholderiales	Betaproteobacteria

Peptostreptococcaceae	Eubacteriales	Clostridia
Peptostreptococcaceae	Eubacteriales	Clostridia
Lachnospiraceae	Eubacteriales	Clostridia
Lachnospiraceae	Eubacteriales	Clostridia
Turicibacteraceae	Erysipelotrichales	Erysipelotrichia
Turicibacteraceae	Erysipelotrichales	Erysipelotrichia
Lachnospiraceae	Eubacteriales	Clostridia
Lachnospiraceae	Eubacteriales	Clostridia
Lachnospiraceae	Eubacteriales	Clostridia
Lachnospiraceae	Eubacteriales	Clostridia
Oscillospiraceae	Eubacteriales	Clostridia
Oscillospiraceae	Eubacteriales	Clostridia
Oscillospiraceae	Eubacteriales	Clostridia
Oscillospiraceae	Eubacteriales	Clostridia
Eggerthellaceae	Eggerthellales	Coriobacteriia
Eggerthellaceae	Eggerthellales	Coriobacteriia
Eggerthellaceae	Eggerthellales	Coriobacteriia
Eggerthellaceae	Eggerthellales	Coriobacteriia
Eggerthellaceae	Eggerthellales	Coriobacteriia
Eggerthellaceae	Eggerthellales	Coriobacteriia
Akkermansiaceae	Verrucomicrobiales	Verrucomicrobiae
Akkermansiaceae	Verrucomicrobiales	Verrucomicrobiae
Rikenellaceae	Bacteroidales	Bacteroidia
Rikenellaceae	Bacteroidales	Bacteroidia
Oscillospiraceae	Eubacteriales	Clostridia
Oscillospiraceae	Eubacteriales	Clostridia
Bacteria_unclassified	Bacteria_unclassified	Bacteria_unclassified
Bacteria_unclassified	Bacteria_unclassified	Bacteria_unclassified
Bacteria_unclassified	Bacteria_unclassified	Bacteria_unclassified
Bacteria_unclassified	Bacteria_unclassified	Bacteria_unclassified
Bacteria_unclassified	Bacteria_unclassified	Bacteria_unclassified
Bacteria_unclassified	Bacteria_unclassified	Bacteria_unclassified
Bacteria_unclassified	Bacteria_unclassified	Bacteria_unclassified
Bacteria_unclassified	Bacteria_unclassified	Bacteria_unclassified
Bacteria_unclassified	Bacteria_unclassified	Bacteria_unclassified
Bacteria_unclassified	Bacteria_unclassified	Bacteria_unclassified
Bacteria_unclassified	Bacteria_unclassified	Bacteria_unclassified
Bacteria_unclassified	Bacteria_unclassified	Bacteria_unclassified
Bacteroidaceae	Bacteroidales	Bacteroidia
Bacteroidaceae	Bacteroidales	Bacteroidia
Bifidobacteriaceae	Bifidobacteriales	Actinomycetia
Bifidobacteriaceae	Bifidobacteriales	Actinomycetia

Clostridia_unclassified	Clostridia_unclassified	Clostridia
Clostridia_unclassified	Clostridia_unclassified	Clostridia
Clostridiaceae	Eubacteriales	Clostridia
Clostridiaceae	Eubacteriales	Clostridia
Clostridiaceae	Eubacteriales	Clostridia
Clostridiaceae	Eubacteriales	Clostridia
Eubacteriales_unclassified	Eubacteriales	Clostridia
Eubacteriales_unclassified	Eubacteriales	Clostridia
Erysipelotrichaceae	Erysipelotrichales	Erysipelotrichia
Erysipelotrichaceae	Erysipelotrichales	Erysipelotrichia
Coriobacteriaceae	Coriobacteriales	Coriobacteriia
Coriobacteriaceae	Coriobacteriales	Coriobacteriia
Lachnospiraceae	Eubacteriales	Clostridia
Lachnospiraceae	Eubacteriales	Clostridia
Erysipelotrichaceae	Erysipelotrichales	Erysipelotrichia
Erysipelotrichaceae	Erysipelotrichales	Erysipelotrichia
Erysipelotrichales_unclassified	Erysipelotrichales	Erysipelotrichia
Erysipelotrichales_unclassified	Erysipelotrichales	Erysipelotrichia
Eubacteriaceae	Eubacteriales	Clostridia
Eubacteriaceae	Eubacteriales	Clostridia
Eubacteriaceae	Eubacteriales	Clostridia
Eubacteriaceae	Eubacteriales	Clostridia
Lachnospiraceae	Eubacteriales	Clostridia
Lachnospiraceae	Eubacteriales	Clostridia
Eggerthellaceae	Eggerthellales	Coriobacteriia
Eggerthellaceae	Eggerthellales	Coriobacteriia
Lachnospiraceae	Eubacteriales	Clostridia
Lachnospiraceae	Eubacteriales	Clostridia
Muribaculaceae	Bacteroidales	Bacteroidia
Muribaculaceae	Bacteroidales	Bacteroidia
Muribaculaceae	Bacteroidales	Bacteroidia
Muribaculaceae	Bacteroidales	Bacteroidia
Muribaculaceae	Bacteroidales	Bacteroidia
Muribaculaceae	Bacteroidales	Bacteroidia
FGB9508	OFGB9508	CFGB9508
FGB9508	OFGB9508	CFGB9508
FGB2838	OFGB2838	CFGB2838
FGB2838	OFGB2838	CFGB2838
FGB2838	OFGB2838	CFGB2838
FGB2838	OFGB2838	CFGB2838
FGB2838	OFGB2838	CFGB2838
FGB2838	OFGB2838	CFGB2838
Pumilibacteraceae	Eubacteriales	Clostridia
Pumilibacteraceae	Eubacteriales	Clostridia
FGB28439	OFGB28439	CFGB28439
FGB28439	OFGB28439	CFGB28439

Clostridia_unclassified	Clostridia_unclassified	Clostridia
Clostridia_unclassified	Clostridia_unclassified	Clostridia
Eubacteriaceae	Eubacteriales	Clostridia
Eubacteriaceae	Eubacteriales	Clostridia
Lachnospiraceae	Eubacteriales	Clostridia
Lachnospiraceae	Eubacteriales	Clostridia
Lachnospiraceae	Eubacteriales	Clostridia
Lachnospiraceae	Eubacteriales	Clostridia
Lachnospiraceae	Eubacteriales	Clostridia
Lachnospiraceae	Eubacteriales	Clostridia
Lachnospiraceae	Eubacteriales	Clostridia
Lachnospiraceae	Eubacteriales	Clostridia
FGB77305	OFGB77305	CFGB77305
FGB77305	OFGB77305	CFGB77305
Clostridiaceae	Eubacteriales	Clostridia
Clostridiaceae	Eubacteriales	Clostridia
Lachnospiraceae	Eubacteriales	Clostridia
Lachnospiraceae	Eubacteriales	Clostridia
Lachnospiraceae	Eubacteriales	Clostridia
Lachnospiraceae	Eubacteriales	Clostridia
Lachnospiraceae	Eubacteriales	Clostridia
Lachnospiraceae	Eubacteriales	Clostridia
FGB9633	OFGB9633	CFGB9633
FGB9633	OFGB9633	CFGB9633
Bacteria_unclassified	Bacteria_unclassified	Bacteria_unclassified
Bacteria_unclassified	Bacteria_unclassified	Bacteria_unclassified
Bacteria_unclassified	Bacteria_unclassified	Bacteria_unclassified
Bacteria_unclassified	Bacteria_unclassified	Bacteria_unclassified
Bacteria_unclassified	Bacteria_unclassified	Bacteria_unclassified
Bacteria_unclassified	Bacteria_unclassified	Bacteria_unclassified
Bacteria_unclassified	Bacteria_unclassified	Bacteria_unclassified
Bacteria_unclassified	Bacteria_unclassified	Bacteria_unclassified
Lachnospiraceae	Eubacteriales	Clostridia
Lachnospiraceae	Eubacteriales	Clostridia
Lachnospiraceae	Eubacteriales	Clostridia
Lachnospiraceae	Eubacteriales	Clostridia
Lachnospiraceae	Eubacteriales	Clostridia
Lachnospiraceae	Eubacteriales	Clostridia
FGB77359	OFGB77359	CFGB77359
FGB77359	OFGB77359	CFGB77359
FGB9639	OFGB9639	CFGB9639
FGB9639	OFGB9639	CFGB9639
Lachnospiraceae	Eubacteriales	Clostridia
Lachnospiraceae	Eubacteriales	Clostridia
Lachnospiraceae	Eubacteriales	Clostridia
Lachnospiraceae	Eubacteriales	Clostridia

Lachnospiraceae	Eubacteriales	Clostridia
Lachnospiraceae	Eubacteriales	Clostridia
Clostridiaceae	Eubacteriales	Clostridia
Clostridiaceae	Eubacteriales	Clostridia
Clostridiaceae	Eubacteriales	Clostridia
Clostridiaceae	Eubacteriales	Clostridia
Clostridiaceae	Eubacteriales	Clostridia
Clostridiaceae	Eubacteriales	Clostridia
Clostridiaceae	Eubacteriales	Clostridia
Clostridiaceae	Eubacteriales	Clostridia
Clostridiaceae	Eubacteriales	Clostridia
Clostridiaceae	Eubacteriales	Clostridia
Clostridiaceae	Eubacteriales	Clostridia
Clostridiaceae	Eubacteriales	Clostridia
Clostridiaceae	Eubacteriales	Clostridia
Clostridiaceae	Eubacteriales	Clostridia
Eubacteriaceae	Eubacteriales	Clostridia
Eubacteriaceae	Eubacteriales	Clostridia
FGB9658	OFGB9658	CFGB9658
FGB9658	OFGB9658	CFGB9658
FGB9659	OFGB9659	CFGB9659
FGB9659	OFGB9659	CFGB9659
Bacteria_unclassified	Bacteria_unclassified	Bacteria_unclassified
Bacteria_unclassified	Bacteria_unclassified	Bacteria_unclassified
FGB9827	OFGB9827	CFGB9827
FGB9827	OFGB9827	CFGB9827
Eubacteriaceae	Eubacteriales	Clostridia
Eubacteriaceae	Eubacteriales	Clostridia
FGB77303	OFGB77303	CFGB77303
FGB77303	OFGB77303	CFGB77303
Eubacteriales_unclassified	Eubacteriales	Clostridia
Eubacteriales_unclassified	Eubacteriales	Clostridia
Oscillospiraceae	Eubacteriales	Clostridia
Oscillospiraceae	Eubacteriales	Clostridia
FGB30328	OFGB30328	CFGB30328
FGB30328	OFGB30328	CFGB30328
Oscillospiraceae	Eubacteriales	Clostridia
Oscillospiraceae	Eubacteriales	Clostridia
Oscillospiraceae	Eubacteriales	Clostridia
Oscillospiraceae	Eubacteriales	Clostridia
Oscillospiraceae	Eubacteriales	Clostridia
Oscillospiraceae	Eubacteriales	Clostridia
Oscillospiraceae	Eubacteriales	Clostridia
Oscillospiraceae	Eubacteriales	Clostridia
Oscillospiraceae	Eubacteriales	Clostridia
Oscillospiraceae	Eubacteriales	Clostridia

Oscillospiraceae	Eubacteriales	Clostridia
Oscillospiraceae	Eubacteriales	Clostridia
Oscillospiraceae	Eubacteriales	Clostridia
Oscillospiraceae	Eubacteriales	Clostridia
FGB77153	OFGB77153	CFGB77153
FGB77153	OFGB77153	CFGB77153
FGB1791	OFGB1791	CFGB1791
FGB1791	OFGB1791	CFGB1791
FGB10290	OFGB10290	CFGB10290
FGB10290	OFGB10290	CFGB10290
Oscillospiraceae	Eubacteriales	Clostridia
Oscillospiraceae	Eubacteriales	Clostridia
FGB1765	OFGB1765	CFGB1765
FGB1765	OFGB1765	CFGB1765
FGB10349	OFGB10349	CFGB10349
FGB10349	OFGB10349	CFGB10349
FGB10667	OFGB10667	CFGB10667
FGB10667	OFGB10667	CFGB10667
Lachnospiraceae	Eubacteriales	Clostridia
Lachnospiraceae	Eubacteriales	Clostridia
Lachnospiraceae	Eubacteriales	Clostridia
Lachnospiraceae	Eubacteriales	Clostridia
Christensenellaceae	Eubacteriales	Clostridia
Christensenellaceae	Eubacteriales	Clostridia
FGB10299	OFGB10299	CFGB10299
FGB10299	OFGB10299	CFGB10299
Oscillospiraceae	Eubacteriales	Clostridia
Oscillospiraceae	Eubacteriales	Clostridia
Oscillospiraceae	Eubacteriales	Clostridia
Oscillospiraceae	Eubacteriales	Clostridia
Lachnospiraceae	Eubacteriales	Clostridia
Lachnospiraceae	Eubacteriales	Clostridia
Clostridia_unclassified	Clostridia_unclassified	Clostridia
Clostridia_unclassified	Clostridia_unclassified	Clostridia
Lachnospiraceae	Eubacteriales	Clostridia
Lachnospiraceae	Eubacteriales	Clostridia
Lachnospiraceae	Eubacteriales	Clostridia
Lachnospiraceae	Eubacteriales	Clostridia
Lachnospiraceae	Eubacteriales	Clostridia
Lachnospiraceae	Eubacteriales	Clostridia
Lachnospiraceae	Eubacteriales	Clostridia
Lachnospiraceae	Eubacteriales	Clostridia
Lachnospiraceae	Eubacteriales	Clostridia
Lachnospiraceae	Eubacteriales	Clostridia
Lachnospiraceae	Eubacteriales	Clostridia
Lachnospiraceae	Eubacteriales	Clostridia

Lachnospiraceae	Eubacteriales	Clostridia
Lachnospiraceae	Eubacteriales	Clostridia
Lachnospiraceae	Eubacteriales	Clostridia
Lachnospiraceae	Eubacteriales	Clostridia
Lactobacillaceae	Lactobacillales	Bacilli
Lactobacillaceae	Lactobacillales	Bacilli
Muribaculaceae	Bacteroidales	Bacteroidia
Muribaculaceae	Bacteroidales	Bacteroidia
Oscillospiraceae	Eubacteriales	Clostridia
Oscillospiraceae	Eubacteriales	Clostridia
Oscillospiraceae	Eubacteriales	Clostridia
Oscillospiraceae	Eubacteriales	Clostridia
Oscillospiraceae	Eubacteriales	Clostridia
Oscillospiraceae	Eubacteriales	Clostridia
Oscillospiraceae	Eubacteriales	Clostridia
Oscillospiraceae	Eubacteriales	Clostridia
Sutterellaceae	Burkholderiales	Betaproteobacteria
Sutterellaceae	Burkholderiales	Betaproteobacteria
Peptostreptococcaceae	Eubacteriales	Clostridia
Peptostreptococcaceae	Eubacteriales	Clostridia
Lachnospiraceae	Eubacteriales	Clostridia
Lachnospiraceae	Eubacteriales	Clostridia
Turicibacteraceae	Erysipelotrichales	Erysipelotrichia
Turicibacteraceae	Erysipelotrichales	Erysipelotrichia
Lachnospiraceae	Eubacteriales	Clostridia
Lachnospiraceae	Eubacteriales	Clostridia
Lachnospiraceae	Eubacteriales	Clostridia
Lachnospiraceae	Eubacteriales	Clostridia
Oscillospiraceae	Eubacteriales	Clostridia
Oscillospiraceae	Eubacteriales	Clostridia
Oscillospiraceae	Eubacteriales	Clostridia
Oscillospiraceae	Eubacteriales	Clostridia
Eggerthellaceae	Eggerthellales	Coriobacteriia
Eggerthellaceae	Eggerthellales	Coriobacteriia
Eggerthellaceae	Eggerthellales	Coriobacteriia
Eggerthellaceae	Eggerthellales	Coriobacteriia
Eggerthellaceae	Eggerthellales	Coriobacteriia
Eggerthellaceae	Eggerthellales	Coriobacteriia
Akkermansiaceae	Verrucomicrobiales	Verrucomicrobiae
Akkermansiaceae	Verrucomicrobiales	Verrucomicrobiae
Rikenellaceae	Bacteroidales	Bacteroidia
Rikenellaceae	Bacteroidales	Bacteroidia

Oscillospiraceae	Eubacteriales	Clostridia
Oscillospiraceae	Eubacteriales	Clostridia
Bacteria_unclassified	Bacteria_unclassified	Bacteria_unclassified
Bacteria_unclassified	Bacteria_unclassified	Bacteria_unclassified
Bacteria_unclassified	Bacteria_unclassified	Bacteria_unclassified
Bacteria_unclassified	Bacteria_unclassified	Bacteria_unclassified
Bacteria_unclassified	Bacteria_unclassified	Bacteria_unclassified
Bacteria_unclassified	Bacteria_unclassified	Bacteria_unclassified
Bacteria_unclassified	Bacteria_unclassified	Bacteria_unclassified
Bacteria_unclassified	Bacteria_unclassified	Bacteria_unclassified
Bacteria_unclassified	Bacteria_unclassified	Bacteria_unclassified
Bacteria_unclassified	Bacteria_unclassified	Bacteria_unclassified
Bacteria_unclassified	Bacteria_unclassified	Bacteria_unclassified
Bacteria_unclassified	Bacteria_unclassified	Bacteria_unclassified
Bacteroidaceae	Bacteroidales	Bacteroidia
Bacteroidaceae	Bacteroidales	Bacteroidia
Bifidobacteriaceae	Bifidobacteriales	Actinomycetia
Bifidobacteriaceae	Bifidobacteriales	Actinomycetia
Clostridia_unclassified	Clostridia_unclassified	Clostridia
Clostridia_unclassified	Clostridia_unclassified	Clostridia
Clostridiaceae	Eubacteriales	Clostridia
Clostridiaceae	Eubacteriales	Clostridia
Clostridiaceae	Eubacteriales	Clostridia
Clostridiaceae	Eubacteriales	Clostridia
Eubacteriales_unclassified	Eubacteriales	Clostridia
Eubacteriales_unclassified	Eubacteriales	Clostridia
Erysipelotrichaceae	Erysipelotrichales	Erysipelotrichia
Erysipelotrichaceae	Erysipelotrichales	Erysipelotrichia
Coriobacteriaceae	Coriobacteriales	Coriobacteriia
Coriobacteriaceae	Coriobacteriales	Coriobacteriia
Lachnospiraceae	Eubacteriales	Clostridia
Lachnospiraceae	Eubacteriales	Clostridia
Erysipelotrichaceae	Erysipelotrichales	Erysipelotrichia
Erysipelotrichaceae	Erysipelotrichales	Erysipelotrichia
Erysipelotrichales_unclassified	Erysipelotrichales	Erysipelotrichia
Erysipelotrichales_unclassified	Erysipelotrichales	Erysipelotrichia
Eubacteriaceae	Eubacteriales	Clostridia
Eubacteriaceae	Eubacteriales	Clostridia
Eubacteriaceae	Eubacteriales	Clostridia
Eubacteriaceae	Eubacteriales	Clostridia
Lachnospiraceae	Eubacteriales	Clostridia
Lachnospiraceae	Eubacteriales	Clostridia
Eggerthellaceae	Eggerthellales	Coriobacteriia
Eggerthellaceae	Eggerthellales	Coriobacteriia

Lachnospiraceae	Eubacteriales	Clostridia
Lachnospiraceae	Eubacteriales	Clostridia
Muribaculaceae	Bacteroidales	Bacteroidia
Muribaculaceae	Bacteroidales	Bacteroidia
Muribaculaceae	Bacteroidales	Bacteroidia
Muribaculaceae	Bacteroidales	Bacteroidia
Muribaculaceae	Bacteroidales	Bacteroidia
Muribaculaceae	Bacteroidales	Bacteroidia
FGB9508	OFGB9508	CFGB9508
FGB9508	OFGB9508	CFGB9508
FGB2838	OFGB2838	CFGB2838
FGB2838	OFGB2838	CFGB2838
FGB2838	OFGB2838	CFGB2838
FGB2838	OFGB2838	CFGB2838
FGB2838	OFGB2838	CFGB2838
FGB2838	OFGB2838	CFGB2838
Pumilibacteraceae	Eubacteriales	Clostridia
Pumilibacteraceae	Eubacteriales	Clostridia
FGB28439	OFGB28439	CFGB28439
FGB28439	OFGB28439	CFGB28439
Clostridia_unclassified	Clostridia_unclassified	Clostridia
Clostridia_unclassified	Clostridia_unclassified	Clostridia
Eubacteriaceae	Eubacteriales	Clostridia
Eubacteriaceae	Eubacteriales	Clostridia
Lachnospiraceae	Eubacteriales	Clostridia
Lachnospiraceae	Eubacteriales	Clostridia
Lachnospiraceae	Eubacteriales	Clostridia
Lachnospiraceae	Eubacteriales	Clostridia
Lachnospiraceae	Eubacteriales	Clostridia
Lachnospiraceae	Eubacteriales	Clostridia
Lachnospiraceae	Eubacteriales	Clostridia
Lachnospiraceae	Eubacteriales	Clostridia
FGB77305	OFGB77305	CFGB77305
FGB77305	OFGB77305	CFGB77305
Clostridiaceae	Eubacteriales	Clostridia
Clostridiaceae	Eubacteriales	Clostridia
Lachnospiraceae	Eubacteriales	Clostridia
Lachnospiraceae	Eubacteriales	Clostridia
Lachnospiraceae	Eubacteriales	Clostridia
Lachnospiraceae	Eubacteriales	Clostridia
Lachnospiraceae	Eubacteriales	Clostridia
Lachnospiraceae	Eubacteriales	Clostridia
FGB9633	OFGB9633	CFGB9633
FGB9633	OFGB9633	CFGB9633
Bacteria_unclassified	Bacteria_unclassified	Bacteria_unclassified
Bacteria_unclassified	Bacteria_unclassified	Bacteria_unclassified

Bacteria_unclassified	Bacteria_unclassified	Bacteria_unclassified
Bacteria_unclassified	Bacteria_unclassified	Bacteria_unclassified
Bacteria_unclassified	Bacteria_unclassified	Bacteria_unclassified
Bacteria_unclassified	Bacteria_unclassified	Bacteria_unclassified
Bacteria_unclassified	Bacteria_unclassified	Bacteria_unclassified
Bacteria_unclassified	Bacteria_unclassified	Bacteria_unclassified
Lachnospiraceae	Eubacteriales	Clostridia
Lachnospiraceae	Eubacteriales	Clostridia
Lachnospiraceae	Eubacteriales	Clostridia
Lachnospiraceae	Eubacteriales	Clostridia
Lachnospiraceae	Eubacteriales	Clostridia
Lachnospiraceae	Eubacteriales	Clostridia
FGB77359	OFGB77359	CFGB77359
FGB77359	OFGB77359	CFGB77359
FGB9639	OFGB9639	CFGB9639
FGB9639	OFGB9639	CFGB9639
Lachnospiraceae	Eubacteriales	Clostridia
Lachnospiraceae	Eubacteriales	Clostridia
Lachnospiraceae	Eubacteriales	Clostridia
Lachnospiraceae	Eubacteriales	Clostridia
Lachnospiraceae	Eubacteriales	Clostridia
Lachnospiraceae	Eubacteriales	Clostridia
Clostridiaceae	Eubacteriales	Clostridia
Clostridiaceae	Eubacteriales	Clostridia
Clostridiaceae	Eubacteriales	Clostridia
Clostridiaceae	Eubacteriales	Clostridia
Clostridiaceae	Eubacteriales	Clostridia
Clostridiaceae	Eubacteriales	Clostridia
Clostridiaceae	Eubacteriales	Clostridia
Clostridiaceae	Eubacteriales	Clostridia
Clostridiaceae	Eubacteriales	Clostridia
Clostridiaceae	Eubacteriales	Clostridia
Clostridiaceae	Eubacteriales	Clostridia
Clostridiaceae	Eubacteriales	Clostridia
Clostridiaceae	Eubacteriales	Clostridia
Clostridiaceae	Eubacteriales	Clostridia
Eubacteriaceae	Eubacteriales	Clostridia
Eubacteriaceae	Eubacteriales	Clostridia
FGB9658	OFGB9658	CFGB9658
FGB9658	OFGB9658	CFGB9658
FGB9659	OFGB9659	CFGB9659
FGB9659	OFGB9659	CFGB9659
Bacteria_unclassified	Bacteria_unclassified	Bacteria_unclassified
Bacteria_unclassified	Bacteria_unclassified	Bacteria_unclassified
FGB9827	OFGB9827	CFGB9827
FGB9827	OFGB9827	CFGB9827

Eubacteriaceae	Eubacteriales	Clostridia
Eubacteriaceae	Eubacteriales	Clostridia
FGB77303	OFGB77303	CFGB77303
FGB77303	OFGB77303	CFGB77303
Eubacteriales_unclassified	Eubacteriales	Clostridia
Eubacteriales_unclassified	Eubacteriales	Clostridia
Oscillospiraceae	Eubacteriales	Clostridia
Oscillospiraceae	Eubacteriales	Clostridia
FGB30328	OFGB30328	CFGB30328
FGB30328	OFGB30328	CFGB30328
Oscillospiraceae	Eubacteriales	Clostridia
Oscillospiraceae	Eubacteriales	Clostridia
Oscillospiraceae	Eubacteriales	Clostridia
Oscillospiraceae	Eubacteriales	Clostridia
Oscillospiraceae	Eubacteriales	Clostridia
Oscillospiraceae	Eubacteriales	Clostridia
Oscillospiraceae	Eubacteriales	Clostridia
Oscillospiraceae	Eubacteriales	Clostridia
Oscillospiraceae	Eubacteriales	Clostridia
Oscillospiraceae	Eubacteriales	Clostridia
Oscillospiraceae	Eubacteriales	Clostridia
Oscillospiraceae	Eubacteriales	Clostridia
Oscillospiraceae	Eubacteriales	Clostridia
Oscillospiraceae	Eubacteriales	Clostridia
FGB77153	OFGB77153	CFGB77153
FGB77153	OFGB77153	CFGB77153
FGB1791	OFGB1791	CFGB1791
FGB1791	OFGB1791	CFGB1791
FGB10290	OFGB10290	CFGB10290
FGB10290	OFGB10290	CFGB10290
Oscillospiraceae	Eubacteriales	Clostridia
Oscillospiraceae	Eubacteriales	Clostridia
FGB1765	OFGB1765	CFGB1765
FGB1765	OFGB1765	CFGB1765
FGB10349	OFGB10349	CFGB10349
FGB10349	OFGB10349	CFGB10349
FGB10667	OFGB10667	CFGB10667
FGB10667	OFGB10667	CFGB10667
Lachnospiraceae	Eubacteriales	Clostridia
Lachnospiraceae	Eubacteriales	Clostridia
Lachnospiraceae	Eubacteriales	Clostridia
Lachnospiraceae	Eubacteriales	Clostridia
Christensenellaceae	Eubacteriales	Clostridia
Christensenellaceae	Eubacteriales	Clostridia
FGB10299	OFGB10299	CFGB10299
FGB10299	OFGB10299	CFGB10299

Oscillospiraceae	Eubacteriales	Clostridia
Oscillospiraceae	Eubacteriales	Clostridia
Oscillospiraceae	Eubacteriales	Clostridia
Oscillospiraceae	Eubacteriales	Clostridia
Lachnospiraceae	Eubacteriales	Clostridia
Lachnospiraceae	Eubacteriales	Clostridia
Clostridia_unclassified	Clostridia_unclassified	Clostridia
Clostridia_unclassified	Clostridia_unclassified	Clostridia
Lachnospiraceae	Eubacteriales	Clostridia
Lachnospiraceae	Eubacteriales	Clostridia
Lachnospiraceae	Eubacteriales	Clostridia
Lachnospiraceae	Eubacteriales	Clostridia
Lachnospiraceae	Eubacteriales	Clostridia
Lachnospiraceae	Eubacteriales	Clostridia
Lachnospiraceae	Eubacteriales	Clostridia
Lachnospiraceae	Eubacteriales	Clostridia
Lachnospiraceae	Eubacteriales	Clostridia
Lachnospiraceae	Eubacteriales	Clostridia
Lachnospiraceae	Eubacteriales	Clostridia
Lachnospiraceae	Eubacteriales	Clostridia
Lachnospiraceae	Eubacteriales	Clostridia
Lachnospiraceae	Eubacteriales	Clostridia
Lachnospiraceae	Eubacteriales	Clostridia
Lachnospiraceae	Eubacteriales	Clostridia
Lactobacillaceae	Lactobacillales	Bacilli
Lactobacillaceae	Lactobacillales	Bacilli
Muribaculaceae	Bacteroidales	Bacteroidia
Muribaculaceae	Bacteroidales	Bacteroidia
Oscillospiraceae	Eubacteriales	Clostridia
Oscillospiraceae	Eubacteriales	Clostridia
Oscillospiraceae	Eubacteriales	Clostridia
Oscillospiraceae	Eubacteriales	Clostridia
Oscillospiraceae	Eubacteriales	Clostridia
Oscillospiraceae	Eubacteriales	Clostridia
Oscillospiraceae	Eubacteriales	Clostridia
Oscillospiraceae	Eubacteriales	Clostridia
Sutterellaceae	Burkholderiales	Betaproteobacteria
Sutterellaceae	Burkholderiales	Betaproteobacteria
Peptostreptococcaceae	Eubacteriales	Clostridia
Peptostreptococcaceae	Eubacteriales	Clostridia
Lachnospiraceae	Eubacteriales	Clostridia
Lachnospiraceae	Eubacteriales	Clostridia

Turicibacteraceae	Erysipelotrichales	Erysipelotrichia
Turicibacteraceae	Erysipelotrichales	Erysipelotrichia
Lachnospiraceae	Eubacteriales	Clostridia
Lachnospiraceae	Eubacteriales	Clostridia
Lachnospiraceae	Eubacteriales	Clostridia
Lachnospiraceae	Eubacteriales	Clostridia
Oscillospiraceae	Eubacteriales	Clostridia
Oscillospiraceae	Eubacteriales	Clostridia
Oscillospiraceae	Eubacteriales	Clostridia
Oscillospiraceae	Eubacteriales	Clostridia
Eggerthellaceae	Eggerthellales	Coriobacteriia
Eggerthellaceae	Eggerthellales	Coriobacteriia
Eggerthellaceae	Eggerthellales	Coriobacteriia
Eggerthellaceae	Eggerthellales	Coriobacteriia
Eggerthellaceae	Eggerthellales	Coriobacteriia
Eggerthellaceae	Eggerthellales	Coriobacteriia
Akkermansiaceae	Verrucomicrobiales	Verrucomicrobiae
Akkermansiaceae	Verrucomicrobiales	Verrucomicrobiae
Rikenellaceae	Bacteroidales	Bacteroidia
Rikenellaceae	Bacteroidales	Bacteroidia
Oscillospiraceae	Eubacteriales	Clostridia
Oscillospiraceae	Eubacteriales	Clostridia
Bacteria_unclassified	Bacteria_unclassified	Bacteria_unclassified
Bacteria_unclassified	Bacteria_unclassified	Bacteria_unclassified
Bacteria_unclassified	Bacteria_unclassified	Bacteria_unclassified
Bacteria_unclassified	Bacteria_unclassified	Bacteria_unclassified
Bacteria_unclassified	Bacteria_unclassified	Bacteria_unclassified
Bacteria_unclassified	Bacteria_unclassified	Bacteria_unclassified
Bacteria_unclassified	Bacteria_unclassified	Bacteria_unclassified
Bacteria_unclassified	Bacteria_unclassified	Bacteria_unclassified
Bacteria_unclassified	Bacteria_unclassified	Bacteria_unclassified
Bacteria_unclassified	Bacteria_unclassified	Bacteria_unclassified
Bacteria_unclassified	Bacteria_unclassified	Bacteria_unclassified
Bacteria_unclassified	Bacteria_unclassified	Bacteria_unclassified
Bacteroidaceae	Bacteroidales	Bacteroidia
Bacteroidaceae	Bacteroidales	Bacteroidia
Bifidobacteriaceae	Bifidobacteriales	Actinomycetia
Bifidobacteriaceae	Bifidobacteriales	Actinomycetia
Clostridia_unclassified	Clostridia_unclassified	Clostridia
Clostridia_unclassified	Clostridia_unclassified	Clostridia
Clostridiaceae	Eubacteriales	Clostridia
Clostridiaceae	Eubacteriales	Clostridia
Clostridiaceae	Eubacteriales	Clostridia
Clostridiaceae	Eubacteriales	Clostridia

Eubacteriales_unclassified	Eubacteriales	Clostridia
Eubacteriales_unclassified	Eubacteriales	Clostridia
Erysipelotrichaceae	Erysipelotrichales	Erysipelotrichia
Erysipelotrichaceae	Erysipelotrichales	Erysipelotrichia
Coriobacteriaceae	Coriobacteriales	Coriobacteriia
Coriobacteriaceae	Coriobacteriales	Coriobacteriia
Lachnospiraceae	Eubacteriales	Clostridia
Lachnospiraceae	Eubacteriales	Clostridia
Erysipelotrichaceae	Erysipelotrichales	Erysipelotrichia
Erysipelotrichaceae	Erysipelotrichales	Erysipelotrichia
Erysipelotrichales_unclassified	Erysipelotrichales	Erysipelotrichia
Erysipelotrichales_unclassified	Erysipelotrichales	Erysipelotrichia
Eubacteriaceae	Eubacteriales	Clostridia
Eubacteriaceae	Eubacteriales	Clostridia
Eubacteriaceae	Eubacteriales	Clostridia
Eubacteriaceae	Eubacteriales	Clostridia
Lachnospiraceae	Eubacteriales	Clostridia
Lachnospiraceae	Eubacteriales	Clostridia
Eggerthellaceae	Eggerthellales	Coriobacteriia
Eggerthellaceae	Eggerthellales	Coriobacteriia
Lachnospiraceae	Eubacteriales	Clostridia
Lachnospiraceae	Eubacteriales	Clostridia
Muribaculaceae	Bacteroidales	Bacteroidia
Muribaculaceae	Bacteroidales	Bacteroidia
Muribaculaceae	Bacteroidales	Bacteroidia
Muribaculaceae	Bacteroidales	Bacteroidia
Muribaculaceae	Bacteroidales	Bacteroidia
Muribaculaceae	Bacteroidales	Bacteroidia
FGB9508	OFGB9508	CFGB9508
FGB9508	OFGB9508	CFGB9508
FGB2838	OFGB2838	CFGB2838
FGB2838	OFGB2838	CFGB2838
FGB2838	OFGB2838	CFGB2838
FGB2838	OFGB2838	CFGB2838
FGB2838	OFGB2838	CFGB2838
FGB2838	OFGB2838	CFGB2838
Pumilibacteraceae	Eubacteriales	Clostridia
Pumilibacteraceae	Eubacteriales	Clostridia
FGB28439	OFGB28439	CFGB28439
FGB28439	OFGB28439	CFGB28439
Clostridia_unclassified	Clostridia_unclassified	Clostridia
Clostridia_unclassified	Clostridia_unclassified	Clostridia
Eubacteriaceae	Eubacteriales	Clostridia
Eubacteriaceae	Eubacteriales	Clostridia
Lachnospiraceae	Eubacteriales	Clostridia
Lachnospiraceae	Eubacteriales	Clostridia

Lachnospiraceae	Eubacteriales	Clostridia
Lachnospiraceae	Eubacteriales	Clostridia
Lachnospiraceae	Eubacteriales	Clostridia
Lachnospiraceae	Eubacteriales	Clostridia
Lachnospiraceae	Eubacteriales	Clostridia
Lachnospiraceae	Eubacteriales	Clostridia
FGB77305	OFGB77305	CFGB77305
FGB77305	OFGB77305	CFGB77305
Clostridiaceae	Eubacteriales	Clostridia
Clostridiaceae	Eubacteriales	Clostridia
Lachnospiraceae	Eubacteriales	Clostridia
Lachnospiraceae	Eubacteriales	Clostridia
Lachnospiraceae	Eubacteriales	Clostridia
Lachnospiraceae	Eubacteriales	Clostridia
Lachnospiraceae	Eubacteriales	Clostridia
Lachnospiraceae	Eubacteriales	Clostridia
FGB9633	OFGB9633	CFGB9633
FGB9633	OFGB9633	CFGB9633
Bacteria_unclassified	Bacteria_unclassified	Bacteria_unclassified
Bacteria_unclassified	Bacteria_unclassified	Bacteria_unclassified
Bacteria_unclassified	Bacteria_unclassified	Bacteria_unclassified
Bacteria_unclassified	Bacteria_unclassified	Bacteria_unclassified
Bacteria_unclassified	Bacteria_unclassified	Bacteria_unclassified
Bacteria_unclassified	Bacteria_unclassified	Bacteria_unclassified
Bacteria_unclassified	Bacteria_unclassified	Bacteria_unclassified
Bacteria_unclassified	Bacteria_unclassified	Bacteria_unclassified
Lachnospiraceae	Eubacteriales	Clostridia
Lachnospiraceae	Eubacteriales	Clostridia
Lachnospiraceae	Eubacteriales	Clostridia
Lachnospiraceae	Eubacteriales	Clostridia
Lachnospiraceae	Eubacteriales	Clostridia
Lachnospiraceae	Eubacteriales	Clostridia
FGB77359	OFGB77359	CFGB77359
FGB77359	OFGB77359	CFGB77359
FGB9639	OFGB9639	CFGB9639
FGB9639	OFGB9639	CFGB9639
Lachnospiraceae	Eubacteriales	Clostridia
Lachnospiraceae	Eubacteriales	Clostridia
Lachnospiraceae	Eubacteriales	Clostridia
Lachnospiraceae	Eubacteriales	Clostridia
Lachnospiraceae	Eubacteriales	Clostridia
Lachnospiraceae	Eubacteriales	Clostridia
Clostridiaceae	Eubacteriales	Clostridia
Clostridiaceae	Eubacteriales	Clostridia
Clostridiaceae	Eubacteriales	Clostridia
Clostridiaceae	Eubacteriales	Clostridia

Clostridiaceae	Eubacteriales	Clostridia
Clostridiaceae	Eubacteriales	Clostridia
Clostridiaceae	Eubacteriales	Clostridia
Clostridiaceae	Eubacteriales	Clostridia
Clostridiaceae	Eubacteriales	Clostridia
Clostridiaceae	Eubacteriales	Clostridia
Clostridiaceae	Eubacteriales	Clostridia
Clostridiaceae	Eubacteriales	Clostridia
Clostridiaceae	Eubacteriales	Clostridia
Clostridiaceae	Eubacteriales	Clostridia
Eubacteriaceae	Eubacteriales	Clostridia
Eubacteriaceae	Eubacteriales	Clostridia
FGB9658	OFGB9658	CFGB9658
FGB9658	OFGB9658	CFGB9658
FGB9659	OFGB9659	CFGB9659
FGB9659	OFGB9659	CFGB9659
Bacteria_unclassified	Bacteria_unclassified	Bacteria_unclassified
Bacteria_unclassified	Bacteria_unclassified	Bacteria_unclassified
FGB9827	OFGB9827	CFGB9827
FGB9827	OFGB9827	CFGB9827
Eubacteriaceae	Eubacteriales	Clostridia
Eubacteriaceae	Eubacteriales	Clostridia
FGB77303	OFGB77303	CFGB77303
FGB77303	OFGB77303	CFGB77303
Eubacteriales_unclassified	Eubacteriales	Clostridia
Eubacteriales_unclassified	Eubacteriales	Clostridia
Oscillospiraceae	Eubacteriales	Clostridia
Oscillospiraceae	Eubacteriales	Clostridia
FGB30328	OFGB30328	CFGB30328
FGB30328	OFGB30328	CFGB30328
Oscillospiraceae	Eubacteriales	Clostridia
Oscillospiraceae	Eubacteriales	Clostridia
Oscillospiraceae	Eubacteriales	Clostridia
Oscillospiraceae	Eubacteriales	Clostridia
Oscillospiraceae	Eubacteriales	Clostridia
Oscillospiraceae	Eubacteriales	Clostridia
Oscillospiraceae	Eubacteriales	Clostridia
Oscillospiraceae	Eubacteriales	Clostridia
Oscillospiraceae	Eubacteriales	Clostridia
Oscillospiraceae	Eubacteriales	Clostridia
Oscillospiraceae	Eubacteriales	Clostridia
Oscillospiraceae	Eubacteriales	Clostridia
Oscillospiraceae	Eubacteriales	Clostridia
Oscillospiraceae	Eubacteriales	Clostridia
FGB77153	OFGB77153	CFGB77153
FGB77153	OFGB77153	CFGB77153

FGB1791	OFGB1791	CFGB1791
FGB1791	OFGB1791	CFGB1791
FGB10290	OFGB10290	CFGB10290
FGB10290	OFGB10290	CFGB10290
Oscillospiraceae	Eubacteriales	Clostridia
Oscillospiraceae	Eubacteriales	Clostridia
FGB1765	OFGB1765	CFGB1765
FGB1765	OFGB1765	CFGB1765
FGB10349	OFGB10349	CFGB10349
FGB10349	OFGB10349	CFGB10349
FGB10667	OFGB10667	CFGB10667
FGB10667	OFGB10667	CFGB10667
Lachnospiraceae	Eubacteriales	Clostridia
Lachnospiraceae	Eubacteriales	Clostridia
Lachnospiraceae	Eubacteriales	Clostridia
Lachnospiraceae	Eubacteriales	Clostridia
Christensenellaceae	Eubacteriales	Clostridia
Christensenellaceae	Eubacteriales	Clostridia
FGB10299	OFGB10299	CFGB10299
FGB10299	OFGB10299	CFGB10299
Oscillospiraceae	Eubacteriales	Clostridia
Oscillospiraceae	Eubacteriales	Clostridia
Oscillospiraceae	Eubacteriales	Clostridia
Oscillospiraceae	Eubacteriales	Clostridia
Lachnospiraceae	Eubacteriales	Clostridia
Lachnospiraceae	Eubacteriales	Clostridia
Clostridia_unclassified	Clostridia_unclassified	Clostridia
Clostridia_unclassified	Clostridia_unclassified	Clostridia
Lachnospiraceae	Eubacteriales	Clostridia
Lachnospiraceae	Eubacteriales	Clostridia
Lachnospiraceae	Eubacteriales	Clostridia
Lachnospiraceae	Eubacteriales	Clostridia
Lachnospiraceae	Eubacteriales	Clostridia
Lachnospiraceae	Eubacteriales	Clostridia
Lachnospiraceae	Eubacteriales	Clostridia
Lachnospiraceae	Eubacteriales	Clostridia
Lachnospiraceae	Eubacteriales	Clostridia
Lachnospiraceae	Eubacteriales	Clostridia
Lachnospiraceae	Eubacteriales	Clostridia
Lachnospiraceae	Eubacteriales	Clostridia
Lachnospiraceae	Eubacteriales	Clostridia
Lachnospiraceae	Eubacteriales	Clostridia
Lachnospiraceae	Eubacteriales	Clostridia
Lachnospiraceae	Eubacteriales	Clostridia
Lactobacillaceae	Lactobacillales	Bacilli
Lactobacillaceae	Lactobacillales	Bacilli

Muribaculaceae	Bacteroidales	Bacteroidia
Muribaculaceae	Bacteroidales	Bacteroidia
Oscillospiraceae	Eubacteriales	Clostridia
Oscillospiraceae	Eubacteriales	Clostridia
Oscillospiraceae	Eubacteriales	Clostridia
Oscillospiraceae	Eubacteriales	Clostridia
Oscillospiraceae	Eubacteriales	Clostridia
Oscillospiraceae	Eubacteriales	Clostridia
Oscillospiraceae	Eubacteriales	Clostridia
Oscillospiraceae	Eubacteriales	Clostridia
Sutterellaceae	Burkholderiales	Betaproteobacteria
Sutterellaceae	Burkholderiales	Betaproteobacteria
Peptostreptococcaceae	Eubacteriales	Clostridia
Peptostreptococcaceae	Eubacteriales	Clostridia
Lachnospiraceae	Eubacteriales	Clostridia
Lachnospiraceae	Eubacteriales	Clostridia
Turicibacteraceae	Erysipelotrichales	Erysipelotrichia
Turicibacteraceae	Erysipelotrichales	Erysipelotrichia
Lachnospiraceae	Eubacteriales	Clostridia
Lachnospiraceae	Eubacteriales	Clostridia
Lachnospiraceae	Eubacteriales	Clostridia
Lachnospiraceae	Eubacteriales	Clostridia
Oscillospiraceae	Eubacteriales	Clostridia
Oscillospiraceae	Eubacteriales	Clostridia
Oscillospiraceae	Eubacteriales	Clostridia
Oscillospiraceae	Eubacteriales	Clostridia
Eggerthellaceae	Eggerthellales	Coriobacteriia
Eggerthellaceae	Eggerthellales	Coriobacteriia
Eggerthellaceae	Eggerthellales	Coriobacteriia
Eggerthellaceae	Eggerthellales	Coriobacteriia
Eggerthellaceae	Eggerthellales	Coriobacteriia
Eggerthellaceae	Eggerthellales	Coriobacteriia
Akkermansiaceae	Verrucomicrobiales	Verrucomicrobiae
Akkermansiaceae	Verrucomicrobiales	Verrucomicrobiae
Rikenellaceae	Bacteroidales	Bacteroidia
Rikenellaceae	Bacteroidales	Bacteroidia
Oscillospiraceae	Eubacteriales	Clostridia
Oscillospiraceae	Eubacteriales	Clostridia
Bacteria_unclassified	Bacteria_unclassified	Bacteria_unclassified
Bacteria_unclassified	Bacteria_unclassified	Bacteria_unclassified
Bacteria_unclassified	Bacteria_unclassified	Bacteria_unclassified
Bacteria_unclassified	Bacteria_unclassified	Bacteria_unclassified

Bacteria_unclassified	Bacteria_unclassified	Bacteria_unclassified
Bacteria_unclassified	Bacteria_unclassified	Bacteria_unclassified
Bacteria_unclassified	Bacteria_unclassified	Bacteria_unclassified
Bacteria_unclassified	Bacteria_unclassified	Bacteria_unclassified
Bacteria_unclassified	Bacteria_unclassified	Bacteria_unclassified
Bacteria_unclassified	Bacteria_unclassified	Bacteria_unclassified
Bacteria_unclassified	Bacteria_unclassified	Bacteria_unclassified
Bacteria_unclassified	Bacteria_unclassified	Bacteria_unclassified
Bacteroidaceae	Bacteroidales	Bacteroidia
Bacteroidaceae	Bacteroidales	Bacteroidia
Bifidobacteriaceae	Bifidobacteriales	Actinomycetia
Bifidobacteriaceae	Bifidobacteriales	Actinomycetia
Clostridia_unclassified	Clostridia_unclassified	Clostridia
Clostridia_unclassified	Clostridia_unclassified	Clostridia
Clostridiaceae	Eubacteriales	Clostridia
Clostridiaceae	Eubacteriales	Clostridia
Clostridiaceae	Eubacteriales	Clostridia
Clostridiaceae	Eubacteriales	Clostridia
Eubacteriales_unclassified	Eubacteriales	Clostridia
Eubacteriales_unclassified	Eubacteriales	Clostridia
Erysipelotrichaceae	Erysipelotrichales	Erysipelotrichia
Erysipelotrichaceae	Erysipelotrichales	Erysipelotrichia
Coriobacteriaceae	Coriobacteriales	Coriobacteriia
Coriobacteriaceae	Coriobacteriales	Coriobacteriia
Lachnospiraceae	Eubacteriales	Clostridia
Lachnospiraceae	Eubacteriales	Clostridia
Erysipelotrichaceae	Erysipelotrichales	Erysipelotrichia
Erysipelotrichaceae	Erysipelotrichales	Erysipelotrichia
Erysipelotrichales_unclassified	Erysipelotrichales	Erysipelotrichia
Erysipelotrichales_unclassified	Erysipelotrichales	Erysipelotrichia
Eubacteriaceae	Eubacteriales	Clostridia
Eubacteriaceae	Eubacteriales	Clostridia
Eubacteriaceae	Eubacteriales	Clostridia
Eubacteriaceae	Eubacteriales	Clostridia
Lachnospiraceae	Eubacteriales	Clostridia
Lachnospiraceae	Eubacteriales	Clostridia
Eggerthellaceae	Eggerthellales	Coriobacteriia
Eggerthellaceae	Eggerthellales	Coriobacteriia
Lachnospiraceae	Eubacteriales	Clostridia
Lachnospiraceae	Eubacteriales	Clostridia
Muribaculaceae	Bacteroidales	Bacteroidia
Muribaculaceae	Bacteroidales	Bacteroidia
Muribaculaceae	Bacteroidales	Bacteroidia
Muribaculaceae	Bacteroidales	Bacteroidia

Muribaculaceae	Bacteroidales	Bacteroidia
Muribaculaceae	Bacteroidales	Bacteroidia
FGB9508	OFGB9508	CFGB9508
FGB9508	OFGB9508	CFGB9508
FGB2838	OFGB2838	CFGB2838
FGB2838	OFGB2838	CFGB2838
FGB2838	OFGB2838	CFGB2838
FGB2838	OFGB2838	CFGB2838
FGB2838	OFGB2838	CFGB2838
FGB2838	OFGB2838	CFGB2838
Pumilibacteraceae	Eubacteriales	Clostridia
Pumilibacteraceae	Eubacteriales	Clostridia
FGB28439	OFGB28439	CFGB28439
FGB28439	OFGB28439	CFGB28439
Clostridia_unclassified	Clostridia_unclassified	Clostridia
Clostridia_unclassified	Clostridia_unclassified	Clostridia
Eubacteriaceae	Eubacteriales	Clostridia
Eubacteriaceae	Eubacteriales	Clostridia
Lachnospiraceae	Eubacteriales	Clostridia
Lachnospiraceae	Eubacteriales	Clostridia
Lachnospiraceae	Eubacteriales	Clostridia
Lachnospiraceae	Eubacteriales	Clostridia
Lachnospiraceae	Eubacteriales	Clostridia
Lachnospiraceae	Eubacteriales	Clostridia
Lachnospiraceae	Eubacteriales	Clostridia
Lachnospiraceae	Eubacteriales	Clostridia
FGB77305	OFGB77305	CFGB77305
FGB77305	OFGB77305	CFGB77305
Clostridiaceae	Eubacteriales	Clostridia
Clostridiaceae	Eubacteriales	Clostridia
Lachnospiraceae	Eubacteriales	Clostridia
Lachnospiraceae	Eubacteriales	Clostridia
Lachnospiraceae	Eubacteriales	Clostridia
Lachnospiraceae	Eubacteriales	Clostridia
Lachnospiraceae	Eubacteriales	Clostridia
Lachnospiraceae	Eubacteriales	Clostridia
FGB9633	OFGB9633	CFGB9633
FGB9633	OFGB9633	CFGB9633
Bacteria_unclassified	Bacteria_unclassified	Bacteria_unclassified
Bacteria_unclassified	Bacteria_unclassified	Bacteria_unclassified
Bacteria_unclassified	Bacteria_unclassified	Bacteria_unclassified
Bacteria_unclassified	Bacteria_unclassified	Bacteria_unclassified
Bacteria_unclassified	Bacteria_unclassified	Bacteria_unclassified
Bacteria_unclassified	Bacteria_unclassified	Bacteria_unclassified
Bacteria_unclassified	Bacteria_unclassified	Bacteria_unclassified
Bacteria_unclassified	Bacteria_unclassified	Bacteria_unclassified

Lachnospiraceae	Eubacteriales	Clostridia
Lachnospiraceae	Eubacteriales	Clostridia
Lachnospiraceae	Eubacteriales	Clostridia
Lachnospiraceae	Eubacteriales	Clostridia
Lachnospiraceae	Eubacteriales	Clostridia
Lachnospiraceae	Eubacteriales	Clostridia
FGB77359	OFGB77359	CFGB77359
FGB77359	OFGB77359	CFGB77359
FGB9639	OFGB9639	CFGB9639
FGB9639	OFGB9639	CFGB9639
Lachnospiraceae	Eubacteriales	Clostridia
Lachnospiraceae	Eubacteriales	Clostridia
Lachnospiraceae	Eubacteriales	Clostridia
Lachnospiraceae	Eubacteriales	Clostridia
Lachnospiraceae	Eubacteriales	Clostridia
Lachnospiraceae	Eubacteriales	Clostridia
Clostridiaceae	Eubacteriales	Clostridia
Clostridiaceae	Eubacteriales	Clostridia
Clostridiaceae	Eubacteriales	Clostridia
Clostridiaceae	Eubacteriales	Clostridia
Clostridiaceae	Eubacteriales	Clostridia
Clostridiaceae	Eubacteriales	Clostridia
Clostridiaceae	Eubacteriales	Clostridia
Clostridiaceae	Eubacteriales	Clostridia
Clostridiaceae	Eubacteriales	Clostridia
Clostridiaceae	Eubacteriales	Clostridia
Clostridiaceae	Eubacteriales	Clostridia
Clostridiaceae	Eubacteriales	Clostridia
Clostridiaceae	Eubacteriales	Clostridia
Clostridiaceae	Eubacteriales	Clostridia
Eubacteriaceae	Eubacteriales	Clostridia
Eubacteriaceae	Eubacteriales	Clostridia
FGB9658	OFGB9658	CFGB9658
FGB9658	OFGB9658	CFGB9658
FGB9659	OFGB9659	CFGB9659
FGB9659	OFGB9659	CFGB9659
Bacteria_unclassified	Bacteria_unclassified	Bacteria_unclassified
Bacteria_unclassified	Bacteria_unclassified	Bacteria_unclassified
FGB9827	OFGB9827	CFGB9827
FGB9827	OFGB9827	CFGB9827
Eubacteriaceae	Eubacteriales	Clostridia
Eubacteriaceae	Eubacteriales	Clostridia
FGB77303	OFGB77303	CFGB77303
FGB77303	OFGB77303	CFGB77303
Eubacteriales_unclassified	Eubacteriales	Clostridia
Eubacteriales_unclassified	Eubacteriales	Clostridia

Oscillospiraceae	Eubacteriales	Clostridia
Oscillospiraceae	Eubacteriales	Clostridia
FGB30328	OFGB30328	CFGB30328
FGB30328	OFGB30328	CFGB30328
Oscillospiraceae	Eubacteriales	Clostridia
Oscillospiraceae	Eubacteriales	Clostridia
Oscillospiraceae	Eubacteriales	Clostridia
Oscillospiraceae	Eubacteriales	Clostridia
Oscillospiraceae	Eubacteriales	Clostridia
Oscillospiraceae	Eubacteriales	Clostridia
Oscillospiraceae	Eubacteriales	Clostridia
Oscillospiraceae	Eubacteriales	Clostridia
Oscillospiraceae	Eubacteriales	Clostridia
Oscillospiraceae	Eubacteriales	Clostridia
Oscillospiraceae	Eubacteriales	Clostridia
Oscillospiraceae	Eubacteriales	Clostridia
Oscillospiraceae	Eubacteriales	Clostridia
Oscillospiraceae	Eubacteriales	Clostridia
FGB77153	OFGB77153	CFGB77153
FGB77153	OFGB77153	CFGB77153
FGB1791	OFGB1791	CFGB1791
FGB1791	OFGB1791	CFGB1791
FGB10290	OFGB10290	CFGB10290
FGB10290	OFGB10290	CFGB10290
Oscillospiraceae	Eubacteriales	Clostridia
Oscillospiraceae	Eubacteriales	Clostridia
FGB1765	OFGB1765	CFGB1765
FGB1765	OFGB1765	CFGB1765
FGB10349	OFGB10349	CFGB10349
FGB10349	OFGB10349	CFGB10349
FGB10667	OFGB10667	CFGB10667
FGB10667	OFGB10667	CFGB10667
Lachnospiraceae	Eubacteriales	Clostridia
Lachnospiraceae	Eubacteriales	Clostridia
Lachnospiraceae	Eubacteriales	Clostridia
Lachnospiraceae	Eubacteriales	Clostridia
Christensenellaceae	Eubacteriales	Clostridia
Christensenellaceae	Eubacteriales	Clostridia
FGB10299	OFGB10299	CFGB10299
FGB10299	OFGB10299	CFGB10299
Oscillospiraceae	Eubacteriales	Clostridia
Oscillospiraceae	Eubacteriales	Clostridia
Oscillospiraceae	Eubacteriales	Clostridia
Oscillospiraceae	Eubacteriales	Clostridia
Lachnospiraceae	Eubacteriales	Clostridia
Lachnospiraceae	Eubacteriales	Clostridia

Clostridia_unclassified	Clostridia_unclassified	Clostridia
Clostridia_unclassified	Clostridia_unclassified	Clostridia
Lachnospiraceae	Eubacteriales	Clostridia
Lachnospiraceae	Eubacteriales	Clostridia
Lachnospiraceae	Eubacteriales	Clostridia
Lachnospiraceae	Eubacteriales	Clostridia
Lachnospiraceae	Eubacteriales	Clostridia
Lachnospiraceae	Eubacteriales	Clostridia
Lachnospiraceae	Eubacteriales	Clostridia
Lachnospiraceae	Eubacteriales	Clostridia
Lachnospiraceae	Eubacteriales	Clostridia
Lachnospiraceae	Eubacteriales	Clostridia
Lachnospiraceae	Eubacteriales	Clostridia
Lachnospiraceae	Eubacteriales	Clostridia
Lachnospiraceae	Eubacteriales	Clostridia
Lachnospiraceae	Eubacteriales	Clostridia
Lachnospiraceae	Eubacteriales	Clostridia
Lachnospiraceae	Eubacteriales	Clostridia
Lactobacillaceae	Lactobacillales	Bacilli
Lactobacillaceae	Lactobacillales	Bacilli
Muribaculaceae	Bacteroidales	Bacteroidia
Muribaculaceae	Bacteroidales	Bacteroidia
Oscillospiraceae	Eubacteriales	Clostridia
Oscillospiraceae	Eubacteriales	Clostridia
Oscillospiraceae	Eubacteriales	Clostridia
Oscillospiraceae	Eubacteriales	Clostridia
Oscillospiraceae	Eubacteriales	Clostridia
Oscillospiraceae	Eubacteriales	Clostridia
Oscillospiraceae	Eubacteriales	Clostridia
Oscillospiraceae	Eubacteriales	Clostridia
Sutterellaceae	Burkholderiales	Betaproteobacteria
Sutterellaceae	Burkholderiales	Betaproteobacteria
Peptostreptococcaceae	Eubacteriales	Clostridia
Peptostreptococcaceae	Eubacteriales	Clostridia
Lachnospiraceae	Eubacteriales	Clostridia
Lachnospiraceae	Eubacteriales	Clostridia
Turicibacteraceae	Erysipelotrichales	Erysipelotrichia
Turicibacteraceae	Erysipelotrichales	Erysipelotrichia
Lachnospiraceae	Eubacteriales	Clostridia
Lachnospiraceae	Eubacteriales	Clostridia
Lachnospiraceae	Eubacteriales	Clostridia
Lachnospiraceae	Eubacteriales	Clostridia

Oscillospiraceae	Eubacteriales	Clostridia
Oscillospiraceae	Eubacteriales	Clostridia
Oscillospiraceae	Eubacteriales	Clostridia
Oscillospiraceae	Eubacteriales	Clostridia
Eggerthellaceae	Eggerthellales	Coriobacteriia
Eggerthellaceae	Eggerthellales	Coriobacteriia
Eggerthellaceae	Eggerthellales	Coriobacteriia
Eggerthellaceae	Eggerthellales	Coriobacteriia
Eggerthellaceae	Eggerthellales	Coriobacteriia
Eggerthellaceae	Eggerthellales	Coriobacteriia
Akkermansiaceae	Verrucomicrobiales	Verrucomicrobiae
Akkermansiaceae	Verrucomicrobiales	Verrucomicrobiae
Rikenellaceae	Bacteroidales	Bacteroidia
Rikenellaceae	Bacteroidales	Bacteroidia
Oscillospiraceae	Eubacteriales	Clostridia
Oscillospiraceae	Eubacteriales	Clostridia
Bacteria_unclassified	Bacteria_unclassified	Bacteria_unclassified
Bacteria_unclassified	Bacteria_unclassified	Bacteria_unclassified
Bacteria_unclassified	Bacteria_unclassified	Bacteria_unclassified
Bacteria_unclassified	Bacteria_unclassified	Bacteria_unclassified
Bacteria_unclassified	Bacteria_unclassified	Bacteria_unclassified
Bacteria_unclassified	Bacteria_unclassified	Bacteria_unclassified
Bacteria_unclassified	Bacteria_unclassified	Bacteria_unclassified
Bacteria_unclassified	Bacteria_unclassified	Bacteria_unclassified
Bacteria_unclassified	Bacteria_unclassified	Bacteria_unclassified
Bacteria_unclassified	Bacteria_unclassified	Bacteria_unclassified
Bacteria_unclassified	Bacteria_unclassified	Bacteria_unclassified
Bacteria_unclassified	Bacteria_unclassified	Bacteria_unclassified
Bacteroidaceae	Bacteroidales	Bacteroidia
Bacteroidaceae	Bacteroidales	Bacteroidia

Bifidobacteriaceae	Bifidobacteriales	Actinomycetia
Bifidobacteriaceae	Bifidobacteriales	Actinomycetia
Clostridia_unclassified	Clostridia_unclassified	Clostridia
Clostridia_unclassified	Clostridia_unclassified	Clostridia
Clostridiaceae	Eubacteriales	Clostridia
Clostridiaceae	Eubacteriales	Clostridia
Clostridiaceae	Eubacteriales	Clostridia
Clostridiaceae	Eubacteriales	Clostridia
Eubacteriales_unclassified	Eubacteriales	Clostridia
Eubacteriales_unclassified	Eubacteriales	Clostridia
Erysipelotrichaceae	Erysipelotrichales	Erysipelotrichia
Erysipelotrichaceae	Erysipelotrichales	Erysipelotrichia
Coriobacteriaceae	Coriobacteriales	Coriobacteriia
Coriobacteriaceae	Coriobacteriales	Coriobacteriia

Lachnospiraceae	Eubacteriales	Clostridia
Lachnospiraceae	Eubacteriales	Clostridia
Erysipelotrichaceae	Erysipelotrichales	Erysipelotrichia
Erysipelotrichaceae	Erysipelotrichales	Erysipelotrichia
Erysipelotrichales_unclassified	Erysipelotrichales	Erysipelotrichia
Erysipelotrichales_unclassified	Erysipelotrichales	Erysipelotrichia
Eubacteriaceae	Eubacteriales	Clostridia
Eubacteriaceae	Eubacteriales	Clostridia
Eubacteriaceae	Eubacteriales	Clostridia
Eubacteriaceae	Eubacteriales	Clostridia
Lachnospiraceae	Eubacteriales	Clostridia
Lachnospiraceae	Eubacteriales	Clostridia
Eggerthellaceae	Eggerthellales	Coriobacteriia
Eggerthellaceae	Eggerthellales	Coriobacteriia
Lachnospiraceae	Eubacteriales	Clostridia
Lachnospiraceae	Eubacteriales	Clostridia
Muribaculaceae	Bacteroidales	Bacteroidia
Muribaculaceae	Bacteroidales	Bacteroidia
Muribaculaceae	Bacteroidales	Bacteroidia
Muribaculaceae	Bacteroidales	Bacteroidia
Muribaculaceae	Bacteroidales	Bacteroidia
Muribaculaceae	Bacteroidales	Bacteroidia
FGB9508	OFGB9508	CFGB9508
FGB9508	OFGB9508	CFGB9508
FGB2838	OFGB2838	CFGB2838
FGB2838	OFGB2838	CFGB2838
FGB2838	OFGB2838	CFGB2838
FGB2838	OFGB2838	CFGB2838
FGB2838	OFGB2838	CFGB2838
FGB2838	OFGB2838	CFGB2838
Pumilibacteraceae	Eubacteriales	Clostridia
Pumilibacteraceae	Eubacteriales	Clostridia
FGB28439	OFGB28439	CFGB28439
FGB28439	OFGB28439	CFGB28439
Clostridia_unclassified	Clostridia_unclassified	Clostridia
Clostridia_unclassified	Clostridia_unclassified	Clostridia
Eubacteriaceae	Eubacteriales	Clostridia
Eubacteriaceae	Eubacteriales	Clostridia
Lachnospiraceae	Eubacteriales	Clostridia
Lachnospiraceae	Eubacteriales	Clostridia
Lachnospiraceae	Eubacteriales	Clostridia
Lachnospiraceae	Eubacteriales	Clostridia
Lachnospiraceae	Eubacteriales	Clostridia
Lachnospiraceae	Eubacteriales	Clostridia
Lachnospiraceae	Eubacteriales	Clostridia
Lachnospiraceae	Eubacteriales	Clostridia

FGB77305	OFGB77305	CFGB77305
FGB77305	OFGB77305	CFGB77305
Clostridiaceae	Eubacteriales	Clostridia
Clostridiaceae	Eubacteriales	Clostridia
Lachnospiraceae	Eubacteriales	Clostridia
Lachnospiraceae	Eubacteriales	Clostridia
Lachnospiraceae	Eubacteriales	Clostridia
Lachnospiraceae	Eubacteriales	Clostridia
Lachnospiraceae	Eubacteriales	Clostridia
Lachnospiraceae	Eubacteriales	Clostridia
FGB9633	OFGB9633	CFGB9633
FGB9633	OFGB9633	CFGB9633
Bacteria_unclassified	Bacteria_unclassified	Bacteria_unclassified
Bacteria_unclassified	Bacteria_unclassified	Bacteria_unclassified
Bacteria_unclassified	Bacteria_unclassified	Bacteria_unclassified
Bacteria_unclassified	Bacteria_unclassified	Bacteria_unclassified
Bacteria_unclassified	Bacteria_unclassified	Bacteria_unclassified
Bacteria_unclassified	Bacteria_unclassified	Bacteria_unclassified
Bacteria_unclassified	Bacteria_unclassified	Bacteria_unclassified
Bacteria_unclassified	Bacteria_unclassified	Bacteria_unclassified
Lachnospiraceae	Eubacteriales	Clostridia
Lachnospiraceae	Eubacteriales	Clostridia
Lachnospiraceae	Eubacteriales	Clostridia
Lachnospiraceae	Eubacteriales	Clostridia
Lachnospiraceae	Eubacteriales	Clostridia
Lachnospiraceae	Eubacteriales	Clostridia
FGB77359	OFGB77359	CFGB77359
FGB77359	OFGB77359	CFGB77359
FGB9639	OFGB9639	CFGB9639
FGB9639	OFGB9639	CFGB9639
Lachnospiraceae	Eubacteriales	Clostridia
Lachnospiraceae	Eubacteriales	Clostridia
Lachnospiraceae	Eubacteriales	Clostridia
Lachnospiraceae	Eubacteriales	Clostridia
Lachnospiraceae	Eubacteriales	Clostridia
Lachnospiraceae	Eubacteriales	Clostridia
Clostridiaceae	Eubacteriales	Clostridia
Clostridiaceae	Eubacteriales	Clostridia
Clostridiaceae	Eubacteriales	Clostridia
Clostridiaceae	Eubacteriales	Clostridia
Clostridiaceae	Eubacteriales	Clostridia
Clostridiaceae	Eubacteriales	Clostridia
Clostridiaceae	Eubacteriales	Clostridia
Clostridiaceae	Eubacteriales	Clostridia
Clostridiaceae	Eubacteriales	Clostridia
Clostridiaceae	Eubacteriales	Clostridia

Clostridiaceae	Eubacteriales	Clostridia
Clostridiaceae	Eubacteriales	Clostridia
Clostridiaceae	Eubacteriales	Clostridia
Clostridiaceae	Eubacteriales	Clostridia
Eubacteriaceae	Eubacteriales	Clostridia
Eubacteriaceae	Eubacteriales	Clostridia
FGB9658	OFGB9658	CFGB9658
FGB9658	OFGB9658	CFGB9658
FGB9659	OFGB9659	CFGB9659
FGB9659	OFGB9659	CFGB9659
Bacteria_unclassified	Bacteria_unclassified	Bacteria_unclassified
Bacteria_unclassified	Bacteria_unclassified	Bacteria_unclassified
FGB9827	OFGB9827	CFGB9827
FGB9827	OFGB9827	CFGB9827
Eubacteriaceae	Eubacteriales	Clostridia
Eubacteriaceae	Eubacteriales	Clostridia
FGB77303	OFGB77303	CFGB77303
FGB77303	OFGB77303	CFGB77303
Eubacteriales_unclassified	Eubacteriales	Clostridia
Eubacteriales_unclassified	Eubacteriales	Clostridia
Oscillospiraceae	Eubacteriales	Clostridia
Oscillospiraceae	Eubacteriales	Clostridia
FGB30328	OFGB30328	CFGB30328
FGB30328	OFGB30328	CFGB30328
Oscillospiraceae	Eubacteriales	Clostridia
Oscillospiraceae	Eubacteriales	Clostridia
Oscillospiraceae	Eubacteriales	Clostridia
Oscillospiraceae	Eubacteriales	Clostridia
Oscillospiraceae	Eubacteriales	Clostridia
Oscillospiraceae	Eubacteriales	Clostridia
Oscillospiraceae	Eubacteriales	Clostridia
Oscillospiraceae	Eubacteriales	Clostridia
Oscillospiraceae	Eubacteriales	Clostridia
Oscillospiraceae	Eubacteriales	Clostridia
Oscillospiraceae	Eubacteriales	Clostridia
Oscillospiraceae	Eubacteriales	Clostridia
Oscillospiraceae	Eubacteriales	Clostridia
Oscillospiraceae	Eubacteriales	Clostridia
FGB77153	OFGB77153	CFGB77153
FGB77153	OFGB77153	CFGB77153
FGB1791	OFGB1791	CFGB1791
FGB1791	OFGB1791	CFGB1791
FGB10290	OFGB10290	CFGB10290
FGB10290	OFGB10290	CFGB10290
Oscillospiraceae	Eubacteriales	Clostridia
Oscillospiraceae	Eubacteriales	Clostridia

FGB1765	OFGB1765	CFGB1765
FGB1765	OFGB1765	CFGB1765
FGB10349	OFGB10349	CFGB10349
FGB10349	OFGB10349	CFGB10349
FGB10667	OFGB10667	CFGB10667
FGB10667	OFGB10667	CFGB10667
Lachnospiraceae	Eubacteriales	Clostridia
Lachnospiraceae	Eubacteriales	Clostridia
Lachnospiraceae	Eubacteriales	Clostridia
Lachnospiraceae	Eubacteriales	Clostridia
Christensenellaceae	Eubacteriales	Clostridia
Christensenellaceae	Eubacteriales	Clostridia
FGB10299	OFGB10299	CFGB10299
FGB10299	OFGB10299	CFGB10299
Oscillospiraceae	Eubacteriales	Clostridia
Oscillospiraceae	Eubacteriales	Clostridia
Oscillospiraceae	Eubacteriales	Clostridia
Oscillospiraceae	Eubacteriales	Clostridia
Lachnospiraceae	Eubacteriales	Clostridia
Lachnospiraceae	Eubacteriales	Clostridia
Clostridia_unclassified	Clostridia_unclassified	Clostridia
Clostridia_unclassified	Clostridia_unclassified	Clostridia
Lachnospiraceae	Eubacteriales	Clostridia
Lachnospiraceae	Eubacteriales	Clostridia
Lachnospiraceae	Eubacteriales	Clostridia
Lachnospiraceae	Eubacteriales	Clostridia
Lachnospiraceae	Eubacteriales	Clostridia
Lachnospiraceae	Eubacteriales	Clostridia
Lachnospiraceae	Eubacteriales	Clostridia
Lachnospiraceae	Eubacteriales	Clostridia
Lachnospiraceae	Eubacteriales	Clostridia
Lachnospiraceae	Eubacteriales	Clostridia
Lachnospiraceae	Eubacteriales	Clostridia
Lachnospiraceae	Eubacteriales	Clostridia
Lachnospiraceae	Eubacteriales	Clostridia
Lachnospiraceae	Eubacteriales	Clostridia
Lachnospiraceae	Eubacteriales	Clostridia
Lachnospiraceae	Eubacteriales	Clostridia
Lactobacillaceae	Lactobacillales	Bacilli
Lactobacillaceae	Lactobacillales	Bacilli
Muribaculaceae	Bacteroidales	Bacteroidia
Muribaculaceae	Bacteroidales	Bacteroidia
Oscillospiraceae	Eubacteriales	Clostridia
Oscillospiraceae	Eubacteriales	Clostridia
Oscillospiraceae	Eubacteriales	Clostridia
Oscillospiraceae	Eubacteriales	Clostridia

Oscillospiraceae	Eubacteriales	Clostridia
Oscillospiraceae	Eubacteriales	Clostridia
Oscillospiraceae	Eubacteriales	Clostridia
Oscillospiraceae	Eubacteriales	Clostridia
Sutterellaceae	Burkholderiales	Betaproteobacteria
Sutterellaceae	Burkholderiales	Betaproteobacteria
Peptostreptococcaceae	Eubacteriales	Clostridia
Peptostreptococcaceae	Eubacteriales	Clostridia
Lachnospiraceae	Eubacteriales	Clostridia
Lachnospiraceae	Eubacteriales	Clostridia
Turicibacteraceae	Erysipelotrichales	Erysipelotrichia
Turicibacteraceae	Erysipelotrichales	Erysipelotrichia

Phylum	Kingdom
Firmicutes	Bacteria
Firmicutes	Bacteria
Firmicutes	Bacteria
Firmicutes	Bacteria
Firmicutes	Bacteria
Firmicutes	Bacteria
Firmicutes	Bacteria
Firmicutes	Bacteria
Actinobacteria	Bacteria
Actinobacteria	Bacteria
Actinobacteria	Bacteria
Actinobacteria	Bacteria
Actinobacteria	Bacteria
Actinobacteria	Bacteria
Verrucomicrobia	Bacteria
Verrucomicrobia	Bacteria
Bacteroidota	Bacteria
Bacteroidota	Bacteria
Firmicutes	Bacteria
Firmicutes	Bacteria
Bacteria_unclassified	Bacteria
Bacteria_unclassified	Bacteria
Bacteria_unclassified	Bacteria
Bacteria_unclassified	Bacteria
Bacteria_unclassified	Bacteria
Bacteria_unclassified	Bacteria
Bacteria_unclassified	Bacteria
Bacteria_unclassified	Bacteria
Bacteria_unclassified	Bacteria
Bacteria_unclassified	Bacteria
Bacteria_unclassified	Bacteria
Bacteria_unclassified	Bacteria
Bacteroidota	Bacteria
Bacteroidota	Bacteria
Actinobacteria	Bacteria
Actinobacteria	Bacteria
Firmicutes	Bacteria
Firmicutes	Bacteria
Firmicutes	Bacteria
Firmicutes	Bacteria
Firmicutes	Bacteria
Firmicutes	Bacteria
Firmicutes	Bacteria
Firmicutes	Bacteria

Firmicutes	Bacteria
Firmicutes	Bacteria
Actinobacteria	Bacteria
Actinobacteria	Bacteria
Firmicutes	Bacteria
Firmicutes	Bacteria
Firmicutes	Bacteria
Firmicutes	Bacteria
Firmicutes	Bacteria
Firmicutes	Bacteria
Firmicutes	Bacteria
Firmicutes	Bacteria
Firmicutes	Bacteria
Firmicutes	Bacteria
Firmicutes	Bacteria
Firmicutes	Bacteria
Actinobacteria	Bacteria
Actinobacteria	Bacteria
Firmicutes	Bacteria
Firmicutes	Bacteria
Bacteroidota	Bacteria
Bacteroidota	Bacteria
Bacteroidota	Bacteria
Bacteroidota	Bacteria
Bacteroidota	Bacteria
Bacteroidota	Bacteria
Firmicutes	Bacteria
Firmicutes	Bacteria
Firmicutes	Bacteria
Firmicutes	Bacteria
Firmicutes	Bacteria
Firmicutes	Bacteria
Firmicutes	Bacteria
Firmicutes	Bacteria
Firmicutes	Bacteria
Firmicutes	Bacteria
Firmicutes	Bacteria
Firmicutes	Bacteria
Firmicutes	Bacteria
Firmicutes	Bacteria
Firmicutes	Bacteria
Firmicutes	Bacteria
Firmicutes	Bacteria
Firmicutes	Bacteria
Firmicutes	Bacteria
Firmicutes	Bacteria

Firmicutes	Bacteria
Firmicutes	Bacteria
Firmicutes	Bacteria
Firmicutes	Bacteria
Firmicutes	Bacteria
Firmicutes	Bacteria
Firmicutes	Bacteria
Firmicutes	Bacteria
Firmicutes	Bacteria
Firmicutes	Bacteria
Firmicutes	Bacteria
Firmicutes	Bacteria
Firmicutes	Bacteria
Firmicutes	Bacteria
Firmicutes	Bacteria
Firmicutes	Bacteria
Bacteria_unclassified	Bacteria
Bacteria_unclassified	Bacteria
Bacteria_unclassified	Bacteria
Bacteria_unclassified	Bacteria
Bacteria_unclassified	Bacteria
Bacteria_unclassified	Bacteria
Bacteria_unclassified	Bacteria
Bacteria_unclassified	Bacteria
Firmicutes	Bacteria
Firmicutes	Bacteria
Firmicutes	Bacteria
Firmicutes	Bacteria
Firmicutes	Bacteria
Firmicutes	Bacteria
Bacteria_unclassified	Bacteria
Bacteria_unclassified	Bacteria
Firmicutes	Bacteria
Firmicutes	Bacteria
Firmicutes	Bacteria
Firmicutes	Bacteria
Firmicutes	Bacteria
Firmicutes	Bacteria
Firmicutes	Bacteria
Firmicutes	Bacteria
Firmicutes	Bacteria
Firmicutes	Bacteria
Firmicutes	Bacteria
Firmicutes	Bacteria
Firmicutes	Bacteria
Firmicutes	Bacteria

Firmicutes	Bacteria
Firmicutes	Bacteria
Firmicutes	Bacteria
Firmicutes	Bacteria
Firmicutes	Bacteria
Firmicutes	Bacteria
Firmicutes	Bacteria
Firmicutes	Bacteria
Firmicutes	Bacteria
Firmicutes	Bacteria
Firmicutes	Bacteria
Firmicutes	Bacteria
Firmicutes	Bacteria
Firmicutes	Bacteria
Bacteria_unclassified	Bacteria
Bacteria_unclassified	Bacteria
Firmicutes	Bacteria
Firmicutes	Bacteria
Firmicutes	Bacteria
Firmicutes	Bacteria
Bacteria_unclassified	Bacteria
Bacteria_unclassified	Bacteria
Firmicutes	Bacteria
Firmicutes	Bacteria
Firmicutes	Bacteria
Firmicutes	Bacteria
Firmicutes	Bacteria
Firmicutes	Bacteria
Firmicutes	Bacteria
Firmicutes	Bacteria
Firmicutes	Bacteria
Firmicutes	Bacteria
Firmicutes	Bacteria
Firmicutes	Bacteria
Firmicutes	Bacteria
Firmicutes	Bacteria
Firmicutes	Bacteria
Firmicutes	Bacteria
Firmicutes	Bacteria
Firmicutes	Bacteria
Firmicutes	Bacteria
Firmicutes	Bacteria
Actinobacteria	Bacteria
Actinobacteria	Bacteria
Tenericutes	Bacteria
Tenericutes	Bacteria

Firmicutes	Bacteria
Firmicutes	Bacteria
Firmicutes	Bacteria
Firmicutes	Bacteria
Firmicutes	Bacteria
Firmicutes	Bacteria
Firmicutes	Bacteria
Firmicutes	Bacteria
Firmicutes	Bacteria
Firmicutes	Bacteria
Firmicutes	Bacteria
Firmicutes	Bacteria
Firmicutes	Bacteria
Firmicutes	Bacteria
Firmicutes	Bacteria
Firmicutes	Bacteria
Firmicutes	Bacteria
Firmicutes	Bacteria
Firmicutes	Bacteria
Firmicutes	Bacteria
Firmicutes	Bacteria
Firmicutes	Bacteria
Firmicutes	Bacteria
Firmicutes	Bacteria
Firmicutes	Bacteria
Firmicutes	Bacteria
Firmicutes	Bacteria
Firmicutes	Bacteria
Firmicutes	Bacteria
Firmicutes	Bacteria
Firmicutes	Bacteria
Firmicutes	Bacteria
Firmicutes	Bacteria
Firmicutes	Bacteria
Firmicutes	Bacteria
Firmicutes	Bacteria
Firmicutes	Bacteria
Firmicutes	Bacteria
Firmicutes	Bacteria
Firmicutes	Bacteria
Firmicutes	Bacteria
Firmicutes	Bacteria
Firmicutes	Bacteria
Firmicutes	Bacteria
Bacteroidota	Bacteria
Bacteroidota	Bacteria

Firmicutes	Bacteria
Firmicutes	Bacteria
Firmicutes	Bacteria
Firmicutes	Bacteria
Firmicutes	Bacteria
Firmicutes	Bacteria
Firmicutes	Bacteria
Firmicutes	Bacteria
Proteobacteria	Bacteria
Proteobacteria	Bacteria
Firmicutes	Bacteria
Firmicutes	Bacteria
Firmicutes	Bacteria
Firmicutes	Bacteria
Firmicutes	Bacteria
Firmicutes	Bacteria
Firmicutes	Bacteria
Firmicutes	Bacteria
Firmicutes	Bacteria
Firmicutes	Bacteria
Firmicutes	Bacteria
Firmicutes	Bacteria
Firmicutes	Bacteria
Firmicutes	Bacteria
Actinobacteria	Bacteria
Actinobacteria	Bacteria
Actinobacteria	Bacteria
Actinobacteria	Bacteria
Actinobacteria	Bacteria
Actinobacteria	Bacteria
Verrucomicrobia	Bacteria
Verrucomicrobia	Bacteria
Bacteroidota	Bacteria
Bacteroidota	Bacteria
Firmicutes	Bacteria
Firmicutes	Bacteria
Bacteria_unclassified	Bacteria
Bacteria_unclassified	Bacteria
Bacteria_unclassified	Bacteria
Bacteria_unclassified	Bacteria
Bacteria_unclassified	Bacteria
Bacteria_unclassified	Bacteria

Bacteria_unclassified	Bacteria
Bacteria_unclassified	Bacteria
Bacteria_unclassified	Bacteria
Bacteria_unclassified	Bacteria
Bacteria_unclassified	Bacteria
Bacteria_unclassified	Bacteria
Bacteroidota	Bacteria
Bacteroidota	Bacteria
Actinobacteria	Bacteria
Actinobacteria	Bacteria
Firmicutes	Bacteria
Firmicutes	Bacteria
Firmicutes	Bacteria
Firmicutes	Bacteria
Firmicutes	Bacteria
Firmicutes	Bacteria
Firmicutes	Bacteria
Firmicutes	Bacteria
Firmicutes	Bacteria
Firmicutes	Bacteria
Actinobacteria	Bacteria
Actinobacteria	Bacteria
Firmicutes	Bacteria
Firmicutes	Bacteria
Firmicutes	Bacteria
Firmicutes	Bacteria
Firmicutes	Bacteria
Firmicutes	Bacteria
Firmicutes	Bacteria
Firmicutes	Bacteria
Firmicutes	Bacteria
Firmicutes	Bacteria
Firmicutes	Bacteria
Firmicutes	Bacteria
Actinobacteria	Bacteria
Actinobacteria	Bacteria
Firmicutes	Bacteria
Firmicutes	Bacteria
Bacteroidota	Bacteria
Bacteroidota	Bacteria
Bacteroidota	Bacteria
Bacteroidota	Bacteria
Bacteroidota	Bacteria
Bacteroidota	Bacteria

Firmicutes	Bacteria
Firmicutes	Bacteria
Firmicutes	Bacteria
Firmicutes	Bacteria
Firmicutes	Bacteria
Firmicutes	Bacteria
Firmicutes	Bacteria
Firmicutes	Bacteria
Firmicutes	Bacteria
Firmicutes	Bacteria
Firmicutes	Bacteria
Firmicutes	Bacteria
Firmicutes	Bacteria
Firmicutes	Bacteria
Firmicutes	Bacteria
Firmicutes	Bacteria
Firmicutes	Bacteria
Firmicutes	Bacteria
Firmicutes	Bacteria
Firmicutes	Bacteria
Firmicutes	Bacteria
Firmicutes	Bacteria
Firmicutes	Bacteria
Firmicutes	Bacteria
Firmicutes	Bacteria
Firmicutes	Bacteria
Firmicutes	Bacteria
Firmicutes	Bacteria
Firmicutes	Bacteria
Firmicutes	Bacteria
Firmicutes	Bacteria
Firmicutes	Bacteria
Firmicutes	Bacteria
Firmicutes	Bacteria
Firmicutes	Bacteria
Firmicutes	Bacteria
Bacteria_unclassified	Bacteria
Bacteria_unclassified	Bacteria
Bacteria_unclassified	Bacteria
Bacteria_unclassified	Bacteria
Bacteria_unclassified	Bacteria
Bacteria_unclassified	Bacteria
Bacteria_unclassified	Bacteria
Bacteria_unclassified	Bacteria
Firmicutes	Bacteria
Firmicutes	Bacteria

Firmicutes	Bacteria
Firmicutes	Bacteria
Firmicutes	Bacteria
Firmicutes	Bacteria
Bacteria_unclassified	Bacteria
Bacteria_unclassified	Bacteria
Firmicutes	Bacteria
Firmicutes	Bacteria
Firmicutes	Bacteria
Firmicutes	Bacteria
Firmicutes	Bacteria
Firmicutes	Bacteria
Firmicutes	Bacteria
Firmicutes	Bacteria
Firmicutes	Bacteria
Firmicutes	Bacteria
Firmicutes	Bacteria
Firmicutes	Bacteria
Firmicutes	Bacteria
Firmicutes	Bacteria
Firmicutes	Bacteria
Firmicutes	Bacteria
Firmicutes	Bacteria
Firmicutes	Bacteria
Firmicutes	Bacteria
Firmicutes	Bacteria
Firmicutes	Bacteria
Firmicutes	Bacteria
Firmicutes	Bacteria
Firmicutes	Bacteria
Firmicutes	Bacteria
Firmicutes	Bacteria
Firmicutes	Bacteria
Bacteria_unclassified	Bacteria
Bacteria_unclassified	Bacteria
Firmicutes	Bacteria
Firmicutes	Bacteria
Firmicutes	Bacteria
Firmicutes	Bacteria
Bacteria_unclassified	Bacteria
Bacteria_unclassified	Bacteria
Firmicutes	Bacteria
Firmicutes	Bacteria
Firmicutes	Bacteria
Firmicutes	Bacteria

Firmicutes	Bacteria
Firmicutes	Bacteria
Firmicutes	Bacteria
Firmicutes	Bacteria
Firmicutes	Bacteria
Firmicutes	Bacteria
Firmicutes	Bacteria
Firmicutes	Bacteria
Firmicutes	Bacteria
Firmicutes	Bacteria
Firmicutes	Bacteria
Firmicutes	Bacteria
Firmicutes	Bacteria
Firmicutes	Bacteria
Firmicutes	Bacteria
Firmicutes	Bacteria
Actinobacteria	Bacteria
Actinobacteria	Bacteria
Tenericutes	Bacteria
Tenericutes	Bacteria
Firmicutes	Bacteria
Firmicutes	Bacteria
Firmicutes	Bacteria
Firmicutes	Bacteria
Firmicutes	Bacteria
Firmicutes	Bacteria
Firmicutes	Bacteria
Firmicutes	Bacteria
Firmicutes	Bacteria
Firmicutes	Bacteria
Firmicutes	Bacteria
Firmicutes	Bacteria
Firmicutes	Bacteria
Firmicutes	Bacteria
Firmicutes	Bacteria
Firmicutes	Bacteria
Firmicutes	Bacteria
Firmicutes	Bacteria
Firmicutes	Bacteria
Firmicutes	Bacteria
Firmicutes	Bacteria
Firmicutes	Bacteria
Firmicutes	Bacteria
Firmicutes	Bacteria
Firmicutes	Bacteria
Firmicutes	Bacteria

Firmicutes	Bacteria
Firmicutes	Bacteria
Firmicutes	Bacteria
Firmicutes	Bacteria
Firmicutes	Bacteria
Firmicutes	Bacteria
Firmicutes	Bacteria
Firmicutes	Bacteria
Firmicutes	Bacteria
Firmicutes	Bacteria
Firmicutes	Bacteria
Firmicutes	Bacteria
Firmicutes	Bacteria
Firmicutes	Bacteria
Firmicutes	Bacteria
Firmicutes	Bacteria
Firmicutes	Bacteria
Firmicutes	Bacteria
Bacteroidota	Bacteria
Bacteroidota	Bacteria
Firmicutes	Bacteria
Firmicutes	Bacteria
Firmicutes	Bacteria
Firmicutes	Bacteria
Firmicutes	Bacteria
Firmicutes	Bacteria
Firmicutes	Bacteria
Firmicutes	Bacteria
Proteobacteria	Bacteria
Proteobacteria	Bacteria
Firmicutes	Bacteria
Firmicutes	Bacteria
Firmicutes	Bacteria
Firmicutes	Bacteria
Firmicutes	Bacteria
Firmicutes	Bacteria
Firmicutes	Bacteria
Firmicutes	Bacteria
Firmicutes	Bacteria
Firmicutes	Bacteria
Firmicutes	Bacteria
Firmicutes	Bacteria

Firmicutes	Bacteria
Firmicutes	Bacteria
Actinobacteria	Bacteria
Actinobacteria	Bacteria
Actinobacteria	Bacteria
Actinobacteria	Bacteria
Actinobacteria	Bacteria
Actinobacteria	Bacteria
Verrucomicrobia	Bacteria
Verrucomicrobia	Bacteria
Bacteroidota	Bacteria
Bacteroidota	Bacteria
Firmicutes	Bacteria
Firmicutes	Bacteria
Bacteria_unclassified	Bacteria
Bacteria_unclassified	Bacteria
Bacteria_unclassified	Bacteria
Bacteria_unclassified	Bacteria
Bacteria_unclassified	Bacteria
Bacteria_unclassified	Bacteria
Bacteria_unclassified	Bacteria
Bacteria_unclassified	Bacteria
Bacteria_unclassified	Bacteria
Bacteria_unclassified	Bacteria
Bacteria_unclassified	Bacteria
Bacteria_unclassified	Bacteria
Bacteroidota	Bacteria
Bacteroidota	Bacteria

Actinobacteria	Bacteria
Actinobacteria	Bacteria
Firmicutes	Bacteria
Firmicutes	Bacteria
Firmicutes	Bacteria
Firmicutes	Bacteria
Firmicutes	Bacteria
Firmicutes	Bacteria
Firmicutes	Bacteria
Firmicutes	Bacteria
Firmicutes	Bacteria
Firmicutes	Bacteria
Actinobacteria	Bacteria
Actinobacteria	Bacteria
Firmicutes	Bacteria
Firmicutes	Bacteria

Firmicutes	Bacteria
Firmicutes	Bacteria
Firmicutes	Bacteria
Firmicutes	Bacteria
Firmicutes	Bacteria
Firmicutes	Bacteria
Firmicutes	Bacteria
Firmicutes	Bacteria
Firmicutes	Bacteria
Firmicutes	Bacteria
Actinobacteria	Bacteria
Actinobacteria	Bacteria
Firmicutes	Bacteria
Firmicutes	Bacteria
Bacteroidota	Bacteria
Bacteroidota	Bacteria
Bacteroidota	Bacteria
Bacteroidota	Bacteria
Bacteroidota	Bacteria
Bacteroidota	Bacteria
Firmicutes	Bacteria
Firmicutes	Bacteria
Firmicutes	Bacteria
Firmicutes	Bacteria
Firmicutes	Bacteria
Firmicutes	Bacteria
Firmicutes	Bacteria
Firmicutes	Bacteria
Firmicutes	Bacteria
Firmicutes	Bacteria
Firmicutes	Bacteria
Firmicutes	Bacteria
Firmicutes	Bacteria
Firmicutes	Bacteria
Firmicutes	Bacteria
Firmicutes	Bacteria
Firmicutes	Bacteria
Firmicutes	Bacteria
Firmicutes	Bacteria
Firmicutes	Bacteria
Firmicutes	Bacteria
Firmicutes	Bacteria
Firmicutes	Bacteria
Firmicutes	Bacteria
Firmicutes	Bacteria
Firmicutes	Bacteria

Firmicutes	Bacteria
Firmicutes	Bacteria
Firmicutes	Bacteria
Firmicutes	Bacteria
Firmicutes	Bacteria
Firmicutes	Bacteria
Firmicutes	Bacteria
Firmicutes	Bacteria
Firmicutes	Bacteria
Firmicutes	Bacteria
Bacteria_unclassified	Bacteria
Bacteria_unclassified	Bacteria
Bacteria_unclassified	Bacteria
Bacteria_unclassified	Bacteria
Bacteria_unclassified	Bacteria
Bacteria_unclassified	Bacteria
Bacteria_unclassified	Bacteria
Bacteria_unclassified	Bacteria
Firmicutes	Bacteria
Firmicutes	Bacteria
Firmicutes	Bacteria
Firmicutes	Bacteria
Firmicutes	Bacteria
Firmicutes	Bacteria
Bacteria_unclassified	Bacteria
Bacteria_unclassified	Bacteria
Firmicutes	Bacteria
Firmicutes	Bacteria
Firmicutes	Bacteria
Firmicutes	Bacteria
Firmicutes	Bacteria
Firmicutes	Bacteria
Firmicutes	Bacteria
Firmicutes	Bacteria
Firmicutes	Bacteria
Firmicutes	Bacteria
Firmicutes	Bacteria
Firmicutes	Bacteria
Firmicutes	Bacteria
Firmicutes	Bacteria
Firmicutes	Bacteria
Firmicutes	Bacteria
Firmicutes	Bacteria
Firmicutes	Bacteria
Firmicutes	Bacteria
Firmicutes	Bacteria

Firmicutes	Bacteria
Firmicutes	Bacteria
Firmicutes	Bacteria
Firmicutes	Bacteria
Firmicutes	Bacteria
Firmicutes	Bacteria
Firmicutes	Bacteria
Firmicutes	Bacteria
Bacteria_unclassified	Bacteria
Bacteria_unclassified	Bacteria
Firmicutes	Bacteria
Firmicutes	Bacteria
Firmicutes	Bacteria
Firmicutes	Bacteria
Bacteria_unclassified	Bacteria
Bacteria_unclassified	Bacteria
Firmicutes	Bacteria
Firmicutes	Bacteria
Firmicutes	Bacteria
Firmicutes	Bacteria
Firmicutes	Bacteria
Firmicutes	Bacteria
Firmicutes	Bacteria
Firmicutes	Bacteria
Firmicutes	Bacteria
Firmicutes	Bacteria
Firmicutes	Bacteria
Firmicutes	Bacteria
Firmicutes	Bacteria
Firmicutes	Bacteria
Firmicutes	Bacteria
Firmicutes	Bacteria
Firmicutes	Bacteria
Firmicutes	Bacteria
Firmicutes	Bacteria
Firmicutes	Bacteria
Actinobacteria	Bacteria
Actinobacteria	Bacteria
Tenericutes	Bacteria
Tenericutes	Bacteria
Firmicutes	Bacteria
Firmicutes	Bacteria
Firmicutes	Bacteria
Firmicutes	Bacteria
Firmicutes	Bacteria
Firmicutes	Bacteria

Firmicutes	Bacteria
Firmicutes	Bacteria
Firmicutes	Bacteria
Firmicutes	Bacteria
Firmicutes	Bacteria
Firmicutes	Bacteria
Firmicutes	Bacteria
Firmicutes	Bacteria
Firmicutes	Bacteria
Firmicutes	Bacteria
Firmicutes	Bacteria
Firmicutes	Bacteria
Firmicutes	Bacteria
Firmicutes	Bacteria
Firmicutes	Bacteria
Firmicutes	Bacteria
Firmicutes	Bacteria
Firmicutes	Bacteria
Firmicutes	Bacteria
Firmicutes	Bacteria
Firmicutes	Bacteria
Firmicutes	Bacteria
Firmicutes	Bacteria
Firmicutes	Bacteria
Firmicutes	Bacteria
Firmicutes	Bacteria
Firmicutes	Bacteria
Firmicutes	Bacteria
Firmicutes	Bacteria
Firmicutes	Bacteria
Firmicutes	Bacteria
Firmicutes	Bacteria
Firmicutes	Bacteria
Firmicutes	Bacteria
Firmicutes	Bacteria
Firmicutes	Bacteria
Firmicutes	Bacteria
Firmicutes	Bacteria
Bacteroidota	Bacteria
Bacteroidota	Bacteria
Firmicutes	Bacteria
Firmicutes	Bacteria
Firmicutes	Bacteria
Firmicutes	Bacteria
Firmicutes	Bacteria
Firmicutes	Bacteria

Firmicutes	Bacteria
Firmicutes	Bacteria
Proteobacteria	Bacteria
Proteobacteria	Bacteria
Firmicutes	Bacteria
Firmicutes	Bacteria
Firmicutes	Bacteria
Firmicutes	Bacteria
Firmicutes	Bacteria
Firmicutes	Bacteria
Firmicutes	Bacteria
Firmicutes	Bacteria
Firmicutes	Bacteria
Firmicutes	Bacteria
Firmicutes	Bacteria
Firmicutes	Bacteria
Firmicutes	Bacteria
Firmicutes	Bacteria
Actinobacteria	Bacteria
Actinobacteria	Bacteria
Actinobacteria	Bacteria
Actinobacteria	Bacteria
Actinobacteria	Bacteria
Actinobacteria	Bacteria
Verrucomicrobia	Bacteria
Verrucomicrobia	Bacteria
Bacteroidota	Bacteria
Bacteroidota	Bacteria
Firmicutes	Bacteria
Firmicutes	Bacteria
Bacteria_unclassified	Bacteria
Bacteria_unclassified	Bacteria
Bacteria_unclassified	Bacteria
Bacteria_unclassified	Bacteria
Bacteria_unclassified	Bacteria
Bacteria_unclassified	Bacteria
Bacteria_unclassified	Bacteria
Bacteria_unclassified	Bacteria
Bacteria_unclassified	Bacteria
Bacteria_unclassified	Bacteria
Bacteria_unclassified	Bacteria
Bacteria_unclassified	Bacteria

Bacteroidota	Bacteria
Bacteroidota	Bacteria
Actinobacteria	Bacteria
Actinobacteria	Bacteria
Firmicutes	Bacteria
Firmicutes	Bacteria
Firmicutes	Bacteria
Firmicutes	Bacteria
Firmicutes	Bacteria
Firmicutes	Bacteria
Firmicutes	Bacteria
Firmicutes	Bacteria
Firmicutes	Bacteria
Firmicutes	Bacteria
Actinobacteria	Bacteria
Actinobacteria	Bacteria
Firmicutes	Bacteria
Firmicutes	Bacteria
Firmicutes	Bacteria
Firmicutes	Bacteria
Firmicutes	Bacteria
Firmicutes	Bacteria
Firmicutes	Bacteria
Firmicutes	Bacteria
Firmicutes	Bacteria
Firmicutes	Bacteria
Firmicutes	Bacteria
Firmicutes	Bacteria
Actinobacteria	Bacteria
Actinobacteria	Bacteria
Firmicutes	Bacteria
Firmicutes	Bacteria
Bacteroidota	Bacteria
Bacteroidota	Bacteria
Bacteroidota	Bacteria
Bacteroidota	Bacteria
Bacteroidota	Bacteria
Bacteroidota	Bacteria
Firmicutes	Bacteria
Firmicutes	Bacteria
Firmicutes	Bacteria
Firmicutes	Bacteria
Firmicutes	Bacteria
Firmicutes	Bacteria

Firmicutes	Bacteria
Firmicutes	Bacteria
Firmicutes	Bacteria
Firmicutes	Bacteria
Firmicutes	Bacteria
Firmicutes	Bacteria
Firmicutes	Bacteria
Firmicutes	Bacteria
Firmicutes	Bacteria
Firmicutes	Bacteria
Firmicutes	Bacteria
Firmicutes	Bacteria
Firmicutes	Bacteria
Firmicutes	Bacteria
Firmicutes	Bacteria
Firmicutes	Bacteria
Firmicutes	Bacteria
Firmicutes	Bacteria
Firmicutes	Bacteria
Firmicutes	Bacteria
Firmicutes	Bacteria
Firmicutes	Bacteria
Firmicutes	Bacteria
Firmicutes	Bacteria
Firmicutes	Bacteria
Firmicutes	Bacteria
Firmicutes	Bacteria
Firmicutes	Bacteria
Firmicutes	Bacteria
Firmicutes	Bacteria
Bacteria_unclassified	Bacteria
Bacteria_unclassified	Bacteria
Bacteria_unclassified	Bacteria
Bacteria_unclassified	Bacteria
Bacteria_unclassified	Bacteria
Bacteria_unclassified	Bacteria
Bacteria_unclassified	Bacteria
Bacteria_unclassified	Bacteria
Firmicutes	Bacteria
Firmicutes	Bacteria
Firmicutes	Bacteria
Firmicutes	Bacteria
Firmicutes	Bacteria
Firmicutes	Bacteria
Bacteria_unclassified	Bacteria
Bacteria_unclassified	Bacteria

Firmicutes	Bacteria
Firmicutes	Bacteria
Firmicutes	Bacteria
Firmicutes	Bacteria
Firmicutes	Bacteria
Firmicutes	Bacteria
Firmicutes	Bacteria
Firmicutes	Bacteria
Firmicutes	Bacteria
Firmicutes	Bacteria
Firmicutes	Bacteria
Firmicutes	Bacteria
Firmicutes	Bacteria
Firmicutes	Bacteria
Firmicutes	Bacteria
Firmicutes	Bacteria
Firmicutes	Bacteria
Firmicutes	Bacteria
Firmicutes	Bacteria
Firmicutes	Bacteria
Firmicutes	Bacteria
Firmicutes	Bacteria
Firmicutes	Bacteria
Firmicutes	Bacteria
Firmicutes	Bacteria
Firmicutes	Bacteria
Firmicutes	Bacteria
Firmicutes	Bacteria
Bacteria_unclassified	Bacteria
Bacteria_unclassified	Bacteria
Firmicutes	Bacteria
Firmicutes	Bacteria
Firmicutes	Bacteria
Firmicutes	Bacteria
Bacteria_unclassified	Bacteria
Bacteria_unclassified	Bacteria
Firmicutes	Bacteria
Firmicutes	Bacteria
Firmicutes	Bacteria
Firmicutes	Bacteria
Firmicutes	Bacteria
Firmicutes	Bacteria
Firmicutes	Bacteria
Firmicutes	Bacteria
Firmicutes	Bacteria
Firmicutes	Bacteria

Firmicutes	Bacteria
Firmicutes	Bacteria
Firmicutes	Bacteria
Firmicutes	Bacteria
Firmicutes	Bacteria
Firmicutes	Bacteria
Firmicutes	Bacteria
Firmicutes	Bacteria
Firmicutes	Bacteria
Firmicutes	Bacteria
Actinobacteria	Bacteria
Actinobacteria	Bacteria
Tenericutes	Bacteria
Tenericutes	Bacteria
Firmicutes	Bacteria
Firmicutes	Bacteria
Firmicutes	Bacteria
Firmicutes	Bacteria
Firmicutes	Bacteria
Firmicutes	Bacteria
Firmicutes	Bacteria
Firmicutes	Bacteria
Firmicutes	Bacteria
Firmicutes	Bacteria
Firmicutes	Bacteria
Firmicutes	Bacteria
Firmicutes	Bacteria
Firmicutes	Bacteria
Firmicutes	Bacteria
Firmicutes	Bacteria
Firmicutes	Bacteria
Firmicutes	Bacteria
Firmicutes	Bacteria
Firmicutes	Bacteria
Firmicutes	Bacteria
Firmicutes	Bacteria
Firmicutes	Bacteria
Firmicutes	Bacteria
Firmicutes	Bacteria
Firmicutes	Bacteria
Firmicutes	Bacteria
Firmicutes	Bacteria
Firmicutes	Bacteria
Firmicutes	Bacteria
Firmicutes	Bacteria
Firmicutes	Bacteria

Firmicutes	Bacteria
Firmicutes	Bacteria
Firmicutes	Bacteria
Firmicutes	Bacteria
Firmicutes	Bacteria
Firmicutes	Bacteria
Firmicutes	Bacteria
Firmicutes	Bacteria
Firmicutes	Bacteria
Firmicutes	Bacteria
Firmicutes	Bacteria
Firmicutes	Bacteria
Bacteroidota	Bacteria
Bacteroidota	Bacteria
Firmicutes	Bacteria
Firmicutes	Bacteria
Firmicutes	Bacteria
Firmicutes	Bacteria
Firmicutes	Bacteria
Firmicutes	Bacteria
Firmicutes	Bacteria
Firmicutes	Bacteria
Proteobacteria	Bacteria
Proteobacteria	Bacteria
Firmicutes	Bacteria
Firmicutes	Bacteria
Firmicutes	Bacteria
Firmicutes	Bacteria
Firmicutes	Bacteria
Firmicutes	Bacteria
Firmicutes	Bacteria
Firmicutes	Bacteria
Firmicutes	Bacteria
Firmicutes	Bacteria
Firmicutes	Bacteria
Firmicutes	Bacteria
Firmicutes	Bacteria
Firmicutes	Bacteria
Actinobacteria	Bacteria
Actinobacteria	Bacteria
Actinobacteria	Bacteria
Actinobacteria	Bacteria

Actinobacteria	Bacteria
Actinobacteria	Bacteria
Verrucomicrobia	Bacteria
Verrucomicrobia	Bacteria
Bacteroidota	Bacteria
Bacteroidota	Bacteria
Firmicutes	Bacteria
Firmicutes	Bacteria
Bacteria_unclassified	Bacteria
Bacteria_unclassified	Bacteria
Bacteria_unclassified	Bacteria
Bacteria_unclassified	Bacteria
Bacteria_unclassified	Bacteria
Bacteria_unclassified	Bacteria
Bacteria_unclassified	Bacteria
Bacteria_unclassified	Bacteria
Bacteria_unclassified	Bacteria
Bacteria_unclassified	Bacteria
Bacteria_unclassified	Bacteria
Bacteria_unclassified	Bacteria
Bacteroidota	Bacteria
Bacteroidota	Bacteria

Actinobacteria	Bacteria
Actinobacteria	Bacteria
Firmicutes	Bacteria
Firmicutes	Bacteria
Firmicutes	Bacteria
Firmicutes	Bacteria
Firmicutes	Bacteria
Firmicutes	Bacteria
Firmicutes	Bacteria
Firmicutes	Bacteria
Firmicutes	Bacteria
Firmicutes	Bacteria
Actinobacteria	Bacteria
Actinobacteria	Bacteria
Firmicutes	Bacteria
Firmicutes	Bacteria
Firmicutes	Bacteria
Firmicutes	Bacteria
Firmicutes	Bacteria
Firmicutes	Bacteria
Firmicutes	Bacteria
Firmicutes	Bacteria

Firmicutes	Bacteria
Firmicutes	Bacteria
Firmicutes	Bacteria
Firmicutes	Bacteria
Actinobacteria	Bacteria
Actinobacteria	Bacteria
Firmicutes	Bacteria
Firmicutes	Bacteria
Bacteroidota	Bacteria
Bacteroidota	Bacteria
Bacteroidota	Bacteria
Bacteroidota	Bacteria
Bacteroidota	Bacteria
Bacteroidota	Bacteria
Firmicutes	Bacteria
Firmicutes	Bacteria
Firmicutes	Bacteria
Firmicutes	Bacteria
Firmicutes	Bacteria
Firmicutes	Bacteria
Firmicutes	Bacteria
Firmicutes	Bacteria
Firmicutes	Bacteria
Firmicutes	Bacteria
Firmicutes	Bacteria
Firmicutes	Bacteria
Firmicutes	Bacteria
Firmicutes	Bacteria
Firmicutes	Bacteria
Firmicutes	Bacteria
Firmicutes	Bacteria
Firmicutes	Bacteria
Firmicutes	Bacteria
Firmicutes	Bacteria
Firmicutes	Bacteria
Firmicutes	Bacteria
Firmicutes	Bacteria
Firmicutes	Bacteria
Firmicutes	Bacteria
Firmicutes	Bacteria
Firmicutes	Bacteria
Firmicutes	Bacteria
Firmicutes	Bacteria
Firmicutes	Bacteria
Firmicutes	Bacteria
Firmicutes	Bacteria

Firmicutes	Bacteria
Firmicutes	Bacteria
Firmicutes	Bacteria
Firmicutes	Bacteria
Bacteria_unclassified	Bacteria
Bacteria_unclassified	Bacteria
Bacteria_unclassified	Bacteria
Bacteria_unclassified	Bacteria
Bacteria_unclassified	Bacteria
Bacteria_unclassified	Bacteria
Bacteria_unclassified	Bacteria
Bacteria_unclassified	Bacteria
Firmicutes	Bacteria
Firmicutes	Bacteria
Firmicutes	Bacteria
Firmicutes	Bacteria
Firmicutes	Bacteria
Firmicutes	Bacteria
Bacteria_unclassified	Bacteria
Bacteria_unclassified	Bacteria
Firmicutes	Bacteria
Firmicutes	Bacteria
Firmicutes	Bacteria
Firmicutes	Bacteria
Firmicutes	Bacteria
Firmicutes	Bacteria
Firmicutes	Bacteria
Firmicutes	Bacteria
Firmicutes	Bacteria
Firmicutes	Bacteria
Firmicutes	Bacteria
Firmicutes	Bacteria
Firmicutes	Bacteria
Firmicutes	Bacteria
Firmicutes	Bacteria
Firmicutes	Bacteria
Firmicutes	Bacteria
Firmicutes	Bacteria
Firmicutes	Bacteria
Firmicutes	Bacteria
Firmicutes	Bacteria
Firmicutes	Bacteria
Firmicutes	Bacteria
Firmicutes	Bacteria
Firmicutes	Bacteria
Firmicutes	Bacteria

Firmicutes	Bacteria
Firmicutes	Bacteria
Bacteria_unclassified	Bacteria
Bacteria_unclassified	Bacteria
Firmicutes	Bacteria
Firmicutes	Bacteria
Firmicutes	Bacteria
Firmicutes	Bacteria
Bacteria_unclassified	Bacteria
Bacteria_unclassified	Bacteria
Firmicutes	Bacteria
Firmicutes	Bacteria
Firmicutes	Bacteria
Firmicutes	Bacteria
Firmicutes	Bacteria
Firmicutes	Bacteria
Firmicutes	Bacteria
Firmicutes	Bacteria
Firmicutes	Bacteria
Firmicutes	Bacteria
Firmicutes	Bacteria
Firmicutes	Bacteria
Firmicutes	Bacteria
Firmicutes	Bacteria
Firmicutes	Bacteria
Firmicutes	Bacteria
Firmicutes	Bacteria
Firmicutes	Bacteria
Firmicutes	Bacteria
Firmicutes	Bacteria
Actinobacteria	Bacteria
Actinobacteria	Bacteria
Tenericutes	Bacteria
Tenericutes	Bacteria
Firmicutes	Bacteria
Firmicutes	Bacteria
Firmicutes	Bacteria
Firmicutes	Bacteria
Firmicutes	Bacteria
Firmicutes	Bacteria
Firmicutes	Bacteria
Firmicutes	Bacteria
Firmicutes	Bacteria
Firmicutes	Bacteria
Firmicutes	Bacteria
Firmicutes	Bacteria

Firmicutes	Bacteria
Firmicutes	Bacteria
Firmicutes	Bacteria
Firmicutes	Bacteria
Firmicutes	Bacteria
Firmicutes	Bacteria
Firmicutes	Bacteria
Firmicutes	Bacteria
Firmicutes	Bacteria
Firmicutes	Bacteria
Firmicutes	Bacteria
Firmicutes	Bacteria
Firmicutes	Bacteria
Firmicutes	Bacteria
Firmicutes	Bacteria
Firmicutes	Bacteria
Firmicutes	Bacteria
Firmicutes	Bacteria
Firmicutes	Bacteria
Firmicutes	Bacteria
Firmicutes	Bacteria
Firmicutes	Bacteria
Firmicutes	Bacteria
Firmicutes	Bacteria
Firmicutes	Bacteria
Firmicutes	Bacteria
Firmicutes	Bacteria
Firmicutes	Bacteria
Firmicutes	Bacteria
Firmicutes	Bacteria
Firmicutes	Bacteria
Firmicutes	Bacteria
Bacteroidota	Bacteria
Bacteroidota	Bacteria
Firmicutes	Bacteria
Firmicutes	Bacteria
Firmicutes	Bacteria
Firmicutes	Bacteria
Firmicutes	Bacteria
Firmicutes	Bacteria
Firmicutes	Bacteria
Firmicutes	Bacteria
Proteobacteria	Bacteria
Proteobacteria	Bacteria

Firmicutes	Bacteria
Firmicutes	Bacteria
Firmicutes	Bacteria
Firmicutes	Bacteria
Firmicutes	Bacteria
Firmicutes	Bacteria
Firmicutes	Bacteria
Firmicutes	Bacteria
Firmicutes	Bacteria
Firmicutes	Bacteria
Firmicutes	Bacteria
Firmicutes	Bacteria
Firmicutes	Bacteria
Firmicutes	Bacteria
Actinobacteria	Bacteria
Actinobacteria	Bacteria
Actinobacteria	Bacteria
Actinobacteria	Bacteria
Actinobacteria	Bacteria
Actinobacteria	Bacteria
Verrucomicrobia	Bacteria
Verrucomicrobia	Bacteria
Bacteroidota	Bacteria
Bacteroidota	Bacteria
Firmicutes	Bacteria
Firmicutes	Bacteria
Bacteria_unclassified	Bacteria
Bacteria_unclassified	Bacteria
Bacteria_unclassified	Bacteria
Bacteria_unclassified	Bacteria
Bacteria_unclassified	Bacteria
Bacteria_unclassified	Bacteria
Bacteria_unclassified	Bacteria
Bacteria_unclassified	Bacteria
Bacteria_unclassified	Bacteria
Bacteria_unclassified	Bacteria
Bacteria_unclassified	Bacteria
Bacteria_unclassified	Bacteria
Bacteroidota	Bacteria
Bacteroidota	Bacteria
Actinobacteria	Bacteria
Actinobacteria	Bacteria

Firmicutes	Bacteria
Firmicutes	Bacteria
Firmicutes	Bacteria
Firmicutes	Bacteria
Firmicutes	Bacteria
Firmicutes	Bacteria
Firmicutes	Bacteria
Firmicutes	Bacteria
Firmicutes	Bacteria
Firmicutes	Bacteria
Actinobacteria	Bacteria
Actinobacteria	Bacteria
Firmicutes	Bacteria
Firmicutes	Bacteria
Firmicutes	Bacteria
Firmicutes	Bacteria
Firmicutes	Bacteria
Firmicutes	Bacteria
Firmicutes	Bacteria
Firmicutes	Bacteria
Firmicutes	Bacteria
Firmicutes	Bacteria
Firmicutes	Bacteria
Firmicutes	Bacteria
Actinobacteria	Bacteria
Actinobacteria	Bacteria
Firmicutes	Bacteria
Firmicutes	Bacteria
Bacteroidota	Bacteria
Bacteroidota	Bacteria
Bacteroidota	Bacteria
Bacteroidota	Bacteria
Bacteroidota	Bacteria
Bacteroidota	Bacteria
Firmicutes	Bacteria
Firmicutes	Bacteria
Firmicutes	Bacteria
Firmicutes	Bacteria
Firmicutes	Bacteria
Firmicutes	Bacteria
Firmicutes	Bacteria
Firmicutes	Bacteria
Firmicutes	Bacteria
Firmicutes	Bacteria
Firmicutes	Bacteria
Firmicutes	Bacteria

Firmicutes	Bacteria
Firmicutes	Bacteria
Firmicutes	Bacteria
Firmicutes	Bacteria
Firmicutes	Bacteria
Firmicutes	Bacteria
Firmicutes	Bacteria
Firmicutes	Bacteria
Firmicutes	Bacteria
Firmicutes	Bacteria
Firmicutes	Bacteria
Firmicutes	Bacteria
Firmicutes	Bacteria
Firmicutes	Bacteria
Firmicutes	Bacteria
Firmicutes	Bacteria
Firmicutes	Bacteria
Firmicutes	Bacteria
Firmicutes	Bacteria
Firmicutes	Bacteria
Firmicutes	Bacteria
Firmicutes	Bacteria
Firmicutes	Bacteria
Firmicutes	Bacteria
Bacteria_unclassified	Bacteria
Bacteria_unclassified	Bacteria
Bacteria_unclassified	Bacteria
Bacteria_unclassified	Bacteria
Bacteria_unclassified	Bacteria
Bacteria_unclassified	Bacteria
Bacteria_unclassified	Bacteria
Bacteria_unclassified	Bacteria
Firmicutes	Bacteria
Firmicutes	Bacteria
Firmicutes	Bacteria
Firmicutes	Bacteria
Firmicutes	Bacteria
Firmicutes	Bacteria
Bacteria_unclassified	Bacteria
Bacteria_unclassified	Bacteria
Firmicutes	Bacteria
Firmicutes	Bacteria
Firmicutes	Bacteria
Firmicutes	Bacteria
Firmicutes	Bacteria
Firmicutes	Bacteria

Firmicutes	Bacteria
Firmicutes	Bacteria
Firmicutes	Bacteria
Firmicutes	Bacteria
Firmicutes	Bacteria
Firmicutes	Bacteria
Firmicutes	Bacteria
Firmicutes	Bacteria
Firmicutes	Bacteria
Firmicutes	Bacteria
Firmicutes	Bacteria
Firmicutes	Bacteria
Firmicutes	Bacteria
Firmicutes	Bacteria
Firmicutes	Bacteria
Firmicutes	Bacteria
Firmicutes	Bacteria
Firmicutes	Bacteria
Firmicutes	Bacteria
Firmicutes	Bacteria
Firmicutes	Bacteria
Firmicutes	Bacteria
Bacteria_unclassified	Bacteria
Bacteria_unclassified	Bacteria
Firmicutes	Bacteria
Firmicutes	Bacteria
Firmicutes	Bacteria
Firmicutes	Bacteria
Bacteria_unclassified	Bacteria
Bacteria_unclassified	Bacteria
Firmicutes	Bacteria
Firmicutes	Bacteria
Firmicutes	Bacteria
Firmicutes	Bacteria
Firmicutes	Bacteria
Firmicutes	Bacteria
Firmicutes	Bacteria
Firmicutes	Bacteria
Firmicutes	Bacteria
Firmicutes	Bacteria
Firmicutes	Bacteria
Firmicutes	Bacteria
Firmicutes	Bacteria
Firmicutes	Bacteria
Firmicutes	Bacteria
Firmicutes	Bacteria

Firmicutes	Bacteria
Firmicutes	Bacteria
Firmicutes	Bacteria
Firmicutes	Bacteria
Actinobacteria	Bacteria
Actinobacteria	Bacteria
Tenericutes	Bacteria
Tenericutes	Bacteria
Firmicutes	Bacteria
Firmicutes	Bacteria
Firmicutes	Bacteria
Firmicutes	Bacteria
Firmicutes	Bacteria
Firmicutes	Bacteria
Firmicutes	Bacteria
Firmicutes	Bacteria
Firmicutes	Bacteria
Firmicutes	Bacteria
Firmicutes	Bacteria
Firmicutes	Bacteria
Firmicutes	Bacteria
Firmicutes	Bacteria
Firmicutes	Bacteria
Firmicutes	Bacteria
Firmicutes	Bacteria
Firmicutes	Bacteria
Firmicutes	Bacteria
Firmicutes	Bacteria
Firmicutes	Bacteria
Firmicutes	Bacteria
Firmicutes	Bacteria
Firmicutes	Bacteria
Firmicutes	Bacteria
Firmicutes	Bacteria
Firmicutes	Bacteria
Firmicutes	Bacteria
Firmicutes	Bacteria
Firmicutes	Bacteria
Firmicutes	Bacteria
Firmicutes	Bacteria
Firmicutes	Bacteria
Firmicutes	Bacteria
Firmicutes	Bacteria
Firmicutes	Bacteria
Firmicutes	Bacteria
Firmicutes	Bacteria

Firmicutes	Bacteria
Firmicutes	Bacteria
Firmicutes	Bacteria
Firmicutes	Bacteria
Firmicutes	Bacteria
Firmicutes	Bacteria
Bacteroidota	Bacteria
Bacteroidota	Bacteria
Firmicutes	Bacteria
Firmicutes	Bacteria
Firmicutes	Bacteria
Firmicutes	Bacteria
Firmicutes	Bacteria
Firmicutes	Bacteria
Firmicutes	Bacteria
Firmicutes	Bacteria
Proteobacteria	Bacteria
Proteobacteria	Bacteria
Firmicutes	Bacteria
Firmicutes	Bacteria
Firmicutes	Bacteria
Firmicutes	Bacteria
Firmicutes	Bacteria
Firmicutes	Bacteria
Firmicutes	Bacteria
Firmicutes	Bacteria
Firmicutes	Bacteria
Firmicutes	Bacteria
Firmicutes	Bacteria
Firmicutes	Bacteria
Firmicutes	Bacteria
Firmicutes	Bacteria
Actinobacteria	Bacteria
Actinobacteria	Bacteria
Actinobacteria	Bacteria
Actinobacteria	Bacteria
Actinobacteria	Bacteria
Actinobacteria	Bacteria
Verrucomicrobia	Bacteria
Verrucomicrobia	Bacteria
Bacteroidota	Bacteria
Bacteroidota	Bacteria

Firmicutes	Bacteria
Firmicutes	Bacteria
Bacteria_unclassified	Bacteria
Bacteria_unclassified	Bacteria
Bacteria_unclassified	Bacteria
Bacteria_unclassified	Bacteria
Bacteria_unclassified	Bacteria
Bacteria_unclassified	Bacteria
Bacteria_unclassified	Bacteria
Bacteria_unclassified	Bacteria
Bacteria_unclassified	Bacteria
Bacteria_unclassified	Bacteria
Bacteria_unclassified	Bacteria
Bacteria_unclassified	Bacteria
Bacteroidota	Bacteria
Bacteroidota	Bacteria
Actinobacteria	Bacteria
Actinobacteria	Bacteria
Firmicutes	Bacteria
Firmicutes	Bacteria
Firmicutes	Bacteria
Firmicutes	Bacteria
Firmicutes	Bacteria
Firmicutes	Bacteria
Firmicutes	Bacteria
Firmicutes	Bacteria
Firmicutes	Bacteria
Firmicutes	Bacteria
Actinobacteria	Bacteria
Actinobacteria	Bacteria
Firmicutes	Bacteria
Firmicutes	Bacteria
Firmicutes	Bacteria
Firmicutes	Bacteria
Firmicutes	Bacteria
Firmicutes	Bacteria
Firmicutes	Bacteria
Firmicutes	Bacteria
Firmicutes	Bacteria
Firmicutes	Bacteria
Firmicutes	Bacteria
Firmicutes	Bacteria
Actinobacteria	Bacteria
Actinobacteria	Bacteria

Firmicutes	Bacteria
Firmicutes	Bacteria
Bacteroidota	Bacteria
Bacteroidota	Bacteria
Bacteroidota	Bacteria
Bacteroidota	Bacteria
Bacteroidota	Bacteria
Bacteroidota	Bacteria
Firmicutes	Bacteria
Firmicutes	Bacteria
Firmicutes	Bacteria
Firmicutes	Bacteria
Firmicutes	Bacteria
Firmicutes	Bacteria
Firmicutes	Bacteria
Firmicutes	Bacteria
Firmicutes	Bacteria
Firmicutes	Bacteria
Firmicutes	Bacteria
Firmicutes	Bacteria
Firmicutes	Bacteria
Firmicutes	Bacteria
Firmicutes	Bacteria
Firmicutes	Bacteria
Firmicutes	Bacteria
Firmicutes	Bacteria
Firmicutes	Bacteria
Firmicutes	Bacteria
Firmicutes	Bacteria
Firmicutes	Bacteria
Firmicutes	Bacteria
Firmicutes	Bacteria
Firmicutes	Bacteria
Firmicutes	Bacteria
Firmicutes	Bacteria
Firmicutes	Bacteria
Firmicutes	Bacteria
Firmicutes	Bacteria
Firmicutes	Bacteria
Firmicutes	Bacteria
Firmicutes	Bacteria
Firmicutes	Bacteria
Firmicutes	Bacteria
Firmicutes	Bacteria
Bacteria_unclassified	Bacteria
Bacteria_unclassified	Bacteria

Bacteria_unclassified	Bacteria
Bacteria_unclassified	Bacteria
Bacteria_unclassified	Bacteria
Bacteria_unclassified	Bacteria
Bacteria_unclassified	Bacteria
Bacteria_unclassified	Bacteria
Firmicutes	Bacteria
Firmicutes	Bacteria
Firmicutes	Bacteria
Firmicutes	Bacteria
Firmicutes	Bacteria
Firmicutes	Bacteria
Bacteria_unclassified	Bacteria
Bacteria_unclassified	Bacteria
Firmicutes	Bacteria
Firmicutes	Bacteria
Firmicutes	Bacteria
Firmicutes	Bacteria
Firmicutes	Bacteria
Firmicutes	Bacteria
Firmicutes	Bacteria
Firmicutes	Bacteria
Firmicutes	Bacteria
Firmicutes	Bacteria
Firmicutes	Bacteria
Firmicutes	Bacteria
Firmicutes	Bacteria
Firmicutes	Bacteria
Firmicutes	Bacteria
Firmicutes	Bacteria
Firmicutes	Bacteria
Firmicutes	Bacteria
Firmicutes	Bacteria
Firmicutes	Bacteria
Firmicutes	Bacteria
Firmicutes	Bacteria
Firmicutes	Bacteria
Firmicutes	Bacteria
Firmicutes	Bacteria
Firmicutes	Bacteria
Firmicutes	Bacteria
Firmicutes	Bacteria
Bacteria_unclassified	Bacteria
Bacteria_unclassified	Bacteria
Firmicutes	Bacteria
Firmicutes	Bacteria

Firmicutes	Bacteria
Firmicutes	Bacteria
Bacteria_unclassified	Bacteria
Bacteria_unclassified	Bacteria
Firmicutes	Bacteria
Firmicutes	Bacteria
Firmicutes	Bacteria
Firmicutes	Bacteria
Firmicutes	Bacteria
Firmicutes	Bacteria
Firmicutes	Bacteria
Firmicutes	Bacteria
Firmicutes	Bacteria
Firmicutes	Bacteria
Firmicutes	Bacteria
Firmicutes	Bacteria
Firmicutes	Bacteria
Firmicutes	Bacteria
Firmicutes	Bacteria
Firmicutes	Bacteria
Firmicutes	Bacteria
Firmicutes	Bacteria
Firmicutes	Bacteria
Firmicutes	Bacteria
Actinobacteria	Bacteria
Actinobacteria	Bacteria
Tenericutes	Bacteria
Tenericutes	Bacteria
Firmicutes	Bacteria
Firmicutes	Bacteria
Firmicutes	Bacteria
Firmicutes	Bacteria
Firmicutes	Bacteria
Firmicutes	Bacteria
Firmicutes	Bacteria
Firmicutes	Bacteria
Firmicutes	Bacteria
Firmicutes	Bacteria
Firmicutes	Bacteria
Firmicutes	Bacteria
Firmicutes	Bacteria
Firmicutes	Bacteria
Firmicutes	Bacteria
Firmicutes	Bacteria
Firmicutes	Bacteria
Firmicutes	Bacteria

Firmicutes	Bacteria
Firmicutes	Bacteria
Firmicutes	Bacteria
Firmicutes	Bacteria
Firmicutes	Bacteria
Firmicutes	Bacteria
Firmicutes	Bacteria
Firmicutes	Bacteria
Firmicutes	Bacteria
Firmicutes	Bacteria
Firmicutes	Bacteria
Firmicutes	Bacteria
Firmicutes	Bacteria
Firmicutes	Bacteria
Firmicutes	Bacteria
Firmicutes	Bacteria
Firmicutes	Bacteria
Firmicutes	Bacteria
Firmicutes	Bacteria
Firmicutes	Bacteria
Firmicutes	Bacteria
Firmicutes	Bacteria
Firmicutes	Bacteria
Firmicutes	Bacteria
Firmicutes	Bacteria
Firmicutes	Bacteria
Bacteroidota	Bacteria
Bacteroidota	Bacteria
Firmicutes	Bacteria
Firmicutes	Bacteria
Firmicutes	Bacteria
Firmicutes	Bacteria
Firmicutes	Bacteria
Firmicutes	Bacteria
Firmicutes	Bacteria
Firmicutes	Bacteria
Proteobacteria	Bacteria
Proteobacteria	Bacteria

Firmicutes	Bacteria
Firmicutes	Bacteria
Firmicutes	Bacteria
Firmicutes	Bacteria

Firmicutes	Bacteria
Firmicutes	Bacteria
Firmicutes	Bacteria
Firmicutes	Bacteria
Firmicutes	Bacteria
Firmicutes	Bacteria
Firmicutes	Bacteria
Firmicutes	Bacteria
Firmicutes	Bacteria
Firmicutes	Bacteria
Actinobacteria	Bacteria
Actinobacteria	Bacteria
Actinobacteria	Bacteria
Actinobacteria	Bacteria
Actinobacteria	Bacteria
Actinobacteria	Bacteria
Verrucomicrobia	Bacteria
Verrucomicrobia	Bacteria
Bacteroidota	Bacteria
Bacteroidota	Bacteria
Firmicutes	Bacteria
Firmicutes	Bacteria
Bacteria_unclassified	Bacteria
Bacteria_unclassified	Bacteria
Bacteria_unclassified	Bacteria
Bacteria_unclassified	Bacteria
Bacteria_unclassified	Bacteria
Bacteria_unclassified	Bacteria
Bacteria_unclassified	Bacteria
Bacteria_unclassified	Bacteria
Bacteria_unclassified	Bacteria
Bacteria_unclassified	Bacteria
Bacteria_unclassified	Bacteria
Bacteria_unclassified	Bacteria
Bacteroidota	Bacteria
Bacteroidota	Bacteria
Actinobacteria	Bacteria
Actinobacteria	Bacteria
Firmicutes	Bacteria
Firmicutes	Bacteria
Firmicutes	Bacteria
Firmicutes	Bacteria
Firmicutes	Bacteria
Firmicutes	Bacteria

Firmicutes	Bacteria
Firmicutes	Bacteria
Firmicutes	Bacteria
Firmicutes	Bacteria
Actinobacteria	Bacteria
Actinobacteria	Bacteria
Firmicutes	Bacteria
Firmicutes	Bacteria
Firmicutes	Bacteria
Firmicutes	Bacteria
Firmicutes	Bacteria
Firmicutes	Bacteria
Firmicutes	Bacteria
Firmicutes	Bacteria
Firmicutes	Bacteria
Firmicutes	Bacteria
Firmicutes	Bacteria
Firmicutes	Bacteria
Actinobacteria	Bacteria
Actinobacteria	Bacteria
Firmicutes	Bacteria
Firmicutes	Bacteria
Bacteroidota	Bacteria
Bacteroidota	Bacteria
Bacteroidota	Bacteria
Bacteroidota	Bacteria
Bacteroidota	Bacteria
Bacteroidota	Bacteria
Firmicutes	Bacteria
Firmicutes	Bacteria
Firmicutes	Bacteria
Firmicutes	Bacteria
Firmicutes	Bacteria
Firmicutes	Bacteria
Firmicutes	Bacteria
Firmicutes	Bacteria
Firmicutes	Bacteria
Firmicutes	Bacteria
Firmicutes	Bacteria
Firmicutes	Bacteria
Firmicutes	Bacteria
Firmicutes	Bacteria
Firmicutes	Bacteria
Firmicutes	Bacteria
Firmicutes	Bacteria
Firmicutes	Bacteria

Firmicutes	Bacteria
Firmicutes	Bacteria
Firmicutes	Bacteria
Firmicutes	Bacteria
Firmicutes	Bacteria
Firmicutes	Bacteria
Firmicutes	Bacteria
Firmicutes	Bacteria
Firmicutes	Bacteria
Firmicutes	Bacteria
Firmicutes	Bacteria
Firmicutes	Bacteria
Firmicutes	Bacteria
Firmicutes	Bacteria
Firmicutes	Bacteria
Firmicutes	Bacteria
Firmicutes	Bacteria
Firmicutes	Bacteria
Bacteria_unclassified	Bacteria
Bacteria_unclassified	Bacteria
Bacteria_unclassified	Bacteria
Bacteria_unclassified	Bacteria
Bacteria_unclassified	Bacteria
Bacteria_unclassified	Bacteria
Bacteria_unclassified	Bacteria
Bacteria_unclassified	Bacteria
Firmicutes	Bacteria
Firmicutes	Bacteria
Firmicutes	Bacteria
Firmicutes	Bacteria
Firmicutes	Bacteria
Firmicutes	Bacteria
Bacteria_unclassified	Bacteria
Bacteria_unclassified	Bacteria
Firmicutes	Bacteria
Firmicutes	Bacteria
Firmicutes	Bacteria
Firmicutes	Bacteria
Firmicutes	Bacteria
Firmicutes	Bacteria
Firmicutes	Bacteria
Firmicutes	Bacteria
Firmicutes	Bacteria
Firmicutes	Bacteria
Firmicutes	Bacteria
Firmicutes	Bacteria

Firmicutes	Bacteria
Firmicutes	Bacteria
Firmicutes	Bacteria
Firmicutes	Bacteria
Firmicutes	Bacteria
Firmicutes	Bacteria
Firmicutes	Bacteria
Firmicutes	Bacteria
Firmicutes	Bacteria
Firmicutes	Bacteria
Firmicutes	Bacteria
Firmicutes	Bacteria
Firmicutes	Bacteria
Firmicutes	Bacteria
Firmicutes	Bacteria
Firmicutes	Bacteria
Bacteria_unclassified	Bacteria
Bacteria_unclassified	Bacteria
Firmicutes	Bacteria
Firmicutes	Bacteria
Firmicutes	Bacteria
Firmicutes	Bacteria
Bacteria_unclassified	Bacteria
Bacteria_unclassified	Bacteria
Firmicutes	Bacteria
Firmicutes	Bacteria
Firmicutes	Bacteria
Firmicutes	Bacteria
Firmicutes	Bacteria
Firmicutes	Bacteria
Firmicutes	Bacteria
Firmicutes	Bacteria
Firmicutes	Bacteria
Firmicutes	Bacteria
Firmicutes	Bacteria
Firmicutes	Bacteria
Firmicutes	Bacteria
Firmicutes	Bacteria
Firmicutes	Bacteria
Firmicutes	Bacteria
Firmicutes	Bacteria
Firmicutes	Bacteria
Firmicutes	Bacteria
Firmicutes	Bacteria
Actinobacteria	Bacteria
Actinobacteria	Bacteria

Tenericutes	Bacteria
Tenericutes	Bacteria
Firmicutes	Bacteria
Firmicutes	Bacteria
Firmicutes	Bacteria
Firmicutes	Bacteria
Firmicutes	Bacteria
Firmicutes	Bacteria
Firmicutes	Bacteria
Firmicutes	Bacteria
Firmicutes	Bacteria
Firmicutes	Bacteria
Firmicutes	Bacteria
Firmicutes	Bacteria
Firmicutes	Bacteria
Firmicutes	Bacteria
Firmicutes	Bacteria
Firmicutes	Bacteria
Firmicutes	Bacteria
Firmicutes	Bacteria
Firmicutes	Bacteria
Firmicutes	Bacteria
Firmicutes	Bacteria
Firmicutes	Bacteria
Firmicutes	Bacteria
Firmicutes	Bacteria
Firmicutes	Bacteria
Firmicutes	Bacteria
Firmicutes	Bacteria
Firmicutes	Bacteria
Firmicutes	Bacteria
Firmicutes	Bacteria
Firmicutes	Bacteria
Firmicutes	Bacteria
Firmicutes	Bacteria
Firmicutes	Bacteria
Firmicutes	Bacteria
Firmicutes	Bacteria
Firmicutes	Bacteria
Firmicutes	Bacteria
Firmicutes	Bacteria
Firmicutes	Bacteria
Firmicutes	Bacteria
Firmicutes	Bacteria
Firmicutes	Bacteria
Firmicutes	Bacteria

Bacteroidota	Bacteria
Bacteroidota	Bacteria
Firmicutes	Bacteria
Firmicutes	Bacteria
Firmicutes	Bacteria
Firmicutes	Bacteria
Firmicutes	Bacteria
Firmicutes	Bacteria
Firmicutes	Bacteria
Firmicutes	Bacteria
Proteobacteria	Bacteria
Proteobacteria	Bacteria
Firmicutes	Bacteria
Firmicutes	Bacteria
Firmicutes	Bacteria
Firmicutes	Bacteria
Firmicutes	Bacteria
Firmicutes	Bacteria
Firmicutes	Bacteria
Firmicutes	Bacteria
Firmicutes	Bacteria
Firmicutes	Bacteria
Firmicutes	Bacteria
Firmicutes	Bacteria
Firmicutes	Bacteria
Firmicutes	Bacteria
Actinobacteria	Bacteria
Actinobacteria	Bacteria
Actinobacteria	Bacteria
Actinobacteria	Bacteria
Actinobacteria	Bacteria
Actinobacteria	Bacteria
Verrucomicrobia	Bacteria
Verrucomicrobia	Bacteria
Bacteroidota	Bacteria
Bacteroidota	Bacteria
Firmicutes	Bacteria
Firmicutes	Bacteria
Bacteria_unclassified	Bacteria
Bacteria_unclassified	Bacteria
Bacteria_unclassified	Bacteria
Bacteria_unclassified	Bacteria

Bacteria_unclassified	Bacteria
Bacteria_unclassified	Bacteria
Bacteria_unclassified	Bacteria
Bacteria_unclassified	Bacteria
Bacteria_unclassified	Bacteria
Bacteria_unclassified	Bacteria
Bacteria_unclassified	Bacteria
Bacteria_unclassified	Bacteria
Bacteroidota	Bacteria
Bacteroidota	Bacteria

Actinobacteria	Bacteria
Actinobacteria	Bacteria
Firmicutes	Bacteria
Firmicutes	Bacteria
Firmicutes	Bacteria
Firmicutes	Bacteria
Firmicutes	Bacteria
Firmicutes	Bacteria
Firmicutes	Bacteria
Firmicutes	Bacteria
Firmicutes	Bacteria
Firmicutes	Bacteria
Actinobacteria	Bacteria
Actinobacteria	Bacteria
Firmicutes	Bacteria
Firmicutes	Bacteria
Firmicutes	Bacteria
Firmicutes	Bacteria
Firmicutes	Bacteria
Firmicutes	Bacteria
Firmicutes	Bacteria
Firmicutes	Bacteria
Firmicutes	Bacteria
Firmicutes	Bacteria
Firmicutes	Bacteria
Firmicutes	Bacteria
Actinobacteria	Bacteria
Actinobacteria	Bacteria
Firmicutes	Bacteria
Firmicutes	Bacteria
Bacteroidota	Bacteria
Bacteroidota	Bacteria
Bacteroidota	Bacteria
Bacteroidota	Bacteria

Bacteroidota	Bacteria
Bacteroidota	Bacteria
Firmicutes	Bacteria
Firmicutes	Bacteria
Firmicutes	Bacteria
Firmicutes	Bacteria
Firmicutes	Bacteria
Firmicutes	Bacteria
Firmicutes	Bacteria
Firmicutes	Bacteria
Firmicutes	Bacteria
Firmicutes	Bacteria
Firmicutes	Bacteria
Firmicutes	Bacteria
Firmicutes	Bacteria
Firmicutes	Bacteria
Firmicutes	Bacteria
Firmicutes	Bacteria
Firmicutes	Bacteria
Firmicutes	Bacteria
Firmicutes	Bacteria
Firmicutes	Bacteria
Firmicutes	Bacteria
Firmicutes	Bacteria
Firmicutes	Bacteria
Firmicutes	Bacteria
Firmicutes	Bacteria
Firmicutes	Bacteria
Firmicutes	Bacteria
Firmicutes	Bacteria
Firmicutes	Bacteria
Firmicutes	Bacteria
Firmicutes	Bacteria
Firmicutes	Bacteria
Firmicutes	Bacteria
Firmicutes	Bacteria
Firmicutes	Bacteria
Firmicutes	Bacteria
Bacteria_unclassified	Bacteria
Bacteria_unclassified	Bacteria
Bacteria_unclassified	Bacteria
Bacteria_unclassified	Bacteria
Bacteria_unclassified	Bacteria
Bacteria_unclassified	Bacteria
Bacteria_unclassified	Bacteria
Bacteria_unclassified	Bacteria

Firmicutes	Bacteria
Firmicutes	Bacteria
Firmicutes	Bacteria
Firmicutes	Bacteria
Firmicutes	Bacteria
Firmicutes	Bacteria
Bacteria_unclassified	Bacteria
Bacteria_unclassified	Bacteria
Firmicutes	Bacteria
Firmicutes	Bacteria
Firmicutes	Bacteria
Firmicutes	Bacteria
Firmicutes	Bacteria
Firmicutes	Bacteria
Firmicutes	Bacteria
Firmicutes	Bacteria
Firmicutes	Bacteria
Firmicutes	Bacteria
Firmicutes	Bacteria
Firmicutes	Bacteria
Firmicutes	Bacteria
Firmicutes	Bacteria
Firmicutes	Bacteria
Firmicutes	Bacteria
Firmicutes	Bacteria
Firmicutes	Bacteria
Firmicutes	Bacteria
Firmicutes	Bacteria
Firmicutes	Bacteria
Firmicutes	Bacteria
Firmicutes	Bacteria
Firmicutes	Bacteria
Firmicutes	Bacteria
Firmicutes	Bacteria
Firmicutes	Bacteria
Firmicutes	Bacteria
Bacteria_unclassified	Bacteria
Bacteria_unclassified	Bacteria
Firmicutes	Bacteria
Firmicutes	Bacteria
Firmicutes	Bacteria
Firmicutes	Bacteria
Bacteria_unclassified	Bacteria
Bacteria_unclassified	Bacteria
Firmicutes	Bacteria
Firmicutes	Bacteria

Firmicutes	Bacteria
Firmicutes	Bacteria
Firmicutes	Bacteria
Firmicutes	Bacteria
Firmicutes	Bacteria
Firmicutes	Bacteria
Firmicutes	Bacteria
Firmicutes	Bacteria
Firmicutes	Bacteria
Firmicutes	Bacteria
Firmicutes	Bacteria
Firmicutes	Bacteria
Firmicutes	Bacteria
Firmicutes	Bacteria
Firmicutes	Bacteria
Firmicutes	Bacteria
Firmicutes	Bacteria
Firmicutes	Bacteria
Actinobacteria	Bacteria
Actinobacteria	Bacteria
Tenericutes	Bacteria
Tenericutes	Bacteria
Firmicutes	Bacteria
Firmicutes	Bacteria
Firmicutes	Bacteria
Firmicutes	Bacteria
Firmicutes	Bacteria
Firmicutes	Bacteria
Firmicutes	Bacteria
Firmicutes	Bacteria
Firmicutes	Bacteria
Firmicutes	Bacteria
Firmicutes	Bacteria
Firmicutes	Bacteria
Firmicutes	Bacteria
Firmicutes	Bacteria
Firmicutes	Bacteria
Firmicutes	Bacteria
Firmicutes	Bacteria
Firmicutes	Bacteria
Firmicutes	Bacteria
Firmicutes	Bacteria
Firmicutes	Bacteria
Firmicutes	Bacteria
Firmicutes	Bacteria
Firmicutes	Bacteria

Firmicutes	Bacteria
Firmicutes	Bacteria
Firmicutes	Bacteria
Firmicutes	Bacteria
Firmicutes	Bacteria
Firmicutes	Bacteria
Firmicutes	Bacteria
Firmicutes	Bacteria
Firmicutes	Bacteria
Firmicutes	Bacteria
Firmicutes	Bacteria
Firmicutes	Bacteria
Firmicutes	Bacteria
Firmicutes	Bacteria
Firmicutes	Bacteria
Firmicutes	Bacteria
Firmicutes	Bacteria
Firmicutes	Bacteria
Firmicutes	Bacteria
Firmicutes	Bacteria
Bacteroidota	Bacteria
Bacteroidota	Bacteria
Firmicutes	Bacteria
Firmicutes	Bacteria
Firmicutes	Bacteria
Firmicutes	Bacteria
Firmicutes	Bacteria
Firmicutes	Bacteria
Firmicutes	Bacteria
Firmicutes	Bacteria
Proteobacteria	Bacteria
Proteobacteria	Bacteria
Firmicutes	Bacteria
Firmicutes	Bacteria
Firmicutes	Bacteria
Firmicutes	Bacteria
Firmicutes	Bacteria
Firmicutes	Bacteria
Firmicutes	Bacteria
Firmicutes	Bacteria
Firmicutes	Bacteria
Firmicutes	Bacteria

Firmicutes	Bacteria
Firmicutes	Bacteria
Firmicutes	Bacteria
Firmicutes	Bacteria
Actinobacteria	Bacteria
Actinobacteria	Bacteria
Actinobacteria	Bacteria
Actinobacteria	Bacteria
Actinobacteria	Bacteria
Actinobacteria	Bacteria
Verrucomicrobia	Bacteria
Verrucomicrobia	Bacteria
Bacteroidota	Bacteria
Bacteroidota	Bacteria
Firmicutes	Bacteria
Firmicutes	Bacteria
Bacteria_unclassified	Bacteria
Bacteria_unclassified	Bacteria
Bacteria_unclassified	Bacteria
Bacteria_unclassified	Bacteria
Bacteria_unclassified	Bacteria
Bacteria_unclassified	Bacteria
Bacteria_unclassified	Bacteria
Bacteria_unclassified	Bacteria
Bacteria_unclassified	Bacteria
Bacteria_unclassified	Bacteria
Bacteria_unclassified	Bacteria
Bacteria_unclassified	Bacteria
Bacteroidota	Bacteria
Bacteroidota	Bacteria

Actinobacteria	Bacteria
Actinobacteria	Bacteria
Firmicutes	Bacteria
Firmicutes	Bacteria
Firmicutes	Bacteria
Firmicutes	Bacteria
Firmicutes	Bacteria
Firmicutes	Bacteria
Firmicutes	Bacteria
Firmicutes	Bacteria
Firmicutes	Bacteria
Firmicutes	Bacteria
Actinobacteria	Bacteria
Actinobacteria	Bacteria

Firmicutes	Bacteria
Firmicutes	Bacteria
Firmicutes	Bacteria
Firmicutes	Bacteria
Firmicutes	Bacteria
Firmicutes	Bacteria
Firmicutes	Bacteria
Firmicutes	Bacteria
Firmicutes	Bacteria
Firmicutes	Bacteria
Firmicutes	Bacteria
Firmicutes	Bacteria
Actinobacteria	Bacteria
Actinobacteria	Bacteria
Firmicutes	Bacteria
Firmicutes	Bacteria
Bacteroidota	Bacteria
Bacteroidota	Bacteria
Bacteroidota	Bacteria
Bacteroidota	Bacteria
Bacteroidota	Bacteria
Bacteroidota	Bacteria
Firmicutes	Bacteria
Firmicutes	Bacteria
Firmicutes	Bacteria
Firmicutes	Bacteria
Firmicutes	Bacteria
Firmicutes	Bacteria
Firmicutes	Bacteria
Firmicutes	Bacteria
Firmicutes	Bacteria
Firmicutes	Bacteria
Firmicutes	Bacteria
Firmicutes	Bacteria
Firmicutes	Bacteria
Firmicutes	Bacteria
Firmicutes	Bacteria
Firmicutes	Bacteria
Firmicutes	Bacteria
Firmicutes	Bacteria
Firmicutes	Bacteria
Firmicutes	Bacteria
Firmicutes	Bacteria
Firmicutes	Bacteria
Firmicutes	Bacteria
Firmicutes	Bacteria

Firmicutes	Bacteria
Firmicutes	Bacteria
Firmicutes	Bacteria
Firmicutes	Bacteria
Firmicutes	Bacteria
Firmicutes	Bacteria
Firmicutes	Bacteria
Firmicutes	Bacteria
Firmicutes	Bacteria
Firmicutes	Bacteria
Firmicutes	Bacteria
Firmicutes	Bacteria
Bacteria_unclassified	Bacteria
Bacteria_unclassified	Bacteria
Bacteria_unclassified	Bacteria
Bacteria_unclassified	Bacteria
Bacteria_unclassified	Bacteria
Bacteria_unclassified	Bacteria
Bacteria_unclassified	Bacteria
Bacteria_unclassified	Bacteria
Firmicutes	Bacteria
Firmicutes	Bacteria
Firmicutes	Bacteria
Firmicutes	Bacteria
Firmicutes	Bacteria
Firmicutes	Bacteria
Bacteria_unclassified	Bacteria
Bacteria_unclassified	Bacteria
Firmicutes	Bacteria
Firmicutes	Bacteria
Firmicutes	Bacteria
Firmicutes	Bacteria
Firmicutes	Bacteria
Firmicutes	Bacteria
Firmicutes	Bacteria
Firmicutes	Bacteria
Firmicutes	Bacteria
Firmicutes	Bacteria
Firmicutes	Bacteria
Firmicutes	Bacteria
Firmicutes	Bacteria
Firmicutes	Bacteria
Firmicutes	Bacteria
Firmicutes	Bacteria
Firmicutes	Bacteria
Firmicutes	Bacteria

Firmicutes	Bacteria
Firmicutes	Bacteria
Firmicutes	Bacteria
Firmicutes	Bacteria
Firmicutes	Bacteria
Firmicutes	Bacteria
Firmicutes	Bacteria
Firmicutes	Bacteria
Firmicutes	Bacteria
Firmicutes	Bacteria
Bacteria_unclassified	Bacteria
Bacteria_unclassified	Bacteria
Firmicutes	Bacteria
Firmicutes	Bacteria
Firmicutes	Bacteria
Firmicutes	Bacteria
Bacteria_unclassified	Bacteria
Bacteria_unclassified	Bacteria
Firmicutes	Bacteria
Firmicutes	Bacteria
Firmicutes	Bacteria
Firmicutes	Bacteria
Firmicutes	Bacteria
Firmicutes	Bacteria
Firmicutes	Bacteria
Firmicutes	Bacteria
Firmicutes	Bacteria
Firmicutes	Bacteria
Firmicutes	Bacteria
Firmicutes	Bacteria
Firmicutes	Bacteria
Firmicutes	Bacteria
Firmicutes	Bacteria
Firmicutes	Bacteria
Firmicutes	Bacteria
Firmicutes	Bacteria
Firmicutes	Bacteria
Firmicutes	Bacteria
Actinobacteria	Bacteria
Actinobacteria	Bacteria
Tenericutes	Bacteria
Tenericutes	Bacteria
Firmicutes	Bacteria
Firmicutes	Bacteria
Firmicutes	Bacteria
Firmicutes	Bacteria

Firmicutes	Bacteria
Firmicutes	Bacteria
Firmicutes	Bacteria
Firmicutes	Bacteria
Firmicutes	Bacteria
Firmicutes	Bacteria
Firmicutes	Bacteria
Firmicutes	Bacteria
Firmicutes	Bacteria
Firmicutes	Bacteria
Firmicutes	Bacteria
Firmicutes	Bacteria
Firmicutes	Bacteria
Firmicutes	Bacteria
Firmicutes	Bacteria
Firmicutes	Bacteria
Firmicutes	Bacteria
Firmicutes	Bacteria
Firmicutes	Bacteria
Firmicutes	Bacteria
Firmicutes	Bacteria
Firmicutes	Bacteria
Firmicutes	Bacteria
Firmicutes	Bacteria
Firmicutes	Bacteria
Firmicutes	Bacteria
Firmicutes	Bacteria
Firmicutes	Bacteria
Firmicutes	Bacteria
Firmicutes	Bacteria
Firmicutes	Bacteria
Firmicutes	Bacteria
Firmicutes	Bacteria
Firmicutes	Bacteria
Firmicutes	Bacteria
Firmicutes	Bacteria
Firmicutes	Bacteria
Firmicutes	Bacteria
Firmicutes	Bacteria
Firmicutes	Bacteria
Bacteroidota	Bacteria
Bacteroidota	Bacteria
Firmicutes	Bacteria
Firmicutes	Bacteria
Firmicutes	Bacteria
Firmicutes	Bacteria

Firmicutes	Bacteria
Firmicutes	Bacteria
Firmicutes	Bacteria
Firmicutes	Bacteria
Proteobacteria	Bacteria
Proteobacteria	Bacteria
Firmicutes	Bacteria
Firmicutes	Bacteria
Firmicutes	Bacteria
Firmicutes	Bacteria
Firmicutes	Bacteria
Firmicutes	Bacteria

MetaPhlan Annotation
k__Bacteria|p__Firmicutes|c__Clostridia|o__Eubacteriales|f__Lachnospiraceae|g__Acetatifactor|s__Acetatifactor_muris
k__Bacteria|p__Firmicutes|c__Clostridia|o__Eubacteriales|f__Lachnospiraceae|g__Acetatifactor|s__Acetatifactor_muris
k__Bacteria|p__Firmicutes|c__Clostridia|o__Eubacteriales|f__Lachnospiraceae|g__Acetatifactor|s__Acetatifactor_SGB415
k__Bacteria|p__Firmicutes|c__Clostridia|o__Eubacteriales|f__Lachnospiraceae|g__Acetatifactor|s__Acetatifactor_SGB415
k__Bacteria|p__Firmicutes|c__Clostridia|o__Eubacteriales|f__Oscillospiraceae|g__Acutalibacter|s__Acutalibacter_muris
k__Bacteria|p__Firmicutes|c__Clostridia|o__Eubacteriales|f__Oscillospiraceae|g__Acutalibacter|s__Acutalibacter_muris
k__Bacteria|p__Firmicutes|c__Clostridia|o__Eubacteriales|f__Oscillospiraceae|g__Acutalibacter|s__Acutalibacter_sp_1XD
k__Bacteria|p__Firmicutes|c__Clostridia|o__Eubacteriales|f__Oscillospiraceae|g__Acutalibacter|s__Acutalibacter_sp_1XD
k__Bacteria|p__Actinobacteria|c__Coriobacteriia|o__Eggerthellales|f__Eggerthellaceae|g__Adlercreutzia|s__Adlercreutzia
k__Bacteria|p__Actinobacteria|c__Coriobacteriia|o__Eggerthellales|f__Eggerthellaceae|g__Adlercreutzia|s__Adlercreutzia
k__Bacteria|p__Actinobacteria|c__Coriobacteriia|o__Eggerthellales|f__Eggerthellaceae|g__Adlercreutzia|s__Adlercreutzia
k__Bacteria|p__Actinobacteria|c__Coriobacteriia|o__Eggerthellales|f__Eggerthellaceae|g__Adlercreutzia|s__Adlercreutzia
k__Bacteria|p__Actinobacteria|c__Coriobacteriia|o__Eggerthellales|f__Eggerthellaceae|g__Adlercreutzia|s__Adlercreutzia
k__Bacteria|p__Actinobacteria|c__Coriobacteriia|o__Eggerthellales|f__Eggerthellaceae|g__Adlercreutzia|s__Adlercreutzia
k__Bacteria|p__Verrucomicrobia|c__Verrucomicrobiae|o__Verrucomicrobiales|f__Akkermansiaceae|g__Akkermansia|s__
k__Bacteria|p__Verrucomicrobia|c__Verrucomicrobiae|o__Verrucomicrobiales|f__Akkermansiaceae|g__Akkermansia|s__
k__Bacteria|p__Bacteroidota|c__Bacteroidia|o__Bacteroidales|f__Rikenellaceae|g__Alistipes|s__Alistipes_sp_DSM_11234
k__Bacteria|p__Bacteroidota|c__Bacteroidia|o__Bacteroidales|f__Rikenellaceae|g__Alistipes|s__Alistipes_sp_DSM_11234
k__Bacteria|p__Firmicutes|c__Clostridia|o__Eubacteriales|f__Oscillospiraceae|g__Anaerotruncus|s__Anaerotruncus_sp_1
k__Bacteria|p__Firmicutes|c__Clostridia|o__Eubacteriales|f__Oscillospiraceae|g__Anaerotruncus|s__Anaerotruncus_sp_1
k__Bacteria|p__Bacteria_unclassified|c__Bacteria_unclassified|o__Bacteria_unclassified|f__Bacteria_unclassified|g__Bacte
k__Bacteria|p__Bacteria_unclassified|c__Bacteria_unclassified|o__Bacteria_unclassified|f__Bacteria_unclassified|g__Bacte
k__Bacteria|p__Bacteria_unclassified|c__Bacteria_unclassified|o__Bacteria_unclassified|f__Bacteria_unclassified|g__Bacte
k__Bacteria|p__Bacteria_unclassified|c__Bacteria_unclassified|o__Bacteria_unclassified|f__Bacteria_unclassified|g__Bacte
k__Bacteria|p__Bacteria_unclassified|c__Bacteria_unclassified|o__Bacteria_unclassified|f__Bacteria_unclassified|g__Bacte
k__Bacteria|p__Bacteria_unclassified|c__Bacteria_unclassified|o__Bacteria_unclassified|f__Bacteria_unclassified|g__Bacte
k__Bacteria|p__Bacteria_unclassified|c__Bacteria_unclassified|o__Bacteria_unclassified|f__Bacteria_unclassified|g__Bacte
k__Bacteria|p__Bacteria_unclassified|c__Bacteria_unclassified|o__Bacteria_unclassified|f__Bacteria_unclassified|g__Bacte
k__Bacteria|p__Bacteria_unclassified|c__Bacteria_unclassified|o__Bacteria_unclassified|f__Bacteria_unclassified|g__Bacte
k__Bacteria|p__Bacteria_unclassified|c__Bacteria_unclassified|o__Bacteria_unclassified|f__Bacteria_unclassified|g__Bacte
k__Bacteria|p__Bacteria_unclassified|c__Bacteria_unclassified|o__Bacteria_unclassified|f__Bacteria_unclassified|g__Bacte
k__Bacteria|p__Bacteria_unclassified|c__Bacteria_unclassified|o__Bacteria_unclassified|f__Bacteria_unclassified|g__Bacte
k__Bacteria|p__Bacteroidota|c__Bacteroidia|o__Bacteroidales|f__Bacteroidaceae|g__Bacteroides|s__Bacteroides_thetaio
k__Bacteria|p__Bacteroidota|c__Bacteroidia|o__Bacteroidales|f__Bacteroidaceae|g__Bacteroides|s__Bacteroides_thetaio
k__Bacteria|p__Actinobacteria|c__Actinomycetia|o__Bifidobacteriales|f__Bifidobacteriaceae|g__Bifidobacterium|s__Bifid
k__Bacteria|p__Actinobacteria|c__Actinomycetia|o__Bifidobacteriales|f__Bifidobacteriaceae|g__Bifidobacterium|s__Bifid
k__Bacteria|p__Firmicutes|c__Clostridia|o__Clostridia_unclassified|f__Clostridia_unclassified|g__Clostridia_unclassified|s_
k__Bacteria|p__Firmicutes|c__Clostridia|o__Clostridia_unclassified|f__Clostridia_unclassified|g__Clostridia_unclassified|s_
k__Bacteria|p__Firmicutes|c__Clostridia|o__Eubacteriales|f__Clostridiaceae|g__Clostridiaceae_unclassified|s__Clostridiac
k__Bacteria|p__Firmicutes|c__Clostridia|o__Eubacteriales|f__Clostridiaceae|g__Clostridiaceae_unclassified|s__Clostridiac
k__Bacteria|p__Firmicutes|c__Clostridia|o__Eubacteriales|f__Clostridiaceae|g__Clostridiaceae_unclassified|s__Clostridiac
k__Bacteria|p__Firmicutes|c__Clostridia|o__Eubacteriales|f__Clostridiaceae|g__Clostridiaceae_unclassified|s__Clostridiac
k__Bacteria|p__Firmicutes|c__Clostridia|o__Eubacteriales|f__Eubacteriales_unclassified|g__Eubacteriales_unclassified|s_
k__Bacteria|p__Firmicutes|c__Clostridia|o__Eubacteriales|f__Eubacteriales_unclassified|g__Eubacteriales_unclassified|s_

k__Bacteria|p__Firmicutes|c__Erysipelotrichia|o__Erysipelotrichales|f__Erysipelotrichaceae|g__Erysipelatoclostridium|s__
k__Bacteria|p__Firmicutes|c__Erysipelotrichia|o__Erysipelotrichales|f__Erysipelotrichaceae|g__Erysipelatoclostridium|s__
k__Bacteria|p__Actinobacteria|c__Coriobacteriia|o__Coriobacteriales|f__Coriobacteriaceae|g__Coriobacteriaceae_unclass
k__Bacteria|p__Actinobacteria|c__Coriobacteriia|o__Coriobacteriales|f__Coriobacteriaceae|g__Coriobacteriaceae_unclass
k__Bacteria|p__Firmicutes|c__Clostridia|o__Eubacteriales|f__Lachnospiraceae|g__Dorea|s__Dorea_sp_5_2
k__Bacteria|p__Firmicutes|c__Clostridia|o__Eubacteriales|f__Lachnospiraceae|g__Dorea|s__Dorea_sp_5_2
k__Bacteria|p__Firmicutes|c__Erysipelotrichia|o__Erysipelotrichales|f__Erysipelotrichaceae|g__Dubosiella|s__Dubosiella_
k__Bacteria|p__Firmicutes|c__Erysipelotrichia|o__Erysipelotrichales|f__Erysipelotrichaceae|g__Dubosiella|s__Dubosiella_
k__Bacteria|p__Firmicutes|c__Erysipelotrichia|o__Erysipelotrichales|f__Erysipelotrichales_unclassified|g__Erysipelotrichal
k__Bacteria|p__Firmicutes|c__Erysipelotrichia|o__Erysipelotrichales|f__Erysipelotrichales_unclassified|g__Erysipelotrichal
k__Bacteria|p__Firmicutes|c__Clostridia|o__Eubacteriales|f__Eubacteriaceae|g__Eubacteriaceae_unclassified|s__Eubacte
k__Bacteria|p__Firmicutes|c__Clostridia|o__Eubacteriales|f__Eubacteriaceae|g__Eubacteriaceae_unclassified|s__Eubacte
k__Bacteria|p__Firmicutes|c__Clostridia|o__Eubacteriales|f__Eubacteriaceae|g__Eubacteriaceae_unclassified|s__Eubacte
k__Bacteria|p__Firmicutes|c__Clostridia|o__Eubacteriales|f__Eubacteriaceae|g__Eubacteriaceae_unclassified|s__Eubacte
k__Bacteria|p__Firmicutes|c__Clostridia|o__Eubacteriales|f__Lachnospiraceae|g__GGB20149|s__GGB20149_SGB29430
k__Bacteria|p__Firmicutes|c__Clostridia|o__Eubacteriales|f__Lachnospiraceae|g__GGB20149|s__GGB20149_SGB29430
k__Bacteria|p__Actinobacteria|c__Coriobacteriia|o__Eggerthellales|f__Eggerthellaceae|g__GGB22635|s__GGB22635_SGB
k__Bacteria|p__Actinobacteria|c__Coriobacteriia|o__Eggerthellales|f__Eggerthellaceae|g__GGB22635|s__GGB22635_SGB
k__Bacteria|p__Firmicutes|c__Clostridia|o__Eubacteriales|f__Lachnospiraceae|g__GGB25041|s__GGB25041_SGB36960
k__Bacteria|p__Firmicutes|c__Clostridia|o__Eubacteriales|f__Lachnospiraceae|g__GGB25041|s__GGB25041_SGB36960
k__Bacteria|p__Bacteroidota|c__Bacteroidia|o__Bacteroidales|f__Muribaculaceae|g__GGB27876|s__GGB27876_SGB4031
k__Bacteria|p__Bacteroidota|c__Bacteroidia|o__Bacteroidales|f__Muribaculaceae|g__GGB27876|s__GGB27876_SGB4031
k__Bacteria|p__Bacteroidota|c__Bacteroidia|o__Bacteroidales|f__Muribaculaceae|g__GGB27878|s__GGB27878_SGB4031
k__Bacteria|p__Bacteroidota|c__Bacteroidia|o__Bacteroidales|f__Muribaculaceae|g__GGB27878|s__GGB27878_SGB4031
k__Bacteria|p__Bacteroidota|c__Bacteroidia|o__Bacteroidales|f__Muribaculaceae|g__GGB27918|s__GGB27918_SGB4035
k__Bacteria|p__Bacteroidota|c__Bacteroidia|o__Bacteroidales|f__Muribaculaceae|g__GGB27918|s__GGB27918_SGB4035
k__Bacteria|p__Firmicutes|c__CFGB9508|o__OFGB9508|f__FGB9508|g__GGB28382|s__GGB28382_SGB40962
k__Bacteria|p__Firmicutes|c__CFGB9508|o__OFGB9508|f__FGB9508|g__GGB28382|s__GGB28382_SGB40962
k__Bacteria|p__Firmicutes|c__CFGB2838|o__OFGB2838|f__FGB2838|g__GGB28399|s__GGB28399_SGB40980
k__Bacteria|p__Firmicutes|c__CFGB2838|o__OFGB2838|f__FGB2838|g__GGB28399|s__GGB28399_SGB40980
k__Bacteria|p__Firmicutes|c__CFGB2838|o__OFGB2838|f__FGB2838|g__GGB28411|s__GGB28411_SGB40993
k__Bacteria|p__Firmicutes|c__CFGB2838|o__OFGB2838|f__FGB2838|g__GGB28411|s__GGB28411_SGB40993
k__Bacteria|p__Firmicutes|c__CFGB2838|o__OFGB2838|f__FGB2838|g__GGB28415|s__GGB28415_SGB40997
k__Bacteria|p__Firmicutes|c__CFGB2838|o__OFGB2838|f__FGB2838|g__GGB28415|s__GGB28415_SGB40997
k__Bacteria|p__Firmicutes|c__Clostridia|o__Eubacteriales|f__Pumilibacteraceae|g__GGB28430|s__GGB28430_SGB41013
k__Bacteria|p__Firmicutes|c__Clostridia|o__Eubacteriales|f__Pumilibacteraceae|g__GGB28430|s__GGB28430_SGB41013
k__Bacteria|p__Firmicutes|c__CFGB28439|o__OFGB28439|f__FGB28439|g__GGB28439|s__GGB28439_SGB41022
k__Bacteria|p__Firmicutes|c__CFGB28439|o__OFGB28439|f__FGB28439|g__GGB28439|s__GGB28439_SGB41022
k__Bacteria|p__Firmicutes|c__Clostridia|o__Clostridia_unclassified|f__Clostridia_unclassified|g__GGB28778|s__GGB2877
k__Bacteria|p__Firmicutes|c__Clostridia|o__Clostridia_unclassified|f__Clostridia_unclassified|g__GGB28778|s__GGB2877
k__Bacteria|p__Firmicutes|c__Clostridia|o__Eubacteriales|f__Eubacteriaceae|g__GGB28784|s__GGB28784_SGB41437
k__Bacteria|p__Firmicutes|c__Clostridia|o__Eubacteriales|f__Eubacteriaceae|g__GGB28784|s__GGB28784_SGB41437
k__Bacteria|p__Firmicutes|c__Clostridia|o__Eubacteriales|f__Lachnospiraceae|g__GGB28792|s__GGB28792_SGB41445
k__Bacteria|p__Firmicutes|c__Clostridia|o__Eubacteriales|f__Lachnospiraceae|g__GGB28792|s__GGB28792_SGB41445
k__Bacteria|p__Firmicutes|c__Clostridia|o__Eubacteriales|f__Lachnospiraceae|g__GGB28798|s__GGB28798_SGB41451
k__Bacteria|p__Firmicutes|c__Clostridia|o__Eubacteriales|f__Lachnospiraceae|g__GGB28798|s__GGB28798_SGB41451

k__Bacteria|p__Firmicutes|c__Clostridia|o__Eubacteriales|f__Lachnospiraceae|g__GGB28802|s__GGB28802_SGB41455
k__Bacteria|p__Firmicutes|c__Clostridia|o__Eubacteriales|f__Lachnospiraceae|g__GGB28802|s__GGB28802_SGB41455
k__Bacteria|p__Firmicutes|c__Clostridia|o__Eubacteriales|f__Lachnospiraceae|g__GGB28818|s__GGB28818_SGB41473
k__Bacteria|p__Firmicutes|c__Clostridia|o__Eubacteriales|f__Lachnospiraceae|g__GGB28818|s__GGB28818_SGB41473
k__Bacteria|p__Firmicutes|c__CFGB77305|o__OFGB77305|f__FGB77305|g__GGB28828|s__GGB28828_SGB41484
k__Bacteria|p__Firmicutes|c__CFGB77305|o__OFGB77305|f__FGB77305|g__GGB28828|s__GGB28828_SGB41484
k__Bacteria|p__Firmicutes|c__Clostridia|o__Eubacteriales|f__Clostridiaceae|g__GGB28851|s__GGB28851_SGB41518
k__Bacteria|p__Firmicutes|c__Clostridia|o__Eubacteriales|f__Clostridiaceae|g__GGB28851|s__GGB28851_SGB41518
k__Bacteria|p__Firmicutes|c__Clostridia|o__Eubacteriales|f__Lachnospiraceae|g__GGB28859|s__GGB28859_SGB41528
k__Bacteria|p__Firmicutes|c__Clostridia|o__Eubacteriales|f__Lachnospiraceae|g__GGB28859|s__GGB28859_SGB41528
k__Bacteria|p__Firmicutes|c__Clostridia|o__Eubacteriales|f__Lachnospiraceae|g__GGB28864|s__GGB28864_SGB41535
k__Bacteria|p__Firmicutes|c__Clostridia|o__Eubacteriales|f__Lachnospiraceae|g__GGB28864|s__GGB28864_SGB41535
k__Bacteria|p__Firmicutes|c__Clostridia|o__Eubacteriales|f__Lachnospiraceae|g__GGB28869|s__GGB28869_SGB41543
k__Bacteria|p__Firmicutes|c__Clostridia|o__Eubacteriales|f__Lachnospiraceae|g__GGB28869|s__GGB28869_SGB41543
k__Bacteria|p__Firmicutes|c__CFGB9633|o__OFGB9633|f__FGB9633|g__GGB28883|s__GGB28883_SGB41564
k__Bacteria|p__Firmicutes|c__CFGB9633|o__OFGB9633|f__FGB9633|g__GGB28883|s__GGB28883_SGB41564
k__Bacteria|p__Bacteria_unclassified|c__Bacteria_unclassified|o__Bacteria_unclassified|f__Bacteria_unclassified|g__GGB
k__Bacteria|p__Bacteria_unclassified|c__Bacteria_unclassified|o__Bacteria_unclassified|f__Bacteria_unclassified|g__GGB
k__Bacteria|p__Bacteria_unclassified|c__Bacteria_unclassified|o__Bacteria_unclassified|f__Bacteria_unclassified|g__GGB
k__Bacteria|p__Bacteria_unclassified|c__Bacteria_unclassified|o__Bacteria_unclassified|f__Bacteria_unclassified|g__GGB
k__Bacteria|p__Bacteria_unclassified|c__Bacteria_unclassified|o__Bacteria_unclassified|f__Bacteria_unclassified|g__GGB
k__Bacteria|p__Bacteria_unclassified|c__Bacteria_unclassified|o__Bacteria_unclassified|f__Bacteria_unclassified|g__GGB
k__Bacteria|p__Bacteria_unclassified|c__Bacteria_unclassified|o__Bacteria_unclassified|f__Bacteria_unclassified|g__GGB
k__Bacteria|p__Bacteria_unclassified|c__Bacteria_unclassified|o__Bacteria_unclassified|f__Bacteria_unclassified|g__GGB
k__Bacteria|p__Firmicutes|c__Clostridia|o__Eubacteriales|f__Lachnospiraceae|g__GGB28916|s__GGB28916_SGB41612
k__Bacteria|p__Firmicutes|c__Clostridia|o__Eubacteriales|f__Lachnospiraceae|g__GGB28916|s__GGB28916_SGB41612
k__Bacteria|p__Firmicutes|c__Clostridia|o__Eubacteriales|f__Lachnospiraceae|g__GGB28924|s__GGB28924_SGB41621
k__Bacteria|p__Firmicutes|c__Clostridia|o__Eubacteriales|f__Lachnospiraceae|g__GGB28924|s__GGB28924_SGB41621
k__Bacteria|p__Firmicutes|c__Clostridia|o__Eubacteriales|f__Lachnospiraceae|g__GGB28926|s__GGB28926_SGB41624
k__Bacteria|p__Firmicutes|c__Clostridia|o__Eubacteriales|f__Lachnospiraceae|g__GGB28926|s__GGB28926_SGB41624
k__Bacteria|p__Bacteria_unclassified|c__CFGB77359|o__OFGB77359|f__FGB77359|g__GGB28927|s__GGB28927_SGB41
k__Bacteria|p__Bacteria_unclassified|c__CFGB77359|o__OFGB77359|f__FGB77359|g__GGB28927|s__GGB28927_SGB41
k__Bacteria|p__Firmicutes|c__CFGB9639|o__OFGB9639|f__FGB9639|g__GGB28934|s__GGB28934_SGB41635
k__Bacteria|p__Firmicutes|c__CFGB9639|o__OFGB9639|f__FGB9639|g__GGB28934|s__GGB28934_SGB41635
k__Bacteria|p__Firmicutes|c__Clostridia|o__Eubacteriales|f__Lachnospiraceae|g__GGB28946|s__GGB28946_SGB41652
k__Bacteria|p__Firmicutes|c__Clostridia|o__Eubacteriales|f__Lachnospiraceae|g__GGB28946|s__GGB28946_SGB41652
k__Bacteria|p__Firmicutes|c__Clostridia|o__Eubacteriales|f__Lachnospiraceae|g__GGB28949|s__GGB28949_SGB41655
k__Bacteria|p__Firmicutes|c__Clostridia|o__Eubacteriales|f__Lachnospiraceae|g__GGB28949|s__GGB28949_SGB41655
k__Bacteria|p__Firmicutes|c__Clostridia|o__Eubacteriales|f__Lachnospiraceae|g__GGB28949|s__GGB28949_SGB41656
k__Bacteria|p__Firmicutes|c__Clostridia|o__Eubacteriales|f__Lachnospiraceae|g__GGB28949|s__GGB28949_SGB41656
k__Bacteria|p__Firmicutes|c__Clostridia|o__Eubacteriales|f__Clostridiaceae|g__GGB28950|s__GGB28950_SGB41657
k__Bacteria|p__Firmicutes|c__Clostridia|o__Eubacteriales|f__Clostridiaceae|g__GGB28950|s__GGB28950_SGB41657
k__Bacteria|p__Firmicutes|c__Clostridia|o__Eubacteriales|f__Clostridiaceae|g__GGB28951|s__GGB28951_SGB102295
k__Bacteria|p__Firmicutes|c__Clostridia|o__Eubacteriales|f__Clostridiaceae|g__GGB28951|s__GGB28951_SGB102295
k__Bacteria|p__Firmicutes|c__Clostridia|o__Eubacteriales|f__Clostridiaceae|g__GGB28951|s__GGB28951_SGB41658
k__Bacteria|p__Firmicutes|c__Clostridia|o__Eubacteriales|f__Clostridiaceae|g__GGB28951|s__GGB28951_SGB41658

k__Bacteria|p__Firmicutes|c__Clostridia|o__Eubacteriales|f__Clostridiaceae|g__GGB28954|s__GGB28954_SGB41662
k__Bacteria|p__Firmicutes|c__Clostridia|o__Eubacteriales|f__Clostridiaceae|g__GGB28954|s__GGB28954_SGB41662
k__Bacteria|p__Firmicutes|c__Clostridia|o__Eubacteriales|f__Clostridiaceae|g__GGB28956|s__GGB28956_SGB41665
k__Bacteria|p__Firmicutes|c__Clostridia|o__Eubacteriales|f__Clostridiaceae|g__GGB28956|s__GGB28956_SGB41665
k__Bacteria|p__Firmicutes|c__Clostridia|o__Eubacteriales|f__Clostridiaceae|g__GGB28960|s__GGB28960_SGB41669
k__Bacteria|p__Firmicutes|c__Clostridia|o__Eubacteriales|f__Clostridiaceae|g__GGB28960|s__GGB28960_SGB41669
k__Bacteria|p__Firmicutes|c__Clostridia|o__Eubacteriales|f__Clostridiaceae|g__GGB28967|s__GGB28967_SGB41678
k__Bacteria|p__Firmicutes|c__Clostridia|o__Eubacteriales|f__Clostridiaceae|g__GGB28967|s__GGB28967_SGB41678
k__Bacteria|p__Firmicutes|c__Clostridia|o__Eubacteriales|f__Eubacteriaceae|g__GGB28991|s__GGB28991_SGB41705
k__Bacteria|p__Firmicutes|c__Clostridia|o__Eubacteriales|f__Eubacteriaceae|g__GGB28991|s__GGB28991_SGB41705
k__Bacteria|p__Firmicutes|c__CFGB9658|o__OFGB9658|f__FGB9658|g__GGB29002|s__GGB29002_SGB41718
k__Bacteria|p__Firmicutes|c__CFGB9658|o__OFGB9658|f__FGB9658|g__GGB29002|s__GGB29002_SGB41718
k__Bacteria|p__Firmicutes|c__CFGB9659|o__OFGB9659|f__FGB9659|g__GGB29003|s__GGB29003_SGB41719
k__Bacteria|p__Firmicutes|c__CFGB9659|o__OFGB9659|f__FGB9659|g__GGB29003|s__GGB29003_SGB41719
k__Bacteria|p__Bacteria_unclassified|c__Bacteria_unclassified|o__Bacteria_unclassified|f__Bacteria_unclassified|g__GGB
k__Bacteria|p__Bacteria_unclassified|c__Bacteria_unclassified|o__Bacteria_unclassified|f__Bacteria_unclassified|g__GGB
k__Bacteria|p__Firmicutes|c__CFGB9827|o__OFGB9827|f__FGB9827|g__GGB29531|s__GGB29531_SGB42317
k__Bacteria|p__Firmicutes|c__CFGB9827|o__OFGB9827|f__FGB9827|g__GGB29531|s__GGB29531_SGB42317
k__Bacteria|p__Firmicutes|c__Clostridia|o__Eubacteriales|f__Eubacteriaceae|g__GGB29685|s__GGB29685_SGB42494
k__Bacteria|p__Firmicutes|c__Clostridia|o__Eubacteriales|f__Eubacteriaceae|g__GGB29685|s__GGB29685_SGB42494
k__Bacteria|p__Bacteria_unclassified|c__CFGB77303|o__OFGB77303|f__FGB77303|g__GGB30141|s__GGB30141_SGB430
k__Bacteria|p__Bacteria_unclassified|c__CFGB77303|o__OFGB77303|f__FGB77303|g__GGB30141|s__GGB30141_SGB430
k__Bacteria|p__Firmicutes|c__Clostridia|o__Eubacteriales|f__Eubacteriales_unclassified|g__GGB30286|s__GGB30286_SG
k__Bacteria|p__Firmicutes|c__Clostridia|o__Eubacteriales|f__Eubacteriales_unclassified|g__GGB30286|s__GGB30286_SG
k__Bacteria|p__Firmicutes|c__Clostridia|o__Eubacteriales|f__Oscillospiraceae|g__GGB30303|s__GGB30303_SGB43268
k__Bacteria|p__Firmicutes|c__Clostridia|o__Eubacteriales|f__Oscillospiraceae|g__GGB30303|s__GGB30303_SGB43268
k__Bacteria|p__Firmicutes|c__CFGB30328|o__OFGB30328|f__FGB30328|g__GGB30413|s__GGB30413_SGB43452
k__Bacteria|p__Firmicutes|c__CFGB30328|o__OFGB30328|f__FGB30328|g__GGB30413|s__GGB30413_SGB43452
k__Bacteria|p__Firmicutes|c__Clostridia|o__Eubacteriales|f__Oscillospiraceae|g__GGB30454|s__GGB30454_SGB43514
k__Bacteria|p__Firmicutes|c__Clostridia|o__Eubacteriales|f__Oscillospiraceae|g__GGB30454|s__GGB30454_SGB43514
k__Bacteria|p__Firmicutes|c__Clostridia|o__Eubacteriales|f__Oscillospiraceae|g__GGB30455|s__GGB30455_SGB43519
k__Bacteria|p__Firmicutes|c__Clostridia|o__Eubacteriales|f__Oscillospiraceae|g__GGB30455|s__GGB30455_SGB43519
k__Bacteria|p__Firmicutes|c__Clostridia|o__Eubacteriales|f__Oscillospiraceae|g__GGB30461|s__GGB30461_SGB43527
k__Bacteria|p__Firmicutes|c__Clostridia|o__Eubacteriales|f__Oscillospiraceae|g__GGB30461|s__GGB30461_SGB43527
k__Bacteria|p__Firmicutes|c__Clostridia|o__Eubacteriales|f__Oscillospiraceae|g__GGB30461|s__GGB30461_SGB43530
k__Bacteria|p__Firmicutes|c__Clostridia|o__Eubacteriales|f__Oscillospiraceae|g__GGB30461|s__GGB30461_SGB43530
k__Bacteria|p__Firmicutes|c__Clostridia|o__Eubacteriales|f__Oscillospiraceae|g__GGB30463|s__GGB30463_SGB43537
k__Bacteria|p__Firmicutes|c__Clostridia|o__Eubacteriales|f__Oscillospiraceae|g__GGB30463|s__GGB30463_SGB43537
k__Bacteria|p__Firmicutes|c__Clostridia|o__Eubacteriales|f__Oscillospiraceae|g__GGB30473|s__GGB30473_SGB43557
k__Bacteria|p__Firmicutes|c__Clostridia|o__Eubacteriales|f__Oscillospiraceae|g__GGB30473|s__GGB30473_SGB43557
k__Bacteria|p__Firmicutes|c__Clostridia|o__Eubacteriales|f__Oscillospiraceae|g__GGB30475|s__GGB30475_SGB63182
k__Bacteria|p__Firmicutes|c__Clostridia|o__Eubacteriales|f__Oscillospiraceae|g__GGB30475|s__GGB30475_SGB63182
k__Bacteria|p__Actinobacteria|c__CFGB77153|o__OFGB77153|f__FGB77153|g__GGB30861|s__GGB30861_SGB44083
k__Bacteria|p__Actinobacteria|c__CFGB77153|o__OFGB77153|f__FGB77153|g__GGB30861|s__GGB30861_SGB44083
k__Bacteria|p__Tenericutes|c__CFGB1791|o__OFGB1791|f__FGB1791|g__GGB31312|s__GGB31312_SGB44628
k__Bacteria|p__Tenericutes|c__CFGB1791|o__OFGB1791|f__FGB1791|g__GGB31312|s__GGB31312_SGB44628

k__Bacteria|p__Firmicutes|c__CFGB10290|o__OFGB10290|f__FGB10290|g__GGB31438|s__GGB31438_SGB44768
k__Bacteria|p__Firmicutes|c__CFGB10290|o__OFGB10290|f__FGB10290|g__GGB31438|s__GGB31438_SGB44768
k__Bacteria|p__Firmicutes|c__Clostridia|o__Eubacteriales|f__Oscillospiraceae|g__GGB3171|s__GGB3171_SGB4185
k__Bacteria|p__Firmicutes|c__Clostridia|o__Eubacteriales|f__Oscillospiraceae|g__GGB3171|s__GGB3171_SGB4185
k__Bacteria|p__Firmicutes|c__CFGB1765|o__OFGB1765|f__FGB1765|g__GGB31823|s__GGB31823_SGB45199
k__Bacteria|p__Firmicutes|c__CFGB1765|o__OFGB1765|f__FGB1765|g__GGB31823|s__GGB31823_SGB45199
k__Bacteria|p__Firmicutes|c__CFGB10349|o__OFGB10349|f__FGB10349|g__GGB31853|s__GGB31853_SGB45233
k__Bacteria|p__Firmicutes|c__CFGB10349|o__OFGB10349|f__FGB10349|g__GGB31853|s__GGB31853_SGB45233
k__Bacteria|p__Firmicutes|c__CFGB10667|o__OFGB10667|f__FGB10667|g__GGB32371|s__GGB32371_SGB41694
k__Bacteria|p__Firmicutes|c__CFGB10667|o__OFGB10667|f__FGB10667|g__GGB32371|s__GGB32371_SGB41694
k__Bacteria|p__Firmicutes|c__Clostridia|o__Eubacteriales|f__Lachnospiraceae|g__GGB3793|s__GGB3793_SGB5158
k__Bacteria|p__Firmicutes|c__Clostridia|o__Eubacteriales|f__Lachnospiraceae|g__GGB3793|s__GGB3793_SGB5158
k__Bacteria|p__Firmicutes|c__Clostridia|o__Eubacteriales|f__Lachnospiraceae|g__GGB42598|s__GGB42598_SGB59794
k__Bacteria|p__Firmicutes|c__Clostridia|o__Eubacteriales|f__Lachnospiraceae|g__GGB42598|s__GGB42598_SGB59794
k__Bacteria|p__Firmicutes|c__Clostridia|o__Eubacteriales|f__Christensenellaceae|g__GGB45656|s__GGB45656_SGB6337
k__Bacteria|p__Firmicutes|c__Clostridia|o__Eubacteriales|f__Christensenellaceae|g__GGB45656|s__GGB45656_SGB6337
k__Bacteria|p__Firmicutes|c__CFGB10299|o__OFGB10299|f__FGB10299|g__GGB47127|s__GGB47127_SGB65054
k__Bacteria|p__Firmicutes|c__CFGB10299|o__OFGB10299|f__FGB10299|g__GGB47127|s__GGB47127_SGB65054
k__Bacteria|p__Firmicutes|c__Clostridia|o__Eubacteriales|f__Oscillospiraceae|g__GGB74395|s__GGB74395_SGB43521
k__Bacteria|p__Firmicutes|c__Clostridia|o__Eubacteriales|f__Oscillospiraceae|g__GGB74395|s__GGB74395_SGB43521
k__Bacteria|p__Firmicutes|c__Clostridia|o__Eubacteriales|f__Oscillospiraceae|g__GGB75053|s__GGB75053_SGB43494
k__Bacteria|p__Firmicutes|c__Clostridia|o__Eubacteriales|f__Oscillospiraceae|g__GGB75053|s__GGB75053_SGB43494
k__Bacteria|p__Firmicutes|c__Clostridia|o__Eubacteriales|f__Lachnospiraceae|g__GGB75109|s__GGB75109_SGB102238
k__Bacteria|p__Firmicutes|c__Clostridia|o__Eubacteriales|f__Lachnospiraceae|g__GGB75109|s__GGB75109_SGB102238
k__Bacteria|p__Firmicutes|c__Clostridia|o__Clostridia_unclassified|f__Clostridia_unclassified|g__GGB81440|s__GGB8144
k__Bacteria|p__Firmicutes|c__Clostridia|o__Clostridia_unclassified|f__Clostridia_unclassified|g__GGB81440|s__GGB8144
k__Bacteria|p__Firmicutes|c__Clostridia|o__Eubacteriales|f__Lachnospiraceae|g__Lachnospiraceae_unclassified|s__Lachn
k__Bacteria|p__Firmicutes|c__Clostridia|o__Eubacteriales|f__Lachnospiraceae|g__Lachnospiraceae_unclassified|s__Lachn
k__Bacteria|p__Firmicutes|c__Clostridia|o__Eubacteriales|f__Lachnospiraceae|g__Lachnospiraceae_unclassified|s__Lachn
k__Bacteria|p__Firmicutes|c__Clostridia|o__Eubacteriales|f__Lachnospiraceae|g__Lachnospiraceae_unclassified|s__Lachn
k__Bacteria|p__Firmicutes|c__Clostridia|o__Eubacteriales|f__Lachnospiraceae|g__Lachnospiraceae_unclassified|s__Lachn
k__Bacteria|p__Firmicutes|c__Clostridia|o__Eubacteriales|f__Lachnospiraceae|g__Lachnospiraceae_unclassified|s__Lachn
k__Bacteria|p__Firmicutes|c__Clostridia|o__Eubacteriales|f__Lachnospiraceae|g__Lachnospiraceae_unclassified|s__Lachn
k__Bacteria|p__Firmicutes|c__Clostridia|o__Eubacteriales|f__Lachnospiraceae|g__Lachnospiraceae_unclassified|s__Lachn
k__Bacteria|p__Firmicutes|c__Clostridia|o__Eubacteriales|f__Lachnospiraceae|g__Lachnospiraceae_unclassified|s__Lachn
k__Bacteria|p__Firmicutes|c__Clostridia|o__Eubacteriales|f__Lachnospiraceae|g__Lachnospiraceae_unclassified|s__Lachn
k__Bacteria|p__Firmicutes|c__Clostridia|o__Eubacteriales|f__Lachnospiraceae|g__Lachnospiraceae_unclassified|s__Lachn
k__Bacteria|p__Firmicutes|c__Clostridia|o__Eubacteriales|f__Lachnospiraceae|g__Lachnospiraceae_unclassified|s__Lachn
k__Bacteria|p__Firmicutes|c__Clostridia|o__Eubacteriales|f__Lachnospiraceae|g__Lachnospiraceae_unclassified|s__Lachn
k__Bacteria|p__Firmicutes|c__Clostridia|o__Eubacteriales|f__Lachnospiraceae|g__Lachnospiraceae_unclassified|s__Lachn
k__Bacteria|p__Firmicutes|c__Clostridia|o__Eubacteriales|f__Lachnospiraceae|g__Lachnospiraceae_unclassified|s__Lachn
k__Bacteria|p__Firmicutes|c__Clostridia|o__Eubacteriales|f__Lachnospiraceae|g__Lachnospiraceae_unclassified|s__Lachn
k__Bacteria|p__Firmicutes|c__Bacilli|o__Lactobacillales|f__Lactobacillaceae|g__Lactobacillus|s__Lactobacillus_johnsonii
k__Bacteria|p__Firmicutes|c__Bacilli|o__Lactobacillales|f__Lactobacillaceae|g__Lactobacillus|s__Lactobacillus_johnsonii
k__Bacteria|p__Bacteroidota|c__Bacteroidia|o__Bacteroidales|f__Muribaculaceae|g__Muribaculaceae_unclassified|s__M
k__Bacteria|p__Bacteroidota|c__Bacteroidia|o__Bacteroidales|f__Muribaculaceae|g__Muribaculaceae_unclassified|s__M

k__Bacteria|p__Firmicutes|c__Clostridia|o__Eubacteriales|f__Oscillospiraceae|g__Neglectibacter|s__Neglectibacter_sp_X4
k__Bacteria|p__Firmicutes|c__Clostridia|o__Eubacteriales|f__Oscillospiraceae|g__Neglectibacter|s__Neglectibacter_sp_X4
k__Bacteria|p__Firmicutes|c__Clostridia|o__Eubacteriales|f__Oscillospiraceae|g__Oscillospiraceae_unclassified|s__Oscillo
k__Bacteria|p__Firmicutes|c__Clostridia|o__Eubacteriales|f__Oscillospiraceae|g__Oscillospiraceae_unclassified|s__Oscillo
k__Bacteria|p__Firmicutes|c__Clostridia|o__Eubacteriales|f__Oscillospiraceae|g__Oscillospiraceae_unclassified|s__Oscillo
k__Bacteria|p__Firmicutes|c__Clostridia|o__Eubacteriales|f__Oscillospiraceae|g__Oscillospiraceae_unclassified|s__Oscillo
k__Bacteria|p__Firmicutes|c__Clostridia|o__Eubacteriales|f__Oscillospiraceae|g__Oscillospiraceae_unclassified|s__Oscillo
k__Bacteria|p__Firmicutes|c__Clostridia|o__Eubacteriales|f__Oscillospiraceae|g__Oscillospiraceae_unclassified|s__Oscillo
k__Bacteria|p__Proteobacteria|c__Betaproteobacteria|o__Burkholderiales|f__Sutterellaceae|g__Parasutterella|s__Parasu
k__Bacteria|p__Proteobacteria|c__Betaproteobacteria|o__Burkholderiales|f__Sutterellaceae|g__Parasutterella|s__Parasu

k__Bacteria|p__Firmicutes|c__Clostridia|o__Eubacteriales|f__Peptostreptococcaceae|g__Romboutsia|s__Romboutsia_ilea
k__Bacteria|p__Firmicutes|c__Clostridia|o__Eubacteriales|f__Peptostreptococcaceae|g__Romboutsia|s__Romboutsia_ilea
k__Bacteria|p__Firmicutes|c__Clostridia|o__Eubacteriales|f__Lachnospiraceae|g__Schaedlerella|s__Schaedlerella_arabinc
k__Bacteria|p__Firmicutes|c__Clostridia|o__Eubacteriales|f__Lachnospiraceae|g__Schaedlerella|s__Schaedlerella_arabinc

k__Bacteria|p__Firmicutes|c__Erysipelotrichia|o__Erysipelotrichales|f__Turicibacteraceae|g__Turicibacter|s__Turicibacter_
k__Bacteria|p__Firmicutes|c__Erysipelotrichia|o__Erysipelotrichales|f__Turicibacteraceae|g__Turicibacter|s__Turicibacter_
k__Bacteria|p__Firmicutes|c__Clostridia|o__Eubacteriales|f__Lachnospiraceae|g__Acetatifactor|s__Acetatifactor_muris
k__Bacteria|p__Firmicutes|c__Clostridia|o__Eubacteriales|f__Lachnospiraceae|g__Acetatifactor|s__Acetatifactor_muris
k__Bacteria|p__Firmicutes|c__Clostridia|o__Eubacteriales|f__Lachnospiraceae|g__Acetatifactor|s__Acetatifactor_SGB415
k__Bacteria|p__Firmicutes|c__Clostridia|o__Eubacteriales|f__Lachnospiraceae|g__Acetatifactor|s__Acetatifactor_SGB415
k__Bacteria|p__Firmicutes|c__Clostridia|o__Eubacteriales|f__Oscillospiraceae|g__Acutalibacter|s__Acutalibacter_muris
k__Bacteria|p__Firmicutes|c__Clostridia|o__Eubacteriales|f__Oscillospiraceae|g__Acutalibacter|s__Acutalibacter_muris
k__Bacteria|p__Firmicutes|c__Clostridia|o__Eubacteriales|f__Oscillospiraceae|g__Acutalibacter|s__Acutalibacter_sp_1XD
k__Bacteria|p__Firmicutes|c__Clostridia|o__Eubacteriales|f__Oscillospiraceae|g__Acutalibacter|s__Acutalibacter_sp_1XD
k__Bacteria|p__Actinobacteria|c__Coriobacteriia|o__Eggerthellales|f__Eggerthellaceae|g__Adlercreutzia|s__Adlercreutzia
k__Bacteria|p__Actinobacteria|c__Coriobacteriia|o__Eggerthellales|f__Eggerthellaceae|g__Adlercreutzia|s__Adlercreutzia
k__Bacteria|p__Actinobacteria|c__Coriobacteriia|o__Eggerthellales|f__Eggerthellaceae|g__Adlercreutzia|s__Adlercreutzia
k__Bacteria|p__Actinobacteria|c__Coriobacteriia|o__Eggerthellales|f__Eggerthellaceae|g__Adlercreutzia|s__Adlercreutzia
k__Bacteria|p__Actinobacteria|c__Coriobacteriia|o__Eggerthellales|f__Eggerthellaceae|g__Adlercreutzia|s__Adlercreutzia
k__Bacteria|p__Actinobacteria|c__Coriobacteriia|o__Eggerthellales|f__Eggerthellaceae|g__Adlercreutzia|s__Adlercreutzia
k__Bacteria|p__Verrucomicrobia|c__Verrucomicrobiae|o__Verrucomicrobiales|f__Akkermansiaceae|g__Akkermansia|s__A
k__Bacteria|p__Verrucomicrobia|c__Verrucomicrobiae|o__Verrucomicrobiales|f__Akkermansiaceae|g__Akkermansia|s__A
k__Bacteria|p__Bacteroidota|c__Bacteroidia|o__Bacteroidales|f__Rikenellaceae|g__Alistipes|s__Alistipes_sp_DSM_11234
k__Bacteria|p__Bacteroidota|c__Bacteroidia|o__Bacteroidales|f__Rikenellaceae|g__Alistipes|s__Alistipes_sp_DSM_11234
k__Bacteria|p__Firmicutes|c__Clostridia|o__Eubacteriales|f__Oscillospiraceae|g__Anaerotruncus|s__Anaerotruncus_sp_1
k__Bacteria|p__Firmicutes|c__Clostridia|o__Eubacteriales|f__Oscillospiraceae|g__Anaerotruncus|s__Anaerotruncus_sp_1
k__Bacteria|p__Bacteria_unclassified|c__Bacteria_unclassified|o__Bacteria_unclassified|f__Bacteria_unclassified|g__Bacte
k__Bacteria|p__Bacteria_unclassified|c__Bacteria_unclassified|o__Bacteria_unclassified|f__Bacteria_unclassified|g__Bacte
k__Bacteria|p__Bacteria_unclassified|c__Bacteria_unclassified|o__Bacteria_unclassified|f__Bacteria_unclassified|g__Bacte
k__Bacteria|p__Bacteria_unclassified|c__Bacteria_unclassified|o__Bacteria_unclassified|f__Bacteria_unclassified|g__Bacte
k__Bacteria|p__Bacteria_unclassified|c__Bacteria_unclassified|o__Bacteria_unclassified|f__Bacteria_unclassified|g__Bacte
k__Bacteria|p__Bacteria_unclassified|c__Bacteria_unclassified|o__Bacteria_unclassified|f__Bacteria_unclassified|g__Bacte

k__Bacteria|p__Bacteria_unclassified|c__Bacteria_unclassified|o__Bacteria_unclassified|f__Bacteria_unclassified|g__Bacte
k__Bacteria|p__Bacteria_unclassified|c__Bacteria_unclassified|o__Bacteria_unclassified|f__Bacteria_unclassified|g__Bacte
k__Bacteria|p__Bacteria_unclassified|c__Bacteria_unclassified|o__Bacteria_unclassified|f__Bacteria_unclassified|g__Bacte
k__Bacteria|p__Bacteria_unclassified|c__Bacteria_unclassified|o__Bacteria_unclassified|f__Bacteria_unclassified|g__Bacte
k__Bacteria|p__Bacteria_unclassified|c__Bacteria_unclassified|o__Bacteria_unclassified|f__Bacteria_unclassified|g__Bacte
k__Bacteria|p__Bacteria_unclassified|c__Bacteria_unclassified|o__Bacteria_unclassified|f__Bacteria_unclassified|g__Bacte
k__Bacteria|p__Bacteroidota|c__Bacteroidia|o__Bacteroidales|f__Bacteroidaceae|g__Bacteroides|s__Bacteroides_thetaio
k__Bacteria|p__Bacteroidota|c__Bacteroidia|o__Bacteroidales|f__Bacteroidaceae|g__Bacteroides|s__Bacteroides_thetaio

k__Bacteria|p__Actinobacteria|c__Actinomycetia|o__Bifidobacteriales|f__Bifidobacteriaceae|g__Bifidobacterium|s__Bifid
k__Bacteria|p__Actinobacteria|c__Actinomycetia|o__Bifidobacteriales|f__Bifidobacteriaceae|g__Bifidobacterium|s__Bifid
k__Bacteria|p__Firmicutes|c__Clostridia|o__Clostridia_unclassified|f__Clostridia_unclassified|g__Clostridia_unclassified|s_
k__Bacteria|p__Firmicutes|c__Clostridia|o__Clostridia_unclassified|f__Clostridia_unclassified|g__Clostridia_unclassified|s_
k__Bacteria|p__Firmicutes|c__Clostridia|o__Eubacteriales|f__Clostridiaceae|g__Clostridiaceae_unclassified|s__Clostridiac
k__Bacteria|p__Firmicutes|c__Clostridia|o__Eubacteriales|f__Clostridiaceae|g__Clostridiaceae_unclassified|s__Clostridiac
k__Bacteria|p__Firmicutes|c__Clostridia|o__Eubacteriales|f__Clostridiaceae|g__Clostridiaceae_unclassified|s__Clostridiac
k__Bacteria|p__Firmicutes|c__Clostridia|o__Eubacteriales|f__Clostridiaceae|g__Clostridiaceae_unclassified|s__Clostridiac
k__Bacteria|p__Firmicutes|c__Clostridia|o__Eubacteriales|f__Eubacteriales_unclassified|g__Eubacteriales_unclassified|s_
k__Bacteria|p__Firmicutes|c__Clostridia|o__Eubacteriales|f__Eubacteriales_unclassified|g__Eubacteriales_unclassified|s_
k__Bacteria|p__Firmicutes|c__Erysipelotrichia|o__Erysipelotrichales|f__Erysipelotrichaceae|g__Erysipelatoclostridium|s__
k__Bacteria|p__Firmicutes|c__Erysipelotrichia|o__Erysipelotrichales|f__Erysipelotrichaceae|g__Erysipelatoclostridium|s__
k__Bacteria|p__Actinobacteria|c__Coriobacteriia|o__Coriobacteriales|f__Coriobacteriaceae|g__Coriobacteriaceae_unclass
k__Bacteria|p__Actinobacteria|c__Coriobacteriia|o__Coriobacteriales|f__Coriobacteriaceae|g__Coriobacteriaceae_unclass
k__Bacteria|p__Firmicutes|c__Clostridia|o__Eubacteriales|f__Lachnospiraceae|g__Dorea|s__Dorea_sp_5_2
k__Bacteria|p__Firmicutes|c__Clostridia|o__Eubacteriales|f__Lachnospiraceae|g__Dorea|s__Dorea_sp_5_2
k__Bacteria|p__Firmicutes|c__Erysipelotrichia|o__Erysipelotrichales|f__Erysipelotrichaceae|g__Dubosiella|s__Dubosiella_
k__Bacteria|p__Firmicutes|c__Erysipelotrichia|o__Erysipelotrichales|f__Erysipelotrichaceae|g__Dubosiella|s__Dubosiella_
k__Bacteria|p__Firmicutes|c__Erysipelotrichia|o__Erysipelotrichales|f__Erysipelotrichales_unclassified|g__Erysipelotrichal
k__Bacteria|p__Firmicutes|c__Erysipelotrichia|o__Erysipelotrichales|f__Erysipelotrichales_unclassified|g__Erysipelotrichal
k__Bacteria|p__Firmicutes|c__Clostridia|o__Eubacteriales|f__Eubacteriaceae|g__Eubacteriaceae_unclassified|s__Eubacte
k__Bacteria|p__Firmicutes|c__Clostridia|o__Eubacteriales|f__Eubacteriaceae|g__Eubacteriaceae_unclassified|s__Eubacte
k__Bacteria|p__Firmicutes|c__Clostridia|o__Eubacteriales|f__Eubacteriaceae|g__Eubacteriaceae_unclassified|s__Eubacte
k__Bacteria|p__Firmicutes|c__Clostridia|o__Eubacteriales|f__Eubacteriaceae|g__Eubacteriaceae_unclassified|s__Eubacte
k__Bacteria|p__Firmicutes|c__Clostridia|o__Eubacteriales|f__Lachnospiraceae|g__GGB20149|s__GGB20149_SGB29430
k__Bacteria|p__Firmicutes|c__Clostridia|o__Eubacteriales|f__Lachnospiraceae|g__GGB20149|s__GGB20149_SGB29430
k__Bacteria|p__Actinobacteria|c__Coriobacteriia|o__Eggerthellales|f__Eggerthellaceae|g__GGB22635|s__GGB22635_SGB
k__Bacteria|p__Actinobacteria|c__Coriobacteriia|o__Eggerthellales|f__Eggerthellaceae|g__GGB22635|s__GGB22635_SGB
k__Bacteria|p__Firmicutes|c__Clostridia|o__Eubacteriales|f__Lachnospiraceae|g__GGB25041|s__GGB25041_SGB36960
k__Bacteria|p__Firmicutes|c__Clostridia|o__Eubacteriales|f__Lachnospiraceae|g__GGB25041|s__GGB25041_SGB36960
k__Bacteria|p__Bacteroidota|c__Bacteroidia|o__Bacteroidales|f__Muribaculaceae|g__GGB27876|s__GGB27876_SGB4031
k__Bacteria|p__Bacteroidota|c__Bacteroidia|o__Bacteroidales|f__Muribaculaceae|g__GGB27876|s__GGB27876_SGB4031
k__Bacteria|p__Bacteroidota|c__Bacteroidia|o__Bacteroidales|f__Muribaculaceae|g__GGB27878|s__GGB27878_SGB4031
k__Bacteria|p__Bacteroidota|c__Bacteroidia|o__Bacteroidales|f__Muribaculaceae|g__GGB27878|s__GGB27878_SGB4031
k__Bacteria|p__Bacteroidota|c__Bacteroidia|o__Bacteroidales|f__Muribaculaceae|g__GGB27918|s__GGB27918_SGB4035
k__Bacteria|p__Bacteroidota|c__Bacteroidia|o__Bacteroidales|f__Muribaculaceae|g__GGB27918|s__GGB27918_SGB4035

k__Bacteria|p__Firmicutes|c__CFGB9508|o__OFGB9508|f__FGB9508|g__GGB28382|s__GGB28382_SGB40962
k__Bacteria|p__Firmicutes|c__CFGB9508|o__OFGB9508|f__FGB9508|g__GGB28382|s__GGB28382_SGB40962
k__Bacteria|p__Firmicutes|c__CFGB2838|o__OFGB2838|f__FGB2838|g__GGB28399|s__GGB28399_SGB40980
k__Bacteria|p__Firmicutes|c__CFGB2838|o__OFGB2838|f__FGB2838|g__GGB28399|s__GGB28399_SGB40980
k__Bacteria|p__Firmicutes|c__CFGB2838|o__OFGB2838|f__FGB2838|g__GGB28411|s__GGB28411_SGB40993
k__Bacteria|p__Firmicutes|c__CFGB2838|o__OFGB2838|f__FGB2838|g__GGB28411|s__GGB28411_SGB40993
k__Bacteria|p__Firmicutes|c__CFGB2838|o__OFGB2838|f__FGB2838|g__GGB28415|s__GGB28415_SGB40997
k__Bacteria|p__Firmicutes|c__CFGB2838|o__OFGB2838|f__FGB2838|g__GGB28415|s__GGB28415_SGB40997
k__Bacteria|p__Firmicutes|c__Clostridia|o__Eubacteriales|f__Pumilibacteraceae|g__GGB28430|s__GGB28430_SGB41013
k__Bacteria|p__Firmicutes|c__Clostridia|o__Eubacteriales|f__Pumilibacteraceae|g__GGB28430|s__GGB28430_SGB41013
k__Bacteria|p__Firmicutes|c__CFGB28439|o__OFGB28439|f__FGB28439|g__GGB28439|s__GGB28439_SGB41022
k__Bacteria|p__Firmicutes|c__CFGB28439|o__OFGB28439|f__FGB28439|g__GGB28439|s__GGB28439_SGB41022
k__Bacteria|p__Firmicutes|c__Clostridia|o__Clostridia_unclassified|f__Clostridia_unclassified|g__GGB28778|s__GGB2877
k__Bacteria|p__Firmicutes|c__Clostridia|o__Clostridia_unclassified|f__Clostridia_unclassified|g__GGB28778|s__GGB2877
k__Bacteria|p__Firmicutes|c__Clostridia|o__Eubacteriales|f__Eubacteriaceae|g__GGB28784|s__GGB28784_SGB41437
k__Bacteria|p__Firmicutes|c__Clostridia|o__Eubacteriales|f__Eubacteriaceae|g__GGB28784|s__GGB28784_SGB41437
k__Bacteria|p__Firmicutes|c__Clostridia|o__Eubacteriales|f__Lachnospiraceae|g__GGB28792|s__GGB28792_SGB41445
k__Bacteria|p__Firmicutes|c__Clostridia|o__Eubacteriales|f__Lachnospiraceae|g__GGB28792|s__GGB28792_SGB41445
k__Bacteria|p__Firmicutes|c__Clostridia|o__Eubacteriales|f__Lachnospiraceae|g__GGB28798|s__GGB28798_SGB41451
k__Bacteria|p__Firmicutes|c__Clostridia|o__Eubacteriales|f__Lachnospiraceae|g__GGB28798|s__GGB28798_SGB41451
k__Bacteria|p__Firmicutes|c__Clostridia|o__Eubacteriales|f__Lachnospiraceae|g__GGB28802|s__GGB28802_SGB41455
k__Bacteria|p__Firmicutes|c__Clostridia|o__Eubacteriales|f__Lachnospiraceae|g__GGB28802|s__GGB28802_SGB41455
k__Bacteria|p__Firmicutes|c__Clostridia|o__Eubacteriales|f__Lachnospiraceae|g__GGB28818|s__GGB28818_SGB41473
k__Bacteria|p__Firmicutes|c__Clostridia|o__Eubacteriales|f__Lachnospiraceae|g__GGB28818|s__GGB28818_SGB41473
k__Bacteria|p__Firmicutes|c__CFGB77305|o__OFGB77305|f__FGB77305|g__GGB28828|s__GGB28828_SGB41484
k__Bacteria|p__Firmicutes|c__CFGB77305|o__OFGB77305|f__FGB77305|g__GGB28828|s__GGB28828_SGB41484
k__Bacteria|p__Firmicutes|c__Clostridia|o__Eubacteriales|f__Clostridiaceae|g__GGB28851|s__GGB28851_SGB41518
k__Bacteria|p__Firmicutes|c__Clostridia|o__Eubacteriales|f__Clostridiaceae|g__GGB28851|s__GGB28851_SGB41518
k__Bacteria|p__Firmicutes|c__Clostridia|o__Eubacteriales|f__Lachnospiraceae|g__GGB28859|s__GGB28859_SGB41528
k__Bacteria|p__Firmicutes|c__Clostridia|o__Eubacteriales|f__Lachnospiraceae|g__GGB28859|s__GGB28859_SGB41528
k__Bacteria|p__Firmicutes|c__Clostridia|o__Eubacteriales|f__Lachnospiraceae|g__GGB28864|s__GGB28864_SGB41535
k__Bacteria|p__Firmicutes|c__Clostridia|o__Eubacteriales|f__Lachnospiraceae|g__GGB28864|s__GGB28864_SGB41535
k__Bacteria|p__Firmicutes|c__Clostridia|o__Eubacteriales|f__Lachnospiraceae|g__GGB28869|s__GGB28869_SGB41543
k__Bacteria|p__Firmicutes|c__Clostridia|o__Eubacteriales|f__Lachnospiraceae|g__GGB28869|s__GGB28869_SGB41543
k__Bacteria|p__Firmicutes|c__CFGB9633|o__OFGB9633|f__FGB9633|g__GGB28883|s__GGB28883_SGB41564
k__Bacteria|p__Firmicutes|c__CFGB9633|o__OFGB9633|f__FGB9633|g__GGB28883|s__GGB28883_SGB41564
k__Bacteria|p__Bacteria_unclassified|c__Bacteria_unclassified|o__Bacteria_unclassified|f__Bacteria_unclassified|g__GGB
k__Bacteria|p__Bacteria_unclassified|c__Bacteria_unclassified|o__Bacteria_unclassified|f__Bacteria_unclassified|g__GGB
k__Bacteria|p__Bacteria_unclassified|c__Bacteria_unclassified|o__Bacteria_unclassified|f__Bacteria_unclassified|g__GGB
k__Bacteria|p__Bacteria_unclassified|c__Bacteria_unclassified|o__Bacteria_unclassified|f__Bacteria_unclassified|g__GGB
k__Bacteria|p__Bacteria_unclassified|c__Bacteria_unclassified|o__Bacteria_unclassified|f__Bacteria_unclassified|g__GGB
k__Bacteria|p__Bacteria_unclassified|c__Bacteria_unclassified|o__Bacteria_unclassified|f__Bacteria_unclassified|g__GGB
k__Bacteria|p__Bacteria_unclassified|c__Bacteria_unclassified|o__Bacteria_unclassified|f__Bacteria_unclassified|g__GGB
k__Bacteria|p__Bacteria_unclassified|c__Bacteria_unclassified|o__Bacteria_unclassified|f__Bacteria_unclassified|g__GGB
k__Bacteria|p__Firmicutes|c__Clostridia|o__Eubacteriales|f__Lachnospiraceae|g__GGB28916|s__GGB28916_SGB41612
k__Bacteria|p__Firmicutes|c__Clostridia|o__Eubacteriales|f__Lachnospiraceae|g__GGB28916|s__GGB28916_SGB41612

k__Bacteria|p__Firmicutes|c__Clostridia|o__Eubacteriales|f__Lachnospiraceae|g__GGB28924|s__GGB28924_SGB41621
k__Bacteria|p__Firmicutes|c__Clostridia|o__Eubacteriales|f__Lachnospiraceae|g__GGB28924|s__GGB28924_SGB41621
k__Bacteria|p__Firmicutes|c__Clostridia|o__Eubacteriales|f__Lachnospiraceae|g__GGB28926|s__GGB28926_SGB41624
k__Bacteria|p__Firmicutes|c__Clostridia|o__Eubacteriales|f__Lachnospiraceae|g__GGB28926|s__GGB28926_SGB41624
k__Bacteria|p__Bacteria_unclassified|c__CFGB77359|o__OFGB77359|f__FGB77359|g__GGB28927|s__GGB28927_SGB416
k__Bacteria|p__Bacteria_unclassified|c__CFGB77359|o__OFGB77359|f__FGB77359|g__GGB28927|s__GGB28927_SGB416
k__Bacteria|p__Firmicutes|c__CFGB9639|o__OFGB9639|f__FGB9639|g__GGB28934|s__GGB28934_SGB41635
k__Bacteria|p__Firmicutes|c__CFGB9639|o__OFGB9639|f__FGB9639|g__GGB28934|s__GGB28934_SGB41635
k__Bacteria|p__Firmicutes|c__Clostridia|o__Eubacteriales|f__Lachnospiraceae|g__GGB28946|s__GGB28946_SGB41652
k__Bacteria|p__Firmicutes|c__Clostridia|o__Eubacteriales|f__Lachnospiraceae|g__GGB28946|s__GGB28946_SGB41652
k__Bacteria|p__Firmicutes|c__Clostridia|o__Eubacteriales|f__Lachnospiraceae|g__GGB28949|s__GGB28949_SGB41655
k__Bacteria|p__Firmicutes|c__Clostridia|o__Eubacteriales|f__Lachnospiraceae|g__GGB28949|s__GGB28949_SGB41655
k__Bacteria|p__Firmicutes|c__Clostridia|o__Eubacteriales|f__Lachnospiraceae|g__GGB28949|s__GGB28949_SGB41656
k__Bacteria|p__Firmicutes|c__Clostridia|o__Eubacteriales|f__Lachnospiraceae|g__GGB28949|s__GGB28949_SGB41656
k__Bacteria|p__Firmicutes|c__Clostridia|o__Eubacteriales|f__Clostridiaceae|g__GGB28950|s__GGB28950_SGB41657
k__Bacteria|p__Firmicutes|c__Clostridia|o__Eubacteriales|f__Clostridiaceae|g__GGB28950|s__GGB28950_SGB41657
k__Bacteria|p__Firmicutes|c__Clostridia|o__Eubacteriales|f__Clostridiaceae|g__GGB28951|s__GGB28951_SGB102295
k__Bacteria|p__Firmicutes|c__Clostridia|o__Eubacteriales|f__Clostridiaceae|g__GGB28951|s__GGB28951_SGB102295
k__Bacteria|p__Firmicutes|c__Clostridia|o__Eubacteriales|f__Clostridiaceae|g__GGB28951|s__GGB28951_SGB41658
k__Bacteria|p__Firmicutes|c__Clostridia|o__Eubacteriales|f__Clostridiaceae|g__GGB28951|s__GGB28951_SGB41658
k__Bacteria|p__Firmicutes|c__Clostridia|o__Eubacteriales|f__Clostridiaceae|g__GGB28954|s__GGB28954_SGB41662
k__Bacteria|p__Firmicutes|c__Clostridia|o__Eubacteriales|f__Clostridiaceae|g__GGB28954|s__GGB28954_SGB41662
k__Bacteria|p__Firmicutes|c__Clostridia|o__Eubacteriales|f__Clostridiaceae|g__GGB28956|s__GGB28956_SGB41665
k__Bacteria|p__Firmicutes|c__Clostridia|o__Eubacteriales|f__Clostridiaceae|g__GGB28956|s__GGB28956_SGB41665
k__Bacteria|p__Firmicutes|c__Clostridia|o__Eubacteriales|f__Clostridiaceae|g__GGB28960|s__GGB28960_SGB41669
k__Bacteria|p__Firmicutes|c__Clostridia|o__Eubacteriales|f__Clostridiaceae|g__GGB28960|s__GGB28960_SGB41669
k__Bacteria|p__Firmicutes|c__Clostridia|o__Eubacteriales|f__Clostridiaceae|g__GGB28967|s__GGB28967_SGB41678
k__Bacteria|p__Firmicutes|c__Clostridia|o__Eubacteriales|f__Clostridiaceae|g__GGB28967|s__GGB28967_SGB41678
k__Bacteria|p__Firmicutes|c__Clostridia|o__Eubacteriales|f__Eubacteriaceae|g__GGB28991|s__GGB28991_SGB41705
k__Bacteria|p__Firmicutes|c__Clostridia|o__Eubacteriales|f__Eubacteriaceae|g__GGB28991|s__GGB28991_SGB41705
k__Bacteria|p__Firmicutes|c__CFGB9658|o__OFGB9658|f__FGB9658|g__GGB29002|s__GGB29002_SGB41718
k__Bacteria|p__Firmicutes|c__CFGB9658|o__OFGB9658|f__FGB9658|g__GGB29002|s__GGB29002_SGB41718
k__Bacteria|p__Firmicutes|c__CFGB9659|o__OFGB9659|f__FGB9659|g__GGB29003|s__GGB29003_SGB41719
k__Bacteria|p__Firmicutes|c__CFGB9659|o__OFGB9659|f__FGB9659|g__GGB29003|s__GGB29003_SGB41719
k__Bacteria|p__Bacteria_unclassified|c__Bacteria_unclassified|o__Bacteria_unclassified|f__Bacteria_unclassified|g__GGB
k__Bacteria|p__Bacteria_unclassified|c__Bacteria_unclassified|o__Bacteria_unclassified|f__Bacteria_unclassified|g__GGB
k__Bacteria|p__Firmicutes|c__CFGB9827|o__OFGB9827|f__FGB9827|g__GGB29531|s__GGB29531_SGB42317
k__Bacteria|p__Firmicutes|c__CFGB9827|o__OFGB9827|f__FGB9827|g__GGB29531|s__GGB29531_SGB42317
k__Bacteria|p__Firmicutes|c__Clostridia|o__Eubacteriales|f__Eubacteriaceae|g__GGB29685|s__GGB29685_SGB42494
k__Bacteria|p__Firmicutes|c__Clostridia|o__Eubacteriales|f__Eubacteriaceae|g__GGB29685|s__GGB29685_SGB42494
k__Bacteria|p__Bacteria_unclassified|c__CFGB77303|o__OFGB77303|f__FGB77303|g__GGB30141|s__GGB30141_SGB430
k__Bacteria|p__Bacteria_unclassified|c__CFGB77303|o__OFGB77303|f__FGB77303|g__GGB30141|s__GGB30141_SGB430
k__Bacteria|p__Firmicutes|c__Clostridia|o__Eubacteriales|f__Eubacteriales_unclassified|g__GGB30286|s__GGB30286_SG
k__Bacteria|p__Firmicutes|c__Clostridia|o__Eubacteriales|f__Eubacteriales_unclassified|g__GGB30286|s__GGB30286_SG
k__Bacteria|p__Firmicutes|c__Clostridia|o__Eubacteriales|f__Oscillospiraceae|g__GGB30303|s__GGB30303_SGB43268
k__Bacteria|p__Firmicutes|c__Clostridia|o__Eubacteriales|f__Oscillospiraceae|g__GGB30303|s__GGB30303_SGB43268

k__Bacteria|p__Firmicutes|c__CFGB30328|o__OFGB30328|f__FGB30328|g__GGB30413|s__GGB30413_SGB43452
k__Bacteria|p__Firmicutes|c__CFGB30328|o__OFGB30328|f__FGB30328|g__GGB30413|s__GGB30413_SGB43452
k__Bacteria|p__Firmicutes|c__Clostridia|o__Eubacteriales|f__Oscillospiraceae|g__GGB30454|s__GGB30454_SGB43514
k__Bacteria|p__Firmicutes|c__Clostridia|o__Eubacteriales|f__Oscillospiraceae|g__GGB30454|s__GGB30454_SGB43514
k__Bacteria|p__Firmicutes|c__Clostridia|o__Eubacteriales|f__Oscillospiraceae|g__GGB30455|s__GGB30455_SGB43519
k__Bacteria|p__Firmicutes|c__Clostridia|o__Eubacteriales|f__Oscillospiraceae|g__GGB30455|s__GGB30455_SGB43519
k__Bacteria|p__Firmicutes|c__Clostridia|o__Eubacteriales|f__Oscillospiraceae|g__GGB30461|s__GGB30461_SGB43527
k__Bacteria|p__Firmicutes|c__Clostridia|o__Eubacteriales|f__Oscillospiraceae|g__GGB30461|s__GGB30461_SGB43527
k__Bacteria|p__Firmicutes|c__Clostridia|o__Eubacteriales|f__Oscillospiraceae|g__GGB30461|s__GGB30461_SGB43530
k__Bacteria|p__Firmicutes|c__Clostridia|o__Eubacteriales|f__Oscillospiraceae|g__GGB30461|s__GGB30461_SGB43530
k__Bacteria|p__Firmicutes|c__Clostridia|o__Eubacteriales|f__Oscillospiraceae|g__GGB30463|s__GGB30463_SGB43537
k__Bacteria|p__Firmicutes|c__Clostridia|o__Eubacteriales|f__Oscillospiraceae|g__GGB30463|s__GGB30463_SGB43537
k__Bacteria|p__Firmicutes|c__Clostridia|o__Eubacteriales|f__Oscillospiraceae|g__GGB30473|s__GGB30473_SGB43557
k__Bacteria|p__Firmicutes|c__Clostridia|o__Eubacteriales|f__Oscillospiraceae|g__GGB30473|s__GGB30473_SGB43557
k__Bacteria|p__Firmicutes|c__Clostridia|o__Eubacteriales|f__Oscillospiraceae|g__GGB30475|s__GGB30475_SGB63182
k__Bacteria|p__Firmicutes|c__Clostridia|o__Eubacteriales|f__Oscillospiraceae|g__GGB30475|s__GGB30475_SGB63182
k__Bacteria|p__Actinobacteria|c__CFGB77153|o__OFGB77153|f__FGB77153|g__GGB30861|s__GGB30861_SGB44083
k__Bacteria|p__Actinobacteria|c__CFGB77153|o__OFGB77153|f__FGB77153|g__GGB30861|s__GGB30861_SGB44083
k__Bacteria|p__Tenericutes|c__CFGB1791|o__OFGB1791|f__FGB1791|g__GGB31312|s__GGB31312_SGB44628
k__Bacteria|p__Tenericutes|c__CFGB1791|o__OFGB1791|f__FGB1791|g__GGB31312|s__GGB31312_SGB44628
k__Bacteria|p__Firmicutes|c__CFGB10290|o__OFGB10290|f__FGB10290|g__GGB31438|s__GGB31438_SGB44768
k__Bacteria|p__Firmicutes|c__CFGB10290|o__OFGB10290|f__FGB10290|g__GGB31438|s__GGB31438_SGB44768
k__Bacteria|p__Firmicutes|c__Clostridia|o__Eubacteriales|f__Oscillospiraceae|g__GGB3171|s__GGB3171_SGB4185
k__Bacteria|p__Firmicutes|c__Clostridia|o__Eubacteriales|f__Oscillospiraceae|g__GGB3171|s__GGB3171_SGB4185
k__Bacteria|p__Firmicutes|c__CFGB1765|o__OFGB1765|f__FGB1765|g__GGB31823|s__GGB31823_SGB45199
k__Bacteria|p__Firmicutes|c__CFGB1765|o__OFGB1765|f__FGB1765|g__GGB31823|s__GGB31823_SGB45199
k__Bacteria|p__Firmicutes|c__CFGB10349|o__OFGB10349|f__FGB10349|g__GGB31853|s__GGB31853_SGB45233
k__Bacteria|p__Firmicutes|c__CFGB10349|o__OFGB10349|f__FGB10349|g__GGB31853|s__GGB31853_SGB45233
k__Bacteria|p__Firmicutes|c__CFGB10667|o__OFGB10667|f__FGB10667|g__GGB32371|s__GGB32371_SGB41694
k__Bacteria|p__Firmicutes|c__CFGB10667|o__OFGB10667|f__FGB10667|g__GGB32371|s__GGB32371_SGB41694
k__Bacteria|p__Firmicutes|c__Clostridia|o__Eubacteriales|f__Lachnospiraceae|g__GGB3793|s__GGB3793_SGB5158
k__Bacteria|p__Firmicutes|c__Clostridia|o__Eubacteriales|f__Lachnospiraceae|g__GGB3793|s__GGB3793_SGB5158
k__Bacteria|p__Firmicutes|c__Clostridia|o__Eubacteriales|f__Lachnospiraceae|g__GGB42598|s__GGB42598_SGB59794
k__Bacteria|p__Firmicutes|c__Clostridia|o__Eubacteriales|f__Lachnospiraceae|g__GGB42598|s__GGB42598_SGB59794
k__Bacteria|p__Firmicutes|c__Clostridia|o__Eubacteriales|f__Christensenellaceae|g__GGB45656|s__GGB45656_SGB6337[illegible]
k__Bacteria|p__Firmicutes|c__Clostridia|o__Eubacteriales|f__Christensenellaceae|g__GGB45656|s__GGB45656_SGB6337[illegible]
k__Bacteria|p__Firmicutes|c__CFGB10299|o__OFGB10299|f__FGB10299|g__GGB47127|s__GGB47127_SGB65054
k__Bacteria|p__Firmicutes|c__CFGB10299|o__OFGB10299|f__FGB10299|g__GGB47127|s__GGB47127_SGB65054
k__Bacteria|p__Firmicutes|c__Clostridia|o__Eubacteriales|f__Oscillospiraceae|g__GGB74395|s__GGB74395_SGB43521
k__Bacteria|p__Firmicutes|c__Clostridia|o__Eubacteriales|f__Oscillospiraceae|g__GGB74395|s__GGB74395_SGB43521
k__Bacteria|p__Firmicutes|c__Clostridia|o__Eubacteriales|f__Oscillospiraceae|g__GGB75053|s__GGB75053_SGB43494
k__Bacteria|p__Firmicutes|c__Clostridia|o__Eubacteriales|f__Oscillospiraceae|g__GGB75053|s__GGB75053_SGB43494
k__Bacteria|p__Firmicutes|c__Clostridia|o__Eubacteriales|f__Lachnospiraceae|g__GGB75109|s__GGB75109_SGB102238
k__Bacteria|p__Firmicutes|c__Clostridia|o__Eubacteriales|f__Lachnospiraceae|g__GGB75109|s__GGB75109_SGB102238
k__Bacteria|p__Firmicutes|c__Clostridia|o__Clostridia_unclassified|f__Clostridia_unclassified|g__GGB81440|s__GGB8144[illegible]
k__Bacteria|p__Firmicutes|c__Clostridia|o__Clostridia_unclassified|f__Clostridia_unclassified|g__GGB81440|s__GGB8144[illegible]

k__Bacteria|p__Firmicutes|c__Clostridia|o__Eubacteriales|f__Lachnospiraceae|g__Lachnospiraceae_unclassified|s__Lachn
k__Bacteria|p__Firmicutes|c__Clostridia|o__Eubacteriales|f__Lachnospiraceae|g__Lachnospiraceae_unclassified|s__Lachn
k__Bacteria|p__Firmicutes|c__Clostridia|o__Eubacteriales|f__Lachnospiraceae|g__Lachnospiraceae_unclassified|s__Lachn
k__Bacteria|p__Firmicutes|c__Clostridia|o__Eubacteriales|f__Lachnospiraceae|g__Lachnospiraceae_unclassified|s__Lachn
k__Bacteria|p__Firmicutes|c__Clostridia|o__Eubacteriales|f__Lachnospiraceae|g__Lachnospiraceae_unclassified|s__Lachn
k__Bacteria|p__Firmicutes|c__Clostridia|o__Eubacteriales|f__Lachnospiraceae|g__Lachnospiraceae_unclassified|s__Lachn
k__Bacteria|p__Firmicutes|c__Clostridia|o__Eubacteriales|f__Lachnospiraceae|g__Lachnospiraceae_unclassified|s__Lachn
k__Bacteria|p__Firmicutes|c__Clostridia|o__Eubacteriales|f__Lachnospiraceae|g__Lachnospiraceae_unclassified|s__Lachn
k__Bacteria|p__Firmicutes|c__Clostridia|o__Eubacteriales|f__Lachnospiraceae|g__Lachnospiraceae_unclassified|s__Lachn
k__Bacteria|p__Firmicutes|c__Clostridia|o__Eubacteriales|f__Lachnospiraceae|g__Lachnospiraceae_unclassified|s__Lachn
k__Bacteria|p__Firmicutes|c__Clostridia|o__Eubacteriales|f__Lachnospiraceae|g__Lachnospiraceae_unclassified|s__Lachn
k__Bacteria|p__Firmicutes|c__Clostridia|o__Eubacteriales|f__Lachnospiraceae|g__Lachnospiraceae_unclassified|s__Lachn
k__Bacteria|p__Firmicutes|c__Clostridia|o__Eubacteriales|f__Lachnospiraceae|g__Lachnospiraceae_unclassified|s__Lachn
k__Bacteria|p__Firmicutes|c__Clostridia|o__Eubacteriales|f__Lachnospiraceae|g__Lachnospiraceae_unclassified|s__Lachn
k__Bacteria|p__Firmicutes|c__Clostridia|o__Eubacteriales|f__Lachnospiraceae|g__Lachnospiraceae_unclassified|s__Lachn
k__Bacteria|p__Firmicutes|c__Clostridia|o__Eubacteriales|f__Lachnospiraceae|g__Lachnospiraceae_unclassified|s__Lachn
k__Bacteria|p__Firmicutes|c__Bacilli|o__Lactobacillales|f__Lactobacillaceae|g__Lactobacillus|s__Lactobacillus_johnsonii
k__Bacteria|p__Firmicutes|c__Bacilli|o__Lactobacillales|f__Lactobacillaceae|g__Lactobacillus|s__Lactobacillus_johnsonii
k__Bacteria|p__Bacteroidota|c__Bacteroidia|o__Bacteroidales|f__Muribaculaceae|g__Muribaculaceae_unclassified|s__M
k__Bacteria|p__Bacteroidota|c__Bacteroidia|o__Bacteroidales|f__Muribaculaceae|g__Muribaculaceae_unclassified|s__M
k__Bacteria|p__Firmicutes|c__Clostridia|o__Eubacteriales|f__Oscillospiraceae|g__Neglectibacter|s__Neglectibacter_sp_X4
k__Bacteria|p__Firmicutes|c__Clostridia|o__Eubacteriales|f__Oscillospiraceae|g__Neglectibacter|s__Neglectibacter_sp_X4
k__Bacteria|p__Firmicutes|c__Clostridia|o__Eubacteriales|f__Oscillospiraceae|g__Oscillospiraceae_unclassified|s__Oscillo
k__Bacteria|p__Firmicutes|c__Clostridia|o__Eubacteriales|f__Oscillospiraceae|g__Oscillospiraceae_unclassified|s__Oscillo
k__Bacteria|p__Firmicutes|c__Clostridia|o__Eubacteriales|f__Oscillospiraceae|g__Oscillospiraceae_unclassified|s__Oscillo
k__Bacteria|p__Firmicutes|c__Clostridia|o__Eubacteriales|f__Oscillospiraceae|g__Oscillospiraceae_unclassified|s__Oscillo
k__Bacteria|p__Firmicutes|c__Clostridia|o__Eubacteriales|f__Oscillospiraceae|g__Oscillospiraceae_unclassified|s__Oscillo
k__Bacteria|p__Firmicutes|c__Clostridia|o__Eubacteriales|f__Oscillospiraceae|g__Oscillospiraceae_unclassified|s__Oscillo
k__Bacteria|p__Proteobacteria|c__Betaproteobacteria|o__Burkholderiales|f__Sutterellaceae|g__Parasutterella|s__Parasu
k__Bacteria|p__Proteobacteria|c__Betaproteobacteria|o__Burkholderiales|f__Sutterellaceae|g__Parasutterella|s__Parasu

k__Bacteria|p__Firmicutes|c__Clostridia|o__Eubacteriales|f__Peptostreptococcaceae|g__Romboutsia|s__Romboutsia_ilea
k__Bacteria|p__Firmicutes|c__Clostridia|o__Eubacteriales|f__Peptostreptococcaceae|g__Romboutsia|s__Romboutsia_ilea
k__Bacteria|p__Firmicutes|c__Clostridia|o__Eubacteriales|f__Lachnospiraceae|g__Schaedlerella|s__Schaedlerella_arabino
k__Bacteria|p__Firmicutes|c__Clostridia|o__Eubacteriales|f__Lachnospiraceae|g__Schaedlerella|s__Schaedlerella_arabino

k__Bacteria|p__Firmicutes|c__Erysipelotrichia|o__Erysipelotrichales|f__Turicibacteraceae|g__Turicibacter|s__Turicibacter_
k__Bacteria|p__Firmicutes|c__Erysipelotrichia|o__Erysipelotrichales|f__Turicibacteraceae|g__Turicibacter|s__Turicibacter_
k__Bacteria|p__Firmicutes|c__Clostridia|o__Eubacteriales|f__Lachnospiraceae|g__Acetatifactor|s__Acetatifactor_muris
k__Bacteria|p__Firmicutes|c__Clostridia|o__Eubacteriales|f__Lachnospiraceae|g__Acetatifactor|s__Acetatifactor_muris
k__Bacteria|p__Firmicutes|c__Clostridia|o__Eubacteriales|f__Lachnospiraceae|g__Acetatifactor|s__Acetatifactor_SGB415
k__Bacteria|p__Firmicutes|c__Clostridia|o__Eubacteriales|f__Lachnospiraceae|g__Acetatifactor|s__Acetatifactor_SGB415
k__Bacteria|p__Firmicutes|c__Clostridia|o__Eubacteriales|f__Oscillospiraceae|g__Acutalibacter|s__Acutalibacter_muris
k__Bacteria|p__Firmicutes|c__Clostridia|o__Eubacteriales|f__Oscillospiraceae|g__Acutalibacter|s__Acutalibacter_muris

k__Bacteria|p__Firmicutes|c__Clostridia|o__Eubacteriales|f__Oscillospiraceae|g__Acutalibacter|s__Acutalibacter_sp_1XD8
k__Bacteria|p__Firmicutes|c__Clostridia|o__Eubacteriales|f__Oscillospiraceae|g__Acutalibacter|s__Acutalibacter_sp_1XD8
k__Bacteria|p__Actinobacteria|c__Coriobacteriia|o__Eggerthellales|f__Eggerthellaceae|g__Adlercreutzia|s__Adlercreutzia
k__Bacteria|p__Actinobacteria|c__Coriobacteriia|o__Eggerthellales|f__Eggerthellaceae|g__Adlercreutzia|s__Adlercreutzia
k__Bacteria|p__Actinobacteria|c__Coriobacteriia|o__Eggerthellales|f__Eggerthellaceae|g__Adlercreutzia|s__Adlercreutzia
k__Bacteria|p__Actinobacteria|c__Coriobacteriia|o__Eggerthellales|f__Eggerthellaceae|g__Adlercreutzia|s__Adlercreutzia
k__Bacteria|p__Actinobacteria|c__Coriobacteriia|o__Eggerthellales|f__Eggerthellaceae|g__Adlercreutzia|s__Adlercreutzia
k__Bacteria|p__Actinobacteria|c__Coriobacteriia|o__Eggerthellales|f__Eggerthellaceae|g__Adlercreutzia|s__Adlercreutzia
k__Bacteria|p__Verrucomicrobia|c__Verrucomicrobiae|o__Verrucomicrobiales|f__Akkermansiaceae|g__Akkermansia|s__A
k__Bacteria|p__Verrucomicrobia|c__Verrucomicrobiae|o__Verrucomicrobiales|f__Akkermansiaceae|g__Akkermansia|s__A
k__Bacteria|p__Bacteroidota|c__Bacteroidia|o__Bacteroidales|f__Rikenellaceae|g__Alistipes|s__Alistipes_sp_DSM_11234
k__Bacteria|p__Bacteroidota|c__Bacteroidia|o__Bacteroidales|f__Rikenellaceae|g__Alistipes|s__Alistipes_sp_DSM_11234
k__Bacteria|p__Firmicutes|c__Clostridia|o__Eubacteriales|f__Oscillospiraceae|g__Anaerotruncus|s__Anaerotruncus_sp_1
k__Bacteria|p__Firmicutes|c__Clostridia|o__Eubacteriales|f__Oscillospiraceae|g__Anaerotruncus|s__Anaerotruncus_sp_1
k__Bacteria|p__Bacteria_unclassified|c__Bacteria_unclassified|o__Bacteria_unclassified|f__Bacteria_unclassified|g__Bacte
k__Bacteria|p__Bacteria_unclassified|c__Bacteria_unclassified|o__Bacteria_unclassified|f__Bacteria_unclassified|g__Bacte
k__Bacteria|p__Bacteria_unclassified|c__Bacteria_unclassified|o__Bacteria_unclassified|f__Bacteria_unclassified|g__Bacte
k__Bacteria|p__Bacteria_unclassified|c__Bacteria_unclassified|o__Bacteria_unclassified|f__Bacteria_unclassified|g__Bacte
k__Bacteria|p__Bacteria_unclassified|c__Bacteria_unclassified|o__Bacteria_unclassified|f__Bacteria_unclassified|g__Bacte
k__Bacteria|p__Bacteria_unclassified|c__Bacteria_unclassified|o__Bacteria_unclassified|f__Bacteria_unclassified|g__Bacte
k__Bacteria|p__Bacteria_unclassified|c__Bacteria_unclassified|o__Bacteria_unclassified|f__Bacteria_unclassified|g__Bacte
k__Bacteria|p__Bacteria_unclassified|c__Bacteria_unclassified|o__Bacteria_unclassified|f__Bacteria_unclassified|g__Bacte
k__Bacteria|p__Bacteria_unclassified|c__Bacteria_unclassified|o__Bacteria_unclassified|f__Bacteria_unclassified|g__Bacte
k__Bacteria|p__Bacteria_unclassified|c__Bacteria_unclassified|o__Bacteria_unclassified|f__Bacteria_unclassified|g__Bacte
k__Bacteria|p__Bacteria_unclassified|c__Bacteria_unclassified|o__Bacteria_unclassified|f__Bacteria_unclassified|g__Bacte
k__Bacteria|p__Bacteria_unclassified|c__Bacteria_unclassified|o__Bacteria_unclassified|f__Bacteria_unclassified|g__Bacte
k__Bacteria|p__Bacteroidota|c__Bacteroidia|o__Bacteroidales|f__Bacteroidaceae|g__Bacteroides|s__Bacteroides_thetaio
k__Bacteria|p__Bacteroidota|c__Bacteroidia|o__Bacteroidales|f__Bacteroidaceae|g__Bacteroides|s__Bacteroides_thetaio

k__Bacteria|p__Actinobacteria|c__Actinomycetia|o__Bifidobacteriales|f__Bifidobacteriaceae|g__Bifidobacterium|s__Bifid
k__Bacteria|p__Actinobacteria|c__Actinomycetia|o__Bifidobacteriales|f__Bifidobacteriaceae|g__Bifidobacterium|s__Bifid
k__Bacteria|p__Firmicutes|c__Clostridia|o__Clostridia_unclassified|f__Clostridia_unclassified|g__Clostridia_unclassified|s_
k__Bacteria|p__Firmicutes|c__Clostridia|o__Clostridia_unclassified|f__Clostridia_unclassified|g__Clostridia_unclassified|s_
k__Bacteria|p__Firmicutes|c__Clostridia|o__Eubacteriales|f__Clostridiaceae|g__Clostridiaceae_unclassified|s__Clostridiac
k__Bacteria|p__Firmicutes|c__Clostridia|o__Eubacteriales|f__Clostridiaceae|g__Clostridiaceae_unclassified|s__Clostridiac
k__Bacteria|p__Firmicutes|c__Clostridia|o__Eubacteriales|f__Clostridiaceae|g__Clostridiaceae_unclassified|s__Clostridiac
k__Bacteria|p__Firmicutes|c__Clostridia|o__Eubacteriales|f__Clostridiaceae|g__Clostridiaceae_unclassified|s__Clostridiac
k__Bacteria|p__Firmicutes|c__Clostridia|o__Eubacteriales|f__Eubacteriales_unclassified|g__Eubacteriales_unclassified|s_
k__Bacteria|p__Firmicutes|c__Clostridia|o__Eubacteriales|f__Eubacteriales_unclassified|g__Eubacteriales_unclassified|s_
k__Bacteria|p__Firmicutes|c__Erysipelotrichia|o__Erysipelotrichales|f__Erysipelotrichaceae|g__Erysipelatoclostridium|s_
k__Bacteria|p__Firmicutes|c__Erysipelotrichia|o__Erysipelotrichales|f__Erysipelotrichaceae|g__Erysipelatoclostridium|s_
k__Bacteria|p__Actinobacteria|c__Coriobacteriia|o__Coriobacteriales|f__Coriobacteriaceae|g__Coriobacteriaceae_unclass
k__Bacteria|p__Actinobacteria|c__Coriobacteriia|o__Coriobacteriales|f__Coriobacteriaceae|g__Coriobacteriaceae_unclass
k__Bacteria|p__Firmicutes|c__Clostridia|o__Eubacteriales|f__Lachnospiraceae|g__Dorea|s__Dorea_sp_5_2
k__Bacteria|p__Firmicutes|c__Clostridia|o__Eubacteriales|f__Lachnospiraceae|g__Dorea|s__Dorea_sp_5_2

k__Bacteria|p__Firmicutes|c__Erysipelotrichia|o__Erysipelotrichales|f__Erysipelotrichaceae|g__Dubosiella|s__Dubosiella_
k__Bacteria|p__Firmicutes|c__Erysipelotrichia|o__Erysipelotrichales|f__Erysipelotrichaceae|g__Dubosiella|s__Dubosiella_
k__Bacteria|p__Firmicutes|c__Erysipelotrichia|o__Erysipelotrichales|f__Erysipelotrichales_unclassified|g__Erysipelotrichal
k__Bacteria|p__Firmicutes|c__Erysipelotrichia|o__Erysipelotrichales|f__Erysipelotrichales_unclassified|g__Erysipelotrichal
k__Bacteria|p__Firmicutes|c__Clostridia|o__Eubacteriales|f__Eubacteriaceae|g__Eubacteriaceae_unclassified|s__Eubacte
k__Bacteria|p__Firmicutes|c__Clostridia|o__Eubacteriales|f__Eubacteriaceae|g__Eubacteriaceae_unclassified|s__Eubacte
k__Bacteria|p__Firmicutes|c__Clostridia|o__Eubacteriales|f__Eubacteriaceae|g__Eubacteriaceae_unclassified|s__Eubacte
k__Bacteria|p__Firmicutes|c__Clostridia|o__Eubacteriales|f__Eubacteriaceae|g__Eubacteriaceae_unclassified|s__Eubacte
k__Bacteria|p__Firmicutes|c__Clostridia|o__Eubacteriales|f__Lachnospiraceae|g__GGB20149|s__GGB20149_SGB29430
k__Bacteria|p__Firmicutes|c__Clostridia|o__Eubacteriales|f__Lachnospiraceae|g__GGB20149|s__GGB20149_SGB29430
k__Bacteria|p__Actinobacteria|c__Coriobacteriia|o__Eggerthellales|f__Eggerthellaceae|g__GGB22635|s__GGB22635_SGB
k__Bacteria|p__Actinobacteria|c__Coriobacteriia|o__Eggerthellales|f__Eggerthellaceae|g__GGB22635|s__GGB22635_SGB
k__Bacteria|p__Firmicutes|c__Clostridia|o__Eubacteriales|f__Lachnospiraceae|g__GGB25041|s__GGB25041_SGB36960
k__Bacteria|p__Firmicutes|c__Clostridia|o__Eubacteriales|f__Lachnospiraceae|g__GGB25041|s__GGB25041_SGB36960
k__Bacteria|p__Bacteroidota|c__Bacteroidia|o__Bacteroidales|f__Muribaculaceae|g__GGB27876|s__GGB27876_SGB4031
k__Bacteria|p__Bacteroidota|c__Bacteroidia|o__Bacteroidales|f__Muribaculaceae|g__GGB27876|s__GGB27876_SGB4031
k__Bacteria|p__Bacteroidota|c__Bacteroidia|o__Bacteroidales|f__Muribaculaceae|g__GGB27878|s__GGB27878_SGB4031
k__Bacteria|p__Bacteroidota|c__Bacteroidia|o__Bacteroidales|f__Muribaculaceae|g__GGB27878|s__GGB27878_SGB4031
k__Bacteria|p__Bacteroidota|c__Bacteroidia|o__Bacteroidales|f__Muribaculaceae|g__GGB27918|s__GGB27918_SGB4035
k__Bacteria|p__Bacteroidota|c__Bacteroidia|o__Bacteroidales|f__Muribaculaceae|g__GGB27918|s__GGB27918_SGB4035
k__Bacteria|p__Firmicutes|c__CFGB9508|o__OFGB9508|f__FGB9508|g__GGB28382|s__GGB28382_SGB40962
k__Bacteria|p__Firmicutes|c__CFGB9508|o__OFGB9508|f__FGB9508|g__GGB28382|s__GGB28382_SGB40962
k__Bacteria|p__Firmicutes|c__CFGB2838|o__OFGB2838|f__FGB2838|g__GGB28399|s__GGB28399_SGB40980
k__Bacteria|p__Firmicutes|c__CFGB2838|o__OFGB2838|f__FGB2838|g__GGB28399|s__GGB28399_SGB40980
k__Bacteria|p__Firmicutes|c__CFGB2838|o__OFGB2838|f__FGB2838|g__GGB28411|s__GGB28411_SGB40993
k__Bacteria|p__Firmicutes|c__CFGB2838|o__OFGB2838|f__FGB2838|g__GGB28411|s__GGB28411_SGB40993
k__Bacteria|p__Firmicutes|c__CFGB2838|o__OFGB2838|f__FGB2838|g__GGB28415|s__GGB28415_SGB40997
k__Bacteria|p__Firmicutes|c__CFGB2838|o__OFGB2838|f__FGB2838|g__GGB28415|s__GGB28415_SGB40997
k__Bacteria|p__Firmicutes|c__Clostridia|o__Eubacteriales|f__Pumilibacteraceae|g__GGB28430|s__GGB28430_SGB41013
k__Bacteria|p__Firmicutes|c__Clostridia|o__Eubacteriales|f__Pumilibacteraceae|g__GGB28430|s__GGB28430_SGB41013
k__Bacteria|p__Firmicutes|c__CFGB28439|o__OFGB28439|f__FGB28439|g__GGB28439|s__GGB28439_SGB41022
k__Bacteria|p__Firmicutes|c__CFGB28439|o__OFGB28439|f__FGB28439|g__GGB28439|s__GGB28439_SGB41022
k__Bacteria|p__Firmicutes|c__Clostridia|o__Clostridia_unclassified|f__Clostridia_unclassified|g__GGB28778|s__GGB2877
k__Bacteria|p__Firmicutes|c__Clostridia|o__Clostridia_unclassified|f__Clostridia_unclassified|g__GGB28778|s__GGB2877
k__Bacteria|p__Firmicutes|c__Clostridia|o__Eubacteriales|f__Eubacteriaceae|g__GGB28784|s__GGB28784_SGB41437
k__Bacteria|p__Firmicutes|c__Clostridia|o__Eubacteriales|f__Eubacteriaceae|g__GGB28784|s__GGB28784_SGB41437
k__Bacteria|p__Firmicutes|c__Clostridia|o__Eubacteriales|f__Lachnospiraceae|g__GGB28792|s__GGB28792_SGB41445
k__Bacteria|p__Firmicutes|c__Clostridia|o__Eubacteriales|f__Lachnospiraceae|g__GGB28792|s__GGB28792_SGB41445
k__Bacteria|p__Firmicutes|c__Clostridia|o__Eubacteriales|f__Lachnospiraceae|g__GGB28798|s__GGB28798_SGB41451
k__Bacteria|p__Firmicutes|c__Clostridia|o__Eubacteriales|f__Lachnospiraceae|g__GGB28798|s__GGB28798_SGB41451
k__Bacteria|p__Firmicutes|c__Clostridia|o__Eubacteriales|f__Lachnospiraceae|g__GGB28802|s__GGB28802_SGB41455
k__Bacteria|p__Firmicutes|c__Clostridia|o__Eubacteriales|f__Lachnospiraceae|g__GGB28802|s__GGB28802_SGB41455
k__Bacteria|p__Firmicutes|c__Clostridia|o__Eubacteriales|f__Lachnospiraceae|g__GGB28818|s__GGB28818_SGB41473
k__Bacteria|p__Firmicutes|c__Clostridia|o__Eubacteriales|f__Lachnospiraceae|g__GGB28818|s__GGB28818_SGB41473
k__Bacteria|p__Firmicutes|c__CFGB77305|o__OFGB77305|f__FGB77305|g__GGB28828|s__GGB28828_SGB41484
k__Bacteria|p__Firmicutes|c__CFGB77305|o__OFGB77305|f__FGB77305|g__GGB28828|s__GGB28828_SGB41484

k__Bacteria|p__Firmicutes|c__Clostridia|o__Eubacteriales|f__Clostridiaceae|g__GGB28851|s__GGB28851_SGB41518
k__Bacteria|p__Firmicutes|c__Clostridia|o__Eubacteriales|f__Clostridiaceae|g__GGB28851|s__GGB28851_SGB41518
k__Bacteria|p__Firmicutes|c__Clostridia|o__Eubacteriales|f__Lachnospiraceae|g__GGB28859|s__GGB28859_SGB41528
k__Bacteria|p__Firmicutes|c__Clostridia|o__Eubacteriales|f__Lachnospiraceae|g__GGB28859|s__GGB28859_SGB41528
k__Bacteria|p__Firmicutes|c__Clostridia|o__Eubacteriales|f__Lachnospiraceae|g__GGB28864|s__GGB28864_SGB41535
k__Bacteria|p__Firmicutes|c__Clostridia|o__Eubacteriales|f__Lachnospiraceae|g__GGB28864|s__GGB28864_SGB41535
k__Bacteria|p__Firmicutes|c__Clostridia|o__Eubacteriales|f__Lachnospiraceae|g__GGB28869|s__GGB28869_SGB41543
k__Bacteria|p__Firmicutes|c__Clostridia|o__Eubacteriales|f__Lachnospiraceae|g__GGB28869|s__GGB28869_SGB41543
k__Bacteria|p__Firmicutes|c__CFGB9633|o__OFGB9633|f__FGB9633|g__GGB28883|s__GGB28883_SGB41564
k__Bacteria|p__Firmicutes|c__CFGB9633|o__OFGB9633|f__FGB9633|g__GGB28883|s__GGB28883_SGB41564
k__Bacteria|p__Bacteria_unclassified|c__Bacteria_unclassified|o__Bacteria_unclassified|f__Bacteria_unclassified|g__GGB2
k__Bacteria|p__Bacteria_unclassified|c__Bacteria_unclassified|o__Bacteria_unclassified|f__Bacteria_unclassified|g__GGB2
k__Bacteria|p__Bacteria_unclassified|c__Bacteria_unclassified|o__Bacteria_unclassified|f__Bacteria_unclassified|g__GGB2
k__Bacteria|p__Bacteria_unclassified|c__Bacteria_unclassified|o__Bacteria_unclassified|f__Bacteria_unclassified|g__GGB2
k__Bacteria|p__Bacteria_unclassified|c__Bacteria_unclassified|o__Bacteria_unclassified|f__Bacteria_unclassified|g__GGB2
k__Bacteria|p__Bacteria_unclassified|c__Bacteria_unclassified|o__Bacteria_unclassified|f__Bacteria_unclassified|g__GGB2
k__Bacteria|p__Bacteria_unclassified|c__Bacteria_unclassified|o__Bacteria_unclassified|f__Bacteria_unclassified|g__GGB2
k__Bacteria|p__Bacteria_unclassified|c__Bacteria_unclassified|o__Bacteria_unclassified|f__Bacteria_unclassified|g__GGB2
k__Bacteria|p__Firmicutes|c__Clostridia|o__Eubacteriales|f__Lachnospiraceae|g__GGB28916|s__GGB28916_SGB41612
k__Bacteria|p__Firmicutes|c__Clostridia|o__Eubacteriales|f__Lachnospiraceae|g__GGB28916|s__GGB28916_SGB41612
k__Bacteria|p__Firmicutes|c__Clostridia|o__Eubacteriales|f__Lachnospiraceae|g__GGB28924|s__GGB28924_SGB41621
k__Bacteria|p__Firmicutes|c__Clostridia|o__Eubacteriales|f__Lachnospiraceae|g__GGB28924|s__GGB28924_SGB41621
k__Bacteria|p__Firmicutes|c__Clostridia|o__Eubacteriales|f__Lachnospiraceae|g__GGB28926|s__GGB28926_SGB41624
k__Bacteria|p__Firmicutes|c__Clostridia|o__Eubacteriales|f__Lachnospiraceae|g__GGB28926|s__GGB28926_SGB41624
k__Bacteria|p__Bacteria_unclassified|c__CFGB77359|o__OFGB77359|f__FGB77359|g__GGB28927|s__GGB28927_SGB416
k__Bacteria|p__Bacteria_unclassified|c__CFGB77359|o__OFGB77359|f__FGB77359|g__GGB28927|s__GGB28927_SGB416
k__Bacteria|p__Firmicutes|c__CFGB9639|o__OFGB9639|f__FGB9639|g__GGB28934|s__GGB28934_SGB41635
k__Bacteria|p__Firmicutes|c__CFGB9639|o__OFGB9639|f__FGB9639|g__GGB28934|s__GGB28934_SGB41635
k__Bacteria|p__Firmicutes|c__Clostridia|o__Eubacteriales|f__Lachnospiraceae|g__GGB28946|s__GGB28946_SGB41652
k__Bacteria|p__Firmicutes|c__Clostridia|o__Eubacteriales|f__Lachnospiraceae|g__GGB28946|s__GGB28946_SGB41652
k__Bacteria|p__Firmicutes|c__Clostridia|o__Eubacteriales|f__Lachnospiraceae|g__GGB28949|s__GGB28949_SGB41655
k__Bacteria|p__Firmicutes|c__Clostridia|o__Eubacteriales|f__Lachnospiraceae|g__GGB28949|s__GGB28949_SGB41655
k__Bacteria|p__Firmicutes|c__Clostridia|o__Eubacteriales|f__Lachnospiraceae|g__GGB28949|s__GGB28949_SGB41656
k__Bacteria|p__Firmicutes|c__Clostridia|o__Eubacteriales|f__Lachnospiraceae|g__GGB28949|s__GGB28949_SGB41656
k__Bacteria|p__Firmicutes|c__Clostridia|o__Eubacteriales|f__Clostridiaceae|g__GGB28950|s__GGB28950_SGB41657
k__Bacteria|p__Firmicutes|c__Clostridia|o__Eubacteriales|f__Clostridiaceae|g__GGB28950|s__GGB28950_SGB41657
k__Bacteria|p__Firmicutes|c__Clostridia|o__Eubacteriales|f__Clostridiaceae|g__GGB28951|s__GGB28951_SGB102295
k__Bacteria|p__Firmicutes|c__Clostridia|o__Eubacteriales|f__Clostridiaceae|g__GGB28951|s__GGB28951_SGB102295
k__Bacteria|p__Firmicutes|c__Clostridia|o__Eubacteriales|f__Clostridiaceae|g__GGB28951|s__GGB28951_SGB41658
k__Bacteria|p__Firmicutes|c__Clostridia|o__Eubacteriales|f__Clostridiaceae|g__GGB28951|s__GGB28951_SGB41658
k__Bacteria|p__Firmicutes|c__Clostridia|o__Eubacteriales|f__Clostridiaceae|g__GGB28954|s__GGB28954_SGB41662
k__Bacteria|p__Firmicutes|c__Clostridia|o__Eubacteriales|f__Clostridiaceae|g__GGB28954|s__GGB28954_SGB41662
k__Bacteria|p__Firmicutes|c__Clostridia|o__Eubacteriales|f__Clostridiaceae|g__GGB28956|s__GGB28956_SGB41665
k__Bacteria|p__Firmicutes|c__Clostridia|o__Eubacteriales|f__Clostridiaceae|g__GGB28956|s__GGB28956_SGB41665
k__Bacteria|p__Firmicutes|c__Clostridia|o__Eubacteriales|f__Clostridiaceae|g__GGB28960|s__GGB28960_SGB41669
k__Bacteria|p__Firmicutes|c__Clostridia|o__Eubacteriales|f__Clostridiaceae|g__GGB28960|s__GGB28960_SGB41669

k__Bacteria|p__Firmicutes|c__Clostridia|o__Eubacteriales|f__Clostridiaceae|g__GGB28967|s__GGB28967_SGB41678
k__Bacteria|p__Firmicutes|c__Clostridia|o__Eubacteriales|f__Clostridiaceae|g__GGB28967|s__GGB28967_SGB41678
k__Bacteria|p__Firmicutes|c__Clostridia|o__Eubacteriales|f__Eubacteriaceae|g__GGB28991|s__GGB28991_SGB41705
k__Bacteria|p__Firmicutes|c__Clostridia|o__Eubacteriales|f__Eubacteriaceae|g__GGB28991|s__GGB28991_SGB41705
k__Bacteria|p__Firmicutes|c__CFGB9658|o__OFGB9658|f__FGB9658|g__GGB29002|s__GGB29002_SGB41718
k__Bacteria|p__Firmicutes|c__CFGB9658|o__OFGB9658|f__FGB9658|g__GGB29002|s__GGB29002_SGB41718
k__Bacteria|p__Firmicutes|c__CFGB9659|o__OFGB9659|f__FGB9659|g__GGB29003|s__GGB29003_SGB41719
k__Bacteria|p__Firmicutes|c__CFGB9659|o__OFGB9659|f__FGB9659|g__GGB29003|s__GGB29003_SGB41719
k__Bacteria|p__Bacteria_unclassified|c__Bacteria_unclassified|o__Bacteria_unclassified|f__Bacteria_unclassified|g__GGB[illegible]
k__Bacteria|p__Bacteria_unclassified|c__Bacteria_unclassified|o__Bacteria_unclassified|f__Bacteria_unclassified|g__GGB[illegible]
k__Bacteria|p__Firmicutes|c__CFGB9827|o__OFGB9827|f__FGB9827|g__GGB29531|s__GGB29531_SGB42317
k__Bacteria|p__Firmicutes|c__CFGB9827|o__OFGB9827|f__FGB9827|g__GGB29531|s__GGB29531_SGB42317
k__Bacteria|p__Firmicutes|c__Clostridia|o__Eubacteriales|f__Eubacteriaceae|g__GGB29685|s__GGB29685_SGB42494
k__Bacteria|p__Firmicutes|c__Clostridia|o__Eubacteriales|f__Eubacteriaceae|g__GGB29685|s__GGB29685_SGB42494
k__Bacteria|p__Bacteria_unclassified|c__CFGB77303|o__OFGB77303|f__FGB77303|g__GGB30141|s__GGB30141_SGB43[illegible]
k__Bacteria|p__Bacteria_unclassified|c__CFGB77303|o__OFGB77303|f__FGB77303|g__GGB30141|s__GGB30141_SGB43[illegible]
k__Bacteria|p__Firmicutes|c__Clostridia|o__Eubacteriales|f__Eubacteriales_unclassified|g__GGB30286|s__GGB30286_SG[illegible]
k__Bacteria|p__Firmicutes|c__Clostridia|o__Eubacteriales|f__Eubacteriales_unclassified|g__GGB30286|s__GGB30286_SG[illegible]
k__Bacteria|p__Firmicutes|c__Clostridia|o__Eubacteriales|f__Oscillospiraceae|g__GGB30303|s__GGB30303_SGB43268
k__Bacteria|p__Firmicutes|c__Clostridia|o__Eubacteriales|f__Oscillospiraceae|g__GGB30303|s__GGB30303_SGB43268
k__Bacteria|p__Firmicutes|c__CFGB30328|o__OFGB30328|f__FGB30328|g__GGB30413|s__GGB30413_SGB43452
k__Bacteria|p__Firmicutes|c__CFGB30328|o__OFGB30328|f__FGB30328|g__GGB30413|s__GGB30413_SGB43452
k__Bacteria|p__Firmicutes|c__Clostridia|o__Eubacteriales|f__Oscillospiraceae|g__GGB30454|s__GGB30454_SGB43514
k__Bacteria|p__Firmicutes|c__Clostridia|o__Eubacteriales|f__Oscillospiraceae|g__GGB30454|s__GGB30454_SGB43514
k__Bacteria|p__Firmicutes|c__Clostridia|o__Eubacteriales|f__Oscillospiraceae|g__GGB30455|s__GGB30455_SGB43519
k__Bacteria|p__Firmicutes|c__Clostridia|o__Eubacteriales|f__Oscillospiraceae|g__GGB30455|s__GGB30455_SGB43519
k__Bacteria|p__Firmicutes|c__Clostridia|o__Eubacteriales|f__Oscillospiraceae|g__GGB30461|s__GGB30461_SGB43527
k__Bacteria|p__Firmicutes|c__Clostridia|o__Eubacteriales|f__Oscillospiraceae|g__GGB30461|s__GGB30461_SGB43527
k__Bacteria|p__Firmicutes|c__Clostridia|o__Eubacteriales|f__Oscillospiraceae|g__GGB30461|s__GGB30461_SGB43530
k__Bacteria|p__Firmicutes|c__Clostridia|o__Eubacteriales|f__Oscillospiraceae|g__GGB30461|s__GGB30461_SGB43530
k__Bacteria|p__Firmicutes|c__Clostridia|o__Eubacteriales|f__Oscillospiraceae|g__GGB30463|s__GGB30463_SGB43537
k__Bacteria|p__Firmicutes|c__Clostridia|o__Eubacteriales|f__Oscillospiraceae|g__GGB30463|s__GGB30463_SGB43537
k__Bacteria|p__Firmicutes|c__Clostridia|o__Eubacteriales|f__Oscillospiraceae|g__GGB30473|s__GGB30473_SGB43557
k__Bacteria|p__Firmicutes|c__Clostridia|o__Eubacteriales|f__Oscillospiraceae|g__GGB30473|s__GGB30473_SGB43557
k__Bacteria|p__Firmicutes|c__Clostridia|o__Eubacteriales|f__Oscillospiraceae|g__GGB30475|s__GGB30475_SGB63182
k__Bacteria|p__Firmicutes|c__Clostridia|o__Eubacteriales|f__Oscillospiraceae|g__GGB30475|s__GGB30475_SGB63182
k__Bacteria|p__Actinobacteria|c__CFGB77153|o__OFGB77153|f__FGB77153|g__GGB30861|s__GGB30861_SGB44083
k__Bacteria|p__Actinobacteria|c__CFGB77153|o__OFGB77153|f__FGB77153|g__GGB30861|s__GGB30861_SGB44083
k__Bacteria|p__Tenericutes|c__CFGB1791|o__OFGB1791|f__FGB1791|g__GGB31312|s__GGB31312_SGB44628
k__Bacteria|p__Tenericutes|c__CFGB1791|o__OFGB1791|f__FGB1791|g__GGB31312|s__GGB31312_SGB44628
k__Bacteria|p__Firmicutes|c__CFGB10290|o__OFGB10290|f__FGB10290|g__GGB31438|s__GGB31438_SGB44768
k__Bacteria|p__Firmicutes|c__CFGB10290|o__OFGB10290|f__FGB10290|g__GGB31438|s__GGB31438_SGB44768
k__Bacteria|p__Firmicutes|c__Clostridia|o__Eubacteriales|f__Oscillospiraceae|g__GGB3171|s__GGB3171_SGB4185
k__Bacteria|p__Firmicutes|c__Clostridia|o__Eubacteriales|f__Oscillospiraceae|g__GGB3171|s__GGB3171_SGB4185
k__Bacteria|p__Firmicutes|c__CFGB1765|o__OFGB1765|f__FGB1765|g__GGB31823|s__GGB31823_SGB45199
k__Bacteria|p__Firmicutes|c__CFGB1765|o__OFGB1765|f__FGB1765|g__GGB31823|s__GGB31823_SGB45199

k__Bacteria|p__Firmicutes|c__CFGB10349|o__OFGB10349|f__FGB10349|g__GGB31853|s__GGB31853_SGB45233
k__Bacteria|p__Firmicutes|c__CFGB10349|o__OFGB10349|f__FGB10349|g__GGB31853|s__GGB31853_SGB45233
k__Bacteria|p__Firmicutes|c__CFGB10667|o__OFGB10667|f__FGB10667|g__GGB32371|s__GGB32371_SGB41694
k__Bacteria|p__Firmicutes|c__CFGB10667|o__OFGB10667|f__FGB10667|g__GGB32371|s__GGB32371_SGB41694
k__Bacteria|p__Firmicutes|c__Clostridia|o__Eubacteriales|f__Lachnospiraceae|g__GGB3793|s__GGB3793_SGB5158
k__Bacteria|p__Firmicutes|c__Clostridia|o__Eubacteriales|f__Lachnospiraceae|g__GGB3793|s__GGB3793_SGB5158
k__Bacteria|p__Firmicutes|c__Clostridia|o__Eubacteriales|f__Lachnospiraceae|g__GGB42598|s__GGB42598_SGB59794
k__Bacteria|p__Firmicutes|c__Clostridia|o__Eubacteriales|f__Lachnospiraceae|g__GGB42598|s__GGB42598_SGB59794
k__Bacteria|p__Firmicutes|c__Clostridia|o__Eubacteriales|f__Christensenellaceae|g__GGB45656|s__GGB45656_SGB6337
k__Bacteria|p__Firmicutes|c__Clostridia|o__Eubacteriales|f__Christensenellaceae|g__GGB45656|s__GGB45656_SGB6337
k__Bacteria|p__Firmicutes|c__CFGB10299|o__OFGB10299|f__FGB10299|g__GGB47127|s__GGB47127_SGB65054
k__Bacteria|p__Firmicutes|c__CFGB10299|o__OFGB10299|f__FGB10299|g__GGB47127|s__GGB47127_SGB65054
k__Bacteria|p__Firmicutes|c__Clostridia|o__Eubacteriales|f__Oscillospiraceae|g__GGB74395|s__GGB74395_SGB43521
k__Bacteria|p__Firmicutes|c__Clostridia|o__Eubacteriales|f__Oscillospiraceae|g__GGB74395|s__GGB74395_SGB43521
k__Bacteria|p__Firmicutes|c__Clostridia|o__Eubacteriales|f__Oscillospiraceae|g__GGB75053|s__GGB75053_SGB43494
k__Bacteria|p__Firmicutes|c__Clostridia|o__Eubacteriales|f__Oscillospiraceae|g__GGB75053|s__GGB75053_SGB43494
k__Bacteria|p__Firmicutes|c__Clostridia|o__Eubacteriales|f__Lachnospiraceae|g__GGB75109|s__GGB75109_SGB102238
k__Bacteria|p__Firmicutes|c__Clostridia|o__Eubacteriales|f__Lachnospiraceae|g__GGB75109|s__GGB75109_SGB102238
k__Bacteria|p__Firmicutes|c__Clostridia|o__Clostridia_unclassified|f__Clostridia_unclassified|g__GGB81440|s__GGB8144
k__Bacteria|p__Firmicutes|c__Clostridia|o__Clostridia_unclassified|f__Clostridia_unclassified|g__GGB81440|s__GGB8144
k__Bacteria|p__Firmicutes|c__Clostridia|o__Eubacteriales|f__Lachnospiraceae|g__Lachnospiraceae_unclassified|s__Lachn
k__Bacteria|p__Firmicutes|c__Clostridia|o__Eubacteriales|f__Lachnospiraceae|g__Lachnospiraceae_unclassified|s__Lachn
k__Bacteria|p__Firmicutes|c__Clostridia|o__Eubacteriales|f__Lachnospiraceae|g__Lachnospiraceae_unclassified|s__Lachn
k__Bacteria|p__Firmicutes|c__Clostridia|o__Eubacteriales|f__Lachnospiraceae|g__Lachnospiraceae_unclassified|s__Lachn
k__Bacteria|p__Firmicutes|c__Clostridia|o__Eubacteriales|f__Lachnospiraceae|g__Lachnospiraceae_unclassified|s__Lachn
k__Bacteria|p__Firmicutes|c__Clostridia|o__Eubacteriales|f__Lachnospiraceae|g__Lachnospiraceae_unclassified|s__Lachn
k__Bacteria|p__Firmicutes|c__Clostridia|o__Eubacteriales|f__Lachnospiraceae|g__Lachnospiraceae_unclassified|s__Lachn
k__Bacteria|p__Firmicutes|c__Clostridia|o__Eubacteriales|f__Lachnospiraceae|g__Lachnospiraceae_unclassified|s__Lachn
k__Bacteria|p__Firmicutes|c__Clostridia|o__Eubacteriales|f__Lachnospiraceae|g__Lachnospiraceae_unclassified|s__Lachn
k__Bacteria|p__Firmicutes|c__Clostridia|o__Eubacteriales|f__Lachnospiraceae|g__Lachnospiraceae_unclassified|s__Lachn
k__Bacteria|p__Firmicutes|c__Clostridia|o__Eubacteriales|f__Lachnospiraceae|g__Lachnospiraceae_unclassified|s__Lachn
k__Bacteria|p__Firmicutes|c__Clostridia|o__Eubacteriales|f__Lachnospiraceae|g__Lachnospiraceae_unclassified|s__Lachn
k__Bacteria|p__Firmicutes|c__Clostridia|o__Eubacteriales|f__Lachnospiraceae|g__Lachnospiraceae_unclassified|s__Lachn
k__Bacteria|p__Firmicutes|c__Clostridia|o__Eubacteriales|f__Lachnospiraceae|g__Lachnospiraceae_unclassified|s__Lachn
k__Bacteria|p__Firmicutes|c__Clostridia|o__Eubacteriales|f__Lachnospiraceae|g__Lachnospiraceae_unclassified|s__Lachn
k__Bacteria|p__Firmicutes|c__Clostridia|o__Eubacteriales|f__Lachnospiraceae|g__Lachnospiraceae_unclassified|s__Lachn
k__Bacteria|p__Firmicutes|c__Bacilli|o__Lactobacillales|f__Lactobacillaceae|g__Lactobacillus|s__Lactobacillus_johnsonii
k__Bacteria|p__Firmicutes|c__Bacilli|o__Lactobacillales|f__Lactobacillaceae|g__Lactobacillus|s__Lactobacillus_johnsonii
k__Bacteria|p__Bacteroidota|c__Bacteroidia|o__Bacteroidales|f__Muribaculaceae|g__Muribaculaceae_unclassified|s__M
k__Bacteria|p__Bacteroidota|c__Bacteroidia|o__Bacteroidales|f__Muribaculaceae|g__Muribaculaceae_unclassified|s__M
k__Bacteria|p__Firmicutes|c__Clostridia|o__Eubacteriales|f__Oscillospiraceae|g__Neglectibacter|s__Neglectibacter_sp_X
k__Bacteria|p__Firmicutes|c__Clostridia|o__Eubacteriales|f__Oscillospiraceae|g__Neglectibacter|s__Neglectibacter_sp_X
k__Bacteria|p__Firmicutes|c__Clostridia|o__Eubacteriales|f__Oscillospiraceae|g__Oscillospiraceae_unclassified|s__Oscillo
k__Bacteria|p__Firmicutes|c__Clostridia|o__Eubacteriales|f__Oscillospiraceae|g__Oscillospiraceae_unclassified|s__Oscillo
k__Bacteria|p__Firmicutes|c__Clostridia|o__Eubacteriales|f__Oscillospiraceae|g__Oscillospiraceae_unclassified|s__Oscillo
k__Bacteria|p__Firmicutes|c__Clostridia|o__Eubacteriales|f__Oscillospiraceae|g__Oscillospiraceae_unclassified|s__Oscillo

k__Bacteria|p__Firmicutes|c__Clostridia|o__Eubacteriales|f__Oscillospiraceae|g__Oscillospiraceae_unclassified|s__Oscillo
k__Bacteria|p__Firmicutes|c__Clostridia|o__Eubacteriales|f__Oscillospiraceae|g__Oscillospiraceae_unclassified|s__Oscillo
k__Bacteria|p__Proteobacteria|c__Betaproteobacteria|o__Burkholderiales|f__Sutterellaceae|g__Parasutterella|s__Parasu
k__Bacteria|p__Proteobacteria|c__Betaproteobacteria|o__Burkholderiales|f__Sutterellaceae|g__Parasutterella|s__Parasu

k__Bacteria|p__Firmicutes|c__Clostridia|o__Eubacteriales|f__Peptostreptococcaceae|g__Romboutsia|s__Romboutsia_ilea
k__Bacteria|p__Firmicutes|c__Clostridia|o__Eubacteriales|f__Peptostreptococcaceae|g__Romboutsia|s__Romboutsia_ilea
k__Bacteria|p__Firmicutes|c__Clostridia|o__Eubacteriales|f__Lachnospiraceae|g__Schaedlerella|s__Schaedlerella_arabino
k__Bacteria|p__Firmicutes|c__Clostridia|o__Eubacteriales|f__Lachnospiraceae|g__Schaedlerella|s__Schaedlerella_arabino

k__Bacteria|p__Firmicutes|c__Erysipelotrichia|o__Erysipelotrichales|f__Turicibacteraceae|g__Turicibacter|s__Turicibacter_
k__Bacteria|p__Firmicutes|c__Erysipelotrichia|o__Erysipelotrichales|f__Turicibacteraceae|g__Turicibacter|s__Turicibacter_
k__Bacteria|p__Firmicutes|c__Clostridia|o__Eubacteriales|f__Lachnospiraceae|g__Acetatifactor|s__Acetatifactor_muris
k__Bacteria|p__Firmicutes|c__Clostridia|o__Eubacteriales|f__Lachnospiraceae|g__Acetatifactor|s__Acetatifactor_muris
k__Bacteria|p__Firmicutes|c__Clostridia|o__Eubacteriales|f__Lachnospiraceae|g__Acetatifactor|s__Acetatifactor_SGB415
k__Bacteria|p__Firmicutes|c__Clostridia|o__Eubacteriales|f__Lachnospiraceae|g__Acetatifactor|s__Acetatifactor_SGB415
k__Bacteria|p__Firmicutes|c__Clostridia|o__Eubacteriales|f__Oscillospiraceae|g__Acutalibacter|s__Acutalibacter_muris
k__Bacteria|p__Firmicutes|c__Clostridia|o__Eubacteriales|f__Oscillospiraceae|g__Acutalibacter|s__Acutalibacter_muris
k__Bacteria|p__Firmicutes|c__Clostridia|o__Eubacteriales|f__Oscillospiraceae|g__Acutalibacter|s__Acutalibacter_sp_1XD
k__Bacteria|p__Firmicutes|c__Clostridia|o__Eubacteriales|f__Oscillospiraceae|g__Acutalibacter|s__Acutalibacter_sp_1XD
k__Bacteria|p__Actinobacteria|c__Coriobacteriia|o__Eggerthellales|f__Eggerthellaceae|g__Adlercreutzia|s__Adlercreutzia
k__Bacteria|p__Actinobacteria|c__Coriobacteriia|o__Eggerthellales|f__Eggerthellaceae|g__Adlercreutzia|s__Adlercreutzia
k__Bacteria|p__Actinobacteria|c__Coriobacteriia|o__Eggerthellales|f__Eggerthellaceae|g__Adlercreutzia|s__Adlercreutzia
k__Bacteria|p__Actinobacteria|c__Coriobacteriia|o__Eggerthellales|f__Eggerthellaceae|g__Adlercreutzia|s__Adlercreutzia
k__Bacteria|p__Actinobacteria|c__Coriobacteriia|o__Eggerthellales|f__Eggerthellaceae|g__Adlercreutzia|s__Adlercreutzia
k__Bacteria|p__Actinobacteria|c__Coriobacteriia|o__Eggerthellales|f__Eggerthellaceae|g__Adlercreutzia|s__Adlercreutzia
k__Bacteria|p__Verrucomicrobia|c__Verrucomicrobiae|o__Verrucomicrobiales|f__Akkermansiaceae|g__Akkermansia|s__A
k__Bacteria|p__Verrucomicrobia|c__Verrucomicrobiae|o__Verrucomicrobiales|f__Akkermansiaceae|g__Akkermansia|s__A
k__Bacteria|p__Bacteroidota|c__Bacteroidia|o__Bacteroidales|f__Rikenellaceae|g__Alistipes|s__Alistipes_sp_DSM_11234
k__Bacteria|p__Bacteroidota|c__Bacteroidia|o__Bacteroidales|f__Rikenellaceae|g__Alistipes|s__Alistipes_sp_DSM_11234
k__Bacteria|p__Firmicutes|c__Clostridia|o__Eubacteriales|f__Oscillospiraceae|g__Anaerotruncus|s__Anaerotruncus_sp_1
k__Bacteria|p__Firmicutes|c__Clostridia|o__Eubacteriales|f__Oscillospiraceae|g__Anaerotruncus|s__Anaerotruncus_sp_1
k__Bacteria|p__Bacteria_unclassified|c__Bacteria_unclassified|o__Bacteria_unclassified|f__Bacteria_unclassified|g__Bacte
k__Bacteria|p__Bacteria_unclassified|c__Bacteria_unclassified|o__Bacteria_unclassified|f__Bacteria_unclassified|g__Bacte
k__Bacteria|p__Bacteria_unclassified|c__Bacteria_unclassified|o__Bacteria_unclassified|f__Bacteria_unclassified|g__Bacte
k__Bacteria|p__Bacteria_unclassified|c__Bacteria_unclassified|o__Bacteria_unclassified|f__Bacteria_unclassified|g__Bacte
k__Bacteria|p__Bacteria_unclassified|c__Bacteria_unclassified|o__Bacteria_unclassified|f__Bacteria_unclassified|g__Bacte
k__Bacteria|p__Bacteria_unclassified|c__Bacteria_unclassified|o__Bacteria_unclassified|f__Bacteria_unclassified|g__Bacte
k__Bacteria|p__Bacteria_unclassified|c__Bacteria_unclassified|o__Bacteria_unclassified|f__Bacteria_unclassified|g__Bacte
k__Bacteria|p__Bacteria_unclassified|c__Bacteria_unclassified|o__Bacteria_unclassified|f__Bacteria_unclassified|g__Bacte
k__Bacteria|p__Bacteria_unclassified|c__Bacteria_unclassified|o__Bacteria_unclassified|f__Bacteria_unclassified|g__Bacte
k__Bacteria|p__Bacteria_unclassified|c__Bacteria_unclassified|o__Bacteria_unclassified|f__Bacteria_unclassified|g__Bacte
k__Bacteria|p__Bacteria_unclassified|c__Bacteria_unclassified|o__Bacteria_unclassified|f__Bacteria_unclassified|g__Bacte
k__Bacteria|p__Bacteria_unclassified|c__Bacteria_unclassified|o__Bacteria_unclassified|f__Bacteria_unclassified|g__Bacte

k__Bacteria|p__Bacteroidota|c__Bacteroidia|o__Bacteroidales|f__Bacteroidaceae|g__Bacteroides|s__Bacteroides_thetaio
k__Bacteria|p__Bacteroidota|c__Bacteroidia|o__Bacteroidales|f__Bacteroidaceae|g__Bacteroides|s__Bacteroides_thetaio

k__Bacteria|p__Actinobacteria|c__Actinomycetia|o__Bifidobacteriales|f__Bifidobacteriaceae|g__Bifidobacterium|s__Bifid
k__Bacteria|p__Actinobacteria|c__Actinomycetia|o__Bifidobacteriales|f__Bifidobacteriaceae|g__Bifidobacterium|s__Bifid
k__Bacteria|p__Firmicutes|c__Clostridia|o__Clostridia_unclassified|f__Clostridia_unclassified|g__Clostridia_unclassified|s_
k__Bacteria|p__Firmicutes|c__Clostridia|o__Clostridia_unclassified|f__Clostridia_unclassified|g__Clostridia_unclassified|s_
k__Bacteria|p__Firmicutes|c__Clostridia|o__Eubacteriales|f__Clostridiaceae|g__Clostridiaceae_unclassified|s__Clostridiac
k__Bacteria|p__Firmicutes|c__Clostridia|o__Eubacteriales|f__Clostridiaceae|g__Clostridiaceae_unclassified|s__Clostridiac
k__Bacteria|p__Firmicutes|c__Clostridia|o__Eubacteriales|f__Clostridiaceae|g__Clostridiaceae_unclassified|s__Clostridiac
k__Bacteria|p__Firmicutes|c__Clostridia|o__Eubacteriales|f__Clostridiaceae|g__Clostridiaceae_unclassified|s__Clostridiac
k__Bacteria|p__Firmicutes|c__Clostridia|o__Eubacteriales|f__Eubacteriales_unclassified|g__Eubacteriales_unclassified|s_
k__Bacteria|p__Firmicutes|c__Clostridia|o__Eubacteriales|f__Eubacteriales_unclassified|g__Eubacteriales_unclassified|s_
k__Bacteria|p__Firmicutes|c__Erysipelotrichia|o__Erysipelotrichales|f__Erysipelotrichaceae|g__Erysipelatoclostridium|s__
k__Bacteria|p__Firmicutes|c__Erysipelotrichia|o__Erysipelotrichales|f__Erysipelotrichaceae|g__Erysipelatoclostridium|s__
k__Bacteria|p__Actinobacteria|c__Coriobacteriia|o__Coriobacteriales|f__Coriobacteriaceae|g__Coriobacteriaceae_unclass
k__Bacteria|p__Actinobacteria|c__Coriobacteriia|o__Coriobacteriales|f__Coriobacteriaceae|g__Coriobacteriaceae_unclass
k__Bacteria|p__Firmicutes|c__Clostridia|o__Eubacteriales|f__Lachnospiraceae|g__Dorea|s__Dorea_sp_5_2
k__Bacteria|p__Firmicutes|c__Clostridia|o__Eubacteriales|f__Lachnospiraceae|g__Dorea|s__Dorea_sp_5_2
k__Bacteria|p__Firmicutes|c__Erysipelotrichia|o__Erysipelotrichales|f__Erysipelotrichaceae|g__Dubosiella|s__Dubosiella_
k__Bacteria|p__Firmicutes|c__Erysipelotrichia|o__Erysipelotrichales|f__Erysipelotrichaceae|g__Dubosiella|s__Dubosiella_
k__Bacteria|p__Firmicutes|c__Erysipelotrichia|o__Erysipelotrichales|f__Erysipelotrichales_unclassified|g__Erysipelotrichal
k__Bacteria|p__Firmicutes|c__Erysipelotrichia|o__Erysipelotrichales|f__Erysipelotrichales_unclassified|g__Erysipelotrichal
k__Bacteria|p__Firmicutes|c__Clostridia|o__Eubacteriales|f__Eubacteriaceae|g__Eubacteriaceae_unclassified|s__Eubacte
k__Bacteria|p__Firmicutes|c__Clostridia|o__Eubacteriales|f__Eubacteriaceae|g__Eubacteriaceae_unclassified|s__Eubacte
k__Bacteria|p__Firmicutes|c__Clostridia|o__Eubacteriales|f__Eubacteriaceae|g__Eubacteriaceae_unclassified|s__Eubacte
k__Bacteria|p__Firmicutes|c__Clostridia|o__Eubacteriales|f__Eubacteriaceae|g__Eubacteriaceae_unclassified|s__Eubacte
k__Bacteria|p__Firmicutes|c__Clostridia|o__Eubacteriales|f__Lachnospiraceae|g__GGB20149|s__GGB20149_SGB29430
k__Bacteria|p__Firmicutes|c__Clostridia|o__Eubacteriales|f__Lachnospiraceae|g__GGB20149|s__GGB20149_SGB29430
k__Bacteria|p__Actinobacteria|c__Coriobacteriia|o__Eggerthellales|f__Eggerthellaceae|g__GGB22635|s__GGB22635_SGB
k__Bacteria|p__Actinobacteria|c__Coriobacteriia|o__Eggerthellales|f__Eggerthellaceae|g__GGB22635|s__GGB22635_SGB
k__Bacteria|p__Firmicutes|c__Clostridia|o__Eubacteriales|f__Lachnospiraceae|g__GGB25041|s__GGB25041_SGB36960
k__Bacteria|p__Firmicutes|c__Clostridia|o__Eubacteriales|f__Lachnospiraceae|g__GGB25041|s__GGB25041_SGB36960
k__Bacteria|p__Bacteroidota|c__Bacteroidia|o__Bacteroidales|f__Muribaculaceae|g__GGB27876|s__GGB27876_SGB4031
k__Bacteria|p__Bacteroidota|c__Bacteroidia|o__Bacteroidales|f__Muribaculaceae|g__GGB27876|s__GGB27876_SGB4031
k__Bacteria|p__Bacteroidota|c__Bacteroidia|o__Bacteroidales|f__Muribaculaceae|g__GGB27878|s__GGB27878_SGB4031
k__Bacteria|p__Bacteroidota|c__Bacteroidia|o__Bacteroidales|f__Muribaculaceae|g__GGB27878|s__GGB27878_SGB4031
k__Bacteria|p__Bacteroidota|c__Bacteroidia|o__Bacteroidales|f__Muribaculaceae|g__GGB27918|s__GGB27918_SGB4035
k__Bacteria|p__Bacteroidota|c__Bacteroidia|o__Bacteroidales|f__Muribaculaceae|g__GGB27918|s__GGB27918_SGB4035
k__Bacteria|p__Firmicutes|c__CFGB9508|o__OFGB9508|f__FGB9508|g__GGB28382|s__GGB28382_SGB40962
k__Bacteria|p__Firmicutes|c__CFGB9508|o__OFGB9508|f__FGB9508|g__GGB28382|s__GGB28382_SGB40962
k__Bacteria|p__Firmicutes|c__CFGB2838|o__OFGB2838|f__FGB2838|g__GGB28399|s__GGB28399_SGB40980
k__Bacteria|p__Firmicutes|c__CFGB2838|o__OFGB2838|f__FGB2838|g__GGB28399|s__GGB28399_SGB40980
k__Bacteria|p__Firmicutes|c__CFGB2838|o__OFGB2838|f__FGB2838|g__GGB28411|s__GGB28411_SGB40993
k__Bacteria|p__Firmicutes|c__CFGB2838|o__OFGB2838|f__FGB2838|g__GGB28411|s__GGB28411_SGB40993

k__Bacteria|p__Firmicutes|c__CFGB2838|o__OFGB2838|f__FGB2838|g__GGB28415|s__GGB28415_SGB40997
k__Bacteria|p__Firmicutes|c__CFGB2838|o__OFGB2838|f__FGB2838|g__GGB28415|s__GGB28415_SGB40997
k__Bacteria|p__Firmicutes|c__Clostridia|o__Eubacteriales|f__Pumilibacteraceae|g__GGB28430|s__GGB28430_SGB41013
k__Bacteria|p__Firmicutes|c__Clostridia|o__Eubacteriales|f__Pumilibacteraceae|g__GGB28430|s__GGB28430_SGB41013
k__Bacteria|p__Firmicutes|c__CFGB28439|o__OFGB28439|f__FGB28439|g__GGB28439|s__GGB28439_SGB41022
k__Bacteria|p__Firmicutes|c__CFGB28439|o__OFGB28439|f__FGB28439|g__GGB28439|s__GGB28439_SGB41022
k__Bacteria|p__Firmicutes|c__Clostridia|o__Clostridia_unclassified|f__Clostridia_unclassified|g__GGB28778|s__GGB2877
k__Bacteria|p__Firmicutes|c__Clostridia|o__Clostridia_unclassified|f__Clostridia_unclassified|g__GGB28778|s__GGB2877
k__Bacteria|p__Firmicutes|c__Clostridia|o__Eubacteriales|f__Eubacteriaceae|g__GGB28784|s__GGB28784_SGB41437
k__Bacteria|p__Firmicutes|c__Clostridia|o__Eubacteriales|f__Eubacteriaceae|g__GGB28784|s__GGB28784_SGB41437
k__Bacteria|p__Firmicutes|c__Clostridia|o__Eubacteriales|f__Lachnospiraceae|g__GGB28792|s__GGB28792_SGB41445
k__Bacteria|p__Firmicutes|c__Clostridia|o__Eubacteriales|f__Lachnospiraceae|g__GGB28792|s__GGB28792_SGB41445
k__Bacteria|p__Firmicutes|c__Clostridia|o__Eubacteriales|f__Lachnospiraceae|g__GGB28798|s__GGB28798_SGB41451
k__Bacteria|p__Firmicutes|c__Clostridia|o__Eubacteriales|f__Lachnospiraceae|g__GGB28798|s__GGB28798_SGB41451
k__Bacteria|p__Firmicutes|c__Clostridia|o__Eubacteriales|f__Lachnospiraceae|g__GGB28802|s__GGB28802_SGB41455
k__Bacteria|p__Firmicutes|c__Clostridia|o__Eubacteriales|f__Lachnospiraceae|g__GGB28802|s__GGB28802_SGB41455
k__Bacteria|p__Firmicutes|c__Clostridia|o__Eubacteriales|f__Lachnospiraceae|g__GGB28818|s__GGB28818_SGB41473
k__Bacteria|p__Firmicutes|c__Clostridia|o__Eubacteriales|f__Lachnospiraceae|g__GGB28818|s__GGB28818_SGB41473
k__Bacteria|p__Firmicutes|c__CFGB77305|o__OFGB77305|f__FGB77305|g__GGB28828|s__GGB28828_SGB41484
k__Bacteria|p__Firmicutes|c__CFGB77305|o__OFGB77305|f__FGB77305|g__GGB28828|s__GGB28828_SGB41484
k__Bacteria|p__Firmicutes|c__Clostridia|o__Eubacteriales|f__Clostridiaceae|g__GGB28851|s__GGB28851_SGB41518
k__Bacteria|p__Firmicutes|c__Clostridia|o__Eubacteriales|f__Clostridiaceae|g__GGB28851|s__GGB28851_SGB41518
k__Bacteria|p__Firmicutes|c__Clostridia|o__Eubacteriales|f__Lachnospiraceae|g__GGB28859|s__GGB28859_SGB41528
k__Bacteria|p__Firmicutes|c__Clostridia|o__Eubacteriales|f__Lachnospiraceae|g__GGB28859|s__GGB28859_SGB41528
k__Bacteria|p__Firmicutes|c__Clostridia|o__Eubacteriales|f__Lachnospiraceae|g__GGB28864|s__GGB28864_SGB41535
k__Bacteria|p__Firmicutes|c__Clostridia|o__Eubacteriales|f__Lachnospiraceae|g__GGB28864|s__GGB28864_SGB41535
k__Bacteria|p__Firmicutes|c__Clostridia|o__Eubacteriales|f__Lachnospiraceae|g__GGB28869|s__GGB28869_SGB41543
k__Bacteria|p__Firmicutes|c__Clostridia|o__Eubacteriales|f__Lachnospiraceae|g__GGB28869|s__GGB28869_SGB41543
k__Bacteria|p__Firmicutes|c__CFGB9633|o__OFGB9633|f__FGB9633|g__GGB28883|s__GGB28883_SGB41564
k__Bacteria|p__Firmicutes|c__CFGB9633|o__OFGB9633|f__FGB9633|g__GGB28883|s__GGB28883_SGB41564
k__Bacteria|p__Bacteria_unclassified|c__Bacteria_unclassified|o__Bacteria_unclassified|f__Bacteria_unclassified|g__GGB
k__Bacteria|p__Bacteria_unclassified|c__Bacteria_unclassified|o__Bacteria_unclassified|f__Bacteria_unclassified|g__GGB
k__Bacteria|p__Bacteria_unclassified|c__Bacteria_unclassified|o__Bacteria_unclassified|f__Bacteria_unclassified|g__GGB
k__Bacteria|p__Bacteria_unclassified|c__Bacteria_unclassified|o__Bacteria_unclassified|f__Bacteria_unclassified|g__GGB
k__Bacteria|p__Bacteria_unclassified|c__Bacteria_unclassified|o__Bacteria_unclassified|f__Bacteria_unclassified|g__GGB
k__Bacteria|p__Bacteria_unclassified|c__Bacteria_unclassified|o__Bacteria_unclassified|f__Bacteria_unclassified|g__GGB
k__Bacteria|p__Bacteria_unclassified|c__Bacteria_unclassified|o__Bacteria_unclassified|f__Bacteria_unclassified|g__GGB
k__Bacteria|p__Bacteria_unclassified|c__Bacteria_unclassified|o__Bacteria_unclassified|f__Bacteria_unclassified|g__GGB
k__Bacteria|p__Firmicutes|c__Clostridia|o__Eubacteriales|f__Lachnospiraceae|g__GGB28916|s__GGB28916_SGB41612
k__Bacteria|p__Firmicutes|c__Clostridia|o__Eubacteriales|f__Lachnospiraceae|g__GGB28916|s__GGB28916_SGB41612
k__Bacteria|p__Firmicutes|c__Clostridia|o__Eubacteriales|f__Lachnospiraceae|g__GGB28924|s__GGB28924_SGB41621
k__Bacteria|p__Firmicutes|c__Clostridia|o__Eubacteriales|f__Lachnospiraceae|g__GGB28924|s__GGB28924_SGB41621
k__Bacteria|p__Firmicutes|c__Clostridia|o__Eubacteriales|f__Lachnospiraceae|g__GGB28926|s__GGB28926_SGB41624
k__Bacteria|p__Firmicutes|c__Clostridia|o__Eubacteriales|f__Lachnospiraceae|g__GGB28926|s__GGB28926_SGB41624
k__Bacteria|p__Bacteria_unclassified|c__CFGB77359|o__OFGB77359|f__FGB77359|g__GGB28927|s__GGB28927_SGB416
k__Bacteria|p__Bacteria_unclassified|c__CFGB77359|o__OFGB77359|f__FGB77359|g__GGB28927|s__GGB28927_SGB416

k__Bacteria|p__Firmicutes|c__CFGB9639|o__OFGB9639|f__FGB9639|g__GGB28934|s__GGB28934_SGB41635
k__Bacteria|p__Firmicutes|c__CFGB9639|o__OFGB9639|f__FGB9639|g__GGB28934|s__GGB28934_SGB41635
k__Bacteria|p__Firmicutes|c__Clostridia|o__Eubacteriales|f__Lachnospiraceae|g__GGB28946|s__GGB28946_SGB41652
k__Bacteria|p__Firmicutes|c__Clostridia|o__Eubacteriales|f__Lachnospiraceae|g__GGB28946|s__GGB28946_SGB41652
k__Bacteria|p__Firmicutes|c__Clostridia|o__Eubacteriales|f__Lachnospiraceae|g__GGB28949|s__GGB28949_SGB41655
k__Bacteria|p__Firmicutes|c__Clostridia|o__Eubacteriales|f__Lachnospiraceae|g__GGB28949|s__GGB28949_SGB41655
k__Bacteria|p__Firmicutes|c__Clostridia|o__Eubacteriales|f__Lachnospiraceae|g__GGB28949|s__GGB28949_SGB41656
k__Bacteria|p__Firmicutes|c__Clostridia|o__Eubacteriales|f__Lachnospiraceae|g__GGB28949|s__GGB28949_SGB41656
k__Bacteria|p__Firmicutes|c__Clostridia|o__Eubacteriales|f__Clostridiaceae|g__GGB28950|s__GGB28950_SGB41657
k__Bacteria|p__Firmicutes|c__Clostridia|o__Eubacteriales|f__Clostridiaceae|g__GGB28950|s__GGB28950_SGB41657
k__Bacteria|p__Firmicutes|c__Clostridia|o__Eubacteriales|f__Clostridiaceae|g__GGB28951|s__GGB28951_SGB102295
k__Bacteria|p__Firmicutes|c__Clostridia|o__Eubacteriales|f__Clostridiaceae|g__GGB28951|s__GGB28951_SGB102295
k__Bacteria|p__Firmicutes|c__Clostridia|o__Eubacteriales|f__Clostridiaceae|g__GGB28951|s__GGB28951_SGB41658
k__Bacteria|p__Firmicutes|c__Clostridia|o__Eubacteriales|f__Clostridiaceae|g__GGB28951|s__GGB28951_SGB41658
k__Bacteria|p__Firmicutes|c__Clostridia|o__Eubacteriales|f__Clostridiaceae|g__GGB28954|s__GGB28954_SGB41662
k__Bacteria|p__Firmicutes|c__Clostridia|o__Eubacteriales|f__Clostridiaceae|g__GGB28954|s__GGB28954_SGB41662
k__Bacteria|p__Firmicutes|c__Clostridia|o__Eubacteriales|f__Clostridiaceae|g__GGB28956|s__GGB28956_SGB41665
k__Bacteria|p__Firmicutes|c__Clostridia|o__Eubacteriales|f__Clostridiaceae|g__GGB28956|s__GGB28956_SGB41665
k__Bacteria|p__Firmicutes|c__Clostridia|o__Eubacteriales|f__Clostridiaceae|g__GGB28960|s__GGB28960_SGB41669
k__Bacteria|p__Firmicutes|c__Clostridia|o__Eubacteriales|f__Clostridiaceae|g__GGB28960|s__GGB28960_SGB41669
k__Bacteria|p__Firmicutes|c__Clostridia|o__Eubacteriales|f__Clostridiaceae|g__GGB28967|s__GGB28967_SGB41678
k__Bacteria|p__Firmicutes|c__Clostridia|o__Eubacteriales|f__Clostridiaceae|g__GGB28967|s__GGB28967_SGB41678
k__Bacteria|p__Firmicutes|c__Clostridia|o__Eubacteriales|f__Eubacteriaceae|g__GGB28991|s__GGB28991_SGB41705
k__Bacteria|p__Firmicutes|c__Clostridia|o__Eubacteriales|f__Eubacteriaceae|g__GGB28991|s__GGB28991_SGB41705
k__Bacteria|p__Firmicutes|c__CFGB9658|o__OFGB9658|f__FGB9658|g__GGB29002|s__GGB29002_SGB41718
k__Bacteria|p__Firmicutes|c__CFGB9658|o__OFGB9658|f__FGB9658|g__GGB29002|s__GGB29002_SGB41718
k__Bacteria|p__Firmicutes|c__CFGB9659|o__OFGB9659|f__FGB9659|g__GGB29003|s__GGB29003_SGB41719
k__Bacteria|p__Firmicutes|c__CFGB9659|o__OFGB9659|f__FGB9659|g__GGB29003|s__GGB29003_SGB41719
k__Bacteria|p__Bacteria_unclassified|c__Bacteria_unclassified|o__Bacteria_unclassified|f__Bacteria_unclassified|g__GGB[illegible]
k__Bacteria|p__Bacteria_unclassified|c__Bacteria_unclassified|o__Bacteria_unclassified|f__Bacteria_unclassified|g__GGB[illegible]
k__Bacteria|p__Firmicutes|c__CFGB9827|o__OFGB9827|f__FGB9827|g__GGB29531|s__GGB29531_SGB42317
k__Bacteria|p__Firmicutes|c__CFGB9827|o__OFGB9827|f__FGB9827|g__GGB29531|s__GGB29531_SGB42317
k__Bacteria|p__Firmicutes|c__Clostridia|o__Eubacteriales|f__Eubacteriaceae|g__GGB29685|s__GGB29685_SGB42494
k__Bacteria|p__Firmicutes|c__Clostridia|o__Eubacteriales|f__Eubacteriaceae|g__GGB29685|s__GGB29685_SGB42494
k__Bacteria|p__Bacteria_unclassified|c__CFGB77303|o__OFGB77303|f__FGB77303|g__GGB30141|s__GGB30141_SGB430[illegible]
k__Bacteria|p__Bacteria_unclassified|c__CFGB77303|o__OFGB77303|f__FGB77303|g__GGB30141|s__GGB30141_SGB430[illegible]
k__Bacteria|p__Firmicutes|c__Clostridia|o__Eubacteriales|f__Eubacteriales_unclassified|g__GGB30286|s__GGB30286_SG[illegible]
k__Bacteria|p__Firmicutes|c__Clostridia|o__Eubacteriales|f__Eubacteriales_unclassified|g__GGB30286|s__GGB30286_SG[illegible]
k__Bacteria|p__Firmicutes|c__Clostridia|o__Eubacteriales|f__Oscillospiraceae|g__GGB30303|s__GGB30303_SGB43268
k__Bacteria|p__Firmicutes|c__Clostridia|o__Eubacteriales|f__Oscillospiraceae|g__GGB30303|s__GGB30303_SGB43268
k__Bacteria|p__Firmicutes|c__CFGB30328|o__OFGB30328|f__FGB30328|g__GGB30413|s__GGB30413_SGB43452
k__Bacteria|p__Firmicutes|c__CFGB30328|o__OFGB30328|f__FGB30328|g__GGB30413|s__GGB30413_SGB43452
k__Bacteria|p__Firmicutes|c__Clostridia|o__Eubacteriales|f__Oscillospiraceae|g__GGB30454|s__GGB30454_SGB43514
k__Bacteria|p__Firmicutes|c__Clostridia|o__Eubacteriales|f__Oscillospiraceae|g__GGB30454|s__GGB30454_SGB43514
k__Bacteria|p__Firmicutes|c__Clostridia|o__Eubacteriales|f__Oscillospiraceae|g__GGB30455|s__GGB30455_SGB43519
k__Bacteria|p__Firmicutes|c__Clostridia|o__Eubacteriales|f__Oscillospiraceae|g__GGB30455|s__GGB30455_SGB43519

k__Bacteria|p__Firmicutes|c__Clostridia|o__Eubacteriales|f__Oscillospiraceae|g__GGB30461|s__GGB30461_SGB43527
k__Bacteria|p__Firmicutes|c__Clostridia|o__Eubacteriales|f__Oscillospiraceae|g__GGB30461|s__GGB30461_SGB43527
k__Bacteria|p__Firmicutes|c__Clostridia|o__Eubacteriales|f__Oscillospiraceae|g__GGB30461|s__GGB30461_SGB43530
k__Bacteria|p__Firmicutes|c__Clostridia|o__Eubacteriales|f__Oscillospiraceae|g__GGB30461|s__GGB30461_SGB43530
k__Bacteria|p__Firmicutes|c__Clostridia|o__Eubacteriales|f__Oscillospiraceae|g__GGB30463|s__GGB30463_SGB43537
k__Bacteria|p__Firmicutes|c__Clostridia|o__Eubacteriales|f__Oscillospiraceae|g__GGB30463|s__GGB30463_SGB43537
k__Bacteria|p__Firmicutes|c__Clostridia|o__Eubacteriales|f__Oscillospiraceae|g__GGB30473|s__GGB30473_SGB43557
k__Bacteria|p__Firmicutes|c__Clostridia|o__Eubacteriales|f__Oscillospiraceae|g__GGB30473|s__GGB30473_SGB43557
k__Bacteria|p__Firmicutes|c__Clostridia|o__Eubacteriales|f__Oscillospiraceae|g__GGB30475|s__GGB30475_SGB63182
k__Bacteria|p__Firmicutes|c__Clostridia|o__Eubacteriales|f__Oscillospiraceae|g__GGB30475|s__GGB30475_SGB63182
k__Bacteria|p__Actinobacteria|c__CFGB77153|o__OFGB77153|f__FGB77153|g__GGB30861|s__GGB30861_SGB44083
k__Bacteria|p__Actinobacteria|c__CFGB77153|o__OFGB77153|f__FGB77153|g__GGB30861|s__GGB30861_SGB44083
k__Bacteria|p__Tenericutes|c__CFGB1791|o__OFGB1791|f__FGB1791|g__GGB31312|s__GGB31312_SGB44628
k__Bacteria|p__Tenericutes|c__CFGB1791|o__OFGB1791|f__FGB1791|g__GGB31312|s__GGB31312_SGB44628
k__Bacteria|p__Firmicutes|c__CFGB10290|o__OFGB10290|f__FGB10290|g__GGB31438|s__GGB31438_SGB44768
k__Bacteria|p__Firmicutes|c__CFGB10290|o__OFGB10290|f__FGB10290|g__GGB31438|s__GGB31438_SGB44768
k__Bacteria|p__Firmicutes|c__Clostridia|o__Eubacteriales|f__Oscillospiraceae|g__GGB3171|s__GGB3171_SGB4185
k__Bacteria|p__Firmicutes|c__Clostridia|o__Eubacteriales|f__Oscillospiraceae|g__GGB3171|s__GGB3171_SGB4185
k__Bacteria|p__Firmicutes|c__CFGB1765|o__OFGB1765|f__FGB1765|g__GGB31823|s__GGB31823_SGB45199
k__Bacteria|p__Firmicutes|c__CFGB1765|o__OFGB1765|f__FGB1765|g__GGB31823|s__GGB31823_SGB45199
k__Bacteria|p__Firmicutes|c__CFGB10349|o__OFGB10349|f__FGB10349|g__GGB31853|s__GGB31853_SGB45233
k__Bacteria|p__Firmicutes|c__CFGB10349|o__OFGB10349|f__FGB10349|g__GGB31853|s__GGB31853_SGB45233
k__Bacteria|p__Firmicutes|c__CFGB10667|o__OFGB10667|f__FGB10667|g__GGB32371|s__GGB32371_SGB41694
k__Bacteria|p__Firmicutes|c__CFGB10667|o__OFGB10667|f__FGB10667|g__GGB32371|s__GGB32371_SGB41694
k__Bacteria|p__Firmicutes|c__Clostridia|o__Eubacteriales|f__Lachnospiraceae|g__GGB3793|s__GGB3793_SGB5158
k__Bacteria|p__Firmicutes|c__Clostridia|o__Eubacteriales|f__Lachnospiraceae|g__GGB3793|s__GGB3793_SGB5158
k__Bacteria|p__Firmicutes|c__Clostridia|o__Eubacteriales|f__Lachnospiraceae|g__GGB42598|s__GGB42598_SGB59794
k__Bacteria|p__Firmicutes|c__Clostridia|o__Eubacteriales|f__Lachnospiraceae|g__GGB42598|s__GGB42598_SGB59794
k__Bacteria|p__Firmicutes|c__Clostridia|o__Eubacteriales|f__Christensenellaceae|g__GGB45656|s__GGB45656_SGB6337
k__Bacteria|p__Firmicutes|c__Clostridia|o__Eubacteriales|f__Christensenellaceae|g__GGB45656|s__GGB45656_SGB6337
k__Bacteria|p__Firmicutes|c__CFGB10299|o__OFGB10299|f__FGB10299|g__GGB47127|s__GGB47127_SGB65054
k__Bacteria|p__Firmicutes|c__CFGB10299|o__OFGB10299|f__FGB10299|g__GGB47127|s__GGB47127_SGB65054
k__Bacteria|p__Firmicutes|c__Clostridia|o__Eubacteriales|f__Oscillospiraceae|g__GGB74395|s__GGB74395_SGB43521
k__Bacteria|p__Firmicutes|c__Clostridia|o__Eubacteriales|f__Oscillospiraceae|g__GGB74395|s__GGB74395_SGB43521
k__Bacteria|p__Firmicutes|c__Clostridia|o__Eubacteriales|f__Oscillospiraceae|g__GGB75053|s__GGB75053_SGB43494
k__Bacteria|p__Firmicutes|c__Clostridia|o__Eubacteriales|f__Oscillospiraceae|g__GGB75053|s__GGB75053_SGB43494
k__Bacteria|p__Firmicutes|c__Clostridia|o__Eubacteriales|f__Lachnospiraceae|g__GGB75109|s__GGB75109_SGB102238
k__Bacteria|p__Firmicutes|c__Clostridia|o__Eubacteriales|f__Lachnospiraceae|g__GGB75109|s__GGB75109_SGB102238
k__Bacteria|p__Firmicutes|c__Clostridia|o__Clostridia_unclassified|f__Clostridia_unclassified|g__GGB81440|s__GGB8144
k__Bacteria|p__Firmicutes|c__Clostridia|o__Clostridia_unclassified|f__Clostridia_unclassified|g__GGB81440|s__GGB8144
k__Bacteria|p__Firmicutes|c__Clostridia|o__Eubacteriales|f__Lachnospiraceae|g__Lachnospiraceae_unclassified|s__Lachn
k__Bacteria|p__Firmicutes|c__Clostridia|o__Eubacteriales|f__Lachnospiraceae|g__Lachnospiraceae_unclassified|s__Lachn
k__Bacteria|p__Firmicutes|c__Clostridia|o__Eubacteriales|f__Lachnospiraceae|g__Lachnospiraceae_unclassified|s__Lachn
k__Bacteria|p__Firmicutes|c__Clostridia|o__Eubacteriales|f__Lachnospiraceae|g__Lachnospiraceae_unclassified|s__Lachn
k__Bacteria|p__Firmicutes|c__Clostridia|o__Eubacteriales|f__Lachnospiraceae|g__Lachnospiraceae_unclassified|s__Lachn
k__Bacteria|p__Firmicutes|c__Clostridia|o__Eubacteriales|f__Lachnospiraceae|g__Lachnospiraceae_unclassified|s__Lachn

k__Bacteria|p__Firmicutes|c__Clostridia|o__Eubacteriales|f__Lachnospiraceae|g__Lachnospiraceae_unclassified|s__Lachn
k__Bacteria|p__Firmicutes|c__Clostridia|o__Eubacteriales|f__Lachnospiraceae|g__Lachnospiraceae_unclassified|s__Lachn
k__Bacteria|p__Firmicutes|c__Clostridia|o__Eubacteriales|f__Lachnospiraceae|g__Lachnospiraceae_unclassified|s__Lachn
k__Bacteria|p__Firmicutes|c__Clostridia|o__Eubacteriales|f__Lachnospiraceae|g__Lachnospiraceae_unclassified|s__Lachn
k__Bacteria|p__Firmicutes|c__Clostridia|o__Eubacteriales|f__Lachnospiraceae|g__Lachnospiraceae_unclassified|s__Lachn
k__Bacteria|p__Firmicutes|c__Clostridia|o__Eubacteriales|f__Lachnospiraceae|g__Lachnospiraceae_unclassified|s__Lachn
k__Bacteria|p__Firmicutes|c__Clostridia|o__Eubacteriales|f__Lachnospiraceae|g__Lachnospiraceae_unclassified|s__Lachn
k__Bacteria|p__Firmicutes|c__Clostridia|o__Eubacteriales|f__Lachnospiraceae|g__Lachnospiraceae_unclassified|s__Lachn
k__Bacteria|p__Firmicutes|c__Clostridia|o__Eubacteriales|f__Lachnospiraceae|g__Lachnospiraceae_unclassified|s__Lachn
k__Bacteria|p__Firmicutes|c__Clostridia|o__Eubacteriales|f__Lachnospiraceae|g__Lachnospiraceae_unclassified|s__Lachn
k__Bacteria|p__Firmicutes|c__Bacilli|o__Lactobacillales|f__Lactobacillaceae|g__Lactobacillus|s__Lactobacillus_johnsonii
k__Bacteria|p__Firmicutes|c__Bacilli|o__Lactobacillales|f__Lactobacillaceae|g__Lactobacillus|s__Lactobacillus_johnsonii
k__Bacteria|p__Bacteroidota|c__Bacteroidia|o__Bacteroidales|f__Muribaculaceae|g__Muribaculaceae_unclassified|s__M
k__Bacteria|p__Bacteroidota|c__Bacteroidia|o__Bacteroidales|f__Muribaculaceae|g__Muribaculaceae_unclassified|s__M
k__Bacteria|p__Firmicutes|c__Clostridia|o__Eubacteriales|f__Oscillospiraceae|g__Neglectibacter|s__Neglectibacter_sp_X
k__Bacteria|p__Firmicutes|c__Clostridia|o__Eubacteriales|f__Oscillospiraceae|g__Neglectibacter|s__Neglectibacter_sp_X
k__Bacteria|p__Firmicutes|c__Clostridia|o__Eubacteriales|f__Oscillospiraceae|g__Oscillospiraceae_unclassified|s__Oscillo
k__Bacteria|p__Firmicutes|c__Clostridia|o__Eubacteriales|f__Oscillospiraceae|g__Oscillospiraceae_unclassified|s__Oscillo
k__Bacteria|p__Firmicutes|c__Clostridia|o__Eubacteriales|f__Oscillospiraceae|g__Oscillospiraceae_unclassified|s__Oscillo
k__Bacteria|p__Firmicutes|c__Clostridia|o__Eubacteriales|f__Oscillospiraceae|g__Oscillospiraceae_unclassified|s__Oscillo
k__Bacteria|p__Firmicutes|c__Clostridia|o__Eubacteriales|f__Oscillospiraceae|g__Oscillospiraceae_unclassified|s__Oscillo
k__Bacteria|p__Firmicutes|c__Clostridia|o__Eubacteriales|f__Oscillospiraceae|g__Oscillospiraceae_unclassified|s__Oscillo
k__Bacteria|p__Proteobacteria|c__Betaproteobacteria|o__Burkholderiales|f__Sutterellaceae|g__Parasutterella|s__Parasu
k__Bacteria|p__Proteobacteria|c__Betaproteobacteria|o__Burkholderiales|f__Sutterellaceae|g__Parasutterella|s__Parasu

k__Bacteria|p__Firmicutes|c__Clostridia|o__Eubacteriales|f__Peptostreptococcaceae|g__Romboutsia|s__Romboutsia_ilea
k__Bacteria|p__Firmicutes|c__Clostridia|o__Eubacteriales|f__Peptostreptococcaceae|g__Romboutsia|s__Romboutsia_ilea
k__Bacteria|p__Firmicutes|c__Clostridia|o__Eubacteriales|f__Lachnospiraceae|g__Schaedlerella|s__Schaedlerella_arabino
k__Bacteria|p__Firmicutes|c__Clostridia|o__Eubacteriales|f__Lachnospiraceae|g__Schaedlerella|s__Schaedlerella_arabino

k__Bacteria|p__Firmicutes|c__Erysipelotrichia|o__Erysipelotrichales|f__Turicibacteraceae|g__Turicibacter|s__Turicibacter_
k__Bacteria|p__Firmicutes|c__Erysipelotrichia|o__Erysipelotrichales|f__Turicibacteraceae|g__Turicibacter|s__Turicibacter_
k__Bacteria|p__Firmicutes|c__Clostridia|o__Eubacteriales|f__Lachnospiraceae|g__Acetatifactor|s__Acetatifactor_muris
k__Bacteria|p__Firmicutes|c__Clostridia|o__Eubacteriales|f__Lachnospiraceae|g__Acetatifactor|s__Acetatifactor_muris
k__Bacteria|p__Firmicutes|c__Clostridia|o__Eubacteriales|f__Lachnospiraceae|g__Acetatifactor|s__Acetatifactor_SGB415
k__Bacteria|p__Firmicutes|c__Clostridia|o__Eubacteriales|f__Lachnospiraceae|g__Acetatifactor|s__Acetatifactor_SGB415
k__Bacteria|p__Firmicutes|c__Clostridia|o__Eubacteriales|f__Oscillospiraceae|g__Acutalibacter|s__Acutalibacter_muris
k__Bacteria|p__Firmicutes|c__Clostridia|o__Eubacteriales|f__Oscillospiraceae|g__Acutalibacter|s__Acutalibacter_muris
k__Bacteria|p__Firmicutes|c__Clostridia|o__Eubacteriales|f__Oscillospiraceae|g__Acutalibacter|s__Acutalibacter_sp_1XD
k__Bacteria|p__Firmicutes|c__Clostridia|o__Eubacteriales|f__Oscillospiraceae|g__Acutalibacter|s__Acutalibacter_sp_1XD
k__Bacteria|p__Actinobacteria|c__Coriobacteriia|o__Eggerthellales|f__Eggerthellaceae|g__Adlercreutzia|s__Adlercreutzia
k__Bacteria|p__Actinobacteria|c__Coriobacteriia|o__Eggerthellales|f__Eggerthellaceae|g__Adlercreutzia|s__Adlercreutzia
k__Bacteria|p__Actinobacteria|c__Coriobacteriia|o__Eggerthellales|f__Eggerthellaceae|g__Adlercreutzia|s__Adlercreutzia
k__Bacteria|p__Actinobacteria|c__Coriobacteriia|o__Eggerthellales|f__Eggerthellaceae|g__Adlercreutzia|s__Adlercreutzia

k__Bacteria|p__Actinobacteria|c__Coriobacteriia|o__Eggerthellales|f__Eggerthellaceae|g__Adlercreutzia|s__Adlercreutzia
k__Bacteria|p__Actinobacteria|c__Coriobacteriia|o__Eggerthellales|f__Eggerthellaceae|g__Adlercreutzia|s__Adlercreutzia
k__Bacteria|p__Verrucomicrobia|c__Verrucomicrobiae|o__Verrucomicrobiales|f__Akkermansiaceae|g__Akkermansia|s__A
k__Bacteria|p__Verrucomicrobia|c__Verrucomicrobiae|o__Verrucomicrobiales|f__Akkermansiaceae|g__Akkermansia|s__A
k__Bacteria|p__Bacteroidota|c__Bacteroidia|o__Bacteroidales|f__Rikenellaceae|g__Alistipes|s__Alistipes_sp_DSM_11234
k__Bacteria|p__Bacteroidota|c__Bacteroidia|o__Bacteroidales|f__Rikenellaceae|g__Alistipes|s__Alistipes_sp_DSM_11234
k__Bacteria|p__Firmicutes|c__Clostridia|o__Eubacteriales|f__Oscillospiraceae|g__Anaerotruncus|s__Anaerotruncus_sp_1
k__Bacteria|p__Firmicutes|c__Clostridia|o__Eubacteriales|f__Oscillospiraceae|g__Anaerotruncus|s__Anaerotruncus_sp_1
k__Bacteria|p__Bacteria_unclassified|c__Bacteria_unclassified|o__Bacteria_unclassified|f__Bacteria_unclassified|g__Bacte
k__Bacteria|p__Bacteria_unclassified|c__Bacteria_unclassified|o__Bacteria_unclassified|f__Bacteria_unclassified|g__Bacte
k__Bacteria|p__Bacteria_unclassified|c__Bacteria_unclassified|o__Bacteria_unclassified|f__Bacteria_unclassified|g__Bacte
k__Bacteria|p__Bacteria_unclassified|c__Bacteria_unclassified|o__Bacteria_unclassified|f__Bacteria_unclassified|g__Bacte
k__Bacteria|p__Bacteria_unclassified|c__Bacteria_unclassified|o__Bacteria_unclassified|f__Bacteria_unclassified|g__Bacte
k__Bacteria|p__Bacteria_unclassified|c__Bacteria_unclassified|o__Bacteria_unclassified|f__Bacteria_unclassified|g__Bacte
k__Bacteria|p__Bacteria_unclassified|c__Bacteria_unclassified|o__Bacteria_unclassified|f__Bacteria_unclassified|g__Bacte
k__Bacteria|p__Bacteria_unclassified|c__Bacteria_unclassified|o__Bacteria_unclassified|f__Bacteria_unclassified|g__Bacte
k__Bacteria|p__Bacteria_unclassified|c__Bacteria_unclassified|o__Bacteria_unclassified|f__Bacteria_unclassified|g__Bacte
k__Bacteria|p__Bacteria_unclassified|c__Bacteria_unclassified|o__Bacteria_unclassified|f__Bacteria_unclassified|g__Bacte
k__Bacteria|p__Bacteria_unclassified|c__Bacteria_unclassified|o__Bacteria_unclassified|f__Bacteria_unclassified|g__Bacte
k__Bacteria|p__Bacteria_unclassified|c__Bacteria_unclassified|o__Bacteria_unclassified|f__Bacteria_unclassified|g__Bacte
k__Bacteria|p__Bacteroidota|c__Bacteroidia|o__Bacteroidales|f__Bacteroidaceae|g__Bacteroides|s__Bacteroides_thetaio
k__Bacteria|p__Bacteroidota|c__Bacteroidia|o__Bacteroidales|f__Bacteroidaceae|g__Bacteroides|s__Bacteroides_thetaio

k__Bacteria|p__Actinobacteria|c__Actinomycetia|o__Bifidobacteriales|f__Bifidobacteriaceae|g__Bifidobacterium|s__Bifid
k__Bacteria|p__Actinobacteria|c__Actinomycetia|o__Bifidobacteriales|f__Bifidobacteriaceae|g__Bifidobacterium|s__Bifid
k__Bacteria|p__Firmicutes|c__Clostridia|o__Clostridia_unclassified|f__Clostridia_unclassified|g__Clostridia_unclassified|s_
k__Bacteria|p__Firmicutes|c__Clostridia|o__Clostridia_unclassified|f__Clostridia_unclassified|g__Clostridia_unclassified|s_
k__Bacteria|p__Firmicutes|c__Clostridia|o__Eubacteriales|f__Clostridiaceae|g__Clostridiaceae_unclassified|s__Clostridiac
k__Bacteria|p__Firmicutes|c__Clostridia|o__Eubacteriales|f__Clostridiaceae|g__Clostridiaceae_unclassified|s__Clostridiac
k__Bacteria|p__Firmicutes|c__Clostridia|o__Eubacteriales|f__Clostridiaceae|g__Clostridiaceae_unclassified|s__Clostridiac
k__Bacteria|p__Firmicutes|c__Clostridia|o__Eubacteriales|f__Clostridiaceae|g__Clostridiaceae_unclassified|s__Clostridiac
k__Bacteria|p__Firmicutes|c__Clostridia|o__Eubacteriales|f__Eubacteriales_unclassified|g__Eubacteriales_unclassified|s_
k__Bacteria|p__Firmicutes|c__Clostridia|o__Eubacteriales|f__Eubacteriales_unclassified|g__Eubacteriales_unclassified|s_
k__Bacteria|p__Firmicutes|c__Erysipelotrichia|o__Erysipelotrichales|f__Erysipelotrichaceae|g__Erysipelatoclostridium|s__
k__Bacteria|p__Firmicutes|c__Erysipelotrichia|o__Erysipelotrichales|f__Erysipelotrichaceae|g__Erysipelatoclostridium|s__
k__Bacteria|p__Actinobacteria|c__Coriobacteriia|o__Coriobacteriales|f__Coriobacteriaceae|g__Coriobacteriaceae_unclass
k__Bacteria|p__Actinobacteria|c__Coriobacteriia|o__Coriobacteriales|f__Coriobacteriaceae|g__Coriobacteriaceae_unclass
k__Bacteria|p__Firmicutes|c__Clostridia|o__Eubacteriales|f__Lachnospiraceae|g__Dorea|s__Dorea_sp_5_2
k__Bacteria|p__Firmicutes|c__Clostridia|o__Eubacteriales|f__Lachnospiraceae|g__Dorea|s__Dorea_sp_5_2
k__Bacteria|p__Firmicutes|c__Erysipelotrichia|o__Erysipelotrichales|f__Erysipelotrichaceae|g__Dubosiella|s__Dubosiella_
k__Bacteria|p__Firmicutes|c__Erysipelotrichia|o__Erysipelotrichales|f__Erysipelotrichaceae|g__Dubosiella|s__Dubosiella_
k__Bacteria|p__Firmicutes|c__Erysipelotrichia|o__Erysipelotrichales|f__Erysipelotrichales_unclassified|g__Erysipelotrichal
k__Bacteria|p__Firmicutes|c__Erysipelotrichia|o__Erysipelotrichales|f__Erysipelotrichales_unclassified|g__Erysipelotrichal
k__Bacteria|p__Firmicutes|c__Clostridia|o__Eubacteriales|f__Eubacteriaceae|g__Eubacteriaceae_unclassified|s__Eubacte
k__Bacteria|p__Firmicutes|c__Clostridia|o__Eubacteriales|f__Eubacteriaceae|g__Eubacteriaceae_unclassified|s__Eubacte

k__Bacteria|p__Firmicutes|c__Clostridia|o__Eubacteriales|f__Eubacteriaceae|g__Eubacteriaceae_unclassified|s__Eubacte
k__Bacteria|p__Firmicutes|c__Clostridia|o__Eubacteriales|f__Eubacteriaceae|g__Eubacteriaceae_unclassified|s__Eubacte
k__Bacteria|p__Firmicutes|c__Clostridia|o__Eubacteriales|f__Lachnospiraceae|g__GGB20149|s__GGB20149_SGB29430
k__Bacteria|p__Firmicutes|c__Clostridia|o__Eubacteriales|f__Lachnospiraceae|g__GGB20149|s__GGB20149_SGB29430
k__Bacteria|p__Actinobacteria|c__Coriobacteriia|o__Eggerthellales|f__Eggerthellaceae|g__GGB22635|s__GGB22635_SGB
k__Bacteria|p__Actinobacteria|c__Coriobacteriia|o__Eggerthellales|f__Eggerthellaceae|g__GGB22635|s__GGB22635_SGB
k__Bacteria|p__Firmicutes|c__Clostridia|o__Eubacteriales|f__Lachnospiraceae|g__GGB25041|s__GGB25041_SGB36960
k__Bacteria|p__Firmicutes|c__Clostridia|o__Eubacteriales|f__Lachnospiraceae|g__GGB25041|s__GGB25041_SGB36960
k__Bacteria|p__Bacteroidota|c__Bacteroidia|o__Bacteroidales|f__Muribaculaceae|g__GGB27876|s__GGB27876_SGB4031
k__Bacteria|p__Bacteroidota|c__Bacteroidia|o__Bacteroidales|f__Muribaculaceae|g__GGB27876|s__GGB27876_SGB4031
k__Bacteria|p__Bacteroidota|c__Bacteroidia|o__Bacteroidales|f__Muribaculaceae|g__GGB27878|s__GGB27878_SGB4031
k__Bacteria|p__Bacteroidota|c__Bacteroidia|o__Bacteroidales|f__Muribaculaceae|g__GGB27878|s__GGB27878_SGB4031
k__Bacteria|p__Bacteroidota|c__Bacteroidia|o__Bacteroidales|f__Muribaculaceae|g__GGB27918|s__GGB27918_SGB4035
k__Bacteria|p__Bacteroidota|c__Bacteroidia|o__Bacteroidales|f__Muribaculaceae|g__GGB27918|s__GGB27918_SGB4035
k__Bacteria|p__Firmicutes|c__CFGB9508|o__OFGB9508|f__FGB9508|g__GGB28382|s__GGB28382_SGB40962
k__Bacteria|p__Firmicutes|c__CFGB9508|o__OFGB9508|f__FGB9508|g__GGB28382|s__GGB28382_SGB40962
k__Bacteria|p__Firmicutes|c__CFGB2838|o__OFGB2838|f__FGB2838|g__GGB28399|s__GGB28399_SGB40980
k__Bacteria|p__Firmicutes|c__CFGB2838|o__OFGB2838|f__FGB2838|g__GGB28399|s__GGB28399_SGB40980
k__Bacteria|p__Firmicutes|c__CFGB2838|o__OFGB2838|f__FGB2838|g__GGB28411|s__GGB28411_SGB40993
k__Bacteria|p__Firmicutes|c__CFGB2838|o__OFGB2838|f__FGB2838|g__GGB28411|s__GGB28411_SGB40993
k__Bacteria|p__Firmicutes|c__CFGB2838|o__OFGB2838|f__FGB2838|g__GGB28415|s__GGB28415_SGB40997
k__Bacteria|p__Firmicutes|c__CFGB2838|o__OFGB2838|f__FGB2838|g__GGB28415|s__GGB28415_SGB40997
k__Bacteria|p__Firmicutes|c__Clostridia|o__Eubacteriales|f__Pumilibacteraceae|g__GGB28430|s__GGB28430_SGB41013
k__Bacteria|p__Firmicutes|c__Clostridia|o__Eubacteriales|f__Pumilibacteraceae|g__GGB28430|s__GGB28430_SGB41013
k__Bacteria|p__Firmicutes|c__CFGB28439|o__OFGB28439|f__FGB28439|g__GGB28439|s__GGB28439_SGB41022
k__Bacteria|p__Firmicutes|c__CFGB28439|o__OFGB28439|f__FGB28439|g__GGB28439|s__GGB28439_SGB41022
k__Bacteria|p__Firmicutes|c__Clostridia|o__Clostridia_unclassified|f__Clostridia_unclassified|g__GGB28778|s__GGB2877
k__Bacteria|p__Firmicutes|c__Clostridia|o__Clostridia_unclassified|f__Clostridia_unclassified|g__GGB28778|s__GGB2877
k__Bacteria|p__Firmicutes|c__Clostridia|o__Eubacteriales|f__Eubacteriaceae|g__GGB28784|s__GGB28784_SGB41437
k__Bacteria|p__Firmicutes|c__Clostridia|o__Eubacteriales|f__Eubacteriaceae|g__GGB28784|s__GGB28784_SGB41437
k__Bacteria|p__Firmicutes|c__Clostridia|o__Eubacteriales|f__Lachnospiraceae|g__GGB28792|s__GGB28792_SGB41445
k__Bacteria|p__Firmicutes|c__Clostridia|o__Eubacteriales|f__Lachnospiraceae|g__GGB28792|s__GGB28792_SGB41445
k__Bacteria|p__Firmicutes|c__Clostridia|o__Eubacteriales|f__Lachnospiraceae|g__GGB28798|s__GGB28798_SGB41451
k__Bacteria|p__Firmicutes|c__Clostridia|o__Eubacteriales|f__Lachnospiraceae|g__GGB28798|s__GGB28798_SGB41451
k__Bacteria|p__Firmicutes|c__Clostridia|o__Eubacteriales|f__Lachnospiraceae|g__GGB28802|s__GGB28802_SGB41455
k__Bacteria|p__Firmicutes|c__Clostridia|o__Eubacteriales|f__Lachnospiraceae|g__GGB28802|s__GGB28802_SGB41455
k__Bacteria|p__Firmicutes|c__Clostridia|o__Eubacteriales|f__Lachnospiraceae|g__GGB28818|s__GGB28818_SGB41473
k__Bacteria|p__Firmicutes|c__Clostridia|o__Eubacteriales|f__Lachnospiraceae|g__GGB28818|s__GGB28818_SGB41473
k__Bacteria|p__Firmicutes|c__CFGB77305|o__OFGB77305|f__FGB77305|g__GGB28828|s__GGB28828_SGB41484
k__Bacteria|p__Firmicutes|c__CFGB77305|o__OFGB77305|f__FGB77305|g__GGB28828|s__GGB28828_SGB41484
k__Bacteria|p__Firmicutes|c__Clostridia|o__Eubacteriales|f__Clostridiaceae|g__GGB28851|s__GGB28851_SGB41518
k__Bacteria|p__Firmicutes|c__Clostridia|o__Eubacteriales|f__Clostridiaceae|g__GGB28851|s__GGB28851_SGB41518
k__Bacteria|p__Firmicutes|c__Clostridia|o__Eubacteriales|f__Lachnospiraceae|g__GGB28859|s__GGB28859_SGB41528
k__Bacteria|p__Firmicutes|c__Clostridia|o__Eubacteriales|f__Lachnospiraceae|g__GGB28859|s__GGB28859_SGB41528
k__Bacteria|p__Firmicutes|c__Clostridia|o__Eubacteriales|f__Lachnospiraceae|g__GGB28864|s__GGB28864_SGB41535
k__Bacteria|p__Firmicutes|c__Clostridia|o__Eubacteriales|f__Lachnospiraceae|g__GGB28864|s__GGB28864_SGB41535

k__Bacteria|p__Firmicutes|c__Clostridia|o__Eubacteriales|f__Lachnospiraceae|g__GGB28869|s__GGB28869_SGB41543
k__Bacteria|p__Firmicutes|c__Clostridia|o__Eubacteriales|f__Lachnospiraceae|g__GGB28869|s__GGB28869_SGB41543
k__Bacteria|p__Firmicutes|c__CFGB9633|o__OFGB9633|f__FGB9633|g__GGB28883|s__GGB28883_SGB41564
k__Bacteria|p__Firmicutes|c__CFGB9633|o__OFGB9633|f__FGB9633|g__GGB28883|s__GGB28883_SGB41564
k__Bacteria|p__Bacteria_unclassified|c__Bacteria_unclassified|o__Bacteria_unclassified|f__Bacteria_unclassified|g__GGB[illegible]
k__Bacteria|p__Bacteria_unclassified|c__Bacteria_unclassified|o__Bacteria_unclassified|f__Bacteria_unclassified|g__GGB[illegible]
k__Bacteria|p__Bacteria_unclassified|c__Bacteria_unclassified|o__Bacteria_unclassified|f__Bacteria_unclassified|g__GGB[illegible]
k__Bacteria|p__Bacteria_unclassified|c__Bacteria_unclassified|o__Bacteria_unclassified|f__Bacteria_unclassified|g__GGB[illegible]
k__Bacteria|p__Bacteria_unclassified|c__Bacteria_unclassified|o__Bacteria_unclassified|f__Bacteria_unclassified|g__GGB[illegible]
k__Bacteria|p__Bacteria_unclassified|c__Bacteria_unclassified|o__Bacteria_unclassified|f__Bacteria_unclassified|g__GGB[illegible]
k__Bacteria|p__Bacteria_unclassified|c__Bacteria_unclassified|o__Bacteria_unclassified|f__Bacteria_unclassified|g__GGB[illegible]
k__Bacteria|p__Bacteria_unclassified|c__Bacteria_unclassified|o__Bacteria_unclassified|f__Bacteria_unclassified|g__GGB[illegible]
k__Bacteria|p__Firmicutes|c__Clostridia|o__Eubacteriales|f__Lachnospiraceae|g__GGB28916|s__GGB28916_SGB41612
k__Bacteria|p__Firmicutes|c__Clostridia|o__Eubacteriales|f__Lachnospiraceae|g__GGB28916|s__GGB28916_SGB41612
k__Bacteria|p__Firmicutes|c__Clostridia|o__Eubacteriales|f__Lachnospiraceae|g__GGB28924|s__GGB28924_SGB41621
k__Bacteria|p__Firmicutes|c__Clostridia|o__Eubacteriales|f__Lachnospiraceae|g__GGB28924|s__GGB28924_SGB41621
k__Bacteria|p__Firmicutes|c__Clostridia|o__Eubacteriales|f__Lachnospiraceae|g__GGB28926|s__GGB28926_SGB41624
k__Bacteria|p__Firmicutes|c__Clostridia|o__Eubacteriales|f__Lachnospiraceae|g__GGB28926|s__GGB28926_SGB41624
k__Bacteria|p__Bacteria_unclassified|c__CFGB77359|o__OFGB77359|f__FGB77359|g__GGB28927|s__GGB28927_SGB41[illegible]
k__Bacteria|p__Bacteria_unclassified|c__CFGB77359|o__OFGB77359|f__FGB77359|g__GGB28927|s__GGB28927_SGB41[illegible]
k__Bacteria|p__Firmicutes|c__CFGB9639|o__OFGB9639|f__FGB9639|g__GGB28934|s__GGB28934_SGB41635
k__Bacteria|p__Firmicutes|c__CFGB9639|o__OFGB9639|f__FGB9639|g__GGB28934|s__GGB28934_SGB41635
k__Bacteria|p__Firmicutes|c__Clostridia|o__Eubacteriales|f__Lachnospiraceae|g__GGB28946|s__GGB28946_SGB41652
k__Bacteria|p__Firmicutes|c__Clostridia|o__Eubacteriales|f__Lachnospiraceae|g__GGB28946|s__GGB28946_SGB41652
k__Bacteria|p__Firmicutes|c__Clostridia|o__Eubacteriales|f__Lachnospiraceae|g__GGB28949|s__GGB28949_SGB41655
k__Bacteria|p__Firmicutes|c__Clostridia|o__Eubacteriales|f__Lachnospiraceae|g__GGB28949|s__GGB28949_SGB41655
k__Bacteria|p__Firmicutes|c__Clostridia|o__Eubacteriales|f__Lachnospiraceae|g__GGB28949|s__GGB28949_SGB41656
k__Bacteria|p__Firmicutes|c__Clostridia|o__Eubacteriales|f__Lachnospiraceae|g__GGB28949|s__GGB28949_SGB41656
k__Bacteria|p__Firmicutes|c__Clostridia|o__Eubacteriales|f__Clostridiaceae|g__GGB28950|s__GGB28950_SGB41657
k__Bacteria|p__Firmicutes|c__Clostridia|o__Eubacteriales|f__Clostridiaceae|g__GGB28950|s__GGB28950_SGB41657
k__Bacteria|p__Firmicutes|c__Clostridia|o__Eubacteriales|f__Clostridiaceae|g__GGB28951|s__GGB28951_SGB102295
k__Bacteria|p__Firmicutes|c__Clostridia|o__Eubacteriales|f__Clostridiaceae|g__GGB28951|s__GGB28951_SGB102295
k__Bacteria|p__Firmicutes|c__Clostridia|o__Eubacteriales|f__Clostridiaceae|g__GGB28951|s__GGB28951_SGB41658
k__Bacteria|p__Firmicutes|c__Clostridia|o__Eubacteriales|f__Clostridiaceae|g__GGB28951|s__GGB28951_SGB41658
k__Bacteria|p__Firmicutes|c__Clostridia|o__Eubacteriales|f__Clostridiaceae|g__GGB28954|s__GGB28954_SGB41662
k__Bacteria|p__Firmicutes|c__Clostridia|o__Eubacteriales|f__Clostridiaceae|g__GGB28954|s__GGB28954_SGB41662
k__Bacteria|p__Firmicutes|c__Clostridia|o__Eubacteriales|f__Clostridiaceae|g__GGB28956|s__GGB28956_SGB41665
k__Bacteria|p__Firmicutes|c__Clostridia|o__Eubacteriales|f__Clostridiaceae|g__GGB28956|s__GGB28956_SGB41665
k__Bacteria|p__Firmicutes|c__Clostridia|o__Eubacteriales|f__Clostridiaceae|g__GGB28960|s__GGB28960_SGB41669
k__Bacteria|p__Firmicutes|c__Clostridia|o__Eubacteriales|f__Clostridiaceae|g__GGB28960|s__GGB28960_SGB41669
k__Bacteria|p__Firmicutes|c__Clostridia|o__Eubacteriales|f__Clostridiaceae|g__GGB28967|s__GGB28967_SGB41678
k__Bacteria|p__Firmicutes|c__Clostridia|o__Eubacteriales|f__Clostridiaceae|g__GGB28967|s__GGB28967_SGB41678
k__Bacteria|p__Firmicutes|c__Clostridia|o__Eubacteriales|f__Eubacteriaceae|g__GGB28991|s__GGB28991_SGB41705
k__Bacteria|p__Firmicutes|c__Clostridia|o__Eubacteriales|f__Eubacteriaceae|g__GGB28991|s__GGB28991_SGB41705
k__Bacteria|p__Firmicutes|c__CFGB9658|o__OFGB9658|f__FGB9658|g__GGB29002|s__GGB29002_SGB41718
k__Bacteria|p__Firmicutes|c__CFGB9658|o__OFGB9658|f__FGB9658|g__GGB29002|s__GGB29002_SGB41718

k__Bacteria|p__Firmicutes|c__CFGB9659|o__OFGB9659|f__FGB9659|g__GGB29003|s__GGB29003_SGB41719
k__Bacteria|p__Firmicutes|c__CFGB9659|o__OFGB9659|f__FGB9659|g__GGB29003|s__GGB29003_SGB41719
k__Bacteria|p__Bacteria_unclassified|c__Bacteria_unclassified|o__Bacteria_unclassified|f__Bacteria_unclassified|g__GGB[illegible]
k__Bacteria|p__Bacteria_unclassified|c__Bacteria_unclassified|o__Bacteria_unclassified|f__Bacteria_unclassified|g__GGB[illegible]
k__Bacteria|p__Firmicutes|c__CFGB9827|o__OFGB9827|f__FGB9827|g__GGB29531|s__GGB29531_SGB42317
k__Bacteria|p__Firmicutes|c__CFGB9827|o__OFGB9827|f__FGB9827|g__GGB29531|s__GGB29531_SGB42317
k__Bacteria|p__Firmicutes|c__Clostridia|o__Eubacteriales|f__Eubacteriaceae|g__GGB29685|s__GGB29685_SGB42494
k__Bacteria|p__Firmicutes|c__Clostridia|o__Eubacteriales|f__Eubacteriaceae|g__GGB29685|s__GGB29685_SGB42494
k__Bacteria|p__Bacteria_unclassified|c__CFGB77303|o__OFGB77303|f__FGB77303|g__GGB30141|s__GGB30141_SGB430[illegible]
k__Bacteria|p__Bacteria_unclassified|c__CFGB77303|o__OFGB77303|f__FGB77303|g__GGB30141|s__GGB30141_SGB430[illegible]
k__Bacteria|p__Firmicutes|c__Clostridia|o__Eubacteriales|f__Eubacteriales_unclassified|g__GGB30286|s__GGB30286_SG[illegible]
k__Bacteria|p__Firmicutes|c__Clostridia|o__Eubacteriales|f__Eubacteriales_unclassified|g__GGB30286|s__GGB30286_SG[illegible]
k__Bacteria|p__Firmicutes|c__Clostridia|o__Eubacteriales|f__Oscillospiraceae|g__GGB30303|s__GGB30303_SGB43268
k__Bacteria|p__Firmicutes|c__Clostridia|o__Eubacteriales|f__Oscillospiraceae|g__GGB30303|s__GGB30303_SGB43268
k__Bacteria|p__Firmicutes|c__CFGB30328|o__OFGB30328|f__FGB30328|g__GGB30413|s__GGB30413_SGB43452
k__Bacteria|p__Firmicutes|c__CFGB30328|o__OFGB30328|f__FGB30328|g__GGB30413|s__GGB30413_SGB43452
k__Bacteria|p__Firmicutes|c__Clostridia|o__Eubacteriales|f__Oscillospiraceae|g__GGB30454|s__GGB30454_SGB43514
k__Bacteria|p__Firmicutes|c__Clostridia|o__Eubacteriales|f__Oscillospiraceae|g__GGB30454|s__GGB30454_SGB43514
k__Bacteria|p__Firmicutes|c__Clostridia|o__Eubacteriales|f__Oscillospiraceae|g__GGB30455|s__GGB30455_SGB43519
k__Bacteria|p__Firmicutes|c__Clostridia|o__Eubacteriales|f__Oscillospiraceae|g__GGB30455|s__GGB30455_SGB43519
k__Bacteria|p__Firmicutes|c__Clostridia|o__Eubacteriales|f__Oscillospiraceae|g__GGB30461|s__GGB30461_SGB43527
k__Bacteria|p__Firmicutes|c__Clostridia|o__Eubacteriales|f__Oscillospiraceae|g__GGB30461|s__GGB30461_SGB43527
k__Bacteria|p__Firmicutes|c__Clostridia|o__Eubacteriales|f__Oscillospiraceae|g__GGB30461|s__GGB30461_SGB43530
k__Bacteria|p__Firmicutes|c__Clostridia|o__Eubacteriales|f__Oscillospiraceae|g__GGB30461|s__GGB30461_SGB43530
k__Bacteria|p__Firmicutes|c__Clostridia|o__Eubacteriales|f__Oscillospiraceae|g__GGB30463|s__GGB30463_SGB43537
k__Bacteria|p__Firmicutes|c__Clostridia|o__Eubacteriales|f__Oscillospiraceae|g__GGB30463|s__GGB30463_SGB43537
k__Bacteria|p__Firmicutes|c__Clostridia|o__Eubacteriales|f__Oscillospiraceae|g__GGB30473|s__GGB30473_SGB43557
k__Bacteria|p__Firmicutes|c__Clostridia|o__Eubacteriales|f__Oscillospiraceae|g__GGB30473|s__GGB30473_SGB43557
k__Bacteria|p__Firmicutes|c__Clostridia|o__Eubacteriales|f__Oscillospiraceae|g__GGB30475|s__GGB30475_SGB63182
k__Bacteria|p__Firmicutes|c__Clostridia|o__Eubacteriales|f__Oscillospiraceae|g__GGB30475|s__GGB30475_SGB63182
k__Bacteria|p__Actinobacteria|c__CFGB77153|o__OFGB77153|f__FGB77153|g__GGB30861|s__GGB30861_SGB44083
k__Bacteria|p__Actinobacteria|c__CFGB77153|o__OFGB77153|f__FGB77153|g__GGB30861|s__GGB30861_SGB44083
k__Bacteria|p__Tenericutes|c__CFGB1791|o__OFGB1791|f__FGB1791|g__GGB31312|s__GGB31312_SGB44628
k__Bacteria|p__Tenericutes|c__CFGB1791|o__OFGB1791|f__FGB1791|g__GGB31312|s__GGB31312_SGB44628
k__Bacteria|p__Firmicutes|c__CFGB10290|o__OFGB10290|f__FGB10290|g__GGB31438|s__GGB31438_SGB44768
k__Bacteria|p__Firmicutes|c__CFGB10290|o__OFGB10290|f__FGB10290|g__GGB31438|s__GGB31438_SGB44768
k__Bacteria|p__Firmicutes|c__Clostridia|o__Eubacteriales|f__Oscillospiraceae|g__GGB3171|s__GGB3171_SGB4185
k__Bacteria|p__Firmicutes|c__Clostridia|o__Eubacteriales|f__Oscillospiraceae|g__GGB3171|s__GGB3171_SGB4185
k__Bacteria|p__Firmicutes|c__CFGB1765|o__OFGB1765|f__FGB1765|g__GGB31823|s__GGB31823_SGB45199
k__Bacteria|p__Firmicutes|c__CFGB1765|o__OFGB1765|f__FGB1765|g__GGB31823|s__GGB31823_SGB45199
k__Bacteria|p__Firmicutes|c__CFGB10349|o__OFGB10349|f__FGB10349|g__GGB31853|s__GGB31853_SGB45233
k__Bacteria|p__Firmicutes|c__CFGB10349|o__OFGB10349|f__FGB10349|g__GGB31853|s__GGB31853_SGB45233
k__Bacteria|p__Firmicutes|c__CFGB10667|o__OFGB10667|f__FGB10667|g__GGB32371|s__GGB32371_SGB41694
k__Bacteria|p__Firmicutes|c__CFGB10667|o__OFGB10667|f__FGB10667|g__GGB32371|s__GGB32371_SGB41694
k__Bacteria|p__Firmicutes|c__Clostridia|o__Eubacteriales|f__Lachnospiraceae|g__GGB3793|s__GGB3793_SGB5158
k__Bacteria|p__Firmicutes|c__Clostridia|o__Eubacteriales|f__Lachnospiraceae|g__GGB3793|s__GGB3793_SGB5158

k__Bacteria|p__Firmicutes|c__Clostridia|o__Eubacteriales|f__Lachnospiraceae|g__GGB42598|s__GGB42598_SGB59794
k__Bacteria|p__Firmicutes|c__Clostridia|o__Eubacteriales|f__Lachnospiraceae|g__GGB42598|s__GGB42598_SGB59794
k__Bacteria|p__Firmicutes|c__Clostridia|o__Eubacteriales|f__Christensenellaceae|g__GGB45656|s__GGB45656_SGB6337
k__Bacteria|p__Firmicutes|c__Clostridia|o__Eubacteriales|f__Christensenellaceae|g__GGB45656|s__GGB45656_SGB6337
k__Bacteria|p__Firmicutes|c__CFGB10299|o__OFGB10299|f__FGB10299|g__GGB47127|s__GGB47127_SGB65054
k__Bacteria|p__Firmicutes|c__CFGB10299|o__OFGB10299|f__FGB10299|g__GGB47127|s__GGB47127_SGB65054
k__Bacteria|p__Firmicutes|c__Clostridia|o__Eubacteriales|f__Oscillospiraceae|g__GGB74395|s__GGB74395_SGB43521
k__Bacteria|p__Firmicutes|c__Clostridia|o__Eubacteriales|f__Oscillospiraceae|g__GGB74395|s__GGB74395_SGB43521
k__Bacteria|p__Firmicutes|c__Clostridia|o__Eubacteriales|f__Oscillospiraceae|g__GGB75053|s__GGB75053_SGB43494
k__Bacteria|p__Firmicutes|c__Clostridia|o__Eubacteriales|f__Oscillospiraceae|g__GGB75053|s__GGB75053_SGB43494
k__Bacteria|p__Firmicutes|c__Clostridia|o__Eubacteriales|f__Lachnospiraceae|g__GGB75109|s__GGB75109_SGB102238
k__Bacteria|p__Firmicutes|c__Clostridia|o__Eubacteriales|f__Lachnospiraceae|g__GGB75109|s__GGB75109_SGB102238
k__Bacteria|p__Firmicutes|c__Clostridia|o__Clostridia_unclassified|f__Clostridia_unclassified|g__GGB81440|s__GGB8144
k__Bacteria|p__Firmicutes|c__Clostridia|o__Clostridia_unclassified|f__Clostridia_unclassified|g__GGB81440|s__GGB8144
k__Bacteria|p__Firmicutes|c__Clostridia|o__Eubacteriales|f__Lachnospiraceae|g__Lachnospiraceae_unclassified|s__Lachn
k__Bacteria|p__Firmicutes|c__Clostridia|o__Eubacteriales|f__Lachnospiraceae|g__Lachnospiraceae_unclassified|s__Lachn
k__Bacteria|p__Firmicutes|c__Clostridia|o__Eubacteriales|f__Lachnospiraceae|g__Lachnospiraceae_unclassified|s__Lachn
k__Bacteria|p__Firmicutes|c__Clostridia|o__Eubacteriales|f__Lachnospiraceae|g__Lachnospiraceae_unclassified|s__Lachn
k__Bacteria|p__Firmicutes|c__Clostridia|o__Eubacteriales|f__Lachnospiraceae|g__Lachnospiraceae_unclassified|s__Lachn
k__Bacteria|p__Firmicutes|c__Clostridia|o__Eubacteriales|f__Lachnospiraceae|g__Lachnospiraceae_unclassified|s__Lachn
k__Bacteria|p__Firmicutes|c__Clostridia|o__Eubacteriales|f__Lachnospiraceae|g__Lachnospiraceae_unclassified|s__Lachn
k__Bacteria|p__Firmicutes|c__Clostridia|o__Eubacteriales|f__Lachnospiraceae|g__Lachnospiraceae_unclassified|s__Lachn
k__Bacteria|p__Firmicutes|c__Clostridia|o__Eubacteriales|f__Lachnospiraceae|g__Lachnospiraceae_unclassified|s__Lachn
k__Bacteria|p__Firmicutes|c__Clostridia|o__Eubacteriales|f__Lachnospiraceae|g__Lachnospiraceae_unclassified|s__Lachn
k__Bacteria|p__Firmicutes|c__Clostridia|o__Eubacteriales|f__Lachnospiraceae|g__Lachnospiraceae_unclassified|s__Lachn
k__Bacteria|p__Firmicutes|c__Clostridia|o__Eubacteriales|f__Lachnospiraceae|g__Lachnospiraceae_unclassified|s__Lachn
k__Bacteria|p__Firmicutes|c__Clostridia|o__Eubacteriales|f__Lachnospiraceae|g__Lachnospiraceae_unclassified|s__Lachn
k__Bacteria|p__Firmicutes|c__Clostridia|o__Eubacteriales|f__Lachnospiraceae|g__Lachnospiraceae_unclassified|s__Lachn
k__Bacteria|p__Firmicutes|c__Clostridia|o__Eubacteriales|f__Lachnospiraceae|g__Lachnospiraceae_unclassified|s__Lachn
k__Bacteria|p__Firmicutes|c__Bacilli|o__Lactobacillales|f__Lactobacillaceae|g__Lactobacillus|s__Lactobacillus_johnsonii
k__Bacteria|p__Firmicutes|c__Bacilli|o__Lactobacillales|f__Lactobacillaceae|g__Lactobacillus|s__Lactobacillus_johnsonii
k__Bacteria|p__Bacteroidota|c__Bacteroidia|o__Bacteroidales|f__Muribaculaceae|g__Muribaculaceae_unclassified|s__M
k__Bacteria|p__Bacteroidota|c__Bacteroidia|o__Bacteroidales|f__Muribaculaceae|g__Muribaculaceae_unclassified|s__M
k__Bacteria|p__Firmicutes|c__Clostridia|o__Eubacteriales|f__Oscillospiraceae|g__Neglectibacter|s__Neglectibacter_sp_X
k__Bacteria|p__Firmicutes|c__Clostridia|o__Eubacteriales|f__Oscillospiraceae|g__Neglectibacter|s__Neglectibacter_sp_X
k__Bacteria|p__Firmicutes|c__Clostridia|o__Eubacteriales|f__Oscillospiraceae|g__Oscillospiraceae_unclassified|s__Oscillo
k__Bacteria|p__Firmicutes|c__Clostridia|o__Eubacteriales|f__Oscillospiraceae|g__Oscillospiraceae_unclassified|s__Oscillo
k__Bacteria|p__Firmicutes|c__Clostridia|o__Eubacteriales|f__Oscillospiraceae|g__Oscillospiraceae_unclassified|s__Oscillo
k__Bacteria|p__Firmicutes|c__Clostridia|o__Eubacteriales|f__Oscillospiraceae|g__Oscillospiraceae_unclassified|s__Oscillo
k__Bacteria|p__Firmicutes|c__Clostridia|o__Eubacteriales|f__Oscillospiraceae|g__Oscillospiraceae_unclassified|s__Oscillo
k__Bacteria|p__Firmicutes|c__Clostridia|o__Eubacteriales|f__Oscillospiraceae|g__Oscillospiraceae_unclassified|s__Oscillo
k__Bacteria|p__Proteobacteria|c__Betaproteobacteria|o__Burkholderiales|f__Sutterellaceae|g__Parasutterella|s__Parasu
k__Bacteria|p__Proteobacteria|c__Betaproteobacteria|o__Burkholderiales|f__Sutterellaceae|g__Parasutterella|s__Parasu

k__Bacteria|p__Firmicutes|c__Clostridia|o__Eubacteriales|f__Peptostreptococcaceae|g__Romboutsia|s__Romboutsia_ilea
k__Bacteria|p__Firmicutes|c__Clostridia|o__Eubacteriales|f__Peptostreptococcaceae|g__Romboutsia|s__Romboutsia_ilea
k__Bacteria|p__Firmicutes|c__Clostridia|o__Eubacteriales|f__Lachnospiraceae|g__Schaedlerella|s__Schaedlerella_arabinc
k__Bacteria|p__Firmicutes|c__Clostridia|o__Eubacteriales|f__Lachnospiraceae|g__Schaedlerella|s__Schaedlerella_arabinc

k__Bacteria|p__Firmicutes|c__Erysipelotrichia|o__Erysipelotrichales|f__Turicibacteraceae|g__Turicibacter|s__Turicibacter_
k__Bacteria|p__Firmicutes|c__Erysipelotrichia|o__Erysipelotrichales|f__Turicibacteraceae|g__Turicibacter|s__Turicibacter_
k__Bacteria|p__Firmicutes|c__Clostridia|o__Eubacteriales|f__Lachnospiraceae|g__Acetatifactor|s__Acetatifactor_muris
k__Bacteria|p__Firmicutes|c__Clostridia|o__Eubacteriales|f__Lachnospiraceae|g__Acetatifactor|s__Acetatifactor_muris
k__Bacteria|p__Firmicutes|c__Clostridia|o__Eubacteriales|f__Lachnospiraceae|g__Acetatifactor|s__Acetatifactor_SGB415
k__Bacteria|p__Firmicutes|c__Clostridia|o__Eubacteriales|f__Lachnospiraceae|g__Acetatifactor|s__Acetatifactor_SGB415
k__Bacteria|p__Firmicutes|c__Clostridia|o__Eubacteriales|f__Oscillospiraceae|g__Acutalibacter|s__Acutalibacter_muris
k__Bacteria|p__Firmicutes|c__Clostridia|o__Eubacteriales|f__Oscillospiraceae|g__Acutalibacter|s__Acutalibacter_muris
k__Bacteria|p__Firmicutes|c__Clostridia|o__Eubacteriales|f__Oscillospiraceae|g__Acutalibacter|s__Acutalibacter_sp_1XD
k__Bacteria|p__Firmicutes|c__Clostridia|o__Eubacteriales|f__Oscillospiraceae|g__Acutalibacter|s__Acutalibacter_sp_1XD
k__Bacteria|p__Actinobacteria|c__Coriobacteriia|o__Eggerthellales|f__Eggerthellaceae|g__Adlercreutzia|s__Adlercreutzia
k__Bacteria|p__Actinobacteria|c__Coriobacteriia|o__Eggerthellales|f__Eggerthellaceae|g__Adlercreutzia|s__Adlercreutzia
k__Bacteria|p__Actinobacteria|c__Coriobacteriia|o__Eggerthellales|f__Eggerthellaceae|g__Adlercreutzia|s__Adlercreutzia
k__Bacteria|p__Actinobacteria|c__Coriobacteriia|o__Eggerthellales|f__Eggerthellaceae|g__Adlercreutzia|s__Adlercreutzia
k__Bacteria|p__Actinobacteria|c__Coriobacteriia|o__Eggerthellales|f__Eggerthellaceae|g__Adlercreutzia|s__Adlercreutzia
k__Bacteria|p__Actinobacteria|c__Coriobacteriia|o__Eggerthellales|f__Eggerthellaceae|g__Adlercreutzia|s__Adlercreutzia
k__Bacteria|p__Verrucomicrobia|c__Verrucomicrobiae|o__Verrucomicrobiales|f__Akkermansiaceae|g__Akkermansia|s__A
k__Bacteria|p__Verrucomicrobia|c__Verrucomicrobiae|o__Verrucomicrobiales|f__Akkermansiaceae|g__Akkermansia|s__A
k__Bacteria|p__Bacteroidota|c__Bacteroidia|o__Bacteroidales|f__Rikenellaceae|g__Alistipes|s__Alistipes_sp_DSM_11234
k__Bacteria|p__Bacteroidota|c__Bacteroidia|o__Bacteroidales|f__Rikenellaceae|g__Alistipes|s__Alistipes_sp_DSM_11234
k__Bacteria|p__Firmicutes|c__Clostridia|o__Eubacteriales|f__Oscillospiraceae|g__Anaerotruncus|s__Anaerotruncus_sp_1
k__Bacteria|p__Firmicutes|c__Clostridia|o__Eubacteriales|f__Oscillospiraceae|g__Anaerotruncus|s__Anaerotruncus_sp_1
k__Bacteria|p__Bacteria_unclassified|c__Bacteria_unclassified|o__Bacteria_unclassified|f__Bacteria_unclassified|g__Bacte
k__Bacteria|p__Bacteria_unclassified|c__Bacteria_unclassified|o__Bacteria_unclassified|f__Bacteria_unclassified|g__Bacte
k__Bacteria|p__Bacteria_unclassified|c__Bacteria_unclassified|o__Bacteria_unclassified|f__Bacteria_unclassified|g__Bacte
k__Bacteria|p__Bacteria_unclassified|c__Bacteria_unclassified|o__Bacteria_unclassified|f__Bacteria_unclassified|g__Bacte
k__Bacteria|p__Bacteria_unclassified|c__Bacteria_unclassified|o__Bacteria_unclassified|f__Bacteria_unclassified|g__Bacte
k__Bacteria|p__Bacteria_unclassified|c__Bacteria_unclassified|o__Bacteria_unclassified|f__Bacteria_unclassified|g__Bacte
k__Bacteria|p__Bacteria_unclassified|c__Bacteria_unclassified|o__Bacteria_unclassified|f__Bacteria_unclassified|g__Bacte
k__Bacteria|p__Bacteria_unclassified|c__Bacteria_unclassified|o__Bacteria_unclassified|f__Bacteria_unclassified|g__Bacte
k__Bacteria|p__Bacteria_unclassified|c__Bacteria_unclassified|o__Bacteria_unclassified|f__Bacteria_unclassified|g__Bacte
k__Bacteria|p__Bacteria_unclassified|c__Bacteria_unclassified|o__Bacteria_unclassified|f__Bacteria_unclassified|g__Bacte
k__Bacteria|p__Bacteria_unclassified|c__Bacteria_unclassified|o__Bacteria_unclassified|f__Bacteria_unclassified|g__Bacte
k__Bacteria|p__Bacteria_unclassified|c__Bacteria_unclassified|o__Bacteria_unclassified|f__Bacteria_unclassified|g__Bacte
k__Bacteria|p__Bacteroidota|c__Bacteroidia|o__Bacteroidales|f__Bacteroidaceae|g__Bacteroides|s__Bacteroides_thetaio
k__Bacteria|p__Bacteroidota|c__Bacteroidia|o__Bacteroidales|f__Bacteroidaceae|g__Bacteroides|s__Bacteroides_thetaio

k__Bacteria|p__Actinobacteria|c__Actinomycetia|o__Bifidobacteriales|f__Bifidobacteriaceae|g__Bifidobacterium|s__Bifid
k__Bacteria|p__Actinobacteria|c__Actinomycetia|o__Bifidobacteriales|f__Bifidobacteriaceae|g__Bifidobacterium|s__Bifid

k__Bacteria|p__Firmicutes|c__Clostridia|o__Clostridia_unclassified|f__Clostridia_unclassified|g__Clostridia_unclassified|s_
k__Bacteria|p__Firmicutes|c__Clostridia|o__Clostridia_unclassified|f__Clostridia_unclassified|g__Clostridia_unclassified|s_
k__Bacteria|p__Firmicutes|c__Clostridia|o__Eubacteriales|f__Clostridiaceae|g__Clostridiaceae_unclassified|s__Clostridiac
k__Bacteria|p__Firmicutes|c__Clostridia|o__Eubacteriales|f__Clostridiaceae|g__Clostridiaceae_unclassified|s__Clostridiac
k__Bacteria|p__Firmicutes|c__Clostridia|o__Eubacteriales|f__Clostridiaceae|g__Clostridiaceae_unclassified|s__Clostridiac
k__Bacteria|p__Firmicutes|c__Clostridia|o__Eubacteriales|f__Clostridiaceae|g__Clostridiaceae_unclassified|s__Clostridiac
k__Bacteria|p__Firmicutes|c__Clostridia|o__Eubacteriales|f__Eubacteriales_unclassified|g__Eubacteriales_unclassified|s_
k__Bacteria|p__Firmicutes|c__Clostridia|o__Eubacteriales|f__Eubacteriales_unclassified|g__Eubacteriales_unclassified|s_
k__Bacteria|p__Firmicutes|c__Erysipelotrichia|o__Erysipelotrichales|f__Erysipelotrichaceae|g__Erysipelatoclostridium|s_
k__Bacteria|p__Firmicutes|c__Erysipelotrichia|o__Erysipelotrichales|f__Erysipelotrichaceae|g__Erysipelatoclostridium|s_
k__Bacteria|p__Actinobacteria|c__Coriobacteriia|o__Coriobacteriales|f__Coriobacteriaceae|g__Coriobacteriaceae_unclass
k__Bacteria|p__Actinobacteria|c__Coriobacteriia|o__Coriobacteriales|f__Coriobacteriaceae|g__Coriobacteriaceae_unclass
k__Bacteria|p__Firmicutes|c__Clostridia|o__Eubacteriales|f__Lachnospiraceae|g__Dorea|s__Dorea_sp_5_2
k__Bacteria|p__Firmicutes|c__Clostridia|o__Eubacteriales|f__Lachnospiraceae|g__Dorea|s__Dorea_sp_5_2
k__Bacteria|p__Firmicutes|c__Erysipelotrichia|o__Erysipelotrichales|f__Erysipelotrichaceae|g__Dubosiella|s__Dubosiella_
k__Bacteria|p__Firmicutes|c__Erysipelotrichia|o__Erysipelotrichales|f__Erysipelotrichaceae|g__Dubosiella|s__Dubosiella_
k__Bacteria|p__Firmicutes|c__Erysipelotrichia|o__Erysipelotrichales|f__Erysipelotrichales_unclassified|g__Erysipelotrichal
k__Bacteria|p__Firmicutes|c__Erysipelotrichia|o__Erysipelotrichales|f__Erysipelotrichales_unclassified|g__Erysipelotrichal
k__Bacteria|p__Firmicutes|c__Clostridia|o__Eubacteriales|f__Eubacteriaceae|g__Eubacteriaceae_unclassified|s__Eubacte
k__Bacteria|p__Firmicutes|c__Clostridia|o__Eubacteriales|f__Eubacteriaceae|g__Eubacteriaceae_unclassified|s__Eubacte
k__Bacteria|p__Firmicutes|c__Clostridia|o__Eubacteriales|f__Eubacteriaceae|g__Eubacteriaceae_unclassified|s__Eubacte
k__Bacteria|p__Firmicutes|c__Clostridia|o__Eubacteriales|f__Eubacteriaceae|g__Eubacteriaceae_unclassified|s__Eubacte
k__Bacteria|p__Firmicutes|c__Clostridia|o__Eubacteriales|f__Lachnospiraceae|g__GGB20149|s__GGB20149_SGB29430
k__Bacteria|p__Firmicutes|c__Clostridia|o__Eubacteriales|f__Lachnospiraceae|g__GGB20149|s__GGB20149_SGB29430
k__Bacteria|p__Actinobacteria|c__Coriobacteriia|o__Eggerthellales|f__Eggerthellaceae|g__GGB22635|s__GGB22635_SGB
k__Bacteria|p__Actinobacteria|c__Coriobacteriia|o__Eggerthellales|f__Eggerthellaceae|g__GGB22635|s__GGB22635_SGB
k__Bacteria|p__Firmicutes|c__Clostridia|o__Eubacteriales|f__Lachnospiraceae|g__GGB25041|s__GGB25041_SGB36960
k__Bacteria|p__Firmicutes|c__Clostridia|o__Eubacteriales|f__Lachnospiraceae|g__GGB25041|s__GGB25041_SGB36960
k__Bacteria|p__Bacteroidota|c__Bacteroidia|o__Bacteroidales|f__Muribaculaceae|g__GGB27876|s__GGB27876_SGB4031
k__Bacteria|p__Bacteroidota|c__Bacteroidia|o__Bacteroidales|f__Muribaculaceae|g__GGB27876|s__GGB27876_SGB4031
k__Bacteria|p__Bacteroidota|c__Bacteroidia|o__Bacteroidales|f__Muribaculaceae|g__GGB27878|s__GGB27878_SGB4031
k__Bacteria|p__Bacteroidota|c__Bacteroidia|o__Bacteroidales|f__Muribaculaceae|g__GGB27878|s__GGB27878_SGB4031
k__Bacteria|p__Bacteroidota|c__Bacteroidia|o__Bacteroidales|f__Muribaculaceae|g__GGB27918|s__GGB27918_SGB4035
k__Bacteria|p__Bacteroidota|c__Bacteroidia|o__Bacteroidales|f__Muribaculaceae|g__GGB27918|s__GGB27918_SGB4035
k__Bacteria|p__Firmicutes|c__CFGB9508|o__OFGB9508|f__FGB9508|g__GGB28382|s__GGB28382_SGB40962
k__Bacteria|p__Firmicutes|c__CFGB9508|o__OFGB9508|f__FGB9508|g__GGB28382|s__GGB28382_SGB40962
k__Bacteria|p__Firmicutes|c__CFGB2838|o__OFGB2838|f__FGB2838|g__GGB28399|s__GGB28399_SGB40980
k__Bacteria|p__Firmicutes|c__CFGB2838|o__OFGB2838|f__FGB2838|g__GGB28399|s__GGB28399_SGB40980
k__Bacteria|p__Firmicutes|c__CFGB2838|o__OFGB2838|f__FGB2838|g__GGB28411|s__GGB28411_SGB40993
k__Bacteria|p__Firmicutes|c__CFGB2838|o__OFGB2838|f__FGB2838|g__GGB28411|s__GGB28411_SGB40993
k__Bacteria|p__Firmicutes|c__CFGB2838|o__OFGB2838|f__FGB2838|g__GGB28415|s__GGB28415_SGB40997
k__Bacteria|p__Firmicutes|c__CFGB2838|o__OFGB2838|f__FGB2838|g__GGB28415|s__GGB28415_SGB40997
k__Bacteria|p__Firmicutes|c__Clostridia|o__Eubacteriales|f__Pumilibacteraceae|g__GGB28430|s__GGB28430_SGB41013
k__Bacteria|p__Firmicutes|c__Clostridia|o__Eubacteriales|f__Pumilibacteraceae|g__GGB28430|s__GGB28430_SGB41013
k__Bacteria|p__Firmicutes|c__CFGB28439|o__OFGB28439|f__FGB28439|g__GGB28439|s__GGB28439_SGB41022
k__Bacteria|p__Firmicutes|c__CFGB28439|o__OFGB28439|f__FGB28439|g__GGB28439|s__GGB28439_SGB41022

k__Bacteria|p__Firmicutes|c__Clostridia|o__Clostridia_unclassified|f__Clostridia_unclassified|g__GGB28778|s__GGB2877[illegible]
k__Bacteria|p__Firmicutes|c__Clostridia|o__Clostridia_unclassified|f__Clostridia_unclassified|g__GGB28778|s__GGB2877[illegible]
k__Bacteria|p__Firmicutes|c__Clostridia|o__Eubacteriales|f__Eubacteriaceae|g__GGB28784|s__GGB28784_SGB41437
k__Bacteria|p__Firmicutes|c__Clostridia|o__Eubacteriales|f__Eubacteriaceae|g__GGB28784|s__GGB28784_SGB41437
k__Bacteria|p__Firmicutes|c__Clostridia|o__Eubacteriales|f__Lachnospiraceae|g__GGB28792|s__GGB28792_SGB41445
k__Bacteria|p__Firmicutes|c__Clostridia|o__Eubacteriales|f__Lachnospiraceae|g__GGB28792|s__GGB28792_SGB41445
k__Bacteria|p__Firmicutes|c__Clostridia|o__Eubacteriales|f__Lachnospiraceae|g__GGB28798|s__GGB28798_SGB41451
k__Bacteria|p__Firmicutes|c__Clostridia|o__Eubacteriales|f__Lachnospiraceae|g__GGB28798|s__GGB28798_SGB41451
k__Bacteria|p__Firmicutes|c__Clostridia|o__Eubacteriales|f__Lachnospiraceae|g__GGB28802|s__GGB28802_SGB41455
k__Bacteria|p__Firmicutes|c__Clostridia|o__Eubacteriales|f__Lachnospiraceae|g__GGB28802|s__GGB28802_SGB41455
k__Bacteria|p__Firmicutes|c__Clostridia|o__Eubacteriales|f__Lachnospiraceae|g__GGB28818|s__GGB28818_SGB41473
k__Bacteria|p__Firmicutes|c__Clostridia|o__Eubacteriales|f__Lachnospiraceae|g__GGB28818|s__GGB28818_SGB41473
k__Bacteria|p__Firmicutes|c__CFGB77305|o__OFGB77305|f__FGB77305|g__GGB28828|s__GGB28828_SGB41484
k__Bacteria|p__Firmicutes|c__CFGB77305|o__OFGB77305|f__FGB77305|g__GGB28828|s__GGB28828_SGB41484
k__Bacteria|p__Firmicutes|c__Clostridia|o__Eubacteriales|f__Clostridiaceae|g__GGB28851|s__GGB28851_SGB41518
k__Bacteria|p__Firmicutes|c__Clostridia|o__Eubacteriales|f__Clostridiaceae|g__GGB28851|s__GGB28851_SGB41518
k__Bacteria|p__Firmicutes|c__Clostridia|o__Eubacteriales|f__Lachnospiraceae|g__GGB28859|s__GGB28859_SGB41528
k__Bacteria|p__Firmicutes|c__Clostridia|o__Eubacteriales|f__Lachnospiraceae|g__GGB28859|s__GGB28859_SGB41528
k__Bacteria|p__Firmicutes|c__Clostridia|o__Eubacteriales|f__Lachnospiraceae|g__GGB28864|s__GGB28864_SGB41535
k__Bacteria|p__Firmicutes|c__Clostridia|o__Eubacteriales|f__Lachnospiraceae|g__GGB28864|s__GGB28864_SGB41535
k__Bacteria|p__Firmicutes|c__Clostridia|o__Eubacteriales|f__Lachnospiraceae|g__GGB28869|s__GGB28869_SGB41543
k__Bacteria|p__Firmicutes|c__Clostridia|o__Eubacteriales|f__Lachnospiraceae|g__GGB28869|s__GGB28869_SGB41543
k__Bacteria|p__Firmicutes|c__CFGB9633|o__OFGB9633|f__FGB9633|g__GGB28883|s__GGB28883_SGB41564
k__Bacteria|p__Firmicutes|c__CFGB9633|o__OFGB9633|f__FGB9633|g__GGB28883|s__GGB28883_SGB41564
k__Bacteria|p__Bacteria_unclassified|c__Bacteria_unclassified|o__Bacteria_unclassified|f__Bacteria_unclassified|g__GGB[illegible]
k__Bacteria|p__Bacteria_unclassified|c__Bacteria_unclassified|o__Bacteria_unclassified|f__Bacteria_unclassified|g__GGB[illegible]
k__Bacteria|p__Bacteria_unclassified|c__Bacteria_unclassified|o__Bacteria_unclassified|f__Bacteria_unclassified|g__GGB[illegible]
k__Bacteria|p__Bacteria_unclassified|c__Bacteria_unclassified|o__Bacteria_unclassified|f__Bacteria_unclassified|g__GGB[illegible]
k__Bacteria|p__Bacteria_unclassified|c__Bacteria_unclassified|o__Bacteria_unclassified|f__Bacteria_unclassified|g__GGB[illegible]
k__Bacteria|p__Bacteria_unclassified|c__Bacteria_unclassified|o__Bacteria_unclassified|f__Bacteria_unclassified|g__GGB[illegible]
k__Bacteria|p__Bacteria_unclassified|c__Bacteria_unclassified|o__Bacteria_unclassified|f__Bacteria_unclassified|g__GGB[illegible]
k__Bacteria|p__Bacteria_unclassified|c__Bacteria_unclassified|o__Bacteria_unclassified|f__Bacteria_unclassified|g__GGB[illegible]
k__Bacteria|p__Firmicutes|c__Clostridia|o__Eubacteriales|f__Lachnospiraceae|g__GGB28916|s__GGB28916_SGB41612
k__Bacteria|p__Firmicutes|c__Clostridia|o__Eubacteriales|f__Lachnospiraceae|g__GGB28916|s__GGB28916_SGB41612
k__Bacteria|p__Firmicutes|c__Clostridia|o__Eubacteriales|f__Lachnospiraceae|g__GGB28924|s__GGB28924_SGB41621
k__Bacteria|p__Firmicutes|c__Clostridia|o__Eubacteriales|f__Lachnospiraceae|g__GGB28924|s__GGB28924_SGB41621
k__Bacteria|p__Firmicutes|c__Clostridia|o__Eubacteriales|f__Lachnospiraceae|g__GGB28926|s__GGB28926_SGB41624
k__Bacteria|p__Firmicutes|c__Clostridia|o__Eubacteriales|f__Lachnospiraceae|g__GGB28926|s__GGB28926_SGB41624
k__Bacteria|p__Bacteria_unclassified|c__CFGB77359|o__OFGB77359|f__FGB77359|g__GGB28927|s__GGB28927_SGB416[illegible]
k__Bacteria|p__Bacteria_unclassified|c__CFGB77359|o__OFGB77359|f__FGB77359|g__GGB28927|s__GGB28927_SGB416[illegible]
k__Bacteria|p__Firmicutes|c__CFGB9639|o__OFGB9639|f__FGB9639|g__GGB28934|s__GGB28934_SGB41635
k__Bacteria|p__Firmicutes|c__CFGB9639|o__OFGB9639|f__FGB9639|g__GGB28934|s__GGB28934_SGB41635
k__Bacteria|p__Firmicutes|c__Clostridia|o__Eubacteriales|f__Lachnospiraceae|g__GGB28946|s__GGB28946_SGB41652
k__Bacteria|p__Firmicutes|c__Clostridia|o__Eubacteriales|f__Lachnospiraceae|g__GGB28946|s__GGB28946_SGB41652
k__Bacteria|p__Firmicutes|c__Clostridia|o__Eubacteriales|f__Lachnospiraceae|g__GGB28949|s__GGB28949_SGB41655
k__Bacteria|p__Firmicutes|c__Clostridia|o__Eubacteriales|f__Lachnospiraceae|g__GGB28949|s__GGB28949_SGB41655

k__Bacteria|p__Firmicutes|c__Clostridia|o__Eubacteriales|f__Lachnospiraceae|g__GGB28949|s__GGB28949_SGB41656
k__Bacteria|p__Firmicutes|c__Clostridia|o__Eubacteriales|f__Lachnospiraceae|g__GGB28949|s__GGB28949_SGB41656
k__Bacteria|p__Firmicutes|c__Clostridia|o__Eubacteriales|f__Clostridiaceae|g__GGB28950|s__GGB28950_SGB41657
k__Bacteria|p__Firmicutes|c__Clostridia|o__Eubacteriales|f__Clostridiaceae|g__GGB28950|s__GGB28950_SGB41657
k__Bacteria|p__Firmicutes|c__Clostridia|o__Eubacteriales|f__Clostridiaceae|g__GGB28951|s__GGB28951_SGB102295
k__Bacteria|p__Firmicutes|c__Clostridia|o__Eubacteriales|f__Clostridiaceae|g__GGB28951|s__GGB28951_SGB102295
k__Bacteria|p__Firmicutes|c__Clostridia|o__Eubacteriales|f__Clostridiaceae|g__GGB28951|s__GGB28951_SGB41658
k__Bacteria|p__Firmicutes|c__Clostridia|o__Eubacteriales|f__Clostridiaceae|g__GGB28951|s__GGB28951_SGB41658
k__Bacteria|p__Firmicutes|c__Clostridia|o__Eubacteriales|f__Clostridiaceae|g__GGB28954|s__GGB28954_SGB41662
k__Bacteria|p__Firmicutes|c__Clostridia|o__Eubacteriales|f__Clostridiaceae|g__GGB28954|s__GGB28954_SGB41662
k__Bacteria|p__Firmicutes|c__Clostridia|o__Eubacteriales|f__Clostridiaceae|g__GGB28956|s__GGB28956_SGB41665
k__Bacteria|p__Firmicutes|c__Clostridia|o__Eubacteriales|f__Clostridiaceae|g__GGB28956|s__GGB28956_SGB41665
k__Bacteria|p__Firmicutes|c__Clostridia|o__Eubacteriales|f__Clostridiaceae|g__GGB28960|s__GGB28960_SGB41669
k__Bacteria|p__Firmicutes|c__Clostridia|o__Eubacteriales|f__Clostridiaceae|g__GGB28960|s__GGB28960_SGB41669
k__Bacteria|p__Firmicutes|c__Clostridia|o__Eubacteriales|f__Clostridiaceae|g__GGB28967|s__GGB28967_SGB41678
k__Bacteria|p__Firmicutes|c__Clostridia|o__Eubacteriales|f__Clostridiaceae|g__GGB28967|s__GGB28967_SGB41678
k__Bacteria|p__Firmicutes|c__Clostridia|o__Eubacteriales|f__Eubacteriaceae|g__GGB28991|s__GGB28991_SGB41705
k__Bacteria|p__Firmicutes|c__Clostridia|o__Eubacteriales|f__Eubacteriaceae|g__GGB28991|s__GGB28991_SGB41705
k__Bacteria|p__Firmicutes|c__CFGB9658|o__OFGB9658|f__FGB9658|g__GGB29002|s__GGB29002_SGB41718
k__Bacteria|p__Firmicutes|c__CFGB9658|o__OFGB9658|f__FGB9658|g__GGB29002|s__GGB29002_SGB41718
k__Bacteria|p__Firmicutes|c__CFGB9659|o__OFGB9659|f__FGB9659|g__GGB29003|s__GGB29003_SGB41719
k__Bacteria|p__Firmicutes|c__CFGB9659|o__OFGB9659|f__FGB9659|g__GGB29003|s__GGB29003_SGB41719
k__Bacteria|p__Bacteria_unclassified|c__Bacteria_unclassified|o__Bacteria_unclassified|f__Bacteria_unclassified|g__GGB
k__Bacteria|p__Bacteria_unclassified|c__Bacteria_unclassified|o__Bacteria_unclassified|f__Bacteria_unclassified|g__GGB
k__Bacteria|p__Firmicutes|c__CFGB9827|o__OFGB9827|f__FGB9827|g__GGB29531|s__GGB29531_SGB42317
k__Bacteria|p__Firmicutes|c__CFGB9827|o__OFGB9827|f__FGB9827|g__GGB29531|s__GGB29531_SGB42317
k__Bacteria|p__Firmicutes|c__Clostridia|o__Eubacteriales|f__Eubacteriaceae|g__GGB29685|s__GGB29685_SGB42494
k__Bacteria|p__Firmicutes|c__Clostridia|o__Eubacteriales|f__Eubacteriaceae|g__GGB29685|s__GGB29685_SGB42494
k__Bacteria|p__Bacteria_unclassified|c__CFGB77303|o__OFGB77303|f__FGB77303|g__GGB30141|s__GGB30141_SGB430
k__Bacteria|p__Bacteria_unclassified|c__CFGB77303|o__OFGB77303|f__FGB77303|g__GGB30141|s__GGB30141_SGB430
k__Bacteria|p__Firmicutes|c__Clostridia|o__Eubacteriales|f__Eubacteriales_unclassified|g__GGB30286|s__GGB30286_SG
k__Bacteria|p__Firmicutes|c__Clostridia|o__Eubacteriales|f__Eubacteriales_unclassified|g__GGB30286|s__GGB30286_SG
k__Bacteria|p__Firmicutes|c__Clostridia|o__Eubacteriales|f__Oscillospiraceae|g__GGB30303|s__GGB30303_SGB43268
k__Bacteria|p__Firmicutes|c__Clostridia|o__Eubacteriales|f__Oscillospiraceae|g__GGB30303|s__GGB30303_SGB43268
k__Bacteria|p__Firmicutes|c__CFGB30328|o__OFGB30328|f__FGB30328|g__GGB30413|s__GGB30413_SGB43452
k__Bacteria|p__Firmicutes|c__CFGB30328|o__OFGB30328|f__FGB30328|g__GGB30413|s__GGB30413_SGB43452
k__Bacteria|p__Firmicutes|c__Clostridia|o__Eubacteriales|f__Oscillospiraceae|g__GGB30454|s__GGB30454_SGB43514
k__Bacteria|p__Firmicutes|c__Clostridia|o__Eubacteriales|f__Oscillospiraceae|g__GGB30454|s__GGB30454_SGB43514
k__Bacteria|p__Firmicutes|c__Clostridia|o__Eubacteriales|f__Oscillospiraceae|g__GGB30455|s__GGB30455_SGB43519
k__Bacteria|p__Firmicutes|c__Clostridia|o__Eubacteriales|f__Oscillospiraceae|g__GGB30455|s__GGB30455_SGB43519
k__Bacteria|p__Firmicutes|c__Clostridia|o__Eubacteriales|f__Oscillospiraceae|g__GGB30461|s__GGB30461_SGB43527
k__Bacteria|p__Firmicutes|c__Clostridia|o__Eubacteriales|f__Oscillospiraceae|g__GGB30461|s__GGB30461_SGB43527
k__Bacteria|p__Firmicutes|c__Clostridia|o__Eubacteriales|f__Oscillospiraceae|g__GGB30461|s__GGB30461_SGB43530
k__Bacteria|p__Firmicutes|c__Clostridia|o__Eubacteriales|f__Oscillospiraceae|g__GGB30461|s__GGB30461_SGB43530
k__Bacteria|p__Firmicutes|c__Clostridia|o__Eubacteriales|f__Oscillospiraceae|g__GGB30463|s__GGB30463_SGB43537
k__Bacteria|p__Firmicutes|c__Clostridia|o__Eubacteriales|f__Oscillospiraceae|g__GGB30463|s__GGB30463_SGB43537

k__Bacteria|p__Firmicutes|c__Clostridia|o__Eubacteriales|f__Oscillospiraceae|g__GGB30473|s__GGB30473_SGB43557
k__Bacteria|p__Firmicutes|c__Clostridia|o__Eubacteriales|f__Oscillospiraceae|g__GGB30473|s__GGB30473_SGB43557
k__Bacteria|p__Firmicutes|c__Clostridia|o__Eubacteriales|f__Oscillospiraceae|g__GGB30475|s__GGB30475_SGB63182
k__Bacteria|p__Firmicutes|c__Clostridia|o__Eubacteriales|f__Oscillospiraceae|g__GGB30475|s__GGB30475_SGB63182
k__Bacteria|p__Actinobacteria|c__CFGB77153|o__OFGB77153|f__FGB77153|g__GGB30861|s__GGB30861_SGB44083
k__Bacteria|p__Actinobacteria|c__CFGB77153|o__OFGB77153|f__FGB77153|g__GGB30861|s__GGB30861_SGB44083
k__Bacteria|p__Tenericutes|c__CFGB1791|o__OFGB1791|f__FGB1791|g__GGB31312|s__GGB31312_SGB44628
k__Bacteria|p__Tenericutes|c__CFGB1791|o__OFGB1791|f__FGB1791|g__GGB31312|s__GGB31312_SGB44628
k__Bacteria|p__Firmicutes|c__CFGB10290|o__OFGB10290|f__FGB10290|g__GGB31438|s__GGB31438_SGB44768
k__Bacteria|p__Firmicutes|c__CFGB10290|o__OFGB10290|f__FGB10290|g__GGB31438|s__GGB31438_SGB44768
k__Bacteria|p__Firmicutes|c__Clostridia|o__Eubacteriales|f__Oscillospiraceae|g__GGB3171|s__GGB3171_SGB4185
k__Bacteria|p__Firmicutes|c__Clostridia|o__Eubacteriales|f__Oscillospiraceae|g__GGB3171|s__GGB3171_SGB4185
k__Bacteria|p__Firmicutes|c__CFGB1765|o__OFGB1765|f__FGB1765|g__GGB31823|s__GGB31823_SGB45199
k__Bacteria|p__Firmicutes|c__CFGB1765|o__OFGB1765|f__FGB1765|g__GGB31823|s__GGB31823_SGB45199
k__Bacteria|p__Firmicutes|c__CFGB10349|o__OFGB10349|f__FGB10349|g__GGB31853|s__GGB31853_SGB45233
k__Bacteria|p__Firmicutes|c__CFGB10349|o__OFGB10349|f__FGB10349|g__GGB31853|s__GGB31853_SGB45233
k__Bacteria|p__Firmicutes|c__CFGB10667|o__OFGB10667|f__FGB10667|g__GGB32371|s__GGB32371_SGB41694
k__Bacteria|p__Firmicutes|c__CFGB10667|o__OFGB10667|f__FGB10667|g__GGB32371|s__GGB32371_SGB41694
k__Bacteria|p__Firmicutes|c__Clostridia|o__Eubacteriales|f__Lachnospiraceae|g__GGB3793|s__GGB3793_SGB5158
k__Bacteria|p__Firmicutes|c__Clostridia|o__Eubacteriales|f__Lachnospiraceae|g__GGB3793|s__GGB3793_SGB5158
k__Bacteria|p__Firmicutes|c__Clostridia|o__Eubacteriales|f__Lachnospiraceae|g__GGB42598|s__GGB42598_SGB59794
k__Bacteria|p__Firmicutes|c__Clostridia|o__Eubacteriales|f__Lachnospiraceae|g__GGB42598|s__GGB42598_SGB59794
k__Bacteria|p__Firmicutes|c__Clostridia|o__Eubacteriales|f__Christensenellaceae|g__GGB45656|s__GGB45656_SGB6337
k__Bacteria|p__Firmicutes|c__Clostridia|o__Eubacteriales|f__Christensenellaceae|g__GGB45656|s__GGB45656_SGB6337
k__Bacteria|p__Firmicutes|c__CFGB10299|o__OFGB10299|f__FGB10299|g__GGB47127|s__GGB47127_SGB65054
k__Bacteria|p__Firmicutes|c__CFGB10299|o__OFGB10299|f__FGB10299|g__GGB47127|s__GGB47127_SGB65054
k__Bacteria|p__Firmicutes|c__Clostridia|o__Eubacteriales|f__Oscillospiraceae|g__GGB74395|s__GGB74395_SGB43521
k__Bacteria|p__Firmicutes|c__Clostridia|o__Eubacteriales|f__Oscillospiraceae|g__GGB74395|s__GGB74395_SGB43521
k__Bacteria|p__Firmicutes|c__Clostridia|o__Eubacteriales|f__Oscillospiraceae|g__GGB75053|s__GGB75053_SGB43494
k__Bacteria|p__Firmicutes|c__Clostridia|o__Eubacteriales|f__Oscillospiraceae|g__GGB75053|s__GGB75053_SGB43494
k__Bacteria|p__Firmicutes|c__Clostridia|o__Eubacteriales|f__Lachnospiraceae|g__GGB75109|s__GGB75109_SGB102238
k__Bacteria|p__Firmicutes|c__Clostridia|o__Eubacteriales|f__Lachnospiraceae|g__GGB75109|s__GGB75109_SGB102238
k__Bacteria|p__Firmicutes|c__Clostridia|o__Clostridia_unclassified|f__Clostridia_unclassified|g__GGB81440|s__GGB8144
k__Bacteria|p__Firmicutes|c__Clostridia|o__Clostridia_unclassified|f__Clostridia_unclassified|g__GGB81440|s__GGB8144
k__Bacteria|p__Firmicutes|c__Clostridia|o__Eubacteriales|f__Lachnospiraceae|g__Lachnospiraceae_unclassified|s__Lachn
k__Bacteria|p__Firmicutes|c__Clostridia|o__Eubacteriales|f__Lachnospiraceae|g__Lachnospiraceae_unclassified|s__Lachn
k__Bacteria|p__Firmicutes|c__Clostridia|o__Eubacteriales|f__Lachnospiraceae|g__Lachnospiraceae_unclassified|s__Lachn
k__Bacteria|p__Firmicutes|c__Clostridia|o__Eubacteriales|f__Lachnospiraceae|g__Lachnospiraceae_unclassified|s__Lachn
k__Bacteria|p__Firmicutes|c__Clostridia|o__Eubacteriales|f__Lachnospiraceae|g__Lachnospiraceae_unclassified|s__Lachn
k__Bacteria|p__Firmicutes|c__Clostridia|o__Eubacteriales|f__Lachnospiraceae|g__Lachnospiraceae_unclassified|s__Lachn
k__Bacteria|p__Firmicutes|c__Clostridia|o__Eubacteriales|f__Lachnospiraceae|g__Lachnospiraceae_unclassified|s__Lachn
k__Bacteria|p__Firmicutes|c__Clostridia|o__Eubacteriales|f__Lachnospiraceae|g__Lachnospiraceae_unclassified|s__Lachn
k__Bacteria|p__Firmicutes|c__Clostridia|o__Eubacteriales|f__Lachnospiraceae|g__Lachnospiraceae_unclassified|s__Lachn
k__Bacteria|p__Firmicutes|c__Clostridia|o__Eubacteriales|f__Lachnospiraceae|g__Lachnospiraceae_unclassified|s__Lachn
k__Bacteria|p__Firmicutes|c__Clostridia|o__Eubacteriales|f__Lachnospiraceae|g__Lachnospiraceae_unclassified|s__Lachn
k__Bacteria|p__Firmicutes|c__Clostridia|o__Eubacteriales|f__Lachnospiraceae|g__Lachnospiraceae_unclassified|s__Lachn

k__Bacteria|p__Firmicutes|c__Clostridia|o__Eubacteriales|f__Lachnospiraceae|g__Lachnospiraceae_unclassified|s__Lachn
k__Bacteria|p__Firmicutes|c__Clostridia|o__Eubacteriales|f__Lachnospiraceae|g__Lachnospiraceae_unclassified|s__Lachn
k__Bacteria|p__Firmicutes|c__Clostridia|o__Eubacteriales|f__Lachnospiraceae|g__Lachnospiraceae_unclassified|s__Lachn
k__Bacteria|p__Firmicutes|c__Clostridia|o__Eubacteriales|f__Lachnospiraceae|g__Lachnospiraceae_unclassified|s__Lachn
k__Bacteria|p__Firmicutes|c__Bacilli|o__Lactobacillales|f__Lactobacillaceae|g__Lactobacillus|s__Lactobacillus_johnsonii
k__Bacteria|p__Firmicutes|c__Bacilli|o__Lactobacillales|f__Lactobacillaceae|g__Lactobacillus|s__Lactobacillus_johnsonii
k__Bacteria|p__Bacteroidota|c__Bacteroidia|o__Bacteroidales|f__Muribaculaceae|g__Muribaculaceae_unclassified|s__M
k__Bacteria|p__Bacteroidota|c__Bacteroidia|o__Bacteroidales|f__Muribaculaceae|g__Muribaculaceae_unclassified|s__M
k__Bacteria|p__Firmicutes|c__Clostridia|o__Eubacteriales|f__Oscillospiraceae|g__Neglectibacter|s__Neglectibacter_sp_X
k__Bacteria|p__Firmicutes|c__Clostridia|o__Eubacteriales|f__Oscillospiraceae|g__Neglectibacter|s__Neglectibacter_sp_X
k__Bacteria|p__Firmicutes|c__Clostridia|o__Eubacteriales|f__Oscillospiraceae|g__Oscillospiraceae_unclassified|s__Oscillo
k__Bacteria|p__Firmicutes|c__Clostridia|o__Eubacteriales|f__Oscillospiraceae|g__Oscillospiraceae_unclassified|s__Oscillo
k__Bacteria|p__Firmicutes|c__Clostridia|o__Eubacteriales|f__Oscillospiraceae|g__Oscillospiraceae_unclassified|s__Oscillo
k__Bacteria|p__Firmicutes|c__Clostridia|o__Eubacteriales|f__Oscillospiraceae|g__Oscillospiraceae_unclassified|s__Oscillo
k__Bacteria|p__Firmicutes|c__Clostridia|o__Eubacteriales|f__Oscillospiraceae|g__Oscillospiraceae_unclassified|s__Oscillo
k__Bacteria|p__Firmicutes|c__Clostridia|o__Eubacteriales|f__Oscillospiraceae|g__Oscillospiraceae_unclassified|s__Oscillo
k__Bacteria|p__Proteobacteria|c__Betaproteobacteria|o__Burkholderiales|f__Sutterellaceae|g__Parasutterella|s__Parasu
k__Bacteria|p__Proteobacteria|c__Betaproteobacteria|o__Burkholderiales|f__Sutterellaceae|g__Parasutterella|s__Parasu

k__Bacteria|p__Firmicutes|c__Clostridia|o__Eubacteriales|f__Peptostreptococcaceae|g__Romboutsia|s__Romboutsia_ilea
k__Bacteria|p__Firmicutes|c__Clostridia|o__Eubacteriales|f__Peptostreptococcaceae|g__Romboutsia|s__Romboutsia_ilea
k__Bacteria|p__Firmicutes|c__Clostridia|o__Eubacteriales|f__Lachnospiraceae|g__Schaedlerella|s__Schaedlerella_arabino
k__Bacteria|p__Firmicutes|c__Clostridia|o__Eubacteriales|f__Lachnospiraceae|g__Schaedlerella|s__Schaedlerella_arabino

k__Bacteria|p__Firmicutes|c__Erysipelotrichia|o__Erysipelotrichales|f__Turicibacteraceae|g__Turicibacter|s__Turicibacter_
k__Bacteria|p__Firmicutes|c__Erysipelotrichia|o__Erysipelotrichales|f__Turicibacteraceae|g__Turicibacter|s__Turicibacter_
k__Bacteria|p__Firmicutes|c__Clostridia|o__Eubacteriales|f__Lachnospiraceae|g__Acetatifactor|s__Acetatifactor_muris
k__Bacteria|p__Firmicutes|c__Clostridia|o__Eubacteriales|f__Lachnospiraceae|g__Acetatifactor|s__Acetatifactor_muris
k__Bacteria|p__Firmicutes|c__Clostridia|o__Eubacteriales|f__Lachnospiraceae|g__Acetatifactor|s__Acetatifactor_SGB415
k__Bacteria|p__Firmicutes|c__Clostridia|o__Eubacteriales|f__Lachnospiraceae|g__Acetatifactor|s__Acetatifactor_SGB415
k__Bacteria|p__Firmicutes|c__Clostridia|o__Eubacteriales|f__Oscillospiraceae|g__Acutalibacter|s__Acutalibacter_muris
k__Bacteria|p__Firmicutes|c__Clostridia|o__Eubacteriales|f__Oscillospiraceae|g__Acutalibacter|s__Acutalibacter_muris
k__Bacteria|p__Firmicutes|c__Clostridia|o__Eubacteriales|f__Oscillospiraceae|g__Acutalibacter|s__Acutalibacter_sp_1XD
k__Bacteria|p__Firmicutes|c__Clostridia|o__Eubacteriales|f__Oscillospiraceae|g__Acutalibacter|s__Acutalibacter_sp_1XD
k__Bacteria|p__Actinobacteria|c__Coriobacteriia|o__Eggerthellales|f__Eggerthellaceae|g__Adlercreutzia|s__Adlercreutzia
k__Bacteria|p__Actinobacteria|c__Coriobacteriia|o__Eggerthellales|f__Eggerthellaceae|g__Adlercreutzia|s__Adlercreutzia
k__Bacteria|p__Actinobacteria|c__Coriobacteriia|o__Eggerthellales|f__Eggerthellaceae|g__Adlercreutzia|s__Adlercreutzia
k__Bacteria|p__Actinobacteria|c__Coriobacteriia|o__Eggerthellales|f__Eggerthellaceae|g__Adlercreutzia|s__Adlercreutzia
k__Bacteria|p__Actinobacteria|c__Coriobacteriia|o__Eggerthellales|f__Eggerthellaceae|g__Adlercreutzia|s__Adlercreutzia
k__Bacteria|p__Actinobacteria|c__Coriobacteriia|o__Eggerthellales|f__Eggerthellaceae|g__Adlercreutzia|s__Adlercreutzia
k__Bacteria|p__Verrucomicrobia|c__Verrucomicrobiae|o__Verrucomicrobiales|f__Akkermansiaceae|g__Akkermansia|s__A
k__Bacteria|p__Verrucomicrobia|c__Verrucomicrobiae|o__Verrucomicrobiales|f__Akkermansiaceae|g__Akkermansia|s__A
k__Bacteria|p__Bacteroidota|c__Bacteroidia|o__Bacteroidales|f__Rikenellaceae|g__Alistipes|s__Alistipes_sp_DSM_11234
k__Bacteria|p__Bacteroidota|c__Bacteroidia|o__Bacteroidales|f__Rikenellaceae|g__Alistipes|s__Alistipes_sp_DSM_11234

k__Bacteria|p__Firmicutes|c__Clostridia|o__Eubacteriales|f__Oscillospiraceae|g__Anaerotruncus|s__Anaerotruncus_sp_1
k__Bacteria|p__Firmicutes|c__Clostridia|o__Eubacteriales|f__Oscillospiraceae|g__Anaerotruncus|s__Anaerotruncus_sp_1
k__Bacteria|p__Bacteria_unclassified|c__Bacteria_unclassified|o__Bacteria_unclassified|f__Bacteria_unclassified|g__Bacte
k__Bacteria|p__Bacteria_unclassified|c__Bacteria_unclassified|o__Bacteria_unclassified|f__Bacteria_unclassified|g__Bacte
k__Bacteria|p__Bacteria_unclassified|c__Bacteria_unclassified|o__Bacteria_unclassified|f__Bacteria_unclassified|g__Bacte
k__Bacteria|p__Bacteria_unclassified|c__Bacteria_unclassified|o__Bacteria_unclassified|f__Bacteria_unclassified|g__Bacte
k__Bacteria|p__Bacteria_unclassified|c__Bacteria_unclassified|o__Bacteria_unclassified|f__Bacteria_unclassified|g__Bacte
k__Bacteria|p__Bacteria_unclassified|c__Bacteria_unclassified|o__Bacteria_unclassified|f__Bacteria_unclassified|g__Bacte
k__Bacteria|p__Bacteria_unclassified|c__Bacteria_unclassified|o__Bacteria_unclassified|f__Bacteria_unclassified|g__Bacte
k__Bacteria|p__Bacteria_unclassified|c__Bacteria_unclassified|o__Bacteria_unclassified|f__Bacteria_unclassified|g__Bacte
k__Bacteria|p__Bacteria_unclassified|c__Bacteria_unclassified|o__Bacteria_unclassified|f__Bacteria_unclassified|g__Bacte
k__Bacteria|p__Bacteria_unclassified|c__Bacteria_unclassified|o__Bacteria_unclassified|f__Bacteria_unclassified|g__Bacte
k__Bacteria|p__Bacteria_unclassified|c__Bacteria_unclassified|o__Bacteria_unclassified|f__Bacteria_unclassified|g__Bacte
k__Bacteria|p__Bacteria_unclassified|c__Bacteria_unclassified|o__Bacteria_unclassified|f__Bacteria_unclassified|g__Bacte
k__Bacteria|p__Bacteroidota|c__Bacteroidia|o__Bacteroidales|f__Bacteroidaceae|g__Bacteroides|s__Bacteroides_thetaio
k__Bacteria|p__Bacteroidota|c__Bacteroidia|o__Bacteroidales|f__Bacteroidaceae|g__Bacteroides|s__Bacteroides_thetaio

k__Bacteria|p__Actinobacteria|c__Actinomycetia|o__Bifidobacteriales|f__Bifidobacteriaceae|g__Bifidobacterium|s__Bifid
k__Bacteria|p__Actinobacteria|c__Actinomycetia|o__Bifidobacteriales|f__Bifidobacteriaceae|g__Bifidobacterium|s__Bifid
k__Bacteria|p__Firmicutes|c__Clostridia|o__Clostridia_unclassified|f__Clostridia_unclassified|g__Clostridia_unclassified|s_
k__Bacteria|p__Firmicutes|c__Clostridia|o__Clostridia_unclassified|f__Clostridia_unclassified|g__Clostridia_unclassified|s_
k__Bacteria|p__Firmicutes|c__Clostridia|o__Eubacteriales|f__Clostridiaceae|g__Clostridiaceae_unclassified|s__Clostridiac
k__Bacteria|p__Firmicutes|c__Clostridia|o__Eubacteriales|f__Clostridiaceae|g__Clostridiaceae_unclassified|s__Clostridiac
k__Bacteria|p__Firmicutes|c__Clostridia|o__Eubacteriales|f__Clostridiaceae|g__Clostridiaceae_unclassified|s__Clostridiac
k__Bacteria|p__Firmicutes|c__Clostridia|o__Eubacteriales|f__Clostridiaceae|g__Clostridiaceae_unclassified|s__Clostridiac
k__Bacteria|p__Firmicutes|c__Clostridia|o__Eubacteriales|f__Eubacteriales_unclassified|g__Eubacteriales_unclassified|s_
k__Bacteria|p__Firmicutes|c__Clostridia|o__Eubacteriales|f__Eubacteriales_unclassified|g__Eubacteriales_unclassified|s_
k__Bacteria|p__Firmicutes|c__Erysipelotrichia|o__Erysipelotrichales|f__Erysipelotrichaceae|g__Erysipelatoclostridium|s__
k__Bacteria|p__Firmicutes|c__Erysipelotrichia|o__Erysipelotrichales|f__Erysipelotrichaceae|g__Erysipelatoclostridium|s__
k__Bacteria|p__Actinobacteria|c__Coriobacteriia|o__Coriobacteriales|f__Coriobacteriaceae|g__Coriobacteriaceae_unclass
k__Bacteria|p__Actinobacteria|c__Coriobacteriia|o__Coriobacteriales|f__Coriobacteriaceae|g__Coriobacteriaceae_unclass
k__Bacteria|p__Firmicutes|c__Clostridia|o__Eubacteriales|f__Lachnospiraceae|g__Dorea|s__Dorea_sp_5_2
k__Bacteria|p__Firmicutes|c__Clostridia|o__Eubacteriales|f__Lachnospiraceae|g__Dorea|s__Dorea_sp_5_2
k__Bacteria|p__Firmicutes|c__Erysipelotrichia|o__Erysipelotrichales|f__Erysipelotrichaceae|g__Dubosiella|s__Dubosiella_
k__Bacteria|p__Firmicutes|c__Erysipelotrichia|o__Erysipelotrichales|f__Erysipelotrichaceae|g__Dubosiella|s__Dubosiella_
k__Bacteria|p__Firmicutes|c__Erysipelotrichia|o__Erysipelotrichales|f__Erysipelotrichales_unclassified|g__Erysipelotrichal
k__Bacteria|p__Firmicutes|c__Erysipelotrichia|o__Erysipelotrichales|f__Erysipelotrichales_unclassified|g__Erysipelotrichal
k__Bacteria|p__Firmicutes|c__Clostridia|o__Eubacteriales|f__Eubacteriaceae|g__Eubacteriaceae_unclassified|s__Eubacte
k__Bacteria|p__Firmicutes|c__Clostridia|o__Eubacteriales|f__Eubacteriaceae|g__Eubacteriaceae_unclassified|s__Eubacte
k__Bacteria|p__Firmicutes|c__Clostridia|o__Eubacteriales|f__Eubacteriaceae|g__Eubacteriaceae_unclassified|s__Eubacte
k__Bacteria|p__Firmicutes|c__Clostridia|o__Eubacteriales|f__Eubacteriaceae|g__Eubacteriaceae_unclassified|s__Eubacte
k__Bacteria|p__Firmicutes|c__Clostridia|o__Eubacteriales|f__Lachnospiraceae|g__GGB20149|s__GGB20149_SGB29430
k__Bacteria|p__Firmicutes|c__Clostridia|o__Eubacteriales|f__Lachnospiraceae|g__GGB20149|s__GGB20149_SGB29430
k__Bacteria|p__Actinobacteria|c__Coriobacteriia|o__Eggerthellales|f__Eggerthellaceae|g__GGB22635|s__GGB22635_SGB
k__Bacteria|p__Actinobacteria|c__Coriobacteriia|o__Eggerthellales|f__Eggerthellaceae|g__GGB22635|s__GGB22635_SGB

k__Bacteria|p__Firmicutes|c__Clostridia|o__Eubacteriales|f__Lachnospiraceae|g__GGB25041|s__GGB25041_SGB36960
k__Bacteria|p__Firmicutes|c__Clostridia|o__Eubacteriales|f__Lachnospiraceae|g__GGB25041|s__GGB25041_SGB36960
k__Bacteria|p__Bacteroidota|c__Bacteroidia|o__Bacteroidales|f__Muribaculaceae|g__GGB27876|s__GGB27876_SGB4031
k__Bacteria|p__Bacteroidota|c__Bacteroidia|o__Bacteroidales|f__Muribaculaceae|g__GGB27876|s__GGB27876_SGB4031
k__Bacteria|p__Bacteroidota|c__Bacteroidia|o__Bacteroidales|f__Muribaculaceae|g__GGB27878|s__GGB27878_SGB4031
k__Bacteria|p__Bacteroidota|c__Bacteroidia|o__Bacteroidales|f__Muribaculaceae|g__GGB27878|s__GGB27878_SGB4031
k__Bacteria|p__Bacteroidota|c__Bacteroidia|o__Bacteroidales|f__Muribaculaceae|g__GGB27918|s__GGB27918_SGB4035
k__Bacteria|p__Bacteroidota|c__Bacteroidia|o__Bacteroidales|f__Muribaculaceae|g__GGB27918|s__GGB27918_SGB4035
k__Bacteria|p__Firmicutes|c__CFGB9508|o__OFGB9508|f__FGB9508|g__GGB28382|s__GGB28382_SGB40962
k__Bacteria|p__Firmicutes|c__CFGB9508|o__OFGB9508|f__FGB9508|g__GGB28382|s__GGB28382_SGB40962
k__Bacteria|p__Firmicutes|c__CFGB2838|o__OFGB2838|f__FGB2838|g__GGB28399|s__GGB28399_SGB40980
k__Bacteria|p__Firmicutes|c__CFGB2838|o__OFGB2838|f__FGB2838|g__GGB28399|s__GGB28399_SGB40980
k__Bacteria|p__Firmicutes|c__CFGB2838|o__OFGB2838|f__FGB2838|g__GGB28411|s__GGB28411_SGB40993
k__Bacteria|p__Firmicutes|c__CFGB2838|o__OFGB2838|f__FGB2838|g__GGB28411|s__GGB28411_SGB40993
k__Bacteria|p__Firmicutes|c__CFGB2838|o__OFGB2838|f__FGB2838|g__GGB28415|s__GGB28415_SGB40997
k__Bacteria|p__Firmicutes|c__CFGB2838|o__OFGB2838|f__FGB2838|g__GGB28415|s__GGB28415_SGB40997
k__Bacteria|p__Firmicutes|c__Clostridia|o__Eubacteriales|f__Pumilibacteraceae|g__GGB28430|s__GGB28430_SGB41013
k__Bacteria|p__Firmicutes|c__Clostridia|o__Eubacteriales|f__Pumilibacteraceae|g__GGB28430|s__GGB28430_SGB41013
k__Bacteria|p__Firmicutes|c__CFGB28439|o__OFGB28439|f__FGB28439|g__GGB28439|s__GGB28439_SGB41022
k__Bacteria|p__Firmicutes|c__CFGB28439|o__OFGB28439|f__FGB28439|g__GGB28439|s__GGB28439_SGB41022
k__Bacteria|p__Firmicutes|c__Clostridia|o__Clostridia_unclassified|f__Clostridia_unclassified|g__GGB28778|s__GGB2877
k__Bacteria|p__Firmicutes|c__Clostridia|o__Clostridia_unclassified|f__Clostridia_unclassified|g__GGB28778|s__GGB2877
k__Bacteria|p__Firmicutes|c__Clostridia|o__Eubacteriales|f__Eubacteriaceae|g__GGB28784|s__GGB28784_SGB41437
k__Bacteria|p__Firmicutes|c__Clostridia|o__Eubacteriales|f__Eubacteriaceae|g__GGB28784|s__GGB28784_SGB41437
k__Bacteria|p__Firmicutes|c__Clostridia|o__Eubacteriales|f__Lachnospiraceae|g__GGB28792|s__GGB28792_SGB41445
k__Bacteria|p__Firmicutes|c__Clostridia|o__Eubacteriales|f__Lachnospiraceae|g__GGB28792|s__GGB28792_SGB41445
k__Bacteria|p__Firmicutes|c__Clostridia|o__Eubacteriales|f__Lachnospiraceae|g__GGB28798|s__GGB28798_SGB41451
k__Bacteria|p__Firmicutes|c__Clostridia|o__Eubacteriales|f__Lachnospiraceae|g__GGB28798|s__GGB28798_SGB41451
k__Bacteria|p__Firmicutes|c__Clostridia|o__Eubacteriales|f__Lachnospiraceae|g__GGB28802|s__GGB28802_SGB41455
k__Bacteria|p__Firmicutes|c__Clostridia|o__Eubacteriales|f__Lachnospiraceae|g__GGB28802|s__GGB28802_SGB41455
k__Bacteria|p__Firmicutes|c__Clostridia|o__Eubacteriales|f__Lachnospiraceae|g__GGB28818|s__GGB28818_SGB41473
k__Bacteria|p__Firmicutes|c__Clostridia|o__Eubacteriales|f__Lachnospiraceae|g__GGB28818|s__GGB28818_SGB41473
k__Bacteria|p__Firmicutes|c__CFGB77305|o__OFGB77305|f__FGB77305|g__GGB28828|s__GGB28828_SGB41484
k__Bacteria|p__Firmicutes|c__CFGB77305|o__OFGB77305|f__FGB77305|g__GGB28828|s__GGB28828_SGB41484
k__Bacteria|p__Firmicutes|c__Clostridia|o__Eubacteriales|f__Clostridiaceae|g__GGB28851|s__GGB28851_SGB41518
k__Bacteria|p__Firmicutes|c__Clostridia|o__Eubacteriales|f__Clostridiaceae|g__GGB28851|s__GGB28851_SGB41518
k__Bacteria|p__Firmicutes|c__Clostridia|o__Eubacteriales|f__Lachnospiraceae|g__GGB28859|s__GGB28859_SGB41528
k__Bacteria|p__Firmicutes|c__Clostridia|o__Eubacteriales|f__Lachnospiraceae|g__GGB28859|s__GGB28859_SGB41528
k__Bacteria|p__Firmicutes|c__Clostridia|o__Eubacteriales|f__Lachnospiraceae|g__GGB28864|s__GGB28864_SGB41535
k__Bacteria|p__Firmicutes|c__Clostridia|o__Eubacteriales|f__Lachnospiraceae|g__GGB28864|s__GGB28864_SGB41535
k__Bacteria|p__Firmicutes|c__Clostridia|o__Eubacteriales|f__Lachnospiraceae|g__GGB28869|s__GGB28869_SGB41543
k__Bacteria|p__Firmicutes|c__Clostridia|o__Eubacteriales|f__Lachnospiraceae|g__GGB28869|s__GGB28869_SGB41543
k__Bacteria|p__Firmicutes|c__CFGB9633|o__OFGB9633|f__FGB9633|g__GGB28883|s__GGB28883_SGB41564
k__Bacteria|p__Firmicutes|c__CFGB9633|o__OFGB9633|f__FGB9633|g__GGB28883|s__GGB28883_SGB41564
k__Bacteria|p__Bacteria_unclassified|c__Bacteria_unclassified|o__Bacteria_unclassified|f__Bacteria_unclassified|g__GGB2
k__Bacteria|p__Bacteria_unclassified|c__Bacteria_unclassified|o__Bacteria_unclassified|f__Bacteria_unclassified|g__GGB2

k__Bacteria|p__Bacteria_unclassified|c__Bacteria_unclassified|o__Bacteria_unclassified|f__Bacteria_unclassified|g__GGB2
k__Bacteria|p__Bacteria_unclassified|c__Bacteria_unclassified|o__Bacteria_unclassified|f__Bacteria_unclassified|g__GGB2
k__Bacteria|p__Bacteria_unclassified|c__Bacteria_unclassified|o__Bacteria_unclassified|f__Bacteria_unclassified|g__GGB2
k__Bacteria|p__Bacteria_unclassified|c__Bacteria_unclassified|o__Bacteria_unclassified|f__Bacteria_unclassified|g__GGB2
k__Bacteria|p__Bacteria_unclassified|c__Bacteria_unclassified|o__Bacteria_unclassified|f__Bacteria_unclassified|g__GGB2
k__Bacteria|p__Bacteria_unclassified|c__Bacteria_unclassified|o__Bacteria_unclassified|f__Bacteria_unclassified|g__GGB2
k__Bacteria|p__Firmicutes|c__Clostridia|o__Eubacteriales|f__Lachnospiraceae|g__GGB28916|s__GGB28916_SGB41612
k__Bacteria|p__Firmicutes|c__Clostridia|o__Eubacteriales|f__Lachnospiraceae|g__GGB28916|s__GGB28916_SGB41612
k__Bacteria|p__Firmicutes|c__Clostridia|o__Eubacteriales|f__Lachnospiraceae|g__GGB28924|s__GGB28924_SGB41621
k__Bacteria|p__Firmicutes|c__Clostridia|o__Eubacteriales|f__Lachnospiraceae|g__GGB28924|s__GGB28924_SGB41621
k__Bacteria|p__Firmicutes|c__Clostridia|o__Eubacteriales|f__Lachnospiraceae|g__GGB28926|s__GGB28926_SGB41624
k__Bacteria|p__Firmicutes|c__Clostridia|o__Eubacteriales|f__Lachnospiraceae|g__GGB28926|s__GGB28926_SGB41624
k__Bacteria|p__Bacteria_unclassified|c__CFGB77359|o__OFGB77359|f__FGB77359|g__GGB28927|s__GGB28927_SGB416
k__Bacteria|p__Bacteria_unclassified|c__CFGB77359|o__OFGB77359|f__FGB77359|g__GGB28927|s__GGB28927_SGB416
k__Bacteria|p__Firmicutes|c__CFGB9639|o__OFGB9639|f__FGB9639|g__GGB28934|s__GGB28934_SGB41635
k__Bacteria|p__Firmicutes|c__CFGB9639|o__OFGB9639|f__FGB9639|g__GGB28934|s__GGB28934_SGB41635
k__Bacteria|p__Firmicutes|c__Clostridia|o__Eubacteriales|f__Lachnospiraceae|g__GGB28946|s__GGB28946_SGB41652
k__Bacteria|p__Firmicutes|c__Clostridia|o__Eubacteriales|f__Lachnospiraceae|g__GGB28946|s__GGB28946_SGB41652
k__Bacteria|p__Firmicutes|c__Clostridia|o__Eubacteriales|f__Lachnospiraceae|g__GGB28949|s__GGB28949_SGB41655
k__Bacteria|p__Firmicutes|c__Clostridia|o__Eubacteriales|f__Lachnospiraceae|g__GGB28949|s__GGB28949_SGB41655
k__Bacteria|p__Firmicutes|c__Clostridia|o__Eubacteriales|f__Lachnospiraceae|g__GGB28949|s__GGB28949_SGB41656
k__Bacteria|p__Firmicutes|c__Clostridia|o__Eubacteriales|f__Lachnospiraceae|g__GGB28949|s__GGB28949_SGB41656
k__Bacteria|p__Firmicutes|c__Clostridia|o__Eubacteriales|f__Clostridiaceae|g__GGB28950|s__GGB28950_SGB41657
k__Bacteria|p__Firmicutes|c__Clostridia|o__Eubacteriales|f__Clostridiaceae|g__GGB28950|s__GGB28950_SGB41657
k__Bacteria|p__Firmicutes|c__Clostridia|o__Eubacteriales|f__Clostridiaceae|g__GGB28951|s__GGB28951_SGB102295
k__Bacteria|p__Firmicutes|c__Clostridia|o__Eubacteriales|f__Clostridiaceae|g__GGB28951|s__GGB28951_SGB102295
k__Bacteria|p__Firmicutes|c__Clostridia|o__Eubacteriales|f__Clostridiaceae|g__GGB28951|s__GGB28951_SGB41658
k__Bacteria|p__Firmicutes|c__Clostridia|o__Eubacteriales|f__Clostridiaceae|g__GGB28951|s__GGB28951_SGB41658
k__Bacteria|p__Firmicutes|c__Clostridia|o__Eubacteriales|f__Clostridiaceae|g__GGB28954|s__GGB28954_SGB41662
k__Bacteria|p__Firmicutes|c__Clostridia|o__Eubacteriales|f__Clostridiaceae|g__GGB28954|s__GGB28954_SGB41662
k__Bacteria|p__Firmicutes|c__Clostridia|o__Eubacteriales|f__Clostridiaceae|g__GGB28956|s__GGB28956_SGB41665
k__Bacteria|p__Firmicutes|c__Clostridia|o__Eubacteriales|f__Clostridiaceae|g__GGB28956|s__GGB28956_SGB41665
k__Bacteria|p__Firmicutes|c__Clostridia|o__Eubacteriales|f__Clostridiaceae|g__GGB28960|s__GGB28960_SGB41669
k__Bacteria|p__Firmicutes|c__Clostridia|o__Eubacteriales|f__Clostridiaceae|g__GGB28960|s__GGB28960_SGB41669
k__Bacteria|p__Firmicutes|c__Clostridia|o__Eubacteriales|f__Clostridiaceae|g__GGB28967|s__GGB28967_SGB41678
k__Bacteria|p__Firmicutes|c__Clostridia|o__Eubacteriales|f__Clostridiaceae|g__GGB28967|s__GGB28967_SGB41678
k__Bacteria|p__Firmicutes|c__Clostridia|o__Eubacteriales|f__Eubacteriaceae|g__GGB28991|s__GGB28991_SGB41705
k__Bacteria|p__Firmicutes|c__Clostridia|o__Eubacteriales|f__Eubacteriaceae|g__GGB28991|s__GGB28991_SGB41705
k__Bacteria|p__Firmicutes|c__CFGB9658|o__OFGB9658|f__FGB9658|g__GGB29002|s__GGB29002_SGB41718
k__Bacteria|p__Firmicutes|c__CFGB9658|o__OFGB9658|f__FGB9658|g__GGB29002|s__GGB29002_SGB41718
k__Bacteria|p__Firmicutes|c__CFGB9659|o__OFGB9659|f__FGB9659|g__GGB29003|s__GGB29003_SGB41719
k__Bacteria|p__Firmicutes|c__CFGB9659|o__OFGB9659|f__FGB9659|g__GGB29003|s__GGB29003_SGB41719
k__Bacteria|p__Bacteria_unclassified|c__Bacteria_unclassified|o__Bacteria_unclassified|f__Bacteria_unclassified|g__GGB2
k__Bacteria|p__Bacteria_unclassified|c__Bacteria_unclassified|o__Bacteria_unclassified|f__Bacteria_unclassified|g__GGB2
k__Bacteria|p__Firmicutes|c__CFGB9827|o__OFGB9827|f__FGB9827|g__GGB29531|s__GGB29531_SGB42317
k__Bacteria|p__Firmicutes|c__CFGB9827|o__OFGB9827|f__FGB9827|g__GGB29531|s__GGB29531_SGB42317

k__Bacteria|p__Firmicutes|c__Clostridia|o__Eubacteriales|f__Eubacteriaceae|g__GGB29685|s__GGB29685_SGB42494
k__Bacteria|p__Firmicutes|c__Clostridia|o__Eubacteriales|f__Eubacteriaceae|g__GGB29685|s__GGB29685_SGB42494
k__Bacteria|p__Bacteria_unclassified|c__CFGB77303|o__OFGB77303|f__FGB77303|g__GGB30141|s__GGB30141_SGB430
k__Bacteria|p__Bacteria_unclassified|c__CFGB77303|o__OFGB77303|f__FGB77303|g__GGB30141|s__GGB30141_SGB430
k__Bacteria|p__Firmicutes|c__Clostridia|o__Eubacteriales|f__Eubacteriales_unclassified|g__GGB30286|s__GGB30286_SG
k__Bacteria|p__Firmicutes|c__Clostridia|o__Eubacteriales|f__Eubacteriales_unclassified|g__GGB30286|s__GGB30286_SG
k__Bacteria|p__Firmicutes|c__Clostridia|o__Eubacteriales|f__Oscillospiraceae|g__GGB30303|s__GGB30303_SGB43268
k__Bacteria|p__Firmicutes|c__Clostridia|o__Eubacteriales|f__Oscillospiraceae|g__GGB30303|s__GGB30303_SGB43268
k__Bacteria|p__Firmicutes|c__CFGB30328|o__OFGB30328|f__FGB30328|g__GGB30413|s__GGB30413_SGB43452
k__Bacteria|p__Firmicutes|c__CFGB30328|o__OFGB30328|f__FGB30328|g__GGB30413|s__GGB30413_SGB43452
k__Bacteria|p__Firmicutes|c__Clostridia|o__Eubacteriales|f__Oscillospiraceae|g__GGB30454|s__GGB30454_SGB43514
k__Bacteria|p__Firmicutes|c__Clostridia|o__Eubacteriales|f__Oscillospiraceae|g__GGB30454|s__GGB30454_SGB43514
k__Bacteria|p__Firmicutes|c__Clostridia|o__Eubacteriales|f__Oscillospiraceae|g__GGB30455|s__GGB30455_SGB43519
k__Bacteria|p__Firmicutes|c__Clostridia|o__Eubacteriales|f__Oscillospiraceae|g__GGB30455|s__GGB30455_SGB43519
k__Bacteria|p__Firmicutes|c__Clostridia|o__Eubacteriales|f__Oscillospiraceae|g__GGB30461|s__GGB30461_SGB43527
k__Bacteria|p__Firmicutes|c__Clostridia|o__Eubacteriales|f__Oscillospiraceae|g__GGB30461|s__GGB30461_SGB43527
k__Bacteria|p__Firmicutes|c__Clostridia|o__Eubacteriales|f__Oscillospiraceae|g__GGB30461|s__GGB30461_SGB43530
k__Bacteria|p__Firmicutes|c__Clostridia|o__Eubacteriales|f__Oscillospiraceae|g__GGB30461|s__GGB30461_SGB43530
k__Bacteria|p__Firmicutes|c__Clostridia|o__Eubacteriales|f__Oscillospiraceae|g__GGB30463|s__GGB30463_SGB43537
k__Bacteria|p__Firmicutes|c__Clostridia|o__Eubacteriales|f__Oscillospiraceae|g__GGB30463|s__GGB30463_SGB43537
k__Bacteria|p__Firmicutes|c__Clostridia|o__Eubacteriales|f__Oscillospiraceae|g__GGB30473|s__GGB30473_SGB43557
k__Bacteria|p__Firmicutes|c__Clostridia|o__Eubacteriales|f__Oscillospiraceae|g__GGB30473|s__GGB30473_SGB43557
k__Bacteria|p__Firmicutes|c__Clostridia|o__Eubacteriales|f__Oscillospiraceae|g__GGB30475|s__GGB30475_SGB63182
k__Bacteria|p__Firmicutes|c__Clostridia|o__Eubacteriales|f__Oscillospiraceae|g__GGB30475|s__GGB30475_SGB63182
k__Bacteria|p__Actinobacteria|c__CFGB77153|o__OFGB77153|f__FGB77153|g__GGB30861|s__GGB30861_SGB44083
k__Bacteria|p__Actinobacteria|c__CFGB77153|o__OFGB77153|f__FGB77153|g__GGB30861|s__GGB30861_SGB44083
k__Bacteria|p__Tenericutes|c__CFGB1791|o__OFGB1791|f__FGB1791|g__GGB31312|s__GGB31312_SGB44628
k__Bacteria|p__Tenericutes|c__CFGB1791|o__OFGB1791|f__FGB1791|g__GGB31312|s__GGB31312_SGB44628
k__Bacteria|p__Firmicutes|c__CFGB10290|o__OFGB10290|f__FGB10290|g__GGB31438|s__GGB31438_SGB44768
k__Bacteria|p__Firmicutes|c__CFGB10290|o__OFGB10290|f__FGB10290|g__GGB31438|s__GGB31438_SGB44768
k__Bacteria|p__Firmicutes|c__Clostridia|o__Eubacteriales|f__Oscillospiraceae|g__GGB3171|s__GGB3171_SGB4185
k__Bacteria|p__Firmicutes|c__Clostridia|o__Eubacteriales|f__Oscillospiraceae|g__GGB3171|s__GGB3171_SGB4185
k__Bacteria|p__Firmicutes|c__CFGB1765|o__OFGB1765|f__FGB1765|g__GGB31823|s__GGB31823_SGB45199
k__Bacteria|p__Firmicutes|c__CFGB1765|o__OFGB1765|f__FGB1765|g__GGB31823|s__GGB31823_SGB45199
k__Bacteria|p__Firmicutes|c__CFGB10349|o__OFGB10349|f__FGB10349|g__GGB31853|s__GGB31853_SGB45233
k__Bacteria|p__Firmicutes|c__CFGB10349|o__OFGB10349|f__FGB10349|g__GGB31853|s__GGB31853_SGB45233
k__Bacteria|p__Firmicutes|c__CFGB10667|o__OFGB10667|f__FGB10667|g__GGB32371|s__GGB32371_SGB41694
k__Bacteria|p__Firmicutes|c__CFGB10667|o__OFGB10667|f__FGB10667|g__GGB32371|s__GGB32371_SGB41694
k__Bacteria|p__Firmicutes|c__Clostridia|o__Eubacteriales|f__Lachnospiraceae|g__GGB3793|s__GGB3793_SGB5158
k__Bacteria|p__Firmicutes|c__Clostridia|o__Eubacteriales|f__Lachnospiraceae|g__GGB3793|s__GGB3793_SGB5158
k__Bacteria|p__Firmicutes|c__Clostridia|o__Eubacteriales|f__Lachnospiraceae|g__GGB42598|s__GGB42598_SGB59794
k__Bacteria|p__Firmicutes|c__Clostridia|o__Eubacteriales|f__Lachnospiraceae|g__GGB42598|s__GGB42598_SGB59794
k__Bacteria|p__Firmicutes|c__Clostridia|o__Eubacteriales|f__Christensenellaceae|g__GGB45656|s__GGB45656_SGB6337
k__Bacteria|p__Firmicutes|c__Clostridia|o__Eubacteriales|f__Christensenellaceae|g__GGB45656|s__GGB45656_SGB6337
k__Bacteria|p__Firmicutes|c__CFGB10299|o__OFGB10299|f__FGB10299|g__GGB47127|s__GGB47127_SGB65054
k__Bacteria|p__Firmicutes|c__CFGB10299|o__OFGB10299|f__FGB10299|g__GGB47127|s__GGB47127_SGB65054

k__Bacteria|p__Firmicutes|c__Clostridia|o__Eubacteriales|f__Oscillospiraceae|g__GGB74395|s__GGB74395_SGB43521
k__Bacteria|p__Firmicutes|c__Clostridia|o__Eubacteriales|f__Oscillospiraceae|g__GGB74395|s__GGB74395_SGB43521
k__Bacteria|p__Firmicutes|c__Clostridia|o__Eubacteriales|f__Oscillospiraceae|g__GGB75053|s__GGB75053_SGB43494
k__Bacteria|p__Firmicutes|c__Clostridia|o__Eubacteriales|f__Oscillospiraceae|g__GGB75053|s__GGB75053_SGB43494
k__Bacteria|p__Firmicutes|c__Clostridia|o__Eubacteriales|f__Lachnospiraceae|g__GGB75109|s__GGB75109_SGB102238
k__Bacteria|p__Firmicutes|c__Clostridia|o__Eubacteriales|f__Lachnospiraceae|g__GGB75109|s__GGB75109_SGB102238
k__Bacteria|p__Firmicutes|c__Clostridia|o__Clostridia_unclassified|f__Clostridia_unclassified|g__GGB81440|s__GGB8144
k__Bacteria|p__Firmicutes|c__Clostridia|o__Clostridia_unclassified|f__Clostridia_unclassified|g__GGB81440|s__GGB8144
k__Bacteria|p__Firmicutes|c__Clostridia|o__Eubacteriales|f__Lachnospiraceae|g__Lachnospiraceae_unclassified|s__Lachn
k__Bacteria|p__Firmicutes|c__Clostridia|o__Eubacteriales|f__Lachnospiraceae|g__Lachnospiraceae_unclassified|s__Lachn
k__Bacteria|p__Firmicutes|c__Clostridia|o__Eubacteriales|f__Lachnospiraceae|g__Lachnospiraceae_unclassified|s__Lachn
k__Bacteria|p__Firmicutes|c__Clostridia|o__Eubacteriales|f__Lachnospiraceae|g__Lachnospiraceae_unclassified|s__Lachn
k__Bacteria|p__Firmicutes|c__Clostridia|o__Eubacteriales|f__Lachnospiraceae|g__Lachnospiraceae_unclassified|s__Lachn
k__Bacteria|p__Firmicutes|c__Clostridia|o__Eubacteriales|f__Lachnospiraceae|g__Lachnospiraceae_unclassified|s__Lachn
k__Bacteria|p__Firmicutes|c__Clostridia|o__Eubacteriales|f__Lachnospiraceae|g__Lachnospiraceae_unclassified|s__Lachn
k__Bacteria|p__Firmicutes|c__Clostridia|o__Eubacteriales|f__Lachnospiraceae|g__Lachnospiraceae_unclassified|s__Lachn
k__Bacteria|p__Firmicutes|c__Clostridia|o__Eubacteriales|f__Lachnospiraceae|g__Lachnospiraceae_unclassified|s__Lachn
k__Bacteria|p__Firmicutes|c__Clostridia|o__Eubacteriales|f__Lachnospiraceae|g__Lachnospiraceae_unclassified|s__Lachn
k__Bacteria|p__Firmicutes|c__Clostridia|o__Eubacteriales|f__Lachnospiraceae|g__Lachnospiraceae_unclassified|s__Lachn
k__Bacteria|p__Firmicutes|c__Clostridia|o__Eubacteriales|f__Lachnospiraceae|g__Lachnospiraceae_unclassified|s__Lachn
k__Bacteria|p__Firmicutes|c__Clostridia|o__Eubacteriales|f__Lachnospiraceae|g__Lachnospiraceae_unclassified|s__Lachn
k__Bacteria|p__Firmicutes|c__Clostridia|o__Eubacteriales|f__Lachnospiraceae|g__Lachnospiraceae_unclassified|s__Lachn
k__Bacteria|p__Firmicutes|c__Clostridia|o__Eubacteriales|f__Lachnospiraceae|g__Lachnospiraceae_unclassified|s__Lachn
k__Bacteria|p__Firmicutes|c__Clostridia|o__Eubacteriales|f__Lachnospiraceae|g__Lachnospiraceae_unclassified|s__Lachn
k__Bacteria|p__Firmicutes|c__Bacilli|o__Lactobacillales|f__Lactobacillaceae|g__Lactobacillus|s__Lactobacillus_johnsonii
k__Bacteria|p__Firmicutes|c__Bacilli|o__Lactobacillales|f__Lactobacillaceae|g__Lactobacillus|s__Lactobacillus_johnsonii
k__Bacteria|p__Bacteroidota|c__Bacteroidia|o__Bacteroidales|f__Muribaculaceae|g__Muribaculaceae_unclassified|s__M
k__Bacteria|p__Bacteroidota|c__Bacteroidia|o__Bacteroidales|f__Muribaculaceae|g__Muribaculaceae_unclassified|s__M
k__Bacteria|p__Firmicutes|c__Clostridia|o__Eubacteriales|f__Oscillospiraceae|g__Neglectibacter|s__Neglectibacter_sp_X
k__Bacteria|p__Firmicutes|c__Clostridia|o__Eubacteriales|f__Oscillospiraceae|g__Neglectibacter|s__Neglectibacter_sp_X
k__Bacteria|p__Firmicutes|c__Clostridia|o__Eubacteriales|f__Oscillospiraceae|g__Oscillospiraceae_unclassified|s__Oscillo
k__Bacteria|p__Firmicutes|c__Clostridia|o__Eubacteriales|f__Oscillospiraceae|g__Oscillospiraceae_unclassified|s__Oscillo
k__Bacteria|p__Firmicutes|c__Clostridia|o__Eubacteriales|f__Oscillospiraceae|g__Oscillospiraceae_unclassified|s__Oscillo
k__Bacteria|p__Firmicutes|c__Clostridia|o__Eubacteriales|f__Oscillospiraceae|g__Oscillospiraceae_unclassified|s__Oscillo
k__Bacteria|p__Firmicutes|c__Clostridia|o__Eubacteriales|f__Oscillospiraceae|g__Oscillospiraceae_unclassified|s__Oscillo
k__Bacteria|p__Firmicutes|c__Clostridia|o__Eubacteriales|f__Oscillospiraceae|g__Oscillospiraceae_unclassified|s__Oscillo
k__Bacteria|p__Proteobacteria|c__Betaproteobacteria|o__Burkholderiales|f__Sutterellaceae|g__Parasutterella|s__Parasu
k__Bacteria|p__Proteobacteria|c__Betaproteobacteria|o__Burkholderiales|f__Sutterellaceae|g__Parasutterella|s__Parasu

k__Bacteria|p__Firmicutes|c__Clostridia|o__Eubacteriales|f__Peptostreptococcaceae|g__Romboutsia|s__Romboutsia_ilea
k__Bacteria|p__Firmicutes|c__Clostridia|o__Eubacteriales|f__Peptostreptococcaceae|g__Romboutsia|s__Romboutsia_ilea
k__Bacteria|p__Firmicutes|c__Clostridia|o__Eubacteriales|f__Lachnospiraceae|g__Schaedlerella|s__Schaedlerella_arabino
k__Bacteria|p__Firmicutes|c__Clostridia|o__Eubacteriales|f__Lachnospiraceae|g__Schaedlerella|s__Schaedlerella_arabino

k__Bacteria|p__Firmicutes|c__Erysipelotrichia|o__Erysipelotrichales|f__Turicibacteraceae|g__Turicibacter|s__Turicibacter_
k__Bacteria|p__Firmicutes|c__Erysipelotrichia|o__Erysipelotrichales|f__Turicibacteraceae|g__Turicibacter|s__Turicibacter_
k__Bacteria|p__Firmicutes|c__Clostridia|o__Eubacteriales|f__Lachnospiraceae|g__Acetatifactor|s__Acetatifactor_muris
k__Bacteria|p__Firmicutes|c__Clostridia|o__Eubacteriales|f__Lachnospiraceae|g__Acetatifactor|s__Acetatifactor_muris
k__Bacteria|p__Firmicutes|c__Clostridia|o__Eubacteriales|f__Lachnospiraceae|g__Acetatifactor|s__Acetatifactor_SGB415
k__Bacteria|p__Firmicutes|c__Clostridia|o__Eubacteriales|f__Lachnospiraceae|g__Acetatifactor|s__Acetatifactor_SGB415
k__Bacteria|p__Firmicutes|c__Clostridia|o__Eubacteriales|f__Oscillospiraceae|g__Acutalibacter|s__Acutalibacter_muris
k__Bacteria|p__Firmicutes|c__Clostridia|o__Eubacteriales|f__Oscillospiraceae|g__Acutalibacter|s__Acutalibacter_muris
k__Bacteria|p__Firmicutes|c__Clostridia|o__Eubacteriales|f__Oscillospiraceae|g__Acutalibacter|s__Acutalibacter_sp_1XD
k__Bacteria|p__Firmicutes|c__Clostridia|o__Eubacteriales|f__Oscillospiraceae|g__Acutalibacter|s__Acutalibacter_sp_1XD
k__Bacteria|p__Actinobacteria|c__Coriobacteriia|o__Eggerthellales|f__Eggerthellaceae|g__Adlercreutzia|s__Adlercreutzia
k__Bacteria|p__Actinobacteria|c__Coriobacteriia|o__Eggerthellales|f__Eggerthellaceae|g__Adlercreutzia|s__Adlercreutzia
k__Bacteria|p__Actinobacteria|c__Coriobacteriia|o__Eggerthellales|f__Eggerthellaceae|g__Adlercreutzia|s__Adlercreutzia
k__Bacteria|p__Actinobacteria|c__Coriobacteriia|o__Eggerthellales|f__Eggerthellaceae|g__Adlercreutzia|s__Adlercreutzia
k__Bacteria|p__Actinobacteria|c__Coriobacteriia|o__Eggerthellales|f__Eggerthellaceae|g__Adlercreutzia|s__Adlercreutzia
k__Bacteria|p__Actinobacteria|c__Coriobacteriia|o__Eggerthellales|f__Eggerthellaceae|g__Adlercreutzia|s__Adlercreutzia
k__Bacteria|p__Verrucomicrobia|c__Verrucomicrobiae|o__Verrucomicrobiales|f__Akkermansiaceae|g__Akkermansia|s__A
k__Bacteria|p__Verrucomicrobia|c__Verrucomicrobiae|o__Verrucomicrobiales|f__Akkermansiaceae|g__Akkermansia|s__A
k__Bacteria|p__Bacteroidota|c__Bacteroidia|o__Bacteroidales|f__Rikenellaceae|g__Alistipes|s__Alistipes_sp_DSM_11234
k__Bacteria|p__Bacteroidota|c__Bacteroidia|o__Bacteroidales|f__Rikenellaceae|g__Alistipes|s__Alistipes_sp_DSM_11234
k__Bacteria|p__Firmicutes|c__Clostridia|o__Eubacteriales|f__Oscillospiraceae|g__Anaerotruncus|s__Anaerotruncus_sp_1
k__Bacteria|p__Firmicutes|c__Clostridia|o__Eubacteriales|f__Oscillospiraceae|g__Anaerotruncus|s__Anaerotruncus_sp_1
k__Bacteria|p__Bacteria_unclassified|c__Bacteria_unclassified|o__Bacteria_unclassified|f__Bacteria_unclassified|g__Bact
k__Bacteria|p__Bacteria_unclassified|c__Bacteria_unclassified|o__Bacteria_unclassified|f__Bacteria_unclassified|g__Bact
k__Bacteria|p__Bacteria_unclassified|c__Bacteria_unclassified|o__Bacteria_unclassified|f__Bacteria_unclassified|g__Bact
k__Bacteria|p__Bacteria_unclassified|c__Bacteria_unclassified|o__Bacteria_unclassified|f__Bacteria_unclassified|g__Bact
k__Bacteria|p__Bacteria_unclassified|c__Bacteria_unclassified|o__Bacteria_unclassified|f__Bacteria_unclassified|g__Bact
k__Bacteria|p__Bacteria_unclassified|c__Bacteria_unclassified|o__Bacteria_unclassified|f__Bacteria_unclassified|g__Bact
k__Bacteria|p__Bacteria_unclassified|c__Bacteria_unclassified|o__Bacteria_unclassified|f__Bacteria_unclassified|g__Bact
k__Bacteria|p__Bacteria_unclassified|c__Bacteria_unclassified|o__Bacteria_unclassified|f__Bacteria_unclassified|g__Bact
k__Bacteria|p__Bacteria_unclassified|c__Bacteria_unclassified|o__Bacteria_unclassified|f__Bacteria_unclassified|g__Bact
k__Bacteria|p__Bacteria_unclassified|c__Bacteria_unclassified|o__Bacteria_unclassified|f__Bacteria_unclassified|g__Bact
k__Bacteria|p__Bacteria_unclassified|c__Bacteria_unclassified|o__Bacteria_unclassified|f__Bacteria_unclassified|g__Bact
k__Bacteria|p__Bacteria_unclassified|c__Bacteria_unclassified|o__Bacteria_unclassified|f__Bacteria_unclassified|g__Bact
k__Bacteria|p__Bacteroidota|c__Bacteroidia|o__Bacteroidales|f__Bacteroidaceae|g__Bacteroides|s__Bacteroides_thetaio
k__Bacteria|p__Bacteroidota|c__Bacteroidia|o__Bacteroidales|f__Bacteroidaceae|g__Bacteroides|s__Bacteroides_thetaio

k__Bacteria|p__Actinobacteria|c__Actinomycetia|o__Bifidobacteriales|f__Bifidobacteriaceae|g__Bifidobacterium|s__Bifid
k__Bacteria|p__Actinobacteria|c__Actinomycetia|o__Bifidobacteriales|f__Bifidobacteriaceae|g__Bifidobacterium|s__Bifid
k__Bacteria|p__Firmicutes|c__Clostridia|o__Clostridia_unclassified|f__Clostridia_unclassified|g__Clostridia_unclassified|s_
k__Bacteria|p__Firmicutes|c__Clostridia|o__Clostridia_unclassified|f__Clostridia_unclassified|g__Clostridia_unclassified|s_
k__Bacteria|p__Firmicutes|c__Clostridia|o__Eubacteriales|f__Clostridiaceae|g__Clostridiaceae_unclassified|s__Clostridiac
k__Bacteria|p__Firmicutes|c__Clostridia|o__Eubacteriales|f__Clostridiaceae|g__Clostridiaceae_unclassified|s__Clostridiac
k__Bacteria|p__Firmicutes|c__Clostridia|o__Eubacteriales|f__Clostridiaceae|g__Clostridiaceae_unclassified|s__Clostridiac
k__Bacteria|p__Firmicutes|c__Clostridia|o__Eubacteriales|f__Clostridiaceae|g__Clostridiaceae_unclassified|s__Clostridiac

k__Bacteria|p__Firmicutes|c__Clostridia|o__Eubacteriales|f__Eubacteriales_unclassified|g__Eubacteriales_unclassified|s_
k__Bacteria|p__Firmicutes|c__Clostridia|o__Eubacteriales|f__Eubacteriales_unclassified|g__Eubacteriales_unclassified|s_
k__Bacteria|p__Firmicutes|c__Erysipelotrichia|o__Erysipelotrichales|f__Erysipelotrichaceae|g__Erysipelatoclostridium|s__
k__Bacteria|p__Firmicutes|c__Erysipelotrichia|o__Erysipelotrichales|f__Erysipelotrichaceae|g__Erysipelatoclostridium|s__
k__Bacteria|p__Actinobacteria|c__Coriobacteriia|o__Coriobacteriales|f__Coriobacteriaceae|g__Coriobacteriaceae_unclass
k__Bacteria|p__Actinobacteria|c__Coriobacteriia|o__Coriobacteriales|f__Coriobacteriaceae|g__Coriobacteriaceae_unclass
k__Bacteria|p__Firmicutes|c__Clostridia|o__Eubacteriales|f__Lachnospiraceae|g__Dorea|s__Dorea_sp_5_2
k__Bacteria|p__Firmicutes|c__Clostridia|o__Eubacteriales|f__Lachnospiraceae|g__Dorea|s__Dorea_sp_5_2
k__Bacteria|p__Firmicutes|c__Erysipelotrichia|o__Erysipelotrichales|f__Erysipelotrichaceae|g__Dubosiella|s__Dubosiella_
k__Bacteria|p__Firmicutes|c__Erysipelotrichia|o__Erysipelotrichales|f__Erysipelotrichaceae|g__Dubosiella|s__Dubosiella_
k__Bacteria|p__Firmicutes|c__Erysipelotrichia|o__Erysipelotrichales|f__Erysipelotrichales_unclassified|g__Erysipelotrichal
k__Bacteria|p__Firmicutes|c__Erysipelotrichia|o__Erysipelotrichales|f__Erysipelotrichales_unclassified|g__Erysipelotrichal
k__Bacteria|p__Firmicutes|c__Clostridia|o__Eubacteriales|f__Eubacteriaceae|g__Eubacteriaceae_unclassified|s__Eubacte
k__Bacteria|p__Firmicutes|c__Clostridia|o__Eubacteriales|f__Eubacteriaceae|g__Eubacteriaceae_unclassified|s__Eubacte
k__Bacteria|p__Firmicutes|c__Clostridia|o__Eubacteriales|f__Eubacteriaceae|g__Eubacteriaceae_unclassified|s__Eubacte
k__Bacteria|p__Firmicutes|c__Clostridia|o__Eubacteriales|f__Eubacteriaceae|g__Eubacteriaceae_unclassified|s__Eubacte
k__Bacteria|p__Firmicutes|c__Clostridia|o__Eubacteriales|f__Lachnospiraceae|g__GGB20149|s__GGB20149_SGB29430
k__Bacteria|p__Firmicutes|c__Clostridia|o__Eubacteriales|f__Lachnospiraceae|g__GGB20149|s__GGB20149_SGB29430
k__Bacteria|p__Actinobacteria|c__Coriobacteriia|o__Eggerthellales|f__Eggerthellaceae|g__GGB22635|s__GGB22635_SGB
k__Bacteria|p__Actinobacteria|c__Coriobacteriia|o__Eggerthellales|f__Eggerthellaceae|g__GGB22635|s__GGB22635_SGB
k__Bacteria|p__Firmicutes|c__Clostridia|o__Eubacteriales|f__Lachnospiraceae|g__GGB25041|s__GGB25041_SGB36960
k__Bacteria|p__Firmicutes|c__Clostridia|o__Eubacteriales|f__Lachnospiraceae|g__GGB25041|s__GGB25041_SGB36960
k__Bacteria|p__Bacteroidota|c__Bacteroidia|o__Bacteroidales|f__Muribaculaceae|g__GGB27876|s__GGB27876_SGB4031
k__Bacteria|p__Bacteroidota|c__Bacteroidia|o__Bacteroidales|f__Muribaculaceae|g__GGB27876|s__GGB27876_SGB4031
k__Bacteria|p__Bacteroidota|c__Bacteroidia|o__Bacteroidales|f__Muribaculaceae|g__GGB27878|s__GGB27878_SGB4031
k__Bacteria|p__Bacteroidota|c__Bacteroidia|o__Bacteroidales|f__Muribaculaceae|g__GGB27878|s__GGB27878_SGB4031
k__Bacteria|p__Bacteroidota|c__Bacteroidia|o__Bacteroidales|f__Muribaculaceae|g__GGB27918|s__GGB27918_SGB4035
k__Bacteria|p__Bacteroidota|c__Bacteroidia|o__Bacteroidales|f__Muribaculaceae|g__GGB27918|s__GGB27918_SGB4035
k__Bacteria|p__Firmicutes|c__CFGB9508|o__OFGB9508|f__FGB9508|g__GGB28382|s__GGB28382_SGB40962
k__Bacteria|p__Firmicutes|c__CFGB9508|o__OFGB9508|f__FGB9508|g__GGB28382|s__GGB28382_SGB40962
k__Bacteria|p__Firmicutes|c__CFGB2838|o__OFGB2838|f__FGB2838|g__GGB28399|s__GGB28399_SGB40980
k__Bacteria|p__Firmicutes|c__CFGB2838|o__OFGB2838|f__FGB2838|g__GGB28399|s__GGB28399_SGB40980
k__Bacteria|p__Firmicutes|c__CFGB2838|o__OFGB2838|f__FGB2838|g__GGB28411|s__GGB28411_SGB40993
k__Bacteria|p__Firmicutes|c__CFGB2838|o__OFGB2838|f__FGB2838|g__GGB28411|s__GGB28411_SGB40993
k__Bacteria|p__Firmicutes|c__CFGB2838|o__OFGB2838|f__FGB2838|g__GGB28415|s__GGB28415_SGB40997
k__Bacteria|p__Firmicutes|c__CFGB2838|o__OFGB2838|f__FGB2838|g__GGB28415|s__GGB28415_SGB40997
k__Bacteria|p__Firmicutes|c__Clostridia|o__Eubacteriales|f__Pumilibacteraceae|g__GGB28430|s__GGB28430_SGB41013
k__Bacteria|p__Firmicutes|c__Clostridia|o__Eubacteriales|f__Pumilibacteraceae|g__GGB28430|s__GGB28430_SGB41013
k__Bacteria|p__Firmicutes|c__CFGB28439|o__OFGB28439|f__FGB28439|g__GGB28439|s__GGB28439_SGB41022
k__Bacteria|p__Firmicutes|c__CFGB28439|o__OFGB28439|f__FGB28439|g__GGB28439|s__GGB28439_SGB41022
k__Bacteria|p__Firmicutes|c__Clostridia|o__Clostridia_unclassified|f__Clostridia_unclassified|g__GGB28778|s__GGB2877
k__Bacteria|p__Firmicutes|c__Clostridia|o__Clostridia_unclassified|f__Clostridia_unclassified|g__GGB28778|s__GGB2877
k__Bacteria|p__Firmicutes|c__Clostridia|o__Eubacteriales|f__Eubacteriaceae|g__GGB28784|s__GGB28784_SGB41437
k__Bacteria|p__Firmicutes|c__Clostridia|o__Eubacteriales|f__Eubacteriaceae|g__GGB28784|s__GGB28784_SGB41437
k__Bacteria|p__Firmicutes|c__Clostridia|o__Eubacteriales|f__Lachnospiraceae|g__GGB28792|s__GGB28792_SGB41445
k__Bacteria|p__Firmicutes|c__Clostridia|o__Eubacteriales|f__Lachnospiraceae|g__GGB28792|s__GGB28792_SGB41445

k__Bacteria|p__Firmicutes|c__Clostridia|o__Eubacteriales|f__Lachnospiraceae|g__GGB28798|s__GGB28798_SGB41451
k__Bacteria|p__Firmicutes|c__Clostridia|o__Eubacteriales|f__Lachnospiraceae|g__GGB28798|s__GGB28798_SGB41451
k__Bacteria|p__Firmicutes|c__Clostridia|o__Eubacteriales|f__Lachnospiraceae|g__GGB28802|s__GGB28802_SGB41455
k__Bacteria|p__Firmicutes|c__Clostridia|o__Eubacteriales|f__Lachnospiraceae|g__GGB28802|s__GGB28802_SGB41455
k__Bacteria|p__Firmicutes|c__Clostridia|o__Eubacteriales|f__Lachnospiraceae|g__GGB28818|s__GGB28818_SGB41473
k__Bacteria|p__Firmicutes|c__Clostridia|o__Eubacteriales|f__Lachnospiraceae|g__GGB28818|s__GGB28818_SGB41473
k__Bacteria|p__Firmicutes|c__CFGB77305|o__OFGB77305|f__FGB77305|g__GGB28828|s__GGB28828_SGB41484
k__Bacteria|p__Firmicutes|c__CFGB77305|o__OFGB77305|f__FGB77305|g__GGB28828|s__GGB28828_SGB41484
k__Bacteria|p__Firmicutes|c__Clostridia|o__Eubacteriales|f__Clostridiaceae|g__GGB28851|s__GGB28851_SGB41518
k__Bacteria|p__Firmicutes|c__Clostridia|o__Eubacteriales|f__Clostridiaceae|g__GGB28851|s__GGB28851_SGB41518
k__Bacteria|p__Firmicutes|c__Clostridia|o__Eubacteriales|f__Lachnospiraceae|g__GGB28859|s__GGB28859_SGB41528
k__Bacteria|p__Firmicutes|c__Clostridia|o__Eubacteriales|f__Lachnospiraceae|g__GGB28859|s__GGB28859_SGB41528
k__Bacteria|p__Firmicutes|c__Clostridia|o__Eubacteriales|f__Lachnospiraceae|g__GGB28864|s__GGB28864_SGB41535
k__Bacteria|p__Firmicutes|c__Clostridia|o__Eubacteriales|f__Lachnospiraceae|g__GGB28864|s__GGB28864_SGB41535
k__Bacteria|p__Firmicutes|c__Clostridia|o__Eubacteriales|f__Lachnospiraceae|g__GGB28869|s__GGB28869_SGB41543
k__Bacteria|p__Firmicutes|c__Clostridia|o__Eubacteriales|f__Lachnospiraceae|g__GGB28869|s__GGB28869_SGB41543
k__Bacteria|p__Firmicutes|c__CFGB9633|o__OFGB9633|f__FGB9633|g__GGB28883|s__GGB28883_SGB41564
k__Bacteria|p__Firmicutes|c__CFGB9633|o__OFGB9633|f__FGB9633|g__GGB28883|s__GGB28883_SGB41564
k__Bacteria|p__Bacteria_unclassified|c__Bacteria_unclassified|o__Bacteria_unclassified|f__Bacteria_unclassified|g__GGB
k__Bacteria|p__Bacteria_unclassified|c__Bacteria_unclassified|o__Bacteria_unclassified|f__Bacteria_unclassified|g__GGB
k__Bacteria|p__Bacteria_unclassified|c__Bacteria_unclassified|o__Bacteria_unclassified|f__Bacteria_unclassified|g__GGB
k__Bacteria|p__Bacteria_unclassified|c__Bacteria_unclassified|o__Bacteria_unclassified|f__Bacteria_unclassified|g__GGB
k__Bacteria|p__Bacteria_unclassified|c__Bacteria_unclassified|o__Bacteria_unclassified|f__Bacteria_unclassified|g__GGB
k__Bacteria|p__Bacteria_unclassified|c__Bacteria_unclassified|o__Bacteria_unclassified|f__Bacteria_unclassified|g__GGB
k__Bacteria|p__Bacteria_unclassified|c__Bacteria_unclassified|o__Bacteria_unclassified|f__Bacteria_unclassified|g__GGB
k__Bacteria|p__Bacteria_unclassified|c__Bacteria_unclassified|o__Bacteria_unclassified|f__Bacteria_unclassified|g__GGB
k__Bacteria|p__Firmicutes|c__Clostridia|o__Eubacteriales|f__Lachnospiraceae|g__GGB28916|s__GGB28916_SGB41612
k__Bacteria|p__Firmicutes|c__Clostridia|o__Eubacteriales|f__Lachnospiraceae|g__GGB28916|s__GGB28916_SGB41612
k__Bacteria|p__Firmicutes|c__Clostridia|o__Eubacteriales|f__Lachnospiraceae|g__GGB28924|s__GGB28924_SGB41621
k__Bacteria|p__Firmicutes|c__Clostridia|o__Eubacteriales|f__Lachnospiraceae|g__GGB28924|s__GGB28924_SGB41621
k__Bacteria|p__Firmicutes|c__Clostridia|o__Eubacteriales|f__Lachnospiraceae|g__GGB28926|s__GGB28926_SGB41624
k__Bacteria|p__Firmicutes|c__Clostridia|o__Eubacteriales|f__Lachnospiraceae|g__GGB28926|s__GGB28926_SGB41624
k__Bacteria|p__Bacteria_unclassified|c__CFGB77359|o__OFGB77359|f__FGB77359|g__GGB28927|s__GGB28927_SGB416
k__Bacteria|p__Bacteria_unclassified|c__CFGB77359|o__OFGB77359|f__FGB77359|g__GGB28927|s__GGB28927_SGB416
k__Bacteria|p__Firmicutes|c__CFGB9639|o__OFGB9639|f__FGB9639|g__GGB28934|s__GGB28934_SGB41635
k__Bacteria|p__Firmicutes|c__CFGB9639|o__OFGB9639|f__FGB9639|g__GGB28934|s__GGB28934_SGB41635
k__Bacteria|p__Firmicutes|c__Clostridia|o__Eubacteriales|f__Lachnospiraceae|g__GGB28946|s__GGB28946_SGB41652
k__Bacteria|p__Firmicutes|c__Clostridia|o__Eubacteriales|f__Lachnospiraceae|g__GGB28946|s__GGB28946_SGB41652
k__Bacteria|p__Firmicutes|c__Clostridia|o__Eubacteriales|f__Lachnospiraceae|g__GGB28949|s__GGB28949_SGB41655
k__Bacteria|p__Firmicutes|c__Clostridia|o__Eubacteriales|f__Lachnospiraceae|g__GGB28949|s__GGB28949_SGB41655
k__Bacteria|p__Firmicutes|c__Clostridia|o__Eubacteriales|f__Lachnospiraceae|g__GGB28949|s__GGB28949_SGB41656
k__Bacteria|p__Firmicutes|c__Clostridia|o__Eubacteriales|f__Lachnospiraceae|g__GGB28949|s__GGB28949_SGB41656
k__Bacteria|p__Firmicutes|c__Clostridia|o__Eubacteriales|f__Clostridiaceae|g__GGB28950|s__GGB28950_SGB41657
k__Bacteria|p__Firmicutes|c__Clostridia|o__Eubacteriales|f__Clostridiaceae|g__GGB28950|s__GGB28950_SGB41657
k__Bacteria|p__Firmicutes|c__Clostridia|o__Eubacteriales|f__Clostridiaceae|g__GGB28951|s__GGB28951_SGB102295
k__Bacteria|p__Firmicutes|c__Clostridia|o__Eubacteriales|f__Clostridiaceae|g__GGB28951|s__GGB28951_SGB102295

k__Bacteria|p__Firmicutes|c__Clostridia|o__Eubacteriales|f__Clostridiaceae|g__GGB28951|s__GGB28951_SGB41658
k__Bacteria|p__Firmicutes|c__Clostridia|o__Eubacteriales|f__Clostridiaceae|g__GGB28951|s__GGB28951_SGB41658
k__Bacteria|p__Firmicutes|c__Clostridia|o__Eubacteriales|f__Clostridiaceae|g__GGB28954|s__GGB28954_SGB41662
k__Bacteria|p__Firmicutes|c__Clostridia|o__Eubacteriales|f__Clostridiaceae|g__GGB28954|s__GGB28954_SGB41662
k__Bacteria|p__Firmicutes|c__Clostridia|o__Eubacteriales|f__Clostridiaceae|g__GGB28956|s__GGB28956_SGB41665
k__Bacteria|p__Firmicutes|c__Clostridia|o__Eubacteriales|f__Clostridiaceae|g__GGB28956|s__GGB28956_SGB41665
k__Bacteria|p__Firmicutes|c__Clostridia|o__Eubacteriales|f__Clostridiaceae|g__GGB28960|s__GGB28960_SGB41669
k__Bacteria|p__Firmicutes|c__Clostridia|o__Eubacteriales|f__Clostridiaceae|g__GGB28960|s__GGB28960_SGB41669
k__Bacteria|p__Firmicutes|c__Clostridia|o__Eubacteriales|f__Clostridiaceae|g__GGB28967|s__GGB28967_SGB41678
k__Bacteria|p__Firmicutes|c__Clostridia|o__Eubacteriales|f__Clostridiaceae|g__GGB28967|s__GGB28967_SGB41678
k__Bacteria|p__Firmicutes|c__Clostridia|o__Eubacteriales|f__Eubacteriaceae|g__GGB28991|s__GGB28991_SGB41705
k__Bacteria|p__Firmicutes|c__Clostridia|o__Eubacteriales|f__Eubacteriaceae|g__GGB28991|s__GGB28991_SGB41705
k__Bacteria|p__Firmicutes|c__CFGB9658|o__OFGB9658|f__FGB9658|g__GGB29002|s__GGB29002_SGB41718
k__Bacteria|p__Firmicutes|c__CFGB9658|o__OFGB9658|f__FGB9658|g__GGB29002|s__GGB29002_SGB41718
k__Bacteria|p__Firmicutes|c__CFGB9659|o__OFGB9659|f__FGB9659|g__GGB29003|s__GGB29003_SGB41719
k__Bacteria|p__Firmicutes|c__CFGB9659|o__OFGB9659|f__FGB9659|g__GGB29003|s__GGB29003_SGB41719
k__Bacteria|p__Bacteria_unclassified|c__Bacteria_unclassified|o__Bacteria_unclassified|f__Bacteria_unclassified|g__GGB[illegible]
k__Bacteria|p__Bacteria_unclassified|c__Bacteria_unclassified|o__Bacteria_unclassified|f__Bacteria_unclassified|g__GGB[illegible]
k__Bacteria|p__Firmicutes|c__CFGB9827|o__OFGB9827|f__FGB9827|g__GGB29531|s__GGB29531_SGB42317
k__Bacteria|p__Firmicutes|c__CFGB9827|o__OFGB9827|f__FGB9827|g__GGB29531|s__GGB29531_SGB42317
k__Bacteria|p__Firmicutes|c__Clostridia|o__Eubacteriales|f__Eubacteriaceae|g__GGB29685|s__GGB29685_SGB42494
k__Bacteria|p__Firmicutes|c__Clostridia|o__Eubacteriales|f__Eubacteriaceae|g__GGB29685|s__GGB29685_SGB42494
k__Bacteria|p__Bacteria_unclassified|c__CFGB77303|o__OFGB77303|f__FGB77303|g__GGB30141|s__GGB30141_SGB430[illegible]
k__Bacteria|p__Bacteria_unclassified|c__CFGB77303|o__OFGB77303|f__FGB77303|g__GGB30141|s__GGB30141_SGB430[illegible]
k__Bacteria|p__Firmicutes|c__Clostridia|o__Eubacteriales|f__Eubacteriales_unclassified|g__GGB30286|s__GGB30286_SG[illegible]
k__Bacteria|p__Firmicutes|c__Clostridia|o__Eubacteriales|f__Eubacteriales_unclassified|g__GGB30286|s__GGB30286_SG[illegible]
k__Bacteria|p__Firmicutes|c__Clostridia|o__Eubacteriales|f__Oscillospiraceae|g__GGB30303|s__GGB30303_SGB43268
k__Bacteria|p__Firmicutes|c__Clostridia|o__Eubacteriales|f__Oscillospiraceae|g__GGB30303|s__GGB30303_SGB43268
k__Bacteria|p__Firmicutes|c__CFGB30328|o__OFGB30328|f__FGB30328|g__GGB30413|s__GGB30413_SGB43452
k__Bacteria|p__Firmicutes|c__CFGB30328|o__OFGB30328|f__FGB30328|g__GGB30413|s__GGB30413_SGB43452
k__Bacteria|p__Firmicutes|c__Clostridia|o__Eubacteriales|f__Oscillospiraceae|g__GGB30454|s__GGB30454_SGB43514
k__Bacteria|p__Firmicutes|c__Clostridia|o__Eubacteriales|f__Oscillospiraceae|g__GGB30454|s__GGB30454_SGB43514
k__Bacteria|p__Firmicutes|c__Clostridia|o__Eubacteriales|f__Oscillospiraceae|g__GGB30455|s__GGB30455_SGB43519
k__Bacteria|p__Firmicutes|c__Clostridia|o__Eubacteriales|f__Oscillospiraceae|g__GGB30455|s__GGB30455_SGB43519
k__Bacteria|p__Firmicutes|c__Clostridia|o__Eubacteriales|f__Oscillospiraceae|g__GGB30461|s__GGB30461_SGB43527
k__Bacteria|p__Firmicutes|c__Clostridia|o__Eubacteriales|f__Oscillospiraceae|g__GGB30461|s__GGB30461_SGB43527
k__Bacteria|p__Firmicutes|c__Clostridia|o__Eubacteriales|f__Oscillospiraceae|g__GGB30461|s__GGB30461_SGB43530
k__Bacteria|p__Firmicutes|c__Clostridia|o__Eubacteriales|f__Oscillospiraceae|g__GGB30461|s__GGB30461_SGB43530
k__Bacteria|p__Firmicutes|c__Clostridia|o__Eubacteriales|f__Oscillospiraceae|g__GGB30463|s__GGB30463_SGB43537
k__Bacteria|p__Firmicutes|c__Clostridia|o__Eubacteriales|f__Oscillospiraceae|g__GGB30463|s__GGB30463_SGB43537
k__Bacteria|p__Firmicutes|c__Clostridia|o__Eubacteriales|f__Oscillospiraceae|g__GGB30473|s__GGB30473_SGB43557
k__Bacteria|p__Firmicutes|c__Clostridia|o__Eubacteriales|f__Oscillospiraceae|g__GGB30473|s__GGB30473_SGB43557
k__Bacteria|p__Firmicutes|c__Clostridia|o__Eubacteriales|f__Oscillospiraceae|g__GGB30475|s__GGB30475_SGB63182
k__Bacteria|p__Firmicutes|c__Clostridia|o__Eubacteriales|f__Oscillospiraceae|g__GGB30475|s__GGB30475_SGB63182
k__Bacteria|p__Actinobacteria|c__CFGB77153|o__OFGB77153|f__FGB77153|g__GGB30861|s__GGB30861_SGB44083
k__Bacteria|p__Actinobacteria|c__CFGB77153|o__OFGB77153|f__FGB77153|g__GGB30861|s__GGB30861_SGB44083

k__Bacteria|p__Tenericutes|c__CFGB1791|o__OFGB1791|f__FGB1791|g__GGB31312|s__GGB31312_SGB44628
k__Bacteria|p__Tenericutes|c__CFGB1791|o__OFGB1791|f__FGB1791|g__GGB31312|s__GGB31312_SGB44628
k__Bacteria|p__Firmicutes|c__CFGB10290|o__OFGB10290|f__FGB10290|g__GGB31438|s__GGB31438_SGB44768
k__Bacteria|p__Firmicutes|c__CFGB10290|o__OFGB10290|f__FGB10290|g__GGB31438|s__GGB31438_SGB44768
k__Bacteria|p__Firmicutes|c__Clostridia|o__Eubacteriales|f__Oscillospiraceae|g__GGB3171|s__GGB3171_SGB4185
k__Bacteria|p__Firmicutes|c__Clostridia|o__Eubacteriales|f__Oscillospiraceae|g__GGB3171|s__GGB3171_SGB4185
k__Bacteria|p__Firmicutes|c__CFGB1765|o__OFGB1765|f__FGB1765|g__GGB31823|s__GGB31823_SGB45199
k__Bacteria|p__Firmicutes|c__CFGB1765|o__OFGB1765|f__FGB1765|g__GGB31823|s__GGB31823_SGB45199
k__Bacteria|p__Firmicutes|c__CFGB10349|o__OFGB10349|f__FGB10349|g__GGB31853|s__GGB31853_SGB45233
k__Bacteria|p__Firmicutes|c__CFGB10349|o__OFGB10349|f__FGB10349|g__GGB31853|s__GGB31853_SGB45233
k__Bacteria|p__Firmicutes|c__CFGB10667|o__OFGB10667|f__FGB10667|g__GGB32371|s__GGB32371_SGB41694
k__Bacteria|p__Firmicutes|c__CFGB10667|o__OFGB10667|f__FGB10667|g__GGB32371|s__GGB32371_SGB41694
k__Bacteria|p__Firmicutes|c__Clostridia|o__Eubacteriales|f__Lachnospiraceae|g__GGB3793|s__GGB3793_SGB5158
k__Bacteria|p__Firmicutes|c__Clostridia|o__Eubacteriales|f__Lachnospiraceae|g__GGB3793|s__GGB3793_SGB5158
k__Bacteria|p__Firmicutes|c__Clostridia|o__Eubacteriales|f__Lachnospiraceae|g__GGB42598|s__GGB42598_SGB59794
k__Bacteria|p__Firmicutes|c__Clostridia|o__Eubacteriales|f__Lachnospiraceae|g__GGB42598|s__GGB42598_SGB59794
k__Bacteria|p__Firmicutes|c__Clostridia|o__Eubacteriales|f__Christensenellaceae|g__GGB45656|s__GGB45656_SGB6337
k__Bacteria|p__Firmicutes|c__Clostridia|o__Eubacteriales|f__Christensenellaceae|g__GGB45656|s__GGB45656_SGB6337
k__Bacteria|p__Firmicutes|c__CFGB10299|o__OFGB10299|f__FGB10299|g__GGB47127|s__GGB47127_SGB65054
k__Bacteria|p__Firmicutes|c__CFGB10299|o__OFGB10299|f__FGB10299|g__GGB47127|s__GGB47127_SGB65054
k__Bacteria|p__Firmicutes|c__Clostridia|o__Eubacteriales|f__Oscillospiraceae|g__GGB74395|s__GGB74395_SGB43521
k__Bacteria|p__Firmicutes|c__Clostridia|o__Eubacteriales|f__Oscillospiraceae|g__GGB74395|s__GGB74395_SGB43521
k__Bacteria|p__Firmicutes|c__Clostridia|o__Eubacteriales|f__Oscillospiraceae|g__GGB75053|s__GGB75053_SGB43494
k__Bacteria|p__Firmicutes|c__Clostridia|o__Eubacteriales|f__Oscillospiraceae|g__GGB75053|s__GGB75053_SGB43494
k__Bacteria|p__Firmicutes|c__Clostridia|o__Eubacteriales|f__Lachnospiraceae|g__GGB75109|s__GGB75109_SGB102238
k__Bacteria|p__Firmicutes|c__Clostridia|o__Eubacteriales|f__Lachnospiraceae|g__GGB75109|s__GGB75109_SGB102238
k__Bacteria|p__Firmicutes|c__Clostridia|o__Clostridia_unclassified|f__Clostridia_unclassified|g__GGB81440|s__GGB8144
k__Bacteria|p__Firmicutes|c__Clostridia|o__Clostridia_unclassified|f__Clostridia_unclassified|g__GGB81440|s__GGB8144
k__Bacteria|p__Firmicutes|c__Clostridia|o__Eubacteriales|f__Lachnospiraceae|g__Lachnospiraceae_unclassified|s__Lachn
k__Bacteria|p__Firmicutes|c__Clostridia|o__Eubacteriales|f__Lachnospiraceae|g__Lachnospiraceae_unclassified|s__Lachn
k__Bacteria|p__Firmicutes|c__Clostridia|o__Eubacteriales|f__Lachnospiraceae|g__Lachnospiraceae_unclassified|s__Lachn
k__Bacteria|p__Firmicutes|c__Clostridia|o__Eubacteriales|f__Lachnospiraceae|g__Lachnospiraceae_unclassified|s__Lachn
k__Bacteria|p__Firmicutes|c__Clostridia|o__Eubacteriales|f__Lachnospiraceae|g__Lachnospiraceae_unclassified|s__Lachn
k__Bacteria|p__Firmicutes|c__Clostridia|o__Eubacteriales|f__Lachnospiraceae|g__Lachnospiraceae_unclassified|s__Lachn
k__Bacteria|p__Firmicutes|c__Clostridia|o__Eubacteriales|f__Lachnospiraceae|g__Lachnospiraceae_unclassified|s__Lachn
k__Bacteria|p__Firmicutes|c__Clostridia|o__Eubacteriales|f__Lachnospiraceae|g__Lachnospiraceae_unclassified|s__Lachn
k__Bacteria|p__Firmicutes|c__Clostridia|o__Eubacteriales|f__Lachnospiraceae|g__Lachnospiraceae_unclassified|s__Lachn
k__Bacteria|p__Firmicutes|c__Clostridia|o__Eubacteriales|f__Lachnospiraceae|g__Lachnospiraceae_unclassified|s__Lachn
k__Bacteria|p__Firmicutes|c__Clostridia|o__Eubacteriales|f__Lachnospiraceae|g__Lachnospiraceae_unclassified|s__Lachn
k__Bacteria|p__Firmicutes|c__Clostridia|o__Eubacteriales|f__Lachnospiraceae|g__Lachnospiraceae_unclassified|s__Lachn
k__Bacteria|p__Firmicutes|c__Clostridia|o__Eubacteriales|f__Lachnospiraceae|g__Lachnospiraceae_unclassified|s__Lachn
k__Bacteria|p__Firmicutes|c__Clostridia|o__Eubacteriales|f__Lachnospiraceae|g__Lachnospiraceae_unclassified|s__Lachn
k__Bacteria|p__Firmicutes|c__Clostridia|o__Eubacteriales|f__Lachnospiraceae|g__Lachnospiraceae_unclassified|s__Lachn
k__Bacteria|p__Firmicutes|c__Bacilli|o__Lactobacillales|f__Lactobacillaceae|g__Lactobacillus|s__Lactobacillus_johnsonii
k__Bacteria|p__Firmicutes|c__Bacilli|o__Lactobacillales|f__Lactobacillaceae|g__Lactobacillus|s__Lactobacillus_johnsonii

k__Bacteria|p__Bacteroidota|c__Bacteroidia|o__Bacteroidales|f__Muribaculaceae|g__Muribaculaceae_unclassified|s__M
k__Bacteria|p__Bacteroidota|c__Bacteroidia|o__Bacteroidales|f__Muribaculaceae|g__Muribaculaceae_unclassified|s__M
k__Bacteria|p__Firmicutes|c__Clostridia|o__Eubacteriales|f__Oscillospiraceae|g__Neglectibacter|s__Neglectibacter_sp_X
k__Bacteria|p__Firmicutes|c__Clostridia|o__Eubacteriales|f__Oscillospiraceae|g__Neglectibacter|s__Neglectibacter_sp_X
k__Bacteria|p__Firmicutes|c__Clostridia|o__Eubacteriales|f__Oscillospiraceae|g__Oscillospiraceae_unclassified|s__Oscillo
k__Bacteria|p__Firmicutes|c__Clostridia|o__Eubacteriales|f__Oscillospiraceae|g__Oscillospiraceae_unclassified|s__Oscillo
k__Bacteria|p__Firmicutes|c__Clostridia|o__Eubacteriales|f__Oscillospiraceae|g__Oscillospiraceae_unclassified|s__Oscillo
k__Bacteria|p__Firmicutes|c__Clostridia|o__Eubacteriales|f__Oscillospiraceae|g__Oscillospiraceae_unclassified|s__Oscillo
k__Bacteria|p__Firmicutes|c__Clostridia|o__Eubacteriales|f__Oscillospiraceae|g__Oscillospiraceae_unclassified|s__Oscillo
k__Bacteria|p__Firmicutes|c__Clostridia|o__Eubacteriales|f__Oscillospiraceae|g__Oscillospiraceae_unclassified|s__Oscillo
k__Bacteria|p__Proteobacteria|c__Betaproteobacteria|o__Burkholderiales|f__Sutterellaceae|g__Parasutterella|s__Parasu
k__Bacteria|p__Proteobacteria|c__Betaproteobacteria|o__Burkholderiales|f__Sutterellaceae|g__Parasutterella|s__Parasu

k__Bacteria|p__Firmicutes|c__Clostridia|o__Eubacteriales|f__Peptostreptococcaceae|g__Romboutsia|s__Romboutsia_ilea
k__Bacteria|p__Firmicutes|c__Clostridia|o__Eubacteriales|f__Peptostreptococcaceae|g__Romboutsia|s__Romboutsia_ilea
k__Bacteria|p__Firmicutes|c__Clostridia|o__Eubacteriales|f__Lachnospiraceae|g__Schaedlerella|s__Schaedlerella_arabino
k__Bacteria|p__Firmicutes|c__Clostridia|o__Eubacteriales|f__Lachnospiraceae|g__Schaedlerella|s__Schaedlerella_arabino

k__Bacteria|p__Firmicutes|c__Erysipelotrichia|o__Erysipelotrichales|f__Turicibacteraceae|g__Turicibacter|s__Turicibacter_
k__Bacteria|p__Firmicutes|c__Erysipelotrichia|o__Erysipelotrichales|f__Turicibacteraceae|g__Turicibacter|s__Turicibacter_
k__Bacteria|p__Firmicutes|c__Clostridia|o__Eubacteriales|f__Lachnospiraceae|g__Acetatifactor|s__Acetatifactor_muris
k__Bacteria|p__Firmicutes|c__Clostridia|o__Eubacteriales|f__Lachnospiraceae|g__Acetatifactor|s__Acetatifactor_muris
k__Bacteria|p__Firmicutes|c__Clostridia|o__Eubacteriales|f__Lachnospiraceae|g__Acetatifactor|s__Acetatifactor_SGB415
k__Bacteria|p__Firmicutes|c__Clostridia|o__Eubacteriales|f__Lachnospiraceae|g__Acetatifactor|s__Acetatifactor_SGB415
k__Bacteria|p__Firmicutes|c__Clostridia|o__Eubacteriales|f__Oscillospiraceae|g__Acutalibacter|s__Acutalibacter_muris
k__Bacteria|p__Firmicutes|c__Clostridia|o__Eubacteriales|f__Oscillospiraceae|g__Acutalibacter|s__Acutalibacter_muris
k__Bacteria|p__Firmicutes|c__Clostridia|o__Eubacteriales|f__Oscillospiraceae|g__Acutalibacter|s__Acutalibacter_sp_1XD
k__Bacteria|p__Firmicutes|c__Clostridia|o__Eubacteriales|f__Oscillospiraceae|g__Acutalibacter|s__Acutalibacter_sp_1XD
k__Bacteria|p__Actinobacteria|c__Coriobacteriia|o__Eggerthellales|f__Eggerthellaceae|g__Adlercreutzia|s__Adlercreutzia
k__Bacteria|p__Actinobacteria|c__Coriobacteriia|o__Eggerthellales|f__Eggerthellaceae|g__Adlercreutzia|s__Adlercreutzia
k__Bacteria|p__Actinobacteria|c__Coriobacteriia|o__Eggerthellales|f__Eggerthellaceae|g__Adlercreutzia|s__Adlercreutzia
k__Bacteria|p__Actinobacteria|c__Coriobacteriia|o__Eggerthellales|f__Eggerthellaceae|g__Adlercreutzia|s__Adlercreutzia
k__Bacteria|p__Actinobacteria|c__Coriobacteriia|o__Eggerthellales|f__Eggerthellaceae|g__Adlercreutzia|s__Adlercreutzia
k__Bacteria|p__Actinobacteria|c__Coriobacteriia|o__Eggerthellales|f__Eggerthellaceae|g__Adlercreutzia|s__Adlercreutzia
k__Bacteria|p__Verrucomicrobia|c__Verrucomicrobiae|o__Verrucomicrobiales|f__Akkermansiaceae|g__Akkermansia|s__A
k__Bacteria|p__Verrucomicrobia|c__Verrucomicrobiae|o__Verrucomicrobiales|f__Akkermansiaceae|g__Akkermansia|s__A
k__Bacteria|p__Bacteroidota|c__Bacteroidia|o__Bacteroidales|f__Rikenellaceae|g__Alistipes|s__Alistipes_sp_DSM_11234
k__Bacteria|p__Bacteroidota|c__Bacteroidia|o__Bacteroidales|f__Rikenellaceae|g__Alistipes|s__Alistipes_sp_DSM_11234
k__Bacteria|p__Firmicutes|c__Clostridia|o__Eubacteriales|f__Oscillospiraceae|g__Anaerotruncus|s__Anaerotruncus_sp_1
k__Bacteria|p__Firmicutes|c__Clostridia|o__Eubacteriales|f__Oscillospiraceae|g__Anaerotruncus|s__Anaerotruncus_sp_1
k__Bacteria|p__Bacteria_unclassified|c__Bacteria_unclassified|o__Bacteria_unclassified|f__Bacteria_unclassified|g__Bact
k__Bacteria|p__Bacteria_unclassified|c__Bacteria_unclassified|o__Bacteria_unclassified|f__Bacteria_unclassified|g__Bact
k__Bacteria|p__Bacteria_unclassified|c__Bacteria_unclassified|o__Bacteria_unclassified|f__Bacteria_unclassified|g__Bact
k__Bacteria|p__Bacteria_unclassified|c__Bacteria_unclassified|o__Bacteria_unclassified|f__Bacteria_unclassified|g__Bact

k__Bacteria|p__Bacteria_unclassified|c__Bacteria_unclassified|o__Bacteria_unclassified|f__Bacteria_unclassified|g__Bacte
k__Bacteria|p__Bacteria_unclassified|c__Bacteria_unclassified|o__Bacteria_unclassified|f__Bacteria_unclassified|g__Bacte
k__Bacteria|p__Bacteria_unclassified|c__Bacteria_unclassified|o__Bacteria_unclassified|f__Bacteria_unclassified|g__Bacte
k__Bacteria|p__Bacteria_unclassified|c__Bacteria_unclassified|o__Bacteria_unclassified|f__Bacteria_unclassified|g__Bacte
k__Bacteria|p__Bacteria_unclassified|c__Bacteria_unclassified|o__Bacteria_unclassified|f__Bacteria_unclassified|g__Bacte
k__Bacteria|p__Bacteria_unclassified|c__Bacteria_unclassified|o__Bacteria_unclassified|f__Bacteria_unclassified|g__Bacte
k__Bacteria|p__Bacteria_unclassified|c__Bacteria_unclassified|o__Bacteria_unclassified|f__Bacteria_unclassified|g__Bacte
k__Bacteria|p__Bacteria_unclassified|c__Bacteria_unclassified|o__Bacteria_unclassified|f__Bacteria_unclassified|g__Bacte
k__Bacteria|p__Bacteroidota|c__Bacteroidia|o__Bacteroidales|f__Bacteroidaceae|g__Bacteroides|s__Bacteroides_thetaio
k__Bacteria|p__Bacteroidota|c__Bacteroidia|o__Bacteroidales|f__Bacteroidaceae|g__Bacteroides|s__Bacteroides_thetaio

k__Bacteria|p__Actinobacteria|c__Actinomycetia|o__Bifidobacteriales|f__Bifidobacteriaceae|g__Bifidobacterium|s__Bifid
k__Bacteria|p__Actinobacteria|c__Actinomycetia|o__Bifidobacteriales|f__Bifidobacteriaceae|g__Bifidobacterium|s__Bifid
k__Bacteria|p__Firmicutes|c__Clostridia|o__Clostridia_unclassified|f__Clostridia_unclassified|g__Clostridia_unclassified|s_
k__Bacteria|p__Firmicutes|c__Clostridia|o__Clostridia_unclassified|f__Clostridia_unclassified|g__Clostridia_unclassified|s_
k__Bacteria|p__Firmicutes|c__Clostridia|o__Eubacteriales|f__Clostridiaceae|g__Clostridiaceae_unclassified|s__Clostridiac
k__Bacteria|p__Firmicutes|c__Clostridia|o__Eubacteriales|f__Clostridiaceae|g__Clostridiaceae_unclassified|s__Clostridiac
k__Bacteria|p__Firmicutes|c__Clostridia|o__Eubacteriales|f__Clostridiaceae|g__Clostridiaceae_unclassified|s__Clostridiac
k__Bacteria|p__Firmicutes|c__Clostridia|o__Eubacteriales|f__Clostridiaceae|g__Clostridiaceae_unclassified|s__Clostridiac
k__Bacteria|p__Firmicutes|c__Clostridia|o__Eubacteriales|f__Eubacteriales_unclassified|g__Eubacteriales_unclassified|s_
k__Bacteria|p__Firmicutes|c__Clostridia|o__Eubacteriales|f__Eubacteriales_unclassified|g__Eubacteriales_unclassified|s_
k__Bacteria|p__Firmicutes|c__Erysipelotrichia|o__Erysipelotrichales|f__Erysipelotrichaceae|g__Erysipelatoclostridium|s__
k__Bacteria|p__Firmicutes|c__Erysipelotrichia|o__Erysipelotrichales|f__Erysipelotrichaceae|g__Erysipelatoclostridium|s__
k__Bacteria|p__Actinobacteria|c__Coriobacteriia|o__Coriobacteriales|f__Coriobacteriaceae|g__Coriobacteriaceae_unclass
k__Bacteria|p__Actinobacteria|c__Coriobacteriia|o__Coriobacteriales|f__Coriobacteriaceae|g__Coriobacteriaceae_unclass
k__Bacteria|p__Firmicutes|c__Clostridia|o__Eubacteriales|f__Lachnospiraceae|g__Dorea|s__Dorea_sp_5_2
k__Bacteria|p__Firmicutes|c__Clostridia|o__Eubacteriales|f__Lachnospiraceae|g__Dorea|s__Dorea_sp_5_2
k__Bacteria|p__Firmicutes|c__Erysipelotrichia|o__Erysipelotrichales|f__Erysipelotrichaceae|g__Dubosiella|s__Dubosiella_
k__Bacteria|p__Firmicutes|c__Erysipelotrichia|o__Erysipelotrichales|f__Erysipelotrichaceae|g__Dubosiella|s__Dubosiella_
k__Bacteria|p__Firmicutes|c__Erysipelotrichia|o__Erysipelotrichales|f__Erysipelotrichales_unclassified|g__Erysipelotrichal
k__Bacteria|p__Firmicutes|c__Erysipelotrichia|o__Erysipelotrichales|f__Erysipelotrichales_unclassified|g__Erysipelotrichal
k__Bacteria|p__Firmicutes|c__Clostridia|o__Eubacteriales|f__Eubacteriaceae|g__Eubacteriaceae_unclassified|s__Eubacte
k__Bacteria|p__Firmicutes|c__Clostridia|o__Eubacteriales|f__Eubacteriaceae|g__Eubacteriaceae_unclassified|s__Eubacte
k__Bacteria|p__Firmicutes|c__Clostridia|o__Eubacteriales|f__Eubacteriaceae|g__Eubacteriaceae_unclassified|s__Eubacte
k__Bacteria|p__Firmicutes|c__Clostridia|o__Eubacteriales|f__Eubacteriaceae|g__Eubacteriaceae_unclassified|s__Eubacte
k__Bacteria|p__Firmicutes|c__Clostridia|o__Eubacteriales|f__Lachnospiraceae|g__GGB20149|s__GGB20149_SGB29430
k__Bacteria|p__Firmicutes|c__Clostridia|o__Eubacteriales|f__Lachnospiraceae|g__GGB20149|s__GGB20149_SGB29430
k__Bacteria|p__Actinobacteria|c__Coriobacteriia|o__Eggerthellales|f__Eggerthellaceae|g__GGB22635|s__GGB22635_SGB
k__Bacteria|p__Actinobacteria|c__Coriobacteriia|o__Eggerthellales|f__Eggerthellaceae|g__GGB22635|s__GGB22635_SGB
k__Bacteria|p__Firmicutes|c__Clostridia|o__Eubacteriales|f__Lachnospiraceae|g__GGB25041|s__GGB25041_SGB36960
k__Bacteria|p__Firmicutes|c__Clostridia|o__Eubacteriales|f__Lachnospiraceae|g__GGB25041|s__GGB25041_SGB36960
k__Bacteria|p__Bacteroidota|c__Bacteroidia|o__Bacteroidales|f__Muribaculaceae|g__GGB27876|s__GGB27876_SGB4031
k__Bacteria|p__Bacteroidota|c__Bacteroidia|o__Bacteroidales|f__Muribaculaceae|g__GGB27876|s__GGB27876_SGB4031
k__Bacteria|p__Bacteroidota|c__Bacteroidia|o__Bacteroidales|f__Muribaculaceae|g__GGB27878|s__GGB27878_SGB4031
k__Bacteria|p__Bacteroidota|c__Bacteroidia|o__Bacteroidales|f__Muribaculaceae|g__GGB27878|s__GGB27878_SGB4031

k__Bacteria|p__Bacteroidota|c__Bacteroidia|o__Bacteroidales|f__Muribaculaceae|g__GGB27918|s__GGB27918_SGB4035
k__Bacteria|p__Bacteroidota|c__Bacteroidia|o__Bacteroidales|f__Muribaculaceae|g__GGB27918|s__GGB27918_SGB4035
k__Bacteria|p__Firmicutes|c__CFGB9508|o__OFGB9508|f__FGB9508|g__GGB28382|s__GGB28382_SGB40962
k__Bacteria|p__Firmicutes|c__CFGB9508|o__OFGB9508|f__FGB9508|g__GGB28382|s__GGB28382_SGB40962
k__Bacteria|p__Firmicutes|c__CFGB2838|o__OFGB2838|f__FGB2838|g__GGB28399|s__GGB28399_SGB40980
k__Bacteria|p__Firmicutes|c__CFGB2838|o__OFGB2838|f__FGB2838|g__GGB28399|s__GGB28399_SGB40980
k__Bacteria|p__Firmicutes|c__CFGB2838|o__OFGB2838|f__FGB2838|g__GGB28411|s__GGB28411_SGB40993
k__Bacteria|p__Firmicutes|c__CFGB2838|o__OFGB2838|f__FGB2838|g__GGB28411|s__GGB28411_SGB40993
k__Bacteria|p__Firmicutes|c__CFGB2838|o__OFGB2838|f__FGB2838|g__GGB28415|s__GGB28415_SGB40997
k__Bacteria|p__Firmicutes|c__CFGB2838|o__OFGB2838|f__FGB2838|g__GGB28415|s__GGB28415_SGB40997
k__Bacteria|p__Firmicutes|c__Clostridia|o__Eubacteriales|f__Pumilibacteraceae|g__GGB28430|s__GGB28430_SGB41013
k__Bacteria|p__Firmicutes|c__Clostridia|o__Eubacteriales|f__Pumilibacteraceae|g__GGB28430|s__GGB28430_SGB41013
k__Bacteria|p__Firmicutes|c__CFGB28439|o__OFGB28439|f__FGB28439|g__GGB28439|s__GGB28439_SGB41022
k__Bacteria|p__Firmicutes|c__CFGB28439|o__OFGB28439|f__FGB28439|g__GGB28439|s__GGB28439_SGB41022
k__Bacteria|p__Firmicutes|c__Clostridia|o__Clostridia_unclassified|f__Clostridia_unclassified|g__GGB28778|s__GGB2877
k__Bacteria|p__Firmicutes|c__Clostridia|o__Clostridia_unclassified|f__Clostridia_unclassified|g__GGB28778|s__GGB2877
k__Bacteria|p__Firmicutes|c__Clostridia|o__Eubacteriales|f__Eubacteriaceae|g__GGB28784|s__GGB28784_SGB41437
k__Bacteria|p__Firmicutes|c__Clostridia|o__Eubacteriales|f__Eubacteriaceae|g__GGB28784|s__GGB28784_SGB41437
k__Bacteria|p__Firmicutes|c__Clostridia|o__Eubacteriales|f__Lachnospiraceae|g__GGB28792|s__GGB28792_SGB41445
k__Bacteria|p__Firmicutes|c__Clostridia|o__Eubacteriales|f__Lachnospiraceae|g__GGB28792|s__GGB28792_SGB41445
k__Bacteria|p__Firmicutes|c__Clostridia|o__Eubacteriales|f__Lachnospiraceae|g__GGB28798|s__GGB28798_SGB41451
k__Bacteria|p__Firmicutes|c__Clostridia|o__Eubacteriales|f__Lachnospiraceae|g__GGB28798|s__GGB28798_SGB41451
k__Bacteria|p__Firmicutes|c__Clostridia|o__Eubacteriales|f__Lachnospiraceae|g__GGB28802|s__GGB28802_SGB41455
k__Bacteria|p__Firmicutes|c__Clostridia|o__Eubacteriales|f__Lachnospiraceae|g__GGB28802|s__GGB28802_SGB41455
k__Bacteria|p__Firmicutes|c__Clostridia|o__Eubacteriales|f__Lachnospiraceae|g__GGB28818|s__GGB28818_SGB41473
k__Bacteria|p__Firmicutes|c__Clostridia|o__Eubacteriales|f__Lachnospiraceae|g__GGB28818|s__GGB28818_SGB41473
k__Bacteria|p__Firmicutes|c__CFGB77305|o__OFGB77305|f__FGB77305|g__GGB28828|s__GGB28828_SGB41484
k__Bacteria|p__Firmicutes|c__CFGB77305|o__OFGB77305|f__FGB77305|g__GGB28828|s__GGB28828_SGB41484
k__Bacteria|p__Firmicutes|c__Clostridia|o__Eubacteriales|f__Clostridiaceae|g__GGB28851|s__GGB28851_SGB41518
k__Bacteria|p__Firmicutes|c__Clostridia|o__Eubacteriales|f__Clostridiaceae|g__GGB28851|s__GGB28851_SGB41518
k__Bacteria|p__Firmicutes|c__Clostridia|o__Eubacteriales|f__Lachnospiraceae|g__GGB28859|s__GGB28859_SGB41528
k__Bacteria|p__Firmicutes|c__Clostridia|o__Eubacteriales|f__Lachnospiraceae|g__GGB28859|s__GGB28859_SGB41528
k__Bacteria|p__Firmicutes|c__Clostridia|o__Eubacteriales|f__Lachnospiraceae|g__GGB28864|s__GGB28864_SGB41535
k__Bacteria|p__Firmicutes|c__Clostridia|o__Eubacteriales|f__Lachnospiraceae|g__GGB28864|s__GGB28864_SGB41535
k__Bacteria|p__Firmicutes|c__Clostridia|o__Eubacteriales|f__Lachnospiraceae|g__GGB28869|s__GGB28869_SGB41543
k__Bacteria|p__Firmicutes|c__Clostridia|o__Eubacteriales|f__Lachnospiraceae|g__GGB28869|s__GGB28869_SGB41543
k__Bacteria|p__Firmicutes|c__CFGB9633|o__OFGB9633|f__FGB9633|g__GGB28883|s__GGB28883_SGB41564
k__Bacteria|p__Firmicutes|c__CFGB9633|o__OFGB9633|f__FGB9633|g__GGB28883|s__GGB28883_SGB41564
k__Bacteria|p__Bacteria_unclassified|c__Bacteria_unclassified|o__Bacteria_unclassified|f__Bacteria_unclassified|g__GGB
k__Bacteria|p__Bacteria_unclassified|c__Bacteria_unclassified|o__Bacteria_unclassified|f__Bacteria_unclassified|g__GGB
k__Bacteria|p__Bacteria_unclassified|c__Bacteria_unclassified|o__Bacteria_unclassified|f__Bacteria_unclassified|g__GGB
k__Bacteria|p__Bacteria_unclassified|c__Bacteria_unclassified|o__Bacteria_unclassified|f__Bacteria_unclassified|g__GGB
k__Bacteria|p__Bacteria_unclassified|c__Bacteria_unclassified|o__Bacteria_unclassified|f__Bacteria_unclassified|g__GGB
k__Bacteria|p__Bacteria_unclassified|c__Bacteria_unclassified|o__Bacteria_unclassified|f__Bacteria_unclassified|g__GGB
k__Bacteria|p__Bacteria_unclassified|c__Bacteria_unclassified|o__Bacteria_unclassified|f__Bacteria_unclassified|g__GGB
k__Bacteria|p__Bacteria_unclassified|c__Bacteria_unclassified|o__Bacteria_unclassified|f__Bacteria_unclassified|g__GGB

k__Bacteria|p__Firmicutes|c__Clostridia|o__Eubacteriales|f__Lachnospiraceae|g__GGB28916|s__GGB28916_SGB41612
k__Bacteria|p__Firmicutes|c__Clostridia|o__Eubacteriales|f__Lachnospiraceae|g__GGB28916|s__GGB28916_SGB41612
k__Bacteria|p__Firmicutes|c__Clostridia|o__Eubacteriales|f__Lachnospiraceae|g__GGB28924|s__GGB28924_SGB41621
k__Bacteria|p__Firmicutes|c__Clostridia|o__Eubacteriales|f__Lachnospiraceae|g__GGB28924|s__GGB28924_SGB41621
k__Bacteria|p__Firmicutes|c__Clostridia|o__Eubacteriales|f__Lachnospiraceae|g__GGB28926|s__GGB28926_SGB41624
k__Bacteria|p__Firmicutes|c__Clostridia|o__Eubacteriales|f__Lachnospiraceae|g__GGB28926|s__GGB28926_SGB41624
k__Bacteria|p__Bacteria_unclassified|c__CFGB77359|o__OFGB77359|f__FGB77359|g__GGB28927|s__GGB28927_SGB416
k__Bacteria|p__Bacteria_unclassified|c__CFGB77359|o__OFGB77359|f__FGB77359|g__GGB28927|s__GGB28927_SGB416
k__Bacteria|p__Firmicutes|c__CFGB9639|o__OFGB9639|f__FGB9639|g__GGB28934|s__GGB28934_SGB41635
k__Bacteria|p__Firmicutes|c__CFGB9639|o__OFGB9639|f__FGB9639|g__GGB28934|s__GGB28934_SGB41635
k__Bacteria|p__Firmicutes|c__Clostridia|o__Eubacteriales|f__Lachnospiraceae|g__GGB28946|s__GGB28946_SGB41652
k__Bacteria|p__Firmicutes|c__Clostridia|o__Eubacteriales|f__Lachnospiraceae|g__GGB28946|s__GGB28946_SGB41652
k__Bacteria|p__Firmicutes|c__Clostridia|o__Eubacteriales|f__Lachnospiraceae|g__GGB28949|s__GGB28949_SGB41655
k__Bacteria|p__Firmicutes|c__Clostridia|o__Eubacteriales|f__Lachnospiraceae|g__GGB28949|s__GGB28949_SGB41655
k__Bacteria|p__Firmicutes|c__Clostridia|o__Eubacteriales|f__Lachnospiraceae|g__GGB28949|s__GGB28949_SGB41656
k__Bacteria|p__Firmicutes|c__Clostridia|o__Eubacteriales|f__Lachnospiraceae|g__GGB28949|s__GGB28949_SGB41656
k__Bacteria|p__Firmicutes|c__Clostridia|o__Eubacteriales|f__Clostridiaceae|g__GGB28950|s__GGB28950_SGB41657
k__Bacteria|p__Firmicutes|c__Clostridia|o__Eubacteriales|f__Clostridiaceae|g__GGB28950|s__GGB28950_SGB41657
k__Bacteria|p__Firmicutes|c__Clostridia|o__Eubacteriales|f__Clostridiaceae|g__GGB28951|s__GGB28951_SGB102295
k__Bacteria|p__Firmicutes|c__Clostridia|o__Eubacteriales|f__Clostridiaceae|g__GGB28951|s__GGB28951_SGB102295
k__Bacteria|p__Firmicutes|c__Clostridia|o__Eubacteriales|f__Clostridiaceae|g__GGB28951|s__GGB28951_SGB41658
k__Bacteria|p__Firmicutes|c__Clostridia|o__Eubacteriales|f__Clostridiaceae|g__GGB28951|s__GGB28951_SGB41658
k__Bacteria|p__Firmicutes|c__Clostridia|o__Eubacteriales|f__Clostridiaceae|g__GGB28954|s__GGB28954_SGB41662
k__Bacteria|p__Firmicutes|c__Clostridia|o__Eubacteriales|f__Clostridiaceae|g__GGB28954|s__GGB28954_SGB41662
k__Bacteria|p__Firmicutes|c__Clostridia|o__Eubacteriales|f__Clostridiaceae|g__GGB28956|s__GGB28956_SGB41665
k__Bacteria|p__Firmicutes|c__Clostridia|o__Eubacteriales|f__Clostridiaceae|g__GGB28956|s__GGB28956_SGB41665
k__Bacteria|p__Firmicutes|c__Clostridia|o__Eubacteriales|f__Clostridiaceae|g__GGB28960|s__GGB28960_SGB41669
k__Bacteria|p__Firmicutes|c__Clostridia|o__Eubacteriales|f__Clostridiaceae|g__GGB28960|s__GGB28960_SGB41669
k__Bacteria|p__Firmicutes|c__Clostridia|o__Eubacteriales|f__Clostridiaceae|g__GGB28967|s__GGB28967_SGB41678
k__Bacteria|p__Firmicutes|c__Clostridia|o__Eubacteriales|f__Clostridiaceae|g__GGB28967|s__GGB28967_SGB41678
k__Bacteria|p__Firmicutes|c__Clostridia|o__Eubacteriales|f__Eubacteriaceae|g__GGB28991|s__GGB28991_SGB41705
k__Bacteria|p__Firmicutes|c__Clostridia|o__Eubacteriales|f__Eubacteriaceae|g__GGB28991|s__GGB28991_SGB41705
k__Bacteria|p__Firmicutes|c__CFGB9658|o__OFGB9658|f__FGB9658|g__GGB29002|s__GGB29002_SGB41718
k__Bacteria|p__Firmicutes|c__CFGB9658|o__OFGB9658|f__FGB9658|g__GGB29002|s__GGB29002_SGB41718
k__Bacteria|p__Firmicutes|c__CFGB9659|o__OFGB9659|f__FGB9659|g__GGB29003|s__GGB29003_SGB41719
k__Bacteria|p__Firmicutes|c__CFGB9659|o__OFGB9659|f__FGB9659|g__GGB29003|s__GGB29003_SGB41719
k__Bacteria|p__Bacteria_unclassified|c__Bacteria_unclassified|o__Bacteria_unclassified|f__Bacteria_unclassified|g__GGB2
k__Bacteria|p__Bacteria_unclassified|c__Bacteria_unclassified|o__Bacteria_unclassified|f__Bacteria_unclassified|g__GGB2
k__Bacteria|p__Firmicutes|c__CFGB9827|o__OFGB9827|f__FGB9827|g__GGB29531|s__GGB29531_SGB42317
k__Bacteria|p__Firmicutes|c__CFGB9827|o__OFGB9827|f__FGB9827|g__GGB29531|s__GGB29531_SGB42317
k__Bacteria|p__Firmicutes|c__Clostridia|o__Eubacteriales|f__Eubacteriaceae|g__GGB29685|s__GGB29685_SGB42494
k__Bacteria|p__Firmicutes|c__Clostridia|o__Eubacteriales|f__Eubacteriaceae|g__GGB29685|s__GGB29685_SGB42494
k__Bacteria|p__Bacteria_unclassified|c__CFGB77303|o__OFGB77303|f__FGB77303|g__GGB30141|s__GGB30141_SGB430
k__Bacteria|p__Bacteria_unclassified|c__CFGB77303|o__OFGB77303|f__FGB77303|g__GGB30141|s__GGB30141_SGB430
k__Bacteria|p__Firmicutes|c__Clostridia|o__Eubacteriales|f__Eubacteriales_unclassified|g__GGB30286|s__GGB30286_SG
k__Bacteria|p__Firmicutes|c__Clostridia|o__Eubacteriales|f__Eubacteriales_unclassified|g__GGB30286|s__GGB30286_SG

k__Bacteria|p__Firmicutes|c__Clostridia|o__Eubacteriales|f__Oscillospiraceae|g__GGB30303|s__GGB30303_SGB43268
k__Bacteria|p__Firmicutes|c__Clostridia|o__Eubacteriales|f__Oscillospiraceae|g__GGB30303|s__GGB30303_SGB43268
k__Bacteria|p__Firmicutes|c__CFGB30328|o__OFGB30328|f__FGB30328|g__GGB30413|s__GGB30413_SGB43452
k__Bacteria|p__Firmicutes|c__CFGB30328|o__OFGB30328|f__FGB30328|g__GGB30413|s__GGB30413_SGB43452
k__Bacteria|p__Firmicutes|c__Clostridia|o__Eubacteriales|f__Oscillospiraceae|g__GGB30454|s__GGB30454_SGB43514
k__Bacteria|p__Firmicutes|c__Clostridia|o__Eubacteriales|f__Oscillospiraceae|g__GGB30454|s__GGB30454_SGB43514
k__Bacteria|p__Firmicutes|c__Clostridia|o__Eubacteriales|f__Oscillospiraceae|g__GGB30455|s__GGB30455_SGB43519
k__Bacteria|p__Firmicutes|c__Clostridia|o__Eubacteriales|f__Oscillospiraceae|g__GGB30455|s__GGB30455_SGB43519
k__Bacteria|p__Firmicutes|c__Clostridia|o__Eubacteriales|f__Oscillospiraceae|g__GGB30461|s__GGB30461_SGB43527
k__Bacteria|p__Firmicutes|c__Clostridia|o__Eubacteriales|f__Oscillospiraceae|g__GGB30461|s__GGB30461_SGB43527
k__Bacteria|p__Firmicutes|c__Clostridia|o__Eubacteriales|f__Oscillospiraceae|g__GGB30461|s__GGB30461_SGB43530
k__Bacteria|p__Firmicutes|c__Clostridia|o__Eubacteriales|f__Oscillospiraceae|g__GGB30461|s__GGB30461_SGB43530
k__Bacteria|p__Firmicutes|c__Clostridia|o__Eubacteriales|f__Oscillospiraceae|g__GGB30463|s__GGB30463_SGB43537
k__Bacteria|p__Firmicutes|c__Clostridia|o__Eubacteriales|f__Oscillospiraceae|g__GGB30463|s__GGB30463_SGB43537
k__Bacteria|p__Firmicutes|c__Clostridia|o__Eubacteriales|f__Oscillospiraceae|g__GGB30473|s__GGB30473_SGB43557
k__Bacteria|p__Firmicutes|c__Clostridia|o__Eubacteriales|f__Oscillospiraceae|g__GGB30473|s__GGB30473_SGB43557
k__Bacteria|p__Firmicutes|c__Clostridia|o__Eubacteriales|f__Oscillospiraceae|g__GGB30475|s__GGB30475_SGB63182
k__Bacteria|p__Firmicutes|c__Clostridia|o__Eubacteriales|f__Oscillospiraceae|g__GGB30475|s__GGB30475_SGB63182
k__Bacteria|p__Actinobacteria|c__CFGB77153|o__OFGB77153|f__FGB77153|g__GGB30861|s__GGB30861_SGB44083
k__Bacteria|p__Actinobacteria|c__CFGB77153|o__OFGB77153|f__FGB77153|g__GGB30861|s__GGB30861_SGB44083
k__Bacteria|p__Tenericutes|c__CFGB1791|o__OFGB1791|f__FGB1791|g__GGB31312|s__GGB31312_SGB44628
k__Bacteria|p__Tenericutes|c__CFGB1791|o__OFGB1791|f__FGB1791|g__GGB31312|s__GGB31312_SGB44628
k__Bacteria|p__Firmicutes|c__CFGB10290|o__OFGB10290|f__FGB10290|g__GGB31438|s__GGB31438_SGB44768
k__Bacteria|p__Firmicutes|c__CFGB10290|o__OFGB10290|f__FGB10290|g__GGB31438|s__GGB31438_SGB44768
k__Bacteria|p__Firmicutes|c__Clostridia|o__Eubacteriales|f__Oscillospiraceae|g__GGB3171|s__GGB3171_SGB4185
k__Bacteria|p__Firmicutes|c__Clostridia|o__Eubacteriales|f__Oscillospiraceae|g__GGB3171|s__GGB3171_SGB4185
k__Bacteria|p__Firmicutes|c__CFGB1765|o__OFGB1765|f__FGB1765|g__GGB31823|s__GGB31823_SGB45199
k__Bacteria|p__Firmicutes|c__CFGB1765|o__OFGB1765|f__FGB1765|g__GGB31823|s__GGB31823_SGB45199
k__Bacteria|p__Firmicutes|c__CFGB10349|o__OFGB10349|f__FGB10349|g__GGB31853|s__GGB31853_SGB45233
k__Bacteria|p__Firmicutes|c__CFGB10349|o__OFGB10349|f__FGB10349|g__GGB31853|s__GGB31853_SGB45233
k__Bacteria|p__Firmicutes|c__CFGB10667|o__OFGB10667|f__FGB10667|g__GGB32371|s__GGB32371_SGB41694
k__Bacteria|p__Firmicutes|c__CFGB10667|o__OFGB10667|f__FGB10667|g__GGB32371|s__GGB32371_SGB41694
k__Bacteria|p__Firmicutes|c__Clostridia|o__Eubacteriales|f__Lachnospiraceae|g__GGB3793|s__GGB3793_SGB5158
k__Bacteria|p__Firmicutes|c__Clostridia|o__Eubacteriales|f__Lachnospiraceae|g__GGB3793|s__GGB3793_SGB5158
k__Bacteria|p__Firmicutes|c__Clostridia|o__Eubacteriales|f__Lachnospiraceae|g__GGB42598|s__GGB42598_SGB59794
k__Bacteria|p__Firmicutes|c__Clostridia|o__Eubacteriales|f__Lachnospiraceae|g__GGB42598|s__GGB42598_SGB59794
k__Bacteria|p__Firmicutes|c__Clostridia|o__Eubacteriales|f__Christensenellaceae|g__GGB45656|s__GGB45656_SGB6337[illegible]
k__Bacteria|p__Firmicutes|c__Clostridia|o__Eubacteriales|f__Christensenellaceae|g__GGB45656|s__GGB45656_SGB6337[illegible]
k__Bacteria|p__Firmicutes|c__CFGB10299|o__OFGB10299|f__FGB10299|g__GGB47127|s__GGB47127_SGB65054
k__Bacteria|p__Firmicutes|c__CFGB10299|o__OFGB10299|f__FGB10299|g__GGB47127|s__GGB47127_SGB65054
k__Bacteria|p__Firmicutes|c__Clostridia|o__Eubacteriales|f__Oscillospiraceae|g__GGB74395|s__GGB74395_SGB43521
k__Bacteria|p__Firmicutes|c__Clostridia|o__Eubacteriales|f__Oscillospiraceae|g__GGB74395|s__GGB74395_SGB43521
k__Bacteria|p__Firmicutes|c__Clostridia|o__Eubacteriales|f__Oscillospiraceae|g__GGB75053|s__GGB75053_SGB43494
k__Bacteria|p__Firmicutes|c__Clostridia|o__Eubacteriales|f__Oscillospiraceae|g__GGB75053|s__GGB75053_SGB43494
k__Bacteria|p__Firmicutes|c__Clostridia|o__Eubacteriales|f__Lachnospiraceae|g__GGB75109|s__GGB75109_SGB102238
k__Bacteria|p__Firmicutes|c__Clostridia|o__Eubacteriales|f__Lachnospiraceae|g__GGB75109|s__GGB75109_SGB102238

k__Bacteria|p__Firmicutes|c__Clostridia|o__Clostridia_unclassified|f__Clostridia_unclassified|g__GGB81440|s__GGB8144
k__Bacteria|p__Firmicutes|c__Clostridia|o__Clostridia_unclassified|f__Clostridia_unclassified|g__GGB81440|s__GGB8144
k__Bacteria|p__Firmicutes|c__Clostridia|o__Eubacteriales|f__Lachnospiraceae|g__Lachnospiraceae_unclassified|s__Lachn
k__Bacteria|p__Firmicutes|c__Clostridia|o__Eubacteriales|f__Lachnospiraceae|g__Lachnospiraceae_unclassified|s__Lachn
k__Bacteria|p__Firmicutes|c__Clostridia|o__Eubacteriales|f__Lachnospiraceae|g__Lachnospiraceae_unclassified|s__Lachn
k__Bacteria|p__Firmicutes|c__Clostridia|o__Eubacteriales|f__Lachnospiraceae|g__Lachnospiraceae_unclassified|s__Lachn
k__Bacteria|p__Firmicutes|c__Clostridia|o__Eubacteriales|f__Lachnospiraceae|g__Lachnospiraceae_unclassified|s__Lachn
k__Bacteria|p__Firmicutes|c__Clostridia|o__Eubacteriales|f__Lachnospiraceae|g__Lachnospiraceae_unclassified|s__Lachn
k__Bacteria|p__Firmicutes|c__Clostridia|o__Eubacteriales|f__Lachnospiraceae|g__Lachnospiraceae_unclassified|s__Lachn
k__Bacteria|p__Firmicutes|c__Clostridia|o__Eubacteriales|f__Lachnospiraceae|g__Lachnospiraceae_unclassified|s__Lachn
k__Bacteria|p__Firmicutes|c__Clostridia|o__Eubacteriales|f__Lachnospiraceae|g__Lachnospiraceae_unclassified|s__Lachn
k__Bacteria|p__Firmicutes|c__Clostridia|o__Eubacteriales|f__Lachnospiraceae|g__Lachnospiraceae_unclassified|s__Lachn
k__Bacteria|p__Firmicutes|c__Clostridia|o__Eubacteriales|f__Lachnospiraceae|g__Lachnospiraceae_unclassified|s__Lachn
k__Bacteria|p__Firmicutes|c__Clostridia|o__Eubacteriales|f__Lachnospiraceae|g__Lachnospiraceae_unclassified|s__Lachn
k__Bacteria|p__Firmicutes|c__Clostridia|o__Eubacteriales|f__Lachnospiraceae|g__Lachnospiraceae_unclassified|s__Lachn
k__Bacteria|p__Firmicutes|c__Clostridia|o__Eubacteriales|f__Lachnospiraceae|g__Lachnospiraceae_unclassified|s__Lachn
k__Bacteria|p__Firmicutes|c__Clostridia|o__Eubacteriales|f__Lachnospiraceae|g__Lachnospiraceae_unclassified|s__Lachn
k__Bacteria|p__Firmicutes|c__Bacilli|o__Lactobacillales|f__Lactobacillaceae|g__Lactobacillus|s__Lactobacillus_johnsonii
k__Bacteria|p__Firmicutes|c__Bacilli|o__Lactobacillales|f__Lactobacillaceae|g__Lactobacillus|s__Lactobacillus_johnsonii
k__Bacteria|p__Bacteroidota|c__Bacteroidia|o__Bacteroidales|f__Muribaculaceae|g__Muribaculaceae_unclassified|s__M
k__Bacteria|p__Bacteroidota|c__Bacteroidia|o__Bacteroidales|f__Muribaculaceae|g__Muribaculaceae_unclassified|s__M
k__Bacteria|p__Firmicutes|c__Clostridia|o__Eubacteriales|f__Oscillospiraceae|g__Neglectibacter|s__Neglectibacter_sp_X
k__Bacteria|p__Firmicutes|c__Clostridia|o__Eubacteriales|f__Oscillospiraceae|g__Neglectibacter|s__Neglectibacter_sp_X
k__Bacteria|p__Firmicutes|c__Clostridia|o__Eubacteriales|f__Oscillospiraceae|g__Oscillospiraceae_unclassified|s__Oscillo
k__Bacteria|p__Firmicutes|c__Clostridia|o__Eubacteriales|f__Oscillospiraceae|g__Oscillospiraceae_unclassified|s__Oscillo
k__Bacteria|p__Firmicutes|c__Clostridia|o__Eubacteriales|f__Oscillospiraceae|g__Oscillospiraceae_unclassified|s__Oscillo
k__Bacteria|p__Firmicutes|c__Clostridia|o__Eubacteriales|f__Oscillospiraceae|g__Oscillospiraceae_unclassified|s__Oscillo
k__Bacteria|p__Firmicutes|c__Clostridia|o__Eubacteriales|f__Oscillospiraceae|g__Oscillospiraceae_unclassified|s__Oscillo
k__Bacteria|p__Firmicutes|c__Clostridia|o__Eubacteriales|f__Oscillospiraceae|g__Oscillospiraceae_unclassified|s__Oscillo
k__Bacteria|p__Proteobacteria|c__Betaproteobacteria|o__Burkholderiales|f__Sutterellaceae|g__Parasutterella|s__Parasu
k__Bacteria|p__Proteobacteria|c__Betaproteobacteria|o__Burkholderiales|f__Sutterellaceae|g__Parasutterella|s__Parasu

k__Bacteria|p__Firmicutes|c__Clostridia|o__Eubacteriales|f__Peptostreptococcaceae|g__Romboutsia|s__Romboutsia_ilea
k__Bacteria|p__Firmicutes|c__Clostridia|o__Eubacteriales|f__Peptostreptococcaceae|g__Romboutsia|s__Romboutsia_ilea
k__Bacteria|p__Firmicutes|c__Clostridia|o__Eubacteriales|f__Lachnospiraceae|g__Schaedlerella|s__Schaedlerella_arabino
k__Bacteria|p__Firmicutes|c__Clostridia|o__Eubacteriales|f__Lachnospiraceae|g__Schaedlerella|s__Schaedlerella_arabino

k__Bacteria|p__Firmicutes|c__Erysipelotrichia|o__Erysipelotrichales|f__Turicibacteraceae|g__Turicibacter|s__Turicibacter_
k__Bacteria|p__Firmicutes|c__Erysipelotrichia|o__Erysipelotrichales|f__Turicibacteraceae|g__Turicibacter|s__Turicibacter_
k__Bacteria|p__Firmicutes|c__Clostridia|o__Eubacteriales|f__Lachnospiraceae|g__Acetatifactor|s__Acetatifactor_muris
k__Bacteria|p__Firmicutes|c__Clostridia|o__Eubacteriales|f__Lachnospiraceae|g__Acetatifactor|s__Acetatifactor_muris
k__Bacteria|p__Firmicutes|c__Clostridia|o__Eubacteriales|f__Lachnospiraceae|g__Acetatifactor|s__Acetatifactor_SGB415
k__Bacteria|p__Firmicutes|c__Clostridia|o__Eubacteriales|f__Lachnospiraceae|g__Acetatifactor|s__Acetatifactor_SGB415

k__Bacteria|p__Firmicutes|c__Clostridia|o__Eubacteriales|f__Oscillospiraceae|g__Acutalibacter|s__Acutalibacter_muris
k__Bacteria|p__Firmicutes|c__Clostridia|o__Eubacteriales|f__Oscillospiraceae|g__Acutalibacter|s__Acutalibacter_muris
k__Bacteria|p__Firmicutes|c__Clostridia|o__Eubacteriales|f__Oscillospiraceae|g__Acutalibacter|s__Acutalibacter_sp_1XD
k__Bacteria|p__Firmicutes|c__Clostridia|o__Eubacteriales|f__Oscillospiraceae|g__Acutalibacter|s__Acutalibacter_sp_1XD
k__Bacteria|p__Actinobacteria|c__Coriobacteriia|o__Eggerthellales|f__Eggerthellaceae|g__Adlercreutzia|s__Adlercreutzia
k__Bacteria|p__Actinobacteria|c__Coriobacteriia|o__Eggerthellales|f__Eggerthellaceae|g__Adlercreutzia|s__Adlercreutzia
k__Bacteria|p__Actinobacteria|c__Coriobacteriia|o__Eggerthellales|f__Eggerthellaceae|g__Adlercreutzia|s__Adlercreutzia
k__Bacteria|p__Actinobacteria|c__Coriobacteriia|o__Eggerthellales|f__Eggerthellaceae|g__Adlercreutzia|s__Adlercreutzia
k__Bacteria|p__Actinobacteria|c__Coriobacteriia|o__Eggerthellales|f__Eggerthellaceae|g__Adlercreutzia|s__Adlercreutzia
k__Bacteria|p__Actinobacteria|c__Coriobacteriia|o__Eggerthellales|f__Eggerthellaceae|g__Adlercreutzia|s__Adlercreutzia
k__Bacteria|p__Verrucomicrobia|c__Verrucomicrobiae|o__Verrucomicrobiales|f__Akkermansiaceae|g__Akkermansia|s__A
k__Bacteria|p__Verrucomicrobia|c__Verrucomicrobiae|o__Verrucomicrobiales|f__Akkermansiaceae|g__Akkermansia|s__A
k__Bacteria|p__Bacteroidota|c__Bacteroidia|o__Bacteroidales|f__Rikenellaceae|g__Alistipes|s__Alistipes_sp_DSM_11234
k__Bacteria|p__Bacteroidota|c__Bacteroidia|o__Bacteroidales|f__Rikenellaceae|g__Alistipes|s__Alistipes_sp_DSM_11234
k__Bacteria|p__Firmicutes|c__Clostridia|o__Eubacteriales|f__Oscillospiraceae|g__Anaerotruncus|s__Anaerotruncus_sp_1
k__Bacteria|p__Firmicutes|c__Clostridia|o__Eubacteriales|f__Oscillospiraceae|g__Anaerotruncus|s__Anaerotruncus_sp_1
k__Bacteria|p__Bacteria_unclassified|c__Bacteria_unclassified|o__Bacteria_unclassified|f__Bacteria_unclassified|g__Bacte
k__Bacteria|p__Bacteria_unclassified|c__Bacteria_unclassified|o__Bacteria_unclassified|f__Bacteria_unclassified|g__Bacte
k__Bacteria|p__Bacteria_unclassified|c__Bacteria_unclassified|o__Bacteria_unclassified|f__Bacteria_unclassified|g__Bacte
k__Bacteria|p__Bacteria_unclassified|c__Bacteria_unclassified|o__Bacteria_unclassified|f__Bacteria_unclassified|g__Bacte
k__Bacteria|p__Bacteria_unclassified|c__Bacteria_unclassified|o__Bacteria_unclassified|f__Bacteria_unclassified|g__Bacte
k__Bacteria|p__Bacteria_unclassified|c__Bacteria_unclassified|o__Bacteria_unclassified|f__Bacteria_unclassified|g__Bacte
k__Bacteria|p__Bacteria_unclassified|c__Bacteria_unclassified|o__Bacteria_unclassified|f__Bacteria_unclassified|g__Bacte
k__Bacteria|p__Bacteria_unclassified|c__Bacteria_unclassified|o__Bacteria_unclassified|f__Bacteria_unclassified|g__Bacte
k__Bacteria|p__Bacteria_unclassified|c__Bacteria_unclassified|o__Bacteria_unclassified|f__Bacteria_unclassified|g__Bacte
k__Bacteria|p__Bacteria_unclassified|c__Bacteria_unclassified|o__Bacteria_unclassified|f__Bacteria_unclassified|g__Bacte
k__Bacteria|p__Bacteria_unclassified|c__Bacteria_unclassified|o__Bacteria_unclassified|f__Bacteria_unclassified|g__Bacte
k__Bacteria|p__Bacteria_unclassified|c__Bacteria_unclassified|o__Bacteria_unclassified|f__Bacteria_unclassified|g__Bacte
k__Bacteria|p__Bacteroidota|c__Bacteroidia|o__Bacteroidales|f__Bacteroidaceae|g__Bacteroides|s__Bacteroides_thetaio
k__Bacteria|p__Bacteroidota|c__Bacteroidia|o__Bacteroidales|f__Bacteroidaceae|g__Bacteroides|s__Bacteroides_thetaio

k__Bacteria|p__Actinobacteria|c__Actinomycetia|o__Bifidobacteriales|f__Bifidobacteriaceae|g__Bifidobacterium|s__Bifid
k__Bacteria|p__Actinobacteria|c__Actinomycetia|o__Bifidobacteriales|f__Bifidobacteriaceae|g__Bifidobacterium|s__Bifid
k__Bacteria|p__Firmicutes|c__Clostridia|o__Clostridia_unclassified|f__Clostridia_unclassified|g__Clostridia_unclassified|s_
k__Bacteria|p__Firmicutes|c__Clostridia|o__Clostridia_unclassified|f__Clostridia_unclassified|g__Clostridia_unclassified|s_
k__Bacteria|p__Firmicutes|c__Clostridia|o__Eubacteriales|f__Clostridiaceae|g__Clostridiaceae_unclassified|s__Clostridiac
k__Bacteria|p__Firmicutes|c__Clostridia|o__Eubacteriales|f__Clostridiaceae|g__Clostridiaceae_unclassified|s__Clostridiac
k__Bacteria|p__Firmicutes|c__Clostridia|o__Eubacteriales|f__Clostridiaceae|g__Clostridiaceae_unclassified|s__Clostridiac
k__Bacteria|p__Firmicutes|c__Clostridia|o__Eubacteriales|f__Clostridiaceae|g__Clostridiaceae_unclassified|s__Clostridiac
k__Bacteria|p__Firmicutes|c__Clostridia|o__Eubacteriales|f__Eubacteriales_unclassified|g__Eubacteriales_unclassified|s_
k__Bacteria|p__Firmicutes|c__Clostridia|o__Eubacteriales|f__Eubacteriales_unclassified|g__Eubacteriales_unclassified|s_
k__Bacteria|p__Firmicutes|c__Erysipelotrichia|o__Erysipelotrichales|f__Erysipelotrichaceae|g__Erysipelatoclostridium|s_
k__Bacteria|p__Firmicutes|c__Erysipelotrichia|o__Erysipelotrichales|f__Erysipelotrichaceae|g__Erysipelatoclostridium|s_
k__Bacteria|p__Actinobacteria|c__Coriobacteriia|o__Coriobacteriales|f__Coriobacteriaceae|g__Coriobacteriaceae_unclass
k__Bacteria|p__Actinobacteria|c__Coriobacteriia|o__Coriobacteriales|f__Coriobacteriaceae|g__Coriobacteriaceae_unclass

k__Bacteria|p__Firmicutes|c__Clostridia|o__Eubacteriales|f__Lachnospiraceae|g__Dorea|s__Dorea_sp_5_2
k__Bacteria|p__Firmicutes|c__Clostridia|o__Eubacteriales|f__Lachnospiraceae|g__Dorea|s__Dorea_sp_5_2
k__Bacteria|p__Firmicutes|c__Erysipelotrichia|o__Erysipelotrichales|f__Erysipelotrichaceae|g__Dubosiella|s__Dubosiella_
k__Bacteria|p__Firmicutes|c__Erysipelotrichia|o__Erysipelotrichales|f__Erysipelotrichaceae|g__Dubosiella|s__Dubosiella_
k__Bacteria|p__Firmicutes|c__Erysipelotrichia|o__Erysipelotrichales|f__Erysipelotrichales_unclassified|g__Erysipelotrichal
k__Bacteria|p__Firmicutes|c__Erysipelotrichia|o__Erysipelotrichales|f__Erysipelotrichales_unclassified|g__Erysipelotrichal
k__Bacteria|p__Firmicutes|c__Clostridia|o__Eubacteriales|f__Eubacteriaceae|g__Eubacteriaceae_unclassified|s__Eubacte
k__Bacteria|p__Firmicutes|c__Clostridia|o__Eubacteriales|f__Eubacteriaceae|g__Eubacteriaceae_unclassified|s__Eubacte
k__Bacteria|p__Firmicutes|c__Clostridia|o__Eubacteriales|f__Eubacteriaceae|g__Eubacteriaceae_unclassified|s__Eubacte
k__Bacteria|p__Firmicutes|c__Clostridia|o__Eubacteriales|f__Eubacteriaceae|g__Eubacteriaceae_unclassified|s__Eubacte
k__Bacteria|p__Firmicutes|c__Clostridia|o__Eubacteriales|f__Lachnospiraceae|g__GGB20149|s__GGB20149_SGB29430
k__Bacteria|p__Firmicutes|c__Clostridia|o__Eubacteriales|f__Lachnospiraceae|g__GGB20149|s__GGB20149_SGB29430
k__Bacteria|p__Actinobacteria|c__Coriobacteriia|o__Eggerthellales|f__Eggerthellaceae|g__GGB22635|s__GGB22635_SGB
k__Bacteria|p__Actinobacteria|c__Coriobacteriia|o__Eggerthellales|f__Eggerthellaceae|g__GGB22635|s__GGB22635_SGB
k__Bacteria|p__Firmicutes|c__Clostridia|o__Eubacteriales|f__Lachnospiraceae|g__GGB25041|s__GGB25041_SGB36960
k__Bacteria|p__Firmicutes|c__Clostridia|o__Eubacteriales|f__Lachnospiraceae|g__GGB25041|s__GGB25041_SGB36960
k__Bacteria|p__Bacteroidota|c__Bacteroidia|o__Bacteroidales|f__Muribaculaceae|g__GGB27876|s__GGB27876_SGB4031
k__Bacteria|p__Bacteroidota|c__Bacteroidia|o__Bacteroidales|f__Muribaculaceae|g__GGB27876|s__GGB27876_SGB4031
k__Bacteria|p__Bacteroidota|c__Bacteroidia|o__Bacteroidales|f__Muribaculaceae|g__GGB27878|s__GGB27878_SGB4031
k__Bacteria|p__Bacteroidota|c__Bacteroidia|o__Bacteroidales|f__Muribaculaceae|g__GGB27878|s__GGB27878_SGB4031
k__Bacteria|p__Bacteroidota|c__Bacteroidia|o__Bacteroidales|f__Muribaculaceae|g__GGB27918|s__GGB27918_SGB4035
k__Bacteria|p__Bacteroidota|c__Bacteroidia|o__Bacteroidales|f__Muribaculaceae|g__GGB27918|s__GGB27918_SGB4035
k__Bacteria|p__Firmicutes|c__CFGB9508|o__OFGB9508|f__FGB9508|g__GGB28382|s__GGB28382_SGB40962
k__Bacteria|p__Firmicutes|c__CFGB9508|o__OFGB9508|f__FGB9508|g__GGB28382|s__GGB28382_SGB40962
k__Bacteria|p__Firmicutes|c__CFGB2838|o__OFGB2838|f__FGB2838|g__GGB28399|s__GGB28399_SGB40980
k__Bacteria|p__Firmicutes|c__CFGB2838|o__OFGB2838|f__FGB2838|g__GGB28399|s__GGB28399_SGB40980
k__Bacteria|p__Firmicutes|c__CFGB2838|o__OFGB2838|f__FGB2838|g__GGB28411|s__GGB28411_SGB40993
k__Bacteria|p__Firmicutes|c__CFGB2838|o__OFGB2838|f__FGB2838|g__GGB28411|s__GGB28411_SGB40993
k__Bacteria|p__Firmicutes|c__CFGB2838|o__OFGB2838|f__FGB2838|g__GGB28415|s__GGB28415_SGB40997
k__Bacteria|p__Firmicutes|c__CFGB2838|o__OFGB2838|f__FGB2838|g__GGB28415|s__GGB28415_SGB40997
k__Bacteria|p__Firmicutes|c__Clostridia|o__Eubacteriales|f__Pumilibacteraceae|g__GGB28430|s__GGB28430_SGB41013
k__Bacteria|p__Firmicutes|c__Clostridia|o__Eubacteriales|f__Pumilibacteraceae|g__GGB28430|s__GGB28430_SGB41013
k__Bacteria|p__Firmicutes|c__CFGB28439|o__OFGB28439|f__FGB28439|g__GGB28439|s__GGB28439_SGB41022
k__Bacteria|p__Firmicutes|c__CFGB28439|o__OFGB28439|f__FGB28439|g__GGB28439|s__GGB28439_SGB41022
k__Bacteria|p__Firmicutes|c__Clostridia|o__Clostridia_unclassified|f__Clostridia_unclassified|g__GGB28778|s__GGB2877
k__Bacteria|p__Firmicutes|c__Clostridia|o__Clostridia_unclassified|f__Clostridia_unclassified|g__GGB28778|s__GGB2877
k__Bacteria|p__Firmicutes|c__Clostridia|o__Eubacteriales|f__Eubacteriaceae|g__GGB28784|s__GGB28784_SGB41437
k__Bacteria|p__Firmicutes|c__Clostridia|o__Eubacteriales|f__Eubacteriaceae|g__GGB28784|s__GGB28784_SGB41437
k__Bacteria|p__Firmicutes|c__Clostridia|o__Eubacteriales|f__Lachnospiraceae|g__GGB28792|s__GGB28792_SGB41445
k__Bacteria|p__Firmicutes|c__Clostridia|o__Eubacteriales|f__Lachnospiraceae|g__GGB28792|s__GGB28792_SGB41445
k__Bacteria|p__Firmicutes|c__Clostridia|o__Eubacteriales|f__Lachnospiraceae|g__GGB28798|s__GGB28798_SGB41451
k__Bacteria|p__Firmicutes|c__Clostridia|o__Eubacteriales|f__Lachnospiraceae|g__GGB28798|s__GGB28798_SGB41451
k__Bacteria|p__Firmicutes|c__Clostridia|o__Eubacteriales|f__Lachnospiraceae|g__GGB28802|s__GGB28802_SGB41455
k__Bacteria|p__Firmicutes|c__Clostridia|o__Eubacteriales|f__Lachnospiraceae|g__GGB28802|s__GGB28802_SGB41455
k__Bacteria|p__Firmicutes|c__Clostridia|o__Eubacteriales|f__Lachnospiraceae|g__GGB28818|s__GGB28818_SGB41473
k__Bacteria|p__Firmicutes|c__Clostridia|o__Eubacteriales|f__Lachnospiraceae|g__GGB28818|s__GGB28818_SGB41473

k__Bacteria|p__Firmicutes|c__CFGB77305|o__OFGB77305|f__FGB77305|g__GGB28828|s__GGB28828_SGB41484
k__Bacteria|p__Firmicutes|c__CFGB77305|o__OFGB77305|f__FGB77305|g__GGB28828|s__GGB28828_SGB41484
k__Bacteria|p__Firmicutes|c__Clostridia|o__Eubacteriales|f__Clostridiaceae|g__GGB28851|s__GGB28851_SGB41518
k__Bacteria|p__Firmicutes|c__Clostridia|o__Eubacteriales|f__Clostridiaceae|g__GGB28851|s__GGB28851_SGB41518
k__Bacteria|p__Firmicutes|c__Clostridia|o__Eubacteriales|f__Lachnospiraceae|g__GGB28859|s__GGB28859_SGB41528
k__Bacteria|p__Firmicutes|c__Clostridia|o__Eubacteriales|f__Lachnospiraceae|g__GGB28859|s__GGB28859_SGB41528
k__Bacteria|p__Firmicutes|c__Clostridia|o__Eubacteriales|f__Lachnospiraceae|g__GGB28864|s__GGB28864_SGB41535
k__Bacteria|p__Firmicutes|c__Clostridia|o__Eubacteriales|f__Lachnospiraceae|g__GGB28864|s__GGB28864_SGB41535
k__Bacteria|p__Firmicutes|c__Clostridia|o__Eubacteriales|f__Lachnospiraceae|g__GGB28869|s__GGB28869_SGB41543
k__Bacteria|p__Firmicutes|c__Clostridia|o__Eubacteriales|f__Lachnospiraceae|g__GGB28869|s__GGB28869_SGB41543
k__Bacteria|p__Firmicutes|c__CFGB9633|o__OFGB9633|f__FGB9633|g__GGB28883|s__GGB28883_SGB41564
k__Bacteria|p__Firmicutes|c__CFGB9633|o__OFGB9633|f__FGB9633|g__GGB28883|s__GGB28883_SGB41564
k__Bacteria|p__Bacteria_unclassified|c__Bacteria_unclassified|o__Bacteria_unclassified|f__Bacteria_unclassified|g__GGB[illegible]
k__Bacteria|p__Bacteria_unclassified|c__Bacteria_unclassified|o__Bacteria_unclassified|f__Bacteria_unclassified|g__GGB[illegible]
k__Bacteria|p__Bacteria_unclassified|c__Bacteria_unclassified|o__Bacteria_unclassified|f__Bacteria_unclassified|g__GGB[illegible]
k__Bacteria|p__Bacteria_unclassified|c__Bacteria_unclassified|o__Bacteria_unclassified|f__Bacteria_unclassified|g__GGB[illegible]
k__Bacteria|p__Bacteria_unclassified|c__Bacteria_unclassified|o__Bacteria_unclassified|f__Bacteria_unclassified|g__GGB[illegible]
k__Bacteria|p__Bacteria_unclassified|c__Bacteria_unclassified|o__Bacteria_unclassified|f__Bacteria_unclassified|g__GGB[illegible]
k__Bacteria|p__Bacteria_unclassified|c__Bacteria_unclassified|o__Bacteria_unclassified|f__Bacteria_unclassified|g__GGB[illegible]
k__Bacteria|p__Bacteria_unclassified|c__Bacteria_unclassified|o__Bacteria_unclassified|f__Bacteria_unclassified|g__GGB[illegible]
k__Bacteria|p__Firmicutes|c__Clostridia|o__Eubacteriales|f__Lachnospiraceae|g__GGB28916|s__GGB28916_SGB41612
k__Bacteria|p__Firmicutes|c__Clostridia|o__Eubacteriales|f__Lachnospiraceae|g__GGB28916|s__GGB28916_SGB41612
k__Bacteria|p__Firmicutes|c__Clostridia|o__Eubacteriales|f__Lachnospiraceae|g__GGB28924|s__GGB28924_SGB41621
k__Bacteria|p__Firmicutes|c__Clostridia|o__Eubacteriales|f__Lachnospiraceae|g__GGB28924|s__GGB28924_SGB41621
k__Bacteria|p__Firmicutes|c__Clostridia|o__Eubacteriales|f__Lachnospiraceae|g__GGB28926|s__GGB28926_SGB41624
k__Bacteria|p__Firmicutes|c__Clostridia|o__Eubacteriales|f__Lachnospiraceae|g__GGB28926|s__GGB28926_SGB41624
k__Bacteria|p__Bacteria_unclassified|c__CFGB77359|o__OFGB77359|f__FGB77359|g__GGB28927|s__GGB28927_SGB416[illegible]
k__Bacteria|p__Bacteria_unclassified|c__CFGB77359|o__OFGB77359|f__FGB77359|g__GGB28927|s__GGB28927_SGB416[illegible]
k__Bacteria|p__Firmicutes|c__CFGB9639|o__OFGB9639|f__FGB9639|g__GGB28934|s__GGB28934_SGB41635
k__Bacteria|p__Firmicutes|c__CFGB9639|o__OFGB9639|f__FGB9639|g__GGB28934|s__GGB28934_SGB41635
k__Bacteria|p__Firmicutes|c__Clostridia|o__Eubacteriales|f__Lachnospiraceae|g__GGB28946|s__GGB28946_SGB41652
k__Bacteria|p__Firmicutes|c__Clostridia|o__Eubacteriales|f__Lachnospiraceae|g__GGB28946|s__GGB28946_SGB41652
k__Bacteria|p__Firmicutes|c__Clostridia|o__Eubacteriales|f__Lachnospiraceae|g__GGB28949|s__GGB28949_SGB41655
k__Bacteria|p__Firmicutes|c__Clostridia|o__Eubacteriales|f__Lachnospiraceae|g__GGB28949|s__GGB28949_SGB41655
k__Bacteria|p__Firmicutes|c__Clostridia|o__Eubacteriales|f__Lachnospiraceae|g__GGB28949|s__GGB28949_SGB41656
k__Bacteria|p__Firmicutes|c__Clostridia|o__Eubacteriales|f__Lachnospiraceae|g__GGB28949|s__GGB28949_SGB41656
k__Bacteria|p__Firmicutes|c__Clostridia|o__Eubacteriales|f__Clostridiaceae|g__GGB28950|s__GGB28950_SGB41657
k__Bacteria|p__Firmicutes|c__Clostridia|o__Eubacteriales|f__Clostridiaceae|g__GGB28950|s__GGB28950_SGB41657
k__Bacteria|p__Firmicutes|c__Clostridia|o__Eubacteriales|f__Clostridiaceae|g__GGB28951|s__GGB28951_SGB102295
k__Bacteria|p__Firmicutes|c__Clostridia|o__Eubacteriales|f__Clostridiaceae|g__GGB28951|s__GGB28951_SGB102295
k__Bacteria|p__Firmicutes|c__Clostridia|o__Eubacteriales|f__Clostridiaceae|g__GGB28951|s__GGB28951_SGB41658
k__Bacteria|p__Firmicutes|c__Clostridia|o__Eubacteriales|f__Clostridiaceae|g__GGB28951|s__GGB28951_SGB41658
k__Bacteria|p__Firmicutes|c__Clostridia|o__Eubacteriales|f__Clostridiaceae|g__GGB28954|s__GGB28954_SGB41662
k__Bacteria|p__Firmicutes|c__Clostridia|o__Eubacteriales|f__Clostridiaceae|g__GGB28954|s__GGB28954_SGB41662
k__Bacteria|p__Firmicutes|c__Clostridia|o__Eubacteriales|f__Clostridiaceae|g__GGB28956|s__GGB28956_SGB41665
k__Bacteria|p__Firmicutes|c__Clostridia|o__Eubacteriales|f__Clostridiaceae|g__GGB28956|s__GGB28956_SGB41665

k__Bacteria|p__Firmicutes|c__Clostridia|o__Eubacteriales|f__Clostridiaceae|g__GGB28960|s__GGB28960_SGB41669
k__Bacteria|p__Firmicutes|c__Clostridia|o__Eubacteriales|f__Clostridiaceae|g__GGB28960|s__GGB28960_SGB41669
k__Bacteria|p__Firmicutes|c__Clostridia|o__Eubacteriales|f__Clostridiaceae|g__GGB28967|s__GGB28967_SGB41678
k__Bacteria|p__Firmicutes|c__Clostridia|o__Eubacteriales|f__Clostridiaceae|g__GGB28967|s__GGB28967_SGB41678
k__Bacteria|p__Firmicutes|c__Clostridia|o__Eubacteriales|f__Eubacteriaceae|g__GGB28991|s__GGB28991_SGB41705
k__Bacteria|p__Firmicutes|c__Clostridia|o__Eubacteriales|f__Eubacteriaceae|g__GGB28991|s__GGB28991_SGB41705
k__Bacteria|p__Firmicutes|c__CFGB9658|o__OFGB9658|f__FGB9658|g__GGB29002|s__GGB29002_SGB41718
k__Bacteria|p__Firmicutes|c__CFGB9658|o__OFGB9658|f__FGB9658|g__GGB29002|s__GGB29002_SGB41718
k__Bacteria|p__Firmicutes|c__CFGB9659|o__OFGB9659|f__FGB9659|g__GGB29003|s__GGB29003_SGB41719
k__Bacteria|p__Firmicutes|c__CFGB9659|o__OFGB9659|f__FGB9659|g__GGB29003|s__GGB29003_SGB41719
k__Bacteria|p__Bacteria_unclassified|c__Bacteria_unclassified|o__Bacteria_unclassified|f__Bacteria_unclassified|g__GGB[illegible]
k__Bacteria|p__Bacteria_unclassified|c__Bacteria_unclassified|o__Bacteria_unclassified|f__Bacteria_unclassified|g__GGB[illegible]
k__Bacteria|p__Firmicutes|c__CFGB9827|o__OFGB9827|f__FGB9827|g__GGB29531|s__GGB29531_SGB42317
k__Bacteria|p__Firmicutes|c__CFGB9827|o__OFGB9827|f__FGB9827|g__GGB29531|s__GGB29531_SGB42317
k__Bacteria|p__Firmicutes|c__Clostridia|o__Eubacteriales|f__Eubacteriaceae|g__GGB29685|s__GGB29685_SGB42494
k__Bacteria|p__Firmicutes|c__Clostridia|o__Eubacteriales|f__Eubacteriaceae|g__GGB29685|s__GGB29685_SGB42494
k__Bacteria|p__Bacteria_unclassified|c__CFGB77303|o__OFGB77303|f__FGB77303|g__GGB30141|s__GGB30141_SGB430[illegible]
k__Bacteria|p__Bacteria_unclassified|c__CFGB77303|o__OFGB77303|f__FGB77303|g__GGB30141|s__GGB30141_SGB430[illegible]
k__Bacteria|p__Firmicutes|c__Clostridia|o__Eubacteriales|f__Eubacteriales_unclassified|g__GGB30286|s__GGB30286_SG[illegible]
k__Bacteria|p__Firmicutes|c__Clostridia|o__Eubacteriales|f__Eubacteriales_unclassified|g__GGB30286|s__GGB30286_SG[illegible]
k__Bacteria|p__Firmicutes|c__Clostridia|o__Eubacteriales|f__Oscillospiraceae|g__GGB30303|s__GGB30303_SGB43268
k__Bacteria|p__Firmicutes|c__Clostridia|o__Eubacteriales|f__Oscillospiraceae|g__GGB30303|s__GGB30303_SGB43268
k__Bacteria|p__Firmicutes|c__CFGB30328|o__OFGB30328|f__FGB30328|g__GGB30413|s__GGB30413_SGB43452
k__Bacteria|p__Firmicutes|c__CFGB30328|o__OFGB30328|f__FGB30328|g__GGB30413|s__GGB30413_SGB43452
k__Bacteria|p__Firmicutes|c__Clostridia|o__Eubacteriales|f__Oscillospiraceae|g__GGB30454|s__GGB30454_SGB43514
k__Bacteria|p__Firmicutes|c__Clostridia|o__Eubacteriales|f__Oscillospiraceae|g__GGB30454|s__GGB30454_SGB43514
k__Bacteria|p__Firmicutes|c__Clostridia|o__Eubacteriales|f__Oscillospiraceae|g__GGB30455|s__GGB30455_SGB43519
k__Bacteria|p__Firmicutes|c__Clostridia|o__Eubacteriales|f__Oscillospiraceae|g__GGB30455|s__GGB30455_SGB43519
k__Bacteria|p__Firmicutes|c__Clostridia|o__Eubacteriales|f__Oscillospiraceae|g__GGB30461|s__GGB30461_SGB43527
k__Bacteria|p__Firmicutes|c__Clostridia|o__Eubacteriales|f__Oscillospiraceae|g__GGB30461|s__GGB30461_SGB43527
k__Bacteria|p__Firmicutes|c__Clostridia|o__Eubacteriales|f__Oscillospiraceae|g__GGB30461|s__GGB30461_SGB43530
k__Bacteria|p__Firmicutes|c__Clostridia|o__Eubacteriales|f__Oscillospiraceae|g__GGB30461|s__GGB30461_SGB43530
k__Bacteria|p__Firmicutes|c__Clostridia|o__Eubacteriales|f__Oscillospiraceae|g__GGB30463|s__GGB30463_SGB43537
k__Bacteria|p__Firmicutes|c__Clostridia|o__Eubacteriales|f__Oscillospiraceae|g__GGB30463|s__GGB30463_SGB43537
k__Bacteria|p__Firmicutes|c__Clostridia|o__Eubacteriales|f__Oscillospiraceae|g__GGB30473|s__GGB30473_SGB43557
k__Bacteria|p__Firmicutes|c__Clostridia|o__Eubacteriales|f__Oscillospiraceae|g__GGB30473|s__GGB30473_SGB43557
k__Bacteria|p__Firmicutes|c__Clostridia|o__Eubacteriales|f__Oscillospiraceae|g__GGB30475|s__GGB30475_SGB63182
k__Bacteria|p__Firmicutes|c__Clostridia|o__Eubacteriales|f__Oscillospiraceae|g__GGB30475|s__GGB30475_SGB63182
k__Bacteria|p__Actinobacteria|c__CFGB77153|o__OFGB77153|f__FGB77153|g__GGB30861|s__GGB30861_SGB44083
k__Bacteria|p__Actinobacteria|c__CFGB77153|o__OFGB77153|f__FGB77153|g__GGB30861|s__GGB30861_SGB44083
k__Bacteria|p__Tenericutes|c__CFGB1791|o__OFGB1791|f__FGB1791|g__GGB31312|s__GGB31312_SGB44628
k__Bacteria|p__Tenericutes|c__CFGB1791|o__OFGB1791|f__FGB1791|g__GGB31312|s__GGB31312_SGB44628
k__Bacteria|p__Firmicutes|c__CFGB10290|o__OFGB10290|f__FGB10290|g__GGB31438|s__GGB31438_SGB44768
k__Bacteria|p__Firmicutes|c__CFGB10290|o__OFGB10290|f__FGB10290|g__GGB31438|s__GGB31438_SGB44768
k__Bacteria|p__Firmicutes|c__Clostridia|o__Eubacteriales|f__Oscillospiraceae|g__GGB3171|s__GGB3171_SGB4185
k__Bacteria|p__Firmicutes|c__Clostridia|o__Eubacteriales|f__Oscillospiraceae|g__GGB3171|s__GGB3171_SGB4185

k__Bacteria|p__Firmicutes|c__CFGB1765|o__OFGB1765|f__FGB1765|g__GGB31823|s__GGB31823_SGB45199
k__Bacteria|p__Firmicutes|c__CFGB1765|o__OFGB1765|f__FGB1765|g__GGB31823|s__GGB31823_SGB45199
k__Bacteria|p__Firmicutes|c__CFGB10349|o__OFGB10349|f__FGB10349|g__GGB31853|s__GGB31853_SGB45233
k__Bacteria|p__Firmicutes|c__CFGB10349|o__OFGB10349|f__FGB10349|g__GGB31853|s__GGB31853_SGB45233
k__Bacteria|p__Firmicutes|c__CFGB10667|o__OFGB10667|f__FGB10667|g__GGB32371|s__GGB32371_SGB41694
k__Bacteria|p__Firmicutes|c__CFGB10667|o__OFGB10667|f__FGB10667|g__GGB32371|s__GGB32371_SGB41694
k__Bacteria|p__Firmicutes|c__Clostridia|o__Eubacteriales|f__Lachnospiraceae|g__GGB3793|s__GGB3793_SGB5158
k__Bacteria|p__Firmicutes|c__Clostridia|o__Eubacteriales|f__Lachnospiraceae|g__GGB3793|s__GGB3793_SGB5158
k__Bacteria|p__Firmicutes|c__Clostridia|o__Eubacteriales|f__Lachnospiraceae|g__GGB42598|s__GGB42598_SGB59794
k__Bacteria|p__Firmicutes|c__Clostridia|o__Eubacteriales|f__Lachnospiraceae|g__GGB42598|s__GGB42598_SGB59794
k__Bacteria|p__Firmicutes|c__Clostridia|o__Eubacteriales|f__Christensenellaceae|g__GGB45656|s__GGB45656_SGB6337
k__Bacteria|p__Firmicutes|c__Clostridia|o__Eubacteriales|f__Christensenellaceae|g__GGB45656|s__GGB45656_SGB6337
k__Bacteria|p__Firmicutes|c__CFGB10299|o__OFGB10299|f__FGB10299|g__GGB47127|s__GGB47127_SGB65054
k__Bacteria|p__Firmicutes|c__CFGB10299|o__OFGB10299|f__FGB10299|g__GGB47127|s__GGB47127_SGB65054
k__Bacteria|p__Firmicutes|c__Clostridia|o__Eubacteriales|f__Oscillospiraceae|g__GGB74395|s__GGB74395_SGB43521
k__Bacteria|p__Firmicutes|c__Clostridia|o__Eubacteriales|f__Oscillospiraceae|g__GGB74395|s__GGB74395_SGB43521
k__Bacteria|p__Firmicutes|c__Clostridia|o__Eubacteriales|f__Oscillospiraceae|g__GGB75053|s__GGB75053_SGB43494
k__Bacteria|p__Firmicutes|c__Clostridia|o__Eubacteriales|f__Oscillospiraceae|g__GGB75053|s__GGB75053_SGB43494
k__Bacteria|p__Firmicutes|c__Clostridia|o__Eubacteriales|f__Lachnospiraceae|g__GGB75109|s__GGB75109_SGB102238
k__Bacteria|p__Firmicutes|c__Clostridia|o__Eubacteriales|f__Lachnospiraceae|g__GGB75109|s__GGB75109_SGB102238
k__Bacteria|p__Firmicutes|c__Clostridia|o__Clostridia_unclassified|f__Clostridia_unclassified|g__GGB81440|s__GGB8144
k__Bacteria|p__Firmicutes|c__Clostridia|o__Clostridia_unclassified|f__Clostridia_unclassified|g__GGB81440|s__GGB8144
k__Bacteria|p__Firmicutes|c__Clostridia|o__Eubacteriales|f__Lachnospiraceae|g__Lachnospiraceae_unclassified|s__Lachn
k__Bacteria|p__Firmicutes|c__Clostridia|o__Eubacteriales|f__Lachnospiraceae|g__Lachnospiraceae_unclassified|s__Lachn
k__Bacteria|p__Firmicutes|c__Clostridia|o__Eubacteriales|f__Lachnospiraceae|g__Lachnospiraceae_unclassified|s__Lachn
k__Bacteria|p__Firmicutes|c__Clostridia|o__Eubacteriales|f__Lachnospiraceae|g__Lachnospiraceae_unclassified|s__Lachn
k__Bacteria|p__Firmicutes|c__Clostridia|o__Eubacteriales|f__Lachnospiraceae|g__Lachnospiraceae_unclassified|s__Lachn
k__Bacteria|p__Firmicutes|c__Clostridia|o__Eubacteriales|f__Lachnospiraceae|g__Lachnospiraceae_unclassified|s__Lachn
k__Bacteria|p__Firmicutes|c__Clostridia|o__Eubacteriales|f__Lachnospiraceae|g__Lachnospiraceae_unclassified|s__Lachn
k__Bacteria|p__Firmicutes|c__Clostridia|o__Eubacteriales|f__Lachnospiraceae|g__Lachnospiraceae_unclassified|s__Lachn
k__Bacteria|p__Firmicutes|c__Clostridia|o__Eubacteriales|f__Lachnospiraceae|g__Lachnospiraceae_unclassified|s__Lachn
k__Bacteria|p__Firmicutes|c__Clostridia|o__Eubacteriales|f__Lachnospiraceae|g__Lachnospiraceae_unclassified|s__Lachn
k__Bacteria|p__Firmicutes|c__Clostridia|o__Eubacteriales|f__Lachnospiraceae|g__Lachnospiraceae_unclassified|s__Lachn
k__Bacteria|p__Firmicutes|c__Clostridia|o__Eubacteriales|f__Lachnospiraceae|g__Lachnospiraceae_unclassified|s__Lachn
k__Bacteria|p__Firmicutes|c__Clostridia|o__Eubacteriales|f__Lachnospiraceae|g__Lachnospiraceae_unclassified|s__Lachn
k__Bacteria|p__Firmicutes|c__Clostridia|o__Eubacteriales|f__Lachnospiraceae|g__Lachnospiraceae_unclassified|s__Lachn
k__Bacteria|p__Firmicutes|c__Clostridia|o__Eubacteriales|f__Lachnospiraceae|g__Lachnospiraceae_unclassified|s__Lachn
k__Bacteria|p__Firmicutes|c__Clostridia|o__Eubacteriales|f__Lachnospiraceae|g__Lachnospiraceae_unclassified|s__Lachn
k__Bacteria|p__Firmicutes|c__Bacilli|o__Lactobacillales|f__Lactobacillaceae|g__Lactobacillus|s__Lactobacillus_johnsonii
k__Bacteria|p__Firmicutes|c__Bacilli|o__Lactobacillales|f__Lactobacillaceae|g__Lactobacillus|s__Lactobacillus_johnsonii
k__Bacteria|p__Bacteroidota|c__Bacteroidia|o__Bacteroidales|f__Muribaculaceae|g__Muribaculaceae_unclassified|s__M
k__Bacteria|p__Bacteroidota|c__Bacteroidia|o__Bacteroidales|f__Muribaculaceae|g__Muribaculaceae_unclassified|s__M
k__Bacteria|p__Firmicutes|c__Clostridia|o__Eubacteriales|f__Oscillospiraceae|g__Neglectibacter|s__Neglectibacter_sp_X
k__Bacteria|p__Firmicutes|c__Clostridia|o__Eubacteriales|f__Oscillospiraceae|g__Neglectibacter|s__Neglectibacter_sp_X
k__Bacteria|p__Firmicutes|c__Clostridia|o__Eubacteriales|f__Oscillospiraceae|g__Oscillospiraceae_unclassified|s__Oscillo
k__Bacteria|p__Firmicutes|c__Clostridia|o__Eubacteriales|f__Oscillospiraceae|g__Oscillospiraceae_unclassified|s__Oscillo

k__Bacteria|p__Firmicutes|c__Clostridia|o__Eubacteriales|f__Oscillospiraceae|g__Oscillospiraceae_unclassified|s__Oscillo
k__Bacteria|p__Firmicutes|c__Clostridia|o__Eubacteriales|f__Oscillospiraceae|g__Oscillospiraceae_unclassified|s__Oscillo
k__Bacteria|p__Firmicutes|c__Clostridia|o__Eubacteriales|f__Oscillospiraceae|g__Oscillospiraceae_unclassified|s__Oscillo
k__Bacteria|p__Firmicutes|c__Clostridia|o__Eubacteriales|f__Oscillospiraceae|g__Oscillospiraceae_unclassified|s__Oscillo
k__Bacteria|p__Proteobacteria|c__Betaproteobacteria|o__Burkholderiales|f__Sutterellaceae|g__Parasutterella|s__Parasu
k__Bacteria|p__Proteobacteria|c__Betaproteobacteria|o__Burkholderiales|f__Sutterellaceae|g__Parasutterella|s__Parasu

k__Bacteria|p__Firmicutes|c__Clostridia|o__Eubacteriales|f__Peptostreptococcaceae|g__Romboutsia|s__Romboutsia_ilea
k__Bacteria|p__Firmicutes|c__Clostridia|o__Eubacteriales|f__Peptostreptococcaceae|g__Romboutsia|s__Romboutsia_ilea
k__Bacteria|p__Firmicutes|c__Clostridia|o__Eubacteriales|f__Lachnospiraceae|g__Schaedlerella|s__Schaedlerella_arabino
k__Bacteria|p__Firmicutes|c__Clostridia|o__Eubacteriales|f__Lachnospiraceae|g__Schaedlerella|s__Schaedlerella_arabino

k__Bacteria|p__Firmicutes|c__Erysipelotrichia|o__Erysipelotrichales|f__Turicibacteraceae|g__Turicibacter|s__Turicibacter_
k__Bacteria|p__Firmicutes|c__Erysipelotrichia|o__Erysipelotrichales|f__Turicibacteraceae|g__Turicibacter|s__Turicibacter_
