# Supplementary material for: Non-invasive Vagal Nerve Stimulation as a Potential Treatment for Repetitive Blast Trauma
Source: bioRxiv. 2026 Jul 19:2026.07.13.737563. Preprint. [Version 1] doi: 10.64898/2026.07.13.737563 (PMC13405043; doi:10.64898/2026.07.13.737563)
Supplement: Supplement 18 [file media-18.pdf]

			95% Confidence Intervals of Blast effect on outcome (at specified VNS conditio				
			VNS (–)			VNS (+)	
**Exposure**	**Mediator (Species-level featur**	**Outcome**	**ACME**	**ADE**	**Total**	**ACME**	**ADE**
Blast	Clostridiales_bacterium	EtOH_pref_24h_final	[-0.039, 0.418]	[0.008, 1.327]	[0.147, 1.516]	[0.068, 0.758]	[-1.109, 0.053]
Blast	bacterium_1XD42_54	EtOH_pref_24h_final	[-0.582, -0.066]	[0.356, 1.818]	[0.149, 1.477]	[-0.263, 0.019]	[-0.585, 0.48]
Blast	GGB29002_SGB41718	OFB_Clatency	[-0.064, 0.188]	[0.208, 0.851]	[0.228, 0.948]	[0.124, 0.497]	[0.41, 1.204]
Blast	GGB3171_SGB4185	PD_choice_filt	[-0.434, -0.021]	[0.594, 1.863]	[0.377, 1.66]	[-0.5, -0.012]	[-0.454, 0.877]

			95% Confidence Intervals of VNS effect on outcome (at specified Blast conditio				
			Blast (–)			Blast (+)	
**Exposure**	**Mediator (Species-level featur**	**Outcome**	**ACME**	**ADE**	**Total**	**ACME**	**ADE**
VNS	GGB28883_SGB41564	OFB_Cdistance	[-0.028, 0.236]	[-0.475, 0.565]	[-0.412, 0.675]	[-0.024, 0.307]	[-0.681, 0.272]

n)					
	Taxonomic Information				
Total	Genus	Family	Order	Class	Phylum
[-0.74, 0.321]	Eubacteriales_unclassified	Eubacteriales_unclassified	Eubacteriales	Clostridia	Firmicutes
[-0.67, 0.375]	Bacteria_unclassified	Bacteria_unclassified	Bacteria_unclassified	Bacteria_unclassified	Bacteria_unclassified
[0.605, 1.52]	GGB29002	FGB9658	OFGB9658	CFGB9658	Firmicutes
[-0.769, 0.777]	GGB3171	Oscillospiraceae	Eubacteriales	Clostridia	Firmicutes

n)					
	Taxonomic Information				
Total	Genus	Family	Order	Class	Phylum
[-0.56, 0.356]	GGB28883	FGB9633	OFGB9633	CFGB9633	Firmicutes

Kingdom	MetaPhlan Annotation
Bacteria	k__Bacteria|p__Firmicutes|c__Clostridia|o__Eubacteriales|f__Eubacteriales_unclassified|g__Eubacteriales_unclassified|s__Clostridiales_bacterium
Bacteria	k__Bacteria|p__Bacteria_unclassified|c__Bacteria_unclassified|o__Bacteria_unclassified|f__Bacteria_unclassified|g__Bacteria_unclassified|s__bacter
Bacteria	k__Bacteria|p__Firmicutes|c__CFGB9658|o__OFGB9658|f__FGB9658|g__GGB29002|s__GGB29002_SGB41718
Bacteria	k__Bacteria|p__Firmicutes|c__Clostridia|o__Eubacteriales|f__Oscillospiraceae|g__GGB3171|s__GGB3171_SGB4185

Kingdom	MetaPhlan Annotation
Bacteria	k__Bacteria|p__Firmicutes|c__CFGB9633|o__OFGB9633|f__FGB9633|g__GGB28883|s__GGB28883_SGB41564

rium_1XD42_54
